# Differential β-glucosidase expression as a function of carbon source availability in *Talaromyces amestolkiae*: a genomic and proteomic approach

**DOI:** 10.1186/s13068-017-0844-7

**Published:** 2017-06-23

**Authors:** Laura I. de Eugenio, Juan A. Méndez-Líter, Manuel Nieto-Domínguez, Lola Alonso, Jesús Gil-Muñoz, Jorge Barriuso, Alicia Prieto, María Jesús Martínez

**Affiliations:** 1 Department of Environmental Biology, Centro de Investigaciones Biológicas, CSIC, Ramiro de Maeztu 9, 28040 Madrid, Spain; 2 Genetic and Molecular Epidemiology Group, Human Cancer Genetics Programme, Spanish National Cancer Research Centre, CNIO, Melchor Fernández Almagro 3, 28029 Madrid, Spain

**Keywords:** Fungi, Ascomycete, Glycosidase, Lignocellulosic biomass, Saccharification

## Abstract

**Background:**

Genomic and proteomic analysis are potent tools for metabolic characterization of microorganisms. Although cellulose usually triggers cellulase production in cellulolytic fungi, the secretion of the different enzymes involved in polymer conversion is subjected to different factors, depending on growth conditions. These enzymes are key factors in biomass exploitation for second generation bioethanol production. Although highly effective commercial cocktails are available, they are usually deficient for β-glucosidase activity, and genera like *Penicillium* and *Talaromyces* are being explored for its production.

**Results:**

This article presents the description of *Talaromyces amestolkiae* as a cellulase-producer fungus that secretes high levels of β-glucosidase. β-1,4-endoglucanase, exoglucanase, and β-glucosidase activities were quantified in the presence of different carbon sources. Although the two first activities were only induced with cellulosic substrates, β-glucosidase levels were similar in all carbon sources tested. Sequencing and analysis of the genome of this fungus revealed multiple genes encoding β-glucosidases. Extracellular proteome analysis showed different induction patterns. In all conditions assayed, glycosyl hydrolases were the most abundant proteins in the supernatants, albeit the ratio of the diverse enzymes from this family depended on the carbon source. At least two different β-glucosidases have been identified in this work: one is induced by cellulose and the other one is carbon source-independent. The crudes induced by Avicel and glucose were independently used as supplements for saccharification of slurry from acid-catalyzed steam-exploded wheat straw, obtaining the highest yields of fermentable glucose using crudes induced by cellulose.

**Conclusions:**

The genome of *T. amestolkiae* contains several genes encoding β-glucosidases and the fungus secretes high levels of this activity, regardless of the carbon source availability, although its production is repressed by glucose. Two main different β-glucosidases have been identified from proteomic shotgun analysis. One of them is produced under different carbon sources, while the other is induced in cellulosic substrates and is a good supplement to Celluclast in saccharification of pretreated wheat straw.

**Electronic supplementary material:**

The online version of this article (doi:10.1186/s13068-017-0844-7) contains supplementary material, which is available to authorized users.

## Background

Second generation bioethanol represents an efficient alternative to conventional energy supply, involving the exploitation of renewable sources, usually disposable wastes from other industrial or agricultural activities. In this process, polysaccharides from lignocellulosic biomass are hydrolyzed to glucose and xylose units that are further converted to ethanol [1]. When raw materials with hardly available polysaccharides are used, a pretreatment is needed in order to facilitate its accessibility. Steam-explosion is maybe the most commonly applied method to perform this pretreatment, generating a biomass slurry that usually contains by-products which adversely affect downstream steps like enzymatic hydrolysis or ethanol fermentation [2, 3]. Although all stages are relevant for the process, the improvement of the composition and dosage of the hydrolytic enzymatic cocktails remains a subject of great interest to industry. An efficient hydrolysis of carbohydrates, especially cellulose, the major polysaccharide from plant biomass, is indispensable to recover fermentable sugars from feedstock. Cellulose is a homopolysaccharide of β-1,4-linked d-glucose residues whose enzymatic conversion into free glucose requires the coordinated work of the cellulase system, consisting of three different kinds of hydrolases: (1) cellobiohydrolases (CBH) named also as exoglucanases, which cleave cellobiose units from the chain ends, (2) β-1,4-endoglucanases (EG), which hydrolyze the polymers internally, reducing their degree of polymerization and (3) β-glucosidases (BGLU), which convert cellobiose and soluble cellodextrins into glucose [4].

The main industrial producers of cellulases are *Trichoderma reesei* and *Aspergillus niger* [5, 6], but the enzymatic crudes released by these microorganisms, in particular those from *Trichoderma* sp., are deficient in β-glucosidase [7], and the saccharification cocktails must be supplemented with this activity to increase the efficiency of cellulose degradation.

The current approach for production of lignocellulosic bioethanol relies on combining different cellulase cocktails, which is very effective but involves a significant increase of the production costs. Then, many efforts are being devoted to discover microorganisms that secrete high amounts of glycosidases and to search for novel robust enzymes, which efficiently transform cell-wall polysaccharides, even in the presence of the undesirable by-products found in the process streams. In this sense, *Penicillium* sp. and its perfect states (*Talaromyces* or *Eupenicillium*) have attracted much attention in the last years for their high cellulase activity [8, 9]. In addition, these fungi could have special interest for cellulose and hemicelluloses transformation in the context of bioethanol production or other industrial applications, since they also produce high levels of xylanases [10–12].

This work reports the characterization of *T. amestolkiae* as cellulase producer, and the sequencing and assembly of its genome. The cellulolytic activities secreted upon fungal growth in several carbon sources were quantified, and the pool of extracellular proteins in each one of the secretomes was analyzed by massive peptide analysis. The data obtained were comprehensively related to the number of genes codifying for members of different glycosyl hydrolases families (GH) in *T. amestolkiae* genome, showing that β-glucosidases are highly represented from DNA to proteins secreted in this fungus. The role of these enzymes in saccharification of wheat straw acid slurry is discussed.

## Methods

### Fungal strain and culture media

The fungus was isolated from cereal wastes and deposited in the IJFM culture collection at “Centro de Investigaciones Biológicas” (Madrid, Spain), with the reference A795. This isolate was identified on the basis of molecular and phenotypic analyses (see Additional file 1), and hereinafter, it will be denominated as *T. amestolkiae* CIB. Fungal cultures were grown on 2% agar-malt Petri dishes at 28 °C and incubated for 7 days to obtain spore suspensions. One agar plug of about 1 cm^2^ was cut from actively growing mycelium and deposited in 15 mL Falcon tubes with 5 mL of 1% NaCl with 0.1% Tween 80, shaken, and 200 μL withdrawn to inoculate 250 mL Erlenmeyer flasks with 50 mL of CSS medium (28 °C, 250 rpm for 7 days). CSS medium (pH 5.6) contained (L^−1^): 40 g glucose, 0.4 g FeSO_4_ × 7H_2_O, 9 g (NH_4_)_2_SO_4_, 4 g K_2_HPO_4_, 26.3 g corn steep solids, 7 g CaCO_3_, and 2.8 mL soybean oil. Two mL of these cultures were used as inocula for the production of cellulase activities in Mandels medium [13]. Its components are (L^−1^): 2.0 g KH_2_PO_4_, 1.3 g (NH_4_)_2_SO_4_, 0.3 g urea, 0.3 g MgSO_4_·7H_2_O, 0.3 g CaCl_2_, 5 mg FeSO_4_·7H_2_O, 1.6 mg MnSO_4_·H_2_O, 1.4 mg ZnSO_4_·7H_2_O, and 1 g Bacto Peptone. The medium was supplemented with 1% Avicel, glucose, acid slurry from wheat straw (acid-catalyzed steam-exploded wheat straw provided by Abengoa), maltose, fructose, glycerol, xylose or cellobiose, or 2% beechwood xylan (reported as the best concentration for xylanases’ production [14]) as carbon sources. In all cases, the cultures were carried out in 250 mL Erlenmeyer flasks with 50 mL of culture medium and incubated at 28 °C and 250 rpm. Samples were taken periodically from three replicate flasks and the mycelium separated from the culture liquid by centrifugation at 13,000 × *g* and 4 °C for 5 min.

### Enzyme and protein assays and other determinations

Proteins were quantified according to the Bradford method using the Bio-Rad reagent and bovine serum albumin as the standard. Avicelase (total microcrystalline-cellulose hydrolyzing activity, as indicative of exocellulase activity) and β-1,4-endoglucanase activities were measured by determining the release of reducing sugars by the Somogyi–Nelson method [15]. The standard enzymatic assays were performed in 50 mM sodium citrate buffer, pH 5.0, containing appropriately diluted crudes and 1% Avicel (Merck) or 2% low viscosity carboxymethylcellulose (CMC, Sigma), as substrates for Avicelase and β-1,4-endoglucanase activities, respectively. Linearity of the enzymatic assays was checked by performing the reaction at two different incubation times (5 and 10 min). β-glucosidase activity was assayed spectrophotometrically following *p*-nitrophenol (*p*NP) release (*ε*_410_ = 15,200 M^−1^ cm^−1^) from *p*-nitrophenyl-β-d-glucopyranoside (Sigma-Aldrich), in 100 mM sodium citrate buffer pH 5, using 1.4% sodium carbonate to stop the reaction. One unit of activity was defined as the amount of enzyme releasing 1 μmol of reducing sugars or *p*NP per minute under the above conditions. Direct quantification of glucose and cellobiose was accomplished by high-performance liquid chromatography (HPLC) on an Agilent 1200 series system equipped with a refractive index detector. Aliquots of 100 μL were loaded onto a SUPELCOGEL C-G610H column (Sigma) equilibrated with 5 mM H_2_SO_4_. The column was previously calibrated by injecting 100 μL of glucose or cellobiose in a concentration range from 0.5 to 60 mM. From the area under the peaks, a calibration curve was calculated for each compound. Peaks were identified from their retention times, by comparison with those of the commercial standards, and their concentrations were calculated from the calibration curves.

### Genome sequencing and assembly

DNA was extracted with the DNeasy Plant minikit (Qiagen). Sequencing was performed on an Illumina HiSeqTM 2000 system. 90 bp long reads were obtained by paired-end sequencing of a DNA library composed of 537 ± 396 bp inserts. The Illumina GA Pipeline version 1.5 was used for a first removal of adapter sequences, contamination and low-quality reads from raw reads. Then A5-miseq pipeline [16] was used in order to carry out a subsequent filtering of the reads, the assembly, and the scaffolding. Assembly was performed *de novo* and evaluated in terms of N50 and L50. This Whole Genome Shotgun project has been deposited at DDBJ/ENA/GenBank under the accession MIKG00000000. The version described in this paper is version MIKG01000000.

### Gene prediction, general function annotation, and CAZyME prediction

Gene models were predicted by AUGUSTUS trained with *Aspergillus fumigatus* [17]. The obtained open reading frames (ORFs) were used as query to interrogate the KEGG and KOG databases using KAAS [18] and WebMGA [19] servers, respectively, in order to assign general protein functions profiles. The specific annotation of CAZy families [20, 21] was accomplished by submitting the predicted genes to dbCAN and filtering the results by applying an *E* value <1·10^−20^ as cutoff. Then, the assignment of β-glucosidase function was carried out by doing a BLASTP of the predicted glycosyl hydrolases from *T. amestolkiae* against the characterized GHs from the CAZy database and annotating the function of the best hit with an *E* value <1·10^−20^. The presence or absence of signal peptide in all the predicted proteins was analyzed using Phobius, a combined transmembrane topology and signal peptide predictor [22].

### Shotgun analysis of the secretomes

For differential proteomic analysis, samples of the extracellular pool of proteins from culture supernatants of *T. amestolkiae* grown in the different substrates were independently analyzed. Two mL of the liquid supernatants from 7-day-old cultures were centrifuged at 14,000×*g* for 5 min to remove insoluble material and freeze-dried. Then, samples containing 5 μg of proteins were first dissolved in sample buffer, denatured, loaded in a 12% SDS-gel and allowed to run for 10 min in order to remove non-protein compounds before the proteomic analysis. The protein band was horizontally divided into two similar portions and each one was excised in small pieces and destained with 50 mM ammonium bicarbonate/50% ACN (acetonitrile), dehydrated with ACN and dried. Samples were reduced by adding DTT to a final concentration of 10 mM and alkylated with iodoacetamide to a final concentration of 50 mM. Then, gel pieces were dried, rehydrated with 12.5 ng/µL trypsin in 50 mM ammonium bicarbonate, and incubated overnight at 30 °C. Peptides were extracted at 37 °C using 100% ACN and 0.5% TFA, respectively. The peptide pool was dried and then cleaned using ZipTip with 0.6 µL C18 resin (Millipore) and reconstituted in 5 µL 0.1% formic acid/2% ACN (v/v) prior to MS analysis.

All peptide separations were carried out on a NanoEasy HPLC (Proxeon Biosystems) coupled to a nanoelectrospray ion source (Proxeon Biosystems). For each analysis, the sample peptides were loaded onto a C18-A1 ASY-Column 2 cm precolumn (Thermo Scientific) and then eluted onto a Biosphere C18 column (C18, inner diameter 75 µm, 15 cm long, 3 µm particle size, Nano Separations). The mobile phase flow rate was 250 nL/min using 0.1% formic acid, 2% ACN in water (solvent A) and 0.1% formic acid and 100% ACN (solvent B). The gradient profile was set as follows: 0–35% solvent B for 120 min, 35–45% solvent B for 20 min, 45–95% solvent B for 9 and 5 min isocratically at 95%. Six microliters of each sample were injected. Full-scan MS spectra (*m/z* 300–1800) were acquired in the LTQ-Orbitrap Velos in the positive ion mode with a target value of 1,000,000 at a resolution of 60,000 and the 15 most intense ions were selected for collision-induced dissociation (CID) fragmentation in the LTQ with a target value of 10,000 and normalized collision energy of 35%.

Mass spectra *.raw files were searched against an in—house specific database of the *T. amestolkiae* genome (10408 sequences, 5662098 residues) using the SEQUEST search engine through Proteome Discoverer (version 1.4.1.14, Thermo). Precursor and fragments mass tolerance were set to 10 ppm and 0.5 Da, respectively. Search parameters included a maximum of two missed cleavages allowed, carbamidomethylation of cysteines as a fixed modification and oxidation of methionine as a variable modification. Peptides were validated through the algorithm Percolator [23] and only those with high confidence were admitted (FDR 0.01). Unless otherwise specified, protein identifications were accepted if they contained at least two identified peptides. Results were inferred from data obtained from two technical replicates from two different biological samples. Relative low precision quantification of the proteins in the samples analyzed was estimated from the sum of the number of peptide spectrum matches (PSMs) corresponding to each protein [24–26]. These values were used to calculate the percentage of individual proteins or categories (% of GHs,  % of BGLUs) in the whole protein pool. The data provided in tables and figures correspond to the mean value of the %PSMs from the biological replicates.

### Wheat straw saccharification

Celluclast 1.5 L FG (Novozymes) was used as commercial cocktail for saccharification. An amount of Celluclast containing 0.5 U of BGLU activity was added to 66.6 mg/mL of acid slurry from wheat straw (40% content in cellulose) in 1.5 mL sodium acetate 100 mM, pH 4, at 50 °C. Samples were taken periodically and the glucose released was quantified using the Glucose TR kit from Spinreact.

Celluclast was supplemented with 0.5 U of BGLU activity from N50010 (Novozyme), TAM377 (*T. amestolkiae* enzymatic crude produced using glucose as carbon source) or TAM3821 (*T. amestolkiae* enzymatic crude produced using Avicel as carbon source).

## Results and discussion

### Cellulase production by *T. amestolkiae* CIB

**Fig. 1:**
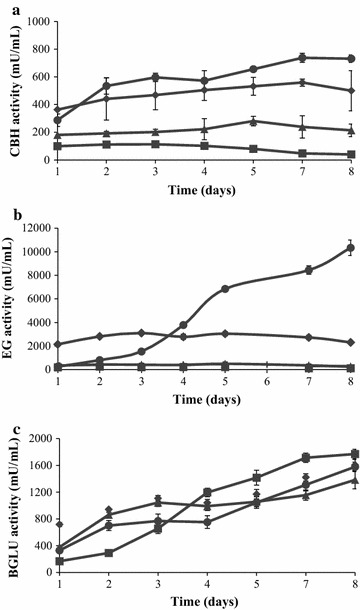
Extracellular cellulase production by *Talaromyces amestolkiae* grown on Mandels medium with different carbon sources. *Circles* Avicel (1%); *squares* glucose (1%); *triangles* xylan (2%); *diamonds* slurry (1%). Samples were taken each 24 h, and the different enzymatic activities were quantified in the supernatants: **a** exocellulase, **b** endo β-(1,4) glucanase and **c** β-glucosidase

**Fig. 2:**
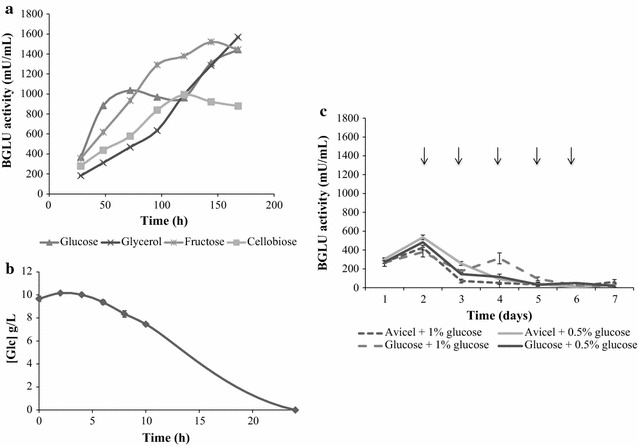
Effect of the carbon source and glucose addition on BGLU production. **a** BGLU production in different soluble carbon sources. The fungus was grown in Mandels with 1% glucose (*triangles*), glycerol (*crosses*), fructose (*asterisks*), or cellobiose (*squares*) for 7 days. Samples were taken daily and BGLU activity was measured in the supernatants. **b** Glucose consumption measured in Mandels + 1% glucose cultures. Samples were taken daily and glucose concentration of supernatants was measured by HPLC as described in “Methods”. **c** Effect of glucose addition on BGLU production. To study the influence of glucose, 0.5 or 1% glucose pulse feeds were daily added to 1% glucose or Avicel cultures. *Arrows* indicate the addition of the monosaccharide. Samples were taken daily and BGLU activity measured in the supernatants

Different carbon sources, namely Avicel (microcrystalline cellulose), beechwood xylan, and wheat straw slurry, were tested as inducers of the cellulase activities. In addition, a culture with glucose, which has been described as cellulases’ inhibitor [27, 28], was grown under the same conditions. According to the results shown in Fig. 1, Avicel seemed to be the best substrate for cellulase production. Cellobiohydrolases and β-1,4-endoglucanase activities (Fig. 1a, b) were mainly detected in media containing Avicel or acid wheat straw slurry. Nevertheless, as this last substrate is a mixture of lignocellulose components, the production of cellulolytic enzymes was slightly lower. β-1,4-endoglucanase activity was strongly induced by Avicel (over 10 U/mL after 8 days), being also measurable in cultures containing slurry. BGLU activity reached similar values regardless of the carbon source used (1.4–1.8 U/mL), even with glucose (Fig. 1c). It is well known that, in general, Avicelase and β-1,4-endoglucanase production are repressed in the presence of glucose [29], but little is known about BGLU production in the presence of easily available carbon sources. Non-inducible BGLU activity has been described in some fungal species [30, 31] but, in most cases, the secretion of this enzyme is induced by cellulose [30]. In addition, the regulation and expression of cellulolytic enzymes can be affected by the presence of inducers, derived from cellulose metabolism, as oligosaccharides or transglycosylation products, or experience catabolic repression by glucose, as reported in *T. reesei* [32]. Then, the effect of small molecules as cellobiose, glycerol, fructose, and xylose on BGLU production was also assayed (Fig. 2a). In virtually all cases, the released activity was very similar (around 1.4 U/mL) and comparable to that obtained before for polymeric cellulosic substrates and glucose, getting lower values for cellobiose, the only disaccharide evaluated (1 U/mL), which needs to be transformed in glucose before being consumed by the fungus. These data demonstrate that this *T. amestolkiae* strain does not require specific inducers for BGLU production.

Monitoring glucose consumption in cultures containing this monosaccharide as carbon source revealed the complete depletion of the sugar after 24 h (Fig. 2b), which may indicate that, in this case, carbon starvation is triggering BGLU production. Daily addition of 0.5 or 1% glucose to cultures in Mandels medium containing either 1% glucose or Avicel (Fig. 2c) caused only basal production of BGLU, confirming that the production of this enzyme is repressed by glucose. Thus, when easily assimilable carbon sources are exhausted, carbon starvation might induce BGLU production in *T. amestolkiae,* while the production of CBH and EG is induced only when cellulosic substrates are present.

However, these data raised the question of whether the BGLU secreted under carbon starvation and in the presence of cellulose are the same enzyme or not. Preliminary results from zimograms after isoelectric focusing suggested that they could be different proteins (not shown). The production of basal levels of cellulases, usually Avicelase and β-1,4-endoglucanase, has been described and interpreted as a fungal strategy intended to take advantage of any cellulosic material present in the surrounding medium [27]. On the contrary, the production of BGLU in a carbon source-independent way could be due to its role in the regulation of cellulase production. In this sense, BGLU from *T. reesei* can convert cellobiose into sophorose, a strong cellulase inducer [33].

In this work, the analysis of cellulase diversity in *T. amestolkiae* and its differential production in specific conditions was tackled according to several strategies, as reported above.

### General features of *T. amestolkiae* CIB genome

In a first approach, the cellulolytic potential of the fungus was analyzed through the sequencing and annotation of its genome. The draft genome sequence of *T. amestolkiae* CIB was based on high-throughput sequencing system (~65-fold coverage) and *de novo* assembling. The 24,330,860 pair-ended reads were incorporated into 215 scaffolds, among which 132 comprised more than 1 kb. Genome size was determined to be 33.7 Mb and N50 and L50 statistics displayed values of 1,486,010 and 9, respectively. The assembled genome resulted in the prediction of 10,408 ORFs (Additional file 2). As seen below, 342 of these ORFs were supported by the secretome data. The prediction of GH enzymes was performed by submitting the putative ORFs to the dbCAN server and filtering the results with the selected cutoff. By this way, 325 glycosyl hydrolases were identified, a number close to those described for *Fusarium verticillioides* (332), *Aspergillus flavus* (334), and *Aspergillus oryzae* (317) [34].

### β-glucosidases are highly represented in the genome of *T. amestolkiae*

**Fig. 3:**
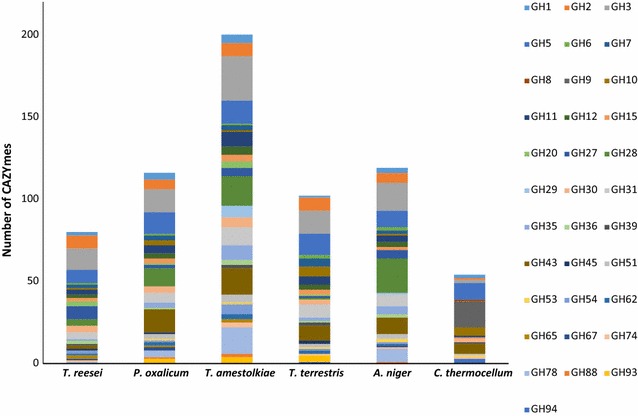
Comparative analysis of the number of CAZyme proteins predicted from genomes of different cellulolytic organisms. Data from GH families that include cell-wall-degrading enzymes have been selected for this representation. From *left* to *right*, putative GHs from the genomes of *T. reesei, P. oxalicum* 114-2, *T. amestolkiae* CIB, *T. terrestris* NRRL 8126, *A. niger* CBS 513.88 and *C. thermocellum* ATCC 27405

Glycosyl hydrolases involved in hydrolysis of lignocellulose are widely extended across the CAZy families [34]. Figure 3 depicts the profile of the main families of CAZymes implicated in plant cell-wall degradation encoded by the genomes of *T. amestolkiae* and six microorganisms of remarkable lignocellulolytic interest. The selection of these species was made attending to the criteria of relevance and availability of accurately annotated genomic data. *T. reesei* is the main industrial source of the cellulases and hemicellulases added to commercial cocktails for lignocellulose saccharification [6, 35]. Other fungi, as *Aspergillus niger* and *Penicillium oxalicum* (previously classified as *Penicillium decumbens*) are also industrial producers of cellulolytic enzymes [36, 37]. *Thielavia terrestris* secretes thermostable hydrolases [38], and *Clostridium thermocellum* is a well-known Firmicutes capable of directly converting cellulose into ethanol and other value-added products [39, 40].

Data analysis revealed specific CAZyme profiles for each species although, as expected, the bacterium displayed the biggest differences. Distinctive CAZyme profiles among plant cell-wall-degrading fungi have been linked to nutritional mode adaptations and may imply different strategies for attacking lignocellulose at the enzymatic level [34, 41, 42]. Regarding *T. amestolkiae*, the number of encoded CAZymes was significantly higher than in the other organisms. In particular, the high amount of genes encoding enzymes from the GH3 family, which comprises many of the reported fungal β-glucosidases, was intriguing and it could be related to the findings of BGLU production reported above. The presence of this activity in the culture supernatants of this organism, regardless of the carbon source, may suggest a central role of these enzymes in the metabolic strategy adopted by *T. amestolkiae* along evolution.

**Fig. 4:**
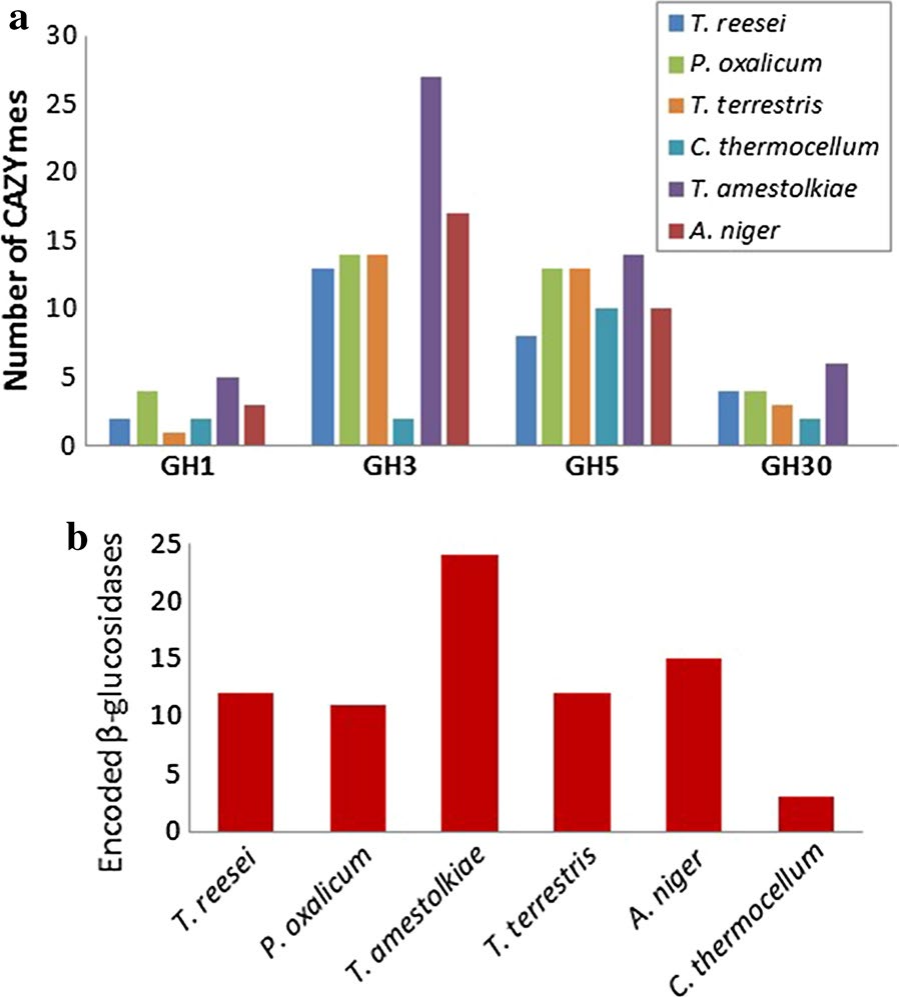
Comparison of the BGLUs predicted for *T. amestolkiae* and the referenced cellulolytic species. **a** Number of CAZyme proteins from the main families encoding BGLU (GH1, GH3, GH5, and GH30). These proteins were manually annotated by running a BLASTP against the GHs from CAZy database, and those similar to previously characterized BGLU were selected, and represented in **b**

To assess this possibility, we carried out a deeper comparison focusing on GH1, GH3, GH5, and GH30, the main GH families encoding fungal β-glucosidases (Fig. 4a). The total number of genes for these enzymes in the genome of *T. amestolkiae* was annotated by running BLASTP against the characterized GHs from the CAZy database, and compared with those reported for each of the cellulolytic species used as reference (Fig. 4b). According to our BLASTP analysis, 5 BGLUs from GH1 family, 18 from GH3, and 1 from GH5 are codified in the genome of this isolate (Table 1).

**Table 1:** Putative BGLUs identified from analysis of *T. amestolkiae* genome

ID	CAZY family	MM (kDa)	Signal peptide
g8961	GH1	53.9	No
g5848	GH1	56.1	No
g7413	GH1	55.5	No
g4650	GH1	55.5	No
g8384	GH1	243.6	Yes
g2731	GH3	66.7	No
g1119	GH3	230.5	No
g377	GH3	88.7	Yes
g3618	GH3	84.3	Yes
g3821	GH3	78.9	Yes
g7527	GH3	92.2	No
g7273	GH3	92.9	No
g3126	GH3	86.7	No
g3139	GH3	93.6	Yes
g6753	GH3	81.8	Yes
g4056	GH3	97.4	No
g6367	GH3	89.7	No
g7815	GH3	84.3	Yes
g9082	GH3	181.2	No
g9183	GH3	87.6	No
g9861	GH3	150.1	No
g6857	GH3	109.0	Yes
g9398	GH3	81.4	Yes
g9150	GH3	86.5	Yes

The presence of signal peptide was analyzed using the Phobius software

The CAZymes types and abundance represented in a given fungal genome may condition a certain strategy for hydrolyzing lignocellulose [34]. The high number of β-glucosidases annotated in *T. amestolkiae* pointed them as key factors during lignocellulose degradation by this fungus. This is especially relevant considering that commercial mixtures based on *T. reesei* crudes (the main industrial source of this type of enzymes) are deficient in β-glucosidase activity [43]. In this sense, this unexpectedly high number of cellulases and hemicellulases in *T. amestolkiae* opens the possibility of improving lignocellulose saccharification through the synergistic addition of several CAZymes.

The expression of β-glucosidases by *T. amestolkiae* in the presence of different lignocellulosic substrates was validated, beyond the genomic level, by proteomic analysis of the extracellular proteins secreted by this fungus.

### Differential shotgun analysis of proteins secreted by *T. amestolkiae*

The enzymatic pools released under the four assayed conditions were compared by sequential tryptic digestion and LC–MS/MS of the whole peptide mixtures produced (Additional file 3). The number of proteins identified in each secretome differed according to the complexity of the carbon source used for fungal growth. The maximum number of identifications came from samples produced in the two polymeric substrates, xylan (184) and slurry (144), while only 119 and 104 proteins were recognized in the secretomes from glucose and Avicel cultures, respectively. Xylan and slurry are structurally complex materials that contain heterogeneous and branched polymers. Hence, it is expectable that the amount and variety of extracellular enzymes required to accomplish their degradation is higher than those needed to metabolize simpler substrates.

Table 2 lists the ten extracellular proteins identified with maximal confidence (with the highest scores) in each condition. Mainly CBH, followed by xylanases and BGLU, was produced when the fungus grew in the medium with Avicel, and the same distribution was observed in cultures with wheat straw slurry. The presence of xylanases in the secretome of *P. chrysosporium* grown in cellulose has already been reported [44]. β-xylosidases were detected only in xylan cultures, as previously described [14]. Surprisingly, a glutaminase was expressed in all conditions. This protein might be released upon induction of protein catabolism by early carbon starvation [45], and its production in *Thermomyces lanuginosus* SSBP cultures in corncob has been previously described [46]. Other extracellular proteins with different functionalities were also detected in glucose cultures. Some of them are probably involved in the metabolism of fungal carbohydrates, as an hexosaminidase [47]. For example, cellulase regulation in *H. jecorina* proceeds through deglycosylation catalyzed by glucosaminidases, which can modulate enzyme activity by this mechanism [48]. Under starvation stress, the increased production of other enzymes involved in fungal cell-wall degradation, as β-1,3-glucanases, has also been observed. The heterogeneity of the proteins identified in the extracellular pool of proteins from glucose cultures is in agreement with the high variability detected by KOG functional analysis (see below). The PSM values of two independent experiments were taken as low precision, semi-quantitative records for further evaluation of the shotgun analysis. These values account for the number of scans identified for each protein, which are roughly related to the amount of a given protein in the sample [49], being analyzed from different perspectives: functional analysis (KOG attribution), GHs diversity, and relative abundance of cellulases and hemicellulases.

**Table 2:** Summary of the ten extracellular proteins identified with maximal scores from the shotgun analysis

	ID	Predicted protein function	GH family	Score	UP	MM (kDa)	pI
Avicel	g2234	Cellobiohydrolase	GH7	49,485.86	14	55.8	4.81
	g5707	Endoglucanase	GH6	15,441.61	8	48.3	4.79
	g9427	Endoxylanase	GH10	4256.38	12	43.5	5.25
	g3995	Glutaminase		3534.39	10	76.4	4.53
	g4058	Swollenin		3179.49	4	41.6	4.54
	g3821	Beta-glucosidase	GH3	2764.60	14	78.9	5.22
	g2018	Endoglucanase	GH74	2689.52	10	78.2	4.94
	g1296	Endoxylanase	GH11	2684.90	3	22.8	5.39
	g2158	Glucoamylase	GH15	2148.19	9	65.2	4.56
	g6537	Endoglucanase	GH5	1555.14	8	62.0	4.81
Glucose	g377	Beta-glucosidase	GH3	20,095.77	24	88.7	4.91
	g2140	Glucoamylase	GH15	18,940.98	18	67.7	4.92
	g3995	Glutaminase		10,898.10	14	76.4	4.53
	g2158	Glucoamylase	GH15	9928.19	12	65.2	4.56
	g9324	Exo-beta-1.3-glucanase	GH55	8297.36	12	84.3	4.79
	g8295	Alpha-glucosidase	GH31	7988.51	19	98.6	4.77
	g3279	Alpha-amylase	GH13	6080.68	11	66.2	4.51
	g1839	Endo-1.3(4)-beta-glucanase	GH16	6050.23	5	30.4	4.82
	g2234	Cellobiohydrolase;	GH7	5462.33	9	55.8	4.81
	g40761	Hexosaminidase	GH20	4795.60	18	67.9	5.19
Slurry	g2234	Cellobiohydrolase	GH7	37,368.64	14	55.8	4.81
	g9427	Endoxylanase	GH10	7497.84	15	43.5	5.25
	g6537	Endoglucanase	GH5	5378.70	11	62.0	4.81
	g3995	Glutaminase		5355.00	13	76.4	4.53
	g5707	Endoglucanase	GH6	5154.87	5	48.3	4.79
	g2140	Glucoamylase	GH15	5064.97	13	67.7	4.92
	g377	Beta-glucosidase	GH3	4539.30	19	88.7	4.91
	g8295	Alpha-glucosidase	GH31	3690.24	18	98.6	4.77
	g5915	Hypothetical protein	GH55	3061.95	13	68.8	4.61
	g3707	Arabinofuranosidase	GH62	2450.28	6	41.0	4.81
Xylan	g2234	Cellobiohydrolase	GH7	49,601.87	14	55.8	4.81
	g2140	Glucoamylase	GH15	37,213.51	18	67.7	4.92
	g377	Beta-glucosidase	GH3	31,858.88	25	88.7	4.91
	g3995	Glutaminase		26,983.15	14	76.4	4.53
	g2158	Glucoamylase	GH15	22,043.67	12	65.2	4.56
	g8295	Alpha-glucosidase	GH31	17,081.31	19	98.6	4.77
	g9427	Xylanase	GH10	15,562.62	15	43.5	5.25
	g9324	Exo-beta-1.3-glucanase	GH55	11,780.55	12	84.3	4.79
	g8981	Beta-d-xylosidase	GH3	9394.38	23	83.3	4.84
	g4068	Arabinofuranosidase	GH51	9345.18	15	60.3	5.07

*UP* unique peptides

### Functional analysis of the proteins secreted in the presence of different carbon sources

**Fig. 5:**
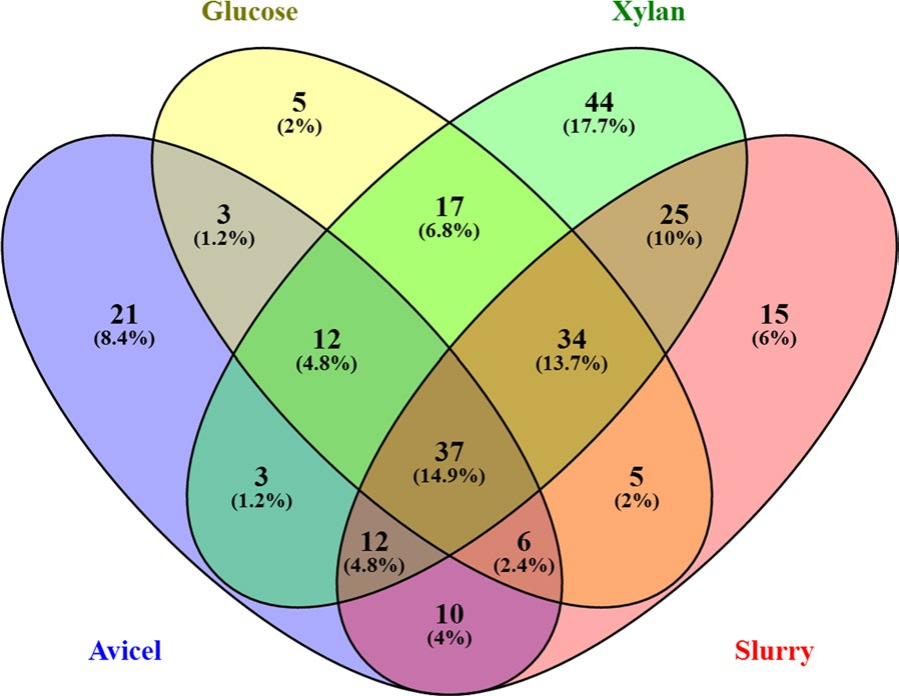
Venn representation of the number of proteins found in the different growth conditions. Numbers in brackets account for protein percentages

Functional analysis based on KOG categories (Table 3) showed that most proteins detected in all conditions tested were implicated in carbohydrate metabolism and transport (70.5–91.8%). The second most abundant group clustered enzymes involved in amino acid metabolism and transport. The rest of proteins from each one of the secretomes studied belonged to a different number of KOG categories. For each condition, the categories that group more than 2% of the total proteins identified were considered to be the most representative. These outlines describe perfectly the results deduced for Avicel cultures, in which more than 95% of the extracellular proteins are categorized in the two main groups. The enzymes in the other three secretomes studied share significant amounts of proteins involved in signal transduction and transport, energy production and conversion. The extracellular proteins from glucose cultures had the lowest representation of enzymes involved in carbohydrate metabolism and a significantly high amount of proteins related to cell-wall biogenesis, as compared to the other samples. Similarly, the notable representation of enzymes engaged in post-translational modification, protein turnover, and with chaperone functions in the proteins from glucose, xylan, and slurry supernatants merits especial attention. As commented before, the number of proteins detected in the shotgun analysis of the last two secretomes is considerably high due to the need of using a battery of enzymes to metabolize these complex and heterogeneous lignocellulosic materials, and this fact would also justify the over-representation of enzymes involved in the synthesis and secretion of the proteins required to accomplish this task. Although these are mainly intracellular proteins, they can be detected in the culture supernatant due to fungal autolysis. In fact, when the sequences of the proteins identified were examined for the presence of signal peptide, it turned out that more than 30% of proteins in glucose, xylan, and slurry supernatants were intracellular, which contrasts with the 14% of intracellular proteins in Avicel supernatants. These data coincide with the results from semi-quantitative analysis (% PSM) that indicated that more than 90% of the total protein amount from Avicel supernatants corresponds to extracellular enzymes, against 74–79% of extracellular proteins in xylan, glucose, and slurry. Similarly, the classification of the carbohydrolases identified into GH families reflected 88% of extracellular enzymes in the medium with Avicel (95% PSM), and 74, 72 and 82% (89, 78 and 89% PSM) in the supernatants from glucose, xylan, and slurry, respectively. More than 5% of the proteins detected in slurry supernatants were categorized as energy production and conversion enzymes, and may have been secreted due to the presence of lignin in the culture medium, an heterogeneous substrate that comes from plant cell wall and is catabolized by oxidases [36]. The number of different proteins identified in the extracellular medium recovered upon fungal growth in the four carbon sources was analyzed by a Venn representation (Fig. 5). According to these data, 37 proteins were produced in all conditions evaluated, and 21, 5, 44, and 15 enzymes were exclusive from Avicel, glucose, xylan, and slurry supernatants, respectively. These data agree with those accounted for total proteins, since the maximum number of total proteins was also found in xylan cultures.

**Table 3:** Functional classification of the proteins identified in the extracellular pool of proteins of *T. amestolkiae* cultured with several carbon sources

	% PSM			
	Avicel	Glucose	Slurry	Xylan
A—RNA processing and modification	0.03	0.22	0.00	0.11
C—energy production and conversion	1.48	*4.90*	*5.44*	0.11
E—amino Acid metabolism and transport	*3.53*	*10.38*	*7.47*	*9.92*
F—nucleotide metabolism and transport	0.00	1.42	0.14	1.07
G—carbohydrate metabolism and transport	*91*.*84*	*65.16*	*71.73*	*70.52*
I—lipid metabolism	0.62	0.17	2.02	1.53
M—cell wall/membrane/envelop biogenesis	0.00	*3.61*	0.89	1.13
O—post-translational modification. Protein turnover. Chaperone functions	0.51	1.71	2.15	1.64
Q—secondary structure	0.06	0.91	0.48	1.12
R—general functional prediction only	0.63	1.89	*2.56*	1.67
S—function unknown	0.37	*3.71*	*3.16*	2.31
T—signal transduction	0.40	*4.86*	*3.42*	4.03

The categories containing over 2% of the total EPP are marked in italics

In the present work, the proteomic analysis was carried out in samples from 7-day-old cultures. As already mentioned, glucose was depleted after 24 h growth, which corresponds to severe carbon starvation in these cultures. On the other hand, the scarce solubility of xylan and slurry components may lead to an overall pseudo-starvation, as oligo- and monosaccharides are presumably slowly released and quickly metabolized by the fungus. Similar cellulase induction has been reported after depletion of the carbon source and during early exposure to cellulose in *Neurospora crassa* and *A. niger* cultures [45]. Our data are consistent with those obtained for the extracellular proteins detected in carbon-starved cultures of the ascomycete *Aspergillus niger* that also cluster into multiple KOG categories [50].

### GHs are the main extracellular enzymes secreted by *T. amestolkiae*

The major enzymes secreted in all conditions tested are GHs, which constitute a very complex group of carbohydrate-active enzymes. Their current classification into families is entirely based on the similarity of proteins’ sequence, and two enzymes with the same activity can be included in different families.

As explained before, low precision quantitative data for GHs were also based on the number of PSMs (mean value from the two biological replicates). Total GH abundance was first calculated as the sum of PSMs from all GHs identified in each individual sample, and then the percentage of each enzyme was obtained. The GHs identified in the proteomic analysis were categorized into 31 families (Table 4). Proteins from family GH3, associated to degradation of plant cell-wall polysaccharides (β-glucosidases, β-xylosidases, arabinofuranosidases and exo-1,3-1,4-glucanases), are produced in all carbon sources tested. In terms of relative abundance (% PSM), these enzymes are the main extracellular GHs found in the xylan-containing medium (>25%), representing 16.3% and 13.7% in glucose and slurry media and less than 10% in the Avicel crude. GH7 proteins, which include endo-β-1,4-glucanases and reducing end-acting cellobiohydrolases, were profusely produced in the two cellulose-containing media, Avicel (47.1% of the total GHs) and slurry (29.5%), while GH6 endoglucanases and non-reducing end cellobiohydrolases were especially abundant in the secretome from the Avicel medium (15.1%). Proteins from these families were induced by xylan to a lesser extent (1.5% for GH6 and 5.6% for GH7). Family GH10 groups enzymes with endo-xylanase activity and is well represented in Avicel, slurry, and xylan crudes. In addition, α-carbohydrolases belonging to GH15 and GH31 families were identified in the samples analyzed. The huge amount of GH15 glucoamylases/glucodextranases detected in glucose cultures (28%) can be related to fungal autolysis, since α-(1-3) and α-(1-4) glucans have been reported as cell-wall components of several *Talaromyces* species [51]. On the other hand, GH31 proteins act on α-linked xylo-, manno-, galacto-, or gluco-oligosaccharides contributing to the final breakdown of oligomers released from plant hemicellulose or fungal polysaccharides.

**Table 4:** Glycosyl hydrolase families identified in secretomes of *T. amestolkiae* grown in different carbon sources

GH family	% PSM			
	Avicel	Glucose	Xylan	Slurry
GH1	0.2	*2.2*	0.4	0.5
GH2	0.5	1.7	1.9	1.5
GH3	*6.3*	*16.3*	*25.1*	*13.7*
GH5	*4.2*	0.5	1.7	*4.5*
GH6	*15.1*	0.3	1.5	*3.3*
GH7	*47.1*	*2.6*	*5.6*	*29.5*
GH10	*3.5*	0.0	*3.8*	*5.3*
GH11	1.2	0.0	0.0	0.0
GH13	0.2	*4.3*	1.8	0.8
GH15	*3.6*	*28.4*	*12.9*	*7.8*
GH16	0.4	*3.5*	0.0	1.5
GH17	0.8	1.7	1.7	0.7
GH18	0.1	0.6	*2.1*	*2.0*
GH20	0.3	*4.8*	*2.8*	1.3
GH27	1.8	1.7	*2.7*	2.0
GH30	1.0	0.5	*2.1*	1.3
GH31	1.7	*11.8*	*9.8*	4.0
GH35	0.7	1.8	*3.6*	0.4
GH43	0.2	0.1	0.8	0.4
GH47	0.4	1.1	0.9	0.2
GH51	0.0	0.0	*3.3*	0.9
GH54	1.2	0.0	0.0	*3.2*
GH55	1.0	*3.9*	*2.5*	*2.4*
GH62	1.5	0.3	1.1	*2.7*
GH71	1.0	0.1	0.2	1.0
GH72	0.2	1.3	1.5	1.1
GH74	1.6	0.1	0.5	1.1
GH92	0.4	1.1	1.2	0.4
GH95	0.5	1.2	1.1	2.4
GH125	0.8	1.7	1.3	0.2
GH127	0.5	*3.2*	*3.4*	*2.1*

The most represented families, containing over 2% of the total extracellular pool of proteins, are marked in italics. Percentage values refer to % of all GHs

**Fig. 6:**
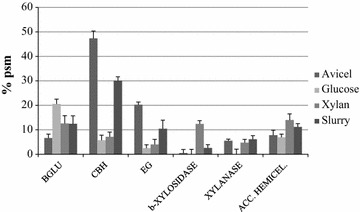
Relative abundance of cellulases and hemicellulases as a function of the carbon source. The sum of the number of PSMs of all GH proteins was taken as 100%. The putative proteins were annotated searching against entries in the CAZy database using BLASTP. Numbers account for the arithmetic mean of two biological replicates. *Acc. hemicel* accessory hemicellulases

The percentages of the different types of cellulases and hemicellulases secreted in each medium are summarized in Fig. 6.

### β-Glucosidases produced in different culture conditions

**Fig. 7:**
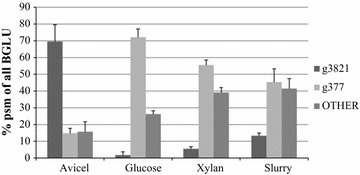
Production and percentages of different BGLUs as a function of the carbon source. The putative proteins were annotated searching against entries in the CAZy database using BLASTP. The sum of the number of PSMs of all BGLUs was taken as 100%

Further analysis of the proteomic data allowed the evaluation of the β-glucosidases induced in the carbon sources tested (Fig. 7). An approximation to the relative abundance of each one of them, calculated as the percentage of total β-glucosidases, was analyzed. The number of hypothetical BGLUs detected varied from 6 different enzymes in Avicel and glucose media to 8 and 10 for slurry and xylan cultures, respectively. Protein g377 (see Additional file 1 for sequence) was the most abundant in media containing slurry, glucose, or xylan, representing 72, 55, and 45% of the total BGLUs, respectively, while in Avicel, the main BGLU was protein g3821 (70%). However, low amounts of this enzyme were also produced in media with glucose (<2%), xylan (5%), or slurry (13%). These findings suggest that BGLU g3821 is strongly induced by cellulose but constitutively expressed in basal levels in the other conditions studied. On the contrary, the production of BGLU g377 seems to be carbon source-independent, although its synthesis and secretion may be triggered upon starvation. Preliminary RT-PCR experiments indicated that g3821 is overexpressed in Avicel cultures and repressed when glucose was used as the carbon source, while g377 is overexpressed in both media, which is compatible with a constitutive expression, independent of the presence of cellulose in the medium (data not shown).

The enzyme g377 could be responsible for the BGLU activity detected when *T. amestolkiae* was cultivated using non-cellulosic materials as carbon sources (mono and disaccharides). Similarly to this, one of the two different β-glucosidases produced by *Stachybotrys atra* is non-inducible [52]. Its role could also be related to cell-wall remodeling, as seen in some plants [53].

### *T. amestolkiae* crude supernatants as BGLU sources for saccharification

Preliminary saccharification assays were performed using Celluclast 1.5 L FG or *T. amestolkiae* crudes from Avicel as the main sources of cellulases. Released glucose was 33% higher when Celluclast 1.5 L FG was used, probably for its high content in CBH and EG compared to *T. amestolkiae* crudes, rich in BGLU.

**Fig. 8:**
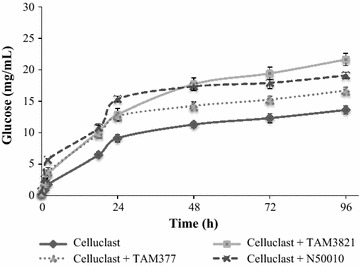
Saccharification of pretreated wheat straw with different enzymatic cocktails. Celluclast was used as the base enzymatic cocktail to perform supplementation studies adding N500510 (commercial Novozymes preparation rich in BGLU), TAM377, or TAM3821 (*T. amestolkiae* enzymatic supernatants from cultures grown in glucose or Avicel, respectively). To measure the saccharification efficiency, samples were taken periodically, and glucose was measured in supernatants as described in “Methods”

For that reason, enzymatic hydrolysis of wheat straw slurry was assayed using Celluclast 1.5 L FG as the main source of cellulases. As BGLU supplements for saccharification, 0.5 units of this activity were incorporated to the saccharification mixture using *T. amestolkiae* crudes from glucose (TAM377) or Avicel (TAM3821) culture media, or the commercial preparation N50010 from Novozymes (Fig. 8).

The treatment of wheat straw slurry with only Celluclast released 50% glucose after 96 h. In the same time period, glucose release enhanced significantly when Celluclast was supplemented with TAM3821 (80%), N50010 (73%) or TAM377 (61%).

These data show that these enzymatic crudes from *T. amestolkiae* are very efficient as supplements for lignocellulosic biomass saccharification, especially TAM3821 that provided superior glucose solubilization than N50010. This could be due to a high affinity of BGLU g3821 for cellulosic substrates, since this protein was only induced by cellulose. Nevertheless, the existence of synergistic catalysis between this BGLU and the EGs and CBHs induced by cellulosic substrates cannot be ruled out. Further purification and characterization of both BGLU will be carried out in order to ascertain their different catalytic constants, substrate specificities, and efficiencies.

## Conclusions

A strong extracellular β-glucosidase activity was detected in *T. amestolkiae* cultures grown in four carbon sources of different composition and complexity. Genome sequencing and annotation disclosed a high number of genes encoding BGLUs, which suggests the relevance of these enzymes for this organism. Differential semi-quantitative proteomic analysis of the secretomes recovered from each culture revealed the production of two main proteins with BGLU activity. One of them was constitutively released regardless the carbon source used, although it was repressed by glucose, and showed to be less efficient than the commercial BGLU preparation used for comparison in saccharification experiments. The other was strongly induced by cellulose and revealed to be an excellent supplement to Celluclast for saccharification of pretreated wheat straw.

## Additional files

**Additional file 1.** Identification of the fungal isolate. This file contains three figures, the materials and methods associated to the information presented, and a brief discussion of the data presented. **Figure S1.** Maximum likelihood phylogenetic analysis of RPB1 (A), ITS (B), and BT2 (C) regions from different *Talaromyces* strains. **Figure S2.** Agar colonies of *T. amestolkiae, Penicillium purpurogenum var. rubrisclerotium* and *Penicillium rubrum*. **Figure S3.** SEM micrography of conidiophores and hyphae from the three fungal species.

**Additional file 2.** Gene prediction from *T. amestolkiae* genome. The file contains the amino acid sequences of the proteins encoded by the genome of *T. amestolkiae.*

**Additional file 3.** Proteins in secretomes of *T. amestolkiae* growing with different carbon sources: 3A) Avicel; 3B) Glucose; 3C) Slurry; 3D) Xylan. The file contains four tables with the list of proteins identified in each condition.

## Abbreviations

CBH: cellobiohydrolase, Avicelase, exocellulase
EG: β-1,4 endoglucanase
BGLU: β-1,4 glucosidase
LC–MS/MS: liquid chromatography coupled to tandem mass spectrometry
GH: glycosyl hydrolase
CMC: carboxymethyl cellulose
HPLC: high performance liquid chromatography
*p*NP: *p*-nitrophenol
KEGG: Kyoto Encyclopedia of Genes and Genomes
KOG: EuKaryotic Orthologous Groups
CAZy: carbohydrate-active enzymes
ORFs: open reading frames
PSM: peptide spectrum match
TAM377: culture supernatant of *T. amestolkiae* growing on Mandels plus glucose, expressing more g377 BGLU
TAM3821: culture supernatant of *T. amestolkiae* growing on Mandels plus Avicel, expressing more g3821 BGLU
