# Supplementary material for: Differential β-glucosidase expression as a function of carbon source availability in Talaromyces amestolkiae: a genomic and proteomic approach
Source: Biotechnol Biofuels. 2017 Jun 23;10:161. doi: 10.1186/s13068-017-0844-7 (PMC5481877; doi:10.1186/s13068-017-0844-7)
Supplement: Supplementary file 1 — Additional file 1. Identification of the fungal isolate. This file contains three figures, the materials and methods associated to the information presented, and a brief discussion of the data presented. Figure S1. Maximum likelihood phylogenetic analysis of RPB1 (A), ITS (B), and BT2 (C) regions from different Talaromyces strains. Figure S2. Agar colonies of T. amestolkiae, Penicillium purpurogenum var. rubrisclerotium and Penicillium rubrum. Figure S3. SEM micrography of conidiophores and hyphae from the three fungal species. [file 13068_2017_844_MOESM1_ESM.pdf]

Additional file 1. Identification of the fungal isolate.

The molecular identification of the cellulolytic fungus isolated was initially carried out from the sequences of the ribosomal DNA including ITS1 and ITS2 regions, which have been described as suitable targets for analysis of fungal phylogeny [1]. This isolate showed 100% identity with *T. amestolkiae* (accession number JX965247.1). To corroborate the identification of this new cellulolytic strain, β-tubulin (BT2) and RPB1 genes were also sequenced and showed to be 100% identical to those of previously analyzed *T. amestolkiae* strains [2] (Fig S1):

A

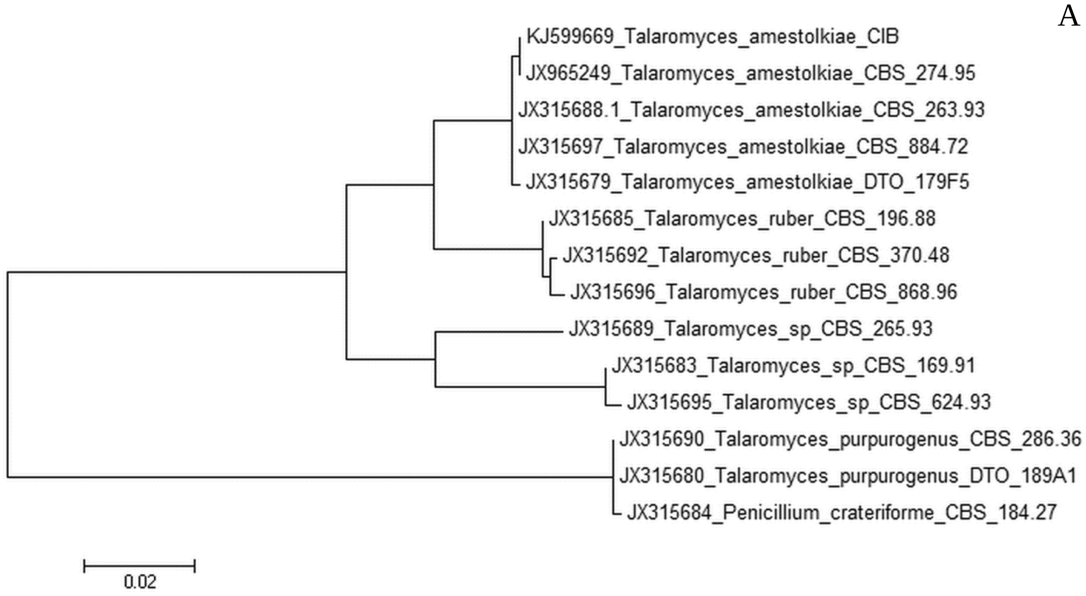

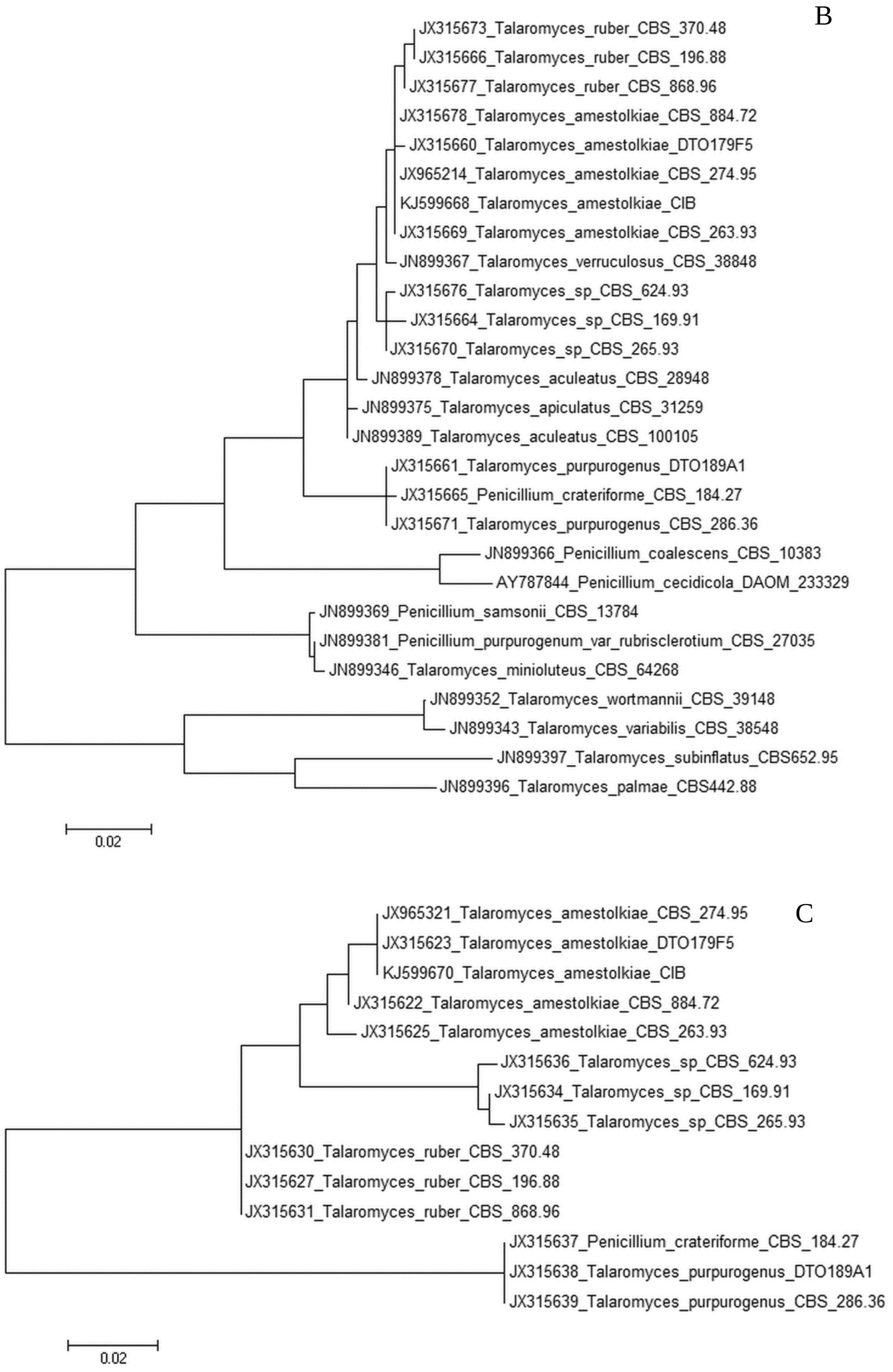

Figure S1. Maximum likelihood phylogenetic analysis of RPB1 (A), ITS (B) and BT2 (C) regions from different *Talaromyces* strains. The unrooted neighbor-joining phylogenetic tree with 1000 bootstrap replications was generated based on a previous

alignment using MEGA6 software. Sequences accession numbers are shown preceding strain names.

The morphological traits of the novel strain, grown in agar plates on several carbon sources, were also examined and compared to those reported by Yilmaz et al. [2]. Cultures of *P. purpurogenum* var. *rubrisclerotium* and *P. rubrum* from the CBS fungal culture collection, which have recently been reassigned as *T. amestolkiae*, were grown in parallel under the same conditions. Growth on CYA and YES after 7 days was similar for the three strains. All of them produced a red pigment in MEA plates, although it was more abundant in our *T. amestolkiae* isolate. In spite of being the same species, small differences in pigment production on YES medium were observed between CBS 263.93 and CBS 274.95 strains (Fig. S2).

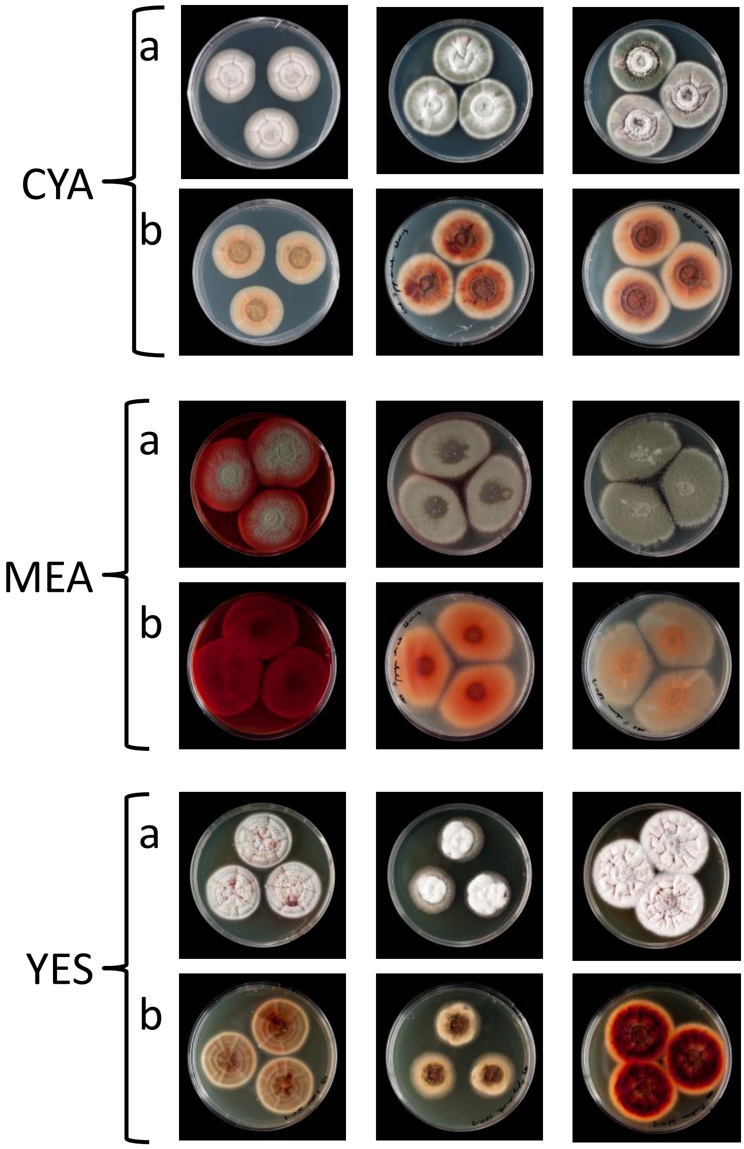

Figure S2. Agar colonies of *T. amestolkiae* (left column), *Penicillium purpurogenum var. rubrisclerotium* (middle column) and *Penicillium rubrum* (right column). Pictures of the different fungal strains in CYA, MEA and YES media: obverse (a) and reverse (b) plates incubated at 25 °C for 7 days.

The microscopic observation of the three strains also showed similar features, with biverticillate, vesiculate conidiophores, and rough conidia (Fig. S3).

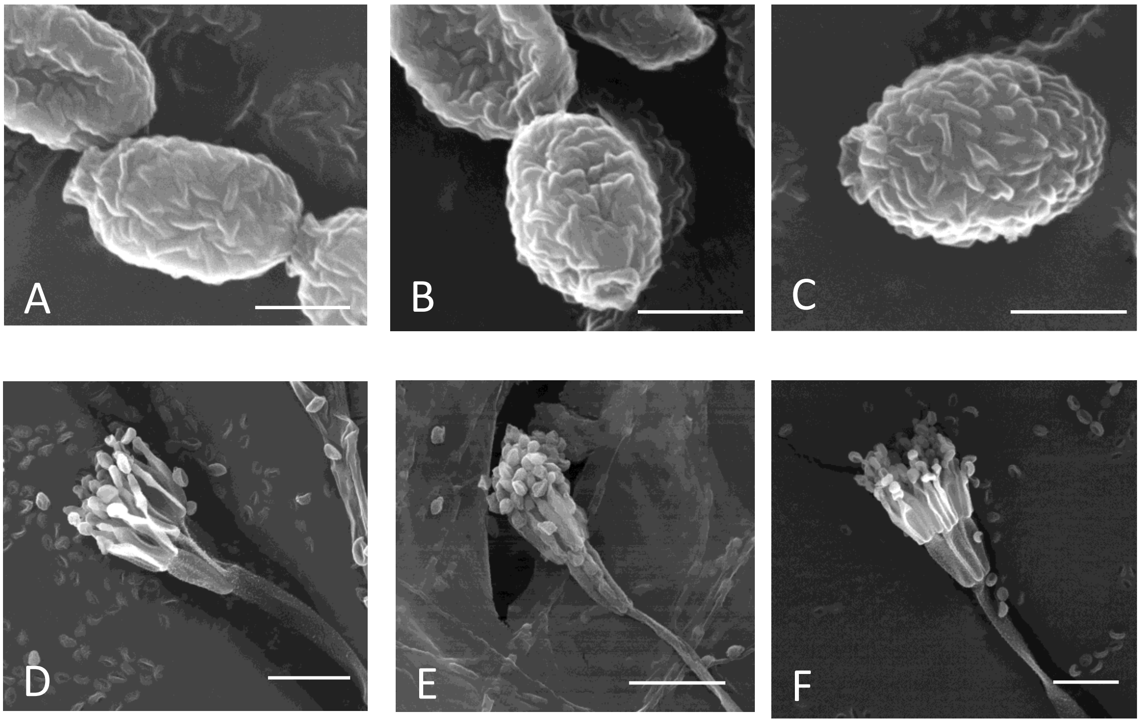

Figure S3. SEM micrography of conidiophores and hyphae from: A and D) *T. amestolkiae*; B and E) *P. purpurogenum var rubrisclerotium*; C and F) *P. rubrum*. All strains were grown on wheat straw and samples were treated as described in Materials and Methods. White bars represent 1 mm (upper row) or 10 mm (lower row).

Materials and methods:

Genomic DNA of the selected isolates was obtained using the protocol described in Romero *et al.*[1]. The extracted DNA was used as the template in a PCR to amplify the ITS1 and ITS2 regions and the 5.8S rRNA gene. The primers used for the amplification were its1 (5’-TCCGTAGGTGAACCTGCGG-3’) and its4 (5’-TCCTCCGCTTATTGATATGC-3’) [21] for ITS1 and ITS2 amplification, Bt2a (5´-GGTAACCAAATCGGTGCTGCTTTC-3´) and Bt2b (5´-ACCCTCAGTGTAGTGACCCTTGGC-3´) for β-tubulin amplification, and RPB2-F (5´- ATTTYGAYGGTGAYGARATGAAC-3´) and RPB2-R (GRACRGTDCCRTCATAYTTRACC-3´) for RPB2 amplification.

For PCR amplifications, 1.2 U of Taq DNA polymerase from Applied Biosystems were used, according to the manufacturer instructions; nucleotide primers were added at 1 μM (final concentration) in a final reaction volume of 50 μL. After template concentration optimization, 0.2 mg DNA were used. The PCR program was composed of 35 cycles: 94 ºC, 1 min; 52 ºC, 40 s; 72 ºC, 1 min. A final extension of 10 min at 72 ºC was added to finish all reactions. In all experiments, control reactions without template were carried out. For detection of the PCR products 1% agarose gel containing GelRed (Biotium) were run in TAE 1× buffer (Tris–acetate 40 mM and EDTA 1.0 mM). Gel bands were excised and purified using the QIAquick PCR purification kit (Qiagen). DNA sequencing was carried out using an automated ABI Prism 3730 DNA sequencer (Applied Biosystems), and the nucleotide sequences were compared by BLAST search [3] to those of National Centre for Biotechnology Information (NCBI) databases.

Macroscopic characters were studied on Czapek yeast extract agar (CYA), yeast extract sucrose agar (YES) and malt extract agar (MEA). The strains were inoculated at three points on 90-mm Petri dishes and incubated for 7 days at 25 °C in darkness. All media were prepared as described by Yilmaz *et al.* [2]. *P. purpurogenum* var. *rubrisclerotium* CBS 274.95 and *P. rubrum* CBS 263.93 strains used for morphological comparison purposes were obtained from The Centraalbureau voor Schimmelcultures (CBS, Fungal Biodiversity Centre, Royal Netherlands Academy of Arts and Sciences, Utrecht)

For electron microscopy studies, strains were grown in sterilized wheat straw for 7 days and fixed in 5 mM glutaraldehyde for 12 h, 4 ºC. The aldehyde excess was removed by washing in distilled water and subsequent dehydration with increasing concentrations of ethanol (up to 100%). Samples were prepared onto small aluminum

stubs, dried under low vacuum conditions, covered with sputtered gold in a Bio-Rad SC510 sputter coater and observed under high vacuum conditions using a FEI Inspect-S scanning electron microscope (SEM).

1. Romero E, Speranza M, García-Guinea J, Martínez AT, Martínez MJ, Garcia-Guinea J, Martinez AT, Martinez MJ. An anamorph of the white-rot fungus *Bjerkandera adusta* capable of colonizing and degrading compact disc components. FEMS Microbiol Lett. 2007;275:122–9.

2. Yilmaz N, Houbraken J, Hoekstra E, Frisvad J, Visagie C, Samson R. Delimitation and characterisation of *Talaromyces purpurogenus* and related species. Persoonia 2012; 29:39–54.

3. Altschul SF, Madden TL, Schaffer AA, Zhang JH, Zhang Z, Miller W, Lipman DJ. Gapped BLAST and PSI-BLAST: a new generation of protein database search programs. Nucleic Acids Res. 1997;25:3389–3402.
