# Supplementary material for: Differential β-glucosidase expression as a function of carbon source availability in Talaromyces amestolkiae: a genomic and proteomic approach
Source: Biotechnol Biofuels. 2017 Jun 23;10:161. doi: 10.1186/s13068-017-0844-7 (PMC5481877; doi:10.1186/s13068-017-0844-7)
Supplement: Supplementary file 3 — Additional file 3. Proteins in secretomes of T. amestolkiae growing with different carbon sources: 3A) Avicel; 3B) Glucose; 3C) Slurry; 3D) Xylan. The file contains four tables with the list of proteins identified in each condition. [file 13068_2017_844_MOESM3_ESM.pdf]

Additional file 3: A) Proteins in secretomes of *T. amestolkiae* growing with Avicel as carbon source

Avicel	% PSM Biological replicate 1	% PSM Biological replicate 2	Predicted protein function	GH Family
g2234	35.32	48.74	Cellobiohydrolase	GH7
g5707	15.82	11.41	Endoglucanase	GH6
g3821	3.67	4.60	Beta-glucosidase	GH3
g9427	4.26	2.12	Endoxylanase	GH10
g3995	3.10	2.34	Glutaminase	
g2158	2.34	1.31	Glucoamylase	GH15
g6537	1.37	1.98	Endoglucanase	GH5
g8295	1.94	1.06	Alpha-glucosidase	GH31
g2018	1.82	1.06	Endoglucanase	GH74
g2140	1.73	1.04	Glucoamylase	GH15
g4058	2.42	0.34	Swollenin	
g3707	1.49	0.97	Arabinofuranosidase	GH62
g1296	2.16	0.09	Endoxylanase	GH11
g2384	1.40	0.81	Alpha-galactosidase	GH27
g9148	1.30	0.77	Catalase	
g377	0.85	0.90	Beta-glucosidase	GH3
g1408	1.33	0.29	Endomannosidase	GH5
g5407	0.78	0.77	Probable rhamnogalacturonase	GH28
g6554	0.52	1.01	Mutanase	GH71
g6658	0.92	0.47	Hypothetical protein	GH125
g10182	0.90	0.50	Endo-1,3-beta-glucosidase	GH17
g4348	0.62	0.77	Aldehyde reductase	
g3706	0.78	0.54	Endoxylanase	GH30

g9312	0.73	0.54	Arabinofuranosidase	GH54
g4603	0.43	0.65	Beta-galactosidase	GH35
g6753	0.45	0.54	Beta-glucosidase	GH3
g4362	0.71	0.27	Cathepsin	
g173	0.47	0.38	Arabinofuranosidase	GH54
g2003	0.43	0.43	Dioxigenase	
g4461	0.38	0.47	Alpha-fucosidase	GH95
g3394	0.64	0.20	Endoglucanase	GH7
g5915	0.43	0.38	Hypothetical protein	GH127
g1569	0.31	0.50	Carboxyl esterase	
g3554	0.31	0.50	Alpha-galactosidase	GH27
g9324	0.45	0.32	Exo-beta-1,3-glucanase	GH55
g9330	0.26	0.47	Serine protease	
g4567	0.17	0.50	Hypothetical protein	GH2
g4633	0.40	0.25	Alpha-1,2-Mannosidase	GH47
g8077	0.47	0.09	Endomannosidase	GH5
g727	0.12	0.43	Endo-beta-1,6-galactanase	GH30
g8462	0.31	0.23	Trehalase	GH65
g4076	0.21	0.29	Hexosaminidase	GH20
g2236	0.17	0.32	Polygalacturonase	GH28
g3077	0.07	0.38	Exo-beta-1,3-glucanase	GH55
g3357	0.05	0.38	Endoglucanase	GH5
g3187	0.05	0.36	FAD/FMN-containing isoamyl alcohol oxidase	
g9511	0.17	0.23	Peptidase	
g1839	0.26	0.11	Endo-1,3(4)-beta-glucanase, putative	GH16
g7045	0.26	0.11	Endo-1,3-beta-glucosidase	GH64

g571	0.24	0.14	Hypothetical protein	
g7641	0.24	0.14	Beta-glucanosyltransferase	GH72
g2989	0.19	0.18	Extracellular cell wall glucanase	GH16
g2283	0.05	0.32	Hypothetical protein	
g881	0.21	0.14	Aldose-1-epimerase	
g6145	0.14	0.20	Alpha-1,2-mannosidase	GH92
g2733	0.12	0.23	Glucan 1,3-beta-glucosidase, putative	GH55
g2288	0.05	0.29	Glucose dehydrogenase	
g8384	0.05	0.29	Lysophospholipid hydrolase	GH1
g9487	0.14	0.18	Alpha-L-rhamnosidase	GH78
g3279	0.12	0.20	Alpha amylase	GH13
g5245	0.05	0.27	Serine protease	
g3553	0.12	0.18	Arabinofuranosidase	GH43
g9673	0.07	0.23	Chitin synthase	
g2448	0.17	0.11	Aspartic endopeptidase	
g8979	0.14	0.14	Rhamnogalacturonan acetylesterase	
g3373	0.07	0.20	Beta-xylosidase	GH39
g3362	0.05	0.23	Alpha-L-arabinofuranosidase	GH62
g9163	0.14	0.11	Arabinogalactan endo-1,4-beta-galacotosidase	GH53
g4704	0.05	0.20	Beta-lactamase	
g4028	0.14	0.09	Glucan 1,3-beta-glucosidase	GH55
g8294	0.14	0.09	Carboxypeptidase	
g4139	0.12	0.11	Oxalate decarboxylase, putative	
g7759	0.12	0.11	Serine carboxypeptidase, putative	
g4952	0.09	0.14	Endoglucanase	
g4882	0.12	0.09	Alpha-1,2-mannosidase, putative	GH92

g3149	0.09	0.11	Beta-mannosidase	GH2
g545	0.09	0.11	Polyamine oxidase	
g5098	0.05	0.16	Isopullulanase	GH49
g7406	0.07	0.11	Beta galactosidase, putative	GH35
g4225	0.05	0.14	Alpha-N-acetylglucosaminidase	GH89
g9061	0.05	0.14	Alpha-1,3-glucanase	GH71
g2125	0.07	0.09	Alpha-galactosidase	GH27
g8981	0.05	0.11	Beta-xylosidase	GH3
g9076	0.05	0.11	Putative Beta-glucosidase	GH3
g9658	0.05	0.11	Mucin family signaling protein	
g3685	0.09	0.05	Hypothetical protein	
g9063	0.07	0.07	Chitinase	GH18
g10020	0.05	0.09	Hypothetical protein	
g416	0.05	0.09	Elongation factor 1-alpha	
g2024	0.07	0.05	Similar to extracellular dihydrogeodin oxidase/laccase	
g1751	0.07	0.05	Endoglucanase	GH12
g2762	0.07	0.05	Putative aspartic-type endopeptidase	
g5719	0.07	0.05	Cellobiohydrolase	GH7
g1079	0.05	0.07	Hypothetical protein	
g3185	0.05	0.07	Hypothetical protein	GH92
g4333	0.05	0.07	Hypothetical protein	
g4745	0.05	0.07	Glucan endo-1,3-alpha-glucosidase	GH71
g8531	0.05	0.07	Cathepsin	
g35	0.05	0.05	GPI-anchored cell wall organization protein	
g5993	0.05	0.05	Hypothetical protein	

g7811	0.05	0.05	6-phosphogluconolactonase	
g8103	0.05	0.05	Tripeptidyl-peptidase	
g8624	0.05	0.05	GPI anchored protein, putative	
g9455	0.05	0.05	Chitinase	GH18

Additional file 3: B) Proteins found in secretomes of *T. amestolkiae* growing with glucose as carbon source

Glucose	% PSM Biological Replicate 1	% PSM Biological Replicate 2	Predicted protein function	GH Family
g377	10.50	10.64	Beta-glucosidase	GH3
g2140	10.13	9.88	Glucoamylase	GH15
g2158	8.59	6.16	Glucoamylase	GH15
g8295	7.04	5.74	Alpha-glucosidase	GH31
g3995	5.35	6.24	Glutaminase	
g8259	4.29	1.54	Glucoamylase	GH15
g7301	4.13	0.29	Aminopeptidase	
g216	2.98	1.71	Neutral/alkaline nonlysosomal ceramidase	
g5915	2.53	2.01	Hypothetical protein	GH127
g3279	2.24	3.52	Alpha amylase	GH13
g8384	1.98	1.11	Lysophospholipid hydrolase	GH1
g4076	1.80	4.61	Hexosaminidase	GH20
g7148	1.80	0.77	Xaa-Pro dipeptidase	
g9290	1.49	1.48	Alpha-glucosidase	GH31
g2234	1.42	2.23	Cellobiohydrolase	GH7

g3307	1.40	0.25	Hypothetical protein	
g4355	1.35	0.25	Hypothetical protein	
g8017	1.31	0.27	Formate dehydrogenase	
g9148	1.31	1.71	Catalase	
g6658	1.18	1.26	Hypothetical protein	GH125
g10182	1.07	1.29	Endo-1,3-beta-glucosidase	GH17
g7031	0.95	0.22	Endonuclease	
g10153	0.93	0.40	Isoamyl alcohol oxidase, putative	
g290	0.89	0.07	Aminotransferase	
g1839	0.71	2.84	Endo-1,3(4)-beta-glucanase, putative	GH16
g4461	0.71	0.89	Alpha-fucosidase	GH95
g7641	0.71	0.25	Beta-glucanosyltransferase	GH72
g1180	0.69	0.81	Exo-beta-D-glucosaminidase	GH2
g4633	0.67	0.89	Alpha-1,2-Mannosidase	GH47
g8480	0.67	1.01	Phosphatase	
g2149	0.64	0.55	Transcription factor, putative	
g3006	0.58	0.05	Glutathione oxidoreductase	
g4603	0.58	1.66	Beta-galactosidase	GH35
g2313	0.56	0.02	Mitochondrial heat shock protein	
g3728	0.56	0.12	Peptidase	
g3202	0.53	0.13	Cathepsin	
g4965	0.53	1.16	Thioredoxin reductase	
g6145	0.53	0.89	Alpha-1,2-mannosidase	GH92
g7540	0.53	0.05	Hypa-like protein, putative	
g3993	0.51	0.15	Protease	
g7845	0.47	0.08	Peptidase	
g9324	0.47	3.98	Exo-beta-1,3-glucanase	GH55

g2384	0.44	2.01	Alpha-galactosidase	GH27
g2575	0.44	0.22	Protease	
g727	0.44	0.29	Endo-beta-1,6-galactanase	GH30
g9150	0.44	0.52	Beta-glucosidase	GH3
g3077	0.38	0.81	Exo-beta-1,3-glucanase	GH55
g395	0.38	0.27	Hypothetical protein	
g4318	0.38	0.60	Peroxidase	
g5497	0.38	0.05	Aldehyde dehydrogenase	
g9487	0.36	0.29	Alpha-L-rhamnosidase	GH78
g5215	0.33	0.25	Glucose-6-phosphate isomerase	
g7045	0.33	0.30	Endo-1,3-beta-glucosidase	GH64
g3821	0.31	0.18	Beta-glucosidase	GH3
g5548	0.31	0.17	Alpha-rhamnosidase	GH78
g6228	0.31	0.50	Amine oxidase	
g4362	0.29	0.39	Cathepsin	
g4704	0.29	0.25	Beta-lactamase	
g5860	0.29	0.12	Endoglucanase	GH5
g2283	0.27	0.10	Hypothetical protein	
g3149	0.27	0.35	Beta-mannosidase	GH2
g6249	0.27	0.07	Mannosyl phosphorylinositol ceramide synthase	
g1410	0.24	0.02	Beta-mannosidase	GH2
g2465	0.24	0.13	Transpeptidase	
g1266	0.22	0.89	Alpha-glucosidase, putative	GH31
g2159	0.22	0.12	Alpha-amylase	GH13
g2989	0.22	1.17	Extracellular cell wall glucanase	GH16
g4403	0.22	0.07	Ksdd-like steroid dehydrogenase	
g5707	0.22	0.20	Endoglucanase	GH6

g9063	0.22	0.23	Chitinase	GH18
g466	0.20	0.13	Chitinase	GH18
g571	0.20	0.82	Hypothetical protein	
g881	0.20	0.89	Aldose-1-epimerase	
g1408	0.18	0.02	Endomannosidase	GH5
g3187	0.18	0.08	FAD/FMN-containing isoamyl alcohol oxidase	
g3707	0.18	0.23	Arabinofuranosidase	GH62
g5245	0.16	0.22	Serine protease	
g5407	0.16	0.15	Probable rhamnogalacturonase	GH28
g7406	0.16	0.10	Beta galactosidase, putative	GH35
g4226	0.13	0.02	Aminotransferase	
g4348	0.13	0.03	Aldehyde reductase	
g7037	0.13	0.03	GPI anchored protein, putative	
g2448	0.11	0.02	Aspartic endopeptidase	
g4390	0.11	0.02	Lipase	
g7668	0.11	0.02	Pyridoxal 5'-phosphate synthase	
g3685	0.09	0.10	Hypothetical protein	
g4734	0.09	0.07	Hypothetical protein	
g559	0.09	0.10	Putative FAD-linked oxidoreductase	
g9061	0.09	0.10	Alpha-1,3-glucanase	GH71
g10078	0.07	0.74	Endo-1,3(4)-beta-glucanase, putative	GH81
g1079	0.07	0.02	Hypothetical protein	
g2573	0.07	0.02	Transaldolase	
g4411	0.07	0.10	2,3-dihydroxybenzoate decarboxylase	
g6921	0.07	0.02	Arabinofuranosidase	GH43
g7811	0.07	0.05	6-phosphogluconolactonase	
g7892	0.07	0.02	Carboxyl esterase	

g8103	0.07	0.57	Tripeptidyl-peptidase	
g10375	0.04	0.12	Hypothetical protein	
g1853	0.04	0.03	Ubiquitin	
g2018	0.04	0.12	Endoglucanase	GH74
g2394	0.04	0.03	Hypothetical protein	
g3394	0.04	0.03	Endoglucanase	GH7
g5993	0.04	0.40	Hypothetical protein	
g608	0.04	0.84	1,3-beta-glucanosyltransferase	GH72
g699	0.04	0.13	Carboxypeptidase	
g7527	0.04	0.05	Beta-glucosidase	GH3
g9385	0.04	0.10	Alpha-fucosidase	GH29
g9490	0.04	0.54	Hexosaminidase	GH3
g9673	0.04	0.72	Chitin synthase	
g10240	0.02	0.17	Carboxyl esterase	
g1569	0.02	0.18	Carboxyl esterase	
g1751	0.02	0.13	Endoglucanase	GH12
g2392	0.02	0.97	Hypothetical protein	
g3185	0.02	0.15	Hypothetical protein	GH92
g7482	0.02	0.35	Conidial pigment biosynthesis oxidase	
g8294	0.02	0.03	Carboxypeptidase	
g9337	0.02	0.15	Alpha-mannosidase	GH92
g9455	0.02	0.05	Chitinase	GH18
g9511	0.02	0.25	Peptidase	

Additional file 3: C) Proteins found in secretomes of *T. amestolkiae* growing with slurry as carbon source

Slurry	% PSM Biological Replicate 1	% PSM Biological Replicate 2	Predicted protein function	GH Family
g2234	20.81	22.75	Cellobiohydrolase	GH7
g9427	5.55	2.43	Endoxylanase	GH10
g2140	4.32	4.14	Glucoamylase	GH15
g3995	4.17	2.19	Glutaminase	
g377	3.92	4.50	Beta-glucosidase	GH3
g5707	3.90	1.09	Endoglucanase	GH6
g8295	3.34	0.49	Alpha-glucosidase	GH31
g6537	3.30	1.82	Endoglucanase	GH5
g5915	2.65	0.49	Hypothetical protein	GH127
g3821	1.86	0.61	Beta-glucosidase	GH3
g3707	1.78	1.34	Arabinofuranosidase	GH62
g2158	1.73	1.46	Glucoamylase	GH15
g4461	1.56	1.09	Alpha-fucosidase	GH95
g9076	1.46	0.49	Putative Beta-glucosidase	GH3
g1839	1.24	0.12	Endo-1,3(4)-beta-glucanase, putative	GH16
g9148	1.22	0.85	Catalase	
g4068	1.20	0.12	Alpha-L-arabinofuranosidase	GH51
g6554	1.20	0.12	Mutanase	GH71
g6753	1.20	0.85	Beta-glucosidase	GH3
g4076	1.18	0.12	Hexosaminidase	GH20
g2018	1.05	0.61	Endoglucanase	GH74
g9290	0.98	0.24	Alpha-glucosidase	GH31
g9312	0.98	2.19	Arabinofuranosidase	GH54

g4362	0.96	1.09	Cathepsin	
g4567	0.96	0.24	Hypothetical protein	GH2
g216	0.88	0.24	Neutral/alkaline nonlysosomal ceramidase	
g10182	0.83	0.24	Endo-1,3-beta-glucosidase	GH17
g7301	0.83	1.46	Aminopeptidase	
g9324	0.83	0.36	Exo-beta-1,3-glucanase	GH55
g40	0.75	0.85	Arabinofuranosidase	GH54
g10375	0.68	0.49	Hypothetical protein	
g3385	0.68	0.85	Chitinase	GH18
g2384	0.66	0.12	Alpha-galactosidase	GH27
g1266	0.62	0.36	Alpha-glucosidase, putative	GH31
g3077	0.60	0.85	Exo-beta-1,3-glucanase	GH55
g4355	0.58	0.61	Hypothetical protein	
g6857	0.56	0.36	Beta-glucosidase	GH3
g1945	0.54	1.09	Acetylcholinesterase	
g8981	0.53	1.46	Beta-xylosidase	GH3
g916	0.49	0.61	Peptidase	
g9063	0.47	0.49	Chitinase	GH18
g2989	0.45	0.36	Extracellular cell wall glucanase	GH16
g3554	0.45	0.73	Alpha-galactosidase	GH27
g7892	0.45	0.24	Carboxyl esterase	
g2743	0.43	0.73	Endo-1,6-beta-D-glucanase	GH30
g8428	0.43	0.12	Glucan endo-1,3-beta-glucosidase	GH55
g7641	0.41	0.24	Beta-glucanosyltransferase	GH72
g7148	0.39	0.49	Xaa-Pro dipeptidase	
g4148	0.36	0.49	Tyrosinase	
g6145	0.36	0.24	Alpha-1,2-mannosidase	GH92

g8172	0.36	0.12	Helicase	
g1180	0.34	0.36	Exo-beta-D-glucosaminidase	GH2
g3202	0.34	0.61	Cathepsin	
g3394	0.34	0.12	Endoglucanase	GH7
g571	0.34	0.12	Hypothetical protein	
g727	0.34	0.36	Endo-beta-1,6-galactanase	GH30
g8077	0.34	0.36	Endomannosidase	GH5
g3553	0.32	0.24	Arabinofuranosidase	GH43
g10078	0.30	0.24	Endo-1,3(4)-beta-glucanase, putative	GH81
g2125	0.30	0.61	Alpha-galactosidase	GH27
g2149	0.30	0.12	Transcription factor, putative	
g3279	0.30	0.36	Alpha amylase	GH13
g3336	0.30	0.36	Beta-xylosidase	GH3
g3357	0.30	0.12	Endoglucanase	GH5
g9511	0.30	0.12	Peptidase	
g3362	0.28	0.61	Alpha-L-arabinofuranosidase	GH62
g3993	0.28	0.36	Protease	
g4603	0.26	0.36	Beta-galactosidase	GH35
g881	0.26	0.12	Aldose-1-epimerase	
g10240	0.24	0.24	Carboxylesterase	
g4704	0.24	0.12	Beta-lactamase	
g8384	0.24	0.49	Lysophospholipid hydrolase	GH1
g4411	0.23	0.24	2,3-dihydroxybenzoate decarboxylase	
g608	0.23	0.12	1,3-beta-glucanosyltransferase	GH72
g8017	0.23	0.61	Formate dehydrogenase	
g9487	0.23	0.12	Alpha-L-rhamnosidase	GH78
g290	0.21	0.85	Aminotransferase	

g5548	0.21	0.49	Alpha-rhamnosidase	GH78
g6658	0.21	0.12	Hypothetical protein	GH125
g7110	0.21	0.24	Tyrosinase	
g4633	0.19	0.12	Alpha-1,2-Mannosidase	GH47
g1569	0.17	0.49	Carboxyl esterase	
g7045	0.17	0.36	Endo-1,3-beta-glucosidase	GH64
g10153	0.15	0.24	Isoamyl alcohol oxidase, putative	
g10298	0.15	0.49	Hypothetical protein	
g3307	0.15	0.73	Hypothetical protein	
g466	0.15	0.36	Chitinase	GH18
g5215	0.15	1.09	Glucose-6-phosphate isomerase	
g2733	0.13	0.24	Glucan 1,3-beta-glucosidase, putative	GH55
g5245	0.13	0.24	Serine protease	
g5495	0.13	0.49	1,3-beta-glucanosyltransferase	GH72
g5497	0.13	1.58	Aldehyde dehydrogenase	
g7206	0.13	0.12	Glucan 1,3-beta-glucosidase	GH5
g7527	0.13	1.46	Beta-glucosidase	GH3
g2159	0.11	0.36	Alpha-amylase	GH13
g2283	0.11	0.36	Hypothetical protein	
g3187	0.11	0.12	FAD/FMN-containing isoamyl alcohol oxidase	
g5849	0.11	0.61	Hypothetical protein	
g7783	0.11	0.24	Putative epoxide hydrolase	
g8539	0.11	0.12	Peptidase	
g3030	0.09	0.24	3-isopropylmalate dehydrogenase	
g3139	0.09	0.36	Beta-glucosidase	GH3
g3487	0.09	0.97	Malate dehydrogenase	
g4745	0.09	0.12	Glucan endo-1,3-alpha-glucosidase	GH71

g5238	0.09	0.73	Chaperone	
g7486	0.09	0.61	Hypothetical protein	
g7845	0.09	0.24	Peptidase	
g10174	0.08	0.24	Dienelactone hydrolase	
g4058	0.08	0.73	Swollenin	
g6132	0.08	0.24	Cytochrome P450, putative	
g6249	0.08	0.24	Mannosyl phosphorylinositol ceramide synthase	
g6784	0.08	0.73	Lipase	
g7540	0.08	0.24	Hypa-like protein, putative	
g9602	0.08	0.24	Peptidase	
g1751	0.06	0.24	Endoglucanase	GH12
g2003	0.06	0.24	Dioxigenase	
g3373	0.06	0.24	Beta-xylosidase	GH39
g3604	0.06	0.49	Aldehyde dehydrogenase	
g4952	0.06	0.24	Endoglucanase	
g6615	0.06	0.85	Hypothetical protein	GH95
g8462	0.06	0.24	Trehalase	GH65
g9402	0.06	0.24	Carboxylic ester hydrolase	
g1410	0.04	0.24	Beta-mannosidase	GH2
g1895	0.04	0.24	ER-associated protein catabolism-related protein	
g2573	0.04	0.49	Transaldolase	
g5840	0.04	0.24	Lipase	
g6625	0.04	0.24	Alpha-galactosidase	GH27
g7031	0.04	0.24	Endonuclease	
g9163	0.04	0.24	Arabinogalactan endo-1,4-beta-galacotosidase	GH53

g10215	0.04	0.24	Alpha-amylase	GH13
g10326	0.04	0.24	Protease	
g1853	0.04	0.24	Ubiquitin	
g2313	0.04	1.22	Mitochondrial heat shock protein	
g2336	0.04	0.24	Cell wall glycosyl hydrolase	GH105
g2465	0.04	0.36	Transpeptidase	
g2727	0.04	0.24	Hypothetical protein	
g3685	0.04	0.24	Hypothetical protein	
g4318	0.04	1.34	Peroxidase	
g4734	0.04	0.24	Hypothetical protein	
g4735	0.04	0.24	Carboxylesterase,	
g6430	0.04	1.22	Mannitol-1-phosphate 5-dehydrogenase	
g7037	0.04	0.24	GPI anchored protein, putative	
g8672	0.04	0.24	Hypothetical protein	
g9490	0.04	0.24	Hexosaminidase	GH3

Additional file 3: D) Proteins found in secretomes of *T. amestolkiae* growing with xylan as carbon source

**Xylan**	**% PSM Biological Replicate 1**	**% PSM Biological Replicate 2**	**Predicted protein function**	**GH Family**
g2234	5.40	2.98	Cellobiohydrolase	GH7
g2140	5.10	4.06	Glucoamylase	GH15
g377	6.48	4.59	Beta-glucosidase	GH3
g3995	5.88	6.49	Glutaminase	

g2158	5.05	3.77	Glucoamylase	GH15
g8295	4.56	6.15	Alpha-glucosidase	GH31
g9427	3.74	1.98	Endoxylanase	GH10
g8981	4.98	3.78	Beta-xylosidase	GH3
g5915	2.33	2.72	Hypothetical protein	GH127
g3336	3.52	5.42	Beta-xylosidase	GH3
g9324	2.19	0.18	Exo-beta-1,3-glucanase	GH55
g4076	2.13	1.42	Hexosaminidase	GH20
g5707	2.12	0.18	Endoglucanase	GH6
g4068	2.06	2.80	Alpha-L-arabinofuranosidase	GH51
g3279	1.83	0.79	Alpha amylase	GH13
g2384	1.69	0.45	Alpha-galactosidase	GH27
g2743	1.42	0.03	Endo-1,6-beta-D-glucanase	GH30
g4603	1.42	3.02	Beta-galactosidase	GH35
g9148	1.23	1.33	Catalase	
g216	1.19	1.42	Neutral/alkaline nonlysosomal ceramidase	
g6537	1.14	0.08	Endoglucanase	GH5
g1266	1.10	0.01	Alpha-glucosidase, putative	GH31
g9076	1.07	1.67	Putative Beta-glucosidase	GH3
g9290	1.07	0.61	Alpha-glucosidase	GH31
g4461	1.05	0.60	Alpha-fucosidase	GH95
g8259	1.04	0.62	Glucoamylase	GH15
g10182	1.00	1.54	Endo-1,3-beta-glucosidase	GH17
g3707	0.84	0.77	Arabinofuranosidase	GH62
g3821	0.81	0.28	Beta-glucosidase	GH3
g4965	0.74	0.79	Thioredoxin reductase	
g6753	0.74	1.68	Beta-glucosidase	GH3

g3385	0.71	1.19	Chitinase	GH18
g881	0.70	0.04	Aldose-1-epimerase	
g6658	0.70	1.25	Hypothetical protein	GH125
g6857	0.68	0.09	Beta-glucosidase	GH3
g6145	0.61	0.88	Alpha-1,2-mannosidase	GH92
g3077	0.57	0.61	Exo-beta-1,3-glucanase	GH55
g3554	0.57	0.23	Alpha-galactosidase	GH27
g8384	0.57	0.05	Lysophospholipid hydrolase	GH1
g4362	0.54	0.03	Cathepsin	
g4355	0.52	1.10	Hypothetical protein	
g571	0.52	0.43	Hypothetical protein	
g1180	0.50	0.85	Exo-beta-D-glucosaminidase	GH2
g4633	0.50	0.85	Alpha-1,2-Mannosidase	GH47
g7892	0.47	0.42	Carboxyl esterase	
g7148	0.46	0.30	Xaa-Pro dipeptidase	
g727	0.46	1.22	Endo-beta-1,6-galactanase	GH30
g608	0.46	0.51	1,3-beta-glucanosyltransferase	GH72
g2801	0.45	0.09	BNR/Asp-box repeat domain protein	GH93
g4148	0.45	0.45	Tyrosinase	
g8480	0.45	1.08	Phosphatase	
g7301	0.42	1.71	Aminopeptidase	
g6228	0.41	0.20	Amine oxidase	
g9673	0.40	0.15	Chitin synthase	
g7641	0.39	0.64	Beta-glucanosyltransferase	GH72
g10078	0.39	0.35	Endo-1,3(4)-beta-glucanase, putative	GH81
g916	0.38	0.04	Peptidase	
g2018	0.37	0.34	Endoglucanase	GH74

g2392	0.37	0.05	Hypothetical protein	
g7482	0.36	0.03	Conidial pigment biosynthesis oxidase	
g4348	0.35	0.54	Aldehyde reductase	
g8103	0.35	0.28	Tripeptidyl-peptidase	
g2149	0.35	0.95	Transcription factor, putative	
g4567	0.35	0.16	Hypothetical protein	GH2
g5121	0.34	0.49	5-oxoprolinase	
g5407	0.34	0.04	Probable rhamnogalacturonase	GH28
g9063	0.33	0.23	Chitinase	GH18
g412	0.31	0.83	Alpha-L-rhamnosidase	GH78
g10153	0.29	0.22	Isoamyl alcohol oxidase, putative	
g7406	0.28	0.03	Beta galactosidase, putative	GH35
g3202	0.28	0.12	Cathepsin	
g395	0.27	0.18	Hypothetical protein	
g4318	0.27	0.28	Peroxidase	
g4704	0.26	0.14	Beta-lactamase	
g8428	0.26	0.08	Glucan endo-1,3-beta-glucosidase	GH55
g10298	0.25	0.12	Hypothetical protein	
g2125	0.25	0.81	Alpha-galactosidase	GH27
g9490	0.24	0.19	Hexosaminidase	GH3
g9511	0.23	0.03	Peptidase	
g3149	0.22	0.53	Beta-mannosidase	GH2
g5245	0.22	0.14	Serine protease	
g3187	0.22	0.04	FAD/FMN-containing isoamyl alcohol oxidase	
g3993	0.21	0.15	Protease	
g5548	0.21	0.23	Alpha-rhamnosidase	GH78
g7045	0.20	0.28	Endo-1,3-beta-glucosidase	GH64

g9150	0.20	0.28	Beta-glucosidase	GH3
g5993	0.19	0.04	Hypothetical protein	
g699	0.19	0.14	Carboxypeptidase	
g2794	0.18	0.07	Hexosaminidase	GH20
g2575	0.18	0.33	Protease	
g5215	0.18	0.28	Glucose-6-phosphate isomerase	
g8017	0.18	0.70	Formate dehydrogenase	
g9394	0.16	0.45	Probable beta-galactosidase	GH35
g3553	0.15	0.22	Arabinofuranosidase	GH43
g3307	0.13	0.38	Hypothetical protein	
g2143	0.13	0.18	Glucose oxidase	
g4411	0.13	0.28	2,3-dihydroxybenzoate decarboxylase	
g466	0.13	0.08	Chitinase	GH18
g2283	0.12	0.07	Hypothetical protein	
g2476	0.12	0.03	Hypothetical protein	
g3728	0.12	0.33	Peptidase	
g3185	0.11	0.08	Hypothetical protein	GH92
g9236	0.11	0.16	Beta-glucuronidase	GH79
g2159	0.11	0.04	Alpha-amylase	GH13
g5620	0.11	0.47	Small nucleolar RNA-associated protein	GH43
g5860	0.11	0.08	Endoglucanase	GH5
g7487	0.11	0.14	Conidial pigment biosynthesis oxidase	
g5840	0.10	0.03	Lipase	
g2573	0.09	0.08	Transaldolase	
g7031	0.09	0.43	Endonuclease	
g290	0.09	0.07	Aminotransferase	
g5006	0.09	0.24	Hypothetical protein	

g1079	0.08	0.11	Hypothetical protein	
g2465	0.08	0.08	Transpeptidase	
g3954	0.08	0.08	Alpha-fucosidase	GH29
g10174	0.08	0.07	Dienelactone hydrolase	
g2313	0.08	0.08	Mitochondrial heat shock protein	
g2024	0.07	0.05	Similar to extracellular dihydrogeodin oxidase/laccase	
g3373	0.07	0.05	Beta-xylosidase	GH39
g5495	0.07	0.08	1,3-beta-glucanosyltransferase	GH72
g7206	0.07	0.14	Glucan 1,3-beta-glucosidase	GH5
g133	0.06	0.07	Amine oxidase	
g2448	0.06	0.03	Aspartic endopeptidase	
g3139	0.06	0.03	Beta-glucosidase	GH3
g5497	0.06	0.84	Aldehyde dehydrogenase	
g7110	0.06	0.03	Tyrosinase	
g7845	0.06	0.05	Peptidase	
g3685	0.06	0.05	Hypothetical protein	
g5238	0.06	0.03	Chaperone	
g7527	0.06	0.14	Beta-glucosidase	GH3
g7783	0.06	0.04	Putative epoxide hydrolase	
g3006	0.05	0.27	Glutathione oxidoreductase	
g6249	0.05	0.24	Mannosyl phosphorylinositol ceramide synthase	
g9337	0.05	0.04	Alpha-mannosidase	GH92
g1197	0.05	0.05	Peptidase	
g3030	0.05	0.04	3-isopropylmalate dehydrogenase	
g3271	0.05	0.03	Phosphatase	
g7540	0.05	0.12	Hypa-like protein, putative	

g7811	0.05	0.03	6-phosphogluconolactonase	
g1325	0.04	0.09	Asparaginyl-trna synthetase	GH106
g3454	0.04	0.15	Hypothetical protein	GH39
g6921	0.04	0.03	Arabinofuranosidase	GH43
g5398	0.04	0.04	Hypothetical protein	
g5705	0.04	0.03	Hypothetical protein	
g9455	0.04	0.30	Chitinase	GH18
g3487	0.03	0.04	Malate dehydrogenase	
g3578	0.03	0.12	Hypothetical protein	
g4225	0.03	0.03	Alpha-N-acetylglucosaminidase	GH89
g7486	0.03	0.23	Hypothetical protein	
g9163	0.03	0.26	Arabinogalactan endo-1,4-beta-galacotosidase	GH53
g9264	0.03	0.05	Pectinesterase	
g5103	0.02	0.03	Hypothetical protein	
g6132	0.02	0.03	Cytochrome P450, putative	
g8624	0.02	0.04	GPI anchored protein, putative	
g1410	0.02	0.12	Beta-mannosidase	GH2
g1895	0.02	0.11	ER-associated protein catabolism-related protein	
g2003	0.02	0.12	Dioxigenase	
g3369	0.02	0.03	Oxidoreductase	
g3604	0.02	0.14	Aldehyde dehydrogenase	
g4403	0.02	0.07	Ksdd-like steroid dehydrogenase	
g7595	0.02	0.04	Conidial hydrophobin	
g9990	0.02	0.04	Glucan endo-1,3-alpha-glucosidase	GH71
g10053	0.01	0.03	Chitinase	GH18
g1105	0.01	0.07	2-methylcitrate dehydratase	
g3500	0.01	0.03	Hypothetical protein	

g4474	0.01	0.23	Glucan endo-1,3-alpha-glucosidase	GH71
g4950	0.01	1.04	Hypothetical protein	GH31
g6020	0.01	0.11	Hypothetical protein	
g8248	0.01	0.04	Arabinofuranosidase	
g9243	0.01	0.09	Arabinosidase	GH43
g9398	0.01	0.35	Beta-glucosidase	GH3
g9689	0.01	0.07	Trans-2,3-dihydro-3-hydroxyanthranilate isomerase	
g1234	0.01	0.03	Membrane bound C2 domain protein	
g2069	0.01	0.07	Ketoreductase, putative	
g2727	0.01	0.04	Hypothetical protein	
g3824	0.01	0.03	Lipase	
g4733	0.01	0.03	Phosphatase	
g5406	0.01	0.03	CDR ABC transporter	
g5738	0.01	0.03	Pectinesterase	
g6430	0.01	0.03	Mannitol-1-phosphate 5-dehydrogenase	
g6580	0.01	0.05	Triacylglycerol lipase	
g7668	0.01	0.07	Pyridoxal 5'-phosphate synthase	
g8178	0.01	0.03	Inorganic pyrophosphatase	
g9447	0.01	0.04	Hypothetical protein	
