# Supplementary material for: Differential β-glucosidase expression as a function of carbon source availability in Talaromyces amestolkiae: a genomic and proteomic approach
Source: Biotechnol Biofuels. 2017 Jun 23;10:161. doi: 10.1186/s13068-017-0844-7 (PMC5481877; doi:10.1186/s13068-017-0844-7)
Supplement: Supplementary file 2 — Additional file 2. Gene prediction from T. amestolkiae genome. The file contains the amino acid sequences of the proteins encoded by the genome of T. amestolkiae. [file 13068_2017_844_MOESM2_ESM.pdf]

Additional file 2: Gene prediction from *T. amestolkiae* genome

>g1
MRKVFTALGVTLYVAAIAAQNISTSNNYRPSNVTANLTDLYLWVGSYYNATAQVDFIPFGGLAASI
PSNCPPAFSNQTARGNYSARLAITQPGSYDSVGDLANVFLTLWPLDYDFTKLPLGQQITYIPDLDF
GLFSSDPLYDNGGDPKNNWIWSLKNTTSPPYNMSSTIEDRYRGFGVFHVNASTPCVETSQWSFLIW
SDPVNGTNFTNPTIDLQFDDSTANLTLQGYAEAGAVYGTGSTATSGSTVVWGEFSLTLSGVIDKYH
SDILKNDTNTPAWVPTVGYNNNSANFGYTTQSTGSRIAEPLLLITIVFALWLIIS
>g2
MKLCTFVALAATAAAQQIWIQAPGLNQEVTAGSDLVIEVQSQDTASPYQDIAAAIGIQPWDTGYVS
TLELGEWLLYQGNWSIEGFAYPQGGLYTNVTVRVPSSLPAGPALLSVALFQVVGESNEPSVQTANV
NVTVLSSS
>g3
MFAPGSGGQAASSLRSSRRRQRTSLEDSVKPPAAKRQRSSLRREPKQPAADKNVTTAQEYGNKPSA
VTRDYGVETGPQKERSLAIRSSEKTSKATSQTDHAVVLSSNDYYTVTQLPSGSDQPGETISGPTRC
IFNSESGFALLLSQSRALVWPYHTNVPSPGGGGLLSVSLSEWCTNDGEALMGTLISNATCTVPGLF
IIVPATGKMIYWETASNATFMGIAKQKQSGLQGSITGLFYGEKVTDVINAEPAGVITTFSSGRVAQ
ITFRDPQGKPALSVNFFQSTAKLGAGGLFFGIRSVLGSSAWRKDIAAVKAGNSLFRGQRDIIVLST
SGLLEIWDSHWNGVNKIRAEVDLQKEICSYLGFESNGKPENCLKVHDFVATYREAKSPSNSEEQHN
ETSLFILIDISRNTPAKSLAVIKARIQNDNFEVLSLNTLENMTLAQGFEKQRPRLHVVEPWETAFI
LMGRKLFIMSLQEIEASPSAQLLNGIVPKPFQDRLQFQEGDKYEILGIGSEETSSETQTPGCNVMI
RGFGLIGISATSPGSDTTEYVRVTAKQKLEQAVFFGTMSNTPLSFSSDGEPAFALEEYEQAALEIC
NDILRSTSQYIPRAGISLDQTLRRRSKALGDLATELIRRRLPLSKTVRWELMWAGEKLAAQRSMWN
LEENFRERWASETFLSRVISLMTDKYKTEYVPSHNDKQNDHVRQWFDCDTFQMQHIIPWIFNAIRG
LKGPSARSSPEFLSQVYQASELSLTVLEGAYKFREDHALQYGFAEEDFDNGVLLGSYSDIPEFWTS
ENMVYNETLHMLDFELESSRWVHQSVSDTSAEDHDMIASIGENSCRRLRVLNMMLTERVRWLNAQN
DPKFADEAIALKDSSVKQRKWQLYKMGGIGQLQGAIDLAEEFRDLEALVELMVELQYLVRNKSSPV
ETSITINSSGVRDESGYKQKIASYFDHFGEPFADAFFSRYIGIHQPGVLLTMKDFQNHLTRFLRKR
PSYAPLSWINDIVGEGDYQTASSALEQLALDHDDNLWDQRVQLSLAKLTRLASLEESNVSDDATPT
KLQELDDKLEIGNLQERLYEHVLPALHSAIDEKAEVELAMDQFGGRVTDQPALKDLLEELLTRLIR
RQPLDAEQLINILTLIDDVRFLEGDESSVSGREFYNALRVLRLSGYASSDPVKYDFLERLIWRRCF
IRDDWHSISKTEFRSDDKNRSLFLNTTLARTLLACVEDGKLGLSQSYTISQPKAYKTTETEENRGR
GELYPVPNISDVLSLGSLELIRSRFRPEQWPHIERDLRTEVDSLERLLEGTNLKEWLSGLLLHAEE
EFGGTNVKGERLVPESTKAKFSWV
>g4
MAQPSIACIAFVAKDDHPLHVTVFPPFRESRLRFLILINSCLDILDLRTRNTFIDQDLGLLQALDE
RLAVYGWLTNTGVKILVIVDLAGRHIDGTGQKKNITPVTGMKHSDLKPAFRALQTAYIGLLQNPFY
NPDDQNESSRIETTSDTPMGINDKKFVTEVKRIGDMWSPSLTII
>g5
MNPLARVGRIRPASLHSIGITKQLEQRAWISRSAAMPATAIERQTESSSSLTASGKVRREVPLPSQ
EKKEGAMQYALTTLDQIANWARQSSLWPMTFGLACCAVEMMHLSTPRYDQDRLGIIFRASPRQSDV
MIVAGTLTNKMAPALRQVYDQMPDPRWVISMGSCANGGGYYHYSYSVVRGCDRIVPVDIYVPGCPP
TSEALMYGIFQLQKKMRHTKITRMWYRR
>g6
MTDKSQFEPGHQIPVHHQDQPGWQHKIPDPKPISDMLPTDEGGYQLYKAAGKLKGKRAVITGGDSG
IGRAIAVLYAMEGASSLIVYLDSEEKDAQDTKQHVEKYGAKCFLFPTDLRKKENCKAVIDKAVEQL
GGIDILVNNAAFQNMLPDIKDLSEEQWLKTFDTNIHPYFYLAKYALPHLKRGSTIINCSSVNPYIG
RGDLLDYTSSKGAIVAFTRALSNQQISKGIRVNAVCPGPIWTPLIPSTMTTEAMEQFSGVPMGRPG
QPSEVATCFVFLASADSSYMSGQSLHPNGGVVVGS
>g7
MDASSLCNAISGSPHGFLVPSNELHTASRLATKNFLDPLASSLTTLQDRWHKSQNNGQRVKKNSSK
NRFRVQKLYLDGFSVGQVWEQVVRILDETNDQLSNDIEETEKSLQKSVGKGRLHSPQSIASDQDDS

ALEDDISRSELDSQLGAEDSNLDSEDVEDDMEEDVEEVSDEDESDADIEERSTVSEDATSETYVED
KFGLNDGFFSIDDFNKQSEYFERMDTRGDQDEPSDEEEVDWHADPTSATTSTARLSKSSADKDEIE
DSEDSDAGPTFGDGDFGDGDSDEELEENEMGADWLDTNDIKYDDFFAPPPRKASKKKARPLPKTQP
TAAPTDDDIERAISDVRRDLFEDISDQADSDVDMDAGEAEGGRSTHEKQKAKIADEIRRLEMANVA
KKEWMLAGEARAPQRPLNSLIEEDLEFERIGKPVPVVTSETSEEMEQLIKRRIIAKEFDEVVRRLP
DSVRQHDISRGRVEVDQTKPQQSLAEIYENDHLRATDPSYVDQKNEKLKKEHAEITQLWKSTSSQL
DALSNWHYKPSVPQPNINVITDVATVMMEDARPGGSAVVGEQGTLAPQEVYHPKDGAGLGGEVVLK
NGVSVAKDEMSREEKSRRRRNEKKKFKATSQQPGKQKKTDEGQQILSDLKKGGVQMVGQGGELQSL
NERRKSGTATAAQSDSLKL
>g8
MDDAQITHTYSSSTESGSPADDSDNSQLLIGESLIPVRERKHAGTTVVTIDGLLKEPLRLKEDLKQ
GCGGQLWPAGLLLSRYMLEEHATDLVGKTIVELGAGGGLVGLAVARGCQTDKPIYITDQEPMMQLM
QDNISLNDLSTKVKPALLDWGTPSPAEIPEHPEIVLAADCVYFEPAFPLLISTLQGLLGSDTICYF
CFKRRRRADLRCIKMIKKLFNVTEINRFSTCEGYNRENLFLYKIQSRQKSKE
>g9
MATLHYLPPVKPSAIALGTVFTHTASLGILAPVFGDAYHRAQAANTKEEFLKSKETSGAVAAWGSS
LVGSALQTYGVAALINATGTLTYKGAAYLGSLIFFASSAPGLISAIITEKRPVDTVAIGAVSRVFE
TVGLSLFLTWWGTRTNPFD
>g10
MSREVAKAAKSASNAIAIHKKYTVQSTGIWEVIRRTLAVDPTRSTGVPLNPQFRNPPPGALPPQSY
DDPVTLPAADLADNPYWKRDVRRNYPQLSVFSQGDVAGLLTFGNKQAPKEDVLQLGEAGEKQLVAA
KQEGEDKGLAAYFEKDKSSVKSILGPDGLPPLPARLNNSAKYELGSGQGYPEKYPCRTFV
>g11
MPVELRKRKAAPSQPEPAQKKAATAKTTKKAAPKAEKATEEVKSKSTAAKPAVPKVGDVISLEGFG
GELQTQDGASTTLQTLIASSKAGVVLFTYPRASTPGCTKQACMFRDGYENLTSTGLAIYGLSTDSP
KANTTFKTKQNLPYPLLCDVNATLIGSLGLKKSPKGTVRGVCVIDKAXKVLLLEPGSPAGTVEAVE
KIVRESPIKDGDKEESKKEAEEAEEEKE
>g12
MKVFSSDCTFDYSWGEVSTANWRKYCPWNDKSTHVKAVDTLSRAIEPSTGILRTERLITCYQSAPK
WVLNLFGGSEVSYVYEVSYVDPITKKVTMCSTNLTWANVLNVRETVTYQPSQSLPDKQTDFRQEAK
ITALCTGWQKMKNKLEEVSVERFRENAKRGREGFEAVLEMSRRVFGEQKELEKQLQTS
>g13
MSDPEVPLPGQWPVDPQEDVPISKDRIWVDGCFDFSHHGHAGAMLQARKLGKELYVGIHSDEAILE
NKGPTVMTLSERVAAVEACRWATKSIPHAPYVTTLPWISHYGCQYVVHGDDITSDSNGEDCYRFVK
AAGRFLVVKRTPGISTTDLVGRMLLCTKNHFVKSVKDILEGNEGSDNAEDRKAHGADLMQRLRDYA
TDESGHRPGPEVWLWNGSDTAKINQVVEQAGKFEALINGVAPKPGQRIVYVDGGFDLFSSGHIEFL
RRVWDLEEAEGRKSGWYEESAKQKRIEEQGTDYGPAYIIAGIHDDSVINHWKGLNYPIMNIFERGL
CVLQCKYINTIIFSTPFTPTESFLQAMPWGVPDVVYHGPTTFIPLTYDPYVAPKQMGIFKEVDSHP
FQHVNAGEIVERILKSRSAYEERQRAKLQKGVVEQDARDKELASQETNPGPQ
>g14
MRMPYFDPKRLSRRAINFLLLGLSLPPILDAHSTPMEYLRALHALLLEFETFQQVHNPDGNFSSNL
VRARLPQMFKRATYTGNKARRASSANEIGLPMHFSDSSDVKAMTGNLASSATAASAVTSFPNTDSS
DLLPGEEYTYLLTPSLPFDPDFFETFVTLCDVLIDCYNRLTSLISSPAVCTTALGELFTKTDAKLR
KVMVAGVVRDFEDASRSGAKSEIGGVSRVVLGGLLG
>g15
MFSRFALRQPSRAVTAATKAVRAPSPWSLASSSPAAIQFRRLLSTETKAAIDKAVESAPVVLFMKG
TPETPQCGFSRASIQILGLQGVDPKKFTAFNVLEDPELRQGIKEYSDWPTIPQLYVDKEFVGGCDI
LMSMHQNGELAKLLEEKNVLVAAEE
>g16
MPLLAKHMPMRSPLHNAVRTLRFQSTRCPTNFYYKIRYSAAVAARLHCTSDHRHPRLPSLEAPTSY
GPNTVAKIQTRALRQSSYNKSSVQLAAPASAPALYSPPQTGLLSYLPKSWIPYAELIRLDKPTGTY
YLFFPCLFSTLLAAPLATPMASPAEVLAYSGLFFSGALIMRGAGCTINDLWDKDFDPHVTRTRFRP

IARGAVTPEKAIVFTGVQLLAGLGILLQFPLQCLYYGVPSLLFVATYPLAKRVTNYPQAVLGLTFS
WGAVMGFPALGVDLLNNTDALLSAAALYSSCVAWTVLYDMIYAHMDIKDDVAAGIKSIALRHEHNT
KTVLSGLAVTQVSLLALAGWAAGAGPLYFAGTCGSAIMSLGIMIWRVRLKDVKNCWWWFKNGCWIT
GGGIALGLAGEYLAQYSGYYQNGDDARKEVDVVRS
>g17
MPAQRPQAAFEPIPPNIDVPALVESTPNFEWVVRIPCDAIYEQGLENFEKLVRLHVITGGKPLVVE
GYDTRFEKWIFAEQWLRDNLSSKSEVARNLTAKTNIPLSIGHYLSNMPMLAEQITPQNFTEPTRQR
IYLKDIDCPDVWHEKLRDIIPPFLFYMNESTLDPTYLSQDFAKAGDLMSSLPTPMRAENLMCYIGH
EGTYTPCHQEMCATLGHNIMVETSTGTFEHGKRTKPGSSIWFMTETKDHHLVSEYWSSILGHDIDL
EDHFAQISAWKAAPFTTYVVEQRAGDFILIPPLAPHQVWNRGTRTMKVAWNRTTVETLEMALEEAL
PRARTVCRDEQYKNKSIIYFTLEKYSGLLAAAERLNTKTTKVRHLQKDFKKLYAMFTAIMLSESFS
NEFSEESKVEFLPYDSNVTCSYCRCNIFNRFLTCTHCVGKFANGEDDCYDVCMECYAMGRSCGCIS
NLQWVEQFPWQELVRKHETWGKQISRMQNLKMQNPKEDRYVSLSTERLHLGHRTLAEVCQLELMRR
PWVDITKHTFDEREGLDSQDDEDDQGSGDERRVKKRKVHPRDKLAKEQARCHICKALEPTWKLAFC
LTCSLHYCYGSLFRAFEIKPQEVMQTYRWECPRCRKICSCGACRRKFTTQPYEPKGLTLGHDTRKV
ADPRSVEALVNFSHSNIGWLKKAGDGTSDSTRRLQRHQAEAEQAKMQEAFAVQDDDALFVEQPVQN
GYSNTESTFHDGVPIDPALGGPPAAPSHNFDIYDPSVHLPELAARVHQLTQSSAVHGLTRSEEEIL
RDAAAIRFEMPSGNRLHVDLTQDVTTDGNATYGIDPGLLQTTIGGENGALPRAANHIPVVQSKSRP
RKNKGQLVVRLRLGQEHMHAIAQTYKDQPSPNAATIITSDILPFQDEPLPTPENSRKKRRRGEKDD
DFKVWRKGRNPPQTLETDHHPENGDKRRRAVRIANYYENSDLELSDGV
>g18
MNSARVLFPSALRASTRRTTPLKNVSPFRNQRRSLIPAPSANSGPLLDRRADRELPALESQFRWAR
TLPIFAVLVGAAMFGIFNYQKSSSSVVSSTLYALRTSKQAREILGDEIYFAHKIPWISGTMDQLHG
RIEISYWVKGTKSKGKMRFKSIRPDRTSYFRTEAWSLETEDGKIIQLLETDQDPFNLSD
>g19
MPPRLPSEAFCLNRSSWTAQFLLDTTTKRFFGIRSLTPPKSTNYNAGPDLPLLKSTPQAALERRLK
ANTMPLRTGLLAIKKGMTGLYDPETGKRTPCTVLQLDRCQVVSHKTLDKHGYFAVQVGAGFKKPQR
LTKALLGHFSVNEVAPKRYVHEFRVKDETGLPEIGRTLSADWFQVGQYVDARSNTKGKGFAGVMKR
HGFHGQDRSHGVSLTHRSMGSAGPSQGGGSRVYPGKKMAGNMGNLQNTLQNLKVLEIDAANGIIVV
NGSVSGPKGCLVQLQDALKKPWPKVESTPLPQTMDK
>g20
MPPVALTISRLLRQQAQSITTRSLVRQPLLQQQQRTIISQQFSQSSRRVAQSRPSLHTRKPFNHPS
KQPKQKRFSSSTSTNETAHHQSQQQQTKQQSQSFSQRMKTLSREYGWXALGVYLALSALDFPFCFA
AVRLFGVERIGHYEHVVVEEAKKLFYSVVPIERATLEGETADADAAEAREKLSDEGASIWTQLAIA
YAVHKSLIFFRVPLTAAVTPKVVKTLRSWGWNIGKKTPKAAK
>g21
MSAPASEDYANLTFATIFQSRLLKFGKQIQRRQLDLPEYAASFVNYKALKKLIKHLSATPTIAAQG
APAPPADLDPQSALRANKEVFFFRLEREIEKVNAFYVQKESEFSTRLKTLLDKKRVVQSRAHADKK
APTYFVSLFEGFLQFDSDLNKLQQFVEINETAVSKILKKWDKTSKSRMKELYLQRAVEVQPCFNRE
VLRDLSDRATTVRLDLEAWAEGENIRFDVARTTERTETALKDEDEQTDLQVLHMAAAGNIIALQEW
ITKLRTTPDSRDRMTRLFLAAVTDFSDEILGAILESGLVDLHAEDDINGRNCLHEAAISGQTFVFE
AGLKNEVDISTPDVYGRIPLHYACIHGRVDMVRKLLASAPDSVNSLDHDNFTPLIHSIVRNRLECA
KLVLNNNARINPTSDTDHIPLNLACQHGTVAIAEILLQKGATLLPDAEGLYPQHLVARSGRAPEML
ALLKKHGADLDQTDKLYQWTPLFHAASEGCVDCLHTLLELGVDTSVLDEKGLSAMYYAAWEGHLEC
MAQLWSKRDPSLPHRPLVSHQVVPQIGGSTTGYDGFRTDTGPSAFSSEGPDGIPDLELPPPIIPLR
RYGHNFLDTKAFVQIYFDISKRGPIVFDQSGRYPASRLTMSSKLSDVIPRTIMLPVQEDSRIVTFQ
VDTLENFVIEFEIFPTFGSKVIAKTVALPSLFSAENSSAGSCSLPLFDPRLRSIGQIRFDFQVIKP
YYGDPLEITHFATYWKATNAIDADHNGLITGSSLSGDYVQLFVQLTKDRVPILYPMFSINHFGIDI
PICTITYEQFTTIARERGIDTQAVLESLHSKTANDMASIHKQLAHSFLSLQDVLLHLPTGIHVNLT
VLYPSPAEEKELSLGSLADINTFGDCILHVVFDHARASREKSPDFMRSVVFTSYNTNICTALNWKQ
PNYPVLLCNDLGQIGDFTREASTSTAIKSDGHTSMSIKEAARIAQSNNFMGVICRSSLLNVMPALI
ETIKEQGLVLIADTSDESATSSLTTTTTRAATDWAYRAPEGVNGVMKATGVLRFNESIDM

>g22
MPHRHGMHHHVHRHIPENPAQLEVVPLEPAPTVSEDPSIVRRASSSTSTCSGSSCETSSSSNLVNT
LPVVLGVVIPVVLAIAVLLFLHRRHVRKLRQEDANDKHKSLDFGMEVVRAGGENPKPEMGEKPHKH
GMSLDIIGSPYLLPPGLHGSKESLRSLSKVISPDDDKYRLAAQSDTASLRSYRSHPRTGHDDASSF
GGSTRHGPLPDDMNQGLLQNASRMSRSPPIDATSPLSVNETIHEEQFDHSRAMGNQDPNHRAESPP
MADSLNNHNTPEPSGRGDDLFFGSFPQRNPNRLTLNPSKGLQAPNEASHEPHPSLGGFDFGTGNFD
SVNDHHDAPVMQETSAESHHVGLPRLSLPMSETSDYGDEPKAHVPPSLNMPEAEDYHQESEDFSHP
PNNRMTQSFTKFDEGFDPHRLTVGIRPLPPEDPTDNAEQRANRIRSFYKEYFDDSKGGGHEEYYED
FGPEFYEDPGVFDPYQSDYFTGPPKPFAQPMGRRAMTPPPRFQGPPRNIMHPAAGSRGAGPRAFSS
ASGRMPPKGPAKPAPPPEPLHVLPSPHLIKDDMSILPIDYAPPTLKERVEGRPESPRGGLRPYTSM
SRAHTPLVSSFDDLAAIPSPHALRKSGIYTSLDFAPPARFKNDTGSDAGSIRSNRTGISRVHDHNI
RTGAYRKHPKDFLQDVSSFDVRRANGQLDYIDCPLQHFIHLICLLNLDIQFFQHNQLLDIVIQHKL
LDIDKLDIKLHQLFEFYFYNKFVNLNDSFSDSTRANDNLCDIYIYGFSNLRDRIINRHFYYQLIDH
GLSAGGTIAVAVVVPVASVALIICVLLWWWRRRKANKLAEEERRKEVEEYGFNPNNDPGLGGSVVG
GAMSSTTYNDQYQSQDGNSGYRGWGTTSHPRKPSTNLSSIPLSETSAMQYNDGPMRPDSGELGAMG
AGGVAGAAAGAQTGDIRRGASNASSAYSAGVHSEGSDDAHIGGPGPNQYYDEGNPYYHDIHNQQAG
GYGDPAYGLGQPVIRDVQARRNTRIENPGVYPQQGNAGIAQNF
>g23
MDEVSRDLEKAEIDASHPGQAVEARDEPALSPSRTSSSLGSISSSSSSEVPVEPIMSRIQTSYTLE
RHPTALSRIATQRSQHSGTVGATLRSRVSKKPLPPFGGGKSYPPALPAKEAYVVEFDGPDDPLHPQ
NWPTKKKVLTSVMLAYTSLVSTFGSSIFADATTSLASIYHVSTEVTLLGLSLYVLGFATGPICWAP
LSELRGRKLPIIIGMTGLTLFQFAVATAKDLQTVLICRFFAGFFGACPIAVVAAVFSDMFDNRFRG
VAITVFSLTVFMGPLLAPFIGGFIVDSYLGWRWTQYLVAIMASAALILDVFFLDETYPPIVLIEKA
AELRRRTRNWGIHAKQEEIEVDFRELVSKNFSRPIKILISEPIVLLLSIYMAFIYGLLYLFMTAYP
IVFQQIHGFNKGIGGLPYFGMAVGELLAGIFIIAMQPSYNKKLEANNNIPVPEWRLPPAIIGGVSF
SAGLFWFGWSGYRADIHWIVPTLSGLLTGFGLLSIFLQCLNYLVDAYLLFAASAIAANTFLRSLAG
AVFPLFATYMFNGMGINWAGTLLGCISALLVPIPIFFYLRGHKIREKSKFAPTFAPERPAEEDEQ
>g24
MEHVSTLCAVSTVGIAATGFALARREQPTGLQSVPSSRDSALPRNSSSDIVNHKLSHHQGASERSS
QQRSGFQPVATQSGQSLVLSQRRRERRRRQTVTNPLTKAVDESSTQDSGAASSRPSSSWLRRISII
SSTTTSSLSSPTTATSSVNISPTRAHTRRDSTVKAPNKLVKRSNSQHSIKQAGSGTGRSRSNTLGL
RRPATSHQRSGDIARKSSLGQESDLSIPPAFLESSLARENDLVDLDSQSWRPFFASTFGRRRQSST
SNAIVTIKPNRISHPRYHINYPTLMLSTSVKKLSHASDMAAKQNTSQSPSTDAVPFIDPFSRHTDA
SNPKLTKHTTQEKRRTVRSSSISDLSVASRKPRVSPMKGDVKAPLSAARFHGKDRIVSNPLPRDSG
KIGSPRLRRKRNFTESDTLNRPQTAPSANYFHNKDPHIDEQFPPDLALSVDLLKSTDSSLAYRKGS
YDNKSFPINYPPSGTSPPIIRYSRNKRLSGATSDWASTVIGSDDTRVFTSADEDDQSDSVFDSFRT
RISSSSQTGRRGPNIETIFHDASADLNKGGSVAVEELDFENLRLSQSTPRSSLSDLDSNITPVALS
IAKNTDATPTPPAKNSLKTPLHSSPLKLSLAPQTEELVFSSDEDAVLEDGDDEFDTSFGENLNFFH
PYSDPDANSPSLTRKSSAIGIKASVFDWSEQTWTERDPQAHEVRPSTVHGKQGPNARSTRRPNRKA
PSAIHLRSQSVPVSREGPVPTESRQPSAKFGTWGLGQKGVSEDWDGFDFGDEDDGDDLGLGDGTD
SVMRIQTMKVPQAIMESQASVHGQYGQVQELTLLVEELKRLRIQGNTLQVIDGSANDLWKEADGIV
NLATFEDEDANWQIPNSPSSCGSFDDFENSPSPQQKPWRNNDSDTKRPPLTLWSDMDHNSKSSSKK
EPTAKAKSVLETIYQQRESSEAPSVEIATQPPQKLPFDTQSLKELVARAGVVTRALKDVIRRAEGV
TAGDESIPSSDPPFSQIFAQRS
>g25
MEAELQAQIPGIDRVISEYSVGYLTHASHAYVEEADANALSPLAEAADTVTEILVSASGDFSSENE
LAIRNLVEKFISSLNAADGIDAERRQIPFAAKKLDQTINVGAQRNMSSTLGLVGASVDLESAATRK
VESRVDRKKLEKAERKIRAKQDKKLVKNVEYEASRLLEQPDSTQSYEEFFMAVNPLQLGADAQSKS
KDIKVDGIDITIGGKRILTDASLSMAYSRRYGLVGQNGIGKSTLLRALSRREVAVPTHISILHVEQ
EITGDDTPAIQAVLDADVWRKHLLQEQEKITKQLADIDVERSSMADTSKDAARLDKERDGLDITLS
DIHAKLAEMESDKAESRAASILAGLGFSAERQKFATKTFSGGWRMRLALARALFCEPDLLLLDEPS
NMLDVPSITFLANYLQSYPSTVLVVSHDRAFLNEVATDIIHQHSERLDYYKGANFDSFYATKEERR

KTAKREYENQMAQRAHLQAFIDKFRYNAAKSSEAQSRIKKLERMPILEAPESEYVVHFKFPDVEKL
SPPIIQMTGVAFGYTKDKPLLSNVDLDVQLDSRIGIVGPNGAGKTTVLKLLIGQLQPTSGLISQNP
RLRVGYFAQHHVDALDLNDSAVGFMTKNYPGKTDEEYRRHLGAFGITGMTGLQKLGLLSGGQKSRV
AFACLSLTNPHILVLDEPSNHLDIEAMDALSEALQKFEGGVLMVSHDVTMLQNVCTSLWVCDNGRV
EKFDGDVKAYKKKISAQADAAGVARAQS
>g26
MEHHDADQPLLANDSNNREDVVYESSDESASGLGGLFIWALTFAAGISGLLFGYDTGVISATLVSI
KTDLSGKLLTTMDKSIITSSTSLLALIASPLAGVYADSIGRRKVMLVADALFTLGALCQALTSTVF
GMVIGRSLVGLAVGAASMVSSLYISELAPSHLRGRLVTILALFITGGQVVAYITGWLFSSTPGGWR
WIVGLGAVPAMMQFVILIFLPESPRWLVKAGYFVEARQVLTKVFGSDSQAAYKTRTVLRDIEQEVA
DEAAQLYNKADNSFRRALQTMAELYSVDCNRRALIIAMMLQGFQQLCGFNSLMYFSATIFESLSFS
SPTLTSLAVAGTNFLFTLLAFSVIDYIGRRRILLYSIPFMIAALLGCALAFSHLRWEPSLLSFEND
QRQPLSSTSSMPVVILICLTVYTAAYASGLGNVPWQQSELFPLSVRSLGSAFATATNWGSNFIVGL
TFLPMMEVLTPRWTFVIYALVCVLGWIGVWFVYPEMSGMSLEEVKDLLSDGWGVKDSLIERIAIHI
ESDDDMLSFALTCRGVANCILSSTSGVWRARFNDKYDAGWKYTSDTLHVEYQMRAMVLGQPVYIQP
NETIREQVWLEVISTLLTETYVTKRGEPRSPRSKNLLRIAEACLSHLCFDPNGPGPCLRSNYDITE
VYSWGQEAHESLVEDGQIRLDTLLHIRNFWQRHLLSTREQSFATSYEENVQRKPREWLRPLEDPIP
VMGYWHGYYSCIHPYPTDVDTLMEWQTCASHSLILIDANRTIWPKRFDDQFPEHDLLCPRKYFIGS
EGFEGDDSLHYHIRGFYQDIPETQGGIYGWMRVCFVSYLLSEERDGQDEPDTTSWETYDIYNDFDR
IYGFEGILLPGGKILLGKYFDLQFEGEEDCERGPCIYWEHTSMGNILDILTISQSIKDPFLTCFSP
PREEQA
>g27
MVRVKLYCLCDSIVIEILANHHVQLQAQVIANSGHDDMIHDASLDYYGRRLATCSSDKTIKIFEVE
GETHRLVDTLKGHEGAVWCVAWAHPKFGTILASSSYDGKVLIWREQPSSAPTNGSPWTKVFDFSLH
TASVNIVSWAPHESGCLLACASSDGQISVLEFRDNSWTHQMFHAHGMGVNAVSWAPAASPGSLISA
NPGPGQQRRFVTGGSDNLVKIWDYSPESKTYNPVQTLEGHTDWVRDVAWSPSILSKSYIASASQDK
TVRIWTSDASAPGQWTSTVLEFDSVLWRVSWSLSGNILAVSGGDNKVTLWKENLKGQWEKVKDIDE
>g28
MADALCGPSNALQNFQKHTAVDRTLQQDRIVNRHPSTQNFRSQHHNAAVLDPEFEAFEKNFVGNAP
PQLIPSGPAFAAPVHSQSLHRFTAPEADWASDFQKLQVSGPSANIQQNHAMPNQAFRPAAMNGWQN
EFMQHQQPQQQQSDQPQFRQGPMTSFQPSFMPSYQGMNGVAAPAPVDQGLMQTTSTAPAAFDESAF
EAAFNQAQADMHEQEIALSEQHQISEEDLQSKEVEPTLEAEAAEQIRIGSDLIGTTEESTAQEHKE
DPDELARTAGHLLDSLSHDTSQKFKESSFLALMRRIRDREVHIEGDEFRETQQPLHPGGKDYPGKR
RPGGLDSDQIRPE
>g29
MSTASPIRGYISSYAPRLRQYSNALLTPIIPPTQVGPSRTTKRGTTAINYAEDGFDDDDFDESDGP
RRPTGLRSLRREDSSLDRVPLSEKVGKEIFAPVEVQGIFRDWMVRRLFRADNADQLQAQSHLPLTL
VPIRIDLEVPAHQPLEPFPLPRNAADPGLNSTLPGYRRPEPVPAYRIKDTFLWNLHEAHATPDEFA
INFVRELDLPEPARLADAISTQIRSQLEEYAGVALHPLFQQGQIAPNPTDQINGLSRDVSITPAPV
TAVTPSTPGRNTIVTATKDPLVNDSLLNPDDAYRCVVNLNINLQNKLYTDKFEWSLLHPPGMAEEF
AKITCADLGLSGEWVSAISHGIYEAVLKLKKEVCESGGMISGVGAYGNEIDNQAANGAEAGWRYDP
EGLGDEWEPKVETLSKEEIEKREGDRERQIRRLRRETARFSSTAGITPELSRTNYFDVDSETPLGR
GERSKRKRRFRSISPVGRSGTPGGRGTPDTGSGAGYGGXGGTLLESERAHWRCFNCGVFGHAVWTV
RDGPAGPRTLCPNCGLLYERDKVPPEWARGLHRHDVPLNRHM
>g30
MDAQTKQHYLADSPPSVVRLEIKQHFEALKDESLKRYAHFMSRAAFQGTRVTLRQVSPESEPIYDL
ILSLYKAVGGNWHSLTEKTSVEPQDLQYFLEYSAQFLGNCGNYKGFGDSKFIPRLSPDAFKKLASI
SPETQAAFEKANSTGGGIYETSDVGLMHLGYPDKGHLTTYYPESSSITKDEITAVGDFLEKKGLPV
ENTRLRKTAQGDFELLIASGLSSPPSRDRDLGDEDSWTLEAAPVAGKKLSLVFGDYQNEMSKIAHS
IKQAELNAANDTQKKMLEQYAKSFGTGSIEAYKESQRLWVKDQKPAVETDLGFVETYRDPHGVRGE
WEGFVSMVNLVRTRTFSDLVNGASQYIVRLPWDKEFEKDKFLSPDFTSLEVLTFAGSGIPAGINSP
NYDDIRQNLGFKNVSLGNVLSAKAPNEPVPFIAEKDLEKFQKYRDEAFEVQVGLHELLGHGTGKLL

QETAPGVYNFDVKNPPVSPITKKPVSTWYKPGQTWSSVFGAIASSYEECRAECVAMALSCDFDILK
IFGHGDGQTDINNDAGDVLYVAYLQMARAGVVALEFWDPKSRKWGQAHMQARYSILRTFLDAGDDF
VKLVHTKEDLSDLEIHLDRSKILSHGRPAVENYLQKLHVYKSTADVVAGTQLYNDITSVDDWWGTK
VRNIVLQKKXPRKVFVQANTQIEALTISYEIGGAPPTALPSIEPAATVEDFSYISPPAHPPLSEAS
SSSHASPRPPPFSSIYSAPDPEVDRFKAAITEDPGASSLPAFAPPPPSPSLFLPPPEDLDESSSVV
AETKAALESVEKKGESSGKPPDDSEPPPPYTEGYSPLQSFSYVMAAAGGAASIITQVQQTGGPPIN
TLGDVNGDEHITLDLRGTRFTLSRDELLTLPEFVLLSLFPNGLLPDGHMGGFHDGDIYPVDYDPNS
LQYMLDFFRTVAQSIPSASPSPSGSPDMDASDSMYSSTRDMLQDRAGIIVLREDLDFYVIPPRPNI
GQAEMMTVKRAAAQVLLRQDGIFSGLRKSDEVGSTEQHLIEMLTAGGFNHEDSWGHRAPEPNKAVI
CSLALAKLRTDIRGDINSNASVGMAQKLLLFWRKPARRCWWEGIELDGVDGVEGKLKVWIRRVWTL
EMSVIGLR
>g31
MFSSALKSFSSNISSHYQISPEPAFISGPWKVHDGTKKSTGTAASIFIFERKTIEPRTGGFGRSAS
ASSLKKIHEEVVERLKREVSSLTRLRHPSILQILEPVEETRNGGLMFATEPITASLGGLLKEKDSQ
EGGGGGGGSGSRASRYMIESPDGTRRRREVEIDELEIQKGLLQIAKGLEFLHESAGLVHGNLNPEA
VYINIKSDWKIAGLSFAGPPDGSTTQSSLPPLALSEVLYQDPRLPPSVQLNMDYTSPDFVMDSNVS
PAADLFSLGLIIVALYNSPHTSPLQAHSNATAYKKLLTTPSTTPSQSNSFLCTRPIPKDLLGSVLP
RLITRRPAQRLTAREFQQSAYFDNILVSTIRFLESLPAKTQNEKSQFMRGLQRVIPEFPPSVLEKK
VLGALLEETKDRDLLSPILQNIFHILKKVPNGRRVCPDLIIPKLKEIFLAPAGKGAAQERDTTRDA
GLMVVLESLNVIAENCTGKDFKEDILPLVQLAMESSTHSLVDAAMKTLPVMLPVLDFSTVKNDIFP
PIASTFSKTNSLNIKIRALEAFVVLCGGIDEKDAEPVDDLTGIVEDKKKTKKSSPSILDKYTVQEK
LVPLLKAIKTKEPGVMMAALGVFRQVGKIVDTDFIAIEVLPTLWAFSLGPLLNIQQFEQFIALIKS
LSTKVELEQLRKLRELSSREDSSMTRQTIGGALEISASSGVQETSNDFERLVLGRKGTSNVNGDGN
WDSLTAGNVPIKSATQITSPTFSWSSNMSPTANRPSISGLASTGHTSRSITPDSNVGVFPSLEPNR
QKASGTSAFGTLQPQQSQPSPTSWNTSPQINRPSLAQSLGSSPSMMTATNTTAFPSPGGTASNYSA
FSIPPPPSTQTQYQSKPASVVQPNPWASGGVPSTAQATQPRPPGEQKGGLDKYASLL
>g32
MIMSSDDSAPRPLVRPEQQEYHEHHASTHLPSSMTTSTPYGKMLTRLEIASTHIPSFYITPFCGAM
AGVASGIVTCPLDVIKTKLQAQGGFLRRRAGHRGDTAQLYRGMIGLTPALLGLSHVAIQFPLYEYF
KMAFTGYGIGEHPDEGDSHWVGISLATFLSKVCASTATYPHEVLRTRLQTQQRHPPASSPEEIAFR
GGLDHPADRGRPPGAASSDGMPNRPRYAGVWRTCQTILREEGWRAFYSGIGTNLIRAVPAAMTTML
TYEYLRKTIHTLQHEGQKKLDDVNVEGDTLI
>g33
MSVPSLTSYVVKRPYLKRWLQPIAEWYTNAAGYRRLGLRADDLIPEESELVQQAIKRLPPKEAYDR
VFRLRRAFQCSISHTLLPAEEQTKPAEDVEYLSPIIRELQKEAQEREDLDNLIVKRK
>g34
MAPRRSARISNVSDLKPAEVLKSTAKDSKSAGTSKEKTNASNGNGVTKKSRKPPTRRTTREKQTER
DTAGSAPEEAWATPQTPSKHRDVTSIADNLILTPPQPPRLDRPVEPHTTNATLVTPRGSSVVAYPS
IEDISPSKTGLPRPTATTGTLLEKALEHLIAVDARLKPVVEQHHCHLFSPEGLAEQIDPFDSLVSS
VIGQQVSGAAARSIKNKFLDLFDTETGDLQADATTKAKRRFPTPAQVAKLDIPTLRTAGLSQRKAE
YIQGLAEKFSNGELSTQKLLRASDEEVMEMLIAVRGLGRWSVEMFSCFALKRTDVFSTGDLGVQYV
FFFFALRVMY
>g35
MAFSKYIVPALMASGAAYASSSSCGKSGSVTNITAQADADALSSCTTVEGDVAILSQAANGITFNG
VQEITGSLTAAGATNVTLIAAPDLTSIGNEFSLSSIILLTTLNFPELTSVGSINFQALPNLQALTF
TKGVSKAGSVSITNTGLTSLSGIELTSVGDFDLTANTALSTINVNDIKNITGTLIISANKNSLSIE
FPNLVSAQNLTFRNTSSISVPSLANLTGELGLFGNYFSNFSAPNLTTCADLDFDGNADLTEINLPL
LSSLTGGLAISNNDKLDTISFPKLSKVQGAIDLTGDYTNVSLPALTEVKGGFNAESTGNFSCSAFD
SLRSNNVIQGTYNCTQTSNPTTMDGGSGSSSSTSSSASHTSSGAAVANMAVPSMGVTAVLGALLSL
LM
>g36
MFEDAWGNDVSRRCSLPTLNSFTFRCQDERQEDTPMRRLPPLPPVEPLPPGPFNNPFTPSTHAQSP

ASTQDTFPASSKISTSSWLATEQQHTIVSPPSPVSSVDFSLKDTFSTQKAFGWPNDLSYTEIMALV
QPRNINRKPPLQQLCGRKRKNETMMSSASDQDDQREKHRIAEGNRRKNLSQLHRELDNRLHDFFLE
RAGWNPGKSLPQSKEHIVQGAIYLIDFLLAIVVYLMGQEKNIPPQLSENLQGQLRSMQLQQLVSSL
QSQNQAMQYQMRALKQEYDDLLDRHRALEFKLKSYDQVFRSPKPEVITPPPLAAIPQETSTPVTAP
SSSHQLVKKTLPGLRVLCNEIPGLSPDCSFSSDASNATFSTTSSFNLDVVPRTVISPMATRPSSPM
CLTPLSSSFSHSSLPSSRRESLNHQL
>g37
MLLPKGGVNWKSARSRLPPWRAVLILLTRTRFLISIAVTGLVVLLWRGISSSAAEMQSFYCYGPSK
SPMHMTINEMNEWNAHLQTPVNFNHHEPYDVNSTSIKFVNLNPIKSTPKAAANGERVLILTPLRDA
APYLPKYFDLLSILTYPHELIDLAFLVGDSTDDTLAVLSSELDRIQSQVEQKVAFRSATIIQKDFG
SDIDMNVKEKHSFEAQGPRRKSIARARNFLLYSALKPDHSWVYWRDVDIVDSPEKILEDFMAHDKD
ILVPNIWFHRYENGHDIEGKFDYNSWIESDKGRRLADSLPKDTILAEGYKQYDTGRTYLVKLGDWR
NNKDEEIELDGVGGVNILVKADVHRAGINFPSYAFENQAETEGFAKMAKRAGYQVVGLPNYIVWHI
DTEEKPGNLGGRKAY
>g38
MNIAPEKREWLCIIPDRPGMLEKRLEIRPIHLANLKPLIESKKVVTGGPMFHDAHPTEGKPANFKG
SMMIFRAATEAEVREYLSNDVYGKGNVWDLENMQIIPVAIVV
>g39
MIELGLSRISRLVQQSAFSWKAIHVAGTNGKGSITSYVSGLLTAAGVRCGRFTSPHLIDRWDCITI
NDKTVPESLFRQVEEQIEQRNRSLGVDASEFELLTATAFEIFDRENVEIGVVEVGMGGRLDATNIL
SNVLVSVIAKIGRDHEAFLGNSIQSIANEKAGILKDGVPCVVDGTNEPEVLQVLQSRIRELGIDSI
TATSDVAIQHFPKLVSLFNDLDLEPHQRSNIACATLALQKALEQTHSPLNVNDLVPNLSKIEWPGR
LQHLSVKSLINRNEPVLIDGAHNGQSANVLAGFVNHLSEILQPLLHPGDNAAVVAFGPVDGMPWVK
ATDPERVVNHIRSITGIGKVEAFGDDIPSALTWASQSARQGPLVIAGSLYLERHVFVFCVRRMVSR
SNDAEESFLPSSRTPGTPVKTRLARSGTSPTKRDDRSSKSLSKDDSELTDYQLGDCLGKGAFGSVY
RALNWNTGETVAVKQIKLADLPKSELRVIMLEIDLLKALDHPNIVKYHGFVKTPEMLNIILEYCEN
GSLHSIAKNFGRFPENLVALYMSQVLQGLQYLHDQGVIHRDIKGANILTTKQGLVKLADFGVASRT
TGLHESSVVGTPYWMAPEVIELSGATTASDIWSLGCTVIELLDGKPPYHKLQPMPALFRIVNDDHP
PLPQGASPGVKDFLMQCFQKDPNLRVSARKLLKHPWISNAHRTGSVVPKKPTEYEEAVKSVQEWNE
ALRSPDSTTMRRPGRTDLQGSNHPRLDPINTALRPKDVTSTRNMSGKFRTPDPEDNWDDDFDSAIS
PRALELPHLRPHDHLGGILSSERLKAFASLDGTTMRDPLGSFLESDTTLGAMDSDPLETIRPLPRR
VVEENKPHVKSRSRHRSSQSVPVVSGVQVLNTKPGTSPSKPLRQPRPTAFYTESADEDFSDLIVAN
DDVLERKLGMFSDVDENVPKTALLIPAQRKESIDSFDSEDSKASFPIRLKARGSFKRSKSSIEIQR
FTEDEHDEDFSDIFGNEETGPKPASEHGSEKSALMLQSKLSNNSWLGDQDDEDDPFAQLEEGFDEM
DLEANIARDKYARLRNQVEGLVSSLKTSQDDDVLADISEQLLVIFCDLPETKAIIIGSHGMLPMLE
ILEGCRRRDIIFNLLKIINSIIYNDYEIQENLCFVGGIPIINEFASKKYPREIRLEAATFVQQMYQ
SSTLTLQMFVSAGGLNVLVDFLEDDYEDERDLVLIGVNGIWSVFELQGSTPKNDFCRILSRNSVLD
PLSLVLSRVLDEEEELAKISEGRIANIFFIFSQAENHVKEMVAERTVLHRVLKELRRMTPVHQITM
LKFIKNLSMLSTTLESLQNSNAIDVLTELLRSTMKQPHFREVSNQILNTIYNMCRLSKPRQEDAAL
NGIIPLLQKIVKTERPLKEFALPILCDMAHSGKVGRRELWRNKGLPFYISLLSDPYWQVTALDAIF
AWLQEETAKVEEHLLEDRADGLSFTDSIVKCLTISKANAFENLLEPLQKLLRLSPPVASTLARSDL
FTRIRQKLHHNKAAVRLNLLRILSSICDSTEEQSDLLARCGLLDAIRELENDPAILVRDMAGKLLK
SNEHLESLSSAKRRPLMRRTSTSALSQSSGGNLSRPTTPNLRPGQTRGLFDVAESPRHQRKSLRPV
TRDGSTSAGSSDSIGHTAARSRVPRAMNSRLSLLGSLPSEEARSPSSLNRPPSALNARRRQTNND
IEHIPAQRGFHSTPAQRQDAPQLKNASGSAGSKRSSTRRTAIRLAAAGGTVGVAVIGFWDDIKHGY
AAAERSARVATALAICINDYRTTLNQTGGTPEEQEAMLKACHKRCAERTLVVLEKNGSIFIKLGQH
LSSMGYLLPLEWTSTFIPLQDKCPVSSFESVQEMFLADTGKRIDEVFSSFSPTPIGAASLAQVHVA
ILRETGQKVAVKIQHPALAEWVPLDLALTRLTFSTLKRFFPEYDLEWLSREMDLSLPMELDFRHEA
ENAMRTKAYFKQHSDAPLVIPQVMTAEKRILTMEFISGARPDDLEFLDKNGIDRDEVSAAFAHIFN
EMIFGDNAPLHCDPHGGNIAIRKNDSRRKPNFDIILYDHGLYRDIPQDLRRNYAKLWLSVIEGDEK
KMRKYAYEVAGVTDEHFPIFASAITGRDFTVIAQNKVVSSRDSEQEKSNVTSALSQGLLQQLVDML
GRVPRIILLILKTNDLTRSLDENLHTRQGPVRTFLILARYATRTVFEEEMEFVSQNGSLLRLGNLY

RFLSAFTAYVRVELKLLAYEKWLSVKSTFGIAA
>g40
MFPRIKPERTSLFALGLIASSSLVTATGPCDIYSSXGTPCVAAHSTTRALYASYSGALYQIKRGSD
NTTTTISPLSAGGVANAAAQDTFCADTTCLITIIYDQSGRGNHLTQAPPGGFDGPDANGYDNLAAA
VGAPVTLNGKKAYGVFISPGTGYRNNAASGTATGDAAEGMYAVLDGTHYNGACCFDYGNAETSSTD
TGNGHMEAIYYGDATYWGSGSGSGPWVMADLENGLFSGESTGENSADPSLSYRFVTAVVKGEPNFW
AIRGGNAASGSLSTYYSGVRPNVSGYYPMHKEGAIILGIGGDNSNGAQGTFYEGVMTSGYPTDATE
NSVQANIVAAGYATTSLTSGTALTVGSAISLHVTTSGYTTRYIAHTDTTVNTQVVSSSSSTTLKEQ
ASWTVRTGLGNSACFSFESVDTPGSYIRHYNFELLLNANDGTKQFYEDATFCPESGLSGTGTSLRS
WSYPTRYFRHYNNVLYAASNGGVQTFDATASFNADVTFLVETAFA
>g41
MGIPLWRPPSPPEPKSSVKSDPCAHSRSSIRRRGAVRHVVSQSSSSSISPRNSLSASQLDDALQQL
DNLLENRPLAQSTVFGDVALVDSTRNAAALSDSARREEGSRLLRDALRHRQPGRRLRIPRESSLRF
EMPTPPWSPSEALHRSSHGDSARTRQAAEGFPYSPRFAPAYGPRHDSSGPSARTETRSPPPAVVRP
PVQMTEDGPAPGVRYLRRVGHRSVSDNNGTASSQQGIDGLGDRQRSASPDDDDLGNAWETLLTTLT
PDPNLPSAESSFTSNAAINTSRAAESGRRSQSSQTQRISSGPTATVHMILEPYPDFFNPCDIPDHA
TDSDTETESELGLSRSGSSRQRRHPRSRLLESTSTVGSTQDSQPPVPRLPRSLGLPSISFISHDLS
SGGDMQAIIDRLAQREHVPDDWWAAAGLPRTMGRQLDVGDGSSEHVNEGRTNAADES
>g42
MIAISGIPGSGKSSLAAVIANRINELYTQHAPGAPSIAAFVPMDGYHLTRTELAAMPDPVFAAARR
GAAFTFNPDKFLQLVKLLREPVTATTNTIFAPSFDHAIKDPVENDIPIFATARVLIFEGNYLSLNR
EPWNQAAALMDELWFVEVDFETARQRLIKRHVEAGIAKDAEDADKRARENDLLNGQEIVDFRLPVD
EVIESTFDEKFSA
>g43
MFVFKRDGRKERVQFDKITARVSRLCYGLDPDHVDAAAITQKVISGVYQGVTTVELDNLAAETAAY
MTVTHPDYAVLAARIAVSNLHKQTKKQFSAVIEDLYHYVNPKNNKSLPMISDEMYQTVMKHAEELN
SAIVYDRDFNYQYFGFKTLERSYLLRINGKVAERPQHMIMRVAVGIHGENIEKAIETYNLMSQKYF
THASPTLFNAGTPRPQLASCFLVDMKEDSIEGIYDTLKTCAMISKSAGGIGLNVHRIRSTGSYIGG
TNGTSNGLVPMLRVFNNTARYVDQGGNKRPGAFAIYLEPWHSDVFEFLDLRKNHGKEEVRARDLFY
ALWTPDLFMRRVEANQDWTLFCPNEASGLADVYGEEFDALYEKYEREGRGRRTIKAQKLWYAILEA
QTETGNPFMLYKDACNKKSNQKNLGTIRSSNLCTEIIEYCAPDEVAVCNLASLALPTFVDTARGEY
DFGKLHEVTQVLVRNLNKIIDINYYPVPEAKKSNFRHRPIAVGVNGLADAFLALRLPFDSPEAKQL
NIQIFETIYHGALTASIELAKEYGTYETYEGSPVSQGILQYDMWERTPTDLWDWDSLKAQISQHGV
RNSLLVAPMPTASTSQILGFNECFEPYTSNIYSRRVLAGEFQVVNPWLLKDLVDLGLWSDNMKNRI
IADGGSVQNIPNIPADIKALYKTVWEISQRSILQMAADRGAYIDQSQSLNIHMKEPTMGKITSMHF
AGWKMGLKTGMYYLRTMAASAPIQFTVDQEQLKVEDTNVARANGISKKRSPAPAAFSNSYASVPRP
MYDQKPVVSNGTGVPDPLQPVSASRVNGAAQPIEIAEDRAEHEIEEAEAGENGESEQDIYAQKVLQ
CSIENKEACIMCSG
>g44
MVQVAIIGGGLGGLVLALNLHKRNISTAIYESRSETDIHGGSIALAPNALRVLDRVGAYEALRVRG
FIYEQIHFVNGKGAEIGFVFHGNRGLYNFPALRIKRSVLREELVRLVKREGIPIFWDKKCVHVVSE
TAATDPESSGSATVEFSDGERVSAGFVVGADGIHSRVRPFIAPDVGDPTFDGLMGITGTIHADELG
DLLEKAGLHIPCMLFGAKGSIGILPSSFDGKEIEWFANLEAEDRSREEWTRFGSDGQGMKDLILQR
FLYDDKIDQWPEMVRQLMLKTRPEAFTNWPFYTLPHVEHWSSPHKRVLLIGDAAHAIPPTGGQGAA
MAFEDADTLSYVLTRAYSPDFNQATNLSDLVTKWEHHRQERIVEITTFTSQNGRMRRSSTYVYEQV
AKEWIIWAALKIKGPQGGAAWIFSYTPEDILAAISL
>g45
MVFSKAFAIAAIFATLTAAAPHQKHQKHQKRAVVYETVTEVDVTTIDTTITVYPGEATPTVASVYT
TEVAVTSTTPAEQVVVPTSTVAPAPVAETTTSSTQAPAAPETTTTAAPVPTTTVAPPAPAVTTTSS
SSTSTSSTSTSTSTAPAATSTGTSGSSGSGPSGTGDGTYYDTATSLSAPSYCDTANDGTTENVVALSQ
AIMNESLCGASITVTYDGKTATGTVVDKCMGCDAESIDMSRAMFGDLASLDAGRITVSWSV
>g46

MMHSRATPTLVVTIVFFVLASVFVALRFVSRVGIVRKVVSHDYLILLAWVLDFGFVFAVCYGTTKG
LGLHQQNVASSSNAALNTSEYVANVLYNPVLMVLKTSILTFYLFLSKTNKIFRWAIFATILVVNAA
GIALTMVNIFQCNPVSAVIWFPADGNAKCFSIVTIYLSSAPVNLITDITIFFLPLPILTQLRLPRK
QKLILIITFSFGFFTAVVDVIRVAYLQSAATSNATEQMDGFSNDGNSKYHQDFSWYGAFTFMWSAV
EINVGIICACVPSLKPLAARLLPRLIKGSTESSSSQLGSAVRPNFPRAETAPLAVPQIQNPPASRM
APRAYTDLGPAAASLPLNSPSRELLEMSDFLANSGPGPSEDTESSAATYPSPPLHVSTFFDFVNLK
KPKSMLAMSKKESIPPLALTTILFFLWGIGYGFLDILNGQFGQITGISTWGSLGLHAAYYGGYLVS
PLTIGRWILKRWGFKSTFIAGLFIYACGALIFWPSAVLSSFPPFAISNFIVGCGFGVLETAANPFI
SLCGPQENAEIRLNLSQAIQATGSVLSAVLAEKVLFRSVTSAAKLVRFQWTYLAIALFDLLLAAAF
YYLPVPEATDEDLQELADQRKDDVTARVFGLPVVWVTFGLAAFSQFWYVASQEVLVFSFPDYVQAQ
LPNGSSGLTPFDFLLLGRGLFALGRFVTALTNLLFKPRWILLVSYIGLIAFAVLAKELHGMSGVVI
GLILFFFQSGVFSITFAMSLRGMGRHTKTAGAIMATTISGGAVYPFFQEIARIDNHGSVPSSYVVL
AVGYAAAAIFPLYLMLVPQAKKQVDPVLNDYLHRRLTLRGQPSRRKNRGGTENGNGNGRDTPNHKG
DHYSLGGVLSRPRSLMDEPLSTIDFETAQPAPGVSVHDLAYEHDHT
>g47
MFAPPGAPDVLTLAQTAYNIYDAYHGAQDRYKELCDDIRALEITLQRLGNRLATHPSTDPASDKNG
GGYNEAKLEEIVHLASTLLEDLQRKIPAGTVPRGLYRLKWSQNEANAFRARITGISAMIAAFNSSL
LLDYVDSSTTQLGETQEKILRTLEDVLISLQSETKTKLKRRDSETSTLVEIQTEIQAEIEIEKEEG
GGRTDLWVDLVTEMQKHGISETQVEQHKSMILQWAGRAIAAGXLNQPDGEETETGTETYWEVISAV
YGPRNVTSILQRMIDTHRQHQQHLRSRPLQFIITNEAFGGDPFPGHVKAFSMAWRKRVVHRHGRQV
STSYSAVQKAFAQEDDVVVIDFDQGVNLEESPVESAARRGVEIVIASWHNLDMTQHITSLIQNGQT
AIMATDMQLLTHDPSPGYIKFLSVTYTYTSAKDNNNEHQYFQVSFAEEGTELDIPPSLDILYANWG
GLDITATLEAKIDPNTQTLTLDTNRMVHIASPDPWPGFYKSIVVIYRYNTIYKSKCGVEGDGMELL
VVGEGMGTTVIKPYQSGAHRSQSWRLDLPFPSDDENGSKARILGIIWGLQPVSTTMWSPRKELRDS
VLLSRRIVCTNNLFGGDGWFGKVKACLVLVQNVETREIVALVGRENTVLELVSPWQS
>g48
MSNLPSEPEFEQAYKELASTLENSSLFQKHPEYRKALKVASIPERVIQFRVVWEDDKGELQVNRGY
RVQFNSALGPYKGGLRFHPSVNLSILKFLGFEQIFKNALTGLNMGGGKGGADFDPKGKSDNEIRKF
CVGFMTELSRHIGADTDVPAGDIGVSGREIGYMFGQYKKIRNQWEGVLTGKGLSWGGSLIRPEATG
YGLVYYVQHMIQHASKGKESFEGKRVVVSGSGNVAQYAALKVIELGGTVVSLSDSKGSLVIANETD
GFTPEEIYAIADLKVARRQLSSIFSADAAEAKLFSTEKFKYIENARPWLHVGKVDVALPCATQNEI
SGEEANGLIDAGVKFVAEGSNMGSTQEAIDIFEADRTSKAESIWYAPGKAANLGGVAVSGLEMAQN
SARLSWTREEVDAKLKDIMKASFENGLETAKEFVPSSGEELPSLVAGSNIAGFMKVAAAMKDQGDW
W
>g49
MATSKSTYSLSSTIKLANGKLMPRLQLGVYLTSGRETEVAVKNALEAGYRGLDSAQMYHNEREVGR
AILRFLETEQDKPNGLKREDIFFTSKLASNVSYDATRQSIKKSIQASGLGYIDLFLIHSPYGGKAK
RLECWRALEDAYKEEEIRAVGVSNYGIKHLQELLATNPKVFPAVNQIEIHPFNTRTEIAQFCQENG
IVVEAYAPLARAYRFRHPTIVSLAEKYGCTPAQLMVRWSLQHGYVPLPKSVKKDRIVANAQVEGFE
IDSADMDTMDGLDEYLVTDWDPVDAP
>g50
MAGLSEASQGSEDANFDIFEWYPRYQSCQRYFVDYAQHSVPVQALSAFLNIQLPFQKQPYPVFNSS
TPAPGTGPSLSNQTPHAVSSVSLIPYIRRLVATGMDFPGVLHGFFGDDWTAGVGPLHEHERRNYLF
AAKSGGWAAVKKDYDMGPSETVPFLRPLQGPIDSELEAAERSWSEWLAMEDWMVGPRFPDTLHDSS
SFHSRNRGSR
>g51
MLMTDTRIVQDLIAPLSSSSLSLNTKDIQNIFAITAEWILDVARWHASNIVDEHQMGGLMSSPDAL
ALFESLGILLVALSATTKAHDALTSESTSDFKITLGQALTAYLSTSATISLNLRNRLDSLQKGYQL
YGEPPSKDLDVQMMDGMNMNALQFEASVLDGPVINSRAGLYVYINALLIGRPLFDDEMLLSYLSNR
YGGQQEVLIQELITATFDVLSNAMYRNESTRNMFIFRSFLVNKLPLFIAGMVATSMEQIPMEMCIS
HALNRVDPNAFPSFSQMFEMSGNTVLSDVRQEFLFACALHRLIPEASIERLLGENPMQTLPVGGRY
VKDNIVAQILSNQGAADRLISEIEMMEGNAGAVVAAVTDVIHSLCEQKETVTLKSICDSLSRRPQV

LDAMLLFRSPKSILQPLCTLLDSWKWDEDQGESQPVYDEFGSILLLVMAFKYRYDLSQYDLGVTAS
SSFVLKLFERGSTSMKLTGLDAKQNKSVGDWISALFMSEGISEETCQTLGACEMGMLELDTMKGGF
EYLLQPFLLPSLVFALKWLGNYIWESSEADLLLLLKVLHALVKPSSISGEAEACHRTVLNIAARGL
EEQLKDVRTRLPGGRSDNQSDIQPSPHQDIQQEIQAILDILDPYLSFQCNGNSRRSELDSWTTHAD
GICGAIRVTFQGLVLWSASAEMSMSPHAYTHRLILAGIRLSTASNVLSVLLDELKAQAEEASGSLD
LAIDIAATLICAPIPESFAQEQTIYHPLDTTKEAFPRCLLLTLRQALMIQHQNVPKLSEKDPTRAE
VIVRLTRRVNALLTPPAHVGALDVSNIIDDMNLEAAAAAAAAGHDVMDLSNGNGTPNDQGAAPGES
IDQILNNAVAATANDHHGAGNEAHDLDTTGMDTSLDDILNAAHMGNPEFLDLDMDGMF
>g52
MEQSNHDVAPLNIVKGRARPPVPHLNFSSSSSTATIGRTQGRMTPPSTPHTSEDVVMSEPIEDDRP
IFHNYLRAFYPFHPSGAVSPSTVTLPLNQGDIILVHSVHVNGWADGTLLETGDRGWLPTNYCEAYD
QIPMRPLLKALTDFWDIIRGGCGSFLRDFGNQDLMRGLIAGEKAECLTREAPLVQRYDGLRRNRKA
LLADLSSLVKSAKRLQELANGAPRDEEVEQVLDDMLLKSFKIVTRGVRFLDVWNEHVGLTRAILEL
EPNAEDTYGSPVQTPSTETFSISDRSTTASTERNWSRPIHSQASNYGENTACSGYTRPPSSSLKSD
RGNRISVSHRVSYTGPAASQRNADLASVRLGAAYDAFLGVLGSFIGLHLQSRSSTELIVTTQQSVT
SCQVLLNLVEAIWEHDFQRSLELGEAKNTMYDRIGELVYAARDIFRPNSTGDEVPFMPDEGKRLVN
AATDCVRGAGSCVLLARAVLEQIGDFEVNLLTTTPSIAEDSSEAPEQADVQMEDTEPRQSSLRLLP
PPISIPQRDLAPATPALTTGVTPSSILSSTGLQSPKLDVDINTAPLVMNDISPSDSTYQDHVAQDD
PQALYRYETRGTTSTGSSFTYKSTIRDSEMSGVSQTSTRATSPDIGGTDNIRSLKESLSRSTLAEE
ADETEAAILGRTYAHELMFKDGQVVGGSLRALIEKLTAHESTPDALFVSTFYLTFRLFASPMDFAE
ALADRYDYIADTPNAAGPVRLRVYNIFKGWLETHWRHDCDIIALPFIIEFSKNKLMEALPNAGNRL
VELAEKVSTADGAIVPRLVSSIGKTNTSIAQYVSPETPLPAPVISKSQLNLLKQWRNTGSTISILD
FDPMELARQITIKESSIFCSILPEELLATEWMKKSGSLAVNVRAMSTLSTDLANLVADSILHLEEP
KKRALIIKHWVKIANKCLELNNYDSLMAIICSLNSSTIVRLKKTWELVSQKTKTTLDSLKEVVDVS
RNYAVLRQRLQNCAPPTLPFVGVYLTDLTFVDHGNPATRNLQDGGMTVINYDKHVKTAKIISELQR
FQIPYRLTEVPELQTWMQDQFVRVRSAGEKSFQNYYRRSLILEPRETPRAGSTDAAASAPSREVTK
DRFDFLTWSHNSKAKSVATTS
>g53
MALSQRIPILLCGKKLSNGIQVTELLKPEFEVIQFINSFDYAKSEFANLLSGQAPKTPSSNAIGTH
DYTQPLRAVMFGRGYSYDQVSELYELFKGKSSRPIAWIAGDPDFKLPPELPPDYAVVAAENTKRAF
KRWEDAGGKREEIVLY
>g54
MADSDTPILPLPPEVAKKVQSSVKITNLNGVVVELIKNALDAAAGSVSIVVDYRRGGCIAEDDGYG
IPAAEFKDGSGLCRAHFHGTAVILKLIQSSFTTLKSSLASFLLQPIRNYEDMVPE
>g55
MVYPAEAQPRYTYLLWHIFNYPTAAGHEAAFAVTKNWGDSINSLYSVLLEVILVQAFHLLILAFVY
VGNRSSSDSIHLATKVFYEDEPAAVLLRVLSRFTGGRVSQNPTSPSNRAIKRYEQTLLLVVIGVTV
LFVPKIVPAFAIHGLLIGTSAPVDPLQVYVPESPWFTTGNVGLQIPLEALAQPRYLRAVGQADTAS
IDLQNKVTVTAPVTLQNLGKGEQILEIGYNYSLTAQDLGLQNFHGLQLNVQGSCHTEYGWLNNTES
EGTSTDVYNIFGDDSINWKASPYYGYVPIAFSDFPKASGTFRINNTYAIMISSMQCDSFTIGTDPW
YLTNTTGTPYNGDTVYNVLRGRPGLSCWQSDEWTYKDQSSSTWNLSSISGLNFPQSLRNVFYNLIG
PPSIFSTATALGSIALASSTTAQLYTIDAASSSIYNDMQRLVLTTYISTANLLVDTTLYNKTNTYG
LNSIVSSGDLDQVAQFVVSDQNITTVSLAYAIGIPIGTVLIYLIVKLLKIYVQPRAFDGVSRTPEA
VVRINEAEKA
>g56
MTSLTHDRYTIAWICALSLEAAAARVMLDKSHSPLSQPSTDPNAYELGELNGHYIVIACLPAGVYG
TVAAADVVSHMCSTFPRLQYGLMVGIGGGVPGKNNDIRLGDVVVSKPVGQYSGVIQYDYGKAVQGG
KFEQTGILNKPPNTFLTHMSQLEAKKWTEGEAALATIVSEALEQNPNMQERFSPREQHTDLFFHSS
YPHAAGEDTCEKCDKEQLAKREPRDTPTPHIHYGLIASGDQVIKDSETRDRLAQPHGILCFEMEAA
GLMDRLPTLVIRGICDYCDSHKQKQWQGYAALTAAAYTKLLLSVMPVEITPETTAQSKSDLTSEDQ
RCLRDLLLSNPDDDRRRIEATKGGLLDDSFQWILDNAEYQRWWNRPGSQLLWIKGDAGKGKTMLII
GIIKQLLKPELSKLPAYFLCQGTDPRLNNSIAVLRGLIYMLIIQQPHIISHLRQRYDTEGKRLFEG

VNAFYSLSAVFENIIGDLRQSTVYLLIDALDECEVDLKSLLGLIKKTMSMPSVQVKWIVSSRNVNH
IEQILDADRGANKLSLELNADRIYHAIETYINYEVSRLGFLEHDQGLQEQVRDQLRQKSNGTFLWV
ALVIKELHKCLFESEVLDTLEDIPTDLSGLYDQMITQIDRLQNRRRNICIMVLSMVVLAYRPLHLS
EMRHLTGMDKNMELERAVGLCGSFLTIRDEYIYLIHQSAKDHLDTIYRTTSILRDRSEIHHEMYSQ
SLKTLKSKLRRNIYNLQDPGVSASEMANFRPHPDPLFDLRYSCTYWLDHFIDSKPAGKATSVQSQI
QLISKFLRGYHLLHWLESLSLIGEIRHGIVGLRKLSAAYAQHQSVFKEAERFASVNGSIIRETPLQ
TYGAALVFCPQNSESKKLYWDERLEFIEQAYITQESWDPCMQVIEGHTLPVNAVAFSPDGQTVASA
SWESIIQLWDTATGAAKQTLQGHTDGVNAVAFSPDGQTVASASGDKTIRLWDTATGAAKQTLQGHI
YGVNAVAFSPDGQTVASASGDKTIRLWDTATGAVKQTLQGHTYPVNAVAFSPDGQIVVSASRDKTI
RLWDIATGAAKQTLQGHTGGVNAVAFSPDGQIVASASKDKTIRLWDTATGAAKQTLQGHVNTVAFS
PDGQTIASVSKWYNTIRLWDTATGAVKQTLQGHTGGXNAVAFSPDGQTVASVSEWDNNIRLWNNTI
RLWDTATGAVKQTLQGHKYRISAVAFSPDGQTVASASDATIRLWDTATGAAKQTLQSHIGGANAVA
FSPDGQTVVSASWDKTIQLWDTATGAAKQTLQGHTDGANAVAFSPDGQTVVSASWDRTIQLWDTAT
GTAKQTLQGHTDGVNTVAFSPDGQTVAAASKWYNTIRLWDTATGAVKQTLQGHTDRVNAVAFSPDG
QIVASASKDKTIRLWDTATGAVTETHDLDTIPMTLSFSADGCSLNFDHGSLSSNRKGRHSFRNEIY
VVEKWISRNGQRLIWLPLQYRATDVLTRDNTTVLGHSSGGLTFLRLN
>g57
MSTSGEKVSRLLASNEKFASSFTGAPTMEQMRSGLVGNGPLFILTCVDPRCVPEAFFGPDLRAAVH
RNAGGRATADVVRTLNALRALVKMDTVVVVHHTDCGMTHVTEEEIREYSKSKNPEAAAIVDNIDFD
LWKEEQLEESVKKDVRKLREDKSLDGIEVFGFVLDTQTAVVTEVHV
>g58
MAMAETSNPPHDRHSRRRPFSTWMKRLTNLKSSSSSDSQSGSRASGKRNNNVKNDKRPNGGAQNNP
YPLSGTIRDIGSLRSYNDTVSYTESTDPGQHHQRASQQSLQASTSEANIPGNSAKSTAPTLSTNGD
TARSEAGYSKAGTTVTGNGGVYGGGEGSTFSSPAPSVRSLTTTLTTVQSAAPSAHIYGVTQNNQTA
NGPTSAAAQNNLNQQNQYSYQFPPSPASAVPPHLAGHPTTYSSATANNILTDNASILTLASSSKRR
RRNSLDTNASVRALAPSSVFGGSRESLPLSVLSANVGVDASNASITNASGVLSRQSLGGLASAERI
SVYSASGTVPPLVHGGDRSSLIASKQGDNGSVRSGTHSHTRNDSAAGSVAGTIGSTLNNSTSAAAN
AGITSVPLPGRISRRSSGWGEISGEGEGEGEEGTHQGGETEESKQENSHANGDPGPSVDEDKQ
>g59
MSNYGGRRAPNFAQYLEDLNTIPSPYDQSAQQQRSTEPFNLDAELALFTNTEFLDFGHFGDMNMPL
GFSPAEEEHKPQEEKHVVEKHLDMNYMDMLNDLGTIPDYTTTFTSAEESRNLQQMNSTFHSIPAVQ
DTTARPAEKTNTRVPKTEIPSPTDISAFVSTPMQVAGTKRKQSTDAKSLDESARAAAEEDKRRRNT
AASARFRVKKKQREQALEKSVKEASEKNAVLEARVSQLELENQWLKNLITEKNGSESGEGKQRSET
DIAHMFKNFLASHKAGKEQSSESKIGVGTA
>g60
MAKKRSIRAPTTSSLPSNLRISSNTPSLVKALGRLSRQSLLDLIVIWLSPKNVELYPPFLSQSSTG
NDVPDQDESPYAAADSVEEIREAYAEFGLRKGGKREVIDRVLEGDWRHGITLGQLAMVDIQYLEDH
PKGQTWSAFRLGLVDKTQQPDKEEDGDLLACLPRLHAATFLSNLQREIAPLVKAHYHLARSKAYPL
IYLRIFITDSPYNQPRHSAGTYLDSARTLYIAFPDSSPYVYTSMSAPTSAKTGPSTAPLTNDPRAL
RGIVRDALPKALSKPNERYKLEATALTAKSLETMLALRGAGKANEANGAFSIFADAVLEGTPIDPR
QAATVAPEEFLASEDKENAPSDQQNTKRKGSDTNAMAASPATKRRKLAVLSRFGTSGTSSSQALLN
RLDIRLLDRPGDDEDHVNDEDSDAGDGQYTMSLSFAGNNVVAGFRKLAELGVVDPIRMPSWMTGEE
GRIVAIMPPFTPSPYRARKRFLEDGHDNSPKKRRTLQPTSTYTPQNTYGSEVIDLTRDSPEPYTLP
PISPSAKAKKSPKTPRSSQSSQYNVEKRRRKFRSGPPRSYLEKLHRATTQRMFVISRRRSENGDIT
EENVDIVGTTGNIYTVTVSYEPSCTCPDARKGNQCKHIVYVLVNVLKARDDLRYQLAFLSTELREL
FENAPIETEEAHADKDSDSDTSGKRKPIEGDCPICFMEFEPLKEKIVWCKVGCGNNIHKTCFDQWV
ATTRNQGVRCVLCRTDWPADADAPLDRTSLLGTARKGADGYMNVAGAVGLSGNRDYSTYHRFWVNR
HLGRQRRGGHTDKMARPRAHSGADGPKEPHSVSLKVLRLSRPSLSYQYPLAREDTRISPKASLAYP
TNEFDPHFILSPNVTLPPAFGSAYVGETFACSLCANNELPTTDSTKRVTSVRILAEMQTPSQVFPL
DLKPAESDDDNMIKSAGEGLDYGQSLQKIVRFDLKEEGNHVLAVSVSYTETLLADTHDGLATTHAA
SGGRVRTFRKLYQFIAQPCLSVRTKASELTPAEVDNKSLGPYGKTRLLRFALEAQLENVGDGTVVI
EKTNLNPKPPFKSTSLNWDVQQLPENENENAQQPTMNPRDILQVAFLVEQEVGQQDGMDTLLKDLK

KDGRATLGQLSIEWRSAMGDRGFLTTGNLLTKKR
>g61
MLGLISKYTLVSIVYLGAFSRFTHGRYTPSFYRYQLDRAPNDASTRIIPVFDTIFATLVLFPATRA
WTAMVCGLIQGGAIVPRVRQGKSVAGDVGLFLTTVVVAWTSWYGVP
>g62
MPLATNTPVESSGPLADFFWIAGVDGKEIEDTYWKLRNDYMSNRESTPGPAVSDTIEEDTELEEDA
STTTNNVGGGVKRNSYNRLSRLSNDARMSIRSVDSRVSGTNSNRSSMTIRATPTVTLENSETSLGD
FDFDKALLKFASERESFLSDLSLSAGAITPNVKPPKPRPKVQKIVSEDRPVSSNPLKSGIGSVRRH
MSFRDMNSMRRQPSVARQASVRTSRRLSNYNSVIPVPQPIDTSPAMHPLKRRFEPVLLDRYPQKGM
PDEHAQRGTFPDFVPMFAFPNDINIVSSDQKPRSTWHGFVMTGGDGSKLHGVCVILWIPLGQKAAE
ALEECCEEWRKDNMTDEERELAASLGGRLAVERAKLSTLLAQLPGVPSGSPEREKLEDDISAVEEK
IGLMTDLLRPVRHGAASKIEGLTDDTGFWIPRAYGILGKNASMTTFWKEWLKAVSVPRTNGGVLRV
PASSPTVGIWQPLERYVMNLCTEAFSPISSKTQVELSVRELRLFARKEAPNELPGSRNTDLYALFR
SLSISNIIVLFEYALSESRIIFLSSHTSMLYLATRALIDLLFPFTWSGVLIPVLPARLIQALEAPC
PYIVGIERRYEKVELPSEDFVLVDLDADKIESDVQPTPLPRHQRKKLLSLLQLAAPHHSRYGVQPG
PPSYAIEAYPFDSFPTENSSIFTPRAQSTQLARYVSLNSTSFGNEYSPTPAPSPIYNVFLHARDEH
PSSRGLSRGNNERPTTSSTSKTGSPPSPRTSSPTSGFFPPPIPGTPVSRNDSGMALQASLREKRSG
HFDSSSRRSSSFGTERRGVPRRPSAPFLGHASNLSVTTLNTDYGAGSTYAPSVYATSTIAASTIVP
QAIAQPVFNTDSTCWIEGHCLQVQPRDDKAICSICDERADEKMYKCTGCKTHVHGRCTPLICLVCP
AAFHPEQIRAAFVRCFASLLYTYKKYLQPATKEQKKSGLTYHFNMDAFLRSLPHEHAEYMTGLQQT
QGFNEFISERERVQPNVKDSKVTLFDEIILSKRNRGRTSIFSGRMTTDFLSNTSDHLWRSAAATSF
PPTSRVQNGISEDYSEVIKRAPAKLDHSFMKEPRMVQGVPRVSKTANNARRKPLPKLMNGLSISPP
>g63
MASVASVTLTEEVPTTPTAGITVTGSHEPTPTASPPTKRTVVIVYGLGQEKILQVVAEVLGKAWTT
VTALSKVVLGSDAVVVGILNRDFESTAPETGVDDFSKLLIVNTYCIDDGSFPDDRLSEGCHYEFIY
SQTPFFRRDLTRFLSLILGQRRPHEDLAIKRRTNLISTTFPDVRAALSNLDILSVGSDAIELRVDL
LEEPLSDGSRNSIPSLKYVGQQVMLLRQHTELPIIFTTRCVHENGKFPMEDPELFHRYLRRAIQWG
IEYIDVELWLPEEIRKDLCDHRGNSIIMSAFHDFSGTWKWTSPEAQQVFKEGARYADIVKMIAMVN
TIEENYELEYFRSTIKANYSYPLLSAVNMGPQGQLSRALNTVFSPITHPLLPIIAAPGQLTAAEIN
RALHIMGQLPKRDMYAIGXFRSTSQSMFMEKCFNELGLPHNFSCIDRGPRGSIESVIMQPNFGGAY
VNPPLSSSAPYIPTKSDAARAIGLIDTITVNGSDGQHKFIGENATWKGIRATLTRDYIPSAYRGRA
VIVLSSSESDAAAAIFALRSLDVGTIYTVGFKASGPLAAGLEPFTSIQSVKLVEQPFVIVSALPPE
KSLLVQPLLRHYRSNGHASPPSTRGKVFLDLTSGQRKGDPLGVATSAGWTAYGIDDVNVWTTVETM
RLLVGQNVPFDFVRMASGHGLY
>g64
MSRPQVSIDRLTPRRVMAEPHAVPKKRGPKTDVLEALLKRVDGLERRLQEEGSTVSPTSENPPSTT
KRKGKGSSAGRSNSADETGSSIQSSLSIAEQEDPNQMSFADRRGSMLLTSTYNGFSDHPINSFRHG
QQTNTTNRQDNAFLSDTILDTYFTRLHGKPFYILEESSTRQRHHMGQLPGPLMMAIYAITIRYAQS
AEGQEGSTRIGLEYALKARQEIDIDNPTIEGLQTLLLLSMVFFAYGLGKKTYMTISSCSAMILALD
LYREPSPKLNLSHTEKETRRRLFWACYMMDRFVACGSRRPCLISNDSVLLRLPSWSSSTSSHALPV
EGELFNNSGINIQLSADPRRRGQPAIVLLIDIIRILGITNRYLATGGVKGDSHFPWHSMSNLSKIR
QELDLWGAGVQDIFISVESLFTHPESTTLFLSKLIYHLIHCLIYRPFLPLDLAELRGTGQHQSWQI
EATNLCFLHSNAIVELVEFGRNSSLVEWPAFISHCVCTAGTVHVHGVHYKGLEGEVFSSSADFLAK
EMQQMSWLRHCWAGVQHQRELLQTVYTCHAELVRNLANSTMRFSPVFHLEDFFDRYMGISVDSSHV
RLVDEVTDTSNDNMSVPLAIDSHSLYNTQAMSTPSLNSSQNFQNQQFSRMSGTASHQPQFQQHTEL
PGSQFDTSSQSQQLQPALYPYNLNDSSPSYSITQQDQHQQQPHSQQSQQQQMPRVLPSFSPTLGLS
PSAFISESLNTVTAPTPPSNFHYATFPFDTPHTAQARTPYTNGYAAQTPSGQSQASDSHATMSESG
PPSEKDPFLSLLEQLAENEHLEGDGPSELDFLLGGIHAPNPAESIAPGAEGDGMIVAGGEVPESVN
NERS
>g65
MTSSATTNLSNKSQQEQQPKSENNENKIPPLALPSTSDSTNQKLDVNGEGTTVSLDHLGPIVVNQD
GTMSRISNWDKMTEIEQKNTMRIIGKRNKQRLEALKAAGVGAEES

>g66
MPDHEPEPKRFEPKNPVQLDPPKDDPITIDELSKCDGTNPSRPTYVAIKGTVFDVSKNAAYAPGGQ
YHVFAGKDPSRALASSSLKVEDCRPEWEDLDDKEKTVLDEWFTFFSKRYNIVGKVVS
>g67
MVKMADDSNKRRRLSEDGSFEAKIVEEVLNEKNETKGESTELPAKPQMRRELFVRSLPASATTEKL
TEFFSQSYVLKHATVVIDPETKQSKGYGFVTFADIEDAQRALEEFNNAEFEGRKIRVEVAQPRKRE
IDEKGGRSVPTAESTRLKAERTKQREQTAPPRLIVRNLPWTIKESDQLAALFRSFGKVKHAVVPKK
GNVQAGFGFVVLRGRKNAEKALEAVNGKEVDGRTLAVDWAVEKSVWEEAQQNAEKKDVKDAMDVDV
GDDKETEAENDEDESVEVGSADEDEEEDEEGDFDDEDEDEDEDEEDEEDDRNASTIFIRNLPFTST
DETLYEHFKQFGPLRYARIVVDYETERPRGTGFVCFWNAEDAIECLRGAPRQVDAGKEDANTKKNS
TSIRQSVLQNDMLDPTGKYTMEDRVLNVTLAVSKTEANKLTEEGVSRRSERDMDRRRLYLLNEGTI
NTASSLYKKLAPSEIKMREDSLKQRQNLIKNNPTLHLSLTRLSIRNIPRSVTSKDLKALAREAIVG
FAKDAKEGRRQPISAEELARESEETKGDDKRRREKGKGLVKQAKIVFEGRDGSKIEEKTGGGRSRG
YGFVEYFNHRHALMGLRWLNGHVVQAKSNDGEAEKKKRLIVEFAIENAQVVKRRKEFEERARTKKE
DGPEKGGDNDKRGMKRKRPEGRGMNKGDKGDKGGKGKDKKADAGDDEDDDEKNKIAKRNRIIARKR
QQRRSRKGKA
>g68
MADASKMDVDMSDKPATPTPKVQQPIPQALPNRAAPSAILVSSRQKGNPILNHVKLVPWEYADIPA
DYVLGATTCALFLSLKYHRLHPEYIYSRIRLLAGKYNLRIVLVMVDIPNHEETLKELSKTSIVNNV
TLILCWSAPEAAHYLELFKSSENAQPTAIRSQQAQSYRESVIEFITAPRSVNKSDAASLMSTFGSL
QAAVNAQPEQISAVPGWGEKKVRQWSSAVREDFRVESTRRKVAPIRDLSTGYVADPKPVVEDSEDV
DVIEGDEDIEEMLFASARKAREFQTEPQASSEPPAKKKSQQEESMSEGIMDALAKLRKQDG
>g69
MHSSTSLMASSALTLLLALPTLISASCSIQGDVTTTFYGYPDNSPPGADIAYTQCGRSAAGGTGTY
DDPLTFASAEGEFQICDVIYAPYLKKYLRFEDECDQCTSDWNNDGTWHIDVWTGSSTQDGGQNQID
CEDNLTPDSQSIILNPDSGLEVDTAVLYDPNSQTCNTGDTFTGYDASQYCS
>g70
MAKSSSRSHSPPDRLENLNACVSSPSPQPRSFQNKDATFREWVVQNQIGISLTTLSTLLAAHNLYP
SVQSFTAPFFQMSYYHPESGKYKQGLDDVYFVISAVFAFTAVRGICLDWIFTPLARHAGMKKKASV
RFAEQAWLVCYDLTYWSYGMYLWSNSRYWGDFKAIWADWPKQEVAGEMKWYLLTQLAFWIQQIFTV
NVEERRKDFYHMLSHHVLTSALLSAAYIYRFYNVANVVLSLMDIVDFLLPAAKILKYFGYEKMCNA
VFVTLILTWLVXRHILYPMLCWSIYQNVPEVMSYGCYNGKTAQLYTTNGYPNRFAYMFGPYLSEEG
PFCMNFTIKWIFLSFLLAIQGLSILWFGMILRVAINAVRSGSAEDSRSDDEDEGEEEDEDENADIS
VSPNNESLSRAAAEKASGSEGSWRRANAPAPTRRGGGILGDSDRKALLGRIGCDKPTHD
>g71
MSNNITSLLTWGNLKSLLLFFGPFLLPRLINHYRAFRVQLAHRPPPRPIPADANRALNFLFGVIVV
FLTLSVPSNPYAPAQNIFTQTGSRINTPTDVVFARLARHRPDNALTDYDKLLRGNLTTIAQKKIYL
RFGPDALLSCQFCNLEDTFSYLLYYLPLNILVPHLVHLAIIGIVTSAPFAGVEAGRWRSWFVYAAL
GLAVLDCYVMTSYDPIAAAAPAVRAGQIPPLSLYTSMNMIRFLSMTIFDAVCAALIYISATHRFFY
SPPSPSDQVNQVIAAATAALTSANSKLHSLSVTRNAVVRDQSLKARDDWYWRTAVAMEGSGDADGA
GGGSIWEEEEVARAMSQVMNGRNRGRQLDMAQLGVNAGEYVDKVTAGLENGPSN
>g72
MSRLSKALAGPGYVILNVLRAINIITFLDIIAASVVMLIKISINNNFFFFEAVSHAFTTIMGVLLI
LSELPFFDKFFTRWVPQLGETASFVPLSLVMIIMGVSVLGNLNNDDYSEDNMGMPFWRIVASAGIL
AMTMGIVNFIASFVFADSENSLTARHVRSYGAVADKVLDSSKSSQRSFRLGTTFNSQADGLPTYRQ
VSITRSAESLSNSRFPVNISSPVREKTATTPTAPYGEEVTMPDLAHHPAMQGGNFV
>g73
MVSFKSLCILAAASLLSTATASPSRHHNSTKNWAVTDDVSSELAVEVGSGTEIIIIAANNGGSSST
KYYNSPSMAAGTTHMVTVGGTAGLVYTPSSINASVGDIVQFNFESKNHTVTQSSFAAPCVKTVGGF
DSGFMPNINNSLSPAPMLQFQVTVSTPIWMFCGQTGHCGEGMVFSINPTAAKSQDAFLSAAKAQNG
TATAAGTGAGAAAASSSGSATSGSTATSSVAAVAAATSGSVVAAGTGTLTSGGACSCSCLCGAAAF
PAGAGLNAMGGWGGELSMVAAAAKRRSLLDHTV

>g74
MSHEEIWDDTALVRSWDEAVEEYNLYHSIHARGENVEEVLDRAEKEGVDAVVAGMEKLQESMDVAE
DGDAAAQEDVQENMTISDEPRVQTGPSISAGIVPPTAGGAPTAPDALLGQVQDPALKNLMMAWYYA
GYYTGLYEGRQQGAQTDGASNQK
>g75
MAPEKKDKKRKATSAAAVATPDLPNKKSKKTVGAEATPAATPKPILKKKNAEEKANGTAKATVTET
ADAPARKIKPRKRASDFLSGDEEDNESAEETKLKKQKESAKNVEKKEAKKTAPAKAKKVEVVEQDQ
SEEEGSEGQESENDEVDDQTAELIRGFESSGDEDASEDEGFEAGKAVPRIPDSKKAKRKIQKKLKE
NNQPDEPGTVYVGRIPHGFYEHQMRAYFAQFGDITRLRLSRNRVTGRSKHYAFIEFASSVVAKIVA
ETMNNYLMYGHILKCKYVPQEQQHPELWKGANRRYKRVPWNRIEKQRLERGKTRDKWAKSIEQEEK
RRLAKVEKWKKLGYEFDMPELKAVDSVPVQEKLPAPEAAADAAIEEAAPAKDIAVVEETAPVAAEE
ETPKKKSKKAKKGKEQIETEVETATPVKQVETAAATPETSSAKKSKAKKAKKSAGKA
>g76
MISFLLLIFLIQLAIYIVNTIGASTIDDLLWILYLRLPVPISKDARKNAELKRDVVQLKREMNATS
SQDEFAKWAKLRRKHDKAMEEYEAMTRWLTLNGPRLFIQFYYTKTPVFDLPAGWFPYPVEWILSFP
RAPLGSVSIQVWSSACATVISLAGDVVISILQRGQASSRQAQAVPAEKTK
>g77
MARFNLFYYLFAVFAVLALVANAAPTSTHPNTGSVAGMPGMATEFDHDNNADADSTQHKQVSSDSE
SEYMNEMENALEAAAKAAAKATPTSAGPSRPTPGVPTTAPSPTGAKAQASPSASAAAAKPKGMLSS
LPLVGGLMDGLPI
>g78
MGKQQQPAYVLGVGMTKFIKPRGKVDYHEMGFEAGVKAMLDAHITYDDVDQGVACYVYGDSTCGQR
VFYQFGLTKIPIYNVNNNCATGSSGLALGRTIVSHGAADCVLVVGFEKMNPGSLQSYFNDRENPTG
LFGMMMAETRGVTNAPGAAQMFGNAGREYMEKYGAKAEDFAEIARINHEHSKRNPYSQFQDEYTLE
QVLKAPMIFEPLTKLQCCPTSDGAAATVIVSQAFLDARPHLKSQAILIAGQTMATDDLSLYDRSSI
DLMGFQMTRHAARTALAEAKVDVKDVKVCELHDCFSANEMITIDALELSAPGKAHEMVRKGDITYG
GHMVINPSGGLISKGHPLGATGLAQCAELVWHLRGWANNRMVKGTSAALQHNLGLGGAVVVTVYKR
ADGKEATPVSDQEIAKVTGLGYNPAVSAKGFTAAQAKSVLSKNISEYAQGDVQEKVLARF
>g79
MSTSSPWAFDTKKRNRMLKKYRTQVASATSTICATLAVTPLENLKTRMQTHNFRNLKECARYIWRT
EGFRGYTAGFLPPLMSVTFVRVVSFSTYQTVKYRISDEYERVTGVSPLQYYNQKGSIPSLATITTF
TVAGMCAGLAASPFACPFELAKNVVQTSVLMSYRSQASPDAVRDPRVRSLPRLGTIQAFRKIISRH
GFRGLYTGYHLHAIRDTIGTGLYFGIYETVKQVIAKEMGKPAQTPFGPPMVAGALCGTIPWLVTYS
LDTRKTRAQSILLGRTKEIGEASMAVSRSSMYKGLSVSLMRTSFQNMILFSLFEYTKAQINNLET
>g80
MLTYNPSSSRGNILQLPFRRTLTVSLSDAITQYISSKYDQRPDMFADDLLILDRLRSEAVNVQEPH
VSGVSRLVTYAAQLKWLGGKFPVDVGVDFSWYPAFGFNTSMPIVQNNLRFELANILFNLAALFSQL
AYSLNRTTSDGLKQACNYFSQSAGVIAHLRKDIIPDLRASPPEDMDEMTLQSLEELLLAQAQECFW
QKAVKDGLKDASIARLAAKVSDFYADAGDFAVKSNAISTDWIHHMSAKHHHFAAAAQYRQSLDCLD
KRKYGEEVARLRDSLKCVNEALKETKWINRIVLGDLNGLKSRVTEDLKRAEKDNDMIYLMPVPPKS
ELKPIDRAAMVSAKAPTQVTDAISMLGENGPLGQPLLAKLVPYAVHIAASIYSDRRDRLVNETIIG
ELESMTDKLRDLLRSLNLPGSLQALEKPLGLPPTLVSHAEELRQQDGLNRLYRSLEDTAQVKNNDK
ALYTEGVDLLAAEKEEDDRARAKYGTDRWNRQSSVIAGKKVYQTASDINGYFSSAQSTDELIRGKL
RDAEKVLRILTGTNRDLESYVPSSRRATITPDLERETSRLRSCLNEVSRLETRRKRRVQVLKEKAR
ADDINSALLKETARLEREFPMQPIEASQFENLFEEHLHLYDSDIDMVAQERTEQEQLETQVREANN
NFNRARRGDTSSKEREKALQELENGYAKYKELIGNIDVGRKFYNDLAKIVGRFRDDCKSFVQERRM
EASQLENDITNATAMAALNISSHHPIHQQQPIQPQPPAPQPPAAQPLTAPQPTRANAALPPPVTNT
GGIWSPDMGIRFGGPLPAPGSTQPGRQGNAGASAGTWDPSKGMRFS
>g81
MISARSMRVFRLLQQQSSNLSTTTTTTTNWIPPDTLPSENDIDHFRQQYFAAEKPVVVRSAESART
LPGYQKWFVQHETGGSSVGLNYEYLSQFGDAVVPLEMTATTSSGEGGLGKDGQVQFKRLHAPLSMF
LAYTQFASPPSLVPQNQNIAMTTTTTIYLAQAHISDLPPTLSSDFSPPPRLVTETGRNDIYAANLW

IGSPPTYTPLHKDPNPNLFVQLAGGKRVRILKPGDGMRVFTAVRNHIANSNGGNDGGGAAIRGEEM
MMGSERKVLKDVIWGDHHSDPAVDSVSRGMVGYDVLLERGDALFIPKGWWHSLKGVGEGITASVNW
WFR
>g82
MASIRTYSTYDKHGYTYYPVAIIGAGESGIAMGCQLKQQLNFDQFRIFERRSGIGGTWWSNRYPGV
ACDIPAVLYSFSFAQNPKWTSTFPSGKEIAEYLADVCNKYEIVDKLQFHTEVAELKWLTDEEEWEL
TVIHLVPGAGDWSEAERRDRIAAEGPKSVYLATEKIRAKIAISAVGGLVEPQVIPKDIPGFDTFEG
ELTHTARWNEDLDVNGKDVVVLGAGASAAQVVSSISSTKYNIKSVTQLLRTAPWVRPDDDSGQLKD
EQLQQLSWYFQYIPGFQRVYRTLVFFLVEHEFFLMFSNTSYTEKHRPLEQERYRRFMERTAPAKYH
DLLNPDYDLGCKRRIIGGAWLRSLHRPKVNLTSRELKSVNARSVTLGAPTYPRAAKTVAENDEVEL
PADIIVLANGYRTNEYLQPLLVIGKDGRSIHEIWEERGGPQAYLGVAMDKFPNFFMIFGPNTVTGH
TSVILASENAITYTLKLIKPILKGTVSTWEIKENAEREWTKKVQDGLKQTPFNNGSCTSWYTNDKG
WNSTAYPFSQIDYGLRCMFPVWKHWQSKMTAEGLKQHNKRTWNRRMALALTVALYLWGRRNSSWEQ
TIRLNIAQAVSKVNTLFTSLNELSTFKTSLDSVPFDQYSTDPSRTGPISTSQEQPXPASPPQTRHP
RFHGPTSSAFNFGVARVSLRDMGLTSTENSIHDDLAAVAEESSLPTALTRLAAMHPTKDPIWAITR
EEAVRLCRVYEEEIGLMYPFFNIEKVISQTNLLYNFLEAATRTGLTQTQLPGLDGLQDDDSLNLKM
ILATTLVLEGGGQSTLARQLFDTVKQVFYTKILEPANIKTIQLFSIMAMYYFHTDNELMCYRITGM
NARACLELGLHRWDAVIKAFPTESDWTEVVRLFWAIYNLDRRFSFGTGLPFAIPDSDIDPSLPEPD
DSRPYSKSMVAYDRLSSKIWYSGVGFEGSTDVNREEIGFLDYQVCQWYKNIPESMKLSVESPGNDN
SGNRGLQRLRVLLYLRMNHLRILIYRPVLHSASSIVENQGHARTVVGIARDTIRILARLNQTSDIY
RTQQITFNWFLVAALAVLFLAVCHAPGEFSREVRDEFYLALDLINGFSAHSYISMRLWNSIKGLRK
IAEKLGVFGRNNGTDARDPHSNAAVAMAGLAGHPMENMSVYGPTMGTISELGNSPMNGLQISHELT
NLFEAVGATNSFGADNLTGFISNDGDLQNSGEGLAGVLSSDAEFSRILSGLI
>g83
MSSSDGAPESWISSFCSLMGHEYFAEVSEDFIEDDFNLTGLQSQVPMYKEALEMILDVEPEDDEDE
EEEEEEEDDDDELLGDEKTLGYRRAGDRRHMRVASDLSMIESSAELLYGLIHQRYITSRPGIQQML
EKYEVHHFGVCPRVNCYGCKVLPVGRTDTPGQETVKLFCPSCYDIYTPPNSRFQSVDGAFFGTTFG
CLFFMTFPELDVGGRAESILSPASLAAGNTSNQSRSSSLNTSVALTTPSNADQQPLANQPAQINGI
LSSNFAPGVGFGRIYEPRIYGFRVSERAKSGPRMQWLRMKPRDINELDESAIYQATHGIDDEGGYE
EGDAEMGSTTAAASQQTAIGMRRKAPMRKRRHVPPSNTEI
>g84
MDRGGFPGHNSQSYQMTSIAAASASLPATSRFTAIHGTSSAFSANANPNEDWTKISDLAERRRIQN
RIAQRNYRKKLKRRLEDLERRAGSASASPETDQAETAQQPKSRAKKSQFPKVKTTATRKRKNPAPE
SDNADWSPSDGSLPLQQHETYGTVPEAGSIYTEQLHSRQIAASPPTDSFFYQSYPPYSEVYGHHSS
YPSPQPLQHDVFQNMPQSQYDQRPGQIASQHHLNHLSQPKQTNTFPDGQDLDPLYLNYASIAGLDI
PASHQPQYGQQTQTPPLTNTYSDGYSSSTSPARSPDEHSPLTPEHAGLTPEHICYM
>g85
MRLSHQFARNTLLRASKTQYTTPSLGSSLWKRAYSIKPISASAELPGVDPSQLEITKTSTPKELTP
NSQLQFGKTFTDHMLSIEWTATDGWLTPRIVPYQNLSLDPATCVFHYAFECFEGMKAYKDKDGKIR
LFRPDKNMERLNKSSSRIALPTINGDSLTKLIAEFTKLESRFIPNEKGYSLYLRPTMIGTQKTIGV
GPPGSALLFVIASPVGPYYPTGFKAVSLEATDYAVRAWPGGVGDKKLGANYAPCIVPQQEAASRGF
QQNLWLFGEEEYVTEVGTMNLFMAIKNKETGQKELLTAPLDGTILEGVTRDSVLGLARERLVPEGW
VVSERKVRMSELAEASAEGRLLEVFGSGTAAIVSPVRKISYKGQLVDCGLQENEEAGEIALRMKNW
IEGIQYGEEKHPWSYVVE
>g86
MPPQPQSKSGGLSLYANLLDPASESSTAPGTIAKAPVLFTQSSEGGIDTDETSADKQQKSAASLRF
QPIKRPQLPNQKPKAKPAFPKPASTPSATGYTTAATTSSTRLQVKTTLADWASTADDDDVNGFYGQ
EKRQRGGRKKRKKNRDGPEMITNWDDIYDPSRPNNYEEYKRSDEKIREVREWKDRLYAHRMARRRS
NDYDSDDESPLPRNRQFAPPSYNFAPPANLNDTSKVLSNDSGEDVYAQRFQRTREQDHEVDMEDID
YQPPPPPAAIPNEATGDDAYARRLQMSQQPPPPPPPEQPQVPSKPSMSAFQPASATISRAPVRYTL
PPAPEDIPATEEELEAVFAEEEPVQDEGEEAPRSLRPGQKGFAERLMAKYGWTKGSGLGATGTGIV
NALQVKVEKSKKKPDSEGGGFATPAGRGKIIASKKKNEEEGKFGPMSEVIVLYGMLDGMDLANELD

NSQGGGLMQEIGEECNEKYGRVERVFVHRESNDPIPVFVKFTAQLSALRAVNALEGRIFNGNKITA
KFYDTEKFEKGVYQ
>g87
MSLCLNRLTEERKQWRKDHPFGFYAKPNRNAQGVLDMKNWECGIPGKSSTLWEGGLFKLQLIFPEE
YPTKPPKCKFVPPLFHPNVYPSGTVCLSILNEEEAWRPAITIKEILIGVQDLLDDPNPESPAQAEA
YNLFKKDRAAYEKKVRQIVRENPAP
>g88
MCGIFACHHHPDVQKFKPTALRMAKAIRHRGPDWSGSFIKNKTILVHERLSIVGVDSGSQPLVNED
SSIALAVNGEIYNHRILRKSLDPSYKYKTHSDCEVIIPLYMKYGVDAPKHLDGMFSWVLYDATEDR
VIAARDPIGITSFYQGWSSETPGAVYFASELKSLHPVCDKIVAFPPGHVYDSKTDTMTRYFEPTWW
DPKRVPSTPVDYKAIREMLERSVRKRLMAEVPYGVLLSGGLDSSLVAAIAQRETLRTRDLQMTGSS
VASTNGESELVGIDDENDLSTVTVLAQLHSFSIGLPGAPDTAAALEVANFLGTRHHAFTFTIEDGL
NALSDVIFHLETYDVTTIRASTPMYLLSRKIKALGVKMVLSGEGSDEIFGGYLYFHAAPNREEFHA
ETVRRVKNLHLADCLRANKSTSAWGLEARVPFLDKQFLETSMNVDPQDKMITKDRIEKYILRKAFD
TSDEPDVAPYLPDKILWRQKEQFSDGVGYGWIDGLKDNAELHVTDEMMKNPKPEWGDDIPATKEAY
WYRMMFDEHFPPSCANTVERWVPTWSKQTDPSGRAIATHVAKYENAA
>g89
MAAPAAFSDIAKAANDLLNKDFYHTSPASIEVKSNAPNGVTFTVKGKSTHDGPIGGSLEAKYVDKP
IGLTLTQAWTTANALDTKLELDNNITKGLKAEILTQYLPTSQSKGAKLNLYFKQPNLNARAFFDLL
KGPTANLDVVLGHEGFLVGAEGGYDVQKAAITKYSAAVGYSLPQYSAAITASNNLSVFSASYYHRV
NAQVEAGAKATWDSKAGNNVGLEVASKYRLDPSSFAKAKINDRGIAALAYNVLLRPGVTLGLGASF
DTQNLNQAAHKVGASFTFEG
>g90
MPPRLTTLRRLSPSTLNTRNKLNVHLRSPGSSSVFSSVFFSTTTRFHARSHEPTYYEILNVPVTAT
AAEIKKQFYALSLKHHPDRNRSDPKATERFATISSAYHILGDSHKRARYDLDHGIGSTSTHGGTHT
HPMGSHSSAGATYAGSRPASGLSKRRTAFRGPPPSFYAHGGYGARQQQQQGAPGGHYTSTGGPASP
GAQNSEDPTSFINDNPVWHFNAKGHYKTQAAEDARRKERRSQQMQREREILEAERGGSFVLRFVIV
SGILLVTAAFGALVSEGSTAAAGPSSKTGRSQHKGRNRET
>g91
MAAQASEELDKLKLNGQNGHAQEQVNAADDAADNDDSEDDEKEEEGGEGAATGAAKKKKKRKPKKK
KKGGAKKQSSPPRVPISELFPNNSYPEGEIVEYKDENNYRTTNEEKRYLDRMNNDFLQEYRHGAEV
HRQVRQYAQKNIKPGQTLTEIAEGIEDSVRALTGHQGLEEGDNIKGGMGFPCGLSINHCAAHYTPN
AGNKMVLQQGDVMKVDFGAHINGRIVDSAFTMTFDPVYDNLLAAVKDATNTGIREAGIDVRMSDIG
AAIQEAMESYEVEINGTTYPVKAIRNLNGHNIDQHVIHGGKSVPIVKGGDQTKMEEGEVFAIETFG
STGKGYVRDDMETSHYAKVPNAANVSLRLSSAKNLLNVINKNFGTLPFCRRYLDRLGQDKYLLGLN
NLVSSGIVQDYPPLCDIKGSYTAQYEHTIVLRPTVKEIISRGDDY
>g92
MASVTSTATPTLRVNAAKGVTENQQPPHGSRTQPPDGSASYDHFFWTYTEEPHRSRRQAIIKAHPE
VTKLCGPEPLTKWLVLAVVSLQITCAYLLRDTSMFSWKFFLTAYVIGATSNQNLFLAIHEISHNLA
FRSALANRLLAIFANLPIGLPYSAAFRPYHLTHHKSLGVAGLDTDLPTAFEAFLLDSLLGKAFFCT
FQIFFYALRPMFVYSPPFTSIHVLNLITQLSFDYALVRVCGGXIQPVLYLLFSSFLAGSLHPCAGH
FIAEHYFFSEVKAGGTESLSELKNEGKKSPLDSLQPPETYSYYGPLNILTYNVGLHNEHHDFPAIP
WTRLPEVHRIAREFYEPLPCHRSWVWVIWTFVLDKNVGLWCRVKRAQGGRIVGGGSGAGLSKKAGR
GGEGISASSMKEEEEEDGWKESEIQN
>g93
MSVDLEPGDLGFRRPFNREVSEVLHIRNPQSDPIAFKVKTTAPKHYCVRPNSGRVEPGKHVEVQVL
LQAMKEDPPADAKCKDKFLVQSVAITGDLEFSNVTSIFEKAPKSAIQERKIRVTYLPAGVANGPTQ
TEEEPPAYSSPGGAFETPAPTAKKIVDDSTPIPAPNFEKSPKPETPDSKEADSSLFENAPSGAAAE
TKSDFTDNVKEQLSDALAQIQKLKAQLTEQGEGLRQRKVGEGSSISKASNTLQQQVAQPAAELGVP
VQIVAALCLLSFLLAYFFF
>g94
MTASDLEQLLEMGFEKERAELAVSKTGGLQAALEWLEANQDKSLDEIKSAATNTTEEEGAPALKEG

EEPRSLVCNECGKKFRSQAQAEFHASKTEHTDFSESTEEIAPLTDEEKKARLQELREKLAAKRAVQ
SEQDKADQKRNEEIRRKHTKESQDIKEELQKKERIKEAAQKKKEKQDEIDAKARIRAKIAADKEER
RLKAEREKAERAGQAPPPQPAAPVLPTTSGAVTSKPASAYTETRLRLQTSKGNIMKTFPVETTLFE
VVAALNKDDGVEVHSFTQNFPRKTFDSEYFGESLKDLGLTPSASLVAK
>g95
MPAVGDQHREPPSTVTGTANPFDEGPRRISEYTAREVATLQSRLDKQLGPEYISSRSGPSGQKVHY
LAAEKCINLANEVFGFNGWSSSIQNIQIDFVSMMVDENPTTGKISLGLSVIVRVTLRDGTYHEDIG
YGHIENCKGKAAAFEKAKKEGTTDGLKRALRTFGNVLGNCIYDKEYLAKVTRLKIAPSKWDADNLH
RHPDFAPTKKAPIHDKKTLDELELPPRPIEYNNRKETSSHETLTFDGDAEFGSDVFDEADFVANTA
GNPDEVVLDVDTPQPIQRPQARPQLPPAQQRPQEYQQRVAPPNPAMTTPSKPERQWNGPAAATNAA
FSNNSNLNATAQRGSSVGNPQGMNQTNPLVNNRSSQEFQRSNNTAAGIVKTEQSANAANTMGNQST
MNNNGPAEPAVGFYSARAVDILRDNPRASPMAPKFDPHAESPSIRKTAGIDHTKSVPVSKPMLFGT
SQSPAGPGAAAAAGAGGGRDFINPATDVHRRIGAPGGVPGGIASPVNKGFTTSSYRPLTRPAVDNR
NAANNNTNMNNPTNDPSLKRPPLNDVTNAPLPGALPGSGDIKRPRLSSEAPQHQQQPPQQ
>g96
MPKDEVGTARNSERRGSNDEESRALLSNSSFHDGHHDSAGDSDDDLVVLPGTADEYQHELQQSQTR
HPARPHIPRQSSISQPAPDGQRRTPRTPHRVRFDLSEHVAEEETLTTNGHTQEHQDGDDLWIDEED
YMVDGSERLRSSTGQMAPLLTGIEAPSVTLATSDDFFPEDHLESARPRSGMRMAFMNMANSIIGAG
IIGQPYALKQAGMVTGIVLLVVLTITVDWTIRLIVINSKMSGADSFQATMQHCFGRSGLIAISIAQ
WAFAFGGMIAFCIIVGDTIPHVFAALFPSLRDMPFLWLLTDRRAVIVLFILCISYPLSLYRDIAKL
AKASALALVSMVVIVVTVVIQGFRVPSELRGDLKGNLIINSGFFQAIGVISFAFVCHHNSLLIYGS
LKKPTLDRFTTVTHYSTGVSMVMCLVMALAGFLSFGSKTQGNVLNNFPSNNIMVNIARLCFGLNML
TTLPLEAFVCRSVMTTYYFPDEPFHPTRHLYFTTVLVLTSMFLSLVTCDLGAVFELIGATSAAALA
YILPPLCYVKLSNTSHTAKLPAYACIVFGTAVMVISLFQAVETALDPSFFYTVDLGLDRRIEITTA
EDKRDPYARFTEKDLEDLRKSVQDPLTARLQDENKNHLDAWSNFHRKVETEEGAFTGEPQLMNGQS
HRAHLHFDMRHKERLVSRAGGHSHHTQAGRKHHLDPXLGPAKSTVGMHKQPAPGLGITRPAFGKPP
GGNRFDRSKALKVQRRPIRAPPKPVASTPQNYEVMLADPGSWLSSVRNKTSQAGAPASPSTFQVET
ASNANSSLESEPVVTEPAMATKPVATSQAPVVTKPIVKIPAAVTSKPTATTQPAGITQMVATTSTL
GLTKPVASQIDVITTPRAATTTKSASVVQPVATPKPTGIPKPVQNLESPFFDLNLPQPQTKTSTLR
AQIRPTKDADLLLDLDSPPKKVGPEPIKSASVPDMMALDEDGDISDGNTAAASVLGKKLAWMRELG
FINDTLADFNLSDSKQKTLLEDRQKKLLDMIHAASNPKNSKPQKMVPPPEKLIEMSPTPLQKAVTA
RPFVPTAKPTFVEVQAPVKGGLQASRWADADVEMADILKESPKKTFKPRNTSNNSENPVDYWQKTL
PKLPPLPADFEKKYASTALKSPAPPKATKSLYESRYAS
>g97
MEEETKQPVSTLKYVDIGINLGDPVFRGSYHGKQAHEDDLTDIIERAREIGCTKFMVTGSDLKESE
HAVQISKDYPGFCYATVGVHPCQAKHFDEHPSGPSKLLQDLRDLALSTKQSGHTVAFGEIGLDYDR
LFLSPKASQLKWFEAQLDLAIEVQLPLFLHSRAASEDFERLLKSRLDKLPKGGLVHSFTGTKEEML
RLVDLGLDIGVNGCSLKTEENLEVVRHIPLERIQIETDGPWCEIRPSHASAKYLKDAPPLPKAVKK
EKWQKGCMVKGRNEPVAIAHVAHVIAKIKGVSVEEVCEAYVLTPVSLYFCGY
>g98
MTAEKLVSVAEIQRHNTPSDCWLVVDGQVWDFTEFHQEHPGGSAIILKYAGRDATKAYNEVHSPST
IRKNLHRSKLQGILDKSTINDEWLKEPPTENARVMFDNEKPPLDTLINAHDFEIVASKTASKKTWA
FYSSAATDLLTRDANKSYFDRIWFRPRVMRNVRHVDTRTSVMGVNCSLPLFVSPAAMAKLIHPEGE
RAIAQAACEKGILQGVSNNSSFSIEELAQTAPNGNFFFQLYVSPDREKSAALIRQVSSLPQFKAIH
ITVDAAWPGKREADERVKADESTSVPMSDAKAKNDKKGGGFGRLMASHIDPALTWEDIAFVKKHTH
LPVCLKGIMSADDAILAMKAGVDGILLSNHGGRNLDTSPPSIVTLLELQRRAPEVFDCMEVYVDSG
IRRGTDILKAVALGATAVGMGRSMLFATNYGQEGVEHLIDIMRDELETAMRNNGITSLEEASPELV
HTGDVDHLVPTGRLHPYARKVAKSRQRVAAKL
>g99
MAAPRSTSLRALRAVSQHHTPATTLHCRRRLHITGAFSAQPVEGTDKAAAYAARTLADLKNECHRR
GLRTGGSKTELVDRLSNNDVLYTRAFNIAKSRINGSVFGGPAETRSFNTSRASKSVKDSSTLDFVY
MPSIASIEAELTPAQRIPILGNMSFDYNASDLSQSEYPPMKPQIHAIGETGPDLAASPMSEVVDNH

AVDIDPFKLTETVGKSRAGENEMREKRLLAGQADKGVIGELFGGMIDDIWGKGSSTVTISTK
>g100
MSYDRLNSLEAQPTTWRRDDDPQYQDDPEFARFTENLSNQLFTLTSNISNLSRQIALLGTKRDTER
VRERVHNLLEETRSGFKDAGEAIKKVQTWEDVNPSQKWTQQKLSSEFKAALDEFQTVQRRALEKQR
ASAVATRTALHGDSDQQATGDLVEGQDQQQLLQQQEQXPRLANQDEVDFQEALIIERESEIRNIEQ
SVGELNELFRDVAHIVHEQGSQLDIIGENVENVTSDTRGANVELRSASRYQKNARNKMCCLLLILA
IILTVIILAAVLG
>g101
MHVGPRTTASPLAALRNVFPLQVLQTLGWYLFSAWWFSEIYLWSAPEDADLGWVKLSRPNSRPALN
ERSIYLHTYHMILAVAQTAIHLYYDYDRLPIPIAKRTSKDDRTHPMEPTIKRLQYGIIGSVIDALR
NGLIVTLASPFIYVIFLRRPAWNFSLYFAKLFWNFSRTAAEPQGAVPPAFYTLFYRQIISGGCLVF
LWQTANLFFTVFLTKEPLKRGQPLTNDSKDPTGSLINGLKAKKDIAKNFAFWELCFISQRFPDRRK
AIFEDLDRVNGEAWKQVLQASIDTIEGVSGRINKFKTPPAAKTAKDTSIAYPQIQSLPQLTDGPKQ
DNIFAPSPKATGFGQSFGATAKSFGQSKDWTPAARAKAKEAFSQASDALLSPERKKKLLGAPDDVK
LLTGPTGTSKSSFNYSSIPFVSQFLRSPIGKPFRQTYAQRLRGIVFGEPHGQVACVIDAIESITRL
LIASLEEDSFGQVSKDVPTIVRLYTSTIITLEDFVHEDTGLGIHWSDVTFPSANSSPEVRRKAREV
PEVDTVLYMLRTSLSELLTSFERYSRDVGLQAKDIRLAKAAAGLVGDDNNDNDDPFTS
>g102
MRRRRDLDPKALEEKAKEKLSLGGWHYASSNAGLSTTHSVNRQAFNRHRIIPWMLIDTNLRDTTTE
IWGHKVSAPIGFAPIGINAIYNHEAERPVAKVAGELNLPYCLSTAGSTPIEEVGAANDSGGNGPRF
FQLYMPHDDELTLSLLNRAWNSGFDALILTTDTWQLGWRHDDVANSNYAFYRGIGAALGLSDPVFQ
KRCREKGIDIEKDPVAASTLWIDSIWHGRAWSWDKLPWLIEQWKRISGGRPFALKGIQSVADAKKA
VEYGVDGIVVSNHAGRQVDGAIASLDALENIVDAVGDKTYIMFDSGVRGASDVVKALALGAKFVFV
GRLWVWGLSIMGEEGVRHVMKSLLADFDIFMAVAGFTSVNEFDRPILESYPKSYSIPE
>g103
MLAPARRIALSSCKAASPRTIIFTEQATRCIKPAHVAKRHFHRTPANDFYKALWQSMRVKVPWVDA
LAQSRQQAGQATIEDGEPVKPDLTPRRMSDSYFAATLPLAEDKWLLDEYLNASGRIRLGTLFMDLD
ALAGVVAYNHTGDTVATVTAAVDRITLVNPLKEICNLQLSGQVTYATGRSSMEISLQVAKAPAEGE
KVKDEDVLITCAFTMVSLDPKTKKPVAVAPLLIETEEERRLFQKGEENYNAKKALRKRSFLEQAPD
QEESNMIHSMWTTGQPYKVPGAAIQRSPRIQNMDRTVLRSAMIMQPEDRNRHNFMIFGGFLLQQTF
ELAFCCAASFSHTRPTFVSLDPSTFENPVPVGSVLYLKATVAYTEPLVRTVDGESSKRTPYTKVHV
RVDSKVRDVEHATKNPTGVFHYTFLVPNEVQVMPTKYAEYMMWLDARRRAQDLDPIIASRFTEDET
SARMDGVTE
>g104
MVCYKPSTSVLITPDNKDFFYICPSHLKDRGFCSPIGASEQEAKKKKDEELAQEIEKVKKEYEEKQ
KRKKEKEKEKDKEKNDDKDKEKTKNEKKDSEEEDVEKEKNDKINELRKAAETKPTDDDSPRIFELH
KNFYQMRINRLRNIDLAKRNAERLRNPASFPSVPKGDL
>g105
MNSVAVTIANQQHPNTEAARTRTTATTATGSTRKQPPAVSDDDQIHDSIYNIRSQTSPLQSSIRKT
ASIDQPDEDPGLRKPGDYRQKQVFKGRKLLWLAYQSIGVIYGDIGTSPLYVYSSTFTEPPTRQDLI
GVLSIIIWTLFMMVTVKYVLIILHADNDGEGGTFSTYSLLGRYMNITNRDPREASMIQMKRYQTGD
LEVAGQHVRHTLESSRVAKTILQVMGVLAVTMVISDGLLTPAQSVLGAVQGIEVVRPNISRASIIG
VTDGILVVLFCIQPLGISKITYAFSPIILIWLAFNAVFGIYNLAKYDAGVFAAFNPGYGFEFLIRH
GQEGWKMLGGVLLCFTGVEALFADLGAFSRTAIQLSWLGYTFPCLLLAYIGQAAYISVHPDAYSNP
FFHAAPPGTVYPALIIAILAAVVASQAIITATFQLLTQVMKLSYVPQIKVIHTSKIFHGQLFVPVA
NWLLMIGTILIASIYKNTTSLGNAYGVCVMFVTFFDTCMVALAAMFVWRLNPFIVFLPWLIIACLD
GTYLSSALTKVPAGAWFTLTLSAILASLFFLWRFGKEQQWAAEAEDRFPTSHFIQYNKEGQLRLSH
RYEGTIISTTRGLGVFFDKAGETTPIVFSQFVLKLTAIPEVSIFFHLRPLETPSVSYNNRYSVSRL
AIPNCYRLVVRYGYNDVIITPDLASVIMEQVRRYLTDDLQSLQAKQCTTASSSNNVSAVNAQTQFP
ETDETRSGEYQDVQLRSRELELLKFAFEHRVLYIMGKEQMVTKSGTNIFRKVLLSAFLWIRENTRN
KMANLRVPADQVIEIGFLKEV
>g106

MLARLRMIPHSALEEYIGISNRVYGRPRTFHCRRFPPLWSPERKYSTYGLERAIRDLTSVYNRGAY
AGNLEVVPLTSDLDICRTIVVARGESRADPDKRHILRSYSHYPNKETGNAPLNPGPSCGASLWEVG
HATMAAPTYFEKQELGSWSFKYGGMRASNPTEDAVHELQLLEPMQPPTVVVSIGTGARHHTSMGNG
GVANMLAPFRGLLAAIGDTDKVNRKVEKQLGRGSSYFRINDRSGDWNRVIMDQWIPLFENERKQSP
GQQTMGEMRNIVEKYLKGGGQEEMERCAEKLVKLRRRRLLADPDRWERFALVSTFHCPSCPNIDTF
TSRAAFERHFENNHPGAELNIDRYKNIWSYEGQRRSSWYIP
>g107
MGKSRSATSALELIRKTRPLAEPNDDFMRQLWLYHEMGCPDDVTSDPRYLRWTSHRQIELSAACGR
APEIDIVRFEDELPRDISSYEGEKVTEIRCRKCRRMLATTPFINPHDQDTKKSTTPSTGSLDCAHI
FLHPLTWMRPCLFPGSAASAASQSSSDDISGLTGTATDEAPLSGRLTCPNPKCEANVGKFAWQGLR
CSCGKWVVPAIGVARARVDVTETKADSTAPVASNNNNASSNGMNSRLGAMGIRLPPHMRGMTQDNN
GQSPRNNL
>g108
MRIPTKTIEDFAPPPLPPPQRINDLEDGHDAGWLHANGMGRPDVTKLAPINPSSSLFGGHHAPEPI
PRIERMSLDDPIGRSSGGFSQAKIELSRRDAPSFHTSTHFSEPILQGERNFSRKCVMSSSDNYDRH
LLSKIGKPHSPPRSSSILGSDLRNSSLGSSFHPSSLYDSSYSLGNYAVSPGGLSPRSKTSWRDPSD
YRSPSVESNAPSTSIDYDSLPIRRRTGTLTPQSEDTFSFFPRSGRQYQEQGVFPDVECEDDHMMEQ
NHGDYMSGQHGMKRRASSPARGPVLHKAPSNGDLASRRTSAHHFTNHVSPISRHLPSHSSISSMSS
ASWRTSTSLSSSAGLSACTSASSYDHMSAGGLSPKSEGDHYHDKAALNQPSPNGSVSGFSASKAPY
YMESKPVSNRKISLQSGISSSKGANSKMGGLYICDCCPKKPKKFETQELLRCHELEKQYTCQYCSN
RFKNKNEAERHQNSLHLRRHSWSCAALSNFQAAFHPSTSPTTQTSAGPTHDTCGYCGEEFPNNPEP
DWDQRFEHLTTMHKFGECNNVKKFFRADHFRQHLKHSHAGTPGKWTNILENACMKEEPQVESRLGS
ISEKGGKRSEMGDAPLTSSTIDEVMDES
>g109
MHLTQYVLPAVVLSTGVSAFYPYIRPSTATPSLTTRLVERFYPFHLGVSASSDDGPLTLPLRKRST
VNRRENKYTIVQGNPATGPNSVTVNEDGNDFSYFFSMEFGSNNQSMWILFDTGGTNSWVFGTDCTV
TACELHNTFGKNDSTTLQTTGNTFSVGYGSGTVSGFLSSDTVRIANFSLELGFGLANNASNDFTDY
PMDGILGLGRSNSSVTGPSTAMEAIAQAKLLPSNVIGVSLQRNEDGAKDGEVTFGGVDTTKFEGGI
TYTAVSTATDRWEIPMDDAVVAGTHLNFTGKSAIIDTGTSYVFLPPADATAVHVLIPQAIQSGSEF
YIPCSTNLTLQFIFSGVAYSVSPKDYVGSPTDGTGVTCFSNIVGTQTFGSDDWLLGDVFMKNVYSV
FDYDQNRIGFAARNGTTSTTSTPSTVAASPSSGTSDTTTSTTTTSTAGANHTSTYTGSATSFTLPS
STKLATLVTFLGLYFSFSL
>g110
MGANASTQASATPASPSPDESKCPVDHKTREIWLQQGGSAGGPPHPHPISAGNATNEDKANPKLQR
QLSNDREVSTIPRAYDGPTXPDTRSSIPSPYAASPAASHATPSNAESETGHDEATGNWIYPSERQF
FEALIRKSNVPGSTQSAKELATSVASIIPIHNAVNEKAWQEILKWEKNSPSSDPGSSKCGGPRLYA
FRGLGTESQFVSPRARLNGLMGYQLPFDRHDWVVERCGGERVEYVIDFYQGKTAAVNPPQGEGSGG
IMGATGPGKLSFYLDVPPTTRLNVYVFGEGSAGELGLGPKNATDVVRPRLNTLLDADKVGVVDIAA
GGMHAVALTQDGHVMTWGVNDNHALGRETAWDGGVREIADGDDDASEDESEDGELNPYESTPTAIP
AESFEKSARIVQVTAGDSASFALTDKGFVYGWGTFVSNEANHGFYWNQSQNQVIEKQKKPMLIPGL
ENIVQISAGYNFCLALNSAGRVYSWGMSEQNQLGRRLLLGRQLSKAVKQDNLDKLYLCVGLVPGIV
PFSARTKIVSVHAGSDHAFAIDHNGNTWAWGLNNFGQTGIKVGAGANGTTVIAPQKVSSLKGRNMK
MIQGGTHHSIGIGHSGECLVWGRMDGAQMGLDLSTLPLDDPQIVVSERGKPRILLEATALPISGCS
FVAAGSDHNVLITSEGKAYSWGFNAGYQCGQGSVDDDVVVATLIDGSAVRGKRLSWAGAGGQYSML
AGPCG
>g111
MADSNVFRATTTAPVNIAVIKYWGKRDATLNLPTNSSLSVTLSQKSLRTVTTASCSPNYPAADGDT
LTLNGKPENINASKRTLACLSSLRALRKELESADPSLPKLSSYPLRVVSENNFPTAAGLASSAAGF
AALVRAIADLYELPQSPTDLSKIARQGSGSACRSLQGGYVAWRAGTKEDGSDSVAEEIAPASHWPE
MRALILVVSAEKKDVPSTTGMQTTVHTSPLFATRAEHIVPKRMIGMEEAITKRDFAAFADLTMRDS
NNFHACCLDSEPPIFYLNDVSRAAIRLVNDINRVAGKVVAAYTFDAGPNAVIYYLDEVSEIVGGTF
KAILGGELEGWEGPFGDRLKSISAGVDLNSLDSKAIEVLKTGISRVILTGVGEGPEKIDTHLVSEK

GDILSL
>g112
MRNLKNVRLAEVELRSKLPLTATAWDTASDSIICTFGPEQDNQVIELRRKRHDVVYTSPASQEDFE
TIASWDAPSPLPDLPCDRVLSLQYFADNLTAILVLEGGDIIIVREDPQPGEDKIEILGSVDVGISA
AAWSPDEELLAITTRANTLLYMTREFENVAEITFSPDDLKLSRHVSVGWGKKETQFQGKRAKALRD
PTMPEKVDEGKFSDFDDSRTTISWRGDGAFIAVNSVGNGVRRVIRVYSREGTLDSVSEPVDGLEGA
LSWRPSGNLIAGIQRLDDRIDVVFFERNGLRHGQFTLRLSEKERQTWASDIYLNWNIDSTVLAVHF
KDRVQLWTMGNYHYYLKQEIALVGEGKKSVRWHHEKALRLIAASSNLVIDCEWVSDVAHGSTTSPD
DYGAVGVVDGRILKLTPLRLSGVPPPMAHCEINVDSNIVDVAFSHSGARIAVLTTDSFSIFSWPMK
TRPIPLPLLESSHPLPQTPSSRPRQIAFLHDSEVFILIHDGPDRRVERIRLDTRESSVAYVISAEE
QIQSIFPNIRHDKLWISKLSLKSKGTAYLQGVLSAEELEISAFDTAPTLEAQWAASTYISDDQDIL
VSMTRTGALYANRRLLAKNCTSFILTPAHIIFTTTQHLLKFVHITNVEDMEXPADTPETDERCRSI
ERGGRIVTVTPSNFAVTLQMPRGNLETIYPRALVLAGIRSFINAKEYRSAYLACRSQMVDMNILHD
YAPQQFMENVPLFIAQVKRVDYIDEFLSRLKEEDVSQTLYKDTLTVSKAEADAAVKTGVSPAASLA
LKASKDSKINRICDAFLVALKNKMDTNLQNVVTAHVCKFPPDLDAGLQLVADLRVRSPTQAEEAIE
HMCFLTDALHLYKNALGLYDLELTLLVAQQAQMDPREYLPFLRKLQTLPELRRKYEIDNYLARSAK
ALKHLHALEAFDEVKQYAVKHSLYRETLELYKYQAEQLSEMTRLYADYLYEQSNYQEAAIAYESLG
IYDEAYKSYQIAHRWRESLYCALMVPLSQTELENHASSLVTTLLEEDKDYLSAAQIQADHLKNYPA
AATLFCRASRFADATRILAINGLQDRIPELVDSGLAEAMGSTTDFLADCKAQLNAQVPRIGELREK
RLTDPLAFFGGDPSLDAAAGGVDIPDNVSLAPTDASTAAGRTMFTRYTGGTNMTRRTSKTRRREER
KRAAGRKGTVYEEEYLVNSVRRLIERVNSTFDETTALIEGLLRRGMRERAVAVEKALQEVLGLCSS
ALGEVFDSPTEEQQKQPEDAEQVAEQTGSAVGGDSVFLESIYAGEKRAAPVMKEFKKLALLG
>g113
MIAASATRARLANAFVTRRAFSTTRAQLASPYHYAEGPRSNIPFNPKTKYFFFRYWAFMITGFGAP
FAIAVWQTYKTRP
>g114
MDDLFDVFEDKPGAYVSDDESAKPRPKKDKSKKRQANGDVKAKKQDSKLEDDDVLPDAPAATNGTD
HSVEENGRPEAKRQRIQDVPAAVVADSFETEQEREVAASAGLQATQDTGAVVLSHQVRHQVAIPPN
YPYIPIAQHVPPEEPARVWPFTLDPFQQTAVHSIQREESVLVSAHTSAGKTVVAEYAIAQSLKKNQ
RVIYTSPIKALSNQKYREFAAEFGDVGLMTGDVTINPTATCLVMTTEILRSMLYRGSEIMREVAWV
VFDEIHYMRDATRGVVWEETIILLPDKVRYVFLSATIPNAMQFAEWIVNLHHQPCHVVYTDFRPTP
LQHYFFPAGAEGIHLVVDEKGVFREDNFQKAMSTIAENKGDDPANALANRKGKGKDKKFNKGANKG
PSDIFKIVKMIMLRSYNPVIVFSFSKRECEANALQMSKMAFNDDSEKEMVSKVFNSAIEMLSEEDR
QLKQIQNLLPLLRRGIGIHHGGLLPILKETIEILFQEGLLKVLFATETFSIGLNMPAKTVVFTSVR
KFDGIDTRWVTPSEFIQMSGRAGRRGLDERGIVIMMVGEEMEPAVAKEIVRGEQDRLNSAFHLGYN
MVLNLMRVEGISPEFMLEKCFYQFQNTASVAGLEKELAELETKRINMNIPDEPTIQDYYDLRKQLT
RFTSDMQEVISHPDYCLPFLQPGRLVHITHQGNDFGWGAVVNYKQRRSKNPNEILAPQESYIVDVL
LQVAEGSSTGTRTHTELPPGIRPPKEGEKSHMQVVPVLLKCLQSISHVRIFLPKEVQTVDSRASVK
RSLDEIKKRFPDGLALLDPIENMNITDDSFKKLMRKVEVLESRLLANPLHNSPRLPELYDQYSEKV
ELGVQIKAVKKKISDAMSIMQMDELKCRKRVLRRLDFINKEEVVQLKARVACEISTGDELMLSELL
FNGFFNNLTPEQIASVLSCFVFEEKAKDTPALTRDELVKPLKEIQNQARIIAKISMESKLAVNEEE
YVQSFHWELMDVIYEWAHGMSFAEICKMTDVYEGSLIRVFRRLEELMRQMAQAAKVMGNEELEQKF
ETSLTKVRRDIVAAQSLYL
>g115
MNSLVATPPVPPHFYEHSRFSPSRSMSTPVQQNTNRKRKADDDGNDVDVRMSASPTNSPAFTPRPL
PNRQMKRSRPNVSGRPLSLPRLLETLDTEALRSVLQAVCQRHPELGDEVVHTAPRPSVASTLQVLR
NYQSTLQTSIPLGSGEYLSDYAYNRVKQHLFSLLEALSDFTPHFLPPNESQPSVSLTYLDGATDII
HSLPKWQTPRHNIGRDSAYEEIGKAWVLVIREAAKRGGGIQLQYGGWDQKLAKHNETSGGKLQDAV
NELRNSLGWMGSSALPGAGAPSASGGDQTSIREQLLSGTYGMGMPLKVGPW
>g116
MEPSPSHIDFVPEIPQPSDHQGNIEADNGRRYHAFREGSHLLPNDENEQARMDXAHHIYLMLTGGK
LHLSPISQPQRVLDLGTGTGIWAIDFADEYPSSTVLGNDLSPIQPSWVPPNLRFEVDDYESEWSYS

SKFDFIHGREMEGMVQDFDRLFAQSYQHLNPGGWAEFQSIELNCFCDDDSRQKAAAWVRWSENLHR
AARTFGKNMRTVRTWPDKMREAGFRNVQSVAYPLPFNPWPKDPKLKELGRYQQLHMFEGMASYSLR
LYTNVLKWPKEEVEVLLNEVQNILKDRSCHIYTLVHFVYGQKPDDATD
>g117
MGFKDQRIAIIGGGLGGMSFINAALHVGLTNVELYERAAEFSEVGAGINITRNANTILDSYGLKEA
MLQKSSHDLPYYMEYRGYRNGEYLGHVEESEDPKARLLHRAHLLDTLKERVPDSKIHLNKKLVSVR
RSEENKGYQLEFQDGTITEADIVVGCDGIKSAVREYLGIIDHPDYSGQMVYRGFVEYDNLPNETAQ
LLRATTNFRGPQRHILILPIGNEESGTAKAAIIGFMSELLEDWTSENWMSRAEIDRFHDHVKDWCP
AVQDIVAGLRKSSPDGKMMKQALYVRKPVDKWFEMNESDTSSGIILLGDSAHSTLPHQGQGSCMAI
ESGIALATILRYWKNDDLNAALQFYKDLRKPRTDRITQTSYEVGRLASAQDPDSLDDKFNPGTMRE
RMRWIMDYDSYRPSSHSPFRSRRSHPALNHISLAPLTPRFPIDDLPDQDDYFQSAHGQSTSYLSSS
SVPSTPPILSHSRASSRTRHHRRTRSSNHALSDTDLRSLGSRRELHQQQIQQPRTSKSGSQTPSAA
KRKSQDSEWLLRAGLALTSSTREEKGQSWLVKRESSTNLVAEVEPDVIRTTRHHRRGRSTPFVRSA
RSSAVSTPAALSRRPSRSREHSRYASRADFAMTAMGPESGGASRHSRKSSIGSYHATLPDFVDERI
RAEIASIHGRSLDDYDDDYDDDHLSGSSSYDSEEGTDDEMDENEFQRLTERGYGLGTWVDQFVGW
TLFGVEEDPAVSPPPVSEDVSSRHATVAFEPAAALHQDETMDDDSGSVINHDEATESVEKPGEKGG
WADASWFLRLARKAII
>g118
MATSLNIGAVRTGLAPRLKQAKPRTSMLRRTYTSATATTPPVSPFAPRHFLSIADLTPAEFTTLVR
NASQHKKTIKSGSVPRNLLGALSGKTVAMMFSKRSTRTRISTEGAVAQLGGQPMFLGKDDIQLGVN
ESLYDTAVVVSSMVSCIVARVGKHQEVADLAKHSTVPVINALCDSFHPLQIVADFLTIHETFSARP
TAGVNSLGMEGRKIAWVGDANNVLFDMAIAAAKMGVDLAVATPKGYEIPSTMLEIIKQAGAGVQSP
GKLIQTNVPEEAVKDADVLVTDTWVSMGQEEETIKRMKAFEGFQITSELAQRGGAKKDWKFMHCLP
RHPEEVSDEVFYSRRSLVFPEAENRLWAAISALEGFVVNKGKIE
>g119
MADDSTAPLLSRASTSSRDTSYSHKHHHQRPDSPSFQLSSESTPLLRHEGSNGVSYGTDNRRRSSA
SSTTHSIEDQTKHPRSRQWPIVIAVTFLLAAVLLTLMFGFAAPAAVKQYAQEAVVFKPHRIALDSA
TSEGVSIRVQGDFSLDGSRVKHPFVRSTGRIATWIAKEVETGETQVNVYLPEYDNVLLGRAFLPAV
KINIREGHSNIIDIVANLQSGDVAGIRQVADDWMNGRLAQLRVQGTADVTIRSGILKFDSQAITAD
LAFTGNLCPALSEYSITDLKTEQDIPHLPSFSIDKLNVHENPWNPQDPGISVEVAVSISNDYALSV
VIPPLGFDILLPNCIPTDPHILVANVTTETVNVEPALPAVVDVSGIVRRLPSELTRVCPGKKDSPL
DLIISAYLRKHKTVIYVRGGDPPSSSTPTWMVDLLKSVTIPVPVTGHDFGQLIKNFSMTDVHFSLP
DPFAEPNSPEAQPKVSAMVKVMVELPEEMNFDVDIPHVRANVDIYYEGDQFGILDLSEWQPSNATR
IDDPDSTRPLLLVTFEIKDGPIQVTDSNVFSDVVQSILFGSDPVELDLQALVDGELATTLGQFIIH
DIPAQGTIDVKPPFGSSIGGLIKPRLESFEILHTTPSTISLQATVNFTNPTPYSASIPYIDCLMLF
NGTALAHVTGRALSVVPGDNIGVVFEALWSPLAAGGDIGIIAGRELLSSYISGFNTTVTLQAHEGS
LPGLPDIGKALSGISVEFMVPKMGAPGDDGDGDGDDDDDDDGSPHFIKDATLHLWSSTAVFTLISPL
NHSTLYIDKLDAIALYNHTEPIGRIDYDLPFAVPPGESETPKLPVTLDFGGVGYDAVRRALGGTLL
MDAKAKVGITLGEYSTTVFYQGKGIGAKIQI
>g120
MAFRRTLAKPVAAASQISASTTASARPAAKSFLARNGTRSTSSSTSALAYKAMHRRSPLKLPVSDN
APQWDAPAAVSSILYETPLPSTNPPKRHILNCLVQNEPGVLSRVSGILAARGFNIDSLVVCNTEVA
DLSRMTIVLRGLDGVVEQARRQLDDLVPVWAVLDYTEAPLVQRELLLAKVSTLGPEAFEELLKHHR
EMTSDEAAGAENEHGVAIEDSHFHPRNIPASQALRHKHEHLDAITRLTHQFGGKVLDISTNNCIVE
LSAKPSRIDSFLKLVGPFGILESTRTGLMALTRSPLTEVTEEVEKEAADVVDASTLPPG
>g121
MADDERRAKRSRFDQTEPEPRRSRFDRRSRSPTSRHSDSTRQRSPLSREPRSPGGDASARSSAAPD
PTAAAAAAAAKINAQLQAKKGIQHVDVPPIRSTGSPAAKISDSTEGATKLDNDVYIADGDYIKDID
VNSLRNRYALTRGSTQRMIKEETGADVTTRGSYYPDRSMATPSNPPLYLHVTSTTREGLDKAIAKI
EELMKQELPNLVDERRFRRREQPEVERDEFGRRKWPEERIPIDLEPIQGFNLRAQVVGQGGAYVKH
IQQKTGCKVQIKGRGSGFREHGTDRESDEPMYLHVAGPDPNQVQVAKELCEDLLSNVREQYQKFKE
NPPQHGYGGYGQRGDRHYGGGQGGGYGGGYGGGYQQHNQHQHQQSPPAGASMSPTGAGTPGESTPG

AATDYAAQYSQYYNGQDPYAAYGGYQNYVAYYQYYQQWAQQQQGGAPGAPGADASGAAPPPPPPSG
DAPPPPPGAGSGSPPPPPPPGGGSYNAVPPPPGL
>g122
MVGISKGVSAALLAASVAIASPITETSSPLERRGTIASDEIVGFAQAVPSNITGEVYLAYQPYLYV
VNGCVPFPAVDAEGDTNAGLKPTGASNGDCSSSAGQIYSRGVSYGDYYAVMYAWYMPKDEPSDGLG
HRHDWEGAIVWLSSSTSTTAANILAVCPSAHGKWDCSTDGYSLSGTQPLIKYESIWPIDHSCGLTS
TVGGLQPLISWEELPAVAQAALDTTDFGSAIVPFIDAHFLTNLGNASF
>g123
MQVDQVAALEAYGIPVATINGTTPLSERKLIIEDILSGHPKIRLLYVTPEFCCTETFRRNLKRIHA
QGELNRVAIDEAHCISEWGHDFRPAYKELSWFRETFKDPLVPITALTATATPKVRNDIVRLLGLDK
DSMKWFHTPSARPNIHYEVKYVEELDDDIMSAEEERLHDLLSWLETVRKRRESRMNSEETGDPNDT
RLRLPPISGIVYVPFRSLADELARQLNKSGRNIRAVAYHAGLPAGDRKRIQKMWASKDPAPPEQIG
PCPSFSIVVATNAFGMGIDNPHVRFVIHWAPPRSFEGLVQESGRAGRDGRGAVSIIYYNRHERDRV
LERVKNDGQTGRTSNRFAPQSATSKTRSMEARIESFNKVIAFCESTDRCRHEIIREFSGDLELETM
MSSQTTRSNPTSTSPCDFACDVCKYGTAEIKQKKMEMLKATGTLETMGACHNSDMHTFMYYWMTYF
VRH
>g124
MGKYNLTALRVRQTALRQKASAKIDKLPEWVDIVGDIPPAQVVVRHQPIQHEYFRQRIRTVPGTSK
SEVFLESVQQKKISHNKKPSKLFQPLKIKYEEDQLRKEFFRDHPWELARPRIVLEKSGKDFENYDW
SRLQQPGRRLDGESVVQRQLWLLNNVPDMTKTAAYDIARREFYRLRLRQEIEQRVAAEEEAEATGAV
FGTRMLDVSMALEGKVFEDWKVWAKTQAQILDQRQAAFVGAPEVAIPADDSRVSAIEAEVEVDAEP
>g125
MFRLEDTVKLSHPLRSQTSMEDDMTIATTTPGSSFSSAGSTQSAPSSIDDEPDYIQKTAKVARENS
EVRKAPLKQLTPEETIELTRQAVENGIQETKRSLAGSEAVSDVVRPKLTIDLGHSCISQIPEGVVD
IIKDEVERLSLSNNQLLHIPYRFAECAHLRYLNIRANNFREFPKGIYKLPLLEILDLSRNKISRIP
DEVRKLTSLRVFSIMQNRLDDLPAGLADMNKLQIVKVSGNFLKYPLRRVLENKESEVASLPMSTNE
KEALVTAELKRFLKSRRQSTTPEPESGSEASEPMPSIDTPKPVKRGGNSRFPVIPSTSDGSSDPRS
PNLSSRPPPIPLRSHQRLASGQSPFQPGFSRPGIAPLPGANERNRSNSEGIIPLSARNKRMGLISR
KTDLATLDEMRPYRNSHLRGLSHGSLLRTNSSSGRNGSASSSPGSPRGERRRARDGPSRRMSSLPE
HKEETALTPIVQGAKGILFSLFQIHSHMSTLINVIKGDDARRHSLEIVFYNASTHVEQLNEALETV
VEMDPDNRDFARVSSDVKRECVTCLMAYTHVGSQLRQGTRRIVEQGDPRYVRSLMLVIYGSLLELR
NACISLGVSSLATRKGHDTLRNKSTDLGAEQVQILSDGLPAVAVTPTREPTQPTRRLRSDTTIQHP
QNGLGASIPSAAFHTSTASPGATTPSSTTMMFGSRSRSNSRATTALSSTAASSLATPRSGESFPPI
PTMQGTRINPVTGLDEFVEEQIFEKIFKQLTAAYNAALHALPSASHQFSRCLEIAETTREPEEIRI
MWNNLIRRCNACFESSEALGQKLLTMKVKEPGGGARNQKDFWQLCKVFMQSFVDLVTDMREVRNMQ
LLPPDIVTILRPVQKASREAGRLIETSPWSYFADXPAAPHIPYVYGPPLQTSQHHPSSSAPAPYLS
SSGRLPGASPQSSAIPATPLSAALGPAVQATIPSTPASAYSDRFFAGDVFQRADTLLSMQNQAPYL
YRR
>g126
MDLQKPKKSRPDTHDQDDSSDYRVNGYGPAGVMENDDTDPRQDLEHEVTVKLDPKRDISSSSTLQP
LVKTPRLMTLPLRRRVSRITAARIPKGIPLDSRQKTRLRYADMIPPVTKETLSELDLERIMQNIQL
RVDANFESGLHFLLDLNGEKGRQKKLMTDEYWEALSIEITIYTYVNMNRPRTSEIDMVHVPDMMTL
MKDENLFQPRLPIMFETLHGILMTLVPEKDHPSVSQNLDISHLMQQVHKGVLDLVSLSKWLADLLK
MHCAPMRDHLADEMASEIGTGSTQADVGTQDVANHQLGAYQIVLIENTIPFLQDYFSRRITAREFK
VDETKAWYLGLQQRMPESLKNVTNRSFYPMSVLLYGLSEQILLSDHEVILPEPFEFDLARLWRLRS
ELQRIINVHVCWSILESLFQCLNVKRPFNSKIHSSFVMRILALLEEDNDSQSKSSSLGTRLPSDSA
VAMEIARTVCVAIGQVDEVSDDVLQQVEEAVKKSFASDSVPLQLVRNYVHQHLLQATCEVAQRYVS
MTPLEICESQRRQAYLQSSSSDFSFIATKLAHIGVLHWRVWAPLVYVPQAAVVHELSAEP
>g127
MSDEPDLPKAQGGAASSPHQERAPKFFDYLRVFSYATRWDVCIYILGSLASIGAGIMMPLMNVIFG
QLVGQFTDTIRDSSSSTTADDFQKLVDKQVLYIMGLFFGRWFLSSINKFCFRIMGIRLSAAVRLHY
LNSLLAQSIQVIDSIPLGAPATAITATTNTLQIGVSERLGTMLQSISTVCAAVIVSFIWSWNITLV

TSSMLLYVLVVLAVVTPLIIKGFNAMTKADEEGIALASEAIEGIRLVAACGAQDLVAFRYNDRVQE
ARRRVQHTAPFIGLQLGLIFFGVFGSFSLAFWYGAQRYAVGAISNAGVVLVVIMSVMMIFMAIQSI
SMPLIDISKAIVAARDLFVVIDTPQAAPGSLKPDLSSQDIMFENVTFEYPSRPGVTVLDGLNVCIR
SGQNTALVGPSGSGKSTVVALLERWYSLKTPNKLPSESKGEKSETDAKTKISGSIAIGGFDLDDLD
LKWWRGHIGLVQQEPFLFNDTIFNNIAKGLIGTQWEDESEERKREMVRNACAEAYADEFITRLPNG
YDTRVGDGGTKLSGGQKQRLAIARSIIKKPQIVILDEATSAIDAKSEKIVQAALDRLSKNRTTITI
AHRLSTIQKADHIVVLRGGQAVEQGTHQSLLANESGVYSALVRAQSLHFSNHKTADAPELVTDDHK
SQKAASISHLIETTTSPETVKSSRYMLQTIVQLFKDQRAQWVSFLGIVISSMAVAAGTPIQAWLIA
KVINIFLLTEAEIRHQSNFLGLMWLALAGGVGIGYFFQGLISVRLQYYVNAEYNLEYFNNLLHQKL
GFFDEDANSHGTLTSQIAGDAKQVGELFGVNLAFLLSGIFVVIGCVVISLIFSWKLGLVALFGTMP
ILLATGFWKVRYEVQFEQMNSMVFMESSQFATEAIGAIRTVSALGMESSIITRYQKLLNGHIQEAY
RKALWTAALFGFADSATLGCQALIFWYGGKLLSRGEVSLEAFFVSLMAMVQGAEGAAQSLALTPNA
TRAAAAAKRIYDTHDSIVRRSDEVTTNVIPNTDGGVEIDIQNVSFKYPTRDVSVFDDLCLHIKKGQ
YVAFVGPSGSGKTSIISLLERFYDLEPGNGTILCNGVNIHDLDVYEYRKHLSLVSQEPILFRGTVR
DNILFGVANPDSIAEEKIHEVCRDVFIHDFIISLPEGYNTDVGHKGIAMSGGQKQRIAIARAMIRN
PSILFLDEATSALDSESEKIVQASIESVEGRTVIAVAHRLSTIQNADIIFVLDDGRVVESGNHSEL
IEKHGIYWEMCRNQMLNQ
>g128
MVLATKVMQVLKSYSINCNGPGVGSRDVEAFLPTIATFIENQEPVRMVLPAFPFKSPNSQDKVMGI
LPDLGEELALKHLNALCQNVTQVYEPGAEVHITSDGLVYNDILGVTDETVWEYGEALRRIALEQGL
HHLRFLRLDDLLGHEELSSRSSQSAKEFYLLHASCFRRELMLRFGNPSFCPRAAIKNDSDIRATYR
GYIKFLTKDIAPQTNSMSKRARGEHISQVAQSMIVRGQMFAAAIKAERGDYVRLSIHESTKSQKVS
VGLNPQDQHAIGHTPWHSCVVVGVDGSYRAAHVDQVRDTHDLIIKNGQPYCYRERSELFDWSKDGL
KVDFEHLYPCGLIVRPRTGSGEPPSIRSIPMSKLRQMACNLSPVVCRGFKNTEGEGLFIEKAGELG
KIATWNQDIIVKVKDSKRIDKENNNVKSNEAMPMHYDGMFKFREETDSITGETTKVQTAPRYQFFF
CRATASEHDDGTTLFASSRLFFRYLPLPWNRERLEKVTWSMENDGFWDAKVYKLPLLVTHPTSGQP
CLRWHQPWDSTKTKFSTCDVTIDNDDASLTSIIDNLSYDYRVCYRFKWEVGDLLSSDLKPGEHVQT
PKESTSLRLSHQRRPRTSTEKVRTGCITCKRRHVKCDEAKPQCKNCLRSRGHCEGYASGGNNKSKS
SGPAQIVWNSTQIVPAAARTVPLYVQPDSDFQGDVDMRYFDEFVTMTQAPWATAATNGDLWTISMP
QIAQGTPSLRYSAMAIGALSKWISQTKVRQLRAVTAPSVSSTEADTHYFNAVAYYCRALKVQNQQA
SLQDAVFLSVLLLFFEILRGNRKAALDHINHGLALLLILTTDADAQNHVAKFAPNPLPVLSSVADV
FTYLAPQARLILRGKSGQSPPALPNFLKGLRRQKQTMESFMVLLSQISKSSPILDRIPLTFESLDE
YEQLWISFQREQMALGLIMEELVHESKSQGATAEDFPDTFHLKIIGDSRMKEFCKGSREVLEAFDR
AFTPLFNKIMMSDPDSTAYLRAIHLRLQHLGMYIFTNTSIFLDMELLQAQTPSFREYLSLAEVLLR
TAQRNSRNPAHLLSLQCVLALHLLTIAMHCRDPLVREQAVWMLGDYPGQDGLFNTHAMYAIAVRNR
TAESINASEGTPEEQLRRLWRREFIFEDGGDRVVFRYMDKDEVAQTWLLVEEVADVQSDIENTKWT
RRVSSSNVVPLLIDLYST
>g129
MGIFQMVNDIGQADFTIDEIASYSGADPVLIERIMRSLIVSGLFTVTPGNSYKPNDSVKGLCEGGA
LKNMITFIHDVTALISQKLPEFLAITGYQNPKNVNLCPFQYSFNTEDRFYDWLAAKPMLYKAFCGM
MEATEKLVLRWLDIFPASKRFESYRGPGATRMLQLVDVAGGIGHNLQYLLDEIPDLKANLVLQDVP
EVLKNKVDTNKGEIQIMPHDIFQPQPIKGADVYVLRRVLHNWPDNECQQILGHIRDAMESNSILLI
HDRIFPENGGEISSADVSLDLSMMMLFGSMERTEKQFTTLLQSVGLELVHIWKAEKEARCQEGVIE
VTRSKG
>g130
MSVFDVIVVGGGNAALVSALSAHQAGARVAIFEAAAEEERGGNSRFSSGIFRIPHNGLSSVEPLLD
ADAVTDVKYCRMDPYTTEMYQRDIERTSKGQCNKEQVAVMLRHAFDTVMWMKEELGVKWQLPLGKF
FDRDNVIQAQKVVNMPPWDTIMAIGEGTGLMEMLWKKVESTDITVFYGTPVTDLIATGDTIRGIRV
RCQDKTCSYFGQVILACGGFEASPRLRRQYLGEGWDLVSLRGSRFNTGTMMEKAVNVGAGVAGHFG
GCHATPHDLNAPKVLKPTDADKMSRYSFPYSVMINSNGDRFMDEGENHFSLTYAKTGSIIAQQPGA
SAFQVFDQKTLHLLEPRYAIASPVQADTLEELANKIGIDVKVFCKTIQEYNAATSKDATERFDAFA
LDGVATESLAIPKSNWALPIDKGPFVAYGVTCGITFTYGGLKTDDAAHVLNDEGLIMPGLWAVGEI

AGGFFAFNYPGGSGLTRGAAFGRVAGRAAAQKAKDISMLERLTRMVRLVWS
>g131
MKPGIVKKFDLMPIQKKAVKLVEKLKKDQTQSGPHTGIFVMPWLAKFTQDVMSLCFFSLDLQALDE
PLVSYERLLSEIIPSMFNRWYMYFPILDKVGRFLEPRKSAFKNIENFDRLLDGVMSHASRNPEKQS
DVVSFRLKQALDSGQINLDQYQANLRMMFMVGHDNTEFLLTSAMWELGKRTDVQDRIRNEASGIPH
TGSGVEIMSSLPYLTSVIYELLRLYAPVASMINHSVSQPGYLGGKIFVQPGPYIGWNSYAIHTNPH
FWGPDAHKFKPERWGSTISEIQTNVRRHTAKGNYIPFSMHSRKCLGQGLALSETKIALFELAKRIK
WTVDPTYQLKMSGVS
>g132
MDTILREALQESLGTIDPSIVVTAAVGLLMILGLVTFRSVLYENILPGVPELRGVPILGAMPIYLK
HGMPQLLGRLIAIGEDGISYAKVVNNVLVSVHDPAMVKEVLSYPEEIASREGDIGRMSWSPFWTLR
RLIGESLFNYVGPDTSHHRNVFIREFNSTKSNAEKFSTISRIAQEHANMLTGQAKVADVDDIRYSA
DNFAIALWGETLYGNPNHYVGGRVLELSETIITLAGNPWPSVWYSIQLFFKMVTPGEPTRSESKLR
QKVAKVVEANIMKLEAYEKTNPEAPLKTIRNLSVMTGGGKTGPLSKFASEFTNLNLFGGHHSIGLN
VTWXLIELDNNPECLKKLMAEIDSVDTNDFNSVNSKMPYLDAIIMEINRLHPTVHATLRVINHETK
LAASKNPVTLKPGMLIYLSYLHLHTSPKFWGPDSYQFIPERFLGNYDKSQPFMSFGYGPRNCVGYK
FATLAAKVYLLTLLKTYDLQVKEHDHEMKLGVIGDAQEGEKAQHSSVNTLQKREDGTDYPSGINLF
LIVLALCLSVFLMALDNSIIATAIPKITDQFNSLDDVGWYGSAYMLTTASLQLLSGKFYSFFSIKW
VFLGSILLFELGSLICGVAQNSLTLIIGRAVAXMGSAAIFSGALIIILAHSVPLERRPMYSGFIGSM
YGIASVAGPLLGGVFTDKVTWRWCFFINLPIGAVTLFVIIIFFPDPQIKRESEIWSKRFRRFDPLG
NLVFMPAIICLLLVMQWGGTKYAWNSWRVILLFCLFGILLIVFLFLQYWQQDFATVPPRIFLKRTV
WSAAIFTFCVGAAFLSSVYYLPIWFQAVKNASPVESGVMNLPLLISNVVASIISGVAVTTFGYYTP
FMLLASIITPISYGLMTTFHPDTGHSRWIGYQALAGFGIGFGVQQPLIAVQVVLDIADVPTGTALM
VFMQVLGGALFVSVDENVFSNKLVQYIVEYAPGVDPAVVLGAGATGIKQAVDSANLPGVLLAYNDA
ITQTFIVGAAMAGISIIGALIVEWKSVKGKNIGM
>g133
MPFPVHFTAFVSGFLSLHALLGPASAADIDLKSFNPEDIISKDVAIIGGGSSGCYSAISLKDKGKS
VIVIEKKDRVGGNTETYIDPATGTPVDIGVLVFHNISVVTDYFSRFDVPLTLYEEDTADESAYYDF
RSGTELNISFPTLNETAVAFNKYAQFLEEYPNLDNGLFLPDPVPEDLLLPFGEFVDKYDIGASLNT
MYELDPAVGNFTTVPTVERIRTMGLSLVEQIATGFLTTAHHNNSEIYSKIQTELLSASSLLLHSEV
VLTQRQNDSEGVQLVVNTPEGRKLILAKKLLITIPPKPRLLKNFDLSADETAIFSKFIDVGYYTSI
VKNTGLPDDLSVFNYNPDTPYNFPILPAVYNVQTTPVPGLHLAIYGTPRSIESFPLSDEEVKSDII
NGIKTLQKANLDKFNQTEPEFVVFSSHTPFYLQVSPDDIKDGFYGRLYALQGLRNTYWTGAAFRGQ
DSSDLWRYSEEEVLPGLLESL
>g134
MRLTLPLLARPKAAKLSPLNKPETLESYLSTLKATQLRSLARETGIPNSGRKDELRQRLNDVFLTS
VFLDDGASRHQARPRKKDEGKGLSVLSIDMGIQNLAFAHLIVRAPETVLETRDADKKQKGLVTLNA
WHRIGVQELGAGSPAPGPATQDADTEVKLKSLLPPSAFRTFTSTTKNQALEEFDAIEEELPTMKIA
AANKNFEPDTLSHNAFVLLSSLLQAYKPTHILIERQRFRSGGQSAVLEWSLRVGVFEGMLHAVLRT
MRELARVDKSRSCEEKDFQVHPISPARIMQFWDESKSSIEGDDSSEKGKGQSQRGVKKQKMDIVGH
LLSASSSSDLGKRGLLCWDVDINTTDTDKQNVSDVVDAYLGRWNPQLLKIIPSSTTSKAVRESKSD
VNKKAYKIEKMDDLADCLLQGLTWLEWQARRARIVRGDRQVDV
>g135
MFFTKVSLAAIAALAAVSEARHHEYGLLHPKRSLSSAPVLPTVTVYPVPLNTGVATAPASSASATD
LTLTYTLGTGTSTQVVTTTIHRTETDTLYVTATAAPGGNEQVASEATTTVFSTSTSTSTVTVFAAS
STGGSGPAATGTGSEGCSGQATVTVTQKETVTVTATPTASVGLNTEVISVQPTETGSAGPTDTAVV
EPTETAKVPPSPSAHGSGHTSVAPYPTNVPGQGQATSSGFLTSTRPLHTGRPRPTGYF
>g136
MANNSLNILRSRYASSLKEFISSPRTFEVLDRVPTESHERRPAQNARSLFILDSSFNPPTAAHLRI
ASSALEESPHSSRLLLLLATQNADKPSKPASFEDRLIMMTLFAQELRTHLQSSLPSIPTADLPQVD
IGVTKQPYFVDKAAAIEQSGEYPSDLEQVHLTGYDTLIRIFNPKYYPPTHTLQPLEPFLSRHRLRV
TMRPDDEWGNLQEQEEYLRHLAQGGREQEGGKREWAERIQLIPGRKIDEIPVSSTRARNAAQSKDA

LLHSLVPAAVGSYIMAQELYQSSDI
>g137
MATSDGTEQAKKSVYQIARSWGENPLPPTLLATLITAQHMRPFQLLPMSFVPVLLFTSYTNVNGFK
KDSAGISAAWSGLYLLLASRRKQKFMKKWSPRGIVRGTSMGLALANMVAGGLTYALGEREDED
>g138
MPSATTSGADSSGSARSREHTQGNQDRKYTPEQKAAVIRVRKCSPTAFYEILAVQKTATDSEIKKA
YRKLSLLTHPDKNGYDGADEAFKMVSRAFQILSDSDKKSRYDKFGGDPDSRFSASGAGAGPSAAGN
PFGGFGGGGGFPQSRAGGPMFEEEISPEELFNRFFGGGFGGGGFGGPFGGGPQFVFNMGGGPGFRV
HQFGGPGVRRRPREANAQTQPASPGWASLTQLLPLILLFIFPLLSSLFSGGQSTPAGPLYRFDAVS
PHTMQRTTPELKLNYYLNPQDVDDFSARKFKNLDIQVENDFVNNLHYECEMEVQERERRMQAAQGW
LFPDVEKMKEARAMELKSCRRYDDVKMKLKRRRY
>g139
MSEPLSDADKIRNKRLAKLGGPATSTSSSAASPTESSSTPRPPSNIPTPQPQPELSPDQNDARTGE
SAQGKRIKITPASATPNVPSPSVSPAPPKQRAGPKAEESLETFEDRTLRALFSITLDENQQKTIHG
QKLTFLPGVLSELKDEGSEIRISTGVLDQAILEAASNTGRDTPLDYLLPCWKRVRRLIKGFRKASD
NDPRFAVVSEAKRLCISYAVFAVTMPEMFGQTPTGRSPLIPNLLLDAEDDRGVDLEFLAEVVKLFE
EQDDLKSAIITTVEQMSQELSTKTMNDDYKPYVTALRNLVHNAAIGSAIAESPRFLNETDAASFEV
NTLLGPWFRLSPLQAPVTSTYFSSPKTRDQGFILNSQRSLRMTQQLLSSDLLDVINHLIRASKEAR
EKVLDWFAAAINLNHKRRAMQVDPKTVSSDGFMFNITTCLDQLCEPFMDAAFTKIDRIDADYLHRN
PRVKMDDETKINADQHTSDEFYARKAEGTSNFISELFFLTVASHHYGSESLTSKLEQLEKDLRHME
TQINKFELERHKWIHNPMQLRTFEEALKKYKDRLDLGLSLKYSLQGLLFDTVWQTRSMQFMRYVIV
WILRLVSGTDFPKQKLALPLPEEPSEIFKCLPEYFIDDIVSNFKFIMWSMPQIITTAQGDELVMLC
IAFLESSQYIKNPYLKAGLISILFRGTWPRPGGARGILVDLLNSLPFANEYLLHAAMKFYIEVEHT
GTHTQFFDKFNIRYEIFQIIKCIWGNPAYRNQLSDSAKENLDFFVRFVNLLLNDVTFVLDEAFTAF
ITIHDTQELLNREGNTMEQTVRQEKEEALSAAQRQAKSYMQLTNETVAMLKLFTEALADSFTMPEI
VQRLADMLDYNLDAMVGPKSSNLHVGNLQEYGFNPRGLLSEIIDVYINLMNKENFIYAVAXXGRSY
KPQNFEKAAEIIRKRALKPDEELAKLVELAKRIKNAKEADDQAEEDLGEVPDDFLDPLMYTLMQDP
VILPSSKVTIDRATIRSHLLSDPNDPFNRAPLKIEEVIANTDLKKQIEDFKAERKAAKLQSMKQDV
MDTTTD
>g140
MPNSALNLNGRFAWSVMEVVGPINLLYMLSTLPAEFNFSFSELPLWNKVAGALYVIHYFNRSILNP
LFVAPSISPVRLEVFALAFIFNWFNSSCIAGWITGYDLTQGLGYTPLPTSSTTLQQCLPYIGLATF
AYGMIGNIRSERTLWSLRREEAQRQAAKKFDDKAGSDNERNIYHKVYVIPPAKGLFRWVLYPHYSL
EWLEWAGFVLVGTAVYPAHAFLPAISHPGLPAPSVAPAPWLQPFAILAKYLRVPFPWPALVFILND
IFTMLPQARRGVRWYTTKFGSDAVAGRSAVIPGVPFL
>g141
MCIKNMAFLKPHLRRKSSERSRPESLGTSVRHRKSESSPSRTASVPDTEKKSEIDVEVEHLFQQAT
DTNRGHTSDQREDPKEDSSLLHKLMRRKDPNSKSTGLTVGIRRSFSQRVRPKWSNEPKPVDDTVTR
GSSLDFASETGYDSDAAFITSPRFSEQSWDSRPHSSPFTLDRTMTSLNHVLADDMKIRPGFNDDNE
AIVLPSTRHLPNVLSISKRRRGKTEHPVDRNGRCSRWVPSIHLSSEGERPKETPALTKSVPKKSRF
MELLDHPGLQGKASIQQPRKVSIGWMSEGKRCGYGYSFVDKDENHASGATDGPGDNSLETEERKSV
ADDDDASNASFTTAVPADNLSETESGSGTDATLVDNGRGASLRQSTVEAFSELEDKDSIQVPEKVP
EKNLSQSNAKDSSKPDFVSSSYRRQRWTLFPSYTRAERNKVTDPNDAVIVRDFCPKASPDRPQRED
NDDEKKHDLVVKAIRQGFHDTFLWVIGLGKREIARYHAGFQVQAARSHENVDLIFEPTVPFWDHVP
KEELGDYHIEAWRAAKRRPKKRSRNLRKFRVSVTHNFDAASKKRKRSMPSPPSSSLSQKFREDIRC
ELENGTKYVGRRHSAKF
>g142
MAAADVRDMLDLPADGQPRPTKKQKVAEKRPEGITRELFALLGEKAPPIALNENKYKLKKSNRRAI
PWRMTEFKNEARSDGLVLRHWKKMEPVNKLDDATMEVEQTTDGAQEEHGQYAFAKYNIKAQVPSRY
TDEQYQRHLQSDDWSREETDYLLGLVAEYDLRWVIIADRYDYQPKTSETSDANATALVTAKSVRTM
EQMKARYYTVAANMLALEHPPSEMSEAEFALHEKMMKFDPDRERQRKELAALQLNRTADEVREEGI
LLEELKRIVTNEQNFIAERRELYARLDVPYHVSNTTMYQSSQGLSQLLSTLLQADKSKKRRSILGP

ESLPSPAGQTPSQTLPNGLRDVETPTAALSNKKGGAAAAAAASAAATPTQPTIRTLTKEEEQRYGV
QHHDRITPGVHFRNDKATKLTQAKSNIQTQKLAAALTELDIPLRLLMPTEKVCKEFERLIHQVNTL
LDARKVSEKVESEIRVLEAAREERQRKEREANPTAASSGTTNNETGIDQAEKQIKLESVEDQPQSQ
GGGAAAEEEETHTNQQNGDTGNTTAETSSAGNKHKRSASVLSAQSDKSSKRQRK
>g143
MSASRTSPSWLLTLFTVLTVLSAQARALHVYMEGQQRKCFFEELPKDTLVVGTYKAEILNRESNTW
IQDSSLQVLITVEETFDNDHRVVSQRTPSDGRFTFTAADAGQHRICFTPDSQSQGWLSSAPTVKLA
LDIAIGATSKIESEDSGKISDITQRVKDLNSRLEDIRREQVFQREREAEFRDQSEATNSRVVRWSL
IQLAVLAITCAWQLSHLRSFFIKQKLT
>g144
MQRHREPRRNLMSDPVKEEEVESNLLVYQERDDQELLDQQLQDTLFGQLNDTFSERVRSPQQRIQS
DDGLFISGDDMMDEPFAAADNTRSMGFEIDTKYRKCTVSVSSTNINNTNYTCWTGFISVNRPAHNP
SVRDGIPHDPNGRPAANPMAVVYHRKKKYPRETNLKRSVNDAGLKVWSVGYTVKEEGSKSGIQEAR
VLIAVLQVQQLKGVVLQHGVIVKGLTIDNVSRTERREGRFYRWN
>g145
MATDRGRKERPYPLQTPTSPASAYTDSSAVSPALSTFSSSSRLQNKMFSSSVSSLLPSPGMGITME
RTGSLPIQLADVKEERNKDIEEEYFGFDQVFEHYGIPSEVYDLTDHPVYYEIDSPKRLRSESQSSR
GLPRLGTRIPSMSSRWMSTARSSEGTYVTDILDDEVRSRANSAASSTSQITSISQEAPASARSSQD
KSRFSGHSLGAISIEKANSAVADDEDEAGAKASTPLLPPVWTEYPFTTANSEVASPLQSPTVADVF
DEDTLPAMTSTRTSMHRSPPLSTKPSVASLTTRARGMTLRTTISGEGAPPLVLTEGDDEWSVKLGH
ANFTIQPEPYLPETSDATTFRQFRDDWDLARTNYAKHLVRTGEHYGETSMIYKFTREKWDAIEDEW
KENLETIVTNDVKFGAILGLTKSNMSPTEAIKIPRLHDNEKFPELGDEEIVGPMSVAPAIHSTIRC
KSPRRRNVFRFLHGLVNRPAPGIAAMRA
>g146
MKRFNSSGGVSPPPVRRKVESTTTKTAVASFFTPISQKKPEKITWRVVNKSLIAGRYTSGPAGENA
QTQSKSVKRRKVAAFDLDSTLIATASGNRFARDASDWKWWNNKVPEKLKQLNADGCAYLSKKNRIP
LLTATLERYQIIILSNQKKISLQTDLKAGRSESKSLANFKEKVLAIMRQLDIPLSIYAATQYDVYR
KPRTGMWKEFVDDYDLDVDDTKVDLEGSFFVGDAAGRPGDHSCVDRDFAANVSIKFHTPEEFFLDA
SPEQVIRQFDPKAYIDVDPDAPVVSTFSKLHPVELVIFCGSPGAGKSTFYWKYLKPLGYERVNQDL
LKTRQKCIKVAKENLSAGHSVAVDNTNANTETRAYWTEIAKEFNIPIRWLRILLSSVPFFSPPLIS
ESDTDFLISWGEEQNPESRTLLPGIAFADFARRFEEPTLAEGFEDITRVEFRFEGDEDSRKVWGQF
WV
>g147
MQPRHRLERRLIFKANRKATRINTRLGGGSQDVQGGEIQRLFKMLNGSTYYVRVVGVVNEADFGAK
AVDEKEDGYDYESQFWRMEWRDIPEAGTRSGVTSRLMVNAVLPQGDIISVMETWGYTFVNEYVIEG
DVFIYNDTTIFLHRVLNYPEDPQDAPSPRTTLPPLKDFTLLDPSGSYLLQASISVQDGASPESATQ
RLYGLRDQLRTAVKLEQADRLSLDTRVK
>g148
MIRLRNSSKVAQLYKRSMSSFVLRRKERGWNKKDTSNDEACQSVLAQILKAKKPRVVLCCHTSYSY
ENEVLKCLEVKVKNYGLRRKKVVFAEGHETDLLQSFHPACAVENTECRPEFRALLIHHFVAAFSVL
SDKFTFPDSVEEIRKLCLRTGNRLKELIPKLEDAEAANFISVALQGKYEDAQIEPKYPGFAYDTEG
EMEVFDKMYARFQRLIGSGTNTFGTLGIAITITFYWKKHFKKDPMKEQLRHWLLIRGSEQEDWFPS
PGHCETHNTHDPLKVDEITSLEYSLSKMSFAPPSDSTLQEMRSINDEITHLASAFLALIRLELVAS
TMPDNLKNLVYRQKCLIEGYIKQSPVANISNTLRIWRLMQRCEFLADMVANSIADSKECERVLEDL
LASVKQLTPILQSLSV
>g149
MPSLYSPKDEALRFIQVLTGYAPNNPSLIWEALQVAGNGNTPGNKDGNKDLALVGDAILSLHVIIE
GRQRHQCRERINKVESSLGANSSLARKGFKLGLESYIQGNQSQRGEISDRTMATTVEAIIGAIFVD
SGCDYSVLPDVMARLGLGWPQ
>g150
MRTTAVLKNAARTPLIKFLGKRSTPASVDHTPHAHPASPSQSLPDSFASYRAKAQQHGPLNTPGRA
ASQSYGAIGGAPGVTLGPVEPRKGEFFDRQELPTRFHRLPWSELEIEAIETGGASLYN

>g151
MGSRPLSLHIPAGNFPPARPTLDEVLHNTAPYPYTLSAFMAYLSQNHCLETLEFTMEAKRYRDSYR
SIARMLGQSMVPPDAPQTQQLIMLWRRLISAYIAPGAPREINLSSAARDPLLANAHAVMAPPPELL
DPAVRIMHDLMEESIFIHFVNSQTFGTQMPGMESPYAEDRSHRVRRTTSRSHRSGSRDDSTYHRMS
SSARVAALVKTGSHLSGYSSSGDSGSPVFTDDSLSSSPSAMDPMTPPTTPPGSEHNYSPRNRSDTT
WKKMGAKLGFKKRPGSSSRDGRWEE
>g152
MADFQFIRLATKPTAQISYNFQPSVSTAKPALLVFLNGLGLPQVTWLQTIAQLKTLRNNANIPAIL
TYDRFGQGQTTDRDPKDEGAADPTHGHDCLEVVHDLHQLISQIIVEKMGVSDSTNVTLFLVANSIG
CALARLYAQEYPGTVAGLLLLDSVLANSDFVSVFPDPDATEFDAASLPAGVTAEALRESRESVRRI
FHPDNGSKEGLSRKNLASLLPASDGPVLQGPDGHGPYVTVVGHDFEAFAEESAKMGHQKLLTTTYT
NPYWHQYNEGLAKITEAARSKGPLQAPGSGHFVQKDNPAFVAQELDEILSKVL
>g153
MASSSSNVVGVHYRVGKKIGEGSFGVIFEGTNLLNSQQVAIKFEPRKSDAPQLRDEYRTYKILVGC
PGIPNVYYFGQEGLHNILVIDLLGPSLEDLFDHCNRRFTIKTVVMVAKQMLSRVQTIHEKNLIYRD
IKPDNFLIGRPGSKAANVIHVVDFGMAKQYRDPKTKQHIPYRERKSLSGTARYMSINTHLGREQSR
RDDLEALGHVFLYFLRGGLPWQGLKAATNKQKYEKIGEKKQTTAVKDLCEGYPEEFNKYLTYVRNL
GFEDTPDYDYLRDLFTQALKNTGEVEDGEYDWMKLNNGRGWEYKPYASQHHPHATGQMQNASARDL
HSTQARQPSRPGITADRLNAAQPPPPSPAKPGAGKTRDRQSGSGGLQGKRQSALGQEISTPTASTQ
AQFQNSNANLPGRIATPNQATTNAQQLSGLQGANEPEPTFMQKAMRALCCGTGRKS
>g154
MQKVIQRTTLARNQAARKARILKEKQERLDVVKFFRERDEHQRRQIDTHLAAVKARHEDWFKGPLA
PQRDSGVNAGTYGALDAQDTFMPRIPKHLRRQFINIAPGDRVVLLAGPDQGKICEVNTVDKESESL
TVKDRCQAAFRTPEYMKDENVADLMTNPVPIPIDDVRLVCTLEDNGQQYEVVAKHVHGSGPFLTRS
NTSTPRHTRYISGENIRIPWPEENKVDTLTDEDGDTSRKEVEYETWTPSLSAMPFKPSVMDEIRNK
YSKYRTRHDPAWLEAKKLEDLREEFLKSRKLLTPAGEYKARLAEEKAAIRQSKKDENGNYIMDQTT
SDFISRFINMASHLLAAKDSKPSITLVKPYKTVLVTPAKVPIMAFAAINNSSRNSSTIPIVNQQSP
ATVNYAVIDNAFAADLSLRKRVYEAISASPQHGALFQDIAQYTSRLLQSQSSQQQQLAKAVSEEPA
IKKRKLEGNNGVSGASPATSVHDLKADSPLQFYVQDVSFAVPQRKKLTLEMTAAGGYLRARNQTSK
EVEFGIPVKEIQHILCLPVPEKAQRQTNFCIIPRCGDGVTPVPDGALVPDQMVFTIADGPAKAAFT
GAGQRVESDENGEALLRGWLNDAIGHTKVIRPDDREFVSATPEAHRKGEKACHVKAHRGSKDGYLF
FLSTGILFGFKKPLVFFSFDNIESVSYTSVLQRTFNLNITTRVDEEQEPQEFEFSMIDQADYAGID
AYIKNHQLQDASLAEARRAKKYNVNGGKAAGADGEQNGEEATAADGEEEEESELQKAQRELEDQED
EEEEDYDPGSEGDSEGSGSSSEEEDEDEDGGGDDDEDEEDEDGDIDLVKQELGSEVEDVDEE
>g155
MAEIRRKLVIVGDGACGKTCLLIVFSKGTFPEVYVPTVFENYVADVEVDGKHVELALWDTAGQEDY
DRLRPLSYPDSHVILICFAVDSPDSLDNVQEKWISEVLHFCQGLPIILVGCKVDLRDDQKTIDELN
KTSQRPVTFDQGEEVRKKIGAYKYLECSARTGQGVREVFETATRAALLTKSHKPKKPCSIM
>g156
MVDESLSVVPAQLAFLTIYNPSLGTTDETLQDQIVFYYSSSQQASRISNSANDSPNIPSTEQQGIK
DDTNGRLRKIGLAQGMVNFAKNFSNGQPVDSVETEKSRIVLHELESDWWILASIDLTRISSPNTIN
ASSETSPSVEYSSREIYPPRSLIQQLRRAHSIFLLHHGDSLSTIYQRIGRDSFCKLLGRFWNKFVR
HWLVLLNGNPAVDVFNGVKLAVGGELGIGVGEEEWGSGEREVLEDYVSRTNGLVDLVVSRFGDAPS
QPSASPSKGTPGRDQVEPDNWLGLDQTPRPSDGVIFSGVGAISRRSLSSISHWMEWIYRYGEAAYG
VDENPSSGRSRKQRRARHKAATASPQPTGLRKLDPKPEPPSGRTSPGIPPPLVVGTPPTVQSLSTN
DQTTEEGGISAETVMKYLTLGYGSAWKIPGISANASNDATISAKAGAENGASQDTTKKSDGDATQG
KFIIGLREDLEEDLSDEDNPNESVFFLPQNPEDKNRRTLLRTLQVEVAESASYDNGEDTFTDQVHK
KLQVLVYYVRQPFMFTFLFDLHTPSLTLPSFYRSIHHQLGPLQRPLLTSTSPLNVTRRIYFSDSDAG
EKKRSSSVINNPIYDLVYDPGNLTVRSSIPNIPEPGSLSPGHNHERQTHWSRLEALNVHSQFLSTY
IETRSRPLETERTCKTSRGWWLIWVRIHDYHSLPDQSQNNSSPPSKEAFLIRRASDALSSTGSGGG
ISGARHGREGSSGIRFLRDLSGASSSSPATGAAAAGGSGNDSASAPGRLTEGLGFDAHRYIESLLS
LNR

>g157
MVSLIHTALPILRSQLQCARAIGRRWYSDKTIDPLRILFCGSDEFSIASLEALHTEQLRRPHSIKS
IEVVCRPGKRVGRGLKQIREVPIKSTASELGLRIHELDTFKGWTPPDGPVDLIVAVSFGLFIPSRL
LTAAKYGGVNVHPSLLPDLRGPAPLHHTLLSGRTTSGVTLQTLHHKHFDQGSILDQTPAPGILIPN
PDSCTVPELLRLLAPRGAEMLIRGIQNRVFVPPLQEVKSQFEGFDAKNLTHAAKITPKDRHINWKD
WSWTEIQRRQRVLGSLWSVARPNFTRKVSDKEISDPFFVKSLEKRVIFQEIEEVSPGTVPEGALSQ
TGPGLPFTLGFAQRKLHDKALYVYSADNKLLRLNQLKFEGERFNDALIAAQKARMFALDDLGNGDS
ELIMFSNKLV
>g158
MGYSLKDRNVLVTAGSRGLGATIAEKFAAEGANVVINYVSSEDRAKETEAKINSQYAVKTAIVQGD
LGIKEDCVRVVKTAVEVLGGLDIVISNAGWTKFAPFGDLDALDEDDWDKCWAVNVKGHMHTFREAL
KTFNANPDGGAFIITASIAGLMPGGSSMPYSITKAALLHLTKCLAKSQGPKVRVNAVCPGLLLTEW
GSQFSEEYMKNHINQTALKRVAELDDAADLYISLAKNASITGQSLAVGECMPF
>g159
MGQEKISGLPDVIHREADDNAEKVGHTAISSPPVASTSAEDWFAWLQVLGAFSLNLNTCSADISWI
GSTQSFLMFLVSVIAGPIVDTGHLKSLLALGSLLTVLGMFMTSLCNAYWQXFLAQAVIMGLGFGCL
YVPAPTIVSQYFHARTALAMGASSAGSALGGIVYPVIFTHLQPSIGFGWATRVIAFIILTTLIPPL
VLMKARSPPMSNYRLLDTNAFKDTPYILLNLGLVFGFMGFYIIFYYIQLYALQETNISGFVASYLL
VIINAGSLPGRLIPGYYADRVGSINVQTTVALVGAILTFCLMANKSSAGLIVYSVLYGFSAGAFMG
LPAAGVVNLTADKSRIGTRLGMTLACVGCGVLVSSPIAGAILNGKGGWVGLVVWCGVLLTASCIAM
SASRICKVGWRVRRII
>g160
MTSTSEDPISIIAKLHSLAASLNDGNNHEARKKCLQLSKNLTSQLEQPENVAVDMAFSPMIAVTAR
IAVDLNLFQHVVNEGPVASARLAELCGAEEILIIRLMRPLSAIRFVEETGPRTWKATPITAAMATE
EIAAGHRMISQLIVPAVQKAPEFLLRHGYSCPIDPREGLVQHAFQTNDTTFERITSSPSLLKDFNT
FMGNTMGARNYWVDWYPVQARILGGADPEKPLIVDVGAGKGHDLLAFHSKFPGAGKLVLQDLQPVI
ETLDNLDPVIEKVAYDFFTQQPVQDNYCREILQRVVSAMMPGYSKLLLHEIIVLEEGAPQFQAQLD
MTMMAFNSGMERTAKQWRELLESVGLRVVKIWESVEEGADGIVEAMKD
>g161
MTTNLNFGQYSAMPALTVAAFAVLSLFFVAFALIISLHLILSSSQHSAVPKLRHRGTPTHVLIVLG
SGGHTAEMLSMLRRMPLDPNTYTFRTYIVTSGDSFSASKAVEFEVILHAQYKDSKHTQRHNYDIIT
VPRARRVHQSYLTAPLSTIQCFWACLQVLRGKHSDQQSSKRDVKFQYPDLILTNGPATATHKDSSA
NTLPLRTVFIESWARVTTLSLSGKILLPFVDRFLVQWPNLEGKQAWKGMRKAEYVGTIID
>g162
MKSFSQSSPPAPATRRAARRRVISCETCRRAKTRCDLSPKASSCNRCQKLRIKCSLSSSDLFSHGN
GHDEAVNVGERQSLEKRIANIEDSLQEMKSTLASMCCFMQNLDNRPMDNVVMTEASSSTAKNHATG
QHADFPHLPSLRTETTKLAPIMMVRKFRPDSRRLYRDPGNDIVEKRILSEEVATCLVSEFLNHRGS
VIQIEKSPTDGHLRHRSPLLFAVCALHGLRFSNSYDTLLNSPTHRHLYEEVRDMLGQAVLASPLPL
DELYAILIMSTFEAAPRPVYEYIESWLLSGICAKQAILSIDFPRINQRIDSGNAERKDKQLMSLWN
NICLINFRFAVGTGKPTTIPANYIDQCPAILNHPEASVEDDLVLAEILLYRTLHNKLANFSLGDDH
GEEDHCTELRTWENKWKRLLDSPQAITLRFSYDIAQMVIARRATEDSIFSGTTASQLRKRKRNNPR
KVHEKTNYHILAYQSALSVAERFIHASVTGVQALPKFHHICIAYCALVLSETACEHNAVTSRVMEG
ISARDVLLMLKRIRQHYHMSFGGEIPEAMNVAVARVAAVVSDTTDSAGYASQGINYNDSSLSGNLP
TIGIHDGTENQYVRRFPGGNELMDMNDFDHATLDENFLVQDLLFDGSNMSFVDYFNSTW
>g163
MSADDSFKITPNLEANVSVRDDLKTTEVVIDESTALSRGLRARHTTMIALGGALGSGLIIGTGSAL
ASAGPAPILICYSVVGFVVWIMLTGLCEMGTYLPIPEGFTGYASRFVDPAMGFALGWSYWLKYSIS
TPNQLTAVALVLQYWVPAEKLNPGVFIAIFLVVIILINYIGVKFFGEFEFWLSSAKVATLVGVILL
SLILACGGGPDHDAKGFRYWHTPGAFKEFDAKGDLGRFLGVWNTMGTAAFAYLSTEFIGVTVGEAQ
NPRKTIPRAARLIFYRILFFYVLSVFFLGMLVPYDSKELAFATTQSTSAAASPFVVAIVLAGIKVL
PGILNACILIFVFSAAVSDLYISSRTLYGLAQNGKAPRFLAKTDRRGVPIPALAASAFIALLAFMN
VSSDSKVVFGYFVNCITMFGLIVWCCIILSHIKFVQARKAQDVPVVHYRAPLGIWGSWFAFCFCVL

VALTKNFKVFIHTKSTGNFDYKNFIVGYIAIPVFLFFFTYYKVVHRTKGVRASEADLYTGLAEVEL
HEREWAEKERADELREKSKVAVFYRRYVSWLF
>g164
MALDMSHGLGAPDYVSPNNVEFYNPSWSYKATGTWSLASKRGNTLYISGLRGIDPLTNKLIPIEGL
ETYNGPSTKDSPAYLRVLKSFENMRMIAESAGATVGDCLRLVIYTTDMYRFRPLVNEAQKEIWGDV
PFPPRSIIEVSRLNQDDIVEVENENGEITDPPTAVTANPAISNDSLQSPFESAGFDQTGLFCGKCV
RNQHLLMKALSSYLPDTDDPTYPIYEQQYPQYRRKMEETYPQVCENCEQKVNNRIRQTGYEAKADH
LRRTMERSKMSRAAQKARRRNWRSILVVAGASGYWSSVAGQLVSDLIGTGDLLNELIGQYWILPST
YFPVVNELASYVGYSSQDLASISLIIGALSIWWNPKIHHKVEGLSGRILGLNQYYECQLIVMVARF
AVWAWLKDTSRMEPSLPTAVHAVMILFMGVATIFSQRLVHYDTRPLVLWSNLTPVTSPRQQKNVPQ
SAPMTKQQGAPFQQDMFQHFPLEKLSMPRESPRSNVRLATPPLEPDDSMDWTPSVAKEIRPMFVTS
KPEVAPEAASHKSLFQGALPAAPRPPAWQLRNPIAQKASSAVPVNNIFQTSRTTTLQNPVFNEQND
TLRTPDAFFAPPKFFAPSDYHATTGLENLFDQTFTIKSPEDDTDENAALQSKVKEIRKNIRKEHAQ
SALLYSSIRLLLLFMSILAWSIDWLKLVPIPHDAVLMGTLIMNILFTGFGLLASLNQPVIVVQRVI
YSLIKFVATIVLALCLSYNFSSTFHQGGHLETCVKSLTALLAIEELLVLFSAMPKSRTQKGMARKE
SEAVTHPLTQQTTETQFIPPVHYSSPPPTSPTLSYISNQPTSVYSQTSSFSQSQPYNYGLYQRDMR
RNQFLDDGSDASEMSGGDSDAETTISSYTNKDIRNMNPFNNNDRDYRVPRAGLGSGLQGLSLDDSP
VSKRVQCVYEGDFHLQREGQTASYIHELETKIEELNERLREVTLSSQSLNKPSTSAAAWTTPATAA
TPGRSILNQELSPESIIGENETPSSDALEEEIREVNRHTNAIEFHGNTSSAAALGHLQRAREPVKQ
HKVTPNRYMHAFADHPPASITGHGHAGNGKPKSSLISTLHNPNFYTSQLQTPLHDGDKYLADYTQG
ENTSDNRFYFEYAHVFMEGYFENLHFIHPIIDKENFVLRAHNLWFGRNPQTDSSFIALYLSLLSLG
ALIRAWDQPTLGGLTRFEWSRKLFAEAQGYLNTWCFTNNLEIVQALYLMAKICQNELAPHLAYMYL
GLAIRTCLAAGYNRETPDVKTDQHDWISRTWXELSFSLGRPDTLGMDEYHNRCQPNRDEHSEYTII
PIMVDLAHITRRVSVDIYHSRISLQQRMKHSLEIEQKLNNWLDALPKILQPDFGQSGGKADAPSSI
SVLRDPKWSRRQRLVLGIRYHNVEMFIFRPFLAYLTLTPRGEGGKNRAPEAQRVLEMAAKKCLDSA
RKTVDIIYETFRQYSFFRCWWYNTTYIMFAVTNILLPLSRKSLTFLNLMETNLLIESVNKSIEILE
AMDESVVAQESVEIVKNHLRDYGAEMNHDTNHGSSHSNDTLNGMSQEPGLVDMRSGTSHSATLHHD
TLDDPASMLNLGDMPFDEFLPDLTLSMGSSDYPFESIAGLFGDITGPPILE
>g165
MSTWGDYFRVTTYGESHCRSVGCIVDGCPPGMALTEADIQPQMTRRRPGQSALTTPRNEKDRVEIQ
SGTEYGYTLGTPIAMIVRNEDQRPKDYGGSTMDLYPRPSHADYTYLEKYGVKASSGGGRSSARETI
GRVAAGAIAEKYLKIAHNVEIVSFVSSVGNEHLFPPSPEHPSYSTNPEFLKMIETIDRETVDKFAP
VRCPNTEASERMTKVIETFRDNHDSIGGTVMCVIRNVPIGLGEPAFDKLEAKLGHAMMSIPATKGF
EIGSGFGGCEVPGSIHNDPFVAAPEVAENKAGAGIANKARLTTSSNNSGGIQGGISNGATIYFRTA
FKPPATIGQAQTTTTYALEEGVLEAKGRHDPCVVPRAVPIVEAMAALVVMDALMAQNAREASRSLL
PPLPKGLPIKPETETSNGN
>g166
MSIAASMLLLRHSSCLLSVRSASSRLAATRTLTSLASSAFAELVSRSSAKHQIYQSLSSDPFVNLS
IEQYLLQKAPEDSNVLFLYVNRPCVVVGRNQNPWLETNLKALQKISDTTESSTRNVLYVRRHSGGG
AVFHDEGNLNYSVICPRDVFTRDRHAEMVARALQRIGAVNAMVNERHDIVLTQQPRESARQKHATG
IEDPPEESTPQALKVSGSAFKLTRNRALHHGTCLLDSPNINNLGAFLRSPARPYILAKGVSSVRSP
VGNVSAGLVDSAEPFSMQRTIVSIMDEFARLYKTGDEVRDCANRLNMNESEFSAGDNWVAGTVGEV
QLQQVPEIQNTLQMMKSTDFKFLQTPEFTFSTFPTEDDPRPRPPLPSSLPPSTKIFIRAKKGIILE
ATISTSTDAYTMQKQERHSAASLTNKPLHEMDEQSWRTIADSVVAIASDSPEQQRPADEVVDDLTA
FICEKFGV
>g167
MEPLWGHLGQSHNGQRQKARAYVPSAAKMPFALSHTFLAWTMWAAVSVAHVTIPYIPTHAFTSSQV
SNTSDLVYIFQPISDQSAVQLLALNLSSTINPSSPEYTVLSDDVPFSAGGEAYVPMLNTNGSIMVF
SGNCNSSQEAPRLWSFQLDDNSSIGNGTWSSVAVNGSGTNITGPGYLASGFAYNSSNDTSSEYYSF
GGMCPFADGNESDWTSNAVYSNEMTALTAQNTSTNSFNYILQQSDSSNSPIAEAGYTVTPLQKTSS
TTSGGVVVVQQESFLVIGGHTQTAFLNMSTLALFSLPQASWSYISIGATVDTGRTDLAIRDTTDIEP
RSGHSAVMSSDGSKIIVYGGWVGDTSTPADPQLAILEVGEDYGGSGEWTWSIPESTGSGPASGSGI

FGHATTMLAGDVMLISGGYVIPASSGTTKRASSEGFQSSSQAYLYNLTSNSWVSTYTAPNPSDTNI
SSHSSGALSSSGEKAGLGIGISAAAIAVVCIGYFYWSRKRWTHRRQRDQELRKLALGAERSNIWGE
PGIESSYRGPSDAVLRNSIIENVYSSGPSHQYSAVPVPGSNNKTEAERSGLLYDVPSPSRGLRRSL
STRPQRVQSANWYEDNRMSYRAGAIHPIDEQEEYETPPEEASERRPESGSSESSTNNRFSDPFKDP
SPSPTRAPLIAFHEESTHQHEGADEASIRSGESRGTSPDKSERTHSDLSESSRSGISEFSIQRSNI
GSQRRSTRLFQSTIPTLEPSHYDDNGSGRTSPEKSSQHAVEGSSMRWPFERSGTVDSLSTSASRHR
RTPLEGDTLLGSGPEWSTPPESPSRFSDRENRPNSNNSQTWMGSIRKTFSNARKVVLSSYGNNFPE
DERASPAMFEFNSPVDFDDDNGLSPQISRRASSASVVAHHQRRKQGPKDWAVDGRSSRQSATGMSS
SSRRQSRRTNDLLDDNEAEEDDAFDDRLDVIDDDPDEHHRGGGSSIGATDDEDWDVELAAEGRLVQ
ITYTIPKERLRVVNAGAGDRVIGDDVSEVVSRTSQEGR
>g168
MREAADQPADTVMMVDVHGRIISKPQSTIEEDTLDLRNEGILSQRTQGVFFDPTNASLCRSDRTYF
DDAATALPAEREEDVPIAYKRQLVASFNIMGQFERRPSHAGLEDVERTSETHDQDRSEQQQTGLEA
GPRAPQNHHPRRGEPTESNRHSSGSNGSNGPTHPTSSSTTSESGLSFANRMLASKHNLKLSGFRGD
SHSSHHLIHFMPQSSNNSRASSAKTSSSSLHAMNEDTVAEPRPEPSSGLSRSSLVRRLSQQLELSF
GQSLVPKANPVNTTADFPVYPDQSYAVLQSQIHPSPYQPYLRGRSSYPSPTENIRPYEHAFTATGA
RTVGNTPISTPGLFTGRSARTSPSVGSDDEGYTFSPYLHPTHLQPPKETHTAEVERDLQTGNKLIN
DYEVLEELGRGEHGKVKLGRHLITKQPVAIKIVQRYSKRRRLGKLGNPEDKVKKEVAILKKARHPN
VVSLLEVIDDPNRQKVYIVLEYVENGEIKWRKKGVREVVQMDKMRMEREKLGQPDSPYFEEEGQSI
INNLQNQRKRRELARERHRAQSQTNHRHLIPAWSLEHGGESDDESNPELALARSISQNLALHHDRT
DHAEHGQSPSQSYNSTASEFDRHMRDSVLAAVEGSMYGAYSDYPLERRYSTASSLFAYSSSEADQF
SDDDDLSYVPCLTIEEARNAFRDAVLGLEYLHYQGIIHRDIKPANLLVTAKGQVKISDFGVSYLGR
PIRDEDEEQVTETDAAELDDARGELSKTVGTPAFYAPELCYSGDDYVDANGRGPKITGAIDIWSLG
VTLYGMIFGRLPFVSDDEYSLFQNIGKNDLFIPKKRLKPVEVDSSKISADPRHGVMNSNKRLEDEF
VYEDIDPMLDDLLRRLLNKDPQNRITIKGIKTHEWVLQDLKEDPADWVQKTDPQSKGNRIEVSKEE
VTSAVSKVPFVDRVKSLAKWLNPRREGRSHPKRPSSAAGSSADSVASSRDSSGASGGRRASLRGDE
DILRPFRAVREEHPLSQSVTASPDSTPDRSSYFDQTPHYDVADETPRAYPSERLSILSTTASAKTV
RPIPSNKSLTPPAETTETLTEQHSIPAPNLAGIFTGATHHRADKTDDDRLLSFDGPASETDGHAEP
SVAWSTASATGQVQVPVATVWEQHERPQSQGNDVTRSLSSAGHRRNRSEQIPPEVSEQSFRSAKEA
LLRQAADYTRSEEEQASETTPVDNNERSVEPSPTTSTHHLPSDGGLMTSPPSAATISTTSVDEYAS
EMSQSVSHPSLPSIVSGASSFSGDGLHTHIEERSKELDTTPYISPILRTGDTITPRNIKAARSANE
EGYDYDCDEEDIDSEDEGIVFGRKKPST
>g169
MFREEGARLIYEYDAEQLYIEPWGPDAFRIRATKCATMPPEDWALEGQQPPVIHPQIAISSTSAKI
TNGKIRAVITKFGKLTISNSQGRTLLEEYTRTRRDILDPKCSAIEVEAREFKPILGGDYHLTARFE
SIDPEEKIFGMGQYQMPFLDLKGMDLELAHRNSQASCPFALSSLGYGLLWNNPAIGRAILGRNTMS
FEAYSTKVMDYWVAAGNSPAEIEELYANVTGKVPMMPEYGLGFWQCKLRYQTQEELLGVAREYKRR
GLPIDLIVIDFFHWPKQGEWKFDPVYWPDPVAMVQELREMNIELMVSIWPTVDKKSENFEEMRERG
LLIRVDRGIRTGLDFEGETIHFDATNPKAREYIWKKARDNYYANCGIKTFWLDEAEPEYTAYDFDI
YRYHLGSNLAIGNIYPRDYARAFYEGMTSEGQKNVVNLVRCAWAGSQKYGALVWSGDIASSWSSFR
NQLAAGLNMGIAGFPWWTTDIGGFHGGDPNDPQFRELFVRWFQWAAFCPVMRLHGDREPRQPQQGT
TGGATCCTGADNEVWSYGPRVYDICENYLRIREELRPYTRELMREAANKGTPLMRTLFYEFPDDKE
CWNISNQYMFGHQFLCCPVLSAGIEKMTVYLPRLPVGNKWKAFHGDNVWGGGQWIEVECHIDTMPV
FVKE
>g170
MSPCIPRPLPPRDVMNRHTRVHSYQQPEIVTGRTQRNAAWLPCEPRVIPLENQGTGSEEHVVAEAH
TTVDPQGSQPEKSPTTSLEPSATLPVDNQTWAQDPGFWRDSPVYGSQGRNLLDTGMTFDPGTYGVF
SSDQLAPRHSSIINWLSPDDNILQEWASQLAGIPDGGIFPLPGPVPDDPSLPQLLCTEPPLGWAPT
NKSTPDQTRVETAAAMASYPKPTDNEPPETLMDGQTSHSSETTRGKYYADGVAWRAPFQSRFRNGQ
VTDSVDRAGVTAAAPSTESVGSVATMDGTTSSLSKWMAEDVHARIVRSVEEETQVSLNLPSLQVFR
RCVHLYFERLHPTFPFLRKATFINERPHWILSLAVAGVGAVYLRSPEGLQWKDSLMQVLDKVSSRR
LHQLQHSVNTTVPANNILETANQMEEFLPLIQAKVLHLLCMLHSSTTFIAQHAMFERANLVQWCSY

LSLVPDSADVFPPGTGKDTQQWVKEQSSLRTGMMIWLLDSIVAYELSCNHLMKLGDIRGWLPCPEI
AWDHPLPENIAFAESVSISLLDALDLVYIEKRLPPQLSQFSQILLIHAIYRRTTEVADQSRMRLLS
WSPTDSIQISSPDLASNQVTWPPSSSIFIKWRNAACDALDVLHCTANSRAAESCREEPTIFYLHLA
RLILLAPCMQFQILAQDPLLRARSGLSVSEDYAGTDQSDAEPRLIYLDRPLDDELVQMYIRMGNKV
SAQLKGVGDITSDGAPIKILKQGFYLLAGEKYSGRNSASSASGNTNFQTWGIQQTWAATIRCLIEA
S
>g171
MRPGNEKDHGPVAHVENAPTDEPLKSDELAKEAAHEEHQLTLASALRKYPKAVIWSILLSTSIIME
GYDIVLMSSFFAQPAFSRRYGQYIPSSDSYQITASWQNGLSNAVSVGTIIGAFANGYFTHHLGYRK
VLLASLAAITGFIFIPFFAPNLPVLLVGEFLCGIPWGVFATMAPAYASEICPMALRGYLTVYVNLC
WAIGQLISAGVQSAFNGSSTEWGYRVPFAIQWAWPIPLFLILYFAPESPWYFVRRGDYVAAEKSMI
RLGYSSSSGEVKQNLAMMIHTDELEKQVSEGTSYWDCFRGVDLRRTEIVCLVFAAQPFCGSAMGGT
PTYFFVQAGLPTSISFKMSVGGLGLASVGTIISWYLLHAFGRRTLYLWGLGLLTIVLMVVGFVSVG
AGNSVGGNYAQATMMLIWLLVYYLTVGPICYAIISETSSTRLRSKSVCLSRITYYIAQIICNVVNP
YMLNPTAGNWKGKTGFFWAGWAFVFFIWTYFRLPETKGKTFEELDILFARKISARNFAKYRVDAYT
VGDAALSKVE
>g172
MIVDPKKRRKFSTTVNHSTEEPSEAKIETPAGTSKPASGSSLLHHDEPEVQHKTSLSFQPQIIVPR
FREIITTLVEKLSKDHWKDQRFSKYSQLIAIHGGIEAVVQSAMESIAPEILGLQDQEISLPMLDDI
REKQGPFEQWQQQSGYCDYVTDNNKPGFWRSYVGQSEEPGIRLHQHLAAVRRKKQDTLHYYFILMG
NGCRHSNFIRLWSLPPQLLADRNARIAVSMLSNILEMVMCYAFQTLPQAVINTFFDDNSTCTLGLN
VLPPLFQDFFISGEEREHQQKQEANLRIWSGDDHRSALLKALSTTAMSTQSVDLDTNTGIVTDVDI
KVLLDNIKASLGLSSECPPLVAPFGDPNKTRIGVASIWTADLQQCFSPRFISTRDSWPDTYRIALA
QFHTTFIQSSNMRVIMLCGENAKNYSELEAYVESEVINNSLTVKRLYICIDYIHDGWRYGDLNSLR
EWSDVLAFITSITKTTNVRNHALMCHQVSRLIILRHHAERSGTIEPMTISDLDVKIRTWLALKGLK
SDDQILRWEKKPTEKIRTARPKLVGGPDNYAPLFSKDIITESKRLYTLITGIKQDSTAAPKRTKNS
GRQASVAGDTFEEALASNKYPPGTVVDDPLVFEEFESLIGGDDETEDLTVEELSEVLKCTEVQQHL
FDKNREPLLAEVIPITKQETERVTKQSYNNRKSTKLIRKELLGDGHWYAGYEKRKNLYRIDHWTCL
VLSFEQQDVNQTEGFVVQAEFSPPGIRHPKIFAKTARDSDPASRLAIKVTGKLNSGQSFERYLIIT
ANEPRRLTERERLKWAKNSQAEGDE
>g173
MTSKHSFERAGILALGLVATSSFVAAGPCDIYSSGGTPCVAAHSTTRALYDAYSGPLYQVIRNSDS
SKTDISPLAAGGVANSAAQDSFCSGTTCLISIIYDQSGKGNHLTQAPKGGWSGPGPNGSDNLSTAT
SAPIYLNGQKAYGVFIAPGDGYRNDKTSGIATGDAAEGMYAIFDGTHYNGGCCFDYGNAETSGTDT
GAGHMEAIYFGDCNVWGTGSGSGPWIMADLENGLFSGYNAKQNTNDASITWRFVTAVVKGEPNNWA
IRGGNAQSGSLSTYYSGIRPSGYNPMHKEGAIILGTGGDNSNGAQGTFYEGVMTSGYPSDSTENSV
QANIVAAGYSTSPGSHTTTSTTLITTTTTPVSGAGQTHWGQCGGSGYSGPTSCVAPYACTTANPYY
AQCL
>g174
MESHGQYANRGRSPSASVHSRNVSPSPHHGQHSPYQNPSAAGLMLDASTAGTGYQPNLAYTAAPPP
SSLAPDLNNPDLYNNFLSATTSAQQHDNLAAQNDQFASSFAATFQDQLDPSATLQDASYSNLINPN
TNDYDFTQYAVAGDNTGMQPTFDTSLLLDQQQQQQQQQQQHNAQNAQLMGQGDMGPMGSPHNLLSP
EHHSSPGNSHTSPPISSGPFYSPAHSRSASLDPMSAAYMSNHNQTQEWQNMLENPSFQSHRRAPSE
HSDVSSVSHHSPYAGHHESFDALEGTSPSLGAQNDPVLYDNTLAMDSFTLSEQQQQGLSPHHSPYV
SPQIPSQDINSDAFMLSGQQNMTQFPALSQDIYVGQPDDAMLAGMQVTDMSGLDPNQMSNMVPPPS
INVEFAPPSRVPSFGPGGENDFDALSPPSRGSRGRSKSDPFGRPTPIARPHSQSVSSTSSLDPAVG
SPGRSLSPFDPMGGSRSNPGSRGVSPASRSSIRRQSTSSIERKVILDLADPQRPGATPGESKRTQK
HPATFQCNLCPKRFTRAYNLRSHLRTHTDERPFVCTVCGKAFARQHDRKRHEGLHSGEKKFVCRGD
LASGGQWGCGRRFARADALGRHFRSEAGRACIKALLDEEAIERNRVFMEQQAQQQHLQPVPQPMMV
PGLDNGQGFTLPAALLAQYPALQNLQWDQIATTGDDVSDMSARNSFDASSGGEFGFDDDDLSISSF
TGASGQGAIYGGSGSHPNSSPNFGLEATDPNYTGQEWNQ
>g175

MASLTTIAIITLLVPLVTFFLIPAAVGYILKLALRSLGWSLKNKTNARRELILSQVRIEEERYLEA
AKSAASSSAGPEDDDWEKVDGTVPSVAGNGKPIGEDWEGIMGFFHPFCNAGGGGERVLWAAVKATQ
ERWPKAVCAIYTGDHEVTKAAMLERVETRFNIKLHAPTVVLLYLSTRKYVQSSMYPHFTLLGQSLG
SLIVAFDAFNLLVPDIFIDTIGYAFTIALSKYLFPSVPTGAYVHYPTISTDMLTSLDDNTGIKGLN
SGAGTGWKGMAKKQYWRLFARLYGWVGSKADVVMTNSSWTSAHINSLWGPARRNKKLACKDASVVF
PPTAVSDIKSAIDVNDSTEASREPSILYIAQFRPEKNHALILRAFARFIHNRKKSPSSATEPKLIL
IGSVRHSSHDETHIYHLRLLAHELQIRDQTTFICDASWPTILSHLRTSSIGTNAMWNEHFGIGVVE
YQAAGLISVVHDSGGPREDIVINLDGKGETGFRASTEEEFAAGFEDALSLPAAEKVGMRLRARESA
MRFTEEEFARKWIAEVGKLVDLRRRK
>g176
MRPLLKPRWSQLRRPVSPRTILSQRVRNFHATRPAAFIPEAIIAATSCLHSVHAYTGLPWVASIPL
AAFGVRMCMTPLLIWFRIRRRREEDIKPIIQCCVKHHQDAMRAEEVEARIQMKSDEASKRLYKQMN
EKTAAIRKEWGVSRWYSLITWVQMPIWLFFMEGIRNICGINMGLLRYLVPMSSKDGVPSFELPGVE
PSLATEGALWFPDLLAGDPTGALPAMLGLTILTNVSLGFPTSTAAEASDKRDLQMYFSTALIGMKK
FLMILAPYIAFSAWMSGMPAGMMLYWIVSTNTAMLQNKFYDRFLFVNKPLEALPRMHVRALKPGET
PPPTKALLARK
>g177
MHVRNFSLAFLSTLVASGAWYAYKGTQQQSSFLNGTTNLQSRGIADTTTETATGEATTSTRRALLV
ENDQFYTAVLSGDQPIAKQSDDSDRRVLEMLTPEQATQKLRKNDESYLVSRGRGVVRYDITQVPSN
SPIEDDHAEKIVEVPLATTQDGSANSDWMFWGVFDGHSGWTTSAKLRNVLISYVARELNTTYKAAA
TDPSIKTPSPEAIDNAIKQGFVRLDNDIVYDSVDKVMKSNSRRVAAEILAPALSGSCALLAFYDSQ
SQDLRIAVAGDSRAVLGRRGPSGKWVATALSEDQTGGTPSEIERLQKEHPGEQYVVRNGRILGQLE
PSRSFGDAFYKWTRETQDKIKSRFFGRTPHPMLKTPPYVTAEPIITRTKIDPKQGDFVVLATDGLW
EMLTNEEVVGLVGQWLETQRVGDSGNRSWLQGLFGSSETKQLPVEKATETKTAGQRPPIRQQQYDI
SGAAQRFVVEDKNAATHLVRNAMGGKDRDMVSALLTLPSPYSRRYRDDITVEVIFFGESANDGSVT
INKEATASAESAKPKL
>g178
MSARALRKLQKQRELEVQLQLAEDHESSNDESIPAPPKKKLNAFDLLNAAGGDDDDDQTSEDETVS
KETSNAQIETSTVTTTVETAKKKKKKKKKKKATQKSAAEPEKVAVEELDDIDRALRDLAVKNGTAV
VSSEKQPALSAREDAFAKSPAELLAIEPKSLNAMNEMRKLFGNIVLESFEEQDAGTGRRRERQREM
LDLGKALSGRYSPASRGQSLAGVTMRKNILMQGKDEWPRTTSGGLGMELVQRLPDGSSQYRLVHNK
AYQDVQMQFDMCVESMDPQRLIQLLQYNPYHISTLLQVSEIAKHQSDHAVSADLLERSLFNIGRAA
HSSFNTQLQEGKAKLDFILTENRELWLTIWRYIANLGMKGTWRTAYEWAKLVLSLDIKDPYCMRLL
IDNLALRGRQYEHFISLCTETVFKEDWESYPNIQCSLALALLRLNKPKEARQQLRAAMARYPWIFN
RLAQELDVQPIPKRIWGKMPPAQSHELLAELYISRAKDLWNTPEVLALLVEIAETLSEESVAIEPP
EITLDIARHVVLSDIPSVTTHLPTRFTTGRISASDPLPPYESEAYRQQSNPVPSYISQIPEAAQPQ
WLRNLLGRANPDEIEHMDDDDDHSGIDDLPDLEPVDEDPTASLPPPSSDQGAVRQWLLGPGILPLQ
RFVRQHGIDPGNWEDTEPVPVNNYVDGLIRIYPFEDREELLYGPIQDILGEMVVDLIEWQGHMRPE
YWEHPNN
>g179
MSNLIFSLAKDASKSIPLTSREAASGVLGSISLTCWVFLLVPQLIENYRYGSAEGLSMAFLVVWFV
GDVTNLVGGLWAQLVPVVLAIAVYFCIADGVLISQCLYYNMRNARREQRRNSVESTLDSPTPTTPL
LGRRFSDELSIPGSRRRRSSASQRDLHQRRASHPDSALAKIVEESESGAKAWFKNVLSVIAIAVIG
AGGWAIAWQTGLWRPAPVNHDGEGGPMAPGAQVLGYFSAVCYLGARLPQIYKNYSEKSCEGLSLLF
FVLSLLGNLTYGAGIIAHSTEKEYIVKNLPWLIGSLGTMVEDIAIFIQFRIYARPALDS
>g180
MAHIKDVPLLIDPSQRSIQELLLKYGIPPIIAKARAQVEFRNFSTGELQACFSKLHRHEADLKTFE
FDSTSSWAAGFTSLLRRKTQEVVWLKPQLELLRESAIKKLALCINAKGEWSLRENAYLALSHVWEE
GIQADPQNRGIPLAHVQQIIARIHATGAEWIWLDGLAIPGSSRSLTLEEEEIKIAIINNLDGIYRR
ADSIIIFDALVMQLQSTDLVDVAICLSCGKWMRRLWTFQEIYLSRKAVILTNNGLVDYLTMAARLR
SLSGLDDEVPQMYASMSCKDLMAVQSMHKNPAKYQELYLQLVRVLGIDGKKPSLTQLAMACQDRRT
GNDIDYARAFFPVLGLTWNSTLSREEGMELIYRDQEYFSKRLLLMHGVPRSSVNKENPSPRDSIMA

LEVDCEGACELCACLIGSNERPETMEGFRQAISTKEAYILTNNELNMVSRSGLAYRAILVERDTAV
ANLNEAWVYMAVEVAPIHKSLPAKPKSWLLLHESPISTHERSGKDWTKIARMLSDEDTLHGGKTPL
HEAAEAGIQDLITELLSENAALLEAQDERGWTPLHNAAFRGQASSILLLVQHGAPVDALDSSERSP
LMIAADEGHTEAVKQLLDLGADVNLSSQNAKCPLNQALLTEHVEVVRILLEAGANPNGRDKFGYAP
MFIACRNLETANLLISAGADPSADLVGGATILHFAARAGNVSLIKRLLELDMPVDLTEGNVPTGIT
ALYRAVEEQKLEAVEILLQHGANPNHVIQTCWTPVLLAAKLGNYEITKVLVRNGADAQTTCQPEKW
TALHVASQAGHRLVVRLLLDAGWDVNARDAAGFTPLKLAETAVHRAVVEVLKQGGGL
>g181
MSTAELAVSYAALILADDGVEITADKLNTLIKAAAVPEVEPIWAQLFARALEGKDVKDLLTNVGSG
GGAAAAPAAGGAAAAGGEAAAEKEEEKEEEKEESDEDMGFGLFD
>g182
MDSPAVLVPLDPREQPILERLLRTRDALLLLKQDKSSYIKSRDVLPLYEEVIGQVETLNAVRKAQE
RDRRLPHNRLDYVLDDCFQLISLLFLTVGRNNEAPATYSLTTTVQRLLDHLEEAGFYSAKDLESIS
RTLATIRETLDRGKDTHPPHLLTLLGSRLEECQIVLDRLNKGLEPLSPELVKLHETLVSILRSTSA
VNTRSKFSSSEVLDLQEKLKEIQNNMKDGNFVGSDGQILSGQEAVKSLFERCWRWTEIVLERKGRI
DERFQEQYARLLEIRNQLERLSMTQAWSLRETDLFQFQRKLDRIDEARVNGNFLDATNQPADLHAQ
RTLLYLIRRSYAYIYALLIASEPVSEALLPIYNQLQTLRRCLLEVKDSGGVSNARELYPYSMKLNS
IDNMRIDGKFYIGNDIPEGQGSVNSLLAECYELAYDLRAAVAEDKEDRGE
>g183
MRLSESVFFSLFGIIFGAVFTGFGINCIVRPDHALSFFYFEDLRVPGDQQLVDYLMIVYGARDIFM
GIALWITLFFGSRKALGGTMIACGAIAIVDGAVCKMHGFGEWDHWGYSPMLVVTGLVTMGVLDW
>g184
MVRQLVWQRAVAARRVSCLNSRRTLATAASSSRMPPYPKILRNLEEVRRVLGSQRALTLAEKILYS
HLDNVEDSLLSNTNNGRDIRGQANLKLKPDRVAMQDASAQMALLQFMSCGLPSTAVPASIHCDHMI
VGERGAETDLPASIQGNKEVFDFLESAAKRYGIEFWPPGAGIIHQNVLENYSAPGLMMLGTDSHTP
NAGGLGAIAIGVGGADAVDALVDAPWELKAPKILGVRLEGQLSGWTSPKDIILHLAGKLTVRGGTG
FVIEYHGPGVDTLSCTGMATICNMGAEVGATTSLFPFSPNHVPYLESTNRAAVAAAAAEIAAAGSQ
SLLRADSKAEYDQLITINLSTLEPHINGPFTPDLSVPLSKFAETVKENKWPETFGAGLIGSCTNSS
YEDMTRAEDLVKQASAAGLKPKADFFITPGSEQIRATLERDQTLSTFSAAGGTVLANACGPCIGQW
KRTDGVQKGEDNAILTSYNRNFPGRNDGNRRTMNFLASPDIVTAMSYSGSTTFNPLTDAIPTPDGG
SFRFQPPVGSSLPSAGFEEGNPAFMPSAAVPDPSVEVVVSPTSERLAILEPFAPFPERELAGLKVL
YKVKGQCTTDTISAAGPWLKYKGHLPNISANTLIGAVNAETGETNVAYDEAGKQHSIPELAEQWKS
QGSEWLVVAEDNYGEGSAREHAALQPRYLGGRIIVAKSFARIHETNLKKQGVVPLTFADRADYDRI
SACDQVDTVGLYDLLKAGGQGEVSLKVTKHKTGETFTVPVKHTLKTIRPAFANRDEPRIAYGVPFP
EATASQAATYFHASKVYVICSGSLSRNTDALERLKTALGDKFAGVRIGMKPHTLWSEVVEIINDAK
SVGADLLVTLGAGSLTDAAKIVSFALANDVTTFDGLYALTTNVSKDVKQPAEKDSTIKASVIPIIS
IPTSLSAGEYSDFAGGTNDKTHRKHSFQKPLRGPRLIILDPELTATTPDSIWLSTGIRAVDHCVET
LCSTVAYDETDELAQKALGMLVPGLLRSKHDKKDFEARLDCQLGSVDAMAACTHPERVVELGASHG
IGHQLGPLGVGHGETSCILLPSVCKYNALKGANNARQARVLKFLLEQPTVQDVVKKYNINTETADL
GDVLDGIIRELGMPRSLKDVGVGRDQLDKLAEHSLEDRWCKTNPVPLERKEQVLEILEMVVGA
>g185
MADFKPRNILLIGATGNIGRFITRSIVSARGDFDRVAILTSAPAAGSEKEKFINEELKPKNVEIIV
GDIGNEEDVLNAYKGIDTIIFALGRGAILPQIPLIKLAASSGSSVKWIFPSEYGTDIKYGPSSAGE
PTHQGKLKVRAYIEEDEEIKNSGLKYTYVVTGPYPEMFLKGDSEYTGGWDVKSKKANLLEKDNKIS
FTTMKEFVPPSVHFLRAIDPDKYSTGDLVLAALRHPGPATFNKALKVNSYTATPAEIQAEFERQVG
GGWTVQEVSLDALKEFEKNAWKDGRPDAAVFTLRRIWAEGGTLYEKRDNDAIGEPETLTLKDTVAW
YIKAST
>g186
MESTNDPSQQQQHGINIHQTDSLIGLWCGILRIVRADDILMIIAWILQAVKIITSTIAIYWGLGRH
DWELSSKHAFQNAMRFEMFSMIFGTLCGIFGRVAFAVFLLYFIQRVSRLRTYLVWAIIVLQVVINV
LFLAIVLAQCAPSVTGSWDSGSCRTSESVLVIEYLQGAINAASDICLTILAVGLVLQIRAPGYIKA
GLGVLISLSLIATVAEIVDAVEGSQTRQEGSDLAYRLNSWNYWYA

>g187
MSLLAPEIHSALSQLLQALATPDNTIRSQAEDQLNNDWVQNRPDVLLMGLAEQLQGAEVAATRSFA
AVLFRRIATKNTKDPRTGDTKECFSNLSPEQRVAIREKLVGCLSSEQLPDVRNKIGDAIAEIARQY
TDNGDSWPELLGVLFQASQSPESGLREAAFRIFTTTPSIIEKQHQEAVLSVFSRGFKDDHVSVRLA
AMEAFSALFRSIPKKQHAGFFSLAPDLLNILPPLKESEEDEELSKAFISLVELAEYSPKMFKNLFN
NLVKFSISVIAEKDLSDQVRQNALELLATFAEYSPNMCKKDPTYATEMVTQCLSLMTDVGADDDDA
QEWGTTEDLELEESDLNHVAGEQTMDRLANKLGGDIILPATFAWIPRMMSSSAWRDRHAALMAISA
ISEGCRDLMISELDHVLALVVPALQDPHPRVRYAGCNALGQMSTDFAGIMQEKYHAVVLNNIIPVL
DSTEPRVQAHAAAALVNFCEEAEKSILEPYLGDLLQHLLQLLRTDKRFVQEQALSTIATIADSAEA
AFTQYYETLMPLLFKVLQEEQSKEYRLLRAKAMECATLIALAVGKEKMGQDAINLVNLLGAIQQNI
TDSDDPQSQYLLHCWGRMCRVLGQDFVPFQGAVVPPLLTLAAAKADIQLLEDDEQAEMVEQDEGWE
LIPLKGKLIGIRTSLLEDKNTAIELLAVYAQVLEGSFEPYVAECLEKVAVPGLAFFFHDPVRVSSA
KLIPHLLNAYKKAHGIQSPGFAEIWSNVAGKIIEVLSAEPAIDTLAEMFQCFYESVEVVGKNALTQ
EHMQAFIDSVQSSLEDYQSRVKERAQEEAEAEDNDEENMSIAYAIEDDQALLSDMNKAFHAVFKNQ
GSTFLPTWQRLMPIYDSFINSTDPTQRQWATCIMDDVIEFCGPDSWAFQDHILQPLIRGLQDSNGP
NRQAASYGVGIAAQKGGPAYADFVAAAIPSLFQVTQHPHARTEEHVFATENASASIAKILRFNNEK
VQNPQEIVANWLNTLPITNDEEAAPYAYSFLAELIDQQNPAVLSNADKAFGYIVQALDNETLQGET
AKRVADAGKRLVQATGINAEAILAGVNPDNQEAVRKYFQ
>g188
MPDFDINNSPSSASHIPRPKPDSAVGQSDVGSSATAASRQRQNQSKRDEAIRRKMEADLNKKRHHV
QRPRHNRKAPPGTVLALKPSQALQIKPNTTVAEAAQLMAAKREDCVLVTDDDERIAGIFTAKDLAF
RVVGAGLKAREVTIAEIMTKNPLCARTDTSATDALDLMVRKGFRHLPVMDENQDISGILDITKCFY
DAMEKLERAYSSSRKLYDALEGVQSELGSSQPQQIIQYVEALRQKMSGPTLESVLDGMPPTTVSVR
TSVKEAAALMKEHHTTALLVQDQGSITGIFTSKDVVLRVIAPGLDPATCSVVRVMTPHPDFAPTDM
SIQAALRKMHDGHYLXLPVMNEAGEIVGMVDVLKLTYATLEQINSMNTGDDEGPAWNKFWLSMDHE
SDSMVSGGGSHRPHTPHRSVLSPDPTKPGFDRGDSVLPNESASHHGDDMHSEILEQHVDDVNTPFA
FKFKAPSGRVHRVNVVLSEGVSALVASVVAKLGQETEAVGGEPSVEDGKLSSTGFALSYLDNEGDT
VSITTDQDLSDAVHQARKLHHEKVDLFVHDPSQXPIPATVEPQPALSKPPTPPESVLKERAPKHIE
EDEEEEDFSRSTRATKHAVPQQRLPEEQLIAGVPNDLLLPGAIVTLAAVIAGVFILSRPSGR
>g189
MVKKRASNGRNKKGRGHVKPIRCSNCSRCTPKDKAIKRFTIRNMVESAAIRDISDASVFTDYAVPK
MYLKLQYCVSCAIHGKIVRVRSREGRRNRAPPPRIRYNKDGKKLXPQQAAKTA
>g190
MDAIPPSYEHAIMKDALAIIADYIPSADLCSASLVSRRWHAIFNPHLWGDPASHFGTQNDAVYVAL
TRFKRTLPHARPEVRNLTHTLHLPPALSEIYGGPRAPWLKDLLDYLPRLQSLIVSKLPFFDHNATI
SLKVPSDPEQTQAYRPYNLRLLLAEGEPNTTSIGIATALSRFPALVYLDLSRTSPARDRLVLSVLS
ELPDLRVLKLRGIGLKDTDAEFLANAIGTRVRLLDLRNNVLTDRSVRSLLQACFAPKGTTEATSNA
DEYHPTHAPVLLQNPGLDIDFLDLLTRPLTGRSIFENLPKSGITHLYISDNRLTVEGVAALLASKR
LHLLDAGSVDTAQTLKDDQPLLSPPGTKTDFNTVPGAEKLVPILGAYAKDGLTYLRVNHAIVTKDA
PATSEASITELLPELPAEESNIHHYHAELDTAHEIYEMPAEDVPLFELEDTSIPSSSLTRPELVAK
SNQYPDEPLLPRRGSAFAAEVAIPELPADEDLLEDYHTRNEGGSISTQRSRKIKELLAKRPKTEVL
TLRHGKNRTTFPYLHPSHIRHVQTLILTDVPSHVSSSSPILSSLTRFITACSDEMLLAKLQARADY
SFPPGRDRAKAEQQHARSLFALECLVLEITPSVKRNVDPPRALSPWKPHQNGQISWKSTTGDSDSE
RLWSAATNDFSFFGNEECGVPEPHEEIATNILNERVRLVSDDDSIHSGVSELEGSDSSNLRRQQPI
PRSSVQDEPEIDLVSALASFRRTKKKEFEDLKQASRRHSTSASSPCPSPFDSQPAHYALPLHTEGY
WPGEVKVIRNPTPKGRSGVVDIYGNYFEKGYLYP
>g191
METSQKSIRVMHYLSEFLDLDAQSFTNIAEFIALIQAIREGLSRIDPTFSVSDFHINLLFLSKLQA
HPKWNDWARNMLRNQRVNVSDSSKTMSFSELAELAIQHERDIKGRSRTRAKHAENNTGRLQNYTQE
EINSFVIRQMRHTHNSNNPQRSEPATQFKRHSKRPSQEEINEFVVRQMREEQERKTRARSKSEPKP
SNRTAAATRIEAHRTTKSADDDDQFTKCDTCGRVTHAGQEKCHHHQTTKMDTKLPVVQVPRANFFP
KKVEFVKRTAGGLPTYRTGFALT

>g192
MEPTRPQFYPNTLQPSEISKASLESMLQELQLAIKTSVDIIAKNCQRLTDASTYGNIYSGTPGIVL
SLLRLERQRLSIQQQTDQDYFQIASDLIIQTPTDIELVDGRLSALASNVGPSFMRVLAYCEQLNLR
PEADLTPDPEDFRLFTQAVERATLHGPVYTFKGFRLGGDELIFGRAGLLWALLTLKKHVTGTSGPI
TAHLDTASQSIPKLVDAIIAGGKQGSQDFVKLYGSRDALPLMWQWMEGYYALGAMHGATGILTMLL
SCDLSDEQQQDIADTVTSLCKICIGKEGHFPMSIPDRPDAATRSSPFVQLCHGPPGLLVLLGMIYE
KASFMERFWRPEWDRAIRLGTEKIWEEGLLSKGGNVCHGLTGNAWSLLPLHNAYEYHGHSLQAAKN
KRMAELAKSGIISETERNEGLNGDYFLSRALPFMMLARESPPYSQEVDLKTAFDFRTPDRPYSFGE
GLAGQISAWAETCAVIKARLRKMEXMSGDVPYERDESLRESLTEQLGFFGLAVNGPKTI
>g193
MPGVHEDIGSEVIPNPQEYAGTICPRQFTLRDRVTVATLVPFISVAQVPHGLLGYLSDQLNKEIEK
GDTYTMIDPIPLEKFAPWWFANIGAVMLLGDIQDVHDVQLMEHSGTDWTKVCLGSFNIRPNYPGRS
SHVCNGMFLVTDASRNKGVGKLMGEGFLEWAPRLGYTYCVFNLVYESNIASCRMWDSLGFKRIGRV
PGAGHLKSYPGQLVDGIIYGRDLSLEGDESITQERFDKIRYYLKHAKYPRGADRAEKSRLRSAATH
YKLIGGEDGEPERLMLKDKEVVSDPQQQYEIAREVHLEQHGGINKTTATVALKYHWVRIKETVSRV
IRDCPKCKEASKAPPVLNGGRTSEPPSIKPISEIETAVTNSLISHTEHLMADATHVHASPFTSTHT
TEVHTMADDSLPDYTHLPLDPQMIDMNSHLSRFQHHHSMSTGYDQSHGLPHAVFEDELRAHHHNEY
QSMVTDDSEAEALQGDALNLVNTQLTDSQHEQEMLSRYVDRSDDELDFT
>g194
MAFAGQTPTIIVLKEGTDASQGKGQIISNINACVAVQSTIKSTLGPYGGDLLLVDSNGKQTITNDG
ATVMKLLDIVHPAARILTDIARSQDAEVGDGTTSVVVLAGEILKEVRDSVEQGVSTQTIIKGLRRA
GAMAVNKIKEISIDMLEASGSEEKKIETLRRLAGTAMNSKLIKRNSEFFTKMVVDAVLSLDQDDLN
EKLIGMKKVHGGALQDSLFVDGVAFKKTFSYAGFEQQPKHFKNPKIVCLNVELELKSEKDNAEVRV
EQVSEYQAIVDAEWQIIYNKLEALYKTGAEVVLSKLPIGDLATQYFADRDIFCAGRVASEDMERVC
QATGASVQSTCSDIQERHLGTCGAFEERQIGGERFNLFSDCPAAKTCTLVLRGGAEQFIAEVERSL
HDAIMIVKRAIRNPTIVAGGGACEMELSGYLHGFADRNVPHKQQAVVKAFAKALEVIPRQLCDNGG
FDATDILNRLRTEHRKGNVWAGVDFDHEGIRDNMEAFVWEPSLVKVNAIQAAVEAACLILSVDETI
KNEESAQPQAPTRGLPPGAAQRALGGGRGRGMPRR
>g195
MSYYPPPGPPQYGGGGGXPPQQQXPPNGYGHPPQQGYPPQQNYGGYPGQGQQHYHHTPPPAHAGYG
GYGTPSPQPYNNGGYDRPPPQAPGYGAPSHSPHPSHGSGYGRQAYGHPPPPPNESVAFGQGAPQGY
NFRYSQCTGKRKALLIGINYFGQKGQLRGCINDVKNMSSYLNQAFGYAREDMVLLTDDQQNPMSQP
TKANILRAMHWLVKDAQPNDSLFFHYSGHGGQTPDLDGDEDDGYDEVIYPVDFRVAGHIVDDEMHR
IMVKPLQPGVRLTAIFDSCHSGSALDLPYIYSTQGVLKEPNLAKEAGAGLLSIVSSYARGDMGSMA
SAAMGLIKKATKGDGAYQKTLQTKTSPADVIMWSGSKDEQTSQDATINGQATGAMSWAFINALKKN
PQQSYVQLLNSIRDELAAKYSQKPQLSCSHPLDTNLLYVM
>g196
MVRHKKDNFSRGGKKSGPPRHRPSPRGGADENNDDASENGATRPAFKAACWDLGHCDPKRCSGKRL
MNFGLMRELQIGHKHAGVVISPNAKHVLSPADRDLLDQYGAAVVECSWVRVKEVPWSRIGGKCERL
LPYLIAANSVNYGRPWRLNCVEALAACFYICGHEDWAEQVLSHFSYGEAFIKINQQLLKRYAACSS
EEDIKRTEEEWLDKIEREYNESRAEREAGGADDMWHRGNTNRLEVPDSDDEDNEDDDDEEDGEGEA
EEEEDKDPFAISDDSDDEEEMAEIRRKILNSKSFQNPTSQPPPQEQQPPPSSSAPQPTSISTSSKQ
LPIDSDSESGSAEASDDDDEAFDNIINATPVTDRTGIVAAKKQRKANDSLTASFSRTVISAPKKW
>g197
MAARDRFGSFADPSLSPLQRAIHNACEPTNYEPNLALNLEVADLINQKKGNAPREAAIAIVHLINS
RNQNVSLLALALLDICVKNCGYPFHLQISTKEFLNELVRRFPERPPIRPSRVQHRILESIEEWRQT
ICQTSRYKEDLGFIRDMHRLLLYKGYNFPEVRHEDAAVLNPSDNLRSAEEMEEEERAAQSAKLQEL
IRRGTPQDLQEANRLMKVMAGYDTRNKTDYRAKAAEEVSYIQQKAKILEEMLQSIQPGDTISAGDV
FEELANALQSAHPKIQKMCEEESEDTEAVEKLKQINDSIQRTIERYKLVKNGDFEGAARIPKGTLG
TTTGVGKNANNELSLIDFDPEESAPSNGQEQAATNSLEDDLLGLSFADRSTPGPVNLGFDAPIAAP
SNVPSVPAQGAASGFQRNYDILSSLSGSRPTSQSSTPAPVVPQASKPTSPPPPVDPFASLISASSR
SASPMISNQKPSSSSALVDLMGGGSPSKTAGGIATPPADDEWSFTSALPTESALPTTNRVRVLDSL

LAVDFEARRKPGEPRTIQIMALFSNKSSQPLTELHFQVAVERAYHLKLNPQTGRDISPLQSVGVKQ
EMQIENVDAGKGNSIKIRFKVSYRLGGQAKEEQGMVPPLGDLDLDASPVQRSKEENQERAFVAASR
RKDRSLDARLESANRASMLHKKRTGKALHITKEIVEKEAMYEEVDERYQEKRLTLLKAHTTQLEAQ
FHRHLMATMAMSRQQQQQQQARISPTGGIQKMRTSSVGSAVSYFEQQNHSMNSQAETPVSPTATSS
TVPSPTSPVFDMYGVSSSASSDTSSFAQTPSSGCYGPTPMTCPQYIDTGLMSPSVSPRNSTYQQCM
NPMQTMHMRSASMAQGATPRAVPYPRQRFASYPDAFALQATQMVTPIQTVATVNYRASSEPSSELS
TTPAATVPVMTTMRENNGLLPSPALSPPSMTANSSQSNTCGDNTQFSAQVAPPVQVQFNDTDLFKQ
QPLLGSPENPDPDYNEFLNFATNMEDQWQFPMGNTPFEEFMNLETLDYELTQSGIYR
>g198
MADQPTHAGQDPPSSTESAISSPRTSTDSRSSSATTTRQQQPPLRLPHISPSPNTSHRQSFGESLR
NTPQSPRNRRQPSLTQAAIQGLIDNPPARNPADPAFLGRDWRHISIGELVKPADLRFVEVDTGIEE
ATNKLIDNDTTVLLVRETAEDKSAVGTFGYNDLNAYLLLVVGLAKPTESQLTSFQEVAQKAREGSK
IPLKDIRDLVPREPLTTLPQSANLMTAVEKFGGGVHRLVVLKEGSDEVVGIVSQSRLVKFLWENGR
SFPIIDQLYPQHLKDLRLGSQQVISINGDKPLYHALQLMNDEGISSLAVVDNHANVVGNISTVDVK
LLTKSTALPLLHNTCIHFISVILSTRGLVEGKDSFPVFYVTPTSTLAHTVAKMVATKSHRLWMTDP
QSPSSSGPPTPALAASYFSPNPPSVNNQPPPSNHGNGGGPAPPPSYTYPHHPIPPQAYQSPTPGLA
PSPAIPASALPGASLSGRLVGVVSLTDILFCYATISGLNPNDPAETRNRRRRSSSSSLNVRRSGDV
ARELFRGGVV
>g199
MGSLVELVLGSGMSGGKGDFFLTGRSISLFKESIFTMENPLINALGVLDEAWVKSQCLWTRKLSTP
TDLVGQEVSWKKMATALGFIKSIAWEIDSPFLDVDYEEPNAFGVSTLKHLATKKVLSDQRNIDQSH
FRHIPWPIAEELWQYLTRSGKRTLYMWKIFCAAYPVEFRHLSQHCDSRVGHPRYSLSGYISLIKSS
SDSWATRLNIWTEFARVPELVELADVTNLVSLEVNTSSHLLELQDHEVASLNDRILRTWSELAKTT
GAFKHLQVIRLYQQRELTEQSFRYLSKLPALEYCVLAMCDRMTQESAIKTAISEGWNVLEDVFDQK
ILQFSGRPSSKNRRGQIKWRGDRLDGHLSASLPSDIPILDFSVGQRHEKLRDRDVVVMQRKDTHKG
NKRSLVETASVENDWGAEKRAKKPAMKERGRDLSGMLADFL
>g200
MPLYDGRVRRYHLGPTNEHGVITQTVSLPQSHMADRSVITSELSGSNGQVCMQTRFAIPHGWLLIS
DIDDSIKITLTSEAAGILRTTFVTDPEPILGMPELYEFIDRELDPTWMYLSASPYNLYTFLRSFLH
SFYPPGTLLLRQATWKDLREFVRSYNTGTRTYKMQQIDKIHSWLPARRVICVGDSTQSDPEVYAEM
YKKHEGWIKAIFIRKVTDIPHMAKKNSSLRFQKAFAGVPHGIWRIFEHPSELYESIARLSFELPTV
RS
>g201
MTSNTMSEQTRIPKYMQALHLPSSFTNKATPGGASSLKYENERPVPKPGSNQYMIKVRTACVSIDE
LEQTCTTPSGQEANVRDSAMFLERPTSDGIDKIHGLIPGREFCGEVISTPREDYASSKGPLFKVGD
EVIGVLSNIEGRDGAAADYTLATENELAYKPTNLSAAEAVTIPHAALIAWQGLFGYATLDPDDGPC
NKPADGPLRVLVTNAAQSDVGRYVTLLLKCQALFPHHVMGLMGGASMGEMQRAVWVVATGAKHEHE
YLLKELGADEVTASTDIASAFRENSWKPVDIVFDCTGAGGALFREAHSPVVVKDHGHILTPRRPQS
GHPEKPSERDERTEIMTRNLTSEVIDVRPNAQQLAKIVQLVEKGVLKPDVSYQVEDILHGQQALVY
AEKAGEQKVVLRIDPVGSQF
>g202
MFRSAIVRSLRASVPRTVRAAAPRIQITPAVRPSQFVPVFNTQTPRFYSAPASLSKDEVEGRIVNL
LKNFDKVSDAGKINGTSHFTNDLGLDSLDVVEVVMAVEEEFSIEIPDKDADAIHSVEQAVSYILSQ
PDAGGLTRRRGGRVGGEDNEEGSRVSSPVPNNRTAVDARTPETSYTDGENGHKIAFDPRDISENEE
RTKMPKLTLMDEVLLLGLKDKQGYLSFWNENISYALRGCIVIELAFRGRISMQKDPARRRFPLADR
VIEVVDDTLTGEVLLDEALKMMKTSEKMSVSTWIDLMSGETWNLMKIGYQLKQVRERIMKGLVDKG
VLRTEKRNFLLFDMATHPVADSGAKEELHKRVRNVCSSRTVILSPNQWLPEDVEFRFLRTISMVCA
AYAANVLENALVTMSHESRERAFAQVDELLAEYSQWPFARKSGAGQGIGANLAQAINDEVNKSKDR
ELQLEIVAACLSVFTRLDSLL
>g203
MASPQSIARLVASRRPFVAVPSTTLRAVVTANFSSSAYRAATPSGPPPSGFRLPPPKRWDQGDESS
LDKATKYFLMAELFRGMYVVLEQFFRPPYTIFYPFEKGPISPRFRGEHALRRYPSGEERCIACKLC

EAICPAQAITIEAEEREDGSRRTTRYDIDMTKCIYCGYCQESCPVDAIVESPNAEYATETREELLY
NKEKLLANGDKWEPELAAVARADAPYR
>g204
MSLRGKVYAVTGGASGIGLATAKIISERGGTVCIADVNQDALNAAESYFGSKTPCVPYRVTQVDVS
NKQQVIDWIAGIKNTFHRLDGAANIAGVIGRDHGIKAVAELDDDEWDTIIGVNLTGMMYCLRAELK
NIADGGSIVNMASIHATTGVANHGAYAASKHGVLGLTRVAAKENGHREVRVNAVAPGPIYTGMMQS
HWDRMGRPSDAPFDDPIAFRRQGTPEEVAKVVVFLLSPESSFRVTMADIEERLRSHSDAFSGLMSL
IPAKYYYEEDHSDQWQRKKQTKEEAKNAKLAKLDPDSAKSAKDVMDENARKRKREEGNDNDEDNQS
QSAESDLGSELPKEGLKRGELKSKKQKQIDAAAEKDTQASNRNAEAEERRKLKAEKNAAKKAAQRE
KKKAKQAAKSDKKQNEEDEKLEPAESKPTLTQKSSSDTKSEKKPSVKENDAEQEELEIVEGLDLET
KDSALETISTTSSNPGSSTFEMSNNNSGTSSTSSIIPPSEAPSQTKPTLKQLKTTPDELRQRLQKR
LDELRAARRADGLNGKPARTRQELIEARRRKAEERKQHKKEVRRKAKEEEEQKRDEEMQRRFSPGG
SGSLLASPRSPAESFTSNSNNNFAFGRIAFPDGQHADPTLSKLLDDKSKQGPRDPLAALKIAEAKK
TRLSGLDEEKRAEIEEKDMWLNAKKRAHGERVRDDTSLLKKALKRKESAKKKSEREWNERIEGVAK
GKEFRQQRREDNLRKRKEDRGSGGGGKKTGGNNNKGKSKARPGFEGSFKAKNGSGKKK
>g205
MSDYNQYNNNQQYGHQGGNYYNDQQQGGYPSQHGGXGGQEQYGQQQPQYGQQPQYGQEGQQYNHQQ
QGYGQQDYNQSGYGGQQPTYGEQGHQQQGYGQQDHTQSGYGQGQQHQYGQEGQQYGDRQGYDQSAP
GGAQDGERGLGGALAGGLAGGFAGHQANHGILGTIGGAIMGSIAEDFIKDKKHKNDNNPYQGGQGG
SGSSFGGFFNK
>g206
MIVRQFGLRQGRNVSQRLLRFSSTNTTSTTTTTTTITAEEAFPISQDSQLSSTATQTTAVSGTEAT
ASPALKSRAAFMQAARQRQKQKSIGTTISQTYDPNHLLKSPPQPSEVTLELLLASQTHLGHTTSRW
NPQNSRYIFGIREGVHIISLDVTAAYLRRAAKVVEEVARRGGLILFAGTRPGQKECVVRAAEMAKG
YHIFERWIPGSLTNGQQILGNCDIKVVNALDQELKDYKQYISTSTNADAPRPVLKPDLVICLNPVE
NEVLLHECGLHNIPTIGIIDTDADPTRVTYPIPANDDSLRSVQLIAGALGRAGEEGQRLRLEEAAR
GKFTYTPVKERDIKPSTEGEQ
>g207
MPQREFVPKKYELSKDGNNSILALRSVGGKQSFDFTFEAVRPNLFRVRFTSDAHPLPPYPSVPEPT
KDDSISSSFTTTDASATTEVGGVTATVEWKSTPVVSLSFSGSKQPLHRDLPGRSYVSDSSGVSHYS
VLDREALHVGLGEKPAPMDLTNRNFILSASDTFGYDVYRTDPLYKHIPLLIKATSEGVVAIFSSSY
SRGTWSVGSELDGLYGAYKVYRQDYGGLEEYFIVGKTLKDVVRTYAELAGFPLLPPRWAYGYISGG
YKYTMLNNQALLDFAAKLKHHDIPCSAHQMSSGYSFSDSEPSLRTVFTWHPKRFPDPKKWTSQMHA
AGLRLLSNIKPFVLSSHPDFPKLKDANALFTDPDTKLPGYMDVWSAGGGEGAEGCHIDFSSHDGFK
WWFEGVQKLANAGIDGIWNDNNEYTLPNDDWTLKLDNKDVKIGNRADAPNSVGLWGRNMHTELMGK
ASHDALVNLRPNMRPFVLTRSATAGTLRYAASSWSGDDVTSWENLKGANALSLNAGFSLLHCYGHD
IGGFEGPQPSPELLLRWVQIGIYSPRFAINCFKTSPADNLLGDVIEPWMYPEITPLIRDTIKRRYE
ILPYIYSLALESHLYASPPQRWIGWGYESDPEVWASKKLKEGEEQFWFGDSLLVGGVYEPGVTSAK
VYLPRKSDGLFDYGYVNLNAPYEYLASGQWTTIASEWQNSIPLLARIGGAVPVGKSVVTRTPGEEN
SDDAAGGISELDDYRGIEIFPPQASSHGHVFSTTWLEDDGYTQNAGVSSFTIKYSSTTEKVTVTFE
RGDGNKYVPAWKSLDVVLPVGGERHVVSSKSGQPLEYVGVDARGRRVYRLSDI
>g208
MTVLDHIPLLRQTLSPGSDVLIKPEDSEFQELLARWTDIDRKEPAAIILPSSENDCLKAVQWALHS
SIPFVARSGGHSEWSTIDKHGVIIDLSRFSGVEVDAEAKTAVLRGSILSKQVAVSLAEAGFFTALG
NGNNVGAIPYMLGGGVAITTSTTGFGSDQILAARIITANGELLSVDEDNHPDLLYAIRGAGQFFGL
VTELTIKALPLSLLGNDAGAIWVGSFVFPIDRAEEVATVMKDLMDDGRYATAGLMMVMAPPPDRRP
ALVIAARYEGNPDDAEKAYAPLYKLSPVIANGGPVPIQNTSDSRAAIGAKGDFKRFGVVGLHGFNV
NSFLETVELYKELIAECPDAINTAFNFQWWSTPPRKPDFESAMSLHDIRYWQNNLIWHTDARNRRK
VDEYNDRCIAIMRGPNPIELVNYQNGTRDGPIECRYRGIERLARLRQLKKQWDPSGVFTRQLLD
>g209
MGDIEKVEQALNAGWVRTAKDLFAGASGGITQVLLGQPFDIVKVRLQTTSQYSSALDCAKQILKNE
GPLAFYKGTLTPLIGIGACVSVQFGAFHQARRYFEEQNLKKSPINPGLSYAQYYLAGAFAGVTNSV

ISGPIEHVRIRLQAQPHGAGRLYNGPLDCVRKISAHNGVLRGLYRGEVVTIIREAQAYGMWFLTFE
YLMNQDAKRNNIKREEISSLKVATYGGLAGEALWIFSYPFDVIKSKMQTDGFGADQKYKSMTDCFK
KTLAAEGYAGFWKGLGPTLLRAMPVSAGTFATVELVMRAMG
>g210
MHRRVFRRICRCPSIDSAYPVARRSCREFHKSSSWLQGETQTPAGNGENNGKSTPSQSTSRPLFKP
KLGGAWGAKLSTPRGPATLTAAELASRHALKAAAPPPELLKPEEDTPAASQTLVSGRVSWGGLKST
TRRAPASLTAAESASRKAISAAPSTPPLEPSIPKNPISRQRKNWRCPLCQLVCYGQRDVCPKCGTP
HPNPPPPPKLDPETLGENVLNEWRKGNVRSRKREGSIKLKRIAIDRLPVERYEESNEDQTPLEESP
IAQDEIKGAEPVEQAEVQPAKEEKGDINDFDKWLASLEELKKAELQNSQQPQRSDKAKGVKKRRSR
DGEEDEYDPEERQKRKEKRKHKQRPAEVAEPQRSPLYLPEFISVGNLADVLGIRQADFIDHLEDVG
FEGVVHSHVLDAETAXLIAEEYNFDPIFETSEQDLVAAPEPEDKFDLPQRPPVVTIMGHVDHGKTT
LLDWLRKSSVAASEHGGITQHIGAFSVVMPSGKSITFLDTPGHAAFLDMRRRGADLTDIVILVVAA
DDSVKPQTIEAIKHAKQANVPMIVAINKMDKEGVNPEKVKQDLARHSVDVEDYGGDVQAIPVSGKT
GLGMLDLEEAVVTLSEMLDHRADVNCNAEGWVVEATTKKAGRVATVLVKRGTVRPGDILVAGTTWT
RIKTLKNEAGALIKEATPGMPVEIDGWREQPEAGSEVLQAASEQRAKDVVDYRLERSDIKKLGQDT
TAINVSRKETLEQRRRSSDDESAEDTETPTGPKQINFIVKADVSGSVEAVVNSVSGIGNNEVLAHI
LRSGVGQVTEFDIKHAATSEASIVNFNSAVDPMISRMAEAQGVSLMNHNIIYELIDDVKAKLSEHL
PPNITYRVTGEAEITQQFEITIKGRNKTLIAGCKVRNGVISRGKKVRVLRDKEIIYEGSLTSLRNV
KKDVTEMRKDTECGLSFQDWFQFQVGDHVQSYEEVVEKRYLH
>g211
MAIPDDIPSSITTPHLFAHIYATHAQKIIDEHIATDQKDKISRAKIEFDIIHVMDDDPDLPLKYKM
SCTSMILHTDTFEPFWGDQKLSYSLSAKSLKRLKPDYRLIDRLPCPTVEDSTALAPLQSLTDKQGN
SRNQPTTAKNKGKGGRGLAAPAKANGKARRKVTAAAANRKRRTVAQKAPKTAKAATGNGVSKPNAN
ANANGTVGTTTTAPATTALRTRRPVGKTAREAYVEHMAKGDDMHLRGPTSDEEESTADEDGSDSE
>g212
MYDPAHLVRGVSHTPKMQKSQAGRKKRKLKGKKRGDPTVKDKDGDVSFGLEKQDPEELADFIDFIA
SEVFEALPATLQTLSYSKIQNDASLAKQYSDGTELTDADLETLTAPIPVSVSESLSVYGILPESLD
ITAAFLRPVLTDYITSVTAAPPAWATTKTEACEICERDWIPLSYHHLIPREVHAKALKRGWHEEFM
LNSVAWLCRACHNFVHKMATNEELAREYYTIDHILERDDVQDWAKWVGRVRWKAR
>g213
MAHSAVLAFDLYGTLLSTESISKRLSEIIGSQEEGTQIAASWRKYQLEYSWRLTXHYDNFFNITRN
ALRHALSEAGVQLDEEQVKQLMDEYDRLSTFPDVIAALQNLHSRPDIKSVIFSNGTEEMVSNSVLR
SKSLSPHAELFADLVTVDAVQKFKPAPEVYEHLAASTKTKMENIWLITANPFDVVGATKCGIKAAW
LDRNGRGWEDAAAPALRPRVIGKSLDEVIKTILSDLGK
>g214
MPGSPRLRNATWLHPLRGIVYFASHPFLWPLLRSRLLPIFLLSGFIYTLLFFFAYLPQVAFLAIFQ
GHSAWVNGAFLVLGEGSAIVALLFEAFFVDETLVDVFDAVLINEGFEEMINKERVIHPDGVNPVQR
LGKPTRSAVYAPFSFRQILEFILLLPLNFVPVAGVPMFLVLTGYRAGPFHHWRYFQLLEFTKDKRK
RFVRNRQLKYTAFGTIALILQLIPIFSMLFLLTSAAGSALWAAEMEKHKAYLETHRSETGEEYHDD
PA
>g215
MDGKSRLQKACDACNIRKVKCDEGGPPCQSCVALDIPCTFDRPKKRRGPPNKHAEALKKQKAEGTT
IAAPSTSPPETVPSVSVTAPSPIPSTAPPTALVPFTAPTTIIAGGAGPSDTPISSQKILPLSGESI
CPLPTLQLLVDDYFTFIHPLIPVPHEPTFREAFARREDLTNGRFLALLASVVGSLVASFPRRPRLH
LRTDSAGSLYSNSMALAKRCHDVAVQARGLGYLDTSTTVDDAAISYHLSLCSSYVYNPQRSRLYLG
ECMTILRVYTLYKPSRQGATNTGGIATGSPVSEPRGQFDDLAETTENLLVQEQGRRLFYVCLAGLQ
TLHQLGSADGRTYIPPETPTNRHPPLPLEVDDEYISATQVAPQPTGIVSQITGFNANVRIFQCYNP
LLALEIAFHDSEVVSWERQRRLIYDSLQKAKSVTAELPPELSLNYSQQFSELSSPSVIGGWPLSFG
AGMTNRDQERRKIQYEIQKVNIYISQLSTRSYLVEKYWTLFEGHIRLQKAKAGSTDTRPTLIPGTN
VAMQGEPTTSTPDAELHAQTDHIGQTMRQERGLVIKDLLCLLKSVQEIHIEPNGASFCHKVRQIAS
TLLNLPQEMSTTLPLTVDPVTGTPTTTTTTTPPTGPQPLSTSDAEKYLRTFLNILVRLEGVSVTGG
DPLQSINTVTDNLNIKPEDLDMTINPTATAGNTTLLDPTNTNIADGSNNISNAVTATTALLNDPLL

AAAAYPSPNHNEAFLLPMSYTGDGVSSQEEEEMRQWATLKEFQKTFVEDGGLLYPI
>g216
MAQRWWTRDLDEGKAQVFSHLIERVMPRDQFEPWDPDTREAYMYALAALGNLKESADSMRLPRALV
IFAHYFAFMAGHTQMWMIGKTPQKEIKGISSLVPEEWQPLMQWPLPVVEVALNGYANLSQIGTGLR
QRIYSKAFIIADPDDTNNTFIYLVLDAQSGDTAVRNGVIQGLANLGGEYARYGEHNIALSGTHAHS
GPGAWMNYLLPQIPSLGFDPQSYQALVDGALLSIQRAHESLTPGQLSHGTIDIPDGNTNRSPYAYL
ANPEDERAQYEYDTDKTMTMLRFDRTSDSKTFAVLTFFSVHGTSLYENNTLTAGDNKGVAAYLFER
SVANDTRFADGFIAGFSQSSVGDTSPNVLGAYCEDTGLPCRFEDSTCNGQTELCRGRGPYFLENDA
GTKSCFEIGRRQYTAAKELYEQLDTNGTQIVGSSSVSSFHVFHDFTGYEFPSPFNGSTLTTCYAAL
GYSFAAGTTDGPGDFDFTQNGTGPAESNPVWAVARAFIHEPSAEQIACQAPKDILLDAGAVNLPYA
WAPNIVDIQLLRIGQLIVIVSPSEVTTMSGRRWKNAILNASSEVLGITDPLVVLGSPANTYAHYLT
TEEEYGVQRYEGASTLYGPNELAGYINLTLTXLPYLGSESAVAKLAPIPAGPNPPINTNNSLDFIT
AVVYDSAPIGQSFGEVTASSPNTTYGLGDTINATFVGANPRNNLRQEGTFAAVEFLNPKTNEWETV
RTDADWNLIYQWERTNTVLGYSSVILSWQIEDSYYAIDDPNPVQSGDVSSAKPLSIPHLRGRDAQP
SASSSAVQENSVVPVSSVGATSIDPTSAAPTSTDPTSTGPESIHPTSAAPTSTVPTSFLSTGTSTA
PASSLSPDPTTTSPPHATDTVATSLASTEASTAVVGISSAPDTTPTSSPPLATSTVATSQATSKAD
SSTPGTTTPAATGTAGAAEMTILPSTELTGTPTTIAPTTPVSFAASSAVISSTPLTSPSSPPSAKS
SSSAVSSAITSTFVTSPSSAPIQTYASPDASSVAIKSSSTPAVSTSSPTTVTTTSPTKIPDIGSIK
TLTTPISPPSSKGSSTTTTLTSLSSAPAISSVTSSTHTPTRPLLSTYETSGTGLITIGAATAASSV
VKPTTSPFHSSQPAVASSAVTQGTTTHSSKVLHTASSAIVSDISVMSSQPVTTQWSLPSTTSTVRP
LSQASVSSVGSSSVPAVTGTPGFSSAASAAAGTVYSASIPAIPATSVVPTVTAHSVPAVPITIVPS
ILEPSTTLTTYSVPSTAVVPTSIPSTTTTARTTRSSSAAIVPTTTSPAVAGEGAGSAVTSDTSVPV
IEPTILSTTQPENTVKPVPPISDVGTELPKTSAHGIPSVAIESSTSTTFVAALPTSSAYNPDTDVV
PTTTEPELVTTMTETLTSTETSAEPTTTLMMTFTSTVFATVTAHTPSSTPDQNKVAPAPNPFITTP
TPDVNNAAAFPTVTVTAISIQQITTTVTSTTTQISTYTPPAVTVLLPTTIPGPQVTTTRTHMVTTT
VVLKQTPSPSSSPAPAAGMGAI
>g217
MPLITEYHSSLPYIDGEPGEQARASAQKLISAELSSDHASTLHPAIPALPESRFSPLIEQELARKE
AGQPMTGGIDLSRYEAPEAPEGPSGNQDDASKMLQEWKDTLRKAYISSSHLSIRHNNLALLEEYGK
NAWLIGNSQLEEILRSVEKELAETKEAVEGVHKERKLAQESGHGELAGLEDTWRRGVGSILEVEVA
AEGLRQQILERRRQQAQQSH
>g218
MSSDPKSTQGEGNNPEKWPQDEKPTHSHQHSQQQRQQSVDSSESESLYEIQPVRSERQVHPELRGT
GDGMLTPQISRMSHNSLARRATTRGTTGTTDPNFEVDWDDEDDPQNPWNWSLPYTAMCIAFLSYNT
LVIVLFSTSYTSGTAQIAKEFGISETIVTLGLTFYLLGLALGSVVMAPLSEIYGRKPVTMASMIVF
NVMIIPVAVAKSMPVIIVFRFIGAIAGSVMISSAPGMVADIIPPQKRALAFSIWSIGPINGPVVGP
ILGGFVTQYLGWRWTCWISLMLGGVALAFSCIMRETYAPVILRKKAARLRKETDDTRWWCRYDYKV
PLKETMKTNLSRPFVMAVLEPICIFWNLYIGVVYGILYLCFTSYPIVFGEIRGWSLGVSGLAFLGI
GIGTIVTIVCEPFFRRMIQSHKKDPETGEVFPEAMVSVVCIGSILIPIGELWFAWTCSPASIPWPA
PIAAGIFFGAGNTCVFIYSSNYMTFSYGIFAASALAGNSLIRSIMGGIMPLIATYMYNSLGPNWAG
TLLGLLEVACIPIPFIFWKYGYKIRQKSLFIRSMQEDQRRLKGKREAREARNATLEDVFEGSAEPQ
DIEKCAHVKVDE
>g219
MWSWFGGQSAQKRRDAPKEAILGLREQLDMLQKREKHLENQISEQEAIARKNVTTNKNAAKAALRR
KKLHEKNLEQTQNQVMQLEQQVYSIEAANINQETLYAMDNAGNAMKRMHAGLTMEKVDNMMDKLRE
QQALADDIATIITAPPLGEAVDEADLEDELANLEQEAIDERMLKTGTVPVADQLNRLPVAGNGELI
KGKAKQTEEEEDEEAELEKLRAEMAMS
>g220
MSSLQTMTAPAVGHNRPPDPGISPTSATNAAARTSDAIVDNTTSNNADDVSRHDFSRAVNANASAN
ANTEINIDIDSKNRDLSTPNTDVATTKQNGSVDPVPRPQEPEVNKDESSDTVVENRIEEIQEKEGE
RKIKEEPQDAVQQGGEQNPAENDQRKDVPDDMPKVKEDSTADKPAENHNQPQLQDSEKQSAATSLL
AQLLGNVNAQAATPEYLPRTPLPEPLTDASTPVAPNEDISLPMINGQTEKMVTSTQPEVQNGDITV

PKHEDNLLTQTQTQPILQTDTPFDIPDDSAATRIPDMVTGVMEDPLSKTSHDHSALFAPFDTDGSI
KNGLDALQQHDLLSNAFLSQEINMSGLPDISTYSQYDVAASIAGSEPRIQAFAKLEFDDGHFYVNT
YSFILGRDVRAARAAFQREVQVRQAMRPSKNKSSSGGNTSHTPNRVKRDGSAYVGSVVSDRGGIMG
FDPDVPHHISQISRRSSVSSQPDSVLHATPAQLQANHTDYNALAMQSLYDGSADAKPVDMLALLPS
PDACPTIPIHPPATTDGNSAGHKGISRKHVKISYNFDKNFFEMEVMGRNGAFIGADWLAPGQVRPL
HSGDYIQIGGVRIRFLLPDVPIGETGADRMEEPLLEDGTTVTREVTEAATGDMDVMEEDSDDESQD
GTKRPRVTKLILKTKSQDESANVDSVDGNGDSQPKRRGPGRPPKDGIMSKRERAEIAREQKLAAKR
EANGGVTPPPPINRPKPPTTTKPRKEDSVEEVVASTSATPATPVTPSKTVEKKSIKRKQPDGSIVD
VPIESIEGGEQQQQQPLPEQQPVVEYPKPPPPKKRKPSRSPSPDYPPESAYTQDQMAKPPYNYAVL
IFDALTESRTPMTLKQIYRALKLKYPYFRFKCETEGWTSSVRHNLNGNRQLFEHAERDGKGWSWQL
RAGASVEKEKKRRPSPPPPSSHIHQPSHHYMPPPSHSYTNQSLQNQQFQFSAMPNTFNPPPPPPAP
TYRPPVPPAPPIQTPAPNPYKNINLPPALTNSGSAKYVSPYPCALPPDILQLQQENQPKPPAQSAP
SHQNQPPPMQMHQPPPPPSQPQQHPQYNQAQHQHQPPPPTHYTPNGPPYQQPANNQISQYPTQNQG
PSPSPSQQPAQPYQPSQQQQPPPPPQPQGGAPMINNGSNSGNNSTNEQESSLSFLERANKAIDDFE
AVLTEDYDDKNYIREVLKSARARALGHADKSSFADGEPKDEAVLLDVLRNLIGSLKDE
>g221
MPAQKPKKKPASATHSKPQPTPPNWPPLRPLVPSTDLYLEPLLTDQIYIIRNFFTANLCKSYVSFL
TSPSSAIELITTPLKPKSRDHAVRVNDRFQVQDASFAEMLWGMTCLKDLVCEICEGREEDRKKIWG
GTPLGLNPNIRIYRYTPGQFFAQHYDESNTIQFPDQSGKSTSAHTTWTLLIYLTTTEGGETVFYPE
DGSAPISVAPEVGMALLHRHGEHCLLHEGKEVRTGEKWVIRSDLVVG
>g222
MFDHDAPAPLAGVVATVIVFAAVGSAALAIYRLCFSPLALARVPGPKIAAITAWYEFYWDCVQQGR
YLFKIEQMHQKYGPIVRINPWEVHIQDPSYWDTIYTNNKIDKDAWFYRAFGDNRGTVGTGPWDLHR
RRRAAMAKFFSAANVARLEPKVLARVQKLLDRVEEFRQTGGGQAAVLPISHAFRCYATDVISDYAA
PHTRDFLSTPDFSATFNQVLRDFSEIMLWHRHFPIVFPIFGAIPRWLISMMDSTGAQIAVLDNQAS
LLKQAQTVIATKGVPPEKSSPTVLDAIYTSDVIGPEEKTLPRMMAETQAILGAGTETTGNTLSNFV
YNVLSNKSIVQKLTTELGEASSASALLDYKTLERLPYLQACIKEALRLGMGVVGRLPRINQTESMT
YTTKISNGESSRTYTFPPGTVLSMSIIDMHLNDDIFPKPRLFKPERWLDSSPTHLRQMEKSFVPFG
KGVRGCLGIELAKEELTLMAGNLFRRYGRNLELYETSERDISIVHDYFAPFGPKDSKGVRVVVR
>g223
MVEYTPGWRDPNENTMSTPIFTNNDNNNDTLIADTTTIAPAGFYYHTQDDDDNDSALGDDKASSTT
SLNDSIFNYQYENGRRYHAFRAGSYPLPNDELEQDRMDMQHHIYLLMFGGELYRAPLPPKINKALD
IGCGTGLWAIDFADARPDTQVIATDLSYIQPTWVPPNLRFEIDDAESDWSYNQLSTDDGQKFDYIH
IRSMAGSIADWPRLLSRAYNNLAPGGWIELTEFETWASTDDNSLPTNSAYHEYQVALCEAAASFGK
EMNIAPHIYRLVCDAGFANVREDVQKCPLSPWPRDRRLKELAMYMNLQMMESIEPYGLALFSRVLK
WDTPRIYTLLDGVRKDLRNLNYHMYSRVYVSLSLSLFALG
>g224
MASNSSFPGTNPFAPGSASSFAKISEEEEYGVTSPTDPSFRDGHAGTTSRSPFLSVAELDDDDKLH
NENNNSSQNHANIFASPFRDSGEGFSAADLNAARPAAGQGMPNNYALGRRTSVSAESLNPTSSGSD
SWSPPFHPKSDDQVSRLKKAVSGNFLFSHLDDEHLKTVLDALVEKPIPAKDIKVITQGDAGDYFYI
VEEGTFGVFINPTGAAQAGPDGLGHQVGSIGPGGSFGELALMYNAPRAATIMSLDPKSTLWALDRV
TFRRILMDSAFQRRRMYEAFLEEVPLLSSLKPYERSKIADALDTIKYAAGSTIIREGDPGDAFYLL
EAGEAEAVKAGTTVKEYSRGDYFGELALLDDKPRAASVMAKTDVKVAXLXRDGFKRLLGPVEQIMR
RTDYDVDPTKAT
>g225
MGTYVFRWPREANEVFVTGTFDDWGKTVQLEKKGSVFEKEVHLPAIEEKVQYKFVVDGAWVTDSDA
RQESDGHNNINNVLLPEDLKPTTKPTTEFESLTGGNTAAAVMAGVVPEATTAQLAGEVPKENGSSV
PGGXPETPATEVNELSVNPLPATNGAANPVKLAPGEPVPEPSTIHTNTVESTVRTDKEGYEADASD
PSFLKKEEPKADVLSVPPVPAGGLIPESSLPLNPPETSEPGVTIQSAHPTSTTSALAGQVPLESQK
NAEVASDVPEVVKESLTKAHQDPEAAANAEAVEEKAEVEKELQSKVGVAGASAVPASGVPEVVKES
LEKAHQDPEAAANAAVVEEKKEVEQELQSKVAPTEQSGEPAPTTSAATAATAPTTAAVDSADVSPN
TTSPPATATQTQPTVTTGVASGTTAEESKAVGASTQDNAKEKKKKNRASGFFQKLKEKLK

>g226
MAKNSIVPVIILLLVVFLFAVIGFVVYTIVQDISDKTKAKMEKKNIKFSKDGLKVSMKEVRDEDYK
DQTQSVLVKVWNNTSFPAYKSRLWDNGVAKDKENAKRR
>g227
MSETTTSDQDTVTSLRELATKVQDLCNILTPEIPDKSRAKLKEKDTVVNMLHEQINNGRPEKILDW
EKVELMAVDDIEKENEQYTQKLENFWKETDESTQTEKEKTXQAYASAFSRFAEIYHLLGWRGAADD
EADAMFDESVLTDSRSLLLESIFRIEVFQMSRFEEMSERSRALFELSRLPRLLLILVRIASRQVYI
VSACGADKVIKERSFDASTVIKVLALVARTLKGGHLVSSVSLEKLLYSCYERRLLDQPNIDDVETF
QKQCELVYDCITALDFTKRSESIQELFDRIRTHEEATVGIEGFPELPLQESTGSTFHPEIFSIRYL
QDFGGLKIEWTDSLKEHLKIYANRNAIRIFAHPTFFYNAIDLHRCIKSDAHDYTHPTYFELSLTYA
LLFRPTSSSNIRRLKRYTTAGEEQEIRWHNMVTFDGRPIHHFTPPWDDKPVDTTAIKQLDINPLRN
PTRDILSESFYRCSLPPSIQVALNIASPDKTVKGITPSWDQHKLTSTSMQQLTTTSLPPTYPEDIK
FMILSIMENDMTETESFVRFPYFESRLRRVKMYLDSRQPTSMRELWVDRRDFRAWWVFWGGAFMGV
VLVGILGLQVASLLVHLKMNLLKLSTIVITVYLLYKLLSFIRFYIIGLRTGFPLILTPIFSRSLPW
QVLAPALMPVLRHKLPPWILIRLDTLVHGWEFRWRDGEVQRWVGADTCSNPALGSAILSRRKDFVQ
TPTVKQFIGIFGENVLQSNGEDWQRQRRIIAPHLNEGIMATVWKESCLQAKSMIDYLVHHPGGETL
DGLRSVAINVLGSAAYGYSQPWSPGFAESLSGGSSNDEKNWGSGRIAYFKTVALVVDSFISAVLIP
NRIKILPFMPESLQFIGRQMERVPQYVREIFEAHQNTKNTAEPAEGNGSRKENVLDMLLQYSQNRE
KAAATNGLYLTEDELSGNLFVFTGAGFDTTANTMGYAVTLLTAYPEYQDWIREELCALDGANVSAD
EYEKGFALCPRTLAVMWETLRLFTPVLHSIRCVNGTQLLAGPEGKTHLLSAPMDVFVCAQIMHCDT
SIWGADAAEFNPKRWFDVNNQLIKPPKDTFLPWSGGPRVCPGMKMSQVEFVATMATLFRHARCEVL
PLTIDEKPEEGRKRLVDMMEDSISKLTLQVKDGKEVKLRWVV
>g228
MSFSSVKALLTQYSPVSESSFSDAEKEAQIPLGQDKDLELHSDGRSSAAPSSRPEKKSFVDARVIS
DAIIGLSDGMTVPFALTAGLSTLEDTKVVVFGGMAELIAGAISMGLGGYLGAKSEEQSYRTTLKQT
REQTLTDFDATTTIISDIFAPYDLPAQTVSDLTKHLVDSPNLPEFLMNFHHTLPEPSGSRAVTCAL
TIALGYFIGGFVPLVPYFFVGPDDAFSALKWSIAVMAVALFVFGYGKTCFVTGWKGRENVKGGVYG
GVQMVIVGGVAAGAAMGLVKLFQVLAR
>g229
MMNPTISAPSWKDRQKSQFSKVQLPTMPWRSFKLLMPHRMRRRLRSKLRSRISPATSIASLQTSFS
PKDTLRSLQSYRWSVYDFQYLLLAIIGIFSLCMIQSADPFGKTFVASALLLSLLLPITRQFFLPFL
PIAAWLIFFYACQFVPSEYRPGVWVRVLPALENILYGANISNILSAHQSVVLDVLAWIPYGLCHYG
APFVCSAIMFVFGPPGTVPVFAKTFGYISLTAVCIQLVFPCSPPWYENTYGLAPADYSIKGDPAGL
ARIDALLGIDLYTSGFHASPVVFGAFPSLHAADSTLAALFMSHVFPKLKPLFVTYTLWMWWATMYL
SHHYAVDLVCGGILAAVAFYFAKARFLPRVQPGKRFRWDYDYIERGSGSDEEGYDLAPFKRHMHAD
SDEWTVGSSSSISSGSLSPVEDQHLWEGETLASISDIESGR
>g230
MSDLRRRAVKEPPRDEDNNGDTKADAQVESETKKQKHHETERNEDDEKPTLSPIVRLKRYITRPRG
KRRNSFVFLLGGLFGIFIALFFANHNEVISLDALMDLNLDSLIDVIPTGILSDAKEFTKHERDTVS
YDSFSVGLQLKSEGIQAKHPVVMIPGVISTGLESWGTDTESRQYFRKRLWGSWSMMRALVMDKASW
KNHIMLDRETGLDPPNIKLRAAQGFDATDFFITGYWIWNKILENLATIGYDPTNAFSAAYDWRLSY
INLETRDQYFSRLRSHIEMTVNTKGEKITLISHSMGSQVVMHFFKWVENDQHGKGGKDWVNRHIDS
WVNISGCMLGAVKGLTAVLSGEMRDTAQLNSFAVYGLERFLSKEERADIFRAMPGISSMLPKGGDA
VWGNETWAPDDQPSTTLSYGNFIRFRETNSSWXSRNLTMENSLTYLFNQSEEWYRNQVAHNYSQGV
AHTRAEVEANENDPRKWLNPLESRLPLAPDMKIYCFYGVGKPTERTYFYQEEQDPLTRLNVSIDTT
VTEPNGGPDHGVVMGEGDGTVNLLSMGYMCSHGWRMKRYNPSGIKITVHEMLHEPDSFSTRGGPNT
ADHVDILGRASLNDLLLRVAGGKGEMIEEHYVSKIREYAERVKIYDDDD
>g231
MASASNDTSRPSALRYIQYDPSRENEYLTAMRQLISKDLSEPYSIYVYRYFLYQWGDLCFLAMDQT
DKLVGVVVSKLEPHRGGPLRGYIAMLAVREEYRGQGIATKLVRMAIDKMIERDADEVALETEVVNT
AAMKLYERLGFLRSKRLHRYYLNGNSAYRLVLYLKEGVGSMDANYDYPVPVSGAPPIPTGQPQVQI
PGYPVV

>g232
MITRGKSCLRRGNLSTFLDNVAIPTSNEPLHFLYPPLAKIHLIRGHTSESRACFSTRRPKSRDHTC
FREHNQSVERKSRRWLNQESYLSRGGNVEDVEVRSRTVISDPEVKRDAVLDRFEDGQRTSRLHTNS
REHETGAHSGSEFENLPDLEYSNELVERKNLGIEDVEYEDVENENLDTTDSITIRTVQSNAPPPEE
RERRALMRKRDAEVARMSAQVKTSALSKKMRYRQFIVSKPKAQHKRSPHWTEFMARLERMHNETIV
TPMKGLRKEILLREETVAYFSGTTRSMENIWYVRIRNGCKIQVLDSLESEGIYRKVILSGTRRVIE
LVEKEFQQYDEQIRQSGLPPVVPSMLALRQQGVMTPLVRSWWYAAPNNNNRTVVSEPAPGTVVNSI
RKFNRLVEQWVYFEPPVKGYWHATRVKDELVDLFTRPGNKLYLSSNALHLALTWLTDHEFLDSARM
VFEAANVVSTTNTFNIFLRATAKRQDPWYFRHVLTEMEKAGLQPNGHTWMALLKSLVSPGPRRQLM
RRMKELGLTKDIRVLHDMIEHDLSLMVSTFLEEGNDIQAFLESLEQNYGSDIITTNLFNELLNAVF
LRRDFDLAADILKSYERFGLAPNARTFTAALPVFYRIGPAVDTMTPYLRDHEHLLHHRHYDMLFLD
AIASNAINACRVIWRYASMRGETSPTMRYIVLSSLLGKATGRGAKNVINPEHIHHHIGALVIGLNY
PQTQPMTKAASLVPLKYQSNPVIYLLHNRDKMTGSEKNKLVKALMEDDIDRGRQYQPLEPFTVMLD
AAVRLDSSPEYSWDRPKEENYAGMKNLPTMEILKHVIDVPVEQALKSLADKAGL
>g233
MNAYSLQFLPAQGERIMATDVAKGIDLIDNTTPSQPLERDLTRSETRSSHFLLRKKLTRDSVKNGL
QNRKYAKWQHERLNASDTESSSQSPSRAATKNTTTDTQSYGKGIDDVDILNIAPVQTKATDNLKTE
DNLEPGSELELLYENQRGWFFFGIPFYSSRSLLQFDPPAWVNKDFRESPVNITNAQLPDPSWEWAW
PTWYVDMSDDVDEEGWQYSFSFLPKFGWHGTHPWFHSYVRRRRWVRLRVKKKYAKLKNGDDQTDFH
MAHLLNEDYFTIHSQALASVEPSIAPPTTTNVPSSSYIRRDLGRSPERNAEDNIDNLPRLIEAMKN
AIVDREKIEALRNFLAQGGEELYYLPDKVPEVLGLFVFRTSRWQFLKLLQDALDDIAVDSDPDVEK
REVDTVNRRRNNISRTIDAVRKEVSDSDIFDISKETLEGLHESELQKAYESSRSKGKERSWTEIKG
IPKEVAVGKEGHIY
>g234
MQSEHYRKIELQSPADLTYLYTNAVAESRQKLDLHFPPSAYSNGDQPDPMKERVKELVDDFIRQTY
TYASDSLTINGLEFDSASISASTSKNNGNNNSTGDAPFPFPSAFSAPNETIEYEPYDGNLASRVSS
LYAQLESLTTTVAQLRRDAPGKAAKMYAANLRGVLEREDEEFDEQQQQQKRKRRRVEGGKDVDVDM
EGHDDGEVQENIDAEFRLDIPFGTQAERERWQSGEMAEVYTDTLRTLLRLQGEESSTEAASHNDSD
KKAISTTVATAERAEKAVDVVERMV
>g235
MSEKSQNFDPKYDDYDFPTTSPEARPGHPGHTTEEQDAKIFQLRAMLEQEGYTERLDTLTLLRFLR
ARKFNVEAAKAMFIECEKWRKEFGTDELVRTFEYTEKPEVFAYYPQYYHKTDKDGRPVYIEKLGKI
DLNAMYKITTSERMLQNLVCEYEKLADPRLPACSRKAGKLLETCCTIMDLKGVGITSVPSVYGYVR
QASAISQNYYPERLGKLYLINAPWGFSSVFSAVKGFLDPVTVDKIKVLGSNYQSELFAQVPKENLP
KEFGGTCECEGGCELSDAGPWQDPEWTRPPKWAAPAAAATPAEAAAPAPAPAPAETAAA
>g236
MSSHDTRSVRASKRMSVTALYLSMSAKDKDLEISDELAKAQKHLRELKAKISSQSKKNFVLEKDVR
YLDSRIALLIQNRMALEEQNEISSRLEESLESSQEGHFPNDEKTQKYGNLLFLLQSEPKHIAHLCR
LVSMSEIDSLLQTVMFTIYGNQYESREEHLLLTMFQSVLTYQFDNTPEYSSLLRQNTPVSRMMTTY
TRRGPGQSYLKQVLAQRINALIELQDVDLEINPLKVYESMVQKIEESSGQLPDYLAKSVTAEVAAE
NEQVKAIIEPRLKMLTDIANTFLTTIIDGLEDAPYGIRWICKQIRSLSRRKYPDAQDQTICTLIGG
FFFLRFINPAIVTPRSYMLIESVPSDKPRRTLTLIAKMLQNLANKPSYAKEPYMAALQPFIHQNKE
RVNKFMLDLCEVQDFYESLEMDNYVALTKRDLELQITLNEVYATHALLERHSTALAASDQHSHLHT
ILQELGPAPSQLPRKDNRTINLPLFSKWETSVDDLTAALDITQEEIYFMEAKSTFVQILRSLPPNS
SVLRRPLRLDRIAEAAATLKNDAVMVRKGIRTMELLSQLQEMGVIDRSDEFGLLRDEVEQELVHLG
SLKEKVAEEAKQLDGVFRTIRDHNAYLVGQLETYKSYLHNVRSQSEGKSRKQQKNQELGPYKFTHQ
QLEKEGVIRKSNVPENRRANIFFMFRSPLPGTFVISLHYKGRARGLLELDLKLDDLLEMQKDAQED
LDLEYVQFNVSKVLTLLNKRFARRKGW
>g237
MDNSADFSSFQEKVTAALIKSTQTVKKIGDEDVDFHRTSSTEFAENLDEQSTRILSLTSSLLKVAT
GGTDINPPVLEDEDAIEDNWRGIVDVIDNLLEKADACLDEFTGIIKKLSPPQQDRIREASTRQQEK
KFPTIYDYGPSKIPKPQLQFRNAPNNHDTAPFRPLLKSKPHAIVPLEKSLQLVETEKKPAFYPNPY

EKEIRDAKYPESAYVVAPPVEFGPVETTQAVWVDTPEGVTEMVKELKKAKEIAVDLEHHDVHTYYG
LVSLMQISTRDKDWVVDTLQPWREDLQQLNEVFADPNILKVLHGSTMDIVWLQRDLGLYVVGLFDT
YHAAVALGLSKRSLKFLLEKYAHYEADKKYQMADWRLRPLTEEMLKYARADTHYLLYIYDCLRNEL
LERSTPKRNQIDYVLERSKTEALQRYERPVYDMAGGQGAGGWYDLLSRNSALFSKEQFAVFKAVHE
WRDRVAREEDEGLQCVFPRHMLFKVAVAMPVDRHTLFKTLSPVTLIVKDRINELLALIKKAKIEGA
TGPELRDVIKPRKTVEEAILSATKPSPLLTGEPTVVSVVRADSSQFWGAALQAAESAVVPPNYKVA
ASLEALRLSVPIPPMPVTVSEVRQMVQETTTPAVTSVEPSPMNTPSTNKADKRSDIFTVKEVAPRH
KRKAIEVVEDSDETSSSGGSSASESESSSSSEEEESEEEIQVQKPPTKKSRKEKKPQRKPQEEDTI
PFDYNAASSVLHSEDMAAAAAAANLPNQKKYFNPYAKSLNAPSGVRKQKKETSGRTFTFR
>g238
MAHGRTWTLDQMIYLVVMKLASSYGWKRVAEAFRARFDSPATHKDVESKFNKDLKKSKIHQIVVDW
MEAQIIPEDDPDGVCILLDALMMIGEIPLEDRLA
>g239
MINGDGLLRVFWPNDIPRSSSSGVIIGWRNSDLDLFVVTVLEDVEAKSVDNALRTGILFRNCPHPI
TQLFVLCGKSAMHVLGSTNPAEPPTAFDAGHLSVTTNTSYKIPRVFCPPETNVSIQVIMFNRPHPT
PLVDKQSNHGKGVSFAPIDAEEEEEKARSKKLIEKLKWHTVVRHVPSQKEVALPIILNQVNCAFEV
GQLIEKNSRLIGTRPKRSMSVSERVVESATTIWDFFVDIFWQWIWPVLTRCFVFGLVCHRAVAELV
LRMLEWRARPDAAALKDISATAQQVDIRLQQFCYWPIQYVKLRQRKDNWESVTTSHPDYIRFYNSL
WLVANDVIIGIALGSYIIDNAQWVASQIDYLITDWTVEGLQRTISWLMGWPAGLKLNNELAAFLGD
LFLWVIEHWAACIANLQPYLPHLIYIIGCSSFAGASMPIALFSDLLSIITIHIYSFYIASARIFNW
QLSIIISLFHLFRGKKRNVLRNRIDSCDYDLDQLLLGTILFTVLFFLLPTVVVFYLAFASARMLII
SLKAALDTWLAFLNHFPLFALMLRVKDPGRLPGGVYFSLREEVIKPSSKQKGAEATRISYIHLKSM
PLSLRAMFDQYFQLGHRLRKHYLSPSVILCLVTGRFVPPIHRRNLYSMQYSMLPAHRAGMFEVWNR
LTEKKSTSNGSSGGRGMNGSMGNGLVKVPSNYVSNGGGRRGHR
>g240
MENIQHSTSVLQALNVWGRRDYKIANYDKMSSRPELKVDDENGFIRFFRSLPSKEVVNESNPTTIR
LFDRGDWYTAHGTDAEFIARTVYKTTSVLRTLGRSDSGGLPSVTLSVTVFRNFLREALFRLNKRIE
IWVSAESGRGQWKLGKQASPGNLQDVEEELGGAGGNAMDSAPIILAVKVSARSSEAKNIGVCFADA
SVRELGVSEFLDNDVYSNFESLVIQLGVKECVIQLDSTKKDAELAKLRAIADTCGIAITERPMADF
GTRDIEQDLTRLLRDERSAATLPQTELKLAMGAAASLIKYLGVMSDSTNFGQYQLYQHDLSQYMKL
DASALRALNLMPGPRDGAKNMSLYGLLNHCKTPVGSRLLAQWLKQPLMDQAQIEKRHQLVEAFVVD
TELRQTMQEEHLRAIPDLYRLAKRFQRSQANLEDVVRVYQVAIRLPGFVRSFENVMDEQYQTPLDE
QYTTKLRNLSNSLAKLEEMVETTVDLDALDNHEFIIKPEFDDSLRVIRKKLDKLRYDMDLEHRRVA
KDLNQDMEKKLFMENHRVHGWCFRLTRNEAGCIRNKKEYQECSTQKNGVYFTTRTMQELRREHDQL
SSNYNRTQSGLVQEVVNVATSYCPVLEQLAGTVAHLDVIVSFAHVSVHAPTAYTRPKMHPRGTGNT
ILKEARHPCMEMQDNISFITNDVSLVRDESSFLIITGPNMGGKSTYIRQIGVIALMAQIGCFVPCT
EAELTIFDCILARVGASDSQLKGVSTFMAEMLETSNILKSATSESLIIIDELGRGTSTYDGFGLAW
AISEHIVTEIGCFGMFATHFHELTALADRYPKSVKNLHVVAFIGDAKEGESNKKREVTLLYRVEPG
VCDQSFGIHVAELVRFPEKVVNMARQKAEELEDFTTATSDNTEKKEASALDGYSQEEVEEGSALLK
SMLLKWKAEIEAPGKENLTAEEKRQIMRDLVAADSSLQSNKVFQGIKAL
>g241
MSSLIPARDTYTLLKRFSMPHGNGTIDAVAVTSSDGLVLISFYTIIMKAIITQFWMGIVLFGVGLF
LHRNSSHDSNSSDLSDSSATATTIGLWNIRHSPRQVVYYMTQRTFSASPFRDIWYPLIWFAVAAAA
LAASLAVPIVKTQNLKVGIVAPVNPESVFVPNFDDPNSTAHELAQQLTPTVPAALRAVGGAAASIP
PDTVYISNISTPSSNGTVVQFNYTYGSCITDYTWYAGFDSSIKADVYYRWNDPDNATDVYLSSDDG
PAFASPWFGSASSTVNYTFGLIVSSLGRQSISSSTDPWYLTIDQQDSIWPYEVNAGRPPLSCWETD
VWSFRGSTSNASELRNLTDNRLSLPISIALSQNLFEPMILSTTQRLGRSSLASSLTAYGAGFDAAK
CSVVADMTRLVNSSYIATRNILSDSTTISAEGQEGLLNTYRGDNGTILPGSDEFVISSPNIITLSV
RILIIVPAVFFGVLVLLAVLACLPDPWRSSKELNATRIHALKRQSTTTKTRNVRVSKGNGNDKQDG
GDGTKDNGDVTEESMANDHPYLDLPIPKYAPIELRPTPGKGWGVFATRNIKRGSLIMKEKAMFVIP
KPSESITEQDIEKAIEKLSVSQMHIFACLSGNRPRELFNGLMDSGPLDLAGIWGKNCFSLESGVEQ
RLFGKFVVQKRWGLFPCHSRFNHSCQANAGIPETPSESQVGKNSMSSYAERDIAAGEEITISYKNG

FHSRTLYERHRLLGFRCRCTVCDRATKDKTFRQVSDMRRRLIRGLIYLLDGIDENSEYDDNDTHSR
PVICDPAMRKAADKCDIPLSARLIYDLLLVALKEAEGTLDVEEAARTWTHVWRRACSFGTEENRKI
ARLAVQQKTWTERLGMAFQLDGRADAADYLHTLAYRRVKGMEI
>g242
MAPFTFVLFAFSVLYRGVLAVTVFEALQSSNTGLFARWVEQDPGLAPLYNSSQVQTVFAPVDDAFQ
TFNQSGKLSSLRRLLVRQNLPSDQGQLQACHNANLLNGQSAGDDPLGRQLDTLFPSSNPDRTQPVI
MPPASNNGNGTSSQRKRQHSSSTTQSFHFYSGLGKSVSVVQADIPYDGGVIHTLDGFFTLPQSFHD
TFANTNATSFASGLNRTGLASIIDNPTGVTIFSTSNEVYKGSIQSNVTTASLTSTLSNHIIPNFLG
YTPSLANGTVLTTQSGNNLTVKFSNGEWYINNAKIIAPNQITSNGVAHIVDSLISTSTSPSVPVST
GMATTHRKVGKVGITASIAYALFAWLM
>g243
MAAKDEITSQAETVTQSHDSEKLSETKASEKIASVASQGAVPSTPQLEVQPITVLQPPLQQQQQQH
SQTLSSTQINASISGNASPQLFQVQQQPGQAPQTMALINGQLVPVSTVHVQVPVSGSGSNSVVLPQ
VQVQVQGPAAVNMQASIVQKQSATEGSTSTSTQQKPTVPESKQEKTGLTSNVPATSTQPQTNNNAS
PASRKTRDPGCGCGFWAAILRSFQQKGDNKNDTKENQTTTADSNTQNDKTTSQPLGTTAVNMAIQA
INPASTSAPAAHGDSSQANRSTGVTDAGQNTNANYYPLGTSDISTGNTMNNQGGYVIPTNTMIPDT
GQYSVAAQQPLYVVDPSQAAPAPYTEQAPGQATADGTYYDSGEGDDDPVDDVYDDDADDM
>g244
MAILSAYSSLATSRHSRPKPLWETTSQDDAMSSGVSKFLCRERAGLQPPVFEHQFSRKRKEINDDE
DNFPTKKAKFSIYEVMEQDIPVDKVLDMISTSDLKAIVKTLLTHSVAGPIVKEELLASSEESIVYS
IEQAERLKQCIMKESADAIKKVTEEAYAYGGMEDGSAWSNALSVFLPRIRELADQGRLVYGPDLAW
EALLRVAKKCILPKQGGEIRLRTGEEDCDDFHKEVDELMLHICHLQEDDGNTAWLQEFARKNEIWD
LQSSAEPPLEFQGPDCRYRYQKTLRFLEYFDTEKMIVDN
>g245
MSTATRSRTRTRSHLPRSQSHQAGIQQFAKTSKPGALPSSKSLLLDGEKPSCQLTASPSKKRKLNE
IAATTINENSTQSRPDLSVEDTPSKTLKFSDLSVSTPRSSRTRPVATPTRSKTTRTRSLPKQPIFK
KSTSTPNIAQVTATKTYPAALGEFLDLHSAFLKALSLHSAHNEYSASADLRELCPSIEKIWKKRKV
TAKDVQRLVQVWDEACNEKNNDDQTEKKSDSSNLRFRLANYGLGKVCIERLDVSGSGSQGMFDENQ
FQERFVQIIEKKWRDQIDAQKDGENKDETDTPFEIENLPLAPIQACLTPFNTFRKGQQRLQDLKGG
VIKVKTAMMKAASITPGEPGTSSVARDPTADRRKGLFERIKNKELLQSKLGPAPTKEQLLRRSAAQ
RAEDVARVLALLRPSYTTTANNDTIVAQRKPFSWQTLIQTIRDSLRNPIPAEEIAACLDMLAQKDV
AGDWIDVVTIGSLKSVVLMSARDISPKEIGLKVSKVQF
>g246
MFWRVDPEPDLERSAGQEDLQFDDPSWIIDHHRRNIILKIFKPEGASSTRSGDTPSSLNAQVHVES
KNSRIHDQREREYSYRINFSELQRLYLRKLQHKLVQNAVDLRYNASEPAGWAEDLREYVQALQDYD
YMGKHTRLSKDPFYVTGERFIERCMLHAAMKNRENDIDPLQYSDSVGEWEKEGIQPVSIGGTRDRN
FHQAWVKDFRKRIGVAMVGGIFLIAPMWLMVLHNTLYTSLIATTVCVALFGLLMASVLEKPIDVMS
STAAYAAVLVVFVGLTTSKSG
>g247
MSKSRLPLYLGLTAAGAGGYYLYKSGGDVDAATARAKADAEKARAKLPGTSEAEHKGKEIGHKTTA
YVDEIVDSAHAKAKEANNRFGEGAKESLEKIEKIRQDTAKQLGTTVDKLDRTVEEKASEAKKSVSG
WFGGSK
>g248
MHEIFSIPQNLELATNQTMWNRDIMAQLAHFLKMLLETGAFIIVWLLLQGFQRHGYRKGSWPRQHA
NLTGHMETINYSQKKLDFDGVDPDLGMHLLDLHWNRQHHAFLVTYRPAFMRDMACNGTYYSKLLLN
AIYFGASKFSSRPEVRRDPDDVRTAGWLYRQRVRELLGGALDRSDITTIQALLVMASSLFALGDER
SAAWLYAGTAFRMIIDLGMHVDGSRLSNQRRFSDEDLEIRRRVFWGAFVVDKIQSLYQGRPVSLQE
LECNVSIQFMDHYEELEHWKPFAYSETQKYPGSPAYSVSTFTQLCKLSLIMNRILNKVYGERSSKR
TQSELTQDLKSLHSDLDQWRIDLPAHLTYDPADTSKPIPPPHVLSLL
>g249
MAGGKNFLGLRGTQLRVAMVLLVVAPSFILFGYNNGSTGGIATLESFVHQFPTIDTVNTTGAQKSH
NSTIKGVVTGSYDLGAVVGSLLCIGYSDRIGRLRTVLIGLLLSIVGLVLQASAFSLAQFTVGRLII

GSAIGTISSAIPVWQSECSTTAHRGAFVVLEGLCISSGITLSEWVSFGCSFSSNKSVQWRLPLIFP
VVFSLFVIPFLFLMPESPRWLARKGRVDEARSVLAALEDESEDSPKVAQEMAEIERSLSLVTGSLR
ELAHNGEERVLHRTLLAACGQMFQQMCGISALVFYTSTIFEDLGFKGDKSKILGACLTTFQSFCAI
IPLFLIDRYGRRKLFMITGTGLAICTAVIAGTGGQPKGSNAANAAVVFVFLFDFFYPIGFLGQTFL
YATELAPLRLRVPITAVANATQWLCQFVVAQITPPGTTNLGSRYWIIFAVLNASFVPIVYFFFPET
NGRSLEEMDVIFRQSDGIWDVVRKARDHSSAVTRHDEDTEESDSVEREVIGGLKPTAQQKEQV
>g250
MPTAYSPVSVETQKPRVSSHLLVKAPDTALDLSRQKRYRLKSVLMRSGTSRGLFLHRHDLPRSPSD
WDSVLIGAMGSQNNDPRQLEGVGGATSTTSKVVVVAKSDRPGIDVEYTFAQVTVGQEKVDMTGNCG
NMASGVAAFALEEGLIVAAPGQMEISVRIFNTNTRSMLVETIQLDGNGHFLEEGDYRIGGVKGTGS
KIKVSFIKPGGSMTGKTFPTGNRQDELVVRSSGSGSPLQEPFTVRATLIDVSNPFIFVDSSSLPPE
FYVAGPDSEASLGIIEAIRCEGSVRFGFASDIEAASLRKGTPKIAIVSSPVVSNSSIVGRDTPDIS
VLAYSMGKVHPSVQLTGAVCLGAAATIPGTVVERLKMRKSRVGSLDQMETPQVQPFDGKNSDCIVI
GHNSGTIDVDVKVTEDSDVESVTVFRTARKVFEGTIFVSA
>g251
MQDSSPKSTTHIIDDVEKGNIRQTSSVVVDAERERALVRKQDLRILPLSAGIYLLCYLDRSNIGNA
KTLNANTGNSLLDETHMTNYQYIVALMVFLIAYALFEVPSNYLLKKLRPSRWISILMLSWGAITMG
LGGTHSFGEVAGVRFLLGMVEAGLFPGLVYYLTFWYRVSERSLRVAIILASATLAGAFGGAIAYGV
GHMNGSSGLSAWRWLFIIEGAPSCASAVLVWFFLPDYPETCRFLNEEDKALAKQRLSVEGTKGNAS
AMTWADAKEILTEWRLYAHYIIYFGISTPFSSLSLFTPTITAGLGYEGLLAQLMTVPPYGVAYVVT
LAVSYSADHFNARGLHSAVMSFIGAMGFLASAVLPADAYLHRYGCLIVAASGAFSCIPPLLGWLSS
NLHSTAGVGLAIALNISFGAPGQIVGVWIYKSDQATTGYPTGHWTNAALLLLVSAGCIGLRLYYGW
LNRKSLVQSTVYAY
>g252
MRQGAAILTKEPTVRRDVVERLERRLRQTEATNRALTERLAALNAKSTPSVASNFSTQESPNENDC
RNVISDEISFLSLNAGGERHFLGSTSGVLLAHLIRSATVEDDNSLAEQLNTQRPDAALGSRLSARP
TQLLTSDKELPSCPPESVARNLHQAYFEHDHLCYPILDQSSAMASLDQIYAEPSALERNPYEAFVF
YMIVAISTSSVQNLESQSLPDAKIYQKQAISGLNNILASGGLKALQAILLLCQHQLISSTHETSTS
LWHMIGMAVRMCFEMGLHREETYKYRTNPNTPAGTDLGIRRRCFWCVLAMDRVVSITLGRPLAIHL
EDVDLALPDCGNAQLSNGSQMDEMALPKEQFQTAVFVHITRYRILCGKIMVSLHRSRHSSNESLAL
AAQTELANELEEWRSKTNTLNLNSYSPYGPRPSSFLTFDWYELLYYNALLMLYRPSPALPLTSSRA
LVAVPIIYGAAKQTISLYSHLHSLRRLNYTWITLHSVFMAGLSFVYAAGGHFRAKKTSYDQRQEPA
MLKADPSILEIVNVSRSCSNALVAVSERWNVPLYCHRVFDRLSDAVLKDAIDYHVSRGVATCPQPA
PSPPLLDSLPPVVASSHFTMDGPTPNGWPIHDAGASPLLAVDNAFRDCFKDLQHFYESSFGEDPIG
QLSQDWLGQIGGPPDIWSVNDR
>g253
MPFKRPIRIGNVSGATGDSPHAMKRMAQDATVDVIVGDWLSEMNIAWNAIAKDNDPSQGYEPGFLK
QLTESIDLITEKGIKVVTNAGALNTRALAVKVQEMCHSRGHKDVVIAMALGDDISHIVTDPEKRKS
LLHLDHSEWALKDWHLEPYCGVAYIGAWGIVEALNAGADIVICGRVTDASPVIGAAAWWYQWAKND
WDRLAGSLVAGHLIECGAYVTGANFSGFKSLPSPLVDLAFPIAEISEDGTCIITKHESHMGRVTKH
NTIAQFLYELQGEEYLNPDVVANMHEVSIEEITQDRVQVQGITGSPPPATTKAMIAAKGGYQAETT
FYINGLDVMEKADMMRNQLQYIFRDHRFTKLSIELYGSAALNPISQQAGTVFLRVFAQATSEEDIC
AEKFKIPVYALRMQSYPGYHMDLDFRTMDPKPYMEIFPVVIPMTALDQQIHILGSKITSKIDIGPP
VITAEYPVIRRSYETANPVNLADFGPTKLVPLGTIVHARSGDKADNSNIGFFVRHNDEYPWLKSFL
TVPQLKTLFGDDWTKGENHAERRVERCEFPNILAVHFRVLDFLDGGIASSSRIDGLGKGIGEYLRS
RLVPIPSKFLERGHI
>g254
MKIMDFSRYGTASKEWLDFVSRNPQASRDGYNDNDIQLASQLRESANKAREATSQRLLSQHDLHKK
VNISTLSIPSRGSHSIPLRRYSPSSYNQNGERAENLARCLVYFHGGGFLFGSETTDDYLCATMADS
LKIVIISVIYRHTPDWPHPSQNQDAEDAMLYIEKSATNLGIDIPSGLGVLGISAGAGLAAAMAIRD
RSHLRVISSVTLSIPWLIHVENYPFELFESPENSSKIQCRDAPVIPWPRLKMFSNLVDGGNNNSLN
KDPLLNAALRADEELRGWPKTAILVAGMDPLRDDGLLLATRLKALKSTPGINVLNATVLEPEALNA

TGILNIYTLCQVQGTITYAAGGNDTPDPKGKNTLTWELYLPINSEYNGRFMVVGNGGYAGEIVDSA
MMAQLNLGYAVAGCDSGHSLADSGNTTYAPFLADKAMVKAWIHNSIAMITPITKALTTEYYAKSPE
FSYYYGCSTGGGQGYALAQYHPDLFDGIYAGSPGNWYSHLILSFLWNGLHTAGSGYMSQDALNFIT
DSVVAACDALDGVVDGLIENPLNCNYNITQLECKSGRNPISASGTVVCLTVDQIKAVQAIYAGPKN
LVTGGEVYPGMSLGSENGWIYQETTLYEYYSRLILQELVFDDLAYNVSTFNWGTDVRKVDELASPL
IDEISPDLSAFRKSGGKLITTQGWADQFNAAIWPILHLDQIRARMTGVDIDDFIEVFMVPGGGHCG
ANPEYPHVPGTYDVLDVLVPWVEQGTRPLQMLSTTPPDGTNTTRKLCPWPSTAQYVAGNIDDWTSY
ICA
>g255
MALTTSTTNSEAPIRPSRNLADRVALVTGAGSVEDEIGNGRAISILFAEDGASVVCVDRDLSAAER
TAAIIMAEGKGGAVAIQGDVTSEDDCRRIIDGALTRFGRLDILVNNVGSIGARGAAPEVDLTDWER
SLRLNVTSMMLMAKFAIPAMQKNVPGDGNIRGSIVNMGSVAGLQGGTPSLLYPTSKGAVVNMTRAM
AAQHARDGIRVNCVCPGNSSP
>g256
MTTKVGHYEEEQVEDVELKVPTTNARARLVPRPSEDPADPLNWPMKLKLLILFEVCWLAFLGTWNT
SSINPAYSLLAKDLNVTAIQASYQTTIAIALNGVGPFIWIPLANVYGRRSVYLLTTFIGFVTALGC
GFVQTYGALVGIRAINGLFPVSMALGPATVTDLFFYHQRGRALGLFTVMLTSGAHFAALFGGPVAK
FVGWRYIFWITAAMNFATLVVLIFGLPETVYYKPRTYSDTSTEAPELTIAQYRQLLRPWTTFPGVR
LKARHVVIPAFRMAMYPSVLFPSLYYAAQYVFTAIFPAVTYSIIFQKRFGWNPFQCGLAYGGTMTI
GSLAGELVAGRIIDDVIRREAKRRNVDSPPPETRFKGLWTGAILIPAGLLIFGFTMRYETHWSGPL
AGMFIGIFGIQVVATVCYTYSIDSYRVESSEVAQFFNFCRQITSCTIAFYGIKLCEAIGYQFAFIM
FALVGSVLAFLPIIWLMWKGQSVRERLGMPTNVSVGEEIITDYNAAYRNESIE
>g257
MFIISPMSACATEDKRDQFLDYISKIAPVTYATEPKCHGYAWFRSAEENDTVPQHWVKGLEVYEDI
EANTQTHRASTEYKTFRAAVGSEKLLERPSDLRFWRPLLGFMNRKENDPEANQFFAHNRLLSAETC
QYIVVDELLPKPRYKGSLLESVSKLAQMAEQNQNILSFWVLKHEDKDEDPGLLVFARYVNRRAWTD
FEDREEISAAWKEANYSCQWPRRTVWVDSGIGFLGR
>g258
MASLISIPPFNELPLHEMDPPHSAWGLWGDGHSSALGSLNYLTDELVLRAIKEEVKTGQRVSLNLP
LDMFDPPLLGRAAFKQEVINKDPLVVNDDVITFNTQGSSQWDSLRHFAYQQDKRFYNGTTQDQIHA
TSRTSINGLQAWASRGLAGRGVLIDYASYAQRRGIDVNHFSGHAIPIEDVLAIAKEQNVEFRQGDV
LFLRTGYVAAYKGLDTSGREKVSGVREWCGLGQSRETTEWLWERQFAAVASDSPGFEVRRILIFDS
STDGKRMASSPDYVGWMGYTFGGIV
>g259
MGTPGVLMRLSNNASSSKLTIPGIEAVYTLNANDGSTPSYNSLYFLNDIAPIVNSRSAYDADNKNP
SASEVSWIICSFINGRDTAGLSQEPQMRVNVLPNPPAIGSILVINGSTPRADKEDDYHAWYDQEHG
GKLTKVPGWNAARRYALAAVYGDVEVANFYGFNFYDEENGLGGPEWKAGVTDWTLRIRSNAAKPNI
RRVWKVESV
>g260
MSSLKVQKGSTNGQTLRKSGTCKTCRECRQRKIKCDGNRPRCSECNTCQRSCIYPQDARREPRPSR
SRVQNLEATVAVMLQHMKTSGIISPHLQAGDLLPDGGEQQMEQNTTEAEIIEDNSGRMGLPYQENR
WENTTSTAAIASPFQTPATSLSGLSRREISPGNCFSFPAEAADRFMERPKPFSTVGVDEASGNANE
VTIERGMDNRLEMLKSSTPIHQHSPSMTQNASSNGSGMSPCEARVAGAFHEHGCVSSVHGLATWTG
EVAIAASKARLISNAALQRQREARLVRQPANMIELDGCEPELAWHLLDLHFNRQHYAYMISYRPAI
MDSIATGGGPWVNQLLLNAIYYSSTLYSDRPCLKLDPQDPNSKVGERFYKRFRQLLVDEIDRPSIP
TVVALLLTSATLVSQGRSSAGWNLSGTAYRMIIDMGCHLMLGPDYSSNMGHGQLLRRDLEQEMRKR
LYWGAFVTDATQALYLGRPCMFAATEARVPLHFLDTFEELEEWEPYNDSQSLPENRPPAFVPQPAF
ALSTFSWLVRLFQISTHITDLYGIQVMKCTSEYLQEKKANIERELECWNTSLPNHLKFDGSGLVTP
PPHQITPHTTFHALTILLNRAFLEEGHLRRYCDDEGRRRSEEACIRSALAIQKLVRAYRTAFTLRR
APFLLSYAVYSAVIVILRQERHERGQFMEPISFFWTCLSELQRGCNFGLEKPLNILRDMVQEFQVS
IREGGAASAMTNDAYQFPSLDETFFSLPLGDTSGMLDASANSEQQHTTPDYYMGAGIDQPDLNFGT
SSPGFLLSLNQLESEISEDTLFGLFAPPQLNSK

>g261
MAPSIIPTDPADFVYKEVQKSDVKITANGTESHLNGFYSSPVHLDPAYQIVEQPIGTRRPIRIACM
GAGYSGLMMAIMFSETMKDKNAELVIYERNKDLGGTWLENRYPGCKCDIPAHNYAYSFAPNPEWPN
YYATSQQIHEYMHQVADKYDCNKYIKYEHSIKSAIWNENNAKWAIRVQKGDGSIVEDNVDVFINAG
GVLNNWKWPNIEGLHNFKGKLIHSARWDEDYDFTGKRVASIGIGSSGIQIVPQLAKVVNSMDVYIR
TQTWISPAPGINEPTAKDPDMDPDYNFTKETLELFKDPEVLREYRVAIMDRRIENFKRAIADSDVQ
KRAQEMFRKSMIERLGDSEKGRKAAEYLLPDFPVGCRRQTPGPGFLEAVTQKNIELRWDDVVRVTE
RGIVTRSGETKEYDVIVCATGFDTSFKPSFPLVGRKGVDLAQKWNTEQPKAYFGFSVPDMPNYFCF
IGPNSPISNGSLILGIQATAVYIYKWLEKLQTEMIRSFEVRDDANEEYNQHMQRYLERTVWTRGCR
SWYKRGTVDGPVVAIYGGTSFHFMEAIKNPRWEDFVMDRMPEARFNRYAYLGNGFTRRERKGESVG
ATQTLDFQEYWRLFVHPEIHD
>g262
MEYELDHRPSDIDSPNNDNASDNASNETETITGIEQQYLHPADRGLHAYLSLLSSFILEALIWGFA
FTFGIFQQYYSTHPPFNKDVSNIAVIGTCSMGIIYILSPLVFGLLLGFPKLKRYSSAFGLCVMVPA
LLGSSFANTTTQLLVTQGIAYAIGASFAYAPLILFMEEWFVKRRGFAFGIMWAGTGVSGVVLPLVM
QWLINTHGFRTTLRVWSTVLLVLIGPLLYFVKPRIPIAQNSSTRPFNLKFLRNKSFMIFQLGNMLQ
ALGFFLPTIYLPTYATNTLGVNAVVSSLTVILFNLASVFGCIVMGAMVDRYQATTCILLSTIGSTV
SVFLIWGFSSSLAPLLIFCVVYGVFAGSFSSTWPAIMREVKIQETSVEPAIVFAFLAAGRGIGNVA
SGPLSDGLLQGYPFEGVLGGAYGTGYGSLIVFTGVTALLGGLSVLARPLKLI
>g263
MTSFLPVNRTPTPNNMKMEDVTSTTPRPDSSDSRLSPSKTDSSQTHSLSSHRRAPSTASTGDETTV
ADDEPDRDQSDNEHDNEENAPPSKKKKGQRFFCTDFPPCTLSFTRSEHLARHIRKHTGERPFQCHC
SRRFSRLDNLRQHAQTVHVNEDIPGDSLAATGTRFQRQIRTDRVRPPQGRARAGTTGSQSGHSRGH
SRNLSTSSIASTASSLSQAPDLRRRPPPLIMANDGSARAQLSLGAMAEPTTPPSQIARSIPGPSPG
HGYPAFSAGGGLSSHYASPMAATSHQPGYWDARGHARRLSVPSGPNPFTNHETYPPSYVTSVPPSH
APYTPGSSVYASPTSSSFGPSRDEGLSPSEAELRRRTWHPSTYPRPATSGLTKYEESANTLRPAFG
AGNTAGQTTRLPGIESFDKVVQGRPLTPPLRKPSPMQIDTPSRGPPYTPGFAGQVPSSRPPPAMSG
LGHRRAQPSWDLHYNLTGLNLGSSNPSYKEASTPWGQQVLGEIHSVGSRPMSSGQPFTSTPRQEPA
TPQNTQRYSIQAATATPGSHVTRTSPEDSSSSEGVATPSTVSMEYHPAIVHSNGHVEPAVAHEAPN
CAPAQQNGYTPTNRPGIFENPPAGASGMGRLEALVAVATSEGKSAARLF
>g264
MSTGLDSSAVWLITGCSSGLGKAFAQAVYNQGHNVVATARNTSSLSYLPDDSPRVLKLKVDVTSKE
SIVNAVSASVEKYGRLDVVINNAGYAQMGDSEGIPEEDARTEVETLFWGPVFLMQEAVRVFREVNN
PRHNVGGTIVNITSMGGTITIAGNSFYHAGKFALEGFTKAMAQEMKPEWNIRLLLVAPGGIRTNFA
GSETASLKVAPRHPAYNTGSDPLSQVLGYINSAGGVETWSDADLCAGLLVDMLVAAGGKERKYNGK
ELPRKVLIGSDAVQYVKLDLEAQLKEIEDWKSESESVTMKGGANLDFMKSA
>g265
MEKDRIDDSSFVVITGPPQKDKSKRISHVRSMIRKSQLRRDGQLQARTIVRNKSQEVHVNGLGSPS
RELTWKMDLPWLTVSETVNKRLRYFFRQYIESSAYLHPFFRAMIPITVLHPPLMSTKLVNATAWDD
LNGPSPEISALTLFQRDIAESMLLDSIKEVTGPNANGANIDVTLISSLSLLSFEIMNGDEVGYLRH
KRNIHRLLDLRGGIDGIDVSLKISILV
>g266
MATSEASYIDYDTFLDPDFSAASFANTLVTNTNNINDTQVDLSTPLSRVLFDVQEIDTHIHNLTTK
SALPLLEHTQDRSQSSQRILSQVEEQVSSLAEGYQRLEKEVLRKWASAEEARVAAQNSLQTLRLAR
AVARCISLGRQLESQIAEVTGRVSSSTNTSGAMAAAKDDHRAMVRAATTILMLRQMFSAIGEGEEG
QGLDRVKVIRTLRSDLVIPAENTTKARAQQVINRFSVSSLIVDGSSRTSNGTQSPPPATSSSSSST
YAQTEEAKARLFSAITTLYLLSPTPKNGVSAEDFQPELLLSTLQGYIHNSLTSSLSSLARGLATLP
TLDRALAEVSGRCQDIAALEALLAGLKRPSHPLLSSSTITTTAQTTEQTSNLLKPLLHVLDTSSLP
SYYWRSLASSLPSRVQEIISRGGAAARSLRSGRDRLRNDLRECVLRGSQLPANVAKGRAVSGAPGA
GGASVVVGNWEREAAVMVSAVVGALGR
>g267
MSSTVTETQTLPTRVVGVSDAPAQHSFPSIVPAEKDEKFQSLVRGNRAGTLKLRGIPIHNDPYAKR

KWMKEHMAAAFRFFGKQGYGEGISGHISIRDPVLNDHFWINPFGKHFSMMKASDLVLVDSEGYVCE
GGNQEVINEAGFMIHSEIHKARPDVIAAAHTHGIYGKTWSAFGKNIEMISQDACNFYGKCSVYNDH
GGIALAQDEGRQIAEALGDNMACILQNHGLLTVGTTVDEAAFLFYSLDKACHSQLMAEAAAANGIP
KIIISDEVAAYTASSVQNPHNLYTEFQPEFELIVEESNGRVLE
>g268
MPPRRRPPRPGALTELPPLKIVRKILLLQALYYVCITALVIFTTLVAGTVFSLDLIFGWDSFRGDT
TVGWMLSLVWILNSFLCVIFLLIFVSRSKLIPDFALTIHFIHLLVVFFYTHSVPRNLLWWALQLAS
AALMTFVGIWACQRRELAPITFGLGLGLGGPSESASGGDGTTQAAETGESHQPDSLGDDLENGMPG
FSRGRRGRNREEYEMVPMKEVEGHTA
>g269
MSWLFGSSTAGPQSAKASDGGRIAPDRSSREVCYNGRDLFFSCLDRNDILDAIKHDSEARKKCGKE
IAEFESACSKAWVKYFKEKRVMEYNRDQTIAKIQKEDAEKAAAEAQVKAR
>g270
MTSNSPDEAIHFRGKTLTPESPRPLHIPEPSNIPVLKNQMDPVFNDTSTYENPSPYQDDEQPSQPE
SDSELYKSANEDENKKQAALLGTVNVHSTLDAQSAAAQIPLSTQPNAAPYHQMGVPSTVASTNMLH
PSMANPEIVAAPSNSAQAEAGVDLQNLLDNLSPSTSTAPSGPVVTATTSSPADNPLLQDHKNPQSS
AVPDHSYALSAGLPPRPPTQQMPELNPNYPSTADISDYHPLSIQGSNTSYVPQPQGHDQHSNASIF
VPGVAPGTSSGANGLPPPPLATFQQFQSQPKSVSAPEATAQSSHKSSRSERYNARTGGSPDDDVFW
GDEIQKIYDEFLREERAYVTEGLWDRFPYGSRLFVGNLPSERVTKRDLFHVFHKYGKLAQISIKQA
YGFIQFLDAESCHQALQAEQGGLVRGRKIPPDQGQAILSLDVNRCQGVLGHHRTTLAADPPMDVDC
AIKAMTATIVPMKTREEVSVTSEMIRPPDVVTITDLLGHHLLVLSVTGTVVFVQEIEPQTDLSDVI
DADRDLLTVEIDDIEAPAHEHARDLQILAADDVDMGFAYHVENSFKTRGLRTDILVLSRVPVDVAI
QRQMEEGVFAVIRLTRSQQYSGKVTLLMFDRSAGGASKPRSMAVEMTVQMHAVRRAGVAPNFYTPN
PTYPVPSLPNQFPQPMPHAQPAMPAPQSNQFANLIQTLDGPALQSLLNTLQQTQHVPQAAIPSLPM
APNASNPVDLASLLSNAQRQQYPLAAAQNQGPQPQATNPFGLPLSAQPSNRLDPNLLALLAKGAAN
GGPIQGGQAAVGPHVQNIVNQLAKWKQ
>g271
MASGKVSKKRKAVTRDVEPDTSIVSGEELNNLDLSDGAHDLSSDESDSEVEIINEFSDDEEEIESE
IDSDEVPSDIEIEAKKRTEDEDDFDQPNYRVEIDANGNERYVYDEINPDDNSDYSDADDNANTIGN
IPLSFYDQYPHIGYDINGKKIMRPAKGEALDALLDSIEIPKGWTGLTDPSTGKPLELSQDELELLR
KVQMNEIPEEGYNPYEPTVEWFTSKEEIMPLSAAPEPKRRFVPSKHEAKRVMKIVRAIREGRILPY
KPPAEEDEADDGLTRYDIWADEAPRPDHIMNVPAPKLPPPGYEESYHPPPEYLPDKKERKAWEEAD
EEDREREYLPTDYAALRKVPGYESFVKEKFERCLDLYLAPRVRRSKLNIDPESLLPKLPSPDELKP
FPTACATLFRGHTGRVRSVSIDTTGQWLASGGDDGTVRVWEILTGRQLWFAKISDDEPINVVRWRP
GQDAVVLCIACGDDLHLAVPQIVNPELEKASLELLDAGWGYAANKSSTKNGDADSKSTPTNWIRPS
SELAESGVCITIPLRYVAKSVSWHRRGDYFVTVCPGSSTPASQAIAIHTVSKHVTQNPFRRRLKGG
GPPQAAHFHPSKPILFVANQRSIRAYDLSRQLLVKILQPGARWISSFDIHPHSSSTSGGDNLIVGS
YDRRLLWHDVDLSAHPYKTLRYHSKAIRAVKFHPSGRYPLFADASDDGSLQIFHGSVTGDMLSNAS
IVPLKVLRGHKITGELGILDVDWHPTQPWCVSAGADGTCRLWC
>g272
MPYRNKGRPSLSHGLDKEIYQIVRKIADEQQAEAGKTRLTTYSVFDTIKKSNSSLNRKPKKLLEDS
IERVLEVIQSDLAGGDEESDSVNGDFEGLEDEKVDQVVESNGLNRSIVSAWSTSAKQSPAPRKGNE
SSTVEASAQSTGSKRRQNAGESVPKRRKADHTIDKSPPTHVSLSDLGGMDEVLQELGDLIILPMTR
PQIFTSSKVQPPRGVLLHGPPGCGKTMLANAFAADLGVPFISISAPSVVSGMSGESEKALREHFDE
AKKVAPCLIFIDEIDAITPKRESAQREMEKRIVAQLLTCMDDLAMEKTDGKPVIVMAATNRPDSLD
PALRRGGRFDKEINLTVPSEPVREQILRTLTRKMQLADDLDFQTLAKRTAGFVGADLNDLVSTAGA
AAIKRYLELLKSNTGEEMDIEDVTEQEVTEKVKEIRRLIKHAKEAPIGQESNPVLVSNADFFTALP
KIQPSSKREGFATIPDTTWADIGALGGIRDELVTAVVDPIKNPEIYAKVGITAPTGVLLWGPPGCG
KTLLAKAVANESRANFISIKGPELLNKYVGESERAVRQVFVRARSSIPCVIFFDELDALVPRRDDA
LSESSARVVNTLLTELDGLGSNRQGIYIIAATNRPDIIDPAMLRPGRLETLLFVNLPGPSERAEIL
QTLIRNLPIEFNDQMRALAESCEGYSGADIGSLLRRAGYSAIKRRDMIRFEDFVAAREHIRPSVTD
MRKYERLRRDWSSGV

>g273
MGNTGSTNKISPQDRAILNLKIQRDKLHEYQKRIIVLTDRETAIAKECLSRNDKKRALLALRRKKY
QETLLTKTDAQLAQLEQLTSQVEFALVQKDVLFGLQQGTEVLQAINKEMGGLAAVEKLMGETEEAR
AYQEEISQMLAGQMSNQDEDEVEDELEALQREVEGTVAMPNVPTAALPELTEEEKEQKEQKVRARA
KARAQERAAMLAA
>g274
MSGHHHHDHGHGDHCHDEGHDHSNDITPALQSLLYSQIQFDMVSTLNESVPRAGTAILQKSWAERL
NEQPELESDADEQLLMHIPFAGQVKLHSLLIYTAPTPSAPKTVKLFKNRNDLDFSMASDLNPTQTI
EIPQPVPGTDVYDLPLSRALWNTTTSITLFFEDNWGEEETTKVGYVGFKGQFMALNREPVSVMYEA
AANPSDHVAIQGVNGIGGRIMPGQ
>g275
MADDFSLSSTFIPALYKPSSLLPIARYREKLLYLIETFPVTIVVGQTGSGKTTQLPQFLDQAGWTT
DGKVVGVTQPRRVAATTVARRVAEEMRCKLGEEVGYSIRFEDVTSSATRIKFLTDGLLLREALVDP
LLSRYSVIMVDEAHERSISTDILLGILKKIMKKRPELRIIISSATLQAEQFLKFFCGDKSTVEGPD
EELGGDVGRIISLEGRMYPVDVLYLENPAEDYVERAIKTVFDIHSQEGDGDILIFLTGREEIEQAV
QLISERAAMLHPKAKSLLPLPLYAGLTTEQQMYIFEPTPENTRKVVVSTNIAEASVTIDGIVFVID
CGFVKIRAYNPKTGIETLTATPVSKASATQRAGRAGRTKPGKCFRLYTQKAFEALPETGTPEIQRS
NLAPIVMQLKALGIDNVVRFDFLSPPPAELMTRALELLYSLGAVDDYAKLTKPMGFRMAELGVEPM
LAKVLLSAPSFNCLSEVLTIAAMLSLQGSLWVQHDGGKDEAESARRKFAVEEGDHMTYLNVYQAFI
TKGQKDSKWCRDNLLNYKSLTRAVSVRAQLKRYLERFGLEVDSTLPISSASKEINKSEQICRCLTT
GYFAHAAKMQPDGSFKTVGGGLTLHAHPSSLMFNRKADWVIFHEILETGAKTFIRDVTKIERSWLV
EYAPEYYRVT
>g276
MSDEGKVQTPTKYSGGANNSNPESPTTARPLDFDDEPQETGVLSTPSKSENTAEVPPPKPPRPADP
RQQAEETLKEAFPSIETGVVKAVLTASGYNVERAFHALLGMTDPTAQEELPPPPPAKPPRPSYPAT
STTQQQLREDEIYARRLAEHFSDSQPRGRQGQEQSYNYGDEYPEERERNFFDDDLPVIRDNIRKGF
LETQSKVNSWVQNFKKRLDGDEEDEQPASDGYLQSHGETQTYGRARRSGDSTRRSGDRERYDADPQ
VLGDDFSALELRDAEAPPPRPARPLANIRPANTSQERRKVSFQDGPPEEIDNLYDAPKTKTPPTGG
KSSKWQPLSTTSHSPVGENDPFSLGDSEDERDNKPKDVPAGEDNDLQKLTAEAMESEIGSDSKDSK
SSQVPFIDLRFLAAIVVYTYLEVNDICNIMTFSLNMLTTLTLILLVALLDTVCATPTPNYPINSQL
PPVARISQPFNFTFSSGTFTNGGTGLQYSLSNAPSWLKVDSASQTLYGTPGAGDAGAPQFLLVATD
QSGSGSMSVTLVVSSDLGPQPGKSLLPQLAKNGPTSGPATVFMHPGRSFSIDFDPKEIFANTQANT
VYYATSAPYNAPLPSWMYFDSSSLKFSGNTPSNPSSVPQSFGFNLIASDAAGFSAAMVSFEIVVSQ
HILSFKETAQTLNFTRGQPFSTPYFIDGLSLDNHSVTADNLSHFSLDGPSWLKLDNDTLSLSGTAP
KDADNQNITVTVGDIYGDNAKLELYLRVSQLFAKGVESCNATIGDEFSYTFDKALLSDDTVQLQVD
LGAASSWAHYDTATETISGDVPNDLTPQTFPIKLKATQGSVTEERNLNLSIFKSGKGGVVSDNQTS
SGTNHGTNKRKAGIIAAAVLVPLAVIAAASILIFLCCRRRPSKATTTKQIDEENRAEDRHLESTPV
AHPEKNLAEAEVAEIPRTFSNSSDSSHDSAPPQLQLDPLWETASLEDEQQRHTQSPSAHVLPQNFV
MDWGNVSIRTDKTHASPNVSPTRSTSVSQTSPFTRRGSRRYSRREPLKPIQARSLKRDSIMSSKSK
RNSRRSSGISVAAGLPVRLSGAGHGAGGFGPLRTDMVGTSWYTTQVSLQSDDTSIENLATMFPRPP
HVRNRDGSISTRHRSYQKRVSMNSNRPLSAEPPEPDSLEAFIQGRARSRNSGNPLFSARMNSSGST
GYRALEKARRSSSIAETTVSTSTFADDRQQAELRPISTISASVYGDDNRNSVIQGRRMSQISEVGA
FPVNRNRTSQGLVQRYTEAIAELPRFWSQASMGSQPRFESPGESLTGSDDYYDLIDERENPEGGRQ
WYPLNTQTQQHSGTEEVTNAADSTRQSGELSPISDAGGSRVRRMSLLRTGGQDSSPTAIRHWRLAN
TQERRPSIEDSDGLQPTTNSSFRGDLAFV
>g277
MQGWRITMEDAHAAILDLQAKYITKSTESTSPDQRLSFFGVYDGHGGDKVALFAGEKVHQIVAKQE
AFSKGNIEQALKDGFLATDRAILDDPRYEEEVSGCTASVGVISRDKIWVANAGDSRTVLGVKGRAK
PLSFDHKPQNEGEKARISAAGGFVDFGRVNGNLALSRAIGDFEFKKSAELSPEQQIVTAYPDVTTH
EITEDDEFLVIACDGIWDCQSSQAVVEFVRRGIAAKQDLYRICENMMDNCLSSNSETGGVGCDNMT
MIIVGLLHGKTKEEWYNTIAERVAKGDGPVAPPEYGKVTAEFRGPGVRPGARHQFDDSPDDYDLDT
DNTRRGFGVKSGRIILLGDGTEVHADQDDEELFDATEEDKGDQNQVRASTANAGHDTGRSFREDTP

GPELTLRNAIQSSETGQQVSASPAAINADTSPEKTEESQATSKPSDS
>g278
MSSPSSSAGKRKRSASTAHNNTIPNTLKPAELLQPSSRDASGEEGDDSTAPTDERPLSRHKKNPSL
SIDPNHAHPSKRARTRSGATAADAQHTTSNGDSAAVNNEDPGEPSETTEASADIETKSKRKSIAAN
GDVDKDEFLQPPLRAGLQDPVGYHTNPPPTGRAVRVYADGVFDLFHLGHMRQLEQAKKAFPEVYLI
VGVTGDEETHKRKGLTVLSGAERAETLRHCKWVDEVIPNCPWIVTPEFLEEHRIDYVAHDDLPYQA
DEGDDIYAPIKEQGKFLVTQRTEGVSTTGIITKIVRDYDKYIARQFKRGASRQELNVSWLKKNELE
IKRHVVELRESIRNNWNMTGQELSKEFRQFWGNSRSNSPSRGSIKNGLDMHSPRSSFSISKTHLSR
GLESPTGRPESPGPAGRNEDFATGYSLGLIGGVRAWMSRTRTPYLSGPTSPVASDDDRDAPSYLES
GDERRRPSLLRGDTL
>g279
MSNSPTLVFIPGAWHRPTCYDKVIQLLQAQHGLKCISITLPSTTGSPTATFKDDIDAARKAVSQET
NHGHNVVVIAHSYGGMVGNSAIRGFTQPPGTISSQRSQTTGYVIGLILIASGFTLTGLSFMDPLFG
HPLPFFRIDNVTGYAELVTSPRELFYHDVPAEEAEFWVSQLTGQSLKSLFEGGEHAYAGWKDLPTW
YIGTIEDRGLPVLVQRIQVGMAREMGGSVEHRELQTSHSPFLSQPEETVDIVLEAIRAFTWELGEN
QSLTSGRSGAVAVPKTTVWQPSTGSILRVDLLYHYIYHIEYKMRHFDAWILRDPYSIWHYYSTGRR
WQESVRDMIHSRQICAPLVDSSNSAPVDPLACSISSQSNCPLLQRLPPEIRLMIYGYVFGDEAVHL
VQLKGKIRHVRCFQHKSASDIPSHRQCCPETMARWRSSSFAAAAGNRSDSHLYPHTHKSLPDKLSN
SSLSLLRTCRAIYLEAADIPYQTLSFDVDDLHTFIAFSLTISSEHLARIKRLTVQWTPIWQPMTGD
SSSYSQTGSNKSSIYSHTHNDHLWKIFWTRVQALEGLQGLHLNLDLGRFMGNAMNTVPGAPGSVLQ
GGANSTKISLDVAEPWLQPLMAVRGLKEFDLCVSARCDASARNSLVEQIGAEAVVLRDCLREVMCL
PREPSTTTGCTVPVSFLKDISASCGMDEYLLEGNVQRPQLMITAA
>g280
MSQRRQYSSCDSCRRSKRRCFFSGPSEGDMCTNCRRLGHACTFEFAKSRRAMRERLRRSQSHSHLS
INRNALPEIADDGLSIGSKSQIEAGPLTFPDTLVPWVTFDEGHSNEVNSHDNVGLDLIDLFMSDTV
PPAQALSEAQLSLYNRPLNRGRSITPILPGSSPSSPIRLLNSKLDATILDDRLVRIHNVIITGCAS
RFADYNCNLFATASRYRIEGWDGASTQDRSPQPDLGVAPTGVACTLTAIGAVRFLDHFGGLYGNQL
SPAARKQSDAALKAVLRTFSLQWLSTSEGMPGPNSIRDASRNAFYDSWFEAREFLRDVQSVRSFRI
VYAILLFEGIATPTEASAGSPHEFLDIGLQTLRLLDALVKKYCTTLGVHSTYGMLLETSMTLVRWG
GYIRDIGASLTTYRQCTLPPVSCHGKELFTMESDFDCLKPQNFPSDLDARIPSICQKAVAEAFSLW
RQIVEVKNSLHRGLHSACLETIASTVAAVSKFNQLFRPFINHCIDNLECLSIHSRVSSVSIALFWE
LSVLILDESLDSCTRENDPVHDLPIINMHAHRAEVVSSVANTVERVLSLPTEQIFNLENGLDAEVP
ILAYHITPSLTATVFQKTIERLIHAENLELESCATNSLGMDPVRQRQIDIIMKGLSSLTVTAGGSE
AAAAVLQSIMSRYGDILSEXQKQEASVAMFYTAAISAACSLLWLFLSISWVLLFHPLAGFSGPKLW
IAFPVFRYISLVRGRLDVDLRAFHRKYGEVVRFGPDEVSFITSQAWKDIYAHGHGDRQLPKVLHST
SNPSDIISANNADHRRFRSALAHAFSDKGLQAQELILRSYMDKLIQRLGEIAASPNPKANMVKWYN
LTTFDIIGDLAFGEPFRGLDLSEYHFWVTTVFQAVQGMAFVKLHDAYPLVFRVLSPFLGSKSLMEA
RKRQIEYSSLVVQKRLQSAIHRPGDFIDSMLRHRGDKEKELTDKEMEANANILIIAGSETTATLLS
GVTYWLLRTPDALRKVTHEVRSSMTSESNITFQSTLHLPYMLACLNEALRVYPPAPGGLERITLPP
DPVEISGYMIPPGTKVSVHQSAAYRSPLNFHRPDEFLPERWLSPAKKDPSSPFYTDNWDVLQPFSV
GPRNCIGRNLANAETRMILARILWNFDLELCAESRGWEQQRSFLLWEKKPLMCILKRREGRGGL
>g281
MSDDSSFSEEPEIGSHLRMFALCGPGGMGKSQVAAEFVYRSNESLLFDAIIWISADEPVKLARSFD
QAAVALGLVEAESIESRDHILTRDAVLGWLANPIKSYKQRENAQSNEATWLLVFDNVEDLEMLDEH
WPAYGSGSILITSRDPLVKSYGYSLKRGLSMPQLDTSDGEKLLIKLTDRRMLSSEEQASATAIAEV
LGGLPLAIVQMAGVITRQDLTFSEFLLRYQKESSHAELLNLKLGPSKARVGYEHTIASVWALEHLE
EGSQALLQAMSLLDPDSIPEYILEENPAAGTWSGYPLTSAEYQNARTELIQRSLIVRQKDKKTLNI
HRLIQDAVRARMTDDEFSKAISSMLSFVSAVWPYEEFSFGNEIYRFKLPTELTQHHLDGPKLFIDT
ASNTNMWGRFDDTIPLLYLAESMCDAFGDENEEDPSKQNLNKQLVVLYRTIIYERGVRALHTNDPE
GALLNMKKFNQLVRDIYHDKPPGTDQTLGVSWNELGNAYLQNNNGIEAESCFLSSKEAFEALDGAT
RISISMPLINLGFAALAQGREGEADEIFSQALADREKEYGRDDKISFV
>g282

MSPVYPGETCGSWLSSVIQVFSDRSEPSSQSTVPPTTAKRRLGTLHPVGISARVDRVSPREYVEAI
KFYSKETIQDTLYPGNIFEDLGLYLKSGVQNFGPSSNIQGTLHTIHKQGGRSLDVYSDPLRCALKL
EEDLENIITLRFVTLGSREQRSGRCDQARVDALRLDGENDMDRYEHDLLLGSGLEAGDSIVRSFSV
LDEKNFVIEQEMSICIHKYGEGWIIAIDAGSDLSNGPQGPWITPEDRRSSSPWTIFAPTIRQNPKI
WMKSNPAKSAASTRFQEKFPQSARLLAQSSGEALDTQTMVLDPFYAITDLFQFCAYSENQFMNLME
AKIKDDTNHNSLRRENPTLSNLLYCQEIVQKHLTHLRKTVKIIECSGGPLWPHVEKIHPHQYQKAH
RAKDTLLKDYEQLVERAEVILERCSKGMDVIMSNVMLAESKRAILQAQSVAKLTLIAFFYIPLSFT
CSFFSMDIVSFEKTIGQIWIWFAVSVPVLLISISFLSLDRLKLGRAWLTVKSVGRGAEKVK
>g283
MPPPRKRGRGSFRGSSRGARGARGARGRGHARGGRNSFQTSRVDEKRELESSDGEDQFNGFEDDVS
SADEPMQHEEVASESSSEDEEPSTERPYNSLLQLLNAKSETTHARKRRKLDHVGEKRDTTSRELEI
QVTTEELEELDVLENQEASDEEDNVGADKAGESDDEDEDEDASDPFETHISSPNGTETAKRVKEIT
ASKWRSIKSTLPETFRLIYSIPDGENGSWSMPSAVRNTRSLKLKKRLVSTAAERIPSFQGVYQNIA
SLVFNYSDVLFGDRSPSNAAQMRDILSLHALNHVLKTRDRVIKNNARLSKDTNEDIELRDQGFTRP
KVLIILPTRQACVRFIESISKFYQPEQQENRKRFMDEYNAEDNESWESKPDDFRDLFGGNDDDMFR
IGLKFTRKTIKYYSQFYNSDIILASPLGLRTIMDKEDEKKRDYDFLSSIEIAIVDHADGLLMQNWE
HVEYIFEHLNLQPKEAHGCDFSRVRTWYLDDRARYIRQTVLLTSFLTPEINSLFSQHMQNIAGKAK
VLPQYNGAITEVSLPITVKQTFSRFDSTFALKDPDLRFKYFTTAILPALARSVTGKGEGNGAGTLI
FIPSYLDFVRVRNFFSTSSQATNISFGAVSEYTEQSEMSRSRSHFMSGRLSVLLYTERAHHFRRYN
IRGVKHVIFYGLPENPIFFREIVQYLGLDPGAVAGSETLDVRAVFSKYDAFKLERIVGTQRAGNML
KEKGGDTFRFMGDVSRTNESEQSEVEREDQTTTDESEVTRTDTISKPDELQYEEPGSIYLNDLRPT
ADRLFGDNLSLYAGNVSNGQSYGQSPTQPGPSPTSVPESQPASRWKRSMVYRVWIQGKGMLMVVLS
QFFGSSMNVMTQLLERDGSHGKAMHPFQILFSRMIITVAASFFYMWYAQVPNPFGSRGIRGLLMLR
AAGGFFGVFGMYFSLLYMPLSEATVLTFLSPIVACYACSFLMPNEPFTRKQQLAGVISLLGVVLIA
QPFSGGNVENLASEIAPLAGDGNSTLAEIVGGELSDNDMADGVSAIHHLIAVGFGIIGVFGAACAY
VTIRLIGPRAHPLVSVTYFSAYTTTVSLIAMIAIPSVSFRLPGNLMEWGLLLGLGATGFTMQYLLT
AGLAYQPPSIGGKQPQKGNGTRATSMLYTQMLFALFYDEVVMDSSPSAISWAGSGLILGSALYVGV
IRDSGSNNNTCRSAGQDIPQSQGGEDEPDTGRVKDTSSRDIRDAEEGRALLAESDDIDAEASSSSS
SS
>g284
MAETDSFLHLARPLGPVTVGSAPTTAPLNVVIQPQAIFSILDHSLRRNADQDRVIGTLLGTRSEDG
TEVEIRSCFAVGHTETTDQVEVDMEYQKQMLALHLKANPKEVLVGWYATSSELNTFSALIQNFYGQ
SDGTWPHPAVHLTVSTEPGKDIETRAYISAPVGVTVERAADSAAFIPVPHEIRYGEAEKNGLEAIA
NARDAEDRQASIFTDIEALERAVEEVIGMIDRVSKYVEAVIDEEAPASTALGQFLLNALALAPKVE
PADIERDFNNHIQDVLVVSYLANTIRTQMELSNRLATAQLTLGGGEGATTGEGAQRNQRGGKGGRG
GREQRNQERNTEEARA
>g285
MAEPRARAARHKGQMNFASDLRTLLSGYGDRDAHPFCAPGPLPETVRVLDEVVTDFILEMCHEAAA
YASYARRQKIKVDDFRFALRRDPHKLGRVQQLLQMDRELKDARKIFDHDDDQVGTGRKVAEELDVS
VDGTVATGTGKGKGSTKGKGKRNAGRRGSDATEETVAKKRKVDG
>g286
MPNFPKTLARCDIVAQLSTSSRGTSQKWIPKGVEPTRRAPGPYTITPDLIRTNRTITNGLVLSSCR
IQETRRLSSHVLRHLIHTQSSLSTSLETESSEDNLTTNIQNDGFQFHPLPIRQVKIDAPRITRYES
QSIERINPKRQKAALQVREKKFLWVDPKLALKQIKNPMKHYTKEEAGIPFKVKDSVRRAELALRKQ
GRFITKQAFDHDIKIRLSTMFSVEGVPKVWISYFNYLFQCKQDGKMDSWQPPPVIDTGTHQLFNLL
REKGLESFREQWEVYGERAKYRQWPLVALHILSTCPSLMPDFLIGTDCAPYPPFAMVSDCMVYMHL
FHKEVDHNARKAAILRCMDPDRWPVVNLPQRGVRLYAMTAGKENAYRAMEIMRSRDSQVSAYTLLA
FMNVCTQAGDVDKALECLRAIALVEDRQGLSMNSEGILRHCCKLLELDEVVDENGVRNFKILPQIL
KLGVTPCLEMMNVVLSNAFKTGDPHLGLDMLDYMKEQGMEFSSYTYNILLTDAVAXXDRDRXDALL
HEINLHPELKDNRFIASKVFHSHYVFGAKDAYRSDNPGRHLMDMLDVYCRYYDPTPLKELGIIPER
FNTMDIPAEDQPSTIILVIMIATYLRCNPRHVATMRLYKRFCELVAQRHPVIAPLVQHPQVYNEFI
ISFRPYGSDLRDCVSIVEDMICAEPFEIDDTVIHPAKPNDFTWNTLMSVFVSHRDMEGVASVREIM

RQSGIKFTDVTWATIINGTVRNQDXPATIAALKEMDEGGFAPDSYTVKALRLAHDPERLQAAIEAL
DKETEQLVEQEMKVIERENDELMDRGLRRLALASKKY
>g287
MRLSTAIAALLTVGLAAAHSRRATCAAQNILDACLATENAQLSSCAATDWDCLCEQSNNVLTCYNN
CPGDDGQFGAQQTMTSYCNAAKAYGTSTSSTASSSATASASSSASSTGSETSATQTGTATSGHSSS
TSTATGHFTTSSQSAAAAAGTFAQGGFVAALALGLGLVF
>g288
MATEAADIPEEQLNAQPQGVEDEEDIAAMKKRVAEMESEAAKLREMQQTLDAESESLREDKEDIDA
RSIFVGNVDYGASPEEIQAHFQSCGSINRVTILLDKFTGHPKGYAYVEFAEPSLVAQALVLNESVF
RGRNLKAWPEVEAEAVVAVAALVAVASHTAAPIVEVASRSAHRATLHEEWDQIFQLSPIYGLQVSI
RIPAQPVYENTRPNANALTYTVIAADGLYKRDVFRFPDPFAVATIGGEQTQTTTVIKKTLNPYWNE
IFDLKVNEDSILAIQIFDQKKFKKKDQGFLGVINVRIGDVIDLQMGGDEMLTRDLKKSNDNLVVHG
KLIINLSTNLSAPAPNQNGANRPQQPSASLSGLVPQVQTPPTTQSQSFVDGPTPASIAASSTNSFT
PTRASSNARPTSGLSAPTEPQPNGQATQGSRTNLSSFEDNLGRLPAGWERREDNLGRTYYVDHNTR
TTTWTRPSANYNESAQRSQREANMQMERRAHQSRMLPEDRTGASSPNLQEAQQSTTPPPGASSASN
AANAASMMATGATTAGTGELPAGWEQRTTPEGRPYFVDHNTRTTTWVDPRRQQYIRMYGQNATGGN
TTFQQQPVSQLGPLPSGWEMRLTNTARVYFVDHNTKTTTWDDPRLPSSLDQGVPQYKRDFRRKLIY
FRSQPALRIMSGQCHIKVRRSNIFEDSYAEIMRQSASDLKKRLMIKFDGEDGLDYGGLSREYFFLL
SHEMFNPFYCLFEYSAHDNYTLQINPHSGINPEHLNYFKFIGRVVGLAIFHRRFLDSFFIGAFYKM
MLRKKVTLQDMEGVDEDFHRNLSWTLENDIEGIVELTFSIDDEKFGERDTIDLIPDGRNIPVTNEN
KHKYVELVTEWKIQKRVEQQFNAFISGFNELIPQDLVNVFDERELELLIGGIADIDVDDWKKHTDY
RGYQESDEVIQNFWKIIRSWDAEQKSRLLQFATGTSRIPVNGFKDLQGSDGPRRFTIEKSGDPNAL
PKSHTCFNRLDLPPYKTNEALQQKLSIAVEETLGFGQE
>g289
MTSRLVLVIGDLFIPDRAPDIPARFKKLLTPGKIGQILCLGNLTDRDTFEFFRQIAPDLQLVKGDF
DVDSPNLPLSKVVTHGSLRIGFTHGHTIIPPGDADALLIAARQMDVDILLWGGTHRFEAFELEGRF
FINPGSATGAFSTGYFPDGQEPVPSFCLMDVQGDVLVLYVYQLKTDANGAETVAVEKVSFRKQGAT
AS
>g290
MSSQPAHSTLLIPGPIEFDDAVLQSMSHYAESHVGPAFVKTFGESLTLLRKLFQTSDPASQPFIIS
GSGTLGWDIVASNLIERGEEALVLHTGYFGDSFATALETYGATATQLKAPIGDRPSLAEIEEALKQ
KQYKLITITHVDTSTGVLSDIKAVSELVHRVSPDTLVVVDGVCSVGCEEIDFDAWGLDVVLTASQK
AIGCPPGLSILMLSGRAINVFKTRKTAPASYYASIANWQPIMQNYEAGKPSYFATPPTQLVHALHT
TLTSITAKPVSERFAIHTKTSDKVKAAVAELGLTQLATKPENQAHAMTAIRLPEGVTAPDILPGLL
KRGVVFAGGLHKAIAPTYIRFGHMGVSVTDPNRKDIDNAITALQESWAEVKKAKGL
>g291
MNSLNILSSRVIGQAPNSSTRSRSHSQGEASPANLRNDLAKARSYSEQQFHYTLPKDDKEIDARGS
PAQGYSYDGTIGYDEKTQLLNQLQKTAPNRFFLVAQRLFDAIYETIQAILSTIAAPGVYVAQTFRD
DDGYYSPIVPFRKVTRYVAFRNAESEHSRSKRTTLSHRHKDNEKSRRNGGSTRRLRSTASQSSIAS
SQSESDGDHDSAKPSTDKETRGSKSRSPDKESAATDTNNTPRRSIRIKLNNDESLKRQKRRSQLNE
APHDSTGGENTSPGAVITAESLKSPMSQSASHHRFTKYPHAPAPPRPLVPVRQPSYTMRTTGRQAP
TRVPQKTLVLDLDETLIHSLAKGGRMSSGHMVEVKLAAPVSTTGDPTTTLAPQHPILYYVHKRPHC
DEFLRKVCKWYKLVVFTASVQEYADPVIDWLEQERKFFQARYYRQHCTLRNDAFIKDLSSVEPDLS
KVMILDNSPTSYIFHEDNAIPIKGWISDPTDNGLLNLIPMLEALQYVTDVRAFLALRRGEAES
>g292
MVSNSAALTPGLSSFLKSLKTNPIETSIDNLVSLLKRRQIRLSRSCAIATAYLLLRVVSAFRTTDA
TKLIERVQSVGRRLMVAQPRELVVGNIVRRVLGLIRDEAEDERDADFFLASEAGSDSRPHTPHGVE
SHSPPRSHIADDLAIPVHDKDFLSLRAEGSERSSSRPPLSSTPSQAPPVISMFSLLQHPEADSPQP
SSPGTVSPSGRFQGQGQHKDIRAEVLDGIGEIIDELGQVDDQIAGYALEHIHSNEVILAYTSSTTV
QKFLLKAATKRKFTVIHAESFPNNHVATHSRVSGNNDNDDQSLSAENFTKPLTALGITVIVIPDSA
VFAIMSRVNKVILGTHSVLANGGLVAAAGTRVIARAAKVHQTPVVVVSGIYKLSPVYPFDIESLIE
YGDASNVIPFEDGDLVDKIDVENPLYDYVPAELVDLYITNLGGHAPSYLYRIVSDHYRKEDISF

>g293
MGKGKPRGLNAARKLKDHRRDQRWADLGFKKRLLGTAYKSSPFGGSSHAKGIVLEKVGVEAKQPNS
AIRKCVKVQLIKNGKKVTAFVPNDGCLNFVDENDEVLLAGFGRKGKAKGDIPGVRFKVVKVSGVGL
SALWKEKKEKPRS
>g294
MSLAPRPKPASRISSQRLPPPSLFQGPPSRNESSASVHVGLLPGNQDPASAQRPGVQRARSRMSGL
SPNGNRSLSPFLSRTQSPRETDSAEILWQEMQNTLGEVELSAFKGEHVFGEEHSRALEELRLKQLK
LAQTWTRNEADEVDENESKETNSPSDTKSQKASTVPHDNGTTTTTDGAASYKALEAETEKDLQQAR
KRRLANDQYFDRVNNGVLDVVASLEEVAQAMRAVRKESEDIWNDTASESLNVTADSITG
>g295
MGFMAGFFGGFTLTASVLYLSVQVHRSTRLAQRDAIREQVEILNDISSPLGAYYRRFGQENDLQKS
AIAPLPPPPQKKPSAEELLKQQWNKEVEALAKKTVTLRWQDVTNTATEVARVISDLVKRQ
>g296
MAGGAAKYRHLSRSSSHRQALLRNLVTSLIKHESITTTWPKAKEAQRLAEKLITLGKRNTEHARRR
AQSIFYTPHDLLPKLFGPLRERYAERQGGYTRVLRVEPKKDDQAPSAILELVDGPKDMRFAITAKA
IARQREQGIQTLNELTALNVRKVTRYRKDGVETLEKEIEKMTLEAGKKKNKKNQDEEEGGEDKGEG
RRAK
>g297
MSSNNASLEAAATERKARLAKLAALKRKQPEPEPLTEVFAEKDQQELQEASGDNITKTYLSGRNYD
AETRGPKLGFEQAPIEGQITLEQQASEIARIAAEQTKREEEEAAEQPIDLFKLQPKKPNWDLRRHL
DEKMRVLNVRTENAIARLVRQRIENAQREEKAKKGRSNGEDQGEEVGIEGEALVEGIHVREQEAAD
DREDEDLV
>g298
MASQASGSNLNRPSFANIVAMPALPKSQDHPDESEHHMNAEFQNPLQTGLGLGESHDVPLDAYVQG
IEDGVERLSMTEKHGLEFSQSPDEVRGTGMLQREASFEDDQTHLSNSSTKPTSFDSKSMASVTTFA
MDEKDSLRPDDSASVQAADEDDSLSGPASGAPNSLTGSDAGVRAYRDNIKDGILQRPRGVLPMPIQ
RYPDGDIGVTGAIPPDSVANNFIVSNQADFVPGKSLNGYALEPDEKLLEAMGSPKDRLFLLQLEEM
IREFIQDSKEQSLELPPSNAFGRLLAHKLGDYYHLTHFVDNNVTSVRLHRTPFCRLSVYVLTTSSS
FMLTNFSPTPLSLLRPVESHNTPPPNAPAMKIMRRNDDRNVGENGTTNSSGPSKPVSEADGEGGND
EDRGGSSAGATPAKDRSAMTREEREAKYQEARERIFRDFPESKSVDSSAGDNSANISRSSSTSGRK
KNFRQKTPHDDSFEARSQFNAYYPGMSYANNTAPAPMNNGATYGQNHYTGXPNVSSPTMNYPQGTS
AGNVYQNNVSQYQMNTMPMGQAHTWQGPAVPQQSPYQGYAALNQPQTMMGQQTQSGPMAAMNNYPV
PNPAAAYPPNTSTPWTGQQYQNNYPQAVQRNAHWPNYPSHPMATNTNPYSYGQQMPNQQYPTPQGH
NSHQMMGNFNRTMFNPQTRSFVPTGNPTTGRYAAKGVNHTGGVAYNNPQSNVQRQKWSGQGDPSGQ
SMAYGSPYPNNSRSNSNATSPTMPRVTQPIPNDSIAKWGTPAHLPPKPPPSEVPSRNLAAPTTPFP
NNGASQHNQSGGPLIISGGSSNMPKSGAD
>g299
MSSTKPLLVVLGATGNQGGSVVAHFLSLSSSPYALRAVTRDPSSAKAIALASQGVEVVAGDFDDPN
SLDAAFREKAATSAVSPGLYIRDYEAQQNRNIIDAASKVRTLERFIYSSLPYADRLSEGKYARVYY
YDSKASAEEYGKSTYPELWEKTSIFYAGFYLENYIWGTKDLYFCPKWNKGKDSLIAWGMEPLTTFN
WPWYSVVQDTGPLVAALIRAAPGKKLIGVNEWLSMKDISKLIAQILGKDIEFVDSGPNFNQGDPDF
QRSRREMVGFSMEFGYYGDKVDTTVLKPSELGVSVQLSPVKEWIEKQDWEKVLPAE
>g300
MTTKPTHYSHPAPAPGQGQGQGQGQGRHVSFIIPVYSYTHGTQWGFNRLIPTHPGISQMDDPTLTL
ANTLAYLDGDTGIPRSLLMRAGRSQRRWNLDGEPEDVSPEETKIASDLVGVLSDADHLQNAIDQLC
FRGALVRTTYPGHGDCDIYRMDAATRAMYKQSSLVDPQHGMLQALLLVCHAFPVDIHLDSDFRELG
SAYVPQLQHVVSQHYDALQALGALTNDMRKTLAYVLVHSSCLSSTAWRLEAIERAGKIASNLVTPD
RCLERMILLQTIIQNCSNMSFEDYSECAGFSHASNKLNALSGQVFLARCNQHLRNEKGIAAAFELL
LQIRPINLNHISTMEELVLQHKALAWGRLLRFQGHFEDACELLEAVYDARAYPEGVVSIGDSNCEL
LSQIAETYCELEKPVRAEILVSAKLESMREKRSNETNLGQCKTDILKLAFAQAEAALQQREYWRAF
DLYRDMNRYIWSTRGLQNWVRAVCRMHIGLARVHHLQGEWALALKYWERALRMFEKHPGSQDFGAA
VTYASIAYVKLRLNDIDSARSFATKAQDLFKITGRQHWYTGLGSRWYDEMLAGLKDVLQSV

>g301
MEDNKSTYPSFAPGDITPATFTQLLDLYPATLKESYKRKLVATHARKHRKHPERMNKEDPIFDKQT
DEYFKLDEWRYQTIPRVLRDREEGKEDGEEKKKNGAIHLKQGESYGPLFMHKDELVQLMEWKLKHG
QYRPALAGMIKTNKPDVVRKTTCDAFKAFVDKTPTRDTLEETFPKKSQDILMKPLRAVGTATASLI
LAVATEGKQNEIPFYSDDIYWWVCLDLFPGSEKNRYNYKKATNRTRDDGRLDVKYNMEEYRELYEE
VFKLRDRLNNGEDDEDSKEERRQFSCADVERVAYVLKNFGVSGFPNAAEILEQYETTVDEARKEFD
QNKHGKKKKSRDDDSDEEHTLGVEPSQSKKRKAGFAETGRGKRNKA
>g302
MSVILCTAGYDHTIRFWEALSGICSRTLQHPDSQVNRLCITPDKRYLAAAGHTNVKLFDIKSSNPN
PVMTFEGHTNNITGVAFHCEGKWMVTSSEDGTVKVWDTRSGSLQRNYVHKAPVNDVVIHPNQGELI
SGDRAGTVRVWDLGESVCTHQLIPEDDVAVSSVSVASDGSLLCAGNKKGNVYIWRMIQDTEVTRIV
PIAMFQAHRDYLTRVLLSPDVKHLATCSADHTAKIWNLDQAYPPAVAAAETAASRPNNANGTATPT
PPTNGVPNGNGVGSSATTIPQPTRSTSTDLAASDFSMVSSAASLNPLLSPGQSQQQQQQSNPFPTQ
PDGPPIDPSTGTPFLETTLSGHQRWVWDCAFSADSAYLVTVSSDHYARLWELASGTIIRQYSGHHR
GAVCVALNDYSEPR
>g303
MRHAPTACESESETSTNVVQQQACDGALLKASSYDHTQVATWNSSIINSVLKALIVATTPEQSSTT
TTTPTTPPYRFTVNSTIVQQGVIDKAAAGSSGEIGKRGMHSASGAYWDVNRDGMWTFKYPGGEERG
LDVVVSVTWFAMG
>g304
MSTQPLLQTVPGKRIALPTRVEPKVFFANERTFLSWLNFTVILGGLAIGLLNFGDRVGRISAGLFT
IIAMAAMIYALFTFHWRAQSIRKRGQSGFDDRFGPSLLAISLLAAVIVNFVLRIRETNKA
>g305
MGSAPLQLLKRKRSDNHEIFTVIDEPSAKRLQSSNQPPLTELNGSNVANLPTPPSQDEACHESAPS
APSKTNVASKSTTTQRTTHEQENPSKRASLPPKPANIDINVLRQSLDAQLSLEVLLKHNELRLIDQ
EIAKCQVALEQLRRCAEVPYPGSSVNGISQSVSNGSGFAVPYSGTGPXPASPAPWGVADGPYSRHY
AKWLLPDSRFDGGESLDLPPSNASSAESRYTRGTFTENGYVAGKSRAQRGSMSTRLAALPNGYPVV
KEKAGPMVIKRKSDNKQVKLVCLDCRRFDFSSTQGFINHCRIAHSRTFASHDAAAEACGEPVDVDE
AGAIIGGTTPNEPPVVVNPGSVHPLIRTAHTIEKTVAPLCSETPKVARLKKGHSATSSSPFKASPA
TPHLSSMLADLGSKLNLDQIVGDAKTTVDLSEFMSEGESEDEDASEEPQVGTGARLPARTTMPQAA
SQRPTSSKRHEMPMSRDMDVSTPTKVSHAPVQYQTFLPSALPLEESGATDLMHMDDNLSPVTVESH
QAPSLVSDNEDDEFEAASDSDNFSSRSSDDGHDDEEDEDDIEVEDDEASTTSTKTTHEVTSFRRNQ
DKKRDRVLPPAIVSLNPGGKRQNSKSKKAGNQKKGN
>g306
MASEKNIQSSRRHSTVVWHRLVRSLIYLLAWIFLLLVMIGNISNKPVLRETYFLKLDTANVIPQSI
PNAVFINSIARSIGLHDFYQVGLWNYCEGYDDSGITSCSHPKALYWFDPVSIILNELLAGATIALP
TEITDALKIARVASHWMFGLFISATVLSFIAIFLVPFATSNRPPQSISADEHINETVHPHRRPTFI
LFRALPMAILTFLIALFTVVASVVATVMFVIFKNVFMNNTADLNIKAYLGKPMLAFMWIASALTLL
GFIVQVGSCCCACCGGRKARKQLKAQRKARQKEQSVSSAGTDVNARRRFRWGRRST
>g307
MLSLWMAVALLTGALAQSTTSSSSSTTSASNTETAAIVTLSGTVDPLSIDGATGSVTYPSVTTTVT
LSSDSSTLTGTVTRNATDAYTTTSGTVTMLVGSQGTTTLAPNATASRNSTTTTSTTSTTPLPTNTQ
PCNGYVEFCARNYSNVTYVAAHNSPFDRQGNIASNQKYPVTTQLNDGVRMLQFQVHLQNGTLYLCH
TSCDLLNAGTLQDYLTTVNKWLNNNPYEVITILMGNYDLIGVGNFTDSIVNSGLSKYAYQPPKIPM
GLDDWPLLSDLILTQKRAIIFMDYNANQTEVPYILDEFTQMWETPFSPTDPDFPCTTQRPPNLSEE
SAKSIMYMANHNLNVEISFSGLDILIPNTAVLNETNGVSGYRSLGLMADNCTATWGRPPNFLLVDY
YNDGSFPGSVFEVAADMNNVTYNGHCCGSTTSGALKLQVPGHWWVIMAVGAFLFY
>g308
MDLFTRGNDALNVNPATGVAETLSQHGSDWLWAVTAIYIIAFFGTLIPCFTAPESDRVFHYLLTMS
LLVGSVTYFAQASDLGWIALQDAAHNTTRQIFYARYINWTVSFPSLALALGLLSGISWTTIILNVF
CSWFWVLNYLVSALVTTEYKWGFFAFGTFAYVILAMSTLNESREAAERLGIARDYMTLSLFVNGLW
ILYPIAFGLSDGGNVIRVTGGFIFFGVLDVLLGPVVCGLFVVMTRKWDYKKLNLAFSEARHPVAES

VKEPALVGGERNEA
>g309
MVDILPLSSFPSYMXLLPAVQTCNITNLPENYFLKYYLYHALTWPQLSFVAVVRPRTSKTGARQVD
YDYPKVVGYVLAKMEEEPSDGVAHGHITSLSVMRTHRRLGIAERLMRMSQRAMAESHRAHYVSLHV
RVSNTAALHLYRDTLGFEVEKIESKYYADDEDAYAMRHDLTPLWLDWSEDTTEEKKAVEEGDENAD
EGEAVGDLGAKEKEKKIRVKVGRGLGVGDLVERNEAAGR
>g310
MDPDHPHEAPPPYSAVDPMQLQEQVDGHNEDLPPPQLEASVASLNIQSPSPTSTSIVTPTTETPEI
SITDSGPLSEHPAPTVPAQFASAAGYFAERSQPVSVTEDAKRELLIHHMTIYARSHGKDFPRRPRC
WNSRTSEVNQHDWDTFLNFLFPSHLNPAASSNRLHRQIRAEIERDRKDRAQETDEQRSARIKEVIT
EWNDHFFAIRAVHITWTYVADLEEGPTSSLCPNCYPQATKVSRDRGLGRNDSTNAPIPVSTEQPIK
QIIKRRPVAQNSWGSDSQTSSAGVELSPVVVSGGFLGQGRNQTRMINPESYQSSASWKSHPVAWAA
QMTSIAQQYATKISNQAQEYGRAMEESALARSRQVEMYSKKVEQDALARARQIEAMTVAKGQRIEQ
AGDRIASWASNMGKRAVNPRPGPMPSDHSYDLSTYDFTYNNLIRQQQPSSRRQSTASDTSISSVSS
IDTVSSVSDLEPDDLASIRAQLSSLDDYHHHELYDAAVSLRSQLEAMKKSRCTGRFRSHHHHRPWG
RWESPEESANREQRRVAMKQESKLLREKFNEIERRAKREIHDMQKARRDMKKRQQRQQQRQRQRTC
GSMVSEQSLHVEPPRSRGMPDRTVSDTNTDLYKEVHMGPPESLDRTISEPITQVISKNTSPSITSS
TSTSTASATTVAVNPHEAAKAWTEAQRLRVKEIQKANKERIKEIQKSYKEHEKQQRKAMKKLSKQK
HTTTSTFGGVYSSSTASLETGTARMGSGQAVEDLDDVAVKPPQETGTENSAVTREMPSQERPPSEI
AGTFIAELDGTSVERK
>g311
MDFLKSAVASAISKGSSLPFSLGDRVDSNESLWTLHNATKRDDGSNCSVFTFDVNANKSRLPLAKN
AMRKFRTLRHPGVIRVLDTIETESHIYIVTERVTPLSWAVKRKSITEETAKWGLYTVASTLKFINA
DASSVHGAIRVSSIYTSESGEWRLGGFDILSSMNDDDAVIYTYGSLVPDSARYAPPEINKGGWEII
KRNPLAAVDSYGLGILVFEVFNGSFTGPDQVGKTTNIPPSMHQSYRRLSAANPKLRLSAANFVDQG
KKIGGFFETPLIRLTDDIESLGLKNDEEREEFLNELDSISEDFPEDFFKMKILPELLKSVEFGGGG
PKALTAVVKIGTKLTDDEYSSRLTPVIVRLFANPDRAIRVSLLDNLPLMIDRLPQKLVTDKIFPQM
TSGFTDVAPVVREQTVKAVLTVIGKLSDRVINGELLRFLARTANDEQPGIRTNTTICLGKIAKNLS
PGTRSKVLVAAFSRSVRDPFVHARNAGLLAFGATIESFSEDDCASKIMPALCPALLDKEKLVRDQA
NKTLDLYLQRVRKHGSTMADTVLPPPNAVDPAAQNNPNAARIGTSNDTSWAGWAISSFTNKLTAAK
GEIEPTSANNAPAVTELARPSSVPQPTTTPMAGPVKTASRLQVEAPALVRAASDTAAQTAIDTLEP
LDDDDWGADAWDQGDEDATKADEDQFFDAGSSPAPTSPATTSTSAVPYDDGGEPDFAGWLAAQSKA
KTKKPLPKGLNSNKTSAAKQTLPVRSAPVSRSASSRPAAKPAKIIDTKPKDEGEEDGWGDAW
>g312
MRPTRSSYILYGVLVLLASTAASTSTSAGINSTDIKYGGQLPSVIRKMSDDEGEKFYFDYWSWNDP
FLPSDSSQREVVNTSVTGLVEPQPAILLHTSTDADLLSSTEEERDYARSARRALFMLLGRDFQCPT
VQDTGLGVVGCCPAGETCGDTSTTFISSSTVSTVPTVPTSVSTTSSSTHTTISTSTTSSSSSSPTS
TETTSTVSPTAPVRPTTSSQTTITSSATTTSSTTVSTCPTGFYACSAVYEGGCCRTGRNCDTTSCP
ATSSTTLINTDGITIAVPTGSAATTATESSGSCATGWXSCAASAGGGCCPNGYVCGSSCTASSSGT
ATATVVKEQPGVAGRYVADVYFSGVMAVFVTVWSIQPTMSSSEKPLPFAYQFAAGAIAGVSEILVM
YPLDVVKTRIQLQSGAGTGEEAYTSMLDCFRKIVRNEGASRLYRGITAPILMEAPKRATKFAANDS
WGAFYRNLFGVEKPTQSLAVLTGATAGATESFVVVPFELVKIRLQDKAQAHKYNGMIDVVSKIVKE
EGVLAMYNGLEATLWRHILWNAGYFGCIFQVRQQLPKPEPGNKSQQVMTDMIAGAIGGTAGTILNT
PMDVVKSRIQNSPKVAGSVPKYNWAWPAVATVAREEGFSALYKGFLPKVLRLGPGGGILLVVFTGV
MDFFRTLRAE
>g313
MASQPSGPRSSSGSAQSHASRSGVPGKQISQIEASVTHLLVATKQLLETLTQWSRRTATENEVSDV
YVRLGYEFNLACRAFGAIGVDTSDLGPVPDLLRTILEDTLSQDASPQSLDRYLPRIRDIIINLLHG
LKKKQARLRSRQPRDGMPPRQASFGSLAGSEAGPSQPDEGTPSSPQKRPSSARRYGSSGSLEDQPP
MPRDNAVPDREASRRDADRTLISPTPTSSLSASALQGQSSQNDKSTVPPYSTPTPPPPPPKQDDAL
GALQRSGELERRASRRFSAYQIQKHLGTSSNGVPVLPTQTTPIPNRGRDVRESLNAVRLRGSYHGR
QKSANRQESSPSRTTYQRSTSVIPENEPSQPKPQVVPVSEMPANDDSPMSRTPTDKLRSAVEIDGP

DISTADSSILFPQPGQGLPVSNDIEPTKTQPTDAVPRTPKAQEPPITLGTPPQVQQFIPEQSPSPG
KELTLFLQYKSKIKKYVIPEGSTGLTIGRLQLAFIEKFAWNTQQNGVDLPEIYIQDPVSGVRHELE
DLSDVKDRSVLVLNVEVLDEVKRHFDDGIGSIRRLIEGVKSTIDGQETAMQRVSERQLDAAKEMAR
LAATPQITLPPIADTTRKVQSPVPYDGSRVAEIQSLRRDIAVLRQTYSNFSSDIAASMSKIRAKAS
GVKTAAAVATTPSFEGDAGRARINSGKKDLGDESERLVGRVDDLQDLVEDLRKDVVQRGVRPLPRQ
LEAVSKDISTVTKEVKKMQDFLKREKPIWTKIWEKELQLVCEERDQLTMQEDLAADLEDDLEKAAQ
TFALVEQATRQQNVQTTDGGPVLRSTSRTLAIDQSIDPIKAKDGVLGEVRALQPNHESRLEAIERA
EKARQKELESRRVGLFQKELGSFVEEGKLKKSGGFEETERLRRAKDERIRKEVWERQQARAAAMEE
AEKTAPDATSPEGEKGTDEPPEGEAESAQEDFSSPLAAESTETSTPNELENGTTEXPPAEPNVI
>g314
MASTANSVSAANRASGNNLNPSQNNGARQSLRTNTSFKGADATRRQSASPGDGAQRRSSSQKAWTQ
GINPITQKASSQPNGNHAPSKSTPSPKPTTSTSEHNAHGRLVFLMTSFIGLSATITIKNGEKYSGI
FSSSTLESDNASVLLKMVQRVASADTNANGISETTTLFVGTTPDHSMKFKVDEITDIVVNNVSTAE
VVAKDTNGPQTGFRTDTDISGSLAMRERNLQRWEPSAADPSVDMSLEGSGSTGWDQFEANERLFGA
TTSYDENIYTTQINRNDPLYKKKQAEAARIAREIEGTVTDNPHLREERGHAPEGEGHDEEERYSGV
RRDANYLPLQTGQPNKYTPPARRQQAAQQPATISAAATDPAIISAQLAKPAAAPEVPVSSKPAVPT
ITLEKQNTAPPVSDQKTPVSTTATAQVKRPGAAENATSNVETEVLDHFRQFANNEKMRMQERRRNQ
ASHDRTVKLNELMKFSQNFKLATPVPKDLVPILAKDPSKQEEIIERAQRQADEKAAIKTVPQPAAP
VTEQKPSSTTPRTTAPRSEAAPANIPPSAPADRQTHHRGRQPYPPQGPHFGMGGRVQTFHGGRGNL
STRLADNFRQQKNALGTVPAPLPIHDSRMPPSGPSGDNLVKSQLQTPTSTKFNVRAMEFKPNPAAN
TFTPGGPGSSSGAAASPQLSSRGRSVSRASTPSAFFGNKKPVPAAERPSLKNQCSPIERLKKEGAE
QKDKDFTFNGGVPPPYRTAPTWDVAPGNEEKTYKDMFKSAPPSISPQSRSGSSSHIPHPSQHPFNT
QQNTPTLHTAGGPIHGPPHLHQQHPSSHFEDPHRMQLSASASHMFPSPRLQHAYPSPMAPHAQLAY
GQPVPQFYMNQGGQQPGHMRQYPGGPQFINPQNGMGAPMMVQQPSSGPYMGVPQGMNGHYNPQMQM
YSPNPSYAYPQHGGPPPQPHSGYPSPSRGAPMMMHQGSQPGQPPQPMMYMNQGQQGPVYAPQQPGG
HAQWAPRLLIFKVNKRRPQQPVLQKPQMTLNEHTGLLFTIIRNTWQVLELIPCLSWEAFAIIEMNF
VYPFSASTMPRQKRALTGVEPNITTPQEPTNAKRTKVTTETSSTDNAASNAQPNGGNKTNAAAPKF
IAVARPNWDIRAEAYDKEEVDDDDNSESNDTEYSEAPNPLRNQDELDPEWPWTMSSSAIDKYIELI
KQVECRDQDNHNVYMYNDFTAYGVNEVVDNWLKGFHREVSRRSKTPYTVWAELEAVSWFLQLDYID
IWFSGDDCHGFAETLALVGKALLAGIEILKTNKLFKPYNPEETSGIRNISTVLALFVRFANTWITI
GGDHYGETKWVSKVGKVAKDNGIDIKGPYKFHETAEKLFKPDEENKPREKKKLVRFDHGTWGVPKI
VELYSDEDEQAIRDNGDYSIKGWKEAFKEYSEAHGKGPNKTIGGSSFAITKRSESKKKQTMKKQ
>g315
MSINIEASHRDILNAFTKATANLKLCPNRVWAIAKAELPTLIPADDAVSWRGSQDNSEFDNHEHCT
FDFCEYSRRDFTAVEQRHECLDKRECVQLKYMFPRDTLTRAAEAGESTAWNLSGESLTTFPQPYMA
ISHVWSDGTGTGSWSEGEVNKCLYDFFRGIANQFQCKGIWWDTLCIPREKSARNKAIKKIQSNYQD
SKITLVHDCFLRNWKWTGAEAACFAILMSPWFSRGWTALELAKSRRVKVVFGGPDGPLIKDLDEEI
LKRDTTTFKAHAKASEIIRKLRDTEIKEIDDLLTVLGPRYTSWPKDIAVISGLLVGVEIVPEEAER
DVWQQNIYKAILRKIPRISHQHLFHNSATMSHGFSWCPTSLFSMPIASSPASLRIEANGNVSGMWI
PICLDRISHERYNAETHVYY
>g316
MVKRLLHRGADYEARCTYDGQTPLHRAAWGGSKELVELLLKTADNANLRDKDGNTALHIAAEKGYG
SIVALLIDKENVNSKGRDGMTPLHSATMYGHQGVIKLLLDRGVDVNTCDSVGWTPLYYAIEKGHEA
VVKLLLDNGADINAKDNKVGWMPLHFAAMSGHAAVVQLLLNKAVSDPASTRDEAGWTPLHFAAVKG
HIAVVKLLLAKNADAKLKDEKVSWTPLHNAAMNGLDEVARLLLDQYADVSLEDNDKHWMALHYAAD
KGYKKVVKLLLEDKSTNETVRILLEEKGYNQAAKLLLNQGVDVNLRDEENGRTPLHYAAQRGHEEI
TKILLNNGANVNVKDGDEHFGHIPLHYAAANGHEAVAKLLVDKGADVNAKDGKIYFGQTPKDHEVD
INTYIGRTPLHYAVMNGHGKVAKLLLDADADINTRDGDRHSGLTPLHYAAENGHKELAKLLLDRGA
DMNSKNGSWNGNECSNWTPLHYAVNNGHKDMTEVLLDKNADVNLRDDQERTPLHYVAKKGHTVIAQ
LLLDKGADINIQDSKNGYGQAQAPLHYAAENGHEELVKLLLERGVDVNIRDGKESSNRTPLHYAAE
NGHEKVVNLLIVNKAEVNLKGIYNGSCGTPLSYAAGNGHEEAVQLLLDNGADVRISRAGNMDTPLH
GAAWGGHLAIAKLLIEKGANVNAREFRERKGPNRMPVHYAASWGHEEIVKLLIDNGADFYTGDENV

RYVRPGNASARYYDLKDDRHFGPTPLHYAAMYGHEKVVELLLDKGVPVSLKDREYHSHTPLHYAAE
YGHENVVKLLLNRGAEINIRAHSEPISFREYVNGTPLHYAAAYGHEEVVKLLLDEGIDVNAKDSFL
PFSSLTPLHYAAKKGSVAICKMLLERGATSSLNNGPYGKTPLHYAAQHGHETVVELFLDNGADINR
RENMFSRQTPLHLAAGWYGKEHEAVVKLLLERGADTNVRDDIFSRQTPLHYAAKNGYAKLVELLLE
KDVNIDAKDGITSRQTPLHHAARNGHEKVARLLLEKGADISATDGIFSCLTPLHLSAIYDHQKVTR
VLLDKGADPNVKAGIFSLRTPLHQAARNGHEEVAKLLLDNGADINAKDGIFVHQTPLNYAVNKERN
DLERLLLGRST
>g317
MANIMTVAPVPKSLLGYHRQLAPNAAVKVSPLCLGSMGFGNSMKDLMGECDKDASFKILDFFYENG
GNFIDTANVYQHGESEQIIGEWMKMRENRDEIVLATKYSSAWQLANPKVKIQSNFGGNNRKALLVA
FEASLANLQTGYVDIFYVHTWDFTTSLAELMHTLHDLVSSGKVLYLGVSNTPAWIVSKANEYARQQ
GLTPFSVYQGQWSAAERDVERDIIPMCNDEGMAFMPYGVLGSGYFRTSAQRAEEKQNPAAKREGRN
IAFIDKPEKAIAADTLDVIAKARGTNITSIALAWARSKQPWVFPVIGGRKIEHLKATIDALSISLT
EEEIQKIENALPFDLGYPQTILGGPGGATKPADVWLTKRFGHFDWVSTPQAPKSHQK
>g318
MPSQAASVSALLLGLTTCASAALYDQVIQTQYGPIQGYAAFNSPFTGNLTHWKDITVWKGIPFAAT
TAGENRFKPPQPVTGWTETFNASSWGDICPDSSSSSSYTVSEDCLSLNIWSAANSTDAKLPVVMWS
YPGLSTAADALFDGGGMADQGIVYVNYNYRASSFGWLALPELSEERYEETGYNSSGNWGMLDQFAA
LQWIYENIANFGGDPNHITVMGQSSGSAATYHILNSPLTKGLIKGAIIESGVRDPQDPLCTSLAES
YTPLNESLQAGKEFMASLNVTTLAELRALPMEDLESSPSNPGSIEFTATLDGYAMPATYYDTLVQG
LAHNVPIITGNTKDESGAAYGLNITVDTYLSDFNSTFSGEWLDKFLALYPADNDTAASGAVNSQWT
DRSKVGTWLWTQMWLASNVTTAPVYNYYWDHAPPGMDQGAYHESEINYVLNNLYDTDLSWEEEDYN
IARQMNGYWANFIKTGDPNGEGLTTWDPVNSSQLVQHVGDGWGAIQVAPAAKIELFKAWFATLEKY
>g319
MSQSGYTMANQQFDNHFGNMNGNDAVDPSNLTMQNGMFMPYSFGSQQNMSASFNMGNSGIDTDELL
DLDLNGQNGMSRADGMNFMHDQRQNQGLSMSHQSQMTQMYSQTPDGAPMHSPFLQNSFNYDQFQMP
NQGTPSHLSAPNHFDHSFLNGKTRPSLQTMDRSNSDRNPMTPKTPAIGALNLNTPDSGSFPSQPIR
ASNLQHRHQKTLSNQWDGTPGSAQSFIESPIASPNLHSHHAGISEILKSGKHASLPAKVDNPSAQA
LESQEAKRRRRRASHNMVERRRRDNINERIQDLSHLVPQHRLEDDKVRKQLLNNSTLSPPGGSMSP
PNATSLLAGGNGRRATAGNITMGLPIEEKEKGPNKGDILNGAVGWMRDLMWALHVKMQQESELAEL
ISNLGGTWPFEQTEDDKRMRTELLDAMEKNDPNTFTYSRAPGSGLRVPKHTNIAGETVQNGAGLSP
QSLSPAFHSGGSGTNSGAGQPAYWGSSNNIFKEEEEYTMDM
>g320
MASASFRENMNSLGWSRRDQDTPVRTDANTPILSRLRSLNPFGSGEGYLRLPTQEPGAPLPAPSRR
EEEESFFAPSWAVLMGPITYIRHLLSGPRLPFTAAYFGSIAMTLYFAVGLQKSFLTLFAAIFQLVA
LVWYLVSYFPMGSTGLQFMGRFGVQRVAAWVSG
>g321
MVEQDPHEVRQMVQSLDAATKRSRSESRKTFSCKKSTFVVAGSQNISVHSWRFMDWDYKRSDLPTY
ARGLFTTKLKDGRDEICIRGYDKFFNVDEVNETRWRNVETNTRGPYELSVKENGCIIFISGLEDGT
LLVCSKHSTGKRADTPISHAQAGEQWVERHLSKVGKTVKELALELRKMNVTAVGELCDDRFEEHVL
AYDEDAAGIYLHGLNYNLPEFTTLPGSEVHRFADEWGFKKAEFVVMEDIKTVKDFLDKCAETGSWN
GRDTEGFVIRCQLGDGKSDGYRDWFFKYKFEEPYLMYRQWREATKAVIAGRVPNIRKHKKITEEYL
IYARRQMAKDPKIAKLYNQNHGIISMRQGFLDERGLKGSEIIAMENEGDLESEMPARNFVLVPVAS
IGCGKTTVALALANLFDWGHIQNDNLVQQKNKSQKFALMVTNSLAAQNAVIADRNNHQKRERKQLI
DDVTKIIPDAKFIALHWVHEPKGLLLDDIRHITRERVLTRGDNHQTIRAGSKDSGEIIGIMEGFLQ
RFEAIDTDREPDMYFHEVIDLDVRDSSRENLEKIVTRLHSVYPTLVKRIPDSAELDAALASALNDY
RVDTDLSWGTSKKTDKKAEKASKKNDTAISANKAQDPMVSAKHIEYIGILVPPTDIQQVLTSIFND
NSGPAERNKLYHHLKNSRRIQPSFHVTLIHRAASREQPQIWEQYTDLYINRMNERVSQGLPINPPT
KLADARVRVERLVWNDEIMAFVVRILVPEGNNNSQEKLPSSWPCANAVPHITVGTANPDVKPKQSN
DLLQKWLEFGSGGDTGIWEAEIPGVKVLEGSILPVMMRGK
>g322
MSQPNLYILAADNPSAVLSHLRSDSSRASNQDEHGYSLLHAAASYGHIELLRALVQEFHVDPNLTD

EDNETCLFVTEQLDIAKCLVEELGVDAKHTNDEGFTAQEMIESEGSFMDIAAYLGEVTGNTRSEAQ
LQALRDVHPPPPLPHNISVNLGTMSQEDVDGEAGEVDIEFRRRIAELAAREDFHTEAGQNQLRALV
TDALRGVGAQTEDRDVRRRTE
>g323
MPVQTGPSLVYASLPSFARDFSDSANYTSNNSPQQENRSLFTHSLGPAQENNFAPTTEHPISFEHR
ESVSSSGLSESTDSSPTTTISTFDTTSVTDTSPSSSPESPSALQLPYPKSIRIPEITERMAMFSEA
PASALAPLPSMIASTTRPVSPGRRARNLKNLSLKMPCQISRPAISTASVVEATSQNYSAPPSPVHR
PAKTARRKPANLTIRTPAFDQTTFARDVTDIIPPTPSIPQRSLRHFESSPSLSIFSPTAAPLNGMQ
LPRPVTQDGTPLMPGSWQESPHPSRQSQAISGTSLQQVTEEDDYHLDSRESTKRTEQSYPDGPIKI
YDSGVYLYLEPTREEASKFDVVINVAKEVLNPFADDIEAKPETVVSTLRRPVSLAKRLSMAEPMTA
ISEESFHSAFESLPSESNSPTTPKVEKSSTPEYIHVPWDHNSEILEDLATLCQLVDSRINQGKSVL
IHCQLGASRSASLVIAYGLYKNRDLDFNDMYSIVKGKSRWVGPNMSLIYQLTDFRSRVQRDEPVKA
PNPDWFKTPHLSRPVQQDSLPASVEKETSELAPVQQVESQVQPTPSTISEMIFSPMQTTFTKPFVK
PFPPLQKQHSSNGRGLLPRPLPLREKYPSSESLAKPVNKHETRQVSHIRYPTVQMDLVMQDVPSSP
SILSPRAAPYMCSSISRTLAGDLAGDAPVTLGFGQSLFDPRSPPQRQELLITRSIDEVL
>g324
MSALSPSALAVMRLDLENDPNWTRPRRQTNPDLIHRPSVLLRQATRRSADVSSLQNNSHSSNYSNP
SSPLADQRPRTSGSITHTSAASSSATVTPTKRTSTSSHRSSLSASALSNPALAEIPSRKYNAARLS
AGSTDGYAENYRQHSNNNNNNLGGEPCAAAPRDPAATNXPATSAVNSLSPISIATPNSNYSSSSYF
PQTSPGSELIPPSSTSSSTTKNPLPSSRLATATSPASATSTAFNDRLLRPTPKQATTPQTARLNNS
QDSPSIDAVLKNKSILKKVGSSTDMASSPSRRRRESTPLSPRSAATASNMRSLAVDERYQSGSSAY
GDSNTDEQKEYSTDDTRRSDDVFLNIAKSSVNSRRNSLDRRRKLGLSGISTRSSLAKEQTPSPEQL
KYQSSPLYSQHASPLFNSNYAASVTTSAHPLDESARLRHHSSTAGSRSVVGVPRSRYNQETSPELP
HIDFKASLADARLRYSQISNNSSRTVRQASMSDAAERARLDGEKAKHDGTTESTLSTTAPSTVWDE
LDDLKSRIKKLELTGKFPSSSAAAMSSVSGERPRTAATTATTLSSSPKQKHVRKSSISPEVLAATA
TANSIQTLLQSALAKAKATVGPEVYNALEATATDALTLTNMLATAATTVSGNSSTVTGTGLSERQA
KRKADSLCRGLTEVCLALSEEQTMTQPSSQQQPQEQIRPRSRRQPSVTGDTSDAMSSISTRFRRSM
SHEPEDLAQPDSGARIFSRFEGHRANTINLGSTGRRERLSHDESASSPHTPSLTAPTSRLHRLSGS
HRIKREDDSEDRSSVFSRTLTGRAMTEVNGYTESPSSRYSTPYQQTLSQHQPKVSPSVSSITQRRS
YATPPSAGTGIPTASSLKIQPGFRRYGASTITGLSERGSSEPPQTSDSFSPSPGLPQTRISTPSSK
MATSYTAIQQPRLRNETLGSRRLLRTRPSTIGTSNDH
>g325
MTLDANQIVSIVEIVVYVPALVLSLIVCNRHGFSRSSGWYYTFTLSLIRIIGDVLLLLTYSNDSTG
LLIAAVVFDHIGXTXLLLATLGMLSRLYDLPLPPKNSNFSHDVPPRVDLINAGTQTSITIKYFRLV
QLAVLVGAILGIVGAEQAQSSSSPSTMTYIAVLCYVAAYAVIVLIFCLALPYTRDYIPDSSERALA
PAIGVALPLVLVRLAYQVLIVFVHRGVFSRLGQGSVGVHVAMAVVEEILVVVIYIYLGFRLDRLEV
NEQGPILSRPWKRSKGGRRNRSRRRGRSMRGERDGGYTRGTQLHFIGVE
>g326
MLVAAKGERFCDQKRPGCSQCARKRALCPGYRDMSSQIFRDLTQVTVRKYHAKRSQSAIASSSQNK
SPKLVKAKPSLSLENVALNFFIRVYAPSSPSAWLTYLESVLSCTDANSQLSLSAVLAPAIAIFSHE
QKRPSLMYIARRYYLTAIADTGLRLTSIQHAVRDSTLMSINLLLTLFEARAFQGRSPPTDWTTHIEG
AVELIRLRGNDQFKSVLGQALFLDTVNNIRTSCAQRRIALPAPIQDLLQTLLQKDSPPLSYGILEI
GVGNVVDNIIGLVAGMQTGAVYCNDYKAVILEGLRLDSQVSELSRQLWSIQSYRVLPTDASGAWRW
SGLAHDYDNPQSARLWNLLRMLRLYLNKWICRAYKKAVFEQQQQQKEEKLLEMGQEFQHIPETAAS
NTVLMAEYILSSVPYFCSLILSLEASSSSALSSTVRWLIWPLSVVAVSPLSPVSMQIHARDCLRKL
GEEMGSAQAVEIANMLDNSETLEDWYASHPRMIVDQKAWF
>g327
MALPLPPILSPQEAAQYGRTLAPYVFSSQILELPSRVIDAANSTDVLHSFQELYLGTNPMLTAIAF
ALALCPFFVLAAEIRNNYSQVDCWWPILPSIYNLHFYAWAYGNGLPTDRLQTVGVISVLWTVRLTY
NYWRKGGYSWGAEDYRWPILREQVNNRFLFFLFDVTFIALTQSLLLCAVTAPTYLFTLLAQLPKTG
STFDVADLVFSRLLFFYILIEIVADEQQWRYQQAKYKYRDTGIVTEGYDKEDLERGFVVSGLWSYS
RHPNFAAEQAIWLTLYIWSAYKTETYVNWAGVGALGYLALFQGSTWLTEKLSAGKYPEYAEYQARV

GRFIPRWSVNAKGQKKANAEAKKIEGKKKE
>g328
MPGNRILCVAEKPAIAKAVAQHLSGGSFQTMNIHGQQYVKNYVFSFNFGGAWGNCEVTMTSVIGHL
TGLEFGKEYKGWTSCPPSLLFEVPVFENVDIDKLSIAENIKKQAQYCKALFIWTDCDREGEHIGTE
VKKQARLGNARIEVKRARFSNTERAHVLRAAXEPIELDDKQANAVAARIELDLRVGAAFTRLQTLQ
LQTLGSPLDRGPISYGSCQFPTLGFVVDRYLRVQNFKPEPFWAIAVIHKRDDINVNFNWRRVRLFD
RPAVIVMLERCLGAKNAKVTKVTQKPTSKWRPLPLTTVDLQMMGSKYLRMTSHQVMQVAETLYTRG
FISYPRTETDQFDKAIDLKKLVEKQVSDRTWGQYAQGTPRAGKNNDQAHPPIHPICAVSPSALKDD
EKRVFDFVVRRFLACCSEDAKGQTSEVEIQYGDEYFHAKGLIVLERNYLDIYVYDKWESSTQLPHF
EVGEVFEPTEAKITEGKTTPPTYLTEPELIALMDANGIGTDATMAEHINKIKERQYVAARPRTGGA
NNEAGGRGRGRGRAAAGNTGGNTGVQELIPTRLGVALVEGYDNVVAGLADCPSLSKPHLRKEMEQL
MRDICNGTRTRAEVVEQSLDMYRGVFIQTQRRIDMLKEACRKYVFQQATVTVTTTNTNGRRR
>g329
MASNKAAWLHKAGTSLEVGDAPMPIPGPNEIVVQNSAIAINPLDTHMQDAGVFVQQWPAIFGCDVA
GTVYEVGSEVERFKKGDRVIGHAINLVTGKPRDGAFALYTAVPEDKAAILPDAISFKDGVVVAFAI
EAAVCALSVKQPGVAMPGVSTPALGLPYPSLDSVSSNKVLIVYGGSSSVGSMTTQLATAAGIKVIS
IASERNFDLCKSCGATEVFDYHEPSFVENVIAAASETRRELVGIFDAISTPETYANDFVILEKLGG
VHLACVHPPPADVPANLKAGMIFAVDNVATPVWRDYVTPALQAGKLKCLPPPTVVGKGLEYIDEAL
KKCKAGVSATKLVVEL
>g330
MNAPVGAGDLLHYNAQHKLLICLECKYAIQKHAVDSHLLRHKIYRGERQQLLSAISELEIPEAGGV
RFPTEPCEPVDGLPVIDGYKCTARGGCGSLYASVKRMRRHWSESHGLTDLPEGYASAVHMQTFFRG
TKVRGVLLEHFSNLPYQMAEKLGRPNAPIDFLAVMSAIGALIDCCRQTYDANDAGSAWMGLEKWLE
KVSGRFTELLVTEKSPAALIVFAQWLLLVAMAERFCWFLRGVAMKMLHLIMAELEEHGTFRDLLKQ
LMGWALESV
>g331
MNKLYDVIIVGGGPVGLFLACELGLARASVLVLEQDTKLENPFKDAPLGFRGLNTPSVESLYRRGL
LGELIDLDKRALGPEKTAGFQFGGHFGGIGLNANKLELDRWKYRLPGPGLMPXPTTLQQVEMLLNK
QAEKLGVTVLRGHGVQNIAAQDDKSVTVEAGESHESFTGKWLVGCDGGRSAIRRAAGFDFKGTAAK
FTGYAVHCELDHPEKLKPGFNPTENGMYVSIRQNSMHLFDFDDGAYDRTQKITREHLQEVFNRVTG
ITDLKIEKVHYASSYTDRCMQAATYRKGRILLAGDAAHIHSPLGAQGLNLGLGDAFNLGWKLAATL
RYEASNDNAAANLDLLDTYETERHPIAAWALDWTRAQVSILQPGPFGRANRNLISEIIDTPDGMNM
FLDRFWGLTGRYNLDANDGKTVHPLVGSSVPDFEFEDGSRLGSKMAAGRGLIIDFQDNVALKELAE
GQYGAGIDYLNLSAKDRRGIGAVIVRPDGFAAWAIEDDEKPDIDAAKAALDKWFAF
>g332
MSQRPSKRSRLHGSDSATLSDASSSSQDDESRVYKVGYVAKLASRGCFMRRSFAGPIQADIALCER
LLHESVDIPPGVSFEDEFIDRFRKSSRDQPKEWVLTEVHPLLVPPIEEQYVEETVILEKTVDVYCK
EWLNVDSIYGPRPVPDQARGLKWSVFSDSQRQKLKYHPEETSPFTAREDMLFPYLTTQVECGNRAL
MIAERKNMQCMCIAMRALCSVAQTANYLEKVHRKILGFSVSYDSERLKINGYYPEIEDGEVAFYQW
PIASLDYWNKHDKWASYRFVKNLDCKFLPIHTKRVMDLLAEIPVPKDFLDEADNEDDIDSQERSIA
SQDLSNLQNMLHTLKRQSEDREARHAKHLAQFSREVIGNLGRQEDFLTQLQQQKEREEEVLSQIEE
NIVERKARHEKLLAQFAQTMSEKRTLCQNLLPIANEKMAEAMAHDDSNLAHMEGQEEQMIRRIETS
S
>g333
MSRRGGGRPMPREVVLSKALSHLLRHSAERENLKISKEGYVNVADLLETRKVKSLNATLPEIIQAV
SSSDKQRFSLFYISPETIEAGKQESQEQQEAVVATAASEEKQKNATAHALSVSDNDPSHFLIRATQ
GHSMKVVDTALFLERLSLEDDATTKLPHTVVHGTYHGAWPLILASGGLRPMSRLQVHFATGPTLDA
VFPNGRDAPLATLDPRSKATVISGMRSDAEILIYINLRRALEAGCPFYRSENGVILSEGMSLGDDG
ETKTVPIEFFDLVVERRAGLGVLWEDGRVVQELPSHLAEKKNPKALRGGDSTITLKYRYSKRREIE
DNIKMTGHQYDPTFTDRVIDAMGPKTNPRLRQLMSGFIRHIHDFARENELTVDEWMQGVQMINWAG
QMSDAKRNEGQLLCDVIGLESLVDEITYKVASEADAPTATAILGPFFRADTMVRQNGDSIVVTGVD
DGEMVFMQGQVVDYKTKKPIPGTTVEVWQASTNGLYEQQDSNQVEHNLRGKFTTDENGRYWFYCLR

PTPYPVPDDGPAGKLLQLMDRHPYRPAHIHIFATHDGYKPLVTQIFDRKDPYLTNDSVFAVKDSLI
VDFVPRKGDPKASTELTYDVKLVARDS
>g334
MPYIEVNQKRLNYSDSHPNGPPAGGLTYIFIHGLGSSQNYYGGVIPHLTPNHRCIAIDTYGSGRSA
FTGLSQSIDTIADDVVKVMDGLKVSKTVLVGHSMGGTTVLQCAAKYSDRVDAVVAIGPVHPTASTG
QVFEKRIQIVSEDGMEPMANTIPNAATGSKATALQKAFIRELILGQSPEGYISLCHVISEATAPDY
AAIKAPLSIIAGAEDKAAPLEGCKHIIANVSSPMKTLDVLPGVGHWHCIEASDEVGKLILKFTRSH
RD
>g335
MTPVIIHCNGTFPCANCSKRQVQCFFVAPVPATGKPPGSASTASDQPTNNKKRKLDDVAGYTDATN
RTGYSTTPSGVNHDQPPHGGYTANLSSTAQYSPDRAAKVLQNMTQLQHEQQQRNRDSLLNMSTAVS
AQVDQTTGDEASDETDEAELQGMSRMLDDGKGRMLYIGDSAPLSYLQTIRQLVGSVMGTSMFTVDP
HRHNILEASMQAQPVTFQYAITLPDREAALYLVDSFFTNTKGMLHLFDEQVFKQRVERTYLNPLAA
DQSWMCILYLVFAVGLQLRCVPAQPSPKEAAILKRLLSDNVDRSEMFFLGARHLKDSASGIEDGDF
ASIQALLIMTLYMLSVGKRNTAWAYIGMAVRLAYALGLHRAETQRVYDETDKHSMSLPRVLLWRSL
YIMDRFLASCLGRPTSIQDEEISEELFPHKDRFTNLSPGAVENFEFEALFSSVQAAQIHGNILHQI
YRSRKVSLKIARNVAVQIHEWTQSLPEILQWRPTPMPDDNPGKAIGQLHVWMTYFHTIILLTRPFL
LLHVKKIIAQERRRRNPPPAHNSPDTSETVASSPSEIRVAENPELKKYSAACVRSATHMIRAVQTI
RVKGCLPRRNPFIINWMFIAALIVLTNSFFNVYENPENDSIAQMAIYLHQHFAESDILAARYLHIL
KSFQKTIADRRSMNGLLETTNKPYTMKDPIEDLFSGKAHLSQRRGRNNSGATATTMAPADMSTQST
QLPVSWDWPRAATSLPQANNAVGESTLPNIATTAGLDLSTIPGLSGDHHNHFGDNVEGLVFPGAEE
LLGPDPGPILEEVIHFDMLWPLNQDTGLYSGNIPMYGIDSYL
>g336
MAHNIQSTNIPSSGSSPVSSERASTLKSFMSSFYTTSDNPTAHDEYANFFTKNATLIMGGKRADGF
DDILAFRKSMWTAVTSRKHVVTKIFFGDDNDMMLHGTVTYVMKQDPQKKDVKFDWAARAEFEDGQD
GSKMKYYQVYLDTAAQQALAAAVQKS
>g337
MGSVNVPYSPTNEAQRILGFLCKQSQALNLPDAVVENQDAVSFNSTTNSIYFPIPFKETETLAALK
AVEGLVAGEIANLRYGAPSQRKRCVEISLEAATYFACQAYMAKVGGLGKLDPGVKTKLKDTDLLAA
QSNGYRRMSANLYKTKDERFFHIHGSLEATTTLNMIGLEGHRPELTDYEEIIKTIEEKVQKFTAAE
LETMNAERRQAGVTAYKYEDFVKTPHGQINVKESPWSVNPLKGTIPPTKFPPAPAGNYKILRGIKV
LELCRIIAGPTITRILAEYGADVLKITSPNLSDVPFFQVDGNMGKHAADLDLKTPEGKAEFEKLVA
GVDVFVDGYRPGALEKLGYGPEKLAELAEKRGKGIVYVNENCFGYKGEWAGRPGWQQIADCVTGVA
WAQGQFMGLNTPVVPPFPISDYGTGCMGAIAALTGLYHRATKGGSYHCTSSLMQYDLLLFAIGQYS
AETQDQLRKEQLPEFFALRHHDSVDRISATTLQMLRKRFPDLFVADSSKSSPYTEKWFAEKYGEEI
EVVKPVAKIEGVENGFVRASRPNGSDKPLWEDFEVDEKDYRI
>g338
MASQMAVSNIGMALAAKSASMDIPIPRDAVKDSRGAFAVASARLGQPTMLPTPPNSISPTLPPHGF
KNKAHHNVGNLSFSPPVAPVQVDSDIDLQDAVDHAKSQDHHPHVSNSAQNVDSVGAITPAMLAKYH
LPDILLAHGPLAIRHVMGHLTTTVPGFAGIPPAKARRLVVGALEGRGNGGEGGGIDGNIIFEKVGW
GRWDARRSGQPPRERQAYNSSYASTSPPSSLPSAAGGMQIPQHSQWRTTNNRPAYGTSVADSAIFS
HSDLDFGDHEADRMSLDGDDVPEYCSSSDIPEDAIPDDEWNEGDVTDEEDWAGIGADALRARSLNG
GGIVNTNHSVHTRVQRHPGGPASSALAKSVPGGITIQQLGFSLPDGIGGDREERAAVEALLRLGSM
>g339
MPPIRTFKSAPINSHSAANTPTPTSETQEQKQQHDNDVGSGETIGNEGAPAWTPATPTAAASIGDT
RYPAARPGAPAMPAPTSSTSVNTYTPTRTYPTSTLNSADQTSSPPAPQPGSRPVPPTSSSSSSLSP
SPNRATATGAVPPPPKVGEKVQPASYYTPSPLQQQHQQIIPNSTSTPYLSTYQPPSSYTSTYVAGS
ASGLPLSQPEQSNSTSASSIFDGALGEGTTGQDILNTARSWMASAGNKLAEAEEEVWKMVNKKS
>g340
MASQGDKTVFGMPGFVVDFLMGGVSAAVSKTAAAPIERVKLLIQNQDEMLRAGRLDRKYAGIMDCF
RRTAAAEGAMSLWRGNTANVIRYFPTQALNFAFRDTYKSMFAFKKERDGYWWWMAGNLASGGAAGA
TSLLFVYSLDYARTRLANDAKSAKGGGDRQFNGLVDVYKKTLAADGIAGLYRGFSVSVAGIIVYRG

LYFGMYDSLKPVVLVGPLEGNFLASFLLGWSVTTGAGIASYPLDTIRRRMMMTSGEAVKYKSSFDA
ARQIAAKEGVKSFFKGAGANILRGVAGAGVLSIYDQVQLVLFGKKFKGGSG
>g341
MEPPSKKPRKLMEDSDSDSDSDSQGGVSVAPGADFKINEEYARRFEYNKKREERQQLEAKLGKSST
SRKPANGDEASDEEDSSDEEDEDEDGELATEALDSEIFATLNAIRTKDPRVYDKDVKFYSEVTESE
IPGAQEKKTEKPMYLRDYHRQNLLNGDAAEEADDTPKTFAQEQADLKKNVLKEMEEAANEASSDEE
DGDFLVAKSKPDNGGATHKKPEVEVDVENADKDPETFLSNFLSARAWIPTKVSQFQPFESDDEEEE
KRAEELEEAYNFRFEDPNKLNEKLMTHARDTANQFSVRRDETSSRKKKRDAEREKKEAERRERENQ
KNRLRKLKMEELEEKVAKIKQAAGLKVADFTEEEWAQFLDDNWDDEKWEKEMAKRFGEDYYAEDDA
EIDEVEDESSEKKKRKPKKPTWDDDIDIKDLVPDFEDEEAVPGISLIDDEEGEEEVDAEDGEESGR
AKKKSRTQEKHDAKRAARKEKRRIEEAVERNLDLDPTLLPGATRKNASQFRYRETSPQSFGLTSRD
ILMADDSQLNQFVGLKKLAAFRDPERKRRDLKKMGKKARLRQWRKDTFGTEEEPEFVLPQAPAVEE
ENDDDEMKVDIREGGSRKKRKRSKKH
>g342
MAASSQPTTVPQVGKLVSVVPVGLKEAALDSPTFRATTVHFADQLEVVERWLDGYAKAAGKLASEL
GVFEQSMISFMSYMTNPLNVSEAVIDHDYTLLAIKRHGDCMKDTWGSLLGSIKKADRLIVDPIKTF
IQGDLRTFKETRRTLEHTQRNYDHLLSRYSGQAKQKEASALREDAFQLHEARKAYLKASLDFSIQA
PQVRNTLDKLLVKISFDQWREAKLIHDHNTTNFTKWGQDMDRIKGWVHEMENSERYSLKELLAARK
QIEDAAELGTRPSRELDDYSVSTVPYLGQALSVKSDGEKPSKAEKQGWVYLRTTTGKPTRTLWVHR
WAFLKNGIFGCLVQSSRTGGVEETERIGVLLCNIRPAFQEERRFCFEVKTKKNTIMLQAETQKDLM
EWISVFEAAKQKALENPASSVAGKLVVQDPAFSISHPPAPEFTSDSTEFLTSTAGDDPSNVERTPT
LPVPERDPSSVRASGDFANSARRIPGLDGEAGSGRDHASRIIQKFDIHRKSTTPAPLTPLGNSSGG
IASLITASHSVLPYSPAGVMPRSQAEIDPNQAGRDEATTLAPVTLANPPAPTSMSKVAVAVSNERG
IGVGLADSTGGMPSGMMANLWGSTNWGFVNTFHTEGIRLRDPKASGEGSASQGSSSAAADDTKPSP
TPAAARHRQTVSLDGDASKLQRSIIGITHEYPSYYPYRLRFQDAQFRLLFPNVKREESLVMVFRAT
WNPNDNQEFPGRAFVTTRNIYFYSHHFGLVLTASQPLDTITEVTAAIGRDCDFLFLHIISLPGADT
PGRVTIKTFLEPLRVLQKRLNYMIKVATSEESTDLETIFKALTKMENESLPRSVSLDSWEDVSLDV
PTDIRSRSGTGGYRDVKIPVYVEKDLSANFERNGNGRDIQKFKLPNQPVQYVPQGNLHVAAEKVFD
ISPKALFHVLFGDKSIKQGPWRTLDSGHMRRDFDYFIERNDLLGRPNKTKVSDYQIIDVLNDHLCY
VVTDKRTPWHLPFRRNFRLVSKIVITYISKSKCKLAIYTKVEWLWSPYVLKSIIDRQAMNDLEQDA
LDLVDLISDQVRKLGPRSRTKRAITIFGLIGHETGVFQFSAADMRAITQAHKSRKQRSLTYLMFES
TASLLQSAISSLMMWFLALVRWTWKTCSAHSLILALLGSSLLINGFYSTRDTYDWWHERNAAKLLT
RLGVHSEHVMSKAIFVKDLEEATAFTDSVWVRESDGNASYCFSTFHEQMMFNQNNSLSFASEIPTD
DYVEWNANRRLQATRQRLGMYRHDLVVALRVVNRIENEVLKGEWERWLGRETQRCDRVSGILEEHT
GDLNTNESVLSGHEEDVKRWYDEYCSSCHEELQRLDSPLV
>g343
MTSSKVEAPKTSALTTLISTTSKTESPTPLHALTAQVYHNLQHQHIWTSLKIHDPSEDASLPSLSM
PLISGIPPQRMYTHPDEQLYLLQIGLREEDLPSERLWVLPSTQGQTWTLGKLATVFDALPLRTTGT
ESSTNAGEESKETFDKGKEEKLAKYFEKKREADKENKWGGKRLLLAVVDRLVGGDGTVVYYVVQDG
HVKPRQN
>g344
MARTKQRAPLQKTPSSDVMERLSDLPDTNANIQNGHSTPSNSSANGSANPKAEASEIEEHSPGLIQ
LAICVGGIYASFLLWGLLQEAITTTSYPVDGAKGEAEPNERFTFSIVLNTIQSCFAAVTGSLYLLL
ATPKGQAVPSIFPNRQIMFPLILVSISSSLASPFGYAGLQHIDYLTFILAKSCKLLPVMLLHLTVF
RKRYPLYKYAVVMMVTLGVATFTLYHPGTSKKMAASTHSGQTAYGLFLLFINLMLDGLTNATQDHV
FSSPSRYTRYTGPQMMVAQNFLATVLTTAYLLFTPYLTDNGPIVSMLPFQIPPSAGLELSSAISFL
QRHPQALKHVLGFAACGAVGQVFIYYTLSKFSSLLLVTVTVTRKMLSMIISVFWFGHSLTHGQWLG
VLLVFGGVGAEGIVQRREKKAKEAAKLKKKL
>g345
MDSNPAPPTATDIFRYRYQHGTNLGSIFVLEKWLHPKMFDKDSKGSSELEAIKQSLKKNGLLATRQ
KWEHHWQSALTEADLIWLTDTAKCNSIRLPIGYFTLGPQFCKGTPFEGEPSQVYINAWSAVKKIIK
DCHDHGIGVLIDLHALPGGANINAHSGTNTGKAELWTSEQNLKVSKDCIKFVLQETLSNRLPNVIG

VELCNEPSRAASSAVFKWYEDVLAMVRTVDSSIPIYIGDCWDLPTAIKYALTKNNLEDTSNPVIVD
THKYYTFAAHDHAQAPQQIIERVKNSLDDIVKNQGHIASHKTALAVYIGEYSCTMDGKTWSKVDAA
HRPALTQQFGRAQTDKWQDVTSGSAFWTLKMSWMDGGDWGFKKQVKTGAVTPPRSLTFSFDEIKSK
CREANACGEQLRAAALMQHANYWDKQSSSTKYEHWRYERGWSLGFDDAMAFLCSHLDGNLPLIRQV
EGGDKIGALELWIGKRMVETGQLGSPLGWEWEHGYRKGVKDFEKLIYT
>g346
MDRTRVAKVEDVTLSRRGEQVTGTLHLTPHHLIFSHTPHVSEEALASGTPIRPRELWITYPIIAFC
TLRTAPTVSRHPSSIRLRCRDFTFVCFYFSNENKARDVYDTLKQWTCKIGRVEKLYAFTYQPPPPE
QGLDGWQLYDPRKEWYRQGVGREGSTANWRISAINADYSFSPTYPDTLVVPCNISDNTLNYAGRYR
SRARIPVLTYFHPVNNCSITRSSQPLVGVRQKRSIQDEKLLAAIFSTSQSDRPLAGFTPPSLEQES
TNSSWEEGASLQSQEFDLTNAEELEDEVVSAARGDGPEKPSVYGAQQRNLIVDARPTVNAYAMQAV
GLGSENMDNYKFATKAYLGIDNIHVMRESLNKVVEALKESDVTPLGPNKELLAKSNWLKYISVILE
GASLIARQVGLQHSHVLIHCSDGWDRTSQLSALSQICLDPYYRTLEGFIVLVEKDWLSFGHMFRHR
AGLLNSEKWFQIENERIGGDTNRSFGESSEPRKALENAFLSAKNFFNQKNTSRGSLVDDDGEGSNL
DNDSPLKQPSSTPRSTTSEKETTKVNETSPVFHQFLDATYQLLYQHPNRFEFNERFLRRLLYHLYS
CQYGTFLYNSEKERVDCNAKTKTRSVWDYFLARKEQFINPNYDPLIDDKQRTHERLIFPRIDEVRW
WSEAFGRTDVEMNGTRTTANTAFPHRSGQTTSRNSPVLAGVETTDSTXGDVPXKPLPAGITSLTAE
LSNLSMPKFSHNADNSTKKEVELEMQ
>g347
MAATQGAISKRRKFVADGVFYAELNEFFQRELAEEGYSGVEVRVTPTVTDIIIRATHTQEVLGEQG
RRIRELTSLIQKRFKFPENSVSLYAAKVQNRGLSAVAQCESLRYKLLNGLAVRRACYGVLRFIMES
GAKGCEVVVSGKLRAARAKSMKFTDGFMIHSGQPAKEFIDSATRHVLLRQGVLGIKVKIMRGSDPE
GKSGPQKTLPDAVTIIEPKEEQPILQPVSQDYGAKALAAQQLAEQQRLAEQQEAEGAEGAASAEYA
QE
>g348
MPTVSVDKYALFKELGRDYTTEEFDELCFEFGIELDEDTENQPRAENEAPQLKIDIPANRYDLLCF
EGIALMLNVFLGRKPFPNYRLVQPTSGQLEKIIVKEDTTKIRPYVAGAILRGVKFDEARYQSFIGL
QDKLHQNIARQRTLVSIGTHDLDTIEGPFTYEALPPKEIRFTPLNQTKEMNSEELMAFYDKDKHLG
RYLHIIRDSPVYPVIYDSKRTVCSLPPIINGDHSKITLDTKNVFIEITALDKTKLEIVNKIMVTMF
SLYTSEPFTIEPVEIVSEHNGETRVTPDISPRTTQAELSYINQCCDLNLSADEVSRLLTKMAYKAR
PSKTSSDLIDVDIPPTRADILHQVDIMEDVAIAYGFNELPRSFPSKSGTIAQPLPVNKLTDIIRME
AAMAGWSEVLPLILCSHDENFAWLNRKDDGNTAVKLANPKTLEFQVVRTSLLPGLLKTLKENRMHS
VPMKIFEVSDVAFKDLSLERKSRNERHFAAAWYGKTSGFEVVHGLLDRVMAMLKSAFITGEEGLEN
TAVNDSRYWIEELDDPTYFPGHAASVHVNIGGKEQVIGSFGILHPTVLENYELKLPVSTLELNIEA
FL
>g349
MDSTAESDELYPIAVLIDELKHDDVLLRLNAIHRLSTIALALGPERTRDELIPFLDDSVEDEDEVL
TALSEELGNFVEYVGGPEYGHVLLSPLENLAAIEEPLVREKAVESLNKICDQLSSKQVEDEFIPLV
LRLSKADWFTSKVSATGLYCSPYRKAAAGVQQTLRQNFGGLVHDETPMVRRQAANNLSKFVKELNP
AVIIEEIIPLFQYLTSDDQDSVRLLTVEILISIAEEIPKEQQSSHGILLTSLRSLFEDKSWRVRYM
VADRFEQIAKAVDEEVVTRDLVPAFVKLLKDTEAEVRTAIAGQIPGFCNLIDRDTLLNEIMTSIED
LVSDQSQHVRGALATQVSGLAPILGKEETISHLLPMFLQMLKDEHPEVRLHIISKLDIVNKVIGID
LLSQSLLPAIVQLAEDKQWRVRLAIIEYIPLLASQLGVKFFDEQLSDLCMGWLGDTVFSIREAATQ
NLKKLTEVFGVDWANQSIIPKVVAMGAHPNYLYRMTTCFAVSTLAPVLNLEILENSIIPILERLVT
DDIPNIRFNVAKSYAVLVDTLRRLPASGTLQDLEKAGESGTPAPHGQELIQQRIIPQLEKLQQDDD
VDVRYFATTAAGGQDEAMQTSP
>g350
MAWPYRFILSLSDEELERRRNILDSRGHYAQISGFILLGAFFLYGLGRGNRFEQNRGKSWWDAPAI
RGGSETRKQYAVTLSWLSWLVSLSIWRTGDDYIHLTKALGHVGLSQLPFLVLLAPTSFIFTSNSTL
PSIISYLTSIPQPVLTAYHRLFGRFVIVPLVCSHAALFLLFYVQVPHPVFGTVFAKRIRDPDVQFG
LAGTFFGILILVLGRANVWKLRDLISVSRPESRRQLFYVVHFVLVAIFFSLAYSHVRYARPFVVEA
IGASVVNLACCKLLASR

>g351
MRVSRTIYLLCFITIAALTLFILRKPDIDTGPKAPFEKSLPADFETLLQPTTEDSHPIARLINDAN
TKFDEKLARQSKTLTEAVEEYRRRYGMPPPPHFDKWFSFAKKRGVQLIDEYDTIYHSLLPFWALSP
SVIRSRVHEALGYDNALIGLLIRDGIVTKVEGGGDGYHWQREATAGMLESFLKYLPDMDLAFNIHD
EPRVVVPHDDLQRLISYAKDTVVPATFEKTSLQNKWSPRPADLNKGDRIEEVRTTRFNKFAHQPTW
THSRLSCPVDSPARSLDENVADNMEPYATSDLGFVYNTTAFSDICLSPSLRKTFGFFDRPNAFNIV
HDLFPIFSQSKISSFQDILYPSPWYWIGKVTYNASRDYVWEDKIDQMYWRGSTTGGFSRDGGWRRQ
HRQLFVKNINSAADASIFTQMEDGRWITSNVPRQDVKSLFNVTFTYVGQCDPSDCRAQEEYFDISP
SVDQQDAWAYKYLLDIDGNAFSGRYHAFLRSNSLIYKMAVFREWQDEWIDAWVHYVPLGMKGTDAV
ESVRYFAHEEEGKIQAPRLAKQGKEWAQKVLRNEDLEVWFFRLLLEYGRLIDDDRENIGYMP
>g352
MDKEYPGALSSLLIQAIGFMFMGYYFILTVISGQFFKKPTAEYRSELTLARDRFWNLSKKPFDLHH
RFLTLRDGFKFHYVTNAIPDPETPASKPLVIFVHGFPDSWVVWRSVMASTWLRESSNLVALDLPGY
GGSDLLKRYSATEVLEKMTEFIISLREAYGIDNPASTGAQQRVIIVAHDWGCVISMRLAADAPQLA
DRFILTNGPLISLVRSNVKRLISSSVKMLKLFYRSPLRSRPMLRKALQTMNPIIRQAVASGYIFAL
QLALPIVRIQGSGGNTALLRAIHRMAHGVNTTPSAKEIAEGVAGCLGPSLKQEPTETSTSERYSKD
VVKQSDAEKFVSMTHYYRHGTSLGRWKKSLETITGLYSISDGGEIGRKTSHAGVFDEGPEGSLKAK
ATIVWGMEDVALNAHLNLDGIADYLSRDSQVVTLPRAGHHTPVEKESLVALQKVVEWAVAGEEGDV
GSIANAVYPGATVTVRI
>g353
MAAPGDAVYCTLLTSDQYLPGAVVLAHSLRDNGTRAKIVALFTPETLKESTVRELRTVYDEIIPVQ
LHSNGTPANLLLMDRLDLISTFTKIELWRQTQYSRIVYMDADVLALRAPDELLSLEEDFAAAPDIG
WPDIFNSGVMVLRPNLQDYYALRALAERGTSFDGGDQGLLNTYFKRWHRLSFTYNCTPSGNYQYMP
AYRHFESTISLIHFIGPQKPWTQSRHAFTSGTPYYQLLGRWWAQYDRHYRPLPTSLPPAPARVSFQ
SQSSYNASSAPRGASSYTSSWEESPFPAQGGDTAHQHEYHHHAPSTYHERPAQIAEDLHVLRHSPI
SDGFVDTTPAFTPAPALAHPQPIHPPSITRDLRGHQAPVEAAAEVRSVVPLYVHGEEQSSVYVTVP
SGGIDNFTELKAQYITQKAFEPYHQHQTSIETTPITQPKAPSPPPPPPQQPEERPFSPPRAEWDAS
REPPPRHSRPEALSLETQTYTMSEDTHLFRPPPSYPEAPKNMWYEVPSAKREPRKLAPIFPWETRA
SKPTRVFAEEETRGPSISGVMEHERHPKQTLSTEQTSHVEHIEHIEHTEQTQQTKQPEQPEQPPKG
TATTFEYTTPNFTPAESWETFSRGNAWDEVPEINKYVDAIQQRSKKANGRGGKAGGAKRPESLKIT
DFPTEVDRPSLPVTPAPMRRTFWGGQDGQQESSEFPAAEGVPNQGDWNPLDRLEELRQRQLNLFMT
TSPQGPTESSDETEQPSEADK
>g354
MPYLKSDPEQASNNDNAFEKLVQDLSAALGPSSGLDSEDVDPHDIERLMEKYVSDETEWGVYAMSD
VSRNYTRNLVDEGNGKSNLLILVWSPGRESAIHDHANAHCVMKILKGSLKETLFSWPEKGQAPNAP
LQITKETVYGENQVTYMSDKLGLHRISNMDPNNVAISLHLYTPPNAANYGFSIFDEQTGKSSHIKQ
CDFYSRGGEKV
>g355
MATTVDKIKEIEAEMAKTQKNKATSFHLGQLKAKLAKLKRELLTPSGGGGGGGVGFDVARTGVASV
GFIGFPSVGKSTLMSRLTGQHSEAAAYEFTTLTTVPGQVMYNGAKIQILDLPGIIQGAKDGKGRGR
QVIAVAKTCHLIFIVLDVNKPLTDKKVIENELEGFGIRINKQPPNIVFRKKEKGGISISSTVPLTH
IDNDEIRAVMGEYRITSAEIAIRCDATIDDLIDVLEAKSRRYIPVIYALNKIDSISIEELDLLYRI
PNACPISSEHGWNIDELLELMWEKLNLRRVYTKPKGKQPDYTAPVVLRSTACTVEDFCNAIHKTIV
DQFKIAIVYGRSVKHQPQRVGLSHELADEDISLSTPVF
>g356
MDTDAPNGQESDYGSSPWIELGAFESPGGSHHSPPLPDFHGFSYGSSPIMPLEPAYSMSIPPPYST
INLTMPAHPWPSMLTTQSPFPETSLSPAPTPTLSIPQPVPIRPLHTTPAPSAPTPRRTLTDEDRRR
MCLYHEENKSAKQTDIGALFGVERSTVSKVLRQKEKYLSMDDGSRSPIKRSKGKVPDIEKALANWA
RNYQRQGFVLTDAMIKEKAHFFATTCASPDGKSKILTASWLEKFKQKNNLSGSKSRKNSTDTIASD
REDLPHIHSPTGTLISTGVSPISPTGLTPSPLSPSMSQETLKKELGESALLDFSRDPVKHGHSQST
TSLDTIPSLSAGVASPTSPLVSESPYTPTVRSRLPSISSAASRPRSQTFPLAGDAESLNGQGEHHK
VTNSKHSMNGSLSVSVVDSPLDDDSEHKSPNDTGDDSNSSMKHHRSNSDLKSISTSMQPPPVPPSR

SSTVSSPVSPGSPTQDEARRALELVMNYIQNQPSGLGLQMQDYVTMGKLMEKLDLVHNNQSISGRL
HRIDEDTDGPQMTKKRSIHSLT
>g357
MTTSLPPRGPSPFNPGATTSQPKTSNVSFASSQPGRTKSRTENRRNTADSESNDTSGDRSTVALIR
RVLCPDVSTYGSSAPRPLQELLPPLTSSNDVDLQLYALTAVIIKEFVYSWYARITPDHFFVDEVLQ
LIAHCTRALEQRLRRVDVGQLVLDEVTGLVHAHITAYRIATQGSDLVAVKPSAREIYHHLNPHPGL
SPVPNVSTPSRSSEQQNNENLYRHLLAQGVLAVLLPTEDLENVCLRTLVEDVLSDLILGNQVSGRI
CEGWFIWTAISKVITMAKQRDSVTTVTVGQSLDAVADLNRLERFGLLSEDGNEDRNAQKHRRSTAS
LWLWKILYSVYLTYVTMRFVIGGLLHTALSNPVFDIPIIGGTTENAMAKWFALVDSEFVSGRSWQD
SNDVSPSFQQPQQGQPLNGTDHQTVIDNSDIKDASTPAPGGNTPSPSEIAVIKRQCATDILSLIPE
SVARAFFRGNAENQVNDYRPHDRNESSHNETSPFTSKGSSQSSTADTEDLLETIESDLLDPFSDAY
CNKHLIYAIVELVLIKLIPELSEQSVSSLMEERGIVK
>g358
MSSSTIPLWLDCDPGHDDVFAILLAAHHPSLNLLGITTVHGNSSLANTTANAGSVLEAIGRPDIPV
FPGSKKPFSRAAVHAPDIHGHSGLDGTDLLPKASRPPVLDANPILAMRNALLAQPPNTAWLVATGT
LTNIGLLFATFPEVAEHIKGLSIMGGAVGEGFTDVPMSRLPGEESRVGNTTPWAEFNIYCDPEASR
SILSNPILSPKTTLIALDLTHQVLATVPIQERVLKTSTSQASSASELRRMLYELLVFFANTYHVTF
GMEGPPLHDPLAVAVILSNLNPDYEQAHPKEVLKFDDKNGERFVVHVVTDGEHGSTPEVTGQLGRT
IAKPTEGQGETPGVPSAALANRYFTNELQMLSDPVPSLAATLTNLPTEAEDANHENVNFLRTRPHS
PSDSSSRARRRRLALLPEVDLESMDIDEQRRFLPSSAEATPRLPTSRRSHEVYNTNLSSEGRISGR
RSLYGWAPGSEGEPGNDYLGYQHTSQNEAPDSIWTVGRGLDREANRNTSLSRMARRDATGIALPSY
TVDPAEATDPSSRHWSDHNSDTFTEALLQSVRRGARYASRARTLQNYILDRERLARQEANETEREG
DREREREREREREERRNATSSSSSRALRMSLRNISGDLRDRLNSHRHLLSENPPSAKLRETIKYLERVR
TSGSLSESIETALKGGVVQYDHLKGPYNDSDFILDTSFIKPPASSSWLRPGTVFSGSQKAVNSIAG
HNFDMLSHRVSQPTEPSADDSTNRISVSTSSGRRYWANSLSHFPSDHHHSPNSKHELWPVKVTIHS
VDYATMTLTGTMEAYNIPDKTSPTRDAHIVTFLEGEIIDLNAHSLETTSFQADADIDCTYWRELRP
FRGLNEDQMTKNLLSKKWVIDELNKNWILMRWKERCFITPTDARQGLTISGFYYISLHRATGEIEG
LYYDPGSSPYQQLSLKPERPTMSFPSVEFR
>g359
MSDADVPPSSSLDPVGTDGPGVGHKDNVALKSSPSPHGPDENQPVPQDNATADPEEENAKADSEAE
TIIQSGRESLSPEKRRKHIQHALHVHADKGRNGDGMADGDLSPNSRKRKRVEDDVPTNIQEAAPAT
KSRSRTSSLSSVVKKERTEAVAPVKARRDSIRSTSDDRQTPDAYSRSRKRSPSGSAMEADFTDNDS
HVQERRRRSSIKERERREKTHPATNSTASLSKHRSVSPSYRSHKRVSSGPHQGSGEITQRKRKIPA
PLTNSHRRQSSEDRLSVSSSASASPMPSARSRRLTSEGVHASSAKPSNQKKQRDQNGRTRLARACA
AQEYEVAVARHAERPEDLNVADNAGNTPLQIASLEGCAPIVKFLLDAGCEIDTRNIDKDTPLIDAV
ENGHLEVIKLLLDAGANPRLGNAEGDEPYDLVPSDTEDYDEIRRIISHAKAXPPRKRRSEDHGPPS
AKGSIPRATSGASPRDSPPAHLMMSPPPGSSAFSRRKTVRSEATRNDLLWTKATPENLCNFAAKGD
MAXVANILNVIQKADTESLIAAAKGGHEDVMSLLLGMGDADADPEPIQNPNCRPGFNTPMLAAIGR
GNLAIIRLLLEYPGFNPARRLYRGKTYYELAEERQGDGWEEEADLLKRAYDKYKGMKKARKAEAKS
PKRPREQEKDPKKAARRSSSSPVSTLXKPVRSPTSSRSVDVVGKEKIRKRDPSLQSKERRIATSLH
KAAAQDDALSEHSVEHETAKSKKSLVQRRQSDASSVNRGDDVPKRRRLIAGRPPDRDRRRLSLMST
DSVSSREELTKSHPRALKDAKDMKDTLEVPVKLKRPRNSVSPERSRSRGSERGRESEEILQKKKRR
LQHEENSRKLLNGTHKKGDDTSEATKVPASKKDNPETGRISKDAIPEKRARTTEASVPVKQEHKKQ
DTQELERIPMDESASKEVEASAARRKEEQQQKERDARKAQEEEEEEEEEKKAAEQLVKERIAKEEE
AARKAEEQARLSREQGEEEERKRKELEQRRVKQQEEERQRRAEQERLRIAKLRREHEEQEQRRRDA
LPNRLRISANLVGANDPRAKSHSWLKQFMPVVTARTVQLDSSCVPEVAEEVWVPNFLVAPLLATND
LQLSQYASWEKRPATYTQRLNLWRVTRRILVDGDDMEVPCSSFGEVIQRDGETRPKYFNMEHVFWV
KLSDFMDLVPHIPHLNGLDIDFLKMHIDQEPSSHGDFSSPLDGPKVNGNGYYPDHHEAAATVNGLT
NGFGHTRPNNIITMSDGEETISNPPVAEAEDVEVPAESSGGQMSVNDALKGVLKIALIHDGLARGL
REASKALDRRQAHMCVLNESCEEEAYKKLVVALCSEHDIPLIKVPDGKVLGEWVGLCQLDREGNAR
KVVNCSCVVVKDWGEESQERSVLLNYFQTSQ
>g360

MGPKKDTKGGKSKGKESKDTTDGDDKGKGKGLKAANSINVRHILCEKHSKKEEALEKLRNGAKFDE
VAREFSEDKARQGGSLGWKVRGSLHGDFEKVAYDLEPSTTANPKYAEVKTGFGYHIIMVEGRK
>g361
MAEQEDATPWAVLPAADVKEDQQNLLDGRAHFSILGVVRRKPGRADAEPTISKSCTDKLALRQVTS
ILSYPASVLIAPTRSAYITALVLPEEEISQIGCERAFGGGPTGRMRALVGQTFSAPNVDVEYHFRP
FEVLSVSMDVVKPMWSFGKYRSDVARRAVKPGNISAIWIASGSTLNPYQVNDMSRTEYTPRINPES
TAVVECIIGGAKQGTPLPKRQLVGLSSRYSNFSSIADNESSPNQPDDDHKSPSAADYEDEDPHIER
YRNHNSVRTQKLDVDALGKPGQILVVPLRKRRRRTIEQQNDQKSDDSPKKVVSPVSAELFGSINRG
NDNNEYAAYLDEIRASYSPGDTVSREEFIELQAKVEEGFTLSQLSKYYDSFPQQSTPLPPKEVIDF
NPNDNEVNDDDEQWQATATREASEDNELSSEEVSEFQKTKKAKKLRKDILTERIIRDCWQLEVHDE
IGSLKVNIPSQVISVILSSQQYSLEELAHSHNTKIEPFVSLNFIRVSGNRSACESVREIVKAYGAK
IHTKSFEQPLLNDKSWLIDGKAAKSGFLDSIARTLGIYINDPGRSSVASAAYTAKHGAFVRMKRDL
ELANYSQSANEQISFCADVPSSASGFMAPISKKRREAMGLLERGKPWSRWAIPTSLQDYNGESLPP
LFSQHKPTFSSEILQVLREKSRKNGQHGLEEKISATIGQCLFRASDVMDSVTKINATQLGEHSFPR
FFHKWLHPKGESLVKTRRIFPGDRTQIRLHRFRMTPCEPNDNKLPVLEVEISVPWITAPEAKFPFA
GPELLSVKAIVEETSIDYLLPEVNYDIRFTRTLTQEIYGVNGTEQPEALRNALLSSLNEVLSDPKQ
PFPPSCQVPVSGKLLRDILSDQNLSESPKTIEGNYWFPGVQKALGTMVRTYEYSGERLCYSPNFKR
SLDSRSDQLNLEMFVNATPSYYSPSYYTQLQGRKNVDETLDPASDSLVQEFRAFYMTACSLPLHTS
GTTLTASFSNIIIPTLSSPKTQPNITIILLLNINNPIFQHHPKILHTNNLIKQPLLTVHHLIQPIL
NLAKSTLHIELLYRLHELRECGDVLLELFEGFLVGVPFLEEALVPVCGVEVVPSFEVLEALH
>g362
MADKLRTLQNLEALQTRYVGTGHADTTKYEWTSNIVRDSYASYIGHPPLLGYMAIGMGESKEKVRT
MMIEKMIRGAGNPPEVQD
>g363
MAELHEALSYLKPILWEEVPASSSSTSDLRQYVQEILTKARLIAETVPEQTPSSASLGDTGTGAVS
ISVSQSQLASLRKEWGKPIKMNNAKENPLNIPIYKLSGKDGKGAWFARRSVHKGLPFSRWKSKMQV
EMDETLKARQEELKQGRIPATSIRGIGGDRRLENVEIKESETEKALGKVEVYELSAQFPGPTTPRD
FVTLMITSDAMLSDGADSDLVLPPTYMIVSKPCQHPDAPERDDYIRGQYESVELIRELPIEKTTAR
EAHTKRPASTRSATTYDSPSLHPNSVLDGESSKSTGRPRGQTEPPVPVDLDGSSGNEAVEPNPVEW
IMITRSDPGGSVPRWMVERGTPKSITGDAVKFLNWASEPDQIGEGEDTADIGEVTESVEAGRRHSA
DGIDEVRSSSKHGTTVSDQEPDAKGTGDKGIDQLDQSAQRGRYDGDDEEQRTDSNLLSSVAKVVQG
YTPKAVLNYIPASLSPSPGEGKAGRTVAMRDAKAGRSEEVLSDEPGKEMGDTEVDDDTSSMASDDS
FASAGSHMSPSVSRDLRPPIYIGESSNTNISPQFASGVSSEAALISTPQSDSAKSKPSQKEKELAK
LNSRKREVEVKLATIQSEIRELVGQTGSKSVKSDVDSTRGSMLSVESDAKIEEESGSSTTATTTKE
AAQNQKRIDHLARTESKLVSRLNKIEAQQLKVIRKLEADQRKAADRDEKIRSKSEVDSLKKEVSSL
RKEVQELRGERTKWLDIVGRLQKENTELIAQMEKNSQGASGTKDAV
>g364
MPTALLPASAAAFAPRGNPNVVLGRKVEQWLTLTLKRVNRVKRPLNNPTQHTRCLTETLSSPNAIW
TLCSLMFPKAPDSELQKDENPLVEAMFNYQMIHIEAYVVHVDMVSQNEVAFKLTPETIESLVDFHK
DIFSVDTAASTWDWPEKETQLKKLQAEFVQSANKFIYRTKVQALEGMEEDGAGELLGGRSEDAKTA
ILNLFVPLLPPPPPRLPDVVRPPLLPSSTIAEDWWSSPVQQPVMPVEAWKVVPSSPSPVSSCDGAP
NMWTTMSMSMAEAQLPSPAPSFSQPYTTAPYDAVQYYSAPVTMAALAALPLPSMLIQPCSTAASMN
GFGGGFGGYPERYQEFPALSYGTTM
>g365
MTEEIVLAELQPGDPPGDSKTELSLRATGEVSPEDPPEYPHGYRFALISASLMITVFLLGLDATII
TTAVPKITDEFQSIADVGWYGSAFRLASSMSQLLQGRVFDYFSVKWAFLIHLIIFEAGALISGAAP
SSAVFIAGRAISGLGFSGISQGCMVRIIATTRPLDKRPIYIGAISASEFFATAIAPIIGGAITSAL
SWRWCFYLNVIVSIIPALITTVMFKAQSGRAIHQVGHLQRLKQIDFTGMFLFAVALLSLLLSIQWG
GDTYSWSNDRIIALLAISSVFFIIFVLVQVKKRDLAMLPLRLLKQRSILFGALFSLALQAVNSIAS
YYLAIWFQVVKDASPVQSGVMLLPMVIAILVASVASGYAITYMGYYIILMIPAGFTTLGGAIMMMY
FEKDTPAKFWIPALALLGFGIGLGISGPFLAAQAILPERDRSVGLAIMTFSQEFGVALSLGIAQCL
FLNGLRASIIRITPELNPETIINAGATSLAGSGLEKYASQIDEEYSQALKATFDLSLGMAALVVVT

GCLMEMGKFYETIPETLSTWILTQKIFWVATAPLSGTGHVNVSPKGGPYFGLLDNKTFWYLDMTGS
GSETISHIYEPGNGRVTIMFNAFEGPPRIVRLWGKGRVLENGGKDFADFVERNHVQLVSGTRSIIV
VDVHQVGSSCGFSVPFYEFKDFRETLLDHHVRKEKKYKEGNEKESFPRYWAANNTWSMDNLPGYDN
GVFSGLTTDELFLTTMGSPNSTLLGFIVAVYELGCLVGALASFFWGEALGRRWLTVSGAVWLILGT
VVQCTSYGQAQMIVGRIVTGFGMGCITSAVPIWQCETTPAHLRGRTMAIELSCLIVGIVVAYWIDY
GCSLYTNELQWRFPIAFQIVFAIMLIVMCFFLPESPRWLASHGREQEALEVVCLLRDGNPEDENIR
AEMAEIKDAIALEEEAAGGWKDCFRDGGVMGWQRVAIACSAQALQEFTGTNIITYYAPYVMVNSVG
LNSHQSLLLSGGLQLWFLVASILPWFFLDKIGRRKLFFLGSAGMGACMLISGLTIRANTHNTGIGA
VVALYMFQAFFTWGWMSNMWAYPSEILPIRLRQTGSALSVVWQWLITFLVVEITPVGIQNIGWKLY
IVFCILNWASIVIVYFFYPETAGKSLEQIEFLFASQSSLRQVVKLSQRKDLDEMAVIRETQAQAHQ
QKSDTKQVEDTDAGETVE
>g366
MRVFTTIATAAALANLAVGSPAGNGSSSGIVQGAAAGIVSTSVASTTTAAADAPNEAVASTAPAAA
STTAPAADTSAAGSDTGAANADSATSTPIVSVSVPAAADSTAAASTAPAAAASSVSDAATGVASSV
AAAATSTTSPSGTTGASDASSTTSSTSSSTTTSASAGDLASLLSGLLGGSSSTSSGLGSLITDLEN
YLSGITGLLSPTFLKNLESFVNNLALLLDAPTTQQTKEVLSVVHTLVNNGTASSLVSLLSSAEQLL
TPTFVNETSNLISKVSPLIDELSPLLTGDTVTNIESLLTNGAALLTPTFVKDTQALITDVAPILDD
LKPLLSNQTISELEDLLTNANGLLTASNTKAIEDLLTNANTLLTADNTKAIGSLLTNANDLLTPEF
VKDTQSLITDVAPILDDLKPLLSNETISELESLLVNANGLLTTKNTQAIETLLTNANTLLTADNTK
EIEALLSNANSLLTADNTKEIEALLSNANGLLTPDNTKAIESLLTNANTLLTADNTKEIEALLSNA
NSLLTADNTKEIEALLSNANSLLTPDFVNETKSLIGDVAPLLGDLGPVLAAAAPLLTTKSIDNIET
LLTNAGSLLSTSNTQEIENLLSTASGLLTPENTQDIEYLVTNATKLLTPSFVSETSGLIGDAAPLL
SEVGPILXLVKPLLTNTTIAELEGLLSGAVSLLTPKFINETRDLIDEVGPILSTVSPLITPDYIEE
LGWLLTNASNLLTPTFVNETSGLIGDAAPLLSEVGPILTLVKPLLSKDNIDDIEYLLTNASNLLTP
TFVNDTRGLITEVAPVITPDLLAQVGGLLINADNLLTPTFVNETGSLIGDVYQLLPLLSSLINAL
>g367
MSRLPSPPSEEENARRQSRAFEPVPLDSSIRTTPIHEQLPEIRVPQNDTVPSTHYHPVTCELLNIE
EIQPHLQHLRKEHPSKTAMLKAQEDAVKEIRQKLDEKDKKRDAAQKALDAKAKEWEVEYKVLTKYQ
ASKS
>g368
MQAFVPKNRRTKFELKLRILDLNNIPLVAGTSYVKWHLPSSIAAEHRGHTDRAVIQDHRASWDYEK
ELPVKLTIDRNHMLQECDISFEIFQEFSSGSRGDRITLGNIKLNLAEYVDRSEDDNGIVRRYLMQD
SKINSTLKVGILMRQVEGDKNYITSAPHPIYLEGFEESLLRLRRPPLKSAMVFGGIAGILPHEHGE
TEEPGSLPSINVSRETGDLQDMYRRTLAASWTAQSDELLPDQLIEDLFAGGRGVRSATRSGARSER
ADDDDGNISDTGSRTTVQESRSDNKRPKSALSSRSRTDLRDTDSTMSGRGSLESSFQDTKEKAWKS
SKASHEVSEFDIRDDLRTWEITVND
>g369
MGPKKGPKATVPVQIAPEKRGYEFGGPLGATGIVFGLPILVYCFAFFCNDVSGCPAPSLLHPSTLT
WDKLKSEIGWPENGIIGLYDTEVTLWVLSYYALLLFLQIFLPGEEVEGVELACGGRHKYKFNSGLA
AGTFVYGAEFAVWTFLWDNYVQVLTANLLITIVISVWVYARSFSIPEPGRPNPDLRELAPGGWTGN
VIYDFFIGRELNSRVTLPVPFVSETARTIDIGVFCEMRPGLIGYIILNLSNVARQYRTHGYVTDSI
ILITIAQSFYVLDSSYMEPAILTTMDIIMDGFGFMLCFGDLVWVPFLYSTQTRYLAIFPVKMGLQG
IALSLAVVGLGYYIFRSANNEKNRFRTDPNDPRIKHLKYMETKSGSKLLISGWWGTARHINYLGDW
LMSWAYCLPTGFAGYTIIEAINSTTGELEKHAVQTPESRGWGSIFTYFYILYFGILLIHREGRDEE
KCHRKYGKDWEKYTSLVRSKIIPGIY
>g370
MKMLTRPTLLATFNVLLLLPFSQTIEAASDTPKKDVNPPCTVQSVTTGAFFDLSTISLSPPELKDG
RKVHKNDRDESWEARGHDYGTNFTINICAPVIESVVDVVGVDKARWQNVSAYYEQKGKVYSLGEQN
SEPIFRGKRLVLNYTNGSPCPSSDASYLNSRYYSDDDDDNDDYAAAQTYKNRKTAVATTAASDSKT
TRRKSTIMSFLCDREAAASQATVSFVGTMDSCTYFFEVRSFAACGGVAAAPEGGLGPAGVFGVIAL
IAIVVYLVGGCAYQRSVMHQRGWRQCPNYSMWSDLLGFFKDFAIIIFSSLTRCFTRKGSPSSAGYS
RANGQSGLIGAIGGRRGDRGGRGDVDAENRLIDQLDEEWND

>g371
MATERPANGRQNSMTENHDDNMSDTSTPDRDQDDRNDYGQTEEGAPGRGQNSNGATGAAQGEDESE
KPAWTELKTKAGKERKRLPLACIACRRKKIRCSGEKPSCKHCQKSRIPCVYKVTTRKAQPRTDYMA
MLDKRLKRMEERVIKIIPAGETRDMPTIGRSQVKPTPPGQTSKQPKAAQQKKRTADEAFASKLDAW
VNPKQASAADHLFPMTREAKSHEGTELLTEGAEFLPPMEIQEHLSEVFFDCVYGQSYLLLHRSSFM
RRLRAGTVPPVLILAVCAVSARFSTHPQINTEPAFLRGENWARPAARIALSRHDEPNITILTVFLL
LGLHEFGTCHGGRSWSFGGQALRMAYALQLHQEVDHDHLGKKSKLSDLSFTDREIRRRTMWASFLM
DRYNSSGSQRPPIGNEKFIQIQLPVKESHFLMEIPGPTEDLYGDVPNPVPRGQGQVSDPRANMGVS
AYIIRAIVVWGHIVDYLNLGGKKKELVPLWDPNSGYTRLRQEIDNFASSLPSSLVFNPENLQIHAA
ERIANQFIFLHIIIHQNMLFLNQFAIPLTPGGRPPKGMPKSFLSDAGRAAVEAAHHISMLIDQASG
YSLTVPFAGYCAYSASTVHIWGIFSKNAQLEARSKENLRHTYRYLNKMKRYWGMFHYMVESAKDRY
RQFADAAIRGLPAQQNATEEAPSMFQYGDWFDKYPHGVSSMHWEGPEADKRREIGDDAVMGQRPDL
QSVEDFFASLSPSQSTTEPRKHQRLARRSSKQVSAQAPSQPSADINQQSMTSRQPQQQQQQLSPLD
SAAPIGSTPTTVMDTEPTYPDFNSQNLFAAPPQNFGLPDFDYGMMPDFLPQLDRHFVHGAYTGTDL
SSLASGELNFLGAQNDTNQLGNNIPENNNNNNATNNNSVSTAIWTDAFTPSGTGEFHQPSAWFLPF
NLDPFSTGMLGDSTASAAEVGHADISGASNAAESMNGANDLSYGFT
>g372
MTQTKHRTDLTTRKNRTRRLNMTAKQIITLITGANQGLGWEVAKNLILSSASYHVVIGSRDPSKGA
KAVADLQALPDIKGTLDTLEIEVTDDKSVDAAAETFAAKYGRLDVLVNNAGIAGILGIPRELAAVR
RDNLREVLNVNVIGAASVTEAFLPLLRKSDEPRLIFVGSSMGSIIGASDPNSPYYRPSATEYRASK
AALNMLMVQYHHILGLEDKKWKVLTVDPGVNATNFVGDVEALRARGAPEPHVGGAVIAAAVKGERD
ADVGKMVGKYGVSPW
>g373
MVSTRHHPKDFPPPATPSSASDSTKYVVHSPSAPAHASRKGVHTPSIIVLAWLIISVPLVIWDTGY
VLLRPHSMPGGKFHSPIWTPYALYGTIDYIYGWPAFDARNGFTAAQGLLNLFETAAYILYLAVVYF
YGTTATSSGRASTKKITKSLPWFLFEEKVVPGRVGSLALLIAFSSSVATFSKTVLYWCNELFSGFA
NIGHNRLFDLIFLWILPNGLWLIFPAYMIYVFGGEILYALETSTPRPLKAGRPKSQ
>g374
MSCKYEDADERLNRLARVRENQRKSRARRQQYIEELEQKVAVCNAQAQQREIEHLIALQKLEAENA
KLRALLQRAGLAPGLVEDFLRDVSQPTVSEKIAIPRLKAPISPQSTSSHHSIPSIKTDEPYTPASS
AGVATACNNNATSASAIHYPVAMQKSAGCCGPASSTNVNAVYNINNSSQKPAGCCGPASSTNVNAV
YNNNNPCAPPSQYAPTVQRVEEPYPPTLANFDIVCNDNISCTQASECQDNCTTSKPLEPPAVAPAD
PVPTQQQQNIKLPSIASLCDCGPGSASSWPRLTTPVNTTLCDVAQDLIDQYNTKGVDLNVIRQRLR
AGFRNDDANGCRVQNNVLFEVLDEISGDAPSSGGT
>g375
MYQLERRAIFSRHWILLTHRIRFQKTGDYQSFDIANFPIFLVRDHQGRINGFHNACRHRAFPIVQQ
GSSGTASILSCKYHGWSYGLKGNLAKAPRFETVSTFDKTQHSLYPVHVHVDKAGFVWVNLSAENDT
DWKSKWEEQYKSAVDDNPRLQMFDFDDEFRFDHAWDMTVDANWKTLIDNYNECYHCPTSHPLIARV
TDLNQYRVEPNEKCAACLEHTIINKVEEREGQFRRSITYFFPTTSVTITDNFFYIQRMIPLSPTKS
IIENEVYRHKNAGDEEFDNITAFYRQVLEEDKQLCDGTQVNLNAGVYTSGMLHSDKEKGPIHFQHT
VRDIVMQHRQAEKQEKTEIWPATPKRTAGAATTKLAEEEEFCASLNSGLCGSRELEW
>g376
MDSTGPKSDSSPASIQLADEDSAIRDFYGSAISQSYRLKSELVAEHLAKIGMGKFQYLLFIVTGFG
WIVDNFWSQGISAVRPPVSNEFTDISRLSFSSVAYYVGLIIGATFWGTTADIIGRKPAFNTTIFLG
GIFACAVAGSQNFVTFCALWAVIGTAAGGNVPVDSIIFLEFIPKDYQWLLVGLSGWWNLGQLIVSL
LAWVFLANYACDSDEAPCPRSDNMGWRYNMITLGAIAIAFGLIRIFIFKIPESPRYLLSRGRDAEA
VEAVNYIARYNGKPETLTLAMLEDIDRQVSVNNPDAKISPTETTKQSLTDILKESFSDFTADKYRK
LFKGRTMAQHSVVIFLIWLTVGIGYPLYFAFITSYLETKSSYQTDSSLSYTYKIYCIVSAVGIIGP
IAAAFLVESRFGRRWMMGLSAVLTGVFLFAYTAAKTQTADVGFQCATGILGNFEYAVMFAFTPESF
PGPVRGTGTGVAATLQRTGGLVASLISTYGGYTVTPIYVSAALWIVVGVLCFGLPYETHGHASI
>g377
MRNSLLISLAAAALAEGKAYSPPAYPAPWASGAGEWAQAHDRAVEFVSQLTLAEKINLTTGVGWEG

GQCVGNTGSIPRLGFRSLCMQDSPLGVRDTDYNTAFPAGVNVAATWDLDLAYRRGVAMAEEHRGKG
VDVQLGPVAGPLGRAPEGGRNWEGFAPDPVLTGQMMASTIEGMQDTGVIACAKHYIGNEQEHFRQG
SQENYTVADAISSNIDDVTLHELYLWPFADAVRAGVGSVMCSYNQLNNSYSCGNSYSLNHILKGEL
DFQGFVMTDWGAQHSGVGDALAGADMDMPGDVAFDSGTAFWGTNLTIAVLNGTVPEWRIDDMAVRI
MSAFYKVGRDRTQVPINFASWTLDTYGNEYYYAGEGYKEINQHVDVRGDHAKVVREIGSASIVLLK
NVDGALPLTGSERFVAVFGEDAGSNPDGVNGCSDRNCDNGTLAMGWGSGTANFPYLVTPEQAIQAE
VLKNGGIFTAITDSGATNTTATTVAAQASACLVFANADSGEGYITVDGNVGDLVLHTVGPVLVEDW
VNHPNITAVLWAGLPGEQSGNSLVDVLYGSVNPGGKTPFTWGKQRSDWGTDIIYEPNNGDGAPQQD
FTEGIFIDYRHFDKYNITPTYEFGYGLSYSTFSFSNLQVTPLAASPYKPATGHSGPAPVLGKVLNA
TAYLFPNYIKRIEAFIYPWLNSTDLRTSSGDPNYGWSTSKYVPDGAQDGSPQPVNPAGGAPGGNPA
LYDPVAEIRVTVKNTGKVAGVEVPQLYVSLGGPSDAPKVLRGFGRLSLGAGEEAQWTATLTRRDVS
NWDTVSQNWVVSNYTKTVYVGNSSRNLPLQQTLALKIGH
>g378
MATSTAYPRSGSPSQLKRTNTNTRTHPPVTVLAVYPLTLLLGSIYSVISPTARSSQNFGSAAPLTP
TIATDINLPAEHRPNPVNYFARKNNIFNLYFVKIGWVWITLAFLALLLTRPYYTKAPGNLRGKRSV
QGLFRYAIATFAWILTTQWFFGPAIIDRTFVITGGKCERLPAVTPDDTWEEFKVALTGAACKASGG
LWRGGHDVSGHVFMLVLGSAFLALEALGTSPSHTPETSQVHDKDDLDGEHTAASDPVTKYSTKFVW
IVIGLSWWMLFMTAIWFHTWLEKLSGLLISLSVVYITYFLPRSLTQWADIIGIPGYEPAHRRAGLQ
RHWPSSHSNSSSVQSRMDRLEQLIVTMMSGKEASAADASNDRVVAYRNGQVPRHEASKEELSRPID
FVKSRMLRIDADYGNSYTVGEAHWAALLNEIHEVRCYLQTQQKQYEDQSQKISRLLKKAPDDPGPT
LLFGTSQLLGRGEILAHIPSRYTCDMFVERYFKTLDPAIHIIHPPTFQKQYEEHWEDPGRTPLVWI
GLLFAMMRIAAISWNRDGDEPLEFQGKSQDLSRSYRTRVADCILLADYTKPHEFLIETLVLHLFGE
YSSVKDANSSAWVLLATIIRLAMRMGYHRDPDRLPSLSPFQAEMRRRVWTFIWQADIFFSFQNGFP
SMVRIEPPENILPRNIYDESFGPHCVELPASFPPSEPTQISYLICKARLALGLARALKELNREDTP
PSYEKVLEIDRSIRETYAAAPEYFQLRSMAEQQHDQVCLIYARFALASIHHRAICVVHSRFLEAAR
MEPLFVNSRRTCLESAMALLSLQAIQHQEVGADGQTRKLTKYVNSLVTHDFLLAATVLCTELSIDH
GRDPFPFPVTGPTRAEMIESLDRSADIWSQMRDESIEAYKASDVLGMLLKKLRHPNENMQDAQPRN
YPAPTSMSLEAGPVPNSTQKDLGAAVNTGTLGLPSPQRYGSRDIFPTGYGYTVPMNVSSARHPSHQ
DPVRLPSYLC
>g379
MEALSTSRLNQFEKSMESLYGSFSNIKDPEQWTPPTNSGGHRGRYLWTDAFGVVNFLTLHQEHAKI
AAAAGSASVSGSGSAPGINTADKYLILARRLVGSVHDVLGRTRDGKTRLPGATDENPLGGGLRIGK
LDETGVDGDGQYHHYLTLWMFALNLALAQAIHPRFFVNRTSTRPRMVWKMSIDLATPLVPSEGNLD
PIDGYVVYRLLQASAMAMVGGGEGSNVGSDKPRVLDEEITDYKRVMERKGEHFVSSDTLDLGMTLW
TCHWFSDKEKWASKLAERCFQQIYELFEIDRYLARNIKYRLAFREFGTSLGIGCHYGKSGERERSI
DLRTYSDSIIESWEPYMALSLRGDSDLLADDLRPITRVMYSSALIPGVLSDGSFSDWIPWTGAENN
TMS
>g380
MPGQLTQLVQAAAKPLPDIRDPLFGQMFDTFRNYKVVLLGDGSHGTSEFYSARAEITKHLVDFHGF
NMIALEADWPDAEAIDRYVRLRPGPKGRIGGRSKIEPFKRFPTWMWRNREMQDLVEWMRDRNAKVR
PEERAGFYGLDLYSMGESIKSVVDYLDRVDPELGKNARKRYGCLNPWVDDPASYGLAALQGMENCE
SKVVQILKDLLQKRLEYSASAHDGEALHSAEQNAHVVHDAEKYYKAMYYSSASSWTLRDTHMFDTL
DRMFQFKPPGSKAIVWAHNSHVGDARFTSMGQRRKEINIGQLCRERYGRDNVAIIGCGTHTGTVAA
AHEWDEDMEVMRVNPSRTDSWEYVAHSTGIPNFLLDLRPDFASSQLREAFAQEPPKLERFIGVIYR
PDTERVSHYSQAYLHNQLDAYVWFDRSSAVNPLERVQPRTALGKEETYPFGL
>g381
MAPRRSARLRGNTPVTEDQFPAVPTTTPAKLAPVVENNATQTKDNAAKTITTPKSQPSVKKSILSP
IEKPSMKTPSTIGSARPPREEMHPSKAQKSTTKKLDSGLILGFQPVQRDSSGNVIKETLTSNTPSK
VLSSPPSNLGTPSKFGFKFSSDDAELSEDARKLMESVREDVARIKAQMILDRGEQKRKAEAGEQTL
EGRRIAKPKGKAGRYSDAHMAEFKKMDSIAGHPSAFRAESARFTPVTKSLKRKNSKASLDEPETQP
SPSKSPLIEAKRVKRTEIKTTTTTRQLGNIGTPCKMAIPQPSRSTFRSSSLMTPTRSSAARFASAR
PMKTSMIPSLADSLVSRQALPQTPKTEFNPRLKKSLPSLGNLKSILRRRQPLFSDDPAKIAAGTHI

TAPNFNPDINLKDLPTANFSGIEQTPSPKKRVEFTSSTKSRRESVLATPTAIEFPPEAIAASTSDI
VYPVLPETMTPEKEVIDTPTIRRVRESIPAAPYSPLSTIPAIPHGIPNKERKHDSKKEQAVNFSSP
ALKTAFSVPSAASPFPQMPTVPHGISNEKRSREEHGKKLEAKSTPILKTTSSPLKAKSPSASLLAM
PHGIKNKKRQRADMSDDEDTENIPPTANSVAERNAKRLKVSPMKLNPVSPSPIKNRSIITPGRMTG
NKGRVSTPGTASKKGVLSLSRLNMLSKPKLRS
>g382
MASRLDRLVTLLETGSTQLIRNTAAQQLADVQKQHPDELFNLLGRIVPYLRSKSWDTRTAAAKAIG
LVAANAELYDPNEDDGLTIKKPDGDDDDVEIKTEVKSEDSIPLAEDLLRLETLDVGSILKFGRKLL
GSAGKEYEYTIGGMDPAARLQHQKKTLTARLGLAGEYLEDDLVDEADFTSTAKIEKPVPVPSRQNS
VINGSSNEPQNGEESGLSKRQLNQLKRKNKQNAKLGANKVRVVDLSSRKPSDSMPTPSVATPYPIK
SEIGEEENEKKPDYFSLDRSSADDDSKIVSEFKGPIVPEKPLIQTEAEEQGKEWPYERMCEFLEVD
LFDPNWEIRHGAAMGLREVIRVQGSGAGRLLGKSRKENDLLNRQWLDDLACRLLCVFMLDRFGDYI
SDNVVAPIRETVGQTLGAVLSHLPSKSVIQVYRILHRIIMLRELGLERPIWEVCHGGMIGLRYLVA
VRKELLIKDAKMMDGVLEAVMKGLADFDDDVRAVSAATLIPIAEELVASRQGSLSALINIVWDCLS
NLQDDLSASTGSVMDLLAKLCTFPEVLDAMKANAADDPEASFANLVPRLYPFLRHTITSVRLAALR
ALTTFLRLEDQDSNAWVDGKAMRLIFQNLLVERNEGVLKLSLEVWYDLIKALDARNLFTAETLSSS
VQPLITLAMGPFGVPRYPIPMNASLFIKPSGVAYAFSHPQTPGRKSSPVNAPESGKIRRRKSEKKE
VPPPSAHNVDGHMLSGDIDLVGLDTMVRSKIFAAQALGQFLFMWDSKDLPSLWESIVSGLTLSGSS
SQLAAAMIIEEYAKRAGATGNYTSLLCENLRPIIETDRPGWYSDIVSYLQITRAQCHSLLNAFRDH
AHVPSGRLPTLAVVVQGDPEAGPNAFSINDAEKVVGPDFERLKKSLTPAQRVTALQVLNDTRSSAE
TSIEEAKYIKDQRDLRIRAATAGALVALQDIPKKPSHIIKGMMDSIKNEENSELQQRSALAIVNLI
NYYTTATKRGPVDKVIGNLVKYCCVDTSETPEFHHNVHLEKFILSLRKEEDRRDHVDAAKFEREAR
AARIMRRGAKEALEQLATKFGSELLEKIPNLAALIERPLREALEGDLPANIREPDNELGQEAVDGL
STLRALLPKFHPGLHSWVIKLMPVIAKSLQCELSVIRYAAAKCFATICSVITVEGMTMLVENVLPT
VNNALDVHHRQGAVECIYHLIHVMEEGILPYVIFLIVPVLGRMSDSDNDVRLLSTTSFATLVKLVP
LEAGIPDPPGLSEELLQGRDRERKFMSQMLDPRKIEPFEIPVAIKAELRSYQQEGVNWLAFLNRYH
LHGILCDDMGLGKTLQTLCIVASDHHMRAEEYARTQKPELRKLPSLIVCPPTLSGHWQQEIKQYAP
FLNCVAYVGPPSQRSQLRGELEKADIVITSYDICRNDTQIFTPINWNYCVLDEGHLIKNPKAKITL
AVKQLISNHRLILSGTPIQNNVLELWSLFDFLMPGFLGTEKVFLDRFAKPIAASRFSKSSSKEQEA
GALAIEALHKQVLPFLLRRLKEEVLNDLPPKILQNYYCDPSDLQRKLFEDFSKKEQKDLQNKMGST
EKSAKEHIFQALQYMRRLCNSPALVIKEGHKQYDDVQKYLSTKNSHIRDISHAPKLTALRDLLVDC
GIGVDPNTEGELDTGASYVSPHRALVFCQMKEMLDIVQEDVLKKMLPSVQYLRLDGSVEATKRQNI
VNQFNTDPSYDVLLLTTSVGGLGLNLTGADTVIFVEHDWNPQKDIQAMDRAHRIGQKKVVNVYRLI
TRGTLEEKILSLQRFKIDVASTVVNQQNAGLNTMDTDQLLDLFNLGETAEGAEKPVPESAAGNEAD
MVDIDGEVKEKGKKGWLDDLGELWDDRQYQEEYNLDSFLATMKG
>g383
MASGFGLNGGPSRCFAFWQELLGCYVVNGSEGVEGKKKCLFALEDYNECLHHSKEIVRAKRLQAAY
RKAEATFPRENAPKAEDIRGLGLLGKESQSAKVLGLE
>g384
MWIGLSIFYWSAVLNVDSAATYTTTTTELIAGPRTAVLHGGLQSRMTNLDSLSDVQAILLACELCA
ASNVAALPHLQQHFPTSLTAVRLFRILLSFLPETTPPQQYTTVLDEIDRGTSLTPNTGDIDITNVK
NIEESVAWKRVRRLRLLPLKYPQANKTTETTDPLTEFLIHRAHRIDAETGLQTYILELILPFYERS
ESLRQWLVSKILPLLRSNYEYYPDKEDTISLSVLESMDQTTGINVLLSMTGNTASATDLTRNLRGL
VGPWMCGAGQTKRRRLNHDLETNNATDEATTANWDDVNDWLLSRSLVEFDSVASALDNWDGPEDVD
LGGYMDVDFSESERLRKSYGQAGLAIIYASDPARGSLERLYQITTKIARLLQIDTATDMNLDDPSL
SALDLTLTAISTASKASLLQNGLLRGDNALTAPSRHSISFLQAILISARILRDYGYITSCRSAASV
CLHGNAETQSWEVNAVLDMIVKQPKPGQEWKKIREQILWLRDWRGSLNTSEKANECHGLFWRVSRV
RVEIEILKAMVTTGEYRLATDIYTQSSSPLDSTQVETAVVDTIISFYDTASNGNKTRGKMKKAVEI
LNAFQPRFTNSKRFKELTALIAATHALSFYSLTLQHGVPFQPVSIRVHEDPLALIEKVLDQNPKSY
TKLDDLISIGRNFVTAGLLPNLSEDTEQSSLVIAERRIISMAVSSALASDDFGTAYSYILTRLTPS
SLLPSSTSDTITTTSPVEEDISWRAAYNAGRYRSPTTSTEADLTSQINNLSQRMELLSLALILAPS
ADPLPEVLGAWRRCDEEMSILRNREFQEAEEWDQRGDLQSAGASAGGPATVPGGFGPTDRELDAYE

NAQRQQRKSRASYYDGANNRARDGAEAPMGLFDVARGAARAFSKNLTPLQQLQQQQQRQQRQQLAT
EEVEGEEGELSRSTESLERVRKRDVVSNMVTGGLASGIGWVLGAQPVNNR
>g385
MSSPAPLLRPPVPGGRNGNGARTPRLTLGIPPSPSMKPVNPPSEDPRISDIPLPQASSRPAPPQLR
LATPMGGSNHQSHESGRPTPYLSSISTSSNSLSDASNGLSTSTTHLDGKASGPGSASSSSYSTLSF
AMGLRQPTVGTPDPSSAISSVYSDRDGGVSMERENSVNSLLPDLDKLSLDKGRPLDVEDLDDEGWL
AASEQKKIVELGSLGEGAGGAVTRCKLKEGNTVFALKIITTDPNPDVKKQIIRELNFNKDCASEHI
CRYYGAFMDKTSGTISIAMEFCEGGSLDSIYREVRKLGGRTGEKVLGKVAEGVLNGLTYLHSRKII
HRDIKPSNILLCRNGQVKLCDFGVSGEFGTKGDANTFIGTSYYMAPERITGQSYTITSDVWSLGVT
LLEVAQHRFPFPADGTEMQPRAGLIDLLTYIVRQPIPTLKDEPENNISWSDNFKYFIECCLEKDPP
RRATPWRMLDHPWMQDIKNKKVNMANFIKQVWGWTD
>g386
MRFSIAALALFAGASVAQITVTSVDVETITSCAPSVTNCPARSSATAPAAVSSGVSPVSVAPAPSK
AVTSSAPAVPAPVSTSTTLSPVAPYPVSSASLSTSVITVTTCVPTTSLSTITFTATPTPVVPVGTG
SPKPVSSSTAVPGKSTTTPSSPKYTGAANAVSGSIMMAGAAAVVAAAVFLA
>g387
MEDLSSLEGPPIQDGRFTPISFPMSPVSPENTYIAYNPLRDVPQGSVSPLSSARSAYARSSISSVQ
GSNQVPPLRNSRSHSALRDTTDVPPRPASTDNSNSNTSAPFWLLVNGRLVWIEDLQLWVLWDDVVA
SQNLGEYYTQQQQRGQQQQQQQQQQSGRGIRTWKSLPPLPHEIHHLLPMPMDIGPSFDPNDRIQRR
DHYYSQTSPTNARIQLQWASLAGQLENTRSHQRNWNRQEPYQDNLNLSLNTNLNTNLSTNLNTSLN
TNLNTNLNTNLNTNLNTNLNTNLNTNLNTNLNISLNTNIIRTNTNLNINNSITIP
>g388
METLGIRLPPPGFRAQDAPQPTTTYQQPWRHDVPNVHITPPNPVNQSTTQPYEQTYDSLSASYAQF
QPDPDHLEECGLSYTPSLSGHDLAEGESHLSTYSSTMPGQDEHYPAISGYMRYPEIDPEALYPFDY
NNLIPHSSSLSDPDPTMIRPLTAEELNLPGFSMTSFCDPPYQGTLGDFFIPTSAPSVQPIPPLHVQ
RPPYDMTKDFRQYHESHHAPSNKRAATDSPLMESHVSKKPRMILTPTLRTPSGRIQKPASVQQSVR
AVQTQNETLSKLIIPPTTAAGRPSISPPLPLPPQYQQYQQTHQVAPPFLLPSNADSSGILMKPTHP
SMRRTVSTSSSESHSRNAESNKKLYANPLKPPDLLGPLYEEQESPPIKDMTPDDPEMIPHKQNLRF
ENDLYTPRWVRGHGNKREGWCGICKPGRWLVLKNSAYWYDKSFSHGVSAVTGKAFEGPREIRRIKR
MRNEDIGNNGGAGEAASESGQVLTQRFGGKTTTTWEGLCGSCNEWIPLVSSKKKGTTWFRHAYKCQ
TSPDKDKEKKKDKERKVKSREDSSPKRKDTKSRGRPQSSDLSLDLDSITIPAPDSPPPTFSPSSLS
SSPQKSASSSPRKSSPTKTTLSFPNLRPPHSRSSSTTTSKPAFFSSNRNSRSEDSHPLNLPPDELR
RHLSAMAAARDDSMRSSMDIDRDTTASPQPEATPMSTNGTQNGDTDRSPTPPPHRSPPPNSDGGES
HKLAGNKFYKQGDYERAIQEYNKALEVNPNSSVFLSNRAAAFLSANRFIEALDDAQRALDLDPENS
KIMHRLARILTSLGRPADALDVLSKVQPPASAKDRANAETMLRHITQAEDSLKNGKGGSLVVFAIE
QARQMLGPGVKTPRKWQLMAGEAQLKIGNDNXFGKAHDVAISLLRENNQDPDALLLRAKAYYGQGD
NEQAVKYTRMSLQLDPDNKKAFTLLRLVQKLVRTKEEGNAAFKAKDYKRAVELYTQGLEIDPTNKD
TNSKLLQNRAQAHIALKEYEKAIEDCTEALRLDPGYTKAQKIRAKAHGAAGNWQEAIKDYKNVAEV
NPSEKGIQEDIRHAEFELKKSQRKDYYKILGVEKDASDGDIKKAYKKMAILHHPDKNPNSSDEKFK
ELGEAYETLIDPQKRAAYDNGDDLIDPSEMFGRGGSPFGGMGGMGGMGGMGGMGGMHGVNIDPNIL
FNMMNGGGGXGGFAHAGGNPFAGAQGRGGGFQGFQF
>g389
MARKYWGGSGDALTVWISIAASTVIIFYGYDQGVFGNVIIGENFLKTMDYPSSNMQATMTSVYNLG
CFVGSMSTIWTGDIFGRPRQVIIGSTVIAVGAVIQTTSFSVAQMMVGRVVAGLGTGMNTATAGVWQ
AETSKMRSRGKLIIIQMANCISGFSLSNWLTLGFSFLSGSIAWRFPLAFQIVFTLVIYVLCPFLPD
SPRLLIRKGKYDEAKEVIAALQGDGATPESPSVISQFNIIKDILDREHMTTYTWGQLLTGRGPSGV
LRRMILGAWMQAMNQISGINITSYYMSYVFINALGISELLSRILAAAGSVDYLLFSCLAYFVIERY
GRRKVMMYSAAACCTCWVVIAIVLAISDTGRGDSYQLGIVAVSFFFVFFASFGLGVLGVPWLYPTE
INALEMRTKGSSLAMATNWIMNYMVVQVTPPGIANLGWKFWIIWACICFSFIPVTYLFYPETANRT
LEDIDRFFETKPGIIVAWNKTATQLERPEEYIRMDEEIERLATANNEKLSDNDDRIEKTVQAVDSE
TDHREHV
>g390

MAKDPENMKTPLLSDDDLERSDSFEHIAYSDSDSTSLSGSTVYNDPLVPEGKGAANLDAKADTKSL
PGYTVYETTHLEKGPLVGEEPGQGDGEENKCKRRGCFRRRRCRRASGQNDKKRRIRRRIVMFIKFM
FLIGLFSFFVTRMCVKHRRGTHSRPPVFSDHSSEDLQAGREIILNKGSTAVYGRYPLYDLLNIKTT
SGSVNIIVDPQPADPKKPDEPARLIIETEAGAVSVAFLQYSIDTIATFENGDTKKVDNIIDLDYLK
TSGATSISQVKGRIPYRPYEVEIKTQSGSINGRLVFTTSAKLETQSGYISASFVPVVSPDEVVHAT
LTTATVSGGQDISVGEPLIIEASDKTYPAGTGEMSTSSSSHTSQSGSLQIKYPHSWAGHVEAFTKS
GWIGLGGDGVEVIEQTQGHAVGVKNPDRDHQKHGWWGSRGDMDVVIESTGSGSIQFHVKDGLFPVE
ESEDFEVYILGEN
>g391
MDGPPPPPPPHGANPNTTEGTPRYRKSTDLPDGPYDIFVIPPHSAGGGFIYLPSLNCHRNSFIAGS
LSTLAIVVLWIQLQPLVKTWLAAMSKSNAPGGMGVILLVVAVGFAGWYFGTLQNDAGGMNGNSSGR
PRPGAGFANGFQYGGAQPNPGASYPGGGPAPNGQYNNTGGQYNPGGASQPPPFNNHSAQPNGAAQD
DTQDNTPKNAQNSTPKEDPKKAEAEWERAREETRRKEEIRRKMEAYKKKREAEEEAERRRQQEREAM
EKELKQRKEELEREAAAAKAAAEKEIRERAAREEAERAERVRREAEEREEKARKEAAEKEAAAKEA
AKKEAAARFAAAKEAAAKKFAAAKEAAAKAKEASKAPPPSAAPAPSAASAPPRTPSPKKPPPPSAR
TADVDDDAYSFRPYDRPRRPYGTSAPSVYSESSYAPSQSTARTTPPPSHRGPYMTKDPDKVIIRGV
YLFNNAFQKTPIAQLVSGQGSVTDGLVLRITTEGLFVDDDVRGVAQREWDVKAWTLKSVETIKLRG
LQVVRASVRDQEGKRYVFVLTEAEVWKVTQGFKKLKSGSQARHLSQSELSKNEANTYLTNLGY
>g392
MASYHSQAQSRSAPQAYPHQAVNAISMAASAPAGTFVPGTKVQVGNHRVIIEKYLSEGGFAHVYVV
KLPHPIDGVETAVLKRVAVPDKTALAGMRTEVETMKKLKGHRHIVTYFDSHASQLKGGGYEVFLLM
ENCQGGGLIDFMNTRLQHRLTEPEILKIFSDVSEGVACMHYLKPPLLHRDLKVENVLISLSSGKSP
LYKLCDFGSAAPPRPAATSAAEGRLIEDDIQRHTTMQYRSPEMIDVYRKQPIDEKSDIWALGVLLY
KLCYYTTPFEDVGQMAILNASYKFPSYPSFSDRLKRLIASMLQEDPQKRPTIYGVLKESCNMQGKE
VPIRDIYAERTQSEARRNQELPPPSTEAPRVGAVFSPPMQETQIIPEIAPMRRGRPSKTSSQQNSA
HPSPSPYRARSSNDPFAALDGNKRDSQNADEIAARFPTLDQFSLLHEKGGKFEFEPTAEKKPEADD
LSQRVTNALADDAFVKPASPYLPAKEEIESHRAVSVSVAEQAQKLQSPSLKAEESSSRKSSSQRPT
MVSTGTMTESPPPATSENRASISRPSSSRPIYKFPPPSQDRQRPERPWASEIDRPSSGLGTVPSQT
TTIQVVEPKSRVSSEDVSRAQSSARPSLETSRPSFRELGDGLNRPKSANSRVRPSSVHLGGRFDPT
SSRDSPLRSPALDTRRPDYEGGEPLKHARTDGDYDAERGNITSDVGYLRAREEEEAARKKEKRLSG
GXKHVKRSSLSSLAISGKGLLAGRFGDAFRRFEQSTSDNKSRTPSPSEVDKQLTPIAGSEATDDRS
DDDALGSEATDISPEMRRELERRRLSQEEKRVANAAAEYRLRVAGRGGGGREGTKSPAIQNRVQSL
LQDNSRPAPKTASGYGKYTNSESESEAAQASESDREYTPRLPTRPKPAPKDIRGAPALSKINSPGSST
QPTKPSPVDTIPSQRTAQRPAAPPKPKKLQTGGSRPDERVPPSSSVGVDPTSPTEDWERNFSRRYP
SLSGLEMVETEIDVPKLSSIRTREV
>g393
MAPVVLSTVENEVKDVIQQLFEIQSAVHGYLGSETQIELVRKIKNLTIALNTLSNDTKPDPSINPE
TYNIDNSTANRDDPPVASIQLPPEIIDYVDAARNPDIYTREFVELVQRGNQDLKGKKEAFASFRDV
LATEMRSAMPECRREVDRVVANTSGPNRGA
>g394
MASTANSAPSNGITGGYTGHDLGLQIAMGTFIGIAWYNAIELIALIFVTFRNYRGLYFWSLLISSA
FGVLPYSVGFLMKFFQLTSATWLSVTLLTVGWYTMVTGQSMVLYSRLHLLNRNPKVLRPVLCMIIV
DAIILHIPTTVLTYGSNFASSNHHFVEGYNVMEKIQMTGFCLQEFVLSGIYIYETIVLLRANPEHG
RRKIMYQLIAINMIIILMDVALLVVEYKNIYVIETTLKGAIYSIKLKLEFAVLGQLIRLVRTHTWK
PQSAIPGARDGPVGLNIINDDALPDFVDATRVTSDLTHATPPPRTSSHHRPFDDMDDISIAMFEHS
TSLQDQDEMREIRPQSTNSTGKSWSGKTRMNGNTNGANHQPPDSETVDFADEAHTTRSNSHDQGRY
LSIRLSNGHQPG
>g395
MWWHLLPLSILAALGAAAAPSCSSAPSTLHLPDAPYDNFFYSDCNTAAQVVVTTPQPDSNLSLISP
RLVIAWPAGNSGVCTYFAPQNGVNGSLSIEVVNSTVGNPLAATYDDSNPTSGNDTTPFYGVTGTIR
FNSSAVLTVPILGSIRTIRDFVEGPSLLQPQIQGAINASSLSNGGALLERLWLDNMTSTYLNFTPT
STNSTVTVGKNNTLTFEAGDYIFTAKFNYPQLKQLSPAQVLTNASTDLIKQDSERTSALSFLSYST

KLLAGAWRFLTYFGRDSMISALLLEPILSKGENGAMEAVIAAVLERINRTDGSVCHEETIGDYATW
LNEEQYGLYSTEAQCDYKMVDSDFYLPVLMNKYFVEDSVGRTRAGAFLNTSAGAFNTSNGDLTYQQ
MAAINAERIMKLAAPFAGHKNQTKDNLVHLKDGQIVGEWRDSTYGIGGGRIPYDVNTALMPAALRA
IASLARNNFFANQTHWAKLADRYAQVWEDETLSFFRVTVSESDAKQLVTSYAQTIDFAGPNQTDTI
DGDVTYHALALDGNNNQSQVLVMNTDDCFRHFLLNTTRNQDQLTAFINQTANNIRRTFPAGLLTDA
GLIVANPAYGQDPVYASNWTSGAYHGTVIWSWPLAMMAKGLEQQLGRCETVNSSISHSQHTRIRIP
NFCKDATVYDNVKAAYNTLWDSIEANQAQLSQEVWSWTYDSSNDTFVPTPLGVMPPPPGTSSQTGM
LSFHLYMVNIDTDYGAC
>g396
MELTYYLILLGLLVPLGIITYWIVPYFTTYKELQAIPGPFICKFSNIWIALGARNGQKYAWVDWAH
RKYGKIVRVGFNHVSIATPEGLHTVYAHGNGFLKDHFYEAFISGVPGVFNVRDRVQHTRKRKIIAH
AFSPASVHAFETHMSNNLRRWTSQLDKLAAQTPKDTYSKMNIMPWCTYIAFDIIGDLAFGAPFGMV
ERGQDLCESRLPGEPVVYVPGAETLNRRGEVSSTLGLLPDLRPYARYLPDPFFSKGLQSVRNLHGM
AVAAVEKRLAAAQQTDEKSSKSRNDILDMLLRAKDANGNPMPREELVSEALTQLIAGSDTVSNTTC
AIIYYILAGERAAPGTILPKLLKELDDAIPASEEIAQFSQVRGLEYLRRCIDEAMRLHSTSSIGLP
RIVMAEHGIQYADHHFPTGTVLSVPSFTIHHDADVWGDDVEEFNPDRWLPENLTPRQKICFNPFSY
GPRACVGQNVAHMELALIVGTAFHRYDFELHQTQLKSHEGFSKKPLECWVGIKRRH
>g397
MDARIDVFRALGLKEGDAHVIRNAGGRVSDALRSVIISQQLLGTREIVVVHHTDCGMLTFTDDHLR
SKIKDDLKQNVDHIAFLPFKDLKQSVVDDVQALRNSPLVLDVPVTGYVYEVETGKIVKVDV
>g398
MARPRTAGRWNPFAFYRVPVTVFVTLIYVALLAPLVVVQNILPTVPESELEGLDLHEAWQDLQHLT
SGFHPYNSRKNDEVRSWLLSRIEDILSKDAKQQDGVQTYVFDDNQSNLTVVQSNLGVYFEGTNIIV
YIRGQEDDKREWWKEPGLSPSGKGGVLVNAHYDSVSTGYGATDDGVGVISCLQLIKYFTSPGHEPT
RGLVLLFNNGEEDFLNGARVYSQHPISKLPHTFLNLEGAGAGGRATLFRSSDTEVTKFYKRSPYPF
GSVFSDAGFKLGMIRSQTDYIVLEGDMGLRGLDVAFMEPRARYHTNQDDAKHTSQQALWHMLSAAV
ATTEGLVSDTSHNFEGRPQGPGKLPSGTGSGAVWFDLFGTAFAVFEIHTLFALSVTLLLIVGPLTLL
ITSIILANQDRMYLFGISVSVDDGFGSVPLRGWRGFFRFPFIFGTTTASVVGLAYLMAKVNPMIAH
SSEYAVWSMMISAWFFVAWFLSRIANFARPSALHRIYVLTWMFILTWVLLVITTAYENRDGIASGY
FVIFYAFGTFMATWISYLELFSLPKKGDFANKVSGQISSRRTSLGGSRLLTPSGESVGQHQEDDEP
TESTSLLRGQRTSFANYTRSTEDDGDGDLLSTGVSTDVSDARDLNVYGYEQPWSANLPKWTWILQF
LLIAPIIIILVGQLGLLITSAIHQTMQDGSSALVPYLMIAILTTLIFMPTLPFIHRYTYHIPTFLF
LIFIATLVYNLVAFPFSGNNRVKLFFLQEVDLDTGANRVALTGIQPFVGDAVASIPSTHGQNVSCV
PKLDRTECSWDSNLEPHVITHPGSGKEQNPLNQWVKYKAVRLEPGRQNSNRAQIKISGLNTRGCLL
HFDKPITQFQVAGSAIDSRFPSNPSLEDGTEDGVQEIRLWSRTWGNQWVVEVEWASDEDIQNEKDT
LTGRATCLWSDANTQAAIPALDEIIQYSPDWVAITKLDAGLVKGSRSFEV
>g399
MHRSRAAARSSSKTPSESSTSSSPERAADDDTDFFAQANDSQSSIGVGTFREQIGSNEPDIIIPPI
GRLPPEILISIFSKLSSPSDMLHCMLVSRKWAANCVGILWHRPSCNRTENLRSVVTSVGKIESFFP
YSELIRRLNLASLAPKITDGELSAFTQCKRIERLTLTNCSKLTDKGVSDLVEGNRHLQALDVSELH
SLTDNFLYTVAKNCPRLQGLNITGCSQITDESLVVISQACRHLKRLKLNGVNRVTDRSILSYAENC
PSILEIDLHDCKQVTSRSVTALLSTLRNMRELRLAQCVEIDDSAFLRLPPHSLFESLRAMDLTACE
QIRDDAIERITDAAPRLRNLVLNKCRFITDRAVLAICKLGKNLHLVHLGHCSNITDAAVSQLVKSC
NRIRYIDLACCNLLTDVSVQQLATLPKLKRIGLVKCQAITDWSILALARSRALPHSVSPSCLERVH
LSYCVNLTMEGIHALLNFCPRLTHLSLTGVQAFLHEDLTAFCRDAPPEFTQQQREVFCVFSGEGVM
RLRDYLNRNVGPFPSETEATMYDDEDELDEEENHVTGLMSAAAIRDDDDDMGDSVIEVGPPTG
>g400
MSGPKSRLSGLLGHFLPSHVDHDVNKVCNYHTLSPTFFLPRAAAIEPEAEAIYHLTANNKVLRRSY
IETADRARGLAYYLKKHKFKRVGILAPNTPAFLESIFGIGAAGCVNIAVNYRLKPEDIAYIFAHSD
ADLIIVDEEYVSLLDLFRSEHPNIPLLIDTDTDATEGQLSGPFDAAILEGLGYDKDTGAKGWNGLE
TQAADENSTIALAYTSGTTARPKGVEFSHRGSYLAAMGNVIESGLNYHIGRCRYLWTLPMFHAMGW
TFPWAITVVRGTHYCLRKIDYPYIWQLLKNEHVTHFNAAPTVNTLLCAAKEAERLPDPVRVTVAAS

PPSAHLFEQMTNLNLHPVHVYGMTETYGPITKGYHMPVWDTLPDKEKYEKMARQGHGFVTSLPARV
IKTDVKGSAIVDVQRNGLELGEIVFEGNICAKGYYKDPIATSKLFEGGVLHSGDLAVWHADGAIQI
MDRAKDIIISGGENISSVALESMLVTHPDILEAGVVAVPDSHWGERPKAFVTVQSGKHIEGSEIIT
WAKTASGISKFMVPREVEVVPELPKTSTGKVKKNVLREWAKGANREI
>g401
MQNPGTAEKKQHPEGWQSLDEPGRQYIRAAGWATLASLKKRNIRTCLGNLVTSTGRSSQNQTACAH
CDPLPQSYPPTPVRDAVLTFSRGLLGGKEAKRSEEKLPTKRERNSSCQASPPKVDFKLQTDNPLRQ
QPALRLCWIFFEYQSGDKSRMSTFEPVVVIDGKGHLLGRLASIVAKQLLNGQKIVVVRCEALNISG
EFFRAKLKYHAYLRKATRFNPTRGGPFHFRAPSRILYKTVRGMIPHKTARGAAALERLKVFEGVPP
PYDKKKRVVVPQALRVLRLKPGRKYCTVGRLSHEVGWKYQDVVSRLEERRKVKSSAYYERKKAIRR
QLAQAQKTAKVDDKVKSQLAELGY
>g402
MSINIPDFDPYTVLGVDKDAPNSAIKTAYRKLVLKCHPDKIQDEKLRATAVNEFQKLQESYEILSD
EGRRQLYDQEIYLNKLRKEAAAQKAYASREYRDGQMYEERVPQYFNSSDEHLYTEEPMSYSQKYAD
GSKRPHTKATEERKKSKGPSVYTYSTARPAKQQSDRDHAKAAHYDRARTRTKERRRDASDKYERTA
AGFDSDSEDAFSDFERDQAHYRAKVESEIRYSSPRESVPPRRKADTVPSYSSKHHRSRGSSTGSDS
EDNHDDYPSYSTSKLDGQEEWAKKYIGRTTDPVIRPHTSRHAGYEDRERRHSETPRYSTRSSHSHK
DSVHVETSSPRTNRRSNERLNSDERAERSHDYERAYERDRRRDRDFERDLDREFDRRERERERERG
RTVPSLSTAASQKVPSSPRAPPTITRSATAPYPPRSRREGSSRSESKGPLSGIVNGLASMVVDGFA
GDTKASSRSSTGRGVSRGASRPRGTERNDSGYSSGPSTPEMPSNASAKIKQYKVDITGDRPRVESV
DVYPPPSSSRSERARDRSAAPRPKTTTRTKTYSFEPEIITRQPASASSKTSSSGRPLYGEIPMDGH
RYTRQTVA
>g403
MTSLKQFIRNVRSAKTIADERAVVQKESAAIRASFREESHDSNVRRNNVAKLLYLFTLGERTHFGQ
IECLKLLASPRFADKRLGYLGTMLLLDENQEVLTLVTNSLKNDLNHSNQYIVGLALCTLGNIASVE
MSRDLFTEVETLITTANPYIRRKAALCAMRICRKVPDLQEHFLEKAKILLSDRNHGVLLCALTLAI
DLCEHAEEFDEGPEDVVESFRPLAAPLVKVLKSLTTSGYAPEHDVSGVTDPFLQVKILRFLRVLGR
GDAATSELINDILAQVATNTESSKNVGNAILYEAVLTILDIEADSGLRVLGVNILGKFLANKDNNI
RYVALNTLIKVVAVEPNAVQRHRNTILDCLRDPDISIRRRALDLSFTLINEGNVRVLVRELLAFLE
VADNEFKPAMTSQIGIAADRFAPNKRWHVDTMLRVLKLAGGYVKEQILSSFVRLIATTPDLQTYAV
QKLYSSLKSDITQEGLTLAAAWVIGEYGDGLLQGGQYEEEELVKEVREGDIIDLFTNILNSTYATQ
IVTEYITTAVMKLSVRMSEPGQVERVRRFLASRTSDLNVEVQQRAVEYSNLFGYDQIRRGVLERMP
APEIREEQRVLGEPTNKRQSRMLKDKSKKPVKQSEDMLLDLIGGSDVSTVSSPTGANGVSTNTADL
LADILGGSGTTXPAPPAAPSNTAAIMDLFGSNGTPPPARPQTQSPLPTTASQDLLGGLGGLATSTP
PPPAAAAGHTAFNKNDLVLTLQVQRNASGSGAQILARFRNQSDFTRITSVGLQAAVPKSQRLQLSA
ITKADLNGGEEGTQGMRIAAVSGALPPKLRLRLRVTYGIDGASPVTEQVDWTEP
>g404
MSTTATSNGSMTSQVLRARAIKSLQAADEQLGNTNSILERESIDSSGASTPIPDDAPPSVRSISTA
RKQVRARRRLFHTIDYVPRVSHFDPQSDYHNFRGFFTLFWIGLFIMVVTTVLRNIKDTGYPLRVKV
WNLLTANVWQLAISDLAMVVSSGFALPLQKLTRKSGNLLRWYRTGILIQSLYQIGWLTLWIKWPFM
LHWTWTAQVFFTLHTLTILMKVHSYAFYNGHLSETERRLSELDKPGQGSMAAAVRYPSSPARAETM
NGTFNFKQEEPLSRLRDDLATELTSPLGQVTYPQNLSLSNFVDFLFCPTLCYEIEYPRTPTIRWTE
VFFKTLAVFGCIFLLTLTSEEFIVPVLNEASISLASVNTWSDQALILAETTSMLLFPFMITFLLVF
LVIFEYLLGAFAEITRFADRRFYSDWWNSCDWLEFSREWNIPVHHFLRRHVYFSSKSYFSAPVAMF
ITFLVSALAHELVMSCITKKLRGYGFLLMMLQLPIIAIQRTPLIRQAKTWNNIVFWFSMILGLSMM
CALYVLV
>g405
MAREAFIPFLVAMMLVTGVCNTILNKYQDMQCVRDCDSANPAHHKLFEQPVIQTAQMFVGELGCFL
VVLLSYLHTRFVASRKSSTAPLFAGGYRPVPDDDHLPEYHDGEEDGENEAGANQNNHIPEAAIAKP
LDETRRVLNGWRILMLAAPACCDILGTTMMNAGLLFVAASIYQMTRGALVLFVGLFSVLFLRRKLY
LYQWFALVIVVLGVAIVGLSGALFGHDQGHDPDSGVSDSMLKLARDVDAAARTPEAVRVIIGVLLI
AAAQIFTATQFVLEEWVLENYAMDPLNVVGWEGLFGLSVTVIGMIFLHLTIGSTEAGRYGYFDLKE

GWRQVTHNRAIAMSSLLIMISIGGFNFFGLSVTRTVSATSRSTIDTSRTLFIWLVSLGLGWESFKW
LQVLGFALLVYGTFLFNNIIRPPLKACLPSLREEAREELLPEEPIEHI
>g406
MNSVNFSNVGTGAMLTGANPQGPMQPPQNAQVQRHIAVALQNQGPFTGWRAEVTIPDRAGKILQMI
TSLRLIQPRIEIQNALQAALQYEEKAFREANQRDEYEKECASKLHAIKETRSRQQAVYNQSGLMQH
GGTNGGMPSVVHPNQMPPQFTPQMNRPMQTSPIPGQMPMHMGMNDPTMIQQQQQQQAFAQNQMGQQ
RLASGMPVIDELSMLSQPDFEQISRLAAQMMSKMTPDDLEKRRAAATSKMSPEQKQMISQKYGDPL
TWMMRLQILKNMKARRSQQIARAQGLDPNASMVGGDTMMNAAQRQMAPNMMGLQRNSALPMNNQQG
LDPSSFINVENLQGQQADGLRSQEAGQLVVPASTAPMSSQPSFADPTGLFQQGAPQQNTQPNLNRS
INPQQFLAQQQLQNAQSAQQIGMQQTSQFQAQNQAQAQARAQAAHNAKMAMSSQAGGQTTNQMNQN
MPHQSPAMPMLNRPVGPNMSPAQGAGQIRPPSRQLGVNGQQPGNQQPAMRPQIPPNLPPALKERLG
QMTPEQLNAFLIAQQRRALANSQAAQAAQAARAANSQQMSMQTNMPQLPQGGQFNGPNIGTSMPMQ
QSLNGLAGGVQNNAMLQGQQLSLQQQQQQQQQALLRHQQALMRSQSQSIDMNAEQTREMDRMAFP
PSMINSNANMNPPVPKTVKTWGQLKAWVVQNPQVLGNVDSAKLLDLQRLHFAHTVSQQRESGRNNE
QAGQPNWPGNNQPFMPGQNVTGRQPQSHPPGPTITPEEIMNARKRLGPQAQNMPDEQLKAFLLKNK
QKMWMQQLQARGLAMQQMNPAQNIQNLNQTQPPQVTPVPTPMPAKQTVPPATQAPPQPVQTPQPTN
AKVQPTSSAGKGAKGGAAGKSTSKKRPHSDDVVEIPNPKAQPPSQAPPQAATTSAPARLPLNLTRE
QLAALSPTQRSQLEAHMKQQRQPRPQLSKIAAEENWNATLPDQLKHWYNEMAKAVIPNSGPMDISA
EDKAIMAQQLRESTDMLSRLDTLVQWLIKLPNQEKNVKALLSMRIQLMKQFKSTEWTVNDEFTIAP
DYLKRSIGIIKNMFTSMITKVQSQQLQQGQKLRPPIPPSGGQPPLNASNLQQLEEEAKRARRTQNQ
NVPAAPTATQPPFPLGDPSPQGVPQAYGPGGFSPDKLKIPPTKRRKQSHASVSAAQGAAAAKAASK
ADAAKASLFKCAVADCEYHVKGFSTQAALSKHVEDEHRVKEEITDPLKYMVDSLDVALGVPETGKG
VLEGEVDKKTMPSETSVVKQEVKTEGITPAISGATPMSRLVSQTEAKIISPASTSTMTPRLQATKT
TKATATKQAKAGARAEAAKAVLEQPPITENAVAKDGWADSKVSLDAIQETFDIPMTDSFPGMGGGE
FFEMFLNSDMFSTQNEDTPDSIDSNAFVTQTPNDGDMARDDSAIQIKDVEEDGSWPADWFSHPGPM
FSGNANDDPWMDWETVNKELEAAPLGKGISFSVS
>g407
MYSLRSNARYQLTVLCCGIVGLIYISIQNGFDFTSIKSLVMALAYVWGLVLAIYLMGHGLVSIPRS
FFRKANNAEALRRIQAHAPKIHDRLTDAVAELEEVEAQVAQLQKRKTGTARMFEDWIEELADYTNI
PDSRPAALHSLAEPTVIPAVITERYLAGLSRRLQRARHQKARFVDEWDRLVRLAADYQTILNSTAS
KKLDFGPVGGSEAGSSSRRIGLVTPYMRYVIYVRVLPGLRLVLGAVFAAASFCVVWSEVVKALAPK
LCLISLSLVPNSDKAQVGLGGQLIASMWLLYMCTAALKGVNDAQVWGNRALVRRNTYGESACWYAG
QVAKLTVPLAYNFLTFLPSELRHDTTFYQFLGQYINLTPLGKGFDYFPVFLLIPVCATMFNLYGRI
KKVFGFGFVDDEEDPEADPGGFGTGGWREGRALIERELVGLGSLGLASRSVDAGRAAGLASSSSST
SLNISRAASPQPGWSSSSSVPKTSSRVPLIGSSSSANAITPATVIDDGVAEEDNIFQSFAHRVRNT
IDTASTPRWLQGGFPNISTPKWLNSDRADSGANNGSSQGGNKIFGGLFGQRSSDGHVRL
>g408
MSWRDTFGFFRRASSYGHDVPAAPPQVSDSDYSYLTSDDIVEPPRSYQQSHHDGRRVVRRDANRLE
DESPDILLLKHRGVTYPLHFTPFAIDDGDLTIGEIRRLAAEKTGTADVKRIRILYKGKLLRDDAKP
CKDEGLRQNSEVMCVVSEVRPGESNSSFSGSEGGSRSEALSLDSRSNQSPPSGGGGKKKKGNKKGK
KKQQHLAPEPTIAETRPPPSKDNLAPPSDIRPSSAGRSSAAPPPAPSLQQFATPNERVDALLRYLR
TELAPLCEKHFSYPPTDPKARSYEHKLLNETILSQVILKADSIDSEDARPARRALIKEAQGLLNKL
DALAPQSS
>g409
MAGTATAISSTDALTSPPNLTTAWDLAVPLGSEADTITPKRTSNESSSLYNKPNYRHRTPNGNPRS
RTSSQTGSRRGSNAHDTYGREIGPMTKEMADSYSNGHVWKGDQPGKASVDTDGPPNDDNWIHRDKL
ARIESQELQQAAMRIQRQVRTGSKSSSMRGRSHDTQSLNGTVATPPEQTESWPGLQRQQLESPIPF
EDSDEQEIESERVNWDLRRPEEIAASSFEGNDDASSKFYKSPALKKSSSRIPVLASSPHQASDSND
RESSNQRIRTRTVSSGDEDGVSHIPSRRASESAVIDTPEASPEPGYSTTPPLSTSRPNSRGGMLSQ
VQSSPTKKAPTKGTSTARKSSAPPNNRKPSASVKPRATSGSSTKDRPVTRSGDNRPSTSANRPEGD
PPWLATMYKPDPRLPPDQQMLPTHAKKMQQEQWEKEGKTPTAYDREFAPLAVRPDEPVSVPTTEMK
NEAATTDGDNLAADSGSPWPLQPPRSPEPTRPGTSGTNYRRRISPSIKSIQPTISALFSRLFGSLA

ARPISPGTMSAAKIKAQSIIDENKVVVFSKSYCPYCKSTKSLLTGLGAPYYVLELDQVDDGAAIQD
ALEEITSQRSVPNIFINKEHIGGNSDLQARKNELPQLLKDAGAL
>g410
MPPGRKRRAHPEQQNNANSDDEGRPTQRRRQSQDNVSASDASEDEGHRAPSSTDVMVKKMVRLALS
SEYARLPIRRTEISAKILGEQGARQFKLVFDEAQKVLRHRFGMQMIELPAKEKVTITQRRAAQRVE
KPSTASKSWMVMSTLPPSFRTPEILPPSKAPTSFLDSTYTALYTFIISVITLSGGSILEQRLDRYL
KRTSIEQYTPIDRTDRLLQRLCKEGYLVRNREMDGGEEVIEYMVGPRGKIEVGESGVSGLVRKVYG
RDAPAEGEAIDEFEARLARSVGVKRTAQVTEAASTTVAEESVKYSCNKRRGSWMIVTNHVMSTDLR
RVVIFRVKASSEEQMIKVQVRPRLIQRTANRASRQVSTGPNLRASSKPVGEVNPLRSSARKPNQAQ
PEAAIAHYRRRMALSAAGIVLCGATMYGMVKSNSFGLPEDAAKKEGEPNPNNLNNGNIKLDGPSEF
PSSPSVIRIQGQDGAEQVETGNSSVPLFPTTIRLPKSMSDTRLTPGQDLPIAPESEEEYQLLGFGI
RSVSFLSIQVYVVGVYVAKSDIGTLQRRLVHTGVQAPAIGADQPGTVGAAAAATSLVSTERDALKK
LLLDPELGEAAWTSILKEGNIRTAIRIVPTRNTDFMHLRDAWVRHMTNRAQKESARIKELAKTKGD
QPVPLRSEFDDETFGQSVSEFKTLLGGGSRKNIPKGQVLLLLRDQQGALDALFQRDPKEPIQWLGR
VADERVSRCVWLNYLAGKQVASEPARQSIVEGTMGIVERPVGTVTQKVI
>g411
MHWLRNLVPLTICSTALAAAASKGGVSQKGNVCTVTALGGKRNDVPNILEAFSRCGKDGTIVFPED
QNYWIATRLNPVVSNVVIDWRGVWTFSDDLDYWRNHSYPITFQNHHAGFVLTGDHITIEGHGTGQI
YGNGNAWYNVEQAVTQPGRPMPFVFWNVSEVLVEDFTVKDPPLWSLNIMNGTNMVFNNITCNATAV
NAPYGSNWVQNTDGFDTMDANNIELNGFTYQGGDDCVAIKPRSYNVRVSNVLCHGGNGIAIGSLGQ
YLEDASVKNVVIDNVNIVSYNKDMHNSAYIKTYMGGRVLQSSYESAGLPNGGGWGTVENILFSNFN
IXGAXAGPAMTQDSGDNGTFKGTSLMAISNIAFVNFTGYLNGASHSASVSCSNVHPCYNIDFQNVE
LLPSQNATAYGNGTCSYTESGGVHGLVGC
>g412
MVLLSKISLVHVWTLALSAAAEAGSCWRNTTCTGPADTSFPGAWEKNIYAPASRLVRPQSILETSD
SRTDFKVGSGHNVLKGNGSQIVFDFGIEVGGIVSVDYTASAAGSALNLAFSEAKNWIGKFSDSSNG
AFKQGDGYLTYNITEAGKSVYTIPDNKLRGGFRYLTVFVTGNATLDVNDITLEIGFLPTWSNLRAY
QGYFHSNDELLNRIWYSGAYTIQTNMVPVNTGRQIPAVAYGWDNNATLGPGDTIIVDGAKRDRAVW
PGDMGIAVPATFVSIGDLESVKNGLQVMYNTQASTGAFAESGPPLSQKGSDTYHMWSMIGTYNYVL
FTNDTDFLQLNWAKYQLAMDYIYGKVGSSGLLNVTGTRDWARWQQGFNNSEAQMILYHTLNTGAQL
ADWAGDTTGLSSNWTERAKNLAVAVNADCFDGAYGAFKDNATATTLHPQDANSMSILFGLVDEDRV
QGISQRLTENWAPIGAVAPELPENISPFISSFEIQAHFVAGQPDRALDLIRRSWGWYINNPNGTES
TVIEGYLQNATFEYRSSRGYYYDASYISHAHGWSAGPTSALINYVLGLSLTGRAGSTWQLAPQFGD
LTSVEGGFTSSLGQFQASWTRESTKSSSSYELSFKVPKNTQGTVVLPPVNGKKSKVTLNGKNIQWG
SDNTSTISFTKGGSYKITVRSK
>g413
MDAIPPSQSQKRKPWIETPLVESAALSKAAGCRIFLKLDCLQPSGSFKSRGIGNLILSHLTDPSNN
DKKLHFYSSSGGNAGLAAVVAARDLGCACTVVVPHSTKQHTIDRLRAAGANDVLQHGASXFQADTF
LRETFIDAPKQEETGDVKNVYVHPFDNPVIWEGHSTIISELAEQLPAREDGDNTQFPADVVVCSVG
GGGLFNGIVRGLSDYNKSHPKDGPREKKVDVLAVETKGADSLAYSLQNGELSSLDAITSIATTLGA
LCVAPKAFQNASEPPEGVRVTSVVVPDAEAARGVVTFADELRILVEPACGVSIDVALGARLREAIA
ERELGYDSRVVVVVCGGSAVTPEVVAEYRTKLALGWV
>g414
MSEKSNEEVAEFFNNVKDGMGNHALHICAMYGSYDVMDALFDIQYFECDPLTRIDQDTPLHVAVRF
ANEKDPNLGLEMIEMMCEAGCDPRVRNKHGQKPADLVYGDHKDIKSALQKAEYILSENIQVNEEEA
EGDAGSASDSD
>g415
MPLTFCPHCSNALTVSRADPSTAYPMGVNRFQCRTCPYQFVIDRLYVEEKVLKQKEVEVVFNDEEM
FKNADKLPVQCPSDTCNGEYAYFYQLQIRSADEPMTTFLRCTTCAKTWRD
>g416
MGKDERTHINIVVIGHVDSGKSTTTGHLIYKCGGIDARTIEKFEKEANELGKGSFKYAWVLDKLKA
ERERGITIDIALWKFQTAKYEVTVIDAPGHRDFIKNMITGTSQADCAILIIASGTGEFEAGISKDG

QTREHALLAFTLGVRQLIVALNKMDTCKWSQDRYNEIVKETSNFIKKVGYNPKNVPFVPISGFNGD
NMLEPSPNCPWYKGWEKETKGGKVTGKTLLEAIDAIEPPTRPANKPLRLPLQDVYKIGGIGTVPVG
RVETGTIVPGMVVTFAPANVTTEVKSVEMHHQQLTAGQPGDNVGFNVKNVSVKEIRRGNVAGDSKN
DPPAGAASFNAQVIVLNHPGQVGAGYAPVLDCHTAHIACKFSELLEKIDRRTGKSVEDHPKFIKSG
DAAIVKMIPSKPMCVEAFTEYPPLGRFAVRDMRQTVAVGVIKSVDKSVGGTGKVTKAAQKATEVAR
QRAPPPAFNELMEAPWTGTIDFGVYAKLIFAEGAIDPETDNHWVYVGSATAVNAGLAARQKQHDRP
SEAEKRKSHHFRLVFGPIKRKTKFVTLFRAPNPTTISDEDVFLHRQLCMVVEAVYAILLGAICPKL
DDRIDEACVYGLPYEVFTSRGACRRLCLVEPQRPIKYTLPDAEKPSKIKKAGSKAQFWAQFWAENK
KMLDQTSEEEEEDEEDEGEEEEEEEEKEEEEEEEEEKEEEEEEEEGEEEEEQEEIVEDDEDDKTLLD
PPIKKATKRESPEQPRRYREKNPERERKKKQLARQKPEQRQKARDYSRRYREEHAEEIQARKDAMT
EEERQAQRDKRAAQQRARRAEESRKNPKPKHIVPLRVKQAPEPKAPKEESXAQNAYDNAKAAKADS
SKLAALELKLATAMVDRFNLNQGYGSKNLAFVPRKYFQLVEDHRNKQTAEKSLNQKPNLETSPGGP
PMPIIPEAERDQNP
>g417
METVRQKCRPKHQVLILKCFPRYQKGVLEVKPNGSELSYLLYYASTRRSKLQKVGAFLEKKAARDV
SRGRIGNVQVTLQILSALIEKLPRELPLYGHSVLNVLDTVLQSHDIAMVEETLQTFETFCSHQDLA
VLAAEQRYATQYESIVQSYAAFASSKSSISSKVAATSLPIRMRWKNIGLKAIKSMVSSEGLSADGG
KQLACIVPVILENVYDGGEELLDSLHRTAQNNDKATPEQARRRRASTVTVQNVDTADGNVAEASGT
TADADKVAEMEVRLLAIKCLERIFVVSSNRPQIRTAAGLVLHFIVSRDGNNKFETAPHDLGSWAGS
LMDVITKWCPVQDRFIILFTAVEILRGISADGQPISQQIVMASLIDSLLKSPVNMIGLSVIDVLVG
FVQHILRLYQAPLQSSQPTQIDTTASMDPNTNDAVQQESMLTAEHVKLASLLKQCIADLATHTYYA
DQISDMVRTLLRRIRPLAPTDVSTATLSKIITEPNASTNNISNLTGERQAENYFYLAASRVVALEA
VKDILVVANQGKTINGPEIESRNKVGLQVWEGTQWLVRDSDRHVRNAYAEAFASWLELETTKNDLK
VPDSSGRLSRAQSKRESDGYKRNASMAAVDKTHTHASFVFLQLLHLSLYETAIEYADKQEDILVIY
VLLVQMIDKLGVNAVRFGLPMVLKLQSEALTTATSQVNIGSLVYGYLTALTDKFGFGSSQLGSAIT
GEIFKRQKNGTWLEMVQLPPLSVSQIPSSANGSLDRPGLQEPLRAFSAVGELVKQIEEAYNTLLAA
PQSPPGSPGKHITSQTTGNGYLDSAQTSPTLPIDVKEDMLSTWSKESFIAVLEREKARASSVAGSR
NGTSRRSHGSYYPNGYGNNTSNDGGSPISLNRNNQTAAGAGPGSSSSSQGYQRHSNSRAADGASGI
SSRDSTIRVNDLKRMLTVTGNSNVRRSSPLRGRLDASNNSVHTSSSESFVSGTFSASDFDAESRPQ
SVREGSETPKATPATTTSSHSHSSTSINNYDNDTXPPVPPLPSGLVIPGGFPDTSSSNLSRQNSSR
SDGRSSVSSPNHINSGGSIKGKSNGTSLNRSLSGKRTRSTTSLHKAASLRQQQEGSTGTENNVRL
DIEKLLDGVLPQNNSATLSVVDQTNGHRRTSGGIGRPPY
>g418
MENQPPPPSLTNPRFTLELEFVLSLANPYYLSHLAISFPNLLGTSTVDEKGDDDANDETTSSASGK
EARAFQAYLAYLYSYWKTPEYAQFLTHPGATLRALRLLQEDSFRRDIIRPQVIESLLAGGDEGQAA
ATGAENEDTAEHNNGVQAS
>g419
MSLAPLSVPEHKPAQLSINLGSNNPFRNRAVSPSSTSPISPSPRPERPRSTNPFLDNTDMMSPQSA
PAGAILSPSSEKQTFTGNTAELFENLSLKPSSKERRPAPPPPDKPSSSRPAAARPPTSRPRQETRD
RERNRDQSNKKREEDPFDIFADPPKKESSRPSRPREGRTRPRRNSESSIMERPKAMDPEDERRRRE
RRHRERERERDGKPRSKRAPGYRLDVIDKLDVTSIFGTGLFHHDGPFDACNPNRNRKGSKQAPMQA
FPKDSKNMALGGAGPNNSRLNLELIHGHTAEGYLDYSATGVKKEEAFDSARAEVIHGTESMGLGTS
TFLEGTPASRAAIQRRQSESDQQPPAGGLQRTKSLAQKFKGINRTGTVRVTSPDSTQRSPASAGPT
SGSSRTQERNPFFQDYDEAYDAKTGKIDEARIGGRNRASSSPRRAAGLERKTTNGSIGAEENRQNG
GGFMNRMKSLRRPRPERRVPSE
>g420
MGVNPYEADPKRVPKKDPYLGRSPHYGRYAPQADDFEPEFKDWHSSESETQREYWAEIVRQHCTPK
NSLNVVGSRAAFAIGKVLIHVDGDRAEGTAAERYSCLNANELIASRKAEEHLKDLNVAVPTIFFCG
TIDQKNIVVETRIPGVSLEVAWNYLTTSEKNGFKTQCRRILQRLTAIDDHFVTPSYAFEGLNGVPQ
PDIADVEQDILFASRDEDEVLCLTHNDMVRSNIIVDNGQVVAITGWRQSGYFGHERAKKIHRLLRI
PERSHIFSSGERSTQDQAWADLYDFLDASGEPIQDRNGHDSEPRVKTEIAAASIEKVPAAQSGART
PQYDGADEHPTPKKIHDLKRGSTSRASSIDRSSPSAPLIPSKLGPNARKSSSVSTKKASIATKIPS

KKRKIDALDTESVGDRRSNSPASSVKGPTAKKRPTSSVNGSPAPESKKSKPTKKKPSSKQSAATTT
STNGQQLEEEDDEAGSDVENPDEVFCICRRPDNHTWMIGCDGGXDDWFHGKCVNIKQEDEELIERY
ICPNCNEAGKGQTTWKPICRLKGCRKPARITKPRISKYCSDEHGREFMRQMTGQLRSTHSGPASTL
TPSALDRVREINRNRDSRSVDGDGDSVMDTNDEDDEHDQRGRPVLEELGSRGGILTVSDLKAVIMG
VSSSVEFRRLGNTLITPPPSSSPPARQIETTAKVEDEDDDEDGAKSKRKKDGKGNTAVQDEDDLEQ
EELFKSDKLGLDINPPGVNYSAAEEAKLQELRARREQYRHQRRMIARRNGFLGLVRAKAKAVLDRL
KAKEPKGGWKDICGWDARMSWNEEELDDWRQNTETGRKAFEEGKLEAEPIDNLASNGVAISDDEDD
DDDDDNGKNKKKNKHNRNNNTTKDAEFAEFSRGVCMKKRCDRHKQWVKIVQQDIAFEEAMLKNEFE
ACEKEANAIAEGAVLRTHAG
>g421
MRFVPNRLPRETQSIVLKPVDLRSRSRSRATTMPPSRWQFIDLSKDSADNLTQVKRHVMQEYMRQK
RIETQQQERPNDDKDSDAKNSIKKTAKRSRGKATKPTKESNIGGKAQRRTATRDADKKEEESSLRI
VPVPHGNTHRYHSSDYSEFSSSSPSSSSSSVRYFDPTIDGENYGLDFGAGFQSTPPQSTRSTMLSP
PRTVLSAARTDPFDTLPWNSSKEDQRLFDFYVNEMPAVSYGHHFRTKRAHNWYTEIFVPEAMKGAL
TFVNTILVHAANTWAWVRNENETDSTLRYRQLAISMLRDHMQKYPYDTTDNVITACMSAAALEDFD
PRPGHKEISWIHMRQAQKLIRRRGGPIAFQNTKLARLINWQDYILAGYVSDADAFHFEDERYHNYA
IKSSPIASSTTSFPSPPNSVSPTTLDRTPSPTTHHFTNTAKEEIALQCEEFIDFLTRCEHLALSQR
MKLSSKDAPVRYSAFGPGSTLHRILASPAEKRKTLTGDRKQFITRLAALMMLNAALWDYRYSVPRT
EAFIEHAILQLRDSEVNVDASIEALLQILLACKDFAFNDSVPFAMHSLSLPPASRPIGDFSQYSET
ATSPFDRPWFVGRMLKVAKRLGLQSWLRLNNFLFECLTFRAQEHTMRSWENDLRREILQAPLTSYV
MPALQEAVFLGT
>g422
MNEQFNSSQTANYAREENGKNTPSDSQPHMHDDGGDADTHDESLEDNHPSLTEPGKLPIPMQKRRR
VTRACDECRRKKIKCDGKQPXCTYDQPSNRRRNPTPQYIEALESRLQKAEEILRILAPDLNLNDPR
LLAANPEQIVSALRRDDSREATVAKPQADEQSKQEPESSCPDGSLLETMMENSGSLDLDDQGHWDY
HGHSSGIIFMQRLRKQIGNVISMPIRPLQNKTTPLFQMLESPKSQSESPQELSNPNSPPTQDLPSK
DVARRLCRSALDHACILMRLVHEPSFWATFDRIYSTPWDQYTNEEHTFLPLLYVAMATGCLFSDNV
ESTLDKSGYEGAIGQGFQYFKAGRQLLDITDCRDLTTLQAICFMILFLQSSAKLSTCYSYVGIALR
ASLRLGLHRTVNANFDPVESELRKRIFWLVRKMDIYVSTLLGLPQMVSNEDIDQEYPLDLDDEFIT
PTGILSMPTDRTSFMAGANAHTRLADIMVKVVKYIYPVKLTKSQSKSDHTYMVSHSKIREIERDLQ
AWMEDLPPALRPGSEISPELERMRQLLRISYAHVQMVMYRPFLHYVSNSFQAQGIDKRSYACAAAC
VSVSRNVVHITTGMYQKGLLNGSYWFVMYTTYFAILSLVFFVLENPDLSTAKDGILKDALEGKTTL
GGLAKKSMAADRCAQSLTGLFKQLPEKLKNRQSSAAGHVNLKRSSPSDTAAKSRMQRPASMSLPFD
TPQRSNSFPTKLAALQSRPSQEPKSAVDDTRLPRRSQTWLWEGHSDSADSTASPSSTSAFSHDQTA
SPASNQYLTQPSTQQPFTSSQQLGASQILPDLMPIMFPSGDQFAYPAQPLSTLEDDHFKDELPNSP
MQYTQDSFTSQGSNSLNGNFSRPMVNGFDGLAGLQGLSGPFQPAHAATLAPQLQHLGLRTPSTHSS
TPDNIQSPDLVSLPHNYMWQSFSLSNPGMPNEQIMREQSMPGGKMTMTENGALDGMSSLGMGFDMD
VNFGEILGNIGANPGTGTATNMNDEWSQWMNTGV
>g423
MPTPSNLNISTCITWLFYLVPIYIFLIAPGLRVIFPEQQKKDANSVSGRFEFDDEADYDQLVPGLR
IEDDNFISPEEDSGQLTCREDDYRVHIFSHKPLIIYVENFFTDAEADHLVTVSEDKWSPSIISDGT
TERVDPSIRRSDRALLDRDNVVRCAEERARAFQGWRPWLYIERMWTQRYNTSGHYRHHYDWRGSAA
HRGGDRLSTFMVYLDANCTGGGTNFPRLRMPRKSQWCQFIDCDDSQNEGTSYEGVTFKPIKGNAIF
WENLRPDGTGYPETWHAALPVLSGTKVGLNIWSWYQPPVRGDKK
>g424
MADTAPATAAPPAAATTAPAAAPSQHQFQTTTQPQASETSSAQSPNASTATATNGQSQAPAPAQFQ
SQTQTQTQSQAQAQAQAAQAQAQAAAAVAVVGNQSNTNGNAGIEEFPCMWQGCTERCANPETLYEH
VCEKHVGRKSTNNLNLTCQWGNCRTTTVKRDHITSHIRVHVPLKPHKCELCGKAFKRPQDLKKHVK
THADDSVLIRSPGPTDVRGQEIPYGMMNNPKGFPAGAHYFEQPMNPVHAGQGYHPQAQYYQGQPHP
QPPNAAYGNVYYTLNAGDNHTVYDSRKRIFDSVNAFFGDTKRRQFDPTSYAAIGQRLGGLQGLQLP
LNAGPGADYHMGGGIPVGPGGGYGPALAAPAYHLPPMTNARTKSDLLDLDRMLETIHTTVYESDDH
VAAAGIAQPGATYIPRMSHGGTTHQTPPITLPSSHATATTTNMSNASIIAATSPGSTPALTPPSSA

VSYASSHHSPGSYSHRVSPPHDHDMAMYPRLPSTTAHEMAGGYTASANAAPPSTLSNSFEYNDRRR
WMGGVLERSRPDDYAKSSIDDMLEDGERTPTRKDPLDHDSNESDDKFNHNLIDPALQQQETRSSSS
PDAEAAKRAAQAATEATRRGEEEWLENVRILEQLRAYVRDRLARGDYEEEGESKSDNAASSGAASS
EDDHMEGVEHTEHSHSTEERQKSEDSNPLAALKELTTSSPSSTQGLYPTLKGIEDDAESTKME
>g425
MPLSSRRRTRRKDIQLQETSDAEAPDSSPSRPASKKRKVEARPSSRHVHTPDPASADDADRSSPDN
DLSANQDLVETVIASLNVSRDEVNVLRDYSNTETENKQSIQAYAKIAGRDWTYYVKTAHVNIGRPP
DREQKLDEQSSPVSIAARALPEVHLDLGPSKFVSRLHAEIFFYGEDEESSAWHIRVNGRNGVRVNN
AMLKRGSDAILNCGDIIEIANTQMMFVTPGDKAVIHPAFVERAQRIAAGEEIPWDASQHAHPTTYN
AQQPGSAVDNGVYAANTGVQPGKPSLAPAPQFLKRQVTPPPRSPDTVGGQTATKQSPLYNRGMMME
STEEIDYSQDAAKDLKPPYSYATMIAQAIFSSEEEKLTLNSIYQWIMEKYAFYRHSQSGWQNSIRH
NLSLNKAFQKVPRRTDEPGKGMKWMIAPEHRQEYWKKQLRKGNNSSAPSSPATKEPPSSRGTNGHA
GSSYEGIFSATKTSPQTTSPGFSSFSVAPVEAYTPVRGSRLGQRGTDLVRSGANTDFDEQSPLPTR
IRNTQHENGNNSQNNSASNANDGTTSRAYGLSGNATGSPPVLSSSYYDEGPSSMITPAPQRHQPRL
PPPSTAQIPSKFMPMSSPAQFWKFADIGSTPARLVPDMSPLKGGTIRALGDSLMPSSSPPPPNFAS
PSKPSTSNMRALGPIKKEEEPEQIRHHRLAAPDDEEEEEEGFDLARGFQPIGSYHRQLNNSARASA
TS
>g426
MAPPSVLMVGTGEYTTGFVGGGASGSDKKVGVVGLTMFDLRRRGKVGKLSMVGVSGRKFPAIKQHL
HKNISEVYNGLDVSFESFPADDQTDPDAYKAAIDALEPGSAITIFTPDTTHYPIALYAIQRKIHVL
ITKPATKLLEDHLKLLEESRKHGVFVFIEHHKRFDPAYADARAKAQKLGDFNYFYSYMSQPKFQLE
TFKAWAGKDSDISYYLNSHHVDICESMVPEYTPVKVTASASTGTAWDLGCVAETEDTITLLVDWRK
KSDPSRVATGVYTASWTAPQKAGVHSNQYFHYMGSKGEIRINQAKRGYDVADDEQGHLVWYNPFYM
RYAPDEEGNFAGQTGYGYISFEKFIDAVTALNEGRVTLDELDKRGLPTLRNTIATTAILHAGRKSL
DEKRSVEIVSDGSDNGWSFK
>g427
MASRAPLRPGVYCPTVTFFHPDTEELDVSAIRKHAVRLAKAGLVGLVTMGSNGEAVHLTREERKTV
TRETRSALDEAGYKNVTIIAGASEQSVRGTVELAKESAEAGAEYVLIVPPSYYRYAVGNDESIYEF
FTAVADQSPLPVILYNYPGAVAGIDMDSDLIIRISQHPNIVGTKFTCANTGKLTRVARALDAITPK
SPFPTKPVGPITKTAANHPYVAFGGIADFTLQTLISGGSAIIVGGANVIPRTCVQVFNLWSEGKIA
EAYELQKILSAGDWVLTKAAIPGTKQAIQQFYGYGGYPRRPLGRLNETSIKSLESNLREVMEVENG
LPDFA
>g428
MPYPEEAQGFMIDSPDTWLSFQKRHYKLKPFEDYDVDVKVEACGICGSDVHTISGGWGSNQNWPLC
IGHEIVGTAIKVGPKVTNVKVGQRVGVGAQVFSCGECKQCKNDNETYCPNIIMDTYGSKWPETGII
SQGGYSSHVRAHEHWVFAIPEKLKTNSVAPMLCAGITVFSPLVRNGAGPGKKVGIVGMGGIGHFGI
MFAKALGAETWAISRTRAKEADARKIGADGFIATCEEGWEKEHQFSFDLIVNCANSSKNFDLAKYL
SMMDVHGRWVSVGLPEEEGQVIKAQNLISNGVLIGASHLGSRKEMIRMLNLAADKGLDSWVETIDI
NEENLKEAMQRMKKADIRYRFTLTGYDKAFPDA
>g429
MASNEIALYYRVFFLFIEPLFALFGAICAGLYPGRYLAMTEHLPSSIGILPLGIHVSLRQLANMYL
LFALNEALVLRVTTDRRVWRMLLLNLAIADIGHLISVAPLGPGIYCDITQWNTMDWGNIPFVYLGL
TSRLCFLMGYGVKSKRE
>g430
MTLHDSYPLRPTRRRPSLLLRARPKTAGPGEMPGAGVPWMNPGPWALNPSFNTGDDSQISLCQQEH
ECNGVFEEISVFPEPTPRHRTAKSFSSIRHSVDGLRALGRRLSVTIRGKAPKHNLHVSQGTNRYVD
EEKSSGVAPEGRHRNAWFKGHSINRRPSLHSVSALHSFYAPTANVANFIPGMGFEPPVFSDNLSGG
AAARAAAAAQNEMAKTGRILSPGDSKIFDTESGIGFDLRDRSDLSDSETAVVRLDPVDYLPAELIS
HVFSFLNPASLVQLEAVSKSWNKAASSHHVWRNVFRQNYGFRSSSSTVTRKIQSAGLGKTLPNQDW
KKMAIVRRNLDQRWKDGKAAAIYLKGHTDTVYCVQFDEHKIITGSRDRTIRIWDAHYPWPCLKVIG
APATHQQRQSPSNNANPQPVDNPPFFSICAPSQQWTDVADQSIPLSDCHNASILCLQYDDEIMVTG
SSDFTCIVWDVKNDYKPIRRLVGHRSGVLDCCFDDRYIISCSKDTTICVWDRETGRLVKRLLGHRG

PVNALQLRGDLLVSASGDGVAKLWNIASGLCVKEFSSKDHGLACVEFTEDGRTILAGGNDKVIYQY
DANSGDMVGEVDGHNGLVRSLHLDSLNNRLVSGSYDFSVKVFDATTGKMTIDFPGWTTSWILSAKS
DYRRIVATSQDASVVIMDFGYGLDGIELLEE
>g431
MSESRQELLAWLNNLLQLNITKVEQCGTGAAFCQIFDSIFMDVTMNRVKFNVNTEYAYLSNFKVLQ
NTFKRHSIDRPVPVESLVKCRMQDNLEFLQWAKKFWDQHYPGGDYDAVARRKASGGPATSAAPITA
ARTSSAGARRGTTPTGAGVRPRVAGAGAGPGVAALNQEIAAQKEAIAGLEKERDFYFAKLRDIELL
LQTAVEADPELEKDEDSLVKHIQGILYSTEEGFEIPAETEEGQEELETF
>g432
MKGFSCGAERREEKEAHRPKTAAAAIEALPDELAADVVEVDKGNCVPLEVVCPELENEVTEEPVDE
PAVDVKDERLVVEGIEEDDVVEDSEDVSEAVVVDEEEEEEEEEEKEEDELDVSDCVEVVEVVEVVD
TALVGDEVDKVKSD
>g433
MPSSHASCTEVSADCPVSATTLGYYPNLGANVFFVVVYALCAIFTLGIGLWKKTWVFGVVVFAGFA
LETCGYIGRVLMHSNPWSDPGFKLDLVCIILAPTLICAGIYLTLKHVARTVGPDFSRITPRLYTWI
FIPFDVFCLCLQGVGGGVDAAASDKTPIDAKTLKTGNDIIIAGIALQVVNLAVFGLFSLDFFIAAR
KHFKNPSEKVRNSAEAQIWYSRRFRVFCSAIACAYAGILIRCIYRVAEMAGGWGNAIMRNEVLFYV
LEGAMVLYPAILLTIFAPGLYFPEMGSKAPLAPSPATDSENAVAPNEESKPVTPEEGMMATH
>g434
MLSAINSMFRMTDDTDDSGGVIFEGPFDPDAQATVTDFIDYTEYLPSDLIRSLTLIRGLDETYLAA
TQKVHELTKIYGQLPELPVESRRNPQELREQISIHLNRAINAREASYAEACRIYDVVDRHHDRLGN
IKRKLELLPKPASRDATPPPKRSKGGRKVEKGATRLTLRVDGQVPDKNGLPKSRSRRSVIAGLDPD
SPIASTEQSDNETPKARSRRGGHVTLPEQETLQPKKDKKRAKPRVGGVGTNVHSSVAGISTSNALA
LLKPPPEGAKLGSEDLPWLRLTDWEMTKLRKKMKKNAIWQPSEIMINRELATGGRGWEGYRAAKAK
AQATGEELIDCDDIENTYVPGKLIRKSEATADMSAAEEIKTSNRGMILNEAKRLKRENMAXEQEAA
SAAAAAAAAEAELAAKRISDIGSTFKRLFSTQGENDHVAGTDSAKANGAPVTPAKGRAKTTRKRKA
EEDPSVEGSDQPTSAKPPSKKRNTGKILSTEATSVAADLSQPVLDTTTKVLKPSSSSPPPTRARSA
ASTTLENGPETISMTLKVAATPTPPTTRPSSRRRSAATSIEPGSAMNAALMGVSREHLRRKSVTPA
SKTPGPDSGTTAVGTGARRKKRPAPGPVSSSLEGGATLSHGKRKSKPKRRAPGKETIKGEDYRIDE
DGVLEKIDPNEPRYCLCKDVSFGMMINCENPDCEGEWFHLECIGLKEPPSRRAKWFCPECRVKLKK
APDGIERVGGGRR
>g435
MEKATQRLNLLFLNNEPLLEKDTGLRLPVIKTKPEILTTANPTVANKNRNLSHGRHPEPSASVVVN
NQIVAPDKVDTLQTRLNYGRNKEDGDGFDERTAFQSGLEASPSVVAQSFCPAAAIMKLPYKYMRGT
TAEIIGRRFFDKGQFWAREWDLYYIFPPRDLAPGPLLLLPASQVQQFLNLISKDYEMRLTIPMDSE
LGLLLSFQEEGIPKPKYLGRSTNRDVKDDLVSTISSLPRDSVDFIDQPSDGLVIAIDIVNAFREKL
EAGLNATRNKSKASKEKRRAAKIERQRKWVRDLKRAQCYFGLHPRCKGYISHSSAKDELTAPLPPL
LLDIVAPYSFADHPIFISIDVEWNERQRNQITEVGISTLDTLDIAHVAPGLGGKSWQQWIRSRHIR
IKEYAHIVNCEFISGCPDHFGFGKSEFVPLQDAARVVESCFKPPYSAQVPVKFVAEESNGDTPRVT
IEKAIEGYKLRPRNVVLVGHGLHADVSYLCALGCDMFAENVSKIQSPNSSQVLVKEPPDHNAKLLF
LDTLDTATMFQGLKRDVHSRSLESVLASLGILGWNLHNAGNDARYTLEAMVRMTINARVALDKGPQ
ELSGNYMDWALRPLANKDSALHELAWQTEVDRRVSEVAADREEQIRDDCKIWETVTGWDSGNPPTS
QDIDGGEPRELIFN
>g436
MATMPLTSLLRITSKSAFEKATKHPFLQAAGKGELPKEKLSQWLAQDRLYAQAYVRFIGGLLTKVV
LPTNDPHSRPKRAVPIEQHVFDILVESLNNIQRELRFFDEVAMEYGLDVTALSNQQSEDQHFESGY
FEPLYTTRAYIDLFMSASSSGVTLLEGMTVLYATEYVYLHAWKHAADIMSSTATSHETLLSPASFS
TGAERDLDGGALRRKFIPNWSSPDFEKFVIRLGEVVDELAGCVKGADVIEKTRSQCIGWYKHILWL
EAQFWPDVDED
>g437
MAQINYRTINVDVLDPESSINFPMDTLLPGNLPEPTNSGAAASVAQQVRQLLRGGDPEGALRHVLD
TAPLGGDAQAKEVHLATVIDVLQGIRQGEMLKVLEGVISGEGGTERADCLMKYIYKGMAVQSSTSG

AQSPSSRKGLSPQATGSGFSQVQSRNFGEGGGGQQMSVLLSWHEKLVELTGHGTIVRVLTDRRTV
>g438
MSAPCTFTKLSSRWRLSLRLPTHTRNYAVQAPGRPTFEVFNRTVKHMQKDRAARNVEQSRQVDYIK
DEVAKRLCERLLDIKRSFPNTLDLGANSCNIARALTAPNPDPAVETSPPLSDRIGALTCIDTSHAL
LHRDAEDEFNNQISIQREVIPDLESLPYEANTFDAVLSSLSIHWINDLPSLLAQINNILKPDSPFI
AAMFGGDTLFELRSSLQLANMERRGGVIPHISPLADVRDVGGLLTKAGFKMLTVDVEDIVVDFPDT
FALMQDLQAMGESNATVQMGTLSKDVLLANEAIYRELHQEEIDAEHGQSGIPATFRIIFMIGWKEG
AGQPEPLKRGSGEVNLKDILGGGDFEPK
>g439
MWTTLDVGIKFSKPTNIRSQLSRIYEEKGHGGIVPTGDSKSSLSMGGNLDELEGPTTAEAEDREET
LPTQGQNDQIELDTNARFAALKRFYTKQIEDTSVSATDPLASDFGAAFSSPSALSRIMSLYTSSSH
LYGGGAKKAKSKPPVMREALTPGEGVKVANGAKERSIINRMASEDCAWDDMLSKEQRAFQDPEARF
MEELRPLPVLLRTKRSKRCKSCKHILVKPEIKPASTRFRIRLIALSYIPLPTLRPLMPAALPGQSV
SATSLLQPNLDSLTPLRPIQYLLTLKNHMFDPVKVTLATPSVTPGRIGSKVTILCPQFDLGANSDV
WDEALQAGPAAPTSSSIATLTSSAGRAGNVSGYEKVAEAGKVWDKGRNWTTVVVEVVPEVLPGTQQ
PGIPSTPSKAGKKKDSDVAEDEEEDADTPMREDEDVLEIPIFVHMEWDAENQIDQDDGASGDKTHL
RERVGGDSETQKSDSIKRELAYWMVLGVGRISPKLF
>g440
MAALNKIAANSPSRQSPSELESSLANALFDLESNTQDLKASLRPLQFVSAREIEVGHGKKAIAIFV
PVPLLQGFHKIQQRLTRELEKKFSDRHVIIIASRRILPRPKRSVRSRNTLTQKRPRSRTLTAVHDA
ILSDIVYPVEIVGKRVRTKEDGSKTIKVVLDEKERGGVDHRLDAYGEVYRRLTGRNVVFEFPQSSA
AEY
>g441
MPESAPAGSKPSSSVKLVLLGEAAVGKSSLVLRFVNNDFQENKEPTIGAAFLTQKCSLPTRMIKFE
IWDTAGQERFASLAPMYYRNAQAALVVYDLTKPASLVKAKHWVAELQRQASPGIVIALVGNKLDLT
DSGAGGQENEDDATDAENNGAPGPDAEGDEEVENNDLSNTTDARKISTREASTYAEEEGLLFFETS
AKTGYNVTEVFTAIANAIPETSLKGAKSGPGGGQTNLGGGRSGDDSSRVNLGDRAAATPKDGCAC
>g442
MAKIILSVNAGSSSVKITFYKSQHPPSPIVDAQISGITSPPQTLKYSKSSGDKKEKLDENLDTPPK
AFKYLLERCLSDTEISEIANVDDLACICHRVVHGGDYEDSIEIDDSTYHHLEKIEDLAPLHNFSAL
EIIRACRKELPNVRNIAHFDSAFHRSLPLHVRTYPIDQEIAKKNGLRKYGFHGISYSFILRSVAQF
LGKPRDQTNLIVMHIGSGASICAIKDGKSIDTSMGLTPLAGLPGATRSGDIDPSLVFHYTSDASSL
SPASTKEMHISTAEEILNKKSGWKGLTGTTDFAQIAVANPPTENHKLAFDIVVDRISGYVGNYYVK
LQGKLDAVVFAGGIGEKSALLRQAVTQQCNCLGFDIDSEKNNKGVANDDPVTDISNSDTGSRVLVC
QTNEQFEMAYHTLEELKMV
>g443
MPGEVIDQPNPPPLPSDLPEELDKFAVQLQNISLDQKDYDDLAKFRRAACYIAAAMIFLQDNVYLK
REIKPEDIKPRLLGHWGTCPGLILVYSHLNYLITKYDYDMLYVVGPGHGAPAILASVWLEGSLEKF
FPEYSRDTKGLSNLITRFSTTAGLPSHINAETPGAIHEGGELGYALAVSFGAVMDNPDLIVTCIIG
DGEAESGPTATSWHAAKYIDPEESGAVLPIVHVNGFKISERTVYGCMDNRELTALFLGYGYQPRFV
ENLDDIDTDMHTSMVWAVGEIRRIQKAAREGKPLSKPRWPVLILRTPKGWSGPKKIHGQFIEGSFH
SHQVPLPKAKKDKEELQALQDWLASYGPEDLFNEEGGPIDAITSIIPKMDSKKLGQRIESYGNYHP
LEMPNWKHFVVSKGSYESSMKVIGKFIDEVFVKNPHTVRLFSPDELESNKLDGALAHTGRNFQWDQ
FSRNQGGRVIEVLSEHMCQGFMQGYTLTGRTGIFPTYESFLGIVHTMMVQYSKFNKMARETRWHSD
VASINYIETSTWTRQEHNGFSHQNPSFIGSVLKLKPYAARVYLPPDANTFLSTLDHCLRSKNYVNL
MVGSKQPTPVFLSPEEAAVHCRAGASIWKFCSTDEGLSPDVVLVGVGSELMFEVIYAAAILRKRMP
ELRVRVINVTDLMILENEGAHPHALSIDGFDALFTPDVPIHFNYHGYNTEIQGLLFGRPNLDRVSR
FHVAKAAVRGAGKRNEKVRLRCQELLSELDMNISETSKYIVEHGNDPEDMYDMPKFD
>g444
MFEKTHQSYGVNEVLLASVLGSVVFSIAAAQPLVIVGVTGPITVFNYTVYDIMAPQGTNYLAFMFW
IGLWSLIFHWILAITNACNGLTYVTRFSCDIFGFYVAFIYLQKGIQVLTRQWGMAGETSAYLSIMV
ALLVLMFGYVCGVLGESTLFQRYVRKFIEDYGTPLTIIFFTGFVHIGHMRDVSVETLPTSKAFFPT

ADRSWLVSLWDISVGEVFLAIPFAILLTILFYFDHNVSSLIAQGTEFPLRKPAGFHWDLFLLGLTT
GVAGLLGIPFPNGLIPQAPFHTQALCVTQQVSDDDETNKGRPILITDHVVEQRVSNLAQGLLTLGT
MTGPLLVVLHLIPQGVLAGLFFIMGVQALQGNGITQKLVFLAQDHTFTPGSNPLKRLERRSAIWAF
VGLELVGFGATFAITQTIAAIGFPVIILFLIPVRSFLLPLWFRPEELATLDAPTASPFTMESVGGT
YGHVESDENPAQASGGRVASSVVKAECKEEEYN
>g445
MSWRNSQGITGSNNIPLGKRRFGGDDEGASEASTPTVAASDNGATRGRSPTRDPPADGVKRRKKRN
RWGDAQENKAAGLMGLPTMIMANFTNEQLEAYTLHLRIEEISQKLRINDVVPADGDRSPSPPPQYD
NFGRRVNTREYRYRKRLEDERHKLVERAMKVIPNYHPPSDYRRPTKTQEKVYVPVNDYPEINFIGL
LIGPRGNTLKTMEKESGAKIAIRGKGSVKEGKGRSDAAHTSNQEEDLHCLIMADTEEKVNKAKQLV
HNVIETAASIPEGQNELKRNQLRELAALNGTLRDDENQACQNCGQIGHRKYDCPEQRNFTANIICR
VCGNAGHMARDCPDRQRGTDWRNNGGYAGRGPQRAIGGGDAVDREMEQLMQELSGGGSGPNGEAPR
RIEGGPGGYDQDSNYKPWQQRGPPAPVSDVAPWQQRGREQHGRRDDYESRDHNDAAPWAQGRGGGG
NYGYGSHQGNYSAPGAAPGGAAPWQQQAPPPPPGGQAGYGYNYSAFPPPPPAGGHGAPPGLSGAPP
GLESMYYGGAAAPPPPPPGEGPPPPPPSDHPPPPPPM
>g446
MAAQRTPDITLYTAQTPNGIKISIALEELGLPYKVKKLEFSKNEQKEPWFLEIXPNGRIPAITDKF
TDGKTINIFESGSILQYLADNYDSEHKISYPRGTREYYEQTNWLYFQNAGVGPMQGQANHFTRYAP
EHIEYGINRYXNEXRRLYGVLDKHLSTSKSGFLVGDHISIADISHWGWIASAGWAGVDIEDFPHLK
AWEEKLAAREGVEKGRHVPDRHTIKEVLKDKDQIAKHSASTLQWVQKGMADDAKK
>g447
MMASLTTTIIMAYMATTVYAEQQPASGGGGTAAPTTGGATAQATNTQYLNNLQDEYTHFMLVAMAA
SVALYFVWKSALRLTSHIRRLHGLGSDKQRYFTRDDFRMAWLKRNIVDAPLFRVRHNREFQLSRAV
NMGTLPSRFQAICLAGLIAMNVILCVLHIPFSGNIESYGKLLRNRTGTLATANLIPLVILAGRNNP
LIPLLGISFDSWNFFHRWLARIVAVEGLAHFLSWMFTSAAQSGWAKIIEEFHTSQLIMTGLVAVVG
FVALVLFASSALRHAFYETFLHLHILLVLMSLIGLWYHLDGLPQQVYLLGALILWGLERFTRLAIN
VYRNFVGGRTTAKAEALPGDVTRITLKMAXPWTFRPGQHVYICIPSVGLWTYHPFSVAWNEKETLP
IDEKGLVMTQQDVMSVQKETVSLLVRRRTGFTDTLYKRAAKGVDSHIYLSAFVEGPYGAIHDLDSY
GTVLLFAGGIGITHHVPFVRHLVQGHADGVVAARRLTLVWTIQSPEHLEWVRPWMTSILQMDRRRD
ILRIMLFITRPRSTKEIRSPSATVEMFPGRPNIQTVLDKEIEQQVGAMGVMVCGGGAMSDDVRRAC
RERQAISNIDFIEESFTW
>g448
MDDIHTTAKWANRMLRPLTSLYHRLGKHSESINAQEEEINKIVAAATNTEYTKRDASGDAEYDSEY
GDRDPSWIPQNPELKRVKHKYVARGERLRLQPRARVLSVLQSPEATKTLPGAIEIATPLITGKSTD
VFGTNNKGLGPGHRYNFPSGFNPWRPDTPGRDPIRLLQRYWQVSNNKSYVFKEIELLLLRNIMPPE
WLVTITLKDCVIAACTSQSKWDNEYAAAISLIKTILITAGDAHRGRKDVKKTPIKKRTQTKKQTQD
TSYSQADSLDVNQGRCPVHLQDATSNLVSSLNAALCCMYGVRMGQPDGPDYISGERIVKTYTDLAL
TIQRSLPLSGFGVEDNEASRASLLRKGYTLLAHFVLRSQQDLLLLDDTDVKILDVVLSQNCEPFFG
LLEQNVDIIKNLSVMVCQIIRLSERGDELAQCQQARRLCKHLLKYERSGLEKLTLFLGKIAIEVAL
DFAQYSQDAEDHTFAADIQEAVATIRRRIKSHSNCAPTPGKSSMQSFRWEEGIGEWIAKTPLDRTK
ATMPRVLSQPIVEIASTGPSSSASATSPSLSIDSESSNTSIALSSSTNLKRRRTSIVSLQPNVKRP
RRIYKKKVFKKIRQSTRIATRLSDISVVAQGKDYKDSSSHSDYESDYEQDAEDAEAASESEEWLPP
TETGDTPDAAQGTRTVRELQGSVAEAQTQRMRAGFDVIIHNAAAAPVAPIKRGRGRPRKQVARPAS
CSASQTNGPQAHAHVSRRSVIPCSEDEDSDDELSFL
>g449
MSISVAILLYQDVDPLDFGGPLGILSSSVLHDAFTFTIIAQHELTTTCAHLRVPRDMSIDEAHGKL
SQFDILLVPGGSVDSLAALAQNTDSPEVNFVKAFSQQSERPSGSRRRTIFSVCSGALILGAAGALA
GMNATTHHLCLDLLRGQCAKHEERTNVVHSRYEVGNLSPNGTRVLTAGGVTAGMDAACYLVSLELS
MDDALASAKMNEFTWQTKDQLIYPSY
>g450
MAADNAVSENAPEKLTNGDHAAEATEENNGGGVFQITVKXPXEPYKIQVMVSSQEQVQDLRQSIVE
LPGTFQYTCFHLEFNGQRINDYIELSEVTDLKADSEVTLVEDPYTEKEARMHVVRIRELIGAAGDR

VDNLQGLSTGXSLHDSVAXEXDNSESETHPLTGYDPNASGTFSTILPRADEPAPKTIKAISLSPWN
PPPYHLRQKGHLLYLQVTTNEGEQHQITSHVSGFYANKCSNAKFDPFPRPAPKNHSAHSLLALISQ
ISPSFESSFRALQESNSQRDLLTTFPFQNAIPNSPWLVTPTAASLNAHQPDVARSQENLLIAGVDN
SETLRDWNEEFQSTKELPRETVQDRVFRERLTSKLFADYNDAAARGAVLVARGEVAPLNPTEGRDA
QIFVYNNIFYSFGADGVGTFASEGGDEAARVAVGKDVLGIKAVNQLDIAGLFTPGTVVVDYLGKRV
VGQSIVPGIFKQREPGEHQIDYGGVEGKEVVAEHPEFIPVFEKLSKALRVKKHAVWDKEGKRHDLE
GSVETKGLLGTDGRKYVLDLYRIAPLDISWADEESDHDAYPHRMSILRLELVESYWRSKMSQYVKD
EVEKRRSKKAEENAAKSEAETQPEDAQKAAEEQERVDISGFNLALNPDVFSGQVPQTEEEKKDWAE
DEQEVRNACDYLRSRVLPELVQDLYNGDVGFPMDGQSLSQLLHKRGINIRYLGHLAGLSQERGHRL
QSLTTLVTQEMIARSFKHIANGHLRSLPPPFAASCVAHLLNCLLGSDVNAKPQADIDESLREIYPE
ADFSFEKITPASLQAEIEKQVKIRYRFSLPSDWASSLKHLQLLRDISIKLGVQLAARDFAFTKAQA
ASQPAVTNGVDETKHEETNSKKKKKKAAGDNASPTRSTVVKAFTTFLADDILNIVPLIKDAAPRSA
LAEEALEAGRISLMQGQKQLGQELILESLSLHEQIYGILHPEVAKLYHQLSMLYYQTDEKDAAVEL
ARKAVIVTERTLGVDSADTILSYLNLSLFEHATGNTKMALVYVKHAMDLWKIIYGPNHPDSITTMN
NAAVMLQHLKQYSDSRKWFEASLTVCEELFGRQSVNTATILFQLAQALALDQDSKAAVNRMRDAYN
IFLAELGPEDRNTKEAESWLEQLTQNAVSIAKHAKDIQARRLRKVNLSTRVSLGTKVQPQVGQSAS
SAAGAQNGPAAASQLDNRSIDELLKFIEGGETSSPRGKQSKRGTANPKLRGTKQSK
>g451
MYNLRTLVRKSAVVSGFGRVRPQRPGQLPAVIFSSASQTRFWASGLQDPVEEGSQDKSDSNWKSTA
FKMLETAGATMASIAILGYYKYLVLHKIDHAFEPGDPALDVAGASPGKEAPAAEHWIVRDEQDRID
HIISGNSKGHYYLLIGEKGTGKTSMLLNSMRKINGARVAMFEAHADLEIFRVRLGKALDYEYHEDY
IGSLFSIRGPRDTTALLDIERALNKLEKVALQRRKKKETPLIVIINSTHLIRDDEDGRDLLELMQQ
RAEQWAASNLITMVFNSDDYWVYERLKRYATRMETIPVPDLPKQKAMAALAKYRHRYYGEQVSDEI
LTHVYDQVGGRLSFLNRVAKSRDMLQTCKDICEAEKSWLLNRCWILGAEMDDDVMDEQKFSSAAFV
LAKALVDQEQEMDSIYDDEHGHILPQMPLHKARQVMTRADFIQGYDHDNIFTIDSNAMVRADSVPM
QHAFRAICSEPGFEDHLEKTLTRIGDIESLGRTRELTIKDLWDQGKYKVIVRDNKGRESGTVEFSV
KGKEEEIDD
>g452
MAKKVVVDVVDANDIEVVSVEGPSHFSFRIAKMPRTVRFSLVILSSLVLSSGLLTVFSLQTAGHLA
GISKHLEEWWEIVGLILWRATELGLAWVFGFDSLDVAELTLLTHAPIYFLLYSFYGVEPSAVLTTF
AIEIVSKTVPFFVFRGPNSVHALSSPRGAEVSNREILQDYPTAIFASGGAAAIYTIVMYLSFNTWL
PVHLVTYFDGISDISLVHSGPAGFPKVLTQLFLTGYAAYDFLFASSAGWSAKPDSTEKSVAEGEYL
ITSLYNRTWGKLSPKGKILANRSFILAIITLLNTIIAGTGSIKGIELKGSVGWGSLWAVASLIVSS
LFAWIEAVPGV
>g453
MATALQIQLQNQCHAYDPALEDDTNYGYVPSLAAGIVFATLFGLSMIAHTVQFIWTRWWWCSVFSI
GAMVEVLGWAGRTWSSQCPYMLTPFLMQISTLIIAPAFFTAGIYVLLGRLIQLLGRSSSILSPSLY
LWIFCTCDIISLVIQAVGGGMASGQAGKVGGNTKPGTDIMVAGIIFQLVSITVFAVCAVDFLRRVL
KQRLLQNVQGSVTPLLLAMVFSIVLIYIRSIYRVIELVQGWSGYLITHEAYFIALDGSMMVPAVGI
FNIIHPGWFMPKRKTVNDFDELSLNERLA
>g454
MAKPQNIEEEYSDDTSSQEFQEEGSDVPSINNSAAELADRPAKRRRVSVSSNDSEDSNTENYTPIA
PLPNISRIKKKSDVSSISNVPSAEEPVSSRDARDIGLQVTDSTFATLNVAPWLVKSLSTMAIRRPT
AIQKSCIPEILQGKDCIGGSRTGSGKTIAFAVPILQKWAEDPFGIYAVVLTPTRELALQIFEQFKA
ISAPQSLKPVLITGGTDMRPQAIALSQRPHVIIATPGRLADHIRTSGEDTIVGLNRVRMVVMDEAD
RLLTSGQGSMLPDVETCLSALPPSSERQTLLFTATVTPEVRALRSMPRPANRPPIHVTEIGTENHA
PIPPTLNQTYLQVPMTHREAFLHVLLSTEQNSTKPAIIFCNRTKTADLLERMLRRLSHRVTSLHSL
LPQSERTANLSRFRASAARLLVATDVASRGLDIPNVSLVINFDVPRNPDDYVHRVGRTARAGRTGE
SVTLVGQRDVQLVLAIEKRVGRQMIEWEEEGVSVETRVIKGGVLKEVGEAKRDAAREIDEGRDVLG
RRTNKLKKIR
>g455
MQAAIPGNTPQKARRRVLGEKTANASLTPATKRIADANAQKFQTATIPYIASVKKSSPTKTRDISS

GACHAGQKRSIDQVVENIKEAESEPRKVLMTSQTEVEGEFEILSDDNGTQSRQRKAPEVPTSQETE
EESPNEVSSSRVPTEPAARKLFIQQRAALARSRLQSAMRRVSEKSLDCALSQFEANSRPFVRSLSS
SSASTPYQQPQEQRSQKPSPTKKKRVLLPPLPLQPRSKPVRSSAQTTAASPSSKSLSIVDLPETDS
EVEAFGESNEDNDKTPRQSDIREQDDIVESHDLGVTMSQESDSVMDKLIKWTQSSTDSVRAET
>g456
MRWSAVALAGPLLQAALSAASSLTPPVLPLTVRNPYFSTWLANARDVPWSKWPIFWTGEEIGFSLL
AQVPSAGIVYPLLGRPQDSLRKDSGAKYAIDYPIYEGATYDASTTNLTYTLPIAHRELEIVLSFLS
PITPTSTLRQSIPASYLNVYVTGNVDVNVYIDLNGQWVSGDRGSQLVWNFDHINAQSGAKKLKKWQ
FSRQNELLVSEFADRAEWGTLHFVAPEDVRHESGTSAILRDRFARTGTLEDAVDTRFRSIMDEEPV
FAFSKHFALNTTHSSTKSDSVQFTIAHTQDIVIQYASARGLTYMRPLWKSWFNTNDELLEFHYDDF
ADANTLASNYSAQLAVDAYKSGADDYVDIVTLSARQVLGATSFAGTAENPLLFLKEISSDGNTQTI
DVIYPAFPFFLYTSPRWMAYLLEPLIEHTLSGQYPNKYAMHDLGTHFPNATGHSDGRDEYMPVEEC
GNILILGLALVNSLRYSDDQESISLWAAQGAVTPETIYDQAFPFTLADLQTISEIDHQDGKWGGEG
RGAKHARQWVQRSYNLWKQWTGYLVDFSLEPENQLSTDDFAGWLALQTNLALKGIIGINAMSQIAD
IAGEASDAAYFKRIADDYIIKWEEFGMSRDGSHAKLAYDWYGSWTTLYNIYADAQLCFHLDGADDI
YDGLLQTPLSTQKRGFVPRHIYQKQSNWYHFVRQRYGLPLDSRHLYTKTDWEFFSMAVASEQVRSE
ILESVATWVNETATDRPFTDLHETEGNGGYPGIFFIARPVVGGHFAFLALQRACGGRAMDGLSFLN
DVDPESDTVAAAAASEFNRMLRPSLNGEL
>g457
MADVRSKNLYELLGNDPELDSDREPEPPTKTIDKPAQRHGKRDAPKEAPAAPPAAPRGGARGGRGG
REGGNEGAFRDRNAGSYNNRNRPVDEVKEAGRRGYRGRDDRGARTRTDRHPVRTSHTDTEKQVGQG
WGAKKGDAEWNDEKAGEAIAKAEEEAEPQTPLEGGEAAAAAEEADKAKSYADYLAEQAAKGRGELGV
KEARAPNAGSKDKKWQNAKELKRDGEEEDYIKPQEEKGKRERQRKEKNFLEVDMRFVEQPRGRGNG
PRGSGGRGRGTGGRGGRGDGPRREGGGRGGAATGPTVQVDEKNFPSLGGK
>g458
MVSVVDTRPMNDDDISMDTIVPKTEAAQDGHVSMSSSVSTPEPEEPNQDVAQTQKRKGGRKPIYAT
SEERKQRNRQAQAAFRERRTEYIKQLETTIKRNEESLQSLQQSHRSAADECLMLRYKNSLLERILM
ERGIDVQTELRLKAANSSAMPPVKPNANPLAPKQPLPLERAAIGRTSTQRRQTSGAIAPKPDHSGL
PQSRDGVYNANSPQPQPTPTSHVSSPSTGRSPGFALQGAMSPKGADSQYQQQRNRAPLLPNPRDFT
QQNPLGVNPMRPMDPYPAAQPVIPGGMPSHMQSYYSAPFQKHYDQLEQEYDAQADMLDDADNAENG
SDANAYVPDFNRPPVPTGQGRMGLPVPSSMQTSDVDQSMYNAGGSLFNQYEPVLDSDTFGLSASMH
FQTPFSYEQEMLRH
>g459
MSGHVNSGVNVESLRQIVDLDILSEQLLRSVGTSPHVNSPTSGIYAASVELPDIFVSTLLLYHTAF
NDFAKFWHCSFSIQTLEKSTGDIPPNKMASTEQAAETADKRDERETTPRAEEPVVSATPNMVTDDE
DSEWEDLDEVLDDFSGPQQSTKSTTSSKQPDTQNNATEEAFIKQLEADMLAQLMGGKASGKDKAPA
PAPAPPPKTNTEEPSLSNLIKMFQDMSEEDAVKFEEELKKDGGMQNLFKNLADSEGTGAGSSTSAS
AGVAGESFPDTIQKTLERMQESGEKITIEAKTSQDKLIDASISRLMEMAGEAAGDGGNLDLEQVFS
DVIKEMSNKEMLYEPMKDYDTKYGPWLEENRATLSAGDYANYEKQAGVIKKIVETFESKDYSDDKP
ECLASIWDLMQQMEACGAPPSDLIKGSMGEGILPSLVPFMDAIPASGNLEDIDPGQVPPECNQQ
>g460
MLETIPQQSLQLQYTPQTPQQGYDCRQYSGRFPYTPTSPSPLGFRNVNAQAAIFPTMTERDLNKSE
GFQDQENASYSPFDKQHSDSNNSKPFSFSAAYNTITPPNRRSTYEERFRKSGGSTNAYSRIFGSPS
SASNTPNNDTSTSSKHRQLFLNRVKQSRDDARFGSRGESLMMMEYRSEQQSWDERMRQRADTLSQQ
YHIEDGEILNEELNNDYEDNDMMLDSEQSALEEFIMQEEGVDPALIENLTNGDDHKRSPAKSVRSD
GSLFGSDDCDYEDIFMDLVD
>g461
MRSSLFLAGVYVLAATVAGAAEPEKRDSLTSLGDILSPTVATTAASTTSDPTSTAPDSASQTTAAE
TTSADPTTSAEPTTSSSSSSSSSSSTTSTTSSSKTSSSTSTTEPPSTTTDPATAKTSATSEPPSST
TTEPSTSQTETVIYRTTTISGVATTQASTSTAPLTSSTSSSSPSLSSGSSGSSSGLSKSAKNTIIG
VVVGVGGAILLAGVAFVAWRVWGRNRHNNDNDEDDLMSAGTAVGSNLREKAPSPSSTGTPFRSTLD
QYHNPGPVNAASNF

>g462
MSRAQTCLLDICSPHFFWRHTVPRAFNPPMDVANHLMKGGTAQKLFGKMSKDSLAAKSPRLLGLTV
QRITIEPLHLVRLGRLQRPTAAPPTSTTGILDTPKSPSSSFSCPDLTSLQITSQHRGPPSPVSLNP
PGDDSFSERGASRASSPTTTSGPRGRSRKIAATGKKASRKALSVLNRFRALHHKSSGKMAQSPIRI
DTDIHGTKRGLPYDGSEGSDFLAVPDEDQFDTQTSASIPGLPSFLNLPYTEIRKKFEDLEWLQRSR
IAEGVMSKDPSHRWAIDTSPEVKSRNRYQNVQAWANSRIRLKVPEGECDFINASPIILKDSRTGDE
TRYIATQGPKVGIHLAHFWHMVFHESADVAVIVMLTQTVEAGREKCAQYFPIDEDDAVMNFPAEAD
DSLASDQDTQEEEAEEEKEEREDGSSSELSGSITLREHYFDDACRSEIRKLELQIGNESKIVWHFL
FAGWADYSKPEGEERNALLQLLQRTAEKSSPDNPRIVHCSAGVGRTGTFIALDHLLRELDSGELLD
SKYFDEDPIYDTVNQLREQRMMMVYNEMQLQFIYEVIREQSIRKLGGTLDDEMTLDLRSPKVARLT
SDENYTVSNKPELEPETKEDTLGAESPEQSPAVSDDE
>g463
MGLLPHSTSNTIPTNLQPNDKFLHFITFFLLSLVFYWXPDTSRRRAIQASLLVCTFCLGIGSEIVQ
GLLPNDRLFDPFDILANVVGSVGAISLCGWYHRRMLERRRQARFGALSDDLGGEPHDDDIELGLSG
SESARLHEDTGPQETGIITNLEREVDNWDENAVDNWDTEDGPGPDEENGNAKTRND
>g464
MSLGQNLAWMPPGHLRPPDDHHPDARGVNALSRVSSGTRTPQMRFSNSEQGSQAGNLISDVDTAAE
EDARITLFRDLYNRSEAKINDLFSAKRSESVPSEEDAMVLEAPEITPARPQDSERPAKKAARKIDD
DDYDEYDDEEDSEAQEDINASPLKAKSAATPFHDATPAQRPSPAIGTPADSGKDTKKDTLEGIRKK
LEEGKKATEEAARQSFQTIFYTLENDHDAMKEQERLEESERLVEAEMSGQGVNSGGNNVTSGANGY
GXLSNANLGASSLTLKNLIARIDMKRTMVQASDAELRSLMSEVRKNRSKWASEEKVGQEELYEAAE
KVLSELKAMTEHSSFFLSRVNKREAPDYHTIIKHPMDLGSMTKKLKTLQYKSKQEFVDDLNLIWAN
CLKYNASPEHFLRKHALYMRKETEKLVPLIPDIVIRDRAEVEAEERRLQLAEMDGAEESDDEPIIS
SRGRKAPGKTSQKGAAPSRQTPSGSEAPSGPSGSQPKNSVGPDADGLAEGSQNGLSTPPPGTLTPL
GVNGLGAAGSGSQLDAMEMDSFVAPALTIAGSSAIGFDYDDPEYQVWKQVTKKDRALIAAERHRLF
KGDKLNVEEPALLRTKTGMRRWLKNQKQAGPESDKAVESQSNALQPGTSETLAEGIEEEDDRVIPD
YYDVMSGIPDIPAHLAWREDAEGNVVDNLEEFLHILPKGSFTQPDSKLSRRMDANMRQMQETRKIC
SKVSIVKQMQLQSQVYQNQFQKYQPEPFVEQDVSTHVMNDEGPVINPWISKAVLQRSVGKILYHTG
FEEYQPSALDAITDIASEFFVKISRTLKEYMEAPKIAVAEASDTSNPNTAQYKSQYTQQEVILHTL
SAVGTDVESLESYVKDDIERVGTKLGVIHERLRAHFTELLRPAFHDATGDGSNAFNDGSEQFVGGD
FAEDIDEDFFGFKEMGLDREFGLASLSVPFHLLQNRMYNAHQSQNTSTSQTTSNLFPVPPPYPHVT
MESLPSQIGLVQNFFLQKLQANGEEPITEDLELPLKQRPSRPKLPGSGKIVPPNNSNLTTSPQKRP
LPPSIAQISSSKGAINFAEPSKKKAKKNNGTAADASSSTTMAPPGNEDSFALSNGDSKLLTENKSA
DDQARLKQSAELAKSDFGATHDGVGMTDSGATLTGELDHHHDKLNVISSANNENAVGTNSMMSPES
INGHS
>g465
MPTQAEISEIKGSIGSRKADEIYRQFLEGSTSVPRPGFNTTGREVDLVLNAYPITGFPTQNVHQYD
VTILKIGKEHEQPPPRSRKFAWDTTARKNTWKQMIYDGNKLAWSSNSYDEMKSVEVNLDNKTNDDK
PPAYRIIIRKAKTINLQVLQNWLQKKGSFDERVLEALNFMDHLLREWPSTKYSPVKRAFYDPKEEP
RNFDSTLDLRKGFYQAIRPAFGGRLVVNVDSVLCAFWQQVTLVSLADGLLKHFNWDKTAQALKRKN
VNPQNPFAGYIYSKAHVYVWNKLRHLEVKPAFQGCTVNRKLRIHNILTANSHEKXFTWTDPATGKE
ENISIFNYYKRRYNLTLYCPELPVVEMQSSPTKAIFYPMECLHVAGLQRFNHKLDDKQTAEMIKHA
VRRPNIRFGDIETAKAKLGHSTDPILKHFGMKISDQSITTKGRLLPAPEIQFANAKHNPGTQGRWD
LRGKKFLETNKIPLKSWGVGVFKQGRNDLTLQKVDEFLDLFRKQYAGHGGTIVGRPVIMDITGDTA
EGVYRLYNGTGQNFNQRPQLLLFIVPNKDTMVYHRIKKSCDCRFGVPSQVLQQAHVERKQPQYMSN
VAMKVNAKLGGVTCKAAPRQGSLNRPGCMIIGADVSHAAPGSTASSLTAISVSADQNCVKYMGSCQ
TGYSRVEMIDEDNMKNMLTPLVDEWTKTVGNGRRPQCVYYFRDGVSTGQFAQVLEKEVPIIKDVIC
RGSGEKTIPKITVVIANKRHHIRAAPRPNDKAAADRNGNALPGTLIERDVTSPHDWDFLMISHVAL
QGTAKPVHYHVIRDEMNHQPAQLQNMINNHCYQYVRSTTSVSLFPAVYYAHLISNRGKCQSQDEFA
DSSEESGDSPTDQPGAPKALLPMRTEGRNHLQMWFV
>g466
MSGLKSVVYFVNWAIYGRNHNPQDLPADKLTHVLYSFANVRPESGEVYLTDSWADTDKHYPTDSWN

DQGINAYGCIKQLYLLKKQNRKLKVLISIGGWTYSANFAQPASTEAGRETFAKTATKLVLDLGLDG
IDIDWEYPQDDAQAQNFVALLQKCRETLDQAAGPNRRFLLTIACPAGASNYQKLRLPEMTPYLDFY
NLMGYDYAGSWDQVAGHQANLFHSNQNASATPFSTAGALDYYIKSAGVPPSKMVLGMPLYGRAFQN
TQGPGTSYNGVGEGSWEQGVWDYKALPRPGGRDQVDPEIGASWCYDGNTMVSYDNVQVGEIKANFI
RNNGLGGGMWWESSGDKGGSAAAKADGSLIGTFVDTIGGVGALDQSPNNLDFPESKFDNIRTGMQG
A
>g467
MQIFVKTLTGKTITLEVESSDTIDNVKSKIQDKEGIPPDQQRLIFAGKQLEDGRTLSDYNIQKEST
LHLVLRLRGGGKKRKKKVYTTPKKIKHKRKKTKLAVLKYYKVDGDGKIERLRRECPSAECGAGVFM
AAMHNRQYCGKCHLTYIFDESK
>g468
MLISPSSGMQRHQRQHRRQNSIPVALEAAKAPLLPAAAMQRYSMHKRGMSLNQPTANLQPNPISTS
QGQYTNVLRETQQQRTSQLNQQSYFDESQHQIISNAVNTDGQRLDTRGLNVYTNQYSNENSPINAC
MNSGGLNIKNTNTKSRRLKPAIQPIQHQQQQQQPGQETMIFAEPQLIGDGTWNPYLVNQSMLAQMY
DLRRASVQSDISQQSYIPTTPPKQLQSNYVPITPDTTPFRRGTEFAPFLQNVHTSPIKNIAYQTQP
AYMQRTKSLQGVPGSNYAEPKIDAPSPPNTAPVDYDCYDLLTSQESDFESTEFQVVSKSMSIKSED
QETYNTQILSATHSFQSSPEIAYMPLPAPSPIKAPKVPISAATPSKPSSIRSNSPPADLSPSKTKL
SPKVASIDSLNLDARVQASITETGISIDEIAAYISGPDPIDGKWVCIHPGCDRRFGRKENIKSHIQ
THLGDRQYKCDHCDKCFVRGHDLKRHAKIHTGDKPYECLCGNVFARHDALTRHRQRGMCIGGYKGV
VRKTTKRGRPKKSRPDMEERQDKAARTREKVSNSSASMSVSSSDSSCSSPPSDGFESMSIQASSPL
EDMAMFESTNYCLPSEVFSFTPPASPGFSTGHNSSPGQSSRSYSPLSDIGSVSRTSSRHPLEDISE
DIPDLPPISEAAGCFDPEPESSDLPSSSIVPALTHSTAGSEIDIFINNNSSFSDFGHESGIPNDMD
MFSGKNESDFFLDFNDGNATDVFF
>g469
MELVPFSQYIPSPQRILKAITTGALPFLSSGNPAKLASKQFSSCPKPELSCEAKFDGQDTCCFNYP
GGHFLQTQFWDADPAIGPEDSWTIHGLWPDHCDGGFDEYCDSHRRYSNISLILVDGGRSDLLDYMS
EYWKDFRGDDANLWEHEWNKHGTCISTLETKCYSDYVPQEEVVDYFDKAVNLFKALPSHETLAEAG
IVPSYDVTYKKADIEKALNDAHGARVTIRCHGGAFNEIWYHYNVAGRLQTGNFEPADAVGGGSNCP
SSGIRYIPKRSPPPSKPRPTTTRQPGQPEPTDPVEPRRGNLRVYTGDDQQQRGCLISHGTWFTSGT
CATFKAKDVTGGFNVQTRRGSCGFVKDVFTCGHQIRNPALFEISEDNKLTYSGNTTFFADKAPKGH
VQSKVFAHGGNGEHAVELSIAWFET
>g470
MNHLRRYSAKPGIVQAFPSQFGPSYVCSTCRQIATQPLHRPSRTIVPLLDPRQRRDFSYLRARNSQ
KHGATESPPEKDAGSSGNQSQRRKLARSPAANSSLRRVAVEAQRTRSGILSRTQLLEQGLGELKTV
TAYAVAEQFNVTKVRDILQEKGYEPDPFNTGLYPQVVHIQVPIDSVRRMTNPEATNLGSDETGDVF
VFPSGTVVAWSLPDGFTSYLASRTLLPAAENPHTDNLETEDMDFIEDPHRESSFIKGDTIVLGTKA
SDGSPPSAPNRQSIDTVLTKIAFSSGLARSTKLAVLESLLSDYFESTRNIPTLLSKGSRLPYTRDF
ILRKTGQLLSVRAQLNIYSELTDSLPDIFWDSRHELGLEGYYEEAGRALDVGIRIKVLNERMDYAQ
DIANVLRERLSETHGLRLEWIIIILLIAVEVGFEVLRLWKEREVESKEKKEATN
>g471
MDLPGAAYGSRDADSLKKKXPPAESFSHPPAVDSSRDNSEEEHDARKQNDFADDDEDEDEGEDDDE
GDDDEEEDEEPRLKYVYLTKCLPSLYRAGDATSALLVGGDRMIVGTHNGNVNVLSLPTLRALRVYT
AHSASVTSISISPSLLSHTIIRRNAINRSPEEDNAPSPNASLRSKSKPAPTPGLPPTPSNSIHIAT
SSIDGNVCIYSPLDPKDVLLRNFGRPVQTVALSPEYKSDRQYLSGGRAGQLILTVGGRAGTTEKST
TLSGAASGAGWLGSLGLGANSGKDTVLHSGEGAINTIKWSLSGKYVAWVNEEGIKIMRSNLHLEAG
DAEFAWTRIRHVDRPNRPQWEEMASVWKAHAEWVDMKAFELPENSDTESILGQQSTSGAPEVEKLV
VGWGGTIWIINVYPDRMPPPGTRAGNRKLGDAEVVTILRTDSIISGVSMYSAKLLLVLAYLESEKS
DLEEQPQSSRRHKAAEPELRLIDLETQEEVSADTLSVTRYQNLASSDYHMSIMRPPRITAAAVQRG
ALGALGTGLLDATLYPARLFTSSSSIRSSGSNGDRGSERVAASFMSSSVSGSSTIPKEVQTLVTAK
GLKVFVHSPYDCIAAMSRDLSDHLAWLESHQKYEEAWILVDQNPSILXXPAEPQTEAFTRSQTSLV
DFFADDSSSVMTAGQPLSANARIEKRRIGELWIEQHIQNNGWATAGEICAKVLDIPERWEHWIWKF
VEAGKYDEIAQYIPVNTRPPLPSAIFDAFLEHYVSHNRQTFEHLLSKWPPELFNIGQAISNIEDRL

ESEKIPSDSNDWQILTGSLGKLFLANGQHREALRCYIRLQDAEEALRLISEYRLADAISDDVLDFI
LLRVSKHQLAKAPISELEQATAESISILVREAYNGIVRPETVISQLYIASGRLYLFFYLRALWKGE
AHPSKAEAKTQLRGRGRHARDAAEKLAADEGRSLVEPFADTTLDLFADYDRQLLMDFLQASTAYSF
DEACRICEEKHYTSELIYLLSKTGQTKRALNLILSDLNDVSQAINFAKSQDDPDLWEDLLSYSMDK
PIFIHALLTEAGTSIDPIKLVRRIPSGLEIEGLRDGLTRLLRDHDIQASISQGAAKVLQGEVAIGM
DALRRGQRRGINEDAGAVATGTDNDDVLSSITGPVASMAQNSDRAEEVRKLLSAVQDLLIPFISSA
DSLPSSNGHTANGIDTDLGTSSTSLVDYKSPEDLQKLLKLDLPSTGQGQDGFLQVLQQVLRYSVNT
WHQGFLDKLYASTNAPGVAAELILAALNTNVHVYQVSPVLTLIEKHTAKQLALLFGLNGPHSGGIS
VQGGSASNTTSIVIARNVLYPITKTEGNGSYRFVIFTSGHGHYSIEKAAQMLGFGSNAVWGVPIDK
QGRMIPTELEKLVEKAKAEGRTPFYVNATAGTTVLGSFDPFHEVAAICKKHNLWFHIDGSWGGSFI
FSTRHKHKLFGAEKANSIAINPHKMLGVPVTCSFLLAADIRQFHRANTLPAGYLFHSNPEIDQTNG
KLDGDLTADLPVDSPEVWDLADLTLQCGRRADSLKLFLSWTYYGXKGXEHRIDEATEMASHLATLI
ENHPDFILVSENPPPCLQICFYYAPGKRFAYEADGSIDSDDVRAAKNSSVTEEVTRAIVSKGYMVD
YAPPTGGDVDLRGKGKFFRCVVNISTERRTVEGLIRAIEEAGPAIIQKLKVSAI
>g472
MAAPLASLSSQEYARVEDYLNDKLQSDEDLDSLDSLLQTLRTQHELQRRQLVEAQEALEEATKASN
DHRDAVRKQVETFNQEQAEIDRRLMIITQSETSDDAVRIFESSMQKLQRLDVAKGYFSQLQEIETL
SKSALEHMNSDPTSAIPLYKALRNISRSLQAAQVAAEGAAPHLVDHAEQLANSVKEQLQDRFSSQL
QEALDKLKWPSRELVLNEAFVQEWVTSVDLLLDLQEPDLKSYSESSSFRSADIQVLLPLEIMMHPL
ELRFKYHFSGAKPTNRLDKPEYFLSHISDLISTYNGFFTTYLQPVLDRRADIVDEELRSHFLSATN
AFITSLLPMVRRKIDTFIPQVETSPQLLSHFVHELMKFDDDLRQTWNYLPNPYSAENWKGLTSEIL
GQQNWFERWLQVEKEFALSRYKDIIDTPESGQIDYDGVDANVTKPTSAAIRVNDLLETVTDLYRPL
SSFSQKLRFLIDIQITIFDQFHERLRSGLEAYLAMTSAIGRTVQGNDQGTLEGVAGLDRLCRIFGS
AEYLEKKMQDWSDDVFFLDLWYELQDRVRRKHGDGKNVVGPMSVAEVAQRTSQAVAEAGQGRSSEG
ALFDETASAYRRLRLRSESIIVSTFSSEVQSSLRHYSRVSMWSSLSAPSASFPHPLTSELTPTLRI
ISSNLSFLARVLGTAPLRRINRQVLLALQSYILDNVISRHSFSAAGVSQLMSDIDHLCGVIDSALG
HHSSSAAYQGEAGRVMRKLSESLVLLGLRIKPQSTKSDENNETYGVELGLWEVERRLFANNESARE
VLSELEIETLTEAEARAILEKRVEIRS
>g473
MPSPAKKRKINGSTSTRSIESFFSNQNAQPAATSKLSTEIIEDSKQFLTDEELARKLQDEWNQVXP
PIVQESLPLRTEESKPPGKGLDEAQSPISKAPEITSQIQISKFHVTQKDTLSLQSSLGSEDTIVSN
IPLDESPLTFDPAKYLPELQKHWSQDGGDASYAFLTQAFILVNSTTKRIKIVDTLVNLLRILIEGA
PDSLLPAVWLATNSFAPPYEPAELGLGGSVISKAFKNIYGLNAQGLKTLYNKHGDAGDVAFEAKKR
QSFTLKKPKPLTIKGVYQSLVKIARSKGTGSQETKQRIVERLLQDSRSAEESRYIVRTLVQHLRIG
AVKTTMLIALARAFLFSKPPDAVFTTKGMDSLLKLKKEELNELYLRAEETVKASYARHPNYDDLVP
CLLEIGVTEELLVRCGLTLHVPLRPMLGSITRDLSDMLTKLQGRDFACEYKYDGQRAQVHCDSYGK
VSIFSRHLELMTDKYPDLVSLVPHIRGGSVSSFILEGEVVAVDQETGDLQAFQILTNRAKKNVEIG
SIKVNVCLFAFDLMYLNGEPLLARSFRERRDMLRSLFTEIPKRFTWVKNLDATSGDSEAVLEFFKS
ATDVKCEGIMVKVLDNDLNSGNGGTNPGVEDRGNLEVDDEAQDKRLTKKKDKSTRRKALLSTYEPD
KRLESWLKVKKDYSTSSDTLDLIPIAAWHGSGRKAKWWSPILLAVRNPETGVLEAVTKCMSGFTDK
FYQSNKEKYAEDSENVISRPSYIDYAGHPDVWFAPQEVWEMAFADITLSPTYTAAIGLVSDERGLS
LRFPRFLRVREDKSIDEASTSDFLAGLWRKQMERAREEEGAAVDPAVDDDIEEQDEQAYGELDE
>g474
MKDRTVNPTTRKNIAQVCMSLQDRLGLAKVKYQQGRLGASDLRDLHNLDKPSGSSSEIAYSRYETP
LTSSPPGNATFSHELPRSSRNKHAATFNPEVMQPMLNASRKRVRSNSITDRPAKAPRLSWKASLHL
PESSPVHARPLQIYSNHPPAFDAPSSAFLGTSDEENDPELPVPRAHNASSSMVSSSPPRTPTLRSA
RLPRTDKDMKNEDGADLLLFLANSPTPARVSSRTQIRDFPPSTPPSQYAALPSMTPTPGGGLFGNL
GTPSQQFNFADFVNVTPSPAQRPWGGRTPGSLSKTPLLRTARKSLNFDNLVPPDMHSPVRRSRDAG
LALQLGEELRP
>g475
MVQSHMTALEAFLNEHPEIKCTLPSSSEYAKDREIFLLSRKANPLAIAHPESAEQVKALVNFVRYN
GIKFTVRGGGHNLQGLCIEEGALTIDMRAFTSVKIAADRKTATVGAGILQDQLGRILWKEGLSTPA

GAIPSVGYLGWAMYGGYGPFSSNWGLGVDQIVAATVLDASGAIIEADTTLLKSIRGAGGAFGIILN
VTIKVYPLKTLFAGAIIYDSKDMEKAFIEYNAGYRKLSREGIPKELTLQPMVFNSPHGRLFCATFT
WSSEDLDEGRRWSEKVASLTTVLMNTVAVTTIPDWYDGNAALIPTNAYGQFRTLNLHEITLETAEC
LGRHLAKMPSDLLREPHFMLEIIGCATTEEKSAESEQWALNLWNDVGEIERKNVLDGAYISLHYAE
EPHLSTLSRYFGSHVNDILATKKRYDPENVFDLTVPRLGAYI
>g476
MSIFKELRRRSKASFRTTTSADSQSNGNAVSGQSSSTIGTIESSTTPPSSIKPSLSSPNLSTLNEA
VNGSVPPLPTPLPQRPVPITSQSNRHSYYSTNGSPANGSIRTPVAASPYAPRIISVSDNSWVHQKV
LLVSGHIGDPRQHPIDGTITLHHNQDSFPATTWPVSDSHFKALVHLSPGPNRLRFDFNSPKLSSSS
SSGHSSWHSINYLPLTNSPPLDLVILLGKDSPGTFDSTPERIQSEGNDLDLAVKKYRMAAYLWQAY
TGEQMFRNTFGRRCFRLEEEWQTGTLSQRDTFAGQIRNEAKVHVVRSEKTVAELRDLNIAQQYGPA
TKKDELFNIAKEAVTAYFRPHPEQKRYVSVLLLDSHWDTSSQVITAHAALGSDGGNIKMAIFGSHS
LHSYPSCLEEVVSAFSDCTETDTRYVANDCGEAGSNWEAANIGIGAHLHEVGHLFGNPHRESGVML
RDYTRLNRTFTTREPYSTRTKGQGLKLCLPADECQWHRLDVLRFRFHPCFRLPSDPPTRPDDSIQA
WPVDNGKILLTAATGVAYLELYPEGDDLCHKYLEFINNESPNSGIPKQVTITEAELRNLLPPEQQK
RKLRVEIFSGNLGSHTIDDLGALKSKKSIVKLPKGQTGFRSSKLGMSQLEGSESQEVILDSVFVQK
KLLTSIKVYHGYALDGLEFCYEDATSQIFGKRGGKPGGDEFVFDTRRGEHLLGFYVRAGLWIDGIE
IITSFGRKSGVYGNATGGSGHALIPPRGFSIAGVSGSCGAWVDGISLIITR
>g477
MFRNRRTSQKPDEELISKFKKTFPDVNSTGSTSEGRHSGSANPAAVDGGISGQTDEDEIKVNDPTP
RNLQDLRFTPSLMDPNSFQFMALANQPPGYYTPTPGGMTTLYHHQAGDLHTPLGYNLVTPISLPNT
LSAGFPAGPNGELHVNHFPAQQQQFMPQHYNPFAQQTAFAPSAFMHRDSVYDPMDRSEESPIDQMT
VPGAQPMNLMMPGVGFSDQMEVSSETDGEKFRFGVTLRAPTAMVKHADEIPVTYLNKGQAYSVSVV
DTSPPPPGAQPRKYRTYIRVSFEDEQQRSKPSTCWQLWKEGRGSSEAHQRGGKLLAVEYVDPNQGA
DSDQSQRQIELEKASFDGFCVTWTANSTSASADCAISVRFNFLSTDFSHSKGVKGIPVRFCAKTEI
VSPSDTTPDLPRSEVSYCKVKLFRDHGAERKLSNDIAHVKKTMEKLKQQMAQLEMGGVKVPKHKRT
WSMDSQENDAKLSPEDDLHLKYAMMQDMFSSTRPVSVLALRGDDQDDPDLFPVVLPGDLQEFKFEG
LSRQSTRESLYSNDAASGISNILSPSNSSFSNTNSPRRPPERQLSNQTFMSQEEGMEWSNAALLTQ
PVKVKKGTTGYIEAIDIDPTYRPPAERPPKPIACFYIRFPDEQNGDYYRAVYLTERTVRDLMTKIS
EKYKIDPDRIVRILRVSREGLKIMVDDDVVQQLPEGQDMVAEITETSKLEVTTPDTSTPPTAAVEV
KLTY
>g478
MPREKQKRGRRAQEKENKRKLEDDHEEPALKRQKPTEDQLEAADYIPLTSGEQEEQIEQYEQDAPV
ADQNDTPFYGLLDTEEQEYFSRAAEVLELNQFQTAEEQSIFVESVYEEAKGKELKIACSQSCSRLL
EKIISLSSTDQLRRLFGKFLGHFLHLVQHRFASHCCETLFVKVAPALTHKASTTKKLIIEEEGSDE
PSLSLADMFLQVVSELEGNWGYLLTERFASHTIRVLLLVLAGEPVDLASHGSLVASKAKEGVEVHR
IEGQEQVISAKKHTVPDTFLNVLKKVMADMTAGLDDTYLRALATHHVGNPVLQLLVSLELSHFGKS
TAKDSRSVLRRLIPDDTMEEGTQSAIFLTGLLYDPVGSRLVETIVRSAPGKLFKNIHKNIIRERIA
SLSRNEIASYVLVRCLERVGRDDLQTDLEMIIPEVPNLIERSRLTVPRALIERCLVRNIDTTPLAK
TLQESLDENPVKRLQQLLEVNNPDGPAEEKAPSERPVQGKVKASPVLLHKSLFVQKMLEAPGALSE
LVYSGLLATSTDAIINMACNSVTSHVLQKALTASTSTIQFRRQFVPQFSSHMKQLALDTSGSHVVD
TLWDATKDIYFVKERLAQELADSEHELRDSFIGRAVWKNWSMDLYKRRRGEWKGKAKGLDDQVKNL
NIGDEKPRTKSKLDLARERYDAAQKSAAGRSSIVTKS
>g479
MVIGSSSRLLRAQLRARNFRRNYSQETSRTSGGSRKWAGPAVALLGTFTAATAAYVWKSQQGPPEA
QPSRRVEKVTFEKSKINTSVTAERNREILSAQHAQIKKMSANPGVYVWGSNSHRVVDPDSKETIIK
EPRRLRYFEGQFLRDIKLDQTSGAAVTDNGDLVQWGKGYSETDFQPTPTLVGKNLVKISMSRDRII
ALSSNGNVFSIPISKNDQQSGSKPSESSWLPGWSSRARVSYRQLVPQLGLGEKVTSISSGLEHVLL
LTNAGRVFSAAAATEAYPARGQLGVPGLTWATRPKGPVDYCHELSTLKDSKIVQIASGDFHSLLLD
KNGRVFSFGDNSVGQLGFEYDPASPVIDTPTQLPMKGLYSTKNWSAQVTSIAGGYTSFITVDAVR
KLLMDDTSTSSNQGVPSVDTWAFGRGIHGALGNGKWTHFQDSPSKMKALSGLSEFDEKTQKVQPIR
PVQISVGATHAAAVLGNNAHLNNGKKSSIADITWGQDTLWWGGNESLQVGTGKRSNVPTPAHILPP

PDVATSTNLDEARFQIIPSKQRIECGRQVSAVYSVKI
>g480
MLPQSAGRLRSPSDPSGIQRKKPRAKAPAQFSQIEEEIGIAAINDEVEFGKWYHDVEEDLLDASHE
AYQSCLHELEISRAHLDSLLADTSSTLELLSSLSKSFQAVDLQTSTFQKQCEGLLNTQSKNTHLAN
DIRDNLVYYEFLDPASRKLNAPGAGKTVRNSEFSDMLRKLDECLDYMESHPEQKEAETYRSRYRLL
LTRALTLIRGHFVAALRETSSGVSKRIADKQLNDTTMSALLYAKFRIGAAEMKQIGLEIQKRAVPP
VDPEQGTEAEYQSLMNELHTSFAATRGKLIIPLARKKLTSIAQAPSTSKDLVAFARASIGYIRGVC
LDEFELWGEWFHGQAGLYDFLESICEPLYDHLRPRIIHETNLIKLCHLCTLLQTRYMSDPEDETEY
NDPNQLDFSALIQPALADAQTRLVFQVQGILRSEIERYRPKPDDLDYPTRIRQTSSSTKGVPLSGR
KGSALPKTPTIVDEEIDSPTEKDALWDIETQATFQAWYPTLRKAIWLLSRIYRLVNSTVFDDLAHQ
IVHQTTFSLHQASTQISSKYSRTDGQLFLIKHLLILKQQIVAFDIEYVTPDISLDFSGVTNTFWEL
RERGGLFNPGNLMRLVGGGLLPKVVENMLDAKVELDGTLRTVINDFTNHFATRMTSTLPTKFIEKG
NMADGVAISPVCRSIEKEVPLLRKALDDYLEDTRTKETLVGAVQDRVIQIYEEFYEGYTALLAKSK
GVAANVKGKGREDAVWDVDTFEEWSEGVFRVGVAGLRSIDNGEDDDHDSVVSRRS
>g481
MEQRQQYLPPPPPLSQPNQSHIINLPPPPPRPSAQQHGMIPPPPPGPPPQSAYPGSNQWQAWRTQN
FPPPPPPPPVLHPSLMPANQAYLRQQPTPLSIPILAPPSEGQPLTSATYIPSSETFGPGVGIPPLYE
NYGHSAITERPRPGATTWDSTSSDNSYRKEGSIPPTPSRNLPPSLVLGAYNDNMSPGPATATIQNH
GSEPARTTSHKHNASSTSLGGISAGDAAVQWPVERVVSWLARNGFSKDWQETFKSLELQGADFLEL
GHGSNGRGNLGKMHQVVYPQLAKECQKSGIGWDQARERDEGKRMRKLIRQIHDXGSEVKFTDRRRE
SQTLPSASTDGGVENSPNLGWDSPFPPFGQAAVENTYSHPAHRLPPALISKPSTQIRPVTAPSSQR
HDAGDSDPAINQRSEYSRSALANLDEHRRQSPSSTSDGGMFPPPSLKAHEDSPQSGSPAAQHAFPV
QSGLTSSSTGDLTSRFEHHRGNSTDSLSGRGNSMPRFYENRKQTQESSRLTPQDTGKDPGKGFFQF
FKKKSRDHHSNPSPEEQLLDSPTSPSIMRHNGLNSPFNKPSYNNSDLSLAERPLSSSFSDYERSTA
RLKSAKQRYAMATLDGWNYRLVDITEADTAENLRTVICQNLGIVDWHTAQIAVTEPGQIEHEDFLD
DSMLVLNRRARADHLGSLKFYVRGNVSPGSTGLGVSFAEKATSSPMSAHRQVQRKPLEEDSTLRGT
QGVSGSPLVAHQSATATSSDQLSPSLPEKARADAGADLDSASRLEERQLDVDRKQRTLSRPKQPPE
LQLHSGNYSENGYRSRGVIDFDQPRVSPYEDKRVEGLVPQRKPPSAPSESNTLTKVNSLSKKSTDR
ARMSLRMQHQSPKYRLIQEEESPETSISASLNNSDPVYTAYGQSTGMATSSSTSSQSTLTENSFNT
VRRNPSTASSFKPPSLQSRKSFGPVHEFEETNVTFNAPRTPQMESEESEDDSDDGLFAVPLTTKKD
TSKGKGPVPEVTSPKSQKPSLTVNTSTRSTKGLSVSFRSPNPNDEISAGPSYPSSAVMPADLRSMQ
DKNGVSSANANPSPDDESYRRRDSFARDDIWASRPPVEGVINNLDDFFPNIDLDEPYLEAPGTSPP
GSPVNTVMKDKPGSSIQAPPNPSHQGSDQQIPSDPNARYSVAQRNLIKSGGLSRMKSIREVAKGAQ
QVHRNRSIAGPASKKSGDILRRKSTKMFGAKIMQISPKPGSRLSQLNNVPKREQTFRIIRGQLIGK
GTYGRVYLGMNADTGEVLAVKQVEVNPRVAXQDKDRIKEMVAAMDQEIDTMQHLEHPNIVQYLGCE
RGELSISIYLEYISGGSIGSCLRKHGKFEESVVKSLTRQTLSGLSYLHDQGILHRDLKADNILLDL
DGTCKISDFGISKKTDNIYGNDASNSMQGSVFWMAPEVVQSQGQGYSAKVDIWSLGCVVLEMFAGR
RPWSREEAIGAIFKLGSLSQAPPIPEDVSVNISPAALAFMYDCFTIDTFDRPTADTLLNQHPFCAP
DPSYNFEETELYAKIRDVL
>g482
MASDIVTRELKQPINLVEYLYRRLHEVGVRSVHGVPGDYNLQALDYLPKCGLSWVGNCNELNAGYA
ADGYARVKGISAMITTFGVGELSAINAMAGAYSEYVPIIHIVGQPHTASQRDGMLLHHTLGNGDYH
VFADMGAKVSCAIARLNDPVDAPTLIDNAIRECWIQSRPVYITLPTDMVLKKVEGQRLETPIDLNR
KPNDPEKEEYVVSLVSRLLHEAKDPIILVDACAIRHRVLEEVHDLVQQSGLPTFVAPMGKGAVNEA
HPNYGGVYAGDGSNDGVRERVEESDLILSIGAIKSDFNTAGFTYRIGQLKTIDFHSNLVKVRYSEY
PNINMKGVLRKVIERLGEVKVGKIPHISNKVPNHEADADSQTITHAWLWPNVGQWLRENDIFITET
GTANFGAWETRFPKGVVAINQILWGSIGYSVGACHGAALAAEEELEENQRTILFVGDGSFQLTAQEV
STMLRKNLKPIIFVICNDGYTIERYIHGWDASYNDIQPWRFVDIPSTFGGQQGQYETHQIRTRKEL
LDLFSNEHFSSNECLQVSLIIH
>g483
MQYYEKSLGNQDSSQATGQDADDEDDDDIEAQIRKEVEGLKPRSTKPRLFQKVTSNLPCIVLYRID
KSIDPVKLVHDICEEAKANPDQRKSRWIKRMTPITQIRKVLSVDLTAFAKEVLQPVFHVDDVPKKY

AIRPAVRSNNTLNRDVIIQTVAAAVGSGHKVDLKNPDYTILVEIAQNVIGMSVVGSDYEELKRFNL
MEIYSPTPKPQQTPVS
>g484
MRLPSIEDFDGHSLGKASHDVSFSTQMLMGLGTPETDCFRNVVQLLFPPTVHLSSTQLLSTRVHRV
FHLKLSNGHQLIVKVFPPPAIPLLRRERNALETEVRALTVLARINHPSIPRLFRYVPHDASTSVSF
LVRQYLHGVPVSELQNRLSAKDLDGLDRHLAMLINVIGQQFSTAFGPLGKVAAGTGSCHWREAFVT
LFEEVLRDAEDMFIHLPYSQIRRELYRWSFALDEIKTPRLVIFNFGRPSEVLLDPETRQLSGLLEF
GSALWGDVMLAEIFESPSDAFLAEFGPIPSSGQSQFIRSKL
>g485
MPFFPDQDNILQLNDQQFDFNIQFEQLFFSIIPSVLFIVTSLWRAASQAQKPTVVNAPVFQLIKLC
AITAYFGLDLSLLILVVVGSFQVTSIFIASSVLKLVSTLFMIVLSLLDHSKSPRPSMLLNSYLFLT
LLLDATQARTFFLLSGDKPELTYSSIFVAAIAFKAGILLLEAQRKSKWVNWDEKDHSPEETSGIFS
LGVFFWLNKIFLDGYKKVLTIKDLYPLDSSLSAESLHEKFLENMDYSNLKGDKFGLVKVLFRFTFA
QPFFIEKLLKXLAEPEADSNYGYGFIGASLLIYLGIGISTALYWYFHHRMRAMARSILVTEIFQKA
TKVRMGFDNDSAALTLMSTDIERINNGLRSIHDIWASIIQAALASWMLYDQLGVVFVAPMGVVTLC
SIGLAILMNFTGDSQRVWMAGVQKRVGLTATVIASMKNLKISGLSNAVGDFVQKLRVEELAAGTRF
RLIWIVAAVFAFVPLMISPPLTFAFSQRTLDVSRIFTSLSWLLLLTNPLSQVFQSIPEVLSGLACL
GRIQAFLESEDRYDFRQVLADAKRKFEAVPPDVVVSPDSNTESAHPVVIANGKFGWEANKFALRDI
NTKLSKDSLTIVVGPVGSGKSTLCKALLGETPFDEGTVTLSNEFRHVGFCDQTAFLSNGSIRDNIV
GFSPFNQDRYADVIHATSLDFDFNTLPQGDRTNIGSDGITLSGGQRQRVSLARALYLQTNLLVLDD
VFSGLDADTEEQVFRQVFGPDGLLRRWRCTVVLCTHSIKHXPAADYIIVLGEGTIVEQGSYAQLVT
RQGYIHGLGLSGSLDSSASSEGSISENSLREANPQLLQTTMTNTSALEPETDGLRQVGDKSVYKHY
FKSMGVFLAALSILFAALLGFFTNFPTICVAVFVVSVSRAGANLHNNALQTLIRAPLRFFTATDTG
VVTNLFSQDLNLIDTELPSALLNTLYSVFQAIGEAAIILTSSPFMAISYPFLAIILYILQGFYLRT
SRQLRLLDLEAKSPLYTHFLDTLKGIATLRAFGFLSNDVRKNIRLIDSSQRPAYLLIMIQQWLNLV
LDLVVMIMAVTLTALAVRLHSNSGFIGASLVTLMNFGESLSGIVIFYTRLETSIGAIGRLKTFNES
VKPEDRQEEDIVPAEQWPQSGVIELNGVSASYTAEDQTDGTSNLALRDINLKIRSGEKVAICGRTG
SGKSSLIALLLKLLDPTSESLENVIIDDIPLYRIARPALRERIIAVPQDAVFLPDGTTFRANLDPL
DASTAEECQNVLVAVGIWGFIQERGGLDAGMSAGSLSAGQRQLLSLGRALLRRRIRARNAGLGAGA
SESGILLLDEVSSSVDHETERVMREIIRTEFRGYTVVAVSHRLDMIMDFDRVIVMDTGEVVEVGNP
TILAKEEGTRFGGLVRAGAK
>g486
MAQFLRGKQAGIQKDLSEGLSPTLFALDEYTRYGINSRISAITYDPVQSLIAVGTSDTQFGSGQIY
VFGQRRVSVIFNLPRKASAKFLHFCADKLVSVDSKSELSIFSLETKKTLVSYAPPSHVSAVAVDPS
IDYVVLGLQNGEIIAYDMDREGLTPFRLPNLWLEKNPRVRMAPVVSLAFAPRDIGKLLIGYPEGAV
TFSIKQNIPQNFFEYEVPAGAPGGDSDPTLSRDIRRPKLTQALWHPTNTFILTVHEDTSLVFWDAK
DGRLVMARTIEEANINQPGTRAEKPTSPHGNFSVKDPIFKVSWCAKENPDNTGLLIAGGHPSSDRD
KGLTFLDLGPTPNYQTSSWQILASYLEKPRSRFTLSTPPGAEVVDFCLIPRTSPFFAGAQDPIAVI
ALLSTGELVTLSFPNGHPISPTNMLHISLSFVHPFITKAILTPVSRNNWLGLKEKRAQGPKFLLGG
AEGKKAMKRFEDRNIAITANSDGVVRLWDAGHDDEIENGEVIQVDLTRAIARNVNTQASQISFSGA
SGELSVGLRSGEVVIFRWGKNQNAGSEKAPGANAGPGKITNISHRADPNLSTGLLPLSLLEMQNGP
VTALKHSDVGFVCAGFEEGHLVFIDLRGPAVIHSADIRDFLKANNKRGSLRRSRTGDSTPLEWPTS
IEFGVLTLDGEDYSSICCFVGTSRGNLATFKILPSNNGTYSVQFVGSTSLDDSVIGIFPINADSGE
PALATQMAVSGLRSGVRVNGVVVAVTQSDCRIFRPATNKGASKTWGDFLCDSAGFVKIEGRGYSLV
GLFGDGNVRAYSIPGLKEIGCTQISNILDVRRFSEAVISPSSDVIGWTGPSEVAIINVWGSGTPIP
PTDDKLYNPDAMLPPRPTISNFQWIAGTQYVSPADMDLLIGGPDRPPSKRMMEQMKYEERERRQAE
RQGRSVPPSASRNDEGYWSYMQRQVQERTERLGIMGDSMDRLEENSSNWANDVSKFVSNQKKKAVL
GVLGSKFGL
>g487
MSVVSLLGVKVLNNPAAFSDSYEFEITFECLEQLQKDLEWKLTYVGSATSSEHDQELDSLLVGPIP
VGVNKFIFEADPPDLKRIPQTEILGVTVILLTCSYDGREFVRVGYYVNNEYDSEELNADPPSKPII
ERVRRNVLAEKPRVTRFAIKWDSEESAPAEYPPEQPEADQLDDDGAAYGAEEVELENALIKELEET

EQQDGEDHEMEGAPVAAAATAEDDDASDAGSEDLEAESSESEEEDLEEEDAAEGDDDVEMADDANP
GSAQAPQAQPEVMVH
>g488
MAEEDAEQAFFXAXAMSNEYDPAGVAQNIEASDSEGDYDPSNTLHDEYSITVAESNPNETIAQSND
ASSATQSIPQDSYQPDQVDTAQTSQPPSRPDSQTSVLGPSSTMHVQPKTKTIGGFVVDDDEDEEED
EREDEDKDEAEYEPPGVLETIEHGGHISAGASEQLPFSEKNANETISTSGVSIQPLVPEMASSKDV
SNNPVSFHSGHNLYTEALQQAGETTDSNATTPIPLAANSAASRSRLPHDRIGLLEDRIKEDPRGDI
NAWIELVAEHRSRNRLDNAREVYERFFKVFPSSAEQWVAYINMESENNELQRLEQIFNRTLLSIPS
VQLWTVYLDYIRRHPLTTDTSGQARRTITSAYDLALTHVGLDREAGSLWTDYVEFIKTGPGVVGG
TNWQDQQKMDLLRKAYQRAICVPTHSLNTLWKEYDQFEMGLNKLTGRKFLQERSPAYMTARSSYTE
LQNLTRDLDRTTLPQMPPAPGFVGDVEFQQQIGLWRRWFNWEKGDPLVLKEEDISAYRTRVLYAYK
QALMALRFVPDIWFEAADFCFQNGMDAEGNELLTQGLEANPESCLLAFKRADRLEIESESEQDPQK
RGAKVREVFDKLLDALYALNGKAREREEQDIARIKEQFANESDDYQPVADDEDELGRSQEAQAKEA
AKESQIEAMRKAHATQITMTSKLISFAWIALMRSMRRIQGKGKPGELAGSRQVFAEARKRGRITSD
VYIASALIEYHCYKDPAATRIFERGARLFPDDENFALEYLKHLFDINDVTNARAVFEMTVRRLTSK
PENVFKTKPIFSFLHDYESRYGDLGQVISLENRMRELFPEDPTLSQFAHRHSDANFNPVAAQPVIS
LSQVRPRTVPLIDQQQGSVQGTPTRYLDVPPTGSPKRPFPSDDFEEDRPRKFVRAESPLKGIPARR
PDQLKRPTPQVNGGQYRPQPSAAPLPRDVVHLLSIIPPASAYNAGRFSAEKLVDLLRRIDIPSSTS
QFRQY
>g489
MAFSDHITHRGASSSSRRWDNKQQAQQDLLSPHLHRAHLNLDLDLDRAYPSDRKYSRRPSAVAKDA
PRPRSNFRLTRTLKDAFDATSRTINMDTNVGASSVPPVTTTDRTPSPKHRHQRSYTLPSPYTEVTS
PPPVEILETYQRITDADDLADLVSEDDRDFPDHGVSRKSSSERRRRDSPGSLNNRRTRNEILDPGF
SYLDDAPDDYVRGNLSTYKRDEERLSRVTTSQSPVFSRARVGSRAEGLQRRDSDPPAEDTGQDLGG
DDGVLGLNVPKTWGNKSRSHRQWLTSITGDTPRADTSARNTERETRARIGSRSRSPHFTDRSPSRL
DRKTSQELASRSERSRLGAPPADGLHNSARSRSRSLGRNGLNGDTIPNTPITIYKSTGKDPSQKQR
SDSRELLRKLARTESPPQSTPAQQKIEKTPLPQKTPVVIGAWVDTPMTVRNTEKNGEDDTKEIESP
SKTASKSGVIPPTKDSQPGITANLPKDKPADKTMTKPKIQEPSSKSNVLTEKSANIDNKKQPKPEL
VKPKLPKSALETFIEDAKANGNPLSLGDDTIASLEGLMDSTDDKSPSRFTNIKKEELEKEVDAINQ
SSDRYTNSLDHLGSKLQSIIHSIQDARSGLTVIEHAIVPGYGKAEGKLCESCGNPQHGHGDGRVYL
AIPIPRLWKRDSKSKRLRPTYLGWATAILGLWYFIESIMCDIYCHPTIAEVCDGYCLQPDAPQFPF
VLPTMLWRWSHLSSILTPIITICVAFGRLIAQLLGLWDGYVDDSNVDFKWPASSSSYQNDIPIGSF
EHHGVKNGFFGLWPGGSQKHSTSSVVKSAFTTTASTSIPTILQSILHDPHGDNADESFDHDEIVS
>g490
MAKATKRTGKGKQPSTKSTVKPPTPFVKAPSVLEPFLETLSPNEIYLVHIDRESPGFKKQLFILPV
LLNAAIVLFFAYRIYNGFYVYPEIFASALGLSSESTVDLTSQPIKTAAATVLRRTVTFSIDYFLVS
IFLSWPIRFIQGPIYWRRKVGFRQREVVVRQSRSSWSAELERNRWIYDDDKVVQERVVPAVAPQRV
KKSGYLLIDADWDLDYNAMVRAHELISQISKGTGVQLDEFRTAIFVNTDDEGWLIWRVADEDTKER
EEQRDQLLAFQEKLAAMGKEDLFFRWVELVQYESSQPGGFTPERQRNAMLQAQEMFESEGVDFSHF
WREVGGMQGGINLS
>g491
MSAAVAQRAPLGRTSSPMDTKKNIMKSEDSKDQKPATDTDSLPPXPRPAASGPGDTPDYFNSLHNP
FSLEPNPFEQSFGNPSAETPGKSLLPPVASITSPALPGASSAGYNWPNSLRSGPLSPAMLTGPTGT
DYFDSIGRGFPTPNESSLRTGLTPGGGGSMFPAPSPNTQAILSQLQSGGATPSTLEFHRTALNAAK
RSGFNVPTSNPTSEPEQLQNMDKKTAPAVVDQFTHHDAADAANGLFMLAKGGQPNNDAFAAANKPT
DMSDTKRSTRNAHNSVSSGRELTAEGSDSQGEQSKPASKGKGKKNTPVKQTSSINGRRKAEEAPKG
SNKRAKMNSGPMEVTPEEESDDEDMKDDLNGKDPKKMTDEEKRKNFLERNRVAALKCRQRKKQWLA
NLQAKVELFTTENDALTATVTQLREEIVNLKTLLLAHKDCPVSQAQGLGPLIMNGMSAGFDPHPYN
LASNMGMQPGAPMPQAIRR
>g492
MSARRIPARSRRVEDAMSQLVDSLLPPISPSAFGDEFSGDEEDAVAAAEEQRLQEHLRRAWRILDV
VGAEKEQAGIDGSGGSAAENINNASDLIKRKLLRENASPDKAKWAILYLLYRLSEQEDSENNREED

AVTNEGGRSRSPLLDDGNLDNLLFKSRAGMRGALVDDIDEGPAITEPCLLYKELSGFVDSSEGGLV
SQALRASIFNELRSYLGLVATLEGEIRRALTAAASDDPKGMVKGAVTLKRCVVWTRDATMALRLMS
IMVEEARDKKGGQLISLIHGYSTSHGDPFVGNFAEKLLSHITRPFYEMLRQWIYDGELSDPYKEFF
VIESEFRPSADPRRIASSVWEDKYKLDDEMIPSFITQDFAKKVFLIGKSLNFIRYGCGDSAWVEAY
SKETSKELRYGDTARLENSIDEAYKTTMARLIHLMDTKFKLFEHLRALKKYLLLGQGDFIALLMES
LASNLDRPANSQYRHNLTAQLEHAIRSSNAQYDSPDVLRRLDARMLELSHGEIGWDCFTLEYKIDA
PVDVVITPWGSTQYLKVFNFLWRVKRVEFALESTWRRFMTGARGVLGSVEDKVGSDWKLARCVIAE
MIHFVCQLQYYILFEVIEASWDQLQAAISKPGCTLDDLIEAHTKYLNSITHKGLLGSSGTSRSSSS
STNKPEESFLAQLHQILKIMLNYKDAVDGLYSFSVAEFTRRQELNAKIETRTARGQWGVTEHDFNH
PSPFLSNQENANLTADDHMLTSLQTRLRDLSVDFRARLNILLGDLAYQPDVDMRFLGVVMNFNEVY
EPVRKGRREKTSAAAAGASSSTRDRDKDKKTRRNVVPPSTSAATGGADPQESQGDAQ
>g493
MSVESKHDLRVSELKAKKKEEKRRLKEAKKEKEYVQDVEYTVKETSEKTSKKKSKKRSRDEVDSEE
KQKDEQNERRNNDKIEEDGVSEKKSRKKRKSVSFADNVDVQTIEHKDEMNGAGTETKTDDHDDKID
ADNEEQNRERRKKEKREKREQRRKNSEKTATTTKGDKSMTESADKTIIAYLWQYHHDRTSWKFQKI
REAQLLKHVFSLEHVPSEYNPALLAYLKGLKSEGARTRLRKSAQDVIKFDEKHTSALSADESTVTE
ETSDIAQIPVLPESWREEYGNAVWRFKGNLNAGVKDLNEGVTLTAPDGDNNENGEIDPNVLTRLEH
RKRAEMVLWTASGKIGKFSSATSKPEKSAEAEAESEPTETTSVTKKETKQKAPARKKRKNRTMIVD
ISSSSESSDSDSD
>g494
MSESIATGSAGYDRHITIFSDTGRLYQVEYAFKAITAANITSIGVRGKNCAVVLSQKKVPDKLIEP
SSVSHIFKISPSVGCVMTGSIADARACVDRARGEAAEFRYKYGYEMPCDVLAKRLANINQVYTQRA
YMRPLGVAITLISVDDENGPQVYKCDPAGYFAGYKATASGPKQQEAVNFLEKKLKNKDHVEGDWEE
VVELGITALSNVLSVDFKKNELEIGIVGGPRLDGEEGTDVSFRALTEEEIDERLQAIAEKD
>g495
MADGLNDARAMRVAELINDYRTLQHHISQQLASVPMGNTQQEGYRVLAQSSASAQRLLAAGFSSMP
IEDQGSDPEMERAQLRQVILDASVRRFQAHKIYLRVAAAKRWVINRNELLSRSFKGQSTQLREIDQ
LLRQELDSITDHTIFSDLRQADSRAGLWVSEDPPLAAIQLWINNSRR
>g496
MLSALGGNLSNARQSIAQVLNFALVLSTAFMLWKGVSIASNSSSPIVVVLSGSMEPAFQRGDLLFL
WNRGERTEVGEIVVYNVRGKDIPIVHRVVRSYTEEDKKLKAKNKKTGLPTVAPQKLLTKGDNNLAD
DTELYARGQDFLDRKEDIVGSVRGYIPGVGYVTIMLSEHPWLKTVLLGVMGLMVILQRE
>g497
MGSMKRISKELAELMETPPAGISVQLADESDVYKWKVSMKGPEGTPYQGGTFLVNLTLPNEYPFKP
PTVSFATKIYHPNVSNDDKGSMCLGMLKSDEWKPSSRIAAVLEFARQLLVEPMPDDAVEGRIAEQY
SNDRKRFEEVARDWTRRYAREEK
>g498
MVTQHGGPPLDDSTSGDDVVQRRRVPPPRLNLDNNNSNTSDSYDRHHKSPVSPTSPSNPTKYIPYS
PRLARPNLDQLSYNTDTSFQNSPSRNEEPLFHPHVSDPMSSRHDVLVASSPPTKTPNRTSSQDEEE
QEAAWPLPMTDTAQSINSVPRMRSARTGLRISMSTSSLNGRRRSPLLDRQYSDMNANNRPLPRFPP
RTTPDFLQYNDPLRSSQTSGQTSYSSFEQMSGTERSSILTKSSSITDMSPDTPNGYFGEDEDMTVE
DAIGMYLDDIVDVPEDSASAHSLSLGGELQANAENIYSPTKHTSGTSLTPGPEDPAMEPQEPASEQ
RQSGFSFTNDHRTYEVPAEGEKTVEGPNEQNENHEEVKPSRLSAEPAESVGNEIASPKSKVSRQLS
PPNLSIDTSAAVEMDTTKAANQPIPSPRYSTIVRVAGKVPPSFLPGDDDRDRYGFRKATHHVSREQ
FEAWNKPYAALAESRRLKWIKLMESSGLPTENPVTFPPKSNAIKRFVRKGIPSEYRGAAWFYYAGG
YQHMHPKPGHYASLVHQAMSSPINTDKEHIERDLYRTFPDNVHFKPEMSAEGGGDNAESNIPKHSS
VIVETQMIRSLRRVLYAFALHNPRIGYTQSLNFITGMLLLFLPEEKAFWMLHIITSELFPGTHEIS
LEGANVDMWILMVLLRDNLPGVYTKLVTITPTTSKGKAPALTVNMRLPDITLGLTNWLMSMFIGSL
PLETTLRVWDCFFYEGSKTFFRVALGIFKAGEREILSVSDPMEVFQIVQTIPKKLLDANALLDESL
SRKYRMGQGRIDALRDQRREAVRQEKTRLSLFTSKGLGLDRPATRTGARSPLPRLDGWRAIKKNTF
N
>g499

MRSFVTLLAFAAAATAATSTLAAREDAQTCAMRTLLTSSACNTGDAACLCSDAARQSVMEEIKSAC
GAEQSDLTNSMATEACSAQKVRRATQPSATVVHMGLATPSASAAGRVNGAQEEACTCANTNTKSNT
PSAPSENNTQDPKEEESEMTGTTSHGSNTPSGNSAQSSRMNAPSMMATPAASSARHGAMTTPSAAS
SSRVARPSGADAYSSFKGAGSQIIPSLTVVAGGVLAGVVGVFAAL
>g500
MTRSSLVGLIHDKTIYSPAVSYDNGQAITLMSTDADSLDDVAEMIHETWAQVIEVIIGITLLATQV
GWIWPLPLFLIYLCSYMSRFVAKNLRPRQKAWNIATQGRMAAISSMLSTMKVVKMLGYQNHLMNRI
QELRKEELWAASRLRWVMVYYNMSANALGIFSPAITLVISALISVARGGKLDTETAFTTIAILSMV
THPANMVMTIVPRAVAAFAGFERIQTFLLQPSLQETRQILSENTRNEVLWDQSSPAIQIHDLAAGS
FAIISGPTGSGKSTLLRAILREVTPVRGSIALLTRNISYCAQRPWLPNDDTFSGLDGGTEQTIFNN
LFGPSGLIRHLKTTVILVSNSXTDQYFHAADHIVVLKDHEILDQGKWQDIQSKNTFSTKLPSSYRN
RDSTILSANFEKLNNQLLAKDETELDLARQTGDPTLYGYYFSYVGFDNLLILLGCTISYSFFITFP
QYWLQLWTDDGTRDLFYACGFLLLSTLSWASTSTQMWSILIRLAPQSGSKLHERLLYIVTSAPLSY
FSKTDVGSILNSQDMELVDKKLPSALQTVVTQIFKLLIQIILLCVAEKWLALSLPACVPLVYVIQK
AYLRTSRQLRFLELESRAGVFSNFLETVEGLETIRSFGWSKAMIDANVRSVDYSQRPEFLLLCLQR
WLKIVLDLLAAAIATSAVAIAVTYRDRISGAQVGIALNIMLVANTTLLRLVGNWTTLETSLGAISR
LKSLEETTPFEGGHTETLEPPEDWPSRGNVEFKNITASYQSGSVALRNLSLNXPAGQRLVVCGRTG
RXVKPYSTNDFDRGLTGLISGKSTLLLTLLRLLELQSGKIEVDGIDIKTVGPNILRERCFIAVSQD
PLLLPNETLRFNLDADGSVTDDDLVDALTETGLWSHFFKGDTSYDVEGATTIEISEDSDEHPVLHR
RFALFQELSVGQCQLFALCRALVKARFLRRAGLKPVILLDEVTSSLDIETESTVHDIVDREFTHRG
HTVIIVAHRVGALEKHMEVGRDAVALMADGKLQEVVQE
>g501
MAAKASFQLFPSPAKNKKNPFRTIPRREETRNISPVISEPDESIKSGIQTESVIIKIIEDTNTDTI
QPQSTALSNVSPSETLSDATHFNPEQRQRTPVSPGKPINSMFPQYXPXLPLNKQAYFPRNRDSNNT
SQESTIFASGPYRETQVPPEVDAVLGPKTVPASVFNFPSGALSPRVHYSTAEDLVTLWESANGQEL
RETLGTFNLRAERVDTTTFTFGNPQLPFYTLHTGADGFSLLRNHPLKRNRGVQVMALDFENPARRQ
PPNDGLVTVIFSKLAAMLAVEQAAELTRHHALAPSEAMEVETNAVNRAASQESCRLIWNERQHRYE
LQHPSILKQHSPALLGDDGNPLPRVQTAKPGILHITVSLTAERESEHYQPPVIMVTSPRSPNSIQA
GDMEATPRTSTLPQADIDEPLASFDLNTMSLSICAGLTTSIIPSLYAIDSLIVAILAVAVTDESTY
PVLANTELYVSGLDSSRTPRQTPQKHRKSRLFRNSIASTAAKTNTTYSGRYFATFAEREEAEEEAK
LMAQLRASESKERSKKKKHSTSEKKKGGMFSFLGSKKSQGKPDGKTKQIIVEEFDMEKYGHFGQGS
SREGQDLPGPAKMLVSLLVFGLQIVVTLLSTGVKCAVWMVVNVTRCVTSEKF
>g502
MDAEGPARALEPSSPAELILQQKSGALQGLAQPGGNDGGILSQLLSNPFFTAGFGLAGLGTAAALA
QRGLKHGAALIRRRMLVDVEINVKDDSYPWFLHWMTLYQQSQLNSARSVGTKGGEKLGIVETLLRK
FTPAMRQLSIQTQKVEHANGAIHTQFTLIPGPGRHVLRYKNAFVFVNRVRESSSRDLQSGRPWETV
TLTTLYAHRHIFEEMFTEAHAVAAKSHEGKTRIYNSWGAEWQQFGHPRRKRPLESVVLDEGIKERI
VEDVKDFLESGSWYYDRGIPYRRGYLLHGPPGSGKSSFIQALAGELDYDIAILNLSERGLTDDRLN
HLLTIIPNRTLVLLEDVDAAFSNRRVQSDEDGYRGANVTFSGLLNALDGVASAEERIIFLTTNHVD
RLDEALVRPGRVDMTVRLGEATRYQVAALWDRFYGEFDTKGVYKERFLDRLADYSLIEDKTGNKAD
MTKAVSTAALQGLFLFNKGDMEGAIRTVGELAESMYDQQA
>g503
MSSRQRYMSYSSSALPTLTVTNGYEELTYQPANSRPHSQCISALDFDRNAHYMDDRVSIGSSLSDD
MADEEEIFVNLNTRAMAAIPNRLQKIMCEIQQERYDRLKKENPNLRIMPADYWIKPVEPVRKKPVR
YDRAPTPLYLAMPPSANKRNAFQQTTNHAARGHPTLQSLGPGDRYRWTAHPEIVDYASRPTTSHGS
APTREFRVLNGYRQPVPPDLVLKQKFRKPQNRRPSGIRSLSVKPVASNLRARLKHSLSRLNKWMKN
EN
>g504
MQAPVVVMNTQQGDRQVGRQAQLSNITAAKTVADIIRSCLGPKAMLKMLLDPMGGIVLTNDGHAIL
REIEVAHPAAKSMIELSRTQDEEVGDGTTTVIILAGEMLAQALPQLERNIHPVQIISAFKRALTDA
LQIVEDISMPVDIDNDKAMYSLIQSSIGTKFVSRWSELMCSLALKAVRTVSFEVGGGKREVDIKRY
ARVEKIPGGEIEDSTVIDGVMVNKDITHPKMRRRIENPRIILLDCPLEYKKGESQTNIEVSKEDDW

NRILEIEEEQVKHMCDAILALKPDVVITEKGVSDLAQHFLVKNNVTALRRVRKTDNNRIARATGAT
IVNRVDDLQESDVGTRCGLFEIEKIGDEYFTFMRKCEDPKACTILLRGPSKDILNEIERNLQDAMA
VARNVIFHPRLAPGGGATEMAVSVKLQQLAKSVEGVQQWPYKAVADAMEVIPRTLVENAGDSPIRV
LTRLRAKHVEGNYTWGINGDTGAIVDMKEYGVWEPEAVKLQSIKTAVESACLLLRVDDICSAKSAQ
QAGNVGGGGEE
>g505
MSEPGKESIPTSADPRSKRPTKRRQLTPVSEQANQINVLFKDPSKEVHLAPLSKPRTLDSLAPPPE
IVANVQGSSAGAGSGEFHVYKASRRREYERLRLMDEESKREKQTEEFEKKRAETLRKDQEKTDRNR
RKREKRKAAAAKNKNGVSSSTAMEVDGDAEKKAKAPSTRLGPLPPRDRDHDTHANENNGEQQVYEE
QGVIIHDDD
>g506
MGSYRIEESPNKRAGCKVKACKDHNIKIQKGELRLGTWVDTERYQSWSYRHWGCVTIKVLANILEA
IEEDGEPNYEMLDGFDELSAENQAKVKEAILKGELDEADITDHSLLNGGASEDVQNEANGSPEQAK
ADTKAKAKKRSRAESDAEEEAPSKQTKKAKGAVKEEPTDGKPTKRSRARKAVKEESPEEEAIPTKS
AKKAKGRTTVKEENPEEKPVKRSRARKVDEEETAKPGKTATKSKKKAAAAKDESDEEEADVGEEEQ
TGEVHLPAKKARSKAAAATSEKPKRGRKKKVTDDEE
>g507
MSKTIALISGANQGIGLATATRLAREHGYQVIIGSRNADNGLKAAKELQSEGLSVDSVQLDITSDD
SIANAARYIDEKYGKLDVLINNAGILLDIGARGEVTSKMSLRQLWDLTLSTNVTGTACLTEAMIPL
LRKSSQRPRVIFVSSRMASLAESLNPKTLYYKIDYKMYDSSKVAVNMLVSNYARILGEEMKARVNA
VCPGLVQTQLTNYNAYGGKPEDGAQRILELATVGDDDETTGTFSDSNGPIPW
>g508
MKRIAAGTKAESGRGLGLCTRKEIDCVYEQPRKELTASDAPIAVSNHGSNSNNDFLESAPISNVLE
VLDGELAGFDRSRHVLGDYVENFLPTLATDYELDGFRIHKDLSTIGGTSMPATLSSEMDNIMSVSQ
PDSFDLSSSISIGKGLNYIPVHPPTYSSTSGTEVTPTTIHFSKKQIVTPAQQLFTSMLIDMIRAYP
LMMTRRETFPPFLGSMNKFELFSALQASLLYLIMRAIDIGPQHSAHDFELVRAHEMICDYTLQQIG
HHADPEVNDPHRQWKDWVFDESIKRVAWVWFSLSVVYQIRSGVPGVPCRVADSTEGMPLPCTKTEW
EAQTEEDWRREYYKARDSQSTASIQTFGDLANAYKGSPADRATKATQLEAWNAGIDNLGVLLNLAV
YMTRN
>g509
MNPSTKQPSSQTSLFQVFLRLRPPITKEEAAHAHRGERYLNVEPVEITKDNDVSSPQNPHPTHITL
QPPNDSRKRAVEKFAFTRVFEESSKQLEVFENTGVPNLVKGVLKEGRDGLVATLGVTGSGKSHTIL
GTKTQRGITQMSLDILFRSLADTIRSADNFEDPTLLASLSAADSSEAQIFSARNFIDSIYGDPTER
GRASRAHTPMSRGQTPLMESTHTPMIPRRQLNQRLNALPSLPNTNHLVTPMDSQAEHVVVVSMYEV
YNDRIFDLLSPSQVNGTASRGNNQKEKRRPLLFKSTEGSPDRKIVAGLRKVVCSTYEEAMMVLETG
LTERRIAGTGSNNVSSRSHGFFCLEVKKKVYLAGSERARNARTAGATLAEAGKINESLMYLGQCLQ
MQSEISEGNKTVVPYRQCKLTELLFSNSFPSSHSASNNRYPQKAVMIVTADPLGDFNATSQILRYS
ALAREVTVPRIPSVSSTILSTSSATSTTQSRPPTSTGRTTPYTASTEDLQEAAREIVRITDDYEAL
IVRLAEEEIARQEVELRLRAAEEKCLMIEQEVREECWAEMDEKMEEERKRWQEAMDRQAGHHEEHL
DNKLEIASRGIQVYEDPEPTADERVDELERENDFLRSKLAALERQLNSRSSPTRKSKPRPAMSNND
AGNNQNHLLSSDSSDLENMFQRVTAFKVAETYKSPVKPKAALVHGARNPRRVTSRKKDLGSEDDF
>g510
MASFTALNIXPAEDIQEEVDDTKEIQIEEALKLYQNALKLHSQGPAFYPQAKDAYDALLQSEVFKY
PESISDYRRNTLADGDNDEVAVDALGEIDVNDAAASALPQTIFLSYKNNGQFLLDVLKETLRKQPP
SVDEQRAAITKESRSAMSMFADALERDDTDLDLWRKSARIGGAMNSYRLARYCLESVLVGDDNEVE
VRTEQLGLDEAFAAEDLRKTIQALKDELSSIQAPPLKKPRSSLFRFFGKHSQDPYPYLPGLSRDIS
DTAPSTLKQPLNRHIVKSPERTWDAVGKAIMQLLTELRRNPAVLEPGAAVGIEIPSEEALPQSPGE
KNQPVPANDDVTEKTTAMPEVSNTIEPVDDQSSIDQRADAQLRESLEGFSGQPANAQDTTTEDKNG
ENVESTSANVNSRKRSSTSVANDEMADNTRTKSRRIRARESIVESLQPTEDISLDNRYYDDRLEEF
AHADEWMFGTVGDLLSKAGIEALGTVDDLKQQMALSNSPNSQQSKDIDSTLVQDLQNALRTWNEER
ARSAMQGDSSTALQDSRGRQRSGLAIFLDHSRKSTQKASKRKALFESTGLAEFTSSINDCWLHVRE
VSFQWLSSLLQPGRAQFFDAVSGELSNESSYVSLPWPESLKETVVKLLIQEDDYIYERLADVVCGS

TQRALKQAVSNTSLTCADDAHAKVDMIQAIHELHLDVYAHMNNPNSQIDQATRALQRDRLTRWGAL
AQTAVNNLLDTEPESEQLDRISLRHLWSMTFYSNMAEDVSRDYIMLCLEDLKEALVSFGDPEITLI
NNAFMPQVSAAALDQEITRLNSMGFFMRIFGSEGEDPVSLIESIEPVLDPASVTYVRGDGANDETT
SVPIHFRELISFLDKGDATLRLFLWRRLQDAYRAIDYIPKVVSCQLRSIEAIVKELRGRVYLETQD
DRASILLRWLKSTNNILTKLIPQLIEDPVKSFECIDVDHLRSSMTAVAHLSRLLHSFALYEDAVKV
GQRPPVELRGALGKSLESFKEKLRDMQVRCWVLQYTLLKEFMIQEKEKFSTPSDYQIQYLRAVHNA
LGIRLACKYADKVLLRLMKSELLSLETQEDCEFDIAQVLLDLHGFKFSNQLGTSDHGCPTEKPNAQ
SAAMMIDFVLMQVNRLNMKDLSKSDLKTTIEKVQQAIGPVKHSSLSFNKRIISAYLKSPINPSELF
ELVRGVGGLALRSLPITYAENAKKGWYFLLGHASLTKFRSQKRLNVVPTNDLDDAIGYFRQDLEYA
TERWESWFRLAQTYDTKLEEDIIWSSDKINNNQAELATLQRSAIHCYAMAVAGAMRAAEDDSKTRQ
TISDLFTDFGMRLYASSREPLNMAAFSLEDIERHFSRFESQELYTGKPFKELGVYSVWSLAGHMFK
RAMVDKPNNWVNHYMLSKCRWKMFNSDEATRGSAGPVSIDEVLDPLLDAIEALPQRKERGSFPDRI
LEPHFRLASVLHKLVTRKQLEPTKAGEILAETPWSKNTSPPQNIEEWTPYILDVFKKLKHADKSNW
HHRILSRMAKIHYHGQDDADAAAAAKNELMPQIFTKTMVLQVWRPEYERPGRHYVYTTQYVSYFTK
LLDQISDHVNLEQLLRRVRKKQGDYINHSKLWEDMCIVYAKLIRRAGNISEHYEEGVFKQLGWDEF
VTNSARLDDFKDLATTSGRTMDLLREAVEFKKLNNNLMKVTMFEDLTADIYARLYEENKHRFIEQA
NEENRGRMKVDHLLMNTDGAADVPTPPTSAPASEAPVPRGRTKGISRRDIQKRAEAIVMRHARPAI
TKTAATPVEEERFPAAPIVRVPSIGSIRGLMTGTASAAQDSVPASLHESADDESELSEIDDEKLAK
LKAEQSLLFPNLKSMRSPEPVSELSTAVSIDADRNEDAMTADDEANEHDGEDDLEGETGIVEEGED
GETELQAAPEDDEDTENADEEEDVAGAEDVDMDVEKFEEAADETIMTIDEGDETEEDESMFKTNGK
GNENIDRKEKEKEDEDKLAAFNEAKTFGDVETSKTGLGETDADEVDES
>g511
MVLPAVPEIYEEEDVFTAVDARTESLQNLRELGPPDLVYLVKQSKTNPTRQTGVYHHVTGIDASSS
ASLAAYVNTLTYSPVDKQNKVVSGVYCCYNAFSHLDMRVEVKIPGSLESYCIDERGDKRVATEALW
LETFLCAVLRSYTYADDGTGDAIKKIVGVRRFNPVTNTEMEHKFLDAAERLFFMGRQLSSEPETQV
PNTVANHLTSGLLKYIHTTGRYASGINLFEKLRTKDVEVSSLLARVLISADEEVQAVRLMHDALQD
VPMDYALLDCQAAFCLSKGEGELALECAKRSVTAAPSEFSTWARLAEVYVTLEQWDLALLTLNSCP
MFTYQDKDIPRMPPPSRVLLPILAESALDEIDEGQPKQGDPHDFVHPSLRKLHATGYQGTFLKAYE
LLTKIAAAIGWDQLLKIRSDVFVMEEEYRVERQQTTKPSHSHNASTVALHPPPSPMGDAASDAETD
EDHSGEANGTENGNGSLEPVEPSIEKPEQTVASETVKSGNEDPDPSHADYAQFQNKRLCERWLDNL
FMVLYEDLRIYTIWRSEMAHSRHQSLEYKKTATEWEILGELAERLHHFDEGIEAYRQCLSIRFSPK
AMRGILKLYERQGDTRGMLNALIRLIAWQYRWYSEFSPDLLLLVRKLIEEEGAVKVRSIVQATNLP
QPVLDLTHQYCQVCATFRSTGSDV
>g512
MSTRKRKQEAEEEEELQALPSDESEEEEEYQSSDAEGDDYDESEEGSEEEEEYDEGEGEEENKEEA
PAPKKRKTAHDNKAKDQDDDGEGEDEGEDGAGEDYDEEDDDGTNDTADKSGPAQAAKKQKGGDVPK
EDDLSEVEAED
>g513
MSTNVTPLKRSAIEAGLDNDDSVIDVDTKASVPKVSKARACAECKRHKIKCEVKPGETSCTKCLRS
GIKCVVNDFSQKFVDDDLAWKSQANSTIQQLQAAVSHLLRHNNLPDISTFSPSESNVDRTPEATVT
PSVRLPSHDTKRLSVPAINSSTATEGQMMARETSQDGNYEEQDLVSAPMRSLYEVTKLRNFGNHMV
EKPKSTLLEEDFISRGYVSLHEAQELFAYFSRTMNQLLWGGIAVPHRDLTSVRRASTLLLAAVLTV
AALHIPNRAETLNTCYNEYVSLVSNMALTRSHTLDDIRGLCAGAFWLSDLSWKLSGHAVRIATEMG
LHQSFHKMTRGYADQYHYERAQLWYLLYVCDHHFSIAYGRPPVIHEDSAIKNCEMFLQTRFVVPGD
IRLMAQVALFHILTEAYHTFGSDAEQPLREEDFGQLRVHNVAVDQWRLLWQPRSADSPYVRTYPSK
GVVLHYHFAKFQLNSLALRALSPTNTPVFSMDRKECANAAISAAMSCLNMVLEEQDVRDAIVGVPI
FTHTMVTFSAVFLLKVAVNWNSAYLNIDGRQVRHTVERVIELMNCVSAGDRHLTRHIARGLSKMLD
RFNAWEGWFNNHGTRNPSTAVPVGNSNAGAPTNGYDNPAASTATITNSNDLQPSSTVVNTGNNVST
GVRRRSIPGGANAMAQGLPPPDLIYTMVGTYGFGLDEQLMDPTIPGFEYMSQ
>g514
MNILITGAAGFIGQLLAKELLNHPAHKVILVDIVDPPVPAGVKYPQNARSIKTDLVDGDIESLVTE
SAPDAVYAFHGIMSSGSEANFELGMKANVDATRALLEALRKKAFPGVRFIYSSSQAVYGQPLPKIV

TDDVIPTPESSYGAAKIICETLVNDYTRRGFITGFTLRFPTISVRPGKPTAAASSFLSGMIREPLN
GQEAIIPLEDRYFESWLCSPRTLVRNLIITLGLPADCVPPHIRQINVPGICVTIQDMMDALEKVGG
KDKLALLKEQEDPIVKKIVHSWPTRFDNSQAIKLGFHRDESFEQAVRDFKEILEQ
>g515
MVLDNAKPATASSGQRPSNTIENVTNDINKVDILKSELNAPTDSHALAHAEVEEASLIHTSPDDEA
TTDIGWQQQPHEFDEPIVGGISNEDLWMLIRRFNKQIYHVRDTPTLPLDNLDLNPVEDEEFSPDKL
RSNIERLYMTVIVGLMNSYKHIVRLRSWREQRRTAAFCAVYFAAWILDLVVPVLLSVVVVLILYPP
SRSILFPPAPIALVDSQTGGVQKPRAGVLGSKDSLTGAPERYRGEAVEREASNLVSSVAGVAIGSA
AGKHDPAVPDDAPMEDKVPDVTDVASQVGDLQNSAGGGIPSTKHDKTKKPMSDAVWQKMKPVMHIL
GDITDTYERFGNALSPAPPFPKYAARLRLAAILLPMALASLFITSYMVVKGTGFMFGFVFFGDPIL
VRGAKYLNRRFPHWQKILELRNSILKGVPTNAQLTITLLRLGEVNGTPIPPPPTSLDQAPSKPASI
HEDIPLDASQAEIESAVRPDPTITKQVTEPNSHLNEKSKKKGFVPAMLRFFKGTTAKGVETKLATN
YALAGLGYRNAKNHLGILPKKGLEVSPRGPVDFQCRYMGKKGCLVLDCSKEEPILYFTSETGTQPH
NLEEAKKVLFAILIKDIRHLKKLGGMGWKGKLVVGWAEAEKEVIDGLRIIGKDPKQNFHITAMKTR
DQLFNRLIAMGDQVWDIC
>g516
MPEPPNKKKTQKRQSGDDLTAQDQPAKKKKAKPYVPALRSGAYALILGLSTIDENSSQGMSKAQLI
EVAQAHSDTSFTAPSDPTKFYTAWNSMKTLITKELVYEQGRPLRKTAFTKSVGLPTSSRTFQTNDK
AFTSLDDDGITVLDEIGPISATRSKESQLSNSNRPSSPIILPPNSFTIELVLDSREVRAKNDRDYI
SKELETKGITTQVRALELGDAMWVAKCKDPDYLSRYGEEGDEVMLDWIVERKRLDDLIGSIKDGRF
HEQKFRLRRSGISNVVYLIEEFNVSHPSASAAGNVPNYQDAVASAIASTQVVNGYFVKKTKNLDDT
IRYLARMTFLLRKMYGSESLLASSLGSSQPSRSSVKPKSVALIPSSNLSSSESYLTTLRELRCGRS
SETTTYGVSFSTFSALTSKSDTLTLRDVFLKMLMCTRGITGDKALEIQRHWRTPRAFMEGFESVSS
SAATPQDAAKARERMLTDKMGNLIAKKKIAGTLSRKVAEIWGEKE
>g517
MTSHKPALLTVLLATNIHCASCVALVKEILTFYQSITSVDVSVLQQEVRVRHGPEISASDLTSTLI
DAAFEVQLATTQDESTGQILESIETSSWVTSPPVVRSPLLSSGTHTRVTRERRLYESQRRKHIENC
QACQTERQYALHEAKNSPPKYDRVWTEKLVTTTPTLKKNDPEKETPTLESVQVTQPKLPDVFHARI
AISGMTCGACANSVTETIQQLDFVKEVSVTLLTNSAALIFTGPRENIDKIIDEIESTGFDATVDTV
EKVTETGNDVIPVWEAQFGIGGMTCASCVNAVTNHVKQMDHVKDVSVSLLNNSATIVFSGDQSYSK
TICDEIEAIGYDASLIEVVQQNKPPSDARPRSNRYIANISINGMSCGACVGKVTQAVQSISHVNDV
VVDLLSSSARVEFEGKDNTQQIINEIEDIGYEATLIDCKPAEVDTTSKPTERTVMIRVDGMFCHHC
PDKVLLSLKDLDDSIQISKDPTLKDPIVKVTYTPKQPNLTIRRILGIIDSSHENFKASIYHPPSIE
DRSRAMQLRERRRFLFRLAFVLMVAIPTFIIGIVFMDLVSSDNRAHQYLSEPILGGSVSRMEWAMF
FATTPVMFYGADVFHIRAMKEVQALWRPGSKVPLLRRFYRFGSMNLLISAGTSVAYIASLGVLIAG
ATTKSSDATNVDTYFDSVVFLTLFILAGRALEAYSKSKAGDAVSDLGKLRPSEALLANPTTSESVD
SDSGVQRIHVDLLEVGDIVIIPHGASPPADGVVTSSGDYQFDESSLTGESRPVSKSTGDEIYSGSV
NVGQQVSIKVTEVGGASMLDKIVSVVREGQSKRAPMERVADVIVAYFVPVITLIAILTFIIWLALG
QSGSLPLGYLDTTLGGWTFWSLQFAIAVFVVACPCGLALAAPTALFVGVGLAAKRGILVRGGGEAF
QEATRLDAIVFDKTGTLTEGGNLRVSEHEILDSTNSQKVAIAWTLARELEQTSNHPIAKAIVEFCK
DKLSAENKIGVKSSDILEISGQGMKGTFTLLIDGETASYEAVIGNERLAKSVVTQTSDSQYFFENI
LSRYQSAGRSTAILLLRKAQSSINKKDKETGVDMDNSDHYTPLIIFATSDPIRAESAQVIAQLQKR
NIAVYMCTGDNEKTAHAVASTLGIPQTMVLANVMPAQKAEYIRKIQFSSLSSSDTENQTKQQVRKI
VAFVGDGTNDSPALTAADVSIAMSSGSDVAISSSSFILLNSNLTTIFELVKLSRRVFNRIKLNFFW
AAIYNVILVPVAAGAFYAIPDGTQSITVAGYTQHVSGHWRLSPVWASLAMALSSVSVVTSSLALRI
EGRDIKRWIMKVFKKA
>g518
MPLVKPPVRPQPRQPVSRLQSIKPPQSGAIPLKRSVGIQVPRVGSMSSSSGLSHAAAPPPKRPESK
KPAETCPNPNCPDPRIIRDEDMKVCETCGAVVSEDTLVSEISFGETSAGAAVVQGSYVGADQTHTR
SSGPGFHRGGGMESREITEQNGARYIQQMALALDIPESAQKAAGQVFKLAVGLNFIQGRRTKTVAA
VCLYIACRRQPGNTVMLIDLSDVLMINVFKVGRAYKMLLEELRLGGTVFTMNPVDPENLILRFAKQ
LEFGNRTMHVAKEAARIVQRMNRDWMTTGRRPAGICGAALILAARMNNFRRTVREVVYVVKVTEVT

INQRLNEFSSTDSGGLTVDQFRSVHLENAHDPPSFNPKKKEGRKRKSKVLETAAEIEDSETETETD
AQPTPPPLPKQPRIDADGFAIPEIPIDPALTAIDPALSAGNYQSTTAQNTVSSIEAELLAQSVLDS
EREQTSKKGKKKQTVPEPTPEELASEEALENEMNQFLANGSDMVEAAATNRTATAAASNTKPKAPV
SDNREIEASEFESDPEVANCLLSPAEVEIKERIWVHENRDYLRAQQAKALKQALSQGEDDGTLQKK
PRKRRKGRLGDVTYLQGEDGKGSRASTPAEATRLMLEQRGYSKKINYKMLFDLYGDEGAAEIEKAE
SQKQSRSQSVASEAASSANLKPVVSATPKIAVPGATAGGFNEADYDDEEEEAEDAVGEEPDDDQED
IENAFAGNYDEEDDYYDQGSDDGY
>g519
MGSVYNNFSDGPTMILPSSQNHPSCQFPSQSMQGFQAYDSGSWSMDAVYANPHKAPPRHSSSWTPP
SSSKTLFTNPSRKRSRNESEDEDLPSSKQSIAPLAPVVEAPIYGEGMVLLNPRTGMAVSAESQTGT
WYEEKAAKKAAQLQQCSSGNKLSRKSQRLDTSASSTDDVDLAWIQNQMQTTTNDDIRRSLGKSSSF
NMNDEPQVDDVTLLLGISWQRVSHDDNMAPAVTGWEKYINNHFYKYMRDARIILKSRSLNAYLVAA
PPAHEIPLQDTARHSDYAAMPSEYQFDPSSYFFLFKEDLTEAQLVSSTLDNCLRNIQSTPIQFEGS
EVLRASERSPERTVIPDQVNCVVGNGIPIARLSRDDREKVNLNNDMAMSMGTDMDVDL
>g520
MSSDNEKARTSGEVSRPEPVLPTVNPEAPKSEPPKAALHPAFYVGTWIALSSSVILFNKHILDYAQ
FRYPIFLTTWHLAFATLMTQILARTTTLLDGRKSVKMTGRVYLRAIVPIGLFFSLSLICGNVTYLY
LSVAFIQMLKATTPVAVLFATWGLGMSPVNLKVLMNVSAIVVGVIIASFGEIKFVFIGFLFQIGGI
IFEAIRLVMVQRLLSSAEFKMDPLVSLYYFAPVCAVMNGVTALFLEVPKMTMGDIYNVGLLTLLAN
AMVAFMLNVSVVFLIGKTSSLVMTLCGVLKDILLVAASMAIWHTPVTGLQFFGYSIALGGLVYYKL
GGEKLKEYGSQAQRSWAEYGSTNPGQRRLVIIGAVVVGFFLFVGALAPSYAPESVDHIRGALGGAS
AGRV
>g521
MSHPDISVDVLVIGAGPTGLGAAKRLNQIDGPSWLIIDSNEIPGGLASTDVTPEGFLYDVGGHVIF
SHYKYFDDCIDEALPKPDDWYTHQRISYVRCQGQWVPYPFQNNISMLPKQEQVRCIEGMIDAALEA
RVSNTKPKTFDEWILRQMGVGLADLFMRPYNFKVWAVPTTKMQCAWLGERVAAPNVKAVTTNVILN
KTAGNWGPNATFRFPARDGTGGIWIAVANTLPKEKTRFGEHGKVTKVNPNSKVVTLADGTTVGYEK
LVSTMAVDALCEQIGDKELIDHTKELFYSSTHVIGVGIRGERPERIGDKCWLYFPEDNCPFYRATI
FSNYSPYNQPDASKKLPTQQLADGSGPASSEAKPGPYWSIMLEVSESSYKPVDLENIVKDSIQGLV
NTEMLKPGDEIVSTYHRRFDHGYPTPSLEREGALTQLLPKLQEKGIWSRGRFGSWRYEVGNQDHSF
MLGVEAVDNIVNGSVELTLNYPDFVNGRQNTERRLVDGAQVFAKKK
>g522
MFRNPPTRVLLSDYEVFQTIHAICTQRLMTLSLAYCPPLSEEDESEGEAEFHQVSPSMSYTRSSSV
QYPMSEGDRRACDVLKCYQDTKVYINSSTPDSDDLPAHSAGSHTALDTRSTNPSETYQSEDQDFLE
IPDSQMSSTSQESSTDTTPPATIIFSPRSPEPTPRPRRMHRSVYYGNFPVLQVQVPEIYECIPGMV
ISDIMSGSGSMNLHGKSDPQPNSPEMYIPVFRSDEINMRIGVGTCLHSLPKKTVRRRS
>g523
MDGDLSLSQSLGGLRIANPDDSSLNSEDATAPTTATPSIPSSSQTILPPSDHDPINKHVETSVHSL
PLQDEYKSGYVPDSTPELSSSYRSQILPPTDSAPSHRNATYRSSVAYLPENPSSSQHLQNIPRSAS
THVHNHYANGSASAAARDLNNYRIRTDSSASGPDRMSRTDSRGGSVALQAGVPSRDNSHSDRSYKQ
AQYLAGNGPIPHRKGSRMGHSNPAGSPYALENGPPASSDDWQDRGAAVGIRQEVDANGRTVSRYIK
KGVRDFSFGQTLGEGSYSTVCLGTDRQTLNEYAIKILDKRHIIKEKKVKYVNVEKDALNRLTDHPG
IVRLYYTFQDERSLYFVLDYCKGGELLGVLKRMVTFDEECTQFYGAQILDTIDYMHKRGIIHRDLK
PENILLDSQMHTKITDFGTAKILDTSKQAENSLGDIPSLDSTDPPVQERASSFVGTAEYVSPELLT
DKNACKASDLWAFGCIIFQLLAGRPPFKAANEYLTFQKIVALEYEFPTGFPAVARDLVERLLVLDP
ARRLPIEHIKNHQFFDGIQWGRGLWKQKAPRLKAYVPPPREPIKLSGGGDEDSFPPNISSAPSGMA
TAKSTRAYPRVITELPPPSQLDIEWSPVLKKDNERILKLGILTVMSSHASHSPGSNGNGDSETHRK
FSRFFGGSTTKKKQRLVMITSQARIILTAAGGDEKRAKLEISLLAPKTTYRSTLDAKGSASWIVDT
RDKHYVFEEPKTSSSGAASSANSIQEWIDTLDRARELAISQQSSAYSGDEYRELSSGFSSQANTLD
HSADLQTEVQPSVRSTLQKNTGDNESLKGKKRFSKRHSKNGLAAVF
>g524
MYALVDPVFQDLNETSRFYVDIFNRLSVRCLALYDAVKNPYRELIPYINGSSVLADSLAAIGAIQF

AYKSDNHGQLLIDAAAGDETSFKAGMTLMKFRDSQAYEHFLRLKQRALHQLSAEVSHPLTRIDDRT
VAAMFVLILLDVIESGNTSWMYHLEGAKNILRSRFSDSNRVPSTDGIDAFVVDACLITEIMGSTLA
RPGVLSRPFYSPSMGSAMLKRLEKTSWVGCPAYLLDVIFFVHAQRYSETGETAEYSLSFLTPFGEV
TRAESPLAVLRHIDAFDADVWAEEMQSYLTLSDLSQRLALARAYKAAVYLYARRVLSRTTAFASDC
AVTDFSTSPLQPRKTVENELIQNLLLIPPEDEHFKCLIWPTFIAGAECTSREQRSITLRLLGLLWN
GFYSLNLQNAACVLKVMWDKQDERLQATALEEDVDDEGFDWIQELDRSSTDWLFI
>g525
MAMVSYPDTVDRTNPPPPPASASSRLSHSSRRHRHGRSHHGGSSYTPQNEFPVFAHTGDVEIVIAA
GGQEKRYLLHRLILSQCSGFFEASTSEEWSRYQAQMESASAAIDSDPSLQSVAEDGSSILSRRGSA
QGSATLPKLRWRYELDWQHRESDEEPILVQKPPSSDPVFAMPPSYSNPPPQPITKPVAPRAGFFRS
VANLTGMQSAVHIPSNAVVPDTQTQPLIRDYDNLFRTFYNFTPILNSVNIATAYSECKALLGLADM
YDALGVVGSRIDHHLLRFSSRLFKQIAKYPPSYLKLGYLARSRVIFTEALVHVVGQWPAAQAQLNN
GSFSPLPDTVLDLIEDKVDDLEDLKARVDAKLFRLTLTTSRGERVSPSNAYIDWLAVSLFRQWFVE
NTTPPPAPILKNSSGGQEGRTVSAPRHSASTVISSGRVYRLIGSSSPEAYLPHDELKRFLKLRASS
ASESLYNRDNFKRFERKMDEIKRLAREVVKPLMRNFLELDLKGSDHNGGSGGSGVGDGGIGGLPYL
TCTRIEEADLPWR
>g526
MAGDNDLSITRDGTVNPPSENIEPKRATEDISHIERVLSSDSNHAEENLKGQMDLSRVDKEVQQYA
SMGQIEIDEETNKRLLRKIDRRVLTFMVATYFLQAIDKGTLSFTSIMNIRQDTHLVGQQYSWLTTC
IYIAVLIVEYPTNWVIQRVPIAKYLGVNVICWGIVLACHAACTNFTGLVTVRTLLGIFEACCQPSF
VILSGMWYKREEQAARVSYWYMMNGGQQIVGGLLAYCFSLIGNHHAIKSWQAIFLTYGCISVLYGL
VLITFMPDSPMRAKCFTEEEKHLMVERVRSNRTGLQNREFRAYQVKEALLDPQTWGYCAIQIFTTL
PTSGLGSFANIIITSLDFTVLQTQLLAMVLGAYIIIVLLTSTWLVNRYHQNLLVMAAYLIPSFAGT
IVLMTVVNHNKGTQVGLLISYYLTLSFWSAQTLTLSMVSRNIAGQTKKASVVAATFIAWAAGNAVG
PQVFLARDAPRYFIAFSVHLVCYSLMLISIFALRWWLKRENARKEKLKAELGDDPNMVHAFEDRTD
RENIHFRYVY
>g527
MSATKPTAAAVHSAIRLLIDNLVNIKDETGKFLLYLEDGRVIDTKSWAGWEWTHGIGLYGVWKYYE
MTGHESLLKIIEDWFAARFAEGGTTKNINTMAVFLTLAYVYEKTGNVAYLPWLDSWAEWAMYELPR
TKYGGMQHITYNSINSEQLWDDTLMMTVLPLAKIGKLLGRPEYVAEAKKQFLLHIKYLFDPRTGLF
FHGWEFKGENGGHNFAQALWARGNSWLTIVIPEILELLEFDLNDPIRLHLIHTLDAQCGALKQFQH
SSGYWRTLIDQADEGSYVEASATAGFAYGMLKGQRKRYIGSEYRETAEKAIQAVLSAIDSHGELQN
TSFGTAMGHNLDFYREIPITSMPYGQAMAIMALVEYLRTYI
>g528
MKYIAAFAALLSVVVADEFGTASVYCGSSCTDGTLIWTGNATTRPEACTDLGATYEYCYFEVDPAE
YTANWALQIGGNGVCNVAADPVVDAGDCSAAGSYDSYDVYFYL
>g529
MTSADYTEDGITNPILNEEEPLLGPNGSVTQNDTTPIYHNFLTGTAVVAQAGIWVLFAVIWSAVFS
HDLIFFTAHPLLNSTALLLQVQAILVLQPTSTPRQKSVGTYIHSALLTLSLAGFISAFVVIELNKD
SEHRLSSIHGVLGFITYILIIIQALVGVAQFWVPKWVFGSVENGKKIYKYHRVFGYVLLVLELAAV
AAATQTTYNISVLNIALWAVLVTSVLILLGVGARVKLSKFGF
>g530
MAPTNLSRIPLTHCRFILRRNFSSSCRRCRSAELSRPPXXPKPVPDVKLIREHAELFARNCVNRNY
ASHEGYPHQIKELYAETKRLEEALLDPRRKIKELEKEIGRVAGNREVNGEEKKQKLNELRKEAQNL
KKNSQDTLLRKETLDSEIQKLALSLPNLTSPEAPVGDEPRVVGYINYDPDESSTWLRNKQPYRSHV
DIGTKLELLDFTSSATSTGWGWYFLINEGALLEQALIQYSLNVALKRGFKXVAPPSIVYSYIQEAC
GYQPRDQNNEQQIWHIEQSEKDKARPQRSLAGTAEIPLAAMYAGRDIDEALLPLKFVGTSRCYRAE
AGARGVDTKGLYRVHEFTKVELFGWSNNPRSDTPDPLFEKCTPLKELFNELGEIQVEILTALNLPC
RILEMPTGDLGASATRKRDIEALFPSRLRAVETETGNSGYDLETGWGEVTSASICTDYQSRRLGTR
IRTSQGEKSRYPLTVNGTAVAVPRVLAAILENCWDEEREVVVIPEVLRQWMGGTETIGKK
>g531
MEAPTIPLEKAAELSGLSSNGVTRVPNDEIDKTFARTTNLASSALNARVLSRSNDYFASAENLLTP

GPPVHKPGVFVHTGAWYDGWETRRHNPEPYDWVVIKLGVSSGVIEGIEVDTAYFVGNYGEKAELQG
TYAPAASAETDEQIASPEYKGWKTILPLVECGPSQRRAWKIDSYDAANPTPYTHVRLLMYPDGGFA
RLRLYGHAIPPPVTTAATEIEELSSALVGGLALSASDQHYTPASNTILPGRGKDMGDGWETARSRT
PGHVDWAIFRLGLPGTVHKIVVDTKDFRGNFPREIRVHGLVTSKDEKEDPTNEHPGWVELLNGDKK
TQADTEHVFEGVELAAXLDNKTVFTHVKLTLIPDGGIKRFRVFGKRAI
>g532
MEEHYILGDDLPLPPARLFERLALLPGYIWDQGIEPVHSTYDNWHVAGIKQASDHDVVTPAAASSG
TPSTTRESPRIESRPPFRHHWRSSLSESSSEISSSRIDQESYFLPVIARVSSHVVRLEREFHMLRS
IVQVSDPDCNHTVRPVDLVRLPPEPGDTGPILVSIFESPGNNALKDLIAFGPAWYSIGTLSESSNN
TTPGEQVSLPVFLDFAIGACDCLEILHYGQKTIHGEIRPDAFHFNRATGKVRLVNTGNGARSFDNA
LSEGWSALSREVGVKTKLQFIAPEQTGRMPTEPDSRTDIYALGVLFWMILVGKPAFTGSTPMEVVQ
NVLGRKLPPISSKRMDVPDAVSAVVMKMTQKGLHDRYHTITAVKKDLEDIVKLLGDGDFNSLKTFE
VAQNDVSSFFTLPSQMFGREDEYQKVVNIAQKVHRRQQALYAKMAAQANASGFAGSASSISESRMD
SFDIAEGSSDSGSLNHVTSSRHNSIAGPFGLNHVPTRDSVHSTESSLSTQRGFGVFNRPKGSASAE
LKSLGDSGDRDSAHISVNTTQSPMETLNPMSRHKAATKIRRSGHTEIITITGNPGIGKTDLIQRVH
PAIRKLGYIGVARLDRARRIPFEPFAKILASLLRQIFSERDVTTEYHNNLRTFLRPLWPTLHQVLG
LPEQLISNDQKEKSVSPKMVVAQHLLKESSKVEITKRPSFLHGQFPTDLFSSTSTPSKTMRLMEMY
LEILRHLCSQKLICVCLDDLQYADDESADLIGNICKTRIPCLLVLAARKQEIESREVLSLFNAETA
SITKIELRALSEEDVARFVSATMQLTPDPRQTPLSVVVIEKSQGNPFFIRMMLETCYRKNCIWYSW
RNSKWEFDIDRIFTEFVSPEYGDNLGTDFILKRFQEFPPAALSILIWATFLGSPFSFSLIQRLTEG
EFWYEAAEDTKCKITPICKALTPRSEAEVVSGLQFLVQGYIVLPGETDDEFRFAHDRFARAVSTLS
ECHDVEKMHFVIAQTMMKYCTKEDEMYPKSHHICQAADLIRKRVRNRTRYRKALRNSARIAISLGA
RPTALWYSRHAIKLLQPSCWDEKAPDVDYQETLELYLSTAELLSYHGDKEEALRLLTQVFRHGKTA
ADKSRGWIIKSKISTHAGDFNGAMDALLSSLEELGIHIRQPTTYAQCDIAFTELRDYLQPQDLDAL
VLQPISEDPRVIALGTVLAEAMAVAFWGDDLTYLYMGVEMMKLHLFTGRFSQVGLACCHLAMTAYS
RHKDLEFAASMSDLSLLIFESYAEPSSRGTGFILQSFMVEHLRVPLRTILPFIETSVEYAFNSNDP
YLMLTSFGLMAATRLYLGQDMSEIETFCTDTPDEIVDWTRDVRAGVLLVAVKQVTRALQGKTSWRH
PNLVMTDDQHSTLEYMDHIRQRSMRSDRPHDIYWSYAMIPLYVYGHYDKIIELASSMMDSIERLWC
MRAAHLTYFIVPLAILTKHIDNPNAGSLESRMELVLKCKEVIDFARTACDVNHAMWSLLIEALMHE
HEKEFNSAVQAYEAAIDHCEVHGFPLEEAMALELYGDFLVRRGAKRPARAMIREAIAAWMGLGAIG
KAGHLSEKHEWLLKTATAPRHNDAGTQTIDSLANINQNAAVETTAAVQTNLEDDRKKHWLENSGNH
VETGPLDIPSVGLDIIDLSSILEFSQVMSSELQIDKLLTKMIEIILESCSGSDTAVITTEFEDTGF
AIAAIGSQDDGQKAFLDGLPFSELEDKMAQQITHYTLRTKQEVLVHNVLEDERFSIVSEGYSARYP
AGRSVITLPIVQADNLLGVIHIEGKPNSFTQRNVVVLRLLCNQVGISLSNAFMFRKVRKVSATNAA
MVESQKRALAAAREAEQKAKIAEAEANHNVKLKEDAARAKSVFLANVSHDLRTPMNGVIGLSELLK
GTNLDKEQDNYVESIRVCADTLLTLINDILDFSKLEAGKMKISTVPLGLKETISEVVRALRYTHRD
RGLETIEDLDKVPEDLVVMGDPVRLHQIFMNLLSNSYKFTPKGHVKVTALVVREGKGRVRLECSVA
DTGIGIPEEHKSRLFRPFSQADNSTARSYGGSGLGLSICKAIIEDVLGGLIWLDSTPGVGTTVTFQ
LVFQRAPKDSAVTAPWMQSFNQADKYDVPRITQVVARDLTLIPREQIRVCIAEDNPINQKIAVKFV
RGLGLECEAFSDGQQAVEALRQRSKEGRPFHVVLMDVQMPVLDGYDATREIRKETDANVNQALVIA
MTASAIEGDREKCIEAGMNNYLAKPVRSSVLSEMLDQYLSPVKRSKLRRRPITQQSIEGLRDGAGT
PGSQSSSSSASQVIALTPDDERLHPRLSSSPVVSPPAVSPPILPPPVISPSADQQLPQRLRKPLLD
QSSPTKPDEKPPLPRRKGPDSSI
>g533
MRRNPARLTPTKAPSPSTGPTSHSRNPSRAESLLHITATISDTSPTQDGGLNLASDATTDASHTAQ
SNARRLEELRKQMEQTLVNQQAQHQNNSAKISKPGTDASRSSHHMQVPSPGSAPRTAINSKPHSPF
DVSASRTGSNGSATTVKGANLTSRTPDLHAPKTPSYPFPQMPVLRGQHVTSDALLQSDVAQIANPR
MKEKSKRTVSKAPGTIDMSPPKMKTGSHTAFFIPESAAMSDDPMYPSPNLYDVILKLNSEPGLESW
WNNVIDIFQTHYGIERASLAVPGDATDLENVPWGQKASFNPNELFEVTNPTASSSDATTAFNEGAA
TQEDDTSSKNTGNTPTQKKHSSSKRPSLLARHSFAGFTKDKKSIITPPISVTGKSAFEISPAASSD
GNTTSSSAESPPSSASFPASQDNTMKIGLGPSGLPISRASSSMVVFAVPRDLEAENQPLVKRTGVV
KLFGRSKPAVLTREYAFDSAVPSSTDLDVTTAVHSPSDVFQATPAAEPSPSFQLDPIKVDKDNTMP

PPTQRQRSKSSAHNPNVLQNKRDSLSHYAIPIYEEYEQVPSSPWSQSPAPSPAPRVHPEQNPFFMN
HAVDEAAFAQDPPSHDYAQVQPVAAIGVDRSKSVVHVPLLHANSTKESTWETIRFPVAVLSFLSPV
VPYPANLRSSLLYLMPHLTASYLLAHQYSQLERQLIPGVYKPRFGHLLGLGGTFSDASSELELVAG
LSGQAMTDGGSISARASISSPDDMTNNSKFSPSLSIMGTPGMDNMSTGPSSEPGTTPAMGQNVDKI
DGYFNLHQVRSSAGPSGTRTRVSKSRQNLFPSMPVSPSVSRSKSAREEDWSNRDSSATDQFHDNGR
PTPASIPTRSSSRNTSTTSVIAHLQREGISISEHIFQLMLNSVPLHLFLAKAPSGEVLWTNSKFDA
YRRTQPQDQSIRDPWQNIHPDEVESISRQWHKALQTGSQFTERVRVKRFGDDNTYRWFIFRANPLL
SQTGQVVSWIGSFLDIHEQHVSELQAAQEREVFATNVKYRALANSIPQIVFEATELRGLISANEQW
HLYTGQTTEEAMNYGFTAYIHPDDLAKCGVLSTEIVRVRATNDVTASQPDVAPLKLFANGVTPGLL
DLVNQGIVNSQQDENGRIFYTTELRIRSKGGGYRWHLVRLVKVEASNFGDGEASWYGTCTDINDRK
LLERELNKAMQKLNREMESKTKFFSNMSHEIRTPLNGILGTIPFILDTALDNEQRRMMDTIQNSST
NLRELVDNILDVSRVEAGKMNMVKSWFHLRSVLEDVIDTVASRAIDKGLEINYLMDTDVPDMVIGD
RFRIRQVLINLMGNAVKFTGQGEIYTRCSVYREPEVPLRDTELLLNFEVVDTGKGFSDTDAERLMQ
RFSQLGGNGSQQNAGSGLGLFLSKQLVEMHGGKLTPSSRKGQGAKFSFYIKVDAPPQVSDPSLTRN
SSDMSDTATATSQGTTQPRIPLQSNNSTDSRIFNFHDMSPALDMSPMTEVSGSPDHSIRPAFYYPS
GSALSSAVPTPDSSAPTESAPKVGPSPLAGEFFTAEATEISEPLVQSTPTVEKASASSFLPPVIYS
VLIICPLDHAREAVRQHIEQVIPLEVPATSTAVADLEDWKDLKETSKSVTFSHIVLDFPGGDEIME
ALQHVLTSDMKTKPSIVIICDLYQKRQMNGKIEELIAAGFRVDTVPKPVKPSSFSGIFDPDNRRDL
SKDRNQDMAREVNNNFKTMSKMVKEVIGNKGYRILLVEDDETNRMVSVDQLLAQLHACTNMD
>g534
MPIKNGYDTCREIRAWEAKNHFHQIPIMALSANAMTDQIDDAARAGFNDYVTKPIKHNELGKMMMS
LLDPNTSGTLLRDRKDWRQQKP
>g535
MAPTTNNVKIIFGGAVFSTQYGFTVDKVNEVLSWLEKEDIKTIDSSEVYGDSEELLGAANAAAKGF
AIDTKIGGGLAHVASSGERVVEAAEESLRKLNTDSVDVYFIHTPDKRVPLIDTLRGINQVHKQGKF
KRFGLSNFAPAEVEEVCQIALENDFVLPSVYEGNYNAVGRRVETELFPILRKYKIAFYAYSPIAGG
FLTKTPEDVANAKGRFDSKIIFAKMMNTIYNKPSMLQFLTEFGKLAAQEGITQAELAYRWIVYHSH
LDGEMGDGIIVGSRFGDHLTATIEGLKKGPLSGAVAKRMDELWKGVEKDSILDNWNDFISKNGGL
>g536
MSNATPVTFNATLYNNTDLCTLDTCPLDWAQVDYVPSLPGNLIYVAIFGLALLLQIAFGVYYKTWT
YMIAMIGGLVGEIIGYIGRVQMHYNPFPQTPFLEYLVCLTIAPAFLSAAIYLCFSRIVIVYGGRIS
RFRPKTYTYLFIAGDFIALLLQAAGGGIAASASGSTQQTGINIMIAGVSWQVFSLAVFAALCMDFA
LRVRRTQSVDLNPQYDALRISRSFKIFLWSLGIATLTIFVRSVFRCAELSGGFNGSLANNEVTFMV
LEGAMISIAVIALTSFHPGLIWKREWRELARSMRARKDGKDYQAGALMSASDADRESGYQMR
>g537
MGPLPTQDSKNQAGDDAGTIRAPVRIAPNLHQTNNAEQDKSNVMFDEEKNSVGFQPEESKQKTATT
STTTDVPYCILPERQKIFLMMLCSFAAMISPISSSIYFPALNSIGSDLGVSTNKMNLSITMFLIFQ
GVAPSFIANFSDIHGRRPAYVIAFVIYLAANIGLSLQKNYAALMVLRCLQSSGSSCTIALGSAAVA
DLSTRAERGKYIGYATMGVTLGPALGPVIGGLIDHFLGWRWIFRFLIIILAGVYASLVVMFLPETCR
AVVGNGSVPSARWNRSGWQILSSRFRHGRKPPEPNYDTVQIGKRRPNIFASALIATEKEPAIILAY
GALLYCGYMAVLSTLTSQLKSQYNFNSIQIGLCYLPLGCGSLTSRRTVGTLLDWNFKREAARQGMP
IIKNRQQSIEKFNIEIARLAITIPFVYGGALCLIAYGWVMQFKTSLAGPMVILFFMSFLTTGAFSS
LNTLIIDTNRESPATAVAATNLWRCLTGAGAVAAAGPLIERIGLGWTATFIAFLWLAFSPLVWAVY
KWGHGWREDLRKKREDVEDTA
>g538
MRLFRRQYLGLVPIATLFFIFGTIYSTHSWTSTQQLNKDLWSQRPGDQETQQLHDGQNYDDPDCAD
FPRDRLRDVQIVLRIGSTQPFDQISSHINNITNCISNLIVISDRVEEIGNGFWSHDIIADLPKVYW
EGIDQDDDETDQTAGLKAYADINKGDSSWKQGKHASDRLTHRQGWLLDRFKFLPMVEYAYNVNPQA
KWFYFIEADTYVVWDTLFRLLDRYDFKQEWYMGSPSPGRELDGEKTWFAYGGNGFILSRTAIQRLV
SKEPVKSTDESSNANRTELSLTERWADLVKSDCCGDSVLGFALANKGVFLSGLYPIFNPHPLHGIP
FGPSGKPYWCQPVLSLHKTWPGDVPGLTNFVKDHTSERPLIHADLFNYLGLEQVAQRDDWQNSDWN
GFEEAPESPVHETIEACAAGCHAHGECFQWTYWTETSWKYKSIPKKKCTFVRSIRLGTRKDREESW

TTTAVWTSGWDIEKISKWAKDNDCGEPEWVEPSLQRIY
>g539
MLLLLLSLLLLVLLFLVYIVYYPPFSIIQYFQRRWPDVLWHVSTSEKVIALTIDDGPSIYTAEILE
ILKANDATATFFIIGSHVSNMTHEGDEILQKLIRGGNELGNHAMYDEPAYKLPDDELVSQINKVDE
LNRKAYEAVNGASTIVEDDTALPRYYRPGSGWYTTRMRNIVNRLQYKLIMGDVYPHDPQIPFAKVN
AAHILRMARPGGIIICHDGRPWTLPMLMAVLPELKRRGYRVVKVTELLQCANS
>g540
MGIIGRVLKLTTTGGVATIGAHLIWTRNSHVEPLPRTDYLFTSPWYKKFNPNENAVLRDDCIRKVP
LSQIDPKLLEKKGKLVEHFCAGVWGGLGYAYQRRYLAKKYQGPETAHQLWSTNELLSSTYEVGTEI
TDHFQVVEKTDNRIVVRCGDSPLKRDVRESDGLFEMSVDVKEDEGVAEFHLKSIFFNGLSGTKVEG
SIMPWHVELLHREYAKIWMESALRNVYA
>g541
MSGLSPTAASATSAVTCISSASTETLKPEERMTTNEVQPATTQGNPPPKLYKIHSNPWFQILLISG
ICFCCPGMYNALTGIGGSGQVDTTVAANATVALLSAMAVTSLFIVGPVFNQCGPRICLLLGGWAYP
LYSGSLLCFNKNGNGAFVIVAGAILGIGASFVWVSQGAIMTTYVPEQQKGRAIALFWVIFNLGGGV
GSLASFGLNYRSTTGTVSDATYISLLVVMAIGWLLGVLICPPSSVRSHSTVVESRRKSPAKLLNIC
LMMVSDWRMLCLIPLFFSANVFYSYQQNEVNGSNFNIRTRSLNGALYWLAQMVGGLVMGVLLDLPG
VSRPQRARIGWVFLFTTGMCIWGGGYAFQKWSSHRINVEHRKQDIDFTWTSSSVGPMFLYIFYGAY
DAFWQSFCYWLIGAQSNSATVTAVLVGAYKAFQSVGGAMAWRINALEVSPTVQFFMDWGLCMGSLV
LVIPTVLAVTLTSADEDTVDTEEKDQSSSTMQIS
>g542
MEDPETRPIASGAIAPALTGNDGPHKGDVLVIEQTRGITIAEHETTFLEAIRQWPQAVGWAFLFCI
AVVMAGFDAQIITTFFALPAFQQRFGYEYNGHYIIPAPWQMALNMGNPIGQVLGALACGWPLEKLG
RRLTLALCCVWSIAFVFVQFFSNSIGMLCAGEILGGLAYGFYVVIAPTYASEICPLALRGVLTASV
NLAFVIGQFIAQGVAAGLESRLDEWAYKAPFAIQWVWPVVLLVGLVFAPESPYWLVRKDRIDDARK
SLLRLSSAKNRPDIEGMLVMIEQTDFLEREIEATTTYWDCFKKANLFRTEISVMVYLIQVIGGNPL
IAYATFFFETAGLDPAQSFNMGVGNTALGFVGTCLSWPLMLRFGRRTIYNSGMILMTIILFVIGFL
DLGRNNTGVVWAQASLMDIWTFIYQTTVGPICFVIISEISATRLRGRTIAIATAVQAAANIVFTVV
LPYMLNSDEGDWRGKAGFLFGAISFACYGWCYFRLPESKNRTFEELDILFERKVPPREFKNYDLLA
SEGEHVAWG
>g543
MQATQDNVEDPFADREDAPFLDPSQPKNEQAFRKTDNNVSREKLRIRLMVTLYAILLLVEMGSAMT
QGPYTRIFESVACKHWYRDHDPSQLGSDGEVPEEACKSSVIQSEVATIKGVLEFFDGVTSVLLAIP
YGLLADRIGRKPTIMLSIPGFILNMAISLAVLWWSDIFPLRAIWFSALSWLFGGGLVVASALVWTM
MADVTTEQQRSAMFFQSGVVIMGSEFLSNSIGSWLMLYSPWRPLLIGWSIVIVGLCLGLTLPETKN
AFAPTKGPEQEYSHEMSDLSTLADDEEEEEGTLPFTKTTAAQQPPTTAWMKVKTSFIAYSFFFKDR
QVVLLSSAFLVYKLSRGTSWFLIQYVSIRYDWRLAAANMITSLKSILMVLLFVAILPLASWYLQKK
RGVDSRTKDMILTKTSVMCLLVGTLGMGLSPHISIMFFFLVIQTMGAGFVYTTRSVITTMIHRDQT
ARLYTLIEIIQALGMILASPVMTGFFNWGLQLGGXWIGLAWMVAAGLFGIVGLIIWRVRLPALSTR
SDD
>g544
MATPSNQAAWINEKNAKPLEVKEAPYTKPAANELVIKNAAVAINPMDWMLQETGHLAFTWIKYPFV
LGADMAGEVVEXGSEVTRFKPGDRILAMGCGSDQDRNRAAEGAFQKYAVVLDNFASPIPSTLAFPD
AAVLPLGLSTAAAGLFQEDFLNLQLPTVPPAAPTEKALLIWGGSTSVGSNAIQLAAAAGYEVFTTA
SPKNFDYVKKLGASQVFDYNSKTIVKDLVTALKDKTVAGAYAIGVGSLSLCVDVISKTKGVKFVTD
VGSTDIPTDKKLAGFTLIPFLLGTMSRGAALWVKTKRTGVRTKFVYASDIKKNSVSKSIYADFLPQ
ALAEGHYLAAPEPQVVGHGLENVQEGFEQLKKGVSAKKLVVTL
>g545
MPNYKHLLLVPLALASPVLGGSKQTCEKTTVAILGGGTAGITAAQALSNNSVHDFIIFEYNSDIGG
RMRHTTFGQDANGDPITLELGANWVQGLGTAGGPENPIWLLAQKYGVNTTYSNYSSILTYDETGYT
NYSSLFDDFENAYSVTEELAXTVLSENLQDRSARAGFTRGDWQPKKDMKMQAVEWWEWDWEYAYEP
EVSSLVYGIVNYNTTFYQWSDENNFVWDQRGFNTWLKGEASTFLTKNDKRLRLNTTITNVTYSDTG

VTITDSEGGCYQAEYAICTFSLGVLQNEAVSFQPEFPEWKQDGIDNFDMGTYTKIFLQFPSDKVFW
PKDTQYFLYADPIERGYYPVFQSLDSPGFLEGSGIIFVTVVHDQSYRVEAQTDDETKDQVMAVLRD
MFGADNVPEPTAFMYPRWSFEPWAHGSYSNWPYGVTLEMHQNLRANVGRLYFAGEATSAEYFGFLQ
GACFQTPSSKQPRGAVDRLQEILQHSASSITKQIWRSSNSPSNELAATSQARVCVDRLNHVLSEES
RGPAPHPCLSYASTSQIFVTVTKLALTYRDEGLVRSSAVFFNTLIDAEVDGIVDNRVFSRALIDLV
RRNSVRTDDTESRLVELLFGVANNIRLRPQILTAWFIPKQAELPERELEEDEQFAGVTRRDDFPLF
YLLVDYVHREGRAGDFARTGLLYLIETATRSRILEKWLIESDLATLMATGLGAVYSQLSRNVYPTF
VEGSVPTIVALSDENDIQRNFDGVVRPDVESFISYLLFWQDTVEHCQSKEVNDTLLDHFEVLFLQQ
LLYPSLLESSDVKGGSTSTVLMYLCRLLESIDQPKLVHRTLRFLMAAPTDTEPSVDVSQTLHKKHL
SVSRRKSLDMLATLAEAEEQQPSPSLFNLADLILLSIKSQNKETIVATLRLLTVIVRRHHPFAASL
IRTKEPMSAPQRTVGALNEELRRYLSFATTIIDDPSLNDSFEHYSKDAALVLESRLFIPSPRSSIL
DGPADQPLDLSLDDVIFKEILTLLDRFFSNSVTVNLGLTELITSLASSNLISLNGWLLVDPANYDY
ESDLSPTGMKTSTVTEDNDSDVNDENIETDPLAAIRKSLAPATWSDGHEALIAKSLRKLTEHLENW
RRDIPDFDILVAARRDLLHSEDDDDVSRNQRSINTSRQPSQPPTTHSIEPPLSPRGRSISNSVNQD
GPLSSTPGSLPHSTIGSPLREPSIQPPVSSSPAHRPVAVEDLRQRLASSYRIDKAAEQPTSTPQEN
KFTNENRTDGPVDGSEQPNQEQSPAQNETVVTLGHILTNAVILYEFILELAAMVQVRATLFEEAGY
A
>g546
MAKDILVVFGATGQQGGSVIDYVLGDTSLSAKYHLRAITRDVTSSSASFLRTKGVEVVEGDINDKA
SLHRAMRDTQGVFAMTLPLFNDVEAKELEITQGKRIAEVAVEENLSFIIFSSLPDVTSISKGLYQG
VDFFDAKAAIELYIRSLPITSIFLIPGLFMQNFSQHMGPQRKPDGSFVLSNVVPPTTRLPLLEVSR
DLGKFVGPVLLKPETFNGQTIYAFTRYYTYEAVADAISRATGQIIEYQQTSTEVFTAXMPPKFAQC
LVEMMLFVEKFGYFGEQPSVITEKHANFAAPSALTSFEEYCSKVSWQNSLS
>g547
MSPLQNETVLVIGGSSGIGFGVAEKALIEGARVHIASSSASRVQESVASLQQKYPGGQVTGHVIDL
SSSEVERHLENLLSGIGSTLDHIVYTAGDPLPTLSIDQIDLEAIHRAGHIRFFVPLLLGKLAPRFL
KAGYQSSLTLTTGAGSQKPFPNWSLISGYLTGLHGLTRNLALDLKPLRVNLVSLGIVKTPMWGSNG
VPENIQSSMPLGKVGTPLEVAEAYVYLMKDTNATGSVVSTNSGSLLL
>g548
MTSSVVNFLSSSSLLLQVSVIFTALISLSVLVNILFQGVAKSNGNKPPLVFHYFPIIGSTISYGKD
PMVFFERCRQKYGNVFTFILLGRRVTVYLGAQGNNFVLNGKQSELSAEDVYAPLTTPVFGTDVIFD
CEHSKFLQQKKFVKFGLSQSALESYVRLIAKETTDFFQTYHDFNIKPKSKTGTLEVASAMAELTIY
TASSSLQGAEVRQQFSSAGSRIANLYHDLDMGFSPINFFLPSWVPLPRNKRRDTAQREMTRLYTDI
ITKRRQHEQEQQTNQPVEAEAKDMIWNLMQNCTYKDGSPLPDKEIAHMMIALLLGGHHSSSAVIAF
AILSLASNPKVQEELYSEQIRELRGQELTYESLQRLKLHAYVIRETLRLYNPIHSIMRFARKPLIV
REEQRGNEIVVPQGDILLASPAFSARDRAYFGEPLKWDPHRWGQKLDTIDEEDDDDNDSLGKGSNS
PYLPFGGGRHRCIGEKFAYVQLGVILAVFVSSFRVTAVDESTELLGIDYSSLFSRPQSPSKVIWER
RSKV
>g549
MTDPESSYRPRSPDLSSFPSSFSNTFNNNAYQPQPPFLASPHGQRASYDASPFFAGYQPPVANSRI
PQQQYQQPFYNPVYDNNPYDDDMARRSARLSAQQPQTQAPVHAQVQPQLQAESHSHIHHHQPPHAQ
IQVHHQAPLPHPSEQNFVFDQHVAAVAPKEEPHETRPKPWDGIDVKTKFPVARIKRIMQADEEVGK
VAQVTPIAVSKALELFMIQLVTKAAQEARDRNSKRVTAAHLKQAVGKDEVLDFLAEICSKVPDQLA
RKNEEDGSDHNEGGKRKSGGGRRKKDVDSDDF
>g550
MMASTELSQSLLDIISSLTQDDIPFKLRCAICNNLAINAFRLPCCDQAICESCQTSLPESCPVCEH
NPVSPDLCKPNKALRTTLKAFLRTEEKKREKDRPSTTPAVTTAPTSNVLVETQGEPAIPATKTADT
VVSTETVPTESAVAVATDKPSVDENEAPPGAPPVQGNGDLVPPTGENGIDPVSANSASIDPEATEG
TEAKHAQSESEPNQVFPNMGAGMAGMMPGMNWGGQDMSAMAQFMPNAMGNFANTMGMPGMGPMNAA
QGMFGGFGMNMSNMSNGMNMGYGTEQQMYNNWDNSQNNMYNANPYSNGMGQDYGTSGYAGYMSQPN
GNYSQMQQYSNQNFQNGYYGNGPGYMRGMGRGRGRGFYRGRGGYDHANYYQQNYQNNVSQESVNQG
GGQTEDDIQKFNNELAPGGSDDFSEGPKDSTIIGPEDANAADGTDRTDKPLESDINGEPTSRDEEP

TSQLQGIPTIDSIDSAPPQAMSPMGYSRGPVPVLDQGYGRGRGFGRGGFMSHRGGYYNNVQGPPTG
QGVVGAPLAPRAMREGINRGGLLSGSRRTASISQTAENSSRGATPTIAQENVVAHSPSVVDHKPRS
YSPSRERSPRPSRPQSPPSPESRQSRIRRRDRSESARGSVDVALTLTLRAALIPSFCRSVSRHRDR
GSAAATNGDDKYDLARRIANAQRLGKGSSSRHEKDSRKRDRSEREKRHDNGSDRDTDRDRDRRHER
DKPRDRDRDRDREKDRKRSRRDRSLSGDESDSSRRHSRRSKRSHREGRDVGEMERPSERGTTSTKD
PHTLEREARNRERLMKEKQRRDAMTTERDSKSSHRQHDRETNSGRRLNYKYEGDDDDMSRVEEERE
AGRWR
>g551
MAGNSALSIVTDEKQQLRPNLNFLLLLQIILSALLAYLIVYTHSHYPEESTIWFLQCSPVSGEATS
LAVRNPDGTGVAEYVDQAELVKRVNVKLFATALGALGGISYTACAADIEPTTKAAICAIAVIGTVI
SAVSGVIRSLVGRDGLAIDPRLPSQLVHHFPQQDGLSTLNQLYSAYQDTPILVAHSDCFGPGCHQV
HYSTRKSTIGDHIIHSFSASPRHNNSSVQRRDWSGDDSGAGNDDGLYGVYTFINDNPTDETAEAYD
GDFPDHMEKASDMDIFANQGTFCYNIEDADDNDNYGTAGYLTLQQDEPYYDPDQEDAWIDQCNNSH
>g552
MLNIGAAAVSTPTPSSQSLRGVPSVVGMKEWLEALGHSTNDINALNIIHVAGTKGKGSTCAFTRSF
LHAHGLRTGFPNRIGLYTGPDLQSIRERIQIDNQPITEDLFTRYFFEVWERLSPDSCDATGENKKS
RLPRYLQLLALLAFHTFIRERVQAAIFETHHGGEYDATNVIPHPVVTAITSLGMDHVAQLGPTIDD
IAWHKSGIFKPGVPAFSAPQDPGPVEVMRRRAEEKNVPLKFVSNYEMLPKNKKVLAVPVQRINCSL
ALEVARCFLQAKAPECVIDDDLIREGVENFSWPGRFEIIQDGQIRWFLDGAHNPLSLRQAAQWFAD
NTNHSETTKCRVLIFSHYSEERDGMTLVEELARSLPENDARPYKVIFTSYNERKDGVRRIDKTLKV
PETPFPNLGTHYATRWEQLDPKAQLYVEPTIEDALMLARDIAVEKKGLDVLVTGSLHLVGGALSLL
RP
>g553
MATTISKLAGKNILLIGGTSGIGFAVAQKALQEGAHITISSSSKDKIANAVERLRVSSPENASNVR
SYAADLSVKDQIEANVEGLLQFTAKDSTIDHVVFTAGNVPPMAPLAESSFEHVDAFLTVRFYGALA
IGKYAHKYITPAKTSSITFTTGSQARKPISWLPPIISNAVEGLMKGLAVTLAPVRVNVVAPGFIIT
ELLEKLPKEMAHAGAERAKAKSLTQDNGSPDDTSEAYLYFMKDTFVTGVVLDTNGGVFFS
>g554
MAPSVEWTNVFYALTGTWFIYLFVGAIYRLYFHPLAKFPGPKVAALTGWYEAYHDMYRRGMFIWKI
QEMHDKYGPIVRINPHELHIRDADFYEELYAPASKKRDKYKNWVILSGTPDASFSTVSHSHHRLRR
NALNPFFSKQAVQRMEPLITEKIERLSSRLTDAAKTQRVVRLDAALMALTMDIICHYSYGESYNYL
ALENFKEEWKAAMISALENGIFLRNFPWALPLLKSIPYSVLKVLQPKAASLAEWAMLVKKKVDILI
QEHSMGNKSSGTIFQAMLDSDLPPEEKSAARLIDEGQSVVGAGSETTANTLTIIMFYLLENKNLLR
KLREELAPISLDETPLLPKLEKLPFLTAVITEGLRLNIGVMSRSPRIAYEPIQYQDWVIPPGTPVS
ESIYFVHWDPTLFPEPSKFRPERWIKAQETGVRLEKYMICFSRGSRSCLGINLAYAELYLTLAKII
PRFEMDLFETTVEDVEPERDFFVAVPKLDSKGVRVKVVGVN
>g555
MLLKGELHSAPITKTPQRVLDLGTGTGIWAIDFADQYPSAQVLGKSIFFLICPLTRTHKKHTNTLV
GNDLSAIQPQWTPPNCSFEIDDFEADWPFARPFDYIHGRELAGSVKDFDRLFTQAYANLKSGGFLE
MQSFRIEIFADDDSLDKAVYTKQWVEFLQDAAIKHGKPMANMDEWPDKMKKAGFEDVTMHIVKVPM
NPWPKDPKQKAIGRYMQYEQEQAAPGYTNALLSRILGWSKEEIDVLLAHVISELRDRSIHQYAKAY
LIYGRKP
>g556
MAEKAANLQTTVDDDSTSSTPIVAQKYADVTLEFVELYGDSTPPLTEKEVKSLSRRLFWYIILLLT
LTNLLLFIDKSTLAYASLLGIFEETNLNDTQYNNLNTLFYVGYFVGQFPGHYLMQRLPLGKYVSGT
VFLWSLIIFLHDTARSYGGLIPLRFFLGFVESSLVPAMEITLGMFFPPKEQGALQPLFWVSCMGSN
IPTGFIAYGLLWSQSSIRPWKFLMIITGGLSLLLSVFCWFFYPDNPAHAKFLSRAEKLHTIKRVHD
ATKGSIEQKQFKRYQAIETLRDPVSWLFALQAFTLGVSNLGSTLVGVASSGFSVVCSIVAFFLMKK
FPNNNAYWSVLWILPAIAGGIGGTFGISYITALGWTSSSASGYTKKLTRNVMFMVGYSISNIISPQ
IWVARNGPRYYPAWIVQIVVSWVGTPVILLIIRYILSKRNKERREWIAQQQASRALGFVEKVDDDG
REVQTTVDLAFLDLTDLENKYFIYPL
>g557

MAEIYYEDTVRFKTTVDGATKYVTLAVVPPSETCWLTLTDEPTEDSNFLIREWNYDNTSRLYFSGN
LRADTPDSHGNFRLLHRDTRPPDHNVGDDYCVSMAMYEPGANMGYWGISWDAAYDLETFALFATAW
PDSDGGVCSGNSVKIRSLIGSKKPPPVGSIGQFLTINNINGQWRITGISDHATYPVGAEFVIEKVV
>g558
MTVSTDLSSLLITEKLSLVPINLNDPDQHAEFKQQRVVCGWDHSDKALQMFIEKQEKGLKSLFWIM
IQQKAQANGKQQNGGNVSVTQHTNGHAADGNAVSPSATLFTIRAGHISLDSYTEPADPELAREDRS
IMTVQTFFILPEYRTGGVGRAAMDLVEVLAMQEPYGSPHCHTLALTTVSKKHIEIDAPEWRGLWKR
CGFNVPKFSNEIWYEKLGYVYWKEEPRYDAVALDGSPSHIIASFMRKKLR
>g559
MATRSTPSYYIGLAVIFAYGVRAYTPGPTACANLTSILGTSIVNSNTLSPAYIESRTDYWNTLQDV
YEPSCIIYPSSAQDVSVTLQAVRAAGSRFAVKAGGHNPNTFFSSVDAGVLIDLGSMTGMSYDPDTT
LATYEPGSLFGDLYDYYVQYNRTVVGARLAGVGTGLALGGGLSYLSPQYGMACDSFRELEIVLPSG
DIVTASNTSEYSDLFFASRGGGGNAYGVVTKYTVQSRPIGELYAGNIIYLFEQVDAVASAIENFIQ
YNEDPKASIIGTYEILGTPDLELDLDQAVIMFLVYDGTDPGDAFSNFTSIPYLLNLMGNKTYPEVA
NMPIPLSTELSRGENIFRVGVHRIGDGGYAAALDAWRTWAEDNKGSYVLTSLDFQPIPLSLTEASK
SQGGNAMQMPDGPWFWVNYLISSSPLLTSSEYDAVQASFRDMVANVSNAEGLPLFINDANKDQNPL
TTFSTYSTLQQIKQKYDPDDFFSNYTGGWSFD
>g560
MPLLAFGIPVVRSGPQYPLDDPFYVPPDGFESTAPGSILRYRTPPYPIAAFGLVKVNLAASYQILY
RTTNSFGDAVATVSTILIPHNADFTKLLSYAVAQDAAYADCSPSYAFQLEAATDGVIGLIMPQLEL
VFITAALEKGWVVTVPDHLGTKSAFLANXLSGQATLDNIRAALASTEFTNISSDATITMWGYSGGS
LASGFAAELQPTYAPELKIAGAALGGTVPQILPVIYATNKGVFTGLIPAGIQGLANEYPSAAQLIA
ENVLPSKAAAFNKTQTLCLAGDITTYLGQDVYSYVRDPNIFTGPVATALLEANAMGQHVPEIPLYI
YKSAGDEISPVNNTDDLVSQVYCPGGARVQYLRDELSEHATMAILGAPDAFIWLEARMNGEPVDAG
CSTSTVVTSLTDPSTLLALGVDVVDVLLSLLSVPIGPIMIG
>g561
MKAVVVHEPGGPEALKFETREIPQPKVNQVLIRVKAFGLNRSEKWTRQGLSPNVQFPRILGIEATG
VVEEAPNGEFEKGQIVMTAMGGMGLFFDGGYAEYTVVPSNQVQAITNPTKLGWEILGALPEMLQVS
YGCVHRSLEVKHGDRVLIRGGTTSIGLTAITLAKSLGAHVTATSRRADRESMLREHGADEFLLEDG
ELATQLAAKEFDQKFNKILEMIGAATLKDSLKCITQGGTLCVAGRVGNEWIVKDLNILSDIPWGSK
LTVYGGMTPEFMSTPLNELIQQVENGSLKVKIGKTFHIDQIVEAHRCMEDSAAEGKIVILTE
>g562
MSKLSLSEPKDTPIEQATLANKARKIPLPSGNTRVPLRQAKSTCQLTYTPPRRKPKSLQKTPSAIV
PFLTKSSQLKAWEHDTRLQELETFADTFFSKISQVGQNSDVMKDAINLYKSRVEELEKNRDEIMAN
NCTLRIEMESMKNKLSTAEEALKIAETEHEIATDEHEQRLRIETDAVREEGQTQLQNLISQHQNEI
EDLRRRHERELDNERSTYQREISQVTSQSALDTQKVHLEVANRDRQIEALQRDLRTARDDIEAEKL
KNRELRRNLDTSSSNTLTLESSVRALKARIEFLESGSQEQSQAFERLQKQLEDALAETNEAKEKLR
KEEMLRRKLHNQVQELKGNIRVFCRVRPTLNFEPESDIAQIAFPDGGEDCKEIAVMGPEERSSLGT
VSRKQNAFSFDRVFGPGNQNADVFEEISQLVQSALDGYNVCIFCYGQTGSGKTHTMSSADGMIPRA
VHQIYDTAKSLEEKGWTYTMEGNFVEVYNENLNDLLGKADDLDKKKHEIRHDMQRCKTTITDVNTV
TLDSPEMVETILKRAAANRSVAATKANERSSRSHSVFILRLMGHNKITGERCEGTLNLVDLAGSER
LSHSGATGDRLKETQNINRSLSCLGDVIGALGTGKEGGHIPYRNSKLTYLLQFSLGGNSKTLMFVM
VSPLQAHLGETLTSLKFATKVHNTHIGTAKRVSKIQN
>g563
MSGRFVRSSKYRHVFGQPTRREQCYDNLRVSRNAWDTNLLKVNPQYLAVNWEAGGGGAFAVIPLEE
RGKLPEKIPLFRGHTAAVLDTDWNPFNDSLIASGADDGKVFLWRVPDNFTVRPDVDVDQVQDISPV
GKLSGHPKKVGHVLFNPAAENVLATASGDYTIKIWDIEAGAAKYTLKIGDIVQSQSWSANGSLLVT
TSRDKKLRLWDVRQERPAQETAGHSGAKNSRAVWMGEHDRIVTTGFSKMSDRQLALWDVRAVREPI
SGFKTLDSISGVCVPFWDEGTQCLYLAGKGDGNIRYFEYENDKFEFLSEYKSGDPQRGLAFMPKRG
VNIHQNEVVRIYKSVADSYIEPISFIVPRRSENFQDDIYPPTTGITPAMSSSEWQAGKEAIPSKIS
MEGIYDGTGLKEVTPTQSKPSSTITADAPKPAEAAKPAATEPVRAEPTPVVVRPAPSMKDQGASMA
AMVDKFADDDEEKEEEIDDASSFEEVTKPVERAASTPVITKSPPSPIEEKEATTPVASTPTIETPK

TTTQNPAAPSTSVSDDIAEIKALIAQQTKTIASQAEQMQNLTAEIESLKAKINN
>g564
MPSQTFYLLGQSPSSAREVQIDASQTFEGLQLLVAGHFAIVEPSGISFQGPDAVLTEVSEVLAAAT
PVPITIDGHPVRDPPCPKELPFVGTFFEVYPDHLGNHQRLFEKYGPIIRTSNLGRVTYQTNDPKIA
AIVFSESDFFTKKINESHPLHALKTPAAGVFLGDTDTPEWRVAHKFLPPALGPKAVRHYAPTMQKT
VEDSFKVFDKLDEQDEAWNVYQYMLKLGSQAVGKLTLGIDFQHFDAPDAPVHEMVHNIAEVLSLNK
KVTSKGNWYSSLPFGDPKRLRNLRARIEEMVEESMKKASRGGVEDLPLQDAALTASNMVDYAIRAT
DNKGEKLPKSSVVWALVVATGAGFTTTSSLLSWLIYGLCQYEGMQERLLQELIDHGFDENTEMTAD
FTDKLEFLDKYIKETQRRHNPSFQPGRTAKVDLILPGGYKLPKDAVVIPALHHIHNHPGLWDNPAR
FNPDRWDTEEVKGRHKAAYIPFAMGPRMCIGFNFALQEIKVFLPKLVYRYKFSREGEEPIEASNQM
ASKVDNSFIVRFYENIRHALPNIEDGAGPKTDKEKQVMIFNFASLAPLHVLIFVPESITIKIFETV
AQLGVFDSSITPKVVPYHLHFQGSNEKKLRFYDQTTFVCAMEGKLFSSDELSETDLKWKPSNKQLL
EVVVECQAIETRESREYEVSENISSWRDELAARQLNNNTSTIQNDPLPQRDPESQVVPATNTREGV
AGEHNKEKEITADPQTLCKSSPRARTVPDTPCTPIAENAHKKTDLKANTTLNFDTPDAVSIVEIDE
ED
>g565
MREFTLEEVEVHKSRADLWVAIHGKVYDVTKYVKNHPGGVDVLVDVAGKDATEAYEDVGHSEDADE
ILEAYLIGTLKDAADYKARTTVRVVQEPVVQQPRKGSSSAVRTVGVTAGSILSLYFASISLRPHLN
LHELSKPLLQMARLPTNKFGDILPHGGFINGAVAATCIFAMLGSVLVSKLSKLTEIDSGFRRFPPH
IKRNTVHKIDPHLIRGFLQPKEYKTLPLVAKELLAPNVYLFVFQLPRKNEAIGLPIGQHVSIKATI
DGQPVSRSYTPTSNNLDLGRLELVIKCYPDGILTGKYLANLKVGDKVAFRGPKGAMRYKKGLCKKL
GMVAGGTGITPMYQLIRAICEDDTDTTEIYLIYANRSEEDILMRDKLEAFALAYPRTFKIWYMLDN
PSPKWQYGKGYVTPAVMREHLPPPSPETKIMLCGPPGMVNASKMNLAGLGFQTPGPVSKMTDQIFC
F
>g566
MIPNWQPPQNYRERPVAILGAGVLGRRVACIWASAGYKVQVRDPIPEQRADCIAYVKQNVDSYAEK
TGARPGDITATEDLEDTVNNAWLVIEVVPEKIQLKIDTFEQLDKLAPKDCILASNSSSYKSSEMLD
KVSDATKSRILNLHYYMPPQVMVVELMTDGFTDPSIIQFMFERSKEAATKPYVARKESTGFIFNRL
WAAVKRESLTILAEEVSTPSEIDSIWKEIFIKGGMAPCKGMDSVGLDTVAFIEKHYIAERGLPSEK
TVDFLQRKYLDYGKLGSKSPKGGLYEPSDEVAKSDAASRSLLVLDLGLSSKEPSFEAGEILQISAD
GHVEKALTTGQALPDGIVVDPSSKRVFWTNMGIPGKEDGAVLSANLDGGDVKTIIAPGAINTPKQL
ALDAESKKLYFCDREGLRVIRCNFDGTEFEVLIQTGDPSAPDAGDNMKWCVGMTVAPSLGKFYWTQ
KGPSKGGKGRIFCANISTPEGKSASTRDDIQCILSNLPEPIDLEIDEETRVLYWTDRGEIPFGNSL
NRLYLDHFGLPQPHTSCLGYDILSRNLNEAIGLKLDLPNNSIYLTDLGGSLYRCDRDGKNRMKLFS
DENRALTGLALV
>g567
MESSSQSTPVAKPARRYRSTVACDACRQRKDGADCVVTARKPKVTPKARKHASNRSQRSSSRVRGL
AVDSDTMTTDPRQPAPRREEDLVHDQNSREEEENGLEIASAALGQSQTTGGVPFYTGEKTGPTSAL
ELCSPGTALQRHLLIPSRARKPIPEEDRKFLLAKGVFALPGNDTCASLLRAYMSHVHPIMPIIEID
QILNHYHDGRLHEYNILLLWCIFFAAVNFIPASVVQKEGYKSRKEMKSVLYSRAKCMYSNGGERDK
IALLQASLLLGFWHSELDEHMQPWYWTGIAISLCQVLGLHRNPDGSRYNPNFTEQQRMLWRRLWWS
CFFRDRWLGLTYGRPLRINIQDCDTPMPLASDLLYDVAAVPGFLSDAFIPKEMTQLGRYWVMLVDL
SKVLGECLMNYQVMKPMPSLDEVEALETKLLRCQLPDKYEKGLTRLATFNCYHVHLHYQALAITFY
RPFSSETADTLPSNYPEDWCLRMRLKADAAASQTTGILETLAQNNLLDFAGPMTLHKIEMLMLIME
ELQKTYTVASLYRGIFVKAIQQLFPTYRPCTSPSAQAAGVNQENATNPSASLNAEPITGVPFEDSQ
YGMGVVFNDGLVDALMDHASLFNIWEHWESS
>g568
MEAGSIPSTPPPPYQTLPPTPVPTPVPTPALNHSSVHVPSTVIAPSQSTPDIAYHCAHPLPYQQPV
NPVYYAHHAGGHPPTPAATVLARPQQRPWRSYLQVGAIAATLNDAGNLAVEVYDSIANPRPTREQY
GGALDAVALRLDNVLTSMDDGTYQVNSADLAVAENQVFANADATASSLTLADIPPSSSENHKQSRH
RDHRRRPSTSATKINVPTVARIPGSSTNPFSKVYNYANAYTSSTLPPLKLYFATWPLICLASTYSR
RAYEKPSSKERQNFVSGDWRRGTKAVVLKSIPLDEKNTVVLAIRGTQNFQDWAVNIRTEPTAPTNL

LDDEGNLCHAGFLSVARKMIKPVAAQLRDLLQENPRRATCSLVLTGHSAGGAVAALLYCHMLSQTV
TSELTELQDLFKRVHCITFGAPPISLLPLQKPNSKTRYDKNLFYGFVNEGDPVARADKTYVRSLLE
LYARPDPAASLKLPSCSNDSKVTITGGKSKPIWNVPEATLSLAGRIIVLRTRKHSRSSKYHPSEQR
KDGKRKTAFSSSSISIEAVTASDAELRGLIFGDPLMHTMDLYAQRIEHLATRAVVGSSVA
>g569
MEKFSQFRDRGSGIAPFLPIPTPRSGYALPLHIFLFCFRLPLLIFVCLSYFAVLQWLPIGSLGKKA
SLWCILGVPSLWWIDLQVDGVKRGRLASQRARLPISKCIIASSFTSPIDPVYLAAVFDPIFTASYP
NTRKVERLSLLQAILRSFSYPLVNPPSDTQLVELSELLEQYQSRPVVVFAECTTTNGRGILPLSRS
LVSVPPRTKIYPVSLRYTEADVTTPVPGTYVTFLWNLLSKPTHFIRVRVAEYVTTSSKNLSPSSQA
QSTSTDFFEDDEPTSFLEEYAGSNDDSELTKSELAVLDYVGESLARLXRVKRVGLGVKEKQDFVDI
WARHKRSR
>g570
MLTKTIVATLLAIAAVALPTKTARDSVTTPDWILSPQPIDENHRPGDAVKRDPNSLVHFYIPGAPA
GDAEETAGPDDAPVKRDPNSPVNFWIPDAPTSGAEETADLDKRFYIYHRPPTPGAETVEKRHDIDV
IPF
>g571
MFFATVLLVACFADIRVAGLKSGDSLLEIREPYPTAHFQIYDAPLEVRPSRRRAIDNTDESHSIAL
AYLQNSHGIRPENVKITDAYTNKDTGISHVYVRQTSDGLDVINSLTNINIDKYGRVISSSHAFAPT
HELRKIVRSSRRSLTTRSDQYASLKSALMTLSEHLNAEVDDNVLQNLRISTLSSDEAHAPTLVIEN
IPTALAEDGAAKAQQSIMQRSDSTLAHVWDMTLRQANHRWNARVNTATGDIELLADKMIRVFGRGS
DAKSSTRNTLQKRLSYQVVPVTRKDPTYGFEFVVDPETVSSPNGWVYSNNTSGNNVLAFNGAQTNV
AVETSLNVFAYDYDETASPATPQNLGAAITNAFYVVNTLHDIFYIYGFTEATFNFQDDNFGKGGQE
NDRVTISVQDNHGTNNVNFYAPPDGQNGEMQLSLFDYTSPDRDSALENDLITSSYAQGMANRLTGG
GTASCLASSISSGLLVGWGDAVADWVEQSDTVPDFTLGNYVMNNTVGIRHYPYSRDSSVNPLTYTD
GPPSAARDDIGEVWANMLHNVLASLVDNRGWSSSFLTDSTGDSGNVVYMHLVVDGLSIQPCEPTFI
TARDAIIQADQNRYNGENYCSLWRSFASRGLGFGAAEDNVNEYSVPPGC
>g572
MPSPLLKMATLALLASPAVATSSYNLLHKFDETNFFQNFSFYSDPDPTGGFVQYLDYDAAHTAGLA
KIQSNGNVYLGVDHTNTYTTGGRPSTRVMSDIQFDQGLLIADIAHMPGNACGVWPAFWTTGANWPA
DGEIDIIEGINKQKNTQVALHTLDSCDISTTASELTGDLTATACSTSVGSGGCTVQGTDGSYGDDF
NNLNGGVYAVEVTSDAIKVWFFSRANIPGCIASGHPDTSSFGTPMAVFQGSCDFEKDFTAQQIIFN
TDFCGDWAGYSYAASCSLTDGLTAIKSCEAYVGANPSAFTEAYWEIVSVALYQKGNAAVSSVAPTT
AATSTAATSTVPATIPTSAPTSAPTSAPTSAPTSAPTTAPTSTGSALPTSSPARLSPSGKPSSRPV
APSSGAGTGSGSGSGSGSGSGSGSGNSAGSSSGIVGSGARISPAKTSCSSKSTVTVTSVVAPTSSAPV
VPTTSPCSSKSTVTVTSFTSPAPSVQAVSPASSKLTTTTVIVTSYVDVCPTGYTTKTITQTVTYCP
ADVTSSSIAPAFTTTTKLCTSGCAPSPTTVVVVVPVETSTSPAKATGTALVSGSSPASGSETTILP
ALAVSAPGTSQTGVSTAVSPVRTGIYSTGGLVPVAPSTTSSPASAKPSSSLPGKGSVTTTGVSPAF
TGAGSRITPSAVGFIAAVAGFVLFI
>g573
MSNREEREAEDLYERENDASPVPGGEVDNSYVGETNPNLRNVVPVQNDEADIDDPMQPPYSNSDQQ
LAQDEREAIDQSNILGGDRLRHAKPRTRNAYNEGPDEDDLPAEVSQGQDGRSATGRAVE
>g574
MSTPFQDLDPAGVSPEDRVNALRGYKATTNNPRVSEEAKQHAREMIDALGGDQPRAEMKQFDREKD
QTRVAGGLKAATQNPRNSEAGKGRAQEKLQQMGGPSE
>g575
MTKIFLTGATGYIGGEALYAIHKKHPDFSFSVLVRDASKNDTIKAAFPSVRIVNGALDDFELLKKE
AADADIVVHTADASDHVVAAQAIAAGLAEGHSASKPGYWLHTGGTGILTFRDSDRKVFGEYDDKIY
DDWDGVDELTHLPDHAFHRNVDQIVLEAGTKNGDRVKTALLCPPTIYGPGRGPINQKSRQVYALAS
MTLQIKKAPLLGAGKSVWNNVHIHDLGNLFVLLTEAAIAGRHDNGLWGTDGYYLVENGEHVWGDVS
RSIAEIAAKEGYIPAPTVETVDVETCEKFAGYQALSWGMNSRGKARRAAKLLGWKPVAPSLEEELP
GIVKLEYNDLKK
>g576

MDTDYEYPIYGWTATNHYNETLDLFYHRWQTGREAQEIFLEQNPLYLEAESGQPQQVQAVTGLITE
DDAQVAASWNSLWDEEEDSDNTEQEEDNNSFCYCLSPERVCSMESVTSTGGTVVSFSFIGDEGGRE
FPSTGGQNNEDSPRFDRPTDRSEVSAQDLTMPSPHTSPDPSRSSRVTNTKHLDKGKGHDLSGLDLD
RRLSSDVHAQDDPQNDKPTHGMRWEPVTVMLGNWEKEYSVLKPYADYTDVPSPIILPGSPPQRPPK
PSLSAFYKVPTVRDAYGDYLHFRRRNRNVKEVSPPAPPLPFPEHIAGQNQNLRRRQRSNSDISFLD
LGDPYQPLDANHLAGDASTTISLSMSTSVGPSASGHLTTTSIYQPLVSPPPVTLNPVQGLRRRRGL
DGTSLSVQIPAGSDPRRSASGSPQSKVASTTSSLGATFQLDEELNASMPFPLSYLF
>g577
MWNDEDNNPYGAFDRPDGHLSDSLHSGAVSPPPYDHDTSEPSSPVSTQPPDYVSRLTDSEDEDDND
FEIRQPQPSGRKKGVYDSRIEQILYENPEMPILITDAGKNHEGGGGYIVYTIRTGDLEVRRRYSEF
ASLRQTLVSLHPTLVIPPIPEKHTMADYAAKPTKAKEDSAIIELRKRMLGVFLNRCRKMKEVREDG
VWWRFLDPNVSWSEVLHSHPASSVPKNNLKAPPLDPANPTPAHAWLPVPSASAKLRSSSTTSASGT
PASPSEYSQTPSTAAHSTPGVQVLGRFPPTSKTLSEQDLDPYFINFEASTRELELLLQGNIEKVNR
RTLSHLSSLSADLMELGARYNGFSLSEQSPTVAAAIERIGQAADTSYIETEELSAALGANFAEPMR
ESAQFASVVRSVLRYRVLKRVQEEMTRDELAKKKALLESLERSEQEAKRIDQYLNQANSTSTATRS
SRSASASSATSSGQGEHDTSRTSEDTTSIDSDFPPTHGDISPQGSPSQANPYRDVPPAHRKSPSGN
FVTNKLFGRISHAIHGFADVDPERTRRDQMGKTKESLVQLEQALRVSETDVKDASAGVLQDLKRFQ
KDKEEDLRRYMVAYARCHLDWARKNLETWTEAKDELTATTSSTKRRKVVEEPRRHPKAPSSSGSGA
RHADSEERRHGQPTKEPHHQHRKSNDSVSNNYNNIQKTSAHRQKQFGSSSPSPSPSLATSTSSAPP
SLPPHLPPSSRSTAALAPSNKPRSSGSSPRYIPAHLQEEVLGVANSRTASARAPTPEHAVSPSAAY
AGLTLDSDPATDMSSSEKDQQREVRSSSPGVKRRMSQMNQDERSQEDVEMDNTDHQMEESDSRPAQ
RRATSVDMIGANEDAEMADAVDSSNNNDSDPVYPTPSSMSTYKSSSATKEGAKIHDSASSSELPPI
DEQVQQVLELAQKLPEDKQKGFVLSYNWLNRVLARSSKERVLKAIDKKFAEGEIGPVDNTDIILDT
DPSLTFQDEAGEPFVPLRPGLQMNEDFEIVPQAAWDLIMKWYGLAEQSPAIVRYAHNTSEGDATNI
QYEISPPIYTVHKLPGSAGGMSHHALRDKSAAPVRFLASRQTNFQKWLKKAKELAHIDMATKVRVW
RVLDGLSSTQTSGVITPAHSRSNSPAPGATLVANAGNSLVLDVNTFSSLSDTHRELIEDAKDQTNN
EKYNGNSTVDHFGLGDNAVVVLEEQIGGPGGGEWVSDASRSTLNRLAIPSATRPGANKVKSKAVTT
TSGRTTPVSEPIRGRKKDGRPKGNTGFTNLGNTCYMASALQCVRSVEELTLYFLNDYYKKDLNPSN
PLSHNGDVAKAYANLLHAVYDDSPASFSPKHFKHTIGRYGPAFSGYGQQDSQEFVLFLLDGLQEDL
NRIQKKPYIEKPDSTDEMVHDPAALKAFADRCWEIYKARNDSVITDLFAGMYKSTLHCPVCDKVSI
IFDPFNNLTLQLPVENLWTKDIFYFPLHKPPVLIDVEIDKNASIKALKELVAQKAGTDPSRLIMAE
VYKNKFYRLFDNTSTIADSNITGSDDIGIFEVETVPTNYNPDRPKKSSYSMSFNSDHEDVPKIDSP
RADRLLVPVFNRVQRSTGRIGSKQNFGAPSFIVLSREEAQDYDAILRKVLLEVATLTTRDFLHESA
GVEEITQDDNTDSDAVIMNEDDDDSDSKIKAASVEGEEGLVDVSMEDASDGKELPDDSSKPETKKS
PPMRFRNLFEMGYVRSRHEVIPLGFSSVDDHTNYPLISSRAPKPRPKPKPKPRKVETKESPDSPVS
SEDEINTIRSVDDKRPSRTSGSSTEFGKLANGDSSDSEDEINTVSKRLTETTRKEEPVYYIRPGEG
IVLDWTETNHDGLFGADQKDRNELRGAPTWTNVEHLPDPELAAKRAQRANRRKKGLSLDQCLDEFG
REEILSENDAWYCPRCKEHRRASKKFELWKTPDILVVHLKRFSASRGFRDKIDELVDFPIEGLDLN
GRVASAEPGESQIYDLFAVDNHYGGLGGGHYTAYAKNFLTNEWNEYNDSHVSKAINPAKVVSTAAY
LLFYRRRSDRPLGGQLLQEITEASTRANSDNEDDSRATSPSGEGRRLVDSSRNGSTSALAGAGAAH
QAGVGGSQAGLRVRSVEVDSSDNEEELPPYESKHQHSSSNFSIMDQPAWSFDNITGPMVSDDDDGL
FDDDDDNDSNKAVGGGDMSDLEQHFNGDADETAKFHSVIDDIGTVSDGDEDLPVVELRVNEDDRVV
>g578
MTTMASVVTTRRTFGTPFSMLRSSRLVFKQYAHKLYATLHVGAKHKGDTMDKTPRMYTSKRLKFDS
DDFKKDVSQLCATLNSGYEEKVMNAILDTYKKNFERGAVLWKATSKEDGVAFRFYERERVDVVGPA
INRGLIPCSHPMIPLITSWADFKENAIASCDFDPAKGLNKTWIWLXXRFSLEELLAAPNVPDTLRS
LEPKLKDAGLHTVRHAAVDWRSSTVNIYFWVPGPIALTAVNRLIALSGSEPIDEEALAAMKPFFKP
NGFTFATTIDSATGQFKRAAFYALRLDSNNLPVMGDRLATFFKEAPSLDKKEMNIVAWSFAGGPSG
SSNYIKGERSYVGDLEQLLNGWGSPI
>g579
MAYSSQTWLAVFEVVLIRFDDTTPKPMKQVLNGLFKFLKKHPDATEAQRIEAGVMDKIMPSIILAD
KSRLKSSLVSLERLLRESEADIKAFRSFIADLPLDPVLTGNMEGSGSVDEFILLFSALGVGKKIGF

VHEDQFEKRTAATRNVDVADILILKSDVLGRFLLHNEPRVRLLALSLLTTAPVMSKPISAIALNSI
LDGLYSINADSDAFIRQETMSYMRRLMLRLRGPGILKDFMEKEGKDSKIFMEHYVDFLEKELLPTA
SYQRHISALKSISLVLKTGIDPNATHVPEEEQVLWRYKVNILRPSLFRLLVDLLLDPFEEVRATAL
ALIVVAEKLVTALSRAEALASGTSRADHADTVARLYHALYSTAKDGHETGAVDSWYSTKIGVANEI
LRRLEVKLSQETGLFQASVRDAPLHGNTSALRYIVATPNFYATISGSTDEYDSSWRKVHHRIVSIC
ENIWTKVTPVLCIDSPEGHTDELIEDFMVGPKDLLSYSWRALKDSSLLLHAIVSNATYAPNDGLRR
ADFETIGTLSFVQLSELRHRGAFSTVAQTFAACCLRCAENKNSEISSLPDMWYQNSLKMIQHHASK
LTRRSAGLPALVTGICISKSGDPLFGKIMDDLQNIASTPAVQVSSYNELSLPQVHAMNCLKDILAT
SKLTSCTENYIMPVLRFGADSLSSPIWAIRNCGLMLFHASMNKMCVRRPGVPFGFSGDSGVEPNMS
LAFPKYEGLTQLLAQLLASADEARDGGREPEQGLLASNETEKVFPALGIIAEKVPSTSNNDDDLLR
GLVIHHIKSTIWAVREQAARVYASLLRYGDMLSSINALMDDDLAKLSQINLHGKMLCARYTLLRAW
HSTYWRDNVDEVLATVRKVFAGFFPFIRSPFGQGTLLDILVDVLESNVSSGKEVISKTTQFVSEFI
SDYSLEHGLAELVNSGPQSPDRISAKRAESLLRRSLAWCLILNNTLSIVVSAADTSPAGLMGPFFD
LSASDSDATCWILERIRLVFPSYEGQREFLSLYLLVLLQSFHEENVKARTALNLAEILEDALERGI
EVEFSSKWARVSDHLSLQSEENIWSRELGENVLRLQGSLLTLKHMHAESADPTTETNGGLDSLDLR
RWAVSLRSALSEETVFSARSSAIMSLKTFSRILRKPNQAADTTPDTLDIYLILYDMLNDDDDELRS
LSAPIASWVLSHSAIFPNQAVTLGALPASESLVKFIIENYATQPALFQHVITRLMKETLSQKKQTD
FKSFETLFEDYSKESLALFEEEKQNLFIDEVREIDIWTKVLRHLDKSAYDQNTINKLAAWVSNGLT
YLNNKVTSSAVDGQEDDVLGWTSKPEVYTLGCLLFNVASLLIATSEMEVAGSFQRALTTLYEKGQM
ISLHSQWLDRIQATISLSRKI
>g580
MAIWDSFSGRKATTSQDAFDPTSAQDVSSFLGQTSFPDPSQLHPLAGLNQDTLDYLSLEDSALDAI
PGSRSALPSRGWSDDLCYGAGSTYLAGLTIGGVWGLAEGLQRTPANAPPKLRLNGVLNSITRRGPF
LGNSAGVVAMVYNGINSMIGYTRGKHDAANSIVAGALSGMIFKSTRGTRPMLISGGIVASIAGAWT
VIRKAVFE
>g581
MSTPESQRQTVGSEDQSQHANPRTLLPAEFQGRTTKTSTPATSEPVCNVSHSHPYNNTIASVWASY
NGAHYAPRSNPFPPLYVHTDLPPNHRPVTPLNDNDSSLRQSLSSTSLSRRTPSYPVRVNTMSSTES
LSPSSMRSSTVSSPALAAMADITPLPSPIGYLSPASWRSGGRTISSHSLSRTSSTLSRSGSFLNLR
LSDSTHLLRTASSRSRSRRYSSTRLTGSEVRESGLGGHEEVRGHSRNRSLSEHIPKRGPASLQRPD
GLPRTGHPSDLSSSSSADSTTDPNRLHREQYLGEKRGLALPTARPPTPPRSTRNGDEEADSKPIIQ
SDASDGSQNETYFVRSIRTKQPRKYRKLRQLGQGTFSQVSLAVRIPDDAPMKDGHVRMPSLMSQKL
VAVKIIEYGPAGGADEERVEVSLKREVDILKSVNHPSLVQLKAFGSDDKRALLVLDYCPGGDLFDF
ATSDMKSLMTPPLIRRMFAEMVGAVRYLHANFIVHRDIKLENVLVNLPPSAMGQISDWRTYDRAVI
TISDLGLSRRIPEPPESPLLHTRCGSEDYAAPEILMGQPYDGRSTDAWALGVLLYAMMENRLPFDA
LPGTRGDPAKLRARTPHRIARCEWSWYRYANEDEEWDPVKGAGLEGARDCVEGLLKRHSRRKSLDQ
IAKHPFVSEALDLEGELKRGDKERARIGWSGRCRTFALSSEMDLVSPDRTTSSKISFSHSPPTFVN
SFYNQYSIEXGRLPSALKTFQNTQFASRSLSTTLPRRSDDEGLNKVSRAITQPISQGASQAMLYAT
GLSEADMNKAQVGISSVWFTGNPCNMHLLELNHLVKKGVEKAGLIGYQFNTVGVSDGISMGTKGMR
FSLQSRDLIADSIETVMGGQWYDANISIPGCDKNMPGVMMAMGRVNRPSLMVYGGSIKAGCAATQN
NADIDIVSAFQAYGQFLSGEFTEKERFDVIRHACPGQGACGGMYTANTLASSIEIMGMTLPGSSSN
PADSKVKQLECLAAGEAIKTLLKEDIRPSDILTRQAFENAMILVNITGGSTNAVLHLIAIADSVGI
KLTIDDFQAVSDRTPLLADLKPSGKYVFNDLYQVGGTPSLIKFLIKEGLIDGSGITVTGKTLAENV
KDVPDFPEDQKIIRPLSNPIKNTGHIQILRGSLAPEGCVGKITGKEGTIFKGKARVFDDEDDFVAA
LERKEIKQEDKTVVVIRYCGPKGGPGMPEMLKPSSALMGAGLGKSCALITDGRFSGGSHGFLIGHI
VPEAAVGGPIGLVKDGDDITIDAESRVLDLHVEESELASRRKQWEADRAAGKLPATGLTMRGTLGK
YAKLVSDASHGCVTDAL
>g582
MTAPTQQMLNGLVPMPVNYDGGYIALSYIVSVMGSTTCLELLHRRSSRSGLLNWYLLLASSLSMGG
IGIYSMHFIGNRAIVLGDGSNAQQISYNIQFTIVSLFLPILVLSTAFYAISFEEKPSLVRLLAGGI
GTGAAICAMHYLGQLGISNYHCSYGPAYIVGAALIATVSATIALSIFFRWKAAWTNSWWRRLVCAA
LLAGAVSGMHWTAAVGTQYHRIPEFLGAGQLSRSQTVIICAALSFAACITLTICAIIAGGRRHQST

TRARQLVLSCAFFDPAGRIMVSPQALLPTRKVVDRYINKSMKDDDFSRTHPAFLWAFRASRNWALV
KGIIPSMKNTLQSNELEIKRLINQRGIVTENDQDTQFSFDTIFKHFFCITAQNLADELRLPLSGLG
ELYHQVLSTVTQVSRFELGSRALPRIGKGQLMFTVRQLDEHEAVKFMASGYRFAPIERVTDILSRR
VHLSPTDLAIHLHEMRDYASSDRGYPPGVHLVAFGLRPTLREHFEILTRTGGGMTLPSSTLPISSL
TKTDLELLNSMDECGFTACLKQLLALRSTIGGSNGSVSSVDTLSSTAAEESFPTHLYRAMLELMTI
LPENIASAARFSAKPLLAPCYRQSPSDPEQVQEQVNDKISERERFIRELNAEFASYSAQSTANHKE
EENSINPSQKIIVFPSKILSRLQSSVSSKKIQLLPGRLTRATSQESLVRQLSDIHTKSAFLSAPPP
KSPPTSISPTRNVLNPFINEILVSKEVTVDVARLDDVEMTDIPSKRKSSIQPHASSAPQNNDAISE
YQVDGHVSYSMASLAGAERRTVIEGGLARFGTTYVDELYGLCLGREVRMKPGFYNYDTSEK
>g583
MNHKYSLPGWTYPRSGRIGRLAYFATVTVVALFVFSLAYVYVEEPPFWKSGPPPPRLDDMDTGSAH
IMVSPGSSHVEPEASPLPTLPSLPRPALGSTTGSSHDTTSSEDDLASAVSSLHKPEGLRVVGIIFY
GRRDRSSILECYLRQNLVSSGGWLDEVIFAANTDNKEDLAWLDRVADASGGDYKVVRMEEKGYGNV
YEASFKERNTIYVKIDDDVVFIDPQAIPKAVTTLINNPNALMISANVVNSPALGWWHYHSDVARSY
KPELEPNETALASRGNGAWKTSELPTWSGDHNLDFTKFENFTDYFNVDKPEDIPKHRWLPTRNETD
LFTSSIAATNAEGGPHLTQWEIGAQNHYGFFEHLENNELGRYYLSKDYGKGTTWNMRGYRLSINFI
VMHGADVLDYMDVITGHPQGDDEHQLTVEMPRLLRRPVLVESQSIVSHFSYGPQRWLHKTDIMERY
FNYANDNVCPEQSLIDPMNPTKTWKLPSNPATSPQSSPSSASTTKPTLSLRFFRR
>g584
MAIVVDVSEARRGRSRVPSEIERLEERIRELENQLQTPSDPFNDELVKAAAPPENGNDRWIQAGLG
KPREGIYITGAQSQQSQWYGLLSNTYYISRLRSDLTIALQQPQSDDHIQPKSASQIFVSPTSSKPS
DSSLEETALSADLGASGSYLTPTQEDYFLSLYWESYHCTFQILDEGEFREHYRSLWKTSSSTRKPS
PLVDIILAICMQYGLAFLPREASSSEPKTVVDGNDATIAGRWYYHRSQMLLNSQLETPSMMTLQCQ
ILAVKYLCNASYQNMAYSGLATAIRTAHLLGVHLEPPADMPREQRELRKRLDRPWSAPFSLISCQL
PADDRQLALTSGSNFASYGEDLTWLTYTLQMTKMTLAVRSIFIALADKCGDVVASGRGIYKDPQTL
EKCATFLESQMEVFHSFETQLPDGLKTKRKSGGLPFSTDRKMLDIELYAPLWLQRQRLMFELLYHN
LALNLYRPFLCFPSTFAAEVPPQTSPAFTNLTPVTNSHANSCIEHAMAITRMMHQILTGSEILSGM
HEAFHFQWNATVSLIGFILAYPLSPLTPEVRENINNAIAVFETFGKHFAVGASAANVTRNLVAKAD
YLKERFHARLTSLETSNTPDLLRPYLGTLSVDNMDPSQFPEFMELMNFPMSGTTDLGFSLDPFTGL
EQFYSGSANPADTWVLDQN
>g585
MSLPKTFRQAVFKEAGGPLVIEEASFTPPAQGEILVQVEACGVCFSDTITQHDYMGGGFPQVPGHE
IIGKVVAVGSGVSGWTVGDRVGGGWHGGHDGTCTACRTGHYQMCDNQLVNGVTRGGGYAEYCTIRS
EAAVRIPPDVDAATYAPMLCAGVTVFNSMRQMQIPPGSTVAIQGLGGLGHLALQYANKFGYRVVAL
SRDSKKEKFARQLGAHEYIDGSKEDIGEALQKLGGASLIVSTAPDKASITPLLSGLGIKGKLLILS
IVNDLPVNTGILMRYGSSVHSWPSGHAIDSEEAIQFAGLQDIKCMVETFPLEKANEAYDAMLKGSV
RFRSVIKMN
>g586
MSEIVSSVNFLLNESDIVVHDPENVLRKYTDKNTANGGVIERQFCGTCGSPILSLVLAAPGTVFLK
ASLLDIDDIPQPKKENFPHKKPHWMREVKYLLKR
>g587
MPDEHSTLQDAKESLFNPTPRKQGLPEDNMMTPYNGVRSIFDNSSGSHQLTDDNTNQERALSKAKL
ADENIAPFLAKHIPSQYAPLGTDTSKSAGPPPKYMNSRYCYRHRPDLKCRRQADEPSMDHLQWELQ
SLPQSDQQGIAHVWSLFSAAPAKQRELMLRGILAQCCFPQLSLISSSVRDLIRIDFITALPPEISF
KILSYLDTASLCRAAQVSRGWKCLADDDVVWHRMCEQHIHRKCTKCGWGLPLLERKRLRASKEQIE
KRALGVPVSPESSATAQAVDATSGVKRPAEDLEASDSQIVKRQCLPTEEDTDLYKTNFRPWKDVYK
DRFKVGTNWKYGRCSVKVFKGHTNGVMCLQFEDNILATGSYDTTIKIWDMETGEELRTLTGHTSGI
RCLQFDDTKLISGSIDRTLKVWNWRTGECISTYTGHLGGIIGLHFQNSILASGSTDKTVKIWNFED
KSTFLLRGHSDWVNAVRVDSSSRTVLSASDDCTVKLWDLDSKQCIRTFQGHVGQVQQVIPLPREFE
FEEDHDAGHEEDSNASVSGDESPFSQISCSPNTTFFEGDRPAPPRYILTSALDSTIRLWETYTGRC
LRTFFGHLEGVWALSADTLRIVSGAEDRMVKIWDPRTGKCERTFTGHSGPVTCVGLGDSCFVTGSE
DCEVRIYSFKN

>g588
MSQSSAETSSPPQSKSQSINPGPQREAAHDSHPLAEEPKSQLDTLISYLVAAKRSLSSIYHVSRAD
EIVNTARNALEESVVLSAKTGFLRRGQKNQIRLLYNVRGEIENISHRGRADFAAVLTQLDDVDGRL
RQTLDKLRQTVVHPSFRPEGEEPKSLHDFVDERGVEELQVILKAAIDRTTAAQSQLDTSSSSFDEE
LRALQHALGRYRTTMELASSRSSLSASSPSASSPALVSPSSIPPMLRSLESHAQEMADLLESLVHH
FDRCVTAVKHTEGGGAVARSITGDMPQGLNVPHTEETADDADPSANDTVDPMTDSDYEDMLGVLTK
DAAEAEDVVLEIQERSSEMETILESILSHRDSVTTVYNATTEVFDHLSTLSTSRLPEFISQAHAFT
HVWNEEHERIRTGMADLVDLRSLYLGFLDAYDGLILEVSRRKHMRQGIERVLRETRIKLDQLYEED
INAREAFRVEQGDYLPSDIWPGLSRAPMRIKFSKLSGGKXPAVTGQDDGDEVPVEGTAIEDEESID
GDDYIPDLPKHIVDEAIGRLKARAKEDGLQ
>g589
MYIKQIIIQGFKSYKDQTVIEPFSPKHNVIVGRNGSGKSNFFAAIRFVLSDAYTQMGREERQALLH
EGSGSAVMSAYVEVIFDNSDERFPTGKPELVLRRTIGLKKDEYTLDHKNATKADVMNLLESAGFSR
SNPYYIVPQGRVTTLTNMKDSERLVLLKEVAGTQVYEARRAESLKIMNETNNKRAKIDELLDYINE
RLGELEEEKDELRNYQEQDRERRCLEYTIYSREQQEIANALDSLEGQRQTGVEDTDVNREHFIQGE
KELSQIDSEIAQCKQQIEFLKVDKAQLEDERREASRALAQVELQEKALKDNQSAAQKLKARYENDL
NAVQTAISQRESELQTILPQFNAAKEQEDAVKLQLDQAETSRQRIYAKQGRNSRFKSKSERDKWLQ
KEIQETKNSIKAVNAVKAQTTEDIEDLQKTIESLEPEIENLRKQIDGRGDAIQSIEQEIQNAKDER
DRLMDQRKELWREEARLDSVLSNSSQEVDRAERSLSHMMDNNTSRGMAAVRRIKRQHNLEGVYGTL
AELLEVNERYRTAVEVTAGTSLFHYVVDTDETATKVLEILQKEKAGRVTFMPLNRLKPRPANVPKA
SDTIPMIEKLQYDPQYEKAFQQVFGKTIICPNLQVASQYARSHGVNAITPEGDRSDKRGALTGGFH
DSRQSXLEAVKAVTKWRDEFESKKNRASEIRRDLEKLDQQVTQAVGELQKLEQRKHQFHGNSGPLR
QELRSKRELLQNKNDNLDAKQRALRNIENNLTALDGQVSALEAEISSGFQKALTADEEKELESLGV
TAQSLRRQYADLSSQRSELEARKSVLEVELRENLYPRLDQLSSPDVDMGDDNSQGTLKEAQRQMKK
LQSSLGKITQRLAEVDQSISEANTQVGQLEGRKTEVRNNLEALAKSIEKHQRRMEKSMQKKAALTK
QAAECSANIRDLGVLPDEAFTKYNKTDSNTVVKKLHKVNESLKKYSHVNKKAFEQYNNFTKQRETL
TKRREELDASQKSIDELIMVLDQRKDEAIERTFKQVSREFHNVFEKLVPAGRGRLVIQRKTDRALR
TDNEIESEDEDRRESVENYVGVGISVSFNSKHDEQQRIQQLSGGQKSLCALALVFAIQACDPAPFY
LFDEIDANLDAQYRTAVAQMLQSISDSTNGQFICTTFRPEMLHVAEKCYGVSFRNKASTIDVVSKE
EALKFVEEQKS
>g590
MESGGATHGLADGQLPPHQFVPNTGYINNTPVDPAVDNAQDTNAPQAGEEVDEEEDEYYDDIFEED
LEEDFSSANPNDFTKSYNRQRRLNEAMADANVPKSSYPKTNPQKPKANVAAAVDDQITSLSKHAAK
IKLDDQQSGFRGKDGRTVDKSDRATSEQVLDPRTRMILLQMINRNLVSEINGCLSTGKEANVYHAV
TIRDEEGDVQTLQRAIKVYKTSILVFKDRDKYVTGEFRFRKGYDKSNNRAMVKVWAEKEMRNLRRI
HSAGIPSPEPLYLRLHVLVMGFLGNSRGIPAPRLKDVEFDIPDPETRWQELYMELLGYMRTMYQTC
RLVHADLSEYNILYHKNKLYIIDVSQSVEHDHPRSLEFLRMDIKNVSDFFRRKGVNTLSERVVFEF
IISSQGPEMVGGDNESMVTAIQKLFEIRDENGGDAEGVTDDVDAAVFRQQYIPQTLEQVYDVERDA
EKVQYGESADLVYRDLLADKSKAAIPSTKAETKAQLGEDDESDQSGGVSISDGSDESADGSDDEDP
FAKKAPRGKKHEDKDAKRLHKQKVKEEKREQRAKKMPKHVKKKIINATKRK
>g591
MFYQPGVTDHGLPHDPFKACVIPRPIGWISTRSKDGQDNLAPYSQFNNLXFDPPYVMFSSNMLPPD
NIQRKDTVVNAEQTKHFVWNMATWDLREAVNITGEYLAHGVDEFERASQYISKEPATLMDVSMVKE
SPVKFECEYHSTTRLPGNPPMGTVDIVIGRVIAVHIKDEVLTDGILDVKKTLPIARCGYHQYTVIR
ETFEMVIPGDNKLMRLGLEGNAKAARQATQQGDKNEA
>g592
MATSAVEVDIQRISAYGPARSTVKVDKPLDKDEVRKMDEYFKASLYLCLGMLYLRDNPLLKEPLKK
EHIKNRVLGHWGSDAGQSFTWIHMNRLIKKYGLDTLFISGPGHGAPGILSQSYLEGVYSEVYPDKS
EDEDGMRRFFKSFSFPGGIGSHATPETPGSIHEGGELGYSISHAFGTVYDHPNLIALTMVGDGEAE
TGPLATSWHSTKFLNPLTDGAVLPVLHLNGYKINNPTLLARISHEELEALFIGYGWTPYFVEGSDT
QSMHQSMAATLEHCVLEIKEIQKKARESKKAFRPRWPLIVLRTPKGWTAPKDVDGKRLEGFWRAHQ
VPITDVLTNPDHLKLLESWMKSYGPDKLFADKGKIIAELRALAPTGNSRMSANPVGNGGLLRKPLD

LPDFRTYGVEEIDPGVSVLGNMTNMAKFLRDVIAKNQTNFRLFGPDETESNKLGEVYKAGKKVWLG
AYFDEDKDGGNLATEGRVMEMLSEHTIEGWLEGYILSGRHGLLNSYEPFIHIIDSMVNQHCKWLEK
CLEVEWRVKVASLNILLTATVWRQDHNGFTHQDPGFLDVVANKSPEVVRIYLPPDGNSLLSVADHC
LRSVNYVNVIVADKQEHIQFLSMEEAVEHCTKGAGIWDWASNDQGEDPDVVIASAGDVATHEALAA
TALLREFLPDVKVRFVNVVDLFRLIPEYQHPHGMSDQQWKALFTEDKPIIFNFHSYPFLVHRLTYR
RPGQKNLHVRGYREKGNIDTPFELAVRNETDRYSLAIAAIDNIPRLHNKASAVREKFINLQIAARN
TAFEDGLDSEEIRNWRWPFSKK
>g593
MAEFEQWVTAQDGLDKLRLIKAPLPKPGPNEVLVKVHSVALNYRDIEVAKGEYTHHNYTGIPTDLV
PCSDMCGTIVEVGEGVGKDQASLKRGDRVISTFNQDHLTGQITSKELATGLGLPLQGVLAEYRVFP
THGLVKIPDYLTDDQAALFPIAGLTAWMSLFGLRPVKEGDYVLLQGTGGVSIAGMKIAKASGCKVI
ITSSDDQKLARAKQLGADHTINYRTTPDWEKEVLAYTQDHGADIILEIGGSQTLRKSFDCIAWGGL
IAAIGYLSGKQDDEARDLLNVNILALRRNVTLKGILNGPRDQLEELLAFCEKSRIAFEVDRVFDWG
DAKEAFKYVNAGSHFGKVIIKVNKTT
>g594
MSTITTTTESNAAEAFFRQPFYAKDGKTRLPQYVAHRGFNKIYPENTLSAFQAAIDVGCHGIETDV
QISRDGVVVISHDFTLTRCFGIDKKINECDWEYLSTLRTIXEPREKMIRLSDFLEFISAKGRDHIW
VLLDIKRNNPANVIIKKIAEILKSGPYSSQPWHLRIALGCWAPTYFPVCKEHLPTFQPVLIGYNVV
AARKALRTIPEVSFNTHYTSIKGPLGYGFVEAVHRNKNLKDPREAKLVFGWTINAARDIRWAIRKQ
LDVILTDDPTVYKQISDAWDEEYSKRHEKEDNKMTLRERLYLTFWNLWFVFFGWVFPLLYPYVQRF
LVEKPAVKTNVKTSSART
>g595
MANDTDFAAMLPTIKCSNCNMNVEISAMGEHVCAPTQQSSSPPPSAGMKALRPGPPPRIDPTIANQ
PFLSPSYGSPSSSLGSHPLTPRMSPLRSQTSPYPLTPSSLEFNDEDNNPAATIRAALSPTMTTKSP
LRSTTISEAPTINSDTAISDGSEGLHTRSPSIESKSTYRTSVSSKRYRESTSTYSSRGVSISMPRS
PRNFMDEVPPIPTGPLTSFRPSSQMSNVDSNISSSPPKTKGASYSGFDFGLMDEPQSYEDVPKQNT
LIRSQTMPNTVETNLDINDNFLTRWPSQPSPTVPGRPSLPRESSIPDQAFPKPPALEPIPQQDNLS
GLPTSNFNAGLGLEKTAYHATEGSTSSSNSSRSETGSGSSISSPPSDFDRKPSDLTQIDSMLQELE
LDQKQSPVERQPPPPPLDGASLEPPRGMGNLDRGPDSPTDPTLVGGSYAARNFHPPELTPKSPLRE
KTPAEHAQQISRPNTAGGQKRRCRGCGQGIVGKSVSSADGRLTGRYHKACFVCFTCRSPFETADFY
VLDDHPYCSQHYHELNGSTCATCKLGIEGQYLETIEQSTYGTGNPQKFHPDCLTCRTCRVVLRGDY
FEWNGQVYCERDARRAAMSAPPPNRQRPTMPSSPLAGPPGFPPGPPPPGYRGPPRQGGPGGPMGP
GGPMMPRGPPPPGGRGMGRPPPSPRQGGSLAPPGGARRFPERRTTKLMMV
>g596
MAPHANETGAVNSAVNGVAGNSNEKTPLFTVDSPNVVYTEETIETKYAYHTTEVTRAGDKLVATPK
AQHYNFKVDRKVGKVGMMLVGWGGNNGTTVTAGIIANRRGLEWDTREGKRAANYYGSVVMGSTIKL
GTDAQGQEINIPFHDMLPMVHPNDLVIGGWDISGLGLSEAMDRACVLEPTLKELVRKEMAEMKPLP
SIYYPDFIAANQEDRADNLIPGSKASWDHVEHIRKDIRDFKAKNELDKVIVLWTANTERYADIIAG
VNDTADNLVEAIKSGHEEVSPSTVFAVACVLENAPFINGSPQNTFVPGAIQFAEKHGAFIGGDDFK
SGQTKMKSALVDFLISAGIKLTSIASYNHLGNNDGKNLSSQKQFRSKEISKSNVVDDMVAANSVLY
KEGEHPDHTVVIKYMPAVGDNKRALDEYYAEIFMGGHQTISIFNVCEDSLLASPLIIDLVVVAEMM
TRISWKKADGAEDYKGFHSVLSVLSYMLKAPLTPPGTPVVNALAKQRAALTNIFRACVGLQPESDM
TLEHKLFQ
>g597
MADTVGKTITCKAAIAWEAGQPLSVEDVEVAPPKAHEVRIQIFHTGVCHTDAYTLSGKDPEGAFPV
ILGHEGAGIVESVGEGVTNVKPGDTVVALYTPECKECKFCKSGKTNLCGKIRATQGRGVMPDGTSR
FKARGKDILHFMGTSTFSQYTVVADISVVAVTDKAGTDKTCLLGCGITTGYGAAVVTAKVEEGSNV
AVFGAGCVGLSVIQGALKNKAGKIIAVDVNDSKEEWSRKFGATDFVNPTKLGDQSVQEKLIEMTDG
GCDYTFDCTGNVGVMRAALEACHKGWGQSIIIGVAAAGQEISTRPFQLVTGRVWKGCAFGGIKGRS
QLPGLVDDYLKGDLKVDEFITHRETLDNINTAFEQMKAGDCIRCVVDVRA
>g598
MEGLNASEQRELAARMERKQMKEFMQMYSRLVQRCFDDCVNDFTTKSLVAREEGCVLRCVDKHLKS

SARLGERFQEQNAAMMQSGQLPGR
>g599
MTTGTPQPLAAHAPVQNFTTNNHANEPAPPAAPTKRDLASWWKNFKRNTKKEDENKGGPQVGGIFG
VPLNVSIKYANVAISLTNDKGESFIYGYVPIVVAKCGVFLKDQGIYTPALSDDIKLTIVSIATEVE
GIFRLSGSARRIKELQEIFDSPERYGKGLDWTGYTVHDAANILRRYLNQLPEPIVPLDFYEAFREP
IRNHQQQAVGSTEAVDIGDFDYEKAVATYQQLIRELPPLNKQLLLYILDLLAVFASKSDKNRMNSN
NLSAIFQPGLLSHPTHDMAPLEYRLSQDVLIFLIENQDHFLFGMNGTAADPQTVKEFSSTAAAAAG
TPSRSSGIRRSASNASGGADSLRKYETLRRNVSVSSRNSGNTQSPGTPTSTVSTVKRSNTVPSKRS
PGLVAPRLTRPGESASPSSAXLTPPAEASRNPAPETRPPAVQKSHSEAPKPAQPAFEEGQFLEAQS
AIASPPTPLLTPTKERKISSFFAKSPPASDKEGRQPNRLKKKRIPGSANASAQSSSHSLSAATSNE
SGYFPSTSQAVTPMAPLPNDPISTIPENQGFPAGVPEGVNSHHQHQPSESTLKPNRSRTPSMHSKS
SYTDHSDFDQYDDVSKAEKRDHRRSWRFPLSRHNHGNPTSSPPMVGTNAGAEFSTSSIGSLSAQGQ
SFTNDSYNPSTDVTSVPDNEVTRSGSASREGGVSSENEKLSLFEKIKAKVAPKKDGSERAKSPPQS
DSQFESIAPNRSQVEETNKLKEPEPRSEQAKQLEPQQQPTVVVQAEDTLPQPATDAK
>g600
MASSPTVSSPSKSSKAPPSQQSSTTATSQPEKAHAIDILPTPVAQTYSHIHPTILLALYSLRFSAV
VTDPVSTLWSDLPLYTALQTAHGEAGTGEVSADVKKTPSSSGKRKRQAAGNKSGTLSQKLTAAFIA
LTLTFLLGTPALSLLLILFGAPFTTHTAHTVLCAAHMALLTVTPLVYVHGVDGPVWKQIWAFARPA
DAVWGGALGACVGAWLGAVPIPLDWDRPWQAYPITILTGAYIGFAAGQLIGRSPLLYGKRVEFD
>g601
MPPYVVPELSVDSVVVSDGTKTLVARASTQTAVAGGKGVINPDHINMQGLLALFAILGAAFVMATI
WFFFWAKNGGCVFRKGDWDDYKSTVLRRKGPDGRTLSNATTSTNLGGGTVRGYDDDGLTYTDITET
ATEFTDEKSKARGGRKKNKKNFKETAKEKLLRRTKDEKWEGEADDDMRAYRQEKPARVGGLNREAE
GTYYGTEYTPSSPPTAYTESEVYHAPPPEEERRRRDTRNASGFSFTAGSEDVISQATEEHLIRDPA
QTRRDTRRQRREREEERRSRQNSPRKQQPSNNRTSMPGGYTEPLDFSSRGTNSEYQYSTVDTEDDLG
TRSYHHPIPGLSKGYRRDRDGGGRRRRDSLSDSE
>g602
MAKKVPQTPSAAYLEDYDDQTGDVVDGTRQSANIAARRSKSDIRPGELVHLDFASDSGYSSRTATT
GASGQSRSGGRPGAGPSTVRTAAHSRAPGPPPPPPQLHRVKESRKEKGKQREDKRASVDMIHAQRL
ANGMAAPVRSPSKSNRRESVSQQQPEVCWDCMNGYHNVHGIHPTAAMDPRTGYPSYYYPQAPPVQ
NIPPASPQSTQFGYPYGPDVMIAQSRQRRPSRSAAYHSDNRPMSYHSGMQEAGYNPGALQMSQYEQ
RPPSTYSGYFTQPATPRQQYYAEPDAPYQAIYERSRTSSMSRPPERARRGSVYGRSTLEQITPKAS
YEEGSFLERQTSREHRPRRMSQSQRRPETYDEDEDFYRMPPPPPPKPKVVQPKVVQPKANPPKIIQ
KRPDLTHKPATTGSMPPPTNRRMSHSRDSWDMKDLMEALPHQQIRKISAAPERSRSMKARRTSYHE
SESGRQIAVEDSRRRRTLYHEEEPSIRDIREVRERPAPVSEMEQKHRNAEEYQAAKSGRTGVPLTE
DALRMKAKIAQRVDSDSGSQKTRSNSSRGSDARTRDGSGVGSRVDEDGGFTMTMNGMTIGFTSESV
NGKRIHLRTNDHGSIGLNIEGRRPKRYIMPASSEYTSTSSRKEIEDGRRAREDRRSDRASRRSSRS
TYSGRFD
>g603
MAAPSQALAEGQSLPSIEGLTLHSTNETSKFAGCYPSLNPVDIYREHISEKLAEVTGIDPQTIYPK
LAWTNTLDKGDLNLAVAALQIKGKKPQDLAVELADKFPESDLVEKPTAFGVHLQFFFKPGPLTKTV
IGRILENKSTYGTNGNLGLRDPSDPSKGKKKMIVEFSSPNIAKPFHAGHLRSTIIGGFLANLFTIT
GWDVLKMNYLGDWGKQYGLLANGFERYGSEEALQKDPINHLFDVYVKINKDMAEQEVPIKELRETI
KNKKEKNEDVSAEEAELAKLVDASDDEKARRYFKSMEDGDPEALALWKRFRDLSIEKYKQTYARLN
IDFDVYSGESQVLPESLQKAYETMEKSGVSEKSEGAVIVDFTKHKFKKLGKAIVVRKDGTPLYLTR
DIGAITERHEKYHFDKMIYVVAAQQDLHLAQLFKVTELMGYKDIADKCQHINFGMVRGMSTRKGTV
KFLDDILRDVADKMHEVMKKNEDKYVQIDDPLATADTLGITAVMVQDMSGKRINGYDFNLEAMTSF
EGDTGPYLQYAHARLSSIIRKAGISPGDLPKADLSILTEQHAIDLARYLAQWPDVVINTVKTLEPA
TVLTYLFRMTHMLSSSYDILRIVGREEKVALARMALYASARQVLYNGMKLLGLNPVEREAKYRPSK
QLSQEKRAIIYEGRYNDIFKMGSVTPAPSSRFVTTLERKYGRNALPNMFDAKAKEPSARPYAYVLK
TPNPADGFEEVTYTMLANAVNRASWWLVNEASMAEGEVFGYMGPPDLRYLILSLASAKTGRRIMLP
SLRNTISAQKTLFERTNVSIILYATELKKNIQPLFDALPEIKTREAIGYKEVLDSTVVPHYDAREI

TDENFNEPMFFLHTSGSSGNPKPVGESPAFWLATCSFLDVPEDGGDIHNTIHLLRNANLLQFFPPF
HMAGLFFFMLPAILDSSLVINHTAAAITAEHVINIINQGLATSLAAADSILTDLSRTEEGLQALEK
MDKIIYGGGPLSVQTGSIIAPRVKNLSSAIGLTENGLLHCVALRGTSHWDCLRFNTRVGYRFEEIS
PDVYELIISLSPKYRMFHPVALLFPDIEEYRTKDLYTRVPEIDNCYRYQGRRDDLIVLSNGEKINP
VPLENIVASHSAVKNALFVGEHQFLPSLLIELREGYTVNNEEESREMIEKLWGIISEANLEAPRFS
RVPKSLVYILKPGETFNRSGKMTVQRQLTVLKFAAQVDALYTAAGEGLLSEGLELSDPSDPGAIKS
LAKKLYAQLLDSDESAPVVGDDDNVFELGMDSLQVTIAVQKLKAALRAQNLKVDTSKIGPQFFYTS
PSSNQLARAIDELINGASANDNRSNRQIYMQAMIDKYTAGLDVELAPRKKRIDNLTVVLTGSTGSL
GSYLLHSLIEIPRIAKVICLNRSTDAQKSQTAKNKQKDLPTPWESSDAQTSPVEFLTADLSKADLG
LGEETYSRLLQRVDAIIHNAWKVDFNHTIESFEKGHIAGTRHLIDLSRKCTYRAPILFISSISTAL
NWMQKNPAQDVPEAIIEDLDSPEFLGYGESKYVSERLIEAHSSSSGFTSSVLRVGQIAGPVLSTAG
IWNVQEWFPSLLCSSKHLGLLPDSLGTMNSISWVPVDLLARIIVQLLSHTYDNDGARSGALKVYNL
VNPRTVPWSALLDTVQNGLGGPEKIRIVSLAKWVEALEQSAQENHGFVVESNPAIKLLEFWKIISE
KSDRSRTAELLTSNGENGLQNDEKVSNLQQNKADRRKSWLKQWTGKLFSRKDAPDKLQQTTSYEPP
TSDGLLQTEGGLQHEFEVTNLLKDSSEASDLRSVSSHWMKIWLKQWAF
>g604
MTNKRQTAISRSIHRALLREHDQTGTRRYNVLALIFAFLSIWQIRLVRISAQLFKSLRNEVWDIDE
VDYIDSFVYDKKKADGAGILPMGDLGYSGSTFFKTANSKFLIKSLPRRSEHSFFREDFLRPYYDYS
KSNPDSLLVRITDFLGTAYPAIGTLCQCTPSHHVIMENVLCDKDDDARGPDKWETYDLKPVDYFYP
ERDLLPEPLTSEETMSRLFDEFNDKIRITKQQYSEFRKTIEEDTKFLRSVNTVDYSLFLIRYPAHL
RPRTPTDRKSEWREGALSTDGNWKYRAVLLDFFWAKHKLQAQAMTGIIQTFNVIGRKGPMSITTTA
EEYREKFLEMVDGLVELPDER
>g605
MSSPLSTETIGSVLVPSAILIAGTFFVKQELVPYAVAVAAVFSAYRILTARPRKVLDPKEFQDFVL
KEKNLISHNVAIYRFALPRSTDILGLPIGQHISLQAQIAGNPTPVVRSYTPISSDHEAGYFDLLVK
TYPQGNISKYLDDLKVGQTMKVRGPKGAMVYTPNMSRHIGMIAGGTGITPMLQIIKAIIRGRPRNG
GNDTTKIDLIFANVNPEDILLKEELDKLAAEDDQFNIYYVLNNPPEGWKGGVGFVTADMIKEHLPA
PADDIRILLCGPPPMISAMKKSTEALGYKKASPVSKLQDQVFAF
>g606
MGSIEGEQPTEQKPTPFNLTEVDRQVLSQTDEEFVFHDWEDLKGIIARNDLAILRRKPSELRRYIA
WTNDIKSTFGSITKYICLKRLQWWSPDPSVNLIETPIPEIPYKNVVPFADPFDYKVLRNDWPYGVT
PDITHIVVWSKTPIATKPENGDVTDESRAFIEGFVDRTFVQRLQKDPAFENSEKEVIRKEHVLWFK
NWAALQSVRSLEHFHVLVRGVPDAVITEWTDERDVVR
>g607
MSVHETTADVENEAVDNKNARVLSTTNESTALGLKHSFPETARVIDHEAERALCRKFDFRLLPILA
FMYLCNALDKGNLGNAKTDGMDKDLGMTGSQYNLLLSIFFVPFVIFAPPIAMLAKRFSPAKVIACM
MFSFGSFTLLSASVSNYSGLFALRWFLGMSEAAFFPLVIYYQTTFYRRGELARRLAIFYAASNIAN
AFSGLLAFGVFQIKNPTLQGWKYLFLIEGAFTVCFSAFAFFYLPASPQQARFLSEEEKQLAFYRIQ
VDSSAVVNEEFNLRHALRILQRPTSYAFLAIEICLGVPLQGVALFLPQIVARLGYSTVKTNLYTVA
PNVTGAVMLLVLAFSSDAARLRSPFIVLGFIFTLIGFVIYAAINDVTAQIHLAYYACFMMTWGTSA
PSVLLSTWYNNNIADEGQRVLLTSIGVPLANLMGLVSSNVFRSQDAPKYQPALVTVACFGGAGALI
TACLGVYMWYDNMRRNRRAGRKIDARDVPTERLWDGPKMEDFRWFL
>g608
MKGTLASLAVVPLVAAIPQNLHIRDGMTPVTVKGNAFFQGDTRFYVRGVDYQPGGSSKLVDPLADA
ATCKRDIAKFTDLGLNTVRVYSVDNSVDHDECMNALADAGIYVVLDVNTPKYSINRADPEVSYNDV
YLQNVFATVEMFAKYDNTLAFFSGNEVINDGPSSKAAPFVKAVTRDIRQFIRSRNLRQIPVGYSAA
DIDTNRLQMAQFMNCGTDDERSDFFAFNDYSWCDPSSFQISGWDQKVKNFTGYGLPLFLSEYGCNT
NKRQFQEVSSLYSTEMTAVYSGGLVYEYSEEGSNYGLVEINGNDVTEKDDYTELKNALKQTPNPKG
DGNYNSTGGANGCPASDPPNWDVSNDALPAIPSPAKKYMTAGAGKGPGFSGSGSQDQGTKSTGSAT
AGSGTAADSDSVSTSKGAAANLRAPEITFTPVICSAVVALFTLFGASLVIL
>g609
MLATGPTGSEKKPAKRRVPDSQRRRTLVSCDRCKLRRIRCCRNNENEACTSCLNGGVKCESKMPRK

QRVYGSVERFSVRYRALDALVRGLFPNEDTDNIDVLFQLARNHNVPMPSPDDESHAPEAFSQDTTN
ATPKPSVDNSDLQAVEDDCSASEVVYERMIPGPRRILHYVGPSSSSEFASIIRHLVTRFKKITTPT
PYDQIQMHDKNYMKAMPDVHPLSGVAQTLANGHSLATAMGEEQQIDTKNEGPRRTTPHQQSQISLP
SSKRTSQDLSSNSETTNDLRSLLRDLLPDRSLCDVLVQAYLDHLHGAFQVFYKPIFQLRYNFLWDN
KIAQTGTSDIGWTCCLFMVIILGADILGPEHCPDSIIIQTKYMKLVRDSFQSLVFTATLENVQALL
LLQLYEQNSGERNTSWLILGLAIRMAITLGMHREGTYASFDNAEANTRRVVWWTLYQVEFNMSMIL
GRPPSINAKEVNVSLPADDILDGAGYPQGFEEHYLKLTNIGHRVRRLITTVTAKFADESALLGHQG
HVKMLLHELEVWKANLPHHFAPESPSVTLRHRRAVLLLQAIYYHHRSVLTRTFLVCKSSRNIDRLS
QEGNQTFGPVIPVTESFSRECRASAIAVLDCFLQLSKHGLLEGVAWIDFYYICHSVLVLGLFELGQ
MDESAPELEREQSSSKIAEMNEIISQIRLAPTYKIFTKVSLGFARTVGLGIVKSAPAEEIDTHQQY
IQPAQTSTLDFPYTSAPYGQMPWSTSEMNTTMFPPTTETTMSPPGLLGWFWREANSGSHMSWDIFN
LGNIQLSGTDYMEEPPQYSTMYTAQQQFEHWPS
>g610
MSAADYYQQDQVQPAAPRGQYGGQDQSHINYNGKEEKNNDYQQGYPPQQPPPQQYPPENPPSYDEL
FKIEKPKFNDIWAGLLLIAVFLGYVAVSGITINKYASTRGFNGGSIYGSSNDFGLDTNTLILFIFV
LCVALVLSWAYFLGARYFTKQFIWITGILNCVFAIGTAIYYLYRKQWGAGIVFLLFGVFAVICFIS
WIPRIPFSVIMLQTAIDVSRSYGHVFIVSAVGGLIGVALSAWFSVTLVSIYVAYEPNGGGTNPSCG
TGGCSSAKVIGLVVFVTFAMYWVSEWLKNTIHTTVAGIYGSWYFGRGAPPKGATRGALKRATTYSF
GSISLGSLLIALVNMMRQACSIAQQQEAAQGSMIGSIAFWILGCFIALLDWLVRFFNRYAFCHIAL
YGKAYIPAAKDTWTMMKDRGIDALINDCLIGPVLSMGSVFVSYVCALLAYLYLEFTRPSYNSSGTF
TAVIMAYAFVIGLQICQIFLTPIGSGVDTIFVAMAWNPEILMRDHPDIYHEIVRRYPRVQQMIHA
>g611
MDYIERILSFILQKRRGHDEGLIPRTAAFAHCPKPTITLECPNIGPSGSKIPSKYGFFEQGMFPRL
KWTLPANIQEQEVKEWFLAVEDPDAPLAIPVAHGLYYSIPAYRRSVMDTDFEKPPHPVPGDGSYSE
DEFELNGGXKYGLNRRKVVYIPPRGMLGHGPHRCFYEIVALSEPIDKTKLSSTGATREELVKQLEG
KVIAWGEWVGVWERT
>g612
MAEVNVNPQFGAELKDAXKPVNSWVSNGIAWLDDIQQFYRERSAIEREYASKLSALCKKYSERKSK
KISSLSVGDTPTMTPGSLESASLTTWSTQLTTVESHAAVRDKFGLDLISHVADPLKNISARYEELR
KSHVEYHGKLEKERESQLSELKKVKGKYDGVCQEVENRRKKTESSFDYNKTKAQTAYQQQLLEMSN
SKNTYIININIHVANKLKNQFYHEYVPEVLDSLGDLNETRVQKLNSFWSLAAQLEKGASTQSTELMTH
LENEIPRNNPKLDSLMFLQHNASQSQEPPNLTFEPSPVWHDDDQMITDESAKVFLRNMLMKSKSQV
RELKAEAEKQQREVESAKRIRDNVRQGKDKRDEVDVVKAIFNLQEKLHETERKKMTAEVETLTILA
VVGDLSLGAKNHNFRSQTFKIPTNCDLCGERIWGLSAKGFDCVDCGYTCHSKCQMKVPAECPGEQT
KEEKKKLKAERQEQAQSAPAVSEHAPSNGNNGTVADMPALTRRDTMNSLSSGYAHSAHRSVSGSIS
SSKPSGEEPAELSAAPPRSSTSTTKRNRILAPPPTQYATPPVAAEAPTSKKNEQRGKMVYGYQATG
DGEVTVSEGQEILVLEPDDGSGWMRVKAGSQEGLVPSAYAELAPAPSPALTERPASTYSNSSASLA
GSTTAKKVGPAVAPRRGAKKLQYVEALYDYEARSDAEHNMSEGDRFVLVTKDSGDGWAEVEKGGQV
KSVPANYIQEV
>g613
MASENANEGKSLADRITKPGADSTDAPLNAANKDGASEALGGSNLQEPEYDVEVKLSDLQADPNNP
LFSVKSFENLGLSPSILEGLYAMKFMKPSKIQERALPLLMQNPPTNMIGQSQSGTGKTAAFTLNIL
SRIDLSTEEMQKSPQALVLAPSRELARQIGGVISEMGRFVEGLSVGMAVPTEGKRPARLEHSVVCG
TPGTVMDLIKKRIMIVNKLKVLVLDEADNMLDQQGLGDQCIRVKAFLPKTIQVVLFSATFDDQIRL
YAAKFAPNANEISLKQEELTVEGIRQFYLDCVDDNDKYSVLVKFYGLLTVASSIIFVQTRSTAAEI
EKRMTAEGHTVASLTGAVEGSVRDKVIDDFREGRAKVLITTNVLARGIDVSTVSMVINYDIPEHHK
PGQPRTADPQTYLHRIGRTGRFGRVGVAISFVSSKEEWQMLMNISKYFNTNIEGLQTRDWDEVEKL
MKKIIKNPRARGDFPQSTAA
>g614
MSSPLPANARVSTNPFLESKLNHLRDRSLGSSAVRATTAEIARILAVEATQNHKPDHGKIALIVVL
RSGLGMSDAFLSQFAPDTDVVVYHIGMFREKYSLQPVEYYNKLPNNNTEIKRVYVIDPLIATGGTA
TAVIETLRDWGVVEHITFVSMLTSQQGLEHAAKVWPEGTEFVIGAIDPRLDASGYIEPGIGDIGDR

LFGTGLSH
>g615
MPESIPIATTTDDVPPGEDTGSSPKSPKSQFSKHILLTTYPGQNGVDPVLLQWGAPDAKSRGPIVA
SRHPSQLKRRNAMGAHGGSYSIYNALAIAAGDLPTDFKPDFNNTEPTFDFPVQPAWSDPKKIVSLD
PFGHDIVKHFKSYLDVGWDLRPSMAITRANMRLSEIEKAVSEGQIEVDGSIVVGKNGDVRVTKVAV
EPVWYLPGVAERFGVDEGTLRRTLFEHTGGSYPELITRPDLKVFLPPIGGLTVYIFGPPERVSDEN
VKLALRIHDECNGSDVFQSDICTCRPYLAFGIQEAIKEAQNGGSGVVIYFRKEGRALGEVVKYLVY
NARKRGGDTADKYFERTENIAGVRDMRFQALMPDILHWLGVKKIDRMLSMSNMKHDAIVAQGIKIL
ERIPIPEEMIPQDSRVEIDAKINAGYFTTGKTFTMEELAQVKGRGWEKWEDVTSLTTVNPQTQPND
IHAGRIFIASLHWNNEAILRADWNDAVVQLVSHLGAENVFVSVYESGSWDDSKGALRDLDARLDAL
GAPRNITLSEVTHEDEINAQPGEEGWIETSRGRRELRRIPYLARLRNWSLSILEGLAREGVTFDTI
LFLNDVVFTTQDVLTLLHTNGGSYAAACSLDFSKPPQYYDTFALRDSQGNEPLMQTWPYFRSSESL
NALLSMAPVPVTSCWNGMVAMPTTPFLSQSNPLRFRGIPDSLALHHLEGSECCLIHADNPLSATHG
VFLNPRVRVGYNRAAYEAVHPVPALGESFASLVFDAGILEDGYSGEA
>g616
MPVHYLILLSRQGKVRLAKWFTTLSPKDKAKIVKDVSQLVLSRRTRMCNFLEYKDQKVVYRRYASL
FFIAGCSATDNELISLEIVHRYVEQMDKYYGNVCELDIIFNFQKAYFILDELLLAGEMQESSKKNV
LRCISQQDSLEDIEYLPLPQIEEVSNALNFDTPDLHVVGGCDLYITKAASDDRKLYNKIEQSLESQ
YESLLKLSASLSPPKASAPNNVALSLNLSRSSPFGPLTEHSSRRTFAYLIATLNASHSDYDYSHVL
RPTDFRRERSLKKVMNTLDMTLHNLRPHMRDDGTPSPPSDSFSGSYSSSAGTAWGPRMWKTIDEEM
SLNDCAIFSYSPEDDINDDDDGAIWSHNYFFFNKQRKRVCYIYLRGIPIMSHDDDEIMAMWEEDGV
ATPVGKRAPEESYFTPDVTAMKRQRYWLGDLASDDDPISSTTRRKGAVRAMSEEMVDSMEV
>g617
MPIGNIYVIAATAVVGGGLFGFDIASVSAQLTEPAYLCYFNQGPDGPPFNDNPNCSGPTSLHQGGI
TAAMSAGSWLGALISGPVSDRIGRKWSIIIGCIVWIIGSTLTCAAQNIGMLVVGRIINGLAVGFES
AQVPVYISEISPPTQRGRFVGLQQWAITWGILIMYYISFGCSYIGGQTAQDYNTAAWRIPWGLQML
PAIFLGFAMTFLPESPRWLARQDRWEECHGVLTLVHGKGDPNHPFVQVELQDIKEMCEFERRHANV
TYLDLFKPNMLNRTVIGLFTQIWSQLTGMNCMMYYIGYIFTMAGYTGNAALLASSIQYVINVIMTL
PALIFIDRLGRRNLMLIGSTLMAIFMFANAGIMGAHGVIVPGGIKGVAAESMSVSGAPAKGLIACT
YLFVASFAPTWGPASWIYPPELFPLRLRGKGVALSTSGNWAFNTALGLFTPVAFANIRYQTYIIFG
VFNVAMTIHVFFGFPETAGKTLEETEAMFEDPHGIKYIGTPPWKTGVAFKRMAAIESGALDIKSTL
ADTHLHVEETPAGEEKAVEHSAAV
>g618
MPQQAPDESPVNNGIRSLMPAPTEMPATYTHPILSPPNTGRDVSSSPPESLSRPPLSVLQSYGSSI
SSSASVSKLLLRDYARTTDSSTTDQEIHVRETSVVEPQSAGTIPVQQQAEAQRLAPAATDKSVTSA
PTSPFLPLRSFRTHRQGNLSLDTIPRQTIMKALASRPPLNGTTPSFTLTSSLNGVLNGSTRPNAEE
TEQQEKDGTSNSQKLSAALCNLQSQSTGYSDKSSATARLALQSPCFFHQRFDDAVNIQKVLEEIAD
DEWLSHSRLVQTATGVREVSKQLQRRPIKRAVKTVMIVTKARDNRLVFLTRELAEWLLSTPRYGKD
TGITVYVDSKLRSSKRFDTPGLLAKDPRFGSMLKYWTPDLCWTSPEKFDLVLTLGGDGTVLFTSWL
FQRIVPPILSFSLGSLGFLTNFEFDKYKEHLNQVMGDGGMRVNLRMRFTCTVYRADRSKGAIPGDV
EEGEQFEVLNELVIDRGPSPYVSNLELYGDNELLTVVQADGCIFSTPTGSTAYSLSAGGSLIHPSI
PGILLTPICPHTLSFRPMVLSDTMLLRIAVPNLSRSTAYCSFDGKGRIELRQGDYVTVEASQYPFP
TVVSGGGEWFESVRRALRWNVRGAVQKGWNGDVDENAENGLDEENVDFDIDFDNATTGTDSGVGPS
EDGDTYGNGAGTVSEDLGLGHYTASLLSLNDLDISPDQPHADSSSFAAGASKDEVYAQVLEQATGL
VTGQRNWVTNLANVASLLWHGYASLPKPSSAVNWAGFYVRDDLFPLLASPKRSNSISGLTTTTIST
SYASSHDKKLLLGPFQGKPACQEIRFGRGVCGTAAQNKDTVVVPDVLEFPGHIACDADSRSEIVVP
IMFDGETVAIIDIDCTEPAGFDDVDKKYLEQLAQLLAESCDCDHGQAFDITAATSDTSGPALPGKP
TTSAPRITIVEDLSSACSPANMSDGGSVLAPSVDLTNMNMDHFYSPTVTSVASTSMSSLPMTSIPS
QDFHPLVTQFHQQHQQPLLAPKQHTPPSSSVSPALSHIHSIDLDNSNSKNTKKRSRAESAGLDLSS
NNSAQDSEEERIVEKRRRNTMAARRFRQRKQDHVSDLQSQLGKVTKERDDLKLQVAKWEGEVMALR
KLLEMKK
>g619

MIEPTATISPLGRSDGSAVYTCPATGFQILGSVNGPIELPARRDAQKPEEATIEVLVKPATSQSAI
GERYVENILKALLGKIILGREKGFARRGIVVTLLIQGSSSGGKLERGDSYLSTLPALLHAALLALL
SAAIPLSMTYTSTILAVDFSNQIIREPSLNDCAKAKSQHVLAFSSKGHLLLNESQGSFDFDTWNRV
YEQALSICRESEVAGQDGDVSMEAKQRLEKFVRETVEHKIASDYSWSDMAA
>g620
MTVIWGLDLHEMQWSKFKSSYMFTRVYHLRRVKMIVYQLAMILCVCSESVGTAALSDYVDQQNFIE
KQNHLAAEYNDDFVGIASYNIFVGIAVATIFGAAFFFDLFWPERHESPSVRLSWKISAIVVTVMCF
ADAIALTVIVATRHAYISGVDADEATRLLNASIKNPNLYYRHNPKCLASVILLWPGLIATIGSCYL
LITSQNHDDKHGPISRHGQDEQEAVRRAKTETQPNGEINGEKTTV
>g621
MGRKLRILGLHGYGTSGVIFSSQTTSFRRYLQQQHDQTGNGDTFDFEFANGFLDSGPAAGIELFYN
PPYYSWWPADPSLEDMHKARERLKQHIKQNGPYDGVIMFSQGCSLGASLLLEHYKNSPQNPPPFQF
AIFICGGPSLKQLEAEFGFTIDPELWEVDAASRRALARRADTAAILAQGSDRWSNDVNDITDYSIK
ELVQMVRGPYQIKIPTVHIVGSKDPRNLSGHQLHALSHPDITRIYEHDGGHDIPRNEATSTTIARL
VRWVSSSITAEKAKQII
>g622
MATKHQGYIKHSHQKVPESSPVDMALVYVPIMVLAGCALMVFFHRVWTEVTNNQRLKVAIQHDFKV
SINRPNRTKSWLNRHLLYAPLFDRRHNREFRIFRGHVNMGAIPTRLETLLIACYIALNVLFCVVVI
HWNHPISHSLHSLIGTTGTLAIVNIFPLVITAGRNNPLISVLRVSYDTFNLVHRWLGRIVVAEAVA
HTIAVLIRVGIIKGWDTIPDHLFHTPIFTYGFVAIIAFVAIFFQSLSPFRHAFYEFFLHFHIVLVT
ASFVALWYHLHKFVQRWIFLAAITAWGTDRLLRLAIVAWRNCGKTLTSATIQLLPGDVARVDVTVI
RPWKFKAGQYMYLYIPALGLWTSHPFSAAWESKDKQHGYFSATNSERSSNSSSRSILSGNRQEERR
TVSFLIRKRNGFTKKMLKSIGIEFGLQRRVLALAEGPFGAIHSFDSYGSTVLIAGGIGITHSISYL
RELVDGFARGSVATRRITLLWVVRSIDHLQWVQPWMAYILNHPVVQGSVAFPNTSTTPNSRNPYSL
DNGTHSERTLSLSVSVHLTTYYSNSNTNIFNTDPLPARYSALICNQEKSNNPYNNEIAPVLPAHTN
TSPRETPSFSRPHPNSRPPLVELSTFNASSSSSTLNKTIDQYFYQYDDEKARILTPVTDTNTNTNT
STPTNTNATTNSTFQDWTQLSTPTAPVTINLGKPLFRQVLEREMAQQIGAMAVSVCGPGGMGDEVR
KAVREVQGRKTVEFFEESFSW
>g623
MTQPSQFNGNRYLQPAPMEETVGPGVHPQDQDWSQWMQWDWDDTNPGAVPKREIDSSSLSPKFSNA
AVPAIDRVDFASDSLFPATNARKIAAEPRTQPDAQGLASLARRRNTGGAKPDNHSSRKRKTSSEDD
EGSVEAGDDSPPDSKPQASKKRSHNVIEKRYRANLNEKITELRDSIPSLHGLTRDTNGGSSIHDDD
FSTGNKLNKASILSKATEYIRHLEIRNSRLEEENKALKTRLRQLDKAVIIEQSASTSVGSASSPDS
YSGTIDSLESPPSVFSHADESPNFRKASTSTSPPEGMIKVPDYIKNMRAKAPTQGIFAESYIKDDP
KPQQQIGQARRGMTSGSKLFLGTIAGLMVLQNVQTEKESDEKGLLAVPLKMLNDFLANTFASTQRT
FLVWNQEMAPWSTWQFKAILSLLLTGGLAVASAFFVFLYLFYARPKSENDSAKIASAAGIRMTPAE
IKRQAWLTSMQRLGVPRHRFFLEWFAVTSRWVEYSVCCLIGRSVYSWITGITEEDEKGRIKTWDIA
IDAQLAGGDTEVSKSRLVLTIFASGTLPRSPGRIMLKAFHCRVVLWRVGEPGSFVSAVANKVGRVL
ATYQWNLARELNRSLPKDHPDALPSHLAALLEMECNDILLDPIVQRAVNLTWSRPTQEGLDEDEAL
LDVVAEDPAIQIFADSIAAWWSSHLLQNALLNSFDIEADGIYGRKGLEKQIDLALKVAPRLSAAHT
RAAAMRAVLFEQNRLHDIKTVLSALPSKKNHQQSQEVSNFLDSSIPMSVRNELAISVRCAMIAAII
KARMTNDTSFPSHLTIRKAINWLNELPLDPLELTLISFAAVYHLLHTVVAYGELLSSSSSASSSSP
SATSSDFSISTTKSAPPAASSLTKIDSSSSPPPPTHKRVRSNSASEPTPQYSRIANDLVYWARNAY
NPAFYGLTSTMVDTVEAECVSICKNAGIDLLSGDAKTLQSSIRRLIRKDDQKQRHKRRVSVSEKRK
GQEEEGQDISRRKSLESNDTGYASDSR
>g624
MEDPKSRASSQEKPAVSPNSTPDSAPKPLRRRRRVPIACASCRLRKTKCEHIVPRNNLQKHPGQDA
GAYDYIRSLEDKVKQYEAQLRSVHNNHHETSYQPNPPGPLLPAPAMHNTQQPITLPPLARPSLAPD
RSPAAITAASPSLFLGGDGAISFTQMILNAMDPDAPSRVQSTTQLFSSPRDPSREMPDAKLHDLPP
DVRELVQRYFDFHHILTPIFHIPSILPRLEQVLNMDRTRRYEDPLMLAMVNMICAISAAHRRNGFE
TSTVQTRKYYDRAMALVGSTVLFDWSVEKVQILLLGARYLQSSNFPDESWTLLGLAIRVAQGLELH
RPPPDNLDCIQKEVRKRLWYACYVTDQLASAIYGRPVSTASNTFTTPLPEDLDDDRISPSQLLYPP

TPTLSTMSHSIQGVKLCRIMESALTLVDPPLEKIVELDEQFEAWDAQLPSAFRVYEHEPVLDDKKL
IMAMRANMTRILIHRHSVVKSLGLLSRGERLIPLSDGLRANMMQNSRHICVRSAEDTIQLVGRRHD
TTKAVTGPSWFNLYYLFNAILIIASHVVDPEFRDDKEALAHLEDGMRMIAQMSANHTTARRAHIFL
RQLLDLVEKILPQQSRNLARKSSLSTTSTSSSMGPQTQPSVPTTTEGATNGFGSYSQPSREFMQLW
DSTVDLTTALGSQLDYYSSMGSGMWSWGNQAPDTRPYAAMPQSIAP
>g625
MTALKPGDSFPSDVVFSYIPYSEESGEITACGIPISYNASKEWADKKVVLFSVPGAFTPTCSVSHL
PGYIQNLPQLKAKGVDIVAVLAFNDPFVMSAWGKANKITDENFLFLSDPDAKFSKSIGWADAASGR
TGRYAVIIDHGKVTYADIETERGSLKASGADTVLAHLKMADDEEKAKAEKLAAARKRVAQMQKQKG
KKGTKKAAAGESSKDETVNEKPAGDDKPAVETHETEAADQTPEPAVEQEEKKEEEPLEDTPTEPMP
DAPTSPDPETSPDETKELPLRGAHKRQPSLSVQSKMRSSSFRNSISTAVSPGSSIKSPPLPPLAPG
DEQIHEVFRRQTARVEELEKENKRLEKEYSTANARREKAEEELEDLRESSVEVIELKGRLAKAEKQ
VAELESLKSEIASLQRQNSLLQSKTHRSSSGHAESPPSELVQQLESKSSTIEAMEIEISNLRAELS
TSSTSIDGFKSQITALEEKLSQSESALQKTQGELSDVKQSLSRASEKALKEGVDKTSTETLIKSLQ
REVEELQEAKSNTEKKAEGLDKKLQALSNLHKESESRHQSRLKDHEKVEKEVALLKKKLASVENEN
LRLREEKDRERKRNADVEGNEGLDELEDEERSRLERRIRDLEGENFELRRGVWKERKKELGVGADD
ENEGIETTSSPGNAFDEVDLVGGAPGHSRRRSLMAQSRQQQQHSSFTTVLSSGLAAFTGGNNNWDR
RPSTQHPPAARGSLELLAEEDNEEFDEDAFARAQAEEEARKRVEWAREVKRKLRDWKGWRLDLVDS
RAGAEGAGVAMGEIFEV
>g626
MGDSAEKLLADLEQSKTLPLWAQMSRLNPPEPNPHCIPFVWEYEKIRPNLLRAGNLITEKQAERRV
LMLVNPARDAPYTTDTLYAGLQLVMPNETAPAHRHTAFAMRYIIEGQGGFTAVHGRRIQMQKGDVI
LTPTWNYHDHGKDGSGPMIWLDGLDLPNFRHFPVHFVEHFDQLRYPAENIDTNSSPIVFPWVQMKA
RLDTEPGDWAVQRYLKADGREVSRVLGGAAERLNAGSSSPSRRETLSSVYHVISGSGYSTVGDSKL
KWKKGDTFAIPSWYKYQHFASPGETVYLYRFDDKPMITSLGFFREEGMDVEKLVSD
>g627
MRSYDVPLTLPVSLDVNFCLPPEQVTDKCMVSLNIILVGAGLGGLATAIALTQNGHRVTIYEQTPV
LGEVGAGIQIPSNSTRLLFKLGLKPYLSPYVTEPERISFRRWQDGRVIGNTELVPDFTKNYQAPYY
VIHRAHFHSALCERAKDMGIEIKLGAKAVDYDPIAGSITLADGTMHTADLIVAADGIRSVARKTVL
GGHDMPFHKPGFAAYRATVDVDRMRSDPEVSWILEKPSLNIWIGDGRHVMTYTIGAGTSFNMVLSH
PDSSDPLTWDPERAVEDMRREFQDWDPVLTKLINMVEKTMKWPLMTVSPLNRWISGKLLILGDAAH
AMLPYMSQGAAMAVEDGVGLARALSKMTSPDQLTDCLQLFETVRSLRSNQMQEASLLNGRLWHFPD
GPLQRARDAAMDIVLQSGERQIIDIPSPDADGYTSFQGATKQLAVQAAESSARAFASWSKSKPTER
RELLQKLAHIFKNKVKEIERICVEEIHCDPIYAQNIAQDGLDLIEECAALTTSAVLGSIPSVRGDA
YGLVFKEPLGVILGIAPWNAPIILGLRAVIAPIAAGNTAILKGSELSPRVHYFLASLFREAGFPPG
VVNFLLHGPSDGAEIFDVLIKHRAIRKCNFTGSTQVGRIIAAQAAMVLKPVLLELGGKNFVIVLDD
ANLDLAAQEIIKGAFLNNGQICMSTDLIIATKHVAVQLQENLLRLLQTIKRSGTVITAAAKIKLDA
LVKDAVDKGAKVYTAPVLDYSTNDSALSFIPTIITGLRQEMAFYETESFGPLCGMLTVEDEDGIIN
ITEQATYGLSAAIFSQNHYKALKLAGSIRSGAVHINSMTVHDEATLPHGGRGDSGWGXXGSQWGLE
EFLQTKTVTLHQ
>g628
MAHFPDLPQFSGFMKPCRFEGEVQHLEVYGDLPKDINGTFYRVMPDPHLPPRVENDPWFNGDGNVS
AFRITHGTVSFQQRYVRTEKFVREREAKQALAGKYRNKYTDAVEFAVRSTANTNIVHFNGQLLALK
EDSPPYAMDPETLETKGLYDFDGQLPSLTFTAHPKLDPVTKEMICFGYEAKGEGTLDICYYTVEPD
GRFRQTVWLVSPVCGLIHDFAVTENYVLFPVIPQLCDIERLKAQGEHWQWSPETPFYLGVLPRNGA
KSSDIKWFTYKTSFPGHTANAYENERGYIVFDLGLSDKNVFYWWPDAQGNSPDPHTIVTQLTRFTF
DPKTKDLNLPQPEILHPGNSEFYRIDDRMMSKEYSHSYFDLMDPSLGTDFARIAPVMGGGYPPYNS
LAHFNRKTGKTDVYFPGTTYLVQEPVFIPRAGSSQEGDGYVMALVNNYSTMLSELHVLDANDFSKA
KAIILLPLRLRPGLHGNWVENEQ
>g629
MTLAWKRLIRFEAIDGRILRGEPLLPEDKDIDLGFITETDQLRARILEGDDIYDTTGKTVLTKNTA
QVKRVLSPLAQSDVPILRCVGLNYAKHIKEAGRKPPPFPFIFFKANTTVLDHGEPVVIPKIAQNDQ

ADYEGELCIVLGKDAKDVSVEDALDYVAGYTVGNDISSRKLQRDPAYAGVIPQWGFSKGFDTFAPL
GPVLVAASEIPDPSQLHLKTIVDGEVRQDESVSDLLFDCKYCISYLSQGTTLQKGSVIMTGTPGGV
GAGLNPPKYLVPGTQMDVTISKIGTLRNNVVFA
>g630
MNYLHPTPTDSSGDPLRWPFWLRCTAVVVTSLANFVGNMAXAGISVAIPDLMQEFQKSQSNATQVL
TVHTPLYSGDASASAYDANHPSKFNFLFLGIGNIFWVPVAMKFGKRASMLSSMAMLAGMLAWSAAA
QTFNSLVAARCLIGFASAAGESIVPDIVADTSFTHQRGAMMAIYVLLISGGSAIGPLISGFMVANT
SDTWRSSVWLCFGMAILDLLLIFFFYPESNFDRPQDSFDQSQILQEIHRSAKNGNTQHEEFVEGSF
PSLPMETTYTIRTPPISEILRPVSYNPSVNLIQAMFEPLKLLAHPSVVWAIFTYAICLSPQVILIF
TMSPLLQAPPYLFTTSDVGLVQIAAIVGFILAFCGGGMLSDYINGVIVRRTASRLTIFRAEHRLIS
IIPGMLIGPAGCILLAFACGHQLHWIVIAVGFGLVSFGTVYTPNIAITYVTQRHQRDAAKCLILIN
ILKNLVAFVFLYVAVDWVNKNGYVQIYMIMFMLNILVLGCALPLYYFGLRNHEEFQ
>g631
MAQFQRKHVSRLVPTDHRLLMPLLLGVTIVNSATLGYDSSVMNGLLILPAYSDYFKLSTATEGLNN
AAMWMGGILGAFLMQPIPDHFGRRRAILIASIVTIVGLILQTASQNIGMFVVARIIVGIGSAISNG
AAPTLLGELLPPRQRARILGLFFSCFYVGSLASSIVNYGSQNIQGTWSWRLPSLLQLIPSLLAIII
VPFVPESPRWLIAHERNDEALEVLIIMQGKKKTDTQDGSEQLREIRDTLIHEAEEYPRNPWREIVS
TKGNRRRLFILCTFGPMINMFGNFIISYYLTKILSQAGITETVTQTQVQVIINCWSFAVAIIGSFM
LDILGRRVQLLIGISGMVSMLLLIGGLIKRYGGSSDTSGIYGTIAAIFLFQGFYAFSVCPMTSLYP
TEISPFKLRTTGIAIFRMLDSSFGLLASFAMSYAMADLGWKFYFINASWDFVFLIIVYFTYVETSK
LKLEEINARFERTQVLAGLVDDSSSGVVRRDTKDGEVVEKTREV
>g632
MPAPTRQWPAWTEYTHGGAKESGELEFLAVKKAIIAEYGTEALRRSWIKVCNELQKITDDIREKGN
KIIPVYNTADILKKGFTDAQRKEIRSIGACVCRGTVPEEDTNKLYHDLKQFLSDNEGHIKGWPKES
PSMLMLYNSPTQNTIRSHPNHLKLQRSLNELWHDSTGETSSDPLVYLDGLRDRAPGQPFLGLGPHI
DAGSLCRWADPTYRKVYDSIFSGEPEKFDPYDLSVRKDANQTLYAGEAHSQVLRTFQGWTALTPTA
PREGTIMFYPNVKAVIAYVLLRPFFQPPTGPGLDVMDAEKWTVDDSSGWFPGTFKPASQRLSRSSH
PHLKLEECLVYAPPVKAGDTVWWHCDLCHAVDTEHLGLNSASVAFIGASPTTKVNKDYVRTQLIST
LEGRPAPDYSEGNELDETKLKGYVGLDQLDTDARRAFGFDLLETS
>g633
MGENEAAHYLVHYHIFMARTAAAVYRFRTNLRFALGILETIKAVKTVDDELAEVIDSLPRLLQPET
NLEDEELHQLELANPWIKWQRYDVTLVLLHLRLRIHRTLQSHWLSAPDQYNWARSVSVSSAMSVIW
INRNWDQPASMRKQWYLCLFHVFLPVVTDRFRTLSYHIFVSAILLLRECQNELTNAEEYYEAVCTA
LSLLEEVQDWNAVANHAATILRVQMNGSASAINTV
>g634
MNGYTKHELDEDAFGEKENVGAGLKTFDAFPKTKPNFTTASRRGGQWTVIIFAICTFLTFGEFVNW
YRGTENQHFSVEKGVSRQLQMNIDMVVKMPCNDLRVNVQDASGDHIKAGVLLTKDGTNWELWNEKL
NQQSSNGVPEYQTLNEENVKRLMDQEEDVHARHVLSHTRRNPKRKFPKTPRLSSKYPTDSCRIYGS
LESNKVHGDFHITARGHGYNEVGQHLDHSKFNFTHMVTELSFGPHYPSLLNPLDKTVASTETHYYK
FQYFLNIVPTIYAKGNNAVEKYTANPAKAFKKSRNTIFTNQYSATSQSLSLPESPYNVPGIFFKYN
IEPILLFVSEERGSFLALVVRLVNVVSGVIVTGGWLYQLTGWATEVLRRRRRGGYSQGVLNGRTHS
AEDEE
>g635
MPPPPAPSARSRRQSHVPPPSSTSVSNRAPRKSVGPGIFTMDTPGESMPKRRPSLSMRKASVDNNR
PDPRYRLRSTNNNDSDSIGTTSARSLKTKSLQPPSRERLTQDSLLTPTAGDRTNGLLTPVKPAAGS
TNTPSSSSSSSKRMSTMPHATGLGARTISPTDARRMKRMSMMPQAPPVPITPPTAQPAPPEVLTLR
PRSSQSPSMIPRKSVTPSSSRTTPDPSRKSYSSGVSLSSNTSYNSARNSSGSLQPRLSQTFSSSRL
PTPKPRMENSNNGTEEVPPVPAIPKAYESPKTEAEIVFPTSARKSSLPLDIESSRGSDSSRPSTTV
NSTSESSAPAAPWMTGTRTPEAKGKQSAALGRKSLQPLKLPPLNLLPLSTPMAAKIEALNNNNNHQ
QNPSTPPPRTNIPKTPSTPMTASKASFFKNDEDEVEGTQLPRSSTSHFLVSTSNTTGFRAPSSSSA
IVPLEMPSLSRNMSPYISSSLPKSSSDFNFMRQHLSGEYSMAQPGTKLMGPRPQTQSSAFSLNTDT
GTRLSSTPDADASGATLKSKLPSTLRHSVSKPILASDTKVNAAKYDSMPPPRLPASATWNGLSAVA

TRTTSPTLKTSFMSNHRKSSLSSLNTKQSSFNNETTTYTMPSPAITDPGESETADNHRPASSILSP
VHKMINSAKSNAGPRPRNADPNVDLVELAADEEMRRLGSKRKDFEVAAKELDELRRRASPKERVGP
AQALKMASLNIFERGEIIDFKDIYFCGTQKAKKHVGDLKSQATNFGYDDERGDYQIVIGDHLAYRY
EVVDVLGKGSFGQVVRCVDHKTGQLVAVKIIRNKKRFHQQALVEVNILQKLKEWDPNKKHSVVHFT
QSFYFRGHLCISTELLGMNLYEFIKAHNFKGFSLKLIRQFTKQMLSTLVLLHSKRVIHCDLKPENI
LLAHPMRSEIKTIDFGSSCFDHEKVYTYIQSRFYRSPEVILGMSYGMAIDMWSLGCILAELYTGYP
IFPGENEQEQLACIMEIFGPPEKHLIDKSTRRKLFFDSLGKPRLTVSSKGRRRRPSSKDLRQSLKC
DDEPFLDFVARCLRWDPARRMNPNDAMNHEFITGVKPTGRPRAYLSSSNLVSKRLTTSGVPSSRPL
PEPPGIGGKVGLGFHKATSSSPVKPAAKRLSSVNNNMQQPAANKRISNSGTVSNGVGSALPRRNPS
GSLAAAAAATSLSNK
>g636
MNEYTADAVVNRDEPIPVVSFDSQNPSQSKLDKAKSRIPGLRHTGHGSKGDAQSGWSIQDRLFSKL
LQQVIPSEERDGEDVDNADKPPSKSVTRPPFSLPVMTNNFRRFNARIGIAFVFQARMIHLFGWRYP
TQTWSFLAVYSFICLNPYLLIVLLPASVLLYIMVPAFLARHPPPPSNSTLGTTPYYSFGGPALAPA
KTIKPASETSKDFFRNMGDLQNSMADFSNLHDALVASIAPLTNFSDEALSSTVFLLLTIVTASLFL
IAHLLPWRLIFFLGGNAAIISSHPTVHEFLNQIQRHKEEQVPDQKNQKPAKGGTAPKQPSPITQVL
GPLANISLDSAPEEREVEIFELQHRSISQFAPSSEWETLLFSPVPYDPLSPNRIAGDRPRSCRFFE
DVQAPSGWTWKGKKWELDLECREWVVERMITGVGFEVPGNGTSGNSVDEQIGGWVWDLPQVETEGS
VNDDEVSMMAYGDLPDASPSNASSVAKGKSKVQSGQGRDWDETMQAQRAGSWRRRRWVRVVHRVGM
SGSEKE
>g637
MGRELQKRKNRAKVPKLKKKRKLLRNGDKKINVLGNQLIADNWNKNETLIQNYRRLGLTTRLNAPT
GGVEKPHGVDAVTADKLTSDTLHITGNTAAAPNGTTLFDPEEVQVERDPDTGKILRVISGRNEEEA
DVIEVAGQKVKRSNPLSDPINDIENGISSLHKKAGLGTSSTSASEFVRQLEMRALREEEAVKKRRP
RQQSKREEEWVAKLVARHGDNILAMVRDKKLNPMQQSEGDIRRRVRIYKERRAA
>g638
MARQSMLFSWRACAFSMVLVIALSSFVSAKSQVPDKAAIASYDAAQIEEELQHCPLVQALNEHKLA
SRPETASWTSQIFAVLFPGSPAVNALLATLYISGPPNFLLALCPPNIDPSSLSIMVAFAVGGLLGD
TLFHLLPEIFLGEDSPEHARFVLVENNKNLLLGLGIMVGFLTFVAMDKTLRIATGGEGGHDHSHSH
NHDSAVATGSSPGKGNNGDVKKRKGKSSKSKAETGEKKGEINPSAKLGGYLNLIADFTHNITDGLA
LSSSFYASPTIGATTTVAVFFHEIPHEVGDFALLIQSGFSKRKAMGAQFVTAIGAFLGTFIGIAVQ
EFGGNTMSSSTSPEISRGLWGTGLSWGDMLLPFTAGTFLYVGTVAVIPELLETGKDKGVEIRKTIA
QFAAIAAGAGIMLWISWS
>g639
MMAAANDPRRTKDSNRHPVVFTTAASYTSCSSSHSSASYYPSSCSSISPPRNYYHQHLNTASPPSR
RSQANEHNSAREHLASLIRKHGPPTPVSSSSSLRKLTSTSPINTSFLPSDSVETPPASPAPIEEPY
ESTAGSQPIPIPRANLSRSFEDLPLTPLTGRFDHQYLAERPFKDSHSSSAHQRHSRNEKTSRKKKS
QPNSSSSASEHISDNHRSSRSKKSPMSGPSLTMSHQSGPVSPKPTAKDSPMYLGNLPRFHPAVYQSP
NSTPNASLSPNTSNHHPRSFGYRVSSAGSGSSREALRQYRELVAASVSLSRNATDISGAGISAPRL
DPLGSPGPVTPLALEEAESYLSGRSGDYTSPHQADGERERHGSARTKETKGR
>g640
MLFQLSAVTAVAFASAALAAPKNPGQCTSSTLPTQIRLAYAGDHGMAVSWNTKQQLSNPTVYFGKD
QRLNRVAQSDVSTTYPSSSTYNNHVVLTGLEPDTAYFYKPNCGNVTYTFTTARRPGQETEFSFAMV
GDMGTFGPDGLSTTVGKGAANPLKPGDLTTIQSLAARQSSYDFIWHVGDLAYADAWIKEEKSGYVS
PYNLTDNGAEYDRILNDFYNEIEELSSAKPYMVGPGNHEANCDNGSDLSVCVPGQLNFTGYRQHWN
MPSTESGGLENFWYSFDHGSVHFVMFNTETDFPNAPDEPGGEGAENAGPFAPSGAQLAWLEKDLAA
VDRKKTPWVVAAGHRPWYVSTTVCTECQAAFEPILIEYGVDLVLHGHKHFYERHSAVANNVTQEIT
DNPTAPWYVVNGAGGHYDGLDTPSTPYVSTSRDVIVAYGWSLFTVHNCSHLTTQFISSSNNTVLDT
ATLIKDHCHDDDSVGRIKMSTSSVSQTAASVLPENDHGRTITRTASSETKVNTILPAENVSVKNTA
KSKRDYKGFVAGVFSGIAKLSVGHPFDTIKIRMQTSEHGRFRGPLDCVMQTIRKEGFGGFYKGATP
PLVGWMIMDSVMLGSLTLYKRLLLENVFSNPQLRNSIPFTSSQRDLHTLPSFGHGIAGIMAGCTVS
FIAAPVEHVKARLQIQYAADKKQRMYSGPIDCSKKILRSHGIPGLFRGLYATLIFRSFFFFWWGSY

DILTRMMQKKTNLSAPAINFWAGGISAQIFWITSYPSDVVKNRLMTDPLGGSHGDGERRFPRWKDA
AVAVYRERGWRGYWRGFLPCFLRAFPANAMALVAFEVLAAILAIGFIFFLFDPTSSSTPITAQQRQ
EDAASHPLSPPTKPFPKKHAQGRNGQQAPPPVVHYKMNELTTSDASAAKGERVLILTPMMNFYQQY
WDNLVKLQYPHELISLGFILPKTNDGNAATLALQEAVSKTQSGPIDDRFASVTILRQDFEPPLQSQ
DEKERHKMENQKKRREAMSRARNSLLFTTLGPSTSWVLWLDSDIVETPPTLIEDLXSHNKPVIVPN
CYQRYFNSEKGEMDVRAYDYNSWIDSDMALQMAAEMGPDDVLFEGYAEIPTYRSLMVWMVSQDPQD
ADPHNEVELDGVGGTALMVKADVHRDGAMFPPFPFYHMLETEGFAKMAKRLGYTCWGLPDYFVYHY
NE
>g641
MVFFINTLAPLLLAGTALASPTSIPAARRFRGGGSTSYKNFTAPADEASAATLGTYTFTNWAGAFL
DTTDVTGVTGTFVVPTPSAPAGGDASTSYCGCAWVGLDGISNFCPNGALMQAGVNWCVENGVPSFV
AWYEWWPAEAQQYWDNISVSAGDSITVTVSAASTTGGTTTLTNNSNGQSVSFTWTGESPALCEVNA
EWIVEDYSVGNNLVAFADYGSVTFTGNSAVVGGVSTSDLSNSYIANMVSESDSSDVISASTVSGND
ITVVYQ
>g642
MAIENGHSNGHANGNGAVAPIDESIAVKANAIDKVPQLLEEIAKQGKALTQDGAQNRRQLLESVRS
LSYALETPREAMIRYCWSQPTIFAAIELGINLGLFKVLAKDDKPKTAVELAEATGAEPVFLSRILK
HLGAMGAIYEVGPNTYQPTNFAKTLTIQKYADSFPCMNDCINSAILAFPQWVKKNGYRAPTDGKNT
AMQLGFNTDLHFFEYLATNPEFPVRFMNHMSAYHQGRPSWMDPGFYPVQERLAAGLDTSAPLLVDV
GGSTGHDLQEFRRKHPEVQGKLVLQDLPEVIEKAKLLVIPEIEAVEHDFFKEQPIKGARAYYMHSI
LHDWPDNKCKEILTNLAQAMKPGYSKILINENVIPDMDADWQTTSLDVVMMTLFASQERTERQWHA
LVESAGLKIINIYTAEKGVESLIECELA
>g643
MSSSTSGYSAAYLKESKGVNILAPMWTLTMITTSMVIARIYIRIFGLAYLGLTTANVIVGYGRHAV
ALDLPHLEKAILLNTVGFFFGIFSFTIPKIAVAIMLIRILNPSKLHKYFIIGLVSFAACVVSICVV
LLFTECDPPASQWDKTITNYTCRDPWILIDFAVFTGALSAFIDLYLSIYPAIVLWKLNMSVKKRLA
LMCALGLGSIAAAMAIVKCTQLKGLADTADYTFGTWQLVIWTNIEADVVVMASCIPTLQPLLEYIL
RKSRGTSYNNKYFNRLEKDSSGMNNSQSIEMRAHRSNRSKQHSISITNVERGDGSQESILPPEAAQ
NQIRRTDNVTINYESRSEEPASSKMRAW
>g644
MVPPPRVPPAGIWAPAVTAFNSEDDTIDYESAKKYYAYLSRSGLVGLVILGTNAETFLLTREERAQ
LIKTAREAVGPDYPIMAXVGGHSTKQVLEYSQDAYDAGANYLLVLPPAYFGKQTTPAVIKRFFADV
ARKAALPVVLYNFPGVTNGVDLDSELITDIVNTSAASSADGTSNVVGVKLTCAQVGKITRLAGTFG
PDKFSVYGGQSDFLLGGLAVGSAGCIAAFANVFPKTISHIYKLYKEGKYAEALALHQKAALAESPS
KSGIATTKYAVAIYSAPKAGIENTEEKFLPRTPYEPPTEATKQLVKKVMAELAAIEDTL
>g645
MNTEYIPPPLPPPFAHTTPPPTLLAQGAEARLYKTTFLTSDTPAALKIRPTKPYRHELLDRRLTRQ
RVLHEARCLMKLVREGVSVPAVLALDWDPATPADGTRSVGAWMLMEWIDGLAVKHILERWEKWMKK
SITSNDSQFNKEEEEGKVKELMKKIGRTVGSMHKVGVVHGDLTTSNMILRPLDSAPSGVTESPSMM
GDIVLIDFGLASTSSAEEDRAVDLYVLERAFGSTHPMTEDFFSEILDGYAESFKGAKLVLKKLEDV
RMRGRKRSMLG
>g646
MKLILSTGNIMTXGPSVVRFQAEKSNVELINSLKSNFEAAQQTLPPQDEQSNGTKSTSWTKQEGDT
LYVPATDFALSGLSEERDQYDITVKLFYLPGIPASRRQAHTRDAIDLVLKELHVESIDLLIVSFPG
VSFDAEDEETEADDDEAGSRTGSESKSGSDSTEQADDFESMVKTWHALETLVENGKIAQLGVAEFG
TERLARXIQQTTVRPSVDQINVKDCCVVPKSLILYAKQEKIRLLTHNDCTNILPPGTTRDLLGSGK
DGAGVFASTPGAVDLQGEIEPQWVVKYTAVVKDRGVIENKGYFALAEVGSCIKTDDRS
>g647
MAWYSLLPDSMSTWETWIIRLFIFLGFLTIGPWAGLVIFDIVYYFARVIAYELPFFGGRANGEHKP
RAPTLTERPSGRRRAFSLRGEELASQASQTTTLEKEMMRRLVQTENEKTVI
>g648
MEQPESQVEIAKKLLETLHSLIVHFPENARNNVDKLMEMIDWPKQSQLLISVLLGCGLLFQIFLKY

RQVKTNKKSRFLESPGFPLPESLNDFDWATEEPVQLRPFKPKYHLTMALENLDPSELILMDKTYKE
RILYRRKILKEHRETVIAMHNESDPRAIAAVKEFYRYIMATYLPVRYPTMFRLHETAFETGKAYML
ENLVFNELYPAEVTEFTSPMRALEILYKTIDEDYLILLPDETDDNDPKNAKYRLVAYETCYPAGFN
PREKLGKLLADIHGPVPGYQEKLERSMDRHFANVEVGKYVKRVNWSISTNTELFAAFGGLHSSMNE
TQVEEKIKEGTLNVDSTLLRSERQTLHRLPTTRAMMFGFHTYTYPIKKIKEEGLGEDLATAIDGLK
EGSVPKIFGYKRGPVWGDAVKAYLRS
>g649
MLKSTGVGGVGTAFLQQLSKLPNAPSLILLARSSQTLLAPTPSXSPAIPAADWATASSAPSITTTG
TLTPTEVISYLSSAPGRSVLVDNTSDPKLASAYPDFLKAGISIVTPNKKGFSSDLSLWKDIFASAA
AGKALVYHESTVGAGLPVLSTLRDLVATGDEVTRIEGVFSGTLSFLFNTFAPVSAASGPSAKWSDV
VSQAKELGYTEPDPRDDLNGMDVARKLTILARIAGLEVQSPDSFPIESLIPAELASLPATSAGVTE
FMTRLPEFDGQMATLKEEAEKAGKVVRYVGSVDVGKKEVKVGLQQFPKDSAIAGLKGSDNIISFYT
KRYGSNPLIVQGAGAGGDVTAMGVTADLIKVLERL
>g650
MTSNDAALYGIQRPKNGPSQKDITSSSTLAFTSHLSSLISKDAAFRPSSSKSTKDDNYKLARGRAR
PSKGGKPDLFAVHNKGALKRAAKDEATGEEYDAHGNKNSRQLHQSSDTIGTVDAFTLQKSKRRMQE
KADLYENLRKGHHFAGGSSSDEDAEGGGGDEYTSRLRRKERNALVDFDRKWAEDEEKDADMQQEED
GDDGVGPLVDYEDEFGRSRRGTRAEAAQAARFRRQTQDAATVEEEDRSRPSRPSNLIYGETVQAHA
FNPDAAIASQMAYLASKRDRSATPPESLHYDAEAEVRTRGTGFYKFSKDAKEREQEMHDLLNVRRE
TEKEREERGQRKAERDRIKDERRAKIRELRGKRQADQFLEGLGVPV
>g651
MDLQVHRIISFTEDVAVGLRQFREQLPEYSAEITNVIAELYAISATLTSLEGLTRQFPRNFGPVKA
DFEQVLVSLRYTLNEIINSFGKLDRRRTAENYRQIWHDLNVYFHEESGYSLKRRLNKYKLFLEELQ
DVVQNKAIDYRFMHNLRKSILAILEEQDGRFAARLAGLSLGGGRPRSSTSSSNMSGNSFEAEGHGI
EKRRSYERTRSGFRPQPSPLHSPTSTNDSAPPWAPDVPGSPISTSTTTQSNLSSAVLNDHWAKDVF
SYRGPLMKFPVEGEGSKCLGEEVQDIKEWLHEQGFDEVAYLPFDEDVSIYFYVREDDSRARILCKA
RRNRRAKFYCLPLNMLEVRRIGPCLQLCRRRRSGTELVPWLNLHFESIEKMVLFFCTFIALRSQDG
HKRVQEIRDYELEAEKELFGGLIDDDNFIHALRVYRDRMSGAIRLQASVHEGDMRRAPVWTAFITH
HIDTPHWVRRVKTMVLLRDAKRVIFFPEYSPPKNNRGEHILKFTSEDDAKDFVDVIERLSITD
>g652
MGGFRAVEDRPTPKEVYNWRLYTEAIIIAFGSMLFGYDSAFVGTTIARTSFKHDFGITAANSSSVS
SNITSAFQAGAFFGALFCYFITERLGRKWALQISVFVFIIGAILMVVATSQLSFIYTGRVLTGLAC
GAITATVPSYIAELSVSSIRGILTGLFEVAYQIGSVIGFWINYGITQHIPATSSTSWRIPMAFQLV
PAGILFIGGFFLHESPLWLMRKEKYEEAYQALEDLRHLPKEHQYVQEDIAAMQNRLAEEAAIAAKY
GNGSWAFFRGAMAEFSRRGMRNRIFLVFCAFTLANLSGASAINYYSPTLFGSIGISDVALYTGIYG
LVKAIASMIFYIFLVDLVGRRRPVIVSSIACSLCLWFVGSYVKVGHPADIIAAGKQLSDSTVKGGQ
AATGMIMIYSIFWSFGLNGVPWIISGEIFPGALRNLSGTWAALVQWAIQFPVTKALPYIFTSFGYG
TWYFFASWMLVASVWAYFLVPETKGLTIDQMDYVFGYNTGAPYMIPHTSRTTKEVPRLIEDTQIEK
VVSV
>g653
MPKKHHATLYEVYNHVSNWRVIRPAKEKTQPYILGNFNSFALHPGGRQAILSHAGKEVTELFSMLH
PSDALDNLPPEACLGFVDPSTVPAQDKVLSEFDLQRRAAREIMPPAQSMLLLRDFEEWAQRVLSET
AWAYYRSASDEEHTFMENQNAFRRYFFRPRVLVRDMSTGSIETTFMGIESSMPIFISPAAMAKLGH
PLGEVNLTRAAGDWDIVQAISSNASCSVDEIFEASSEGQPLIFQLYLNKDRSQSETLIRKVEKLGA
KAIMFTVDTSWDSKXTLDSRAKAPTSSGESSATVATLPVNQAISGYQDRNLTWDDIAFIRRNTKLP
IIVKGIQSLEDVELCISHGVEGVIISNHGGRQADYAPAPIDILYEIRALRPELFEKTEIMIDGGIR
CGADVVKAIALGAKAVGLGRPFLYANGTHGEEGVRRVMEILHEEITNTMRNIGANTIEQLKPEMVG
PSGPWVGANRPPYSITLVFDNYVIVVALVFVIALTAAATEMITNGGVGLQLGVVALADMEYMFKLL
LVLQLLYAIGLLLVKTSILGLYWRLFGVKLGFRRAIYVVGAVVWAWAISSILETFLLCTPFEYNWN
PAVEGGKCANRNAAFIATGVLNLVTDLMVLSLPLSHIWALHLPLGQRLGLLVIFGLGMLISAISMI
RLVSLTQLDFANPTYTATMPLLWSVVETQLAVITANLPFMRQIFSLLLPQGWLGSHKPGYDQTTHP
YQRNESSRTAHNSYQMTAMYQGPKHGEVVSMAQSKVSDGGQGDDSDTELAARGVLPDGIYNTMAHS

VKSSEQLLVPWIYDTLLSIFRFCLSIFFREVYAPGAWRIPKKGPVIIVAAPHANQFVDSILLMRIL
KRYAGRRISFLIAEKSMREPYIGTLAAQMGALPVTRAMDNIKPGVGTIYLPHPEGDPTLVCGKGTN
FTDPMFMERGTIILPKVGQDAPEQQAIAEIVGPEELRLKAPFKKFARDHPLFESLRTGTSFKVAPH
IDQSQMFDAVYRGLESGGCIGIFPEGGSHDRPRLLPLKAGAAIIALGTLARSPDCGLTIIPCGMNY
FHPNKFRSRAVIEFGPPVEVHPDQVAAFKLGGNSKRNAVGSLLETIQEALDSVTQQAPDRESLMLI
QATRKLYKPLRMKLPLPVVIELNRRLLKGYTTFRHEPKVMQLAKDVRAYNRQLKALGVQDHQVEWG
NVRRRPWWLVFLTLLYRIGELLMMGLATLPALALFWPVFVTTRAISHSKQRKALANSSVKMDGRDV
VGSWKILVAMGFAPTLYTWYTVIVTGWLYYCRHDGHYASVVPWWVNARSYVPDSIPLRIFAFFFFG
LMISVSFTGLRIGEIGMDVFKSLPPLFIALNPSSSSALIKLRNQRRSVVAQVINVINTFGPEIFPD
FEHEKLVDDNYDYDGEMGVDMDYATYKSRLKSMPPSEPQTPNRSRSRSSGRRPSDSWQGFSSGNFL
KPLTSGVSGPSKDELSELNRRIGTFSESEQEKTRVVD
>g654
MASLSNLSDTIASTLANLPPQDQIQDGERMRLLKLVEQLQTALEPPFLHLQRFCFSHYEIVIIRIA
QGMGVFDAFVQTQGADLSLKELSSKVKGDEKLLKRVMRFLCSYKVFTQAVNDVYKPAPLAMMLGGL
PGDMVKHCHSIMQISAKLFEYFENCGYKNPEDAFDGPFQFAFNTKQHYFDWLQTQPNAQSEFNSVM
TFSQQLRGKDWFEIYPVAEKLNSSSGRALLVDIGGGVGHDVIAFKKRFPDVIGELVVQDLPQVIQS
INGALPDGIIAIGHNMFEPQPIRGARAYYLRTVLHDWPDKQALDVLARIREAMVEDSVLLINEHTT
PESLEVPIVSASLDFQMMGIFSSLERTEEQWISLLRRAQFKVVKAWKIDTIMARRVLGGGRVLGSG
KNLSPATASSTSSPQPKGRLSPSPSSVSLNSQASVSQYSPDPQDLTSRISLDNGETSISAAPAATG
AQLACPICNEEMVTLLQLNRHIDDAHQNLEEVKQDEVKDWFKAQMDKAKKFQPLAVLNQKLKGLDV
FESNDNPQPPSIAIARQATGPVEVKPPDPDEVVTRLIRDHTEDFTKFRKPTIDKAFLEVSRLEKRL
TRLTQALANLPADQIQSGASKRWPLGRQSDQRREIEQSIVSWQDDTSVPQCPFCQQEFTSYTFRRH
HCRTCGRVVCADPSTACSVEVGLAVASKRQTPSEKSIPSKINVDVRLCKECRATLFSRRDFDEQRL
QKPPVARSYDNLVQFERGIRLLQPRFQKLLTALQDPHNPPTPAQLTEASKVRKRLIDSFAQYDTAA
RRIRDLPTNSPTQQKLQKAIYQQASNFLHLHMLPLKSLPKILKHATPGGRPLPNSHSRSSLHADPS
STAKPFSSALASIKYGNNTPNGSSQSITSDNSSAISALEDEEKALRDRLIVLEEQKFFVSEMIADA
NKRRKFDEVSALALNVEDLSKEIDRINGILNNMDFESLYTGNAQS
>g655
MGLWSFFENRANRLHGEYRPLLPAYSADEQSPALPAKEVTKIALRLKYQIEQVIPYELQQSLITNP
NSSVITKDVIRTAKEAGGEEYRACVLFCLLVCQKWFHTEGLNALWDSPLCDCRALACQVIAKKLIE
SEEDQEWLHKEALLKRYSILRKGEVTPPVNVVERAVDMHALYIIGSSGYQKCIKGLWRGWLVQDDQ
EPFRFVDFENKSNISYWTHFDPDRMRVPLYQNALLIFFSLLYLALYTNVINTVNPDGDIDVAEGIL
YTMTLSYLCDEISKIIKIGKYYIGFWNVFNFTLFSLLAVSFVLRMAALAQSSDVNDAQRRHFNQMS
YNFLAFTAPMFWMRLLLFLDTFRFFGAMLVVLKVMMKESLIFFALLFVVLVGFFQGFIGLNNTELA
VPVTSKILKGMVNSIMQSPDFDAFEDFAPPFGILLYYVFNFIVMIVLLNILIALYNSSYEDISGNA
DDEYMALFSARTLQYVRAPDENVFIPPFNLIEILFLIVPFEWWLSTKSYERLNDIVMSIIYFPLLL
ITAYIETRDARWIRWNRRHGEADDTVRQEWEDLAHEVGFDGFDQSDEWVVAVGKTKPNVDVTVAEL
GIRELKEQVRQLTEMVEALTEKRRQDSTEAGS
>g656
MSLTNCRFYEEKYPEVDSFVMVNVKQIAEMGAYVKLLEYDNIDGMILLSELSRRRIRSIQKLIRIG
RNEVVVVLRVDKEKGYIDLSKRRVSPEDVVKCEERYNKSKLVHSIMRHVAEKTKTPIEELYQNIGW
PLNRNFGHAYDAFKLSITNPDVWNDVTFSNSIIKEELQSYISKKLTPAPTKVRADVEVTCFGYDGI
DAVKDALRCAEEKNXPDTQIKVKLVSPPLYVLTCQTLDKSLGIKLLEEAIQSIETKIKAANGGLVV
KMAPKAVTEHDDAKLQELMDKRERENMEVSGDESQSESDEGVPE
>g657
MSNGQPPQREDSSSLRERIVDILSPGSPSRRPSTDHNHGPSHGTSEDHDHDGLNETSRLLEDYDRR
HSVCGLPDCNHGTFSPKPEQQEPSSLAGSYLWGTRTPRFIPSGLQTPATDGTSVTNRLVAEMGLKN
SRRMYLSYYIPFVNWITQYRWEFLRGDLVAALTISSVYIPMALSLSSNLAHAPPINGLYSFIVQPL
LYAILGSSPQLIVGPEAAGSLLVGTVVKATVESGHSHEGDALMHAQIVGLVTCLSGAFILIAGISR
LGFLDNVLSRPFLRGFITAIGFVIMVDQLIPEMGLMERARGVNHASTVEKLIFIIDHGKYAHKLTT
IVAFSSFAIIMVFRTLKQKLSKRLPQVVYFPDRLIVVILSAVLTWYFEWDKKGLEVLGNVKPDESG
GSGLFQFHWPFRPSQMVHIRSSLSTSFMIALLGFFESSVAAKGLGDGDRDGIKGAPVSANREMVAL

GMANVTGGLFTALPAFGGYGRSKFNSSAGGRTQMSGIFLSLITLGVVVFFLDYLYYLPKAVLCAMI
SVVAYSLVEECPHDLRFFIQVRGWSELVLMILIFLSTIFYSLTMGIALGCGLSLLRVIRHATKPRI
QILGKVSGTPNQFENAELYPEKVEFIEGCLIVKIPEPLTFANTGDLKSRLRRLELYGTSRAHPALP
RVRPPGSDRNVIFDVHGVTSIDASGTQVLLEIVENYVNRGVRVFFCRLPSLNGSVFQLFERSGIVD
KCGGPTHFVPGVDEALRLTEIEDLQEGLV
>g658
MVIATPSQLFNTSWTAHRLSPLHHSKDHRVLIGNQDALKTYSKRLRDLLTGDFLRSMQVTLTKTVA
DDALSKAGALVDCRWEFISTNDYLDHNEEDDEDGNESQIEMGSCMGIFVTLEYEHIAYRAALLTTP
DGYAPESKDKRRKQSTHLPLLLTRMPTALRNTFTEFLTSSFDAYCSTLQLTPKFLSMILEATITYL
ARSGSSRQASSVILEQVLKETQLTLSFGREIAPGLKTLDVNLPRETLSTFARGARDKTTSHFSTSL
AQYMDQHLAMKVDLGNTKNDADDVQHVWLSKVATGTFVLNTEGRFKLIDTSTPQNQEEGQTNVQRL
TRSANEQILTSLVRRAVGEDMIT
>g659
MRRLFSSPKKASEGGHSSKEKSYKFGGLRKTKPFKIIEEDVQPTPTASYWDNLPVELQISIFMECG
IRDFLSLKLVCRGFNEVLTVHELAIAQKYLRRRRHGTLPSTTNNERTYTMNPEDDVVLLSDLFPPA
KSAKERDLMFERGTAQLQFKLIPLTYCVAYFLETYASARKEHSNHLLREYEAGRLPVPIPLRVRRD
MYIQLQTNILRSPPFNDTSTLIATHHCMYLLIMSLLDTLVGPRGHSERVDPRWIGGLLTVSGFGRI
VEYFSAEIGDGAQTRTQRKEFMTNFERDMDLNAREEMHPCVYEPPPSGQPHHSIVQEVWLDAAGRE
LSSRDVNPHDSERFVIWDEIPIFIGCNQCRDADGWMSF
>g660
MAETPNTSSTVDGLKAHACVLCQRRKVKCDRRQPCATCAKAWLVCEYREPLPPRRRKKKDSETTLA
ARVRHYEHALQKAGIDVSSIDAEEPLRMNTRNTVEAASIRNTPVSRDNHSIISTENSPGQGPGRLV
SRKGRSLYLDNHLWKSVSHELQDADDILPEDVSEKSAEPRIQNGEEFNSSFLFGGGKSRQVLTHCH
PNTIQIFQLWHTFLDGINPLTKIVHVPSLQQQILDAASDLGSLSPEMEALMFSIYSSALLSMQVDE
VQKYFKQSKSTLLARYRQYAQQALVNAGVLGTSELMVLQAFILFILSVRLVYNPHVLWSLSGICIR
IAQRIGLHRDGSKLGLSVFEAEMRRRIWWNLTVVDATIGHLAGCESHMLPLADTRLPSNINDSALD
PDMKEIPDESSGPTEMIFCLMSYELGAWLARQAESKTSSFDGFWEFLGSTSIPIQQKDGMIDELEQ
IFERKYISHCDSSIPLHFVTFVVAKSAVTSSRLRVHHPRQFQERGQPLPPAERDLLFNLCLRLLEF
ADPIFRSQKVQKFHWYLDFHFPWEALIIMLLVLRYRKTGQEVAKAWQLIDILFRRQQKIMTQRKRS
PLHLAVANLAIKAWSAHVTEAERSQVDTFPQPEFISLLWRYAHQDVPLPSTFSISSEATGERQQTS
SEHDIPTPTLTSSRSHINNSAQSTFGSFPISTEGNTYKQADANDGDDATNGAMEVEANLFNDSPFD
WDQWDALLQEFQDYNANDMNMLFPPPLS
>g661
MAEKSYRQLSTPDGTTPINEKPIQETNAAPKNAPDDHEYPGTLSLVAIMTALYLAIFLVALDRTII
ATAIPRITDDFHALNDIAWYGSAYLLTSCSFQLLYGRIYTFYSPKWVFLAAIILFEVGSAICGAAQ
TSTAFIVGRAIAGLGSCGIFSGCIVIITDAVPLQKRPMYTGLMGSLFGIASVVAPLMGGAFTDNVT
WRWCFYINLPIGGITILVILFILKASPPPNPSPANSWKERLHQLDPFGTATFLPGMICLLLALTWG
GETYPWNNGRIIALFILSGVLLIAFVLIQAWKGETASVPPRIAKKRTMMAGFFFTFCLGGSMMVVV
YYLPVWFQAIKGASAVKSGIMNIPLVLSLVLGSIIAGASVTNLGYFTPFMIAGSVVMSIGAGLLTT
FTTTTGHSKWIGYQVILGLGIGMGMQQSSVAAQTILERKDVPTGASIMMLAQSLGGSIFLAVAQTI
FTNGLSDNLRTEVQGPDAPDIINSIIAAGATGFRSLPELTSENLAAILSAYNKALTNTFYVCVGLA
SASIIGALATEWVSVKKQKHDKQANLKQVEGSCELDTLEKQKESV
>g662
MLSAALPVLSTAFPTQNTEKTPLPLIIWHGLGDSYQADGLKEVAQLAEEVNPGTYVHIIHLDESDN
GDRQATFIGNVTEQIAQVCEQLTSEPILSTAPAVNALGFSQGGQFLRAFVERCNFPKVHNLVTMGS
QHNGIFKFQSCESTGDWFCRSAEALLRFGRWSDFVQSRLVPAQYFRDVNEYDSYLEHSNFLADINN
ERELKNVVYKSNLESLNRFGMFMFSEDTTSVPKESAWFAEVNQTSDEITTLRDRSIYKEDWLGLRS
LDEQGKLDFKTIPGPHMALTEKVLTEVFTEYFGPVEVPDNSADLPRLVVQGA
>g663
MDSNDSAEGPHIIFRAENEAEVYRSRWIWSLSQFRETFNEWQNGEEDQTQDWWIASTAIPLVAATT
APLANLMSVVALVTSWRNEIHPNQLDSIGRTLQTGIKDSSWCMGLNASSLVFGVLGNLFLLLFNFTQ
KMRYIFALPASIGLWFLATGILAGIIASVHIYXPPVPPGELFSQGYWSAVIAAVLYFILAVMLMIN

MLGYVLGHYPEHFALTDDQRTLILQTTSFMIWLAIGAAMYQQLVDISFSDALYFSDVTVLTLGYGD
ITAGNDVARGLIFPYAVIGIIILALIVASISRFARELTNANVVKGHLHRRRVEVLKRSTTIDEEYE
RTPEGRKRKFQEAEKLAGRRRPLQNMVSNFVNPNRSRTIIMREEKDRFNAMRAIQDETLRFRRWMS
LFMSLVAFAIVWCGGAGVFSRLENITYFNALYFGFCSLLTIGYGDITPQSNGGRPFFIVWSLIAIP
TMTILISKMSDTILAGVDNATNYIAEFTVLPKEGRYVEFISQFPKVREWLERRAERIRIERGFTVG
EQDPENIDEASGKKKATTDYERAHPRPRTIEELEKHRRSPTRLARDLIFAIRRVTQDVVAGDIKRY
TYEEWVEFTQLIRFTNPNTKQQGPEVNIDENEWGLIEWDWMGENSPMMAEQTESEWVLNRLTESLL
RYMARIESQQDGKSQWRRSISIGDEHWGKDDEGRAESSSSSDLKSRKTASEPGNI
>g664
MPTQDAGLNSMAPSKVYKSSRRNRVPLSFRDERASCKYESPKNEPATFQHHGTKQGHSMQQRINHL
EDLVRNLIAQNHNAPITPEGVETGGSFKDSVIDASESTHSPGKTVIDGGHSVYKGAEDWYDILQEI
NKLKQVWSESQDDPTYNVAPTLSNMVDGSSLLFGQVQRTDKIELLTTLPPKHQVDKLIHHFFDRDN
FPITIYSEHWKDPTKTSVIWLGLLFSILSINMLAYQQFGEPPDYEGMSEPLVQLYRLRTAQCLIIG
DMAKCLPYTIETLRLYATAELNRKDDNSRGLWIMTGVIVRAAINMGYHRDPSQSPSLTPLQAEYRR
RVWISVMEMDDMASFVVGFPRMVSGNDFADTMEPRNLYDWELSDDLTVLPPSRPMSEVTPVAYLIS
KTRLFRALGSITDFNNTPTQGPYEQILDIDKNLTKAYQDLPADLRMNGQQWEYTVSGKKATGSNTQ
LELLYHQGMCQLHRRFIAKGRMDPQYNHSRERCISSALTLLRFQHIMEPFWYNFSRARKIIALSAM
ILFLELECRRKNSESNTFFPDTGELLTALSTSCALWEKAQTVCDDAQKTYKILSGMLSSFQTSVGS
SSTSQLETPRPLFEFAGMGSSIQPGTANFSDGKEWLPMSEDMNIDWASWDAFIEGAIFEDEV
>g665
MYDSTPHMTFVGSALYDVAQYPENIKDDVFTYYCTKSHVKVMNQYNISKLLLLYAVIKLSSSVDDS
IVINTLDPCFCKTGLGGESSGSAKVARAIFASLFARTAEEGSRLIVKAASTGRESHGGYMRAGELQ
AYAPVITSAEGVQRSEHVWGALCSKLERLQPGVLANVTAV
>g666
MSEIEKPVEEAPAVTSAGASNGAAAGQTTTKSPTFLDKLSMFTVGKNRGQLPTEKDVATESDENAA
PPPPLTFWQKVKLHFRRYWICHLIVLIILLAILLPCLFLLIIPAIAQDLLNSGSIVIDSASILQPT
NNSVILSIASHIYVPGPFTVHTDEEHLQLYVPQVGSDYPMALLNLPAASVHKNTSIGENGQYTVFE
NYTSWQNFVHNTIFLSAGSLGLKGKIGTQLGKIKKFTLDLDKQVPSNGLAQFSGFSIDSASLVLPA
ESDGTNLIANATLPNQSVLTLEIGNTTVDIKAGNLTIGKGIIENLYLKPGNNSVQIRGTANLTTIL
DNLSSLIAQQKEYIKNGYLSLTTQVTDISYNGSTVPYYTEEMGNLPLTAQTPLLGLLLNTLSGFLH
SGAANTTNLTSILKNYTSNRVTRDNTDDILNNATQLEVVARHHLERLVKL
>g667
MSFSSTATLPIRRASSRFTESYEFYSSDFVRLTGSLDSDSENEILQSLEKLQQVPKILQEGILLTG
GAVAILLQAAAHGMARTASKTGAGGKVKTLAEQLYQSIHSTALYLYGLTFGTRQQRKQILDRIYAS
QQQQQQQQQQQRRISHVARNGISHLSPYPQDPKLRLWIAATLYATGTEVYQRIVHELSFEEAEQAF
SEFTVLLNLTLNIPKGLWPANRAAFWDYWDEHVEKLDVDTRARPVVHDLEKFHNVPSWVKMSMPFM
KNITPEMLXPHVREQYGFQSSPTSRFRYKFSMGMGRAIYPVLPRAIRSIPKKRVMKEVEEMLRQ
>g668
MFLLRLLSFFVFLPFLTSTVTLAQSTTSTGLTVSSSALADIENLISGAKTLLSTDSINNIETTLTG
AATLLSGTTANDTKTLLTEVSALLSPELVSAVGQLVTADTVNKLGDIVNNAHTLLSAEFVNQTTTL
INDVTPLVDDLSKVLGGLLSALLG
>g669
MHSTILSILLLTFAVLSSAAIEKRDSVAPTTTSGSNFASISAILGSLASALSTATPAPGVTPVAVQ
TANLSNIAEPPAGFIPAIMTAVPLSVLGDLLDSSSRSSLAAEFQNGSTPAWYQSLPASIKSYIAEV
NSQIHAGALYANTSVAVATSSSTALAAQRAVAPTGVAMGVNVLGALGVAGLAMVL
>g670
MKIPILDAYKAGLGVPTVPLTTSGLLFEEIVDTHQNALKKPQLARYPKHGMQNETNIVSMGPGVIP
KHLYPFEDVSFESSIGMNDKCLAHNIPRSEKERIPNSCDLNSALSYGQATGLPQLAKMIREQVKIY
HDPPYADWGCVLTTGSTSSLDIALRMLTERGDSVLVEEYTYPTMIETSLPLGIKPVPLQMDEFGIV
PEAFDEMLRHWDSSIQGKVPRLLYTIPTGQNPTGVTVPPERRSLIYEIAQNWGIYILEDDPYHFIQ
FQKLNCDADIGQTLPKMVAKTLIQSYLNIDSDGRVLRMDTVSKTIAPGLRIGWITAPLEIIERVTR
IQETSVQSPSGFSQIFLLKLLDAWSDTGFANRILYLQSIFRDRRDEFEAAVQRHLPPGIITYAKPT

AGLFIWIKVNLQYYQYFRQRVPAVIENEIYDKAIEKGALVIPGSWFRASTDTEVQEVTFRLSFAPI
PAADITEMVKRFGAALRSFDPEQLQVGRIEQT
>g671
MAPSLSKLRSNLKSNEAKKKSHAIQKNAKTYGDDSKAPMNWPKSKKLTMVCLVILADLNDAMGSSI
FAPVVHEVSRDFHASSSSVASLLISVHVIGYIAGPLILSPLSEVYGRCPLIHGSNTTFVAATIICT
TSVNIPMIIIGRILMGFAGSVPATVGGGVISDIIPVDERGYAYGFLVFRRTKLTEDTQVQLAARFG
ELDDVTPWIKPGTVRRLNRTELMDMSNIKLDGTLASQDDMTTKLQKGNLLFHVDSSYNPRRASYSF
LLGKEIPPSGHGGQTAFADTRTAFDELPMDLKHELLENEYVACHSIHHSRKLAAPEYFGHIDPSKF
PMGRHRIVQQHEPSGRSNLYIASHIHHIEGLEFEKSKELVDRLMQHATQDKFVTQVEWKNIGDLII
WDNTCLMHRAVGGTYINKYKRDLRRAIVHDNSSWAWGLNDHREERQSLATLSQAS
>g672
MRECRSLEMMRTWHQDYGNTFQTILGRTLIVTIEPENIRAILSRKFEDFDLGHFRNKSLEPLLGTG
IFTSDGEAWKHSRALVRPNFQRNLVADVEVYEKHVSKLISCLPKDGSMVDLQDLFFRMTIDSATEF
LFGESVDSLDCSSSEGSDLFAQNFDISQKGLAIRTRLGPLMFLYRDKKFFQSTVEARQYVHRFVQK
AIAYRAAVDAGAESEDLAPRYVFLHELSKQTQDQKALTDQLLNILLAGRDTTASLLTTTFFILARR
PDVWKKLRAEVLILKGRRPSFEDLKSLKYLKWVINESLRLYPPVPVNSRTANKDTFLPVGGGPGGN
EPLFVPKGRKVLYSIYCMHRLPELFGLDATEFRPERWENLKPTWEFIPFNGGPRICIGQQFALTET
AYTVVRILQEFRDIESRDPQPFNEKLTLIITSKNGCKVSLTPS
>g673
MIDKTQSKLFQPLLIGDGKIRLDHRVVFAPLTRNRGEPSHRISTAQTPNRIWYPGDLMVEYYVQRT
TKGGLVISEGIPPSLESNGMPGVPGLFTQEQADGWRKVVDAVHKKGGYIYCQLWHAGRATIPQMSG
SPPVSASASVWDSPDECYSHIPVDETKPVRYADHPPVELSIEHIKKTIKDYCDAAEKAMHVGFDGV
EIHAGNGYLPEQFLSSNINKRTDEYGGSPEKRCRFVLELMDKLAKRIGEGNLAIRLSPFGLFNQAR
SEQRVETWTYLCKSLKYAHPALSYVSFIEPRYEQIFSYKEKDKFLASWGLADVTLDPFREIFGDTP
FFSAGGWDDTNPWGVIESGKYDALLYGRYFISNPDLVTRLKNGIPLAAYDRTRFYGPFKDNTIGYT
DYPVAEQIISE
>g674
MSGTDPPALDTPSGMTPDFDHPTDVLRTILLVTQCLCIPIVSIIVVLRLYVRIRFQQQLGVDEYFS
FAAWILFMAYCISAILLSSHGGGCYQWELYRSELVGFLRISYIATLFYAPMALFVKVALLTLLARV
FKPYRKWVILIHIILGVVLAYYVPAFIIKARICSPVSAYWNGTASGGSCLNQGRVIVADSVMSVVT
DLAILILPLPLTWSLQMPLEKKLKVMGLLSAGGLATGFSLYRLVMIVQDGQSPDQTMVFTRVVLTG
NAEGAVGLICACLPALNVLISRTHKGSSYLAKPESHSGSRLQLRKIRGSKSDYRPMVDKNVEDGTA
FGSDERVLISNVAASYSGNDFSDHTSFDTGIMKSVAVSQSVEVTNK
>g675
MRLCTREAKLHSLPYDVLYQVVEDLGLSDFNNLSRVNRSLYNLLQNDQLAKRTIKKHVPHSKEAQW
ATTGRISFRQAIGRIHDMQHAVAMAEPYSVAVLSYAATFIYKDGFLSYIIDNEIRVLNVHQPDNTE
QVVDIHNILRRIIPTYRQQEAADRSAQITFLHYSSGVLAFLVEIDGDEHAWLILLDVTPGCSKRLK
FIRQLEDTRRLFVRHNGVHLFYGVHSYVDASGELLWAIQWVDLTRNTKKDMPPTVLDNLAGTEIGH
NVCFEIFDDHLYAVSNTIDSEEEALNWRSFYEWIRIPPAFNRRRVRMQRIWRRDHLEGPINDTWTE
MSLRMDEATGQPMILECRREWREGRSDHVRTYYAEPLRPDEKAINGEVEVEAELAEPSVTQSPSSS
SFLTHPFPGSLIGPSNITLPAPLPLNGPSVEDLARHQRIGKHVHSEYTSSCCNRREFIMAKTKFRT
YNFSARSFVDLVNDPEPNNRVGGATDRLRLRINSRKRKCTSSDDHTAKRSRLDTTPLTSASPATGD
KDESDNVRLWPPDNADEDLINLLCPTRQATNIHAVADERSLIYSVDTEEPHKQAIILISFDPQIRL
PCMHSRRNDGAPSASTPVGISLLGPEDTKKSSRRQKCSIFSPCSSASAAAASSGNSKSSVRTEAAM
HLSIDKGYWFDVKRW
>g676
MASDPFFDALIARRTIYGISDASPIPDSRIEEIVTKAIKHIPSTFNVQSARAVILLKDEHKKFWDT
ADQFVKSALPDAVYQSLGPRIAGFRGGYGSVLFFEADDDLTALAAQHAAVAPFITEWSDTSSGMHQ
LAVWTAFAAEGLGASLQHYNFLPPLVEKIKEEYNLPKDWKLKAQLVFGTPVGGPLDKTTKPLEERV
KVFGQ
>g677
MKSLTEHSYRTENYPPPYTSLPPTPPQTPQSFGHGAPSSYQFQYSNCSGKRKALLIGINYFGQANQ

LKGCINDVTQMSIYLNSAYGYRREDMVILTDDQSNPMSQPTKANIIRAMHWLVKDAMPNDSLFIHF
SGHGGRTPDLDGDEDDGFDDVIYPVDYKVAGHIVDDDMHDIMVKPLRPGVRLTAIFDSCHSGTALD
LPFIYSTQGVLKEPNLAKEAAQDLFSAFTAYGKGDLGGVAQTAIGFFKKASIGDSARERRMMTKTS
PADVVMFSGSKDTQTSADTFQGGQARGALSWAFIETLRQYPKQSYLQLLNNIRAKLERNYSQKPQL
SCSHPLDVHLLFVM
>g678
MADNHNDDDLGAGNLFADPEGFYPEEEQPTFSSHQMLSGQTIRVRLVGSHPLYGNMLWNAGRISAE
YLEQNAPRLVANKNVLEIGAAAGVPSIIAAIKGARTVVMTDYSDPDLVDNMRRNADAAAPMIPEGS
QLHVAGYKWGADTDEIMSFLPGDSTTTKAFDTLIMADVVYSHREHPNLIKTMQETLKKSRDAVALV
IFTPYQPWLLPKTEKFFPLAEENGFVVTKVFEKVLDKVLFEDDPGDETLRRTVFGYEIRWAEENL
>g679
MNNNRYNLRSSAQPQIAPSFYPTSSTTGTATTGAGSTSTTTSTTGVSSSSFLPTQSTFQSGQAQQF
QAQPGQFIGQQAQQQQHLFADTQAHAHAQAQQNMNTILPTQAQVQPGQMTLGGPAATAPFLQDFTL
VAEAVKRVQMDAVMTEMESITL
>g680
MADESETMMSATLRFPFQASLDGYCMVETDGNLYYGLDISLEPAELPAQDCSHPSSEPDTHLGLGL
DSDDDPEDEDKQQQEEQPSESKALLHPPCWDSQVKSQQQEQAPDVAPRPVSIVMHPHRIDTSDRQR
FATDHEMAAAEEVEEENIICESPSSSTLDETEAEESTIRVSAPSSSVQRLIPSRYYATPLIEDDLL
STPVAVYGLEKPSGHVXKKKXPAVLRQRGSPGAAQARPSGANQALYVIYKETVNVFGENTYTNRGV
FNWCEQTNYRINIVVRVFGYYCNNEVSSICXTNVVFPIYDPRPGPDIIIRGDARRNSVFNFSMPKL
SPMMHHRAGIQSSENLTTIRAVRDDDQVTITNHPLHDPSPEASSPSSSKEMPFSPDSFVSPHTNAT
PQSHNTTTNPPSDSIAYNNTTSFSQINRPHTSHHDQRPPPIPHTKNEQQPPHSKLRKLKKSDHQQH
RATKLFTRIFVTDIKQKA
>g681
MPLIILTGYPCSGLSYRAKQLAQLLEETQTQLFDALDNENNATGDDGNQEKSKQKRFTFHIVPSHD
PGHPRTVYDHARTEKEARAVAYARVKRALAKDAFVILDGMNYIKGYRYQLWCEAKAVGTTCCVVHI
GTPIDKCVAINNSRFKNQQSAQDTLPETNATTDDLAEPDTEEPYPSDLHQNLIFRYEEPSTHSRWD
KPLFTVPWSDARPPVEEIWTAATGIEIEKTFLSDAPETTPTSTISPIAALLHTDPNNPSPSPLTAA
IQQQASDTASIAYTIRTTNTQALNGGRLGRPRIKSHQATVLPTQTDSGALYNYEKKTSAIVAAIRN
YTSSTPSAEAALAQSTKQEGGVKRGDEEGISISVPDATIPVFIPAHIVRSAPTDDLAGAGGLLTLP
RLQRLRRQWISQNRTYSGMTNKQAKGALGAEQIGDAFVRFLNFELAGNTPE
>g682
MESSPIGTQPETPEVTMLSYSFSQQLIPGATGINDSAAMFHSLSDIQAAGGLGEHTEMDYSHPHHF
AQAFASSLFEYNYENDEATQRCMHDDSASLGHNSPHEDGQLFSTQDTWNLTLDTTNLVGAQYDQIS
HIYSPPVSPPLTEASQVSVTSACSQPAYYQVNEEKINIQDSFHPMSPPLNDQDPNRPTKQSQRPLL
SASSTRPTRKSDPEAYALPIGESVPGAKDKTEVRTPRDHPYYSLSTQSDGKYYCPFATGENPCNHT
PTTQKCAYHKYLDSHLKPYRCKVATCAAQNLHFSSNACLFRHEREAHGFHGHGDNPHLCFYPGCER
AVPGYGFPRRWNLFDHMKRVHDYTGSEQPSSPESNAVGQVLKKKDSAGVRKRKVTKPVSATTMKRT
KSASSQSNAVKVNPAQHEQRLRNAEQNYYDCRTRLLEKLASIKPEDTVMHEKANASLQELITLGLN
YRQLEASSAATTFSNV
>g683
MSSNENSGSSTLQSYVDSAVGAAQNVLSSITGNPADQAEANASKSQAEQEYQNSHTTARVGPFTAD
PNTGAAVSDNQDRTSGSWNQTVGAAKESFGNLIGNENLRQSGIQQNADGKAQQAKGQLSDLGQGVT
DRARGAVGAVGSAVLGDQEGQQKWQDVHDEGKTRQRGVEADLQKTEQR
>g684
MLPPIPVLSDYGIDPQRGFLPPELPLTSLPDPYYAKWETVIEKVQALILSRRIRTSVERLPVLSTA
YLHTEAEWQRAYVVLTLLLHAYVWGGDVPEEIIPPPLSIPLLEVCEHLDLPPVATYAAVCLWNYKP
IFPDESADDLDNLAVINTLTGSTDEQWFYLVSVAIEARGAPMLPLMLEAIAAARRDNSAVVTSCLQ
ELAQCITEITVIMQRLFEHCDPHVFYHKVRPFLAGSKNMGDAGLPRGVMFDVGTGATEFRQYGGGS
NAQSSLIQFFDIVLGVEHRPTGVGRDGAGAHSDTEGVAPKPRHNFIQEMRQYMPPDHRRFLEHIGS
VSNIREYVDAHRSDKALCLAYDASLAMLRDLRDKHIQVVSRYIIVKSREQRRNSLSNPQPPTTTTR
PIVNLANVKSGNNKKLRGTGGTALIPFLKQARDETGEPAIDAWARRLLSNGPADRSFATLGKVGEH

ADGQIEIVGMAGTWAMDESEGGICHW
>g685
MSHMASNALRQASRSCASRRLSSSFLRVSSPLAGRMVTGRRYVSESKVGNAQVSVDTAIKAEQRKF
MESTGIQPQNAELSASSVSGDAAMSPTAGILKQATIMDEGNXPIYLDMXATTPTDPRVLDAMLPFL
TGLYGNPHSRTHAYGWETEQAVEDARAHVASLIGADPKEIIFTSGATESNNMSLKGVARFFGRSGK
KKHIITTQTEHKCVLDSCRHLQDEGFEVTYLPVQNNGLIKMEDLKAALRPETAIVSIMTVNNEIGV
IQPVEEIGKLCRANKTFFHTDAAQAVGKIPIDVNKWNVDLMSISGHKIYGPKGIGACYVRRRPRVR
LDPLITGGGQERGLRSGTLAPHLVVGLGEACRIAKADMDYDTAHVKRLSKRLLDGLLAMEHTSQNG
DPERHYPGCVNVSFAYIEGESLLMALKDIALSSGSACTSASLEPSYVLRALGNSDENAHSSIRFGI
GRFTTEAEIDYVLKAVTDRVSFLRELSPLWELVQEGIALDTIEWSQH
>g686
MSLKGLGKSLSRAPQTFKARFNLGENTKDPVYIDAERRFQELERETKKLHDDSKKYFDAINGMLNH
QIEFSKAMTELYKPISGRASDPSTYTIEGNPAGIQACEEYEAIVRDLQETLAPELEMIETRVISPA
NELLDIIKIVRKVTVKREHKKLDYDRHRATLKKLEEKKDKSLKDEKALYKAENDVDQATQEYNHYN
DLLKDELPKLFQLEAEFIGPLFQSFYYMQLNVFYTLHEKMQGINIGYFDLTRDIEEAYEAKKGDAK
EKAEELTIVHYKTTGGRKTSRFASAKDKLASENRLSSSSTYRRSTDTNEKPPPPYSPSGAPGTTSI
GRANSTGSALAAAAKGKPAPPPPKPKPGYLAKNVETVTALYDYEAQAHGDLSFSAGDVIEIIQRTS
NENEWWTGKVNGREGQFPGNYVKLN
>g687
MILVDAMQKHDVLLETLFACLLLIVCEVLRGSDVAAQYHLDGAIRLICMPSVAKSQLFLANNNSNS
NRSALSPFGLLKEVSLIFQQLDLQAAAFSGPRVPINPEDIGSITQYTKINHQGAQIAGKDVKSKST
IKELRQSLQSIQAKISRFVDSKKVRECKYRPRLKQPQKQKDELNAAQAMQENLHEQLEHWKTNLDT
ISPSPKEGLNSIKQCATIVMLLSYLTSYVLLSTSLSPDETAYDSHSATFARIIELSEIFSQQHLAQ
DTPLFFTFFRIDMKVVYPLYITALKCRDRTMRYRAVRLLSMCGREGVWDGRMMASIARSVITCEEE
FAAKLASTSETDDMAIDSIPERARIHGVGIVEMDRERGDVKLICSKKTGMMAVDNQIGWEKVRLHS
KF
>g688
MDIFTSGFEEPVPPVIKSTYNTSFIQHKWNSNLSHITSGYIYNLPDQAKVIATEAYAGNLATSTFN
YANISQDGLVDNAVIYIPIDGDDTNREMFRDYVNPGFPLIKDDFLVSNGAAFGGLVSRELLPGKVA
TWNIIYQNAIPVTVYVDNCNVVVGYDFFSPNERTYVITMYFNTETS
>g689
MDKESDTSIQVPPLKPQPDYVDFDGPDDPLHPWNWPIGQRRLISVILALSTMIVAGASAIFSSAIP
SIMVIHGISREVGTLGVSLYVLGFATGPMIWASFSELKGRYLPFTISMFGFSVFCFATARSTHDLA
SIFICRFFSGLFGAGPLTLAGAVFTDMYMPRERNTMIMLFSLTVFIGPMLSQPIGGFIVSNASLGW
EWTEYLPGILGSAAFIACVIFQKESYGPIILARKADRLRRETGDWSIIAKHDTILLEPKTIANEFL
FLPLRMLASDPIMLFMCTFGAFVYGLLYLFLTAYPAVFQGVHGMKPGISGLPFLGIVVGQLFFVVI
NGLFQKVWLXPRMVANGGQMEPEWVLPIAIPGAASFSAALFWFGWSGYKRDVHWMVPTASGIFSGF
GLLAMFLPSISYLVLARPQRAASAVAAHTFLRSLFGAGFPLFATYMYQNLGIEWASTLLGCLAALM
IPIPLLLYIYGAKIRQRVNIDY
>g690
MPFDPYEQNITLTASDGSTMDIPLPYFDDYVQEGVVSCINYGCQLGATIVTFVLLCLLTRSKKRSS
VVFCLNVTALLLNMFRLICEVLIYTSAWFETFAYFTGDISRVTQGDFANSVLVSVMDALLQVVMEA
SLVVQTRVVSTNMSCWQKNSILVVSIGLAVSSIGFRMAQMVLESMLIVKNKPFNSDIWLQKLNTIL
TTTSIIYFSVVLICKLGYTIYRRKQLGVHQFSPMQIIFIMSCQTMIVPAVFSILQFSSVNIHHINN
DIVTFVVVSLPVTTLWAATALPGHAAVHDAESGMPLWNKLSTSRLGSVGSDRKKSASTSETITFPS
TVNFQGLEMAKQRGSCSHVNNRDFSNSSSYNQCVSGNMAAVPQSTLTWLWNVLTKNHHDPKQTYRD
PNRTYHDVARALAQYPSFGPRTDVYTYENGTPSLLLHLVGTLPVSFRGNSYNIPIDTWIPSTYPLE
APIVYVTPTPDMVVRSGQHVTLEGRVYHHYLAHWHEAWDRSSIVELFGVLRDIFSKEPPVKGRPQS
RPPQPETPRSGPPPVPPLPPELSTSPRPVEPPSRQHQQTPPRPPQLPPKPGQTARVHSPPTAHTPG
PAIPPLPPKPQNGHIQEWRTPINAATAQLNRSSSLRTQPAPVNPHYQQDPYANTSPVSVQSHGAYP
HHQIQSPRQYSTPQSAHGYQQNVHQQQYPHQAPVPYPAHQNQQPPYQTPPQAMTAPKXPKPQTPDL
LTSPFDVELPSITPTGPPPPVPPNPEKDALIHTLSRTLTENLQGNVSQSHSAVTSLQSQSQALSSA

MMTLQGELATLNQFKVTIQSNISILQQSLHRADAVIADAKARTDQNGTGTTTASGLPAIDDVLVAP
TVVGKQLYDLVADERGIQQAIYALQAALVKGVIGADTWSRHTRSLAREAFIKRALIRKIALGMGLD
LGEDALRPES
>g691
MPTWFLPPDFTFTSEGPIKLGTVLEHPSRPTLVLASLPSDTGIVLPEESIIMESNHSHERSTSRSG
SLNVWTKFLEMASASVSTTAGHSFVESYSAVDHEIHSFTGPLTVSTAAAITALHDVKKYIDSGLFG
KKSVYIVTGLRIAKDSFKVMKEVASNFSGEVSGSALPTGGTAPIEAGSDLSGSSERRVTDSYETAP
GIVFAYRLSIIRTKRAGVEVELFSDKGAFLTGDGTHAELPLVVVEATKEELEEDLEEQISFEASIV
GEYEYCISF
>g692
MAIKNRALSSELLSPVAEEVWKPIFEVARTSKPPSSEGANIAIHLHLVAEAFKRQSLYHLTSEIQR
QCDLVLRDIFNICTWPLSSSSYHNPKETSAQYLYTELSRKFRHQPVQDEMINSISKFLLQGRSLIR
RYSQRLENLSVELDRIDQNDADESAVSSQYLSPRNGDEYPDHVNRYLIDALKEHSTCYPESHAVKY
RDMAKWHHTRISLSNGVLADERFAEVELMIASQGMTLWQDISLYIPLDHSMNSNDETDHDMDLIPI
TQGAVCQMLEEKSYNRIRLRLDMNHQLHKFRDPEFLRYFISPGDGVQLSYILHHCNLSVEHKITLA
WTSDSIWFMPLDNHNKNQIPLRAYVSIPFHQQDQSLAEYLETGDYTHRYPRILFLGIILLEIGLGK
SLGIEPFETSNLSLVAHTNKAHSKAQMRLNEFKKISWTDFSYKDVFVEAPLTANTSIPKERRIALY
RKVVAPLCWLANVGFEASREVPLITVQNLQRPKSTVLEDETTRAFWTSVKDPAFDTTNSVSQAGSW
IDRLKNATISDCFKDWKDFAADSQISVDTFGHGTFMACLLLQVAPIVDLYIARIAVAQDQLELSED
NVVQAIKHAGFTWKADVVSMSFSFPKPSVAISSAIESVMRKSEKPIIFLGSAGNSSSDRGEAFPAR
HPDVIPIYATDHQGAFLRSNPTRSGDGPRVLGTYGDNIPELIYAEMKQFFPLGDFSPGTSVSTAVA
AGIVAMMLSYSAALPALIQATLFEEILEKLRTPKGMTNMLRVMSQNTDHRGDFVNPIRFWSDKSKD
KEVFLAICKALAE
>g693
MASRRVEEASTIKATKSCFEHFTHCIRELEGAQNTHIKNRCADFKLWADSVGAGAQGQASLDWRFH
DRPDDIFLVQGLLGMLEEFLKECSAVLEDQKEVEKVLHKIDSIMHDLISMGVAIRRSGRKSRLHKA
DSSFDRDRKKYCDLQTHLAIVIVSRPSEGPRSLSESDSFADIKLSPIQNRLIEANLRRRHRFMEAQ
RHSNLLKSPTLGIFESPSLQEVSDILATVGADSSLEHETHPNEPALRQLLPVTAARAETLTMSGTI
ASAPETDFKGLQHDGHAGSTATRITAITAAARYPKAKTLGTQHFSFKCPCCCQAIPAKEAEENRFR
KHLASDICPYTCIFDNCPTPYELFVTQKEWKEHFMHVHPPKFQCFYCSKAPLSSLAEIMNHIEIDH
PSISDDELADALAESPIHVMGITQCPLCDSEGSPDSPDLIEHVLEHIHDFSLRSLPWPKDPVPHLN
KAVGTFNIAVQNVGTIIEWAEESSPENECQLQLCGIDMKSLTEEEVAYNESQLDYFDQNDYFMEES
SDGRLSSLSGQSYLSRSESQHSSIRGDDRQNASHDSAEYLHDGATIRIKNAYHGLVQVDYQFSEKT
TAGQF
>g694
MEETWYQLCVQIRDITNHDIEEFGKRIRKDVINPMRTVFNLLKGAQKVMNERERRLTDWIQFMEIK
RRGDLPDKKITEKGEKFIVLNNALIVGLPKLFRLVEDLMQTCLKIFVAIQQEWYLTIHRKLCSLVA
DIPWDFSEVVRSWSARFGTVNAQIESLRLFENLFKQI
>g695
MTAADFASTSTLSAAEKDNLAAQIRAAIDAKASELWKINQQIHQNPELGFEEFKAHDNITNMLESL
GFQVTRHAYGLDTAFVAEYGQGGRVVAFNAEYDALPGIGHACGHNLIAMMSIAAFIGVAENLKKLQ
QTTGQVVPGRVRLIGTPAEEGFGGKILIINAGAYKDVDACLMTHPGPFTECPGFTGDAYMPTLASL
KLSVEYTGKTAHAAMAPWEGVNALDAAVLAYNGIGILRQQIHPSNRVHCIVSKGGDRPNIIPGNAA
LDCYIRSPSVKIAEELLERIKNCFEGAALQAGCTMDLKMKNTYADLRPNKALAVVYAQNMANLGSQ
VRCDLSSAGVPGSTDQGNVTYVCPAFQAYVGVPAQPGCNNHTPGFTAVAGTRESHDLCLQAAKGMA
ITGWNILTDEVVAERIWADFEEDKAVRDVDSHTGMDRRILSGNAGFC
>g696
MSQITNVAAGQDVQSTIDLGTVHGDMKKFEDTDVGVTVLNGMVDELNYDSAEARKVVWKIDLFLLP
LLAVTYVIQFLDKSCVSYAALWGMRTDAHLVGDQYSWLTTVFYLGYMVGELPSNILFQKFDIARTC
GIFITLWGVVLLCMTAANDFAGLLTARLFLGILEAGVSPCFVLLTTMFYKRSEQPLRTCIWFSMNG
VANIFGGLIGYAIGYINAAIPAWKFPFIIFGSLTIVWGVVFTLLTPSNPTNARWLTQREKAIAVLR
LAENETXIDTKVWKWEQVKEAFVDPRFWLMNLLTLANTIPNGGVSAFAPLIVNSFGFTKFQSTLLG

MPSGASQVLGLWISGYLAMKIKGIRHFLMIGGLLVALLGASLIYALPDSNRVGRLLGYYLLVGFST
TFVLSISLVQSNVAGRTKKTIYNTAFFVSYCVGNLIGPQLFFAREAPRYKSGFVSWIICFVVQIFI
VAVMYIVNLRENRKRDRLYEEGNYGEGPGQNGHEDRIALGLSDLTDIQNKHFRYVL
>g697
MSEDLALQSADIIAEVSAAIDNASEELRRLSLEIFNNPELIFREVKACKLLSDWCESRGWSVKRGA
YGLETAFEARFSTGSGGRTVCFNAEYDALPGLGHACGHNLIATSSVASAIAVETIMKRHSINGTVI
LMGTPAEESGGGKWIMANHGAWKGVDACVMTHGMPTFSTPLCLTKASWKIRAKFHGLAAHAATAPW
KGCNACDAIVNAYTAISMLRQHLEKGQSIQACILEAGKASNIVPEYAEGLFSIRAPTMKKLDELKA
RFEPIFGAAADATGCTVELEWTALYEDVVSNNTLAEQYRQYMLRHLGLTPEQMPTLAESRIAHDQP
GCSDFGSCSYICPGIQPMFEIKATDFPHSIGFRDAASGDFAHQEALRAGKANALICLDVLTNDGFA
EVMRDEFIEAMKERGRWGESGESL
>g698
MHGVELADDLMSDPIRLGFIQRALDSASLIIQTQFESETFRQSFHYTMDYNGTPTYYAIGFILKAL
PIAHEFVDCKRLLVMLQQAARMFEQAGAIDAANELRTDGERLAALTQTVVSPEVFTNSEAPFPTLF
DIPSFFDEMVWDDDFPALGTLPLD
>g699
MLVQSLISATLVATGLLAEGALAAGYHVPIPAKAQRRYEDHYGAMLGKRTSSASKNATQDFRYLTK
ETKPYRVESLPDVNFDLGEMYSGLVPIESNDTSRQLFFIFQPTIGEPVDEITIWLNGGPGCSSMEA
FIQENGRFLWQPGTYLPVENPYSWVNLTNMLWVDQPVGTGFSTGTPTATSEEEIAQDFIKFFKNFE
KLFGIKKYKIYVTGESYAGRYVPYISDAMINENDTEYYDLSGALVYDPVIGAFDSVQMQVPAYPLL
AENNNMLNFDSKTLAELEKIHTSCGFDKYIEKYLTFPASGVQPPMSYSEDCDVFDAALTYALAINP
CWNIYEINAVCPLQWDILGFPTVLDYMPAGAEIYFQRKDVVKALHAPTNITWTECSSEAVFIGSGG
PESEGDLSADPIQSVLPKVIEHTNRVLVGNGDYDFIIMTNGTLLSIQNMTWNGHLGFSEKPSTPIN
ITLVDLEYAEVFAENGLSGVDGGQGIMGIQHYERGLMFVETYMSGHMQPEYQPRSSYRHLQWLLGR
INEI
>g700
MNMQCLTLNPNQVQPPTRDLPSGKPLPDSLPDAKEVKVHPRGSDGTENASLFFVGTATTILLVKHG
LPNFLHAGDHVHLGPGVSAMRQTNPAVDLHDLPRIDLVLLSHYHGDHFDQSVEASLRRDLPIITTP
HAKDHLTSKGAQDSFSNVHALDFFEQMLVDISGNTSSRNEKKPQVRVTGMPGKHVPTKPLQVLNDL
VQAVPPTNGWMVELGHSTSSSDFKVGYRIYISGDTLLVDELKEIPKRYAGQNIDLMLIHLGGTTIP
SPALSPMTVMVTMDAKQGLQLIQLIKPHLTIPIHYDDYDVFASPLDDFKAEVEKAGLADRVVYLER
KDQFKFRVVS
>g701
MNRFVADQYVFMIETQRDLWKSRTEHAALCIARGDVYLGLLAVSACLFLLHYSLYLAKRLWTKSEN
DDESPPVFAQFYTPIAYDVAQIVFATASFAISVAILPQGGSWKRSLLLGYATVLYIGRFAGNSSTQ
SRIFRHVNTIIVVTALLAVIQTFVPVFIVGATYRPGTLESALEDESNENLSFTYKASLEETCSLFS
YYWSYEWITWLIIRGLRNELTIEDLPILPSYDAPIKWFKGYQKQHIQKMIFWAALLAFGEFLAPSA
LMRLLGYLQDPANAIVHPLRYIFVATRLLVRVNMTLVQEIYHTAIRSHIYDSTVAERKVNHGGSVV
DKELSKGRQADITTLMSSDVQAIYNAREVFFVPVVVPITVIIAVIYLYRLLGWPSLFGVATLFLLS
PLPTLASRRVSRIQRSVMQATDARISKITEYLGSIRTLKYFGWEPAMEKEVDVLRQVEQSRVWKRN
LTAAIISLAGDLLPMMSLLISFSAVVLLTKNSLDAPKAFTALSIMEILRNQCLWVSNIVRNASTSL
ESLRRLDRFFDSAVEVKRHPQGPPAFHNATFRRTPVAAFRLQDINVAFTESALNVVTGPTGSATCP
RDVAYVPQTAWLQNDTVRQNILFYSEFDKTRYENVVAACGLLQDLQQLPEALDTHTTAWVYDQCFR
KGILAGRTVILVTHLPSALEDAQTIVHIENGTITSVQTNEDSRLASSESSGAVTPLNNGEAEANKK
TDDEESTDNDPSERKVSSRLVAEQSSKGRIPRNIAFQYLFNFGGFSFVILAMVSTLAVQIAYFSVT
YWLSIWADAYDRSDSIPNVGYYLSIYAATILIYLALQFGNVVIFQAGGWNAARKMHRRLLHAILHA
PVSWFDQNPVGRAMNRFGNDVRSMDAVLPEYLNKLIDNCLRFLFRVASIASVMPIFIIPACIVCSI
GLLIGEMYTRTQIQVKRLSSVNASPVFIHFIDSIAGLSVIRARGGMDEKFFSLLSEKVGVHARAME
AQLNSNRWVAVRLDLCAATVTATVGLLAWKVGGDPGLVGFSLSHAVGLGQTILTLVRTMNDLEVEL
ISFQRVKEYAEIQQEEQEEPLAIASKRDQVPAHWPNAGQVEFSNVTAKYHDGPNILQNINFTINPG
ERVGIVGRTGSGKSTLGLTLLRFTELVSGRVAIDGIDISKIPLNRLRTSIALIPQDPVLFSGDVQS
NLDPFGELGETELQAALSACCTSIEVAASNATPDDGNGNTPRSLRLDTPVAANGENFSQGQRQVLG

LARAVSRRAKVVLLDEATASVDRETDEHIQRLIRTEFPESTIIAIAHRLRTIVDYDRVIVMGGGEI
LEMGSPAELISLDGIFCDMLQKTGEYEELVQLIRNEV
>g702
MATEKTPVSTHQRSDSSAPSSRASNSDDSLRSAHETRLRHEDGEDRVSHCSEQDHDHEGSRDIEKN
LPSLDQTPTNQSRNNNLSILDTVRSHVSHQDMHVSDNVYREQNAEQYLRFSPARKVAIVAILSACS
FLSPISSTTILPAMPEVANTFNTTGSVINASNAVFILTMGLSSTFWGTFSQIYGRRPMSFWSSFFF
FVFSLATALSPNLAAYFIFRALTAFQGTSFLVVGSASISDIYTPTERATALAWFLSGTLIGPALGP
FLGGVIVTYRSWRXIFWLQTALGGFAFILVALFIPETIPHKTISDLADLSPLEKTVAIWHRMNPIR
VIKLLFQYPNLFFAALASASLVWNQYSLLTPIRYVLNPRFNLTTPIESGLFYLAPGCGYVFGTFFG
GRYADHIVRKWIRKRDGVRVPEDRLKSCLLFLGVSIPGWVLIYGWTIDQKVGGIPVPVIALFLQGV
SQTFVFPSLNTYCLDVMQSKGRSAEVVAGNYMFRYALGALGTGVVLPGIGAMGVGWFSTISAIFVG
VSGFFMWLTTIKGATWRQRVDEKLAQRAAA
>g703
MATKDEASAVPAASKTSWTSFLKSIASFNGDLSSLTAPPFILSTTSLTEYSAYWCEHPSLFVAPAR
EPDPAKRALLVLRWFLSTLHQQYCSRSEKLGSEKKPLNPFLGELFLGKWIENEDVGETKLISEQVS
HHPPATAYSIVNEKHGIELQGYNAQKASFSSTIQVKQLGHAYLSLTPPGKDATNEADREHYLITLP
NLHIESLIYGTPYVELEKTTKIASSTGYISKIDFSGKGWLSGKKNTFSAVLYKESEGEKHPLYTAD
GQWSSSFTIRDARAKKDVEVFTVSNMKTTPLTVAPLEEQDVWETRRAWRDVAAAIERGDMEATSVA
KTKIEVAQRELRKKEKEEGEEWERRFFKRVNEKDDHTFMRLATMLDLTQGNGAGIESDRTGGVWRF
DASRAVDAXPPYHKIGGEGLGL
>g704
MSEVLSRPSAPRGRGSARGGRGGYSSRGGRGGPRSAKAEFADNAAPTSYEDEGEIGQLKLKYATEL
PTLKELFSEWTDEDLVFALEDANGVLETAIEHISEGNASQWGEVKKKTTDRSRSKAKDVPKSQTTG
SETVPLASRGVRGRGGFEGRGGRARGDRGRGGRGGRVGGARDEKPAAPTEQVITSISTEPATAEVE
TAKPVESVEAVKEPQQEAPKAEPAKKGWASLFAKPTPPPPQKKPPPVAAPPAVPPVEAPVETKPEP
IIPSVPATVATPEIPKAQTPEQPPVPLDEPAHEPPSTQSFEAPTDDLTKTNLEQIPDVSVPPATAT
AASTIASVQDPNSLPPVTPQSRGPVSGYAASAFKAAGTQGRSASFQRRVLEQQEAVVMPANHAVDR
AAVQFGSMGLNGNESVDVDEQREEAETRAQPPQHSPVAPRASLPPPQSQPSTETPAVARPAPGLPP
VPQTDAAPFNDFSRYAEAQKPYDPFTQHPAQTQAQAQEPFSHQAPTQAAATTGSEYSPFYAADQQR
LPYNYYGYPSQDTTGQRASGFATSGAEGQAQATQTPSRYGHTEAPGSGQNTPQPTLPGATQSQQPS
GQHIPGGQGAHAANYGYGYPYYSNPHYAQTYMNMNQQHQYGRNRPMYDDARRYEDHYMPHSNQYAY
SNQYAPYGGKGAMYGQPFSYDHSSSPAGSGFNQALPGRDSVYGRTGSAQPENQQSAAANTTFGTAV
PDVFGRSQSGFGQNQPISQQQPVSSEESTKGYETSKTGGPSPSLAQANRPGSATSSIPGQSQSQTG
LPPLQGQQGQQGFGGYPHLNPQYSGGLGGLGSHQANVSQNHHQGSAYGNYGGAAGFGNYYGNSGRG
GGWGGNYGH
>g705
MKNNTAFLKLKGWPKIHHPLPQTPRESQQLLSALTSSFRRQLDAADTNKPAQSASEHLDAILENPL
FRVVPSKPAYPTIESHQNRPETEETFSIVKDPMRLLDDNVAAGTLTGRILKEVLRSQLSLIHNAST
DPERALQNTGAGARVAKWFWASDSTSRKSFFKIPGATKHAIIFMVAEGLHDELRAFLRLLYRSDLG
GTDGKIPKRIADKIFATFLNNYVAAEITHGRGMASAMEIFAEVADSIAHSEDLQSTNFKESLLKPT
VFHFGRFMTRDMNAGVFKDVPASVFDRYCNILEALPGLRPYGLAMRIYHPVQQDARPFMEYVRELR
KSKSPPRTEMGQDVLLQTSLNGLRLLIDQKDYRDAAKYSQHVKNMLFEERVTDRHNDSYSPQLNQL
MDQLSLGQNALSFRWEPVP
>g706
MSGTKIWLRAETKPQEARSALTPTTTKALLDAGYDVTVEKSSQSIFDDEEYAAVGARLVEEGSWVK
DAPKDTYILGLKELPEDDFPLEHVHISFAHCYKKQAGWEKVLSRWPRGGGTLLDLEFLTDEKGRRV
AAFGYSAGYAGSALGIKTWAWQFTHGKDEPLPGETPYANESLLINSVKEILEAGIKKAGRSPKVLV
IGALGRCGSGAVQFAKDAGIPDEDIIKWDLEETKKGGPFKEIVEDADIFINCIYLSAAIPPFVNAE
TLASSKRTLSVVCDVSADTTNPHNPIPIYNITTTFDKPTVPVTNISTLGNPDLPLDVISIDHLPSL
LPRESSEMFSTALLPSLLQLKDRQNARVWVQAEELFKEHVSTLPAELRKRD
>g707
MGVLEKLSRKTGVIVGDDVLELFKYAQEKQFAIPAINVTSSSTVVASLEAARDQNCPIILQMSQGG

AAYFAGKGVANSNQEASIAGAIAGAHYIRSIAPAYGIPVVLHTDHCAKKLLPWLDGMLDEDERYFK
QHGEPLFSSHMIDLSEEPVDWNIETTAKYLKRAAPMKQWLEMEIGITGGEEDGVNNEDVDNNSLYT
QPEDILAIHNALSPISPYFSIAAGFGNVHGVYKPGNVRLHPELLSKHQKYVKEKIGSASDKPVFFV
FHGGSGSSKAEYKEAISYGVVKVNVDTDMQFAYLSGVRDYVLKKKDYLLTTVGNPDGADKPNKKFF
DPRVWVREGEKTMSQRVKEALEDFNTAGQL
>g708
MPDKPGTVAAYAAGASLAAITLFYVFGPNFTIDGDESSSSDRKKGVVGLSNPANDCFINSVLQALA
GLGQLRIYLIRELHRRELDGPDVYNQLPGPDEATGKRSLPPDRLRELQQGTVTLALKEMLDRLNER
PIYKKTISARSFIQALEFAFRTRISRNQQDAQEFLQIVAERLCDEYHAAVKARQRAQRAAGADVDG
SSIAQAESDANSGEVSVRIDDGSKDGLPAIIDNKLKEIDNEHGFPLEGQLESQIECQTCHYQYRPN
RTSFVNLTLQVPQKSSATLSSCFDGLLKTEYIDDFRCDKCGLQHALETKIIAKTKAKSAAEIEKLD
EQIRRIQHAVDTDPEQGLDGVDLPAADLAPKRRIARHMRITVFPKVVAIHLSRSIFDQSSSTKNAA
KVSFPERLPLGGILNRKWYKLLGIVCHKGSHNSGHYESFRRSHLYPPFSTPDVFRSYAASRAGSEN
PSIAGSPRLPATKKANYXPSGGVEPSPLSISPVESSLSLSSTNQNNPPSSTSTPSTAQTASSFSSS
SSQETANPPRSSLQLQPSVNQSQDNLPRRSSSVRLGTPKLNTSSSAGGSGGGLSAQRLLRRRKTAD
RWWRISDEKVKECKTSDVLGMQKEVYLLFYEMDSQTPSS
>g709
MAGPVHHMAYAMAIRDDSDPSPADQFLRLIQNPFKETIQLNAFWVSLATSLGISAMLALLFSIFRP
YHNAIYAPKVKHADQKHAPPPVGKGVFAWVPPVLSVKEENLADRIGLDAIVFLRCAKMMRNIFLIL
SVIGCGILIAVNITQSNGSAVPGTSAFTLMTPLYILTEAVWAQVVCAYAFDIVIMFFLWQNYRHIL
ALRRRYFDSSEYQMSLHARTLMITSVPPNLRSEEGLMRLTDGVNPTSSLPRTTIGRNVKDLPSLIR
KHEEAVRELESVLAKYLKNPDRMPINRPTMSVPRKSRGEGGSGKVDAIDYLTDRIQELEARIKDVR
QSVDKRNPMPYGFASWEAIEHAHAVAYTARKKKPQGTIIKLAPRPNDIIWENLHLSPQTRRWRRLX
NVFSITLLTLLWVAPNAMIAIFLSDLNNLGLVWPAFQTSLEAHPNTWAAVQGIASPALTSLIYLVL
PIFFRRLMRRAGDLTKTAREQHVIHHLYFFFIFNNLIVFSLFSAAWTYVAAVINAKNNNESAWQAI
KDGQFWYKALSALCQVSPFWVTWLLQRNLGATLDLVQLVTIVWQWFMKTFMAPTPRQSIEWTAPPP
FDYASYYNYYLFYATVAFCFATLQPIILPVAALYFALDCYFKRYLLLYVFITKNESGGLFWRIVVN
RMLFGAFLSNIVIALVATSKGTWTMVYCLAPLPFIILGFKWFCSRTYDDDMVYYNKGVLNDAEALG
ASMGKGPKRAGDRLNSRFGHPALFKPLITPMVHARAAETLKSIYRGRLGNLDSAAEYSDIAMQSMS
TSAPGKPMDSAPFEVVAENQLDFSYFKNRADFRDEFGGDIYGRPEDLISERSQTPRAFMGDSPNSS
RASSPVSAFTRKQTEGYDLGMQRPLDMDHPAFRPQMSRVGSNVSDPNVPSLYMQSNESESRLLSHA
QLPAVEGDNTIHTVDRWRTRGYGPVAQDDNNPPPTSYDYFRGKR
>g710
MRANYGQRLLVTTLEEKSRSQPEQAFCLLPRSANLQDGFYEVTYKQIQMSVDYTTQWLQNNLGPFS
PNETIAYMGLPDLRYNIFFYAAIKLHLKVFLPSPRNAPTTNASLFEQTQCTKLCYSVDSPGIVASC
EAVLKVAKALRSFEFLSLDELLSAQCXPLPYLGDFAQLRDEPIVILHSSGSTGIPKPVTLTNGSIA
CTDNDRNFPTVSGRRNQDLTMWDFPPGSYIYVPFPPFHMGGFTFNTMVPIFTDTVPIFGPATRIPS
GGLVAQILRCLKVSGCLLPPNIVADLYNEPDGPELLKRLSLLCYTGGPLPEAIGNELIQHATIRQL
LPQKENWHYLEFHPSENLELQALGDGTFELVVNTNDETATFSQFNHNFPGVREYRTKDLFLPHPTK
PGLWKFYSRKDDIIVLLTGEKFNPVPLELGLQAIPGVSGALIAGDGQPRVALIIELQSDHNFGPDP
EDALWPSIQGLNANTAGPGRVTRSMIMIAKKDKPFVRAGKGTIVRKLTLQAYEEELNKLFEGNDVK
ALLRSIIENTLDGAVVDESINLYTQGLDSLIYNHSSLNELATVLLNWLNDGTYPESIDKVSRTQSL
LSYYEKSLPIKSGVLRTWGVNLERKLNIALIGSTGYLXHPRAQETWEDHYSTKQAFKNKEWLXKIT
FRQIDFTKPKFGLGDSLYNDLKKECDLIIHSAWQVNFVLPVGSFKDSFSGLMNTIELAAASERNAR
ILFVSSVAASGMFSPHSSIETKVVPEDRITDPNEAMHTGYGESKHVAENLICLASAKSGITAKKDA
LTGLLRTSKSLRIVPSDLLDIDWLPVNEVANIVNSILHQDRYSREDIRFYNLNWCGQTSSAASLKD
WIAAVNTREKEEQGSLHNLPSIPLLGFFEILTQRGPAHKYSQQNLLSIKNTALIKPIDANQLKIWL
DALS
>g711
MQAETTQTSLPFYNRGDVYRCLVEQNPPQRIKDLRSLCLVSARLNNLLKPVLYRNVCVRKNGFRSQ
PLNQLKWSLENDPKLEEYIISAVIPCNDSIYDVYRFFWFPNIEALSIHRFNDWEPLEFEDDSHIGT
SPITVLNLIDCGAHEEALATVLSWPAALEVLHYDAEQIEWEGHYGDEPAKSWTCAAFVRALQPQKA

TLRELTLSRPWLEHEGLGNGPRIDLHDFTALTTLRIYQVFLCGLEDPLEAWSSLPRSLERLDVFYD
DTDLTRFDEDDFLRGLLAHKKEHLPHLRTVSINSPEQIWDSDTEEFKSTGQWTPPYPLAQAFKTAG
MDVEIWLGPVERPKVDKLDIPQFLEPPRKKHCLDIDSS
>g712
MTSITPFKISISDERLKRLQQKLTLTDLPSEVTGLNNPHSRGVPLSEIKRLAFHWQHNFNWRAIEA
KINEIPQYTAHIAVDGFDTYDIHFVHERSQINNAIPLLVLHGWPSSFLAVQPMLPLLVEEGIDEPA
FHVVAPSLIDFGFSSASNKSGFNIEQHAEAYHKLMIALGYNKYVVQGGDIGYLITRAIAIKYGPQH
CRAYHLNNVAPAEPPSKEDEPSTQLSASDLKGLARTQEFTTGGGNAYYLLQTTKPQTLAYSLTDSP
LGLLAWIYEKLISWTDNYRWTDDEILTWVSIYYFSTAGPAASLNLYYEMEHGADGTAFDKAKKYIS
DVPLGVARFEKDLILLPRAWNQTLGPVVWESVSEKGGHFPTWECSEVIVSDLRGMFRKNGILFGCV
EGRNGYAHAE
>g713
MTREKTLRIVLENVDFRLPTQVTDDNSVVALKSLVFEPLLRWQAHGHVKPGLFDTWEFSPDGGTWH
FHIRDGAVFHDGKPCNAAEVVTYIHGFLHSRDYFGMSWSYSRYFAQTKFTAEDDRTVKVENPEPIA
DILDIFCEFWPSRIAEDGKPVLGTGPYRVTEFERVDGIGKATLRLVDRHSFTGPDVIIATHERDGE
KRLRLLKDGEVDVALNLERADNLGVIDFNPSLRWGRTTSTLSAMYYLNCPSGIFSSPEARLAANLA
IDQKSLVKKVYQGFALPSATIVSPFHLGFEESGIQPIPYDPSKARELLHGLDLSSPILLRTPEYMP
EHAQKISQFVASSLEAVGFKVTIELETNRPEYARQIGLTKRIGDLALFDSTPNSTFRVLDDKISSE
SHATWWLGYHDTEVQRLFKIARRQVGLDERKQAYAKCLQRLQQNPPWLYVAHPDVVWATTTDIKLE
IGPSGVLSLS
>g714
MTNSKDIDVVAVPDASSSDDGHASKEIGDIAVTEVNTGIEVKNTSRWRKIVGYVWDSVEGDPEYRQ
YVQRLDLFFLQRICQCLGIMVGSVPAMMTQLSFIRPSILLPSCELVWSALVIGMGFAKDIKTMYAL
RFFIGLFEACAFPGYVAMLGSWYGPKELTKRTAILLEIESIASMFSGYLQAGLYTSMNGHAGLAGW
RWLFIMDGIISIPIAFWGFFGLPDTPHTTRAFYWSAEHVRYGIERIEKFGQKAQTKLTWKEAKRIY
LGWEIWAFVAPYTMIAACHTATSYFNLWLKAVGYSVVEVNYLPTGGNAVAIVVTLLWGILADRTGR
YYWLIVGLVLAMMLSNILLSVWNIPKGALMFAYYLSYAGSATTPVLIAWGTKLNASDPSLRKLLVA
TANVVSYAWVLWVPLVLFPTYDAPKYKYGYQILILFGGLAIIFVSSMCQKQTQEGGQGVEANREET
VVEG
>g715
MPIVTDYKLPKSAEDLDLPADSNSPFFISFHASRDPKTGTPWCPDVRAALPPLETAFSADTAPRVA
FIEVGQRPEWKDPKNVYRTKWNVHSTPTLARYERVDGKVKEVGRLEEAEILDSKKLKDLITRPSAS
L
>g716
MTVISLLPADAPAFGDQTLFFLPGGTSSVRLKDGYIPLLANFTHEDKAAKGLRKRSADDKHEPSVY
FSALEVVRDTRFLFLSGKPWSGKTTLAKYLCYSGATGGLEKARPVIRNESGFVEEESWADCKIDPY
YYIIDCFETLKRLIDKFIPETLEKFVKNTSIQQQEALIVLDGIEKAGEQATSLLPNILSFAESLKN
VRILILGDTQTCERWILPAPFIRHEILPLLEAQRREALGAILGVKPSEVDIATGAAAANPGIFCLA
VPARHQGDLAENTIDSWLSCAVVPPNSPDRLADADFETFSLDVPKDAEPSLSAPLLRRQLLAARHL
AALPTTDAALELFSREPLVAQPIIRSLLIRLSSSERCQSFKTLCEDLVRGSPDMAKRGALLVSEFM
DQVPDLREHVATHMLAIIAGGTFSVRERVQAGQVLSQLGDPRDLTALADIPAGAFIFGSRSHPNSQ
PQGELSLGSYRIGVYPVVNRDYTAFVNDTKRKWLSPDHSNIDKLNAPATDLTWHDARAYCEWLTRK
WRASKKIGPTEQVRLPTELEWERASNGDLNRLTDSTDPVYPWGLEWKHDAANSEETGLNTTCAVGL
FPEGKSPYGCYDMAGQVWEWCSTLWGEDMATPTYAYPWQDDDREAPNAPESVRRVLRGGCFSSGKL
KACGTYRGSLEPAGFWRGNGFRIVVSE
>g717
MRLSALLSAAAIWHATTAQLIGPVGPTTDLSDKNIECNILNYGAKADNKTDISTALEDTFTDCVLK
NPGSRLIVPEGDYLINRSVVLSNATNWALQLDGLITAAYGGNWTVDRELILQGFAGVQVLNSTING
EGDNKFLLDVLVIVNAVDFEFYSSNGLGAFQGQGYIYRNLNNTDRPRLVRLVSPTNASVHDLILVD
SPKFHIVLDFAVNVEAYHITVRGANLGSYDGIDAIGTNYWIHDNEVTNRDECVSVKSPSHHALVEN
LVCNQAGSGISIGSLNVSAEISNIEARNISIIQGNNIAFIKTYPGGSGYVTNVTFENFRSLGSLYG
LDINQYWQNTFTPDTGAVTLSNLLFKNFSGSVANGALRPPLYLIANDLTFATNVTVEDFSIWTETG

TQVLNRISNIFGHGDNSYGTNDGIPSLSAGKAPYTYTSTYTITASPTSWQAPSTPTWAVASTGYGT
ASPIPVYTPAPLWRPGGVDYDLHYWGSF
>g718
MDYAEEIDNWEPFLKLFRLREETVANYTQEVQNRCSKALSASNIRHPPITSRIKSWESAKGSISRR
NRERVRRRMRDLKESRDRRRDGYAHETESGEEIEPFKGPDEMFSALHDFGGVRISLYFPGDIEKVA
GIIEEQFHVDREIPKGHGSQRCVGSLEERLESLNNPGRSTQDTVDDRALQRSVRTFTGYKATHFVV
KLRYEHINEDRKVAWEDIVVEIQVGTLIMHAWSEIEHDMIYKPLDSQDGEVSEDEKRILDLINGIV
LTGEVALRQLEASTAQRINKRAEVENAMASSQYELATWIEKHFNQHKMILIGPEWRYLEQLFAILK
VTGTHKHSEVIKLVDDAAQQTLNRYYPTHKHSEVIKLVDDAAQQTLNCYYPTHKHSEVIKLVDDAA
QKTLNRYYPTEQILSVLIRSPASSEWPPLLSNPVSESDFARNARLLALRLVHGMNLAIYFGVIDEI
LDVYSNSKFGLYTLDMHYRPSITSFLDILHPERPQYSDKETVMRIFNFCKEIIGNRRWALEKMVKV
AMDLPMTHLVAXFAEGVSRTIPIPAIITRLLPLEQINEPQEDSEDPLGEMNETFGIIGLIDRFASS
HGRRDDPIVREQPVDSTDNQSIQTRFFVPIMHSDDISFGHWKLRDQIVDIEKMNAEDINGFTKSPT
KTRPRFEYSDGVLQLAYRLYSEEEWVRIREAWKLIKPLKRKRTTSVSSSSSERTFPIRTTSARELA
NKKRRVAEEKMKKKGKEISM
>g719
MGSSYSDYVTTISRQRPSLKKLSDFLRHQPRSACNSLISYVEIETTGGAGPPQEISTSKLVARITS
GLKKSMVLVVEDIHADDIESLGSCLDIDPLFFCGHIASSYGDIEKDPLPPLLSLPPSRLVSGAFIN
IHHQKVLDLGDEVTLGHVPYDLVLLANVTRSVRRLPALSSRSIGILRACTSLIKIDLPGGHWISLI
LIDPTSDIVVHPTRTAPRRLVGPKLPQIPRRIAIETISQTPTYVQYQATDTASQKFQATNSREELL
RFFHTSPPGWRMGNHSILCLTYYPIQTIIQEWIVYALLMGRYVKYYEYSSQTAHSRLERFEKDDIL
DLHRWRRRSLQSLHKLDILKRFLDHWVLKEENLDQDLLQAANTTTREGLVTWNLLKGDINYLEEQI
MQHARSLEALNPIITALVQLVDSKRAILQAEDTRRLTYIAIVFIPMSFITGIFSMSEPYGPGSDRF
WIYWVPATYRVMTHADEDLLDRIKRSRECLSACLVEAILDPDDDGDVREFWFGQYTNGFRQFVPRV
NSVSVDGFDDDDDDDGSVDSLEPEMLDDGFDNVFND
>g720
MVADKNNVRRKVVCFSDFDGTIFMQDTGHVLFDKYGCGAERRQVLDEQIKTGERSFRDVSEEMWAS
LNVPFENGFDVLADTLEIDPDFREFHTFCAQNDIPFNVISAGLKPVLRKVLDTFLGEEDSARIEIV
ANEASINEDGSEWKPIWRHDSEVGHDKALSVNEARIQARDNCGEDEVPLIVFIGDGVSDLPAAREA
DVLFARKGLRLEEYCQEHNIPYIPFDTFADIKREIEKILAEDQAKTGGHGKPLRFNPRANMWRRVS
SKQAVPRFVAMTPSKEERMFLWPETFSELKAGQPDAAALKTPGVTTSAVA
>g721
MLSEKSSSEPVAFGEMTERARTPTERSETTNVAESTTSDYVPNNMMEDIEKFEIAQTATNDIKERD
EQPAEDPNIVWWDGEDDPANPLNWAPLKKWTNIMLISAITFIVPLASSMFAPGVSQVASEFGESNE
ILEALVVSIYVIGLAVGPLVQAPMSEVYGRWIVYATCNVLYVVFTIACAVSTNISMLIVFRFFAGC
MGSAPVTIGGGTIADSFPPHQRGRALSIYTLGPVAGPAIGPIAGGFLSESEGWRWIFWVLAIASGV
ITVGQILFTQETSAVVILQRKVKRLQKETGNMNLRSKLDRQIPGSEVIKRAIVRPAKLQVLSPISI
LMSVATAVVYGNLYLMLTTITTVFESTYGFNTGTSGLAYIGLGLGNIIGLLMFSFTSDRYLQQKSA
KGLQLKPEDRLPLMLGSCPTIAAGMFWYGWSAKAKTHWIVPIIGSAFVGLGNMFFFMPMMGYLVDA
YTIYAASALAANTVLRSIGGGLLPLAGQSMYAALGYGWGNSLLGFILIGFTPLEYRIVSILKGKPI
LAPDDPGRSNTPTPESTRASTPVVTSSASSLSAPTISAPTQSPSPRPAVKGPFVNPKPDEVVYEDV
GSKHRLMLNKFCLYRPMLVLPTKEFALQSDDLDASDITAAWAILHSYHTFEPIVIYNRGVNGGSSQ
GHKHLQLFPVPPLAPFYGKADNLWPAKATSSTEVSDRIPHVPFKHYVLRISPQTSAKAVVDMYLRL
LQLTRKAHVEAGYDADGDYNVTMTRDWIAVIPRTTAGPSDGPFGTSTVGMLGIPAIRDERDREKWR
KLGDAKFLAKLGIPQVN
>g722
MTAPRGRLQGKNAIITGAAGGIGLETTILFAREGASVLMADISEPALAAALKKVKSLVPDSARVET
IKCDVSKESDVQAMIESQDSWGGTDVIFNNAGIMHADDADAVDTPDKIWDLTHNINVKGVWYGCKH
TVLSLRRHKKTKGSIINTASVVALVGSATPQLAYTASKGAVLAMTRELAIVHAREGFRFNALCPAP
LNTPLLQDWLGDDQAKRHRREVHFPTGRFGEAIEQANAVVFLASDEASFVNGTDFVVDGGMTKAGL
HLLEYETLANI
>g723

MSPPSEEAITAENIATLLAHDTKVKLAGVDADGILRGKLVAKKKFLSIAQDGFGFCSVIFGWDMHD
RTYFKELSISNKENGYRDLLAIPDLQSFRRIPWEDNVPFFLVSFFDPETKAPISACPRNLLQTSVD
KLAKLDYKAMAGAEYEFYQFRVPHDASSSERNSSATASFLKKNPVESLPPLTEGMFGYSITRPVHN
QDYYYGIFDACEKFRCDIEGWHTESGPGVFEAALQFGEIKEMADRAGLFKYVVKSIGSKHGITPAF
MAKPREGLPGNSGHMHISIATKDGKNLFARETVDPSPPYPDVAHLSDIGRHFLAGLLEGLPDIMPI
LAPTINSYKRLVENFWAPVTVSWGLEHRAASIRLIAPPTASAKATRFEVRVPGADTNPHYVLAAIL
ATGWRGVEKKLEISLPPLGKGEDTGSASDKGARLAKSLKEANEIFRRPDSVAREVFGDEFVDHFSG
TREHEIVLWEEAVTDWEVRRYIETV
>g724
MTAIETQLQGVSILGAVNNEHRKILTKEAVAFLALLHRTFNSTRKSLLERRIARQAEIDRGHLLDF
LPETKHIRENDSWRGAPPAPGLVDRRVEITGPTDRKMVVNALNSEVWTYMADFEDSSAPTWENMIN
GQVNLYDAIRRQVDFKIGNKEYKLRTDRTLPTLIARARGWHLEEKHFTVDGEPISGSLFDFGLYFF
HNARELIARGTGPYFYLPKMESHLEARLWNDVFNLAQDYIGLPRGTIRGTVLIETITAAFEMDEII
YELRDHSSGLNCGRWDYIFSFIKKFRQNANFVLPDRSAVTMTVPFMDAYVRLLIKTCHRRGVHAMG
GMAAQIPIKDDPQANDVAMENVRKDKLREVRAGHDGTWVAHPALAAIASEVFNKHMPTPNQLFNRR
EDVHVTANDLLNTNVPGSITEEGIRKNLNIGLGYMEGWLRGVGCIPINYLMEDAATAEVSRSQLWQ
WVRHNVTTAEGKRIDKAYALRLLQEQAESLASNAPKGNKFQLAARYFATQVTGEDYADFLTSLLYN
EISSPGNAAKL
>g725
MSSVQVSSDEALATHINDYENPRIFQRNRLPARFYYLPETSLCLNGTWDFNYAPSPLHAPEPNASL
GSDKSVIESAIETTSSDDEVQSLSMLDLKDSSNAEWTTISVPGHWQLQGHGHPHYTNVVFPFPACP
PYVPSENPTGTYRRSFQVPSRWEKSSQLRLRFDGVDSAYHVWLNGVEVGYSQGSRNPAEFDVSSIV
KWDGINELFVRVYQWSDGSYIEDQDQWWLSGIFRDVHLVAFSSQARIEDVFVKTELDSTYTDATLE
VSLSLHAESPCLVRAKLYQPGDSDEVLKSTELNIETGQQTAEIVMPVATPQKWTAEAPTLYKLEIS
LFKSTDGSPSLQTIVQKVGFRKVEIRNGNLTVNGQPILLRGVNRHDHHHLFGRAVPLSFIREDLLQ
MKRYNINSLRCSHYPSHPRLLELCDELGLWVMDEADLECHGFYDAVARPMDIPESMDYEERKKLAF
GKAAEFTTNNPEWKDAYVDRMAQLIHRDKNHASVIIWSLGNEAFYGQNHKAMYEYAKKVDPSRPVH
YEGDAHALSADMYSYMYPSLERLIDFATAEGDDFSKPIVLCEYAHAMGNAPGGLVEYMDAFRKYRR
LQGGFIWEWANHGLWVEPKEPGQKGYYAYGGDFNDFPNDGDFVMDGLCYSDHSPTPGLIELRKAYE
PVRAWVEDDLIVIKNDYDFIGLDDILAVFKVEAFSEKSAKILSSGTLNLPHIPAGQQGTIPLPTGI
SIPSSGEIWLSVSFLSKTAHSWADNHTEVSWFQHCLRPNKSLTLPIRTMTNTTSLEIHTTGTSQTI
IGQSFSLTFSRVTGNLSAWLVNGHSLLESDPLTNAALSVGFWRPPTSNDIPFDLPEWRRFGIDTLT
NHQLRSFHIARRSDGSIQVSTKTFICTPVLAWGFRATSSYTIAVDGTLTVSIHLVPEGPAPKTLPR
IGLDLYLADPLDNATWFGRGPGESYADKKSSQKMGIYKAGTAQLHTPYEVPQENGNRLDTRWLRMA
DGRGWGLKAIRTDASGGTSSPFQWAASRHSAQKIEEAKHPRDLVAENAVRLRLDVASAGVGTGACG
PRTLEQYQVPCEESVISFCLQPSFDDFDI
>g726
MEDTKKVSETIGEGKTVLSVTPKHDKPWFLVPHLLKLNIFLMAGVLAQVVSGFDGSMMNGLQSVVA
WQDYFNHPAGSRLGTMSNGITIGTLIVIPFVTFLCNYLGRRRSIMLGCLIIIIGAVLQGVAQNFSM
FMGARILIGVGGCLASTAASPYVTECAYPTHRGTITAMLIASWPLGAFFAALITWGTYQSSLQTST
WSWRIPSILQGFFPLIQLILAFLGPESPRWLVDKGREEEARQFFVKYHAGGNEDEVIVSLQMAEIT
ATIEQEKLQNLSSWLKWVQTPAMRHRLFICFFIPAMLQLEGNALISYYLHIILDNIGITDAVAQLK
INLGITVWSLVTSVALASIIDMYKRRKVFLTGLTLLCLVYTIWTILSALAQQSNFQNHGLAIGVVV
MIYLYQPCNLPALIGIPYVMEICPYSMRATGSMIYQLSGNIAGLFNNYVNPIAMEAITWKYYIVWC
VNLLVLWVIVYFALPETHGKDLEDVGEAFGSDXLXGXAAVEHALADKGEKGFLGVEEDHVERITEK
DKTEAL
>g727
MKRTMSREWILGLALTTAMVPIVSADLTTTISSTTNWGTWEGFGVSLAWWAKAFGTRTDLADIFFS
TKSTTYNGQSIPGLGLTIARYNAGACSWNTVNGSAMVVSPDIIASRQMDSYWIDWSSTSPSSSSWS
WSVDANQRNMLSLAKARGANIFELFSNSPPWWMCLNHNPSGSNDGSSNNLQSWNYDQHAVYLATIA
EYAAQNWGISFQSVEAFNEPSSDWWNGETGTQEGCHFNSSTQATVIGYLRSELNSRGLSSTLVSAS
DENTYDLAVSTWNSLGSTATSNVGRINVHGYQYTGGRRDTLYSLASGAGKRLWNSEYGESDATGEE

LVTNLMLDFRWLHPTAWVYWQAIDGSGWGLIDGDNDNVSLGAVSQKYFALSQFTRHIRPGMRILDG
GSNYTVAAYDSTAHKLVIVAANWGTAQYLNFDLSKFSTPGVSGALVPRWITQTGSGDQYVSYNDTY
ISGTKFWSYFPTGAVQSFEVSNVVL
>g728
MARHQNPPFRAEQLGSLLRTETLVKKRDLLTEGKATREELIPIEDQDIKDIVAKQQELGFHAITDG
EYRRHMFWGSFFPNLEGFTQIMTPSADMFRLYMPDIAAFVEEKVEPGETVVCDGKIKHVGSSYVED
FKYLAGLVKPEEVKNIKITLAAPNWYHLRYKEGYAYSKDAYSSDEEYFGDIAKAYQEELRILYEAG
CRNVQYDDPNLAYFCSEKMIEGWNADPLNKGTTDDLLDKYIKLYNDCIAKRPADLHVGVHLCRGNF
HNSRHFSEGGYDRIAVKLFKELNVDTYYLEYDTPRAGGFEPLKELPVTKNVILGVVTSKFDDMEDK
EEMKKRILDAAEFIAKGNNISVQEALKQCGVSPQCGFXSHHGGNAISREGMFKKLKLVREIANEIW
PNEP
>g729
MPQYKVNDSVRYKPVGGPQSHTSETVGVIKEVLTSPGNLTGRQVQASEKDPRYEIENSHTHKRTAI
KEANIIEHC
>g730
MGGSVSKLMGKIFGSKEMRILMLGLDAAGKTTILYKLKLNQDVTTIPTVGFNVESVTYKNVKFNVW
DVGGQDKIRPLWRHYYSGTQGLIFVVDSSDTARLSEARLELHKIINDREMKDALLLVFANKQDTPG
HLSPEEITNALQLNKLKDRLWYVVPSVATEGTGIFEGLAWLSNNVKTPAPPK
>g731
MHQFQRRGSVFQKDLNQNVADRCAWQGAPFDNLYAGISLQFDDSAAAVLAIAIRDTTYLVDFYEHH
FDSRPEAKLARTDIGDFIIDRLREYSEEHLERFTGLGMPQDIADRCPHLCSRLWRELDIVPIAFTE
GVPLFATSPKDQTKWNARCMDELAEAMSRRCVRFFGPSNNPILDVGYQGRVEVDCASRIRISTLDD
YKPTVGPTTWEAVNHYASELKKRNIKIAFFSATPQGGGVALMRHALVRYARELGVDLQWYVPKPRP
GVFRITKTNHNILQGVSAPEERFTEDHQETIEDWLHDNARRYWLSSCGPLQDPSEGGADIVIIDDP
QMPSLIPIAKNAAPDRPVIFRSHIQIRSDLVGTPGTPQHEAWTWMWDRIKHADLFISHPVEAFVPA
TVPKEKVGYMPATTDWLDGLNKTMRDWDVASYGRIFNTLCYHNRAPLINYPDDEYIIQVARFDPSK
GIPDVLESYARFHELLKEHGVENKPPKLLICGHGSVDDPDGNLIYDATLQHIENRLPHLKDLICVM
RLGPSDQILNALLSKAKIALQLSTREGFEVKVSEAIHKGKPIIASRAGGIPLQVQHEKNGFLVEVG
DPEAVAKYLFDLWTDQDLYDRMSKTALETVSDEVTTVGNALCWLYLASHLTAGKSVKPNERWINDM
AREEAGIPYKKGENKLKRAVIVENMG
>g732
MPPKAAKGEYIETDTGNKISRRSLIHGTQHIILGGKTVIQADAVIRGDLFRVQPPSSSSHSHSNDG
ENPSSSSTSTNTPPSVAITVGRYTYISRAAILRPPSRLHRGLHSYYPLKIGDHVFIGEHSIVEAAQ
IGNHVHIGNDVVVGNMAILKDFAYVLDGTVVPPNMVVPSFCVVGGRPARIVGEVGEGYGVEGAEGG
LARERYRLVGR
>g733
MTSTQRGLESHSEVSNDVLMATPSSEDRHHLHEKHERQDENEISSSEAPNSSDDDFPGRFEPVRPG
DREELTRIASSLSTSRRPSTLGRRDSAPITRMDTVDEMALEDPRIDPGNDKFDAYLWSRKRLRLVQ
QEGMGFPRASVTFKDMNVSGSGEALQLQETVGSVLTAPLRPGKFFSFAKKKEHKTILRNFDGVIKG
GELLMVLGRPGSGCSTFLKTLHGELHGLDLEKESTIHYNGVEMNKMHSEFKGEVLYNQEVDKHFPH
LTVGQTLEFAATARAPSHRLKGQTREEYVKEVTQVVMAVFGLSHTYNTIVGNDYVRGVSGGERKRV
SIAEMALSRAPIAAWDNSTRGLDAASALEFVKALRMSSNLTGSCHSVAIYQASQAIYDVFDKVIVL
YEGREIYFGPCKRAEQYFVNMGWAKPARQTTGDFLTSVTNPQERQARDGMDKQVPRTPDEFEEYWK
KSPEHAALMQEIKDHETEFPIGHVAEKELAEKKHEQQAKHVRPKSPYLMSVPMQIRLCTKRAYQRL
WNDKATTLTTVIGRIFMALIIGSIYFGTPIATAGFQSKGAALFFAILLNALISITEINSLYDQRPI
IEKQASYAFVHPFTEAMGDIVADLPIKFVASAAFNIVLYFLAGLRYEPSQFFIFFLFTFIATLAMS
AIFRTLAAATKSLAQAMALAGIMVLAIVIYTGFVIPGPQMHPWFSWIRWINPVFYAFEALVANEFH
GREFICSAFVPAYNNLVGDTFVCSATGSVAGRRTVSGDDYIRTQYDYSYSHEWRNFGILIAFWIFF
MFTYLTCTELNSATSSTAEFLVFRRGHVPAYMKKSDNQAKHGAIEAPPTAENEGATEEELGILPEQ
RDIFTWRNVCYDIPVKGGQRRLLDNVSGWVKPGTLTALMGVSGAGKTTLLDVLAQRVSIGVVTGDM
FVNGKPLDASFQRKTGYVQQQDLHLQTSTVREALRFSAALRQPKSTLLSEKYDYVEDVIKMLNMED
FAEAVVGTPGEGLNVEQRKLLTIGVELAAKPALLLFLDEPTSGLDSQSSWSICAFLRKLADHGQAV

LSTIHQPSAILFQQFDRLLFLAKGGKTVYFGDIGKDSTTLLDYFERNGARKCDAAENPAEYMLEII
GAGASGKATQDWPTVWRESQEAKNIQTELDSIHAHHAATAPNGTETSKSTSDEGREFAMPFTAQLK
HVVVRVFQQYWRDPQYIFAKLFLGLASALFIGFSFFLPNSSLQGFQDVLFSTFMLTSIFSTLVQQI
MPRFVSQRSLYEVRERPSKAYSWVAFLIANIIVEIPYQILLGIIVWACYYFPIYGAGQTGEQKGLM
LLFIIQFFVFASTFADLVISSMPNAEMAGTIATFAFSLTLTFNGIMQPPNALPGFWIFMYRVSPLT
YTIAGMTGNGLDGRPVVCSSSELSVFNPPSGQTCGEYLKQYLAVAPGKLYNYDATSDCHYCSLQSA
NQVLASSDIYPDQRWRNWGIGWAYIGFNIFGAVALYYAFRVKHYNPTSIVRGVCDAGRFVGRLFKR
RSEAPKGRKAQDGRVF
>g734
MLQTNIFAVLIALAPSAVLASPDANDVPPIVGSVVSSALSLASSYASEYSTIPPDAQSAVSSALSL
ASSYESYGNSVASSAQSLASSYASEYGGDGSDSTTTTTASVTSSTGTGTSSGAKTVTKTNDSTGAT
ATQASSTGSASYNRAPFTPIGGGLMAAMGILAGSIGVLAVVL
>g735
MKISSILERISLPVPVGVPGSAVPAILVGLFVALGGVLFGYDTGNINGILAMKQFRDQFSTGYTDT
IDGQDNQPDFTAGQKALIVSILSAGTFFGALFAAPVADKVGRRYGXMVSCVIFIVGALLQVAASTI
PVFAVGRCVAGLGVGMLSTLVPLYQAETAPKWIRGAICSAFQFAVTFGLFLAAIVDNATKDRTNSG
AYRIPLTVQLAWAVILILGMIALPETPRYLIKRDRHEDAARSLARLRRLPMDSRYLAEIAEIAEHH
EHELNLGGSSYLDCFRGNLGKRLMTGCLLQAFQQLTGINFIFYYGTSYFSSANMGNPFIITMITNS
VNMLATIPGLFMIERWGRRPLLLFGGIGMAISQFLVAGLGTGLTQSDATNKASIVLICLFIFFYAC
SWGPVVWVVPGEIFSLKVRAKSMSLSTASNWLINWALAYSVPYMVGSGTGNLNLQAKMFFIWASFC
VLASIIVWCLVYETKGLSLEQVDEMYAEIDKAWESHDFQAAVALRNSQRRSVNSQAQPWNRHSAKS
ASYTVHIEASRSLTTEDSERHQSRSSRGTIIEQEPSIRLRQFP
>g736
MSGDLAKEVKLVVPTLTSSPNSLQTPDQSATQNTSQQTCEQRKDGDYHSNVDEDIEAQKTKRKSKL
RAYWNFLRYTALNVYRRLFSFIFLANLAVFIAIMITDQSSLLAFVNATAANLTVCGLMRQPLVVNT
IYLVICSIPRSAPLRLRRIASKAFHLGGVHSGCGVASFMWYVGLVALISKHYWFPEEYGNSSSPDA
AQQQVTIIPTAVVVIAYIILVLLLFIMIAAYPAFRFKHHNGFELTHRFSAWAVVALFLALLLAFSD
QARRAEDLSLGQYLIRLPAFWLLIVVIAAIVHPYLLLRRVKVEPEPLSRHAIRLHMNHARTRFGLG
IQLAKNPLWDWHSFATFPDSAAEKRRGSALQSSNSFSCLISKAGDWTKDTIEHPPQYLWKRGVLVY
GFAYVMRLYEKIIVVTTGSGIGPCLSFLGDGNRPMMRVVWQTRSPLKTYGQGIIDLVKQMDPDPLV
MDTDLTGRADMVPIIERMARDFGADAVCVISNPRLTKKLVYELETRGLVAMGPIFDS
>g737
MGGQTSSMAHNTRFQRPVYKDLLSFALDEQPVAYDEQRPLFVDAENPALSLNARQVRVFVRTVIAG
LRKRAGVETGDCVLVTLPNNVLYSSIFFSIIGAGGIYMGINPSSQYAELEHFLELSTPKLIITAPA
GLNLLQEVTEAKGIPQNRICVLDDYAIAYLSAILSSRPSSPTEAMQEDNINHARHPETLNFSALLR
HGQEDWIKFDDKVRSMTTPAAMYTTSGTGGLPKGALLSHHAIIMHHQSLYYETPYDTTRLICIPMF
HLFGALYTHIWPLRYGETCYVLPRFDIAQFVQTVYLYRITETFMVPAMVQALNKYPDLELSQFFRS
LRYIGTAGASLDSASAQRLEAKLHPEAQISQIWGMTEVGVAFQMRYGCRNETGSIGRLLPNYEVKL
LDVHGLEITSDDTPGELYVRGPGVLMEYKGIPEAKDEHGWFRTGDIVSVRAGMYYVVGRAKELIKV
RGWQVAPAEIEGVLLKHPCIADAAVVGIKKEGSSHQLEDELVRAFVVRRRTTAAARLTGDEIYRFA
RHQLVSYKSLTGGVVFVDEIPRTPSGKVQRFKLVEMCNYRDIVSSLQLFFGPPQVVDNPIRLAAVH
SLGLRSVGHRKSGVGTPRIELRS
>g738
MPSMHERIQEDLKLKERQLWTALTSADPAHAVNKLCSPSANLLFPNKDIIALNPDQPDKNEKTLQE
VLAPPFHRFDDFSLMDVRPMVIDLMAGTITYRISAYRGKREYNATGSTTWAQGSDGEWRIIAHQET
LL
>g739
MRPSTSLIPLAISLAPDHASAQVTVDRATTYQIIDGFGFSEAFGFGVAVQDAPTIQQNQALTYLFD
RTDGAGLTILRNRIAADPDNTIEPNSPGSPSADVYEDGGYLCGVTGETCTTGDWRQAFANYLVKYL
QDYESEGVTIDFIGFLNEPEFAASYDAMLSDGTTGIVCCDAEGWNDQVTYTEQIIAAGAEEYLSRI
SSHWYTSQGTSPINTTLRVWETEYADLDDAFSTTWYSFGAISEGFTWAGYIYQGAQSDSNAAGLVT
LEGSGSSTTVTPSATLWAFALFSRYIRPNAVRVATSGTPTNTKSAAFTNTDGTVAVVMLNSGSTSQ

NVTVSGVSVASVEAYVMDNSVSSPAALSASSNGDVEATLPAYSLVTHNLSATSIRKKVSFDLADLT
DDSDPEDYRDDYKSDWSPRSAITGITPRRLRKYVIASLVFFVILYITFHRSHPPRGLSPYLNYDAI
DWTQYAYTQYATDETYLCNSVMVFEALDRLGSRADRILFYPKDWDLKVNFADDRLSQLLNLAHDRY
KVQLEPVTMDSLHQHESEDRATWDESINKLWAFRETTYRRILQIDSDTLILQNLDELFFLPSSTVA
MPRAYWNSPENIKLTSLLVLLEPSYKEYKALMDKVQGVEAXQLGTGRDALYDMELLNQRYGGSALV
LPHRQYGLVTGEFRTKDHQKFLGNDYETWNPDKVLSEAKLVHFSDWPLPKPWILWPTAQLAKMQPV
CVHNPGTPQESGCRDREXWKQLYEDFRLKRKDVCKLLSYPAPEWPPRPKDMSNLPNQPPPQDLPPQ
APSVDYEVDANANIDATDESNAKVDTIAKAEVGG
>g740
MRATTVSVVALTSVFAALTSGSTTGRPQNGPACCRALKGILPADTFVSNSTEYNAEYHDYWSSTED
LTPSCVFLPDTTDKVARAVQLFTRLNCQWAIRGGGHSPIPGAANIDGGILLGTDHLNTIEINHQEG
YVRVGAGNRLGAVYNATDPQDLVPIIGRYEDVGLGLAVGAGFSFLCNREGLTIDNVLNYEVVIANG
TVVNANKTSNPDLFWALKGGNNNFGVVTHFNLRTFPTNGTVYGGNIVHPESSFDGVAELFYDYFTY
QAIEDTLTHVMPSYEYIGSTDVTTAVSLVVYNEAVDELPPILQPWIQVPYTSNTFSRRTYGSLAQE
EGSGIFDGFAVDQRLFSVFPDRKFLKEAWDIQLQWLRDRKDVPGLVGVQVPMPITPNQVAQGVAKG
GNALGLDTTNEGKSIVVILMLLNFSNLSDLPRLRVEHNALMEELVDLAKKRGVYHPYIMLTYSGAG
QQAIASYGADNVAKLRKIARAYDPTGVFQHLVPGGQKLPSD
>g741
MEREDCRKRWIKLDSKINKGLWSQEENKRLHEGVAKYSTVWTEVAQVVGTRQPDQCAKRWQHFLDP
DLDRSEWTPEEDNILLAEVEKRGRNWAQIVDEVLHRRSANDAKNRGNPSSSGQRGEARVLISTTAN
SELGHEDLHIDSDMFSQSANSAGKQPSNTASPSELPHNGSTPEILGEMEIEHTGSLESWVDYVMGT
GILMGTDCPTPALCNDDSRINTAQSEERKMQLDAITRTTTTTTTSNTITTDPRLTVDELQTSLDW
DWDQVPDMDLTPPDRESIQSRTMILQQMQPEQISRVMDFLLSWNTPIDVKIVNRE
>g742
MSYIASHEKLVDQTMDHFFRRLDELFVDTNDVCDLGTWIQYFAWDVIGNLTFSKPLGFLELGGDVE
NITESIGKFFGLNGMITQMPWLDYLFFKNPILRRVKNEKASPVVTFAMARAKERLEELEKNKAETM
NDRDFLSGFINAMSKDPSIPKSALAAWATSNMTAGSDTTSILTRSVFYQLLKHPEKLQCLLYELDQ
AEQEGRLSEYVSWQEAQQLPYLDAVIKEAGRLHPPFGLHLERYVPPEGAIICGTHLPGGTIVGTNS
WAIHRREDTFGKDADEFRPERWLCDEAERRKMGRAILTFGAGRRVCLGKHISILEMYKLIPSVFRR
YKLEFAVPNGRSYHVHNAWFTHQTGLDVRFEKRTASN
>g743
MISFIRFGSILGLIAQLAAAAGSYNISPLVRKLSPGAEVFYPSAANWSEVTPRWSTNDAPTFFAAI
KPATPGDVQNIVRYASRNQIPFLGVGAGHGFTTTLGELKEGLEIDLSQFNKVSVDAKNNRMTIGGS
VRFEEILDPLYAAGKEIRGSLGAGVGKYQGVHGLIIDALESVKLVTATGDLITVSKHQEPDLFWGL
RGAGFNFGIITEATYQVYDLTNNGXVMNADFEFAGNLNQTFFEFLASFNGKLPPKLALFGVLANDA
TVGVSLNVLVAQPTILLNAVYIGPQDEGLAYLQPLLELPYLRRNITQLTWNTVFDAHMFGSIAATC
KGGNPQNNWSQGLAGIDVPTHVAYYNALADLWTQHPAAANAILSIEVFSPQTALSVPDGETAYPWR
DTAVQIILNFPVNDDTVTNFAEKWVPEFTETGGFNESRXYVSYAHGDESLETKYGKEKLPRLTALK
TKWDPKGLFSFNNGLPVY
>g744
MVDGYPENAVAVIGMACKFPGADSLDDYWQLLDEGKSMCESAPESRFATTENRRHQEKSIYNGNWI
SDIDAFDHKFFRKSSREAASMDPQQRLLLQVAYQALESSGYFGPVEPRTDVGCYVGVCASDYNDNV
AAHPPNAFSTLGTLRAFLTGKISHYFGWTGPSVSVDTACSSSMVAIDAACNAVQLGNCYSAIAGGV
SLFTSPYFFQNLSGASFLSPTGPTKSFDADADGYCRGEGIGLVVLKKLSNALADNDNILGVILSNA
VAQSSNLVPITVPYSPSQTSLYRRVLKQANVSPEEVTYLEAHGTGTRIGDPQEYDGIREVFGCQGQ
REQPFHFASVKGNIGHTEGASGVAGLIKTLLMMERRVIPRQGSFKQLNPKITLEPNLMIPTSSVPW
TAKTLIACINNYGAGGSIAAVIVKEAPKLSTQKKSSSLAIQARYPVFLSAHSPQSLSELASKLRTY
INHLPPSLTRNTLADFAFNLGDRQNRSLSNIVATTVSSMSELDDQLRILSSVPDSAPAQTAVVDPK
PIVLAFGGQTNRSVGLNPAVTEASGLFRSYLDECDRILKSLGSNSIYPGIFESNPRDDIVGLQTMQ
FAVQYASAKTWMDSGLRVDYIVGHSFGQLVGMTVAGILSLEDGLKLVHGRAALMQQGWGPERGEMV
ALDADLKTTQELIATSSPNDKLEIACFNGPKSHVVVGSNVAIDGLIDKLRVSSAVKYKKLNVTHGF
HSRFTDAILPALESLAETLTYCSPQIPFEACSDGSSWSAPTPQLIAQHTRIPVYLGQAISRIVSRL

GSCIWLEAGSNSSITSMARRAVGDDVQSHSFIPLNLSSPGAIGSLADATVQLWRNGHHRIQFWPFH
RSERVSYTHLSLPPFPFEKTRHWLEWVEAKPVAXPAAILQDTDKDNHNNQLLFSPMWLDANRQTAR
IRVNPRTDEWHRVVEGHAVLNNPLCPAPLYIELVARAMKILGLGEVSAFHSLEIIAPLGLSQDGDL
ALQLEQQTNNNWSFSFVSQPLNAPKQDLAVHATGAVTIASDNNSVKAEFDRTARLLPYSRIKEFLS
QEDGEAMHGALIYKVFNRVVQYDDYYKGVSHVVAKSGQVAANVVLRNPPLSWAAEQVCNPLALDNF
LQVSGLHVNSLRDCADTDVYVCTKVDRLSISPSFFTSSSQPGHGCWGVFSNLNVINDKQVENDVYV
FDATTGELVLTVVGAQFSRVLINSLSRVLSRANNSSISTTAAATTTITTASVLPVPVTAPFSESPF
ASTQSPTAPSSSATKARVVRKNKTPNIDKELRQLLSRITDVPEEEFSGQATLEDLGIDSLMITEIV
SEISTTFSISIPQEDLQDLVTFKSLSTYLARRGARGNSADEEEIVEKDDSPNLVVLSDIAPAVPN
TVRADSKRDESLRLQLAQLVASHLEVPASNFVRTTNLADQGLDSLLCMELASDIERSFGVRIDVTL
LTTESNFGDLVDMVFGRGNGSEKPLLPSSILSSSGTESLGTSTPAGTDTDYEQISLPDSGCNSIQA
FEKIQFDYDQYAKELGFIGFWKNVYPTQARLVLAYVVETFRDLGCPLDALQPGQTIPLLPILPKHD
RLRGVFYEVLRDGQVADYTGKDYIRSEKPLDSETSAEIYSEIIRAHPQHAKEHELLHLCGSIMAKL
VSGERDPLQLLFGTKKNKELLEEVYSNGPMYRAMSQLLAKFLKQALSNRSSPVRILELGAGTGSTT
KWVVDTLVRSGISFHYTFTDISTSLVAAGRRKFSKYDDCMEFQVLDIEKDIPQKFFRSFDTILSTN
CIHATSNLPNSLSHIYQMLRPGGFVSLVEFTKNMFWFDLVFGLLEGWWLFNDGRQHVLADEAFWER
CMRNVGFQHVAMTEGPSLESNTVRIITGFTNKATEPPRTASWIERKRDETEIETVAFNTTPDGVIL
RADIYHPSSSSITGTSGLSSLKKWPIALLIHGGGHVMLSRKDIRTKQVQHLIRHGILPVSVDYRLC
PEVNIVDGPMRDVREAFVWARTQLSSLKLSASPYLQIDDSRVAVVGWSTGGTLALTLGWTPEVKAP
DAILSFYCPTDYEDPCWQAPNFPNKSEAEAQNEDYDLLEGVYNQPITSYNVPPSKRATGGWMCPSD
PRSRIILHMNWRGQMLPVLANGLPSKDASRKLSTEEQKQIARLPQPSSEQIARFSPLAHIVKNEVH
SPTYLVHGTEDDLIPWQQSQRTYEALVAAGVDAGISVLEGQPHLFDLFSDGDGKKWERILEAFDFI
FRHLGIASKQ
>g745
MATLTEIAQNILESARALDEYTASQGLEPTSFTQHNLPTLSGDLEVVRKKLVDSTTELKALVQGPI
GQLYDVLFAFTDLMSIRFIYRHNVPHYVPLEGDISIQDLAEKTKLDPIRLRRYLQHTMMYRVFVES
RPGFVAHSSVSRLLKVNPAALDTVGFLVEELAPAGTKVVEAEAKWPNSAEPNETGFNVEFNTADSF
YLEIAKTSERARRFGAAMRFMTSGTFYDISHLIRGYDWKSLDVPGSIVVDVGGGQGGVSQALAGAT
SNTRFVVQDLEGTVLEGQRILPQHLQERVSFQAHDFFTPQPVKHADVYFFRYILHNWADKYATEIL
RNLIPAMKAGSRVIIYEFLLPDVSQPEWTKKQWR
>g746
MTAFLVGRTICGLGGSGMYTGVMMMLSFLTTPEERPAYLGLTGLTWGLGTVLGPIIGGAFALNVSW
RWALYINLIFGAVAAPVYFLMIPELQTAKGSIISRWKKVDFLGTILIVGMLISCLMAINFGGILYP
WDSGQIIALFVVAGVLAVVFGVQQALCIGTSTEYRSFPVQFLRNPQVLLIFAAEICAITLTFIPIY
FLPLFFQLVQTDTALQSGIKCFPLVIFLVVTIVGNGMVTARYPWFQPWFLVGSIFGLTGAVLLYCT
DETTSDARIYGYSVLIGFGAGCFVTLCFATAQTQVKIAEIPHAVAFMAFAQQGGAALTLAVSNSVF
LNVAIRKISEILPDEPRSGIQRILSGLGSDILSSLSKTDRYRVSQAVAASLSNAYIVTIICGALAV
ILSLFIVRDRDWEKQARLAVGKVLGRPKETDREEELGIHK
>g747
MSEKVASDTELGDVTAPTPTRSIQGLRWFLVVLAILSSTFFYGLDNTVVADIQPTVVQRFDAIDRL
PWISVTFLIGAATTNFF
>g748
MSPSVIVAEESTTTAGTAVSKATTRIDSGDIFEIPTVDISAFLTDPDSPEAQAIVPIVRSACRSTG
FFQITGHGISKDLQQSVFTGAKKFFSLPFETKKALDAHSNQGLRGYDVLASQSYEDGVLPDLKEGF
FTGLDLPKDDPRVQARRFLMGSNVWPDTSLLPKDDFCKPAEEYHAALMKLSISVLKLIERTLPYGS
GIFDNFIANDPIAPMRVLHYPPARPEDEKNGKTQYGASAHTDFGAITLLLQEESPGLEVLDSRTGE
WKPVPPNPDVYVVNIGDMLSFWTKNEYKSSVHRVINREPGDRYSIVFFFDGNGDTKLSPLDGSEED
NPLTVEQHMIRRVVESYGKK
>g749
MTRTLLTGGSGFIASHILDVLLKRGHSVVTTVRSQEKAEQIKTSHPTVSKENLDFVIVPDVSAPDA
FDKAVVSDPPFEAVIHTASPFHFRIQDVKKELIDPAIDGTVGILKAVKNNAPAVKRVVVTSSGAAV
MDFTQPKTYTYTEADWNNMTWEEAVSNPLNAYRASKAFSEKAAWDFVEKEKPNFALTSMNPPMVYG

PIIHHINDLNGLNTSNERIRDIAFGKAKDGLPFTGVFVWVDVRDLALAHALAIEKPEAAGQRFLIS
PGQYTNKDIAEVISEEFPELRDGLPTGDALKGGDFPPGGWYNWDTTKSKEVLGVTYRPFKECIVDT
VNSLKPFVK
>g750
MAMNRLETGFTWPTISLRENEGDARSLPELIDFHARENPGHRFCIQAQKDGDAPLLDITYTQLKHA
ILRCQSWLCENVAEIQMQNGIKDRPVGLFMDSDLTLVLYLFALMGLGIPTVLLSTRLSAEAVKHLM
QNTRTSAILVSARLNGTADEALSSWDTNDSIPPSKYFPAAYEQFLTEEVSPSPKTVSSQNNVSREN
YYVSESDCNVLILHSSGTTGLPKPIYTSHRHYLSFALCHEFKNKEEMLSPVLSTSPLFHGFGLLPP
CLSLGVGKPFCLPEAGTIFTGPSTTQLLRSSGAKSLLTVPSILEEIALLPDNEGINALKELHFVVF
GGGLPKETICDKLTAAGVKLLNHYGATETGPLAPLYVPQTKYDWHYFKLRTDIQDALQVRLEAIGD
EADQWKLSLRPVGWNERFPIQDILVPRPSPDSDNEYTVLGRSDDLIRLATGEKVRPTILESMLAQN
EAVKAAVAFGDSQFEIGVLIEPTFTISSVEEENKLKSSLWPIIQQAGEKMDSHARISSPEAIAVVQ
AGSLPRSDKGTVLRREVNQRFEKEIAEVYRRLDAVIDYSIPALRIDSLEDDLRKLISDNLKWTNNS
ELTDERDFFELGMDSLQATILRRLLISSLRDGLSKVSIGRDFVYQHSSVAKLAKAVREGCQDQAMA
SIDGSFLEGFIDTYSLQLPHVNGDRNAATVLLTGGTGSLGSHFLAHLAQDPAVRRIVCLNRPSKPG
QDPYERQVMALTSKGLSVTPEEWSKVTVFGTNTNSPKLGLSEDVYTWLCERITHIVHNAWPMNFKM
QVSSYESQFRVIQNLLLLAREANSHQLSRKRMRFLFISSISVVGRYGKFTGETIVPETPIEDFRAA
LDLGYAQAKLVCEKIIERARADFYDEIEAGYIRVGQIAGAQRGFWNADEHFASLVASSETLGQLPA
IRGTLSWLPVDLASAVLKEIIFNPEPLELVYHLENPVRQPWNEVLDILASELDVDKQRRKPMAEWL
EEMRRRPVDESNPAAALADFFQEDFEWMSGGSIVLSTETSRRYSPTLRRAGPVQEETIRRYLQFWR
GIGLIHQRKNQVEPSNPSSCS
>g751
MDNQKTQPKRVSIKVEPITTAEDLGRFFDIAASAFARQAKDNVWITLHPGWDTPEGRANAISGNTK
RWSAPTKDRNGYPNTIFLKATVSRVDEPGKEDIAGVAIWAQASMVEGYGDTPTNDIDKIMDVNALY
PGNLAEQEYARQLLRSFYARRVEVANEIASSSSPALMILDLCAVDPAFQRLGVATELVRWGLDEAK
RRGGLEATLEASSMGRHVYKKLGFQQEGGELHFRVDQEFEPRDRPSVVFMRTGRPGL
>g752
MGSPSPVIAPEHILQLLSKLHRISLEQEAAISNLDKGKVYSSKVLDDLEDKHGPKGSSKAFDQLML
DKFIALDEDKCQFMYQLINAMGATNVIEAGTSFGVSTIYLALAVAQTKAVTGKSGTVIATEKEPQK
AKIARDYWAQCGELVEQQIDLRVGDLLETLKFGLPQVDFLLLDIWSSLALPTLKIVQPYLRHGAVV
LADNTISSAEGYADLLAYMRNPENGFRNMTLPFTNGFEMSVYMPSTK
>g753
MTSTTSSPRLVTVAACQLGPVHLADSRQDVLKRMLSLLEQAAQQGAKLAVFPELAFTTFFPRYLLA
ERDLDQYFDVEDPSKGGIEQSPNIKPLFDRASALGIDVYVGYAERTIQKYGTHVDYNSSVYYSANA
NKIIGKYRKVHLPGTVEPRTEPGAVQQLEKRYFRPGDLGFPAFRAPGLVEGALKKSSGIYNATQST
GKGDPIIGMLICNDRRWPEAWRVYGLQGMEILCCGYNTTAFPTTATGHQLDLTPEAAEEMVLLHHK
LSCQGNSYMNACFSINVAKTGAEDGNPLVGGTIIVHPLGHIIKEAQTKEDELVVATIDLADCHRPK
NTVFAFEKHRRPEHYHLILDRTGVIEPDLL
>g754
MGLDTIVRNGLIVTESGVLPSGQEIGISNGKISLIGYDLPEGPETNIIDAQGAYITPGGVDSHVHL
AQRNAPTGDTWETGTRSAVAGGTTTVLAFASQTKEMASLFPALDEYHGRARGQSYCDYGFHFILTN
PSPTILEQELPRIAKDEGITSVKLYMTYELYKLSDRQLLDTMLACRSLGLTTMIHAENSDMINFLI
QGLERNENTAPFFHAISAPKIAEDEASYRAIRLAELVDAPVLLVHVSSDGAMKHIREAQTRLLPIH
AETCPHYLFLLSDKLANHEHDAFHGAKGVCSPPLRHNPDDLEALWRGISNDTFTIFSSDHAPSKYD
HPQGKKAGIIDGIPKFSSIPKGIPGIETRLSLLFSESEGCLPKERARLSLARFVQLTSGNPARLYG
LTQKGSIMPGFDADLVIWHPHSQGEKIITQERLHHGVDYTPFEGMRVRNWPRFTMLRGEIVWNADK
DRVTGVKGFGNFLKRENGRVLVGKLGQLPAGMIEDERDLWRLKSVT
>g755
MSFYVVAGAGVVGLTTALELKDRYPSANVTIAAKYLPGDSFADYASNWGGANWFPASADNGRQEKW
DSITYKKFKQLSRLRPDSGVRAMNIRFHYDRPIEEVGIITPSTGKIWFEDLVGGLKPIEKDELPDG
AAFGFEMASFVLDVQRYLPWLQMEAQSRGIEVHRKIFTDIREVFSIYPEVSVVFNCTGLGSLTLGG
VEDQMLYPARGQIMLVEGPQEPIPTMYFQAPHRDADATHIFPRGRDGMGGVILGGCREKNNWDGEV

DMEFAEIIKRKCCALVPQLGKPEDLKIIKHGVGLRPSRKGGARVEREYIDGKLVIHNYGAGGTGFQ
ASWGLALEAVDLISERARL
>g756
MATDHADVILPQVSGSSGFRLINILLVNPNSSEYMTKNCLTALGPSLPSDVIVHGFTAPYPAPTAI
ESQTDAVLSTEACIRAIKPIGDKYDAFLVACFRAHPLISVLKEEFSQPVLGIMEAAMYAARMLGAN
MGIICTSERSATAHGRTVSEYGFSDYFAGCEAAKLGVLELDSQPRAEVHAVITQKLNRLVHERGAD
CVLLGCAGMAEMRAICEATVEGSGARVLDGVGLGTQILIGLVREGLRTSKNGIFKDSSLDRVKRMQ
DWL
>g757
MPVSAGDQPSSLPVEFLTKFHTALRRWQKSWETSSDPSITPSSPKGPLGFNATAIFRIACIRLHLN
LGPHRSLRTGDPEVIASAFCNALRPAQTPQIYQAVLQSIHALSIPVRLGVEYVARTQTLTWSTIHA
LSNLECAVFLCKWLETLASDPSHVSKDTRRILRIITSVLREADLAPPVNWAGDFQPDQLRRMGAML
VRLLSEILKGPHVFEMMYVFCDSLRTYANLLENDLDSNMAE
>g758
MSSISTTSDDGRLEKPLVERKTVGVQLQEPPFGLYHLRHKVLDYALFLCRGRKGSRLNSNKHNAGT
DDSESNTRLVVLDESATMEFDEHVRTLSARTPAEGGPDADRERIDRIMSRDEDTFLEDKSKAFELF
EQYWNIDMDDVALFTSELVSSYHSRIVKVNGDICLDISYNIREDILYDETNISSLESTITVNVIKS
TDLRVQHEYESLYFRHNITTRQTTYMLPNASREYRGLLHSVGSGLSERASRMHPFSLHAVILFQSL
AARSAQIDDLYRRLLWIETQIYQGSIFQGTDSAKFIRYIQLSHKLSRNLITLEHRNERDRSHIEKL
LEDHKRLRRLIKQSSPNPSKQIDMNTHEHVRDSLLSLKDLAHDQDRQIVNAQRKTQLFITLLYNLI
TGHDSGINLRIASETAKVAHEAKKDSTSMKIIAGVTMFYLPATFVCSLFGTNFVALNTNTSEPTFV
VSKLWWVYIAFAVPLTAATMMGFLIWRRWRRGQVIVEDRYEV
>g759
MNSESTNIDAINTSTRTNTDFTDGNNTMNNLNNNNTDYNNDYTQNKTDSFNYNADNKRDNLRQGVS
QPAETATTNTDSFERDSAPRESVGRGFEDSESGNRNFENRDTQSRNFEDRNVENREVEDREIDNRD
SNRDVNTDSTNQDDVPKKGVAGVIHSNFGADKTTTSTFEKSQGEKYGENFDETGAKIKGRFSVDES
AAASGSGGATGGGEYSMGGKQGAKAGKDTNVPSEHGSHGSGTQKESFGHKLMDKAEGIMHRHHHDK
AESRTDVQ
>g760
MDEPPEYIPDAEFDHGAERASLEGRDYNRPLPSSLVLARTTETQATIRAHKIVSAYIAACPPFADM
ALINEWRIVCRINHTDTYEISLXGLPCRTDGACDVNETYIQLAPGPAVMTNGRLEGMAKTLKISVK
SGSTIGDLLDEIFEKNLNQFQLLHGRGNRYWLWGILQVATRTFHALDENVVGMTYLSQGWDQEFPI
RPGDFRAPAKGLWLDSSYWRE
>g761
MTALPDPTVDLDWSGYVGSIQDIFGKNAQTHPDRTCVVETASAEGPERRYTYKQIYEASNTLAHYL
HDAGITNDDVVMIFAHRSVDLVVAIMGTLSSAATFTVLDPAYPPARQKIYLEVAQPRALINIGRAT
DEAGVLAPIVRSYIDDELNLKAEVPSLRIGDDGTLSGGEIDGKDVFSEALRKASAPPPTQVGPDSN
PTLSFTSGSEGRPKGVLGRHYSLCKYFAWMAERFGLSSESKFTMLSGIAHDPIQRDGKSIRPLQIM
PSRSDIAHERLAEWMAKYSPTVTHLTPAMGQILVGGASATFPSLKSAFFVGDVLTTRDCKALRALA
ENVNIINMYGTTETQRAVSYYSIKSRAEDPNGMDHLKDTVPAGTGMQNVQLLVVNREDRNKMCTVG
ETGEIYVRAAGLAEGYRGDQALNDLKFVQNWFVDNNKWVELDQKNNKGEPWRKYYKGPRDRLYRTG
DLGRYLESGDVECTGRADDQVKIRGFRIELNDIDSNLSQNPMIRDCKTLVRRDRNEEPTLVSYIVP
EMKLWPQWLESHGLEDVPDEGTDFGVTKVYLKKFRAMQTEVRDHVKGRLPSYAVPSLFIFLKALPL
NPNGKVDKPNLPFPDISEQTEVASADDLKRWESLTETERTVATRWATLVRGLNEKKVSPDDDFFTL
GGHSLLAQQMLLDIRKETGAQVSINILYEHSTLRAFSAQIDKQLHGGETQEESQEDPAYAKSLDHL
LTTLPEKFQAADPSTVRASASPTVFITGATGFLGGFIIKDLLERNTRQLRIIAHVRAKDAESGMAR
LERSLKGYGLWQDQWKSRLSCVAGDLAKPQLGLSDEEWQRLAQEVSVIIANGATVHWVKRYQEMMA
ANVLSTIEAMKLCNIGKPKIFTFVSSTSVLDTEHYVRLSEKQISTGEGAITEDDDMQGSRRHLGTG
YGQTKWVSEHLVREAGRRGLRGSVIRAGYILGCSQTGTCNTDDFLIRFLKGSIQLHSRPRIVNTVN
SVPVNHVARVVVAAALNPLEGVNVVHVTGHPRLRMNEYLSILEYYGYDVPEISYEDWKHDLEKYVS
AGGQEKDQEQHALMPLYHFCVNDLPGNTKAPELDDRNAVKILKLDADHWTGVDESAGYGISREDVG
RYLRYLCETGFISWPTGRGRPLPEVRLTADQLQVVGAVGGRGGVSQ

>g762
MADTVDKAVLKQVQIALQNADVELQDINKKIWSNPELAYNEYMAHDNICAMFENLATARSMNYQIR
RKTYQLDTSFEIEYRKGNGGRIVVFNAEYDALPGIGHACGHNLIATSSIAAYIATVEALEALDTSS
LPGYTVRLLGTPAEEGGGGKLKLIEAGAYKGVDACLMVHPVTQGNNDESIPSIHIAGPESLLANNK
IKINYTGSTAHAAGAPWEGINALDAVVSAYVNISLLRQQILPSQRVHGVIANGGERPNVIPGSASL
HYYIRSPDTKSLNTLTDRVFKCFEGAATATGCKVEFEWINNYQELKNNKPICEAYMETLKAMGHNC
VLSDREVKGSLDGASTDMGNVMHQVPGFHALFQIPTEDGAGNHTHGFTAGAGSSEGYRRSIVCATA
TDIPFKNENAFAQGRPRRPRKRIGIVTKPQSGNFSTYKEPTPLNLQRVDFPIEIYAFQYWIENLAS
WPEDLFDVGQEYGAGARYHSRCVDETKSSLHLAVSAFSLAIFGRANNAYEILQRAEKNYARSITEL
RKELKVLSNNIIDELLIATMILSKYDNIMYGFRVRKSLKPLPPARYSGLPLLGSYAYHYKGTASLL
QVRQERGYTQNVPLEKAVRRTIIRAHLLSGDPVPEWLQDDTYGEPDPALALDYLMIKAASLRSRSI
IVINQGNRGMPSFIRHGEALKMVLAEAQMLDRLFAEWAENIPQSYKFITHKIPQHNDKKVPLEHHN
LFFDRMFHSYATHGHAVVWHRYRAVRLLINSIEMDLLSQVAQYRPENPGKYLELSKVCQETINNLI
TDLCRGIPFFYLLNSCLNHGQRHTERAVELDDSMTIISSQFPPLMMAVLLAWPITIAVGIEGVPPD
QMEWLKSRLQAVTQMVGSPMLQVTRK
>g763
MSLKFKVPASLQQSLDATKVEYVRLGNSGLRVSLPILGCMGLSMTGTSPYANWLLNKEESLKVLKA
AYDNGINTWDTSNVYSSGQSEEVIGAAIRELKIPRSKVVIMTKCAFAVGEDETVLGQGFPDLLLQS
KDYVNQGGLSRTAIIKCVDDSLARLGTTYIDVLQLHRFDKNTPIEETMKALHDLVQSGKVRYIGAS
SMWAVQFAQMQFVAEKNGWTKFISMQNYYNLCYREEEREMIRFCKETGVGLVPYSPLFAGKLARPV
GYNVSARSKGPTAHINGLTKADEEIISRVEKLAADKGWKMSEVALAWNKSKGTIPIVGLNSAERIE
EMAGLRDRVLTDEEAKHLEEPYEARAVAGHF
>g764
MEVLAAEQYTLQVPTERILHTNVPYSAFRQTPQALEQPKRKVVAIDCEMVGLWKNTDCVALLCAVD
VLTGETLMNTYVDPVSKVLAWRSTVSGITRRSMTDAIEQGQTLRGWAAAREALWEYIDAETIIVGH
ALQNDLNVLGIFHPRIVDTAILASQVVFPETENKKFPESYGLKRLLLSWLNMNIQTGKKGHDCLED
TLATRELAIWCARNPDVLKSWGLQMRVAYEARKLAIQQAREEAKRRAKEAAKKDAEEGAEKVVIAD
VKKNVKYTIKPEARKEVRRDPRKDIQKFVKEDANVDAKNDGRKHTRRHPRKNNRKQGSRNEVRDKQ
EAKQSIKVDIQQDANKGVKTDGDT
>g765
MRFAEISLILALSTAAVAAPHPDGEKTLYKVGTSPRYILARGSGEKVLHKGSASQLLARQTSGESG
SAGNSDTCYGVDATCASCFGTNWAPCSDGVTCYDKTDPTSTCTSSGSSGSGSGSGSGSGSGSSSGG
SAGSSDYCYGVDATCVSCFGAGYLECSDGVTCYNPSDPTTSCPDGTSGSGSGTGTGSGSGTSTGGA
GSGLIGISTTATAAGSTATSGGAGXGGSSXTSAAPTTHSTTSTTSTASSTSTTSTSTSSTSTAAPS
PISGNKLGGAAGFSIPLGLSAGAGAAVLVAAALI
>g766
MPRKSASETFHDAKSNVRAKATSIKAWELPKQPTTFAPDGTWTNIDNDVTPVERRIWGHWALLGYW
MSDILSAQSWEGASTVISGGLTYKEALLCLIMGTFIIAIPISFNGSIGAKLRVPYPVAARSSFGYV
FSRVPVVIRMVTALFWHAIQTYAGSTAMTQVIRAIWPSYLNIPNHLPESAGITSQQLLSHFIFWTI
QLPFLLTPPHKLRWFFVFKGIFVTAAAVGTVIGMCKLAGGSGDIWDQKPQYTGSTRSWMILSYMTS
LSGGWATMATNIPDYTRYLKKPRGIWLQALLVPAVCTFIGVLGIISTSASKVLYGSYIWDPLELAS
HWEGPGGRAAAFFVGLAWVVAQIGVNVSANVVSFANDLTSLCPRYINIRRGAVVATIIGGWVMVPW
KIVYSATSLINFMGALSIFLSPIAAILIADFWVIKRQAIDVPALYRPHARYHYVKGCNWRAAAALL
ISLGPNLPGLVNAVNPAVDIGGAEKIYNFNYLWGFSSAFVVYVVSSYVFPVPETLISVTIHDDGNV
FVGQHVSEKEILTQEALPEKQV
>g767
MAFTSFAGRSYNVVSDEVKQSGSFDEIPMIDLTQPQEKVVESLRDACTRVGFFYISNHGIPQDIID
QVFSTAEGFFGLDDELKQQVHFNKGTALCGYEGFQNVYTDETKKPDLNEAFSWRYTPDMDSFNEKA
ETTRVEEKNVDNFLTSGQNLWPNAKPEMRRDISQYYSHVLKLARKLIRLFALVLKLPEAYFDQMMQ
HPGSMGKVLHYPPQPAKGADPPFGIGAHTDIECFTILCQGGDVPALQVLNPKGEWILAPPIRGTFV
VNIGDMLARWSNDTFRSTIHRVLNITGRERYSMPVFIGPNYNTVIKPLETCLADGAKYDPIPAGLY
VWKRLAISRLDKDEYERQRVKMESQLTLPIGA

>g768
MTTSSGMLSPMTMRRVHENVFFQMTPDVEVSQNLRKGDIWDEVSVDDVESLVRPHGATLVKLYFRI
VHPSFPILHKNVFIEKYNRTYREITAHLLAAVYALAVCWWSYDPDLLLQEKINEAALVRIAHHAVQ
DAMHRPRLNTIQAGLLLLQRHRNPLFMDNSWMWSFTSSLLGLGQHLGLHLDCSNWDIPEWEKGLRK
RLAWALYIQDSFSALVLGRPCLISEAADWIVKPLESTDFSEGAGDEEVSDQIGSVSIENGKVLFME
LVSLSRVTARMLRELYSVRSLAEVLEPQQVLAIAKPLGTDLAQMLKNLPEVLSLENIEPGKLCVNG
FLHLALHATTAALHRRLLWAIQQSKSEVDPQFVQFIRRMLRTRAISLVKFMSYLRPEHMEAFWFFA
AGGCATILGGFLGLLRATSVTLEESEDLQNLMKEFEWQLRMKAKMSEWVSYTLTRLRALGWDDWKN
SKVALPEPFLNTLSVDGNGNAETFVDLSLVLEDDVASSPLSNQSLSSAFIDLDNLMLWTIPGLNYS
GYDFIPSEPT
>g769
MVAAQSIKRIAGGVEENETVSFEAQPYAFRINPSATALVIIDMQRDFLLKGGFGYIQAGDAGVAKV
QATIKPTLAVLRMFRERGIHVIHTREGHRPDLRDLPTPKLLRQARAPNTRHSMVIGDVGPMGRLLT
RGEYGHDIVDELQPVAGEYVVDKPGKGSFFSTTLHEHLVDRGITHLIVAGVTVECCVTTTVREAND
RGLDACILSDCTDGFVPTFKSASLDMIHFSEGLFGFVSESQPLLAALSSLPARSGKSASEWNGSMN
IRSLKNAYLGGLSPVTVVKRVLEKINADRSQNPAVWLNLASTDDLLRRAESLEQSADRHLPLFGIP
FAVKDNIDVSGLPTTAACPEFEYVPEKSAFIVTKLEAAGAIVIGKTNLDQFATGLVGTRSPYGACH
CAFDSTRVSGGSSSGSAVAVALGQVSFALGTDTAGSGRIPASFNDIVGLKPTKGTVSTTGVIPACR
TLDCVSFFASTISDARSAWLAAKEHDPEDPYSRSSPSLASLNSRSLLHEESTYTVSFPPEDILKST
LCPAYHEQFAKVLSLVRGLVNVDEVNFDWLSYLSASDLLYKSAFVAERTAALQEVLSSKAKKITLH
PVTQKVLDLASSMSATDAFRDIYEAQRLSSAIEGEFDKCDILVVPTAPNHPTIAEVEQDPIGPNLK
LGIFASAVNVLDLTAVAIPAGHIEGLPFGISIIAPAYREGVILEVAQRIQARLKHFVL
>g770
MARKNDAKEQDLRYRSFNIAALIERALKAKRERAYGLTCVKILKLLEGDHNKVLLLTMSDGDELIA
RLPNPIAGSNWFTVSSEVATRRFVENICNVPMPKVWDWDFRDRNTVGAEWIIESKAAGEPLRKSWF
NMSRQAQLGIIEQVVDLETKLASLKFSSHGSIYLERWLYAKKLLCRRMASPKVIVNRDEASALMSE
RFYKQFVIGPSTDRRLYRGPGDLSKQFRGPFTDLFHYASILSRNERRYILNNPWAKADYITSTQDK
QDQSEYMELIYKYSALQEQLIRPEFNNDPCTLSHPNLSLDNIWIDPRTEKIVSLTGWQSTAITPPL
LKRPYPRFLDTEFQTQSGDRSQILPKERYRELVKISEPLRYDRIFSNPQKYELLVGPMSSIFNTWN
NSGIFRLRESLLAVRNLQQLVLANPVPELEQFSSAELESHAKEKYARQELDMLFNMIQNVQHTVNI
PRDGRILAEDFERAQKLNEDYRQQYINLAAGGRKTQLRKAWPFDSRDDKEAQNKASIPRESSLVKK
HPSIGKSPTVVRKIIVD
>g771
MASISSEPWTLVQHTIPAFQSRAYRRAVRDPNTSRLYLHVNEYRITNTTATTDPKGVTIIFAHGLE
STKEQYEPFFSDLLRTVGSGTTIKAIFAADVYNHGQSFLLNRNEIGDEAEWFDPARDIVQMINQFR
SQEDGSISRAYMAPPLVGMGQSFGAVHVLAPAAWHPRLFHALVCIEPVVENGSWHDDGEGTRHSTQ
FKYRLHQLKHSWKNMAEAREHFAKSPYYGAFESRVFEKTLKYELWRPDPTSERVTLVTPILQRIVW
YMKADPPYEGIAPSEDWATRSEKSIIPGGFYRAECDKIKDLMPGIHCKTLYLWSRNADFISDAAYR
KRLLDCTGTGRGGGGGMATGQVSEAFVEKGAHALNFENPLGTAVQVAKFLDVVWPAWLDEEAQRSR
EPVPRAVELPKDLVERLDRYLLESTKVRKARSNL
>g772
MVRHNEAPRDSIDSTLPQMSDLVPADGSLFRPSHARQPSHPNVFSDDYSIDSIDAPLFSNHQRNHS
FSSFDSTSTVEPRRCDTPKPVAENSTRVNLLDDQQPNRRSLASVHQQDESLTLGRTVSRQSHASSAS
IEDLNPFADQPNPSFAESARPSSIRKGITALNRQSTVSTTSTRSGYTAVPRAMSPYRGATTGPSHP
YAMYPQVGVGRSPSVATVSTIRHADSPLRGPTGPQHPYGMYPQNIDYEEDLGDQPIPVGFPPRGIQ
PQGATTQADEVGDIIGPDGHLEQLPPYSRYPDGVIRPTMGRGPASIASDIRGRWERYQDEPHTRSD
SSSGSGTLINVDSSEPLRSPTTSSGRSSTLVAPVPLEEKLRQKGQRKCCGAPIWLVVMSSMTMVIC
ALLGGVIGGVLGERAAEHRAQLAAAKHSSHPASVVTVTYTSDVSPYTSTPTGIAALPTGNYQVPWT
VTNVSKFCVSTSKQQSWNCQIMDPLPIDIQGSSFMSSSLTVQQPSIQDGHFNYGAQLPYDINPLQQ
QLNIMIDKEDPQLGPALFFQTMFNKLVIVQDSQDMQSKRSLDERDTSGFVNGQNQNTNYPKPWFCW
WNQTQFETFIYINQTQDATGSGTTSTTSSTTQPASITTAPSPTSGSQKRDLDLLLRDSITGNYPRK
VKIKENRVNPTAPQAYCQQMQVNPDNSITPIVGQRFNINETVPTGEHGGPVPCYCEWMT

>g773
MQSILRISRSTTVKPMCPSLLRGRMPIANQTLRYPSTIKRYSHQSYGGDDNPQGDRPASRKSRDLE
HPGPPPPDVSKDSAESNPSSKKQSEKQEQEQEQAKHEDLYQKKQPSNKARPTLSSGRESPNVDEQG
NVRDDVPEDVKRHNREMEQRHDKAYNQIGDEGKVNKGFWKGNGESLTEEQGGGRAQR
>g774
MGSVSPQCQDLELPHGVSFNFILSLLILVGILVSYLPQHIRIIQLKSSFGLSPYFVLLGTTSGTAA
FANILVLPRSARDVACCREISGVSCFAGLLGIFQVGVQCLSFYVILLLFLIYFPRATSLVNPTTSA
ETENESHGNENVENDEKQPSYSTAVIVSITCLAHAVIVLIVSLVVAIKHPSNLQTWANVLGIMSAV
LASIQYFPQIWTTFRLQKVGSLSIPMMCIQTPGSLVWAASLAQRLGAEGWSAWGVYVITALLQGTL
LVMGIYFEYLGQKDVIEHPFMADETPAANAEQDQQATENTPLLSEQR
>g775
MRFRWATGLLVVLATSEAVGASNWFSRSVYNSWHETELERWLADHDIPFPTPADRKDLEKLVKNNW
NAKVQAPIADVSDTASDHLSNVKEWIFDSWTESRLKAFLDRHSIPAPQPRKRDTLLRAARENYETV
AKKLGETAAYPGNWLYSEWSDSDLKEWLDARGWPVPQPTTRDRLIAAVRRQSRLASLAAQQSAASL
SASAAATEQTLSEALFDAWSDSNLKKFLDEHGVKVPQGSKRNELIALARKNRKYLTNQASSISASA
ASAYGAATSKAGNEYAQATEDAQLKAEDAFDAAAAQWSDSRLKAFLDARGVPVPQHSKRDELLAKV
RLHKHKAATGYSAWTFDTWTIDNLKKYLSSVNNKYAKKADATREELLKQAQEQYAKASKSGGTHYA
SATSYLAQATAAAKKDTFDEWSESDLKSYLDSYGIPTYQGSHINEFRAAVRRNAQYFRYGTSTPTQ
TIFARIQQGAGWLLDQLKFGAAVGRAQGQDAAESAKSKAAEATQKIREEL
>g776
MTYSVLPIGEIHYPRYPEKPPYDHIQPGWLHDIDITNVQFWLQPGVPDIKFYLEIEQAIDIHNARY
ALGVRVFQDISAMETMLKWVGIEGKIKKKVLKNWYLRVVRMYDYIEEERPAMPSLALPCDFRRQDE
DEAYNSEQDGEYELDEEYDAALVAGINRINASTATITDTSTADVVRSTTNKLKEVNQMETNEHNKI
IKSAIIAHNFWPLFNRMCNRDMNFFRLQMDEVCPHISNEINWSFLLCEPCTDVSAMIFLYVMSVWQ
SRPDGKEDELAVPLIWSLAIQLRILVPSEQVLRVLEHTRATDHIKGLKPISQWR
>g777
MAHRQTLATPINLILASVFIVVVYLQFRPKKPVTLPKAPAPVVFRTFTPSTLLPFNGNDGAPVYLA
VRGRVFDVTPGRHFYGPGGAYENFAGRDATRGLACQSFDESMLTQDLKGPLDDLSGLDEEQLENLQ
GWEERFLEKYLIVGKLVAEGDPEAPKA
>g778
MWSRINKNRDHARHIGGHASVESPKGQPVLQTTSGNPLPSRSMLKDLPALPRESTQSPSNDRVTSS
IYSRDDSGVRFTDGNMPISPTRDYAEYSRYAEQQQEPEISPPDTPVDLPSAQAHSDVSPIDDDKHF
FPEMPKHRASQLPVPRRNITNDIPVNVADQSSSLQAKRTNTEGFEGPSNKPTTSRNTTSPKPRSAS
PPVGIADARSKEATRLFSAGKEKLQMFTKTREHKQRDAKQPREQWKGASGRFALVPPIDTKSTVKH
GKKHVHAQRVTEDPTREYLNSSSDTYTITTITAGPPPEEKLRSKTSQFRLRSRNASPDKKDSPKEP
ERKVESTVSRKASPVIDVPGIDPATPHDVRQFSGSRANTPTLDAPNESNISDMFHDLSLPQEPRSR
FSASTYAPTEAATTPPGSSHADVPPMPSLEYSPIMTRRRPIPSSAASIISMKRKPVSADTPEVDIK
NLSPEEQAQRRVDSLEGRRRQLSLRKESIKTMLHELTEVIQPSSVAYDMATRDEVKKTVSSLKSEL
DDIGKEDHEIGLKLVRLYKNLNNVSEFEQSSLWVKRITS
>g779
MGGQGRDGGKAKPLKAAKKEKKELDEDDLAFKERQRAEAKAKKDLMDKAKGKGPLNTGAQGIKKSG
KK
>g780
MASRRSRSLSTPSEGEIVESGSETKATTSQTSMNDTNVNRRSRLSPSFSPRSPASGAARSPPSRRR
SRSRSRTRSPYRRGGKRRRYDDYDDYDNYRHGSSSSYRYESRSEHDRRYDSRRQRTYHDYDRDDSY
NGGLRYMDDYDRRDKRQKTRSRSPFRDVRKPKKYDDADDIGEPRKVAFRNQSVSERGVSPVVAQDV
KPHTEPGRQVESSTEVSTGLNGSSPDLTEAPEEILDEAARLEARRKRREAIKAKYRGQDALRIQAL
HQGADPDLTPVTPMSAAESPKSPALEVSTETPESEFTVAKDEDLANGTQPMEGVEKDEPSAADYDP
TQDMREERQRHDQQFSEDVSSAAYDETKTTKQDVLIPEPQPEAPAKDPYDMFAEDDDDMFAPEPEE
QPEHASAKAVPKAKELDMTMLDNWDDPEGYYNVRLGELINGRYHVHQNLGKGMFSSVVRATDSKTD
KYVAIKIIRNNDTMRKAGMKEIQILEQLLEADPDDKKHVIRFERYFDHKGHLCMVFENLSMNLREV
LKKFGRDVGLNLRAIRAYAQQMFLGLSLLRKCNILHADLKPDNLLVNEQRSVLKICDLGSASSASD

NDITPYLVSRFYRAPEIILGVPYDYAIDIWSIGCTLYELYTGKILFTGRNNNQMLRSIMECRGKFP
PKFLRKGALTGAHFDDMLNFHSTEEDKLTGRVVTKVLDFKKPTRDLKSRLMGTGSGGKGSKGMTES
ETKELAQFVDLLDRCLSVNPEKRCTPVEALKHPFISRK
>g781
MLPPNPELKTAELADQSLYNRVWQSPSPTLELAQSPLFKRPERLLTIEERTRLTYERARKICDVYG
LTIEDIVTLGPKFWKLQTDLISAVDGGALTLISIQYNLFVGTVAPFAAKRPDLHWILQRALSFEIS
AQFMLTEVGHGLDARNLETTATLLPDGGFELHSPSWGAAKCMPPTTPRGGLPVVAVVIARLVVDGE
DRGVRPFLVPLGDGREMCKGVVAKALPPRTGANPIDHALTLFNHVVLPPTALLGSLDKPQNEREHF
FNTIHRVSAGTLFLSGTAIPVLKVAVYNAAQFSMRRKVTGHDGKPMPVIKFRTQHLPILHAIAQFH
VLQAFLVHAATIFRNRKVDPRVRHAVATAFKAVAIQNFQKSIKPLNEGCGWHGYYEHNQTLQSEME
FRAVGTAEGDIRVLAIRLASELLIGRYQIPPPTSPNSPLAQHEAALMTEAQQHLLLIGGMHRSEEF
NRNILPLSLPLIQAIGHRMALEAAKEAGIDSKLIDLYESGVILDDSAWYTEQGGVSRLAQKEMEAQ
AADALLPEMEKLLYDTGAAPYSNAPMASEKGWNVFVSELETFEGEASFETGISVV
>g782
MRHVWLAQCVRRAETFVGSAIYNLWFSPLAKYPGPLFARISAFPDFYWSLTGDRHMWIAHCHEIYG
DVIRYRPDGLLFRTPQAHRDIFNGKANVKRAKFYDMMTRNKHDTSTITGTDPELHAQKRRVLNSVF
SDKSLRSMEPFLVKHVSRWCELLVDGDGAAWSAPRKMSDVCDYLVLDVLTDLCFGQSVETKEPGEN
HYREIPHAITSFLRIIYPIGHSPWLNLIVWLKPRGLDWVFDAMTPRNIRFLYDFVKQSLAQRLKLE
DESPSSESGCRSDMLHYLIHAKDPATGRVGYTPEALEAEAIMLTVAGSDTTSVIMAGIFFYLVRTP
RVYRKLVNEIRNTFTSLDDIRGGPKLMSCQYLRACVDEATRIVPAGPSELPRFVLPGGLTVDGNHI
PEGTTVGVAHWTFYRNEEYFPDPNVYRPERWIVDDAEEVGVTAEEVARARASCLPFAAGTTGCAGK
NFALLEIYMTIASTLWQYDMRLLPGDLTGAGGQGQGWGKRNPNTFHVKDSYISVRDGPMVQFKRVS
>g783
MYPTMLPTPPPSPPLLRCCTPEERLGLLLANRLELTSILGVGAYGVVYTAIDIHTHIPYAVKALNK
IGLDPRQRKFQQREIKLHHLASQHPNVVSLVRIMDSVDCTFVVIEFCPEGDLFSNITERGLFVGND
PLAKRAFLQILDAVDYCHSIGIYHRDLKPENILVTDGGMTVKLADFGLATTDYLTSDFGCGSTFYM
SPECQQTTPRPGSCYVSPVNDVWSLGVILVNLTCGRNPWKRASNEDATFRAFLKDPEFLRSILPVS
PELNVILQRTFECDPFKRITISELRQLILECPRFTMPAPTTIPAASTRKPGHPEVLPGYSYYAYPP
APRVDLLNAQYSDSAVSDTSLTDDETISSVSSDSDYASEGETEGFSFVDPQAVPEFWEPVHISNVP
EKPFALPQHIEPYIAVY
>g784
MAPQDRLSQVSSHLNFPQGLFHNQTAIVTGAGQGIGAEAARLFANEGAKVVVADIDQVKAEAVTKS
INDISPNRAIAVAGDVMDAAYIDTLVKKSAEFGGGKIHIIVNNAGFTWDGVIHKITDKQWDTMISV
HNTAPFKLIRAAAPYFRVKDKEPRVIINISSTSGTHGNAGQANYSLAKAGVVGLTKTIAKEWGPQF
GVRAVTIAFGYILTRLTAAKEDGATFVAADGTKVALGIPGRKPAAAQGTGATAYTDIPLRRPGTPE
EAARAIVGAASPWFSYVNGETIEVTGGRNI
>g785
MYFTKAFGLLAAFGTLASAAPTSMNSTNSTNSTSTQGTANTSGGSGGSVNIHNNMQDTIYYWSVSQ
DAGSMKTLDPGASYTESWRTNPDGGGISIKMAMKPEQVDVLQYEYTLQGDTIFWDLSLIDMGTNSQ
FTKVGFAVTSNDGGCPSATCAPGDTACADAYLVWNDDHATHGCPSGTQMNLNIGPSS
>g786
MSSFTVFKGVKDGFPKKSTTAKPDSLTGDKVYLKVTASGVCGTDLHYRHSDIVLGHEGIGIVEEVG
PAVKHLKKGDRVGWGYETDSCGLCMQCLQGTEVFCEQRVLYGSGNTDQGSFAHNAVLREAFLFKIP
DNIKDEDAAPLQCGGATVYTALSDLKPNEVVGVLGVGGLGHLAIRFASQMGARVVVLSGSDRKKDE
AFELGAHHFIATKGQDQLDLKGAPKISRLLVTAATQPNWDAIIPIMAPGSRIYPLTVSGDNLSIPA
MGLILNGISVVGSLVATRATQLQMLEFASFHNITPVIEKFPMTEEGIKEAIEKLENGNVHFRAVLI
PQ
>g787
MKHSSEHLSRLQTPLRSRQKRNQVARACDECRLRRVKCDSFSPCSNCKTRGSKCTNNANKIISLPH
AHQEIDNLRSRVEDLQRELQQLRQQTDKQSQIPSPVTSVPTPELSVSESIHSNNDGVSRGTNWSGI
HFRTARSPNETWYGPSSLFYFMGRITNFLNLNLDQAQSKDQMVPDSVSRQLSGPLTGFENRLRNGI
EDGHYLTPTQEDYFLESYWSSYHTSLFPIIDENEFKQHYRTLWTTDGNARKDSALVDIVIALGMQY

GMSILPNMKHQLADDFDATIAGRWYYRRCQTLLAYEVESPTLATLQCHLLCSVFLCSASFQNTSDS
TCAMAMRTAHMLGLHLSPPQSMPRKEREVRKRLWWALYNLDSKLGMKLGRPFLLYQTSTTPTLPDD
DIEAAMLSGSSFAPLGNNATWLSFNLQNMTMFLAARDAHTAFYNRDLNLKEGQTLWDDFNNLEGQA
VSVYPFVKKLENWTNGVPSPLMTKRKDGSSPFSTGGTELEIEQYAPLWLQRQRVVLELMYHNLSAN
VCRPFISFSLTPPLTVAEELAFKCAGHAIALTSITHQVLASTTILSGWHEAFQWQWNSAMTLVGFV
LAYPQSSMTVAARDAISLSVSVFDIFGHSFAVANSAATIVRNLITKIDFLAKRAWQRQSVVEGHGR
TSDQAPVYSNTTYSKPRTDMDDNMFQPSDGILDFGDMNLESMQDMFHMAFDIDQWSDLNGLWSQTS
KV
>g788
MQSIDETGKPRYRAACDACRQSKVRCSGGGVCVRCKKHDYKCRYSIAHRAGKPKGSKNKATLKKLE
TLQAAQQGKLALGGTGASTRKILRPLTDAEISQRKRRKSISTHDIRARYNFIHPAQLHMRNASHPM
ARKYAFASPMFPVYTSPTSPAEPRVCEIGLPLTDMVFYGHLETTYAHVASPAMVPYLPSPSEEPCT
GFGLQSNSSTVAGPCNCVDRLEASQRQLSTLESDLTLWRFDPTMELVTACLSSCHIFLSCVVCPKI
SGGSSWLLISVLDRSFDILNHLVLYRANRNDHGFGSGHQQQQYHAMPYNYALALQDCILEQSITTS
HQIVRALRDAIDTELGISGGVLGNISMHNHGLSSSSSSSSSSGYPSPASMETPARDFMVKHEQLHT
TECSALKKGKAKSNNALSSNKINFLLQAIHRYNILIGNLQAVVAHNTAAATAQAHHPASWPIGMSD
TATNQCGGGVTSYQPATTMEGFPQQPHPEHTPRFASYDVIGLP
>g789
MSDPRLRASTRKHESSNDNKRPANENITPEMVVKHPAKAIRKLLHWDELPPWLQDNHYIHDGYRPA
SSSFLVSFHSLTYLHNETVNIYSHLLPALVALPAGIRLYQVLAPRYQSATQADISAFACFFVGATF
CLGMSATYHTISNHSPSVARIGNALDYVGIVGLITGSFIPSVYYGFYCAPSLQLIYWTMICTIGLG
CIFVCLSPKFRTPEWRAFRAGMFVSMGLSAIFPVIHGLWLYGPEQMTDQIGLYWLLLQGFLYIVGA
TIYAARVPERLRPGRFNIFGSSHQIFHVLVVCAAVSHLTGLLRAFDYRHSGLAEICL
>g790
MSYRGRNPPFRRPDTGTSIRNNTAGSRLRPVSDPLEAVGFPSKGDKTLLDHKAQRTYYDIVVDRYM
RFCSSHSQDLEAAWASLPASASADATKNPPSNLPALPIGTPSTKPASRDDSAEDLVAKTESLSLST
IKTAERQRPPAAELSTLLMSLRKLREGILATSFMTPIDFQQQVYIFSIRFSILARHPPSYFSSLRY
LVDSLHTVSHPLTPHVLAEFTSYLILDYACRQNSLAPAYTLLIQAKEKYGFKSCVVDRVLHALTHD
NWIAFWRIAKEVDGYVRAIMGWATDHMTKQALKAAGRTYLSVNVSWLLRTSTGEEGWTWQRLAEKE
KLGWQLEGDSVVIRKPKVKT
>g791
MASSQSQKHQHNTSRTTRPRASTKGPLDLSDDDTVSSEDLLSSPRSPQKSQSQHARFSHDAGSFSP
SNPLDLSPLKVELDSILANYELSTVVTKDFSFLLRPEIYHPLSQLEIPPMFRSSFYNPSIGETIGA
SMEILNKQLAQGHFLLAAHLSAKLLTSPLVHSSDANIIFTLLYTRLACLELTGNSAFAAQESKALE
DLNSTFYYVDADFKVDSDVHDRARRSYHIVPWPLRVLAVRLQSIAFGDSRRSITGLYELGFEARRE
LMQPGLDKTQKQTWRDRLYDLGVRTVNALVEMGDLDAARRSLSNLGDSRNVDNQARSILLALRIGD
LALAKDILEKSTVLQDGILKPLTTMAEGFYDEATVQWKALLENPAQLDRPMIVQNLAVCLLYTGQL
NESRSLLESLVSENHGFQSLTFNLATIYELCCDNPQNRKLGLGQMVSQQTHNGDMNLDRANADFKL
>g792
MAKQNKYSVILPTYNERKNLPIICWLIEKTFREQNLDWEVIIVDDGSPDGTQEVAKQLQKVWGTNR
IILKPRAGKLGLGTAYVHGLQFATGNYVIIMDADFSHHPKYIPKMIEIQKESNADIVTGTRYAKRG
DLKGGVYGWDLIRKFTSRGANLIADVMLMPGVSDLTGSFRLYKKSVLEKVITSTESKGYSFQMEMM
VRAKALGYKVEECPITFVDRLYGDSKLGGDEIVGYLKGVFMLWLKV
>g793
MGGFHGVVGTQDSGKVHHITESLVAFEYITSRANEKPHSLVFIGGLGDGLCTVPYLKDLAAGLEST
EWALFSFIPSSAYDMWGTGRLGQDVEDIAQCVEYITKYKKQSLPTSQTQQSPKLVIMGHSTGSQDV
LHYLYSPNPLPIDAVFDKGLRHINRPVVDGAIMQAPVSDREGILAGLTDKDGNFHDAEVEGAYRQL
VEMAKTCTYSSGNAYDVILPPSMTSKIGYPPAPITARRFLSLISPDAPENPGEDDLFSSDLSDKRL
QETFGMIATRDLLRSKLLVLYSGSDEHVPKDIDKVKLLERWRAASDKSYQKVWDDEHSGIVPGASH
NLGGDEEDDARQDLINRVKLYLEDVRASR
>g794
MFNHSFLSLRRLIAGISALIVILTTLRLLHPMRGPQPGLLGGSGSALVPDPLGPPVDYLEKNPDPR

SCTERLGLAYLEGFRENRAQYCSPASPSQLMCFHSQTTPDGRVDSFCVGQNAVFSSSLGKFQFSCE
RVEPRGVESNQTAPTLEEFPSYMYKTGPRHIFDDLVNLDSQIADNKAMEGITILVKREGADNIWHS
LMEIMATSMTLDVLQMTRSAGSLHPIISTEDALNTQVVIVDSHNNGPYFDLWRLFAKMPVTRLPDL
STDFNTSLVIIPLPGGSNPIWQGDWEPNICQHSDLLRTFERRVRQHFNITEPIKLPDQVVVTFIKR
EGTRKLIDQDARIEALREQYHEDKVLIQVIDFAVLPLTQQIQIIRSTDVLVGVHGAGLTHGLWLRE
GSAMVEILPDSFEHKGFRNLAGALGHNYFSTHASTRKTSRRSDNTWQMSDVTLDEGRWLQLVDVAI
RSMYNKGRHNFDIN
>g795
MGLMWFFVNLNVATLFSVLVSAVEAQSSTSSQCIPSPIAIAIQNVTVANQPNGAIVRGLNIGVGTP
PQKIAFMPGYFDSKFLAASRFANFWGRFFNNSFAYTNNDACIVVGSQYSGNGCITTRGGAYEASQS
TSNEPGNTSNFSPDSPRPSPSPVVDTFALNNNTSLSNYTFGVSQDDLGYQLYNPQASLGLDRNSTF
LRALKDAGKIGSRSWSFFWGLTAPNNSLDGHIIVGGYDKEKITGNAANYTGTIDYAASSACLSGMV
VDITNITILDLNGTVNSLLSNSNTIPACLRPDWPGALNLPWDPLFNKFAELTNYNSALLDLANNTV
RAFGSNYYDMRYLSTDIPFGGDLEITLEGGLAIRIPNDQLVVPDQYLDQNGIWQDNSTGPDLLINV
ETGSYQLTSLNRYFFSAAYLAVNQDAGQFTLWKAHTTPNQNLVGLDEKNQVASAFCSPTPSSIASS
STLAPSPQPNGLSGGSIAGIVVGVIGGVALVSGTGFLFLRYQKKNKIKKQQHTRQVADPSREDKST
TQTLTEGYNHPQHYEMPVGEPPNIAQFELDASPSANR
>g796
MLSGTTSSIIFFFCFSFLFQSRVSLGNNNCTPHPISVKLGNVSLDNTTARGVSLSIGVPSQPFAFL
PQWPLNDTFVYGLDGYCGDGWSEAACQTFRGGIYDPSKSSTYQDATNAPHLTEASPYPSFDWAADN
FTLNPNITLSNFSIGIARADWGEQGYHPQVAFGMGQNSTLLNTLYEFGHIASRSWDMVLNFANGTD
ASLFGGAESSAMSACIVPDYPVLLTLPLDPYFSAFQDLTGTLITDRSFGIYYYGMLYSNTSIAYTG
DLTIKFSSGLSVRIPNDQLVVPNLTIDKSTGSILANDSAPELVLNSIQSINADDLPQLGRQFLTSA
YLMLNQDANTYTLWEANPVTNEDLVAVDQGGTPTATTNAAPYNKNSGDKEDLSSGAIAGVVVGCVA
GMTLVVTMVFCIIAKRRRERTKANDNGIALKSPEVVYRQGGSPLELHPDSFDPTAPHELYGKQQPQ
STPAYELP
>g797
MPTFTASLDPLDDDAVLVLVAMVEVVDIVTEAAAGAGEAELVTEEMVEDNKVEVVVMEEVVEEDEV
DELVVVTELPNVVKIEMEPARKKAPFPVWQSHVVTLADSQQYLVPPQATNAGVR
>g798
MHFSTTLLFLSAAISLPCRTKYFFFFFFFNDYRPTNNNFINNTCLTNFDANELLFINTRCKLHILH
IQRADTNHITLLNLYTYHDDTVAIILTVMDPFHGTPISETHGTD
>g799
MSFKTSYFLKAITANSQDHPPSNTAQNDKTNNENPDGRYEALVSASANIVLTMLHRGRFLEDELKR
ALDHIRTSNEFLQKFVELLDSVDEERCDGKEGVTSWKEVAERVKSVVKGARGQDDEKDGSIVVNEE
KDGLDGEPLAE
>g800
MAPRRSIRRAATRKSYTEDAFEAIGLEDDEDVEHVAKPSASEESKGKSRKRQRDESDDEFVEEAVA
AEENDEDEEDEIVKEEEEQEADDDDFEDEADAEESKKAAARLSNLSITARGSRTPNSNAARQRRDD
GTILFKPTDTHTRGAYNPSEHVGKSMHIKLSFGTDDRDLLATAAARTQWSLGVDATFPTRNALENA
PSADEFGPGAAHGTSAEELEREATEAWDWYYDANYGPAFRKRQRVEKIGKQRAEQIYLHRPSKSSH
KIIYGPSENLSTAHLGQLESLNFGDAWKYRDETHKKGKTSDTAKKGRGRKKIGTSSSSVNDDKPSD
IPAGPRRKNHYGWILNLSGKIQALGWAPNQDGLTQYLAVSVPISSEQEKENDPPEPPVASRAFSAS
PPYPGAVQIWSFDAEDRKDGMIKTINVNSKPKLRQXLCMNFGHIRKIYWCRFSRRRRDEEVVGADL
GLLACVFSDGRTRVFDVKVNKNAESTEYLHVKSAAFTAIPSPTISTCMTWLSPTDIAVGCANGWVA
VYNIAMYQTTPRPGGQEDEPVPYIYVPIHSTYLTNIESAYPQHPHLISTVSMDGNTRLTSLLDTVK
DVVETQRQRLGTMQISYYPHLQAFVSSDENDFVRGLSXRRFYTSTLIGKIPSTITTIASGSRWHPS
VMVGCTEGSIFCFNPLRRFLHAKEAYYHTRWFLHEWAAGKDEQTPNTTRFTDDLEVENLSLVRNVE
GGKVVNGVLTITIYDEQQHVIATNWNPNQHCAGWAAAGLGSGLVRVEDLAMFH
>g801
MSTTPTPAEDQGRLLEEALGVVRQQSHMMRKCLETPGKLMDALKCASTFVSELRTSSLGPKQYYEL
YMAVFDALRHLSVYLRESHPVNHLADLYELVQYAGNIIPRLYLMITVGTVYMSIEDAPVKEIMKDM

MEMSRGVQHPVRGLFLRYYLSGQARDHLPEGTGDGPQGNIQDSISFVLTNFVEMNKLWVRLQHQGH
SREREKRTQERKELELLVGSNIVRLSQLVDLDTYKNTILPPLLEQVVQCRDVLAQEYLLEVITKVF
PDEYHLHTLDLLLSSIARLNPYVDMKKIVIGLMDRLSSYAIKDTEQTDDPAKKKENEEEAVARLLE
NFKLSEEKKTEKASAEQTQENGAAPEEAAAAESSTKPSEQQDTNGVKAPAETKLYETFYEQVVNLI
KSRGLPIQDTIALLVSLINLALNIYPERLEYVDQVLDYATQKVAEHADQADLHSAPAQQNLLNLLL
APIRLYVSIFTGLALPHYIPLLASQSYPTRRAVATEVIKIILTNKTAIISSENLDRILQVLKVLIK
EGAPHPAGYPGVHPQRRGETDETIEEQGWLARLVHLIKGPDNDTQLKLLQAVRTAYAEGNERVRYT
TPAIVTVSLRLARQLKSREHYDDNWQSQSSTLHRFMHQCISNLYQRVNSPGCADLSLRLFIMCGEV
ADQTGFEEVSYEFFAQAFTIYEDAISDSRAQFQAVCIIASALHNSRGFSKENYDTLITKVALHGSK
LLKKPDQCRAVYLASHLWWVVENSQREEDPKSLYRDGKRVLECLQRALRVADACMDTAVSVELFVE
ILNRYVYYFDQQNETVTTKYLNGLIELIHSNLQSNDGEASPSLENPKRHFQRTLEYIRSRDYEGVV
TEARQ
>g802
MSAAFHRPPAATQAPATSKTKQPSAVRTTAASVPVQLDRSASENSSRPESDTMAPTVNRKKQKRRQ
KQAAKLAAEQQDHSADNSTNPSDQMHYSNKDQSDADDGDVYDQDEIDDEDDTHDHSGPMTNGNGTT
TTSKSKKKKKSKTPSTSQKLAEESSTSLSAPSATGGRSQPQLQSMPSNQTIKKPKDRIWNTSTQEE
RENIKKFWLELGEDERRALVKVEKDAVLKKMKEQQKHSCSCTVCGRKRTAIEEELEVLYDAYYEEL
EQYANTNQGTFENGAPIVPPPRLYHRNLHALNRHAHRHSHAHHSHHSHSHSHSHSHSHSHIHDHHH
DHHHDHHHDHHHDHYHDHDHDHDHDHDHDHDHDHEHDHHDHYHDHDHDHDHDHDHDHDHDHDHD
HDHDHHHHHHHHGRVHEIPEDEDDLEDDYDEEDDDEEPYSDDELDDDSHGERADFFAFGNSLTVKD
GILTVADDLLKNDGKHFIDMMEQLAERRMQREEDTQYGMVAAHQSYHAGHNHGPLDEDDYDDEEEE
DYDSQEEEDYEDDEMDTMTEEQRMEEGRRMFQIFAARMFEQRVLTAYREKVAQQRQELLLQELMEE
QTLNEQRSAKKAREAQKKKDRKKQQKQAREEEKARKEAEKAAEEAAARALEVKKLEEQKRKKEELR
KKKEAEKKAQEEERLRKEAEKQKRLQDERERQAEIERKQREQKEKERKKREDAKRKEKEEREAKER
ELKDRKAKEEQDRKLREEQGTGKQRSQSQSSSQPTVAGPKRASQPTAVGFVPPVFPSPQVAPVASS
PHFQVATPIIHKASTPARPRQPSQQGSYASSPRSQATSGPEPLSSISPQSLPLSQPSSTPLSSSGK
IGLPQQPLLXHPQPSAPLSPMGGAGRSHPPGYPPVNGLPAHPPPGMPGLATRPMGNENMAMYPPHS
GPIGGQYRGFPAPNGVLLPPGIGGTRPMPIPNRGFPLDPGHIAFSSQAPGSGVMSTVQLTGRPIAH
SRQASGSYDRPPVDGQPKNAPISRPTPIKRPSSPQKGDSDVDSLSAQLGSSALLDDSDVPFSTHMS
QSLPGAPIPSGFGLSRASFGAPPFPDPLSPAKHPSFSGQGNSSWATHSPFGVPFPTSWNVAAGSSW
PNRPFGSGGPHRTHASRPVAIRLLVIQAIKHLSSTASSEFAPGFYEVNRLLDQVEQLKSPNDPAIT
LDEMMDICDTEGSSQNGGGSFAVQEDGSGNRYVKFEPDSTSPLSSHRGSFVPGDIGSPVPASSMPA
PFGAPGVRQYSSPTTF
>g803
MAQQPTDNVMRRKLVIIGDGACGKTSLLSVFTLGYFPTHYVPTVFENYVTDCRVDGRSVQLALWDT
AGQEDYERLRPLAYSKAHVLLIGFAVDTPDSLDNVKTKWIEEANERCPDVPIILVGLKKDLRDDPV
AIEEMRKKSLRFLTPKEGSETAAQIGARKYLECSSLTGEGVDDVFEAATRAALLSFEKERNGCCVI
L
>g804
MRPHSPILASLANVFRIPITRPQVTPSTSTILQTLSAKNSQQTSSFSTTPSLAKRKSAYKPDRRIT
LIRYFLYHPLTPRPLRFSRNRYLRHWTIHRAWQLYQANLRTQQETELYRQQQAMSAACEELRTSCG
ERGAKLFRVSMNKKGVFTDLFPIEYARLQTDSPGREGWNHGWKRM
>g805
MHLVFGSDEVHHIFDRGVDDAWSPVRNVGIPFIPIVLIFSRMSWADGVLPAIPVLFFATHNPTGFE
PQGTLWPPSATMTFAALPYVKSIYSAVYERVFGKLERKWIAEVQPRRGEEEGQQGDQQRGDQRAAL
GIGGNGGILMEIDLELQIGMDDDRQNIDEPVNAPDPNNVGQQADQDGQAGVQNGQQDPEQQILGRR
RQELIHDTSNLADTVLGALLFPAISAGMGGLLKLALPKSWTAPQSAFASGRAGLLQSRWGRSVVGG
XLFVLLKDALVLYCRWRLAQTHRKRRVLNYDRQKKKVV
>g806
MMPIRSSRSALRTLPSQRQLVSNRRHFSTTLVAGAVSPHRTFKRGQSTATASSEARPVPSPAFNQE
TRNVSPLENRKLPELDDSFVGLSGGEIFHEMMLRLDVKHIFGYPGGAILPVFDAIYNSKHFDFILP
RHEQGAGHMAEGYARASGKPGVVLVTSGPGATNVITPMQDALSDGTPMVVFCGQVPTSAIGTDAFQ

EADVVGISRPCTKWNVLVKSVAELPRRIKEAFEIATSGRPGPVLVDLPKDITAGILRKSIPMNSTL
PFYPTTASEAARALGRHNLENTLDRVADLIGVAQKPVLYVGQGLLARPDGPKLLKEFTDKTSIPVT
TTLQGLGGFDELDPKSLHMLGMHGSAYANMAMQEADLIIALGARFDDRVTLNVARFAPQAKAAALE
GRGGIVHFEVMPKNINKVVEATEAVEGDCADNLALLIPKVKAVPERPEWFAQINDWKKRFPLTLYE
EQTTDGPIKPQALIQKLSDLTAHMKDRTVITTGVGQHQMWAAQHFRWRHPRTMITSGGLGTMGFGL
PAAIGAKVARPEALVIDIDGDASFNMTLTELSTAAQFNIGVKVLVLNNEEQGMVTQWQNLFYEDRY
AHTHQQNPDFVKLSEAMGVQAQRCSKPEEVEEKLKWLIESEGPALLEVFTDKKVPVLPMVPAGSAL
HEFLVYDEAKNKERKALMKKRGVTQMLN
>g807
MAPQQLLVPQTENIADVYATDDISAQSVAPEIKARWQNLVKQFTETYGKKPDFVARSPGRVNIIGE
HIDYNLYDVLPTAVSVDVIIAVKVVPTEGSEATVKISNVNSQKFPSREFGVPFDKDVVIDPKKHEW
INYFKAGLVGALKFLRKDNPAVQPASLEIHLDGNVPPGGGISSSAAFVCASALAVIKANGHDVSKE
DLLDLAVVSERAVGVYSGGMDQAASIFSLRGFLLYTKFFPKFSVEHVPIPVADEEIVFLVAQSFVT
SNKAETGPRHYNLRVAECTLAAVALAKQHGIVLEKDNSSLGYSLRNFHEELMRKQGRLQDPLEYQI
DSVIQTTTETFTQEEGYTREEIAKLLDITVPELESRFLSSFPVEAERFKLRQRALHCFKEARRVLD
FKACLSQADKLDTKRIHYLGQLLNETQDSCRDDYECSCPEVDQICEIARRAGTWGSRLTGAGWGGC
TVHMLPKSKVDAVSKALHEEYYSKLSGVTQEQLAQAIVISKPSNGAFVVHGAALEA
>g808
MPSTRSKPATEGPSEETKSSSSSKRNTPQSVDVPNRTFILPSKRSDAARFLSLPNPSSGVLNRYYF
DPQGAGLYEFTAVASTHFTPRSILFTPNKDDGEPETKGYIAKKAELLIATPIDVLFFMLSVIAPAD
KTSTSNMFQPLDDILDSQDELSAHLRHVLYDNSFRSTLERRMQAICDTMEAADEKLYRFSERKLLN
ELIAKADRMIAQGLPASLEQTFIHKALAPPLMSVKREDTVTATATVVENEDSQDLEVVETQSTSTT
TMSATFSEQQSGISTPATQTYTVEEISSSSESVARLLRLRIALAFMKQSYLPQNLSTRIDEILKSP
ESPFDFSPLDDRLKELAALRAEAFASRAMGGDFTRKRGFEDEDGDSERAEAKRRKEEEKKAKVAES
RAVRDLKKVNTSGMKKMSDFFKKKS
>g809
MSFQCDEMVDPASWPPKSPREALLASPGGRKRYIELRRERETNEQTSPLKRSRTTPNLFSRKLAQH
ENSNEDVGVGDGDDMEEDEETLRLQLAAIEAKLKLKKLQSARLNAASPRKDDTSLQGSFTSSSSSG
QPASRMRAMRERLATSEQADDVQVPLSPSKRTMSQDPMSPRRALINFKKASEVSLKRPSGARPGTA
QSKASSIRPGTSRSFNNQDSPPDIWSAAADDLKKPKSFSERIAETRNADRAKQQRTEQIQRSRNSA
FQVDNKEIEKYKEEAASRPKPASPDKRPAETFSREQVLQSYGNPQVRIKKSTTAPSLLASPPITSR
HLNPVSIGNASQSSSKEPDISKLEPLSGLNLSNRILPQSFLSRTLENKTVLRIPQLLKSVKAPEFE
LPETIDGDYVVFGIVASRSDPKQHKEPKNSTKEKDMYDDGLNNTEKYMVITLTDLNWTIDLFLFDT
AFPRYYRLSEGTLIAILNPAILPPPPGKIDTNRFSLCLSSSDDTVLEIGSAQDIGYCKAERKDGKI
CRSWIDSRKSEFCDFHVDVQIRRTQASRAGVNGGTGLFAPGGRNASRIAEFSASKGQRGGRRGLKP
EGSRYDWATQSTYFVAPAPKSASSQGASMHPRYGMGRGASAAALIDGIDDNPFTDQVRGNANKEER
TRRRLVEQQRERDIAQKLSSRGGSTGSEYLRAGLEQAASKASTTRLGTSSNPGLSTPGSQAPTASI
FKFRKADEVNLGPMKKRARAQSDDKVSNSGVPASVKKTRFITAKGIREAGRDSLGASTGSEAHKSA
PHMTMNNDDDDDDELEII
>g810
MSFTTAIRDTLGSPSTAYGFWLTLPGAGVVKTILRSTVGSPDGGFSWVLVDAEHGLISDKDYYELN
NAIGSEGASPIIRVPWAEEWMIKRALDSGAHGIMTPMCHSAEDAAKVVKWCKYPPTGSRGYGPMFA
AHSLPRGTNYDEGADKALTVVVQIESRAGVENVEEIAKVDGLDVLFIGPFDLAKQTGVVRGGEEHE
AMIQRTLQAAKAAGKKAAIFCVDGADAQKRAQQGFDMVSIITDVGVLAQAMAQQLDIAKGKGIEGN
KQRDGY
>g811
MSGAYKLYDYDPSKGAAVAFAIIFAITTLLHMFQMIRKRSWYFIPFVIGGLFETIGYAARYSSATQ
TPNWTLGPYIIQTLLLLLAPSLFAASIYMILGRIILLLNGASRSLIRPSWMTKIFVTGDVLSFLIQ
SGGGGMMANAKAQNTINLGNHIIIVGLLVQLIFFGFFIIISVVFHRRMLASPTEASMVVTVPWSRY
MTIMYAVNALIMIRSVYRVIEYVQGAQGILQEHEAYLYAFDSSLMVICSLIFNVFHPSQVITGGGK
NYEKMQEDLEMLNASRQNLAYEGGDR
>g812

METPIEFTNQLRCASGMTCLGGGSKFWKDWKEEKTSPHSDHQTPPQGQNDDIFVTLERWTGSQGAD
AMPAMEVNVVDLELMMHWCNSAHRVLARNEATTWIWRQLVPQEAFSHRFLLWGILSVSALHLSCFK
AEGHQSIYIKKAAFHQSRALTLFRETLQSVGSWNGKAVFAFSSLVAIYAFGSLYNQEMSDPIQDIY
QILVLIRGTNQIVARVRQTLVESDFAPLLQVDEYHNDIPADTKTAIARLHALNDLLQEEPESHAAY
RDAIDDLEEMFVLFYGGVNSMTPSGKWAIKLSPFHVVLFQTVQATGNFEICLHSSIMAWXLYYYTP
STAAAGIFIALFGLSTVYHFYQLMRTRTWFMIPFLIGGILETVGYIGRILSASQAPNYSTGSYAMQ
SALILIAPAFFAASIYMELGRLIQMLRAENNSVVPVRWLTRTFVAGDVLSFLMQAAGAGLMVTSSS
NPSTGEHVILGGLFVQIIFFGFFMITTLIFQIRINKNPTPATTGLESLWRKHLFALYVTSTLIMIR
SVIRVVEYIQGYDGYLLSHEIFLYVLDALLMFVVMAFFHFIHPSEINCQIGRGERFFQKGFEVRKI
ESISTWELENPTTATNTVAV
>g813
MATTPPPSDFRELREPRAPRHGAGYDSFEPYPTRQSARLAGQRSSESHTTPPPPSPKKSARRNEID
XLSPPSTLSPRKKPVHGKASLLSETLDLEASDLSDGAGVSANRFSRSAILPGHSLLLPTPAKTPRH
QKSIGNLGAAARSLFPVNASSSRHTKSTKKASGFSLDSFNEDSTGNNSSIEIFTDSRDRIPVVNKS
HDNPFLSKTEPKMSSDSETPKAKRQRVSSRRVAPEVDPKEAVKRTDGMLYTFRGKKVFRKFDYQRS
DSEDEQNEEDELGFFAVRPDLLDSSVNLDVPRLTRSAIKGRRLFAPISETQQSGSVDENEEATTDI
EDPVDSVESTDIVHSEGDLETTPKRSLRTRTTGLHSDDKTEPVHPAVADTKKSDKRSSPFSVWARK
KTQPADVSVSKKREADPFTGSPAPAAKRTRAH
>g814
MATPNATEEMERLQLSDEDTEELWNSPSQRRARQVPQRTPASGDRLQNQDIEPSLDQEEARELALR
SELQTVRNVNLAIEGVLESLERARGNMETVSRTVDSASTLLNTWTRILAQTEHNQRLLLNPQWQGA
SHDIAEMESEAVAKQQAVERRERELQERREAAARKAEQEEKARAAATTATGRGRGTTRARVYIENS
STSFGGINATSSQRDCPWFQCCHSWACASSTIIMNFAPYQDESPEVERALSPPPPFSTDPTNRTRS
PSQNQYNNNNRQQQQQQQRSTPPPYQASPTSHFYNPSNGGYGYQESTAASASWTDPESQQQNSNGY
GGARADLDAFETSLPLRVDYEAMLAYLLLPPAGGVFLLLFEHKSDYVRFHAWQSSMLFSAIFVLHL
IFSWSSIISWLMFIGDIVLIAFLSLHAYQDVYSLDHYEVPIFGRLANSFVDDE
>g815
MPSINGKEVGPIGFGLMGLTWRANPCSQEQAFKAMRTALRNGCNFWNGGEFYGTPEYNSLVLLERY
FEEYPEDAPKIALSIKGGINPQTGRSVGSPDNTRRTLDDSLAQLKGRKKLDLFEFARRDPNVPFET
TFGTMDRMRAETIHEAVKVAKVSAVEVELSMFSTEVLENGVAAACAQYGIPLIAYSPIGRGMLTGQ
IKKIEDIPADSRLRHFPRFQPSNFEINLQLVAQVEQMAQTGACTPAQLAINWVRALQRRSGMPTII
PIPGATTAERVAENSVVIDLTDEEMAEIDATLAKFTTAGELPCPGPHQHRVTPSTRWSKLRNTETL
ESIEGQFIMPGTMVDGPTVSMSFANNFWGKDDAGVIPLLERMHNAKVTCDEVKSFYNTRAAIEDEY
ARKLLALCRKSLGSNETGTLRVSLDVVRGETEAIAKAHAAISQQMKSELEEPLAAFAGGYLAQGHM
VMGQEERKNKAKLEKTQIQLASNSNEYEAAVKVLQETTGRWNKEWKAACDKFQDLEEERLDFTKSS
LWAYANIASTVCVSDDASCEKIRVSLEGCEVEKDIGSFIQDRGTGQEIPDAPRFINFARGDINDTS
SEASDEDNYSVAQFQRAINPAFRSSSPQPSTYESHHDPSSELAAQMGLPNLATTESREAAMNPQRS
SPQPMPQSQPAQPVQPIQTAQSPQPVQPSPLDLRRGGQLPPNYDPHQHGEIGNVPHNSYPTDGMTM
FCRTGPASERSSATSAYRPSSREDNASDISNPTSFSSQEPPSGKQSPVKQPTNGVAIPGMTPEKQI
QKKRSAFFSNSPFRRKSRHDKDRQSQSYSRSPTKPTYKQPSPEPPRSTSPEPVDPRANFQLNIGNN
VFDVASPDKMPAQASQPPEDDLDPIARALADLKGVGKPSAGRVSADRYHGIATPAPSATSSTYGNT
GNADAPPPSYNEPTKRLDAPQPAFTSAQMQKTTQKYVGQAQNMFGGSNSPQKGSTRPQASAQEVAR
SRSPAPSFGGPQRTTTRAAPAQDIPRARSPAPTLRRSASPQPAPGPVDTRANQYSTGAGAQGGSAS
RAPTTYQSRNYNVRNTQSPTTNTRPGSDRGYSPQQYSRHASPVEATRPISPRPAFAQETRPASANG
MELQLSGGHVESYRGGYDSYNGRGGQNAGRPQSMYYGNGADSVSRSRSKSITVADPGRHLSRDGRP
ILHFARALYSYNAAIPEELGFSKGDILAVLRLQDDGWWEAEVTGSRGHPGLVPSNYLQSI
>g816
MTFRPPSPSAQSSIASGTEKSSAVRSSRSQHSVWGAPSSHSGQRRGLTPLSTTNISSSTAPPLSSP
FSGILSSSIRSTRKPPSPATTPSAFNFQHPGSQPLSHTAHSLSSPPKSRAITPSAGAHLAYSASNA
LGGGGSGGGGGLGSSRGVNFSPLLSGPTVNSPTGFASDKPGSAVSSSQSSLTKISIAQVFLLLDSI
TEKEGKEKWEVKAAQIYKLVESNGMEVFSKYFRRLLTGNAPQIFPGINKVVENAGNYPLLVQEMQK
VSQDLDQAQKIAETIDTSEGDIFRDFDLSTFLDHFKLDAIVKVSLALAFKFASRSDLRAKADAILT

NTVPHFLHSLLNPTETTKDFSAAYIGLTIERFILFPPRNFDDEVKSRLVQAATMRYRNQALELPLD
VFNALQMFDLGSSRMAVIRQLHSRGPKATASLEAVNEVMASCGSESWGEEHVGNALVFMLLSQYGQ
SYSLEKFLRAVKSHYSDKAINWSLVFRVFDRESLRITPGQFNKLYSALLSIAGDDTSLDVQRLWGG
DWKNRQTQISFLTAYLNSRIDPSQIPNFRASFPQDLFEDAPDAVRMQAELAEKSPIRSVDAIKAIF
DLALFSQASWNSPESAPLIKIVLSQDLPVFLLSALAVPQPWTAIQQSLVLRTLAFFILRQEEGYQL
ALYGVWKQDRQWLAEQLFTMFTQDPTSTAAIYENAVEYGWLEYLLSFANGLALDLASLAHRKGDFD
LEAWVRSAAQKGQMDMGSLLSKFLRIKAEDELQAHRPEQAAPQMVSLAVKTVYTLLTILEEYVGDR
ENLTPVQRICVQTYPRLINYGEGFDDIIDANGEKSNAIPKAIDEKMQELFGRMYHEELSLREMLEL
MRRYKTSRDPAEQDLFTCMVHGLIDEYHCYHEYPLEALTKTAVMFGGIINFRLIDGIPLKVGLGMI
LEAVRDHQPHDPMYKFGVEAIEQLIPSLHGTPISQKAEEVLMEQGHQPANDTGPARVNGDGATNGD
LEDTDSSRVFRSLHVGPPLRPEIYEDPDEEVQDKILFVLNNVSEQNIEDKLRDLRDVLRDEHYQWF
AAYLVEQRAKLQPNFQQLYLDLLELIGNRTLWAEVLRETYVSSIRLLNAETTVNSSTERSHLKNLG
AWLGLLTIAKDKPIKHKNIYFKGLLMEGYDNQRLMVVIPFTCKVLLQAVKSTVFKPPNPWLMDIIG
LLIELYHYAELKLNLKFEIEVLCKDLDLDHKSIEPASDIRDRSQQGEEVISAANIPEGLEVFEDMT
LAGINPAIRTERLSPAAILSSLPSLDKILVFPSSASSMVDPSILRQIVHTAVERAIAEIIAPVVER
SIAIASISTVQLISKDYAMEPDEEKVRHAARTMVRQLAGSLALVTCKEPLKMSMTNYIRMIQQEYS
EQPMPEGLILMCVNDNLDAACGIVEKAAEEKSVPEIEKIIEPQLEARRRHKAARPNDPFIDPSSMN
RWGMLIPEPYRQIPGGLNKEQLAIYEDFARQTKGPASSHLQNASTDSGRQLPDVLQESYPAIPNLS
TPAEPPAVPHQATQPQQDSRLPQPSAVGAQSQLNGFLDSQNPREKVEILITGLQQDARSASEEHIK
DLGRDSPVIQDYNQILRTIISSPSGEDFARLTCLKICTSLYSQTESPLEIEVLVSLLAKICDMSSI
VARYTWAILSDVDDEHMFNVPITVALIDAGLLDLRRVDMLLNKLILQKNNSALDLLASLMDRVLFN
DEPSALRSDFSGSLEAMNQWLVEEPDLAVAVDIIRKLPKRDQMEYIFSEWIGVYKFAGATDRTFQS
FLKDLHNRQVMNSQEDSALFFRLSVDISVAMFEHESQNPNGSLDEAFLYIDALAKLVILLVKFQGE
TTGAVKGSKPAYLDSVLSLLVLVLNHHQVMRGDAFNQRVFFRLFSSILCEYSLSGLHMSDQHQEMM
FAFASRFLSLQPKYVPTFVYGMLNMRDEAGWAPYCEIMQVLLSYMGEQLKPGNISYVAKDLYKGVL
RILLILHHDFPEFVAENHFQFCNVIPAHCAQLRNLILSAYPSSFHKLPDPFREGLKLDRLQEMREA
PKITGDIVTPLQQANLKNVLDTSFQSNVVNDSTIQAICDALYKIADKETGLFMTPIHVDVVLINAL
VLYIGEQAVASGGLKGNTRAAFEESPHSMLLDKLAKVLGPEARYYLLSAMANQLRYPNSHTYYFSF
AILQLFGIDYAEQQESDIRQQIIRVLLERLIVHRPHPWGLIITLQELLQNRSYTFFRLPFIQAAPE
IGRLFDALLQHIQQQSPRVLS
>g817
MSTNTSTTLIAHSRRPAILTYTSPLVDTAQTLSHMPLYLFGWKREAETLDVPMFENLEFARSWRNV
PASLRLEIQSREQMQVYGVEVKFRAKFRGLRWRLLSFFAFTSMFWSVSMISSSIVWLLVSQYLGST
AHDDQRIKNEEDTEDGVKAIKKESSERDDGIVKQEEIEETTNIPPLGTQSGNEAEDVESIRRIHDS
SDSGRGTAFDGHDTLGAQRRRSLLSE
>g818
MHVLINYPAVQVTNDDGPPSNQSSPYVHSLVHSLQNAGHTVSVILPNQQRSWIGKAHIVGASVKPT
YFRPGTLHHDDGTIHPLPRGSEEGDDEEGDEWVLIDSTPASCVQIGLYHFFQDRGPIDIVVSGPNY
GRNTTAVFALSSGTIGGAMEAAVSGKKSIALSYAFTSRHHDPVVIEEASKHSVQLIEYLHANWDDA
VDLYTVNVPLEPGISKAKVVYTHMLHNQWSSGSCFEAIDASASGDEPGLQERDLREQSEGQLQDGG
SSVLNKKTALQHKHFKWAPKLTDVYRSVEESKDGNDGWTVKEGMTSVTPLRANFMHAAGIQGEIKL
GTRKNDNFDFYCILNYEDKYVQPLLEELFDKLASENSCKIVKSVADLPDPFQPLLQYRAYEEIDFD
HIMSHPSTSLTNSFIIRKALIRKHYLSTTIANWVSKYPDSILKNHFKPAFDFELDFAEFLDEALLE
AYELRESLDQNESSADDEKEWWILKPGMSDRGQGIRLFNSEAQLQAIFEEWDVEESDEEDEEGNGG
DGTQDESGSRLNPNNEQGVVTSQLRHFIAQPYIHPPLLLPSASGRKFHIRTYVLAVGSLKVYVFKE
MLALFAEKPYKAPWEEDDEIRDLSRHLTNTCLQENGSSTEGTVRRYWSLDDDIPSLEPGWKDRVFD
QICAVTGETFEAASRGMLVHFQTLPNAFELFGLDYLVDAAGKAWLLEVNAFPDFGQTGDDLKHAVV
GRLFEATIKVAIKPFFAKSHATDPDLRLVTDLDLGRKA
>g819
MVYVPVQLGMITNTLTQEKTLPWQQITTFVLLRLLDSSGGVSALRAYMWMPLEQYTYQRIAVNGFN
QVMDLSCDFHDDKSSGSVWHAINRGTIIQNVVRKVLFQFVPMISDLFIAVTVLHVTFGPYMGLITA
AMIGLFLWSSSTISKFQKAKRKRYISSIRKEVNIRCEATNNWLTATYFNKIDYEKHRHFSAVKDRL

GSELSVRRWLRTENFVQSSILSLGLIFACFIAIYQISNGTRPIGSFLMLLGYWAQLSSPLEIFSNG
LTSITLDLVDAEEFMALLKRVPSITDSSGASKIQAQAGVVSFSDVSFSYDKKRHALNGVTFEAQPG
QTVALVGETGSGKSTTLKLLLRLYDPDCGSIEIDDQNISHVSLQSLRDAIGVVPQDPALFNDTLLN
NLKYADESATLEVIHRACTAVRLHERFLSFPDGYETIVGERGIKLSGGELQRVAIARVLIKDPSIV
LLDEATSAVDSETEAYIQQSLAQLTKGRTTLVIAHRLSTIVKADQILVFKNGSIVERGTHQSLIKR
NGYYCRLWKQQVGADTKTNGSALKPEAPEFVPMGMMK
>g820
MDRTSHGERIAERQYGHRSHTRSVSPARLGSSNRNRYRDRSPNRRHHHRSSRSPAHDSKHGDKNRE
RKRYHHRERKELSPVSAAPVVLPYNARHLSKFDLSLLEPMFAMYLDIQKGLVIEDMDEQEVMGRWK
SFIKKWNRGELAEGWYDPATFEKAKGSLNDARVDHNKEVVKKLDQFGERSDNDMMPYNKDDPPVAE
TDDDNGDDDDECGPKLPRGPVTSRGALSGPTIPNIQDLELRREQALADAEAARSEARTQHKHETSS
HKTNLRAFEDEVAPRAEPGTHERKLEKKREKAAANREFASGRRGGSPTMEAAPDSELMGGGGDDDL
SALKRAKEQEARKKNEREIRREEILMARKAEREERLAEYKKKEEETMGWLKALAKQRFG
>g821
MAPREELISSAITFLQDPSVASSPLEKRVAFLQSKNLTQEEIDIAIARAGEDSVQTTTTTTTATPV
GYAQQQQPIYRPTAAQPGYGPGPYGQWQPPPEVPRRDWRDWFIMATITGGVGYGLYVLAKRYVVPI
IAPPTPPQLEQDKQSIDEEFARAFTLIDQLSTDTAALKSAEDARTERLDAALREVESVLSELKSST
RRRDDETRRISDEVKSLKDSIPKALEGAREGNEGRLKELGTELRSLKTLLNNRLSAGSPATTPVTG
RPVGPTATPEPALSPATALPATTPNTNTAPIPSASNSQATSSPASTNPLSQLGKSASIPAWQMAAA
NRSKTPSQPAQSATVEESQSS
>g822
MSQDTGLFSIKRPRETLASVQNFSALPQPSSALKRTSMIGSHQNPPYTSQHSRSTSLFNSHGRPHQ
PNFQRSSSGASFGPEAGLSTVRRSVSNNLFQNTAVGRLSYAPNSFPTNSASQQNLQRRSSVFSRPS
MAGGPMAHQSFFTQGPVPAGAPKDPRPLRDRSFQARIGQELLEYLTHNNFELEMKHSLGQNSLKSP
TQKDFTNIFQWLYRRIDPSYKFQKSIEAEVPPILKQLRYPYEKNITKSQIAAVGGQNWSTFLGMLH
WMMQLAQMLDHYSVGEYDEACAAAGVDISGDRIIFRFLTGAYHDWLQGGEDEDDDVAEKRLVPHVE
AMAAEFEQSNEKYVRELEVLEAENRALRDQLEDLEKNAPDMAKLDKDFRILEDDKRKFEDYNSNVQ
GKIDKYKSRIQFLEEELKKTEDELQTAEEERGSLQASVDSQGITIQDIDRMNTERERLQRSLEDTV
GRLEEAHARVMEKETEANQKLEDLEQVVKAYNTLGYQNSLIPESAINANGEDYEIRLNVNEASFSS
SQMGVSQDQGSPEGYRLLADANNGYQPVHLVKLDLRGVVRANLISLRKEINERRKKVAEKDMADRD
LLNDIKEAMDEKRSEIEALEHRRRAAEEEFERTKEITTTQKVQSDAQIEKMEKELAKMRASLIESV
QLMEQREMNTDVEYEQLTLRANQLREELHTGVESMLNDIIRFKVHVQKGLEDYESFVVEEVEQELG
GEMQSMEDTELAEAHAASDGEKISAILSRARRLHRLDLLAYLGFTLQRWPAVYALITTMLDAADKL
QMIGHSFEGTPSNLQWELLDDDQIVTGISNNSAAYVSMEALTDEPRYLLARMAIMGQIWQSLGFIV
LTAANKTSQESELAMSYVYRTLAHLHQSGNISDAIYKFTNRTDNGDTFRPPGMHLLSTHIMNVLSD
AAWLDHEAEATRKAKAAGEDSPFRPFKMGIRHLGSQIWLEFILWACIEQGHIMEGLWILKHLRGSN
YAAAWKVASWSPLLENPELIKNTKIDIHDFWPHPDIPRTPDELQNKSGFFHAASYVQINHESDNNA
KDLIELLASVMEIKQSYSDKEIEQLDHLLHALPPPIPPWNDAVPTDRQGLSAFAEQNLYETSSLYT
GLLQQNLRLYAHHQQIENAMRIFDTLLEIIDSSKLDRIQDFWSKGQDNEGTSIAIDQSPIPTLPNG
ALGDLLDLVRISKAYAFGKWLLFSKDADGPIITFSSYDNQELGDLVIQQIREPISRNTYNAVANFY
IAFGQWEEAEGIFKLLIEYRKKAWGETTLATLASTILRLEQRVWEEPWDEQLQTSLLQAQTMLRKF
LWGRYNPTKVYNESVNLYYKQTIYRWHCLLSSVPGAVARVANDVKPVYTNPLGRDKFSFIPSPAFN
ILLTRLAVDEKYRLQMSADPANTENLDIEYDWPWHYERQRKAVNPNLNTLRIISRAAVMELKQSQE
VAPDRKNMTPSQQQFRDQDEIGRETGGHLRRIRMWQSRKRRNIVRWVYGDLGEDDTGGVEELLAKT
GNEEKDGVY
>g823
MDPSADPSQLNPQSNSDNLAMSSRTTSRSQETQSTPEKTDAVAIPAQQANQGYLAMAKVAIPRSRA
GASLRHMEELSQKANDYESLLKDLRSTVNEAVGDRIAATLNKYAIDGEQNTTDHPSSSSRTPLDDV
DLEPSSPSSIGSLEAIDRVEEDLNRGENVRATGFIGKNSEISWMQRVQRESVQRARGEPGMYEGEP
QTRQYEDFAINSVSYHLDDLDINVSGPIDKYKMPSREQADRLFEDYLTTVHPFYPIISRPLFSSQF
KYFFENSARPGDRWLAILNMIFAIAAKHSHLVQASWKSNDTQDHLIYFTRARYLSMTDDDLFSHPD
LQQVQVEGLMAFYLLSTDQINRAWRISSLAVRSAIALGINMKSSSESTANTSKESRYRVWWSLYSF

EHLLGVMTGRATCILDGVCTTPMPLPFEEERFNEPDVQEIMSNSSLREEKIQNAVASSLIRLMPQN
PVGGQNVDRREKTDRFAWIRSIPFNSGLLFLLYMDLTVITQEIVNKVYTSDAVRVPWSHIENRIGD
LKSRIDLWFNQLPEAFKFTQKESSSPEQVRAKLGLAFQYYSARITLGRPCLCRRDATSNKSTSFSH
KMALVSLEAAARMLDLIPDAPDPFQLYQLAPWWSILHFLMQATTVLLLELSFANVHAPDEEKNTFL
SAKKAIRWLYAMSEHSTASLRAWQLCDSCMRRIAVGMNYDVSDLPPLPSDAILEQQKQYQARDPLQ
TQPAVTSGPSRNTPQHSEAFDWSTGMESLATPSTEAARRRELLSDLELEQNAALTAAESVTYPLSF
SPFMMNPGVSGSSISDSHFPYDPLSGEFMGSFFPPDADQHEWDN
>g824
MFDTTYRPSSAQRLANQMEQLNEGPAPNCTMDDLSVGDVFSSQMDIDPEKNGVKDNASSRLGHAQA
GEDPALRRTNLPPAGAQVFQLHEKAASRRKKKKGKRADVEVGHGNPALSTVRGFTPMASGDEDSDM
STAGSRVMSPQLNAVPTTNMPSGISALKLQLDALSLSERIPRSGDSVISSNASVSSDSDRTEVMTS
YEVPLEHDFISEEAAAEEKLSSTPKNDLCRKMTSRDFEQLMCLGKGTFGTVLLVRHKPTGRLYAQK
QFRKASITVHRKLVEQTKTERMILESVNRHPFVVKLFYAFQDHEKLYLILEYAQGGELFTHLAAER
MFSEDTAAFYMAEMVLALEHLHQTVGVVYRDLKPENCLLDSEGHLLLTDFGLSKVSVSDDDRCNSS
LGTIEYMAPEVVQGKPYGKACDWWSLGALGYDLLTGSPPFKANNHAKIQEKIVKQKLALPYYLGPD
AKDLLIRLLRKEPHKRLGYHMPKDLDIIKKHRFFRKIDWKALARRELEAPIQPLITDPALAENFST
DFTGLALSPTLGDDVHIGEMLKNVGLSADPSNSMGETDPFHGFSFVASSSLLNGGFSLAT
>g825
MAGIANLRPATRPSIVDNVSLRNINLHFPAAPDPWHRPGKSQPCTAAVKLSYSSAVAAANADDVSL
SLDYGKLYRRIETAVRDSTKSQDGGRLSAAAEAIVSNDVRVIAGRIANCGLGLLDETIAGVRRMDH
LQAHPESPSRRRASQASRRLSGVSASVPKELQSATSPEVLNGMFGECEVLLHLPNAHLRAEGGLSF
RTLQTWVYADDSASLENAVESSRQVAIVEQEFRVESIRCHCILGVNSHERIEKQAVIITLDFRGSG
EAAWASTFLKTYQEMVRTIAERVENTSYQTVEALATSVARTATMDFGNDSVTVLVEKPSALAFVER
AGVQITRSKAFFAEQDFSKARGGI
>g826
MANKVLATDPIPNESTLLIPKSPDLERSSSPCRRANDDFEDAKYSNKRLDLTEFALLLRGSTPVIL
AYTLQNSLQTISVLIVGRSSPENLATAAFSYMFAMCTAWLIALGGTTALDTLASSSFTGSTNKHDL
GILLQQPVFLFLGQDPNLSRDSARFLTCLIPGGLGYIYFEAMKKYLQAQGIMRPGTYVLLITSPVN
AALNYLFCYTFDIGLLGAPIATSIAYWLSFALLVLYTRFIAGSECWGGWSHTAFQNIGTFARLAFL
GIIHVGTEWWAFEIVALAAGRLGTISLAAQSVIMTADQVMNTIPFGVGVAASTRVGNLLGARNAHG
AARAAHTAAFLSILLGGVVLAILMVTRNSFGRIFNDDDHVVRLTAEVLPYVALFQIADGLNGSCGG
SLRGMGRQHIGALVNLVSYYGGALPLGIWLAFHGWGLKGLWVGQCIALYLVGALEWIIVYLSDWEK
EVDNAFKRLDVRLQLEDGDVDTLV
>g827
MYASLLTFVAFSAIASAHANHDQSPIPGPHKQLWYNSLPGDGGTQADSVFSGISTFGRLPFFPCLA
SEDEKYDIAFLGAPFDTGTSYRPGARFGPSGIRQGSRRLNLYGGYNVPLEANPFSSNIKILDCGDI
PVTSYDNTWALRQIEEGHNSVMMRKPFTNAEKKGLSKAGKTLPRVITLGGDHTITLPLLRSINRAY
GPVTVIHFDSHLDTWKPKVFGGAPSEIASINHGTYFYHAAMEGLLRNDTNIHAGIRTTLSGPSDYD
NDGYVGFEIVEAREIDTIGTGGIIKRICDRVGTINPVYLSIDIDTLDPAFAPATGTPETGGWTTRE
LRTIVRGLDGLNFVGADIVEVAPAYDTNAELTTMAAADVLYEVLTIMVKKGPLSLSEREEL
>g828
MTTDKEKRQPKDLPSGTEPSNLDNEVVVVDDDGTAVNASGWKDQLHRQYGLLSLCGIALTVDNAWV
ALGSSISVSIANGGPPGLIFSLIVAGAYYSFIGLNLAEFASAIPSAGGVYHWATVTGGPRWGRILG
FYAGWINFFGWMFDLASLVQITANITIQMYATYHPDYVQQAWHTYITYLLVLWISAFIVIFANRLL
PYSQYAAAMPKTHATSYFVWGSFDENNLTGWTGGVAFLCGVLNGAFTIGTPDAITHMAEELPHPKK
DLPKAIGLQIGLGXLYAFCFAIALCYSITDLNALLSGVNSYPLATIYAQATGNNAGATFGLLFIIF
CSSLLCTIGTVLTNSRIYWSLARDNAVPISSLFGKVNESLSCPVYATLLCVIFATGLGAIPLGSST
AFIDLTGSFIILTTVSYAIPIVANMLTDQKYLPKGPFQLGRGATSRFVGWGAVVLIMFFNVFYCFP
YSIPTTSAAMNYNSVILVGVVALTSIWWMVHGIRNYAGPRLTHLYIHEGIEQVNHVAGTPLQSQSE
EKTASSE
>g829
MASKGAARFLLKREIQSSSRTRPSSRAISAVNPGRRLSPLTPHNRRQHCNATDPFNARIQPGQTRA

FSRSARRCYAKEDVDPNQVERESDEVDVCIVGGGPAGLASAIRLKQLANEAGNEEFRVVLLEKAGE
IGAHIVSGNVLQPTALNELLPDWLDKSNPSRFENVTPAKSDKMRFLTKNMSIPIPAPPQMNNHGNY
IVSLSELTKWLGERAEELGVEVYPGFAASEVLYKPDGSVRGVATNDLGIGRDGKPRDNFERGMEFH
ARVTLLGEGCHGSLTKQVIKKYDLRRDSQPQTYGLGIKEVWEIQPEKFKAGEIVHSMGYPLPSDTY
GGAWMYHFGENLVSIGLVVGLDYPNPWLSPYQEFQKLKHHPLFKDVLEGGKCISYGARALNEGGFQ
SIPKVAFPGGALIGDTAGFLNVPKIKGTHTAIKSGMLAAEAAYSALQGSNDGSVFLYDYEKTLRES
WIWKELKEVRNMRPAFSTAGLFGGILYSGLEAFVLRGKAPWTLKHHSTDSGATKLASQCNKIEYPK
PDGMLSFDILTSVSRTGTNHEEDQPVHLQVKDWDAHTAEAWPKYRGIENRFCPAGVYEYLEDPSKE
QGVRFQINAQNCIHCKTCDIKVPTQDINWQTPQGVTKKDIEEYFGSHGTGKITEIKLMNGFGFIEY
EDALDARDVVPAFHGSDFKGERLTVQFARGPRHKETFNGPSDRPSAPRPRRTVFRMQVSGLPTETS
WQDLKDFARQSGLDVVYSETTRERDGRGFVEFESHADLKTAVEKLDGRELKGSQVNCVADKSELHI
ATLTDPVRLRVADILQGMITIDEDLHVVTVPDHTIVNAPHKLCDVITTTGMATDGGALHGHAWRIT
LRRDDHTTTLTHHRRHLRLAIMTILTLQEGHTDAHEARPEAIMLGTNDARTGSM
>g830
MDSLTTHPSNAQQAKAFTSPASLSFPGGAGDLTPPSSEKEGNLAGLNGQQQGANANGNGVTPATPA
ATPGANNVGSGIVPTLQNIVATVNLDCRLDLKTIALHARNAEYNPKRFAAVIMRIREPKTTALIFA
SGKMVVTGAKSEDDSKLASRKYARIIQKLGFNAKFTDFKIQNIVGSCDIKFPIRLEGLASRHHNFS
SYEPELFPGLIYRMMKPKIVLLIFVSGKIVLTGAKVREEIYQAFELIYPVLSDFRKT
>g831
MELPETILIVGGGVFGLSTALSLSERHPNKKITLIEASSTIPNPHGSSVDTSRIIRADYSNPAYAK
LAADGIKKWRSTEWGQEGRYTENGLALLYTEGNSDSENYTKKSYENVKRLLQEEGESLESIAEKLV
YLPNQAALEKVVPRYAAGMNISGGYLNRGSGWGDAEAGVRFAKQKLDQTGKVDVKHGEVERLLFEE
TSSTSQAKVTGVILKDATGSTSIIKADLVILATGASTGNLVDLRGIVDATGQVLAYIKITDEEQAQ
LADMPTILSFSTGMFIIPPRNNLLKIARHAYGYLNPSDVPIPGATTTSSSSHTMKISLPVYDLAIP
AEGERACRQALREILPLFAERPFARTRICWYSDTYVIQPPFHIPELYFYIHETKDSPMNSPTGDFL
ISYHPSHANLFLATGGSGHGYKFFPVLGDQIVDALEGRLDAELQQLWKWPEQRHITEKHGGEFVVT
KDGSRSGKIGMVLKDELAKSSRL
>g832
MTAPTAGRRMSPRMMTIFAVGMLGISTYCGYLYSSYSQAVAQSKTLKVPEDVSDRYNSTAMGYDAE
VELGEKFMRLGKRRKELVQMARGDVLEVSCGTGRNMEYYQLGEKRGLDERGRPVLQGCRSVTFVDL
SSQMVEIARHKFRRLHPEFAKAAFRTEDAKKVGPVADTSAKFPEWVYSRSHFDTVIQTMGLCSTPD
PVGLLRHLGSITEPQRGQILLLEHGRSHYDWLNKILDNLAPAHAHRHGCWWNRDIGQIIEQSGLEV
VEIQRYHLGTTWRAVLRPRTQT
>g833
MPYNTRRKSLSLPSLGIHLPNASRSHRSPSASKSSTSGSTTLDELTHPSKKVKRSHDAVDADKSQK
IATGRIRNSRGAYEHTPPPSPGDAVDVAMKIDTEGINDDVVVAAIEQLEKTGNKPHLVRELAAVLV
KVNDTVANSANPAALLSSRLSTYMKRSWTALSPCPIGKELINVHPRKVFYYLTNTPHQPLPENLED
MVAMDEKRLTPSLSSGSVDQDEDDAMARERSRLSPSPEIDLSSPEFEEHDTFNARHTSGAHANHNN
SHRLSHNHRAASPPLEGDEREFTQTASSLRERASEEKASRQSSLTALSIPVDESQEAQVVRFPGSP
MDEDLLTASISEQNQYEDYFSHGGSRDQEQDPDDAAVATLFGTSPSPSLTSVASSLSSGTTALSEA
ELADVPPALDSASGAYTSDPARPITTITTTEPSLKRSFDVVETGFPTKSVMDLKHSQTFDFEMVID
SWADLRSPETVEVDELDAMFGDF
>g834
MESTSQHPPRRPVPAPSNNVIVESQKQWYFTDEELTRTPSLLDGMSLEMEHMQRSKGVNFIVQVGI
MLKLPQLTLTTAAVFLHRFFVRHSMVDIPRRPGLHPYSVAAGXLFLASKVDENCRKIKELVIACCR
VAQKNNNLEIDEQNKEFWRWKDTLLAYEDMCLEALCFDLQLEQPHKICYEFLCYFGKNDHKGLRNA
AWAFLNDSNYTVLCLQFYPRTIAAAALWAGARLCDVAFEDDEEGRPWWVQIDVNLSEVRRAVSRMV
QLYERNITVHRQAHEYPTMPTDGDNGQEMTRIVNPNPHNVTESLSAGESNGRKRSREPEEGGRPSP
ARNLYPTESNNHTQEPSPKRQRLTPDPTRTSIKSESPRSLANGHHQSSRASSRIPSAAMDDTHRRH
PLPPPPPPPPDDAVQQRIDEIVQQGMSRGDKGGPSAGRNDTYYGRHREWDRDRERDRDRDRDRDRDR
YRDLDRYMDRDRDRDRERSRDRDRDWRRSNSYEADYRERNSRSRDYRDERNRNPNNKDRLPQRERR
RSSTTNESLPQRPPPPASTTSSQRPSEERRRGEPNEEGEISEEGELVTNNHNHAHNERSHKPDENM

GNGDDDGGGGSEEGEL
>g835
MALSASAPPAGMMIDPTKQGDYPILLGDQLAKKNGGHASTFINITYNHKSKSATANQRTKITRSPT
SQDIYNLTITDKAGNAEQTSLEYKYKGSIDPSIPVQGSEARNLVLVFDPSRKAFILESVSTSLNFN
LRAAPGKNKDVIEQYEQLHTLGDDGDHAPRDESDDEKPEDADQDNPYDFRHFLKRPELEKAKSTLS
TTTTPDPHGAISTANTPAMQPTKVEVKKMAPRPKQQTNPLRQPRRAPKAKPTSVSSTTKGKAQPKS
APRVDPEDDIAISEAEQSEKEEDDYQHPKSSQAVPSPSSNIIVDGDLIIDMGSPPTRPAFKVDPTH
FSSSDQSAHGTDEEGNEEDDEEIEDFRLPSPARHDARVATSAAAAEPEVADEDEDEDEDALAAEME
AAFEEEASRTQSLQQTQRYVPSDDESEVSEEE
>g836
MTDAILELLDPHLNSNNNASSPSTSRPLRASSTDATTTTKYLNRLTSLSLEQIRNSEPQSISQSAH
STLLSLQALSNRSHKALISSSDHLSTLRNLIPSLGQDVKALQERIPILDSEAVRFSTTYSKSSDNP
NLDRRKKAMQVSRNVDRISDVLELPALLSTAVSTSSANAGPNAGSAASGNYSTALDLYSHIKRLQT
LYPDSPLIKDIILQAEDAMKTMTTNLIAGLRTQNIRLAAAMRTIGWLRRVAPELEDPRRGHETGTG
SGEGSLGALFLVCRLANLVSTLEALDPLRELADQETQRRGEAAAGERAASSKWSGGQQTERYLKRY
IEIYREQSFAIVSLYKNIFTPEPVENGSKPISVPGLDLKSLNLKTQPPAKPTKKVDPLQALPPAVA
TFPLYLVRLLTDTLRTYLPNVRDKSSRESLLSQVLYCAASLGRLGGDFSMILTELEDEEVEDETIV
PEWEEVMRKHRALAGRLEQLTSGQAAGGATKSISRVTSPVH
>g837
MGKPRMIILIRHAQSEGNKNREIHQSVPDHRVKLTPEGHKQALEAGRRLRELLRPDDTLHFFTSPY
RRTRETTEGILESLTSDDPSPSPFPRHTIKVYEEPRLREQDFGNFQPCSAEMSRMWQERADYGHFF
YRIPNGESAADAYDRVSGFNESLWRLFGEDSFASVCVLVTHGLMTRVFLMKWYHFSVEYFEDLRNI
NHCEFVIMEKNPDNGKFILQNKLRTWSALRQEKEEERKREESAVSKGSQVQSPEAEVPVRRKWGGC
PNGCTHGEAVSSTWRPTRRDEVDVFRDDDGGETTDANGRKRGPSTDRKKTFKRMPSLKDRKTLTHI
DKSKFHLTIEPQNPDDIVSSPNQTPSYIALGGVVNPREPGEHEILRVRDNVAPKDISTQGGPSAQT
LPNIHLLQLAGRDGGGSTSGVNSVEISDSESDGLFPEKSESLAALTSSNSTTNLQYSTSYHSVQDA
EDDGDDEGAGTASRRSRRSTKAIYQNHTSHHNHQHQNKPSTTAYQRKANALGDMADDDNDDNDDNE
NDGNGNADTVESIPDTEIEQFRREEESVSGSVY
>g838
MPSFYNPGLLGLPPTPPHLHGNGKMELDPPFFVTGHTSFPPRYPQTGCEFIEQYSQATSFVKSAPA
NSQQTTMSIRGGRDYAAALHQQQQQHQHSLAQSQLGGPQSLYGVVSNNVPTLPPIRSNLHMPASS
ESAIPPQYRHHDAHIPAETRIKEEKPTGGVAAHLDYEMDQMSDFVAETAQGMYALYSSGINLDDID
MLGSVYPGQTIXPPPFRKYVHQILSSTRLPSSTILLGLFYLATRMRMLSAKGVYATGTGQVYRMLT
TALLLGSKFLDDNTFQNRSWAEVSNISVSELNTMELDWLFAFEWKIHERIHNKKDGFGIWLARWET
YKTTMASQNKVRQKLSPIDTNITRQLTIPKPLMSPDGPIPPQYQRTTGYENTWLNPTACEYSPPSA
PHSGPNTPDYYNGGAWMYSNPPPPYTRTWASQTEYPSFPHPPRSQPPSYHHTPVYTAPMNHSVWTG
HGSSCGCSYCLKHHEHYFSGHTFSTLQPMVAG
>g839
MSGTAPPSLPDASDDEMVDAPPSRDPSVDVQTADNQDDDPDVEPEEEPEPEQQNVQEDDQEQAQDQ
EQQQQEQTGADETGVEAQAETDAEPKAEGASSPAAPEPDTPSKFSGTPRGGGGLAGYPRKRRPGRP
PKNPRPELDDGTEQKIVVSTPVKRRRGRPAASGGRWGRRGGNPHVTQVAVDKEGTMMDVVNDEVAL
PDIEEGEKKVDKMGHLLEGREYRVRTFTILNRGERLYMLSTEPARCIGFRDSYLFFQKHKMLYKII
IDDDAKRDLIERDIIPHSYKGRAIGVVTARSVFREFGAKIVIGGRKVIDDYDEKAALERGDTEGEL
AVPEDRLPPAGEEYNRNQYVAWHGASAVYHTGAPSVPLPVGKAVDQKKRKVTVTGDNWMIEHARET
SRFNSVLANVRRENLEGVYDVHTNYVQYPKIMQPTHARWEVVPPSEDSKSSNQLVENMSSLQIMSG
NTPKADEDDEAVNDENGESGENQLSQATPAVASMMPPVPSIFTRRFAITDIHYETPETSTLGRPGP
DGDVQDIGSNGIISMANPQHPEFVSAEILAELPPECKEALMEAAMKEYEWKSKWQTESTDAARGHI
LQSYNWHP
>g840
MRQAVQQNSAAHEDDHPDNLITSSGSTPSXIITPQPDPADKRLPSIIHYFSQVGNSAFPLSSLKAR
LSSSFTTSVANATPAPVESNEQSNVETMKPDVDGVSDAVSPSSGANATEGQLQNPEQTETTETFSK
PTSLPTPPYSSACSLSQKDMEEMESTAPTTEKTAVSIYSALKDYMSSTTSNNVSELHSSRRPTCDP

VSSVSDDPVLASHFSNPSLPDASDLPPPPAVPLLEHEKPHHISKSSENLAKLTEGMANPSHLKNTP
PLTPRAMSQENGMSQPNGSTPVPSNSSNQRRSHTETPSEVSDGKSSDSRSSSEGPAVAPLKGKLSV
KISRARGLRPSADPYVVCVFEWNEYISKGTQSGAQSPEGKNSGKDYLDNDTGRPMAIPMKSRQSSH
NSQLDNVDLKGRAPVTDPQWNHEAVFDVMGEQSEIDVSVYDRYNQEAFLGHVRMSLNLREERSSLE
GWFPLAARGQGDFQVSGEIHLEMHFERTEKKQVGPNDFQILKLIGKGTFGQVYQVKKKDTQRIYAM
KVLSKKVIIQKKEVAHTLGERNILVRTAMAASPFIVGLKFSFQTPTDLYLVTDYMSGGELFWHLQK
EGRFQEARAKFYIAELILALQHLHDHDIVYRDLKPENILLDANGHIALCDFGLSKANLTKNDTTNT
FCGTTEYLAPEVLLDEQGYTKMVDFWSLGVLVFEMCCGWSPFYAEDTQQMYKNIAFGKVRFPRDAL
STEGRNFVKGLLNRNPKHRLGAKDDAKELIAHPFFHDIDWEALGRKEVIPPFKPKLKSESDTSNFD
PEFTTALDHSTSLNDRAAALANGLMPGSTPLSPGMQANFKGFTFVNESSMDHHLKDNSADRMDEDT
KEDESWHRHQRSVDSTDRRMSGVVKTGEEGGVFNNFDI
>g841
MSVITATSQRAPVTFRDYDNQYSVAIQDVPPRRGSAASVSSYTSNYSSSTYSSTPPTVYSPTSPKP
AYRVFKQQTAGAQQQPPPTPTIPPSFLTLPRQPNEPAIKCLPPAVYECILKQLQDLHEAPENYQYG
CTTCFQRDLHALALTSRTWERNVRSKLYNYIHIHGADSPAQLKKYRLKRGSRLKLLRRTLRERKLL
ANLVLELRIPEIDVSPTVANGKMNPQWEDYRDLVASVVMVCPNLERLIGFNMSFHHEFDRLTYALS
TRKRLREHQWILGEPLDSTVSIDDKNSRNTSSSSPTKMSRTNGKMLEPQQAFEFLHYHSCWSNLET
LMLHSLNMGGVLEHGVFLRIFNRLPALRNLCISSFDADAFTDRTLQFLPPLQSLRLENLRGVTDGG
IAQYVSRPEARTIQSLTLIEQNISSLLVISKIFASLRLLEKFNFVQSTRLPTLPSEAMVFQPILAS
ASLKRLHWDVSGPESAAGLGKLLDLPFVTPHKPYDSPNFHLAQSILYSGFPHLAELRAPSDIEPPG
ILQSVCRPVPHGQALIPSDRYSLPRSSHGSVSTRPLALPAGNNLTSARIRAQTFIDMAAKDSEAGM
KVLVTDHSDSYIPDSAMDGESDDGSDEEDEANKDLFGGSYPGVQNPALSDQVDESNPNGPKKIYEF
RMPAIMGIVRAVTPHDTTGLGSIPRFDIQPDVRGPAQMSSSNTAQQQSFSSLSHTSSFSSSSTNTN
PDHLKRSESDLSTTDSIVSPTTTSATSRLASWSGFGSRSNNNSSTTTPTITSPTTTAYPPTPISYS
FTPFF
>g842
MSTLEDLDDLEREDHSKKEQQGGDDNGDKKPSGDGDAEMQDAEPKKDEIDELLDAELVGASTDDIK
KRRRLLDNELRIMKSEFQRLTHEQNTMREKVKDNQEKIENNRQLPYLVGNVVELLDLDVEAEAAEE
GANIDLDATRVGKSAVIKTSTRQTIFLPLIGLVDHEKLKPGDLIGVNKDSYLVLDTLPAEYDSRVK
AMEVDEKPTEKYTDVGGLSKQIEELVEAVVWPMQEAEKFKKIGIKAPKGALMYGPPGTGKTLLARA
CAAQTNATFLKLAGPQLVQMFIGDGAKLVRDCFALAKEKAPSIIFIDELDAVGTKRFDSEKSGDRE
VQRTMLELLNQLDGFASDDRVKVLAATNRVDVLDPALLRSGRLDRKIEFPLPNEEARAQILQIHSR
KMTTDDGVNWAELARSTDEFGGAMLKAVCVEAXMIALRKGMNKVNHEHYVDAIGEVQSKKKDTNMG
IYV
>g843
MVSSFEIPKKIYATSVYEDPPGHIKVNWSDNHLGEYPKSWLESHQFPSELRMSNKVLTFRKYRHSR
DSELHPCVDYEAVMNTNEGLSGWLQHVVDWGYCLVKGVPVTPEATKELLERIAFIRETHYGGFWDF
TSDLTFKDTAYTDEALGAHTDNTYFTDPARLQLFHLLEHTQGEGGKTLLVDGFYAAWRMLLEDPQN
VEAFTDYAQPWHSSGNEHVSIQPYRYFPVFERDPTNARLMRIRWNNYDRAAKIDWTSKMAMHWYKA
ARYWNALIHRRNHQKWLQLEPGTALLFDNWRMLHGRSEFTGKRRLCGGYINNDDFLSRFRLLKNGR
EKVLNDIGTYNTRQSL
>g844
MSGPGAGFEYPRRAVTWTKRDALLFANSIGCKSDELHFLYELHPDFAVFPTYINILPFKLTNTDVI
DFYKASTSTPIPDVPKFNPQRVVDGERRIIFLKQLPTSSEGKQFEIRTKVIGVYDKGKASVVETEV
SLVDKETDEVYAKMIGSAFYVGQGGWGGPKGPSAVNYPPPKGKAPDAVFVDQTSEETSLLYRLNGD
YNPLHATPEPGTKMGFGGIIIHGLYSWNATAHGVLKELGGSDPKNLREFQARFASPVRPGDKLTTE
IWRTGEVDKDGFEDIRFSTKNQNGKAVLANGRALLKVVTKQSKL
>g845
MRSSQKWSVLAILFVIIPVSLAIKGTYKVPTVDVLNGTYYGIHSQHYDQDFFLGVPYAQQPVDNLR
LQIPRSLNSSWTKPRNATEYSPSCLGYGQSEGASESCLTLNVVRPSGTKLEDKLPVAVWIYGGGYT
SGSSADQRYNLSFIVDQSVQMGTPMVAVSLNYRLHCWGFMWSQEIKDAGVGNLGFRDQRLALHWVQ
ENIAAFGGDPSMVTIWGESAGANSVGTQLIAYGGRDDHLFRGAISESGAPSTYDRYQTPADWQPYY

DAVVEAAGCSSAADSLGCLRGVPVNTLYDIFNNASIVPVHTLSGLSGPQFVQVIDGDFIQESATVQ
LQQGKFAKVPYLIGGNADEGTSFAVPGINTTEQFKEVISNWGLDNATTDTLAALYPDIPEIGIPAT
MTGAPPAGYGAMYKRVAAFQGDVNIHAARRLASQIWRQYNVPAYSYLFDVINNGPDVGANHGSEIP
YVFNDVNGVGYDDSHNPLLGKPTSYTQLSVIMSRMWVSFVTTLDPNLPYEGSRWPMYELDNPEIIV
FDVNVTSLSYVMPDLYRAQGIKYIGDNLASLFGH
>g846
MAAEGGITSSADRMVGADHAEVRYFTSYDHHGIHEEMLKDDVRTRSYRDAIYQNKHIFKDKVVLDV
GCGTGILSMFAVRAGAKHVIGVDMSSIIEKAREIVAVNGMADKITLLQGKMEEVNLPFPQVDIIVS
EWMGYFLLYESMLDTVLYARDRYLAPGGKIFPDQATIYVAGIEDGEYKDDKIGFWDNVYGFNYSPM
KDVALTEPLVDTVEMKAVVTDPCAVLTLDLYTVTTADLSFKVPYSLPVKRNDFIHAIIAWFDIQFT
ACHKPITFSTGPHAKYTHWKQTVFYLRDVLTVEEEESVSGVLENKPNAKNKRDLDIKLSYTLETRD
PIRYAQGECEYKMC
>g847
MLQPIVQFHGKWPFYRVLGMQEIFSVLFSLLNFLAHYYGLKWVESSIPASYPLRKYYKGFAYFGYA
SWTFSMLFHTRDFPLTEKLDYWAAGASILYGLYLVVIRVFRLDQERPRFKPTLRRLWTYLCVGLYI
AHVSYLTFWSWDYTYNMIANVIVGVIQNVLWTAFSIYRYRKEPKKPWMAWPAIIVLWISLAMSLEL
FDFPPWWGLIDAHALWHLGTVIPTAWWYLFIVEDAKNDLAAERLKA
>g848
MASPAIHQAITEAASKYRFPEGRVFEYGTAGFRMKADLLNTVVFAVGLLAGLRSKKLNGQWIGVMV
TASHNPAEDNGVKLVDPMGEMLEAEWEAYATKLANAPLDKVADVYNDLIKEIDIKMDNPARVVFAR
DTRASGSRLVGILNAALTATEVEFVDFKFMTTPQLHYIVRCKNTLGTPYEYGEPTEQGYYEKLGEA
FKKVMKSVKIQGHLTVDCANGVGGPKLHELIKYLPSAAEGGLDIKVVNDNVINPDSLNFECGADYV
KTKQRAPPSSKAAQLDRCASLDGDADRLVYYFLDENNVFRLLDGDRIATLAASFIGDLARNAGIAQ
KLKIGVVQTAYANGASTEYIEKVLKLPAVCTKTGVKHLHHAAMRYDVGVYFEANGHGTVTFSENAL
KTIKSAEPQSPAQQHALESLVGLTDLINQAVGDALSDMLLVEAILAHKGWTTKEWLGTYTDLPSRL
VRVEVADRSIFKTVDAERKLESPAGLQDKIDALQSRYNKGRSFARASGTEDAVRVYAEAASRSEAD
DLATRVANAVKEAGSGGDA
>g849
MPALKKRKISHEKLVEKEEVSEDASSSGEEVHNSANEDEAAKAPKTFKELGLIPQLCEACETLGYK
APTAIQAESIPLALQNRDLIGLAETGSGKTAAFALPVLQALMDKPQPFFGLVLAPTRELAYQISEA
FEALGSTISVRSVCLVGGLDMVPQAIAIGKRPHIIVATPGRLLDHLENTKGFSLRSLKYLIMDEAD
RLLDMDFGPIIDKILKVLPRERRTFLFSATMSSKVEGLQRASLSNPLRVSVSSNKYQTVSTLLQYY
MFIPHKHKDVHLIWLLNEHIGQTTIVFTRTVHQATFLSLLCRALGFGAIALHGQLSQSARLASLGR
FRSRQRDILIATDVAARGLDIPSVDLVLNYDLAADSKTHVHRIGRTARAGKSGKAISLVTQYDVEV
WLRIEKALDKKLPEYKVEKDEVLVLSERVGEAQRQAVTQLKDMEEKNSGGRGGRGGRGSGMRGSRS
GNGGKRGRDNMDQEEG
>g850
MARRRTKKRTQGQGQNEAANSKNAASQSPKSMVIRIGASEVGSSVSQLAKDFRLMMEPDTASRLKE
RKANKLKDYVVMAGPLGVTHLILFSKSSTGNTNMRLALTPRGPTLHFKVENYSLCKDVAKALKHPR
GGGQDHRTPPLLVMNNFNTPDANENSKIPKRLESLATTVFQSLFPPINPQATPLTSIRRVMMLNRE
KSPNDDSYILKLRHYAITTRKTGVSKRIRRLDPTLQRHKDPKKSLPNLGKLEDVADYLLDPAAGGY
TSASETELDTDAEVEITESTTRKVLSRREMQRAKAGENATDKQPGKPNVEKRAVKLVELGPRMTLR
LKKVEEGLCGGRIMWHEFISKSEREVKRMDDEWNRRRKEKEERKKQQRANIEKKKQDKAKAKANGQ
GGDNADDDEDVSMDDDYEWDSDEYDQEVGVEQDSEDEGGDEDESMEE
>g851
MFTGIVETIGTVTSLEPLDTSASGGGGTSLTIGNCAEILGDAHLGDSIAVNGTCLTVTAFDKDWFK
VGVAPETLRRTNLGSLKKDSKVNLERAVSSETRMGGHFVQGHVDTTAKILSITPDGNALTFRLQPS
DRSILRYVVEKGFITLDGASLTVTKVVDGEDGYWEVMLIAYTQEKIVTAAKKAGDDVNVEIDMVGK
YVEKSVQAYFSQNTGGDFTILERMVAKLVDEKLCQAKAI
>g852
MTRVLAGPYCTQILGDLGADVIKVEHPQRGDDTRAWGPPFAKYQDASKKGPGESAYYLAVNRNKKS
IGLSFAHTSGVEILHKLIKECDVLVENYLPGSLKKYAMDYETLSKINPKLIYASITGYGQTGPYSN

RAGYDVMVEAEMGLMHITGSRDGPPVKVGVAVTDLTTGLYTSNAIMAAIIARGRTGKGQHIDACLS
DVQVATLANLASSALISGEKDSGRWGTAHPSIVPYRSYKTLDGDILFGGGNDRLFGVLSDRLGHPE
WKTDGRFLKNSNRVENRVQLDGMIEEITKTKSTQEWLELFEGSGMPYAAVNDIQGTLNHEHVRARG
MVTEIEHPECGPMKLVNTPIKYSHAQPGVRTPPPTLGQHTDEVLQGLLNYSEDEVLSMKKEGIIS
>g853
MALLRNRVIGYPALAPAPSPALTQPVPTAKALVEASTPSVVQIAPGTATSAFSSLAQSIPSSSQLV
ANAQLQGIPLPAPDLSINPTITPKQPTPLNSSPSKTSVKSRKRRNKRKRIHDSEDEIKAHDTSSDD
ELEDQLPSARQTKSGRHVNRPTFFAPSPEPVPISRQKSSPSGSTTTAPAKRRRKVYRKNGKEINVT
CRHCQRGHSPVTNMIVFCDECNDAYHQYCHDPPIKQELIDDKDAQWFCSECRPETRHRVLLKLKLP
PLQKDIPSIETPVLSIPLVGGEQFTAEEQRGYLSSLSHAALVDMLVTLSKANPALPIFPENLKDLQ
HSKFAVAAVTATNPVDSAEVAMEAKDATSPGVDEEDSDAESDSGSEYEVEEHRLYPRSGNGFHLPP
EEDDFDILLDDPACPTFSYTLHSYPGSNPHVDTTPVVQVGG
>g854
MEKIATTSRLVTPHPAQKWYEHFNLDLIIKVLNQTFLHPFIAWIVVLCLRAQVTPTDHPAWIAAVA
YASFLTLLTAANMLNRRLAYGLPREVEPSHEVIVITGGXSGLGQLIAQIYGMRGASVAVLDIKEVS
EVDGWDELSGVEYYQCDVGNRKALEMTAKKIEEDLGRPTVLINCAAASVHGVPLLSLTPASMTKTI
QTNLLAPFHLMQIFLPAMLETNKGGVIVNVSSVLGHLTAAGLSDYSVSKAGLSALHKSLEAELRAS
GHDDKIKTLLVETGQLSTPLFNGIETPSRFFAPVLEPIKVAYEIVSAIDNGQSGVIRLPAFAKLVN
WYAVLPASIQRLARHLSGIDTAVAKAYNRNILQPESVPESGSESDV
>g855
MKIKALSRSAASQQAPGSSVARLQRNLDPAQHPFERAREYTRALTATKMDRMFAAPFIGQLGRGHV
DGVYTMAKDPGSLERFASGSGDGVVKVWDMETKDEVWNTQAHENIVKGLCWTPGRKLLSCASDKTV
KLFDPYNSPPEAPPLATFLGQTPFTSVSHHRDENAFAAASSVISIYDLSRPSANASQTLHWPTSTD
TITSVAFNQTETSILGSTANDRSVVVYDLRTSTPVAKVILTLASNAISWNPMEAFNFAVANEDHNA
YMFDMRKMDRALNIYKDHVAAVMDVEFSPTGEELVTASYDRTIRLFNRNQGRSRDVYHTQRMQRVF
SAMFTPDNNYVLSGSDDGNIRIWRTNASSRAGIKSAKQRQKLEYDQALIRRYSHMPEIRRIKNYRH
VPKAIKKAGEIKKEELAAIRRRVDNVRKHTKKGSMPQRAEREKVVLATEQ
>g856
MASTSASERIEGAKSLASKDPTKAEQIYRDVLSGGIGKTESASRDYEAALVGLGELYRDNKKAHEL
AELIKTSRDAFSSFAKAKTAKLVRQLLDLLSEIPNTIEIQVAVIKSSIEWAVSERRSFLRQSLETR
LVAIYMQKQSYYDALTLINSLLRELKRLDDKVVLVEIQLLESRVYHALGNQPKARAALTSARTSAS
AVYTPPTLQAGLDMQSGMLHAEDKDFNTAFSYFIEALEGYSSVDDTEKATAALQYMLLCKIMLNLV
DDVTNLLGSKQALKYASTRLEAMKAVARAHANRSLEEYEKALSDYRYELGGDVFIRNHLRRLYDAM
LEQNLIKVIEPFSRVEIDHIAKMVGLDTQQVERKLSQMILDKVIVGVLDQGAGCLIVFEETERDKG
YDAALQTIEKLNNVVEVLYANQASMLE
>g857
MAMNAPMQPNYPRGYPQAAQRXPAAPRRGAPVPGPGVPMPHQQVAHPQYMAAAAAAAAHQRGMPPV
SDAHLRRSRKPTEKNMPDGIEEYVIGDGVQEYKKLQNIEKRLDSSMVRKRLDIQDSLGRSIKRYQT
LRLWISNTTEGQEWQQGEQNGNGEGPGAGRYKVRIEGRILDDGDVDVTVPAEDSDSEPEEEDTAGG
EEGEDSEKPKSKQRQQRPRQKLSHFFKSITIDFDRTNSVKEADLSPITWTKPQIPPTAVSLPPSAD
FDEISFSRAAQENVNFTLTLVRDEQPERLKLSQELQDIIDMEEATRSEIISALWDYAQVKGLQEDH
EKRLVRCDAKLRNIFGRDQIFFPAIVDVIGNITAPLDPIKLPYTIRVDQEFHNNPTPTIYDIRVAM
DDTLLQKMVTLTTNPQYTTSLRNISDLDDRLATIIQAITHSKARHSFYTSLSRDPANFVRRWISSQ
RRDLETILGEAVRGGGEDGSGPEFRRGGTSSAWNAPVAREAVRYMLARPEANTVYR
>g858
MLDPFPPPPAWLQDLVTPWAEYLRLYTLPYHVHEVIFAFVFYQVIHTYISPWLSTRLFPRHYPQLP
KRTRLNWDIHVVSFVQSVLINVIALWVMFADEERKRMNIDERVHGYTGANGLVQALATGYFIYDII
VSTLHINLFGIGMLFHAISALCVFSLGFRPFVNYYAPTFILYELSSPFLNIHWFLDKVNMTGSKLQ
WYNGMMLLVAFFSCRLVWGTWQSVIVYGDMWHAWKQSRSMTHSPFLDGVSTNAPVFKLGEGGLLCA
DDSCLRANAEISQFAHYFTDVSLPQWLPIAYVVSNLILNSLNFYWFSQMIDAVLKRFRQQPVEKKE
TAPKEPTYVFDAAEKLGKEQGYFETGDAAELAIPTATGVETTEGGNALKQRKVVIA
>g859

MELIPSYTNYRWDSSDPERRPPPLPMNPGAGSPKTRSNASPGIQAVTASLAEKMRENAPSPYTINP
MPLKSSPEKSLVKGQYHRRMQSLQPTTDARTEFRNFVENRSPERKTRSSTFNDDYFDKSPTRVGTS
GSQDSGRDSPSLYVSNRYLSKPIIGENTPPSATMLALQNMQLPGDELKSVQVSVSKPATATTLPES
NLSEQIQSLTNIATSLQKEMTSLTRRSKDNATDLLSLKAATNARDEDIRKSIRDLANNISSKLLDP
ESASTRSGAFGSVRSGYSIDSKPFDSPPNHRKSFNLPRVASPSSFAAALERDLCGSPAPISDGSAS
IALLEKVLREMATKEAQEKLLELVDEVKKRPAKDGSTKEADQNVTKMLEEILNLVKENPTNRALIR
SDAEEPGSLERTRSGPIVLSYDSNGAEKTAISEDIIGLLNRIKNSVAEGGGLTSEVKALVRELRGE
VLGMGREIGRKLEEASISKSEEDAPRSLGADEVAEIVRAGLAELKEQMYSMIQDHQQTLSTSLTRS
GADADDVFTAVRSALEEYSPQHLAAPAPSTVGMDKEDILEAVREAWEVNKPEIELQNFGLEREEIL
ECLTEGLKAYQPKHDEAMTYDHVLAAVQAGMQSFVPPPIEAPPAITREEVAEAVKECLESFELTIP
EPNITKEDIYAAVSEAMDNQQRALPHAEDMPKITRDDVFEAVAEALGRSTLAESLNAAGLTRDDVH
GVVMEAMAGQRGLTMDEDAPKLTRDDVFEAVAESLNAAGITRDDVHATVIEAMAGQRALPMDEDVP
KLTRDDVFEAVAEAMARSTLGESLNGTSLTRDDVHGAVVEAMVGQRAITQDESDTGLSRDDVFNAV
TEGLAAHFAAAKEMGESSVTREDVMQVINDALASHSNALVTQEPALTREDIVSAISEGLVNQSSIS
REIELNKDDLFEAVTSGLQEAAASSQLNVGDQVLDRLRDLVQEMKDEFKQYSAASGHDTEQVLDSM
KDGLQVLRTDIESYVDKAADVTGKEEIISTVKEGFRLLQADLEHSINEAALRSSGSRGNPDTPELL
DAMEKEFEHLRSTLSGLLIRTETSGDKAEILDAIHEIGTSTSNEVDTSQLVQAVREEFENIRESIG
MSLVKSEPSEKDEIISALRESFENLQAETMQKRDGNESTFSNTSELLEAFNDGVDTIRADLETLIH
KSDEQSNSEVLDALKEGLESIKFEMEALRTSQKEFEETSTTRGQELILANESNISSDIESLKVLIT
QLQIKVESIEANPAPAPPPEDALKREHLDEVLSAVRDVHGSVAEANTRKEDAEIIETLLRDTVAKF
DEFNIPSTEDLAKSEQVMTLEVVVSELKDAIAEVAARLETESCTKADFGTLETLLKDLWVAVEESK
SQAKDLPEGEEAPEPIFKSDLQTVEAMICEVKTSIEELKLPDVETLPAKSDIEALSELINAFKEKV
ETESDLTAQAFEARKVEHGGLAEKIEEAKVVVADLRDELKGKLDGSEEGLLELKTLLEGLAVSSEA
FTTVESVKELSDLVNREFERARGEQEATKLETEERDAAMMVKQDETRAAIVADLEKKVDERIDHIL
SKYDELQENLDKKFSETEERDSLNVEHLTSTKGLAEDIKLIIGAMGNSVTESCERMGDDTKTLFGK
ISESYDKMEEMHNEIKEFNDLSKVELEKTSAATDRVESQILEYHPQILGAIKDILMLVGQHYEHSQ
KSNEELSRNLSAIPASIPTMLPALLPPPPEPREIIVPDKYDDTELHSKLDLIMNHAESTKNIIPEK
YDDSELRSKLDMIMSHTESTRNIIPEKYDDTEIHSKLDLIMNHAENTSKTMAGMDKLDQIHEKVIE
TSREITEMVATQSRLVIEDYERKKREAEEAAYALERRLAQKEKVESEILSLNDEKESLLKMIQAMK
TEKEQLTKQNTKLGKELHGLETALDIRQHEMELFEERAQGLEKRILAGVFDHARTMLLKESGRGKS
RMSLRRIPSYGSNATRTSRATSRATSTNSAAKDTRSLVSNGVEMVLKRRQPAKTPAYNGSTVSSNG
GKERRILSLSHVTGNRGPTDRHVTAPVGTGGLTNLKRSHSVRSNIPSRKTSWGTSRDFEANKENES
FLEEDEQFSAGHSDAGTERTADTGHYLESVSHDSESTISASRQPSRQTSLQSSRQPSYASTTNGLV
AEHTGSIIEEDNEDEDEVEHHDESYEQESEQESERSDAEDPEDHEEQHDGSVDDEGDYQTTIDEHE
EHSAIEEGLSELEAPPGFKIPGQNDSGLGSDIQRPINV
>g860
MKFMKVGRVAIITRGRYAGKKVVIIQPQDTGSKAHPFSYALVAGIERYPSKVTRRMGAKKVEKRSR
IKPFIKVVNYNHLMPTRYTLELEGLKGVVSNDTFKEVSQREDAKKTVKKALEERYTSGKNRWFFTP
LRKYRYLVKKRIEVRY
>g861
MAQVATRRYPGPLDPSYVPEVSITRLPATAPIEKILAILDRDGGIILEDFATAEELQKIEDEIKES
HREEDNGKTHTGLPIVPSETRVVPGLVGRSKTVAELCERPVLNQLREEILVDKFSVTREQFTDHYR
IDPLLSISLSFQINTGAPRQSLHRDDAIHGTKHSAPFDLKKASQFACLIAGCETTRANGATMFVPG
SHRWDDNRIPRVDELCFAEMKAGSALIFMGAAYHGGGHNSVPNFCRIVHGLFFVRGTLRTEENQFL
TIPHSKALTMTPGMRTLLGYKKPDTALGLVENRDPNDDLEDVFKHLAA
>g862
MSSIRSTATANEIDPNQLAQTKARFEEEKTKRLRSDGNAQFIEIAKVAQFARFADDPWVDPAAIQP
LRAKFPDGRCEMLIVGAGWGGTLNAVRMIESGIPAEDIRIIDPAGGFGGTWYWNRYPGLMCDIESY
CYLPYLEETGYVPKHRYSEGEEIREYVELVAEKWGLTDCAVFQTQAQKIVWDEDAKEWVIDLIQRR
KDQAPETLQIRSRFVTIAAGVLNWLKLPNIPGILDYQGDIFHSARWAYDVTGGSPKDPSLTKLKDK
DVVIVGTGATAVQIVPQLAKWCKHLYVVQRTPASVDVRDQRETDREWFHKEVASSKGWQRERMRNF
HEHFTLGEAPTVNLVDDGWTRAPGLLGLTGYTEGPKKPEDIPAYTARLIEVDAPRLKRIHARVDRE

VKDPAIAAKLKPWYPAWCKRPLFHDDYLQAFNQDNVTLIDTNGKGVDRITADFVVIDDKPYQADVV
IFATGFLAPPAGTPAEKADMSIIGLNGVSMSEEWPRFGPTTLHGVIDAKFPNLFLSGPQQASTSGN
YRFNLDEYAKHISYILVESKRRANCAPLVVVPSAEAAEDWGLQVMMHSAPMGVAIGCTPGYFNLEG
DLARVPVEQQEILARSGLWGSGIEHWLGIIEDWRATGNMKGIMVR
>g863
MPHAERDTMGEPLVNGEKPHSQFISHLASYPVVSDSITTFKNNKYGATSIKYADQGYERLAKPFLP
YLSTPYAYVAPYVAKADSLGDQGLSKVDERFPIVKEDTEKIKNTIVDTAFFPLRLAGDARKHVFDT
YGAEYKKCGGDGYVASGKAVITTGLVISQESLGWLSEFLADKKERTKEVVNEKVNH
>g864
MASSISASTNGHTNGNGKAPQTPGNVPATSLVQVQPARVEDLQPRYAQQIKHDDDNPDAHGWYASM
IHGLGECIGCLGAIPCCIICPNPFKPVAQGEVGLIQRFGRFERAVDPGLVKVNPLSERLRTVDVKI
QIVEVPRQVCMTKDNVNLNLTSVIYYHIVAPHKTAFGIADVRQALVERTQTTLRHVVGARVLQDVI
ERREEIAQSISEIIEDVAAGWGVKVESMLIKDIIFSNDLQDSLSMAAQSKRIGESKVIAARAEVES
AKLMRQAADILSSAPAMQIRYLEAMQAMAKTANSKVIFLPAPGQTVSQQLSLAENLGEGPSRYQNQ
ENSYENDDGFQRAVNARVVENI
>g865
MPRKQFILDLEKASTSDAFPHIQNIRQGDDNESLCFSFFNPGDPQIAYELQVAVSDLTEYPHTHQY
LIFSTSATVPPAIIGVLEGVQAAADGYPVSDLLNTLHDALEKTLAAGSHEDPSKNPSGVDYPTDDD
AMSADSEAESENEVHWDDGFFDEDQQFFGSVHKIVSKSSTLRPDLRTTKAAGFKVGYLGDPKGSLI
ISISCRISKLGISEEAMQAWDVKSNQYLVLLLRFPNRYVNADYLLEASNDGSLVQMHVGLCASYKP
SVTEALQAFARTSTSYLPTLELMSESDNLGKLLPLFLEIPLGSLLNERFITILKLRHRYALSWSGA
ESLFNDSQGLIAVDQNPQENHALYRCKDEWSETVPHLVQDDHFTSANDPESLSFPLLAIQFTLRHF
VKCTEFCLVCHCKIDTNFEALKPYVCSKPLCLYQYMALGMGPSLEWEITRQPLVIDLLISFAYASA
TAGAMEELPVGLRMMVPPIHKPNSWLSGSFYRSNKQLTVDDHDQKVTIKEGDWILIDMSGAGQYCY
CRVERTDRWPIVEILEPVTYNTSDTATPGDNESLLVKFALHDVNFDDIESAAQKQTVIVALLQSLP
DVLSMKSWLESAPPGSRSLAAWKNVPPAALDLLRWIVASNRSCIMQDGDITEQAEGTSSHNLVTGM
KGYLQFRFAQGSPDKEEKFVSAVSDNSNDTKHPTIFAWHGSSLSNWHSIIREGLHFKKTTNGRAFG
HGVYMAQQFHYSLGYSGRAGFSGIPGMRTCSKWPKSILNVNVAVSLNEVVNRPHDFVNSSNGVYVV
NRIEWIQTRYLFVDCQKPAGNVNAQPTTPVDTTFEGDDAYYVQDPKHTAMGSDGKQIRIPMTIFSR
CRRNLALESHQHKESPKTKGKSKKCKKASSRKRKIGTPEIIPNDGPEYDDDDVASVATLAEDLAVL
FSDAEEDSESETLKAIKKLKASTSTEKAKVKAKNSALTPFEPGTLTESSVTLLEPPAYASSSATKT
LQQRLLQALKVQEQTPPDELGWCIDPQLINNPYQWIVELHSFELSLPLGQDLQKAGLQSVVLEMRF
PHDFPMSPPFVRVIRPRFLEFNAGGGGHITMGGALCMELLTGSGWLPTFSIENVLLSIRLALCSVD
PKPARLANSASRFGSSSARGGDYSVQEAVAAYTRACRAHGWEIPKNFEAMAW
>g866
MAQPSQASAGATTQSQLDSSLRHFTESSFDPVDFLNDHLPPLTLSTSQAHATRAPSSVNLADLSSR
SQSLVSQLGTQNVRLSGNLTQLTEEILRSGGRLAYEVEVLRGEAISLADTLSENLHEDIIKFAPEG
QDASEQKEDTPDEEGEKEAIPRLPNEPEFIAQLRILGQVRTRLEEVIQTFGDAMEWPLPPSELSIT
SSFISVSAPEPGSESYSREEKGQELVKKLRTEITELLDSNGGGDAGLAAATERLEKLRNLATIWKG
TAEEKARSRFLDSLARIIEDRRKALEIQRDRASKKARSSMDQIAEERDSGPGAGLFRGLQRIRDEI
YLE
>g867
MALIQLSGPGGQSWSPVPDFNSSENSFHILISSSPKNAQLCRTITSSMILEYPPFTLLRPKPETRK
GATAKQAAVEKVESMLTFLKTNQRIGERDLLLIVDSDDTVFQLPHDVLIDRYMEITRRKNEHLRSK
YGTKVLINPENENDRYTVQKYTQRALFAARKDCSSNLTDSAACASVPQSPLPPDIYGAKTDKKRDG
TLTRPRWIESGAVMGQTSDLIPIYERVLEEMKKQPLRANEQLIFTNMFGAQEYSREIERRKTVSPF
KEWLWDYMGILEATNVTKVRAHIQPGAQHEFGMGLDYEGQLFLSTRKTLGDIEWLRYRDSRKVSSV
QIQHGIPREVRLNLPLDVEQNELNPFTKVKLSNSLSSKTANMTSIDVLPPSWNFTWNDLPLVTYIQ
TAAVPALIHTGGETNVRQTFWLNSWFHPWARALLRNYVRSAPKERPVQVPTLGTTDLLRHPNRKGG
MWTDNDEWMSFEDLCSGLEVPLFDDELGPWGSEKPSALRAVYNLWGKLMAGKGPKYRNLGEMRSEA
EEIERFILGIEDDEEEDEETE
>g868

MKIVDPQTATLTNIEVLSYFTANPPRKSAEAPPGMRNFIPKPDLRDHCTVVTEIHNYVERTSPHLL
NYPRYTHDSSPNPTVHTLQQPSTDQVEPYMQPTEPTPADNAIRKVVQELAPYNLTKGEVMMLINLG
VGIKQKKTTDGDAMDVNGDEDAAADENGEGGEEVDDMYGHKALLSTIVEEMEQRLSEDDMMDIFRI
MDKNLGGGQ
>g869
MTLFILTETSAGYALLKAKDKKLLKRDDLDIPTEAATPEGVSNLLKLKSFQKFDSAATAVEEATAI
VEGKVTPRLASLLDEIKDEKKVSLAVADPKLGNAIGKLPGFDLKVIANSSTAETSPTAEIYRAIRE
HLPTLIPGLVPEDISAMALGLSHSLARHKLKFSPDKIDTMIIQAIALLDDLDKELNTYAMRVKEWY
GWHFPEMAKIINDNIAYARVVLKMGMRTEFESTDLSDILPEEIEAAVKNAADKSMGTEISAEDLEN
IQALAEQVVGFSDYRQQLASYLTARMTAIAPNLTALVGELVGARLIAHAGSLMNLSKSPASTLQIL
GAEKALFRALKTKHDTPKYGLIYHASLIGQATGKNKGKMARILAAKASLGIRVDALAEWEEDVAEE
EKAALGTEARFNLERKLAGMEGKPLKPRGVNIAPNGISSQPKKFEINEARKYNADADALSGNEEPA
SKTKKEKKVVEEESEDEDEEMEDSEDSDETEKLAAKAGLSVKRYLRKLERGEIEFDAEGNPTAISK
KDLKKAKKEAKKAAKEDDGKKRKRDDDDADDKAEKKKKKKKSKD
>g870
MEGLFFNVNGGYLEGIVRGYRNSLLTGQHYANLTQCDTIDDVKLQLAPAYGDFLAALPPNPSTSSL
AAKMTDKMVAEFRYVQAQATGSFAKFMEYLTYGYMIDNVALLITGTLHERDTRELLERCHPLGWFE
TLPVLCVATNIEELYNSVLVETPLAPYFKGSLSHQDLDELNIEIVRNTLYKNYLEDFYNFINTNSE
LKGTPTQEAMAEILEYEADRRAINITLNSFGTELSKQERKKLYPEFGKLYPEGSLMLSRADDVEGV
ALAVSGVGDYKTFFDAVGLSQGGPGGIGNMAGGGSSDGKSLEDLFYQKEMELCKVVFTRQFTTAIV
YAWVKLREQEIRNITWIAECIAQHQKERIGNYISVF
>g871
MTDSRSRSPRPERRRRSRSPDFDRSRRPRNQGGFRWKDKRPRDDRNRDDDRRLERGYREQHRPRSP
RRDRSRDRYGDRERGRETNAPARQDEKKDEKLDGNKPKPAPKKAPVVAAPAEEMIIVNVNDRLGTK
AAIPCLASDPIKLFKAQVAARIGREPHEILLKRQGERPFKDQLTLQDYGVSNGVQLDFAAFGWAGG
VNGFAATLVPPFPIRPFFQHERDRAKPGSAFAGGESFFSMQIKIVDIEPRPDVLYYLLQPPSSGTT
ARPHRQTWIFDSRLTLQSPDRRLLRLHRKFFRPSFRLSVISNVLLSKFAVGETRVEIQESVREKDV
YIIQSGGGKVNDHLLELLITISAXKTASAKRVTAVLPLFPYSRQSDIPYKKSGAPLVKSSIQTQFT
FDSTPSTPHPGKAESQGMNNDVDSLQKSLARAQIESESNGSPXKRHPIANGGKRSGTLESARTESS
LTNGVNGDEAASTSSGVSKVPEFEPRPGYRQWVAQAGTLIADLLTCAGADHIITMDLHDPQYQGFF
DIPVDNLYGRPLLKRYIQRHIPDYRNCIIVSPDAGGAKRATAIADSMGVEFALIHKERRPTKISDR
QNATMMLVGDVRDRTAILIDDLADTSNTITRAAKLLKKEGAAKVYALLTHGILSGDAIERINASAL
DKVVVTNTVDQTEHCKRCPKLEVLEVGNVFAEAIRRVHHGESISVLFQYD
>g872
MSALQEPKPGPGQGQDYSTWSTSSLVERIMELERQLNAQTNEFGAVPQTIPKSAEATAKAAIKPQF
QDITPVRSPSPEGANTPKKQFREMDPSKYHTRHIALKFAYLGQRYNGFEHANGNTTPLPTIEEEIW
KALRKTRLIFPTTTSPMELNLDKQDRTQPFDLSWDGCQYSKCGRTDRGVSAFGQVIGIRVRSARPK
RKVETATSTTVTSTTAGETTVQQVDVEASIVDSTKDDAEPSPDVETQEVDEDNGWDDIRDELPYIQ
ILNGVLPQDIRILAWCPNPGPDFDARFSCRERQYRYFFTQPAFNPIPGELGFRRSAAHHRGIAKKE
LRDGWLDIEAMREACKHYVGRHDFRNFCRVDTSKQIDNFERIIYRASIDLVDPSKNPLGYVGSKEF
QPTRNPVHSANGSNANEEQPSTPLVYTFTLHGSAFLWHQVRHMVAVLFLVGQGVESPSIVPELLDI
SKNPCKPTYEMASDAPLVLWDCIFPDEESGSREDSLNWVYCGDPRQGNPSSGKGDGKFGFGGLMDT
LWQTWRQRKIDEILAGSLLDLAAGQGSPSFIQNELPAKPFKSQKIFFGGDEGKLGGVYVPVMQKGR
TATVEVQNARYLASRERRAERLKLEGKDKTSSIQV
>g873
MAPSNLPPVFNATSQDIEMLLAAQCHLGSKNLQVHMEPYLWKTRPDGINVINIGKTWEKIVLAARI
IAAIDXPADVAVISARPYGQRAVLKFASHTGATAIAGRFTPGNFTNYITRSFKEPRLIIVTDPRTD
AQAIKEASYVNIPVIALCDTDSPTEFVDVAIPTNNKGRHAIGLVWWLLAREVLRLRGTLATRETEW
DVVVDLYFYRDPEAEETKEIADEAKVPGAEEVGPAAIESGFVGDSWDAAAPGAAAPGTAFAAASAT
AGATWEAEAGGDWAAESAQPNPETKW
>g874
MPPRLSLQGISKPFSGSSHLSNPLTIVKREPCKVDIRAGSFEARQRRRHDPFLMAQSRQRKAANIS

RQQALFEERQAALGDPVKSEPTPFIQQIDAIQSGNIPPQTVGSANVSTWNHFIKADEFDSALEYSK
TLTEPLPPQDANIADPHAHAQQIQRHEQAHQNALEAMKRIVQLSSGNTADRTRVNIEACIETFGRH
NTDKVLPPKPAAVTTTEHPEKTPRAGPDTGSPEVQVAILTTKIMNLSRHLQTTNKDRHNKRNLRVL
VHKRQKLLQYLRRKERGGPRWQNLMTSLGLSDAAWKGEISM
>g875
MPRTRAQAQAQEVESPLVSLESPRRRRRIPTTTPTKASADTKKATDTKTKRGRKPTKKATAVATEE
ITQEKIQNKSEIATQETHIQTPTQESHWTQETPQNQGEVPILQTPRGINAAQDNGSTTSPAPATAR
SASESDASSITSLGSVSSLGSPVKKASVRNALKFFKKSIKQSSSTVRRRFHELKSRREPRENLFET
GVPALPTDVDSYPTYQTNYSLAEDEILEAALKIVLHRQTTDGTKPFAESIRCPCCYDGVVKMECVR
GHDLRSLMSNETIHALNIVLTNICAEGILATAKAKEDRLRVEKEAEATAKDKAAQVAEQQKKELSA
KQKGKKRALESNNDENSTDGPPMAQRRRTRPPPGQTPRNKRRPIPTTNPGKKLSFMEAARQRATNG
ERLSAQSSIFQLDEAVTNLREQEQAQQAAREAAEEEARNAITQKAMERHMVLGPIPENMQSSFQVP
AFEDEFDVEDHEDEKQPVETPTSNNWGLRSFLGSVRRRLVPFVRQAPDQAVPFAAGGGATIVRVDG
AAVPSTESHSTQNADVDMTYSLFPPIPSPLGIAPSSEEKAKAGEQEDKGGKTTATVEENSQVPGTE
QTDQNKTNGEPQNGEPQNADTQAEPETGSHKRKRAPSPDVIPNPAGCSYGMDLDYFTYSDDEIEEA
EEYSRRELEREAAVAANSTVSAPTARPPEKRARLLPPEPESLNVQLPSTNTTTQTHWQAPVPPGWN
RPTTTVYSGQMFQGMTHAAPSFHLNPYHPTPDLRTQPPPLENPFMQPMQPVYPMQPMHPMEPMPPS
PKTFPQSGSISDLRKAAVERRLWNNLVNNGLGTHTNRFPQPQVSQPAQQPVAHPSPVRTPAQQSIL
QPRVEPPAQQIVQQPRQQPSVQKPAEHPTQQVFQQTATGASSNGVATNEPRDRSLYAATKQSVNDE
TIHLPPIKPISATARGQPVIERSRPKTPSRLRNHIPLSSSPASQASPAQTLTAMHRQDQDQEDLIQ
FFGDDEHGQQAFELYQSCPSGDLSKVQWPSFDPLPSNNIQETTLSTSVSAGQQRASHHLFKKSFAQ
FQNELDQGLIDTDEILSDL
>g876
MTTVTAITQQPVEAFNTELGENEPAVFATSYPEGGRDAWLVAIGSWCSMTAALGMVNSVGVLEAYV
STNILTSSSTDSTGWIFGIYVFVSYFCGVQVGPIFDARGPRELMLIGTVFLLVGTFTLGLCTEYYQ
FILAFSILSGIGNSFLFTPAMGAISHWFDKRRGEASGFAFTGSGFGGVLFPLMIQSLLPRVGWAWS
TRIVGFVFLLLCTVGVALCRSRLPPKRGAAASWRVMMPDLSIFSKDGTGAMAVTTAGVFLLEWAYF
VPLTXLPSYYLTRQGLADTAAVTGAAAFAYQLLAILNAVSCVGRYLIGHIADRAGRYNTMIVSNLI
CLISVISLWLPDALADSPPSKALLIWFVVVFGFASGSNVSLMPVCIGQLCPIEDYGKYYASAYTVA
SVGCLTGIPIAGNLITATEGGRRGYWGLILFTGLSYVAAFACFLWVRVRAKGWNLRTVW
>g877
MAPKPRASTGRLWENLDPPLSEWILEAMSTMGFARMTPVQASAIPLFMSHKDVVVEAVTGSGKTLS
FLIPIVEKLLRLDEPMKKHHIGSIIVAPTRELASQIYNVLLSLLEFHGPSASAIXPADETQEGVKK
YPSSTLKVIPQLLLGGTTTPAEDLSQFLKRSPNVLVGTPGRLLELLSSPHVHCPQSSFEMLVFDEA
DRLLDLGFKDDISKILGRLPKQRRTGLFSASVSEALDQIIRVGLRNPVKIAVKVRGASGVEEKRTP
ASLQMTYLVTPPTHKLPAVKNILDILEPRPQKSILYFSTCAAVDYFQHIVPLILGNDFTVIPLHGK
HPANVRQKNFTRFTNSVTPSVLLTTDVAARGLDIPSVDLVIQVDPPSDPKAFIHRCGRAGRAGRRG
LSIVLLSPGREEDYVPFLEVRKTPVTLYENPAFNITDEDARNATEIVRKTVLSDRSYHDKAQKAFV
SWLRSYSKHQASSIFRVADLDWEALGHAWGLLKLPKMPELRNFKGDKFLGVALDWDNYAYADKQRE
KHRLEALKEYQASRSEENTKKRPAVSGESVPWSQNLEKRKEREQRRERKRARRDRDKWDKMTEEEK
QKVRETEAMIENVRKEAEKRREXAKAAQNANADGSEDEFKGFD
>g878
MSSPRVPATPRVISPSPTPSDASEQVDGYFAPVTRSAARRQRLNNDTQSIPEEDQNDDSDYQSRSG
PRNPSSRSGAPGTARRRGSTRKTPKPANSPVVKSQPLVGANGKQSSSNGYLSPYTSLQDRWRDLSR
SPSPLGLIPLHQDYRVFIHKHEIPRKLLHVSIGFMTLEFYRRGIQTFEITPWLLSLLIPIAATDLL
RHRYERVNQLYIRSLGALMRESEVKGYNGVIWYLLGAFIVLRAFPKDIGVMGVLLLSWCDTAASTF
GRLYGKYTLKLRQGKSLAGTVAAFFTGVATALFFWGAFVPSVGAFPNDPEDAFMFRGRLNYFPESI
RNLIGWAGSDDQSSIITGPLALGVMSVVSGIVAAGSEFVDLFGWDDNLTIPVLSGLGFWGFLKFFG
>g879
MGIPGLGKAIGNADRIALSRFAIRHLEVSKRPIRIAVDISIWLFQLQAGRGGQNPELRTLFFRLIR
LLALPIHPLFVYDGKQKPPFKRGKATTARSYGSAPIINLSKILIDLFKFPRHDAPGEAEAECARLQ
QAGVVDAVMSNDIDTLMFGSGLTVMNFSKEGSTGTSAATHIDCYTTESQLDVEANVKLSRAGMVLF

AMLSGGDYLPSGVTKCGPGLAGEIAKAGFGEDLFDIIYSSEDEVEAKLAEWRERLQYELDENESGY
FQSKHKAVRIPDTFPDRQILSFYARPIVSENRDIELLRQRLVGAWDHEINALELRHFVANYFDWKY
RSGARKLVRNLAEPLILTRLRLARSPIAPFGYGSYVPNPDLPVLQRIYRSRMHFSTGCISQVQVEM
IPADVVGLDLEAEEPNPPPEWSQLTEPSTQPVEDEDDAGGEAAPVEDQAPKFHAPKNPYNPFEPEK
IWVFETVAKMGIPDVVERWQKQEAEKKAPKKPAPKKMTTRKKVIDPSMKPGGILRYTTVTKPGSDM
NSVKKAHILDAAGLSSSPQQSSSQSTYSSQYNAISDIGIFSSQSPQQSQQSMKPSDLAIDELASQF
SATCSIGDGYSDVRDSGFRHPPSIRGFDSNSFDFDSELEGSVTFSPLHSPSPTKFKISYTAAGSLS
PLAIETIRVNPLTSAVSPNPKLRRSNRIKAKQSSSEKSLEATFELLSLSPAVKSPESETHNDTQKL
RKPRSKAAAKSNEPLSVPSPKAPAVRLKDEHKPSALQEQEGEPKVKKFIEVIDLEVIEQAPPKVDV
EVEEPDQSQSQPPKTLTEVIRTHKGFWTLDLCEQDETHPATHEAQQEHDEDTEKGKDKKKRIARVS
LLDMR
>g880
MATTESRHASKSSISALEAARARIRKDDQSFEPRTPPHHRNRSMVSSFGSSSSPLSSFRNEEDAII
LEMGARSLRAGFEGESTPMCSVSFGPEESRKVGDYRGWAKLNKSTTEGEGVSFLDAEEWVKNHELW
RMDLREVDLGLVEDKLERAVREIYNKYLLTDAGNARLVLVMPSVMPHPLLSVVLETLFDRWRFPSI
TLLPSAAMVAAAAGVRAALVVDIGWAETTVTAIYEYRDIASKRSTRAMKTLMQHMGRMLSHLDNST
QEETKKAGKISVNFEYCEEVVSRFAWCKSTAEDENLNKHVSIPSPLSPDSGYTEVPFSQFAKPVEA
NYFATGTDEHEFDDDETPIHSLIYNALLELPPDIRGACMSRLIFVGGGSRIPGIRQRILNEVSSLV
AEHGWDATRGRAVEEQRKKLQELRIGRELEHAPEQKVDTTDEAHKASPSEEKKDEEIDFVEQRIRR
NQARNSKPFIQGQLRQVESLGAWAGASLITSLKIRGLVEVEREKFLRDGLASASRDLDGHHQVADR
RSGVRSSVISDRSSWTLAGWG
>g881
MPLATGQGRFSQLSSFISFYFKKLARPRLSLAALIIYALVFLGIAVTVIYKMRFTSYVSAAALFAL
PALGGSNPKSNSTTDPFKIYNITATNITASFIGYGARLVALYVPDSNGTIQDVVVGYDDPKTYLHD
SETNHTYFGPVVGRYANRIKNGTFTIDGETYHIPTNENGGADTLHGGTVGYDQRNWTVAASANDSI
TFSLYDPGWEGFPGDVLTHVTFTVDALRTAVNPKGLPQLTSRIVSLSLTEKTPIMLANHIYWNLDA
FQEPTILNNTLHMPLATRVVAGDNILVPNGTIFDVDTAFDGAIDYTAGKEIGADFDHALGVCGFNC
TGVDTCFINDRTPYYLDGATVPVIHLSSPATGITLDVATNQVAHQIYTCNGQNGTIPIKASQKARN
EAAGKGGVDYVNKYGCIVIETEGWIDAINQPEWAQKEIFSPADAPVVNLATYQFGTL
>g882
MSSVSSSASSPDALGPEGDALYLISSPVPEFSGRQSWVPPYTIQTPRRQERVPRRTPRQQSSQTMR
FNDIILPTSPHMRQRSMSPTKMQSEGNLSPWRIRVTVEAEHEDDENNQDGPRKKLKPSTVTTKVPL
KDEESNPPSAKKRRGRPRKSDAREKSLSPIKGSPGRTPKQQPPDSVKRPRGRPRKSMQSEPVAQSV
EAVESPVRNNMKSATLDGVIEREPPAPFMTADMGPPSDASSEPDPFLDMAGSGAFEEQSPHPNSYR
FSPPVVFDLADSHSAKPAQLDMSAQQATPQASVSPIRGHTGITPQNTLYAGHTPRKVRRSYPTPTS
SSLVDGNAEHSTTMRQSNTTHSIADPTDVHHDYDTIMESEDFSMVSLDTLPSAKQAGLSSILGSSK
GSRKPFAEKHNGAFTENTNRRTTKSPQVVTSHVLEPSITPQSKPVGLLSGHESDDQVVPVAIDTYA
INASVQEPSIQPVVEKDRKNSLSLARTVRLGLSLHDTLIHQQRFDTEDSDEEEEDLASAADRLKRI
FGDLDMESQRHLQAGLRFGELVARGTMRSRRLQRQKERQMIEARERESSVGEPIESLQQADTFSDD
LPEEEGLSEEEEMSRTQRLEVEWQRERELVSRQIDMAGSDNVIVLDSDDVSVGNRVEDQVYKQNEV
NEEEESEKDEDYGDVWQEEARNHDSSSNRHSTFASSMDEKRASTTNRRTGSSYKNTSQRYDWDPDN
GEIPPLGRSRTAQLREEEADLSLLLKPRDTPKSRQYYGNGPRTASSQRLRSDRSSRYGGTSRDASS
PIKRSSLGTSRVNDEPEDIKNDIGRDNEILDDDDLLKEQEEVEEKDEDDEDDEEQEHGEYEDEEQE
HDQDDDRERDDVFITEEREATDEHTSFGHSVTTPDQSMPGPKQSWFRRITNNFTPGWWTASKEVPL
DESQSPPFPIDERTPSLKRSRAARDAEEAMEMEQITPAISTIQSRIKGRRLSSFSSPKALATSGYF
TDGHYVALKRLYWEAKRYPERFPYHPTSHRDEMLGDSLWTSDGEHGLPIAKIQFGILDRFVSELIS
ADIRNGGRGQIGWTEDDLHKRLFSIIVGDEIRRERRQQRLLNEMR
>g883
MSEVSSLWWTDERIEATLCRQYVFSQLQPDHQSRLFEPLPWGEGLTSETYLEWILTKAGKLFLILN
ELGIPHRIFDLADDFVDDSDLPFAESRVPDLPLVARDESVSSSAAASLNNKFFLTQWRYLVRGIAE
GEHVRYTENEGVPVEVLKTSLVGSNNNRDGVEKVVLSGATCRIFLRTQVGINAAPYFSEEDEVLEE
IRSLRQLAHEHVLSIYGXYTVDNSINVLFSGVYERSLLSFLSDTPQNFKKLPKPDRREILVNWPHC

LVSGLAWLHANGHPHGSIRPSNVLIDSDYRIFLGHFEGLDTLLDSPKINDIEAYQYAAPERWIRTT
AIQETNVAKTSFHSGGRTMRGPGSSSSSGSSASTKTPRASWMTAGSTQSSESRGTVIRVGSRNSLV
SSSSSSASGGSGGSHGKSAHKLFRTKPILYTPSIHSSNLASDSRTRPVTAPESAASIPFVPHNTAL
VRTWQSQQVDAQLSDVFSLGAVITDIFTFFCERKISSFANHRSAKNRTPGRGGGVADASFHLVTNL
PQVTSWLTLLTADSKKRKGLAFRAVEPMLAVVRDMVARAPERRPSAEEVEARFAHTIRQLEGIIIA
HCKQAPSNTVASYVKKPSTPPAPVDMPLPPSPNLYPREYNSLRAGSRPRKQEILDDEDLEPTIPNF
ADLSNKRAKPKDQQRPLQRLDIGNHDFDFGFASRQRQQQQQQYRDDQSPTSTDSSSTPPPSSGNSY
SSFHHQEQMLGMRQYHQQNRQNNRDKKYDENRDKKDKNTTNAATIDETILHLRKALEEKLNREYDD
EHHNDNNDDNDDDENKRRRRRRRDDRRRYLDDLALTLNGTNINLPRYEA
>g884
MVQRMFWHVKGVIENSVEFEADFTGLYDYCSHREQDVKETQQEEAQGTYQCMEVHSANMPTQDLCE
QYRWLKDATCILRVSGQFGYQGVIEIYQSDNVDTSVWNDEFTISQGDDSMNLDSPAPTFQTSPVQF
FEFPIDGTHIGKAMLDTAIGGEPRGHYIYLDVRADPREDATAGSNGSHSSPSYAFSLDWFPPSSDD
EGDNDDGTSSSHYIVDGQRFSGPSRFMNHSCNPNCKMIPVSTHHSDQKIYDLAFFARRDIPPGVEL
TFDYNSGWT
>g885
MSFQSPQFSSAEDWLSPSIFSLNSKLFVRNSSLALRSSYHLVLGSQSIAAQLCANLVKAPFRANGQ
LVKQVEQNSLRKPPRGSERYAATAPKTPYSLRCQPGESVFILTKAKNEFLISAILLYEGLAKSKYI
QSFKAFCQEFIPALRSSYQLVHGSQYVAAKLCVNLVKALFRVDKQGEEVPSTHSDQAPAASRIAQS
SAALNTTVTVHDDTSSDSSEDIDIITGTEALISNEINVPSTAPATSNEKYTDRVIPETQDGQMLDV
SNAKNIDLKEYLTNAFLNNFKIAYDDIAAVTAAVTSHSGIKLANCFYL
>g886
MSFSLMRWASLVMLGMQASQVFAISQISAVGAKFFNSSGDQFYIKGIAYQLSDNDPLVNEAQCKLD
AALMKELGANTIRVYHVDPDGDHDGCMSAFSDAGIYLFVDLETFNSAIDQSAPHWNSTMLNAYTKV
LDTFHGYDNLAGVFVGNEVLTMTNGSWAAPYVKAAARDVKAYRDSKGYRQIPVGYAAADIADLRPM
LQDYLACSTNSSEDLDFFSLNAYEWCGDNTFTGSGYSELQKNATGYSIPIFFSETGCNTSPPRTFE
DQSAIFGSDMADTWSGAIIYEWIEEANNYGLISYGPSVAATATGTNIVGGYTRGGTPTPVSPDFSN
LKSVWATLNPTGVALSAYSASASKITPLACPAETSGIWNVNPTAALPTVGQTADASGTGSSGTTTG
GSTGTASGASSTATSSKGAAPVGIVTPGSAGATSAVVIYGVLVGFTGLMAWWL
>g887
MDQLPMEILDMIISNVQSCDEHHRHSPHTSIIYSDADRLRLKEIGLVNLAFRRSVARVLFQHVIIP
IDSQLLFLSRFGDLAGSGFATRVKTIQFDIVTSELLQSWCGDDEEEEEEEDDDDDDDEEEEEEDNS
EKAITKISLILEKFPNLQTVVLKFGEAGEADLFRMLIAPLIISLARARLMKMAELRLPDFSRVLEQ
FVNTNNDGVDQFLLKIQHLHLMESDDRLVDFNILSQGIPDLRCLTVEGQSTYTICPEIQNHFHNLE
SLEFREMQISARNLLAVIQHCRQTIKYIALHRIILSSASWLHVLHQIKIMSLCLKAFDCHLTTYPM
DDNHNKIWTLYGRFLRDQRYIPHALGEIQRQVNVNRLTMGLDPWHATIFQYLDHQPLEVAMGPRLY
QELVSQSQRFT
>g888
MKSDVVPEPTVRKTEINSDIMKEPTVRKIETNSDVVPEPTIRKIETNLDVVPEPTRRNTETKLTVA
PEPTSRNTKTNADVVPEPGIGKTETETFVVKEPTVRKTKTESDVVP
>g889
MEAHVWKTDECLRAPPGTLAGDWKSREAYVQAPWRESPRIVMDECEKVVSVHDRMVKENTRVSMYT
DGSGYQGFIGMGDGDSSVAATINRVYRYGGHVNGVRGGGMWNSVRFTHIDERALMWTRIKGFRRTT
SLQALLDEKNAAVAIARFIHDTGILAQFVGADLQAMDTTEQETDNQGTDLGTHMTTVPGDASARSA
HLSVRTNDEADTSLGPMEIESVDGDECEDERGGVNVRERMIARTRHRFVGLSAHWH
>g890
MALSSPDYERMTRDELRQVDGVIKELDKRKPFDGSNQPRTEARIIEYMKKKGLSDDERWKLLQMTR
DNTFSPRNFRDLLPGMEAPYAYAQKGSYNQEWTTNARIIYLRKELVRKTPIETILAYRTISGIGGS
NGGTPGIGGSSGGTPGMGGSNGGTPGMGGSTGSTSKPKAPKKPKAPKVPKALSVIAREESKFISDN
IFTPEYGLPPVAAEFFGTGLVIKHCADEHARNWKPFATSPSSSLGPFKITNNALTPLDKLEAIAGP
KSSLWNLIPNT
>g891

MSLSTDISSPFKVPAEAMAMLEHDDAFQALRPRCSELGYVGLCPSTKQQSNNSSLCSFHNEECSEQ
QLAVFQLHRDVIYTLLQPLFEIHNQATQIATRVLGRRQGAEPERAFKGEARGAFTWLHCILTEERD
FCLAQGCPACIVSHVLSSEPTIRLVTVACLLCDFLSSSDFADSKSNKPQRPDFSFWLKAMETAVRE
DPLWGDAFWPEIQHRAESLAVDIKYLIMQCVELHNPTELQRTSSTNTSSSSAKSSCSMTMRSDRHA
YRVHVRSIPVQPSALARRQLNMMWEEQELVSKFLQGYWNQLCWSDRRQKMLEFAKTGSLYLPQGTI
>g892
MSTWSWQIPSLLQGVPAALVILAVIFGLPESPRWLYSKGRIDEARRLLTLYHANGDESSDLVIHEL
EEIEMILQAKRQAKGLSAWSMLSKSAANRKRITLVSVISYYFSPILDNIGVTSTQQQTGINGGMQI
WNLIWAIACAVPADRVGRRTLWLTSFALAYFDQHGSKLAAYLVIVFLFLYNAAFNIAKSIVVLLSD
GDSALCD
>g893
MDLTDSSSPRSMSTSAPYFLPPRSLSSSPSPRPRRRASQGSVSTQRLHYPLSRFLASKEKAALHIP
SFGTPRASSSFASSKFPMAVDTAIPRETLQTEHPHSLPLTPPADDEDDVHVRWAIDSVEPAINTEP
KPQVAMDENRDRHSASPAQRQRDSRSSFGAVPGRRSSSDDDVNMSDQDMHDQPPPENWLDNGIKTA
LSSVFDSDRSLDAVRLVSQTLPYPRAPEQNVRLPTQSTVFGAIIENIQDRVRPERPPYIHVVHAVP
EDFSLSNLPTSPPSTPRHLMQVDNYFDATVFASASTAPVYHDVHGGVRPPPVSWPHPIVPPYSIHM
SVLERYIPPSTRQEYRDMFSHGRPSVLTDRLIELSPSGGCLLFIYPTRVGANTFKSQYLGPILDPL
LRQLVVVNGFSADVSRDLGRLEAVNYMDDFDTMKMNVEALCRKLSKETAKFTVVEAVKGRAPLDRS
LWTEWYIQQERPRMKSLLSRSWQTTGGRHTPTVNSPETSGGQLSSAMLLSEIIEGIKRRQYETFPR
SDIELGVFVIRRSH
>g894
MVYSPGPASPMAMGDRSSGLHARNRSLSPPAPLPGSSAPQQSKRDKRRTQLQERLHDLTITFNQNR
DSQFRQQLHALQCDMTLINNADPYEAGPLPDSAEEIAQLIETTVGGGKFGKEMANISGSWYSRFVQ
EINEIKEDRDADLVAVMNRHREAVKRYNHEFSWRQHFAREEFRLLSGTLRERLVQKVSANKTRLMR
EKEQMDISDTNALLLNPNQFTITNPSSPGGIHGNRKTRHTRHRIGIDEFGNGIGLDNRRKRKAPDE
DTGSPGRDGLSTPAERAKATAEKQQIDKIYSINSLFTDKELALHANQAHIATAHFFSTSRHQEGVS
TAVNGNNSEDADDADSAVPGEDGTPAASDMARTASQSYHATRSTRNQGNAGLNLLAELSDKPATRP
NLPYHILANHNSRTNQSAPPLNSLMNEEIDDDISRMDRLQAKPHGWIDKGLIDNMTEPVEEEVDGV
PTNSDRFSLLHADFPTEIGIKWYPRNNNGGYEMFPQVSERASKRRA
>g895
MSTTTTTTATTTLTSSDPSYIPRGPISAELSFFEPPADGSIPHNYVEEPPKGQPRTNFERSSHTVD
INDIRGREAEYNLDKDAFQVYSGVQSATTYETFNDDAEIKRVYYPEVEKLILDKVEGAHKVILFDH
TIRRQDPNAHRQPVNAAHSDQTDRATEWRVRLHVEDPEEAERLLKGRYRIINVWRPINGAVQAAPL
AFASAASGNPDKDLVPIEHRYPTRTGETMGVVYNPEIRWNYWSGVDNDERIFLKCSDINPPADKEV
GKRCPHTAFTDPRTPKGAKPRESIEVRALVFG
>g896
MDEIDTSPLLEGGAIRHKYPAPVSRATYSRVGASSQYDRDFDHDEFEMRSVEPSSVLHKSAFRPPP
AYDHGMSAFHEEIDEPEPEPMPLPGPSSAMQSPRLENSRLIPRLSQRLSMRRPTNVRSAINNKRDL
RHSNSILSSASPLTPSGVLSFWPVKNKQPANNQELENLNEEDFVTVDISFLEGPDSDVARHQDAFP
YSSSIGNNAHQRSESTPKPDEVVVQHTAREYGQQLANQKNMIVSVKEAAVAPVVDLSSLEGTERRS
TYRVASSLDEETSFFYPTDPEKPNWKPFSMTAPYLLMLITVSLGLAGVQEYLYQQSKSLESKGKGL
IQFNDVAEIPTAQFFAWKYLPTMVMVTYGVLWQIMEYNVKRLEPYYQLSQPTGNIASKSLNLDYTT
LFSYFVPYKAVRNRHWTVLVASLGAVLATTAAPSLQNASVMPIQNPNCPDKKCTSSDQFLYFVHID
AAWSRLVTICLALVAVMALVLIFQLRRKSGLLSDPKGIAGIASMATKSHILNDFQGMDTATDNDIH
KRLRHRRYVLYKSSIWQGEHIKYTEEHAVQPFSTASPHPIALRRIPGIVFLIYLTFLVAAIPVVTY
TNANVVAVKLPWLPVLLAVIAKQIWTALEFAIKMLEPFYALSQGNARPENTLTLDYQGTPYGVLPF
QAMLNKHYLVSLIGVGSILSDVLTVTSASLSIKDETEESFMTSSAISTVVVFFLLCSAALALVCRR
HYFVPRPPSTIASILAFVHQSHMLDDFVNTERFSSDQMRSMLVSKNKRYGLGWFRGRDGKLHCGVD
EEPMLSRYVHGISYKDAQAAPLADF
>g897
MALQYITNVASKASPWSSKPVLTDEDEAFLQRVTSTPDDGNKNIDTPEAINPLGRDAQTALMDGAQ
DIPLPMSPSEDVERRLPEQVTETKEEKEELNNVTITTTKKRRPWSIWTKKGSSEVHTKKDKGKDPK

IDADEIATKPTAGDGIPIANKEQKEEQEDLAIIMERLNLSAENNRAFSVSDETQQLLSKFKLIFKD
LVTGVPTAYRDLESLLTNGDKQLKDAFGHLPGFLQKLVQQLPERFTESLGPELMAVAGERASRTGV
NMENAGKAAAAAQKMGFKAPNLKELVGKPTAIAGMMRSIITFLRARFPAVLGMNVLWSLALFIVLL
ALWYCHKRGKEVRLENERLVTEAEITKMNEEYEQHIRPTETLSTTAPRDAPISDVRDGVEQVQKTR
EQALASDSSTNLQKPQAETVSPSPAATVAVTAAPVSSNTNATPTSASTPPATGRRNSERSRFSLLR
TFSKRTKSEETSNIQPYPGT
>g898
MPKTSSQSPKRSPLTMGKYRRSEMSQLTSARNKGLKHPAGKKTVTAKSLNRKRKPSFSSISTISSV
SGFEDDDEQDEADSEASSDEDDESAHRTPSYGGKSRRGRKTGSKTKSSTLSIKKRKLSYDEGYEGQ
RSSTGESDSDYAAVDEIDDDEDMDVEKLEEQMILDSEDDSQMIKSTFSTSDAPDFDEWEGFGNFDD
HVLFSTEPFLEDTLYSAMETFGETDFTSEAVETPVQRHVHFVVEEDDDADSDTSSDSRDGSTDDEI
PGDFLQQDSLDPTLRRMIDSEYDTSNSQRYEDIFGESDISHPANIYHVESDAVSDGSSGYETDDGE
TTDEDLPPPATITHPRSILRRDSTASLTATGDSTSQRSNPPRRRGPIMGTFVADPKKPVALVDCTG
KHLVIIPAYASSRHDWLESAANSMCGTAHNSPRTTTMHLVDESDTDALTSPNGFELNPMLSSGNLM
MTALGNDATPGGQVMGPPEAFYPSQNYGGDSSYDEDDEDDERMLNVDDFLDFGNGSSDEEDEGMNK
GSDDDEGLTTSPAVATSVQGLTTPTPNRAHDNDSAERFLNHLDKGIVTAFRRNHTRYQTLIRLPQH
REFMPANSPSRVASVFKHSRYADPRTPPRKRKSNQYSGGEAVRRKLIDGHRRNQLSF
>g899
MLLLKSFPYLSLFAAAVSVFGHPSSHHTHKRDQIEYDYVIVGSGAGGGPLAARLAIAGYKVLLLDA
GTDEGNATQQMVPALQLQSTEYGPQRWDYFVNHYTDLERQKEDSKMVYNTTSGDLYVGLDPPTGAT
PLGILYPRAGTLGGCTGHNAMITVTAQDDDWQYIADITGDTSWEPSNMLSYFERLEANRYLPSSIV
GHGYDGWLMTSLTSLGLVIEDQKLLSLILASCTAAGKSILGMIITTVTGLAGVLLRDLNSPDRNWA
TGPFQVPLAVKLPDSSRTGPRDFLVNTIAAKNSDGSRKYVLDIQLETLVTKVVFDQSGTTTPRAVG
VEYLQGQSLYRADLRSSTAGPGVPGAVNATREVILSAGAFNSPQLLKLSGIGPKAELDQFNIPIVV
DLPGVGTNLQDLQWQNNKIDPGTYATNGIAIAVLRNTSTSSSSFPDIFVSGAPAKFKGYFPGYADD
SLADARHWAWIVLKSHVRNNMGTITLRSTDPRDTPIINFRYFDEGVTAGGADEFDLQAQYEGLMFA
RQAFSDLIPLDGSFTETWPGPNVTTEADMKDFIKREAWGHHASCTCPIGNDTDPNAVLDSNFRVRG
VDGLRVVDASVFPKIPGYYIALPTYQISEKASDVIIAAAKAS
>g900
MNNTTENRPYVRVSFTIRNNQDAAFFVVVDGGDFDCFAMSVSGNSEVQLYSDEFAMHLHKPAAQRD
DHATKNVTSTLCGAPIRDRTAVVSVAGLAGGALAIVFFLLRMVTRLPRFGVSFGLDDAVLTFVIGL
AIVLTYFSYVLASHGLGKDMWTVPFDNITHILYIYYIDEDIYLSILPLTKISILLFYLRVFPQRSF
RITTYIVIAMNLCYLIAFVLVSVFQCHPIDAAWLRWDGEHPAKCQNINAQGWASAAINMFLDIVTM
VLPLRELSKLSMSLKKKMQLMIMFTLGLFVTLVSILRLHSLVEFANTTNLTWDYVQLGYWSTIEVD
VGIICACLPAVRKLLRGVFPNVFASTVHSGAKTPAYSHTRSAQSGGGKRFIKNGEYELHHRDAALE
EGDAMPLVQLVPYTLIEIHLLVPVKAVRHVDHFVNKKDGVYSQDFNSRLSKFSS
>g901
MRFENIMLSALLAASAVSAFPTAENLGKLMSIKPTTGDKRCPFAEIQNGIEKAIKKRSLLDPLSSP
IQVTGEHSFVPPDFASGDQRGPCPGLNALANHGYIPHNGVVSLAEAIPAINEVWGMGADLGLVLSV
MGTLWVGNPLSIDPRFSIAGESPAVQSLLGNLFGIIGVPRGLNGSHNFIESDSSPFRDDLYVTGDA
WTLNLTKFRDFSDMSTDGTFSMDLIAEFAKNRYEESITTNPNFYYGPFTGAIARNAGYIFAGRLFR
NHSVENPEGVLTKDVLKSIFGVSGSDDALQYNYGYERIPENWYRRPTDFGLVDLNLDLLNFATQYP
ELLNIGGNVGAVNTFTGVDISNLTGGVLNAADLLKDNNLLCLVFEIVKTVSPNALTNLYSTIAAPL
ELATNLVATTLLDLSCPAFEDMTYNGEPLWDGLQSVFPGAGWAKAAL
>g902
MKFSSVSAVAAFLAVQQVSASWDTESCFPSPSNCDNKCTSTQNGGWDWSWLSPGQSLQNYDGFGFS
GFSCSERSSGKGKRTSDKCISGSISKSGKGSSPSISCDGDVDAFSITHFSVYTDKDTDVNFNFGMP
DGSKCSFTHKCGSEGTDVSNDQCGGATSVSWTIPDHSDVDDCGFGIYSIGFDCSPSTGGHTTTSSR
GSHSTVPVSVPVVSVPTTTPVSIPVTVPGVSTEAAPTTAPAGPSTTPCTTTPGATGVPGTTAPGTS
VPASTPGSSAPGTTAPSTSIPASIPGSSAPGTSVPASTPGASAPGSSAPGTSVAPGTSAAPTSVVP
GTSVVGQSSPAVSSPGAVSTAPVQSFSTSTVYTTTEVTITSCAPTVTNCPAESKTVVTQTIAVSTT
ICPVTATETGVPGAASTAPAGAASSTSPAGTSPTSSPAPGSDSCPSVVPKCINTWLPSTCTDNTDV

SCYCPSSNATSAIISCIQAWSSDSDELSTALSYFTGICSGYIPGNPGIVTAVPTTITLGPTPAPSG
TAAATSVPCTTITYSSSTWTVPQVAFTTATATGVGATPTVGLVGVTSVPAPANTQPVAAVTTLATK
TSAVPGVAGATGSKTPTASSTLKPFTGAAVPGAIVSRQGMLLTGAMGMLTLFL
>g903
MFKRSFTAKNRVGKAVKPAKRTYRDKAKEKMEESLDSPPMGSPITSPIVTIVVGHERRLFAAHEDV
LSRSTYFEAVLKDQFLEPNGKKVDLPDEEPEILSCILEFLYKGDYFPRLLHHKRRHTLELEDAQDV
YKTGGRGSSMATMFHSGADAELLRDTVVYCAAEKYGLDELKHLALRKQGIQSGIPVDVILRSARFA
YEHTPDTESRLRAHYLALIIRSRKTFKRSGTMQMEMEIGGKLFFDLFVAMSNHIDDIVEIGLTPID
EDTVLQHGMSFVVKWRNPGTNRVDFWIGIYVPEELSFTTRKEDDGKYMMYLPGRNIYRRIEIKSLY
SLSELPPLEYNQAGHEYPDVYKRIYGLAEETADIRYWSSLAKKERKFKDRKDKVPSIDFVPANGDT
GGAQGYEADSDMDDDDEIIGSAIRHPNGSIIFYSPVESQGQMEPAKRQREGSVITPSKKAKVTKQH
RRPQQQEQQVPEPDGEEVVDIYIGNGTDQKVFHLSRNNINKSSFLKGLIQGKFPYIFHPFLHHMSP
AEFEPVYTFLSQNESPDSDLVSVYGDEVGDDDDDWEDILGDVGRMGTYWKLKDVHNTEELKAYILQ
LRPTFAQACLLGLSEMAANVTANVQVAWNVYGRVDQLPIYLDFIEGVIQDSARGRAPGACHSDQFS
YGFGASSQSWVVRFLAETFVLCATGVPQRFWSLMEKYPSLRAAVFKRRAILDDANILELEKKFRQR
ARLAEADFAIPAKTVKRIEQEAAGQDEEAMEQVSEPMAESAEEEATEKDGEGVEQVAEANE
>g904
MSTLNNPVALLKALLARIPLILKTLVYHGLQVTPASKKQDLRTEMTVAIIRSFLDMSVSAGTQQRR
SMNDPGIKGPMWVSKVTLPAPGDAVADAVLHAVKTLSEGDEYQLLQLPPVKAVEAEWTGYRSGVDK
NAPLPDISEQEKYKALTAEAKSDLVIFYIHGGALYLMDPCTHRVPVAHICKLTGARALNVRYRLAP
QNPFPAALVDLLTAYLTLIYPPEGSYHEPVPANKIVFAGDSAGGNLSLALLQTLLTLKRTSTTVQF
HGREVSIDLPAGLSLISPWCDITRSMPSIRDFAVYDYLTPPPHEPSETMYHPTAKTPDSIWPSNPP
RSDMYCSAWLVDHPLVSPLACPKQLWEGAPPIYITTGEEALTDEDLLTARKMYHAGVPVIVEQYEG
MPHVFGFIMIATPAGRLFFQGWSQFLRDVLAGPLQTSRGDLGKVIYHHAGWYSTKVVSLDKVHKFS
DAEVEKVLRDSRDHRVEWETKLNEEWQAKARL
>g905
MESKKELDIAEESGGQTTNAGILGQDIVPTTLSFNAVSPVDVWVHNLTVQIDTTPSIWQASPAQSW
HRLLRPKFKSDDALSYKTILNGVGAHMPSGSITAIIGSSGSGKTSLLNVMAGRMESSRLKISGLTT
FNDDSNIAHMRSSYVMQQDVLIPTLTVRETLQYAADLRLPPPITQAERKAIVEQVILELGLKECAD
TRIGNSAHKGCSGGEKRRTSIGVQMLANPSVLFCDEPTTGLDATSAYQIIRTLKGLASKGRTVIVS
IHAPRSEIWSLFDHVILLSRGSPLYSGPVSESLPYFSELGYTMPTFVNPAEFLIDLAAIDNRSSEL
EASSLARVNFFRDSWQTKEKKGDVNEVGETASQENLPIAPNDTNEITKKVPFSREFSVLTSRTFKT
TLRDPLGVAGTIFEAVGMAVITGWIFLQLDKSQAGIRSREGSLYTASSLNGYLVLTYETFRLTIDI
RLFDRERNEGVVTVPGFLLSRRAARFLLEDLPAPILFSIIFYFMVGYRLDVAAFFIFLVISILVHY
IAVSFAAVGVGIARSFSGAGLVANLSFTLQSFACGYFVQSNQIPVYTRWLKWTAYTFYAFGALCYN
EFIGTSSPTYGQLYDCPYSNDPLDPACKQYTGVYIIESLGLPSNWIWRPCVILVAYVVGFYTLAGL
LLQYNKFAMGIAQARKSDGDAQTGKEMAVVTRSTEDIRRVLISLNKYTLEIKKKDYFWKAPRTIPI
LQPITTEFQAGQLNVIMGPSGSGKTSLLNSIARRLHGSIGTKYRLGGTMSYNGAIPSESVIRSVTS
FVTQDDDALMPSLTVRESLRFAAGLRLPRWMSKEEKNRRAEDILMKMGLKDCADNLIGSDLIKGIS
GGEKRRVTIAIQILTDPKILLLDEPTSGLDAFTATSIMEVLNGLAAEGRTLIMTIHQSRSDIFPHF
SNLLLLARGGYPVYSGSGANMISYFGSFGYHCPPQTNPADFALDLITVDLQQDGREATTRKRVHHL
ITAWQSTDHVVTRQTSQIATPAELGSLKKQMHPLRVTFPLVLHRSTINFFRQPDLIMARTSQIVGI
AIIMALFFAPMKNDYAAVQTRMGFVQEFAALYFIGMLQNIAVYPPERDVYYREEADHCYSAETFIL
SYTVLELPFEILASILFGVIAAYAVNMERSVLMLFVAAFNCFAIINAGESLGIVFCTLFDHVGFSV
NVTSVILSISTIMGGIMSLNLNTVLQGFNHLSPIKYAVANLAPYSMRNQHPRKNQVIDRNKRVKIS
DDMIAESLKKPRFMPPYFEHDALDPNLYAGEWAKPCAQISTDHLPYDIESGPYEEDNADEEEDDEE
EINRVLDSMLSGTEDKVTIESSTAPAPPSDADRRVIGTRPVEGKGKKLCGLDDMDKDDRLIYYLKL
AKWSERAIHEKLVAEGRIGYSQKTIGTRFARMRRFIMAENDQRLKDGTVVWLAAEKNLLPDAVAYA
TQQIDKERKALRQRKWELVSEFIQKREPLALYSGEACREEYETMLTRDDDYDATSNDPKVSKLEKE
RQTHAEMLEHFKQSESL
>g906
MFPDTDDQNRRRIYSRSRSPVANDSRRKLERSQERSRDYEKEHTRRRRSPDRSRERHSGRRDDHRR

RRRENSVSRRDSDRPRSRERRDHDTRRRRHDSSEPDERSKQRDRHRRSGSKEITRRRRSGDRRDRS
RSAGAPVRSRGPLPSQNDAFESTEATKTGSPAPVKEKPNFANTGRLAAESNTIAVNGSSVVLKYHE
PAEARKPPAKDDWRLYVFKGEDLLEMVPLGERSCWLIGRENLVVDFPIEHTSCSKQHAAIQFRYVE
KKNEFGDKTGRVRPYLIDLESSNGSMVNGNPAPGGRYIELRDKDVLKFGHSTREYVLMLSQS
>g907
MPTLRKIKQYGLTEVYISPGRPAQVDVVLVHGLNGHPKDTWTSKSDVFWPTDVLPEFLENSSLRIL
TYGYNANVAAFTDGASKDRVHHHAETLASELHANRSLRGCLERPIIFVCHSLGGLIVKRCLIMCRS
QENEKLRHLRSIYISTFGILFLGTPHTGSDVAKWGLLIQKICSAVFPKKFMDTSPQLIETLKSNNE
VLQNINRLFNDFFGRFHVYFFHETKPLDMKGTREFIVDESSAAPDVQGAERMGIEADHSSMVKFED
DSSPGFEAVAEAIIRYAREAPPVITGRWKEERAHVHLQVHSMADELTRSSVDFSQEPPQPEAESRM
GNSVPNLPLPPIFPAEHTPRMAQSVEILPTQRGPLFVVPPGFHPNATFIGMEKELNELHIRLFNTK
RREERAAAVLICGGPGSGKTHLAREYVHSHRKDFPGGIFWIDAKSVLAMSTCYWDVAQAAALIDGR
KEYPTSTDATIFVEQVRHWFESREEWLIVFDGVNFDEDSQLNAFKKYLPFRKNTSIIYTSVDRTLA
KKQRLFEPYRLNVRALKAEEARQLLFDDLEIENPTPEQYKKATEIVHHYQGLPLAIHAIAHRLTAT
RKPLERYHLNSHLTDQRLXEPFIGIMQDLRAHEHFEAMNLINLLAFFGHHVPVGMISWGKSALEGH
NLPILTSSRPGEPGDLDTTLGVLIQYGLIERISDRYPGKSLLQRDDFKVESPESEISGSESFTESM
AASGIYQNAIDVVKIHSVVQAFFRDELRIVDGKNKQLQSDSTDSGTSQLPDSQTSQPITSFYTWWL
LNATRVFCKSYEIARYKMSRDRSGCLFVKDLREYENHAERLMSYYRKVDSRPSITIVKDTKKLLKE
AVKDISTEIGKISPNAFEESFRLGKSIFDRSSSSSSGPSSSLEDSMASHRSTWNDDWESIKVQSPV
ELANPQQNPFQFPDASLSQEGLHEHIPFQFPLADLYRERLQEQNDGYLSDGEGGLRVKSRRSSADS
SRYSENTEIPPPHPTAPTAPIASVDXPAATSTTVPSPAPSPGELGWEIVQRKKKGSKLSDVPKKGP
RFRGRLKDLGSFRPSPITKMSSAQGEGSMSRILSGSSDRNTTPTNAKELLASFQKPDARKQVGDAD
QKEIHSAWTSPVISPPAAKRPSSEIPLPAPSQSAPSVLGHGNNPSPKPRPKSTIENRSDLRSELRP
SSAQTTSSRPSPTLVEFGRNHRLNPMSHSDPSLVRGRGQSQSNPNSAPGSRNHSRHPSSSVKSTLG
RESSKASIPGPQAFRLNPTHQAFIPRLSTNDMGSRYVPVPVPVPEGGMVIDVQKRNTRRWDEEIVY
PASNLSTSPRQAIPGGYTSEPLSADMSRDPSGQSRASWQTDPVPATRRSSQQLTQPQPPTAFGPQL
PTSPTYTVVYAPEGSSRRQAAAYTVPLAAPNSIDYLGLSNRFEFGGEPDTPIVGGRVLENQSIFFG
EHEVDVDEARRRVMDWNLRQSPALMYDPRTGQPFLAAPRMRAQRSDPGTTIIPVPVAVPVHMPIDT
SNTLALITMAFVTRIVPARSLMTGASFQAYRSFSTTLATQRGPVEATKDVLKKADRTVSDAAVKGI
ETGETLTHKAQETLGVKSKEAEGKAKELAGEAKGKAHELEGKAKGKAEELRSGR
>g908
MPPRGAIVFSILRPTRSTATFSRATTHFQIRTNSTASTSEATGNTINVQQVPAPGSGYVRVLLLNR
PKARNAISKELLNALRFQVDAISAEQGNGPTRALVLASNTDSAFCAGADLKERKGMTKEETNAFLT
KLRGTLSRLETLPIPTISAVSSVALGGGLELALSTQLRVFGSTSVVGLPETRIAIIPGAGGTYRLP
KLIGLSRARDLILTGRKVSGAEAYFLGICDRLVEVTPEEEKQEGVARNKVLHEGIKLALDICEGGP
LAIKAALQAVNHYENGEEAENAAYNTVVDTEDRLEALLAFAEKRKPVYRGR
>g909
MAAHSPVIPVNRSGYQAIMAVQLAHEKTPAERIFAKQKALSWPPWKEARMGIDSERGNEGMRSRTM
QVLSQMIEAGYAPSPWDDVAAILAGWDTDGTPTIQTRSLARPQKSLGPGTKESKVTSTVWASRIRA
TRTLREAWACFLSYETRCGHPSEPVYHAMARKLIYRMEGSDDINSKNESLPGDGFEVYPEPSSARD
VTYVPTEPPTLEELIERMISQGILPGVDFVALLLRFSPDFSTGLRYIAAFLSPKQTKAVSALYVGD
LEGENLETLKAMKPSLFDSIITFFCTKSHLPCPGHRFRKSDLFPILMQRPSQLVVDTSTFFSDEDV
LREGNWNGHPQTLSHAVELLLARKPRRVAAWIPLLKALASPRGPSEERLVKPHVHWYFAWYEVSEV
IHWMQELGLEPGMEGFIVLCQIFAHAVGVGIQSIAAAEESLYLAHRMKRQNDKLHVNIADDFESFV
YQGLRVLKSYFDRLVLPIHPMLFKNQSPSSETGQSSSLELPKLPGMIVPDMIEVPTPAALHSFVRA
LGTAEDHDGILNLLEWMSRYASEIADAADLKRGGRKMLRRTLTAMRVYLEDLNALKLPMREPEPEP
MVSGKASLDSYYQLGEKKDDHAEARPIFVDGRVEEAYEIIESTAIWGSWPSDDEVRAYVAHYMHPD
I
>g910
MASEIAGEAATMPLTNGVLHPPSDESAAPSTPGKRRRASTPAATPTQEKKSTVKTSEEDKAELNTN
LRYIIQIAAQDDEHLSVFSSSFAPSSPSKPRSKRPKLSAESYNLDSIESRVAGGQYLSYQGFLSDV
DQACAAIFERQTAVDNEDVLSVQTKVAALKHRLNGLVHQALSQGSAQIKVEPISNESPRDITRRDR

KVLTLYGGSSSNAKQLFSSLQKLNDTESERSATFPEDRLPNGITTXRAIPYNLEMDEKPVKTFGEV
FAPRSTLAQLEPPRRGRPWARDPSSTWIDAFDAITDYDNILGQKHHHSFAKLPSGYWLHYGGDSES
PSYWDRREKDDEGEASSQNARYINEDTAVIYGAYSSFAPSFDSSHSVMQSDAKSSMWWAKRGSRRF
NAMLALHKAARLDEDQPLTGIDETTLEQDVKSFIENGSLEAKSEETSEAAMDDKDVDAVLQDISEL
LQTLNSFRRNRNLESPSQGESKTDDNTGTPISPSAAELATYETLKAGLAAIISNLPPYAVAKLNGD
QLAELNISQKILVEMPTYNGTMEEDDYSLLQRRIAATAPAATPSRXGSYQATPGYNQRMYSTTNAR
PQQPAAGPQYYQGRQSSSTPYTPAASTPQQNYVGLRPQASPSQRPSNVPTYSPGQYQPQPRATPNG
YAPYSAQSAPSPGPGYQQNPAFTAGRSASPQKPQGYATPQTAARTAYLNPAPGNAQRYYPQQTGPS
PAVYANYAPSATQTPGQYAMSPNVAAAHYRTAAAEQAMALARHKAQFEASRQVSGTPNPPQSITQA
SSQARSESPATKQSATPTPAT
>g911
MPASISGRRKGSRATRSNKPPLAASKAPASPVSLPAVKELSHPVHPEPQKELGMQKKAQVGAELIR
TQNDAPSNKPNVFQFLQEGDSSSTTSEDTDSDSDGDQEEEGLQALAQSQLPRDTRDANSSYLISPE
SSFRASSPEQTFSVTSRDSVITDPTTSPDGSPATAYLRLANRQNLQKIAQARSRRDIVHQRQSPPP
TDDEDDDEHSDYSVPEDYYLNERMHYHHQKQQEQQHQHRSHTSRPHIHRQRKSRDRSQKSRRDPSI
SSERSAGQLVKTDTSDKNNKIVKTSTAEPRLSSGYALLASKLDSSTVLSTTDSKNGDRRLVPVYRR
FENVNHRILLHLQDEISQLEEELQMMDEYEAKHRVAMAEKEGPEQLEPSSRRMDVEAAGRYSAFHA
RRXELVDRLAYKVNQYNDVLTNYAKLTQILPKASPKEIQMYRSWMIEHTPIAKNETRFLDHDLDLV
SLKAAIDAIPAAAVRGDSSTGILFFIIGVLSTALLLPLLAY
>g912
MPRLWYSRYLASARLRCRPLLLSEQPPNRLXQPLLCRTVNTGTNDHRPNDFVPLRKQLKEEARRAK
SQSRDGRGKKQVAANDKWELTVGIEIHAQLNSEAKLFSRAPTSTVAEPNTNVALFDLAFPGSQPEF
QAATLLPAIRAAIALNCDIQHTSRFDRKHYFYQDQPAGYQITQYYEPFARNGYIELYDYDGIAPED
GKFVRVDIKQIQLEQDTAKSHEHPPSAHFLDFNRVSHPLIEIITMPQIHSPATAAACVKKIQAILQ
ATSAVTTGMELGGLRADVNVSVRRRDAPPGAGEYYGVRGLGQRTEIKNLSSFKAVEDAIIAERDRQ
IRVLDAGGVVEVETRGWSIGSTETRKLRSKEGEVDYRYMPDPDLNPLVIDSGLVSALKDTIPALPD
QLFTMLIGPAYGISLEDAKPLVELDDGARLEYYQDVVDALQALHSGEESDATRKLLGRTAANWVLH
ELGALHTKADVTWHADSIPAQTLAELIDQLIRKKITSSVAKQVLAMIFDGDQRAVHQLLEEENLLL
RPLSRDEYIALANSLIEENPQMVAQIREKDRLGKISWFVGQMMRQGEKGRVEAQRAEEIIRELILG
Q
>g913
MTAPSTIKQRFLSQSNDLGVVAVGFSGGQCKAGVEAAPMALIESGLLDQLHEDLNYNVHHDNIVHD
YADLMPAEDPDHRGMKKPRTVSAVTQKLSQQVYEHAKQGKMVLTLGGDHSIAIGTISGTAKAIRER
LGREIAVIWVDAHADINLPEESPSGNIHGMPVAFLTGLAKEEKKDIFGWLEKDQLLNVNKLVYIGL
RDVDRGEKRILREHGIKAFSMHDIDRHGIGRVVEMALAHIGNDTPIHLSFDVDALDPQWAPSTGTP
VRGGLTLREGDFICECVHETGNLVAMDLVEVNPSLEATGAGDTIRTGCSLVRSALGDTLL
>g914
MLLRLPPTLHYPITVKSLLKRPGDSVERGDHLFWYVYKTTVIEGDGLGNDIEVQKEFPIKFESSVD
GTVVEWKISKGDIIDEPVNVVEIDEPCAHEVQFGGMCAECGKDMTAANYNTDVMDAMRAPIQMVHD
NTALTVSQREATRVEEDAKRRLLASKRLSLVVDLDQTIIHATVDPTVGEWKEDKNNPNHEAVKDVR
AFQLTDDGPGMRGCWYYIKLRPGLESFLQNVSELYELHIYTMGTRAYAQHIANIIDPDRKLFGDRI
LSRDESGSLTAKNLQRLFPVDTKMVVIIDDRGDVWKWNPNLIKVSPYDFFVGIGDINSSFLPKKQE
LAPGKTKGEKPAVTPRSGHHVNGTTQAKEAGMEVSALEQLVTMGGGDNPTMLQEQTTQQEETIMHQ
VEDRPLLQKQKELDAEDDASESQAGADSETSTSSMEDSQDLTRQRHHLLEDHDQELFYLQDRLENV
HKQFFEEYERRRKRNLEKGRISILRGERTIRDRDIDLELLPDIKVVMPQIKAKVLGGVVLVFSGVL
PLGTDLQNADISLWAKSFGAIISSRIGSKTTHLVAGRNRTAKVREATRYPRIKIVTTQWLLDSLTS
WKHMNEEPYLIPVHPEDRGEPVLGSSPGSKLGSSWLSSSDDDTGVSLTDDDGVNEEIIRSSGMTEH
SPVGYTAAEQAAVHDELKEFLGSDDDDSDDEEDEDYVDDGEEDDDADNDSEEELAVPASPESRKRK
REGDEEPEAEGEGAAIAPLTQRLKRSGGDDGEDTSKQDIVITTGTGEEDESYNQSSYTENADYDED
ELEREMLAAFENGEYDENAEDAIGADNG
>g915
MDPNQQQQQGNPQNEDYVDKGLDFAEKKYGQGKIDPAKMRSTNEKITDGARNLFEKVTGKDVPSKF

SN
>g916
MKTTSVLAAAALVGAATAKVHKLKLDKVPLSEQFDKRSMNDHMRSLGQKYMGAVPEGMYQDTSIQP
EGGHDVLVDNFLNAQYFSEITIGTPPQNFKVVLDTGSSNFKYDSSSSSTYKKNGSDFAIQYGSGSL
EGFVSRDTVTIGDLSIKDQDFAEATNEPGLAFAFGRFDGILGLGFDTISVNKIVPPFYNLLNQKTL
DEPVFAFYLGDSNKEGDDSEATFGGIDKSHYTGELVKIPLRRKAYWEVDFDAIAFGDNVAELENTG
VILDTGTSLIALPSTLAELLNKEIGASKSWNGQYTVDCAKRDSLPDLTVTLSGYNFSITAFDYVLE
VQGSCISAFMGMDFPEPVGPLAILGDAFLRKWYSVYDLGNGAVGLAKAK
>g917
MADVAVENPANSVAVHKRPTPADSVPIIDSLEGTGNDSGDEYATLKRLQRHLEYIHLQEEYIKDEQ
RSLKRELVRAQEEIKRIQSVPLVIGQFMEAIDQNTGIVQSSTGSNYVVRILSTLDREKLKPSSSVA
LHRHSNSLVDILPPEADSSIAMLGAHEKPDVTYADVGGLDMQKQEIREAVELPLTHTDLYQQIGID
PPRGVLLYGPPGTGKTMLVKAVANSTTASFIRVVGSEFVQKYLGEGPRMVRDVFRMARENSPAIIF
IDEIDAIATKRFDAQTGADREVQRILLELLNQMDGFDQGSNVKVIMATNRADTLDPALLRPGRLDR
KIEFPSLRDRRERRLIFSTIAAKMSLSPEVDLDSLIVRNEPLSGAVIAAIMQEAGLRAVRKNRYNI
IQSDLEDAYSSQVKTSQEADKFDFYK
>g918
MNAWLTDASNIPNHDNGTFNGTIDPSMAFLQPSQAPQDPAQFQRMFQNGVPRNASPGFHNPNQVIP
SKRPRPEDGIPISPRQAPGGVPVSRSQTPQVSYSGYQGSANGNPQFAHPTAYQHLQQPQQRGPINT
SQSPSLQEFDQHGVQKMQTSSPSPFSPAGPVANQISPPQSDHGSRVNTPQNNTYIQGQPFPQGIAP
QFTQQAAMTSGATPATMQAQMANMPQGFSPQAMAAQQQRLYHMQLQNQARQMQVNNPAMVGRQNSA
MNPMVNPQMAALRQMQHNMAQPNISKANSPEIFLKTLQKFMMNRNQPLDLNPVISGRPIHLMQLYA
TVMKMGGSKKISTTNMWPAIAQALQFPPQQYPNAALEIRDIYAKNLAAYEHAIIMSSHQQKQMDSM
QQNALQRQSTDPNSMQFQQSPVKALPEFGAQGSEQFTQSPQVTATLSNSMPAGATNGIATPQQPRQ
AKQQTHPQQPQPPHGQSNQRAGVHRQAQIPASPADPSLQAQVAPPGASAKSVAATGKPAAEQEAAP
AKTIKHHIDKTFKATVLPDNHRHGPVVIDEIYQLGEEITRLKPNVPSFPELGVIDIHALTMSIKSG
IHAEIRVALDTLMTLSSEASVLSLDNCDDLVETLVDCAEEQLDLLAENAAEVSDEMLLPSYEEVIR
GCHVEMASLLNVPEFGSLGYELDRAADRVLCITALLRNFSFSESNFNILGSAPVITMFSTIIRYLG
TRNMLLRTNQNILDFMKDTVTYLSNIAQSVLLPGKEEASCLLHFLLSFAPLPTPVLGPDGVTFTQY
SPSIHKYTPAAIDGLAKLLARDEPNRMYFKAIFQESPCSPQNELLTRAFGMAICPVPDQPRKPLSF
ADARKVFLMQGLLAAEILTSLVDGITARTWLQSTDRFAIHLLRLCCLLSTDRLAMPPNPRHPQARN
QPDPEHHAYVSIINRGLSILRKLAEKSKQEDSLSGRPFPLGVLPKRESLLGALLTPNLESSVVKQL
IAYAGLGE
>g919
MESQSNFPSIHHHLDRRYQSNKPEHFSQNSSAIGAEAPSAQAQPTPLQLTNLPYFVRRTPSNKLPV
YLETKAGGTKRLTKIQKTEGDLEALKSDLATALNLETGSKKPEVTINSTTGHIIVKGWRKDEIQKF
LIDRNF
>g920
MADNAPPSAETLLNGAAAHPPKTAEEIAKEYDLLPKLIPYLDRHLVFPLLEFISGQDDDNLEITRS
KYELLKHTNMTDYVANLWQEINNSDTIPEEFVKKREGVLAKLQQYQEDSETITELLQDEAVVGNLR
SDKVANLKFLEEQHGVTVEMVNTLFNYGRFQYSCGSYGNAAELLYQFRVLSTDNDKVSSATWGKFA
CEILTTNWEAAMEEVQKVKDSIETRLFNNPLGQLQHRSWLIHWSLFPYFNHEPARDVLTDLFFSPA
YINTIQTNCPWILRYLAAAVITNRSRAHKNTSMYQKQLKDLIRVVRQEGYEYSDPITDFIKALYVE
FDFEEAQKKLGEAEDVLRSDFFLVSAADAFVDAARHLISESYCKIHQRIDIKDLSTRLGLGQDEGE
KWIVNLIRDTRVDAKIDYKEGTVIMNHPPQSVYQQVIERTKGAFFRTQVLSAAVAK
>g921
MRTIAPIAAPATAINNPSRILPALLSVDFVGTAAPLVLVPLLPAAEEIDGAGVVAGMVLASDEVTR
TVVAPVELPVEDAEAEPVNVAEDPGAELVVEVADAETVVDVAETDAIVADAAIEGVQQERYEVVRT
AVPE
>g922
MSSTALIHDDEALEPTLQSILDQKTLRWIFVGGKGGVGKTTTSCSLAIQLSKVRKSVLLISTDPAH
NLSDAFGQKFGKEARLVDGFTNLSAMEIDPNGSIQDLLASGGEAQEDPLAGLGMGGMMQDLAFSIP

GVDEAMSFAEVLKQVKSLSYEVIVFDTAPTGHTLRFLQFPTVLEKALAKLSQLSGQFGPMLNSILG
SRGGLPGGQNLDDLMSKMESLRETISEVNTQFKNADMTTFVCVCIAEFLSLYETERMIQELTGYQI
DTHAIVVNQLLFPKKDNPCEQCNARRKMQKKYLEQIEELYEDFNVVRMPLLVEEVRGKEKLEKFSE
MLVKPYVPPE
>g923
MSLHIYIAHTGEHLLADPVSFASPEALRSWIARNTSIAIQRQILMTARGKNVKLQTLATEDEIFVY
DRQLVSEGGPVELPDIPIPPPFKPKNPPDTLADQNDLQSWRNLYMARRTWALALAEECVPVDQALQ
EHNERIDIIYRGVGVALENLKSHVASLETKFQEAQTWADDLLKEQKSALDGWQAALDKLKAIPARK
DISFLGRPSTPKKGAKDRSNGTLQDFLDVDEVRRAASKGAAVSQSFSRRVNEVQAAVSSISSDANT
LLEQAQPPMIDGGHGLLEEVETLAKKVSSDYEHVLGLPSNQKSLANISRMALGHTKELLPSLREIS
LELHTALDQAVQHRITAMKAAVNHMRTVSSLESRLADVQAQIAKLDVDADIFDIIFEVYHLPVAYG
SVLVEAVRRREWXEKLKTDSLTLAEEMAVFRDEEQRRRKKWVKSIGELVTLPDDTTPSLEINMQGQ
GQEWPEVHRKEIELYIDALKARNDMSAIVEEVSQLYADLDKPTRQQKRRAKAFKQGSVIDMGRSSL
LIRGDDMVRSLQDEKYKLEEKVKSSESRIRKLEDLLHRQTHIARPLSGNFSTDFPISPASPRPDPL
SRRSSVSSRRMSSNQTAEDRSLVQRIVGLEADLATERETVQRLQKEARMERQSSTDRMQEAQSTKE
DLIKNLESRQREFEDERKFLEADAKQLRHRIEELEEELFNVLDRRDHEKQELEERAQKLEEQLLTV
ETXTAKELESAQTEYKALQDNYSRARQRASDLEAAIASDRDQLSNQEAKISELQAQMETLHGQNQE
NLSTLQAAHTILSPERSAPSDFASLVKAIEILAEGLTIHARNTEKSHATLVEEKKDVEKELNSQQA
ELNQLREIVETRKAEITKVRNDLIQEQSQVSVLKSELADERSQLHHLRSKFSAGETGSEVLRERLA
EEEGKVASMSQELIQLSAQTRTAEDEAKDFREKLALAQASVEQLTVLVNARGSKAYEVSQRLLLQA
GSIGHMLEQLGFAVVQTDDTLTVQRASKVNSASASGDLLASSTVASLRPDPTLSTWLHDKPDYEDA
KFKEFMDSMAKFDVEVFGEAVVKREKEIEIVARKWQKEARNYRDKYHRTQSEAHDKIAYRSFKEGD
LALFLPTRNQAIRSWAAFNVGAPHYFLREQDAHKLTTRDWLLARIGKIEERVVDLSRSMNGGNQDR
RSITSDGTSFDDENPFELSDGLRWYLLDATEEKPGAPSTPGLGKSTVASAHVDAKGSIRLKRTAAG
GNVAKTLTKSLDSRRNSSASKKGTPVPTLAAAESVGDNVQPADADAGVQPRESDPIFDESLDALTD
PQAAEQFQAAPQSPVVRAELDDLHSGASSPSKRPSRLRPWEKIWSLDYRLEGDKQ
>g924
MASNDKDRYASNTSGLTEAETALLDLAADNRQKVLSLSSKEELLLQLYDQIQELELERAILEQDSE
ETTAEDAEEQLSVAERELLEARATFTVQRKATESILMTDPILKAVHSKATSPAERALLPLVNCRDV
LSLVYENLSIARTATVEALSNAEVENIRLMEQNKGLASELLALTAQETSWRDRVSDKAVIAQLEDI
EQEYKKTRAQRDTIQSMISALVVGSGVDWARDDRLRNLVLDELD
>g925
MPKEKTTRKTAKPRGEKRKKDPNAPKRGLSAYMFFANENRERVRDENPGIAFGALGRKLGELWKGL
SDSERKPYEDKAAADKKRYEEQKASYLAAGDDEEESS
>g926
MPAPVIPPGKTWLDTLQKSFVDVPVDAANDNAITTKDFLDATESFTTLFDVLAVTAFSAVKSDLLG
NVKXLRERYNAAPAESETLQALVLNELKTKKHTATEGLLWLXRGLEFTGEAIRDSLDTPNKELVDS
FRAAYGNTLKPHHSFVIKPIFNAALSATPYRKDFFDKLGFDASAEPEPALKEYVSALQNQVKILKD
FQSRKEAQPKLVADSQIESASLHHPLPVFLHTYVWPFLIAWPIFFAFYLNPTSYDTYIQGQEWTWW
SVNLNTLFTTTPASEVEIAQLIKVIPITNAGAPEICQLLRDNTRGKETTSFLFQKRRFLWYPEEGK
FAPLSYALDEEPKPAIKSFQKSRGLQSKSEIERIQHHYGDNTFDIPVFCVGLWMLDEYWYYSLFTL
FMLVTFESTVVWQRQRTLNEFRGMSIKPYDIWVFRENKWQEITSDKVLPGDLVSVNRTKEDGGVAC
DILLIEGSAIVNEAMLSGESTPLLKDSIQLRSGDDLIEPDGLDKNSFVHGGTKVLQITHPNLGDLS
EKAASAVSGVPKPPDNGALGIVVKTGFETSQGSLVRTMIYSTERVSANNAEALLFILFLLIFAIAA
AWYVWQEGVEKDRKRSKLMLDCVLIITSVVPPELPMELSLAVNTSLAALSKFAIFCTEPFRIPFAG
RVDIACFDKTGTLTGEDLLVDGIAGLTLGQTGAKVDKDGAHTGLIKVAEVGNETTLVLATAHALVK
LEEGEIVGDPMEKATLSSLGWVLGKDDVLSNKATGSQGSESVQIKRRFQFSSALKRQSSIAHVTTT
DKASGKRHKATFVGVKGAPETIRTMLVHTPPQYEETFKYFTRNGARVLALAYKYLSTDADLGQGRI
NNYSREDIESGLHFAGFLVLQCPLKDDAIKAVRMLNESSHRVVMITGDNPLTAVHVARQVEIVDRE
VLILDAPENDNSGNRLVWRSTDDKINIEVNPAHPLDRDILQTKDLCVTGYALAKFKDLPAFSDLLR
YTWVYARVSPKQKEDILLGMKAAGYTTLMCGDGTNDVGALKQAHVGVALLNGSPEDLTKIAEHARN
TKMKELYEKQVQMMARFNQPAPPIPVHIAHLYPPGPTNPHYQKAIEREAQKKGQPVPPVTGAVAAP

TAPAQQALTPQQLRQSQAQATAAGLADKLTTSMLESELDDEPPTLKLGDASVAAPFTSKLAHVMAI
PNILRQGRCTLVATIQMYKILALNCLISAYSLSVIYLDGIKFGDGQVTISGMLMSVCFLSISRAKS
VEGLSKERPQPNIFNPYIIGSVLGQFAIHIATLIYLSNYVAKIDPPSGDVDLEGEFEPSLLNSAVY
LLQLIQQISTFSINYQGRPFRESIRENRAMYWGLVGASGVAFSCATEFIPEINEKLRLVPFTEEFK
ITLCVLMILDYGGCYIVENVLKQLFSDFRPKDIAVRRPDQLEREQARKLEEQAEAQAEAEKNRKV
>g927
MTTYDDNQHHNDAIIQVEPDQYRGLDATSYTARRLHHASAHHLYLTSRRHFIGPIPEDWIHSHRKA
WYRSRLSFKNYTSRTLTFSAKTTSFNEAPRSLSPEPPTAVNEALDDDRDDDEAPRSFTIRHDSSSH
EQINENETVDTTGESESRPSDADTGVASTFVTARETLPGEDETLSPSITKNTTSDEPRAPRDFCTA
SSSPERSSHVPSTLNLRTDAGSTASLLPHRNDVETSSAISTKTLRPQEPQPHNVQSAKPASGPQDR
VRAKIARFNLDDNVLNQQQRIRSRIERTQDKVSANRPRWSKESEGEMVRAQKMLVRIEETSQELPS
DYSENASLRTETREIKKWREYVVVCRRALEDNAPFTLKMYKTRVIQDVEKSRKSAYYEISMDKKTT
KVNLYSALDKTWAIWHPFKRGWHRPTSLLINVPDLDISIIFKDPFEQIRLRNEESREKEILAAESI
VAHCIDVLKTWKRFDRLEWIHGSNEEHMYGTIGMQTSHDLELRPKHHYPSMIKTDGEEEEEPAPIE
GFLPSKALPPHPPDTGYADTAIPTYQEILERNPLQYDILPYPVQNGDISWLVSGNAEFVRRHDEEA
IAQFGRNIANLRNAEGLIDLCQVNDVRTSTGIGADSDRNDSRFELVLSNDLVVRFKAYNDETRNEW
VKRLSALVRYWKNRVKADAGELKTIRQHNLEILNIDERMESLFGQFASKWEVRRAEASPHLYNMCH
LSGCRSIKMSGYLYRKPRRRSTFHRCQVICTSGQLLIFQDTLRKHNGVEIPHIHKERVATLDLQDC
YIYSGLLTENDLLYTNQTFDNNYPGHHALPRIYLAQDGWTSRDEDTAICFVIWHPTRKSLFRASEV
KEGKTNSMLRRVSALGVPGRTVVFKARNRLERDRWVLCIESEINRLQEERGEDVRIV
>g928
MADSKAKQTAARIISGAIKSKPRVLSRLELKQKTSFNNTKNPQSSPIDISDFTIYKERPSRWSSFR
SRFRARYAALPVSVRWTFRALRTIAPIVPVGIFFSEHILQLMWVAGPSMTPYLNENYEQTQTESDV
ILVNLWPWGTLWPWNRTRTLERGMIVTFRSPANPENVAIKRVIALPGDRVITREPCPRPSQIVPFN
HVWLEGDADDPRKSLDSNTYGPVSINLITGSVVAVLYPRMRLLKWWEWDPPAETQADSGAQKEMGE
EYGSTVRDRVVKQAVKIEVPGLD
>g929
MASNALSLKEIPTLTQLYRARLLSPAKHTMGSAPSKALNDILSHIRHDITKLQVDCIVNAANRSLL
GGGGVDGAIHRAAGHRLLDECRTLNGCRTGDAKITNGYNLPATKVIHTVGPIYDDDNHQLSETLLR
RCYRRSLELAVEHGQRSIAFSAVSTGVYGYPNEAAARAVLDEVDEFLREGDNLSKFERVIFCSFMP
ADVRAYERYLPSYFPPTDEDIEIANASLKTKTTNDTMIVDVNEDDSYDSISDGEQDWEELSRDDGD
KNHELDEEPVDLNSNSQAASVTDVQSLQSSSAEFDELSKSETENKS
>g930
MDAGGLTQMTQNSSFNSASLGVPGTGFASRGKGSHIKRLSVPPQISTINETGPATGVATPRTSRSH
LLAGLRTAPKSATVPSTHNQQFQGHGRVGLEGSRYASNRTNDRVPQTATGATFPQYSYGLDSQSTN
MGYNTGRPLYSLPEQVLAPPAIDLGDADGMDEKLYAELMTTNLFLAAQQQRLQQQLLSVTAAAQQF
QNLSLGANQLNQQFQQQFQQQHLQSQQGQFASFVPGMSVYQQQLQQGMQPVVQPVSGQPGLYSVYN
PMTGQQSFIFDNSTPSPQEEDPLPTYQESIQSPIGYQQQQQQQQQQQAPFRAEVSPPPESARSSVH
ASSRSESPKDTPSPPTNATPLSALSGNSLRRNHKKSHSFVPGLRTNTELARTGGSGPRSAVLPQTP
LTGTFGPGQGRAGEHPSRQPRGPPALEELVAKPTSKHEGSKNFATRQRRAVHNLVRAGIERRGES
RSNHGSGGSNTPASETEFVFSVSDGDDSRRGSGSLSSKPSLGSLRAFANGAIGSEIKEKDRSSRER
VSAENMYILSAAGSDESFPNVLSPVGPPSTAAIVAGQRSVTQTTERRKTPMLVLSSAEKRKSPIM
>g931
MAGSTEGIVYSATYSGVPVYEFKMEGECLMRRRADDWINATHILKVAGFDKPSRTRILEREVQKGV
HEKVQGGYGKYQGTWIPLPEARLLAERNNILDKIRPILDYVAGDRSPPPAPKHTTASRPRAPRGSA
ANKRVTQEEPFNMRAPRMSVPSFPPPEQYEMPPPSTFEEEESIEQTTLESSSMMAEEDMVPMSQHS
THSRKRKRGMGEMNVMSTTEQEHILYGDQLLDYFMTVGDAPEAAKVQPPVPPVNFQVDRPIDDQGN
TALHWAAAMGDLEIVQDLLRRGANIQAISVHEETPLVRAVLFTNNFEKKTMPSLVNLLQDNLTFRD
WFGATIFHHLAETTRSKGKWKSARYYCEVLLQKIRDTLPPQELDMLLSCQDSNGDTPALVAARNGN
FRLTSLFLLHCRRSGDLVNGKGDTAIAIMDQANAQRTVPHAPSSVTYVNERAENEALEVMETDKSL
NLPPTSSPVTSAMASKIARLMDEAQRKLSLAYGNAKVGQQSAGDATTPQDLFEQLEKDRQALQKEK
VSLLSQEDTNESFDLQMNRYKEIESRCASLIEQLHRKQLSDRFAVGAPKEQAPASNEPVLPEELLQ

RFELVRRLVSAQEGRREAVRDLLQQKADAGVSTKLDMHRKLVALATGLKEDELDPMSAELADTLEF
ERMNGKESDDGPSDRIPLLSHAVLSGPTVPVDA
>g932
MRIPALTFRKPLRQLGTALEWPEARKNADKVRKWGIEVGLVARKKRRFRMIDTQRQQLLAIWNRAK
GKERDVLLWGDEVILPDLCLGIHRKVRLSLAQADILKSLARDAALHKVGGDVSDMKANHEGETLPT
FHPEFGRFMLEATPGRPWGINLSDLLKVESNMRWRREIAKGHMADNEYPITLTTFPRLGSPDDYIQ
PYYPPSGPALRSQFVPDEIANPHIRFPTLAANIRSRRGRKVEINVPVFKDTNTPWPFNDPTVNYDL
HDWPEDDDVRNGAAKEGHVYMDAMAFGMGSCCLQITFQAKNITEGRTLYDQLSPLGPILLALTAAT
PIYKGFLVDTDVRWNQISRAVDCRTPEELGEKPLKNDRWRIPKSRYASNSTYISQDRRLRKEYMDP
NLVIDEDIKKRLIEGGMDDLLATHFAHLFIRDPIVIFSEDLKELDLNEVNHFENLQSTNWQHMRFK
PPPPDKDIGWRVEFRSMEIQMTDFENAAFSIFIVLVTRAILSFDLNFYIPIQRTTENMETAHARNA
VLEKKFYFRKDPFPPRSSRQSHLHNSGTEPSSAGSSATVSAPSSPPLGPVDSEYALMSIADIINGS
ADGTFPGLIPLVESYLNSVNVDVETRCSLARYLDLIRKRANGTLWTGAKWIRDFVDNHPDYQHDSR
VSEGITYDLVRAVQAMEEQEGKAGSIGWQMLTGQK
>g933
MATGVSVNPAPIGPPSAMYPGPSHQSYPTPTMSSIPPRLPGHNNPPHALRRSMDLDQEPSPVQPPR
QSLPSIHEALGNDNPLPYPGPSPSGPPSHPHTTPNLLSRPSTEGPAGPPNPFSSNNNSNPAPYLRE
PQYNTQQGSTAAVDQQRVSMVSLSTQGSRKQSLQSLSGKSPTSSSKTAPTTISQGSAPYSPNSYGS
YPSQPYSWPSQQPAPYDGRAYNGGPWKPNVDEKNGHRPGMPPHSDSVKRQFDSYDVESSLAEIIDG
STKTLDFSRHYASIAHQNQRSGPILGTLPSLHELDDLIQQQQRNNEALLRIRNAVVSQEHALAEQR
IQQRHPKVENGYDDEHVGLYQDGFKSPGGFTGGDAKKRRGVTNGYFSNRRQLLQDDAIVVIALRLL
NGAAVQTALALCATHAVFTMPS
>g934
MADGFLAISLPSSDHVDDAGKNFSRRAPRGLIRTTSISSQSFSHTSALKSPNGVTSNKQTPGPGDA
QSKPKNVGILQVTNSVEYTINQRSLQVEQKQQPMSTSVAHTQEGVSHLQGSTIAANIPYADSLRGD
SSHRLSSDDSSSPSSYDSTSSGSTSSTNECSTEITDDDYDDGIGTEEKLKAADHAAQEQARWPFCL
CFPKSKFLDVQGLSVILPINIPIVSKVREDLPLVVLTEKLYDLLNEDEEGSAAKERETGRSIKLPK
ALLIVQQMTRRGLNISPRVRASSPGLHSGQSTPSEDHNPDDCLASRSHNNFGVLANLLRLHDRRHA
ATNYGPSPEYVSYPDSLHLRNGSFDDTDSSERSLSQDSGIVKPPKRKWWEKNSGNSSPPLSWTNSS
TKSSFSHPSGQRPPLRRSRSSGGLTPAIQRLLKMRAQENARDEMCITVQLAETLSRQRYLLRLCKA
LMKYGAPTHRLEEYMRMTAKVLEIDGQFLYIPGCMVISFDDAATHTADVKLVKAAQGVDLGRLFEV
HQIYKEVIHDVIEVAEATRKLEEIMKRKPRFNVWFLILMHGCASASVGPFAFGARPIDMPIAFLLG
CLLGILQLVLSPRSYLYSNVFEISAAVLTSFLARAFGSIPYQGGRLFCFSALAQSSIALILPGYMV
LCGSLELQSRSIVAGSVRMVYSIIYSLFLGFGITVGTSVYGLLDPNATSDYYCPPSPIENPYLQRL
PFVIMFTFCLTTINQSKWRQIPVMLLISLCGYLTTYCSTKRFGSNTQVVNALGAFVIGVMGNLYSR
LRHGLAAAAILPAIFVQVPSGLAASGSLVSGITSADEINHNTSFYSIINNGTQGFLEAQQNMSLYG
SSKMYSGVVFDIGYGMVQVAIGITVGLFLAALVVYPLGKKRSGLFSF
>g935
MVRLPLPQRFISSKSTTPIQQSRNPSPLRSPMDSTLTLKVNVIKGRDLAAKDRGGTSDPYLVVSLG
SARESTPTISKTLNPDWNVTFELPVSGVPLLECVCWDRDRFGRDYMGEFDIPLEEIFAEGETQHQP
KWYTLESKRRRGKKKESIVSGQILIQFSLVDTANLGANPADTYRKFRSIVCAAEEEDDTALDSVDQ
DENDKDEETSDETDDPTKPEIVEKRRRRLRIARLKRKSLAARAYQFSGASNGVQGVIFLEINKITN
LPPEKNVTRTSFDMDPFVVTSLGRKTLRTRVIRHNLNPVYDEKMVFQVMKHEQSYTMSFTVVDRDK
LSGNDLVATTELPLQTLIAAAPEADPETGLYKLQEPSKPGQPLQSQRSRFRLPMSRSNSASSLSKS
SKPTLTSQSSNPSLLTTQTRSDQSPPTPPTTVPSEMSPPLLDVPSTSSLSSKIDETPPSPQGSLAS
EDLKTYTIPLLLKNKERWEDKYSPELYIKAKYMPYKALRQQFWRVMLKQYDADDSGGLSKIELTTM
LDTLGSTLKESTIDSFFSRFSAENESGEFNDLTYDQAVICLEDALQKVQKSNFGGLDSKLRKLVPS
SLSSTGDRTPDTSSGADETTDLEGMESQPQQLDLSGFPEGEQPSPGYGELAPDDLADERGEEHIIE
IGECPLCHQPRLSKRSDADIITHLATCVSQDWRQVDNIVMGGFVTSSQAQRKWYSKVVTKIGYGGY
RLGANSANILVQDRITGQINEEKMSVYVRLGIRLLYKGIKSRDMEKKRIRRMLKSLSIKQGKKYDD
PASASQIESFISFHRLDMSEVLEPVENFKTFNEFFYRKLKPGARPCSAPDEPRIVVSPADCRSVVF
DRIEDATKIWIKGREFSIERLLGKAYPEDVTRYKNGALGIFRLAPQDYHRFHIPVDGILDTPKTIE

GEYYTVNPMAIRSALDVYGENVRVLVPIDSVAHGRVMAVCVGAMMVGSTVITRKPGEKVSRGEELG
YFAFGGSTVVLLFEPGKINFDSDLVDNSKGALETLIRVGMSIGHSPDVPQFEPDVRQKEQEISQEM
KEEAHRRIVGSLALGQLRWWGRSSNQKPRSGASIFGSATRAFQSKLLTLGRFIFDRILRMGTVSRE
SVICGGQTARKLLSKTTIDPSGKRGVRVIDPLKTTKYHDRANKTNDSNNVFTTFTKTCEFSPTHIE
LPAMTTTVLTATSTRTARREPLKVIDMAASQGRPASSSSVRGNSEKGKGTRTSSRLNAKDDGDDEG
AAPPKSAHGRPTRNASKRNAAEYDEDIGGFQFSRATVKKLKSAPAVNNVAIEEPVVHSPRRGRPPK
SHPAESAEDTTIEDVGAGTKKTKATRGRPKRVSAELQAEVPSRQTRKRDEEPPFPIEKPAKKGRPA
KTKLPVDISLRSPEPSQGATSKIALPVADTPVIRRNKEMREGRDGKGQRRNSLGMRGRRASSLIDS
GASNALPHKEVPTSEFYKHIASDGLPEPRRMRQLMTWCATRALDDKPSGSRSDDDSARLAARVIQE
ELLQDFSNKSELTNWFAREDAPAPAVIVKKPNPKNVQNAEKIKELEEQIRKLQNERHALNALLRQP
SIPPLGPPPSEPTAKDDATSKNLPVIDEAILDLSQRTIITTLAPDGLRIHREGGPSDENILPTSTT
NPPLPPATISRRLSRITSHLAPTLDAFASGLHDIDIYRSTADRLSHQILKVCSDRLEERDSRIAKG
ILEDDAPSKADSHGDGNSKDTRQMALRTRQARQQKEDISLILGALSRVERR
>g936
MSTASKQSHVTVFLIYLVFVSTLGPLQFGFHLAELNAPQAVITCEKKSITSVSARLPQCLPMNASQ
FGLVSSIYTLGGLVGALVSGPLATRSGRLLALRATTIFFVLGSVAEAASPNIAVLSIGRFLSGVGA
GASIVVGPIYVSEIAPPSSRGFFGAFTQVMTNVGILLTQSLGYFFSRDNLWRIILAVGAGLGLAEF
VGLLLVPETPSWLAEHRHTDKARQVLQRIRGKDADIEEETQNWKVGFPDDNGVDEEESLLSQHTHQ
PSAQQPRVTILTVATNPQYRPAIIAVVIVMLAQQFTGVNSIIMYSVSLLQTTLPTAAPLLAVIISA
LNLVVTLVCSPLPDKIGRKTCLLLSIGGMGSASVLLAIGISIGQKIVSALASLLFVASFAVGLGPV
PFILASELVGSEAVGAIQSWALSANWIATFIVAQFFPILNAALGKGQIYWIFAVLASVFSVLIYLR
VPETKGKSTPDEVWGRVDERRID
>g937
MTSKKNSQIDEDVTTDVETPIDAEKPQSPESTDPFLVTFTEPHDPENPKDWPISKKWAVTSVMSAT
GLNRIMVSTIIAPALSDIASDLNMSDIESVMAMSVYLLATAFGPLLIGPLSEVYGRRPVLHATNVW
FLVWNLICGFTNSKGLLFASRLLAGFGASAIYALAGGVLGDIWRPEQRGRSLGLYLLIPLLGVAIG
PILGGLISGATTWRWTFWSTSILQGFMVIGSFVVFKETYAPVILEKRTLQLRKSTGQDQYYCQSMK
QSAGRSELDVIRNSLTRPMRLLMFHPIIQMQACLSGLDYGITYIVIATYADLWTSQYGESVTISGL
HYIGISLGEIFAALIGGPIMDAIYQRLKERNHEQEGRPEYHIPLMLPGSILLVTGLFMYGWCAEYH
APWPVVDIGAAILFIGGTMAGQPLQAYVIDTYSEHTSSASAATQFLRSMAAFGLPLFSPAMYSSLG
YGWANSLLGFVAAALFLPSVLVIWLYGEKMRAKMKSSY
>g938
MPSILSDADKETVKRTVPKPGNKILAVGVARLYVAHPNPQKWTYTGLQGAAVLANDLVGHTFWLKL
VDVSPANRGVLWDQELYDGFYYNQDRVFFHTFELEECPAALSFSDEKEAKTFLKKMHEREKHASKE
TKATPFGSTRGQGPAIVSSGKSGRSLFSGLLGRSSSIATPTIPATLPPVAAPVQPPPPPVLSPAPV
HETSSSIDLTDPEIQGVLAELRQMGITDAQIKDNEGFIKTYIQSQAAEASNRSSPSPEEQRKAKAP
PPPPPAAAPQPKVATISPQNTGGSGGGRRGPPPPPPPPSRRRADAAPTPQEPEAPAPPPPREPSPP
RPRFRAPPPIADAGRFAQPVTAPGRPRASSGATPGATPGPPPPPRPPKTPMEEAASPPPPPRGHGV
PPPFQGERKVSAPPAPPSRGPAHAPPPPPPRDVPQLPPKIPHAVAPASIPPPPPVRAPVAPPHLPA
SNTRPVPPPPPPAPSSGAPPPPPPPPPVAGIPPPPPPPPPPSAGAPPPPPPPPPPPPASSGAPPPPP
PPGGAAPPPLPSVGGGGRDDLLAAIRASGGGGGGLRKVNDTEKRDRSAAMVPGGSNETAASTASAA
PAGGMASAIQNALDKRKKKVSGSDDEREDEDDXRWWHSWLFKKESYQKWKNSTSPPITSFTDDGAE
GNLFNLGRTLASKTLDEPRLRCTEFDENGNVTLVNGEFRKSELIAKYGLLNRDLRKIDSSVLPHIL
VRPSTILISLLHLRVLIKADRVLVFDAYGSADSYTQSVFMYDLEGKLRQRQAPGSGALPYELRALE
SVLISVTSGLESEFEGVRDPVVRVLRALEEDIDRDKLRHLLVYSKKLGSFEQKARLVRDAIDDLLE
ADDDLAAMYLSDRAHGQVRAENDHQEVEMLLESYHKICDEIVQASGNLVTAIRNTEEVVKAILDAN
RNSLMLLDLKFSIGTLGLAAGTLVSALYGMNLKNFIEESDFGFGAVSVTCFISSIIVSLYGLAKLR
KVQRVRMWGEGMDTAGMGPLQSRSLALTNRGNWRSDAIEPMLAGLPGEGRSERLKRFKEAVKSTAK
GNSATDAARRMAISQARRTASQSQDKNATKPEDSASDETTTKA
>g939
MQLPRLPSGVPRYVLNTGIAIFVLSLVLWSINALNITSIDTLRGKLTQPLPLEQNQGQDGINNDEF
LEPPPPLQLIPSPTPSPTPRPDAKLDATTDRRCNAVPNAPDVAIIVKTGANVLYDKIPFQLLSAWR

CAEDPLIFSDLATTLGSYQIYDVLDNITESIKNGPDFDYYRKLQDYKKSGQDVRELRTEGEAGWKL
DKYKFIPMLKKTWMMRPNHEWYVFLEGDTYAFWTNILLWLQQFDPNGLHYLGEQTYVNNEGFAHGG
SGFIISRGAMARVLDDDPDITIRYDQIAQNEYYGDYVLMKALKEKGVELGLYKPLLQGEPPSNLRY
GPGRYEGERYWCQPLMSLHHVTPLDVDAVWQFEQQREDLSQPILFADMYQHFMAERLPEGDSLDDW
YNHSDDANIRAPDQEEDKPIPPEEMTPTQEEAYKSPEHCAAACAEYPRCMQYAYEFMAKECSYSFS
YRFGEKRTRKSDGTRYKSGWVRSKIEKDIAENPCPSPVWKGFHTP
>g940
MVDLSFRRALWLLVWSLLWSLSLQDDTYTFTNPPPDSSQISNPSYVVGSDIDIEWVGGNDFVSVRL
VHVLPNDNYDEFTYVFRNVTNLGGHYTWAIDLGGMNLTSSAEFFFNIFVEGETSPRAITHYFNLTQ
AVTSTTAPASTSFTTSASTSSSTISTTSSFTHSTTSAATATNTVVPSSGGLSTGAKAGIGVGVAVG
VIVLLAAAAFWWRSQRGKNGAGGPTGGAESGQATGPPYQPVNQQPPYEYYKAPGAPSEVPGDYTAS
ELAGSAPEALEMADTSRTHH
>g941
MADLEKVASAASRRSDRAPSLSRVYSAPHMDNQNAFYATSKEANGQETDSVVPSYTGRDDDNADLE
NGNGSSDGHEDDLQPTQSSRSRRSERDPKLVTWKGPDDPEDPKNWSMRRRWAAVFVVSCYTFISPV
SSSMVAPGLTAIAKDLNITNEVELEMTLSIFVLAYSIGPLFLGPLSEIYGRAIVLQIANLFFLVFN
IACGVAQTKTQMIVFRFLSGLGGSAPLSIGGGVMSDCFRAEERGRAVSIYSLAPLLGPAVGPIAGG
FIAENTTWRWCFYSTSILTGVIQFIGLFLLQETYGPALLSRKAKKLRKETQDPSWHTEYEDANRTF
TTLLSTSLRRPFILLATQPIVQVLALFMAYIYGLMYLMLASFPGLWTGANHYNESVGIGGLNYISL
GLGFFLGSQISARLIDLIYRKLKMRNGGVGAPEFRTPLLFIGALAIPIGLFIYGWTAQYKTHWIVP
NIGAVIFSVGAIMTFQCIQTYLVDAYTRYAASALAAVTVLRSLAGFGFPLFAQYMYNALDYGWGTS
LLAFVGIGIGVPAPLLLWKYGAKLRAMSPFAAG
>g942
MSLHATADINHIEAPVTWKAYLLCAFASFGGIYFGYDSGYINGVNGIKMFIDMIDPGQTSLSSAHS
SLIVSILSAGTFFGALIAGDVAEKIGRKWTVICGCWIYAAGITIQLLTGVGGSALGIIVAGRLIAG
FGVGFESAIVILYMSEICPKAVRGALVGCYQFCVTIGLMMASCIVYGTENRKDTGSYRIPIGIQYI
WAIVLGTGLLFLPDSPRYYVKKGRVDDAINALCKLRGQPRDSEYIEAEIAEIVANEEYERSVIPDA
GWFGSWKNCFTGSLWEQKSNLRRTILGTSLQMMQQWTGVNFIFYFSTTFLQSTGAISNTFLMSLIF
TLVNVFSTPISFYTVEKYGRRPLLIFGALGMLICQFIVAIVGVTVGFNKTHPDGSGGTIADNIPAV
NAQVAFIAIYIFFFASTWGPGAWILIGEIFPIPIRSRGVALSTASNWLWNTIIAVITPYMVGTDHG
DMKSSVFFVWGGLCTACLVYSYFLVPETKGLSLEQVDRMMEETTPRTSAKWRPHTTFATEKGMVHA
EHEETKQQTV
>g943
MSSPLRPSNSAANRGLGSLGRRKRDREPDDETSSVAPPSSPPPSSPPMLPFDDELDRDEEDEIEGD
IDDIEELAEDEDGIDLFADGFERDYNANQEEYRGAYINDDEEQEELDMATRRQLDARLNKRDRELA
RRRRMPAAFLQDDDEDTNMDLSRQVRRRRHHYDEDRDDMDMQEDILDEELSLEELGDVKSANLTEW
ILQPQVMRTIGREFKAFMTEFIDASGRSVYGERIKTLGEVNSASLEVSYDHLVAAKAVLGFFVANE
PTEVLKIFDQAALETTLYHYPHFADIQNEIHVRITDLPLCYSLRDLRQSHLNCLVRVNGVVTRRTG
VFPQLKFVMFRCNKCEVTLGPFQQEAAQEVKISFCQNCQSRGPFTMNSEKTVYRNYQKLTLQESPG
SVPAGRLPRQREVILLADLIDIAKPGDEVEVTGIYRNSYDAQLNNKNGFPVFATILEANHVVKSHD
QMAGFHLTEQDIEQIRLLSREPDIVDKIVRSIAPSIYGHEDVKTAVALSLFGGVRKEAQGKMAIRG
DINVLLLGDPGTAKSQVLKYVEKSAHRAVFATGQGASAVGLTASVRRDPLTSEWTLEGGALVLADR
GTCLIDEFDKMNDQDRTSIHEAMEQQTISISKAGIVTTLQARCAVIAAANPVGGRYNGTIPFSQNV
ALTEPILSRFDILCVVRDTVQPAEDERLAKFVVESHSRANPAKPLRDEKGRTINKEGELIDEEGYR
VDKAGNRLPLTPEEQQSRAEARRKAEEEKEGEIPQELLRKYILYARERCHPKLYQIDQDKVARLFA
DMRRESLVTGAYPITVRHLEAIMRIAEAFCKMRLSEYCSSQDIDRAIAVTVESFIGSQKVSCKKAL
SRAFAKYTLARPKPQSKRKAGIPVAKPYMHREIHAPIDDDE
>g944
MAPPAGGDRPIEAQNGGIQTTRRRESFASQESQTAKDFIESQLQLEADAREILPYSFDSCTQELGP
LRQTLFSCLTCNPPHNNNNNNNNNNNNNSNNNNNNNNNNNNNNNDDDPYIPAAVCYSCSISCHGEH
TLVELFTCTLRVDRKTKAKGVRSQPPADGNKYNHNFRNRFCACNEEYDPHKEKGTMFQCLGLGTVE
TGGCGEDWWHPECLLGLPRGRQDTAKETEATPKNEEAEANVDKPAEDNVDEDEIPLPSGFPSEDDF

ETFICYKCLDSNPWLKRYAGTRGFLQPVFRSPSTEKFETSEKGGEAKLQELTQSRKRKADDEDDKN
QDTKKAKVDGQDGLETIQEEKTSTEVSKHKHELLPSENPSGTFSLFLKEDFREHLCHCPDCFPHLI
PHPQLREEEETYEPPLSEDGEDENNGAGSQGTTSLLDRGEAALSNIDRVRAIEGAMVYNHLRDKVK
AFLKPFAESGQAVSAEDIKSYFEKLRGDDMAIREAGGQASASHNNGGSGRDSDQEGGGSNRKEQSG
M
>g945
MSLTNPPSSTGGPLTTSSSIRPNVSSANLNLKRSVQAAFDDSGRSGSGYTSKVHIRERYHIVGFIS
SGTYGRVYKALGKNGKKGEFAIKKFKPDKEGELIQYTGLSQSAIREMALCSELNHPNVVGLEEIIL
EDKCIFMVFEYTEHDLLQIIHHHTQQQRYAIPAKMVRSILFQLLNGLLYLHTNWVLHRDLKPANIL
VTASGAIRIGDLGLARLFYKPLNSLFTGDKVVVTIWYRAPELLLGSRHYTPAVDMWAVGCIFAELL
SLRPIFKGEEAKMDSKKTVPFQRNQMMKIVDILGFPRQETWPGLAAMPEFNQMQSMMQSMKMSSRG
ALHYNKPSGLDTWYQGCLKTGGYSSSSNVGTPGADGFDLLSRLLEYDPAKRITAEEALEHPYFTNG
GPISGNCFEGCETTYPPRRVSQEGNDIRTGSLPGTKRSGLPDDSLMGRVAKRLKE
>g946
MTERPAAPVKLSLPLKYQQDLFNELRNEDELVILARGLGLLRIVTNLLHAFDAAGNNLIIVVGADE
RENEWIGEALAEHYAISKSPLARGLKVINTDRATVPMREKTYNQGGILSVTSRILVVDLLSSKQAE
PWVIATALEAFIVRVYRQFNRNGFLKAFSDSPEPFTTGFAPLSNMLRNLFLRKPSLWPRFHVTVAE
SLEGRRKAEVIELEVPMTDKMRDIQNAVMECVETSVSELKKANSGLDMEDWTLDSALHKNFDVAIR
RQLDPIWHRVSFRTRQIANDLTVLRAILHSLLTYDAVSLLKYLDTVVAAHSAPPGSTRHSFSPWLF
LDAAHVLFETAKSRVYRGKITGAETLTTPSAFSAALQPVLEEQPKWHVLAEILQEIEQDAYLNPTQ
RGDSNSTILIIPLAGTDEEDEALDEKKSPEDSTSGEILMRRKLREYLNWKQKFSRVKDNLYVPKQN
STDATNRGQSSASVPGRAPPNKRRRMRGGGSNSLVSARAPNSSVQGQDEAPIQVSELLEDLQPTSV
EEVLKEDNVIDNLEDMEDYYELYDMNDLVMIHPYDGDMDERILEEARPRYIIMYEPDAAFIRRVEV
YRSSHSDRNVRVYFMYYGGSVEEQRYLSAVRREKDAFTKLITEKGGMAVTLTHDKSLEDPEEQFLR
TVNTRIAGGGRLAATAAPPTVVVDVREFRSALPSLLHGRSMVVVPCQLTVGDYVLTPDICVERKSV
RDLISSLKNGRLYNQAETMLKHYKNPLLLIEFDQNKSFTFDAFASASLPSTNFLSDYVFTSSGNPA
SAASSSLVNPSTPKSAQHMLVLLTLAFPRLKIIWSSSPYQTAEIFAELKNKNPEPDPIKAVQIGLN
VDIATSSSPGDVMAAAGIEHRVFHQLPQDMLRSVPGVTPVVLERLSLETNSIHEIANMSVEELDPL
VGKETARKIVKFFRKSVFEDETEVS
>g947
MPKNKGKGGKNRRRGKNENDNEKRELIFKEDGQEYAQVVKMLGNGRLEAQCFDGEKRLAHIRGKLR
KKVWINQGDIILLSLRDYQDEKGDVIMKYSADEARSLKAYGELPETAKINETDTYGHEGFEDNVEF
DEDRDSEDEKEIDVDEI
>g948
MPMHARHTIALRPQKMSSQQLARLIPRYDVSIHAETAKTLYSQPDMILLPLPAQPIRPAYKVFGHH
HILFKYHVGSFLTNWTIFRSRQYEQPHVLKPKIHEFYDNRDPNTLWWKVATQPMKVKRVVRSHWSR
RLRRIFREELYSRGYDETGKPLPLEKLPKGVTLHTAVKRLVKIQLDRVMEAMKEWPIDKRTGAYQN
SPP
>g949
MLVSLTVGKVDAGVAVLLTQDNRLIEFPSVLLPKNISSGSIVDIDVSRNHAAESKSIASFQSLQKR
ILNTYGLETPSPPVLRLRNATQTSLVLEWEPIKLATASLKSLSLYRNGSKAGSIPRPLEMMSTKIS
GLAIHAEYTFHLVLRTTAGTYHSEKLTCRTHKMTDLSGITVTPGIMPQPQREALARAIDRIGGKLI
DTVRIDTTHFVCTEGRGPAWEKAVEMNLPVVRPEWLDACEAEGTIVGVRGYYLNADPKHRQLGPRQ
VPQQQQQPTPESVVSEATXPPASSHSNLPVQNAPAAIPEGPPTPFPGGPTEVTAAEIQPEHEDDHS
EPPAPPPKDEPAEGVKDDEKDDADESDGHDEAEDTKETDVKEGLETVPAVEAETLKEIDKVQPEGE
GELEEVSL
>g950
MSIPEEQPTGEYRQYLPDLGIPRFTTMKTQDAHEYAHAFKTKHIPPWLYALFEHWRDLYEEPYQGI
STDGTIRQGLFPIQDEGLPVEDIVSSVEGLTKLLNDDQRKAISFHIDSPEWRTWSNPEFLLVHKGL
RLDEVDAPVREAIMKVLETTLSPEGYDKAVKAMRINHYLGELVESSKVMNEFSYNFAIFGRPSTTR
PWGWTFYGHHLCLNIFIYKGQIVASPWFTGAEPNEIDSGPYKGTRILQVEETLGIQLMQSLPAEVQ
QKAQTYKKMNDPEMPPGRTNKDDQRHLCGAYRDNRQIPYEGVLVSGFTSAQKELVYSILEQYLLYL

PKRSRDLKIEQIKKHEAETYFSWIGGYEDNDPFYYRLQSPVILVEFDHHSGVFLTNEEPKKFHIHT
LLRTPNAGDYGYALRPLIPPVESTIAKPITW
>g951
MLNGPAAPSPVIPTYRPSKKTFSTTPRHTIMPAAITNYQQFPLIPKIRKARPRSAGASSRPMRNEN
IADLVRFFQAQEDTEEANPPKKSTNGSMDLFKAGQRRLRYLGQTKPEKTPGQGSNASGSSSEKLTV
QRQQLTALQREGLLPGLGDSTDKSSPSRTKKDVEAIGRPWLDHALGSTTSNGDRSTSQTTLPLHDL
RSESLSLGDLAALVEFSVSFPELYPHESSPPPYQARAQSDSQPQPESQQPSSLNTVNSPLVSATDR
RLIQATHNGNEDDQPGTIPSGGQSRDSRVGGASLAPNKSVQGHRVAGAVSTGRQAPQSKSNIDKDD
DDSCCGSNNCECDDAKSLAVLLRATERLGVEKKKSDTQNPREPAPTSATISTAATTNRTAPTKTTI
STVIVNAATEENNGPPHSLNQGVTDTPLFGRKLVEEKIDGVQAAHELSTKASTSRRTDQPTLLSPA
EPGLSKPLNAGPIPLKLVAECMSPTNSRLVTSPQQPLSPHDPRPQAGFQGRLTGDSALVHPRSASS
IGPSLAIFPRPQALLSPMPIQSRRSRSATPDVAASDSTSKKGKKVKRTPLYVITNTAPLPPPAKPL
PLIPQSAGANIGNKPAPRDRNISTPVHPFSAPLEYREEDRDSPILGMNTQKLRLRRSWIGPRPSSR
YSSRSNDASDGSSINDDQTPRTGSPALSRPESRRGRADQVHALRMRDLDASKKKEAFVSEPDLDAT
QIKPSNTVIAQPSSPSWKEEARKSSRKHRVSAVPGIPLPRDPPVNAQGTPNHRRRNSATSMPGSIA
TTNGYEGGLSRSVSVTSGSNESLSIRHGKAAYPARDTTKHMTQRQVQPNSPLLPSSDEERQKHLSH
PAEDSLLEARASERKTAGKQKVVYDHQPLSSISQTTQDAVLTGGSQTNYLHLVRSLESRVASLERQ
NKMLQAALLAALDMGVSHDADSIHSRSASPSLGSMRIPTISERSMLSPHGLGVDTARHVQGKSSQR
HHKSRNASYAHDDALQESRGSFETTSSRSDGSLREIEVILSDVDVGDSTRKSRQ
>g952
MEVLDTIRELQTIPDGIHLLCPRQRRDDYGRYDDTDGEYEAQRALERRDKALSCLPILAFSQDGLG
QLQDWLWQKFNDTLESCDFCIRQYHRGKVTLVENLKESYDEEDIEKLMRLLEEWDIKRMKRNLTTA
LEELNAVPPEKIGLDALRTSSLLAIFECLSSEVMQKNAALLREYFDAPFRMIQTKRTLKLTDYVPG
LTNFLFDPDPIRNHWALHGWSRFRRPPTSDEFEWAVKDGLTRALISASPQQQYLDIAAVQRLWIGL
RIIIMRLDKDQITHHLRGLDIDPCRLSIEHLGIQSPVLRVLLNSIQLLLEKAPVDFWDAMQAISPQ
AIAEQVFNNPQFDAYLLEAKDDVTLSKSPLNDMLSWISPFMSSLKSINQTPACRYIVSQLFKRFQD
KRLPSVARFQCFLCGIEVLLCTLRTFTDNESSRGSVARVVLSETLEIVKEHIDIIVTPELDGAGDR
AQSIINGCMDVVSNTLALECQSLKTDYEVLLNHSSMQHGKSTYSPEIWDAVIARLSQSNRDLSTAA
LKGILELVGLEKFPTRGEDSQEKTKYNVIYGHLTHLSALIFERLAEFDPEHLDALFASQDTACALI
SALFSADINTYQAAVDLIKNVSAPRMIHTGTDIIDVLCDTQTGILRTRKLANKKEFTEIQNLWERQ
WSSLTTIFDMTEDWHNRGNDKSVMLEFCRDTIQFADVLFAQYPVFASAMSGGDASQEDTVRETLLQ
YPTGTMKGMVKWLRLKDEYLATTLVGLVSKILRRLGELNITTVERSALQTIENVAVTGATKTLLTA
REKAELADSSGRSTPARSTSEDEYGDSSALDETLLQLSRSVELNKARVASQPQKKLPTTTKSPLVL
SKPPTRPGVSVESFREKREKEREAKKKRDLAELQRLKKNIPGLGVAEQTAGQGSGVSGGIKEESDD
EVDAELFGKKGASTPAAVREYQESKRRALKQQGPVKKVRQWRSAKDMRARLVPDLSSLHHTLLEWD
FFANGDLPPNSGRTDYSLITSTFRDPIEYQRTFEPLLVLEAWQGFQSAKEEANFKPFEITVATRLS
VDSFMEVSTTLRSAADLKEFGLGDADIVLLSKSRDPANDNAMVSALSPGSTLWAVRISSLTPLERE
YGALKALQYYDLCEEIIKAKPSPLLKYNDASLKPLVDNYNVNQAQAKAIRSALDNDAFTLIQGPPG
SGKTKTIVALAPTPKAPTTATKKLLVCAPSNAAVDELVMRFKAGVKTLNDELVNARLNQAVQKNSE
EKNPQKIFEDHKSTDTAFKEIRSKLDQFRAKGQVPPDDLLREFELLKKKRTQLSQEIDNTRDRNNE
IARNNDLNRRRVQQEIVDSAHVICATLSGSGHEMFQNLSIDFETVIIDEAAQSIELSALIPLKYGC
AKCILVGDPKQLPPTVLSKEASRFQYEQSLFVRMQSNHPNDVHLLDIQYRMHPAISLFPSITFYDG
LLQDGPDMAKLRARPWHNSQLLSPYRFFDVQGLHQSASKGHSLINIAELNLAMQLYERLLTDFKAY
DFSGKIGIITPYKGQLRELRTRFAARYGNSILAKVEFNTTDAFQDIRRMNVGLTRAKSSLWVLGNS
QSLVQGEFWKKLITDARQRHVYTDGDVLSMLERPQFTGYRNVDMADADANESSTSTVHTPTETRSS
NEMESVSARNTPTPVLSGPSGGSNGLDDTKTCANCGSFDHFTRNCDNAEALELAKGMCFRCKQPGH
TKIRCTAPRCLMCGEIGHLAEACRSKEVLPQKDLNRIAQEESRHKRAQQAKRDEIKQKHTLARDPG
IPVVKATRSPSPPPQRQQQQGKRKLPPSGPFAKNTKRKIDAETPQEVPSNLVRPSREGPGYQHIGH
NKNPMNDNGGKVLVDNNLIQAPRGPKGVRPGPGPGPGPGSTNLPPRPPMPMRKKKEADPFIRPKRR
>g953
MSRIQFEDGGEGNGNSLHPTNSEVSKPTRRQRWATRRLGNSSGLKKRVSIVDRLHKRNVSGAEEKR
NSAGSSAGDGNDGATNEGAGADGQQETRRIFFNMPIPDEERDEEGYLKAQYPRNKIRTAKYTPLSF

IPKNLWLQFQNIANLYFLFIIILGFFPIFGVTNPGLNAVPLIVIIIVTAIKDAIEDWRRTVQDNQL
NNSPVYRLVDWTNVNVSEEDVSLWRRFKKACTRFIIKTYRSMQRNKNKKVEEDSAEEDDRRFSMAT
YRQSVYSQRQSYYGDGAEDGIQMTPVASPHPDPQVEGNGWPNEPNDGNQHLQVAKAVRDSVAPGGK
RAGTILDRSRQTPQTARFKREYWKSVKVGDFVRLYNGDQIPADIVILSSSDPDGACNIETKNLDGE
TNLKVRQALNCGRLVKHARDCERTEFFIESEPPHQNLHNYSAAIRWQQRDENVPNGPPKDMVEPIG
INNLLLRGCSLQNTEWVLGVVVFTGPETKIMLNSGETPTKRPALARAMNFQVIPNFTILFILCLVT
GIVNGVSWASQGSWTFFEYGSYGGSPPVEGIVAFFAGLILFQNFVPISLYITLEIIRSVQALFIFY
DLDMVYQRLNMACVPRSWNISDDVGQIEYIFSDKTGTLTQNVMEFKKCSINGVKYGEAFTEAQFGM
QRREGVDVEAVAAKAQQVIAQGKVRSLELLRKIHDNPYLIDDNLTFISPDFAMDLSGQSNDTTQKQ
AIESFMIALALCHTVITEHTPGDPPQIEFRAQSPDEAALVATARDCGFTVLGRNGDDLIVNVLGEE
RAYTVLNLLEFNSTRKRMSAIIRMPDGTIRLFCKGADSIIYKRLARGQQQELRKTTADHLEEFARE
GLRTLCIAERVLSEEEYQVWNESHDLAAAALVDRDDKLEEVANVIEQELMLLGGTAIEDRLQDGVP
DTISLLANAGIKLWVLTGDKVETAINIGFSCNLLNNNMELIVFNVPADKPEAAASELQRYLDKFGI
QGTDEELIAARQDHTPPAATHALIIDGDTLKLMLDEDLKQKFLLLCKRCKSVLCCRVSPAQKAAVV
QMVKNGLNIMALSIGDGANDVAMIQEADVGVGIAGEEGRQAVMSSDYAIGQFRFLRRLLLVHGRWS
YRRLGEATANFFYKTLVWTFALFWYSIYNSFDGSYLFDYTYIIILINLAFTSLPVIFMGIFDQDVSD
KVSLEVPQLYMRGIERKEWSQRKFWLYMFDGLYQSLMCFFMPYLLYNQARFQRGDGLSLDDRQQFG
ILVASAAVISSNTYVLMNTYRWDWLTVLINAVSSLLLYFWTGVWTATTASAQFYNHAAEVYGTLAY
WTVLFVTVVLCLLPRFAIKAFQKVFFPTDVDIIREQVTQGIYRRVEKTDNDAFGSSQANAGGSPAG
SDVSAVSSVVKPIEPQVSRDPRVLEDERPIYPPSIAPTNTTHNPRSQNGSNGTTFTADSFDFGTKH
PAVNSWEQPEAGLTIHHGQNPSVTSTDFSNGTPLLRIESANGKPQTPHSPLRIPHDIHAV
>g954
MVSVIRISLSILALALLYPFLAERARVVRLLVDNAPHKFTAVNTFAASSVKFADRIRNCEDGLMID
EDHVAIFSCDPGRDVWNTVMGTFTPDLSTIPHGNLHLYRYSQKDASDEEAKLTTIELRDFPGAKEF
HPLGIEYHRPTSTLYVCNHGLNGSRIEIFALDFSDSSKTPVATYQRTIISPLIRSPNAITVLSEHE
LYVSSDHYFLKRYNPVLAWIETYLGLPLGGVVYVNLLTNEFRTVARVPFSNGITLLNETTVAVAST
STAAVRFYEMSLDRSLQQTGAVNLDFMPDNLSIDKDGVLLITGHPHPPTLDKMRVERLGCLLNSTV
GNPDCVDGKAPTKVVEWSADGNIKTLYATTSEIVGGCTSLRDTVYNIGITLGLYGKGVLIWNQ
>g955
MSQPRTIKDYFKRPAYAPPPTVNLPALSQPSSPLSDPPSDLPSHFSSSQQLPDPLERQTNSSAHDV
RSPNRTVPSYTSDFTSFRPSSSLNASFNSSQRIVKNGKEVVIDTDAESASSEELEDVDELLNKFLG
RAPSSTSTKIAESRDLKPKTASKPPNRRKQHEFRNFRAKVGLDEKKYKFSLESLVQRSVDDDEIET
NVAKAKAILETQDQTIPTVNGFSNKSRPSNLRDDILASGLSTDDTGSDFKRLLNAVKRTEALEQEK
SWLFFGDGTKKQGLPEFPRDSILASSREAFLRVSSEPRDELRYAYCRAIKNAKRERIESLIGQTDI
DQLFFSLGGHPTAVDANESIQADVSYYQAETSRNWSFLLSVLQMLRDIAVCLHADTREHTLHILLR
LTLDTAVSHNDMVIASLENTIDYLLQSIPREEFENVVQPIGKTLLKTIKDTQFQSKLLNHILSSNR
EIAIFKCRLAMAFLTKDESLLDNFREEVIPFRQILNYLRTARLDSQLHKARLDPDYDYMEVKAVTS
LLNIVIDTGRTTLVFPNKEAERKFNEVVDSLAEQVKRIFSAMEDSGASHLKRTEAKQGLEALQYRI
LYSIRTKRRVKSMFGYSSPIKKLGDYAGFKNTVEQS
>g956
MSQRHALSDDQVAGELRKMTAFIKQEALEKSKEIHIKADEEFAIEKSKLVRQEIAAIDALYEKKFK
QAAMSQQITRSTLSNRTRLRVLSARQELLDELFQRAREEVTTSTKDAKKYQSILANLILEGLYYLN
EDKVAVQARKKDNAVVKKAIEDAQKEFKEKVGRDVTIDLDESDPLPDGSAGGVSIVGGGGKIDINN
TFEERLRLLEIDALPAVRETLFGKNANRKFYD
>g957
MGLLGDISPEGSIAIGVIVGVISTSLQAIGLTLQRKSHLLEDEKAPYEVRRPPYKRRRWQLGMLMF
VVANIVGSTIQLTTLPLPVLSTLQASGLVFNTISATLILGETFTKYSFVGTVLVCIGAVLIATFGA
IGEPAHSLDELLELLDQRPFVVWMVMTGLVVLLVLFSSRIIKTISTPGSSKIFRSIHLPRSFISPH
QGKFYRGLAFGFSSGVLSAHTLLLAKSAVELLVRTIVDHVNQFNRWQSWMILIGLVLLALTQLYYM
HLGLKLCSTSVLYPFVFCIYNIVAILDGLIYFRQASQLTGLHAGLIALGTVVLLAGVLCLSWRLEA
NDTHPSLTLPNTAQTPLGAGMGIVEEYAEPEEDEEFMDGERQPLLSKGTDTSHQRAPSLPLIPTYR
YRSHTLDAHTFNNESAQIWAELDDSENEGAGRRRTSTESPSSSTTRKRILQYPHRRTFSVLPSETR

GQRRPSLGDNESHSYFRVSKTRDAKQQADPPSALSRWSPRRLQYQRGYTGSSRRTSAPVSLGKKPG
FGHAPSPIRNNQDELPRTYGATSHSNEQGNTETVVDNNDSDSGTILSAGWKRGVVLLKRWTGISWP
RRNRRDSTHSPV
>g958
MADLSANIVSDEHASPSQDELQQMGNGANVDRKRSRMSAAGDDDDDDDDKPGRERRKIEIKFIQDK
SRRHITFSKRKAGIMKKAYELSVLTGTQVLLLVVSETGLVYTFTTPKLQPLVTKPEGKNLIQACLN
APEPSAENGVDATDVPAESPEDVSHNNVNAQQAHMARQPGIQPGYMTQEQQQQMAYYQGLQQQQAQ
AGGQYPGMPVGGRMPPQHQSTA
>g959
MRVDKILLSYAGANFLFMAGGIILLVGSLLFSQAVNSTPTMNSAPSILLLKMVPEKVALVNAIIVF
ATFLLALPSMIMRDSRLWLQLQGWLVIVSGVFTLVIGLIIWIETLRTRAALNTSWGQQTSAVQSLL
QQRFDCCGYYNSTSPPFVTDATCTTPLVAAEKEGCVGPFSSFVNSTLDAIFTAIFGIVALDMILLI
CVAVLSKDRKEKERYRLIDAKVGLQAI
>g960
MPNDTEIASSEAGAAGAGAPRWRISKACQQCRRRKIRCDGGEPCQHCKQKKLECEYRGFVRQRKRK
HELLSKNEDAADAGETGETDDGEDGDDAAILDMTASASARGKGIAVNRKGSRSISGPSHGAAAEGG
SRRNDLVDYSVAATHVASPSCVIQLYYGPHSNFSLMQLMYRQLVDGLGASESNNTSSSRRDEVEEV
GPGLDLFSFRRLFFGDLAGNQDASLSGGLGSGLNSLFFIHPSTASKYLERFLSTIYYFCPWKSKER
YRQMLNGLYQTNNDMSADNPQTTLLILMLALGAAEMRDDEMVNLPCSDASMLTLDYAHYQSEKARP
NSAFLCLGTATRKALAAGLHKNTRFKVEQTTEDAEEKRLTFWSLFFYDIWICFGLGRSMTIPAHEI
TIPNPKEQPMLLALVEIARIMAXAAHNMYSQRHDSLLLMWKSAKEIRKDAQAFARRVQGVLNFGLD
ASPKTGEVGVCQIVLQLLYHNTLLLTFRPFLVFRARWRRDGIMKQNENPSASSDKSEQSATPPWLD
EACESCLEAARCMIRNLSGACEVNQYVREINYHGFHLETACFTLAFDMMHSTPQAAEKHLPWVRLG
LRCMSIMIPEGEYKGQTYLTMNAVQQMLIAVFPELKTATGPDLSSPDDSNRFDSEVYDRSSLGDNV
SSSLSERNYNLPTRNPMSNDAHFASGQQQQYARQQQIYNPSPTALLDDYNNNIYTNPSTFFTSLGQ
NLMATPVSSTESSANNANTISSADDIGMVDFTHADIPIDWKDFDFSTMDLDTFMSIDPVNSSAANI
NNHAVNYLNTFGGGTGHGPGGWGGNDFGXGFMSAR
>g961
MPFFRLKTLFPLVAILSIGFFFWCVDRYDRAALSSKIKHPIEQISNTANSITSNKSPPAQVQLPSH
SSFGNCEFDTASAPPLPYSEWLIRKNFTRAFIRPNQVSAQTQFRSLEDIDTPVLPPFRALERGLII
SDENNDRTVPCPTVANVNVALDHDVEETSKLLFGLATTVDRLERLLPSLLYSFAGTKASLLVLVPE
SDDNLSAQEAYFRSRGLDITLESSPLEFTARYFGLVEACSLYIKTQRPQTEWVGFIDDDTFFPSLP
SVALQLKQADSSKKHYIGSLSEASWQVDTWGHMAFGGGGIFVSKPLLDILMENYDECQSWGEQPGD
QKLGQCIERSSNTPLTLWPSLHQMDLTGAPDGIFESGLQIDSLHHWSTWFTKDVVKMGAVAAAAGK
RSVLRRWVFDQQEFVDQSTGESYRTFWVLTNGHSIVRYTLMPGTPNDAINFDHVEKTWEEDMVGYT
QRFGAMRERDQDGVIKERYLLTDAIVIGDNVHQFYTREEETSHSLIEIVWLGPKSGSSTTTTGSVA
SAGGSRNPN
>g962
MESLAETPWDVTVSGTGLAQSLLALALSRSGKKVLHVDKNTYYGGPEAAFSIQEAQEWVVSLQKAP
GREPFEDVSIYSPSDASEDGKKLSFSRAYTLSLSPQLVYTRSKLLPTLVSSRVYRQLEFQAVGSWW
IYRKSANPDDDVSGLRRVPSSREDIFADDKISMKAKRMLIRFLRNVXQPQQTDEGTEIPTTEEDKN
MFLSEYLNTKFNAPSELHDPIHSLSLCQQAPHKTPTNLALPRIKRHLGSLGVFGPGFGSLVAKWGG
GAEIAQVGCRALAVGGGVYVLGRGIESVDPSKDNEGFHHLTLSNGEKIRSKSVAGTLWDLPDDAGS
PLISTQKVARSISIVSSPLKGLFPITAEGGPMPAGAVAVIPGEALTDEASEATPVYLLLHSSETGE
CPDGQCVVYGSVSVPGQEGQRLLETAIDLLLDSFSDATGKPAVLWSMRFTQVGTLNDASASPVLYK
STRSDQILYFAPPSLDLAFDDSIVESVRECWKAILGEEAQDDTFMNFEDRETYDDD
>g963
MATDILFDTTMGSFVVELYNDHAPKTCKNFATLAQRGYYNGVIFHRIIPNFMIQGGDPTGTGRGGS
SIYGEKFEDEIHSSLKHTGAGILSMANSGPNTNGSQFFITLAPTPWLDGKHTIFGRIKTGMRVIQR
MGLVKTDAEDRPQDTVKIIKARLIEEGAED
>g964
MADMNSIFESLQPIVEQLKDMQPFLPTYAHLIVSALIPIYTGAHASLSRPSSAAKAEKKSKAETDS

EDEEEDEGKDTGSKMEGLEPSDALMFPLMXGLTLGSLYLIIKWLKDPSILNKILSFYFSQMGLFFA
VAAIVHEKSSDTASPIERTSPLPGFFGAIPLPVTIRNSFWGLRAVAYQKVHLRIKALNSFKAKVRF
DMLDVTAAVTALIAVTYFAFFAKPWWLTNLLGFSFCYGSLQVISPSTFTTGSLILASLFFYDIYFV
FFTPLMVTVATKLDVPIKMVFPRPTGPNEDPNELSLAMLGLGDIVVPGMMIGLALRFDLFLYYKYK
SMMLSKKESSVEGTEKAIYQRATGGWGERFWTRSNSSSFPKPYFYASIVGYISGMVATLVAMQFSQ
HAQPALLYLVPGVLIFLWSTAYFRNELDSMWSFSDMMEDEDPKENTEEDKEKERKKTTTKAGLFSR
IWSGDLKPQLDNVEVKRDDEQATEKSLVEKPSSSEETPEGSPDDSVPPKTDEEDEKCLFSMSISVP
RQKA
>g965
MASSTKTTNPALVGPSSPSLALPPSQFARLQPHAYLLAHLXXPPSSQQQSPLRANGRAPLEFRATS
ANLGSLSHTNGSAVVRIGDTAAVCGVRAEILHTEDIASWGVSTSSGSRKRRKIISDDDEDIKQEKE
NDEEDDDYIKNLNLIVPNISLSTGCAPGFIPSAPPSALAQALSHQMLSMLHTTHLIRSEDLQIWHH
PPDLNALANEEQGDDAMETESVDQSFTPEAEIKGFWVLYIDVMMISLAGNPFDAAWASVVAALQDT
KLPKAWWDIDNDMVLCSDKVSESRKLNLRGFPVSCSYSVFEADAAADWRAVAVPDADENGGVKQTS
QQPERWILADPDGFEESLCRERVYMMVDRDDKNGKTKILGMEKNGGLSVGREDLRKLVDISTQRWK
HVKTIYDGLH
>g966
MPKNEAVMAKMQMNERQEIIHILPKHEKQCWKLQWIEKYIEENDRQTENIAVASAIYLCNLLLVKM
EYEPHSTAAAKFGSILMKHPVLMNKDKLEKKLTPIGSAHFRALDRTKRAAAAFQPRRLGTSRDRNR
HWPSLVVEVGFPNLIESSRKMR
>g967
MAGNMFESLVSLAKRINVPITASTTAAEQAEMQRDPWSQSGKYALGWIYFSVVIVVLTSLVRVYHL
LSDRVRVAAHREINLLSPNGNSAGTSEQELTSAATDSSTRKFFPRTGPLPSSSRQHESSLLRPMSV
VIAVFRWVFYRPIPTLGIGKMRIVFPALSTIVLVSAGLVFITLYCFLPQPLYYTSIALGSPPLAIR
AGMIAVAMIPWITALGTKPNVLAIVTGISHERLNVIHRWLGYLCLFMSLVHMIPFYITPVWSDEGA
YALFSQNLIPQGVQMYIYGTGIAAFVPLAVLCLHSLSFIRSKAYELFAYLHGPIAVVFLGMLIWHT
KNFLLSWNYLWASIAVWFFSYCIRGIYLNWFYPLRMSWLIGEESSVTILPGNAVKVTIPTEMRWRP
GQFVYLRMPGISPLQNHPFTIASLCSDDFPSNYGPEYRDMVLVFRPFGGFTKKVMETAKRKGPYKI
YRSMLDGPYGGMQRELAAFDDVVFFAGGSGITAIASQLLNLIKKMRDGHAVTKTVRVVWALRRPET
MEWFKEELRICREYAPPSSVFCNFYLTGTDKDNAYATETVNEILHGVSNKRNSAWIREAAGGDLQQ
EKELRRENEDDIAPLPNAHLAGSVGSSSYPPQAAYVHYDPYAPYTYGAPPQNVPFGASQAQHQGFN
FGFQQNQSPQQQQYNYDQYQQYSQMMQQTPHXPPQQPHPISTTALNTRNTLTRFAFLPRQKQDSWR
TDYARPNISELLQEQSKSWGRRTCVFVCGPPSMRVEVATTVARLQHIVMKDPSKDEIFLHAENYAI
>g968
MAYQVFAKAQAQQRTAANTTSTTATSPPLSPPSLLAKQPPLHPAPLVLPQSASSSSSHRPPSIGDP
NFTPTPFSEPLITYQTPHPHVTIDPVRTAHIPSLSRITGLLLPIRYPSSFYTACITDPVIASLSRV
AVYHDHPVPSGPSTETTFGATRQSPTSTAGGGAGGLCGTDKVIGGIRCRLERLFPEFAGLNGQDSR
SPPTNLYIQTLHLLSPHRGNGVAASLLNALLFAKPPSVSSKSYRVSSLVRHYNIHTVTAHVHETND
EGLRWYVARGFHVEDGVVKGYYRRLNPGGARIVRLDLDWDNDEQEEEAGKQQQDSENSLQSSDVTK
ATISANEEQSDDEDDDWEKLEPEEDDADDHGVVHLTDSQILDDNSPTSTASLSGKRKRDLEQFQKR
HK
>g969
MAGLDSNKTAETGSRVLTSASFSELSKDILNATFYNIIHDIVAKVHRDEKLARMRSAVVLARQKAE
AEAEAGGVPEKKVQVETEGAICENGKVYLKGNPLVTTKEIVCPFCRLPRLLYPTMGVGARPPPDPS
KEYCTKHPLVSRPGYDVHGNPFATDKTKKKKKNTNTNTPISSPPSTPDANGSSKQNAPDYPTIKCP
NCPRYCQTNRVSAHLDRCMGISGRAAARNREGGSATPTTSGPPKRPFPDDDAAPMNFKKKKPNTPK
KLGTSKPAPPSKLKKAATPDTMLAEEAAEAAEAAEAAESAESFHDPSVDGEEKLES
>g970
MSTTSTKPVLHYLDIGSLGRGEVVRLFLKDAGIDFEDVRYAYDHTWPATSEELKGKGITITGNVPA
LEYNGTILTQHIPTLRYLARELNDYDGQTSLEKWVVDAVADIYIDWRFQWVANLKNASDEYKNNHV
PKYYKIIAHYYSQRGGPFLLGDRVTYTDFAVYQSIENDQKTGTLPETLPSVIETFKKAFESRPRVK
SYLESGRIANVNQ

>g971
MSNSLVTWALPKLSNFLPLDEESLKQIVTYSAGLSKEESAEHLKNLLGDAPPVLEFISSFNSRRAD
SGTSTPQSGATTPRTTRDDAGPSERNHVKKNKRTKAPLHNAGPPRRPENYGNVSGGYKKADLEQDY
MTISGRPASLQPDIGSGYGSSSADSRNPSPAPKSNSTKAPPSAAGPLISDYLPNVKSKTSKAHRPA
GTGSGSGHSTPGKGSVTTTNIADLTSAIAALEVSTNPTSAKKRQKCDCNASIHPLFTPAPNCLSCG
KIICSLEGLQPCSFCGAQLLSSQEIQSMIKELRAERGQEKMRAHNEGVHHTGGPGVPDSGPPSKLD
AAVAHRNKLLAFQAQNAQRTRVVDEAADFETPNIGSTQWMSPAQRALALKKQQKILREMEEKAKPE
WEKKQTVLSLDIKSGRVVRSYHAAPSAAVTPEEEEKDVDDTAMVDAQRNETGATGAAFSKNPLLAS
GGLMRPIWKSRTDGEVGDKNKEVRRTWRRVQDDNDDNEQWILDGGAHGYSNDSGA
>g972
MISEYFGLARFVAAVGIAGIVIATIGYVAYNRFFHPLRHIKGPFLATVTPWVQLYHGIQGDRHLWL
HKLHQQYGSHVRVAPNFVSINTAKGLRDIYGHGKQFKKADFYHGFAAIKGVYNTHNAIDKAIHGKK
RRVLSHAFSDHALKAMEDVMLIHIRQLCAVLGEEPQYMSDVILPNEYKTEKGQIVRNMSNWLGFMT
YDVLGELCFGKSFNLLIDGAKRNMVHLVDRAAYRHYVCGLWMPLDRWGLDKIFIRKLTMDRWNFIM
KSRAEATERAKERVEAGHGAKKDFFYYLLNAKDPETGEGLSMKELWGEANVLMVAGSDTTSTSIVA
TLFYLVRHTAVMEKLKKEVRSSFTDVEQIVSGPQINDLTYLKACIDEAMRLSPAVPGSIPREAMEG
GAEVDGVFLPAGSECGTPAYSIHRSPDYYREPLSYIPERWIEGATCQTRSGRQSWVAAKPEIEVAR
NAFCPFSIGPRGCIGKSMALMEMRITLSRLLFLFDIELADRTGEDADGFLALTDHFVSAKTGPNVL
IRRRQDL
>g973
MTSGISSLTRVDVYPHDFPFSRVVVVDVFLGVQYLLIEEEEEEEEGGGRWWMSAIFLSLTARSSMG
ITTPAASPLLLPAVVTEPEEEEEEEENQLKDGKPGKRDSIHRCQ
>g974
MAATAPSKRKYPLIHSSTDSKPDNAARKRAKNHDARALAVQSADAALSATGELDVAAYVGAREYEI
RALEAGIDKSKGALASRAFQKVPRSLRRRTASHNVKRVPKRLRARAKREMIEDNTPTVTSRRRKPT
KILRIRLETARRLQNLNQKTKEKRAALKTKRDEEAQGKASTEGSHTHKIAPRVPKIKKNKLSQPLP
PASKYKKRQQSKTWLPTHMFHAKRAHMTPPEEPLWRFAVPLTPTEKSYRPTHRAAGARGAIAWDSS
YMSTIQLEGTFFALEIVLKSLRIEGYEYWGSKGRKWRQGTRVLQKWVYEAGEDKKPIAPVTLIWCA
KKPKVEEDQKMSNSGDTSASKKRQARDKLFIRVHPSAFLQLWNTLLQVCKQQNPPVSVQDLRFDIG
SIEITGPGSTEALLATMRPFKSNEPEEKDGDNHSTTWKSLLGVTNPSSLPKGALLSFSISDPRLHY
PVKTLKPSTSESDMNNLATILSSWPPDRYRSSPDLFDRSARLTASRRLPSQKALNRRRTLAGPGVP
LKAQNTDPQIPVMVYASRPESGSNMNNHGTWTVLLPWRCVTPLWYVLMYYPLSSGDKPRFGGIKEQ
RQLAFEYGQPWFPGDYPGTRAGWEWNAREDEERKKEWERRPKGKRIEYDSLDLVQKMQSDIMDETQ
DTESSGLKETLDSKSDTLPQLSIEHWRLDARTASELNTSARTWKFPDDKPETPVQFSANLVEALQS
SKETDSSRAKSLELHIQARVAEELERLRQREKQTLEEIEKRVAAELPDRKVSGLTAPSLSTSFAAP
AGSLDLDAPRIPFAGREFDAPLFPIDPATXAPPQAPSQQTPAVVDPSREAVLKDIERLRTKLESRK
KLSALDENVERAKTEVVNCLRINDRRPLDCWKEVDSFKREVAKLEQAFVDKCHANCTLHSAYPSLA
VFAVTCQSLSPLVNRILIVASLGVAIWRSHRPSLHKRVRHSTTSTILHACLMTAKFSESDSQSASP
REDATWTHLVQSSVPGLGFLFEVTGKPWTAWLGVLGLVFAATIAGLLGFGGGMHHRYANDENYYAD
FLGSSGTTASSCPSSAAATEIIAPDTFYSSFNPLCDAPSACTSSSLTSTCANPLNILSSSLLPSST
SSPAGMIMAAPGPSLDAALRVAREFDLPSDQVQRGVDEFLSQMGEGLAKNGTTLSQIPTYVTSVPN
GTEKGLYLAVDLGGTNFRVCSISLKGDSTFDLTQNKVAIPRDMMVAPSYKELFKFLASQIEAFLEE
HHNDHFEATVRRRRMTGTTDQYRREEIFDLGFTFSFPVDQTSINRGTLIRWTKGFDIPDAVGKDVC
LMLQDAINELRLPVRVAALVNDTVGTLMARSYTAPGKTETLIGAIFGTGTNGAYVEKLDKVTKLDV
KNDKTIDNNKNQMVINTEWGSFDNHLSVLPNTIYDQQLDAESVNPGIQMFEKRVSGMFLGEILRRA
ILALSKDASAKLLQADGSSIPSDSPLYQIWGIDTSFLSIIEADTTSDLSATKAALSEHFKVTNPST
DAAQAIKAIGHAIAKRAARLSAIALAAILLDTKRIDTDETVDIGVDGSLVEFYPKFVDYMREAMRE
IKGIGEKESKVRIGIAKDGSGVGAALIALVAHKGYAIGPR
>g975
MAIAVEQVDTRWPYRAGYPRLLRPEIINADDEEEASPIYKSEYKELESRIDTIIGTYNIPKGSHPP
EVCLRAQKFGMSKLWRVLVDCTYEKGKSDSKMWAKAVTEIHTAAKEVAKEGREVGVELCDRAFERS
SHICTPPDSSSLEANWDVGRDYCHQILQLFENRPTMFQIMTPVGRCSRGWHEYEWTTVVLFEAIDA

EDVAWDFLEERMRALLPDDIGIEIRQRANPLFCFDGAPGIIAYRQLDLKAYVRPPKPGCEFSQHNQ
NTRAGTMGGYIVTEAADTKMRTTFGVTNAHVALGSYKEMSLNTSADYDINFQSPSGSRRIKHETVL
LNEIAKAESRIQTQHEIIESIQEQDPVLFQRLGKSLSINQANLAFLKKDLQLVQSDLHIGSVYKVK
LGHTHEKPHEIHWKENFEDPIIEKDTTCHFWEVDSLGEHIKVVSRLSRSGSYTMGTVSNFQAYCVE
HEEEGRSGEFKGFAWVITCPRRHGNPDEPDARRPFAAPGDSGSFVIAAADWKYDGKENKITMLPSR
EAAVTNDPSQHPFTVGLLFGASDSSDIALFIPFDAVKSEIESMTGEKLVWPQKRTASLKEAKASLP
SWMKDGQDNLCG
>g976
MADIKPPFTAETARKKVKAAQDLWNTQDPERVSKAYTPDTIWRNRDSFLSGTDEVKAFLVKKWQKE
KSYRLRKELFAFTDNRIAVQFWYEYQDSSDGMKWKRCYGLEDWTFDYETGKMRKRQMSGNDLVLGS
DGNGEGRWFVEGVDVDQAEISEQHXIPTIDILPLTVLDSSILDNTCNTKSVYNLQETKKTTKLKWN
DIEPLNTGLRHLVATTDVLAGYAMDNAGNRSAQGRQPLADAAARSNHGPTIITPGVDPTTPRHERL
KSIETTQPQITHNSPVVVADTARLSVATDSPATSNRNSAISTTSTASGKGKRKTHVGPWQLGRTLG
KGATGRVRLAKHAVTGQAAAIKIVSKKSAAMVQSESIAAMDRNASILGDQATRPMPFGIEREVVIM
KLIEHPNVINLYDIWENRGELYLVLEYVEGGELFDYVSTHGPLPEEEAVRLFRQIISGLAYCHRFN
ICHRDLKPENILLDPNHNIKLADFGMAALQPAGHWLNTSCGSPHYAAPEIIYGRRYRGDKADIWSC
GIILFALLTGFLPFDGGDLSNTLHLVKKGNYHIPTWLSVEAADLIQRILQKRPEDRISIQSMFNHP
LLKKYEILHQAMSQHQLGPPPPLSAKDCGPAVPSRADIDLELLRSLQTLWHSANTESLIERLLSLE
PNHEKMFYNALIKFRDEQLENYHGQPLEYSASDYHHISRPPAATRAVAKHSKNGRMQGHSRRRSQF
SILTEASRRSNSGREPGSSASYDPFRASRTPVATSTVQYAKVTIHRDDAIHKTPEDVVVEVEDDAV
PPSSPPRADIIASSSLATSRRDYSKSQGSIAHLLANDKSSNLLTAAPSSVRMSSPVLPAQPAIRDN
FWGEEARKVSHELSQICEEAFNRSSVSTGHTAVSTATTATSMSIHEEAAERSHAGESSEALDKIAD
LPASYTVKELAETRRKLIEHSTKAEAAGLPDYLCEVIAHLDRLIEQDIARSKAKSEFVAENPNRRI
LSEPLPKPVVDTGYLPSISEEIFTPLETSSGYDLHRVDHHATPEPSPRVSQNLETKSTIRVVPKDS
SLPSIDDIKPLTIRKRSAVTGIPAPISRSRHSSADSVIHIRKQGVASEQRTSSDSVATSLRYNSRL
PMVLETISEGPDSRRSSAKSSNEKKWSWFGKHKSYVHDENDQEATLPSAKTAPAGSISIEAPLDTE
QKKIPRKASAEKSRSSFLKIFSKRKIDKTDSDSLTGDSSHDQDDALRQIESNDTALSHVPVPSTNR
TRRLSGANQNWFARFFHVKPAVRIIAFKIPKVKIRKEIYRTLREWKVYGMDGVFLDRAENVVRGRV
CESNYLNLRPVEFSAEFFTVLEHGRHANLSLVRFRQERGAASSFHKVVDTLELVLKRRSFVVEDPV
RSKKMVKILDNVP
>g977
MSLQPGDQFPENVVFSYIPWVEESADIKSCGIPINYNASKEWADKKVVVFALPGAFTPVCSAAHVP
GYIENLPALRAKGVDVVAVLAYNDAYVMSAWGKANGVTGDDILFLSDPEAKFSKSIGWADDEGRTY
RYAIIIDHGKVTFAKKERSKNVLESGSVATIKLPLIISALRNHANLAIRVVLTKSASYFLQGQSAE
QPTIASIAKLPNVEAVYQDEDEMTESWVRGAGILHINLRKWADILVVAPLSANTLAKIVNGISDNL
LTNVIRAWDTSGLVDGGARKRILVAPAMNAAMWLQPITKKQILVLDKEWGIEADAGNLEHQGWFEV
LKPIEKSLACGDVGVGGMMEWTNIVGIIERRLDLIAPTK
>g978
MANPRLLLDIDDALVSDLNPARTDTEGLDYERCARLHNYLVAYGWMAYHQRSPEDLDELLARPKYF
ERQHDDSEISRGRLDAGLISYLESIIMPVPNEGLSYWVADVAVIPADELFFIEENSLDNKERFVIL
YGSWSEHGGHSVGLVYDQQRHQVAMTLCQENIESVSPVDEHLDMWFPLETMLTNWIYMLRIGKVTA
GPQRVWDDDGRSGTSAADQLGPWMWQPYSLAQVDSAVAAIEKLSAAIEARMPSDSLLTITPDTPLL
AHADLDAASVPRDCFIRSVLTKVRTPRFRHIAPGLEVPHDATKFVAQQQFTALQAARNEEGDEIIP
SVLIFAATDKRRTVGFNKEIRRTFVTANDLQPNVPYKDGQPILAGLYSELIRRGDGDIAEEGFRLL
LPFGLRSNFNEDQYAVRRSDGSLVDAGSHTQLFQFGAFHPFGGEWRAQRLEKLMDRWRELVENGIW
SVGSDGVQGSIDVFADAEVGAWRDYWIEPEW
>g979
MAAVHSQVQGSQDPYHRLPPPPAPGPYQRPPDMYAQPPPGSQVVYQAAAPRQRTAIACRYCRRRKT
YRFVVPVSRAPLMDAAPTVFDSTRNACSHRCPRKRKPSFPPMQPTLTCCVRKPKEGAVVAIPAITS
CYMELMANLSRHNSKRCLRAHFLHHKVLIIKLPMAVLLIWMIEFRLLLYSTMCLTINASREVDEAQ
VQASSIPSPSTSRQSPQALKHQVSLQHPTIMARHRIDVRLHNLMPTIVQAIHQCQPMELRLQLRLQ
ALHVED

>g980
MPRRSTRLSLGERPATEVDTKRKRLSAGSTMKSTAKKSKYFEGSGTDESASDVDGDSGSSYEEEEA
AHEDEDSVDEEEAFEDEYEEEKPKKKGRGRLSSSAKTKQQDSGNDAEDSPVEKMAKGKELWREGXK
TGLGPGKEVFIKKPKARDLGGIDYKDDTIHPNTMLFLKDLKENNERQWLKAHDADYRASKKDWDTF
VETLTEKIVEKDETIPELPAKDLVSPVRPFGYSPSDPNDRQVFRIHRDVRFSNDPTPYKTHFSAAW
SRTGRKGPYAAYYVHLQPGHCFIGAGLWMPEGSKLALLRRDVDRNSTRLKSVLRNADLRREFFKGI
PDDERKAVKAFISHNQESALKTKPKVS
>g981
MDAPSQYGDATVTPGFVARKSKILSDLSVPDAEYTDLSPKGSVDEGIRDLIKDINALHGLVTTSSC
AGRVSIFVEGSKKTIRKQKTEVTSQATEQAAGQVSNQIDNGEINGQSQNENEDEDGLANKEGDAGW
KFALSGGKGEGRWLYVSHDPVDNSIKSSYHELFGLVPGNGIPKSSTFGDASMRLIRFHYDPMILHV
MAATLYHAHPVLAAASHAGFRESGLQSLRCLEVLHSGGPGQHSSSTDHASHSPIVAVRSAGLALES
VIGYCEDSNDDSEPLLRSLVTEEYLRMLVALANERFEVNKERVQRFRSRLLDLYKAALDTAPGTAQ
RKKPSDWEDPQVRKERKRLEGLKRQAEKAQQKDNDSRPDDTDVDMNISF
>g982
MYHQVPGFSAGRGPFQAKGQSRYGAGPYMPPSMRNQVFASGSQFTQANHQQHHAQPQVQLQQQVHS
PLPTQINHRQLYGHQMEQFLNEQRKQIQQQMEQRMAVQRKIDQQNMDKSEISKVQARLGAGACDTQ
NFPFKNMAAEQQPKDYGVVKISNLKLPYAVTRHEIHQLMGRHAGIVGPDLGGGIHIIMERSTAKTM
DAFVEFNTQKDAEAAARRLSFTESGRYPRLGTRHVDIALSSQDELMHDLFPRAKCVEWKNGMPQLL
PNNDPYSAGFQGFLTSEEIRGLVSHALVPARSPFTERCRQRTYECLISTLWKFPWFATSMYTVADR
DELYQAAIQQILELSEKVKQRRIIGLNESLLVDLLRAGFKCPSFNERQRFCLIAAAEMPAFSPVVH
RNHFQYWPFDCLVKSNSASEYEVMYYAQLTRKALGTCDKDNLIVRNNWRNRAGLMKESPWGHLWVE
WNFHDKTTIFEIAAKLEIQLASDLIRVALHAEVQNATRPFAGPMILSSPISTQHSLIHQSITTLDP
WAKEVTKREVSNHDSPSPESCDQKILSNSNSDDESLVSALRGVRLRSGTSSTLSGAVSQFGSTNGS
LVTTESGIYGVSLENKPKYRHQHSQSFSAAVPLTVKPDLKGLVPARRRESISIPPTIQSISGLSDN
HDHSLLSRSASRMSQAAALFPSKVSADQDVKSSDSHNVNTLMLQHGIWSTSASDRVPLTTPSKNAF
ASAEGDIVSRSERSSRHASNLSVSSQQRGLTILSEDEEDVFSQGTPIKS
>g983
MPPELPATTSPLASIIQCYDLAQHLNGRVELVQDIPLADYHYGKTECIHIPNLDTKLTIQSQSLRP
YLQCVRSSLTAALSVSNFASQTSERHNVPEIEAASSPEVLLNPLTVARNESERVLIEPSVNSVRVS
IRIKQADEIENILVHKFTRFLTQRAESFFILRRKPVRGYDISFLITNFHTEAMLKHKLVDFIIQFM
EEVDKEISEMKLFLNARARFVAESFLTPVSI
>g984
MATIPVIVKHQGKRYDVELDPSSNGETFKFQLYSLTGVEPERQKILVKGGQLKDETELSTLKAKSG
QTFMMMGTPSGADSSAILARPKEAVKFVEDMTEAEAARAEGATPAGLQNLGNTCYLNSTLQTLRVI
PELQQELSRYNSTGSDGTSRLGDLSTLGLGGLGASSDLTGSLRDLYKQMSETQEGFPPLMFLNVLR
NTFPQFAQKAQNGHGYAQQDAEEAWSQILTQLRQRLKVKDGESETSFVDKYMSGRFESTLECDEPA
AKEAGEEPVSSSDVFLKLDCHIDKDINHLGDGIKAGLQEKIEKRSAVLDRDAIYTKKSSIARLPKY
LTVHFVRFFWKRESQKKAKIMRKVTFPAELDVVEFCTDELKKRLIPVRDKVREIRKDEEDAERARK
RQKLAHRQEEDRKADGESGQPAEPLQKKKKAEEAETSKGSDKDGDTAMDEVFKSDEEYEAERRAAI
LKAKKELFELIDPSLATDDGSNQSGLYELRGVITHQGASADSGHYTAYVKKQGRLVDDARAPNGKR
REDDGKWWWFNDDKVTEVDAEKIETLSGGGESHSALILLYRAIDLPSKDELES
>g985
MFHTSRTQHNALYFLHGVPRSFRRHVVPTQLTSTPLRHNTTVALETRRVPRFKWTRRIFWGLLFGG
LGYFSTDSSLKRFFHPAEPGSKEDAAKLGYLRKWVDRLPRVQELRLRPNYEEWDAYEDTTDKERRL
TSGALKGSRALAVQKIFWNKAENACINVVQFGNSTDGWPTVVHGGAIATVLDEQLGRVAILSVPAH
TGVTAHLDINYHRPVSSGQFYTITATLDKERSTDRKSYVTAVVFDQEENIYCTASALFVVPKNIPL
KPLERF
>g986
MDTKEMSPSFSHGTTNSIHSSPKSNLEAIPIVEVVGDSSSEAPMTMKKFLGLTAMAFLWTGSQIPI
YLFGGIPPYIYASIGGADRWIWFVLANLLALAGICPFVGSLSDLLGRRYVAIIGATLVCLGLIVSS
TANTMNTFIGGMAISGAGAGINELTALSATAEMAPTRKRGTYVAALILTILPWSASVMWAQLIAYH

SNWRYVGLLAGIWNGIGLICTVFFYFPPKRDNSQGLSKKEIISRIDFVGGFLSIAGLILFLAGMQW
GGYQYSWKTAHVLVPLILGFFILVAFGFWEAYGARYPMIPKKIMQEPRTLGLTLLITWISGANFIS
VILFWPTQSFNQFGHDPVGVGLRSLPVGYGILAGACITLCLLSYMKGQIKALMIGSSVLMTAGCGA
MAVARTDNLSTLWIILIIGSLGIGGIVVPASIMTTIICPDDLIATIAALTLSIRVVGGAIGYTIYF
NIFINKFTTNSVHYIGGLMATELGITNVTYITEAIELTTASLLEELQTIPGIAGNTTAYEMVVAAG
QVAYAESYKYVYLASIAFGALSIGASLFLDDITKYMDDHVAVQYH
>g987
MSSPADSKAPTSAFESPTFGEDSSFHVEQPVGSASISPCGRDVVLASREGLHIIDLDSPYSPPRYL
PHHTPWEVADVQWSPFSHRDSWVVSTSNQKALVWNLSMKSWENSIEYVLHAHSRAITDINFSAHHA
EILATCAVDSFVHCWDLRTPSRPVVSFSDWFAGATQVKWNRQDPHVVASSHDKYLRIWDDRMGAIP
LKSIEAHSTKIYGVDWDRTRREGIVTCSLDKTIKVWNSEGEADVPEKVIETRFPVWRARNTPFGCG
ILAMPQRGDNDLHLYGLSSTEDPSVHTMPLVHSFPGHKGQVKEFLWRPRGNVVNGLDHREFQLVSW
GADRELRLHRVDADILRDVGYENGKTVDYSMRFTRQGATYKTFRDEPNDENLEDSLYAETVISAGT
RPQVGAGVGTNNVSTRYSRVINHKIHIESRIGMQGRTQIRANTNPIAWMRGVKISVWDVETLGDEI
THVGEKFSKVDFETVDVRQRKVTISLHGPWAAEGTSLFLKLDIKFPEDYPRSAIPTFKIQKTSAMT
NELSEKIVSELRTISETYMSRKRGCLEGALRYLLGESSLEESIALVLGETIDTIKSPVAGLEDDES
SDEDEDVGQFDKQDLSMSSELLRPINANVMVPVAKVCGATWSHDGRLVCFFPPKKDKPGSLFDSLG
FKDISRLTRNDRVFEGFGRLHTSSPGRLPTRKALGTMSTTDDGGSDYSDDSDTASSSSSDSSGMIL
GLPNRYQTASMWRSVGTLGTYRTRSADNSQLSTVGGPAVTIGTVKSSDSAHNIIAIHDFSDLLPAK
RALAGQYKIFGRGADVCAHNAAVAADSGYHHLAQVWGLLKLVLQNHGDSRSQHINTDVLAGRLGGQ
VAYNSIKTKQASKGVLHRFGFNNSAFGGRWLINALFDHFEQVGDVQMLAMLSCILHEQSLRNRFGR
RRDKEVSFSQQAHSPIALDFFSSKVPDSKSQAATPMTSYSRDSVNISALQSPEIQAEQWNSMGTTP
HSSSASPIATHSRLLGGKTPALSISASPDSHGPSRLGSGVGTALASSLSRSFTFGPSAASSPPTTS
AKKKTSPIGSLNIPAAVGWSASQLLNRAVSAIPEYLNSSMVITSRSHSEADSEKKKDARKPMRLHV
SHKNRTAFDADDSPASPFIDRKREALFQTYRNSYANLLFVWELPIQRSEWDNVKIKHPSSCRHSKL
VVKKIRQVILSMSNDTVLSAGMPSSDLFSLLLSQKILVAKW
>g988
MAGLPKRIIKEXERLMXEPVPGINAVPHEDNLRYFDVTIHGPAQSPYEGGIFRLELFLPDDYPMTP
PKIRFLTKIYHPNIDRLGRICLDVLKNNWSPALQIRTILLSIQALLGAPNPDDPLANDVAQRWKED
EPAAIQTAKEWTRTHAMA
>g989
MRTYDDSFSGQKIYPGKGKLYVRGDSKIFRFQNGKSESLFLQRKNPRRISWTVLYRRQHRKGISEE
VAKKRXRRVVKSQRAIVGASLDVIKERRAQRPEARAAARQQAIKDAKEKKAASESKKKAEKAKSAA
SASAGKNRIQSKQGAKGAPIKVQAKSR
>g990
MTTDLYSVPASNGIESTNLLRTTDETDATATTNNNTHNASNGIIQKLSQQLENVTTKDNAEEEEPL
FSPSLIFPEILSILPEGYTARPLRRSDFHLGYLDVLRVLTTVGEISESMWNERYEYLFKRNDEYYM
IVICDGTGKIVGTGSLIVERKFIHALGLVGHIEDIAVAQDQQGKKLGLRIIQTLDYVAEKVGCYKT
ILDCSEVNEGFYIKCGFKRAGLEMAHYY
>g991
MAANTRIETDAFGEIEVADDKYWGAQTQRSLGNFKINQPQDRMPPPVVKAFGILKGAAAAVNMKYG
LDPKVGEAIQQAAAEVAEGKLLDHFPLVVWQTGSGTQSNMNANEVISNRAIEILGGQKGSKKPVHP
NDHVNMSASSNDSFPTVMHIAAVLELEGTLLPSLKSLRDALQAKVEAFNHIIKIGRTHLQDATPLT
LGQEFSGYVAQLDSNIKRIENTIPDLRLLAQGGTAVGTGXNTFKGFDAEVAAEITKLAGTEFKTAP
NKFEVLAAHDAITEASGSLNTLATSLFKIAQDIRYLGSGPRCGLGELRLPENEPGSSIMPGKVNPT
QCESLTMVCAQVMGNHVATTISGMNGQFELNVFKPVMIRNLLHSIRILADGMASFEKNLVHGLEAD
EKRIDSLLHESLMLVTCLNPIIGYDMASKVAKNAHKKGLTLKESAMELKALSEEDFDKYVRPELMI
SPKEKK
>g992
MYNKRKSNISTPVRIGVESQWALSSVTVAVCPEIVEIDPSAKDILLAITHPYRIMSTTNSSLTFGS
NYAILNLDLMAILINTVSDTPQGQVMIANCTKWNDAVHLKTPRPLTIFSTLSFSPSQHEVASDSPF
SRLIGPFGDFRSGSSEVQICDVFQVDEKDFIMQKTRWSATTGSVLEQILKAQNIKAVVICGLSLSG

VAMSTIYRLFDLDYEIYVIRDNVLELPVEQTEAVASMMLDILLPKMGLHVITLAEALRALGNS
>g993
MKSPEPGINEPPYSIFDKKQKWLIIIIVSTAATFSGFASNIYFPALPTIASDLNVSLELVNLTVTS
YLIFQGLAPSIWGPISDVKGRRTAYICTFLVFLGACIGLAETTNYATLIALRCLQSTGSASTIAIG
SGVIKDITTRANRGGYMGIFQAGLLVPVAIGPVIGGAIAGSLGWNAIFWMLTIYSGVFLCLLVILL
PETLRSIVKNGSQLPSNLLIRYPLKFYQTTTQVDWQRRASDNQHAPKKHVDVLGPLRILLSNHAAP
IIIFLAVYYAGWQMSITAMSSLFKTQYGLTEIQIGLTFIANGVGSMVGTLITGKILDKDYHRVKEK
YEASLDIERRESSQEEDFPLEKARLRLVPTFSLLQCLSILLFGWTIQSAAASASLNLARCLFAAGG
PLIQYKFAAGWRRKNREKAEQRRRERTSDM
>g994
MPIPKDKKIIIIGAGVFGLSTALELSKRGYNNITILDRHVPPVVDGSSVDISRIIRFDYADPLYAR
MAKEAYQGWQKEYSDFFYQSGFVMLAKDASNVYVNKSMENVRSQGGQVDVFHNANDVKKLYAGITS
DFSGLYATHNASGGWADAEKSIQRLASECSLAGVSFITGPQGTVHSLRQEGSRIVGVNTVNGGTIL
ASQVILCTGAWSNRLLNLTHTASASGQPVGFIKLTPDEARELENTPVMINMTTGVFCFPPTPGTNI
LKLARHGHGYATEAVSETTGRVISSPRREGSNAKSGYLPDDADEALRDGLRQFFPKLEGRAWMRRR
LCWYTDTPTGDFIIDHHSALDGLFVATGGAGQ
>g995
MLIQDQSAKSTPKASSFSNLPDAPVDEIFSLNHAYVADPNPNKVNLSLGVYRTEEGQPWPLASVEQ
VEQELYTQNDPARHEYLPIEGDSVFLGHARDLMFGFNKRNADVGKELEAKKRIASVQSISGTGANH
LGAAFLAHHFKPKHVWVSIPTWANHHTIWELQGIERKGYPYFDAATCAFNFDGAIAALENEAVEGD
VILLHACAHNPTGVDPSKEQWKKIAELCQRKRLFPFFDSAYQGFASGSADEDAWAVRYFFNLSPPL
EMCVAQSFSKNFGLYGHRTGAFHLVTNGANPTETELVRKNLCHIIRGEYSMGPRYGSTIVKRVLGD
ETLRAQWQDDLTVMSSRIKRMRQALYDELKRLNTPGSWEHIINQNGMFSYTGLTLKQVLTLRDEFH
IYLLKSGRASVSGFSEKNVAYVAQAIDNVVRRDIQG
>g996
MSLPSPRSWRLEYSVSNKKFLIEQLGVTTNTTIHEDISVETLWQVLKHVMDTKKVISGKGSIPFWG
GFLGYFSYEMGLAGLSHPKTSPIVASHVEDSGSTQKNESSVPDTSLLWVERSIVIDNVLQTAFVQS
TREIDNQAGGWLDTIYQKIEQFQSRASQVVDIRHKSAALEQYSLLTSAERELVDQLLQGSTVKIPD
ETRYKSQIAECQEELKAGESYELCLTGETTITLPAISDARLRKLRPWLIYKKLKTYNPAAFSAYGS
LGKVKIASSSPECFLIWDRTSTLEMKPMKGTVRKSPGMTLEKAKEILATTKEMAENLMIADLIRHD
LYGICGSGNVHVEKLLDVEDYGRVYSMVTHVKGIVDRTTAKDTKHDSMAAYGLTTLQRTLPPGSMT
GAPKERSCLHLDRIEGWKRGIYSGVMGFLDLGGSGSFSVLIRTAFSCSDHEDAASATKPEVWRLGA
GGAVTILSTPQGEWDEMITKLRTVVSIFEPAK
>g997
MAHSIDFNALKARTMGSSNDEEAVTVDTRVPLPNSADQTATIKHVISHHTLRRLMISNNGHAFTEK
DWARLKRIADGNPDETKIGAFGVGFYSVFDDCEEPFVSSGNAAMAFYWKGNSLFTRRLELSGSDAS
TDTTFVLDYRNNSSPVPSLLELAKFLATSLTFVGLENIDLWVDNWKLLELTKKVAPSEKVTIPRDV
ETKTTEGLMKVTSVTREVAQVDAAWMKIIEWSPQTTLAARLEGMRDTTTTLRSFFSKFTGTSITSD
KPPTDELERATRKPPPKRTTIAILTPSYSTDDSSKNSKILSSVLPSKSGRIFIGFPTHQTTGLNAH
ISAPSVIPTVERESIDLNTRGPKVRKDDIMPFISEAIHTANQFSFRESTPSATLGQTIEDSFWMCN
KNASIEILSTCGVLPSHHVRIAPKDLSFMDGIPSLPDALVETAKDFIKKLIDVGLVTEVTVSDIKR
ELENSALTSAQLVEFLSWIGKRAASGELDQKTTLSLLSAAVANEEPSEGQPGRLLTLNGIDLIPFK
YTKSLEKRQLDAIGWXELQIVPWLRWLVENAGNRNVLSVEKDLTHAASFAAQILPVLSKQWDLLSQ
SSRQTVIDLLQPQTVIPVKKGMKRPEEAYFPSDKFLAALGVRKTVELGVIFERLLNEPDPTVSTES
VKPKWSHVDLIKYLTSICLAENSSSRKRKEAQFLSTLGLKRHPSAIEVIEIMAKAALAEDTALQAK
SMSYFITEYHANKYNQVNMEVVTIPFLPTEGNKKLSAPKDCFTHEGAALFGFDILRRDLHPHSTIF
GVREHPPVMESVFAYMAVRIPELKGADTTRLGDAAIIPIPNGTTEDFKDIFDFVHFGQMGNLFLQA
VGSKQEPTKVEVAQMLVKEPARISSRFQSPEKYLNLLRSLADNLSTLKRHKDLFREMMKAPFLLAN
EASDIFEDDEQEHIFAAPQEESLEKFYAALGTPILSTLVEERANWGAKSSDQTSAAKLQKIISERT
RLFLHEHSPDSVKHDAKWLEKHLGIVVVHSISLRRSLKNMGATHSQKRHAIVTSQGSDRTLWISPG
KYDFYEISQALVHILLARPKLHSILTLEMLLKTDLYELRARGYNVERILKKKQQEARMAEDLRQKE
LEEERRRIQEREAERLKLQSQREVEDRNSQQEQHAMPGVFPDSPSNQRHGEEEHTGIDQDFNPRGL

FSNLSKRFGLDDNNRGQNPFQSLFKNRTTQGDGGPGPSSEVSAPPPYSETDPRGQNQGSSSNPNEP
KPVTSPIQLHNNLLSAISACRPHGESGVYSRPATNQVSETKSYCDERPSHDLEYVATLPSRLHFLS
AKSVADRSAFLAENSAGINAFAKVLVECASVFSVRLESISVFYDPAGKTIAFNRSGSLFCNYLFFK
QLHQARLSQDPIGGRADALVYWWVILCHELAHNLVEDHSSDHSFYTEGFVTQYFSKVAEKLLTNTP
QPS
>g998
MGLDLFSPVAPVRLNILLLPVGRIKRSRFLSFATRLQAEKLVRLGDISPDERPNRNMFTPLAFPNG
LILYNISFSVPPTSHLELFPFEIFREPLVVIAIADGAELQHDQDPEKETNAKRNSGVDIPPNPSGL
EDLKRELSWVKEQNPRALVNQLLIFDYEGLEKSISGPDDVIWVPAPQFSRPTTIKTVLCDITSLLL
GEMDGFAKLMQSIPAIDSPKASSWGPRRGPELRPRPIDKLMHRMTMPAQFPVSNGDSQSATSSGRS
SPAPGSHDSPTTFDEITRSIQVSNRSATGTQSKESSRDRMSIQGLSAADRTKNRIKGRLGVVVGTL
YLQTGRWPDALKELVEAATMARASSDYLWHGKALESILLCLLMLGWAGMDFQVPQICYPVAEKSSS
KSSHSTPVHSVTDVVGSGQSAAGNRLISLQNLTNLLPDLCNNILHLYNRAAVITDEPLPPLVFSET
VIRLSRVLCAIRLRSGTLDDNALRHIVMNETLTPSHTEWPRGAPVLKKNDIATFLFRAMPISLGVE
LPITDAAPILVGISSVLSILDLPRKKALVLRELILLMVPGLVEARKIGAAEIGIHPAAGLSTLSEA
AFEINALDVGRGDMGESTRALLTMIGEIYGVQPSIYQEHKKSELSSRPSSAKGSGMNEKEYDSIAS
IVERAFRHVALDGYGDLSLKIAILRVSINLCEALPDFEGVLQYTVELLQTIRGHLMLPEFGRKPPI
LSSDEQVRLLNTIKRTVGAAHKLGATHLESEYWDDFLVRGVTLLEFPEFKHYKTSEALMIAKELAS
FKVTLQNPYDFELEIESLQLQGDNTTLDARTYNIIVPPLSLYDVMVPAIINEVVKVIHPQPSVVVH
STSLSQSALMLLEGETRTFDITLHNVSSCAVGFIFFTFQDSTTRQLQNALSNKDLLPAEIYELDLQ
LSSKPPLRWVRHGSKPDDVTIEAGEKATFTIEVFGKPGLQDAAVQIDYCHLGDKPDSIPEYFYTRQ
LSIPITVTVNASVEVARCDLVPLSGDISFFNNQVKDSSMSSQVDKVYSSPLLANHTAQITSTLSHL
SSDSHCLAVLDLRNAWPNPLSVALDVFREKDQDESWVTVKGTIQPGQISRFILVLPRIFLDNPHAA
IPILHSVRRQFVVSANKLTFEAEAATREAFWFREELLRRLRGTWREESTGREGSVDFRSVRFTPRM
VDAMRLEDLEVTFALTSSDDQQSVAQVKSSKYTAQTNGFLELTVEIQNRSSRPVHPILRLQPGLCN
QPSTIALDLSKRLAWTGTLQRALPVVPGGGKTAASLGLIFFCRGEYEIGASVEEVRLMKPVTGSKD
TSHVTRDIDEIFIPDTFPADGGARKRRIWHAKDHCVIAAYD
>g999
MPPFKPRDRKQRARRQDGKSTAAPVDTNVTEIIPVSKAEKEAKRQQLREQLREQQPKMSSKKKKRL
DKYIDTKLRKDENLEFMKKLAETKLDLSEFRSIKDMSKKERKRQGNVDSTLQSLQAGLSGDESDEL
EAGLSEKNASAPDIPKPAPQVQVGSGLKRPLDLGADGFPILKKRKRTKKAQPVVQEVTWEGFDSGE
EEADDMKDDDVSDVDEVSDSEGRKRNKLDENENENENDSSHGEDKETSDASDDDEDDSDEDDDDEE
ETDIRIRPRNSAFKSWATQQINEAVGFKPSSDLPLSEQLPASSEIFLKAGKLPRNTVYEEEPLPLE
LQVTTGDPNRKAFTVNVERREEIQEARLKLPVVGEEQKIMEAIYNNPSIVIWGATGSGKTTQLPQF
LFEAGYGHPDGPNPGMIAVTQPRRVAAVSMSKRVGDELGQCSDRVSYQIRFDTNVSSNTAIKFMTD
GILLREIAKDFSLSNRIVDLRESMRKEDPSVKPLKMHLFRHGTPPLVQAEGRQYPVTIHFARKTRR
DYVEEAFRKVSKGHRKLPPGAMLVFLTGQNEIKQLSKRLKQAFKPTQREDMELGDTGAGPQGGDDS
DVDIIGLDDDEDSEDEDFDIGEESQLKVFETPPEGSRLIVLATNVAETSLTIPGIKYVFDCGRSKE
KHKASASQRAGRAGRTGPGHCYRLYSSAIYEADFEEYTEPEILRTPIEGVVLSMKSMGLHNVINFP
FPTPPSRQGLAKAEKLLRYLGALKADGQVTEIGRKLSLYPLSPRYGKMLQIVGDLFIQESELDLTL
VKQNKDMEKIYTNADRLEDTQRESRRKDFNRVQRLLSKHDDTSDALKYLSAICAYAYSSGSPDEEE
SFCEQMFLRGKGLKEASQLRGQLTEIVRTNNPGLLGPYEARLPEPTAKQVKALKQIVTAGFIDNIA
IRADTCPDPPEMPRKPRRAIDVPYYTLFKSREGRAAEISEKARSTASRVPISALANGTPLLEYGKP
IGKVESLGGVPERRQCVVVPSLVGEAGTTGWPLPAKKVVQVKDSKEGWIVEKFL
>g1000
MSSNVGLSTPRGSGTSGYVQRNSAFLKPRNAGYGAPYPPVSSSDDKGLGNFRQRKPDQKILDHDRK
RAIELQVLEERDRLEEENDELEKDKKTVLTEEEIDERCDALRARLLKELEEGGEKSGSRRQQQRGS
GGGGQYEKDRRQLKSYQVHELAEAKIQESERLRKALGIKEDWEKKDAGSATKEKEKVERREEGEGG
RDRERDERSRRR
>g1001
MASTMATNRAAVHNNVRPNRIGTSTLNGDRTNSTHNAGMANGFPASKDRWNNNIWSRNTFGSDSES
EGTVTRENAFQGKSGSSSLLATSESDGWAGRASLPWNTVGSASTLNSIKTSPIEARSGDRSAPASA

GSGDNNSYFSLPRSAAIGQNGSTGASKAYLGSNGENYSVPATEGLSLSNFGNFRNDEHSVQAQFPS
FSNNQTTGGFQRKSSLVPNTQRSEEVLVSMGASAFSHVGSESISAXSTRHVGSNNYPHSAYQSYQA
EAQNLGSRLSSGQIDIGAGLNQLHLNDNGYQNAHRNAYGQQDSFDISSGNRFNHVQTDDVNYQDYV
SEFSNDGPLAPYQSLSRLGERDQSPGDYGRGLGNSFYSSARTPPVAASQLRPSSGHRMSAQISDGQ
AALLDRRLRGLQQDQDFSHAAGTAIQQRISIPNGYDLSGYSTARLNSFPQYLQMPYNNLPPSIVQR
VSHREPDPSQVVRSPLLEEFRTHNKSNKRYELKDIYNHIVEFSGDQHGSRFIQQKLESANSDEKDQ
VFREIQPNCLQLMTDVFGNYVVQKLFEHGNQSQKRILANQMKSHILALSTQMYGCRVVQKALEHIL
TDQQAAMVKELDQHVMKCVRDQNGNHVIQKAIERVPTQHIRFIIDAFKGNVNKLATHPYGCRVIQR
MLEHCETADRESILAELHVCTESLIPDQFGNYVIQHVIENGEEKDRSVMIKSVVKNVHNFSKHKFA
SNVVEKSIEFGEESQRREIIRLLTVHNDRGESPLLALMRDQYGNYVIQKVLGQVKGSEREMIIDEI
KPLLSQLKKFSYGKQIMAIEKLIVDPNAPTVAANSSTTPPASHKSSPQPSRRSLADMRPLGGVAPP
XPPPTDTQSLHEGSATDGSVDGPIETSKGLATTSIAAIPDSTTTTHQTGAEVTVAVSVPSESRA
>g1002
MANMRVLARDDSDGSLTPKTINLLISLLVLILLAIVLSGSLLLLRQRRRKLEQQSQLPLYKSQAHQ
RRLTITQNSRTGSIHVYNEKQDLTQSSLSPPQSPVPEIRITFPEEHDEQTGKPKAGRVVVVHISEN
GGVGLSPAHDELPPYQTNESGRFQSLDLERMGGLKEKEERWS
>g1003
MLANKPQKSPGSHAARSSDQPKANSSDSLQSSQSPQNEMRGNEGKAGLTTSSQPSKAEITKALERD
FKWKHLGAVGILLWICFLHIVGVYFFTKGFLLTRLVLDNKSTCDVVPLDVVATSGDSEKGCWHPKT
FDKAVFLIIDALRYDFTVPFHPQFEGDEARIYHNNIPVLYETAVQYPERAILLPFIADPPTTTLQR
LKGLTTGTLPTFIDAGSNFDGSAIDEDNIVSQLRAAGKNLVHLGDDTWDKLFPDYFDSELSHPFDS
FNVWDLHTVDNGVNDNLFPLLHPTNSSKWDVVFGHYLGVDHAGHRYGPDHAAMAAKLQQMDQVIRE
VIDKLADDTLLIVIGDHGMDSKGDHGGESDDEVEAALWMYSKKGIFGRTNEDHKMPPATAKERAVP
QIDIVPTLSLLMGIPIPFNNLGSPIEEAFAGPYGADIKNLAIVNRITSGQIKRYQHHYSVARGVND
SQTSGPLSLLADAELQWQSLSKLSSNSEKYRTAYNAYREYQRDTLNVCRALWARFDVPSMIRGIGI
LAGGIVILIVYARGIRNESRPDFDSHLLRRIGLGAGFGSVVGLIGKREDAFWADIVALSAAGGSIA
SVVSGAAFASSNFKSPLPTSLWSWFAVIVTVSQSIGFASNSYTIWEDEILLFFLNTFGVLAGISSM
RQKLTSDRVLGVYHSIIFIVMTRLASLSRLCREEQMPFCKSTYYASASSSTSAPWQLAIPFAVALL
LPGVVRSFYAGSKSYEGSATLWIGVAFRVGLFISAAFWALDAADNGEWLSFGKGAFKSFQIILAQL
VLAIAFAAGTTTFIWAKPCISINVTPGTSTDQAGNTQQPQPPQRTTITILGFGNVHGTRFFLLLIN
FVLAITLLSKPMGQGAIAIETLQILSLLEILDTNGLTTTNSAIGPIVLGLLGSFHFFKTGHQATLN
SIQWESAFFALSSIRYPWSPLLVILNTFGAQILAAVAVPLTVLWKRPVDTHGRLPVQIFKDEDTSS
SSSDGTITDTKPKTDSPMLRLLSDIVQAVSTHILYYATINLATTMWAGHLRRHLMLYRIFCPRFLM
GAAVLGVVDVFVILVGLGFGVRANMLSVTEVFGW
>g1004
MSRGSISNSSLDHAAVPSYEAALENLKNPVLAIDEVEVLAKHSSKFTPEQIISLWEASSYLLCQPD
FDDARQRSSRFLDTIVGRPDLSTDLKTQLCKSILVPTPADVIPTRVNALITLSDNGRKLDIMQVNV
LPALAVALFTLFKAVANLRSKAKKSKQPRTIELEESHLENLFDFILNVITLQNVPLEENDTASLLE
EVLKVCRKTASPDDLKSALSIFDAIISCAEVPDDSFVPLLEVLCSIHASVKTLAGRTSQVMRNIVK
SSKRLAMLELLYSFLEEVPEDHGRNLNVVRGAVETFTDIIRSHDRKKLPELSFTRLINCLQAVVPQ
KSGRLHAATLDLCANLLDGDFVSICIEEPDWKSFAKLLIECSRGFGQEVTESAQSPVVSPTRSVTH
DDHLAASVSRLSALIEQLWEQLSSEQKSEISLFLRTSHRYLSPTQARLLINHIKMKKSYFPGTDNW
DQDAKWLVDSFIQAPGKVSDVRASALEALEDAMFQDNNLINFEQSGVIDLLLRNFADEKDTIYMSS
LVSVMIEIAARANSDLFHVIIDALSSPMSSDLLEEDDPVVLSGQLLQNRRNSSSVLGPSLSNVCMM
GLVRLFLRFLNLSAQRAALLFETFIFVVKTSRKRPADAMLTVLKLLFRLRCDSSGAIWVTTDIEND
FLVAVLSRSLDVGVKQSTTTEGSILERSSVNSDDGLTASTGRLSLRNQSVSIETQRSMPRMSSSYL
RGSTKSLPPSWVQLDSSLPNEPSTAASPFVYAYKTVAEGDTELSDPATGVLKINVYLETIIDLLQK
RDTPWDVYSYILAHLGPQLSNRDFFSSAVPQIQMLRNILCGQIKGEMFREPPAIAGIKKADVAICI
FDCLAMLVGFHQYFAKSEQDELVRAFMLGIGSWDGTSRGCIHALSVCCHEMPLSVTKSLNTILDKM
SKVITMAHVAVHILEFLALLARLPDVYVNLREEEIRTVFGICIRFIQTSREQRLKSESAAARASTI
PARLSGGLREIAAMQPESSEANIWQESMSNYVYTLTYHVLVFWFLSLKLQDRANHVNWITNRLVFT
DELGQEVVEEQTEVFIDLMQRVTYSDLGDTIPYETFPPSPEDGPVSSKSWVVGMSIITVETAVVSG

LTQITKRMASGTTYAEYRQRTAPVLPHQIPATPDTLSTFDAASRTAILPSHVLLQMVTSAFPTPKV
SQPIPLPDDAMTRRAISSFDRNDIVDGHKVGVIFIDKDQTTEAEILANTAGSPDYNHFLNGLGTRV
SIRGAQFNTQGLHNDIDGEFTYAWRDRVTEMVYHVTTMMPTNLESDPACVAKKRHVGNDFVNIIFN
RSNRPFNFETIPSQFNSINIVITPVCRIAESDFSNDPDTARDYSKSFYVVRVMSKPGLPELSPASA
PKVISGNSLAAYVRILAINASVFSLVWNREGGEHISSWRNRLREIKRLRDRVMTSRASSVDATESG
FLGHRRNTRTNIHVEDEMASQRAHIRPDVSGEWNVDNVSQALDFSRWTR
>g1005
MANNLGAVAQLLEASLDPRQNKQAELALRQEEKKPGFSLFLLQITASADFPYNTRLASALCFKNLI
RRSWVDEDGNHKLPQDEVVTIKRELINLMINVPGGIQTQLGEAVSVIADSDFWERWDTLVADLVSR
LDPKNPVVNNGVLTVAHSIFRRWRPLFRSDDLFTEINHVLNTFSTPYLALFEGLDAYIEENKSNKE
TLSQGFKQLDLMIKLLYDLNCQDLAPLVEDNAQSIANLLLKYLLYDNQLLHTDDESEAGVLEFVKA
GIFEVLTLFVQKYSDVFEPHVGQFVGNSWNLLTTVGQETKYDILISKALQFLTSTTSMPEHAKIFE
DQGTLSQVIEKVILPNIALRESDEELFEDEPIEFIRRDLEGSDSDTRRRAATDFVRQLATKFEDSV
TRVVSQYTDHYLAEYAKDPASNWKSKDTATYLFSAIAAKGAATASHGITTVSKLVDIADFFQKHLA
ADLVSDGAVNPILKVDAIKYLYLFRSIITPQQWQEVFPLLVKHLGSDNYVVYTYAAIAVERVLAFH
DSAGQPVISPANITPLAKELLEHLFQLVEKDPSPPKVQENEFLMKCVMRVLIVIKEGVVPLTDAVL
EHLIKITRIISANPSNPRFYYYHFESIGAFIRFAAPAKPEKLEQALYAPFAEVLQADVQEFMPYVF
QLFAALLEANSSATLPEYYQNLIAPILMPVMWESKGNVPALVRLLSAIIPRGAQFISSNNQIEPIL
GLFQKLLSTKANESHGFDLLESVIGSFSANALEQYFTSIMQIILTRLQNSKTEVLQLRFVRFYHFV
SARDDQGYSADFFIQVTDKVQAGLFTPLYLNVILPESQKLARPLDRKTAVISFTKTLANSEAFAQQ
YKKGWGFTCEALLKLLELPPVPVTRDDIITEHDVDDMAFGVGFTQLNTVRPRPKDPWPETGVDLKA
WTGKYLTEANSKRGGRISGFVQERLSPELQKILASYMTA
>g1006
MRNFWSKTPNTIYTACRCTSRQLTSFSTWPALAVKQLPPRPKLDDKEITGSYLKGTGPGGQKINKT
NSAVQLIHKPTGIVVKSQATRSRSQNQKIAKEILAAKVEALEKGEQSREAIKNALKKKRKASSMKK
KRRKYRALEESKQEQEGQVEVEEDGSYYEEKSTATTTTR
>g1007
MATPPSPSVFTSFLRWLRLKNYQYEVTFALYMLTPTEKFIFNFIVLTIVSLFVTAAYVYLPDHVAQ
IYGHVHYYFTADVSMIKEYIPSASSILQGNGGPATGALNVVYDAASTAGLDEL
>g1008
MSNILRRLQGGNLEVFKFGMYILFPIGWMYYFGTNLEERFSVPGFWPTVEQSHKIPETKEDIEAEL
SRMRTLDAVRVKRRQQQEEEARQRQQEIMGAQASGEGTA
>g1009
MSTVRYFDDIPHIGRFGMRDDDKGTSWTFFYRNGIRFTIHVDEKDVSGTTFHSLWRPLLGEDLGMG
KSILHNYNKFFELIVSHSMSTLQEISPVKPHWITLRDYLHTPAYHLKLVADEKGTDGVRAVVIEGP
EVVAAYELQPAATFKNMPQTIPEYPSSDLTVLDKEADWRRPPHKLRTTDGLICYFKGREQSSTNFE
TGEINNSSLDSIEAHLRLFKYGQEKGDSYATRRSKVLGIVTDVASASENSDPADEQHNAETNQALV
AGILLSSPTISSHTQSLADVVAQSETTTADFTEKTQRWRTQIEDEASRLHDLKLYWGGREDWFYIN
QYTVLIDAESGNAYLSLDTATFLDDDDATSQDKRTKLARMDNSAIENLFEKWLPGELSGKRSEVS
>g1010
MDLEPLDWNTGENSGLVSVGTHNLWLSVYGPDRKDSEPLVILIPGLANNTTSWAAVRRGLATSTRV
LQYERSGYEKSDISPAKPTATTIATELNLLLKIASLHPPYVVVAHSWGGILSREFLALRPKDVVGM
VFIEANQERTLEVLDWRLLAQSSLLAGVNYLDATGISETHKLTHEEWHVYRDTQSTESHQRQAALE
IAEYANSFPTLKAKSQLHRKPPFLGDRPVCVIRGNNKADFEKLLKAGLARGNGDGAEQAAFEGMLS
TWDEKDESLQSEILSLSSCTRYIETKNSGHNVQLSEPDIIVEGVGWVLSQTIG
>g1011
MATPRLPFLYPQLFRSVRAYENSARSFRPSSSAFNATRHRNQSTVSRRQAQSIESKLPPPTQPKKR
SARQASSSSANRNAPTKGQKDVSKENSAQRVDAKDGNKDTTKPAEASAESQSPAKDEAKPDASKAA
DSMPKEVPPPKNPMDAVLQMPHPSDMGLARLPSNGQEVPHLEPAPYIHHFDTYTLVKNLEEGGFSE
KQAITVMKGIRGILQEKLDLAEATLTSKSDSENEEYLFKAACSELRSSLEASRHLEVERQRSSRTQ
LQHEVDILNMRITQELAKMNDDLKGMWNDHKMTTREQQQGQDTGIQELNYKIAVSLASDGKSEAEG
LRWVLTRRAAFAIVFSAMMALTFLKFWSARAHELERKQALAKEVAKVEPVKDAAVQTEPSLSDSLA

SETLG
>g1012
MASFNTDVDFNESRGVEINVIGWVFTGIAIATVGLKLFARGHMAKNLGWDDFFIFFSMGLSIIATA
FVSYAVTLGLGRHSAAVVAEKGMDGYAAAAKWQIIAFPFNIGSFSFPNISIAILINNILDPNKFRT
IALYVMAVLQVVFAMISAVLVFVQCKPTAILWDPRLLSEGSCWSADVFNDFTYWVSAYTTMTDIVL
AIVPVTVFWKLQMRTSTKVAVCIMMSLTLLSAIVTIVKATYLHLFTDHTDPLYNVVTLVLWGLIEQ
NVVIVAACVPTLRPFFRKVFTNKSSRSGNSTGSHLHSSQAFKLSSKAVHSGFQRPESESEMPLDNR
QGIWQTREVTVESEDDNS
>g1013
MSPVSSIIENLVAAGKAYENNEPGAREALIDNSRALVTSLEIPSEFLQRVFWAEPAQSAIIRIAVN
VKLFQHLKEAGDAGLSAEDLSAKTGMDIVLLQRLARHLVAMFLLTFRNGAFHATEMSNGLAEENYQ
HSIAFCYDTARPSFNGFPEFFQRTEYKSPGLGSLDGPFQSAHNTQLPFFEWLVATPPNLQHFDSFM
SAYRAGKPNWYDPGFYPVAERMVTGFDAAESDVLLVDVGGGRGHDMALFCAQHKASSPGRVVLQDR
EPVIAGVLAATQDDLPFEAQAHDFFTPQPIKGARAYSLHSILHDWSDEDGVKILQNLVPALKQGYS
RVLFNEIVVSEEKPTLAATSMDLMMLAHFAVRERTEAEWRGXLEKAGLKIVNIYTYPGVAESLIEA
ELA
>g1014
MATDTPDVRNLKSWKEAFQYPIPTVRKVEQELRRDIASNREKLRSLVGTRYRELLGTAETIIEMNI
ESSEVESRLASIGIRCNTNLIGKKSVNLTDINRESTGRTEGDKAFAGQLALLHRCTTVTGRLLRKH
DSPLLASKLMVISRLLYKTLSQHQNTPPFLESLRSQLSYVQKLIRRRIDRRLGSADSSVEDIIDSM
AAFCLSNNASSLDVISHFHNIRLNYIGKRLDRDNGSECIPKALSLYIQTLQDTKVLFSRRLSDALG
KLQAQPLLNDHDIQKLDDIDLDILGRWVVPDVKNFTPWIKSDALTKQSAEQTLKAWSKKAFQAFLE
RGAATLESSVDFAAVLALRKKTLDIWLAAVSTTLTHSSTSVLEGLRGLFNTQLKNILLKEAQALLS
VSDSVKSHLKGWQKTEASGTQSLWDPDLLSLDVSEGAATFKKAITETLLGQDAKITSVLEVYHSWL
ATIETSRQLIEDLKDTKWEDTLDEDEDEEFQGSTVDLLTKDDPYLLREEQIKTVRQALLDLQGSFE
DGIQTFDSNSEKAAFVLRLIRDLRRESPNNLQDDELEFAQGIVPELHEILVEEILSKVPASNIIPP
VKNIARLPGRSLWEKTPELPIQPLPSTFRYFKRLVEAMEDLGSDLWNPPAVNILKTKLQKTAIDSF
ASKLEQLKSADSTEDKNDADEAEEKETSVKTDPNTARDWKIQLLLDAFYIRGVLSVQIDGTNPLDD
LIETLQGEIDEDGIAGTLQESAHEFWNRTQLLFGLLSDH
>g1015
MASVDSVPVEPVEAQDSGPKRLPFPPVTRSHILHCSYHYWHPLYRAITPKARLIPLSDAFLSYLRA
DGIVLPPENPSRAAMMGEDSGIETTDSEEDDEEEDPSVEWRDIHTQVEETIRELDGKVTPKLNWSS
PKDATWIAVTNDLQCQTPNDIYMLLKSSDFITHDLEHAFDDTDPEPEGSNENLDTNNIPYHLVLRK
YFNLNPSLEFRCFVRNRTLLCMCQRDLNHFDFLFPMRDMLVSRIQSFFDEKLRDTFPDANFVFDVY
VPPPHERVWLIDINPWAIRTDPLLFSWLEILTMKDLAPLEDEVVRLSIKDDGPSATADSEVEVDDS
EEEIEDLSFIPELRLVERDDPEAYGFTSPQYSAHKLPREVVDASQAGTGGMASFMQQWKDLLAQQT
QQQQADSSDDE
>g1016
MAERVTLRYEGRIAIITFNRPEVLNALDGDCYYLLGERLREIDKRDDIYITVLTGSGRFFSAGADI
RGNGVNRQVDKSLSEGEVRREIMRGFLVNNLDVTRTVYNHSKILVVALNGPVVGLTAAVISFADFI
YAAPHTFLLTPFSSLGLVAEGGASRGFVQRLGISKANEALLMSKRITCEELVATGFVNKVISAPSG
KKEDSDGFLKAVLAEVDDKLGVHLNQTSLLGIKRLIRQPEREALDRANLLEVFGGVDRFVAGIPQE
EFRKLATGEKKHKL
>g1017
MESTTYTVRAEVSFEGVYPLSPSDLLIPPRAIRQAFIYRNDNQVETKEHTLIAALQESLKTFLKPL
DKDKEPTETAYPQLLGRVVRPKDGSPPHVAVDESSSIPFTVVRRPDIIFADLSPAQRFPEERIQKT
NFAIGLDDSDLLSKHNFAVQLTFIEGGYVLVVQIHHAITDGYGYAGFVRQWLQMTKALIADGLYEE
AVPQMSIHDKSELLRDIDESPTAVEELNGLKTWEALYKEASTTAKANKNPLMGNNGIQSKIFWLSP
ESLESLRAKMHSHTEDRPTIFESVMAHAWQCLARARADSNTVVETTISNGFFAADIRNRLVPRLAT
DYFGNAVTPVVARVPLSTLLLQQHRDIIKPIQSTLRTDTTDRNLRAINALIFRQTKAGIFPPGNLL
EQDVFFNSWEHLYPSLEAMEIGVGKFCIMRNLMDSPITPSYVLVMPSYGMRQTSSSDGASYRYPGG
IELKVHLLQHQMERLVEDDEWLHYVSI

>g1018
MGEADNSENSIDGMGAMRFTDEEERGFFGSSSNIAFMGYISRAMATVNPDRASFSEILPSIQRPGG
VVSVTRSQAPPIDEAAMSDPSPAPTLVKIYALPPEDRAWNLIDQYFQKTGQLLPFIHELSFRETYS
QMRREGFNKVSRTWLGLFNIVLAISASLSAKDELPPEERIKESDIYYQRANGLCDRDSKRSTSLEM
VQYLLILAISTAYQLGLHSPDANQGYPALECEIRKRTWFGCVLLDRTLAMTFGRPCSIPESYIKLD
APSKDIHMLNSSDEKASYSPFDGAFYTAAIQLYSILYKVLDVCYGQNLSLKNSIHASDWMSHILDG
QHQLSQWRTKLLPSLGLQIQPDLMTPEDVMKMNTRFFILDRFNIVLSMRYHNLRILLHRPRLESFL
EAFWSTDDVGDQEKRIIMQMDIASVQNCVESAVSIISIAHSITTASSSHRELLGAWNYSLYYTFNA
ALVIFSSLVVASKEREGNPSAWSVVDESRPYHDKAIEVLHRLDHGNRVVGRCIEYLSQLSLVLNTL
STSDQFNSNNNGLSNLLSGYTSDFLDAQSTNPLPAMDLGEFMIDQDLDFLGKVFNTAVK
>g1019
MSASQGNRRKQIVVAITGATGATLGINLLIALRHLNVETHLIMSKWAESTIKYETDYHPSNVKALA
DHVYNINDMAAPISSGSFHTDGMIIVPCSMKTLAAISSGLCNDLISRAADVMLKERRKLVLVTRET
PLSDIHLRNMLSVSRSGAIIFPPVPAFYIKPSSVEDLVNQSVGRMLDLFNLDTGTFERWSGWEKD
>g1020
MSTETLPAALSLRTFLEESKAEGDVVEIDMEXDPYLESGAISRRAYETGSPMPLINNPKGKDGPEG
LFRILGAPVGVRSDRETRYARLAKSIGLPSNATGHDIIQKLLAAKKAEPIPSVEVYDAPLKEHKLF
GDEIDLLKMPVPKNHASDGGRYLLTYGLHSVQTPDGKWVNWAITRCMVIGKRQLTGLVDVKQDIGA
IWAMWKALGKDTPWACALGVPPAAAVASGMPLPKFVNEPDYVGAITGVPVEVIKCETNDLFVPAHS
EVVLEGTISANETAIEGPMGEYHGFIFPAKKAPQPVMTVNAMTYRNNPIVPITVAGRAPDETYTVW
AVAICAEILDLLQQAGLPITKAWCPYESQAIWYVVQVDRKRLVEMKTTPEAFCRQVGEIVFSSKPG
RFVPKIFVVGDNIDPSDLHEVVWAEATKSQPQDSDFFFVGNYPTYDLVPYATHGFNPXQPQAKVVR
LCMLPAEFKTLDRPWVEASFRGSYPKEIQKKVQDNWLAYGFKENPNDPYNHNMLLTIHPNASSLTT
AIRLFLLFLPLTTTAASCAGVLLQTIATRWDSLGSAYQSEICARGCEPVMSTWDDWTYNNAFLPLI
DTMASEMSLSDNFKTTFTRIGQDIASGTKSECSALLSPGQHFCTGGNTPAGDKLAQWNWCFKKQAA
KQASRNAFTMLRLVSSEICKGEAYKYLEDDKLWMEVLPQYMRKYADTCQLPPPKEERKQKEMKDEL
>g1021
MSAPEPAPAPAAAAEKPQEPRRTELDVAKLHALPSEQQDLYLLTFTSELVQHVSALTKEEITLEQA
SLKQELFKVINLSSPSVTRVIRNNLGKCFGTILGKGDRIPLYDTVLDLLAIVNGGKSVELKTKLAA
GHCLGEVFAAAGDSAYSHASAATPALFKLLKAGSNHAGVRGSVFAALRKVVGGLGGPIEEPIARDI
WKQARNAASSDKSTLVQINACRCLEQLLITTQYFDNQNDSETLKTLVWKVIDNPVAPVRHAAASCL
ARALVKSYSPQSGTESSTKSKKSKRQSKKPPPKANDADEDEPADDSPSAKKAEPRLSLSLSDILVQ
LSTQYVRSSTGNRARAGIAVCYKYVIRNLGENVVEERYGQIASHLLVTLLNHPTVTYNRFRLLMTR
RFIKNILEDTLGREILRENSQVNAANWLINSILKDYPQVIKERSEPSKYTLASTLSALSSLISSLG
SAVTTLADACREALIQVLPHPSYTVQIQTAQCLRNFVLACPNQLISCVTICSNSLNREIGQLTTPR
SSSRRCLGFANGLSAMLGTSRLQPLYGSVDVFARVLSQATDLLKNSGSSELRVASTQIQVAWILIG
GLMPLGPSFVKIHLSQLMLLWKNALPKPLGRANTAEQGPLETSFLAHVRECALGSMLSFLEFNAKL
VTADGARRIAAMLQNTVAFLDEMTLPKTAEDISQRLFPSIQMHDFAVMVRRRVLQCFTKLVKIHHT
GHAEIVSQSSLLSIAISSFADPEIVHPNPIETSIVASTGQFESLWDLWDNFGFGVTGLARTYVNET
LTGKRAGGATAWNANDSTDQEIDDILNFPVCQASEHDSVLLYGSRDGFSTFYTDPPVTEAVNAAID
LFAVALPLQTPKVQESSVEQIVTLLSSHSLQRNPGRKVAMTINIAIALLHTLKVAVRETIAPPGKL
EASTEKIIQELLQRFVMDPDAIIRTIGFEALGRLCNSAGNAFTNTQINWLVDTIVANREPSARAGC
AAALGCIHSRVGGMAAGFHLKTIVGVLMSLCSDPHPVVHFWAIDGLERVAESAGLTFSPYASSSLG
MLAQLYVADTHNEEAASLATSNLEVSFPTPFVISRSVDSLINVLGPDLQDIAKTRNLILTLVRQFQ
LEESPAMNTQSSRCLDHLSLYAPSHVDFTAFVKRLHAGLTSKSSLMREAAIRGLDNLMKRDAEAVI
GSNTSTLEDDIWLALDDSPGTTSLRSLIRNWLQQSGLSHTQLWIQRLHNVLTKTRAKKDEIQPKKL
ARAATASEVQDDEVAGFASAVAGGDQEEGDSSANTGQELLKWQTRNFAVSCLGELLLMVNDKILPD
QTIPAEVALQQKIGDVVRMAFSASTANVVELRVWGLKILDQVLKMFGKTPDPDFQEASLLEQYQAQ
IGSALTPAFAADSSPDLASEAINVCGTFVAAGIVTNVDRMGRIFRLLVVGLEQFANDPNITEIGDL
KGLNSNARVMVKMALFSAWARLQMASLEQQYLVNVVQPYTAMLTPLWLSSLQEFARLRFEPDISGS
LGTPSTGDLDEVYAALNRETLLNYYQDSWLNLVDAIASLVEKDSDFVFDALDNKSKAQGDDVDENK
AKGVVPSGKGDSINYRDEPVAFFFVLFGLAFEALVDQSVSPSQRLEILQALAKILRPSVAGNAIYQ

DAVFSETVDTLDRLVLTEGHNIQTVIVEMARNLSLDHPSAKGGQDRAENLSDDIEQLFELTRNIIL
VLAGLLPNLGDSSSRTFNVSSDETLTLIRLALSALVDVAAIFPAIIRHDLHACILHIFSTILATGI
CQAEVVPHALPIFRRFVQGMTSQGTPTNEAVSLQVRGCLTRFLTTLSIAQRRESETSLPCAKNTLL
AMTIVLSTSSHVIPPQDPLVARVLDEYLDCLQDVGLASVAAGCIRSVLLRSGPLSTTDSAIGRYLF
PRLIAFVAGTPNDRGEPPIDPENSRAAISQTLVLYISNPIAFPPPKVSTAIPLVVSTLLARAKNEG
SAIYQEMAARLLEIGKVNPVIFRALIAGMDPERRVLVEDVLRSIGAGSAGEARDAGAQEQKATPSI
ELRMDF
>g1022
MASAFGLRLTRRPGYNSRWICDTCRSFHVSAATQSGHSRWSTIKHDKAKNDKAKSKERGILTQELI
NASQLWGPDPKNNPRLQLAIAKAKQSQMPKAIIDNAIARGQGVTASGQALEAVTIEAMLPGTVAAV
IECMAENKARILQDVRFTIKDNGGNVTPTTYLFEKKGRLVFENKEGTNPDDYLDQAIEAGAMDIDT
DAEGRLVVYTEPTATKSVGEALSQATGLVIAQSQIIWDPNKDTMVKVEDPEQIDQLEEVLAALREE
SSVQDIFLNTTI
>g1023
MSSPIASSVASEQFGDIQAQLRSLRNNVDHLEAVANLPAKQRTTSVKELAGEIASDAYNIGISTDI
LEQLVDILTKPNHLDQTTTTTLIKNLYPQERVSSNVVTKIVCCLGPARVKPSPATQTLLLQWLLLI
YEFLEEPAILSRLYSVLFNMLDMISLRRPLCHILSLITRRPNVKPFRIQAIMELLRNAGDDEKELM
GLLRVFKNYYPDIIIGDIGRSKFFFKHPDPEWTAKLKQLQERNLERIQSGNGHNNVFQVVRRGASS
KRRKVAGIIPDVQTSRVKPSFTSLEELRNVDGFVERLEKLELPNQIVSVLENRMAHKYMALVGPET
AIHRLDDWLDAFLKDEVEMAKMPDSADSETFRYVLGAICGYVQSTKELPGVVQRFLDNYLRTWNGQ
DYRQEILNILKFIPRGSYDDLRDHYLERLDTTILDHTASSRTDLLDFYANLIRKWGISLRSETFSV
TSGRFKPLVSLITHVELLLLSLMEIPFTADAKDTGDFIPVTTSILDLYSDLAELYSHASSNGNIRL
TVPLAPAIYSLTFTSNLAQISRLSNILSIYKNSFETSLTSQTLQSPDKSAGGFYAPEMVGLYNGYI
MDLCNLIWRNRALNSEDQNAHGCLVPKATKESFVEYINDSNEISKHRRAARSEGPAFNHSLGLMFS
LSYHVALASHSAACFSALEEQAXLTGDRPTLQRPVTQKALEALEKEGGIKLSWSEYRLKMLDWFDD
LGSDGIGQLMRSTMKALRKDG
>g1024
MPEQRQQQQQEEGGFNFRTIIQGIAIFALVQFAMGQFFGGNKNQSQGGASTGPTGQIPAYTDRPAR
NEVENWNAIPSGIAPMWPSNSSLDISIYVSSSVHMPALSAVPKESLVMEEKAFSMANWSESREIDT
TFKVPKEVQQNGTLWAHFYVALSGHQLDPAAKDYSPESAVHFLRPLNQYLPKKKVAKRKNLLASAE
DAADKEGDTTPKYSVSSYYHPNFTVSVIPDFGVANYQQMXPAVRQYVHLESTGARDITGQNTWYYP
VLYLNTFWQLKSHMTELNSTVETLPLRIVLNNLSNWKFNILASMDEGAKQNARNAASGQPVPGGGD
GSEFEMIKEVLLNTNIYLLGTTAIVSVLHMIFETLAFKNDIAHWRKKKDNIGTSVRTIIANIFMQL
VVFLYLIDNNENTSWMVLASQGFGIVLEAWKVTKTVDVRLRPPPQNSFWSFLPYVVSFEDKHKLSE
TEKKTQEYDEIAFKYLYIIALPLLGAYAVYSLMYETHKSWYSFIIETLVGSVYAYGFLMMVPSLYI
NYRLKSVAHMPGRALMYKFLNTFIDDLFAFTVKMPWLYRLGTLRDDVIFFVWVYQAWKYKVDYKRV
NEFGQGGESDEEVEEEVVSETVKKVEADTAAKSTGAAKAGSSTRRRK
>g1025
MTETNIPQLRFPPHSDRRVWLISSGDNPIGXNVARQVLAHGDCVVSGVIPSNIVRDENRRTYFEDF
LAEVERNEEHGWKDRFRTFLLDIRKMGECQAAVAEVVNLFGRLDIVLCCTSQAVVGSVEELAASSR
TLSLVRDQFEINYFGPVNIIKSALPQMRSQRAGHFLVLTGISKTVYIHLIEGVILTSQAAHLGTPG
LGVYCASEWALEGFCDSIAYEVAPFNIKVSILQCSMEICILTNLISSVPPIQPAYSVSENNAPLFR
NILDGLVSRLPQAAENLNDSNEITTPKSNNSQSKQGPFSSPHDVITMYAPFSAAHLEALTLETVHA
ITAIGGHENPPSRHIIGQEGVAAVKEKLKTVSEELEDFVQSSYSVNIHESTVEEDFNGDIV
>g1026
MPPKRKAVSAADVKSDGPPTRTSKRIRDKQLQAAAKLVSDAGHLETKAMSSAPKQNAARKRGPWGE
DYLMTSTKSALAHADLVRLFSNPKAWECLEEDEKRELLSLLPAHIHPNPDPDPNDPEAKIPPLPET
FLRYDNNWRAALRNFQSDLESGRYRPDWQRQAKQAVQERAQGKFDDYKEQEFEQFWGQKQKINHGL
IAGESSKVKLETLIAHDVVRVGDVWKYCRVFGGKKAQILVEKEVKITAIDGAKLSFLMPTGQRVFL
SAGSDLKSADGKIENERERSAGEGTEDIKTEGKIVGFERIENSISDGKTENADEVKDNTQMDIDLH
PASQPGEDPPSQALDEHTQGHASTQYNLKGLLDYALPSVEATAEGEKDKIDSKESKPTAHQQEAPE
SPVAEVGIRRKRGRPAKRKSPHDEVQHTPIEVNGATEIEAAGLSKEDVEEPAAITENGVNADPLQE

SAPEQEHKNADSSQQSTTPTTANLRETALSPKFEESESAQCSATATPLEVEIHDIAGPNALMKKIL
ETDGRVKNPPNGNAWKEIRCYRNNQDMGSLWEVRQAWYVKYQQD
>g1027
MVRSTQIARLDGLMLAASVDDEQAENELAQVKSQSKMIFRRLNRNSAPEASIESGQYTLHYLIKDD
VCFLCICDKSYPRKLAFTYLADIATEFTTTYSSQQYNSPNLRPYAFVEFDTFIQRTKGTYQNSRAA
ANLDKLNDELRDVTKVMTKNIEDLLYRGDSLERMGEMSGRLREDSMKYRKAAVRINWELLLKQYGP
FAGVGFIMLFFIWWRFF
>g1028
MSSTSSLSHRRTHNLLLISKLLSLRDTASPLTLVLDSLEQPAKPLLNEYIRRAQISKVHVTFISFE
NLKRPNGVDSFILASRKKPADVAKEVVAGYFNSLHKRRLIIIDAINPLLSKPGIHLPSYLGSLIAP
VNATPGSVPIQTSLVVTYHRDVVSTKPQQSYAPSDLSMLSYLSTTIITLHSLSHILAQKAAHDRSL
AAPVFGLEEQQEGIIIGKTDQSKGQEQYVEGIVIEMEHRRKSGRGVVEWYFLPPASRYSAQEVKEV
ITLLEDHPLYRRPEDDIDAAEQDEPGSTFELKLTERQRRDREGVVLPYFDAQSGDGPGEGGRILYD
MGEEDDFDEEEDEI
>g1029
MATYKPLLAAATHFTRRAARRRMPIISFILTISLIFPVLLYLLLGNILANDPRLVPHAVRNAQNVL
FITAHPDDGALYFGPSILQSLGRNNVNRYMLVLSSGVNEYETSHLETKASCLEYSIPSKNCLLLQN
KDLHQDKNWDDHIIQRILERHITKWEIDLIITFDAAAFSDQNHKAVSNAVQRYAILHSEQRNPAAY
ALQTTSELRRYLSVLDLVTTSVPFGFRILTAMVCGVPEGYHTSLEGKGVVPPPTDGDVYADKALIV
SDWSGHAGSKVALAKHASAYSWNRALYASLSRYMWFNDLRRM
>g1030
MGVFATFRRRWQRLAVASVVFCMIAFSVLSHRGEQFFTPQQYSEAQPEFSSDTPLIWKHINSSTVR
GGAFWIPPEWLSPSTPKPTTMIEAARLVSQLTNISNTTHLMENTEIPLVAHQTWKNTHVDTWPDLI
RDSVERWMENVIETPMAYFLWNDEGIIEFLEEYEPAIIEHFSALPRMVEKSDIFRIMVCKYIGGVY
GDVDTRPLRSPATWIEKTDLAPWRDNETDILYNSTKPVRAIFGIEADCPPDTDTYWRMGYTNPVQL
TQWALASAPGHPALTWFIQKIYGILDEVASHHDGNLTTPEAYEELDYLEPLLFTGPDAITVSMRSW
LEDRIGLRWNALTGLHDNGKSKLADDILILPITGFSPGRGVYGNMGSKPVAHHDARLQHLAQGSWK
GFDLRLEFGKMCRTFFGMCRDWPKS
>g1031
MAEPPTKRARRTDSSAMWDTNDKRPRLSHTPEADAEQTHHSRHGSSRPEDPRTSTKDDRRYRSRSR
DRYPRKRDRSWSRDSYERTRERDRNRDGGKRDSYKDGRGVRDRDRSTSRDRYNRRGTTKSGRPDRS
RSPRNGTRSRDYSARDPKPDQRDGKRDRKSQPDQRHTNGASKKVVKDEEIEDADKMSDIEDTDDIE
AMMRKAVGFTRFRSTKNTKVPGNDIYGVRKEKKTEYRQYMNRVGGFNRPLSPGR
>g1032
MASEKERNITIIGGGIIGSCSAYYLTRHPSYDPSIHKITLLEASEIAGGASGKAGGLLALWAYPSS
IVPLSYKLHAELAKEHNGKDAWGYREVNCGQLIVKGRAVDKKDNAGSVSLQKRDAAAIKNLRAAGI
PTDLDWLLSDGIRGYESMSEPGETAQVHPYQFTTSIAKLAEEKGVKIVLGTVTKINQDAGGVQSVT
YTDKASGESHTLPATDVVVSAGPWTKRVLPSAPISATRAHSVVIKPTRPVSAYTLFTNIELPSKHN
PKRSTVETPEIYARPDATVYACGDGDHIVPLPDTTADVEVDPKRCQDIIDSVGSISDELRLGEIVK
QQACYLPNVDARGGGGPLIGHSRTKGLYVATGHTCWGIQNAPGTGKLISEFVFEGSAKSAKIAALD
PQQFM
>g1033
MHLQVSFYLLWSSFVLGCVYAANDVSVLINEVGKANNQSLLWGPYKPNLYFGVRPRIPKSLTAGXM
WARVDNYATAQANFRHTCEQNEGMRGYGWDEYDVRKGGRQSIHDVGNSIDLTIDFVKVPGGLHGGS
WAARVKGTPREDAPEDLITSLVFYASLEGLGSLHVDNAVEQQMGVKGDVKLKGMTPELGDFKISVT
TGPDSNKHPEFNHPSSSEKSLDRPLVASVETPQEHLWQAKALMFTAMKPEVDNMLAKYGTDTPPPP
AQLFTVPNHPGTGNIHFVQHVFEGAFEFDILFESGSASEPVTSETLTEKIDEMSSSFXSRYENVFA
PQAPFDKPEYSEFSKSMFSNLVGGIGYFYGSGLVDRSEAPEYEEENEGFWEEAAEARLRVKPTEED
PHELFTSIPSRPFFPRGFLWDEGFHLIPVVDWDLDLTLDIVQSWFNLMDEDGWIAREQILGMEARS
KVPPEFTVQYPHYANPPTLFLVLEAFVDKLQANKSTLVEFELNRADIADSLRSAHLESRELAESYL
HAIYPLLRRQYFWFKKTQWGDVKSYDREAYSSKEAYRWRGRTVRHILTSGLDDYPRAQPPHPGELH
TDLISWMGLMSRSLRKIAAAIGEAEDAEEFAIYEYAITRNIDDLHWDEDAQTWCDATIDEYEESVH

VCHKGYISIFPFLTGMVGPDSPHLKATLDLIRNSNELWSDYGIRSLSKSDEFYGTDENYWRSPIWM
NMNYMIVKNLYEIATSQSPHKAQATEIYNGLRKNLVENVFRQWKDTGFAWEQYNPETGKGQRTQHF
TGWTSLVVKIMNMPYLSSSGRGHDEL
>g1034
MDLVAGIRKEGSRGGRDSFKWSDVKESSHRENYLGHSIMAPVGRWQQNRDLNWYAKSGDSADKAKQ
QREEEIRRIKEAEQDAMAVALGLPVAPRASNNANLTPLGGGEVRKAVQEATEGDGEDDGGRGGIGF
GSFGGVAGNDAEDEILAPIDRGTMTGDEITGSIAIVTIANIVNIVKPANTGTTGLGLARCRDLRDR
NVAIEDQRRIVVVTLVVWSAIGRVLHDVIAIVVIMKAVGMRTIDIIVDRRWLVIVYIRAMHGNGVS
VFCCYELELER
>g1035
MFNCSLKQTALSQRRNLLFVAYSHQIYVWEPAGPRQVLGRKPAMIITPVMRDPDAEGYISSRFPHG
INNILVDDLGRDEILLLATDSGNVCGYHVENIFSYIKAAKSRGEKFPILEPRIDPFFCESVGLSAW
GLAVHKFARLIAVAANTGTITVFAFALAEADAESDDDPAGFWADLSMSFEQNWVHIQNEEDFATLH
ELYRENRHRSRNVRISFQGHYTNIPSVSFLSSDLDSDGMWMVSTDIENKVFVWTIWDNLDPVRVLQ
FAEGEPLSGILREEERGWSVLALDPRSFRVHQSYFEAFGGKVYLRPFQPSNAVLDVTNLALDVPDS
SSVFVPVFPIVDDSPEEPVLPDMFDRHSRIRSDSSSLSSEESLSPISITDTSIAEQFSQSKKSAGA
FTQNQETTTNTDQLPQQSGQHLSNEYFTSLLRSIMAESSSQDANDEDTGMVSANSDSENELDESDE
DVSIGSEAVYRDTSNPRDPSNLRPLLNLDLLDTDVYPLNPYSSSEHICSEFPILHFSETDIRLLEG
PFVDHPSVVSGGPLRQQLPRSFWSLHSYDRFNMVKYLPEAGIVIAATQKGRAAVITLTQVANRIYA
LRINWMIPFASQEKRAERPQVGLLGLAVSPMQGFEKQPDVVHVPXGAXSHNDFSFHYRAHGAESID
ADQNWEWQYNSETDSEESEEDGSFSSSTCVPELSFAESHGTANWNYMPQERWHGVMSSRHYRVLLT
YADHTVMSYEFWYDWTDIAVGVSSAGRDEAKKEDYERELEERSESNRQDHLLL
>g1036
MAPTPDSYDALDHSTVLRRSLTNVNHTQAVTLGVIAVYVVVIALLWNLPYVRWSLWPFKMLVIAFH
EFGHAITAVCTGGRVKSISLDPHEGGVTHMVGGASAITLPAGYLGSSLIGALLIFCGFDIVASKVA
SIVLGVCFLLTLWWARKDWLTIGTILAAVGLLIGFWFIAHAEALRFLVLFIGVMSCLYSAWDICDD
LILRKVNTSDASVFAKRYGGSSQCWXVIWSLISVAFMAVAIVAGLAXFNQSFAQQESDSSHFIPTK
I
>g1037
MAGPAKLTPPPSPPSPSASYYDISDDEESEYNTIAHSSSGRGVKLLFSKSKVYIHPTPSAKDNIPG
FIALLQQKPVTVPISQNVSNNTSGSSQKDNASSYLLAWVPESAMGDAYDTYVKVDLSENNSPPRQS
YLVPALPTMTTYSDTIGMYAFAVPLSEIYSLWVRPPSLGWWYGSLVICTKSGDNFPALFFHDNECE
STILQKRRMTRESFDPFSADGSMFWGGDEVLRWVRRYADVQRAGADPSKYLINPSEEDKLSFGRAL
GSKSDAAKGPSSKEAPRDAAMDPITKALKETRWKVLEGFSRISTFTRRTANDLAENPIIPTQVRRL
MKNPEIQTLQNEFDSARIYLARWAMNIAEQSERERNQRIWTAQDVLASEDSSVGEFEILELETGTM
SLQERRKPVTLQEWNSWFSSHDGHLQMTVDEVKERIFHGGLDPNDGVRKEAWLFLLGVYPWNSSED
DRRAVMNSRRDQYVRLKGAWWERMVDGDASSSEYESWKEQKARIEKDVHRTDRTIPLFSGEDIPHP
DPDSPFADAGTNVHLEQMKDMLLTYHEYNPGLGYVQGMSDLLAPVYAVMQDDAVAFWGFVGFMERM
ERNFLRDQSGMRAQLLTLDHLVQLMDPQLYLHLQSADSTNFFFFFRMLLVWYKREFEWGDILRLWE
TLWTNYYSSSFHLFIALAILEKHRDVIMDHLKHFDEVLKYINELSNTMELIPILTRAESLFRRFER
VVQAVDKKNNFPMPGGVSTPSDSQSQKGKLPSRPTDTERKERVISPELRQLLSRENITVASLSQTE
QAGSSTTKP
>g1038
MSLSAEYLHGIDHSDPPSEDDLDLEELDPESARYRQGSRTSRDNSRQGDGYGARIALRNLRTRKRN
RLWNRDHQGADHDLDSLFDDRDDIAFNAHEDDAPLLSGQRRSNTSFDDRRPAKARLPSLRSNAPSR
IPDAPSSSASAREVLVGQTQHLKFPANAVSNAKYTPWSFLPRTLYNEFSFFFNLYFLFVALSQVVP
VLRIGYMSSYIAPLAFVVAISLGKEAWDDIGRRRRDAEANAEEFTVLSFGPPAGGNSRIVSDSRLG
ENVYEITKKSRDLKVGEVLKMKKDQRLPADVVILKSTVTESTVQPDAVDNIQTPEVDLLEASSEAT
TTTETNGKAGNKEEAVQASGASDTFIRTDQLDGETDWKLRLPSTLSQTLDLRDFQRLKVHASAPDK
RINDFAGKIELLPPLYSSYDPPVNKHTAPESNDPNENAESESDIKSSPLSIDNTAWANTVLASNTI
TFAVIIYTGSQTRSAMSTSPSRSKIGLLEYEINNLTKILCILTLLLSIVLVALEGFQPTNDKKWYV
AIMIYLILYSTIIPMSLRVNLDMAKTIYGRFIERDLDIPGTVVRTSTIPEDLGRIEYLLSDKTGTL

TQNEMELKKIHVGTVSYANEAMDEVASYIKQGFVVSDSSRQSLITPSTVFGAQVGTGAATRTRREI
GTRVRDLLLALALCHNVTPTTDEEDGRRVINYQASSPDEIAIVKYTEEVGLRVAYRDRKTILLEST
NTNQVVVRVQILEVFPFTSESKRMGIIVQFDHATAGTAPSETEPEIWFYQKGADTVMSSIVAANDW
LDEETANMAREGLRTLVVGRRKLSLEQYRGFTAAYKEASMALQDRDTGMAKVVGGYLERNLELLGV
TGVEDRLQRDVKPSLELLRNAGVRVWMLTGDKVETARCVAISAKLVARGQYIHTVSKLKEKAAALE
TLDFLRHKTDACLLIDGESLSLMLSQFRSAFITVAVTLPAVVACRCSPTQKAEVAELIRQHTKKRV
CCIGDGGNDVSMIQAADVGIGIVGKEGRQASLAADFSITQFHHITKLLVWHGRNSYKRSAKLGQFI
MHRGLIISACQTMYSIASHFDPKGLFINWLMVGYATVYTNAPVFSLVLDKDVDEELANLYPELYKE
LKSGRSLSYQSFFGWVFISVYQGAVIQGLSQLLLNTTTGKRLISVSFTALVLNELIMVAIAVTTWH
PIMIFCIVGTALVYAASVPFLGDYFDLEFIITVDWLWRVVAVCAVSVIPVWVGKVIKRSWKPPSYR
KVQA
>g1039
MRPEDAPDASAAESISVPATKPPSQTDETSADHVMEENTVPEVLSRPLDEPNVAPASTDEHLESPA
TSKEITDKLPKENKPFDINWGEETEGATWDVHSQNPFEREDVAARTNSFPPIETAAEPVDEEVSTX
PAVPQTYEDSQIGQQEDPFLASQPDASQNNSNNSWEAEAAEDEDGFFDQLKTQTKPIYVPPEAESR
YEEGIPLVENEVDFQADEADKAEPQLDVFGDDDADDFFASTATKDASTFEKPAILRKSTYDVLGSL
DNGIPDSPVADSPAAFQTQEDDDLAARWQAELDDVEEVPGAEEPKEEEDLAARWEAELDDDDDDLL
LEDNTLNEAQELPEEPSFFGGDSAGDAWSTQLPTPSNYANPYAPHQPSSSELFQSLPTIPPPGAMT
ATPFGIPQAQPLNEASRASSFVDQAKDGYKSPYDLPDSLAPRPRRAVATTRKTAPPPPSSTGMPPP
PPRSSSIPTSVSTPAVGSMPPPIAENDIPGSVRGTVPVAQNFFEELPAPAPRQRPASRTSVYNPSA
PAVSNAPVAVPVPTESMLPPAVPSSSLPPSQIAPEVAYQNQLQSPERIGPYANLTVPTASAGPGVG
PRYSPKPPSTTGKSPLSARYSPAPPSSQPPVNGRNRYVSQPNNLAFLPRTSSPLAHHEKAAYEAQQ
PQQRPSTSSGSSSLSYPVASNHTRGSFSVPANELASDVVPQLPSETQAAPPFSPPANPYAPVLPSE
SSRRPSTESPYLPNGGPIQSNILPPQANDLGFPPPRRSQTQSPGRQSFGPQLVRTSKEPVQRPASV
HAPSSPTKAVNSYAIPQVTSSVRDVPELNFIPPTDGRELDPLQRWKGAPVFKFAFGGLVSSCFPQH
VPRYIAGQMSPMIQPAPGEPQVRQVKQILPSDGDIVRYPGPLKTKSKKKDVVAWLSSRIAALENEG
VPSSLYQEPDAFRRHDEKILLWKLLRILVENDGVLEGTVDIEKSIRSVISPELESTEQSVDYDSRA
TSGSYQPTDGSVSPEPVSSKVLGQIKTNLLSGNRDKAVWLAVDNRLWGHAMIMSSSLDKALWKQVV
QEFVRREVRSTGENIESLAALYAVVSGNTEESIDELVPPSARLGLQMVSKEGQGINKNAVDGLERW
RETLGLIINNRSPDDHQALCAMGQLLGSYGRTEAAHVCYLFAKAFSQRSIFGAADDPQASIVLLGA
NHFNFPTTFFRDEDAIALTEVYEFATSVLTGTTAAVLPYFQSYKLQHAYILADKGLKSEAMQYCEA
IGSVLKASTKPSPYYHQRLFAEIDELATRLKQAPGDGSSSWMSKPSMEKVSGSMWARFSSFVAGDE
SDAASNGSNIDADVGPFAKVTEAATISRPPSVSEVYGSYPMAQPATNAFSRYAPANQFAPSSSPEQ
YRARTSMDSQRSPSIGVTYNARRGSQEPATPVESSPYNFGANAYGSPTSFPYHSTPPQSSYMPLAP
VEEDLAPQAQQAYAAPQQSALTSGLQASPERFGQPLHEVKETAPAEIPHYGGYEPPAMSSYEPPSY
QPDLGMSTDGPGQEDEADEAEKPKKKSFMDDDDEDDLAARAAAVQQAEKARRDREADDAVRKAAEA
DAQRPAAAKKGWFGGWWGGKKEGEATGGPIRAKLGEENSFYYDPELKKWINKKXPNSATASARATP
PPPRGPGPQSRSVSVGSASSGPPMLQRPPSISQLGTTPDSRPSTGAGASSLGTSPAVGFAPNGLPP
RSVSTTAATAAGSSPAASGPSLGPAMQPPLPRPSTTSLSNASSIDDLLGAPTARKGGTVKSKKKGR
GYVDVMAK
>g1040
MEHSQHVEAGEKVPDIDMDDVKGNSNAAQEAAMDAEIARLEESSQGLKRPRFELSFSNPATFTYVL
VGFASLGGLLSGLDQSLISGANIDLPIDLNLSANQASLVNSGMPLGAVAGALILSPSNEYLGRKNS
IILSCILYTIGAALEAGAIDFGMMFAGRFILGTGVGLEGGTVPIYVAESISSSLRGNLVSLYQFNI
ALGEILGYAVAAIFIGVKGNWRYILGSSLVFSTIMFIGMLFMPESPRYLMFKGREQEAYSVWKKIR
GFDTYESKAEFLGMRETCQIENEEQAQTKRFPWLDFFTNPRARRALVYANIMVFLGQFTGVNAVMY
YMSVLMEYIGFTAKDAVFMSLVGGGALMLGTIPAVLYMERFGRRYWANLMLPGFFVGLIFVGVGYQ
INLETHRQLAEGLYLTGIILYMGFFGSYACLTWVIPAEVYPTYLRHYGMTTSDANLFLCSFIVTYN
FTRMMNSMTRIGLTLGFYGGIAVLGWVYQILFMPETKNKSLEEIDELFSKPTSVIVKQNVKNTVQV
MRDLAAFRFKKVFMPE
>g1041
MSSYLASLASSTTISXLGTRLSSLRRAIGSGDEKDDPDNEDSSHISNVLRAYYTEKGRPFPEWLPP

DPKAPVPQARMVATQASLQQGNTSAYGQPQAQYTGRGGGLGDLWGESGPAQPPQSQTASLRSRGGS
RTPVAAIGNNNTTLRPEHQQQQHHSQTPSPHSVSRRPLPSQRGESYQSSHSSSGMSDRGASSGGSA
SERLRARLQGGLGSSSQSPPPSRSQPQQYQAYTSDGGQGHGSSGGSGGYKRPGGLPSGPRPGGYR
>g1042
MASDEIVWQVINQQFCAFKLKTTKGQNFCRNEYNVTGLCNRQSCPLANSRYATVRSDPETGAMYLY
MKTIERSHMPSKWWEKIKLSSNYTKALAQVDERLIYWPKFLIHKCKQRLTRLTQVNIRMKKLIKEE
ERLGERVVPKLAPKIRRREETRERKAEAAAKVERAIERELIERLRSGAYGDRPLNVEEGIWKKVLR
GLERQXEGERDEDLDEGEQWDEEDEEEEGVGEVEYVSDLDEDEEDLEDIEEWLGGDSADSSDDYDD
EDEDEDSDDDEGSDESAAEDKKKPATGDKRKRAAPPPKPRKKGARVEIEYETEGPAKESVLA
>g1043
MDFPNVPPSFMPPSFPLIPIPRPCPHVHQAVKLSQFVNTASGHFIDGHQQFRTYPLKDSLSPGVPV
HMSPTGETICARCKVSYCCRDLFMLHLETSLDHNLCVLCHYTKDYPTFTELQDHLEQEHLWCEPCN
WFAPSYEGLMQHFQHFHFMCSICKECFRNVNEYTGHANTHRPLTVACPVCPEKFALRSAAFNHIES
GTCTGGATTEDICFVVRHFWANLTPDLKGYWVSDSLLFTCQPCHRHYQRLSDLLQHKETKSCPEGY
GKGNSMTDRLVQYVQQQLPVLIEKRKAEGKLESTVQKPKLVAPAAVVHPRVHMLRRGRLF
>g1044
MSTHHFEADEPEQVIIKADEVDQTGTDISPVPDEELLAEVVDGLTLEPSPVIERQMPAGPASGNSR
PTGRPGLARDAAAPPPPMQPPPPAPGQNAIELAPDSLSLAQLRRIVQDIPKVEQQVYAFRYADTQP
FPEELDEWFQYSEPERMMLVGSKVSFHQQWKEFCHRHSLDTGTSWLDADFDTRKKFLKHLLTGLDD
SDLYTRIEALEAVCYAMCGVWGLTGGRPVDAYPENPTAEEESDNPRTKSLQIKWIVDNVLLIHECG
GIPRLYEYMKSIYDKESSPLETDPVDLDNEKEHATYLIAREREANLILTCLYFVVEVARRQETLDK
TNVKLRDAVSALQPSLLITLVKVISGLRWEDSAAIPFTRIALLFWKTIILIFGGTQSLEDAKRALE
PKMDIPTDPADRREPFLTASPLDYHIFRQEVTSKYPAYNPPPLVVPLELDHKSILPPFANHPSRLA
STHGVSGPPAVGSSGSILHQPVHIATPAPSPPPSPIGPSGKAAKKQNYQTNQHFPFMYPPLDDSSN
NIGGKGTSEMQDTLVGKKWEGSDVPASIIEAGKLFSSHVKMTRATRQLWEERERFMKYERGWNADD
NVNEGTNDEEHSDTSSAPSESRRDSVRQTTPETENEDVQRRLDAVENFYKQAMPHLQAIVIVLLKV
ILTNISSMINQANGQGGHGAGMTNGYPYGGTREDFLNDVEELDIIRLREITGKAISGALLLLLKWF
KRSHILKFEYMTQLLLDSNYLPLILKMFAHQDVDHLIAQQNDRKEYSFFHFCRFHSDDPPEEDASI
ENESMDEEEEAIPPPILRRRASVNGSSSVRGPSPVKNEQGYTDGTTRPEVDELGYPTGPVPEDPIT
AYSFRNFFSVINYLHIMQKITRDKAHRCLLLVQYRSSTILRKGLKIPDPHLRLYTLKLFKSQVPYC
GRKWRQTNMRVITAIYLYCRPELRDDWLAGSDVDAEVEDALPLEQALRGLTHWWHLRQYKDVLNIE
EEGTSLFEEERDFFTRELDAMGWGLYGEDMLNDVDERDFGMPTSGTTEWDGGPLQMEGW
>g1045
MSGPDQPLASLSLTHVHYNPNDLLSHASAWLALVPQALCIIYVTLIWASREVEVLLMFAGQMGCEA
LNFGLKRLIREERPQNMHGKGYGMPSSHAQFAAYFSCYAILFLLLRHRPNYSLTPDAVTFFQRVAL
SFLACVGASAVAISRLYLNYHTKKQVVVGFTAGIIFSVVWFSFVSYLRSSGWVDWGLDLTLSRALR
IRDLVVSEDLTEAGWQRWEAQRKLKRREHSDAHKSD
>g1046
MMAVPTSIPEEDDRSMELSSIAAIYPEIRIDERYPFRASLDIPVAPSVPIRVRFELGSEPFQAPMT
PPTSLDTNGTSGKVEIFNLNNANVAENVYDDVHAISHLPPVTLDIELPLGYPSEKPPIFNISTSPQ
WLSPRQLATLKEDGKRLWLEAGKDMVVYTYIDHIQQMTDDVFGIARDHGDEIKLPRGLKIALLDFD
MKAKREKFEQETFECGVCLEPKKGVNCHRMLLCSHVFCIPCLQDFYNTCITEGDVDSVKCMAPDCG
RDTNRAHDAAQEPSGATSRKRKRRNKDRLISPSELLQIPLEQETVQRYVFLKRKKKLESDKTTVYC
PRQWCQGVAQSKKHPKPVNPMADDFDDSADEDDAAAEFDPLGEESQLPPMADRVAICEDCLYAFCC
VCKKGWHGELVRCSPRREAELTAEEKATEEYLKLYTTQCPTCKTPCQKKMGCNHMICFQCGTHFCY
LCTAWLXEDNPYKHFNTLDSSCYNRLWDLEGGDGIDPDGAEELHRPPENVADFDSEPESDDDNPPD
DPDDGHDQPVWEFSPDNSDSDSDDEDDLGRRRPPPPAPVPPRVIPVAGNPDGEPGFNLRGLDAAGR
AVAAERQAQALAMAQIRDRPGAREAFPRPPVRPGAGLPPRRHPPGNQPVRLEGLQRFLELVQNDQE
DEWDSDELNE
>g1047
MSDSRSFSFSTVRIPPPPSPHSPLRSPKRRLHLQHLPAHEDPLLGNLSPESILDALAAINALARHE
GYANDILTRSISQASPEDRLLGARAALAAKKLREWLKELQAWQWPKGLGIQQGKGFISPLDNQSEY

WGSLPARQVVEYEARIEQIRDGMDGLNVDELKEHIMNVHVPGRSRPSSAHSALSTISAPPFTHMQL
SDFTAVITATILRALPTLARLNMLLDTWTVRLLVLRQIPSLLSSLETARASLDSVLEATKESQRNK
RYSMLDSNYRREALIELIGTAGSRMDTVLDALEGREDSIPENWIDRMDALETDFASWVQLTGKMAI
EKVWVSSELEAPKQHTIKEDQLQHDATDTGQPPHEDATERDVQICEDPAPPTVHEIVDSSPNVSTT
SHSITVSPEESDSPPVNFTDSGSTVTADTISLLANEEDVQVLSTKNENHTPILMVTKPHPPSESTD
ENPDLGIATYTAEFERTEDDVYSPIPVMEHSHDHEQKAIEVDTATNYEAEESTIPLRVKRSIPVLE
VIEESTSEESAPSVKNHTKPLESLTPLPVSIPGTTDSNASPAMHAEASHAITEPKTNPLAILAQPT
IVSQDEPQVAAFEYVRSSGQFEDQELQHQEISTPNLEEEDVTKEIPDQISGEHLSKREATSTLEPA
TRTVDYSSTVDGGNLDQIRGRNKEISSSDASIPDSDAGESSPRRADSESPHSIMTPSLALEDSAIK
ETPQSSLIESPIKVVATENQSFGPFLEQTIPLHPDIPVQSIEQDIPSRLCQNHEAAQPHSRKTSGQ
SYRSSVPFSPGVFSSTSDRTIREKSSPQLSSSALQDLEAYKHSNDASLPLQRFINDKYDGSYSASH
EMHSDSASSRDDTSQYLLEDTEILRFRDRSESPPPNAIPRRAIRGPSSSLMRGTISSLNKVVGTGN
RVESDPFSYESAVESAERVRSRSRLGSPYDVDSEPTPWRRKSSTSDLLQAPKHHLQNQPSMESIGS
YVSSNGTGDMRRRYSFSTDGGSFAIRPVHEADSDLQEKIHSILTTIPGKIRLSNKVARDYDQHSVI
SSMSSKPRLKARSPFSTPSRAGTPTPSEGFTPSSRQRRTVSHKSEDKTVKVYHLHHRGKTEPTKLF
VRTVGENGERVMVRVGGGWADLGEYLREYVMHHGRQTPSSSHVEVKGLPATTTSPGSTTSAAATVI
AQTSPKGPTTIPTSNRPASSLSVHKTRRISKPSELPELAIDDVEPTSDNLSLPLFPSIGRRTSISS
LNSVSVSSILGDGSSVYSPHPGSARTPLSHSSTPLGLAGPKPRTRHVSMTPESEAWVEDVIGQARR
TSSITVKPPTQKHADHRSEREVHNSSRMSMRSVSDFPSVGRNKRVVLKGLGTHDRS
>g1048
MASSQAPIAIIGAGPAGLTLARLLELANIPYMIFERAESALWADEHSSSGTLDIHKGSGQAALEEA
GLMEDFQRIARWGVPVKFVDSQGEIVFATVSGDSNEDKPEIDRKDLQKLLLDSIKASTIRWGCKIE
RVQKESDGAHSICYANGGVESGFRLIVGADGAWSKVRKLLTPAEPQYLGTHFFTTFLKPDNPYYSS
LTSMVGNGNYLALRKGRQFFLHYLGDGSYHLAVGMKLPENWNPDPTRLHDPAALWQSLLQYEYAEW
SPEIKALIQSATHGYRSWPLYSIPRTSVPWEHVPGVTLLGDAAHLTGPGGDGVNSALHDSVELARQ
IIKHGIDDLDSAVVEYEQAMFPRALEAINKGQWAAEHLFNAESSQSFLQAAMQQ
>g1049
MVERNQYADTSETPSVASSVGPPKALGEKILADVPTELLTELKRLKEMFIVDREKLKIITQHFVGE
LEKGLTDNGGDIPMNVTWSFGYPTGHETGSYITIDMGGTNVRVCNVVLTDDKGGVDLIQEKFLMPE
GLKRSNAETLWDFLADCTAQFLEKHNPGTKEVLPLAFTFSFPLTQPNIRSGILQRWTKDFDVDEVE
GEDVVPQLEKAFKKQNLPVTIVAVVNDTTGTLIASAYQDPHMRIGSIFSTGVNAAYLEVCGAIPKI
KHYGLPPDLEIAINTEYGAFDNSQKVLPRNSFDRIIDEKSSRPGQQLYEKMVAGLYIGEILRLVLL
DLHESNYIFRGQDVSYLRQKNSIDSLFLSTVEEDKSDALHDIAFIFNSALNIKAIDYELKVTRYLT
ELIATRSARLFSCGIAAISKKKGLETCHVGVDGSVFTKYAHFRNRAAQALKEILGWPEDSSDPISL
HSAEDGSGVGAALIAALAMQRSDGVEASKCGR
>g1050
MRFRSQLTNVGTFAKLAASLNSLGKICWMRLERETVHFTIIPDQGTQVWAVLPAEAIFDESYVLEA
AAGAVNLEVPIAALSRALRSAIGDKSAQLRLTRKGNVPLLALTVVSSSLIPGNIPVGSEVDEFGEI
DHDQVQSNARANAPRERETVITQEIPVKVLHQSAVEGLHEPRCRDPDVHIILPNLAQLKAISDRFT
KLAASNGKSSGGASLASGTTGPKLQLSANMHGSLKIGIATDSLRISSVWTGLTXPPLDPRQMQGTD
IDQLPSERMRQLGNSADDDDGAGWAKCKDVFPSDEAKEAHENLHQTHELTCVQCGQKCDTPYELRL
HYDYHDDMEFRQTRKEVSSSKSSHRRASSHPQINHGGSISIPLSPTENAIRRIHKRKSQRLLDVWS
LKKAGKGEEGEIVAMAFSLPEDMERVHAIDGRLQDVEARAVVNFPITQFTTWLDMRDNDEKCLDSP
TCSEKDSLSEKGRPSLDEDKAAVAPRRMSLLKSVLGLRLPKRLSRSDTSASQPRKLMKKENSVRFS
FVSKPKKSGETTRSESRISNRILEPSEKTMDLGLPRMDETGGSDGNPFIHVRISADGAIVEDKSGR
RRVLQMEQIAHIYSVVSADTSLASIKSRITSLQFRAETDLEGENPRDAILAYKEILDSLSRYPALD
TNLRIRSGIYHRLGSVYSSLGAAGESEYYFLKALAIYRRIYGRDQSVIYALLNDIAKLCEKDGYAT
EASALYERVLAGRLRVLGHNDPETLHSMQELASIKTNLGDVESALQLLEDAVPAFETVLGLQNEST
LVAMSHLSILYQKLGLKEKSLAASRKILPYCKNVVGYDSILTRNTVIRYLEESESFDFPTDVKSIL
DHYRRSRSAESFRVLQTLGRAYMDSGLNRDACEVFLSLLDETTGAKELDSLEFFDALSALCVALEH
LGHFDEAIKNYGQLLQSAHKMPSDHPSRSRMDYARSRVTDLIHRREVLTAERRAWEMFEDGPCTYC
QSKTKSVAITPRCPPAVQNDALSMILPAERGSTPPSITASHTVYLDPRNFTTFRMKLSSNVNTLLV

FSPEADIRYTIIGNLSESGSDQVSLPASASVSISAGKKPSNRTTSTLTGHEWLTPAQQESVSITPM
EQSAAIYLVVAPGEQMMKSLVEKRVRARSGDGEKEYFEALDIPDSKLIEFAQGLMMNGYMGEAFLY
IVGWM
>g1051
MTNLLSTWETSASLSSLVSIGTHRLFVSVSGPLRHARPQSNASNTDPIAGYSTTWTNDPIIIIFPG
AGESSSSWPRVQENVSRFARILVYDRTGLGKSESRTNDTNVDNKGAAIIAAEEVCTLLNALRITGP
YVLVAHSYGAIVAREFLHLRSNQVAGMVLADASTERQPEYFRLPDPNMNAVMGNLKFSAVTGLRAN
AQLSSDEWRTRAIEMSQSAPAIQAEVAGYEIVCAQLGEKNQFKGQVLENRPLSVIKCQSFRDYEKM
YEAGVEAGNGTVEQRQAFKGLLDKWDAWSEELQREQLQLSSNARWIEVSGCGHNINLLRPDVIVQE
ILWVLGKSKDDLRL
>g1052
MAELTRTLPASWYCSSPLYQLERRAVFLKSWYLIGPVVRFLSVDEKVSFEIAQQNIYAVRVSATTE
FPGNDDLKVYEASTGNQLPSHVTPTGLLFAALSSSAPTFHEFFPNLEKLLAKVDFTKRPWRRSIKY
EGNFNWKTMVDGYQECLHCQYTHPSFSVYYPPTFYTVYNHQNFSQHVADPKKPDDGLFLYFFPICT
LNVYGGGMSSFRVCPTEEPGVTRMEFDYYHVEEGAKFEDYYQFTRQVAMEDFELCEKAQTNLEKGV
YGEGILNPEKESGVAYYQERVFDLVRQQFEEERNKHDTDGDRSVVQSMPTSRVIEAEA
>g1053
MGAAGSSKPTVLHIGDAIKYNHDFYEHEFASRFNVVQATETDRESFIEALKSKKYGTFSAIFRPHF
QTGGFMGQWDSELIPLLPPSVRIFASAGAGFNWANVDLLGAQKIWYANGAGASDEAVSDTALYMVL
SVFRNFTRSQIAARTADVETFNATHKVIATISRNPRNHVLGIVGFGNISKKLAYKAKTALGMKIHY
FDIVRAPAAEEEALETTYHSSLDELLKVADCISLHTPLNKHTQDLIGSKQLSLMKPGSRIVNTARG
QVLNEDALITALNNGHLSAAALDVHYHEPQVSPVLAKMENVTLTTHIGGGALETRINFELNAMKNI
IAVVGEDGNFKGEPLTPVNRKAFEAASA
>g1054
MPSALAAKAKQLPPGVYVPVISLFKDTPRQELDLDAAYKHFSHMIRGGVHGIVLAGTNAEAVLLSP
NDRIELIRVAKKAATDLGVPNYPLVAGITGHSTNESIQLAEDAAKAGAGWALLLPPNYWAKAVTKD
VILSFYREVADNSPIPVVIYSFPAVTNGVDMSSDVMSELAQHPNIVGTKLTCGNAGKVTRLSYQYT
PEQFSVYAGSSDWLLPCLIGGGVGCVTGMGNVFPKSTSHLYNLWAAGKTEEARKLQGVVAEAEKVC
KEGIALTKFASYYFNGRDMGISEKVYYPRKPYQPAKKDREEWAIKSMAELAAYEKTLPDVLGA
>g1055
MAPKRQRLQADPPRGGQLSGTRGGSPPDTGPSSSFNDATRGDHGFLPQLHADNTATVVSASPGTKI
DIPSGNGLSSHTHQQVQPAETPMSAPARNSSNPANVYGSLPGITRKITACAACRKNKIKCDMPASG
PPCTRCRRRSLSCVLNRSLQSLLEDTKNTDNLKTDIQNLHRTLERVCNYLKLDNPEPLFSINGDHQ
APPVNQARSDDQAANDTEGCEISPPESPSASHAPIDTFLGMAKMGKPPSSAGSPQNRKRPELLRED
LISRSIISTTVAQTLLDRYFTRLDHYIYGTAGGLYDIQKLRKKSPSLLAAICTVSALHDSHDSQVY
EICNRELRQLVSRSMFEKRDINHIRALCISSFWLADASRILSSDAIRRAADLRLHRLIDNTSPSPK
NVPQGNHHPPEIKMTDRLRLWYLLFICDQHLSILHNRDALLRGDRAVNIDWESYLSHPDTTQDDIR
ILSQVNLLIIMSEMRDTLGGDYETQVPQVLFSQINNYARRLDRWFNKFSLLFQPNQQIANFPRKGL
ELHYQFSKLYLGHHVFRNLNGQSVPSHYLTAAVAAHDAAVSIFDMILTDTTLHSSLVGMPHYFHIM
IAFAGNFLLEICKNYHDQLSIRVQNDFALIEQVLGVFQTIPCLSQHPIHRMTLGLGRKLYDCAASV
GLTADITRQNAYGFGDVSHQSLMHGGAGGVGGSMGLQHDQSEYSTMGGQPLSQLVAAQTTPDEFFL
FPDLNFGFTDMTAMMGME
>g1056
MTAVETFTLNTGAKIPAVGFGTWKSPPGEVAKAVEAAFEAGYRHFDCAPLYFNEAEIGEVFKKTKV
PRSEYFVTTKLWSSDHRRVKEALNKSLSDLCLDYVDLYLMHWPVSLDPSTSTEDYGKENRKVHATG
WDFRDTWREMEALLSTGKVKAIGVANFSTINLEKLLQTAKIMPAVNQTEIQPLLPQNKLHAYCKSK
GIHQTAFGPLGGAVSERSLHQMDVIKEIAEKRSTGTANVMLSWGIQKGWSVIPKSVNVERIKQNLH
GNFVLSAEEMEKIDSLARPKGKRFNRPDWGTVIFHDDADVDLED
>g1057
MSMKILITGAGGFIGQALTKELLSDTQVSSITLTDVMKPPVPSSENNPISSPEIDSLAADLTSLTT
CQELCSSKAFTHVYLLHGIMSGAAEANLELGLKVNVDSMRHVMDTLRTVRPGIRVIFPSSLAVFGP
ADPLKPEKVTENTIPLPQSSYGAQKQMGETLLNDYSRRGLLDGRIVRLPTIIVRPGKPSGAASSFC

SGILXEPFKGEESELPVPTSLNLWVCSTRTVIKNLIIARDIPTEAFDKVVARGSSRIVNLPGITVT
VQEMLDALLQVGGEEVRKLVKEVPDSSVESIVGSWPAEFDISRATQLGFQQDGDLVKTIKEYLEDY
APKKL
>g1058
MAAAALDVIVISGEELRKHRTSKDCWIAVHSKVYDVTEFLEEHPGGSAIILKYAGADATQAYDEVH
SPGIIHETLAGEHYKGQIDPFDTPKVEEPVKTTTNVEQVKVAEITAVKSEIPHLSTLLSVTDFEAV
AQKKFSAKTFAFYSSAATDLISHRDNIESYRSLLLRPRVLRNVKNVSIKRRILGYESSAPFFVSPA
AMAKLAHPDGELAIARACAEDNIIQIISNNASYPLQDIVAAGKPGQIFFLQLYVNSERQKTEQLLK
KAREAGIKAIFVTVDAPIPGKREADERISAENFVSAMSGAVATNDKKGGGLGRVMAQYIDSSLNWE
DLAWIKRVSRLPIFLKGVQTAADAKLAVEYGCAGVLLSNHGGRSLDTVQPAIVTLLEIHKECPEVF
GQLEIYVDGGIRRGTDILKALALGATAVGVGRPYLYSLAYGQEGVQHLTEILKDELECTMKLAGIT
NLDQANPGMVNTIKLEHLIRTGGERHPWIKWEPRFRL
>g1059
MVLEIFVEICIQNPKAVNKARAELDSVIGPKRLPSFSDKDNCPYINAFILELLRRHPISPFGVPHM
VTQDDEYMGYRIPAGTIIVANQMGMNMDETIFENPEKFESDRYMQNVDLPHPATFGFGRRQCPGHH
IARANLFIVISRLLWGYDIHQHEQADTDNKFAVSSDKALFVVRSDEHRKTIENAAVEEK
>g1060
MAEFISVTFPNASTELNPIPNAGIDRDYLIRYARNLDDYGFNYTLVPYDSSSYDPFTIGATILAVT
KQIKVIIALRPNTLPPTVAAKALATLDQLGGGRVVVHFIAGGSDVEQAKEGDFLTKDQRYARLEDY
IRILRRAWESAEPFDWDSQFFTFKQFSNRVRPVNKTIPVSVGGSSDEAYRIGGSLGDIFGLWGEPL
KETKEQIDRIYAEAEKAGRVDRPRIWVTFRPIVAETDELAWEKAHRTLDALTTNRANGQGKXPAVA
PAPQNVGSQRLLEIAARGEVQDRALWYPTVTATNARGASTALVGSPQTVADSILDYIKLGADLISI
RGYDNLNDAIDYGRHVLPRVRDGLKSLES
>g1061
MNSERDGPSDIEGHKTEQAFLLNDTVPSFSWQQLIVTVKDRETKKAKDLICDVNGSVEKGELMAIM
GPSGCGKTTLLNLLARRNPTSSAKVAGKTLVGGSDIDTASFQHISSYVEQEDTLIGSLTVEETLKF
SAELSLAGSTSRAERNDRVQLLLKAFGIQEQAKTLIGTPIRKGISGGQKRRLSVASQLITSPKILF
IDEPTSGLDSTASYEVISYLKKLARRNNLIVIASIHQPSTSTFELFDKLLLLSKGRTCYLGPTAAA
RNHFANIGYPLPMNVNPAEFILDIVSTDFSRDDESGAYGEKRNSSQPTRTAIERLEYIQKSWQESK
QAHITNTQVMNTSDEKDKYRQMKHIAESAASSRPAVYRIVFVLLHRSFIKSNRDVIAYGIRIAMYL
GLAIMMGTVWLRLHESQEYIQPFINAIFFGSAFMSFMAVAYVPAYLEDRSIFVKERANGLYGAAPF
IIANFLIGLPYLFLISLLFSIVAYWLSNFQPSAVAFFTWVMWLFLDLVAAESLVVLMSSIFPIFVI
SLALVAFANGLWMSVGGFLVTPTILNPFWKYVFHYIDYQAYVFQGMMVNEFSRRNYSCGSSCQCMY
QTDLASQCQIRGSGVLEQYGYAEGRTGKWVGILIGIIAVYRLFGWIALTLKRT
>g1062
MLSDYNRRGSAGSSTLESEIFDIDDRAAAALYRPYFLFPRRTRWRKYICRSLRRVVYVLLLLLVAL
ISVTFVFFPSYTHXPPHYRSLEHTINYSQESGRGNPRHEKVFIANNLYDASGELARGKWGSSLLEL
IDLLGEENTFLSIYENDAGDEAGRALAELDAQVNCNKSIVFEPHYDFSHAENVTLPDGSVRLRRVP
YLADLRNRALRPLEEQNRKGIQYDKVLFLNDVYYNPLDIVHLLFSTNADQNGVSSYRAACAVDFGN
LIKFYDSFAVRDAHGYPIGVPFYPWFVSTGAATSRKDVLDQKDAVRVRSCWGGIVAFDAKYFQTST
NTAQQVSKSVPRETLADRPKEQERPAVTTSTPVRFRTISDVFWEASESCLIHADLQDPYTNIDQVI
DTGIYINPYIRVSYEYRAFTRLGFVRRFERLFAWPNWIANLFSHHPEHNSRRTNIAGETVTQRVWQ
PNASYDEGGYWKDETRVASRGGFCARRSLFVLNNKRQEDDSGWEPIRVPPDPDSLDWTL
>g1063
MVSSESQPIVRRQSAQHYQTFNTPPPKSRGRPPSNDSERSHSFGSLHANGDDEDESAAAQNKLPKK
QMAVLALIALAEQTALNSISPYLPDMTSRFPEVHGENVGVYVGLIASAFALAQFVTNYYWGWLSDR
VGRKPVILLGTACTAVCFVLFGFCTTLWQAILVQALMGMLNGNAGLVSTCLGEITNRSNQSRAFTY
LPVLYGIGGITGPLLGGSLIFKTNPLNKGQPNPYPYVLPNILSAVILIIDFIFVICFLEESLEDAQ
NLPTFRDRFKELFSWMWQFTTSHRPTYLRARSGPVYHHPTARRGRSFSDLTQDESDLDSASETADD
DDQPSELTREQIFNRDTILLLLTYLIFALVNVSFNSLYPIFAQASEPAGRDLSPQEIGLSLGFSGI
VTIVFQICVFGKLRDKMGNKWSYRAGLLGFVIAFLLMPLIGYKGSDENGRLSKKSALLAVELCFVL
LIKTVAAVGGLTSALLLVTNSAPNHSVLGALNGLAQTLSAAGRSVGPFLSGGLFSLAQKLPKGEML

AFGVFAAVSFVGFILSFGIRSSALEAAGWSSDEDDDVDKSDDETEHGA
>g1064
MVLPPGVYRADHVGSLLRPESLKIARLQFKNGEITSAEIKSQEDIYISDVVKKQLSHGLRSVTDGE
FRRWSFHIDFLTQLGGVEYREDLATIKPKRDSPPRLFVTEKLYHPRAIQVNDFKFLQQAVRENSSS
IIPGSAKVCIPAPTALHFRGGRENISREAYPNMEEFFGDLAKMWQLEIMDLYAAGCRFVQLDETNM
VYLCDPFFRNAALERNEDPDQLPLQYAELINAAISQRPADMKIGIHLCRGNYRSQWFATGGYEPIA
EVLFQKINVDVYFLEFDDERSGGFEPLRFLPADKIAVLGLMTSKRPSLEDKEIVISRLHEAAKYCP
GGLDQLCLSHQCGFSSSVEGNELTIEDQWKKISLMVDIAKCVWNEDLSR
>g1065
MRMSGVRSRYGKDPEFYLLARLAPGKENLASLTDESLHGRHKAKLAPAYTQDVNGARELTIDAHLS
DLLDSIRTNYLATSTSTNTMDLAKEMQYFTTKAIIELTFGPAFDDLLTDEVVGSLVDMPEDAFNFP
GFLYAMRLTKFMGSPIISRLLSPKENPEVGMGKFLSVTNQLNDEKFLASIPKNTLGMIQVYIQNGL
DSEQVAHDALTQVLAGQVTVSAAISGIILLLMSNPRVLCTLRKEIDDAWDRSSTSEHEGKKIVTWT
ETLELPYLQAVIREGLRIFPPTVDPLPRITPPEGDSVEVGDRNVFIPGGTRVLTSTWAITRRTDVF
GEDADIFRPERWIGLDTSTSVRMQKVADLIFGFGKGRCLGITIARIIISKAVFELVRRYDMALTKP
WSPMTNVLNMGAWERSKMMVIVTEREAV
>g1066
MEVFESFGFEQEIIKLWDPITDDAVWMRDVDGRFYRAERETRPLPLRTRWVGGLLQQAVIENIIKQ
HIKSISPIQVEYESVPTSLWFDEAEINDPDGYPCIINLRRRKSTKEDAATNDHDQLPQNTETVRAK
YVIGADGGKSWTRKQLGFAMEGNKTDSIWGVIDLIVISDFPDIEGGNLFFFRRERGMTRFYVQLNR
GKVTENLCRESVTQETIIEKIRYMLRPYTVAVKNCEWWSAYTVAQHLSTGMTKNGRAFLVGDAVHT
HSPLVGQGMNVGMQDSYNLGWKLAGVIRKQFDSKILDTFEIERRPVAEQLVEVDQIYLQLFDAPKG
KEPEWLLERAAQLEPFLMGLSVNYSTSPLTALSSTSTQATNLLPSDLSERIIPGRRFPEMTVYNHA
SGKLYPLQRLLRANGNFHVIVFAGDISQLEKRNLMNRAGTGLESIQKGLQLKLNIFAIHRTTRAAV
ELANMHDIFFPFDETMGRDYDRVYCDLDVTYEEAGIDIGGGIVVVRPDQYVSWTGGLDNTDGLREY
FEHVFRT
>g1067
MNSDPAKVGRAPITATEKLIECLSKDGCVIATDFTSVEDLAQVQDDVNPYMEGYTEIRAHLFPPQY
RWCGGIFGRSETVRNRWLAEENLDLVRDHFLTTTLTPPGGYDGATFESKPVLAFSVTLDIGPGAKQ
QPLHRDDYLYFHRHRDLTKSGYEIGTDMMMLMFVPAVKTTIENGATLVVPGSHLWDDVRTPVDNEV
CYATMTPGEVLLVLGSTWHAGGGNVTTDVRRPQHTFFFTRGMYRPEENPYLSYSPQQVLSWSEKSQ
SLAGMNRDTLAFDRTNYLVPSALYAESTKAQIPDK
>g1068
MRETCMLSLTPNEVDQVGEYCVANTTPLPHCLLEHSVTTANKTERNGEMAGSLAQCAYLMSTARGL
RPKRVLELGTFTGVMSLALYEATRDIGSEIVTLDIDKEFIAIAKDAFQKYQAVDRIEIIEGDCTES
LSQLQGDFDLIYIDADQFQYEAYVRAILDRELLSPQGIILVDDGNQIPPVHRSCPVNEITVLLRGI
TVNPSMSPTVSDEDREWCREVAVNMNKFNQFAATDPRILGTLLPFFNGIMHITWR
>g1069
MIVPIVPYTLEERVGVDDADVLKWTSIFLAVFGFTNVISSPLIGLLADKFPSRRILFTFGLLVMAI
STALYWIGTTIFMLVAARALQGLSATAVWVVGMAIINDVSGPDDLGISMSYVWSANTLGLLCGPII
GGTLFQFAGYHSVFAVAIGLLGVDTVLRLLMAEISNEDVSPSSNIVRAVDTEGELREENPLLQAPV
AAKHSYNDWNMNPVPENEPTADPSLRVRSAFSILLCYPRTATIVVASIMNAGILSSFETVLPLYLV
QLFHIDSARVGLFFLSLSGSTLLSPTFGKIADKYGTKWITVISTICVGGAILMMSLVNRNTTFHQS
LLYVLLFFCGIGRTGQRVGMSTDTGLALRALVEEPGVVDQQLTVAGITGQAYGLLSTACGIGVTLG
PLLRSLTLSLAVPSAMYTGGDLRRKKEKQALVGV
>g1070
MLAVLKLGAAFVLLDSGLPVSRLQNMVEQVNAAIVLCSGSSVQLAEKLASTVVKVPVVTTEDNASS
TEFCVPANIDPLLTIAAIVFTSGTTGTPKGVLVSHANLASTFKYTATTVGLNERTRMLDSASYGFD
AAIYQSLGTLCNGGCVCVPSDSERLTAMASAIRRFNVNTAILTPSVARLLEPESVPSLQRLKLVGE
SASLTLIEKWLSHNVKIVLGYGPSECAVCSNVGIFQRDVTAVNNIGHTLGCLAWIVDLDDHEKLLP
VGATGELLLEGPNVGAGYLKDRERTSAAFIKAPKWLQRGFDAVAGRNSTLYKTGDLVQYHRDGSIL
YIGRKDQQKKLRGQRIDLKEIEYAIAQASAEKLDVIAEICTNALSENELQLVAFCCLRSFENTIKN

IPQQSKTDTESLILPASREFMTIAEQIRKKLQSEVPSYMIPSIILPVSEIPRTKPSDKLDRKRMIR
EVRKVDQSHLRSYFPVTSKALSTAKELKLQQLLKQVLRSDLNISADDHLFSLGGTSITAIELIAAA
RQSGFFLQMQDVLYHDNIAALASKMSALDDTKDMSTSLPPFSLIAEKLRTEVFREASLRYGIYSTS
IEDILPCTPMQEGLMALGEQRYGGHIAQQIFDLPPETDLKCLKSSWETVSLQNSIMRTCIIDTPAR
LLQVVTREPLLIPEYENVELGFVTRTLISQIRLGSPLVNLAIISQSRGKSSCSQLIWTAHHAACDG
WQVPRIFDQVELAYRGTKLPPQCEFNLFVKYIVDGDRERHSAYWTRQLAGPIPATYPIHHEGEGWA
TESKRIQVHLGDSAPHRHLLATKINLAWSLLVAAYAESSDIVFGLTLTGRNAPIPGIADMTGPTIT
TVPFRQQVDFSLTVGEALDRVQKQLIEMIPYEQTGLQNIRQLSDEAAAACDLQSLLVIQPSIETQS
EGRYKVLIPAQKYKESRLAFSNYPLTMECTICSNENLVNVSAQFNDRIISSDQMTHILHQFGHALR
QIHAHPDMKLSTVNLLSPYDIERLIAWNGTCQTAVNQTLNELIERRTDQDPNAPAVDAWDGNWTYQ
ELNTLSSQFAFFLQQLGIHSDEVVPVCSEKSRLTPVIILALLKIGAGCTLVDARLPQGRVNQILSN
SRARYFICSEMYQYHFDVADTSAKKIVASVPWIQELRPVLVDVQWNNNPDTIAFVVYTSGSTGRPK
GVVLEHRALSTSIFAHGSVLGIGPSSRMLQFASCSFDINLYENMTTLVLGGCICIPSEDARLNKPA
QYARESGATVTLSAPTIMQALGQEEIPELKTISLGGEPITAEVVDTWKDQAQVFNGYGPAECTICA
MHPVEKNTKGEGQANPLATIGRGLGCTFWAVDIVDPSRLAPVGVVGELYIEGPVLARGYLNDFERT
MSSFLSSPSWLNRFREGDTSQSRVYRTGDLVRYNTDGTVVFVGRRDTQTKLRGQRIELSEVEYYLH
KAFFESPKVVVDVATLLGKNQLIGFVEEKTDQGSLCKDCNRVDPSTSEQKQSEQFVFKSLSEYQAE
NAEATRMLFQALPSYMVPSLILHITAIPKTLTGKTDRKALREAASNLSAEEISLAMQGETQHRSPS
TEAETKLRDLWSDILRLPKDCIGADDSFFARGGDSITAMRLVSSARALGLHLTVRDVINSPKLSSL
AQIACPDILNGLHDQTRERKTRQVWEPFHALECEIGSRQNFIQDIIRPFDIPDSNVSDVLPATSLQ
EFALSNCPTFFIFNIGDRLDLDLAVSSWLRIIAEHDILRTLFISHNDKHYQVILRDLSRIEQSFHT
ITENTDEAVDTLLREKEAASVSLPVGNSPLKFIIITSPETRYLVLRMSHAQYDGFSISELWSDWRS
AFDGLPLHPRCSYADFVYSARALGENGGYDFYKVVLKDSTMTYLTPLPTAEALGDDKANENSGVHE
VETVVENIHGLPNVTMATFVQAVWIMTLVRWSKKDDIVFGLVTTGRRSKRHDFDTVMGPCLEIMPI
RAKLRPGWSLYDLCEHLQRQDADSMEFEGMELFKIIKECTNWDPAAPLGTWLQHDNTDWNAELVLG
GMNYGGPRVYDTPYGQDDVGIETLYLGDGTLKIKLSSPAHILNASMLHDLQILTCETLKIFAEKPE
TNLADYYCQQTALF
>g1071
MKSSTGDIVQQLDQVRVNIDAFTPDDAQARRDLRASALALASTLSSPEDIFADAFYQPALAVAILV
AMEAQWLQCLASESAPLSATELSQRTQTSRSTIVRFMRVLTSHRIVTEDDTEKYIANSNTRWLTTP
AALAGAQILYPATAEACLQLPNWYASRKFAEPTSLKDGPFFATFKKTFWDSLRDDPAKHAAFSLSM
EGVYEEDAIKSDAELIGRKLKQAADMQVGDVNDVLLVDIGGGTGHLLRTFRKLHRELPGKLVLQDR
ASVIDKMDSKIMEGIDLIAYDFFDPQPIKYAEAYSLRHVLHDWSDEDCRKILANTCAVMRKGYSKL
YIMDILLPDQGCSVTSALQDIMLLALFCGKERSKRQFEELLGSVGLRITEVFVSDASTAEAGKIFG
VPTPLGLHVFLPPKMVPELTEFSDHESNLEAHFALHTLENKTKFSRTEPLFAKVTHKGINHHGRET
VQILEHGICEEIRKVIGPCGQWKQFNASRIIKQVAMAGCNLVAHGPMLGSNQAWLSEIEGYLDDAM
FGFFDVMEYPQFLHETLALFNKRMRRLPGRLERIKETLKPVLEDYRQSEKKHIDGIQWASDYASDE
EQRGIEYQALTHIIMYYFNSIFIMPTIVILFYDLLSHPDCIDPLRKEIREVFGKDMPADFTPNKLD
KLLKLDSFLKESQRIATPTLCNFLTMGRLALTEIRFSNGVVLPKGTPFGIPLWPLAHDPDLWPEPS
AFDPWRHLRMGEAEGNRVDNLMTAARPRWLLWGRGRHACPGRFFVVHVIKITAVMLLSSYDMRFAE
DVTPAGEYIGSGFMPDMKAEIQFRDRKDTSVQF
>g1072
MLVSSTVSPHTLLNNLESLLDTNSIPNLRNSPCADEKKLHTYFPWQDQAAFSGDKLLDILLNTPSG
EKAREWSSYYTHESHLPGQGHQQAVWTKEKWDEFGLPENNIVAFDVIVPEPRGQRLALLGHDDEVL
HEVSMFEDESQIKNQEKDKKPFITAFHGFSAKGNVTAQYVFVNFGTADDFADLAKANLTVEGKIAI
MKYGRINRGEQVKNAENMGMTGVILYNDPQQDGGITEANGYKPYPEGPARALTSIERGSVGNMEYD
ATEKPVRNGPSITSLPISYNDAIPLLKALNGCGPTADSLAPQWHGGALGYKGVDYNVGPSPPWLTL
NLYNDLEHVQRKIYDVIGSVKGTLMDDEVIILGNHRDSWVSGAGDSNSGSSVLMEVARSFGAAYKQ
GWRPLRSIVFASWEGEEFGQLGSKEWVRENLSWLDPTSVAYINVVMGASGSKFHAKASPLLNKAIL
KATGETLSPNQTVPGQTILDIWGGDILPGAPSDAMRFLDTACISSVDFGFSPAPGDPVLHYHSRFD
TVDWMERFGDPGWEYHTTTARLWLLLASSLIEDPIISFNVTDYAITLDEAVDLVRKDSSQIQTDFD
WTQILDQIEQLRQVAMKFDAYAASVSDRIDRSPAQHWTQYQYHSEPSLATIRSINKIYITFERMFK

YEPGHGNNLWMKHVVFGPSAWYNRGAVFPGLRQSLQSRDVNATEVS
>g1073
MAELNHGDNEPTATETTPLITRKFRDGQSNILPRKKLLLVFPALALVHFTSFIDQTAVSTTLPSIA
SALNAGSTISWVGTSFLTTSTSIQLINGRLSDIFGRKTCLITALTLMGLGNLLSGFSKTAGQLYAT
RAFSGFGAGALNALVQITISDITRLDQRGYYFGILGIAVALGNGLGPVVGGLLTEHISWRWAFWFI
CPLTMAAVGLLVLVLPGSSTEGGIGKKLKMVDWVGVMTSMAAIILMLIPISQGGSALAWTSPIVIG
MLVSGIILFIAFIGIEWRLVELPILPTMNIGIGWVFWGNLFYIPLYFQTVRGMSPATAGSVILPMV
IAHGITSGLSGIITAYFGRYKPVICTGAAFWAVGAMVKSTYGQSTPIQWFFFVGIFEGIGVGCGLQ
PVLVGLLAGSDNVDRAVITGLRNFIRDIGGSIGITISGAILNNVLHSGLKGRFSPSLISQLTSSAF
LLGELDLSDEDRNEIMAVYMRGLNTVFISYAGLTAIMFVASLCLYDYGLTGRRDTKRQVFSHLEDG
NRDSEPGAGSSE
>g1074
MTIVQDVLLLQAPSVDCAQERDHINFLNSSLERFGVVKIDIGFEDDQCRYLEQVIRNLHQYHGHGL
PIDHSATRGWFWDIRPMAPVKQEDDSTASNSSVPRDQARSETARDFPWHTDCSYESSPPRYFALQV
LQNDRCGGXTLSILNVERLLGHLSEASRSSLYHPEYSISVPPEFIKNEGQRQIVGSILCDDENGNT
GLRFREDIFTPLTKAAASAIEELKSVLSGPEIEEEILHLHSEYMSRGSIILVDNRKWLHARNEVKD
PERHLRRVRWDACAFR
>g1075
MERTIQSIVDALSSRRINTLTELRRMERILLAAVQPHVTDLRESSLVGVLAAAWSNYVQNNNLLSE
LRNLTRSYPFSSELLDEAKALVMADPERSRSWNYAWLVLAKIEEENLIDKHARTLATSPDMWGGSL
PHEEMISMLEEKCREDWKRAVEIMLRHWETQPVYPIEEVEQE
>g1076
MEQSLLKYQSTGGLRKIATSAGDFESTAMRLSRSESALHRMALHRSASDELVSNRKPALSKLETGR
SEHLNDRESQQLDVVSAVLSAGEDATSSNFPTAAVSPLSLEMPMMGPTFGVEIFRLEAQTETDIGN
ISETSASTSSSPEMWEEKPRYPSSASSNSTLRSDFDTETSSSDMKPLQRPSTPPRSERRPLNSSGK
KNSPEDKAKSVKFSKGKKRGEEQYFKQKPLPDIPPTLSIVNPQSAQHIARLTSKYSSSRSEXPIDR
LIVLQATEKTLTQAANDLERELEKIGDVHPALRPEQPLSKPADDDENLVAPLNISPKPNRTAASSM
NTQKENVPQGKQPRTYRVFPPSEKPDTTAKDNTIQHNRYNSNSSTESETIGVSVKQNARDSPSSKG
VSRKVKSSVRLPTLRRSRNSNQTAQAKPDGGNKKSDISTRSPVKSLQDEQAALARSTDRYIDAEIA
KALNMDHEDDEDDDGENNKKDAEESEWSTMYPLKAVKKLGIPLLTNSHIPRLSSYDAERILRQQLP
KLDTNSIVSAIDCQKLPPSSIALNKRYPETPDDIGIQARSFITDSTPIQTPTEEKFNTGNVESHND
KVFVGFDRMETVPEGMSPKTEEVPLIMGTPTMSNSPATVFPPRKKSLYELDEYGSTPISQTTKSFF
HIKLPMPTHMTDEVVLALMKNVDNLDALFKYAQVNRQFYRVFKNNELPLIKNALFNMNPPAWELRE
MSPPWSDNSDGLKDPDAPVPEYTASSYLHHYARDVFALVRLKALIYARCSSIVRSETIGGLTGEND
VRAAEIDEAFWRIWTFCRIFGCGKNREGDLNGQIDWLNAGYLAKKNKSGATVITAEPFFSMNNVLF
DPPSGFGDGNRDGLTCNQLYDMLEIWNCLGTLVQGFNRECKDARVAGIFDGLDIPVGDIAKEEAML
EEWTHYILTLGPSALVGLSSLSPDHSTEEIFTKAKQMGMTKWEAPEDGGTRSSFLREAIARAYDSR
VSIQQKLSNSPVSNGGSVDSAATRRERIRNLTINSTRNTGQPENSSWNDGDDASTHRVPPYTCVNN
DGTSMAPLLSPPPRYSVTEARLIDPVDKAIHRMVHELGFSERDATWALKITDTGDMLDINAAVQLL
VREREKRNRFKASDSLAESTASGRDDCLVDISRHRGTVSWRWA
>g1077
MAPAPNCENKHHESQQAFLEIAIFNASSAQIAYTSCADRLELCAEGSSVNGGTTPSLSTLESVITS
LQKIAQRTTISGEDHITNPVKIKKRSIPIFAMIRPRGGDFVYSDEEFKVMKEQLLSLKSATYHQTT
GKKNDSQEQNLFGKVDGFVFGILTNHKLVDRERNEELVKLAAPLPCTFHRAFDEVITSFERSDISA
IRQDELLPRGIIDECEAVINAGFASILTSGGSGRSAVDGARHIAALTEVAKDRIEIIAGGGVRSTN
VGELRRQSTHTRFFHSSAILAKSDGQVMDVADADEIVKLKKELMAPLG
>g1078
MSSTVAGGLHKVQEVIQSAAGQDKKLIDLSRDTADVHKRTKFTTDHGVPVSDTDHWLRVVNDKTSG
PSLLEDQIAREKIHRFDHERIPERVVHARGTAAFGNFTLKEAIPELSHAGILNDTSRKTPVFLRFS
TVQGSRGSADTVRDVRGFAVKFYTEEGNWDLVGNNIPVFFIQDAIKFPDFVHAVKPEPHNEVPQGQ
TAHNNFWDFTFLHPESTHMFMWAMSDRAIPRSYRMMQGFGVNTYTLVNKEGKRHLVKFHFIPHLGV
HSFVWDEALKLAGQDPDFHRKDLMEAIDNKAYPKWDFGIQVIPEENQDDFEFDPLDATKIWPEVLV

PLRIIGELELNRNIDEFFPQXEQVAFCXSHIVPGIDFSDDPLLQGRNFSYFDTQLSRLGINWEELP
INRPVCPVMNHNRDGAMRHKITKGTVNYWPNRFDAVPPTKPEDGGFVSYPQKIQGIKQRSQGPKFR
EHHSQAQLFYNSLTDYEKHHVAKAFSFELDHCDDPVVYQRMALRIAEIDLELAQKVAVMVGAPTPE
KAGMQNHGKKARGLSQIDFKPRVPTVASRRIAILIGDGFDSVAFNGVYTAVKAAGALPFVIGTKRQ
PIFADGVDPQTGKGVTPDHQYDGMRSTMFDATFIPGGPHVKSLAKIGQIRYWIAETFGHLKALGAT
GEAVDLVKQALAGVEAVQFATQSNTEAVESYGVVTAAGPQKPESFKEGWKMIKGATDFLGKFFFQV
GEHRNYQRELDGLADTIAF
>g1079
MLMSKVVLSLGSIILAQCVPATATWPTQSFNSVDLLPPVLEITKNAATAPGYLFFGPKGKGALIMA
DDRSGGDLIWAGPSELVFAFDMAELDSEPILVYWTGSAFPDPNGFGYGMIKVFNSAYENVYNVTLP
QSEGSFYTGTTTTYNSYIDLHESFVTNRGSILVTATNVSQTDLTSVGGPSDGWLRQSLFYEIDIKT
NEIIYQWNSWDHRDQIPLTGTLEELHEEGSASSSAFNYFHINAVSLFQGYYVISSRYYCAVFLIDP
KDGSVVWEIQGQDGGDFTLPSDAQFCYQHDARIVSMTSDNVVLHLHDNANSYWANGTTPTSGLLLS
IDLKAKEITKLARYQNPADPLFAISQGSYEALANGHVFMDHGYLPIMEEFDEHGNCVETVQFGVAG
TTSSYRGFRYEWEGHPKTAPAVVGCLDDSSNSVNLYVSWNGATQVQEWEVYATYNHSKGQQQVECT
AKTGFETTITIDYDPHISDFVVQAKGFQQSSTLSLASLNVC
>g1080
MVVIYTSTTVPINSFNDIDPLTSAEFWKVLEHKCRNPVPFVKPITRCRIISEDHDYETGQLTSLTR
MVMVNGGEAEIEEVAVLKKPVMVSFIIPSTGTCVTNILSRGADEADLYMVSTYEWHHPEVQAGSDE
HKKLTEYYWQMSSETVKNTVTVAKQMSREGKLDNM
>g1081
MAPLQIGLLLGSSRPNRISPQVGNWIKTFLESIRATSPEFSISTVDIETFGLPAFNKPVHPALISD
LSTFISPSVRAWNHEVAKYDAYIVVSPEYHAGIPGTLKNALDFLYHAWAGKSAMIVTYGIMGGASA
SEGLRVILEKGFRMKVSSFVPRLEFPGRDPTKNNSSPALMEAMQGKLSVDTVKSWESETEEILQGF
KEILEFATAK
>g1082
MKNMTLSRNKYISGDFWTNLGGEVNGLRSLLEQPVDDDDDESNDDDEHFKTTPSITGSTDPPVHSS
LFDFVFHGADSRLEQQTGFPVSPLHFKALSDTYFTNVDPIFKIIHRPTTLSCIANCTRNFSAPPNN
NITELLMLCMYFAAITSMSPKDCQNTFHQDSQILVTKFRRSAELAFARENLLITDDFAVLQAFAIY
LFTLRVYSKTQSTWTLFALVLRIAQSFGIHKDADGTGLSFFEAQQRSLLWWQIMILDLRCAEDRGT
QPMIFDSSFNTSMPLNICDDDYGPETSEAVPEFSGLTDITLSHITHKLAFIFRKLLFPNQISFTEK
EQMVKNLSHEFESLFFQKCDVNIPIQWYLSTVGRLLILKMWLSLRYPLQVQDQPIYPNTGTTRNQT
LRTAVLILETMDMYENDPKSVRFKWVGRTWVQWHPLAVVLAELCIQTEGPLVDQAWVVVESLWNTF
GERAADTTEGILWKPLKRLLKKARAAYQEAALKRDFHRIGDITANGVAQPLSDPIFDLSQMEIDEY
NPSLTFNSCAPADAQMPTLWNNSDTSYNMNTTPQNIINTDYSNQANWAVWQGFLLDSEVPYSIEEN
AFEAYPLASDTA
>g1083
MRILIIGGNGQTGRFVIDEALQRGHKITALVRNPSTLSAKEGLKIVKGTPLEASNIETAFTAVPDD
IPTAVIVTLGSSKEKGARLMADTHENLIVAMKRHGVSKIATLSSFGVGSSLPNITVIMRWAISNTG
LAHSFADHNHVEEILRKSGMKFVLLRAARLTMGRKAPVRFLGDDGKGLGVFAGMGGISRISVAVCL
VDAVEKSTWDGRTPVITH
>g1084
MTTISFDSLLLRKDGTPGNAWGRFGNDDELGTLNLLTPKVTAEAAKEIVEGVRVSVDWPLDTFVVA
ADECRIVWVENGGIVGRGVLLDYVSYAESKNIPIDCFSTTSISPSVLREIASVQGTIFRPGDILFV
RTGWTEAYGRMTRAEQEQLAATYPPPAIGLESSKETLRWIWDNQFAAVVGDQPSFEAWPCQNTDFM
LHEWLLAGWGTPIGELFDLKQLSEECQKRKRWSFFFSSMPLRVPGGVASPPNGVAIF
>g1085
MSHEFSQEVMEFLSLWHLRLGHSPLEAIVAMADGAATGMNLPANMPSMADLDPYREHLNCSACLSV
HGSASGPDPDQA
>g1086
MGKGDTQSNSHQLLPHVLKNRAQQNPDGAWAQYPVSEHTYAEGLRTATNQQVFHAVNKVAWLLHNQ
LGPSSNFETIAYIGPFDPRYFIVVIAAIKVGYKAFLPSPRNSKVAQLSLLSKLECTKLVTVEPQPP

AIGAVLEEVPYTVIAIPSLEELLNGNESDAPPEYPYEKSYEEAKDEPIFILHTSGSTGIPKPLIYT
HRFVTRTVNLTSLPAPEGFSSGNDTIRSGHWFTFLAPFHISGIGFGLMVSAYNDCVPVYPXPXQPL
STAQFLDAVKHVDMDWAFVLPFILEDLSKDLDALELVSRKLKHLYFAGGSVPQAAGDLVASKIPVY
QTTGSSEVSLLSQVHSLTDGSKNEDKWAYINIHPAINIEYRHHHGELHEMVIVRNKENEELQPVFL
HFPELQEYETRDLLTPHPTLPGLWRYRARKDDIIVFLNGEKTNPISFEQEVSRHPEVRSAVVVGAQ
RFEACLIVELHRNDPLSPEERERVIDRIWENVQTANKDCPAHARVAKENIILADPSKPLPRAAKGT
VQRAATLVLYEEEIERFYAEREAQNLFAPRPALSLIDMTDADAVSTKLGDLVSEITRWNTSLSYDT
DFFSLGMDSLQVLQLSRQLNIPPSAIYSSPSIDLLTKAVMSQTSESTSRDIVLTTLTDYEKRIDDI
AASPAEASMQRAPTEIADGAVVALTGSTGAVGSYILQKLLQNDQVAHVYCLNRVSDAETLQRDRNA
KRKIKTDLPSDRVTFLKVDLAKKNFGLDDAELYRRLQSTVTHLIHNAWPVNFNQTLQSFGSSLDGV
LGLVSFATHASRLASTVYLSSVSAVSNYHETSGANIDFVPEQIITDTKCPAGMGYGQSKYVAERIL
DYASKKLNLANIRVARVGQIAGTSTPEQGPPHGWNRHEWLPSLVISSHYLKAIPESLGYKGDGNNA
GVLDRIEWVPIDQLATLLLEISFGTEANSPVSKGAQIFHPINPNWVSWQSLIPAVVETLEESSSSG
SAISVLKYEEWLALLQSSANSTDALSDNPAVKLLNFFESLMVNTNEEKPLAIQRSVKMCPSLQSLD
AIQVEWLRGWIKDWVLGSDNA
>g1087
MESLLGTYLTHVQRLWADFDIDSESLRTGSVAAIVSVIALYVGYRYILSLAEAPVTFNVPLPKQLR
TDWNGTKWEDLEGEGKRILEDQAQGKWNTERIVSYCPADGRILSTNIPLATRESIDKAVQDAKRAQ
VEWAQTTFAQRRRLLKTLLKYVLDHQDDIVAACCLDSGKTKVDASFGEILVTAEKLKWTIDHGEKA
LTPESRPTNFLMMYKKNSVRYEPLGVVSACVSWNYPFHNLISPVISALFAGNAIVVKPSEQTAWCS
GFFVDVIRGALISLGHSPDLVQLVVCLPQVADSLTSHADISQLTFIGSRPVAHKVCESAAKSLIPV
TVELGGKDPFVVLDDSTTVRGLESIASILMRGVFQSAGQNCIGVERVIALPGTYDKLVEIVTPRIK
ALTLGSILLDTKNDSFTPDVGAMISPAGFARLETLIAEAVEQGARLIHGGKRYQHPKHSRGHYFTP
TLLVDVTPTMRIAQIELFAPVMVIMRASSVSNAIEIANSTPYALGASVFGHNSRDVETCVRNIKAG
MVAVNDFGAFYAVQLPFGGVKGSGYGRFAGEEGLRGVSNLKAVCVDRFPKIVGTQIPPRVDYPIYK
GETPRTNGTGAWEMCKGVVETGYVYLL
>g1088
MPVSSSAARMAARNAGNDHVKDGDDARDDGLEDCADAVDDGHQAATDCAQDGLDLFQYTXMWQIDV
YRDEALQREGDIFYKILAADVTSLILQSQSRNDVERATLGRMLCPSLGLASHLTSETQGLGLFGPQ
ALAIDIMTRTCTPLLPANDVSLSLFPRLSLLHARQVAPSSSFKLLTSKHVLSPPINIRKYVDTTDM
TTLSSTLPPNTEPQRQASQNPESAYNDDLTAPKRRKRRPGRPLLSFAQLRARKAAREADRRSKSRW
KFASQQANPSASLPEEAQSKRRNGSSALEQLPVELLERIFLYALETNFCRASPFLASAVSSERIYR
TLIRLAFFKDGSFPQVSVELRHQIKTAHDAIADALKPADYETMRLDETDRVNLQVTILRCRWCTKK
RILTQFPALLQMIVWKHWVGAGIVLDPRQQPDLDYILRGSGEDYIRNFPYRDPLEGIGPDGVTYHA
LTVPLNRVVIYGQRPGIVGIKDTHQVMNIRVIPDYLLRGRRKVDGDGYQFTDEDIDLLEIFRDVYG
DDGNGHDVQFSRDALHSGIRTAISTWHERALTSLLRLDEYMLRLRLENAPPPGYPNLSTGIFYAIP
QQHFLQAIQLPMPDAMYCFLLLLRCNAESMPPDSSELTQWAMDLSSYRCPPEGNTSDSDVNLARKL
GKWLLDFMIELPGSIEESRENPQEKAIFYKGSLNRHTQLGRRFLEEVCPYISRDFQSWENEDKHWV
RLVSYNMRKQWFIDVQQ
>g1089
MDPSSPPLARHHHHHLSKLECLPPELLEKIFFHSLSINLPRSSLQIAAVLSKPIIYKWLIRLAFSS
TNPSSRHGFFTEDFLPSPLGFWALSNVERTLLQQEILTCRWSTLPLFRECQREYVKHVLRQKGAGL
VFVSKEDQAGFESIDEMFSRPMDFDKAESGRRGSGDLILKAKRATEGGREVDMRVSFWFHFGGIQI
REPSPVSHEIDMFRLPCPPSPNGRPRMPDKLLHAPWTSEKIEFLTLLSQETYIDEHNNSTERSHQV
LRQLIRDRDYVTFATLLELNVTTRDYSYPQKWRXKTRHFKEALRHVTGLNDPFVRLLYEKRWDCLK
DRKVRDEVLKCIEL
>g1090
MAAPRSAALKIDWTKVTSSLGLRGQTAASLQAFKKRNDDARRKVTVLSEQPQSVDFAYYRKTLKNQ
AVIDEIENHFKTFKPATYDVSRQLKAIDAFEAQAIKSAEETKGRVEAELVNLQKTLENIETARPFD
ELTVDEVATAQPEIDEKTASLVSKGKWMPAGYKEKFGDLSIL
>g1091
MANPRVEELPDEEIPKAGVEDAGSDSESEAGEEPTIPGGAAVTIHSRNEKKARKAIGKLGLKHVPG

ITRVTLRRPKNKLADFCVDTMANNRQILFVINQPDVYRSPSSNTWIIFGEAKIEDLNAQSQVNAAQ
QLAAAEAAASSDPHAGHDHSHEGKGKAPETEAKKEEEEDDGEEVDDSGLEAKDIELVMAQASVSRK
KAVKALKENDNDIVNSIMALSI
>g1092
MAANIDIKTTSWKLVEVGRVVLIRSGPYTGKLATIVEIIDHKRVLVDGPSTQEEKIVPRHSLALSH
ATLTPFVIPKLPRAAGTGPVRKLWEKEEIDNKFASSSWAKKNAQVERRKNLTDFERFKVLRLKKQS
RYEVLKAAAKVRASA
>g1093
MQRSPFAPLPAQSPPLHHPVPQHVSTVPMMRSPPPPASQSGYDNPYQPAPAQGGSGTFAPNFGGFM
IDPTAQMGFQVGKTAMMAGQEYMEQNINRYVSIPALKHYFNVSNSYVLSKLSLVLFPWYHKPWSRQ
QARMAATAGPDGQLQQQHYASVFLPPREDLNSPDMYIPTMAFVTYILLCTVMAGLRGSFHPELLGS
ITTTALAVVIFEILCLKVAMYILAISNDSQLLDLVAYSGYKFVGIIVILLTAGIVTPARGTGSWVG
WAVFVYTFLANAFFLLRSLKYVLLPDSTGDAPMRSGTMHTVARGQRNRRTQFLFIYSYVVQFIFMW
VLSREGPPAATAAAS
>g1094
MLDAFEIITTSGVVLWSRSYAPVASHIVNSLISEVFIEEKVAPTDGASTAFRKETYTLKWRRAKDL
GLIFVAVYQSLLHLSWVDKLLDNIAIIFVDLYKDQLKSTRARIYEYPFDKYFDQQIRELEDTTGPS
IDEYTGSYGDIKKNPLVASDNGGPPPPSDASLLIAQQQAAHPAAATSNESSPVATPQSSRPGTPST
NHLLTAKSGPAGRVSRRARKAANASANVSSGDESVRKGKTGKEKKARVWTAEGLADEDDGVNLDYS
VAAADEATPAPVVEAVAQDTWGRKTAKGQFVLKDLGDEVHSILEKADSEKAKSGGSSGIVGSSFGA
IGGLFRNIVGGKTLTEEDLEKPLKAMEDHLMKKNVAREAALRLCEGVKQELVGKKTANFQSVDAAL
RTATEASLRKILTPTSSLDLLHEIQAVTAPTAKGQTPRPYVISIVGVNGVGKSTNLSKICFFLLQN
NYRVLVAACDTFRSGAVEQLRVHVRNLTELSSRENIGEVELYEKGYGKDAANVAKDAVAYGAANKF
DVVLIDTAGRRHNDQRLMSSLEKFAKFANPDKIFMVGEALVGTDSVMQARNFNQAFGAGRNLDGFI
ISKCDTVGDMVGTLVSMVHATGIPIVFLGVGQHYGDLRGLSVPWAVGLLMK
>g1095
MISLPEAIASNERIASTFPPGLVAVFVGGTSGVGEYTLKAFVKYAVSPRVYLVGRSQVSADRIIEE
CTQLNRGGKFEFIKADISLLKNVDEVCNQLKSKETKVNILFETQGTMAFEKKTSEGLPFAIAVGIH
SRIRFILNLLPLLRKADHLRRVVSVGAATCEGSIDLSNIPGVGIPLLPWRNQIASIQTLLLEEMAK
RAPEVSFVHTVPGVVKSGIFRDSEGTGMAIRIAIAKLLMPLIATSPDESGERHTFVATSAVYPPGQ
QGAAHAGVPLHGILTTARGTDGKPGSGVYSVSNKCEVASPKVEELLATFRKDGTAEKVWQYFAADF
KRITGTDVPL
>g1096
MTITEIQTTIPKGSWILVTGASGFLASHVVKEFLQRGYKVRGTVRDLNKYAWLVQDLFKTDADKSN
FESVQVPDLAAPHAFDEAVKGVSAIVHIATIGFDPNPNNVIPKVVAGTKSLLEAATKEPSVKQFVY
TSSVAAMISPQLGDTARLTANTWNNEAVEQAWAPPPYEPIRAFTVYAASKTQAEKAVWEFANEKHP
HFTINSVVPATIMGECLSKSHAELGHMFLRILYEGKPDAVAAILTMPGPIPHNVRDCAILHVAAVL
DPDVKNARLIGWGQPCSWNDIVAIMRRLCPDRQFIDEIPGLEAYKITADDEQSLVILKKWTGQDGW
TSLEQSIAEHLPNIVKWYP
>g1097
MESATKFRLVVFSAKQYDRFFFDIAASESHKTLYDIEYLDFALNSETAPLARGYDAVCVFVNDTLE
KDTLKILHTNGVRAILLRCAGFNHVDLVAAGELGLFVAHVPAYSPEAVAEFAIALVQTLNRKTHRA
FNRVRESNFNIEGLIGTTLNGKTVGIVGVGRIGLATARIFNGFGCKMLAYDVNENPDFAQYGSFVG
LEQLLAESDIVSLHCPLTDSTRHLISTKTLSIMKPGALLVNTSRGGLIDSTAVLESLKSHHLGGLA
LDVYEAERGLFYTDHSGKIIDDDVLMRLLTFPNVLICSHQGFFTKEALTEIANVTFANLSDFIERR
PCPRSLVAGGHIDSSIGVQPVRL
>g1098
MSVHQALGFDRHESVFQHDQVKSHLVRCDWSGPTIDTLYAGIAGTFADGKSQIGIALRNETYLLDF
LEQEFEGSDSEIVICDFILTELQNYSNIHAEKLLGAALSKSFHQHCPSLCRRLWLELDIVPILLHE
EPGTERTVGTEAVTHPNGKEIDEQAESVSRKCIRFFGPNKLPFLHVDFLGAVEVDSGFHINIADLE
SFSRTVRPKTWSGVQHYVKDLTSRKVKIAFFSATPQGGGVALMRHALVRLAHLLGVDVKWYVPKPR
PGVFRITKTNHNILQGVAGPHERLGVHQQRILTDWIEDNAHRYWTRKGGPLTRPSEGGADVIIIDD

PQMPALIPIAKQADPIRPVIYRSHIQMRSDLISTPGSPQSEVWDYLWSYIKLADIFISHPVASFVP
RSVPLAKLGYMPACTDWLDGLNKDMRDWDYAYYGRIFNHACRNAGMPPINYPDDQYIVQIARFDPS
KGIINVLKSYKEFRRLLTQHSTNVRSPKLLICGHGSVDDPDGSLVYDDVLKWIRQNASALSDDITV
MRLGPSDQVLDTLLSNSRIVLQLSEREGFEVKVSEAIHKGKPIIATRAGGIPLQVEDQVNGFLVDV
GDVQSVANHLLRLWTDSDLYERISSCDSSYRVSDEVSTVGQALNWFYLCSLLSQNMSLEPKGRWIH
DLARESLGQEYTKADGKLKRAVDETVLSV
>g1099
MPDTSLEKYDHVPVTKEDLDWAELITLDLSLYDQPGGKEQLVKQLDHAVQHVGFFYVKNFNISQEE
VDFQFALGREFYGLPLEEKLKFHNADDLARGEYNGYRPAGHRILGNGIRDNVQVYNIPKFDGHHYR
AQPEVLQKHIAKIEAFSRKCHTEVVEKLLRLFAILLELPDEDQLVRDHQYDIQGEDHLRYMHYAAR
SAEDNKKVGELYSPGHTDLGTITLLFRQPVAALQILNSEGQWKWVRPQDGTITINTCDALTALTSG
FVKSSIHRVHAPPQDQAHVDRLGVLYFARPNNHVVLDPIQNSPVLNRLGLTKNAFTELGQHLTTEQ
WVKVRQTQQQRRNRDVKITDDGKYTYKSKDLEIIPGLQAVIYN
>g1100
MSEIKPKQPKLTLFGVIKEIFNWYPAEYPSHERKLLFKLDVSILVFACLCFFVKYLDQTNISNAYV
SGLKEDFSLNGNQLNYFNVCYYTAYVVFQVPGLLLLSRPKLARWLLPTLEVLWGICTFAQSRVTNA
NQLYALRFLVGMLEAPVFAGTHFILGSWYSGPELFKRAGMWFICNPLGSMVSGYLQAAAYTNLSGV
GGMPGWRWLFIIDGIFTIPVALIGYFVFPGIPDSPKPFYLTDEDIALAKERSKRFKIRRPGKLDLD
VFKRTVKRWHIWIFVFCYMYVPPLLAISWKVAYENSCMIISSYPFSYMNLWLKAEGTYSVAQINKL
PTVTYGVQIVASWLGTTLAAVYPSWVIFGIASVCCLFSTLCMIIWNIPTGLKFFAWYLFGLTGCLS
PILYSTVNSIVKNDSEERALILGSMMTIGYSFQIWVPLLIFPTAGPYGAPRWKKGWPVTFTFLSLL
LIGFITSLVLHRRETKKTAIDASSDVGSNDEEDTGEIVVSEQYDKR
>g1101
MRVTRSMMSTTTSDKPAAHVKPTKATSKNKIQESTSSTLKSSFPVSKSTNASKKRRKSAIHEIDID
ALPHNLGKPATQITRDALDVPQDIKTKTASQPQTKRIKLENGSQEAETEDVDLAKKIKKAAAKYGL
TPGVSPFPDFPHPTAQECEEVNRLLSTVHGEVKIPTKVPQPSLTVTGCGEVPSVLDALIRTLLSGA
TTGANSARAFKGLVERFGILESGIGKGSVDWNAVRIAPIKEVFEAMKSGGLATTKSKYIKEILSMV
YEENLARKAAHLKSEEEPGTSGTAGADHESKAQKEVEIALKDENVLSLDWIHALDKEEAMLELIKY
PGIGPKTAACVVLFCLQRPCFAVDTHIFRICKWLGWLPSSDTKKVTEIMAFSHLEVRIPDHLKYAL
HQLLIRHGKSCPRCKAITGEKSEGWENGCVIDHLVKRTGKRKGGF
>g1102
MPKSNSTITAPLREGLFETFRAATRLVRPERRRQLILVGGTASIAHSSSLRTEDVDVAAPPSVLID
IWEGVTAGAPNFSIEPDGKIAFDAPQGFRVRIDLIQIGDGCIETIHETESFFEGSVASMSDLLRLR
AVTVVDRGSDGEIEDFRWLLWEVAKAGQVLPKLGVEDRMYIRQAGESCLGVLDRLVLAAVLGDNNL
DVALSFLSLA
>g1103
MSEYIKTTFLIMSDSHGHQLLSGPVTGHIDVAIHCGDLTNESKIEEFQKTLQYLQELKADLKLVIG
GNHDFTMDVPAFKKLVANAKQTLEPELVRKTYGDYGEIRNILSANAAAANIIFLDEGSYEFRLRNG
ALLRVYASPFTPSLSGDWGFQYHLNEGHHFEIPETVDVVITHGPPKGILDYTDGRRAGCPNLFQAI
ARAQPLLHCFGHIHECWGGKLVTWRSNMPSNSMPSHITHIDNSKSTVLSNISRLEAARQTSDLACF
SVDFDVTRSGSQTLFVNAALQGTSEITQVPWVVNVPLPRV
>g1104
MVGEKRTNDMVTGNQQRKRRSSGPVINISATVNQNIVVQQGLTQPSTTRSGRPIEKKVEHIDLTFV
DDAELSSDTEPGSIDEPLEMGRFDDDDYLDDDQLQHLLERWDTDTNKAGPAGEHPATVVLKDYNGL
VPGMAVELTGDHLMRIVQIIRLVNGNIYLRGLYFKRLTDMESPFPQKEMELCWLVEKDAEGKLHLE
KEVPVSAVLDIVNIVLTNHPWRFHFRQPYISYVCRLVWQKTAEKGEWIIRYLSFEEADHQARTPAG
VIRQKWRGKTIPYGAEAVDINQLFKDQREKAEKSLKMQRQYSFGDAFCGAGGVSVGAWSAGLRVKP
GERLPTFPTPTHGPGALPYVTIKDAIDNIPRLADNHDPDNIGFQDGKTRAPYDENTLAKTITCGGG
EGNYHPSGLRPFTVRELACLQTFPVQYKFPTAYAKKQVGNSVPPRLAEIIYRAAIKSLQETDEKEY
MEYKEAIVIH
>g1105
MSAGAAALRRTSAIGHRAPRTLALSSRISQAPALSRSLTSTPSALRTSTLAKSSTLSRQISKFSTM

SPLQAAPVLEREYDPEIKDMADYIHNYKVDSELAFDTARLVFLDTLGCGLEALRFNECTKLLGPVV
DGTVVPNGTKVPGTPYQLDPVNGAFNIGAMIRWLDYNDCWLAAEWGHPSDNLGGILAVADWISRTN
RAGGNIGQGKILKVRDVLEAMIKAHEIQGVLALENSFNKVGLDHVVLVKVATAAVTSKLLGLDEKK
TADAITQAWVDGQSLRTYRHSPNTMSRKSWAAGDACQRAVNLVLKVLKGEGGVPTVLNAPVWGFYD
NLFKGKKFEFQRPYGSYVMENVLFKVSYPAEFHSQTAIECAQAVNKKLAELGKTAKDIKEITNRTH
EACIRIIDKQFKAMDNFADRDHCVQYMVATMLVFNRLTAQDYADGSEAATSELLEDLRKRIKCVED
PQFTKDYHDPSKRTIPNALTVTLNDGTVLDEIVVEAPLGHKFRREEAKPEIFAKYKRHIEAHFDAA
RVKHLLELGWNQKELENTDVDKYVDLYVTDSIVAPYGQ
>g1106
MAEEKETESQSQTRYLIRQPLALQWFDNGKLVKRCEQERQAGRFELFLDLLYVAILANFAESLAED
VTGVKLVKYILILAPSWHVWSDLRELMNSFYNDDLLQRILILWIMAVLIVYGNNATLVDEEISAMR
STVGAYMVARMTANLAHLFYSFSSYHHRAQQRLWFVMSSVALCVYIPLYFESVSLRNKIAVAAVAE
VLEESIWMFCYSPLAKRMLRARYTTAVDIAHEIDRFAAFYIIALGEFLYTIIVGSPAAVGFNLSLL
RAVWTLVIAFCLNWMYVHNDCAIDYTHPIRHSVGRAFTWVSLHLPLIASLLAGGHVAAASAKSYDS
TTTTTDSSSSSSSSEKRATSEGQTQAFDDGQRWLLCASLGVGLFVLYAMALLYSSNDGDCQLLLPK
HFRIIMRPVVGLILVLLPLAKSLNLTETLSVIMALVVFCLIWENVSSLQRGAKFWEVWRDTGYPEG
GNRAHKHDHAASEDREVNAELGRGSEGVESASADNNGFKG
>g1107
MDATRLQAIEYKPRVFIITDIGNEPDDAESLTRYLLYSNEFDTKGVVACTSCWMKSRVQPELIEKI
VHAYANVRDNLNAHVHPSNPYPTAHNLLSVVRAGPAVYGREALKQPLSPGAELLVQQLKESSEGEP
LWILCWGGTNVLAQALQYIHQTMDATESAKLRSRLRVYAISDQDDTGLWIRVSWPDIFYICSVHAW
KDYNQATWIGISGDRLSHFDEGGPDFTKVSKEWLKENIQVGDFGGEVYPDYSFAMEGDTPTFLYLI
QNGLGSPEHPEWGSWGGRYALIDLGGATRHYADTHDTVVGKDGRQHHSNQSTIWRWRDQFQDDFAA
RMRWTLSADLSSANHAPVVIVNNIASGVKPIELDVPAGSEIVLDASQSYDPDGDELSFTWIFYKEA
TSAHATIQWIVPDLEWETLPNSPNGSTIRVKMPPPEKCTVDLINGQALEKGQAFHLILQVRDSGTP
RMTSYKRIILQSTNLQLLGGTGKRFDTFTDVLHSGLLGDVLH
>g1108
MELPTVDDLKDQGESCIEHIPLDDGNDSTESQSLLLTLWEYKKVVLFSIXVNVITLSLPGFKLVFG
YEYEGELIVGAIWNSLWGAMTSLGMVVGGVTSGFVADLWGRRAAILAGAILSAVGVAVEYIAATPS
VLLVGKIINGIAMGIFLSINPAYISEISPLTLRAPLISLTAFFIIVGQMTAIGIGNTRFGIMDSSA
YKVLFAAQWAFPGAIVLFLLFLPESPRYLVKRGRNEDARKALSRLHSSKFDISAALSVIQETVTAE
LELNNTSVSYLACFKGADLRRTRIACAMFFIQQFTGIALYGQALYFLTMTGFNVNLAANLAIAGFG
AGLIGNIAAWIFMSYFGRRQILLVGTVINFTILTTLGIAGFYSSNKSASLYVAIVLNFIQLIHAPS
VSAVSWAISGEVSSVVLRAKTQSLCTIMNALVTWVFNFITPYLINTDEANLGTKSAFMWAGLTLVS
MVWVWFEMPEIKDLTFDQIDEAFALRTPTRKFPRPVKSNVVVPGSKWAAG
>g1109
MTSTRPRDEQDENVSQNLPDLEDADLSEDNIVKLLAQIVRKQNDEINKILISQNEQAKKQAQEQRR
LSGMIEKLNSRLTSFLESQQKCACDKDSEVDLIPSLKARVDALEDAERDQEQFKDEVLQSRVDRLE
TRLDQVQYQISKDEPQSSETADGLNNEDLQSLTEALATRQLSMQFHIDELADTVNRQIITNEQKSI
AQNQASSMRFSHRSSHSTTTLPTPIERACLVSESAYNSPHRHTASHSQDLTSARSHDSGFSIESFH
QQATSLTNKATQQDISLSSHKKDLLNLKRRLAHAEKLIGARYRIGXFSIHGXSFYQDAAALRYISG
DHAARDSFKMFYGNRIGAIEYVDIAPSAILACFDIISRIRCRHDLWIDDDKFEAKQRIRCAAEQIV
DDWINSLPLRDGGEHVHVSAQELRNRFNQLKVLCYGAGVVFDFTWPTID
>g1110
MDQISVPLVAAAVAGTATAAAYLNAKWHVTKDVSAVWFVKSGERSVKRAVANGRLSNFFLFENAAL
SFPDKTAIWSRERTYTYRESLEIVSQLGHYFLSIGVRPGELVAVYLMNSPEFLFIWLALMSIGSAP
AGINYNLNGDGLLHCIKVPNAKFVVVDDDAACRERIEAVRPTLENEFGITTLSLSEVMRKATTSFP
KTTPDEKYRLNVKESFPLMLIYTSGTTGLPKGCAFTTSRFYAGASVTKPTDDRWYCCMPLYHGTGA
VWTLARLVSGTSVAIGRKFSTSNFWNDVRDSESTWFIYVGETVRYLLNNPPSPRDKEHKLYGMLGN
GLRPDVWEKFQQRFGVQEIHEFFNSSEGMLGLMNVNRGPFSAGAVGHHGALLRLLMHNQYIPVAID
YETGDIWRDPKTGFAKRQSYEEGGEILVAIPNKEAFQGYWENDAATNKKFAQDVFKKGDLYYRSGD
ALRRLDDGRWYFMDRLGDTFRWKSENVSTAEVAETIGRYPGVTEANVYGVLVPNHEGRAGCAALDL

AVTEKEFDWKDFTRFVRSQLPRYAVPVFLRIQNGLRHMHNNKQNKVPLRQEGVDPALKGTKEAEGA
DDRLLWLVPGADEYVEFGQKEWDSLVSREAKL
>g1111
MLSPSRTARYGWKLAQASSRPLIVSTRGYATAEPDLKSTLKEVIPEKRELLKQVKARGDEVIGNVT
ISSAIGGMRGLKAMVWEGSVLDANEGIRFHGRTIKDCQKELPKGTSGTEMLPEAMFWLLLTGKIPS
TTQIRAFSRELAEKSYLPQHILDLFKSFPKDMHPMTQLSIAVGALNTESKFAKAYEKGLPKADYWE
PTFDDSISLLAKIPRVAALVFRPTEIDAVANQKLDVNQDWSYNFAELLGKGGKENEDFHDLLRLYL
ALHGDHEGGNVSAHATHLVGSALSDPFLSYSAGLLGLAGPLHGLAAQEVLRWILAMQDDIGTKFTD
KDVEDYLWSTLKSGRVVPGYGHGVLRKPDPRFEALMDFASTRPDVLANPVFQLVKKNSEIAPGVLT
QHGKTKNPHPNVDAASGVLFHHYGFQQPLYYTVTFGVSRALGPLAQLIWSRALGLPIERPKSINLL
GLVGK
>g1112
MSSSLPPVVPPVSTSLPSDSFQLLSTAEKAGDAENALFEEQVKEVEAWWKTPRYEGIKRPYSAADI
VSKRGALQQTYPSSIMARKLFNLLNERAAEGKPVHTMGAIDPVQMTQQAPNQEILYISGWACSSLL
TSTNEVSPDFGDYPYNTVPNQVERLFKAQQLHDRKHWDARRKLSPEERAKTEYVDFLRPIVADGDM
GHGGLSAVIKLAKLFAEKGAAAVHFEDQLHGGKKCGHLAGKVLVPVGEHINRLVAARFQWDLMGTE
TLVIARSDAESGKLISSAVDVRDHEFILGVTEDVEPLSETLQAMEAEGVSGAEIDAFESKWVKNHK
LVTLDEAVDAHLKAQGASQDAINTYWNQVKANPDLSMTKRRDLANTLTDKPVVWSCDVPRTREGFY
HYKAGFPAATKRAKEFAPYADLLWVETGDANVENAATLAANVHEHFPGKKFVYNLSPSFNWMAQGF
TEATLKSFIWDIAKHGFVLQLISLAGLHTNATITTELSRAFKDEGMLAYVRLVQSREKELGVDVLT
HQKWSGASYMDGILGSIQSGSSSSKSMGEGNTETGF
>g1113
MPIAVSKNSSSVPPTEDEVASLVHTIFSAKTSQESLDASYALTNLFINSVGYRGIIAYNLIPQIKK
AAADKKDGAKRESAMLILGALFELFPRKDPLSEVVFLIQDGGILNIALDALADKGAVVREAAQYAV
DALFAGLKPESLVNALLPAVLRYLGKSSGKWQGAVGAYALLEKMANKAKIGTGTKDEERQKDLLRE
AMGNTLKDVIPVVESGMHDLKNEVAKQAVKTMTAVTTLITNEDVIPRIPLLITAMEKPSSESLQKT
IHALSQTTFVAIVTSPVLALLTPLLERSLNTPTTSQEVLRQTVVVVENLTKLVHDPTEARTFLPKL
KPGVQAVETRASLPEVRELANRALSVIKKAMGDDNGNVADGAVVKTTSDDVLKVLDAKIVAHGGPA
QPGEAKFFDIVKSYIADMVREDINVRMLDRITECVGPYLATFVQDGQNDAVASEVQEHFVEEDHRR
FGAPVQEDDGEVEIVNADFSLAYGGMLLLSHTNLRLLKGHRYGLCGRNGAGKSTLMRSIANGKLEG
FPPKEVLRTCFVEHNQGESAELSILEYCAHDPELQNVSKEEISTVLSEFGFTAGPEGRQSQKVGSL
SGGWKMKLALARAMLMKADVLLLDEPTNHLDVANVKWLQEYLKAHTDITSLIVSHDSGFLDEVCTD
IYHYESKKLVCYKGNLADFVKVKPEGKSYYTLSASNVQFKFPAPGILTGIKSQTKAILRMTNCTYT
YPGASKPSISDANITLSLSSRTAIIGGNGAGKSTFIKLLTGETVPQTGRVEKHPNLRIGYIKQHAL
EHVEMHLEKTPNQYLQWRYANGDDREVYLKQTRILTAEEREQMEKPVDLGGGQGPRRIEALMGRQK
WKKSFQYEVKWVGLLPKFNTMISRETLTELGFSKLVQEFDDHEASREGLGYRILEPKVIAKHFEDV
GLDPEIANHNQISGLSGGQKVKVVLAGAMWNNPHLLVLDEPTNFLDRDSLGGLAVAIRDYKGGVVM
ISHNEEFVGALCPEQIHIADGRVTHRTSTAVAADRFEDSQPGSPAPNSTVPSTVASSVVSSAAPSA
VNSGAEDTGELKFKARKKKKLTRAQVKEREARRRLRHIEWLNSPKGTPKPVDTDDEE
>g1114
MSTGKWLKMLVAGTIVAVGGPLFVQSIRPSDEELFSRYSPDLKKAALENRDRQEKEYNEYVNKLKE
WSKSDKSIWFAVKEEEAAKLKGQAPTTSTTSTTASTSARSKEEERIQREEMRRELQGGK
>g1115
MAAAPTVSGINPTTGPTSGGTPLLIAGTNLSGATLVTFGSGTSSVTATPSSSTATTVSVTTPVWPF
APGTARVTVTTPGGSSTQPVFFNYQSITAPTLTSLSPNNGPVTGGNVTTITGTGLLNTTNVSFTQG
TITVNAPSFAVLSSTQVAVVVPPAPVGAGPASVNVTNGAGTSATPLTYTYNIVRPLPVVTSAVPNT
GPAAGGNSVSLTGTGFTFATQVNFGTTPALSFTVINDTSITAIAPPGSGTVTITVTGPTGTGTGVP
YTYNPAPVPSITSLTPSSGPISGGNTVTITGTNLNTVTGITFGSVPATAFVILSPTAINITAPAGT
APGPVNLTVTSGTFVSPPVPYTYIALPAPTAIFPTAGVISGGTPAAITGTGLAGTTSVLFGSTPAI
ITGVVGDTEVDLIIPAHAVGTVPITVITPGGSDSSLSFGFQPPPVITSSSPTQGPTTGGTTVSING
AGLINATGVFFGGTPAASFNVVSDNLITAVSPPGTGAVSVTVNTPVAFSNGTLFQYVAPPTLTTIN
PTSGPRSGGNNVTLTGSGFTGTSNVFFGGLPASFVVLDDNTITAVAPPVGTAGPISVTVQNAGGTS

PGITYTTT
>g1116
MKFISIITPAVLAASAMALPTSQKRDGSTGPFSVIALRSASPIHLQPVNAAGERFWIGGQAATYCP
SEVVDPCPPGTETVWANTNALDVEVPGGQEVYVDPSGALAFTQAHSANIPTGASLGPFTFTPGTQF
GTYSTSAFGATGFMACPDDAANPTKWQVFAAFQGASVPTGNVGDCLGFDAGAPAYTGDIPAWQYI
>g1117
MLAKKLPPNLSTVDQNDLQGLEERLLDLDRKILKYHDRLLEKSDYLVTATNHIRDELQILTRHLNN
QTSVINSTRHIISAQARTISAQARKIRSQQEMIDVMQRDIFRLGTWYSEFSGRMNSHDEILSNRAG
EVRELWESLERHTRFTEQIAKETYWMHVYTKQVMLIRASEMALIGASAGCADDGDDDGDDDDDERS
DSDTAASVCTESTLITPQQHIMLDTIVVRMIEDAMPDRLKDVCDGFEVVHGCSAFIQGREAAQPPE
SEFDVDAEMLREDSDHGSMLV
>g1118
MSSRVGLRFLANSRAALRNFRQNVSNNKGFRFSSTEASAAGTGESQNAFQRMWNSPVGFKTVHFWA
PVMKWSLVIAGISDLARPAEKLSLTQNLALVATGTIWTRWCFVITPKNMLLAAVNFFLALTGTAQL
TRIFLWRRSQEGSAKEAAKDIAADTVESAKLVTDSAKGAVKAAEEKVKSS
>g1119
MADSKYQPVPHHDTLDSPPQSNSTLHHQTRHHLDPADVSDLSFQDTDDENDMMDLDEEGGADETVM
APLNRRASRSSSSSKKRQQLSSRRWPTNSKFRGWLSRLTKSIVRHRLALVAAAXLIFIFLPLIAYQ
RSIRSFFWADRVYESPPWYPSPRGGTSETWAHSYRKAKTMVQNMTLLEKVNVTTGVGWSMGLCVGN
TGPAMDAGFPSLCLQDGPLGIRFADHITAFPAGLTTAATWNRSLIEERGYLLGTEARGKGVNVLLG
PSMGPLGIAPAGGRNWEGFSPDPVLSAIAAADTIRGIQRTGVMATAKHFVLNEQEHFRQGREWIIP
DAMSSNIDDRALHEIFIWPFAESIRANVASVMCSYQMVNNSYACGNSKLLNGILKDELGFQGFVQS
DWLXQRSGVISALAGLDMSMPGDGASWADGKSFWGKQLTIAVLNGTMPMERLNDMVTRIVAAWYHL
GQNEPGDNPTSPNFSSWTNEENGHLFEGSGDPASGRVNHFIDVQGSGENAHSIIARRVAAEGTVIV
KNEDDILPLSSQGPSSHGKPYRVGVFGEDAGSGKGPNFCVDRSCNQGTLASGWGSGAVEFPYLVTP
IDALKQSFDETEVEFQGYPTDKYEDKDLDNHDLCIVFANSDSGEGYLKWDDVNADRNNLFLQKQGD
GYIQRVANKCGGEDGVTIVVIHAVGPVVVESWIEHPRIKAVVLAHLPGEESGNALADVLFGRVDGG
RLPYTIGKSLEDYGPSAQILYFPNALVPQVNYTDGLFIDYRYFDKHNITPRYEFGYGLSFTTFEYS
DLVISSVREKSPLPSPRPNDPVSPPEYSGQTPDARSALFPAGFRALNKYIYPYLSDLSEIKQRHFD
FPKGYKEKYTLSPAGGGEGGNPSLYETFVVVTANVTNTGTRTGQEVVQLYVSFPNDVKDEDVTFSD
YIEFPVRVLRGFDKVELAPGETTKVELALTRKDLSYWSTRRQNWVMPACIDKQVGIEFSILAFGYS
KVERMRIPIGLQLGLLVLVTSLVGLMIISVATWVNNYNFVTGVKSQGLVLAASLKAGQVASNIALI
EANCQTVTTRVLIQSALRRFYQGNRTDANWVTAISDVQSALGSGGYVSLYQAIIYSRNEAGDQDGL
LNVTTTSLTENIVLPYTYDNGTSVVLGDAGLGYPPSLYPNLTYVVDDGVDPTNATSEQATAYAFSD
FPINVTSALLLGPLYVNSSFTLISITLPIINNTSNTDILGYMTVIASAESLRSTLADNNGLDTSAS
VMLVGPATADNMFTGDARAATRTSPETNKTALQEAQARYVFTPTLVSGFEDRHSKYRATESTFPLA
DYPTVLDALQTPRSTFDNSSSDLDTTNEQGYRVAVGVARPQSTLVDWVLVIEETHTQAFAPVSQLR
KIILACVFGTAGFIAIAIIPLAHYSVTPIRKLKAATEKSIHPSPYHHHNNNEGAVLETLNAQNEKR
AGIFGAFSNIVHGRWGLSQGTASEDGRSKTFKVPGKVKESKHIVTDELTELTSTFNEMADELMIQY
NRLEERVAERTRELEISKKAAETANESKTLFIANISHELKTPLNGILGMCAVCMGEDDLPRIKKSL
KVVYKSGDLLLHLLNDLLTFSKNEIDSAIRLDEKEFNLAEIKSQILTIFEKQVIEKHVHFTVKFIG
APAAAETIVINDEKHALTMASPSTSHAHGPPGTGRMKDMILWGDQHRILQVLINLVSNSLKFTPEG
GRVEVRIKCLGDYEKPEGSTKVSFDSRSNSQMRSRTPSFQSTQIGGSVAGSTFDHHFREREMLLNY
RTLAFSFEVEDTGPGIPPHLQQRVFEPFMQGDLGLNRKYGGTGLGLSICSQLSRLMGGTIHLDSTV
GKGSTFRVDIPLKFLKEMAPSTRSSSTAGSRPQSEIISLDEAANINRTSGANSPVVKPVETQDLQP
RLVGLSQPFFAAAPPSSPPINNITTTASTTNNPSSDTKRHVQLKSTLSTASDTSTSAKIRVLVAED
NAVNQEVVLRMLKLEDIYDVTVAKDGQEAYDTVKATMSEGNKFDLIFMDIQMPNLDGLESTRLIRQ
MGYSAPIVALSAFAEESNIKDCIESGMDMFLSKPIRRPALKQVLKKFATIVEEEDGEAS
>g1120
MNLTYVAELVVRQCSYRPSAAPISAHPILYVATGVRWVGTRMDRLEVVCVEAEVGPFEHLEVAVRD
YQMADLFLRLEAVLFHILLRRRPLEIGLRIQXXGHQALHKLHEKEVDETLVVLDVILRHSQDLLHH
NHHMQVHLDHNLQVPAIGGPVRGEDLSEEAFVAGAYEDDRRARELFHYFQPDNPALLPAGHRDAGL

TNPPSSPTKASPNLVLTALTQLAAIKLGVQRAIISLIDRETLYVVAEASKSLHLGNNDVYEDDGDG
LWMGCSHGPVAGTLCEKTITLKPTPEDRYPLFVVRDLRQHPIYNNIPCVCGPPFFRYYAGTPLTTT
NGITIGSLYVIDPRPDITLTPSHVESLGTIADAVMEYLETSRQSLESHRLAKVLSGLNSFVQGDDG
EDSPIAAVKHDSLSDRASSHMSSPQESPKAEAHNFNLHATSDTSSLRSASPEHNADVTDIMLDSPH
SSKSTNPEVPAPLSAKRRAKKTRSTFQRAANLMRESLDLGQDGGVVIIDSSEHPESDSLDDIDRDK
ERKSASICALSTKGSFEDSSASSQSLIAPQLDLHFVRRMVRRYPRGGLWYFYQDGTAFSSDDDAVS
SGSEIAVSNSTTRSQQSVHPQSVGTLREKDLQTLRKYFPRATRIVFAPLWDSFNSRWFGGCFGWSA
VETRVFSAHVELGGVFGFGSSLMVEHSRAQSQESDKKKGDFISSISHELRSPLHGILAATDFLSEH
VDSKFAKSLLDTVRACGQTLLDTFEQILDYSKINSLDMKRQLYSSRLRERGRNEKSRIESLRLLKV
VHVAAVVEDVIESVYSGYIFSNNLASRVWQLEPGDEHNQNSDGRNISPIDILIDVAPHNWEFMIEP
GALRRIIMNVFGNALKYTREGFVSVRVDLQKKSAGTDSTSSDNSESEVLVLTVSDTGKGISSEYLH
TNIFTPFSQEDTLSPGTGLGLSLVRSILRSLNGTIKIKSQVGAGTVVKISLPLVVLHESNRQPDTQ
PASRIRAPHISDTVEPSVSKSYSVTLTDVSKSVQGSEIAVKEAPDTGIRILLVEDNPINMALLKKF
VHRKSPRVLHTAVNGKIAVERVQQTTEDYHYIFMDMSMPVMDGFEATQAIRSIERSRGTTNPATII
ALTGLGSSEHLAKAFAAGINVFLTKPFSFNDIARLMDERNIHAN
>g1121
MGNAPPPIKIPQRSMTTPNSSSPYTPPLRSPKYPGQNTYPEPPAEYGEPETVAAGNLLAPTSSHGD
ELKYKTFSTYVHHNVFADYFAEQDEEEGGEVPGAGVGAGAIKEEDEDELDMPNFDAMPTSEPVHRR
NESFENSLSAEKKPGGAYAPYKPTAPAEYTNQFADAGFQFDLPGDGMHNGHNGNFDGSQRTPSMGS
YNAHPIGSPESQNPDALPVHPSPYRAGLDQATGDRPAPVRQYGGPPPQNDAPQPAPSAPPPGGPTG
RPTSPPITQTELERLQIIVRRNPDDYKTALRLAKKYVEASTRLIGDEGKVDPKTRARNKDKYIHDA
YKIVKRLVNDAFPDAMFYLADSYGHGGLGLQADPKEAFQLYQSAAKAGHAEAAYRTAVCCELGQEE
GGGTRRDALKAVQWYRRAAALGNTPAMYKMGIILLKGLLGQQKNPREAVTWLKRAADGADEENPHA
LHELGLMYESTAQSDFIIRDEAYARELFTQAAELGYKFSQYRLGAAYEQGLMGLPIDARMSIIWYT
RAAAQGEHQSELALSGWYLTGADGILQQSDTEAYLWARKAAAAGLAKAEYAMGYFTEVGIGVPSNL
EDAQRWYWRAASQNFNKARERLEELKKGSGRMQKTRVSRSAVAQQQQQGGDCIVM
>g1122
MRTSLILAAALTALGVQADSSSTIIGFFAPNWDVTVPAYGGYTSTAASLAAVNAEAATYHVGCVND
APKTDCDYPSSWTIVQGPATVSVTAVYIASTSGKVTSYDLTVTESYECSLKSSTQSASCTMSVSIS
GSIEGSKWSSSTSTSATYATAPISDSYDQLTVTAGLSSLTAPEATKTGGAVAATGVVGSMITAAPV
VAAAVAALL
>g1123
MSTATIATINPVPYQERAGIKQSLQASLIPAEWRLPSIPPVEEVPDALEYIRKSNLLSERELSITE
TSDVSVLLHKLASGQLSSLEVTKAFAKRAMLAHQLTNCCTEIFLGDAFERARQLDDYLEQTGEIIG
PLHGLPVSIKDLFNVKGVDSCIGWVGLTNKPAIRDGNAAATLRRLGAVLYVKTNVPQSMMMSDSYN
HVFGQSVSALNRNLISGGSSGGEGSLVSARGSILGIGTDLGGSIRIPANLCGLYGLSPSTGRQPYE
RSGLRQDIVRSVAGPMAMSLSTVEKYMEALPAARPWEIDHQVTPIPWRTELCTTTKRLRIGYVVDD
GVVKVQPPVARAVDEVVQALREAGHEVFEWDASSHAHAYQLWEKAILSDGGEACRILCELSGEPLI
EGMLVGKPHNKLTTSQTHQLISDKYAYERQYLQRWTSASLDALIMPITPWVGYKPQTWIKSHQYVG
YTSIWNLLDYPALAVPVTKADRTKDDPSVDKEWMIHQPRNKSDEFNHQQYDIDLVHGAPVGVQIIT
PKLQEEKCIGVAKVIEQSLAAQRIAEMTQRAKL
>g1124
MHDRYTTVNQYSFIKRLAAFYEQIKDANTQASGGFLAPEAIELVSRIWDFIRVRMKGQTASNVSTD
RQSVDEVQYHVDALMRLTEAIDKAHPIALFGIATFAFFEVCDGSFGEWQCHLNGARSLLDFHCRNK
ADLDRLSHELTGLNEIVAHLVWFDTMGAIIRGSNGLIFDDWHRETLSEEFFANVGCPVDTFHLFVT
LAKAGNHINTLDLSFEALDQLLLRAEPGSSDMTLVENAWRCTAAIAIISRIGDAGSLSRRKALSTA
VDQACRVIAIMPPTSGIYIHLAATAYLAGMHATHPRQCEIVRAYWRHCRASDFPRYPDGQAKCEAK
WRAEGLA
>g1125
MEYNLTENPDTMLPPGTVHLVTEQSDKIILVPKPSADPDDPLNWSKTRKWLNVTLLLFYVFTTGIG
GTSVYSVFTPISKETGISIAQLNNGTGYLFLMAGWTNIIWQPLALTFGRRPIILLSLLFCVAISEW
TAWIDQYPAWAAARCLYGLACAPVEVLPQICIPDVFFAHERGAFIGWYMFVLCASNFIAPLIAGFM

NDTFGWHWVQHWAALILALNFVLAFFLYEESMYTRTSIEGEKLNGLPDRDDTRTFQQSDVTAGYYK
RKTFLGKMKLWSYNGNSFSQIIRIAYRPILIFFQFPNIMWAGFMYGFALAWYNVYNATTSSILSAA
PYDFSAAIVGLSYVAPLIGATVAGVVSGPVADWLTLRLASRNGGLREPEQRLWGLVIYCILMPAGL
LIWGLGAYHRVHWAVILLGGLLCGYCNVSGGAYALAYAVDCFKELSGETIVSVILCRNTLSFAFNY
AITPWIDAQGLQKTFIAVAILSWAFGGEIYLIYIYTPTSNKGPSSNIDFGAISEDRISAIPT
>g1126
MSSSEDFRLRRKVGQLFAVGFHGYTPSTEIKTLIRDYGLGAIILFRRNIKDAAQLQALTLALQHEA
KEAGHEYPLFIGIDQENGLVTRITPPIAAQQPGQMTLGATQSTANAYDVGKCTGEMLNFFGINMNY
APDCDVNSEPRNPVIGVRSPGEDPNFVARIASATARGLRESGVVPTVKHFPGHGDTAVDSHHGLPV
ITKSRSELERCELIPFRRAAAEGIEVVMTAHIALPKITSGPGSEGLPATLSSEALGILRDDMKYDG
VIITDCLEMDGIRGTYGTVEGALMSLKAGSDSVMICHTYNVQVKSIERVVEAVQSGKLPESRINES
LRRITSLKGKSLSWKHALRSADMSSLDELQAMNQRHQQIAERVYSQSTTVVRNNLGVLPITKPVSD
VLLLTPGGKIPAGGAVDDGGPEHKTYIEVLRQDIGDMTSSVTQVDYPDGGELLEEHWRMIENDDAI
VILVTRNAKEAEGQRSFALEVAKRRKDVIVIATCSPYDFLDDEKSVQTYVTTYEATVEAFKSAIEV
IFGRVPAKGMLPVSSTSNKQRDPSFKTLPLCESQKGPMIDGILKVWDAALPNYPLPADNLRTVIDQ
SHAHHFIAQDTQTATIIGFCLSYTSQNQGQTSAEIAVIAVHPTEQHKGIGTKLLSATREYLYENHG
CKNASLRSYFPRFWPGLPADLAPEVEQFFVHRGFRLRRGGDADLYQDIRDFVPPQQYIDKALAAGV
TYKVIEAPEEFEDCLLGQKRNFAHYTGWVETYISLHPSKHPSSIMAAFDKHTGTQIAWTLMLSPDD
DFVAHNWAFPPLASGNLSLKTGIIGCVGVDELHRSRGVGLALLCHAIQDMKKRGVEAVFVDSTDKV
DCLNAQDHALSDHVHRYLNYHEDQLAARLKPKTVNDGAKKMTDSNDRYDAHLDHETQHDDQQQHSA
HKNPTAP
>g1127
MDEKFEKGDALEYVENGIQHPYTEAEKQKVVRKLDLHLLPFCFLLYTFSVLDRSNLGNAKTIGLAK
DIDISGNKYSWLGTIFYIAYIIFQFSTLGWKIFKPHQWVSAVVVFWGLASXLQATVTSWGGLMACR
FFLGWAETMFGPGVPLYFSFFYPREMLGLRFGIFLSGAALANVYGNLLAYGLGQAHSSISSWKFLF
IIEGVPTVLLAVATYFFLPDSPTTAKFLNEREREVARQIATSQPDDYEHEGLQLSQVGEAFKDYKN
YLFAIMNFSNNVSFASLPLFLPTIVSEMGSFTTVESNGLLAPPYFLCFLLIIAISILSDRVRLRGP
FAACFAFLSAVGFILLGATTSVTSRYIGTFFAVLIFVTTSIVLIWNANTNSTSSKRAGGLWIIMTI
GQCGPLLGTNVFPSNQSPLYREGSWICCAFALLSGVTALVLSFCLWQENKKMDRLYGPLEELDPIE
RPENDEVPRFRYII
>g1128
MSSDFSILTTNPMLGYGYKLEHFWYGIEKFSPKAIIVDSGSTDGGPYKLGLNKMTCGRDSYIRDLT
PMLQACFHYKIKVLIGSVGGDGSDKHVQEMLEIVQEISQKHGFSFKVATISAGFDKSLVKDRIVNS
RVGPCGPVEELTIDSVERAIDIVGQMGAEPYLKALEMDPDIILGGRSYDPAPFAAFSMFHGVKPGT
AWHMGKIMECGGICAVPKGRSMIATMRKDSFDLTPLSPKERCTPTSVAAHTLYEKTRPDRLPGPGG
VLILDDASYEQITEKTIRVRGARFEPTNVYQIKLEGVEKLGYRTIFVGGIRDPILISQIDEFLADV
RAYTQNLFPQLDQSPECRLIFHFYGRNGVMGPLEPDPKFAHELGIMGEVVAPSQEMSYTIANNARA
SILHMPYKNQVATTGNFASPLSPHETAAGPVFRFNVYHLVDLNKGEEEILFPIKSVTIDNYPPEEK
SIPGLTTAQREQLTSETLEPLRFKEIPEGECQMLDIAKIVRSKNSGPFELTLDIMFDNKGAYERVK
KANILTNERIQTLYHLKPEDIITNMFFEPALAWKCTIRRPWEQGTVGERDTLGTQQHAPLLTIKVP
KSENSTTISPKSKTHFQDRSHFSARDSVAYIWKELDLPADSLKSLQMTGEGLGLPSSFKIGHIAQA
SISLSALLAALIYGHRKKAPTPKVTIPLLHAAIEFKSERLYKLASASMPDPWGPIGGLHKTIDGYV
RVHDNFPNHRETAIELLGCSPDADRKEVAAAIAPWRAIDLESAASDSKSVIAALRSYSEWDLLPQA
RAITDFPIQLRKLCDGPVGLPKEMLDANPDKCLRGLRVLELSRVIAAPLSGRVLAAHGADVLWVTS
PNLPDIPAVDRDFGRGKRTIQLDLNDEKDSLDFHGLLDKAHVFTQGYRPGSLAAKGLTSEALVDTF
KDRNIICANLSAYGPDGPWKNKRGFDSIVQTCSGMNVSEAEHYGAGEAARPLCCQALDHAAGYFLA
AGIMAALYKQATEGGSWEVNVSLAGAMKYLRSLGQYEGRNGFDTKDYTCTEDVSSEFLETRETGFG
KMTAVRHSASIEGVQVGWDMMPKPLGSDEKEFL
>g1129
MSRVPQRTGTTLYHVPLIIDGREETGETTFEVISPYTNQVCWTAACATPADAVRAVESAQKAFPAW
ANTKPTLRRDILLKAADILESRLEENGEFMQTEMGADVPSSTGFVVPKGISMLRDIASRITAICGS
VPVVETEGQSAIVYQEPMGVILGIVPWNAPYVFGVRAAASALAGGNTTVIKSSEYTPRCYHALLSA

FLEAGLPAGCFNLISTRAEDAAAVVNIMIEHPAVRKVNFTGSTAVGRKIAAFCGQHLKPCLMELGG
KNSAIVCGDANLETAALAVLMGAFLVNSGQTCMSTDRIILHSAIAEKFKSTVERLLDDNTSAAPPT
LVSMSSKARVTGIITSALAAGAEVVHGAFSDATTSQPDSSAPRVSMTPVVLGNVSRDMHIWKEESF
ATVAAYMIVTSDDEAIELANEGGYGLSVSVFTEDLRKGLAMAKKIQSGAVHINSMTVQDEAALPHG
GVKNSGWGRFNSEEGIKEFLVTKSVTWMD
>g1130
MGSPETANPVATRAKGRKYGFACLSCRRKKIKCDGNKPACINCNKSKESCVYRDNPAFVGYLADEV
RKSKIRIQELESVIKELSILDSKSRQSRLAALVADLEQRSSSPSPGSSAFLESDEPHVGLSVSYGG
SVEYNIDENGRQQYFGFFRDMAIGGPYFSLFLLNVMFAHAMRHMPEDDPRFKPFDKGEFFMKRAML
LLLDEIKESKPKIPTIQGLLILGGRQCAVGLNSEGWLYTGMAIRMITDLGLHLRKGRSAMKEYEPD
DLEVRKRLYLSCYAWDKYATVAIS
>g1131
MLILIFRPFFIWRWNTHLRDHPLALRAQVVCTEQAADVNEIFRAYGRLFNFQYQTYLVSYCVYTAA
TIDVRLIHHADRNLAEKAADRLVVTLRMLETELEQTPGIKRSIEIIRSHLGKQRLPVPNENVADAR
ETKPAYHYQQQQNRGEKWQGHEISPSPSTSASYYSMHQQQRQRQKIPLISALPHHPHPHHTSQLHQ
TTEIYEQHNSSTILQPSHPDIRTPLPNSDINSNNDIHHQSTYIDAQIQRMDMPWFDWNVDDSGGGF
VPDMAYWGGIGDGGMQYNHM
>g1132
MPCFKGLAVSIHTPDGPIAEFSVQRQSRQSRIACYIPVPAPKITESGKAQPSTFAVSITLLNAGQS
VPYSTPKPTPDNPNPKPQLVGVSETDEKGQTHWINPYLPLTGSVNETVAAYIYFDGRQKEEVATLL
RRGEETWVNSRWVSVPDSEGGGLAEREFLFREVGLERWLNGLDLEGKDAAAKIERRRQKMEKRRLL
REAQDNGDMEMEGESSTKRDKGIMRYGADAKAPLENLSDEEFSSDSDDDPIPESAGQIKVALFRVL
ASGEIRRGEYSPQFDAHDDDEEGGQGGDGNADVDHTTSFAKPKTLDPTTISTQTVTGIDPTDKPYA
VFTFMYRGERQLQKMGILKSKAVEETPAKRKSTDFSKLAPLKPGGAVGFVGYREGDKASSSKAGKK
SKKSSESDMDSDEDEDEKDPVTAKADEDESNDTTHLSPDDLRRQGDLAEGVRKIKLKRQHSMDPMG
DSTSNANTPASVATPPAATNEASTPPPHASASAPAPADANINDVPNSLPDNLDSADYVGSPMKKAR
ASVSTADEDGLRKRLGSNLANNISEILSGEKTDSPTTFGGSGSHFGGNINPTAAPAQPINTQQVSQ
DEDEEL
>g1133
MSDTGPQDEVHGVGTAPNAGASGVKTVIAEKTADSSAVSDSEQGHFERVNLNNNVSAKIKNPLADL
SPHHVIRDVTEFAQIHGLTHITPVLQRGALVARDPANYASVEGLTEDESLAIQDEVLHKWKQPAAL
YFTIILCSIGAAVQGWDQEGSNGANLSFPDALGIPESGPDAASNLWLVGVVNSAPYIGAACIGCWL
SDPMNRLLARRGTIFLSAIFCVLAPIGGAVAQTWPQLFITRILLGIGMGLKASTIPIFCAENTPAS
IRGGLVMSWQLWTAFGIFLGCTANLAVMNTGNISWRLQLGSAFIPAIPLLIGVYFCPESPRWHIRN
GNMRKAYESLLRLRNHEIQAARDLYYIYAQIQVENEIIGTSNYLTRAIELFTIPRVRRATLASFVV
MIAQQMCGINIVAFYSSSIFSKAGASAVSSNWASWGFGLVNFVFAFPAIFTIDTYGRRALLLFTFP
NMAWTLLTAGLCFLLPDGSTKQLATVALFVYLFAAFYSPGEGPVPFTYSAEVFPLSHREVGMSWAV
ATCLGWAAVLSITFPRMLDAMTPTGAFGFYAGLNVTAFVMIFLWMPETKQRTLEELDYIFAVPTRR
HMSYQLNTALPYWFKRYILRQNVTLEPLYHFDHVAEEIIRVTK
>g1134
MTPKTDDKPEGVTRKLCQWIQDINLEDVPDDVRLRARYLILDGLACAIVGSRLPWTETAAQTVFAM
EPTGNCKVWGYNDKAVTPLSAALLNSTAIQAFELDDWHLSAPLHSNSIILPALFAAARQQAKSTST
KVSGTNFLLSVIVGCETGPRVGLGLYGAHMLTRGWHSGAVFGPSASAAAVSKLLSLPTDAIEDALG
IACTQAGGLMSAQFGSDVKRMQHGFAARNGLFAALLAQNGYRGIKNVYEEGYGGYLAMFGQGSGKE
PPYLPEEICKELGKTWQSRLIRVKHHSSMAGTHCTIDCVQALQDKYADELSDPDAIASIRIGMTEA
AFNHGGFDVKRPVATTGAQMSAAYTAAVQLVDGQVLPNQFRDDQLDRDIVWDLIGKTECFHDLDLG
EDRYAQRVTITLKDGIELSETLDNPRSVSPGLSNEEILEKWRRLTQGIIDEKTRDQVQQLVLDIEA
LEDVCIVDDLMSISVSNPLLS
>g1135
MTRLGILKPQALKPRLRPEKYRPSLFAFPQARLQSNMALQRSSAPLVWVDCEMTGLDPETDQILQI
CCFITDAQLQLLEPNGFEIVVHQPKSKLDAMSQWCIDTHGKSGLTRAVLASTTDAATASSQLLAYI
KQYIPEPRTALLSGNSIHADRAFLAKPPYSPILEWLHYRLLDVSTVKEAVRRWGSDELLSAAPPKR

EVHLARDDILESIAEMKYYRSKLFDGK
>g1136
MSVQTVSLTPFQDQKPGTSGLRKKVVVFQQPNYSESFVTSILLSIPEGVEGSFLVIGGDGRYHNSE
VVQTIAKIGAAYGVKKLLIGQNGILSTPAASHLIRKRKATGGILLTASHNPGGPTEDFGIKYNLAN
GAPAPESVTNKIYETSKTLTSYKIAQLPDIDITQIGTRSYGSLEVEIVDSVTDYVDFMKEIFDFDL
IRDFLNTHKDFKVLFDGMHGVTGPYGVRIFQKELGLPASSTQHCEPKSDFGGGHPDPNLVYAHDLV
EAVDKNGIQFGAASDGDGDRNMIYGANSFVSPGDSLAIIAHHAKLIPYFRDQGVYGLARSMPTSGA
VDLVAKAQGLESYEVPTGWKFFCALFDNKKISICGEESFGTGSNHIREKDGVWAVVAWLNIIAGVA
KQKPNETPSIASIQKDFWNTYGRTFFTRYDYEKVDSDGANKVIADLSKLIDNKDSFVGSDVSGRKV
TDAGNFSYTDLDGSVSKNQGLYVKFDDGSRIVVRLSGTGSSGATIRLYIEKHESDASKYSVQTQEY
LADNIKLATELLKLKQYVGRDEPDVKT
>g1137
MAINTLPTTTTTKQLRFLVIGAGSRGHRYAEAVTESTSALIHAVAEPRPWNRQEFGERFIWGTNNK
PSNGQEFTDWRDWLKWEQERRQALANGQQNVPLGVDGVFVCALDEMHVEIMCAIAPLNLHVLCEKP
LATSLADCLAIYRAFVPDHKRDVGPSKVFSIGHVLRYSPHNMRLRQLLLEERVIGDVVSVEHVEPV
GYWHFAHSYVRGNWRRETHAGVGSLLTKSCHDIDFLVWLLSSPPPGSPKDMPPHEPRTISSTGRLT
QFIGKRKPKEAGDATNCLSCPIERKCNYSSVRLYKERQLDKGNTDWPLHIVCPDIEDTFKVAGADA
AEKLLMTALAEDYDKSSAPDAEIKSRSWYGRCVWESDNNVCDDQFVTLTWDDEQPPSTDEGKYTPR
TSKTASLHMIAPTEKQCERRGRIYGTEGEIEYDSRTIKYFSFATNEFTTIEIPKAKNPKEEQAHGG
GDWGLTRMFIGAVQAVEERGWDVRDAQREFIGCTLEEAVRSHAVVFAAEEARREEKVIRWQDWWDQ
HLKSLV
>g1138
MISDDSSEIDHEVNSSILADKTDHEFHGISHSILALPPAPNGTTVQMTTHAEVYDIIIIGAGPCGL
AVAARLREETPSAMFTDEEHQRYHWIKKHQGKMSLARARTRRLHGTPLTASTYTKPNNCCSGENDR
GGQYSTLVLDSSGSQWMARWNRAFKALKIDHLRSPMFFHVDPADRDGMLAYTRETGREKELYELSG
CVGQELSKHKRKKIRNLSRFPREAEINERDRKDYFTPSTGLFADYCESIIARYGLDDGSARIEQSE
VCDIKFGEVEGSKDTSKLFTIVTTHGQSFHSRAVVLAVGPGLTKLMPWSLSPKEETGACHSSEVGV
RFPSPVLARKIENQQTTHIVVVGGGLSSAQLADMALRRGVTKVWHLIRGDMKIKHFDVSLNWVGKF
KNYDKAVFWSADDDQERFEMLQTARNGGSITPHYQKILKQYTAKGRLSIHTQTVITSKSFDTETHT
WKITTDPPIENFPEQIDYICFATGMKVDVNEMSLLQSMNRDYPIESINGLPCLTDDLMWRSDVPLF
VTGRLASLRLGPAAPNLEGARLGAERVAWALQDVMGEQSDVDFCSRDGKRLQKAFCGLGNRYESLA
QYREIDV
>g1139
MLVLSKYAVALATALSIITATVARPTELHPGGASDLIINAQDTINGTISASASKSQSVAATNGTSL
TLTITNNIAGSVNCYVNGQDPNQNNAPVMLTPDFSWYYPSAPSTTDGTPAPVSETIAIPLGAQGES
TTITIPSYVISARVWFAVGELQFFTVGNADGTFSIVQPSQSNPADPSAGVNWGFIEFTYTADGGIY
ANISYVDFVGLPLGMSLTDTSGGVQTALGLTSDAVTSVCNDLTTQAATDGLPWDALCQTINGTVMR
VLSPNDYMAANASSGFETYFDDYVNEVWSNYTSSTLTINTQNDAGNVSCTVSGDTLECDGDGINYS
KPSTADIFGCNSGPFVTNPSDTAVHQAAVPRLCAAFNRGTLLLAGGDVQPSLGADSYYTTAPSNYY
SAIVHKYELDGKGYAFAYDDVNPSGDDENASGTVSSANPSVLSVTVGGAST
>g1140
MVDEKVRYEELPIPTYEEATASQSGSSHLGPEHSSDDLERQGLLQNEESNDSLPNRSRTTRNGGYQ
PPTVESARSSIDYLASSASGSARQSEEDLRREIHQMDVDDSGSSSRTRRPLFSRRFSKHFANPFSN
LTKTLSSIQLPFRNAFPHFQFPWRFWRVRINEDGFACMLFLRLFGLFILVFLIYLVFVSDIFAVGS
RFNMGQSYTAASVESFVQSHINETNIANNLQKATQFAHIAGTEGSYVLAEWIEQEFINAGLEDVEM
EEFQVYLNYPRPDGRRVAIVDPPELAWQAAIDEPQIYNDPRRQQTLVFHGHSKSGNVTGPLVYANY
GSQDDFKRLADSGISVEGAIVLVRYYGTQGDRALKIKAAELAGAAGCIIYSDPAEDGFIKGPAYPD
GRYMPSLGVQRGGVSLMSWVVGDVLSPGFASTPNEKNRLSPEDSPGLVQIPSIPLSWRDAQRLLQV
LKGHGSKVPADWVGGVPDISEWWTGDLSSPKVNLMNLQDEVERQPIYNVLGRITGLEQPERKIIVG
NHRDSWCFGGVDPGSGTATFLEIVRVFGEMRSVGWRPLRTIEFASWDGEEYNLIGSTEHVEKEIDR
LRANGFAYLNVDVGVSGNSFEAAGSPVFQRLLNTAMGRVGDEAANKTLKEIWDEQGKRLEGLGAGS
DYVAFQDIAGTSSLDFGFGGAPFPYHSCYENYEWMAKFGDPDFRYHRMLGELWGLLLLELADSPIL

PFDLEAYSADFRGYIDNLQEYAASKGVPLKTASDTSQARTETVDLKSLYDAAEVFKTNAADFQAWE
QVWYSAVYGSGGFESNIMGARRIEHNNRLAKFETNLLDLSQGGGVPNRTQFKHVLFAPQTWSGYDE
AFFPAIRDAIDAGDWNATQLWVNRVADIVTQASVNLNV
>g1141
MATRPQNIGVKAIEIYFPSQCVDQAELEKFDGVSTGKYTIGLGQTKMSFCDDREDIYSLSLTALSS
LMRKYNIDPKSIGRLEVGTETLLDKSKSVKSVLMQLFEESGNTNIEGIDTVNACYGGTNAIFNSIN
WVESSAWDGRDAIVVCGDIALYAKGAARPTGGAGCVAMVIGPDAPIVFEPGLRASYISHVYDFYKP
DLTSEYPYVDGHFSNRCYTQAVDECYKAYNAREKTLQEKFGANGVISEEQVPLDRFEHVLFHAPNC
KLVSKSYARMLYNDYLTNPSHPAFAEVPAEVAEVDYEKSLADKVVEKTFMGLAKKRFNERVQPSIE
VATQCGNMYTASVYGGLVSLISNVTFDTSKPKRVAIFSYGSGLASSIFSFKVVGDTSTMAKNLDLR
NRLESRRVVPPSDYDALCLLREHAHLKKNFKPAGNADNLFPGTYYLTEVDDMFRRKYAIKA
>g1142
MTNITTPRTSTSPNYQHGPQLLPKIRTQDVVLEPVSSVGPRRHRRVLSNTRNPPGYMPYPTARPSA
LSRNVIDASAAADCSGLISPISPASTHPAMISSGLSSPIALTTSAASAANKRKSMPSAGHSRSGST
SSIDEVTLSRYGYPTYRQLPKYVSQSEASPISPVDPSSFVFPAYNRQVPSVQVPSVQVPCPVQLPT
QQAAYSALPSPQNYDYLRVHSAQPSPVGFLAPPXPXPASSTTLLTYLTNTTPAVNLVRTVNYGPTR
GLNDYFWWDIRQLRSWSSFSLNTFNDINGLTQLLKTPIPSHLTPQPMIASARLTPESENALINLVG
DIYAPRINAALRVSQGSDHMRLYVSPDANSSGSRGANGPHFLANYASDTDVTASGVRRGRVVGIVK
SFDRWNTGMRNEPPHRRVEYLRGLAHLQRCMREHSCRYGFIISEIELVCVRAGCDDGDDVPYFGYL
ELSSPIATKESSSNHASRCADQTSRRRSCTPPHSSASSSYGDGRSTHSRSSSLSSLSSHHQDDNSA
ENGLGGVPLTATLALYFLLMLSKSMPLPSQPSGHLNVGGPGALSRQRILPEPKDKWIPEPQLGEKR
EAKRVRGWIMPQDAWHRREGSRSKNNTNVNGDGGRGKRGRKELAMQ
>g1143
MALKVDRIQVQPFPPFQQEQHDAPVQQDVKSPTLLHRAASFESTNEEDVQDSPVSPGVKRNFSDTS
LSSKNESSTVKENFAAGKDILRRVTLRPSNKPKXLXRRISTNEIPTTANSVSGVDNAAPRERPGSL
IEENKDMPSQDSTPQIVVRPSKSRSVSGKIATLARVSWMSSSRSPSPARDVKRGASSSREQSPTPR
SHPASTNGLSLSSETKEPRPNTNDDMQSKRPTSSKRSRRPLSAFVPRSKSADPPSPSLRSRKSVDQ
LGARGPELPPLPNPPIPSSIPALEPGRKKDELWNVFRGLEADLQKFHAKSTNLKVNVVRSSLLPFL
QRYASHPSFNELKPEDLDRRVNILNKWWTSLLGLLHGKHNQSVSGTDRPVFLEALSGIMMRKEWRI
PYQPQNFFAHTSNSSLESSSSDFLAESIFHNVRNIFNQNLLSQMSFVVERMSLRHAPASLVSFCGK
ACAYAFYFCPGVADMLVRLWNTPSDAFRRLLADPLGYKGLGGRAIAQNLAARFPLPVRPLAFCSHI
ALSRYLRQPLILPLSATQIPWHGPWVSRWCGRDSDLFYVFVKYFHTLNCDFIPPETDTSHQLFAPG
MLSVQGHLLMVLEDTLYRQSSPQAPENPFAPSSVTFDDFIEGPNAAMSTMHIGMANCHRSMAENRL
IILLRDFLSEPSPDSAPARRMYIDSFMRVLKTAARKTSLFNHNACLVLCDFVEETAPLIDRYARKA
KEDIFHWPFWLDVCKQMTLSQNSMTEVRLFSFLFSMWKPWTSNKERYYSLCSQFLLHDDYFYTYFS
HWNPMVRAYFQRLICWRVARFSANPTPVESDVYEMLMDKLEQVWAFYLTYNLRARKEMLPLLDAAA
GNPAPARRLLIIRSDNQQPSPSSPFMSFDRLSTPSFDQSAYKSHGTLKFDINGMPSESDSPSAPKR
RWNILKSVFGTTANPKPGEVTPPGSVSDENEINPIDSLMKKQEENGNHVTEEDDSIYESQPPHQPY
NFKFSLEWYQHPHKNHNPHHKPPKPRPLFEPGLPRKTLYHVQHQRSAKALRHSTTTTEESIDPRRD
ADASTTSTRNSYATTKTSVDEETDSTIFAHIEDSKILRDERIVSSKYAGRALAEWAAIITEFDNFF
DRRLKEGVPSDELVETPTLSVENLKK
>g1144
MLSHSVRASRSLLTRVNRQSVGVSYRTFMTTATRRADPVQDLYLRELRAYKPKPAKAGEAEAQVQK
FTAPKPPPSPEESSLAGDLQAYETQAVEVEGQAVEGEEAPKEESWFEEDEEEAHPAAH
>g1145
MATTTAPTGVAAAKWEKNLSAHVICPECKEFPPNLEFPGSHETVCGXXGLVLADREIDIHSEWRTF
SNDDQNNDDPSRVGETSNILLNGNQLETSIAGGATGAARQLYRAQNKLSNEKNNKALMAAYKEIGA
LCDGFNIQKIVADTAKYLFKIVDDHKAFKGKSQDVIIAGCIFIACRQCQAPRTFSEIFAVTKVSRK
EIGRIYKSLEKFFTAQNIERTAIVGSDAGFKQTSSTKPSDLCERFGSFLGLDYRTWHAAADLSDIV
TKAGDLAGRSPLSIVAACLYMVSHLAGVPKSAKEISAVVGVSDGTIRGAYKQLYAEKDRLITDEFV
AKANGDRANLPQS
>g1146

MPDDTKAKAFTDISSKLSAPPKRSLFERQKAEAEAKRAREKAETAAVYEDFVKSFDDDGGDSGSRR
TSSNFRSSGPGSLGGPPPKRHFTSASSTRNSGPGSLGPAPSFGRKRTYDGLYRERDRNNREPGSHG
IFGYDDNDAPAGFRASDDEVDRVVEDKEAERAVAKPTVYLSSLPPGSSPAVIKALIPGNLTVDAVK
IIPPPAQPSMERRSFSAIVTLAQETAASDIDAAVSALQGKYLGWGYNLSISRHLSSAAIHSGMPVT
VGSSSKSSLPFGAKPVSQAPIGRLNRAPPPGPHRGGFAPPASYGSQYGKPASTAEIEVKPPSDLKQ
LRLIHKTLENLLKYGPEFEALLMSRPEVQREEKWAWIWDARSVGGVWYRWKLWDVLTRANTGHGRH
GRNTGPASLIFEGGASWVTPEMPIRFEYATTLDEFVSDEEYNSSDEEGSDNEESKRRHAADDSIGG
QDGIGYMDPLQKAKLTHLLARLPTANTRLRRGDVARVTSFAIEHASAGAEEVVDMLVTNVLRPFAY
SRANPDREEVRSALRESEANAAAASGDKADAGPATSGRTVEDTSAAKLVGLYVISDIFSSSSTSGV
RHAWRYRQLFESAFRSYKVFEHLGRLEKDLGWGRLKAEKWKRSVNAMLHIWEGWSVFPQSAQDHFT
QRFENPPLTEKEMEEKKKAEAEQAQSAAGNKGKSRWKTVDEDAPAGGRFDPSQVPLPEQSVAAPDH
DDQDGEQMSDIDGVPMEDSDLEADELDGQPLDMDVDVPETQEEREQPDQTLESTSKPEETTPVGRA
RKPRPKAEDMFADSDDDNN
>g1147
MADFNTIAQQFVQFYYKTFDEGRSNLAALYRDNSMLTFESDAKLGTQAIITKLNELPFQKVQHQVA
TLDAQPSNENGGILVLVTGALLVDEEQKPMNYTQAFQLLPDGQGSYFVYNDVFRLVYGA
>g1148
MTLAEEFRTRNFSIYGQWCGVLCIFLCFALGVANIFAPIGRIPFSIVCLICAFIIVFIEVPFLLRI
CPTSSKFDDFIRRFTSNYMRALIYIGMSAVQWVSLVTGASSLIAAAVFLLIAGLCYLLAGVRNQEF
IGSKTLGGQGIAQMIV
>g1149
MPAFVDSAKKNETSASNYTRHLTSIWIAVLPTTFNFFGPIFSEDTFMTAFNDPNTILDAKLPWTIW
GATTEVANFIHNTELRRLPMIRTQAVRHELNQEPRLFGAIFTYRQLKLRYIAHSRAVDDAPITREH
QSEVLSEARFCYCEYP
>g1150
MSAPSAPVIPPRPARSPRPLGADIPSIPPRPINRRLDRSVSPGRDSYAPSPLNGPPGLSRTTSNDL
PARPPSVTIPSLGEEGIEYEELSSEPAETRNVGSDLKLHAPKPSLPTSSAKARVQAVTRTDSDQAA
AAGFGKSSSPALEEHDQRSRSIHSKPSGSRAESTASSTRRQSMQFEEEHGIPEIGQRVPMLAYAGD
VQAPSPAPPRSGSASGQRVSRHHHRTRSGRDVFLPPGSYGLHGHGVHAADRFEKAWYDKHPDELAR
EEQGQYGPAIAERSAWALSSDDLNRLVKSSDADTSVGTSPAFAGTPAEEVGYIATDELTRQDTQPA
VESPLRHSTFATSELTRTKSAMSTASSDGGRVIHVDEPYHSLHHPDGFARTPEPPSVQYYDEAVED
DEPILAADESRPGSAYLHPAVSPRRASTDYHDLERVRSRTPSAPNSRPTSLHGAPAGLTRWSSRGE
EHDDVHTPLEDVEEYEPLFPEDEKSSKPLSAAERFQKRTELLKHRFPSQDIWEDTPDSLQLHASVS
TPDLPEDLSKTKFETPEAEAQRKREADQIDSHKVASHILHGQGTQGRQARPDTVKHRFPSRDIWED
APESHQLMTTIEPSETDTTSPDKAKAPGIPSRPSVPPRPAKVVKPFDGSTDEKKAPVIPDRPKPQI
PIRPSKPVSGDAAAPPVNRNKPVIPARPNAGKIAGVKANFLSDLNSRLQLGPQAPKPAPEKKEEEV
PEEKAPLADARKGRARGPARRKPAASSSSEKRLPSIPEIRIMDAWNVWELGVDGNLTVGLSEKKEP
IVEIKPQASDADTESDQTAMAPPIAKNMAGEDADPVVVSPEKSVEPEPEIHEPAKTETVEHHEDAQ
TSEHVSPKHSVDQPSESIDTQVMSIDPEPTTDQIIEQLAATADGKHKSDGSIDAEETVA
>g1151
MAAQLQALLRFLSQDAKVPLASAMGKIKELQEAGLQNVDDISKSNLDSLQSIFKDGKVAKQVLNAA
KRVTKGGAQKRGASSALDDKSTSTSPKKARRSNDLEGAHRTPYEIESSLSLHLASEPEETLSSTIL
RTNRAPLVLAFAVAVLKYTMPEQPMSSRLSLGQAVVSANSRSKAISLGIEPASSLDNGDEKDRKLE
EGQPKVRVLGREIAVLKRWDYDPAEGGPGRKYEEGDGAEERVGTGLPPLWGLDLEVLRRNDSIGSA
GMTSGRARNQTMSTLPVHTPESARSYLLKSFTLFKENDDESSTKSKKKIADSTAERERCLGLLLQA
LDTLFSSWASTLSAEELDRRAWSWYVRVRPEIQSGVAGWGEKGSLKLADILSLKR
>g1152
MQLLTKILLAFSGLLVVNAAPYDAEKRDGNYRSVAYYVDWAVYARNFHVQDLHDMVPYLTHILYSF
ANLDESTGEVLLGDKYADIQKTYPGDPTNDNGHNVYGCVKQLYLLKKENRNLKVLLSIGGWSWSSR
FAGATGTEANRANFVRTAVGFVQDLGFDGIDIDWEYPSNYTEADNYVTLLKELRAGLDNYSAAHTP
GYHYLLSASVPAGPSHYEILHISPMNQYLDMWNLMAYDYSGSWDNVTGMDANVYQKTAYPNSTPFN
TDQAITYYTGNGASASKINLGLPLYGRAFQNTDGPGTTYSGVGPGSWQDGVWDYKALPRPGGTLYH

EDSSVSAWTYDHAQRYMVSYDTPAIVQTKASYIKSKGLGGGAFWESSGDKTGSDSLIHTLVNALGG
NGVLQRADNELNYPASQYDNIRKGMQ
>g1153
MASLWNYLSLPTAFFTSPVAAILTPVTAGALVGYTTTSVSDTQSIYKALRQPPLYPPGWVFGPVWT
MLYSAMGYASYRATIAGLSSPSPTIRELAYTSQTLYTIQLATNLIWMPLFFGMRKPLTALADILVV
GGSVLALANNYRKIDDVSFWLLVPYLGWLGFATYLNFGVGYLNDWDITDEKIGRSKRK
>g1154
MRLIFVFSLLAANVLAFESNTANLPDVALLVKRQDGGSGNSSFTSVAAATTTAADTTTTAAATSIS
TTSVAPTTTAEPTSVAPTTSSTSSATSTSLPPSVVTTTSTSTEPPASTSSSSTSTSSTSEPPATTS
ATTTSEPASSSTSSSTSTTSTTSTSAPSTTFTSTTSRHASTTSFLTSTYQSTTTLPNGSLSTVTAV
TVVVATGAADTTGTSTTSASSTAHLQTGGAASPSRGVAMGMCAMIAGAAAFAISF
>g1155
MASSRSTTTRLLKELQDYTSNPNEALLHLGPINDEDLLHWEAVLKGVNGTPYEGGLWQLHIEIPTN
YPLSPPTIRFKTRISHPNISFTTGEICLTLLTTEHWSPVYTLSTTLSAIHQLLTDPRPDSPLNVDV
AALLRDGDVAGWESIVRYWTEEERWQGPPGNAKPAAR
>g1156
MSPTPVPISLFADAQRKLLQKEHEAEKLSSALATSSTTTISPSTRRTLQATGYALTGIVLEQCRTG
MGGRIVGEFGADAAYATESTEDGDGRARFGAHGIRVGDVVRVLEISSSRKGGKEGKEAGGNKPNGA
EGVVIKASEKGLSVAFGQSGGGTNAKAEEESVDELWGKKLWLVKLANDVTHRRLNQTMEKMEKMSD
SEHTYFMRVLFGHTTPSSPDYDAIGPLDFFDPTLNDSQKEAIRLALASKEIALIHGPPGTGKTHTL
IELILQMTRRNMRILVCGPSNVSVDNIVERLTPHKVPIVRVGHPARLLPSVLDHSLEVLTQTSEAA
EIVRDVRKEIDQKQASIRKTRNGRERREIYGDLKELRKEFRERESKCVENLVTGSKVVLATLHGAG
GHQLKNQKFDVVIIDEASQALEAQCWIPLLTASRAVLAGDHLQLPPTVKSSNMDAKKIGKSKTKEA
EKDSSESLNGVSLEKTMFDRLLALHGPSIKRMLTTQYRMHEKIMQFPSNELYEGKLTAADFVKDRL
LKDLPYGVEETDDTKEPLVFIDTQGGDFPEKAEDEDTTIKGGLLSESKSNEMEARIAILHVEGLID
AGVRPEDIAVVTPYNAQVALIAQSLREKYPGIEIGSVDGFQGREKEAIVVSLVRSNEKHEVGFLGE
KRRLNVAMTRPKRHLCVCGDSETISKGSKFLKNWMAFLEEHADLRYPDASELY
>g1157
MRTSRVSQETAKLVHAISPPKRQTRSSLAASALRNFSYSDNRVAPEPGDDSSLSSANTEDIEDLIE
PLAKKRRRNSETPTTSISSVSTTKRIKKEVTVKTEIPATKAKNTSSKQRRQPAKKIKREDGSIEVQ
PPSNWETMYDIVKKMRAANPTAPVDTMGCADLHWRTSPPKEQRFHTLVALMLSSQTKDTVTAVVMQ
RLHTELGPEVEQTSTTVSPSKNFIKKEEYDDTDGIKLGSTKTGSTLTVQNIIAVSPERLNQIIWAV
GFHNNKTKYIKQAAEILRDQYDSDIPATPEELMKLPGVGPKMAYLCMSAAWGKHEGIGVDVHVHRI
TNLWGWHVTKNPEETRMALQSWLPRDKWHEINKLLVGLGQTACLPVGRKCGECDLAGTGLCKSEIR
GMLVKTKREVKEEAKVKIEAV
>g1158
MGTKSNSNNNDSGSIEQQSRRPQIDSLKSDDDIPACQACRRKKARCSREQPCSQCLKLDTPCIYDG
RRLKPGLRAGVIDNLSRRIETLEDMFLGQEMLWKQMWQTLHPNLEFPGVPSAAGNDLNGNGTDLRG
RRDQLKNSLLDLASTHRRNDNSFAIGKLSASVDMLQPPPAKRLRRDLSSSFQEVEHDIPQYMTAGD
IIDSDLMISFVEFYFQNIHHWIPILHVRRFRDQIQHPEGRQKAVHILHAIIAVGIRFLQDPRLGDD
ETKAKTAEASRQNVVLCSMESFSVQSLQALVIIAFDTIARGRGPSSWSIVGSMARTVEQLQLSVEE
DDLRERNQTGEALIRRMAFLRPSTCWVEIEERRRVFWTVFLMDRFCSLSTGWNVSLTSADVKRRLP
CEGALWEREDEARPSYFGISDTKEATTPHRSLLVESPTMAHPAEQESIGGFAYCIEATESLSLVSN
FFVRQPLDVRDNEKAQLWLMRFKELDLRLVQWKLFLPSKWREASTLNADGIMDPNLTLAHITHNAA
VILLHQAIAYPPLHWKSSPVKLPSSSSAETCIEAASDISTIGQQFLLHSPIFVSPQFSFCLFIAGR
MLLTHSSRYSNANPVIAPALDTLIANLFEISARWKGPRDFDDAGTENLASAFAKRLVQAKDNNISP
MAKSSLDIRQTVYSETSQHEDEEPVMHTTGPTTTTSTIAMNQPHHHHQIYQQYGEYQSPDGANTFD
PSLTLAFPPLPLSFQQNFAPFMDSEADVFGLQLGGSGEYQLPSHGTANFSARQQQNQMPEQSGWTN
RNSTEQSSISSPYYTRLSNHRDRMSNSPRSAETMQR
>g1159
MEDIEFQNKSTVSEHNETILNTEKREAMSKYGVDQETAEYANGPPVFIDPATNRRLFWKINGRILL
AMTGTYFCQSLDKGTLGFSSIMGIQKDAHLTSNDYNWLGTILYIGILLGEYPTNVLIQKLPVGKYL

AANVFLWGVVISCSAAATNFPALMVVRFLLGVLESCVQPIFVQMTSMWYTRQEQLMLLAFWNCMTG
LQLMVGGVLAWGTSHYVGHHIHSWQLLFLVLGVATVAWAIFLAWYVPDSPMKAKCFNEDEKRLMVE
RVRANETGIQNKVYKKYQLLEAFSDPAIWLYVGVQVTSTLILGGLGTFSNIIIKSFGFTTLQTQLL
NIAQGGVVIAVMLGSASLATKTKQTVLVMHLWTLPPIIGTAIIYSIKPTASTRVGLLIAFYCTQFI
WSMGPMLFSLVSRNVAGQTKKSTTLAITFIAWSAGNAAAPQVFQSSDAPRYEKGFTAHFCLYGLLN
VVLLVLRWVLVRRNKLKETNELSAQSTSETTHEYAFMDMTDKENPNFRYVY
>g1160
MAPSIGVPSKRPLEETLVKSPTINGSAHQDNAHQQSKTRQRRSKTKLVPQALMQALFSESVAAKLW
NVAQKALGSVEPPTLYPEYTDKEYFQYIYRKLDFWTSGFFPGSLYLLLERQMRFSWPAKSGAKPHP
LQLQHLCQWWTVNLHQNATIKNTHDLGFMIAPWAMKAWSLHRDSQAYSSLVLAAHSLASRFDARVQ
SLRSWDVCYTKRYSYDDPSKEFLVIIDNMLNLDILFWAAKHTGDTSLREIAIAHAKTSQKHHIRPD
NTTYHVVNFEVETGFPKSKFTNQGFNDESCWARGQSWGILGFMQTYGWTQDPSFLTTARKLADFFI
SKLPEDNVPYWDFDAPMNEGSPRDTSAAMVACCGLVLLYKALKDTEESIAAQQYLDTALRIVSGVV
TNYMTPSTPQFNVDTSASTVPTYPENLSKQQQQPGILTVTSENSQRGFAKGGSASNCPDTILTGAT
INNYEFAPRRWSDHGLVYADYYFMLLGNMLLEMGLVDVNDLDS
>g1161
MRKLFGIKRHSNLDGEEENESPSTVRHQPMATPTGPNRILFETTHQSDSSSRGSVSKRKQSIPTSH
LSSASSDHIVIAQDNRIEIPAHTVRRRATLPSLILTEDDDTKGSIYSNLSPTSTRPVSVVIESHED
REHQEDHEDTKSQPSFRAHRRSRSADALRDLVRHHRMSPIQWRRRSDEIKFWRTSVLEMDEGDELG
GVQPTDNGQLKPRMDSLPECEKTKSANDDSLPLQDVSTSAPFQIESLIASMADPDATVEQRLTTIE
VKLMDLEFAISKIQGTDNNVFSKPPVSSPEPTKESLSPIPAPRSPNDASSIVSEATTQSSASFGGG
EDRPISTATLRPNMVYSQQPPPWQNNSWSSMNLHGISIEQYSALVTLVRREQTARKALENQVTQLQ
EEMQQFRRASGLPASPPGTLYPIPSPDSDDGKFRRRTGSSRKDSTTSGDTRISDSKRRDSMKYRLT
RERHDSNMI
>g1162
MLQGFSRKDHATELDMTLPCRDVDQGTQDMWAMSENDSNTAGTINAPMELHPLVEKYLSEIGEVRM
YQERLGELLLEHAEVTGREASLALVNMSLDEGSRYFLDNFESQRLELEEIISRGMSTAASIREQCE
KEGLSLPTNLQWGVETELDIDIKEGLPQDRDLLWRSELDEPFNFFEQAQARDFNIYKFIDGWILHH
LRHSTSQIYRYKSNPRLQELNMDGDELSDWVMKLWFRDDVSKSLPFGDISREQ
>g1163
MKRKLDANDVPSTDATEGDNESQTTFENLALDPRLRQALVKENFSKPTLVQSKAIPLALEGKDILA
RAKTGSGKTAAYVLPVLQSILQRKSNDPSLKATSALILVPTRELSEQVQRVITSFSSFCGKDIRSV
NLTQKVSDSVQRAMLADIPDIVISTPARVVSNVNNSALSLHHLTQLVIDEADLVLSYGYEDDINAL
AKAVPRGVQTFLMSATLTSEVDTLKGLFCRNPVILKLTEAEDEGGGISQFAVRCAEDEKFLLTYVI
FKLQLVKGKCIIFVGDIDRCYRLKLFLEQFGIKSCVLNSELPANSRIHVVQEFNKGVYDIIIAADD
QEVLGTKKIKKTAAITDADNNENGEDAEAELEESSDDEVPAENTQTKSNKKQKLSGKQKDYGISRG
IDFQNVACVLNFDLPTTSKSYTHRIGRTGRAGKTGMALSFVVPKDEFGKHKPTSYPTAKHDEAVLA
KITKRQAKLGHEVKPYHFEMKQVESFRYRMTDALRAVTRLAIQEARVREIRQELIKSEKLRRHFEE
NPDELRHLRHDGEIRAVRIQPHLKHVPDYLMPSKGKKGLMAEDSQRRVGIGKTSENRIRKARERNK
AKGRKAGGGKKSDPLKTFNAKGRKK
>g1164
MPKKHNKYPSIKPASTVHPSLESSTTKRQYGGASRSTADNQSVNDLIQHLRRTQVTSPNDGPSNTS
RTVTQRSLHPSLRNILDVPETPPPRPRSNVRPVGGRFFRRTPGPPPPSSWLQTQAPSHESADRNQG
VSLGHDLVLYRLERLPGVQFPPKDSLQHAVMKSMAINWAWHLEYDNEFLMEVPSRLKLLILSYIAT
HASRFDSVSPTHTLDFLFASPEASAAEVGYVTRLDLSGAIGNWISMKALSKQLLLTRKETNDPVTK
SETIVPASWDDSEGSIDDDELEAHSLSSPQSPLFKSPTEVLRFPNLRYLSFAHPRRTAVSWGSLLN
LLSHMSTLTHLSLAHWPVPTLTPNLINERITHPTMRSVSFAAGGTDTYSADENNWVEAVNILRRLS
RATYCLKWLDLEGCGEWFEALSWDGRTLHDDEPFQTLSSAWNGSWRDVEWIGLGPGWIPSQKVDMD
DLGPSFIPLAASRHSPASQRQPGQARNPDDDLTSVLTEQDLREAAIAREQQRRVNERKAYEETLRK
ARRVEKHIHRVRREGGGKWIHFSFGGEGVEEQIVLEKVLAKIDS
>g1165
MSLGATTDRPSAWWRACSSLTMFEIGALCRGFLLGLSKLEVNGLEKFTELLDARRDPSKRMRGLIT

VSNHISVMDDPLMWGALPFKYHFNLPSYNRRWGFGSHDICWATRAGAAFFTLGQVLPTHRLAYSPY
GGLFQPTMTQGIRLLSKGPFPADPHFANIERQRWSLQNVCVDPFSDLPTAYTTTGEDSIIAPSSYA
CNSYSWIHIFPEGMIHQSPKKVMRYFKWGVSRLILESSECPDVVPIWIEGTDEVMHEDRAFPRFLP
RLFKRISITFGDKADTEAVFGDLRQRWQKLKEKAAKRNASVAQLPLGVLNEELMFGEEAVELRKEC
TSRVRQLVVELRRSRGLSDEDPKASLAQTWAQEGPKREGKMLDDSWVRDT
>g1166
MFKVTSPLLGGLLWKIPWRLSSRQKFRQRKRLRAVDNVVDTVAAALERNGLPQVKAVERWYAEMPR
EEEMLPKDKYSIYDRKEKTYRKGIHKLPKWTRVSQRVNPPGF
>g1167
MAPLPFPEPRFRSPPPPASVKSAASAAIHQLGRFRVLVYILLGTSAAFSFGFLIWKLGSLFRQFTL
GRILRERKPVETRYIKTWYGWVPQDRYDTKKLKWMGLYSNFKDWFSWDDKDDYSKLWWDSDHGGDQ
IRRRSRPGSLRSRLRRTSLVNRKSGRIVVNRVTTQQEMPRAPSAVNTYHLSHRPSSAYLTYPLCLK
PNNQVLSVDHNSGFPIPRVASVLQRGFREHTSFRYQVSKGDSLTRSMVRKTRQLNHLRIWATRLEL
GSLQSILPHQSGTLGRPGSPLIIMHSNSDSSQQIDLGKISFPRIASFSEIVIDENDIPRKVQPVHS
NPSNPPGNLLPNSKQIDLDQVAVSEARAEKESKIEDVEWRFVDTLYRHLEWVDNECRPGQRGFKFP
ILPKNWANNGKWLVYTNPCGASVEYMRRYAQCDFGAGYLEEIDRQKGLAIVHRKRLRAESIETWRA
AINKARGQHGMARVRSAEIFTSSTEDTPDAVIDPANWMLRRPPQGFEMPSRQKTAYWYGGIGEWAK
LEEWQVVGKLNDDPADAWKKRMDVFASSQEAQREFLRNHAEVVCS
>g1168
MELTSASEGHLGSKSHIKKRIENIGLNLLNRNASVQNLERKIASLENSLFRSGLIQDQGSSGPSYA
SDHRSDEIATKTLGSTSEFGFGDVRPLVNTYGRSSTPPPHTEECLTSWNVGQAYEKSISGSDLPTL
KSSIGTNSEISAVREKFFASLPGENTARRLRDQIRRGPFISSIFTDLPPKAHLESLFELSFNNINC
QWPLFDPSILIPLLDEQYASDSSSIHKIPARWALLNSAIAIAIQSRAAEDSYSDMTDLSWHFFKNS
FSVFPAIVSRGTDILALEALLSMALFMQGSSDARTTSLLISDAARLSTTLGFHKSSFYVGMEPITA
ERHKRAFWIAYILDKDISIKTGMPSAYNDDDISLGYTSSDCLGCPGKIVVSEIRVEATVFRLRAQL
AVIESAIQNQLYSEKTTTCGAERCLQAVAELDYRLGDWKGQVPSNIQPGHINWSTVRPLREPILSL
HFVYYRAMSAVHSFAMHLMPVNDVKFLSQSISSDSIHATMAHESIQLLQLLPLQAPGDLWQTLFHP
LSAFIALVGIVLKHPTDVRARSHLRHISDFVKLLVNVEKCGVLHVEPMLHLCIQLEKLAFETVTAE
TNVPQSTESSMGSQKLHAKAQRLTSHEAFYGNHLQLAHGLIGNMPALQAIAADVFYDMLPAGARSS
AAAGILAPVLLNPETYQFAFT
>g1169
MDRKKVLVLGATGPTGICVLRELLSRNVPALAFCRNPSKIPKDLSDNALLEITKGDMTNSEELSKA
ISKSRAIISLLGPSAERLPRDTFADIYRIIVPLMKQHGVHRLVALGTTAIYRRDDKKSITRALMAG
LIRXIVNSAYQNVMAIQEYFESVNDPSIEWTVYRLGWLSGTSEPAAWSEDRTKGEAYAGPVAGPGF
TSGVNRSILAKWLVDTSLDPNAKWAHQMPAVSNLHN
>g1170
MSPIRKVALIGKGILGTAVLEQLSKNGFEVTVLSRNPSSVGTLPAGVSVSAVDYTSIESLASALQN
QDAAVATIGSAGILGQKVIIDACIKAGVKRYIPSDWGSITTDPAARHLPTNFALVQIQDYVKEKAE
KGEIEYTILSVGGFFDLLLDVPFLLDLPNSSVEYFDKGEHPFSVTTLDGVGKAVVGALKASTETKN
QNIFVHQAIITQAQIVSIVKKHCSPGRQWQETHLDAETKFQESLDLATKNPGDFLSIMALLKATLL
SGKFNSAYSTVDNNLVDLPMLGDEELESILAGKLREKGQ
>g1171
MQFWYKEGIRLHHLLNSFATGLTLKSSQRDNNSILICDKSLHPAVGVVYSAQMLLYDMHTCADFDH
LSGVGIPEQLEMQKIALEGVRVVCTAVSSFARGIRAGGVGNAGTSPLVINCLYEAAKYHFWYYREV
NKPELLHEVNEIILTLQAIAGTWAVADRYLSILNGDEFRPHAMPGESLP
>g1172
MVNNVFSVDSLPDLTGKLYVVTGGNAGIGKTTVAGLASRGARVYMGSRSAERAENAITDIKRELGN
PQADIVFLRLDLTDFKSVVEATRELRRKETALHGLINNAGIMGVPFSRTKDGYEIQFQTNYLSHWL
FTYHLLPLLESTALSQPGTARIVNVTSDGHERFTLKEAIRLDDPNLESESAMVRYGHSKLANILHT
RQLNAVYGPKGKKSSGIIVAAVHPGHIDTNLNKQATGMAPAAVLRIMTPIMRCTGILDHEAKGAVS
SLFAIADLNFTESGAYVVPYAKTGKPSQKAQDDQLAERLWEWTFELFQERGYL
>g1173

MSEPTDIPAGSTVLVTGVNGFVASHIAKQLLLRGFKVRGTVRDSGRTSWLAKDTFKAYVKNGDFEL
VTIKDFTAADVYDDAIKGVSAIAHVASVVTFDSDPNTVIPQTVEAATRIMEAALNEPSVKAFVYTS
SIVASTFPAPGNDTRVSRNTWNDVAVELAWSPPPHPPTQGSIVYAASKVAAEKAVWKFVDEKRPKF
RVNSVNPAMIIGEPLNDSHLQTVGAWIKKLWDGDISRLANFSATYHIDVKDVAVLHVAAIVDPKIN
NERLQAWAENCNWNDMLAILRRLYPQRHFIDDLPDQQRLSISTDLTLPLHLLKKWENQTGWRTLEQ
SIKDNVRLLGENVKYSA
>g1174
MSDSDISPTTTEAQPYTNGNPVPIDVVLKVDAPEQVQPGSWPYPLIVEVAWRDPVITHGSPIICFC
EGFKYGSEDVLFKKDGQEIDAISFDELKAVVKKRDPKKTDAEIKKIFETLDFDDNKKITFDEFLIF
DFALDAYGYDYSNAIII
>g1175
MSVRRHYSWPFSGSASGARAEELRYNPRKVGSLLSTSNPQRSTKRFTAVDQPFGPALSLYSSSEEE
VILSSAGILDSKIEKRQLRRQRRERRAEHKRRRHERRQRSAEANNNEDGATEEIIISSITQANEAP
IAIMRRFVWPHLEKARHFFLSLHTFQYLTGSWTSHLRRVGPTDKKSHEIKARYAAKLASSSKPSLS
PKQPSADEGGMSLSIPDRNEWMLRPERRHRRCHSEQPRAWREPNPGLWTLAEE
>g1176
MPDSLPVDSDAPAPKLDGLSPSSSLSSDSSSSSPATKSHHRRFLSLDNIEETSSRILTRIRSRSPL
PRSSSPTADKKRRRSPPPLQPSSAASTSPISPSSVDRVYSPSEMSPTSQHSQRPSMGHRDASSSTI
DSSERPRSDSYKRYSGTINHYGRHSNDWLFGGFSVRDTLRDGIEKLKGHNERDS
>g1177
MADLNSGFVLDTPTLLSIGFALSFMPIAYGLGTSLIPSNQLRNRALFMWHAYDALTHLFIEGSFLY
ECFFSYVDIINPADTLLSKDHSSYFLGRKDRVYGAKFGTGPSARLWQEXAKADHRWAVADTTVISL
ELLTVFLAGPAAIYICYLLHKIASNKNSSKAEAQQLGATKGKLWLVASAVATGELYGGFMTFAPEW
LSANSQLDGSNPVFLWLYLVFFNMLWVFIPFWVLVEAYKELKGAFVDAEVLSLSVEKKRA
>g1178
MSYNRPPPQPPAGYPQGHSPYPQQGYGHPPPGAPPGAPPQGGYYPPPQQGHYPPPGGPPPGQYGGY
PPGPPPPGQGYPQHGAYPPPQXGAXPPQQGGYGAPPPGPPGPYGNPAPPGPYGAPPAQPGYGAPQL
GPPAQPSFGYIPGQTAPGDFKPQADSLRKAMKGFGTDEATLIRVLAPLDPLQVAAVRETYXRHINR
DLYKDVKSETSGYLEQGLLAVIEGPLNHDAELVREAVKGLGTKEWLLTDVLVGRSNADLREIKNAY
QRKFNRSLEKDVEDDLSAETKNLFKRIISATRHEESTPLNPATIESEVKSIHGSTAGRIVNNVAEV
CAIFAQSSDNELRAISHKFHERYQVELEKHIASQFSGHMKAVLIHMLRTATDPAHRDAALLEEAMA
GPGTKDFMLVTRVVRLHWNKQHLDQVKKAYQHHFKRDLHSRVKGEVSGDYQKLMLALLGY
>g1179
MVLVANPERDRTAEEKEEIAKKYKPGDQVLVSPGGSDAESDGDGRLLADGTNSRRRRASTSRSGDG
HRQRSADNRDHHHHNVARHAARTGAIVGINNESTRRIEHQSSLRSLLSASDVEDNTQEEIVRLIME
EGLLDGIDLSGLDQSQEEELSDRLVQIFLSRHPDRSRQPRRSSDQAERTIAPEHRRSRSQTLQSQP
QNSTPVENTSRRPPVSRPHLLGVSSDPHNHRRRASDETRRRRTSPTPVAPASTSETALRPALRSSS
DISSQRPRASPSRPRTRDSSTASVTRRSTEPEGRASDLWAGITGSQQNVASRQVVASQLTNSPSTT
TPTERPFSASATSLPGVSTNDTQSATSNVQSSSRRNTATRSGPARQANRVPSTHYAEPSISCERCG
KTDIQYELHKRCLKCKDGNYHVCLHCYRVDRGCPHYYGVGSIGEMNFNKVHHPALRSAVEMPHVMK
SQRYRRPKEASTRGTSDGKQMTNDNPSGRLQEGMFCDVCHSAANGCFWQCDECNESEWGYCRRCVN
QGRCCNHPLLPIRRISSESPTPSPTDVHPLGPITPPVPTQKALPGGATFQVLSFSTECDICKYPIA
PSSTRFHCLQCNEGDYDVCTNCYLKSVVSGKISKENGHSGWRRCLKGHRMIVIGYEDHEKGQRRVI
VRDIVGGYTLKDEQVNNQSQSSSLSSSGSVASPELGTGDWLWKEGNTKRKKAGRARTALDSNSPSS
SPSSQTPAPTTSTTTPADRRGAATIPPDGGYGLVLGANWSYFAEESCTDELSFPRGAEITEAENIN
DEWYWGYYAGKTGLFPGAYCSLVREVG
>g1180
MRSISSIAAIITAILAVGHAVSTPPAIVSLPGQTSKIPGWSLQSVLHASNDFAGLSQPGASVANWY
RVGPRTTVMAGLIESGVYSDSNLWFSDNLNEVVDRSVFNTPWIYREEFTVEPSAGQHYFLVTHGIT
SKADIWLNGKKLVDKDFQEGSYGGHKYEVTPYLQSGVNALLIQAFETNYLLDSAMGFVDWNPYSPD
NGTGVWRDVEISQTGPVSLAPIRFIHDWTPGKTSTKVTIKVNATNLESHSINGTLTGKIVLGSQQL
ALTKPFTLAEHEEKTVSITGELQDPQIWWPWLWGDQPLYNVTVTALVKNEISDIAEPRRVGIRYVE

SGLNDHGDRTFSINGLRFLPLGGGYSPDMFMRWDTEYVRKKFELMIDMGMNTVRLEGKQEHPELFE
LADEMGLMIMPGWECCDWWEGWTYNTDVPEYVRFTDHDYWVANYSMLHEASMMQTHPSVLAFLVGS
DYWPNDRAASIYLDALKSWDWPNPIIASAGELGFPDALGSSGMKMDGPYDYVPPNYWYGGQVGAAF
GFGSEEGPGVGTPEIRSLKKFLSEGDLNDLWTQPDKGLFHMSSNVSSFYNRSIYNDALWNRYGPPK
NLEDYLLKAQIMDYEATRAEFEGFTSLQDASRPSTGVIYWMMNSAWPNLHWQFFDYYLNPAGSYFG
SKVGARPEHISFSYDNGTVHLINRLNFLEHGESGSRWVTIDLIDTAAISKIKDVGFLRLILSSDSK
TEVVQSRNVYWLASQNDVLNWDDSNWFYTPISTYADYSSLQNLPEANVVTTVKQNSSSGSTTTLQV
HLENKSTDTPVFFIRLVLIDSNTNDSINPPFWSDNYVTLFPQESLDLTVTFNSSLAANPAVEVSGG
NIEQRVITV
>g1181
MIHRSARVVPRSAASRRLSSPRRSISLMPRFFPPSPFFSNRSGPSGEFSSIFRFLDDYTDHVTRRF
DDSFPDFSRNLTSTFAPKFDIKETTEAYQLDGDLPGIEKEALQIEFLDENTLQIKGRTERNFSSGN
PSSDAEDVTMTGAGAESDVENKNTVDSASTQTPAETSATEGAETTKAVAQTAETSSPAKTENTGNE
RYWVTERSVGEFSRTFSFPAPIDQEAVKASLKNGVLSITVPKKVVEEPAARRITIE
>g1182
MLRDIIIIGSGLSGLSAALAFSHYLTPLIPDLRITIFELHPVPSTSGGALSLSPVALRHFDHLGIL
DELEEYGPESGVDVDAVEIFSSRTGKPISNLDFSGKQGLGYGDAETGKRYRARRVMRINLSLAMMA
ATEKRGNIKIVFGKKFLKAVQLEDENRIEVHFQDGARATGHLLLGCDGVWSATRRKFIDPGRDAKY
TGFSLVQGTVKTTSLKVNPHFRNSSMNLSKNGSLLITFHEKQHQEIFTSAMVECREESVKDHTDMV
KWKRDMINDSLRNEISSRFKDSVIPFVREVATSPEVDWMMYPIYQVPLGGNWYTKRAILLGDAAHA
MLPRDESAAYAIDDAILLARIVSNHIDYPLETSFQIYDSLRRTDIAHAFKDSAKLWQRRNTDAGPI
ETWFRERLVPFQIKHELVSRKAAFEYDASKAAIPSLSRSLTPLSSLRPDSKSGSSFSSHDTITSSG
GSSSHNGHLRTHSSCAQAEMTKLMSGLVVNEK
>g1183
MEGKLTNIDQQEEVATHSRRTFPKINSYRFKTSSRLLRRGKSPNPENENQKNAQITTEANTATLEV
APVDSKDIWSGAKTVTVDPIKDNDMWAIAEEKLRKDPRKREKLDKYNGILEAYFKTKLQPVGTPER
REQFLEFLNSEIEQLNSATNSGTRLGRCSNKAKRFLRNAADSIIATKGIITAATTPCLPASVACAG
VTVLLSFCVQAADERQVLFDGLNTISGSIYRIAEYEKLLCDHGNQQTEEVKVSITTACCNIIEFQA
RAACFLQTNPIESALRNMFKWDGWDKLLSAIKEAEVRVKDCAERKSMNDICQIFLKFDDVKGEIGE
GFSKLAITQEVIDQKQRVDAFLQLLYDNACSYEDSKNRNRKRVTGTCDWFTNHDLFREWNMPTDSH
LETSHLLFVTADPGCGKSVLSRYLIDDILPDDGRTLCYFFFKDDFENQKSSLGALCTLLHQLFDMN
RHLVTPDILNKHGSRGEKFIESFSELWKTFIDVASRQETVCVLDALDECQSSDRNQLIDVITTTQV
PGLKFLLTSRPYEHIRREFSRNLKAHMASIHLQGDRGPTADAIVQEIKLVVDSRIEETADFFRLEP
DERRLMRKQLESVPNRTYLWITLVFDGLMEKKSSITRKDIVNLTKKLPQGVYDAYEKILKKSRDQE
MSKKLLHLILGAKRPLSLSEVSVALAFNGQRSWDDVAEDMIPEDRIQDTIRDLCGLFVTVVDKRVY
LLHQTAREFLVCDFSDKITTETWRLSSSKDYNTGAIIFIAQGLALLFATLLHIMRSILHSLWLSLT
KSWQHSMDPTYSDSVLADTCISCLELGFAKENASLFEYSAIFWADHYHQSAKNYKVTAAERTKYLC
QAESRTQWTEIHNEHDSVPLNGSPLCLASALGLEMAVKMLLPEKDSERDSRTPLSWAATYGHEAVV
KLLLEKGAELEIKDNRYGQTPLLWAAQSGHEAVVKLLLEKGAELETKDNDNSQSPLSWAARYGHEA
VVKXXXEKXAELEIKDNMYGQTPLSWAAQSGHEAVVKLLLEKXAELEXKDIHGQTPLSRAAENGQE
AMVKLLLEKGAELEAKDIHGQTPLLWAAMYGHEAMVKLLLEKGAELEIKNIHDGSTPLLWAAKNGH
EAVVKLLLEKXXELEIKNIYGQTPLSRAAESGQEAAVKLLLKKGAELETKDNDGRTPLSRAAKNGY
KAVVKLLLEKDAELATKDNDGRTPLSWAVDNRHNRXXXXXXXXELETKDNHGWTPLSWAEKNGEMA
VVELLLEKGAELETKDNDGRTPLSRAAENGQEAAVKLLLKKGAELETKDNHGWTPLSWAEKNGEMA
VVELLQRRNRD
>g1184
MFFQLKSFDESLTDSEISTQAGILQGAFTAAQFATAVAWGQVADAQWGGRKFVLLVGLLGTAVSCL
GVAFSTSFGQAVFWRFFGGAINGTVGIIRTMISENVKEKKYHSRAFLILPIGFNIAALFGPVMGGW
LSNPVETYPNIFGGPSAVRWLVKYPYALPMVANFFFMVFCATLVAIGLEETLESCKNRPGLRLYML
KLLQRALGSTNTPSPQQYTSIPIHNPDDEEGPLLNQQGEEGGVDLEDSPVPEKSRRILPFRRIWTK
NVLCTLCAQAFFDFQMGAFNNLWLLFLSTPRYDSNNPNSPARHLPFIFTGGLGMSPQTVGLATSIL
GVIGMILQFTIYPRIQSRLGTAKSYQYFLIMFPAAYALAPYISLISSSYASQAQSNGLWVWVFIII

TLFFQVTARTFTLPASIILLNNCCPHPSVLGTVHGIGQATSSAFRTVGPIVSGWWYGHGLDIGVIG
FSWWLVALVSVLGAVAAIFVYEGSGHEIFLPGEEDVELKQH
>g1185
MSLGHSSSLTAKPWTDIFTSDTCIDRRQCHRTVPMKVLVLGVGRTGTASIRAALQRLGYNKTYHMM
SASVENPPDCLMWHDALCAKYDGVGKFGRAEWDQLLGDCQAVCDWPACAFAKELIEAYPNAKVILN
TRDVDSWYESVKRTVYWRVRDPEHFLASKVSWAAGMYYPMLNKFFDTFFRGDFLNEGKQVYLDHLQ
EVRSLVPPENLLEYRVSDGWGPLCKFLGDDLPESPFPRVNDINNFQKRCSTRNRMQMLNAALQAAV
FSGSFLATGWVALTFLQRHL
>g1186
MILDQDGHLVWFKPMGETYNMNVQTYKGQDYLTFWSGDDSIVGHGEGTYYMLNKTYQTQYQFSGAN
GLRGDLHEFHITLNDTAVYTVYDKVPADLTSVGGSQQGWLWDGTFQELDVETGELLFQWRASEHFG
FEEAYRTREGHGNSYEDPWDFFHINSIDKDEKGNFLISSRYYHSLTYIDGRTGDIIWKLGGKDNRF
EDLSNGAATNISWQHHARFREGGKSITIFDNSSRGQGAPIHTSRGLWIDVDQVNMTAKVRQQFWNP
TPISSQSQGSVQVLENGNVVVGYGYNGAWTEFNQKGEPVCEVHMGPEKWFGQGQIESYRVYKHRWV
GKPTSIPVWLVPDPKQGIGYVSWNGATEVKTWMVEGSYAKDPEHTTGAFGFVAALAKTGFETAISI
PENNTYPQLRVVGLNEKGRRLGASQFVLVNPDFDSNLYETSGKTEVPGFVFFLIGFFTCAMFMICG
WAPNLIRFQRQKWESLNPKKNRWWNKLRFGPFKQSKKDLDDVEDTLSERDELLVAHADDFELPKME
DDEAEEQIHVPNTIERGHTLLGNTYLVE
>g1187
MPSLFSSPAVIFSLAALMRVGLLFYGLYQDNHSAMKYTDIDYMVFTDASHFMAEGQSPYLRDTYRY
TPLLAWFLIPTTWEPNWLWFSFGKVLFAIADLVTGWLLLLVLRTEFPGMSEKARLSTRGSSEGLLG
VMVIALLWAVLNRHIVFASVLLGLGVHFKIYPFIYAPSIIWFLSATNTPSTSLVKRLLNFITFERV
KVTVISLLIFIGLNVWMYSLYGHSFLQHTYFHHVTRIDHRHNFSPYNTLLYLSAATDDIDAASRGI
KFESVAFLPQLFLSAFAIPLALAKKSLPGAMLAQTFAFVTFNKVCTSQYFLWYLVFLPLYLPSSSF
MSNPILGIGALIMWILSQAFWLHQGFQLEFMGLSTFLPGLWLASLGFFIVNCWILGIIVSDVGKWP
AKALQTKREDARTVTQKDK
>g1188
MISTVPPPRPVSVNSPILPLSSPVHYHNSTSIPTKDAYQTYDDVVSSTYSLTASLDRLPSMDLEME
DSDDSVIFSNHASQSEIQFDDLPFEIHEAILDHLFGERTSGSGSIVPGRPDARSWIRALRHPRRKA
LSNLALISPIWRDLVQERIYRHIKVKGTTEGLAECENWFRSHPHLVRHVRHIEFWIPVWGNRAHKS
VAPHLREIPPIRRYLNEEAGLFGHNNGYLQQQIVAPDWDLSDNRNRTPNNFAFHVASHNASLEDIF
RHVKELFPETRILTLEAGHCKKPPLMKHFANDPSGLSGEYQLEQLPNIRTLFMRGAWNIMRDYQHW
TTISEALPSLREWHCAYAKPKIEAYHTICGALSSLSSSLIHLDISLEGFTSKDQSYWLGGKIGHHH
MCRMLGRLAPQLEHLSVTGYICADLFNIARAVTHDRPQDARLRSVDLVVKTCCKDEKNELLSPLLD
EPPGITNMGFVDSFEKLVLSSIRCLDTYLDLSYIRIRFIDLDSQCAVLNPYFHLVDNKCTGVWSHD
IVETLSQVRPAAHFVELADGIFTQYGPNNVIIGAVYPRTRPLSINVNTYKILSDAAKS
>g1189
MWLLESDGDLLAGKRVWLKPGKKYLFGRIKSEGVRHVIQHQSISRKHLVIEVASVQPGDGAHVHKK
SKLIVTDQGSKCGTTIDGVQIKGSSSVLTEDEHAIQLGKYPQALKIKWYPVVLSFSFSTKELKAKD
PLGPVRNRLEELDIKTIIPYVEKTTHVVQNKRNTAKGLQALINAKYIVSDSYLDALVYAATPGDLE
ATESLSPLEIDFDAHWPDANQHLPPAGKEPVRRPAEAFAPNTDRVRVFEGYTFVFCDASQFENLSQ
PINNGHGKSLLFNVQNGETTAEELLQFIRSAGGEKGIDNQRDGEGGVVLVRFRAKGYEDWSIELGN
QVALRMDQRVIEQSEFLDAILNVDATSLCRPLPHEESSGLYEPAKRGNARQGVVEDVSGPEVDASQ
GVATQQEDTTMMETQTQSKRSARSQSRPFVSRMKKFDDGFDMEAVPAYLSDPIDAAEDTSEPFPNN
SPGPSRQPSASSIRVEEQEEEDDMAGLLPGASAMKRRRVESGSRSLREQGVKEEAPKPKRQKVDVI
AEARRHREEEEAAAQERKQEADITFQEAIEGMTVEDMKKLVIVEEYEIPLHEKESVIVDKTDRWDE
RWNGRKNFKKFRRKGDQSVARSRIQAVIIPLDEVKRREYGVGDHYWGSNEREDSPEPIFSRTSQQQ
TTQKSSSRTGSQVSMAISEEPATASSVNRARSEVSRISATPTTQPSLRAGQKRTRETRPKSDDEEG
HSDDDEELRFKFRRRRK
>g1190
MLRAEESLLNSKDDLLEKTERIYAHRTNTQRCWQAASRVLILTPLLLSIVLFVVYFRDVAKPLDLV
AGNTTTKPQITANKGMKRDFGIFLHPEDHVLREPNVLRLSWNITKAIIAPDGVEKNVFLINGQFPG

PTIEARPGDILEITIEIDKEMFILSNPALTRIQSFLLEADSHLQQSIGDQTDAELRRLNLDELEGF
PLETSLPEPDVKFMIYTTVQILNALDDDIRALINRTYWETPSVPLIASQRDTWSSHQLVPWTGDKP
VWVELTINNIDINGHPFHLHGFDFYVVGLYEGHSGWNAYNPFDLWSSPRGGPFNLVNPIRKDTVLV
PKSGYAVIRFLADNEGIWALHCHVLWHQASGMTMALQVLGDETKGFSEYASGRDALEQCMR
>g1191
MPTPSTNPDKHNNGIPAKPFTPTLSSAFRPTAKSPLPLTPRLAHLGNTQTPKRPESSVVPPPREDV
ATPDPTYLGINITPRSGPRSSRRDGYISSPEITSAATHAGSYTSRPTGSVTPSYIRSERSPVRGYP
RSEISKSTVSRGSNIDGPGSRTVPRPPSSSEQLNSTSKFFHADEARSTGSHEAEARPRTLSKXAQT
STFFYADGSHDQDSHTDDSSKGVLPKRRSTGSSRLPVGAKSPSVLSPRLRTAQLVDPISRSSSEGS
LQPSPNIEEVSMSVTTIPAEHHSPGLITRTPQLPPHHTHRKSCSVDSTTNIASPRVTSRRVTPTSS
SPFLPEPLSSPPEQFSHSPLIISNPLIQLTDHRDQPYTVPQSPVKIDGGSNAFGEDAINARTERKV
LDLEISNSSLLAINRTLERELRKQNAELRRFRRLSRSGRLSITTSLRSTSGGGLSVVSETGDISEL
SSTYSNDEMSDESDQDSIDDGTVSPNSLAEHDSRHRVDDEKQFMLDLAKHQELLIDSQKLNQSLKR
CIGWTEELIKEGKKALEYNVHVNDIEIGGRVLDPDELGEELGRGHGLLSPAAEVRGDFEFSALDAL
DDVLEENGLLAEPVDLKVESEQ
>g1192
MEDVGDSASPKERVEDPDPPPSQEHKSLKYSLLGPSLLKAGQDAVDQRKVSEIIYNASKGSKFFNH
EEARDKVLTEKIEQILARKARLEKLDLSADLRRADEYIAELELSRDLSQTVVHVDCDAFFAAVEEL
DRPELKHLPMAVGKGVLTTCNYVARTFGCRSGMAGFVAKKLCPDLICIPQNYEKYTAKAEEIRAIF
VGYDPCFESASIDEAYLNITAYCDENNMDPQEAVQQMRARIAEETKITVSAGIAANTKLAKIASNQ
NKPNGQFFIASERQAIMAFVKDLPVRKVNGIGRVFERELRAIGIETCGQIYPQRAFLKKLFGEKAF
QFLMQCYLGLGRTKIQPAEDYERKSVGTESTFSEISDPDAIRAKLRWAADELERDLSRTQFKGRTL
VLKIKLHTFEVYTRQVAPPKAVWLADDLYAYSLPILTKLQKEIPNMRLRLLGLRCTHLVSRKKVGL
DFFGFQAVPKPSLNPTAPAGSAEDAAVEDASTDLDNQQLVGDVQATTEQEFEDAARQEREDDMEEL
EQLSQEDAIESTTSLPLGRKPTGTTALTPEPETEITQHWTCPICSRPQVADDKKFNQHVDYCLSRE
TIKEAVQTSSSSSSSKAVESENPIRTPNDLANTWSKEQYSRKRKTNAETDLRQKRLFFS
>g1193
MSDQCDIKNAKGAASSQGAATDCSKPKDELPKLTPAEFRVYNRLAEHMDYFHNHFRHSWNELYTAC
CDNKRPSGVSIRQFLNMGTQFLSQLNMHHSIEEQSIFPVLAKKMPEFKREVHLLKQHKEIHKGMDR
LEAYLMDCRSGEKELRLDEMKKVMDSFGKVLWDHLNDEVRTLGAENMRKYWTQEEMRRLPM
>g1194
MGLLWNIAYYTFHPSELRSIIQWKVWHNPPHERDASQDPETRKKCFQFLNLTSRSFSAVILELHPE
LLVAVTVFYLVLRGLDTIEDDTSVPLDVKEPLLRDFKNIIEKDGWTWNGNRPEEKDRQLLVEFDNV
IAEYKALKPAYRKIIKDMTDKMGNGMADFVRKAETEGISTQTIEEYDLYCYYVAGLVGDGLTQLFV
EAQFGNPGLLSRPNLHKSMGLFLQKINIIRDIREDHDDQRHFWPKEIWSKHVDKFEDLFDPQYKEA
ALNCSSEMVLNALEHVDECLFYLAGLREQSVFNFCAIPQAMAIATLELCFRNYSMFERNIKITKGD
ACRLMFESTQNLRVLCETFRTYARKIHQKNTPKDPNFVKISIACGNIEKFIETLFPSQTAAEAKLK
LSGQSTEDEEKKKQQAKESWEDIKMMMIIMGSIIAFITVLLFGVAWFLGARFDLAWAELTKGNFRP
PNKIEHGEL
>g1195
MVVDTTYYDALGVPPTATELEIKKAYRKLAITTHPDKNPGDETAHVRFQAIGEAYQVLSNDELRKQ
YDKFGKDQAVPGGGFEDPAQFFTMIFGGEAFVDLIGEISLMKDLTTTMDITMQQMEEEELAASAEE
KLNIHDEETHGETPSAPSVPSAPSAAPAEAATATPIPESSNDSGTSTPRRNWGQQAIMEKSEEEAR
MEAAGLTAEEKELRKKEKKKGGLTREQQERLAQYEEERRVARQERVDTLVRKLIDRISVWTETDKG
PEVTHAFEEKIRLEVENLKMESFGLEILHAIGQTYLQKATSFLKSQKFLGISGFFSRLKDKGTLAK
ETWGTISTAIDAQMTMEEMAKLEEKGGADWTDEKKAEYERKVTGKILAAAWRGSKFEIQSVLRDVC
DRVLGDKSVKLEKRVERAHALVLAGNIYQKAARDPDEEGDYLAFEQLMAEAATKKNKDDKKKKKEK
ESKASKSPEPTEEKPSTTV
>g1196
MATLVRYSNDAVAKSDAAKGFPWEAPIPANRFWNSFKYCIVRSILINFPDEEELKQLPVDPNSTAD
EPTKLHFLLHLLRDKLAREERATSPPGSLNTQNYTQWNQLMQGIYVIENELDLPEAEQTVRTLVER
RPETSNVVPPHMLSEYLVKHGKYEEAEKTARPVLAWMDARPHLGKSSPQALNSRRIIARALWFQGP

SRRTEAISLLLTEIHELVEGMGGDKFEVYQEEERQFNEELIAELQRKT
>g1197
MKASIVLSHILLLAASAAAAVTRGHGLAARMAARGEMTRLTRPKIPAIDVDADEVANRTNVDYSSN
WSGGVLTAPPSGTTFTSVTAQFTVPKPSLPSGATQDSYASAWVGIDGDTYTNAILQTGVDFGISTS
GQVTYDAWYEWYPDYAYDFSGIDISTGDDIKITVTSTSNKAGSAVIENLTNGQSVSQSLTAPSSTA
TLGGQNAEWIVEDFESNGAQVSFANFGKVAFTNAAAGTADGDSVGTSGVDLLDISQNGKVLTSASV
PSDSEVLVEYTG
>g1198
MASEDTRNEEGPVIHLIFENFRSELDEHHDRRERIIKASRDITALSKKIIFALQRVRTINAPLPPN
ITKENKTRFTQIMDLFKSIAPELTGTNSWRYQRQISGGIQEFIEAISFEHYIKTQSLITRDEVAAQ
LPDGIIVTEDDYLMGIYDLTGEMMRFAVTTLSTGGQVKKSDVKDESEMDVDTENQSGPAQKYPIFP
PEKAGIVVDLRNMRAMLEKLNVPRRHSSHMMKDMNKKMDVMQSSVEKVERAAYGLLVRGSERPSGW
TPDLSSVTTGAGATNVEVESY
>g1199
MSSAIDDTPISPILNSTTNQQRRDSLEKHLQTRPELQDLKDRHILLNTSVAPGLQAAQAELARQQA
TDSLRKHLEKRPERDELVDRNILPVRPDVAPALQAHAKELEKHMLADNLEHKIKERPDPDQLIEKG
ILDNSEDPRSPSE
>g1200
MSSINVFARWRPLSQPEDKHGEIHRKTLSDCPPLLAVSVNPQASAEGRSWVSSPAFHAIFEPGDNN
HTVYNDVVAPNIPKILQGENCSVFAYGHSGSGKTHTILGYDYDCQNSQKLGLCLAAAKQLFDALHG
LNEKSEGEKLGIGLSLFELRNKSAFDLLNHRNQCHIREGPDGKVHIRGETEILEGGKVRVRPIVQR
LCWTFESFQHELIQAISQRTTGSSTVHEQSSRTHAVFELEIISQSLIDARHALFDRQSELVPVGKR
ATDIRIEEESKGVIRTADGCWAPNPNSRIDQERIDVAEAEKAQYEALVTAAEQNIEAILEASAFPC
LGVKMVFVDLAGAEFYDDNGSRPQTVRQTPQERQEGRQINTDLLALKQVMGAWAEGQKRIPFRSST
LTMALREHFMSAGKGNSTIIVTISPATEQYKATLNSLKYASLVGAARV
>g1201
MSITNESKPYIPLAGLASDGWSTEDEATATCFCGEVQLVFPTHGPGLVDTFVCNCTDCRKLTASMF
ASNFTVDDQYLKHVRGQENLKTFGQSKTIGSGKKMTNYFCSNCGTLMYRRGEAFPGLSILRIGTVD
DFNLHETKLKPRVEQFTKTRVGWFHGMEGIRQVEGSAYT
>g1202
MSSDSVSFDVFRGVSGSIVADHATKSLGYNEIFIETTHSGLCGTDLHYMHSGKVLGHEGVGIVRKL
GPGVTSVKVGDRVGFGYTRKVCGRCQHCLSGWDQFCENVVDFETRGDPDIGSLSNGVVWDAGTVVP
VPDGYDSVDAAPLLCAGGTVWTVLTEYGIKATDRVGILGIGGLGHLAVKLGAALGCHVVVLSSSDS
KRQEAFEFGAKEYYVLKPGKKPEGLKPLDHLLVCGSSSSMDYTFLIELMAVHGSIYPLTVSSDKVP
VRLHDAVLKGVRFQGVLTASRRSLRKLYEFAAERDIHPTVQKFPMSVEGIEAAAKTLDSGKMRYRG
VLCWN
>g1203
MRLSLAKLLALSCLYEVSQGSPVPLGNEYQLQPYNIDLSSRVPRMLDLIKGTKLPAQPVYINTGTS
AGISLSLLESFQTEWQTIFDWNREQEEMNKFNHYTAVIEGLKIHFIHERSEAEDAIPLLLVHGWPG
SFLEFVPVINELTQNATTATGQNVSFHVVVASLPGFAFSSSPPVNWTVADTARVFNTLLTKVLHYD
TYATFGTDWGAGIAYSLYSQFNNTVRALSLDFLPFQPYTPAQLAAENITLSPLEEFEQAEATEWEN
TGEGYFIEQTTKANTIGLALYDNPVGQLAWIGEKFVNWSDPNAGQSPSVLTHNEILRSVSLYYLSE
CFASSVVIYQQNANGFSTVYTKANNDAPLLFSAFKYNVGFWPPAVVAKTGNLVLYTNHESGGHFPG
LDNPPALLEDLREIATYWSN
>g1204
MDADASPAGTRPYRSKLKRPCDRCRSRKIACILPESGPCRNCWNVGKSCTFDKPPNPRNREIVNRR
ASTKPTDHSSYFAPSPDRSSEYMRKQAKPAASFGSIDVINNIRDTPKIQHIRSLDQLNASTAQLFG
TSAESDPWLLRHCKYDDNGMRHLHGIHFRNVGGVPVEGLVPVHFLVTEDSLLVPSKEATSCSAEVD
VLKIRDELNEMIPPKFGVRLVPLFMKFVFPVMPVISRSQMGFMSKITEQSLQNIPAHLLAALYASA
LPFKIHDPQLVLCGVYDNQICDKLWRRVYQLIMQEIHTPHLSVLQACLLYLQRLPVGSQSALADSP
FLWSFLGTTVGLASSLGLHLEPRPWGIPAWEKRLRRRLWWAVYVEDKWRSLLIGRPPFVDREEWDV
QSLDENDFVVDGQDESLENGATFRSFTKLAIIADDIHQTFYTLRASQRMCEDFRISINAARPIREE

LQAWYASLPESLRIKQKHENNYQAPRIEGVSSLHFAYLILELFVYRAILRPLARSPPPPPIADDKY
PQDISWLLEDLGYDGQGLDQLPVVDFSELGDAAEATLNAAEKCAGIIVNFVGALTPVDFDTFWYPW
TRISFATVSNFILLLLIQAPTLQHASRTKHLLDIWLQNLSRQYKNHENLMSLGLVRLRTLMADGLA
RNFIVSPHLAEVLNTGTTGHGEMPTDNTWNIQQ
>g1205
MVKRIRLGILTPSSNTSLEPLTQSIISQLPNVSVHFSRFTVLKISLENDALAQFQNEKIIEAAKLL
ADAHVDMIGWSGTSSGWLGFAADEKLCEEITAATGIPSTTSVLALNRALQVLGEKELGLVTPYKDD
VQEAIIKTYATIGIDCSKERHLGLFTNRHFADVTESTLDDMVAAVAAKDVKAISTFCTNLYAAQRG
AVWEEKYGIILLDTVVTVVWEMLQLCGVDSSQIKGWGRLFSVPLKTEK
>g1206
MASSRTPFTHRTQQILDKLKVSNDSDKSEESGQFGKWSNKDLDPSPPSQRIWSAWSFFAFQFSIAF
SPTTYNAGASLYAIGLNWWTIFIASIIVSIMIAILLFLNARGPARYHIGYPTLVRASAGIYGSLVF
IFIRGVVAILYMSIQTYYASEFLAVMLRCVFGHRWTDFPNHLPASAAITSANLLAFGLLWLIQFPF
AFVHPSKMSRVFQIKSVIAPIGLIVTMIWALVRKKASSHGADFNGLSSKTVSGAALGWSFMKAINS
IVSNVIPPLVNIADLARYVQKPRDTLPMPIGLVVSKPLVVFLGMVITAAGYKQFGEAYWNLWDFYS
SVLDHYWSPGARTLVFLAAGIQAFATFVTNFTSNSIPVGCDLTGLFPRHFTIVRGQVLCFILAWVC
VPWKLTHSATSFLNFLGSYLCFICPIVACMIVDYWLIRKGNLHVPSLYKPSDDSIYHYTHGFNPRA
FIAWIVAIALVIPGVASVLSPGSIGPAAVKIYNMGFLLSTIVAGVVYYLACRIWPVAIYPAEMDAR
DKSWEAMGYTEGFFAEDEVVPEYLREKVFDRGNDGRVYVETKEAALDDGDGHPTRWV
>g1207
MSAARKVFHCAVDDTVLTTNVSEIKRWASNGAITLFVPLYTLERLQEPKRGGSQVAINSREAVRFL
DRVTSGKDSIPADRVVLQGPMEQYDNWADAEQFFLPEFEEEVDEYAAPRDGDITDITTPKTEIAKV
NGISAKSAPEQKGPGLPGLPSDLSQMLLSKLNFKGPAAVGAQNDIKSSVSIPSVGTQSDEGSRASS
RSSQTSPEYANGNAKRGHNRSTSTSSNAPPTPAVLRPLLSALLWRLHHDEGSIGVPQNLTLISNDR
DIQVWAQKFGIMTKNIHQLRTAIQYEEKEFKNRVKYAEKTQHVAPSPKPLFSYENDSEEDELVFVP
RGGRGKGAGRGSGSRGANGRKTRAATAAAIAQADTTVEVPSQPIDPDSFSRSIGATTVVKQPPLDL
SSQSGASRGLAAASRRNGDEHNHGQANGGNGGSSRRRQRGAGSTRANTSTRGGRGQGKLWVP
>g1208
MTQTQQRFNSFSSYLVTPHELNNALKHYPSSEISTAPRVVPLCAAWFMPNDPEGRTGIQAFRKSHI
PHSRFFDIDGVKDEDSPYPHMLPTKERFAEAMSELGIHRDDEVVVYDTAEQGILSAPRVGWTLRFF
GHPNVHVLNNFKLWVEQGLPTESGDAKPFTKTNYPIPSYDVDRVVHFPEMKEIAKDYGKEGADEVE
ILDARPYGRWSGESPEPRAGLSSGHMPGSKSVPFGELLDPKTKAFLSPEELRKVFESKGVDPSKTI
ITSCGSGVTAAVIEAALTVAEFGDPAKRRLYDGSWTEWAQRVTEDSGLIKKLTT
>g1209
MKVTIKEWNAVASWRWDMPEDEVCGICRVQFDGTCPTCKFPGDDCALLIGTCGHSFHMHCLLTWIA
QESSKGLCPMCRQKFEWKKNNEESQDPGQPVGPAS
>g1210
MSSLPKQNFVQPSSSSRQSPFRAANTTSANPSTATSRPRPIAPSSEYNKRSDASYTQLGESPQQHD
DHQPAPERPFEDVESGYNNDDHDNRPKPTPANFKPFFTLIEDANSADYHHPTVHYIFSDDDAELMT
EASLRVLEAGHHDATSPPPRNDSEERLDSADQQSSHKLENDRPSLLPPPIPGVKERFIVLDVEQSA
TAIGXPSSAPDALLAGTSAGTGTTVVSSSPAQRQQQPYGYRIVSAHSLTPDWQVLDASLSPAPTF
DTPTGDQVSVTNTLMLRIEGTGGYARDIPVGKDKEGPQTLEEMMEQFDKRMVELRRVIDTNGDYSP
LGATATATATATAPAPAPPGDNDVSRAEEVRHEEADEEAVAND
>g1211
MPAPYTRPAAGAATRKPTLRQPTRRIAASATERSQSPAVALKPTTTNIMRLQRSPSVQSIAGAKRK
EREYEPTGGEDTSIHVVVRCRGRNDREIQENSAVVVSTEGTNGVELSMGPNALSNKAYHFDKVFSP
AADQTTLFDDVVTPILNEMLSGYNCTIFAYGQTGTGKTYTMSGDMTDTLGILSENAGIIPRVLYSL
FTKLEDRESTVKCSFIELYNEELRDLLSPDDKANLKIYENESKRGHNSTTLVQGMEEHFIHSATAG
IKLLRGGSYKRQVAATKCNDLSSRSHTIFTVTTSVKRTTEAGEEYISTGKLNLVDLAGSENIQRSG
ADNKRAAEAGLINKSLLTLGRVINALVDKSSHIPYRESKLTRLLQDSLGGQTKTCIIATVSPARSN
LEETISTLDYAFRAKNIRNKPQINSTLPKKTLLREYTMEIEQLKSDLIATRHRNGVYLSAEAYEEM
KIESESRRIINEEQRAKIESKEASLKHKTEELFALTSNFNNLKKDSEETRAALNETNDVLEQTEIV

LRDTKKLLDQEELIRKAHEQTEEKLYGIGSELLSKLDQTVEHVNGLHQKLHRKSDLHSHNRDTWEA
STGDVVDVTKLIEDRVEIFESEHSALVQGLSTKIAEFITTELQTVTTGNSQINTSRYFLAETENEL
KEQSTKAHDEMNDVLEEIKVLREDVKGKVGQGLNGLSAAAERISKEVIDEMTDFHTQLHSSYSTLG
RDFKAMFENITTHIESQKTEIEELRAQLLEANREAIQGNQSAATDLEKALEEERQAAETDRADLLS
QISLLIEASSQKQATRLKGRVDNVTSDLKYSGDILQKATDKYQEGMEKWVDRENQLIEDVIGSRDA
IKGRMQEDWEVFEQRNESIQKSTEAVHQETIRIVDEQVRQMAVQMEALDDFVTRARSQNGSHHESH
MANLEGLTTNIKGSFASLEEGFGSMNTQLHAFQETVTEKENSLKQAVTPFVEEVRQPLVELRNNIQ
NAPMTEYKPTGTTPPRTSYEYPRTLPRTASHSALLDKLKQTTQPILTPLEEETLDPGLKSPIKHRM
GSPKKTRVYNDAEDEVGEPTITTSTIITSSNTGLREVDLNVAAKQLNSSSPERDFSKSIIDVLEDP
AEPPPKKRQAASESKLPQKALMRRAPPGIALDGRENAPLDACIGRRLRNRPSIG
>g1212
MSTASAPGAMQVFPGKGLGNLTLGSSLHNVLSRIKSRPQTYPAIDLAYSSTEPLIEPVVLNLPKNG
LRLRFDGPDQRLRLIEVLDFSKAPLVYKNHEVVKIPKPQEQPLPTGPSFRHVYNRLFGPSYPGEYI
PPADGSPFGTYVLSYPGVAFSFPLQDSAWSNNCDFVALLSSSAASPATSMAIFQGASWSEARNKLF
TAPPQLPRSPALIGKGKEAVPDEIEEIRVLGAGRIELLRRSSPSLVINLSETTPQDLIAELGPPDA
IYRKNDRRISIHNARGVNQPRLLSPSPGRGLDTPDTDHSSNNSPEDSDDETFSSSKVDPSSLPSEC
FFNYFHHGFDAFISYPTPSGPALPGSELSDXXPPATSSLLTVTKLIVHGNVPGSYPFNRYRRSRWK
ILLDDSGREVLTSETHYDEVAERLQNVWKGSYASSSEETALQRGMVLNRGWGDSPESSVEFLGGWE
ESVGGKHRAASSGPDSGQGLGNTELYGFPGLLFEVLKNGAVSSMTIY
>g1213
MSAKYQAWRVYPKASTHDEKSVEILDNLALEEVEKPIPGPGQILVRIHAAALNFRDLLITAFSPKY
PVPSIPGLSPCSDGAGSIEAVGPSKNNNWKIGDEVIFRVTNSWDDGDVSNFKGNGLGSGDIHGTLS
QYLLLDESWLVRKPAHLTWEQAASIAGAGGTAIQALFHNGISNGLDLSGKTVLTQGTGGSSMFCAQ
FAVAAGARVIGTTSSDTKSELLKSLGVHEVVNYKTHPSWADEVVRLTDGKGVDLVIDVGGSATFEQ
SLKAARFGGTVAAVGFLTEPRPSDPGLIHAIIFGAKTIRGQMAASLEMYHELVELMEKHRIVPVVG
QVFEWTEAKDAFEALMRQSVSGKIVIKVV
>g1214
MTRSWTSTLKLPRSTFPARVPVADQTKYLRRCSDELYAWQRENRPASDTFVLHDGPPYANGELHVG
HALNKILKDIINRTQLSRGKRIHYVPGWDCHGLPIELKALQAQQKDGNDFSKGLGTAATIRKAARK
LAEKTVKEQMRGFQAWGVMADWEGHYKTLDKRFEMKQLGIFREMVEKGLIYRRFKPVYWSPSTGTA
LAEAELEYNEKHVSTAALVKFPLVKVPDQIRNNPLIDTESLSAVIWTTTPWTLPANAAIAVSESLN
YLIVQSSSHGQLLIADSRLSYFQDMLKEELQVLVPSIQGSELCQGTTYRPLFPREGTEEQPIIAAD
FVTADSGSGLVHCAPGHGMDDYEVCLAQGIQAFAPVNDEGCFTDEAMPDDPSYLSGKSVLDEGNGL
VLQYVQSKNQLLNRHKYEHKYPYDWRSKKPIIIRATEQWFADVANIRESALASLEEVNFVPATGRQ
RLKNFVQNRTEWCISRQRAWGVPIPALYDKTTGQAVLTKETVSHIMNVIDERGIDAWWTDSPEDPA
WIPQHLQQENSPGYRRGTDTMDVWFDSGTSWTQVADESLGKERPADIYLEGTDQHRGWFQSSLLTY
VSHQVVSGKVEPSTIKAPFKHLITHGFTLDQDARKMSKSIGNVMKPDAIMNGTLLPPLKQKKSKGA
KEQKPSSPVYDALGPDALRLWVASSDYTRDVVIGQQVLQTVNTSLHKYRVTFKLLLGALGDFDPKV
NILQYEELHKIDRQALMQLTRLVDTCRNAFDNFEFYKAVNALNRWTNLEFSAFYMETLKDRLYTEA
EDSASRRAAQTTLFHIYTYLQELLAPITPLLVEESWEHTPELVKDQLEHPLRRIARTVPAEWVDEA
ISNDFIDLMAANTAVKVIQESARSKKQMGSSLQSFVHFELPDNASLDIFQRYLNELPDLFVVSSVS
LSVKGSDLPNEIASAEWSYAEEFALPNSGKKATAHVYAPNQAKCPRCWRYVAPEPVAEEALLCERC
DNVIGQLEHN
>g1215
MIVPGMEEILKHTATLPTSTPTVGPIPTVLPGDWPVVLHIHGTGKRTLWVVVALMAISSIAFYTLA
ARVRVQTRLLHTLTALITTVSFLSYLAMATGEGVTYKHSIVHHTHEHVPDTHQEYLRQIFWVRYLN
WIITTPLILINIALLGGLNGANLVVAIAADLIMFAAGLTATFAHDQRRWVWYTIVIISFLTVGFQV
GYNGVRSVRRGADQHRALFTSFAGANLLVFLLYPIILAASPLSQRISVDAETVAWAIHDILTQGLF
GYWLLLGHDRSETGQLFVDGFWSHGISHEGALRVGETDGA
>g1216
MTTSSSDSTISFALYRYTPSIAAASIFAVIFLALTIAHCIRLWRCRAYFFIPFIIGLLFECAGYIA
RIFSHFNTLALGPYIVQTMLILVAPPLFAASIYMTLGRVIVALNSQQLSIVPVRFLTKVFVIGDVL

SFLLQCGGGGYMAAGTVAAMNTGANIVVGGLVVQLLFFGFFVIVSAIFHWRVKRQNYSSIQVPSPS
FSRSKSWESIMWALYAACFLILVRSIFRVAEFVEGNDGYIMKREYLLYIFDACLMALTGIVLAVVY
PGMSLGRDDGKRESGLRLTSTDEEMQDSTHIGHTTRNANNSHLRPKQRISHTKSRNGCYMCKQRRV
KCDEVRPTCNACVVRGNDCVYPSPRGALDQRRRQRSVSRGQSSESSHNDRRSVPFRPLNFNLSVPE
EPQNVGSGLNMSDLNLLQHFILHTSKNMTLHKIKTVVWERVFLDLAATNEFLMHLILALAGADILI
TEGSPISWRPSLHLIMEHHQQGLAGLQQALCATAEPNEEALLAGSMLVTGFAFASLGIKDLDPSTE
PTLQNLTTHGGSSIPLQIQWLHLVRGTTSIVGQFWSTLRRCRLRALFFFNNANDDWKLCESELRSG
LAAVDQNIKSKRLRKFASGANRALSDLREICVALRDSEPLQDGSDDSPLTDTPRSEHGGKTSLHAC
EQAIDVAEYIYMRILYTLKMEPLNSASPMDLEIQTDLEDAAIFAWPHDLPEAFIWILDSAQHQGNV
DALLLGVSLTILAHLYVTIAVLEDVWYLGKTCDAEIRKIDGLSQEDWYVSFM
>g1217
MSRFKRNNEHKSGTPTEGTTGLGKDASQETVESSALSHATTSSRSSFLIQNSRQDTSLLTLENKPQ
ILEDAETQEPLVRRRSMSDTDIEPIYLGVDFGALLRLRGSIASQDTLWIDMWQHFPPSKTVVPLDD
QYLSCLTAFKNLRCLWVTGMLKSYQKSLFQAIWKMEHLANLQLRMAEEPRLSPEGERTFRPIEPGW
SPLVNDDMNHDSPGDRKGRILKQYGDAEFLENQVIENAKSDPKISHPEHNVWSTPHLPIVHLTLRG
FVVNAMPFYSCFDANKLRTITFKDCYDAGFYLPDDMKHVELKVDSKYEHRATNGRGVSTDQEVKCL
TYKDGKKVGQSRFNSSSSSSSLQDPAEQSTYRMKQVESPSSIPRYAASSSPTPSSYSSSTTMVSGQ
GLRKSRLSLDFRKRPVSSVIEEDEGEE
>g1218
MADAAPRRGGFGSRGDRGGDRGRGRGRRGRRGGAKSEEKEWQPVTKLGRLVKAGKITSMEQIYLHS
LPVKEYQIVDFFLPKLKDEVMKIKPVQKQTRAGQRTRFKAIVIIGDGEGHVGLGIKTSKEVATAIR
AAIIIAKLSVLPIRRGYWGTNLGEPHSLPTKESGKCGSVTVRLIPAPRGTGLVASPAVKRLLQLAG
VQDAYTSSSGSTKTLENTLKATFLAVGNTYGFLTPNLWKETKLIRSPLEEFGDVLREGKRY
>g1219
MLRWYAARMAQRPLLTSSITTATLFGAGDVLAQQVVDKKGFDKHDYARTGRMVLYGGAIFGPAASA
WYGVLQRHVVLKSTAATVVARVAADQLLFTPVNLFCFLSSMSIMEGTDPMEKLRKAYWPTYKTNLG
VWSTVQLGNFALVPLEYRVLVVNVVSLGWNCYLSFVNSKA
>g1220
MATATENGTAPAINEKTAAQNGDVVTVFHDPENFNVKHPLMNEWTLWFTKPPSGKGDNWNDLLKEV
VTFNSVEEFWGIYNNITPTSELGLKADYHLFKKGIRPEWEDQQNKHGGKWSYSFKDKRAVPIDELW
LHAQLAAIGETLENDDDNEVMGVVVNVRKGFYRIGLWTRTVGKSLPGDKSTEARTAAKGKEVLESI
GRRFKEVLRLKEADVVEFSGHTDSAHSGSTRAKAKYSV
>g1221
MAAADHEVEVAQLHHTFDDVEGVSHPTKDITGGSLPEVDEKLPYVTPDGEVPTEEEKHRLKHVAEN
LPLSAWLVAMVELCERFTYYGVSGIFQNYIQRPLDGSEGRGALGMGHQGATGLSTFFQFWCYVTPI
LGAIIADQYLGKYKTIAMFCGVYLAGLFILLFTSLPTSLEHGAGLGGFVASVIVIGLGTGGIKSNV
APLIADQYTRKRMAIKTNKNGERVIVDPAVTVQRIYMIFYGCINLGSLSGLATPYMERDVGFWSAY
LLCTCMFAVGTAVLVIGRKYYIVRAPQGSVITDAFRAMGMMIKHFNMDAAKPSWQAEHGGNSNLRW
DDHFVDEVKRALVACQVFCFYPIYWVVYGQFSNNFVTQAGQMEGHGIPNDLMQNFDPISIIVFIPL
LESLVYPLLRKLHIDFKPISRISLGFVVASLAMVYAAIVQHYIYKAGPCYESPLCAASEIDGVAQG
NHIHIAIQTPAYMLIGISEIFASVSGLEYAYTKAPPNMKSFVQSMYLLTNAFGSAIGEALTPVAYD
PAIMWMFTGLACASFAAGIIFFFTFRHLNAKEEEMNALDVDEYPPVEETDRK
>g1222
MAPVAEQDIAPPNIYSVKSWPKPTHPGLIQVVRSDKPFGSGAVSLVSLPAGAVFAHIEPDVISVVP
EQRYTTVQVDRNAHIELNSDLVYCNHSCDPSVVFDMHRNEVRVVEGRPLKSGDALTFFYPSTEWHM
DQPFECTCGSSKCKGRIDGAANMQRSQLEEYWLNDHIVQLLKERDDVN
>g1223
MTVDKRIITIFGGTGQQGSSVGRSLLLNKDKRFFVRVITRNPSSEKAQAIAALGAELVKADAFTEG
EIEAAFQGSWAVFINNDSGNPVLNTETFPTNYDIEATIIDAAHRAGVQHVVYSSGQSICAATKGRV
HQEGFETKYHVEQYARTKPFTTFTAVLPAAFWENFRDENFCYGLGGFPFYRTWDGKLQLRAPLYGG
KEDMPWLSVKDDFGDIVHGIFLNPQKYNGMTIQAFSQQLSFAEMAQSFTDACVDTAAMSTVPDDQQ
NQQGNSGETDSDGSTQIGGQNSFRPIVPGPSYPGPGRTPADLRRTMGPGWNKRVSTACLACKKSKR

KCSGTPPCDNCRALHRECIFDESLDQRRRVAAKRTAEELEYHRDMLNDLFKVIRIADEKQAQRLME
MIRSDATPEEIRLFIDEVLVQLQTFEPPSKAKRETAAQLKEIRSRANMQGSAPSFRRQVMDVNFLC
VIPPIQVPASPWTTATDDDAFVSHLVSLYFTWDYPFYSFLDLNVLINHMKSRDLHSEFCSPFLVNA
LLAHACSYSEYSEAYGIPGDITSKGAKFLKEAEKCLEKRGDDVSLSMLQGTMILYERYSMTGQDDL
GYAMLYKGIDIADKLGYTGGEGQTIDLSEKSKDLYNSAVKTVWGLFQLDTVAHAAFLKPCRIKNVR
LCRTPALSLERDADFWTAYPSQKPPRKAFFVTYFDKACTLSEISRDISTTLFADEIDDDKERLSAA
VDSLYTRMKKWHESLPAEFHMSKKPAPHILLLHMFNCPRGHNYHHKATEIGTPAKAPGSQMPEGGE
DPASIAITSARAIARLIRVHQEEYGISRSHVFALFAVNLALFLLLDLDVYDISDPDCLSLTSAFAI
ITTRSMLGREVRYIFYRSVSKSKKNGVSKWDQLPEGLREILGEDADDSSDSEGNHNDEHKHDDIGD
GYNHYGNTSTRTTPMTATEEDEEQAQKPKKGWGPQEGSKGLCEMLCRYETMTLGRKDHVTGKK
>g1224
MAPSGVPKFINKLAGQRVLIIGGSSGLGFTTAEAAIENGASVTIASSNPQKLEKALYRLRTAYKSF
LQPGQTIAGQVCDLSNQQTLEQNLHELLRVAAGDSKIDHITITAAPMLWPPPLKDTKVEEVEGHGF
VRLVAPVMIAKLIPQYMHMTHKSSFTMTSGSHTVKPDPGWALHAAYCGAVEALMRALAVDLKPLRV
NVVSPGAVLTEGVIEILGDVYEREVERCRRNSTLGVCGKPEDLAEGYVYLMKDQYVTGTIIESNGG
MLLA
>g1225
MDGKLVLQARFDENLILEKQAGRFVEQMAHLVQQIQYAATLEGETTKLGKLNIASQSDLASIQKWC
AAQNTEPVAQLVHDVIHGSALTTPGAPALISWDGELTYKELDELSSQLAGRLAHHGVCRGSIVPLL
FEKSIWTMVGQLGVLKAGGVTLVLDANYPDQTIADVITQSESILGIASKAQAKRLRTHMPSVVEVS
SHQSKNWGNEKPHDHTSERDRLLLHPEDPAIILFTSGSTGKPKGILLSHRALVTTIHHHASAASIH
RNSRVLQFSSYAFDMSIYETYTALATGACICIPSETQRLNSICQFIRDCKVTWAFLTPSMLRNFEP
EDLRGLVTLGMGGENVPADLASRWADELELLNIYGPAEATLMSTCRIVKDSWIPGTFGSGVGCRTW
IVQLSNVDQLAPIGAIGELVIEGHLLASGYYNDPQKTSKAFILPPKWRQLFSPAATAPVFFRTGDA
VQYNPDGTIRYVGRDDAIVKIRGQRVDLEAVEHAIREIDPSLDVCVEAVKPPGAAQDESKLTAFVT
CTLAVADQASSVTAESLFRHLQLSLSQKLPRYMVPTVFVPLSSLPLTRNKKLDRRQLQCLFAEQMS
LKITIPALCSEEQSFEQLGEKEECLRLLTGEILKVDSRSIDMKKSFIEVGGDSMTAIRLTARARRK
GLKIRTEELLQSSSTLLDIAQTIGDCSAALVLPPTEEPFAFFSDQKTRDSILSTAEQQYQIFRSDI
SDIYPCTPLQEGLMTTTTTRGSLVYQDQFVYKLHPGIDMECFHRAWMAVVSANPILRTRIIQISSG
QTIQVVEIPKPTPFMEHKMKSLDSYLINDQKEQMGFGTELVRFAYTEDSTEDANILPCFVMTLHHS
LYDAWSLSMIPHQIEAAYYGRSPERSSFSSFVRYVHSGASTDTLSFWTTQLSNVDAAIFPELPSST
YNPRATAAVEYTTQSTAPTHKRKCPFSDSIRIHLAWSIMLSLYTDSDDVIFGTVVSGRMASFPGIE
KVFGPTVATVPFRSVLNFETSIRATLEQMQTQFTAMLPYEQTGVQNIARMGGDTKAACAFQNLLVV
QPPAPSSTSVVSNVPSSSPSGLFSSNVEEKTGAINSFPGYGLILLCYPGTESVKFEALFDENLLDE
KQVTRMMAQLSHIFDQLDSELDRTLRQIHLLAPSDQCQLMEWNNYMPPPAEMCIHEMISTQCRLRS
KERAICSWDGDFSYEELNSLSSAIAISLLKRGVKAGNITPILLEKSKWIAVAILAVMKTGAAFVLL
NGNQGQQRHLDICHAVNAGLIITSAAYSKRLDGVVEHQTIYIIDDEKSINTKVVTAAGVALDDARG
GLLSAVKPSHDDLAYAVFTSGSTGKPKGVLINHGSYCSSVLAHRDRFLITPATRVLQISSYAFDAF
TVEILTVLCTGGCVCIPHETEMQGDLAQAIQRMDINLILITPAMLRLIHPDEVPDLQTVIAVGESM
TPAQVALWSTKVNLICGYGPSECSTAASCLHIRPSSSYSNPQNIGYSMGCHFWVVHKNDHNVLVPI
GTIGELLIQGPIVGSGYLNDIERTREAFFSTTATVGWRTSYYGLPSQGRFYKTGDLVRYNVDGTCN
YVGRKNQQTKLRGQRVEVGEVEQHVQQALGPGYHVVAAVIRPHEHDAKPLLVAFVHITSSGNSTIE
TPKTTFCPVAGNSDFTKRATVADKILRSALPSIMVPSTYIQVEHIPLTPSKKINRRALEIGAAALT
TPELFKLGSIISQEQEICTLDKNDAMVWQXSDTISSFGSSKISDYQERFHGKDVPLPFLGLDSIEL
MALCRNLSRQFDVHLTVAMLSDETLTVARLARVIENCKKEAARSSSHHQEANDSSFNLYEEFMASA
TLLPQPNRQKTGRVFLTGGTGYLGTEILRQLVQNPQIVSVTVLVRAESKEDALGRIKQSAETAEWW
EGDRHARKIIACPGDLSSTRLGLFQTDWDNLCQGTFDWIIHNGAVVHWTKSYRSLEAANVTSTIQF
LQILAHPSCSFTGFTYLSALVPSVENKSDRQIAQELSSVDGYTQSKFTSELLIKEFKKSYSNPISI
IRPGLIMGSSANGICNVDDYLWRVVCASLRLGAYNSEESANSWLFISPVNRVASLVVEESLSFIYR
SRNHHDPASSLHTIPVLDCLPVPEFWKIIQAASSRKIKAWTTNQWLDAVKSDVDQAGTLHPMWPVI
QFLEAANGCLGTPQRSSFTQAPAISRSIVSALQENIRYLTRIDFFRVSDSEYIGDCGIRALPKTVF
RRSHNVASTSAKITVPMGKGVR

>g1226
MLTSSPGSTVWNIELRRPPELPFVPQNGKSDSNIRVCIIGAGISGLYLGMLLDELDLKNVSYDILE
SNERTGGRVYTYKFGPGKMEYYDVGAMRFPRIPPLARTFDLFMRCGAQLIPYNYGGEQCPNLFNTI
GLAPGNEMATGHDPFKFSVSEGGLVPDMTVMEGPDGILKRAFDPFISGLADNFTEGFDELMKWDHL
TTRDYLNRKMGLDFYSIWYLETVSSASGLYDQSLSEAVLDAMDFDYPTTTGAKVEWYSVKGGTSEV
VKNMEKRLSRKPALGHRVVSLSYEPASDPDYPMLVQVDGETQPRSYSAVFNTASLPCLRRMNVGEG
ILSPGQKAAVQSIHYDASTKIAIRFKSAWWRKYCNIQGGQATTDLPIRVCVYPSASSTSSATEGEE
EDTPTVLLCSYTWGQDAQQLASLVNSKSESARRELKQLLTHNLTLLHQEYLDAASGLPYGYARMLH
IILDEYDSFHGYDWYSDPACSGAFAYFGPGQFKNFYPELVKPAANGHLFLVGEACSAHHAWISGSL
DSAYRALLQLLYKLKYQGRLGPDVLERLQTVWGTLDEIPPEVLEWQAFLAMVRETSPVTGGG
>g1227
MESPKKHVVFDVVGTCVSFDAFFDAIEQSIGQKLRTHNVTAKFLGFAWMQAAELEHTMLLMSGRNT
PYKEVFKALFYRVLFMAGIEDPRSFATDEERDRCQAGYSQLELRPGCREMMEKLKDNGFTIWCLTT
GDTERVGGYFRRAGYDMPADKLVSCHEFLPQDSSPSGAALLAAMKPSMESYKPMLDKFAPEDQKWF
AAAHMWDVSAAVKAGFHGAYCSIYEKESCIEIFNTEMAVIADTLPEMADKMIAMSTNI
>g1228
MAVVDILPAKDDGKHQIEQQIKVSSSWCGPGPAAYDFRSDTITTPTLSMLQQIAQSTLMDDVYQED
TTTTEFEKFMAKLTGKEDSLLVMSGTMGNQIALRSLLTQPPHAILCHVSSHILNSEAGSCSSFSQA
HMQPCTPANGQYLTLEDVKRTAVISNNIHVAPTRVISLENTLAGVITPLEEVRRISAFARSNGIKM
HLDGARLWEAVAAGAGSLTDFLECFDTAQLCFSKGLAAPIGSIIVGRKSTLEHCRWVRKSIGGGIR
QAGIISAAARVAVEENFGTDPSGKGNRLRATHDKAKVIEKMWADRGGSFNRPVETNMVWLDLPHSG
ITVDEIISAGKEEGVKLMSGRLIVHHQIADEAIVRLEKVFDRLLSSKEKRNGEVQEAQTVYR
>g1229
MSSINSYVEGNANEIVHKSGQDEKATPHNIMTLVPLADEATGEFEEVESTRQGLHQRHIQMIALAG
TIGTGLFLSSGRAIAHSGPLGAFLGYLVMGCVAGAVTLAVGEMGTLMPLNGGIVRYAEYFVDPALA
FANGYNLVYSYLVSIPAEIVAAAVLVQFWSDLNSAIWITIFGLLMLCTALVFVRVYGELEFFFSML
KILLIIGVNIMALVITCGGGPNHETIGFQYWRNPGPFVQYLGIGGSLGRFLGVWTSLNNALYAYSG
IETITVAAGETKSPRQAIPQAAKRIFFRILIFYIISIFMIGLVVPSNDPDLGHSSGTASTSPFVIA
ATRAGIKVVPSIINAVVITSAWSSGNSNMLGGSRVLVGLAKHGRAPKFFARLNKFSVPWIAVSLYG
LFMCLGYMSLSASASTVFDWLQDLVSITTLTNWITILVTYLRFYYGCKKQGISRKSLPWATPFQPY
ITWASLVILSLFLITGGYSTFIKGHWDNETFVSSYINIPLFLILYFTYKFVCKTKIVALEDIPIQP
FIDIANRNIEPPQKPKRGLAKLNILWG
>g1230
MPKVFLITGTSTGFGHDLVQEVLDRGDVAIATARKPSVLKFEKATEDNYLALKIDVTDPQSIKTAF
SKAVERFGRIDVVVNNAGYGLTGCFEEYTDEQIRMQMEVNFFGLVNVTREAMAVMRTQSPSGGVIQ
QVTSVGGQRGSACFSMYAASKWAVEGFTESLSKEVKPEWRIKFTCVEPGGFRTNWAGASMAFTDRH
PAYDHLDAKAAMIARNGSQPGDPKKAAKAFYDLAMMDSPPLRILLGTDAYPAILARLQGEIDNFKK
YEKISLSTDVE
>g1231
MDLKSLANSLPASCLPVKNSTIPFWHQGLHELHNHRTTEQLPESSDVVIIGAGYAGISTAYHLVKG
DSADKKLSVTVLEARGVCSGATGRNGGHLRPDMYSPMPKFIDRAGIERAVEVAEFEVAHLNAIKSL
VEKEKIDCDFTLTRSIDVWCNEEAAIKAKKLYDALVSRNMNYMKDVFFVFGKDAEGISGVKGAKAC
ASFTAATLWPYKLILHLTESILQTGLVNLQTNTPVTSITRTASGGFIVTTPRGTTLARKVVYANNA
YVSGLLPQYSQAIVPCKGLCTHISVPEGTTPPLLNNSYIVREKDEVVSYLIPRADGSIVVGGANTK
YRPFLEQWYNNVDDSILIEEVKDYFDGYMQRNFRGWENTGARVDKVWTGVMGYSWDSNPHVGPVPG
GDGQYILAGFNGHGMPVVFLSALGIAKMVRDGIGFEDSGVPRLFQTSQERLDNAKSGPVGGDILTK
NS
>g1232
MTKRRPPPQASNFETTAYEEPTDLERSVESPHTNTEYSLPSAPDTGLGLSSNGYKAASKRNSLFQK
EVGQHETTRFSAVFFENQDSFGPVIDDVTNSSFQEPNQESGQELDARRREYWPRMELAVDTLLNFP
TARTCEKLVEGIHHIYDVWVSPIMIQQCLEQVWREYGDCLGDHRTRESVAIMANDLFLNDKKPRAM
PETNDFERAGWMNWFTGPLLRWEMVGILFSWAGMAFRHRQDWDPVFDLPEQQGRDRNSAAERMREC

ASACVRLCDDHSEISDIMVICMKNNVKLQSIIISDESDRIRVDYGTVRSAFITAGLHRLPQLRKVT
PYSQYRACIASSMYYLDKCESLFNAQPPMLSRHFCQYSLPLDLCEEDIYGGRERLDAALARLDSNG
WNTDGRISSTTWLRALVMLSPIREGILELSLRINQQFTKIEVEDLIVQLRRIVASYPPHIRYDRNL
EWPLKSGVGSLARSAHQVYIINRIQLDVLQCHFLLQRLLVSRRFNGGQDLFDIAQETLSIIVSLWL
HRDELQDLTHTFDWISVSYGMPCAGILCIELLRASNLAPLTPXTESSTTAAIHSPVHLSRSQVIQN
LTMFKALLDWTRPTDNNVQLSSKFSKVLQRIIDTIFDSLEPLYVAQSQQKLDDQRIQRQYEIDDQQ
PQGQNLSSATIGVESDLNLDPALIASNDMDWLNTIDWTQGGWLEQSNQTFLH
>g1233
MAAKAIPSHLRAQAESGAEGESFQRKHHGKSQSHMAFENASTSVAASQMRNALNALSETVEDGSTK
KRFEAEMDNFFALFRRYLNDRAKGNAVNWERIAPPQPSQVVDYNELGNSASVEFLNKLAVLKLNGG
LGTSMGCVGPKSVIEVREGMSFLDLSVRQIEYLNRTYNVNVPFVLMNSFNTDDDTQNIIKKYEGHN
IDIITFNQSRYPRVLKDSLLPAPKSFDSQISDWYPPGHGDVFESLYNSGILDKLLERGVEILFLSN
ADNLGAVVDLRILQHMVDSKSEYIMELTDKTKADVKGGTIIDYDGRVRLLEIAQVPKEHVNEFKSI
KKFKYFNTNNIWMNLRAIKRVVEENELEMEIIPNEKSIPADKKGEADLSIIQLETAVGAAIRHFRN
AHGVNVPRRRFLPVKTCSDLMLVKSDLYSLQHGQLVIDPNRFGGAPIIKLGSDFKKVSDFQKRIPS
IPRIAELDHLTITGPVNLGRNVTLKGTVIIVASEGSTIDVPPGSILENCVVQGSLRILEH
>g1234
MKTILILGGSFTGVSTAHRILKQASKTGPAVKVILVSPNTHLYWNIAAPRALLPGQFTDEKIFQSI
PEGFKQYSKDQFEHIVGFASNLDAANKKVEVSADAEGKKLVRTIRYDFLIIGTGSRSKEFDEDVKA
PFKGLGSTEATKNALHEFQELVTKSKTIVVAGAGPTGVETAGELGYEYGKTKKIILVTSGNTVLET
AISSVSKTALGMLRDLDVDVKLQTKVTGXSXTKXEGPNQFEISLSDGTTLSADLYIPTSGIVPNSS
FLPDKHLNANGFVKVDEFFQVKGLENQHVWAIGDVNDLESPQLMVADRQSGHLAKNIGLMLNNKAP
LPYKGGLRESTTMAANSSEAETKQQGIIETAQAAASDPQSKIQPELIEEKLVEESRKAGAVAYQFN
PNDSPEEKAKAAAAFMVRTYKFQDITGPAQYDLPSTDKATEEVNQAPTLNESDKKSAQNGHLTEDQ
RWARDRTGWAPQFVKPEEKREEAETLLDHRTFLEEKLGDKFFGDWYHNAAVIVFACLSSWIIAVLG
GGLGWVFLVMAACGTYYRTSIRRVRRNFRDDIHREMAKQRIETDSESLEWINGFLLKFWPIYAPVL
CDTIINSVDQVLSTSTPAFLDSLRMKTFVLGTKPPRLEHVKTYPKTEVDTVIMDWKFSFTPNDTMD
MTARELKDKINPKVVLEVRIGKGLVSHGLDVIVEDFAFSGLMRVKMKLQIPFPHIERVDICFMERP
EIDYVCKPLGGDTLGFDINFIPGLEGFIKEQIHGNLGPMMYQPNVFPIEIAKMLAGNPVDQAIGVL
AVTLHGGYNLKGSGRIGNTVDPYCSISINNRDELARTKTVRDTSEPRWNETHYIIITSFTDSLTLG
VYDYNDVRKDQELGVATFPLDKLESEHEHEGLSLDISYSGRSRGVLKSDIRFFPVLGGRKLEDGTE
EPAPELNTGVARFTVEQAKELDGSKSLIGSLNPYAVLLLNGKEVHVTKKLKRTNNPIFQNSSKEML
ITDRKHAKLGLVIKDDRDLATDPVIGRYQIKLDDMLKMMDKGQEWYQLSGAKTGRVKMMIDWKPVA
LRGIVGGAGYVAPIGVARIHFKGATELRNLETMGKSDPYARVLLNGISSGRTVTYKNNLNPVWDEI
VYVPVHNVREKLTLEVMDEENMQKDRSLGEVEIALSDYIHEDENGEYEVDDEKQDIKSGLRMNGRG
AAKGILNYNIAFYPTLNVYDPEEDTEEEEEEGELASLTSRGHSKQNSVDSTRKSSDAPRPSTDVKS
NGRSSIDTAVSKAATNGGLTSPTSPKAVVQERPKIHIEAADLTKYESGLIVFKLIDGQLSHTNVQL
EVLVDDHMFPSYTSMKIKSREATFQDVGDAFIRELDMSQLTLRLVQKSNDKDEKHVIAKLTGDTLS
TLQRCLYTPTELSLRADNGAVSKIKVSLRFIPVQMKLDPRESINNSGELRVDVLDAADLPSADRNG
FSDPYCKFKLEGKDVYKTKVQKKTLHPAWNEFFETSIKSRIGAKFRVDVYDWDFGDKADFLGGADI
NLEMLEPFQPQEINLDLDGKSGAIRLKLLFKPSYVTKTRQGQSTMSGTFAPAGKIVGAPVKGVGMV
GGGVVKGASFLGRGLKSRFTHKESEGNGSSTPVREESPPPQTPSKSEGGLARAPGLVIEPSSPSSS
VDAPESLNRRHSRTKSVKSLLGDKGTPGSIGAEAGSAMFTIVSASGFPPSANVRCHVRLLGAKGAK
EVHKTKAVKSSSGSVQFDASHETFKVPHVPADAQYQLKVVDHSTFGSDTSLGEALFFVDDQGTAMS
KEKNITVGSGSVVIKSSFAASDVSNGRPSTSHSVNLEAPDANGDTPDKKRRSFLSKRSVSGV
>g1235
MADKPAIYKHADKDGHFRRSDSAFRSSISRSPTAEFPAEPNRYVLYLNYVCPWAHRANIVRFLKGL
EDTIQQVVVGNDLGPDGWYFTGVDGAAERDPLYGGKYLSELYFKANPNYTGRYTVPFLWDKKKETI
VNNESSEIIRMLYTEFDDLLPPDLREENKPGGGLYPAHLRAEIDEMNEWVYHKINNGVYKTGFATT
QEAYDANVYPLFEALDRVEQHLGAPEHQPYLFGEHITEADIRLYTTLIRFDPAYHHVFKCNIKMIR
HDYPRIHRWLRLLYWDESERTRGGVFKKTSYLHLYKINYLQALGRKQGVDGGGLIVPAGPIPEILP
LNE

>g1236
MDRSSSPFFLVPIPECRSFVTSSSDRSSSPFILEPSDSPTLPPTSVVIRKKVAPRPTLIPPPPSGQ
LRQRPLRPASRLPVPVIRQVQPPRTVTPPQSALVSQSLMDRRWARQQRERPWASATRSTPIRLTSA
LARSIISRSAVRSAPHKPLQGADSYRSVNSAKAPAKKVSFAEVKTVKVVSRWINDVPADYSEWRHH
>g1237
MSLGRVSYLESWEHHPPAFIRRPSFGDGPASGDEESINRHSRTQSPDPHFSPSSLPSSFPSRAIHH
KLSFNPIAGRGWAHSSLTAPDEEQRPLFRPDVSPDLTIQDAGPARLAEDGIAAENVFQDPKWDTAE
TTPAFEVIVAVIYCLLAAGPVFGFAAIKPVFIREGVYRNRCTSDELEHGFGLCYGQETRLNLMFTI
AAVATNICALPVGTLLDTYGPRVSGIVGSVLIAIGAVLLAVASKIPFDVYIPGYLFLALGGPFVFI
SSFQLSNAFPSRSGLILSLLTGAFDASSALFLVFRIINEKTEGTFTVQKFFTIYLVVPVFILLAEL
FVMPGASYKTAGELVLQAEQVIVAEANDTIDESLPEPDEAERQRENRRAERQDIVNNIQDLLEGST
SRKIDDIIFHENAPAAVNYQTQESIQKPDTQPPASNDIKHRSGGVWGAMHGASALQQIRSPWFILI
TVFTVLQMLRINYFVATVRSQYDYLLGSPELARQLNEFFDLALPVGGLIAIPFIGTVLDNASTAMV
LFALVTTSTIIGILGCIPGSIGFGYANVVLFVAYRPFYYTAVSDYVAKVFGFQTFGKVYGLVIALA
GICNFLQTGLDALTFKVFDRNPTPANVVLTVLVAVVGSFLVTYVSSKARELAHSQTDMTGEEQALI
SEGQNGRQSGGSYGAV
>g1238
MENPNSSGGDYWGTLINADKSPTPLLEELCIALAQLMIKHDAGNGSTDLTPDKLARFYREVGGNYD
TLFLETKGSALSFIYQSLGCFHTLQPTTNPFEPPCIPALLPTGFVRWQVINLLLCPDEHVRYLQEA
VSRWDVPNPCGGTFPKVIPREAFPQAPDKDMVEWHENVSKRLQQDHADQNSSRRSPPVDFVRYYSA
ATGARKYRHPFYERTHRFQDGSPAPNARDFSDHADETRHHRRRHSPDGRGYRARVFGDGADIRPGS
RTSSRNPSPRQTASPASAARVPMRSKTTSARHRDFASERLSSYRKHRPMNSSDSSNNDDGLLPQRR
HGSIDDRRHGRSTHLSPARPSRPPRRHSHDATSPMAGDSDVSPEKSRKSHRYDRHAAPRTQFHEDM
FDDTPRPGRKADQPLPYEINPNIPNIPRVHVRYDGSPTEPRASKRGSGSSGSERARSSSNGSGGGG
GGGGEASWKRNISPHRNGRKLSHS
>g1239
MTSTTPAVKRRACVACTIAKAKCTPQTVNMCQRCARLGKSCTYLDIPQTKRKHRAAPSRVELLEKK
VEQLKSQLAVLTRQNGQTLPETFTPLTDNDLGSSRTSDLDSADISTLLEVAKDPAHGIHPPTSSVL
SGQLSIVERGLMSEAEAERLVATYRLEFVHRFPFVLLAPSETAVRIRHREPFLFLCVVAAAMSSAH
PLRKTITEEIMKHITLRIVTCSERNIELLRGLLVHIAWYSYPAERHHPRLLLLIQFCVSILYDLNL
HQKPDINPDEHRALLGTYRLSNSLSYSLGRPTGIEHDSRIDERIASLEYMEHLSDRCITPFIRLQS
FVTTMDEVYASVQASGGRTAVQVIRNSLQRQFDSMRALVEVDLSSCPSSTGNTVLIEIKRAEMRLD
ELSLREKLWNTEPASPVRIAMLIGIIQRSRELIHMIRNLPSSEIDHLTVSTSANLCAAIGYMPIAV
LSLVNLISTSSVDSAIEAQVQAVADVAEYPSIVTELANTLEMKFEGMSAADKEADIVGSICTKMRL
LARCYPYQIRAIGGNKHLSQDARQHTSTMAVDANEGIMTQAWPSIYGDLDDIFPIDDIQWDQLLSD
FTGFS
>g1240
MSSYAITGASKGIGLEFIRQLAANTDNTVLAIVRNPKASDINEVASKYPNVHIIKGDVTDPRSILE
AASGASTITGGKLDVLIHNSNAVDSATTALSPSQFPFDAEAVQKMFEPSLVTAIYGGLWATNAFLP
LIEEGTQKKIVHISSAMALPDLINKTGISYAVAYSVAKAGMNVQVAKYAAELAPKGIKVLALSPGW
VNTYDGEKPAEVVQGEEFMLKQFQIADPELKGQIQPEESVQKCLQVIDHLDEKSSGLLLSQNGDQE
RWL
>g1241
MLPPFDYFRYTQIRNTKRRQREARFSSLSPDYHSPISAADKAIINKPIEELVGEVQNGTVSPVDVI
RTYGKVALRAQEKTNCITELLLPEAEQWAISEVDLKGPLAGAVPYAKTALPMTLLSFESANGLWGT
CRNPHVPGYSPGGSTGGEGALLALGGRIGVGSDVAGSVRAPAAWSGIYSLRCSTGRWPKTGVDTSM
PGQEGIPSVFSPMARTLNDLSYFTKSIIGMQPWKYDFTVHPIAWRDDEKQEASRKQLRIGVLKSDG
VIPPTPAIERAIDTTVAALTAAGHTVVEVTPPEAADPFTGFNLASQLLNSDGCVTFNSFRYSFEPT
DPGAAQLSRIAYLPRALRYAYYLYVRYIKRDVKWATVIRGFGPKSAAELWQITAKREAFRRTWHSW
WDSEPQQYDFILCPANATPALPHGAMHDAVSSCGYTFLWNLLDYSAGVVPVSHVDPKLDALPYSGK
RGYKTALKELGQYHAMARGAWMHYDAQKMAGLPTAVQVVGRRWEEEKVLGYMAAVEGALEKAGSKY
TILDVD

>g1242
MANYLASIFGTEQDKVNCSFYYKIGACRHGDRCSRKHVKPSYSQTVLLPNLYQNPAYDPKNKMNPS
QIQNHFDAFYEDIWCEMCKYGEIEEIVVCDNNNDHLIGNVYARFKYEDSAQAACDALNSRWYAARP
VYCELSPVTDFREACCRLNSGEGCVRGGFCNFIHRKEPTPELQRELDLATKKWLKERGRDERSVSR
SPSPEPTRRRY
>g1243
MSFATASVLTVTKSSSENISVTPFENPSLVFLPGLFNLRRTYAWVELMTAQSYPHLDVPSTRTSRP
SNLKSTSSASPVNCNMQQSNLSQLSFPGREPESPATLTNLTITNSIKSDVSLVKDPEFWRRFSVAI
HQDEEQARQPERKKTESESWLARQRKKNHRSKVWGFLIALIIVLIACGAGIAIWLLSKNNWLRKET
PHADHTQS
>g1244
MSSRPSFTHSPLDPFPSYPTDFAGSSNSSPVYRGDTRPIVPFLPSSPEDADTSYEQHRAHLVNMDR
KRRLTATANDDATLHRRPSALSVPSSLAHGRLQQDRPLPSLPVGDDSNDLIPSDMPGSSRENAIDL
TSPPSVVANTSASLSRQHSRSLPTVHQRHRHNYVEYTLPRWQPDSEVTHCPICNSQFSFWYRKHHC
RKCGRVVCAACSPHRITIPRQYIVRPPEPVEQATAEIPPRHRRDSSRAVVDLTADDIRDTTARLSN
RPHLETYPSNPALGGGEEVRLCNPCVPDPNPAPPLNYGAIRSSFDRLPGQDWDATMPTVPISQRFH
QVRRSLSGAQMFGSRRDLENEAENNTHRNQVVAEHLLLLRRLLSRFMASSRAPLAMLPLRVSVIRH
INPLPGCNLIHSHLIDLDLIPRVGLFPLILPAPAREEYDPWQTLTVLAFLVLHTERTLMKEISVRF
AARSYHPVESTVVRMSEKRTFAIVLPDHQGPRPEWPHHNRRTCYACYRLRRPKKIVSVKTARRKSA
LFAWRNTKLAINSPVWNAYANSTSLAFGMDNDPRMRQGNDNTKQNSISFSNRDVFSRPQHYPTSYA
HNQMLEYSTRTEHGPGYGQIKPDSTNEQQHRSASTPSSTGNNAVGIDEAFGTLLNEMSRGLPGDEH
DPDDHLADLKRPRACDACRQLKVRCEPDDKNPSGSCKRCAKANRRCVVTPPTRKRQKKTDSRVSEL
EKKIDALTASLQASRRVDTITSPEGRSQEQEVSATRRWLGGGPPPASLPPASSISSASPGTSTKRT
ASGDTKFSAPPGSLPQSASFTGPILTNPSNSESVVDTANEFTDIIDRGLIDVQTAVDAFDYYVKNI
APGLPVVVFPPGTTMASVRRDKPTLFLVIMAISIGHFRSELQMPLVHEAHRLFADKIVVKGEKSLE
LAQSIILACIWYLPPDQFEELKFFQFIHMAVVMALDIGMGRVTRRKGNKQYGFLRDIMGKNQNRTS
FDPDAVETRRVWLGAYVMAVNASMALRRPLLCRWLPYMDECIEILQTSPDAEPSDRNLIHWAKLTH
IAEEIAFQFSMDDPSSSLTLNDTKVQYALKGFEKQMDEWRREIRTEDYTPILQHSECIISIFMHEI
AMHTEHNIDDFRTPFNSDFKTDVKLDRATAAQIDALTTCLTSIHTCLDCILSMESEVVVNLPTHLY
ARSAYAFIALLKLFSAVSSDNGLERVFSLADLKVEEYFDKMINHLKVSGMRPGGRTASRFGMVMNL
LRNWFLNRKTEKTGATEEPASSRPKEAKEDSQGLSRSATNVSQQSLPTTTDASDKPITPLTNTTSD
PQQWAQYSVSGPETTTAHLLRLQQYSFNQAPGQDIGNPVITTGPLSDSQTDFASLGPDFDFQMPFN
AEGIFSMGGGILPDDVFDLPFDENMNFYLPQ
>g1245
MATDADIEASVLSQLASTKYACSSITRLNGGTANFVYRGTLTDPESIGSTERENPNVIIKHTTDFV
ALNRDFKLDGERCVFEALVLKGLNNFSAARDAALSPPITVKTPQLYDFNPSTHTQIIEDLPDSSDL
KTWLLSPEGMRIDKPAANAIGHALGSWLASFHTWVSHDEQIALRNKLAENKSMQQLKYVINYDRLL
NMIDIYPHILGENRSVFEKVRAHAAQEISTHTSSSASIGDDSWGPIHGDFWTGNILISPNQHKLFI
IDWEMTQSGMRALDLGQVLAELYEIKQFRNIDAGIWIMQGLVEKYHLLNEEQAYRTAVHMGVHLVC
WSGVPGWATASRVEDVVRMGRDIIEKAWERDREWFAKDETLSFLLR
>g1246
MSAIALVAEHLVRRSVSDTPNEPSDPEPKQPFPFMAVILFYITIALLSVVSGCVGYVYGKVVTTLA
AVEDPNPDVYVRIDDDHAPLNPENTGDETADVPPQKPVTSSLKGTISHLTTRAGRLAPFRGLVLYI
LTGIAINIITSPISASFRIFGSAVARFVAEILLANLRVAWIHIVISEPSTKSLWSRIPSWRKTFMK
IAPAAALRSLAAQIVTFVPLTIAYGTKVLEYGSEPAARSPYAALSGGFGLLALFLVLYVLIQXPAE
VTFIRVAASMLPEDDETIVPFDRSFGGKVTPEIVGGQGKIGIVDAWKSFGWGSRMRFLTVVGKVFL
LQAALGLLFGVVLFAEILIFFGNKGFVDVSA
>g1247
MATTPAGRMLTRQLAQMQSDKDIPGISCGLVDDNNVFEWEVMLMISDDCKFYGGGFFRARLTFPQE
YPHMPPKMKFETPIFHPNIYPNGDVCISILHPPEEDKYGYESAAERWSPVQTPETILLSVISMLSS
PNDESPANVEAARLWRDDPKEFKKRVRKCVRDSLGED
>g1248

MGEALDLNLYPSCYNCLSWSDDGELAVAAGDTVHLQLPQWKPKYVHPLFEKEKSMPWTSTKVRTNV
FTHQEWKVMMPTGRDEFSIGAEQSLSSVSQLAWSNEGLGLHRRCVLAVLTSNLLLSLYEADGQRRT
WSRVFVVNKALEKYFESKERPKSISASRIRSFAWCPPLKNDARGEDDRADFRWGEQLLTIATDSND
LVLVRVRRNHSSKVSGWRFSIDVIDHIELGNLKRQYSPVPQGSLWADALNSKARVLNISCGPWIRA
DDQQNGTKDSRLKYHAMIALVLGGSLHLVNVQARMSLEDRQTISVEGQLTHNTTVLSSRSLECINF
TGLLQWIPVDETRVLLVSGYIGGXATLRFDHNSYLKSSMQGKVDEKSVIFHQRSFHDQGYSSMGTM
YSQYNEPISGVVAPTSSTVSHDTRLIYLTTLGRFSEATPLSTIGTLLEETLSKTQTPWLEQVERYR
QRYSIQYELGDMSIVRTWGLATFDGWTAVAFTMHPGDVLEYTIRAEESTIILFTPPEPGAVFPKPA
DLSDERMAMKRDEVLHYTLSSASRLQESNLRSARLIYSACVCAINANYGAQQEISKNDVLLNLARQ
ALTHLSTTFDLSIDEELSYCDIHKPSTDPVLGKTPKPASVLEGPIGWIYEKCRTCFRRGKGDVGLS
WRDQNTTICANGHTWNRCALTFLAIQEPYISMFCSVCDMNVLSRDEGIDYTLVGHEQVAEDENRKE
LYDSLYERYDICAYCGGKYQDE
>g1249
MLSMGGIALLRRQRAGQSLQNGYGFQLSIGSAQKALLRVLSLILPIYAGMVIGGTMVALALSLAYA
AGIPTIFQVEWNGGKLREKLGNKKASLFVLSLFIVLNVLGYDFPVDTQPFRGYIALIVSIFVLRPP
FATPRDSIPTLQKSDITSASQLFDGFMALFGQQASALSXIPPIASPEDIQLTLASGGVVGLFCVLV
APFRAGNIPFGTYDLIKTIFIALAFAFSMTFSSPAGLRSRQKIGFIATALVLVLTSAVPHEESSRI
AHIAWSILAALCYLAAAFDDPKPGEVTPAKIELSSMTKFLLKHGESWPILHSILSKEDSRKIFYFM
CLNFGFMLVQLTYGFVTGSLGLLSDSIHMFFDCLALVVGLSAAVMSRWPPSVRFPYGYGKVDTLSG
FANGVFLMIISLEIIYEAVERLISGSEMRRIEELLVVSIAGLAVNLVGIMAFDHGHAHGHDHGHGH
DHSHGNENMHGIFLHILADTLGSVAVVISTILVHFYGWSGFDPLASCFIAILIFASAVPLVSSTAS
SLLLAMPADVEYNLRDTLAGLDTAMDTGTIMDMDIVTITTTAMATLTITMITTIITIMTIMTTVMI
IVTKSIPPNPKSLA
>g1250
MTDAAIEPPACVSKFSYEGCQIMLSMPTVQAQMHKPRAWQMLERTPSVAQPISSEKQARSPATRSG
CENAPNLPWATKVELVEIYLENVHDRPHSIFHPATLRSNLRSGAVGGALLCAICAIGSKFSTNLDH
RSLESRLTSEAKRLLQADLENVCLENIQASILVATLSSGNYPASSEALFVRIATSMAEILKLDSSF
SSSGTITQLETARRVWWSLYTADRWCFSGLGLPHRIEDLDGSCELPMDEATFRSLSPEQKTLDVPW
KPGIWAHLVTLVRLSNPIQDLNRRVARGNADTAELDQDFAGLEQKLECWREMLPADMQFTIQNLHG
YQHNGLGGPFISLHLAYHHSSTLLYFRFLEDKQNPQSNYCSYVARCKHHASSFSSLLQLSRQLEGC
VSNCPTIGHMTTVSSSVLLHTMLFGDLEELPRARRELSANFEALVELQQCWPATSALVTTTLTIIW
SDN
>g1251
MSSLLEPVTIGGKLSLRNRVVMGSMTRNRCVDSYKPGPSQVKHYTDRARDGVGLIVNEGTFVDWTG
CDWEYAPFMITKDHAEAWRQVTDAVHKEGGKIFFQAWHAGRVQHDQMPIMKEMKGQVLAPSSIKAE
GGKYRDLPGSPGHTENVEEIQEPRTVVETYRRSLVLAKKAGFDGTELLAQGGYLPHQFLNSRANKR
TDTYGGSVENRCRFLLDIVDAASEVFGGPEFICVKLCPTDFLNDSMIDFGEMKQTYTYLIKKLVSH
KVGIINISRRGADCPGNTGDFYSLFPRPEVYPVPKGYDPVLDFGPFVKYPGSPSMLMANHDYTVEE
ADRLIKEDKLDLITFGRPFIYNPDVISRIRHGIPFAENDRGNEIFYGPYQTPDEGYNDWPAAAL
>g1252
MIETAISIAFWLSSCLTVLILALPSQYIPRRAKEGKGDDGKEKVTVQILVLGDIGRSPRMQYHALS
IARHGGQVDIIGYQESDPHPDITSNANISIVPLPPHPNILQTSNKLLFLAFGPLKVLFQVLSLWIC
LAYRTKPAKYLLVQNPPSIPTLALASVVGFFRQTRLIIDWHNFGYSILALKLGDQHPLVRISKWYE
RLFCGWATAHFCVTNAMARVLKEEXGITKAPILTLHDRPASHFVPILDEDERVQFLASLAEVTATQ
AELASGDVRVLVSSTSWTPDEDFSVLIDALCRYSTVATTENTALPRVLAIITGKGPQKEMYLKEIS
TCEKAGKLGKVTIKTAWLSTLDYAKLLGCASLGVSLHTSSSGVDLPMKVVDMFGAGLPVVGWNRFE
AWPELVTEGVNGRGFGSSEELAIHLTDLFGNPKKLENLRGGAQNESLRRWDQEWDPIAGRLLELVK
P
>g1253
MQVSRATSRLALHAAHLPQRSFSTSRTVAKEIQDAYILSAARTPVAKFNGSFASLSAPQLGAVAIK
SALEKSNVPVEKITDVYMGQVLQGNSGQAPARQAAILAGLSSSVEAITINKVCSSGLKSVVFAAQN
IQLGLAEAQVAGGMESMTQVPYYLPRASQLPAFGNMKVEDGLIKDGLWDVYNQFHMGVCAEKTASK

HNVTREEQDDYAIQSYQRAQAAWKENKFADEIAPVTIKNKKGETVISKDEGFESLQIEKLKALKPA
FVRDGTGTVTAGNASTLNDGASALVLTNKELAQQYGSGSRVLARIVASADAALDPVDFPVAPAKAV
PIALERAGITKDQVAVWEFNEAFAAVIKANEKILGLQHARVNPLGGAISLGHPLGSSGSRILTTLL
HQLQPGEYGVAAICNGGGAATAVVVQRLDQVE
>g1254
MSTSARRRLMRDFKRMQTDPPAGVSASPVADNVMTWNAVIIGPADTPFEDGTFRLVMHFEEQYPNK
PPGVKFISQMFHPNVYGTGELCLDILQNRWSPTYDVAAILTSIQSLLNDPNTSSPANVEASNLYKD
NRREYVKRVRETVEKSWED
>g1255
MASSSSPNTIKVVARFRPQNKTELAQGGEPIVEFEANDTCKINSREGNSSFTFDRVFGMNSKQTDI
FDFSIRPTVDDILNGYNGTVFAYGQTGAGKSYTMMGTDIDDDMGKGIIPRIVEQMFASILTSPSNI
EYTVRVSYMEIYMERIRDLLVPQHDNLPVHEEKSRGVYVKGLLEIYVSSVDEVYEVMRRGGAARAV
SATNMNQESSRSHSIFVITVTQKNVETGSAKSGQLFLVDLAGSEKVGKTGASGQTLEEAKKINKSL
SALGMVINALTDGKSTHIPYRDSKLTRILQESLGGNSRTTLIINCSPSSYNDAETISTLRFGVRAK
AIKNKAKINAELSPAELKQLLRKAQGQVTNFETYISNLEGELQLWRAGEAVPKERWTPAISENVAP
KPPRPATPSRLQTELIRAETPSRPDSRIGDRSSTPSIVLEKDEREEFLRRENELQDQIAEKESYIS
NVERSLQEAKEELKNLKESSARTGKDNEKLNTEMNELKMQLEKVSYESKEAGITMDSLKEANAELT
AELDEVKQQLLDARMRAKETSAALDEKDKKKAERMAKMMAGFDLSVNVFSDNEQRLKALIEKVDSL
HRVSAAGEAVAPDDILELRASLLETQGFVRQAELSMNDRSELHELQDGKRVYLERRVVELENEYEE
ILKRNLGEDDVDEIRERLEKLYVGRRDAETKAAAEIKIELARKDEELLQLRQNLADSQVKAATNGN
TGGAVAITNKTLQQQIADFDNMKKSLMRDLQNRCERVVELEISLDETREQYNNVLRTSNNRAQQKK
MAFLERNLEQLTHVQRQLVEQNSSLKKEVAIAERKLIARNERIASLENLLQDSQEKLTAANHRFEA
QLTAVKERLEAAKQGSTRGLVTSMEGAPGLSFGGSRIAKPLRGGGGVDANGATHQEPAAKRGSWFF
DRVK
>g1256
MGNTFAPQTRNIPVPTYLAWVNAWIEVLAHPKQDDTVLSLQEQTKTFIQATSCSIPDYASLKRVAF
SFQRRLRNGEISLGGKAAPSCTETDPASGDYRPVSKCSCRGLYPIPENADIFAVLAQTNCVSVRNT
INALQAVTERENEWSSKSWYTPTALKLAVDELILANSDIQPPPDTCQGPASVTQIPPVLAPDRRPN
PRTDSYQDVHRQIYPLAEGIKFCVDAKYYFVLGTVPNNPAYDGIVRAIADSGNDILIGDYCEVADE
TTLNLLQRTGAAAVAFLKLCVLSGSFSPWAFDNMMSSILHFRVLGYYRDHAREHNLVPPGVYGSRM
KGITAHRCVDLGLFVAVAAASLVTGKTIGVSEKDMLIEACTLINDLIDLRSDTARRQRENVVLRGV
RGNLCEYLDSLIGLCLERSAAAIRTNKTSALVVMAYCNWAVMGSHHKLFELSTQVSKVTNSTGCNA
CVYHSVDNRHQYQNLLSALAPFGTLGQDGPHVGRTRAELDIGFGICRLSEKQHLAWLADVTRSLLE
PLTMRRIVDVVHYAWTGSVGDANYCP
>g1257
MSVLLTLATILTLYGILTALRIGSRDERLPPGPPTLPVIGNIHQVASKGLYRQMKKWGDKYGGVFS
LKLANATMVVLNDREAIQELTEKRGALYSDRPLDEVVSIVGQHNFAFWDANALWRSERKVAAAALS
PSRLDGELGLVQEAEVAMFIMDLLTTPDNFFFHIKRTVSSIANIIVWGFRASSYDDWWGHTVHDVS
YILFKFLEPGSYPPVGQLPFLRYLPDFLSPWKTRASQISQVISDTWAEARRRLEARRLKGDKRSSL
ADRLLDGTQTMDLPLDTERENCFLGILVEGGAETTASSLETAFLFLAVHPEVQRKARREIDQVCGP
DRVPTWSDFSKLPYINCIIKESLRIRPIAPLCFPHRVRENDWYRGMLIPKDSIIIIPTWALHHDED
LYPDPETYNPDRFLGHPGLAPDYAGKGDPMTRDKLSSRRICPGIHVGERSLWHMIATTLWAFEIGP
KTDPITGKTEDLDTSAYVETLNHHPKPYEVDLKVRSEGHAETIKRKAARASEFLSQFAE
>g1258
MTTLKLYPVVVEDCSALADIWLAAFSDPASRVLFPNTLGVRQWLESAISYDLLQRPFQHYVKIVDS
DATTNKGQRLAAYAKWDLSTAEERGARYPAWHADMPAEQCEAFVMRGESNRKRIMGDQKHIFLDIV
LTHPDYQRRGAASMLLKWGCELADSQGLPIFVSASQEGAPLYAKFGFVDRGAGGTTLMVRQNE
>g1259
MSYAGKVVVITGGSKGIGXATAEQFIALGAKVAINYSSDSKAAVEVLRLGADNALIIKADAGSFSG
IQTIVDQTVAKFGKIDVLIPNAAAVETANLQTMTEKLFDTHFTLNVKGPLFLAKAAVPYMPRGSHI
VFVSTTLCHSNTVKPEHLLYVPTKGAIEQITRVLARDLAPNGIIVNCVAPGPVATELFLKGKSEAL
VDEISKGSPFADTMATDGLSTRMATTVKVFLPVANALSARGKIGSDDIDLANGENNLMKLEMVDMC

KKAMSDSITANAFANPVGFVGDMPMKEALAQYFTNYFHPAESVTADNIVPTAGSGNALDALIFTLW
GYYPYARFHAEAHIIPAFVKPSSSLSWATESLSSAIIPAMEAAYSAAPDPKRIKAVLASNPNNPMM
NCWPKDVVRQMMDFCHERNIHYISDEVFANTVFDTEADQFASALSLLKSPNSDKDGVEMKSLIDRG
LVHVLWSTSKDFGASGVRAGCVVSYHPLVRAGVSFATAWQVSSLAAHFTEKLLTSDSLPDLISSVR
FQLAQGFQICREILSTLELSGRIELLHASAGLWANALVTLKKDEVLQDIILKARQNGVIVGEATEF
APFLQKEQGVFKITFAMPRNVLLQGLERLKASLL
>g1260
MKVFATLAAIVSVTTAQATLGYWKLYCGDSCSTGTLINQGNFITNVTTDCTSLGATYEYCYLEVPE
EYQAIYHLSLSAGTACGLPTILGGGECTSAGSWDSYEMVYSA
>g1261
MGQTTSKIDEKEAVAGMGRLSIDKNAYVFVDNDTLPPPYEASDRISTISLDQARQWEEKLLSSARN
RLAISALSQNPVSAILTNNNAAVTDSQIFNIKIPFEGAPITNQRSSGRCWLFASTNVFRVAIMKKY
NLKEFELSQSYLFYWDKIEKANWFLEHAIQTAGEDLEGRLVQALFASPVGDGGQWDFVVNLVNKYG
LVPQTLYPDSYNAKNSSKMGSLITTKLREQALILRKLVATGATDLIAVHKNHFLQEIHGILTIMLG
PPPSPDEEFTWAFYDADNKYTKVVTKPTQFASTLSDRQTVRACGGADVNELFSLVNDPRNEYNRLL
TVNRLGNVVGGVPVRYVNVEMETMKAAAISMLKAGIPVFFGSDVGKFSDSASGIMDTDLFDFNLGF
NIKLGLTKEQRLKTGESAMTHAMVLTAVHVENGKPVRWRVQNSWGESAGDKGWFVMTDKWMDEFVY
QAVVDPRFVSKETRDVLKQDPIVLPLWDPMGALA
>g1262
MENPVREIQTVIKLLTQSPPTLQASTIEKFFTPNASFSHPFCRTWSFNGSRWLIQAIYRFYKILSP
RIDLEVKSVAFDQDNLKLYVTISQIFSIFIIPFYLAPVTLTTVLTLTTDPNEYRGYKDFEKTGRQL
QNSDHSNEETKFDGETTKYWISSQEDLYQTTEFVKFILPFGIGVFAVFVWHVLATAGCVFGSLVLS
PVTWAEERFSLKREVRQASGHNVSEVTGHVVEHYAHDAAGLVSGVAESVQERLKEL
>g1263
MSPKIAIIGGGPAGLTLASILIRNGITPTVFEHDASPEARTQGGSLDLHPGTGQAALAACGLTSQF
EKYARYDDQDFTAVDKHGKVAIQLRDRDVGRPEIDRGQIRQMLIDSIPEGVIKWDHHLVAATEDSL
QFKDHTESGWDLIVGADGAWSKIRPLCCYVPPFYSGISAYTMHLTDASTKHAAISEIVGKGTYYAI
GEDDGMALAYQRNDNDSILVYAFGRRSENCVKNSGIDINDQDSIRATLKKDYAQWEPKLCAVFDEC
DGDIAYSSLYMLPVGLRWPSHKKVTLIGDAAHLMTPFSGEGVNLAMTDGMDLANAIISNPDDIAAA
IAEFQPAMWERAKIAGNAAWRNSMERFEPGALDSLVKRLQSTLEKQGNLQLLELTEQKK
>g1264
MATPRYSIPEEFKDQLRFVEAADARSDDEILASLRKAAPLTSSEKNIWAFWDSGIDKMPSWTRRNV
INWARLCGPSWTIRVLDMVPDSPNNALNFVPPAMLPQAFTKGTLKGPYIGPHSADFLRGALTYLYG
GVFMDVGIILVRSLDKICWDILSDPSKPQQISVPWMYGTTMANHFVAARQHDPFIKRWHDLFVYLW
DGKDIYEGLSSNPLLAFTAKLDFSASRSRGFAWDFQVGPMVVFDYITQVTAWLRLCMLEDAGDGFS
CADYAQKNILWFDALEEDWAAEKVIGYQGEKLFEVLTTRLDEDPESDRYKKAYEAVWRILTNSSMQ
KVTHGKKLTKTAALGVLLDKPENANKDIEPGTFADLLRYGSVHFEQTRSQITIIQTEKPAETMKKG
VFEP
>g1265
MASSQSTRISSLIKELFAIEQESAGYIYENENLRTDAAHLVRKLAVALERPQEVILMNSSMPAQTA
AVRIAVDLNLFLHLGDGPESKDISELAMKTGADAELLIRILRLLSAMGWVDQVGNTSFKPTAITRT
LKESESLQAGVKHMYLSLPVYGKLPKYFAQYDYKSPDDVHGGPFQYAHGIKETTFDYWAKDPIVTK
IFNTFMTGVRGSRPHWVHWFPIQEELINGSSGKDEDVLLVDLGGGKGHDLNKFITTFPDAKGRYVL
EDLPVVIDDAYDRNPRIEALKHDFFQEQPIQGARIYFMHHVLHDWPDDDVVRIFSQVAQAMKPGYS
RLLLGENVLQDIDCHLYPALLDWGMMVLHSGMTRTVGRWRSLLEKAGLKLVKYWAPPGDGDGIVEA
VLEKNIA
>g1266
MKGLVATGCLIASASAAALATRDAASCPGYKASNVKTSAGSIVSADLTLAGAACNVYGTDLDNLVL
QVEYETETRLHVKIYDAAEQVYQVPTSVLPRPVSSNIKPAKSDLKVTIVNSPFSFKVTRKSNGEVL
FDTAGQPLIFESQYLGLRTSLPENPYLYGLGESTDPFPLPTNNYSRTLWSRDAYLTPQYTNLYGNH
PIYFDHRGSKGTHGVFLLNSNGMDIKINQDKNGQYLEYNTLGGVLDFYFLAGSTPKDVAIQYSETV
GKAVMMPYWGFGFHNCRYGYQDVFEVAEIIANYSAANIPLETQWTDIDYMDLRKVFTLDPLRYPVD

LVRQIVSYLHEHDQHYIVMVDPAVAYQDYAPFNDGVDAGAFLTVSNGSVYQGVVWPGVAAFPDWFA
SNTQSYWNNQFSTFFSPDNGVDIDALWIDMNEASNFCTYPCSDPAAFAQSAGDPPAPPAVRISSPR
PIAGFGPDFQPQCVANVNFTVYAETYYGENIYVLGNSPTLGDGVVSGAVAMSANNYPEWQLTVQMP
ANSTFSYEYVRKESDGSWIYEKSNRTITTGDCNTGLQSVSDTITTSSGAQKRGISPVPLVRSPVAA
ALQKRDGSMLGLPGRDLVNPKYNINDTAGSISNLTIQTDLIHENGLAEYDTHNMYGTMMSATSRNA
MLNRRPTVRPLVITRSTFAGAGRQVGHWLGDNHADWDHYRWTIAELQEFAAIYQVPMVGSDICGYD
GTTTDELCSRWVFLGAFSPFFRDHSDNTSPPHELYRTEAIAQAARAAIDIRYRLLDYAYTALWTQT
QTGXPMINPMFFEYPSDLNTATLPYQFFWGDSILVAPVTDENSTSVSVYFPKDTFYDFYTGKPVTG
NGAAVTLNNIAFDSIPLYYKGGSIIPQRIASANTTALLRQQNFEIVIAPNTFGQASGTLYLDDGDS
INQPKTSVINFEYLDGLFSMTGTFGYDTGNVVISQITVLGQNVQKHPVNLKLTGAHVGVYL
>g1267
MADTVEPQVEPYPNEKATHEAVINEAWTPDQLPSGWMYRHRGRIPWYASPAIQLGLVAFVCFLCPG
MFNSLGGLGGGGKTDATLADNMNIALYSTFAVVGFLAGTIVNRLGVRYSIGFGGLGYSIYAGSLLA
SVHENVPGFNIFAGAFLGVCAGVLWAAQGVIMVSYPTEAQKGRYWAWFWAIFNIGGCIGSLIPLGQ
NINVKVAATVNDGTYIAFIVLMVIGAFLALFLVDADKIIRSDGTKVILMKNPSWWSELKGLYTCIL
AEPYIILLFPMMWSSNWFYTYQENAINAAYFDTRTRALNGFLYWFAQICAASVIGPLLDNAKIRRS
LRARVMFVTLIVLTVAIYGGGWAWQKNYDRADVVTDSAPFFQAWDWSTPGYVGPMFLYFFYGAYDA
VWQGCMYWIMGALSNSGRRTANYVGFYKGLQSAGAAVMWSLDKNKLSYKKEWGTNLGLLLGSLIIA
APLIFIRIKDHVEIEDDLVDVDETIEDVVPTVVLEKTEKV
>g1268
MSRAFSSATRLVTSNDELVNSLEGIECTMIESMYLNNAGNLRRAWLANRRAMVMAQMMGLHGSVKS
SSMFLEEKTRDRIDPDYMWFRIVCSDRYLSLMLGLPQGSPEDTSSDALENFMPVDRMERLESIASG
LILQRNRTERTDLVATRKIDKLLLEAAESMPPDWWVSSNFGTIAGNDGKAFEETIRFMMQIAHHHL
LVQLHLPYMLLPSSISSNYDYSKLAAANASRAIITQYISFYDSISAPAYCRGIDFIAFIASTTLCL
AHIESRRQFKDSVTFFQSLEHQRSGDRGSLERLLAIMERMAGESSDVVARKISSILQPLLDIEDNS
FKGSCYQICASEAVEPESRCIGQTKEAVHELRIQIPHYGIIVIEHRPQSHTLPHTSIINPSVLEPI
PSHDVVPGQAAPWAFGSLPGLEFDEHDWALQGVDMALFGNLTGQGLS
>g1269
MEQVSNRSPLKVAVAGGSGQVAREIIDALLAAKKHEIVILSRSDTPVHSIASEIRWQKVDYEDKKS
LVETLRGTHTLLSFVQILSDPDQMSQKNLIDAAITAGVKRFAPSEYGSKDIVNMSWWCGKGVIREY
LEKVNGTENVLEYTLFQPGLFLDYLAYPFKTSKYLDPLQTFLDFANRRAMVVDDHEYAAITFTTVS
DLAAVVARAIDYDGKWPVTGGIRGNRVTFSQIIDIGRRIRGSLDVTVLKVEDLEFGDLKSPWNLQA
VHHAVPDDQAQALLKQVAIGFLLSSSKGAWDVSDDFNRIFPDFEFTSLKDFLTGTWEDKP
>g1270
MGIFSSSRPPPEIIPGDRRIPLSFWDDQDYARGMSLDITLTFNDVLDPEKLRDALDTLLKKKGWEK
FGARLRKNQYLTIKQKGKELEYHIPEQFTEKRPAFLFSTDKHDMTLSEHPEMRALAFGKLEKPTLL
GPFDPLRPYLRGARTPERLDDWIFSDAPQLAVHVVVLKDVTFVTITFIHLLMDAMGFTALLKGWTA
ILRGREDQVPPIMDAGEDPLATIHRMTASSKYVLADRMLKGWAFIVFVIQYIFELVWWRKDEERLI
YVPGEYLKDLRDTALGELSADPSQAAKPAFISESDVLLSWWTRIFIKALNPSPSKPITILNVFDIR
GVLAEMGLLPSADTALIANVTWPTVILTTAGEIVSKPISFLASRIRHAIDTHRTVEQVQAIAATQR
EAKEKTGQPAVLVLNTESHTSRSTSSKQDANEIVQPHSSLLRAALICASVIFHIFDAEDSDGDSWM
EVVEGSPGPSMQNSKASLLHDRDDKVDFTHRTASWVADDSWVSEIEEEYEDVNWNYDAGRGSESTS
TNAEALTLYDSAKDKTQLLDPHHLSNSAHSHFDQELHLDRPEIGDVKRHFNEVQTLATSQPRKGLY
YRLWLCWMYRMEWAKIEHMVQNAPNYIIQVVNYWFGLRNHPFWTASDKCPYQWNVSAFRASSYIET
TYIDEKYETTENSLDTSHRPRRAGNRTSASRLLAVARWTIMTASVVMLATYHFYPIPDSPSADTTS
GPDLCLEPECVHAASEILYNLDPHYKDIDPCVDFDQYVCGGWRDRHDMRPDQGSVFAGTLMAENAQ
MRLRHILEAPEAPEESTLSSADKDNFKKLKSAYDACINVDALKKRGSEPLEEILKNIETIYSSHGK
HSENNLTDALLYLMQTDVSALTDFSISPDDRDPDNVVIFVTPPRSIGLPSREYYNDTKVVDDYRSL
ASKMFGNFNLHHGKSSEVETPFQLAKNVVLFEKRLADATPDTQTQEDVTQYYNPKSLEEAGSLVPE
ISFNHILTELSPKDVKTDRLIIGSPSYLKEVSKIIKDTPREIILAFFKFKAIQRFYSDIEDPKVTP
LREFNNRLAGKDPQATQDRWRKCINDLDSGVGWILSRFYVLDAFPEESKELGDQIISDIKEQFVYV
LDGTKWMSADVRQLGKQKVANIIQKIGYPTRSPDLMDAEEVKKYYSSLDISSDRFFENSIGMANFE

FKRMWSQLGRPTNRDEWGMTAPTVNAYYNPPGNEIVFPAGIMQPPTFYGPRAPLYLAYGAFGAVSG
HELSHAFDSTGRHYDQIGNYTNWWDDKTIEAFEERAQCFVDQYSNFTVPGPDPDSEPLHVNGRLTL
GENIADAGGLSASFRAWKKRDSSHSDKALPGLEKFTKEQLFFISYSNWWCSKTTKEAAVQGIYTDP
HAPKPARILGTMANSREFQEAFQCPHKEPHTFGRVYPPSRYTQSVPQESAGKIDCALRAFAIAVCG
NAELHDRVINPASSARVTGLLANISPPWLSTEQREARSRLPHLFGGTMTTFYDESTDKPATVTEDE
FSLHKPISRASTLAFSNTSSRPLRAQPNALQRAHTVTQSGSTKRAGRAMAADQGRRPHQRASSLLR
TKGGGNESFPQRLLAHDMVSAGRVDNTFAVANVGNNGKLYLRPLVESGSRRRHGKSLPKLSLPAPV
NATKLSPPRPTAGPDTRHSIWSSSQFSELRPKFAREEIAEVNGDASDTEPRRRRAHSFSTISEYGQ
SEAGDEFRIVIDRFDTHDSVNVTSMPALEVPIPHYRLGTMQFNAEGTPVLRSSVYTHTSDNTNPSV
YLKDNTGNGGQNRIPSTYDSPGLSHPVGTLSVAMSLFSGVAPSATKARNSNSTYRSGIYSVQHPIE
PSVFDLLAENMDDPALVKYLPGTSDISAATPARIVAQISSEVFMDYELVSDFFLTFRSYLSTTDLL
GLLLARLEWAINRLQPDGRIIRIRTFAALRHWILNYFVDDFVSNRDLRVQFCDTINHMYDDLKARR
SAGVSDIKILVDLKRCWHGRCLMHWDSPLFTSPDNPALPGEVLDSQDNYAASLCEIANTINEAVME
NPLTKIQTQQFTQSINALENVRNLGHPVMHARTISSGTTRSMPGSSSEQSVQALSCSFPGKSASIP
ISAVDAPHPVVVAIQPSPNTPTGPRSPTASSTFRYPQTHAHKRSGSFSDSIRDDRAPLSRALADRQ
DQEQALLPSSDGGNGSLIRGNLFPPIDAKVAGPSPPSPTISQASDGLQEHHARSASEGNIRPLGAS
PALRTVIGSIRRALHTRNLGQNSAAPPGYPSLRSQRAQTATLPVNVAFKNDIYRDRRIPPKPEAPL
RVDCISEQSVRDYREAIGLKPPTPSISEGSDRLGSQPGHPRPQMLSPQRLASEVTMGSKSIVIVDD
TGLDIPVVSGGLEWMGPTQTSNASSPSLVSRHDHLPPGDIDVPFAASSELLRHDKSHLKPQPLDIS
MERFSKSQSISSRLRKYASFHSGTSHSFDANSEAGDVESGKKPMSRLLRRRPGGDLRKIQNVHDLA
ADQRPQSFATDTSLTESFTSSARHYLRRSFSRSSQARAQSTPPRFDLINTHSSQNLRPSFEAAIAE
FSRIPDDIDGGIESTLLKLEGKWPSPTLSDSQGTAAQPFYYGGDSSNTAMTAASNSERLPMRSDET
IFAGQSGSQSQVYTESVVESEFSDNSIPLLERGLSDDSMKKPIRDVRAHVTPRQSIQHAPVNAIVA
SIDSPRPSIQLIDDNDRIRQITNDMEEYLEVDPHSEQYEDVDSRSELSSELSVEFIEKDDALGASS
SMDSLATQSLEIPHPLAHPPSPPMTIPQAAPTIAHRKPETPVNPPATLTPGPSPTRMGDQYRGRHT
SDEIPSIEPKLAGLHIDIPHMPSHFPFVLAYNSRLLAQQFTLVEKSALDEVDWKDLVDMKWNNTSP
SIVSWVHFLAEKDRKGIDIVVGRFNLMVKWVLSEIVLTRDMSERARTISKFIHIAAHARQTCNYST
MLQIAIALSSVDCTRLQRTWNLVSLSDRRLLDSMESLIQPLRNFHELRVEMETANLQNGCIPFVGL
YVHDLTYNAQKPSQVAGTRDGDALVNFERYRTSARIVKCLLRLIDASNKYNIEPVPGVVEKCLWIG
SLPEDQIQAFSKSLE
>g1271
MDIGDPHAGADFESENEGFSPDGTPKKPRELPADLPTSLDDRRRAPEYAVETEMYDGWQGQSQYLT
TPITAKPLSFSLALDDHSHDEEHELRARYAQPFYEREDDDTTARLEDSDARLLEMLAAQAAHREMD
SLGTDDDDIALDDKLSEEEKKSILQKSLNMAASNGDVDRVRKLVNGHAHQYVDVNKPDEEGAVPLI
YASCFGHQEVVSALLDAGAFVDKQDRNQWSALMWAMTNRHKTIAKVLLDHGASPDIKSSSGGTALD
FLQPGTDISQYLHDNGYNIGPVGIEDDFYDSGFSHGRFEEEMAQNEMKRRMMMEESALNLEVDLSS
LGLDEKIEEPDDFEDDQQEFVWDKCLNDQMFVFQDNELEKILNIIITNMTPQRSPSQKPVPANLLF
LSARYAHYHASPELLAQLLMSATDKINDVVERHQWDMTILAFWISNATLLLHYLKKDPGLMMSTVE
FQLHLAELINEIFILIIRDAERRMNRVLDQAMLDHETIPGLSDVTFQNEWKLFKSKPKPKVEPPEK
RFRPPSPKRRAQVSPRNITSLLSSTVFVLDLYDVHSIITSQIISQLLYWLGAELFNRIMLTKKYLA
RTKAMQIRMNISALEEWARNNNRKPEHYENGSTTTTGDTTVDSARKHLGPVIQLLQWLQCFSSLGD
DHEALITTLLQLQQLTPNQLLQAVRSYRAEVGEKGLSKNATKFLVSLQHDPDLLYREHVKLQKLAI
AASTQPSPSPDDGNKPTSLPPSSPTKTEHPETPQTPTIATAPDQDHDEQTNGPSTPRSSVFLNSPI
MSSPSYHFSSSAPDSYLLDPSMTLPFILPTSTDMLISYGAGFGGTNKERARKYIPTVPTEVLSRFD
RV
>g1272
MLLTLKPLPQNGTNGPHEYSSVRQAVPSRRSPWTTFRASSAPSASSSKQSRDPDPEMDNHPRRELP
PPSAMTRPAESPMPAVGSQGQLPTLDPAYYSEDSMRQWLRTKAEEDRRRQEEERTRQETLKLEQRR
IEQSMLRDAFQAGVPPQMVPLIFVGIGGGNLSMSPDVAQQIDSSIQQAHSWQQQHRAHTVQQQVVS
TQINPRLPPPGTDLSQSLPLDVRRDNNRAIPPNPYASQPAPVRNTSPPQHXPASPTQTQLYRGSLQ
PARLHSFGDSQPQQSAMPRLNTSEIQFSHSSHNIATTQPQPGAAPSYVPVSASAPPPATTKPETQP
TQSPSIYFHHWVPPSQSQPSTPSTRGRQESSSRPHGQGEAHASPGRKRKAVGSHPPPPPPASQQEQ

PSPAFSHASPRRGSTARMGQGASHFRRQSDLSHGSRQEHSESDSDNFSRFRPGRESILNPIQPSSR
RTSSDAPPGQPPGR
>g1273
MQLDQPPVNGLPEEPTSSLTLSPVMETQEEDSTLEEERFADALEEHTVSEEKEEKTEESKRDSKFE
DDFEEVPQIVEIKEVPVMQDVVIKQEDSRGTKQETNNNDNNTQKTQTRPQHEDENTSNDKPVYPSQ
PVLILLITGLCLSVLLVSLDRTIITTAIPAITNEFGSTSSVGWYGSAYLVTACALQPTYGRIYTLF
DVKWTFLHSVAIFEIGSLICAVAPNSTTLIIGRAIAGWGSAXILTGAFVVVAFTVPLQKRPIYTAS
IGMMYGAGAAAGPVLGGVFTGKVTWRWCFYFNLPVASVTLIVVFLFFKSVTGVSTRVGYLQRMTQI
DLPGSALILISFTMLFLALEYNTTGHAWSSSLIIGLLCGFGVTLLIFLAWTWYRQDKALIVPKIIL
RRTVGAACLLAFFIYAVLILHGYFLPIWFQAIQGTSTESSGVDMLTYVLPNSVFSLLTGIFITRVG
YFTPPAIIGCAITTAGSGLISTLQPTTSTATWAGFVCLVAAGVGMAIQQSFTAVQTVLPLEHIPIA
TAAVTCFQSLGGAVFVSIGNSILANEFRQASQANELPGIDIAAVLSSGATQFRSTVPADQLPILLS
VYNNALQKVFIAAIPMAGIAFVSTLLMEWRSVRVPKAETSMIEARNNDEPPVLPDVEVETRISPVT
TRVGFISRSNSSRGSDH
>g1274
MAPTTEPLDLVNFWLSLTWSLVWKILVTFLVLKNFKNFPFAWHIRLLRGYRWLLRSNRSKDGPSPA
QLFQPLILSSGNALCELDYNGHKSNSTYFADLDIARAHLMCTLFSSGVDYARNAPRRTGILGVSDD
LIGLALGAVSCTFRKEVKPFQSYEIWTRVLSWDEKWIYTVTHFVRKDTVRPRSFTLLPQGYSSRQG
KLNDLTRKEDAIFASALSKCVWKKGRRTVSPEIMLQVSGLLPPRPGQEDFVASLRDEPLGKLTVTK
QFEEYYDLPFKLVAKMEDAWDALKAHFSTEIEDVAEEGRIRSGKPPHPIRNNQQQQPREEWTWER
VDQERRRGMEIARSLADMDRLHGEFTADTDALAMRRDLW
>g1275
MSTTRIDAILGPVLLKTSPWSLFDQAWSAIMGYPADEFRFTQEKTPMSTFKETGTMIAFYYLTIFT
GYQWMKNREPFKLSALFKIHNLILTVVSGALLVLFLEQLIPSLWNNGLYKCICSTPGWTDKLVVLY
YLNYLTKYVELLDTVFLVLKKKPLTFLHTYHHGATAFLCWTQLVGRTPVSWVPITLNLTVHVVMYW
YYFQSARGIRVGWKEWITRLQIIQFVLDLGFVYFATWDFYADEWGLDGLHVGRCEGELVAAVTGCL
TLSSYLLLFISFYIATYRKPSARGRKAVPFKKDQAVTATGRAAETLKSARSRFGTATMDSVQSSKG
QWAVSRD
>g1276
MATQSTSSVAPFKPRFMPLMLTATLAAGVISRASSLSTAAALVLLYAVTIPSYVVYWSWIYPYYVS
PLRHVPTVPGNPLYGQVFEIIENEVGVCQRQWHKQYGPIVRYFFPLGTERLSIADDDALKQMTVKN
PYNYPKPVGARKWMTRILGEGILLAEGNAHVHQRKALTPGFSIGSIRTLAPIFWRKSFLMARLWDE
EISRSKDKKTGTFEVLEWLNRTTLDIISEAGFGTDVNSLENPETPLREAYRKVFVFDSLSRLMHGL
QTLFPLTKYIPAKMNRDMEASRCIILGSATKIIKEKQATANPADKDILSLLVRDNLKLQAAGEAGL
SFETMRDQVMTFLGAGHDTTATGVTWTLHLLSKHLGIQSKLREEIRTHYPFLFDLDTREGYDLSTI
DPDHLPYLDNVCRESLRYIPPIPMTVRELVDDDKLGDYVVPKGTTVLVYSNAINRLPEYWGPTADD
FDPDRWDSLPESYTTNAFMTFLQGPRGCIGRKFAETEMKILLCVLLSQFTFARDESVQDPEELKMW
RLVLRPRDGVQLKVTKL
>g1277
MPEPVPSIHAIAYEETAPATASNSIKPISSSENTAASSNDDKTQRETDDPTLDQSNNSTRAPNGNG
LTNDEQSQSVQNSLAIPGFAEDQSLTPFTNIPSIKVDDASSRPSSRWSERKWGVLKSRKSTESVRP
KSANTPNSSGQPSFEGITMSIPTTDFQDLTPSSMEFSKRGSLVTAKPATPAMSLPSSDPGPSSSPR
KRLSNSLRVRQSVLTSRAVSADEDMLSRRVRLMYEKGEDNVTDADVTRAIAEENGILWEEQTPVEG
PSDQLFELNGSTTDFRSITSVGATNSIKRESKELAGGIEDWQNVEAADVDRYGFIVPQIKRGDSAE
PQPIQRVSTSLLLASESPRRKNTFRRSAATVRSSSRSLVSKSPTRDRKMSDSSTNRPSSSQSAYSP
TLKRSPSKFRYATNRLPHNRDRRVRDEAGDMLTLPFEVPEVAAEDTPVSMAMKKKEWEREDKWMKM
AKPTKKKHDGSGMTFEFDTSSSKLIERTWKGIPDSWRATAWYAFLESSARKHEGSPSAAELISAFH
EYQNVSSPDDVQIDIDVPRTISSHIMFRRRYRGGQRLLFRVLHAMSLYFPDTGYVQGMAALTATLL
AYYDEENAFVMLARLWMLRGLQHLYREGFSGLMEALGDFEKEWLGNGEVAGKLSELGIPPTSYGTR
WYLTLFNYSIPFPAQLRVWDVFMLLGDAEQPSSKASSNGTHVSNTRPDQPVSSFGQGLNVLHATSA
ALIDGMRDIVLESDFENAMKVLTSWVPIKDIELFMRVARAEYKVHHHRKKNG
>g1278

MALVNGLYLEEMDSAPALVRSLFDSVIREDLLFQLAHSIRKLPFEARKDTQTIVTHILRYKPNNGP
GDPPAIDYIVHQRPEVIIELCRGFEDPHSALSCASILKEAVKSDVIAALILYDQSNEYEPAVRLND
INAEQPQTGDGIFWKFFEWIDKGSFEVSADAFTIFRDLLTRHKTLVASYLSANYSLFFGRYHQILI
QSDSYVTKRQSIKLLGEILLDRTNYNVMTTYVDSGDHLKACMNLLRDDRKMVQYEGFHVFKVFVAN
PNKSVAVQRILINNRERLLKFLPKFLEDRTDDDQFTDEKAFLVRQIELLPSEPIEPSYSSTSRPSV
NAAAA
>g1279
MSAFLQDAHRGEDSYSDSDSASDSLNIVNGNDEGWEDIEPEEESQPVVGLFSADVYPDVRALLKDT
KERYNFDLVKVQRDLDLDFLGTIRLVNYLRSEIKKGNKEPSVSSSALFDDDAYLKPVLDDDALLYS
LDDLTDDVEVEDGKAPSEKRILELQEELEKLKNQFSEYRLAVQKSLGEQLASVSVDDSAAGPGAPK
KGKIEEADADYFTSYSFNAIHETMLKDTVRTDAYRDFIYDNKGLFKDKVVLDVGCGTGILSMFCAK
AGAKMVIAVDNSNIIQKARENIYKNGFEDVITCVRGKIEEVSLPVPQVDIIVSEWMGYGLLFEAML
DSVLWARDHYLVPGGLMVPSHTTLRIAPYVDSDFLDSHVTFWKSVYGFDMSSMLEHIHDEAIVTTT
KPETVVGSSAVFLPLPLHTITVAELTFLKNFEVTLTEDIPGLDGWNIWFDTFFLPSPTFKFDENTE
PVELKKHGLVAFTTGPFDTETHWQQCVLLANHGGKEPTALKKGQVIKGSVGYQKKEEGSRALDIEV
SWEIDGDKKRQLWSLQ
>g1280
MPLHLLGKKSWNVFNPDNIARVRRDEEQAKAREEEEERRMQEVDAERRIQILRGDRPPSLSPPRLS
TPGEVPKSEHRRSEADYGTQRHKKRRRITGEDDTDRDIRFAKEEAAQYGAKRDDLLSSHREWKGRD
EHISITDSAGHINLFTEDMTKNQRAEKNVEAEAEKHKKQRAFEDQYTMRLSNAAGFKESAGKTPWY
STGPTEEVNASNAMRNTDVWGNEDPLRQERERARIDANDPLAAMKAGVRGLKNAQKERKEWQRHRL
HELEDLERATRSESRQRRRRRSHSRDSFNSLEGFSLDRNNARHLTAELPKLINASVGNVLREASQP
FRQIDAWVGLCIPQDW
>g1281
MATHQYYQPSMAMNMPSKAPVPAGLYPVGRVAVQPADFSDSSTTGGSRTTGYSGTTSSYSGSISGD
YESSTMSGVDVVDMLNERVHGSFDPMPLDRGLATQAQTSGHLNAKQQELLELQALAQRRLKGVRSS
FNEGLKAAKETKRDLEWTSKRVSALKAKAEKAHPEEYRRATSKYVFDDDY
>g1282
MKFFAAAAALAAGANALTIRGESCCFTLTASGETSGTVGQLNDGQNRIGGGLPAATFCITPGGTIT
DSSGRGCILTRATADSGFSVSSSGQLTSNGSPDFVACQTGDNGALNIYTNPSPSDVTGCVHITLSA
DQCATSAISSVQSSTPAASSPTASETSPVSPPASLPASSPTNPSGPGSPPASSQGSPSVVVVTATE
TVCTPGPSTASPASIQTNAPGPSGTSVPCSTDLVSGNFEFPHLIVPIDSSSPGTAGGTQFKAEVTS
TISTIFNFDIPASDAGKTCTLVFHFPEQKDLQTSSFTFSGDGNVYFAELSSPATQTTTFNNAPSVK
TDFGVTTIAPGNSYVISTFACTSGGAVAFELKEAGTTDFTFFEDYNPSPIGLFITTC
>g1283
MMTSSQPDLHRKTIEDVETDVLQACESSLSTHESVEDAEGLDNTAIRNLVWKQDLRIVPLSAFIYL
LCYLDRSNIGNAKILNADTHNDLLTETHMTNYQYTIALMVFLIAYALFEVPSNYLLKKLRPSRWIA
FLMLSWGAITMGLGGAKNTAQVTGIRFILGVFEAGLFPGLVYYLTFWYRVHERSVRVALILASATL
AGAFGGAIAYGVGHLNQSHGLSAWRWLFIIEGAPSCFSAVLIFFFLPDYPETSHWLKAEEKDLAKR
RLRLEGSQGNAKAMTWEDAKTVLTEWRLYAHYAVYFGISTPFSSLSLFTPSITAGLGYEGLKAQLM
TVPPYAVAYVVTISVAGSADYFNARGIHSAIFSFIGAMGFLASAVLPPDAYQHRYGCLIVAASGSF
SCIPPLLGWLSSNVNTTAAAGLAIALNISFGAPGQIAGVWIYKSNEAKKGYPTGHWTNAGLLLFVA
AGCILLQIYYAYMNRRLRRQPGAKLYAY
>g1284
MADKTESAVYTHGHHASVLRSHTWRTALNSAAYILPHLKPDMKILDIGCGPGTITVDLASYIPQGH
ITGLERAEGVLDQARALAKEKDVKNVDFVVGDANGLSYEDQSFDVVICHQVLQHVRNPIGILKEMY
RVAKVGGFVAARESDYGGFVWYPSLDGLSKWSHLYDEVARNNGGEPNAGRMLHSWARQAGLADIKC
STSTWCYQTKDEISWWSDLWAERIVVSSFATTAIDAQIATKGDLENLAKVWRKWGDEEDAWFNLLH
GEIIAYKH
>g1285
MASVLRISSALLLGAALVNGQTTTATTTSIAPAQQTVSSDSDSGVPLNTAETVQLTTEVLQNLTTQ
LNETTLSYFDFGNDTATTTKRSTTGCKVFPGDKAWPIDLLWDIFDLLLGGRLIKTVPSASSCYSGW

GDQNAAECAYVNSEWTDSHFHMEDPTSIMWPLFQGRTCLPTSYNITGTCTLGGYPSYAVNASNVAQ
IQLAVNFARNLNLRLVVKNTGHDFNGKSSGAGALSIWTHHLKSLSYIPNYSDSTYSGPAVKMGSGI
QAYEVYEFANAHGISVVGGEGKTVGVAGGYVLGGGHSPLGSVYGLAADQVVAMEVVLPNGVFTTVT
ASTNPDLFWALRGGGGSTFGIVTSVVAKAHPKIGVTVAQFTFGTSDVISADTFFEALKTYYSYFDA
FTAAGTYVYFWVFPLGNDQYLFEFNPFFAVNHTLDQFTTLIQPFVDDLSLLGISVTPGGTYYDNFY
DAWMSAFPLETVGSVTMLTGSRLFPRENFVNATLLNQTVAAHRYTLEQGYPVLAFQMKADAPADAV
ANAANPAFRTMLMHAITSTSWAANATNSEILSDMDFMTNTILGSWRDTCPDSGAYMSESDILEPNF
QQAFYGSNYARLYSLKQKYDPYGVLYAPTAVGSEDWTVASADGLPDQNGRLCHV
>g1286
MGQVWNYANQLLYNPILSFVKVSVLLFLLRLESRSPVVRYMIFGTMYFTIVLGISILIADIFQCTP
VAYVYDTSIAGGHCFKQGAFYVATATMTLFTDLLVVVIPMIIVYSLQMPIRRKLMVVGILCLGLVA
TGVGAWRLDQLVEAFFPTSVNLDYTYSIGFVSSAIEVNVAVISACCPALKALITRFAPRFLGTSGR
GTYGKSSNTRYGHGTYIRSNVDPNDSYELGQKNYHNKQQTEISGGNSHGGRLPPRSKTAESLSLSD
DDGIIAGAMGSIAIVKTTNVSVQRDRRDDHPEKASSVDSLV
>g1287
MTRLGSVILALPLLAAAQSYNLSALFQSVLSPGAEIYYANDSNWAQDVTQRWSTWNAPSYMGAIKP
VTERDVQNIVKVASAHNISFLATGGGHGVSQGFANIQNAIDIDLSNFKTVDLDIEKNQVTVGGGAT
YGQLYDPLYNAGKEIPTGNAPCVGVIGATVGAGVGLLQGLHGYTSDALISARIVTASGVLFEASAT
NNTELFWAIRGAGANFGIITSATYRVYDATNAGQAQNADFLYPETANRSVWGSLQTLDETLPAELS
VTISSTYNQTSGQAAINVNLVYFGSYDDFQPYVDQFLAINPTQWQNVTVPWNQLIGAANFGTPSRG
CETNSHINFFSIALNQTDPLTFQGFFDDLILFSQQNPSYNGAWVIERYGTQGPLAIPEESRGVYPW
REAKMQLLWESDYPDSSLDGTVRAFYTQQRQHFNEFSGFPTFAAYINYAHGDEGPNVWYGDADNLA
RLSAAKRKWDSNNLFGAAKPVPL
>g1288
MSTQSRLSDITVAVLFQDIEPPVINGVHKPKKPSGYKDSGADIAFTLQRSNVKVLTPQKNPNPLLD
SDWCFSDTEEGILCAIRQGATHIWANTVVFEAHPLQTSSRLQQFASQIYIVGQPPRLVEKFDDKAY
LNDKLRALESYTLPQSWLVNPXTIEVIVRSIKDYPVVAKPVRGRGSHGVKVCYDLIGLKNHLDTLF
QESPTVLVEEYLAGEEATITVMPPSRERGEYWSLPPVTRFNHLDGIAPYNGVVAVTTNSRVVTQSE
LDADLSYYAIMDECRNVAKLIQATAPIRIDVRRFSPGSNFALFDINMKPNMTGPGRPGRDNQDSLT
AIAAAGVGWDYVTLLQTILQTSQTLGALRTYVSPL
>g1289
MAQDGMVSLAGGLPHPSLFPIDRAAFTVTNPESFKDVTVSGNLDLDVNNLSNPKLSDFIQYGNCTG
NARLRQWCLEFTQKVHRPLYKDVETLLHPGNTNAWSKVVGLLCEEGDYILCESYTYPSAQAMWIPL
GNFAAPIAMDGQGIRDDELERILRSWETDHPGIRRPHVLYLVSVGSNPSGVTMGRERRQNIYDLCV
KYDIIIVEDDPYFFIQYPQYDLKERLAADESKSSNEFLTSLSPSFVQLDHQGRVIRLESFSKTLGP
GLRLGYFVANSIFTERLLRTTEVETQDPAGLSQVFVLSLMQRWGMDGYLTWLQSLRNQYQTRRDWM
IKAFAQNFELLAASEVKSHGLKDDDGLVGCLRDASGHIVPIFSFVSPTGGMFIWSKFYLSHNSDYE
QLRKSQEVEDPEQAFAEKLWMQFAENLVLLTPGSYYHPWQGAHRVTTKARGAELATTFFRFSFSMT
TKEDMELGIARLAKVIRKSWKFAASPTASDPKVRVWRACRECRKKKIKCDGKEICHHCVAHSRECQ
YPPSNDNASANRQSVATVDQQCKVLSQLCDKVDQLANKFTVSMETLERLTTETRSLPIIDCADLDG
SRAFPVDAGQDEAGALVGYHEEDETGSSSDSEVDEIGNKSDGHMALDSYGQLRYIGGPANMILVKT
IQDLAATSATERNTNPLELLNTRPGETPGSASSTGISSTLPFFSQGLVWPKLPHLPAPDSVPRPPR
YVSDLLVNLYFSHIHYTFPVLFRPSFMRKYQAMLDAKSSVSSSARFMSVFFAVCACASSLLPHDFE
KIGSFPGLEYYEKSLLLHYRCVGQGNIEQVQCLGLLSLCSAGWNTLTQSWKFAGQAVRAAQDLGLH
VDLHRVTPDPWVLVDCEHGDIDDSAMYHAVGAIAASGSSPIVRIAAAEPYMIKRALDSGAHGIMVP
MCETKAQAELIARASHYRSTEFSTGIRGTGGLFAPANFNISAASYMNHANSNITVIVQIETETGVE
NAQEIASVPGIDMLFIGPNDLCCSMGYFAFDHKRIPQVQEAVIRVRDAAKREGKYAGYFCLSAEDA
AQRAKMGFEFVNCGADIVAISSWMGSEMAKLKDLLASDQQTNGV
>g1290
MTTKIGQNADITKASPTDAPPESASIHEGKFPDAKAVDIVHAAEEEFTEEQYRKLVRKVDLILLPL
MWIASGTQYADKASISTQSTFGITQDTHLVGQQYSWLTSIFYIAFLVAEAPGSYILQKANINYTVS
TCMFIWGILVLCIAFAKNFTDLMVLRFLQGVAECTTYPALLIMTAAWYTREEHAMRAVIWGSANSG

MDILTSLINYSIGTRAKSHPGGLAPWKGISFFLGSLTIVISVFAFFLFGTPREVRWLSETEKRMAY
ARVVKSQTGSDAQKRNISWRQVLETFKDPQTYFFFFIVLINSIPNGGTTSFGNLVYVSFGFTALET
ITEGKIPQQALSIICLLSAGYLTLKKPGLRLYISMISVVPAFVGMLALALLPKRGHLWTRWGMYFI
TIVGNVAGPRTIKGVQYDNKARRYAGIPYALPPTGDHRWRKPRSLPSSYTYTQPDGTPLNASGFRA
VCPQATFHSGAEQGLDQEKYTEDCLILNFWTPVPRDEQQNKRNWPVMLWLHGGWFQMGNPCQELGM
DPTEMISTGKLNAIVVGIGYRLNIFGFLAGEALLQESNGECAGNMGLWDQRLAMEWVKEYIGAFGG
DSDNITLAGRSAGAYAVHAQTLHDFRTKNTQALYHRIFMCSNAISAQPKTVAETQPQFDEVCKHFN
VPSTLTGLEKLSQLRKISSDDLVNAIKNLQHHTFRPVTDGIFIHAGLIDYQTGPEFAQEFKQRNLK
LLIGEVLNEETLYSTYNAPEPNKESLSLQVSNYYAPATTERILQHYSFPKSTDPNEWRTTYGNIVA
DGQVRAPSRSLANSLFEHGVAMSDLWRYQIAYRLSFIDDKVAPLSFGVAHAMDKPLWNFAIVHGPT
PKDREIMEEWVAILVAFVNNDPTYRFGTTSIRELKVLTSEGGAPVSPWISSAIPAVVKNATATQVP
RTTWTRDEVRQVYETPLSQLTHAAAIVHRRFHDPAAIQMCTLMNIKTGGCSEDCSYCAQSSRYNTG
LKATRLSPLEEVLQAARIAKENGSTRFCMGAAWRDMRGRKTSLKNIKEMVTGVRSMGLEVCVTLGM
IDTEQAKELKDAGLTAYNHNLDTSREYYPSIITSRSYDERLQTLSNVRDAGINVCSGGILGLGEAD
SDRIGLLHTVATLPSHPESFPVNALVPIKGTPLGDRKMISFDKLLRTIATARIVLPTTIVRLAAGR
INLSEEQQVTCFMAXANAVFTGEKMLTTDCNGWDQDHEMFRKWGFYPMKSFENEEVKGTKSAVITE
SDSGMVAASI
>g1291
MDGDTAPLEQIAPLVQKYFPERNAYLIVDEAHATGVFGPNGAGIVQDLGVEDQIFIRTHTFGKSLA
SQGAIVLCDLLTKEYLINYARTLIYTTAMSRPSLIAIRSAYELMAEGQTQLFQSDLQNNISYFQSR
CDEMVHRIESPVLQIEYNHRSPIFSIRTTLPRELARYCQERGLIVRPIMAPTVPLGKERVRVCLHA
GNTRQEMDKLLDTIEKWAGRYHQLKAKL
>g1292
MTPVGAALWRQLRAHQVYGANTDVGKTIVSTLLVKALSERSKVGFLKPVSTGPLDEADDRHVKQFT
AGNNNVMTKCLYQFDDPVSPHLAAQSSQRPDDNEILRSVHDTLASWSAKGVKNALIETAGGVHSPG
PNATSQADLYRPLRLPIILVADSRLGGISSSISAFESLFIRGYDVHSVLLFHDDYYQNHEYLDAYF
SKKDIPLLAFSKPPLRPKGNVTLDSRAEDQEVMSDYYKSVQRSFDFDSLLKRIEEKSEQRIEELEK
MATRAHQAIWYPFTQHQGMEPQDITVIESAHGDYFQTLKTSPGADKKQESHLLQSTFDGSASWWTQ
GLGHGNSELALTAAHAAGKYGHVMFAGAVHKPALSLAELLLQNLGNPRLRKVFYTDNGSTGMEAAI
KMALRASSLRYDWNSSTEDIKILGLKGSYHGDTIGVMDCSEPSTYNKKVEWYRGRGHWFDFPTIGM
KDAQWHVSVPDMLVQELGEGEHFESLNDVFDLDKRLQSSTGQRYMKFIKETLDKLVNKYGCKFGAL
IMEPVLLGAGGMLFA
>g1293
MDLNATDIVGDDKRQAEETVKSYSNDNDEGGVSIRRKSSVLNVVVAGLALFSDGYNAQIIGYMEPF
FSVLYGSAMSSTIKSRLSNSYLIGEIFGMLFFGVLIDRMGRRTGIVAATAFLVLGVVLATAAHGTS
DLGMFWMMIVARGVAGFGAGGEYPVCATNATEASDETSELRTKRGFLVALTTDFAIDLGFVIAGVV
ALIVLACYHQATSPGVWRVSFGLGFVLPVVICFFRLRMIDSTQYRKHSIRSKYPYWLILKRYWRPM
LGTCLAWFCYDFVTYPFGIFSSTIIGQLNPNNTTVQNIGYGTVVNCFYLPGCLIGGLLMDRIGRKQ
TMTLGFMLWAVWGFILGGAIQPIQTVFPLFVVMYGIFNALGEMGPGVATFLCAAESFPTPLRGHFL
GWAAAVGKAGASIGTQVFTSIQNSFSSTEKGQQAVFLIGSAFCVVGGLISWFLIPDMSRELETEDV
RFRAYLEENGYRYEDGAIVSDRDTS
>g1294
MDSPQVVAKSLLGIFEKYGQGDYIGESISQLEHSLQAAHQAKQAGAHQELILAALLHDIGQIIPLE
KTKEVRMTLSQGDAGNVGRIGHETIGAQYLHSLGFSETVCRLVNSHVAAKRYLTAVNQSYYESLSS
ASQKSLAFQGGPFRGEELKAFEQDKLKDEMVSLRLWDDAAKIVGIENETPRAHDYLDLMVAHLSRN
ASGN
>g1295
MAASSIYATYPSLSDRVVVVSGGATGIGASFVEEFTAQGARVIFLDIQEQASVNLIENLKLQTPKH
TPIFMQCDVTDVEGSIKPVAARILELFPNIDGLINNAANDARQPTLDITLDQWDRGVAVNLRHVFF
LTQALMPGLIKSGSGSVINMGSISWAIPSTGLAPYVASKSAIVGLTKTLAHEFGPRGVRVNSIMPG
AIATARQRHDILTPEYQAVILDRQAIKRILQPSEVARLALWLVADDSAAVSNQSIVVDGGWI
>g1296

MFSFTTAFVAATLAVGVLAGPVAAPSALTKRNTPNSSGTNNGYFYQFWDDGSSGTTTYTNGPGGEY
SVNWQNVGDFTAGKGWQQAEPRNISFSGSVNSGGNFYLAVYTWSTQGENYILEDYGTYNPCSGGTS
KGSLYSDGSEYQVCLVDRGNNYLQNWSIRQNKRSSGTVTTANHYNYYQSQGMTHNPLSSAAYQIVS
TEGYGSSGSATITVS
>g1297
MPLLKLCGCGGGSSEEEASDPPRYSNGIQGKQSSSVPKNPQDSTPDDESSPAVPNGAAKPLTQKQE
PTLAERLWDQAYNNLRDGQQSKLVREYEKLLSCDLKDGDTDSDTAVLSLDGVENAIDQDNPDRRRA
QMRDVIKVGLXKTEKXADTKRVMGAILGGISPVTDIISVAISSSPQAALPWAVVTTALKLLENPVA
EVASNRTGTAYVIRRMDWYWNLSGILLKKKNVLNDGPSDGLRDILEADLIDLYQELLLYQMKTALS
CYGSQTLNFLKDIIKANDWQGTLTGIQDAEQKFRENSQVYDNQKITASFEKSLELQENQSRKQFRE
DKKEILDWITKADYSTRQSDHFEKRNKGTGLWLMQSDEFQSWLRESGKALFYHGIPGAGKTILTSV
VVEELSTRYYTDNTIGIAYLYCEFDQQDILRPVDLLLSILKQLTEGLHFIPTELEELHERRRNERP
TFADVMKVLRAIISQYSRVFVFVDALDECPVANGNRRDFLLSSLLPNIFTLQDECGINVFATSREI
PEIMEAFDRNGSTNLPIRASDEDIRIFLGDQMNSLHPRVLDRPSLQEQIKNKIVDAAQGMFLLARL
HIDSLKGLSSPKDITSALASLSVGLGKLDETYERALKRVDNQTSNDQTLAKRALSWVTFSTRPLSK
EELDHAVAVEIGSSEFDDDNLSGVDYISSVCAGLVTVDASSNQIRLVHYTTYDYLKRTLPTWYSVG
HKEIARNCVTYLSFDTFATGPCSKSDLKERLKTNELYEYAATNWGHHTRISSVDGEDIVANFLESQ
AKVLACIQVMEPSVKTPLLSNETPQEVIGLHLAVFHGLLHSLEVLLSRRPDIDLEAKEDFDRTALI
VAAENGHADVVQFLIEKGANMEAQDHDDRTALYTAAESGHEAVVKVLLEKGADKDGPEGPLYSPLG
MAVENGHSEVAKLLLHNGANPEGTLHEYGKTALLSAAEWGDADMVRDLLNKGANPNAAHIDYGWTA
LTKSTENGNEKIVKILLEHESVDIDAYDDYAGATALFLAARYGREGIANFLLEKGANWKAENEDGE
TLVDVAAESGNEEMIKRLLEKHGKNLE
>g1298
MTQLIHKIETQSPVITRPIAPANLPCLVLRQSLNPITAFSKLNDQDHTSLADDPAGVVPRYFGKTG
PFNADPNKTKKDGGGKGNWGRSGEEVQDYDYNFTNARRRSNSSNHGLSDFKTKFETVEPEPVFEEE
FPDVNEKQGNGVIREVEADTDANGVSEA
>g1299
MSFSTLVSDIAFRDTAHEDRDSQISRARSQVTARSLASTRATSVSISGDISSQLHAGYSHPLARSW
QAERQLTKEMLIYPLFVTDNPDEETPIPSLPNQCRRGLNRLVPFLTPLVRKGLRSVMLFGVPLNPS
AKDPLGTAADDPSGPVIQAIRLLRAQFPNLYITVDVCLCEYTSHGHCGILCDDGSLNNTQSVERIS
DVALAYAMAGAHCVAPSDMNDGRVRAIKLKLIEAGVSHQVLLMSYAAKFSGSLYGPFRDAAGSTPS
FGDRKCYQLPPGGRGLARRAIIRDVAEGADIIMVKPASNFLDIISDAKELAKDMPIAAYQVSGEFA
MIHAAAKAGVFDLKEMALESTQGILRAGAGIVISYFTPEFLDWLST
>g1300
MATVAQPASISNGTYVQESDQHTEHGPPRFTAVNGKDTTVHNSNAAASTASPPQSNENTAIGGGVN
GNTNGSRQAESQRELLNASAEQVDMYVPQESSPQTTPNGGNKNKRKRSESLERQSQQQDLPSDAQA
ISARSSELGSEPPMQSESSNGAKLFSAGHEYSGQSSTGGYMHAEMKDAQAINGTGGSWQDYDSHLI
SQAQKAQNLDTSDAQLVEALQRETHSADSIEQKPWPTGPPSVTGESMSPYTQEKSQSTVQVGPKRK
RVFSNRTKTGCLTCRRRKKKCDEQHPACNNCIRGGFVCEGYSSRSTWQKPSSSKGPVPLQSKDGYS
EAXNQYLQDVNARHHERIQTTAQQAEVNKVRPVVVEDDNAAAQYLGTPTNVSGSRNAWPKRGWPSA
SHPPYMAEHMAKSTDYREVPPIHELSREGQPKAEYHVVPSIRELSQQGPHPKPGMPMFQGPVDQRP
PHPSTIDTSSPQAQARLALSIEHQLSARHNTGEDTEREKMIHGELYRPFDMQLVEDRDRCKSALWR
FNNACNPLSGVSSKEQNRLLREIIIPAAATTPSGSPAPRPMGSIGQGAVVEAPFRCHYGFNIHIGE
DVMISENCSMVDDCPINIGAHTWIGPNVTILGSMAHANMQERKGSQSRYQGRPVTIEEDCYVGANC
TIYPGVRLRRGAYVAPGEVVKSDIVAYGFQGLKPSYM
>g1301
MSFSVQELDNTVRAFYEGKGDVQKQAQQSLTEFKQNPDAWLSVGEILQESSYSQTKYLGLQVLDEV
IMTRWKVLPRDQCQGIRNFVVNLIIEHSKSEEKLKSERAFLNKLNLVLVSILKQEWPHNWPTFINE
IISSCHTSLSICENNMAILRLLSEEVFDYSQDQMTSTKAKNLKTTMTQEFSSIFQLCSEVLNTANQ
PALIKATLETLLRFLNWIPLGYIFETPIINTLLERFLDAPETRNVTLKCLTEIGGLQIGPQYSYDE
KLVVMFTETLTRVAKIIPLSLDLKSTYMNSNSRDQEFVLNLALFLCNFFSVHLNLIEKLPNLDYLT
HAHFYLIRISQIDDREIFKICLEYWTKLVQELYEEMQQLPITDINPLVSMGVTGLSNGGAPHPSTL

ANYPLRKHKYQEVLSSLRTVMIEKMVRPEEVLIVENDEGEIVREFVKESDTIQLYKTTRECLVYLT
HLDVVDTETIMADKLAKQVDGTEWSWANCNTLCWAIGSISGAMNEETEKRFLVTVIKDLLGLTEQK
RGKDNKAVVASNIMYIVGQYPRFLKAHWKFLKTVVNKLFEFMHETHEGVQDMACDTFIKIANKCKR
HFVALQPGETEPFIEEIVRNMRKITCDLSPQQIHTFYEACGYMISAQGQKGLQDRLIESLMALPNS
AWDAIIAQANQDPSILQDSETIKVVGNIMKTNVAACSSIGTYFYPQIGRIYHDMLNMYRASSQLIS
DAVASDGTIATKTPRVRGLRTIKKEILKLIDTYVQKADDLEMVNQNMVPPLLEAVLLDYKRNVPDA
REAEVLNVMTTIIHKLHNLMDDKVPAIMESVFECTLEMINKDFHEYPEHRVQFFKLLQAINLYCFP
ALLKLDGNQFKFVIDSCMWASKHDNREVENTGLTMCLELMNNMAETDLQTSSIFFRQFYLAILQDV
FFVLTDTDHKAGFKSQAMLLSRMFYFVESGKIQEPIYSPEQAPAGTSNKDFLQEYVASILQSAFKN
LQEIQIKNFVIGLFAYNDDFTKFKTHLRDFLISLKEFAGDNAELYAEEREQALQDAKAAERDRAMK
VGGLLKPADMDQDDDL
>g1302
MDESSSESESSSSSSDDSDTDDDSDMRARRRPVRREGCGHDGHDLEHDHDHEIKKAKRKRPSPNAYE
RQPRNERRPTQQPKAAD
>g1303
MARLNEIPGPAESVEALKRRFVRQNREIARVNSLQSLRIQGLEAELSHLLKENAALKEQVISLTHD
AEKYAAGRAFHKEIHQCKDKLAAKVSELNKLVSDLGLIPESFMKKTAHLEAESETQRSADPNRRPL
TWNDNGDRLPTIMEDKYFPRRTLDPQELAESQDIDSPSIGSPPVAHFDINQSPLARRRHSDFLDTD
EAILPEAEDNGHNPAESVSNINEELPIQEQQVSPAKSASPPVLSGSKRKFSATEDELTFSPLPVTN
TSTMDDDFQFTRAVELRTDRPRVIVEIVNESVVQEKKQPPKKAPAAKRKVLEPKTTNMASSTKKQT
KETAQQAEGKPEKSMNKQGGDENSRRAEPAKNASQFRSENTKKPKRAATATIYEDESIKPLVPTMP
KPLESGHIQKKSFNANESAVTDSMPLPGLGSRPSRRARGAVSYAEPNLRDKMRRATNELVDAVIIA
GGRRTSTTVTGTPGREHDHETATLNNIPEEHESSTDSITATITTQALTELPKHMVTERKRRTLSAS
TNDIIRLNTLESGSTSKQTGSIDDSSAILSPSAGKSASIDNTRRRSANPTAQSRRHSSNPSVSDRG
KTSLDDEISGYDSEFHDDSLEDSQSSIKDIGTIKAKTARTLTEENSILQTKRGQRISTRRRSMMV
>g1304
MAHPTINASLRQSSAYPTTSQQAMALTMRIEAKKAELENLTQLRDLSNALASQMQALEAKLATLKD
GTEXKTAAGVEGLATGENRPATLVRIPTATIDTLRDA
>g1305
MASQHSRRFVRPLLYTSAALATGTGLLYVAYRPRNIPGSEPAVVPPPGYRSGKLVPPSFPRIKSRE
EQIADLRRSAGFGAQNGEEEPYDLLVIGGGATGSGIALDAATRGLKVAVVERDDFSAGTSSKSTKL
VHGGVRYLEKAVWELDYAQYSLVKEALRERKYFLKTAPHLSSWLPIMVPVQKWWQAPYFWMGCKAY
DFLAGSEGIESSYFLTHSKALDAFPMLKKEDLFGAVVYYDGAHNDSRMNVSLAMTAALYGSTVVNH
LEVTGLTKDASGKLNGAYVKDLVAEKNGQEAKDFHIRAKGIINATGPFSDSIRKMDEPETKEIVAP
SSGVHVILPGYYSPSNMGLIDPSTSDGRVIFFLPWQGNTIAGTTDRATQITAHPQPDEEDIDWILK
EISGYLAPDINVRREDVLAAWSGIRPLVRDPKAKNTESLVRNHLITISAAGLLTCAGGKWTTYRQM
AEEAVDEAIKAFALQPRKVQDVPDVSGTGLKTDNFNLDGTCQTHQVRLIGAHGYSKTLFINLIQHF
GIETDVAKHLTISYGDRAWQVAALSSPTNARFPLRGTRVSSLYPFIDGEVRYAVRHEYAQTAVDVL
ARRTRLAFLNAQAALEALPSVIDLMAEELKWDEKRKNLEWKDTVQFLTSMGLPQSRVNITRKEVEE
GKSRILIEGKPSSASTDSPADILQGDLTSIGKKDPGMSPESPVNK
>g1306
MVYYFVSNVVSPSATIYVGKDKFENEELIKHGLESDVCAHVYLRLREGETWENIPKELLEDCAQLT
KANSIEGNKKDNVTVIYTPWANLLKNASMATGQVSFHNPKQVKKVFVAVRQNPIVNRLNKTRVEKF
PDLQAEKEEVLKKQRKEEQRAREDKKSAEKKERQEREQKKWQKEHAYDDLFSEENMMQSNNQDRGD
DFLDDFIYVYHNHLDNYTTAKQQSTMSPDKQSSSAKSKKNKKKNQANKQHADSGKAAENDTQNESV
VEEELRDNEGAENEAQDVNGESKAIADPATEEQRLDEDNDDDNKPPEPETKTSTKPADSEDNSKFD
DLVRDRDSLRAEVTELRKSLEQIQHKHEEEMEALQQKLNESEDKKTQAETQFQKLLERVNTIKSQL
GERLKEDAEELSHARSRIEELEDQNTALTAEVSEKLTKIADLEKDAREHSNELSSLRNRANLSQQN
WQQEKEELIEQESYIRSEFEEAKQAMHNWEVLAMEERSIREGLTEKVNDLEEQLSSLRDEYNSAVS
DNNTQSNTIDGLQRALQEIQDARKQELRELVQSSDAQLEELREKLQQVEKRAEEANTELKQAQAEL
DRLLPFEKEVKEKNLLIGKLRHEAVTLNDHLTKALRFLKRGKPEDNVDRHVVTNHLLHFLALDRSD
PKKFQILQLIAALLQWDDDQREQAGLSRPGTSTTISNITTALKVPSFYVHRTPSTPSLMSDYMENG

NNAAGGKETLAELWSNFLEQEAEAAGNGNKRGSAAGISPQ
>g1307
MATSSVHKYRPVVVSGPSGTGKSTLLKRLFEEFPDTFGFSISHTTRSPRAGEEDGREYYFTTKEDF
LKLVSENGFIEHAQFGGNFYGTSVQAVRNIASKQRICILDIEMEGVKQVKRTDLNARFLFLSPPSI
EELERRLRGRGTETEDSLSKRLAQAKNELEYSKQPGAHDKIVVNDDLEKAYIELRDWIVDGELFGT
VST
>g1308
MASAPTVAAPPSTVFPRSHVGFDSITSQIEKKLLKRGFQFNVMCVGQTGLGKSTLINTIFASHLID
SKGRLTPNEPVRSTTEIQTVSHIIEENGVRLRLNIVDTPGYGDQVNNDRCWDPIVKYIKDQHSAYL
RKELTAQRERYIQDTRIHCCLFFIQPSGHALKPIDIVVLKKLSDVVNVVPVIAKSDSLTLEERQAF
KERIKEEFTFHNLKMYPYDNDELDDEERVMNVQIKDIIPFAVVGSEKNIVVNGKQVRGRQNRWGVI
NVEDENHCEFVYLRNFLTRTHLQDLIETTSQIHYETFRAKQLMALKESSAAGGGHTGGSRPISPSA
DRELSRNSQRVTMNGY
>g1309
MAVRHISALPFLSPFTSPVLRQSRQWSNLYQRAVLLQGLPLLSGYIANEIPSFLAGLWESVLRAVP
KKKTSHMKKRHRQMAGKALKDVKSLTKCPGCGEPKKSHVLCPECVKDIKGQWAQAAIDKQKSKQYL
SWGSADNAHSVDIFSLAVTSNQIISASGASALKIHSTKDTDFPLTQTIEAAHKIGCHHVVTSRNGL
KAASIGFGGEFKIWNNNNGEWSEESALSNEIKEPDTWAICLSEDGRYLAGTTNNGHIKVWDLSNGE
QFRDYETKGAFGACIDMSGDGKYIASGHQNGSIYMFSNETGRMPFSLSGLIKPVRAVAFSPGGKLL
AAAGDSKVIVLYDTESGEQVTQLTGHSAWILSLDWSHTGEYLLSGSFDGKVKVWSIERRTCVATHS
ENDKAVWSVKWLPKIGRTEGFATAGASRSISFYREATGG
>g1310
MSSFEPSLSTSAMRPPLASAEAPSIADTLPSLNFGFEDLRSRMAQFTAKFDAFIEKGRKQVLQERN
QFHSNLAELQKDYDMKQNDIKILTSKTQDHQQTIQREGAEAAEIHAHISSITLERDNRLSKRDRLK
RQIAETQQAIDQKLQAQKAHAQYLDSQARLNVPELEFWQDYLCMRIEGAGREDRLKIVYTHLLEKD
WEKEAWFELGTASRDYEIYHTKPKLEREAIERELDIVNEDRDFGALLKRMRRLFLESMK
>g1311
MDDHTFGLALQYGNPSPATYTSASQEWHFARQLRPSQIISYTGYERRVVAPSAANFPVAREKNKDI
NALLEDYPELITIAPSFAREEELSHTVQEADSHFNPQISSALEFGNAQLSEYVRTRRGRMRTVPIA
AFTSGQNQNVLSLRTIEAEKVVSNLDQDAAFRVPTIGNSKGVDWVFTQTPIRQIKFADSIESPGGL
LAVRLLSSTAILQPIYHREAQLIEPPELFGTAYSRSQLSHIDPCVVAEVDVSQTGGFTHVDVTFNP
WYLKQFAIVDERGHWSIWQLQDIVRLGSGVVPELQHSGLLPCDAVESDDGDSLTDEVKYDTWGRIE
WVKDVNTFIVCGRRNAMLYVMQGTTTTSYGIELNWQSSTEWILDVKSFSSRISLVFVLTTSRILCF
DFKTAGQEAQLIRPQFAWLHDRDADDLTLRLTSLIIGEEYYLLLYSRLDSCILSFKVPVEGYEDGN
YSMSDPFFVYVPSKLSTNGLKTIRYETPIKQILFKEVEQQIFLSKRVALDHCPRFMKLFILDGAMA
IHEYLYSMPSGLSSTEELEVGREALLLSAVSLLPNKKKRKGGRETSVELSDNDELDGVTEMPLCFS
KPVETRPARVSVDFTRIYSLLSSGDWHKLIRPSNSRPEYQSLRDYVEHLITAVTDSAEEGPPFVHT
MLDIIDNPPVLSDIDDSSHEIDWFRSRFAPMESSAETALCFLPLNLLSERSQGQVNVLRSFQPFGV
LVLYEKLVRDWLSKLPRQLPSRIRISKEKLIRQVSVELSLAGTVQIDTSIPDSTSVVSSQSSLPGR
RSSTVSQRNSVNEVTDFSEEHNVITHALLDLRTVSPPSMSHSLGGAIQDVLSFWDVGGNPDAFDLE
RALAAETTDGNFSSRSRSRSKQRTRTRSRSRANSVNARTIRSSPVVPSIQFSGSQPQFDSSKLPIR
SSQFLPSSQITDSMPMTQVERGVFGSRQAGGNSNVKARKKKRAAGF
>g1312
MAEHQYKFNVSMSCGGCSGAVERVLKKLDGVKSFDVSLESQTATVVAEDSLGYERVLETIKKTGKT
VNSGEADGTPQAV
>g1313
MTRLPGRPGPGRAQNSSARTKFRKHKVILESVTQKRKKLRTILSFEAKAPPGYTFIPAGNPTFTTA
CKEFCREEGLRIFTVTVKTTPHQNTHGLSQQVHRVGYHFPSAVVAQKCMELGLYISENGNVVPFEQ
FGTSFPTTDEVVEVPQITINTEARDVIRDLFPRIPDDDINQIIKTAFQKGQRKVGTAVELPLARRA
QLAVVAHIRHVYTDYDKLLKTTSFQEARRSVEDSTLEKLVQWRGDDENGMPELEDVFREVIVISDD
EEGDTEESDFIGNENVASAGRDRGLSVEVLSSQVAADNLDVITNDDSNHIVSPFPIRVGRKPLFSD
YNIGAGVTNLASSKTKVDRRGFSRYQAWDRAMDRYREKSLSRYGESSLSRAPAVTRDEAIITSRPL

HRHPNQVTGRPLTPIVERTSGPSFKGAAYDNRPHSSDVLQHKGQYASQEGNRLQAGPPTQHQAPDI
LHFPDGSVFSKITTTSSHKRNSFPIDVSSAPVFVAGPRLFSEGNNGTTLKRVHPTAHRPPSGNWSS
DSPRGHTIIPSIERRDSPISAGFPLQHKTSDPNMLPSVARGASASQYRRPLDELSKRIDLIDLTED
TLEIPKRRRLESQEAPSNVLRSSYRDEFRSSEAPGSRAQPAPSMKHRRYHESVPQGKRPGVAPIMR
TDPASYFVNRPRQETDQAAYPGNKLHAVPSSIPQVLLDSSEAFNGSGSTPTGRPPVDTLPQYRNMS
SSGDSCVAQPPKEAPPRLDHPVSSHHSRGTGSDDAMWRSKANSYGSQRRLLAAQDPAWLTGSTTVP
QPSLPVSRTHRIERATHSSAHNQENFVEPLVDLTRDATPDTTPHYYGAQRRISSQPPFDGQRKRDA
LPASAVRRYEVQLEKLNPHRYGSAPYQERQ
>g1314
MSDYISSLLIEPILRHARRFSQVPELSVSDDESVPPSPRNYDFDEPAPSTLFQLSIPSLQKLDNDD
SHSVTRSTLLDADLDCAQRRVVACRTSGLNDDLRRTMDDSQSQPVSGNQDTRETQSRLAMDTDIGN
YTDGLSVAGTAFTPSDAISDRYRSSKLRELPEDDGMADLRRRIHAIRDNDASGTQKARLIHSLMIE
SYEAARATFSERPTSSSQSPTRVRSREQSPASSPLRFRRLSNRSFDDLIEQSTIASVRAYYNVSPE
DLEPTFAPKDIDVPTDTTEQSTVANVVPSSGNEDNLSNSDYADEAAQVLGCPHYKRNVKLQCYTCK
KWYTCRFCHNQSEDHALERPKTENMLCMLYRTIESQPMPSEFEDTRAMIYCNDCGAKSNVPYHWLG
LKCDLCESYNTAQLRLLTGTDETTESELALRTRAIPVNILDTSYDVISATSLGVEPFDSADANPRW
RPSTATSTDGRPRQRAVSPTVGNYFEMSRDSTWAASIFSRRRSEGGNEEGEPGFWATSPLRKYAFF
RKHSVSSDEQSDSDNESGTTDEIEDEDDNEVEDDEEDEIDAIEIFGHR
>g1315
MPVPYIDTPRTEIDGNATYLTNGLRSAYRGNLSALDSVENSFQTPSKDGDIIKTLEARRHATNGAT
TQTPRVSGTGARISRNSLGDKRTLPANAPPKGEFTPMMRSVTRNNYLRNMSAVRGSGVPKTPAYLK
DGYRSNGNTPGLPPIDATNIYEDGGTSSMAMDDITPLPQVASSSPNGTPLPSFSRKGGAGGVLDSD
QNMLSLKEQENAINKLDKENFGYNQAALKENTELKVARITMQQEIARYKKGLHQAERDLEAYRLQL
QELREKAKRRQADEAIQREIEYMREEIATREAQVNNLQEELRNVKDRDSDEVERLRDEIVDLETTL
REKDRIIDAKDEEIETLKEDEGQDSNAVAELEAELERAKQQLEEFQDDLEKAKADAREANRNREQA
IEQKDKAEDNLRELQDEMANKSFSTKGLSRQFEERAEDLEKELTQLRDEYNDLKEDYAAKERREEM
LEGQLEEIQQERAVELDSLRDKADSAEQYLAERDEALGRLKEVLDDLDRKTDEKELLQTRHHALTD
ESASLQRELANAQSTIRRLEQEIEDEKQRGMDNNHELRTQHREEIERLSEEIVSLHQEIEDKEGQF
ALEQDRWESAKRTLQSQKDRAEEQAAGFKRTIEKLQDVELTVSGRAAKLQDVIDSEKDRHLQEEAV
LSRQIKELGDDIASKRQIVSEQRSEMLTIKEELRVSRREEEVFREKVQALEDEIAILRASLQEEKE
YSKAKTLQGVPGQEGQLQKVISERQNLRDQLASANVELHNLRTSTAEAEAERDLLQNELNQIQSKS
NGSNDFDHEKVELRKAKLRLENELQRLKEEKTSLVAARKSIEDALNAEIERSTAEENRLSSELVQL
QDQLRASSTARDRELTMAKSQVRQLERRISELEAMAEHQQPVNVEGSSVAADIPLLRQNLDDARKR
EKSLLEREGEHKKSIRELRNRISELEEALHEAQMQKLNIPSPARSSSDGLHEDLRAMRNRLKEAHK
IVMELKTKNRELERTALKAEDRRDLHELLKSSTLEAESLALQLSEKELQVNELKASIRRIREERSL
AIKKSDKTSQECKALQERIDSLMEDVNAKTNRKTRHDKEIRGLGKEIIWLRTRLRREEKFRRDLAW
SKGLMELGERVRAACNEADLRMIAEMGINAPEPKKVYDARRKIRTAGLAVMATVRMQRMSQEWTKV
KKIGESLRRAKNELAMKRDGQRRREGSLSLREISSS
>g1316
MPPMSEEDLEWFKSTFRPIPRPQLPEDAIEYSLYLLSADPAPASTDTVVHTRARLQEVQKSASELV
KDLLKDYIWQREAFSLEIAKENDVTYLRGRTNFGDSIEDEWVIVYLLRELTKKHQDLWVRVVDSDG
EFLLVEAASSLPDWLEPEIADYRVWINNGHLVIINPKPDRKRVTEKITLEESLKIAPHENDRLLRS
SSIEEEAFYRLRKYPEQIKENLHCALVSVPRKVAYLLHQKPAYISAAVEAFYTRDPISLRTLKARG
KDDLLFPPGDFVTVSVRFTRVGYAQVKSQDFPAPVSWAQNMPAPTDVEDFTKVETGMKVSCGFEML
LNDPQNQDKPMVREIKLLLEDLDTGDEVLPTVDEMQRSWSMQQDDESWLDISFEDLDRELKGEGGK
DKKSGAFGDQSAQENLQRIVARFEEFLKDDSANFDGVDLYNSDTDDEPDDEEASSEDEEADLKFDE
EEFIKYMKEMRMDPSGVDLRTRFDKAMKGRVEELDSTDEEDDIKEIEELSRQMEAELRPTGVLNVN
NRDADQNPAAVKGKGKGKQRADLEDFDFDEEDPANVNLVKNLLESLQSQAGMPGPAGNLLGMMGMQ
MPPDDRKSGKSRKS
>g1317
MASPAQCFYCFESLAASYKDEEPPSLAATERLYWNYLQSEQLAAVEEKALAMEEDDDEEPDNNTNR
PKDIKLPSISRLQQDMSSDSSSTTPSSATSSRSMLSASTNVTTPDEDLSSQPPFSADEEYPLFVTW

NLISRHGHSSLRGCIGTFEAQKLSHALQEYALISNFEPCADPMDWELGTHGLRISFIHRNRRYGAT
YLPDVAVEQGWTKEETIESLMRKAGWSGSNSGGSGVARRILRGSNTVGTNSKPWEEVSDFKTTRYQ
GLGASADYSDWQKWRKWVEADKARLDLLLA
>g1318
MPSRKPSKYGNNTKFRSSTASFNPKRAKTVEFNALRSTEATSQDEKFEAIRLANTIDETMGFPRFE
AGEKRAGWLVNMHSTTVEDPNIPGGRAGVDFYFLDDDGNNFKATVEYDPYFLIAVKGGREQEVEEW
CRRILEGVVKDVRRVVREDLQMPNHLMGNRRTFLRLSFANVGNLLEARKLLMPIAEKNKKNVTAMD
TYAEMARTSAGFDLFDDELDESRPNAALNASDFIIDIREYDVPYHVRVSIDKDIRIGKWYNVEAKH
GVTKLTCIEERLQRADPIMMISYMIDGNGFLITNREIVSEDINDFEYTPKPEYSGPFVIFNEPDER
HLLERFFSHIKEAKPTVIATYNGDFFDWPFVEARASVLGIDMYAEIGFKKNSEDIYQSDYSAHMDC
FAWVNRDSYLPQDELDPELMTRYASERPQTLAEYSVSDAVATYYLYMKYIHPFIFSLCTIIPLNPD
DVLRKGTGTLCEMLLMVQAYKGEIILPNKHKDPPESFYEGHLLDSETYVDPKAIDELLHDLDAALK
FSIVVEEKKSLEDVTNYEEVKAQIAERLENLKATPNRSERPLIYHLDVASMYPNIMTTNPCDFNRP
GKTCDRRLPWAWRGEFLPAKRDEYNMIRRAVENEKFPGKHKNAPMRHFRDMQTEEQAAIIKKRLQE
YSKKIYHKIHDSKTIEKEAIICQRENPFYVDTVRDFRDRRYDFKGKQKVWKGKTEALKVAGASNAE
IDEAKKMIVLFDSLQLAHKVILNSFYGYVMRKGSRWYSMEMAGVTCLTGARIIQMARELVERIGRP
LELDTDGIWCMLPATFPENFAFTLKNGKKMALSYPCTLVDPKSFKYETTSDNSIFFEVDGPYKAMI
LPASKEEDKNLKKRYAVFNDDGSLAELKGFEVKRRGELKLIKIFQTQIFKFFLDGKDLGETYQSVA
RVANRWLDVLYEHGATLADEELIDLICENRSMSKTVEEYGAQKSTSITTAKRLAEFLGEQMIKDRV
TERAIPVTIFSAEENVKRFFLRKWLKDDPNDMDPRSIIDWDYYLERLGSRRINTRDDKFKQKKMTD
MFAKNPLAEKSANIVDHRVPHNADIEDMAAQNMDKKLSPSGKMVQKRKLPEGPTTTSLDPYASLPA
VMPSITEDYEGFIKYQKQKWKIQKQARIRRRQLFGEKSKASDSLSHYFRNQAQMLFINTWTLLQLR
ETDVPGEVPRQLFINFKDDSLPEVELPSXEVYLGESEKMAVLLNHPSVEGVYERHVPLTLRAVMKL
GSTCTFDESQRGVLGDGLDRGFELSSLLHTTSETPYLEVFALFSTAKSEANIILLNRNRDMSSLPN
IDKMYMELLGRKIAADSDASTGAFDYQEKIHFKTTQVTTHRKAILEVGDLIKQLRNEESHPVEYPM
LSVKPEVSDTDMPPLGWQSFIAKRLLTHYLYLGSWIQHLKMLARYGDVPVCNLESDDPRYLIDVSY
ARRLEKSNVVLWWSAGPRPDHAGYEKDDILGSLDTVEMPSINVPGTYTTVSIELEVRNLAINTILT
SSIINDLEGSDTLLASTKESADGVLYSEKAFASAGVFVLREMVKQWWSEACTGNTMADIMVQHLVR
WVESPSSCLPTRLLLQTTKQEVGTAYAYSQYILKSIRANPVFHFIDLELKEYWDFLVWYDEFNYGG
KGCQEVVEAESQQLDTVMHWQMNRFLPSPMQQVFTDWVVEYIDLMHGLKRGEEGDEDSSTPRLTQI
PIKSLTEITDDEVTNFLSDKFSKPLKKQIIGLIRQQRDELLHPELASDYNFPVLPGALVDPNTENR
NAVLELVKALMQVLSLSKITTLETRILRRELLALFEVREFSKEGRFENPSASLKLPELTCSACCLI
RDLDLCRDEDILPDQDTDGKKTAPKAWRCPFCQTEYDRLAQEERLVGEVHGMIVSWQAQDLKCSKC
NSLKVSAFMEHCSCSGKWTTTLDRTAIQKKIRVMLSVARYHDLKLLEGVSEEVRNEIAVLKRVSMG
HQNILTLVDYFETMNNLYLVVELALGGELFDRICRKGSYYEADAADLVRAILSAVAYLHDHGIVHR
DLKPENLLFRTPEDNADLLIADFGLSRIMDEEQFHVLTTTCGTPGYMAPEIFKKTGHGKPVDIWAI
GVITYFLLCGYTPFDRDSNLEEMQAILVADYSFTPLEHWRSISQSARDFINRCLTIDPKARITAHE
ALQHPWISNDLPSKKQGEDLLPTVKKNFNARRTLHKAIDTVRAINKLREGGGLMMDGAASIEPRPE
RVVGNDVHEEERYAPHNDDMMKVDSRGNARGQTEEQIREQERKVKEVVTGLWAKAAGNRQ
>g1319
MAEETQGGDVASIKSPTNKPKHELPQTQVGKLWEAFGNPDERINVHPGASYNPTHTPKPKDITISE
SLKTIKLEDFSTFHKKPCARDSLLLGLGAGFGIGGIRGIVGGMSAMWPASNWAVGAFAIASLASYE
FCQRRRVNEMKGMQKAVELMAELKAKKQKEKEQLQAIREAEAEQLRKRKSWTNLSNYKFW
>g1320
MVPGAISYRSPKRKRDIVEEAEDNTPPASPTSTLSVASYPELRLSEGEELGRCSPRTAVAGRFKEL
AINEGVPHTIPIAQWHGSGSTAENPETFGQAESLQLSPKKSLNKTNQSEISKNEVDVAQDQLQPTT
QSINGPSRNKVKTASPTKSRRRQSPPPDDSAILTSLTWDDSEITGHDPTDPNDDGYGINGIGFRPT
AAIAWARSQQRQKQVAEWKNREAREARERRRERREGSSAVKPEHDPGGAVQKKVKFDLG
>g1321
MADYLLFEAPMGYSLFKAVHKADTVGNSLKEVQEGVLDLAKFGKMVELVSFLPFENNKQALSEIND
ISEGVATETLISFLELNLPKAGKKKKVSLGVYDRVLATSIKAAFPSVDCETGDTSEVVQDMLRGIR
LHSTKLLKGLREGDLDTAQLGLGHAYSRAKVKFSVQRDDNHIIQAIAILDQLDKAINTFSMRVREW

YSWHFPELIKIVSENYRYAKLALFVQDKKSLTDDRLHDLAALVDDDEGVARTVIEAAKHSMGQDIS
ETDMENVISFAKRVVSLSDYRKSLHSYLVAKMSVVAPNLAALIGEVVGARLISHAGSLTNLSKYPA
STVQILGAEKALFRALKTKGNTPKYGLLYHSSFIGRAGPKNKGRISRFLANKCSIASRIDNFSETP
STKFGEALKKQVEERLEFYASGAPPTKNEIAMKSAMDSILADMDVDEPEDSDVEMDDEEKPKKAKK
EKEAKKEKKEKKDKTDKKSKEEKKRKRDSEVGVSKKKSKA
>g1322
MSATQLLNPKAESRRRGEALKVNISAGEGLQDVLKSNLGPAGTIKMLVDGAGGIKLTKDGNVLLRE
MQIQNPTAVMIARAATAQDDITGDGTTSVVLLVGELLKQADRQITEGLHPRVLTDGFELAKNETLK
FLDNFKIDREIERELLISVARTSLSTKLNNAIAESLXPAIVDAVLAIYKAPAKPDLHMIEIMKMQH
RTASDTQLIRGLALDHGARHPDMPKRVENAFILTLNVSLEYEKSEINSGFYYSSAEQRDKLVESER
RFVDAKLQKIVELKKQVCGNDPKKGFVIINQKGIDPLSLDVLVKNGIMALRRAKRRNMERLQLVCG
GIAQNSVDDLTPDVLGWAGLVYEHQLGEEKYTFVEEVKDPKSVTLLIKGPNQHTIAQLTDAVRDGL
RSVYNTIVDKSVVPGAGSFQVAAAAHLLSEEFSKTVKGKAQYGIRAFADALLIIPKTLAANSGHDI
QDSLAKLIDEQRQGHVVGLDLSSGEAMDPVQEGIFDSYRVLRNCIASSTGIASNLLLCDELLKARQ
MSRQGGPGPGMDE
>g1323
MTRSTAATIAKQPGNRAGVKRVVLGDLSFPTWYQSIYPEELVTKDTEVLYVCRWCFRYSCDAVAYA
GHVKLCDRRATPPGEKVYEHGGYAVYEIDGEDDKLFAQNLSLFAKLFLDHKSVFFDVSSFLYYVLT
FTDPDAPDDYYVLGFFSKEKLSWDLNNLACILIFPPYQHKKLGRLLMGVSYKLSAWEWEGGLIGGP
ERPLSELGHRSYSRFWEERMARYFLLGPPGGDPEEYIQQAQQKRQAAMKTAASKKNFPPRESMTVR
EIGQATGMLPEDVITALKGMGVVEPEQQTKTTTNKRKNTTSTDGETVIVRKSNVLEWTKIHNVTLR
DPVREDGFIGEWAPEKLQMQEESED
>g1324
MADTVLKPFQIRNLHVYPAEENLYDSSTESVISSSPTDESQIRPSCLIQLSAGEYDKIISDHPNGT
LTYIDDDDGDLITVGSSFELEQRLDESSQYKTMISRDQPEPSPIHLFDIRRRRSIIKLWKDIEKNS
KIRWARLEDEFLEEPKSSEAGTSFEPRGLDVNESTDSRQRWLDACRPPRLQSQQTLEKESPYPTLK
DIDSSSPTIMSLSAITDEGRRQAEEAGSRIRSMWNAPASVSHAAPDFIRNQENPWAASVEPVTDDN
DSNHSSEAQLSNIEVESQPLLSAFEAEMAKILENSQKPVNDANSPXPPPEPQNQXXEPIEQIWPVN
LVTGVLQAVVDGIENMGSELKTRIPEMERHLANAQRTIPDDIGTKMQTALSAFESQVQNLAQIVQQ
ASDASGEAAERVRQADLRSTEQMLNGLGDIAHEFEDFGKTLFAAFEAEFGQRKNGNEADVTDSMNA
SQDANPAQSQAQAQEDVLSSTKVDLPQESLDKASEAAAPAAYQSPVPPAPLSPDLREQLPPHLRES
LLSELKTATSQVLLSDAKDRSVQQHQPKPQLSTSTPRESILSEIRSRATSETSLLPEIISRATQQD
SQHPEIKSRVTQQESLSSEIRSRAMQNNQPQSQQQETEKPLFSSWPNRPAFVPPPFIPTTTRPPVP
PKAVNDKPTPHMDARPAGPFDTRDERSEDMTRSLFMGNIGFEVTERVIEEVFASHLFSAKVHLPID
SSTGKHAGFGYARFPSVSSAKAALARLQGVVIDGHSLNLEHCDNSPIDDLQTQIRPNDSLPSVFSG
NARSTNSRISRPTFAQSSSLNPFDHPEETPEFAARYPSLLPTYKPKNDSLAIPPRSMTLSPNSEMA
RFPPVSQIDAHLLANNQRRDLTETSQDRPRPHSVTGPQQQETRRVGEEIRLRKPRRVASTIHRGST
SQLDAQTSNAHTLRRRATEANTLRRRAHENPFRDPRPVSLYDRPAGSIPGSFPVDDTPAPVTTNEE
SNSDGEFRYAMRQSLIDQCVDHLLKLGYGTYIDGGRDRLRIIAEAANLQVSDAIDMIEEERMAYER
YTPNA
>g1325
MRVWRRSFHTSVVRRYTPSSPLAGNRLRCAELLNATTTTDTTDEKTKAHKYENEEITLKGFIRSVR
KQKRFAFAEISDGSTVKSLQAILTPEQAADLSTGMAIELSGIWTACPAGKEQTHELQTSAIRIVGD
ADAETYPIQKKYHSPDFLRQIPHLRLRTPFNSLLARFRSETIYQLGQVFRSVPQGEFVQVQPPLIT
SSDCEGAGETFTVSPQSSAQAPGHSKGSEKELEYAHFFRNPKYLTVSSQLHLEAYAAELGNVWTLS
PTFRAEKSKTPRHLSEFYMLEAEVNFTNELPALLDLVEYLIRDLARRLYDTPVAQEVLSAKRTGES
GLDSSVDIQGVLRRRWEVLLSQSINWPRITYTQAIEMLQDAQNNHGASFIYPPSWSEGLQTEHEKY
IVDTIFKGVPVFVTDYPQEVKPFYMAPSVGADAAHRDQVTVACFDLLFPEICEVVGGSLREHRLPE
LISKMRDYGLIKHDSKSPAQLQDENDDDEHILALSKNYPCLQPGERLGHLQWLPDASVDPDLLIDD
IVQLGARGAGGIEFLNYFNYGGGEGPAATNWTIYGYATPDYRQVLKAALQAHKDHGMFMDYSMGPQ
SGQGVPAEPDNPGLAWELVSFNATIGENETYIGQVPGWGYGDLVSAVTFAVVNTTNLSIPAIVIPG
EPLYYTVIENTISSDSLIEVTKLISPNGTIQVTASSVSGAESYYLYASYARRSYARACIASSDNPQ

NILQNGSFAVDHFSTTGAKVTTSFLEDYVLIDGIDELLKEVGHYIWEDSVEIPTYVYWTPDLPTVF
EERHGYSMTKYLPLLSGNEGELSAAEGPIRFISDASDQGYGFVSDFRSTMTYLLTTYLGYLNDWSL
RKLGLQFSQQVGYNLPVDMLAAIPSVSAPETETLSFRNIIDGFRQFSGPADLAGKSVISIELGADF
GQAYYQTWTQLLMEAKHSFVAGVNQVVIHGATYSHEYANTTWPGFTSFDYLFAGQHSRHQPAWDVG
YKQAGDYLARCQHVLQTGTPKVDLVFWDKQTAQAAYPTSLYESTDLENVGYTYQYLSPENFDLPAA
YVEGGVFAPQQQEFRALILRGNDTLTPGGVEVLTKYAREGLPVILSGGISTIWASSDEDAIHQAEN
SLSSILQLDNVHQVSYTGLASFIKAIGITPRAEISSNGTWYTRWKEDAEGETYVFIYNDGNFSTGY
ISFETVGVPYLLDAWTGGKTPIVEYAEQDGRTVIHFALQETETRIVVFSASSKCGNEAEHVVSSSD
SVLGYTSTSSGIWAKVAAGDATASVTLSSNETQLLHANPQPAFSLTNWTVVIQQWLPPVNLYDVES
LANKVNATFSVPGAVLSSWSDLGLPNASGVGYYTSSFAWPDGKANNTGAYLVIPPVAQGLIVTING
RELPAVDITNPTTDVSEYVVGGKNTVLITISSTLWNSLIPIWEDLLTGGTGPGFTAADSEALGYGP
QSYGILGEVQIIPYQLVQIK
>g1326
MFSKALGLFAALSAVATALPAVHNLNNVRHHAHSMHRHLMARQESTNITSDGVQIVNNMEKTVYLW
SVSDVSSDMYTLNSGETYSENWRTNPNGGGISIKISFSPEQTNVLQYEYTYENPTIWWDLSCINMG
DNSDFTTMGFAVSSTDSTCESATCAAGDVACAAAYLLPDDNWATHSCESSDLLKLDLGSSIL
>g1327
MAEYGTYHSSGYAGAPGEDPNRQQSAAPAPYYNANAPPGQAIQHPGIPPYGAAQPPQFPGQPGVGY
GPGPVPSPPQALGGPALGGLATQMEGLGISSDAGARGHKKKHRHAHHDIGGPNAQGLNTFASQTNL
QSQFLNTGLNQPEQQPAPSAAFPGAPIGQTPPSAAPGVAPEMGGGAGSVPTQGKIDPEQIPSVPRS
RDVPAQYYFNNVYPTMERHVPPPASIPFIAHDQGNSSPKVARLTLNNIPSSSDFLQSTGLPLGMIL
QPLAKLDAGEQPVPVIDFGDIGPPRCRRCRTYINPFMTFRSGGNKFVCNMCTFPNDVPPEYFAPVD
PSGVRVDRLQRPELMLGTVEFTVPKEYWVKEPAGLHQLFLIDVSQESVNRGFLKGVCDGIINALYG
VEEQAEETESETRKVPEGSKIGIVTFDREIHFYNICSRLDKAQMMVMTDLEEPFVPLSEGLFVDPY
ESKAVITSLLEQLPTLFSRVKSPESTLLPTIKAAISALQATGGKIISCLASLPTFGPGKLVMKDKS
QAPDGENKLFAIDNPDYKAVATKLTEAGVGIDFFVAAPGGSFMDLTTIGYTAAISGGECFFYPNFH
SPRDSLKLSQEISHTVTRETGYQALMKVRCSNGLQVSAYHGNFLQHTFGADLEIGTIDADKALGVI
FSYDGKLDPKLDAHFQAALLYTAANGQRRVRCINIVAGVNEGGIETMKCIDQDAVVAIIAKEAASK
AGDKTLKDIRASITEKTVDIFSGYRKNFSGSHPPGQLVLPENLKEFSMYMLSLLKSRAFKGGSETA
DRRVHDLRMLRSIGCLELSLYLYPRIIPIHNMSAEDGFANEQGQLQVPPALRASFSRVEEGGAYLI
DNGQGILLWIHSFVSPNLLEDLFGPGITSLQALDPNTSSIPVLETHLNAQVRNLLQYLSTVRGSKA
VTIQLARQGIDGAEYEFARSLVEDRNNEAQSYVDWLVHIHRQINLELAGHRKKEDGATSAGEGALS
SLAGIRAPYW
>g1328
MALNSTVQYPEGVVVEDKSKATLETRPPDDSRPSGDMATIHDDDERLLARIGYRQELRREFSRWST
VSYAISILGVLGSVPATFSYPLAAGGPAAAVWCWFIGSCMAMCVGSSVAELVSAYPTAGGMYFVTK
HVVPKEQVAIFSWIQGWCNLLGQTAGVSSVAYTVSQMLLACVSMNSTYEDSGRYSYSPTAAQTVLV
SIAMLCVMGIICSLTTKTLHRVILWFAPINILATIAICTALLYLTPELQSAKWVFTTVTDGSGWGS
KSFSFLLGFLSVTWTMTDYDGTTHMSEETHNAAIQGPRAIRTAVVVSGIFGWFLNVAMCFCINDLD
AVLTSPTGMPAAQLFLNAGGRIGGTVMWFFVILVQFFTGCSAMLADTRMTYAFARDEALPFSKFLA
KINGITHTPVNSVWFVVFFSTILNLIAIGSTETATAIFNVTAPALDISYISVILAHRIYRNRVQFI
EGPFTLGRWGQPVNWISIAWVIFISIILFFPPTRPITASNMNYAICVALFIALFSLSWWWLSARNK
YIGPRTKELMQSVPEDDYDDVDHRNSTDV
>g1329
MSSPRIAILFYSMYGHILKLAEAEKAGIEAAGGKADIFQIPETLSEDVLAKMHAPPKSNYPVATVD
TIKDYSAVLFGIPTRYGNFPAQWKAFWDRTGGIWAAGGYWGKYAGLFVSTGTLGGGQESTCIAAMS
TLAHHGIIYVPLGYKTVFPMLANLDEVHGGSAWGAGTFAGADGSRSPTALELEIATSQGKAFYETV
SKVAIGGN
>g1330
METYHGLVRTPADAIILFEACRLGLLPRVQRRLSEKERQAIKSGSVFVWDEREAGMRRWTDGKSWS
ASRVSGSFLTYREMEGKRGGGVSTSRSEKTPDSRGNSDDDRDGSEDGPDGYRYKPDGLMKQSFSIT
TSAGQHLHLISYYARSHPTAPNFNQPSTDPALRHIRPQKGLYPESTVNDQQNLPVVTRGPMPGTMT

LSPHMHMAGFPRNGATHPQSYAPLYSWPPTPSATPPTATVPYHPGQSYLPPLGGPSPYGQLPPHPQ
AVAGPPHPAAHHQLPTPSERSLHLDTTLPPPVASGTVSAPGYPGYNRSPRSGPMSIEAQRVHSPSY
AIKANLPGQTDPRLMSPRSQKQPGSNNENRASPRQPITPNQSHLPPPQGHEAPATGATVPSIVTFM
NGPATSPYSKLNGSPSSLRVEGPKDIPSDKIGFGGEDMRALRQLDRVFTA
>g1331
MEISVDQDAPPPRKRRKTEGDIPKSLFWFGQGPGVTQFGVGLLQPFSQATNPHIDYTSLHLPAIIL
SPAKTTMSQSLPDGSNGPQFQGLQMMHTDPHSVARFASQQKTHEPLKTEQYQNPGEYPYNDDIAAK
KSLAFGSACSLLPPPRVHVRSSSAFPELNDIHVESISAAALFDKRRRVNSQPTLTSGTKESPHEYA
SLHSAGGVWDRCQRPSEDYSSEGSSERAPLLSRSRQLDLGMAASHSRKHRSLGLSTDDGVNETLQH
IGKRKGLXIDQYPYQPCILSSNVYRMLQPGADKIANEDQKAILTEKLMDGILSGKFDPDVMFSTVH
YQKRVQAKIFLEPESAVCFNGVHNMLQLALAISAIKRGDCAELQGLFDQGLRPDLWTEELILRPLD
VAASVGNEAIHKVLIANKSPVSNSRGFFEPWSNILTTAARCGNVEAVEEWTRAIQSMNYSYRDWMG
NAICLNKPYDTSMALIEAHHSAARGGHVEILKLLGGRIKHMSAENRFHSFVEAIKVGQCDVVEWFL
SQGSVDLQMRTIPNEIFSPTGRTLLQRALEQDDSETARLKGADRMYGGLCEKKNLKERDIGHVEKN
LISLGWNDKELRDKVLECFALNFEGIRNSK
>g1332
MFTFTPLLGAQFSSSRASQSILELEGGIKILVDVGWDETFDVLELAELEKHIPTLSLVLLTHATIS
HIGAFAHCCKMFPLFTQIPVYATGPVISLGRTLLQDMYTSAPLAATFLPKASISEPGASISAAAAT
AATASAEGDDRSSKKLATSGRILLQPPTGEEIARYFSLIHPLKYSQPHSPLCSPFSPPLDGLMLTA
YSAGHTVGGTIWHIQHGMESIVYAVDWNQARENVVAGAAWFGGSGTSGTEVIEQLRKPTALICSSK
GGDKFAPPGGLHKRDALLLDMIRSSLAKGGSVLIPTDTSARVLELSYALEHAWRDAADSADGDDVF
KKTEIYLAGKKAHSTMRLARKGGDAATARGHKRSDSQSRNAGAKPGTKLGPFTLKHLKIVEQKRKL
EKILADGIPKVIIASDTSLDWGYSKEAFRTLAQGSQNLIIILTETLPIRYQTDDPEQPDKMTLGRMI
WHWYEERRDGVAMETASSGELLEQIHSGGREISVVDVERTALDADEQGRGDANIDSTADALDDGAS
STSSEDSESEQQGRILNFSTSLAHTNRNKIGVRDEDLGVNILLRRKNVFDYDVRGKKGRERMFPYV
APKKRGDEYGEFIRPEEYLRAEEREEADAQQQESGPQSEMKLGQKRRWDETGPNGRRQSGGAHKRQ
AMSPDGGLGGDIGGIREDLAAGSDGPDSESENEVDEQPFEGPAKTVYRHSTVTVYARIAFVDYMGL
HDKRSLEMLIPLIQPRKLILVGGMKEETTSLAAECRNLLAGKDVGDRSAVVDIFTPKNGESVDASV
DTNAWVVKLSNNLLKPPEIVSAEDEVIESTNKKQKLLETDPGTVSTPVGGVNDSSISKADAYPILD
VLPASIAAGTRSMARPLHVGDLRLADLRKLMIAAGHRAEFRGEGTLLIDGMVAVRKSSTGTIEVEA
SAQSSASDRRGQAGSFLAVKRKIYEGLAVVAGG
>g1333
MGLFNKSTALVYTALLLLSSTLVAGIQYCHTGADFCFAATTQQNISTESHNVYLTISATPSKKGGW
TGVGLGREMKGAVMFIIYEDQESKSLITSIRSASGHVMPKVVEDADQRVKVLQATVSDDKYLAQFV
CYSCWSSDVEIDGLSPYIFAGNKEQAFNGADKYSSLMYHDFKGKVWGDMSVTAKDTTETSAPDIQG
EETIGITEKSPEPETKKPEGFFTAVRVHGTIMTFSFMGLYFAGSVAIRSPLIRAFKYHWMVQAGAS
VLALGNGLYMFLRSKHFGPHKIIGLIILCSLTIQAAAGYKHHIDFVRIRRQTIFTLVHRWLGRGIL
LVGTMNVGLGMYYRHWSSAGLFTWFVIWCVEIAGYGYVLLRHQRRQSQQRAHAIPKEDLEDIADAE
VFDIGDDFDEDEDGVEAVPLMERQNKHEKKR
>g1334
MADIALSDIPVTRTSTSKVHYQNAVSESAYQSDRDTNLLSTDRPTTAASLSSLSSDADISVWQLAN
IVLAVSGISFLGSFTTGLLTIGLPQTAADVGLSEDLILWPSSVYALASGSCLLLAGSIADVVGNRI
INLIGCFLVGCFVTACGLSQTGVQLIMFRTMQGVAVSLCLPTSVAIVSQSVPAGRKRNISFSVLGF
VQPAGFSAGLVLGGVMVNTIGWRYGYYISGGLTLALFVVSIWSLPADRPGAAGKISWARLGKEVDW
VGGIVSCACLAMLSYILAALTNDVENIKTPGNIVMLVICVILVPVFIFWEQRQERLGKPALIPNSL
WRNSVFTNVCLMVMFSWAVINVMELYCSLFFQKIQGLSALQASLRILPNVVTGSICQLTTGLLVHR
VSAFYLVVGSTLLTAGSPLLMALINVEWPYWYDAFFAQALSPISADILFTVALLVISDVFPPRMQA
LAGAVFNTVSQFGNSLGLTIMAVISSVATKHAAASPADDQSTGPLMVGYRAGFWAAFAWMMCAFFI
GGYGLRKIGKVGLKTD
>g1335
MLAMHSHQSSVQNPSPVVNKPSVAATSVTSGAGPFRCAPTSSSLFHSHVHAAQSGSAIESPSSSDD
SQSHSLNDDTSDELLNDWHHQDEHHLSSANAYRMRRESDQTSPSSATSATFADPQSNNVSDAIDPN

SPDALAPKIEELDDDGEVLMDLKPGDDRPADDVSNAIASLPRKRGRPRKHPLPMPGGQMKVAKGRS
KTGCITCRRRKKKCDETKPACLNCQKNAVVCEGYPPKEIWKSGKQKMADAVRRTSISILPRGLPIL
IDGIETDVDRRFLDHFVFDFSRVLTLINDDSNPFKEILLPMATQHRGLMHSLMCLSGSHLSARDPE
PRFKERKHYHFDRAITDLRDSINAAPLAEDAQEPSLLVEDPIIASTLALCLNTICEGETAGEYRSH
MDAARYLLLNQKPKNEKFRQFIIEFFQYHDVSNSLTSLDRRPVQLTGDLRLPDFVPHAQAGTFLGI
FDGLFNYISEITRLRDRIRVRHSEGYEPAVDYQILSEAVTIDSALRIWEPSYTPETPNWYASQLYR
QSTWVYLYRTIRPSRPSDKISQVVDDALKYLDLLPQDAGAYSIMLMPLFLIGCSAFLPRQRDRIKK
GFEAMKEYSSLRNIEPALRVVERVWEVMDTKMEESWDWEKIIEEMQMDFLIT
>g1336
MAPKFRDGDAVVTLNGKWISYAHTLFAAFAFIGAFVVGVQLHFHKIVQNEHFGYPDEWFPSVSATI
GDRYPERSVFQVFIAITSGPRFALVFLWYLMTARPGSTLPKVVAFTGVFRTLTCGGWTYVTSTDDH
DWHDIFMISYLVATLPWTLGCLALSPANRRAVKYRKVLASLFFGTLVPLVYYFIQHKVHKVAGAYT
KYAFFEWSLIIFDVGFDAVTALDFDAFELVVRDVKGVSRGQLKTTADNVIEKEKDKPVGNTFGTGF
SWPEAFDVAADVYNGFVFWSNYTAMPLLVWYFPLWHMGISGYEVIIVTTISPLILAIPSVRSLVVR
NRALFQFLPLTGLMAFLVHDPVYRLFITGFSVAMGCLVWSATFYGERALNVRLESRISAFAIGLVM
SNVAKFAFQTNNPVWPTMNAENGGWNKFGLFLGVLAAIRSARQPSVSGGDYYPPSGKKGSAILAGV
GIGGVLFGVHSLLSDSSTMISWVWEGFPIRGPIQVPHGALTIFVMGAGYLFGLYNPRLAGSWTAFG
VGSLGAAVLTCYSHWFGFYGGLTIAFYLMAVTPVLFASAVHHSPASTFGTGFCVWVFLTLFHVWVV
AYAFVPGGPLVRERTDWLMTITMLCVGAGVFSGSVTNSGPSKKNFVNPNGRRQRSYYVYALAVLQL
LAVAIAYLRFPTNDYAPYHKEDKVATAGIWTIHFSLDNDMWSSEQRMINLIREAELDIVGLLESDT
QRIIMGNRDFTAVLAEELGMYVDYGPGPNKHTWGAALLSKFPIVNSTHHLLPSPVGELAPAIHATL
NMYGEMVDVVVFHSGQEEDPEDRRLQTEYLSKLMGSSPRPLILLSYLVTKPLEGNYNTYVSDVSGM
KDIDSTDWDRWCEYILYKKLKRTGYARISRSTITDTELQVGKFVIGQPESEEEVRIPEEMVPEGQR
FPALFRGEGVRGHRYHVFDEPRYFQ
>g1337
MAAEKARFYLEKAVPELKEYEQKGIFNKDEIRSIAKKRSDFEHKVNTPGVTESDFVRYIEYEMNLE
ALRKKRTKRFGIKATPQHAGRRVLFILDRATRKLPGSLGLWIQYIEYCRRQKMYRRLSDVFSDALR
LHPANADLWVYAAKYAMEDHADMTQARSYFQRGLRFCKSQRNIWVQYGRLECIYIAKLFARRRILG
LDESGKNAEVAGTAADDDTDMIALPAITEEDINPTGKKDGEIDEGALETLNSTPALTGAIPIAIYD
AAMKESGHNIILSHEFFNMVFEFEDLPCLRKILDHIVTQQTTKSPLHYRTAICHIKAPVAGLKVTS
PEFPQALGISLSNMKKYQLDPNLAQELIKWLEPLSEKEELDPALHKVLEITLARARKTPI
>g1338
MGRPRKKQKLDGDINDNPDGTERNLGAISEIEKDWFSADLPTDLPTICPQVYLSHYNGVVPAKDPP
FSSGSTXPHPPQLLEPIAATLDPWPDFSTTSAAGSMLSNVPSSAATGDDAPTCPCLSYLYLCLSTL
SSLNSFSITRETTTSLYTAARTAQSVIRCEICPLRFATGMQNVMMLGTLLSVMADAWYKISLVDAG
SLGRELASPEFKQLVINNNKSLHADEELTWDHWLRRFLRRAIIGTPMPPNLCPSSVACSITPDLYS
LIRELENRQRYWHSIRISHPLDPFDTSRAEYGSLPTPPLSTRTCASEKECNADTISTSCSTMANKT
LGEAASVSATSSPPDPAELQQNAQEHDFLCLKIVGAAKRVIRKFGFKPDEYPEDVPPIPLSE
>g1339
MSSSEDEAVRRPGRGGRAYNGDVSDDDNVNISAPENDQADDNDDKDLFGSGSEPDLDERPHRSLDD
SDLDSGDDEGRYDRKEDRMDLGEDEEEGFGEVKVMDLSLGRAPEPRSTNADVYTMEMPNFLSLETE
EFRPETYVAPPYSTAATSLCWRYDPNDQTSLQSNARIIQWEDGSLTLQLASNPKEQYRISSKPLAP
LNKAGNYDPTLDTHTYLAAAAETASVFKITSHLTHHLKPLPTNAETDDAIQRLQENLIAAGRGGKA
NANGNGIAIIDIKEDPELAGKRAEAAERLKQREDRKRQQYADREQNRTARRGTYRPSRGGLTAAGL
EDDDGMFATRPAKRRHRTNRHGEIYSDDEEEHGRRRQDSYEVDDFVADDDEELEEVEDEESLIDDE
MDAEGEDDDQEIERPAPSPKRREVAAEGGSGSPPTRKRNRYRVEDDDEEE
>g1340
MATFFQSVRQGFGRGGNNKNANQPKMNAGQGQQVAQAQQTVPGAQGLHNSPSIASNLSADIVSNNE
DAPKYFFQEKYAPLNVKGNFLTLCACPKNVELGEWLAHQIVEQYRLLHGMLQVIQEVNTVNGYPIC
NESTCPTMSAGRLTYTWLVDGRAAKISAPKFINRVEKWIVSKIHDPVMFPTDPVQGAPHTFASGDV
STPPATTPIAAGPTNLNASLTTLAGQDWIGKSSGFPPTFYKDCQGIMKQMFRCYAHLYHGHWLDPF
WHINRHEMLNMCFVHFVTVAKYYKLVADKEMEPMQPLIDLYMKQEKIPPEAIAGHWASQEAAAAGT

PAPAANAPPS
>g1341
MSSEAQPISPSAFAEAIQELPLSSLYAKVSELRNSISHLTRSNEQLRTYIIESDEGPDSPDNKELE
TYVLENEEVMQAMAERIRLLKAEVEGRGQRWMEEVLEDGEEEGEYFDGEIIESENPPLVNGVQSHE
TNGDTNGHVVPRVNGLTNPSNPQNGDQERRNEDDDDEGIYL
>g1342
MGNRILSILHGRRLQGTLDLNLPADIKRTVRPRLIEHGLQWLRANHPIDEDAAILARIEREEKEEE
ERLLRYVQELGPQSGHWNAQLGEGGDIYGRSTFQETRKVNEARLLAEEKKKRKEWLEGEMKDREKI
EKQMKENTELQKYNPSAVVEARPRADPQERPFLAWAQKHYIRAENTDSDFSTLTTARRILPALGVT
LLVLGLCYYYAETYQPPVYYKRMWKDLPPAAATIMGLMAANVGVWLLWKLPPAWRMLNRYFISVPL
YPYAMSVVGSVFSHQQFKHLLTNTVILWLIGLRLHDEIGRGNFLSLYLSSGVIGSLISLTSHVLLQ
RLTVTSLGASGAIAGLVAAWCMLHSDDKLVPSFLPHDWRDSISANGSTVLAAIVAFEVFNLVSPFK
VVGKLDNYAHLGGYFAGFVWAILYKSKREKERQRRRNERGFLDRFGSV
>g1343
MSTEKVQEPAPASESLIAKDTNGTGQAEGLDKLGYVQELTRNRSLFTLLFQTLAIAAIPYGEGGPL
MSAIYGGGPLSIFVGWIVVCILDECVALSLAELASRWPTSAGPYYWSFQIVPQRAKVVLSFINGWV
WLIGNWTITLSVNFGFASLLSGTISMYHPDWSANDWQLLLIFYAICLASFVICTFGNSFLPMIDTI
CAAWTAISILIIMIALSVKADVGRHDASYALGHYDTSLSGWGGFTFFIGLLPAAYCFSAVGMISSM
AEECANAVVKVPQAISLCVPVGGIAGLFFIIPICVTLPPLEDIILAPAGQALPYIFQQVMGSPGGG
LGLIFLVLAITLFCSISITVAASRTTYAFARDEALPLSKLWAKVNSRLGVPVWSLVLVTVVQMLLG
LINLGSSSAFTAFVSVGVVALAISYAIPIGASVFHRRQEVNNAKFNCGPVVGLVVNIIALLWIAFE
LVLFCMPTVLPVTAVSMNYAAVVFVGFMAISAVWYGIYARKTYKGPPASDGL
>g1344
MTPKRVRTVEGSCWACKDRRVICDLQRPQCSRCTSEDQACEYGKLRLRWCNGVAARGRFAGQNVPV
SPTDVDFNEEKSMQRDEFRKSSVNGQVSPSALTPPSPSPSLSLSSPPLFSPRETSLDLVKSDDGDE
EQANQFPDYKKACQFFEAPSRVTADDLMLYFEHIVIDRFNLSTEPVLVDLVAVNNYPALRYSVTAV
AKAHQQLSTCPGHHDAFLSKRSARHKAIYNFRTQLENPTHHKKSRRLLPLDLFIANVLLCILDGVI
DPNDESAACHVHYRGGRAILSQWKLHQQLLQRHKRGLPALMLSCFATMDLTYSLLTGEEQYFRDTT
WAHFAGSDGWWGVLPPYDPFLDIMRNLSRLAHLGHVLHRGGEVAPFSDELEALTMALKGYTTLTDG
LEPLFMTQQNEFFAIKSSRNTQAPLAPHLLVPMNSCAPQLDSDESWTVFCNAYRLAGLIYIYRVFH
RLDASHPLVQQTTSYGLRAICESRLKGKLAHCLLFPALVIGAHCRTTPQQKANLLAMRSTAAFLNF
GSLRVMETFLQGLWARNPSSYEEEGESESWWGCFKPIAEKTFLF
>g1345
MASYNEQAQGQFAPPAQNYETNGTVSAGSVDVIAGPLLNYRRMDVNDTSATYYGSILIVTTPGQAQ
PHLSLRHVGPVSQASANGQFGSQVPSSYEVDKLYEDPVKAFWRFQFSFPLTEYEARWAYAIPGFKY
VNESASNASEWTFTVPAWNQSMRIMFHSCNGFSVGTDTDSWAGPALWNDVLRVHEKRAFHVMIGGG
DQIYNDGIRVDGPLNDWTNIKNPIKRQAHDFSEKLRAECDEFYYKNYVRWYNHEPFKTANGQIPQI
NIWDDHDIIDGFGSYTDHFMRCAVFRGIGGVAFKYYCLFQHHVAPPKSTFTTDSTEPIDPRQLVDT
FVLDEPTPDPRWIMGKTPGPYVEERSRSLYMRLGRRIAFAGIDARTERTRMQINYPETYDLIFQRL
HQELSQANGEIKHLILLLGVPIAYPRLAWLENIITSPLIAPVRLLNKRFGVAGGLFNKFDGQVDLL
DDLDDHYTARHHKVERRELIHRLQQFSKDHSVRVTILGGDVHLAALGRFYSKLDLNIPVENDFRFM
PNIVSSAITNKPPPKTVANLLARRNKIHHLDHETDETLMTLFDSQPGGVEKSAAWNKVTMPSRNYA
CITEIEXPATNGSTAQPPSLAPSTAPKDGHSPLHLGEEGTGTSHPAADGYSNTSQLAGGLDVAIRV
EIEQGNRDGNAQGYGLSIPQLSVAV
>g1346
MILDVVQFWNYRWMLWLRSRSPKDQLSYALSRADTFEEWEENAFRLDELLSADLWRQNPTSKHYDY
RLILQRLESILGARESEDILTLVSILRSGLVRNLGNITSMKLFNHAYAGTKLLIDDYITHVALSIQ
YVTSLQTIPMHAFGRTSLVLQGGTVFGLCHLGVVKALHLRGLLPRIITGTATGALVAALVGVRTED
ELLDFLDSDILEFCAFNGEAAKGASKEKLIAGKTQYERPRIGFGWLRRYFTEGNFLDESVLEDCVR
TYVGDMTFEEAYAKTKRILNITVAASGKGAFPNLLNYLTAPNVLIRSAALASNASSGTIYCKDETG
AIVPWPHTQDVTFQSWRQVNLSGRELPLARLAELFNVNHFIVSQTRPYIVPFLYPDTXPGQRLSNH
ASLDQPLMRLIMLEIRHRLKQLDYLGFLPMGLRRLLMDENIPGPSLTLIPDLTSSDFLRLFQAPSK

AGLDYWVRKGERGVWPAVSALRVRCAIEIELDRCYQMVRRRRRRSDVSQQPVVVPHYRHGANEGVP
RKRRSIGHDCVD
>g1347
MRYIHSEETLPIPENGKLRFLFFGFDGCVCEGEVSSTVRMLGRKDXIRDDQEYGVVTDFTNFTVNI
RSRIVTVEGPRGKLTKDLSHISVSFGRPDKNTISIELHHGARKGVATLRTVRTLINNLIIGVTRGF
LYKMRYVYAHFPINVNIEKNGETGNFEVEIRNFLGEKIVRRITAQPGVEVITSANVKDELQLSGNS
LEAVSQSAADIQQICRVRNKDIRKFLDGLYVSERTNIVVE
>g1348
MRSQRRLRLYVVVVVVFLVAFTYFTTDTGGGIQSHRFYKKTVTAIDNAHTATTKKNNEAKEAKDRS
HIQRLDLENNNNNNNGGTRKTTNSPPSSYNENQEKPIGASHGKIEDSEEISVAGRTKMSVPKSKED
QSQLSEKKPEVVDKPAGSKSDIATAHEHEISKEEEEEAEASAELDSILKRSPIIVFSKSYCPFSKK
AKSILSQYNIVPAPYIVELDKHVLGPHLQKLLGKITGRRTVPNVLVNGVSIGGGDDVEALDRDDEL
ISKIKSIAGKSIMEIERLPVAEEHENEHEI
>g1349
MALSRRQLLLAGFSGFVSWGLVVNWVPVARYLGYAFVAGVLVTCVCICSAILLTVKTKSEFIDRRT
TVPNLRFLAADTWSEEIGVYRKATEYEAKSLYPQSFVVSEAIDELLQLASRDFIRSWYGHISKNST
FINEIDRALRIALENIRDRLLSEDVTSIAVSRIVPILTAHIKDFDRAERAVRGRNLARSVTEAEEL
GLAIANKYRDGRLHPAVAVSLADQKATQQDYVRKLVMGLLPQLLPDNLLTSRSVSILIREIVACAV
LLPVITLLSDPDTWNQILETYGRTALNDRKTVRKLRAALDKHASPSKPKGAPSFPRLSPNDSERAF
ERFVRAIRRCNNLSDARQFRAQVASQLKRESMVEGQDQVYLRRLETGKRVLDQKVSKLAANASIRR
PPASLKPEVRRDSVPNHKDVSLVDLMHDASGLSYFMEFMDRQNKMSLVQFWIVVDGFRNPLEDDFG
DESTAASAAWTSADVSDMILINENYLSKPELNVLGESRDAVKAFLRAGKRASPEQYLKARTAILTT
QSTILEEMESKYYPKFRDSDLFFKYLASDETSLAPTEQETPLRASTPEPQQRERRPLPPLMTRTQS
QPSMQKPKDLRRAAVSNDVQSNGKLFDDVESSPRRSVDSDRSARLFDDDYDTDPLSHSFSMSSQNG
DAEAKNTEVIENMEQALNEIIVDQPNNGKPEDSRLFSPMSSSSLFPDAEQSSARGSLENFRNEVRL
GEKNKPSISSLGLIGNSSRTGVFEDELFPDEKKFIEDEYADPEGEEKDPEDEIHQAAPGDLGLTEA
IVALTEEIEKLNAQESVLDTLTRKAELTNNTAELRILGKSKASIQREIRRKEMQRQQYVIQESDNS
LYGRSTIHIPSIMVGKEEDGNEFAIYVIEVRRNAGEQMPAASWAVPRRYSEFHELHQKLRMRYPSV
RQLEFPRRRMMMKLQKDFLQKRRVALETYLQQLLLLPEVCRSRDLRAFLSQQALLPHSAPASDGDT
KDIVSRIYNSVADGMDDFLGNITMLDQLSTAGQNLISAATSQLTGPEPALATEDAMTAAEAEAELN
AFEDRELEPFIKPICDLFLEAFELNRGNNWLRGRAVVVVLHQLLGGTIERKVRDSAKSLIQDDNLL
RYLTLAKDTMWPGGVMRKPVVRTSSQKSKSKNEASFMLAALIPDLAGNVVGRANAQAASRKIYDIL
NNRRLNAHLVFTILDEIVLVLFGATDPGRSRQQSTV
>g1350
MSSITDYIDLSQPSLQISALAIAFNPIFWNIVARQEYKNHFLTRIFGSPYYGCYFLAVAIFSLGIF
RDHLYLKALEDQPFYEPVHQPYIAYGLFATGNVLVISSMWALGVTGTYLGDYFGILMDAPVTGFPF
NVTGSPMYWGSTLSFLGTSLYYGKVAGLLLTLEVFVLYWFALQWEDPFTAEIYAKRERERAKVAKS
S
>g1351
MAAQAALIEETIIGLKKALRRENENSGPDEAITQPTNRGNKLRANAKYVHEGSLGFSNPRDFYKQK
IDHAGYTRYILHRNPPRFDSEGEEIDGDEEPDTEAEAEAAEDNPFAGIALEHILCPLKQASELPDH
PTLSQPYRSKALPTMIKAIDEKLRRERALLARAHNLHRLFLGDSVWMSCGAVESKEDPFIFRTRDA
EASHQQHRDNGSQIARSHGEHVDADDTTQPGAQSSDKNGGDDVEMVEAPVSEQDKIAENGELKQED
VPSSLRDVSRHSEGAEADSTARENNSSDNGNLNNKDQGPDTTQSSPHALGAEGGDTAMQDASESKS
NEGTSSPEPPRRMTTRAQANASTSNENGATSSSSRYPSPDAATQLSTAHPMFLTSDEIRPDKDFGL
PSNEAEDTRRLLWSYIQKQEETVRNFTYMLNSLLRADHLKEDVFEWCKAEGHIGEMSDGEDWYDRE
KWGLAEGEDLKKGADDDEVETAVVEERTTGKRGRRRQ
>g1352
MDAYPDDYIVHNLPLVLLSGIAHEDDESIPNSQYPSLQERGTTIESDFPLLTGPLVESLRTAFIDH
DASDAPWRPSYDAGRSNGIPLRSYRLPPQKAEFPSDVDFSSSSVVPVVHSPISPLTPGSPTFPDGI
LTPLWMLKHQDLIPAATINVFPLTSDPKLRDLKDSWNASGYRSRFVVILVAEDGISAQEVEDRLAN
IRRATNLDPRSLHVLNPDLSPVEEMEAAQRNFEAAYETLFGQEVFESIAGWNPSQPTAAVRFWLCH

KTNTEDIVSRRGKGTKNYGWEAWVARWSAVMAQLISRAELPAFSAEFLQNPERLYDSIFHPPEKGF
SSDENLLAWEYLHHQGYWLDRSARHTVRRRELAEQIPEEHRVAADQAPASQAVAQSQFYDTYLAPE
PAAEAPNPDGSGFDHNSLILQTLKASLQHFAARDQQWQEAYDILKPLWRHLTWRKGQWWDLMSEFA
WTLRSCALELHDIDILIWVHWELMSKALPSRATWDYNFHQCLDGLSVGSPKPTIVLKAEDVVTCLS
TAMVFQKGEGNVGQALQLQLAITSHAHQHAAPLKLSEVKIVFEGGLRPITLHAKDVTETTNDPSSQ
IHNLKLRDSATSTESSTLLSPTGHMASMIGQADLTFNPSETRVFNMTAIPREPGESRIASITLAIE
EENFNFLYVITNHERDRSFWWRDGPRGSYKKRIGKGRDTASCKILPKPPNIQIQLPNIKQHYYTNE
RVSLTVAIQNNEDDAAEVAMQIRVLGQPDSPIKISWSDDAGDTTEFGGVSVETTNHLKSRSVGTLS
QAAISEIPVILSNTVDALEYEVEISVNYYLVSDPETPISKSISANLVFVRPFEANYDLVPRIHPDA
WPNLFQVDIDSDGDQTDRPDGLQQRWCVSPKIVSFASEPLVIEGVRVGLLEAHGGALCEIGPERLT
TPEAAQILPEELRASEFILNVQKTSLDDRRSVTLDLSLDIEWRRLSSPDQDENTTTVTSLAMPRFM
IPMGEPRVLASASPSKETPGLIYLDYTLENPSMHTLTFSLTMDASEHFAFSGSKTRALQLVPLSRH
RVRYNIFAFKSGMWIQPHLVVIDTYFNKTLRVLPTDGMRADKKGILVWVDAYDS
>g1353
MSAKSVASESGRSTHTFSTYYVYHEGEEHFLLSPVRKPPKVKKKKNKADQDDNADPEKGKAEAECK
EVVADEDCYFVHQPYVSFHSPPRTLRRGNSKHGIPICLMQNSWCWKRWKLQFGERLADVIDARGVV
SWEYNGNDTEEDKALQGYKVRSWRLWGESGKEYHREVNEMRKREALGKESHSPSLSNACIPTHPVV
EEVHYLDWLSPMSKDVRCYRFQYAGLDFYWKGTHLESDGAFKAVFLRHNHLKLVVRLPNQTLLQQE
SNSEESSELCLAKFTSSIDPEMTGTLELFESVITGRLQHHLLSVGHTTPIDTHDVLIATAMCMIIG
EWQKRKWVKGVAWGLIGAGVESADP
>g1354
MSITSPRDSDTIAPDITAAKNLDQGLSYELVSKAKFQAYHIIRPGPQATSIYYIETHETLKKNKFD
LVLHEGHSKDGEVLGVGKMHLQGFTIGLGDPAREIEXKPMIWERLERLEKYRHKVYHFDFGTSAHR
MTYTYRKTYGVLGRLKSMELRSGGIGKEDGELLAKWVGSSSWTMKAGSLFIANMAKSSQDDNAHLD
RDAKKWEIMVCLTAFAIIESQHRRSKG
>g1355
MSAFIRAIHLLQTTAIEHTSESPSGLLISRQNADTEPLSSSTKAGFIAVGICGLLSFIAALGLLLF
LTYRFIFWKRYYKQPLAHNQYVVLIYNLLLADIQQASAFLLCLHWVSQNEVYYPSAACALQGWWIQ
TADPGSGIFVMAIAMHTGAVVLRGRQLPFSVFVYGVIGIWGFIIILGIIPVSLYKSETFVISEAGW
CWLSPEHETERLWGHYFWIFLAEFGTIILYGIMFFYLRRRMFQAAILHQNHQKNLKRLNRVVIYMV
IYPFTYILLSLPLAAGRMSSARHIVPSRAYFAVAGSLMALSGLFDTAIYTLTRRQLLVDTNGSASE
GQYNAYSGSHVYETHISTVAGGGKQKRTNARSKLKRGLQTLNDTVNDYRNESSEEIVRPDEWEMRN
IDGGVYQETTIEITHERADQEEDLHNHKQGEYRG
>g1356
MATPKLDHVLTLRAYGVPIAVPAGKVRDGPLRTCHPLNGGFLRGVDGTETEGLSVELTEGGSDWIS
YDPSTNIAHVDVRTHGTDGAGDGFYIRYSGYLQGEEIGQKFLGDWSAEVESTNYGDHYWWTHAVIE
TGSEKYKSLTTTFLVGQGRWYAGEGKRAVEYKFFKVSNGV
>g1357
MAPNEDTVPPELPRTVGSQDANTSRRRRSLPGRSTSVPRQRPATHAPPMPPASPEIISSLISSLSS
ISTPARSHFENLPHIGAQTAPSSPGFHQTEFSQPQGPGQGFGVDYGAYKPPSDAFKSPFLHPDDAA
LSPVVRMAPAPTPSSPRSPRSPKFKTSFGRDRSPLRPRSTSSFSSSQAAMDEQSLSGFGIITTEPG
TRVSRTGSVASTSSDGRKSLRSPLSMLRRGSKDSWQDKEAERSVKSTAHSDNLKPNFARSRASLQS
KNSMADVAEEGGTLNPAEDLSFKSGKVTTDTNALTQTTPNTPLPSPGGIGSGRIIPTRESSLRHSM
NNKPNRKSRHSRPPEERSVKERSHVESKTSEARAATEQTTKRIQQVKEQQKRIKNELAKAQEVPAG
RTSIETFNSRAKTQPVAEPASARQSQDDALISRYLIHAETDLFEESAPSPAIQTRRTREAKDKKRQ
SLDKSDIPLSPSVSKAHKRHSSGAFTMATRPSIAEERPSSADSIDFAVESYIASPRLTQKVPHPRS
GRMIAFSEVGDPQGHVIFCCLGMGLTRYLMAFYDELARTLKLRLVTLDRPGVGESDPSGEGEGTPL
TWPDDVAIVCNHLKITKFSILAHSAGAIYALATALRIPQHIRGRIHLLAPWIPPSQMTNLGTHKEP
LPNNAVPYSQRILRALPTPILKVANSSFMSATSSSITTSLPKSPRRNKRKSFGRDTAAPTVTELTS
PTPNTTANQAKFDRNSTLTTATNATSTPLISHDQRNSTMSQASALNRPSAAEEAAILASQQKERQT
EYDTRLTYRIWELATTNANPAVDLLICLERRQTIGFRYVDINREVVIHHGTKDTRVPVDNVRWLGK
TMRRCEVRVLEGEGHGLMASAVVMSNVLTEMAKEWEDWTTVVQGRGRGRRTTVSHHYPSSRSGVVA

V
>g1358
MTEELTRIDSAVAGLSISPKDEKTQTKTKTKTVEKEKKHKRAGSTVEGVRNIKDLEKDQIPIEIAI
ETQKTGWKLNTSPSTIDDKDILKEFLVTPPVKKIDLHFPLGLEVTARNLKGVTIKDALDAIHKQFK
KKADDELGENPYLAGFEWDPEESWTRLIVHQKKAGAPATTSKKSKKKDKAEAEQ
>g1359
MSGLFGNVGTAAATTAATNTTGDISKDVALNSPPEDSISDLAFSPVSNHLAVASWDKKVRIYEIND
QGQSEGKALFEHEAPVLNCCWSPDGTKVVGVGADKAARLLDLGANATTPVQVAAHDAPIRCCEMIP
NPTNSSQPLLITGSWDKKVKYWDLRQQTPIAQVECQERVYTMDVKNKLLVIGTADRYINIINLDNP
TTFYKTMQSPLKWQTRVVSCFADATGFAVGSIEGRCAIQYVEEKDSSSNFSFKCHRETPPGSRDVS
NIYSVNAISFHPVHGTFSTAGSDGTFHFWDKDAKHRLKGYPNVGGTIPCTTFNRDGSIFAYAVSYD
WSKGYTGNTQQTVNKVMLHPVTQEEVKPRPNTRKPR
>g1360
MDTDILDNILSLEEQFYKEGYDLGVIDGARAGYTEGSVFAVEKSFEKLLQLGRLYGRALVWNQRLI
NSGVSEESKTPKVEEREHAITTDIVTSMPPLHQGSRLAKNIQTLLALVDPATLVLQNTEDAVSEIE
ERLKGAALKVKLIQRALGETAFGDGTAADASAVGEGRGGGDGSGSIEDISSLHVRH
>g1361
MWILRVFSSATYLTAIVLSIPLAFDVGGKTCGLAYSLSLASFYFLFSLLKLATPDTSRFRRLLVIL
VGSTQWLIVPALLIWSLNLFSVDSKSRSNANRWVDKYTTHSSYAKEVNNGVWSGVSTWIFGPGGLV
ESFAIGSWDKLLRWSSPFFQLVEGFCSLLVIQAAGQITRWLVNRSGGGDTWMISLLVVSASIISSS
VYFLWRVLQFPEISNVDAALIGVAITSAVILCAWGIGSGRGNAVESSLLFAYIVLCVYQIFTDYQP
SAPVEQATPAQAADFPPLPPIIMASYSTLINLLSLLPSILHAAFNVIAAAFSAVTPSILITLAYRL
FVFYASTRIIPAVRESGARALSQEASLDDDDGANKLLGLLSWFSPSILVAVYTSLLMQHFAANSPG
IAGGVAAGQWWLSQGDVGSNFWRWINLACTMALYAFELWIGQEDDIDSGLTGHWKTD
>g1362
MLGKALLLLLLSSPICALAQSAADYYVKSIPGQPDGPLLKMHAGHVEVDPETNGHLFFWHFQNRHIA
NRQRTILWLNGGPGCSSMDGALMEIGPYRVKDDHTLIYNNGSWDEFANLLFVDQPVGTGFSYVNTN
SFLHDLDHVAAHMITFLEKWFAMFPEYESDDLYIAGESYAGQYIPHIARAIVERNKSIQPRQQHWA
IKGLLIGNGWISPRDQYPANLQYAYAEGLVKEGSAMANQLDETEKSCNEQLNAPGAGDLVDIRQCE
GILNRLLDLTRTSDDQCVNVYDIRLKDASCGNTWPPDLDQMTRYLRRTEVGIALNLDHGKANGWTE
CNNQVTANFRVGHSGIPSIQILPGLIESGVKVLLFSGDRDLICNHLGTEALIHNMKWSDGTGFETK
PGVWAPRRDWTFEGDAAGYYQQARNLTYVLFYNASHMVPYDWPRRTRDMVDRFINVDIANIGGTPA
DSRLDGEKLPQTSVGNTSSSTSETDQIDDAKLKDAEWKAYTKSGEAALIVVIIGVSVWGFFIWRAR
QRAARDGTSPTKKGYRSVYRGGVDNNSSSDGAGLLSRFRNQSSNGGSRDLEARDFDEAELDSLTPD
LDRDHYVIGEEDEDEDNDIGNGAKSNLHG
>g1363
MAQTPSTRTLTFVVEHLDPELGPWSALEYACIARESKESGAQFLLTSVPEELQMPDNLARMEELKV
ERRSVEEIFADRKSRVCLLDPAAKTELSPADGDDFDVFLFGGILGDDPPRDRTSELRKKGYTGRRL
GPVQMTTDTAVRVTRMISLDRIPLDKIPYVDHPELRINEHESTEMPFRYVRDSQGKPIMPDGMVEL
IKKDADKSLDDMF
>g1364
MNPHQANKVDISSLSPDEQRLLRLYGKVPTKKDLLQNKLKERKYFDSGDYALSKAGKASDVGVTSI
GSRHPVPENIPHLTATSPSANNPSNGNNGHIGGPGAASVTSQSIPGSISGHPGSIGFPQNRGSPVK
EGSFVQRKSSLNHDSQNDENADEPAEEDRGRQVEKDDQ
>g1365
MRSYYVTMIRRNSDLEIHLPDADAPSSANIIDPADIEMLTSDDEKQILALPPHIAARFYRRSASAR
RASDKLSRRSSVSSIHSHHSTTSAHGGPQSDHLAQHLRRASILESRKARLADRAAHAEKVRLRAAV
AKAATRNLQREERALAAQQARERLLAEITAKCEDEVRRAKKKAEETRELKAAKYARLRLEMAEKFA
DAEKRRLQYQQMHRRPRTASLPAGEESKMVKTVLNSLTRDAAARIVQKAWKNYVARRVMSTFQSLN
LSRQKIGSMSFDDVSALLSEETVLETMARVLRLCGLRDMEGGALGERGAVRTFLSSYLILSHPSSV
MSSDGEQEQDLIEKARDLVVSFEQITKRLLKHQSITSMSSELQVLGEAYSVFFSAFHAWKTHDSTV
LIEIMLAQFVELELIWQTVKEDRAGGAADEYYQGIRHNQILLLARLKRLAGPDKALDMVKQALRKA

KREKKRSNSSKQSIPRSAAVSTSADAHADSVSTPISETFNNVESAVLHELDKQRLSPHESFTKALT
ALPENRVLVHELLINREFRIEQTQYTEPRQQIMKHMTDMMRSDVDAGLGTKWIVAMATVIQDRLLR
SLRPGNSLHVLISEALDPKLIENQCDAGTFSYDSFFDFMCRILPQLCAPFRDPEVKAFAENKSGDP
IERLSSLMGIIDLLSLDHTNFMIQVAAPQLIQEAPGYEQRTFQKGLDDGTISLEKTKRFWQKNHNL
VVDDMLRRNLEIADRRQVQPPAGKVYAQGLVDLVLNNAPVPSDLVPETLELDNARLETLHMTAFKI
AATAAILLTAKNLLKRDSRSQWKAEADRILSLDFTEIKPERIQSIIECTRPMPAAARSQLLSTMRR
VLSPVATATTSVDTTTSSQMPSVEYVSGPISSSASTSSASTSTATDSSATSSSFTDPVARLILSRL
RAHVLSRLSAASASERVRATTTASQSLAAAGMPEFVNEIGKLVDELGKVRDVDWTCHGMTNIFRKM
WKTSLISDLTPPSLERAWQSTPHPSLPLVATSSADKTVRIYSLVNYKLLSVVSGGHKRSIRSSAWK
PTASAGVTKGESVLATGSFDATVGIWRRWDDYNNNENHDHVHDHHDHDHDHDHDERQEDEEEWRFA
VLLDGHDSEVKSLSWSASGSLLATCSRDKSIWIWEDLDDGDNNFETVAVLQEHSADVKCVAWHPTE
ECLASGSYDDTIRIWREDIDDWGQVACLKGHKGTVWSVDWEGVDFSPFPSVSLPADKQAKIKEEYR
DSFALSGPRIASCSADKTVRIWRRESKPQSERSAFSASNTGIPSIIRPTGLDETWHEDALLPAAHD
LAVYAVAWSKRSGLVASTGADGRIVIYAEFFVENAPKEGGSDEMDTSEDTKFKTDWKVIATIEAAH
DIYEINHIAWAKRGDRRGGQRDEEILISTGDDGSVKVWSIERE
>g1366
MSNTDFLGRAIDTVKKAIEHDNAGEYEKAYQGYYAALELFMLALKWEKNPKSKEMIRAKTGEYMDR
AEKLKNHLANADNKKKPSAVGANGKVANGGGKGKEDEDGEDAEAKKLRGALQGAILSEKPNVRWED
VAGLENAKEALKEAVILPIKFPHLFTGKRQPWKGILMYGPPGTGKSYLAKAVATEANSTFFSVSSS
DLVSKWMGESERLVKQLFNMARENKPAIIFIDEVDALCGPRGEGESEASRRIKTELLVQMDGVGKD
SRGVLILGATNIPWQLDAAIRRRFQRRIHISLPDINARMKMFMLAVGSTPCQLTQADYRHLAEISA
EYSGSDISIAVQDALMQPIRKIQTATHYKKVLVDGEEKFTPCSPGDNGAMEMNWMEVESEKLLEPP
LVLKDFIKAIRNSRPTVSKEDLDRNAEWTTQFGSEGS
>g1367
MEQAQKALQALSEEYQNLQAELETIILARQKLESQQQENQAVQQEFASLDDDSNIYKLVGPVLLKQ
EKSEAVMAVDGRLEFIEKEIKRIEGQIQEINNKSDKKRTEIIQLQSQIQQQQAAAGAIAA
>g1368
MAKVDQKVALIVIDGWGIPGPDSPPEGDAIAAAETPVMSSFADPKSKTAQGYTELDASSLAVGLPE
GLMGNSEVGHLNIGAGRVVWQDSVRIDQTLKNGKLGEIENIRKSFQRAKDGNGRLHLLGLVSDGGV
HSQNKHLYGLLQVAKDIGVPKVIIHFFGDGRDTDPKSSTKYMQELLDKTKELGIGEVGTVVGRYYV
MDRDKRWERVEIGMKGIVTGEGEDSTDPVKTIEERYAKGENDEFLKPIIIGGKENRVQDNDTLFFF
NYRSDRVREVTQLLGGYDRSPRPDFPYPKDIHITTMTQYKSDYTFPIAFPPQHMGNVLAEWLGKKN
ISQSHIAETEKYAHVTFFFNGGIEKQFPGEERDMIPSPRVATYDLEPKMSAWAVGEKVAERVAEEK
FEFVMNNFAPPDMVGHTGVYEAAIQGVAETDRAIGHIYEACKKHGYILFITADHGNAEEMLNEQRK
PKTSHTVNKVPFVMANAPEGWSLKKEDGVLGDVAPTILAAMGIEQPEEMTCRSLLVKS
>g1369
MASRSPVSAVSQQQKISSTPDIAPTSPQPSAILPTGPQAAIPFPSPQTFDFIPPLHALILRLHSST
NTSASTAAAGAVTSQSTIGGNASVEGVVASQSEAGLQTQPQTQTQQKQSQSQLKSQLPLGGGGATD
SATPSSSSNLPPLEAKDLFTAASAVRIRIQKARAIVESLPDVDRTTEEQDDEIEELVARIASLRGV
IADFGRRAGMQQTIEVDKMDVGV
>g1370
MATYALSESHKDLMEVHLADFDPEIAEIIKKEIQRQRESILLIASENVTSRAVYDALGTPMSNKYS
EGYPGARYYGGNQHIDAVELTCQARALKAFGLESDKWGVNVQTLSGSPANLQVYQALMKPHDRLMG
LDLPHGGHLSHGYQTPQRKISAVSTYFETFPYRVNAETGIIDYDTLEANAQLYRPKILVAGTSAYC
RLIDYARMRKIADSVGAYLVVDMAHISGLIAAGVIPSPFEYADVVTTTTHKSLRGPRGAMIFFRKG
LRSTDPKTGKEILYDLEGPINFSVFPGHQGGPHNHTIXALAVALKQAATPEFRQYQEQTIKNAKAL
EVAFKEYGYKLVADGTDSHMVLVDLRPNGVDGARVEAVLEQINIACNKNAVPGDKSALSPGGIRVG
APAMTTRGLGEEDFKRVVGYIDQAIKISKSVQASLPKEANKLKDFKAKAASESIPEILSLRKEIAA
WASTFPLPV
>g1371
MASHDDPEKKRVSHRIRSLSKSLHRLSSNHVPDRLKNNDDEQEDFTAPPKILTARDIPYMHQSVFS
LIAAAGSRSDFHSRFDDSSDSDGDDSEEKKRQDPLHRRLVGRLSNKPGKEVSSSQVIPEETGQEPR

PSKIPSTSSVSSSKRTDKEGKSDQAAEAAEAAQSPRNEVQPARETTTTPRTAPVLSRMIEAQSRFD
PAALSTEEHAQAEKETDDKQSDEQKSKVLLPIRLKEIFGFERPEPVIGEYPCWLMQNVALQGYMYI
TEGHICFYAYLPQKSNVAVKSGQLSDVFSYYADPSKLYFPSGHIDLRYGISASLAEKEKDSASSAN
EWVKALQKVIFRSQNEGGRTFKIRVVDIHETYAIDEHFFSFFNFGQDAFEVLKGLINDSTARRIAQ
DPNATQVLNSSMGQTESSLSLQSHPDTIVTASQILTRDDVFNSPTIHSLRPNTSLDGVSFESPSRK
VLTEPIKKTGKEVSRDPVQDASIERKSYVTAAPTIHELVKAGTYPLQRAAGFAEYLKSRSRQMSSL
LATESMGYIEKVSGMWIGGGKHYAEGEDPALEQYFDGEDAETSGTYGDRFRAHFALPPTEKLQATY
YGYLHRVLPLYDIENVEKEKGFRFGYQGLVLIIRGHEELFFEFNAADSRDDCAVTLHHRLESXRFL
AQSSILMQKDKDEAAAAKAEQQLLQEAMQGTLVEDSQEVHPIFDDPRASIVNFKPTESLRITCLTI
GSRGDVQPYISLCKGLLAEGHRPKIATHAEFGPWVLQHGIDFAPIGGDPAELMRICVENGMFTYSF
LKEASSKFRGWMDDLLTSAWNACQDSDVLIESPSAMAGIHIAEALRIPYFRAFTMPWSRTRAYPHA
FAVPEHKMGGAYNYITYVMFDNVFWRAMSGQVNRWRKMELGLRGTTLDKMQANKVPFLYNYSPSVV
PPPLDYPDWIRVTGYWFLNEASTWTPPTELTNFIKKAREDGKKIVYIGFGSIVVSDPAALTRTVVE
SVQKADIRCILSKGWSDRLGDPKSTRSEVPLPSEIHQIQSAPHDWLFTQIDAAVHHGGAGTTGASL
RAGVPTIIKPFFGDQFFSGSRVEDLGVGICMKKLNVGVFSRALWEATHSERMILKAKLLGEQIRKE
NGVATAIQAIYRDLEYATTLTRQRASLSTTPLTATEESKPADEGEEADDMEGEESWTFVDDYADPE
LLKNENLFYQKNFTSSPASSPTLTNK
>g1372
MFGKSLTVAILLSLNLVSAGPIVKRSVLTDLQAVYSEVQVFDSAITSWNGQLLTALPLLNDVSTLE
TDIKTATTDTNNSAAFDDADSASIATETQSLSTLITKTLNDLVAQATKVKAVGLTSTVESSLEQLK
TLSDGLITALESKIESSYLPTATSIAAAIDSSFATAIAAFAS
>g1373
MKFSTAISIATLGFASGVLANPTPVINEPSLVKRDLASIESVVSGITTQVNQLDTDIKAGSLNTVL
SDSTALVSIINAGITTVDASADLSENDALTLANYIGGLINAVNTTVTDLIGISSAVQAAGLVSTTL
TNLQDQYTASQGLADAITAKVPSDLQSLSAQLAAYITTSLQQGIDYYSSLPATSTTSSSSVVSSTS
ATSATTSPTTAPTTTPTTAPTTTPTTAPTTTPTTAPTSTSPATTTPTTSPTDTVPTSTPTSAATST
ASGSSTVVPTSTSAPTTTSSFSSTGVSTTKTTTPSSRASSQPVSVSSSWSSSTAGPGGSNGSTTTS
QPGSSPSGTNTAPGGGVVPGGSPSGTNTAPGGGVVPGASGTSKTSGTAGSSTKTGSSVPTYTGAAS
INGLNIPAQGLAILAAVAFAL
>g1374
MDFFRRIMYMLLLLLECVCIIGVDTTGQTPLNSEDKATSRNVAIIGAGSAGASTAYYLRKFSDLAG
VPVNITVFEREAYIGGRSTTVNVFDDPSLPIELGASIFVSVNYNLMDTAKELGLKISGAGGDRPAE
SVNMLGIWDGFRFLYLQGHSLSWWDMAKLLWQYGLAPIRTQRLMKNTVNKFLQMYKPPYFPFSSLS
ATVQALDLHEATGVPGAQFLESNGISESFSNDIIQSSTRVNYGQNLGLIHGLEAMVCMATDGAVAI
EDGNWRIFSGMLDSSRADLRLNTTVTAIKQNSDRSMTLFHSSNGGSTSRHTFDEVVLAGPFQYSNI
EVSPSLDRSPEKIPYVRLFVTLFSSPHQLSPEFFDLSDQKDVPEMILTTLPRGFNLGDREDGVGPA
KFWSISLLRIVEASAEDSRQKHYVYKVFSPEKLTAGFLASVLAVEIPASDQNGTIGDISRNDISWY
HEKTWHSYPYEYPRMEFEEIRLASNVWYTGGIESFISTMETSSLMGMNVAGLMTNEWHDKLHLGKP
VSWSGWTQQRNNEL
>g1375
MDPYDSDSSLEDAGEYTETGVLLGYSSTEQTDDTISHLGGWPTWLDENTPPPGDLAKCKVCNNPML
LLLQLNGDLPERFPNDERWLYIFGCPRKACSRKPGSIRGLRGLRKVKGQPETQKKEESKPKDTPPA
PKQDIGAALFGATSQGGLSSNANPFSTSSSPAAQNNPFAPLPPVSTLAAKPPQNPANNLSETFAAK
ARISTPETSSEPVAAGPVVPWPDKSAFPPPYTEYYLDADYETLSRPPTPTIPTNTTVENVEEDAGG
AVDVKDAFESSMDKDFLRFSTRLEHNPEQVLRYEFAGTPLLYSGTDAVAKAFPSHQTQGRGLQVSS
NAGTNRIPRCPSCGRERVFEVQLVPHTITVLEDGREGIGLGKNDAGMEWGTIILGVCAGNCGTTKE
GDLGWKEEWIGVQWEETK
>g1376
MAKEIDHMRTSLDTEEHSLQSPITEVERENDQNISDAPEGGTRAWLVAGGAASIFFCTLGFANSFG
TFEEYYITHQLQGESASKISWIGSLGSFLPFFAGMVAGPLFDRYGEKIIRPATIAYVFAMMMLSLC
KTYWQFMIVQGVLMGILMGLLQFPAFAAVSQYFQKKRAAALGLAVSGSSIGGIIIPIVLSKLLNGT
SLGFGWSVRIIGFIILPFMVFACASVKARLPPRVSQFWITSAYKKTNFLMLILSLFFMFIGMTTPL

FYLPTYAVSQGMGVTLSGYLLSILNAASTFGRILVGILADKYGRLNMFAIGGVVSGVVVFCMNSAT
SNAALIVYSVAFGFASGTIISGASAAFSVCPRDPRDIGTYMGMGMAISGLGALIGPPINGAIVGKY
GGFFEVSMFSGTMCLFGGLLVIAAKTLTKQGLLGRT
>g1377
MTRGAFGHDGIGRPAQTASSILCLVQKEKDKMQPNQSSSRIDGSVGPSTDATHTSQQHSARDDESV
RLKLRIKELEEQLSKAALGPVKPPVVPLRLNIDTTTSHIGGTYHIQNEILPGQNVSIARSITHKSR
LFGQSHWEVNVILMVRDLLETIESHLPQAALKVYPGMEMCKSLARTIKARRAPPWPSPPTSELPPK
EVCDALLDCYLQSSESVNRILHISSFRRDYEAFWVSGNAPDKGFLVQIKLVLAIGTLTYDDRFSLR
TSAIRWVYEAQTWLSEPKFKSRLTIQFLQTYLLLLIAQEQVGAGGDSMWISVGGLLRKAVYMGLHR
DPIHLPKRTIFAAEMRRRLWNTILEIALQSSLFSGGPPLISLNDFDTAPPGNFDDDQLIADDPIPK
AEDQFTQVSIAIALRKTFPARLAVVKFLNDLPASGTYEETLQLDTQFRTVYKALNRTLQTFNRSSL
PLQLSASSLRFEIRVVDLIMLRYLSSLHLPYFGPALHQAMYAFSRRVVVDSSLKIWRLIHPLPSHI
LSTTTTVAAAANLHHQESNPLARLSSFSSGFYPVLAIHAAVLITIELQTQLQQEESLGPVSVRPDL
LSVLEEAKTWTRRAVETGETNIKGFLLIENERAEALVKAAEYSIETCLPMLEEMAAELRRDAGGGD
EQQQGLMLATPGDMEDWSFMTPDALFSLGDDDPMNWMLKDGMDMVPSLW
>g1378
MKRKADKQQSSAPRSAVSAVAARRARQHQLQVASSHGNAPIDNKEAEPPAKKARPSPDRSLKHAAP
SNVVRANGNRLAAPVKAVSADLAFELSSIDATEALKSETSEDGKIVAXDENDDEEEDTDARNNADH
ATPSRIHSTAENFTLSRNKLRRSDIVYSDEHMLCVRLKEKTTLVLIGIYDIWVKRGVISLMGAKLH
PSTETYRVYAPSTHSLPVIKCVSGVEGFAEIELQSCSSDLQGLKELSPHYSRIWNSSRTVGDSATL
GGSFQRSFSILYTSSDDPLNRPLRPLHLEKKWSTTIKSLSRRAHSLRALICGPKGAGKSTFGRYLL
NHLLTPAPDGSSDNLNGIAFLDLDPGQPEFVPMGNIYLAHLREPCFGPPFSHPNLNNSRYGKVIRC
HHIGATSPKEDPDNYASAIMNLLDQYRVLTATYPRCPLIINFPGWIFGLGLEIATWIIRSAGLSDV
VYMSEKGPTEVIEPLRHAALEAGVSLTTLPSQPVDMVTRSSAQLRSMQMQSYFHMTQSEGLLDSFW
NESPLIHSKPIHVNYSGPQPGIAGIMVLGNHYSPQVLRDILDGSIVAVVAIENASAIPNYSEIKAK
LDGDSRAEEQTDAMAEDQSEDNEKPNVDELSANGNQRVKDFLNDSITRCQKSGLPYLVWGAGSSTP
LDPKASRSLGLAFVRSIDVSAQRLELISPIPGSSIRDAINQGQSLVLVRGMIDSPDWAFSEEYYAA
RNAEQDFITQISSTNKSTAKSRGKEDDETKREQNEQLRRLQERVRAASKMPYLKVIEDASALHQNE
GRQENGTWRLRKKVYISSDSELD
>g1379
MGKKLSEDVIVSDSDESMEDAPETTSVNTKNNAKVSYNQGASHSSSESSDEVSSEIQNTEFSKKRR
FVPPSGFRPSTTKSRPSAAISSALSNLEDKQVFHITAPSSLPLSEIKQIAFGKATSGEPILSHQGV
DYGLVETTRQQKQSSETLLVYDEKSNTYLRKPELRKIESYSIQEIVRLPEMDTLVPSSRGTTKKQP
AARPQPKHLKMRFHPVGSTTLPPETVGSSSESEADEPTFRAPVTSSKKQAEKEHKRKANEQNNTEE
KAKKSKKDKTHSSRAESQPESRRDKEKKSSKHRDETSQERRARKEEKKKRKAEKANK
>g1380
MIGSARSMGDVNGSPTNTSLTAFSEGHTPTNSKSNIPRHVEEPDFTILELICPINVEDISNRWLNS
YVPFPGQKQKDYPASVTAFMYRILKSYAAITAHGRGLPPFVHPLQVTPKSTKLPLSTCLSLVRVCD
QPRPGSEDFAADILRREMGTIYDQRRKYDDMTLLAAFQAYMIYCMALFFTLNQTTDPFLRQAMMNL
QELACATSSQGLVCTAERQHTRPRWEAWVVAEAKRRTLYVMYLFDSLLFAQDGLPTFLGTELYGLP
APSAGVLWKAGSRQEWEREYNVHLADWIVAGLHINELWPMPAEMDEAGVVEQRARVDMWLEGVDEF
GTMMYAVTNCTHGG
>g1381
MFTSKTNSYTPLPDGEQKSEIVDFDPEIGLFATFINFAEKENHCNIGPNPAWKDFAHPELDFLPTD
YDQHVIFNGTLDFPSIYRGTPRPELDAAWNRVIDLGPLDIQLSTDELERLGVDAATAVRLPPENGG
GFYVNLEFQHQLHCVNYLRMATYMSYYETKSVEFTDSKPTQRIHLGSADLSTPVQLDAYTESYPRP
IEPRHNSSESTSSQAMFEFTTGPSVESIFSPESKQLGGTSDLLFSLEHRVPEITDEVINLLGNDEG
NTPLHEAVIYDRHTVVRTLIDRGADLLATNVFGKTPLHLAAEFGRHNVIEMLDPGQLGLDIRDKFG
LTPLHLAAKSGHEQVVALLLDKGAGIDAKVKRG
>g1382
MRAHSALIVLSLFTTTAFAAAIPGIDTGLNVRDGKSKRDGEAALGNYEIHKSYEDHAIAKRDDEAA
LGSYEIHKSYDEKTKRDNEAALGDYKIHKSYDEKAKRDDEAALGNYEIHKSYDEKTKRDNEAALGD

YKIHKSYDEKAKRDDEAALGNYEIHKSYDEKTKRDDEAALGSYDIHKSYEDHAIAKRDDEAALGSY
DIHKSYEEKTKRDDEAALGNYDIHKSYDEKTKRDDEAALGNYVIHKYYDD
>g1383
MCHRTPSTSHETTSSASSHSLRGKQRRRLSSTPDNSALSSSLTASIQSNSANASQNIEDHPYFLKA
QKSQSQIRVDPVSERVNRVGGVNGDTCASCSISLPEDVSKKLPTSVPGQSDGESGRGAKNNSPVLR
SREVVHSCGGGSNRHSDICAEDESHEHDAQHASLPDSVHSVSVTSSSSDSSCHTHILTYLSLHGPP
NPNDYALLRQSSIRTLSCELLPRGLSSGPLCFGDPVAGYTVAYVFRLPDPMARGKRRSYALIALAG
KDAGRAFRASPIIWRIFQRIAAGIVTAAEKHQEEEKMREEQAFRSSNASVATGRHDYTPVSSFLTG
RTLDANGNPRRAGQIRARNLAEIVGNEYIFAELHAQFVTLLQQLGNMFGGAPISDDRFVCSTVSST
ESAMQSRRTSVAMPTNTMNNNNQKPGHNRRPSDLTLSSLNISSGPQPIPISPRRPIVA
>g1384
MVSAFDKCDTRPAHIDAILNGLDRYNPETTTVFQDYVVQQCEDRTFDCYANLALLKLYQFNPHLLQ
TETTTNILAKALTVFPSPAFSLSLALLPAHTQPFGNTSDGSTQSSDFVESVQKLAHLNTLLESAQY
GSFWSTFNSDDLYADLVADVAGFEELVRVRIAVEVGKTFREISADALAQWFDLKSREALQKFVVDV
CGWEVEGALVRVPKNRENEARSEIKSERVGMDMFGRVIRRGFEQPA
>g1385
MTTTQKPGEGTNPRGIPYAPFVDNVSDYVSSRTEVEATLRSFQEMISKYQFMEMNTQRRSVGLKEK
IPDIKKTLDMVRFLKLNRENAEDVESEDDEEEDGKGAIQTHFELNDTLYARAKIAPKDTEEVYLWL
GANVMLAYPITEAETMLSEKLAAAELSLENCEEDLEFLREQITTLEVATARVYNWDVVQRRKEKGE
GKDDTGEPDG
>g1386
MVLLTTTPLIAKALNELSASTRLDLNLPEHIPPTGAPISHDQLISLGRQRNGYTLNNLLKGTKVYI
PPPPPKPEPTPEYLALKARLQALADEQAYNALIAPNSTHLNPYKPSSIFADTPTVIENDENVEDMV
TPSLVINIFMSVILCGFATFWALKNFQTPSFLGWILLSPTGRRDGVNTGGGGATRSKDPVFVLIAM
SVGLLVGVAETVVYAAYVRKVKYAKAAERKVKERKEVVSSVELGGEKNDTKVVDDNADGEEIIWGK
GVNGGVRRRVRERWETLNDDSQ
>g1387
MTTVVASSRAVVSGRLTPATIVICHSTGKIIQVHDSIRPASDFPDCESYKDYSPLVLLPGLVDAHV
HLNEPGRMEWEGFYTGTQAAAFGGVTTVVDMPLNAIPPTTTVAGFKEKIHAAQNKCWVDVGFYGGV
IPGNADELKPLVEMGVRGFKGFLIDSGVQEFPAVSAPDIELAMKTLADQPTTLMFHAEMLPPITDS
VGDDVQTSMPPLAPAGPLQSYSTFLASRPASFETYAIAEIIQLASAAPNLPLHIVHLSAIEAIPLL
REARANGVQITAETCFHYLSLAAEEIAEGDTRHKCCPPIRSRLNQDGLWAELERYADDGVIKTVVS
DHSPCTPDLKLLPAHIMQPQPIDSAGSSDSSAVATPHNSSSNSSVSDYFEETKEHEIADGNFFSAW
GGISSVGLGLPILWTEISRRKKLGAAGGTKTINQDLQDIARWCSTNTAAQVGLSSQKGDLAVGFDA
DICIFDDSAEWVVEPSTMLFRNKCSPYQGRTLKGMVRETWLRGQQVYSRSEGFNRVPAGKLLLEKR
SQ
>g1388
MSMFRAKRLDLGGFINARVIRDHTKRKVYEQFEPERQALRYAIRNTALPQRVRAQAQLQLAQMHAY
TRSTQIKNRCVAGGIARGVFRAFRLGRYQFRQQALAGELPGVKKASW
>g1389
MADSLSSSVASLRTSLQLLESSIDILDSGVNDFPRLSKVLQTTRHFELLPEPTLQEAQQSLLDEIT
PSIAHLLSITTNHVERLARREDSLRAKCQLQEGRLSDKRQLPSANTTTTQKTLGAATRGSDAGAKA
AQLRRLVQKKERLKYAVDRLELQSKQKERELRKSMAAQ
>g1390
MSLSCRAAKSCRICSPGASQHIIQLSQRSGVRRFGSFIRNSRPALPSTIYSESKHAIIAGASGRKW
RQGKVRELATNTPPEVPDLTGRKEEIYTLIDTIHEHEDELAELFEDFLPFDEFSHIIDTRSWGMDD
VFSATIGHRNYESLENKVRNARQQFGEYLPEDHLNEPELELYNRLYGEALFRPAESDVIDEREPDI
LLRDDGEGGYEEIDVPPVDAEGEIPVAYAMEQEILDADEEIPMNRAKQVAERLGGILLEPGSEVEE
EESESGPRIHPLTAAGKYSTEPRTVFIPKDSVTGPISVILSNYSNKHIAEAAHKVFGGQRLPHSTT
TLPPSAQIAQTPIPLSASQHQMSDMEANAYLAALYPGIYASTLSVLAEVRKRLGSKWLRDLMMQET
GPRVLDAGGSGAGILAWRDVLRAEYETMVPDHPKDAPIPTGKSTVLAGSDALQMRASILLDNTTFL
PRLPDYLHVRDSSTLDDERAPPKRKQFDVIIAPHTLLEIDEDYLRKQHVKNLWTMLNPNGGVLILL

EKGRQKGFEAIAGAREMLLEKHIASPGSTEYESLTESGDQGSIQKEAGMIIAPCTNHGKCPMYHIH
GHAKGRSDYCHFEQRYIRPPFLQRIVGATDRNHEDVQFSYVAVQRGVDLRQTESMVQGKAATDAAF
EGYEDSFSPEDGESAQTSTINPLSLPRTVLPPMKRRGHVIFDFCTPEGKIERWTVPRSFSRQAYHD
ARKARWGDLWALGAKTRIPRNLKLGSAASTESKKERLQRRAATKIAQMTGDEEAGDELNLGLDEVE
ERKGTAAPALANALQNQARSLERRKKGHNIPTWKRHADKKKIRQAVRKASSNTAEEEFI
>g1391
MATWAYPPLPADQLKREEETALSRELEWLLASLQESLASLRDGLQECASLLAPKEPGSTLVVSSVR
SESVKGFVTRIGTRIVKGDIQLRLTSLPPPRGASSTRLNVSNVPGAPELVLDQLATVRDLINQGLD
VIDVSTFTGDPLNASFISGQLRLLYEHISEAKSTLKGGAETSKWWEQSADTSVFDPPSPPYLSFHL
TVVEAALVLHLRTLEPNSANQTSTAFGSEIGFSLRDRLFGPRLPSHDEQNDVFVWQGEEVKVKEKI
RVETQDPSLIAVMTKLSALEHEVLKCRASLKIVMGDDEADSDLE
>g1392
MAAGRPIFGFLALFFTAGALVLMFLTFLGGVNNHVPLNDIYFLQADTSSIPGAPSTSRWTFWNVCP
VSDGKSQCGSVHPDFPFDPPSPRNFGTTTNVPGAFIGTHHYFLLSRFMFPFILIALFFAVLSLFLG
LLALCTRIGSYLSSLLAWIAWVFQVITASLMTACYVQARDQFNNNGESASLGRKAFGFMWAAVACL
TLSTVLYCMGGSAGRNNSSGYTGRETRRRGFFASRRSSTKSRGSFRSKREDVV
>g1393
MSEETGAGERFAIGISFGNSSSSIAWTGPDGKAEVIANEEGDRQIPTILSYVESEEFHGTQAKAQL
IRNSKNTVGYFRDYLGKDFKSIDPTPAHQSAHPQQFDSTVAFTIQDTDAETPSTVTVSEITTRHLR
RLKQSATDFLGKTVNAAVITVPTNFTDAQREALVAAAKNADIEVLQLIHEPVAALLAYDARPDAVI
EDKLVVVADFGGIRSDVAVVASRGGMYTVLATVHDYELGGSNLDQVVIDHFAKEFIKKHKVDPRED
ARGLAKLKLEGEATKKALSIGTNATLSIESLVNGIDYGSTINRTRYELLAGKIFSQFTGLIESAVK
KAELDVLDIDEVILAGGTSHTPKIARLLESIFPEKTKVLAPSTAPSAINPSXLSARGAAYQASLIQ
EFDTEDIEQSIHPMVTVTPHLSKTIGVQLISADESGAFVPLVNTETVLPLRRVAEYAVPKAGGDVL
IRISEGEREIKVTKPEPKPKAEKTEEDEEDSDFDSDDEEEEETREVIWKSTKPIAEAAVRGVKAGG
KVEVTININSDLGLQITAREVGAKGGVRLAVEAPAATANGSA
>g1394
MSTTSIVLPGDQVPSSILPSTASTTPLRLGPGLRLLEQQSQAKTKTNSSSAPVIQATQAGLLSIDA
NRKSVSVQTFPNRRYIPTVNDLIIAQVQRSSQDFFHCIVTPHAPLAVLGQLAFEGASKKTRPNLKQ
GDLVYARVLSTGVGAGTEVELTCVNPATGKAEPGGLGLLSGGMVFDISTGLAARLMKASSSSSSAE
AASVVILEELGKKFESKGGFEIAVGRNGKVWVDCSGSGDIAIKITIAIGRCLKELDQRHLTSIEQR
KLVSRVLRELNIDA
>g1395
MARKSDVPMSEPAKRTKRKRIWEDNDGEATPPPSAQRVKIKDRLSINQLSNDKGESENGAYTQGST
PSETPSGKTKRTNRGKHLPPGFRPGHVSSTNDKRTGLFSPEEIEALEQYKLWFCNYYHITADVFDA
SIQSAKSSKNGIFLQDRPPVDERSFWQEIYGQIPDRDRRSIARFCRRHFSNAYKPHQWTTADDDEL
ERLHSMYGTKWAIIARDMERTYDDVVQRWKNKVEHRHTMKMGPWDDDEIDKLIQNLEDTRKAWLRL
GRTKEDIGKDIYELPDELIAWGKISERFEHTRSRQQVADKWRKIKRIVLERRADGYEDAVWESSLQ
PKGGKRSKSRPVSVESEKRVFISNEMVDSSDEDEDQNKKETEDGKKKRSAKANGTSQPKVNEKVNE
SADESTDESSDASSEDDSDDSSSDEKSKAAKNSDKDDEDDVVDESAGDETPLPKTNGYHAASDNES
VVPESQAEESEASPSRSSSPEAETNGHTAAKGRQPSEELGLDPVSAPEAPEVTKADKDTRASEEEE
EEEEDDDDDDDDEEGSESADQEVLKPLTTSSQPKPSPASSPAKPTKLHLIKQEEKHSSRPHDSGSE
SESDDDESHGRSSSGSGSDDDDDDDEEGDDEEELSPEEIEKKIFKFKQEPEDD
>g1396
MNMMRASLFSLATCLLVLLQPRSAVASPSVNVALQASFGAGPYLVELLETAAQENATAYFPLLDRI
ASGIFEDATNDKDLYESFLQVLIQDNHLASKESLDSFKFALAIRSAAPRIQAHYQYYNTSVTSTLG
TAQDAACPVWVHMDGKQYCSSTLERAQQDVSGDRDPRELPFDRTLGDASATPAVLYADIASPIFAD
YHRTLKGLADEGQIAYRVRYRPAHDGVSQPLFVSGYGVELTLKRTDYIVIDDRQAEERETTKSNTK
APSAQDLETEEDEAPPDLKPLSSSEVAKLGMSAASFVLDSSDPFTTLVKLSQDFPKHSSSVAAYNA
SNEFLEEYKKNRQAGLPAXRNAIWINGLQIDSRQVDAYSLLDYLRRERQLIAEFQKIGLSGSEAID
LLSYPALAEVQGNSEAQRYDWRDEIEGGEVVVWLNDLEKDKRYANFPTTLQTLLQPTYPGQFPSVA
RDIQNVVVPVDLANVDDVQFVVKYLYAFIKRLIPVRFGLVLTANTEESKAQAKIAHYLHQAYGLAS

LLQYFEASLSSEISRAHKPSFEAAIAERSLRPDQEALSFEEVLQSEALDLIVARTENYLKRLDLKG
ALPPTLVNGVPMSRSETYMQELPMQLSRDLQIIQQGLIQGVVSEDDYIPDYFLSQAAQSRNEWIVP
ERDSGILIADIRSAAEAHNDFANILRFPADAKHPTANVQLIVTGDFNTEHGFALLISALRFRQTHP
EVEIVPLHNPDAEDKVGDNSSKLYDLLRHPEPIDLDHVLAQLQSGNSQQAVQKEEEQTYWINVQQL
ITDIGLSAGATSLIINGRVVGPLPSSVEFTEADFELLFSYETSQRLGPLSAALKDLDIESKIKGPL
NFAKLTSLVMLSSDAPEGVFEQRSKYRVNVWKRWNSTHSAIDASTNTKEASINIAAAVDPTSEMAQ
KWLPILKTLSKLAGVNVRIYLAPNGQLSELPIKRFYRHVLQSEPTFDESRALERPEASFTGLPQDA
LLTLGMDVPSSWLVAPKYSVHDLDNIKLSVIKDGSDVDAIYELEHILIEGHSSDTTLRSPPRGVQL
LLETEKGSFFADTLVMANLGYFQFKAQPGFWKIELKEGRSQDIFQLDTIGGGQKSTGTNEVALLSF
QGKTLFPRLSRKPGQEQEEVLETGPKPGSAMDYVSKGLNFAQGVLSSVGVKQTGKSESAEKHAEIN
IFSVASGHLYERMLNIMMVSVMKNTNHSVKFWFIEQFLSPSFKSLLPHLAEEYGFTYEMVTYKWPH
WLRGQREKQREIWGYKILFLDVLFPLSLDKVIFVDADQIVRTDMYDLVILDLEGAPYGFTPMCDSR
EEMEGFRFWKQGYWKSYLRDLKYHISALYVVDLQRFRELAAGDRLRGQYHTLSADPESLANLDQDL
PNNMQTLIPIKSLPQDWLWCETWCSDEALKTAKTIDLCNNPLTKEPKLDRARRQVPEWTEYDDEIA
ALARRVASTSTEGEDEDDVEAEEVYERKDEL
>g1397
MDEQSPSPQARPKGIAGSDGSAMPFILEHYMMNPTSYEIPLRSLYALNCQTLPLTAGPKKFKESNF
ANPPMQNSTLPLADAASQFKAQLISHISKGPHQYSLPVGFTMSFVRRVFADNLEYVDFPQALTALD
YLKNLESRRKKEIIAAFESLRVEWSISPSQTIRVSEVHKQDLREKYPGVVRWIEMIESKEREAVSI
YIKMCLSLRRWTLINELWLEPFNKVACMSMLNTLFAFQVDDSKVPKPEWDYEDKVETARQRQYFFE
YIRNLERNGKVALESLLKSGARPGDETAWPMVRGFLDKYLNLANDTIHECSRVDRDYIEAEFRSKT
HKRSADSGVSFTSANESFSSANTRAATPSEKSRSPTPDSKPSKSTLERISHQLRKIRSRPDIKDTA
KTKPALSKKKSLTALVSGRSASSSSSETNFDVDEMKRQKMIWEAKERKKALQAQAEAARATEDGKQ
TAQVGYAV
>g1398
MLVYKKRDIPPDPVYPVDLERLGFFVNDEDKIRQIASPDEGFKFKVNANERWNTVRRNAYHECIRH
IVISRLEEVGLATLRLPLNSQPSDKHVPILVSSNLRTATRITLILGSLDQELGVWTWRSIAERGIN
AGSMVEITREILKSKADSNTALIIANLGQLVYHCGSEQAMSQRTWFALPVETAAHPPPRQTWRNII
PRNGNWREHVKCIFEDLLDPESGMVNPEAKIDIIGVEEGGSGAVEYLAENWALWKTSISGICFSRP
QHQKHNLTLDSADDTVPEVGSFAHFISTRSRAYVLSDKPLEWPVPGTLEYGCNTYSGNEPLNHEDV
IVSAWQSMLQWLDKIYADPSYEEVQFVIEETVEDDEGWEKWRKGDVDAVDNMRKLAVVDADS
>g1399
MIAKQFLFALAVSASFLGSATAKGTGGNTGSNTGSTGNSGSNTGGNNAGSSSSLTLDSNLVQTGSQ
QTGGNTAGETASATDNANFINFCQGKTLTNGLQVTAGSCNGIVMGDIPAKTSMVSTVILSPQNLQD
LTANQSFNVSLQVSNLQAGAFTNATSTYYAAPQALNSAGQIIGHVHVTIQDVGSLNPSTPPDPTTF
AFFKGIDDAGNGKGLLQAAVTGGLAAGTYRVCTMSSSSNHQPVLMPVAQRGAQDDCKYFTVGQGNN
NNNANTGSNTGSNASTGSKGNTGSNANTGSNTGSNTNTGSNASTGSKGNTGSNANTGSSSSSTSTS
ATASTSSSGSNSNTGSKGNTGGNSGSKGNTGSNASTGSNTGSNTSTGSKGNTGSNANTGTGAGGKG
KVVASAATSASIDCSSVATSAAASATTSTAASATEVVAAASSSAVAATSSGKSGKSGKSGQSSKHS
SNGKRSNGRFVSRKFIA
>g1400
MASIPPPTTAAPPPTATTTLKTLTFEKKNTHLLHIPSKQNGDSNKKTYRIAKRPIPRAPIASPYAG
SHVPKPVYISSKTPFMSAVKRVQKLLRAAERRAMAPLLGMGGRRRHPYHHQHAGKGGRDVLEKCRA
KLREEKVLIKATGRAIEKALSVGRWFEEGKGGGGEEYEVVVRTGSVCVVDDIVEEDVSISDHEDEE
EEEGEGGGSVDPDITMSMTATNSTTNTHDKDAVTEDKQEKEESKNQRRRRRKRERIREQRLKAFVD
EDGETPESRTRWVNTVEIAINIK
>g1401
MTAVASPPSVQSGPRLGWYSTGNGGQAALGSMNTDDVSRMFMPRKSVQRSNSSSSLSSNASATSTN
TVAHASNGIVSNGESATWSSKKKAGRGLWPAAKSEPVSGVTNVRPQPVSASTSTNGGLTASSAMSA
IHQPSPVPSQHMIQSPQQNGLSAASGMQGSSDPPAILTLIPLNGTFEKKHITVPYMPDVLRIGRQT
NAKTIPTPVNGYFDSKVLSRQHAEIWADRRGKIWIRDIKSSNGTFVNGQRLSPESRESEPHELREN
DALDLGIDIVSEDQKSIVHHKVSAKVEHAGIYNTGPNIFDLNFGDIDPTSGGGLLPSPLSQPVVHL

RGRASSSLSNRSVQSTSSSQMSALHQQRQMNYWLSPITIEHVVKRLTTEIKQAKQQSLDLRQTDEF
LQTLISSDGADKDKLNHKLENHRQMNGRAKMSRIDPIRFSEPPAPPPQQPLPEKPDVARRATEPPA
AGLKRSDTEKPLKDSPTSSPVSKESSQILSLIEALSTTKKELEAQNSRVKELEELLRQERSARETA
EERKQEADNLTAEAAPERIETAESDVPEVEEIAKTFETSNTEPAEVAEPKSLEDPVLDAQTQSLQQ
QLDNMVTELAEMKHQVETFKQRAEKAEGETIEVRKSLAEMIETFRQEREQAAKANETLADDTEAIV
ADTRGIGKSDEKESLPNHKLDPHKIKEIENAVSSLTQQRKPNILENSGPYASMVGVVLIGIGLMAY
LNGWQKIER
>g1402
MPVVAGPPQAHGPSTFEKMKMGAMMGTSVGMIMGFIGGAVSIFQYGAGPNGVMRTLGKFMLGSGAT
FGMFMSIGSIIRTEGPYHEAWRRVHANPIILPRESSERR
>g1403
MKLSITAAGLFLAVSSFASAQSTSTITTTHTITQTIIHASTVTASSRPASTPAVSPSSTPKPSPSS
SVVPASSSVSGTTTTTTPSVPLSTNSNAAVPLAGNVQAALVAGSFAALLAVL
>g1404
MTGDYFYKIGLKIQKEEYPNDARLFAEFRDLVSSWENLAYYYSGSPTLFTEPPKYYHETNSWSNAH
LIEGDPADFEPFEDAKSEASLKRLVAWPHPKYWPELRKDAIVIDDVRAITFPKDTEIPKYVKNLIE
DAKSFLKWYRKYARDHNALFMDSDEHWAVRTKTVTRGGSFVAPDWLGSSDDSEVCLDEDQSDEDGS
SDEPGDEPGDDDGDDDGEEDEDEGSEDYYEEEENGEDYTDTDCEVKGSDEDFSHEE
>g1405
MKILSDNLSSITTXPTNKPIFIPTEIILQIVSRLDRSLRGHERQQTLYNICLVSHQWYSAALSFLY
SRPRLEKGNAYRKFTDVLCPPAGMPKTSSMSKDLGVFVRKLDLSRLVHHSSNSMTARLIGRVRENL
EVFIAPAAGFSVNCLSPLSKCSKLRYLDLSLIHEPLSLKSILKSIRSMKHLITFRSPLSMRINTDE
QSSSTKWPSSLKSLQFSGTIETAMETFHWPSNMTQLCLKNCQNLSLENMTNLVSNPQLDARLKRLT
ISTYNRGLQPECVRLIPAFLPNLLFLSVPGDLVQDTFFSMLDYQRHELALEVLELGHTHTGERLQF
HMQSLIDLLDSRLPYLRAVGFHTMYGEYPDLDDALLERAEALGEEMRVSEETDEFDVGTYYFD
>g1406
MAYMKKDEDADQAMIKLDRTTVFQEARLFNSSPISPRRCRTLLTKLAVLLFTGEKFPSDEATTLFF
GISKLFQNKDPSLRQMVYLILKELATTAQDVIMSTSIIMKDTAVGSDVVYRANAIRGLCRIIDATT
VQGIERLIKTAIVDKTPSVSSAALVSSYHLLPIARDVVRRWQSETQEAASASKTSTGFLGFSSGQS
HAISQTNYMTQYHAIGLLYQMRSHDRMALVKMVQQYAAAGAVKSPAAVILLVRLAAKLAEEDASLR
KPMMQLLDGWLRHKHDMVNFEAAKAICDMKDVTDAEASQAVHVLQLFLSSPRAITKFAAIRILHSF
ASFKPHVVNQCNQDIEALISNSNRSIATFAITTLLKTGNEASVDRLMKQISGFMADITDEFKITIV
EAIRTLCLKFPSKQASMLTFLSGILRDEGGYEFKRSVVESMFDLIKFVPGSKEDALAHLCEFIEDC
EFTKLSVRILHLLGVEGPKTPQPTKYIRYIYNRVVLENAVVRAAAVTALAKFGVGQQDPEVKRSVS
VLLTRCLDDTDDEVRDRAALNLRLMKEEDEVANRFVKNDSMFSLSTFEHQLVMYVTATDKETFSVA
FDVSSIPVVSHEQALAEERTKKLTSATPTLKAPSTAPTKAKANGAAEGAATAAATQKYAEQLMQIP
ELKEYGTLLKSSNVVELTESETEYVVSAVKHIFKEHIVLQYDLKNTLPDTVLEDVSVVATPTDDEE
ELEEDFIVPCPKLPTNEPGIVYVAFKKTGGEHSFPTLSFTNNLKFTSKEIDPTTGEPEKSGYEDEY
QVEDLDLTGSDYVIPAFAGSFDHVWEQTGANGEEASETFQLSNVKSISDATEQLITALSLQPLEGT
DVALSNTTHTLKLYGKTVSGGRIAGLVKMAFSARTGVTTKVSIRSEEEGFAAAIVASVS
>g1407
MDMPVNVPVDHPEADTEWNDILRKHGVIPEKPPSPTPIIEEAILEAQRRAHENRLEDKDLDELDAL
EDEEDEDFLEQYRKKRMAEISTLQKASVYNQVYPLQKTDYAKEVTDASAEAFVLVNLTSSTGGNTE
SRVLSEIWRQLAPKYGEIKFCEIRGDMCIEGYPDRNTPTILVYKDGEIRRQYVTLRELQGVRTKAE
DVEKVLVDLGALKEGDMRLRKQDDSDDERNRAPEKEDEDDDDWD
>g1408
MKSAILIIPFLSHLAASQTANWGQCGGQNWSGDVTCNPVASSTTSTRSTTSAGGSTATGSFPSASG
TSFVIDGNKGYFAGTNSYWLPFLTNDADVDLVMGHLQQSGLKILRVWGFNDVNTVPSSGTVYFQLL
ANGQQTINTGADGLQRLDYVVKSAEAHDIKLIINFVNNWNDYGGMNAYVQNYGGTQTGWYTNSAAQ
TAYKTYIKAVISRYTGSSAIFAWELANEPRCNGCSTDVIYNWAQSTSQYIKSLEPGRMVCIGDEGM
GLSVDSDGSYPFGYSEGNDFEKTLTIPTIDFGTIHLYPSQWGETDSWGSSWITAHGQACKNAGKPC
LLEEYGSKSLCSSEAPWQTTAISSVAADLFWQWGDTLSSGQSPNDGYTVYYGSSDYTCLVKNHVSA

I
>g1409
MERPEQVEEKKTAAALEEVENTTLRVVTGSGAFNEALIQEPPILSHPTTILLCLCLLVGFFCQTMN
GFDGSLFSGLLANTVFLDHFHGSDSGIWAGIVSAMYQIGGVSALPFVGPAIDTWGRRVGMFLGSLL
IVLGTIVCGLTITNASVGQFMGGRFMLGFGVSIAAAAGPIYVVETVHPAYRGMVTGYCNTFWFVGS
ILSSGAVRGAITLDTNQSWQIPIWLQMVFAGLVIVFCWMIPESPRWLYVHGKREKAIETMTKWHGY
GNRDSLWVKLQISEYETHLNVDGSDKKFWDYRSLFNRRSNFYRLACNCAFSIFAQWAGNGVLTYYL
VPALKGAGFTSDVTQANINLGYSCFQFFFALCGAAFVDRLGRRPMMLSGMAGCCLVWVAVLAASSQ
VYDSSGVLNHSASNATLGFIFIFGGVFSFCLTPLQALYPVEVLSYEMRAKGMAFSSLAVNAAGLLN
QFAWPVALAKIGWKTYIIFVVWDAIQTVIMYFFLPETKNRTLEELDQIFEARNPVKASIRGTEIAV
DEDKRVVAIKDEA
>g1410
MAFYQQQLSTGWTFRDRDSEQSDLDTWMPVPAIPSTVQQDLIANNKLENPYIGFNELKARWVNEKS
WVYRHEFQRPKIPTGAKVALGFDGLDTFASVTLDGKTILESDNMFLGHRVDITEALLKSTEGKKYV
LEIEFDCAQSRAREIREKYPDHKWVGFNGDIARLAVRKAQYHWGWDWGPIIMTAGIWRDVRLEVYA
ARVADLWPKTTNIDFDGKRASVTAIAKIDASSSTSKDLKASFRVSLRGHEIARQDDISVNTTDQTA
KVKFEITDPELWWPHGYGSQTLYEVTVSLSHDGTELHNTTKKFGIRSAETVQQPDKHGKSFFFRIN
GVDVFCGGSCWIPADSLLPSIDAERYRKWIELMVAGGQVMIRVWGGGIYEDEKFYEACDELGVLVW
QDFMFGCGNYPAWPEILESIRQEAVYNVQRLRHHPSIVIYVGNNEDYQVQEQAKLTYDYDDKNPNN
WLKTDFPARYIYEKVLPEVVAEYSPATFYHPGSPWGDGKITSDPTVGDMHQWNVWHGTQEKYQIFA
TLGGRFNSEFGMEAFPHLSTIDYFVENDADKFPQSHVLDFHNKADGHERRLATYLVENLRTATDLE
TYIYLTQVVQAETMMFGYRGWRKQWGDSRHCGGALLWQLNDCWPTISWAIVDYFLRPKPAYYTVKR
ALRQLTVGVQREHHDWSVTHAQPPVKSGYELWVASSYRHEVLNATVELRFLSVVSGRDLRAPVVYD
NVTITPNGTTDVLTNGVIDHIAEPEPHVLSARLWVDGQVVARDVDWPQPFKYLDLSDRGLEVRPET
TRSAAGERQQILRVSARKPVKCLVFEERDGVRVSDSAMDIVPGDEQVVTITGLAEGDAPLKYRYLG
Q
>g1411
MVSATQNRQRQDEASGSIDAAAQRREHALHAVNGKLDAMARSHVKLVGGTRRPSNVVIRSGRGKEE
TSSLPDYRGALERLFPETAPENLVDLSRERLLALISKPADGSPYNQHSQHQDSLTIATSASVETHV
SALSMERPGLESLHAIPGEHEQLDENQCASASEESEEHISDDVNALSLPARNLTSYLGVSSIQAAL
KVIAWLHPELNSHLSSPKEQRHPHHRSSISAGPPRTELQLLDAYFENFQPFSPLLDEETCRSTFIS
GRRNDDRWLALLNTILALGSITAAGVDNHNHRAYFERSMSFLNIKTLGNPSLEVVQTLGLMGGWYC
HYISQPNLGYALVGASLRMAVTLGLQREPPFDSHSMGANTARSGYQEFKRRVWWSLCCLETWGHET
LDSQLLQWWNNLPPVLKDYNPCPDALYAVRTVMRWRFYNQRILLYRPRLLNYAMRRIPLIAIKGEE
RIAVQRCREIAEMAIEDISAATAVKLNQMIAWNAVWLIFQASMVPLIYLSTAAASAGAADSDDDVE
RCKAQVHTAIATLDRMRPYGHTAERSLGMISSILETILHTPDNTGLMTTDPATNHDDGEMQTQNCP
PIPTEYQPITRERVLDWTATTAATTGSVNTSFENYSSQHMWEYLSWGENNDFWAEMYTSLNPQEQA
NFFIPS
>g1412
METSFEYNNYPSFHGSSYYPNPGGAAVLRPSPHTQQPQQTPPTSSFPRHNSIEMVPGDDHLGQYIF
SEFLEDPLAVAQRSTRSSSEEKDAITPAQSRRKAQNRAAQRAFRERKERRVRELEQKLTDLQAQSM
TLHADNERLKRELAKVATENEILRATSSSGENISDGNNSPRGMDGEEDKKKPSPTTATINGPKKWV
SLDVRKKYAADFKAGNLPHRIVLSAETGERLLDTKATWDLIQSHPLFQKGLVDIGDVCERLKRITK
CDGQGPAFEEGRVRKIIEESVACGNDELI
>g1413
MTTMDLRVGNKYRIGRKIGSGSFGDIYLGTNIISGEEIAIKLESVKAKHPQLEYEARVYKSLAGGV
GIPFVRWFGTECDYNAMVIDLLGPSLEDLFNFCNRKFSLKTVLLLADQLISRIEYIHAKSFIHRDI
KPDNFLMGIGKRGNQVNVIDFGLAKKYRDPKTHFHIPYRENKNLTGTARYASINTHLGVEQSRRDD
MESLGYVMLYFCRGSLPWQGLKAATKKQKYDRIMEKKMTTPTEVLCRGFPNEFAIYLNYTRSLRFD
DKPDYSYLRKIFRDLFVRESFQYDYVFDWTVYKYQKNAAMIVDASNPKKDKETDEQQRRAAQAAPA
LANQVPSAKPGAISGQRRKVIERGTVDTPDTNRAVGGSDRM
>g1414

MAFLFKSKKAQPGQQPGSQGFPPASRNIHTSEGSMQEKQPFVNGDRPRDMGRIVSPTPSGSINNSL
NSVTTPGSPDPSRMRQRADSETQAPRSQSSAGTGSPTTGSNAALYPWSQRRLNFTSSNANPFPRYG
AAINSIASKEGDIYMMGGLIDGSTVKGDLWMVESSGGNLSCFPIPTVSEGPGPRVGHASLLVGNAF
IVFGGDTKINDNDVLDDTLYLLNTSSRQWSRAIPPGPRPAGRYGHTLNILGSRLYVFGGQVDGFFF
NDLVAFDLNALQSPTNKWEVLVRNTSDGGPPAGQIPPPRTNHTIISFNDKLYLFGGTNGSLWFNDV
WCYDPRTNSWSELDCIGFVPSPREGHAAALIGDTMYVFGGRNEDGIDLGDLSAFRIGNKRWFSFHN
MGPAPSPRSGHSMTAFGRQIIVLAGEPSSAPRDPTELSMAYVLDTSKIRYPNETAPNGEKAPDGRK
TSSEKSVTPTGRVSREAQNAPVEQPRRMTTSYRESMIGPSVKPQSDPSQGPAGSRLPRVSIAQAPA
GPPPPGQAPTPTPRVNGQVTQGSRSKTPTKQDRNFGPQVDTVRAAAVDRDAQSPNLKGSPLDSNRA
PADTSPISGGRRTPNSQPPSRTAARQMEAGEAAPLMSGPTRQRSLQKQRQRASMDSTDESILGRQT
SMDGSVESRSHRNSRILGDEPRSPRLTPHQEALIKELEAARNRNAWYASELALARKAGYNPSPSSS
STLDERPPDAFTDEDRPLIEAFLAMRAELAKMQATVDRQAAIASKRVAEVEHQRDAAISEAAYARA
KLAAHGGSQRSTPQLEGPSRGDDDAGSDRATDLGRRLAIALAAQAEMKSKLEAMTVDLDQEKRARE
LAEGTSEATRKRLAELELQNHTLELESLRAELHQLQAHARQESALRSEAEANHKQVSIDKEELSQK
LEDVTTRLKDHSGNMASLREAVAASAAKAALMEKHLEEERERREGLERKLLQLRADHEERTAELET
ANRRLKEAEEMAESHAREAESHKLAFISGLDRASSIDSDSSIRSITDQRVVALQGQVEAANKLARS
NQAAADEAADKLRRAEERIAGLEAYQEQVSREGLHLRRQLQTAIKDSQTMATENKELKSQLESQQR
DTSALAIQHGALKDLLGERGVHASDSRRSPLLDSPGSRFGTPEQTRLRDLEQQLQASLKAHEETKI
TFESREQEADRAYREKLEQLENDYQSAVHYVKGTEKMLKRMKDELARYKTHAATLQTELETAQKNA
ELSSSSPAPASWESERSELQKSLSELQTNMSTSITDLESQITKLREDLAAAQADSHETRSAHEAAK
EELLAVADRSRAEIEQLKEENSLLESRALHAEQRVTMLLEQMVTSVDNHRRQSQHGQGFSTISRTH
SNASTATAGAFDGGRARGNSNVSRDDSFLDNRSSMALDSLANELDALRSHWETTNRSHRLSSTFDL
EATPTKETHEGNTLSESVSSWRRRLEEEELRASSPAVDLSDGLRTPTAATHAGAAGHSGNNMI
>g1415
MEHSNHNGDTQSGPASAMNGTRRNQNRSVRVSFEPTPDSVISPRDRSPTPLNSHHRSLTEVEPQES
PSAQPARFDGSYDSNGATVTETLAPPNRPSLGGRPGALQRARSDYGPRSVLSSQATPNEEDDFAMR
HGWQEEYTSTEYLKLLHSNFYMYFTEKRHETSGLPREPSGSWAIQEWRMRDRLKTVSAALAICLNI
GVDPPDVVKTNPSAKLECWVDPTSTTGGQNKMMEQIGKKLQEQYENLSLRTRYKQYLDPSVDETKK
FCISLRRNAKDERVLFHYNGHGVPLPTQSGEIWVFNKNYTQYIPVSLYDLQSWLAGPSLFVFDVSH
AGNIIHNFHGFVEKHERENIEAKKRDPNAPIQNYGDCILLAACQKSETLPTNPDLPADLFTCCLTT
PIEIALRFFILQNPLNINLKLEEFKIPGRLQDRRSPLGELNWIFTAITDTIAWNTLPRPLFKKLFR
QDLMVAALFRNFLLSERIMRTYQCHPMSSPKLPETHNHPLWQSWDLAIEMVLSQLSALLDNEEGRR
QYEYQHSTFFAEQLTAFEVYLSSGPTEKNPPDQLPIVLQVLLSQAHRLRALILLSKFLDLGPWAVH
LALSIGIFPYVVKLLQSAAQELKPVMVFIWARIMAVDHSVQNDLLKDNGIHYFITILNPTSPIPVG
NASEHRAMCAFIVSIFCKNYPQGQNVCLSAELFDSCLNHLMDVENPLLRQWSCLCLSMLWVDFPDA
KWMGIRYAAPQRLCELSLDPVPEVRAAMLHALTNFLGIPDLTDQVAKIEEVIAMAILPMSTDGSVL
VRKELLVFFSTFVKRYENKFLVAAYEGMVEEKHSLLRRAQNESNQFALLGETDDSDDNSHSLSHNT
TFGTIWKQLLILSVDPHPEIAQDGSTVVDYIHFALLQSPMATMANKVREEIMDIAARLNPNKPQLA
EPVEFKAAHPAPPAQPQPKNEGYFSLSMRRTASVAASLKNLAFGPATSADQEPLSPTSSQHKASPT
KSRPPITPRGRAPPEWTRPPEVNDQVAPATAYHQAPAPLSRGFESKDLSVPPSIPLKSRFLDWSTE
YFREPQMKPNEPDEPGSADYNERLWRRSRNERIIAETQPLKEKAGSSRWHVSTALLTNDSQPMKMS
FHQFEDHLAIGDDRDGISIWDWQRNICLNRFSNGNPPGSKINEIRYINEDDQALLMTGSSDGVLKV
YRNYEKRKKIEIVTAFRALPELIPSNRNAGLVFDWQQGQGKALVAGDVKVIRVWNAATEVCTNDIS
ARSGSCITSLTSDQVAGNIFIAGFGDGAVRVFDQRLKSTAAMVKVLNTSVMAFLGILATASFGALT
FITYFRHGEHHLYGASYFASFLLICTSAIIYLVSKQGQPFYEALGTTTKTATSFLFGIYTTCVLYR
VGPFHSLHRFSGPFSARISNSWLFFQVTPGKQAHLVLLNAHDRYGKIVRVGSNTLSIVHPKAVEKI
HNTCLKAPFYDLNSPSTSIQTTRDKSLHDQRRRPWSKAFSETRLRDYEPRIKEIHRHLLARIDRDS
SMSQKNEKNSPVDATRIINQYAFDVMGEVAFGSHFDMLSTSKNHKAVDMLNESIGLVGFMCPIWLI
RLAVAIPGLTRKWWQFEQYCFEQMLMCRSTKSSSPVIASTLLEPYEKREPTPFETRILRGEAQLLV
VAGSDTTSAALTCALYELARNPHITTQLQDAISASLPPNEKEVGAHHVRNIEILNGIINETLRLYP
PVPTTLWRLTPPEGLWIDDETYIPGNVTVSTPHYVLGRRNDIYDRPNDFLPERWSSNPELVKQEKA
FAPFSVGAHTLPISIYSRTIPLAMMNLRMTLAQLIYTYDISFPESMTMDEAKIAMEGNMKDNFTLM

PGQLDLIFKRRV
>g1416
MAPSGGPSMTSQARAAELNAAAAARARILNGNRNWELPDNPPRSIPSQSPSSQTNNPSSQKKNAEL
GAVESFRRPNRPVDRYYPPVATSATPIPVYAXPPPHYTDPRAMYVPAPYPVATDYYYPPMYQSMQS
FQPIYSTHMSGYRPPSPLHRGAIVAMTRQNTSRSAGASPTKPGPLQHVKVPATPPVKSSRFSSPAK
SLPRGSSRGQSRGSSRGTSRSPSKSPVKRLRATKSSEKSPVEVLSLFTPDDLSPLKVEVELKRFKA
ELARIASSKEEKKSSDNAEGKASPGKSPVKSSNSHEGDNDHHKDKDPKVEEDGQSTPRAKQPEPSN
VVRAQRINDQLAMEVHPEARRKPPTLEDSPLTQKTYRNSMVDKVRPFSFLPAGLMPRPAVKDNLST
ADQMLRVQEAHNANPDKSMYQDDGIFHVAQKTDDSIGEATPKAVPRRLQSPFQLRPRSPVAFYKPI
VNKYARNVFASQQSRLEEFCKVETGLSWMAFRQKPEVKDLPGPFKGLPRFSLLGENAQRPMDTGIL
VPPPGFAIPGPLLLPRSVHWNPETNPSNRAATYYERVLAAEDWFHQSILERKESSNQFQRKLNEVR
SIIFNDQDPFKDLPPPPTNLKGAQVTELVKRVSEQLISYTDTTENKRRGFDFSDVEFTLYAATGFR
GSKRNRRRNRRSHRARYRGVEFDIAPGSEIFDGGIFASELC
>g1417
MSSIINKVKEAVTHDKHEKPSHSTNAGPHDSNIANKADPRVDSDRDGRAQHTYGTTGATGTTGLNT
GNNYAQNEYGTGSTNAGPHNSNVANKIDPRVDSDLDGRAVHGNTGYSATGMNTGSSTLGNTYGTTG
STNAGPHNSNVANKMDPRVDSDLDGRAVHGNTGYSATGMNTGSSAMGNTYGTAGSTDAGPHNSNVA
NKMDPRVDSDLDGRATHHNTGYNTTGNTTGVAAGGYGTGATGYANNGPHNSKMMNKLDPRVDSDRD
GRNAFGHNGSADTTTKSFEQSQTESTPTSTTAHGAGSTNHGPHNSNLMNKLDPRVDSDRDHRAAHG
GMTGTTGTTMGAGAGGMGAGAMGTSTTGHHGHHGHGIGAGATTTAGPYDSNMANKMDPRVDSDRST
YGTTGQYGTTAGTGMTGTSTTGGHHYNTRSTNAGPHDSNMANKVDPRIDSDMDNRARHQHMGTTGA
PAGSSYASTGAGQTQGSVGPHKSNVLNKLDPRVDSDLDGSRTVGNTQRQY
>g1418
MRPPRLLLLIFCFIFLPILLTFLSLVTAEPRRGGAEGEAPVGDRSSRLRALFSFNTPTSLFPPSAI
ISLTDDNSTFFLARPAAFGPLLPSKGLSGQIWIGSGFSEDTLGKGVGTAGFQGELGCSDVPGWDKD
GQAKPDKALKETHTILPANPATDKETIDKRKNADTEHTAADKSTEPAPNDGTDDHLHHTFSAVKDE
NSSDKPSTHADIQSLQESAEIAGKVVLLSRGGCGFLEKVKWVQRRGGAALIVGDDTRGGSLITMYA
RGDTSNVTIPAVFTSYTTAHLLSSLIPERSALEGQKEIDSKNVSADQKRPSKPTTTTTKITNQPTN
TRVPKKDAIKKEESSKLKDNNFFSAIWSLFGFGRGESLADDSRRPPSSGNIDWVLVDDYEEEEDVN
KLKSAQSNKDKGAKVDKSKKVDNSPVDGDGFEIGVHDWRDPDLVPSKSSGKLISDETESKKKDEKT
DSKADSNLKGGIITPGSGEYSHMVKVPVDKTGKNGADDRDETSSDSEKSWFARHFGWNEGLDDELD
QTLKNRVHSTGNPVAYVREGEHTSDDHPGLWVTLTPTSMSTSPFFDTLLVLVVSPLITLTVVYALL
LLRSRIRRRRWRAPKSVVERLPIRTYHTITQSTSTTSSPRSPSPGSITSVTSTSPLLGSRPRQTHG
RSRQRSQTVSGAGIRTHDLTPVKEGTFSGSDLWRRKYIGRQVECVVCLEEYVDGQSQVMSLPCGHE
FHAECITPWLTTRRRTCPICKGDVVRSMALAEPTTDSQEPTSFVRTASVPIIPDISDDSNLEEGRR
ADEPLLSDDDNNDDESNANAASSSTPHPPNWRNLSSINFSALSGDAMWHSSRNDRSR
>g1419
MADRVKTVAQSEIQRISSLTFRALKSGAYLYPFQGIIYLLSHRTLWAPLRSRLMPTLALGVGVTTA
MFLLTYIPQMTLLAFTSGPLVAPVSAALLVINEASAITSFLASRNMFFGLGQRQSSSTSSVTLDAF
DATLILKGHETLVSGGRELRPARGGNILARLGGFVGMSKRTSFTSNNSTIDNLIRSLLYLPLNFIP
IIGTIAFFALKGKRIGHNALNRYFELKGFSAREREEWLQRHEGECVSFGVVASVLEMVPFASLGFM
CTNTVGAALWASRVETSSKSDTAPGLREDSKKAA
>g1420
MDQELPLPPLRSIRHRPPAKAPEHPFRKPKRLSSRLSQSSLPSSDPALFSSDDIPSSSLENYNGQQ
IEHSRKRRYRGTWWGEMAKEVKRKRADFKEKRNMDSGVWMGSDESSSDCLLSSEASSCEDFMARAW
AGQNQVEPKFTTGIIDTRSNDQDGQIAGPVFAPRNIQNLVESQEHRDARTMINECLEKGQESVDLS
NFGLKNIPTDLLRPLLHLTKQPAVSDGPVTDEVFTSLTPSLRLFLSGNSLKELTSEIFELEHLNVL
SVRNNELAEIPGAIRKLTNLQEINLSVNQITTLPWEILWLIRKGDLKQLTVHPNPFMSIEKEAGDV
EHWHHGNGTEGDLRACEYEGPIPEEAWAPIHIATSRVQRFDAEGKLVSTKAFGRSQSATTQNQTSR
APSLRELALLECNKAPYLDQFLADDATSAESPEPVIRLLRQAISVRNAGGMTCSVCHRSFVIPRTQ
WIEWWDCATYENGLKFPRASGAELRPLPFLRRGCSWGCVPTSGES
>g1421

MAYPSKTRIFMTGASGYIGSVITEFAIAQGYEVHGLSRTETNDAKLKGLGAVPIRGDLETLDVLRH
ESANAQIVIHLADAMTWNPDYSAGLRIDAAAVDAICEAIQGTDKPLLVTSGSLLVEADPNGEETTE
TSPLQKNPVNQRWKSEDHAIRWAKEKGVRVIAMRLAPYVYGRGASGIRLFMQMHARNGEVTCIDDG
SAKTSTIHVDDAARMYLLAAEKGSARNVFNCTSSTDVTALQIAEAMGSVLNLPVKFLKFDEAVAKF
GPFFSKFLSAVNRASSAKAVQTLGWEPKEVGILDDIKGGSYLAVAKELRKD
>g1422
MPLNRIPRSAFFSQSCYNASLRIRVSRHAQYRRGFLTSATEAPEIAFAFDIDGVLLRSSKPIPGAS
DALRTLQERGIPFILLTNGGGKHETERVAEISEKLNISLDPSVIIQSHSPFAELVNGAFGQEALEN
KTILVAGGDGDNCRLVAERYGFKSVVTPGDILNAYPGIWPFAKNFKDYYKSFARPLPRPIDPANPL
ESLKIDAMFVFNDPRDWGLDAQIIIDALLSNQGIMGTLSDKNGKPDLPNRGYQQDGQPPLYFSNPD
LWWAASYHLPRLGQGGFREALEGTWAAVTGGPTQGVELIKTVVGKPYTLTYQFAENQLLRNRSKIF
DSEGLPPLRNVFMIGDNPESDIRGANTYRSDHGSKWHSILVRTGVYSGGTPAYQPTTIVDDVKRAV
DWALKTSEY
>g1423
MADSTLPHRPKPEESSEPAPATENAAPAAEGGAAAPSKSALKKAAKEKAKAEKAAARAAQEQAQKA
ASAAADTAVGLYGKLPENEDLEPAIDFSKLGEEHYEKEVTVVARVDNARSQSAKLGFLMLRQKGKK
LQTVIAAPAEGPVSRQMVKWTTGLNVNSIVQVTGVAKKPAVPIASATLTELELQIKKIYIIVEAAQ
VLPMQVKDAERPPPLTDGIEGEPEVDAEGTPIVTLKTRLDNRVLDLQTETSQAITWISSGVAQLFT
EFMLKSGARWIFSSKLSGSSTEGGSEVFEVKYFNKKGYLAQSPQLMKQMAIAGDMESVFEVGPVFR
AENSNTHRHLTEFTGLDFEKTFRHHYHEVLDFAEELLVFILTELKERYKDEIAVIQKSYPKAGDFK
LPKDGKALRLNYMDGVALLKEAGVDTSEQEAFENDFTTAMEKKLGQIIREKYDTDFYVLDRFPMAV
RPFYTKADPEDPTYSHSYDFFMRGEEIMSGAQRINDVKELEESMIKKGVDPKTEGFEDYLNAFRQG
CPPHAGGGLGLNRIVMFFLGLPNVRLATLFPRDPQRLRP
>g1424
MPEKRKTDSELRDGDYTTPAKRARQQISYDDLDDSMLTPPVVRPEPTRNELEDDVGDAFENESNLG
HDSKNKTSNKHNSRQRHNQQPRVDPVYGQRSAFPGLDDAGSDELLYGPPEDGLEYLRLVRSEANSL
PALFVAPKPKMEKKETQQANEALGKLETTGKEPEQVQSETGYVLIDGVVIATSTAKETTELDVEEK
RDAQSSYYNLLKHRFILLRTTLRCSXPANTITDLDESHPITLPFHVRAARTAWTQLLYTAEPQMVQ
LACMDMESVLGVLRLFGDVMTKCIKDNDANQLRRIGAWAWGLLGRCRDVGELGSEEVGELRVLGRK
ALSLLKKMDRNKTLSGDGFSVVEPDLEDKESSDDEDEMMVTVESMESAEGQEIDELEAAKLRLQSR
FLGEETTAEQAKRQRRNTSSPLXPAIDDEPETTPSPKITRTKSLPNIAVTGEEQSRILLDMIITIV
GEFYGQRDLLQERDIWN
>g1425
MPPPMQLDDPGSPTSNRDPSLHMTISTLSLTERKPDDGHAIPAQRPPPLPPRKDASSSTLSLGPSG
TPQDGRRKLLLIYIHGFMGAEDSFRTFPAHVHNLAADALADSHVVYSKIYPRYKSRRAMSAARDEF
SEWLIPHESDQTDIILLGHSLGGILAAEVALAETEDASSPIRHRHRMLGTINFDVPFLGMHPSVVG
TGLSSLFHPRRVEDSSQEDLNQTTSLQTPETLSSDQSSQYSTESTASSWYEQKPDPNFDPPFVNDV
HRVKRNQLDGALNFLKKNSGNLRTAVKEYANSYFEFGGCLADYSGLRRRYASLKELDGIDDLEPKA
DKHGRLQRRLRFVNYYTASPGFPKKSPSASIESKGLMDKSPPSQTLAMESVENLTKLPSPIISTER
VDDSSDEYLTEVEPSPMEQSPSPRQSFDAVSEQSAAIEDNKIDEVEKDYTIMLKSLPPLPMIPPKP
QALDPSKFYNKDILKQAQKEHARLVKLHRKAQKEHHLTLKEREKLAQKWSKKGKKSESISLRSESR
GDLDNSIIIPDDNHDDVQDRHPAPTSASTEEVSTSSSPPQLDTTEIETEINTISPPYIQEPRRSSS
PATPTSTKPPKDRVFCALPPHTTKSSIDPLWVRIFMPDVDEVIAHQSIFLPNGLYYEWLVNDTAAR
IEKWVQDDCTRRAIWEQFGEIL
>g1426
MPSNVEITIPTATLTGDYTTYNITVRLPLRSLVVQKRYSEFSTFHDDLVHQVGAQPPVPLPKKSWF
HNTNSNEKLREERREGLEKYLQAINSAEDQRWRNTSVWRSFLNLPVQASAGDNTSSARLHKAVTSP
GGLGSAPITDPVLWLDCHRELKSHLHDARLHLTRRDQATTPQKQHESSAHAKSSLSKAGSMIIALE
EGLKNPGNDNWAGSGLGEGEIRRRKDLLASAKKEKDGLENLHNAMLQKSKLDTAVASIQDKDKLMS
SAKPRPGGRVLGKETDKTRELDNQGVLQLQKQTIDSQDKSVEELLKIVSRQKELGIAINNELEVQN
QLLTMADEDVDRLQNKLDVGNKRVGKIS
>g1427

MTQASQIQTSQTDVLIVGAGPAGLMLANWLSRCGVKTRIVDKRGTKIFNGQADGLQCRTLEIFDSF
DFAHRAWRESNHMLEICFWNPDESGTIRRSGRIPDTIPGISRFQQVVLHQGRIERFFLDSIKEHSD
ITVERGVLPTSFDFDVSKAGDFDDYPITVTLQALSEKEATPQQRNQHQKSADGEQTTVSDGLFRSN
LAPDDTDDLINAAKSNNGDGHHIETVKAKFMVGCDGAHSWVRRQLGFSLQGDSTDYVWGVLDIIPI
TDFPDIRQRCAIHSSSSGSVMIIPRENKLVRLYIQLQATEYMQSKGKVDRSWITPDIILQSAQKIM
HPYKLDYSYCDWWTAYQIGQRVGDNFSLNERVFLAGDAVHTHSPKAGQGMNVSMQDTYNLGWKLAH
VVKGYSDASILKTYQSERRRIAQDLIEFDHRFSRLFSGRPAKDVMDEEGVSMEEFKSAFEKGNEFA
SGIAVNYGASVIVAKDGDALEQGDGTDVIGNHLLRSASKPQLAKGIDIGKRMPSFKVLNQSDARPW
HLQELLKSNGRWRLIVFPGNLTLPENMQRMQKLGDKLGASDSFLRSYTPSSQPIDSVIEVLTVHAG
PRTSLELLDLPEAFHPYDSKEGWDYWKVFVDDQSYHEGHGRAYVNYGIDPSCGASVIIRPDQYVSW
VGDMDDYDEMAKFFSGFMKIQVPGAA
>g1428
MLPVLIFTSIMTLFFFGFNGERIIDAAALTGPQIAVRAGKSFCGLLMICGSAYGLQSLLDGEVPTL
QSGEFHIFASNGTSAAYSHDPFRSTILEDPATKFWYSTRQTPVPYFNPDGTLWGHTAPDARKVEEY
NAVQNMMNFAFFGFLIGVFVYICIQILNKIFRLFFEGNWSQIHTQEALGFHTLNVNPISQTPADPS
IKDSLDKILNAVRTTPTRPEVRLGSGDPGSEHHDTLETIRTEITSNNTAQSRASGRLSTRMEGMEE
RIRERLQESDAEARQYFGDIRRLVEAFSRQYVNDQNVNNTNDEDVRTRLQNLDAQIQALNAIVGGF
NLNDTNSRIVSIDAKIERLINLFNTRDLNDRTQSSNETVEELSIDMFGALERRMEQVNDSLRALEA
KYDQLSQLVRANNLDSKITTFGESLDQVHDQTSKALTVDSLEFKTLNDRVSTIQTQVTGVSLDLTG
HNTSTEGFNREMNQKVENLEREIQKVSNKIGKYRMLEAKIDGLQTEVTDSFKLTARQSDVQLVSTN
VKDMQKGLQTVIDIPERVQKIQKDISKFSNLGGDVRSMSEKLEDMQGELQNIMPVTGEFKEIRKEI
SKLSNTAGLRIGYLNEDLGEDVRSSNERLDKMQQQINSMPGEVEKLDRKTQDLIRQNHDSVLSKLE
ASQCNCQNTLTMTQEEMPKINQMHGTVHDLRQEVEDIQEVQKLHTSAVSKLEANLRELDSRLIGQE
TRADEIEKLHEERLDRNEESLSTHQNRLNTHGETMSVLRIDLDRIKEENEKNPEAPRRKIGRADVE
DDSVEELVQILAKQAIDLYVTNHFDVLKPQLETDLRDQIKELDLELWKERFGYVKKMTGTFEEFKC
KCNLGGLEDRVGKLEEGIERSGCTCNPADFAENAADPGPVKELMTENYAKLEKKVEDKLKTMKQGL
ESVERALKAQNCTELEKKMEDRLTILEENLDTFEEARAKDGVQLEDVDGRLRGIEDQTQKTNDFFF
QRFSSTIRTRSGISQPEIADQKIQELANLIQKEGIGYTIRQMMNYVDGLSKGMNFLRKTVCGDLSI
EPVGSIFEQLDIIRTELGALSYVYNLPAVQKVLQEIDQAETQTLAFTQSYDQQIAPAQETPAPSQS
DTPMVPQSSSVSQPILRRQVSDTDEVPTEMRAILAHGPPRADSSVSYASQSKQPNTSTQTALLQQF
SPVLQTTRTPGWVSVSQQASPPYQPHAQDNTVCQTDVTSEEQITEPQAKPVNIANRKIKVPMSRRK
SHQKQQQLSPTTDLTFQSQQQLQQSPATGSQSQSQPQQQQRHQQQPARQDQQDDQTLQPLMFEDII
DESMLADPTTTDAPEITAQPPSPPPQVGPEAQAMLEAEATERAQPHGTYDELFDDFFDDDDDAINA
ALDEEEKKQEEVEEEERKRNYPLSLPGDFQAANVLEXXQAETSRAATQEPSQSQGRSLSQGQGTVS
GIQKDVPPPAEKSKPVKTSQERKKVDESIKQQNKSSNVSSSAPKQTDKKPRSNSKPIPIKAPEGKK
GEDDGYWQKALPPLLPLDKDFKRFAPKEAGNQPQRNTTSNPATTSRETPGPVRPTRPTSSPFLGQS
KSRSQLTERLRGAAPKSQGPSLSASRWAKEDDSAPKDKEVRNSAEASEEQKEVTQAAEQVSSEEEA
DEGWDDPGLRHNAALEVLSSYKGTPLSASRWADESEGNEEPSRKDEPREGVDKKITGQKSLATIAK
QKDKATSDDISSTPSKGLDDQNVRHRTVVEGPEIYKGSPLGTTQWADESEEQMETAPTPKSETRDS
PAVQAKGDEELANETLPGTPDKWNQWASSTGGVKSSPAMTPGERQAERRAVKEDEKKNTEGDIRIK
GEGEDVAAGNNAASSSAEESESKLRKESMEEGSVQELAMALLYRGLGLRGPVKASQRTEPGSQDPI
AEYQNEPTVNGEGGSFADVNG
>g1429
MAEPEKIARGEKVVREDNSVGSEQAENVKTEAPGILDPENIHNRPADKLNVMFENPLANVPRDQLL
KDVTEFCHKYNLTEHIDEFRKGALVSQNPAAAMDLTELTDEEKNILLREHTHKWHQPWQLYWLVIM
CSMAAAVQGMDETVNNGAQAIYLKNLGISSKFSGKQLDDITGLIVGAPYLCCAILGCWLTEPLNRY
FARRGTIFIACFFAAVCSIWEGVANTWVNLFLARFFLGLGIGPKSSTVPIYAAECSPAPIRGALVM
MWQMWTAFGIMLGNIMGVAFMSLSDDLSWRLMLGSTVVLPIIVCVQVYMCPESPRWLIQHGQVEKA
FKSFRKLRSHDIQAARDLYYAYVLVQVEREINKGKNFFTMFMELFTVGRNRRATIGSWIVMFMQQF
CGVNVIAYYSTTIFTTSGYSIQSALLASMGTGILNWVFALPATLTIDTFGRRNLLLTTFPFLAICL
LWTGFSFWIEPDIVDSKKRVAMVTTGMYLFEIFYSPGEGPVPFTYSAEAFPLHVREVGMSWATATC
WCFNFILSFTWPSLLTSFKPQGAFGWYAAWCIIGWFLVLFFLPETKALTLEELDQVFSVPTWKHAS

YQLKNAIWHVRTWVFRQKLEPLPPFYQGIDKLNES
>g1430
MSYLGPFHILSYGTLLGAQIYQSFIGGTTAYKALPRPQFSALQNKLFPIYFSLQSALPLALALTFP
GKFGTSDLSSVYGVLADENRYAVLLPLVSVSVAGLINMFYLTPKVGRVIKERFQQESIDGKKAYDS
PPHSQKMTELNKRFGKLHGLSSLLNMGALIATIAYGVYLGGRLH
>g1431
MAITSRSHDDYTVGWICALPLRMVAAKAMLDEVHDDLPASSSDHNTYVLGNIKEHNIVIACLPIGD
NGLVSANTVAMHLLSSFPSIHFGLLVGIGGGVPSSLTDIRLGDVVVSKPTGIYGGVVQYDYGKALS
AGFQRTGMLNRPPQALLTALSKLQTYHLTDGSRILDFIAEAEQKIPQYASKFARPPQEDLLYFSDY
QHADSDCTSCKACDPTRVVPRSSRDNNEPVIHYGLIASANKLVRDSKLRDQLSRDLGVYCVDMENN
NWQGYASAVAAAYAKELLLYTTVRHSNQVQTVRDSPSNAPEYYVPFDLPGALAVEDFLGRHDELER
LWQHLQPESKGYRKVAVLHGLGGIGKTQLAIRFAREHKEDFTAILWLSGNNRETLLRSLSSALSRI
PGQCSKSAAITDQGLKQNAEQVLRWLSSSGNSRWLVVLDNIDQYSPGTDDGYDIGNFFPTADHGSI
LITSRLQSITELGRPFPLGGLDFDDAISLLWQSRHLPIPGTSTKLDPDVVDLLNRLGGLPLAITIA
GSFMRETGTDISEYLKYYQTSWHDLQSDASEVAAMAEKEATVYIVDVGKSMKQHNNGRDISDLDWA
MRYVWDKITTTVATGRKTATVGVVGLKTDGTKVPLEDEEGYENISVMQDLGQVLMPDIRRLREEIK
PSETDEGDAISSLVVAIQMINVYTKKLKYKRKIYLVTNGKGAMSSDGLEDIASKLKADNIELIVLG
VDFDDPDYGFKEEDKDEGKAENESLLRGLVEDCDGVYGTLAQAIAELDNPRVKVVRGIPSFRGDAR
LGDPTQYDTALRIQVERYYRTYVAKPPSASSFVIKTGGDASQPPTQTLEDKDVDANLTSVRSLRTY
HVDDPTVAGGKRELERDELAKGYEYGRTAVYISESDENITKLETTAVLDLIGFIQADHYDRFMNMS
NSNVIIAQKTNDKAALALSSLIHALFELDCYAIARLVIKDGKSPLIVLLAPSIEPDYECLLDVQLP
FFEDVRSYKFPPLDKVITISGKEVKEHRNLPSEDLVNAMSKYVDSMELIDKDEEGEIVETIPMEDN
YSPLLHRIEQAVRWRAIHPDEPVPPPSERLTRLSKPPKEVQERAQKYLDRMISAADVKKVPPKAKG
RKRNRDVDKPLSGLDIDELLHREKRAKISPNNAIPEFKQMLATVETVEGIKDATNQMQKIIEDHIR
TSFGDSNYDRVVEELGVLREELIDYEEPDLYNEFVRPLKEKILKEKLGGDRQELWWLIRRSKIGLI
DKAASDRSEVTEEEAKQFLSSK
>g1432
MSGEPNAAWPVADEALTQNILDVVQQASHYKQLKKGANEATKTLNRGTSEIIILAADTSPLAILLH
LPLLCEDKNVPYCYVPSKLALGRATGVSRPVIAVSITTNDASDLKPQILAIKDKVERLMI
>g1433
METAIRWSPSSTTKEQRFLYVDVIGKSFKLCRVTSVKGNELEYDVLSTHIRVPGFRAFDWSAADES
LIAVGQSSGEVNILRMDGNSNESWSFPLRNQRYCNAVAFSSHGLLAGGLDKVRNDVCLNVWDVNHR
IMTGSSRGTLASDRQGAEPLRKLSSDPITSIKFFREQPDTLVAGVKGQFLKLFDLRDNSSQAFLTF
STKCVHNTAVDWMDENYFASCSSSADATVCIWDRRMGPRLTTGPANPEGGQVGPSLSLGNTVQNAT
IWSLRFSRTKRGCLGVLSSNGQFKMFDIAKEYLSAGDRNSVDETLGQGSSSSYPEQIYTKNIRDIR
HPYSHQTRGYKKPNRVVSFDFLNMSRSNEPSAITLLGNGKVEFVTVRSSAAPIRLSSQNVLVRGVP
SGERDFKTIGPRQLREKTSNIIQSLRACAKQNGLTEEEEKKCLSSRENREQALFLGRIKPKLKVVD
VLTYLSVSQARCREGYLFDCEKNIRIVSDDADLQEFWNWVRHSREESANGLTIVNRIDMSYLGVCD
VWFNEIGATSKELRQHSSINKIDLAPEQMIKDMVQQLQLPEGKRCETDFWEHRQLCLHICGAIESE
QELEQIVDDLVKKRQHTKAAALAIFQNEAKIAFHALRQNEPTQAHKLLAMAIVGAAKGQQSDRDWE
ETCNEIATELTDPYARAVLALVSKGKWESVLAETTLPLKYRVEVALRWLPDDDLTTYLQETKEEAI
RQGDIEGILLTGLRHSALNLFQSYIRKFNDVQTAVLAMSHTVPRFIDDETSKAIFRTWRETYRRQI
NSWKMQIERARFDVGSRRFAVTWDGRRLIAPAPQQVSLTCNYCTRPLHQAESISSTTISGGLSTST
STATASAVSDLLQSGGNGSSTATSNPAMLVNNQSYLGAQGAILMSGTVCPKCFRHMPRCGVCSLWL
GSPDPMSRAAIAADAKREEEEKLNHEDDDDETKEHTNGDVTSARKRTGPSEDEIIRRFVVFCINCN
HGFHANHAKAWFSRHRVCPVPECSCVCDR
>g1434
MASKEKSTRFHPYDTTKKPKIHRSDNTTPKRRHNNNDKTTQKQPTNKNSTGDKGLSINDLKRRIRD
VKRLLTRSEHLSPEARIVQERALKGYERDLEQEQARRQRSEMIRKYHFVRFLDRKTATKQLRSAQR
QYEKAKQQQDQTSDKLAALQEKIDAAQIDVNYTIYYPLTEKYLSLYPQEKKREVVDGHNQKVASTD
DGEEESGDEDTQTSGDDKIAENEVEKPPMWDVIKKCMANKTLDKLRDGKLNIGFDGKPIQNLEGTA
LSNVGKDTGKEKAETTTANKEKKRKRKEIEVEQPKEEEGDSDGGFFEE

>g1435
MASTLADDTISSGIASPVTPPSPTPGQIPMEQPPKLKGRRRLLQNLQRMSSSPSLLRRTRSLSTGY
QRDRKASLSCVSLSPAQCWGDPTTSLSGGLETASPTSAASGNARIRVIGSRNVSQTTVPLPVDMRP
ASAPRNMQPELSENDATTVNVRGKNQYAKRTLDFWNEIPNEIKTKILAYLTPKEIVRCSLVSKAWH
DMCFDGQLWYQIDTSEFYNRISSSALMKIITTAGPFVRDLNLRGCVQLREKWLFEGEHISSACRNV
VNFSVEGCRIDKVSLNGFFLRNASLKYINVSGLSTVTNSAMKIIAQTCPQLETLNVSWCSSLDTKG
LKRVVESCSKLRDLRAGEISGFDDESIMESLFEKNTLERLVLHRTDVTDESLKVLMVGRDPEIDVL
TDRPIVPPRVLRHLDIHQCASLTDAGFRSLAHNVPALQGLQISQCTELTDDSIVDVIQTTPKLSHF
DMEDLDNVTNNLLVELAKSSCSDVLEHLNVSYCDSIGDIGMLQLLKHCRQLRTLEMDNTRVSDLCL
MEASSLVRKRGYGTDLPKVGLRIVVFDCINVTWAGVREVLSSNAYIPRSLKNQTTVTVKESTNRAG
GAYSSSSSSSSTSLPPVAVVSYYPKEIIELKCFHGWQMTANEHTKRVLRGSLASANNLDRKWADYM
MATEEAGAGGANARRRRRRAREAERLYQFDEEEDLSLDVYVGGRRRARSGGCTVM
>g1436
MDTAASSNDYDQLSRSPLLEPDGLGPSGLDRIRQQPLSRVFTLPNPSTSSIGQHNSPPNRSSASSR
AASVYVPSGGSIPPFEDLHRFPSESLHSFSFAQRSEELLATRQNILKRSIDFMKDRLGWAATSPGL
VNAQARISGDSEFQGMVDLLSKASVLDRDSHGRQLGSLNGPITSPPEADADNVFERSFHSPTLPSH
QTPFPNYPGDAAEAVDDSQLLSPFYDDNVYSRRRDLRSAPASRRVSLKRTYTDLSSLSLQSKLMET
LAQPYAVGDSLSSSGYNFGLGIGSMLHTHSSKWTPVSQAVFRTEAKAPWTIISANDLACLVFGVTQ
SEVRKLSILEVVQEERRQWLESKLQDPTTDATAKFPNSNVKPRFNMPRGNGVTAQLLSKPSSRQKT
GRRAQTDDGYGSSKRNPRKPNHPATKSRGVLLCGDVVPIQKRNGTTGSASFWVMEKRGGLIWVLEE
ITENVAYLTYDERGQLTKVEGDTDEIWGRDVAKSEMSITNLFPEMPAECVSDSEKLLDLKYFGARG
PGKSSIPTTVIKNPNGDLRSLRVSTFPHVAGMMVLSSTTLNIISSNSVFSSALFGQERPEGHHISE
LIPEFERFIATLTEEDKVPLVDGMVIPEHSFRRARDLLLLRENRANAVSIFLESEGLPAKHRDGSR
LSIDVQMRIVKSETVFPDSQSKREDGTSPTRLSENSDHDDDELADTIAVTEVVYALWVTYSRQIHA
GTGALSSEDQATTERKSTTSIHQTRSGQRTPSPTPTLPSHSNTPSLDNKTPLSLLSHKLNEAASTP
LADRSMPEPVRAPPLIKEAPKKRTISDYVIIEEMGQGAYGQVKLARLKKIPAKKMVLKYVTKKRIL
VDTWTRDRRLGTVPLEIHVLDYLRRDGLRHPNIVEMEGFFEDEINYYIEMVPHGLPGMDLFDYIEL
KSTMDEGECRNIFKQVVDAIHHLHTKALVVHRDIKDENVVLDGEGRIKLIDFGSAAYIKNGPFDVF
VGTIDYAAPEVLQGKSYRGKEQDVWALGILLYTIVYKENPFYNVDEILDHPLRVPFLPFSEECIDL
IRRMLDRDVDNRITISEVVDHPWMVTD
>g1437
MKTVAEARRRWETLLGYGRDTYELKQAIISEDGENPCENGLRSVCWKIFLLCDDLDRSKWTDRLAD
TRSAYDSLKDHFLKYIKNPNDLQSTVDPLAEDEESPWQALRHDEATRAEIYQDVERCLQDNFFFRE
PSTKQGMHELLAPILWVVERDAVAQSTKTIPVNATDDESVMLHLLDANYIESDSFNLFCSVMQVAR
SFYEHTDDKPVNGQAEMAPIVARSQFIHNELLVAADHELATHLNAIEILPQIFLTRWIRLLFGREF
SFDDTLLIWDLLFANGLRASLIDHICVAMLLRIRWKLLEVDYSSALTLLLRYPALQDHGPQTLVHD
GLYLEQNLSPARGAFLVSKYSGRPPELARDRHQQPPRESSPKKVDRRSHSRDMSGGSSSGQSPARA
NQRTLESLFQDVSEGLQRRTEGWGVAKAVRGAVSEAKRNMANAEVGGIPSRAWRGGPRFGTPRLGP
SRTSDPVDLVQQVASLKKRDNSLAELLSEAIHDLSHIKESTVGLSLEATEVLDRAFERIKIVQTEL
QGSSGSTRTKSTSITTNDEKVASDKPEILEREEEEEAIQEKPTEKAAVNEKEKADTKAEASKADTD
FEPVATSASPSASDPLQSRSTPTILPRRPLAQSEFSWMLGDNTHRSHFVSSASLPPDQSRRSQSRS
RQGSLFGDGRDEKREQSPRDEDSLSLNSFT
>g1438
MSADLLAEFGTNTSHAGYAVNAQGPSQSQTNDLFFDDDFDSFVSPEPTDTGALGNQENISSPETNH
DRQRYTLDVATLPPVSEGAEVLFDASAEVRSDEADEDDWGTFETXKPATSPAQSGQLLDIEDDHQP
LSTPKYNLDPTLSYVSAPVDLISLEDKPQSPQSIASQITPSSRHLQTLKTPAFTKKAPIKSKSPPK
PSEDDFFNEWDDFEDGTKEKSQVKSNVSVPTKGTKANDAIKSTKSRQSTQSQSIVRPTNIPPPSVL
LQIIPSLLEEFRVQLDSTKRGSPTTTSDFVTNLVQTLRVASRIIAGRTLRWKRDTILSQSMKIGPA
QSGKSSGMKLSSVSKGESIKEEQEAVGVLEVWRHHTSAFNLAIQSSGERPVPVITDKTRVITANAA
QGALRASHACALCGLKRDERLPKLDDDVQDSFGDWWVEHWGHTDCKWFWEENSNKLHQR
>g1439
MGLPERMKAVVFHGPYKVAVEERPVPQIQASTDVIVKVRYTALCGSELHVFRGHQASKTGFIMGHE

FTGTVVEKGDDVKTLAVGDVIVSPFTTSCGTCFYCKQGFSSRCANSLLFGCPILDGGQAEYARIPY
ADTTVSKAPEGIDEKLLVLMADIYPTGFFAASNGFKDYTREQATELTVVVIGCGPVGLLAIINALE
YKPKHLLAVDSVEPRLELAKSLGAEPWNFVKDREGLDKRVKELTDGRGADVVIEVVGLSPALRTAF
DLLRPWGTISSVGVHNAEIPWSGNEAYGKNLKLQMGRCPVRSVFPQALEALKRNQHLLGFMADKIM
PLSEAVEGYDLFDKMKAQKVIFEA
>g1440
MAATTENHDTSVPNDEVMNDAYGRGNIGESGELEDQPKTEEEYAQAILTLRAIVSSKEAGVIIGKA
GKNVADLRDETGVKAGVSKVVQGVHDRVLTVTGGLQGCARAYSIVAKGLLEGAPQVGMGGVVQNNG
THPIRLLISHNQMGTIIGRQGLKIKHIQDASGVRMVAQKEMLPQSTERIVEVQGTPEGIEKAVWEI
GKCLIDDWQRGTGTVLYNPAVRVSVGGSGQLNHNGDRPSGSYGSGRSYNRTGNGADFSEPSSGYNR
RGSGDNANRSIPMVTDDGEEVQTQNISIPADMVGCIIGRGGSKISEIRRSSGARISIAKAPHDETG
ERMFTIMGSAQANEKALYLLYENLEAEKMRRSQQSPE
>g1441
METQHPQYLEPSELGTKEYWETYYERSLEHIASKTRTKPENSTTTTNTTEGDDNEHDEEEDDDDDP
GTSWFSEHDAPQKILRFLTSRKFPLSPRNVHRKKGAEPSILDLGTGNGSMLALLRKRGGFGGMMVG
VDYSEQSVRLARELQRLRLHSAYESGDDSDDDDDDYDEEDDEEEAAEEEEVEEESVVVASSAGNAA
DDTVQCEEGIPLKNQDIQFEEWDILDPANETILEINTTKKLSWFPYESQGFDIVLDKGTFDAISLS
DETTASANTHICHQYPVVASKLIRPGGYLIVTSCNWTEDELVHWFTTATKTTELEVWHRLEYPRFR
FGGMEGQGVCTVCFRRKE
>g1442
MGLTYNVYLNADKIFGCRECKAHLADYNDIVSRNFRGQHGKAFLFERVVNVTEGDPVERNMTTGRH
IVRDISCRTCRVVVGWKYDKAYEPAEKYKEGKYILEEALLCLVDQK
>g1443
MKPALNRSLARKPSQVFKPVQSTISTIDPAPPDQATKVTVPDVGTKVKKTESQPSQSTSLPKKIES
SVAATKPTKVKLDDIDGGIVGSDLWSAAYREAVESMQDEMDITILKGSSVAQLFEKLEEIDKDVNQ
KSAFVRGMEVLKKAKKPLENFKLALDLASPLAELEPVATTVVGVLKGVTAIAISVASADVKFEDQI
VEMLQHISYIDDCDTLGQKADDKDIHKALVVVYKKILEFYKVAFDILTRKGVKLIMKMVMENDRLP
SIVQDFLKCADILHKLIEKSVLRITQDIQAMLYHSEITQWLGTDKIRGQSTYHSELQSLRADRAFL
GDKGHGKTVAMAYLVDQLNGRNTSQIPQPKLCYYYCRDDETGEAIQILSALILSLLEQLPGLQKPF
FEMYKQAQASGNIDPATNIKTMEEFLQKLLKAVDRPMFVLIDGLDECSTESRNDLLKILKTLSQEV
PVLKIILSSRPQEEILEQLDKIPRIDLISDIQRDRIIAEATVERQLTNRSTDVKELVIDELSKLAE
GSAIWTKMTVNLIKIRKIRMKVPMKRFLETILLPPKLSGLYVEIFSRCTSDDHENEGLARTALKLL
AVSRRPLSIVELAWAVTLGVSHGITNVTALTEEVDHQRLMSLIDPFISRTDFSDIKKRQVRLVHQS
VKEFIIEKWTSGYRPALAGDRANSEERIVEDLEACMLDICVRYLLLDDIGNRDLFPEEFVAISELP
QGSDLFDEDKESVQYDPYCTWDTWEDDMIRYDPTERGFGEFFVYASCHWTGHFGAISANSALFPSL
EHLESVCKRGSTRLKNWIEQNRRPICAINPREEFDYTLYDPLSITSLFGSKAMLCYMLENSNFDEE
KYLPNPLIGAADQVLQWGDISRLRILFLNYKGDHQLQNLESFRLVIKNWFYPRKRHNNWEVVYDLV
DSLTDKMVREHWGNELLCIAARAGCMPIIRRLVTNAQQNVELRSELLRESRGEQQPPAFAEPTRQS
IEEAVLGNHVDAVECLLTMKDIEVHLQYQNCCGENVFHLASTICNPAMFRVLVPRYREGVHQVDHQ
GSTALVRVITNALASEHRYEIAKIIISESSPDWTSHYHDGQQNPLRAAVQLGDLEMCSLLIRDGGM
SPVSALSYDKEGRAGLKDRNSTNEDKAGEILQLLSKHADLHPT
>g1444
MGTPVRQPQAQRSHSHSISLGAINANHRVTRRKSMSRRGTESTSIGATSGFSSYLSRGIHAASPDS
IGRKPSPISIDENVAVEGSGHEDKHVATKNRNRRASEGSHLVRGEGKRVSTELRCDQCGKGYKHSS
CLTKHMWEHNPAWAVTSKLLISKHQQVQLLEAATVLVAMNQETADAAAEVTAAAAAAAAASMEAET
HEIESDNSSASPGASSDIHDGISSAETTPPPMDEDDIELEDKPYGIRNYNYNLPSAFSRSYQSVAS
SSYADNSLHSPAFSHFRHSSIDTRPSTAETTGIHEEDSEASDLAAAIRLCNFGTPRSSAIAGSSDV
PPVPPLPARYLSQQSGISNAGSVGVAGSTPAVFGSFSLDPSQSYKVSDERDVKMGDADRESRHRRN
NDVDFGSRQMHDDDDDGVFGRMEE
>g1445
MLIFPLYGICAIALTGLFFPWIEALQEPSIALFYSASAISQDYFKDFSDWLQLSTKDAGRVLSRSF
SSLPLVVYLPTPSSSNSNIWNGTKEFIVIPSDSSLATINQLENSLSLLTPFKVHPPRFPPPFPDHE

SGSAVYNDAGEAFDTEELTNSNDGLPLFLGLVLSVLAAYHLVIYLLWERKLAKLNRRADEAKARAL
NSLEDLSKAKIVLDKTEDLYLKGVLGGLESTSKQSEIRSSSSSEAQIQILEHLYVLEDSPSAAAPT
LFSFKRWTMPKSEFESTYGAIPRASSAERSIVVPQLPIDESSKRKRSDSDSGNGNDDKGPAVPRNV
HFLTRQSDSGGRSTVIRVSDHSLTVVPNLHVERPSERKRRSIPKSARHPRPQRLPSRPAVLGIIRT
PPMVDIHAGDPSDTNLGRREHVIYPSTTVDIRVEGASDRDLGEPQASIPSESMPSVSIKKTAEPSA
MVDIHAEGAPVAKFTGAGSQPEPPIVESGQNDGYDISTSIQPTILAPRTPSAATATATTRVEVQAS
PQSPTESPQTEQPSGRRRKNRMGQRQRRRLREMRENRGCEAIQQSSQQGDESFFDK
>g1446
MSVYPSKGITFVRNDARAFRFCRSKCEKNFKMKRQPRKVKWTKTHRALRGKEMIVDSSLLLSQFAQ
KRNVPVKYDRNLVAATIKAMERVEEIRQRRERVFTKKRLAGKLARERQREEDRRVVAQGEHLIRKE
LREMQEQNVPLGATSAEKEKAVGTVFGEERPRQKKKARVLVDGGVEEEMDVD
>g1447
MASAFLVGLGLATSAFLGRAGYVALQRYRGGVNKVGRAFYKGGFEPKMTRREASLILELSERTLTK
DKVRKNHRQLMLLNHPDRGGSPYLATKINEAKELLDKTL
>g1448
MESFADDSQDGLGYEMPPQIYPNYGMAGGSPFNASHAGQAIQDDERDGDPKRRRIARACDMCRKKK
IKCDGKMPKCSHCINYKTECIFTQVEKKRNPPKGAKYIEGLENRMRRLEGLLKMAGIADSETDIPS
IERRLQQAQHNDLNGFSGATAAARSSIENAGKSRTGSLQSTPPQDSNSSPRVADSPVSQEDKEHDV
EALSDMMCSLVTNNCGETRYIGSSSGFSIFSPRGIQWVNEKTGDTSFHDMIQAAYVDDNKWLYWKP
EIFSDIFARRVFKPLPPKEEAMSLFRDFFDNFNALLPLFHEPTFMHLVERQYSRDPYEGSGWWASI
NIVLAISHRLRVMSHLVPQEEDKKAWLYLKNAMGVLTELTMRNTDLLSVQALLGMSLFLQGTPNPQ
PSFFLVAAAIRLSHSIGLHKRGSLFNLNPVEVEQRKRVFWIAYLLDKDICLRSGRPPVQDDDDMNV
ELPSEIPPDNVGLIPLVTGEGQFNIFRVMCRFAIIQSRVYKRLYSTKAAKQSDGEILNTIGELDKE
LEEWKDSIPLDYRPENEIKTTHTPLILHVMILHFAYYNCLTTIHRMAVHHGYWTSRLSNYAIQGLN
ARPLNPRVFSSANICVSAARASINLIKYIPQGDFACVWLVLYYPVSALVTLFANILQNPQDARARS
DIKSMNVVVNFLSMLGSDESNGSVRRMLSVCAEFERITRVVVDRAEKEALSRRKRKVATEDMNVNN
TQRADTSNRSGPTASSENFMDATAFSPGEQQQQQGTPSGTLPTTQPSIGVESRNPSDMMSSTFGDL
NANMPVFNPEVSFADQPNLHSSATATTTSPSDNQFYPFQPSFVPQDLWQMPMTLEWDWADFSTMNL
PNMDQPDMFQATMHNGM
>g1449
MATLLRRPANIARYSRSAAELFTTSRSTYSHHTRPQLWSLISARQARSYASINPGDPKRPDDDKDK
NKKKIQKSKDEELAEFFESLKKNGPQAKKTFNSAADEQEFLQQSVQFINKLISESGLPQREAELLR
LVQTLLKEAKYMPPEMEEFFRKHVDQKQPMTLEDWDKFSKFWLQHIGEENVRAAEFAKLSPDEIKA
LFKKLSEEKPEGAFTWGAQQQKKTNGQQGQKPKDDKDQKGGPKVYEFKFDPSSFLVTSIISYMLYT
TLFPGEASRDITWQEFRSNFFDKGLVEKLTVMNRSRVRVDLNREAVSGDSPDSQNSQRHVHYYFNI
GXVDSFERKLEEAHNELGIPSSQRVPVAYVDEVPWLATALSFGPTLLLIGFVFYFSRRAGGGAGGQ
SSIFGIGKSRARRFNHETDVKIKFADVAGMDEAKMEIMEFVSFLKEAEKFQRLGAKIPRGAILSGP
PGTGKTLLAKATAGESGVPFFSVSGSEFVEMFVGVGPSRVRDLFANARKNTPCIIFIDEIDAIGKS
RSKQNFGGGNDERESTLNQILTEMDGFNTSEQVVVLAGTNRPDVLDKALMRPGRFDRHIAIDRPTM
KGREQIFRVHLKKILTKEDMDYLCGRLAALTPGFAGADIANCVNEAALVAAREQAESVKMKHFEQA
IERVIGGLEKKSLVLSPDEKRTVAYHEAGHAICGWYLKWADPLLKVSIIPRGQGALGYAQYLPSSE
NYLMSVNQLMDRMAMTLGGRVSEELHFDTVTSGASDDFNKVTRMASAMVTKFGMSKAIGPLHFEED
QQQLHKPFSEETARNIDLEIRRIVDEAYKRCTDLLTEKKKEVGLVAEELLSKEVLSRDDMVRLLGP
RPFPDTGEFTKYFGGGTIAPPEPPVSDEGITGKDGRDETPLPT
>g1450
MDDSQTSHPQSEPSVPAPTTIETENVRSPMSPTRGRRRATTAVSPTGGTSAMRELFSDRASLRQRK
STATRRTGDPIPSLTRRTTLAERTQGNFTMAGPDETSQLHLTHQPFVQPGYAELNPSYEQPANTKP
VWSLAKPLPRVVRPGMVPTTEELLQNRASPQLPAENSQRIGLDVDPNDLEKGQIPKTADPRKMAAQ
VTDARAQRETNFINTVLNGDTTSVGGSRISRQSSRARRPSRWEGPVEELPIVEERESQKYSSESRT
ENVSVDSLHSLPPPLPEESGLEPHLDDDVKPGDETAGINNIPALEEAAYPEDLHPLLQNLVEEEIH
NNHTVWSVIRTHHREALAESLGVFVQLTLGFCGDLGVTLAAAGNPNTTDWVWGFATMAAIYVCGGI
SGAHLNPSITIMLWFYRGFPKRKMPEYFLAQFVGAFCAALAAYGVYYHSIRNYIDNGTGVEVDVVN

SFVTSQRESFIGPATAFFNEFLGMVILSVIILALGDDQNAPPGGGMNSLIIGLVITCLSMCFAYQT
GAAFNPSRDFGPRLALLALGYGKQLFTNPYWFYGPFAASLTGSFIGGFLYDFMIFTGGESPVNYPL
DRTKRSLKKSRAKWSRRLHLTPREVKDKMVQ
>g1451
MAQSYSPKPSARWSSSSFQLPVDSETPPPPPPKPDSHEASRRGTPLAGSLPTPPSQSQGFQSPRNF
ANDHTPPAKPAAQGFGAEQHQAFAEPPRLEDEWLPDIVKNKTTPELQSILSNPSLLSALSTLHPSY
PASQQVLQSHLDSNKALAQHVLDLESRVANLRSTTESLLLNHQSLEVSWRKKQTEMDAALEPWSPK
ALYQRLAAAVAEQEAICHAVEESFLEGDHHMGRATDKEVADWVRRIRTEGAKLESRKEARARWDEG
RVGGWR
>g1452
MPGTRQKLNTGAEIPALGFGTWQDAEAQVDAVKEAIKAGYRHIDTARVYGTEEAVGRAIRESGIPR
HELFVTTKLWNHQHDPKDVPKALEDSLNDLKMDYVDLFLIHWPVAWKSGKDLFPKENGKPAVVNID
FVDTYKAMEKLLETGKVKAIGVSNFSKAQMEHLLKNTSVVPAVHQIECHPWLQQTDFANWHREKGI
HITHYSPFGNQNEIYGAKHLGKLIETPELVEIGKKYGKSGAQVALAWGITLGHSVLPKSKTPKRIQ
DNLQGDFKLSDEDMKKIQSINKKLRFNDSSADFGMEFFEDLEGKGSI
>g1453
MPMNLEGSCQCGGVQFELQSNTPVPYQLCACSICRKVAGYNGSVNLGGLADTLKIKKGKELINTML
WLWDHHWPELIHPFAPAIDTELPVPDEMVCIMDSSKPAYVRWPEGKKTVHKLYGNESLEEWHKKHN
LYYE
>g1454
MSFGAPGGAAANFKPTPPERGSFPLDHDGECKHLIQNYLRCLKSQGGVNNEDCRKLAKGYLGCRMD
KNLMAPDEFKNLGLEFKDKSTGDDPTAKKTSRGGPRYVFHTPANPDLEKLLGPNFRRGRGANDFGS
DEESQSSSDEEDEEPIGPGGGGSGGGGTAGGSSAGGGAGRTSAANVDDGTSTTASSAGGESQRPPS
LNSGKVRKKADLEDSTDLTALGRGSMSTSYSAVFDSVFGVPSEVWEKLLSPTRAWHKRRFEIGIND
LAFVGWPVFVRDDGHWRKKSRRRKRQKPKNSQETKNGQECDDANPTSDNDIPDGTQSSRDEDNADK
TDVEDNADSQTGSPESDKDTMSMFNVVFVMAPPVLEYSMHLKEMYEHVIKKFSKALKYEQDHSDYV
WKEAQHILQIKERARERDVSGENLHDELVSKSSLAAAIHSVFDKISKSDIAFVSLKSRSADFQIPP
TTSIAELPSSSDTSLAGMWLTTANTVNADHGAEIEVPNQVLAKHFALLLLESEASIMEALKGSELA
PALSHYIRSSRPNKSFAQISALSGIPLDTIHDLAQHLVYWRRARAIPPLHQRDTYIVSPNCDLSQL
KKATQSYAATFPTLPSLPKMLSALSGTPRPYATFIPSKDHKETYFAILAWLLRGGXVTQLRTFSRV
KVTPEIKFVVEQKMRKEQLEKYIASGGHASSPQSDRAYGDNAGGRYHDDAASTRSSSSVTSQTSGQ
LTPLAPRRGSDEDHSQLSDSYMERNAHLNMSSLIMFPHRASPLESRWLDEIMSRFPDAIPQPKLAP
KTQQNETEDSAATDSLKVHWPKFVKYFNGTDALEKIAVREGMRRKVVWQLLQRWGLGQSAVPGLDV
REQVLVTVRHW
>g1455
MATTATKAEVPTFSYAQAAKGLAAPATSQPITKPTEQSGQTGQDNEAIKGLAAEQISTSVSAESEK
AESIADHESKSATTGSTKNAVSGASSPSFGTSSTSTLAKEDEIPPITNGDSHWDNKSQTSAPAEKS
NKKKEKEGKKDESGKSSDKTPPVKELKAAPPPAVNIWQQRKEAQEAKAKASAAVLKSVNGATKPTA
SKSASLATANAPDSAXPANKKKAANDXQADASGSINKDRKRVENGKPRDEGPKKSTNRIPREESTD
IAPPPVADATAWPTPQIALGEEKRKAQEKSDKSDKVEKSPSTRGKEKWTPVHYVPTAVFNTPLPTG
ARRGGRPARGGREGGRGAHSSNSTTNSTATPVDARPAPAAQPNQAQPKQNANLPERGRNDGGLARA
NSLPAQARKPVAADGNTQQQEQRRFQANSERPRVDTRSKATEENNAAINGTSYPSADTATKTFREP
RSAKAPEFVPAHKASERSPRSGTSPADAQANGRFVPNHERRFDNGTKSETPREMNGFVPREREFKD
YNREKGEFQRDREHQKERGESRPERGRGGYRGRGGHSYNSPHNQHFQNSQVSQHQFVPKQFNAGDR
QRSQQQNFQNGTQPQHVNHRLSLRSPSMPNSAGLYSTYPVPEINTVYQGYQTIAPGPMSAVPYQPY
MEQYTVFTLIQMQLEYYFSVDNLCKDLYLRKHMDSQGFVRLAFIAGFKRIRHLTEEYEMLRHCGRQ
LRNAEYIIGEDGQDRLRPRDKWEQWVLPVDQRDASAQNDGHHAAAAVQHFSEQNNFAVPMTNGVDN
NGYQATPNGLPNGGQHEQTALSSAAPEFTPYVAVGDHNEVPNVG
>g1456
MEMTSRSASRLIQSSNRGSTRLLLRAGAVNSTYNALTLSRRNLSNAAPSLAPTETSLLGFATGSPS
SSAPQTYFSNRNTLPANTVIRFVPQQTAWIVERMGKFHRILEPGLAILIPFIDRIAYVKSLKESAI
EIPSQNAITADNVTLELDGVLYTRVFDAYKASYGVEDAEYAISQLAQTTMRSEIGQLTLDHVLKER

ATLNTNITQAINEAAQDWGVVCLRYEIRDIHAPEGVVAAMHRQVTAERSKRAEILDSEGQRQSAIN
IAEGRKQSVILASEALRAEQINRASGEAEAIMLRAEATAKGIDAVAKAIRDGKENAQSAVSLSVAE
KYVEAFGNLAKEGTAVVVPGNVGDIGGMIASALSVYGKVTEGQTRALAAKALGVPESKEASHAHST
NKDTEAGIEKAEQTAEPSGRNSVAESVLDSFDQTTQQKR
>g1457
MSNHSGTIHTVRFSPNGKYLASGADDKIVCVYSLDANPPSHATTFGSDEAPPVENWRTIRRLIGHD
NDVQDLGWSYDSSILVSVGLDSKVVVWSGHTFEKLKTLSVHQSHVKGITFDPANKYFATASDDRTV
KIFRFNSPAPNSTAHDQLNNFVLETNIVTPFQSSPLTAYFRRCSWSPDGLHIAAANAVNGPVSSVA
IINRGSWDSDINLIGHEAPVEVCAFSPRLYSAYPIKSGSEHHNLVTVIACAGGDKSLSVWITSNPR
PIVVAQDISAKSLSDLAWSPDGTCLFATALDGTILAVRFEQAELGYPMSLEENERSLTKFGTNRRG
AGIAETTDGLLLEEKSKAGEIKGVEGRMGALMGDGHAAEGTSNGTTTITSTAAVVTNGTSEKQKTQ
TNGTAPNTVSEQEKELEKEKEKEKEKEKEKEKPDPYQAKLERLKQRPTYTKDGKKRIAPLLVSSAGVS
ESSLPQSRLVASSTVSQVGADAPQSVIDLSKPFDGLPKGGLSALLFGNKRKLAQIEGDEDNQVEKR
VASISQNGSTPIMSNTTAGLMPASTQAATAGQQTTPEFIRPAVVNPCMAISQLRLAVPKIRSQILQ
ALDKNGNPTEPSGDSKQSRIDLVFEARNPPPGTLTGRSTDREPVRISLSRGDQPLWQDFLPKSVLL
ATGNTGFWSVACEDGSIYIWTPAGRRLISTLVLEAQAVILESKGPWLLCITAVGMCYVWNVKTISS
PHPPVSLQPVLDAATHAQTGQAILAPAITGARINSEGRIVVGLTNGEGYIYSSAMYTWQRVSEPWW
AVGSQYWNTTDASVSNLQAMGSQNGEESKKKKISAGIIPFLERNTTTETLLRGRAYFLQRLIKVLL
SREGYESFESSVSIAHLENRMAAALSLGAKEEFRLYLSMYAKRIGAEGLRGKVEELLKALVGGIFD
YDDDESGGSSVEAAAQRNLTEAEKDGRNWSESLDRLCGWPRDVLLKEVIIALGKHRDLQRVTVPYA
KLLGVVDGEEPDSEAMETN
>g1458
MTENPWLPFLLSSGVEFLAVIIALIFAPETLPSTKAPLSSINVENEDTPSNKEEMSLLLLVFFVAT
LSRQAMALLLQYATKKYHWSYSKATILVSVRGAVNLAAVLVILPFLSRLLLQRAKLDAPRKDLWLS
RGTALLLTIGTLLMFSAPSPAILIIGLVAVALGSPLPLTARSLITSLVLPNQVGVLYTSLAVVQSV
GILIAGPLLANTFRWGMKLGDTWLGLPFLAASGMFLLSFLLISSVNLSRRPRVHSA
>g1459
MPMSHQIDCSHLSAPHVPGATVLSINATERWNYSVPATPPFLLSPVQNLNICDVTVSLTHAGAENQ
VTVKVWLPLQDWNGRFQATGGSGYAAGEFDLTLGPTVKNGYSAVSTDAGVGLNALSPGSWALNTDG
SVNFDALIDFSYRSVHDMAIVGKELTRQFYGTKPKYSYWNGCSTGGRQGMAAAQRYPDLFDGILAG
APAINWAKYVVAEQWPQTVMQQELTFPSTCELKFFSDAAIKECDEMDGVKDGIITDPENCLYDPLE
SVGQVIECEGQKITITNSTATIVKKTLQGPLDPYGRPLWYGLNAGAPLDNLVNTTTEINGTRTGNP
FFVNDQWIKYFLARDPDYNTEQINYNTFLDLFWQSYTEYDSIIGTDDADLSSFHHHGGKLLMWQGL
SDQLIFPGGTIDYRNRVERLMGGENRTNDFFRLFLAPGVDHCAAGTTIGAIPNDPFEALVDWVEQG
DAPNMIPATINDGTQAERIICSYPQVATYIGGNADCAASYTCA
>g1460
MRFLSLISLPFWLVSALPAIATANDASPSHLTPLKFATTSTLLNVAYYEAGPSNGSAIILVHGFPY
SIDAFASVVPILTEQGYRVIVPYLRGYGETSFLHPNTPRSAEQAALGSDIIALMDAIHIEKAIFAG
YDWGTVSVNVAAALWPERCNGHVAANSYLIQNRSTAWVPLDPDSEALRWYYYVFLTARGAAGLASN
PKAWARSLWQKNSIGWSFPEEYLDLTATAFENPDYVDIVLNFYRNRLLYAPGDPKYVDLAAQLDEQ
PAISVRSVTFDPQDSVVFPPTNGTATAKYFTGPRCHYVLPGVAENVPYQAPETFANAIFAVDGLPL
NGGSYACPHSL
>g1461
MVFDELRGRRLSLIVGLVGAMGFMLQGYDQAVANGLLTLDSFVKTFPAIDAIHATGAEKSHKSTIQ
GTTVAIYEVGCAIGALSCAYLGDKLGRRRTIFLAGCIASVGIIIQASPFTLAQLIVARVITGLGVG
GFTAVIPMYISESSRAENRGRMVLLEGFFAIGGIALASWIEFGLYYVQTNDVSWRFPIAFQLIFAI
IVTSCILSLPESARWLVSKDRVEEAAAVLGRLEDTNGDSEAVAIGLDTIRRSLQDENGSERSRNPF
AFNDTRNFHRTCLAIGVNVIAQMSGINIITFYSDTIFESDLGYSGTIARIISGCLQIWQFICAGVA
VYLIDRVGRRRLLLSAALGMAVSQACLAGLTSVISTNKSAASASLLFYFLAFTFFPIGLFLVPFMY
AAEISPLNTRGKVTAMSASANWLFNFLIAEVTPTGFDNIGWKYYIIYFCINAAGFVIFYLFYPETK
GRSLEEIDEIFIRSKSPFDTVRVAKELPFQLDVLATADEKRDDKEHIEEV
>g1462

MSVSTSDSTAGKRIRHFVGVESSKQLAFIDDLHRLGLSRTVDLPELIVVGDQNTGKSSVLQAITEI
SFPVESALCTRFPIKISFRQTPGSSTSVQAEIIPGKTTQDDEEFIERTKDFRFTSNELSASVMGEI
IQEVIFSIPGFIISKTDQTLSDAILRIERSGPNEMHWTIVDLPGLVQNRGKLSASKKIVANGGESS
HTNNAKIAKDLVRSFLENERNIVLWVVDDTDIERHKTLELFDEIPGLQSRTIGVLTKCDRKQETSD
DWMVKLLRNEPSTKHHLDQGWFGLRNRKPKEAHISDQERDQNESDLFEKPEWAGIRKEQTGIKALM
DHIDKERRRRIQESMPKIIAEIRDNLRKCEFEIEKLGEQRDSTAAQRFYALQFCTDLQKMADSALY
ARYQDIPSIDPRVKLRFRVNNRLESFQKEIAALDKIKDGLQFRPVQDALNYLAEKSNVNPEQWVEY
ISIAEYGNIFSEIIKESKICQGTNLPGSISPEVEGKIFQKQSAHWRDIAFDLVNDIKNQVKECHDI
FLSLAIPDSRTRGEVIAMTSKTNEAWEVEIDAALEELIDDHQRRPPKTLHPYFTSESRRFDLELRK
QIEWIRRHKKAKNTPEQSKLGTRAEDTEDGSSLDDSPSQLSLELNQIFHVSKRLELYYDIAINRFI
DNVAMQVIERHVLGSKCPLLTVNMKLLASLSDEDLQRIAGEDESVTRMRERLKKDRSNYKQALTQW
DRVRYF
>g1463
MAPKKEQAPEKIPLGRPGNNLKSGIVGLANVGKSTLFQAITKSSLGNPANFPYATIDPEEARVIVP
DARYDWLCEQYKPKSRVPANLTVYDIAGLTRGASTGAGLGNAFLSHIRAVDAIFQVVRCFDDAEII
HVEGDVDPVRDLNIISEELRLKDIEFTEKALENLAKQTRRGGQSLEMKKLKEEEATVAKVLQWLKD
GKDVRKNDWTPKEVEVINPLFLLTAKPVVYLVNLSEKDYLRQKNKHLPKIAEWVKEHATGDPIIPL
SICFEERLTRFETEAEADEECKKLGTKSALPKIIMTMRNALQLGSFFTCGSDEVRQWTIRKGTKAP
QAAGVIHTDFEKTFIQAIVFNYNTLKELGDEAAVKAAGKVMTKGKEYVVEDGDILLIKAGAAKS
>g1464
MPKKASLEAVKLPDRTTLYTIDSSPLFYQPFDGPPIPHLKLIHAFPDVVPKIQIDRGAIRFVLSGA
ALMAPGLTSAGGRLPDKENALEAGDIVAVTAEGKEEVCLVGPLKMGTEEMKEKGKGVVMDAGHYLG
DGLWKLNLD
>g1465
MYWLNGVPRVYAVNGPSIPLTSSSSDQEPPTTELSEDESIQRGKPAVVPEDQKPPAAWAEEAIKGL
CNSRNGQLFATMTDSSISVWQTKPTVVVATVARSQTSLKAYGPNVSLLLHPDSTILVAQTQNGYLL
TYTVASDANTRVYQQQFDHSNPHRRQQLARHFGSEEFNGIKDVSIRFRMAIKVESGIVGALALDNE
LVVATLXPAAIQCIRWTPDKNGTQTTSELLSRILNTPKKITVTEMVYDRAMSLLIWITSDGHAYTV
QRKTDAPETEQSAKLFKGHCFHTPTSDDDQAIKAAVNARFSLLAVSCSNGDILVYTAKDYIGNVVL
SHRLQPPASVKATGDLTFMSYSPDGYCLFAGYQNGWANWSVFGKPGGSSFTADRSITENNSEDWLT
GVSMGCWIGGGSDIILSAQNDRRLWLLETARSALTGCYSPANLSRALLQTGTEIILYRGHDLPDLM
TISGKDSLWHHAQYPPAYLHAQWPIRACVVSQDGRYVSIAGRRGLAHYSVSSGRWKTFDDSNVENS
FAVRGGMCWYGHILIAAVESNGSYELRLYSRELPLTNHNILHTEXLPSPAVFIGPSGEDSLLVYTY
DNILYHYVINSTYGSVSLVPVGQIAFYGIVRAPTRVRSISWILPEEQMRNGDPSQDVKVASVLLLV
DGNLVLLQPSVSDSGDLKYDMRIVAQDVEYYVLMRDQLSFNFAPPAEEPAPSSPSAEVVLKAAQSN
LSLRDSLWMFCGRDLVAWSDVQEILQREDVPKPLPVPVDFYPLSVLLNKGIILGVESEMIQRRDIT
FSILKYAIRTHLFLPYFLRYSLASVDMPSALSLSHYYSHLSYFPHALEILLHHVLDDAVDGHSRDE
SQNETRQPLLPSVISFLQASLPADVYLDIVVQCTRKNEIRSWRTLFAHLPPPKDLFEQALKLRSLK
TAAGYLLVLQALDDEEDEEISKARTEDSAVRLLGLAAHKSDWDLCAELARFLIALDGSGEMLRRTI
DRVGIRRSMTPQKGGNPSETGKRNMRGLGLTLGIPAVPSPLSPRRTEHRRRKGSDMSSIASDGTTS
NLSPITSKTDDEGTSVSEGGSSGLDESYHIP
>g1466
MAPSESKKRLALAIVDFLGSSLKDGTLTAEDADSIEIAQNCIAETFGVDPTDSAAVQDALGGQSLF
SIYNVYEKLKGKTAGGAASAAAGEAKPAAQPTSNATPESDKLKSEGNAAMGRKEYDAAIDLYTKAL
ALAPSNPIYLSNRAAAYSASGQHTKAAADAELAVNVDPKYARAWSRLGLARYELGDAKGAVEAYEN
GIEAEGNGGSDAMKRGLETSRKKVEEMNRAESAPPAEEVDNAPGASRGGPGGMPDLSSLASMLGGG
AGGAGGAGGMPDLGSLMSNPMFASVAQNLMSNPDMLNNLMSNPRLRQMAESFGNGGGMPDMSSIMS
DPSLADMARNMMGGGAGRGAGGQ
>g1467
MIQPTRFGPAIAARTATLLPGRAYLSRTSNDQYRRLSTLLSDARRTPQSSTSFETTILSSVLLSRR
ALHSTAIRSQEQKEQAHTNHKKAETETESKSDSEKNKEGEGESSQEQKKKDVPPPPPHGDKTPWQV
FTETLRSEFKKSKEWEEGTKALASSAHTFTESESVKRARAAYEAASTTATSTTSTALKSTGRAIGQ

GAAWTWETPVVKGLRKGVSATGKGIEKVTRPVRETEAYKSVKEAIDDGSSSRYGGWIEKEERRRQR
QLREENELKSGKSSRLEPMEEDPNAGTNLTLHKDSAWKESWKDFRDKSPVMQRLFSLKDTYNESEN
PLISTARSISDRVAGFFAENETARVIKKFREMDPSFQLEPFLREMREYILPEVLDAYVKGDIETLK
LWLSDAQFSVYSALAQQYTTLGMKSDGRILDIRGVDIANARMLEPGDIPVFVVTCRTQEVHVYRNA
KTNELAAGMEDRVQLVTYAIGVTRIPEDVNNPETRGWRMIELQKAARDYI
>g1468
MKARLLNTVEHTIPWLSRGTRSPLSQIGACPQLSTASSSYAPLNSTRDSAVRRQRETDALKPSQXA
TIESSKSDSSVAGIIRLMAAKKPTVGQKAGTYADGLRVRQEESYQAYNNRAPPYNLHVYAHKHNTI
ITLTRPNGNPIMSLGCGNIGFRKSHRSGYDPAYQLSSHVFAQIQERGLLHEIKGITLVFKGFGMGR
EAFIKVLLGNEGRNVRPLVTRVTDNTTIKFGVPTTESLFGSRDRDSNALYSDLVSSEEFLGSHVLR
IPNPPGPSHKSDAPNARDTRAKAKQVTTVNGRTVIVKENSVYSNKGFKSLCQAQIISDVLYHTPNH
SSQPWLIYYISKPLIGTYDPGKIVPAVPSRNIQGNDGEPPNRNAGDTSSVQKRDIKSFAELLAHFP
MIARQMQPGLDRLFREFGKELGKPLPPPPSQSPSSPSKSEQWDPEGTDHSQADRLPFNSDEYFADD
EDLMRRSLETAVTAAIDLFRLVDKQQLSLLGATTDLTGPLVERLIEKYIAQQVHETLLFPRLCSFR
RTEDVELDTRIRQIEHIDVSQVGIAIEGGLAGKKELMNRLSRGVEAFRKMNDATCPQDMLDVLLAT
IKAVTVADESDRSLDSSEKQPPVFTVNADILVSMLLVVVIRSQVRRLQARLLYMQHFIFVDDVESG
EMGYALSTLEAVLTYLVSDYAPLRKASARNKRLWQATKTGNTRDIRSILEPNEDENNDDFSAYDNI
DPDPDALNKKAIRTPPLEQQSIRFALANGNSPEPHADTEVEDSPLEPPPLAHVFPFQTWAHSSSNN
TDKRPRKRVSMDVSSISGSSMISLLSRTTTNASTFSGIEGDTSIETLSHTHDPAGDSVPMMAVESH
QPGALKYLLSLQEYYSIDVILEDTNRDGTTLLSAAVQHANRKVVDIITEFLLTEADIHTISSYLEK
EDIRGRTVAHYLFSAPYLISSLGSLLPWQKKDKHGQTPLFAICRSYDHINYQAMVNEALTVAQRAQ
DDGQPLRIGDHVDLKGNTLLHIVSDPEIITRILAECDCDPNATNDRKFTPLMVASKYGRVDLMRRL
FADPRVDTHITESRGLTAVELAKDDEVRNRIDDMILFLNPPMSSGDITGRITTVVRSLFVEDATVR
FILKSGAQAFSDTTSINGTTYTITTCRRSFADFEHLARCLAVEHPASYIPSITESRSPFQIHSKPS
RAILHDMQGRLDQFLRILLTHPTFATHEMLWEFFLMPELQPDMMIDRSQRKASVLYESIYDDYPPI
TTDGIKDTEQFVAHAQEVVRAVHANTRSVIRRGHSAQHAAFDFGDAASLCASVLSTFKPPSNAIPV
SHIDAFSQYAAAMSTSSSDSSPLLQFLIAITSIQSTTTAVLSALSRPGRLIYTLSSTNRNLSRSRS
SLASSSLPRKFNLNLSVLEESRQKNLRELEQKINESEAQASRLSREISWNKDVVVSELAGWTSWRE
KVGRDAIRAFVRATVVQEKERGHRLERCLRNIKDLPLLPGSTK
>g1469
MRASTILLLGASTISAVPLFLRAPSTLISTINTRDSTDDNNTDNNKVPLAGIIIGSIFGTALLLCL
IAYLVSHDNCITRRGWFKRRREKKISKGSSQHLNVQDDEEALTKRASFASERESIMFSRSRASSLN
FAVVEDVDHSQRRMSSQVYVLRDGKYVPVDQTEAEQRKGLLSGNEMAEVDIVSSSMDRSSERASLT
SIPVIVSPPLEDTHTDQQHDYMSRTGRDNRSSVASVVSAFQEILPDDPRTHE
>g1470
MTRLLRFIALAGITYIPLAYSATTNSPASASNTSGIEVDIIFPIHNATYNITESLPIVFALQNLTA
AAAIGPFTFEWDIMPYGNVGELQEPGGVLNDFASVYFTTGNATTEPYILVNQTDVQKWQYGPYYPH
GSVYALQWNIQWDVDTGPCDSARLGMLGTLFFNINLNDPEPNLTNVTGQCPQLGAVYQINTAAKNS
SCSAVVTNNGTGDPCAVTLDQARVANISSTWKGLVAASTATTSASATATSSPAKKNFAVGSNVPFS
YMLVATLLGSLQMIMS
>g1471
MPLVVPGINSSLTGDKKNDWLDKLMGKKLSDSTSDEVSFAKRDLPSSHRVIGPNDMSTTDHNPDRL
NIHVDNEGTVRDVRYG
>g1472
MQLIFAARRGRRVLHQVNLRQTASLSTRVPIAVSHVQRVFRVADIRPAVQSVPSTLKQLGAALPKN
QYSTTTTTTTTSADPSKPKRRGRPPKDENAKPKTKKKAPKKRKPKKKVKKVLKLSEDALEKRRKQR
LEQKVKDTIKTLIAESLKKEEPKLRSPIAWNLFVSEKLKGVEREAKGQALRDIGAQYRELPESQRE
TLTQEATKLTKENEIKMREWVHSYSPLRIKQANAARRFLPKYMAKHEKAPIIKLSAIHDDRQVKRP
VSAFLWFFKERVATGEYRGLPVTEISERVKAEWLNTSDSEKQLYLNKAEEDRQRYITEYRDTYGED
PIFVQKDGIARLLIWITRLLPLHKSAQWPLKRIIGVAYLKSRSLPLRLQNYQNETTGETIVRYCGK
HSLPHIGTTLQCEGNTPSFTLHEIEGNAIYEHFSAGHSGPVLFYFHGGGYVRPINGKGQLPFVFDC
ARTANASKIWILEYTLAPDLLYPGQLVQAATALRYLVNERLVSPDHIILGGDSAGGHLALSLLAHF

KSPKLGVPSVNDALRTPVRAVYLLSPWVTLKCSTESFRQHSNKDMITPTRVQGFISAWKPVIDDKW
AELLHAGTPDLWSNLPAKRILVTAGTFEAFRDDIVAITRTLQANNMDVSLSLGENEIHVQSAVDLA
VGLPLSRTGLDVLQWLKNM
>g1473
MEVLDSRTSSHSEFSAKYNGLDTHLGCYERQLGLADTGDVYGGEKVKVLDQGQKSIDCESVTFRLA
LLNAYAVVNELIAQNTEPAGYGTPPPPYTDVHDDLPPEYSAFPALAEAKPVAKKSTPPSNDYSRSR
SSKSKPVSSIDFESTHGFRQHGKKQKAKLAAKKAVGGSSGGDGGDSGKKNDETIDPPGGSGLGGSA
GGDDGGDGGDKNDDGWGEVTSNSKKKKSKKQQEEEEEEERKRKEAEEAAAAGTAGNNLSWADDLDN
NNDDSWAGFTSVGKKDNKKKNSLDPTSAAFGEPAGNSFQEINLDDSAPQLDLSFESTKGASGGGGY
GFGGWGKDWNTGSKWGFGSMGNDTSAPKDPPAKTESPWGLGDKKTKKAAGSLGFDFGDLGTSTADI
GASKEVKDDETWGAFNAVGKNNKNKKNSISEEPPSTDDWGPWGPKKTVKKEEPVPESKTGQSEDVW
AAWASKDKKKVPTSSAAKKTETDDIWAAFKKDNKADIIDLDFPSEKAPAPEPEPVIDNVWAVPGKK
DKKKASKKGTEPEPVQSKTEDAWASFETDTKKEPEEESGWGWGIASKKKDKSKGLIEDLSSSSQVD
SSAAPPEKDWLSAWGIGGKKKEVAPEPELEPAPEPEKPKNHEFWDGWDTWTPLQRRKKEKEAVKKG
IPARIEILDPVPEPIIEPEPEPEPTPEPPRRNHEFWDGWQSWGPVTRRKKEREAVKLGIPPLIEDL
APEPEPEPEPQPEPEPEPVIETPRNHEFWNGWQDWTPLARKKKEREAAKKGIPPLIEDAPEPEPEP
EPEPQPEXEPEPEPERPXXHEXWXXWQTWTPSARRKKEKEAVKKGIPPLIEDAPEPEPAPEPEPEP
EPEPEPEPEPVPETPKNHEFWDGWQDWTPAVRKKKEKEATRKGIPPLIEEIFVPEPEPEPEPEPEP
EPEPEPAPPPPPVNHEFWDGWQDWSSSQRKKKEKEAVKKGIPPLIENIPEPVIDPEPEPEPEPEPE
PEPVPLPPPKNHEFWDDWDSWSPSQRKKKEKDAAKKGIPPRQEIVEPEPEPEPEPEPEAAPELEPE
PEQPRNHEFWTGWENWSSKELKKKEKEAKLNGIPALQKDLPPALESEPEPIPEVIPEPEPEPEPEQ
PRNHAFWTGWENWGSKELKKKEKEAKLNGIPALQKDLPPALEPEPEPEPEVLPEPEQPRNHAFWTG
WESWGPKERLKKKKEAKLNGIPALQEDLPPALDPAADPEPVVEPEAEPEPEPEPEQPRNHEFWTGW
EKWGSKERKRKDKEAKMMGIPPLQEDLPPALDPAAEPEPVLEPEPEPEPEPEPEPEPRNHAFWNGW
ENWGSKERKKKEKEAKANGIPPLDVNLKPALDPEPEPEPELEPETQPEPEPQPELIPEPLKNHEFW
DGWDDWSASQRKKKEKEAVKKGIPPRQDLPIIDIPPEAPEVPEIPAFEPAVVEEHKDDYWADLNNW
SLSDRKQKQREAKKKGILMLDEDLAPPPPAPTPPAQGLTPEPHLDDLLDDDAWGGFAKSSKKKAAP
IEETPAKAVRGFWASFGTSAATKTKSKEEKPREVDDLLIDVDEGAEALAAPLVDDIAAPAAPPPKA
TSKAAKPGRLSVAERIEALERSKREKAKEKAKEKEAPKQIIDDFLIDDAPPPPAPDPYEELLTKKG
SSKQKLTKSSSSKKAIPEEIPKPVEEEISHESIPGSFPEPEPEEPETQPEPEPVPEAPKNHKFWDG
WQDWSPSVRRKKEREAVKKGIPPLIEDIAPEPETEPEPQVIPEAPKNHEFWDGWQDWSPSARRKKE
KEAVKKGIPPLIEDIAPEPLEPEPEPEAIPEAPKNHEFWDGWQDWSPSARRKKEKEAIKKGIPPLI
EDIAPEPQPEPLEPEPEPEPEREPTPPPPPPESSKKSKKSSSTKKSSTPVKVSTKVSKRESLSRSM
EEEPVVEPPAAQEPVTEESISEEAPRPLTPPPEPSTKSSKKERTKVERTAGASSWGFWGAAPPPKK
PSKRAEQAAPEKEARPKVSRTMSDATARRRVRESDNEVDIERSSTDKDRRSDSKRQSRSMGFSNFI
MAGAPPSRNKSIRRSGAPSRPTSRRPSVDVADAGLPSPPPEDKPEISDKAAKLMGIKASSSRREGG
ERRKRRSVRDPYALDGDEDIVMVNAEAEGEFAEKSRRRKSKNMSEARPVEEEPVIVEAEQSPPSAE
VVSGPDDIAFVEAPPKESKDSKERRSRRSIPAPASATKKPEAGGLMGLFGSLRKPTKSDPQRRKSR
PRRDEMTETERDEKRAKRDRRRSVKPETDAEGFTTDAGPVNVDTEVDDMEAMRAERKARRRAAEQK
AREVELQEVEERRARRERQREQRERELREEMEREARRREEKQAKRAAREERRAREEQEAREAQMLA
EAKAAERRERRRTREPVEMSGGLSRHASKADRRRSERRSEDEYARYQDERPRVSRRKTAPDPAEPP
RGYGMPGKEKTSSWVNSQVLDPPEAPPIVPTVLDMPPSNENGPSLSSDEEARRRIRRQARRRSKYP
DMADEEVEEMRSRRRDSRKVREGAKSSSGDGDFERENGMRRRDSAYEPPSRAPSTKRTSWFKKLTS
L
>g1474
MEKETPLAAVQVEALVVMKIIKHSSQTFPTVATGSLVGLDVGGTLEITNTFPFPVVEVPPESHFDT
STPNAAAAAPRAKANTAYQAEMLRMLREVNIDAQNVGWYTSANMGNFINLNVIENQFFYQKELNER
TVALVHDISRSAQGSLSLRAFRLSPQFMNAFKENKFTSEQLQKSGFRYQDILVELPVKIHNSHLLT
SFLHQLPSPLPSEPLDLPPSLSALKNGAAAQSNVLSPNFDNLSLSIDPFLEKNCDLLLDAIETHHS
ENNNFQYYQRSLAREQQKIAAWQAKRKAENASRATLKQAPLPEDEWQRLFKLPQEPSRLESMLNTR
QVEQYSRQIDGFVSSTTGKMFAIKGNLLPGEASE
>g1475

MAQPETFSYGPHELQKIHVWKAEDSPKDKDLLWVIYIHGGAWRDNTVLADSFLTTYSLLTQNPLIQ
THVAGFASIDYRLSPHPNAPQDPSNTPSTQLRAAKHPEHIHDIQAALAFLHDKYAFEDRYILLGHS
CGATLSFQVVMNKVQGADVTTFPKPRAVAGVCGIYDLALLRDDFKQIKIYEDFIKGAFGADERLWE
GVSPAKVTGVDGVAAGWEGGMVAVLASSTGDSLINQPQTDAMLQVVKQWQGLVQGRNVIEIYDLKE
EHDDVWAKGEELARVIITTVQTLVKXPAI
>g1476
MDPFQPPMSIFNMVDDDDDDDDDDDDPEPPPRPRQEIHVPPHIVRSLHYLSLAFRPYEEPKINISRP
FSPQFISSRGNEYFCEIDEEYLTDRFNLTGLNTEVQYYQYALDLITDVFDLDADDDLREQIEKSAR
HLYGLVHARYIVTTRGLAKMLDKYKKGDFGKCPRVLCDQHPLLPMGQSDLPNQKTVKLYCAKCEDI
YNPKSSRHASIDGAYFGTSFHNILFQVYPALVPEKSIQRYEPRIYGFKVHATAALARWQDARREEM
KERLAKLNIDPGFIEDSEDENDEFEDDIDDDKSEPTGEEPAPVVDNASARMDTGV
>g1477
MPPTSGHLLLPKIWRAARFAYEKTARALKAKLSEPAQQLQPKLQPAYAHVGRQPISRAAAIRQAQS
RRFSTRAGGYIGSYLRQVSRPASNAVPRSSIRQMVGRMTSHSPFNSALRPNLTGGTLGRTAGGYSV
GAGRLGGARYFSHGPAAPAQVIQNVSVAMRGFWLSGQRARFDGIDPHTGEKRFKTVTPLQDKAQRQ
MAEISRNAPGSYIHFQLSPTITAIGNFKNESLKSEGLMELLSVDFARALKDLAAILNDLKRLGTLG
DLPVSLENKSTLRVRFPGCDAEQVERLCNEVGVERGQIFQDEDFDQRTGAELALLFPFAPSIAPSP
ETELFTIHENPRLPAKHDLDWRLMMSGQTSPGMSNDTNQISFEDVEMFGNNPWVSSPSGFSSVGVS
DLGDREFFAELSPHGGDSHSDYEGVQGIYKFLEQCDLAAGQRF
>g1478
MSSGFVSGGTIDEPTQRDDEWRRAQQELEDERRRKAELGKQNDGKSLYEVLQQNKMAKQEAFEESI
RLKNQFRSLDEDEVEFLDSLLESTRAKEDAVKKETAEHLAAYQRQREEAERALIEGPSGGQNVSGS
ATNPAGDEELWAVAGKKRKRNKKDFLVPPKIRKSSTNLNDTPNAPDPKQKPATTTSRGVTKESIPR
VSQSASVRTEGTSVKEIKSKSNEALPIKDSTEQKAAVGPSLGLVAYDSDEDSD
>g1479
MPLPPRWYQFLIGAFASLGSFLYGYDLGVIAEVIACPSFSSKFAVNATQSGLVVSMFTTGAFFGAA
LAGPTGDYLGRRWTIAIGALIFCLGGGLQTGAQNISYLYSGRFFAGFAAGVLTMIVXPYQAELCHP
DIRGRVTSLQQFMLGVGALCASWISYGTYIGFSPSDSAQWRVSLGIQIIPAVLLGSLIMIFPESPR
WLIDNGREAEGLKVLAKLHSHGDENDSWVRAEFSLIQESITFEHENEAKSYVELFTSRSAFRRLFL
CCALQASIQMTGVSAIQYYSVEIFNQIGISGDETLRYQAINSVIALLGEFSCNLANMVTFIIACIL
LAQFPPEVNNSGAHWGFIIMTWLYNFSFSATCGPLSWIIPAEVFDTRTRAKGVSIATMTSFAFNTM
IGQVTPIAMQNVRYRYWFVFIICNFTNAVFFWLLMPETSKLPLEEMNSLFTNAPWVVVGLKKEDYV
SHDLERHLEEVTGKTEQNTAHYE
>g1480
MTTFTKIDDDLIPTISVNATAQLSKINPNIYAGFTEHMGRCIYGGIYDPGNSLSDENGFRKDVLEA
LKELNIPTVRYPGGNFVATYHWLDGVGPKEQRPARPELAWLGTETNHFGTDEFLKWCEVIGTEPYF
CLNFGTGTLDEAMAWVEYCNGTGNTYYANLRRKNGREEPYNVRYWALGNEVYGPWQVEQTTKEAYA
HKAFQWAKALKLLDPNLILILCGESGTSSWDYYTLKQCLKPTKVTLTSNPTPLIDMHSIHMYTASK
DHLQNATAPLAAERAIEIASSLIDLARIENGVGPDEPRPTLCFDEWNVWDPMRAEGRFGAEETYTL
SDALAVGVFLNVFVRKSRDLGMACIAQSVNVISPLMTSPTGITKQTTWWPLLLFSKYMRGWTIAAH
VACGSYNGDTSPTWLRSVKDTPWLDVSAVLSDDGFVNLVTINVHEQKDFEVKVEGVKGPVEVFTVT
GANVTVTNMKGKEEVFVKETTWDGQGHYVFPKHSLILMRWKA
>g1481
MARRGDTRSPSPVGSSYSSSKRSRRDEDRFDRDGRDEGRSHRRSRSPDRRYRDRDGYRRRERDRSV
DRRDRDGYRDEHNYRPSKRDRSRERRRSLDRRSDRDHRRRSRERDRSRDRDNRSRRDDSRDRVRRH
RDDSTDSRKKARRDSRDRLLNRDSAAASREASKAPTPAPAAQTDEEKKAERLAKLEAWKQKQAAER
ERKQREIAAAGGPRNILTEIDKKSGLAPLSPAPTSPSTPTSTTAPSEYGGKFDFKAIAKKASGVGS
ESSLLGKDVIVPDSIRADSVVKANKVQVATSKVSGEFFLNERPFDDTKANGACSAAPLKASGNVGK
FGLGNKLATETEKSSTTKRTLDFGEEESKRKKLEKLPSPPPEDVEDDTKAVVAEDDDSDVDMENGT
EEENAAAARAAAEKREERLQGQQVEPNGDIAMGDAVITNGEATAEAEEEEIDPLDAFMSGLTEKPS
FKKSNIIKSAKSKQHQPQAMFGDEDDVDMSAVAHGDTDDFLAIAANKAKKKKDIPTVDHAKAEYEP
FRKSFYTEPLDLAQMTEEEVASLRLELDGIKVRGIDPPKPILKWSQCGLGVQTLDVISKLGYENPT

SIQSQALPAIMSGRDVIGVAKTGSGKTVSFLLPMFRHIKDQRPLDNMEGPISLIMTPTRELATQIH
KDCKPFLKALNLRAVCAYGGAPIKDQIAELKRGAEIVVCTPGRMIDLLAANAGRVTNLRRVTYVVL
DEADRMFDMGFEPQVMKIMANIRPNKQTVLFSATFPRNMEALARKALIKPVEIIVGGRSVVAPEIT
QIVEVRNENTKFLRLLELLGNLYSDEANEDARTLIFVDRQEAADNLLHQIDRDSTIEDFKAGIFPV
LIATSVAARGLDVKQLKLVVNYDAPNHLEDYVHRAGRTGRAGNTGTAVTFLTEDQERYSVDIAKAL
KQSGQEVPEPVQKLVDTFLDKVKAGKEKASASGFGGKGLERLDQERDAARNRERKTYRTGDDGEEE
ERDDAKEKKGEEIFAKAASAIQSASAPTPGIPKGIDLDGKITVHKTEKPAAGKSNDPLDKVGSAVA
DIHARLNRAGVMRSGVPIDNKGPDAGAFHATLEINDFPQKARWAVTNRTNVAKILEATGTSITTKG
NYYPTGKEPGPSDIPKLYILVEGDTELVVTNAMRELMRLLKDATIAAADRSGVTPPQTMQQHISAW
GSLNAQCPEFEESSVRRSWGAFAIAAQSTIRKLLDVYFEIVYPIFPLFHRPSFEEKLNQNEHLKDT
GLFASTMAACALASARVRDGALFSARWQSTDLSQPPSEMFFNAAKDLIPKDLGNANRTSFMRACAL
LAITSIQNGQIRSMQQYIGYYHTLMAMDGRHDEKLWPKDLNPIEKEERRRLFWSIYTLDVYSSIVW
GGIIRHREAQCMVHYPTELDDEHVTATGYDMSSNSAVNWLQGWNFTTDLYRILEHAVDAVRGRSSL
STGIVQSLCGVNPRPTEATVMKGVLDTYLSLPPQFHECREITGEADKDLFGFQSANIQATLQLLRT
VLLSAEDDVSVSRKCDVAGELLRVFSIVPVEYLRAISSPLLHHLAGIGNILGSVIEGSLSESSYQR
IRKLLLDLADLLHRLESRLYRAAGASERLRMQVDKIDEYMRNQRANDRQSRQNTHNILIQSNIGGP
QEHAPLNISNVNEDSLLYHEGTNPGDAELSPFQLPPELLMDWPWPFDFSGADSLFTIPAAE
>g1482
MGYRTLWKTLSPTQLNVAIQVFSLISIFFEGYDQGVMGGVNSSPRYVETMGIGLPDGTVTDTTHEG
GIVSVYYLGAIFGCFAGGWLADRIGRINGLFIGSLFALVGGALQCASQSSNFILVARVVTGTGTGA
LTGITPVLVSETSSAAHRGGFLGYVFIANYLGISIAYWLSFGLAFINNGYSVVRWRFLLGFQCIPA
LFLVVFIKMLPDSPRYLASIGRKEEAREILLQVRHGHASQEAIDREYLEIVATAEESKPSSPIQFA
KILMGKGGRPGSNLGRRAWLCVWLQIMGSWTGITAVTAYSPTLLKSAGYSELTQNGLAGGINTIGI
IGTIISAHIVDRLGRRTCLMGGAGALAAVNLLAGALYEGARQHPDKASKFAPAAVCMLFLFNLAYA
ATWGTVAFLIPTEIFPSDLRAQGNGFGITGWAIGVGMTTLVNPIMFATLQNRTYFLFFGLNLIWIP
IVYLLYPETSNRSLESIEALFATSSPFSWEMEKAYNMHGNVLAERGYEKDDASTMEHSERLPHDV
>g1483
MGSISNGADHATGEVGAALDITVLGLNSGTSMDGIDCALCHFHQETPESPMEFELLKYGEIPLEQT
IKKRVMNVILHNKTSPSELSEINVILGETFAAAVHQFCKDSNVDINTIDALGSHGQTIWLLSMPEE
GEVKSALTMAEGAFLASRTGITSVTDFRVSDQAAGRQGAPLIAFFDALVLHHPTKLRACQNIGGIA
NVCFIPPDNKGGIAECYDFDTGPGNVFIDAVVRHYTNGELEYDRDGEMGARGRVDQELVDDFLQHK
YFKLDPPKTTGREVFRDTLAFDLIRKAEAKGLTPDDVVATITRVTAQAIVDHYRRYAPADMEIEEI
FMCGGGAYNPNITKYIQKNYPKAKIMMLDEAGIPAGAKEAITFAWQGMEAIVGRSIPVPTRVETRQ
EYVLGKVSPGKNYRQVMRKGALFGAGRDHLKPVQELINIVDGKVFDNKW
>g1484
MGSVCSKSANKSDDTFSQPGRVLGSSAASGTPNQPARAAVPGNVVSQGPGRTLGGASSPQDTADAR
AKAAEAAQKRAEALNASSKGKLGSQLAAQKARTQTQTLGAASQAEVAARNADSAAETRQWN
>g1485
MARPLEGKFGIVTGGSRGIGEAIARNLASKGCSLLLNYTSESSRAKTEELASSFTESFGIQCTTVR
ADLTDAQAASEIIIDASKKHLGDKPLRIDILINNAGVSQDRKLADSAKGPIDADYFHWHYNINVLA
PLLLTQACIPYLPNDRSGRIVNISSVSSSLGFEGQSVYGGTKAALEAMTRTWARELADRATVNAVN
PGPVIGDMYFLTGEQFWKDIQGWQDNTPLSKVAADASDLEGLDAEKIRLIQEKMGGRRPAFTSEIA
GVVAMLCTEDAAWCTGSVVNANGGMKMTT
>g1486
MLKFVKRLGSRQDLRGWKEISNKNSAHKDVPSTASSDSSPSFRNHKPMAATFEIPSDVAYDIPSTM
KAQQLDDYNTPYVFRNNVPVPTPTHPYDLLIRVDAAGYCHTDYVLASGHMSSVEQPVFPHTGCHEF
AGTVVALPPHSAQSDFKIGDRVGVSCRPYHACGECAECLEEDTPDSDPRGYSVLCPTIKSHGIGIP
GGFQEYVLVDSRQLTIIPPGLSQVEAAPLMCAGVTIYAAIKKTGLKPGQRIGIIGCGGGLGHLGLQ
YATAMGLRVYGVDNADKPLALARGLKNVSGARIIDARTTTAAEAIAHISTEDRRPIPGQFGLDAVI
ILPESQEAFQYGVDMLKNHGKCVVVSFPPEGFHTSSLDLIFRDITYVGNVSGTTKQLREMTAFSAK
HGIKAQINTFPLEQLNRLVEAYLSGHGGKLVVDYHYKNSAS
>g1487

MTPLPSRPLLQVALQLRLSRISITSVQSAASANAALRCYTNTGLSGFRARSKASQTRISSLSKNFK
QSVTEIQHRGYAELKASGGKTEADLIVEELQELYENAKDELEIATDSTNGATIYAASDRESARELL
DQLLYVYNVYTDMTRSPASTSAESPPHENEAALAPNAQQANADRLDAGATAAATDPTPILDTNEAL
SRDSYVGVAPNYDPEYISVDVKEEVKKRVGQRVRELKSAVERLEEMATHE
>g1488
MSYYDSRDSRGYRVKRERYSRPDGGYVEETYVDNRGGYPYDYDTRLVRRRGDSEESVVEEVSRDFP
PGESYYGYPGPRRAATVREGVRRARSAGRDPYYDEEYYRREDYRPRRSKRSDGRRDRYERSTYSSS
RSPSPRPRRRKSLGEEALGAIGGALGIKAARDRSRDRDRDSDRGRSRKYRSYSDSRSRSRGHRAKS
EARIAQAMKAALAAGAAEAFRVRSTPGPWTGEKGRRVLTAAVSAGGVDGLIDRDPNKHGGRHVLES
ILAGMATNHLVNGSRSKSRSRNGTRGRSQSRGGVRDMAAAGILAAAGKQVYDRFSQSRGRDRSGSR
DSRDGGRDSKKRGSSVSRALNKGLAALGLDDKNQSDRGRDRSTRSSDYSDSEDDRSYRSSRRRRSS
RDVRSQRNSSVSGSTSRAVAQKDGSSRGNIECHSDCDSDSDLSINSDDEKREKKKMLRRELVTSGL
ATVATIHAAHGVMKNLEAQKKRHEAVKSGAISKEQERTMQRKADLKNLASIGLAALGIKGAIGEWK
EVQEQRKEHKEFNHKCEERHEKRLRRAQSAGGSNNNSQGSRSRAASAASMSMNTGHDYGRDYGHNY
GHDYGHHAPHYYDGNPYGAGDISIDV
>g1489
MPRPTDRSEILARLRKVISDGGIIVGAGAGIGLSAKSVEQGGSDLIIIYNSGRFRMAGRGSLAGLM
PYSDANAVVVDMAKEILPIVKHTPVLAGVCGTDPFRDMPEFLGQLKELGFSGIQNFPTVGLIDGKF
RQNLEETGMGYDKEVEMVRIAREMDILTTPYVFNVDEAQRMVRAGADVLVAHVGLTTSGSIGAETA
LSLDDAVKIVQDIRDAAVKVNPDIIVLCHGGPIAEPKDAEYVLNRTKGVHGFFGASSMERLPVEIA
IKENAQAFKNLKIERK
>g1490
MSKPRIALLGTCDTKLEETLYLRDQIIKAGEAEVLLLDVGRTPVQNSDIDILSSTLALRKSIDGPS
SMVQMSRADYIKYMIECATPFVAELFQKGEIHGIVSAAGSSGTSLVTAVMRNALPVGFPKLMVSTM
ASGDVSHLVEETDITLMYSVVDIAGTNSVLNQILTNAAAAIAGAATAYYRSQIENASRNTGAEQRK
KRIAVTMFGVTTPCVDAIREYLESEYAYEVYVFHATGAGGKAMERLIRDRQIDAVLDMTTTEIADE
LVGGVLSAGPQRLSAAAEAGIPQVISVGACDMVNFGPRDTVPAEFASADNQRLLYEHNPAVTLMRT
TPQECTRIAEFIASKLRDRAVRPDLVRILLPTGGISMISTPSQPFYEPKSDAALFDTIERQLEGSG
IEVRRDDQDINNKGFALLAADLLVEIIRKKEAEEHSDGSRDVKSRSEMSAI
>g1491
MPDRSGKIRELYLQGKKLFPRRIMEDMWMILKDYKSGNCKNHVFEIPDYIDGNKYVKVSLDLGYHM
HGQGAKLKVMGYIPIQILLLRSWQPITTFETEFDEAYARNLQAPTDDDIKELVDIRENAYRNWIES
CARDSVRATFMRINIDKLKKVKKIIAFGNGSLWYGWKKEPKLISDFVAQHAVIQDIRNLIQQRAGD
QGVACYAQEPKYTPADEKVLLEHFGIESLEEIEALLKVDESTLVFAMNQDIPLLQILADILTAEPF
AMILCGEIDQTGKLEEYDRTQLSASPSSPRVQSLMQNYAVPLAISDTDSLFESENKTRLYVRK
>g1492
MPGRKAPVVRPRTTPLETNNRPDEWRIEQGMQGAKLPIIDQSGKETVFIHPVDPPEITKDQAAIDA
VGDRKKLFAAERKGWKGYVEWENYPEKKAKAREILHAQTFPPTPDYMTGPIPGTNPVLPGDDFTAW
HHAIDGELADAPESSWETVLKEKHKDMLHLLKFPYNGEPPKRLVTSKAITPNPLHFVRNHGGIPII
KKKDFTFTLDGLVANPKTYTLDDIMDESRFPRIEETVTIQCSGTRRIEQIALYPGQGDEVPQAPWA
EGAIGTATYRGISLKKLIKDCGGLVNGGKHLEFYGADTYFKDLDVLNYVVSVPWSKVKANEVILAW
EMNGEPLPAIHGYPLRVVVMGYIGARSVKWLYRIKAIETPSLAPVQSREYLYFNQQTGKHNQKPTS
GIQIQEMPVSSAIMSPWKGHVIIHNGKIHCKGWAYSGGGRWPERIELSSDGGFSWYEVPHEKLSKK
GQWTWRTWEIDLPCDVEGWIEIVCRCWDNSLNTQPLTVRSAWNWGLHVTSSAHRIPVYSINKTKQL
TRERLAKLEKEGIPLAPISRYEIVHTQSDEEILKFYKEHDPRDADNL
>g1493
MSSESHHSGTFDKLFHHKHRDEHHEGEEGAHLENHDQYSGDARPQQESEFKRFDKGLKKEENKFKN
YLQKDEELEEEGKTYGGLM
>g1494
MSTGTAVSIQPVDAPLTASATFLVLSIKNSDDDALQTVRSTLADIANLSKNVAIRDPSAGFACTTA
LGSNVWDRLTGLPRPSELHPFREIKGKKHVAVSTPGDLLFHIRSERQDLCFEFERQLLDQLGDAVE
VVDETKGFRYFDVRDLLGFVDGTANPVGPDMHPAVLVAQEDDANHIGGSYIVIQKYLHDLKAWKTL

STEQQEAIIGRTKMDNIELDDADADQQKAHKTLTTIEDEDGNEQSILRDNMPFGSPGSGEFGTYFI
GYSRRLWVIERMLENMFIGNPPGLHDRILDFSRPVTGTTFFAPAASVLSKLG
>g1495
MPKSVFITGANAGIGFQIVRALYSSSKESYHILLGSRSLSNAHKAIENIKTEFPTSTNNTITPIQI
DIEDDTSIQSAHDTISSLLTGGKLDVLVNNAGAQFEHDLISGKIGSERELWDKTWSVNTTSTQVVT
SKFIPLLLRSDDPRLLFVTSGTASFAGTANLALKVNQPPETKGWPKPGFVGLPAYRSAKTGLNMLM
REWHRILKGDGVKVFALSPGLLATGLGGDTELLKKFGAEDPTVAGPFVKSVIEGERDADVGKVVSR
AGVQGW
>g1496
MSSPQPPAALDGRCSVVYNNTVYVYTPSALLSLPLEQGAEWNSTDAGEAVTDAVCVTGTVDGSADN
QALYVVGGTSTNSTYMGLQRYSFADKKWESIDLGSGLSGLQNLVNHSAIYLPGTTSILSYAGSNNG
NQDASTTTFEIPLTNIGKILNRDGAGIAAGIKPTLLPWDDSTAVYVGGTPTNTEVFLYHGNSGWGT
SQISLASGIPSTAGVAMQSNSDGSKFLEIFDFAVSPNEISYVALLQTGGTAAYPGQNVTFAPSSTK
QKRDWGNIPSYNSTLAPSTTRSSFSLAQSDNGLVVITGGSGTDAVTVFNQTQNGWVNTTKLFYNDD
VSAGLQHPIGSSSTTTAPTTSATATTTTSASASASTTAVAASGTGKISTGVIIGATLGCLLGVAAI
LIVLLIILRRLHRKNGDPTGKGSRRYGQDKDRLSFQDQGIEPLTMAAVPMGRTHIPSAVDSLNMIS
GKFANEYPGTFMADKTPVTTRSLVPPAKPPLTLITSNEADQYRNKTLTPINESSHPRGDRSTDEGW
GKYFQDGGDGLTTDSPRTTMNSDISQITKSDYRGSMWPHEVPERTTIALSALDGPRPLGQVPSGSP
STEYLPSFGSRHIHQAQSAHISSADSASFVSEEEDDHDRHDAFSSGVPQSVHDGTQWTHQPWNLRP
PSSNYTDSFYQSSAREPSGVQEPVDSRSHGRRSSGILHNDMDEYSRAHVNSDMSWLNLHGDK
>g1497
MEISRGPPRVKNKAAAPIQISAEQLLREAVDRQEVGVQAPTQRFEDLEELHEYQGRKRKEFEDYVR
RNRINMNNWMRYAQWELEQKEFRRARSIFERALDVDSTSVVLWIRYIEAEMKTRNINHARNLLDRA
VTILPRVDKLWFKYVYMEETLGNIPGTRQVFERWMAWEPDEGAWSAYIKLEKRYNEFDRARTIFQR
FTIVHPEPRNWIKWARFEEEYGTSDLVREVYGLAIETLGDDFMDEKIFVSYAKFEAKLKEYERARA
IYKFALDRLPRSKSVTLHQAYTTFEKQFGDREGVEDVIVNKRRVQYEEQIKENPRNYDVWFDFARL
EETSGDADRIRDVYERAIAQIPPSQEKRHWRRYIYLWIFYAIWEEMESKDISRARQIYQECLKLIP
HKKFTFAKIWLMKAQFEIRQMDLQAARKTLGQALGICPKDKLFRGYIDLERQLFEFNRCRTLYEKQ
IEWNAANSQAWIKFAELERGLEDIERARAIFELGIEQPMLDMPELVWKAYIDFEEYEGEYDRTREL
YERLLEKTDHVKVWINYAKFEINIPEGEEDEDEEEERPVSEEAKRRARKIFERANKVMKEKELKEE
RVDLLNAWKAFEQAHGSAEDLAAIEKQMPRRVKKRRKLDDDRYEEYMDYVFPADDASAANLSRLLQ
RAHAWKQQQGQS
>g1498
MSAAEDLLRDFEDSDFEDQEENGVENEGQEMEVEPETTEEAEQENVAVKNDFEVAMSAADQLARLH
KDLRDHYSTRFPELETLITNPINYAKTVAILKNGPLDNIKQISTSSDNLVGAPLNAVLDGPSLMVV
TVEGTSTRGRQLSEAELKVVLDTCEKILKLDRERTARIESVQSRMTEIAPNLTALIGSQTAAQFLN
QAGGLLELAKIPACNLAAQGSKKQEGLGFATNVGIRQQGFLYNSPMIQDIPNDVKRQAMRIVSAKM
VLAARADVARSSPDGSMGEELKQQCFHRLDKLTEPPPNKGARALPAPDDKPARKRGGRRARKAKEA
VAMTELRKAQNRVAFGKEEQEAGYGTGDGTVGLGMLGQENDGRIRAAQIDQRTRARLSKSNKGWGA
ATPISGTASSLRGPGNATMLQAKGLRSSGVGTSLNATAGTASSIAFTPVQGLELVDPKVQAELNRK
RKAEEDRWFKSGTFTQVGGNGGFKVPDVPANKKVNTGGGNMAPPSLPLKK
>g1499
MAAVDGTATTTFHPPRVTLRAPSSLFHHELVTKDFGLLQFHAAAALQLQFGTQRHELVATSDDRLI
ASPYNDPGNLLDLKRLETPYQLLAKAFTIFKPIRDDYATAPYVESFNWQAVVDFLRDLSVAEGYQW
KRQRFYVVSFRSTLKPVIDNQRLHDLDYHSHQEAMASGGLLKYWFGAKSEKHQNLATCVWRSREDA
RAGGTGPWHKQARAAAGVMYDSIVFKTLALIIEDDASSWEITDWIDEDA
>g1500
MAAPAHTFKVADISLAAFGRREIELAEVEMPGLMAIRAKYAADQPLAGSRIAGCLHMTIQTAVLIE
TLTALGAEVTWTSCNIFSTQDHAAAAIAASGVPVFAWKGETEEEYEWCLEQQLSAFKDGKKLNLIL
DDGGDLTSLVHKKYPEQLKDCYGVSEETTTGVHHLYRMLKEGKLLVPAINVNDSVTKSKFDNLYGC
RESLIDGIKRATDVMIAGKVAVVAGFGDVGKGCAQALASMGARVIVTEIDPINALQAAVSGFQVTT
MEEAAPHGQIFVTTTGCRDILVGRHFEVMKNDAIVCNIGHFDIEIDVAWLKANAKSVQNIKPQVDR

FLMANGRHIILLAEGRLVNLGCATGHSSFVMSCSFSNQVLAQIALYKAGDAAFGSKYIEFGNEGKR
PVGVYVLPKILDEQVAKLHLEHVNAKLSTLTPVQAEYLGLDAAGPFKSDIYRY
>g1501
MVSAKALLALYAIAGIVQSYPIHRPIEGLSEPHLTSRSPSPRFRNPFDVDSYLRNLDYKIPNNKSV
QQWVKAARKKGALAAAGKFSGDDDDDDDDKHEDKKDHDNDDDDDEDKKDKDNHDHDHDDDKHHKTK
GTPRVNGEKDSDHKDDHDHHQHAAQSASKPDDKHDDEHDDDDDDDDDKDDVNNVNKDEVAQPARPT
PTSTPKTADSTVTSTPKPNQEGDEANNIEVMQPNDTQDAATPTIPSNTVSEGNNAAAPAVQTVYVP
VNVFPTRFPNFPDIDDDEKPSPSSTPLPIFPPPDATTKANLPPTDVSHSPKESANTNGAEVSEAPV
AEDATNTPDVTDAPEVVDAPMNEEPTSDNVDPTNTFTPTPQMPSHEAQLSKISELLDMFTPKDKSD
PSY
>g1502
MPTILGKQVGSTGYGTMRMTWNPSPPSKETCFETLNTALELGANFWNGGEIYGTSKYNSCHLLQAY
FEKYPQNADKVVLSIKGGTVKDGLQPDGSEENIRRSVDDCLKVLKGTKKIDIFECARQDPNTTVEQ
TVTILAKLVEEGKIGGIGLSEVDAETIRRAHKVHPIAAVEVELSLWSLDILENDVAKTCAELDIPV
IAYSPLGRGAFTGEIAKFTDIPEGDFRRHVPKFQDGNYEHNLKLIQEVVSLAKRKYVAPAQIALAW
VRSLSNKPGLPTIIPIPGGTTKDKVAQNLQGVETLTDEEVAEIDALLKKYEVKGTRY
>g1503
MNALKMTTRKVSMNQPSTVAVIGAGSAGLSMLKTLREDGFKVTCYERRSQVGGLWAWSDKKEVTSA
LKTTTANISKYTCGMSDFPMPDEYPPHLTQAQFQSYMQSYAEHFNLLPDIVFDAKLEQVYRNESDS
KWLLQLNINGKPQIEEYDKVAFTHGYQTKAKMPQFAGQEQFECELIHAQAFKSPEQFRDKKVVVVG
IGSTAGDIIETLLPVAAKVYVSHRHGTAIISRWRGDKPTDLIVSYRRRQIRTEFLMKSKWGKLDPE
WNLLPFPSPLLVLPSASEAVIPALQNGSLTSLRGLKGFVGPRSVELTDGTIIDDVDAVICATGYTA
DFTVTPFIETSQPAGPEGPIVRRLWKNIFPPKYADSIAMFCYSAYGKNNGFSFCDVSSMAISNIWR
GVHPLPSQQQMEKEIDEHHRWLVLNWKLNNNIDPSMVKSWEYQSFVHDAAGTGMENLGWGWKGXNF
FFKDPKMSYLMNNGVETAHAFRYFETGKRKVWPGAREAILHANEAAKQISH
>g1504
MAYSPGSPLSSIASDDLTEDTKQDKDGHSPSASSMPPSKRRRTGVASWDHHTPISSIQGDLIPTSP
SSSAISSDTDGEIPNSPSLIALIGGGQDDDYSGLGGDQVTACRWDGCEAGDLGNMDNLVNHIHDDH
IGTRQKKYSCEWIDCSRKGQTHASGYALRAHMRSHTREKPFYCALPECDRSFTRSDALAKHMRTVH
ETEALRPSDPVPKSQLLNNLPNAATPTNKVQRIKLKLSFGPKGEDTESSTNGEGGGGGGSRVGDID
GQEAELLPVPEFTPDLGFDAHELALPPSQLYRLLRRQVHWAEIETSKLKQQWQDLEPKRKTAFFEK
EAALTEVIDAEGRLSKVVSDAQRDADKETGNVQDGVEEVVA
>g1505
MVYPSVTELSSGTDASKPLTNGYNGFDHFSSSEQASDQEDLVMSDAESETSDSSHVNTNNNMESRA
GNGSRNGSSASNPELYGLRRSGRSRTERRQTLVDSDEDDEPKAPRSKRRRIVNSQPPSKESSRSVT
RSSPSDSDTDDIPAEVRFSSRKATKVANYNEDSDDPFEEDDEDATPNDVGTAQYYNGPGIDSVLDH
RLKDGVDPNISDLDVTIRFRRLENYFKNKVKTDLYLNNDPDVAPEEKEKWNLDRERDIESLEDYKK
VERIIGHQIAIDELDQFLDRNDKVVSCDKSEMQPRTRSPHVPITGSPPFLQNGQLKDFQVKGLNFL
AYNWSRNQNVVLADEMGLGKTVQTIAFINWLRHVRGQDGPFIVVVPLSTIPSWSETFEYWTPDINY
IVYTGSSQARQILKDYELMKDGNPRKPKFNVMLTTFEYANMDFDFLRQFPWQFMAVDEAHRLKNRE
SNLYGNLLDFKAPARLLITGTPIQNNLAELSALMDFLNPGLVEVEVDMDLNSEQASEKLAKLQNTL
KPLMLRRTKSKVETDLPPKTEKIIRVELSDIQLEYYKNILTKNYAALNEGANGQKQSLLNIMMELK
KASNHPFMFPNAEAKLLEGEPRREDLLRIMITSSGKMMLLDQLLAKLKRDGHRVLIFSQMVKMLDI
LGDYMRYRGYQYQRLDGTISATNRRVAMEHFNAPDSSDFAFLLSTRAGGLGINLMTADTVILFDSD
WNPQADLQAMARAHRIGQTRPVSVYRLVSKDTIEEEVLERARNKLMLEFITIQRGLTEKDVLPGKH
NRAMGEPTGTDEISRILKRRGQKMFEQTGNQKKLEQLDIDSVLANAEEHKTEQTEGLEADGGEDFL
KSFEFVDVKVDEMSWDDIIPKEQLAEIKAEEKRKADERYLHDVIEQNRPRKRNAPNDALDEREERK
AKRRARAQVNMETADGSDSDTQDPKRSLKEKELRHLIRAFLRYGDIDEREEDVVREARLVGRDRDT
VKAALREITDKASQLVREDREKLEEMERSGKIPTKKEKKAVLFDHQGVKRINAHTIVERPEEMRVL
RTATQGLTDQTSFRVPEASKKADYTCPWGAREDGMLCVGIVRHGYGAWPEIRDDPELGLGDKFFLE
EHRVDKKNERANAEAKGTKSPGAVHLVRRADYLISVLRDKSSNGHSLTARKAVENHHRNKKFQNLS
RRQPSVSASPAPSSGRKVRRESEKPRQRSHTNGPRESIERANTPRAEPTRIKNGHATEKVHKRSQN

GVHEDHSRPRSVSATAPPSTMEDSIFEPKVHLLHKLRDVKKNNPGKEERANEMRRLILKIGNFIKQ
LLRNENYPGLEDRLWGHVAKNCFPPGKATLGGIKTMYEKVVANNRDTATTAR
>g1506
MSSELPSTMRAWVYRTKGLPSEILKLEINIETPKIANLAPDEVLVEVYYSALNNISTQLMQTLRPR
PLGFFISQKNRLAIPEIELGGRLIAAGKNVLSRRPDLKLGDLVIGLTDPAKSMRDGSGALAEYAVL
PVAHVVPYQLPSGEQEQLSLAEWSALGGVGCTAIQVTERAGLKAGDKILVNGSSGGAGTTVIQVAR
YILGPSGHIVATCSASNIDLVKSLGADEVIDYQQCGPLHTYLAMSVHAQTPFDAIIDCVGIQDLFI
HSPTYLALGKNFINIGSLELEHSIKGTLHWVWLAFWNQYCPLIFGGIPRHFEFYSAEPDVQSLEKL
RKLTQERKLRPVVDSVWEFEDASKAYQIMENKRAKGKIVIRVKASD
>g1507
MSDKLTRIAIVNSDKCKPKKCRQECKKSCPVVRTGKLCIEVTPESKIAFISERLCIGCGICPKKCP
FGAIHIINLPTNLETQVTHRYSANSFKLHRLPMPRPGQVLGLVGANGTGKSTALKILSGKLKPNLG
RYDDPPDWQEILKYFRGSELQNYFTKVLEDNLKAVVKPQYVDQIPRAVKGPVKEVGPLIEARSQMD
NKDRIMDVLELNQVENRDINLLSGGELQRFALALVCVQQADVYMFDEPSSYLDIRQRLAAARTIRE
LLRPDDYVIVVEHDLSVLDYLSDFVCVLYGRPAVYGVVTLPSSVREGINIFLDGHIPTENLRFREE
SLTFRIAEAGDEFLVDKGRAFHYPKMEKTMGNFHLSVDTGKFTDSEIVVLMGENGTGKTTFCKLLA
GAEKPDGTVNIPRMNISMKPQKITPKFTGTVRQLFFKRIKAAFLNPQFQTDVYKPLKIDDFIDQEV
QNLSGGELQRVAIVLALGIPADIYLIDEPSAYLDAEQRITASRVIKRFIMNTKKTAFIVEHDFIMA
TYLADRVIVFDGKPSIDAHANEPESLLTGCNRFLKNLDVTFRRDPNSFRPRINKYQSQLDQEQKLN
GNYFFLEEES
>g1508
MNPAGSAPGGSSLWQEARNADGRVYYYNVQTKATQWAKPAELLTPVERALSSQPWKEYTAEGGRKY
WYNTETKQSTWEMPEVYKTALAQVPSPSTQPSAPTPVAGGAPTFVAGGTTSFPPYGQHRDRDDGDR
SGLDRRSGYIGMETNGFVTAQQTDPEYSSFEEAESVFIKLLRRSNVQPDWTWEQAMRATIRDPQYR
ALKDPKDRRAAFDKFAAEVRMQERDRAKERFAKLRTDFYTMLKSHPEIKHYSRWKSIRPIIEGETI
FRSTNDENERRQLFEEYILDLKKAHVEQEAVTRKAAMDELVNILKALELEPYARWSEVQNALQANE
RIQNDDKFKTLSKSDILTAFENHIKSLERTFNDARQQHKAAKARRERHSREKYLELLKELRSQGKI
KAGAKWMQIHPLIQDDPRYVAMLGQSGSSPLDLFWDMVEEEERALRGPRNDVLDVLDDKRYEITTK
TTYEEFASVMATDRRTADIDTDILQLIFQRVQEKAQRRSEDEKHAADRHQRRAVDALRSRIKHLEP
PVRLGDTWEQVRPRVENFEEYTALETDDLRLSAFEKFMRRLKEKEDDAERDRERERDRGSRRGDHH
ERLDRDSRNGSYRSDRRVRPSRTPDPDPYEADRRKAQADRERSYRKVSGLSPVRERRDDRERTRDK
ERERDRDRGDRRGLSHYDRERRNREEERERLYRTRGDPRGSRDELDYGGDTRSTVSSDRRRRRDSD
AESVASRSAKRYRRDEREKERSRDRSHRDREPGRTPAIEEEPKAEEKAVHSGSEEGEIEED
>g1509
MAGPSVHGDFLRHAVLETVVPHNPNVDIEAALTSALEGGAEDLDAVLAAIPQRSLLFFDETVTVRV
VLRLSNCSENALKAHLPRLELGVNALVFNPTPPGPEDPAPAKDVVFAGNVNNKEDPLVVVNVFEGD
EGSGNHVYVIWKVDAFLHRPRIRIQHPCIAFSAAASLNAADTSDQSFHTDEYLPRTVPASANVLQA
LSSDPALKSTTPYLPASRLLRVIPAAQKEAPVYNLWQQSQHPIRIVPAASARIRCSKMNTFSSRPI
TIASLDLEVTPFLSCDVVFEKAEIILSDGQVENISGVPELQPPITLRPRDDVTLIYKLVPEYGPDA
YFSTTALVSTLDISLEGVIKLTEECQPRIFMQWRTNVDFSIPLNPTFGGPSQVMQRNNRPASLSMT
SGQSAAPSTGAVNRSSYRERAHSFTQGGVTISFSGPVNVEIGKPFQWDVFIVNRSRAPRKFTMMAI
PRRKRVDFRRHVARPSSSSISSKKDKKDEELAEAVTDENIIHAMQKNAAGQDAELVCLSTDLRLGP
LLPGTCHSTEIILLPLATGALHLEAIRLVDMNTNETIDIRDLPDIVAVK
>g1510
MVTNAVLGGIADTVAQTITAFRMRSMKRSVDPDSGVISIEIQDFDKEKPPQWGELGYAKNRPAPFD
FERLTRFMAYGFFMAPIQFQWFRFLSRAFPITKTSATGPAFKRVAVDQLMFAPFGLFCFFTFMTLA
EGGGRRALMNKFRDVYLPTLKANFILWPAVQVLNFRVVPIQFQIPFVSTVGIAWTAYLSLTNSSEE
E
>g1511
MSLSNKLAITDVDVKDKRVLIRVDFNVPLDAEKNVTNNQRIVGALPTIKYAIDNGAKAVILMSHLG
RPDGKVNAKYSLQPVVPELEKLLGRSVIFTKDCVGPETEEIVNKASGGQVILLENLRFHAEEEGSS
KDEQGNKVKADKAKVEEFRKALTALGDVYINDAFGTAHRAHSSMVGIDLPQKAAGFLVKKELDYFA

QALESPKRPFLAILGGSKVSDKIQLIDNLLPKVNSLIITGGMAFTFKKTLENVKIGNSLFDEAGSK
TVGDIIEKAKKHNVEVILPVDYVTASKFGADAEVGSATDAEGIPDGWLGLDVGPKSVESYKKAIGE
AKTILWNGPCGVFELEPFASGTKATLDAAVAAAQSGTIVIIGGGDTATVAAKYKVEDKLSHVSTGG
GASLELLEGKDLPGVSALSTATLILDDDVDGAENGLSNGDSSFHKTGKGVVGFLRALLGFEQEIMR
EAAERLSDAETSAYNDQQRVAHTSSAPDAFHSKIYDVGTEYALCQAMAQIMTAVVGVLNESLTESL
KGFYKMRKAYATLDGIVRMEERYLQTRKARTAPAALLVEKSPVRPKPRPAGAHISKSDQDLHKSIA
ELKLTDDEPHSEPASTHSSEPGTPLGESLTHDPELADLFSHPIDTFIHTGASLCLGMLLAMLSTLP
PTFSRLLAIVGFRGDKERGLRMLWQASKSSTLIGAVAGLGILGYYNGFVRLLDIIPDATEGDDLGI
EGYPTDRLKTLLITMRTRYPNSQLWLLEEARMKSGHRDVEGAVDMLKNSKKSPLKQVEALQAFEKS
MDSMFLHNYEDCAKFFLECVDLNSWSPALYYYIAGSAYVILYRRTSDTDPAAAKIYAEKATECIRK
APTVAGKKRLMARQLPFEVFVTRKIAKWEARAKGLKVDLVDAVGVDPIEEMNFLWNGYSRMGAEGL
QESLRRLDQSEEDVLHPEEDEKAISQLLRAAILRAQRKHAEAKEILKQNILVLDKNIFKGHLRDNW
VLPAAHFELAANLWMERPSYVPTHASPWDPEPAEKEASEDEIVSAERKQVQECDEHLEKAAKWESY
DLDTRIGLKVTAAREAMRKWHTSHPEVA
>g1512
MPILQPITTDSSEDSDSSSSQSPRPAFHNTRTRESHYSIQSYGEDAVPIPDHVAPLELEKTNTAGS
TSSRFNQRAQSMVSRIRSRDPGQIAKFTHPLSHTKTGSDVLVDFDGVDDPYHPKNWGFRKKCITTV
LYGMTTMGATLASAIYTPGTQQISAEFHVGTEVSLLGLTLLLLGFGFGPLLWAPLSELYGRKAAVM
TPYFIAGIFAFGTATAKDIQTVLITRFFTGFFGSAPVTNTGGVLGDIWSPQQRGAAIVGYAMAVLT
GIYMLFILTLDIIFLDESYPPVLLVYKAQRLRHETGNWALHAKHEEWDVSVNEMANKYLVRPFALL
STPICFAVALYASFVYGILYLNLAAFPIEFIDERGWGQVVGELPFLSMLVGIMIGAGVNLLNQKFY
ISRFKKNNNRPVPEARLPPMMIGSIVFAAGMFIFAWTSSKSIHWIGPVIGAACMGLGFFTIFQAAI
NYLVDTFPLTAASAIAANTCLRSIFAAAFPLFTDAMYNNLGIDWAASTLGFISVALIPIPFLFYVF
GKRLRARGKWSRASVYE
>g1513
MAIIYKETKVDVRPVEANAIVDIEIPTAENAQRRVRVSLSSGAARADSTPIKDDEDFARRYLATQG
SIYFRQRKIYPRTFLWRVVNDSTVLEIQSADVSKSIVDKHEANLTLRLDFQEAIIPSGVALADTED
HEVLNVFVLTTSRRLHTITLRPEFFRRFSSIDENIQDWCKSFIPSPLTFTNPHRLHACSTTALFIA
LDSGALLXLTRRAGDDGSHWSQITFDEKTWGASLRGYVPWSSAQRISYDGRSLDPMTPNSIATTSD
QTYVFAVCLNHTLKVWNLATNKLVGTKDLLDRPLPKQQSDSTAYFLNPADSVFLRVFNAERALDGG
YRYYVATYTPHEMGQFKFWAVKGGLTTPLTIEDLFPQKVFRPSDPDSTGNMFWTVADFQIRPMEEG
TRMELWVLWRNNGLYQLYTLHFGFESLEQDWSSNWTSTAWDSRRNEPAPALVVSDVVDPTEKWIEY
FFHPGRYTVETLEFALTSYQEALKPRTSATFLKRSAPLQERLCSAITASVSLRKYAEDDMDYAKYR
TDTDHKWRQFWQVVEEVHNRRLEPVSLAYDTYSDLPWVVTTDACALIRECSATELLLHNSKLNLER
ELPMIADRWRHRKLETEVGNRSADSCHIIKVAADFRQRLPATALRTFEEALYSEIFLEPLLSTTER
LASFIDQSELSEHVSDDMYNGLCAAMNKQINIYQLPKEPFQIILDTLFRRFIEKDSELSFTAFGAK
VLTNGSQEAISQIRRLLHDLLILVVFVEGEISQEQGSSFDAAELFPILIDLIKECELLHWLGSHTR
VSPDRSDYESGDVRAQSKRIGAVDTVLRDLFVPRILPHPHVNLPQSFTLTKSISDLMSWVVRAGLV
TRDHALVHIQCDLIAHGNLELASDFLKFQPSDAWSNYIKGRLFVARGEFNTAAIHFQKAAYQLAFG
RPLGDLTYMSSGLLDLLSADQFNNGLPKYYQHILSLFEKAGSFSHVADFASLSLQALESDPHKVTD
DPDYDGLRMDVLSRMFHASLRTCQFDKAYSALSRYTNLPLQKSALGLLIKAILSAYGSGTSGLEQL
LHFPLWLTPNFSSYVDETLLSLARKQTSFSSSLDGDAWESNYTPDYHRILHAYRIARNDLRGAAEL
GYQTVQRLRYARDSPIAQRALIRRGKVDEEESKRMVEEDDIESKEIRHELLSLINLLACVDKSEAY
ILVDLDAVIPATAPAVGEAETPGKRHADDDDDVFMDDGTPSKAPSGSFSQRRRSSGAVSMDSARLT
LTNNNNNKHKPSPRRVIVTLEQLRREYQSELDRVSRIQRGDWEFGVDEYEGIEGGAMDESLLDF
>g1514
MTADIEQQPQEYDDGGLPGPGAPTPLSALEGVAGLTARDIKLIVDGGFHTVESVAYTPKRVLEQIK
GISEQKATKILAEASKLVPMGFTTATEMHARRSELISITTGSKQLDTLLAGGIETGSITEIFGEFR
TGKSQLCHTLAVTCQLPFDMGGGEGKCLYIDTEGTFRPVRLLAVAQRYGLVGEEVLDNVAYARAYN
SDHQLQLLNQASQMMCETRFSLLIVDSATALYRTDFNGRGELSNRQTHLAKFLRTLQRLADEFGIA
VVITNQVVSQVDGGPSSMYNPDPKKPIGGNIIAHASTTRLSLKKGRGETRICKIYDSPCLPESDCL
FAIREDGIGDPSPKDLENE

>g1515
MAFWKTWETWEKMVFILACAMVVVIVAGGFVAWLNRRQVKLYNQAFADEAGRVRNLVSPEGSGISD
VPFGARALESGVQVDGIYTPGRYTPRQGSFQRDAAGSPVLGIPTPLTPTVTRATQYEQYEQHGPYS
PQK
>g1516
MSGTKRKVDSAEDRNGKRTKTKTNVAPATAGAKQKYLPPTFQKKGITRDAKKMNRDKKLHKQAEKQ
KPSKKGHYKNKDEDEEEEDDMNSDISSDESDMSMNDDDDVDDSEPVEEEEEVMKEEDEEQSENSTD
NVDGDKKSSSRESHAKQKALAQERKAAKPNADIIARSKKLWERLRRKSHVPKDERKKLVTELFEII
TGRVEDFVFKHDSVRVIQTALKYANLEQRKLIVSELQGHFKDLAESRYAKFLLGKMLVHGDADIRD
MIVPEFYGHVKRLVRHPEASWIMDDIYRTVATKQQKANMLREWYGPEFAIFRDNDGSQVTAELSTI
LADSPEKRPPIMRNLYELINQLVQKKTTGFTMLHDAMLQYFLNTKPGSSEANEFIELVKGDEEGDL
AKNMAFTKSGARLMSLCFAYSSAKDRKLLLRFYRETIKMMAGDVNGHLVILAAYEVIDDTKLSSKS
IFPELLNQETEESARHEELLLQINDITARVPVLYPFVGDKTKWLLPAGDEDVLNEIFEIRKETSKK
EPAIRRQELVKAASPSLLEFIAARAESLMETSFGCQFLSEVLFTAEGDKSAALNAVAVAAKSKTEA
QDSPFVGRMLKSLVQGGRYNSAEKKIEKVEPALNFDALLYDIIKDDILAWATGPQTFVIVALTESE
DFAQKSELLKTLKKNKKALEKAAAGPKDSSDKTKKAGPSSSGAKLLLSKI
>g1517
MRLSDVLVAAASFGIAAAGVPQPRSSEETCVNAEFTGEVVQYSLSITEYPIIVDVELQEDTTVTID
NTIIIGVTNAPTHLHTTVYAATTSTITKTISTATITASVTTVVEATTDGSNFQAAPTATITSKVTV
PNADNSAVGSGATALSQSASTRSLAPVAGGSAVGSGVNTALYTASTSNTAVRTLTATFTNSHYTST
KTYTTTVASATATLAAGTWTDWKKYKANGVNLGGWLELEEVFDQYWWNQYAPNATDEWTFCETLGS
RCGPVLEQHYATYITTDDIDKVAAVGVNTLRIPTTYAAWIKVPGSALYHGNQKEYLKTITTYAIQK
YNMRVIVGLHSLPGGVNSLDIGEKAGNDGWFFNTTNLDYSYEAVEEVLDFFVDTGYPWAFTIAPIN
EASDNPSAFASPETLTTNGSNWIVEYTEGVLSRISQVDKRIPLMLQDCFLGEEHWSPYFANDTNLV
IDSHVYYFAASGVYSQWANGAICGQASVLGGDGKFPVYVGEWALQTLYSNTFANRKSLFNTQRYAW
SYYVQGGSFWNIKHNSSVVVDGEGTQKDYWSYDLLIDAGVITRPIGNATYC
>g1518
MILPDDGERQSSTPSPPFQLTPSDPHMLQNGVQQANGYYPSNGSTYRAPRPRWTYEGHAPGRPLSD
IGEEDFQPSSPAYAGRYTTTQPQQEKSDSDSVGSGSTIGAGSQRQLNLNPEESRGGNDSGFNSRRS
SYASQSSIDRERAQASDALVSGSSQQTEEESSSAILSSEAERILENAKRRLTLMEGNLTRARSSIR
LGQTPSPSPTSPGILLPTRNLQPAGALYRSISQTDRRGSLLRPRPVYTTAQEGGHSRGHSETNLPS
AASLKSPSLQPSRSMSALASSGSSHYDSRENSFPYNAADGSDSQERRQEPHPEDDYRRSYLSPDVN
GSSGLGISAAPKMSSMEDFNSAYPPDGPPSRAQSQLQVRDLKDQAAGLRTKVALLKVKTQEDNLRR
RSLQSLRTPSPFTAAERWYSSALEHGNGGANVHNNAGYGWVSEPTSAKQDSNGTSTSNGTDEYPID
APLSPQRREPASSKSPHIIDEFAFDNDDQSVLESHYEDAEEGEYDDDEDGDIDRERLNEILNEPYD
DDDQSDVFEDFPSGNAPEATPHEEREDAFDYENFYLHSALGTFSRNKIRRNSYGSTGSTETTRPAR
DQRPGTHNRNASSDSVSTFATFATATENAYDDDYDDDQEDALEAIDQIVDTWNDFEDEDGSTPVQS
RRATVIDRSDDRYLAASRNRDNDGPFTPTSKSLPIDRNLSTPTTPVDAFMSSLSSIRSASTRPPSS
LNTDDTRLLEQVFESLGKVCTELQELTMATAASATTNRTVVTANGSAGTTPTADSKYIRTLRRRLD
AARRVLDGQLDSDA
>g1519
MGRKPNQLILEYFTRGPKLEDASNRYQHTCKACGEKFPKGRIDSLTTHLVKKCQALSMSARQSIVM
RIHDLPALDDSDPNKAAMLAKIQKAGKASDLPYSTRPAFDGLNVLAEASRRVGASDQTKRRGAGAL
RFTQPMTVGGKTIMIDPALEAEAFHPPPTDSNDDLILAGVAAAAASLNANEERGPSASPSLQNNDT
LQPDTNTDERQSSSQLSLIAASASEMASQSLAMGSELTDGPHVGSSWTDHQLSSTDQFLLDSLQGH
DPTLAATQRAASYPRPIAMNPNTTPTKGFVNDFGSSPKPQKPKVRSRFSADRRKEVQEVRKRGACI
RCRMLKKPCSGDSPCQTCASVESARLWKQPCIRTRLVEECELYGAGLHTTLAFHDVNSMQAQTKYE
SYVGRVEAAHFDDGPFFVTFSALIGQKAGMSPIDPQLQALVEDGHLQGSTQDMYMLDGDTDDLPGK
CENYIKRLQSTFIENETSPIMKSTLTLAAHLADEKKDTLLDRVVELWLVTHVLVDLELQWKLFVHP
LLPPTAVDTLAMPTEEGRVPIDRNANADSYNLICSQLRAAVEKRASLLSKNVMNELERRLLQRHHS
GWFETFLVSVILLNCVERACWLFTSWENEDFVGRWPLDKRPQYYANQGDRFSEILHMLLRMRGLPP
KTTINAETGLLKAVENSDPNAILWFDTIQISPLYLEQRQLVQFDPTDSRSLDLKFASKILLPPPTA

>g1520
MPNLALARIRKDLIGTWKLVSYTSKPAGKDGPIEYPLGKDAKGYIIYTADGYMSAQIMRPGSKFYS
SLDPFATADDEAADAARHYLAYNGPFEVIEHEGKVAVKHHADLALVPNWVGSQQLRFCELEGDELV
LMPTQPWILNDQFTESPKRKRCLMSHLTLASWASVVPCSCLDLATASQSSLEISPVTHTLTMLPHE
NPECGVKFVPAIEYFDSADADSFLAKENGYIDWPDFRILKSEEYPVHHEPINLGVTYRSWVLNSPV
YLQWCHKQAKEQGAQFIRANVVSMEEAISIFQGQSQGSDADDIQAVINATGRGLNDPDSFPSRGQF
IIVSNQCDKTISHHWADGSSTVIIPRPLGGGTVIGGTKEPNSWSEDISDTVTKTILKRVAGICPEL
IDRADNGTDEAHGFEIRQVYAARRPMRRGGLRLEKEMLSQGIPIVHCYGAGASGFKISWGVAKQVE
RLISSTQLLVNDPLETS
>g1521
MRKDLEMTGPGINKDDFFRDLDGLGDLSDDAQTTGDFAGLIRRTDTAPTKLARPSTEPKEDDIPTP
AQLKRHSSMVTTSATKSSTSDALEMQSETLVKRQTEISLTEQPKGRKKRRRQSVQIIPESAQIFKG
LKFFFVPNNDISPARRLRIQRSQEYGASWAQSWSSDVTHVIVDKGLDYKEVLKVLTPEQLSANIAI
VNQDYVSDCLIFKSILLITQLRFRVDGTPKTPEIDNAAPAPAAAPDTKAVQQDGYMISAREAGQRE
TDLLDEIMLKVKAAGDLPLDEPDDEVALDATFDNSWETELSGKATAVANVPNWQKNFACMQKHDGK
SSDNNPNAKTIEILQKMADYYDRTADTWRTMAYRKAIAALRKQKTKVCTKAEALSIPGIGQRLXEK
IEEIVSTDRLRRLENINWTPEDKALQLFLGVYGAGFTQASKWVAKGYRSLEDLQNEAELTANQKIG
VERYNDFQQRIPRAEVAQHGEIVRAAVHALDPDMEVMMAGSYRRGAADCGDIDVLITKTTGSIEYI
RTLMMENVVPELTRRGFLKVSLATTSRGDGSKWHGASALPGTDLWRRIDLLFVPGDEIGAALLYFT
GNDIFNRSVRLLARKKGMRLNQHGLYKDVLRGSNQVKLTDGTLLEGRDEKRIFEILGVPWRPPQHR
IC
>g1522
MLSIESRLFSIFRDSRCLRCQTQAVWTSVRHKSTTSAKPSPEEQPDNVQPIELDRPIGLPYPPLPG
QNTGIDNRSLSERRDDFVDYQKHLQRRKELTAHLAKPYFREWTNLKYNEGKTFRSNPRLFKHDKAL
FFPNLYGVTLESPKKPQDTTDIIKGKISVVSLFSSLWAESQVATFTKPENNPQLYETLAANPVTTQ
RIDINLEENRLKAWLVRFFMGNMRREMPRDQHDKYFLVQEGMTDAIKQAIGMLNSKVGYVYLLDDY
GHIRWAGSGSADPAERDSLNAGLLRLIEEKKKRADDISGASMTSPTKRRIVTTPKE
>g1523
MATSTGGGWAQLRQQARSLETQTESLFHTYSQYASMSQLPPKPSEEEQRVEGQIQSLLEKREALIG
QLXRLLDSEATLTSSALKQNNLARHREVLAEHRRELNRLSSSISEARDRANLLSNVRSDIDAYRSS
NPHAAEAEYMLEERGRIDNSHNMMDRVLSQAYAVNENFGLQRETLASINRRIVGAAGQIPGVNSLL
GKIGAKRRRDDDIYLVDS
>g1524
MSSAKVKTAQLWGKSKDDLTKQLDELKTELGQLRVQKISQGASSKLNRIHDLRKSIARILTVIKAN
QRAQLRLFYKGKKYLPLDLRSKQTRAIRRRLSKNEASLITEKQRKKNIHFPQRKFAVKA
>g1525
MPLIMEEGLDVDDLFGDPNTLDLGLSAAQPTKGLAQRLDEMRLLGCCRKIAWSRLGCIAYISQDGL
QVIVRHLACNPIDGKWALTEEYSQSQIPQLHLGQQLLHLCWNETGSDLAVLDASGRISILSISAAL
NSLGISRQTVVDPPDDGSQVVGMMWMNSNRAVHAFHQAAKLNGRWNYSPYRRKPIGPFHPAGKPAL
ICFTRNGIMRLLYQNPDQRWAEVSAELKTTNQSDRILTHAACLPSPKKLSLYRVQIMWNPSNWDQT
QQRQASGVMPFPVPSIRISHCKTELPGRVFSFNKDQSDDLQNLAPNQSFLYQLTNLDLVSGQFDNT
GATTSPPWILAVYSSPFNSAGHDPQQQEPPASVLVRWQLETSTLNLHPVFEESLSKKTNGQQRTKL
EIRRMEDIHFDKHVVSIDQIEFGCVLAITHDDSSVSFFDPKSMSQFAEGGDNNTVTSLAQAGFRFP
SDTPGLNICFSPNSCLAVVLDADWQAQLRIAGYNFEPDGDLHEGKNSNGIIALALTFSRGCAIEYN
TDDLIMTISRHLSTDAQNVFVNEVYRALPINCNFSMEQDKPMQHPYVPRCLSLQAALGFQNFQTPR
GLPYSVPWAILHLRHASLLFAFFFQYSKQPKENESLDHDVLQMVYGNTKWALDFTQYLLDDLFALA
REFEPVLDDQEALAQKGEGLRGVHTSSMASPFAGDGRGYSKELYTLIDSAPFRFNVYEKFLSSVES
AIKNAYQRAGFGDDERRTPEKDLLLNSRIPAVMIPVLVSLFKQIIPAIQTEIDQMSIYLTDYSWLG
FCSDRRTEHFRQTRAVDVVKKVPLRGFNLSNNNIEPNMSSEITSNGKKAGSSSRRRCVRCCETTGD
PSSPRSILYFQLTLRLQLIRSCLCGGMWTFENGPPTTAATHKQVG
>g1526
MANTSSLPTLPMAMDHEPSDYSYMSDSSSISDLSSPPDSPQPPPECYPTPPPTQDDQEERARATSS

QDEPAKKKRRVGPPPRSTQHLDLTNPERLPYADQLPQIKLLTKTLREHRKIVVIAGAGISTSAGIP
DFRSADGLFKSVQKKHNLKASGKLLFDAAVYRDDSLTASFHEMVRTLSQEAADTKPTAFHHMLARM
AQDNRLARLYTQNIDCIETSLQPLITQVPLPPKAPWPTCIQLHGSLDKMVCQKCRHLAQFDRDLFD
GPEAPAXPACTETDELRTTTGQRSHGVGKMRPRIVLYNEHNPDEEAIAAVMNSDIRSRPDTVIVVG
TSLKIPGVRRLVKSLCKVVRAKPKGIAMWINNEPPVGKEFEDCWDLVVKGNCEEVARLAGLKQWND
NTPQIFDECNDLDVERVKREQSVSVVVNTPKKVRKTDAGVLTPVSNNDESGDELPHKNSKQPLANP
ASRGRTVMEALIASKSNNKKKPAPKQQVPKKLVASAPKKKGPTKEQSAKITNYSMVTKGNKGSKVG
KGGKAMKPLPANSILFPGLVDPKGQETISPESIPKDMEALIS
>g1527
MPPRKSTSSMPATDNEDPGSPTSAAAASAQVTEQQAKSQSDGISVEDLLLPRAVTQRLAKSVLPPD
TAIQKDALLAIQKAATVFISYLSSHANEATLKRTLAPSDVLNALSELEFDSLRPQLERELDAHTEA
LADKKKAQKDRKAATTSDTQAEAKDGGEAGGVVPAAAGEEKDATAAGTSTSSVIKGNKRIKRDSAG
NEKHSPDETEVDEDETEEEEEAEPEPEPEEEDDDEEVEEEDEDNDTQEVEDLDRVEDLDGKMQADY
DDSGSDDDDNGPAAQLRGFG
>g1528
MVKLGKKSKRTPVRLRHKIEKAGAAKQRKARKQAKKDPTWRSKIKKDPGIPNLFPFKDKILAEIEE
KKRQKQEEQLRIREEARERRKAEKKAAGIETADDEDEDDEIIDDIDIEDDDEDVDGMDEDGDGSNP
MAALLASARARAAEYEENGDEMSEDYDDDEDEDRMDEDAEQGGVSLQSGDIPTIASQVASKESSRR
AFDKVFKQVAQAADVILYVLDARDPEGTRSKDVEREIMAAEGGSKRLILILNKIDLVPPAVLKGWL
IHLRRYFPTLPLKASNGAPNVHSFDHKQLTVKGTSETLFKALKSYSQTSQLKRAISVGVIGYPNVG
KSSVINALTARLNRGRSNACPTGAEAGVTTSLREVKLDSKLKLIDSPGIVFPNSSKASSKKKKEND
EARLVLLNAVPPKQITXPXPAVNLLLKRLQTTNETLFQKMLGLYGINSLFPMNGGDTTTDFLIQVA
RNRGRLGKGGVPNIASAAMAVITDWRDGRIQGWLEPPVLAVAGVNTAAAASSDAANAADNGPAADV
KEIVTEWAKEFNIEGLWGNGESEE
>g1529
MATTYHGEYTGPAQLLSFDGLLFDFDGTIIDSTAGRELGVDPEAILATSHGRRSIDTLALYDESKA
NWEYVSAIEGALPRKYGQDATEIPGARALLKQLVDANAPWAVVTSGTRALVTGWLEVLQLAHPKYL
VVAEDVQQGKPDPQCYQLGRSKLSNDVGFPQESTNMLVVEDAPSGIKAGKAAGFKVLALETTHTVQ
QLKDAGADWIVKDLRSVQLKSWSQGSKVEVEIKDTLQS
>g1530
MNYLHHPAYTYGHAGIHMDQSNLGHPGMANSMDNYLITRPTYELAEYYTHMPVMEDYEEYAENLSR
PRLTKEQVDTLEAQFQAHPKPNSNKKRELAVQTNLSLPRVANWFQNRRAKAKQQKRQEEFERMQRE
AKEKEEAKSIKDEELAHGLPESPMHKDESSNSPTKSPTPTQASSCTKDRPQTSDSSSLSEPKHHKT
GSDIAQEKTYASLQRAISAAVAARDQYSGPPDDSISAGPTDRAFDVRQTNIPVPSTNNTPQTSMNS
TFSEWGSSRDSSIAWTPSQSPEEGYEYGSLNSVPFASEVAHMDDSPNPDVSASHGFGSMTTRSQPM
WNPQFQGRADIHSSDPMYGSMSFSALQPSSASSSRRPSSSEDLVDTISGIGINTAALSDSMESSMW
RRPEKELDIAARRKRPRPAAIGTAHHRLSTNPSMVSPNARMASFGASHTIRHAKSSHTLGSRYAGV
RKLSANQRSPLAYSFAEAGAAANAASESRQKQRLHTSASVGNLAPPTPLTPEDFQHMLPTTTSDAQ
MSFSSQHLVDTQSLFPVTQSMQINVASPPETPLSLDVFSAMQYQNMAPPLSAPPQYASFTDYSPIT
SEPLTGVSWAVSTPDTSLFPPSLQSQRQQPPIYIYEQDDEQQESKWTLSGDDGSSLYGSTKASAXP
PASMMTVSEEHDPNGMTEFHIHEFPKQQEAHRNVAQQLAPQIPKNYTFSNQTPNDF
>g1531
MYSAPHQASAPMNGMSGEVIDGSGTINPAALNTAASLSAPTAAPSNLSPRGIKRSRSPDQYGDAGF
DGGDHDDHSIHHDEHGRRKRGRPPKTSRPSSATAATTNSNSHYVSPLQHPSAVAGAMQTPQLSVQS
LPNQTSISPPQHSPPAKTTPTKTLVKALPTVRDHTTDTLNEGRDEYLAKEWDDAGENKVDMDGYLK
DGRVYRCRTFRVPGRGNKLFMLATECARVLGYRDSYLLFNKNRSLFKIIASQIEKDDLIQQDILPY
SYRSRQIAIVTARSMFRQFGSRVIVDGRRVRDDYWESKARKQGFTEEDLAGEKRPGAAKARDAAAA
EAAANAGILPGLGQNDIVYSSAIEGMPHLPPGLAGHGGVSLAPLPMIHLAPTTDDPRLREYSSMPR
PRQEMTGQAYQDRSQPSTMAEIRNQATHTAEFNKILNNQRAFRQKGLEEFYTTPREVPTSSPAHLG
QADVIAATAAAPASSLQSPQMTSSGAIMNQASQQHRGVLQQPTQMMTQPSQAAYSQQQQAVSQVTA
QSPLGNNMHGIRPDALHSRQANAAALSSAASQTPYGYQSQAQPIWGQPPPQPQHQQPALSASPHMG
MPQYAGQMQQQSPSPHPSGGQPHPSQSPHNQARPTQAMPQGMQMHPQVGQPGMPSMGYQAGGVAYS

NVPNPRAMYAQAQSPVQQQFVPAQAGGMTMAMGAGVGMPGWPSPAGSGHIQPGQPQQGQSGSPLGG
WSSY
>g1532
MASRGIPGVLESTSLTSLNAINGPTAPLPPLKRRKIFHTDDVEQTSSSQTVFTVKGHASSLSDNEF
KLAPIELLPRSALPFSWLDTSTAIQSNNIFASNDAVLEGMFFPKKGAEQPDLEPSVLATRSLSNGA
IYVVERVKTGIFAMTKLQSNIEEGEIRVAAKAAAAMSSKILRIWSSTMDLFTTSTSANKWDWREAA
RFPDDIPRFDSSATKRKIDISMAFEDCSSNDHRAAAIESEVYTGDSFMALDGVESGMQEHNRTLPD
ISIEAPQFEDQGDAQNTSPTPDVIMENLRSQYLEALYISKTSVAYFAKGPLTRCRNAFAQYSRDEN
VPNLPSQYRESILSVKKMDAKYRETLPVILRNMTLLLSDDERPKKRKSKKRKLGKNGLYTGEDEFL
QRYWKSRQISKNKEGAEESKEEEIKRHISDLRLRETQLQILLILETIHLEMPNVAPSIEDAKGHEA
STKRSKSKKEENLNLLLELFIDRLCIWHAISANEGVISESFKETNKNHLSGKKIESDALRDYCNEV
IIPFYASRLPEQCKTIKKKLGGASEVSPNRPVVPKLKASTSNSGSKSQSGVGDGKKIQPQQKPRRT
LQRVLTDEQTSVSQRSRHPSLMRSSTTPALQEVKRESVEPSLPSLGGSVRGGIQIARRVDKREVDL
NAVAKVHETKLKKMNTLIEQKKELDAAINALRKPNRELVSKEVADQAEKRTFSSSRKSRNPVRNPL
GQGVQVMATPRGTRKKDMSLEHQSQPSLPRGPLARPSMTKHGVDETSPDLGSDISLVPGSTIRTNT
LTSSIASKRKFNSITETPSRGVSRRSGALSLMDDHEAEAISINTPASVIPCNRALFKVPSLPAMTA
RSIDERMTEPENSDVTTGDKIFSIDQTPPAKNKTFFIQXLSAPKPMTMTASSSPVPIFETPVKMKP
KQNMMQQDDSARVVQSTPVKSIYEALGWDEDDDDDLAL
>g1533
MGSVSHKTGTFLFTSESVGEGHPDKIADQVSDAVLDACLAEDPLSKVACETATKTGMIMVFGEITT
KARLDYQKIIRGAIQDIGYDDSSKGFDYKTCNVLVAIEQQSPDIAQGLHYDEALEKLGAGDQGIMF
GYATDETPELLPLTLLLSHKLNRVMKEGRLDGTIPWVRPDTKTQVTIEYAHDNGAVKPLRVDTVVI
SAQHSENVSTETVRKELLEKVIKKAIPAELLDDRTVYHLQPSGLFIIGGPQGDAGLTGRKIIVDTY
GGWGAHGGGAFSGKDYSKVDRSAAYVARWIAKSLVSAKLARRALVQLSYAIGVAEPLSIFVETYGT
SDKSSDELVKIIRDNFDLRPGVIVKELDLAKPIYFQTAKNGHFTNQNFAWEKPKTLKF
>g1534
MSKFGVLVMGPAGAGKTTFCNAIIQHLQNSRRSCFYVNLDPAAESFQYDPDLDIRELITLEDVMEE
LELGPNGGLIYCFEFLMQNLDFLTEALDPLSEEYLIIFDMPGQIELYTHIPLLPTLTNFLSRQGPL
NISLCAAYLLESTFVIDKAKFFAGTLSAMSAMILLEMPHINILSKMDQIKDMVGRRRLKRFINVDV
QLLDEDDNTDEKQPKSITQDDDDMEEHEQDMDLRVDPSSKDELMSGGSFHRLNKAVGQLIDDFSMV
SFLQLDASDEDSVGAILSYIDDAIQYNEAQEPREPRDAQEVDYEDAEV
>g1535
MEYLQDNVLPTLQTYSILLFRKTLSTLTNPKDADKFLSNIQQKYLEPYLLVPLANILDTSTSAFSS
ANILSFITLLLVLYISLRILDYARRVIMFWVMLVVRLTFWVAVIGVGVWIYNVGVEKAMQDAGWFW
GVVQGFLEDFVVNAKAESAASRGGQYYGGSGAGGSRGYGGGAGRSSGYWHDRNRRV
>g1536
MSIPPAALQKLVQEIESQAIAAQQQISIVKTQIATKQRDIRLLQLTSSELGSLSKDTNVYEGVGKM
FVHTPIDAVNTRLSSEDQTLKKDISDLEKRLHYLETTHEKSREHIEQIFRGTGA
>g1537
MSHTEQQKKLHGLLKRISNPRNFFRDRHHVEIENAAESSSPQPPSTTVIPVNALDEVAEVNPLSSS
VSTQVGNDKNPVVAPGSDLASRSTSIHKALWEKAYSNLRSDEKKAEYVTKYEQLLSTVILNQKVGE
QSIGEDQMKEIIAQGLKKIEKYQKAIKNSEDKLEAVKAIKSIIEIPLKNIPQTALPWAIISSTIDI
FLKPLEVGAEFYNGVGFVVGRIEWYSRFTNQLLSQSSFRNESPLMNIRTDIELSITNLYQSLLFYQ
IKSVCCFYQRRQILVLIRGALRFDNWKGDLDDIKAAEKALREKCDFYRLECLNDEIQEISMKHDVQ
ERANNFHKRLQAIRHVDPKNIAGTISKLREDPMADLYEWIFGTDVFQAFAKWEDQNSSRRLWISGQ
AGTGKTMFLLGTIKQIQTNCLFQTGLEEPPALIYFFCQSTDDQLNNGAAVLRSLVWMLLCQQPDLM
KHLEEKFSSSGDKFIHDRNSIVTWCEVMTNMLRDCGRIIVLIDALDECEEVSRRFLASFLEELLGE
EELSHVKWLITSRPIWDLKHSRSGNAPEKHSLLVLDDHDLSPLINKYISRKMAEIRDLAMNKQFVL
TMEKQLCERASNTFIWASLVILGFKNSPEKDWRRILDRIPSDLQKLYEFLFEKLRDPDCKDVLAVA
MLANRPLTLPEIEQLSGLEPGKNAGRNTVTLCGSFLTLRDETVYLIHQTAHDWLVERHYRLRRESL
KELHAYVYENALKGLKAVLKQNIYNLPHFGILTEEVKPPSDDPLSPIRYGCQYWVYHLKESKVVQT
PGILDFLRAHFLHWLEAMALLGLLPEILQIVTELLLLEGHHSKSELSNFLLDSKRFIKKNLPTVTV

APLQLYVSALIFTPTDSLGGIGYHSAYLRKNDPEKLVIGFKYSFVIDVLYVLFINVPKFALLLLYN
RVFPKPLRVNLFVRLLMVYLILHTIGTTIALFVICHPSAIDFDYIHHPHSKHCGNAQALHDLYIYE
SLPNVVSDVAILVLPMTVIWRATMDNRMKLGLAVTFSMGGLGIIVSILRFIYYYRDASTTDPSWAA
VTLEIYTQAEVGTYLICACLPTFRPFLNCSFRERHVQLPSMMYSGNPMHMPAPERNMRPNSFASWE
ERYWADANRLENGPKNQL
>g1538
MSFKNIVVVGGGGNLSPSIVDALVQSPHKYTVSVLSREQSKYQAPTGVKHLKTDYTHDSLVSALKG
QDAVVSVVAGSAIADQKKIIDAAIDAGVKRFFPSEFGSDTTTQLALDYFPGWGSKVEIRDYLKSKQ
DKIEWTVVFNGFFFDWGLRVGFIAFNAKEKTAKIFPKYKDVKFSATNLGDVGTAIAQALSPGIEPK
TANQILRIRTLTTSQSELLAAFEKATGEKFKVTEADLDAAVTEAKEKLSKGDFSGVGTLIQAAALD
ERTGNDFDKGGNVSNELLELPSLPVEGAVKALL
>g1539
MLSRSRVNRYLRDHGAPPPAGFSILLLSMCLITYHQDLXPPSAHPLDQETLYVTTKTLFGLVQASF
PPSLHLIQATLIMATYEYANGKIHDAFASIGLCARMGYAARIDTAQAEQKMEDEEYLEAQEKSNTW
WSILICERYDSPLRLWRKVDMYRTVLCETDLKQPLSTRLPRNTTGHRPKGSPRQRVGSSAKSETDD
SDAGGFACIVQATYLSDQVVKALDMADFESRYNQLYGLDCALRTVLAVIMDQSQGRGGVYCTAIIV
LLR
>g1540
MTETVTGTETETTAQAPATSKEAPSSGPILPPEEGVQGWLCVVGSFLALFATFGFLNAIGVFQTTY
QETTLSSYTSSDISWIFAVQLCLLWALGPLYGRLVDTYGPSPVIYPCSFLCVFSLCMTSLADKYYQ
IFLAQGLVFGLGAGGVFTAAMVCVSQWFVKRRGLAVGIATSGSSLGGVVFPIFVNRVIQDVGFNGA
VRYTALFIGIMLAASCIMVRGRLPKKPWNNKTPWFDVTLFKDKPFAFYTLGSFLVMWGLWGPFDFI
SSMAQAKGFSPTLALYLISIVNSTSIPGRIIPPHLADRFGHFNVITLCCALIGASMLALWLPFNYH
PSHAGIIVFGLVYGFVSGSFVSLLMPCVAKTGSLETLGQRFGTFQIVMAVSCLTGLPIMGAILNRQ
GGTDFSGLQIFASVTALLGSGFLVASTYLLSRVRNTWKV
>g1541
MPRRRWVVDDESESESEFSETDVSFTRPGGRTSVKRRSFSRVRRDSPNSNAFLTPAIIAPPPRRSA
STGGRRRRGGDDPIVVVDVHNDVHAKQDYRDAHRNKIDQRLAQREDDIDEIDIIRSRRHPRIVADI
PASRTPSPRQQQRDWEILMDQRILQKNDVRQDMELAKQQQEIERLERQLAKSREKRREEPHHGSTS
RRRLAEEEIWEDELAERLRKLDRLESSKRTEEEQKLADYRSKVKRLEEIEREQRIAEEQKMADYRA
KVKRLEEAEHKAAEEEEARRLAKIKRLEDMERKAAEEEEAKRIAQEKHLKEIEKREKIKAERDRII
KEIKDEEARKTLEEEQRRKEMAELKRKAIEEWQRAEEAKKMKEIEEQKAKDKEFRERLRVEFGYSE
EEIEKMLKKKHEQEGKGEKHEHHHHGGGAVVVLEPPKEKTTFIRVHRKYLLPATLDAFKLPWDWDD
RDGNYIIIKQWISEDFQEELFAHTRRLRGGKLIEETSTTLTELKVNDRKRDKMYLVRKKNPGKSWI
FT
>g1542
MLKMTASKTPFHFAAGLASGLTTSILLQPADLLKTRVQQQSKRTASLVATIRTILSSPNPIRNLWR
GTLPSALRTGFGSALYFTSLNALRQEVARRGPAAILLDGSSTKNTSSALPKLSHTANLLTGAVART
TAGFIMMPVTVLKVRYESDYYAYRSLAGAAKDIVRTEGVRGLFAGFGATAVRDAPYAGLYVAFYEQ
LKRTFGRLQHSSSTPSSDKTNNTSSTTINFASGALAAGLATALTNPFDAVKTRIQLQPGKYRNMIH
ALRLMIKEDGGRSLMSGLGLRMGRKAISSALAWTVYEELIMRAERRWEAQAREMKI
>g1543
MSIPIVDFSAWTQTSNKEARIQVAKQIVEASKEVGFVYITNHKLADSLLEEAFEWSREFFALSPED
KLKAPHPEGWAVHRGYSWPGLEKVSQAISEGNDEELVRKLREVKDFKESYDMGSDNNSDQPNQWIP
EEVLPGFRDFMTRFYWDCFGAASEVLRAIAVGIGLDDEDHLVRKHSGHNNQLRLLHYPSIPADVIE
SQAMARMPAHTDWSSMTILFQDDCGGLEVEDLAQPGRFIPATPVKNAIVMNVGDLLQRWSNDYLRS
TNHRVTLPILKDRFQGPERMTRERYSIPYFLAPDPTSLIECLPTCVTEGNPAKYEPITQGDYNRMR
ASMQY
>g1544
MKQSLLSLATLLISSSWCAELEVAPFTHPATAAATAYPEFARDFDFFKRDGNCPANYNTCSNEGNS
GICCQSGTTCTVDGANNYACCPTGAKCTGTLGATATGGGNGNSASTTSTGFMFPQGTSSTTTTTSK
ASITGSTVPGAAFPFVYIPTSFSNAASCSSYYSFCQAEYTSCIVSLGGQYGVTVAGGNGGVTVAGA

TAVTSASAAISTCSSLSQEACYGLQQSYCTAFGTSGETTTSGGSFVAGSYAPARKSSLHEILLAAA
AGILGMFL
>g1545
MRSFSAISLLPLLAALSQPAQSQIVTASPYDVALEQRDCSNPCGYYSQLCCTSSQKCITNSLGQAE
CSNSDSNSGSGSWQYYTTTYTQTDLSTVTSVYSSYVTTAPSSTTTCATSLGQSTCGTTCCSASETC
DNGVCVAGVSSEESATGTATPATRPTSSGAETVTATQSATTTVGFIAPVSTNGTTVISEAHQGGGL
SGGAIAGIVIGVVAGVFLLILLCACLCIRGMLDGLLALLGLRPRRRTETTYVEERISHHSHGGEAP
PPRRTWFDLDWVGWDGLQSLLEQWHYVWAFGDGGTKRRRASTVMVLAVLITTTATITVAVANNQTI
DRRDKPATREDLDPNDDRHAALAAFTIHQYPQAEPSWLHQQQHHQAQHHQQQQQHSSLAAQQHAQ
AQAAAAAAAAAQQQHYNRIAMAGGNGSAGGNQAQGSGNNAAGLNADGSSLSAGAGIDGNISEESRK
VFIWVAELLDPNRRETALMELSKKREQVPELALVIWHSFGVMTALLQEIISVYPLLNPSQLTAAAS
NRVCNALALLQCVASHNDTRTLFLNAHIPLFLYPFLNTTSKSRPFEYLRLTSLGVIGALVKNDSSD
VINFLLTTEIIPLCLRIMETGSELSKTVAIFIVQKILLDDLGLNYICATYERFYAVGTVLSNMVTQ
LVEQQTVRLLKHVVRCFLRLSDNSRAREALRQCLPEPLRDATFSSVLRDDAATKRCLAQLLINLSD
NVDAGSAAGAAM
>g1546
MVSRKRAHSEMETATPKREQPAAEEPSLLQRIRNSWEFASLMQYIFFFGKVMKIDEDLGVEDLEME
CLKPEPSEKLMEIGLSLLKFVSSHRGLTFDNFDEYTRRQYNAKAPQHVNPFGYDEEPNKFRDFDVF
LKLKVLHQLSLWTFWNPDRIREKMPEQREIDQTQWRIEEFGWDSKDRIYYVLDDNRLYRRTDPPIP
PPPPAKPKANSKKGRAAARAAKRRRVSAKVEEEDDEEEDAAESEKTSFETTTIEFKWECLATNLPE
YQAFLDTLQKTRDPNEKSLRDRIIEGVLPVVGRAAEAEERKRQKREKELFNMQLLAGAKRSSRLAE
KTDKERRDREAMEAARKHEQELAAARKEQEIQRKMEKERQNRIMTRERRIQERESKRILHEEEMAR
LEEEQKKLESGEGRVSERQIKAELDKRKKEMEELAEEDQWIFDCAGCGVHGENIDDGSHCVACERC
NVWQHSKCLGISQAEAEKDDFHFVCADCKRKEEEAKRPKIPPLKFRIGSASPATTAPATIKEGEQS
PLASKVSLPTPSNATGSLQQPTLPPAQPRFNGGAPASPERRFQPAANGHPFAAPGSPSKASIGPAS
SPLSSLPEPAYIQRQQPAQNSTAALQNILSGNPLSSHRPSSSHSVHSQAHPSPIQNRPSMSPTQGN
RDVGPLAGFPPSNIPNGSLPSTPYGQHRTIVRPQDRGVPSFSSSFDQRTTSFSDSFASHTPPPAGS
QNNNVALSGLSPTKNDSPRPVTASSVSSASILPPIHRLEPSPKLMGRSSVDAPIPPPVKMMTSEQE
GRRQREHQLASGTTLPPLSSWNPPSSNNVQQQHPQSTLPPLGPVPNTQDQNNGMNGH
>g1547
MSRISSLPSYVGSPSFASDAGSQHSSQIDDAPQFEPIGHGSSPENGILTTRSGRTFKFPPALGLRT
MENENKVRVLKTPRVRRRPKTKGEKRPPIDLPAPLSQLTEHLHHIPIKDMEAWVNRSADVRRQEAQ
KKGKIARPMNSFMMYRSAYADRVKEWCVQNNHQVVSQVSGTSWPKEPAEVREKYELLALVERDNHQ
KAHPEYKFAPNKTQTPPRKKRSNNGHVSDVDDSRYRLGSTASPNPHKLMRLKSEFDSRESTPYDQD
TFFPDPYPGRWPIPIGRPLPSRIMASHEQQHPGAYHMTIHGSFPNMIRSSDDGHATARPTGYVINN
GYGTPSSLASIPGSINQELLRPYGSNSSAASTSTANSGSNSHSDGSTVTRTDSSQLDPQLLTSQQA
GLDMRSYNQVNMSTIWPHQDQDLGTYMPMNAPMGNNGIPYDALNTSYTMQSLEEGQQVWAPCAEDE
GIGGSEVVGKDFDQWIHHQNHQPGFGGPQ
>g1548
MYGELGNKLVQHAKRTQSLAHLPPYQTEIVRSVAREVRDLNNDVAHHLAPFESVDGSQQSSFNPSA
QPAIACALLVDHLCMRRNKRCLLAYHRVRAEKLEEMCWKGIDVLEQQQPVNSAGVGGAGANNNGNK
EPATEENNATTNLSGQSSLSPEEEEYFRLYSEMLAAYKGQWTDIDLTGSLEPPKDLFIDVRVLKDA
GEIQTEYGAINLTKNSQFYVRQGDVERLIAQGYLQRLS
>g1549
MDIRFGGRGRSNSRISRNDVLGTDRNHHDNTITDQNDAYDAASSTSEAPFSPTITKWDSVQSLSNV
KTPARSFYHRAFHGSLDPAQYSSHGVREQTAELASLAISDAENNLSAASAPPLNDSIPPGLDIFRQ
QDARRHRVSHGSTDGVSRPTASDFETSARFEPSSMSSPPQLSTSNLTAILRSSPPNTSQTRPETST
STNDSDSRGVDSSSNVDEAQDENQPDESTALIRKRTLSKGYNYGTTAGDIESQPTYIYDGRKSFLQ
EAYSRSKRVFHITTHPKSWDRHVIWQEGIVRPFSLLPAVFLGLLLNILDALSYGMILFPLGEAIFS
DLGSDGISIFYISTIISQLVFSSGASVFKGGIGSEMIEVVPFFHKMAFMILSRVGEDNPRAVLATT
ILSFSLSSILTGVVFFLMGVFKLGSLIGFFPRHILIGCIGGVGWFLVATGVEVSARLPGSLEYDLV
TLRHLFQLDTLFLWTVPLALAIILLVVKRFVKSNFLVGGYFILVAVLFYIVKFIARIDMQTLRGGG

WVFEPPAASNPWWHFYTLYGKFWTIRSMFLFIQLISYADFDVVDWPALVDTIPAMFALTFFGVLHV
PINVPALGISTGEDNISVDRELIAHGITNTLSGSVGSIQNYLVYTNSLLFINSGGDSRLAXIMLAA
ATAGIMVVGPSIIGYIPILVVGALIFMLGIELMEEALVDTWGKLHRLEYLTVVIIVVTMGAWDFVI
GIFVGIILACVNFVVQTSRKSAIRATYSGEFTSSTVRRPPIQQKFLKDAGKQTLIIKLSXFLFFGT
IVKVETTARGLIDEEAFTRQPIRFLVLDFSRVNGLDFSAAEALTRINRILGKRNVQVLISGLDVEG
EVGRSLQNVGLFADEGQVQIFEDLNSALEYCENEYLKVFYNRKEALTEQQQSSRASLEVPTGTVAA
NQPSFLDPYSHSPRRNYLQQAALSTLRENETSASITQKWTTFRQPLPLLLQTFHDLTTYQSEDFWY
PACAYFRREAYPKDTVLYYEGDEPEMFYLLESGMLRAEYELPQGRYSELIVAGRPCGELPFFGGTP
RTATVKVDQDCVAWCLDRERWTELRADEPEIAQELLMVTLKLTAERMDSITSHVLAIAG
>g1550
MSAAEVTKPVEETVAPVAETSVAEPAPAITEPVAEVPAEAAPATEATEAPAAETTEAAAVEAPKEE
AKEEVTPATDGTLGYKGPGLVKSFRFTKRFFYFSEDAVEAKHLTSYLASEKGSAAAQRTAAWATQT
GKGLLFLTKRAEDKATPAGIFNLSEVSDIAKEGSTEFSFKTHGHKHVFQATSTAERDGWVSALNTK
SAEAEADKETVTGSEGYKAELEKITKPAVVAAAVKATPAPEKDAKETEAATAEDKKEEKKEDKKSR
SQSRKRASIFGSILGKKEKEEVKEEAPATTEEAPAEETAVAETAAPAETTEAPAAEATTEETAPVE
AAAATETPAEEEKEEAKPAAKTKRASIFGDFFKKVASPSKEKTEEEVAPVASTAPQLENPVDETAV
KPIEPESVTEAPAVEAAAAAASSSETPAAEAATEEKKEATTPEKRRTSLFGTLGKKAKTESTSDGE
GKKTNKLGGLFRKPSKAVKSEDDKKKEAAPAETIPEGEDKPEPIAKDAPAEEAAAPAPIEKADETV
VAAEPVSVAPTAAPVQAAA
>g1551
MSSSNIAQGNYSLHLHPAPCPTESVPDIDANNYPFPFTDFSLDLFDLPTQSPLLDDLTSPISSMMA
SPLPDSFSAFMTTSSAPVPPGTVSPKDLMSGPPSSFSTELGTPQSAFDSPADLSFGHFTSPVYVAD
GDLPADHNDWSSLFPDSHDMSEDFDMTLATFAQEKTSKPRPSTVSMSAASPESYTDGDASSPKAKN
KRNLPDPKFDPTDPVAVKRARNTMAARKSRRRKLEKQEQLEDRIRDLEAMLAKSEKDVEYWKAMAQ
TSMTDA
>g1552
MAAAQALLDEVHEDLMADPRDNNTYALGRIGKHNVVIAGLPAGGYGLAPAAATATHLQRSFPAINI
GLMVGIGGGVPSKDADIRLGDVVISKPTDKHGGVVQYDFVKAMSNGRIRMGMSDRPPSILHRTISR
MQATHLAHARHVMDHVAAMNEKLGEYATGFARPTQKDRLYLSDYEHVDAGSTDCDACDPRATVPRC
PRDDNVPVFHYGTIASANTLLKNSKLRDELAQEFGAQCVEMEAAGLMNVFPCLVIRGICDYADSHK
NDKWQGFAASVAAAYTKELLLATRFE
>g1553
MAAVHPLDPLTPQEISLAAHIVRNSFPSNNLIFRAITLWEPPKKEIIPYLEAERLKERLPTPPPRI
AQVLFYIDKATQYRRGRIDLEQKKVTDINDLDGHHAYVDAGEMKKCERACLDDSRVQAAIRALQLP
EGAVVVCDPWTYSPDGMNDMTRRCVMCFFYMKLSPHGDANHYAFPLEFVAELSDEMKVMQVLKVPS
GVNDQMITADASTLRPFDRAKIHTTSEYHPDLATERRTTVKPLTVSQPLGPSFHTSGNLIKWEKWR
FRVGFNYREGLVIHDVTYDNRRVFHRLSSSEMFVPYGDPRAPYPRKAAFDFGNNGAGVNANNLGLG
CDCLGHIKYFHFWHHTNEGVPTKMSNVVCCHEIDDGILWKHTNYRTDNAVVTRSRVLVLQTVITVS
NYEYIFAFQFNQAAEISYEVRATGILSTAFIDRDTSVPFGTVVAPGVMAPYHQHLFSLRIDPAIDG
YENSIMVEESHPMPIEDLKSMTNVGYITKNEFVENETPLDTDNRVGRVFKIVNENIRNPITGGPVG
YKLIPHYSQMLLAHPSSYHSIRSEFGDYPIWVTRHYDDELFAAGEHTLQSTTGSGVATWIKSRRDN
PESVRNQDLVVWHTFGTTHNPRVEDWPVMPVEKMTVTLKPVNFFTRNPALDVPISTQADNKSVLVG
DDAEKGCCGTTALIHETASVISDTRQSLNPSKYFIIVPALFGNGQSTSPSNSPHLRDAFPVVTFAD
NVRAQYLLVTQKLGLTKAKAVVGFSMGGAQAYQWAVQYPDFMDVVVPICASAKNALHNNVFLEGVK
SALIAARGGLSLGVGKGQRYPSNEPWTPQQREVGLKAFGRVYAGWGFSQAWYRQKLFSKFFGAKDE
EEFLQTFWEPWGLKNDPDDLLVMLRTWQLGDISRAPEFGGDLQKALQSIKCRVVVAPVETDMYFPP
EDSQFEVENMVTGRGTLAVVPSVWGHWGGGCTDSKDDLQFLDEAMVQVFAETG
>g1554
MNRTLPSKDPPSLSLYSPCIQTLEDQLRQSVVIRIRNIPQLGCGSPGVDAKVAVLFSGGLDCTLLA
RLAHDALPKDESIDLINVAFENPRVAAALKAKHEKENGKEQMPSIYESCPDRITGRAAHAELQKVC
PDRGWQFVAVNIPFREFSAHREEVIQLMRPHNTEMDLSIACALYFASRGQGEVATNDSSDEITTYT
TVARVLLSGLGADELFAGYGRHGIAFARKGFQGLIDEIDLDVGRLGKRNLGRDDRVISNWGREARF

PFLDEEFVSYATQTPIWERCGFGTAKSESASDDELDQISESLDFEKKALRLLALKLGMVQTAQEKK
RAIQFGSRTAKMESGRSKGTQTI
>g1555
MSASDKPRSLLSNEDAKXKDXSHDNIQEPVTANLESIESANAVSMAEPETSQPELLHIDPSPMDEP
AAESSASTHNTNTERLQELSLSSKKPDTLSVETQIDSPPPPPLKDDAYIDPTPKTPQAPRSPMGMP
SEYTEKELPSVPQDEQLEDRKEDVGANDDDQSEIQSIMEQFSDEMKGPNETEIMSPRLEIAEHFQS
QFPQRKSSLEPLPQSASNYPTSSGPSSVPSPIIVPKSAPNAGTDVPDTPQSTSSRTGLPPPEPEPD
QPFDFHRFLEQLRHRTADPVAKFLRSFLTEFGKKQWLVHEQVKIISDFLAFITNKMAQCEVWRHVS
DAEFDNAKEGMEKLVMNRLYSQTFSPAIPPPPSVPRSASRSKRKELERIHGPGRRGQHQEDVERDE
ILAQKIRIYSWIRPEHLDIPSLGNNGRRFINLAQQELSKIKGYRAPRDKVICILNCCKVIFGLLKH
SKNPDTSADSFVPILIYVVLKANPEHLVSNVQYILRFRNQDKLGGEAGYYLSSLSGAIQFIETLDR
TSLTVSDEEFEKNVEAAVSAIAQENMKAESETSSTQATPRVSADTERLSQRRDVGPPTTNEDENAP
VTGLLRTIQKPLSSIGRIFSEDSEVDRRPQQSSANAPPLPERGPAAAQAGNERIDAQEAAARQASA
EAAEARRIQRVEHRDVVETLSGMFPNLDKDIIDDVVRMKEGRVGLAVDACLALSSTE
>g1556
MPVSFASASDKQPTSQNPSEVRSTGNAGAASLEATNYLQQNEIGARTHIDASSDGVVSAPHSQQPA
LPSGNAPSARQLTKDEADRLYEENIEDEYAKREGGA
>g1557
MAQIRGTAGYNLGHQSPFATGPGRQDATSDPSPLEAIREQTSKIEDWLDTISDPIKPYIPAIGRFL
IVVTFLEDSLRIVTQWNDQLMFLRDYRHIPWGITHLFLIVNVVVMLAASIMVITRRHSEYAVGGLL
GVVITQALGYGLLFDLNFFLRNLSVIGGLLLVISDSWVRKKYMPAGLPQLDEKDRKMYVQLAGRVL
LILLFIGFILAGKWTFWRVLVSLIGFVACVMVVVGFKAKMSAVILVILLSVFNVLVNNFWTLKPHH
PNKDFAKYDFFQVLSIVGGLLLLVNMGPGQFSVDEKKKVY
>g1558
MNTTKKVAIIGAGPSGLVMAKTLLHHYPKGYFEPIIFDSKPTVGGIWAVTRPDGSTLRNGSSEFVD
CFMRTNLSRFTVSFSDHPWNGESQDKDGNEENMFPQAWRVVIRTQRNQVDGNTPTARRWTVVWTDQ
SRSDGEEHLSESFDYLVVASGYFSRPFIPSITGLDQFPKDRVIHSSAIRSSSQILTSLGGDGGKIL
VVGGSMSGAEAAASMALDLPSKSIDMERKRPVIHHITTRPFWAVPTYLPANDQPGYFLPLDIVMYD
LSRRPPGDIQYALGPVTAERANMVHAYFESLLGTDQSDVAEELALKAQSQDRSSAPWVAVTDHYPG
FVRAGDIAPTLGRVSGTSWTGEEAGSKGVVEIEQNDGRTLKLDNVAAIVFATGFTPFDSLSFLPEE
ILQFLEYTENDTVFPIVLDEKGVRNPKIPDLGFVGMYRGPYWGVMEMQARSLARQWSGQVNNVNTN
NDMTKVKDLRTTSSRAQFPMGDYVGLMETFAREMHIPRKSLQGHTTERTGPVIPARYFEPTSTGEN
DNSNNSKIASLRNLLLDPTEPSSNHKERQRGLSKAILRALHGHWLYERELLPEMKTVTGQASIRLQ
KASFDKDSGFSMEFIYEEDSGSTTQEEEGKVIFRISEQDGRIDTARHTTKGRFRFVNVEKIEDGDS
SAPYQYRHLAKELDVAPSDIDRNYVFNMKGVTMTSWKVKVENKQTNTATVTKYSR
>g1559
MDLSQSQSSLPLATAARPPPISIPPLVNNISSKLRNRLSFKSARAAYAEREILKRENRAARPLTQR
NMDTFLIEQDLHDARVRRAENLHVSQWLQQLP
>g1560
MWNMQVTKRWTEVVAYKRSKTNSTSRGDDLPRIRHKKCDETQPACYMCTNTGRVCDGYQQTPDRRT
RAFRKGKRDSSYDPRSPIDDLLTEPILVEELQAVEAGEEATEEDFIGTALPQENVPTPSVKTRPVT
RRVVVSSPMLKDRPSVFAKSHRSDLISSAHYLTDPHKASLSQRERWHFEFFIRYTSSQCSVFYGYD
FWQRIVPQYTEAEPGLRHAVIAVGALHRNFEDSQLNSQEPDFSFAIQQCNRAIYNLKHRLIGDQSQ
HMEVALITCILFVSFAFLQGDAGAACRLIHGGVKMMTEWEKNSNSLKDSTIKLTVMRVFSRIQFHA
MTCGDPDFFVEEEYNPKGTMNVLSSNQSLSQPIDSIEAASSLLFDIGWQVMQRKPWLSRPHRNEHY
DVVATLNNLQRWEAQCHIALMGRREEMSIRDRGSWIMLQMWSETIYILAATDWCADGRESRFDGLL
SHFRKAIGYAKELLTTESMRSFLPTFHMGPGIIPPLFLCINKCRDWKLRKEAMSLLRSWPCQEGFW
NTAGSAKCLEKLIEIETQGLLPGEVVPESQRIIATHVDYLPITNTYRFCDADIEAQMAGSYSTFAG
TTNDDSQNRDDTSVRSSESGRCRINPRLISDAILGLSDGLTVPFALSAGLSAIGDTKVVVLGGLAE
LIAGAISMGLGGYVGAKSELESYEATVREVNEILDHPDETRSMVTSTFANYNLSPSTLDEISSSLQ
AEPERLRDFLISFYHRETKPDCNQAYMSALTLTLGYFIGGFIPLIPYFIADKVYTAFVASVIVMAI
TLFAFGYVKTCIVRGWKGGVNIRAGVIGGIQMCVVGGLAAGAAVALVKLINGEGSV

>g1561
MSEKFYRALSAIDEAHSQDPNLTTITEQNAETKSIPYELHYAQKMTSYLNTYNPSASELLRLAIRA
QHLRRWEVPRSSYPTTKVGYLSWRTFLKNRQADQARQICLDAGYSDGEASRVAALIRKEGLKRKTT
TTTDEAAEAEEDETQILEDVACLVFLDDQFAAFEKTLDEEKVLGILRKTWGKMSDKGHRMALEIQM
SDRCRELVQKALNP
>g1562
MAGIQTPFSTYRLLSFDIYGTLIDWESGIVQALSPIQDRLPDSSPLKSGGNDLALGTVFTKHEARL
QSEEPGATYDKILRDAYIAVAKELLENVPGEDQLEEEGVAFADSIAVWPAFPDTIQAMRKLKRLGF
KLVPVSNVDHGSFGKTLAGPLSDLREPLSPGSDPLDPFFDAVYTAQDIGSYKPDLRNFEYLINHVK
ADFGVEKHEILHVAQSLRHDHVPSKQIELDSVWIARGKGGVSGMGGDVEELLGKVSFAWKFESLGD
FADAVEAELQK
>g1563
MVSSENDALNILFEAATCTEPANAFPSTSKTALTGFPSVREVEESPISLQQSTPQLSSIDSIDYDI
LRIWNACRFVKMGWFTAHEAMTLADLFFINMSNRSPILTDFYASHKNQYWLVTQEPMLCCTILAIS
SRYHVLPGPGGKTRGLFIHHRLWQHCQHLLLRLILGQEKASKAKTRNVGTIEALLLIVEWYPLSVH
FPPDSDGWDSDVLLTMPDTRDPPLSHEIPALERWKRDVIEPTKRSEQMSWMVLSSALALAHEVGIF
DKKPALSGKSGAFFDAVKPDAKAYTEHLKIRRERLPKLLYCFINLMSSRLGCTSFMCPESTVALLE
VDKDLSTDLNQQWQSFMNCWVELTKLTKSITDTLFPLMTADDDEKFQNCLQEKQQLLSHWYTTVDF
GNCHYEDLLFIEYQYLRVLTSSTGMQRVITHYSQPQSAHNFRPTFSARPFQSDHEYHLVEEVIDGC
CHIIELTIRKAESNTNNSLHYAPIRMFSRMISASIFLLKALALGVRRTKLAESLQVLEMAISALQT
SNLDDIHLAAQYGELLKIQVDRLRRSFESSGTPHYHGKESAKHSGEGHCSIGREQWQPKLQLPANQ
AASPSSYFPPRLSGGEDLLDDQRYYSSHGQWTETASLDKLDANNQGAVDINTTPAKPNGLSLIVIL
PPGLSRERNSALPIPSFAHRPSQSLISPSLCLKQTEPGEFDQSDTAHYYSRDKAHVRFIPAGRQRL
PIFVFVGAQNYASNFAHTHTSPSHTTTSNGQPLSSPPSSTPMSTQNSQQPTVSVTTSFPTPASSVS
GHPRNTTPADESELADKSWGQGPQNSHATGNTDLGSAENLGHRRTDHDRHRLPEGFDMEGRPSATD
VDMMDIDSKADTSSFRDSSLDALQQDIGTAFHLCKSVPTITGPDPSFDLISLYGLGPIGRSVMRAD
PTTGEKINRLRKSYEGKLKGLGLSGRNKAVKNENQAGGLRHLMMWPEEEWQIQKVHGKEIKVAEAE
SGLQKLQMRAMKLEAGPLPNSEYWEDILGHEKSSKHAVIESGKRAVSTPNALRPTLQANGAPAAAA
APERARPTRGKKRHYDDNSFVGYGEGFVDDEDEGALYSNSEDRSGKKKRKKVFQPSHKSHQTDKLM
DSFQEHISKMSPSMPERTGSYGVGMYGIGAR
>g1564
MTTTKDTQVQGETLHLEDSWSNIGNANTAGLSKDLGLSSLQFSWVLYSFYICYVLFEWTTVLWKVL
PAHIYIAVLCVCWGITAMCAGTVHNMAGLVVDRCFLGIFEAAFGAGAPYFLSLFYHRRELGFRVSI
LLGTSPLANCFASALAYGITQIKHSIAPWRLLFLIEGAPTILFAPVVFFFLPDSPDTAKFLTEDER
TEAVERLQIIDTTRKQQVEWTQVLSGLTDYKNYVHMAIHFCCNYSFAGLSNFLPTIIMDMGFSSVR
AQGLTAPPYLAAFLCCIGAAFVSDRFGVRGYIISAFGCMGTIGYVLLVALQDQTQTGPRYLGVWLA
TCGIFPALALNITWLLNNQGGDSKRGSGMAVLAILGQCSSFVSSSVFPKEDGPTYVKGCAIGCAFT
GMVVILSLGLHFKLEHENRKRDRQYGVVDEDARIDVTDGGDYNPRFRYLT
>g1565
MATSYPFKFESTGYKDIEASNGATRDITLTLKNVRGRIPSLQASRLRSMMLEAHNSPDKILAHCCS
YDGLSSRLVEEAGYPMVFLAGYPVASSYGLPDTGYIAMEEMCHKIQEAVRQVSVPVMADGDTGYGS
PMNVRRTVECYAAAGAAGVMIEDQTWPKRCGHTKGKSVVSRGEAFARIQAACDARNEGKDIFVLAR
TDALILGWDEAMARAKEFKRLGVDAVFVEALPDREAMKACVQEIGIPTFANIIEGGKTENLSAKDL
AELGFCAVAYPWTLVAAKLKSIREALEGLKHSMTTGPPPQILSYAEVCKGVGFNRYWDLEEKYKYE
A
>g1566
MRYYSTLVCLVAVSLCFTPHQRNDETPLGQGKQLIDTDDVRMNQVRGNRDRLIEDVRVCGQFLNGL
NVLSGEFSNDTASLDFVKACERVVAILDQAVDRSMHRHVQSREQRNLPALIRKNRPDLEEIELQKT
FSTEFSEEILARDAISSFPDTARHRLKHKYRRDEICEDRVCEANRLNELCNAEKIDPSVAQTCKMC
YPVRNAQLINAHCQAKWRRERKAFYVVSFVLIGVTLVSGLILYLRRRYLRAKNPRTDELAESGINP
PGELYYYDGPRSILPSERGSSLTVNCSPEASRRENSNKYHNSWDENEGDVIRTSFAQRRLRQLGIL
SRTGRKRIHDLFDLEALQSRRGTVKGPQQHSDDSVRVPVMPWAPNATIRSSSRSSSCVRPADSTSP

ETVRLRGSSLNINKDLC
>g1567
MENGIVDYSQHNSTKIENLGSASNVILIDNYDSFTWNIYQYLALEGASVTVYRNDQITLEELIAKK
PTQLVISPGPGHPDTDAGISKAAIKEFSGKIPVFGVCMGHQCLISVFGGKVDVAXEILHGKASVLK
HDGKGVYDGLPASLDVARYHSLAGIHPTLPDSLEVSSSTNLEQDAKPIIMGCRHKGKKPTELPAGQ
GECDTASHAFLNLSRSPGPETCEKAGKAGTCSGATDEFAKGKERSYVASLEGYCEKLEKRLAELRR
RKEAQGSNHYSGQEVPQSSITAFAAENSSSRAHRKEVRDIDDLVGDFGFLSVNATSRDFHGITSNV
SFAQLLLTLSIVKPLPQLAPQPLPSRREATRFVQYYFDNILTQLPLFTETTFWTSVEAVYQDGGRF
AKPIDQWMVYMVLAIASASLWHQQPSVNQQFALSMVSAAMPFAGEVLQPGSSSGIQAILLLAQYSL
FNPEHFRPRFLIFFASRVMIDLGLHQDPPTEVVVDRERLERRRRLFYALYTLDRVVCTSLGHSFSF
SDLSVDVDLPIVRVLPVDLPNENVFMRKSDCATHMIKIRRILSKGYQQMYFDGRDPVPHALARTWK
LCADAREWYADAPITRTSSIGLLYRLELLYCTIVFLSPSFGDPEICDFSRVVLFDRCIDYVSQLHQ
VLEQPGSLPFMTFVDIRRVYQVGDRLVNILEESYDLLLSNQLPEPPAVPPGTPEPPYLAVEDRINC
RSRAARCLQYIIDILHYGCTRWNMRDELDSFVRESITVRQRLSPDEGSSSQAGSQIPPYTYAAQSS
LPANDHPASQTYPDSGPNYHYQ
>g1568
MQAVRLHPAPATSQAYSPSNPAPSSALHLDKDLPVPKLSKPGEILVRVKATTVIRDMLTWPETYAH
EYPILGNDLSGVVIETFSPQSSFKPGDEIFGMTDGDRPCTWAEYCIVMEDEVALKPKGLTWDAAAA
MPLSAMTAYEALYDHAGISVPVLGDVSRPDGHRSLTSEHKQRLLITGAAGAVGIYLIQLASISDVY
TVAATSSNARNKEFLQSLGADETVEYSALLSEYKKSFDVIIDTVGGSILSGCWENIKEGGSIISVD
SASYDFVADHQKRGLSKEGINALFFIVKGGKRVLKFLAQLAEVDKLRPFVLQTYPLSEVRQAYDHT
NGRYSGRGKVILTID
>g1569
MKLFIATFLPAFAAAATSVSISAGKINGGRCESAEDTVFFKGIPFAEPPVGDLRFEPPKPYNGKFP
EEGLNATTSAISCIQFTHDYTPALPTSEDCLYLDIYAPRNITKDSGLPVKVWIFGGSNTEGTISDS
LYDGCNSPNGDSILVTINYRVGPLGFMALSSAGIQGNQGIQDLILGLEWVQSNIAAFGGDPNQVLL
FGQSAGAENAYVIASLPQAPSLFKSVISESGGGRGLINNATTQMIGASYAQALNCSLTDKACLKLK
TADELTKAYDDDDFLSQGIGYWGFLGILGPVTHSFYPYADGKIVPEDPYTSGVKVPAVFGFTKNDG
IIYSIDWANSSGNIPSLAAYEEFLRKDFGQAASLVEKQYPLSLFESTIGDSPLLGELSAALGLNKT
EAAVVVAMATVITDSTYKCPAYYGAAQATRNNIPAWTYEFTHNSTCTWIDTIPQKYVSIFSATHTA
EIPFVFGNMNNFDLPDGDCTFTQSEKYLSQQMRSLWTSMAKHDDPSTASLTWPRFHTTEKNLTTPG
LIFGDSAVAGTVDYTGCDLWPKVHAILAGSNSTTTWSSPTLY
>g1570
MMAPSELHGSTLPELPTSLYEAFYQNVVKFPNTIALISTQQPSALYGIPSTSIDSAKHGADYLRWT
YQDLSHAVQRLVTGLQTRGLKRGDPLVIFMPNTAEYVIATWAAYELGCTYVPINPKGLSNAQEMRH
MLKIVTGQCQSDFVGIIAGKSDICARIEELTSEQNCMRILVEGERDGWTPFKEVMSDPAPDLQQSV
YRDHDSEFSDRTIFFTSGTTSLPKGCLIPSAYGFSAALRWRESSVPMLPGDRALFTLPNNHGFGWL
CIIGPFLNAATVVLPGPSFVPEAVAKAIREEQVSHAGLVPTMLHALSNVSLASGKLNSLRRIVMGG
SPPTEEVIKICLDTLGASGVENLYGMTEGVLISTDVVSHASDIIKGRDVSIGTPLPGMTVRVCAKD
SNASTKTGEAGEIHFSGPALIKGYIGGMDSNFYNGEDGRPWFRSGDKAFIGTDGRLYLIGRYKDTI
IRGGENIEPSAIEAVLGQIPDINILQPQIVRAPDNIAGEVPVAVVNQKIDDSTADRLKDIVLTRMG
NLYVPAEVISIQTLGHEKYPTTMAGKVQKTKLEELVRTYWERQNTQGGHSIDGPISGPQVMRSLSM
AIEKTMGHPIDLSAGMIELGIDSIRSIAILKRVQIETGVSLPSSLFFTQATVGAIFQQISSIPDTA
GFLDFTPVMKDAPEDRCFSVLLQGTPRAGVPSLFLTPPGSGNAFVYRTLPKFSNDCAVYSFGSPFL
MTKSETSWTIEETATIYANTIQSIDPDGPYMLCGWSMGTATAYETAYQLHEQGKQVLGIIMLDLAL
PRPPPTIPEATVELFEMMGVFPPIRRQGKPDVEIPAFRKKHRVAAYYAKMKYAPRPFNNRGNTSRP
KIFVIWAGHGDHDRMADMVLEATKLAKKEGPGTKLQISNDWLTVPRESFDAGGWDEMVGSENVEWA
IVEDADHDTLIESEELVVLTKDLMEKAMDKWLQQADLKSDIPV
>g1571
MAHRTTSARSKSTVIVGERSLDVADLEVIELEGLTAKDPAEIAKLLKAAESQGFFYVSFDNSLSKK
ISEFLRISYLNSHEFFSKPLDEKMKAFREDVNYGYKRAGMESFGIFRDEQDSMSLPSPFAEHAVAT
LEFLNVCDNIVRTCLHRFSDSLGRGDIDNAHHPDGKSDSEIKFVSYPTKASAADAPDTTHTDSGSI

TLLWCEKWASQMQTKETKEWLWIDPKPDHVLVNIGNYLQGQTGGRLHSPVHRVVPSRIQDRRGQHL
LRPPIAIPLEKTDTEHPFQIPPSMLSPANVEPSLDVAREVVAKSQNSQYPPNVLPLTASIPADLIT
PTVAYLKIAAKSKPSFLYESAATTETIGRYSFFGAVIKTGLGHGPECDPLPILEKELSEVRSATIP
GLKLPAVTGGAIGYVGYDCARYFEPKIARPLKDVLGIPESLFMLFDTTFAFDHFFQIIKVITYIPI
TDGGANLEEAYQKGKETIQDAINTLLHPDIPLPPQDPILQNQEYKSNIGQAGYESHVTRLKEHIKV
GDIFQAVPSQRLARPTSIHPFNLFRHLRTVNPSPYLFYVDCGDFQLVGASPELLVKNENGRIITHP
IAGTVKRGRTSEEDEALAAELRNSLKDRAEHVMLVDLARNDVNRVCDPVTTQVDRLMVVEKFSHVQ
HLVSQVSGVLRPDKTRFDAFRSIFPAGTVSGAPKVRAMQLIGELEGEKRGVYAGAVGYFGFDRSNQ
DGSSKITGDMDTCIALRTMVLKDGVAYLQAGGGIVLDSDEYDEYVETLNKLGANIACIKGAEKKYL
LLEKEAAAAK
>g1572
MSFKFESTIANHQAGFPGNLVLICAFVIAFLSWKLFKGYLKTEGSNDLSTPGLKELKGFKEPRPYT
TATVSSARELEFYKSLYYKLQNLEEHPDIIPVARQAFLVLLSQAVEDYAKAPETNILSVQHFDSDA
LSRFLMNDQDDVTRQYHQYISRRRSGGPRELFLNRQAAEDWLREIAPLKLVDGAWLGHLNKITMPF
SLRHIVKQTWQVFTDELGNGNPDQHHVKIYEDLLRIIEPDLPSATTKAILHPRYKLGSLKYWRAAV
AQLLVSLFPHEFLPEILGFNMHFEALQLETMQAAKELPEVGFDGYYFLLHVSIDNSHSGHAAMATA
SVEDYIQHISESEGDVAADAVWKRVQAGYIFSEWQFRKGNQATNILARLDDDSHGRLESIQSKVLA
IFGSKIQAAHRLHCGSRVKIGERLLTDWLDPEAFSTEQWRQKFIESLSTSKPWIYPGDSQRSKLVQ
ALQWGGKMYGSFTPSELDTLVLWIDSLDSTHRPSHYSYIGQPPMRLALDHSDHVALAAGSRAIEPA
TLASLRKHLGDRTAHLNIAHLLPLWFTQCCLLESFLYAPQRSGDKLGCAVVGFLRAQSGFETDDQP
YSAENGGIVEFGLEMIRQASLPVPENLDGILSVWPSDFATTMLKLASSPVRNIGVLLGMSLAFLEL
QEMMSESSNPGLLCPKSSERLATLVRKQYQYLQICLGEIPAGDARRGDIDHGFSMAKIEIHRLFEH
PTSTPFTPNVN
>g1573
MGQKSHDIASLTTKAVASIDTYQATQTEQTRQDALASAISLVRALETPGDAIYKLFASPAILMAVK
TASDLNIFTTLSDRTSPVSWKELAGPKNADIQLVERIMRVLVCYDFASEVGSGQYMPTELSRKMTE
RKAIGTMDSLFVDFLPIIQKTPEFFQKSNYKNPGDPNGGPLQHAYNTTDSCWDWLAKNPDALGRFN
TFMEGGRDDTSHWADWFPVQERLLDRLSPDRPLLVDVGGGRGHDLEGFKQRFPVEAGKLVLEDLPS
VIDDIQNLDGGIQRIKHSFFEPQPVKGARAYYFKHIMHDWSDYNCQVILRHITTAMERGFSKILIE
DYIIPDQNARLKETLTDMIVMVWCPGIERTRQRWTELLESVGLRVINFWLPDGYHKGIIEAELQ
>g1574
MLRNEKRLDFITALEDKLVNGVPGFEPMESMTSSKKIIHAVTKHSLNRHLGTFIEPLDEESKYALA
QVWTDDPEFHEVMLKDSVWKIFGRIMSRTFINDKDFYRNPEWVNASSEYVELSALAGYELRGYPKW
AKRWVAPFLPNCRKLQSLFKHINKLLEPLKEKLDQEPPGVDSTDPLSFLYQKTGGRLDELASMLIA
LCLVSYDGGGELFTHVLHSVFTNSQLVNDLRAEIVTVIGKEGFNKNTLQNLVLMDSVLKEAQRMHP
ESVLMMQRIAVEEVVLPDGLIIPKGTALFVSACHIIDASVWPDGDKFDGYRFYNLRQKGKPSTSQV
SYNFTSTSPDHFSFGHGSQACPGRFFASYMQKILLCNILMRYEVSVTIPDEGAWFQRGQTHVAHPG
LKARVRRRKEEIEL
>g1575
MTAPQEESGPPQGEQLRNPNDRPECFSSTLQECLFVLTTTMAIAQTSFFEGLVLVVSASIGKDLNM
SSAEITWITAGVSLTSGAFLLAFGKVADMFGRRIMFMASMAGFTVALVIAGFANAAIYMDVFSGVM
GIFCASAVPPAVGSLGVVYSKPSKRKNRAFACFSAGNPLGYVGGMIISGVASQVGNWRIAIWTLAV
IYAIFTFLAVWTVPNDPPMAKAKLNWQSMKRLDPIGMLLAISGIALFSTAISTAGDASNGWKTPYV
ILLLVLGIVLIAAFLFWESRYSTPLMPLYVWRDRNFSLLIASLCLGFMGFISKITARLLPMIINGI
LVNVVCGLILHKVSNKLLMLIGATAYMTAFLIMSFTPDDGTYWVWYFVPLMLMVVGADTEFNVVNM
YVMSSLPSSDQSLAGGIFNTASKFCANTSLGITTAVYNSVRNQGPASPIKPYLSTYWFAAASAGLG
VFLIPFLKLGTQGHEDKTNSSDEDTLDTPNVGANAETKI
>g1576
MSSNGPGIQLKITVIGGGMAGLASAIALSDMNPNHQITVLESNASLSEFGAGLQLFANSTRILHRW
GLTPALEKVAFQCSHLSIRRWEDDAELTFNMNNPSSTWLYGWPQWQIYRPDLQKALFERAKEFPNV
SILFAKSVKSVDAVTGTVYTVDGEVFEADLTVAADGIWSRTRRCLPASKDVSPTAYREHHYRAVIP
RSRMLSNPITAPFIASPEAKLWVGPGVFVLAYTVASKELYNLVVGVPRPSEGVPVGSWNHPANLET

MRGLLSGWCEEVQALASLVQEDCVSWTLGEVPPIPSFVSTSGRVALVGDAAHALLPHAAQGAGMAF
EDAAALSEFVRHLRSKEDLPKVMNAWSHFRQARVDHLRNTSRGNASDMTLPDGELQIARDMKWNTI
RDKQRAQWAELGLEKIREKMIREKPTPDPDCKSTFEPGGRMWVYGYDIYENSRKFCREHLEWQ
>g1577
MTLDHDQPASSESRDHEADGSQDVNAVPDGQTAWAQAKATEEPASDNAGHTLIESHTSHPIVSQRE
DGASLSDTVLRVSTDPDGNTYPEGGLQAWLVVVGSWMALFGSLGLLNSIGTFQAYISKHQLSDYSD
GTVGWIFGVFACLTFFCGAQIGPIFDAKGPRFLVLGGSILIIASLVALAFCTQYWHFMVTIGIFVG
IGTSMVFNPAIAAIGHYFYRRRGEATGIATTGGSFGGIAFPLILEALFPRIGFAWATRVVVLLCLL
TLGAGCILIRSRLPSKPASKENIMPDVRIFRDPIFLLTTAGVFLIEWGLFVPITYISSYSLAQGYS
TSFSYQILAILNAGSILGRWLPGYIADFIGRFNTMILAILGCLVSCACFWLPAGDRKALLIVYALA
FGFFSGSNISLTPVCVGQLCKTENYGRYYATMYTIVSFGTLTGIPIAGEILSRSNGEYWALITWVV
ACYFAGLVAFIAAKLAKVGWKSLWVVF
>g1578
MELAKQNTDSKSIKDVVPEMTAQEIRDMFAKESSKPTSKVYPMISSVPQESLPPHDLTNKLIDLYM
VKGQAMLPLLHEPTFRQDAEDVFRASRDPVQNFQLRMVIAISMQKLSPEYAGLADSYYLAALPFLQ
PSLQRMDTRSLQCLALIAQYSLVTPTRTASYWVVGAAAKLAQELGLLEEANITKSATGEPLNVLEV
DMRRRVAWVVITMEFGLAHSLGRPNAFCISHDHLDIRFPQLVDDRFITAQGILPGAKPIWAKCIAI
HFFKMRLLQAEIRRTLYLNKREAPVNDQDPWFDQILTKIDDWVESCPKEDGGSGLSKTWFVGRKNT
MIIMLYRPSPQIPEPSVDAARKCYEACAFNVGMHMDQMSTGSVDLTWAFTQSLFQAISTMLWTLSY
PDIRIEHDIEEVKSYLQIALEAVAISAQRWPGCESALQLYKSLITACLKAYDTAESFVVHTPSTQP
SPVSVQDVTTPPFISSPSAKSQQSVQTVVTVATSTSDAYLSHSRGPSTEPLKNPTPVQEQPNTPVS
QPSSSPIYMPSQAYNLPNTHLANKHLHRRNMHHNQHSNIQMYAAYGDPNFDPSTPFNTFPSVVPGL
PGWDPNYAAVPVTTSGSYVDMSADSTYWMGPFGDQYSQYSNQPGPWRGRTLSQEEQIELMDSLADN
IPDVSSMLMNDTIIYYRS
>g1579
MLAVSEKELTKLLHSLTHTKCDNLLQTLSTALAAFSAQRKTADIDIHQPARTSIVSEKWSSTTLFM
PVSDHASTGFKVVTVPKVSTPTNGIRGVINILSPEGRLEGVMSAAEITAFRTALASMIPFRRFCSS
AQQQTKGKRVLIFGSGKQAEWHARLALLTVPKPTDIEGMTFVNRSQRRLEESISSTLVPELKVQYP
HLEIHAYSREEHEGKEEDFRARLRADLASSDAIFCCTPSIEPLFTYDDLNPPKRRYISLIGSYKPH
MQEIDSRTLLSGVNGTIYVDSVEACMEEAGELIMAKVEERQLIELGELDTSTASILEGDGNVVFKC
VGMGIMDLVIARNLLDMAREQGLGTVVEGF
>g1580
MNPYVSWAIMLVVAGGLGYYYSNGSKLKNKAPIKAVPEKVEPIVQALKPKKQKARKQPEPPAKKLD
EKPVAVTTQVEDTNEPEEDVDNKEFARRLAAARSGVSVGETKAKAPKEKRNKPLPLTQESASSDFS
NRAPSSNGADADDDLSSTDFVSPSGRNVSDMLEKPAPGPSVIRLTGSMETKEKKAKPQAFKQVETK
KQRQNRQKNEARKQANQEAEAERRKNLEKQLHTAREAERQEVSRKKSATSVPSNAWTSTNGNRAAA
APAPAVNAALPLLDTFEPTASAAASGSSSGAWSGNLPTEDEQMRILGVAAPAADEDWTTVSSKKDK
TKKKTKVDESANEASASESVASFEPETTPQASEWVAPKVTQVPIPRGKNHPLDSDWAA
>g1581
MAKNKIIIDTDPGIDDILALLLAFAASPEEVEVALISLTFGNIEVQSCLRNVVSMLHILEREMEWR
KKQGRPEGFETLRTHKPVIAVGAQQPLDDQKMLADYFHGKDGLGGIHFTHPHLTPGESWEHLFDPI
SDPEVLPKVGPSPSKSHQSFIPSKLPAADEILRVLKENEENTVTIVAVGPLTNLALAAAKDAETFL
RVKEVVVMGGTIDKPGNVTPVGEFNSYADAVAAARVFALTSPKPESTLXPSKRLPAYPATLSKTLT
LKLFPLDFTSQHDLIRGDFDRTITPLLQSGSPLAEWVDAFMAHVFQTLERLHPGHEGAKASLTLHD
PVCVWYAMTSGDTRWKPTPTSPEDIRIETVGQWTRGMCVVDRRNRHKVEDDVESHSDHGLWLSSRA
GNRIIRMGGSPGGETFGMLLLKKIFE
>g1582
MYFQNVLAFSVALLAGTSIAADYNGGEIPSNLRKNRSKLRAADLLSGSSSAQVVTVTPMAAATPSS
SAVADASKVMVPMSSVAKATSSAAPSSSKHATRFQSSVRPSKSVAASSSAAHRPAQGTHAAQMPAS
SPSTVGDNDVSVPVQELAKQADATANKDANKPFWNRPISDSKPRPNAPDFSEAPETRVDDKDSCSL
RCQDLANQCAELLPKDNDFCWENYPACQDRCIKEHVHS
>g1583

MPAHLPPXPAASGEFIVKESSFHAIDAMSEHSFALPPAALSPVAADSGIANSRKGSLVSQSPISPA
SPERKNSVGSAARAGTKRTAAATASTASSTTALDEFNLPPPPTRSRKIIQMKPKTTPSSSRSASTP
STALATKQSPQRKASTSKPSSGSTGTSSKSGNNATAISPVEAALNNEATSPTQQQSKRKQPSATSA
AGRKIARKTAHSLIERRRRSKMNEEFATLKDMIPACRGQEMHKLAILQASIDYMNYLEECITELKN
NASSAAGRTTSVSKKLKLDGPPLAPPSPTSPEMLVPPDMAEEDSAGSASESSSSPELFPQTSSNNN
RTNTSTYYSSPSFSPYTGPVSNSQQQIQTLDGINHPILPSPALRPLYSPQIRAQQPQSELHQMGNA
TSYRVPQQIASTTPSPAILPQQQHHYQQQQQHRQSISSTHTMASPVFVAHDSIIDQEASAALLMLN
MDRRGASISEGSAGKEDKKSPSSASASSASQDRRMGMSVRDLLTS
>g1584
MWLARGVQSAIFYYATCTPCAEAENRRRRKKDALRAQREQAKSSVIVTDQPHLFPQPTPFSTNAGW
MEEIALGPGPPARRHRSKNCPGDGLPRLSSTSSMTVEGSVLSGGKEKATLSDKFHWSRFQREDEVL
WGEEEQEDTIQGSSVGISGRARAGTGHSNKYYIARAPPVNDLHPPIVSGPTSRAETRWMLQPPPSA
KVMAGKARHDVMRESRDPSIRRRSRIDMKIKESEEEEEMIQEGQLSPITDRPGWLINKATNSYRRT
FDNYKKHRPPMLEIDTPLSSPETGDGDRDNLQAPPKAATTVETPRSTSMTNVRAYDEWHLQLSPSF
HSRSNSPSSMGSPDDSLHWPDTPYSRPDSKRTAADGGKGFHPSYLNLAVSSREVDKSIEAIHLEVN
EPRSREQMQPLKWRWSFDI
>g1585
MSLSAYINKKVLVITVDGRTLLGTLLSTDQLTNLVLTQTVERIIRTPEDPEPSSEIEHGLYLIRGD
NVVVCGEVDEGIDNDIDWSKVKGEVIRGTKNV
>g1586
MPPPPPPPPPPPPPGMGGPPPPPPPPPPGGSMPARPAKGAGGGDRSALFADINKGAPRLRKAVTNDRS
APIVGNENKSGGPPIGGAPPVPGMPKAPGGLAPPIPSANRLRSSSDTGPARDDSGSSGATSGAPQL
GGLFAGGMPKLRSRGGVDTGANRDSSYLSDSEATRPXATTASAPRPPVGAAPKPPGARPPPVPPSA
PESPAPNPLVANLRRPPPKPASRPSSTLEIPPRAPPPLPGSGKTPPPPPPPLASRKPSGTAPSVPPP
PPPPVSVSPAPPSAPPPPPSIPAPRLPVSAHPPPPPPPPSSAPPLPNGASPAVASIAMQAARNAFGH
SHNTSAPSAPPPGSAPSLPVSSPPPPPPPPTSPPSLAPPGRPSASSTPVSSRPTSYDDHSLRSQGRS
ALDPSAYTLSNGTSTPVSHPSSPGPTRSTVQDTRFKFQDESLLPKPRPFTGGVRRYRAGRGSSVPL
DLSSIG
>g1587
MGSVFIRPHGAPRLRRAVCQFCERTKRTEAVSYSTLQRAPLRNALQHPTRHASNTPLRQDYVHLLA
SDSSIVKPLQIPRRLLSSTPDITTSLAEIKEKSEALRRSDTAAPEEAVIALLQQCLHHVESFLKVQ
ELHETENSNPAPKVTATKSTAASSLLGLDEKQATPGKLRIPKVESRVVDSISQISTELLVDEKVFI
SPEVLELYTKIHVLLKRADYLPQIFSLYANKPIPEANTSPIKFRQQNPNSLKNAISSKLANEALEI
ALEQKNLSLALAIIDTTFCAPAFTRAKLLKNAAVPLAGLATAPLASYVVATWAANMQNTMDESLAT
GITFTATLAYIGFTSSVGIIAVATSNDQMERVSWAPGIPLRHRWLREEERGALDRVAMAWGFRDPY
RRGEEEGEEWESLREFIGMRGMILDRTELMEGMQ
>g1588
MSKITVAGVRQNVQQLLDYSQNTKKRNFLETVELQIGLKNYDPQRDKRFSGTIRLPNVPRPGMTVC
IIGDQHDLDRAKHLGLESASVDDLKKLNKNKKLIKKLARKYDAFVASDTLIKQIPRILGPGLSRAG
KFPTPLSHAEDMDAKVTEVKSTIKFQLKKVLCLGVAVGNVGMTEEELISNIMLAINYLVSLLKKGW
QNVGSLVIKASMSPPKRLY
>g1589
MATHQTAADFYKSLSSEPAFAEIFTNIDDAHNFRQCQQKLLTSTTRNFVLDFGDQDAWCGFDLEKD
EFMSLMKDQRPKMFGTRWIHLWAPETQKESVNALANQYGLSYRLVKMMCSDPVRQMNTTVAEPNVL
EMSVSADESEEKTQHNVSSGDLEGAYHLDRLDPVAASAEVASLGYLSFSNIINQIWHFCSVDYGPK
YTCVGYNSLYTVKINGKIIENGKGLPEGKRLWCWIILCDDGTVISMQENPYPGPYMPSIEEEKVLI
AATRRNVVSIFSGASKQHPLVSDNESLVSVRVRHFSSIEPDEASIKQEDGPGLILYYMFDDWVSSY
RLVARREHKYGALLDELRRNMLTKPNVDLVDDLHWLGRRLAILKHLYQSYELVITRILQRQRLLRD
EAAQIRRENLHNANMSGDPEYSEAAHMSFSMADVSRSYDFTAGVQLTSAAVGRFERLADRIRLYCL
SEIETCLTEKETLTFMNFNLIALKDSQAVEKLTRITILLAKATILFLPISLMSQYFSIQIPDIEGP
LSLRAYWISFAVLMVLSISVLTLFGYASDTVEGKTIYRSVTRTLFNSSKQKSMELMSQRRVQR
>g1590

MGMVVSKLLDRLWGKKEMRILMVGLDAAGKTTILYKLKLGEIVTTIPTIGFNVETVEYKNIQFTVW
DVGGQDKIRPLWRHYFQNTQGIIFVVDSNDRDRIVEAREELQRMLNEDELRDALLLVFANKQDLPN
AMSIAEITQQLGLQSLXRRAWYIQSTCATTGDGLYEGLEWLAETLRKTGRD
>g1591
MSNDTLRYIPLAYDHTDSQTSALRLVLTIKPEWEGPGNNIEFVRFTDGITNTLLKIVNRKPGLTDE
QIDNEAVLMRAYGNHTEILIDRERETRSHSLLAQHGLAPPLLARFKNGLLYCFIRGHVTSPSDLIR
PSVWRGVARRLGQWHAILPITSSADDSQTPSSSAQDHYDIDKSDGMPIASTITPIQPRQAGPNLWT
VMQKWILALPVQTEEQRSRQKTLQTELERTLNELDDGSGIGENGLIFAHCDLLSANVIVLPHAETN
GAVSSHLDSQDVHFIDYEYATPSPAAFDIANHFAEWGGYDCDYNMLPTRSVRREFLTEYIKSFAQH
GGKGVDADQQEKVVEKLFQDVDRFRGIPGFYWGVWALIQATISQIDFDYASYAEVRLGEYWAWRRE
QDGSKSNGAHEEKPLRERRWAQEG
>g1592
MADIQKEQPTPITSLTGNAVSPIHDTEKGKDTSITKLSPIEAPPELTLEGSDDPYNWPTARKVTIS
LIISLSQLVCLMTTSVVAPALSQIATDLNLSASKAQLAFSIFVLGQACGPFVIAPMSELAGPIAAD
MFRQKDRGKSLALASMFPYLGPALGPIVGGLVSQYVSWPWLFWVISIFSSVIFVAGVLFVRETCKP
VLLRRKAQGTDVATTEVVKPSLPEERLLSRILVSLQRPFKLLLSRPIIQILCLINGIGFGIYILVL
SFYATVFIDQYHQSATTSSLQYIAIALGCTISTQTGGHLMDIIFARLRSRLPEHSPLRDSPPPEFR
VPLMIVGVLMLPIGLFWLGWSAQAHIAWIMVDIGAFIFVAGEFLSSQAAAAYLYDEFTTHSASANA
ANRLMSNVMGFAFPLFAPQLYGRLGYGWGNSLLGFIWIATVVPIPFVLWYWGDRLRARGKK
>g1593
MQDDSISVVHKSSHSSMPPDGEESRLSLWTSHMSRLYAVLALGYLCIVFQGYDGSLMPSINAMPQY
QHYYGLKSAGSSTGLVFAIYNIGSLGSLPITGPVNDLLGRRVGIFLGCCFIIIGTCVQAPATSRQM
FLGGRFLCGFGQGFVNVSAPVFVSELAHPRWRGPLSGLMQTNFFIGSIIASWVTYGTAYLDGEKAF
RIPIWLQLVTASIVAIGIWFAPETPRWLMAQGRRQEAERVLADLHGEGSTEHCYVQLQIAEMESQI
RETGSDKRWWDYRDLFNTRSARRRMICVIGMAWFGQYSGNALVSYYFPVIVQQAGINDPHMQLLLN
ALNPVISWFMAILGAMLLDRVGRRPFLLTGVLGMSICLAIVTGCTKLSVEYSNRVASHTGIFFIYL
FSAIFAFCIVPLIPFYIAETLATETRAKGTAVGQLFGSAASILGQYTTATAIGSIGYYYYLIFIFW
DLVEFVIIYFFFVETKERTLEEMTEIFEAPNPVKKSLEKRSPQTVEHTIYHEK
>g1594
MILSSLLLPSLALSALGQAFTAAPVITNKPPATYTATLSDNPNTPIRGYVTASGAPDGVGVVFRVN
FTGLPPDIGPFPYHVHLYPVPTSGDCYATGDHLDPYSRGELPPCDPSDPATCQVGDLSGKHGSASG
NDFFAEYTDRYLSTDPISNAFFGDRSIVIHAASLARLNCGNFQLVPSPQAASPHIYSHNYPDTYYS
SNLYEDTEFTGRGCI
>g1595
MTTLTERERKENHVISLPKPPRASTENIDPKTIAQQWLTNLETTLSTNEGNFTADQMKLLFHDDSY
WRDHIALQWDFRTIHSATNIASFLQEFQPASQLSNFSLQTSGKYVPHLDTPENGVPGIDFVASMFH
FETKAGRGSGILRLTLDDAGREWKAYAVYTSLQELKDAEEPLGPKRLYGTLDSLPGGSAGGTWKER
REKAVKFEGVEPTVLIVGAGQAGLNLAAXLQSLDVSTLLVDRHERIGDNWRKRYRTLTTHDPAEFT
HMAYLPFPKNWPQFTPKDKLGDWFEAYASLMELNIWTNTSVVSAAYDDHKSIWTVTVRKPDGFERT
LHPKHVVFATGHSGEPKVPTFPGQEKFRGTVYHGSQHRDAAEYDVQGKKVIVVGTGNSGHDIAENF
YENGAEVTMLQRRGTYVISVDKGVFMLHEGTHDEYGPPTEQADIWAESLPYQVAFAFNVHLTRRIS
EVDKEVLDGLAKAGFDVYKGIDESGIARLYMTRGGGYYIDVGCSQLIAEGKIKVHKSPDGIKEFTP
HTLILADGKELEADMVVLATGFDNMRTTVRKVLGDKVADRCKDVWDLDEEGEVRAMWRPSGHPNFW
YMGGNLALCRIYSKFLALQIKAVETGLAPENK
>g1596
MSKTTRYREPNQWSRPRDHLDPEKASSPMAYDSSLSDDEPVYSDTSRVTSGSLSSHIPMLGSPSRR
RRPRRNGIMPHRLMRCVCLAVFVALLLFVLNLFRFTLFGRAEQVPIDVAKPAPKPHAWESFPFLKR
YHGGIRTLVPRNESVPEYPGDGTLDELTLAMEDALRTDKSPGEEEQARKTVQKRSEGLPMTSTAYN
PYPNYNSEEYIHKYGEKVECFLDEDSQVRMPYLQYYPGVPQGFPDAIMGSNEMLGMRDDVCFDRFG
RLGPYGLGYGARKGGSGAGMEGDREGSERVWDDVAPVDFREINWAAAQNRCVIANSHRFAKLGDPH
LDRAFAMPIGAPPKPHMESSASEKEYAKGTDRLPRTAVLIRTWHDFHYTEEDIMYLRALVTELSLM
TGGEFTVQFLIHVKDDNLQIWSDEETYDRVLRDALPEEFQGMGILWSERQMNLIYGGLEESFERGL

PVHGVYRSTFMPVQYFSHLHPEYDYVWNWEMDVRHTGHWYHFFDKAASWARQQPRKGLWERNSRFY
VPSVHGPWDDFMHTVRVQTELGTNSPNNIWSAVKDSEDKSDPNHKDHGRHGEDFIWGPVRPHESDV
MELDTDVIPPTSMEKDKYEWGVGEEADLLVFNPLFDPEGTTWLLRNDVTGYNREDGVPERRAAIIT
ASRLSRKLLLTMHRETSMKRHSMFSEMWPATTALHHGLKAVYVPHSVYIDRKWPTRYVEAVFNAGR
NGASGGARTSVFGDREHNFRGTTWFYSAGFSSNLYRRWLGYKVDNDGGEEEELAGEGRLCLPPMLL
HPVKDVQLVIDNGDVPDEGEVALPVAEEES
>g1597
METEAMASESIDTNFEVHGNGNHQHSSQQQPENNAISRQSTIITATPTQPVSVATASTLKTSRIAV
IMLSLSASVFVSALDITIISTASPAIASHFRSASGYTWIGSAYTLANTATMPTWGRISDIWGRKPL
LLAGLVIFFAGSLICAAADSISLLLAGRAIQGVGSAGLNILVNICVSDLFALRDRGFYFGLLSVVW
AFASGVGPVLGGLFSEKISWRWCFYINLPIIGAVFVLLWFALNLNNPRTPVWEGIKAIDWTGGSLV
IGSTLMLLLGLDFGGVTRPWSSATVVCLLVFSVIVGALFVVNEKKFARYPVIPMDLFKYRSGTGSF
VLCFCHGLVFMGQAYYLPLYFQAVLGISPILSGVYFLPFILAISISGALTGFFIQKTGRYMPTTYL
GLALMALGVGLLINLDEYLNWPKLIAFQIVSGAXIGLNFEAPLLSLQAIVGSLNAATATSTIGFVR
TIACAISLVLGSVVFQNQMVKQGPTLTAALGPSLGGLLSSGDAAANIGVVNSLPPAQQAVARHALH
HALTSMWIMYVGLAGAALVGGCLMSSHPLSREHEAVALGLENNHLSRGDDI
>g1598
MRLTRALLSAQPARLTLFTRAHCGLCDTAKLTLSKLQQRRTIRYTEVDIMVPENKKWKDVYEFDVP
VLHVQPAASEDESGLRKLFHRFSESEVERVVDEVEQ
>g1599
MASKCVHKGCGKVFTDPEEDCVYHPGPPVFHEGQKGWKCCKPRVLTFDEFLTIPPCTTGKHSIVDD
TPKEAPKEIDSAALANLVTEQSAPKTTTPISAAAAALPTPTPNTASAPAPEPDSDDPSLSIPANAT
CRRKGCNATYNPDASRDSELCEHHPGAPIFHEGSKGWSCCKRRVLEFDEFMKIPGCTEKKRHLFVG
KGKPAGEEKVEDVRTDFYQTATTVNASLYLKKINKEKAKVTFNESSVEFDLPTTDNKRFTNTFNLF
GPIDPEASSYNIFGTKMDLKLAKKDGQSWPVLRSDDKWTGERIQIGQAGRVA
>g1600
MEDLTLDGKVAIITGSSRGIGEGLAVEFARRGAKVVITYTSESSSAKADNVLSHLADYKYGSSAIK
VQADLRTLEGPSQVVAATLKAFPDNGITILVNNAGCEVQKRLSEVTAEDLQYVYDLNVRGTMLMTQ
AVLPHLRRPGRIINIGSVGARAGFPGYSLYASSKAAMEGFTRCWAAELGADGHTVNCVNPGPVDTD
MIYQINPAIVEAQKKSTPVEQRLATVDDIAQICTFLAEERSRWVTGQTISASGGWTMY
>g1601
MATRNACQPNWLSGAVDGDDDDTDQELKDFANMKPLLQDLDVSLFAIWMASHEVDQKYLGQLAATT
RHENPFLSSMLYKILGLSVILSKKLVRARKLRRLDPTRETKSLQLYYHIVWLSREGLVIVEQYILP
MVQDYILLKVLAYKLRASFYHIFVLFHNQPAIHTPGISSLPSSTNLPNGVRHTPSDDQSDPKRESI
SQTPPPSEGGPVRRPPESSVKKGPPGLNPYPPPKRAASFLLPALDYTQTATPCFSHAATLADTLLP
GSHPLRLSVKLEYAAYLFDCLHDADACRRLAKQTIADVYNAQEGMDDESFEDAAELVAVLGRMVKR
GGKTSSAGGSSVGRDRDRSRSNDRQSTTKGTPPQQTPHTPVRQTVTNHQKTPAQQSASPTPSRKKY
SPPTPSQMKGKPKHSPSAGSGGSTTPKAPVPVINPI
>g1602
MMNAEHSSSLNRYRESKVRRRLGLPLLFEGGTGPTTVLTCPDSGSDWNVMAFETAASLGYPIEPHG
SSDSTQFVLANGGTVNSIGKITAKCAFTTGATSTELVYECIFFVFKKLAVPIIMGMEFLEKTETLS
KHRDRLVEEIAPILQNFRVQAVGQQKQSLLCRLDNYVGCANPDTGSDLDLVSYQYASAKFNIXPAN
EAVMFADGSIDYTCGVFHTSFTVGDVANGFEFIARGKTLSLAFHVLDGLTSDILVGQDTLEMLNVF
AEHTASFIPGTPRLGGSDINIIQLVSNFESSTRDRLRDAGRWVSNVLGRNTDPIPTSTMSLEQRLD
LEDQRENARAETPTPVTNEFQCTFPNCNVLPFATQYLLNSHSNIHSHARPHYCPVSGCPRGLGGKG
FKRKEEMFRHGLVHDSPGYTCPFCADQKHKYPRPDNLQR
>g1603
MASLNVSNLAPPPSRYLITGYPSLAAFIASDPDRTAGIYKRFDRLAARNLLYLQSELADLQAELDV
LDEKDKADRASKQCARNWHDFKKRAEQEPSRLQLVNDIRATLKEYREALFAEEKLASMPHPDKKTL
NAFRFTFFHGRPGDSMSFPTLGGSSKWVYDDANDLVATKNPAEQDRLSMFIQDNFAFLFKVCYFPF
FKYKWSRVHADRCAVVILYLVPSSNVRLGLISLFTVLFANSVALLTNARRTEVFGVTAAYAAVLVV
FVSGNLERPLSVIMAGFPAIKPAFTVRVLIDAPFPVGSHHRTTSLVIVPMVGGSIISEPGFTPALN

AKFEGTGNDYIRNDPDGKRMRLDAHGVVRTHDDALIYLHYKGTVIMTEGISKALSGQSGDAETPYG
DSFVSFEIETGDERYKDLENGVFVGQGHFVTKTGEQTIVEYKVGQVVVG
>g1604
MLKTILQPIHSVRLPPPGSFQDQTVLITGSNTGIGLETARHVVSLGASKIIMGVRSVEKGERAREG
ILSKIPANKSAKLDIEVWQLDMSSFKSVMAFVYRIKEYFSRPDNRLDTAILNAGLASGVWQLTPDG
WETQIQVNGLSTALLSLELLPVLLSSASSSPSRPHLVIVSSDMHQQALLPQRSASNILEALNDKEQ
WQKLHAKSPVERYAVSKIFNQWTSIEVAKLVPRDRAGREKVIVTSATPGFCKSELLSREEGAPFLL
KVVQAVCARTPADGAKAIVDAAVRQDGHGKWLENQKVTE
>g1605
MQINLQRLLVAAGAVAIAATASPFVPFEQKPLIGELHRPETSDVGETASRKLTGKFLHITDFHLDR
FYVPGSLTDEDNVCHHGKRKGNAGYLGTPGSECDSPELLINQTFGWIKENLRDEVDFVIWTGDSAR
HDNDERIPRTENQIEELNQDIANKFIQTFSHSSTHPDETLIIPIIPNIGNNDIMPHNIFKKGPNRW
TKTLSQIWEAFIPEAQRHTFVEGGYFYTEVIPNKLAVFSLNTLYFYDSNQAVDGCAQKSQPGYHQM
EWLRVQLQLLRERDMKAILMGHVPPARSADKQAWDETCWQKYTLWLQQYRDVVVGGLYGHMNIDHF
MFQDSKDIDLQYLLPEEHMSPFVAENNYENFTIESKGSYLTSLRDLWADLPTPPASVLEDWSISDV
DEDAGVAKKKRKGKKKEKEKKEKFYKKIGGKYAERFSLSLVSPSVVPNYYPTLRVIEYDITEMEKV
PTWADVQHDKAGKKDEQWVDREIEIQKKSKKSPIPDPPSKSAPPGPAYSRQPLTLLNIAQYYANIT
KEEGIKGVVAANDGVDDESSPKGLRVNYGVEYHTNKDDIYRLDDLTVRSIYNLATRIGAGDKASSL
PTETEDDYTEIDEIKADQFEDESDIGSTKKNNTTKKKRKYKTNKTWKAFAERAFVGFLAYDEIEDK
L
>g1606
MATNSPNPLPFAEPPYLRGLPSPYYTASHLAFQKKARAFIYENLSRHALDYERDGIVPQHVFDTFA
KNNMLIPNLPSPLPVQWLKRLGIHDILGVKVEEWDYLHTGIYLDEMGRSGIAGPSASLTAGMAYGI
PPLVKYASPELQERVLPDLLTGKARSCIAITEPDAGSDVANITTVAEKSPDGKEYIINGTKKWITN
GIWAEYSTMAVRTGSPGSGAAGLSLLVVPLKNYPGVSMRPIKVCGTETSGTTYIELDDVRVPVSNL
VGTEGAGMKYIMTNFNHERLSIAVGATRQARVALSTAFDYVLKREAFSKPLITQPVVRHRLAKAGA
ELESLSAWIEQFLFQMTKLKKEDADRELGGLTAMAKAKAGMVLNECSQTAMLLFGGNGLTRTGQGE
LIERIYRDVPTSRIPGGSEDVLLDLAVRQLVKIYEAGRKRLERDAHI
>g1607
MRFSTSAVLVTMLGWIGAATAHNIQLKAHSRECFHEKLHKDDKMTVTFQVGDREFGGSGNLEIDFW
VEDPAGNRQYFKNSVSSEDYSFTAQRDGKFTYCFSNEAWTSNSKEVSFNVHGIVYVPESELAQDPL
EAEVRRLSESLAQVRDEQSYIVVRERVHRNTAESTNARVKWWSIFQLTFLIGEGIFQVWWLKRFFE
V
>g1608
MDPERMNGDDYNGRGKQLLAAIDDTEETTSPAFLDLTSSPSNRPHEHPILADESVQTSVGPSRATS
PAASSVIALGSPSQPRPPVSPESLPSLPKYPGAWDSLSSSSPENRKANSAYFTAAWGSPYATPSPR
RLSWTLSNQPSLNLPSRDNSPSRGRGYTSVRGPGSFRTTSTSSQVPRPIHGSIGRSSVRDFSGVQA
SSSFKQFTEDWIDRYLSGQPRTERTNWLSDDEGSEAPSFFTARNNFGDDPTEGWLGLDEPNEEDLL
RTPTASNSLSVRSRKGLGKQLSRTRKHLSTFSTATLRQEDVWGFAFGDEHPSTAMSDIADTQLPPA
EHDSTTESSVEKPLPLPPAQNATPVDNNESLKTETTPRPPLVSQGSSQRGRRKVAWRGKTCLIAVP
TDDKRGSEGSGYQLLSKADVEKRLQNWLDEGYHVDGFDVFAPEQDSVSSQTGGFSRLPFPDETESH
QDQVSRNFSVRFPNLGEWDAYVQHLQEEKLRALGVFLDDDPPASESPAASMGQMTQMGLFPGHITS
PPIPTSSAASNPMFGSHTFSPHFNQSTNPSTHTESLASPPPQFGGQHSFYGMDANFVPGLPYPYQP
TPPVAGTLTPQSLFNARQASFAGPGMPNLNSIFSPVSPLNADDIFNQSSNGPRDRTDSIRDPSYHD
SSNKRQVRESRAATLILDESQLETEHFQASNVEIAHPTPRGHSHNLSETLQKGLDRYSQADYHLEN
SIQRQLDDNEGDLHNSRWAVGADDVQPSPAHAQFSVPDSFSTGHHRLFGEQQQQPMDIIQDADEID
TNPSLTASPREPKLDMSHHPWHSAKPSSGSFVAGHKSKASLSGLNVEAQEFNPTGSFSTNTFAFQG
AAPFQSNGLGNGSIFSPTSSVFKPSGSIGSFNVAAPSFTPSSNDFNASSASFVPSASKGFTFSTAS
FNADAPAFNPGASVTSNTSDAPSHLKGSIFGDIDFNQINKPSKSSKAIPIVRPDDDEEEEPIERGV
PGTPPRQKRARRGAGEEDKDVEFAVPTPLAEATQAQATATANQTHKSAEGKENELPEQNALRVSVK
ADSPDTKRRFVEDDAVAVEDAVEDIQEKAAHPVDTEPFPTVSDLNQVIDPAEPIIDDSAPKESAEE
NIATPVPEAAPEPVAEHSNAEIPKREPRKSIFSALRSFSFRPSISEFVPIKSPSNEAKSPGPEPTP

SPEPVKAQPEKPKTGLFASRYAVEEEELNVTELAPINNPPEMSKGNAKDTANGHDSPDEEELNAIM
DQLNGDDSDVGVERLSTPLPPQMTIGTPGGHSMEQTQLPHIIRSDAPSPSPARDFNQLLRGTSKIG
PELDVGQSHHSFPQRGFASGAQSPVRQLLSTNEHISDWDDVISSGEDEKLVQRSRFFDRHVNALVS
EVLAERLGPLEQALSIIQNSVVSLHSQSAGRQSFTGRQADVEHSDADDEDDEVNIPRSISPLSKRD
RKLEKMKGAVLEALTAHGLGRQEVPPASANLNVIQETLAELKALTQQRQQPELPDFKALVQEALTV
HAQANARKTPLSEAEEIGAESLKLQLDGVKSMLRTADERTEQEIKARRDVQEALTEVQKMLGHAEG
EVARYRQAAEQAEATVRELQEEKIPQLEKAQTRCDALERQQESWEMTLSELSAKNIALEGTLDEYR
VSHDFWKKKVATTEEENKDLRSSITHLTARIEDSMRARQSLRGKFDKLQDDMANVTKDVTRDQANS
RRKEEELTAKYESLRAAYDREVKLREKLEIDIGELEKSERETAKLKFIFAQSQQENHRLEELVASL
RQESQEHQNTASRFEREFNEARESSRMEIQRTRTSLEADLDAANNQVNYIRAGLEAEINRLESQLE
NVRMDADTTKERYELLLEEARENKASAMAELAAAKDMAMEEQRKTHDRVLNDLRERHARALHNASE
DRQRIESHMNEKLDLSNEKVAHLQDRVAHLEEKLEIAKSAARAAAQAAQAKGVAVPHPVQHHSSPS
LSYNKDSSGPERISPQALRESILVLQDQLQQRESRIEELEQELSGVDKEAPNKIKERDTEISWLRE
LLGVRVDDLQDIINNLSQPSFDQNTVRDAAIRLRANLQMQQQEKERAMSGQAFPSLADIAASPRSL
PLAAAAAWGNWRKGRDSFTSESGASQTPSKSTNAGGFLSGLLTPPSSNVRYTPKNMSAPPGPARLA
GRRTASESRPLRGYNQPRSLSARQMDKLPVVHQESIEPPTTPPLLRRSSYDHDAEQNSYDGSALMD
DVDSMIAADSPEGVRDDGLFARDE
>g1609
MDYFNVTNAFIAIIVGIITYSAYNMLGFGPNQFPVNGKTILVTGASEGLGRSIAIQLAQKGANVVI
ASRSAHKLEAAIADIKAAAVNSSTQRFHFISADLTDPDAADRLLEETTTWNNGAAPDVVWCCAGFC
HPGLFIDVPIKTLRDQMDTVYWTSAYTAHATLRRWLVPVSQISTAAKTEQPPPRHLIFTASTLAVF
TVSGYSPYSPPKAAMRNLSDVLVQEIEMYNGARRNRDADAPATDVKVHIVLPMGMTSPGYENEQKL
KPELTHIMEEADKPQLPEEVATIAIKGLERGDFQIVTNTIGQLMRTSALGSSPRNNVFFDTVVSWL
SSIVCLFVISDFAKKAFNYGKQKGVPSAK
>g1610
MAAKKRKASGRQPSGKKPDSAMRTKYNINETFDNSEDEFLAGRDQVLLEEGPEAKRRRKIQEEEAD
FQPSDEEVLGYDEDEDELDEDEDDGYENDYTSRKRKGGSESELEAGVNDEEEDEEGLEAWGTSRKD
YYNADMIETEADALEEENEAKRLQQKHLKAMKEADFGFDELDWVESGKGAGDSKDDQDGVVTEVLP
QLEVTDDMSSEEKLKVLKSRYPEFEPLAKDFVDLQPEYMNILAEAQKADEAMDGSETSVPVPIIKF
RALASYLGSISMYFALLTSPAEDGDGKTVALSPAELRDHPVMGSLFKCRQQWETVKEWTIPEYSKD
LEDVEAEDSEESVELALAVQLKEKKSTQSAQKDKKLSKSQLAAEKARVEAEARRAEKIKKAEANLA
DLADLVKTSSKKSKTLSKPTSQPQEDDDSDLGDEGALTAREAEEKARKKRSLRFYTSQIAQKANKR
NAAGRNAGGDEDLPYRERLKDRQARLNAEAERRGRAQANETEQLGGDSDEEDHRLAREVRGDTAGT
DDDEYYDMVAARNKQKKADKKARAEEAEAAQKGERYEQVEEVGPDGKRRITYQIEKNKGLAAKRNK
DVRNPRVKKRKKFEQKKKKLASIRQVYKGGEGRGGYAGELTGIKKNLVKSVKL
>g1611
MASLKRKRDLGPRNSFRASSAQRRFATDPVNPIRLRPINPDTFNVTTGLEHVPTHGWCGVPSAINE
DEHGIDDSGEVVMAVDIKEKGTVGCCFYIAREERLGILGDVQFGEENLIDILKLEINPTTILTSTR
AEEATMADVQSSDDALRLPYRIDVRPIQEYSVNDAKVKLAELEIFHQEADLAQFLVPGDGLTQQAE
EMIAENAGFSFEKGKLLRLGGIFATHTLDLQEAIRETHWQDMVSLRERVDIEESQEQQRTVIKQGV
DRDLDLLKDRYNGLDGLLKQVAIDVASTIPASLQIEVNVIYFPQLGFNIAIPFDDTGRAAYTGSCG
TWEQVFTTENRAYFKDERMRELDEKLGDIYGLVCEREIEIAHEIAQSVLKFEEMLSACSDLCGELD
CYLAMAKTAEVYKFARPQMSTENVIRVEGGRHPLQELTVASYISNDVNLCGGYGASDRRHSNGPSM
LLLTGPNFSGKSIYMKQVALIVYLAHIGSYVPAQAAEIGITDKILTRISTPETVSKIQSTFMLDLQ
QISLLLKFSTRKSLIIIDEFGKGTDLNDGAGLACGIFEYLLSLGDERPKVLAATHFHEIFENGFLP
ERQPLTFGHMEVRVDEELANAREQITYLYNFKLGRSNKSFGILCAAMNGVDEAIVARANELVSLSA
RGQDLVAACARISKTEEAILEKAESIARRFLNMDLSTKMGRDIKQVLADLLADGAVIV
>g1612
MLSASRLRTARLGLRRPSAVIPSQPSARSSFYVQASRIRFDPSSQPKSTYLSNFPVRSYATGRGPH
PPGGTHRMNLGGEPEKSALEQYGVDLTAKAKSGKLDPVIGRDAEIHRTIQVLSRRTKNNPVLIGAA
GTGKTAILEGLAQRIVQGDVPESIKNKRVISLDLGALIAGAKFRGDFEERLKSVLKEVEDAEGSVI
LFIDELHTLLGLGKAEGSIDASNLLKPALSRGELQCCGATTLNEYRLIEKDVALARRFQPIQVSEP

SVASTISILRGIKNKYEVHHGVRITDAAIVAAATYSNRYITDRFLPDKAIDLVDEAASALRLQQES
KPDSIQELDREITTIQIELESLRKETDVTSKERRASLEEMLKAKQEEVKKLMDVWNKEKEEIEGIK
RTKEELEKSRFELEKAQREGNFGRAGELRYSVIPSLEAKLPQEGAENENKDTLIHDSVTADDIANV
VSRTTGIPVTKLMSGEVEKLIHMEDTLRQSVRGQDEALSAVANAVRMQRAGLSGENKPLASFMFLG
PTGVGKTELCKKMAGFLFSTESAVLRFDMSEFQEKHTISRLIGSPAGYVGYDDAGQLTEAVRRKPY
AILLFDEFEKAHRDISTLLLQVLDEGFLTDSQGHKVDFRNTIIVLTSNLGADILVGLDPLYKHKLT
DEGNVPDDIKKAVMDVVQSSYPPEFLNRIDEFIIFKRLSKVALRDIVDIRLKELQGRLDDRRMTLQ
VDDETKQWLCEKGYDPRYGARPLNRLIAKEIGNKLADKIIRGEVTAGHAAQVKLDKEKDSLEVVAV
>g1613
MGFTDFVSDAGLAIANNFLSTRSYIVGYAPSQADVVTFKAFSGAPDAAKYPHVARWYKHVATYESE
FSTLPGDPSKAYTTYGPESVELPTNPKDKPAAADDDDMDDLFGSDDDEEEDPEVVKEREARLAEYK
KKKEAKPKPAAKSLVTLEVKPWDDETDLVQLEKNVRAIEMEGLVWGASKLVAVGFGIKKLQINLVV
EDEKVSTDELQQQIEEDEDHVQSTDIAAMQKL
>g1614
MGFFSRIITFVGLVLLAHAGYSAHEHTTLYSNVALAASTASTPTTASNFFAVVPLDITIETLIAVV
LVSAGLVLGAEKLKPVTHSAWAGQIEREGGARNPYKNLEIRSGFWDVRKSRQEFADWMRTQGQEK
>g1615
MRSIIAVAALATVAMAQSSSETSVSPVSVSNPATQYLTETNSLGVVTGQPTAVTSQPSAVTSQPEV
VTSQPAAASIPAGLSSPVVPLSSTTSGSASGSQTGSTSVSGTSTRTASGTTTTGTGSSTFSTGTAT
SSGSSHTSGSSSSTGTAAASTGAAAMVTAGPVGLSLVAGAAFAMLI
>g1616
MVSKLRRLIADHADLRNNELPPNYLFCATDNQHSDDLSQLVVMLAGPHGTPYSAGLWRLQLRIPDD
YPASPPKAAFRTRIWHPNVEQSTGAVCVDTLKRDWESKLTLRDILVTISCLLIHPNPDSALNAMAG
SLLQDDYNEFARQARLMTSLHAPIPFAMKAAVLQAKQRGEDPEMAARIEKDPEFREWRQVVKEKAS
LVMKKRFLPSSQTNADEDMEDVEDDYKENDASLSPELGAALPTSHTTTRRRGLAKRPLSVLPITQF
LDTDLVMLDSTSDDEGMPDDGMTASERNIAANTDSSNSSSPSSQNSSIFSTHTPRQQTSFSSQPFP
SLHNNSNDSTSNSLAIYEDNVEIDSLQALVNSHNSNHSRRPTYGKENMAGSFSSLPKGLSISSLSS
TDTSPYSTPTGVFMKPISASSSSSSLPSSTAATKPLSPTKVTKPAYNSNNSGSTRKGMPSKGKPRL
GLRRL
>g1617
MDHQQGQPGIPGPAGRRLHIAHRRSPSELTPLMMEQLAIQQQIELLQQQQQQIAATHQQYVNMGLL
QPQQPIGQVSGFSPQLGQGTMAGVSPQVNAFQFPQQLGVPTNNGPSHRRNQSALPGMGMGPPPAPS
SGASGSAFADLHQGNQNRENGGPSRGRGGPIGGGHQRRHSLALPEAKKAAELAQQKRTAAGFQFPA
PGSSESPTAEEKPKTPQPQGLGLQRAGNFRGGTHGRSQSMAVGRGATGFQFPIPESSPAEPPRRGH
GRTPSRNFDGNWRQPNNNNNQNQDQQKSNQGGFGQQQGGNTFQPGHRSRASMNQSIGSIGQFQYPN
AAPQLIQLPQGQVVMAPQLFNGQQLNPLQLAQLQAMQQNGQLNGLQASQHAPQQLTAQQQQQQRKT
LFXPXLPQANLPALLSNGQLVAGILRVNKKNRSDAYVTCTELDADIFICGSKDRNRALEGDFVAVE
LLDVDEVWSQKREKEEKKKRKDITDSRSGDKLSRSDSGANGDNQQVGPDGSIRRRGSLRQRPTQKK
NDDVEVEGQSLLLVEEDEISDEQKPLYAGHIVAVIERVAGQMFSGTLGLLRPSSQATKEKQEAERA
ARDGGHGRQHDRQQDKPKIVWFKPTDKRVPLIAIPTEQAPRDFVEKHHDYANRIFVACIKRWPITS
LHPFGTLVEQLGEMGDLKVETDALLRDNNFAADEFSEAVLKNVGWEDWTVAAEGDALLTTRRDFRD
EITFTIDPTGTKEFNDAIHIKKVGDGKVEIGIHVADVAHFVKANSLVDREAKKRGTGVHLMTRVVN
MLPPRLSNEICSLLPGQERLTVSVVFQVDTATGAVDDDVWIGKSVIKSSGKLSYDDVNAAIKGETG
SPLEGVTADDIRLLGAITTKFKETRFGNRATDIPALRLIYQLDDENMPVELNIFDSSPAHEVIEEL
SYKANFFVARKLFAAMPEKAFLRRQAVPNPRRLQTFVDRMNRLGYDIDPSSSGSLQASLFKIQDVD
LRKGMETVLIKAMQRAKYYVAGNVTEAQRQHYALNLPLYAHFTSPTRRYADVIVHRQLDSVLSNGA
IEFTEDVDTLVKTAEQCNNKKDSAHNAQEQSVHIESCRVMDKKRSELGGDLISEGIVLCVYESAFD
VLLPEFGFEKRVHCDQLPLKKAEYRKDTRVLELYWEKGIPSSAYIPEDERPNASSSRAAHAAAAAQ
EAEAARERAREREEAQRKQTETGTMSTDDVDALFDDDDDISEVTEMAAGVSLNSSADRPTQSMPPS
PTRNGHDGQGPQRARSDPKIAISASETPEAKLTDKEKYLKLFKLREEDGEYIQDVTEMTRVPIILK
TDLSKSPPCLTIRSVNPYAL
>g1618

MVGRRSVGKSLDSPSRQLVFDLAKDLEAIRLHHEDLKRVYNYQAQAQEDEEDEIDRAHQEFYYAAI
EKTYDYYDGHRREAEIVLQEHLREEEEKERRRQEEERRRQAEKERLERERKEREEAARKREEEARR
EAERIAREQEELRKKAETEAKAKKDAEEEKARREKEEAETKEQQRQAELQRAEEEEKKRQTLGGSQ
GVTQEEVKAQEQYLELHKYLKELRKFVLAEGKKNPTLKSTIGDYRRGITKCIGQLREGQGTVANKG
QVTEIRDILRKSQDIPIPCDARRFFVNAPEIASLAEDKAQVSAVFVYLLNIFVKAVVLQLINEAGI
KLEYAEPLGVVVAQIFSSEQFCFQGHAFSDVFWAKYRASCPALWGFYGDEKTAAGKQALGWKRIEA
GGPFLSEGDHRQRMVGLGGGFAAVTLRNFGKTNRKNPFPNHIFWRTVARMVSIPPNELQETHFHLL
YAMLRFSAERIIGFWGHAGLALLRSAIVNLPSIATNKQAGQRRKEHLKTLELADKERSSTHAEEVE
RLRRDLEELRAENRALKSSSKLSSPHASSLPSSVIMPSSPSPYPTSPFRSLELYDSAHDSAADDIV
KSPSESQADEVYQEQHEYRTRGHRSRPTTGPDSFCALFPQDIAVVRRDLHLQLAPVLDLNIISNPQ
LHLSTLAAIGPSLPLALQPTLLQLQTPHHAYIDLIPSPTLRDSLIQAGFAVANSFLTEVCTFVYET
EDLGQLTIWGRDYLNVMSWEFSEQVLKAWPNLLTEEWRERANFWRAQRNEPLIIFD
>g1619
MLFTSLNLLFFWMTWTTLVLSHSPLRVELFGTLAIRLVFYLAPSIFFFAFDTILPYAASSIKAQGQ
IALPSGHKRRKISRMELKIIGWSLFNIFLSIAVQGAMEFTLTTLLRRRSLIKVAVRLPYPWGITID
VVRGFIVREILNYAIHRFVLHNDRLRVNRFHETWYHGLRAPWPLTAHYDHPLCYILWKFVPLYLPA
ALFRFHMITYMIFLGLVSLEETWTHSGYSRVPLGFLLSGMARRTEMHVISGGDGNYGAWGVLDWLG
GSTADESETIEEDVRAELADLDIDTKIRKAVQEARDHMGDSVAKEKVKTRRRINGNGKA
>g1620
MSDAQTKKFGKGERTIPHHSQKAQKWYPVDDETQPKKVRKTLRPAKPRESLQPGTILILLAGRFRG
KRVVLLKHLPQGVLLVTGPFKINGVPLRRVNARYVIATSGKVDLSGIDSATLEKVAASDYFTKEKA
QEKKTEEAFFKQGEKPEKKKITSARAADQKAIDQSLLATIKKEHLLASYLATSFSLRTGDKPHEMK
W
>g1621
MSIAAPIITFKAGICDLDTSANPPQVKPKSTPGYIYLYSEDDLIHFCWRPRSAALTDPELDLVMVP
SDGTFTPYRSTQENTRAPTNGRIYVLKFSSSSQRYLFWLQSKSQNERDLAYFSPRDLKLGEIVNTL
LQGEEVDVQTEIANLPSNESGGDDGDDDATMEDVEGSHHTSDHHGGHGSGGAGFDATGGDIREEGE
ESREGGADGGRAASAGQTDASSVIQGLLNSLQGGNQHTQSQSEEYLYTTLADLLSPSSTIPMIESA
DDDQVDNLLNFLPPALLLLAQNIDDIPASDINAETAQAVLLSLDLQQKRDILRRVLRSPQFTQSLG
SLTVAIRDGGLPSISEALKVPVENGGFVRRGGVPLGGGVAVEAFMEGIRKQVTEKPSGSRGDTMET
D
>g1622
MASNNNQGHVPGQDPTQRAIPLQNLSRDESDDFENIDDRRGRRRSSGARTGHGPRRSLLAGGTFKT
YERVAEDSPSPKERVGLRITQPSSARLRENNPYAPDDHDFVDDPGGFAEAMGSIGFGFRPPSPRRP
DSEEYMMSSFPGPSGHGGRATPDDYFSSPIVPIDDTAPLTDKKYLQPISGAKEGDSEGRTSMQSIR
LGNSYSSSRLGDDLETGAGRRRGSSSAGGSPSRSRSLSPSVSGSALHRAGTVMQMMSQRVVNLSNE
PVEQAIMRRESMKRSKHDEPPNSPSQHESDNIPHRMESVSEKGSTQSAWRTNVNPFKGKSLGIFSP
DNPLRTKLCDVLVHPATEPFILLVIVVHAVLLTVQSAHSVWTHPRSDQWGSQAIDYALFVIFVIYT
IELSVRIIVSGFIWNSAEYSTMDRSLGFKKAIAERGKSLLSLQREPSTKKKSVKFEDAQPSYLRKT
VTGLQPFTDSSDDPRHGSRKRLAYRAFLRHSFNRLDFLAVISYWIAFGLQITGYSSKHNIYIFQML
SCLRLLRLLGITDGTSVILRSLKKATPLLLHVGFLIGFFWLLFAIVGIQSFKSSLRRSCVWIDPEG
QSNFTMNDPYNVIQLCGGYLDSTTGEARPWVYANNEPSSFSPKGYLCPQGSMCIEGTNPYNGTVSF
DNIFNSLELVFVIMSSNTFTDLLYYTTDSDYLSAALFFAIGFVLLSLWMVNLLVAVITSSFQVIRE
ESRQSAFAMEKVEEPESDESAHYKVNHLKTLYEKTRWLWITLIAYDLIVQCMRSATMSPSTERFIS
DTETAVTFVLLLEIILRFASDWRHFHQSKRNLVDLALVIITCIIQIPPIHSSGQVYMGLTLFQILR
VYRIVLAFSVTRDLITVVFRNVVGLLNLILFVFLITYLAAIFAVQLFRGVIPAETDNGNTIDMTFF
TIYNSFLAMYQILSSENWTTILYGTTTFTKMYGTSWISASFITIWFILSCFIILNMFIAVIQESFD
VSEDQKRLHQVKAFLQQKELGSSSASNLALTSIFKLGRDNARHREPLDYGPATMDMLLKDAVVQEF
LDEHFESFDTQTPHQPSVDKDALQPGWLSTIWTKISKTFLNKEPNPFYSRLKFSKAYDELDPRTLA
KEVVMASEQRKVAQREYLQRYPLYNKSLYIFSTKNPIRRGCQRMVGPGRGSQRYEGVEPYKPLWYT
FSAFIYACIVAMVLLACITTPLYQRDYFLTREYTPDNWFVWTDAGFAAVFTIEAVIKVIADGFFWT
PNAYFRSSWGFLDGVVLITLWINVFTSLFTAGSVSRIVGAFKALRALRLLNVSDSARDTFHSVIIV

GGWKVVSAAFVSLSFLIPFAIYGLNLFNGQLVSCNDGSLSGSLGGCIGEYQSSPYNWNVLAPRVAA
NPYYSFDNFGESLFILFQIVSQEGWIDVQSSVMSITGLGQQPQDFSSQANGLFFVVFNLLGAVFVL
TLFVTVFMRNYTEQTGVAFLTAEQRSWLELRKLLRQISPSKRSNNAFADNWRKTCYRLASRKNGKW
ARFITGLLVFHLLLLTLEWYPYPVMWDLIRDVLFFILTFFYIADIAIRIIGLGWPRFRRSSWDLYS
LLVVPGTFITIVLAFIFRNVNRMNAVLQLNKLFIVSITLLIIPRNNQLDQLFKTAAASLTSIVNLL
ATWFVLFLVFAIAMTQTFGLTKFGANENNNLNFRDVPNALILLFRTSAGEGWNQLMEDFATMQRPY
CTLNDEFLQSDCGSQGWARGLFIAWNVISMYLFVSLFVSLIFESFSYVYQRSSGLGLYTIDRDEIR
RFKEAWANFDPRGTGFITKEQFPRLLGELSGVFEMRIYDGNFTIGRILEKCRSGPNPRDSLVSFNP
SERAGGNEVDIAKLARILDQIPVEEIRARRKRLNIFYEEVLVSADPERGVSFSACLMILAHYKIIV
DSKSLRLEEFLRRRTRLQRVEEAVRRNTVIGFFDTLYWARRFRKHIENRKSARLTAVPQFSIPEIF
VEDQERQEADDDFFDGESSLQKDSRLTLSSAISQISGMSPTTSPRPSLQIDTSIAADSSSEWSRIG
AALSPRSTAAELGVXPPGRSRSMSGHSSQGSQSQDNSVIVQDMMQSLGDSAWGQSIRRSFTQRRSG
QGPPPS
>g1623
MKTTLITVAALAGLASAWMPGVHKDIVSLSGENLFNKTDPITKRWLPSSSKIRGVNLGSLFVFEPW
LAENSWSSIGCSGQNSEFDCVNALGQDTANTNFQDHWGSWIVEDDIANMSSYGLNAIRIPVGYWVR
EDIVYSDSEHFPQGALSYLAQICDWAADYGFYIIIDLHGAPGAQVAQNADTGQWASTPGFYVDYQF
ERALEFLEWMTTQIHSSNSFRNVGMLEIVNEPVQDSSSVGTMRSTYYPDAFSRIRAAESALNITAN
NELHIQMMNNLWGSGDPTEYLTDNYFAAYDDHRYIKWDTSVAVSQDNYISTSCNDDRGGNTPTIVS
EWSLSVPDDVQYTADWDPSTNQDFYAKWFAAQVIAYEKQDGWLFWSWKSELGDYRWSYQDAVAAGV
IPTDLDSVYDLGVC
>g1624
MQPKRPSSPSKSLEPSPMADISLSLRTAFMDNTHYRRESDSLSTSALSSASGSPTSSPTSGWPWRK
HRFSGSHTTEPNSRDRSQSPYASIIPFSFRRSSPFFLRRRPSAVDLALSEERSRCDEDAIERVGLS
MMEPRPVDPVPIAMGLNTNVLSDIASQRSSYAPSSQGSIRGGSQPRYVMGGIIEVMEGSA
>g1625
MAPLREVKVIDLTQDSDDDMEDEDDADQAQALLARFIKPTFGQSRPLKRKSPVDEVEVRAIPVNHE
AVFSREAPLGPLPEPKDNHKTVEKNISVAVSSPKPWSLFAASGKRVKRESRDQTPKSFAGVPPPPR
PITSLQSHLPSMSSLHSQTNSSNVSSRSSSKRPAFKAKTTSRASTSVDSRVSSRDSSAGFTKKARE
ISTSRHTPSPSVMTKLSKLPEVKVVIPPLQSLPVSDTRSLSKRKINRLPSASSESEEESDPDEPKG
IKQKYYPIEMYDLQADMGRYPKARSTNSDSLVLPTRPATPNGNISRGYPKPPQSRKLYLREMLEKK
LRKIQGPPVTMARDWDGTGSIASNFEFTNTYKLRQGVTRVAEEFNYGCDCGFQCDPSSCTCLSKEE
DSEELMVAYEHRNGKLLLKESFLKRKAMIYECSRLCPCPPTCWNRLVQHGRKIRLEIFHTGDRGFG
LRSLDTVHKGQFIDTYLGEVITSAEAELREDATAGSNGSHSSPSYLFSLDWFPPSSDDESDNDEDG
TSSSHYVVDGQRFGGPSRFMNHSCNPNCKMIPVSTHHGDQKIYDLAFFARRDIPPGVELTFDYNPG
WTPEMNSDDPNAVKCLCGEAKCRGQLWPNQRKSTNV
>g1626
MSAAASTSTSPTVVRSHSSSRPHAHHRPPSSDLPHRTRSTTARPPSSYSQQHSSSRSQSYDRRPPS
NQAAFANIARRDFENSNLARPSTSRRDSSRERSQERPLTSHRPDPARAPQHHGTASRHRRNSVDMP
GTVVDANAANVAAAAASAQQAGHSNPVQPKRRTMISTPTGQWALGKTIGAGSMGKVKIAKNIETGE
QVAVKVVPRQTTEEHRSGRDAERADRSKEIRTAREAAIVSLVDHPYVCGMRDVVRTPYHWYMLFEY
VNGGQMLDYIISHGKLKEKQARKFARQIASALDYCHRNSIVHRDLKIENILISKTGDIKIIDFGLS
NLFSPRSHLRTFCGSLYFAAPELLQARQYTGPEVDVWSFGIVLYVLVCGKVPFDDQSMPNLHAKIK
KGEVEYPQGLTSVTDPKKRASLAEIMNHPWMTKGFNGPPENYLPVREPLQLPLDPEVVEKMTGFDF
GPPEFITAQLTKTLESEDYQIAARSFTRESATPNPSSDKRRGVFDFYRRRNSASRETLSNSSVEAV
QTGSDPLNAYSPLISVYFLVKEKLDRERASKNPGALSIPSDTVLKMPDLAAPETAHTNQYDVPGVK
DTSSRSRPRARTHGDDDLADGIKKLHVNQSASPNTNPTIVTPQGDSPAKKESTAAGILRRFSTRRV
KDRGRESGEREKHFPQTVPSLNVQPPADMASTPHRGFSIRRTRRAEPSPTTLHESSSQTQQDPLQP
PRSAGGPLQETRSNKYLERSTSVNSADYRSRRLARKYGADGNGSNLNEPPLTSGSDGLGGSDIEKQ
KPSLGESAPLNETKPAPRSGAMRAKSLGHARRESIQARRARREEAREATVPEDEDDGDLSNTGNAL
EAPNTGEDFAKPVYLKGLFSVSTTSSKPLPFIRADIIRVLRQLNVEYTEIKGGFSCRHAPSIDLDR
VVDARPSSPERQGQVSAHRRRISFGGLRANEEARDDTRAAHASRYNRRNQGPPDRSFVSNSEASDD

YGQSQNNVVGERVVGETTTRVQSDTGENLVLRFEILIVKVPLFSLHGIQFKKVSGGMWQYREMAKR
ILDALRL
>g1627
MASKRSRGAFEADLQALQSPYAFYGTPLPPLDPGTRDDGSYVPVWKQEVTDERGRKRLHGAFTGGF
SAGYFNTVGSKEGWTPSTFVSSRQNRARDTKQQKVEDFMDEEDIREAEESKQLQTSEDFAGLGGGQ
TQATGRRGIVDLLKTEGETMGVKLLKRMGWREGQGIGPKVRRRANLEDSSNLPQHAIHQLETHLFA
PSDAPMVTFVRKVDHFGLGFEREERLDSNLRLKDTPSRQDEDEDEAGDSFFGGRLTTQKPRKVNQA
PRRGVFGVGILNDTGSDDEDPYSMGPRISYNRTIGGDKKKKKKLQSTEEGKAVSANPLLSNKPVFI
SKKTSLAGFRKCHDGRLPLDGFVLADAVSGLSLSAGRKFTPPDIPPGWKSAKAPAAQSDLSKYMST
ADAAKSSNLDPKSRAELLGEEQLPGKSIFDWMTPEARERLVKATGRTDLPPALGEKAPKGFHISEA
ERRRNLWDLVPKLDKEAAVQALKRAVGGWMPYSEDESKRARYRIFLEISAGLKQGLPDRLPGSTTD
EWVNEMREFTRAAEVFKPISGVMASRFTSASSAPKVTSDQPDSSTEALLSRPPAKPEDPAEEAAKI
GMFGPLTRSTISFYPTRLLCKRFNIKPPAHVHMDPGDHLTGGTGGPTGTAGSTKTSLVSQETMDQL
LLESGIAMSGKHISAPAPEQKPLVIEPERNEALEGERPGEAVFKAIFGSDSEEEDED
>g1628
MQNATQERQEDALSTHQSERHSRQQDSNSSTRRRSRPRRQGVALPLDQHYNQPIKPHVWRSKRRLW
TRTQIDRERDEFFDTRVTGRAEVWAALRAALSLLRESEIETAQGIIDAAGVTVPTGDLCEGAYDEN
GVLYRLPQCIVSDPDNIVDDSGGAAGETDGGDDDIEDGISDRKIVSEESDEELISDDVERRREEKG
KMSERDMIRVKARLSDRGGPDVIMPIGKTQNVGVLARKIHSEAKLANTQRIRIVYLGRMLRENEPL
VDQGWKPGQVINAMVVAKPS
>g1629
MATADIQKTPFVRELASSDRKTRDKAFESLTLFLKARKDLSLLELLKVWRGLFFCFYHSDRPLTQQ
ALARALSYSLVPSLPRESRLRFLRAFWITIGTEFHNLDRLRLDKYLFLIRCYVGVAFEIYLKGKIN
DKSETGKEEEEKQDEGNKKRKRTGEDKTKNGKRRKQSGGNNNEATSAQGGKWTELESYISLLEEGP
LCPINFDPKEQKPGKDEIAMPHGPDGLRYHITDIWLDELEKVVSSYDSDDKSEENVPVKDRLDVPI
ELLLRPFEKLKKESQTKSVRVKVVSEVLDDERLVEWGFKERKKKRSVGSGDEEDDEEEEDENASWD
GFDD
>g1630
MPTPSEVTPPLTASALVDDQIVHTTCPPEIIRASAFSIATEERSVNDGDDGALEEGTVSATVESTA
IEERVGDDATSLQIATTPDSSSMKGRESEDDTDMDSPSPASSPADSKDDSSGSPTSQTSSVTSTSS
TSSSLSHEFSNVRLVPNHTCSFLRAGSRFEGTQESDHQVYNVHVEIKHVDMAESYLCGYLRLTEEH
PTLTTFFEGEIIGTKHTFVTKNETWGANEKTDMQHWARFEAWRPLAKQAKKADFTYRNYAQREHLF
MRWKEYFLVPDHRVRQISGASFEGFYYICFNQIEGTVSGIYFHAKSEKYQKLELKHIPDRGCTPAI
EFR
>g1631
MSDTNEPFYLRYYSGHSGRFGHEFLEFDFRTLGDGRSASARYANNSNYRNDSLIRKEMCVSSLLIA
EIKRIIKDSEIMKEDDSKWPQKNKDGRQELEIRLGTEHISFETAKIGSLVDVTESADPEGLRVFYY
LVQDLKALVFSLISLHFKIKPI
>g1632
MASYLENAYSLVHQDNSADQPSLQELKMQLEKGTDETKQETMRRIITIMLNGDPMAGLLMHIIRFV
MPSKNKPLKKLLYFYYEICPKLDANGKLKQEMILVCNGIRNDLQHPNEYIRGNTLRFLCKLREPEL
IEPLLSSARSCLEHRHAYVRKNAVWAISSIFQHSESLIPDAPELLQTFLESESDHTCKRNAFAALM
SISHQKALEYLSTTFDSIPNADELLQLAELEFIRKDAVQNAQNKARYLRLIFDLLDASASTVIYEA
ATSLTALTSNPVAVKAAASKLIELSIKEADNNVKLIVLDRVDQLRIRNEGVLEDLTMEILRVLSSP
DIDVRRKALGIAMEMVSSKNVEEIVMLLKKELAKTVDEQYEKSSEYRQLLIQSIHHCAIKFSEIAA
SVVDLLMDFIGDFNNNSAVDVISFVKEVVEKFPKLRGSIIERLVATLSEVRAGKVYRGVLWVIGEY
SLETSDIREAWKRIRASLGEIPILASEQRLLDEVPDDNAAVLEQVNGHKAAAPSGSRKVLADGTYA
TESALTSQSAAAAKLEAVKAAQKPPLRQLILDGDYYLATVLSSTLTKLVMRHAEVSQDAARTNALR
AEAMLIMISIVRVGQSQFVKAPIDEDSVDRIMTCVRSLSEFSERKELETTFLEDTRKAFKTIVQAE
EKKRAAKEASEKAKTAVQIDDAIPIRQLTKKNAGEGTDEIELDLIKATGGDATVEDLSSKLSRIVQ
LTGFSDPVYAEAYVQVHQFDIILDVLLVNQTTETLQNLSVEFATLGDLKVVERPTTQNLGPRDFLN
VQATVKVSSTDAGVIFGNVVYDGSSSTETHVVILNDIHADIMDYIQPAYCTETQFRTMWTEFEWEN

KVNINSKAKDLREFLKQLMESTNMTCLTPDASLQGDCQFLSANLYARSVFGEDALANLSIEKEGED
GPITGFVRIRSRSQGLALALGNLKGLKASAA
>g1633
MSADEXPADASAAKMAASDMGELSPDLSLAVGNNVTLTLQSDALFVNDPATKKDVRYCGLQVVATT
ASQSIPYYDILSAQISDSALILKYAKPVVKDDYAPSTLTYALDNDQTSLAIAEAWIEQLLKSAYGQ
AQRNKRLRVLINPYGGKGYAKELYNEYAAPMFEAAGCKVDLELTKYAGHATDIAEKMDIDAYDAVL
CCSGDGLPYEVLNGFAKRANAAEALAKVAVAMVPCGSGNAMAWNLCGTNSVSLAALSIIKGLRTPL
DLVSLTQTGTRTLSFLSQSYGIVAESDLGTDHLRWMGAARFTYGFLTRLLRQATYPCDISFKLETD
SKQAMKERYLEYKNQKPALRPIGGSQEVIGQGLPALKYGTVDDEVPSDWQTISTDTLSNFYAGNMA
IMTADANFFPATVPNDGLIDVVMIDGAIGRLRALSMMTAVENGGFFDYPEVEVRKVHAYRLTPRGS
KDGYISVDGERIPWQPFQVEVHPGLGTVLSKSGHLYEAQGPV
>g1634
MDTADIIMNAPDSDGSGSGDEYSSPPAFIVRSTPDLSDLEGQPTPLATSPPSLVSTLSSGYASTWS
SRSTGRPRGSSLVGTPAFEPEVPLTERDLRLQRPSGPARTPSSTYAPQRRPPHFINLQNDGRRSSS
TKRNARRDPNAQYRAQEKAYVQRLRANPQAWYQQFGDFQGMATIDSDVDEPSPSSEMPFEDDQYDP
DIQLFLAENDNQPTIEELKNPKNQERLEWHAMLASVLKGDVVKQEKQRLIGSTETRRSAAMNGDIW
LGVRAKACGRSVPLQKKLIEDARASLGTVIEDIINFQIKGEKEIGKPATEQVEDVVRKISQCQDLY
PTRKEMELANPRAASEQFHESCAAVCAWHNITQLINTELAILQNWVGNDELDFTKPRDRSTRTDLS
DDGSFLDRIMKEDGLKTLQGEHNMIIGIGEVINKAKVTLIENAEAFSKRHLPPYIEELLTLINFPS
RLIQEIIRVRLTYAKKMKDPAQQSPILVDQMISQFQILMRVAVEIKQRYLVISKVEPGWDLPPCID
ENFDNVVVDAMKYYFRLLNWKLNANKNTFKEAEILEQEWGFSNEIGRRLQSGDIEVAEQFSALTAK
SLQRLMIHFERELSPRSDEDPADMDKRYKSSLDSTRVRQRKLYRFSRFLRQLFENATEYNMGGDIA
DEFFEALLISDHFLITSNDSVGQKGVYLIAHHALWEQPVDIQSILGTSFREEDHPKDGSNFPYILV
IRPERQLSWMGKVMEVEGLEHPTDVRPGKLRLVADGTQQRLVSARHELAQLTNMNLDIAIEQRANL
GRVNAELNKIKKISFKLSMTIMDSVAVIRQQLREKAIDNTELIQACYAFATEFGKRSSNYVDANRR
AMNSARLVELSLEWVSFICDDCDASDRRTFKWAVAALEFAMAITSSNHLLSMDDAQYSQLRVKVAG
CMSVLISHFDIMGARSTLAAQAENKRLDETRGGSRRYGGXGRILSDEEATKLVREQRLAHIATVED
MRVEENAKRQAVGKVLEGSNEADRSLTVLSSSATNVSLRWQQGQFIGGGTFGSVYVAINLDSNYLM
AVKEIRLQDPQLIPVIAQQIRDEMGVLEVLDHPNIVSYYGIEVHRDKVYIFMEYCSGGSLAGLLEH
GRVEDETFIMVYALQLLEGLAYLHQAGIIHRDIKPENILLDHNGVIKYVDFGAAKIIARQGKTVAN
AEAFTNGVGGGHKEGLGGAKEPQRKNQKTMTGTPMYMSPEVIRGDASELVERQGAIDIWSLGCVIL
EMATGRRPWSSLDNEWAIMYNIAQGNQPQLPTREQLSDIGIDFLSRCFERDPLKRPTAAELLQHEW
IVSIRNQVVVEPQTPVSDAGSSTASNSSAASSRHNSSTYL
>g1635
MSDSGDYVPLVPYNGTTATGGDSLTEDLNLYYNAGDIAWMITSTALVLLMIPGVGFFYSGLARRKS
ALSLIWLSIMSVGVISFQWFFWGYSLAFSHSAGTYIGDLSNFGFKDVLGAPSVGSSKVPDLLFAVY
QGMFAAITVALAVGAVAERGRMLPCIVFMFVWSTIIYDPIACWTWNPSGWVFKLGGLDFAGGTPVH
ISSGAAALAYSLMLGKRRGHGTHELNYRPHNVTHVVIGTVFLWVGWFGFNAGSALSANLRAVMAAV
VTNLAASVGGVTWCLLDYRLEKKWSTVGFCSGVIAGLVAITPASGFVPAWSAVVFGIVGAAACNYG
TKLKYVMSIDDALDIYAVHGIGGLVGNLLTGLFAADYIAHLDGATVIPGGWLNQNYIQLAYQLADS
VSGLAYSFFGSCLILLVLNFIPGLSLRSTEEDEIMGIDDAEIGEFAYDYVEITRDVIGATDGSSIP
VSKVNSIERTVEESKV
>g1636
MAEEEQPATTSAGGFPTDPADFDSDPRISFSKLDNKFILETEDGQEYEYDSALKRWVQTIDDALLE
QQRQAYRVEGVDETEEVTPSELRKKKRKQPDEENGQKQKRQRVNTAVYITSIPLDATLDEIRDVFS
KCGVIAEEIDSGRPRIKMYMDESGNFKGEALVVYFRPESVNLAIQMLDESDFRIGQPGPLGPMKVQ
AADFSYKSQQNAPTNTNMRDKKKIMKRTQKLNSKLADWDDDEPSALPDTSSRFDKVVVLKHMFTLE
EIEEDPAAILDIKQDIRDECSKLGDVTNVVLYDKEPAGVVTVRFSDPDAAKQCVQTMGGRFFGGTQ
VVAYIADGSEKFKKTNEKRAALEDLAEGRDPDEEADRLEGFGSWLENQHESERVVESFAH
>g1637
MREIVHLQTGQCGNQIGAAFWQIISAEHGLDGSGVYNGSSDLQLERMNVYFNEASGNKYVPRAVLV
DLEPGTMDAVRAGPFGQLFRPDNFVFGQSGAGNNWAKGHYTEGAELVDQVIDVVRREAEACDCLQG

FQITHSLGGGTGAGMGTLLISKIREEFPDRMMATFSVVPSPKVSDTVVEPYNATLSVHQLVEHSDE
TFCIDNEALYDICTRTLKLSSPSYGDLNHLVSTVMSGVTTSLRFPGQLNSDLRKLAVNMVPFPRLH
FFMVGFAPLTSRGSYSFRAVTVPELTQQMYDPKNMMAASDFRNGRYLTCSALFRGKVSMKEVEDQM
RNVQNKNSSYFVEWIPNNVQTALCSVPPRGLKMAATFVGNSTAIQELFKRVGDQFTAMFRRKAFLH
WYTGEGMDEMEFTEAESNMNDLVSEYQQYQDASVSEGEEDYAEEEPLEAEE
>g1638
MEQFSLSAIRMKIMESTPSYLQETISSVQQLSTSEMALYISLTLGAYVLLVRSLRHRRIRQLEAHY
NYRTRASMAQMTDHEAWEIQRAMAQYEFPFTVEKSLQFALFRTYGIPTISNTLMKTKQFSNNLYSG
KRYVDTCVLIGEFIVHGPATVRSRIGLARTKYLHSGYRKSGSVREEDMLYTLSLFATEPIRFVNLY
EWRAATDLERCAMGVFWKSVGDALGIDYAAFLPSAKAAAEAGEGSNGYTDGIHWFDEITAWAQEYE
KKAMVPAQTNRDTADQTTAILTYALPRPLKFIGNWFVSSMMDDRLRTAMLYPRAPRIVENVFWLAF
EARKLFLQNLCLPRPEFMRHVAFTEKPDTQTGAYHVTTWTAEPFYVKPTLWNRWLSPSAVVARVLG
FPLPGDQGDKYYPQGYDLGDMGPRSFEGKGKEYLEREMNIMEKQRTGQCPFM
>g1639
MSGVSEDGEPSHTSAPVRRARAPTITIDTSAVSPITPTEQNFQPTSPTGPAGAPSLSRDNSDITVQ
SGISPTDERSTTSSEWYRESRPTSPHNFSSPTSKFNGEGHSNFLAVPGTRSRGNSIESESGTNTDY
SFGGETYVQSSPSHTERISQDLSRGLTVLNDEEALRPDPGTEEDFEVEDNKFAFAPGHLKKFFNPK
SLGALHAVGGMKGLEKGLRSDRNSGLSVDEAYLDGTVSFEDAVHATKANQPKAVSRTDTHRTQASR
HSQSKEAFADRKRIFGANELPEKKVKTIWELAWIAYNDKVLILLSIAAVISLALGIYQSVTATDGE
ARVQWVEGVAIIVAILIVVGVGAVNDYQKELQFVKLNKKKEDRQVKAIRSGKTVEISVHDVLVGDV
ILLEPGDLVPVDGVLIEGHNVKCDESSTTGESDVLRKHSADDVMRAIDNHESLNKLDPFILSGAKV
SEGVGKFMVTAVGVHSVYGKTLMSLQDEGQTTPLQSKLNVLAEYIAKLGLAAGLLLFVVLFIKFLA
QLGSIDSANEKGQAFLQIFIVAVTVIVVAVPEGLPLAVTLALAFATTRMLKDNNLVRLLRACETMG
NATTICSDKTGTLTQNVMKVVAGCLGTSNRFSDSQNNESSQSDENGNSDAGEVSPSTLVSGLSADA
KELLLDSITLNSTAFESQEDDGRLTYVGSKTETALLTFARDYLGLGSLNEERSNANMVQMVPFDSG
RKCMAVVIKRKEGQYRMFVKGASEILLGKSTRVINKIENGLSSIPLSDDARTGLLNIANTYASRSL
RAISLLYRDFEQWPPRGAPTQEDDKNLAVFDSIFMDMTLVGVVGIQDPLRPGVTEAVQQCQRAGVF
VRMVTGDNINTAKAIAQECGIYTAGGVAMEGPKFRKLSTKQMNQIIPRLQVLARSSPEDKKTLVSA
LIRLGETVAVTGDGSNDAAALKTADVGFAMGIAGTEVAKEASDIILMDDNFTSIVKAMSWGRTVND
AVKKFLQFQITVNITAVILTFVSAVASGDENSVLTAVQLLWVNLIMDTFAALALATDPPAASVLER
RPEPKSAPLITTTMWKMVIGQAIYQLVITLILNFAGLSILSSMSVFTNPNDPGNATKELKTVVFNT
FVWMQIFNQYNSRRLDNRFNIFEGMFRNYWFLGIQLIIIGGQVLIIFVGGQAFAITRLNGPEWAVS
LILGAISLPVAIIIRLIPDEFVRRLIPSFWKRKKSQSPQVLVSDEERRYEWNPALEEIRDQLTFLK
KVRGGRLKNIKHKLQHPETLLPRSRSGSRSREGSIFAGSNNGEPTETASPPPATPESRSRNRTPRS
RSNSAFGPAAAMAGVIAGSIAGWSPVERNAGDRESWHLSSTSPHSGLDKQQGIDIHPQTAEDDPII
VNDYNLLSSSTPPSQNPDLIPVFEHGPPDRVPSRGRRSTSRRSRSNTMTTFNSRRSTSHSQAPPQ
>g1640
MSSNPITEYGFTAVPASPTQLLNDPTTKPSSPPPAFTVSSFPIPDTALAKRIQAYAKTHLPEPTYN
HSLRVYYFGQAIKRYRFGHIGGSSVWDFSDETYFLSCLLHDIGTTEENLNKTRLSFEFYGGFLALD
VLQNDENDAAVAPREQAESVAEAIIRHQDLCEIGTITAVGQLIQLATIFDNTGAYADLVHQDTIEA
VSTAFPRLKWSSCFVETIHKENGLKPWAHTTALGEEEFPAKVMGNKLMAPYE
>g1641
MAPVFNSPAPTRTAPSFVFGRTNQTQSFNNAPEIIEIDDDDDDDRVGDTENSVDEMDQDESDDANE
SDEDEVIEDEDEEEDEEEESNVDDDREEEEEEEEEEEEELTVESLGKVNGIDSTDVFTTRRTLQSSP
AIRLPQTSMFTSSGIQEALHPLRHTADRVTRQMQAFAEKLDRFKQQGRTQESTYQAAYTLIKSYQQ
HASDAIKDASRQNTLQKARMGWNTNRVAEQPDAKTQEDLRRLQLEVDTWDLFLNLIATGDSESIRN
FKYAQETVFQNLHRYSTDKEIWDQFLSADNYASECAMVLKWLEVKAQSSSQHLDTMIANLEAQADR
GSGLWAHGWLYTKESIKGQKRLRALPQPLEPGDPGLLASLLDSDNKEPLITQLDPDAVIRQKHGLQ
KQDQFYEQATWLTCWKMIRQGETWTKIQEWSQERLESWRAVSLCGSRVETQASGAKKQAEDSMTRM
MNCRSQDTWRAACSALARNPNTGEYEKAVYALLSGETEPAYRVCQSWDDHLYVFYNNILLSRYREF
CSQFSRKLSHSPLASVPFVPGPMDYGAVQRFLESLRAHETIGMEARNPYVTIQSAILGRNFDSFFL
SMARAVSESNKDSRYSSLIPDLGATGVDSSALIAVRDKDALRIIAHSFIITQRLSYTRSDSHFSET

TALIVIAYAENLRQAGLTDWTPLYVSFMPTRLVPDVIVQVLVDVVEPRDKSRMIKLLEKHSIDIVA
VQEALTEYVLYTSPKAETRTITMDHKVKRDQAKGVVVLLPVSRRFTGNEISAKTEELIRCVEWLAD
LQAGWDIVCTSLEFLYKRFFGNGMLLEVRELSLRLPLSELSQKLLGYDIRAGQYLEELSVIDENSV
LRSPTKSPSKSTRFRRPQTEAERQGQYDAALVLLDLEFVIWAYNALDNFAEIWEHFELLKNSADAD
DVQITELSQALQEAIEQATEHVEALFDKEWGTLIQRSKPEEEKKLAHIRVTYFPDIILNYLSAVYY
ASLKLNRGLLTRNMEIAILLAEDNQILDCFRQSGRLRELADAMALASVAMVNAPADAKTRKKADGP
TRLDIWNIEVAQDNNDGRVSTRS
>g1642
MADESGDAGPAPVEHLNIKVTDNHNEVFFKIKRTTQLKKLMDAFCDRQGKQASTVRFLFDGTRVRP
EDSPDTLDMQDGDTLEVHQEQIGG
>g1643
MASDLPAESKASLEKSLEELNIQSKKPDPDISRLSLSAAVEGSNDKRGTKKKKADVADSWEDELSD
EEEDGSATETEKENDPNAQLKSSLAAAKTEGPLAPPSTPSTHHAGDDYGLPQWGRVPSTNTTTPGR
DRDGARARPEKQTAVASRLIAGALGIRAPKRTEEQRAYDRAVKEKEIKRRNKEKEEAAKAKQEEER
AKAAVWDA
>g1644
MSCLPETASEEDISSYPSVLSRDNRKRRTPEPSPSPTTANKRPCDNTLEERDGEAYYNHYYHIESG
IKSTSPIDMTPGRYPLDPVETNPRVLVMTRIVSGSVSNHVSKNVHHFNEVNDVILRNIRQATAGSL
LERVCDAISVAYITDESQPAAFNDNKARACQSAIEAVEVALRHPRQRTLDSTLVAVLMIDVYKLLL
TAHVNPPEASNQGWGWDSNMLWMIRLRGPEQFTRQDGRNLIWAISYCITIPTSQSAISKEQESGYT
TCPFADLLGTANKEGSLSYPEALDFDISSFFNAPNDGVVVNTAGLKPSLADRMSQDMSRSAERQDK
SSGPFQHRNRTSTRDERNGCDCMTHHAALLLQLSNLKNDKDLLSLDDVLVSVQQALTPWNHLNECA
VCESDDDQGVLIFSAMTIRTVLALLQRFCAESIYLHKVDEGSAISSSAQLLDGNQITIGKYKASPE
EHSLVTALLITRALSNVRSVLVSLKVKLDRSTKRIKADYLKKKTGGDNLVNGNNQEEVPSTVSNAV
PKGEESGCIQILLRSLDATVEEIGKTVPKMSFSALSQTNPFAY
>g1645
MLPKPAEMTEKLDSESVPTAAIDALTENDKASTSNAIISGDSTDIEKSEDAQVAASSAAVTEEDPH
PVKGIRWWLLLLAIYSTTFLYGLDNTIVADVQGSILDKFGQIEKLSWIGTGFPLGSVAVILPFGFA
YGSFDNKWVYVMSIIIFETGSAICGGAPNMDALIVGRAIAVFRVLNLITRFTTLRERSHYMGMTGL
TWGAGTILGPVIGGAFADSSATWRWAFYINLVILAAFAPIYFIWLPAIPVIPEKTFVAKFLAMDWT
GMALNAGIYTTWVMALTFGGSMYPWNSGREIALWIVFGVLLIAFCVQQYTCLFTDRKRRIFPAQFL
QRRTLVLCFICTNCAATAFFVIVYYLPIYFQFVHNDTGLESAVRLLPVVICFVVLSIVAGTTMPYV
GYYFPFYIVAGVLIATGGGLMYTVDETTSTSAIYGFSVLIGLGSGSMMQAAYSIGPAKVIPVWEDI
PPVIGFINVAQIGGVVHSLAISGVIFQNYAFRYVSAAVKDLHLSPEQIRSAIAGTNSAVFDTMTTE
QQSLAIGGIVNAMQKVYILIATAGALCVVCGALMHREKLFLESSAGGA
>g1646
MRAKLFPHVAVRLSSARTASFSTSSRALGYADTLPNLKIGAHTKVLYQGFTGRQATANVKESLEWG
TKIVGGVKPGVEGEHLGLPIFPSVRVAKEKSSPDASAIYVPGNQTAQAIEEAIEAEIPLVVAVAEH
VPVHDMLRIHSILQTQSKTRLVGANCPGIISAVGKCRIGFQPLPCFTPGRIGVIAKSGTLSYEAVA
SLTRAGLGQSLCISMGGDVLAGTNFVDALKVFENDGDTDGIVLIGEIGGTAEIDAAEWIKDYHRRT
KKPKPIMALVGGLEAPKGRVMGHAGAWVAPGEPDARAKYDALQKAGVTLVSHPEKFGPGMKSLLGN
TGRVSNTSTTPTSAGQRRGFHTVRQTRDRVQPVQCRFLHIKQHQAFDMLEVAQIQSQAKEASSEDF
HLSITVDRTYLSPCVTVSSGSKSPESSLRIPFNYENPEFATNTNKAIQSEIVSFLGFEAEYQEAVR
KYLQELYKIFKDNEAFALETRASFSAGKKIVVHETRFGFDDAAYKSSGRHEKLHQLRDVAAEVTEE
VRAEKSGIVYVKLDGEGSIGTLVNGAGLAMNTVDALILKGGKPANFLDTGGKATAATVKECFSILC
EDARIKTIFVNIFGGLTRCDMIAEGIMDAFKELDMKVPVVVRLRAHTAVIEDLNYPLGGLCGLFPH
VVHQIYWAGHRWRRAYKSAAPLVQLEYPGSTVPKPRSRIRRVIGFTSLAVFSYFVGSAIPLNRTVM
EIVTGGIPSDEESLTIFRPTDEISQEIETHIMTHPLAQKYRNDPAFTESRPHMKIPEKLRGRHLIT
GTLLGPKRMAVPPLVFLEENGKEMIAITYLGQEVCGHPGIVHGGLLATILDEGLGRCCFPVLPNGI
GVTANLSIDYRKPVTADNYIVLRAATVKHEGRKAWVEGRIETLPEGDEEPIVLVEAKGLFIEPKYA
GATANMSKNTLYQLLSLPTTIVPSHHHDDALEAISSTIGVDGSVSSFPIPEFKIGTLDALVQQAEE
LAKLEGVCQAVVGKVGEALKGVLGNDEEQIQRMKTVNDKPVDQYLRTFQWNKVKYRADRSIGELID

LLKKEAASIDSDIRSKYSQYNQVKNTLATLQRKQTGNLASRSLASIVDPQKLVHDSEYLETHLIAV
PSQQIKDFLKLYETISPMVVPRSANLVAEDDEFSLYAVTTFKKHGAEFTHKARENKWIPRDFKYTE
SGREEEAKEVERVGGDERKLWGETIRLGRTSWSEAVMVWIHVLVLRVFVETVLRYGLPLDFVSSIV
RTTSKGAERAKKNLDNKYNYLAGNAFGRDKKGRVKKDDPNEMQVAGEGGGADYTAYVYYEFEFE
>g1647
MATNITYHAAALTRSERSSLRNQQGLTIWLTGLSASGKSTIAVELEHQLLSTRGVHAYRLDGDNVR
FGLNKDLGFSEKDRNENIRRIAEVAKLFADSTSIAITSFISPYRVDRDTAXQLHEVPTPGEETGLP
FVEVYVDVPVEVAEQRDPKGLYKKAREGVIKEFTGISAPYEAPLKPEVHIKNHEVTVQEAVAQIIA
YLDSKGYLPAKTAN
>g1648
MVVVSSRGLEPHRTPMPSMLDYFKMRLSPGTRHKNQVFPCQMPYTLGLQLRLLKPTTAGKVFLVNN
SPILSMLLHEKIFKVGSQAVKSAESPTEAQGQNIDALPEMEQPAEPTSETEEQNICELPQADESTS
GEVEPALGAEEQEDQALEDKWPDWMSDTSSVYTFTVTILDKVLSSGGQHPIVNFGNFNCVSESENP
IPPEYEAGTEFLNAALKQAPSPRPNTPSTADTRYQPHEDPLDFIKTRLTEKNLEKNRKACMPSIPI
LPRFYHFMKNPRHPDPCQMPYDRNGTPLGCWDGCPDCEVWPSDYGSRIWLVNNWFETYFTPHEMDM
CDFLKNPSHPSLKYPEPDPNDDDDGNVTVDKREHDEDFLMGAEGPDDFPGFKWLYDNEPEDTHQDV
VTLLAQYMQLWECIHADGGRYGFDYYYGERCNAIMNEYEPHVLPNVGFGAEDKMHVLRYPGIDDMS
KPIWHTSGSPWPMQCDYNTRVEYTRKEYNRKMLMFRGQDEARQRYIQQVREAARYEYVQKGHVEEA
Q
>g1649
MAPGNEVDQAPKPVPVETNEKVHDSETSSSTQADVTGTGALDAEKPSRHTHWTADNQTHELEPTPS
VRSDGKVLLLEEDVYDQLGYGFPSWKKWTILTVIFLVQVSMNFNTSLYSNALGGISEEFHISEQAA
RCGAMIFLVLYAFGCELWAPWSEELGRKPILQASLFLVNIWQLPVALGKNFATIMVGRALGGLSSA
GGSVTLGMIADMWESDNQQYAVAYVVFSSVGGSVLGPIIGGFCQEYLHWRWCIWIQLIFGGFVQLL
HLFLVPETRSTLMIDKIAQKMRKSGENPNIYGENELTPWKERFSAKEIIKTWMRPFHMFLTEPIVL
TLSLLSGFSDALIFMFIQSFSPIYQQWGFSPSQQGLTFIAILVGYLLAWFSFFPAIKRNVRERRAK
PDDEVAQYESRLWWLLYTAPCLPIGLIGFAWTTMGPPLPWIASMIFVAIVGIANYAIYMATIDYMI
CAYGPYSASATGGNGWSRDFLAGVLTIPATPFFNNIGGRYHIQYASTILFCISFLLVLAVYVIYWK
GPALRKRSPFAQQLSDARATTDPTGNRLTRLPTGSRANSFARSQQELRLRQALGSRQNSYYGSRAN
SRANSRANSRVNSRRNSVAEVSEV
>g1650
MSGITRSVYRGAKSLQNARQLSTVVAGRIPKSVSSTTAITGLRKSVPAQQHIVRPTIATVPTYTGK
SLRYASSSSSTSLNRTQLYDLHLKYGAKMVPFAGFDMPLQYSDLSHTESHHWTREKASLFDVSHMV
QHRLTGPGALPLLMKVTPSSLDKLANNTSTLSCLLEDGTGGIIDDTVITRQGPEAFYFVTNAGRRE
EDLAFLTAEIDAYRSEHGADSIKWEILSDRALIALQGPLAPSLLQSYIYTGEGEDPASTDLNTLYF
GQSRELYLQLPDGSKTAHRLLISRTGYTGEDGFEISIPTANGATDLPHQVTELLISQPDKCRLAGL
AARDSLRLEAGMCLYGHDISTAQTPPMAALGWVVGKERRDESSPASKFNGSSIILAQLAKPSKTIS
QRRIGLTIEKGAPAREGAIIIDPSSVTEENKTPTQIGVITSGLPSPSLGGTNIAMGYIKNGLHKKG
TEVAVLVRNKLRKATVTPMPWIESKFHRPS
>g1651
MALNMSHNPEKQTENAIYGKHGSDEESQTRRTSVIGDVVVDRATIGKQLELEAESAIKYRTCSWQK
TAALLFSEYICLAIMSFPWAYSVLGLVPGLILTVFVALVVLYTSLTIWRFCLRHPEIRDVCDIGQY
LFWDSKIAWWATAVMFLLNNTFIQGLHCLVGAEYLNTMTGHATCTIVFSCVVPIISFFFSLPRTFS
GLSHMATVSAFFTFISVLLATIFAGIEPHPADYTEELGKPIVTAFPVSGTTFVSGMSAFLNISYTF
IGQIILPSFIAEMKEPEDFWKSVTAVTIAEVIVFSIVGAVIYAYTGNQYMTAPAFGSLGDDLYKKV
AFSFMIPTLIFLGVLYASVSARFVFLQFFDGTRHKTQHTVVGWASWAGILAVLWILAFIIAEIVPF
FSDLESIMSSLFDSFFGFIFWGVAYLRMRAEDHGSSFYKERGIRGWIGVIVNVFLIAIGFYFLGAG
TYASIESVVLSYNEGGFGGPFSCANNGL
>g1652
MAKNKGKRSSTGSVPQAGESKVSTDESFISLDAGALANLTRNIEQKLKDGKNTANNKSQKRTNNSK
TETKKVPVHDFKDKKDTAVKSQNSNQGKKRSRDGEVIEKLAKGGNSKNDQSQDDILRKEILALGGS
KEDFDLLAEVASDSEVEEASGSHIKAGSNDNALRKELAQLLKDAGQYNPVIADDQVEEEQPASEDG

SEEEDEEGEVEEHGDEEIDSASEADDANMPSKEIKSKIATQVPDKKNAAFAETQSQFPKEYSRLTV
MPRADWYSTELPSVTAPKVTTGLPKFLMDRIQQHATSLLEKENQLYSQIQQQSSSSSHKFYSTIMT
SGTLSDKISALTLAVQESPIHNTKALGDLVALSKKRSRAQAVDVLRSLKDLFAQGTLLPSDRKLKA
FANHAELVASFQKAGPKWTERDPLPGDLQPQHLILWAFEDFLKVQFFEILKVLEIWCNDEIEFSRT
RAVSYVYELLKEKPEQEANLLRLLVNKLGDTGKKIASRASYLLLQLCQAHPLMKPTIVKSVEEFLF
RPGQSPHARYYAIITLNQTILSTKEEKVAIHLLDIYFAVFLQLLKSTDKKSWKPDNKKKGKKSDDA
KTQKAQAEHDDQLKEKLISAVLTGVNRAYPFTSSDSDRLSKHIDTLFRVTHSSNFNTSIQALMLIQ
QLTVTHQVAADRFYRTLYESLLDPRVATSSKQSLYLNLLYKSLKNDSSIKRVKAFVKRLVQILGLH
QPSFICGVFFLIRELEKTYPGLLSLVDQPEEDESDEEEVFRDVPDEDDQPEAETSKQVELTKKVND
GYDARKRDPQHSNAEKSCLWELLPLLSHFHPSVSALTSHLLNHEPLSGKPDLTLHTLSHFLDRFIY
RNAKAGGAPRGQSIMQPLAGTDSHDLLVSASGLAGDARAQVPVNSEEFWRKRTEDVAADDVFFHEY
FNRMSKSQTGKKAQKKSKDPVSRDEEAADESDNESEIWQALVESRPGLEDDDDEDDLDMDDLESAY
DNDEDEVDGSEDGGVIFNDESDENVEEIDDEEGDVDTEVKQKKSKADEDEDDEDSFDMDVSDEEAF
RDSDEDLPSDLDIDMGGVALPSTAEDEAANKKKSKKRKLRHLPTFASMEDYAALLADEDDGMM
>g1653
MKDLYTFDYNAEQALVTYEATREAYKRIFDTLKVPYLVAAADSGNMGGNLSHEFHFISDKGEDTLI
SCSNCDYVYNEEVSDGRTTASVTENPLAAPMPSETGVGTDNPLAISMALWTAISKDKKTLVRAWYP
RYSTQDLGSEPREREINSHAVKAVAKSAGVDLDTSVENPVKLWYEHVKNGEIETLGSYEESARAHK
ILDLYDSQVAPYSRPPLSGLPEDFDKVKHPVDFALLNCFPGSQDGFSLTRVIDGEKCPKCSTGSLH
THNAVELGHTFYLGTRYSEVLNAKVFVDDSALKSKEPISNKSQKVPMEMGCHGIGVSRMISAVADA
CSDVHGLNWPRAIAPFEIVVVPGRDLESEAENVYDHLRRHTDTSDVILDDRAKKVGWKLKDADMIG
YPVIVVVGKAWQSSQKLEVHCRQLDGLREDVPVDNLVDWIRSLLEKL
>g1654
MSTPASGEVPPGAVDVTTLSVPQLRSLQTRLNSELEQLTNSHAKLRAAQSKFRDCVRTINDGVVGK
KTPQPQSADNNSILVPLTSSLYVKGKLADREKVIVDVGTGFYVEKTTAKAIEFYNKKVKELEANIT
DLEKIVQGKSTNLKIIEDALRQKVVSGEAVTAPSG
>g1655
MATRNRQAPKLQKQAFPNVTRNPDFGYGGAQSAKYTANSGISDRRNSTKLYLLGLPCPGRFVKSIL
LDIQPEDKQKTKKAKVNFVTTEIRFTGLLIDKAELWSTNINRLLAAFEESFRCVPSLKGVAQMRVT
LGSFSFEQYRYPKNPQNGYSFDEFNEMLSQPMTRGRITSGLSLDQLLNQIRQANEMFVLENDQSAI
STARRSVKVEFPPKDNELIHLEVDFRASEGHYEKVNSRWVRSDLGRSTAGNRPLLQAVMLNPERAD
LEFKIQEKTILDPGQLDQSMRDILEGIRFRPSENDTVDFSFLAPAKRHSKLPIGAKIFRLVEKATV
QYQVRNTDYFLEIARYDIYYPKPSRARQAADSTSYLEFNPPTSFSEVVLYGQSWDNATKAFGRIQG
TESFDLNSALYALFKPEGRETPRDAFERFILIATNVAGLWNPAGSRPMESIPRPSSTSRTTKMSVN
EGFLIDLD
>g1656
MAAQTRPSSGQNVRDTKEKARQWLTKGALVREDSDDELGYDDLPWEWIYEEDTSEDKENKPHVSAT
EEDDHESKTLRRRSGRSNNKKRKIVGARMGTFECRLGHVVLLKSPEAGKDWVGLITEFVEEDDEDD
DEEIIKSANIMWFASPEEFLSAKTKNKKRADALPNEQYLTADFNVNPLTSINGRGRVMSKDAFFAR
YPNGIPPKDKEELAEYQKCIVCRRGVNQLQGKYTEEFLWEDVYKGDEASVYNLIELIKAGLKTAKK
RKAADDDYVDMNDGDEKAPITPRKKQRTTALTTPQSKRKIFTTPTSKRIVVKKPLEFTPLGTRVLD
PTHFTSSPYRQARSLLHVSSVPTSLPCRSAEFETVYTHLSAAIAEGSGTCIYISGTPGTGKTATVR
EVIAQLNNAVLDEEMDDFIFVEINGMKVTDPHQSYSLLWEALKGDRVSPSHALDLLEREFNHPSPR
RVPCVVLMDELDQLVTKNQSVMYNFFNWPALRHSRLIVLAVANTMDLPERTLSNKISSRLGLTRIT
FPGYKHTDLIEIISSRLANVPGDIVDPDAIQFASRKVAAVSGDARRALDICRRAVEIAEQECEANT
SKNDIDDAESLPPTPSKSAIRRTQSQKGNSKSLVKIESPQKKPPTQKTIPRVTIATIKQAIQEATS
TPLQQSLRCLPLAGKLFLAALLARVRRTGISESTMGDVLDEARRIADAAVAVAGLAGSSIKNYLLG
KDNPGARVHAMVFAAMELMNSGVVALETGVGIRVRGGGDRSSKVRLRIAAEEVKSAFRDDIEAKGL
GLGFDQ
>g1657
MESTIAQPLKLKCGLELKNRLVKASMAENMADANGMPTDVHNGVYTEWAEGGWGMVLTGNVMVDRT
YKGDAGDVAVNPSRKTELLSKWKAYASTCRSNDTPIIMQINHPGRQSPAGAGTRPFFAKNLAPSAI

PLNVGPGLLAKFASAFVFGTPREMNQTDIDLVVQQFVECSQLAAETGFHGVQIHAAHGYLLTQFLT
ADTNHRTDEYGGSPEKRAKIVIDIIHAVRAATPEGFCVGLKMNSVDHQSPEALKGCITQLKMIIEA
GVDFVEISGGSYEDVTFIEGTGQSQNPKSAHTVAREAFFLEFANAIRSQVPEIPLLLTGGFRTRRG
MIAAMQNNSCDLIGLARPAVLMPSLPKSIIFNNEVPDEEAQLPYKKITAPWLVRWTGIKVLTSGAE
PAWYSKQIQETGRRKIASSS
>g1658
MDVVPHPLPADQAVRQSAKRTVELFGSDYLMATPSTLGDGSIGVSYRRKAEYEDVKELPKSLAEKQ
AKATASRTKRPKISGQGQQSGSSQALVKTTPAGPSSAENQSKSLIARPTATPQQRPDWHAPWKLMR
VISGHLGWVRALAVEPNNEWFASGAGDRTIKIWNLATGSLRLTLTGHISTVRGLAVSPRHPYLFSC
GEDKMVKCWDLEANKVIRHYHGHLSGVYTLAIHPNLDVLVTGGRDGVARVWDMRTRSNIHVLSGHK
GTVADVKCQEADPQIITGSLDSTVRLWDLAAGKTMGVLTHHKKGVRSLAIHPKEFTFASASTGSIK
QWKCPEGAFMQNFEGQNSVINSIAVNEDNVLFSGGDNGSMSFWDWKTGHRFQSLDTTAQPGSLDAE
AGIMSSTFDRTGLRLIVGEADKTIKIWKPDDEATPESHPLDWKPTLGRQKY
>g1659
MAAEPEAKFAIHEAAREGKTSVVESLLNANPKLASRKDDDERLPIHWAAAYNRLPIVELLVSNKYF
DPDVTDGSGWTPLMIAASLKNAEGDAIIDLLLRKDADVNIKSVSGQNALHFAASKANLSTVKILIA
HKCSARVKDRRGQLALHRAAAVGSTPILKILLDEGKSPVNATDVDGLTALHHAISEGHGDAAILLM
KAGTDLEKRDGDGNLAIDLAPDTKVRKYILQTAEREGIELP
>g1660
MTSNAQPTKSRWGVGALLQQAVSGVESRLDTILANDEESPKTSGPKAEDAATKSALTAAKPAGITR
TSSTARKNDRLQERLARAVAKSQSPTMSGSPRSSIDVPSRSSTPLQPTEARASLDSTRTSLDQSVS
SARNNEAITVTVTRDSLDSVSQPRTSKELSVIEHMPDNNNEEAEGEVPEIVEPEVADDSAKQDTFD
ASDIIVQPEFTKPPSPPTNDPFEELRAEHQAAESRWQEDMHEYIERIDALQSKLMYLAKEAADSAR
DAATAAKPGSIEKRLSEKDERIALLLEEGQKLSKTELDNRTVIKKLRQQLAESNKQETELKRRTAK
LERDISDAEARAKRAEASEKKALESLTSQSRASKDFESITSERDALSSTVEELRSQLSRAVSRAEI
AESKAQAEVVEHSKRRITELEDDLSSAKIERELSEEKLRREIRSLQDHLEREKENARLLEAELKSE
QSVLESKMESLRSRAEEVSSSTAGETQAKLLRQIETLQTQYAVASENWQGIESSLLSRLNGMEKER
DEIARKEADLRRKTREVNIRAKKAEDELETANETIADLDRSLQETKQQLQKLDQRVEKSEEDLNAV
RKELADKEKSVESFWTQKLEEERVRWRDQVASPPPFLQEPRTVSPVASSRRSTGLDPLTIDSRPLS
RRSSTLPSQEMGAPSRTNSFPSFQGMGSPHASNGLPNPNLLGAPPIHTLEPDEYYSGTPATPSAYG
AQTSHSRGNGINEIISVSTVGAGPSVQLVERMSATVRRLESERAATKDELERITAQRDEARKEVVE
LMRESEERRKAESRIEELEMQVAELDQRYQTTLELLGEKSEQVDELQADIADLKKIYRELVDSTMK
>g1661
MSLEPPTYLNSLQNNIRARPIPWDGAVRAGNITDEHLRKIKAVDKVRKQERKQAVEADLNAYTTLL
AGNPPDVKSVLDSASRRTDIVQYILVLAADLINDVPALASALISHPNPYNAFLPFLRNLTNPEDPI
PLLASSFLTNLLSSALISDQTSDTEEAALPPLYTYLADLTKNQDSGLQDIGVQGFSALLRNKVARE
LLWKQRAETLDPLVEILRTAAGGKDNGSINLASSAATSSLRTADISIGGGVGLQLLYHVLLVIWQL
SFEGSLVGEELQSQYDIIELYTQLLRLSPKEKTTRLLLATLNNLLAANRTLLPVATFVRLPALLSN
LSGRHLTDPDLLEDLQSLTDLLEEYTKTQTTFDEYAAELQSGHLRWSPPHRNPTFWRENARRILEE
ERGALPKKLAEIIAKSWENDKQVLAIACNDVGNLVKEVPERRIQLEKLGLKTRVMELMTDRDESVR
WESLRAVGEWLRYSFDK
>g1662
MSRSFKGCKRCKARRQKCDEQHPECGRCKATGVQCRYAMELQWGGRAFSRSRFGDCGPGMQKLEYS
PGQFIYTTVKSEGASPASSPKTTTASSPSPSPSADLSIARRIDPFSSLSPSQKALLHHFISDASRV
TSSHGGMQQEICQMIVPMALQTPSLLYATMALSAIHIEALNNHSGTVKAAPDIARLMARSLEHFRL
ELQDSSRKGSDVLLATARTLCLAEIHSGAIQPNTWRAHVEGAKALMSTTTQSSYTPATAFRKYLDR
WYRSIVALTALNGNGPPIGETWAQTETSVSDDPDYLDDYWGFTVHLADIFREIGALAWQRHQDAIT
TTQLKTLEPTKKDEDAEMNIDEQAARLEKSVLGLIARYNQHGGLVFYPGVKENLSEESVQEFMLCN
EAFQHTALIHIHRRVRKVPASSPDVQYSVKRILACTSQIVPRSGLSPWVMLTTPLFTGGCEALGED
RDAFKQLLTSLHNTTRIPNVLQSLEFLEKYWLHGAHTKNEDWSHFLDRMSFDFIPY
>g1663
MSSMEGVISANHPPPIATGSGGFSSSFLRRQRKDTSSSSLTSTSTSTTTDDHDLPPPILPDTDHSA

RSIVASSSSPVRRKPLPSNASPVILSRLSQGSSLSSPLLSPPPTRIPPPLLPQTPPSSRPLATVPA
GRQSTDSSSRQLETVDEVLPFVPRDLDRYPRGQSPLIPFPIDTSRTYKDFQQKQSNFSYNRVDDDF
YLPHGTPIHSRLSSEPIIPVLRSPPPSTNHKRSETMALQPPQSRPPPLNIDASLSAMPSPGDRQPK
TPGNKITSFFGWKTTSSPGAESTSTEISDTGRSPLPSPLPQSASAPSFTTKATSSFPTSISENNIH
GFPLPPARMGSLSGPASGSNADFMNKISDLEAELREISSELAGSIRREMELEDLVERFQLESHNDA
NRRTSDYFSDSGMSGSIRTGTDGGKVEDIERIKRAAEQERAQLKVELSQKWQEERSKRAACESHVQ
ILENQVQQFRAQRVDNSNLSSKTRELEVALDDTRRKLLEERQLKDNFEDLLTAMRVELEQLRNERD
HFRDEVVPALQAGQAGQKNRFQSIAEEDGVVRNRGSMVGLSRSNSLARVPKRSSMAGGGGLSRSNS
ISAGNNRQVESRESLADRVKDVEAQRDALHKALKSLLDRQAWQARENEKRITILEMELTKAQDIGS
PRKLGYEREVRNLREEINLLRRRADDALEQKWQCEKGLSGLKMDLDRAEQETTSLRMLLQEHDIAL
PAGMDMASNEEGLANMQITSSALESAYQQLQAEMESAEAESWSPEDVNRTENLAGQVRQQLETNNA
LRRRLAEAIAKGEREQKMSASRINEMQNRLRKLEDTVMDAQHHSEDEMAKHEEEVRALKDSHSVQL
MRAKMGIKTPVSLSPNPAASPFLGARSPRLDVTTTGEGVPLSEAVSVAKLEVKVKELEKALRDADF
EMEEVVQRMNKAQIEVAELQSDRDEALRQTRRLEAAIKAEREKVKQ
>g1664
MAEHWPHFPFIPYPPSKAGYWSPVTSTLNWCEEDYYATPYSAEIVNSLTNLLFMLLGIKGILNVRK
HGHDRVFEIAFYGYLLVGTGSFLFHSTLKYPMQLVDELSMIYTTCLMCYASFSYSRSSPVKIFLAV
ALAGLAVFITLYYHYLQDPVFHQNAYALLTTVVVLRSMYTMEVALRPKWRKSREEDRLARERQGLP
VPSKERQEYENARDLKILNTMWFMVVYGLSMFLGGFGIWNLDNFFCSTLRSWRQQIGLPWGILLEG
HGWWHLMTGIGAYLYIVWGIWLRHVLNKEQDEYTLIWPHTWNFPEIIRKPGVPSKKTE
>g1665
MTSPFRIVEHVIPGQYLREYPRATSTSQEETLHLAVKQYIPLDNPEPQPGDVTFIAAHANGFPKEL
YEPLWEDIYGQSQKHGFRIRSIWMADVAHQGQSGVINENKLGNDPSWFDHSRDLLHLINLKREEMP
RPIVGVGHSMGGAQLTYLSLLQPRLFHSLVLLDPVIQNESTQFPDKFEGRFVPMNTILSTHRRDIW
PSRKAAAEGFKRSPFYKAWNPRVVDRWVKYGLRELPTVVHPDESKHKVAGDDKPVTLATTRHQEVF
TFSRPNYDYDREKEKPADRFDTPDLLPGGPNTFPFYRMEPNFVFSQLPRVRPSVLYIFAGQSFMCL
PSMMKDKMDNTGVGQGGSGGVAAGRVKSVYLKDKGHLLAQEAVEECAQAAAEFFGRELQRWRKEEE
VFQKMWGEKSQVEKXTFDERWLKEVGPPPARAAKTTDGNSKPKL
>g1666
MASVDTSSLTDLREQYDYIIVGGGTSGLVVASRLTEDPAVSVLVLEAGSNRVSDPRIAIPGLAAST
YFDPEFDWCVTSVPQVGLNGRSLAEPRGRTLGGSSAINLGMVIYPSRTDIDAWEELGNEGWNWKSI
SHYLKKSQTLTPPSDEIKQELSLQYIDPAVQGQSGXLQVSFGDGPFPAFNGAWPKIFENLGYKLTG
DPMSGVAKGAFCGPGSIDPTTRARSHAGVAYYNVEVAKRPNLRVVTEVLVEKILLEKDGGKDAIAT
GVLYCAKDGVQKTVSANREVVIAAGSIKTPHLLELSGIGNSALLKSQGIKTIIDNANVGENLQEHG
FVPFSWEAAEGQATAEALRNPEIGAAAMAAWQQSGAGPLGLCPLPSAFMPLPESQNGQLSALFDTY
LKEEAISPLLKTQYKVLRRMLEDREEPTGQYTMAPFQILPEKGPSPKGIFGMTEPENFVSIVSVLN
RPFSRGSVHLASADTKALPVFDPAFFSHPLDLELHARHVRWLETLAWTEPLASLLKADGRRLQRSS
PDMSLETAKELARDRIICHYHVVGTAAMLPREQGGVVDSHLRVYGTQNLRVVDASIIPIIPRGNTQ
ATVFAVAEKAADLIRDSH
>g1667
MAITYALNTPLSRNPCIAVTVLGAVSTITTILRFYALRLRGVKPGAPEWLIVAALGIVYVDIAIEF
IHISVTIVGGAGRKMGEVDPSSIIVTLKTILPLEALYGIVLGLIKTSIMLFYFRIFGSKKSFRISV
FITMAIVWAWSVSVILETFLLCRPLAYNWDTTIRGTCGNRNATYVVAGTMNLITDLLVMALPVPHI
WKLQLDIAKKVALSSVFCLGLFTSIISIIRLVALMAIDFTDITYSVQMGVMWTVIEPELAIICANM
PLLKAVLSKVVPGLFSTARTKYGASGPESFERLQDPQYKNTHVLSRLDRGTMNTHLSTTRGTGERE
GSEESIPDDFSDEQRLHNQPEHDKNGIRVDQNFVNLPTGRKKRRIMTERAPLLSDCKICIACSNAL
EEDSVMQLDISISEHGGEVVIHEEPAPFPPVEQFTHIISLTADFPAYQSACDALIPVVKPQWLQAS
IARNKLAXPRQYSPDPRMFFSDVVVTCGDIPDGDKDAIIGGVMAMGGLYSPRVTSQVTHLVDLSME
SEKAKLIVSKSLGVKVVLPHWFDDCLKLGRRIDERPYTLPNPEILSAGPGVPIRTFGNRDVVGAST
PEPTALPTPSRSPNKRSLNVLEGKHIMFSSDLGIGKHLLESIEDLVKQGNGTVTHDLTKADMFICR
YREGFEYRTASRLNKDVGNLSWLFYLITHNTWTSPLRRLLHYPIDRQGVEGFKKLRISLSNYAGEA
RAYLENLIQAAGGECTKTLKQDNTHLITAHSNSEKCAAAKEWGLQVVNHLWLEESYAKWKMLPVSD

PRYTHFPKRTNLGEVVGQTKLDKAVLERHFFPADDKADESRVMQQKNQNVAANVEVTKTVSPQGRA
KSVAKSSPAPSKTTATITPQTNKSTRKTSNLADAKTPIISRFLADEKENVTPSTTXSRKSKEAAAA
RIHDIAPDIALYEKEMKRVGGVVYGGRRKTDPDLVVPAKNKKRRSAEPDEESDVEEKDAKRQKNAK
PPVTMQLMITGFQRWVGNMKKEDADKRQLRNLGIAITQDAKKCTHLAAPSILRTPKFVNALAYAPI
VVHIDYITECLAKDELLDPDEFALVDRVTEKKAGINLKKTLKRAAANKNKLLHDYRICCVETIRGG
FDAFKSIVEANGGECTLFRGRVSMNDRRRESGSESSQDGKGHPMKDDVFLISGDEPEHVKVWPRFR
QLVLEAGRTPRIVNVNWLLDIAMSQEIGWKDEYEYTDRTA
>g1668
MTTNSPATSYLSAPTTAANVLTPASTISSREPSNTSASTRPRTVSPSGYTFPDVYDWPAFFTLQPN
TQTRQAQMRRWANLISDWCRYHRTFRLSLTEAVESPLFYNAKMRRRVGIQEAKTIVDWMVMSQEDG
GGGRRAEWVPAAAAGGSEKTIAWIWWRRPEEWAKVIADWVEETAQKNTVLTVYELIHGEATTSQEF
HGMDADVMLKSLNVLVKGGKAQVFGNDDEKGVKFF
>g1669
MPSKPVRRRDYLNTQKTLRAEGSTIDLPKKRFYRQRAHANPFSDYALEYPLSPAHMDWASHYPAFI
DPDASKLTLSGTRKLLKDVEVADIGCGFGGLLIGLAPVLPETLMVGLEIRGQVTEYVKVRVAALRN
QQLKLRTASSSTSTPLPTAEEPEITSNENHEDLPEDAPALPDSNQQAQTLIPGGYQNISAIRANTM
KFLPRFFAHHQLSKIFICFPDPHFKARKHRQRIISSSLNAEYAFVLKPGGLLYTITDVEEYHYWVL
RHFGVLDHANPATSENGAETTAQEKGSAEEDDEDEDDENSGVARVRELFERVSDEELAADECVKVM
REETEEGKKVARNKGPKFVAVFRRLPDPDWSMS
>g1670
MIHRNRMKDSSRKVSPPGPTYMSDDQVASYLKDLRSSRPSRPNGSRPLPSRSTTSVSKVPEEPHPR
AASALSMNEWRDSPANGPSPTDPYPRSASAMSNSRRSSDMNRGSSAGRPLVQQPNGYSVDSSYAPS
ARITSQYEGPVSPGAVYRESGSRWMEKQEARSLRDALQEMDLQDEERLHSAAQDEATKLVWEHQNP
GAAYKNPHAAYRNPDLHGTNRFRQHLEKGSHARSQTVPGADTAYNHRKSLSESGSHEEAQSPTSSN
ASTKDSIKKKGRVNFALPSEDGSPETHRVAPRTRTVSNESSKGIFRNPEDSIYEEPEDSSMQTHGQ
TSSTNVRSALKVKPRNSLPQGANPPVRIRNSPFDRKNKVDIYKNPPTQTRNPLYTTNAPIQPNRES
KKKDEAEEQEEEEVRMKNGIEVRSDDIRAATSMRLKDRSEKLPMPTAVSDRPGQPIVSFDPSWEPP
EEKNNSRDGPPVINVVDASDGVPTINFPDDPKEDVPSIAVEPPKLETAGATESVASRRRKFDNPQG
MQPTSQGKWYSPFTRAGVPTATCTNCVLPIEGRVVTAAGSRFHPECFSCYHCGTGLECVAFYQEPD
AKREERLSQASPEDDEARVLRFYCHLDFHELFSPRCKSCKTPIEGEVVVACGAEWHVGHFFCAECG
DPFSQETPFVEKDGFAWCLRCHSRRTASRCLGCKHPVLEDVVVTALGGQWHDKCFVCHTCGGGFGP
EGKFFVKEGEPKRTAKGRIIGGPVQLAVCEACEARRLKA
>g1671
MKSLRRDFSSNPQAQNVSSKVFIRSTKSGKVQKIVRELYLRQDIPCSSQLCTICATYAPADASGNI
APFVLSDRPAGTKAFPQGHYIIPDTNALLNGMDVFEHTGAFYDVIILQTVLEELRNLSLPLYNRLI
SLIKSDEKRFYLFFNEFRLETYVRRQNDESINDRNDRAVRAVAKWYTEHLRQAAKGKSIPAIVVLT
DDKENLRKAKEEGVVALSLAAYVSGLEDSDTLIDMINDSREERGKRTEFFYPEYYTMSKMMTGVRA
GTLHQGIFNISPYNYLEGSVKVPAFEKSLLILGRDNSNRAVSGDVVVVEVLPKDQWKSPSSKIIDE
EAVTKNENPDSEETEVIVNEREKRALQEEVKKAHGTVAEGKAQPTARIVGIVKRNWRQYVGHVDAS
STSLQGNSGRRQQTVFVLPMDKRIPKIRIRTRQAAELLGQRILVTVDSWDRDSRYPTGHFVRSLGE
LETKAAETEALLLEYDVQYRPFPKVVLDCLPAEGHDWRVPTNMDHPGWQKRRDLRDLLVCSIDPPG
CQDIDDALHARLLPNGNYEVGVHIADVSNFVKPNNAMDLEASLRGTTVYLVDKRIDMLPMLLGTDL
CSLKPYVERFAFSTIWEMTPNGEVVSSDFTKSVIRSREAFSYEQAQLRIDDASQTDDLTQSMRTLL
HFSKILRQKRMDAGALNLASPEIRIETESELNDPLTDVKTKAHLATNSLVEEFMLLANITVASKIY
SAFPQTALLRRHASPPPQNFEQLAAQLQKKRGLNLDVSSSGALADSLDKCVDPKNPFFNTLVRILA
TRCMTSAEYFTAGQHAEAEFRHYGLASPIYTHFTSPIRRYADLVVHRQLAAAIGYTSAEGLSRRSQ
LEDVCKNINFRHRNAQFAGRASIEYYVGQALKARGEMMAKNKGNDKNLGVDEEAYIMRVFENGVVV
FVPRFGIEGIVRLEDFMLPGEKVLPNVHDPEQMREVAHRRESEYDAEEYTLRVWDKTHPETERSLM
VELFERVQVNVSSVKEEGGRGAGKRKVRMLILGKV
>g1672
MVKIARDRAAMLNSEHGTQPRSVSDALFLPEATDYIASSSAESVSLVRSVQQSEFVLGLQKEARNA
KFPINVGIHEPAQGGIKVKNTLIWIDEHGEIVQRYQKIHLFDVEIKDGPTLKESASVEAGTQILPP

FETSVGRVGLAICFDLRFPEISIALKRQNAQLITFPSAFTVPTGQAHWEALLRARAIETQSYVIAA
AQVGAHNEKRRSYGNSMIVDPWGQVVAKLGGEFKEPEIATADIDFDLIDKIRKEMPLLRRTDIYPE
V
>g1673
MRQATSIALRSRCLLRTPQTSRAFSTQSGLRSADHGNHYDPPSGWLFGVKPGQKYVKEGWENIWYF
GFIGSLVAAGVAYVFKPDTSIQTWALEEARRRLEAEGILEDPEKAQK
>g1674
MTTKQNPYVSASSKSKLNNFQYVPTNNDAIPKKSSSKKSAQAIANADKENHKSWINGVMEPPKSPV
KSPSKSPGKALPSTQTKETKPLSECPKTPANRIPLADLISNAEDVMVMPSGQELTPDDYVSWQPAP
PRSSATRRGTSSRSKKRRHSSSPTSSPLKDGTSKKPKGSFDMKNFQRLLKTPQTDMATDLWNKYIG
QSKLEGTLDIPTKFTNLPSSPQTPASQRTGRDSSALRRSVSCNVDWPSSKTKRRRVEGVDGARTAR
DIFSRSKSNVLDSGSSTKLSFLLNKIQDTLASTKPNPKIMPPSSPPPPLHDYVREDLSPSPSKKQQ
SKPASRRHIPEVTMEEKVPWDRPQIIQKTHNLKSSSSDFGDDDLDDDLLALVNSSTSPIMDSGHED
NAVVDNNAPQHWLVEQSNQPDLPKYEQPPSKDDDDDEFDDGVEFGEGIEELLTQYEKQAPEIQKQA
LPQPSRNLSHEQPMMKVSPDTITRNAGISDENSLSDDEFEDDLDLNNVLQEMEHQSEKPKAKQAIK
RYLIVQIAESTYTTQKGFEQPEQVLSVMDEKTSTQKVIILRESWADSPCTKNSYIHLIGDYDHKDQ
CIVDNAHNMIILHPDHLISATVVADAITCQRRAVLQDRIKATSDINKPQVYGHILHEVFQEAMKVN
QWDLQSMRTLVERILVQYVESLYAIHVSMIEAVEYVMSKVPELKAWADIFLRPKPDAECMVEDRNG
SKANLSINKLLEVEEHVWSPMYGLKGNVDATVQVAYNDGGNQKTLTIPLEMKTXKKDXSHSHRAQT
SLYTLLLSDRYDVEVTFGLLYYLEMKKMFRIRGIRHELLQMIQERNRLAXFIREKMELPPMIQRKH
LCNQCYSKTACFVYHKLVDDGNGETSGLGPKFDEVVGHLDARHSEFFKKWDNLLTLEEKNMMKFRR
ELWTMTSTEREPLGRCFSGVVIEPGSAYEDPSGSKINRYRYNFVKSKTTPGFSFTESQITVGEPIV
ISDEKGHFALANGYVVRTSPKRISVAVDRRLHNARVRCKDFDAERNQSFKGIMEVGQGSSSEYPEE
QMVYRLDKDEFSNGMATIRNNLVSLMENFSMSIPLRKVIIEQKTPEFKEPSSSAEAIFSSSQANLN
IDQKQAIDKVMSAKDYALILGMPGTGKTTTIAHIIRALVSQGKSVLLTSYTHTAVDNILLKIKNDN
IRTLRLGAVAKVHPEVQQFVDLAAILKKTIEELQESYQESKVVATTCLGINHPIFNERIFDYCIVD
EASQITLPVCLGPIRMAKTFILVGDHYQLPPLVQNKAAQEGGLDVSLFKLLSDAQPSSVTNLEHQY
RMCEEIMLLSNTLIYSGRLKCGSFEVASRSVKLPNLQNAIKTHHVTSLADIASGRCPGPTRSRCWL
SDLLSPAAKTLLVNTDTLSNRAPETLVGSRLTNTSEAILCVQLVQSLISSGISPRDIGVITFYRSQ
LSLLRQSLRQHSSELEMHTTDKFQGRDKEIVILSCVRSNGEKQVGDLLRDWRRINVAFTRARTKLL
VIGSKSTLRDGNELLQKYVELMESKQWVYDLPRNALQDHVFESLEDGAGMFSPTQRRSIQYNNVKT
SPSPIKRSMNDIRMTPDKNLKRKYINGPGSPTGPGGKSPLSPAAQQRKIIKREKKMKTPEKRGNRV
PGVEKLVHKRPVLQDLLNEFMG
>g1675
MTSPPSIAIIGAGPAGLLFARLLEENGLRDYVVYERDESSAPGLWQQGGSLDLHGPSGQLALKEAG
LLDEFNKVYARWDASKIHIVNGHSGETIGCFGEGRDAPEIDRLQLRQLLLESIPAHKVEWGHGVRS
IEIKNPGNSGDRDNGCIIHFTNGSSATGFKLIVGADGGWSKVRPLITSAKPVYSGKMYIEGQLSHN
NPVYRTAKELAGPGTMMALGPCKSLALQQGSDGTYRMYFGLVVPEDFYNHRSGNAVEKTEAVRELM
LSSDKFFATWAPQLKSIAANVEGPFRAWSLHHLKADEIGWKRDTAPGVTLLGDAAHLSTPFVGEGV
NCAMYDALCLAKCLVNIYEEGLGFVEASLDKALLSYEEEMFERGRDLIRRSAESEAVLFSENAMEG
VLGFVDGEHEELLYDVKASSDK
>g1676
MNPTFLPGNSLDLYCNASENDPNGFFDDLFTANGDLTGLEASFGSDRLPGISWDDYHLITEDQLPE
GAPYFDMPAGNGPGFSNINTSGAPPTFNVTAGSADIRSGAPSTFNSGAESALANTTIGSPIHTQNA
GINVSGSLPMSNLAASGALSPFDFTAGSANVNMPIDLQTPIEQSSFQGFSYGAFLDGTNGGAYMLN
GGWVSTCQQARQPIGIHAPLALPNTHLKISSSPNTPPTTPQRRPSLAAMPDTYSKNSSNTSSETLS
PPKRSQPSSPRARRRPKRRKGAEDTLVFVHDKLSMSTDFYPNPDNHGRFEYSTTAGWRYLNAPGAA
EKRREERQRHQEGN
>g1677
MLDFVDGASAHEKSYITIGQILEDANPESKKAPFSIISKHTLVHTTEFILPRDVYTATWSSIQEKV
ETPRYIQVIMPLIALIEGDFFNDFVKPGSVSMLSEGRPGVDDLFSLEQGILTLQMSREKYERTGLT
GKPIRSGGRKHAKERFVIELDLRQPSMLHGKKGFERIVWACRNVLNASVTWLLACNALSQTSLDKA

AAALQKYQPRLLDCHLETIAHPVVNVPPLSMDEVRSQSSPADLEDYCNDVSEWLGLVSLQSPRISV
DDVIDTYLSRYAVPRSESLQPQSTALLFTSYLLERRLHTDKLSSWSALSASTFRRVAVENSDGYTV
LSMAESTKPREIGFVAWEFVGASLSA
>g1678
MASKQPDRRLIHKDRFGYVFGNLKTQNYQDPVARGPGSHTIRTLAWNPTGTLIATGSANRTLRIWN
PERANARYSTELRGHTAGIEKVIFNPVRDAELASCSSDGTVRFWDVRSKTCVSKLDVGGEAFTLSW
SADGRVVVVGRKDDTLVPVSVESLSTPAVSNGASSSSVGEQKAASTYTILPSHPQNTQTNATTFSY
HIPTSSAPDLDFLATTGDGTVRIFSYPSFNLQHTLNAHSSACLALALAPTGRYLAVGGSDALISLW
DTTDWVCRRTVSSEGGGTIRGVSWSFDGRFLCGSSEEVGPNGLEIFHAETGDSVHKIAASAGVPAV
AWHPTRYWLAYSLMAEGSNAGGLRIVGAAGGGL
>g1679
MADQDDDYFNDLSRQSMSQDAIQSQSGNTGGNTSPTEDDGNPVPRPKRMACIVCRKRKLRCDGQKP
SCGTCARVGHNCAYDEVRKKSGPKRGYVKQLEARLAQVEILLKNQEPENSWNETSRNALPNTSMPP
TASNPLSGVGNLMSFVNGSTTRVPDAMRQPQGSQNGASTTNSTFDHESSWEVISLGLEEPLPTQDV
IDELFDIFFTKIHPSHPMIHRPRFFASMNLAPHMRPPVCLRYIMWALAASITPKYSFLEEHLYARA
RKYAHLDEMKGHGEHMVSVSHCQAWVLISLYEFKQMFFPRAWVSVGRGARMAAMMCFNRLDGVGLD
VKHCVPPPVDWVEREERRRTFWMCFSEDRYASIGTGWPMVFDERDIMTNLPSSEEAYLTGTPETAP
SLKDVLAGDIANLSPLGSVAVMACIFGLNLTHLHRPDPHDREDDVNGEFWKRHRAYDNILLNISLS
LPHSLRLPQGIADPNIIFSNMAIHTSTICLHQAAIFKAEKHKNMTQIAAESKRRCIIAADQISSIM
KMVSHTDLSLLNPFSAFCLYVAARVFVQYLKSRPEGQAVKSSLQFLLTALGALKQFSALTESFLIQ
LDVDLSGTTFGSSMRSMRSKEAVESNTGSNQYCNPIANIRSSEADALAEVNLNRDAAANNSSPVGT
SASSQSFGSLPNRQRSNNTPRSDQQTLNVSRNTALDSTDSNNTSSQQDDNAMLTNFDMDISPVVSG
GSENMHSENASPQTMNSSSNHSNPAFTPPSMEQTSMGGQLNNVHVKSPMNPSLIDFSTGLDGFSMN
GNVSFSPFFDSNAMDTGINAIGMENPLPLNDGWGGQQQNPGVANNGLGDMMGNTNNFSDRQFDLLL
QNMGWNGWNDQA
>g1680
MAVSFQPRLFLRALQLPIRGVSFSSFRSYSSVIQETERPPVEDTSSFDPSIAFAPPPTRDEAGVLV
RTYKPRTPGVRHLRRPINDHLWKGRPVHRLTFPKRGHAKGGRNHSGRITVRHRGGGHKRRIRMVDF
ARNDPGPHVVERIEHDPGRTAHIALVRAKSNGKLSYILAADGMRAGDVIQSYMPGIPDDLWKSMGG
VIDPGVLAARTAWRGNCLPLHMIPVGTLIFNVGLRPGKGGQLCRSAGTFATVIAKGTEQQKNEERK
QQLEAEAGTGETEQKRITQRDRQKTVRDANHVTIRLQSGEVRLIHKDCCATVGVASNPNYQYTQLG
KAGRSRWLNIRPTVRGLAMNAMDHPHGGGRGKSKGNVHPKSPWGIPTKSGYKTRPKWKINKAVVVP
RVRNQGKRRRGYN
>g1681
MSSFTVLQARSLRQRTVTNENDENATSTRLTRAKAAALTSTDLNGGAIKKPLQSKRANATTATTTV
AQKRRPALGDVSNVTKTDLVDAKDGKKATSKASLTSKSTTHAGGVQKLSRSNTTRAALGVKDTNKK
AASTEVKRPGSGSGVKRSSSQKSIKDDLNQEEPPRKKVDVEKKTEFIQKQIVSENASEVGVDVMDE
KDLEAEGVLDLDTEDLDDPLMAAEYVVDIFDYLKDLEHETLPSPDYIDHQPELEWKMRGILIDWLI
EVHASFRLLPETLFLTVNIIDRFLSAEIVSLDRLQLVGVTAMFIASKYEEVLSPHVANFSQVADET
FSDKEILDAERHVLATLNYNMSYPNPMNFLRRISKADNYDIQTRTLGKYLMEISLLDHNFMPYKQS
HVAAAAMYLARLILDRPGWDATLTHYSGYTEEEIQPVFLLMVDYLRRSVAHEAFFKKYASKKFLKA
SIVTRQWAKRYHSLYLDVESEEAPKK
>g1682
MECVIRFAQSHETFRQAEIEALASLAGVDVEFVLYDKYSPFCIAKFSDLEAARKVISRSVLAQDVF
ELWGRGATHEELHADVRHRTEHRWKDYRDVSFRFLIDTFSVKRSQAEKRSLIESFAYLDLQGPIQL
KDPDELFVILEDYVSDTEEIQQQEQQQQEQKPPKQGKRKDLKNVYFGRWICASSRTMMDKYDLKKR
KYISTTSMDAELSLVTANMAHAAPGKLFFDPFVGTGSFLIAAAHFGAATFGADIDGRSFKGRHKIT
KENPMGLLANFQQYGIEDKFVDALMSDLTNTPIRDVPFLDGIICDPPYGIREGLRVLGVREGKSKE
PAYKDGVLAYTQDDWVPPKRPYGFVTLQHDILNFAARTLVVGGRIAMWMPTANEQNVEFIIPTHPR
LEIASIGVQTFSTWSRRLLTYRKLPEGEVSSVSRIKSATDDPNGVTANELNEFRRLYFATKKPAKS
PTTEQI
>g1683

MDLAPGPDATETLKSSVVELNVVEAADDTNVDDDSTPSQPTIQKSKSPTSDADDISDESCEGEEWE
TDSLYEETLHFVRDDQLSTVPDACTPQEATAYRKLLREIGKVAFVEETIGKSTITAKKLLTAFGVV
PPSFLEDAPDEAYHPLLGIVISREFAKRQKLDEYNSIDDAVDLLKKAKNVIVITGAGISTSLGIPD
FRSKDTGLYSRLEHLGLSDPQEVFDISLFHEDPSIFFSVAKDILPTEKKFSPTHAFIKLLQDKGKL
LTNYTQNIDNIEANAGVKPEKIVQCHGSFATATCTKCGFKVPGDAIFDTIKSGKIPLCKPCKEQIA
LRPQMLKRKRSSNGLQTSRKKGTFDDDSDEEDYDIPTPGVMKPDITFFGEDLPDEFGRRLVEEDRE
VTDLVIVIGTSMKVAPVADVPSIIPSTVPQIYISRTPVSHIGFDIDLLGDSDVVVSELCRRAGWDL
KHSMIPEDQKVEIIQEEGYVSRHTFRVV
>g1684
MGNSRTKNKKNSAKSEKSVLHSVGSTVSKRKMADSSTELLEKATILLQTGQAEAALPLAQRVIEIS
DENSPKDTLLAINLIAEIYVELGEIDAARESFLHAVQLDPEGIIPESQGGGAEKFLWLAQLSEQGG
ADSVAWYEKGVSALRHIIQTLEHNGGTDTPLVLEEKRKKLAVALCGVVELYMTDLSWEDDAETRCE
TLITEALLVAPDQPDCLQTLASVRISQTRIDDARAALSRSLELWQDLPPEDPKIPDFPSRISLSRL
LMEVQMEVEALQVLERLILEDDESIEAWYLGGWCLYLLAEKEKEESSEEKRHESMVASRGWLKQSL
KLYQKLEYEDERLRDHALELVQELNKELGDDMDDDSDDDGPVFPGAEGEDEEWDGEIEAESDDDDE
DHEMEDS
>g1685
MGREEQVEEREVLDSIFPEEITDISDTSYRITINLDLDEDEENDEAPILLLQVSYPEAYPDVAPDL
EILTPPNSAKHTRFDIQEDKARLMEAIQPSIEENLGMAMIFTLYSTLKEAAETLIMERVASERQAR
EREIAEAEEEENRKFQGTLVNKERFLEWREKFRKEMEEEEQREKEEKEAEEKKKKPSAREEQKLTG
RQLWERGLAGKGDYDEEGEDELASEGVEKLQISA
>g1686
MHAAAQGDSHSILRINQLVRDIESRPTPKRNEARIPTKPGLREKKRLENIAFQIQTMYRHPDVEPI
LSRPRLSVAGQRQVPKLVNARGVPFLRIKKPQPQNLSRIIRYKLRFKDKLIERRDRLLVETLFAKD
EEDWDRLLTGQTLTEKVIHAQNHLAWVEGTWLESPLECYKKAYYDNIEFEKKQQALAEKMWEVILA
ERKLAAEEEAKKSGLSDGFWRPPLIGVWKSLIRTLRAKLFA
>g1687
MAKVPRNFRLLEELEKGEKGQGAEACSYGLADGDDITMTNWNGTILGPPHSVHENRIYSVNIQCGP
EYPDSPPTLHFVSKINLPCVDSRTGKVDLSTLPTLATWKRDYTMETILLELRRFMALPQHKKLPQP
PEGTTF
>g1688
MSSQILQTELSNLIQESKRKYSDLKNAAEQSLNELKSLPSTSEAQIKADLIRRPHFVTPFILACQT
RQSKLASIGVVCLQRLATSHAISPARLNDTLSALGDITGLNQDVQLKILQTLPALLHNYSDNLSGE
LLAHVLEVCATLQASKVAAVSNTAAATLQQLISSAFEKVLVEDGLQNALTVQTQVGGSTVDIGVAA
HDALQILSDLCRAVEGEKLEFLHIKALPTNFVLELIESILINNIRLFLNHPEQMQLLQTRLLPLTV
KHLSERHNFPPTLRVARILLVLLRNFMSLLQDECEMALGLLIHLLEPEASPEWKRVLCMEVFRSLH
SEPSLIRLIFTLFDGTQGRKDIVKDHMTALFKIASESPSLIGVSHQSTVPQDASQTRSNTDDQITL
EAGGVDGVIAAAVNSADESVTGISSQWSTVRTPYLELLDKSEPPPPPVSYIYSLVLNCLSALAEGL
ARFIIPLTVTESKSKRRKRIATNDREQGSNDLHRSDSTKSNGSESRRQTVPLNPLDLQEHPQRSAI
TSSAGIIESCWPVILTTCSIFFYAALDADFYHNLVRSFQKLTHVAGLLRQSAARDAFLSTLGKATL
PVDDVTAHLPSTHAPEAKQSRNSDAGHEQSLAAHFDTPKASFEATRASLSTRNLLCLRALLNLGIA
LGPTLDQSAWSILLETLQNADLVINASATASGKQTPTIGNGEQAAQSGNDVPRANIGNEILAVSTA
ASKMFESTADYPDPSFQAILEALLGLLSAKGPGALPTALSPVKSANQHFANSRIQDDEINLVLDRT
KELCEANIERLSVVGEDEDFAWITLNKNLKSIITSGTHHRTLRLKASGILNYVILQTMKHGWFEDD
SRNTLQLRNLQSLQSQISSLYEHRDKLTSVDAEAHGQALDTLRVMLEQYGEAFTSGWDLIFELISS
VFEKPETEQSETSFTRKSLSKARNFKARSPRLVRVAYTSLQLIASDFLTLLPPSCLLELVDSLSFF
ASQNQDFNISLTTTTFFWNVSDFLQSQVGTFSIEESMDTSISEESLASLTDDDDISTSRGALWLLL
LLRIVDLTTDDRSGIRNSAIHTLLRILDAYGPQLTPKTWHLCLNRVLFVMMVDVQNKALEIESSAD
GGSDAVKEWIETSVVVIKGSADLIATFFDIIIQDPRFVNSWKRMLECFQALVRINNYGIKEAIFAS
TYDIVKHATSSEFTKEAIECAWLLWTNNHLAPEGAQLNLDEPNQDALIAYFRFFQQIYRLYRDELS
KELVAQVLHHVRTAIYNAVVSRYSVDIDHQSTLQELVLDCIKTLCTDKDESQSDIVMCLADFSDAA
LTQWSTDRDKSRPSYVAFSKSAIDLLSWYIAEHGIKADIFSDGTLTTALQHLAHPITRKYEWRGKD

REQLIWQKATTAAIKIIRVAVPYVESKYASSQKSLITSFWSTVIDITRGIISADGYDDFALSTSRI
EADEDFDIDSFKTLIPLLIPSLGASNVPDSTRRDLACALFRSSLIYFPQRMDTPMETLLENPLHDL
YKIRRGRTYDPSPTARSRIADVLIDTMFTLAEASSLSSTDESQNASRIAIARSILPYLILRSFLPL
KSYIADQPLRGLLPQPAPAHRELVHILDRLFTLKSEPAAIPDAPCGIVSSPQSTSSPSSKNDKPGS
RSTFHLAYSKHLGWVYPLVIKCVRVAGKQKGRGNNDVVVLHHLTRILDAVCDDEYEDEDVV
>g1689
MAPFFFSTPVDIDVVLEDSDERQTVDMKLDKGRREKAPLYMDGESVKGAVTVRPKDGKRLEHTGIK
VQFIGSIEMFFDRGNHYEFLSLVQELAAPGELQHPQTFPFNFKNVEKQYESYNGINVKLRYFVRVT
VSRRMADVIREKDLWVYSYRMPPETNSPIKMDVGIEDCLHIEFEYSKSQYHLKDVIVGRIYFLLVR
LKIKHMELSIIRRETTGAAPNQYNESETLVRFEIMDGSPSRGETIPIRLFLGGFDLSPTFRDVNKK
YSTRYYLSLVLIDEDARRYFKQSEIVLYRQAPEIAPTPEVAQQQLIQKQQLQRQQQVLVGPSKNPQ
YHQPPPQAQPQGQAPAPAAAAAEASSSLNPTPATEAQAQA
>g1690
MLSANDKPSTTTSNGLTSNLYGGCTAQPQSRHTGPKAYMRNHAMNSESSLQHIWIVTGPAGSGKTT
VAKNLQAEMGLPFLEGDDFHPPANKEKMANGIPLTDADRWDWLISLRDAAIDILTKPPSTETETTA
AVDGTLPPPTGVIMSCSALKLKYRDVIRVAEYYNPSLRIHFIYLKADEKTLMERVNGRASHYMKGN
MVHSQFEALEDPSDEVDVLAVDVSVPPDVVQRSVSDVVKKKLAEYNS
>g1691
MMRSLGLSRRLLCRSVHHGASPIAPAFLNLARHRRAFHGSSAFWGIRSQVLKDVGEGITEVQIIQW
YVEEGAHVEEWKPLCQYQSDKAVDDITSRYAGVIKKLHFQTDDTVPTGRALCDIEVDDALYPDDNV
PTQAVQAESAPEIAESAAIGETTQTTEAQIDVAPEQEKKSQSKHASFATPAVRGMLKTHNLNILDI
TGTGKDGRVLKEDVLKFVSQRDQPKSNGAASSTDFSSTPTSGLRATASNDSQQVESTQSLTHIQSQ
MFKTMTKSLTIPHLLYADELNINTMTALRRKLASNPNNTQKVTSLAFIIKAVSLALEEYPILNAKV
DASDPSSPKLIMRANHNIGIGMDTPQGLIVPNIKNVGAKSIFEIAAEISRLSALGKEGKLKPADIT
GGTITVSNIGNIGGTYLAPVIVPTEVAILGVGRSRVLPVFDENGQVTKGEMVNFSWSADHRVIDGA
TMARMASKVKEFVEEPDRMLIRLR
>g1692
MAQALTIRNFTSTPLELKVIERFAPQEKRKEDGLQAFGTMAKNFTRMVTDAATNVTRASNSAQPVP
QDAQPFAHLDTNIRVDPFATNKTDVKTFENNDKERLRLTFEVDGERHVITVPVPTVESAEMRCLTD
NPRHRFTGVYVTQEGYLAIYSSAQLNAWMRELRDETMLSSLSIPGTHNSPTCHVAPPSVRCQAVSP
KEQLENGVRFFDIRVQPQSPDDPSKDELILVHSAFPISLTGNKYFRDLLNVVNEFLDRNPSETLII
SLKREGTGKATDEQLSRIIRDHYATPESRWYTDPRTPTLGEVRGKIVLVRRYNLXEPQKGEHEGRG
FGIDGAGWADNTPNATCPGGELCIQDFYEVLETQNIDKKIQYVNEHISRAGAARYPFGRGDPNQKY
PFFVNFLSASNFWKTQTWPEKIAAKLNPATVKYLCLDHCNHPDGDWGTGILVTDWVGLDGDWDLVR
CIVGMNAKLKLREN
>g1693
MPPRLRLSSSHLSRPVRTQKRLGQQNGMIHSTRYASTATAAAASSATVPPPSLEQMKKSPSRIART
PSVQPPSHRRPEERRSQLLRQYTSLIRTAPLMVFFQHNNLQSTEWTAIRRELTLALRKVDEQQASE
GRSTTPIAPHVKLQIIQTSIFEVALRIVDYFKPENFADKIAAAEAEEDILLHDLSRTVHGAVRHMK
GKHDLSTILSGPLAVLTIPNVSPEHLKAALSILAPKASGFSAPSRKANPGYHELTVQDGLQKLMVV
AARVDGRVFDLEGTKWVGSIESGIDGLRAQLVYALQAVGAGVTSTLEGAGKSLYLTMESRRSVLEE
EENGGEKKEESS
>g1694
MAEAPKPEPVSISPLLQRLAYPDVAQIQVSAEEIAAAFALIFEDRLSLIQTAALLTLLHSTGKDRD
AKVIALCSHRMREAASTVDRPALKAVVKARGKPEGQYKGGLCDIVGTGGDSHSTFNVSTTSSIIAS
ALLLVGKHGNRSQTSFSGSADVLNCILPVPPKISAIKANDIAKVLEETNYTFLFAPNFHQGMKYAD
PVRRGLGIRTIFNLMGPLANPVDWALEARVVGVAYQSLGPIFIEALRLGGSKKALVICGEEDMDEV
SCAGPTNCWRLSEYPNPAYKPENDDDEEDDDEDEEDSNPRTLAKLDIFQLHPRDFGFPTHPITDVY
TKKMPKQNAEKLMSILRNELDPEDPLMHFVLINTATLLVTSGVCEAETSNMGPGDDGKVITERGPG
GGRWKEGVRRARYAITSGQALESLEKFIEATNRL
>g1695
MSREAYQVPALDNQNAFGNSAGAGGAGGYAQPQVQSVRYICGECDAKFSLPPNATLRCTECGHRIL

YKERTKRIVQFEAR
>g1696
MSAILSADDLNDFISPGLACIKPVETLPQNSTTSTENAYEVTTEDKIQPENQAPTQISLTDCLACS
GCVTSAEAVLISLQSHAEVLNTLDAHRPPPLIVAEDGNVSVSRKVEPDEKIFVASVSPQVRASLAA
TYGISERKAGYMIDQLLSGPQGLRGGGKHGNGFTWVIDTDVLRQMVLTLAAQEVTETLQTSFTKSG
SGKYTSPKKPILSSACPGWICYAEKTHPHVLPHLSRLKSPQALSGTLVKSILRRNLGIAPSQIWHL
AVMPCFDKKLEASREELTDAAWNTGSNHPVRDVDCVITSRELLMLASSRNISLPSLPLKSLSPTYA
PRFPDSTLDSFLFSRSQTSKQSSAYGTSGGYLYYILSSHQEKHPGSRIAVQRGRNSDVIEYSLLSD
SNETIMKAARYYGFRNIQNLVRKMKPARASRLPGANRRGAAGSGASSSGLDYAFVEVMACPGGCTN
GGGQIRLDDAREANASLHSTTSNDPAITTQKHTTHEQREWLSRVDEAYFSAESDSENEANAAAAAA
DNSLCDMELRTQSILQHWTKLTEVRLEDLVYTTFRKVESDVGKDKNVNDTTRVAELAGKIGGGW
>g1697
MPELAEVARIVHFIRKHLVGKTLANVQAQNDDIVYGKVGTSAAEFQKAMQGNKVIGAGQQGKYFWI
TMSKPPHVVMHFGMTGWMKIRNADTYYYRTNNADDKEWPPKYWKFLLETDESPKTEAAFVDPRRLA
RIRLVDCPADEIRQYTPLKENGPDPVTDKDILTVEWLQDKIKSKRVPIKALLLDQANISGIGNWMG
DEILYHSKIHPEQYSNTLTDEQIEQLHTSIDYVCTTSVGVLADSEQFPEEWLFKHRWGKGKKNQPS
VLPNGNKIVFLTVGGRTSAVVPSVQRKTGPVAKEISDEDPEDEKEKKVVATGKRKRAAVKEEEDED
DVKDEVVALKRAASKRGTRSTRSGAKTSTQGRVKKERD
>g1698
MPQQLGSKDSSLFRQVVRHYENKQYKKGIKTADQILRKNPNHGDTQAMKALIMSNLGQLEEAFVLA
KTALKNDMKSHVCWHVFGLLYRAEKNYEEAIKAYKFALRLEPESQPIQRDLALLQMQMRDYQGYIQ
SRNTMLQARPSFRQNWTALAIAHHLGGNLTEAENVLNTYEETLKTPPPRTDIEHSEAVLYKNTIIA
ESGDLERALEHLDTAGKRCSDVLAVMEMRADYLLRLDRKSEAETAYAALLDRNPENSHYYDGLIKA
KGLADTDHQALKAVYDELVEKFPRGDAPRRIPLDFLEGEDFKRAADNYLQRMLRKGVPSLFANIKV
LYDRPAKRDTVQELVEGYAAGKIEPQTNGSSESQKPEDKTSFDASTFYFLAQHYDYHLSRDLQKAM
DYVEKAISLVPKSVDYYMTKARIYKHYGALPKAAEVMEEARKLDEKDRYINTKAAKYRLRNDENDK
GLEDMSKFTRNENVGGPLGDLHEMQCVWYLTEDGESYLRQKKLGLALKRFHAVYNIFDVWQEDQFD
FHSFSLRKGMIRAYVDMVRWEDHLRDHPFYTRMALSAIKTYVLLYDQPDLAHGPIFADMNGVDEEE
RKKAIKKAKKEQQRLEKAEADKQELKKASAANAKGADGEVKKEDTDPFGNKLAQTTEPLKDALKFL
TPLLEFSPGDIRAQTAGFEVYIRRQKYALALKCLLAAHAIDSSDPTLHVQFARFRLALNNLKEPLP
EKISDVVLKEFEAILPKSTNLNEWNDTYLTSHKENVDHVRAALSARLLIAPNEKSACEKEFVATID
LASATLEKAISGLETLDEWKADAATKQAYKEKAHNKWKEASAFVSK
>g1699
MSENANHNPTSGTASASTTKPPTPPKPQNPVFKMMGLPNFRLRLPSRNWMIFLSITGGFTAAITYD
RRQKKRAQQKWADLVAHISKETLPIEQNRRKLTVFLSAPPGDGLRSAREYFIEYVKPVLVSAALDY
EVIEGRKEGDVRAGLAEKIRERRRQAGEPSTVVQEPSKELIIAETRRRMGVTDEPGPLGDIVIGRH
TWKEYIRGLHEGWLGPLDPPLPPPVLIESPSPISASEVPADISAAEISGEPDSSPISTPEEKPTEP
EKEKEKEKPKGPTPAYISTVDYSSRNLPATIPATFEASLPVPFPHLLGFLNTPIRIYRYLTKRYLA
DAVGQQVAAAVLASASRPYYEGLTNSSDSDVGPSTESFTTETQSQQIYEQQTLLEHEEKEWHKSVR
KAAEARKQQAEEDNAASVSDDIQATEKPGILKEEREWIDDVVIDPRIAARMSRYILAPEEEARAKR
IDEGLEWVLGEGERPKKLPFWQRAWINYGYGEDPEVAKRKPILGNIDNEGE
>g1700
MDPNNNRLHLNFGFNNNNNDRNYNAANNRVYPTTPSAFPQPIYQGQDFMDPHGAQNTGYGQGYFVN
GQYAQQAHYPMQQQQYAQHNLPSPQPAYGARAYNNDGTNGLIQQFSNQDLSASPRPGLSPRAASPA
QRPRTAGSTGPQQHGGHLAPGIANRSSKPPGEEEELQRYPERYSENVHKRGKAAKELINVFFQENI
ERARERNMRSVNLDKLVQAHSMPEAKRRSEAEALAKKESNFLRFLRTRESPSNFQTIKIIGKGAFG
EVKLVQRKSDGKIYALKSLIKTEMFKKDQLAHVRAERDILADSKDNPWLVKLHASFQDSAYLYLLM
EFLPGGDLMTMLIKYEIFSEDITRFYMAEIVMAIEAVHKLGFLHRDIKPDNILLDRGGHIKLTDFG
LSTGGKKTHDNAYYQNLLKNSTSKDKNRNSGFFSDAINLTVSNRGQINTWRKSRRAMAYSTVGTPD
YIAPEIFNGQGYTYLCDWWSVGAIMFECLVGWPPFCAEDTTDTYRKIVNWRECLYFPDELVLSRES
EGLIRSFLCDAEHRIGNEGGQHGGASQIKNHTFFRGVVWDQLRNIRAPFEPRLSSNIDVSYFPIDE
IPQEDTSAAHRAQVRAMPEEQEAEMSLPFIGYTYKAFNAFQAN

>g1701
MDYQNRAGSKFGGGGVASQSATNADRRERLRKLALETIDLAKDPYFFKNHLGSFECRLCLTVHQND
GSYLAHTQGRKHQTNLARRAAREQKEGKAQDGVVPGAMGVQVKKNVVKIGRPGYKITKTRDPMTRQ
LGLLFQLQYQEITPGVVPRVRFMSAFEQKVDDPPDKNFQYLLVAAEPYQTCGFKLQARDIDRREGR
YWTWFDEDSKEFWIQIMFKTEREERYSGVPGLAPRP
>g1702
MSQKFNTGDRLSFDGALCTVRYIGDVEGTKGDWLGVEWDDPFRGKHSGEHKGKRYFTSGSFVRPTR
PIDKPQSFLEALHQKYASEFEEEIAKRKAANNDAGFQMNDTIQFNGKVVEEVGFEKIRKQLAELQE
LKVVLLDGLRIAGVLAYDGQDESRYSEELQKIERTCPKITELDLTRNLIHRWRDVHDICKQLKKLR
SLKLKAVAPMSVTGLPEKPMTVDEAFMLTLARLENLTSLNYSKITQQDRINGELYYLSLIGKELSA
SSASDEARILGAHPRYSALCETYGIPTIIRKSEGVQGSNIKPGSVAARLVTFEFYQMSSDSTSETA
IDAASKLCLIPKTFDTYKIKTIVSRLFSLAPLRFRLVWETEEWDPVEEFNILGGEEWDSDDEGDHQ
HKRGERGGQNNDRTEPTVVKADGSKFVRREVELVDSTRQVGFWFDDNTDRVRVRIERL
>g1703
MAEYWKSTPKYWCKHCQIYIRDTAFEKTQHEATGKHQGNLKRFLRDIHRNKEREEKESQRTKNEVQ
RLRTLVGGKPAGGDASQTRPAATAASAAATPRQASAEDRKKQIAQLAEMGVAVPEEYRREMALAGD
WETISVRRITPAEFSKDDAENKLNIGVRKRKLENEEDEEEAQMVAQPRHKGWGNTTRDYPGAGGGE
DGDLDALLMNTTTVKKRDVLDLKPTLQSLEAEDEAEPGTGENTEKDPDVSDMSKLLKKEEPGIEHS
IEAPPLPAGDAPVVFKKRKNKAARQ
>g1704
MSETQPGHVPNGASPTPTATATATPDATTPAPALSSASAAVAAPAPTPNPAASQPAPATAPAPEPL
AAPAATSAAPASLVSDTAAPVSFPPLPHISTDDFERTTDLTSTVPSAHGTQCASCSIGTGTSTYSR
KRTGANILCAPNSSHERSDADIFFSAGISACPSASGGVYNCTRTFASTFVSTPNDIYASAFICPTT
ASSSDVRSFTVSVSYSGGTSSSVNVTIPVNTERSIIESSADARATSYGITASVYCASNAINGSCDE
GHPVCRNCVKSKRECLGYDPGFRPQPAPSDIRPASSTPQPSLVVNPQENHTNNNPYPSAPPGYIPA
SAQPFAPSVQSDSSIQSFDQSNNNRQGPAEPSVEESDTMSIMASVQDTIDGSMSQPVGTPDLSTPA
LMPLPGQISQVGRQEKVKIQDLLALQGIAPPPPYPIVPIQQARLDQIQTVYLHTYAPAIDRFLETR
WFRDKGLSQLLTNAQLMAQYSALIDAFNDPKLDDIAVRGRVESFEASVVWDTMSICRSARNLVRNG
STEPMQEYDLLTTANRFDILEALITDDHLKDNPLRADLARPPSTNLNPLEGQLRTRSLNFWNCIGH
FLTLHDNEASSAVEIDETLARCRSLLDEIENRDVLYSMAIGRHLGQRLSDFHPRNRPENSSDERNA
NTKLIVAQRFIEDESKDKGTTQVVKRLCGMVHRLWELKNLLLPRA
>g1705
MARPKVLLLGEIVHAGKTWEDLSDIADLITTSATNRTEFIQECKDGKLDGVVAAYRTFGSFAVTGQ
IDEELVAALPKSLLFLAHCGAGYDQVDVHACSERTPPIRVSNVPTAVDDSTADVNMFLIIGAIRNF
NTGMHALRQGKWRGDPAPPLGHDPEGKVLGILGMGGIGRNLKKKAEAFGMKVIYHNRRQLSETMSA
GAEYVTFDDLLAKSDVISLNLPLNKHTYHIIGKPEFAKMKDGVVVVNTARGAVIDEAALVEALDSG
KVFSAGLDVFEEEPKIHPGLLRNPNVMLVPHMGTWSYETQTEMEEWAIGNVRQAIEKGTLKSIVPE
HVDL
>g1706
MTLEDFEKSLAEGHERRESDNHKDKDRSGSHRHHHHHRHHHRDSSRTRESHNHRSDKKDEERRHRH
KRSRRDDNGDDESSLKRRHRHDRNRDDDDLGQGEARESAAKVSAHDLKRDSWMQPPSADDIDYIHR
PDKSAQKQPEAKSLRIDLDLKIHDKELNTHLRDLKEGKVSEKVDNQPAQHEVDYTFGDAGSQWRMI
KLKNVYREAEEKGKKVEDVAIERYGDLRSFDDAREEEIEMDRRETYGESYVGKDKPSGELFQERKL
EQGVHRDLRASYSRDIASPVKEQGKSMETVEPTRTTQHLDATALNRLRAKMMKAKLKGSSDAASLE
EQYNAAAGLMANRKEGDVVVLGIMENRMLAGGTRSEVKPVENRRGRERGQLQENEDMSIEDMIVEE
RRTRGQAGGEGLRLAERIAKDVKFDNDLEYMDDNAAKLAKRVQKSELNLKNTAISEFHKMNRILDN
CPLCHHEDTDTPPVAPIVSLATRIFLTLPTEPELSEGSATIVPIQHRTNLMECDDDEWEEIRNFMK
SLTRMYHDQGRDVIFYENAAHPDRKRHASLEAVPLPYSLGETSPAFFRESILAADSEWTQHRKLID
TLAKSKQGLGKMAFRRTLVKEMPYFHVWFELDGGLGHVVEDTHRWPRGDLFARETIGGMLDLPPDV
IKRQGRWSRGGDRRVEPFRKRWRKFDWTRILTEGQ
>g1707
MAAATTPEAPAGESASPVTIVCVGMAGSGKTTFMQRINSHLHSQRKTPYVLNLDPAVHSVPFESNI

DIRDSINYKEVMKQYNLGPNGGILTSLNLFATKVDQIIGLLEKRTAPVDPAHSGGQPIKHILVDTP
GQIEVFVWSASGSILLETLASSFPTVIAYVIDTPRTSSTSTFMSNMLYACSILYKTKLPMILVFNK
TDVQDAEFAKEWMTDFDAFQTALRQEEDAGAFGAEGGSGGFGAGSGYMGSLLNSMSLMLEEFYRHL
SVVAVSSMTGDGIDEFFSAVEEKRQEFERDYRPELERKKNERAEQATKRRDIELGKLMKDMAVSNR
GKNKLADDPATVSEAEDDPHGDDDAIGGDESDGSSGEEDSASPDGGLASRYQQALADTSQNPTQPS
DEDISFMRYLRSSQP
>g1708
MRLSLHKLSGWLRRFYTGNNHRESHPRRQNPKKSDLNKNLESRTSSDTETECVQHHTIVKASTMAP
HMLESDSPIRTRIGIPDSEEYDKMIAELPFRVISCPLLQAKKLEARRFCTRYNVDDEILKDDTIPL
HELFMGLRKERERLLRTVIGTVGQGPVIEPPFHFQYGCNIILGDSFYANVNLRIMDSGLVTIGNRV
LIGPNVTIVTELHEKEIMSRRSGKVFAKPVTIEDDCWIGVGTTILPGVTIGRGSVIGAGSIVTRDV
PPASVAWGDPVRVVEPVSDADAVFAEMT
>g1709
MDVFYLYNYGSSAWLALQGVALVATPKLIITLLIDDTRAASDIEAYLSRAFGLALLAVSILTILLT
GSVPLVSPVGDVDATTPSPEDESARAPYAVPTLLVTTLFQATLAFYNYTWYLANGQKALGLGMLGS
AAVASLGPQFDNANGRYDIAPARGSAHGLTANNNQITPNRAQVWNGAGELEWVAASISSSLPNAKS
RAQQVDTSLHVGGDGLDVSSAAAAEIKDSHLGGLKIVPNPPDLNAWRERLFNVDEPITLTEEQFQV
YFPHVDNVYSHRSTQKYKRKPFVSHYWDCRLKGRPSGTPKSDDPNKKKRKRVARERDLCDVKIKIT
EYFPEATAADIAHHQRSAAESPMLSSENHHGATATGTTMFFTTNGSEAPRGNGNAQPFGMLAPSAA
LSENPSVVPGNGHKLYTIQRVNGNGGNGKTDGVGGGHRHTLEDSDRVKKNSVHRYLLKKSKDEKKI
VLDDLDEGTPLLPPGDARLRAHCRLWSDHINRHIVPSFYKVLMEQTPQMQAKHAAELQEEIEKLVN
ASHVHGPFFLGPSISFVDIQLAPWILRLSRVLKPYRGWTEPAMGSRLGRWIQAIEDNEHVVATTSS
DELYLESYQRYSENRPNTSQLANAINSGRGLP
>g1710
MGGGVCLFLRDREQTLTLKREAPETFHYVESRSAVPLKQDFXPAVTVLSRRPQIASRNSNNINAAS
EGISNLSINGSSANLSDSDDESANKPPQLTAEERQAKALKDREEKQRKYEEARERLFGNSSSAPGS
GASSPGGTPPPSRNRDRDSGYENSGPRGGRGRNRGPGGRENNNSNGREKRDSQGGAPNKQRQLYDP
GYSTKPNTQRRPPQGSTERGDGEQQQTIRPTRNPRNPDGSGRGGFGFDPRGRGTLLSRDAST
>g1711
MSADLDGDSEMLSSSGSESPIGARTPTFDKPIELSPPGSQTASHHESFGGAGNALVSAFGAGTSSF
TSAFDKLAGDGNKSGAQGKDAPGASWNNQRAQEEFNRALENVVDREFSLGEFGDPFDESDMLQGQA
>g1712
MEAIRSRTGPSAAPSRIEESDYVNEISQSANAAYLRAVKRQRVLHAHTERGGSFLETKWELETGRR
KRKLAGRTGDEQLVTNGQEVENEADGEIAEQSLEEISQSVGMSAAVDSGEEEEDTDQSAVISEIVD
LAINAQGGQVAAEVRELIAKKETNLVRSALRMNSLDGEDAALLSEFLSRAQAKRAANAAMAGAEKT
VVRQKKEEVLSSPAVQSRRALEELDVNSPSIKSSTSTPVKAEKLPESPDASETADIDQLTSSPTVA
RRRSGRTKPTEQTRSLRPAVPNQIPVRRANGTEFVFLQRTEAQELALATRKNTRRNKGNALLPRYL
LQAIVKQERKDETTQPFAQIEGKDGSRQARKHTAGRKQVCWKEDQLVEYAPEKESSPATESPSPSK
KKGSSSRHEGNKSASVTTHALPATPSKKVRRINMSTSGTSTDDXARAISKPMPVGTPVPKRKKLTP
RLKSTSVASSVEFSSDHPSRDMLSSAATKTAAKHIAPPAQQTASKMSGIPRSKSLIKPSVPSTLTT
TSSAGATPMVRRMRARRAV
>g1713
MPRFDDSDIGADLTQTRSPRDARPSVGGITASDGTTLPIPADLHHGRRESEVVEVPATRSSISDAS
QYMHNLSLNPSMREKRGSRNSFGISLPIPRSPRPSRLSIVSGIDSAKAAVSRELLASQVQDMAKEK
VAAAKNMAFAFDIDGVLAHGNQAILEAREVLKMLNGDNELGIKIPHILLTNGGGKTEEERCQQLTE
ILESPISVDQFIQSHTPMQALSEYYETVLVVGGEGFKCRGVAQNYGFKHVYTPKDVLAWDAEASPW
RNFSDAERADAHPVDFKRVKFDAILVFADSRDYATDMQLIIDLLLSEDGYFGTRSKNPESGSIPIY
FSQGDMVMPTQFKGPPRMTQGAFRIAIEAIYKAQTGTDLERVVYGKPELATYKYADEVLKAWMEQI
HNENRLPQNVYMIGDNPASDIIGGNMYGWNTCLVRTGVFQGGPGENDPNSPASFGVFDNVLQAVQA
VVRKELGKEFKFKWNEKINPVLHGGDSLSAVE
>g1714
MSDGKTFKLSNGVTIPAVGFGTFANEGAKGETYKAVTHALRVGYRHLDCAWYYLNEDEVGDAVQDF

LKENTSVKREDIFITTKVWNHLHRYEDVIWSLENSLKKLKTSYVDLFLIHWPIAAEKDGQEKPKIG
PDGKYVILHDLTENPEPTWRAMEKIYEDKKARAIGVSNWTIKGLEKLKSFAKIQPHVNQIEIHPFL
PNNELVEYLFKNNILPQAYSPLGSQNQVPSTGEKVNENATLKNIAERRGFTLAQVLIAWGIRRGYV
VLPKSSTPSRIESNFKSVELTDEDFADVNSVADNRHCRFVNMKDTFGYDVWAEED
>g1715
MKQWTTLMDGIHSLQLNTVPQPNEIKDDEVLVKIDRIALNHRDAKIINGDFNDSYPTPSQPLIPAS
DASGTIVKVGDSITAAGKWKRGDRVLSLMRPTHLTGPTTPEHAASGIGIPGSGVLTEYRVFSADGI
VAIPDYMSLEEACTLPTAATTAWMALNWDREIGRPRRGKDVVVLLLGTGGVSIAGLQQAKALGLTV
IITSSSAAKLERARELGANHTINYKTTPDWAAEVLRITAGRGADIIFETGGPATMEQSIRCVAAGG
NINAIGVLSGTTDQGKGAGQAIALSLIRKNATLKGINVGPRDRTEEMIKTVYEPFQVHPVIDRVFG
FNEAREALDYLYGGSHLGKVIIQVD
>g1716
MASPSDIRDVTLTSTFWTSMQTRSRDITIPAIIHAQKSLQHWYCLTWKEGHEIKPHPFWDSDIYKV
TEAACYFLLKNDDANLRQEVEQAVDMIRSAQHEDGYLNSYYTVRDISKRWTNVRDMHELYCLGHLV
EACVAYETLTRSGRLLEPVMKALRHVDSVFGPEEGKLHGYPGHPEIELGLLRLYDLTHDPLPLKLA
KYFILERGQKDENGEIFYDHEARARGGDPYDHMSAELPNPYHYPRYYEYQQAHAPIVEQDEIKGHS
VRAMYFVTAATELVRLTGDAEVKAALDRLWRSTVDKKMYITGGIGTLRQSEGFGPEYFLSDTEESH
ACYAETCATFALIVWCSKLLRQELRGEYADVMEVALYNGFLGAVGLDGKSFYYQNPLRTLTGRKKE
RSTWFEVACCPPNVAKLLAQLETLIYSYQQDLVAIHLWIASDFTIPESEGTIVSQKTNLPWSGDIE
LKISGPKAVRLALRIPDWAASNYTCSLSGGDLKDGYLYLPAVTNTTINLTFPLQPRKVYAHPKTGK
DEICIMRGPLVYCIEDVDNSGVDIDHIALIDGPVADAPAATIADVEDVVTVNSPGRELVNTNWSSL
YSSEGWKYGESTKDLTFVPYFLRMNRGGNGGMRVWVKRISS
>g1717
MLSSGFTNAPVSKSLVIYIVASSIALSIFDVKHLANILVDVHLWRYGQFSRVFLWQVAGYANSTEV
LFAAMLAYHMRVVERVWGPRKLATFVVAVLPYTTIITPLLLALVLRPLSLGTLNYLPSGPTAVMFA
LLAQYHASIPHTFKYRISTTGESISRQPSTSQNDNTNNSSKRSLTILLSDKSTTYLVAAQLALSQF
PASLLPAFVGWAVGTAWRADILPGSKSWRVPAWIVGEKESRRPAGVSSAASGNGDAEDGERYADLR
RRLDAASAAAAQQGASSGVEGSSGQSQRRPLLERFRGVF
>g1718
MTRFDSTDAPFEASASSSITEAEPPYHILSKKQKWNLVIFVSLAGAFSPLSSNIYFPSIATISHDL
NVDSSLVALTITVYMVVQGLAPSLFGTFSDTCGRRLTFAISLTIYTAANLALAFTSSYPMLMVLRG
VQAAGSAATISISAGVIADIASPDERGGFMGTNAGVRMTGQAIGPIIGGLLNSAWGFRSIFWLLFV
MSVIVLGALLVFLPETQRSIAGNGSVPLSGFRKPLIYILKPPQAWQGESYTDPKATTNRASDQARA
KISFKKAFAPMVYLFEKDIAALLAWGAVAYTAWSMVTSSTTTLLLHSFPFLTQWQIGLCFLPNGLG
CICGSLSTGWLLDHSFRHVQARFKEEHGILSHENVIKRSDFPYIKARLRLVPLFSVILMVSLVLYG
PSFEFNDLHKFYSPNLAAPLILQFIIAFTATAIFNINSTVLIDCFPDRPASATALNNLCRCLLGAA
GVSVIQPMINAVRAMKAFSIVSGVVLLCMPLIWVEWKWGEKWRQEREKRLEAN
>g1719
MGANSGDWVGPQFFREISGVSGSVTLQGGDADVHAVDLSGYLAEGGSSNGASSMADSVTAHNAQYP
DSEIVVSGWRQSALVAHKALQNKSPPLSSTASSA
>g1720
MATRSASASYKKKDGTLTVAKDHKSISWIPAAGGAGSAVTVPVQNMTNLQQTPASNPKVMLRIFAQ
QNETQEQYVFTFTSGANARPEADGIKDILSAAIAAAKAASAPAAIGAGTPSAEGGGGGLSAAMAMA
NAISSSGTGLAIWDNDDKLKADAELQQSLLKSDANLQRMFMESLRTRPETLSVGQFASQFWSSRVH
LLRAHAIERAQTRGSYNVLSSLKPRTDNNVTKLNITKEQIQLIFNQHPLVKKVYDENVPKLSEQQF
WSRFFQSRLFKKLRGERISDSDATDAILDKYLKIDEHADRVRDTQVPHFIDLAGNEVNHSQRQGNR
PDFDMRPASIDKVPIIRTLNALSESIMANVAPADGDPSAPVGMDEETYNQLLLRDLKGDEEQSRVV
LNIRDQSRFFGQDKEKEGKEEKEALFAQRDPKEIIVSLQTDLNQTLPADGSFHLGKIVEPDEDEDD
EEDETKASKVVGSKINMQKASSEIFAIIRERRAQADVTSDSGTWGLSSTLFDRLSLTHATTTEFLN
QFWNAFLSGNPERAGELASLVESLNRAMERIKAVATDAEAEKETEINKLKQHAREVFAATGKKIRV
NLAGVGGGEKVVNQLLAPTISALKLAVDKYKEALAEATAISAREDSMAL
>g1721

MVSSPGTSHSSPLALAHPVKGDIKQRESSKSRSRSSSRVRRSSFGSVKEDVEGIAQSFLDTTRPKE
GSSEDNAHLVDMTAAVDQMPDFCCPCGGFMGWKQIRLGGRKMSKSYSDLSMLGQRSSSTGWMWDTT
SKRPTIKEVPVEVEEKRPALTIEDLPVEVLIQVISYLAVELPPNGYTPRNVDLISCLLTSRTLHSA
TLSVLYRNITVPHSVIFSKLLKHLTHFPALGTIVRRLDFSHFTSVGLGRTRQMNAEIQNLTSKTLL
QCLDLLPNLMEGLFQEHLETDIDTAVVQKIFAGLPKLRAVDFCGCSSQTFSTGFMEAFTVNSTIPL
FLPQLKRISLHECSGLPSAIFEKLLPRLVNVTHLDLGRTQVTETALLSLPTTAQITHLNLSKCLRL
SGSQVVNFLATXPAVTESLVYLNLMTDPSRYRLLESDDVTNLLPVLPSTLRSLNLNGARITTEHVP
LLIPLTKHLEELGLSGADLSASDINSFFVPQDLESEKSWQPSALHYLDVSKVTSITLQSLFNPRLC
VLVSPRSLPLEVVELGEKLITPLRDRTKSAKSTGWSLRELGRRGWYVREPIKVEGAEWMIDDGSRS
WKMGARWWGMRKIPVTVGEVGGLYGHYMFKK
>g1722
MASALNGATASNSLSQAPGSRTTARQTRTNPSRVSKTAARSFPYYGSQQGHSLASDGAGQSAANNV
PHGLFPALTHFTDAITALPREFRRHNSLLKEVDAKAWALEDNLYQLLLSASDSRPVPFPQNPAPIV
DGEVRDYAHTLGDAQNVESQESKQRRLLFDRIRRTLSDLMLTADEKNHVLTNANEELDHQLYRLDA
VHPFIFNEVSDEARLGSLTHWAYSNRATAKAAAKTTTTERPRREAATATSHLVQALHEADVAAHRS
EARRDAAVRKPRRNQADSDFDETRPTQRKNAAAKARAAGGDGVDSGAPPVPKRRRVVDKPAAVHTG
SAAMERSTSTVTNNGRTASKDSAGMEVKKRARAPNAVTTAGRKRNNTVTSAVDSPLAPSPPVIGTF
NPPRSAPSPGPRPQSSRAQQATTQATSRQRPPSASSRANNGSIDKPMDAKVNVSTEDSAKEKTPQL
IPGDNIRKDALETKPTPSLDKEDRSEVKPLEAITSAVQMSPALSTIGVTKGRTSKTSTPVVSTFAE
SQTRARSSRNNNATNNSETAATTKRNTHKKSNSTVSAAYKAKIAQQEEEEEESSREGDDEDDESEPR
YCYCNQVSFGEMVACDNDACPTEWFHLSCVGLAKPPGRNVKWYCTECKESMKRGRVSAR
>g1723
MMRALASKKATSGSLLLRGSTSSACSACRRSHFLATSKTTTAALGARSNLQLTARRPLAVIDRVFA
GRREYAQSAEETSRGVDANDSFLQGNTANYIDEMYLAWRKDPSSVHISWQTYFKNMENGDMPISQA
FQPPPTIVPTPVGGVPQHMHAAGQDLTNHLKVQLLVRAYQARGHHKAKTDPLGIRGEAEAFGYNKP
KELELDHYGFTERDLDQEFTLGPGILPRFETESRKKMTLREIIAACEKIYCGSYGVEYIHIPDRKP
CDWIRDRFEIPQPYKYSVDEKRRILDRLIWSSSFESFLATKFPNDKRFGLEGCETLVPGMKALIDR
SVDYGVKDIVIGMPHRGRLNVLSNVVRKPNESIFSEFSGSTEPSDEGSGDVKYHLGMNFERPTPSG
KRVQLSLVANPSHLEAEDPVVLGKTRAIQHYNGDETEFNTAMGVLLHGDAAFAAQGVVYETMGFHS
LPAYSTGGTVHIVVNNQIGFTTDPRFARSTPYCSDIAKAIDAPVFHVNGDDVEAVNYVCQVAADWR
AEFKRDVVIDIVCYRKQGHNETDQPSFTQPLMYKRIAEKKNQLDIYVNKLISEGTFTKEDIDEHKK
WVWGMLNDSFDRSKDYQPTGKEWLTSAWNGFKSPKELATEVLPHLPTGVDIETLRSIGDKIGGAPE
GFNVHRNLKRILGNRKKAVDEGQNIDWATAEALAFGTLVKEGHHVRVSGQDVERGTFSQRHAVLHD
QESEATYTPLQHISQDQGTFVISNSSLSEFGVLGFEYGYSLTSPNALVMWEAQFGDFANNAQCIID
QFIASGEVKWLQRSGLVVSLPHGYDGQGPEHSSGRMERWLQLSNEEPRVFPSPDKLDRQHQDCNMQ
IVCMTSPSNLFHILRRQINRQFRKPLIIFFSKSLLRHPIARSDIEEFTGESHFQWIIPDSAHGTTI
DEPEKIERVIMCSGQVYAALTKHREANGIRNTAITRVEQLNPFPWAQLKENLDSYPNAKDIVWAQE
EPLNAGAWSFVQPRIETLLNATEHHNRRHVMYAGRAPSASVATGLKASHVKEEQDLLEHAFSVHQE
RLKGE
>g1724
MPPSTVFSYWRRDHRRTSPAPGSVSPGAVATGGAYSPTTTNAPPRLPGLPAPTTLSETFIDAHDLP
EYEQRPDSVVYLGAGDKRLSSALSSQHSYNNLRVPSTTSTDNLVRPHSSPGNTAKNDESLLAVDSI
QSPSSNAYGVQPDVQSQHRSNNGSWRLGFNRNQAEDHRGAYPRKTVGKDLRINTNVDGELGFIGGV
TLKELRTDSYYHEQQQQVQPQSQQEHTSQQKHGKTKRHLLNPMTLLARRRSSQNTGSRVDEGSLGG
RNLVPAIPDDYDPRIRGNIVHDFSAPRPRPQLAGRDGSGESAKKSSLPSSAVTRHSEHSPVFKEHF
EEDGKVLQIEKKGFLQSSLLTDADHDIVELPEFARRLPSSLPVHDNQHQPVEVEDESDSQDTISV
TPQPPASIQQTPRSSPRKSVEVAPNQASSIPIRLKSDASRFSFDMNGVGSSVQEKILEEKGKEKEA
ARKEKAQQERSSFSDYDDDDFDYDAMEDDDGFEERIPGINADAEDEYDDEFQNFSGVVRTSEPSTF
VPMLPTIAASPITPSYSVVGVFNAASGTPLASVSDVPVDMTAPFDLNTSLEEEEEEEEEEDGFDQS
DDEIEGTVVNTLNLTTTLSGQPLPLIEEDEDDMYYDDGDFGELPDNEENDKFDESIFDEESGQLYE
RKFYPGTKIPIALQEGDEQQNTESQIRHVPSLASEFRPESWDFRNGNPPAKATGGASENTKAPGGV
LSEQNLEAFHSALARVADEAAAGSLQRNTSLSENSLGQGSNSQLADSHPGLVLDDNRVSSQHSGFD

DEVLDDFNYDDDDGFDDDLIIAEANADALENDDEGFYGQEFGFYARSNTENCIDEPVYGGYFGPSG
AGGLNRSHSGGAGFREPSLTPITERSEWSTRNSIISLTTHGPSAVQQPPPPNPGLSQLVDMEYNEE
DMSLGALMKLRRGAFGGSNGSLRSTGSLSPQPGPVGHSNRGSFLSIPEDSPVDLTPDMGFQQSYEH
GSGLSGVYYHNSNMNQSPLSEGQSLPGSPTLTLDRLTGGERRSSVGNTT
>g1725
MDYNPVAQVDDNDVASQSSDSDIADHPSVKAANSGHKAHESIASVQQGMENLIDSINTGDRNHSSS
YELIEDDDYEETTEQNKPIPVAGTNSAHQTSTVADLSTGAEIAPRSTSPEPLVRTSSDVSIPLRHP
TPDLQSLQGAYVGNIERLEKSAERLSLTSSDIGVELRKLDLEQKRRSSASSAYQGPDSPSTRQMSI
GSLSNSIIAVNSAARSGGYSPGGFLTSPRGSIISGSRFRSTSVTSPLQEIHEPGHEEEMEASAVDN
FDDKDIPILTPRPLPAHARNNEVELHNPYAPEEPFQDVPDRPLTAQSTDTYQQATNLFLDFDGVHF
VPHPKEPGLSRQLSLNRPPLATSAERHKEPQAGKDMVYYPAPIPKFLNLPQRLSKQSAKVNNEKRR
TQLMDIISATDKEAKVEEMNAQRVSKRLSTLPPQLRASVFFDQPSSNLASLEVQLKDHSAVTTLDS
ILDASTHAPVSAFTDHPIVGSAGAKVYGSADKKRASRLLAGKGSKDRIKSSPGPRESALFVPGQGL
HAYSDTDAAEGDPMHPGGSEHEHRSHSQSSSDAESSDEHGSEEHTSDENDDEEEDSTSEPERFDND
ESMLFGRPTTLLAELQMRKEEQKQRNRSAAKAFPNGLHSTLLQLDAVAQAQSNKRRQKHVTLAWEA
EAVGDPDDADDEDIPLGILYGNKNPAEEDRPLGLMERRELEENEPLSKRRARLRGEVIPATMPNKR
ASTMTQLQSFPAAQSDSEDEGETLAQRISRLKQEKKDKTKSVISTEFETEIMSELGKLLPAEPVKE
PEPEPTTKAAEKVVAGEEEEEEETLAQRRKRLQAEAAQAQSKNVRASRSMADILSAYPASNAGFPVS
GNAHARQTSLRPASVHTQRLSTGFTVPPSMTFHTGLAQTPSHHPSMLGYETQPAYGNMPYNAAFMS
GAAAGMNAYGGGMQHPHNAAMMQQNIVDSRQADVIDRWRQSVAR
>g1726
MNAEASEATRNSDFEIGIICALPLEAVALKEIFHTIYSENQFDGKEPGDLNSYTKGSIGQHKVVLA
HLPGMGKVNAAAAGSTLKSTFRNLKLVLLIGICGGVPEGANEERIFLGDVVIGTSVVQTDFGQWTP
KGFDRKDTTRDNLRKPPPEIGSFLAKLESDKSNISATTLSYLAMLLEKPGFETARFPGVHEDQLLA
GSPPTISRERSHSEPNIHFGGVASSDSVMKSGIHRDRIAKAEKVIAFEMEAAGVWDQLPTLVVKGV
SDYADSHKNDLWREFASASAWACTKAIIEKWEKQATKEQYRVVSQSPF
>g1727
MSHISHHLQESENAVLVEHFFNARGSGLEKTFLGLLRSLIYQLVSEVPELYSAFVPIYHKKDMIQG
RWEWRQNELRNFLRSELKRSKLRIIILVDAIDECSLDEIASVVSFLEDLSLDAVEAGFNLRICLSS
RPYPNISMRKHLGFTLDGNAAHDEDIKIYVRAKLKWENLEIENKVLQKAHGVFLWVALVVAMLNEA
VERGRPEDMQKTLEDTPSDLDKYFERIIFGDPEKNPETLLMFQLVLFSKEELTPAELVFAVIARLQ
EVYLKPRRQSRMTDAMLKARIVDSSKGLIEVYRERRVQFIHQTVTDFFLRNRRLERLYATSEPDAI
AXSHDRLRACCLDYILKFPIDSQSHNGWSAEELKNEYPFLNYSAMYLLSHTEAAIEGHQLGIESAK
WLLSPDPWFQTWKRVVSAVESTWYLHGDLNVGLVYVLLLHNFHNSLAVVLRRGDVDVNTQAGFFNN
ALQLACAQGNHSLVSMILKRGADLNASGGHYGTALQAAAARGNIRILKTLLERGAKVDISGGIYGS
ALQGAAHNGNLEAVNLLLEQGALVNSQGGIYGSALHAAMEPRKTEIFQRLLRAGADVNSCGMTALI
WAARNGRRDIIELLLEYGADIEARTSSGQTALLAGSEAGHEGANALFKAAANGHVNVMHLFLGHEG
FHDIQNQNSFQINGGICYFDMNNGLDIRKLEVNGGSFHLMVHSGNRVNFDEVSLLGGSFICNSRSK
IYAEHVKMGNTLSHSSDRQSTAGPLSTGGLPDMYIENSKGRIEQYGIARTGEQNIQIDAVLSPLDV
AVQNGHLEALNDMVIVE
>g1728
MESRRRRKEEAEDNENVGELEERKGRVGDDKGKEMRSTREGGEDTKQNSNNEWQNGTETNERDCRI
L
>g1729
MFNTMNVPRIAASFALAAVLLAPGSAACDDIIPMQVRLAYAGPKGMIVSWNTYSQLERPMVQYGRS
PDALIHEASSDVSVTYPTSTTYNNHVTLEHLEEDTLYYYLPEHSNATEPYTFRTSRRAGDKTPFTM
AVVVDMGLMGPDGLSTRVGTGAANPLGPNDTNTIQSIEKHLDGIDFIWHPGDIAYADYWLKEEIQG
YLPNTTIADGYKVYESLLNQFYDEITPLTSVKPYMVGPGNHEANCDNGGTTDKTHNISYTVDICMP
GQTNFTGYINHFRMPSPQSGGLGNFWYSFDHGMVHYIQLDTETDLGHGLIAPDEPGGPEAEDAGPF
STLKDAQTNWLQKDLASVDRKKTPWVVVAGHRPWYVSASNRSSTICEECREVFEPLFLQYDVDLVL
SGHVHVYERNSPVAKFNVDPKGLDNPSSPWYITNGAAGHYDGLDSLVYPLQQYNQYAQDTAYGWSR
LTFHNCTHLTHDFVASRNGSVLDTATLYKNRKCVGENH

>g1730
MSYYDIDAILTDAQKLPCTFELEVPGLGFLEGNPGENIKTGTQVDLPLWLXEMLSIGARLGTSRLV
TLDLPSALSERVLNALKADPRTVDLRSLAPHFYSLGIRVLELFEADNMADILSDTFRKRASEIADH
AHNSRGALGEGVEFLRGLDETERQLFRIAHDSAKEIRIWSGEAKKSK
>g1731
MSSPSSRPQTPDYENFLGTDSSSPSTTLVNPVSSFLQDAIEERRASRNTKTPQPSEIVDNRPTTSG
SHSKSLVASRSGKEKENERSDHKKPQDMGLLATKEYISKISKLNFDLQMRIALREQQLATLERKVE
RMHQIEQELERMQGLEDELHRLQAVEEHNQRLQEVNDQLKSEIDVRDQAVREAVDLIDELETKNEE
LRAELRAGRGKTSLPRNGSTDDVSEVCTPKAQTFVDIPDRTSSRKGTVRRPRKSPSFLLEKSGSTA
ALRSVFLAEENKSVRSVSASTIATTVDSKADPQSPRLSTLSECSDFFPQLTATGFDQLDRLDVLTL
VNNDHSPVVAAPTENLSQGRLSDRISEWMQFDEHSPRRRNSNRPRALSDVSRVSQVPITPLDEPFQ
PMRKTRRTQRASPRQQHLADFGGNLPPTPDTMSTFRPTFKNGSNASLRMDRSLSRPRSAGELTTRS
ASMSTHSDGIDTAASVISQYNVQNNSIAQNSTFKYYGRGSAKTTRVRGPGSPIDPRISYHGGDLLF
NEEVIENAVSDMDMTRALSLKATTPEASGESLYASALTPQDWLDAAKPEQEKSKMQYSSPELETDE
MSLEDNNPIEFTTEVNTPRNSPLRLHAWANESQPMPEVQQHRRRLSRFFTRNRQSKDAQPESVPTP
KTGPSPIPQKIVLVKHRRTSSGPGLNSDEGTAAIGRASGRALSKSLLDHRPSTASRPITSDSAEPR
SKGSGLLGWMKSSASHNKDNEPSPSGHLRFARSTTERPQSTASLDVSNLGLGLDVAAPDDEPAWRA
RRRSRRMA
>g1732
MSIKTPYPTEDEPRLKLEPDDKLENHTVVLVTGANSGLGYSTCLRLIDEFAKDPANNSKQLTLIFT
TRRPPAQVQTLKDDMRNYAVRLGTQVAARINILAETVDLLNLPSIRALSIRLNRNFPKLDSIILNA
GIGGWSGINWPVAIWNVLTDTLHAATWPSFKLGYVGMVVPKQTQSPKENPLGSVFCANVFGHYMLS
HNVMPLLRRSKSPGRVIWVGSVEARAESFDVEDIQGLRTDVSYESSKTLTDILALTSDLPSTAPWV
RKFLSADDEAETETGTDAESAAPVTYLSHPGVCGTSIVPLILPLYWSFVAIMYLARWLGSPWHTIF
PYPGANSLVWLALSPQSTLDAAEKPYRDRGGGHAKWGSSVDFWGRPLVASTETDGWGYGGVVDGEH
VVEADRVRRRRRGMKVATAEDKVRFEELGRECWRQMEELRVEWDGLLDTLES
>g1733
MRPFLAVILPVWSYVQLVTASSQNTTRYEPDELRSEVSHTFLACLRNTGVQYKVYVDNGATVVIPA
TKREIDFDGIDRKLLECIIQVNHLMHVVAESALYDQDEHGNAAAGHSVTHEWLSTQGANGLMEVML
NQPNNLYYELFL
>g1734
MAPGGSRGFNCSWERCGKSFNRKSDLCRHYRIHTNERPYHCTVPSCNKSFIQRSALTVHSRTHTGE
KPHVCDHEDCRKAFSDSSSLARHRRIHTGRRPYICSEPACDRSFCRKTTLTKHQSRTHQPGGNTRP
SSEEAPTPESMTYQTPVITTMTPNSNQYMIAQQPYYHNGPPPTTVDFYQNSQPAIHFPQPQAMVPQ
PIPTAGLPTPPFEPQHYAQIMQQQQPRYDPNPHPTYIHPQYQSPTPPFVAQQQQQHHMTADVRPLM
ATYHPDQFKPPARILSSQEGTDWSFLGCLDWRCNPGPGTQDYAITFSGNEKRPLLGLS
>g1735
MLEGVVANLLNRFLGMYVKNFDGKQLNIGIWSGDVKLHDLELRREALDQLHLPLNVVEGHIGQLTL
SIPWSNLRGKPVRVEIEDVFLLAAPKVETDYDPEEEEKRAHNIKMDKLESAEILRERNAEGMTQEE
QKRNQSFTQSFTTAIVDNLQVSIKNVHIRYEDSIASPGHPFAVGMTLKELSAVSTDSEWRALAVYW
NTDTELLGIPHAELMAKLRDGIELDENNQYILRPVSGRAGLEMDKTGKLERPRMKARLLFNELGFV
LDSDQYRDTLMLVDLFHYFIRHQEYKKLRPSKNPKEDPRAWFRFAGNAILSKIHERNRVWTWDYIK
ERRDDRLAYIYLFKKKKREEALTVDQNTELNELERKYTYEDLRFWRSLARNQLRKENVGVKKPVRA
QTWSEWMWGAKKQEEDETETMTDEQRQELYNAIDWDEKKALAESVELPRESVKLQVNSSLRAGSLT
LKRDPHGAATEVMKLVFDNFRAKALQRPDSFFVDLNLGGLRVYDGTTEGTIFPQIVRVKDSQGQIE
NSSPDDSQELEYEEESDDGGQDDNLFHLQVEQNPLESDADTAVKVKLKSIEVIYNPRFVVEVARFF
KPPERHMESVGALLDSAGATVQEIRQQTRAGLEYALEAHRKVDAQFDLQAPLIIIPESITEEGALC
LILDAGHIRLNSELVDRGTMSELQNKQKRQYSEEDYKQLEELLYDKFLIKLDSIQLLIGPNIEATK
AQLTSNDPTKDLHIVDRINVDFVLELCIVPKTTALTRTRVSGHLPELHASMSDSKYKNLMKLIEIA
IPRFEEDCEEKSDRPEAIVDKTSEQEARNRARSASFQPSFRRDIPLLDEESDSEDSGKREVAKKDD
QPINLHQRIFEFKFTVGKLRGSLFRSDPREAKSDQLLAELVAEAFELDYYLRPFDMVAELTLKSLS
VDDYIEQNATPEFKQIVSSKGFNAEEDKDLFNLKFVRVRPDSPEFHSTYEGVDMNLDITVSTINII

VTRRTLLTLLDFVLITFTNPEQPKAVDQSADSTAVDVSQGQPESQEVRKIRIKSQLKSIALILNND
GVRLATLSLNTGDVGIFLVGRTMQIQSRIGSLTLVDDVNVGASEHSSLRRLMTIEGENFADFRYQT
FDPDSAEYPGYDSEVFLRSGSIKIQLLEEPYRKIVNFLVKFGKMQAIFNAARQAAANQANQLQENA
SQNKQRDTVTAELGEIYANNTFVPLDDRKDSPAVNMITTGIRNIRLTSDLYYDDATHEQLEMIQKV
NLDFSICYLEHQPNQPRPDIEVEGTMSPIKLRISQPQLKFLLEILKTVPGVFTPDLDEQELEAMQS
LPSLNTQNEEQSPEIQQVESVGLELLLAKENKPVGQIEDSSLSRFSLNNTRVKLRMLSDGALESEL
LIHSFSIRDSRAQETNKFRKIMSLINNDVQQQFMATLQTFASEAFAQDQPLEIEEIDETPEDSDAA
SSAAQMTFSLRADIVDAQVIMIANPAIANTEAIVLGTKQVLFSHQNVSTLQINKVGMFLCRMDKFE
TSRLRILDDFTLEFIDVHVEPLVLRLSLRDILLATQIMNKVIKEIKGTGKKSSKRRSTIGGKASSA
TALKSRSHLVPEQRLAPQQSAVIRREELGAQFDGIRVILIGDLHELPLLDWSVKKFSVDVKDWSST
LNADTSFDTFINVYNFSKSAWEPLIEPWQLGFHMSKEVNPDVFSVEAYSHKNMDLTLTSATIALAS
KSFQFLNTDEDVLSKPRGADAPYRIRNHTGFDLNVWADVEDGENGPAAKLSDGEEYPWRFEDATTM
RENLTPEGNAGLVGIKLEGSGFDSIQRIPVIREGETLYNLKPKKNKVLHKLLVEVKLGSDNVKYIT
FRSPLLLENNTQIPVEMGIFSSEDGHLLKIEKIQPGDARPAPVGSAYLHQLLVRPDQGFGESGQQA
PPFYFQLNASYDKRDPIINNYPHMKLRLSAPVEIQNLLPYDFKYRIYDKNTRKDWTNFLRRGGQSE
FAIINGTSQDFRREDALSLKDERGVELRLKLHYFNLPDSGGAFKVSVYSPYLVLNKTGLSMEVQTG
HAKPYMYSYPTDDQKNRSIIKIGDSRWSKPQSFDAIGSTFEVIVGARTERAEFHAGVSVAEGEGKY
KLTRVREPGTSNVLTMKAGDLVPLHFLRQVPEKQLCLCFPGVNNQWSSPFNIADVGIIHVKLAKAS
QRQRLIKVEVVMENATIFIHLRMETRHWPYSMRNESDMEFIFYQSNPNVDEDEYEDTSSGWRPIRY
RLPPRSIMPYAWDYPAAKSKSLVLNCNGKERHIRLAEIGNLIPMRVPSNNNPNGQSIVDLNIVAEG
PTQTLVLSNYKPSKSIYRQQKGQTSQASVATGFEVKVENSDVTFKAQLRLGGVGISLINQNMKELL
YCTFREIEIKLRESKVYQTLDTTIKWIQIDNQLYGGIFPILLYPSVVPKTGKEMEAHPIFHAMVTR
VKDDSYGVLYIKYATLLLQQMTLELDEDFVFAMLDFAKVPGASWSIEQEGKLCDEDLDIPEPKYEE
NRQDVYFELLHLQPMQLDISFMRTERVNAEDKLQPSNPLMFFVNVMTMSIGNVNDAPVRLNALMLE
NARVSVPLLISNMRKHYTQEFLRQIHIVLGSADFLGNPVGLFNTVSSGVADIFYEPYQGLVTDRPQ
DLGYGIAKGASSFVKKSVFGFSDSMAKITGSMSKGLAAATLDKEFQDQRRMSKARNRPKHALYGVT
SGGNAFASSIVSGIEGLARHPLQGAEKGGFGGFMKGVGVGMLGVVTKPAIGAFDLASNLAEGVRNT
TTVFDSDGLDRVRLTRFIGMDGIVRPYSQREALGQFWLKTTDDGKFFKEDYIAHLELPGRDMIVML
TYDRILLVRSDKLRSEWEIKLTDIQTISKERTGMSITLKGGANGPYIPVQDESSRNWLYRQIAIAV
NAFNEKYNAKN
>g1736
MLYRMSAARAAFRAASSSNASAAIRAAVPGNVFRAQLTSASRQRLTTPKLALAVRKPVTTALVRYA
STATEKAGSSAAGLPEDEPDMLAGVKNDAKVIKETFTLNEVPKEALYFGLAGVVPYLVTSLNTCYL
AWEINNNVINGSGFLLSGETASFLLHLTEPIQVGYGAVILSFLGAIHWGLEWAGYGGNHGFRRYAT
GVIAPAIAWPTLLLPIEHALITQFLAFTFLYYNDARAASRGLAPHWYGMYRFVLTFVVGASIVATL
IGRERISQSDSEEHTLTEKIQALLFLRKKEIEEAKKRAQEAKEALEE
>g1737
MAPHRRRESVINKTTTHDGDRSIIQDETSRTDNLKDKFIGAIDQGTTSTRFIVFDGTGLPVASYQS
EFRQIHAHSGWHEQDPRELVSTVEKCIEETMKAFLALGHSKSDIVTIGITNQRETTVVWDWETGEP
LHNAIAWPDTRTKGMVRELRSKEGADELDKICGLPLSTYPSSVTLMWLLRHRPDVKKAYDEGRLAF
GTVDSWLLYNLNGGVKNKVFVTDVTNASRTMFMNLHELKYDDKLLNFFGLDKNKLRLPKIVPSADP
TAFGKLDGGPLAGTTITSCLGDQSAALVGHCAFEPGYAKNTYGTGCFLLYNVGEKPVISQHGLLAT
VAFQLGADRKPVYALEGSIAVAGSGVSFLMNNMGFFRDSKKVSEVAATVPDNGGCVFVTAFSGLFA
PYWIDDAKGTIFGITQHTQRGHIARATLEAACFQTKAILDAMAKDSGHKLTDLAVDGGMSNSDICM
QSQADIIQIPVERPSMHETTALGAAIAAGFASGIWSTYDELRHMNRANRSVXKPYLDAQESALMYK
QWTKAVEMTRGWLDASEIGMDEDDEDAAGGEEPSHATHETAEHVVRVLQKEESDNSADDVAVGSES
D
>g1738
MAFNATVNLPNIQSATSDIWALMNSHWRRANTVYRAPSTRRQHTEASPVSVEPFSLQEALRPVPLD
KMRSQLALGQVQQQRSIFGGLSQNFLAQKERAANSNPGSANAQNTFYQALLRANMPAIIVERYRTG
QFASNAATETIYLKALQRLGGDGAVQGSFSGSQGLDSEQLQSVGQAVAARTHGSQVGVSTKQAGTG
AKESPLYVVVEESLGSSVFRWVRFLLIFGFFTYMSLVVITLLVETTGVLKNVRGTQTNEATPQQQK

VRFSDVHGCDEAKDELQEVVEFLLNPERFSTLGGKLPKGVLLVGPPGTGKTLLARAVAGEAXVPFF
YMSGSEFDEVYVGVGAKRVRDLFTQARAKAPAIIFIDELDAIGAKRNERDAAYVKQTLNQLLTELD
GFSQTSGVIILAATNYPQLLDKALTRPGRFDRRVVVGLPDVRGRMEILKHHMKGVQTSTDVDVAVV
ARGTPGFSGADLENLVNQAAVHASRHKQDRVGPRDFDWAKDKIMMGAEARSRIIQDKDKLLTAYHE
AGHALVAYFSPSATPLYKITIVPRGMALGITHFLPEMDMVSRNYVEYLSDIDVAMGGKAAEELIFG
PDNVTSGISADIQSATETAFTLVTQFGYSKKLGNVDLSTNYDKLSSETKQEIEAEIRRLVEEGRSR
ATKILTEKRKELELLTKALIEYETLTKEEMEKVLRGEKLDKMQSTPSAPLKLPEALTAVRLNPITQ
LPESDTTAK
>g1739
MGAPSGKLQWDDKDLLQVAVPPFTKLSTTIPSTSNSSGTDSTKWRVLQSAGTARLHHPEWTQDTLF
LDTANLPAKMPRMSDDVLLSQFYEHSLSVLGTSFSSESDNNDTTLPFDEDSDINEITSSGIAGSAE
AVPFPYPCELHNIKDIPNTTYLRSIIPQTPSVNLVVAVIGINPVRRVTARQSKQELDILEIIVGDE
TRTGFGVTFWLPRNHKQLNTLAAEFREKILGIRLRDIVLIRNVGLSSFQDLVYGQSLWKGMTKVEL
LHRQQVDSQDVQGAYSTVAIETATREDRLLYKVCNVRNWLMNFVGTKARCGRHDVGQPLPPDTQ
>g1740
MIENQSEKSGGQILEHNNNSSSSRDIENNFLSEGSTPPISSGATTPPPKPVLPEGGRQGWLAVLAC
WCIMFNTFGYINAFGIFEAYYKSTILPHESQSSIAWIGSIQVFFMFSAGLISGPLMDRYGPRPILI
PCSFLFILSVMLTSLCREYYQFILAQGILGGLTNGLTYTPALAAVGHYFSKKRPLAMGIASTGASI
SGITLPIALNRLLNQTTVGFGCSNAPKRRSGPPLLLKAWKNRAYSLQVAGTFLVIWAVFVPLFYLP
GFGQSIGLSVDLSFYLLAILNAASLFGRLLGGAAADRMGRINTLSFACLICGILILCWLPIRTQGA
IIAFAALFGFFSGAVIAVFPATIAMTAPQPNEIGSYLGMALGVYSFAGLSGTPITGAMINDHNSYR
PAITFAGVSALLGALIIFASRYYIAATRMVV
>g1741
MKALILVGGFGTRLRPLTLTLPKPLVEFGNRPMILHQVESLAAAGVTDIVLAVNYRPDIMVAALKK
YEEQYNINIEFSVESEPLGTAGPLKLAEKILGKDDSPFFVLNSDVICEYPFKQLAEFHRAHGDEGT
IVVTKVDEPSKYGVVVHKPNHPSRIDRFVEKPVEFVGNRINAGIYILNPSVLNRIELRPTSIEQET
FPAIVKEGKLHSFDLEGFWMDVGQPKDFLSGTCLYLTSLAKRNSKLLSPSSEPFVHGGNVMVDPSA
KIGKNCRIGPNVVIGPNVVVGDGVRLQRCVLLENSKVKDHAWVKSTIVGWNSSVGKWARLENVTVL
GDDVTIADEVYVNGGSILPHKSIKQNIDVPAIIM
>g1742
MSATLLRNSTARAALRSGASASRAGLAGTSFVRTKVTLPDLSFWVPEAIRTLATGYKTRPYLEPQK
LNDKLLTKSEDDYGALEPSISGKIMEIHHKGHHNVYVTGYNTNIEKLQEAQAKNDIAAQIALKPLI
NFHGGGHINHSLFWENLAPKSAGGGEPPSGVLGKAIDETYGSFGEFQNKFNTALAAIQGSGWAWLV
KDKETGDISIKTYANQDPVVGQYTPLLGVDAWEHAYYLQYQNRKAEYFKAIWEVLNWKAAEKRFGA
>g1743
MIIKNDKMDYDDDESPARSPFAGQKRKLREVSSSDEEEEATARPSFGFQPFRRAASRSESPPTMTA
GLGGGAARSAWNSTASTPTATMQSRGRGGGTGGGDNKGGSTMGKNSFAARMMAKMGYVEGQGLGEG
GRGIVNPIEAQGRPQRIGLGAVKEKSKQAREEEKRQAAARGEVIEDSSDEGRQRRQKRKAQRHKAD
DRSGVSTPAGPPKTRFRTARELEAEVEGLVVPNVLKSLVDATGREQKVLTSTAGLMTPMEFVNQAE
GEALKIARRARHDLEAFVDEWKGLTERKKFIDLEETQVVEEMDSYQVKSEQLARLVSALEGIQIME
PRETILQRFEEVTSKLELIEVEYRDVIDEYRLSDAAVAAIHPLFREAMEEWEPLKDPTFLVADLSR
LLPLLSRKSQQIEDEQVQRHRQSTSPFESLIYSIWLPRVRSVLLNEWDVYEPSPATSLVVAWKDVI
PTFVYSNILNQVIVPKLSAAIREWKPRSSRRHPDSSQGSRFPWWLFTWLQYLDERHTNPRHATGLL
SDVRRKFRVILDTWDLRKGLLNGADLWKEALGSEFDAALHKHLLPRLARHLRENFEVNPQDQNLTA
FEDVLKWKDLFKPKVFGLLLVAEFFPKWHNILYIWLTNDPNFEEVAEWFTWWRSQIPADLNEIVMV
DEEWTQGLKMMDLASQLGDRAATELPAPEKIPTAAPITAAGLQPEKTVPAKAAKPKAPVKEIAFKD
IVEEWCADQGLIILPLREAHPQNGQPLFRITASASGKGGVIAFLQGDVVWVQNRKARDTWEPMGLE
DQLVERAEGK
>g1744
MQKFLRRGAELLSPRTGSPATTDDDENYGFKTASTQTALFPKVDPAVDGKADWVRDVADEKGSVME
AIDKGGLTPTNGNLKLSASNIPVPDEYHHAEDGARPTTVVLLPAFKIIDHVTPALAPDLIKYFVNP
SITTTTPLRSNPSTTTIPANGISEQDLSSLTPLRSRPLPHAAVILLCSQKTRDARCGQSAPLLKRE

FERHLRTHGLYRDSHDERPGGVAVHFISHVGGHKYSANVIIYRRRDFDWHKKIASSSTAAAATEEE
KVQAQQSQDEGAAQGIWLARVRPEECENIIKFTILQGKLVKPDQLRGGFDREKGLVSW
>g1745
MIFNALSILNSTVYFILTIVLLALSLLTPGDFIYQCRVNHRFTNIFIVAGVYVLTVLLGLLIYASR
IYTNRTALSGIPKAWIPVSKGEVDKKVQRVVEEGLNRSAIIAFQARPRNVSDESSEPVHDVELSIA
ADRPPWGAVSHAGWSAPSSTDLPGLRYESVIEELPHLIEAKAVSLVPSKPVRGFIDPTMPGDDDAP
DPRLVELLQRPSAMGLREYIAHLTTFGLINPPELGSEFLVIYEQSRFSSRPLFEDEFRALMGIFAE
ILRGMKELDYHYLQELGGFESRVDSESFIGPSDEEGETDTLNSEQSIVPLRRERSTDSSTWSSSFH
SVHTAPMEPSGSSPTLARHTDRYASRGLQSPWSRSVSRARSIASQSSGGGSVIRLTRTRSPSELPY
EINLSRLNTAGSV
>g1746
MSFSGRRVSILRPAETRRFSASKDLSQNEKNSETHRHFRSAHEGHKPHAGLDASRASTGVIWCTER
ANEHGFSERPGDWANLGQGAPEADDDIAGSFPRPTTIPIDSAAREYGPTAGIKSLRAAVARLYNEQ
YRQGKESQYTWENVCIVPGGRAGLIRIAAILGNSYLSFPIPDYTAYSEMLSLFKNFSPIPIPLSEG
DHYQIHPDKIAEEIARGTSVLLTSNPRNPTGHVIQNPNLAKIQDICRDRATLILDEFYGGYNYTTD
CDGTTLSGAENVIDVDQDDVLLIDGLTKRFRLPGWRIAWIVGPKEFVEALGSAGSYLDGGANVPFQ
EAAVPMLEPSLVKQEMKALQLHFRNKRDYVVSRLQEIGFRIKDIPEATFYIWLDLTSIEPPLPESA
NISDGLNFFNALLREKTIVVPGIFFDLNPAKRRDLFDSPCHHFVRLSYGPKMEVLKKGLDGIERVV
RRARGENVGPTLEDEVAIEED
>g1747
MDTPLSNGLPVGGPVAEDNIINRRGGESIYQSCVNLKLRLAEVPGFEQHLDEMEAEERHTADPVNS
LWTCLRAGYPLITIFNASQPDEMLEIDPEKVPEAKRPKAATFKFLQACLQDMGFPQQDCFLITDLY
GENTTGFIKVIKMVNRVLDILEMQGRLRRPSAAVSSLQEGPEGSVKLTKREHILKELLVTERDYVH
HLQNLQALKKELEETGAITGDLSHQIFMNLNDLLDSAQRFLIRMEQHYALPEERQDWGHLFVENEE
FLRRYEPFISNQLQCDEVCLREWDKIRVAPRSSDLQQMVAMPATLNSFFVKPFQRLTKYPLMLGEL
RKQIDDEALQSRITIAINMIQSVLDSANAAIDREHLQMAMVDLANRVDDWKALKVDGFGELLRFGT
FTVLKGEGNKDSEREYHIYLFQRILLCCKDINPNKQKTKLMGSKDKPSANLKGKPRLQLKGRIYMA
NVTDVVLLQKPGSYRIQIFWKGDPGVVDNFIIRYVNEDTMKKWYQDIDRLRVESVGLRNPRNTGTS
DSEFTYMQSMTAGMVNPYQQEYDSEEVPHIQSASFSEFPVSRNASSTSLRARSATGGSSGSIGLPA
ARPPPRFPLPEVPPLLQTQLSNGNLSPGERVIGTSYFSPVTETPSTRSSSQSTFNYPRQMTPTNAW
NEESNRYTAPALSRGVSRDGTNGNPYFSTSNGANGRVQRPSLPPMSGSGSSQMSQRMRSASSPDIH
NTPRYMNGHSMQTIDNVPVPPIPPQLASMRAPVNRSQNNSPTNNGLPLRSATQTPSGASRHMQGND
YSDGYFPMMANSQSSLSEHPLANSGTLEPDTQASQEPIMPTQLKAKVNFDDNYVTLVIASNILFRS
LTDRVDAKLARFTNRSIGNRSVRLRYKDEDGDFITIDSDEAVQLAFMEWRDQHQDSLAQGQVGEIQ
LFCQAVEN
>g1748
MHSSEGLLRMRSDKAAYTAASLAASYKLDEGYSDDTRSQLEGEDGVAQNVSMLLPDWILPYSDSER
AELAHSILTSLPTNLVASVVERLTPRLHMDPVLRLPPEITAEIFSYLDPATLFTASLASRAWRNRI
IDPRLWKDMYIREGWRVDIDAVAKYEQLHTERFVSQIRKSRSRLADSDSPEPQLKRRVTEASLQSR
DPSDYNAQSNPPWNEQHPMVEADVPVSPLDADNDREMRDVNAACQPLSSADSPFEPTKSPRQSPCV
IRLPNGTAKINWPYLYKQRRRLEDNWTKGRFTTFTLPHPAYSDEAHQQCVYAIQFVGKWMVSGSRD
TTIRVWDLSRMRLCLPPLRGHQTSVLCLQFDPSPEEDVIMSGGSDNNVIIWKFSTGQKLLQLTNAH
QDSVLNLKFDKRYLVTCSKDKFIKVWNRRALTPMDKDYPSVVRGSVNKYPTYIIDTTQYSPSWLEA
NLANGHIKKLEPYSLLMTLEGHDAAVNAIQINDDEIVSAAGDRLIKVWSIHNGSCLKTILGHKKGI
ACVQFDNRRIISGSNDDTIRIYDHASGAEVATLEGHSNLVRAVQAGFGDPPGAEEALRLEALAVDT
DFWDAARNGNISYQLRTPVFRRRAGPRNTGSRNPNDVMALGAHIPPGGGGSRWARIVSGSYDETVI
IWRKDRDGRWLVGHRLRQADAVTMASSSNSIRARDQAMRHMETLQSQPRTGPIIHNQARNLTAAAV
AANNNNNINNQAPGAVFNALAAPQQANQQAAPAAGNNFAPNPLQQAXAVNDIVAQAQGVAPALQAH
AMAIAAANRHQHAQATTPARIYKLQFDARKIICASQDSRIVGWDFAAGDEDIMEASQFFLGV
>g1749
MGILGTSRLAIPTAPFLWFRRGTWWMATEVRQIWQRMIGLDIVGYVDFSPSSQYLTVVDECNALQH
SAAIPQMNAPSIDTHDPVLLHLLTETAIGDSAGYEILPFEEVEDLKREYATLSSRVESTKRKLALE

TKLRDAAQSLRRLSSLKSNGYTNGVTNGDTNGVAHHPEDEEYTVSSRKCEELSQELWALERRAQDV
HKRLLEHTAGILQLTHRGLKKNANRGSINNGLNGTTFEFDDRSLYRELETDDAGLRSYSGVSGLTA
DVDTLRNTEQKLEELNGRLRDLLLENHSTHDIGPLPLRVTQDTSLTFVQDHLSYLERSLDTLQTEG
IRRGEGEPELSATEMGSELSAINSRLQTLLGQAGSHLSPTLPPPPSPTGEFLSSHIDYLKSGMSDL
QNRVAGLQDQKSILTTQIQQQRELNSKSDAEKDAHVADLAQQIVETRKQYELSERELEAVRDELAL
VMEQLDSVRQQENIQDQQRERGQIAAFENEKEARSKAEAEVQHLQEKLNHLEGEYAREQAEALELE
REARSRAETEVQRLQGEVAQLEGGSTRAQTAEAEVQRLQAELNELEAEYARAQGAETEAQRLQQEL
NDLETEFARAQTELTVVKAELDGAYGTRAERAAEAAASPALYKEIEDLNTKNISLAEELAALRAEQ
VNNGAGQGELQKRVETLQKELEETIEDYEAMTKASIEFEKERERYEGAIDNMRDRIEQLEAQLSDE
RITWLGVSQTAMRDGATETTSTMVLKNEFKKMMRDTRAENMKLLRELLQAEQEERKRLEALVRSLK
MEQTIGKPTLNQSMVT
>g1750
MPSNVSFTGPAGAAAKVSIIDSGFRLSGLATDLLLTPPVEHFDRLPGVGSWSFLIEGSTGRKILFD
LGGPADIDQFPPLVADIVKQADAKVEATKTVADVLVENGIELAHVESVILSHGHWDHVGDITVFPS
TTELVVGPGFREAFCPGYPTKPDVELSERYFEGRNVREIDFSNDKLSLKYGPFRAVDFFGDGSFYL
LDTPGHAVGHMSGLARTKTEPDTFIFMGGDVCHHGGEIRPSPYLSIPSQVQFSRTGSLQESTFLDG
EKFRKLNVERGREPDETFFDPVLAVDIPRAIQSIKETQEADAQDNVFFVFAHDMRILDVVDLFPKT
VNDWKDKGWKEKTLWTFLDDFIPAAMSGQ
>g1751
MQLTILLGLASAAYAQSLCSQYSSYTSGQYTVNNNLWGESSGSGSQCTYVNSISNSGVSWSTTWAW
QNSNSQVKSYANSQLSGLTKKLVSNIGSIPTSVQWSYSNTNINADVSYDLFTAADINHVTYSGDYE
LMIWLGKYGSIQPLGSQIGTATVAGITWQLWYGVNGSQKTYSFVAPSQQTSWSGDILQFFHYLSQN
QGFPASSQYLIDLQFGTEPFTGSQTTLTVNNWSAAVN
>g1752
MASGKSASPDEVTTNMATEMPVYKVYKRRFFGLFQLVLLNVVISWDFAFCVATPVVMYMLNKGGPR
VVFITASVLILVGNWIRYAGTRADNGIFGLVMFGQLLLGLAQPFALAAPTRYSDLWFSEKGRTSAT
ALTTLANPLGAAIGELVDSAWTTQPGDIPNMVLYISIISTIACLPSFFIPAKPPTPPCASAEVSKT
PFLESVKGIFRIREFWLMFIPFSIYVGFFNSVSALINQILEPYGFTETEAGIAGGVLIIAGLISSA
IVSPINDRFKKYLLIIRIAVPILAICYIALYFAPSSPYGLAPSIVVCALLGASAFALLPVALEFIV
EITYPYSPEIGSTILWTGGQILGAVFTIIQTELKASPNANPPANMKKSLIFSAVVACVAAIFPLTL
NVFGPKIVNRRLEADHARNNVNGVELGHRVDGEVKQVEVA
>g1753
MGLTDFFSDLVSSFGLAEAHAEAPPAEQNDDSVAEEQEEKTEESAEPTEEPTEESTEESEEAEPEA
EAEDAEEGEEEEEEEEEEEEEEEEEEPEDIKPKLEEDCANSKTCAPFKHHFDECVERVTRAQEDGDS
SGPQEDCVEEFFHLQHCATQCAAPKLWRSLR
>g1754
MLSSKEGFVRLPNEHIIYTSPPRTALSLKPPSAWQGKENFSISSSSGRLYLTNQRIVYLPTEPNER
IQSFTAPLLNLHDSHVSAPWFGPNAWTVLAQPVSGGGIPASLQLVELKVTFNEGGAFDFHSNFERI
KDRLQQAVDHARDSGTASVQATAGRGVNFTNVHLDELPAYDGPNVSSADRNQSVNVAVEPPIRETA
NTTSPAPVEPPPGYEEVQQQSVAEELEEQLRRAR
>g1755
MAKAPYIPPQIGWLYDLVLWTFSVLVDLFFREVHPRGSWKVPRRGPLIIVAAPHANQFVDSLILMR
VVRREVGRRISWLIAEKSFKRKFIGLLSRGIGAVPVARAMDNLKPGAGTIYLPDPVNQPTLLRGNG
TKFDGPGFEPEGTIHLPTINGNSYSATIAEIKGPEEIVLKKPVKEKDALFQLTGRNDITDDGKFNG
EGSGDKSDFQGTKFKFAPHVDQSAVYKAVFGRLSHGGCIGIFPEGGSHDRPELLPLKAGVAIMALG
SLAENPDSGLKIVPCGMNYFHAHKFRSRAVVEFGSPIEVPRELVEKYKRGERREAVGELLDTIYHA
LLAVTVNGPDYETLMAVQAARRLYNTKGKKLPLPMVIELNRRLITGYAHFKDDPRIVKLKNSITSY
NKQLRFLGLRDHQVEYAKFSILKVVFTLVYRLIKLTLLAIGTLPGLVLFAPVFIATKSISKKKSQE
ALAASTVKLQGRDVMATWKLLVALAFAPAVYAFYTAVFTYWTYRNRIQGYVPEWVPLWAMILLGVV
LFPTITFAALRIGEIGMDIIKSLRPLMLSLNPSSANTLVKLRERRAELSHEVTEVINTLGPELFPD
FDSARIVADPFKESGHVEGEKSAEGYGETRSRPGIQKSYRTQEPLPRNESFHDLASIGFFSTRPAS
RSRSRSTAGGLRLSSSGSVQPLSEIDGHDRLEEVSKRIRGAMRERGKERRQRSEDLGWESVSAGSI

TPPDDGEKKTR
>g1756
MSSSMHFSHLRKWFIASPPVEFVINRVRELLLGALRQGPIPQHIAFVMDGNRRFAKSHGIETIEGH
HLGFEALARILEVCYKSGVKVVTIYAFSIENFNRSQFEVNGLMDMANVKLAQLAQHGDILDRYGAS
IRFLGRKDLLRPDVVAAVDRAVEVTSRNGDKILNICCPYTSRDEMTHAIRQTVVDYSTPLETTRAN
GGRKPFSESHIASNIRAQTLQNMKDQDGSADSDSASAFSQDEDNGXPXVNQANKXYDSPSSFSSST
TLHLNGQAAGSPDYLSSEFITSQTLTERMYTGNNPPIDILVRTSGVERLSDFMLWQCHEDTEIAFL
DVLWPEFDLWHFLPVLWKWQRRMTKAWKGENALDNDEWNSAKNANILVPETKTQVEAF
>g1757
MGGGPLFPERPGAELAEDPSPSSNNLYAVVDIGSNGIRFSISDLSSPTGRIIPTVHAHRVGISLYD
AQYDTESGSKIPVPRSIVQSIIGAILRFQATCVHFDVPSSRIRIVATEATRTAINSAEILESIKNA
TGYEVELLEKTDEGLLGAWGIASGYSDVEGLAMDLGGGSTQITWVVSHGAELHISPKGSISFPYGA
GALSRTLAELEKGKSKKEAKHARDKLRQEMKNNFLDAYNKLEIPDRLADEAKQNGGFRMYISGGGF
RGWGYLLLYLHQTKGRHYPISIINGYRAGRDDFAKTETLKEIARTADRIFRVSDRRRQQVPAVAFL
INVLAESLPHGIKEVYFCQGGVREGILFRQLQPVIRRQDPLEVATTPFRTRSAGSIGHLLLTAIPP
SSSANRFSMPNTLNVHIIQAFANFMYAHAGISKELASTAALYSTSTGLLAEVHGISHGDRTRLALL
LQERYGGHLPPREEKAKSAFQDLLSVQERWWIRYVGKLGLVLGILYPTGIIDSLWPRVLLSVRWAS
DLGKRQDKFGIELTFTAHASSDHMMIKEVIEGAKRKIEKVGKKKNRIGGRQGWGMKVKVVVNIN
>g1758
MGKDISGSKVLLLGSGFVAKPTVDILDQAGVEVTVAARNLDKAQKLASGGKHTKAISLDVNDAAAL
DKELEKVDLVISLIPYTYHAQVIKGAIRTKKNVVTTSYVSPAMQELEADAKKAGITVMNEIGLDPG
IDHLYAVKTITEVHEAGGKITSFLSYCGGLPAPEDSDNPLGYKFSWSSRGVLLALRNTGKFYRDGT
EFTVDGTELMGEAKPYFIYPGYAFVAYPNRDSTPYRERYNIPEAQTVIRGTLRYQGFPQMIKVLVD
IGFLSETEYDFLKTPISWKEATKKIIGANSEKEADLLAAISSKTTFADNDERDRILAGLRWIGLFS
DDLITPRNNPLDTLCATLEQKMQYGPEERDMVMLQHKFEIENKDGSRETRTSTLVEYGDPKGYSAM
AKLVGVPCGVAVKFVLDGTISEKGVLAPMGMQLCAPLIKTLKEEHGIELIEKTL
>g1759
MALHNPNNWHWVNKDVAPWTKTYLQDSLSKISAEEDGVSAKVEKVLSMDGDVDVSQRKGKVITLYD
VKLQLEYEGSSFPSLKRVFSFCKTKDEETVTGTITIPEVAHDTEEDEFVFEISVYSESSSKQPVKD
LVRSKILPQLRVELAKLPKVLVTEHGKDIQHAPGSNPSSGFATPAYHPHKSPSPAAGTPKTTTTST
GKVSVNTTTVTASDEFRTTAEELYTTFTDPQRIAAFTRGPPRQFEGANVGGKFAIFDGNVTGEYVT
LEPSKRVVQKWRLAQWPEGHFSTQEIVFDQNDVDSVTVMRVTWTGVPVGQEEVVQRNWDGYYVRSI
KQTFGYSPPSSFATITTVPRDAPPPSFSTSSSSSNQFSLVTRLLMFFFVLLLGVGLARYSEILKFF
QS
>g1760
MISMTEGNVSWQSTYSVPPPPPAGNSRSSSRADADRYQTSMDSGYGSTTPEPMVSHYLSATQQQAT
YSDLSNMRQFDMPLHAPSSKRAASTGALNTATSSVSLNPTYERSSPRNSWEPGMPLPPPPPGPPPA
ARSRSASTTTSDASSRRALQVPRSRGPPILGTSLGQVPPTPADWVDETVVSRRVADGAQRLHIDTE
VTYNAPAVPAAREREPEPEPAEPSSSRRASSRGGLFRSLAVKRDSSAKGIRERRIENRHRRQQVSE
DLSAISNTSNPWANALTPTNLVLNSTNGAHGTNDTETSEDKTPSGRRSAFPKPLSSARSAKSEEIP
SSSRSRTSSGLFSDQSSYGTPKPESIFSHAEITPTPPFSPDNESPLAKDKQHMLNSKPLPTTPSSP
ASESKSSSRPGTRLEERPVSHLLHIPNDVVSVLPPLSPRRMSTTQTPSLDSVLHKDNDFLEGGLQR
YRATLEKEAAAASEAEALKIFMEFLVTESQLRRRLYVRAIADAEIDVESTRKKLFTQPQPPQPPRE
KPTGIKLDTSFTQPNKAPGNMPTRPESMWWNNYKPCLSPIASISIDADEMSSRGRPPSRWWESKTG
SSSEGGERVSQRSKRESKYMGVPRELREEMQRGYGGPLSVTEEEQYSDQQNFSQGGYGPDEYPPEK
VGLHEPESNSAQYHQYQLEISHASHYRETRGPKKMDISRLVTLPPPYPRHHPAVNNSHPDLVIYRT
TVRSISDLSEVRATREQNKQQMAQLKQEHESKMRENRKFFTANIQQQIQEGSIKYAEAAEAEAALE
AEEQKKERDLAQSEYDTYQKKVVEPLHLILSDRVNKATERINELSGKLKEDIQHENPDRTQEEGDE
KPEILEQLTQLKWLFEAXEQLYREIYDLLGESNEKYRAIVTLPYKQSKNDEKLREADAFFVKDSYD
RRVQYESEALARMERFMDVVEENVKRGVETHLSAFWDIAPSLLTLVQQVPENLSNFQIEIPAQEYE
ENPSYYQFPLQYLYSLLSHAEKSSYQFIESQTNLLCLLHEVRSGVMMTNCRLMAAQQTRHGEPEDL
VRREMQEVQAREERELTADLKEKVSTVEGQWTEALGSQIQSLRTRVKSHLLAEDGWDETSEQLE

>g1761
MAANSNGGSGVGQENINTDIVTLTRFLTEEQLRVPEATGDFTLLCHALQFAFKSIAYYIRRASLIN
LTGLAGSSNITGDDQKKLDVIGNDVFIAAMRGSGKVRLLVSEEEEEAIIFDEHPNARYAVVCDPID
GSSNLDAGVSVGTIFGIFRLPDEILGAGKTVSGKDILRDGTELVASGFTMYGASAQLVITMRGGSV
NGFTMENSLGEFILTHPKMQLPPKRAIYSVNEGNSMYWDDWVNDYFHQLKYPGEGGKPYSARYIGS
MVADAYRTLLYGGIFAYPADKKSPKGKLRILYECAPMAMVFENAGGLAINSHGERLLTVLPEHIHD
RSGVFLGSKDEVQKAIDVYNKHHKK
>g1762
MSSAGSIEQKLKALESLSACDVSDALVKLQKVPPGTPVRAGQLADITPISPFIGRQPNKPKIIAPA
VTFKFIPKGEAAPTVADPEKNGFPPGKHWVDWNQQGTIAVLDQPAEQYCAVLGGIMAARMSYIGVK
AVVVNGRVRDLAELSASGLHVWSKATSTVGTGAEAKAGLRNVPIDFNGVTVDTGDIIFCDPLEGVV
VIPRNLLDDVLELCPKLMTQDDKVKADVEKGTASAVDQQCVPHRCYLVRSFGLRLFWLSRPAILYN
MAPKDTFFRSADMSLTQLYIANEIGREVVSALGEVGQVQFRDLNPDTNAFQRTFTKEIRRLDNVER
QLRYFSSQMEKANITMRPSSEFGNTLTAPSSAEIDELAERSESLEQRIVSLNDSYETLKKREMELT
EWRWVLREAGGFFDRAHGHTEEIRTSVDGDGDDAPLLQDVEHQVGRDQNGETQGQQSFSVMNIGFV
AGVIPRERIGSFERILWRTLRGNLYMNQSEIPEPIIDPATNEQIHKNVFVIFAHGKEILAKIRKIS
ESLGSSLYGVDENSELRRDQIHEVNTRLGDVESVLRNTKNTLDAELTQIARSLAAWMIIVKKEKSV
YHTLNKFSYDQARKTLIAEAWVPTNSLSSIKSTLQDVNDRAGLSVPTIVNQIRTNKTPPTYVKTNK
FTEAFQTIINAYGITKYSEVNPGLPTIITFPFLFAVMFGDFGHGFLMTLTATAMIVFEKTLLRTKL
DELTYMAFYGRYIMLMMGIFSMYTGLIYNDVFSLSFEIFPSQWQWPRNIDEGQVVQATLKQGYRYP
FGLDWNWHEAENALLFTNSYKMKLSILMGWAHMTYSLSLQYINARHFKSKVDVLGNFLPGMIFFQS
IFGYLVFTIVYKWSIDWNARGDSPPGLLNMLIFMFLSPGTIEQQLYPGQAGVQKFLLGLAVLQVPI
MLLLKPLWLRREHNRARGLGYQGIGERAHVSALDEDGDLNGRASEGRDSIASDGEGVAMIAQDIDE
GEHEEFDFSDEMIHQVIHTIEFCLNCISHTASYLRLWALSLAHQQLSIVLWTMTLGGSFGIESPTT
RVIMIVVGFYLWFFLTVAILCVMEGTSAMLHSLRLHWVEAMSKHFIGDGIPFQPFSFKTLLEEDPV
D
>g1763
MPASHELGVNSHVEDRVDSDQDAEGEEDTDLYQLDQELQSAVQKADAGSAVEEEEPMAESDAAEDD
DKSEEEDYAEQVNDDAVSRESENEYDETPRRPRRVRRSAAISSKSDDEESDADVAYGNGDARSDSE
SESNASDREAEDWEAESNGNDENDADKLIRSNCIFCGQDEDHDPSEDFEEYLTCAVCGDHSHRQCA
REQSAFNDDEDAEWWRCTTCVQDNLQPDPETDAASRRLAAESIAGDLQAASADGQHSVFNNLIVDD
DPMDGTRSLRKRKTSPTDSEDHVPALRKRQHRTPSQGATDSADGSLDADGLPRTRPRRARKAEKEL
CRVVESDVGRCIIALHLNFPKMAKILNSRPRPKQRRRKQPKPPPVEQEPLSHFAPIPPSYTTPFYS
FHDREVDELKSKPYGGILSEADADTSRTLPTQADREKFEAARRSAEEDWQRKVMESEPVGELNHRA
SQKVSGPPSKIKWINFGGYEIETWYAAPYPEEYSRNKVLYICEFCLKYMNSDFVAWRHKLKCPAKH
PPGDEIYREGSVSIYEVDGRKNPVYCQNLCLLAKLFLGSKTLYYDVEPFLFYVMTEYDDLGCHFVG
YFSKEKRPSSSNNVSCILTLPIHQRKGYGNLLIDFSYLLTRVEKKTGSPEKPLSDMGLVSYRNYWR
LVLSYQLRDQKTPVSIAELSDRTGMTADDIVSGLEGLRALVRDPVTKTYALRLNYSYFEEYIKSWE
VKGYVKLNPDALVWTPYVMGRSNQSHYDRAQLHTVAPREDPNDEDEEGADEDGDLGLGANGTSRMN
GDSTKPDGLSSYAPAGPPSTAGLSSFGENSTVLSQTNGETRSKSPDPAAGIPPTRFEIYPPVRPPV
FKRRPGRPWGSKTKYNSAYMSTPRNARSTPRRTSTSMFGIGSPASTNAAATSARRGRSGLVSELAS
GPDSPLVNGTEGHDDDEEEEEEEQQLQDQEMDEAAEPDQELTNGNGATPDAAIEAEEPSIDDAKGE
DEPMTNGIGQPDSVSVVAESGNDNATTATNTTNSTGNLTPRRTTRRSMADGEKRVTRTTPVKAVSI
TAAANVLTTNGNGSGDGNGDSDIDADGEIDDDVPMGL
>g1764
MLSNTSPDFELIVRQQPEQARVAGGKEKERKPVDPPPVIQLKVSPENTYLAHLYHATEDHPVHSSQ
PSTALAGTLVSSLHRLKDTDNTDGGFFVFGDLSIKVEGEFRLQFTLFEMQKDNVVALKSIISNSFP
VLTPKNFPGMRESTQLSRSFADQGVKLRIRKEARNAQKRGGASRHEEYAPSLPRSPDRPQMPQMVS
SAFSGYPQHTFYPTTPAPKRVRTSMDMDQRVFEPDARYAPTYQPPAMYSNQPGPYPNPMFSYTGPS
QHVTVPDYGVPVRPQQPLTSTGSSYPPSEDPMLAMRSFNNTGYRYPT
>g1765
MSSNLNNPGHIQLKPLPTLSCEFCRHRKIKCDKLDPCTACQRAGVKCESIRRKRLPRGRHIAKSVA

GRGRGSTNDKMTRHDALKEKIDRLEALVNVALSEADGKGGSGGAGETASTSSAAGMTGKVVSRDGN
QGSDGFEPTPNALVSQFWSSVINEIRESRESRGFAGDDTEGDDDEDDEEEEEESEVENEEAKGERV
HLAQEPARAYLPFMGLSLGRLGHTLPHSLXLRPSPAITAALCDIYLNQIDRIIKVLHRPSLQRHLL
EGIPYPSSLKSKSSREAENALDTAVFYSAVASMTDRQCYKLFQCSRNEVLPEYQSACEMALERADL
MRTTDMMVLQAFVLYLVATRAHDKSRAVWTLLATAVRVAQWLNLHVESLSQTETFFNQQMRKRLWF
TICLLDLQTCFDPDSRPLITLEMTQPTLPLNVNDADFDPSFDNSGSTALQERDEVTDMTFALFTYN
LQFCGRRLYYIPRGAHSAREDILSDFKNSVLPLTRHCNPDDNNYSWLVYYASQNLISSTQLHTFRR
PDFVSQQQQQQQQQQQQQQQQQQQPSLLELCLRNLENIPRIHGDDRGEGFRWYIQPQWPQILLAIK
ECFVTTDAALLKRAWPLIEDVVGHYERTHRGPNTSSRKSMLRKSLRRARRRVDSIIQSAEEDYQNI
QANGFEDLPDIDVGWDELFNDFSFGEFPKPPAGSEVFLYRETQSLVYEDNLARELGKDTACDSFFH
VYASSFERTISEWAIPTDHASSGIVRYPALHNTLYDSVQRLDGKASALIVRRTSPGHCHRAIRALR
LTVMAFASQSNCIKRAVSRDDRTQAYGEVFVSANDFQTLLFQTLWHEARRSLEWFVEEDCLQSILA
KIIFCLTHSPYYYLRYEDSVSRRDPLAYDSPDIMALRKTHFDKALQSLSLWKKELQHLFQDPERLQ
KSRVDIDDEDITSFCLFVKFAIICDETSAVLWNRDLLIAEEEGDVAWPQNGFSKSLQTLSTGFTNP
SPSPSAQSGQVTPQNQNHTSPPRQPLKLPWNNLITADIFLQANFLKASLWRKLGQIRRFARRSPTL
TGNPETYIQETLQLFHEWGTCCASFIENCMASITTSSFDLQALFFGHVMHWHLGALLFATEVELLD
ATSRSDSVRSSIRRSTGLTRELRKDSVYAISDLARAANRLAIEGVSRQQVDTRRGDYYKHPLLVGP
PSAVSHIVFSETCEILMEWRNQLLFKLGGHRPGSSRCQDEEYLADIVGRLGHCINTLDLLGAKSVV
SKGYAHDLRTRLDIDRGLY
>g1766
MPKTLQPYSMRLVIDWFELEDDAYPDVGLPLGITVFVELQVEPDSVFTVMRDVEGVINLESTWYKG
VTKIADRAPASSVEDVEDQDTSLKTEEEDHNLDARGDSYSADDLDEGESFASSTRSSDTVRPSTIR
PKLSRIPIRKSGTDGTFDDLDLELDMPPSPTLSSQDSLPWYSTKDTGDNHMDDENGPTGIDLDDLF
TDLEDIYHPFLRYENGNITLHAAREVEPGFYQVEIAFYRHFVRDTQGWYGVDLCDVVKSCPPIPGK
LVFNIQDHPSLYPDIIGPDDLHDMEDGSREATFDPREEPYLAFWAWTHATYLEFWGDGKVDNNYYD
DLIAPNAYLDSDELVDYVDFMFKTRDDEEVVDGASEMAETPEKVAQSKQYISPIADQEDDDEDEEE
EEQEEKDGQVDGLILGDVTEVNVDHVVSGLEERPMSPIPEEEECESGDDEDVIWEDVPIPGVQNFE
YNPEEGPETAKKGGVEDMLMDNVTTPNVKDDASQSEETVIEEEAHDVILKDVTAPYVDNVTLESEE
LPKPPTFREKAESSTSEDITSSHDDNPPPLSPDGKPPADDSKENNLKLITFYHPLPPPPLPTYAEA
LEGYEKYQAMMTSRAILRRRAIFCLSRILPALFVLVFTICACIGGPPTTISTPTATLQTVRYYANH
LGGMGYNALMQVPTFADFGAYHQRDWYEDGVALLESGNEGEDLGDVEVHLWWKLVDETLEIAGIAD
SPAVPESAEPVVESVVSPSRSELDAKSPPPEIAAEALTPNMSTPVPAPDVKTPVQPETASSFNATT
AALSLAIKPPPLPRLCPNRHRQPNTPYPPDCPLPSPPKPALTILADLTQEIFRHIACSTSPICTIS
RISDKYEHPGDIFARFQCDGKGGWEPETRRDGFDWGLGWVPDECRLL
>g1767
MASLQLPLKSLLRPAFAESNTSALAVSRHVHYQRRSCHHHHSCHPSKSLTNSATTRKRQPQTNHFS
TTPSHQTDKLQSGLEFNKSQWAPSYAEKFASLGSGQQPQILWIGCSDSRCPETTILGLQPGDVFVH
RNIANIIHEGDLSSSCVIDFAVGALKVSQIVICGHTSCGGVNAALGDAKLGVLDTWLLPLRKLRAQ
NLSTLEKLDSKNAAIVKLAELNVLDGIAKIKEKSVVLEAIEQRGLKVSGLIYDVATGLLRTVDGGE
ESQEEIKARLTAFKTN
>g1768
MPLSLGFTADAAAAHRHEYSKTNPAACPMLQITQSLGFVKPLKDGVTSYITPPPPPIITSRPSQTE
LSSSSEGEEEDRGNEQKKAKKYTMSRQVPNRPKTLYQFCHPVASRRRLKLRPKLLLQLHESNPSSR
PFPKYDVLPPDMTTKLVCRLSKPLGNVRAFGSKDLVIVTSDMYEQVSTSDDRSVSSDEACAEQREV
VATICQTGSAEDFPRGSKVELALSSGACWEGTAMPNGSYELVSKSGIADSKKIRWVARDKKPRQGS
GLSGSTVSASSTPGRFTFSVINPNSRRHPVIASMTRKGLTVFDQYSYPIQHDDEQSPPASPSTAST
RSAPEAGLITTDEELRQLIVMTGTWVAFMEGWSTKSPVNGDASRTDSPLSPRIRSSTYLSVEGESS
IDRSLGGPSTPTISRMNSFSRKKMINRRSTHSDVSRERLSEHDASSDNRRRSKSLTTADRGERWTD
VEVDKAIESGQRLSVDPGRREVDHADEEDSKAKTKKWRRLSSMFGRKKH
>g1769
MDDGSKRKAIDPSTDPYYLRHPIRTLTLLFLAWKAILDPIIAISPGLGYDTSTNLLAVSSPSIAFD
ADSLATWFDLLLSIANSSSSGIWNFVRWDAIYFVRVAERGYLFEQEWAWGYGWTGSLSFLGSAFAN

TNGSVPLSAIAVSGIAISHASHFLSALVLHALTKSLFGHETQSQKAFCLVSAALHIISPAGAFLSA
PYGESLFSLLNFTGFYVYSSALLAENARHQARRDILFVLAXIIFATATTIRGNGIISGCLFAYDAV
QGTWQFLNSGLSIDLIRRTAFVVLGGSIVALGSIIPQYIAYTEYCVAATRPWCERLLPSVYSWVQV
HYWNNGFLRYWTVSNLPLFILAAPVLTAMLYSSFRALTGRLGPYHIDHVSISQMNTSQTVVLRLAI
AQGILATMTLTSLHIQIINRIASGYPVWYWYLATSAWASSKPLSSWVFATAIQGMAMYALIQAVLN
TADRIASILTSENFQNGHEMSRATSPGGEPVETDGSKKARVRPRTYPYFSYLPYQAEDDSQREHDL
HEILSCLYVAIKAGDFSPGAVHWTRELRGWLSLKFDPTREERIKLVRLYYELSLAPGIDPNVAERF
ASMFMLLTKRKHYLRPVKDLVLDWKPLFKEIKAFVLPTESGLIQSTNLKRNIKTLTKLCAFAQLYF
DPCEVPAMLEEFLPHFTTSFSEGAFVVVGLMNLLLPTSPAPEPRDDLRPQKFLPTYFHLWSLVNRS
KTFDMNFLDLLSRLARDSLPASHIPFSEYGIFTKDQSSVIFTAILRLLEIPXGXATSPYSALVDIS
SGLGIMLDRDSRKHPVAHHIARWIVMSLSPACLDSPDSILHQLEGLIQAVETFFHPSNSGNWTKTL
AQLVYYLSDFFIMRWNREHNGEMEVPPERKLNEPLRKRFVLCLRDVIFMGIYAKSGTAMNFSLSTL
QSLAYLEPQLILPGALQRIYPSMQGLVEVHRTTSSLRSLQILSRIIARTKGFRCHMTTLLGLALPG
IDANDLEKSMHALSFIQSVCYNIPFEDLTKGRDDVNCNMLAMQWITGEVERMEVEGANVQLNYDTD
LSDETEEMILRSSTTGFGEFLTSFLGRVFTLLENLPDASRVKSGSPEENVVNTLPATLMPLLSALS
PELYDMALQKVVDFVSNHVIHQARDAMAFICNAVCKVNSEKALKRFVPLLIQAIRSEIDENGAAST
RTTGSEVLPRDRALVWNVSMLSMCVVHVGSAVLNHKKELLDIAIYMQEKCKGIPTIHVSNFIHHLL
LNLTVTYTLDFGLFEHEDIKDGIKIEHWGYRPDPRNLNINWHIPNREEIEFAVALFQSQAESALSQ
LTALTDGTSSVKRDGSGKEWSDEVTRKLVLLRLIISGISVLFDPKAASKAGDDTNGSASDVEMTNG
HVTEEEDQDNNDSSLDSSDEEAVRETFTYPTGYSLTEDDPLYHTIHSIRKRAGFILHDVHRFLTDK
QEDDVPCFGALYTAYRSWFIDVGIERSAHVLDRVTRLLAADIHPYKVSGVRKDYPRPLLVRRANVY
HLQRLRHNAAPRPRSKLDEVLFLDLAESSVSLYTDIRRNAQNAGESALKAVWGSRLVVIPPLLKAL
QKAIKENDHARIKGALYSLLYGSLAKTVGRHWKYTPDVVRAFIDASSVDKPSVQKLCSGAIFQIMD
YGRPLERMAVLNEELVNAIAPAENVEDTIIQKRKSISKKRVQIEGKKAELAEELVEMAQTSHWKIA
SRAASIVVSMGLRFDHIASEKLIDLVTKGSIDTHPGLRGMYSQALIALFTMIDVRAICNHSYENYI
LGEQTFLARIQVATKRYEKGWTEEYLASFAKPSAEYYIDHDFPGWLVWSSKMPAYKANIKRDIEYD
DVEWTIRKHMGQLLDRQWFRSFFAYLKQEPRDASADKFRMACAMMLLYTFELMIRDGLTAATFEEI
KEEVELVFEDGSDKHQHRATAEILGALISSVADTDVEKRNMVWEYAFPMVKKILEDGLTPENSGYW
TTFLHMILQCRDPRRAWPLVDWLASFKLDMASNAAFKESSKISLLHQCIIDAGWHFQLEKPIVEDF
VAHLDHPYKGVREAMGHTLGSIFRTRYHESHADVDSLIEAQSSSSSIGTYPYDPSKDFSEMLTTVF
ERLKVWRHERQPGQQTPSSYTSGSKTVLLWLDSTLSSHECTQLVPFFATTFTEELLHMMDVKEDPE
LQSLAYHVFRHLPNIPYPAENESAFIQTLVHIGQRATSWHQRLRVMINMQIIYFRRLFLLSAGDRD
KLFECIANMLQDSQHEVRVGASATLSGMIRCSPIALREAYVAKYQERFTKTLVENPLPKKPKNHIG
RSSATSSRTGTPTPEHTRLVIVRHAAVLGLGALVQAFPYASPPPSWMPDALTTLSTKAAHDPGVVG
SSVKSIISEFKKTRQDTWHIDQKAFTSDQLEDLSGVLWKSYFA
>g1770
MSQAQQRLSQVSSHLSTPKSTSNNILSKNPDDIVVTAALRTPFTKGGKGGFKDTAASDLLVGAFQG
LLKRSGIDPKLVEDIAVGSVLPPGGGATEFRAAALVAGFPESTAVRSLNRQCSSGLQAVTDIANAI
RAGQIEVGVGAGVESMSLQYGPGAVTEFSDLLENHLQAANCKVPMGVLSENMAKDRNITRADQDAF
AASSYQKAIKAQKAGLFDEEIYPLTVKWTDPKSGEEKTITVKADDGVREGITAESLGKIKPAFAKD
GSIHAGNASQVSDGAAAVLLMKRSTAEKLGQTIIGKFVTASIVGVEPLLMGIGPWKAIPVALEKAG
ISKDDVDIYEINEAFASQCVWCVKELGLPFEKINPKGGAIAFGHPLGCTGARQVSTLFTELKRTNK
KIGVTSMCVGTGMGMAAVWVAE
>g1771
MGTPILCIDDIRKEAEKKLEKSSTDFFNSGSTDQVTVRENSTAFAKYRIRPRVLKDVSNADTSVTV
LGRKIAFPLCVSPAGIQALAHPDGELATARACAKRGVHMGVSSFSNYTVEEVIAASKETGLGRTAH
VMQLYTMQDRALQERVIRRAEAAGCVAIFLTADSPVLGFRYNEWRNDWRXPAGLAFPMLEKTTEMI
QSQTHDESFTVFNSDSHSWAYDIPWLRSKTKMQIWIKGILTAEDVLLARQYGCDGVLVSNHGGRQL
DGTPATLDALPECIAAADGKIPVHIDGGFRTGADIFKAIALGAQCCWVGRPAIWGLGYDGEKGVTK
TLDILYDEFKRVMQLAGCWLRLITVSDLLNDDNPTLILSEWRGLDITNPGDSFIPKYATISHSWAA
SDEVQRLSEIADRPLRIDLGNDKYHTISWEGLVQAAKAAQHLGCVFIWLDLTCVHQNSSEDKKLQI
QHMGHVYQRSVAVIVMPGGVASAQGVGNTAPWITRAWTLQEATLSPENVHVLIKEPRWDTTNYDYE

YGTTGPTYQIDQVDDDLALSKLQNLLSCRKRGLEITRVNKSTKVKTRVPFIVRCFGADESLITALE
GVLRGHTDEMKRSAAWRSIYLRTSTKPQDMVFSVMHLLGVSIKVDYDRTREDLILELARKTKDLPS
WLDIGEDIPFDARFGLVPALPIFHPNDMPSYDIDRELVPANRYTESGTYIHEYDIKIQTSPTSIFD
GDLICAKIFPITFQNVSGASITSGDGEKFDFSDVDQKNVTGSHVVVLGEARVYALAHLGLMHFGGP
QVIFVGRSERGIWERHGKRAFIPGKFAENAWRWHLTIGGKPGSEITPLVLQSIPPFIARHGFVIHH
FFLELNKSLNTSLSRTLFYTQRATYASTSSSPFRLSSSPKQPQSQSQPQPTEDEDAYRYEYEAENE
GAYYSPYRPKRQWPPDMSKLSPKHQLRLERKYRRRAALKYARPRWVKFTKLAQWGIIIFIVIYSLL
FMEWGKEGEEHPFEDFRKDFFASINSLFSAPERPIKKKE
>g1772
MSETASEEMKTTLAVTTANADSPKMVKDKQGRDVVLIPQPTDDPEDPLNWPIRKKIIIFASVCLAA
FAAQMSPNSNMLTFMSQVPVYNKTPNDLLYSVSAGLAGWVAGPFFFIPLVGVIGRGAVIFWSLVCI
FAGQIWGGEMTGANDYIPFTISRLFTGLFGGIPAILGSGYIIDMFFLHQRGKAFAVFEIMIIFAVV
GGGTLGGFIANNNPWDLVFWWTLGPIGAAMILVFIFVEETTYNREAAVPRPPLPKNWLANRIATFF
PGTRTQPAGKGKEFVRRAIIPFQITFAPITLLMGTYIFVALGLPIMQASTLATFMEPPVVAGGYGF
SSLQMAFFTMTAWVGILCAQIYGFFFNDKTPLWVARRRGGTWHTEYRLANTILPSIMLPIGLGLWG
AGLQYHLHYMVLALGSFLIWFGALLALPVCYNYIIECFLHNPVEASVSLNAYRVTFGLISVFITTN
WEDAVGVGWMWGMGSFFILFVDIIMIGLILKGHVVREWTTKLSKTLAFTEDGATISTKQDVVA
>g1773
MSSQVTVVDVNRASELLIPTILTTIVAFIVTALRIYVRLRLIKLLDWDDFFNVLAMATTFVVMGLV
IGGTSNGIGRHIQFLENDIHAATYSIMLMRIAEFMLIISTVFVKISISLFLKRLFPTSKRWKAFFW
AFIVFNTLTSLIDAAVIFPQCTPVALNWDKTIEGHCWSDTAINALGIAQGTIAAATDFVLSILPIV
FLWNIKIIWRVKIGVCAIMALGFASGGFALARTVLVPSLTATHDPTWDLVPLFKWAVLEATFGVIA
AAAPSVRPLLGHNSVTESYSKSNSRSHSVPLNTMSRSGQRSRHRSWLGSQRDQMQELPDEEPGSDG
SSQKKLWESKTHGIVKTTDVSVFHSSREGGTDRSSDEMLVSDAVVYPKQSR
>g1774
MERSEESPKKQFTVAIVGGGIGGLTLAIGLLRRNVSVRIYEAAAAFGEVGLGLSIGPAAHRAMPLI
DPQIREIYDSLITTHADSPGFEKFRQTWFEVIWATGGQAGEKLMTLNALPSGQTTVRRSDFLNALV
GLVPDGVAQFGKRLXKLQENTEXVTLSFEDGTSVVADVVVGCDGIRSKVKEALFPDEGDLERFKPK
YSGMYGYRAVLDMETMVEAVGDQRAKVSTWYVGKGAYAITYPIMRAKKVNVGLYTLNDTWDSDSWV
REASRDDMEKDFGYMGDYVNRIIKYMTDASQWAIFEHPHIPTFAKSKVAILGDAAHASTPHQGAGA
GQAIEDAHVLAELLADTHITSPEHVALAFQAYDAVRRERSQRVVTSSKENMDLLCLCYEGIGDDGD
KLKETWQERFRWLWDIDIQEQAEKARTIMLDLIRQQELRS
>g1775
MLSNTQAPTIFDDATRSPTKTSMLRSILPSKAHKRNTSAGDALPSVMPGTSFIGNAGFLPADHPHA
QPLGERAHNRDAAGTQPRRDGLHKKTKSSVSLRALLRDREKKESRSSSDSPDEEGSLGKTMKKMKS
TASLSAILKRSNRGRKGETVAEPKDKENRRPVSEVVSPIWAQFATQPVEDQTGNVYVPESQRTLED
EMSLYTPKQYLPSKQRNFYDNQPTLVQKPRPKSDYISSGTMKVREMLGQIQRVPSGKQRHSEKQAE
PQSAPDARSIHESKRNSTQSGGRVMAVVSALEAKDSTRKDQDSKQIESEFEKLLDARNIPMNMRDK
MRSLDTNIKADFIQKNHAESSIPNSATSSTFDSAGRRGRKNDRSAPSQTRDKSLRSLSRARSPFSS
TRGDTSPKKHQKSNSASSFKRPKSVDLSRPYMMPTASASSTSLHAKNQTDTSADPADFIHYLREVQ
KPELVEVGKLHKLRLLLRNETVAWVDSFIAGDGMDEIIQLLYRIMKVEWREEHEDNLLHEALLCLK
ALCTTSVALQRLTALSKELFPALLGMIFDEEKKGPSEFTTRGVIISLLFTHLSTAPEGEMPARAAE
ILAYLRDPTSNKHQVEFISNIYQPRPYRVWCREVTNVTKEVFWIFLHHLNVIPLSRDNVVVEKEGD
YFKKHFPAPRPPVPAAPYVGGVEWDATNYLATHLDLMNGLIACQPTPKQRNALREDLRASGFEKAM
GGSMRTCKEKFYGAVHDCLKTWVSAARDDGWDFKFVREGPEPGSPKKSPIKTSSKKPVEEPPKLAL
DIDLEKKSPSRICDVDGWI
>g1776
MNQPSRTDDTLEHPGWNQNPSALNADATTRNDFNGIINLRNRRDHSLQTSSQDELAQIAVIPPPPD
PSSSPKAAAGGVLTTTTGNDTPQSRSKALTMSISVPKFLRPIVKFASFIGPGFLIAVAYIDPGNYA
TDAAAGAQSKYALLFVVLMSNLFAVFLQSLCIKLGSVTGLNLAENCRAHLPKWLNIILYILSEAAI
IATDIAEVIGSAIALNLLLKVPLVAGCVITMADVLILLIFYRPQGSMWGLRAFEFFVMLLVLGVVV
CFCIQLSLVKTQSVGEVFRGYLPSPAVIQGSGLYQSCGIIGATVMPHSLFLGSGSVQPRLKEYDLN

AGYIEPSSEHDDEIYRPSIHAIRGCLKYSIIELTLTLFTFALFINSAILIVAAAALYGTPGADNAD
LFGIHXLLSQSXXPAAGTIFGLALLLSGLSAGIICTIAGQMVSEGMINWTFRPWVRRLLTRVISIV
PSVIIAASVGRQGLSTALTASQVVLSVILPFVIAPLIYFTARNKFMTVRNESVDLSELNTNAEQAQ
SQPQETAFKMRNSWPVSVLALIIWAAITVMDVAALVLIGLGLA
>g1777
MNRLFGTKNQAPKPTLDSAISNVDNRVSSIDVKLAALNSELSTYQTRLSKMRDGPGKTALRQKALK
ILQRRKQYEAQRDQLSQQSWNMEQANMMQDNLKNVMTTVDAMKTTTKTLKKQYGKIDIDKIERMQD
EMADLMDIGNEINESISRAYDIPEDVDEAELDAELEALGEESLFENPMGDSEAVPSFLQDEVAPPS
FVDEPPEQGKVKEAAGGVG
>g1778
MFWRFGGYANISTVDTLLDKPDVSLEELLDEPELISELKQHNTKLIEYLREDHILKKLLEYVVAPP
LINDDDNDDDDDDDAKKKSDEKSAAEDDTKKDEDILEPEDLERADKQRSKFAYMACEILSSETWSI
LESIMLNEAYLREFWNFMKRPAPLDSAQAGYFTKVNEVLFDKKTEEMLEFFKSLKGIVPAILQHVD
NPMVMDLLLKIISLEKVEGGQGIVDWLKDEDLIPVLLSYISSEHSSSMQTSAGDFLKAIITISANA
TQNEQSCIGPNSLTRQLVSAPCVEQLIGFMLQGGNSLTVGVGIVIEVIRKNNSDYDENIGGPDARP
SIYDPIYLGTLLRQFSTHIPDFMALILSSKHTVVENGQIKIKDRGQLSSAWGMKIEPLGFDRFKTC
ELMAELLHCSNMGLLNEVGSEDYIRQRDTERENLRAQGAFDLNRKEASGMAFSENAGESEQEQAFR
VEPQSATVENVNASDDDGFEDVGSTKDLGPEIKTDAGEQPQPEKVEFHAQSPGLRIEDSIVEETLK
NSSKTETGLAPVESSVDPISPTASGLTESVNEITLNKSEQVSAVDELKDQAKPTVESHAEDTLTST
TQSELQSELRPEGPSGTNDEGQSNDSQATHIEDEFAPQIQYDHSGQPVVGDFLKIQFVEHKVVPTI
LDFFFRFPWNNFLHNVVYDVVQQVFNGSMDRGYNRCLAIDLFNAGRITEKVVEGQKRSDEAQTTKN
TRLGYMGHLTLIAEEVVKFTERHPAEILSQSVMDKVLHPDWVEYVERTLSETRERDNAILGGTRPD
LMGHRQAVMNAVQGFTGSNALANAGLNGGNGGSSFENFDSMTHGSASSGAFGLGGSNSLLSGFGSS
SDDEDEEMEDQDDDDTRGFSDNLAESGIENEPASSSTSQPIPILAPPPAPLNIVPSRARRQLAARL
ALHKQQANAAAENSSDQSNAEESHHNGQWQSNPFIIADLEDDTRELGSAEFSAMDENDQPSSPTFH
TGFTPPGSPSTNSSDDTSGDPRETVRRKVRIPLEVEDDDDDEMGEMVGPGSGSGSGMMDSDEEEEA
IINESLGYSNLFGAGRYGGFNRAYGRNGQSSFDDDDDDNDSSDGEDGLVEILVPGRKSFSSN
>g1779
MMEAWTHKTPNETLNAHIELEAWVTQHGGRLDDTVQFAHDAQRGVHLQVKPSTTGTTAVAAATKGT
CVMKIPIELTMSYFNAINYQPLSSSTTPQKEQSAKTFSSHGLHLPQKLIEAIGPEETTAFFLMGQY
LLQEKGFWFPYIKSLPRKEDLTTPLFFGEEEDLEWLGTTSLAAERERRLRIWRGNYEHGVRVMKDI
GFEGVELYTWDLYLWASTIITSRAFTAKVLASVIPTLQQEAEADRVSVLLPLIDATNHRPLAKVEW
RAGTDSIGLVVMSDVGAGEEVGNNYGPRNNEQLMMNYGFCIPDNPCEYRVVSLRAPPDSPLAQIKA
QYEQHFPKSGSNNSKDSEEDKYYVFSLPYPLIDRSQPLEYSMFSPDLLHALSVIVANDRELETVTI
DEDGFKISRNQYAHSRNLIASLNQIVVELLSYIRRLEVSGRRLGEPRNLKQVFAKQFRESHVGLSR
TAVFVANWTLMRSRGAMERGREEVLDELLSRIPSQVFDDEKKGKIKERILSRNSLLSDNQSGELFR
FEDLFQLLPSAFQTIFRQCLEDLATRTKQAIAIDDGTGSVSGTESGPHALFAYASFINLVIAVHRH
NPSHLPSRLRTWSQFLLNTYPAPPNDVAWTLPDETDETILSAFDSFINDHLPGEVLAAFTPWVGTP
VPDQGERDVSDWWLSPNWLRWACDLTGSHLLSARSAIPSINLLPAPLSVQIKMMSRAAVPSTSTLA
HISHRAAALPSSAAARLSTATRSFATVQDGAAPKKTYGGLKDQDRIFTNLYGHHGTDLKSAMKYGD
WHKTKEMILKGHDWLISEIKASGLRGRGGAGFPSGLKYSFMNFKDWDKDSRPRYLVVNADEGEPGT
CKDREIMRKDPQKLIEGCLVVGRAMNATAAYIYIRGEFYHEATTLQRAIQEAYQAGLIGKNACGSG
YDFDVYLHRGAGAYVCGEETSLIESLEGKAGKPRLKPPFPAAVGLFGCPSTVTNVETVAVVPTIVR
RGGSWFAGFGRERNQGTKLFCISGHVNNPCTVEEEMSIPLRELIDRHCGGVRGGWDNLLAVIPGGS
STPILPKNICDXQLMDFDALKDSQSGLGTAAVIVMDKSTDVVRAISRLSHFYRHESCGQCTPCREG
SKWTQQIMHRMEHGQAREREIDMLQELTKQVEGHTICALGEAFAWPIQGLIRHFRPELEARIKKYE
QENGKQPFAGGWAPGTRDEGKLIAPGQ
>g1780
MTTIPEKETAMMRDDPSRHSISNDTESSHENSHNTDQATHPEEIEKDEKEIEKEAATPKDELTKTS
TTLSQKPYTSFSTGQKWFIVGMATAASFFSPLSGQIYFPVLPVLTQSYHLSQTLLNISVTTYLIFQ
GLAPSFMGTFSDASGRRPAYILAFIIYTAANIGLALQNSYAALLVLRCLQSAGSSGTVSFGYGVIA
DVTTTAERGKFLGPMAAGVMVGPALGPVIGGLLAQYLGWRSVFWFLVIISGGYVVVYAILAPETHR

KIVGDGSILPAESWRLSFLQYVRIRRRIARMSPEEKKEYDEQQAELRESQKSRKIGFPNPLLSFVI
LLEMDAFLIILYVALMMFGAMVMMTSLPTLYPEYYGLDELQVGLCFLPFGVSSAIGAVINGKLLDI
NYKRIARKLNFSIDRKRGDDLTNFPIEKARLQLTFLWSIVLALAFLGYGWALNYKAPLAVPLVISF
LMGFTLICTMNSLTTLLADIFPDRVSTASAAQNLLRCLLGAVGAAVVDSMLSGMGVGWCYTFVFFI
MIAAVGLLWFEYVWGMGWRQKRWRKVADKAERERQNGVV
>g1781
MSFGGFGSSNAFGQNNQQQGSGFGSSGFGGSGTAGGFGSNTTPFGAANTGTGGSAFGSNTTGGFGS
SGGFGSNQTQTNSLFGGNKPSGFGSTATTGGGLFGNNTATTSSGFGSGGFGSSGTGTTGAFGNSTA
ANTNPFGAKPATGFGTTTTTTSTGFGGTGGGFGQTATPAFGGGAGAGTAFNGQAPATDGTAGTPFN
PVTEKDPNSTTNNVYQSISYMQPYARHSFEELRLADYQAGRRFANASGQAGAFGAPAFGGSGFGQP
AATGGFGTTGGGFGAAATSAPAFGAQTQTTGGFGASTTQTNSLFGANKPAASLFGTSNTATTQPSL
FGANTATTGGFGANTTNTTGTGFGGGSGLFGNQQQQQKPGLFGNTQTTTTGFGSTPSGFGANTNTT
STTTPFGGTTTTTGFGNAQQPGTTTSAFGGGFGQQAQTQAKPGLFGNTQQQQGGTGLFGSGGTTGT
SLFGNQQQQQPQGTTSLFGGGQTQQTGGTGLFGGAQQQQEQKPGLFGGLGTNTAATGTSAFGGLGG
NQPAQTGTTSLFGNTQTQPQQKPSLFGNAGGNSMFGGGTTTQPTGSLFGGQTQQQPTTGFGTSMFS
TSQQNQQQPVQQQAQAPAPGSFQASLLDGNPYGGQSIFSGLPGQNAPSPGPLATPLSASMRQKQRT
PLPIYKLTPNAANRLITPPRRQGYGFSYSTYGSPASASSVSSFSGSLLGSSSLRGGNVNGSFGRSF
GKSFSTSNLRKSFDPDSDSVLAPGALTGSASRYSSGSLKRLTIDRSLRNDLFQRPSASPAPITNGE
DAAQSPDKLKKRVSFEATTNGALVRAETESAEPTAEELGFLRSIRKGEQVNGTNGTAPGKPKKPEM
EQARGSELAVVPETGLPTTNGTPKGVTVDPQPGEYWMKPSRAELSKMSRDQLKQVEGYTVGRQKCG
QVTFDRPVDLTTIDLDNVLGGIVQITVRSITVYPDEATKPPRGKGLNVPSTLRIENSWPRGRDRKT
SSSIISGPLFDKHVDRLSKVTNTEFVTYEKDTGVWVFRVAHFTTYGLDYEDDEGESLNQSTLSAGP
DTPTPKVRRDAGDRAAETTVNSEFDTTISFDDSIADDTFDFKKRKLVPGAFGTQEGQIEGDEMSLG
EEDDDGSTVENDDMTGSISLQDVDDVTESINSDVSIDDGDMEMAGSFPIPPDDAESEDLTAHRTTH
WGTPSKPSLDLSGDWADQLQRTISPRKQNRDLLREKQADAFVDRNSPDKRPESRGVPTAKTKTITS
SIDLMHSLFAHKGVHAGSPGKKQKGQLKAFELSHTKTPKTFASSSFELSADEVAFHRTRKPRWASK
DTMVKVNDGYSNVQGSNQPWSSGISFGEIDVLRFAEAAESIDVLEAQRRQSSIRLIDSAPFARLNS
FDFRSFVQSSTGPERYVWQLVNILFNDEIEDDISAGVPPQLRNNFLHRIKKDRLSRLWENIVREKH
GEDLGKLVLPEEVAIAYLTQHRVEDACKTLVDSGNLHLATMLAQIGRDHIIRSNMQKQVESWRQHN
VYSEFNEPIRALYELLAGNALRSVGRSSGATEDRASTFTLSERFELDWIQAFGLRLWYSITEDEPL
EVAVARFLDDITAGDEPAYPFPAHLERELAGLRQGVNSSFLESPLWVVLKVYAATMSTNVASDLKV
NLPDAISPEAVSGSRLSSRLSFQLHQALATIVGQNDSVTIDKARTDQLTWSYASELAASNAVEPAL
FVLLHLSRATARERAIKETLGQFAASLPSPLSDGKPDNVWNYLVQALQLPESWIWVAKALYARYSG
DFANEVGYLIRAKNWNEAHSTFCRVVGPRTIIEGDWGLLRALIKGFGESPDRKVRGWSSGGAVYED
YLRLVTAQGSRDANVLKRLIASLGHMGEKTKQTSGLEGLEERVAFMEMSRVVAGWXAREEDGVDLS
AILRLPLTGEARVAHTAEIGRRYYSMVMATAH
>g1782
MSEGDVLPPKASVVAEAHEVDTFHVPAAFHKKHPTRPHLSFGEYKKLYEESIRQPDVFWARMAREL
LTFEQDFHTTHVGSFEHGDNSWFLGGKLNASFNCVDRHAIKNPDKVAIIYEADEPSEGRSITYGEL
LREVSRVAWVLKSQGVRKGDTIAIYLPMIPEALVAFLACARIGAVHSVXFAGFSSDSLRDRVLDAN
SKFVITADEGKRGGKIIGTKKIVDEALKQCPDVKNVLVFKRTGADVPWTKGRDLWWHDEVEKYPNY
IAPEIVDSEDPLFLLYTSGSTGKPKGVMHTTAGYLLGAAMTGKYVFDIHDDDRYFCGGDVGWITGH
TYVVYAPLLLGVATVVFESTPAYPNFSRYWDVIEKHKVTQFYVAPTALRLLKRAGDEHIHHKMEHL
RVLGSVGEPIAAEVWKWYFESVGKEEAHIVDTYWQTETGSNIITPLGGVTPTKPGSASLPFFGIEP
AIIDPVSGAEITGNDVEGVLAIKQPWPSMARTVWGAHKRYMDTYLNVYKGYYFTGDGAGRDHEGYY
WIRGRVDDVVNVSGHRLSTAEIEAALLEHPMVAEAAVVGVADELTGQAVNAFVSLKSGNESTEQVR
KDLIMQVRKSIGPFAAPKQIIPVEDLPKTRSGKIMRRILRKILSGEEDSLGDTSTLSDPSVVDKIT
EAVKALRK
>g1783
MSSAEESPAQQAARLRRERREAKIKAGGSARLDKITSLSGRTPASMQDASPSPSPSPXXRPQAVPTPN
PPTQPSPQATIPSAGGAEPDAETIRAQQEYLRALLRANPPEQQAQQQIDQDPMMKVLSSMLGGIPG
AENTAPGAGMAPPNQGDLGMNDLTSALGIPPLLSNMIFGGGASQQTPEQKNRERILKLLHTLFALV

IGVYLVTLVGSAVTTYGVNPPPPATAQNPFVIFMTVELLLIGARVAFGKTQQGTLATGLHIFRTLV
RDGSIAIFALGAASWWNGGWQVAASQ
>g1784
MVSQGSAQHGPAGHWSAANPIPTIHKFVENLDRDKKERDKQIDEEVQRRQQNAQDQGQSFDKEAAD
IQKKGTRKVTDPVTGKEIEIEDVQKHYLKEAKDPKLTVPNANLNKPTNVKTDPNQEYSEYRHNQDI
TAPPEPVAQGTTSDVPIHGEKTNILFXPTPSVTFQPMFDDIEKRANGLVAGIFIGIIIVGRTFGGS
LWGLIPLAACVASGVFLWVKELIRSGRDAEWRSEQLRGETATVNLLPESVEWMNAFMSVTWGLLNP
DMFASVADTIEDIMQASVPSVIENVRIAEIDQGNNPIRILSIRSLPDEHVEDLKHNVREENKKNKD
PQEAAADDEGGDYYNFEVSFAYHAKPTEGSASARARNMHMQIVFYLGIKGLFGVPLPIFAELIELI
GTVRVRLQMTPQPPFAKSLTFSLMGLPHVRAGCVPLVKRGVNILNLPLISNFVNYAIGAAASLYVA
PKSMSLDLSMMLKGDDIQKDTQALGIFWIRIHRAIGLSKQDRRGSEGGGSDPYINLSFSKYGKPMY
CTRVITDDLDPVWEETAALLVTPELIKADEQLSVELWDSDRNSADDIVGKVELSIQQLIQHPGKMY
PQVSKLQGLDEGSSMPGELHWEVGYFGKPRVRPELRTDGKNPELPATLRDEPSLQDDKGVINTEEE
EAITSTPPDPLWPSGICSIVVHQIVNLQLENIYGSQGNRKGREYEPAKAYGENTEEQGKELPTSYC
TILLNDQLVYRTRAKAVSSKPIFNAGTERFVRDWRSALVTVTVRDQRHREHDPILGVVSLRLSDLM
KTSSQVTRWYPLDGGIGFGRIRISLLFRSVETKLPPNMLGWDVGTFEFLSNRIALKNYAQTAKIRM
RTGGSTGKVPRSACKSDGNGGQYFEFSDAITAPRLPVKYRYRSPVVFEFHVASKRGAAAYAVLWLQ
HLVDNEETQIDIPIWTTKRPGRLIQNYVTEQNYKSLNLHCLEDLTEVGRLQFTGRFSAGMDESHEK
FVMDNNSRETFETWEACLAEGIRSREVIAKTPENVQQLHEKSLVQGRDVLRDIDPKERQRWMTKEG
VDWSGAFGEDPKEYVDQEANESSANFYNAGEHTVKSHDHAEDPDDDDDDDDDDSSSDETQSVHARS
IGTATTTTTTSSSSAQSSKSGNKANKRTEQRQQRGLMQWKPARNALFAKHEATFALRKAKKKFGLG
DLTGREPDVETETGS
>g1785
MSTTAPKSDPFQPAARVAGQRQDVWSIVNEAAAASPVQPIVNMGQGFFGYNPPKFALDAAKEALDK
VDCNQYSPTKGRPRLKKAIAAAYSKSFGRELDPETEVTITTGANEGMLSAFMAFIEQGDEVIIFEP
FFDQYISNIEMPGGIIRYVPLLPPKDGAVRTSSASQWTIDFEQLEKTINSKTKMIVLNSPHNPVGK
VLTKEELERIGALAVKHNIIILSDEVYDRLYYVPFTRIATLSPELYERTITVGSAGKAFYATGWRV
GYLIGPPHLIKYVAAAHTRICYSSVSPLQEASAVAFEKADAEGFWEESRNEMQGKIRRFCEIFDEL
DIPYSDPEGGYFVLANMASVKLPSDYPFPPHVASRPRDFKLAWFLIKEVGVAAIPPTEFYTDENAH
IAEDYLRFAVCKNDDVLETAKERLRGLKRYIVKSE
>g1786
MPPTNPEPSHMYPAHPPNTRMPSHSEPFHESRLDLRLNPLLGRPSIQTGLETLTEDAHSRRVHDIP
GQVVRGQSFDINAPVSAAHGIGRDTVPLSGHIGTPGEKSARNGFPYIQDNDVDQADRPRKFQWVSE
NPAETPKGSHKKRDALMQRLTIDAGGSCIFCSKSKKGCDAEMPCNRCKQKNLPCVRGGYSSLCMAF
TVKHDDHYELHPAIFAQAQDALQNLKQNLRPLSFPVVVHWVFDNQRYRETLISAADQLDLRQADTT
LGKSLMDLLVSPQSDLSDDLSRDAQRASRLLAAIKGLSMSDCLTDPVNIRNARVIVFFLMTIYTIE
LYEVSLRLAKGLYDSLKTISSTRKSERPVLLYMKVIDDMVNLQWPNSLMSAIFAQAQPRLQGLQDL
LRLLFFDENFKPTSHANHTFDFGPPEFEILIGVRSQIQGICGSVDFAQIRYIVSDLLCSEFGENMA
QTIDPSLLPLDMTRNDTDADHDDSTTLCSSDDQAIFSNQVGSGITNTTYPPPDVPDLTISRRESTV
DLGVEPSAGEDFAFIGDHLN
>g1787
MVSCPICAKNVKEANINAHIDSDCQNFVIDDSSLSQGDSASSQKNGTAINFFQPSSARKASSTVIH
PSTLSTPSRNNEVSKPSPAQDDLKRKRELEPSSSTAEPEDATVKRSKPTAFQRAAPLAERMRPKSL
DEVCGQDLVGPNGILRSLIEQDRVPSMILWGGAGTGKTTIARVIASMVGSRFVEINSTTSGIAECK
KIFAEARNELGLTGRKTILFCDEIHRFSKTQQDVLLGPVESGVVTLIAATTENPSFKVQNALLSRC
RTFTLEKLTNDDLTSILKRALATEGSTYSPSELVDDELIKYLATFSDGDARTALNLLELAMGLSNR
PGMTKDVLKKSLTKTLVYDRAGDQHYDTISAFHKSIRGSDPDAALYYLARMIQSGEDPLYIARRLI
VVASEDIGLADNSMLTLAISTHSAVEKVGLPEARINLAHATVAMALSKKSTRAYRGLNNAFAALSE
PGVASLPIPLHLRNAPTRLMKELGYGKEYKYNPNYKDGQVVQQYLPESLQERKFLEDNDLGDIVDP
ELFS
>g1788
MAAPHPPFGSRSAYAGAGSKLPERPNPLPFSASAFSRNRGVLGSVNNNINNNNNTESAQQQQQQQX

PPIPPQHSQNHASSSAAAATMMMGTDAGSGANPLSRLTEEQREEINEAFTLFDLDRDRHLDYHELR
VALRALGFTLPKQELISLLTTYGVPRPQVQQQQQQGQPSTAHPNKPNNNSKDPPPHPSSLLMPLSA
FQTVTAHKILERDPREEILRAFELFDEGGKGYIDLEDLRRVARELGETGLEEDELRAMIEEFDLEG
VGGVTRDGFLSICWQ
>g1789
MKLGIGWLSATLTATAIFLQRGDARSQAPPPIQHVSELENVVIHTPSHRIHSLSSFDITFTIHNNA
EAIKLKLEPNHDVLAEDAEIQYLNADGTISHTESIDRRDHRVFKGSAWTEIEPDHWTYVGWARLYV
KRDGPDPLFEGTFSLMHDYHNIKLRSSYMRTRSDSDVIPAEKDDDYMVMFRNSDMYWDEEHTELKR
SVPDPSCHADKLDFNADPNHPVFRPPQQSTNLAAMTFDQLLGLSKRQSDTGNVGGNTGGINLASTI
GDTTGCPKNKLVALVGVATDCNFLNAFGNNQSAARADVISMFNSASSVYESTFNISLGLKNLTMSP
ADCPDVSSTATPWNMPCTSGNISSRLSDFTAWRGDQNDTNAYWTLMTNCPTDSEVGVSWLGQLCVH
GSSNASVAGANVVVKTSTEWQVFAHESGHTFGAVHDCDSSTCQQGLQTTSQCCPLTSSTCDANGQY
IMNPSTSSNLEHFSQCTVGNICSALGRNSVQSNCLVNNKDVVTYTGSQCGNGIVESGEDCDCGGTA
ACGDNACCDPTTCKFKDNAVCDDSNEACCSSCQFKAANTTCRASTGPCDIAEVCSGSSGACPADQF
VADGQSCTSGNTTGLXCASGQCTSRDLQCRTILGAVLGSNDTYACDDSSCTLWCASSKLPSNECGS
MQQNFLDGTPCNGDGHCNNGQCQGSSVGGEIKSWVDQHKPLVIGLAAGIGGLLVLIILSCIISSCR
RRRFRKTLPASRPPGGRGYQGYRGPPGQPPTNAGGWSGPMPPAMQQQGNPVYGAPPAYAPPGYDHN
GAPWPGVQPRYA
>g1790
MSGDKPIIEFTENVEGQQHKPADHVLVDADGLVQQLPVPSKDPNDPLNFSWKEKASIIVSCCWFSA
MSLSCVGGLGAILGVFFEMYAPQGYSSNQIVWLSTFPSLFVGIGNFLILPLGLLYGRRFAFIVSTI
VLLGATIGCALNKSWEQHLALRIIQGLAAGATESVLPLILAEITFVHQHGMVYGLYWAAQNAITGC
LTLAASYEVAALGWRWYYWVFTITIAVGLVMVVFGSFETSYKRSSQFINGRMVLTDQFGVTRVLTE
HETREYLESRGVHPNDEVPDELREKKTYVQMLKPWSAPTENALTFIPQVFLHIAEAYSSPAILYST
LLSAIVLGSSIGMSLTYDTVLETSYNWSASSIGLINLGGVFGGFGGMFYAGFLGDKFIIWMAKRNN
GIHAPEHRLILLIFPGILAIVALILYGFTASGGATWGGPYMGWTLYQITFVSVLILSTSFAAEAWE
KNPGPALVAVVGMKNIVAFGLSYGIIPMVNTYSYKTTMGILAGINAGVFALGIPVYLLNPRWRRYM
QNKEERAAKL
>g1791
MYQDGTPDTQMQVGFIEESQITPSSTEPFVADYSMPQPPQAAFADRNGAYRLSDPSIPQAIPLTGA
LPPRHRDQLPEQQMGIYREVNEIPAFFEQVMLPPDTSPWPWFQGVQQPRGVFDFMCDPEFTSPDGG
LFNFDTITDLDIILGSGASPVAANLDPGVIVDDQTAKQRVAAFRRSLWLWIPEKNQNGFSGEGHIP
LEDGDMTAFITRLHRSRLESLNIRGKLTVQSRDNIFRLIISTARSQLSVPSFPSAESLDALIKIGI
SKRTETDAWIHPYTLFRQDSRPELLTALVAAGCVCCGISSINKTGVLLLEILRVSLAQLAELDNSV
LRDLQYFQASMMWLDIGIFCGHKRKMQIAESHLQPLCTALRRAGAFDRSFYSQFTLYPDLIDDTLE
ETWNDWIRQESWKRLAYHLFEHDAEVTAAMNRPPLTSYTEFTLPFPSSRELWLAPTAAAWKHLLVT
KYAHLKPPTLSLRDLLADPSILNHLPINVDTVVCKSSLLHGLMSQVWEFRQQALLTPNRHSERGAL
TQLWLKSRQEDLSISIQAAKLDMINRPAIATLLAEFATMYLHINLDSIQRFAGQFGELEAREEYPR
LRDWSITREARTAIWHAGQVLRAAREVPPFELRGFDTLSIYHATLVLWIFGLLQCVGKPRDALNLP
PGSSGGGQQTEPLIALDGPENEFTKAFVNGAVGQPGLTYYSASPEGGHVSMSIQLCSPHLIMEVAR
QVLEGNCQGSGDNLPPLVGNLCNLIRELGDLS
>g1792
MTESNPFRIVIVGGGIAGLTAAIALRGPDRQITVLEQSRLNKEIGALISLQPNATKIVYDTWKLGE
ELEDARSMVDEGFRVYSTEGKLVTEVPLTTKIEYGASRIVYHRMDLHDALKKAAQKTSTIRVASRV
VDCDPLNGIVHLENGEKIEGDVIIGADGIHSILRNYVVDHDQTPKPTGHSAYRLMFPTRVLEEHEK
EFCDRINPRSPFTSMVIAHDCRLIMGPGRQGDVFGIVAMVSDERMNEDPNASQSWVSEGSLEKLQE
TFSEFPSWITSIFKHSTDLGLWQLRDIDSLEKWSRGRLILMGDAAHAMLPTQGQGASQAIEDSEAL
GAFFEDITEKPSIKEISSIFTNIFQSRHARASLIQAYSRQAAKPGTAKGKKTVTMRPDEFMAYNCM
YNGAKEWLQKQA
>g1793
MSTTTVTETIAAPAQSLRMRVDGTDPKFGDFRDDLARDGYAVVKGAIPRDRALKYADDMYSWLEGF
KLGYDRKDPQSVHKDKLPVINEKGMCLQYAVAHEKFTWDVRSEPGVVEAFEKVYDDKDLIVSFDAV

NFGFPNRTDLPANKPWPHQDQDPSKPGFRCLQGLVNLLPNGPNDGGLIVCPGAHLLSEEFHRDMAD
EERIPAWTPEWYGYTERGMKWLEDHGCKWKKVCAEPGDLLIWDSRTPHYNLSPKGETPRFCIYTCY
MPVADATQEDLQRKKEAFEKRLGTTHWPNAKHTGSNIAKRDGQECTSNRFEPVNGVDLGERAFKLT
GIPYIKAQA
>g1794
MAPTNGTEQKAGLKILIVGAGIGGLTAAIALRKQGHHVQIFEQSRFATETGAALHLAPNANGILRR
LGLFPEDFGANPTQRTTEYTAGGHQLHTHSVEEANTRWQHPWQLAHRIELHDALKRAVSTPTSDGK
ASIEFKTSSRVERVDPYNATITFADGTQIQGDLIVGADGVHSISRSALPNCEHIKPFPSGKSAFRF
LLPRQKALDDPETAALVQHTGELSMWFGVDRRIVMYPTSNNSILNFVNIHPDEESASETAGSDTWD
QSVNLDKMLKIFSSFSPSMVKLLSKAEPESVRVWKLLDMDILPKFNESRLALIGDAAHPFLPHQGQ
GAAVAIEDAAALAVVLSLDTTTDEIEDRLELYNEIRYTRATKIQSFSRLVGKDLKPGDEKPNIWDF
QNYNFGHDEWDNSTQKLREWTWKRNKNAYWRMPIAFGPMPGPRQTHFGVAQNSQKSTFSTASIKFK
TSRTLLEALFPPMRSGWRFSVHDTVAYATFSQTTLNKLDWLGGSGYNHIGLYIHGVEYTREDGSAV
KGAYLPVLFESLTDPIISGREELGMPKIYSTIDIYRRNTSYRVRTGWEGALWGDFLLQGLEEVDSS
SLTEEKPAEEALLTYKYLPTTGPENKGKAVEEYTVLYDAITDETKAKVHKTYKAQKASFAIDPLDW
EQLPTLHHVISRLAELPIYDIVEAKIVEGEGVPDLAGARPIV
>g1795
MAEEQILDNGLPQMNRYITDHRQDGKAVFNTEIPAPLSWQKLPNGAAFALSYTTNEFPVNLNDGKD
VGKYQSYLQSPPGLTIPGGSVLRIVDMNPDSLSPMHRTVSLDYGVVIEGEVDLVLDSGEVRHMKRG
DIAIQRGTMHAWRNPSKTEWARVLFVLQESQPLRLEDGKALGEDYGHGMDGVPASHS
>g1796
MPNEIKDISSVDSTSFPYIFEQNVDVPLKAASGLVRVNVYRPKTEDGARVPVVVTYGPYGKDIHYK
DFHPQSYSEVNPEHHSDHSAWETPDPGFWTRNGYAVVRADERGLGQSPGVLDTMSRGTSDAFFDVV
EWAAEQPWSTGKVGLLGISYYAGSQWRVAARHPKGLCAMIPWEGMSDYYRDRCRQGGILSNGFIKF
WWNRQVITNQYGRPGRSARNWGPDTIEGDLSEEELAANRNDQTEDNAANKFRDEPYYASKEYNMGD
IQVPLLSVANWGGICLHLRGNVQGWLWAGSEFKYLRFITGRHDLPFYYHEEVEIQRSFLDAFLKGE
DREGWSVKGKVAPVSILLRKGNVGFNDAEAEKVYTRREETEWPISRTQYTKFFLDSTGSLTTSEPL
VKRSEKLSYKALGTIEQPELVKFETKPFEAETEITGHVVAHLNVSVTPYSGSESIPTDIDLFVTLR
YFGPDGKEVFYTGTAGDPVPLTKGWQRVSLRKVDSSHYKNKTWLPHRNYTSADVEPVIIGEVYPID
VEIWPTNVVVEKGGKIIFEVASGDTQGAGIFLHNDAVDRSKEKLDGLNHIHFSPHFSNYLTLPIIP
>g1797
MSQTLRPIREAVIDGRTKNIIWRQIQLENLQRTLIDNADTIQAAIRQDSAYTTTEAAIEYCLTLKC
LNENYESLDVEQALKDEYAVARGENAEDFRDPVGIVYIVPTTHNFFYSIVSAVGAAITAGNCVVVE
LQITGGQKAVSEILQNLLKASLDHGVIEFVSTRASDEDLSPSHIRLFQEKSQNERQPASSKSLFPL
SSATRSIAIVDRTADLTGAAEAIATARFAYGGNSPYAPDLVLVNEFVKSQFLRALIQATMDQLSLR
MSVDAKFSPSRNTQQTASLIAAEIEGKRAISLSSVGEGSLVDVTSRDSQLLRSKIRGYNILLHSVR
SLDDAIDLASQHGELLAAYTFADPASGKYVSQFVPSQVSFVNQIPFEILVGPSFPETSPLNQSLHI
RYPTDLFSIPRPQYILKTPLSQQIQQYFLLKGSSSKNTSPAVWQKTLITRISKRISATKQKRRPGG
QVGFFEQGILLGLGMTGVPILVCVGTLAYYGLGFAWRHFRH
>g1798
MSTTATEDQMVPRGDMLPGTSHPNPNLYQCGKCSQTYKRIDHLSRHVRTHTQEKPYQCHVCAKKFG
RIDLLSRHSALHNADNDSPSSRKRRRGANDRSIPVRTSQACVACAENHLRVASRTNEGSESSPRLE
QNANSVRDTSPSRHDDSSHVQIEPEDSHWLAEQPGQSEQVPDSFPSVSVEPALSIVPPEQMNSLAP
EGHYHHDAFLNTLSSNYPSGIRTPRNLIAFGLETDLDLSNVDLSFLESYNTRNPFEFGELPPEPLP
LPSVTTPEVNITKDDRAITDSTSLGKLMWRFIPAPQDHGLAEHDNLLLPDQPESNPTPQSLVEVEH
RTTNEKLDLSSRDKILGIFLRQMKQPNISHPLSFPSVELLDSFIQYYLTAPFSNVRSWVHLPTLNF
KRSRPELLLGLAAAGAVLAPDLALRKLGYAMHEVMRIQLTAAVEEDNTAARNLEMHQAFFLYLDIG
LWSGNSRKIEIAESIQKLLITMMRRGGWYYQSSYPFIPLPDDQTGHSLEEAWRTWVRQESFKRLAI
QLFQHDAQTSLAILSPPLISYAEFAVPLPATSALWNASSAFEWHDLYCKELASMNSMSVPTVIQCV
ANLDLLDLSQRFIDLKMSCEAVIYAFWGIVREYRQFDQLLKNKPRYWDNGLVLTSRYQELTRILDY
YRIAYENESALSLHVILLNMHVSLEDVQFLTTAEPQTYHPARASLHEWANRKESRQALWHAGQIIK
HIKLLPSQDLRDFGAVALYHAGLTLWAYGTVRRGPILQQPSMISSAPNIDMQQSTAIAPLPPYSRD

SMDLDPIKVYVDGIQADDVNRYIALERGTPVLQGFEGDAMPVHIYHPQEVLDLMIRLMEQNYRWKF
SQQPPLVANLIHLIERQRDGVQ
>g1799
MSAEPTPVHFFSHGSTMMLGEESTSADYWKKCGDEALANGIKGVVMMGAHWDCSGLNKIQVAMNPK
PKKSPVAYVHPSKYVDYELAPDLELGQKCLDLLNKDGFDASANESFDWIHDTYLILIRMFPEKCPP
TTIISANSRFDPHFHMRVGAALRPLREQGYLIIGTGGAVHNLYRNVWAPMLKYSDNFAQLTPPGDW
AMDFRQAFEDAIVNNSGPALRRAITRLMKHPKFREAHATDDHFMAALFVAGAVGSEQDKGVYGKLW
AETWELTNMCNSQFTFGEWSKAPQAVY
>g1800
MDKGADATHHEELALAKEAAQNQATGTVRLIEDGEVILIPTPSPDPRDPLNLPQWHKWTITIVLGL
FSILAVTWTSGMGAILNTIDAYYDGNPRTSDLMTYPTLFMGIGNLIAMPIAMAVGRRPVFLASILL
LTVGAVWCAASHSLGSHTAGRDILSLAAGQSEALCPLIIQEVHFVHERSRRLAWFSAIQSTGSAAL
IIATGYLVNSIGWRWWYGVFSIFSGLTLIVAFFLIPESRYTRPDDAYNGLVHVHNEKTQETTFVRA
TTRNRPPLDFERHHARTLSYNLKIFHGPADWTKFTTCWKQMGQCILFPNILWVVLMNSAVLGIYVV
SATEFAAILASPPYLYPSTSLGLVQGGQIVVSMLMVPVLGYGGDILYKWMAGNRDGMVNSELRLIP
ITLPVVVTLVSAVIFGQAGQNPTQWSSWAVVTTYNGIYFGFIGIILIGYTYSLDSYGERAVPILTL
ICAIRGIISFGISFGVTNFVTKQGYDGAFNICAIIMGVISAFGFIVFFFGAKIRSFTTKYAVDGAK
IEI
>g1801
MAPSRIEVSEAXTVSSNKLDAQTTYGDFRDEFYANGYAVIKGVLSKERAKEYQSKALDWLESFPLG
FDRNDKSTWKKENVPQSFKGGMYLHFRAAHEKYVWDIRTEPSVIEPFAKLWGTDELVVSFDTVNIT
LPAKIVGGYDSKPWPHCDQAPERSGMVCVQGIVNLSESGPNDGGLVVMKRSAPLFDKFFEENPVTG
PTPWRTAKHKDFHPFAEKDLDWYREHGCELVKVCAEPGDLIIWDSREMHWAQFGESDVLRTIVYAT
YTPAAWMSEEEQKLKKDLFENWETTTHWPHTNLYTHGDATLTVDGKEVPDPLERKEPLTKPERTER
LLKLAGVIPY
>g1802
MPSPIPIATRPINEPKVGRNNYQPFGFREEVLPAGWTSQEGSLPLPCDIHASHDVKVTVRDGANLY
IDIYRPNTSEPVPAILAWSPFGKKFNGISMLRMLPWGLGVPKGVISGLEKFEGPDPASFVPKGFAI
VNVDARGAGDSDGNVHIMGTQEAEDGYDVIEAIAKMPWCNGNIGLAGNSHLAIVQWHIAQLQPPSL
KAIAPWEACGDLYREQFVRGGIFDAGLFDLIIDHNIQGHGGVEDFHEMYRRYPKADSLYWKDKRPD
ISKISIPTYITASYTSFVHTMGSLRGWLQLSTSEKWLRICPWQEWFDMWNDKDSAADLAGFFGLYL
KGEKNGWERTPKFRTTALRFTQDPVFDIVEEDFPIPRTDYRKLYFQPEQKLGLEAPVEAYSVSYDS
EKYLDHAGFTHTFFEKTRLMGIPKAVVYVSCADFHDLDIYVLVRKLDAQGKPLLNLNIPWSSIASQ
GASPDKIDEIPPSHKNNLLFHVGSQGILRASRRAIDWSKSIHENFPFHPHDRDEYVTPGEIVKLEI
GIWAMGVEYEAGESVRVEVHGNSPALRGEFKEDNEFASLASHGRHQVYIGGEHASYIILPFV
>g1803
MAFTISVSADSPFTLDNIPFGVISTDSNPKPRCATALGDYAIDLAAYWKDRTYAQLEGSRSLYGIF
NQGSLNEFAALDSSIRSDVRKHLATELAAGNVSETCAIPLKSVKMHRPMAIGGFVDFLCSLEHCKN
CAPLAGGAVSNNFYYAPSVYNGRTSSIVPSPEPVRRPHGIIYHPETKEPTFCPSKKIDFELEMGIF
VSKPVPIGERISIETAASHIFGFVLLNDWSARDLQAFEMNPLGPFHSKGFGTSISPWIVTIDALMP
FTCKPWHDHTSTEFEHQRYSDRTKATFDIKLDVTLVRNGESHKLATSNLNYLYWTPYQQVTHHTLA
GCGLETGDLLGTGTITGETKQELGSLFEATYNGTKPIELANGDKLGFLQDGDEIILGASCGGEEGQ
PRLGFGECRGKILPAK
>g1804
MADKYKPTEHGGKREDGQPDKRTQQTEFAYGKVDPHKAGQQGGSTSGAGGSAPGEFAHGKVDPHEA
GKEGGSN
>g1805
MTLQLSHISPVARESPSSVSHTTAQKMIAITTTATPSVMHNTTITKRPKLSLQTAALPATFGNSNT
GLVARSCITASPTIRNTFSNAYDIRQTLSAIDSPSPSRSAGGSARHGSPFPFSSYRSDGMPYQQPL
GVRSILRNSPLKKMMLRRSVSISAGAMAENRRVYFPAKKQVGFRNQLEEEIKTVRFVARHSDIDSE
SEQEPVSEGEEGNSSESSDSGDSQSDHSSSGEDDGDAQNRPIRRKKRKSAPSERQIRAAALRDGID
NDRIAAALSAVPDSTSQKRRCKWRWTLGNSPSTESDSKLGTIPSTQSPSVPPVASEPVISCEQPIA

FERPVASEPPIGNDTVIS
>g1806
MAALRRDDNRVIIHFDYDCFYASVLEAENPTLKTLPLAVQQKQIVVTCNYEARRRGLRKLQLIKEA
KQICPEVVIILGEDLTKFRNVSKSLYGFLKGFTWTQKAERLGFDEVFLDVTDMVDYNLSLLNHHDL
ANSFFCLDKQDPTVGFQFDAASYCGPTYPEFTENSLSGSTAAGDDNLAQRLILGSHLAAYVRSRLD
NERGYTATVGISTSKLLSKLVGNVHKPNSQTTLIPPYSSTDEISNVNRFIDGHEIGKIPGIGFKSA
QKIRSYILHREATFEPYTERKEENQVKVRDVRLSPTMGPPKLLDLLGGSGAPRDVGARTWGLLNGV
DNSEVMEARSVPTQISIEDSYMGLQHFEDVKKQLQLLATSLIKRMRIDLVDDEDRDDTKENDGPRW
IAHPRTLRLSTRRRSPKNADGSRDHSYANGRISRSGPVPNYMFTLAESAEAVAEKLVDDCLVSMFR
KLHPDKAFGELSLINVAVTNMVESAGDDKQSNGRDIEKMFKRQGSLASAAVETVHRNDQLYQGSAS
GNLPDATTGTQVDETAWEESDEEDVPSTQLCDICGSHIPLFAFRAHALYHAAPD
>g1807
MRLFGRTIFPMLPPYGAHDGSDAPPPYADPDKPTPYLGLKARLSQIWINRWTILILLLVLVRVLLAS
TNLQGNMDNAQSQALSACNSVQSAGSAMASLPHYMAQGVNELAADGVTKAVNGLVQVLELMITGVE
GLVVFYINFLTQMYLCLFTLVARGAAQVALGLAEDVTNFLNQTLPSIGSDLSSGVQTAESGLNAIA
SALNTVKDECNKVSILGDSLCGDIPTVPTLNFTSEINSLNSLTLPSSIDSDIQKVNSSIPTFDEVK
NLTTTAIEFPFEELKKLVQTNLGNYSFDSSAFPVPAKQSLSFCGENDGINEFFQKTEDVILEARKI
FIAVLVIFAVLACIPMAWLEIRSWKLARDRAKIIETGNHDALDVVYLVSRPHSSTYGMKAATWFNN
SRHQILVRWIFAYATTIPALVLLALGLAGLFACLCQYILLRAVEKEVPELTAEVTQFADKVIDSLN
NASAQWALATNHVINNTNTDINNDLFGWVNTSTSALNNTLNTFLDKSNEVINATFGGTPLYGAVTG
IFDCVVELKVESVQKGLTWAHDNAYISLPQLPNNTFSIGATNSVNTGDSFLSDPGSTTSNQITEVV
SEVIDKLKEGIETEAKISAVLVLLWFLLILMGVLRALMLCARRERVRGNGGGQENVVPGGPIEPVA
FHTTSNTTVSRSNDNNAAMMTGGIASNSTREVHMDPPHENNPFEYEDEKVGYAGERDYSSAVHADA
RKSAYVEYGGDEKRR
>g1808
MAPGIVEPAPAVAPEKISNLLHLTQDYHDTIRFYLNGTKVVLDDIDPELTLLEYLRGIGLTGTKLG
CAEGGCGACTVVVSHINPTTGKLYHASVNACLAPVISVDGKHVVTVEGIGDVKNPHPAQQRMAVGN
GSQCGFCTPGIVMSLYALLRNNDGEPSTDEIEEAFDGNLCRCTGYRPILDVAHSFSKPMGCSKSTT
NGGSGCCMDNKESGAGGCCKSNGVSEGDTSKLPNLPKPDFIPYNKETELIFPPILMKHDFKALAVG
NKKKRWYRPVTLQQLLEIKDVYPSAKLIGGSTETQIEIKFKGLRYDPSVYVGDIAELKQYTFKDDH
LELGANVSLTDLEHICDEALERYGPVRGQPFTVIKKQLRYFAGRQIRNVASPAGNLATASPISDLN
PVFVASNTVLVAKSLTTETEIPMTQFFKGYRATALPADAIIASLRIPVAAKGEHFRAYKQSKRKDD
DIAIVNAALRVSLSDTHEVLSANLVYGGMAPMTVPAKNAELYIIGKKLTNPETLEGVMNALEKDFD
LRFGVPGGMATYRKTLALSFFYRFYHDVLSTLEVKASDIDQDIIDEIERNISSGQKDHDASAAYEQ
KIVGKSGNHVSALKQCTGEAQYTDDIPVQKNELYGCLVLSTKPRAKILSVNVEAALDVPGVHDYVD
HHDLPSPAANWWGAPNSDEQFFAVDEVFTAGQPIGMILAKSAKIAEEASRLVQVEYEELPAILTME
EAIEAKSFFQHFRHIKNGDTEAAFENADHVFTGVSRMGGQEHFYLETQACVVVPKPEDGEIEVFSC
TQNPTETQTYVAQVTGVAANKVVTRVKRLGGGFGGKETRSIQLAGICATAANKTRRPVRCMLNRDE
DIITSGQRHPFLCRWKVGVTKEGKITAFDADVFANAGHTQDLSGAVVERSLSHIDGCYKIPNMHVR
GWLCKTNTVSNTAFRGFGGPQGMFMCESMIEEVADHLGMSSDDLRVLNMYKAGDSTHFNQELKDYF
VPLMYKQVKEESSYTERRKAVDEYNKTHKWSKRGLSIIPTKFGISFTALFLNQAGALVHIYHDGSI
LVAHGGTEMGQGLHTKMSMIAAQALQVPLSDVHISETGTNTVANTSSTAASASSDLNGYAIYNACE
QINERLRPYREKMPNATMKELAHAAYFDRVNLSANGYYRTPDIGYVWDENKGQMFYYFTQGVAAAE
VQIDTLTGDWTPLRADIKMDVGRSINPTIDYGQIEGAFIQGQGLFTTEESLWHRGTGQIFTKGPGA
YKIPGFRDIPQTFNISLLKDVQWENLRTIQRSRGVGEPPLFMGSSVFFAIRDALKAARKEWGVTDV
LRLESPATPERIRVSCADPIVERARVVPREGEKSFFVAI
>g1809
MIVQRTTSLIAIIAIVACALFILSKTTNTTFESVSNVKFGPSRYIPTKLPSFEDIRHPFRPVAHKP
PEQAHSTSGESKWFSDWQWLNPFSSSITLDEHRTVLPPLPERPPVYTFYELNSKNDEALQKADGQL
LLAWRRAWYAKGFRPVVLSRAEAMKNPLYEEVQRRQLDTELELDFFKWLAWGHMGSGLFADWLCFP
MARYDDPTFTYLRAGAIPEFITRFDMLDRALFAGEKTRINAAIKAAVLDANEKTSSILDIIPKDLF
KEEQSKALAYYTPDTIATSYHDLSEKYNSRPGAERQALVEMIDAHLHTTFLNSFTSGFTVLKPLPD

HTTALVQPAVQLAQALSNCPTSEFADSCPPNRSSCKPCSKSKAIPINQPQPFKNDTNAYSIGVLPH
PLTLIALQHDSVEIDASYIRRETPRDPWLAAVTSELLGPTRGNSARVVTFKELVAGGEFGSSTFWM
TVETLPAKDGDNLPTALVSELEWHFGFKIKTGDATDEGKKGEEESKEKPSLDHEYELISQARDMLR
DKSGRISLRNVAEAWNLADIEVWRFVQAYRARSVVEREQWEEEEKSFIGAKAD
>g1810
MEPRRTSSRVQKLQADHLPSETAEKTETTTKTTATSAYTNFESSNEQKIAHPKKKDVATSVIPMFD
GELNDDPKCTGGDYLFSNLAPLTDGSLAIAKPDHHFGVRPEDVNRQIRDELNHLIVPSTQSDLAIC
QNFFLELKGPDGTPAVIRRQACYDGALGARAMQALRCYGQKPSYDNNAYVITSAYHSGHLEIYATH
VTKSTRRDGRPMYIMTLLDSFSMIGSLRAFRQGLTAYRNARDWTKETRNELIEAANELVEGPRSRH
SSSSQNQTPELVSNMDNSTSIGSTRPANSLTEDTQWKFKR
>g1811
MGSMVLPHLQNAWQVDQAILSEDERLVVVRFGRDSHPDCLRQDDVLSKIADRVKNFAVVYLCDIEQ
TPDFNAMYELYDPMTIMFFFRNKHMMCDFGTGNNNKLNWVLEDKQELIDIIETIYRGAKKGRGLVV
SPKDYSTRYRY
>g1812
MSSDSQAGQSSANSKTIIIGLSGPSSSGKTTLARLLRPIFSADNKIRTFIIHEDDFYLPDDRIPVA
RTKKGELVQDWDTIGAIDVDFLSSALAYVRDHGTLPPRLRSKEDQNEAKDPGVAKELIQTITEEVK
GKLNQAGLLKGEREVTIAFLEGFLLFAPPATENPGHVLRHVHDNIHLHIFLSAPYDTVKSRREARS
GYVTIGPAPTPSAEREDTNERGDEHKGSSSAPVNLEEEDKSEPQNFWTDPPGYVDDIVWPRYVQDH
AWLLVAEDDEAERQSLADRYAAGSTQTEEELLRLAGQAVNLRSDAGVVVAPGKGEMPMSEVLRWAV
DEILSYLKV
>g1813
MAQRTWIFLMVFALIQGSLAAYLAKRTTITIPDGVTYHPESQDICTQTAWTDVVSFFVGNYLSHVA
TIVKAPGEPVYITGMNMVLVLLFPCFGAGRGLITIYQRASFYKDPLQQALRAQALVMVIRDPRQWK
PQTGDSLRSLSLWPPKKAQGTRARMDPDEYYQAALSKSWTKINSEKFKDYIGQFKIIVALPPWLTM
NYGLSKLHMKTERGYHVSGTCNLPEGYRLAYVPPNAQVEHVMPEVALSRILQTSYSLASGVISIVQ
IFSACSALYQSRGYQIDAYGYAAFGFTVTPYLVMSFLNLLANCFTPTFPNLYLVHTETMDEAISRG
GHFTDLVGKLESEPLLVEDLTLFSASIEPPQVGKDNDELMCHIATRIFEHGSTEPMSSNENTKPER
QDGKPSSISDNIVTLATPLESIASPADEETAQKQATVTIEQNHIPGEPQSQTEDGDDDDDSVPTLI
IPSCYKFKIAYSNTSSTSILWKKRHYGLRGWLIGATTWGVAALPFIPLGIMSKFRTGSSSTVAQRV
WLMAWYVVGIVSVSNPYFTDNLVDIIFHNSRALMKNHEIDRFRRAQILFLRAAXXVGMLIFYVTPA
IGGFAVVGQMLIAYGSCTSL
>g1814
MAPAAVADAGYPFLSGIEAYNDNDGKSSVTNELGLGAITPPQTDTPQPSSLPRIAESLLKAASTVQ
SYPKEESVISDQYVPVGILRRSSAQFPEAELYSLQNHEWIQTRWNIYESYPVWSFVQVYVLPHDVA
RIAVSRTSNTLRKALRVVMANIDRSPAAWNGDFDTNSEEQSKKADDESLWYIFNTLDNPNPTIDDV
RDADGKLAMQELIASSRESRPEVQEIFSVIGLKTPLHPYQCRSAATMIQREVHPRQMLDPRLEVYK
TPLGQEYYYDKEEGTMLRSKRIYEEACGGILAETMGCGKTLICLAVILSTRGHFPRIPTEYQETKN
PTRKSVGSLMQMAAATAGRFSVPWPSHFRRLRETGVNYARCEEECERHRGEYTIPPPPSRYTSRGG
WMSRPPAVRLRLISSTLIIVPPNLVDHWTSEIASHTEGLHVLVLRSRNDKTPSEEELLKYDIVLFA
RTRFEKEAGEPETSRHTPSAFSMSAAESPLKKFHWLRIIVDEGHNVAGHGYKSSTMHMLDQLHIER
RWVVSGTPSNGLYGAELSLASHQRLAGDTSSEDDVPSLVLHSRKKTGTDEELKDVDNLRLIVVDFL
KHKPWANSRHDDPANWTKYIKPVGEDGKRRKAPSLRAVLQGLVVRHRLDVINEELPLPRLHNKVVS
LEPTFYDKMSINLFLFNLTVNAITSERKDQDYMFQSRNRKHLSLLINHLRQAGFWWTGHKPKDIES
TIEVAEKYLTNNMENMAADDIQLLKEGLKIAHRALTSESFSSFCHSDELGVYVQRFPDHAREYWSL
TSSSDEAKPLLLGLSQARLAQTFITSHLMADDPAEGLAGAGISARRELMQRSEKKPARNESNVADN
LTHKNKPTENRSPKKTFLKGISRKLSPDSPLSRTKMVATTSAKLTYLLDRVQELHIKEKIIIFYEN
NNTAFWIAEGLELLGIDFRIYANTLKTSQKAAYLSLFEESSSVRILLMDLRQASHGLHIASASRVF
IVNPIWRPYVESQAIKRAHRIGQTRPVYVETLVLKDTLEEKMLHRRQEMSNSEMQHAEKDMLDDST
MSSIIQHERFYELPEHDDLIEPAYLQEACGFFDRHELPIPDDYVEQSTQISTTPTKKRKVPLLVQG
SLPSPQIEVDLSPDLITPVKKKKVSTFQIVTEQGIVMTPPRRGSSSRASRSPPSASDYQSPAASPG
GVDESSLPIRGSSLFGGDAF

>g1815
MSFDRVLPRPPALYDEPGARLLHRQSSSLLEHKIMNMSERTRAAAMSLVSVGEIQTSRPAIEPDNC
STALSQMQLSAINREQAVQNGQLTASFSAASTRQNTPVLATVESTANGAEDRSASRSPTRLEAAKE
VAAQFCLCQPDPKIPRPRNAFILYRQHYQGAVVAQNPGLANPEISKIIGEQWRSLSPESKDQWKAL
AEAEKARHQQQYPDYRYQPRRYGRDYNSRNGSASGNQGSTTCNRCGGKLMNPPSTPHTPLTATIPP
TNSRSMGSVSPHAGSSSSTSSFSSRGNNDKSNRRPNELLIPTDTDRRMFSNQWKEPDSAPVDNKRR
RLNGGTSQRTESPDTEPYSLSSQKTYVSRPGVAYFRSHVESMSNTGLIHQQQQPPRHEGDGYYDPS
LTLPPLKADGNSPCSPSATTRIQTDSVMTIPVLNKIQLLAKISPPLSGRDNTPSRGAVIAVDGQDA
AQVKVMMKYLGQLLMDDGKYVVRVFEGPDILARNESAPGEMKNAMVEYMSIISNWHRISNEIIQFI
SPKSSGSASSESSTGCSTRSPESSGSCPTASPSPTLSQYSASLIPIALVPRYQLTAADAYACKTPV
NDCYTFLDHWQWMASLWRXCIGPDXIVHIRNCECEEIERHGPGNPNPVEIRLDEARTLVVRRVPDP
SKDIEEKALRRVGFEIEDFLTR
>g1816
MKMRYLTRLNMLGSLAFISILVALSLLYASGNFYTAGNELSLKGTGKVAEEDLVNDVPTTTIPPNS
DHRLVVFGDAWSAIGSRLAAQGQAWPEWVCSMWPCRLETYAQREHVCQSSVCGSVVDETELHTVVS
DAARSLEPLPDLRSQVDQWLAAELNATQTDPSGGGLKARTNNTIFALSFLIWDIWKFNGVSMEDME
KSMKRSLDTIFQQLDRLEKYTDSDDLRILLMMSIDPTFLPAFDPTQGQKDMIDIVTDWNDKLKEKA
HVWGNGSVLIFNTNEFLNDQIRSRQFYMAXMLDGQGLGNQTPWDDVENPCVSSKNTWLPFLGNQDQ
RCENPERFLFWDGLHLGPTANQMMATEIFHEIENLWAKDSNSTEST
>g1817
MKLNISYPANGSQKLIEIDDERKLRAFMEKRMGTEVPGDGLGDEFKGYLFKITGGNDKQGFPMKQG
VLLPNRVKLLLSDGHSCYRPRRTGERKRKSIRGAITGQDLAVLALSIVKQGENEIPGLTDVVNPKR
LGPKRATKIRKFFGLDKKDDVRKFVIRRTVTKEGKKEYTKAPKIQRLVTPQRLQRKRHRIAIKRRR
AEAAREAANDYAKLLASRVHEEKAKRSELRKRRASSLRK
>g1818
MATNSIKLLTGNSHPELAKLVADRLGIEMTKILVLQYSNQETSVTIGESVRDEDVFILQSTKPNDI
NDGLMELLIMINACKTASARRITAVIPNFFYARQDKKDKSRAPITARLMATMLQTAGANHIITMDL
HASQIQGFFTVPVDNLYAEPSMLKWIREHLDVTNCVIVSPDAGGAKRATAIADRLDLQFALIHKER
ARPNEVSRMVLVGNVKDKVAIIVDDMADTCGTLVKAADTIMEHGAKEVNAIVTHAILSGNAISNLN
NSCLRRLVVTNTVPHAEKKEACDKIETIDISATLAEACRRTHNGESVSFLFSHAVE
>g1819
MGRIKKAAGSKHEATISPYLSEFIKLATKTPLAQLPSHLRTLPRTWPFPRGDLYHWIPLLDRFDDI
LASVIERYGLNIGPQVTPFGRYVLEDAVAGQDDNVDEILNGLGYGSEGDRELVEVILAFSQLLQEK
CGNRSLYSSSDRLNDLLNTTSNSLLITTLRLALLLAQRYHARQRNASATHFQQALLAAHYNIDLDR
VQKLSSPFPRPTMSGKPPAAPTAPVVKAKEKAVQTRHDANDAVVLLREKDGWEEWGTIRAVYYPSV
ADQEKQPTETQSSEQSHQQQAPSTPTPLRRSATMPTPRLSRMSSNEESPVYGATPSSRLGESTRNS
KLIEIPAMVVTASPLETILGAKLSEVPTESKYDFLHRARVAQSLVKSDLTREQILTIRILALTNLA
YLYPEAIMQQRILQLDSDQPKRLQITYQLAELAHLGASGDLTVLRSTQTAALYALDALTKHKSRSA
DVCAALNVNVNHGILMFLTRKAIAELSADQDDGEESDDWRDALLLLLRTLPGSSSRTPETLIAAGL
IPMFVDVLNLRTEKARRVYPRVLEFLDTFVHAVRDALGTLANAKGFDAISDLIGFETKSAFDNVNE
GIGIPTEYRTPSVDYDIPYFQQQSLRWLFRFVNHIMQHNGGGFDRLLRNLVDSPPLLIALRLVFEN
AKIFGSHVWSGAVSILSSFIHNEPTSYAVIAEAGLSKSLLEAITLIELKTPEADATVMTEEIDSAT
QPGAPSSASSTAANISHRLSRIPDQQLARGIIPASEAMSCIPQAFGAICLNAAGLELFQSSGALES
YFEIFESPVHVKCMDANLVRSLGTTFDELVRHHPALKKSVMSAVLVMVARVGLLCKKLAYKGAGSK
LWTSDEQGNLKVLGGKYGLFTEIGESYEDTVKRDGQFSYPALREGTLPNGGSIELGPITDSDLGTI
RSAPSDADEWGLTAANYIYPVMRFLGAFFENQVNCTYFIETGGVEFILDFATLPSLTFDFQSRQAN
NELTQLVHMMAETKPHLVIPSIVKRTQDSVECLADFYKEPKPEGFFTSLTKPDLGDAMDTESIEGS
DRLAKERGTYYVKHLVATHVLTDILREIYTTPMYQTRPSQQTSAFSQVNLTEQYTNLVKSLGQLHA
ACVWDEILIQKNLPQRWIEATKVRGYDLGSDEANDALGLSIGDDRRNLPSNVEPTPPTSSTAPTAS
DDKVSDTAPAASAGEGAHSEDDHKPAFKNTQTLRYLLSSLPTTISGFFHLLGHGILGKRRPDTYSR
QNASMVADAIAGVVLRQLQLEAPNKSSEVKDRFSYLIVILSAFTQLLFEAPVERSHSHCLTAILFA
FKRQNGLSMMRNLCGLFLQEINTLAPQAGGVDDPNKSARLASAYGXLKIILTFFSEITSAKESSQA

QALASTERERDRPDYFSAGQFLVDLRMEVLPMVRELWSSDFVDRASSSIVKCLVEILRSILDGEHE
QGAVVQKINGHQFYSEYPKKEFFLNQERLNTLVERGYDLELAHEALFRCNNVLATADEYCHAQTWL
RPPPRLQPESDLESTRRTNATTEEAPVADATSNLSVTDTNYALARIIREGPPRLVQGDGSSNNNMD
HEAGSNAEFLTRALSRYFSDPEALLNESQNDPSSRMHRRAPSESVGQSTELRRREITTIEDLDAER
DKVRKNMIERCLDVLNVHHDVTFELADLIASATKKHRDPEGFRKEVGETLVQSLISLQIDDDVQPA
GKKIAAYANLLALVLQDRSIYQATIEELKECFLTLLEFIKIPVSDKAAKESSPWVGQVLLVIEKLL
SDDAQPPQIQWNAPSTLDEPIDAGPASLKQPLLSTDEKVQLFESLLEVLPRVGKDESLALSVCRIL
VILTRARHIAVRMGEKRNLQRLFVMLKQLSSSSNDKLQGAFMLILRHIVEDDDTVRQIMRNEITAS
FESRSSRQTDTTAYVRQMYHLVLRSPELFVEVTNEILKIQRFEPSQKPQNLALKVTKSETAPESKD
IADKAVDTVEDKEPSETKDKGKVVELKPPVIEHPDGVIHYLLSELLSYRDVDDKDSTSEAVEGSTA
NIPEPQNESEMSVAETSPSSSIADLSAARDTKKQEKPTFKADDHPIYIYRCFLLQCLTELLSSYNR
TKVEFINYSRKADPMATTPSKPRGGVLNYLLNTLVPTGTLEHDESVTSKKRSNTSSCAMRVLVALC
TKTGEFGGNNRRRNVEDEEEEPELLFVRRFALEHALKAYKDAMSSTEPLDMKYSRLMCLADLFDKM
LSGYTFASGDTTFPSSTRQLAKTMFEKHFISSLTASIADVDLNFPASKRVIKYILRPLNKLTQTAV
ILSETSSISTSGETEEYEISSATSVSDIDNGREETPDLFRHSTLGMLEPNHEEDTSEEESEEDDEM
FDDEYGDEMDYDEDLPEDDGEVVSDEDEDLDGRGPIEGLPGDTGMDIEVLLDEDEDDDSDSDDEDD
DDSDMDDDDEDDEIMAGEITGDRDNDSLEGDEDDEWESEDMSEDNEEAAEMLNQLESEFGDLRQVD
PHVEGSRFENLFRVIHEAAGAMDDFPTNSLGGDPQEDLAVDGMNEEEDEEEEDEVDELEEGMDDMD
DDQLSYDEGIDDEDMEPWGWDGEEPPTSRHHHHHHHRHVRGTPAWTSFPSGFNRNGIIPIPPYRSQ
RSQITSRNTDDGTNPLLIRTDRGPDGTGQIRGPGTEAFTDWVHGVDSTSAGRVISMDTPVSFMNAI
MQAIEGQGGPGFGVITRPDGIHVHVDRRALLPGNQLQSIFELGRPQIPSSRSRDDPSQSISFALAT
TTARWQEEARILFSSNHLEKTQRVINSLLRVLVPPAIEEEKKRRAEEEAARRRREEERLEKERADR
LAREEEEREQRRKEAEEAARRQEELARQAEHGAPIVEAEEVDEPMNDVDSAESLAEAAAEAQETGP
SEPAQRVYTNIRGRQVEITGMDIDPEYLEALPEDLREEVIMQQLAEQRSQAVASGEEPTDISQEFL
EALPPEIREEILQAEASARRRRERESARRQAAASGAPAHAEEMDPASFMATLDPSLRQSVLADQPD
EILATLGPEFLTEARALPGRRLAQFGDVGRVEQRQRDEVHEHDTKKPQRRQIVQILDKAGVATLLR
LMFMPLQGNARHQLHDILHNVCENRQNRVETISLVLSILQDGSLDAAAIERSFAHLSLRAKMPPVQ
KTPQSAKRNQAPPASSGANNDVTPLLVVQQCLDTLSFLTQYNPHISWFFLTEHESASMLKLRALRK
GKGKEAKDNRYPLNALLSLLDRDMIMESPNCMEQLAHLLSTVTQPLTILLRREKEKQEETSKGKEP
QRPDETENDSGHVVVEATEVHDTDMPEATDAAGTNPSANEEKSKKRTIEPPVIPDHNLQLVVGILA
ARECNGKTFRETLSTINNLSAIPGAKDVIGKELISQAQALSEAILINLDELLPHIEQASSGTDVQG
LALSKFSPASSDQAKLLRILTALDYLFDPNRVDKVKGAEPESAPKEDVLRTLYENSTFGPLWNKLS
ACLTIIRQKENMLNVATTLLPLIEALMVVCKNTSLKDAPISRLTREHSVATPTSETGPGMENLFFN
FTEEHRKILNELVRQNPRLMSGTFSLLVKNPKVLEFDNKRNYFTRRIHSRGAEVRQPHPPLQLSVR
RDQVFLDSFKSLYFKTAEEVKYGKLNIRFHGEEGVDAGGVTREWFQVLARGMFNPNYALFIPVASD
RTTFHPNRLSGVNTEHLMFFKFIGRIIGKALYEGRVLDCHFSRAVYKSILGKSVSIKDMETLDLDY
YKSLLWMLENDITDIITETFAIETDDFGETQVIDLIPNGRNIPVTQENKEEYVQRVVEYKLAGSVK
EQLENFLKGFHDIIPPDLISIFNEQELELLISGLPEIDVDDWKNNTEYHNYSASSQQIQWFWRAVR
SFDKEERAKLLQFVTGTSKVPLNGFKELEGMNGFSRFNIHRDYGNKERLPSSHTCFNQLDLPEYES
YEDLRSRLYKAMTAGGDYFGFA
>g1820
MSSIELVINPRGKPIRKLPKELTVSTNAPASDIYNTLAQKTGYSIHRLRITKGSDGSVLPNGSETI
DGSGLRAQSVIYVKDLGPQLGWRFVVYVIEYLGPLVFPPLFLYVLRPYLYFNFNQLPEPSWLQQLVC
ALLFVHFLKREIETIFIHRFSLATMPARNIFKNCAHYWALAGANIAYWVFRPDSPTATDDLNPILY
YGGLFLFIFGELANLNAHLILRNLRRPGTTERGIPKGFGFGLVTCPNYMFEIIAWIGVYLLTGLSW
SVLIFLVLGTFQMWDWAKKKERRYRKEFGDKYKRKRFVVLPPLLNTDCAEAMHHALPPRKSSKAPP
FAPRTSNLSLQRRRQLKALGLIVFGLIFVLFVLSRFFSFSVSEQIAAVIPSGTASVVLVTLFDKQV
LSDRYTQWIQTNREDYASRHGYATFFANTSDYLYTLNEKTPRSWTQVPAVRHAMAENPHSTYFFFL
DAHAFIMDPTLPLTSHVLEAKRLESLMIKDHPVVPPDSVIKTFSHLTPKDVELIISQDGEDLVPGS
FIIKQGPWARFFLDVWYDPLYRSYNFAKAEKHALDHIVQWHATILAKLALIPQRVINSYSKDSPDV
AADGTYHDGDFVIHLNDCDAEGRSCEEELQPYYSMWQRKTSSLKSS
>g1821

MAXLPTRLISASTGSLHTAFRGLSLNTTIKRSFSTSLSVQKVKTIPEHIPPYPYEPNRVYKQADSG
LYGGVSISFGNKISKGRNKGKTRRSWKPNVRWKKMPSEALGEDVFVKLTRKALRTIRKVGGLDKYL
LCDKPSRLKELGLAGWKLRYRIMQSPAMKKKFAEEREKLGISKAPDTFEQWLKAKQLTEATSVVEA
VEDAREVTETLEGTPLNEESLPEAEVLKGEKKEQAPVLA
>g1822
MVQISEVKGGSRENRTMAHTHIKGLGLRPDGTAESSADGWVGQTTAREACGVVVDLIKAKKMAGRA
VLLAGGPGTGKTALALAVSQELGTKVPFCPIVGSELYSAEVKKTETLMENFRRAIGLRVRETKEVY
EGEVTELTPEEAENPLGGYGKTISHLIIGLKSYKGTKKLRLDPSIYEAIQKERVTVGDVIYIEANT
GACKRVGRSDAYATEFDLEAEEYVPVPKGEVHKKKEIVQDVTLNDLDMANARPQGGQDLMSMLGQL
SKPKKTEITDKLRGEINKVVSRYIDQGVAELIPGVLFIDEVHMLDIECFTYLNRALESPIAPIVIL
ASNRGNTVIKGTGDVTAAHGIPPDLLARLLIVPTHPYSPDEIKTIIRLRAKTENLNITDPALEKVS
EHGSKISLRYALQLLTPANILARVNGRSAIDVPDVTECEDLFIDAKRSATIVSQDSGNFLS
>g1823
MANPLDTDAGSELFSSYEAELKLVQADLNQKLDQIAETTGEQRKSAIGQAERALDEATELLDQMRM
EKQNIPSNARSKVNVRFRNYSSDIDELKRKLKSLSDDRRALFGDRYTDDPEAGDVHMEQRQQLLSG
TERLERSSARLQESQRLALETEDIGRGTLADLHQQRETIERTRQNLHQSEGYVDTSIKTLRGWLQI
G
>g1824
MDHHTISKRPSSSPEAAPELYLIIRFSASIPDVQLGIPSPSTTTVAGLKQLIRAHLPSELSNRRIR
LIYSGRGLEDASSLRTALKLPSRASTPLSTTDVEDGFLSPSSKDRKGKGVVRDVVIPVDVPRVYVH
CSIGDIVLSATDLSAEAAIASTFLVQQQQQQQRNGSTISLPAKQQIEEQDVDNMATTTPAPRGFDR
LLSAGFTPSEVAALRSQFMALQSINHTPDTMPTGAEMRELEDRWMDEGSSADGAGVGLTGAVGGGG
GDEGGGGGGGGGFGSSSNSALDDMLWGAVMGFFWPIGCAMWLRREEGVWSWRKGLAVFVGVVVNVAF
GAVRVMN
>g1825
MCSYRSFSSHAGKYHSSRRDMQTATAYRPHTLPDAPPPPRNAGIPDTSIAADAPYNAQAAYNAFQS
IDRKPSISVREGEAQKATPTQPSETAKPRRSRLRPRKAAMTLTPTAVSQLQSMLSQPDPKMIRVGV
KNRGCSGLAYNLEYVEKPGKFDEVVEQDGVKVLIDSKALFSIIGSEMDWHEDKLSQRFVFRNPNIK
EQCGCGESFMV
>g1826
MEASPLTQQSRPEVFQPKIVKLYEALFQIDENVEPSEGFWREFYLLPPDRAHLRRLLDGLSPDDTL
LLQTVTQRLFSRSIREAASGLSPNDAHALETLTIFLGSILVKKYTNPSSDIITVLAGLDEVDQVFT
DFVAVLDKIIRSGGQLDLRLKAIRTTIAMISGAYKTSLVAYFMQRDFFPSIMKFVHDLDTNLQILE
PFLLLGLLANYNKFEFQNPYQQRLDDFVNESSIQKIVKGVGLSCAGLRNSYVAIQDDLPEGWTMSN
TLAFFGLRALAGGNKDKATTSTAEEVKETFAAQPEAEAAVLLATYDFANANKLFGYNLVTSIPEKR
NEESPFASFLSLTSYLLQHAYRSERVATYAELNMFSLRIIVEDSVLCKQICSDDNKRSVRLCRQRQ
PHLPLVNGDRVLATVIFDMMIDTITHNLRRRLDTNLYSHTIAILIRLFTYLSSNKTRLAYHWAELW
RTLLSLMRFLTTYSSDLSSAPHIDALTSSLVDLIAFTLSTGDTFLPDPASYDDLFYKIVEAGPIIA
RFRDVYNLSTTTPSTSSLQQQQQGASINTLLTVSTHFLSLLFQTDKPASTTTTESSEVAAATARKK
NLGPREVHQIIKQGYDTLSIQPQEGLSTWEKWREADRKLELKKAARCAVEDARRLVL
>g1827
MAPTSSNYNRDEKVLCFHHEVLYEAKIMDMRHVDPDDRKSPYEYLVHYKGWKNTWDDWVPQERLRK
FTEENRELAAQIRREVTAQMWGKANVSTGKTPKRRGGSDSGRGSEERQSSVPAKGTKRGRDNEIEK
EDQFHLRPSIRIMLPDSLKALLVDDWENVTKNQQVVALPALHSVNEILQLYFDEEKPKRTTTAELD
ILEEVIMGIKEYFDKSLDKILLYKFEREQYRLLRQKWESGAENYTDKGPLDIYGAHHLARLFAVLP
ELIAQTNMDQQSINRLREELSKFTIWLSRNSEKFFSNKYISASNEYLEKATGVPIPALGTATSRLV
>g1828
MAPSEESILSNFLLSPASLPTIVSLQKFTELFPKRLQSHPQVKVLYRELQELRSQDMDMITENILN
EVQASEQPKADLLAAAKASGVHGFGDDENRETKLDLEFFGQTSTTHVVEHHSFPSLLAEMESACAA
LEREIVATEQKVTTTLATVKRTVGDLSDLRYGKLNKPGITPEEYTDEIVRGLHGLRDTCDRANNIK
>g1829
MPSIPLXPPSKPITVNNPLPAILQTPSGLAILELQGTINFPTSTTDDDGDNDTNINTEEKSETKTK

VVETELGKIMFPDYSEFMDPDSTKWMKRVYLYAGRYQRMTGEVKKLAKPLAVVQRRERGTIEADRG
GEEGGDELEIVDVIKYKIIFASRPEPVSLE
>g1830
MSTFERITLFNIPKEEDRALLVEEYKRLVKTAVKVMSFSPNLSSIPKPELINVEQDGKPYISNVSF
GPAIPDERSQGYNFGVKTTFATVEDVKFYDTECPAHQTLKAALGGRKEGDVLTVIFENAA
>g1831
MASHGIPRSSVKTEVDTEQNRKEVQTYRELESSVHEKVRTCIRVFMNVQGLTGCQIAAHIYTHDTL
EQVSELLKKNPEYYTIWNYRRLIRQYEFAQATSSPGQPEADQIIPIIKYDLEFLFPLLRSFPKCYW
IWNYRLWILNEGKRLLPRQLARQFWEGELALLGKMLTADSRNFHGWGYRTFVIDALEDLADDGEPS
MAQAQIDYTTKMIKTNLSNFSAWHYRTKAIQKLLDEKTASNEERKEVLEQELELSHNALIDPYDQS
LWFYHQNLMCVFDPSLADRTLAPGLSNSDRLRYVQDEKELINEMLEDYTDCKWIYQGLVDCNLIAA
KIEGTMSAENRAEVLEWLKSLNELDPLRKGHWKDIENQVTHIYGLPFTQHSSKGSHHASDSLFKMS
SSPEPKDYGEESFEDASEENTHAAPPMPQSTSTRSLTDRRLSNGSVTTPRAEHSPGFPFPSTSTDA
KPEEDDDDSVNVSPTPTPHSENEDTPRRKKYQKSPLLTAHRLSTTSLDDVNLAGNKDDDELNDVGS
PVADSSLDSPGAASIDSIATKQSMSSTLMSSLPSVPWSTASKPVPVPVAVPAPAPPPPAPPALPGR
KLTSPFSWLSRSSISGDSKSSPTNLGNSRRNTAASVSTVNTVEEGDSMSIGSKKPTRNSLKDQFKL
LRMREEGPETDQVSIASGNVSIGAGLASPAEEHALPAPSPVIAGTGGPFPPINPNLLPGTVSGIAT
SATDAAAPVDWELWEGIVNNGPEALSGANSEELNAAIKRGIPQTIRGVIWQVLADSRNAELEEVYK
ELVARGTDKERHMSLSGQGPSGAASEKESLASSRSSIRSDNSGSPSPSSQTTTSPVPHEKEVDPIT
AETARKKKLKEDTEAIRRLEKVIRRDLGSRTSYSKYFVSQGSQEALFGLCKAYALYDEAVGYAQGI
NFIAMPLLFNMDEGEAFTLLVKLMNKYGLRDMFIQDMPGLHLHLFQFERLLEDLEPALYCHLRRRG
VSPQLYATQWFLTLFAYRFPLQLVLRIYDLIFEEGLESTILRFGVAIMQRNVETLLGMKDMSSLSQ
FLKEKLFDVYIDQQPSASSILESGFFGSSGGSDKEIYRADLMVQDACAIPLTAEMIQSYTTEWEDK
VRTEKEREQELEGLKHTVASQAARIRSLEERTEKSDQEHVQLASELVRMKVENEELSDTNESLQVQ
VQELKIVVDKQPAEVEEKLRTEMERIMKRNMEVQNENRNMEEQMAEMEKDLVETKMKWAELSENHE
GLRQKWADLRRALDG
>g1832
MGYRETSEESAVIPGAEVPGEQLAVGDERGSIWYYSIELSPFEALSKVTLLAKVDAHLARVCSISW
SPDKRFLASSGDDNYCLLFEMRHILQNQQYLRSHPEIRNLSTLQLSAIVSQVARIIRISSLIPSYL
HSESQPLEDSRASSTSGNVRSVSMDDDAQANITNRRVRGRALESTSEVPPGGIFQPYRRVASAYYS
PAIRDHGTTALHFLHGNHIHSFRHNSAVKAVAFAPWQPTLLATGGGMLDRTVHFYHAPTGSCLAEI
YMWSQVTGLVWSKTRREIAVVLGYGEFEQGHQYRVVVFAWPSCQQVTAIPWNSQVDENFREYMNVA
DRALSVISIPYFVDSLHQKTSGDVDSNPEDECVGIASSRYIRFYRIWAKPHKVFAGVAGVMRSEIL
EALDGIEDQRDEVIR
>g1833
MGKKSSRPPASKTASAAQPAASGATVTGKKSSIIRAAFSPSEYQLALFASVIQGLDSQHLRIHDIH
TGALQCEHSVAPKESITSLDWGYLGGLRDQGSKKKRKRKSDVNGSAEDKFGREAVVAFGTSSSDIR
MYSPAEDKVLCSLSGVHEGGIKDFKFTAGESTSQGWSIGGDNRLVQWDLNTRQSIRIINIPTTSTV
SALSRPLLSNPPVICATQTPMILDIENSDAESLTFPSLTNPIHTLIPSSTHSVKDANFIAADNERY
ITVFDPKTLGIAYNLVAEQEVASLSLSPPEEEERSSTDKQVLAALVEDGTVELFNRPFSQTIAQNG
TKATSQKARAKQMIRKADAILKILRPDTGKAVYAVQVGFQGPELYIAWAEGGVNVIFERVRWQDQD
TGDLAFSGATEITARKSASALKSAAVIEEQQASKSYVNENSTIIESGLYSEDVEMNDAHVEDDAVS
ISDAEEHSDVEIETTQKRQTKKVNGINGTNKDTDMQDTTAEQDEDEDDEGGELTFGELVQRSSAID
VEAELDDHAGAGALVPGLSNNTNQTVAQIPSGVSLSTVLTQALKTNDSEMLESCFRTSDVNIIRAT
VQRLNSALAGALIQRLAERMASRPGRYGHLLVWVQWTCIAHGGAIAGNTEILKRMTTLYKVMDQRS
RSLPSLLLLKGKLDMLDAQINLRQSMADSRKHGMDDGGESLEHITHYAGVSNDDSDAERRRKRKKT
LSYIKSKSALDGGDDEADEDDEIADEVGGLASDSDEEEGSEEDLDEEGGDMIDIEAEESLDDDDDD
DDEDEEEDDDEDEEEDSDAASDMRDFIADSEDDEDLSADEAPAKKSAPPPSKKSKSNSKSRR
>g1834
MATPPPEGTTAVLKADKPLLPPTVTGLPADSPINRSPDSLPNGISLSEKTGLTSAPVQSTLEEEKE
STPTVNGHSTSQPPLTNGTSPHIETSTPPADDLNQSNDLKESEGLNQGRGEATPVPPVKSEEDSKP
AQPQESAAQPSVMEEPKDLPLRDNVEPQQFTETSAAVTDSLVESAPIKEPSELALPPTDIEMNDAP

LSPTKVSRERELDMEDEPAAKRAKVDDDVPMGDGSFEKADISASTEAAPSAPTQTSTGITPVQHKF
LAKQLTNLKRSNDARFYREPVDPIKLNIPSYFTIITQPMDLSTIEGKLKKNQYNAVAEIVADVDLM
AGNAAKFNGPDHIVAQEGQKLRALFAKQLEKLPRPDEVEEKKPKKIVPPKEPAARRESRVIAQPAP
PKQTSASPTFALNPEGLPTIRRDSTNPDGRPKRAIHAPKRDLLYSAKPKKKKFQWELKFCQEILDE
LHKPKYWAVASPFYFPVDPVALNIPTYHSVIKKPMDMSTVQTKLKAGQYENAKEFETDVRLIFKNC
FKFNIPGDPTYVAGQKCEEVFNNKWAAKARWVEQHDPDSHNQSGSSDEESEEEESEADEDQEKLQA
LQRQIAEMSKQVEAITQKKKKTPPASKKSGKSKSGKKDSKKSSKGDKKSKSGKSEKRHVTYAEKQM
ISNGISTLNDKKMQEALRIIQNNVPSLKGLQETEIELDIDELPNDVLVMLLKFVKKNAPQVMDDED
MSAPVSANVQAKPKKNKPMSKYEQEAQINMLEGSLSRFSGGNGTRSPEPVNSVEDDDSSDDSNDDS
EESEEE
>g1835
MFLDAPCGLNEYESNSLQDRLQEASLLNRDIQTFHDKTINALMLPLSESITSWLNNLRSVAEIGTP
SAQTTTNTSSSSSNIPTIASANQSRPRIKSRLSIGAKRIQPPRLSIQVDERIMKLRQMQVSDLPAS
RNKVAITAANLLSSRGEAMERMIVLLERTKYGXLARGTKAQADYLASVAECMSYKVQVTKFETLST
IYTPENVAALKKYSAHLRETLGRLEETQMIANQTLEEYDQVGTSQSNRSGKTGPMADIARRYGDLV
QEVDAVQREIKKLRI
>g1836
MLSRSTRGTLLRLRRSAAPTSPSRSRILVSQQSISHQQNKQTISQRRTFLSTPAIRKGITPDSSDP
KAPNPQSSNAAVAAGATHVVEATPLSDKNYHEVADQYLEVLLGEIERTQEDGSEIEAEYSAGVLNV
VVPGIGTYVLNKQPPNHQIWLSSPISGPKRYDWVIQGDQMHEKQDTRGFINGQWIYLRDGSNLTTL
LNDELTLQLPEDIYSAAEE
>g1837
MAANNLLPQKEKKPPLTHFLCLPLVSETSLPLLENSLAKFKHNLLSSRKSGGDHPSEWLPPLFPDS
AIRPLGTVHLTLGVMRLTSPDRIHEAVKFLESLEIDEILQEVQSELALDTNESTTLGYGKPISVSL
ESLGTLPKPKAATVLYANPIDPTSRLHPFAVKIRQAFVDAGFIEQDFIKQRPKKHKIRKQISSSQN
AANTNNGDINGTIVVAEAAEETIIQQRPKLRPLLLHATLVNTIYARGERSAHHSSHHGNKAKKGRR
HVGPLTFDARDLLARYRNYYVDGTCMEEKEHLPADIIEQSSPEDDGDDGASRSSEEEAHPSRGVEQ
IQHTQETDMTRYPFIWANNVPIDRLCICEMGAKTLTEEDNHLAARLGQEYRVVAERKLFQ
>g1838
MHLLKTFAFLPLIPLALSSPALSKRDEASVLTGIQKIANATLTLNNTVASYPGGIEGTITAVEILA
DSFAVINAIVSTTNDAKHSANFTDAESESIAEAFIGSLVPVVESSLTTIEGKKADFEDGLLGIASL
TGLVETILKELKSDSDKLAAAIVAKLTPIWAGVAPLIVAQIDDAFNTAITTYAS
>g1839
MHSHHALFLPALASLASAWAPPSYSGYTLVWSDSFSGAGGASPSSSNWNIITGNLGVNNELETYTS
SSANVQLSGGSTIQLVPWADSSVSGGWTSGRVESKYTFTPAAGVITLAEADIRFGTNAIANKQGIW
PAFWILGDSLRHGTGWPECGELDILETVDGLLTGYGTAHCNVNPGGICNEPNGRGGNIVIPDQSWH
TWRMTWDRTPSSWESETITWYMDGKQFFQISGATIGDSGVWATLCHDPLYFILNVAVGGNWPGNPN
SATLGGYGSMMEVAYVAVYQS
>g1840
MEGLDKAAAIPRLAELLRHPEDLDKLPALKLEYQRKKVAVDAQLREGLREQLENVQRSLAVLTEGQ
RQVSKTRDELQGIDKLCAESQETVEDFSQIDKLARVQRNFEAVVMMKKGLENFDADLAKAEALLRD
DDDDLENQPNLLKAHILISQLRDFRDEALDQIRRANDSSSETTLLDYFQGLDSIVEWFDDHVGTAC
MNLIPLLQSDNNGMVVRLAIVIAKEEKNDEHVRALQEAQRDHEELANRFKSMNIGPKTVRGYKEKF
LQAIEFYAQSQFDASKEDFLGDPDSLEKSLKWFFNDLFTVQQGMQQLMPKKWKIYATYTKTYHRMM
HDFLISLVEDPELPPDNLLSILHWTPKYYKKMSKLGWKPTDLTPNVLDDREPELVRQWQSVILNAV
DEWMDRIFAADKKALLERSPDALETNADGFFRTKMLGDMWRMLNEQIGAASASDRADVIEGIVDSM
FRALKARQTAWQTVLDEECAKYNNTNLDQAEGLQPLQDWLVAVANDQIACIDDNEETGQVGYLTRF
KRDIESLVTPNYMDDRASVELDALRNGYVDLSTHCLSQFVNLIFVVDFRTTLPDFFTSKWYGEFAI
KRMISTFEDYMSDYGSVLHPSLSEIFVEELSDELLVRYLSAIRNRGVKFRRQDPFTEKFKDDVLTV
FAFFQKFPDSFQTTIKNKWRLVDWLVRLLEAEKGAGIVNVYEALKTEYWDLQMSWVETVLRSRDDF
ERSMISSIKAKAAELNVERGLETIMSKVK
>g1841

MATKITYFRITLMRSAIGLPLRQNMVLHALGLKKRMATVFYPVSRDVAGQIMKVKELVDVQEVDRP
LTKRQVHLQRKPDPGYYVERRAEEDFQERTLS
>g1842
MASSLIPASMQSKLMGVSSRNLSNATLTYLNLDLIKNVVFLFFVLRYTRKTFDSLRGYGIIGSIKR
VLVAIRLWAYYLFLRAPGVRGQVEKQVTTAVTKLEEKMVRRGPGINSYLTLPKEGWTSEQIRAELT
QLAGMEHAKWEEGRVSGAVYHGGEDLSKLQTEAIGTFAVSNPLHPDVFPGIRKMESEIVAMVLSLF
HGPSDGAGVTTSGGTESILMACLAARQKGRAERGITEPEMVVPETVHAAFFKAGSYFGIKVHQVPC
PAPDYKVHVPSVRRLINRNTVLIVGSAPNFPHGIVDDIPALSRLAVKYKIPLHVDCCLGSFVIAFL
KKAGFPSPYEEEGGFDFRQPGVTSISVDTHKYGFAPKGNSVLLYRNRSYRNHQYFIFPEWTGGVYA
SPSIAGSRSGALIAGCWVSLMTIGEAGYVASCHQIMGAAKQFETAIREDPILSANLEVIGNPEVSV
VAFASKNVGIDTYDIADAMSAKGWHLNALQDPAAIHMAFTRPTALAVEKLQTELTEVVSAELAKAE
ERQRQGKSYARQRGDTSALYGVAGSLPDKSIVSRLAEGFLDALYKP
>g1843
MCPGADSTANGHANGNGTTNANGKVNHDGFTAIETRQNPHPTTTRNPYGHNAGVTDFLSNVSRFKI
IESTLREGEQFANAFFDTEKKIEIAKALDEFGVDYIELTSPCASEQSRLDCEAICKLGLKAKILTH
IRCHMDDARVAVETGVDGVDVVIGTSSYLREHSHGKDITYIINTAIEVINFVKSKGIEVRFSSEDS
FRSDLVDLLSIYSAVDQIGVHRVGIADTVGCASPRQVYELVRVLRGVVHCDIETHFHNDTGCAIAN
AFCALEAGATHIDTSVLGIGERNGITPLGGLMARMMVQAPEYVKTKYRLDKLKDLEDLVAESVQVN
IPFNNYITGFCAFTHKAGIHAKAILNNPSTYEIINPADFGMTRYVHFASRLTGWNAIKSRAQQLNM
EMTDAQYKECTAKIKALADIRPIAVDDADSIIRAFHHNIKLGTDKPLMELNEEERAQFAQKEKEIA
AAQANGENIGA
>g1844
MRVSALVTLLPALVAAQESQFAFMDQAKGWFDQVKSFIPQAPSVAEPVQQAAHHTTSKISEQAVTH
LNINNYKDVLEPTTQPQEWLLFFTGGNKTCFGRCLRAEHAFNDSVALFAADPTAPNLGYVNCEQEQ
ILCSIWAAAAPSIWHLDLPQVHPGEKRPAVPLHVQNLNFTTVTPETIYNVHAEKTWEKEDRYEGWL
HPYDGFLAESGLNVPLGRVIYYFSVLPSWLTMIGISFLSRNVMGRRAANPGAIAGRR
>g1845
MAAPVTPDNVPTTASYVFATAIIAGVVGYFAGQGASLGLFGSSPAATGASKSKRATKEKETNEESE
SEEDEDGAELATFDGNQDEVKLVLVVRTDLGMGKGKIAAQCSHATLACYKKYYFSKDPNSPILKRWE
NGGQAKVALQVKTEDEMLVLQAQAMSLGLCARVIQDAGRTQIASGSRTVLGVLGPKTVVDQVTGHL
KLL
>g1846
MADALSIEQNNKIRIALGLKPLPVPGAEAPSGPVFKEAKDSAEEDEPGSTLESREAQGYQNWQKLQ
DEADARRKRQEKNAAIKRARDLAQRDAKLEGRGLGDASAELDTKAWLSQHKKMTKKIEKERARKLA
EELAERDRAAEYTAADLAGIKVAHEMGAFEDAEDGQILTLKDATIDENEEEGDELENLEMVDKEKL
KEKLELKKKRTAYDPMQADGTAGILSQYDEEIDGKKRKHFTLDAKGSTNEEREAKRQHVSDLLNKQ
VFNLDIIPDAPVSDYMDISEVKIKKPKKKKAKATRQKAIDEDDIFPLPDANGANNGENMEEDSAQT
ITNVAPRKKTYDTSFVDDEDLQASLAIQRRAAFKKRKQLRPEDLARQLREESSQTPMKVDDGDGAE
EPGLVIDETSEFVSNLQRPVISERPSRRATTPPEEKDEEEGSPEAGLADVDMEGAEIKEEGELEAA
EAGTAKISGTGLDEESTLDEGLGSTLAMLRQRGLVQQSDAADMNSLHRDRQKFLQEKYKREAEAEK
RARAQRERDRATGKLDRMSAREREEYARWENKQRDQQESRQIADVFNREYKPDVQLKYVDEHGRLM
NQKEAFKHLSHQFHGKGSGKMKTEKHLKKIEEEKKREAMSALESSQHTGMNSAMGATAKKNRQAGV
RLA
>g1847
MLSTLRVASRQAVSRESNMRVLMTGVRAGSTWANVPQGPPDAILGITEAFKADSFAEKINLGVGAY
RDDKGKPYVLPSVRAAEDKIVASRLDKEYAAITGVGNFTAKAAELAYGADSAPLKEGRIAITQSVS
GTGALRIGGAFLERFYPGAKKIFLPTPTWANHNAVFSDSGLEVGKYRYYNKDTIGLDFEGLVEDLK
AAPKGSIILLHACAHNPTGVDPTADQWRQISDVVKEKEHFAFFDMAYQGFASGNADKDAFAPRYFV
EQGHNIALCQSFAKNMGLYGERAGAFSLVCASAEEKKRVDSQIKILIRPLYSNPPIHGARIASTIL
NDPELKKQWLGEVKGMADRIIEMRSLLKQNLEALGSKHDWSHITNQIGMFAYTGLTPEQMDVLAKE
HSVYATKDGRISVAGITTGNVKRLAESIFKVTG
>g1848

MAPSQTSKEASKKEGPPDLTHYQDKLDYNTFEQILEMDDDEEERDFSKGIVFGFFEQAEGTFEKME
KALAANDFDDLSQLGHFLKGSSATLGLTKVKDACEKIQHCKEDLPDEKDESERERSIEGIKKTLKD
VQVDYKEVANILRRFFGEEPVLDDTPDEKKTKEEKKSESPKAEKK
>g1849
MYYILYLASLCKRRHWSEPHYDSFQSSTGFTCVVRVNNREYKTDSICESEVMARENAAMRAYLICR
NFSVNDGMYPAGHDHGGVLQGVPVAIGTGRGSHRVSSRYGTVHHDESDSTSVSGGSRSGGSSPESH
DGARLMTRDHHDRRHGVPTRALAYRASETNPTRKSENEPDDESSKLDEAVSYVKQNQRKTPWQREG
SQIPPVKRERDASAMTKGKLLTTPSRLLKLILPVTTKDYNDDRKDVEPLALLLHSQQPLSYLERLI
QAEVPPITDEKGKLRPPHVSFLAVEIEDGTIQPRKPKAKNWVDESKDDEQESLDRDEEKSSVATRD
SQIARLGDVSDEKEDVGKSLDAERNTETDKETEALRKGSFVRWSSSTELGDFIRDAARVKEFLVEI
ENSPLGKIPVAVPSFNDRTYYLRMRLRKLSKSLKNLAIIKQECDVLAHRGAQRVALGGLGVMVSWW
CIVYKLTFETDYGWDTMEPITYLVSLSTLMGGYAWFLYHNREISYRSALDFTVNRRQQKLYQARGI
DLQVWESLIEEANSLRREIKTVAEEYDVDWDERGDEEDERVTEALKNERRQKERREEAERKQEKED
ERKARD
>g1850
MSAIHRTIAIEDVDTRWPYRAGFPRLLRPRVKSRPIENEAEIPISEADFERLKQRINEITEAHGIT
HLDTPRMVYRAHSSSVSQARIDLYCVYDKGLSHSKTWAKAVTEIYEEANMLCVPGKDRNEMSMNWE
KGQNYRQKILQLFENRPHMWQVMVPIGLYAEGKRSQDHKMVIYFDALNAEDDAWDELEEAMRSILP
EDIGIEILQSTGPLFCNDNLAPLHNEFKPKFSADNYIRPPRPGCEVSIARNDLSSGTMGGYVMTEA
KDTKKRTTFGVTNAHVVFGKRREATFNTQAGDSIVIQSPGEETRRRNEIELRTAIADLEGQISKQT
EIIEAVKFSDPSLAERLDGHVNLIQPTLIELKASLQLTERDATIGPVQVARLGYRHYERPSSVSSG
PDYTKLMMDLALIRMDCLEREHPREIFLPVNRENPRIPGDTTYNSWEVDSLGNSIDTKERISVVAK
LSRQGKYTMGHVTEFKAEIEFPVDLKNGGAKEIRKRSAWVVSTPPIDGNDHRTQLGDCGSLILAAH
GWEFGGSDNEVAMPKTLNVCAMKPFVVGLLFGGTDRGDVTLFTPFDTVKSEIESLTGEEMVWPPKH
STCMQRWEEGYMDDGL
>g1851
MPNEPSISSLFTTLPPLQDSLETKTSQDQDETVTKCLPFLTGRDGTLKSNLNEFGLSHLMKDRHIE
YLYDSLEDYPEGFVAMDSSRPWMSYWALAGLALLGEDVSKYRERVVATFTAAQNPTGGFGGGHGQM
SHLASTFPAVLSLALVGGEDAYKVVDRGAMWSWLGQLKQPDGGXQLVIDGEEDVRGAYCAMVTISL
LNLPLELPPEAEVRKHGLRTFLDGLPEYLSRCGLSGKPGAAEAHGAYAFCVLACLCIMGQPKDMIT
RYMDIPLLVSWLSARQYAPEGGFSGRTNKLVDGCYSHWVGDCWPLVQSALNGPHGDEDAVPKVPQY
LFSREGLARYIMNCCQNKNGGLRDKPGKHPDSYHTCYTLTGLSTIQYYHYQTEQETTSSRIGGVFA
SSFSWKSVPAKITTTEDDEASRDIVDKCDRLVAFHPIYTIPHKNAEALRRWCEEKPI
>g1852
MMSQHNLSNDGLVDLDYDSRNYLQAQSWPLAVDQNIPRHDDARDIAPLQTSGHTFDQIPTGANLMP
EWQFQQSLHSQVSYPHVETTGPQFTSSYGVPLQTSPVDFLPASQAAASLDASSLLDGSYLSLSAPV
DITMPFAYPDFQGLMTFPNGIPDLTSYGGIAHNIPESSSPTDTYLEVRSLTSSSSDNGWATVDHRA
IDHRAIDHFHHHDHALFINPTQTLHDRSLSESSYSDFEPNRASWGSSWVDVQHPISSPGSDHLDSI
YNPAVDRRISLDHASHGSVSPVAVSPVAIVRPIPVPSKKATPQSHSPRSSTSSSSSSPSSSSPPAR
KNARKSPIAAKTAETKVRKQSQTGKPETTEKRIGKRKGPLKPDQRKQASEIRKLRACLRCKFLKKT
CDKGEPCAGCQPAHARLWQXPCTRIDIKEIGYFMKDWKADYERHVTLGFSVGNIKGFSDQERILFV
THGYGEIMPINAREVFVRDEECFNMDWCETVEREMGHYQVSTMKFSAGIEGISHSKLSDYLDRHID
GNGSYEKFVDDYFDGTPFLTQMLKTAFRYYFRTKLPVIRKALKLILAYNLTLSVTMVEGIGEDEDF
LGKIEGEGSRFRGKIMAPVMINFQIKCAMANMWRELQKDVLEELSALYSSVYSGDRLKNWPTIYIL
ATILLAVWEEMQFDCHYRVPDVSAVDKFCHDMESTPVGVIVGLFQAISQKLPAFTDWETSKHHQLL
ASNPDVCQTMTEVRHHVKQHESYLRNRSNTKFNRTDFDCLSNKFLSRLVIRAN
>g1853
MQIFVKTLTGKTITLEVESSDTIDNVKSKIQDKEGIPPDQQRLIFAGKQLEDGRTLSDYNIQKEST
LHLVLRLRGGMQIFVKTLTGKTITLEVESSDTIDNVKSKIQDKEGIPPDQQRLIFAGKQLEDGRTL
SDYNIQKESTLHLVLRLRGGMQIFVKTLTGKTITLEVESSDTIDNVKSKIQDKEGIPPDQQRLIFA
GKQLEDGRTLSDYNIQKESTLHLVLRLRGGMQIFVKTLTGKTITLEVESSDTIDNVKSKIQDKEGI
PPDQQRLIFAGKQLEDGRTLSDYNIQKESTLHLVLRLRGGN

>g1854
MPSYFYHITLELLTSSSNIAHDLYDDGNDNLSRLRSDRSLANEAVREAFLPFQRHSSKKTSVIAHR
QHRSVGSQSGVGSGFNNLSGVVHDGHYRPASFDAKLSSLSLQDHTSSADIAIQSDLRFSQIFIQGV
DMVAPAHNRHRRPSADTKSDDRDTITTGIGTDILGGLHTRGKYVPLDQTLADSVYGIVHLYRDAKE
TPSLIIDDHPLYLKGSSAAARRPPNDSPHTASSGMVHRTQLGSAHGSSRSRSHGLNDHDSAEYNSS
SSPLQVYSPNDEDCSILCILAVPSYLSPADFLGFVGEDTRDEVSHFRMIKTARANRYMVLMKFRSG
KKAREWQREWNGKVFNSMEPETCHVVFVKSVEIETAKQHEMSDASVQSSALTSNTTRSLGAANSSS
PGISSASLSAKPLAPPTPSLVELPTCPVCLERMDETTGLLTIICQHVFHCTCLQKWKGSGCPVCRY
TLDDFGRRSQVGFFDEGSSGECSVCHSELNLWVCLICGSVGCGRYDEAHAFAHFKETSHAFAMDLS
TQRVWDYVSDAYVHRIIQSKTDGKLVELPAADNSALDPPDWTDAVPREKLENMSVEYTHLLTSQLE
SQRAYFEEIVERAADKASKASAAASAALEAAENATKQLSELQQRYNALAKDNVPGLEKEKDRAERR
AEKFETMARKLEKEWREEKAMNENLVKRVELLTEEVAKLKAENADLSEQNRDLTFFISGSEKLKEI
GAAGDEDIVEGTVSIPEQQSSKRKGKGRRR
>g1855
MVRTKNPSQKGPRRGPIGASNAPKPRFPIQRTEIWDNKVKIFGWPSHGGVIVLDNATLVDLDFLGW
DTVNPPLKRDRDQDAEDEVCQRLLLLGAIWFDSQERYDIAAAVEDDFDPLASKVETGEAPRPTKME
RRWVRVGWPSHGGGFWVAEFDNHVFGPPWQGPGLPGETAHLLLARNMDEKCEILQQLGGQFLANLE
DYQGAGCLNAWNNKETGEVGPLVITRYVEW
>g1856
MRRKSSSSPSTQSYPSHPFLLPTDEITSHLETNIDTGLSTQQVSSLQARYGDNKLSGEGGVTWYSV
LAKQVSNAMILVLVLAMALAYGVSDYIEGGVITAVIIGNILIGFYQEYRAEQKMDALRSLSSPSAT
VIRNGEVVTIPSTEVVPGDIVLVKTGDTVPADLRLVDGMNLECDEKILTGEAVPVAKDAAFDHKSL
VEDSSANAETHIGVGDRLNMVYSSSTVTKGRGKGIVVYTGMYTEIGKIASSMQGKKRPSGTKNRSM
SMKKYGPLQVFRGGFLRSWDALGRFLGLTTGTPLQRKLSKLAYSLFGLAIILAIIVFGVNEFHVTN
EVAIYAISTGIAIIPESLIAVLTITMVVGMTQMHRRRVVIRQLSALEALGGVTNICSDKTGTLTQG
QMVTRKAWIPGVGIYSVNNSDDASDPSRGFVTLDTASTNEEEVEKQNEKTPSTPIDSKEHAITEVN
PEVESFLLASSLCNLATVRYDSTAGKWQTMGDPTEIALQVFAHRFGYGKKTLEADLKWKQIAEYPF
DSAIKRMSVIYSQGADEPVIFVKGAVERIIDLCTSVGAGGHQEKMTAELKENILQQMKSLAEQGLR
VLAIAQRPVPVGYQKGDELPREDVETELNLLGLAGLYDPPRLETKDAIKECINAGIRVHMLTGDHP
STASAIAKEVGIIPRYTESLSAEKAASPVKTAAEFDGMTDDEIDAMPTLPLVIARCAPDTKTRMIA
ALHRRRCFAAMTGDGVNDAPSLQAADVGIAMGMGGSDVAKSASDIVLTDDNFASIVNAVEEGRRMF
DNIQKFVLHLLASNVAEVVLLIVGLAFQDNDGVSVFPLSPLQILWINMLTSSFPAFGLGREKASAS
VMRRPPHDMKKGVFTWQVIVDMMVYGMIMGILTLLTFVIVVYGVGNGTPDLGYDCNRKDSESCHVV
FRARAAVFAELTWLILISAWEIKSLRNSMFNLDPLRAGQENVKFPFFRDVWENKFLFWAVVIGALS
VFPAVYIPGLNTDVFKHQGITWEWGLSFGAIIIFVLGAEAWKGLKRRTGWFREPDSREYASVSESD
FGLKSSLSTARSMTKDSRSTST
>g1857
MPNVGRPSRDCHTCRKRRLKCDLIRPECTQCLRKGLSCPGYRDEMEMRIRMSSASSFQVTDRVRRK
TKSREQKTDSKALLEIDVTQTALEHRISIPSNQVFPLTVPMTESWDVHCLPFAISKLELIYVMGPS
VFDTALSLFRKMEETSHLHMACKAIGSAFLANMTKSPKAIIDQAKAYSSSLVAVKQAIRDSRKITS
DDLFLSICLLAFYEVVSGAHSGNSSFLVDSAGYKAHTKALSDLMCLRGSDMCATRDARNLIGMTHC
HLKIFPYKGLPLSCTNSFVYVTPQNGLLIEADNPAQLLANSSDIIQAIDNVEKQVEPFPEKGPIAS
LRIDPPSQLLQNDAGLNSEESKLRTHQYITRILLSSYAVDFVSYASKIPACTPYQRRDFQLLKQRY
IWEMDVLSKQLPTFTQMPLDMXSLFTESVPRERNGETEQLVIIESNNGVHSSGSGHGLSSQSFCKS
PDMIWKGLLILAGCDTSAFG
>g1858
MYQKMTSIVPPLDYVLDVRAEMKPVKISQNSTPQGKSYYFQFIGGYIRSTNGELDIPLQPTFDDAF
LPADADVVSLRVTAVGYDQKSGDYFQARNNGFLSLSDVKGEANSISEYGNSNCTGNFTIQTGNKEW
AWLNTSVLVGPGRFGYDENGDIVWVEYKVFRVRSTW
>g1859
MTSTDKPLTVVVIGLGQMGHSHALAYHHNPGFQIIGLVDRRRPGKTFPDELKDYPLLSSFEEALSL
KPDVVSVNTHTATHADYAVAAMESGSHVFVEKPLAVTVSDAERVVSTARRTNRKLVIGYILRHHPS

WVEFIHQARQLGPPFVMRMNLNQRSSGDAWAIHKRILEDVKNPIIDCGVHYVDVMLQITDSQPVQV
RGMGLRLSDELPPDQGQVNYGHLQILFADGSVGWYEVGWGPMMSETAYFVKDIMGPRGSVSIVMDE
SKKATADGEDGCGSADINSHTRTSNIRVSTVVDGQARNKMLSMEGEPDHYELCAREQQFLLDAIRE
DRDLSKHMQDAVKSLGIVLAADRSMRENRSIDLE
>g1860
MSHYKVLRPATAAASRLAGSSIRSSGYISRQTQKQTLRHHSSVSANELSHFSGLASSWWDPMGPSR
VLHLMXPTRHDFIASCLSESELQTNTQHGLRYLDVGCGGGIFAESLARTLPSKTSARLHPRTKAGS
IVAIDPSTSLIKIARDHASNDPAVFAHLQSGSFNYENTTIEALSQTRGESDKELFDVITLFEVIEH
VDPSTSSSFLTHCLKLLQPGGWLIGSTIARTVPSFIVNKMVAEAPWPIGVVPWGTHEWSKFVNADE
MRGWVEEGLMRAADGAAYRAGGEALVGMKWKVAGVVYVPGLGWKMVNGGEDWGNYFWAVRKGV
>g1861
MEIPTILNRKASAAVAADPRFQAQLAQAVHLHTQEMSEQANAQPNHAVMGYPPNTQPLHQGQLPGQ
LWPTHPNGAPMMPNNWDPNPFAGAPPQPPQPSHTPPVQQQQPRAEPAPRVFHCSTCQKGFARRSDL
ARHERIHSGIRPHACDWPGCGKQFIQRSALTVHARVHTGEKPHMCERCGKPFSDSSSLARHRRIHS
GKRPYKCPYANCQKTFTRRTTLTRHQNHHTGTIEEAAAQTEASLRQNREKSARASESIYSETGSGH
STPSPGQQAAMGLGSELPPLNIIRSTGEYYIPNGGIPPHVRGDFQQASPRASPAATSPALSSYSSH
PRPSMTSHPSGYGPPQPLEPPATTDHRPGSVTGSPHMTSMGWASPSHASMPSPGSPHEFGYTEPNV
AAYASAMPPHMYFPNSTIRRPGSTEPENYEMKPRIGPDGWSTPI
>g1862
MLMDIPSTSLPKPEPPYVKGSQLTVHLHRPPPPTPVIRNNCINTTTDIKERRGIDSIDKALERCNR
HPPLQGNLEKPCYVRLELTGPVVQVGDGRNAQVVVVQVLHKSEGFNTLKKEKEVQKFQIGDRLVAK
IYDPLYFDDEEGSLNPFLCVDRHYTHESRAYHVLCEMQGKEIPKFYGSYSLELPSVGHTFRSVRLV
LIQLIRGSNLSVMNVRAFSQERRQSIMRSVIDFESRVYERDILLADLFPRNIMMKLPQSTLDANSD
EDQHPEKRLIFIDFAAAIFGRIRDDPVPPEYKPNLFLGQYVSPLLRWKLTPTYNFGDRVNWDWLPW
LKTAFEHTIDTITPEMQLAYRDDYDYED
>g1863
MLKNRVFVSVCVNVFLVWGASNATETLASLFFQYVQGLSALQTSVKLLPAPVGGVLASILVGLFVQ
RLRADVFSILAVMIASLAPLLMAIVHPDWSYWACIFPAMAANAIGADTLYTISNLIVTASLPPDKH
GIAGGIYNTIAQIGRSFGLNISTIIANLVTDRYPSTDQGSPEALLSGHRAAFWYCFAANLVTLSVV
LWGLRRIGKVGIKDD
>g1864
MAPPEAGRRNPTVLVTDSRDQHAQRPSRRAALPATRLITVDNVLQYASDIPSMQQRRPMQQRPQMR
PRSRLSASTGIAGAASGSNAPLSGGIGGVGVGGGGNVIGSSGGGIGGGGSGTLGTAGRLAAQARLP
SRSTKVSEKLVLLPDLEDTTEAETVGRIGGDGGDEEFLAEPVDDELVRRIAEERNMDPDTLRRRLQ
LAAQQRLQGDFGVDNDLAPLLAEEEALRQRFVPPEKAKSYAERLPKARRAEKLPRVTAYCTAQGFK
MGATASFVKEWHGARTKLYDDCLYTAYHLPLLPGRGGYRLRSSPVLKGAGGRVVLDDEIERNEQRD
YHDEYYVEEHEHSVGGRRNQNNESEDSGRNQNDHKSDAQDTDSGDSRPSSSERETSESHKPQPTTS
SSTTPTTPTTANRIPSNVFDYAEMFVFSYGVVVFWNFTERQEKDILADLTFSSSGTGPQISLATLP
LAEEDFETEEFHFEYSTEISRPRVYNDMITLRSRDHMIKLAISHGIAQSTKLCFFEEAMSKEMTEA
KDVPRRLALTGKLGMKRDEVFRILGRLFKSRVEVNLSSNMLDVPNFFWESEPTLYPLYIAVREYLE
IKPRIQVLNERCRVFLDLTEILSDSVADNTESHQTWIIIILIIISILVTTSEVFLRFGILSSNHAA
ENPAGPSFVSNLMMNLLGRESSSSAMSAPYSSMGVDGTCVCPAFNSSV
>g1865
MALTVPDTGLSLWRGDQPGNLSPDPHAKQTQILRLNLAQSTLDELVESLRNDQKARLRLGKHQTLY
YGSKSQQFHSHLETHRSELYKTSEDDRENLYFSGVLSHSLEVQKAKEATAATDEALATLEQSLSAF
ERGKESKKTQLVNLKPDDRKSRSKVPLSKIDLDKERFFKNRSISGSPSLGAIRSPASLPTLTPTSV
PSSQNKRKARQDALKTPFIHLLAVRPVSVKYLAQTTRSSQEDCLELARKYGVENRIDPSKFNLKDK
SYKELDIWSFPYPSESDRASAIENAISAFDRQRVSTSDKLWQMLNSKEERAKGICKSRLKLQTAPP
PPIKVHAAEETSKEGDLTGNETDRTNGRLTPNAMAGDTSSKPAAKKPPTTKAKNSTLTGRITKKTE
KKAPPPKPTGKFKSAEFVHDSDEDTDIPDAPPIPSPPPAAKKPAATTESKKPPEAKKSTENKKLPA
KLVDSKPAKPASKPTSAAQSPTVKPQITLKPQPRTIKTIPATNGNGTGSKTLPGSRATKPTSPVKP
SPLASSPPTNASDIDNSSQNSGQTSSSSSSPLIAQVAKQNKTKTAAPTTVIKPKVPAKPNGTVKPV

IEQTNPLKRKALPDPMPTGRLNRNGEDIKRRRPIESMSPSGTSSGSASPPHNRDYHRQQLRQKSMD
FKKLYSRYRDLHNSLVNQMDPTPSDLSRLKQMHARVQRMKKEIWDEDRRLRGKS
>g1866
MAWNSPKVGILGGGQLGRMLTESANRLNIEVHVLDAANAPAKQISAHDNHTIGHFNDQESVQELAK
TCDVVTAEIEHVNTYALEAVASQVKIEPSWQSIRIIQNKYDQKKHLSKYDIPMADYRELVENTPEE
LAKVGEYLGYPMMLKSKTLAYDGRGNYPVKTKEDIPDALEALKDRPLYAEKWAHFKMELAVMVVKT
KDSVLSYPTVETVQEDSICKLVYAPARNVSDAINKKAQELARNAVAAFEGKGVFGVEMFLLEDDSI
LLCEIASRVHNSGHYTIEGCGLSQFDAHMRAILDLPIPPKSLELRQPSIMLNIIGGATSDSHLRVA
EHALSIPNASIHLYSKGAARPGRKMGHITVTGATMYEAETDIQPLIDLVDNIRAERSDIKTAAVKS
SAAKPTPTVGVIMGSDSDLKTLVPGLKLLRDYFGIDPEVDITSAHRTPDYMAEYASKAASRGIKVI
IAAAGGAAHLPGMAAAHTALPVIGVPVKGSALDGVDSLYSIVQMPRGVPVATVGINNSINAALLAV
RILGSYEPTLQRKVEEYAASAKSENLDIKGTKIKEIGWEKYFAQM
>g1867
MILDEDLLEKVAQDGSMNYDQWPEQLEPLIQRLDEIISQFPIPKVPPEIASSYASSSYAFHDSLQS
SNKENAPPXPAATQTTYNGASTSTSEYATANETIVPNSQPSQPDTNSLPPPLLSLIDSIKSTLRTY
FASKPPHTVQRLAELILRPTRQYRTLPAYLRAVDRVVSVSSSADIFPLPRMGISPDDELANGITNG
ARSSFMVTDESLGSDESLGGALLTPIPWLRNHDLEAVQAEMVAQGAVPLRADAAVTQGELIRQEQE
AGLIATSQTPHVSSRSLTEKEEEDGENSSPQPEEIPHARGPSVLGVEDLGLQDGKGVEMVLSDPNK
SQEEEKSEMDVSDSIVDLIPDEVVSEAATKENGADDEDIILDDQGTTSSTETSTELDTDATRTQDE
TG
>g1868
MLQSNITGDAFDMEFNDLDLVLDDGDEQGIEGTGSADITMKDDDVQEQGPRPDDSGVQKSATSAVT
DFTRRRNWTQRILAEMQDILIVLSPDGKVLYCSPAIEPITKYEPKDLDGRFVSEFVHDDDRTSFIR
EFNESIATGHRLRYHFRFRKPDGDFAIVEASGHAHMSSEKITLGTGKEGNPCDGFFLVCRPYPTAS
NALLDTFLEHKVENIRLNRRIADLKKEEEEELRAQQQWVLQSDNNSGTMSPDEIDAYNSAPGTTTT
TTTDQQGGMMLPPARPSVSSSGQNGYTSMITLDNNVDTISRSDTSSVLDGIEMLTGLRYGEGERSK
GLSTGERDAVLVQEQADLHHLGLSSDDNDKKKRIKTTEEYVCTDCGTLASPEWRKGPSGPKTLCNA
CGLRWAKKERKRQGSIQSSNQS
>g1869
MVLAVLAQAAPSQLPRIIRPKIDKLSILGVRSFDNTRSETIQFHTPLTLIVGYNGSGKTTIIECLK
YATTGDLPPNSKGGAFIHDPKLCGEKEVLAQVKLAFKATSGAKMVATRSLQLTVKKTTRQQKTLEG
QLVMVKDGERTAISSRVAELDQIMPQYLGVSKAILDSVIFCHQDESLWPMSEPSVLKRKFDEIFEA
MKYTKAIDNIKALRKKQNEELAKFKIMEQHSKEDKDKADRAEKRSIKLQEEIEALREETHQISQEM
RNAAELADKAWKESESYAQVIGTLEGKRIEARSVQSTIDNLKRHLVEIDESDEWLHSTLEHFESKQ
LEYQQQEDAQKERYMDIKEQIESTRDQLGLKQAEVGKNENDKDQFERQIGRRENMIKEFARENNIR
GFDDSLDEFKIDEFMQRMQRMVKEHTQALERVRKEGQSEVRETQNILNQIAQRKSAFQEAKNVARR
QISDNDREAASVQARLNEIDVDEGTVAVLESQKEEVQSRLEKLKQASRGATWDKDLQDANTELRSL
EDESTKLNQELIAGTKKAGDLARFAHIKKELKDRERQLETMSGAHGDRLSQLVSEQWSPENLELEY
QNVVADVSNSMALAERSRDGIGRELDQVEFKLKECRTALEQRKKERDLCVKKIRDAIDDDPTEYPD
ILQTRQNHMDQTRKDVEQFAGMHEYFNMCLEALDQKKMCRTCMRPFKNETEMRTFKNRLEGLIKKN
FSSSDEDLKQAEEEYESARMVNADYDTWLRLTETAIPELEKNQEQYQGQKEEILKKLESHDTTVDE
RAEKKREIESLSRTVTSIVRIDNEIKSLRSQIEEVSSKQQQADSSRVLEDIQNDIAAIGEKSRAIK
LTISKLSSEKDQSRDDLNKAELALRDVQSSLANASHQLEKKTGLLARVEEYKKSNVKQRESIEKAD
RDIDELEPEIAKAQTKLDDISRRAESKERELQQALTHLSDRVHQLNLANDEIKSYVDRGGPEQLNK
SRKELDNIQQEIRKLEEQQGEITREINKISAQLKDSDNTRRQYSDNLSYRQSCRLLEEVQDEIRQL
EEQNAEIDRSRFKEESERWTRKHNALAAQQASKMGEMKSKDDQLLQLLADWNTDYKDAAANYKEAH
IKVETTKAAVDDLGRYGGALDKAIMKYHSLKMEEINHIVEELWQRTYRGTDVDTILIRSDNENAKG
NRSYNYRVCMVKQGAEMDMRGRCSAGQKVLASIIIRLALAECFGVNCGLIALDEPTTNLDRDNIRS
LAESLHDIIKTRQQQANFQLIVITHDEEFLHHMQCADFSDYYYRVSRNERQKSIIERQSIAENKAM
DQAHARALEALQPFIHLTTSSTASSPRFVANIISNATSNPNTYVFAELLETPAVQALGSTDTPEEF
RGYLKLLEIFAWGTWQEYQETPGLPALNDEQALKLRLLSLLTLSSTIRPLTYQALMQALSIPSAAK
LESLVTTAIYSSLIVARLSPATNPPTVNVTAVAPLRDVRPQTVSTLISILSEWEGRCGDIINGIEA

EIAKIKDQTIRNGVTDRQRTEALEKAVIAAADADGDDTVAGFRGVGRNSKHGLRSGRGGGVRPSSS
SAAAAAAAGGSGSGSGSNSNKREYSGDDLEDEDNGYFDNGSDGGLDVYGGSRMEIDEGAGAGASAGR
GGGASRQSKRLLGVGRKT
>g1870
MSTVSEDMKRKASEEADTPDSSKHRSKKARTESFNEIKEDVVEKPPLKIVPYPEKPAVIEERNGEI
EFRVVNNDGQRESFIILTGLKCIFQKQLPKMPKDYIARLVYDRTHLSIAIVKHPLEVVGGITYRPF
RGRKFAEIVFCAISSDQQVKGYGAHLMSHLKDYVKATSDVMHFLTYADNYAIGYFKKQGFTKEISL
DRSIWMGYIKDYEGGTIMQCTMLPKIRYLESGRMILKQKEAVHAKIRAFSRSHIVHSPPKEWKNGV
VKIDPLSIPAIKESGWSPDMDELARQPRHGPNYNQLLHLLNDMQNHSAAWPFVQPVNRDEVPDYYE
VIKEPMDLSTMEEKHEKDLYPTPQDFVRDAMLIFDNCRKYNNETTPYAKSANKLEKFMWQQIKNIP
EWSTKDIELHQCQVFGFAKQHPAYFTMASLTSLDLEKRKRLPTLFEVLSRYTLAPVDLFSFYIYMR
DQQRSVDYLDFWLDVSQHMSLCRHYVRELRRSVLVATPEGGSRPSSTMLDNIPDIPTEAGPSGYYA
PSTEKDADSRLSAFLRSENPSQPSPPVSGHSEKHSSEERPPRPSFMNSTPETRYDSSNSPGHTVAR
TDIRRSAEKILYTYLIPDSEREIVLPAEMLTSIIEMVEHDGRDDPEVFDQAKDYVFQAMERDAFPG
FLQAKALGNLVPLSILARLAFGLIVFWGAFWASFYVVLTDKSRATRCWVILPFVVASYFITTYQYK
IDPFMALLGFSEYTFMNWQRIQEPYVRSLLNQRALVTLAYTLLIAAALSILFILVPGTML
>g1871
MSLNTLWETSKAPYYPIVAKEGQFTVGFALLFIALLLTGLFGLNRSFLNIPLLGLPASLAFGFGAV
FMICAVGVYV
>g1872
MEPIFSAVSNDAYHLYTLLRCIGFAEKARIQITPDGIRFSVEESRVMQGLVFLDRALFTTYTFNAP
RPQDRNDDTLVDEESAGDIYPQFLISLTALLETLQIFGIGDASQSSFLPNGSFQNTSGGAFTSPAV
LLGRTCALRYSSPGSSLCITLSESGVTTTCELTTYESDDPSFGSAGGEVDIPLQRDAIIMKIIMRS
AWLYNAINELDATDPTVLTISASSKNAPFLTLSGSGGPFSESSVKFSADKENGESEASYKNLQDEG
AKRARLAPAVTETFQVNPPSSMGSLVKENYRFALIRKASRAMAAASKVSMRGDIQGVLSLQFMIEL
GGGNSDGDVGPARGGTKHVSFVDFRFVPLVGQEDDEEGGEENGDSEGW
>g1873
MAAISSSRAQPTAAATTSLHMATTTVKVDGMTCGACTSAVESAFKDVEGAGEVSVSLVMGRAVVHH
DPTTLSPNKVAELIEDRGFDAEVLSTDMPQSSDKSDKAGTHSSVSTIAIEGMTCGACTSAVEGGLK
DVAGIYSINVSLLSERAVVEHDSTVVTASQIADIIEDRGFGASVLDTKSAEPIDSSPSDNSNKLMS
TTVAIEGMTCGACTSSVTNAFNDVEGLIQFDISLLAERAIIVHDSAVLSPEKIASMIEDVGFDARV
LSSIPHLGVSQKSSKTIRLTLYGLNGAASASSLEEVLSQKPGISSVSIDLSTSRATIVHNPTAIGI
RSVVEAIEEAGYNALLSDFEDNNAQLESLAKTKEIQEWKRAFLFSLSFAVPVFLITMIFPMYLKFL
DFGKFRILPGLYLGDVVALGLTVPVQFGIGMRYYKSSFKSLKHRSPTMDVLIMLGTSAAFFFSVFT
MLLAILSSQHNRPSTVFDTSTMLITFNTLGRWLENRAKGQTSKALSRLMSLAPSMTTIYEDPIAAE
KAAEEWNEKDTSTGGTSQAGQTGLKAVPTELLQVGDVVLLRPGDKVSADGVVIQGTSHVDESMITG
EARPINKKKGDSVIAGTVNGAGSLEFKVTRAGSETQLSQIVKLVQNAQTNRAPIQRMADIVAGYFV
PIIILLGLTTFVGWMILSHVLPHPPKIFNKPENGGKIMVCLKICISVIVFACPCALGLSTPTAVMV
GTGVGAENGILFKGGAALEAATKVNHIVFDKTGTLTEGKMSVAETKLEPTWTSNDWRRKLWWQIVG
LAETNSEHPVARAIVSAAKQEIGLMSDDALDGTIGTFEVNVGKGISAVVEPTSSVERTRYRVLMGN
AIYLRSSGVNVPESVETTLDGNVSTGPKMDDFSGFTQIHIAIDGHYTGTISLRDALKSSAVAAVAA
LHKMGYHVSIVTGDTYPAALAVAQALGIPKASVKAGVVPSGKKEIIESYQAAGDKVAMVGDGINDS
PALATAMVGIALASGTDVAMEAADVVLMRSDDLLAVPASLSLARTIFNRIKLNLMWACVYNIIGLP
FAMGIFLPFGGAPLPPMAAGAAMATSSVSVVGSSLLLKFWKRPGWMKIDSLEADANAGFTKSPTSK
ESRWKSIPFIGTGDNRPGTGSRLRTIFTKLVFGKQSKQVREEEGYVPLQTVEV
>g1874
MARNDSDRGRKTPDHLAQMKDSTSNDGNEPSFSSDLDNSAVSTPSLADDIFSPSVSIVTDTTDIAD
DYGWDTPVKGKSMTRAEDRNILGSPQNETQDKIKRAEDLSKPDKGLDTQTQTTAPRRRTKSASRRD
FEARTLSLGSVDISVTSFEKRKQGSDSPVDGNTLASDQGQIWGVLITPPKSKKPMHLQLIEFCTPG
PDGYIDINFSTDQTWIFEARRVIDFGSAVKPTSKTVVSRTEKQDVDHILTKPTSQSIPEITLSPPD
ANRTDHKIGDANTPPSKNILLGDGTIQHVLSLLSNKAFSASISKKEQDSIDKSLSNILPVKIREWL
EEDTHHCLALTTQNRRCKRIHETSHPKMIQYLDSMATLKSSELPKCLNDMISTALCSTTHKRMARE

ELEKWMVDIEKLCDIHADMKESTSVSADYRLLALANYLDILSGGKGLPPRQNIVSSSAKVEESDHS
ITSLGDYRLLQYFKPYIPKYLQKLSVSEALEGLLLKPLTGKKEIGDTGIIYIYWQPSNFGHLKIGY
TTQAFEKRMKEWIKRCNKTMEIYYPSRDDAQKIIAVDHVYRVEKLIHLELKNLRKIEKNCPGCGKN
HDEWFECSRDLAVAVVRKWMDWMRESPYEERQSGSSKEWVLKAEQRSKLKDLSEPMKGVSVSANAM
EKGKNKHPRQTRRLSTGRPPRMRSKSM
>g1875
MSLILWLIAGIISTCGAMVMLEFGSAIPRSGGIKIYLERSFSPKLLQTCVYLFYCVFLQVSASNAY
TFSSYLLLATGVDQTTWKVRGVATASAVFAVGGHLRIDNPHNFDISTSFQGTSSNGYNIGTALLNA
IFAFQGYDNVNAVLSEVKDPKRTLQIALPSAMAVITFAAVPKEDFISSKITIAASLFRNVFGDSAA
TKALPALVAISALGHLLGIAFTVPRVIQELAKDGVTPFPNTIMQNRPFKTPIFALALHLGVTILFI
CAPPAGDAFNFIVSLSSYPTTFLLTAITIGLVKLRLSNTERWNPTRPTPWFVIALYIAGNIFLLVM
PFVRPPNGKGNTSLPYWLTSVVALGILSLGVIYYVLRFVLAPRVFGYVLQPTVVDLSDGSQVTRYK
TIR
>g1876
MAVKFEPFDVPGAQVVYSNVFPYGLQVKSEEGKLNPPALEDSVESIKTLSASGHLSELLKKHGAVL
FKGIGHPAAESFAKVINAVEKARGSTPFVQIGLAGKRTIVAENIWTANEGPQDRRFYQHNEYSRYT
RFPANIHFYCEKKALEGGETPIAHSALVYEKLEESVPELVQEIKKRGLAMKMLFRAPGKEGRGNEF
NWAGEFSFGQEFLPGDDQATKKEKVEKQVRKLTDDFRWTEDGSIELTQHIPGIRRASAKDPPVWFN
GLVGRFGITRDIGALDPPYIGRDGMTYLPCEYGDGTPIPREHLEKLEETIDKLEINLSLDAGDMLL
VDNLQVSHGRKPWKGDRRILVSMWEGAEPTQAF
>g1877
MSQRPVLITGCSDDGIGYGLALTFQQRGYLIFATARDIEEMTKPEGLPNVALSSLDMTQPDHIAAA
VKAVREKAGGTWDYLINNAGRNHFMPTLDEDLNKTRTIFKTKTWRPVAITQAFTPVAH
>g1878
MVFIPRAYKNHYLALYLMCAEIPFILPVLILTGIASHNTYTTKLWQDGANNGFNSAPNQVVYALTN
GRTYKVPIVWSSLLYNFNLVIGVLSTFVILTKVPIHVLRFFYPPVSAVVHGTLCILYIAAACFQAG
SDTTDSRHPQRGPPWYITKKCTVAYSPSNIGYCQQAKALFALTVIVIVIYFVETILAIMSCFVSQE
EKDAWRRRQEEKREEKEAEDRALREYEDIINSPAFPPPALPGAAYTPGLQPMGSPYHQTHFFQSQG
QYQSLNDPSPSSSSDLPFRNWNTPKTSSFSNGGHVSINISEAAEQPQEQPYFPPPPKKANK
>g1879
MTPRKDSIPHETRTAAEPRQNKLYTVHVARLEQVNPSIRLLRFALPQEAPADETAFDAQSQETFSF
LPGQWLDVHIPSIPQAGGFTITSTPADALPLPIPDPTIIQDGTENLSHQQGEFGTEAAADGRYPYV
ELAVQQSPSNPAAAWLWRPHKEILGXDQTLNPAIPLPERSPNHPLHIRFLYSTQIPASSSVLDEKT
LDQILFLSRLRNILQRTRTVNNRPANIKLDLDLFLTNLSSPTAVAQASEILNKVTDGNNASDSRPF
IRVLGQRISKDDLDPKPQQEDTVYYICGPPAMTDEFVQYLEPVVGKERVLYEKWW
>g1880
MDGDEMTRVLWDDIKKRFIHPYLDIDLRYFDLGLPYRDQTNDQVVRDAIEAIREFGVGIKCATINP
DKARREEFQLRNNLPSANGLIRNAFGGTVFREPVVISRVPPLVRTWTKPIIIGRHAFGDQYSGKDL
VAPGPGILEMVYTPENADEEQTRIQVFKFEGPGIAQTQLNTDDSILKFAHTSFRLALSRGLPLYVA
TKNNVMKQYDGRFVAVFKELYETQYEPEFKKQNIWYEHRLIDDMVAYMMKSEGGFVLSLKNYDGDV
QSDLVAQGFGSLGLMTSVLTTADGKAFESEAAHGTVTRHFRAYQKGEKTSTNPIATIFAWTRGLIQ
RGKLDDSPDLVAFAECLEKACIDTVDLDGIMTKDLAISAGQPETYVMTDEYLDATEQRLKNNGSSS
KEKPPQNAEAARHSVFPKGTRIWIVTMAAICGMVSPLSGNIYYPALNPLSVDLHVSQSLINISLTT
YMILQGISPTLMGNLADTSGRRPAYIIGFIIYLGACIGLALQTSYPALLVLRCLQSTGSSSTIALG
TAVAADVATAAERGTYMGLVNGGALVGPALGPVLGGILAAFLGWRSIFWFLVIFVGVLLTLMVLAF
PETGRNVVGNGSIPPQRWNRDLISIIRARRSLAKSQTQASSHPTHLKDGKRGIRMPNPLATLKILK
EKDVGLLLLYNCLVYASYFSVTSSLPYVFQQQYGFNDLQIGLTYLPYGIGALMASLFNGRALDWRF
RQVAKAAGFEIIKGQTTDTRHFPLEKVRLPIALILILIGNSALLCYGWVVQIQAPLAAPLVLLFFV
GYCISGSFNCCSVALIDYYPMSPATATAGNNLCRCLLGAGEAAVIIQMIDSMGYGWCFTFISLVIF
ATTPILTGVQLSVFGFFVFLIILFIDKQFRVLPNSIHGHLPTHHPGFVVTDVTIVTCSSINLFSSC
TLDPSVWSRVDKDLYLDNTWTSAAYVHFQRKREEELLETDKVVIDLRISRFNPGLLDSKTKNPRGG
APNAIDSNENDWEARPGGIWLRRSSEPHVSDSKQALTSVDVLFGADAVDPRPQWEVKDTFVLLNSL

TEATEARLTVRRGVPPVLKKPELRINDSERFKIMQAADLHLSTGTGACRDPVPEERVPGEKCEADP
RTLDFVEKLLDEEKPDLVVFSGDEVNGETSKDVQSAVFKFVKPLVDRKIPYAAIFGNHDDEGNLNR
AQLMALLEELPYSVSTAGPDDVDGVGNYIVEVMGRSSAHHSALTLYLLDTHSYSPDERQFRGYDWI
KPSQIKWFKTTSQSLKKQHNQYSHMHMDMAFIHIPLPEYREDTNSWKGNWLEAPTAPGFNSGFMDA
LIEENILFIHSDHVNDYCMLNRDMNNKPNIWMCYGGASGFGGYGGYGGFIRRMRFYEFDMGPGRIV
TYKRLEYGDTESRIDEMMIVDAGQVRA
>g1881
MAISIRALLSQGKDVVRLCETHDISSTEIIRNTITDDGQRFDGLWFGGLCQTTYLGIPDTELISPL
QRAALLLLENNFYQQRSKPSLRKPVCAAFDADSGGALADIPALVALLDLMGISMVIIEDKSVSEPG
EKVNSLGESSGAQPQADKHEFANVIRIFKAAAVNRDVMVTARIESFTVRKRRADEAEEKASVQAAL
QDALDRAQVYHEAGVDAIMIHSKSRDPGEVKSFLTEYRSRDSVTPLVVVPTTYSDTLQSDLYNSGA
NVIIYANHLMRAKISAVSKISKQLLAENPDLFSDDHELSACLEAQNFGCLLRILWERKYLGKESKV
SQMYRIIATTYASENMRATVQDLLTGEQSGCEADERIIPVKDLLKINARQIYNPGDLLPRH
>g1882
MGSTDSSNLPYIQSSPKIIFFTDFDGTITLKDSNDFLTDNLGYGQVKRKQGNEDVLTGKSSFRDSF
REMLDSVKPGFAECIQILKENMKLDPYFEEFYNWAKENNVPIVVLSSGMVPIISGLFEELLGHKPD
PKHLAIVANDVESRDGKDINTPGGWQIKYHDDSHFGHNKSLEIKPYAALPADKRPILLYAGDGVSD
LSAAAETDLLFAKKGHDLIRFCEREGMPFTVFEDWSTILATTKDIYNGKVSVENVAKQGLEKVQAG
EAGL
>g1883
MARLLQSFRRILGIATSSAAPTTSTRSSFPRSIDTDFSVFREGDRAIIQGRIPHITKPLKHGNRTE
VARGYLNHSDVIGSRVWNPIQAQKGKEYRLSLPTLEEYVVYTPRLVTPIYGADANLIVSLLDIHAV
PPAEGTGDQPPRLEILEAGTGHGSLXLHLARAIQAANPNPPSLPQKSQVRILEERTMSPYEEHAGE
KRTEEDIAGDRKNTTIQKDWDEWRAQRRAVIHTVDVSPKFSTHAEQVVRGFRRGIYAGNVDFHVGA
VEDWIKDQLQRRRSSSSKGLFSNPGSATEPFLSYAILDMPSAHQRIPHVVPALKGDGLLVVFMPSI
TQIGDCVQLIQKEKLPFVMERVVELGMGISGGRLWDVRMAVKKSRADPSSWAKESRDEAEQSTTEE
TDITESVSTSTASEQESTQDNVLVCRPKVGSKIVGGGFVGIWRRIEDSPKKKL
>g1884
MLAFSGIENTPIVNTDTDRATASNNMGGFTEEEGSLVLPDGTSLYTKTWKPEGTPRAILAFYHGYS
DHCNSFFEFFPTLASSGLEVRSVDQRGWGRSVINNRKLRGNFGSTTTVMADLHFFLQSLIPFTKEG
SIPLFLMGHSMGGMNVLYYVLNPESPYHHQQDSTPTTKVKVAGILSVAPLVAVHPSTQPLKIVEFA
GRIARHIVPKMTMVQKVEPKWVSKNTAVEADIINDKGVLFHSTGTLEGLAGMLDRGAWLNECYKKA
TVHKRDVPPLWVGHGTEDRVTWFEATKKLVEGLEWHGDKTFKEYVGAYHKLMNEPDGVGEGMTKDV
TEWIEARLVKDDA
>g1885
MYSQGHQAQHAAMLNGSQTHQRFAMQMPKFQHQNHHPHHAQQHHHTHHNQATHQLSHQPNFPAGAL
ASTTPHFTPSHIQNGTPGNVEDEIDDSINEHWQQQLQLANEARQATSPHYYARTIAQQTKGIQIAP
STADGNDTSNDNRPSAVASKDSKRQGWTALDFTSQGLRAISTALFNYKFLNKLYLSNNKLKALPPA
LGQLRSLTHLDVSANELTELPVEIGMLTNLRTLYAFDNHIRSLPYELGYLYRLEMLGIYGNPLNEV
LMSQIKTHGTKALIKYLIEEMPVHLPPSDRDWIVLDETKSSQAQADKFTVLSYNTLCDQSATPSHY
GYVPSRALAWEFRRDLILNEIRSHDADIACLQEIDQGNYNDFFREQLAYNDYKGVYWPRGRAMGMH
EEEAKSVDGCATFFKASKYILLDKQLINFGQTAVRRPDAKGQDDIYNRLWQKDHIAVVVFLENRMT
GTRLIVVNAHLYWDPAFRDVKLIQTAILMEEITKLSEKYSKFPPCTDKTAFRFSEAEDGAREATTP
VEPAPSADYTSGDQIPLLICGDFNSAPGEAAYNLLAHGGLTEAHPDLEKRLYGNLSRVGMTHPFKL
KSAYSAIGELSFTNYTPDFSSILDYIWFSSTALHVSALLGEVDKEYLQRVPGFPNYHFPSDHLALL
AEFSVKGKKGKVIEADFGPSRDRSN
>g1886
MNGPSWAGSSAYVHPSLLASHLEAFANAHAPIGSPSTNSRPRSAPNTNSSSLSTPSQTTHNGGPGG
PGAAASTSSLSSSPPAPPDYVAKPFTRINDRTYFRDPENPYPLPCDLPEIHRQSLRTMTLIRVFGT
PYCAPQLKDKRPKKVLELACGSGLWSHACHDYFLEHDVGSPKSPKDTSKDDSNTTSFTGLDIVAIA
PNLRKKGVNWQFVRHDIRKPTLPFPSEHFDFVFIKDAGLCQSATAQQIDLLGEPLRVLKRGGILEV
WDSDHVFRALLPNPTAPPNLSKKDQNHADATATYTISSATPFAAAQNKYLLNYNSWIEKAFEKRKL

TPMPCATIGLSFTTEADAFQSSGSRRIAIPLGAEVRWEQTSGRGKLNADQLALRHTALTTTIQMIE
GMEPMLMEASGKSRDEWDRWWAAMTTDLLQKNGLANGECLEVGAWWGQKR
>g1887
MATQELRRQVIRVYKELLYLGREYPLGYDYFRTRLHGAFAAKMNLTDTKEIEEGIKRAEFVKKEVE
AL
>g1888
MTNNSSTLELTFNHLAFPPKLPGTRDPKVELVERDILTRLRTAVHTTKAYSSNNDAMSVWESLEGS
LEICQFVNENGFINREALESTLRSLNPDHTLILHVTQQNAGLIIRSSGEDFIFESFEASPSAEQTL
AAKGAMQWDFPTVAVSLPRQDLVSPQFQENLTYFLEGASLDAVDEFTAKTHKAGIAISEPRDTTHP
ALITDFLMTLLEVNGTRANLPLLRKRVKDDVCWDNAELPWRRSPFWLILRVAVQRSLYLAFGEQEG
RPRYKFLLCVLMSQLLTDCVQASFNPELCNFLRAKLCRRLAKLETEKLHGNSSVVHTYSELFESIG
PICRKAIDMTTTSIEKQWVAFKAQSLRKIPSLPPFADEDDLHLTLPNSGSYLRGVLNQSYYRPPAN
PSIIDAATLGASTSKATTEFYSSLLARYNKLQDWENQFETTTAEVPQSQSECSKKCQSLAKYLLSY
LEAVGDAYDGNSEQMSIFILNVFETWVYMDQCALKVHPLLSQYHPFFEPGMLDVLLLTRLHDMKRL
QAIQEYISKRCTQAERLLDIFADPQQGYFADQIFTLGETGTITKLDALHAIIETASGLSRATKEGE
LQKTNDEYKNRTENMMRHSCTQRRNPDGTHDIRGCTHCWHVRSRRRLKIEVHEDFLPSEDVKRKNN
LIHKRAILFELDTPPAFSAYRTATWEIIQRLSCPIPQNSSIAPEMLLKDYPQLKTYYHDSGNFSLA
STTKSFLGTHYNFRFLPTTLAKVLMPFGPTFYSYYDSGRKIWAKSVQRPLSLAHHFALRLPKEFPF
STLYSSADFAADGNGPSSYDTIASIAECPSDLTVHEYTAHQSLVSGKHRRWLSILIELGSSNVNFS
LQDTMILFQHLILQAGPMMEHDDLRTVNAVFRDVIFCRKLLDQIEQHIKAISQNWRENYYMETLLT
LVIRVCTLGHKLVLPRAQSLLTSIRNITLTWINTLRHEMRTTQEHDVAERSARYGFLAALLCRRTF
YLAAYDGTPMDQEHFKAFVEATLAMQENLVVDVTRFTDATLNMLIRDIKMTYRMKSAIRHSVHQHP
LGLQSAIDTVWPNAASEARQYGAWKPLDAPYEWWVTSTVSSTENTISQVVHYHLLEGHLLIDGRAL
GKLPPAIRDSTTLKELFGNQRLVAFPSNVYGMSYVLGIQQDNHDIHLGFRGSDLVVQARKGRDTKE
FVPSHVFGSGFKLDLPRSLIDDIVHWVDLRSGILEARRRPKIWRNTMGNWNIDLRTYRARRHKSTL
VNPQSRIFGLISQIFRDFVPAPMLTVYQPLKGTLVVEIKSMDLMFRVNRQKLLHCEQLGSEIDPNQ
DPGTFYGLRSMIVLRDASNPSQRSLLTTLGPVKYRRSGVHVEVYMENDGRYAKYSIDSTLGRLHCP
PEPRLLYTKAQLHAYTSFVVPDPLTGHNGTEEALHCLQSSSCQPWNPLIPPLVAILQTIAELSPTR
SYYPKDLRKQQSVSWNPNLTTTIQHDAYRSVIDSIVKKSERLWLFHAKVAESEIKATEAPPTLLCE
RSYWRRSIYERLESLDLPTSIDDFIYNVRGKWRQPWRASNTREIVSLLRHKPESLPTTHGLKDIMR
TWPLIGGYENSFAPRVVGDWLGADLAQEWGGLVKICGDKSREGYSIMFILGLMAFADGANMEILRT
LSAFSILQDLKALNLPNVSSLSGPRMDQAPLLEELISLIRPFCEVYKNPISSRGKRSRKDIQSHED
ARQAHELKCSEASTSFAKHLLRQWPNRDPSADEFSEGILNKYGALDAVVPYWHTFHDALQFSKHIS
EVQTILDRHHAKRSLEGPMAILSDVEVYGSQKKQNAVIPHLFQLLSNRHGKVSPVAEHLIDNIHRI
NHRSAGSDAKRGTNQKPVTNAEIRELEKIVTTHFVHSDSYVQSSYGRDLTRSIDAFKRLQPESSPE
ASLNLENEIMSARKSMRLHHNIIQDQIQDARYTWLSQANLWPSLSPISILEHLRSHNSFKLQSSTK
KELVDYATSISKVQHLRRMENATLKRDRKRLNAEQGNRGYINWVPGDFPDWLLLEIDANIHIRKDQ
VNVALEMISPTSGSNSVLQMNMGQGKTSVIMPMVACVLANREMLTRLLVPDALLSQTAQILQSRLG
GLVGREITHIPFSRRTPTTENHIQEYRRLHEEMLHSGGIILTIPEHVLSFKLCGLQRISDGKIPEA
KQMVAIQGWMNEVCRDILDECDFTLATKTQLVYPSGTLLNVDGHPDRWKVTEFILDLIAHHLRDLA
QQYPRSIDVVERGESAFPITHILRKDVEQALIQKIVDDICASRTPILSLWEYTFEQRRAIRTFISQ
EEIEETTIRIVNKTLADTPVFRKIVYLIRGLLVHGILLLCLKKRWNVQYGLHPRRDPVAVPFHAKG
VPSEQAEWGHPDVAILFTCLAFYYQGLDQKQLRQSLQLVLKSDDPATEYDQWTQASTSLPEALRHW
NIINIDDEGQVAEIWRHLRFATVVVNHFLNHFVFPAHAKQFSIKLQASGWDVPLFSFDRENTSKGS
RRPGVTTGFSGTNDSRNLLPLTIKQHDLPGLLHTNAEVLTYLLQERNRGYVLATNRDGRRFSEHQL
LEHLNNMKIRVLIDAGAFILEMDNRTLVKEWLQLDYSAHAAVYFGSDNKAWVQYRAGKIAPLLATP
FADNLDGCLVYLDQAHTRGTDLLLPVKAKGALTLGPNQTKDHTVQAAMRLRQLGTTQSIAYIVPPE
VHQSIQDVCNKRTRDLFDSSDVVTWLLHQTCNNIEELQPLYLSQGKDFCNRMQAAKTYKDFLVNRF
HQEAFMEVLQQPEQQNLEQLYRPGISPSSEISQSNLGLTGQLVAFQKDLRKRQLASKDAFSYAKSS
ALEEVEQEREVAFQVEEEREVQRPSRMRALQFAGLHKTIREFVTTGALFGQEGYTKATTVLERTKV
GTKYGNHISRLLGHLYVSTEFLRTVRTKDKDILDNYTRPVNWILWSEVSRIAMVIIPEEAEEVIPM
LRAAWGSPVHLLLYAAPVTRKMLQFDALTYYASPNLPFMWKPPTWLPFELAILSGRLYFNFSDYEA

LLQSINAAEQDEDRPSSSSDDQASAKNVLAFLNEWLAIRRQGQDITHTPMGYVCQGRKLRSDHPFF
VQRSEQVDLASGFIASYQTGVNEEEEDDFFDDDDEIEGSVGGDSCKEEENEVAVKEEEDDDVKGVQ
DGDVKGIQVEYLKREQDEKVMKEEVNEVTEEDNMSVTMDTSSEPSIVYS
>g1889
MELTTEEIVAVTILGFFYVILLGRYALRFLKWCNREKKTTATDLEAQNGTGTTDNTAIKEQLSVFL
REEATDIEAQIKITPTPPTTKGSSSRSDAQPSSSSSIQKPKPSNSAMKNSSLPLEQSPKGKGKGIE
TQNETTTRTTKEPSSPMDHSLSSSKSLLWSPPLREAAEIETQNRIITSTANKSSSPSKQLSSSDVV
QIPKSCAIRT
>g1890
MTWPDKITVYHRLTQNPSDTLNKSYFQQEALILSEYKQRPAARVIEQNYLYDYTQLRKTNTPPEFI
LRQFQETWALQEESKKQWQQQVANIENEVRRLELESWDNPDAVEDMGSAG
>g1891
MPRSAAAARRXHNNSNNNTSSVNGNGRNENGHPVNGKRLNKQKSSSHLNGNASGSSTAHSAHHLEL
RSSSDSSVVTTSRADAAATDGLKADGADNVYTNGTDHKTNGDMAYGHSNGTASADCGSASSKPASA
SKRNNSSSVNPFLLASTILRSCPLYDTIAILIFLLQLPPIVLTLVQFLFASLTFMPPGGGHGSFTS
NFDXFXXPAGTPSFGTMIAMDGFCLLLWGLFMWTWAQNFALDLAHVQVAITLGGGGSGKNGGVNAF
CVGIVLLLHIVRSKGIQDFVIGHLSSAQLINPDILSRWSHLIPQEFRRTEPQTSPSWLRSLLAIHI
LAQAGTAMARRSMARNRTPQPSRSGKRIDAEASAGSQAQIDSAFESTASMTSYGVDGSISGPSALK
ESRERLQSAKKRRRQANQVRSRQPFWAALASTKVTVMREYEHSRAVSKTTRGLPMTEDDLQGITLD
DGLVWITSVDSSTIKFAAGDLATAEDNSSSTRCDDAQLESNEVEPFYVCVNGALWATTNMCKVTDG
PKGTSAVQWRGEISGLAPNCAYTCSFVRNDTEEEICSLSVKTPAAPDTDQSTISMMPTPPRPFHRP
SSPTTTLRNSIANAEAKLNEKRSRLKKAKGDHKIAISRIKKEVDNYTHRLSSGSDENKQKQRSLQL
ERNIRQTEEATAALESQLGSLDKIPEEELEEWQKQKAEFDQEMERLKSAKEELLNARTSVSRDLAN
LESDLASSVQKRDRLQSRKTRLNEQYERIISANAQGLNERERRAAEQFAREQEQAKIEANFNEQLA
SITRAVQDYQLRTNQLWQQASGIEQAIQQQQQQMLFETGPLTPEGILPGTRHLPEEGEEDEEDELD
NGPAYNNLTITPANSRSILGASFSALRSSPQTSSSFLAGSTSQTASPLQATLQQPTGYQASPRVNV
NNYFGGRDFAYRDRSSSNRSARSSLYADLDIDINRYLLDPVDRLSSGSDGKGQTSPTYGPVGSPFT
RAGSRGSGGSSGNSGSGSGSGSPRSTRAEGL
>g1892
MSLNTSFRRATTGTGPSKTLLKSTSTTSPSTYICTQCRHASLLRRPKRPYTFTQLIALSDGSTFLH
RTTSPTPVYRSTRDTRNSLLWNPTSKKLLTVEDDEAGRLAAFRAKFGRSFDAAGTAAEEAAAAVSG
GPPTAKGQNVDMKQAQDAAIDAAAEKALAQEEADLQEEEDSLLDLISSFGQENNGKKK
>g1893
MDLDGGESPWGDEPSTSTRPNESSTTPSTTTQTTTHEPSAVPQSPARRGPRTARKVSAQVTKLEAV
DDTSDPLGPLGESTIIEEAPTPPLKEAVGVRVAQPAYSAAQSSAGAPVDRSEEQQRTVNPPPVQFP
GAEATQKRQTQPSISIEQAAKPTFNITVGDPHKVGDLTSSHIVYQIRTKTTSKAYRRPEFEVSRRY
RDFLWIYNQLHNNNPGVVVPPPPEKQAVGRFDTNFVESRRAALERMVNKIAAHPILQHDADLKIFL
ESETFGADVKNKENREPDLGQSKGMFSSLGISVGGGGKFIEHDDWFHDRKVYLDALENQLKALMKG
IDTVVAQRKGLAEAAGDFSASLHALAAVELSPVLSGPLEGLSELQLRIRELYERQAQQDVLTLGIT
IDEYIRLIGSIKTAFSQRQKSFHSWHAAEAELQKRRHTQEKLLRQGKSQQDRINQINADVADAERK
VHQSRLLFEDMGRLMRNELERFEKEKVEDFKSGVETFLEGAVEAQKELIELWESFLLQLDAGEEGN
PLYPPQPENHEEEPAAVESNNESATDAEEERAAAVAAAVAAEETH
>g1894
MAETSNQVAVANGHEDEDNNDHLIQSSDPQHPANLIPEMCRKFYTWGWVTGTGGGTSIRHGDHIFI
APSGVQKELIQPENMFVMQFPTPKYPPSERKYIRKPKNLKPSDCTPLFLTAFERGAGCCIHTHSQW
AVLVTLLVEREYGREGYFEISNIEQIKGIPKGKGKGMHNYHDTLRIPIIENTPFEEDLTEGLERAI
NANPDTYAVLVRRHGIYVWGDTPAKAKTQCESLDWLFQLAVEMHKLGLPWDINKAK
>g1895
MRFNAALTSALVSSAGLMGHVLADEVDVPAPDATSSAIERPTFTPTSLKAPFLEQFTDDWNKRWTP
SHAKKEDSASDEDWAYVGEWSVEEPTVFKGIEGDKGLVLKNPAAHHAISAKFPKKIDNKGKTLVVQ
YEVKPQNSLVCGGAYLKLLQENKKLHAEEFSNASPYVIMFGPDKCGSTNKVHFIFKHKNPKTGEYE
EKHMTSPPVPRTEKTTSVYTLIVKPDQTFEILINGDNVKNGTLLEDFAPPVNPPKEIDDPKDKKPS

DWVDEAKIADPEATKPEDWDEEAPYEIVDEEATKPEDWLEDEPLTIADPEAEKPEDWDDEEDGDWL
APTVPNPACADVSGCGPWTAPLKKNPDYKGKWSAPLIDNPAYKGPWAPRKIANPDYFEDKTPSNFE
PMGAIGFELWTMQNDILFDNIYIGHSIEDAATLREETYVVKKAIEVAEEEASKPKPEKKPEAGVTF
QEDPVAFIRQKVDLFVSLVQQDPIEAVKTVPEVAGGLGAILLATVLFIFGAISASSPAPAKAAEKG
KAAATATKEKAAQAVSSATDAAKSATKRTTRSSAE
>g1896
MAARRSLARGIPALRTVSPRIGLTTKPVAGTWATSKSPSSLSQSVVSQNTRRHIHITARRSSQQQY
GTQSAASTTTEYPTSHEQITSPLDTTNFIDNEFVTSKASEWIDLHDPATNNLVTRVPQSTDEELKA
AVASAEKAFPEWRATSIIARQEIMFKFVSLIRRDWDRLAASITLEQGKTFADAKGDVLRGLQVAET
ACGITSQITGDILEVAKDMETRSYREPLGVVAAICPFNFPAMIPLWCIPVATVTGNTIVIKPSERD
PGAAMILAELAREAGFPPGVINIVHGAHKTVNFILDEPAIKAISFVGGNKAGEYIYSRGSANGKRV
QANLGAKNHATVLPDSNKNHALNAIAGAAFGAAGQRCMALSTLVMVGETKEWLPELAERAKQLQVN
GGFEAGADLGPVISPHSKKRIEDLIASAEKEGATILLDGRGYKPEKYPNGNWIAPTIITNVTPDMT
CYKEEIFGPVLVCLNVETLDDAIALTNANDYGNGAAIFTTSGSSASRFQKNIEAGQLGINVPIPVP
LPMFSFTGNKKSVAGGGASTFYGKPGLQFYTQQKTVTSLWRSEDASHTRASVVMPTHK
>g1897
MAAPADTTIQNLNGTWVLNKTLSTDTDALLALQGIGWLTRKAIGVATITLAVTQHVEPNADDPSNA
PITKINIEQTATGGIKSTESRTTDWRVREHKDRTFGKCAAQSRLVRGQKGADGKIRPVFEVQTEPK
EEKIQRFLRGEITVDGEEDPEGFLVDDVQEKDGVTYPEGEGLWLQSFVRSEEAPWSAEQIWGFETI
NGQRYHSRRLVVATQEGKYLFGRLVYDFQG
>g1898
MSLNKKYANLPDLDLAPDVYETPDLTDGASTVPTTTVRTNSDSDDESNPDIDRNNLNADEARLHFL
RASGVNAREVDFSDTIATKRKAYKSRSGQRRRRRDDGTEDIGDISDSDAEGQESFERKLARLRRET
EELKAEMERRDQARQNDGPKNEEEDDDGIDELSKALDSIHYSSTRANATSDAILSRKLAAAVNASK
DANTADKKKPPSQATLPAPASGLLEHAASFDTRLTLMEAAMGISSGSNPFFAAKDAQPRLQPVLPA
LEHLTSQITALTGTLGGPTASSTAATTTTAQLESLSSRIKRLTADAENLANARRHASEVAARAVPD
SPSGAGGGDASTSSGGTEVNNNTESNTQREEQAAKIQALYATLPTIQSLHPLLPSVLERLRSLRAI
HAGAAQASETLDALEKDQAEMSKEIEQWREGLETVEGKIRESEVSMKKNIEYVGPWVEDLKKRMDR
LEQF
>g1899
MLIKNQDLAPDNDATAYEDRIQHLQSGLCGSVERGFSNCERGDAATDWWLVNLVVETVGGXSLGAS
AAAASSLQQNTTSSSASVTPLRAGSQRKMVHSKVVIIGSGPAAHTAAIYLSRAELKPVMYEGMLAG
GTAAGGQLTTTTDIENFPGFPNGIGGSELMDNMRNQSKRFGTEIITETITRVDFSKRPFRLWKEWS
DGPDEEPAHTADAVIIATGANARRLDLSGEEQYWQNGISACAVCDGAVPIFRNKPLFVIGGGDSAA
EEAMFLTKYGSHVTVLVRKDKLRASKAMANRLLANPKVTVRFNCVATQVVGEEGPRGLMTHLKVKN
VVTGEEETHDANGLFYAVGHDPATALVKGQIELDSEGYIVTQPGTSYTSVPGVFAAGDVQDKRYRQ
AITSAGSGCIAALEAEKFLAEEEAGEGEEPTVKTEVGHSRIEVAVIEQDPQSAAQKKDNAPAVAEY
KENPLL
>g1900
MATKALEAHFRHLSVHDENDGHEHQISKLATTKLRPSERNQQPKSSTVLRTRKPLSGISINTPEGV
AKTKEHHITIIKQQSTALDHVASQVAQAKGGQALSVAQHPRTVALSEASKAEKHSTQLIKQPAARS
LHLGMFEIGRALGKGKFGRVYLAREQQSGFVCALKVLHKDEIQQYKMEKQVAREIEIQSNLRHPNI
LRLYGHFHDRKRIMLVLEYACKGELYKHLRKADRFPEWKAAQYIAQMASALQCLHRKHVIHRDIKP
ENILVGLYGELKVSDFGWSVHAPSGRRLTQCGTLDYLPPEMADPRKYGKPYDEKVDLWALGVLMYE
FLVGTAPFEDTPAMTHKRIARGDMRIPSFVSPEARDLIQKLLVLDPDQRIPLEKVQEHPWIIKYCK
SVAKHDKSPPPKINARMPRPVK
>g1901
MATTSSQTSPVPFNSAPNGPPQRSGSGLGRPGSARSKRLSIGSAEELAAQNYGMIPPMAPAAPEAP
RGPPVSFRNSPMLDRADIPYRAAVPRSFSVRSRNQTPENIHAPEAVGYDVSNGAPYEQLQYPPSGR
RASTGHPTYASPPPAMTNSPSQASPMNRVRNSREPSEALSRPTSGSQASPVSRSSTVRSTTGDKFN
APTGRSPLQQLEGRLVGMSKEEKRARVIEAERRLREKMAKRESQRASLTVPDAPLNATAADAAKNQ
QDVSQSDSSRDIPDYQPQRRQPSISRKPIAPEQSQYATVQTAQEPQYAHGQNSHRVPGPSAQAPQR

QPSGSQRIPPGNGQPASSAQYRNRNVYSPGIYDPSPIEAGIQRTGSIKAQRPISNYDAQRPITSAG
RQPMTTAQDTRPVTSYNEQTFLDPDTGVRPTHFAGEGLNQAVSGQQHTPEDREVTPALKKSKRNTV
SFDVPPPTPPPLDEWRKAPVARLRLIDRDFERLDVDKGKAWWEKGSSKRRQSRGLPNNYRKPTPKV
NERNRFNPLLFLKCGPLLRYLGIRKTTVDGPQGPTEQEVWRGSVMIVTTDSLSSLETPPTLRLFAQ
PMDLLPPPPRQINDEEGAQLAPEYVDPVAGLVKLGRDGRALYVKPVDHLEEGVDLSGVESEDGLFE
SFPSPIDYGTDKNKGSTSIPSNRLHPVNGENSGLYEEIQGFRLYSDPARDVTFWRFNIEVELGDKQ
ERISYRINEGPAIGFWVPAKGQSMNIMFHSCNGFSLSVDRNKFSGPDPLWRDVLNEHQTRPFHVMI
GGGDQLYCDKVMQDSEQFEEWTRIKNPHHKNNAPFSPAFKAELETFYLNHYAACFSRGLFSLANCQ
IPMVNIWDDHDIIDGFGSYPDHLMRSPVFSGLGNVAFKYYMLFQHQSLPEETEADEPSWLLGADVG
PYIKERSRSLFMSLGKGVSFLGIDCRTERMRDEVVSEHTYDLIWDRCYREIERGQVKHLIVLLGVP
IAYPRLVWLENLLSSRAMDPVKALTKAGLFSGLVNKFDGGVEVLDDVDDHWTAKHHKTERTLLIED
LQDLAAEKSVRITILGGDVHLAAIGQFYSNPTLGISKDKDYRYMPNVISSAIVNAPPPEMLSDFLN
KRNKIHHMDSNTDEDMIPIFTHDVDGKNRNNKRLLPRRNWCSIREYRPGTTPPPTPPTPVETPDIE
PDSARPGLLKRTLSLGRGESQPRPSTGLLRRLSGRGPPPTKDFNLDNAQRRRFSTDATATNRPPEN
GDSYFAASDEPRPSPFHRRTTDLSTKASKKAAPDTEGENFVNLEGGLDITLNCEVNPQDPAGITTP
YKLLVPALWFEGDGFDPEPQPIRKGWKKWLGRGKPKRGQHPQQHDDHHEEHQEQHHAASPQQARDA
NGGSYAYEDDDEYEEEYHQAAAHGVQQSHPYQQDSQQYDDEAYSDEEPETPPKRKKWLGLV
>g1902
MRLSNSLSILSLLLTSSLAELLPRTYETHDFFALHLDDSLTPEYVADVLGARHEGQIGSLPGHHKF
SITQDQSSAVESLLEDLRGWRKLNKRSNIYDKRGDGLDGVLWSQKLALKERLHKRAPVELAARVGT
PVAAAVEARKRLESDLAIKDPEFEKQWHLFNTVEVGHDLNVTGLWLEGITGAGVVTAIVDDGLDLY
SEDLKDNYFAEGSWDFNENYQDPKPLLREDKHGTRCAGEVAAVRNGVCGVGMAYDSKVAGIRILGK
PIDDADEAASINYGFQRNDIYSCSWGPRDDGETMEAPGILVKRAMVNGVVQGRGGKGSIFVFAAGN
GGGSEDNCNFDGYTNSIYSVTVGAIDWQGNHPTYSEACSAQLVVAYSSGGGKNIHTTDVGLASCSA
GHGGTSAAGPLAAAAIALALSVRPELTWRDVQYLLLETSVPIQADGDELQMTPMGKAFSHQYGYGK
VDAYSFVHKAKTWDLVKPQAWYTTPWLRVEKDVPQGDQGLASYFEVTSDMLKAANLEQIEHVTVTM
NVNHTRRGDLSVELRGPQGIVSHLSVPRSKDATQAGYEDWTFMSVAHWGETGEGVWTVVVKDTVVN
DNTGVFTDWRMTLWGVSIDAAIQKPHPLPDEHDDDHNIEDAVYATVSIKPHTQATPAPTATDHAER
PTKPTNVESAPTATPGVATPVDEELSTEPTTGSDTAQPTASTSPGFLPSFLPTFGATPHTQVWIYA
SIALILIFCLALGIYFYVQRRNRLRTNPHDDYEFELVDDEDDAEVPLAGRRGRKRRGGELYNAFAE
ESDDELLSGEDEDEGPFRDQYKEKDGEH
>g1903
MLGAVSFLDHFGTLYGNKLSKEDHQRAETALGHALHAFSSQWITGGINLRLNTRSESPPPLQTECS
HQDIRAYYWHKARSSLAETKRVKCFKIVYAMLLFNMTTPPVPLEKPNLLPNEQQTDFWQEFIAILC
PLRVKVLKYSDNLGSSSTYAGALRVGLGIISWLAFLRDTMRSAISLKPPMFDDVSFESYGELSHKS
QSTSTHIPMDTEDTAIFMKCSSAISQMVFLWRRLHPELHPNLGNYGTEKELIFITTVESHEDRIRR
FLNVASALDDYEMKYKSLMRYTLVNHQGLSPRTKFFSSIWIIFWGYWVLRHADALEQMALSTSTTF
MSKIQQFKWTSAQSIAAITSSDILNTLSSLESSHSEELVSSASVDDLWSDHQSTPVISAHATCGIV
VNAIQKAIESLIHLWIETGPRHLHAPFMGLGIDSDPQTVWSRTLSSLLSSLISLQKVPSTSQIATA
KAGELFQQHGDLISEHWVMEY
>g1904
MASKQHIQRQIVVIIGSTVSTAGTLIQFFATQGVNIAVQVIGTNAPATLDTLKNGRDRVLVGHEDL
ANTEKFVARVNEKFGTIHTLINVADMAFQNEKPFTQVTDNQWQEHLSCHQKSRFKSLKSAWALFRK
ENRGTIIHVAAAKSLRSGSLLESTASMSAYGLVQTLAKEGKKYNMRVNMVLVDETIESGLPFIAEL
ARSSSTSNGSLFEATSKGVSCIRWERSQGSFLNPDSPSFTPMALLKQWSHVRDFSNPTYPDKANDF
ISILEKTKTLPHCEEPNDSISYNGRXVVITGAGGNLGASYSRLFSKLGANVLVNDFNRENAEKVVA
EINNQGGKAVANCDSVVEAANSVVEAAIRHFGRIDVLVNNAGFLLDQTFLKLTEDKWDAVMLVHLQ
GTYAMTKAAWPYFIRQGYGRIINTSSTSGIYGFFGQANYSSAKLGIVGLTQALAREGAAHNIHAYA
IAPVAGSQDAMNRGWNLPPSLKAEYNCPLVALLGSDKAPASTGDYIFEQGCGWVAATRQRIFPLPS
FQMENSVRRSSANLDINRAKQNIAAAKALQSKGVRYTYTPKDVLLYNLSVGARWESLSLVYENAPD
FCAVPTFGVIPGFQSIPLFGNQAPYGLDYLFPNVSSADLLHAEQYLEIARFPIPRSASMVTDIRLI
DVVDKGKNAYAFFELNTRNEQTGELVFYNEMAIFVRGGAGTGVPMELAATRRSSSMAAMDDPSGTR

APDLVVTERIPTEAAALYRLNADLHPVHIDPVMARHAGFQRPILHGLCTLGYVGRIIQERFGSYRK
FNARFLSPVLPGQTLRVSLWREGSQRVWIQAIIAETGKACLGNAKAELLLPGGPLASL
>g1905
MPFNTELTRRLGINIPVVQGGMQWVGYAELAAAVSNAGGLGLITALTQPTPEDLRQEIRKCHGLTS
HPFGVNLSILPAINAPDYDSYARVIIEEGIRIVETAGNNPVAVVQQLKDSNIIVIHKCTSVRHALS
AAKMGVDFVSIDGFECAGHVGESDVTNMVLLNKARTQLHIPYIASGGFADGYGLAAALCLGAQGVN
MGTRFMATIDAPIHYNVKKAIVDAEETDTILVLRKWRNTMRLYKNKIASEAMRVEIENTSGKFDEM
APLVTGKRAKEVFANGDTEYGVWSIGQVAGLIYDIPTCRELLARMEREAITVLTQANVAFTPKAHV
>g1906
MVLFAKLLVLALTAAATPILRRDAATVENDITQQIGPQIATLNSDVNGFPASGSTGAENINSDFGN
LIVVINHAISDIKAAGSFGTLSGTSILAAVQELLPSLSDTLVAIEAQEPSWAAIPGGTALVLKDLQ
SLNTVFYTFANALIASEPILLKAAAVAVQAQLSTDFDTAIAAYSV
>g1907
MAKGRSFIIGVGMTKFVKPTSEVDYPKMGREAITKALLDAHITYNEVQQAVASYCYGDSTCGQRVL
YQLGMTQIPIYNVNNNCASGSSAIVLARNLVSHGAAGCVLVVGFEKMSTGSLGANFHDRADPLGPV
AAMSSRINRNYDGVSDQVPFAIRMFGDAAREYMQLYGAESDDFAEIARVNHAHSRNNPYAQFRDVY
SMEEIKGSPTIYAPLTKLQCCPTSNGASAAVVVSESFLSSHPQLWPQAVEIIGQSIVTDSPATYNG
SAMNAVGYEMSKKAGANALREGGVSITDVKIVELHDCFSSSEMITLDALGLASHGKAHEYVRRGGL
TFGSGAAIINPSGGLLSKGHPLGATGIAQCAELVWHLRGWANNRYIPLKSNEVTLQHNVGLGGAGV
VTILRRADCKPNTKMTDKEVAEITGIGYNPATEARPLTVEQAQKVRALERSDWMFRYKQEGSARPN
I
>g1908
MAPSTSQLAQPSRSRWTVTIPSISVPEMIFQSAAGSVPQCLLYIDAAAPNTFQVTFHSLREWSKRL
AGGLRRRGLQKGDRVLLVSRNTIFLPIIIMGIMMAGGIYVPANPRSVPRELLTQMQDATPRFVLTE
EAFVNTVLSVLESDQVQSLGRDDIFVLQRSFDESKGEAAPEGFKHWFSILLPIEEAASFDWKTVEN
PVGPDDTAMLLYSSGTTGLPKGVEVTHFNLVANALQMSHMWDLDPRNRDPQMQTQEYQSRWLCCLP
MYHGLATVVYLTVAPLRRTPTYIMRDYEINNMLRYIQEFRVTNLLMVPPILVAMSKNTEARRGHFD
LNSVREVICGAAPLGREQAATLEQLWPKGHVNVKQAFGMTEMSGLPIMWDPTLYSDSAAVGELVPN
CEAKIMYEDGLRALPDGQRGELWCRGPSMMKGYWNNPTATKNTIVEGGWLKTGDIAIRDAEGRYYI
VDRKKEFIKVKGVQVAPAELEALLLEHPQIHDAAVVGVKMEDTEVPRAYIRAHFIPTQPRLVRKHS
TMASHDDDFDSVSWRNDPSSDASRPTTSGTDADDSAQPHRDTNGKRRMSVAQDEPQAGPLADAVDL
AGIGDGMLECSVDAPLKENDGTKDAYISYLVTTHTDFKTFQKTDFTVRRRFRDFFFLYKTLYREYP
ACAVPPLPDKHKVESYVTGDRFGPDFTQRRAWSLHRFIKRLTLHPVLRRAPLLAIFLESPDWNAHM
RLHGGRTSTSTTDSSGGIFDNFADTFVNAFTKVHKPDRRFIEVREKADKLDEDLNHVEKIVARVAR
READLEADYVDLATQFRKLVPLEPEVEMPLQVFAASVDETSRGLKGLKDHTDQNYLGSLRDMEAYI
LSVKSLLKTREQKQLDFEALVEYRNKAVSDRDSLVNNPSSAYASNPLTSSPASFIRSKMEDMRGVD
HEQSRRERIRKLELRIDELTREVDSAKTTSEMFDEEVVREVADFERIKAVEFRDSLGALAENHIEF
YQGVINTWERFISEMDSEYGGGDDQFRTRSEVVS
>g1909
MDESKNAPSTAPETVREMHGSTSNDQSINAVKYEQPENINPVPDTHTIEPDLPVNSDGPVPPPYND
SDKPTTFIANNNPYPGYDPSRIRIAVTPLERLGEAPAHINXGVMDKPIVHPNFAQPSSKNASPSPT
SPTASATTQPPHGKTSTSTIQYDHETYLELLAGESNSVEKTASQVYEEERAKERSLRARNPAVLSG
PLELDVDVYRFADDGDDGAK
>g1910
MFFSKAIFASLLLAMTAPVQAWNRIDKNNTALLVIDHQVGLAQLVRDYNTNDFRNSMLAHAEIGNL
FNLPTVLTSSSDTGPNGLLMKEIVAMHPNATFVHRQGEVNAWDNADFRAAVKATGKKQLIIGGIVT
EVCTEFLALSLVDAGYEVYANTEASGTFDVRLANDANRRMEKAGVTLMSMFGIVCDLMRDWRNTPG
FDQVLPFLDKYQFAYGXLARQYAGAVQNGTLLAPEAALINN
>g1911
MRLSGISLFLGLGALAAAHVDGSDDACLSEWEVCPPLEPLVDVLLGLLNVKEVCQQIDPEALGPTT
VTITDSLPAATVTTTSYRTVGSPVSRTSSVVAVVTQSSQAVVTNRFSSTAIQSVRVTATAVDTETV
PITETNTLTQTHQATVTQTFTLTTEDIIFETVTTTVTVTSAGQAAPTDKRRSVGLPAVLQSYSNDE

LVDACWCVEFDTVTVTATLPPTTQTQTIVIPSTVPSSVAVIRTISETDTIVSTSTVDLTAVVTQLS
TVTTTATLTESTTVYTTVDQTEDVTAFQTVTATATFVDEYVTVTVPATTTTTTTPAPTNVLVNPGF
TGGAISPWTRTLNNFYETLDSTCNTLIYSDSNCIMFSDFTGTSVGTFELTQTINTYVGQYYSVEFQ
YYFPYAVPSSSVFRCTAGSTVFPIPTTTIGSFQTVSGGFTATSSTTTFTCSGQAVTDQLYIFLYGF
DIRASS
>g1912
MRLLSQSLLRSYVCPSCRSSLTKGXRRAFSNTPNVRTTPDIYDVVCVGGGPAGLALVAALRASPVT
SNLRIALIESQDLNKSRTWNPPASEFSNRCSSLTLTSVSFLQKIGAWNHVDNSRVQPYHEMQVWDG
QTGSKISFDWSVDTSPFEDIRPVATMTENANLVRGLLRRIQELEETNGSGNGSLSIFSNTMVSSIE
NGSQSADGPNLSAWPVVSLKASPTSTSSVSGPIAARLLIGADGINSPVRSFADITTSGWDYDRHGV
VATLSLSDEFSSLLNTRTAYQRFLPSLGSPIALLPLPNNKATLVWSTKPENAAYLKSLPEEAFLAM
INAAFRLSMTDLTYMLSLDPSATVGSQSQHEEELTWRLQHTPQPPYIPPFVTSIQPNTTASFPLRF
RHASSYISPRIALVGDAAHVIHPLAGQGLNLGIGDVASLSSTIEYAVNHGMDIGDHLALEPYMTAR
YAANTKIGAMCDILHKLYNVPGNGPIPFVRSLGLGVVDRVPWLKGFVMKQAD
>g1913
MQATPGLSCFARRASPARLAARFRRGKAVVGFAATNFGPCKNTTINNTTTSHTVDRRRFLSYTKTL
RSLSDDMGKDKDSKVTFNLKTPKGTRDWSGADTLIRDRIFSSITNVFRRHGGTALDTPVFELREIL
AGKYGEDSKLIYDLQDQGGEICSLRYDLTVPFARWLAMNPEIRSIKRYHIAKVYRRDQPAVAKGRM
REFYQCDFDIAGANFDPMVPDAEVLKVINEVFEDLGWQGKFTIKINHRKVLDGMFEVCGVPSDKIR
PISSAVDKLDKAPWADVRKEMVEEKGLDAAVADKIEKYVSKKGRKDLFEELLKDEELMANAAAKQG
VEEMGLLMDYLEAFGVLDTISFDLSLARGLDYYTGVIYEVVTEGSAPTTSTSAQEAKAVQRSSKKS
KATTGDDDDRSNDPTIGVGSIAAGGRYDNLVGMFSPKAQIPCVGISFGVDRIFSITKARMEREKAS
VVRSSEVDVYVMAFGGKGFTGMLKERMDICKTLWAAGVKAEFSYKLKPKLPQQFKAAETSGIPFGV
ILGEDELAAGQVKIKEMGLPEGHPEKEGVSVDITNLVDEIKLRIDMKNKGVETTTTSDAVESTTAA
AANLSVGGDAAAAQQKTEDAGV
>g1914
MLQEDRSPVXADGSDKFFGMENYGNTCYCNSILQCLYYSVPFRESVINYPKRTPIESLETALSRNL
KYQSPEAFFEAEAAAAKAKHTAGQSSSRNAGVTPVQPQKPEDKDSPEYKKKVAIQTLPLLETKDNS
ASYGMSESLFTSLKDIFESVVGSQSKLGVVRPHHFLEVLRRENEMFRTAMHQDAHEFLNLLLNEVV
TNVEAESMKQIENFPTDNTDSEQTVAVTDSSTTSTGTQVSGSTGSKTPNTTRWVHELFEGTLTSET
QCLTCEKVSQRDEVFLDLSVDLEQHSSVTSCLRKFSAEEMLCERNKFHCDNCGGLQEAEKRMKIKR
LPKILALHLKRFKYTEDLQRLQKLFHRVVYPYHLRLFNTTDDAEDPDRLYELYAVVVHIGGGPYHG
HYVSIIKTEDRGWLLFDDEMVEPVDKSYVRNFFGDRPGLACAYVLFYQETTLEAVQREQEQEGHNP
MNGAADLRNEIPLTSPLNHIHTYTGPPEDEAHRLTPLRAVPTAPQMSTHPEHAFSTSPPAPVLPQI
PVDTGASKKAEAQSRKERAKEDKERKAAEKEKEKADRLKRKELEAKRREDLKREDAEMKAVLELSK
ATKAEEDRKTSTENGENGQHKSLAGGLNRFRRSSKSLSHRFALPKEGGKRAASSEFARPDVLSENG
HPPDMKLHEHVIEEDAPAETPPPPRSAIPETVTQIPAIAPRSKADKTDKADKDKGNKWKGFGIRKK
SMSLLG
>g1915
MSSLPIRVSKNDGEKLMGRTVDIAVGPQGEISSVHEKLIRKSSPFFDKALSGAWQESRERTVRLPE
DEPEIFAIYVHWLYYGTVPVVCDETVEMKGVEYLNLAKAYTLGDKLMDVGFQDATIDAIVDASQVG
LADGSLWYPGMQIVQYVVYHNTKESASIRTMLVDMFLMAGTGTWLRETKDRSTVPQPFLFELAAELL
DLRGGIRSTIEPSKYHIHGSNDALKEESTEKEGP
>g1916
MFSTAIRVAKNEGEKFMGRAVEFLLGKGKERFFVHEKLIRASSPFFDKVMSATCLESMQHTGTIQL
SEDEPEIFGIYVHWLYYNKLPVCCNEPGCAANQEYLKLVKAYTLGNKLMDTGFQNAAIDAIVEKST
SAASDGKRYYPVTEVVEYAYNNTHLLAPIRRLLVDMHVSVGSGKWLDYGNFNSERVPQRFLFELSS
KLLDLRRGTRAEVKASDYHVHVAADMKMETEKTEMEIF
>g1917
MKSSVTKILSGLFIGLLLGAPALGNPVPIEGEKGVEKRQFLEELIIAAITGAGSGALNSLLGPEQP
GCTWKSTHDDQNWWARVYTEISGTNYTAADICWTQGGYTITDNDAQAATCLLNEHINQDFSCISQD
DTNCNYPNSAFGNVRILHDLSTSAHQQLDSFAGQRAINVTNTIPGLEHGFDISTNVNMLIYNNDGT

STGWTVELRGTGDEDC
>g1918
MSKPTIVIVPGSFTSAAAYETLASVLAKQYNYEVIVGTLQSTIRAPPEKAATLQEDAAYFRGVIEA
LLNQGKDVVVVGHSYGGIVATESVKKVTHSERQAQGKSTGGVTKIIYVASIVAEIGETAIQAMGGQ
LGPTIKILGDYMYFDTEAGASYLYDGLSSNEALETGRKYTTIHATTSFLDPATNAAVDHVPVTYIH
TAKDNIVPPERQQQIVESLKSRNARNVSTITINEGHMPQVLAPEVLAKAIVEGISQELNIINAILD
KMAILAGLILYASXPAIILLHLLASPHTKVEESFHIQATHDILTFGIPSPLQLNRSAIAEKFQANY
DHFSFPGAVPRTFIGALALASASGPVIWFQENVDRQLLVRAILGLFNAVSLIVFARGIQKSFGNVT
AYWYLLFQASQFHVAYYASRTLSNMFAFGMTTLALRLLLPEPVRNDAKSVKSTRTKEKATKATRDT
KKGTTVLPNYSRYRLALVLFTVAGIIFRSELALLLATNTIYYFLTRRIRIWQDIIPAGIIGLLVGL
TATVIVDSYFWQEFPLWPEFSAFKFNVVSGQASAWGTDPWYFYFTSALPRLLLNPLTCFQPHKEWR
FIIYIIPALTAAAAQGAANIWTNRGKSILYRLLSFSLVVSTLGSFLFSTFILLPISSANYPGAQAI
QRAHFHGQGSQPRITLYMGNLACQTGVTRFLQQSQNSTFVGGSEKEANIATKWEYDKTEDPVLKST
PEFWSNIDYALVEDQEFEQLKLHSSDADSWEIIDTIVGFGGFKLIKPSEPRQHDDRSSIEIDAIRY
IAGDKGVIYYKIMRDEITRRLTRGYWAEMSLVPRIRVVRHSRAI
>g1919
MIINITIALVGILASFAYFLLISGSRDKRFPTGPPTLPIIGNLHQLPRRRAHLKYTELAKQYGGLY
TVKAGHHTIAVITDRRIVRELIEERGSTASNRPSSYIIQHLIGKNDHLLTLQYGQNWRVIRKLIHQ
HFTDSMCDKAHVHLQHAEGVQMLRDFIVDPDNHMQHPRRYSCSIMTSTIYGVRIPSIKSTYMEHLY
EMLDTFTALLELGATPPVDFYPFLKWIPERFLGNWRTRSYHVKGIMESLHQGMYKHVLARRASVHE
KEKATRIKSPSLMDKIFDVREDLNFSTHKLSFIGGGLVEGGSETPACMILTFIGAMTKWPSIQKRA
QAEIDSIIGEDRSPTWADYARLPYVAAIVKECSRWRPVAPLGMPHSLDADEWIDGHLLPRGATLII
NIWGLNHDETYYPNPESFNPDRFYIPGTAVPLAHEYMNKADYNTRDHYSYGAGRRFCTGIHLAERD
MFIAMAMLLWAFNFEKPFDPTTGLPMEPDLSPDTGFVEGLVACPAPFPCKVSLRSGRRYETIMREF
EEAKIHVFAKYDEKVEL
>g1920
MATGQGDNSARMSAGALKSLTFKHYAFAPASQQLPLPPSKRRRIIETAPTYPCIKGADLEISTQGL
NGSYNVNSLNQSPQNGILHNTVLGDSASFSLQSASEKSYPAEQDSDLTGHNTPNTSVSNTSPWPRI
KSTSPAAATKPANILKQVYAADVEAPNYEEFKPRSSIPSTLPGPVYAQQCITAAYSSRLNPFALHK
NEQNVLQHHLCHLHVTTYLNIRNGILRLWTRNPMISVTREEALGCAKDFRWMGLADFAYEWLLRNG
YINFGCVEVPPALVTPKKGRRRDDGPVIVIVGAGVAGLACARQLEGLYQQYRDKVTSLKIIVLEGR
RRIGGRIYSHPLKSHQKTTLPKGLRPTAEMGAQIIVGFDRGNPLDPIIRSQLALRYHLLRDISTIY
DIDGSAVDEMQDAMDERLYNDVLDRSGNYRHKAAIQPTAEGDREMINHGRDIPIDDGVTVHQYEEA
RAAGTHHLMLPAARFRRGIGHNASRILPPPSTAQIPDLGPDEELPAAMECQSMGWNLRGGVSPRTD
LQLDEIAKTSPTQTLGAVMDEGVRQYQHMLPLTPKDMRLLNWHYANLEYANATNLNALSLSGWDQD
MGNEFEGEHSQVIGGYQQLPRGLWAFPTKLDVRTHETVINITYDAAGKSKNRKTTVHTENGPIHAD
HVVYTGSLGTLKHRSVEFAPSLPQWKIGAVDRLGFGVLNKVVLVFDQPFWDTNRDMFGLLRDAEVP
GSMSQAHYAKNRGRFYLFWNCIKTSGIPVLIALMAGDAAHQAETMPDQEIVAEVLSELRNIFKSKT
VPDPLETIVTRWKADKFTRGTYSYVAADALPGDYDLMAQAVGNLHFAGEATCATHPATVHGAYLSG
LRAAAEIMEEILGPIPIPTPLVPLSSKPNANITPVRYPTVDSFKQSISIPAKQSPKSSTPSKPKPI
NKAPTSKPTFSVPAPMPAISTNTPTTEEQRQQQLYKQALEKHIRAKIGELPAKPAKIALNPFLTFQ
KDYWIRAKQQCEENKRQATNNPEAKAARDEIRAVLGQMWREADEETKRPYQEQMIVNRRTNDETAK
AWGQAMREYEIKSIEVAKGFTFEMWASMNP
>g1921
MLIIGLTGSIATGKSTVSSLLSSPPHNLPIIDADILARKVVEPGTAGYKAIVNYFGSTTPDLLLPA
DAEGKQGLNRPALGRRVFGDSDERKRDRTILNKIVHPAVRWEVYKSLLYYYLRGNWAVVLDVPLLF
ESGMDVICGTVIVVAVKDPAVQMSRLRARDPHLTAEDAENRVKSQGDVQGKVKKALYRNKASEQDL
DKGSRGVIVWNDGDKADLAKEVDKAILTIQDNSPRWWAWVLLLAPPVGVAAAVWNMAVNFGSQKSW
DQKVKKERAKL
>g1922
MLRLLLIVIVYGIVHTGLVVAMKHHENGSSYNVQPFHIDLSSSVPHMKDLIGRTQLPKKPIYYGIG
ESTGIDLDVLQDLQTEWLTNFTWYDEQATMNRYRHYTADIEGLTIHYIHERSASPTAIPLILNHGW

PSSFLEFLPLVDALVQPSEIPSLNATFSAPYTGSYSINVSFHVVIPSLPGFGFSSPPTNINFTTED
TARVFNTLMTDVLGYRTYAVSGTDWGSPISYAMYNSYETSVRAAHFSYLPFPILNGTQLAALNISL
DQVDSINLQAAENWIESGQGYRVEQGTKGSSSAATRPIAANMLYTGAINTGRSSSTRRLRLSSSAR
LSTQKGPIRASPKTKNFASPNWRFGTNSNGRSRQMEAAQAAIPAPGPTSTRGAIKKDLIVASMVGD
NTTWLYDWLPDWHKSIYIVDDEYADLTVSTNKGRESMVYLTYIIDNYDQLPDYMLFIHSQRYQWHN
DDPYYDGVPMIKRFQLPYLELIGYVNLRCAWVLGCPDEIHPMTDTNSDETVHAGAYFYNGFRELFP
GAKVPETVAVSCCAQFGVARWKIRERPKSDYERYKQWLLKTNLDDAMSGRIMEYSWHMIFGMDPIH
CPNAAECYCNVWGLCDLKCTQDECENRYALPPYSTLPDGWPQIGWNGKPQNPSKGGLPLVD
>g1923
MNSTEAPATLTALKEKYAEERSKRLRSDGTAQYLNVRSPEIQDLAQDPWVDYEAIASQDAPLKPGG
SIKFLIVGAGHNGCLFAARLMEAGFRREDIVLVDIAGGYGGTWYWNRYPGLMCDVEGYCYLPLLEE
TGYIPKHKYSFGAEIRGQSERIAAQYRFQAMFCTKVDKQVWDETKGRWIVDLTQSLGHVRQPTQIT
VDAQFVLLAGGILAIPKIPNAPGFAEFRTSHKVFHTSRWDYAVTGGSEKQPDMVNLRDKRVAIIGT
GATSVQATPHLAKWAKHLYVIQRTPSYCGERGQRETTPADWNEIADGPGWQRRRVRNLNSYLTADP
ESVDLVNDGWSQNQARAGLIGSSSRLVDPESVEEHYNKLVEMDLPFAEALRARIDQLVKDPDTAEK
LKPWYPGWCKRPTFNDDYLEAFNKDNVSLVNTDGKGIESYTTRGIVVANQELDIDVLVLATGFSTA
SQYTPAGRQNAPIIGRGGRHIDDKWDNPDFGTIFGIASHDYPNFFSYHSRGTASSFNMTSAFDAAA
RLVAYIVREASQQSDDPGKLIIEASKEAEDRWTDEVAKRAAWFSVVVRCTPSYFNREGEGSASQLS
PQESARKARKAAWGSGINDYERVTEAYMKRVEKRLEGFILTQASI
>g1924
MNVILTGKEYITLYELHAQYGPVVRIGPSKLYDDAATFRKIYNIAAPFQKSDYYQPFRIPGPKIDI
FVEKNETLHGKFRKQFSHIYSASGLKDLDANIDGSIKKFIEQIQRTEGANINLGKWFERLLYCMLD
LTFEQNVGYIDSSPADNPFDAVDRFTKQAHLHGMTPWLFYAAKASNFVSKHLFSQKETPDVVSRII
GTFLGALKDPSNTHIGETQRNIASRLLRVQSSKGSNVFTDEEVTHLLSFGVNAGVTDGGSWLNSVF
YHLINNRDKLDKLMQELDQQLSHGKLDIICTSQQAAACPYLNAVIQEAQRMYPSIGGILPRETPKG
GAEILGQRIPAGVTVGSPAFAISRMPEIFGADTSSFVPERWLEDSQPNMSRFSMGFGLGSRTCLGK
NLSWLLMRKIVPSLLLSLDFEAPDPKSDWTVVSG
>g1925
MTISSITDDVASELRRQFPGQVFDESDSSYKSLQDSYWSGNQKALKPRCFFQPHSAQDLARAVALC
ARSRCPFGVKSGGHGHFAGQSCLDGGVQLDLAKLNTISIDRSKGTVLVGAGSTWGSVYTKLQKEGL
IAVGGRAFDVGVGGFLVGGGLSFYAARRGWAINHVRSFEVVLSNGSVVTASQESEPILFKALRGGG
SNFGIITAFELETYPYRGMWGGRRFIESVHAKQAIDAYAGFVQKLEANPKGHTIIIFTYDEGPLQL
LQYLVHTEPVEDLPVFDDLRKVPATESSLGMTDYSTLASNIANLQDFNGDRAAIATLTFRLDKELL
EFAFNVFVQEAAPVSPHIRGTMEFHAIPRTLSPAGNVFGLDDIKEPLISFLLIFSTKLERYNAEVI
AIQKRIIGRVKDEALKRNRYHPFLFANYAGEWQDVIGSYGDANVKFLSEVAKVYDPEKVFQSLHLG
SFKLSHAIQNTKLEEDSPLHASLL
>g1926
MQSETIFYNMNSDAGTKTKLEGSSSQGMDESSQALRIGSVESGGVEYVSSDANAIDDAILRAQGHK
AAMPRLFSAMSSLGLMFCVMNSWIGAMSTFGQSLRYGGPQCAIFALVTAVFVQWIVTLGLAELASA
FPSSGGQYHFTYILAPEKFKRFSAFVVGWMSVLAWWLATTSGLSLVAIAGTGLAAFVNPSYTPKDW
HIYLCYLAMAVISVVPLFLFPKSLPKIASGSMYLSLSGCLAWFIIFLAMRQHTNDGSFILKSDLGT
SGWGQGTAWALGITNAMYCFQGSDSAIHIAEEMQAPGRRLPVVMNLTMIIGLLTSVPVVVAGMFTM
TDMTEVMNSGFPAVDLMYQATGNRPLTIFLAAWLIIVYASAMPPQFVACGRIAWAFARDGGTPFPD
YFAHVDTKLEFPARATVAALAFNALYGLLYLASTTAFNSIITSAVLFLNITLAAPQTILVLRGRNK
ALPERPFKLGYLGLFCNIFSTLWIPVLIVLVCMPPGLPVTVGSMNYTSPVLIAVTLLIIVGWFVFG
HQFKGPKIDWEMLNTGNTMERKVEKH
>g1927
MKKRRTQDQSASGPSITNVDIIESTAVVTPRHFESGTTPSLSTGTPRQPSGLLSSLQTTSRPVARR
SPAITPPPTRPKDTLPELCIDRILKGDYEPPTYIQQSIFITSNAHVASSTQAFFSERKIKVLSDIL
GHNLLGPLIKGLDDATSAKLKGFSRMADKPLAAGSPLTKANITEEDILHAKVYVESFFQYVHPMYP
FLDRDEFEKATSRTDLEDFLSRNVMWQALYYAILGLGSMYHNGGSFLPGEGLSWKFFQAALNLMPE
LLLIRRTIETAQALIAMAIFAQVHASLPVDEIPVNEAARITISLGLGKRPKAPSSFQNRSKAFWVV

YCMEKDFAFNVGRSSLINDCDIACPLPSHVDPVLGELDWIRAWASFSRLTSKAYDLLFSISATLSE
QDTYFRHTDSILADLDAWKEALPQQFRPGSAIKLHLFNGSVSLTLAIRIHFYYYNLQIAIARLILH
LSDGEHTARQSQSKLMLMHAARSIVSLTNFLAIEPFTPCWMLIQMPMVAVFILFELVIHNPVHRET
TNNLIYLDLASGYFARMQMTQGADRDLSISMTQLIQLARDFVQQSRQTYTHAEEPEHQVETISTIS
SYSNLHGSHGSETRHIAHTPQEAQQTDLNTSINPLNFRQSTDFPIDGAVSIPKLYGKKEGY
>g1928
MAKYQVVPVNHQEWSNPLTLTSINNVDVLGVPLSSDPSLPAYSFKSAMSRVALPSTHLKRATVSFR
PTEGYSCEWDQAGFLFVRPKAELPEPSAEHPGSSGTAPSFIKIGIEFWEGKIYHMICSSHEDLTDW
SLHAIPEAYENEKDITMEISRFGDRLAIHAIYGQGPHTVKRMIRIVPWCFVNESKDDPDIWVAVYV
SRPDAARTTTKPLVVQFHDFEIEDLDGVTRVDR
>g1929
MGSLDHQMPPTQDDFASYEGYSIPDTPMGTARYLKIICIGAGASGLNLAFQIRKNMRNVDFVIFEK
NEDVGGTWYENRYPGCACDIPAHNYQFSWAANPNWSEFYAKSSEILEYLRATSYKYDLRRYIRFQN
RILDCTWDEESGMWNLHIQDETSGQVITESCHFLFNGGGYLNHWKWPSIAGLDTFGGDLVHTANWK
EGIDLRGKRVGVIGNGSSGMQVLPAIYPDVESLVSFSRSPTWVTPTFASEFAGPNGANFGYSEEQK
RQFSEDPEKFLEYRKALEKTMTGNFKLNLKDSEMQKETQQFLKDYMKGVLREDRLIKPLIPEFGVG
CRRPTPGLGYLETLTKPNVRLVTDAISEIVPQGIKLETGEIIELDVIVCATGFDYSWIPRFPVTGR
NNVTLSSLWKDRPTAYFGLAVAELPNYFVFLGPSSALSHGSAIPTIEAVTKYMLRIVWKAQTETYK
AVVPSTEAVNDFILHNDTFHQRTIWGTKCRSWLKGGKEDGKVLTHPGSRVQNIHCLLNPRFEDWTW
TPLAKNRFAFMGNGMTVLEEDGRDVTWYINDSNTGYEGIFY
>g1930
MRYSSAIRSLRTSLDQQVDDFSACAATLFTLAQIEGCYAPEHDEWRKHLLGMYGLLSFFSQDSPWK
MSFDAWVLSQNLALSLEIARTGSGGSFDCFRNTDHLLESISRKSHFGYTIGASSAVLQSIFKVHRL
VEALGSGESLIEVEVQCGKILGELGTTKSDWIITQPSHSRQDYLNSLHQRIFRNAAVIYLHRAIYD
VAPIAVRDHVLNVLCDTISFLDLQGGSVSLWPVFIAAVEACSRKERDLARQWLDYSCKLGISNRHA
ARCVIEEVWKQRDQEALLRDIEPEYVVLDWRRVQYHLKIDILLL
>g1931
MAIHPVSVALTTARDKYIQRNPVSKKLHEESHEFMPGGNTRTVLHVSPFPITFASGKDATLSSVDN
DTYVDFLGEYSAGIFGHSNSRIAEAVHEALSKGWNYGGHTVMEKILAKKVVTRFRPSGMDLVRFTN
SGTESNTVAIAAALAITGRRKILVFSNGYHGGTLIFPMDLINNPATRNVNLPHDFVFAPYNNIEET
EAIISALASQSLAAILVEPVQGSGGCRPATKGFLRYLREKADEQQCLLIIDEVMASRLGPNGCSAA
MGIKADIITLGKYVGGGMTFGAFGGRRDIMEVFDPEKGGLQHPGTYNNNVITMSAGVVGLDIYNAS
EVERLNVLGTNFKTNLQGVLIKYGIYPEHAAGACRDIIEVDSFKNDTLIYPEDLSLTPHEPLPIMF
ITGYGSMVNVRFSGPSAADLRALYYFHMLENNIYLAPRGYTPLNLELNDNDLVKYLTAFEDFVNKY
KALLGL
>g1932
MILLRLITTVAALAATVTAGVTRGLIISLDNETAMDVDTTTLGNLNLYEQYSAAAYCFANDIATPG
TPIRCAAGNCPLVETNLATVSFSFLDVGKGGVSGFVAADNTNRILVLAFRGIRSVETGITTLDTRL
VDTSLCGATVGCQVNEGFQDSWDSVSARITREIANAQAATTFTTLVVTGHGVGGALATLAATLYRT
TPPVAAVTTIDLYTYGSPRVGNAIFATFVTTGNDAINFRATHTDDPIPKFPSRALGYLQWGPEYWI
RAPTGQAVHTTDITKLSGTETPFGNGGTPPIFDIPAHLWYFNEVAKCI
>g1933
MSTVTVTAMQSSKPPIIPKSFDAKQPQTIRLYPLSNYTFGTKENQPEEDPSVLARLKRLEEHYDLH
GMRRTCEGVLKVILVDRPGDYLHHDDDEITGFKTRLNERLAPVGSQFSGEGVNEDWEVGDTLAQWW
RPNFETFMYPFLPGHVTRPKECKKLYLIQLPKKKVLSVPKNMKLLAVPLFELYDNTARYGPQLSAI
PHLLSRYNFEFVDENDNVVAVTPGTPHPGIDELKTKVLAGGAGGAAEGEDTGMEDAAEVKQEDQIS
>g1934
MSEVTHITDRLEKPELDDRSYRVIKLGNQLEALLVHDPDTDKASASANVNVGNFSDEEAMPGMAHA
VEHLLFMGTEKYPIENAYNQYLAAHSGSSNAYTGAIETNYFFEVAATGESEASESTNGTTNGTTNG
EKSGPSSSTTSLSKDSATTSPLYGALDRFAQFFIAPLFLESTLDRELRAVDSENKKNLQSDVWRLM
QLNKSLSNPEHPYHKFSTGNLQTLRDEPQSRGVEVRSKFIEFHKKHYSANRMKLVVLGRESLDQLE
SWVVELFSEVKNKNLPQNKWDDVQPFSEKQLCTQIFAKPVMDSRSLDIYFPFLDEEELYETLPSRY

ISHLIGHEGPGSILSYIKAKGWANGLSAGAMSVGPGSAFFTISVRLTEDGLTHYKEIVKIIFQYIA
MIKERAPEKWIFDEMQNLAEVEFRFKQKSPASRFTSRLSSVMQKPLPREWLLSGNNLFRKFDADLI
TKAMSYLRSDNFRLMIVSQHVPGDWNAKEKWYGTDYKEEKIPQDFLKEIGSALASSPAERVSDLHM
PHKNEFIPTRLSVEKKEVAQPANTPKLIRLDDHVRVWYKKDDRFWVPKATVHITLRNSLVWATPAN
HVKAKLYCELVRDALVEYSYDAELAGLDYNLSASIFGLDVSVGGYNDKMSVLLEKVVATMRDLVVH
PERFKIIKERLTRAYRNAEYQQPYYQVGDMTRYLTAEKTWINEQYAAELEHIEAEDVAAFFPQLLQ
QNHIEVLAHGNLYKEDALKMTDIVENVMRSRTLPQSQWHVRRNIIFPPGSNYVYERQLKDPQNVNN
CIEYYLFVGKITDEVLRAKLLLLAQMTEEPAFDQLRSKEQLGYVVWSGARYSATTIGYRVIIQSER
TAQYLEGRIDNFLAQFAKTLDEMTDEEFESHKRSIINKRLEKLKNLGSETSRFWTHIGSEYFNFLQ
HEIDAATVRTLTKPDIVAFFKQYIEPSSETRAKISIHLNAQSAETDDLPVDTSETAEGAESLHNQL
TSVSISNGDATTIKATTRQPIYITNVPQFKARLPVSPGPSPIVDLSEFGDFDSKL
>g1935
MVTKVGINGFGRIGRIVFRNVIEHGEAEVVAVNDPFIESHYAAYMLKYDTQHGQFKGTIEVDGTDL
IVNGKRVKFYQERDPANIKWSETGAAYIVESTGVFTTTEKASAHLKGGAKKVVISAPSADAPMFVM
GVNHETYKSENTIISNASCTTNCLAPLAKIVNDNWGLVEGLMTTIHSYTATQKTVDGPSAKDWRGG
RTAAQNIIPSSTGAAKAVGKVIPALNGKLTGMSMRVPTSNVSVVDLTCRTEKPVTYDEIKAVVKKY
AEGELKGIVGYTEDDVVSSDLNGNTNSSIFDAKAGIXLNSNFIKLVSWYDNEWGYSRRVVDLIGIN
KSINWPNAKGGGHEGISSRTAWYNEPTSAFLHLGFIWIQDWEDTEEMDQDEDTVMRDPGSSTDMPS
SSTDAAATTFTTGTRPNGDGVQPATLFGMPKLPSSLMSPPAAIPMPMSMGAATEITGLTKSIEDEA
LHTMMMKSSEESDSLIEENSDWSVDDPSAATSAVGLPVASLATGLCYDIRMRYHCEVRPAAEVHPE
DPRRIYYIYKEICRAGLIEDLDIVPAPKMLAPMPLKRIDVRNATKEEVELVHTDEHYQFVSDTQNL
PDETLIMLEETRDSIYFNRLTFASALLSAGGAIETCLAVATRKVKNAIAVIRPPGHHAEHDKTMGF
CLFNNVSIAAKVCQRQLGEACRKILIVDWDVHHGNGIQKAFYDDPNVLYISPHVYADGKFYPGGNE
GNWDHVGEGAGLGKNINIPWPSQGMGDGDYMYAFQQVVMPIATEFDPDLVIVASGFDAAAGDELGG
CFVTPACYSHMTHMLMTLANGKVAVCLEGGYNFRSISKSALAVTKTLMGEPPARLAATTPSNPAVQ
VVRTVMAAQSKYWRCMYPKIPTQEGLFTDRFHDVIRQYQAQQLYNNYKLTSLWIYRASISKSFENQ
VLASANYDRAVPLIVIFHDPPEMMGVHHPVTNKLEPHNCWMADAVKDYIAWATQRTYAVIDVNIPK
HVTADTSDVGKYEEEDVDRPSQTEELAGYLWDNYIEPNEATHVFFIGVGDAFYGVANLLINRDSIY
QRVNSVISFVAENPVRAVASPTQTWLSRWYKDNSLVFVSHTHGVWHNENRRKPSKRYGRLQRSPKT
GLNEMLLQHKGEVFTWIEKRVKGAESDEEEGDEGNA
>g1936
MMQVSKRIPFDNPDVLLGVRALYIVSNVIILGIYLYVQSKINKKKDLTTLKYVEPAPMGSGEEPRP
VTTTVMEYDKQQLRQLMRSQLMGVGMMAVMHLYFKYTNPLLIQSILPVKSALESNLVKIHVFGKPA
TGDLQRPFKAAAGFMGMGQGEIKSDKASIESAEKNWRGXVKEE
>g1937
MRVGAKAAVYVTAVLEYLTAEVLELAGNAAKDLKVKRITPRHLQLAIRGDEELDTLIRATIAYGGV
LPRINRALLLKVEQKKKGKVDA
>g1938
MYHDNSSSRSPGSQRHPQTLHRQPSRQFDAYGPMPTNPYDDPLARYDTSRLDRLNPLHGGTNYAYE
LSGSQTWNPNGFAGGHSLAGIGSATGRMKPSRGRTGLPTPWLDQQSGVPPSLAGLAPGPLAGTQMQ
SSSSQDGDDELIPTAIVIKNIPFAVKKEQLVQLMTEMNLPLPYAFNYHFDNGVFRGLAFANFTSAE
ETATVIDMLNHFELQGRKLRVEYKKMLPLQERERIEREKRERRGQLEEQHRPIAASQLQTQSSMSS
LASHIPATSPSPVSQRGPKLDVDLNDSQTLQFYSQMLLFKEDMGREALIFPSNLTPVQRRTVHTLA
HNMGLSHASRGTGEQRQVHVFRAPPGSNVSPPLSSIPATVHAAEASRRGLNRAATIDFSESRSEGP
GPFSTMRGQTSGFLGVLESPGGFGNPQNLRAAKSFADLRSYTPSPVPSSASFPATLQTNGARLQQQ
YDGAASGTSNTPTLTPTPSGASLGLQRDDGLLVNSLGGLSLGTSLGGPAGSPRRLRGMFSWEQDNQ
GAPAGAIGSNRSIGVGFDNQSQERIPMRQPRGPAPERGSGFRRQNGHQSRGSDELRTGSGVEIIVE
>g1939
MSEVISDINPVPDSKEAVVRDSEIGIGNSRAYRPTKWQSNLTLLSCYIANFSDGYQNGLANPTNVI
FKMLLGAKGYPAEIQTRISNSLLIGAILGVIVFGYVTDMVSRRAGLLFTSFLVTISTLMATLALQV
PSSNMLWFFVIVRGICGFGVGGEYPPSAAAGLEESDDFARRYRGPIFISFTTLMATVASPVQMIIY
LICLKASNNNLPVTFRALYSIATILPAIIMVLRFFMVDSTLYHHSNFKSQKKKPLRFYWLLICRYR

FRLFTTSLAFFLYDFINFPNGIMSSTIISSLVKDGNIQTSAIWQVILAVLPVPGVVVGAWLSNVVG
RRYTGIIGFAGYVVLGFIIGGTYSKLSNHIAAFVVLYGLLQAFGHMGPGATIGLISSEAFPTAMRG
MGYSVSNAFGRTGAAVGTECFTPLEQVAGKSSTFYLAGGVGVLGMIVYWFLPESGDVNLEEEDANL
AKYMAEHGFSMNTDI
>g1940
MATLNTRGNGLASNVLSSSTESSQAVVLSGARGSPNSRRAWLFGLGTLGVILTSVLLSPFTSPQES
IPNIDPTDYAARTKHVLSTTPLIDGHNDLPYLIRTELKHQIYNERFTFNTGLLSNTDRKKLRDGMV
GGQFWSAYIHCPKDSETKKDVPLDEATWTLRDTLEQIDITKRFVDEFPDLFQFCSNSTCAREAFSN
GKIGSFIGIEGAHQIGNSLASLRQLYDLGARYITTTHNCDNVFGTAASSVSAGREDKGLTLFGEEY
VAEMNRLGMMLDLSHVSHETMRDTLRLSEAPVIFSHTGAYALSKVLRFAPDDVLKATAEKGGIIMI
TFINRFLRPDDPDAATIHDVVDHIWHVAQVAGWDHVGVGSDFDGTPVTPKGLEDVSKYPRLVELLM
ERGATDDQIRKFAGDNILRVWSEVEKVAQRIQAEGRKPNEAIWEGRTWVRSEMSPPIMFRDSIVVQ
SHGPFQGLPTYPQTAELSNLTAVITGANGMSGYHMVRVLASAPERWSKIYCLSRRSPPGNFFEDLG
QGAKRVEHILVDFLEEESEIADRLRERIQKVDYVFFFSYMQPQQKGNILGMWSDAEELARVNSTLI
KNFIGALRQSGLQPKRFLLQTGAKHYGFHIGPATNPSFESDPRIPLEANFYYPQEDVLADYCKDTG
VDWSVVRPSYIIGAVRDSALNHLVGLAIYGAIQAHLKQSLAFPGDYAAWDREYCQSTSLLNAYFEE
WAALTQGAANEAFNIQDGLPFTWGRFWVYLANWFGTTWTPPEEDESKYRITKSRHVGTPRGYGPVG
VTRSTFSLQEWAERPEVQQAWKELAEKHGLVIDPFTETNRTQIFAMTDSAVIGGWALSLSMRKARK
LGFLGTVDSYETAFNTIRDLARLKVVAPPVRDKFED
>g1941
MDTSPAAALIYLNQSRPTAILSADLTNTDSTAGVHNLDAQSLVNVSVATSIGHATHATGATDVIVS
SSPQSLRSLQEQRVTQDSDGVLRGPAEETLVELYYENFHRSHPILFPKRVYDSGRRYPSYLNAVVQ
YIGSHYSVISAAPLRELAATELARTIGSENSPQIVQARLLYAIVLHGNGEIKEARTRLHEAIDMAL
AMGMNKKEFSNFYSRQGNDLDAESMRRTWWELYVLDGYLTAIDPKGPSSFRMNSISNDVEMPCEES
IYAEGRAPMPHPSSRQELEARIFGEVDSEDTTDLSSFTYRVDAIDLIARVLAVRDPNRAHRDKAQA
VDNTLASWAHYLPSNKTDIVNAYGEIDEMLLQAHMMVQFAAILLHFPRSNLILDFCADPEANDTSC
VDNLLCGPISHKLPPSSTRHIHGIKATEASKRLSNLFSICTSMQKHSPLFMFGLSLCGVVQCSTFI
AHVRFDGAGKCLEQHRDRVRLLNGLLRSASETWPLSKVIHQKLRRVAAILFQIRSVATSMSSAPPV
TATQQDEDGASSAAYNPAVGFVSQPNDFAFQLDNAWMSIDGQFGSPELAPWVWG
>g1942
MANHLSKMNVDLLSVVNTSLVSYIKAIALVTILIFLLNELYRWSIRVRGIPGPRGLPIVGNLHQIG
KKVIPPEQYRLWSLKYGPVFQIQLGNKPILVINSAAAAKDLLIKESGSLNSRPLFYVFHKFVSKEI
ASIGTSPWDESCKKRRKAAASALNVVQVDSYAPILRLESSEFIHELRKACDNGRTALNFLSYARRF
SMNLSLTLNYGTRADNQKDFEGNSMITEINDVESNIGKYRSTSSNIKNYVPLLRLLDPIMLMIPGN
PLQKALDIGRRRLRYHGALLEGLKTEINQKTDKPCIQGNMLKNPDTRSLTDLERLSVSLSIMAGAD
SNTPTVAWGIIFLAHHPEIQEKAFQEIKKSHILDADPFGNGKVPYVDAVTKELGRYYTILRLAMPK
EVTAPVVYNGVTIPKGTMIILNSWACNRDPELFEDPYDFKPERWIAGSESHQQAHQFAFGMGSRMC
VAANLAHKALYLAFLHLIAHFKILPATDDEPASVADPITGGGGASSVSTPPLHTRARFEPRNAFKI
DL
>g1943
MTDIKVANSPSVLSVPTDPEKDISTRIDEEITGNEYVDPKIERSLVRKFDLFILPMCALVYYSHTL
DRANLGNAKTMGLETDLGLVDNEYSLVLILFYIPYGLCNIPAALLAKRFNPARVIPIMMGIWGSLS
MITASTTNFGGLLAVRVLIGVVEASFMPCVYLYYSLFYTRKELGLRTAAWGFTGFIAGATSGLISW
DVSQWHRSLTGWQYLFLIEGGMTVGLAVIIFFILPRDTNTRWFTDEERRVAQIRLRRESNLEGAGQ
ISWNVAFQVVRKWQVWAFAAMAFLCGVGVASASNFLPTMVKRIAQSTPQGNLYTVGPNLVAAFVQI
VSAWLSDRYQQRALVSFCTLIVSMIGFILLATLDLVHHTHVGYFLTYMITFGTYMPELLMPLWLSS
NLTNASSRAVALGLFIGGQNIGGILSSAVFRAQYAPVYAPALITVAICQGLLLVALIAMRQYYVFQ
NKKLATGELAPPKELENNHDFKYAI
>g1944
MSNSRISQPILTAEKLWPGGIPEFVRVHPTDDLIDGPEEWNWKNWTIECKGWCQFVEEEWVPMTDL
NEEMRQRRKLIERWASADQAFRDSFHARSPERESALDYPAHLLSEEGLDEQPFVCLASVDKELYPD
NYIRFVKNLILLYLHDNQGGGYHPMSRRDWKRDPSPTIPDILIDSSTTPDYIKFFSHHDLLINLYL

HPADFRAVSMTRLGTVVFPHERSNSIYTVIDQDNLDLGRVAVMTFKPNGKIGEYANVFAWNFAWMD
SYHYGLGWGYDLLFNGEDCNYATPYCNEPVKMDDPLMCIVDSLIQRRNLNSFGSRSEEWEDEIQRL
APGYLALEAQGREVEFDLADLWNEEGLCYNHYTLEQKKHYRETGSYFMPPRRFIPPSPKPVPATTL
PPPSGALGMSFHQLFQDKLGVKADKK
>g1945
MVPDLFPRSLLLLNILTSASAAVLGKRTYVETDNVPKGAPTVTLKNGSYYGVHNSYYDQDFFLGIP
YTQPPVGELRLQVPQSLNETWTGTRNATQYSPECIGYGSDQWVLGNDISEDCLTINVVRPSNIAAD
AKLPVLFWIHGGGFFEGGSRDPRYNQSFLVQRSVEIGKPIIGVSVNYRLSGWGFLWGEEVAAAGVT
NLGIRDQRLALHWVQENIAAFGGDPSKVTIQGESAGGASVGTQLIAYGGRNDGLFRGGIAQSGGPT
ALGSYVNASAWEPNYQKIVELAGCSSASDTLACLRTLPTEQLSTIFNSTGVTSSGGVVIDGDILTE
SATTLTKTGKFVRVPLLIGTNFDEGTAFGIPGINTTEEFKESLLYEAPSLTEEVINKVAELYPDDP
NQGIPSTLKGRPPPGSPFGTQWKRSAAYGGDLTMHTGRRISTEAWARYGVPAYSYHFDVLVNGVSE
YIGSTHFQEVAFVFDNTAGQGYENAVAVDPFANEPATFDELASIMSTAWISFAHDLDPNNNAFSNK
KGINKEGLQWPVYGQKNPSNIAFNVNVTGLAYVEPDTFRADAIAYMADNFERYWGR
>g1946
MPTAYLLLRSNGRTPPTQMPTLIYGTAWKKNRSAELVYTALKAGFRAVDTAAQPKHYREDLVGEGI
RKAIAEGIVKREEIYIQTKYTPISGQNPHDMPYDASLSISEQVKASIHSSLHNLGLAQDEANTANG
TYIDTLVLHSPLRAIEQTIEAWIAAESFVPDVIHNLGISNCPLPILKALCSSPEIKIKPAVVQNRF
YPDEDYDVKLRSFARENDIMYQSFWTLTANPELIESEQVQQLSENVGISVAAALYALVMSLGKVSV
LDGTKSEVHMKEDLELFSKIEEFSGEHQQEWQGLVKGFKQLIGEPA
>g1947
MTSQGVPSRKRAAPGTSPVVHAPIGSIPSYTDTTNQLSDDQFLQWGQDPHAAAAVAQPFPIDPNPY
TTMSYVAANQDIPVPTNQPSNQLTRRPVNQLMNRNRSFEQSSVSSVPESGGDGWGESLAELEQRAL
IAKREAQAKRKQIPPFVQKLRSFLDEPQYADFIRWSDDGNSFIVLDEDEFARRLIPELFKHNKYAS
FVRQLNMYGFHKKVGLSDNSMRASERKNKSPSEYANPYFKRGHPDLLWLIQKPKNAPGQGSKGAKG
AKIKTEDGEEHDPDELVDDIGRDERPRLGGQLAITNGQDVMPKDQLAGVYRELQTIRQQQQIISST
ISKLRREHEQLYAQAANFQEQHSRHENSINAILTFLATVYNRSLQGHDGPQNIANSFSGIISQDQG
NFVDVGDDFALSTLGNDLASGTPRFKKQPLLLKAPPQANTTTSDRATTLSPAASNYDTLSNRKYSR
PSTTPSHTIEEVQDATSPQTTASSKPSQDQRKPQRDIMSMIQLSNAANGIPTTFADFPNIVNSLET
SGGKSALTPKQRADMLRQIARDTTVSDPSLASSNNALITPTPPPMPADYSAQLANTRTEIDNLMKM
QAEQDRSVQNLTNLLQPLSPTGQIPGLQEGHVVPPSLDLDQIFNSQDYFTDYNDLENKGNLDLGDT
SANLTTGIPSQAYRATANDQTTTDDLFNFDNLGNDAVDTDLFGDARASNAGAGYFDNYDDYLKPGF
SNDDLSNRNTAATVAGNGNNTKSATSPETGRIIETLTDSESTSPTNTVDENYQSLNASNTSDVNTQ
SPKRRRRA
>g1948
MVLRKSPPPGINVPSRARNGSDPRSTVSRSSTSPRSATHQQSSRKDSWELDTDSIYSPDLRTSPAF
DLMPLEEAQKSPVASSSPWEDELVERPVGMAHMSGASQQMPGSNNAAYQHTGSSIQYSDDGFGKQP
SAGESVLGTSPQRFRSNNPFLRAQNSSPNPWEDNSRRERLPSHSEQGSSTSNLPPDQSERNSQTSG
IIPMTARLSIFDQPEDPWNQPSQQPDGGVSLHNAGTVTQALSDKGKMPSTESGADNTYTLNQTEQE
WDTNNHWQELSLRPESANTSNASSSQQIPRASSPQLIDLEDTPTNTEDNPWSSEKRPDPPPKDNMQ
AQPPTSRPPQTTSDSLPPPPMSEAELKSFQEKQAETYAIRHINWTDYTGQLRESPVLVQSRNGPCP
LLALVNGLIMRSDRKTEPPIVRALKTRENISLGLLIQALFDELVTYSNDELPDIEALSRFLTMLHT
GMNVNPRLTLNSPAALGSFYETKDIQLYDSFKVPLVHGWIATPTSGVHDAMVRVAQYYEDIQLLQF
RKEELEDRVFRNGATLTPDEERMMDDIQIINQFVNVENTTQLTEFGLDHLGKTLPPGSVSILFRND
HFSTLYKHPQSEQLFTLITDAGYADHAEIVWESLVDITGANTEYYAGDFRPVGHAPSAPGPNEPSH
LVSQRTSSRNHATTTSSTPSHNTEQEDADYAYALSLQYQEEAQQQQQRQQQPHARSSSTRVASTTA
RNTVPAHRYSQSSASASTSRLRNPSVSSSSIQSPPRRPNDNNNNQNNIPQDAPGVTDNDDDIADDA
PPPSYEQVARNKAAEMFNRQQQAQPGMPLNTEYGTRRIQQYPGGTPPSSTGRRQPLQSVPDRRGQV
PRDKDCVVM
>g1949
MDSFVRPNQWVAIRLSSDMYKLVKATPNTTILLGKYGSFYTNQILGRPFHLTYELSNESEEDGYSL
RVVAAAELHAEALISEGSGEADGIVEEADSANIEQPIRSNRDINDDGSAQTLTWEEIEELKQNATG

AGKEIIDKLLESHSTIDKKTAFSLAKYKLRKEKKYLKRFTVVPLDVNILTEYMLEQKEASRIMELR
HESIGLLGCWGNVHHSGNLENIDPSGRYLVLDDTGGLVIAAMAERMGILYPTDGDEDNEELGNYSN
VNGKEETTTAPSQTNKKQTGAKPQNQQRIRKQPMTAKQNTITVIHPHSQPNLSLLKYFGYEAEEPD
ETHPLFDHLKMATWLQVLDPEADNIYSDKPEEVDDATLKSYKPRQRGTYHRKHARWARVRAVVDEV
REGGYDGLIVASVMDPDSVLKHLVPLLNGGAPIVVYSPSVELLTNLMDQYSTARRTAYIYKKADLE
KERRSIDPENMDVDLPSIESQLSSEFPLDPTLILAPMLQTSRVREWQVLPGRTHPLMTGRGGAEGY
IFHGIRAIPQSQHVQARGISTRKKRKLDVGDIGTPEDASTPITA
>g1950
MPAYHSIFLDEADLPIIGNFPLLPLRTRTRGPAYVLPALPPSESDLDLDPNSESYDCIDEILSLFR
ANVLFRNFEIKGPADRMLIYGILFLSECLGKVKPTMTARDAEKALINVALEQFAIPGDVSFPLNQA
FERPRDRQDAEQLRQYLSQVRQELAIRLLTRLYPGGEGPSKFWLSFAKRKFMGKSL
>g1951
MSDYDEDVDMDSTAQTAIKFSSDNTNTKGKKIVADLPIGAEDNLPWVEKYRPSSLDEVQGHQDILA
TINRFIDSHRLPHLLLYGPPGTGKTTTILALARRIYGSKNMRQMVLELNASDDRGIDVVREQIKTF
ASTKQIFSVAAPAAKENSLGAFKLIIILDEADAMTATAQMALRRIMEKYTANTRFCIIANYTHKLSP
ALLSRCTRFRFSPLKEVDIRSLVDKVIEAENVRIQPEATESLVRLSKGDMRRALNVLQACHASSIP
LPMRNAPKDQPPPEHELITDATIYNCIAAPHPSDIREIMTTLLSTSDVTSCLNTINTLKTSKGLAL
ADILSALGEQLQTLEVPAQTRISWLEGLAEVEWRXSGGGSEMVQTGGLVGVIRNGCELMGDKGVAM
EL
>g1952
MHYKINLLAAAALVPAIQLSAAETILGAYIFHRHGDRTSKSTPPASLTDLGYHEVLGAGTNFHDRY
IADGSATQIYGIQSETVKLSQLAVSAPLDNVLQNSAAGWLQGLYPPVGGASNQTLRNGTVVEAPLN
GYQLIPISITSSGSGSENNPWLQSSSSCNNALISSNEFFNSDIYLEYLNSTTDLYQSVAPAVNATF
TTSQLSFKNAYVIWDLLNVALIHNSTTTYPALSSVSNETMHELFVLANIHEYNLAYNASSTARAIA
GAQLASQALLALNETITTRSDPLLNVQFGSYGTFQSYFGLANLPAADPIFYGIPDYASSMVWELVT
NATVTSDALPSASDISVRFLFNNGTSATTTNLTAYPLFGGSEIELPWSDFVTQTQKFAITSQDQWC
QVCGNTTGVCATSTASTSSSSASASASSSSSGSGISNAVAGVIGAMVTLGVILGLEVLAMLLGGLRLV
KKKSLAAAAAAGTESAAAGK
>g1953
MPSLAQVSLFLSLTISGLVQAAPAPAPAPAPAPTAPPKLSERASSCTFSGSKGASSASKSKTSCST
IVLSDVAVPSGTTLDLTDLNDGTSVIFEGTTSFGYEEWDGPLISVSGTDITVTGAEGHVIDGDGSR
WWDGEGSNGGTTKPKFFYAHDLTSSTISGLSLKNSPVQTFSIDGSTDLTLSDITIDDSDGDDGSAA
NTDAFDVGESTGIIISGATVYNQDDCLAINSGTNITFTGGYCSGGHGLSIGSVGGRDDNTVSDVTI
ESSTIIDSANGVRIKTVYDATGSVSGVTYKDITLSGITDYGIVIEQDYENGSPTGTPTTGVPITDL
TIDNVQGTVESDATEIYILCGDGSCSDWTWTDVSVTGGKSSDACENAPDDISC
>g1954
MSDGRLLVNRRCIYERKLPGDAYVTAHAERLQHGYFSSKNTSDPEIAHVDFLAVNIVMHPGDSGKH
RFKSATIKASIQNSPVGPSTENPYPYPQENPKFLMHAPHLIYGAVSPETLQWTFSLAGSLGISESP
VAASVTPSGTRSASYKIYEMMKIQGSCRTLRSRDGPEFNVEDGEVVWSLSENGLQRSGLPREFTFV
MLIQKPSADSKITFKIDIDPVIDVWYGRYPGWWINRPIYQPLHKRGVNFRTEFGQRFAPIDDSEKR
FNFATLAQSLDDYVNMPGSTYSSNASLRSAASCAMKDYVSPDGVYNDPTGTKISNPGNAPQSSLPS
GDNSNNNDSQPVYIPEYPQYPGYPQYQYPPSQPTYYPPYPSTGQVWPTGSPFQRGWPRGGPYYGGP
PGNDGNNAESTINVRISLDSAIASNIVAATLGSPIRRPSPFLTAPNKSNNLDSSPLSKAPPSDGTS
AIRTKSLRRTKSRDRLDRLSSSPRSLVNTDPAKHDSFYQRQSDHLNYGNTTTDDTSNGLIASPQLD
SPTMYEINDTPPPNSIRDPTVVNGHDLDTPSRSTNYTGLSNGIKQYTPLTNGVQSTTPPSSGIQRM
RNGMGTGHLRRRSASYAQNGYQNQYNRYSYPMVSNEDDPGMF
>g1955
MSSHYNTEPPPTASVTLNTTIGALHISLFAKQTPLACKNFIQHCLDGYYTGTVFHRIVPGFVIQGG
DPTGTGSGGSSIYEDPEFEYDPEARDPNEKVVFRDEIHSRLRFNRRGLVGMAKSEDGTYGSQFFIT
LGNTEREMNGQCTMFGRIEGDSIYNVVKIAEAELVEGTDRPVFAVKITGXEVGEMGQFEGKLKKRE
KVARVTETEKSDVTIKKKKKAKGAKTLLSFGGEEGEDGEDVSVLKSKKPKFNTKLVSAGDSLEMDP
KTRSIERRSSVTEEKQAAKRLKPSTDIPSTNNHVQKSRPKTPDPYAQIPVKDPEEPERSPSPTPPP

AARQSILERTNAQIASLKASMRRDIVAPTETGREKSALEAMIPETSIRGRKRPAPGSAAMKSAGRN
GQGFGSGAEADALKLFNAFKAKLENSDVQSGPGKKQKDEPKKTLQNTKSEEEEDEEGQLCDLHFIA
NCQSCQNWNENAEGSNAEQDQDEDSTDWLSHRLQFGKDTFGKDLKWKKEHREDVDSLMVIDPREKE
KEYVHRGGKSRKTGRDREREHKRERAGEREWDRR
>g1956
MDLQALIEDNIQSLFLEEYRGDLRRAYNDIIQHESQLDTNPESNPEQRAYFLRSKVQVLTLRGRLN
DAYAALDEFKTFVDDNVLGEPQRLASYWGLRYAASKLLVDYYRRYPPCLRYQHESQTPTNTALIEG
ISGPNEITAELNQNVQKYCANATEEEAANNQTDLNACMLFLVLLQFPQQLRILSLRHSSYPDGNWK
PNDGKTPEEVIVSVAPYLHKFLQMAPLEETTDLTFLYGHRLNIELHYAMESPSLETLLAKLFHAHE
RLSDQAGMANAKLIEGDSLYSPPFTSPIIQNLVPVPGCHAAGENTIWDAPEAKITPKDSDGVSRCY
TEALELFETSNSPRGKAAVYLRQASILHAKSYNYDPTDTERVALLQEASEYLDNAIDLFDMDEANV
QIVKANQILVEISQENANIADLRKRAGEIGKWGRESQNELIAFHIGTVMIRFARREWGKYSRFDTA
NACYECAYECCTELGALVPAFISLFARVGLQNEMSNIAATAVLGDQCITMFDSVVESLSQQIQKID
SQTPIGNIERRHLLTRKFDFMSTFNSSISRILARRDSLDTLENYRRWKEKFLWYEQNDESFAFFRG
ESGPMQSDVAKKYFGQSLDDVMDVFALDTLISDKYWTADMEFNQAILDGNLDKAESFMHDFIKTAR
GFKRVYTQYMYQMIAYGRIGDVTKMREILDSVTDEELFNNRLDEFLADKNVDMFFPSLANNAIFVC
VSTTDWERGLRVLDLITKIRPNYFDESPSESPIDLSIRQANAATIYLKNCRPELAFRMLLKARYLI
ELRRSQASDTDVKVGVLSRGLIETFLQLVIICLQCARLNLPLSILDAYDHGHPQVPWEEHALLFME
EGRARSLIDSLVASAQQQSDEEEKRRMSESIYKRRALTHLRSIQKRTEDEDRELDELVKDVERLDV
GTWSSSAKDLMDTMNSTILPRDLYSHLNDDTIVIEASFNRSGCILMAITNKGIQAYETNALRDVDI
RKWTMELMQIMREMSGLREPEEEERKIRMEKLSEQISGVLLTPFAVIIRKMGHIIFSLSQPLTAFP
VSALIFDGKPLVLHAAVSQTPSLTALYHLSKRRAASAVPTVSVFTKALAGDQPSTSDRATKETFLP
MAGVEAISISNMFSTWPIEASNLSRAQFRDHIQGHNSILHIGTHGTVNAGSPLLSSISIGEDFRVI
DMSPIQSRANLIVFAACLSGLGKATAGNDVLGFTHVVLGTGCQAYIGTLFEISDFASMVLMTLFYR
QIKDAPGLSLAESLRRAQVEFLHFDNEKATAFLDKLLEAWDATSVTDSGSKSPEEFVPDGRYLLTL
QKMMLPQIDWTSPIFWASFALMGYGDFCFSHPS
>g1957
MKKRPRFDYSSFHTPDPEMQDWDPILDSSGTLLYENVWGNLLRRIITDNDVTTLKHYIEKHPKHIF
WPGECGDSDDTICVAASSGSVDAMRVMIDHYHTDPDFHANPTQSKCHRPPNFSALESACSSGRFDM
VKYLLDDDPLRDTVYAKEYPSGKALLAAADSLVHLSWCRDEEPEWLREWIDESEALINMLLDRGAS
VRDAIQFYQQNDVKDRDQVLRTVLGNSITRASAKLISRLIAEGADVHARQWWNDYFRFDEADQTTA
LHIGSLYWNLEGLKVLLDNRGEIDLAEMVSVRNSRGCLPIHWAAGGFEGSYSGHVCDAEDSARCIG
ILRLLIESNPDTINVQDEEGKTALFYVLDHAIYRASCRCLEEVVRFLCEHGADAAIKDPKGQSVFH
HIGTMGGIVGSSPINXAILDLLVAHGGRINDADEEGDTALHVLTRWQGKWLKRTHPNQERYTKQME
MYNTMVKYFLEIGGSMEQRNKAGETPQELLDDGYRNEGKEKPVPKTLRGRGRGQLR
>g1958
MDDDSFFLYGIGEKPDALTLGSLVLEKYWMPLIARHYTHDPLRGEKLESHAWTSKLNNLVLHGRTR
FTPGIGVSGFDIVDLRLAWNKDQERYVTAKSGQKLTLKDPETFLTTQVLSNPQARATLKLWLSSAR
SDYVMNMRWARRPKIWFLTGLYMLSDAHTIVRKSSSSSIDVGLSSAIVGALSGVPVGGSVSLGQGD
AWEMAMGMEGEHVWAAQYRLLDAKYISASSSSSLRKDVEVATMSLYKDILSVRNRRTASQIARGAV
DVDVELGLEPLVDTADVVVAEDAAPMNRTAEVVALVPQEDNEANAAEEDEEEDEASFEEYEKRLEE
AIRMFEVAPPRFLER
>g1959
MSSRFPNSSGYSSRGDRSPPQRYSDRRPSLPPGPRDDPNLTPLGREPPRGPKALIDAPRGGYSGRG
RGYPPRNDFRDRDRDRDRDRDRDRDFRDGPPFRRDDRDRDWPRRDRVDRDFDARDGRPPFRRSRSP
QPRDPRDFRDNRDLGPRDGDIVRMRRNSRDSLVSSSSNPGDGPSEIREIREIRGNQGIGHPPPMRG
GLGRGRGRGDWEGGRGRGRGHFLDDRENFRRRSRSRDGWWEREREREDFRDRDREFDRRDRFDRRDD
ERRPDRDDDRDRPIEPWKKDIIPPRSEARVPSGPRSSSISPAGPLNPSRPEPKPEQTAEPTRKPSVA
ALPPVRDPRRDSDRPDLPLSRTEMLRESIAPKGSPPPSAPQVPAFGSVTLPPSLLSIEKSSSQPSS
QPPSQAPPVTATPNKPERSRADIISSATKEPPKEAPTEPKAYRSDKAQVSIPAPKEHVESSRLDLP
SRASKPAPTGPAASLEQPSRDVSPPTAPASLKRDSIPAEPRAHHDNSATASPPLGPAATSPVQSRS
QLPPAGRGGSPQTSPRMTFGSVPTGPRAGVRQSSRGGHAKSKQWLRPGYNRVPPSTTSLGNRQESH

EDADLDESNPAVEEKRPLEDKHDEELQPVDKVLEKVESHKIEKDKPLDTAPSVKAPTEAAKIQDQS
LEAKHEGDGTPSIPDFTRSSDEEEEEENMIFTEDYLQDRKNRYETDMRALESQIPLPILEDPVIVN
LLMKVQLLGMMIDDSVPEEPEASVVETYVDEISNKAVPAPTYKEEMDVDGTVTAPAKEVTPAEAVK
KETITVDSLPFLVSGPPTPISELDVYQENIKTHERIQEAIRNTVAKQRKDASEKNKRLREEYATLY
RPWRLTVRELDHKKDEERKESTPSAATPPVTPAPAPAPEGREGRRYKGNSELDFQNALKASAISAQ
EEFERRRRMEATARPDLDKEAVVPDMLEKYEIEAGIFKDTNNKIDPADALVVYGFYPPPDDFTEEE
HKLFTDAFMAYPKKWGKIAEELPGRTYAQCISHYYMTKEEIKYKAKLNKRWRSQRRARKSTTRPKS
NALMSDLKADGDDEESVQMTETGRPRRAAAPTFGGDSTTEAENSRRGNTKENEQGEKPASRRGRGG
AGSRGGRXGRAAAQQQQQPQQPPPTQLPQVQPQSLPQQTQAVQPPLHIQAQPQTAPPQQPLPSST
ALAPGVPLIAKQEPHVVESLYEPAVKIKEPEPNNEDIIPRNKPARRNKDSIYVFDASVDYDAPKPS
YEMGYGAMQPTSYWSVPEVRDFPILLAHFGRDFEGISNFMKTKTPIMVKNYYQRKIDSGNNEFEEI
VKVTEVKKMRGEPTGPLPTPNPTVKRRYEATPSAVTPRPLAPHTDMDHPEDGRMGQKKVAAISPPR
TVQELAPDRNVGRYQPLAQATGAPGTSVIMSDDPLRSLRPSGAPPAPRSFTGPGPRMGYFTEDRRE
GRTASGGALSGSRGPELQPSPRVAPTVQSHDVGRMEPLPQQLRARPDFHGPGQYQPGQPPVYMQAQ
QGVQPSAMPGSHSRNASLTGVTAPGSPAQQLRRHTPDISPIRRSSFGQTQPQPGYYGYAAANVLPK
TVSPVKELPAPVASQEPPRQVPAKRSNIMSILNDEPEELPPRKRFASEMVSSPRLAYGQTDAVPAT
SNLRHEEKASYFPAQQQSSHGPPPGRPAYDYPPYAPSLGNPPAGGPPNHDWMARFDPRSQTQQSAE
PVRQAPQGQYGAGYQSSQAGSNSQPPTPQSNAPSGGHRGYQGYPPPHGQGAPTPPLLASNRDPRDI
RDPRDSYRQNAASPPPHQNVMYGSRQVQSPAQSPAPSLSMPQPRQPSGPGPSYGSSAHPQTSSPHP
LAPQXRSSHSGHQSYQQHVQAMVSGQQGQPAPPSSRPSIGMAANAPSTYGSSAPPAQPQQQQSGGP
YRPGSLGNPLPRPYTPPSGVHAPPPAPTLGGIPYPNGPGGAHMHHNAYPHPHEPQHPSHHRVYSQG
GSAPPSR
>g1960
MDPVQEPSVTHSEPAGLAVQTTTEPTNTPDRPEEVGQAPNTEMDTSEDTTQPQESVTAETQSTEPA
VETAPEVSVEAATETADDTTMQTDAPATEVPESNPTATEETIEENAQPTDAETTEQTESTEQADSA
PAPPEEEPATWAGVEEDTSSPDEEELKKIESSDGDYSALEYDYWEKSFYSDVDDPEYRPAEKSRLT
WQIKGVRGTKEKPNRAKVMRSPPAYVGGYWWTIKFFPRGNGSQSLSIYVECSKDMPVVDEKFPETE
FTVRTGAATDNLKESEPKMSIKIPALENSQEWYENYKKCYGYTGVDCTPTDTTTEGSKNVWRVSAQ
IGVIMYNPNEPRTGWMQSSCHQFNPHNLDWGWTYFHGPWDEIHLRQKGQRQALLRNDTLAFDAYIR
VFDDPTLSLWWHPSDSEPVWDSLAVTGYRPIGDSVLNHSAEVAGLAAWVNLAPFRKIIQSVDVQEH
RRNCNVKPRPLCEALQEFLWRLRHQSPESQCVDTDKVTSTLRNLHEYSSDVVEFWERMRRTLEIEL
EGTGAVEELARLFDSPPVAVSDPQGSNVTHTLPTQLITSVRIPVDKEKNIQGALGRYFKEQSGKWS
LPPVLHVELNRQRFDQSSRQWKLLYNRIDLDEVLDLAPYVANGQCGEYDLYGFVIHRGRRTSGKFF
SILRPGGPGSKWVAFDDGSHNRVECLTRKAALESHVGLDDSKLKDANDKTGHDFAVAALYVRHDVA
PEYLPGKMEDWDVAEPLRTYFETESYPAVDLEAESKTEQTVQVEVYGLPAIEESLPSIFDTYDLMS
QAKRTNNVMYLTLPKSTSFAELRKKIAFAKSGDNNQIASHRVKLWKVGHTKKQLGAGLLFDRQDDL
TETLAKEDNIVRFWTHILSEEAAKSYAIPEPVVAKTVEEKPEETVEVRDVQDSDEERSSAEGSGSG
AAVVPEPENSAQTDAATVAEPVEDQQPEVAEAASVPSETVTTDVPTSDVPATIATDAESMDIDTTT
SVEAPSAETAPAETEQPAQDTIMEDSPSVSQENSAESTPDSNEVPADVPEAVPTVPHVYYFIQTFD
TEKQALKPAGAFVSSLSDNVMSEIRKNLGWEDNKDFLMWSRIDNTSVVTVSRNDTFSMSVGDGHCF
IVGDRLNKEQRAKLGGAGLFTNPDRLVQYIWASSRRHPTRAFTGTKTVDATFNGDYYSGEFSKGYY
HGKGTHISDTGTVYNGEFIFGQRHGQGKMEYPSGDTYEGDWVEDQRHGQGTFIESKTGNKYVGGYK
EGKRHGKGISYWEVADEEMDLCQICYGEEQDALFYDCGHVCACVTCAREVEICPICRKNVLKVVKI
YKM
>g1961
MSSQTPTQQTNNASHVDQAPSSATAAVLVPSAPVPDDVRKVRGVDFNDYVDRDITVSELVSNMSTM
GFQASAIGEAVRIINDMRAFRDPETGEGTTVFMGYTSNLISSGLRETLRFLVQHKHVSAIVTTAGG
VEEDLIKCLAPTYLGAFSTPGAGLRAQGLNRIGNLIVPNSNYCAFEDWLIPILDKMVDEQGDSKVV
AAKSGDPDDELHWTPSKIINRLGKEINNEESVCYWAWKNDIPIFCPALTDGSLGDMLYFHTFKTSP
KRLRVDIVDDVRRINTMSVRAKRAGMIILGGGIVKHHIANACLMRNGAESAVYINTAQEFDGSDAG
ARPDEAVSWGKIKAGADSVKVYAEATVAFPLIVASTFARASRDGDAGSK
>g1962

MPHSALSRAEVAKHNTAEDCWVIIDHKVYDLSDFLDAHPGGNVVLAQVAGKDATVDFYQLHRQEVL
TKYKDLCIGTLEGETPEVIDQKPGDLSAVPYAEPLWLRPEFSSPYFNESHRRLQKAAREFTDKYVT
PEAQQKEKDGTYISQEMIDRMAETNILAMRLGPGEHLHGRQLLGGVVDGKEFDSFHDMILTQEMVR
SSARGYQDGNMAGMSISLTAVKQWLKNKPLRDQLMEEVLSGKKKMCLAITEAFAGSDVAGLRTTAE
KTPDGKHYIINGTKKWITNGMFSDYFVTGCRTKKGFSVILVPRSEGVETTLIKTSYSTAAGTAFVQ
YDNVKVPVENLLGEEDKGFVVIMSNFNHERYMMAAAVIRMSRVIVEECLKWCNQRVVFKKPLIEQP
AIRQKLAKMISQVEANQAWLESLTHQMCQMSYAQQAKHLAGPIALLKSYSTQCAGEIASSATNIFG
GRGLTQSGMGKIIENFNRGYKFDAILGGTEEILADLGVRQAVKNFPKAML
>g1963
MNLDPMQLLTEETIEMPPGTSLPTSGTDSSAYCENVPSVKALPFELPLPLSTETGGHLPSDLLTEM
QMVDSTTFAPPSQAQLITAQIKSAEFKQFHQAWPLLHVPTFKAETTSTLLTSALMWMQNANRHHLL
LDEISQKLTRALIVRTTEQDLTDKPGTETPLQTLQALVIALIYAISVDASTSTLNWSAQWIDIAVS
MFRRLGILNNQWLPEDQGQLAEERWVQAEQMKRLVYAVLRIDAYLCIILDRPPTLRYQEIRLSLPV
SDELWRAETREARTRLHWYEPAGRSRSAFSTMMRDGLETQGFMTGYLRMPRLSLEDNHFSLCSFMS
ELWGICRETHEEHHLSYRSPELNRTADRVRTWKGYLQDWRAHIEQTDDLEHTFFSDSAGDVSPFLG
LNLTLHHLVSLKMYANLRLLEYKRCCINCHDADVENVLRAWTQSADGREAVYHAVQLKKIYEHQST
LYKSSDHRLANVLGSAGLLASAVVLCFYSTKVSTVAAGGRGTDSDEAIVAENGIELAQSKLVGTPD
YHTWISQGGLATVNGVPLHVFSVPRLSSWYHDELAPYPAYSSRLVDFLLTLKT
>g1964
MQLLPSIRSVTDRDQLAIVLMLREKEKEEEEEEEEAFAKKHAIASLPSRKTSTDSNSVIRSIKTAF
EVITHTDRK
>g1965
MGVGRRMKKQGPPAPLDQATINLIKKRKAAEAEKAEGGKKRRKAVAESEPEPEPETKKIKTKAVKA
KANGAVANGKSSKEQKSAAPKKTSAKVEKSKKSAKKAVEPEPESEDDDDESWGSGSAPDLDQLMQD
EFGELDDVSDGSDAEDGKFDEGSVFDSEEEDHPREKMFSDDEDESDAEEKLTAANIEGLSRKLDEQ
RELEEEEARQELQDSAMQTNIAGERPDIFGEADTAAKAGIAPNMQLLRTRITDTLRVLGDMASLGQ
PGKSRADYMQLLLDDICTYYGYTPYLAEKLMSLFTPMEAFAFFEANEIPRPVVIRTNTLRTNRRTL
AQALINRGVVLQPVGKWSKVGLQVFESAVPLGATPEYLAGHYILQAASSFLPVMALAPQENERVLD
MASAPGGKTTYISALMRNTGIVVANDASKVRTKGLIGNIHRLGCKNTIVCNYNGQEAFPKILGGFD
RVLLDAPCTGTGVISKDPSVKTNKSERDFLAIPHMQRQLLLAAIDSTDHHSKTGGYIVYSTCSVTV
EENEQVVQYVLRKRPNVKIVDTGLGSFGSEGFTSYMGKKFDEKMTLTRRYFPHRENVDGFFVCKLK
KTGPSPGASKTNGAAASNGVKSDKSASEAGDTDVEVIDKTPITDDNGNTVGEEAFGPFDEDEDAEL
IARAERNRLRRKGINPKAVLDKKAKKDQKKANKEDAAASPASEKEQPSTEEESVDKKKKGSKKEKK
EKKPAAEAEAKAPEEEKPVADASSSPSSSSKSKKTKKVKK
>g1966
MTEASFTRSFLQSVDTKPIRLPYDYVADPVTTRLPVPYTLPRLQAPHPPMAKKIKTSAAPGSAKSV
TIQLKSARNPVLDISLDNVPLTTTSIHDLREAVRQRTVAADGTQPPAADKIKILYKKKPVGGSSER
TLAEILGDSEPEVLGGGKTVEFGIMIMGGASVKPEVTESPTGAGAKVTETEPYPSKPAEGASGFEV
LQTEEFWKDLEGFLELRTKDTVESLRLAGIFKRAWQADAAQSKS
>g1967
MEXPTTPPAPAVQQGQLPRGPITPEQQRRIEINRMKAKAIREQREAESAAAAGQSTNASKVSGVKR
SFNTMTSTQGPSTLRDAANNRPLESIKPARAFSKYVEYEFSKMTDTKGGFLTQEDDPHNKVLHVSD
GKEQKPANMTQKEWERKLLLDDLRRNKAGPFEPGLSVLDEQASQRACRECGSLEIDWKWEEAFKCC
VCNTCKDKFPEKYSMLTKTEAREDYLLTDPELKDEELLPHLKKPNPHKSTWNDMMLYLRFQVEEYA
FSPKKWGSPEALDAEFERREAEKKRRREVKFKQKLTDLKKRTRVEAYRRNRQGPAGGNFGDDLGGK
KKHVHEWGRQVENPETGMTVKTCFECGMEVEELEL
>g1968
MSRLLTMAKTLTSRYPWVSQPFIVSAPMRVMSGPALAVAVSGAGGLGFIGPGAKTEDSMADLREAS
SIIRGSESFSSILSSVDSVLPIGVGFQLWSDDVEIAADAVKEIKPCAAWLYAPRHGQMDLDKWSLK
IRTASPQTQIWIQIGTLAEAKKLLRSSQLPDIIVVQGSESGGHGRAKDGLGLISLLPEVADMAEGS
QIPLFAAGGIADGRGAAAALCLGASGIVMGTRFLAASETRISRGYQNEVVRADDGAVNTTRTLLYN
HLRGTFGWPEEYMPRTIINKSHVEQLAGASFEDLKKLHDEALKAGDSGWGPEGRLATYAGASVGLV

REVKDAGSIVREVQEATQRILGGF
>g1969
MDTQKIILVTGANRGLGYCIVSVAAARHPSAHFILACRSVEAGNKAVDELKKSGITASLEVLKLDV
TKDDEIVAAVDAVTAKHGRLDVLVNNAGISRFSQDSSLPALRAAYTDMLSVNVSSVACVSAGFRPL
LAQSTQPKVINITSGLGSITNCLTKKMHRLPPYGTSKVAVNGLTAHLQTVENDRVTDVGIFGDGGP
KVNYYSAAPGFLKTAFTRFNERGKDPYAGAEVIVQLIQGGPGEYPGGTQWEFEQGKMQRVPW
>g1970
MSHMNDELSPLSSGIDSITPFPQLEDDVGQTPATHDDDEALHMKATSRRNRAGTSHSVIRASPSPE
SKSESQSSTELSEDGRNPNADQIPSPSIEIQEDSEDAEFQSQYYVENESLEGTEDISLDSTGEEFN
DDDDDLQELSSDEDEDGEESHEGGRNSVNMHRPFMQSTSSLTGPNAFAPPFYNRPPTPLPPSPSLT
SLLRPPFSTTTSRPTTPDTSDAETPNDTEAAVAKSARIATTVPRASPKVPTYEYYGFVLYLTSSFA
FLMYLLWSYLPSPFLHQLGIYYYPNRWWSLAIPAWLVMFLVYVYVALACYNTGYLTLPMTSIENIV
DDAANIAVIDGKGRRRLGGSEKMKPGSATSQPMSTAQNRRVPGHASTNSASGSGYLLWKDVWAEST
DAVMDIPVGGNFAFMAWQSPSAMAGNGGGLGGAPGEGMPNHPHPQGTEYTLQGVMRFLQTEWHRHE
RDRNAWEIERAEMKSRIGRLEGDLRTSKRLHESLGKHVKMLEIALKKERETVRSLRNGEKVEEIRD
PKEVARENLKHLSQTNKTQTAFLVPEIDNDSAHADFRQDAEREKSRVYLSKCAQEVSYHVIPASHP
PPEFTGQDLQSNLYGEQQLPQQSLEEAYHQQRQKQQQQQQNNIMVRESAIQNHQSIPANYAENPRS
AISAAHAERRHLASQIPTNTGENFASDILTNNQSHGDDSSIIRSSQAPQQQKPQETDSETGGWNFE
EAAPIPDVQVESTPHHRPDTDAFPKANFVPAKSPPRGSHRRKSSGATRRKSEGALDRESSALQQNI
DTTFKVRFALRGHLDVVRSVIFTGGGSPSEPEICTGSDDGTIKRWILPASYGSFGPGAGNDLDITS
YFTHRGHVGAVTSLAACPASPNFSNGGRAVGDGWVFSGGQDASIRVWERGRVDPKATLDGHTDTVW
GLCILPGTAGSVFGDVCSNYGGPDRILLASGGADGRIIIWAVSAPPQVTSPQPGARRGAGGRRXNS
ISSGSNFPSSPQPHTTTSTVFHYAIVHNIVRTESPSPTCISPLSLAGVNFVVSYTDASILVYDTRT
GEEVVGMASLETYDGTPSTGVNSVVTSTIGFDGTASLDPNRALTEEEAVVHGATGTSGGVEGVIIS
GYEDRYIRFFDANSGQCTYTMLAHPSAISSLSLSPDGRELVSAGHDASLRFWSLEKRSCTQEITSH
RLMRGEGVCSVVWSRDGRWVVSGGGDGVVKVFSR
>g1971
MEKRDPTKPAVDMQEAAQVVPWIKGYPSKGRLRMTLAQAGQEMYIIISKYSDNYLEYINDNQPTAD
FMTMTQYGSYSIDSA
>g1972
MDQFSFSNVANPTKYYAHAVMGMVYFTYVFFVITRESLFYANLRQTYLNSPAYVNRISSRTVLFMS
VPESYKSEKKLRQVFGDSICRIWITSDCKELNKKVDQRDKLAYRLERAEIKLIRGANASRLKTEAT
RKKNGLGICDDCELADPLTATKIKRPMHRANFFGKKVDSIQYYRSRLAVAIKEVEELQQKHRDGDA
KYLTAVFVEFKTQSDAQVALQTLSHHQPMHMTPRYTGIAPREVIWSSLNLSWWQRIVRMFMVQGGI
AALIIFWSVPAAIVGTISNVTYLAHLIPFLGWLADLPGFIEVPPLCRLFARKAGLPSLSRVELFTQ
SAHFCFQVVQVFLVTTITSAASAAVSQIIKNPLSAKDLLAQNLPKASNFYISYFLLQGLAMSSGAV
VQIMSALIFKILSVFFATTPRRLFNRWTQLTGLSWGSILPVFTNMGVIALTYSCIAPLILAFAFIG
LFLVYQAYRYNLLFVYNLDIDTKGLIYPRALQHLLTGVYLAEICMIGLFSIKAAIGPLIIMAFYTI
LTILAHISLNDMLRPLMDFLPRSLDTEEEEIQLNQEAAEAYRHAMTRHGRIWKWFHPNLYRDYADL
RRKVRRDHIQVEYSEKELADAYYEPCITAETPQLWIPRDPGGLSQEEIRHTSAIIPISDHGAHLDA
KNKIQWNKHDEDLHMWERRVLY
>g1973
MKNFAATAIAALLALTANAAPADPTSTTPSASPAAQTSVSVQYDTTYDNSSFPISHVACSDGPNGL
EGKGYNTLGQLPDFPYVGASPTIPGWDSPNCGACYNITYNTVSLYVMGIDSSTSAFVLSQAALDRL
TGGQAVALGQVTAFYDSVDSSHCVT
>g1974
MSYQYSHQDHGHPPLPPDWRAQWVPEENQYMFINERTGQRSWHHPEVPHSGGNYGSGTRDIGGPPS
QGGYGGPPPGGRGYGGGPPPPSNRGYGGGPPPPGSGYGGPPXPGGRGYGGPPPPGYGSPSPGGRGY
GSPPPPQGYGSPHPPQGYNTPPPQGYGSPAPAQQKNHNMAYGIGGGVLGAAVGAWAMHEHDKHKEE
EKEEEWREEEKRREQREHVREREQEREIQEQRQELYDEQRVVQRERDERYEAEALYENERRYDEER
RFDDERRFEERVEDERRYDDERRFEDDRRFIEDERIIDDERRFEDRYDDRW
>g1975

MALFPSANSGSGGSEASNETGPNFCPWDAPPREPTILVQSNEVHVIPLGIHKCHHKLEVTAHDTLD
SFSTAGKRLTDDVIIFSSRSGAIFGRIPNSLSDDAVVRWLESFLHFVSTSEEAAWWHTGHSGPWEI
YAFVPMNANRTKWIYSNTQNDKMRPLTILSMLDRYAEEKPWLVQKPGQEYVESYVAEVHGKCYIPR
QHKRRRNAQRHEEQGEQSESNPTRVSFIWDVVEHSREVILGDEEWKDGLLMERFEMSLI
>g1976
MPPRVNITSDIRAISNLFALAFRDSAPTVYVLRACDSTWPVSSIPLSLLGPKMIEWTTYKQSRGGE
LVEADNFAAAAIWFPPGADLPASANDDERLVEYRAMAGKAKKEYLGDRKYWYLNMIARHPERKDPG
VIRALFEPYIARAKEDGVPIWLEAVTEHSKQVYEHLGFRTVATLRMGPGKASPDGELQDGGEGIPI
YAMILE
>g1977
MNAPEPLTCLICAKGFNNKSEHARLVLKPKHVAMLLFHIVRGVWRISKSRTSHAISQGALDYPVDT
LRNHQVTNLDIVETRAYHDYDPYSVTTNVANFDSVKNDAEEFQLTGGEAWEFSDETESVTQDSLTT
LFPRPLRLFIPQCFAGSKSPLTQRYLLCTLRSYPVMMLRGQNPPFIHPRTIFDEPEDFGPRRKMKA
GPLAVCNELLQMFTMKNRDNVVQIWRAIRMEQERLLSECRGYDYHHTLAALQAISIYFILRIFEKN
EEATDFDVPLVQTMLELAVHLARLEKNRSRLNLSDWEDWILTESTIRGESLRNSRLPSGRNIWQAN
SKAAWLREYDMQDRRLMPHSSKSKLTYADLLDYNLKNDGGLDYWLSEIDDFGNFVMSAATLQD
>g1978
MSQITENKAAWITEKGARPFKVGPGPIPNPAENEVVIKVAYTAMNPVDVIMQESPALELPYPFILG
VDLSGTVVALGSGVTRFKVGQRVTGHCDSLLTKKKTNSAFQEYATCLEVLVCHIPDSVPLVNAAVL
PLAVDTASTGLFSILKLPLPVINPQPVGRKILIWGGSSSVGSCAIQLAIAAGLEVAATASSKNHEH
VKSLGARHVFDYNDPEVINKITTVLQPGDLVYDCIGGDKVRATCTEILQKIGGGTLPTVRWFAPTE
TGNVKVEFVNGLLPGLIDTSIGKSVWGNYITKALELGKFQAKPDPYVIEGGLEKIQDGIDVLKKGV
SAQKIVVEIAKTA
>g1979
MPNISASKKALVGRPCLTCQRRKIKCDRAKPDCQRCQKAGLQCGGYTSDSFVLVVPTRALGDGNNK
VRLRRTKGKGNGDSPTMQEDGTSNNTNTEMKRDDTTKVTRLLPESYINVAPENRMQVLSYFLERYL
PASMTSQRKFITPSSWVRSLPSLLGRWEVLDTALSALCLAYIGDVHQDRVHSHESQRFYNDVLQKL
GSMSLDSLKLSNEGVLTTTMTMAMYELFHSTYGRLQGWMVHVKGAGRLLELRGPPTEERPLNMSLY
NRVRNTVLWDAYGSRKMSFLAHSEWQNLSDTPHDILLDSLTCIPGLLEATDDILSSSQSGKVDIAA
IDELLSFWHAIHVRLTQWYVTFEDDELEGLYECGPLGPNSHPYLDAAKPHLYSMFPQVISFRDTYV
AQNMLMYWFGQLVVHTSMVQLYVAREKHQNRLNSDAFPSEYKGIHKTSRERLQKTGDYYATKICQS
TASLKGGYGFQIVMVPLWAAQQFYDICQDSRYDWXQTVLSNFGAACGFVSGEALGFLKPSQYPGIT
MKGDTIIHNSD
>g1980
MAAIEAKPREGIENTEPETDTRDKDVPKAIANTEYPRGFKFVALAGASIIAVFLIALDQTIVGTAI
PKITDEFHGLNDISWYAAAYFMTFGSAQTSAGKIYTYFNLKWTFLISMFIFELGSLLCGVAPNSKT
LIVGRAVAGLGGAGLSVGGTNIVTLTVAPAKRPMLMGIIGMTYAVAAVLGPLIGGAFTDHVTWRWC
FYINLPIGGVAAVSVFFFFYLPAAATPPKVPFTRKLLHIDPVGITLAMGGITCCILALQYGGNSHP
WNSGVVIGLLVGFVLIVIALIGWEIWLGEYAMMLPRLWKHRSLSATAPYQFFFMGSYVALLYYLPI
YFQSILGASAIRSGVDNLPLVLAAAVFALAGGAVVMSTGRPQQVMFVGSMLSTVAVGLIYTLDIGT
PTSKWVGYQFFVGCSMASAIMHGLTIAQANVDANDLSAVTANLLFFQTLGGAFSTSAAQSAFVNRL
LTTLPKTAPSVNPGQVLITGASDLQTVFPPDVLPGVLQAYMAGIKAAFIVAIAFCGTACLCTLAVP
VKKLPTHTKDDAPTMG
>g1981
MLLSGNTTVSLWSIVDVPEDALLVNNAASNVTKKNRNVRHASVSAYSAGATEQNGADQVSAPRPLA
EPDNAVSFFLGYYANIGRNLESARGFFEILVPVFCSQPQNSALSLAVSAVASRILSIWRYGPDSIQ
SPRGTYTQAITSLRSALQDRIERSNPATLLAVLALHLYENIAAIYGRNSATRIHLDGAMSLLPFSN
VHQVDDMIGAYIRRYILHTEVSSAMRQKRSLRSIAYTWLGSKELIGAPVNPSSALDAIGASVAELQ
AKYLRAVTQNAIVPLALHKLQGWIAEAKGIDEQLLEWVRNVPDYWRPVKLVSAQDIDPSIPTYRSV
CEVYPTCQIATIWNLWRVQRLLLVKIRFNSLYTILYGGSSGSINDQILAENEDFVECKQVVQELVD
SVCYSVPFYLGNRTKPSSMVDFNDPTILFPSDHILFPENKNGLNQRSQDPKMSRDEHRRHIVSQGL
WSIMSPLSRLMAFFLEDDDRAMMDFLRPGQYEWIREQSLRVSVLLRIPTIDSDNKNEERLELNSSS

ILVTTNNEVEQLVKMVRKGAIFMSGP
>g1982
MPYLGLKGNGLLAGIALSSGMGFVLFGYDDGVIGGLLTSPTFESTFHLSSGMQGTVTSLFIIGCLV
GCLFTSITNGRWGRLTITHAGSIILSFGATLQASSFGVPQLIVGRIVAGIGLGLITSNVIVWQSES
APRKVRGMLVASALSLLIVGQLLAYWIDYAMADYSSSVGWRFPLAFQAFIAILTSVMLCFMPESPR
WLFQQDRTEEATQILRLLQTHKGVVDEDALSITISDIVEALEMEKKQAGWRDLLKEDAVCSRRRVL
LACLLNSCQAWSGSTPISYYTTVIFQDSVGLSYNLSLLMSGFLQIWFLVASFGTWYTIEKAGRRKS
FMVSAACMAAVMAALAACIAVDTHASGIVAAVMIFLYQSFYTWGFMGGLWCYGPEILPLAHRTKGS
GLATACLWLSTFVVVEFVPTAISNIGWRVYIIFAVFNLAFIPMIYFLFPETAGFTLEAVDLAFMDK
EKGPVKRANELWGIIKKGGDVGLRGELHGEKKEMGGVEMIEKA
>g1983
MAPVTRTLGKNGPSVPAIGFGLMSIGGVYGAVPSNEERSALLDHAHAIGERFWDTADIYADSEDAV
GLWVKQNPEKRKDIFLATKFGIQFDPATFTQTLRSDPEYIKEACERSLKRLNTDYIDLYYCHRVDG
KTPIEKTVEALVELKNQGKIRHLGLSEVSASTLRRAHAVHPIAAVQVEYSPIAIEIEDPKIGLLQT
ARELGVAIVAYSPMGRGVISGGYKTAEEVHAKDPFLAMLPRFSKENYAKILAMITTFERVAKEKGC
TTGQLAMAWVLNRGEDVFVIPGTRKIKYLEENFATQHITLTPEEDKTLSDIIYATKFEGGRYPEGF
PEGYEFGDTPAL
>g1984
MPPKLYNLRVFAAGPNGGNPTSVVLDAESMSDAEMKAVAADHGHESGFVLPAPEGSGCDFKFVFWV
PEHEMEMCGHATVGAIWLLEKLNRLPTTRHADGHVRVLTKSGIVETRVSETLSREKWIEVSQPRGV
VQKLTDVDSLEEIASILNISTDDLASMPIQNSKTSRVKTLIPLKSVALLNALKPQFHRVKQLCEVI
DSTGLYPYAVLDNEKQIICARQFPKSSGYNEDPATGIAAAALAYGLLENGLVKYADQYITVRQGWE
MGCPSEICVRFQEDTGGCWIGGNAIEV
>g1985
MSIETQSAPIHQAGTNDGWHGVIESGGEFEPEAGRYHLYIGLFCPFAHRANLVRHIKGLQSAIDVS
IVRPYPKGDSKGWPGWKFPANEQEYEGATVDKLFGSEYLHEVYFKDDKDYKGRYSVPLLWDKKTNK
IVNNESAEILRWLPTAYNGTSIISAEAAEIDLYPESHRKEIDSLSEWIQRDLNSGVYKAGFATTQA
DYDKAVIPVFGALNVLEKLIYDKGGPYVLGEHLTELDIRLYATLVRFDTVYVQHFKCNLGTIRHDY
PVLNAWLSHLYWKVPGFKETTDFKHIKENYTKSHDDINPRAITPRGPFPDVENYIPEKPTNYGGVS
LPEVLEYEKKLESEVFGKQKL
>g1986
MALVLQACTFAQQYVASVLHPTPTKSAMGLKLGVISTAQINPAAIIHPAETHPDVILHAIASRNLE
SARQAAQKYHFKKAYGSYQDLLDDSDIDIVAGKHVLCEKPFTSNAEEAHKLIQLAKETGLVCEEAF
HWQFHPAAHLFREILDSGKYGRVLRTNAVMTASPGVPHGDIRWQFELSGGSLMDMTYALSFTRFAV
HGSKLKEIQSAVARPYSRDQRVDEAMHSLILFEDSKDGHLIQSRVYTDMARQWAIKGIVPRVWELP
SIEVETDKAIIYFYNAMMPHLYHYISITDKSTGKTQYKKQYRGGPCWGEAWTTGGKGGKSYWSTYR
YQLEAFTEKVRGKHVPCWIPGEESILQMATIDAIYKAAGLPARPSVSKSDKA
>g1987
MSDATRKSGSRMLRTTDEDRPFTRDFKDLFSTLMVSLELGSRRVRFTKIDFTFTVEEALNNLSSLR
FTQSARMPDPNDASRIITTTSTTTFTMAKEMARSIFQNFIQARFLEPLNATFSSLSKGTVCTMTAK
GIAVLHRFCNRNGITAPHVMRVLQSPRNKMTLLILERDLTTDKLVVDRATIEVIFRRFAGVQAPGA
INGSPAWEDRTDYPNGLVGVEGMQDIRVNGSKYKYAFTGKSAINWLMDCCTTIDERETYRVCEQFI
EHGLVIGVVEDKSYIEENGQAALNFQPTRQAIYSLTPRALKVCGWAVSDRLSDESINRKRESRAAN
NSPASSNPAIPSGATGDVSNKSRLQYILNDPSLRLLFREYLAASFCEENLAFYQDVTEYLDNYNKS
KMSDELKDYHKLQDYIAGVFGLYNSFLAPGSPCELNIDHALRNRISSEMTGLSGDEQRIARNLDEV
VSLCQLAQVTIFKLMASDSVPKFCRQPRYAVILQEHDFETISGNGSRPASPVSAPMRSAER
>g1988
MSDKHTVILQDRVGGGISMQRKFDSLSILALSVTLLASWEGLASTLSSSLLNGGPSSLVWGLLLSL
TGTLAMAFSLAEMASICPIAGAQYHWTAMFAPPGIRPFITWMQGWATVFAWQSSTTSIFLLVAKQV
QGLITINYPEYNPTQWQSTLLMWAFTAFSFAVNVWGIQILPLLQLFGGIFHVVFFIALSVPLILLA
PRSTPEFVFKTVMSEGGWQSDGISWCIGMLTVTYCFLGFDGAVHMSEEVRNPAKVVPRIIVQSVVI
NGVLAFGFVLILLFFIGDVGAALDPASGFPSIAIFYQATGSAKAATAMQCGITAIGLMSSIGVVAS

VSRLTWAFARDGGLPFSKFFSQIDSRFHVPFRAIGLVCVVVVLLSLINIGSTTALNAILALSTCSL
YISYLIPIILLVFRRFDKSGTQINWGPWTMRPWVGLVVNIYSIVFGLFIIVFTPFPSMLPVTAENM
NYSGPVFVGMAILLVIDWFFGAKTRFKGPLKEQMQKVANA
>g1989
MNRGITLWANWGLIGAVIAWAVASIVALAARCSSPHPWSFVDGKCDYQVAMFQGIGAVNIITDVAL
VFLSCAVLWTVQVSLGKKLRIISLLAIRLVVVIAVAFQIHYYIILSQSADKTWAQMNASIWDQAVL
NLSIIATSIPSLGRLAHELQPNVNAFAMTENHGLRSRDKYAISSLANHYPRETAEQNMLGTHTSIH
GSVDGETESMEGLVRDGIQQNVFISRTVELKVE
>g1990
MFAAIKAINARIRSNKTLDYFCSTHFWGPASNFGIPIAAVSDIKKDPEMLAIMSVQKIPIIICGAG
SAGLCAATFMASAGIPADNVKILERSAGPMQIGQADGVQCRTVEVFESFGLSEQLLKESYHVLEVC
FWAEKEDSASSSKQLVRTRRTADTMPGLSHHPHVILNQARMNGMLLKKMHDDAGIAVDYDWHVTNV
TVDLEGDSDFPCVVVAKKGLDGPETTMRAKYVLGCDGAHSTVRRSLGYKMIGDSTDAVWGVMDVYP
QTNFPDIRKKVTLHTKSGNLMIIPREGNSLVRFYIELPAGTVAKSVTLEDLHQTAKKIFAPYTMDI
AETMWWSAYSIGQRLVDHFHKDYRIFLTGDACHTHSPKAGQGMNVSLQDGYNLGWKLAAILKGQAP
SSILKTYCLEREKVAADLIEFDRFFSSIFSSKKAEHKPENFPEHFIKAGRYTAGLTAKYDDSVFTS
AASSRPELASGLTVGMRFPSTQVVRLCDARAMQLVRAFPADGRWRIVVFAGDISTPVCSERLNKLA
DFLSSEVGPVLRVTPSTADRDSIIEPILVLHGEAAQIEQEQIPEYFTPVNGKWRMKDLFKTFADCE
SYNSGHGHTYDTYNIDKSEGAVVVVRPDHCMISILKLDEGYSF
>g1991
MSSRRRGLPSPQKLAHVVFRTTPDKYQILVNFYLEILNASIRHEDPGKIAFLSFDEEHHRIAILAV
PGLTVPASDDLPRAGLDHIAFTYKTLTELAQVYVSLRDRPNDPLMPIWSINHGPTTSLYYHDPLGN
KIEMQFDNFQTMEEADAYMKGNEFAENPLGVEFDPDEWSREVLAKMKPDGSEGLSVEDIRALSKRP
NIGRRSEVPLGVI
>g1992
MFKLSKSRFSTVVSEGSSRISSVQSMDLKRDPSEPSQQKCKELQTQILNMWRRIWLXLYQVHDNHG
PYHVERLTQLYLGAGMAVPDMINKPEDSYGRYKTAKGPLGLLGCALASQGVSRLRLFEGLSVESLK
AVIQLLESYKHEVGALGEVYELLRSLQVGLRETTDHDEKAVSEHFPPLAEPLARRLIHLDARLDVL
VADLEDEHMEAFGDSIERNGWLVKDRVFPKRNLTPTKQSQ
>g1993
MSRATETERSTFNQSNRSSNSTCNSLLPPNHVHASSLLPTLDACQPSTGFSEEELGFVANSWSTSD
WDWTSEDQALDTLASLPIGVTIGAEPSELIPQSLSHPHPILSAALESFDDFYSHERIPPSSSLPSP
SRIDDTELRVEQLSELTSRLYLIYRASCGLTGVPQNEHSGSQITTTVFEAVTTLFQEDAAPMAGNT
IYSSLNETFTACRTLLEILSRLLENVEPGRPGNRTGPPQNQPPLPGQPTNSRANINSIGTRTGGDS
QEPVLYHMTTTCYNLLLLIHVTLISALHRDAISHASRVTNPSLVTSPSSSPNSQSMYSAPSMVELQ
LVLLVQVMTYFLDRLQQTMGAYSAQVAQHQRRRSLDRLVEEPDEMLPYQMVPPDTISALENRARSK
LNQLRRALHDSRSRN
>g1994
MHVFATAATAAAVMGRVVSAQYSLPDSQVPFQGGNVFKRNDSAVSNYTFSYKVPFQPTHYGGYYTW
AVNNNTVAGANFTSPHMPNGYDYIPDGSFKINPYFMVNEGTPKSLNKTKLFGSCKSSRAHARDDSP
CELNDQCEGKVQKIVHVIFENEVYNWTISDPFWKMLATKGKSLTNSHAITHPSLPNYAAIVAGDFF
GMASEDFYNVITTTIYDLLDLKNISYATYSEWYDPIATHRGPNDCNNYITNGPIDSTNPDWNSQIY
RRLDVPALLFSTYTTNYERCTKLYDANNTFYDHVMSHSLPAYTYFVPDMLHNGHDPESNSDYVHQP
ETAAIWFNSWLDLYLPELEAQGALVVATFDEATWTNDDDYNPNNDNHIVTMLFGAGITPDTTDDSY
ITTYGFLRGAIQNFGLGSLGRNDTNTTNGNLYDLIY
>g1995
MHLNNPEPIAIVGSGCRFPGGASSPSSLWKLLEKPRDVSTEIPDDRFELKGYYHPKGAHHGTTNVQ
RAYMLQEDFRKFDTGFFNISPNEAESMDPQQRLLMEVVYEALEAGGHTIEGLRGSDTAVFVGTMSV
DYNDIVMRDIDTTPTYFATGSSRAILANRISYFFDWHGPSMTIDTACSSSMVALHQCVQSLRTGES
RVAVAGGTELLLGPEQFVSESKMNLLSPTGTSRMWDAEANGYARGDGIAAVVLKRLSDAIADGDEI
ECLIRETGVNQDGKSTGLTVPSSEAQAALIRSTYSRAGLDLSDTRDWPQYFEAHGTGTKVGDPREA
NAIQTAFNGPAVNGNPLYVGSIKTIIGHTEGTAGLAGVLKAALAIRNGTIPPNMLLNRLNPDVQQY

CQNLHVPTDAIPWPSLPAGVPRRASVNSFGFGGTNGHAIVESYQSSTKERAAESAADLAPFTPFTF
SAASEESLVALLQSYSEFLKTHNPSYLRDLAWTLQSRRSALPVKTALSASTIEDLVSQIDRKLEQA
KKSPENKIGVRSRPAKPRLLGVFTGQGAQWATMGAYLIRSSAVVRTKLEELDQALATLPESDRPSW
RIADELLAGKDTSRLGEAALSQPLCTAIQVVLVDLLRSAGITFQAVVGHSSGEIAAAYAADFISAK
DAVRIAYYRGVHAKHAKGPKGQKGAMLAVGTSWDDAQELLDLPAFKGRAKIAAHNSGASVTLSGDS
DGIDHAKRVFDEEKKFARRLIVDTAYHSHHMIPCSDPYVDSLRSCGIKVKSDRDTSCTWYSSVKPG
ERMEASPDLCDLYWRDNMVNAVLFTEAVQSAATAEELNMAVEVGPHPALKGPATQSLSDIDKSLPY
TGVLSRNQNDVEAFSDALGFLWTHLGPGTVDFQAYQSLVSPGPTPKLTVALPSYPWNHSQTNWHES
RKAKRTALRGSAFHELLGVPSSNNTDHDLRWTNFLKANEIAWLDGHQLQGQTVFPAAGYVAMALEA
SQFLAGSRNVQVFEVQDLTIHKAITFDDGASFAVETLVALTGISAGGPNARTQTADFAVYSCPNTG
TMSMDLVSSGKVKIFYGNSSFSTLSSSPLDAGEMVEVDSDRFYESLLDLGYGYNGPFKTLTSTKRR
FNHASAILSTYNYQDDDQPLIVHPSMLDVAFQASFLAQSAPGDEQLWSLHVPTSIKCIRVNPELCG
SLPGSSTQLPLSAVLHEPDGISVRSSIDVFDENGQETLLQVEDLVMKPFSPATASDDRPMFSTTQY
GVAMPDGNTAIGYDRPSVEETEAAELCERLAYYYIRKWNSEIAEDEWESSKTDYGYLRNAINQLLM
KVDSSQHPSVKEEWTTDTIDRIESLLSRYAEKIDIKFIRSIGENMPAVIRGQSTDLNNARQHNMVD
QLYSDGLGFSRYNIILAQLTKQITHRYPHIKILELDARAGHATRGILESIGNSFASYSYTDPSPEF
VKTAAEEFKIYGQKLTFKVLDPEQTPSSQGFEENSYDVIIASNALHAKSSLESKLANVRRLLRPGG
FLLASGITNDQSIRMLTLMGGQTEWLAGSNGXGRKKNSARISTWHNTLRKVGFAGIDTIVPEIDGV
AWPFSAIAAQAVDDRINFLRKPLMSPSPECLDELVILGNGTLKTARLAEEISELISPLGTKVTILE
GLPTDDDEIPPMSTFINLMDLEEPIFKNLTEDTMEGLKCLFELASNVVWVIEGARTDEPYHNASLG
FGRTISAEMSHLSLQFIDSAGAGVSASRLISEAVLRLAALEMWDEKGTLHQEILWSKELELYLENG
RLMIPRILPVDDQNARINSLRRPVTKLVDSENAIVRISHGTDAAVVLREECIPPTLEDDMMSVQVS
HSVLSAMKVGQETYLFLGLGADRTTGEAVIELSEINASQTVTSIGQRITIPSGQEPGFLTAVASEL
LATSLLSALPQSSHVLVHESGLDKAVSMALARQAVIYNISITFSTTKLDKSKSWVQLSPWSSSHMI
KESLPVNLTHFVNLATDDEGKRATARIREALPAGCREIDSSDLFRPQSQLQRSSGDSNILATLQEA
VNCVQTAIANSPPPHGMVRPSQLSENTVNHNPFTVIDWTSEKTIPVTVQPINAHRLFSRNKTYVLV
GLSGALGRSICEWMSQNGAGYICLTSRSCKSDNKWQTAMKKAGTEVRFYTMDVTKKQDLERTVAEI
RQTYPPIAGVMNGAAVFHDAAFSEMSLEIMEKVLKPKIDGTRYLDEVFSDSKLDFFIVFSSLTSVV
GNSGQSNYTAANAYMTGLVSQRRKRGLAGTSLDIGRLAGIGFVERAGDTVRQQLIRCGFMAISEGD
LHQLLAEAIFAGYPGSVTGPILTTGCRTAREDDDVLVPWFNDSRLSHKVVIEARSQDAKAEGMKSN
LPVREQLAKVTTKEEALEVLKGCFATKLAMISRLAEGQTVDHNIPLVEYGIDSLVAVEVRGWFLKE
LKTDMPVLKVLGGGSVAVLCQEALEKLPDTYLPALGTKGSSKPFKVATKVLPISKPPASKVEPVSE
RSTAPSAGTSSGSRSPIDTPATSISAMPSNLDLAGLSENQKQDVDHVKKPETPYVRKENISFPQSR
FWFLRLLIDDQTTFNVAFYYRVTGNLRIGDLERAVRLVTNRHESCRTSFVADETEADLAYQKILPS
SLVRLERKKINRFEDVAVEYMAMKATPFDLTSGKLLRLILLSLSATEHYLLFNYHHILMDGVSVQN
FLADLEKAYQRQPLGATPYQLPDLSRRQRVAFENGDFDEEVAYWRSEFPHGDHPVLPLLPMAHVSS
RIPTKSFEVHQVGCDIEPALMARIREASKASRSTTFHFYLAVFKSMLMRFTDVDDLTIGIADANRT
DEDAIGVVGLLLNLLTLRFKRDLAQPFHESVAEARTKTYNALANSRVPFDVLLQELNIRRSSAYSP
FFQAFFDYRQGTHWKQPFGNTQLELLELSPGRTAYDLTLDITDTQDGARILLRTQAALYDMTAAQL
LLNTYVHLLEAFASDTFLKLEDPPLSSDSELRRALDVGRGPTLTTNWPGGTLPHRIEQIAAKNQNS
VAVMDGHGNVLTYATMHDRIQAIAEALQTAGVTQGSRVLVFEDATADWPCSMLAIMRIGAIYVPLD
LRNPLARLADVAASCKPVAILVDKTTAGDAPQLNIANSQIVDISSVPAKPSSPIPNSARADSIAAI
LYTSGSTGKPKGIMVKHSGLRNEIEGYTKTWGLQAERVLQQSAFTFNHSSDQMYTGLVNGGSVYIV
PWSKRGDPIEITKIIRDEGITYTKATPAEYSLWLDYGRDHLKQASSWHFAFGGGESLTSTLTRSLA
ALDLPALRFFNSYGPTEISISSTKMEIAYRDPPPQGRIPCGYSLPNYTAYILDEQRKPVPIGMPGE
LWIGGAGVSLGYLDNQDLTDEHFVPDPYATPEYIAQGWTRMYRTGDIAHLQSDGAMVFHHRVEGDS
QVKIRGLRIELGDIESNIVAAAEGALKEAAVTLRDGDPPFLVAHVVFAPHHTISNTTLYLQQLLRK
LQVPQYMIPVMAIPLERMPLSNHSKVDRKALKALSLPVISATSSDETSEQLQLSPTMTRLRQIWEE
VLKTKELGLKITPNIDFFSIGGNSLLAVRLQSRITYVFNVTLPLVDLLGASTLGEMAQRIEESTSS
ARQIDWEHEIALPDLTLPGALSSLRSVRSTDKVVLITGAGGFLGKHILQQLVASSEISRIHCIGXR
DKPTGKPRELXISSPKIILHRGDLSEQWLGLGVETFMKLVNEVDSILHMAAVRSFWDSYHLLRQSN
VLPTKQLIRMAAAHRVPIHYVSTAGVLPQDVPNKAESTSAYPPPVDGSNGYTASRWVSEQVLERAA

STLNVSVSIHRFVPGSTTTPVSTVTAALEDFITFVDKMSLMPDFTGIRGHFEILPVEVAASQLATA
LYDSKAAKILDFYHHECPIRIEIADMQAFLEARRGDKGLPRLPLLKFVGRMKALGLQYFVTSQTLR
MEGATEDGGMEILESRR
>g1996
MEYIDNGVVKPSVALGAIGTLFLLYLLGNVIYALYFHPLRHIPGPWVNALSMIPYARHMLAGTTAE
NSLRLHEKYGEVVRISPNEVSFISGETAFPDIYVMRISNWKIEGPSKYAKGMQYRFSGKLTESYCL
LQDPAWYVKPSNGVASILQANDDDHARGRRVLAHAFSNTAVTAQEPLVQRFVDQFIDKLKEAASSG
EGAVDMVQWYTWITFDIIADLTFGEPFGCLQNRASHEIIKLLEESARALRLLYILAHYPWLKYLGK
LIIDKDAVAKRKQWLVWVAAQVKRRMERETTRPDFMTLILANNRDKGAKLSQAEIDSNSFFLMNAG
SETTATALSGVTYHLLINPTVLRKLQDEIRGKFATYDEITLTAVNDSPYLIATLSEGLRTFPPAAA
GFGRVCPKGGESVSGYYIPEGTVVSVSHHAAYRSERNFKNPNAFVPERWMGDPEYRNDKRDACQPF
NFGPRGCLGNNLAHAEMRLILAKFVWSFDMELEEKSRDWLDRXRXMRFWMKPELAVRLTEVERG
>g1997
MSPENTVTADVNAAVVKNEEKYIPTIDLRAYFEPESPTSKAELCEQVRRACLDHGFFQVVGHGVPV
ESQRRMLAACKTFFDQPKEKKVRLSLHKYSWRRGYEAPGQQQANDPHQGDLLPDQKEGFFVGNELP
LHDVGFGKGPNIWPPDLADSEFREPVMEYFAHMKQVGLKVMEVLAVSLGYKPDVLDTFTRDIALFL
KLLKYPPHTFTDKRQFGAGQHTDYGGLTILLQQPGKDGLEVWHAATHQWIELPALEDKFVINLGDL
IQRWTGGVYKSTLHRVINKVGGERYAVPAFWHGDFSATNPFDSNDPNPETVQEFIRKKFYKGYGIP
AETVVT
>g1998
MASIPSLPATLSPALTGRDAIADVLYRLVLGLDTNNKELFDSAFVPNSTFAINGRVSEGLPAIHTD
CFEPISKLDTTHFITNVRTTIDESGSKAAVTASALAQHXXGGXGXEPSQTRLLAGSLYYADLVKDE
TSGLWKVKDFKMQSSWAEGDWGVLTGN
>g1999
MIFTSRAVLACYLFLCAATGVLSRPLTRRQQAISDIDILQFALTLEHLENTFYKKALSTFSEVDFI
AAGFDEAFVKNLGFVAHDEEQHVVFLEAAITQAGATPVASCQYNFPITDVLSFVNLATVLEGVGVS
AYLGAATAISSKSILNVAAAITVSEGLHLAVQRASIFDVVSANIAGTPLSPNAIFTIASAFITSCP
SSNAALPFTAFPSLSVNGVSNSAQSLAANTVATLSMTGGNGTAIPSSAFVTFISGIDIISVPAQVS
NGAISVTVPQQVSGQTFGLLTSAPMNGTGAIFDDSKVIAGPAIMEIQPASPTIDFSIL
>g2000
MTAESPELSSIRSTDEDKPVDVEATAEIGNVTEKKDPNLVEYDGPDDPGNPQNFSTVKKVYIAGNL
AMLTCMSTFASSVMSPGVSDIAIEFGVSAEVAILSTALFVLGFAVGPMAFGPASEVVGRKYPMTVG
VFVFAIFAIPVAVAKNAATIFVCRFIAGVFGAAPLAISGGTLADIFDPISRGIAVAGFASATFLGP
VLGPIVGGFITMSHLGWRWTQWLTLILALFFLLVYFFTVPETYAPVLLTRRAKKLRRETGNMALHS
LTSERKIKISDMGRIYVLKPWIMLIQEPILALITLYLGFIYGFLYLCFEAFPITFQEKRGWNKGVG
ALPFLAITVGVLVGVALIIFHTKTRVQRQMNERGAVAPEERLVPMMLGSFLLPIGMFWFAXTIQGT
SNPHITWVPQVISGAFLGCGILLIFMQGLNYLIDVYRMNANSAISANAMFRGLLGGGFPMFATYMF
NNLGVPWAMTLLALLCTTMVPVPFLFYIYGERIRKWSKFSPY
>g2001
MITRDSIPLKCEVVIPNPDTPVRFVLDRWSDIGLNRPALVVVPDTEQDIIDTVLYAKNKGLELIPG
GGGHGPFVSITEKTLYLDLKRFDKVVLDTSQEQAKISFGGGTLTRRVIDACTSKGFYTTWTNSYGV
GMVGSVLGGGSQPLNGLHGMIMDQLISIRLITAAGEVLELDSSSEGEELALFHALCGAGHGLGVVT
SMTLKAFPLAALRMKNDEVWCRRVTFPADAINLAAEKYMQLLPPKPSQYVLLMFAREPVTSTGPGA
PIIILSSLYYGPQEEGEQASKPLFDDAVVNNTNIHPETILTPLSQMNEAWAPFSVHGEYKDISSAW
LRTTPVETMKASFQQYVEFTDHYKDATRSTIVHATHNPKKHVAISETPEGQAKYFESRDRGTCMLV
MPWTNNPETVEPMRAFAKKIKALYRKAQPDLDVPRTFPNNMQSDTPLEELHTVDRIKELKRIKKLW
DESGVFWSPYGDNRK
>g2002
MPSATHILPNHQTAIVAEGPGQLRVRHDAPVPALEPGVALVKTAAVAINPVDAKMVDYSPVPGAIH
GYDFAGTVVALGAGTTHIQIGDRVAGFVHGMSKTRPDIGAFAEYVSSPANLLLRLPADMAFEDAAS
LGLGLFTAGLGLFRELQVPASIDPVNPGPEDHSRADFVLVAGGSTATGTRAIRLLKLAGLRPIATC
SKRNFDLVRRFGAEKVFDYSNPDCASEIRAFTGNTLAYALDCIAAAETTQLCYGAIGRAGGRYVTV

EPYRETVVSQRPLTIQPSWLLAVTIFGRKVEMDGEYARDVRPEDQQFAVKFTAQVQALLDQGKLDP
HPVKVMAGGWEGVIEGVDIIRKQAMSGQKLVYPV
>g2003
MVQLLKTTASILLGIAVLSRAHPGHDVKVEAAERAAALKGKRGLSGCTDNLKARGFEARNIARREL
AVSKLRHKLAAKRQVQLDTRDLSSLNTSHFSSLDVDFDTDPEVIFTSNATCILGPDVTQGPYYITG
EYIRENVIEDQAGVPLYLDIQIVDSSTCEPVPQSYIDIWHCNSTGVYSGVVANGNGNSADATNINN
TFLRGIQQSDDDGVVKFETLFPGHYTGRATHIHVLSHTVNETERLSNDTISGLYTAQASHVGQIFF
DQSLISEVENQSPYNENTQTLTENADDSILLQEADDIDPFVQYVYVNGADVTDGIFAWISLAIDSS
EDSSVTPAGYLTSSGGVANPNSGAGGPGGPQ
>g2004
MGTSITVVEVHQDRTDDTSVLSKPTPTVRPGFTRSESTLVAQELDEKIVKSSLDTKLYTETAEEHN
PEAQKESTEPEIGKLSSWKGALILLVTAGAMFMDNVFITSINIALSDVQEEFSIQSSELQWMISAY
TLAFGGCLLLSGVLSDRYGRKPMLIAGLIWLSIWTLAIGFTSSFIQMTVFRGLQGMGAAFTIPSAV
GIISSYFTGQDRTRALGIYAASGAVGFTAGLVLGGLLSSSLGWRYIFRLSVIVSALLAILGFIALP
STPIKKHSKSRMDFLGAFLSTAGLILLAFVLSGGGVYGWGKAFIVVLLILSVTLLVTFVVYESKIS
NPIMPLSLWKIRNFASLWVCGFAVNGSYTTVLYYTVLISQQVDKLSPLETAIRFIPSGVLGFVVTL
ATTRAIESFDGKYILITGLLFEVLAPLPSCLLSSSNFWVNVMPTSFLSITGISIAYITCSTLMLVN
APMNVKSLCGGMINTAYQIGGGVSLAICSAVVQGIDINKDHDVAQQYTTGLWCTSVIAGIGLIFAV
FFLKNDHSASMKSSSETSRAEILEHMA
>g2005
MNGFSGIPDTHPASAALNHDVSCCHENNRQRIAIIGYACRLPGDVSSPADLWELCTRKRSGWSPIP
NDRFASEAFHHPNPSKVGAFNPSGGYFLQDDISRFDAPFFNITAQEAISMDPQQRLLLECSYEAVE
SAGIPKESLAGRNVGVFVGGNFADYELHNVRDVETIPMYQATGCAPSLQSNRISYYFDFRGPSFTV
DTACSSSLVALNAAVQSLRSGESTEALVAGCRLNIVPDLFVSMSMSQLFNDEGKTYAFDNRATSGF
ARGEGAGVVLLKPLEDAIRDKDPIRAVIVNSGVSQDGRTQGITMPNGHAQEELIRRVYNEAHINPE
DCGFVEMHGTGTKVGDPIEATAVHAALGDGRSPKNPLYIGSVKSNVGHLEGASGVISLIKAAMMLD
HDLLLPNADFKKANDNIPLNEWNMKVVTSTRPWPRGKRFISVSNYGFGGTNAHVVLEKAPLNTNTP
DTLSSDIDDTHMYNDPKHKLIFISANDKDSLQQRIKDFGIYFEQRPEVFEKLLFGNFAFTVGSKLS
HLSYRIALPACSLDDLGIRLAQLKVNASKVLGDPKIGYVFTGQGAQWAQMGCALIDEYPIFRAVIQ
QADGYLRQLHAEWSLEAEIKKDPADSRIDSPELSQPACTAIQIALVTLLESWGIRPKYVVGHSSGE
IAASYAAGVYDLHSAMALSYYRGQMTRLLKETYPSLRGAMIAVGTGASIIRPMLKMLRKGYATVAC
INSPTSVTVSGDLSAIDELESILQTEQIFNRRLKIDVAYHSDHMKKISDAYFDSIAHIKRKSTLQS
DVIFISSVLGRVADSTTFNASYWVQNLTSPVLFPDTLEQMCKQDGGTPNLLIEVGPHAALKGPIGD
TLKHLGITSAKIAYAPTIVRKEKAVETLMNTAAAAYVRGANLDPYAINFPCTGARNYSFLTDLPRY
PWQHHTRYWHEPRIAKKHQIRDGARNDVLGVMANYSNDMEPTWRNIVRLDDIPWLRDHKMQGMVVY
PLAGYLAMAIEAAARRAHHRNITFNAFEFREVVVESALVLSDDADVETTISLRPYDEGTRGASDVW
DEFKIHSWSSQRGWIKHCHGLVGVRNSFHGDASNISGSSLKNKSHIQNRKSKIISSSTNHISEDRL
YQVLTDLGADYGPTFKGLENCYADSHHSHADLFIRDTTALMPKNYQSPLFAHPAFLDGLLHLVWPI
LGHGRMTLETLYMPTLIKHAMISSEIPFAAGQHVRAFGTGNPDLPSPEPTHFDLFAVAEDNTSDPL
IVLDGLVMTPIRDAGLGAGSEVRKLCYKLEWQAWEKKSHASNGHAQPADMSNGRSHELTNGHSHID
VQNGQNGVNGTNHSLANGSNCHHQTNGTSITHDNRRDTSNELTIRDDRVVIVRFSRQRDTDPIATE
IAKDLGQTSYRNLSAAELTTQYTDSNVIILQSPDASLRDATAEEFETIRKILLTAANILWVYPNSS
PDAQMTVGMTRSVRSETMAKIVTLGLHSEDPIIAASTVSKVMDAIWPTDGAEPCKESEFKSTDSQL
LVLRTVEDIHSNAFIHNENSEMSLSVQPFHQSGRRLKLKIAQPGSLDTIYFTDDDVAELGDESVEI
EVQATGINFKDVVVSMGQLIQPYIGVECSGTVTSVGKNVTDVFPGQRVMAMPEGAYSTYARCPSTS
IATIPDSMSMEEAATIPIIFCTAYYGLFDLGRLSAGERVLIHAGAGGVGQAAIQLAQMVGADIFVT
VGSQEKKTFLMERYNIPQDRILYSRDNSFGAAIRRATSGEGVDVVLNSLAGDLLRESWDCLAPFGR
FIEIGKADITKNSRLEMLKFEHNVTFASVDLTKVAKFRPRLMKRLLNDVCRLLGEGSIKPVYPITK
YSISEVEAGFRALQTGKNMGKSVVVPNADDQVKAVSLKTSSNLLRHDATYLIIGGTGGLGRSMAKW
MSTKGARNIVLVSRSGSVSDKLESLIHELASHGTQIFVKACDVSSRADVERLVDEELVHLPPVRGL
VHGAMVLRDMLFENMTLEDFKSVSACKVDGAWNLHHCLAQSSLDFFIALSSVAGVVGNRGQAAYSA
ANVFLDGFMEYRRSQGLPGKSIDLAAVTEVGYLADSDPSRQQEVAKNIGGATINESEVLALLALAI

TSDLNKSSQSQFVTGLEATDSLDSFWLHDPKFSSIREAVESVMNESQKDGKELPLRIAVQSASTRA
DAIHVCYEALAAKLAAVLGMSPEDMDVSTRVSSLGLDSLVAIEIRNWIAREADANVQVLELLSSES
LIKLAELILAKSKLSVPK
>g2006
MQFLCLHGAIGNIDNISIQLDPLRKELESDQAATFHFINAPVSVEPPEGFSEYFGPGPHYRWMDDE
GAGEESMINRIRQLPTGRNPEDVMRSLATSEKTWKNRETVMQYLYDTLEAHPEVEGIVGYSEGSAM
AATLILDEVHRLEKEGRPRRIKCAVFFTGWPPVSPDNKLVLSDETEIVLDVPTLHIVGANDPYRYG
ALALYNVCDADTANMFDTGKXHTIPRTGKVISELGDAVRELISCTEE
>g2007
MTITCFNLWLLALATFVIPSIAQTVDPTSCASGVHLLLLRGEGTGDDLNVLDSIQSVVLEQIPGST
SLGLPYQHGDSDKQFAAYNGSLLLQQYIAEYASSCPSSKIAILGYSLGAVATMDAICGVSSLLVTY
NVNALDPQYAQNVIAVVAYGDETYVPFQPWNVGNCTIGAGIYPRLDPAACEPFASSLQSYCDYGDE
QCCSVFPLDDNAAHHTYFTKYNQDVVTFIQSRL
>g2008
MPASRPVRTAVIGLSSSAVTSWAAAAHLPVLVGARNRDLFQITALCNSSVDAAKSAIAQYKLDSST
VKAYGSPADLAADPDVELVLCNTRVDKHYETILPSIQAGKSVYIEWPIASNLAHIEELVATYRARR
NIDQVAAVGLQGRFAPPVVKIKEVLQSGKVGKLVSTEVRFFGGTKDRQTLTSGLKYFTQREIGGNA
ITIGFGHVIDYVQSVVGDVVPGTDHVHFGLQHPEVGLRDPESGNIIDTVRSDVPDLLSLHGTLPES
DFTSRKASLVAYFARGQPYPGDPSLTWTLNCEKGTIRLVSPSGIALHANAYESPVTISVHQYQTDK
VEQGEWSWSEAQLDVPILSRSVQTCLVSVAEKNQKGYVDLEDAAVRARQISRWLDSFSADI
>g2009
MFDSAFDCVVLDGTVVTAADVGRYDIAIKDGKIALLAPARSLSKVKATRIIDAQGAYVMPGGVDAH
VHLSEPQLFGKGRPADDFTSGEDXSRSAIAGGTTTIIAFAPQDRSDPSILNALDEAQQKARDAAYC
DYSLHLLISNPTEQALEEFAVLRERGVSSIKIYMTYEVLQLRDNQILDVLLQARKDLITTMIHAEN
GDMLTWMTEQLERRRLLAPKYHATSRPQILESEATNRAIALSQLVETPILIVHVSSPLAANNIRTA
QTSGLPVFAETCPQYMFLTRKALDQPGFEGAKCVCSPPPRDGEADLESIWRGIENGTFTILSSDHC
PFIYDDNVDGKKSAISEDAPLGRFRYIPNGCPGVETRLALVLSADRLSPQKFVEVTSTNPAKLYGL
YPQKGALIPGLSDADLTIWYPPSSLEPFPVTNSALHHNVDYIPYEGQIVSNWPRYTLVRGEVVWDR
DNGGIVGRKGYGQFVKRGPSAFCREQPEWDVERY
>g2010
MALSHLHIRTYENTSPPLSEKQKPIQDGSLTVIPILQTENSVFGAEVSGVDWTNPVPAETVSQLIA
LQDKYGVLIFRKTNLDNARHIAFSQQLGEKLEVNPFYYGRENDRLGEPFLFDVGNKGSEKKDTPVI
YLLRIKQDSTYHQQRSKYSILLSHGNPVKGGSWTHFADTRRAYADLPESKKEELENLIVEHDLWHS
RKLASPIVYGNPLPHELAAKPPAYHRLVQTAPDGRQTLYLAAHAKLILGRSFEESQKLLWELIDHC
TQPKYVFSMEWLSGGDMVWWDNRQSMHRANPYTETMTARDVRRSTIIDDGPLAYGVAYEERLAAA
>g2011
MVPVCAGSLGEAVHLSYDERIALIQFIRATLDEAGLTSTPIVAGVGGLSTRETIELSKAAAKAGAD
AGMVILPAYYAASLNTDSQQVVQYYIDICKESPIPLLLYNFPANAGGQDMPSAVISEIINKAPNLC
GVKLTCGGSIAKLVRLTAEIEGNPKINEARAYPFLLLDGLIADLTPWMQCGGHGTVSGIPNFAPAA
SMRLWELLNIESLTAEEATECKRLQAILSNADVAAVPGGIRAMKYALNKLHGYGVAPRKPLLPLKK
LEGEAFMDILREMIDLEAEYTQK
>g2012
MISEKESEIGVQDGAKTPVSTGTVKETYNVDIATAADHALQRKLKPRHMQMITIGGVIGTGLFLGT
GSNLENGGPAGLLISYCIMASLLFSVMVALGEMVSQFPIPGGQFALADRFVSRELGFAMGILYWYN
FQNSYIIVLPAEISAAAVLVSYWTPAGDVNATCTTGICNNALWVALMLLIVWAVNAAGTRFFGEME
FWFCSIKVITIVGLIITGIIITAGGGPNHESIGFRYWNETGGFVQYEGIGGAKGRFLGFFSVLIQA
AFAFIGTEITAIASAETANPRKNVPRAIKGVWIRLVLFYVCSAFMVGLLVSPSDPSLNLSSTAAKS
PFVIAIKNAGIPALPSIINAALLTSAWSSGCADLFVSSRTLYGLAARGHAPKIFLTTRKDGLPWIS
VIFCGAFSLLSFMAASKGQAGTVFGYFSNMTAICGMISWICILWTSLRWHKGLKAHGIDRKTLAYT
APLQPYLSYYGMFVCIMVMIFGGFGSFIHTFNTSSFITTYFPIPFFAVLFFAYKFWNKSKMISYAD
MDFTTGSSMDIPEEPAPQTLWKKIAERI
>g2013

MSSAVMEHKDEVDRTEKSDVLHSEDFTKPDLMNDAFDGENREHEMGVWEAAKSHPWACLWAFTMCF
TIVMESFDMFLNSNFVAQKTFQRTYGVLTDAATDTYAIPTKWQSSLFQAGQCGAFVGVFLAGPITN
RIGYRWTTILGLVLMNATIFISFFADSLTVLVIGQAFEGVPWGLFIANSPAYASEIVPLPLRSAVT
ATLQMSWSIGQIIVSGATYATNKRTDHWAWRLPLALQWLFPTPLLVLVFLAPESPWWLIRRGRKEE
AFRSVERLGSKLEAAPQSFAMIERTVKIEEQLGGAPTLLDLFKGTDLRRTTIICLIYASQNFAGNL
IANQATFFFEQAGISSDGAFKLSLVNSCLQFVANACSWFLSSWFGRRTIYLYGTAVNVTLLMILGI
VASIPQNNNTNLTQAALGILISFVFAGTMGPISYTIVAETSSVRLRALSTGVGRAAYYVAEIPMIY
LSSRMLNTTGWNLAGKCGYVWGGTAAFCWVMAYFFLPELKHRSYRESDILFKRKVSARKFKSTVIG
VQENE
>g2014
MWLVHEAIRSDIPRELDPETIYARLMTMNFAGIHTSTFTTVNMLLDSLSRPDVFNAMKEEINMEYQ
RSGCEWNKPAVDNLRFTDNVLRESMRLSGPIVRLIREINAENGVQLNGVSLPKGTKIAADMHNMHR
DEEIYKDANTFDPLRLNPCTGQPMPPAVETSENFLPFGHGRHPCPGRYFASHEIKLIIAIIVLKYD
VKFLEKRPPNMVHAFFRFRTRKFLNSDLSRCYNRVQYQVRYILDTATF
>g2015
METMFMSSASSKNWDRYLQFALLAPGALFILYLLYNELLRFRLRVPNLPGPRGFPLVGSLPSLRGK
ITSDELRKWARRYGDVFQIQLGNSTIVIVNTTVAAEALFIRQREATNSRPIFYVLHKKVQQNGKVT
SIGTSPWDDSCKRRRKVAASALNRVLNKESRAYLLDILRDCDNGNTALDIMLPARKFAINLSLTVS
YGTRIEDMKEFSTNDVLKEIIYIEEEISRLRETSSNYDNYIPLLRPIKSLASAIGLVNSEHLASIG
KRRHAYHAKLQARLRDDIASGTDQPCIQGNVLRDPESKGLSEGELLSISLSMMAGADTSKRTLMWA
CLLLAHRPDLQQRAYQAIKETDQTLLTNPDVAHSRVAYIEALTKEIGRYFIVLRLALPKATHSEVR
WNGAVIPAGTMVFLNSWACSRDPAVFPDPDTFAPERWLQGENQSHQHQYAFGIGGRMCVASQLATK
ALYTVFLHLIAHFELFPAEDSPNSDAADPLKGLLEKENSRVTPSIRHVRFVPRDRDMTRKMLAEKS
>g2016
MTDLVADRPLSIPEELESNSNQQYETALIVQPRRRISGIWNWRYTLGIAGLAVLFSPLTSVVSWLI
PGEIPIDPTDYALRTRRVLQSSPLIDGHNDLPWLLRVELQNRIYNGRFDPTQRLLGHTDINRMKQG
LMGGQFWSVHVHCDKAQKHFDDPSWLVRDTLEQIDVAKRFIAEYPDVFTYCTTPSCVRTAFKSGKI
SSMLGIEGGHQLGGSIASIRQAYELGVRYITTTHNCDNAWATSASTVAAGGRDQGLTTFGAAYVQE
MNRLGMMLDLSHVSHQTMRDVLSITRAPIIFSHSGAFAITPHLRNVPDDVLRSVRRNGGIVMAVFV
NRFLNMEEPSAVTIHDVVDHIWHLADVTGWEHVGLGSDFSGTPHTPAGLEDVSKFPDLVELLMARG
ATDDQIRLLVGENLLRVWADIERRGREIQAEGSRPIEDEWEGRKWHTHYRSSPYMYRSTRERAIRE
GWGEPNQFNVGKTGKHEQIFKADDDI
>g2017
MDDNNDDEKHVVVNESSQASETVLRRRLLRKTDLLVLPGLCIAYFTNTLDRANLGNAKTDGLEKDL
GLVGNQYSLLLVLFYIPYALFNVPWTLAAKRLNPSVIIPLAICVWGVCTMASTAATNFGGIMATRI
VMGAAEAAYKPCEVYYLTLFYTRNEIGVRVSIIGQMGFIAGAVSGLISWSVFRWNGGLYGWQYLFL
IEGSITIAGAIFLFLFAPRSVQKSKWYSQGEKELAERRLAEDTEERDDRFRWNDAKAQLLHWPTWA
FAFLALMYGVGVASSSNFLPTLIKRLTNNTVKANLYTIGPNLVASVIQISATWASDRLQQRSIIAS
CTIVISLLGWILLGTLDLDSRPGVGYFLTFLTTGFTFIPSNIIPVWLASNVPTTTGRAVALGLNYM
AMNIAGIVSSVSFRTQDAPVYVPALITCGVTQGVFMLASLGLRQYYVHLNGKLDRGDIPHAPGMER
RPDYRYAI
>g2018
MFPITGGGGFTPGIVFNPSAKGVAYARTDIGGPYRLNADDTWTPLLDSVNNSNWHNWGIDALATDP
VDTERLYLAVGMYTNEWDPNPGSILRSTDMGNTWQETVLPFKVGGNMPGRGVGERLAVDPNDNSII
YFGARSGHGLWKSTNYGVTWSNVTSFTWTGTYFQNSSSSYTSDPVGIAWVTFDTTSGTKGSPTPRI
FVGVVDTGKSVFKSEDGGVTWAWVSGEPLYGFLPHKGILSPQEKTLYISYSNGAGPYDGTNGTVHK
YNISTGVWTDISPTPMASTYYGYGGLAVDLQVPGTVMVAALNCWWPDELIWRSLDSGATWSPIWAW
NGYPNIDYYYSYDISNAPWLQDNTSTAQFPVRVGWMVEALAIDPFDSNHWLYGTGETIYGGHDLLK
WDSSHNITLKSLAVGIEEMAVLGLIAPSGGPPLLSAVGDDGGFYHVDLNTAPSQYFHDPTYSSTNG
IDYAGNKPSYIVRAGSSSTLPTVSLSTDYGLTWSADYAASSSTVPGAVALSADGDTVLLMPTSGSP
LISKYTSSFSTVSGLPPLAVIASDKSNNTVFYGGYAGSFYTSTDGGVTFTKQVSLGSSSAVNAIRA
HPTIAGDVWASTDTGLWHSTNSGSSFIQIGSGCTAGWSFGLGKAANTTSYPVIFGFFTVDGVTALF

KTEDKGVNWQIISDAAHGFGASSANVGNMVRRAQWQSTIVNSIWDYDIVNRYHSINVVIQIDFHDY
NYI
>g2019
MTLKYLITGATGGLGSRVLAYLVANVPRSEYAATSSQEANRALFEKNGIAFRVANYDDPATLDTAF
NDVENLFFVSTENFDTAGRIRQHRNVVEAAKRTGVKHVWYTSLAFGGLRSDSKIGVQRAHLETERI
LQESGVTYTSIREGLYAEIFPIFLQWYPDTKTVYTSDSTSMTFTSRDELGEANGKLLIRGGYENEI
ILLTANEALHAADLVKIINETTNRQVELTLTTPEKYVEFRKANDESTKPDSYWQQILNWLDGFAKG
DTEMSHPLMGELLGREPKSGSQAIRELLKENPNATWPETEKGVGWDIAKQQIFPYEAFWCV
>g2020
MKSWSGKQIASVANLKQRKIYLWTGSADTTVGPNVMNQLKTQLDNFDNSANVSYVTTSGAVHTFPT
DFNGGGDNSCSLSTSPYISNCNYDGAGAALEWIYGSLNARNTGTLSGSVLSFDQSASYGAPGMDTA
GYLYVPQSCASGATVCSLHVALHGCLQSYSSIGSHFIQNTGYNKWADTNNMIVLYPQAIPDYTIHT
IWNGGVLSNPNGCWDWVGWYGSNADQIGGVQMAAIVSQVEQIVSGFHS
>g2021
MASSPTHSDTEKPSTSAIENGIHDPLHSRERENKLLRKMDIRLIPMLALLYLLAFLDRGNIGNAKI
EGMLTDLHMTGQQYSLAHIDGNRTELSWPADMPSFFGRGCIYHTEAGLYPGIAYYITLWYPRDRAQ
YRQALFFSAASVAGAFSGLLAYGIAKMNGVGGYAGWRWIFILEGLLTVVVSLIAPFAIHDFPETAK
FLTEEERELVITMLRNQTTNEGYVNSDAIQEESKFRARYVIDALTDWQIYLGLFMYWGITCPLYGI
SFFLPSIIKDLGYTSSTAQLLTVPIYITAAIVAIGAAWLSDRRKQRSPFILFFMSLIAIGFIIVIA
SSGRGVPGVVYLGVFIAVVGIYPAFPGNVTWIAVNLAGDYKRAAGMALHIGLGNMAGAMASNFYRG
QDAPKYILGHALELGFCIAGIIAVLILRRSYQTINRRRDQMDVSQYDASQMAQMGDRSPLFSKRKY
DVLRDVVETGSTSPEALATPETPVDHRRSVENRNMYSMPSSMERQNPPSIADDTHEWLREIYEGIF
EDVFGSWLGNYSCPYIFRDNGRQGPQQLALSVSLSRLCQECDYWMKRVQGQGINLPSEGGESPGEI
DIDRQINHSLSCAVSAFSARWLTLKGLSTSSEASVMDTVESLWREVRRDILRVINRPCYRSALTLF
LFALTPIPARVSEEEEKDGIPAQFCVQVALQQVLTLRAQQKSLEFNGSKVSSISSSALSNTTTSPA
PVTRDFLSIESMIYWAAMTFDTSSSLTLNTKSLLSPGLSPGLDQEPSWRLVRTCTNVFHEETEAWR
AQGIHITEERANQIIASAASWKLFVWKAAALVKEALREGREEETVQMAFDTAVDAINQYSLTYHDL
LAACERRIQFFGHKTKLRWYELLLHYNLSILIMLDAVEIAGRTDLLEKLKVVKADAEGTLFNCLIF
GLSNHYFLPRENRPMREAQYSIQRDEDTEKAVAHSTRKVPLIAVDPYPHHVVAGVRILWKAVERDL
ENGWLDHGVAEHMQDTMLNALRLLPQASKSVRTARQQAEASFLRYRYDLNS
>g2022
MARSKLLQEAVVILGAGTQGRRLAYMWSSTGKPVHLIDRQDKQLSEGVEYVQQLRASPTAISKNWG
KITTSSPHGLSTALKNAWLAIECVPENIELKRTVIGELDALAPEQTIIASNSSSYVISEIIETLSL
NHSSRVLSAHCYWPPETPAIEIMGHDETDPSIIEFMLERCKGHGFSPYHVKSTSIGYIYNRIWAAI
KRETLLALHEGVATPKEIDAIFKDVLKTPRGPCELMDVVGLDVVLDIEKHYADSRKGLPIEPREYL
HTMIENGKLGLKNGRGFYDYGS
>g2023
MKPFLRVTVVGAGLSGLTSAIGLQRSGHKVTILEKASELKEIGAGINISPNATKVFREWGLLEQLE
KICIKPVLAEMHSWSSGQPLSATELHPAFEIEYSTPYLVAHRADFQKFLADEARKLGVSIRLGCAV
EKIDIPLSTIVLGNGELITSDLILGADGERSIVRSNLDGNWNLMKDSGYDVYRFTVPRSRVLENPD
LLPLIEPYSIRMWVGPGAHMILYPLGTHDALNVVFTRQHDPERPMIIYPQPLTIEEIQSDFKGWDP
RLHKIMEMAPHSGKRTMLYADECTRWTHETGHLALIGDAAHAAFPYMAQGAAMGIEDAGVLGTLLE
YIRDLRDLPKLLRTYERIRKPRALEVRRRSEALKDVYTLPDGPQQEKRDRALLHEKPRGEWANFLS
DPVMRPYIYGYDVVCMTRKAILLSEDE
>g2024
MPTIAILPRIYWAVRSYTFSISRATKAPDGYQKSVILINGQFPGPLIEANWGDTIEVTVYNDIKDT
AEGVTLHWHGQPQRLSPGEDGVPGVSQCPIPPGSSLTYRFRAEYFGTGWYHSHMSGQYTDGLYGPM
VIYGPTQVPYDIDLGPIMLSDYSHTSYYDILKSAYLFPPVFPTIDNNLINGKGIFECSLANETICT
PWAGLSKFAFQAGKVHRLRLINTGGEGSQKFSIDGHILKVIANDYVSVEPYETDVVTLGIGQRTDV
LVTASGAANGAYWMRSDIDEDCLNLTSVNSHALAAVYYPHSSTNAVPNTTATTWSSNNCQNDPLNQ
TIPYYPTTPPSQPTTTAYVNITVGQNATGEFIFFINGTSFEVDYKCIPLSLAPYLKST
>g2025

MRSHGIDDYLMVGTWLLWIATIAVTTVYTENGGARHVYYIAPEKVPVVIRDSLIAQALGVLTPALG
KMSVAAVMVRLLGRSTNVWKWQLWCLHIIMAIYMVMSILVVIFVFVQCNPTAALWDTTIKNARCWK
KSIYIDYIIAQSSVGTFIDFLLALAPSYMIRRLNMSLKDKMGLCVLFGLGTFAGVCAAMKTVKLKE
FANVIDFTWNTYEVFLWAQAEVFIVIICGSVPTLKPLYQHMRRGGRKQFGPPGSRYQYRSTSQRMA
NSFGSGANHSTASRHGGSSLIAKVSDDTDGEQLVTYPPKTIDIQVSGAAFIPRSSDHNSPRYPFGN
NGSEHDEV
>g2026
MSSIEQLTTLLNEAAKSLPAQGPINDEKRNAFYAAYENLRNAIETPIDAAVRVLFGGYDAVALRVA
MDTQIFNQLAMAEGQSKSISELAKPSGADPLLLNRFIRVLAASGLVKETGEGEYAAVPLTGAFSDM
SPLSKSAFHILSNANSLAKLPQYLQKTNYQNPNDSWDGPFQFGEPTNLQYFEWIEQDPKHQEAFNT
HMTLQRIERGPNWFDFYPVQERLLDTDKDTSKKFLVDLGGGVGHDLAAFTEKYPDLAPRLVLEDLG
HVLADVDKQEFKLHPQIERVPTDLFKEQTVKGAKVYYLRTVLHDWPDKQAKQILSNIHEAMTDDSV
LILNENALPDENVAGFQARVDILMMSILSGIDRTYKQFHTLLEESGFEVLGTYRPQTLSPGAGTIF
EAVKKH
>g2027
MSLTDSEDSDAVWISPDDIRDFNEDHILPLPPDELENIRTWLQPTPYDLERSEFSRHLASYLQGTG
QWLLSTRNYKEWHQGVDNGLLWIKGIPGSGKSVMVASIIQQLREEKVPVLYFFFRQIINANHEPVA
ALRDWLCQILPFSPPLQVRMRDEYLQEKRSIDSLGMSDLWKDLKFAMSTFPKVYCVTDALDEMDQG
NDDFLHALAKLGRWRPANVKVLITSRPVIAVESPLRHLGIPHLQLQDRLVDMDIAAYVRYRLRNSS
IPHEHWNLITGAVPGRANGLFLYAKLAMDAFVDRSADVELVLEKLPADLNVMYNDLLCDHAKRSNV
PDEFQLLVLQFVTHATRPLRLLELAEMTKTSYVPFRNYTLKEIKDLVRAACGPLLQVLHDETVSVV
HHSFTEFLKGLTRSKHLNDAGYPILEVGPTNKRLAIACMDYLTSGCLDEIEVKMRSEANDLYGPKK
SEQSDSRLRFPFLEYAATNWYIHVRRAVLAGADMSLFYLSLDNFFANKQRFLSWLDIDWPENTIQG
LTPLHAAARIGLTEYVRHLLQERDAQPNVKSHRGDPPLYWAALSGHADVAQVLIDNGADPDGEANQ
GYKPLHKAASCNQADVVKVLLVVGVSPLTPKTKETPGRRCGNAPTSIGHTPWMYACKNGGIETLAE
FLPYIKSSEQMLRGLFWMTESGHAVCVELILQQPSLDVNSKYLGKTALFQACSEGHIDAINVLLKV
GADPNVLCDYSVDAVDDMHFAEMHIRSKDANQSEQPRGYTALHALCGINSRMPRIRSAGDRALGVR
LLLEAGANVHLKSPDGKTALHFACSNEIKVVRLLLDAGADPTVETNDGSTIIHTDGSTDKELLPLL
LGSGRVDVNELMTKGRENPLFLRLQGHYPESIIELLKYKLDVNKARSDGNRPLHISFSSWSFRPKN
IVVDALLAAGADPNLQNAKGETPLHLLGHCAKLEIVSKLVNAGADLELRDMEGQTALFKNVMTDDL
ASDKVTLSNILIELGARLDTRDNKGRTLFHQVVLKRLGNLEYLMSRMDFDPSVVDDKGNTLFHEAA
SKKWQSEKLEIFIHLTKLGVDIDKPNNRGKTILHKMCARECRRSSWNPSVKTAFDYVLQECKILNP
RDINGIQPLHVAAAVSEVYVFKLLEAGAYLFGTTNEGLTVLHVAARARNPGVIGVVLRRLADLEDA
AFKAFINQQTVEGKTALHYACLAGRSESVDLLLDAGADPNLPGKDGYTPLRTCADFEVEQSRWNIV
VQRDDHRKGRFKAQSQAPRQAQRAISMLLHSHDLPTTVDDSGRLNWRSHSIKQESASTHLDGILNS
LVLHGADITGHESSLREAVHTAILHRRDYTAKCLLRLQSRFVPNMKLDDDGFVATRSRLESERSSL
RQENCNNNRQTNDANRRARRAFYLTKLLGLRQYEMVKETTSDMDVLDLEAFRMFGEISLLHTLANF
GLSDVLKSVCTRAVALKFDDHEWCNEAEAANRMNRLKIEPLIMVACNREIPNMAVLRFLVEEMGVD
VNATSRKIIFRDNGKRREFVPGKSVLHDLAEGNSWWNVHEALPYLMQKGADLNLRNEVGDTPLHIA
VEEKRYKGVFYKEAIEVLLEGGADANAVNYHGESSLSKAGTDTGLIKRLLSYGAKISPAAIFSAIE
LQQVELLEFFLAQGDFANLRQPASESSERNAVHVQSVVPTSEKYPLFHAAFYTVRDIRGREKDLSP
ARISMMTALLRHGANPYATFVKLHRVKNKSFKNDVKDESDAGVEWKSETCTIIHEILQSGRVVEPL
FDLPSLELETRDAKGQTLLLAVSRGPIKRLEELITRGSDVTSQDHEGRTIAHNLTQHQPTEENYKF
LKTLFSHHPRLIHMTDNAGDTPLHYVLKAKEIRLDYIDILVAHGANPLQPDSDGNTALHFFAQKPR
TYQSRIEQFQEMGVDINARNKMGDSPIFGYIAHGWLRAGGQSAYFYNKGHENHDDVHYLRYFQQIG
ADFFVRNHAGSSLLHVLAGRKLNVSPFMDKAEITVPIKNVVNWFEFLTGMGLDPMLEDAQQRTCLD
VAAACESEHILKLFQQKAVECD
>g2028
MDDSSNGAAMNGHAHFEPDNNVLHEPIAILGMGLRLPGRVHSPESLWELLVNKKETSGPIPPSRYN
TAGFYSASKRPGTIVVQRGHFLDDSDGLDRLDTSFFSMSKAEVEKMDPQQRMLLEVVWECMENSGQ
RDWRGSNTGVFVGTWGDDWLDLLAKDPQQTGGMLNVSGAGDFAISNRVSYEYNLHGPSMTIKTACA
SAMICLHEACQSLREGSCDAAIVAGTNLIITPTCTIAQTEAGVISPTGECRTFDASANGYARGEAI

NAILIKKLGDAIRDQDPIRAVIRGTAVNCDGRSAGISAPNPLAHERLIRRAYKVAGLTNNAADTPF
VEVHGTGTPSGDPLELQAIAGIFGGDQETYIGSVKANVGHGEGASGITSVIKAVMILEQGIIPPQV
NFFTPNPKIPFDEAHLVVPLEPTKWPAGRPERISVNSFGITGANAHVIIESAASHGIQARLNDSIE
DSTVPTPPPPRLLLFSANTPESLQRRASELQDYAAAHPERNKNLSYTLGCRRTHLVSRAFCITGTK
KIYSMSEKLKEVPKLNFVFTGQGAQWPTMGKDLVKNYPEFKDDLSHLGKVLARLPHPPSWDLITEM
LKPENESRLNEAEFAQPLCTAIQVALVNFLAKIGILPAAVVGHSSGEIAAAYASGAITADEAVTIA
YYRGRCALRSTRTGAMAAVGMGRAEATLYLEDGVSIACDNSPNSVTLSGDREALDTVIEQMKVDDQ
DVFVRLLKTDGMAYHSHHMLKLGSEYEQHLRPLLNTQTPAIPFFSSVTGVSTPATVFGAEYWRQNL
ESPVKFFPAVKALIRSQQATDHVFLEIGPHSALSGPLRQIFQNTASKGRLSYLPTLIRGKNSVESI
LETCGQLYLQTVDIDMKQLNPGAEVLTDLPVYPWQHDVSHWVENRALKEWRTRSFAPHELLGSRIL
EGNDFEPTWRNMLNLKNTLWLQDHKVFSDVVFPCAGYISMVGEAIRQLTMADDFSIRYLNIKTAMI
LNDDKTVEVITTFKKLKLSTASSSEWYEFSIYSHNGNTWTKHCDGQAKGGKNNEMAKPQVPENPAQ
LPREINFPYAIFTRIGLHYGPNFQALRSVSAMPGKKESVALLDPPPTTSSAYSLHPTTIDQCLQLL
GLASVDGRPYLFQNALLPTAVEQLYIQPASKEHAPMQAMAKAVTLPRNTGNVQGNITISKNDEVLL
SIQGCKLSIFEQQEQDQNSDARIAAARLNWRPDLDFIPIDSLMTARVKDTEKLRRIEIYSFLCMLE
IQERIRSCPSNAVHFEKFRCWIDQIIEEGHLGNNGIVTNSNDLVKLSGNDRLAMISLIREQLKDTE
FEPAAELVTRLLNNCVGVFNGETEILDVYLRDNGLTNLYDMNGDRIDSTEFFVTAGHTNPRMRVLE
IGAGTGGTTLIALQALTSLNGEPMYANYTFTDISSGFFSAAKERFFDYPGLEFKTLDISKDPLEQG
FELETYDLIIASNVIHATETLRTTLRNVRNLLNSRGRFFLQEHTPSVAKMVNFIMGPLPGWWLGEA
DQRPTEPIISPGRWDLELRSAGFSGIECLVHDDPDRIHHIGVNMIATPVRTTTNFRSATLLLRESQ
LDSESVKHVKESLSQKGYHIDTCFLGIQQPIVFQDIISLLEVDSPFISGFSSDEFESLRRLIGNLG
SSRLLWVMGSAQIGVQNPDYGLTLGFMRSIRAELAPVMATLEVDHLEHGDAEIIVKVFEKFQDTAS
SVNPEQEYALKNGIVYVGRYHWTQVSKELAESDDKSQKPLRLDVRRVRGLKEIVWTPYSPRPLSEK
DVLIIPACSGVSLEPGSGEGNSLLGLEGSGTVAAVGTQVRTLQVGDRVMGLGQSFLSSRVTVSAVQ
VTKIPDSLTFEDAATMPLSFCTAIYTIRKVANLQKGQSILIHSACGSIGLAAIQLSQIASAEIFCT
VDNDESAEYLINSFDIPRHHILYSGNTSFLPELMSMTANLGVDVVLNSLSGDLLHASWKCVAPRGK
MIELGKHNFVGRAQLDIDLFAGNRSFIGVDTMALQDLYEGLLSETIGLYADGHIQPIKPNSVFDSD
EIEKISTVIHHGVTEEKLLVKILPTVEALSVFPMQPELMLREDRTYLLVGGLGGLGQSISTYLVEH
GARHLLYLSRRAGESEKHQRFFRELEVQGCSVHVAKADVTNLEHVKAAVQEINLPIAGVFQMAMVL
NDSPFMSMTHDEWSTAIKPKVNGTCNLHEALRHTKLDFFVILGSVSGTFGLAHQANYSAANTFQDA
FVQYRHAQGLPASVLNIGAMADVGYVSENRGVEDYLRGAGMPFMSEADFFECLHLSICQQFPVEAG
FEATRGGPGIYNRSQLTLGIRSTKPMDDPSNRVLWKHDRRADIYRNIEAARFEDGTDRGDGGNEDK
IAAFIAQIQSNTSVLDQPEAVVFLTYEIGVNICNLMLQPLEELDVTKALVTLGVDSLVIVEIRNWL
RQRLQVEASTLEILNGGTIETLGRLAVERLKEKYQGNPKTE
>g2029
MMKVYQNVQFKTLDGIYLRGRLYAAAQKGPAVILCPGYNSVVDSFPETVPEEFQKAGITALLYDPR
NTGKSGGFPRNDIDPFKQVEDYSDAFCYLTTLPIVDTKAIVFWGISLSAGIALSAASLDKRIAAVI
AIAPVFKFLPVTEKDARRLKTKMIKDRESQLLHGNPPYVLPIAESLAEIPFNSHVNANRGDDEEQR
KRAEEQQMRQQQERWKEEAVEANQEFDEKLIHNPYGTTIQSYHRMFLFESIPLSMVKGVWPTPVMF
LTPEKDQISPPQHQIEVFESLQDPKRQVFAPGKEHVYVLEGSDVPFLMKLQVEFLWQAVRGRLKAA
T
>g2030
MEGINMLISEVEPIAIVGLSCRLPGGADTPEKLWDLVHEGRQCWQDVPSDRYNWKAFHHPDPDTKG
THNSRGGFFLDQNLAEFDAGFFGIPAAEAAAMDPQQRILLEVSYEAFENTGMSLESLRGSRTGVYV
ALVSRDYERQTYKDPYQIPKHHLTGCGDATACGRISYVFDLKGPCMSLDTGCSGGMVALHLACQAL
RLGESDAAIVGGTNLLLGPDMTIAMSALRMINDEGRCYPFDSRGAGYGRAEGVAALVLKRLKDAVK
DGDNVRAIIRNSGVNQDGKTNGILLPSSEAQGQLTAALYRQVGIHPREVSYVEAHGTGTQAGDAAE
FNSIKQVFVDMETQRDHPLFLGSIKANLGHSESTSGLAGVIKTVMALEKSVIPPVSGLEIVKPSLY
KMLESGDIILPKEPHQWPHSGRRLASVNSFGFGGTNAHVILESAPALSHNMNYANGWDEKNGNGSY
TRMRFSDVSENLISPADGLVNTHDDSHDRNKARVENISSPSLDKVEPQLFVISAKSRTSLEGAVRN
LKNWVSNHCATDGTVLNQLAKTLSSRRSQFKWRSSVIASSHLGLLSGLENVRTTKCSPKNQVVFLF
TGQGAQYPGMGRELLVLHTSFAQSLYRSQDILKSLGASWSLVDEILCEESKSRINSSELSQPATTA

IQIALVDMILETNVRPAMVLGHSSGEVAAAYAAGILNHKDALTISYYKGFVSGWCKETLPSKGAML
AVGLGEKEIMRYLQSCHSGRCVIGCVNSPSSLTLSGDELAIMEIQEMLDKDSVFNRRLKVDIAYHS
HHMKAISDRFLQCLDGLMVKPPQNSVRFFSSVTGSEEFLPLSAKYWVDNLISQVRFAPALETLTRM
HFESSSESLVLLEIGPHAALQGPIRQILTSAGPRSSKWLYSPTLVRNKDAHVAALEMVASLFESGI
KINIPANFSRPENYCLQLRLPVLLNLPPYSWDHSNQYWHEGRLSKEYRFRQHAPHDLLGLRLTGTS
TIEPIFRHILNVDDFPWLKEHIIDGFALYPGSAFLCMAIEAMRQVSKDRGEKSEISKYIFQEVSFS
KALVIPDSPASIEVLLSFKPSQSSSERLDITWEEFRVTSVTANGKWNEHCRGLIRADYRSNLLRDV
VGGAVGLDFKPSLARERLQAMSQDCSESVTTEAIYDEMRRNGIDYGDAFAIIQDLQLGDHQAFGKV
KVPEIGALTPSQYMQPHIIHPAVFDAFMHIALPLYHRHCSQGPVMLVSIAEVSISADILNKPGDDL
LVACRLTEAGRRQGSVEVSILQEDLQGNLKEVGHLLQENFCAIGEGESAENAPSSRSSSAGFPPPC
YHLEWVPASWVPKVHQKDHVMTMKRVSVFCFSDNAIAESLAKRVSSRLGVRWDGRSCAVVPQTPDS
MDPENIHIIFIDENSSMSTLKELFVALSNLKSVLWVNIATPKSPSVGRYVYALARSVQQEVEGYNG
VVLDYHLDASLEEELQLSDVVLQVLLRCFVDAIDPENPIDREYVYWKGDLLVSRLVLHNSSNEWLD
AKVNGKNVEKIAAFHADDNGIKLDFKTPGLLDSGLFVPIENSASDLDADEVLVRIYAHAVNQTDVL
IALGRGQSSDLMVGEFSGEILAVGSLSWDIYKPGDRVCGWGALPYANIARVQCHRVHRVGGSISFV
EAASIPLAFQTAAHALMNIAQLGPSQKILIHGAAGAIGQAAVSIAQYLGAEVYATVGSSEKQQLLM
EDKNIPGARIFSSMSNTFSDAILSQTAGRGVDVVLNCSSGDLNMESISCIADFGYLIDITKSRKPL
IFNSRARKNITVSSVDMELLAKRRPTQVQQMFAKVMALYEGGRLTPITPITTKSITDFANAFRHVQ
GQHHYGKTVLKCDKEVSVKQMARKSSPLRLSADGVYIVLGGDETLNHSLCMFLKDHGAISIVSVMF
FGNAIQADSNDWSAFERIRLDIGNETGVPIVLSSRLGLDRVSVMGVLFVEEIFKASKSFIPIILEY
CIRRDARQDSSSDLFVGLKKITRHVKNPVFSALNDKNADGESRDTKLSPTSISQRLADIETLDDLH
TMVMAALVEQLASLLALDPDDIQVRGAAIDIGLDSILAIEFKKWVVRTIQAPIQTSEILDAPTLTQ
LTQLIVQRSKLCQSLLLPRSRSEDFGRHYEATATPSPNGHPLLIPVSCSTDTNENEKGLTTDLLPA
LPIPDLKVIIERHLSYMRAFATDEEFRNTLEIAAEFQATGSIGKRLYDRLQAIKRADPDNWYHDLY
LRNQYLARKGGLAPYMSFFFTHPTAPMPGSQAARAAIVVSAVIEHKYQLDRKQVKTRYMNEQPLCM
DLSRYLFNACREPKAGLDVMHQYSRNDYFIVLRRGHVFKIDFQELDNFTQRERLESIFETILDASL
GNVDWLGLLTADDRRSWAKNREAFVNFSEANAAYIRTIEESAFLVCLDDGSPETAEERGRHFHFGD
GSNRWNDKPIEYVITANGVSGIVGDHTGLDAGTILDLNQKISEQIRCYQSPEPSQRSFARIPVILK
KITYTEVPSNIAANIEKVRSDYQKAIALREHRYPPSLPYGALFMKTHKIPANSGAQLLIQLAARYY
FGYINPCWETVLQSNFQKGRVELNQVVTTQVAAFINAAADDSITLSTCKRLFIEAARTHSSAVLSC
TRGNGSDRLLSILKEILEEGEEVPRLFQDPVYQRSRPRKFCSNCFTSGMAEDGCCLRDETGIWVHH
EVQPEQVKFSIVGPTGKTSEFYKSLVKAGERVKEILNA
>g2031
MAELSTSLQQFAWVLLWFSLLYTAYGIYLVVYRLCFHPLRHFPGPKLAAATYYYEVYYDWFNGPYK
GRNAWHLIELHQKYGPIIRRSPDELSVEDPDWFDVLFVAGRRDKWSRQGKDSNGSVQSTLSRTLHK
KRRGALTRFFSKRSILNLEPSIQAKVEKLSAGVEKNFLNTGAILNASVAFVALTLDTITDYCFDQS
FGCLDKSDFAPEWRKTFWDMFENIPLLKNWTFFANLLFLVPPVIVRMTNPSLTQFFILEKANRQKV
AEICKEWNEDQIKKAKLSEAGLDAPRKNALFKKKKRTIFYDIMDSAVLPPEEKTPQRMAEEAFGMI
VAGGDTTGRAMANLLYHLHANPEWLNRVRQELDSVMIFPSSPITLSELEILPVFSSCIKETLRISY
LVTDSIMLIEPEAPLIYKEWMIPPGTAVGMTLSHLHMDERIFPEPNTFNPSRWRNTKATGVDLEKY
FAPFSKGNRACLGVK
>g2032
MSSPAEESEEQTSGTPAEKKVVLDIEDKTPDNSSNNRLVLSFYFRIWQYSTPLDHVLRTCAIISAV
VCGAALPLMTLVFGNLIDNFNDWDAGKLSPSEFRDKVSKNALWFTYLFIGIFVLASFNTLCFRFTA
TRCVKALRRDYLRSILRQDLPFFDTSLPGTVAALLSNNADLVEVGLGEKLGVFVEGISQLGAAFIV
AFTRQWKLTLVVAATLPLTMLVVIIAVIFDTKIDSKILAIYSKAGGLAEEALSTIRIVTAFNAGGK
LRARYDVYLENAKQHGIKRGPIRGIMYGVQFAAMFCAYAFAWFYGIRLLAWREISSGGHLITVLTS
VLIGTQSLTIIAPSIGELTKTTAAAQELFQTIDKESKLDSMTSDGATPMEVTGHISLQNVTFAYPS
RPTVKVLDNLTIEFEAGKTIAIVGSSGTGKSTILALIARLFDPLAGTILLDGNPIDSLNIRWLRSH
IGFVQQEPFLFSDTIFTNVCHGFFGTAMDHLPEQKRRALVEKACEEAFAHKFIQNLPDNYDTQIGD
GDGRLSGGQKQRIAIARSIIRNPPVLLLDEATSALDPKAERIVQAALDNVSKARTTIIVAHKLSTV
QRADRIIVLSKGRLVEQGTHQELLVRKEAYFNLVNAQTRDEHRDLQHINHNTVSLKIEEAQNSDLI

PGENDVSVISVKSTSELPTPQIQGRSQQLSLVRCFAILLRGRIQLWPLFLFGLIASVGSGAVFPIQ
AIVFSRAVLIFQLPLPRLADHMIHEGTFWGIIYLVLAAAMLMCYAGLGFFFTVAASDMMSFYRSRY
FGAMINQDVGFFESEGQSAGVMTGWLSTDPQRVEDVISTTAGFLLITIVNVLGSCILALVVGWKLA
LVAIFGCLPPLFIVGYVKIRLELTTHARTTKMYLESARFATEAITAIRTVASLTLEEKVVKMYDER
LARTSPRFIKIAVTSAILFGLCESLYLATLGLIFWYGVKLLSQGEYDVQSFFTVFVAVIFGGQAAG
FLFGYTVNTTKAHAAANNIIHLLRSQPKINTSIASENTSPSDTNISIEFKAVRFNYPTRPKVPVLQ
GLSFKIKRGERVGIVGASGCGKTTVISLLERFYEICSGEILINGVPLQNLDVHHHRARIGLVSQST
TLYQGSIRDNVMVGVSPDLENASDEIMRKACKDANIHDFIESLPEGYDTDAGARGLSLSGGQRQRI
AIARALIREPQFLLFDEATSALDTENEIIVQEAINVASKGPGRTTISVAHRLSTIRRCDRILVLEA
GRVVEEGNHNELMAQRGQYYEMVRAQGLDREVIT
>g2033
MRSLHILAFGFGSLCAAAGTFSVPNVNATFSNSPRPFILQVDPNFIEDTRQRVAHARAPVFLNMTG
DGPSAENFTSVRDFWVNEYDWNKTQASINDKFQQFTTVXEPIINNVTYQVPLHFVHHRSNRSDAIP
LLFIHGWPGSFLEVGHIIGNLTNPPNASVPAFHVVAPSIPGFGFSPAPESPGFGPANAGRAFNELM
QQLGYSKYVIQGGDFGGVTLRYQAHLFPQQVVSALSNFWIIQPGDNDMQVFAEGKATSDEVAYINI
IETYINQWSGYRLIQSTEPLTIAYGMTDSPLGNALWIYSIMAKVIDPALTSWTPEEIITWSLMYYI
QGPYGGMRFYKEVQNDGGFATLNFGVLPQVEVPVAISQFPYDLTYRMPLEWAQRGGNVLKRTVHDH
GGHFAAYEVPDLLLNDIWSWFGDKELSGTEAFIH
>g2034
MRLSHLLQQILFSSYGLMMAFAALHDVVRDAPLGQLLRYFTNNRLFQYPEEHAGFELPDPWAHMLG
TTKLPETKQNGSATPSTVTTLPSDQNTISDTTSEKPDVESGHANEDKTSYSIAKVANDGTILVDWY
SDHDPDNPQNWSSLRRALIGLVICLYTFVVYLSSAIYSPSTEGVMERFGVSNLKATLGLAMYVLGY
GVGPLLFSPLAEIPRIGRNPVYIITFALFFILSFPTAFVNSFAGLIVLRFLQGFFGSPCLATGGAS
LGDMYGMIHMPLAMIAWVGAMSCGPSLGPLLSGFAVAAKGWRWSLYESIWVSAPVLILLFVFMPET
SAPNILLRRAERLRKFANSQRLKSQSEIDQAHLTVSSIAVDALIKPLEITLKDPAVMFVQIYAAII
YGIYYSYFEVFPLVFPVQYGMNLGEVGLVFICIAVGCIIAIILYGLYLYYVLIPRMKKSGIGPQET
VLIPGLPASFGPPIGLFIFAWTARASIHWIVPTLGVTIYAGSVFTVLQCVFLYIPLSYPQYAASLF
AANDFFRSALASGSILFAHPLYHNLGFAKGTSLLGGLSVIGIFGMWALFFHGAKLRALSKFAT
>g2035
MRTKSRPLRRGRDSAIDDAEPTSQPPAHRGTFSINYPVPDRVDFSNTIEALRGLHDHAHTSIIFGG
ELGDSAEIANQPTSLSKVSTLPSGRDVDTNIWRQPLFDVASAETLLKSFKSTFGYLPFIVFPDDIT
ISHLATTKPFVLLAILTVASGSRMVQKHALYDDEFRKALGLKYGSGGERSLELLQGILIYCAWHPF
QLRPKNGQLDHCLRIAGDLVHDLDLDEDFLSSSDPVPRKLTDNELDKIRAYLAYIYISLTYGVIWR
GKRDLPNIHPLWISIATETLQYNAQVDGDSTLVALVRTSSLFADVSAIKEGDSQMAANCQLILRRL
EEEFLEMQTSTKQLMTGLGAIRMQAMFVDIFLDCGGLLVLPVSKTYSKTGFCPPLPQVYRGIKKIR
AFLDFVVNLDDTALLSFTVNDWTRFIVVLTLSFRLSFPLTLCPELDSTYARSEIRLDDFLEKMSRG
TDITGQSDLLSASRYMLGLAKSKYDLRLASLKDLQFAQPVSRFFGCPVMRRNLDKSTENWQSISRG
LSGWINTS
>g2036
MATVQFGILAFAYQVIDTIGPIDLLFSANRIAFQFNREYGPVSDELISQAPQFTFHHIGVTEDPIE
LGTGNMTIIPTTTVDKCPELDILLVAGPYLGNFELHPKHADFIRRHVAAGKLLFTTCTGASVVAST
GVLDGKTATVNNIEFEWVRNRWPKVNWTREKKWIVDGNLWTGSGAVAAMDMVAHWLKENYGLPVLV
QAASTLDYEPRDADGLFNVLPQRYGTKGEKISTHVFKYYTES
>g2037
MQNKTVYCGTVPGPALYLKENQTTWVRVYNDLPEDNTTMHWHGLSSIAPYSDGSPQASQWPIKAQH
FFDYEIRPRIGDAGSYYYHSHVGLQATTAAGPLIIEEADGTRPYEYDEERLLFFSELYNHTDKETM
EGLIAPLATYLWAGDAESLLVNGNSFPALPWNETDSPLPAANATVSDTNATCHPDIIHVEPNRTYR
FRAFGAGSLGTYVFAFEDHNNLTIVAADASYTQSFNADKILFTTGQRYDFLVHTKTEEELKMLGKT
EFWIQIELRNRPINATYYAVLSYTSTNASTIPNTPPDQKPVQGITADLRNFMEYTLEPLLPNGFPS
SDQVTRQVYITQQQLTVTAGQFLALNNHTWTETNQHENDTPFNNTIPVYDVPYLVDIYNRGEQAIP
NYENALRHGGWDPELNVYPAKLGEVIDIIIQYDAIGAHAPNDAHPFHLHGTHYWDLGCGPGTYNAT
ANEAKLQGYNPVLRDTTALYRFDDPNNATIQGWRAWRLKATGPGVWMLHCHILQHMILGMQAVWFT

GNAAEITQGTAPGLVAGYLTYGGDAYGNATHDPLVTHFYE
>g2038
MSSQSDVEEKRGVDDFGNSTLPIAAPAPVAAPPAVGPPPNGGLKAWSQVLGAFFLMVNTWGLINAY
GVFQTYYTTTLLPDTSPSDIAWIGSIQGFLMLILAVIFGWALDAGYFFLLSAFGVVFQVLGLMMTS
LCEEYWQLVLAQGICVGIGSGCFLVSAFTIVGTYFSTRRSYAMGWTATGSSVGGIIYPIVVRRLIV
QVGFPWAVRAMGFIVLGTLAISISLLRPRLPPKKLGPLLDLQALKDLPYFFFLLSMLFTYMGLYIP
FFYITDYALSLGIDENLSFYTLIIMSAGSIPGRVAPPYFADKSVFLFPLLDNDANQILRANRIGNV
NVMLPATFICGILMLCWIAIKSLGSLIVISILFGFFSGSAQALIPSVVVWLAPDLSKAGVRIGMTL
FMCGLGLLVGSPIGGAIIDAQSPERSVYWGVFLWGGLTVLLGAVALLVTRTLKVGFKVAIKA
>g2039
MKRPIANLYSMSSVLAYETLVEDTVRVMIDQLDKRFASTGQPCDLSSWLQFYAFDVISAATFSSRV
GFIDNGEDVGGVMEGIWSRFKYFATIGQMPWLDKFWDKSPLINTLFPAKSSPIAAFAMNLTGKRLG
ELSASENNEKEATKKSDFLSKFLEIKAKHKDLPDWYLTSWTISNMLAGSDTTAINLRAIFYYLLRH
PHSLQRVLDEIKEAEAENRLSEIITYNESKTLKYLDACIKESGRLHPSIGLPLERVVPPGGMEING
HRLPGGTIIGINAWTCQRDYNVFGEDADEWKPERWLVDDARANEMSRAILLVSNSFLILALFSSAE
TSES
>g2040
MGHAHSHDIKLHRKYGDTVRIGPNAVSIGSATATKAVYGIGAGLRKSDLYPVQYSYSNGKLIPGIF
TTTDERLNKDMKRPVSNLYSLSNVLHHEGLVDHTIELFISELKRKFADTDSACNIDDWLHFFAFDV
MSEVTFARRIGFIERGTDVDGTIAGIWKRFRYFGIVGQMPWLDKLWDKSPLINALLPAKSSAIVTF
TFMQIKKRIEDFKNTTEGPIEKNSGQPIDFFSQFLKAKFNHPEIPDWYLTSWTTSNMLAGSDSTAV
YLRSIIYYLLKNPKSYRKVLEELSDHENTGKISKVITWAEAKSLNYLDACIKEAGRLHPVVGQQFE
RVAPIGGITVNGHFIPEGTVVGINPWVSHRDKTVFGDDAEAWNPDRWLVEKSEQHRMESSMLVYFS
SKGFGILAVDLLGYGDTDKPLSASEYKTKKIATEIIEILNLENIRKVHGVAHDFGSLLLSRIANYY
PDRLYTTTFLAVPYSLPGQKFDLAKVNALTKQVAGFERNRIKNYDYDDEITAELDPTLSLPTLVIQ
GSEDKIAVSQLLDFVRSYIPHLRSRPVPTGHWVHLEAKDTVNQYLEEFFSECQASI
>g2041
MAVSEDDQHPRFFRSTLFVIIIVSITAFTCPGLYYALNNLGAGGGTNPTIVNAANAIIFGVIAVSS
PFIGAIVNRINPRLGLLIGSAFYTPYAGALYCADKFHTTWFLLFAAVLLGLSGAFLWISSAAILLG
YCEEDRKGLAMSVKFAMQNLGGCVGAAVSLGLNIDRNYNGGVSRGTYIVFMLLMALGPIFGAMLPA
SKHVRRRDSKPVVVKKQPNIFREFVTLKLLLKAPAVLALIPLMIYVQWVLSYQWQFNYAYFTVRAR
ALNSFVFYFLGLISSLGIGQVLDTPLLDRRNRAKVGFIISTLFGAAAWILAEVTQMRYSKTRPSLD
FVDERYGLGAATFALWGCSDALTTTYLFWLVGTLTNDVNESALLGSIVGSTGCVGSALAFVVTVEN
FHLEGACSINLALFFLSVPSLFWVSFTQISSKTEGLMTVNSEEGGPVQK
>g2042
MVHPTISVSIMLAGLSLTAKASNLQYTVPSSVPRDAIPLDCTPVGVSMEFFMWPSYMTNITPPMQC
ISRFTDIYGEKMPIRIGGTTQDRATYDPSFDGYVSYTVANPLDAPDTLTYGPRFWDLINVHEGETI
LGFNRGDDNQNNTYLAVVDAKERVLSNLWAVELGNEPDVYLLQDKPDAVSPWNASQEGADAADWAQ
GFIDAWQSPLPILTAGSYAVAYTDNPEWPNTDYLIDTAYNDAVKAAVRVYVGHLYAAPPGNNTLAT
EMNHAKTVSDLSVYADRVATAASVNRSFILGTQHPLHPFTAEKETLTDQCWPLCCVAGETNFHPFD
VDMDATFGAALQTLDKSLRAVTLGIKRLFFHQGTINQALFNWWSDNQINAPFYGGYFAALALAGGD
HIVELDSGSNSYAQYVIYRQGRPTKVVLLNTDYYSGSGERTSISITLDGLVSSKVKALRMTAMSSD
VTTNRNGTYPTIGGQYFSDVDCSSIGALKYESTTASRGGYPPTLAITGATLTSKWIEGSNYTQVVE
MVITNSNKQNYLTLADTLSITVESDSLITVVPGTLTRLAPSQSAVVQVGVQNKPGVTPGSTCQAAV
VATYGTQYGPAITTNETISGPCGFGSYSASVSSVDYHWNPDWFNEVKFGIFIHWGLYSAPAYGSVA
PNEDYAEWYWRRQHEPTYKTKTYQYHLQTYGENFNYDQFMSNFTDSNFDPQAWVDLIAAAGAKYFV
PVTKHHDGFALFNFSTSISQRSSIHYGPKRDLIGDLLAAAKQYHPEIRRGTYFSLPEWYNPAYEKY
AFGDFGLPNQNPYTNATIPYTGYVEVEDFVADIQVPQMEQLAYTYDTEIMWCDIGGANDMVNFASA
WLNWATQQGRQVTFNNRCGIQGDFDTPEYTTNSGTVSRKWESNRGMDPFSFGYNYQTPLSSYLTGN
DIIQSLVDITSKNGNFLLDIGPMHDGTIPQIMQTGLRNAGSWLQTHGEGIYSTRYWSVTPGLNNFR
YTTTQNAFYIFYLQAPPSQLVIPDPVPWLPGDTITVLGGSQNGTVLSASKNTSGNLVLNLSSAIIS
GDQYVWGFKIAYTSTW

>g2043
MTISGNASVTYDFHKNIAGIVTFEVESVVSANAQLGITFTESSLWISNLSSDATADSGLDAILWFT
LDTAGAKYTSMPAQGRGGFRYLTLISNSPASITLRSVSVNYTAAPTQDLQGYTGYFHCNDELINRI
WYAGAYTVQLATINPKYGNSLTVLVTWPSATPSKYDDWYNNYTITNGTTALTDGGKRDRLVWAGDM
SISLETAIVSTYDLPSVRNSLEALLALQTADGRFPYASLPFPDQESFTYHLHTLINSANYCRYSAD
YAWLVSYWAQLQRGINWALRSVDETNLASVSPSASSDWLRSGMGGHNIEANAMLYYVLQQGIHLAA
VVQDSVSLAKWTSLAENLKVAANALLWNGTSGLYRDNETATLYPQDGNSWAIKANLTQSQSQRARI
SSELHSRWGPFGAPSPEAGSSTVSPFIGGFELQAHYLAGQGSTALDLIRRQWGFMLQDPRMTGSSF
IEGFSTDGSLHYAPYTNDARVSHAHGWSTGPTSALLFYAAGIQVIEGGGATWVIAPQPGDLISVDA
GYTTLLGSFSVTFRRSERSGVYQYFAFTTPIETSGTVRIPGAKGVLVSSSGSRVPLIDGTASGIQG
GRWTLQ
>g2044
MAAEVFDTNWMWHPSFTESRAETAGLFVHFRRDFVLEKIPPGRCCQIRITADTRYKLYVNKEFVSS
GPVKGDQSLWFYDEVDISPSLRLGKNRIGIYVLRFFYATRFATSFPRLSTGGVYIAHVDSQVPQSF
DIGSGSLWQTAIDQSTILCIDQAEDDFLHIYERIDRNTDVQLDWVPAVLLPFQNSTGVSPPWKLSP
RLIPRLQREIAQFSMIHNIQSCVQRTAWERSLITTPWQEVHLPPGTRHELELEVPYHMTAFLRFCF
QRPQVGGSTVVITYAESYEDPPTLVPYLRRKGQRCNVSKSLFGPQDTYVFGGSSRCLGLCYENDED
VDETFTPFHFRTFRFIRISIEVSSASELVFKGIDVQRAKYPLDVLATLKVNPNESVTAMRLWDISI
RTLENCMHDCYEDCPMYEQLQYAMDTRSSALFTYYISGDDRLAKQAIIQLHNSFQPRLGLTSSRAP
THQPQIIPHFSLYWICMLADHWTFFGDPGFLRPFIPVADAILAYFDSRIDSDTGLVKVADEIGIWH
FHDWAEAWRPYGIPPAVTQTGISTYSNNLYAYTLQQASILQSTCAERDTIAQEYILRSQRVVEAVQ
KFCFDGEFFSDTVVTASSDKCHHRSQHNQVWAVLSGASSGKQGEDLLRRTLSPVTGQLLTPASISM
SFYTLRALSMVGGDLYEESFHRFWGPWNTQIALGLTTWEEDSVSQRSDCHAWGSAPIHEYMAEVIG
LRPAKPGWTCLEFQPRLSLYREIEATVPFRYNGSTALAKVSWKTSPSNEIVVSMKALGIPTAIPVS
VKLPCKPEIFIQSADEIKFRLSKSQVKSKGSEVHLIVRISGNIIEDMQSGE
>g2045
MGGSDPPSSASCERCKQRKTRCDRRHPTCQRCERLGVSCEYAGRRKPGFPAGHRQLLEEKLRKLEE
ELQSLRGGGGGSRSGGVRTNFDMHDIDSSESPHGLRATRSPVDEAPGPTSQRTRQIQERRPPTDLV
MSLTSLFFRHIHPWFPFLDIQRVCIAMSASDDPTLLHYALFGVSLPYSFDSRLDQKSSDSFWKYAK
RQILLEVLEEPSFNSLEALTVLILDLSGMTTGPQVWGPLAIATTLLVKLHNGGAPVFRTSIAESDS
NALSSVDQLYRQKLFWAIYMLDCYIYITTSRPSCLRNEQVDSIFYTRQLIWRMPASDTTLTPLYVC
GYQLRLLDVSRKLHMLHLEYASLRQDSDSFFNWFDQFQRCSTELTDWVQGLPSCIQLNIRDDGTLT
AAALNFVPSIVMLHAYYHALVIHTHCLLSDPPYEALLSQPFDDTRADSQVRCVCSVNILTDIASRW
TAKSYDKLGWPIAWAVWIGARYLLVQETHGRHLAPEKFSILLNCLRRMSPYWQISGKYWRLLKQAT
TELQAGNLPNAHGEGGILRLLADFRISTADLEDQFRVDPILRYESTGENPSSVTNNILDPDRHPTL
PENNYFNLTDPTSDNWFHIPLFSSSAYQQDHDESL
>g2046
MPESKLDIKHWWRHRNLRTLNLLLLIPLLSIFSQGFDGSMMNGLQSVSHWQNYFGTPTGATLGFFN
AAYPLGGIAGVFLISPIADGLGRRVGLATGAALCCLGAALQGGAINIAMFVISRVIIGAGSVVVAG
VGAPYITEIAHPAQRSTATAMFLTFYSVGSIVAGWCTFGTFHINGTASWRIPSALQGFPSVIQLLF
VWFVPESPRWLISKGRNMEALAILAKYHGEGDEDDAVVQFEYTEIQGALSAEHSTADLTLLSFLNE
LVTGSGNRKRLFIMVWAAISSQMSGNAFVSYYLSPILASVGLTSDLQQTLINATNQMLSWFSAVYF
ATLPAKLGRRTLFLASLCAMWVVDICITAGSAMFAKDPANKAASYTVVVFLYLFSPAYNLGFNGNL
GLYIPEILPFHLRTKGLSFFYFVQFSFMILSTFAVPIGLENIHWRFYIIFVVWVIVEFIGVYLMFP
ETKGPALEDIAYIFDGPKAKTSENCEVGEIESKINHNHQEKELM
>g2047
MNNSEKLESYAKDLASAVKNFSDYCRTTEIDSITAALPIIPAETDDQVQQARRNILTTVAKIQALI
SKPEDFLQNLASKVRLPPREPLRIMRLTPYSSSPARTADPAARNGSVPMKDVAELVGVPVSVLTRV
VRVTATVRFLCEPQPNHIAHTTLSAPFVTKPDFLDATKFIAGTAAPAALRMASATQEIRFRELTRG
ISLPLNTAQSFQSLFEQAPKLQRQWITYLGSLAEPEIDEGATDVFRQLDWPRLGAACIIEVDSSPS
IMAAIMAERHPSLRITLQICENGNKLKHGADSSKNNYSNIHQNSITQSNSPFVPMVTGRRQQGPPA
AEANSNIFLHRRAPGQPQTIKNVGVYILCLPSPSPRNGLSFHAIQSRLIAELRAHLGVLRENSSTM

LILRMRFLPEHGAVDPEIECVARTRDMTLLQLANEREFEVSEAIDLIQDINDGMGKLMVVSKIRSK
NNAVVALGVKFQLSASH
>g2048
MSTYTVLGATGNTGRALIETLLQNPDAKVNAFCRNRRKLIQLLPQTESNPQIEIFEGGINDVSLLA
LAIQEASTVFLVISTNDNVPGYRVGQDVAINVIRALEKYKLQSSSGAKPPKLILLSSATIDDQLSR
HTPYILRQILLLSASNVYNDLRETERILRAEQSWLITIFIKPGALTVDRKRGHALSFSEESSPLSY
LDLAAGMIEAADAVDGRFDMQNVGVVNTTGRVATSPSSSSVCADFLIPVNITSQHAILDIEIGDNW
DVEDYIFNSTRRDSLTTFKPIIGEVNASAEYRIGATFCTPKSKINATGTALLLTHGSMVDRKYYIL
SLIPLQKDVC
>g2049
MPSSTQPEVLSIAIIGSGPGGLSAGIALSQLPNVYVRIFEKAPAISDLGGGISINHNGWKVLEYLG
ARKAVTGAATVPTVQRNAYTGEVVDPGFKHNPNDLYQARRTRRSRLQNALYTEVPKNTVHFNKKLV
TLENLEEKGVRLIFADGSQTTADIVVGGDGIRSVVRQHIYPDYKINYVGSVGWRTLVPVSAISHIP
DISLTVTNWWRGAGSAIYMSPVDELSNLEHMEVSCRSVNEPEVPGETVSWGKPVGNEKVLSRFQDY
YPPMVEVLKQVPEGGWKEFSGYAGPFIENLTGWGKLVLIGDASHPSGGGFGSGSAFAMEDSWILAR
TIEYSRSISGGVADALRIYNSIRAPYYKKMFEFRETQQLSLKKSGQKSREGFNERLRELMSKDGLG
GRRSSEFLEFVYGSDVKKVWEEYLEKENGSRSGKLSS
>g2050
MSTDAESGQVSEENKERKSWKSFVWDSLDKSPEERRLVLKLDLTLLTLGCLGYFTKYLDQANLTNA
FVSGMKEDLHMFGNQLNYATTCFTVGYIVAEIPSNLILTRVRPSIFIPTCQMLWTGLTMCTAACKT
PTQLYVLRFFVGVAEAGYYPGMNYMIGSWYGKDEMAKRACILNGSGSAALMFSGFLMTAVINLGGK
GGLPGWKXLTGDLPRFSIHERGTYAVQIFSALVFAWVSDAFLNGQRWGVLLFGAVFNLITMGSLAA
WNIAEGWRWACYYLAGIHVGASGIVFTWANEICTDDTEERALVLATMNSMVSVVGAWLPLLVWQQV
DAPVYHKGFITATVFGAIAVILIPVMKILQDKEVKDERLGFLTKGEDIAGLMKSLDTILHLHTFLS
VFPWLMPMVHFIHGILSKNKEVYHQKFGNEKIKLKRQETADADETGPVCMVKKFIDAQRRKDDNKG
ITDWDISLNAGANIGAGSDTTAITLSSIVYFIYQNPQVLEKVRDEIETARLAPIPTFQSVQQLPYL
QAVIKEVTRVNPASGLPLWREVPKGGAVLCGQYFPEGNAGSKLSTAANLSEKIVISRLGLDLELA
>g2051
MSSLKPITVWGKGGPNPPKVAILLEELGIPYEAIAVPLSDVKKPEYTAVNPNGRLPTIYDPNTDIK
VWESGAILEYLIEKYDKDYKLSFPPGTAESYHAKQFLFFQVSGQGPYYGQAAWFKNFHPEKLPSAV
ERYVKEINRVTAVLEKHLSEQKVPEGSDGPWLVGNKYSFADIAWISWQRLVTKIHSVEEYNPDDYP
HVKAWLDKLLARPAVQAGLGQGGKLQ
>g2052
MLRLVYLMTSLALTQIAAGAEYANLGVPYLYTTNPITPXWNDGVPAVTQCPIAPGDSFTYSFIADT
YGSSWYHSHYSAQYADGVFGPIVIHGPTASDYDLDIGPVLVTDYYHLNYTTINERAFSSNFSVVTV
PGVNSLINGRNNYNCSLVDEGDTSPCVSNAGVSTFQFTAGKKHRLRFVNAGASALQTISIDGHNLT
VIANDFVPVVPYQTQYIKLGVGQRTDVIVEGLPDATGSYFLRSQSPAKPCADTIQPNATAIIYYRN
EYLPEPDPWPAFTDAVQVCANDPLETTVPLYPIASPSNPATTWDLNITLAQNETGSYLWYMNNITF
RGDYNHPILLLSNLGNNSYPNPEWNVENFGSNSSIRMVVNNANPRTHPMHLHGHNYLVEAVGNGTW
DGIITRPDNPQRRDTQLVPGNGYMVISFTADNPGAWPFHCHVAWHVAAGLYVTVLERPDDIANYKI
PSTVAQTCRNWWAYTNATVVDQIDSGL
>g2053
MANTNDTIFARDKGFWENYLRGRPRAPDVFFERIFRYHEESGGQFGTAHDAGAGNGPYAQILRSKF
QRVIISDIVSENVKLAKDRLGSDNGFTYRVAKIEEASDIPCYSVDLVFATNVVHFADQKLTAQAIS
NQLKSNGTFVAAGFGPARFVDPKVQGAWSGIYHQGGRAVLKTSDELEETIKIMERSSNYYNVVPLD
EKFFQPGAKRIHLNMDQGGLTPLLPPEYGQKASEPDYTGPNDIVTFENEDGWSFKTDLDGLKEHFK
TFPFSAIDPSSYNKLWQELEELLKDGRVVEGYFPAKIILAKRC
>g2054
MGRGGYRQINKTLNICAFEDYLDTQVARLPRLNDVEQITPRVLRVLGQNEGKFTLQGTNTYIIGTG
SKRLIIDTAQGIPEWADLIESTLADSHVHLSEVLLTHWHGDHTGGVPDLLRLYPYLENSIYKHSPD
RNQQPISDGQIFKVEGATVRAVHAPGHSHDHMCFILEEENAMFTGDNVLGHGTAAVEILPTWMESL
RIMRSHNCAIGYPAHGEVIRDLPSKITTELGSKERRERQVLHALRRIKSEEQSAGSGKGSVTVKQL

VTAIYGDTLDEHVRQMAVEPFTDEVLRKLAGDCKVAFEIKGGQKKWFTIEPV
>g2055
MTLRPNTTPSDSQGDFVKLKLAYFSNEFPRDDLHDLLRLLWSRSKDRTHPILANFIDEATLAIREE
IRLLPNALRTLIPPFQTIFNFADYPELRKGQLSGSIDGVLLVAVELATFIGYYESHPDQFDFDSAN
SFLAGLGIGLLATTAISLSSTLADIPVAGAEVTRLAFRLGVLVDDISINLQQRDWSSPDTPDSWAY
VIPNVEADVVQKELDIIHAKEKAPEASKIFISALSQTSVTVSGPPARLKNVFRVSDFFRDRKFISL
PVFSGLCHASHIYDEKHVREVVRKSPLDSSFKYSPGIPIFSTSTGRPFEASTATELFERVIHEILT
QKIQWEKVVDGIIENANNSGTVDVQVLVFRVSLPIHDLVEAVKTHLPQANATTDELISWAHEGSLS
ISPRGSLQSKIAIVGMSCRLPGGATDTESFWELLEKGLDVHKKIPADRFDVDSHYDPAGKRVNASH
TPYGCFIDEPGLFDAPFFNMSPREAEQTDPMQRLALVTAYEALERAGYVANRTESTNLHRIGTFYG
QASDDYREVNTAQEISTYFIPGGCRAFGPGRINYFFKFSGPSYSIDTACSSSLATIQAACTSLWNG
DTDTVVAGGMNVLTNSDAFAGLSHGHFLSKTPNACKTWDSQADGYCRADGIGSIVLKRFEDAEADN
DNILGIILGAGTNHSADAISITHPHAGSQSYLSREILSASGINPLDVSYVEMHGTGTQAGDKEEIQ
SVSNVYAPITGNRRHSKQPLYIGAVKANVGHGEAVAGVTALLKVLLMFDKEMIPPHVGVRNGINPT
FPKDLDKRNLHIPFEKTPWIRDPGRKRIAAINNFSAAGGNTTLLIEEGPIREVTAVDPRSTHVVAV
SAKSKGSLKRNLERFITYLGKNPGASISNLSYSSTARRYHHNYRIAVGTSDIQELKKQLNFHLNKV
DSHKPIPNTGPPSVVFAFTGQGSSHKSSDLALFKQSPVFRSHILTLDGLAQKQGFPSFVPSIDGSF
LKDHKHSPVVVQLALLCTEIALAKYWGSLGIKPDVVIGHSLGEYAAMHVAGILSANDAIFLVGKRA
QLLQEKVAIGSHKMMAVRASVEQIQDAAGDKSFEIVCINGPKETVLSGTSEEIQAIYEPLQVAGYK
THILDIDFAFHSSQTDPILDDFEEIARSSTLFQEPHVPIISPLLERVVFDDKTIDAKYLRQSTRQT
VNFLSALRFAKDISIIDETQVWIEIGPHPVATSFVKATLPSVNVTVPSLRRDEDNFFTLAHSLAAL
HVAGVQINWSEFHRPYESALRLLDLPTYAWNDKNYWIQYNGDWALTKGNTFYNAEKGLAAAKTTPV
VSSSLRTSTVQQIIEESFNGSAGKVTMQSDLMQPDFLAAAYGHKMNGCGVVTSSIHADIAYTLGEY
LHKKLIPNTKTVDINVANLEVSKGLVARKNTSVPQYIQVSLSTADIHSGVAVLKWYNVQTNGMIAE
PFATANIFYGNSNQWLDSWIPVTHLVQGRIEALERLAAEGVANRFSRTMAYTLFASNLVDYADKYR
GMQSVVLHQLEAFADVKLTEEKSGTWTVPPYFIDSVAHLAGFIMNVSDAIDTKRDFSVTPGWSSMR
FAKPLIARSRYRSYVRMIPTTEDTNVYLGDVYILQDDVIIGMVGGIKFRRYPRLLLSRFFSAPDEA
GTAAAAIAPKPVISTPAVTVVAEPAKPDPSPEILQPSSTAPALGVNSIDSDSTAAKAMLLVANEAG
LELDDLKDEASFASLGIDSLMSLVIAEKFREELGITVGGSLFLEYPTVGDLRAWLVEYYS
>g2056
MSGESAQEAETPFLTGALTTETGFWAEYIASRPSPSEEFFRLIHEYHTSHGDPTTAIAHDVGTGPG
NIAERLLKYYDHVVGSDVNERALAAAPLLIPVEKIQRLTFVKSPAEGLATDGVVPDSVGRGKTDLI
IVSECIPLLDAPKALEAFHNLLRPGGTVAIYFYGRPIFTDADDSGKLNAMYDRIATRICQFLLPFK
ESPGFPIHQRAAEALISYLDNIAFPAAEWSDVVRYKWNYDVPLLFNSKEGYDFDFAPVDRRQEGEI
TKEITDRTWWQDEWDVDRVKEYLSSVYPSYQKKASDKYREIEEDLEQLRQALGGGKSRRKVTFPVA
LVLATKRKNKLDGDD
>g2057
MATTTTTTQQIRLSGENLGLGRISKNPAGSIETANSLLQKNHDIYHIFWRDVGGHNHIAHSVLSIL
ALGGSPSDLQRAYDDGAEIQRPIPPVNLDIVKGLADPVFFRKHIGDIDQYTNFLVFFEQEIAVKGR
EQVLQEYVFGRAPNAETIFAQLYEGAYHPIIHLGFGVEFELPTIIAEGLAQAAVHDSAGLEAFFNE
TEELAAAAVASGLDRKPLNTLYDEVRVNDKIRTAARLQDGPVRVRDGVLGRAGKEIAALASQYRIK
PTELERATAEMINLSAYSAGAAQRPGKKRKIDFFHMHNVNCSIFFTAFNNQSWISLEDKVRLVERK
ARLDLVWYAASAAAELHLEAIVNYKPTFSAGLDWKGLYDAVNKTHDDGHVAKFVRAIKNGEEVSKP
FEEGEGSDHLPVKGESWFKLAQMAYDTTYNQEVDDKWIWGVGYDPLWAKIPALEE
>g2058
MQTATDLWARPLVIHGPSSANWDVELKTWLITDWYHADAFKLYYYEIETPRAAPPDSMLLNGKGIY
NCTPANDTRCTGHDRLYEVNFNQGTRYKIAIAHTGTLRTQTFWIDGHNFTVIENDFVPIEPYVTNV
ITLGIGQRYEIIVEANADFKNGTNFWIHSQYCNGPTLMDSRVGIIRYDIGNISDPYTPPPQYQDYG
CADPDPNSLVPIVKRKVGNRVNGMDPDDYLKIGLEAWPNASDPNSRIHKWVLKDVPMLVDWEDPSL
KRLALDKNSTFSLETQPITLDFDTGEWVYFVITNNYTVDGVEPARTLPQSVHPIHLHGHDFVILAQ
GEGIFTSDIVPNLDNPARRDVANCPLNGYLWIAFQVNNPGAWLMHCHIAWHASDGLSIQYIEQPSK
IKDLLENADVLGEFSKTCDEWTDWYTNVNEKTGTLQEDSGI

>g2059
MDTASLTDDHGFSEATGSTNFGVTLEWPDSEAILQSIVSSDWNTFPLPPGTLPMTPPPPMPPPLQL
QHAGAAPTTETIISRRNEDPEQLSPEMVFKKRSRV
>g2060
MGNSKSTKVENVPTFQHGDLASPPTESPVRIRTIIAIVAINFAVFGQVVSLVGSGFLAQTMAGFLG
DTSKAVWFSTSITIANITLNPPISQAADFWGRKWFVVIPNAIAIPGCIIISRANNIGTAIAGFCIM
GLAWGSQSLLFSVASEILPRKYRGIGQASVNISSGLGGIFGLLVGGALIRQIPGHFRIFWYITCGI
YAVATVGVLVGYNPPPRALQKTMTSVQKLRALDWVGILLITAGLTLFAVGLQFYGNPYSFSDAPVL
APFISGLCLLIAFFLYEWLGRADGLVHHGLFGDRNFAIALFSVFTEGLSFLTSNSYFAFEVSVLAR
VSLFDAALHFSILFFGAVIFAALAGVLITYTKLVREPLVFGFVALLAYNACMNSITPSSGTGPFWG
FAVLGSIGQGFALTTVTIVAQMSAPPELISVATGLIIATRSVGATVALAVNNVIYNNSMDKEVPSK
VAGAVLPLGFPPKNLKDLIQALLSNNADLVAKVPGITSEISLAAQGGLYEAYSISFRHVWIAATCF
CAAGLLSSLFLRNSKSDFNAHIDAPVDVDRKFGDATSSKSIEHAEHAEIAPVERAA
>g2061
MVKLLIMDGSSNSDIKDRFFDQTPLSVATQNGHESTVKLLIETGKVDVNSKCNTGSTPISIAAAKG
HEALMKLLLQNEKVNVDTKYHSGRTPLSHAANNGHETVVRLLLDTGKVDANSKDNDGHTPLCLAAI
EGHEAVVKLVLETGKVDINARDVIDRTPLFWAICRKHEAIVRLLLETRDVDVNLTDNEGVSPLSQA
IKNCQKTLVKLLLAIENVDVNSRDCIQGNTPFSYALTPALSQRDKEYMELLLETGNVDLNCKDNDG
YTPLSQAALLGDVEVVQFLLETGQVDVNSVDNLGFTPMRWAAYQGHEEVKKLLADATFASRGSSVT
LHGDNQLKRQLETTNIIALAFNKVGYISIQILNSASPSVRYLKPDLTL
>g2062
MPAVATKPTKNQLKRARRKAKKDTSTAAPNEDATQNGDTDKPINAPSQPELKEEQQDAAIQLDDSL
WAMYKDIANKFEASEEETTKLETEKPEVFFDDDDEIPDEEEEPKMSKKKRKELTKLSIAELKALVQ
KPDLVEWTDTSAPDPRLLVHIKAHRNVVPVPTHWSLKREYLSSKRGIEKPAFALPKFIQETGISEM
RDAALEKQEQATLKQKQRERVQPKMGRLDIDYQKLYEAFFRFQTKPELTRYGEVYYEGKEYETNLR
HLRPGELSDELKEALNIPPGAPPPWLINQQRFGPPPSYPALKIPGLNAPPPPGAMWGFHPGGYGKP
PVDEHNKPLYGGDIFGVLQTQQTAQQGEPVEKDLWGELQPMEEEEEEESEEEEEEAEEEEEDDDGM
DATHAQSGLETPSGLVSTVPSEFGTTESIGGEFDVRKHHRGTETEDITQPKAAYRVIPEQASRVSG
FFGGDRVYDLKAPETSIPLLGSEDSHRKRKKADDVEVSMDPELLQNNDGISKEHLKGLYDAEKKQE
FSQWNFQDDLSDMIANERKRVRKEEERRGKR
>g2063
MGFASDGNQLGIDFDTFRKDDHHGVPRYQRLAVEEIYFAGHEAEEESVAFVRDLESANENICQKDD
NFRRLAALQISF
>g2064
MSVSMSASGFRLRVGKVDLPHRTDHRGGFQLMMLPKLQYLQPCRRHLRILRNPYSLRVPQHIVPRM
RLWIDANTLAVVGLRDLTVEAFYLTIGTTVCEIPGQARRVSRKTGISPLRLVRPLYRLLKVVHLMY
PYYLLQRGLGEPLAIPVVTRHGRPLQHQDALRLHLMRHMHRMHRPRDPDIGTHTVLQLMTFIVRRL
IDIAAMPQLLPLAFDFVIEKRLMNLKADEGKLVEQALDKQMLKRQGLRDWDRLQRESALNALRSEL
AEAHLQRMTEEDRLEDLFWTFTDSLYNTVFKILFIGSSTYTIYLMLNDYKPTQDPNTDTFKVEYLL
GFSALLAILFPQQYTISEILWTFSIWLESVAILPQLFMLQRTGEADTITTHYLFALGIYRALYIPN
WIYRFFFEGFFHAIPVLAGIIQTVLYSDFFYIYYTK
>g2065
MSSRFTTIRGIPEHDIESQDSINEQTMSSERINPPIEGFPQVEDFDKLMESYVNELSVKKQDKALI
NAQRAQHIRIVLTDPKDTSVGSAQFRFWVKKMFKLEPADSRLPMNRKWICHEGKPVAIREKLFKIL
TRAHQQCQHGGRDKTSAQTKKSRRLIMHFLKGTQRIDLPLRQDMSNMSSPSRRIASFPSRFEAKLA
PYGTQ
>g2066
MQADTFHLLQSESPPVESWDDDGDLQCLDDIQFHAISTTTSVTGSMLRSGHRDSISSRRSIRSDVE
SNAGDEDWQLLLRDNDEAGTEEAIASARNAGIPIPDNVPKSALLSGSIKKLQSKQHKKSVIDDWSE
DLEFPDTDMELSLKANGQESFPESIHQIHTPLMSSPAKTRGSTEPSPFRGVGTTTPQSTTDAKDTI
QGLGFHDIKQEPQDLSTTTTNTVSNLNLPHTPSIGSHSPAVCLARDADDDFEKDFEFPSQEMTLKL
SPPPNMPTMIPSINDDIWDIGWADGDGNGRFGGTKRDGRSTHSSSVSAFSPSASSCLTAETEDDIL

DGLVLPDGPFNFEDNLKKRKVLRTDTEEPSPKSLPPSNNQKTTTSDNFFDELDVDDGSVFTSLKTN
VNRNVKRKLDARPMTPNRSEKTITFTNKTPSVGTRIPRLSSHNRSRTSLEPVSESGNPVTQFRRPQ
SRLTAHGNRSSVESIHTSPPVTPSGGRRSLGGRSSRESMNSDRDLSSSHAQLLKSKRSAPVARGPE
ESVSAAPFQRPSSRQESASLIPSFARPKTPVDRSTTDSRLGNHRPQVPFLPAGASQSQSHHASLKY
SRHFRRSDSETSTESTIGTKSTSRLSHTLRPDTHGRHHAHSASEGSNIVVKHSVTRPTRRRNYGDG
NELESFDDLPTSATLESQFIKTPVGHGPPKSLRTRLAQSPTALRTETPVPTMTSSAPSRPSSFKPR
FARDTNASRIAREQRIASLSLPTRNRDSAPLAPLSTNWKGPQTPRQTDSMLTARSKKGKASRPGGS
KPQLIKSMGVSEAKNVQGMQYNPSTYRWEGNENEVAEFDAINTPRSPKVAPALITSVGTIKGAQVV
GGMVFDPQRMCWLKLASTQPGADKVAVIHDELEDVFAGLEDLQDRPGPKHRRNISEASVPTEVFDD
QSGDSSEEWPITEEFDVGPEFVKRQRAEEDRWKRKVTKWVSSERKKAGDGWRWAIRDLVPGGN
>g2067
MPAESKDWVIRIPRSDSDNEFVLVYIAPGKAALDLKFIATEGENPYVGSLRPSRLKDIRAKNFRGT
DTELEQILKSVLRVRNDVSSQIAANPDSEIEATATIKAKDDVNDLVINIRKRIDNITQRVATITLS
QNDDQTIELFEWMGLMLSRTDAYEHQISTLTKQLQAAEKSIRDLQTSLDDLVQSKKEHEDMLLGGL
AQVLNEKKLKIRNQQRLLASVNVDSEKGRGLESTIDREEEAPKIRRNKRKAPAHQQEPSDSNDEFE
KMDIDKPKPSRREAAAQVEEDEEEDGDERSETPQPLEEETESEAEEERMNSPPEEEAQSQKSNRPN
LTKAADPPPPPRRDLPFLRRGKGADTSGKEAEIVPERDEAGEATGGETDDDEL
>g2068
MRPQLLRAAARSRQFANARSCRSFATTTRRSAEVELTIDGKKVSIEAGSALIQACEKAGVTIPRYC
YHEKLLIAGNCRMCLVEVEKAPKPVASCAWPVQPGMVVKTNSPLVHKAREGVMEFLLANHPLDCPI
CDQGGECDLQDQSMRYGADRGRFHETEGKRAVEDKNIGPLIKTSMNRCIHCTRCVRFMNDIAGAPE
LGTTGRGNDMQIGTYLEQNISSELSANVIDLCPVGALTSKPYAFRARPWELKHTETIDVLDGLGSN
IRVDSRGMEVMRVIPRLNDNVNEEWINDKTRFACDGLKTQRLTTPLIRREGKFVPATWEQALTEVA
SAHARLSPRANEFKAVAGQLVETESLVAMKDLANKLGSDNLALDQPNGSAPIAHGVDVRANYLFNS
KIFGVEEADAILLVGTNPRHEAAVLNARIRKQYLRSDLEIGLVGESFESTFGFEHLGANVSSLQSA
LSGEFGKKLASAKRPMIIVGSAIAEHPDAKSVFESVGQFVEKHASTFRTDEWQGYNVLQRAASRAG
AYEVGFTAPSPEVANTTPKMVWLLGADEINASEIPKDAFVVYQGHHGDRGAQLADVVLPGAAYTEK
SGTYVNTEGRVQVTRAATSVPGAARDDWKIIRAVSEFLGSPLPYDDIEALRDRMEEISPALRRYDL
IESTSVPALSKVQLVDQNKGSKPTGAEFKKVIEDFYFTDVISRSSPTMARCSAAKATGNPETNFMA
AGEMSPQAVYGNAA
>g2069
MTSAADIPNVKVGGNASVPVLAYGTGTAWSKQTGESDISREVVEAIKTAIKLGYHHLDGAEVYNTE
PELGVAIKESGVPREKLFVVTKVYPNIDDIPNAIEQSLKKLQLDYVDLYLIHAPFKAKSDKELQDT
WAAIEKVKEAGKAKEIGVSNYTKAHLETTLKTAKIPPAINQIEFHPYLQHGDLLDYHKEKGIATSA
YGPLTPVTRATGGPLDPVLAGLAKKYAVTPGDVALRWALERGAIAITTSSKEARLTEYLRTVKFNL
TPQEVEQISELGNQHHFRGFWKDKFAADDRT
>g2070
MTCNRIHQMASQLNLAPAAAAYNTLATVPKSNTFTSKLPPDPAFETPKQSHDAPRETLGPRIVKGA
MYTYVRPEAAEDPELLGVSPRAMEDLGLQPGEEKTEDFVSLVAGNKIFWNEEEGGVYPWAQCYGGW
QFGAWAGQLGDGRAISLCELTNPSTNIRYELQLKGAGRTPYSRFADGKAVLRSSIREYVVSEALNA
LGIPTTRALSLTLLPKSKVLRERMEPGAIVARFAQSWLRIGSFDILHSRNERDMIRKLATYIAEDV
FPGWESLPGVVNLPNEEGSGDVNVDEPPRGVTATELQGKEGQEENRFTRLYREIVRRNAKTVAAWQ
AYGFMNGVLNTDNTSIFGLSLDFGPFAFMDNFDPSYTPNHDDHYLRYSYKNQPSVIWWNLVRLGEA
FGELIGGAERVDDEEFITKGVTEEFGQILIKRAETLINRTGEEYKTVFKNEYVRLMSRRLGLLTSK
ESDFESLFSELLDSMEHLELDFNHFFRRLSDIGIEELETDEQRQAVAKRFFHNEGVSGVGNTEESA
CKRIATWLSSWRDRIVEDWTQDGRTDQERKELMRSVNPKVLSRSPNPCSKRLTNDWQFIPRSWILD
EVIERVEHKGDRQILGRVMQYTLNPFQEDWGVDKDEEERFCGDVPRFKRAMMCSCSS
>g2071
MENQTGSRGCFNCGEPSHQARDCPKKGTPTCYNCGGQGHVSRECTQAPKEKSCYRCGQTGHISRDC
QQSGPAGGNNGGFSRGGFSGGAGGQECYKCGQVGHIARNCSQGGSYGGGQGGFGGRQQTCYSCGGY
GHMARDCTQGQKCYNCGEVGHVSRDCTTEANGERVCYKCKXPGHVQSACPN
>g2072

MPKVSDSFDRMSPVLSMLTATRVSKRFHALAGDSELWKRRYFAQWVLPRARAVPQSKHLKLSSTGT
AYSPRVSKWLGHSHLAEDNNSIHWKTQYRLRHNWSKGTCRLDELEVSRPPLPQILIKLYSGVVITA
DREHGLRAWSTKKPKKQLTELPFPTGNDIPTALYVAKGSPASFIEVTVGFDDGHFSVYELDVIRGE
FAIRFSQESTSDATITAIASAPPYFLTISDHKVFNLYKIPSHVTQNEEPAPNQLRLLASLESNNTF
APMSLSLRCSASEVIASIAYSFFHISNGWTIGIQELRFNKDGEHITSRLATTVAVQPKADENGHDD
PSTDSNSKPNNNYHSRGYTNLSTADIQHKEAPTSLSYSHPFLLTSHSDNTLTMYLVFSSADKLVIR
DSRRLWGHTSSVSSVQVGDRGKAVSVSPRGNEIRIWELESAVSSSSSPLSSRSRSIRGESSVRINY
EKPLSAPRFSLGTTSGAKDWSRHVSYQRDLDALPEEPDGWVGFDDEQVVVLRQRGLRAQFLECYDF
T
>g2073
MDSVVHHLDLISKDLEAARNEAAKQAVKYQEDTDKLNKNIRSLEYQRKTDQQRIKKLQSDIADLQR
YKKATNPGAHEDVVVEEEDDGSDDRMIETKLQKLKEKYDPEKATAISWPEGRLSVQLVSSIQRLKN
SYNDIYKETAALVKIISSLRAQVKHHKRRAEQWQRWCAESSAKDKAPTDTNLDTIGPSIEQCVHQQ
NNTLSTPKSVRTQDEGQYIPSSSAVASTASNSSPIPPISSNEARGNRPDYDSGDQSRITRVKDEPL
SPGSLEGPSLGEEIFPVASEDLDEVGDTVTTPRSRRFAVFEDGVSRLQHDGPITTPNFQGERRAFQ
PKDTNQINQFRTPQESAFKRRKTCDSGASAISTVAEDGEDQNYIPSAIKVATDENDTPSKGPVRPS
DAHRRLDDLLAARPHRQSPALDRPRTRILVQSTTPVSRPGNQPASVSANQTPWSNGPTKAITGYMK
TAEKRPVAKRKFQYETNPTTKTNNDEDWEAIVKSRPFRDLPVDCLDLSHFKLNPARNQGLDYAFKE
VVRGESRKCLPGCMRTDCCGEKFRAMVRATGIKYDSEKHRALLEEHLGDKKSALQQLSEQALNGLL
IEAKARQLANQFGKHRHAYERARSPPGFWRTDMPTTQELAKDRMEADRMDREKIYERVSEARRPDG
LWKFADEID
>g2074
MCRRQNVELLQAEVDEDDQSFFRLLIDGQSIKYVTIESGLYTAEDMCFGPALVALLPEFPPGDWND
GLVAGDPKGDQPYFKHVARTQFPGVRYKWHETDVDYLDIAVGKKLRTGIYEITCPKFDTVVVAKFA
RFHWEIQYLENETIAYEWIDGCEIGPRFLGHLIEDNRVIGFLMERITNARHAGPNDLPACQQTLSQ
LHRLGVRHGDTNRFNFLIRGSKAVLIDFDSARKCDDQDTLRKELEDLEGCLNDLSQKGGGGLLC
>g2075
MFVEMESHRQTLRELKADVLRPSKWISSYENFVTKNVNTVNQVESALRSLTYIIPGRFRESELPSE
CVHSGVQLLSLYHDSLVSRVISQLPSTIPRPIPSPHARYTKYWTTKSPLYRKLALALQIIQYTELL
WEMTARRRGEKVRWRVIAAIEFAKAVCRLLLLRLTNSRPLVSPPLPERETDPRAAEAEKEAAAGDW
NGMDEPIEESSVSSSSEISWTMPRTGLTLPSLPDASDISSYLISKVLTADDVKAPASLLHRVSGQG
QFAEILYILRPVIYALAMQRWSKNKRSWSPWLIGFGLEYGSRQLAKRDLAERTAGGLRGLTGLERD
ELKKRGWAMGWWMMRGAFYENITRPWIGGIADKLKGKPILDIVGGLLDDYEYLWDNYYFSTASL
>g2076
MGRPSNKTKTSANKEPDPTGALSFIPPTINPTPLLHGQSYTVYSPCPQSIQPIDPTAITTNDSKKP
TECVLGVDEAGRGPVLGPMVYSAFYLPKHLEQPLLTQEHSFDDSKVLTPVVRARLMQSICTPGASL
HDSCGWAVKLLSARDISAGMMRPGMNTYNLNAQAMDATIEIIRGVLNEKHINVTEVYIDTIGNPAT
YQAKLQKLFPTISITVAKKADSLYPCVSAASVVAKVTRDVALETCYESSYREPIQNVEEDTDMNEP
ETKTEEPGWGSGYPSDAKCVNWLRQNMDSVFGWGNECRFSWGTAKEMLEGKDSIRVDWPADEDEGA
GLADFLSGPQRSTMSGGNEDLGNWYGTRVTAF
>g2077
MAPRGFAKARDYDYSNVGKQGRRTGITLKEGRRDEHGMEELDGVFSSPEKSPVRFTGNDTMMGSEG
MSIDEDDGPGPTDFLSRSAARRSPFFPPPVARSPMKSGLSGSPRRTPGLRSSPIAQNDVPSSPTSA
RQRRGRLNEDAAETNSRQPLNDRRPNTAPIVNGSRQKGAVSAYANDENFPINEDDEDEDEINDVSV
YQXEPIEEDFDVHEIADDGYGDDADEIPDDTTNIQNEDPSEEEPSPVQAASLRTKKRKAPATTEDV
DSPVEDISQNLPKKKRAGRPPKAQTQKATQEKTTERPVKKAKTSKPQPAKEREPLDPELEQVVENH
VKRTGPLKGRSLYILKREVPNEQQAAHTRSGRVSVRPLAYWRNERCVYGDGEADLGQRYPLSTIKE
IIRAEDIEPEKKGGKKRKGGKKSKSKKQEEDSDDDDDERDKWEVEDGVLHGYIKKWDTDNQVASSE
EEVTEIAYAPTGVETRDVKDSTFRVAKLLSQPFFGSGIVELPAKGVKKPKNSKRMHMVFYVCWGRV
QVDINGVQFTAGKGCIFQVPRGNHYSFANITPKEARLFFTQAFMPNGSDNDNDNEPASSSKRAATN
EPEPGPEVEDQEAESSKAPAKGKGRGRGRKAVK
>g2078

MVYNARWGILATGGIAKTFVKDLLKDASARNASDISHTVTAVASSSSASRAQDFITELSIPGSPKA
YGSYDELVNDPNVDVIYVATPHSHHYQNARLALEAGKNVLCEKAFTVNAAQTKILVDLAKAKGLFL
MEAVWTRYFPLSVQVRDIIQKEEEIGEVVRVQADLSIGNPDGFDVASRMVNLDLAGGALLDLGIYS
LTWVFQTLYHTLPPSQRKPPSSIQSSMVKYPATGADEDTTILLTFPTTTPNNNRRTSHAVAMTAFA
VDSHPKGTPSVRIQGTKGEIQIIGPAFRPVKYRVVPTDESKQIREVEPEFPAGGHGMYWEADEVAR
CLRDGKLESEGMPWEESIVIMEVMDEVRKQNGLVYPEKIESVEYPVAL
>g2079
MAPITQETVDGLKDIIHKLESRVADLEGRLVHGSPASSSKSLAEQFRIILIGPPGAGKGTQAPRLK
EKYCVCHLATGDMLRSQVAKKTPLGKEAKKIMDQGGLVSDEIMVNMIKSELETNSECQNGFILDGF
PRTVAQAERLDDMLAARSEKLQHAVELQIDDALLVARITGRLVHPASGRSYHKIFNPPKKDMTDDI
TGEPLIQRSDDNAATLEKRLATYHAQTAPVVDYYKKTGIWRGIDASQEPGQVWKSLLGVFQPSSSI
LSKVGLSK
>g2080
MPATTAETLSIVTRTVSVAPLVLLSAADHYGRTAKGTRKRVVGVLLGQNDGTNVRVSNSFAVPFEE
DEKDPTVWFLDHNFVESMNDMFKKINAREKLIGWYHSGPKLRAADLEINELFKRYTPNPLLVIIDV
QPKEVGVPTDAYFAVEEIKDDGTTTSKTFVHTPSIIEAEEAEEIGVEHLLRDIRDVAVGTLSNRIT
GQLQSLQGLHLRLRDIGQYLQKVLDHELPINHAILGNLQDIFNLLPNLSTPKSANEANGTESDIEN
SELVRAMSVKTNDQLMAIYLSSLIRAITAFHDLIENKIQNRQQQEELEGKKEEEVATAAAKKDGAS
TSATNGEHAKDEKDTSKEKNSKRGS
>g2081
MAAEENLDFHLRNPTDPAISVEPWPLENRPGLKCYRNAIFIWLFHTPLLLNWIDDNHEKHDGDLCP
LCALRELARRFWASDRDYDTTFNQDVDKFWKQTHNPGLIDLNRQNDAEEYLDSLLGYIISTGANQW
APLIQVQKQCVKKCRECSFHTQDSAQDFEQGFFLQGTISRKDIEXQPLCHTLRFIKSEKAACNNAG
CPSKKRKPTTDGDPRPVHDFFYGLRAAPPILVIHCKPYQFLVESANGEMVGHVSGKVTRLEHSLAV
SSYPDRSLDPKTVNWTFYHLYGVILRKGDTGFTGHYVCAFEQPQTGRWLYFNDYIFKEDSDDYEPP
EEGYEVKNDGTPFEVTVRCTRRGLDDKIEDMLKRDWRIFMVMYRKLFQVPVQLLAKITFPSSVIAL
QDQQSLEESGEPEPEPETEPEESAAADDQEEYFTPEGESVGDQDLFGFASDSGQTPSESQGAANDE
QSQGIPSELETRNAQAEQPMQAGLPAEQNVEVEXNSLLDISSPSEPGQAEQPLPAEQNVEIEANSL
LDISSPSEPGQAEQPLPTDLSTEKGVEAEANPDSSTSTTAARQTVGETQRITDNVSERDTSDSKLQ
TGQAQQPLPTDLPTEQAVEVETNSLLDISSPSENGQARQPLPIELPVEQNAELESIPELNISSPSE
TGKAEQLLPSDLPTEQAVKVESIPELDISSPSENGQAQQPLSTELPVEQNAELEAFSLLDISSPSE
PGQAEQPLPSDLSTEQAVEAEAHPELSTTKPDTTNAGNRAQTEKSTHNQHRVKDENEQTTSLQSTS
EIVRTEVEPSVQPRKKQAKGKAIKRAHDKALDEEEKLTSVPASKRAKTEAKSSTQPKRKATKSKGT
NKTKNDASDDHEDEKAEEPASSTKRAETVVEPSTGYGGRETRSRARAKRGRDEIPDVDKVETEQTA
TSKPASKRAKTTKSAAPGKTQMKSKARRKK
>g2082
MATATITTAIELESHPDNVARTTAAAVHSTSSNYSTERREDDDVMQASLAADSEVPDGGYGWVVIF
SCAVLCFWFVGTTYCWGVFQAALVEQGVGSASTLAFVGSLTTACISFMAVLNARVVRKLGTRLSGM
LGISLFGVGEVMAGFTTGNVVGLFMTIGLVMGLGASLLFMVVSVTPAQYFKTKRGIANGIVFSGGG
LGGAITSFVTNGLLQALGVAWAFRVLGFITLATGLPAAYLIKERVPVRPTSFVEWRLFRDYRFILI
FLGGALATFPLFVPPFFLPLYTNSLNMTPSAGAGLVAAFNFSSAIGRLLCGFSSDRIGPLNTVFSS
LLLSALSALVLWPVSTSLGPLIVFVIVNGMANGGFFATMPTVVGNVFGSARVSVAMGMVVTGWGGG
YLLGAPIAGYLLNAGGGVKGGFDAFHPAIYYAGSMALGSASLMLMARLGIDRRLMQRI
>g2083
MSKPTIVFLPGAWLPQPTYASYLQALEQAGYPVCYISYPSCDSASADCQKDATTIREKAIKPLIEN
QGKDIVVLMHSYASLPSASAVRGYSKTDRERNGQKGGILALVCIGAFLVPEGLSCAGLQGGKLPDW
ILLNQPEKGLNVPDNAIEIFAADVEPALAEQMAAQLKPHSNLAFMSEQPSSAWTESAFDGRCAYIV
TGEGRAVPKAAQYQMIEGTGKEWIVKEIPSSSHMAPFMTLTGNCVQLVNEILSECNI
>g2084
MQFKSTALMALVTLTTASPITKRTTDTVTDSWPVSGFSVGCSPAACVYKFAVTRAAGPYNPGFNTT
CEGNDYTSDWQSCGDSSVSAKIVPKTSPFWEVDVMHTYDTVNKEGWAQALANATVEDTVSNFIVTI
YQTESVE

>g2085
MSFQATPADVSVIISTASATQSSSNEPSLATERRITPSWSISQLKGKLETMTGVPPGSQRLLFKSP
GRPDQWIEGEDRLIGEWGLVRGCEIEVHDTRPVAARLNFTDLSSVEKYEXPAEKYESLNNSVLAWK
KSQKLGRFDPNAQSPEDLLHQQVAKDIEAIEKKDIKLLARAIILPSSPPHIRRGTIRYIGPVAEIP
FAPLKDKKFTTEDGHPLEPFWVGIELDEPTGKNDGSIAGKRYFECAGNNRGVFVKPEKVEVGDFPP
LDLDLELDDDMEEI
>g2086
MATTGESQVSHDTADVKPDAEDYESKHVHEVYQQIASHFSETRYKPWPIVERFLLEQKPGSIGLDI
GCGNGKYLKVNQNVFIIASDRSDALVQIATRHQPHSTVVADILSLPHPESYFDFAISIAVVHHLST
PERRIQAVSAILQTLIPATDNDTDHKANTGGKALIYVWALEQKASRRGWDKGHEQDVMVPWVLRNK
PAVAMKNKYGQEESKDQNKPPEQDKVFHRFYHLYEENELERNIRAAGGVVIESGYEKDNWWAIASR
AAPSPTNQQAGGS
>g2087
MEAAAVPETDPEGEAEVATEEGGALVVEVERDALGVVVAAQVPEVTFLEAFLEAFLEAFLGASPAD
EVVAALMALRPGNFLLISSHFMPLPRNSMIKASSSDDHLDCFFAGGSFPSEDDECRFVPVGVEPSI
VVGFAVAVAVGIIADAGPGGPLGVGAPEANMEALLDEAAEEDDDGGRFVICC
>g2088
MIIESKSDDTNEVEIQAKVESNFEDHPSDMMIVLTDRERIGVPKPSSDPTIEDLKAIQQQVKARIK
TMIAEDIPFFTMDGIRNVHEQVQKCMAQGGKGDVFVKGLDGIIVRFQLHIGKTYDWADFTSVKPVI
EYAAIQHLTSEKWVEHLDPEIAFCNLVVYHRVKHLDKETREVATAKDAFRPKEDVGRDFDKSLHEW
MASRIRKIVAFACGSITHSDERSKGRSSYQHGLIKTLHDILSQARHEDVNLTREQKEKHIQCFIQD
PAYTNIDRELFQDDYGIATILENPEGFLEVDDSTVVLSFSPNVPVRQIVVDIAKPAVMIWDRILDE
DDRDVTDPDSRRMKSVIDGLYDQIEFPEDKAHFGDIAIYGRRA
>g2089
MSPAMATVGAGSAAKDIKRESGTARLLGSGSAGIAELLVFHPVDTIAKRLMSNQGKSNTAAALNQV
IFKEYAAASVGRKFTSLFPGLGYAAGYKVLQRIYKYGGQPFVRDYLAQHHGSAFDSTFGKGTGKAI
MHATAGSIVGIGEIVLLPLDVLKIKRQTNPEAFRGRGLFKIISDEGMGLYRGAGWTAARNAPGSFA
LFGGSAFAKEYIFSLQDYNAASWSQNFVASIFGASASLIVSAPLDVIKTRIQNRNFDNPESGTRII
ANMIKNEGAGAFFKGLVPKLLMTGPKLVFSFWLAQTLIPAFGKVI
>g2090
MSSPVLESRHDVSPFPTDVGTEIPSASEAGDLHEYLLNVACECLLVDSSRFWINPKYRLVASSLAE
LSRFIDAVGFSYCAGLYNVLLSSNPPSVEWFESLPSEIPKRHWGVYLLVLKRPGSDRPQLYIGSGT
AAIGGVFARVSSYHSSGSTSFPRYVGDSIREGYSIVHHTLLAWCPIPAREDLQKFRIAVIIMEAVL
SSLFWPMRRRTSLYGLEYTCPWSQDSFEWDGLCSHNPLFEGFNEAYLFFTREELEQIAIQYKSQFK
ARQRIHNEKQKPGTLLRQTVAVDTKQYYCATCNVPCRDNASLLRHNESPRHIRRVQNNNGPWVCEP
CGGKSFAYLSDWKTHTESITHIRNTPELGSLESRTESRDPNAFQCDPCGYNTPHESSLRRHNTSKA
HIKRLEEGLETARNKKYRYLTTNARALRRLRTACERAKRTLSSSAQTSIEIDSLFEGIDFYTSITR
ARFEELCQDLFRSTMEPVERVLRDAKTDKSSVHEIVLVGGSTRIPKIQKLVTDFFNKEPNKSINPD
EAVAYGAAVQAAILSGDTSSKSTNEILLLDVAPLSVGIETAGGVMTPLIKRNTTIPTKKSETFSTY
SDNQPGVLIQVYEGERARTKDNNLLGKFELTGIPPAPRGVPQIEVTFDMDANGIMNVSAVEKGTGK
SNKIVITNDKGRLSKEEIERMLAEAEKYKAEDEAEASRIQAKNGLESYAYSLKNTLGEGKLTIEAA
DKEKLEAEIEKTISWLDSNQTATKEEYEAQQKELESVANPIISAAYGGAGAPPGAAGASATREADE
VEERPEELD
>g2091
MDEMDEMDKMDRMDKMDDKIQDTTSPSIVVFMHSRSLEEGVTPDEGYHEEGATEEEGAMDADVASD
DIVQYHNL
>g2092
MAPAIGIDLGTTYSCVGVFRDDRIEIIANDQGNRTTPSFVAFTDSERLIGDAAKNQVAMNPHNTVF
DAKRLIGRKFSDPEVQADAKHFPFKIVDKATKPVIEVEFKGEVKQFTPEEISSMVLIKMRETAESY
LGGTVNNAVITVPAYFNDSQRQATKDAGLIAGLNVLRIINEPTAAAIAYGLDKKAEGERNVLIFDL
GGGTFDVSLLTIEEGIFEVKATAGDTHLGGEDFDSRLVNHFVQEFKRKHKRGRQNSIISSPIVVR
>g2093

MGDHSVTDYSDPAANAPDAAAYGKGKGKAVDAYDDVNMGEEEDDESSEEEEEELGAEDDDEEEGDN
LEPISAENIIEGGRRTRGKRIDFAEAAEKAKAEGDDPMDDEDDDDEDFHGEDHDGDHMQE
>g2094
MNSFSHSNLHQRDPRSSSSLFDSYSGDRGSRPXSRXPATNATSSTYANAYPGAVSNGYPNGGGGLY
PGGQGQQHGYRAATPNSRGHYSDAVLSELESQNDNEVEGITAKVKALKELTVAIGDEIRSSSNLAD
TMNEAFDSTRVRLRGTMNRMLRMAERTGVGWKVWVGFFLAVFLLFSYVWLF
>g2095
MMSVPLVAPRDSHELWFGSSRPRYRGSTNGSSEPSAPRRHQLHNGNGNLLTNVPTSTRALFSSTTP
SNSLAMLQLEERALRLRKENIASFGAAWIKPAGIPKTMQGMREEEAEREEGLAAAQQELNVAAMVA
AAQAGGVAGADAFGGETMLQQQQDMMVGGDNAGVAGEGDGIGADELERDLDDDIPEAEDGFADSDE
EGLVEEGEEAYEEELMDRNLDDDIPDGYPDEDDDDDDEEEEDYGDDDEIAGGDEIPVRDLDDDIPE
ANDLEDEDMDDDIPEAEEWQHTDSELEDDDDSDMDGDHTNTHDPFAQYNSPADGAVSTNTQTPAST
NTARRGSRRSSTRLPPPPQAEMRRTRETEAQRRFLQRWSGVGAESDSDNETLPSPMMGVDNEDEDD
DLRASITSASYARGQAARNARMRQAQSRFGRFMRRGGPRDSLD
>g2096
MAASINWNPLHRRTHLFNGLTPRISNEYEQTANTSQATENAAETEAPSIAEIDKALKEKAPITEDS
PMRLRMEKLIKEHQKKITDELSRIDGKAFQADEWTRPNGGGGISCVLQDGNVFEKAGVNISIVYGE
LPRPAIERMRADHKSFVGADVDKLTFFAAGLSLVLHPHNPNAPTVHLNYRYFETSDPKDPINGDKN
WWFGGGTDLTPSYLYPEDCKHFHQTIKDACDRHDSTYYPRFKAWCDKYFYIPHRGEARGVGGIFFD
DLDAHFLQSSSSSSQNPQETLFSFVSDSLGSFLPSYVPILERRKDMSYTPEMKQWQQLRRGRYVEF
NLVYDRGTSFGLKTPNARVESILMSLPRTASWAYMDPVSGTRTDAAAVAKGEEGGEETEEISRQKE
MLDVLKHPRQWA
>g2097
MASEHSVAKVLLEWINSFSLGKTIRDTEELSDGIILWEILQDIDPQYFLDELPERNPSDHWVTKWQ
NLKHLHKLLTGYIRKQFDDEIPSGLDPSPDLKAISESNSLKETNKLLKLLLIAVISSPNAETYVTT
LQKLSTPTQEGLKNIIEEAHNSQHEDLSADEDDRDGAAKRDLAVDPELQFEERVGKVLAENDRLAT
EKKEMEKALEDLHNRLARLQENNDTLQTRLASTEDRLVTLKSGKGDAGFSAKALESRSRQQEELIA
NQEAKISAAQDEIDSLTMSLESMRVKTQRFQKLQDDYDEMKTEREQLVRKANAAEKYRQKLQASQD
FEKENQTLKNQVKDLQQQLKDADSNQKRSSERDVELEEYRRVLPRIEQDRHEIQSLKKQLEFNNHA
LTERLQSAEEQRDRDEALISELRERIRELEGSTDSPSYTPGNASPRLQSTLQKDLDDIGARETQLK
SENEELKKEVHKLKTTNTLGSSLESYSENLVLTAQLAQAKSTQSDEYWKLYDQYADIRQKLADAED
LIDSTKRQLDDARAELQLVSKEKLDMIAEVKDSNSSEITKVREDWDELTRKIHHLEAEIDASQALV
RDMVVERDELRAILDEKQVAMRAEDEEFMKEIETLLAEFATRAKNENPDAPQKSGIELLKHFAEVT
EKSAETLARRAEYIDQQGDLIKSLQEQIKHIEDARSDQDDESIPKQREIELQEIINRQSRELSLVM
SAWYDLQGRLQHSNITVARYRHPATSGTGGGTEPKGWLARQRSLVSAASSGR
>g2098
MKLFHQYLIVLILGLFQIVLAVQVQKPIVVSYPDNTPDSVLNEAKKAITDAGGWITHEFNLFKGFA
AKASSSAIETIQASSANFLPVIEEDKIVTVDGDFGSK
>g2099
MSSGLPSDEVAEDYKNSLEDLTTNDKFQISNLTVIAKENTEHAMAISRVLENHIRTTPPSQKLPAL
YVVDSVVKNVGTPYTLFLGRNLYQTFMNAYTLVDQQTRRKLDEMLKTWKEPVPGSLDPRPVFPVDI
TRGIENALIKARTAALQQQQRHQPQDVLARGRNSATPPSWTHTPTPPQGVQHRYPAGSQAGHPQGH
GSESHTPQQGQNVDLATLHRDIENLIGTARAEFAANPFDVSIQQRLKALLDLQAILQRQQLSPDQL
KLVRDQVSALAPPKPVAAQPFPHPNLSIPNISTPPTQPVPQATPQPNLAALLNPNTLASLIQATAN
RQQSTPPPAVGGMVPQVQPSAVPQPTATPVGDNPLIASLRARGLLPPVSSTGTPPTGTSFSLPLLV
PGQTPPASVSTPQSATDITINVHMSTASIKIPRPVLINSLYDAKSNRCGTCGRRFTNTEEGKQKKA
RHLDWHFKTNQRMTDSIKRGQNRSWYVDERDWIKSREFDDDSGVTGTGAGGSGTAAEEEAAKKQPQ
KQWIRAPNDVTLRNAPCPICQEKFESTWSEDVQDWIWQDALKVGNRVYHASCYAEVTKDGPAARAG
TPSVRTGTPDSVLGKRKAEVIAVVQVGA
>g2100
MRSKFKDEHPFEKRKAEAERIRQKYADRIPVICEKVEKSDIATIDKKKYLVPADLTVGQFVYVIRK
RIKLSPEKAIFIFVDEVLPPTAALMSSIYEEHKDEDGFLYITTNQYRKELAACYRRELGGGGFFTE

ERDSPALKESSTDEFATLHKLCQFSCPPDIKFPMANLETLGYVSDEDETEVANAGSAYESPIMEAT
YRVSYTHQEYSGSRQY
>g2101
MQSEIKVSLGKAQVIDNVPKVIKELKFGALSDNDVISQAVVEVSDRRFYDLERDRVVQENGPLDRR
LGISGKMETCLTCGLGHKDCNGHFGHVKLVLPAFHIGYFKRVIGILQVICKECSAILLPEAERRAF
LKEMRRPRLDNLRRLQVAKRVVERCKKTRDCPRCGAINGVVKKAGTAALKIVHEKFRHFNQSTSAK
KVPPISKIVFDNSLAEARASNPDVDRFYKKAQDDLHALRVMNLFKKITNSDCELLGLDPTEVRPET
FLWQYIPAPPNCIRPSVSQDGASNEDDLTAKLGDIVQSNITLRNNLRKGAPVPTLMECWDYMTLQI
AIYINGDVPGLKGEAGKPTRSLVSRLKGKQGRFRGNLSGKRVDFSGRTVISPDPNLRIDEVAVPEL
VAKNMTYPEIVTRYNLEYLQKLVRNGINKWPGAKKVIKKDSGLTISLKHSARSLDSISKQLQIGDL
VERHLVDGDIALYNRQPSLHKLSILSHFVKVRPYRTFRMNECVCNPYNADFDGDEMNLHIPQTEEA
RSEAMELMGVKNNLVTPKNGEPIIGAIQDFISAAYVLSSKDNFFDRRSFTQICLYMLGPETRFDLP
PPAILKPEMMWTGKQVFNILMRPNKDDPVKVNIDAACREFKAPKDGQPKDLDPNDGWLVIRNSEIM
CGVMDKSTIGSGKKDNVFYIMLRDFGPPAAAEGMNRLSKLCARWFTNVGFSIGITDVYPSERLVRT
KNELVETAYAACDEVIAQYKAGKLEKLPGCDELQTMESQVSGILSKVRQQAGEECIKQLSKYNAPL
IMATSGSKGSSINVSQMVAVVGQQIISSERVQDGFQDRTLPHFPKNARHPPSKGFVRNSFFSSLSP
TEFFFHAMSGREGLIDTAVKTAETGYMSRRLMKSLEDLSAKYDGTIRTSSASIVQFSYGGDDLDPV
DMEGKAKPVHFDRTYNHAMAITHNNNERGLRPSEIRAITEELLSQERKRYVRKGLFGEDLPYNDES
DYGTDQLGGARAFLDSIQEHIEKKVVAMEKAMDNSLSDEEARMTLDHVGKLSEGSLRYFIKSCLFK
YEKAKIEPGHAVGAVGAQSIGEPGTQMTLKTFHFAGVGNLALTQGVPRIKEIINASKEISTPVITC
HLAVPDKLHAARIAKGRIEKTFLRDIIHYIRESWSDKEGYITVKLNWDTIENLQLELTLPQIREAI
TSHRRFKGSDLSVDVLRSHIKVRVDMDPSSKAGLSKTDIAATSADPYLRLKHLKRMLPDIQVLGHP
QARRAIIHSERGLNKLLVEGYGLRACMNTDGVEGVRTKTNNVMEMRAVLGIEAARETIINEMGEVM
KDMDIDPRHMHLLADIMTTNGEVLGITRFGMAKMRDSVLQLASFEKTADHLFDAGGTGRTDRVEGV
SECIIMGKTVSLGTGAMEVVRKLNFYEGHTGAKKTVFEDVWSETHRETRRKKR
>g2102
MPAMIISSPSSTSFQSGACSPNNRHFSPAAASGPSSSGAAAQFAASSRVSPYSVFNSPXPFAAPAA
VTTSASSPAFRQHRPSASRSLLSHSVEELRAALKXHTVAATTTSQDPAGPSGDHSRGCMSADTLGG
DSRQALTNASNNTTSADNNDTVQPAQTEPQSQPGAAVPQMPITNPSTVTSVTAISPSDVKGKRQLG
DGTEQAVSTANREIETTPNGDETEQSRLKRLRPPVPAVKLLPRRYELVHPNDLVILISSMIMELIQ
YNDTIPLQGGRLTRFHSRTPPRISVRDYLQRLTTHATLSPPILLSMVYYIDRLCALYPAFTVSSLT
VHRFLIVSATVASKGLSDSFWTNKTYARVGGITITELAMLELDFLWRXEWKIVPQPEVLVDYYLSL
VERCEGYALEPEQSDLVSAASSNMEGVVATPPST
>g2103
MLADQQRPLPLSTTTPQIQLVSQADEIDLKGRAIRFLDSINSTPHNVPNYPPNPFAIVTFTSPVRR
LSVANTGSSISLSRAHSVLSSIYRSIAAAGIRSTSRHKMAREEGPLLNRLLNATSVSEQTVALRAL
KHEIIGHDQRKESWVSSGIVQILSRILECSSREEYEANKEVVSGGRRGGDLTGRALMTEEQEICLQ
AVIIMGSLAQGGPAFISPIMASEAIPSMLAIISSTDCPQLLTVAILKTLSNIADRYPLQNINWPQD
NRLAEIMFTPETIGCFARIIASNSSSVATQTCATFAANIITRLCTEETHKVALAEFGILDALALKI
ASFVVSQGFVLPGAENHLGQDGALSMLPQPAPPAAKLGPILRATSIIVENSKWRAEHFLSSPGIVT
VFPGRLPEFSPTDIKRNPWGMPYLSGSAVPRQNHTNPMDALLPVVPLAQNNASTNFPPLVPIGSFH
KRSLSFLEAPSYEEDESALVPWLFHMVRTENGMSRVMAARLVTGLFRLGLARKHRIPMFNYLVTPL
LLRMLDKDYDFRDESDPEFDGMIPTAISLKEEIPSIIATLVMDTQELQKHAVEADAIKILSQLLKE
TYNPHSENGKPMWYAESKDKDMLSAVPATLQLGPVGYTALAPFRDEYRKAICENGVVPYIIDSLKP
RPPVETQDITGTKTTAADGNSIPTLLAACGAARALTRSVSVLRTSLIDAGVATPLFELIKHRDIEV
KIAATSVICNIALDFSPMKEAIISAGILPILCDHAHYNNTKLRLESLWALKHIAYNSTNDIKNRIV
TGLGAPWLAEIMSMDPTSQRSLAADFKMGMGTSNSAGEKVDLLNPMEDASRAEESRDDALAGGSSL
DALLADPNRRKKLAMSGEIDQSRQARQDDIAVQEQALDLVRNIMCGNGAAEIVDFFLAEIGHHQFL
SILAEKMRPRPNPTRRDTAANPPPPPMPAEILVAVTYVLVNLAAGPPKQRDLLFTHRELLKNLMWL
FEHPNRVVRVNCVWVVINLTYEDDQSDRSASHDRAVKLKSLGVIDRLHSVEEDSDLDVRERTKTAL
HQIRKYIMA
>g2104

MAPVPPSVLRALSLPLDLTPSKATLSTSGLGSGFSNTGRITISIPKPQTNSGLSEEDEEQRQYFLK
TSPDGEEMFQGEFASLNAIADAVPGFCPRALGWGPLDKGKGYFLVTEFLDLSGRSSRLFTTKSSKT
SLAYRLGKLHSTPICSSSSSAKRYGFPVPTYCGNTRQPNEFYDSWAEFYANERLLMILKESERKNG
SDAELRDLVTRTAETVVPLLLADDHLGYDYRGQGEGIYPVVIHGDLWSGNASQGRIVKPSPSPSAE
NEGDGGGVVYDPSACYAHSEYELGIMHMFGGFGKAFFEEYHSIVPKTEPVEEYEDRVKLYELRGGG
NHPAARNNPHNRPHHHGPPPARAVAEEVAKPYVFEHTIQECLSATGVDPQREDDIRISGVTWIDNV
RKALRLPVKTYNTACVYYHKFRLVHPDSQYSYMDAAAAALFTACKIEDTLKKSRDIVCAAYNLKLP
PSEQVSPDDAIFDQHSRGIIILERLMLEASGFDFRSRHPQKLLVKLLKQYGLKRDNEVGILAYCIS
LDLYRTFAPLKQTTGTMAFACLELASRLLDAGLKDVEAGKDYEDWNLGRAEVMETLLDLLDLYIHH
RSSTVVGPEYPLEAFLAIRIPLNKQSEDEGLPRFTHWRDSRVSVSVNIKATNGTGPKHGKNNKHKG
KGKGKDQRDREFENAAPAAVGPPPNPLTPVSANGEKPGLSDRGRDGTVRFMLDVERAEAEKKVVSS
YFRDTMEEVEE
>g2105
MMGRRNVRFPHRSNGDQALRSSFSEVSDGENPVNKTDGNPQEVSDAPALSDYEKKKQTFITRTIWT
FVMIAGFFSAMFSGHIYLIAIVTAVQIVSFKEVIAIANVPSKDRNLRFTKSLNWYFLATTMYFLYG
ESVIYYFKHILLVDRILLPFATHHRFLSFMLYVFGFVFFVATLQKGHYRFQFTQFAWTHMALYLIV
VQAHFIMNNIFEGMIWFFLPASLVITNDIFAYVCGITFGRTQLIKLSPKKTVEGFIGAWICTIIFG
YFMTNILMQYKYFICPVTDLGSNVVTGLECTVNPVFIPNTYQLPNWPLLPKTIHVAPMQFHILVFA
SFASLIAPFGGFFASGLKRTFKIKDFGESIPGHGGITDRMDCQFIMGFFAFMYYQSFIALHTASVG
DVIETAINGLTVDEQLEVVRGLSKYLYNQGVVTDTALECLTGELFRR
>g2106
MDSDDEFLDDGLDFDAIPTGTLLQLEQSAWQATQAPQHDERSQQPGLQTDRNQLNYESQYNAPQRR
QSGFYGNVEVIDLDADLLGNDEPLEDVVKSNPPIAQNVLHGHAASQRQYTPVQTQNIQSVQDSHDR
EIGAKIEEFSRQYDAINEQLASAREDVTKKTGEVAILRANLTKQAQEFERTIASLRAQLTELTTKN
KGVEQAAINDRQKLETENLFLREELRDEALRVNNLRAKKRAEAPQTPRKQRTLPFRDGFDDDEIGV
GSPTKSSGGRSKRGTPSVSGKRKRKVSDAPIPMAPLQLSQGIGAMEAETLALDSMDTTTEERGHIS
EGFEEGFYMTQILNHRNYPNAESDLEVMAXLAFPSEPDRKISSIVLETMSGVTSNYPVKYAHCLIQ
LWDRALVEKFYKVVPSFMDSIKFILLLDLYSISPQLTTELAGILQASIYVNAATRFEHSAVSSKVK
GESRRTPRTVLHADVDSTFALEIMYLLALACRQDDESHEHLWRNIHFDILLMTLNDHQPLQDIVLS
LKILSTSLRQNSFGTIQPSEAEQRQVEYHFIERVTCLLFQRIEPDEGEEDYLPLQICQMRLEALTF
LEMITFYSFEMTTDASKTAIISYPLVLPRLFRTLYDEMDRLYTLCPEHSIRTALINRLTHLIYRLI
RSGGGKNLHEGLSRLSGAKQKHRLVLSRLAFSEGQYLESGITEETMEMARQLLEEEDMSPEEAEAL
FDAFPTGGRLSGREGTQVEQWHIRS
>g2107
MRNRATTPLSKAKIRQSWSKYNLYNLHRFRPPPTSSRTFFQQKWTAKSTARSYHGEQIRESQWTRM
FSRRLASVVPMNPQQLASDDGSIISAGRGSGLESEGRKFTPKRTPYTNMTFAPLERRLDVAIFRAM
FASSTRQARQFVVHGAVTVNGKQMQFPGYLLNPGDLFQVEPERVMYATGAPKNKLERREARVAKRA
ETKNDETEAENSEESSETKAAEESKEVQSPKETLQRLLAQAKTIMSREKDVLPAKKKQELRGFQKV
VRSVLSKSATSTILADNLESQFSELLGLLKAKKAEPGTSRRNKKDTEAKSDNAVAEEITSGLSEEL
KDAFRQAAENPDSVDASELSEDEVEILKQAIAQMRDNPIDTSKPYATPWRPRDFMNAFAFIPRYLE
VNHKICAAVYLRHPVARPGFSEVPSPFGESIQTPAFAWYLRRR
>g2108
MFPWQGEGKGTSFILATQQREQRQQQAPQQPQSARQDNGYGRWRLPSVMXGASQAVNDKPSSNHTS
SNVANGRASSLSNREEALAGPESISLSASGDDDEEDEEEDGDAEQQGDSEGEDIDYRSSRNESNPT
ATTKNGTEKQATRPPGQRLGERMPPLGYPPQPSFRNNHARRLNTEEEIKLFELCIKHSSTFGERSK
LCEWWKNIANEFIENYGGPYSWHSVRRKVDLVTRQRIKYLADLKEGKIEADQATDPWKRVLDEWIP
TWEKFEVSEKRRIEVRDQRAAMRKRKEPPTSVTGGVAAEGSNPSSSPAPSSSTPAAKRKSTGATPS
AGAGAPGSWQATPSKWTPGPSPYGPPPYAIPAGPMAQPPPSTPGMKMPDGYDTMFRSPAHPTTHPL
YPYPPPQPYYTPPMSAPPAADNTLTSAVLETLTKLNKHLEKADGTSSPIVAALTTNNNNNTIDKPI
ENRTSSPQRDADAEDPQEPPSTTVISVTTLQKLKTELRAEFETELAKIREAFTAKLDALEQTQEMI
MDMLRQEPGREGSN
>g2109

MLSRSLWARQVAPLRRQVVTAPFAARRSVTTDAASAHAENIPEEDDKPFTVRLSDESFETYELDPP
PYTLETTKKELKQMYYDMVAIRRMEMAADRLYKEKKIRGFCHLSTGQEAVAVGIEHAITPQDKLIT
AYRCHGFALMRGGTVKSIIGELLGRREGIAYGKGGSMHMFAPNFYGGNGIVGAQVPVGAGLAFAQQ
YNGEKTCSIALYGDGASNQGQVFEAYNMAKLWNLPVLFGCENNKYGMGTSAARSSALTDYYKRGQY
IPGIKVNGMDVLAIKAAVQYGREYATSGKGPLVYEYVTYRYGGHSMSDPGTTYRSREEIQRMRSTH
DAIAGLKQKLLDWSVVSEEELKTIDKEARSFVDEEVAIAEQMVAPEPNNRILYEDIFVRGSEPLWM
RGRTIDETFYY
>g2110
MMDIDRARTRRERTFVGSACAVCEEPLEHTLRGERILQFSCTHVSHEACFYEYIKEFESQYCPTCN
APLGLDTSRGGNVLDIEKLSNIVRSASVSDAATTRSTLTTPTPWDNRNHNQQPQQESLRSYQMQQQ
QQQQQQQQDHNFSRRDSRDTSSQRERIERLTTNSSRRQTSHSRQDSGNTGVASSGEYTDGQQQSTG
RRHDYDVQAMESDLSPRSGAIKNPIPAPIVTVRSEFPTLNRSRQQQPLTCVVTVEVPDGHWRPDMD
DLRFTPLNAQLPPEDVYSPIRSPTVNSSRSVPAESRENLDEIAEDLRLRVENWHGLEFQRFGKLLL
HGSINVGKDRESWQELECYLFAEMLICVKEKKLASDSAAHESKRKSSRCTLKGSILIKKHLREVDV
VTEEPILTLNLIVSDLPCFHLRFQNRGQLDVWRRALAGLHNPEPVRDFDADSAMPEEEDYRTTKTN
KRQSSATSSYGANKSNNTALTEYTNTMRDTGPATFHVPLDLVVVIPVSSSMQGLKITLLRDALKFL
VQNLGPRDRMGLVTFGSSGGGVPLVGMTTKAWNGWGKILNSLRPVGQKSLRADVVEGANVAMDLLM
QRKSNNPISTILLISDSSTSDPESVDFVVSRAEAAKVGIHSFGLGLTHKPDTLIELSTRTKACYTY
VKDWMMLRECVAGCLGSLQSTSHQNVKLKLRLPEGSPAKFVKISGALHTTKRATGKDAEAALGDLR
FGDKRDILVQLVIQPDNATQEHVPQDPWQSLVSGLEALGGGSDGDEQRVVSVEEIPLIQADLTYGD
ILREGHLTHSPRPSLLAITMLPPNPRAKHGGRPSTPPIPPHPSIVQRRMELLASDMLTRALTLVSR
GQHDRAQHLLHETRSILKGLGKGGLPPLPPGANKPSTSNGLLNDPHSPAGTPNSTSPRSSHFQETN
SSSAASETATITPVAAVDQHVMAALDADLEAALEWINHPAVFSRDSRKAVLQAIGVVSSQRAFTYR
TPSESLWSDRVVGVKRLTDKSKEWREMGDDALTEE
>g2111
MSLSIPGPSQAGLFKPGYQSHDAEDGAVIRNIEACRAISQTVQSSLGPYGRNKIVINHLQKMFLTS
DAATILRELDVVHPAAKLLVMASQQQDSEMGDATNLVIVFAGELLKKAEELLRMGLKTSDIVNGYE
KAQDFTLKTLEDLEVDRLKEMRSATELSKALTTVIASKQSGNEEALAALVSEAVLAVLPKNPANFN
VDNVRVVKIMGGSLEQSRVVKGMVFGREPDGQIKKARKAKVGVFSCPIDTSQTETKGTVLLKNAQE
MLDFTKGEEDRLEATIKELYDSGVRVVVAGSTVGELALHYLNRFSILVIKIMSKFELRRLCRVVGA
TPLARLGAPMPDEMGNIDVVETTEIGGDRVTVFRQEDANTVTRTATIVLRGATQNHLDDVERAIDD
GVNVVKAITKDPRLVPGAGATELQLAERISAFADKTPGLAQYAIKKYAEAFEVVPRTLAESAGLDA
TEILSRLYTAHQRNKKSDSEEEDEDDDSENSAEESEEESDEDDEEPYWTTGVDLFGNSTTGTVDAL
EEGILDLMASKSWAIRLATESARTVLSVDQIIVARQAGGPKPPGQNPNWDED
>g2112
MSISSQAAARCCRQVIRSSASSSAPFLRISTSSSTFQQRRRTAVPSARRWQSTDAAAAPANPKIVQ
IVDQISQLTLLETADLVASLKSRLNIPDLPVGGFAMAAGPGGAGAAAAVEEEEAAPAAAEKTLFNL
KLESFEATSKPKVIKEIKSMLGLSLVDSKKFVESVPKVLKENVAKDECEKIIETLKGLGAKVVME
>g2113
MALSTASTSTEVPSSPPICTLSSLDHLVLTVKSIPASINFYTQILGMAHQSFTSPSDPTSTSRHAL
LFGSQKINLHQAGKEFEPKAATALPGTADLCFLTEEDVETVLEKLKEKGIEVLEGGKVVRRTGARS
ALRSIYVRDPDGNLIEISNPTV
>g2114
MSLQQSSQLLRPLCRSCARSPSFLLPSPTRSVTTISTNRSATRPYTISASRQGKYIQTSPAAEKKS
SGYGKILGPQIPYEEWLKNYREVKAKLEMSYMQMCASAHVREMIDPSITPKIFLEIGRRLIDQAYH
SKAIADSLKNIWKGHPCRIYEVAFAIRNSGPGLPYQHVLHWTMKATSDAGLVETLGVIVSAQIKKK
GAMLPNSREIEMIKKIAYETDDPKALSVWADVARIWGQTEEALTIFQHLNKMVYPSERRPMFTDDI
SYGGLIKPPWSAMFDIYHKSERFDEANEMLKIGALHYRDPQALVSYAFIRKEKDDWEAYEQCLVAA
AMSGHGDACFRLGNYYYLISKDEIPSRDQRLAERHRFRAWVAKIFGYAYKKEDWRRLASNWYEMAS
AHGISEGARNLAVLKREDGHPNAWELMEQLRSDPRLWKSKNVIKLRDQWDNPKFKPTLPKAWLEL
>g2115
MVDYSPPPRLRLHIRQQPIATRACSAGEKDRRTIDPPPILQLLMTDFRPDSRTDLATLQNPRLSVA

CLLYSVGKPGPDGKEVLVHSSQVVETTRRHRRDDGRAHNTYRIDSRTAHHINEQSDPSDQRPVQIL
SGRTYVSPFYTPYDPDPETAPYQYQHQYHTIKPPPTIFFVFADLSVRTAGLYRLKFRLLDWGLAQE
TGRPSPILAEIFSDQFRVYSSKDFPGMRASSALTWNLRKMGMTELKPRDGKGKGVAKKEKRRGEDD
M
>g2116
MSRYRGLVSHLSKRQPCHHRWLSNGSAHIDNSTGAANDSTVAPSQVLRYALSGDPETHTTTLRKTT
RSKINDDLALILAKRKLDRRDPHGRLISLPGGARVPTELQNVSDLQELKLWFEQFIVAGFSPMSEL
DVSLKDALVRCEKSSSSLEILSVLTTVISRHKDTDVSIPKSVIDAGLYYAVQSFSVPAFRYHLASY
RGKLDVRTTKELTQGLLRTLRTVRFADPGYDTRPILELVADTTGPGSTFLTVAQFGEGTYAPFIEL
LCELGASKAHEVMWDKLVEQIVEIQRSSKILTSAMVDDAYRSVVIFFKFGMTDYAMACMDFMSKTV
TDMSPSLDLATELAAFLNENGLEVPAMVEEPIQKALSQALEQQDSASTENTDLGNDMADFSTVRSL
LARINDNGTSVSASKIADLIDALNDCAGLAIPLFIESFENYDIEYAWSPQWIPGVSDSSRISFSLH
DSQTLGLLRAQIASRGIIVHSDRSRNLVQLGHLVRREFYSGASQESTPWTKTGYLVVFDRLSAEYL
IIYMGEDIAIIDPEYRAHPEQSPVTGYEQHSFIGPLSHVAMPTSVQDLDRDISGVITPVKNAASRY
VLDLDPGVNLQP
>g2117
MSSVEAEPVPIKDTTKPTEEDAQVSKSSNSSEEPEKPQASDDGASSKAQERLERFKALKARAKSAT
DSNMKETVAESQRLATDPALLSNLSRKHAFASHNLLKADTEEAGEDFERKRAWDWTIEESERWDRR
MEKKQRHRDDVAFQDYRQDARKVYKRQLREMQPDLDAYEKEKIAAVERAAASGGLEIVETDDGELV
AVDRNGTFYSTADSTDFTANKPDRAAVDRLVSDLRKAEEVRLKKRRDRRGEEDADVTYINEKNKQF
NQKLARFYDKYTKEIRDSFERGTAL
>g2118
MAAKRLVVAGGSGFLGSRICKHASVQGWEVVSLSRSGEPQWDTVTASKERPGWASNVEWAKADILK
PATYKPYLKDATAVVHTMGILLEADYKGVVSGKESIVSGLQRAFSSTKRGTQDPLNRGVGEELRPQ
ERDGQLTYEVMNRDTAIALAQEASFEHVPTFVYISAAGGAPMLPARYISTKREAESIISQSLPDIR
SIFIRPGFLYDSSRKFTLPIALGGMIGSEVDALLGGRLKSLVGSMTEKPLKADVVAQAVVEAIADE
TTRGVVGTKKIENLATQAWRKNML
>g2119
MPAATGTKRVKGVSVYRPFVFGSEAYPFDPENKPANAPPDHTHQWRVFVKGVNGEDITYWLRKVQF
KLHETYAQSVRTIEQPPFEVTETGWGEFEIQIKLYFVPESTEKPQTLWHSLKLHPYGDDIEGKRER
REKVVSQNYEEVLFNEPVEQFYDILTGGGNPPSQLSKGKSGKGKQALTQSGAGRTAEIPDSDSPGN
PYSKATENAELERLGNAMKTVDQMIKEEKDRLVEREKRLAELKETEGVPATTKKK
>g2120
MTVLLQSMEKSNGVLATNLNPNFEDGTMFANDNELILYGGLSRVTDGANPPPAEVILGYEAYEGSA
SIESWKPGFEQKSTNNSVTRYVTNGAGVSVPSENLGFYFSGMHGLMGDLVEGDNGSTYSTATSLIT
VNLAAMRNESWQNSSLPAHIPGRANAEIVWLPVSESGILVVIGGVINPEILSSNDNLTDEDIAASS
QNSPIFMKELPVYDIAFLQTTTGDIPPQLTMLCSVMAPVSDGSTFNIYIYGGYDGINASSAPSDDV
YILSISSFTWIKVYSGQSSHGRSGHKCIKVYPDKMFVLGGAYKNDSSNCLDGGFLQVFNLNTLQFQ
NTYDPNEWSNYSMPLIVTDRIGGNTTEGGNMLLPSSWGDGGLSSIFAETYTKPIATYYPYTSTTVT
PASSTATSMIDDSYSPSQALEGFTGRPARMLIKESHIDVPTKAGGQDGSMRIYIFHPTIPGYPNAR
FPGVVVFSEIYQVTGPVARFARQIAGQGYICAAPSSYHEFTGPEALKYDAEDTDKGNLWKISKKIQ
AYDEDASLSVDYLLSLPTCNGRVGATGMCLGGHLAYRCALDQRVKAAVCYFATDIHSATLGEGKSD
DSLKRAGDIKGELIMIFGKNDNHVPPEGRDLIRKTLHEKGVLFSFYEVAWAQHAFIRDELSKGRYD
PAISKSCFEMLLELFNRTLKLDLGDHDGKKLEIEDVYRADRRFLRRFAIPILCPTELQSEARGSRG
AVVAVVAGRGREYGHDNRPGRRSKAFQFKLISLPRSTFFRQYGFDAIREFPLCNLFVPSNQPPNLA
YGGCALTGIHLSNGRYLGNLGSILVAFIAVVTCILLLLLSERKRAAVGRREIQLFLLGYAIISICE
IFTVGAFPLADNVRKGFTAVHIAAITATSWVLFLNALVGFQLLDDGTPLSVGLFAGSAVVLFIGTG
YIALDTGFSWTGHFDSSLSGNNRNIGLYVLYLLFPLVCLFFFYILETILVLRVLGERRPLIYLTAA
ALLFAIAQVFDFVISPHICNPVHGKINGAFFETLFSLLSVVCVWLFWSSITEDDWPAVSSGYT
>g2121
MSNKQDATSLQNQENILPKTRLLLIVISCISLVLFTAFIDQNGVSVALPSIAADLNAEDTIPWAGTS
SLIANTVFSVLYGRLSDIFGRKSVLLTACVILAVADLLCGFAQNAPMLYFFRAMAGLAGGGITNLA

MIFVSDVVTLEERGKYQGIIGACLGVANIVGPFMAAGFIQAPVGWRGFFWTLTPIVASGGAVCWFV
LPASKVTGGFREKLSKIDVWGCLTSSAGIVLLLIPISGGGLYFQWNSAMVISMLVIGSCALVAFVI
IEWKVARIPMMPMNIFKNQSIVALLLQSFLLGWVYQSYLYYLPQYYQNIRGYSVIVSAALSIPLVF
MQSVGSVVSGQYISRRHHYGVILWSGFASWTVGCGLSILFDQDTNPGVCVVALLLIGIGVGFVFQP
TLVALQAHSPKSQRAVIISSRNFFRCAGGACGLAISATILQSGLRSHLPADLKYVASSIYNLPTFD
DSETTVVILSAYMKATKYLYITNTVVIGVCLCACVSIKDRGLGRNDEPAKKETSTPDAITVTESGS
EIMPAIDQTAVEVTKIGRGDIEKGGTISDLERDDNHNHR
>g2122
MATTKLSRAKEAVSSWSSFKKAIQTDDSPDKEEASSAYYNADLLPTPPSQRTWTTMHFFAYYLTTT
FSPSSYNLGSSLVSKGLQWWHGMIAAVIGSIFLSIVVVLNSRGAAKYHVGFPVYTRASSGIGGAKL
FIFVRASVATIYFAVQSYYGGQLTSVCLRAMIGQKWDSIPNHLPADAGITSKNLLAFFIFWILQFP
VMFLHPTILRHLFVFKAVYTTVALFGVMGWAIHKNGGSIGSFSFQTSSLHGSALVWPMISAINSVM
GALCPILINQPDVARYAKRYSQVTWSQSVGILLSKVLVMFVSCATTSATTAILGKAYWNVWDLYNA
ILTEYWSPAARAGIFFASLGMILAIIATNAGTNSLPVGADTSGLFPRYINIVRGQVLCALLAPLCV
PWKIIASASSFLTFLGSYTVFLMPICAIMVVDYWIIRKGNFHIPSLYTKAPGTPYAYWHGWNPRAL
VAWVCGVVFVVHGVAGSLHPGSVNAASTRMYSLGFFLSFLMGGFVFYLLNLVWPVPIYPTSAEADT
PKTWEYMTTTEGMLDEDSMDMITGGRILNGLEDGSHIRDENVTMSEKAYLSV
>g2123
MVHIEEKYQVPRDFEGFGEEGFDPKWPNGARIAVSFVLNYEEGGERSILDGDNMXEPYLWEKGSSG
GYKENARYLNAEQDFEYGSRSASWRLIRLFKEFGWNFTTYAVAVALKKNPTFAKALVRDGHEIAAH
GYRWLDVWDYNLEQDKEYIKKTLLLLKEVSGEMPVGAYFGRGTPQTHMLFPEVWKELGAEFLWSSE
VYNDDVPYWLDLPWEKSLKEEERTGMLLIPYNYDCNDGKFHMAPGFGSSVAESYENYLKNTFDMLY
REGGKMMNIPLHTRIIGKPGRCEALRKFMKYISEKPGVWVTTRRDIAKHYNEKFPYQPDGKWIETA
KKAGVMAIIPSAEIVKRQPQNVTYIYIKALENRIAELEIRLETPSNVAETPSLAQDVRTQTQAAQR
NALGEIAEFLTLGHFEGPAYVGSSSGFSLALNLREMVQASVWNKALATTDAPEDASIISTPETGPF
PIDQNRAVRDNSVRVRPLTLEEIRKKSAGPPSDEEGLRFISAYFTRVATRYPFMDWGEMEKLHSDR
LRLSTSNNLSRRERFGTFKLYLVYAIGSSLLALTEKDFASSSSSEVSVQTGSK
>g2124
MEEELRATAASLGFQVAAEEVDEYVALLERMEKSLQTVSDMPDYQPTPDHAIYPRSDIHFPTADSN
PLAAWAWKFNATSTAPKSDLLKGKTLCLKDNIAVAGVPCLLGTDTFKNWIPNTDATVVTRILDAGG
IITGKAVCENLSRGAVSVTAATGPVHNPYAHGYSAGGSSSGTAALVASGATDMGIGCDQGGSIRIP
ASLCGLYGFKPTVGLVPYTGIASNDACLDFVGPMTTTCLDNAILLEAIAGVDGLDDRQSAGAPFSG
QVPHYAQILEETKEDGIKEFKIGILKEGLDLPGINPEVEKKFQDAMDVFRQLGATVEDIHVPIHLQ
ARTIIVVLSKMGNHMGMLGKATGRRQMMLTDLFEKKNLPYTPEALDKMSVMIKEALISGEYAWKHY
STLYPKVVNLMRKLRDDYDKALEQYDVLVMPTTITESNKLPATDASPLEHINASKGKLENTSPFNA
SGHPALAVPIGLVPSRENPSIDLPTSMQIVGRYWDELTILKIAYAWEREIGNWKEF
>g2125
MADRSKVRIVFKTMALNIFAFASMIGAGLANSHSIHARIQNGLAQTPQMGWNTYNHYGCSPHEAIV
HSNAQALVDLGLASLGYRYVTTDCGWTVADRLANGSLTWDATLFPSGFPALGQYLHDLGLLFGVYQ
DAGILLCGSPPNQTGSLYHELQDAQTFASWEVDSLKYDNCYSDAATNYPNVNYAPSTSPEARYSNM
SNALSQQDRKILFQICDWGVDFPAAWAPSLGNSWRIGNDISSSWSSIYRLVNQIAPQTNFAGPGQW
PDLDMLEVGNNVYTTAEEQTHFSLWAILKSPLVIGAALKDTTTSINAISLAILKQEDVISYNQDTL
GVSANLSRRYTEDQYDVWSGPLSGQRTVAALVNWADEARNLTIDLPDIGIQYAGTIKDIWGNITVQ
GVKTSYTTEVEAHGTMLLELQNTTPSGQYSASIFGTSSGNSTTFEAIYGLTDSSNYTLSITFSSTS
SASETITVKSSVSTQTSTVTVASNRASVSLPITLAAGTTNSLTIQHQLSISSITITPPEGTYYPNT
GFTTTGYATSTTCGTGYCLPVGTKIGYIGPGGTAVTTISAASAGTKFVEIDYINNEVAFSSGSNAR
NITVTVNGGTPTRLEVPLSGQHSELFGPGLGWWDTATLALPISNAQLTGSVGPLTTVAEKAAIKTC
NVLDYGAVADLSTDLGAPLLAAFNDCIHGGLVYVPEGNYAMSTWATLDKGTGWALQLDGIIYRNGT
GGGNMIIIEHASDFEMFSSNSAGAIQGLGYQFHADGTSTTTRLLRLQKVTSFSVHDIILVDAPAFH
LVLDTVEDGEVYNVAIRGGDEGGLDGIDVAGSNIWVHDVEVTNKDECVTVKSPSSNILVESVYCNW
SGGCSMGSLGADTNISDITYRNIYTWSSNQMYMIKSNGGSGTVENVLLENFIGHGNAYSLDIDTSW
AEMSTASGDGVGYSNITITNWKGTESSGLQRGPIKVLCPAGNPCYDFTLKDFAMWTESGSSQTYTC

SNAYGEGFCLASGDDHVAYTATTTTVVAAPSGYSAATMAADLTTAFGISSSIPIPTIPTSFFPGVT
PYSALAKASTAAAASSVTAAGNVLAVSSSVVTPTVTATAAVTSVAASSTSSATSTTTAAVSPVAVS
VTSSTAPVIVAAVSSVAPSITSSTAASTSTLISSSYQESSSASATVGVISSITIPFASSSVASHTS
FSSSLVSSTQSVPSEASSTGVPEPEEEEGCPSGSEHESHAHDHGHGHLHRRRVARH
>g2126
MSSATTTTRGRETVKRRQTPAPPLSVVNEETSSSVKSNGDANGHTNGHANGHANGKSNGNANGHAN
GHANGHAKGDGHDEKQVIDGWIPGEDPKIDHSNHFEFGGSLGTFLLMIGFPCLMYYMWIGATYYDG
NLPLPEKNQTIPEFLAHLCNLVIEGAWPSFKAWLIYWVFFVFEALCYIWLPGITLTGRPLPHEGGK
QLDYYCSAVSSFYTTLIVAGVLHFTGLFRLYTIIDEFGPLLSVAILSGFLVSIIAYASAIYRGAQH
RMTGHIMYDFFMGAELNPRMFGILDFKMFFEVRLPWYILLLLSLGTAARQYELYGYVSGEVCFLVM
AHWLYANACSKGEECIISTWDMYYEKWGFMLIFWNLAGVPLSYCHCTIYLANHHPDTYRWNRYALA
ALFVAYLFVYWVWDTANSQKNRFRQKERNTQVSRNAFPQLPWQTLEDPKKIVSPQGTILIDGWYGL
ARKVHYTCDLYFALNWGLITGFKSPFPWFYPLFFACMISHRCLRDIQRCRAKYGDAWKEYERKVPY
LFIPYVI
>g2127
MSSSSGQIVRNLSKSTDYSSNVSSSSASFQGLNNSIESGLQRRSGGNGSFGAGSTTRTVSAARNNQ
AKKGQHKKHHRPNLVNEDAYSESAIMRSATSRKGQTSITHLMSFSLPPRPQYHPSTHRPRRNPTWG
MGSGYHAIDKARYVHANYRFIVDPRRNYHAQAANADVHLDWDSVLQVLASEQTQAASCPICLGMPV
APRMAKCGHIFCFPCLIRYMHSTDDDASVPEKKARWKKCPICWDTIYVSDTRPVRWFIEQETNTPV
EGGDVVLRLVKRNPGSTLALPRDGAEELHAQDDIPWYHAAEVADYARIMKGGEDYMVSQYDAEVEL
LNQQRQEDELLFGDDSTWVQKAINSIKDAQEKIRGIGNPPPSARTERKRPQTHFDEGLVQEADKRG
LENLTYNASTSELVDEVIAGVEAVNVDGSDHPESQKKEKYPFAAKTSAARDGGTTTDQPYYFYQCL
PHFYLSALDIRILKAAFGDYSSFPATILPRVEHISTGHVLDDELRKRAKYLGHLPYGCEVSFLECD
WTDVVVPEVLEKFRPEIKKRRQRNRDKEAREEKDRIRAEKEEEEKRWAAARRKRPSFGFTSAERPF
SDQDFLPLSSGSFDASAITDSEFAAISPPNGSLTNQMQSHFEALASPSTSPPVARTVWGTAAVQSD
PDQQPIHRGPVNDGWLQGWEKDFLGHREAELVAQATAESSATASSASAGGGGGGGGGRKKKNKKIT
LMSTNIQRGA
>g2128
MRSPPAYMNYHPPPVNGHIPPMYAQHYPPPWYNGYPQMHAPPPGVPMPPGPPPPRPYQQPPYSHGP
LIVSSSYPHSQPVPAPMHVPSNVYSQPPSTTSTPIQSATSPPPMNEYQMIIDGHERPSSVHSALST
PSGIASSPALVAELPKGGLLPHIPFRAPLPWLSVPDEPFPIRAPHKRRSGRSLQTSKVAVELPAKD
SQKQDLAHVNEKQASTQGDSLSEPQTPTTSQAPSESDSTHPTTPSSTTLASRSRDQIQTPKQTTIV
PVVPILPQVPVTPKQSGSAHEHEQRDSAQVPGEAAGQESPEQATTPAPAAPKSWADLVRAKSARAA
GIVPNVPVESDNLQAPRSESLGDVLASLGPNVDQFGEKIAFLEPRGLVNTGNMCYMNSVLQILAFC
VPFYQFLDYIGHRATHSFKSDVPLIDATIMFMREFRVIDAARSEDQLRMRLKHKELEEYGEAFIPE
FVYEVIRQLPRFRDMRRGHQQDAQEFLGFFLEELHEECAHAFKNGASQVNAASTEPSSDADEWLEV
GQKQKSAITRSSGDVDFESPITKIFGGKIRSEFKVPGNKNSVTLEPYQPLQLDIGSPEVNNIIDAL
KGLTKPETIHGDFSSPRGPKVAATKQIFIETLPPVLILHLKRFQYDSVTHGTQKIWKKVGYPLELE
IPREVFPPHRRNAMAIHSGLPHYRLIGVIYHHGKNASGGHYTVDVRRQDGREWIRMDDTVIRRVRS
EDVAEAGGEEDPKVLAAALEQHKRDNNPNIYEQIDQSNQIDDEAGWSQVNGGPNGHASKKSTSGVV
NGVSSPAASANSPGNRTPLGRYGARDNKVAYLLFYQRISAP
>g2129
MNNHAIAAKTTPSPSPRPGLAAGASPTQTSTSGGATSGTKRKRAAAVKYYAVKEGFRPGIYYNWND
CLAQVTGFKGAVFQSFPSVEEANSFLTGQKVPSRNGTASTNNDNSTRFYAVQRGKVPGVYSNWAEA
QEQIKGFTRPRYKKFDTRKEAEEFVKMVEEIGANKSSLQRPGGLSTTNPADAANGDVTVPAAFAPL
PPGAEDGFDPNVLLNPKTGKVVYKEPEQKLMTKTKPTGPPGMLRIYTDGSALGNGKKIAQAGVGVY
FGPNDTSRNVSEPLEGYRQTNQRAELTAIIRALEIAPRHRPVTIVTDSRYSIECVTNWCRKWSRNN
WQTSNGKPVENKDLIQSILVKINERDSLKVTTTFEWVKGHNRDPGNEEADRLAVNGARRGAGEQVS
AVPELRAADNDIPDELADDNFDDF
>g2130
MPTLEITNFNIVCSVLGGFITLFGLVSYLFKERFYLSEALISTLAGVAFSPHATNFIKPMDYAMGS
QANLDAITMYFTRLVLGVQLVIAGVQLPGRYLQTEWKSLTLLLGPAMAIMWLSSGLLIWGLVPNIS

LIQALAIGACVTPTDPVLSNSIVKGKFADKNIPRPLQRIIIAESGANDGLGYPFLFLALYLMKYTQ
GPGFDGGGGKAIALWVYETLVYEILLSVVYGAVVGWLSMKLLHWAEEKRYVDRESFLVFAISLGLF
IMGTCGLIGTDDLLACFIAGNAFTHDDWFRLETMDDSLQPTIDMLLNLAIFVWFGAVCPWAQFAHN
DIVPIYRLVVLGILILLFRRLPIVFAMHKYIHQIEQISQALFVGFFGPIGVGAIFYLSVCRQFLLE
EMIVDGEVRADAARTAEVTYIVVWFLVICSIFVHGLSVPLGKAGYHIPRTISTALSSASGEQEAIR
LPSSSNTHSTATPQSHRGLRFYRKRGKAPSPREPPRIVFQIAGSPQRGEEQQGDQQAQQQREGVEG
AAAANVGPEETLSPLSTLPTVPTPVTIHEPRRPIRLMSDDGQVTETLNDYE
>g2131
MDSQPSPPHTLTQTVKETAEQYSLFILIAILIFSILSTRTITGLQSQSHADSADGEPRRVRKLPYW
VPGLGHAFSYLWRHREFLLDAQKTMNEPVVALQMGLQTQYLVYSPDMIKSLINSGDARFGVYFPRV
MEYFVGDNGASRRLSPQARQAIDATLIPLTQEPYLKKFTSALAKALERETADFVSFNRSLIDQMIW
ERNTGISVIDSNGIDSQSCQVGLFALTRCFASYHITYVLMGQALLDFHPGIVEDLMSWDQEYVRLI
AGAPRWIPSPGISAAYAARDRVQKTISVLQASFSALEDGRNAPLEFRDLDDVSDLVQERMRCWRTS
GISASLSARLDAWLLWKMTTDASKLVFWNILHIFSNPDLLSSVRKEIEPYVKVSRSDPKVTGLPIA
EPPRLSIDTEKLFTSCMLLKATFAETIRLDANPISYRQLTADLTLTESEEDATVFGRDSEKNPRSY
QFYKNDIIGLASGAHHADSRYYRDSKRFTPHRFLSRAASPPSSKSRENDEKPPVTRFDWQAVQPFA
GDETFSTLIEREVMLITASIISLWDVESVDSKKELKIPGHSLSANAFLPSSDVKVRLTRHE
>g2132
MASNTSLDGCTIVWLCPLEVELRAAIAMLDKVSDECPPRARGQNVVYTIGEIGLHKVAVVGYYQEQ
GLAVSGSIAAEVLRDLRNLQLGLLVGIAGGIPSSTHDMQLGDVAVAVPEGDRPGVVGYDFGKAGDD
DHYQLKHWQNSTHPLLGSVITKIRAHDELRFRRHLQVLDRLPEFRRPESVSPHSTNDHPKVHYGTI
LSGNTVIKSKSKRDELRQLYGGIAVEMEAAGMMTRLQVAVIRGISDFADSSKDDKWQPYAAITAAA
YAKEVLLRLPSEHQEDWKYQDLRLAQVLPEKSAFVGRAEEMAFLEEELGFQDQAPIQKSVVCLWGL
TGAGKSQLAARFVSQQRLTHPKREIFWINGENRESFERSITKDARWLLVIDGVNETPPRSASKDPA
SFDIHGYVGRLNRGYVLLTSRRRDVVERYYPSRELKGLKIQDAVSLLHLKIHPQLMEGAEDLVGLL
KGLPLALCLATSVISRYRCSVREYLEMWKNCSQTSEIMGTDETLYRSLELSLEELEKADPVAAKIL
TLFSFLHHGDLWYDLCYNAIDDTYPTWLQDLGNEKKSFRHYCPLLADLSFIELRITTNGSRFWETH
PAIQVVARQRAKASEQEYIRCAVSLVASEVPRSFEANSWDTIRRLGPHVRLCWDYVKRGAWGSDTN
LTDLESLGRVFRHIGHYSEASIIYRMIEHGLGFLPPTRDNNEFLANVLTNLGLVYTRQRKFNLALG
AYDRSRRLMTKLDILTPIESMSIMYNEAVVFMMTERLNEAESLLQKAVTLVSENTIHGHTWRRNEW
NVLYLRILNDMGEVSLRKGAGEAALKFFRDVYDSQRAWHDELHPTLYSIKLNMGRALTKLGRFAEA
RALLLEVIAVYTEWWGRHHPETMRAIDELAWASMEEGKEKQAANETAGVEIQNAEELWTEVLNFYQ
STDGDESDTVTRIEANLQYLYSLRQEMTGLKRIQ
>g2133
MPPKRSAAAGKKSASGLTRTYLFLYNLLNFTLWATCTIRTALALFEKYQQNDLRTISSIPNDVFPL
LLITQSLAALEILHSLIGVVRAPVITTAMQVASRFVVVWGIMYAFREGSELYDGVLSKYFPDIPFV
RQFTSQGLMGVPGKTQYGDLAFFGCMFAWGITECIRYGFFVLQLGGLSVPGWWQWLRYNTFFVLYP
IGISSECLFMYLSLDHAEKYVHEYYKWFLMLVMGIYVPGSYILYTHMMAQRRRFMRGKSRVD
>g2134
MPSPTTAPTGQAQTSKPKLITNPLRLLILTPSDQSNTTIPALLQSLTGVPVTPPPASSSTADNGTE
IQTFAGYTTHAPLPISNKYYAADVPIWVDEIPTSPSPDSPSATPNTGEEDIETTPQWKTTFLSPEA
REVRDAIGAIVLCVRNPSLSISKAVPREALELATAATTSTTTAGSSLTLQSAIEQDGMDAGDRQDV
RTIKELVGVVSELKAKIEEERNCGEEDEQESDVVGGAEVPGILVLVDEHSSSSSASKKKEYSSDKE
EEEGLEAEEPFSSPWWEELLYEQGVFGMEVIRWTPSSSSGANMDVPETRNIYGELEGLPRLKEVLS
THEWTAASSTDNDDGDDDDEDDIMSFLNGGTRRDESTGFRFEVNQLEREMMGLRFAINGGDDEDDN
DGNDEEEDTELQVENLEALMMRMRAIKGLFRSKYTCKNGQADFVEVDMSSDLPESKRKAFAAKAVK
DIMREF
>g2135
MGKLTSTIGIPIKLLNEAQGHVVTLEITSGQVYRGKLLEAEDNMNVQLKDITVTARDGRVSHLDQV
YIRGSHVRFFIVPDMLRNAPMFRTQLGVNDGVNGSDSMRIFMFGSVSIQQALKIDIARFRSFDMRI
FPIGGYP
>g2136

MAPLNTNLQAFNLTVSTLLKQPSHFLPNLTIPTFLDLPEQIGHQLASSSSATITGQQPKRIPKIKA
LILDKDNTLCPPHTTTIPKPYLDKLEQLRTSPTSPFNLNTNPDGVLIVSNTAGSRPDVEKYVDEAR
TLEDKLAHLRISVFRVQKPSTTTSKIDGAAGPGGRRKPFCGPDVLAWFRERGVIDRPDEVVVVGDR
LGTDTLMAAQMGSWSVWCKDGVTHSVTDQPKMDYRGFLAKVEIVLERYLRESGGLKARVPEGWERA
TI
>g2137
MSSIDNQPAVAGSNTATPTNSTPSPSTTSPAASAPSSSASRRPPRKSTLTQQQKNQKRQRATQDQL
VTLETEFNKNPTPTAAVRERIAQDINMTERSVQIWFQNRRAKIKMIAKKSIETGEDCDSIPDSMRQ
YLAMHFDPHKPGARELXGXANGLGGLGNGFGAESTPGKIVIQHFXCRTLSIGSWRRIGQNAMDLVI
FYSPDKATMTYYINNDSAGYKIEYPFSYIKSVMLDNGDLTPNANGMPTRPGGLVIELNRPPIFWMD
CSNSGGFFQCGDFTEDQQASRVMTHHLGGHPKVLSVQLAKLVSLETFQNRLDFNYSVSAPITPPQD
IHRPASQPNRFTLAQMGMYPDSHLSVNHMPRGHKRQSRSRSVPAAIDLSYLHTPIAPFHIQQPSVSH
MTPNPNMYAPIPQNPNAVHTLGNELRIDTTATFVDPRSYPMSATTMSDFAAASPSFFSAAPSTESI
AITGNPGDQFNMSYMSPSPMMEQKMMSQPPTSMANFSHADPLIANHSPPLSTMHNTISNDMFNFGG
DQQQGLTEDGFVLSEMYAKHHINHFQDGPSFDFSMNAISETQSPMTGDMQGFDMSQTTSA
>g2138
MFDESHQQTTIPSNPTVSIDPTKKYELLGNCIKQRQMEPIDLAAGRLDFDGVDPEIGMHLLSVFWN
RQHASGLVVYRPAFMRDMACSGPYFSKLLLNAIFFSASKNSPRTEFKCSGVVTEAPAAVSPFRIRA
EELLSMSTSGLPTRMKSDITTIQALLIMSEALFAWCDERSLSWLYSGLAINMIVDLGIHTDRRTPA
MNKAFTSEDSEIRRRLFWAAFVQDKVQSIYQGRPARLREQDVNVPIAFMDDYEELDQFTSLSYARE
DISMSFPTRSVSVFEQQCRLSLIMDRILFGIYAENYNSRSTSQLLNTARALHQDLEHWRKSLPEHL
NMNFDASSNPPLTPHALSLMSMYYSLIILLYRPFVSDGHVNAISPSAIRDAFTTCAAAASGIDATL
RVYLQRFCITTVPYFMSFATYVSGTIHVRIAAQSGRESEAYKSLENCLDILSQQQSVCRAPRRARR
ILLGLARRLNVHIDDSSASMGNTRMNSDTNLDAPMHASTLENPQSDFELLMSGLDIDAIIQSFEFD
QSSMMNQAEHMNSSSAATNADDFSQWMLQDPLFGYDSLFPMDSSSA
>g2139
MTSSFETSIQTAISNGGLAGASVMAATKSQGLVYSKSFGHRSLDKGTKSEKLQVDDIMTLASASKL
ITSIAALQVVESGLVGIDDDLGVVLPELGALKVLKEVDGEVRLEERTEKITLRQLLSHSSGLTYGF
ASPLVQKYNISQGLPPFQPFKTVTKSFSEPLVAQPGTIWEYGSGMEWAGRLITHLTGQSLEQYTQQ
NIASPLGIRDLTYFPHKNPEIDTAKLVPMTRRDPSTPNGHGGKLIPDTAPHILSEATEEMGGVGLY
TSMASYIKILHSLLVNDGKLLRGETVDTFLFEPQLSASSRESLQGLFSHIEPGQESGAPPYVGTFA
QVRYDYSLGGLLSLEDVDSAGLRWRRKGYLCWSGLPNIFWVCPLLNILIMKYERIDSVHQFIDRQA
GLCGVFGTQVLPNADEQVREVIVGFEKAMYKDFA
>g2140
MTISRLSSVLCALALGQSVLAAPQLSRRATTSLDAWLATETTVSLNGILNNIGASGAYAQSASAGI
VIASPSTSSPDYYYTWTRDSALTLKVLIDLFRNGDLALQTIIEEYVNAQAHLQTVSNPSGDLSNGA
GLAEPKFNVDTTAFTGSWGRPQRDGPALRAIALIDFGNWLLDNGYSSYAVSNIWPIVRNDLSYVSQ
YWSQSGFDLWEEVNSMSFFTVANQHRALVEGSTFAGRVGASCSWCDSQAPQILCYLQTFWTGSYIN
ANTGGGRSGKDANTVLASINTFDPQATCDDVTFQPCSPRALANHKVYTDSFRSVYSLDSGIAEGVA
VAVGRYPEDSYYNGNPWFLTTLAAAEQLYDAIYQWNKIGSITITSTSLAFFKDVYSSAAVGTYASG
STAFTAIISAVKTYADGYVSIVQTHALTNGSLSEQFDKSAGTELSARDLTWSYAALLTANNRRNGI
VPPSWGAASANSVPSSCSTGSATGTYSTPTGTSWPSTLTSGTAGTTTTSATTTTSVSKTTTTTTSA
TSCTTPTSVAVTFDEIATTTYGENVYISGSISQLGSWDTSKAIALSASEYTSSDNLWFVTISLPAG
TTFQYKYIRKESSGSIVWESDPNRSYTVPSGCGVSTATESDTWR
>g2141
MSIQPTLRRVARASNAHNHTVHTAFANPHSQTFQLHDGRVLGFAEYGNPNGRPLLYFHGFPSSRIE
AEPADDMARRCGIRILALDRPGFGLSTPQPGRKIVDWPNDVRDFATGMGLDRFAVMGLSGGGPFAL
ACAHALPKDMVAGVGLFASGGPWAAGTHHMSLMRRMMRALAVYWPYGLGLLLNVLVRGFRAVATSG
PIVRRVDAWLEAQDKKKKEKEQESAASIENDTSIANPKKTPAERRKYLLDVLINEPFVQGASATVL
EADLLSSPDWGFKFEDVDFDPVRIWHGSKDGNSPIAAIKYLAQRLPHGVLTEYENDTHYTMFSHLE
EALTELARDYDAHASDISSTS
>g2142

MMDIDIAPSAFESDPEKKIQPHVAAERIGDQIPHELLEELAPNGESEYILGIINSMGEEEAIAIVH
ESLEFHADDWNFPSDMRERMKRLLEGPKLYGEFYDRDLRIDATMMRYSSPYPGVRAVAELVDNPDV
PIETIRAYFLGIGWAVIGTFMSTFFNSRFPSIGLSGSVIQILLFPCAKFLEAVLPDWGITVMGIRH
SLNPGPWTFKEQMFATITFNIAIYTTNSYSMILVQKSPLFYNEKFITFGYQLMLTLFVQLMGMGFA
GYLRRFSVYPVKALWPTILPTIAMNRALTKTEPRENVYGWTISRYRFFYIVTLCMIVYYWLPGYLF
TALSTFNWMTWISPQNVTLAIITGSVTGLGLFNPITTFDWNVATSSYAALAQPFFSTITMYCGSVL
GAFIILGMYYSNMYNTAYLPINSSSTFANDATSYNVSKVVIDNNFNETLYQEYSPPFYTAGYILTV
GANFAFYPVYFLYIMGNQWKTMKKAYIDFYQGLRHGKGNYEGAMDVHSRLMSKYKEVPDWWFILIL
LAALVVSIVFLQIYPLSTPVWVTFLMIGINLVFAVPLSFLSATTGTNLGLGSLIQIITGFVLPQNP
NAFMFAQSLGSWALAGYGDNYVQDQKMAHYCKIAPRAVFRSQIGTIIITCFVAVGTQDFVLNNVPG
LCTSDQPSHFTCAGDGYPLYTSSLMWGLLGSDRMFNSLYPLFKWCFLIGFAIALIFLVGQGYGPRY
LPRLRESIRTKVRPNTFETLDRTLFRFIASLLWLNPILIIQGIQHWAPSNLSYKTPGLILSFVFMY
WLPRHRLAWWEKYNYVLSAALTAGVAICALVMFFAVEYHPKTLSWWGNKVSSAGVDGSGTGIFPIP
ARGYFGPDKGTFP
>g2143
MSLSRLALGTLLLHIVNCQNADSLPQYDFIIVGGGTAGLAVANRLSEIPDITVAVIETGDSVFNNH
NVTQTTGYGLSFGTEIDWAYETENQTYAGGSKQIMRAGKAIGGTSTINGKVPVSMLPVLDETLSEV
GVPYDRDVNDGSMVGLTSHPNTIDRDANMRYDAARAYYWPYEDRSNLKIISNTQANKIIWAEGSQS
EAIAIGVEVTGDNGTEVIHASKEVILSAGSLKSPLLLELSGIGNPDILKKFDIPVKVNISTVGENL
QDQTNNGLSYDGREFWLGSPTFSALPSVDHIYGDNVSSVASAVNSSLSDYAKAVSNFSNGAVLEAN
VQAAFQIQYDLVFKHRVPYAEIVFLPIAHSFTTEYWSLLPFSRGSVHMASSDPSHPPSINPNYFMF
EQDLNSQADVARYIRKIFNTSPLNSLVGDELAPGLDLVPEDGPESKYTSWVKSTYRSNFHPVGTAS
MLPREKGGVVNADLKVYGTENVRVVDASIIPFQVCGHLTSTIYAVAEKAADIIKKKYT
>g2144
MVKPPENPSAKYYLGFSMAPAATADLASPAPKILPEPFNTLSNAPINDWRADLRDNGFAVIKGAIP
RERALEYQQKARQWLKSFGNDKLDFENPETWLAENLPVQSTINTFSAYAVAHEKFMWDARLEPGVV
DAFARIWGTDELIVSFDSLNITFPNRKDVPRKGSWEHVDQSPLRRGMHCVQGIINLSTAGPDDGGL
VVYPGSHNYLEEFLTTQTDPSTWESKDLFWFHAEQLAWFQKEKGIQPHKVCADIGDLIVWDSRTIH
YGAEPTEASNTIRTVIYAAYSPSKLASTETLKRKAEVFRNYGGTTHWPHDNVVVRKRGAIFPNGNP
DPRDREVPLELPEMTDGLLKLAGVKPY
>g2145
MADEHTENPKDTKHVLDVTVADADDALRALADVQYQSQEPLSPQEDRRILRKVDMCLIPLLFFSYL
FQYLDKQAMGYSAILGLRTDLHLVGQDYSWASSIFYFGYLVASGIVAVLIVRLPVGRFMSGSMGIW
AAILMLTVLSKNASGLWAARFFLGFVESAIAPSMSLIISMWYKRSEQPIRQAAWFMGNVIGGLVGG
LLGYGIGHVNSIAPWKALFLLFGGITFAWSILCWFLIPDSPLTAWFLSQEDQVKAIERVQDNLTGI
KNHQVKRYQVVEALLDPKTWLLALFQFTQNVPNGGAGSFASIVVEGFGFSRLNTLLVQMIATAYQI
FFIVLSALGSTYLKNTRTYWMALNTIVALIGTVMIREIPAEQIWARFAGYCFIIVFSANFPLTMVM
ITSNTAGFTKKATTTAVVFVAYCAGNIVGPQIMFADEAPSYPSGFAGMLVCFACSAVITMILRLYL
IWENKKRDALGEAPDVVEIDGFPVPASSLNLIDKTDWEIHQFRYVY
>g2146
MRTSTFAASALGMATLAAAQNGLISNSASSRVTTPSPTTFASGTTTIKIFEAAETSVDAAGLAGSI
ININAIATTILVECIDTDICDSQASMTMVAGPSTWGYAYTTVEELYGIPVHITAQLACNVVSSTQA
ATCTATEIVSASADGQKTDTTSTSTTTYASAQIFYDDLLITAGVEKLTSPQATETPKGAAAPVPTG
NLGLSVGGMAAAAVVAAAGLL
>g2147
MKSFLTLLPALFLTALGSPISEDFSKRNDRGSETVSGIGAHKTAILNAGGNTLDLAIAMLEIKTMN
TADYPYGDGKTYDAANFGLFKQNWGMLRVCASRYGFQGQSQDDWNNGARLNSDVYADVASRWDCQG
YYGYDKWFGGHRDGATGLANPYTQDITNYKNAVEWIQQQIDSNDQYKSDDTRFWVDIPPI
>g2148
MSDKKDQFAQPSEQGTAQGDEITTPVAANTPENRPQPAQSHIGRAATLPTRRPVDTANTTHAPGKV
RFSSEIERAPRTNYEPPTGQDVNAQSSPTAGKLKINTDVELFPSVVSPNQEVVSSDNSITNTPLSP
PSPQSRTRDRGYSLRRSLFARNIQTQSATETTGPSIELETRQSSAAKRSDTQEKVSVVAEEEIQPV

ATLETDSQDEDLISLADSDSTPLKYNLSLSAGQNWLKSTACKTKAYERLNQWNETIKEKVFRIKPL
PPTKDGRHIELNVSDVDSLIDERSGSSYVDNSIRSSRYSLWSFFPKQLIAQFSKVANFYFLVVSIL
QMIPGLSTTGTYTTIVPLLIFVGISMGKEGFDDLRRYRLDKEENNRYVHVLKPGGRQHELKKSKSK
DDSPALEDDGDWVAVKWHDVRVGDVIQLKRNQPVPADAVLLHADGQNGVAHIETMALDGETNLKTK
QPCQPVAKACQTTEDIIERKNFIRFTVEDPNIDLYKFDGNVTVNEEKLPITNNEILYRGSILRNTE
RIYAMVIYTGQECKIRMNANTNPRIKKPALQTVVNYVVVLIVAIVLLMAMICTIAYSVWNEQYQEN
AWYLSNAIVAVGPVFTSYLIMFNTMIPISLYVSMEIVKVGQMLLLNDVDMYDEESDTPLEARTSTI
NEELGQVSYIFSDKTGTLTNNTMKFRMMTVAGTAWLHDSDLREEAAREADKQWLIHKKRSVKGKKA
MSRKSGVDSIRASSMRMSMASAHELPSRSRSIMHGKPFYGGNTKQMLEYIQRKPHTLFARKAKFFI
LSLALCHTCIPEKDEAGNITYQAASPDEMALVTAAQQLGYLVMDRQQNTLTVRTYPSGLDDDPWDE
IYEVLDVIEFTSARKRMSVIVRMPDQRLCLFCKGADSTITKLLRQASLAQQKATQIERRASQRKAE
EAFEAIRRHSEHQSPVNIQRPSLSTRRSSGLSGKRDTLRRSIDIWLKDRETDGGILKRDTGSEHYS
PRPSTQLNRNSMASSDGRPSFQEDDDGHLVEESLVVDDHAVFERCFQHLNDFATEGLRTLMYASRF
LDESTYTEWKQAYQEASTSLVDRQEKIEKVGEQIETQLELVGATAIEDKLQKGVPEAIDKLRRANI
KLWMLTGDKRETAINIGHSCRLVKEYSEVIVLDQEAGDVEKTIVSTINSIRKGRLAHSVLVVDGQT
LSMIESDEILMPRFFKLAIMVDSVICCRASPKQKAFLVKAVRKHVSNAITLAIGDGANDIAMIQEA
HVGIGITGKEGLQAARISDYSIAQFRFLLKLLLVHGRWNYIRICKYTLGTFWKEMLFYLSQSLYQR
YNGYTGTSLYENWSLSMFNTLFTSLAVIFLGILEKDLSAATLLAVPELYTKGQRNQGFNIRIYLGW
ALMAVIEAFIVFYTMYGLFGSSLMGIDNTIFPMGLLCFTACITIINLKLQFLEIHYKTIVSAIVLF
VSIGGWXLWNMILSSRYNDAKSGKSIYDVRGNFLTRIGRSLNFWAVLFLIVAAVLLFETTVTLFRQ
YFFPTDVDVFQHLEKDPAIKRRFEEAAASELQQGWDRDSSNIRSSLEMAREEEEQLAREQEVHQYL
NRPRDEDQHNHNNLEHRDELDEEAAIPRSGSVSTRRKSHDIHDLFSKGFGTIKRGHL
>g2149
MGFASKIAASQSGGGVYSGAPPAGYTGGPPPAQGGSQYPGQQQYQAYQPPQPQGQQQQQQTPGGPA
PYPTSSPQPPYPAYSPAPAGQQQPPPSTNPGYPPQQSQYSQQPQQVGYSRPPAPLPQYGGGAPQQS
YGQPPPGQQSYAPNYNAGGSYGAYPPQGQQGHQQGPPPPGPPGQYGAPPQGGAPYGGGYGGAPAPS
GPGRQATPQELSTYRNLLVATIQERNIQSFYPPDKLERVLQGLHNAPDRIQQLMREWNVGQEVAMD
VIKLALFDTVIYVDDSGSIQFEEEGSRLTQLRQILGLISTAASKFDQDGVTIRFMNSDERGDNIRS
REDAEALVSRVRFSGLTPMGTSLKNKVLDPLVAGPARSGRLEKPVLVITITDGQPAGEPHGAVKDA
IRSVTDELARTRYGRGGVSFQFAQVGTDLHARQFLSKLDEDPSLGNLIDCTSSFEVEQDEMSRANP
PVNLTRELWVAKVMLGAIDASYDTKDEKSQAPPPPAGGYGGGYGQAPQYGQPQGGYPPQQGYGGQQ
GYGQPPQQGYGQPPQQQGYGQPPQQQYGGYNSPPPGQGGPYGQPGYPPQQGYGAPPPRRY
>g2150
MAATVQSLRTLLQSSTIEDHDEVLKASNEVLKKSKTDVQAQHSKVVALIKLDRYEDALRVFEDAGD
ALKEKAAVEYAYALYKYGRLREAAEVAAKSAGGRGAKHVEAQASYRAENFANAATLYESLLGEEEE
LGNEYNDLRINTWAIDAQRLWQREGDLTRARKPTREDLESFETSYNAACASLARGDLKQGEILLKR
AKELCKTSEDLSPEEQESELLPITIQQLYALISQGKVEEAEEIASEISLDNIAEKSTTRIAQNNIL
ITQHLNINPYTLHKQFHDASEPEGSDTFFKFQLDTLEANSHALDLRVQKYDGVIRSTTKALAQRPY
PSTEPHDNLLSIYNAAARVRGETNQKAIQDLRSLLDRRPKDIGLVLTIVQLYAGVGNTTSAINVLE
SSLRALEESISESDQAVRYNPGLVSVLIALYQREGRKSHIKTELAKAASFWRQQSSEQQPSSLLRA
AAASLLHSSXPADLAISQELFSTLRQKNAADKFALAGYVASYATSDIQKVQKELSILPRSQDLVSD
IDIAALESAGVPQSQASLAANAAVIAGARKRAATNSQQNARAKKRIRKSRLPKEYDPSKKPDPERW
LPLRDRSSYRPKGRKGKQRAADRTQGGIVNEKAEAEKPKVATPGTTSAKKKKGKR
>g2151
MSTAASCCTRTPIGTAQKVPVVKIANLDTYVAGNTSSKTGIVVIYDIFGFYPQTLQGADLLAAQTG
AVTFVPDVLENEYALQAWFPPDNEEKRNNLTTFFKETAAPPVVLPKVNAWIAEAKXKYPSVEKWGI
LGLCWGGKIAILSSTEGTEFVVSGQVHPGLYDGADAKNAVIPHVVLASKEESTDVNADYKAVFDKS
TLGSYIDVYPTMHHGWMGARSNLDDPENKKEFDRGYKEIGDFFAKYLLKVNAAGLIALAILTVASP
LAVRAARRNGVSRSHNRIISKDGQTKPSSVSYNYAGAFLTSSDFTEVSGTFIVPTPHILGNGDDGT
>g2152
MIKFVYRQAKKYIPYSYQSSIQRLTTPISVEESGATPACRPRGNCTLCAAEKRANYTKSWGLLLGL
LPAYFVASLEVTIVATALPEIASHYSEASSAWLILTSSYSIFKYTDYNLDRFGQLNWPATAFNLTS

TAFIPVFGQLADTFGRYASLQFAAVMSLIGSIICATAPSWPALLLGRAFQGIGAAXIDNVTMIILA
DQASLKEHAVNVSIFQLLNGIGYSIGPVVGGYLTKANWRYCFVLCAVIVFLGLFAIFLIRGQLKPG
KLSVYHPPSGQTPTAAIIEGLSTLDYGGIFMFVTGVCLLIMGTTWGGATFPWDSAAVISSLVVGGI
LILLFCLYEYLLEPGRLLSRWFPRTIPMIPAALLHKKDILIICFVAVGAGATFYSIFYFVGIFFTL
VKAYTASEAGTKLLYYIPGLGIGVYAAIRCCNHWPRQTFWALCLGTIFQTVGIAVLAAAVKFRDEK
LVAITMALTGAGTGAVFMPSNLHVAGMFNDHLASAYSLMRFSLPFGGTIALAMMDSVFQNEIAGNS
TGTSGANGYGSPTSQGLDSINVISSLPNDQQDTIRRQASTAIMFAFISIVPITALATILVMFLGNV
WITTETSSQNAIVQQTGGTNEIACCEEHAGSNVDSSIAMPDAIEMRHAHCTESDSSPAETSRYTDS
VYLLGLIRQQVKDGTRRV
>g2153
MRFSNPSVATAVLLLSFGVDATPIKSNKSLRTRRSLDATIWKPTAFAESSTLSKSSNYVALKKVAG
GSARTSAAHLKGSTNGASTGLISLLEGEEFATEITIGTQTFEVILDTGSSDTWVVESGFECVDVET
EKKESESYCDFGTPYYTVDKTFKTIKNEQFNISYGDGEFLEGIVGTETVTLAGITVKDQTIALANY
AGWYGDGTTAGLTGFAYPALTSAYSSSGTQKVYNPLFTNMYKEGLVDSYFSLAILRDVSGASGYLT
LGGLPPITFNETFATTPILITSITGYPKAYDFYTIEVDSINLKGKALSGGSGAKYIVDSGTTLNYY
PTSIANAVNAAFSPPATYNDTEGAYVVDCNATAPAHSITIGGTEFTINPLDMILYAGDNVCISGIV
DGGSDVSQDLYILGDTFQKNVVSVFDVGAVEMKFAPNVNYTSNDTY
>g2154
MASKQPTKQSVPLSIGKIDSYWSPKIIASLNDAYEIKISKFSGEFIWHSHPDTDELFYIIKGKVLI
RLNEPGQNSGLEDVYLDEGDIFVVPKGVMHCPAVVDNDDGTGEAVVMLMEPTGVVNTGNRGFVQGR
TNEVGDLRLT
>g2155
MSIAKDALFVGPATRQRKRVRLILIATIAAAFFFFSYNFSRNRVLYSSISSYDHEHAGSPATRPSS
FTTGAGTCNVDTGVVERYGLSGDVKYTRREIYVVLTDKEETPITTQMDMNLFDVAPTTLSGDVETT
VNCSDPITVRVPPSLPFRDASHIDFAISTNMERLMDSMDAFSVWAGYTNTRFLVFIEPGADQRKAQ
RKAKSLGLNIELFESDVDYENRYSLLVKLLADNARPETKWFCIMDDDTFFMSMSRLLQMLDKYDVS
KPHYIGTVTESDSQLSMFGIFAYGGAGMFFSRPLMDELGRIWGECDAGTDHGDGKIAHCVYQYTRT
KLEMETGLNQLDLMTDASGWFEAARSVPVSLHHWKSWFEADVVKMTAVRDVCGTNCILRQWRFADG
WLLTNGYSVIHHGAGITDDYGAFAMEKTWDDLNGSTDESFVRTLGPLRSKEEDKISLRLADVIVEG
GQVRQFYIRRVEGGDEVLELVWRLN
>g2156
MCCGTNRSNPSGGSRDNGFTADTCLENGLCQNVWQTRDDNGTTVENVGYFRDQCTSTDWENGGCLN
ICTAETNPNDGGNSNTVTQVTPCNGLANATTWCCGNSTDCCGSTSAITIAQKLAQPTSSSASSSSS
TTTSFRTSALTTTTTTEPSPAATTSSIHSSTSSPSASASSGLSTSAKVGVGISIAVAATAALAALVF
FIFIRRRNRNSADQQPLSNTKLGGSISGSEDWAAATAYSAKQPPPSPQELNPSGGVHEMASNEENM
RYELAGVSSPALSPGGEGVMHIGGDQISWERDIPRVRSK
>g2157
MTSVFKSEIDAASPPPPQQKASKQACDNCRRRKIKCNRAQPCDKCQRLLLSCSYCDVLQRKGPKFR
TLYPLAPLQTLVSQYPTPPQSIADKGSQAELMVPHAMIDPSSSSAYYDSSLALSGIEILTEATNAR
NECNNICEQVTLESLLTSFFLFAAYGNLDRQNEAWFYLSQATSMALTLGLHREATYSAFGEDEAEE
RRRVFWLLFVTERAYALQQAKPIMLRNSIRKPAILSTEDHIIQYGFYNLISIFEKITAELFDWIAI
ECDETFIASMTSGRTNLRDMSRQHHFSQPIPIGSVAEIQNLDIAGTQQWLQAMAWKLSMSDLSQFR
STDALLPFHFPVLVAKAVMDVFQGSTVLQSAGGHDRRMSIIKSESGMQGYPTRDINNEEFLLDIMN
ILSRIRPNNSHQPQAHHSFLSQIPPTSTSPSNQFFR
>g2158
MVVTTALAALNLLSLVQGTLVEFVPRASGSLDSWLATETPIALQGVLDNIGSSGAFTEGADEGIVL
ASPSKADPDYYYTWTRDSALTLKTLIDNFIAGTSDLESTIKEYINVETVIQGISNPSGSLADGTGL
GEPKFNVNRTAFTGSWGRPQRDGPALRATAMIAYANYLIDNSQSSTAKDIIWPIVQNDLAYVAQYW
NKTTFDLWEETQGSSFFTTAAQHRALVEGNALATSLGESCANCVSQAPQVLCFLQSYWTGSYILSN
FASNGRTGLDANSILGVIETFDPEASSCDDATFQPCSAKALANHKAVTDSFRSVYSLNSGIAASSA
VAVGRYAEDVYQGGNPWYLATTAAAEQLYDAIYQWKKIGSITITSVSLGFFQAIYSSAATGTYASG
SSTFDALISAVQTYADGYMSIVEKYTPADGTLAEQFQRDTGAPLSASALTWSYAALLTAAARRAGT

VPASWGSSNGNTVPTTCSASSATGSYTSATNTAWPTAPTSSTRPSCTAPAKLAVTFQEIVTTSLGQ
DIYLTGNISALGSWSTAASEAILLSAQDYRSADNLWYVTLNLPSGTAFEYKFFKNESGTITWENDP
NRVYTVASDCGVSTACINDSWQ
>g2159
MKLSLFASAAVAGLLAQVAEAATPDEWRSRSIYFLLTDRFSRTDNSTTAECDAQLGQYCGGSWQGI
INQLDYIQNMGFTAIWITPVTEQLPQSTSDGEAYHGYWQQDIYSINSNYGTAEDLQALATALHDRG
MYLMVDVVANHMGYAGAGTDVDYSVFNPFNSQDYFHTYCEITDYSNLTMVEECWEGDNIVSLPDLD
TESTDVQNIWYSWIPELVSNYSIDGLRLDSALEVQQDFWPSWVSASGVYCVGEVDNGDTTIACPYQ
NYLDGILNYPTYFPLVRAFESTSGSISDLYNMVNTVKSDCSDSNLLGSFSENHDNPRFASYTSDFS
LAKNVLAFTILSDGIPIIYAGQEQHYSGSSVPTNREATWLSGFDTSAELYTWIAKLNQIRNYALQW
DNGYTTYKSYPIYQDSNNLAIRKGSNESQVVTVLSNSGADASSYSLTISGTDFTAGEVITELITCT
NITVSDTGDITVPMAGGLPSVLYPASKLLESGHPC
>g2160
MAVTYTGGYSAPGSQTLPSFRELLPEHLHNEIDQAAYYSSQSRPTDRHRTPDPNKSRSSLPYPHSS
TTTSGGGRGPSHTLPPLRDLQSSNRHHDRPMSPYDDAPSRSGSRAGRGYRTSTEEDPRYRSVSGGG
GGGALYNAYDPRYVQQPYQGSSYPSSNHSNQSNDYDYPTSSPMGGHSHTIPTSSGNFGVIGASGDS
LDSRGKRRRGNLPKPVTDVLRAWFHEHLDHPYPTEEDKQIFMSRTGLSISQISNWFINARRRQLPA
LRNQLRNSDGEHIPRGHSPMSDVETSISPPHSHMTSPTHSRHR
>g2161
MPAAIAPLATKAGIPTTSTSHPDNPIPEEPRNIPHVSTGQIIPNIIQDSHSTASNQSISAPSESDS
DTAETPTTEHSDDGNGQFDGVALKKPKATRARRASTALISESSEDVQRILGNIGAGGTQLVEKVCC
GGGCCLSQPLSRAERVVLNPIKTPDNPAYRSLNLKIRTLALDSELTNIVPLPEKTVSFGPLSSDLV
DVQLGPKDHPPKFVQPHPPYDVFRAPLHHARELTKAGAEKRTYHFDIDVTDYPVEGGDVDFVVGGA
IGVCPMNSDDAVDSVLNCLGIPKPIRDKKIMVRTTTGRWPTIWGDEKARELVTTRRELLKWCSDIQ
SYPPTKQLLRVLAEYASDPHEKKILTFLCSAQGQSVFCDLRTGVHISIEQLLQAFPSSLPPLDYLL
SVLGTLMPRFYSLSQDPTISCTTRGGDCRRIVEIAVTVHESNDWKRGTRSGVGSGFLQSLADQVIA
AEQQGIDPKTLDLHVPMFRGLMANPLAREFVLDGPMLLIGAGVGIAPFRGFVQRRLKSANCANKVW
VLQGVRDSLLDELYSGEWGVHEEQVKKVVQSRKGESRYVQEEVRHQADLVWFVINALDGRVFVCGS
SKGMGEGVEEALIEVAMAKGNLNREEAKEFWGRKKDAGQYIAETW
>g2162
MPVVRKRLVIPAFILTFLIYIIFDSIEYDDYPPEPPPPPPPDFHNDDLLEETVHWHKIPEHYPVKE
FIELPTGGASSIPKIQYDFPKEKASDRRTRQERQSAVKEAFLHAWTGYKTHAWLKDEVSPISGGYV
NSFSGWAATLVDTLDTLLIMSLDDEFQLALDAIDQIDFTTTTDKSINVFETTIRYMGGFLAAYDLS
DGKYPILLKKATEVGDFVYGAFDTPNRMQMSRWDWKVSLAGTPLRASPKTLLAEVGSLSLEFTRLT
QLTGDPKYYDAIQRVSDNLEKGQSKTTIPGLWPVIVDLQNLNWGGNQYTLGGMADSTYEYLPKEHL
MLGATTDQYRRMYEKSVGPIKKNLLFRPMIKDKVDILFSGTLNGNNLDYEGQHLTCFIGGMFGIGA
KIFDRPEDLKTAEQLVNGCIWAYDAMATGLMPEIFRMVPCESLDKCPWDEKKWLAAVAKTSHVNAL
DDDIIHKFITEAGLPPGMTRVVDSKYILRPEAIESVYILYRITGDKKYQDAAWRMFSSIEKMTRTD
IAHASIKDVRFTDSMQVDKMESFWLAETLKYFYLIFAEPGVVSLDEYVLNTEAHPFKRPV
>g2163
MPHTGVFFIPSSPNAPHILGPLTERLRSVYGDEAIPIGRWGLEHKLMRDTPSCLPASAHAPNPAPQ
ARYVQFLSMTNHPRLGFVYASEDDESQQPATTTGVGKGKTESGMIMSTVPIASSSEIFRHFVRCCE
PIWCHRHTVTVTGTVYDVGDFRVRLGEVRQTQPQARPRGTILEVEWRGPSMVAAALAPVSSTSLGL
EERNGDALAMDVDAEMNSAMETPYIPSEAEIQLEYEQISNLIREFWTRIYSSSAAENNQNKQLPAT
TREAILVPDVGHEIKQRIAQRRQPAWQERENRRRRKRREAIALDRSWXGXSEKQQQQQQDDEEEDV
EAGVDLARQYMELFRFNR
>g2164
MADSLTEEQVSEYKEAFSLFDKDGDGQITTKELGTVMRSLGQNPSESELQDMINEVDADNNGTIDF
PEFLTMMARKMKDTDSEEEIREAFKVFDRDNNGFISAAELRHVMTSIGEKLTDDEVDEMIREADQD
GDGRIDYNEFVQLMMQK
>g2165
MWRDRTNLYISYRQSFAHHPTKKPRYFGPSSGAFTDSPSLSEERRGLIAGADGLEDDGDAVIEMDL

LPPRWVDVQEEVSEVLADIAQKSARLDKLHQKHILPSFGDEGARKEEETIIEQYTQEITRGFHACQ
NAIQRIDGLVREQKQLGAVTKGDETMAKNIQISLAARVQESSSRFRKKQSTYLRKLRELEGMASPF
ERVSTPIQSQYFDPSLMESDADKSFSQTTLQQTSQKHQQQQQLVVGGANDVAIAQREREINDIAKG
IIELSDIFRELQSMIIDQGTMLDRIDYNVERMATDVKQADTELKVVSPSFH
>g2166
MAYNDDSVRAKLSALNETQEGIVTVAQWVMFHRRHADRTVQLWLQKLRDSPANKKLNLIYLANEVA
QQSKARRKDDFIIAFSPIIAEATAVAYKGASSDIQQKIRRVVEVWRQRGVFELPIQEAVEARVNEL
DKNRSSGKKPMLGGSLFSAGSVPPELQPLVQLQLNVSKGSVASGTAVNTANAEFDKMNDPSTPAPP
APVHAARLSQLLKSLANAESSVSEIIKSRQALMEGLEKMLESNRTALSRETALLTQMGERKAETEV
RKREVEDAIMRGLSADESLAPADNAGGLEEPSRPTVEALTPPPVESLTPTGSPAVNDGDAHQPQPH
PFTLSDDHQMDWSSQMDSGLSLEQSAGDESRNGFDL
>g2167
MADRFPSLEDFNEGQTEVVNNDAADTDDFLARERAALGDDADQFATPNDQAVTVEDDDLLGGDDYV
PQQGGSAEISGFESSFPAIDTQNEAVAPGGTITGTGSPFPATGYSSYAQPEEESEAVREWRARRDA
DLARRAETSAEKKEATIKKAQEDIDDFYVSYGNRADKARAQTRKEAEEFLANREDTSAGGTSWERI
AKLVDVSGKGVKGGASGSGKERFRELLLDLRKDGNAPGATGV
>g2168
MVLDYSKWDALELSDDSDIEVHPNVDKRSFIRAKQNQIHQQRVQRRHEIETLKYERIINDGLLERI
DKLLGALRQHEDSSRNPEELVFQSLIESAGDPAKDQPPAPPAGIYTHEKEQPKYSQMMSSLVDQVK
KEITESKTESGFKAYITGVQGHKDKVLGLQQELLQKLAELEKEEASKITSDSIHTGFDSSYVAKSD
PKAKGKAAETKVELLNSPSVPASQGAVAPSSGAEADVEDDDDEEADIKVSDLAKRLAKIPMTDYKA
LLQFISANPDVVAESETDGLLVEAFNSQMDGKPEYARLCVHHGLLLQYCRSLGRDGISLFFKRITT
KDHQAATLFRNDVNETYNKIKTRAAELAKNRSPENDPAGVEQIQLHAVDPNTQINISIPPADSTIP
EEIEARKIFESFPPGLQRALQSASLDEVNKVLGKMSVEEAEEIVEKLGEGGMLSLEEGIVDATTEE
GRKKLEEIEAEGKREREVEEIGEPAEDQLD
>g2169
MLLEIGSRCVPSPFCFNKERPNFQGNEIIAPQAQKDNRDVTPVNENKEDGFELSEKAAQAAQINLT
AKLGRDGKEKKKPAFNEIWRLLKIARPEAKSLGLAFFFLLISSSITMSIPFSIGKIMDLATKGEEA
EGVFGLSMPMFYGALAGIICVGAAANYGRIIILRIVGERVVARQRSKLFRRTFVQDAEFFDANRVG
DLISRLSSDTLIVGKSITQNLSDGLRSIVSCTAGFGAMAYVSLKLSSVLALLLPPIGVAAFFYGRT
IRNLSRKIQKNLGTLTKIAEERLGNVKTSQAFAAEVSEVGRYNNQVKKIFELGRRESLISASFFSS
TGLMGNMTILILLYVGGGLVQSGGITLGELTSFLMYTVYAGSSLFGLSNFYSEIMKGVGAASRLFE
LQDREPKISPTKGEPVKSARGPIRFENVTFSYPTRPAVSIFKDLDFEIPQGSNVAIVGPSGGGKST
IASLLLRFYNPVSGRILINGKDITQMNAKALRRKIGIVSQEPVLFSGTIAENIAYGVPGATKAEIV
AAARQANCQFISDFPSGLETNVGPRGAQLSGGQKQRIAIARALIKNPDILILDEATSALDAESETL
VNSALAALLRGNNTTISIAHRLSTIKRSDTIIVLGNDGRVAEIGSYQDLSTRPDGAFTRLMEWQMT
GGDVTPPPTNPRTISFEEQMEELEREQREKESEIEPDEAEHEDEKLPQRRV
>g2170
MSRTLLSRVFPSSTDPEYEPVENDELLGGTDGTGNIQEGSNSRAEDEGSSSLKKPFSWYDYTVFLL
LGVAMLWAWNMFLAAAPYFHRRFEENPWALEHYESSILSVSTVTNLLCVLVLAKLQRNASYPIRII
VSLVILTIVFALQAMSTVFFRAVPIGMYFIFVMTMVLGASFAMGMNQNGVFAYVSRFGRPEYTQAI
MAGQGIAGVLPCIVQIITNAAGPRRRDHGDDHHEKGGDDFYKSALMYFLFAVLVTLVALFSFLRLM
NRTAGSRWLARELHAIQNAPTMSDPNNPAPELTAPTHKTVGLWRLFLRLKWLALAVYLCFTVTMIY
PVFTVKVQSVHNDPATRSRIFEPELFIPLAFLFWNMGDLIGRMSPIVPALARTTNYPRALFAFSVS
RLVFIPLYLACNVRSGGVTLVNSDFFYLFVVQLGFGLTNGFLGSACMMGAGQYVTADEREAAGGFM
SMMLIAGLATGSLLSFVFSGV
>g2171
MRTVQQRRVYGKRKTNAPRAVLENGSPLKSASNENLSLDEVIEQDVSKESINKLEVQIASITLEDE
APSKIDVDTNEPQSPAKSPPKVQYQTIGTPASAKYANTPHRLLSTPSNLGPSTQSPIKVYSSQKRL
SFTPRQPRAKPVRKAKQIIRLSSGCVEDEKTNEYVHRILDEATSQFAARGVQKFGSWAARSETAFD
PVKIAEGSYGEVYKLRLKEDICKRNMSKSRLAKLREYGEGVFKVVPLRAQKGVGSKKFTTIDEIVA
EVKLLKLLDPIPGFARFREVHVVQGRFPPSFQAAWDSYKAAGKDCENPNPANKRAYSDQQLWAILE

MDDAGVELEKFKWSSVFQVYDIFWGVAMGLARAEEYALFEHRDLHLGNVCLRSTRPDGDMQLLADV
DANQLGASCGFGISSLETTIIDYSLSRAELRLTDDPEKVDVASTDLDNKGLFNAVGRDEAEILQRN
TYRFMRAQLYTGLPLTEEKSPAIPGIWADYAPKTNLIWLLFILKMLAKQIEQKDYTLHNSMPLNQT
RQPLQSCPDKANIQQYSVKPPTKLSEPPVSMKHDNIGEALGKLQTTLTDRLDKILEILDLEHGRDD
MCCAADLVAYAIDQQWLDENDDSWEKTLNDEVSELLSQVNSHELVRLATELNDGIPCIFQSGKHSG
IDSTMGCSNYHCWLIFDTGERWIVRIPRMGFSDVPADLVEYLVESEYATLKFLESVNVPSPKVFGY
GLVSDHSNRVGVCYIMMQALPGKPYGAHNASPAQKERVIEQVANYLVEISKRPLPAIGSFAMINNQ
PQISAVASNRFVALGTYGPFTSRLDYITSVIEQYMDLIADGQVHSKYPLEAFLFYHFLRENKDSLI
NPDVAEQQFFIKHVDDKGDHLLVDEDFNVTGIIDWQFARVVPAAEAFGPSYVTADLESLYSSSLTG
VSADDLLLADALRRKGYDDLATIAEGNEIMRRFHHGLANGLSKDEARELLGGMVHCVMGKKFRMDE
MDAWIAEMRVKCRVDRRWEKVEGLLKEQEEESA
>g2172
MAFNFNWSPLMADAGFYTRAQDLLTAALNKSPKPPIIVDDIKVTELNLGSIPPDLEILEVGDLAED
RFRGIFKMSYNGDAYLTLKTRVQANPLNTFLVTRPSFASPKPLAAAAGLTIPLQITLSEFRLSGFV
VLVFSKQKGITVVFRNDPLESLKVSSTFDSISFVRDYLQKAIEGQLRALFMDELPAIIHRLSLRLW
VPEYRDKESESVNTLDQSSEAGPGQDPLASPPQDPVDASGNALNPSEVASLSLDSGVEIHSLFSQK
NLLRLAALTDSQRTLSLFTPSIKDVVYRAWTASAELGESTTLTSPASPVLSRTHSHIGSLHSFVDN
ASTISMQSGGSSNFSGHLHLRTGRHSRSHTKKRKKRVVDLRRPKTTDDVESVSGDSVFSSENATSA
PTIFSSPAHFSEEKNDDPVTPPLGPQNDLHLPTIHERRRISQGDHTVRRSMLSMSEAAQPSSSRSA
QMIAESWPDPDATPRNTIRLPSNDRANNRLTAAPLPSSAIQYAPAVPANNNPREQVXLSKMAAELA
RRMQEEKVDQSGSRPFPDFWDDHSREEIPPPAYGH
>g2173
MVARTFPRLAGNERVVVRHDVQSWDIEGTFVARENRVPYYQRLFQQHDGKRQWWKTGRSGALLWPY
YISLWGTTAITTYAMIRLTLGHKTFFGKE
>g2174
MRPALLRLLKRPSAISLLNDLVSTSSAAAGGIERLDDIRSGKCANCQIRRSHAKLLPYESSSVASS
STETNVRLQVYDIVGLERRKSSNITGRKDDNQYSTGGVLPISLDVDQLEYESSFHTIEINGGRKLV
DDPARKDDSELWKDLLRYRKRRYGYQGVLEIWTGIVDRIDDIQLPVEGETADYIWETFVEAGLAEE
QLLYDITKYSEKLWSNTEKRWKKLYQTVVGGLFTKGLPEEAVAWHRMLQAIHLTEPNDIVSVLAQA
VPCIPQKPPDWSLYSHETITLGNGRGVRAFGEICLATKGHQIYETVIPFLLQKGLALDATYMHDFL
VKRGDMPRDSGTLQQILDFATTESNMFSQKVKQRLRKIKSDTLSEAFLNSLAASGETEVSGNGQQQ
MEADRPKDADDWIKEKTFRDEFGARLFATKALTVDMITNGLQMFGVQAIGPLSLREIALRAEGPKD
ILEKIQALRKANIIVRDCAFTRLVERLALEHKDIILEDLLHSDQHPDVLEDAAVQESLLCSYLMAR
DWRLHNLTKAILNEVAKDDMDNIQFRVSLSAKEWSTASDVVDRMYLDAKPLTQQSIDHMFYGVLTP
RLPGRLQPYPEDPKIDEVKYAERILKLALRSGTEIDPQYWVELLKRMGMNRHDQWERIQDLCLWLA
QNYVVQENSPFLHSLSRSSGVDPDTILAKNRSIIRRIFNPRMQMALIAWGFKSPLWYQNAVHEIPD
PVGGGMVLLWTRGLLLLRDLEQLGVPIYPSFVRQACRERLQMLFGELYSVKRWNRTLRDKNPFKIS
RILNDMLAIHPTLFSQQDRVDLQKLVDRPLQGLAARKMERNYEWLDQWPGSRHR
>g2175
MSIEQDPDDSSDGEKTFNGYSTTPSITTAIREEGQEREIGPSIGELARKFSNQFVHHSQTPTADCN
PFLPSHNPSLDPNSKDFDPKQWMRALLDTTSRDADRYPSRTSGVSHRNLTAYGFGSNVNYQADVLS
IFSRVIDMAINLMGRKEKRTQILQDFDGLVQSGEMLLVLGRPGSGVSTYLKTIAGRTHGFNVDPIS
SFNYQGIPYDMMHSRFRGEIIYQAETEIHFPQLTVGQTLLFAALARTPKNRLPGVTRQRYAEQMRD
VVMAILGISHTKNTKVGDDFIRGVSGGERKRVSIAEVMLSGSPLQCWDNSTRGLDSATALEFVKTI
KMSTDLGHTAAVVAIYQASQAAYDVFDKVTVLYQGRQIYFGPAGSAKSYFTNMGYYCPDRQTTADF
LTSLTNPEERIVQEGFEEKVPRTAAEFAAAWRNSSERAELLKEIASFEERFPLEGEQLKHFERSRR
AQKAHLMRAKSPYTISVPMQISLCINRGFQRLSGDMTFFFATIFGNLLISIILGSVFYKLPQSSES
MNNRCILLFFIILFNALSSSLEILTLYVQRPVVEKHAQYALYHPFSEAAASIICDLPSKIISTLFF
NIPVYFMSDLRREAGYLFIFLLFGFTCTLTMSSIFRTIGQISRTMAQALTPAALFVLLLVVYTGFV
LPTIAMQGWLRWINYLNPIAYAYESLVVNEFHDRQFDCTQFIPMGPSYQNLTASERVCATAGGLPG
DDFVSGDTYIYGSYGYSYSHIWRNFGILIGYIIIFTGVYFLAAEYVSSSRSKGEILVFQRGLDKKH
ASRPDDIESSNPNNTDRQVVEFRGEEDTSAAIQRQTAIFHWKNVCYDVQIKTETRRILDRVDGWVK

PGTLTALMGATGAGKTTLLNVLAERITVGVVTGDVFVNGQPRARSFQRKTGYVQQQDVHLEVSTVR
EALEFSALLRQPSKYSRKEKLAYVDEVIKLLDMEAYAEAIVGILGEGLNVEQRKRLSIGVELAAKP
ELLLFLDEPTSGLDSQTAWSITALIKKLSDNGQAILCTIHQPSAMLFQQFDRLLLLAKGGKTVYFG
NIGENAREMIQYFESHGSRVCRKDENPAEWMLEVIGAAPGAVAKQDWTDVWRNSSEYSDTHKELTR
LEDQQRSEATHQPASDAFDESTAYAASLTDQLFLCTKRVFQQYWRTPSYIYAKLVLCLGASLFIGV
SFYKTKNTMQGLQDQMFAVFMLLVIFAFLVYQIMPNFILQRDLYEVRERPSKTYAWYIFMLSNIIV
ELPWNTFASLLIYFPFYYLVGLYRNAEPTDTVHERGALMFLLTWAFMVMSGTFSHMMVAGLPTAEV
GAILALILFALCLIFCGIIASPAALPGFWIFMYRVSPFTYLVSSLLSTSLANADVQCASFEVRNIL
QTPSGETCGQYLSQYMASAGGAVYNENSTENCEFCPLASTSEFLASVSVFYADRWRNFGLLWVYII
FNAVAALVLYWAVRVPKKGNSGIPSLRRNQS
>g2176
MSPRSSTNSYEAWSALYYTVLAIGCQYHEGSSFDPGSGTAWKLFECALSHLPDLIMRKGSLMAVQA
LTAMAIFSTTLPAYQFEALLVTEAAKMCQSMGYNKASSTIPSLNEDKRRCTFWVVYCLEKMACFNT
GRTSIIMDSDIGCPIPLVTESIFGNYDWFLSIARYSRLISRIYESIFTVSSGEGSAETYHASIKQL
NAELEAWRLSIPVQFRPLEHFSGRGLPGTLEIYVAVTTQYLYFNALLTLLRTTLYIKAGDLGTASD
TQETKKQLMKTVCLILDSTKYIEVAAYTSIWILALMPISALFILFDLVIHNPTHPQTNNNLALLDV
ASGHFGRIEYATSGTLPGSLVSEFAHIARDFVRDSQREKPNQNAQIITNNVQNNSLDIVSNPPIQT
SKEVLDTSNVSLEPQMSCDYQSQLQCQLNPAAPNTITHQTKPPGVQHQQSSNTYLRHNSNPSVGTS
QPSDSTLPRADAGAEQIFFPLADDPTYAVQRDDGDLQQLLGIDVMDLFDFTAPFNWGCWS
>g2177
MAGAFKSARLTYRAIENNDKDNAFIHSLRLESGSRATNEPTLFKPVNKASTVLAAAEIRDESYMAV
LICLPADDFEPERPIGRIDLHPEPKKLLQHHRNAEVGIQIKEEYQRRGYGTEAIEWILEWAFKYGG
LHRVAMSSFSFSPGARRLYERLGFVYEGLLREVMWFDGGWHDIVCQSMLEDEWRKRQEKTE
>g2178
MVTRRVADKVGVALEEELKITLGETEYLLILNQENFLTIHGNTPSLALVYLTKPGQVISRASLREF
RATKLERDDVIHSAFYSNFVFYGSKQEDLRLESDVIEELVEWDTKSWTFVEGTPSSTVAAGPYVSA
KGRTWQPWRIYHDFNATFMLKLDSPQAGIHGRVVVPSRCYYQPSKMRPLDGARIVVKDNMDVAGHK
TTLCNRSWDALYPPASKNAACIQVLLDAGAIILGKTKLEAMVMDEEPLECVEFTAPFNPRGDGYQN
PSGSSSGSAAGIGSYDWLDFSLGSDTNGSGRKPADYNGVFSIRPSTGIMDTDGVVGHFPKFDMPVF
FGRDIARFSDFISVWYGASPMLRTPSKTQVNILYPSDYLPTTNPSQSQVIDNFVKGLESALQVSRI
NISLAEEWRRDCPDGLQNPDIVEYLKLAGGIPFYHDAYYALADFRDKYKEKFGKPPFVHRAVHRQW
DVAKQITKEERDKYWRRSEIYRHWLLDNIFKANDENSVTIMILPIEKGEPNYRDADPPLHHILHGF
DALNMSPMMRAPELTAIVGEITYHSIVTERAEPRPIGVSVIGTDLILADLVEKGMKVLGGF
>g2179
MDKATQKPSPSKPQHRLRAGDDQGGASQGASPASLRMADAKDPLLETNKPYELLYDTPEIPRGANF
DEITESVPIEDFRPLKGKLSLDKDGFVLVDLPRTIPYEDYFDEATLKAGFVNDAKRVLLDVCGARA
VYIHECVIRKRAEKEKGFGVPIPRVHGDYTIDEASKLIGQLCGDRAEAVRKHRYQMINVWKPLKGP
LKDWPLAFCRLGSLADEDLKKIDLIHPDDTLESYRVHYNLNQQWGYFKEQKASEMVIFKSADSLVS
GTVPHCAFDNPGCPPGEPARESIELRALVVH
>g2180
MTESHILDNFLANNPEVNFIQLRWMSLSAILHTKFVTTSGARDIVSGERQYCTNQSGMVMPIATAP
QCDIVRVMRCPIEPDWKSLRLCTFTPGHASVMCSIASEGEEDRYLQCPRQLLEETVEMIEEHSQTS
FLIGFEIEFTLLDAAFEPAQSIDQVVSYCMHSGLRCNTLKIMEEICYALEKANIEVYHFHTEDPHQ
LEIALKEKAPMEAIDDMVFACEAIRSIAAQHDLKATITPKPLSDASVTNGIHMHVSSHDVENADSF
LAGITDKLRTMTVFGIANYDGFERVKDGKAGQRVGWGTEHRDLPIRRIKGSHWEIRCADVTANFYL
FLATLLRAGMDGVNNSKLLVQKDVTKLSKDITEAAWRHEYGVTERLPRSLQEAIDAAKSDADIAKW
IGRYMFDLYLSIKEVEVESFKGLIPEKRRHRFVRFF
>g2181
MTSRDVLLFCQQWQSRLHSVSGSLERDDFRYVSAFKSWEEAHEQIIRAQENIVHQAISQEFAMFLP
SFANLKSFVENLSVQVSPRNVETSIIWGLIGLITKLCHDEGDGASRIPGMLHDICLKIEILNNYRE
STGRSVPQIQEVSFSIALGLLFFLSRIVTFMRGDQWYESSKGLSKEQWEPLELQYNSLIREIDNSM
SRIERAFSTTKRAAPKFDVDDILQLQSMLALSPKPGLMSPQALDRSNEVAKLPCIIPPPFRIPRFF

NRTDVMAKMEGFFGDQDTDHSFRSIALYGLGGVGKSSVALEYAERRLRNGELDAMFWIPSEKAVTI
RQSFTEIALRLKLPDARPMDHDENYEIVLNWLRHTTEGGIEIHSWDSETGSQFLLHLLSTDIGDEL
SIADIKSADQLAEKLSGHALALSHMAGLIHRRSWSISEFMDIYKKQPQKMHGISGNTSINALWDIS
FNSLDHKSREILGVLSFTAPEAIPQALFEAESEAGLPEPLRFCADNLVFWDAIDDLLSRALIKRDK
KTKSFSLHRLVQTSFKYFLTPEQRQRSFNNAAILVHTAYPKLDGVSQLYQMWSRCRDLLPHVISLK
DEFREEKSTTPAFTAPWQFCDLNNLCERYLLETNLYKELEDLLEVDQMALDTVPIEEQTNDMPSSI
TSYKGQLYVRTGKAIEGMKILKKSYDIRAGAVPEDVRETAWALENAGNGIATTNALDEAITWLERS
IATWLRWAETEGPEKGVYPAVLKKSYGMILVWAGQYKKARTVLEEGIQQIESTTPYNWAMAAYTHF
ALGTLDRLERNYDASESHFMQAQNMWTQGDQLRNDPFNAACIYRMGCVALDQGNLEGSIKHFRDAM
ILTDRRKTTLRAEHARCLFKLSEAFAQEPRHELEADGLRTEAESLLREIDPEIVDCGIELAYDSKV
NIQWR
>g2182
MLPTHVAAFFGLEKMILHLQQTELDLNQKDEMGYTPLLWAARNGYEAVVKILIDGGVALTWEDPYQ
PSPIASAARNGYDAVVKLLIQSGAVVDLADENRWTALSIAARYGHEAAVKVLIRSGAAVNSRDICG
RTALCWASSCGNETIVKILIGNGAAMDSESDLGWTPLFYAAIRGHKAVVNILIENGAAPDSMDEAN
RTPLSYAAERGCTAVVEMLIENGAASDSMDLANRTPLLFAARGGHTVVVEMLLKNGAVPDSIDVQN
WTPLLFAARGGHTSIVKMLIENGAVLDLMDKYGKTALLLAIANKDAVLVRMLIENGATVDVLDEDS
REALHKLLQCHEE
>g2183
MATGIETKTHADYTVGWICALSKEQTAATAMLDKRHGDLPNPPHDSNTYTLGSIANHDIVIACLPD
GRYGTNSAANVVTLLSGTFPSVRFCLMVGIGGGIPSNKVRLGDVVVGRPEGQFPGVVQWDMGKAKQ
GGEFERTGALNNPPKAVLTALAKLKTDNELYESKISKYLDDFKNRYPRAAEKYLRSDSLVDVCYKA
NYDHVTPKRKRDDNEEEDEDEVEGDDEMDPCKHCDKSKALKRKPRSMLVHYGLIASGNQVIKDSKY
RDKLNKDLGGNVLCVEMEAAGIVNSFPCLVIRGICDYSDSHKNEAWQEHAAVVAAAYAKELLVFVQ
PIELRGERRIKDVLQEG
>g2184
MAPNTQTLIEAAKSRRTYYNLGRNSKVPDSEIVELVNQAILHIPSSFNTQSTRLVVALNGDHEKVW
DIAVEAFQPLVASGAVPQEVFDKQTKPKLDAFKAAYGTILFYEDPAHIKPYAEKFPAFGDKFPEWA
EHSNAMHQWFLWAGLETLGFGANLQHYNPLIDASLAKTFNVPSEWNLKAQLVFGSIESPAGEKQFK
PVEERVKVFGAQCANLAYEPTATHNHVVREERRRCFWSICLLRRFHGEDVHVLDFASPENLAKYPK
GTGIAPDITKTANPGVEVRIAKGMKDEGIVAYYLQLSETFSRTAGYVKRRGKSNGFLPWSPESEYS
KIIALQMDSETRVPYIHRFKFSGIPDRSVDDLQSNRDYWGPWFFNQFMYHTILCLLNHPLLLSLSL
RNHRSTIPEIFIQHSSDMISTHTSWIVYFVRYFEEKGFRISDPFLGYCAAVVATIELQLSFTEDEG
IRKDKQERFESCLNFVQDLGKEWPHMAKMAEKLQALNGAVSASYVPYTRTQDKSLVIDLSRFWEIL
DYSSGFSDERSSLFGSTLFSESGNKAHEVSSTTPLPEPTRIRVSRRTSHIPGSSAAVPQEIDHTVD
TGMLPTQMDIYGDQYSMLAENFFAQGQDFLRNTEDWFTTGNL
>g2185
MAPSLITPVSSNSEETTANKMASTKFTPPSYSSDAITKAYAETLDKADPLSSFRSKFIIPSKANLK
ATKLAKPGLSQDEKAQLDTWSSIGVLGHFRDLEDSPLRSWQLLAEQAAGDMSKIVGANPEEVAAMG
TLTANLHMLLASFYKPTESKHKILLDWKAFPSDHYAIESQIAWHGLDPNDSMVLIGPEEGEYVIST
EKILSIIDEHASETALLLLPGIQYYSGQYFDIPTITKHAQSLGIMVGWDLAHAYGNVDVKLHDWNV
DFAAWCTYKYGNAGPGAIGGIFVHERHGVVDYSQGEDKPVFRHRLTGWYGGDRSVRFRMDNKFKPI
PGAGGFQVSNPSAIDLAALCASLSVFNETSITQIRARSLRLTHYLEYLLLAGTTDETRKFRIITPS
DPNARGAQLSLLLNPGLLDKVAQRLEDASIICDKRQPDVVRVAPAPLYNTFTEVWTFVNELKTAIA
>g2186
MLNIPSPPIPLDEFRVSYETGFLPINTTNNRKLPLHRLSNPYFSPWEDVISXLPQLIQTGEIRQKV
LDLPVLSIEYLDAHDEAEWQRAYLVLAFLTHAYIWGGEKAEDKLPPSISRPFLAVSDHLELPPCAT
YAALNLWNFTTINIDNSEENIDLTNPDNIYTINSFTATDDERWFLVVSVAMEACGAKMIRLVLDSI
TTINTTAAADDKDGMTTESTSNRNTILTNLLNGMGECIDELGCILERMYERNDPMVFYHQIRPLLA
GSKNMGAAGLPNGVFYDTGDDTARWHQYSGGSNAQSSLVQLIDIALGIQHIASGEVEGEKTSKDKH
TAFMKEMRNYMPGPHRRFLEYIASIANIRPYALSLPASSEVRQAYNAVVMKLGGFRDKHIQIVSRY
VILPASRPVPESSKYKDVAKKNRVNLASATSDTAAYSWNKPREEKVFYGTGGTDLIPFLKATRDET

KAAARFAD
>g2187
MAAQPIQQETRFDLPGFTLPLNWKPGTSSLDIFPCALDADQIDGGGYLSFLRTKREILMMRMMNSI
TDKPDWNKKIYNTEITDKWTKEIVDSGADVSPKMLEWIWAELKYKAESLKENGGMVTAFNPGVVWS
DAAISKELQEDLKTAVLPLEQVPEEEKDYHPGSDQRVVDLVHPSLFPVVYGRTRILRDEILTRDGF
LSWSGKTETLPKPEDPEGTLSSTGWGHRRVKLYSTSFQWLPCEVEFDEAEGCRIVSYINNLHPQQH
EALYQVIEKVIAKIIPLWDESLHEAVSDPKARIPYESVEYLPPTDPEPDWEEDPERYDAWQAKQEI
IQPEPQGDFQSLKRKHYKWGEQLSLRNLFREKGLQIIVKLANIELTPDKPRYEGGTWHVEGQLNEH
ICATAIYYYSTENITDSVLSFRTRANEYSFDDVGYEQGRHEFLQQVYGFGPETITWSDGPITQELG
DVSCHEGRLLTFPNVLQHRVAPFELLDKTKPGHRKILALFLVDPHIRIISSANVPPQRADWVTERD
QLRATLLERKLPVELQQMVLGDVDDLPITMEEAKQYREELMEERRAVKDEQSDMFAIGSFSLCEH
>g2188
MDVLPELSPAIKLEYHAEGGANIVYRIIHPFAEDQVFEPQPSEIPHYGASTPPPTEIDDTINPDID
ADINITSGKLLRLRKNVRYGLPYRDTAQDFQKKIRPLFGDEELVDQTMVRVPHSVIQRCNAHLKER
EHHRKRPAIRCGVYLAEDEPLGMLVTDMTTSIPPSTANVAETESIVELKPKWLIQSPSAPSKARRC
RTCALRDMKRADTPSPSDSRNSTSPPLPVIIPPGKAQFCPLDLTSPNKKDMQRTIRHIFPFLLPEK
VDMIADALYQHPILQKLLSLQRIHDEVGLNGPLPGSKDTNLSMTLRDCSVFLKIFPSTHPSNEQQA
RIDIRLGDLDLKSAANGKAEYWLNIEKRLIDEGWYMGTQTSQAATEGECALSR
>g2189
MAALFWLFIAAFSTTCYALHRTIGDPNDPSIQALANECLQTTPNAGWSYGTCPYLVRCVLEGLHAE
ISSGMSSGSNIVSLVPTTLALIDHPTQDLNTPTLEELRIRQWTIPVPTPHWQGVFKGKAFAVDLAF
LFLLGVMMWQTVTLNYSSVVSWRCDYPFLLICWPVACVVWLIIAMSGLSMLAQKIRIRYREDTQEE
WSEVPWSSLVRHRYNLPGRFNRENDTTSSTMYLVMEDRQPDRQRGKLSRSRYGAYYSIEITMRYDW
AWQYYEAAMEALAVGIYLYATFVLTSSLFCAVCLYLTMAGVFQNVFGGAAPSGAVKSDDDFADFAA
VPNPSPAGLTAGQSAPAAAVTGASAVPYTAWYRVWERVTPQDFLQEAFIIPFLLLLLGFHFWGTRK
NRSKAKTWAIAHAPVLESEFAVVGFGGVARGPSVEGVQSELTDPEKLLKEKSKQEYQSYATGRQNV
AFLDVSIKLVKRYNPLVFIMDNALGLFLESFPPPVERVESTLYTFDGKEKEIVPTPGGDVSSIKVP
NSTYDGFIWAVVHKNAMRQIRQDRYDASMTFTKDNSKLPAWVTVMTESAEITDFLLTPQLIKAIEE
AGDAFEYLIITDQPIDKPLKIEEAVPKKRIQLSLTIPSNGYASTLSLYSYFLRLPDILVSGAHWRP
EVTRKLRNAREEEIRKLRRADEEEKAEERKYAAEKLKKEERERLLRGMTAEEQRKFLEREKEKEQR
KMMKKSTRRG
>g2190
MLSIRNLLPIFLSCTSATTTELTVRSTACNNSPSLCSKSYGEITHLGAHDSPFLRDASTDYSASGN
QFYNTTVQLSAGVRLVTGQVHKNNNAWHLCHSSCDLLDAGTLESWLSEIKTWLDENPNDVVTVLLV
NSDDATDSELATSFESSNITDYAYTPSFTSATTAWPTLQELISNGTRLMTFVASLSSNSNAAYLMD
EFTYIWENPYDVTSASNFSCLPDRPSTVSGNTASALSSNRLPFMNHFLDTNVGLGIEEPDVNAAAT
TNGQNGTGNLLTAAETCKSAYSGRQPSFILVDWFDKGPAIDVVDQLNNVTDATGRVAVPNTNADDN
NSTSSGTYVGLLELVDEVKNGANPSLGNWIWAGGDWSSILGGGVSI
>g2191
MSTPSDLQALFASMKPRPAQSQDAQAAAYPSSEARAFNPQTDGLRFGLQSHAYPFPHGYHNPSVSS
PVYSPGPVNTPPHHGSDILSPNVPTPRGDQQQRTASADQAMSLLNLLKFSQNKPSSPQPSAAVGSS
PSLSATHHEEVQPSHSRTISASDLVASFMAKPGPASQSPALGASATIAASRAPESSTTAAETQDML
MRLLNRSQIKTEESSRALQTPPAYESSAPATVDQQSMHVFGSVDKEAANFEGPKANQSKGSIFSYV
NPFEQLAAASPRRTPQPKSGNGTPAEAATHRPKDSNTISEEQSSLETLKTTVAADEKLSDQKETVS
KAVDQLAGQLDREVEDALSRANFAEATLRPKAEDETAKEVVDAIVQELPNTAAEVKKEIEKQEKAG
KLNETIPEEFVDTVKEVVDEAAEGKLLKNLATDEKLADSWESAEDGADKAEERVVRVFNFPLRPFI
SITIKPDTGKLSTVRDDGIMDVARLKKEFDQLDRCLTSATAQYIVYALAKNGGLRIIRQDDGRDKQ
VFRFARDRLFNVALSNSASASSNSTKDQAIIAIGVSGTVYWAPVFRGDNDLFEMDTLESECLVFPP
YPASDENTSGGQLKTRAKPSSRHPEFFGIGRGKNIYIVSPQYAAHPSYGVSGSQRIVDTEKFFKER
AMKISTGKAGKDFTFSEDDTVVASLDKTGRLRFWDIQEVGQTMDGSQPPEVRVPLTTFVTGSPNEK
SWPTSVQFIDKLRPFVKAVALRYVLVGLKQNHTLQLWDIGLGKAVQEVNFPHENESDAICSVAYHP
SSGIIVVGHPTRNSIYFLHLSAPKYNLHPMSQADYIRRVAEKDSSLPKPESTACLSGIREISFASK

GQLRSLDVLALSKSMGAQRATEEESGLFEIYAMYSRGVTCLNIRKEDLGWSSDNKIMEAVDALDDG
YIEVKDLQTFPSYVVDEPSVNGDSVTSSAQKPPAKDVARKVSDLAVDTISTAAVSRTASPVKSLAK
LSADEQSEVTSGTDKAKKKRKDKAKDVSKPSGAHDLSKYFTPATNTQPEREETPAVAAEGQRSVVE
TNAQQAPASGLTISSELIKELEKSVSAEVVKEVSREFDGLQKRLDDERRAWDVASASKTDQILRLV
SSTLADNVEKSLSRIIGQSVEKQVLPTVVDSASAAVGKQLEGVISTHFNQNFPRELGQRLPNALAR
AVKQPEVLTSISEAVANSSASRVEGEVAKLIQNVITPSVQAAITRQSEAATRDFEQIFQAQSKHYE
AQRQHDSAKIDQLVSAVHSLKETIDVMASSQAALQGEVAALQKQLGSRRDGVAPQPVPVPQPPAPA
QRSPEELELADIARLASEGNFEEASIRWLQSSQQADVFDNFFVRLNPAYLSTVSPIVSLSVGVAVT
TALSTNVQERLGWLEYVLQNVNLVDPDIREVAPQIMDIFIQRLDGLYRTVVEANPQDPLLRKIPPL
VRRSRDLRGH
>g2192
MGWGWWSSSSSSNNQNGQPQSQQPDILQQPSISSSQPEPKPRRQLTREEQADAELRDFLKSLESSV
TPPSPPSSQPSQAPSSTTTTTSPTSLESIQSETPSSIAPDSLYPDTMSCRSAFDYAFFCQSFGGQW
VNVYRYGELRSCSEHWENFWLCMKARGIKDDNTSKEMIRNHYRKKAIKYKTGPSSEDVWEVRMEPL
KGAFSGDFEKMEREMKLAEEQQQQMPEREVSPK
>g2193
MGLLEEVYGNELIDEQAPQEYTEYKAENGFGWADALPAKQGLYNPELEKDACGVGFAAHIKGKPSH
KIVIDARSLLCNMTHRGAVGSDVRDGDGAGVMTSIPHKFFVKNFARDAGVELPPLGQYAAGNLFFK
PDNETLKDSIASFEDIATSLGLRVLGWREVPRDSTLLGPAALSREPMIMQPFVVLRSAYGEGNKPE
ITDPEQFDERQFERQLYVLRKRATHILGLANWFYLCSLSNRNIVYKGQLAPVQVYEYYHDLVNVDY
EGHFALVHSRFSTNTFPSWDRAQPLRWAAHNGEINTLRGNKNWMRAREGVLKSEIFGDELDSLYPI
VEDGGSDSAAFDNVLELLMINGVLSLPEAVMLMVPEAWQNNAAMDPAKAAFYEWAACQMEPWDGPA
LFTFSDGRYCGANLDRNGLRPCRFYVTDDDRIICASEVGTISLDPETIVQKGRLQPGKMLLVDTVA
GRIIDDAELKQTVAHRQDFRAWLDKELLTLPQVEKGLKEQSHEFSIALDESTLQNDVRLKAFGYSF
EQVSLLLAPMAADSKEALGSMGNDGPLAVLAQQPRLLYEYFRQLFAQVTNPPIDPIREAIVMSLEC
YVGPQGNLLEIDSTQCHRLMLPSPILTLDEYESIKNINKVHPDWNVSLIDITFEKAKGTQGYLDAL
DRICDAATEAITAGDKVLILSDRATSADRVPVSALLATGLVHHHMVRNKWRSMAALVVDTGEAXEV
HHMCVLLGYGADAICPYLAMECILKMNREKLIRKPLSDEQVLLNYKASCDGGILKVMSKMGISTLQ
SYKGAQIFEALGIDDAVIDRCFAGTASRIRGVTFELIAQDAFAFHERGYPSRAITEIAGLPESGEY
HWRDGGDPHVNDPTSIANIQDAVRTKNDKSYEAYARSEREAIKNCTLRGLLDFDYEQRTAIPIDQV
EPWTEIVRRFVTGAMSYGSISMESHSTLAIAMNRLGGKSNTGEGGEDPERSKVMENGDTMRSAIKQ
IASGRFGVTSNYLADADELQIKMAQGAKPGEGGELPGHKVSGPIARTRHSTPGVGLISPPPHHDIY
SIEDLKQLIYDLKCSNPRARVSVKLVSEVGVGIVASGVAKAKADHILISGHDGGTGASRWTGIKAA
GLPWELGLAETHQTLVLNDLRGRVIVQTDGQLKTGRDVAIACLLGAEEWGFATTPLIAMGCIFMRK
CHLNTCPVGIATQDPELRKKFKGTPEHVINFFYYVANELRAIMAKLGIRTINEMVGRAELLKVRDD
IRSAKQENIDLSLILTPAHSLRPGVATYNVRKQDHRLHVRLDNKLIAESELALEKGLPCRVECDVV
NTDRALGATLSYQISRRYGEVGLPQDTVHVNIKGSAGQSFGAYLAPGATLELEGDANDYVGKGLSG
GRLIVYPPRNAVYKAEENIIIGNVCLYGATSGTCYFRGVAAERFAVRNSGVTAVVEGVGDHGCEYM
TGGRILVLGSTGRNFAAGMSGGIAYVLDVNQDFHSKINMEMVEVSGLEDPAEIAFVRGLIEDHHHY
TGSELAARILLDFNRALPRFVKVLPTDYKRVMLEEAAKAEAAKKAEFTLPKLPSTPAPEEEKKEHK
HEEKKADLLDIEDSVSDSKAEKKRTALILDKTRGFMKYNRRSEKYRNPTTRTRDWAELSTRLTEDE
LKYQSARCMDCGVPFCQSDSGCPISNIIPKWNELVFQNQWQDALNRLLMTNNFPEFTGRVCPAPCE
GACVLGINEDPVGIKSIECAIIDRGFEMGWMVPRPPPSRTGKNVAIIGSGPAGLAAADQLNRAGHS
VTVYERADRVGGLLMYGIPNMKLDKKVVQRRVDLMAAEGVKFVTSTAVGPDEAVTLASLRQSNDAV
IIATGATVARDLKIPGRELEGIHFAMQFLHKNTKSLLDSEHADGAYISAKDKHVVVIGGGDTGNDC
IGTSVRHGAKSVVNFELLPQPPPERARDNPWPQWPRIYRVDYGHTEVKTHMGKDPREYSIMSKEFV
DDGNGRVKGINTVRVEWTKSASGGWDMKTVEGSEQFFPADLVMLSMGFLGPEDRLLGEEIERDARK
NVKTAPGQYATNIPGVFAAGDCRRGQSLIVWGINEGRQTAREVDTFLMGTGSLLPVTGGIVRRPAV
DAVPQVAAAVSA
>g2194
MAAPKAKAEAPSTPKASLPTNPQESMSQPTESISTSGFLAEVYKEKPLGSAGVERIVTRFPPEPNG
FLHLGHSKAIAINFGFAEHHGGDCYLRYDDTNPAKEEKKYFTAIEDMVRWLGFSPVKITYSSDYFD

TLYELAEDLIRRDAAYCDIQPPESEIAQQRGGGKGATPRYACGHRNRPTDESLTEFRAMRDGEYGP
QEALLRMKQDLTSNNPQMWDLTAYRVIQPSKSSNAEEHVEDENPDHTAGVHYRTGDKWKIYPTYDF
THCLADSLEGITHSLCTTEFETSRVSYNWLCDTLGVYRPMQREYGRLNLSGTVLSKRKIMDLISKG
HVRDWDDPRLYTLIALRRRGIPPGAILSFVSKLGVSKATTTIEIKKFEQSVRQYLEVTVPRLMVVL
DPIRIVIDDLPENYLEMVELPYSKDPEFGTHQVPFTRTVYIDRSDFRETPSKDFFRLAPGTSVGLL
KVPFPITATSFETDPDSGLVTLVHAQYDKPAEGTKFKRPKAYIHWVSDCPSLNSPVKACVRVHNPL
FTCDNPDTHPDGFLSVINPQSEETFPGAMIDIGLHEITQKAPWPKTKSEEMATDSQATPETVRFQG
MRVAYFCLDRDSTADYKVLNRIVSLKEDTNK
>g2195
MTLTYSDYTIGWICALPLEMAAAKAALDELHPRLPASPNDHNIYTLGAIHGHNVAIACLPFGIYGT
TSAAIVASNITSTFKNLRYSLMVGIAGGVPHPSTTDIRLGDIVVSKPTGNSGGVIQYDYGKTLTDG
VFQRTGCLNKPPAELLSALATLESNHFLSKSDISVTLAQMQARFPAFASPGSQYDFLFEPLYSHVG
TRSEECHLKCESSYLVPRLRRSSSAPRVHYGLIASGNQVMRDAVTRDNISRQLEDVLCFEMEAAGL
MDQFPCLVVRGICDYADSHKSKRWQAYAAATAAAYAKELLGIIPLQQRGQESAAAEEPGRGRLKQV
>g2196
MESLQSGYKPNLVMIFACLGEIHAERHHRTKRKLDLDEAIWATKLALSISSADHPLTAILLGNLAN
ELWRRYRSSGCEDDLQQALDYGFRALESVSQESSHQAAVLGTLGAIRASLYNCRGDFRDLQEAISI
TQKSIDIATRCAGTTPMISFNNLGAYLWTQYCLASQGDDMDNSVVSFAKALECATNETVDRVTCLI
NSAHVLTQRYHRTGQLQDLGCALENAKEAMRLDPQNNDLATLLKCLDRFLDRRRESTSSLHWFQSR
GHANTNRKLWTPLILLGVFIFIVTMYAYHLRTEILLSIVAFPIGIIYHWMASARPTTAQEEKSTQD
YYTPILSMRGLLNSRGRSHQYTPKLLAVPLMPVPFASSLRDIHAPDFSAYASSLYHQRRQSLQSVK
SERKVRSRSPAGYRRDGDERVFEGRHSGHRRASSSRIHQGTPTMGNVTPSGFVSPRSQNNSSPSTL
RNVRPQVEIELSRNVMGLSRRQGEAYVRDSNMQRSVGTDSGYGSSSARHERARDVEHNDENHGILL
SDEQHHDSNASHSSAKPVEHVSSPLIPSQKLWAALASQYSEHRIWEEGVSSPEEEEKEQISLIRQG
HTNLKMEKNSGLAMGNVTSTRAEVVGYDNSREPQSQHQYLYHEEFSMMDRADGVQSQISSQKEEVQ
VVSMPEVAQNTVSAEAFVRNIELRVHAPLTSIDENMVRSQVPRIVEVQDEDPEIEAEDFNIDLVDQ
LAREYLESSTL
>g2197
MARLQVIEDNPWEEEWRKRDKEEEDNDEEEELENIKSSHHNTGILPSRSIPMRETRTQKGLSLIFG
KVDRQIELLDKQVNTVQAELLQARTLVMAVRSTVANLNKPEASNLLEEDAFTNYLRIADRLKAISN
NIFMIIPDHGRQIVRTQKAYTYNPGFVYSNSELDEPDPEPSIDIDEWTARETYLYGYKFDNMLFES
AFNESSSSESVSSDFAPDLNVSPPLPRSPTREWYAPNRARWIRRPSPSLRVGYERRRQPILRNRHT
VHFARTPRSRVSSAFSSRDMYDSPSDSSSMNYWQRENFSRPSRRYPYYPSKGRVVPETRRSHRSSF
IPSTKVEFEPQHWQVNESSQIPLEEPSVLSAMPKVLESTRRAVRAASRSRHPLSYRSESTEQYEIE
PREGWVEEEIEIPPEEPRLVSVDTQERQQVFSLERTLEEPRLISVDPKKKRQAFSLEATQEKPRVV
SVGPKETQRAFSLEAILHEERTLLLEKLYKVQEQLEATQRRPIVKSLISARDDIKGSVPKEKFWTR
LRSKFVDHSKQQDGHDLAA
>g2198
MRSSPRTPVDPDLADLFASRGRLDAHRYMETRDAHDLENAISNMRSAIALTKDTQSTRKNTGLNEA
RQLATWALECLSDCDANQAQVLATLGTILTSQYDRSGRTLDLEEAISSLYIATQVRSLSDNGTFRA
VVLNNLSCVLAKRYEITGRREDLNDAVTSALEATEVNSLTNALFELRIVTEISSEDSKLDTLLSQL
GGLAIEQHTLRAPQMDQGIEMSQSYYPKSLTFYFFLFISTTVFVFLPMAFYPLVVFFGGIVSNWAF
QQYYQQITNRDKSRYLLYLISTWVQEKFLLPYNSTGFTPTLFPTPWIDICPTLIFPRSEEEPYKLD
ISRKQKPIHATRLQSKYDEVQENWARDRSPAPDTFSAFKRYTSKSKSPGLYDIADSKIWDDVNNSL
SDNEHSGTLRSVSEEGKVFNHFDDISGDGPNILTRRVSTTEDVPETDIAYPVKFIECFEQQETISE
TVPTSVGHPSKQAVTEAVLPSDGEDNIMDSTKQGKYIMILSPPIEDSHSTLGKEFIDQKVHSRVSP
TQLEKESSAFDSVAQGNTSNDINKNEGNAMGNIYSEEAHVVGHLSRHYGDKFETKNISAVLPWHNI
PAANQSLXPATLDETDAEDIPREVIPREVIQRAVIQREVIPREDIPREIIQASPESESEASALERR
LRRLQRYISSLEHKQDAASKDHLKSAKEKAANLSGELRNLLEISARRYINGHSAEAQTARVINRPA
ESDVEVEVPSPPTSYYGETNSQASSDRYPKDFTYESPRMRSDHIVPTQRESRAKKKVVKQVRTVRA
DRTSMPPQAPNIKHFKTSREQVEVLYSYFRKYLLPLCDLYVRYTPDDQKTRESQHTKLSETIMSQV
LLNADCIEIDNESTRNARRRLVGEAQATLKRLDDALNSSRKVAYVEDMYDD

>g2199
MSDLLRDTLFGQTVRLLSGRTLLTFPDEQNPDLWKAFVPEGKHYDGTPRSNIGIDQDGEHPANAVN
TAQASSSEEQHSTYRDPVTNDEKDGEVMLVDWYDSNDQENPQNWSKSRKLLVTFQICILNFGIYIG
SSIYTPGEQDIMGEFGVSEVVATLGLSLFVLGYGLGPMLFSPMSEIPTIGRSRIYFWTLFVFVLLQ
LPTGYAVNMAMLLVFRFLTGFFGGPVLATGGATIIDMYPPVEVPYWIGIYGASGVLGPVLGPLVGG
FAAQAKGWRWPIWELTWLCAVVLVVLFFLMPETSAANILYRRAKRLRKQTGKPTLKSQSEIDAAEV
SLRDHLVVLSRAFTMTFSEPVIFFVDLYCALLYGVLYIWFESFPLVFGDIYGFNIGEQGLAFLGIF
VGGLITVPCYLFWVRNYLMVRLADGHFKPEVILPPTFFGAVALPVCLFWYGWTSRESIHWIVPLVG
SGCFTISIITLFMPVLSYLGLPVAWCFRFLLLWFGDSYAVDQVLKRVDKPSYAAADHWAVVKRALP
PDLHINIKQVIPRLSEGGLFIKYSLSDGEKRTKGEVAAAVDEFLKKNPIRPWFNPLDRVGVGRVLG
KPWIEDMYHFPSPKLKVEFLPPSSDSSPAELTPEALYSLFRRYGKIKSIDRQPSDSKVLPKYAMVE
FTRAKYAILARSCTHGVNLPETVSGDGVGGSVLRITYERKIKAHWIRDWLFNHPRIVIPAIAALIA
GITVIIFDPIRTFFIKMKVKYLFNGDDKALWSWIRNEVRRANFLSSFKKRNEKNVLQALSDDRKEE
ISQLQSWLDENGNTFIVVQGPHGSGKREIVIDQALKDRKHKLIIDCKQIQDARGDASTISVAAQQV
GYRPIFSWMNSFSSFIDLAAQGIIGTKAGFSETLDSQLSKIWTNTATALRQIALENKKKDGSNMSD
DDYLEAHPEKRPVVVIDNFLHGGRESNIVDDKLAEWAAALVTGNIAHVIFLTPDTSYSKTLGKALP
SQVFRSVSLGDCSLEVGRNFILRYLQHRNEDGDKEVDESNPEKNAPPQGLEDLDRCIELVGGRLTD
LEFMAHRIKSGESPKSAVKHIIEQAATEIVKAYVLEPNTTHATDTPTWSREQAWYLIKTIAENKNG
TVPYSHIMVSDLFNDADGERTLSALEQKELITVSTVNGRPATIRPGRPIYHAAFKFLTQDDILRNR
LDLGIAREMIKRENEKIAKYENELHLMGDPEKYPMTVGWRLRSVADSLRDANSKLQQYESEKKRLX
KFLKTAE
>g2200
MAPSYSQWSHPDMLIINPPTNCTSHVLRQFHLISATFTLIRRNESNSGTFLNCSKWSFRTLLPNES
SFQLAGKFIETDDDRNNVGGEILDFMLTRMAKYGCVAACGTIAKYDKDDNIVSLKNSYKIITMVLK
ILGFINIDWIEHLYEVRAIFIEGCKKGNSIIGDESEIVIDIEFEDILRTWMMLYSGANTEKLITCT
KGRKHRSLIRSLLSMPRLPDQEDNIVTRVRGRFKEAWDGFWDFALRDNVLEVAIGLIIAAAFTKVV
NSFVSDLFLPIISLLPFLHRNLDDKFAVLRKGKHYPDWGPDGYNTLEQARDDGALVMAYGMSAEDI
KEQIVRREESKKPRFVSYLWDTFDKPLAERRLLTKLDTALISFGAVGYFVKSVDQYNINNAFVSGM
QEDLKLYQNQLNYMQTAWALGYMLGQIPSNIILSRTRPRYWIPSLEVCWAVLTLSLSRCNQAYQFY
IIRLLIGLCESGYYPGLQHSLGSWYRSDELGKRACIFQICSAMGQMASGYIMGGVYKLGGIGGYRG
WQWAFIINGSLSLPMAIMGYFILPDVPEITKSWYLKEEEIKLAQLRMHMEGRKPRSKYTKEKLKRI
FTSWHIYLLSLLYVAYNNGIANMQPIFQQYLRHSTNPRYSIPQINNYPTTTSGVQILMTILYAWLS
DTVLRGRRWPPLVFGGLVSIICYVSLAIWDIPTWWKWTCFISAGASFGLSGLCMAWANEVCGNDSE
ERAITIAAMNEIAYLLQIWLPLLVWQQIDAPRYFKGYVTVSAMSVLFIVMCFVVRNLHNREASAAR
MAKEHATESCIQLQQQHQQADGEKDLSGSDLQLSRFKSLETSSSSGQYDSEAISPSSSSNIISPCD
DEKGPFSRRLNGTFPTESGDVKGIEVQPSRLL
>g2201
MPRRLSNTFSSNSSPSTPSSSTEKGLKSSRTSRLSQFFSTSPKPKAEPPTAHATLLAASKSSAVST
GVPIGSASLSLPTISLSTATADNRNMDEPPKTLFQPPSQEETKRLAKQHAQFAPISHXSHRYTSRX
PGGPFPEPVMDEPPYYYLLTTYISYLILIAFGHVRDFFGKRFKEENYRHLKSRNGYGALNSDFDNF
YVRRLKLRINDCFERPVTGVPGRYITLIDRATDDYNRHFYLTGTTTDTLNMSSYNYLGFAQSEGPC
ADAVEENLRKYGISTVSTRAEAGTCDLHVEVEDLVAKFVGKEASMVFSMGFGTNASVFPALVSKGC
LIISDELNHASIRFGARLSGASIGMFKHNDMKSLEEKLREAISQGQPRTHRPWKKILVVVEGLYSM
EGSMCNLPGLIELKHKYKFHLFIDEAHSIGAVGPHGRGVCDYFGVDTKDVDILMGTLTKSFGANGG
YIAADKAMIDALRAYNAGVIFGEAPTPPILTQILTSLRLINGDICPGQGEERLQRLAFNSRYLRLG
LKRLGFIVYGHDDSPIIPVLLFNPAKMPAFSHEMLKRKISVVIVGYPATPLVSSRARFCVSAAHNK
EDLDRLLTACDEIGNVLQLKFSTGIAGGALPPADGLSPEMEKEYYQRQQQRLNNPNATTVPPRWRI
EDVVPNGIGAFLALCLSAQKENHRIFATSRDTSKIPVELCARSNVTILALDVSSPSSVAEASTSIR
ASGHGLDILINNAGLPYSMPLLDADIASAQRVYDVNIWGVVRIIQTFADMIIERKGRIVNLFISWL
TNGGWWWEAVYASSKAALTVLSETLRHELSPFGVSVLCLQVGGVDTNFDARTIAFELPQNSYYVPI
QDDIAGWATGSIKPKGQSGESFAEAVMDDILGKNKNGLVFRGRNTLAMAKVFDAAEVAKHNTPESC
WVILYGKVYDVTDFMSSHPGGVKVILRLAGTDATEEYDPIHPPGTLEENLKPEALLGTVDPKTLPK

PTADAQQQSSEEDQGPPSMETLFNLDEIEQVATKQVSRKAWGYYYSAGDDLISKNFNREVYRSIFL
RPRVFIDVGKCDLSTTIMGHKVGLPIYISPAAMARLAHPAGEAGIAAACREFGAMQMISNNASMSP
EQIVENAAPDQVFGWQLYVQVERKKSEAMIARIEKLKAIKCIILTLDAPVPGKREDDMRTDNTAKK
LPVPSGKVAEEKSDTLPDGTPVPKDGGGGVGKQLFAGTAYDLTWKETLQWLTKTTSLPIILKGLQT
HEDAYIASLYAPQVRGIILSNHGGRALDTAPPAVHTLLEIRKYCPEVFDKIEVLVDGGIRRGTDVV
KALCLGARAVGIGRPALWGLGAGGIEGVHRTLEILADETKTCMQLLGVEKISDLGPEYINSRIVEQ
QIYDGPSGLAKALRARL
>g2202
MPSSINDDLDAIELWRPSAPERTEMFKFKTHIGQKYNLKLKNYDDLWQWSISQPADFWEEVWHYTG
VRAHKTYSKVLDSNSLLFPRPHFFEGSRLNFAENLLFPANCPGDEAIAIIGATEADREFVSWRELR
ERVRVCANAMRQKGITVGDRIGGFVGNHVDAVVMMLATTSIGALWTSISPDTGVHAVVERLRQVQP
RILFADDASLYNGKVHGTEPKVAEIVKELPCLEHVVVLEAIQSAELDLEKVKPQSGLAARYKDFVR
EAQGSTTPLKFESLDPDHPVYILYSSGTTGAPKPIVHGALGTLLQHKKEHVLHSDIKPGDRLFYYT
TTTWMMWHWLVSGLASGATIVVYDGSPFRPFDVEGGKGEMAMARLIEELGITHFGTSAKYLSMIEQ
ASLNPRKHPNRPVQLEKLRAIFSTAAPLAPSTFEYIYSSFHPDVMLGSITGGTDIISLFGASCPIS
PVYRGEIQCRGLGMAVAAYDYAGNDITKSNEPGDLVCTVPFPAQPVMFWPPGPEGEAKYKKSYFDV
FGPSVWHHGDFVRIDPDTGGLTMLGRSDGVLKPAGVRFGSAEIYNVILKHFSQEIEDSLCIGRRRE
GIDTDETVVLFVKLVPEAVEGGIPPMSPDLVARIQAAIRKELSARHVPGIIDVCPGNEIPLTSNGK
KVENAVKQILCGLNIKTSASVANAACLDGPKKYQLLRQHHPDHATNVLSSYRSLINTPDPADFDQI
TTLKNGIRVASESLPGPFSGVGVYVDAGSRYEDDSIRGVSHIMDRLAFKSTKSRSADEMLEALESL
GGNIQCASSRESLMYQSASFNSTVPTTLGLLAETIRDPLITEEEVEQQLLTAEYEINEIWNKPELI
LPELVHMAGFKNNTLGNPLLCPQERLMEINKAVVEKYRAAFFRPERIVVAFAGVAHEDAVRLTEQY
FGDMKSAEGPALHGKGVESTLSGEKEGELPTIHLSTPTANVTANAQAGTSQPNILSKLPFFKNLSS
SAPDTATVKALDPSLLKPSSVDLSRTAHYTGGYLTLPPIPPPANPNHPRLSHIHLAFEALPISSPD
IYALATLQTLLGGGGSFSAGGPGKGMYSRLYTNVLNQHGWVESCIAFNHSYTDSGLFGISASCSPT
RTPQMLEVMCRELQALTLDKGFSALQMPEVNRAKNQLRSSLLMNLESRMVELEDLGRQVQVHGRKI
GVKEMCDRIDSLTINDLRRVAKQVFGGQVFNPGKGTGKPTVVIQEGELEGYTPRAFPWDEIQDRIS
KWKLGRL
>g2203
MVKLTEVEDEHFTTEKPIPTKEALLVSDNEDDEDFTDTESEISEVDSTYDPINETLYDRIVALKDI
IPPQSRRRITATIAGLSDATKSTFSFSGKALWVISTSAFLLGVPWALAFAEEEQYVQMEREQGMMR
GANEMLTPGTNAAAAALGAAGTEQPKAAL
>g2204
MAGAKGKKKQSRLAFAPIQDSPSPRPTGASQSAFTPSRLRYANPFTGKVTVRGQLQLEDYTRPWGN
SAAGDIKEELSEAGQPSSEVEVKRNLIDNRSNQTSSRSISLPREDSSDDEVIRPSKRRRITRDVND
TEPDQDSQYSLSPSGTNNLHNHDITDDDSDMGRRATKHTPRRRSPRLLASSPTETKARRNSLKMDL
SNIGEPEESDANELASPGTHRKRRGPKLKQKSPSVVVLDDSDADSDVVITSHIRKKRKHNDQPSLE
QGADGDDSDDVIIAPTPSRRGLRKSLQKSVATSNEEAPKTPKKYSQQDELDLEEDLEDLRDTVVRE
KRTRGSAINSARSNRQKHLEMLRRRRAGEKEIAEDEDEDEEEEDDEPPRYASTAGINWDDYSKRRD
ASDSDAPSIIEANEDLDADDSSFVEDDGELGVPGDVTVSVPFEFSRHRTKTTRDCFRDVVEWMVHT
KLNPGFNRNDDLYQFAFVKVKDEVXGXAASQLISTVWNADFVNTLRARPHLEVTAHPLDDDHKCDA
CNRSKHPASSDLKFTGKPYSEETFEPLYESDSEGDSDSSDTCDEDENGPDRDREGHILPSEDKHYY
LGRTCKSNAVLTHTLIHWRFHLYEWVVDYLNHEEELLSNPQKSLDREKMSAKKRTKYANNVVDRMD
EEGEVQRLWMDFHQNLRTVREMKDVRQF
>g2205
MERLPTEVLDHITSYVEDCKTLAALRLVDKAFCRSASRGLFRRVILEVGYKPAITTKFQALASSEY
AQFVREAVFSPDPIFKHTRWRPKFVFAPNILTRLTSVLKKLPKLETVAICPLPWDPTLSWSSVALR
AIADLKHHNVTRLEMSLDNSAYLKRCIECRQEKPVTQLIRQVQHLKLYGKNEEEDDMDDLHLILKT
AANLVSLEVNSYCSVYGSSVKEYNFHQHLQLKSLNLVCVSITSYELLGLLEWCKDTIKSVAIGFLA
LNAGSWLHVLMQIKKNLKLLKFFFNEWNISRAKETEYGNYMWRSLGKFRRDHELIGCAIGDLQRQT
NANRIAEGLRPLSKEEYRELDLPTLESAIDDARYTELISRSWDAEKDATYEWDTGSDDPDD
>g2206

MGEKAAVNRPVNPQQRDADIATKLQLYGIYSAFANGKLPSNKQCDVALNSAIKSKWLSSPSKELSE
DGQILVKDLRDVIDKTKLLFLTKNEGELLQEFIWDAQQITGEEFQGITGPVSKDSARGDADRAAEG
FKTLGTLLITNGEFRKLLSDAVVLLRDIAGDTAMKAASKLRPDEDALAQIDQPAEANVWHEKPDIN
KDSLKAQWREQADRFKPGSKQDVQEAAQAATTSATGGRQDAPISQIDARAGAATAKETLQQRAQQN
VAPEDREQAQQVSEQAQAVSSEYSQRTKDFLASKMPQERREQVVWRLKKMIVEIQGHSDYQQAIET
LLSLAETYAGHGRNVSTQGTTATKGARENTILRKAETNLRTLIERFANSTSTDDFFDSLNTIYRDA
DQDPRLKDWFRNVDTYTRKCLREQGFIMQDEANNQWNKLYDEGRFLLRDRYRNHTDRIADEAKFLA
TQFEEDPQNRAFRLSLEKLFKDLGQDQYGKPTFKPHLIKDITNVILPEIFENASYIPIPRIEVSDP
AVDMVIENLIIESDNLMPNVVELGTDNYWRWGRKKISNIDDHKIMISASGIQADLRDVSYYLKKKQ
GFPSLTDTGVMDILLGGTGFGFKIAASKAQKNDRNAVFKLDSVKVNVKNLSIKLKKSKHKVLFSIF
RPMLLNVVRPALEKVLESQIREAFEKADAFAYQVQTEAQRAQEAVREDPENAKNIFARYADATRQV
ITEKKKQAEAIAQRGTRVQVAMTHQDAMFKDIKLPGSVTTKATEYKELAAKGDRWQSPVFNWGAAS
PTSNLPKPAAVSRKPHTTAESRLQEHPRTDGVDGLGADGVNAGALNGFHGVSTTGAAASGSSGQGL
SPINGVTNQSANNGAFKKEVDRAFDGPATVQSTLGGV
>g2207
MSCITAALGMMPLKGASAPAVSSARLRIAEETGSNAVSVALAHRTPQEILSEDSFLNAITVLQAIG
GSTNAVVHLLAIANRHPKIQGKITLQTFDDVGRKTPLLIDLKPSGANYMTDFHNAGGMLALLHTLR
PLLRLNAMTISGKTLGQVLDESPFKSFAYSREVIRPLDNPLYPSSSLVVLKGNIAPNGAVLKASAS
KDRSLLKHSGPAVVFENTADMARRIDDPELPVTKDSVLVLKNIGPVGNPGMPEAGLIPLPRKLGAQ
GVSDMLRLSDGRMSGTAGGTIVLHISPESALPESPFGILRTGDVITCDVEQRILRLEVSDEEIQRR
VDERKIEIAQSVVEKKSQRGYRGLYIRSVNQAQDGADFDFLTASGAD
>g2208
MSAATMRPRPLPAHAPGATVLTYTPNGRYIITAGSNSAIRIYTVGDDGSPRTVDEGVDTHFGIIAT
NDFFITGAEDGTVWRYDLESGKMDKLLVRCALPVRDLAISSDGKWIAVASDELTVKVVNVDDMTNV
KYLREQTKGSKHVSFDPSGRFIAVSCTDGVLYIYSVASEEPELVRKIDGAVRRLEPEDEATSRVIW
HPDGTAFATAQATREVAIFSASDWSPQKSFTSAHNGDITALGWSPNGALLATAGADGQILLWETST
QNILQKYDDFDNVINLSWHPSRNMLCFSTSDGELYIHDNFVSPEYQSILEKRLEPFSVRSAPLGET
SGNARKQIPSRSKIDDVAKPRRGGSPDSLDDILGMDEDDNFVEDDDGAGYAEEINGHGKRTNGHLD
GPDGRDSKRLASYWEPTIHEAFQPGSTPWKGNRRYLCLNLTGVVWTVDQETHNTVTVEFYDSEMHR
NFHFTDPYRYDKACLNENGALFSSSTEDDGAAMIYYRPHETWTTRGDWRTQLPPGETVKAIALSES
YIVVLTSTNYVRIYTLYGTPFRVYRQKSPAITCAAWRDYVMTIGNGPVGHNGFTALTYSIENVKRD
EICQNEDIVAITDREQLKNVFFSDNGDPCIYDSEGVLLILQHWRTPGQARWVPLLDTKLLDRLSKG
RKEETYWPVAVAQNKFHCIILKGGETDPYFPRPLLSEFDFKIPVSTKPSAPNQDDDEESETIRNDA
ARFEEAFVRENLFYSLFQDLISTTNATRNQRAELARREVEIDKILLQMLAVECREGEDRGMKALEL
VGMLQDRSGKMIEAAVKIAQRYNRSILEDKIRDLGERRLMGMEEDDDELA
>g2209
MPKPFLRSVPANATITRRVASGIKPECHQPSTLNPRRYNAANTRSCRCHCRHFSQLTPKPLARLHD
RRDIDAIFFSRSPLITLHSSRNRQKDERCRNKVASQVGQTGHTVKYDLRPGTEGGEDHKPPDERVL
RLGKTLRRLSPLLPNIFVNPLPTDLLSPQITLHLFPSTHPHLPNVKGRVLYRAALWTVPMAWSSLP
LLGNVKLQILSERMVRADSVLGYSDCRRYEDASNGCGEERLVVRWKTSHNTPSTESTSTSGPRQNG
VSGSEAGKNKSLSTLLGGDAPIFLPGKEGRFEGLFIFAFDEKGRIAAHTIEHADQADGWDRTAKFV
TLTDWLLGKARGSLGEAGAAPALAHARYPVESRQASYRGYSEKEGRLCYHDGMR
>g2210
MASTXPXPPTSSSPSRDQTQRKKVLRVIFISLLLDLISFTFILPLFPSLLNFYLQKDPSPSSLLNR
IFHYLNAYKNSFSKPIDSRYDIVLLGGALGSLFSFLQALAAPVIGRLSDRHGRRTALLWSMFGNLI
SVALWVAATDFKTFLASRIVGGLSEGNVQMANAIATDISDESERGATMAMVGACFSIAFTFGPALG
AALANVSIIKENPYATAAGVSLGLILVETGYLWACLPETHPRLTQGVGKSEKSGDTGESNASTETD
GKKKEERVYTNNPALLNLVHLLFLLPFSGMEFSLPFLTTTLLAGSDSNRNPSAVNGRILGLVGLIA
SLLQGTMVRRLPPLMTVRIGVISCAIAFFSLANITSLQGLQISACFXAITSATVVTGLNSLGSLEA
GPGERGVVLGRLRSWGQFGRALGPVLFCSFFWWVGRQVAYTVGGVVMLGVVGIVLLFLREPVRS
>g2211
MSAAINTNLPNQNETKSSKKKRAKNDVSANVSVSEPTPSNPDIDAKAESIVNGVNGLEDETGIIKE

LQRNLRNASKKLNATAKVDSILAENPGKSLDQLIAEKKINADQKAQALKKPALQAQIAQIEEQITQ
YKQFAAHYEERLISQKTSLDTAHRQELDTAREKAVAEVKESQESTVRAQLLTLSQFLRSAASFRRA
GDAESPESQAFEGVLLQVYGGNQDAVESMLKLINGADDKVTGVDGQVLDVSYAKVKALSQEQAAPA
PEATVEETPAADSAVTDPTIANAGLTELQDTAVSAAVEVDQVARATEPVVAPTQTSAGDGANLLAE
SSWEPQASGTLTSDEWVEVAHPTEGEIEPSTAAAAQGSTSWAEDVPTGATPAEGDGFEQVVHHQRQ
GSVRGRGGRGRGRGDGSRGRGGRGEFRGRGRGGRGGERGEFKGGRGRGGPAGQQGSRREAVATN
>g2212
MAPTTAATEPSTLIESFHNALLKVHENPCPQVPNPPNCKKRASVAVILRVRPQYXXLPQSPVIVTD
KAASTSQQLSDFFAQDWVQHGDPELLFIKRASRVGDRWTGHVALPGGKRDPEDENDKAAAVREAQE
EVGLDLTTEELIYIGNLPERVVTTSFGSVPLMVLCPFIFLLTSNNAPALTLQPTEVASTHWVSLRA
LLSPSLRSVEYVNVSARYAKQHGLIRWGSSRWMTGMMEFSALRLLPTESLYSSTTPGFLPDEAHQE
KPSILQRLNPWAVRQATYGEKNRELLLWGLTLGIMADFLDLLPPHNAVDLWKHPTFTAPDLRLIVS
LLTYSIRKRNATKVRAARRPSQTAADDSTMALSVEDEGEQSPSAENKDGTTIALLTTTCAASSEQS
IRLNFDSSKLSKSVASPQTNMVATHKLYPRGTVKRIVKAHAGRSISKNADVLIFLDYMLFMQELMR
EASIRSRKAGEKGISARNIRRVTEVNMLVLLCVVDLDNTDSQIIE
>g2213
MRFGKTLKASIYPPWEGKYIDYAKLKGLLRERELEGDDSDSEAQPWTERDEESFVQELINVQLDKV
NAFQSEMSQQLRDRTNACEAKLMPLVRKSTGGKDEPSEDKRKEIANELVGELDQITKEVSELEKYS
RINFSGFLKAAKKHDRKRGARYRIRPLLQVRLSQLSFNSEDYSPLLHRLSAMYTFTRQILGLEIPE
NQETSGDSRLGHDAYVSYKFWVHTDNIVEVKTHILRRLPVLLYNPTTSKELDVSQKDPTITSLYFD
NSSFDLYNQKVTRATEAGSLRLRWSGDLNEGPAIYLEKKIVGEDRSREVRVQLKEKHVQEFLKGEY
KFEKKVTRLEDRAQGESQEAAALKKEVEELQSFIKENELQPMLRANYTRTAFQIPGDDRIRISLDT
NLALIREDALDPQRPCRDPEDWHRRDIDDLAMQYPFSSINKGEIVRFPHALLEIKLRNGARNVEWL
HDLMVSHLVKEAPRFSKFVHGVAQLFEDNINSLPFWLGELENDIRQDPETAFKHEQEREAQRAEED
FVVGSFLGTKASPLNTMMGSPISRMGAGRGSSPGRSIIMSGTSPVIERTSTQLAARDIPRTTGVSQ
PSTAAEATPTEHAESQEGATSSSYLGTLFPSFSSSRYAQAHRGTSRLPPGVSAPEVWIKDTGPLRV
ESKVWLANQRTFIKWQHVSILLASLSLALYNAAGIDNIVAQALAIIYTFFAVFASVWGWYIHEKRA
RLIRKRSGKDLDNLFGPLVVCIGLAAALLLNFGFKYKAALDEARRTGYPGVVEYLMNTTTLQPIFT
VQGNA
>g2214
MDVCEATFGEIDIVCPGAGIFEPPQSAFWYPPGSLQSSDSVMESRYRIVDVNFVHPIRMTQLAISH
FLKSAARVEQKKKHILLVTSIAGHMPVFVAPLYSSTKHGVSALVQSLAPLDDGSNIRVVGVAPGI
TATPIWFESEDDKKALVNETVDEWVSPEDVAIVMLALVERDFIAKNLEDIDQDGGTIRIQGGTILE
VSKSVRSISIFNDPGPVGRPGNTLGNMAQAEESILQMLSHSVTEN
>g2215
MTEHQESATKKKQWVLNGFTMSTPGHLAPGLWRHPRNRTAEYTSLKYWTDMARILEAGKIHSIFIA
DVLGPYDVYKGPENIDPGLPGVAQLPMADPFLPIAAMAAVTTNLSFGITASTTYENPFLLARRFAT
LDHLTNGRVAWNAVTSHLESAARNVGLETQIAHDERYRIADEYLNLTYKLWEGSWRDDAVVKDREK
GQYAVPGRVRRINHQGEYFRSAGPLTTEPSRQRTPFIFQAGTSTAGKNFATKHAECMFIPGLDTKG
IRKSVDEIRSLAAANGRDPNSIKLITGILVIVDETDAKAHEKYEDYLQYVDLEGAATLFGGWTGTD
LSQFSDEEDFRFTGPAAIQSLVNSWTETVPGTEGLKWTKKRVLEEITLGGPHGRAIGSPKTVADIL
EKWVDETGIDGFNLSYAIAPADFEDIVKWLLPELRARGLFWDEYVADTTRENFLNDGLGPRLRDDH
PGSKYKWAGDQK
>g2216
MSSQVPPPLPQGAGYGVVVGLGVAFALGMIWVTKTLKKTFNEDANSTETFMVANRTIGTGLTACAV
ISSWLYSSALLGATLLTYNYGLALGVWWGASASTMVTLMAYLAIQAKRRAPNAHTLLEIIRVRYGT
SAHKLWIFLCLLNNTFVFTSMIVGSSTTVSALTGMDVIASSYLLPLGVAVYTYFGGLKATFLTDFV
HTFVIMIIICWFTIKVITVNEIGSIGALFDAVVIAGKEKPVAGNYQGSYLTMRSDQCLFFGILHVT
ANVGAVIMDTGFWQKGFSADFAAAVPGYVLGGVASFSVPWAFGTIVGLGALALEKTAIWPTYPNAM
TASEVNAGLVLPYVAQAVAGKAGAGAILLTIFMSTTSIASAQMIATSSIISFDIYGTYINPTPTNS
QLLRWSHIGVIFSSLFISTLATAFHKGGVDMTWLLYMIGLLTCPGVFPTCLSLLWNRQTKNAAIIA
PIVGMMCGIAVWFGTAYSYFGEITIASTGGTMPCLFATITSFLVPLPITVGISLWEREEFDWAVLG

QIDRVKSDDGIASHGSNEEEGSFFTPERTKYLKRMSSWAAFWAVFTVIGHVLLWPVPMFGARMAFS
KSFFIAWIVISLIWLWFTLVVAIFYPLIDGGLQQMWTVIKRKGLHQKTSESTITNSSSEQVEVTAK
F
>g2217
MLAKRNGRSWYQWYAETDTPQERKLIFKLDLLIVPYAFVGFWINYIDSSNISMEFLWGIFTLLQYR
ATTYSELMAYRFFIGLFEASFFVGVHYVLGSWYRADELGRRGGIFYIGLTLGSLTAGLIQSAASAN
LEGVAXLAGWRWQFIIVALMTFIVAIAGFFIWPGTPDIPNKLFLSDEEVCLAQERMNKNGTGESNA
NTPKFSLGLLRAIFTNWRVYVLTIWNILFWNSDPQPWGGYLLWIESLNRYSTAEVNRIGASAPGIG
ILYVLFINFSSDLWLGRTASIVLAHIVNITSMTILVVWNVPESAKWFAFNIYFFDIAMSSVLYGWA
NDILRHDKQQRAIATDTDTVTTSGIATTTVTPTIVKTVTETWILPTPSAYRGVNYYQYLNDYFYPD
GDQGCLTCGYGGGGYETADWNGNYSYYTNGTTQQINFESENYPSYSTILCQLPGQPAATDCSQWTV
VFQGYLHATQTGNYTLAPDLLEDNALFFWGGEKAYSSYENNNTDGGVSYTQPAGLPHTFAYEMVAG
EFLPITFIYANGYGPALNRLTITSPNGTTYPDDLNFFVPPCPNSPFVP
>g2218
MEDASSYTPTYAEIVFSDAKATSSATSQFSTPDINSVIDELVVLLGRDEAIRSLCSQAVKRESIGR
ERLKKNLRRLLKLYATELKSAAKGFLQNLAAQFVLSHSRYISAGIITLSELAYQPSQEETSAFFVE
TRQAKVKELLQRINESDSEDDEDTGPSLDTLEGVHEFLISGPAFDHLRENIWHFVNGQPIISVQHA
TMSQLNHSRFSQKNAHDNQEERISRRQHLKYDTQVAHIMRPTLFPWVTTLKRQIQRWARPQVKEGL
RRFEWNCTCGKELYRDFDQNIDPETLRDFHHIFEQQKSFVRADATGEQKPTEETVPDRSDNQPHTT
KMPTLRHTSNSLQLSPQVGCKTSPTRQGQPIKPKYLELCINTGQYSTSLGEIDLSHVKSDGELFIK
IRERYREMRPFRLRRIFSKPVDVHFVRFCLQRTYQVGIHGKPMEMPPEEEVRKNHYEFNCPLPPPM
GPREFLHYFYFCEAGAHPHSTYLERLPKKLETSMYQLQPEGLTFGWGIHIIEGPNKAILSWACCLI
LLSSFVVSVTYAVVMKTQEQGFGIGQWMVAVLCAALSALYYQWEDI
>g2219
MSSPAGSLLPSLTGYSLPQHCEPSSSFLAFLSSALHVCIPNELGLISSILGCLSIVSWLFAQLPQI
YKNYKLQSTAGLSAFFLVEWCLGDTANLVGALFTRQATWQVTIASYYVFVDVVLVIQYYWYTHVKK
TDKGKDSVNCSFDEDHAPIYDGIRVRDEDQPANNVKPQTMRPSEPKDVGLFKTNANSPPRFYSPSY
SEKFENRRARGTGFPSGSMPLGVSPRTVLMTSMLCAVLANASPTPSSVASPHSPLPSVFDSIKDPH
NMDIETIGRILSWMSTILYLGSRLPQLYKNYLRKSTSGLSPLLFMAAFSGNFFYSASLLTNPNAWY
DYPPYGGGGWADANGNDRVEWVALATPFFLGAAGVLSLDAFMGVQFLIYGDRGDEPLVTVDDPEDP
GHKRWRRVSGWMRGWIPSGELNKYLRSNSGETQSLLGDHTSRGNDYGGV
>g2220
MFTSKTIIAVLAAIAGLASAAPSKASSSSSSAIPSPSTTPYLTTFYLRNTAITNDSGCVVEVDSYT
GCDEVAVLSGTQNCSDIQGTISQPLCGGSSVTADFQGGDRGTHLSYYDENQTLVAYCILPSNPGAS
AKCATTD
>g2221
MPSFNFRARMKALEKEARNDVNLNKFYSLVGKAVSRAPWAGKTYLSSKVPIVQWIGHYSPYWLGND
LLAGITIGILLVPQSLAYAKVANIPPQYGLISSWLPCLFYAIMDVTAGPTAIMGLLTGEIVADLVK
DGYSAAVIASTAAFWVGIYSLVLGLLRLGFLLDFIPTPVLSGYVSGAAITILLQQLKGLFGQTKTG
SDTAGVIRYFFRLLPQTNWRDFLIGLSGLVIIFGLQFVGRRWGKHYRALWYLSVARNALVLVLFTV
ISWAVNRHRPSAPLFSISKVTGTGILSPKAPDTDLLTKVAGRSIAVFLAAALEHLAIGKAFGRHHG
YVIDQDQELNFIGLVNLFGSFFSCMPITGGFSRTAVNSESGVKSPLSGLATTACVLISIYKLSDAF
YWIPSATLSAVIVAAVWQIVLPARVFWAYWKVSFADFFGSMITFWITLFVSVEIGIACGVGYSLVY
ILLHLAFTKVMLVDNDNLGQLYPSSSRSSEQEGLPDDTMLFMIQDSILYPNAARISRQICDYVYTY
TASVHEQSASSSSLDRQATESHQRLWNDTRTHHATRLRREAFPSSSSSDHPPLRILSKVIIDLTRA
THIDTTGLQALDDMRSQILDWAGPRVSIRIVGANKRVRGRFERHHRLSRRKHAEEGRGDGDAPLEA
GYQVFELLQDALAGRRVAGESGEEGLVVGEENIKGV
>g2222
MKYLDGLERRQVAGEYFAPTGFYEYTKNLPFLGSVNSNIETLVAGEWTEVVIVYEVGASGLADGAW
IKGTFKFYSDWALFQTSDPSADNYVSAEYTANSLLPGQTPATVQSLRVRFDQKGHERPFQKAVIVD
IQDGYLNPGDRIIIRLGDRQFGGKGTRAQTFVESGFRWRFYIDPVGTSRFAPIRPDISWDIVAGPV
KKLDIVLPRLVQPDQPFAIHAHAEDKWGNVARATPGLTMSLAIRQTAKPEWPQHQWHTEVASGKWL

NAIFENIKLGNSDYIVEVKLLTSDGAVIARNETYLTVDFSLNIPKVLFGDLHVHSDDTVGTESSVY
NFSYGREIAGLDVLGYTANDFQITQQKWEATIKLIQSLNEPGKFVIFPGTEWCGNSAAGGDHNVVF
LADPATSPPEFPYDRHGNVARSFEWSEDGPEDLVPGAWPLDEVYATYAHDAENTLLIPHVGGRRCN
LAWHHPKLERLLEIGSAWGQFEWLLRDAVRRGWKLGVCANSDEHRGRCGGGVPGTAVFGTRGGLTG
ILSPKLDRKSVADTLRARHTFATTGQRLVGLVSAKETNAIQGDEIEQSAQSPLVLDYHFLGNRGFS
TIEAFDASGRIWQRHFWSESDKPATVLRVTWGGARLFDRYREAVWSGNIDIGGSATIDRIEPFGGL
DDNPEDQSIQTSDRSVSFNSRTSGDVDGVNIFLAEPSSEVSITASGTIGGYVKVGDALAGNPHKAQ
PSFKLEALWAEASKLGGEQIDIGGSAEAFVRVEAIPDIELPRRVQGTVDFGGEAGQERAIYFVGRE
WSGAKRHAADRYRLRLHRARSAVLTSDELVEMRAAQRTFEGXYIRTALSSFSFALIVLKVFTAEFY
GIGALFAVYGTGVLIISVYRRQQGNRQFFSEIGEDGLHRRKFRTSGNAVVFLTALSVAAYASLIGL
TLKL
>g2223
MDAASSSPPTTSQPLHQTPPSPSQSQSSQSQTSQPSSSSSSPTSTTSNGVAYNNYLNPNIAKRIPT
PNGTANGLSTGIPRSTYNPPKTVIGRALGNELHTEAHKPVQLTSQRRLSKDGVGMALSDTPTSTAP
SSPQIRAQLQTPTSSTAQTPKPRATTLDIPGLTRSKVSPDGRIPERDVGSKLVIVMVGLPARGKSY
ITKKLARYLNWLQHDTRIFNVGERRRIAAGTGSPDPSMMNIKARTNSSLDQQLVESVRRMSVRARS
FAASPPSPPEKADALPPPAMSISPAAEILVNGQHVDAPGPVPLSPKGSLTVDTNVESKRESEQKRE
SIDQSAAFFDPKNREAVQLREQVALATLDELLDYILDQGGSIGILDATNSTLERRKTIMDHIRQRA
GPELGVLFLESRCVDPVLLESNMRLKLSGPDYRTQDPVKALEDFKKRVALYEKSYVPLGEYEEQND
MAYVQMIDVGRKVVAHQTTGFLSSQVVYYLLNFNLSPRQIWITRHGESIDNQLGRIGGDSELSENG
HKYGQALARFIDDQRQRWEAHQAQKARTSHFPPHPGDSTPPNPSYGTQSNKPRNFCVWSSMMKRAI
QTTSYFNDEDYDIKQMRMLDELYAGKMEGLTYDEIKTQYPEEYESRRKQKLQYRYPGPGGEGYLDV
INRLRTVIVETERTIDHVLLVTHRSVARVLLAYFLGLDRDEVAGLDVPLGVLYMLEPKPYGVEFKV
YRYNPETEWFDHIPGFELHQARPI
>g2224
MGQHTSLLSEEQQANALLYSTASFTPGILSFTIPKLGVTALLIRILNPTPRFKVFLWVLVGSIDLI
IFGCVIILFAQCSPTRALWTPSIHDLPGVKCWDPEVLAFYSIGAGALSAFLDLFLAVYPATVLYRL
QMNRKKKIGLMMILGMGILYALLPSIHKGDNANWNSASAISIYKATTLTGLDVLTDYTYTSFSVFL
WTSVEGNTIIIAACIPTVAPLLEGIFGNNVLRGPSAEQSSYYRKKGRRDVFAQHNRGNDSKGPIIP
PTALVRDSALAPKQQERSRRGSAQRQDQKGWMSSTLNSRSSRDSNLKSKLSPTSPSYSYMKSMTSK
TDVESQTSILDYDERHLEEEDDEIEMSHISRHDSFRIQYETVADPNNPRNEHNVIEGGTIDRNGRP
LSIWRRSFSKYGYPGLSGYHHTNSNNHNRNNTGDKGNRPGS
>g2225
MSSILASNPILVNIGTHSLALYAHGPEPSNTKEPVVLFISGVACSSLNWAAVVRLLPPSLRSYTYD
RSGFRNSELSPLEPTAENVALELSLLVKNAPIENPLILVGHSWAGVLISEFIALTGNGPHIAGLVL
VDANHETAPLVLDVNDPNLLAISEGLDYYSGIGVAAEHKLTQEEWDALQSDQSTEKYKLTAYKEDE
EQYIPSFETLRKKGLSKKQPIVGDKPVFVIGSLRSRDWTGLYNAGVERGNGTEEQRSLVRELIRTA
DEKSESLMKEWLKISTRSTLVFARESGHFIQFTQPDVVVDGVKWVLDQSKTS
>g2226
MGKKIECYIDCVSPYSFYAFSYLQKNRPALKNLGVEVEFIPVFLGGINVGSGNKPPWTLPAKAAYA
NFDGKRAQKYFGFDFEVPSFFPILSLLSYSQSTFEAAFYACFDTMWNGQLDISKPEHLTTSLSKVF
SLEKEVQDIIAAAGSPPIKALLEIVTGRAVKELGAFGCPWFWVTNGEGIGEPFFGSDRWHYMWRFL
ELPFEDLRLIPSSEKVKL
>g2227
MSYNPGKKPVIAVVGAGPGIGEAVARRFTSEGFVVALLARTEDKLRTMAEGIDADFGQGTARYYIT
DLRVEDSVISSFRAIRAELGAVHVLVYNAGARRVNGRLILDTTTEEFEGFTKINLFGAFWSTKCVL
PDMLARGQGTIIYTGATGSLRGSPGLSSFSPGKFGLRSLAQIVAREYQDQGIHAAHVIVDSPVDGK
LIGGVRRRQWQREGAVEKLENIEAYLLQPTDLAHIYWFLHTQPRSTWTHELDVRPQKEGMFSKL
>g2228
MPLYEVIHAIPLTEKQQDELAEAITVIHTTAFDVLRLFVNVRFVDASSLNTYIAGKRRKANHIRAA
VRLGSRTHEEFLTLCRSIEEAWNRIIYEPAKSEQNRYKLQTLVLTPFNLIGIEAGFEKPIAGEDGN
WLKKHWDEFRQKANEGDEDFIQVVKEAEERGLLK

>g2229
MAALLPQSAGYATILVGGAFFAALMNAFTWVQRRYTNFDPGKIEEFSSASRSVKTGMLSVGITSAW
VWAAVFLQTGTLTYLYGVSIPWWFGIGGFVEIAAFSFISSKVKVNAGGASTYLQVAKVRFGVLGHW
AYMFAALVANFVVGCEILIGGAGVITGMTGISEYAAIWLLPLVIVAYVLTGGLRATFIADYLHCCI
LYSCLLVLILATYTRGDAIGSPGKLYDLLLDASKAVPVAGNGHESYLSFRSDGGMYYALTATTSFF
GLSFCDQSYWQRSIAARPAGTSKAFIFAGCFFLSVGLGIGSSMGLSARALVSNPAFPGYPDGLSLT
ELNAGLAGPYASTVILGKAGPAMYTIIAFMATTSALSAQLVAVATIFSYDIYREYISPTATNSQLM
RVNHATVIGWAVFLAGINTAFAYIGLDLNFLFYLMAVCTSGSVFPIGLLMCWTRLNKAGAVLGVTG
GLVIGMVAWLVTAVTTQGSISTTTLTDSKVILSGSLSALGTGAIVSIGLSLIKPASFDFELTRAIG
RGSTVPVDASSASTDKEPRINQSASNNLEKDPTTYEPALHSVHGGGSTPESEAQYALEVVKLEQSQ
TKFRIITGFLLLIILVLIPAPLAGTAVVYPKGLFILQCVAAATFAFVSLILIVFWPLVESHKELAA
IFNRIAHNKRLDKGVEQPLDHSP
>g2230
MQLMVAQEFIAVTKRRLICTAQYGAFLMSILAVTLRLVESAKPLLSPSEREQASESYFELAQDLLR
SSKNKLDIRHILALYHLALFSEGRTSSTGPVSSFVAEAIGLAFTTGLHRSTKDFNMDPVTLQGRPP
LIRLSECSVELPAIVDNQYVTKAAILPQPESSPPVAVAAAVKITELYMVLEQVLSAINAPSKTAAA
AFSLDCRPFERNHVFTRAQERLDQIERDLPPFLSQIDSSSNPNSKFFHSSRVRATCQFVRTLMAXQ
ALIDGLEANPPPSEEEIDSATSEACHLSIDIIKTYSRLRHTGHLRFCGFQAVSHITAAAHTLIASQ
LIFCSSSHLPSHRPNLSLSFLFRYPEQWITVMAQLASLKLQRFVSSLGNLHLLHLTITLK
>g2231
MTIATRPSVLIIGCGPAGLGAIEQFKRKDFDVVAYEARDRVGGLWNFDAIPPPCHITFAADGHAIA
LTDLERSGRPAPAPTPMYAGVTANTPRDIMPYRSHPYPKGTAEYPNYEVIYQYQQRHAARYEDYIR
LNRVVTRVRHAPKGSSHNKRWSVEWTPSVTSNSPDVGTKFEEGFDHIVVANGTDSRPFIPYTDGLW
DWKGEIVHSRWYRKAKAFAGKTVMVVGSGPSSADIVRELGMLCVELSSLAPTKVYRSFKSKLRYDI
EQEKGWPNHITNVARVRSIEPPSSHSITGRIVTQADEILDDVDVIIFATGYVARFEFCRESDAPFN
KAPLVHHPDTPPGSRRPPKNSVDQADLEGGHRVHNLDAYQLFYLPDPTLAFLVLHAEAIPWPFSEM
QARVVAAHWSGTPLILTPHPQEDDDSHDVLVLGHPGEFTYAEHLLKLIGEGGPEEIDDQGRWGAWP
AWKKKIRELID
>g2232
MSVPLIIDPTSPLLSDPSVVILDASWLYAPDPPTRNAHDEFVAGPRFPNARFWDLDAVSEPHPDGY
ILMLPSPERFAEFAGKHGISKNSHVVVYDSEGIFSSPRTVWTFKVYGHEKVSVINGGLPRAKKEGV
KLESGPPKEFKETHYPVPTVARDAVVQFDEVLKLAQRPTASAEDKFLIDARPASSYEAGHIPTSLL
LDFPSSLLRDEANFTYLRQPEDLKQHIAQQLGQDKLDQIVAGKATVVNSGFCIGKYYGNEE
>g2233
MSRASVADFVDSFFTRTLFQADDTLAASVLDTDLSPDVEIVINGTRFTKATFVTLITEQFRSTFVA
SVTEIKDLNILTTNDAGSTGVVAQWTSYVTKGKDDGKDIKQSATTVVRVEEIDGKHVVTGLWEAQT
SE
>g2234
MSAFNSFNMYKSALILGSLLATASAQQIGTYTAETHPSLSWSTCKSGGSCTTNAGSITLDANWRWV
HGVNTSTNCYTGNTWNTAICDSDATCAQDCALDGADYSGTYGITTTGNSLRLNFVTGSNVGSRTYL
MADNTHYQVFDLLNQEFTFTVDVSNLPCGLNGALYFVNMDADGGVSKYPNNKAGAQYGVGYCDSQC
PRDLKFIGGQANIDGWTPSVNNSNTGIGNHGSCCAELDIWEANSVSEALTPHPCDTATQTICTTDA
CGGTYSSNRYAGTCDPDGCDFNPYRLGNTDFYGASKTVDTTKPFTVVTQFVTSDGTNTGTLSEIRR
YYVQNGVVIPQPSSKISGVSGNVINSDFCAAEISTFGETSSFTKHGGLANMGTAIGDGMVLVMSLW
DDYSVNMLWLDSTYPTNATGTPGAARGSCATTSGDPKTVEAQSGSAYVIYSDIRVGPLNSTFSGTP
STGGTTTTSATGTTTKTSSTSTSASTTTSSAPSTGTGVASHYGQCGGQNWTGPTTCASGYTCTVLN
PYYSQCL
>g2235
MYKTFRTAAAPAVRCARQQPRAGARFVNARYQSTTSQTASSGNSPALIGGLAGGAVAFASGYAWYH
FSGAKTLVKTSKETQEYIKKAKETVVQNTPEPSEAFRWLRDTVKSYAVLIPGARGYVDTAFDDLEK
IRNNHQEDFDKIVKDAYNETRDIFSKEGFNTSSASKAAQALQKYLSQLFDLAGDAASDVLQNHPQL
KEKVGGSYDQLKQMGDAYGPKAKEEVDKTWQQISDIIKAGVSVDSADKIRSLIQEKKKEKLQSLGDE

AWKKGLEESQSYLEKNPKLKQLIEENADALKKGNFKELWGLLKDKVPSGKTEDVEKYIKEKVDQAK
NTDFSGLDQWLNKLPGGSDLISQLQTLQNTAQKKGSETEDVLKETLEELRDVLKKRMEQVEKLAQE
>g2236
MRSFVGLAGLAFLSSAVLAAPAPAITAPATLENRAASCTFSGSSGAASASKSKTSCSTIVLSNVAV
PSGTTLDLTGLNSGTKVIFEGTTTFGYKEWSGPLVSVSGTDITVEGASGAVLNXDGSRWWDGEGSN
GGKTKPKFFYAHSLKSSSISNLKILNSPVQVFSIDGAQTLTLDSITIDNSAGDSAGAHNTDAFDVG
DSNGVTISNAIVQNQDDCLAVNSGTNIIFTGGTCSGGHGLSIGSVGGRSDNTVNTVHIENSTIKNS
QNGVRIKTVYGATGSVSGVTYKDITLSGITKYGIVIEQDYENGSPTGTPTTGVPITGLTIDGITGS
VSSSATDVYILCGSGSCSNWTWEGVTFTGGKKSSSCKNIPSGASC
>g2237
MSGYYNQEYQHQPGAGQQGYGQGYPDQYQHQQQQPQHQYLDPSAQYNHPPPPRSASGSPYPPQEG
YGYGHPQQHQQQQQQSYGYNQHQQPPPYEDHQKTGTAHEYYNTHDSNVPRIEHQSAPDTTDPSESG
ERGLGGGLLGGAAGMFAGHKANHGLLGTIGGAIVGSMLENQAFVEVVASFASVEGKEFVVEWWQ
>g2238
MTRPQMIRADTIDLQDHKSPSAQDHSSANQRVAEGRTGPHQNQSLRHAEQEARDDMSTSPRASLDH
FDGGDQNHHIDIVYDEADDSMADLKGEDGDIGDEESDDMMDDDMMDKISSSPSIDDEDIDFDFVYA
LHTFVATVEGQANAAKGDTMVLLDDSNSYWWLVRVVKDGSIGYLPAEHIETPTERLARLNKHRNID
LSATMLGDNAEKSKNPLKKAMRRRNAKTVTFTSPTFFEPSDNEYSTEEEEEEDTEHVEEEESRAES
QDTQYDIQDEDMVVEPLRPKQHKEKVSRVEEPEEEEIPIEPSSPEKPRPSDESFERPARSRNGTLR
NTDSFFNDDTAETKKISITPSLLRDDSTSDRSKSEPQEGRASMESIEKSLAAGDKSKRKDKKPGML
SGLFKRKDKKGRSSEDDGEDMEKTSEELSRSSPQPKVSTESLREHTNTRPAGVQRQSSKLHKQPPA
EIALINQSQRTTQEPAISTSEASSPIKEDYGPSIRRVVSPSSGPNVAPLQVRTSLEKNAVAPEQQT
PSSYTSSPVKSSPVKSMNTTSMGTNSKMYDDPIAEEPIQIMVKEEQPSNPFFSPHELRDNAHSDES
LSDSLVEVSPSQAPMISQPPGLVVDTSSQEERSVSPLSSQSSSPEIIERPEPKGDETTPVSTVSSA
ATATWSDASLRTYLDDENDIRDLLIIVHDKSNVQPAGPDHPITGSLFKEESRRLKEMSGRLDDMLA
GWISRKTDHRVSKAY
>g2239
MAYAGRETFSGLRLDTKFARDQSPSTNNRYGEEQFSGREASHKEQDDDEESKVIEELHTATPPMQV
PRSDDNSIKDFDSDVSYSHRPEEFRRSFEEFLRLNQSLPEGKSSEGSLPEFAPRSIPKPRPSALRR
VQIQDDDGFNTKESNGETDEMRQVTSPDSGISFVSTRNENANSALDMDQPTSLTSVSTMSPLEEVR
TPYEYPREGPKDMLLSPLSSSPLVPRAASLDDPNMWTTAVAGSYSGKPRSYTIDRQNSWRQVMRQQ
RSRRSTGSSGKSPASAFLSMWSSPEEASATKPDDEGQMVGTDYVLGKQIGFGGFSTVKEAYKVGAD
GSTRRLAVKIVKKHVTGKNERENDQVQAEFDHEVRIWRQLNYHHILSLEAVYETDYATFCFTKLFI
GGTLFDLIKANRGGLDMKLARKYSHQLASALRYLHEDMRVVHRDIKLENCLLDPERSADGTEGAKL
VLCDFGMAEWMTNDTSGEPLDTYDNPADRPPPQNIGPSGSSTSVAGSLEYASPELLLSSTGLIDPV
VDIWAFGVVVYATVVGSRPFQHGFGPRLQASIINGEWNHDAVLAGHEDDTNRQEALDLICHCLDKD
TASRWTIRQTLGCDWFANLTEPSDEHLDERSWRL
>g2240
MAGRFPSLTTRVSQLSISSITAPTRSSLLSLLAPQFHQQQTRNASILSSISDNPGAYNKRIRRGRG
PASGKGKTSGRGHKGQKQHGKVPYGFNGGQTPESIVHGSRGFTNYWSTDLITVNLDKIQEWIDQGR
IDPKYPITVRELFHSNCVHQFRDGIKLLARGKEEGLKQPIHIIVSRASADAIAAIEAAGGSVTTRF
YTNASIKRILMAKTHPFYSLAWSEKSGSEFLIKALGAANEQEAAELKESKVMERGNFTYRLPDPTH
RKDIEYYRDPAHRGYLSHLVKPSEGPSLFFLSPAERKSSAGVKKEKTLAPNRLW
>g2241
MENGEYLSPQTVINTTQSDGQHATGRARSNSTNSGHKRSVSGSLLSKLSFLRMSQSTSTDNNEPLS
SRTHGDETAGNLSPKDGHNPSNNDGYIGGSRAGGTSRAMATAIQHQRKMRRRRGSLRKTALLGTRL
EMKEKRNNNNAASTARDPVNYGSIENSRGVSPSPLSISAGTDGSRTTPPPSTTLTDEDLTPRGSMD
HDGGGHASVHSGYWPRSLTMDRGVVSTEIKSPTSIGDTTDEEDNISLSDFNNTTPNNNNNNTTSNG
RTASTHARPSSVSSSGSIXPPLTATPSSSSGSYFVSSHHHLHHHHKTSSANEPIDATARSLGRSPT
THRARSPLIAPELSSNVSNTLDPSVGVGTSGGWDYSETEWWGWIILLVTWLVFVVGIGSSFGVWSW
AWDVGETPYAPPELEDDPTLPIVGYYPALMVLTAVMAWVWVVVAWVGMKYFKHANIAADDG
>g2242

MCRFLVYKGRSEILLSKLVTEPSHSILTQSYDSRLRLDTRRPVNGDGFGVGFYPDPKISPEPCIFT
STLPAWNCENLERLASKTCSNLVFAHVRASTEGALADNNCHPFQHNSLMWMHNGNLGGWNYIKRPL
SESLNDKWYLGVKGGTDSEWAFALFLDLMEKEGADPSSDPGPDGFGQAFLRQVMFKTIAKINEFIR
QIPEKHKVTDLETRSLLNFAVTDGHTVVCTRYVSSKTDEPASLYFSSGTKWKEGKKEGHFKMERHD
KGADIVLVASEPITFERHNWVTVPTNSILTIHKQTVLLHPIVDEFYNDDPNHDRSSVYALSKGFAP
PAIVTPKKSTAAAKASLALANGPTKLDSLAQKVTQLELAS
>g2243
MSGRIVSQRLAPQLRRGLLLTRHANFVGSRTGGLLRVDNVAVRGRAWPVGASSIHNNVPAVRNISF
ARILPRLALKLARIPAMFGGAMIAGVAYLQYQATQAGNYAIDLFNQGWETAGGAASNLFQGFQDVA
SQTGRGWQRVKDETELPEWMQKIFRLEESTGGDGGSGGNKPPNDTQVAAAAAGAAAGSAYAYENTD
DGDYQTRREADNDQMMVLTRKMIEIRNILTQVGQSNTLTLPSIVVIGSQSSGKSSVLEAIVGHEFL
PKGSNMVTRRPIELTLVNTPNAQAEYGEFPALGLGKITDFSQIQRTLTDLNLAVSDKDCVSDDPIQ
LHIYSPHVPDLSMIDLPGYIQVAGSDQPPELKQKISDLCDKYIQAPNIILAISAADVDLANSTALR
ASRRVDPRGERTIGVITKMDLVDAERGASILTDKKYPLRLGYVGVVSRAPQTSTLFSRGSGNITNA
IVKNEKAYFSAHPSYFGPDSETSVGVPTLREKLMNVLEQTMARSLAGTRDAISQELEEATYEFKVQ
YNDRPLSAESYLAESLDSFKHSFKAFAESFGRPQVRELLKHELDQRVMDILAQRYWNKPVEDLAAA
LAEADPLKDLPKADPENLYWHRKLDASTSNLTKLGIGRVATTVVASALQQHVEHLVAQSTFATHPY
AQNAIMEACNGILNDRFFSTSDQVENCIKPYKFEIEVEDPEWAKGRESVGKVLKEELTACEGALKR
VEDQIGKRKLKDVMSFVDRVRKGDVVLDGNGAGGAGGFSAALLEKGTSSSHFVNLDTDSSGREGIF
LRDRADIIKMRMLAVRSKQCSSLKNKYYCPEVFLDVVADKLTSTAVLFLNVELLSEFYYNFPRELD
QRLGRHLSDSEVERFAREDPRVRKHLDVIKKKETLELALQKIESLRQLEGRSRNASRESIPKEANG
KERRGWLF
>g2244
MSPALASYPSFSLPFPPASDFHRYAYQPYSPLASLSKLTLNPISSGLPNWSAPLRDGLPTPPADMN
GVALNSQLAPSSAYAYHHHNGQYAAKSSSNRVPLANTVSNAAPNYLPPMNKQVVQVEETEKSHKRS
EKESLPSYSQIPTSIRKNGGNLPEFAAQIACLFWFEKTSKLKTIEESTPQKYALVQEAFPTIGFQK
WVATVLSTTQVSQNVILLALLFIYRLKKFNPGVRGKKGSEFRLMTIALMMGNKFLDDNTYTNKTWA
EVSGITVQEIHIMEVEFLSNVRYNLFVSKEEWTQWHSKLSRFADFFEKALRMPPENEFAPTTPVHQ
ISPTADVRRYNLPVSPASKMPSPPSTHPLQPQTQLQPQIQSRPGWNSTTTPYRLNTSPRALPNLEF
PPYSRKRSWEDDVEENPSKRFAVSNYSAPMPVLPVSATSMPSAAAMSSAAAMSSAAAMSVPVLPPV
LGANMPLSVPSLAIPVPRLSQPTTYQPVSTALIPPTLPAATMPQQLPLPHVRAMSTVFNPSSTWSQ
QIPSATVAPVVPSQTSNPMALYNSAVSLPDPVRRQSPYSATVSSDAVSPSSAFHVHTPQNHLSPSF
FLVNRNSPYRPVRAVNTLLIPPPSASLHQPRHLSMDQMHYQPLGKSFTERRTGVLPYLHHDGLPQG
VIPQQPNFLPSQY
>g2245
MLVRQRIAAAPAAFARGATKSFSSASSSSSSSARLFETRPSPSQLLSKQRSTFARRAYATEAPVPA
PAAPKKKRFRFLRWTYRLTVLGLLGGTGYLGYSIYLLRHPAEQIEPDPSKKTLVILGSGWGSVSLL
KKLDTENYNVVVISPRNFFLFTPLLPSCTTGLIEHRSIMEPIRNILRHKKATVKYYEAEATKIDYE
KRLVYISDDSEIKGDVSHTVVPFDMLVVGVGAENATFGIPGVREHSCFLKEVGDAQKIRKRIMDCV
ETASFKDQTEEEVKRLLHMVVVGGGPTGVEFAGELQDFFEEDLRKWIPGIQDNFHVTLVEALPNIL
PMFSKQLIEYTESSFKEEKITIRTKTMVKNVTDKYIEAEVTKPDGTKELEKIPYGLLVWATGNAVR
DVVKDLMSQIPAQKNSRRGLAVNEFLVVNGTENIWAVGDCAITNYAPTAQVASQEGAFLARLFNTM
AKTEALEDELKKLSVAQQEAKNDEARNEIFAEIKERQRQLRRTKQIGPFQYSHQGSLAYIGKERAV
ADISWVSGNIASGGTMTYLFWRSAYLSMVFSARNRILVATDWIKAKIFGRDVSRE
>g2246
MKTAAILPLLCVLAASAPTPKKAPVEDVHRRQTTDVGDAGLGSVLGGLKARQDAGSDESDPLGGLL
GGLRRRQSSESENGGLGGVLGDLGGLGGLEKRQDTATLRSAITSITDDLPVSANQRRQSSVSGSDN
DDNDDSLLNLLDGSDLKLDGNGQDINVADLRRRQRPSLTNNRDANGNNDDTLLNAVDGSRAGVNHN
AQNISIAKYRRKRQLLNGLGGESESVSNSGNGDNDESLLNLLDGSSAQVDHNVKNVTILRRYQLQQ
HQSRHESVSLVMKARRWLSTLFSRDAPTKTTTDSGNANDDDALVNALDDSAAAVDGNGQDVTVLRR
RQASTPTPSASAVPSASAAPIAPAQDPAAAPGPRPALNAADGDNSHSTNTNTGDNNHSDDDALVNA
ADGSGADVSKNLQNITILKHRRQDIGHNDEGDHNADDDETVANALDGSDLNADDNAQGVDVAPL

>g2247
MVNEMKXPAVVGXCHCGKIKYQSSNAAYQMTFCYCTTCRSLHGAPFAPFVNVRREELQWFRRSPSS
TKEGDDEWRKLGHWDESPALQKLQLSKVATRTFCGSCHSPLTMVYNAVPDEVGLVATTIDEKLSTA
PVPKVEQHIYVKQKPGWYDIPVQEGVKVYDGPTDSLRKWLEDAEK
>g2248
MAGITRSVNLLLRSSRSSLLRPRAANPVHHVLSNERYAARSFATAFERTKPHVNVGTIGHVDHGKT
TLTAAITKRQAEKGLANFLEYGAIDKAPEERKRGITISTAHIEYSTDSRHYAHVDCPGHADYIKNM
ITGAANMDGAVVVVAASDGQMPQTREHLLLARQVGVQKIVVFVNKVDAVEDPEMLELVELEMRELL
TTYGFEGEETPIIFGSALCALEGRKPEIGEQKIDELMNAVDTWIPTPQRDLDKPFLMSVEEVFSIS
GRGTVASGRVERGVLRKDSEVEIIGYQKNPIKTKVTDIETFKKSCDESRAGDNSGLLLRGIKREDI
RRGMVIAAPGTTKAHDNFLVSMYVLTEAEGGRRTGFGANYRPQAFIRTADEAATLSFPGDDQSKQV
MPGDNVEMILKTHRPVAAESGQRFNIREGGRTVATGLITRVLEQKA
>g2249
MASLRGTASTSLLLRQAFAASRYTATRRPLQTPSRIQPILRDQIRTIYRPAGAVTNPIETNRVVKP
QRYSRKINDEIEADVIQIVDQNGQLESPTRKSNILLAMRRSEQVLVQLDPGDSTRVPVCKIMRMSD
FRNEERAKEKAVRMAKHASKTSTKQIELNWSIDPHDLSHRLKKLSGFIDKGRTVEIVLTKKRNKRM
ATTDEIKQLMDSLRAAIEEANAHQTKSMDGEIGKTLTITVEKKN
>g2250
MIDAGSTGSRIHVYRFNNCGPTPELEDEVFKMTPKKEGGSGLSSYKEDAEGAAKSLDVLMDVAMAN
VPDEYKSCTPVSVKATAGLRLLGEDLSKNILQAVRHRLETVYPFPVVSDEKGGIQILDGSDEGVYA
WITTNYLLGKIGGPDDTPTAAVFDLGGASTQIVFQPTFDSPSGGMPEKLAPGDHKYELQFGGREFD
LYQHSHLGYGLMEARSAVHKSIVEAKLATSPTDLSWLNRPISNPCIGPGMQRQVNLTFPEGHALAP
KVSVTMTGPKELSAPAQCRGITEKILHKEADCKLAPCSFNGVHQPSLEKTFAKEDVYIFSYFYDRT
KPLGMPDSFTLHELQRLTEIVCGGKDNWGIFEGIEGALEELEGRPESCLDLNFMLGLLHTGYEMPL
SREVKIAKKIKNNELGWCLGARFVSFVVISWKLSY
>g2251
MIPKRGHTEDDGFSSSKRSRASISPQRQHLGRSKGKSTLHSSSEGRYHVIEIENTNNDIIKDDEDD
YTSSSGTSSSEDDNNGQENERYEISETIPYIPGRPKPQISLPAKSSDLLSRISSFLPQLQAANADI
EQRLARGESLEDMILDDVKDDEAQAAEGEERTYIEMNLGLGVLKEKKKKRQPGDISLGDESSSGSD
KEEDETSPKEGADEDGEGQVLNQLMGLKNRSGQKLKTNPGIQEVDDG
>g2252
MATDLNQRPNLFKKEVTDVPNFIIPESLDTLSNLWAKAKISPIHELEQGYLGSGSKVTQTQYLALR
IIRSSSIRKPEYIKRSSKRFGLDKIWNDATDRVARSTEYKAYLQLIRMGISVIDLASDHPSYSGSF
KPVKRVQEQIAAVQDVDIPPEHRYRQWNQQLYKVAKSDTRKEKGKMAKLRPSFALKLSSRKPSQRD
SARNAKELISAQVSEQSEGPPIPESAVEVDEPYVDKYYEALYETTPSSALILLLQGVSELVEGTGL
EWTFDHIHLKASFDRGSFNAWTDGAFQTRGGKDILAIVEVKKKPRERKEMEILMQEGAEMVAWLMR
ENGRLPDLNGHRVLISQDCDKIWVTFAICHPDYIEYIKRKGKITDNTFLKMQTFGPFDVNNVKHMD
ILAVLITAITLKA
>g2253
MHFPLKIIKEKYQKLFPSSPKEDRSFEGPATQPEEQAKIDSVSDAYTKWLCDLWHILQKQTYHQIK
VDDLEAFGIRHEKLELDTGVDGLLQPLNENTFFLPNFEKVKNLQPDPDQWDFEDLMIDLGEWPEPW
KSPWKSEFYRAVDRDHASSLCSALDVAFASLKWKEYEKKESICLQDQTDWKPAHLDDMYVNHFVSI
DSDFSEAFDFDDWRVSGFWLDSKRDSWPENIWLSRNKPHIMVTISHDCPHDNGSLLLCEIQAIIEL
MIVRLVNNSVPQHNIVPVLLYSYTGDKHGRVLQAYMDDKNLVIRTSKFYDFSAKLDANLKLLLQYM
VGDLVGSTMTLTHPQLESINPRVGLFSDLVGSETTLETPDPKSIDRTEDEDSDSGFGSTGSSGSA
>g2254
MSGPRIQINDEPTPADTTHNTRKTLHQNIFGKLRPVPLQYHWTVWFDKHSPVSNNSSPNSNSSSSD
TQKQEQYXSRLTILYEDISDIATFYRVYNNYPWDKIRLRDTVHIFRKGVKPVWEDPENLHGGCWTF
RVPKAKGQDFFHEVAILAMGNELQAAVENEHDHVIGVSISIRFNTHLISVWNKLGSNERSVKILEQ
TIIARISPELRPADNKNFASSYFYKRHADHEGFQEAVERARSSEEE
>g2255
MADTEMVNGETTPSIDKQQPEEVAKAPTEIAAKADLLRPKKYKTSDLPLTQDQQTAIQSLLVAFKK

KGGFDNYRKKIWADFDNSEFKAKFTNALQELAEKEIEREPAHLSRDRGKAATLIEGAVDRSDIYKN
TEHDLESFMTQHLETMLTSIREIRRKEVGEEIALREEIAGNKTEEDYDRWRSERRAAREKIHQAEL
AEQARIDAEKEKIRQEEARKRREIERKREEEQRERDEKRRAEQRALDEQREKERQERYERRRREDS
RDRYRTYNLPRGYDRYEGSSMSASRYRDHDRRTRSRTGTPKAATPPPPPLPVDDKTLEEAALQLLL
KEGEELAAKAKQKPEFDFEEAEAIESGRKLTARPKSITETRFSATGTRDSSRERRRSSRTRSRTRR
SSRYEPDGRNKSKEGSIRLRDREDDKHSVRRADAAAPGPKLVTESVTGSTAAIDANETGTVIEIGT
VIESETETATEKETEIGIEIVTVTELIGIVIGIVTASWTAGMTATPAVIWIAAVKESRHEKGLQKG
TEIVAEKKTKTATDHGLQMIAHGQDHAAAALHPTLIDRALNIVVIGQVLVLLLADDLNHDEDHALR
LIVMSRSPAPAVDLLVVEHVHLNERETGNETAHVSKNGTESLVVTGNRDRDRIKDGDKGHEKDGVR
DLDRARAQDRDRDRDADKGRARDGDRERDRPRARDPDNRDDRTETVTEIGIGTETGIGIETEIETE
NENGKGKETEKWIVIVIETVIGVATEEIVTVTAEMTDEAAIEEVIVVSHGFGTL
>g2256
MVHKVLFWSGFGIAVRLWQLGIEMRPLLGKESLWVYPLFAGVGGSFGYWLQGVENRQTKILAQRRE
AILEKRRRRDEREGLISRTEEAGVLAATS
>g2257
MTNAEATRLSYLGGRSYRFGASELRFFFSIHPNFHSLSSLGQWSERPDKGPNLEAMASSTTLQIPL
PPQTPSPPPDNLDQHSLAAEPQYDPKALSPMHGMKQNERLKGDMANMGPTSPTSPSFGSFSSHVSN
TNAGSDHNPFNFQPMALAKSPVMKSV
>g2258
MDNRGGFFFFVVAVYILLSSSSRAPLIDQTGERERRLIHEENAIKLLNETVYGDFSPPSKKWLSIP
GLRQNDSYSWDLLPQAQEKARRNIRLALESGGLQSPPELDSPNQAPTVNLSSLSLPVYQNITGKLR
GDWTRHTLPHAPHINTTALMLENDFITQEFGLNITGETGRIYVDFVSTDEQKIKQPGTPVRQIRAD
LAIETDDSWGSTWYMSTYGLHFPDQGAIILTTTSEKYDGLLSLPHLALSQDTFNISHDAMVQVLSD
NIARRRNYETTFFPWSSLPRGAHPMAFATPKCEYIMYIQQRPVTISGSAPDRTLLNQIEQELRFPL
GIPIPPPPMIEMSFVVFSPDCGILIESKGAPDFPPSEDLYVIGPKQEEYRKFASRTIYSLGAIMTG
QLYLLMRQVKEACTPSTRSRISFYSIALMAMGDAMVMTMTLLSLFADTSFIEIAATAFLVFLSVSY
IGMRFMMEVWAVQVPERRNQDRQPDTSTNQDTLPPPVTAAAAVSSGATPVVLPPDQTGPAPTPTTT
TTTTTTPGPAQTGNTMPSQSEIGADVGTMYARFYFVLGCLSIVTLWSFLFPVKVGAIYAKILSFIY
LSFWIPQIYRNIMRNCRKALTWEFVIGESVLRLFPFVYFLTAEGNVLFIRTDTTTALVMIGWVWVQ
AWILASQDILGPRFFVPKGWAPPAYDYHPIIHDTSGSGTGDDLESGTTLPIGYLRAEERDTAIPSS
AAGSSRAQLDKGPRPKDKKKKIFDCAICMQDIEVPIIVSPHGIGASSMADGAHMFGELDEVEVAVS
DLS
>g2259
MSLAAVTRPLASRMLCQRLPASVAARRTIPAFPRGTAMRNFSRSSQFEVKKYTESHEWVELDGNIA
TIGITEYAAKSLGDVVYVELPTADLEIAAGEPVGAVESVKSASDVLSPVSGKVIDGNAVLGDKPKT
INESAEGNGWIAKIEVNDPSELESLLDEKAYLESVEDH
>g2260
MLYELIAIVRPGSLKEVKEIAKNAGVQVLRSGGVVRGYTNWGTFTLPKPTTKHQARYSEGWHFIMR
FDASGPVQSAVRRTLSLDPRMIRFSVIKIGDKLEEINNVPGHVDWNGTKTNITDSIVSRATPRAIL
TENAAIRSSPYSS
>g2261
MAPHKAAYIPSKKARLEIGTAEYTHPRENQIVVKTAAVAVNPLDWVKQDVGDVFSSWIKYPFIMGS
DLAGEVFEVGPGVTRFKVGDRVVGHAVGMDERSNKSSEGAFQEYTVIRTNLACPIPDSLTYEAACV
LPLGLSTASCGLFMKDYLALRYPTIAPPSGSSNSKPVSKKETLLVWGGSTSVGCNAIQLATAAGYD
VITTASSHNHEYLKKLGAIAVYDYKSPTVIADITAAFKDRLSVGAIAIGQKSVGFCIDILSKCQGR
KFIAQASFDWPPSGFPQGALDWPAFSMRMGTSMMSLKAKSALKRVQAKFIWGTDNMANEVGKAIYE
DFLPQALRDGRFIAAPEPLVVGKGLEHIQEAMDINKKGVSAKKVVVAI
>g2262
MSSYGQRNPRNSTNSLSPVSSPRKRAQQDADFDASENRRKDPKVRFPAPTVLADDCIAPMRPNIPA
VGPRRRYRWNDKKARQCGMVDGSDLNKMGTGPPSFSNETXPSRDSPELEIEGQYFDPTSGLTFLHR
AWRKFLVQKVETESHGSSEMDKSQLLTCAGDRPFYLGDHSQELIPDDMTARTLVSFYFDTCVVTYR
MFHRQTVENWLEVLLKDREHNRPISQSLGNAKCAIILTLFAIARFRNEKLKGGYYSSDNEALALRE

NDRLFCAAMNLTDSEKGFPRLESVQARLIQVLYLLQTSRMNKAWYTFGSAYQILSSLGLHRRRSQE
QGVPFKNHSRDYISLQCSKRVFWVAYTIDGYLSVVFGRPRFLHDEDVDQDFPDSINDEDMGPHGPL
GSEASEDCHVDSLTFHAKIARIIGRISRAVYAVGDVQTGERVAAANRLIGELHEWRASLPPHLGTI
KPSTLMPSFRRESCALNLAYYHAVIHANRPFLLGEGNGPSCDSPDVNDLVAECISAARVILELVDN
MANDINLFHSFWWTHYVTFCALAVVYVWEIQRSQNKNKYERHSDESFHAGLFDLAERCRSHLLRAS
SAASPSRRYGIILEELRKEAQGQTVRNNRSDCPLPSPNPTLMQNTETPLIDQGLNFLEIPETSSQY
IAHASARGDFPNAVPSMLNTWQPTDWLDLDSSVWDISVD
>g2263
MAPFFGLRGNSLNIAALLGVVMPAIMTFGYNQSLLGGILTLPTFEKQFPAIDAHGNSHKSTIQGTV
TALYAVGGLFGAVLCIWQGDTLGRRRVIMIAAVIQIIGAILMSSAFDLAQLIVSRILVGAGTGGLL
ATVPVWQSEISPASKRGAHVVTTGVFIGMGLSLALFMDLGFSFAHGSISWRFPCAFQIILSLMALV
FVSFMPDSPRWLVRQNRVSEALEILAVLDDVDTTSPEIEAEIRDAQHSLELSGSASVREIFSIGPQ
KILYRTILACSVLMFLQLTGVNAITFYTTTIFQKNLLLDSVTSRILAAIYQVVSPIGGIIAIFTIE
RFGRRGLMLISAAGNAICLILVAGLGSQSHNKHAIDGAVFFTFFFHFTYVLGFGGIPFLYATEIAP
LRLRAVISGLGVATFWAFNFLMAEVTPVAFDAMAWRYFIIFAALNALMIPIIYYFFPETSGRSLEE
IDEIFALSETILDPVRVAKRLPRRQLGNSEMHERDIEKIRRDFKVHDCEREATFAQVERVPA
>g2264
MVGFDVRGLTPAPITPFTPEGAVDYEAIQRLGSWLGSIDXVKGLVVLGHAGEGTFLTSEEQESVIK
AFVKSVDNKIPIIAGITGEGTEVAALEAKRAKEAGAAAGLLYPSHGWLRFGYQPGAPQDRYKRVYE
VSGLPLILFQYPDNTKATYSLQTMLDISAQPGVFAMKNGVRNMRRWDTEIPVIRRERPNLQILSCH
DEYLLHTAFDVDGFLVGYGNIAPEPLIELIKAGKEKDYKKARAIHDQLLPVTKSVYHRGSHMEGTV
ALKHALVARGILTHATVRSPLLPLEPGAEQEIHAAISAAALSKVS
>g2265
METVLNTDSLNEKNSNEFNDDEHELFNPVLASIRLDKLADYASSIRKTLDHHSANFSAVIVGSPIF
GSYHILYPIEFLDKVRWLLKVPINGTRDKFCDLDARSIRSEASTMRYLRKITTIPIPDVITFSSSC
DNELNCPFILMSYIDGKPLYDIWFDRTQPNDVIEARRTKSLQGVAAAMVQLGQVSFEQAGSLFTSE
LNGDIDANSLEIWPLRLVNNIAMLERMKDDDYDDLPIYLEAGPFRDFQAYFTALLDYREKSPTTDF
TRGQLILLRILLDWIPEPKLRDECKPFVLAHPDLDIQNVLVAEDGTLQGIIDWDGVCSVPRPVGNE
RFPSWLTRDWDPAMYGWNEDMERGVEPVGVWEDSPEILRKYRSIYVGFMQSFARSNNNPLFGLTRN
SIIYENLLIAANDPLCTFGILQKVFGEIQNHVRTDVLQSESSDEEGLEEFDLHNISWALSENRLSE
CHLNLLKQGFHLLLNGEEVL
>g2266
MSGITSLDKFCQTAPVPLCALIGPKSPIQGSNGILPTCYARNIDVGNTIIFQGASDFAHIAALSMT
VIMILHVRSKFTAVGRKEITDFFYLYMLLTAFSLVVDAGVVPPGSGPFPWFVAVQNGLASATCTCL
LINGFVGFQLYEDGTNLSVWLLRASSAGMFVISFIVCLFTFKGWVGLDPANPVGLFVVVYVLNAIC
LALYVGMQLLLVWGTLDDRWPLGHIALGVFFFVAGQVLVYAFSATICNKIQHYLDGLFFGTVCNLF
AVMMIYKYWDSITKEDLEFSVGVKSNNWDVKDLYEEDRRLTIYPDGGSEYAPSTHYRSSTFGGNHS
GY
>g2267
MPIQIVPLQQKDIPGVVECIQEGFADDPYHLWVFDGSKFNKERNKSSLTARCLWGINNALFYVAKE
VADNTDQQDGGQDAEGRVVGVSCWLAPXPAATQTQAQSWYAWYQLQLLSLRQLLTNIRFGGRGGLI
TKRYWIWKAEQTAAQQEIWTDKEKGYYFCNMVTVRPDAQGQGIGRRLFEVVMDRADREGVKCYLES
SKGEPNVKIYEKLGFGLVKEIDCVDGGDVCRLPTEDEKYTTDTTIMKKTLLLVFIHGFRGGSDTFL
KFPDQLTALVRHALPNISVQYVTYPKFETRGDLKECVGRFREWLQNKVIDIEVSNSTPSPTIDPSV
HVILIGHSMGGIVGAETLLLLASEQPIPSLSASQMRDSAGESPFFMFPHVQGLMAFDTPFLGIAPG
VVSHGAQEHYRTASTAYNTFNEVAGIFRYSGGNKAATAGTTTTSTTATSAAGSVGAKSAMLPLPPA
TTDAAADAAATPSWSRWGKMAMFAGAAGAVAAGGAAALYSQRDRFNEGLRWVTSHLEFVGCLARPG
DLRNRVASLADIQQERGIRAINFYTCLGQGAAPRSVDSGTTTDNAARSQTSSLFLRIPRAKDRTFC
HLPEDFDVNDHVLAGDGTSDHPGLKWVLALNDKVADETTAHISMFGPRENPKYYMLAHEAADTIVR
WIDQGWYASATTDGPEGEEQTLKETEEKEGRGKGKDDKDDFIVL
>g2268
MAKSSMRYRPMTTYAVGHQIANDGPSKQKPTPSYSDHIIPRSWRKARRPGFTAPSSTDLVELCEKL

LERPRESAKRFFCLRTLSTQETNSMSLGLPRPGRSRPHSEIFVGADEERVAQFMAAQMRDSNSSNI
SLTGGVSRPISRDRSNSDDSTVISSGTMPTLRNERVVATGNGITVSVALAEPLVFLPGYDHSDPST
KRSAILRGHLHIKTTKSVKVKKVSICFRGQAQTDWPDGIPPKKINFHDKKDIMTSGMIYFNQGETS
IVQNPYGAHYYKVSNTMALSTTKEPKIYSVTTTTRELLKNGSTTTVNRETARDLKRLSLQSNHSRS
FNKNEAPSANQPQAVRNYRLFPPGDYLYAFEFPIDGSMPETIKSELGFVRYDLEAIVERSGAFRPN
LLGSTEIEVVRTPAEGSLEQVEPIAISRNWEDQLHYDIVISGKSFPMGSQVPIAFKLTPLAKVECH
RIKVYVTENIQHWTQDKFVHRLQPPKKVLLFEKRADVPSISTYPGSSMRVTAGGGVHWDQREAAAR
GEEQVNRGVSSLLGNLSNDVGVGPTEMEFSVQLPSCHAMKGKDEAHRLHFDTTYENIQINHWIKIV
MRLSKTDEKDPTKRRHFEISIDSPFHILSCKATQGNIFLPAYSDPSSDPAVIQDEYSCGCPGAPMT
RRSAPTSRINSSTALDQINNPSTDANCPTSTRGLPRSFTSGSGGLSRPEAAHIAHEAPNERDARPM
HLLRAPSFAPPAFEELEPPPPLITPPPEYASIVGNSDRETVLTDYFNRLSYFEEQDDEERGLGRVD
VPLTPGGRVHRSMDVPREWVRLGDATVQ
>g2269
MIEHFSCRASTASRKRSKKKTASFDEVADAYLIWLYDLWNHLETSTEYKTGLEEISNIGIRHQNLI
LGDKSLNPQAIQTTFFISMGQNEVRDYLIDHKQPETGDFLQTYFGDWPPEV
>g2270
MADPLSVTASVLAVITAAANSARSLYETIRRFQDRSKTLKRLQDELDDLANILSSLAEAINSETTM
LVLLQKPIDRCHQVCRDFEETMKTFSGKSKPGFRDWTKMEFMRGDINEFIDTIAEYKSTISVGLGT
ITLTTSKISQQVLQEYNEMIKDTVYNLELRLKRIDEKIAQPILDDRGASNTSIDLKDERAVTKQCI
RVCEDAKSYMDSLISRESFMLPQLSYNDSGHVQASFEAQRQTRQIFDENQNRFAELIDHLQERLRS
LVLDRPSNDPDRAKLQEDIDISRQCLEFVMADPLSVTAGLVGLVGFAFQASKSLYQVIESFKSSKR
AVRELREEIESLNSVLATLSQMAIEYDAQLTSLKLPLLRCGKTCAEFEGVISKCAGHSDGQRTSFR
DWAKLQYRGGDIGDLKTTLAGYKSTINIAIGGATFRQAAVTANVLDDYRRMIEEATSDLQDHLQLI
DERMQSLIQNTASMPSLSPPEVEEIIEEKKSIQQCLAICTQVSQLIEAYQTRRMAYQAPTDSVPPR
FSTSRAETATNDLLMGFKNKLSSNVNDLQVRLQELEDRLGKLSKESNDQEDLSLLQAMKEERDSIT
RCLEICTDASDLTATVRTNVYEDVTSADNAHQLVVSTIGDLISARHIMTGSNSVQWLGQMSDETIQ
KLSDDNKRILPVNQTSTSQAQKSVFNDLYGAGYQLKGEVKMASDKVGK
>g2271
MPASDMSNEDYNERSALRTETLRRLKSQLEENLPDEQVTSSFWAFCQVADITKLDNLATSLSNPGM
QGAAGFIINGCQDAIARWLLSLSIRVDKPDEKTLVLEFHWQREMEWEGNVDLMNEPKSSRGLDSYK
NGGPLLRIPSGVKGQSKIIRSGDSITMTTPDPEKLPLPTWDLLEMQWYLQRVAAMSGVAGDDEGFY
HDEDNVEIWLRDVPDGSAVDDLKL
>g2272
MESDQALLLDLIKAIRRDENKYVMELVNLIRGGTELEEVREYLDDKIRRSQLERTPELIEVYGEVR
RLEKESQRKSLLDVKRLTDIPIFSVPAKPWTTVTDDDNFVSHLISLWFTWDWLFSSPVDADLFLKD
MESGNLDSEFCSPFLVNAILAQACFYSDYPETYAVVGSDSIKGQHFWNEAKRILDAEEGHVSFTTA
QGTVVMFVCACTMGKDRLGWGYAMQATDQIHTLRSNQTCLSSKSSLSAGDFAKAMDRLELGSFIVM
STSALALHKSNGILRPKGLRLPEHESKNDQWSPYPNDEKIPRADLEETVLGWYDRLLEWAKCLPEC
MNVEKTSLPSVVTLHMYYHAVIVTIWARLKEAPRDAQSSSVERSKRAREICLSSSHKIAELASIQK
SRWGFEDMSGNTIQWVCVAMFILMEDFENPGSQSDFLILAKAATSFAKRWPLQKGMFRLVQLSTQR
TVKEIPSDIRRFFKDFESKYWKPEDVQKFSSRYPNFGAIIRQQDDYIPDDMELDKFLEKWDSLTLD
GGK
>g2273
MTTYIIRDATPSDLGQILAINTHYILNTVLTFRQTPPPPSTLLSKFNDIKARRLPYLVAIDSSLKQ
KNEDGDESNDLVLGYAYLSPYRGEMLSYAPTVELTLFVHPSHQSRSVGSRLLAAILKAAESVLHRG
YEIGGIVDETATKAVAEENGLDISVRNVLAVMAVDSDGPDEGDKLRKWYVERGFVERGRLEKIGFK
KGRWVDTVFLQYTVDTREGV
>g2274
MATPRSAATAESLAILSDAVNQTLIDTGRFFKSSGSLQSRAQLKASIPASYQRFQTALDTLTEQIF
IAKAFLEKDYENVTARRLAPKPQPEPAPATASAPPPTEDIAMPEAAAPTVGEQIQETVEKTEPIEN
PIKTEPAAIPDSGIFNEQAIASTEPVTATTTAEPIAALKMDEAADKNIQQLPVSMGDEMNFDQMLA
TTSQAPNDFDLSFNFTNDEIGDQNFLAGADFGNANSALGGDANNDNDANAISSLLPGLESYASNNG

GDSFNFDLPKLGEHGNGGHDDLMAPGESSFDDLFLEKNDLEGDDALLAGGELMDLGELDDSWLN
>g2275
MAENAPPTSSSTLPPPPQPSAGAPGQPPFDSAQGNGQGTGAHMPPSLPPVVIPQKTNPIPTAITSP
MSGQMMSPTSTGGFVRRAAPEPNKRALYIGGLDARVTEDILKQIFETTGHVQSVKIIPDKNQQFQS
KGMNYGFVEYDDPGAAERAMQTLNGRRIHQSEIRVNWAYQSNTTHKEDTSNHFHIFVGDLSNEVND
EVLLQAFSAFGSVSEARVMWDMKTGRSRGYGFVAFREFEDAEKALKSMDREWLGSRAIRCNWANQK
GQPSISQQQAMAAMGMTPTAPFGHHHFPTHGANSYDMVVGQTPQWQTTCYVGNLTPYTTQTDLVPL
FQNFGYVVETRLQADRGFAFLKMDTHENAAMAICQLNGYQVNGRPLKCSWGKDRPPTGQFDGYQAQ
QGGPGFNASPYFPQYGVPGGPMSPQGPAPAGRGWDQQGNNFSGAVPGQGYQGPSNAGGYGRGQNNA
GNNWQQPGNNNQNNNFNQGGYQS
>g2276
MVSSLYTTVKTSISLLEATGCNSLDTIQCRLLLVLYEMGHGIYPAASISIGACARAARNLGLHPGS
SEAAEPTSMEVIEEERRRTWWAVHNLDRFINLYGGDAVFATEDANIEDPLPAEDGSWSQNALPDTV
RANLSTPAAFKVGQFARECQVSHLVGRVVRHVFNPISDANFHEYEAAQLERTLMSLVPLLTEEELK
FRNYCGALAMCISSLFTLYTSPILRSKNRDFDESRTLPAIDALSTRVAELSEHLLAIAQDETNKMT
LSPYLPYVLYQTVVVQNRLWKQKNDVTYKQRAETIATILKCFSKRWHVTGN
>g2277
MSTALEQHILEANSRYANAYNSAGLNIQPANKLVISKLHILFGRKASPLTIFFTTVTCMDCRLDPA
AAYGFKLGDTTIVRNAGASARDGARSALLTTHVLGAEDIYIIKHTKCGLLGVTTEMAHGIIKKNLG
LANSTEDLDNFEVFPIADLEESLKEDVAYLRHHPLAMKKVRVTGWIHDTDTGVLKQVVQ
>g2278
MPTTQIKTISLSKSGVRIFYREQSPENALIPEKIILLLHGFPSSSHQYRNLIPLLASRYRVVAPDL
PGFGFTEAPSSFKYTFETLANTIAEFLDELSISKFSIYIFDYGAPVGLRLALERPNDIEAIISQNG
NAYIEGFGDVWGPIQEFWASKNNAEDRSKVATAMLNFEITKFQYENGTPKTKVIAPESYTLDYALL
ERPGNKEIQLDLFMDYKNNVTLYDKFQEYFRASQVPLLAIWGKNDVFFIPPGAEAFRRDLPDATVR
LLDAGHFAVESDTEAISQEVMEFLS
>g2279
MDTVTFPLRELPLEIRRLIYYHYFFRDGIMCQISSIPPPYIKPLGEQHVGEQRLQATAPSSCTGFQ
ATLRSNIDAMSILLACHQTYDESLDVLYSNAVFHIDDLKALRQFLDLSLRSGTDIFVRSIYFDWVS
FVWFDYGSQIAVQAYVDEYWYPVAQRFSEMRGLRDLWVAIQPGKMYKTAYDFENIDYFQPLRRIPT
LRFVLERHD
>g2280
MASSLDAHTATRASSLSSQTYRDNTESASAVAKDTAISNSDTNNDTPSSLYDEKNERKSVDKDESS
PTELGAEQLSGETEYPKAGKLIFIVVSLVLSMFLIALDMTIVATAIPQITDDFNSLDQVAWYGSAF
FLTLAAFQSTSGKVYKFFPLKTSFLLFIFVFEIGSLLCAVAPNSTAFIVGRAIAGIGGAGISSGVY
IIIAFSAPPSQRPALTGVLGATFSVASVVGPLLGGVFTSDVTWRWCFYINLPIGGLSAAIILFFFQ
TPSTAKPVDAPLKEKLLQMDPLGTTLIIGATICYLLAMQWAGVSKAWSDSEVVGLLVGFGLMVIAF
IFVQVYSGERALVPRRIIKDRTIALGCAYVLFVIGPMFIMIYYLPIYFQSIKDVSASQSGVRNVPF
ILSSSLGAIMSGLLITAFGQYGYLMILAVVIETIGAGLIYTFDIGTGHSKWIGYQVICGFGAGLGS
QIAIIVNQALVDSSDVSNVTAVTLFFQTIGGALWISGAQAGFTNTLMKKLPIYAPDVNPALVVATG
ATDLRKVFTADQLPGILLAYMDGLKVAFIFAIALGGFCCIISVFPKWKSLKGKVQPGMA
>g2281
MSAARFLDSQGPPRKKMRKGTKSCIECRRRKIRCTYDTDRPNICNECHTRGFECIDQEHGTVDPKV
AGPLSGEQSYSLRERVTQLENVVRDILQRMEHTTPSSASASPAAQIDHHKYPPTQTSKHYINEYLE
PNPAEPRVPVSGKQMTVTVASEIVEGAPITDSHQIENAPVLQILNNNIISRTEGTATRNQNIAATQ
AISPKAIAARNELVKLIPPRADLRRIRDVAANWWSVWVQMFPELPERDKSVLVGERDYFEIPNSPG
DVAKFILCHLMCIDQLPAGFDYNSLEMPIVPSEYADRCVNTIDRLIINDEDLCGTLPSLEAILLLS
KWYTSIGRPRKAWLLTRRGVELAQLAGLHISTAKDPHPDDKLYDRRLKLWTVLGLNDRFLCMILGL
PYAVQERLYRPQVERRLKTEPPNMETYCLQLSLVMGPIIDRNQEDPTKMSIAETLKLEQDLQDQAR
SMPDHFWQRRIPEQKISVNEATERLLLPFVFHYMRATLHLPFMLQSHGDRRYQFSREAALESTRNA
LLSYNMLRSADMMSPYICRLIDFQAFSAAMLLIINVIGYSEDAPNYSPEQDEKDWEIIDATTEVLR
HAAMEPAGTVAQQSLLILEGLSKNMETECPSAVSCKVSVPYFGSVTVIPGKKAFKVRNRSSAAHQQ

QQQQPTPSSSSCFSQQPSSVKGCASEQTSPFGLYXPPQSNIDFTSCSVSNCPDQQHNQALNDPVTS
SIWDDSSRVQLENITTLPNAGMNLNAGMSMNHNPEQMAGGLMGGFLGNNENLSLWSNIPLDLDLDQ
GWNLDWTSGGAVMQ
>g2282
MRSHHGCARCKHRRQKCDERRPSCTRCEEAAVNCEYVIKLKWDGRVPRKQEQTSRRRRRRKAACSI
TATELESLHHSPPAQPSIDEESKLMEVDAFKALSSRDKLLLNHFITHVSKLMSHTSVREQACQILP
VALETPSLLYATLAFSALHLSTLIYTNSSNATGGGICDTSVADILACSITHLRQELEDKDPTCNVN
HTPGLLHTVKTLCLFEIYSGKYDSSWRAHLKGARALLKTTSTGLSTNASSGGWLIARWYISIEALS
ALTNDETHDEQQQVIQPMIDSVVSQGSMLDIYAGYSADLHLAFRDIGSLMQKYASKKDESCGDDIL
SDLLRHCDECIEIEQRVENMIHRDKDGGLRISAEMSLSAEEIRMFTACNTAYQYSALLHIRRRLYN
MAPESGQVQQCVRGILDAVCNVLPVTVLSPWILLTTPIFTAGPIQKMTPLHAEWSSFITQNPNVRH
DDDGQRNRDFYDSPAGRDLITEVSTRDTTVPARDGHNIPIRVYTAKGQGFRHGVVIFYHSGGFTGG
SLETEDVICRYMALSGPLTVLSVEYRLSPAHLYPVPINDGWDVFQYIVTNLSSLVANLASGMINLV
VSGTSSGGQIAAIVSQRAQTWMKGNVSCVNITMSGVLLRAPVTVRGTEARYIPPPFRDLHQSWCAE
LETVRLTRQGMEEIHDTLGVPPDERDSPEAYPLWGQFHGLPRTYIQICDVDILRDDAVCYAQGLRD
AGVEVQKSLYTWPPEPISPGPDTLVLSVGTYYVDLRMNRDDGSIYWALAGRRIVVSEEPRKVKFTH
EIDSHDTTTNDNTESKEAGEEEVADEGTFTKLPNGDDLEIGEMPAPHLGGKVAAYREVWRELNAFD
NDIKNEDDRVCWMLESVEVPEEKQERKEKKRAFYCRVGRFFLALRRAETRVSDDDPKAVEVEFSGI
RQEFEGREWVTKYSVGTEADDMLHMSTSKFVLENVKGEDSAWKVDDQVVIINKKDGTVREACVVRA
VK
>g2283
MAFYSVVRLTILWHWLCIFASSAVASITAATDLANLRTQLSPNASISWSTSDAPRWSDYDAPQPIA
IINVAAEKDVQITIQFCNSKSIPYLVQNGAHGSSTTLGQLNHNGVIINLAALNRVTFNSEKTLVSL
GGGTLMSEMIQSAYSNSALVLSGTCNCIGYLGALLGGGTGYLMGQYGLMLDNVVSLNVVLASGQAI
TITPSADAEYDESLWWALRGAGPNFGAVTSAVVKSYPVNSTALNAWLGPLVFEESQLGTLIQTMND
ITLQPRMAMSLTFASSGNASVSPLIILTVFYHGTEAQGKAAFAPIYEIGPVADNTSIISYPDWNAA
QDTSCIKGGNKPSFGAGLAGLEPKSWEVVYDAYKEFLLHPEAQDSVILLDAYSTAQISSISDSSTA
YPFRSTIRFNAQASLVWNGSSFDASALAFGSFCRDKWRLSSGLKKNSTYINNAFGDESLTTVYGQS
LQRLQDLKNIYDPYGRFNYWFPLDGDKK
>g2284
MEQLKKSRPVTSSKYVQLPLHAPYHARHLYSEQDMETILTPSILIVNEKRDYKAIPVISASTGRIN
IPSTMKSALEIAVRDILRDELRLDLVLKSLSTDIIELSDTDKSSIILVPIASSAVNGLAAALQDRG
IDVRLDMDSVLDHGIPTSNHIYQASLTKTAVPTPIAIVGISGRFPSASSMDEFWDVLAKGLDVHKT
VPSQRWNVASHTDATGRKKNTSITPFGCWLDEPELFDARFFGISPREAAQIDPAQRLALVTAYEAL
EQAGIVPDGSPSTKRDRVGVSYGVTSNDWMETNSAQQIDSYLIPGGNRAFIPGRVNYYFKFSGPSY
CIDTACSSSLTAIYIACNSLWKGEADTIIAGGTNIITNPDFTAGLDRGFFLSRTGNCKTFDETADG
YCRGEGVVTLVMKRLEDALAENDHVLAVISNAHTNHSAEAESITRPFFGAQKSAIDHVLAGVNPDD
VSYAEMHGTGTQAGDTTEMTSVLASLAPATHEKGSRHHPLYLGAAKANVGHGEAAAGATSLAKVIL
MMRHDTIPPHCGIKTRINSKFPSDLASRQVFIASKPVIWKRDGVSPRRVLVNNFSAAGGNTTLLVE
DAPPKPKIDNEGSLRRDPRSISVVTVSAKSSRSLMNNLQKMLDFVSSDNQERPFDLPSLAYTTTAR
RMHHAHRVAVTASSLGSLVEELKHALDTKVGKKRTVSAPATIFAFSGQGSQYIGMGKVLFDTFSSF
RTDIHRYDQVARSHGVASILPLITSTTGDISQFSSSTIQVATVSLQMAISRFWRACGIQPAAVIGH
SLGHYAALNCAGVLSETDTIFLVAARAQLLEKNCRSGTHSMLAVKASLTVLENFLNHNHMVEVACI
NSPEDVVLSGLHQDIEDCAKTLQSSGIKAIPLTIRYAFHSAQVDGLLEEFVVRARATSYHAPSVPV
ICPLEGKIIDKIGAVGPEHLAAHCRGQVNIVRAMDVADRGGLLSRNCCILEIGDHYTFSNMFKSIF
QKSNTRPDIKIMPSMIKHINTWKTVSEVLSWLYMAGVDIQWREYHRDFVSTLDLISLPAYSWDAEP
YWIQYVNDWSLRKGDPPIIYQNGEEMSTAVQPLDTTVYDTLQEEIGQTSGLLVVQLDLSRDSMKGL
VQGHRVNGIPLCTPSVYADIAFTLGTYLQRRYQSVENRKVVTICDMVLERALVAQATSPQLLRTTV
EVDWSKETAICSFLSVDPKITDWEQVQGTTLFKHAYCSIKFRQEHILKPQHAKEVQRKIENLREGV
ASDRCYKYNKSMIYRMVATVAEFDSKYRGLKEIILDSNAMAASSIVDFRALTNLKSDSLCSHTHPA
YIDAFSQVAGFVMNANDNSDLENEVFVNHGWESLELYTNIDPNIPYQCYTEMSRFEGSLYQGSIII
INGDSVVGKFENIKLLGVNKKILHSILSLGYKAPSQVSAQPRESKSFIHTPSTVYSDESESLLGSN

VNAKISMMFKIISEESGVGDEDLQDDTPLASVGIDSLLSLIITSRLKDDLGFYTNSGITVFDEFRT
VGELKNEVIKFFSPSSTLSPSSKTSDSSTIVSTGSSSPVVTESCTIETSQERHTQFKDSNDITTSF
ESRSSVILQRPSVASSVEVRGKILFLFPDGSGSALSYVDIPRVNESVTIIGLISPFRKHPEAMKTC
NLDELISIYLAEIRRRQSCGPYNLGGWSAGGILAFRAMTELIQQGDTVENLVLIDSPDPTSGLGRL
PQGFYDHCQEQGIFGQIEKINIENNVKSGFAEEQAGGHSKDYTQVPPWLIPHFNATIDMLHDYHAK
PLMKHGENIVEPRVSIYLATDCVFDGNDYASFPTMPGEAAHNTKFLTEPRTDFSAGGWAQLFLGQE
IQVQLAHGANHFSMMHGKHAEALARFINSALA
>g2285
MGSRINTILIIGATSGIGEAFARRFYSDGKKVIITGRRTDRLAALQNELPGIGTRKWDLSDIAGLE
QHVSDIIAEYPTLDTIFINAGIQKSFSFLDPKTTSLESISSEINVNLTVPALLIHLFVPHLMQIAS
AGNPANLLVTSSTLAYFPFPFYPAYCASKAGVHGLCIALREQLGFLPDNIQKNLNLVEVVPPYTDT
GLDSEHRSAVTAMQGENALAPMPLAEYIEKAYVSLNDLDQDGQMRKEVAVGFAQTAVEMWRGSFGA
SIEGMGIRC
>g2286
MAYGGNGDRGPFLNTINWVFNAIAVVAVLLRFGTHIIRDKHIWEPDDFFIILAMVVNLARSICLSI
AISRGFGQHLAILIEESPTNTTALLHLMVVLQAVGLWTFTLPKLPVASLLVRLFGKFNRLAYIVYP
LVGLLIIFVTVTTITTFQQCKPVAAQWDTSIHGHCWNRSVNIGLGYFVGSLSASLDFGFALYAVVT
VMSLNMTRAQKIVIASSMGLGFGACAVSIYKLTIIKEINDLSDPTWATVPLEVWNR
>g2287
MAVLEHIPSLRNSLSSASEILVDSSSSDFQERLQRWTDINREEPAAIILPSSESDCLQTVQWALKS
SIPFVSRSGGHSEWSTIGKNGVIIDLGRYKSVEVDVTSRTAILRGSILSKEVAVALADAGLFTALG
NGNTVGAIPYFLNGGASITTSITGFGADQILAARLITASGELIEANQTHNSELLWAIRGAGQFFGL
VTELTIEAYPLSALGNDHGVIWAGSFVFPLDRAGEVAAVMKDLMSNNKYATAGLLMVMAPPPTRQP
SVVVAARYTGNPQEAAAAYQPLYDLKPIAASGGEVPIQNASDARSVLGAKGDFKRFGVVGLHSFDP
EAFLKTIDVWKEMIAQCPDAINSGFNFQWDSRPPRKSRLGSAHSLQNIRYWQNNLIWHTDADNRAA
VDNFNERCIAIMRGPNVTEYVDFANGTRDGPIEWRFRGKERLDTLKQLKKTWDPNGIFTRQLLDG
>g2288
MMLQSSWIWYITLWVACTSSFAAALSGSPFSPPDPSFGIPGDDASYDYVVVGGGTAGLTIAARLAE
NGSYSVAVVEAGGFYEESDGNLSVIPSDDIWYAGSSPTDFNPSVDWGFVTTPQAGMNERQLHYARG
KCLGGSSARNYFTYHHGTYDAYHMWATTVNDSSYEYYSFLPYFKKSAHFTPPDNEQRPRNASVGYN
PAAFSPTGGPLQVSIPIWANSFSSFAKKGYERLGLRQVVDFVSGTLIGVQYNMNTIDPRRQTRSSS
ETSYLRMVESNNALRIYNQTMAQRILFNGTTASAVAVRSGNLNYTLFAKREVIVSAGAFQSPQILM
VSGVGPAETLRQFDIPIIADLSGVGQNMWDHILFGTSYRVSTLTHSATSNATFLAEVLEEYMTKNG
SGMLGNPGGDLIAFEKLPSSERESLTPETLASLAEFPPDWPEIEYLALDAYSGNNSNYITGAPKTP
YMYVSPSAALVAPLSRGNVTITSASMDDPPLINPNWLTHPADQELAVAALKRIRQLMNTDIVSEVI
VGEELLPGRNVKSDSDILYSIRENGIQVFHAAATCKMGNSDDPLAVVDSRARVFGTQGLRVVDISA
FPLLPPGHPQATVYALAEKIADDILCADV
>g2289
MAMTGGILAEDELQRLKHSRISAQFVTEPAFLDWALFMTRYSAPTALAFARATERWGDTKEKNETA
YNIAFDTPMPFFNHVSQSEEMTQLFAGYMRAQGRSSGLVVEHLISGYDWARLGPAHVVDVGGSTGQ
IDLMLASKFHDLSFTVEDLPNTIEEAKVQLKDIDPLIASRISLKAHDFMQPQPPEISRIADVYLLR
KILHDWPTATAQKILQSLVSSMKPGARIVIMDTILPLPGTVPAPQEAALRVRDLTMAQSFNSNERE
RNDWIELFASTTPPLRIKNEWQSMESVMAVMELILQE
>g2290
MDSFNRILIIGAGIGGLALAQSLKRANIPFTVFERDDRLDSRLQGYRLKIAXPMQARVRELLTDEA
WAKFKDVLAASAEGEMTLSATSGSIIASRRDRLRPGTPLPWTVDRGMFRTAMMTGIEDSVLFSKQF
VRYELEDHCVKAVFADGSVERGLLLVGADGIRSVVRKQLLPEYRFADTGSCCIYGKTYITPELSER
FPALRMRWFTAILDSTPVLQSIIFGERPFSMIVEPVLFNRNSEQSDLPKDYIYWGLIFSRDAIPLD
DAALDEALRSQPAQITLDMTSEWHSSTRSLCELQEQSLASGMRVYTASPNMEEWAPSSRVTLLGDA
IHTMSPAGGVGAMAALHDSATLAEIIHKDGVSESSIGNYETRMRDFSNVCIQRSFRGGXRLLNTPA
FENCSLVDV
>g2291

MKFKHRVTEATFDESKGLWNLLIEHENHVFTEQCNILISATGFLSRWTWPQIDGLHSFQKGPVMHS
ASWDEAYDFVGKRIAVIGNGSSGIQIVPEMASVGSEVVNFARSGTWILAGLGNRMIDGNATYIYSE
EEKTRFRNDIDALRAYRKEIQQGIFSSFSIFVKESEMQTQAVVATRQLMLTKLNGYDDLAAKLIPN
WPIGCRRATPGLNYLEALTQPHVSLVQEPIQRVTEHGITTTDGCSRDFDVIVCATGFDVSYRPNFP
LRGRDGIDLVEKWKKTATTGPLAYLSICVPGFPNYFMFSGPNAPVGHGSLMAGLGWTADYIAKWIV
KIAEEDIKWVDVKEDVTQEFNAYGDQIMQTLIWSSPCKSWYKNSNNDDDNNAEGRVTALWAGSGLG
FREMVSSLRPEDFVIRYQSANRFRFMGIGRVKQEFQPGTDLTWFMD
>g2292
MVDQRPRAESRSFTAPCLIGINGHFASVGDAAHDPKAYEHGVQVIDEEKEFNPNLSKYLSLEDVTN
AGFNYHLISVFGSQSTGKSTLLNHLFGTQFSVMSDRERRQTTKGIWMSKNKTRHEDPNARMADNIL
VMDVEGTDGRERGEDQDFERKSALFALATSEVLIVNIWEHQVGLYQGANMGLLKTVFEVNLQLFLK
DKNTTHRSLLFFVIRDFMGTTPLKNLETTLLEDLSRIWASLSKPQGLERSTIHDYFDFAFYGLPHK
GYKPDEFAAEAKKLGSRFREGRRDRKEQLVGASIENGVFLPEYHRRIPADGFAHYANGIWDQIVNN
KDLDLPTQQELLAQFRCDEISREVIVAFDEAISPFEEKQAAGVRAGEPVILGGLGPVMRGARTKAV
KNFETEASRYHKGVYQRKRAELEGKIDTRLKALFQGQLSAAHKSGVKDFSDAVSNAVKAGQKKGAS
YDFAEIVKLETKAALERYEKEARASLVEGTSWSNYKQELKLYQKDLAEVSGQLRRDEMRRLATRVE
RWVRSRLSDSVSLEFNSLGSGRGGSGAPESGEKPSESKIWDRIWNLFVETVLDAERRFTDRATSFD
ASVDEVDVGLWRLRRKSWGVLRLKVEEEMMEGNLLLKLRENFEDKFRYDEAGVPRIWRPTDDIEGV
YTRARESTLTLIPLLSKFHLADDNAPPPLDRWIGHTPSSATAADEEDLTPIGGVDEEDGKSLEEEV
TILNDAKRQDLTVRFKKAADGVYVEAKRSAIGGITQVPLYFYGLLLALGWNEIWAVLRNPAYFFLL
FVCAVGAYVTYQLNLWGPMLKMADAASKQALEELKKKLRDFLEASDTGRQAMAMSAGEEYEMSSLN
RGGKKAEDEDENDDI
>g2293
MDIQKTSQPTTAPAILERLDPKGNPLIPTPTSDPRDPLNWSLLRKSTCLFIVVYSYFMLTYFTTAP
IPSFGFLEEQLNINYSQVSWSFALPCLGLAIGPLIVGALADTYGRRPVLIACTALAVVASGCTSIK
TINFGGYMATRFFQGLGAGPSANIGLVIIHDISFEHERGLRVGMWTIAANAGTVLGGVFGGLLATS
GEWVAYHVTILFAVLLVVQCLFLPETLYPRAAILAQESQKYGGGAIISIAPTSSERSKLNFFNIKK
IPGVPHPKVWTTTIQFFNLFRYPTIVISVLGYCFLQYWWICSISTLVPDAYINDSPRTQGLLLIGL
LVGLLFAEVVCSGNLSDKLMSHFARKNGGMRVPEMRLWLGYPAAVVSSVGLVLWGVSVDKNWHWMV
GQVAFFLYTLGLQTGNTVLSAYIVDNYPEHANEVITFYTVIINLSAFINPWFIYYWIEASGYTWTF
AAQCIICSFGLIPLYILLQRYGVGLRSTRPMYTKHIDDMLEVPTAQDVSTGGDIAVKQDFSDVV
>g2294
MHFQRDPGIDKRAWHNETLRLEEQLDTLIQLLPPTYLQNIDVLSLESLTPPSSTSPDEPISILLNT
SLLIQTAAKLFFQATLRSLSPQTPKARSLNTEIIGYTKQLSPNHLRSAHLWPLFVGAVYSTGDDEE
RIWFLDQFDIMEEKSQALVARGVLTRVKGIVGNVWKRRDLDSDVAVGVIGLEGEDGIGDWEKYVQP
LSDGLSLG
>g2295
MNSTQLDVRDRPANLFIGSACVVPRPIGWISTKSKQGQCNLAPYSQFANLTFDPPYVMFSSNQTVE
GKRKDTVVNAEETGIFVWNLATWDLREAVNITAEQTPYGTDEFDLCGNISKEQATLVDAPMIRESP
VKFECQYHTTIRLPGNPPMGTVDIVIGKVIGVHIADDVLTDGILDIKKTRPIARCGYYQ
>g2296
MSAPLPAYEDLPNPRQYWPAAPGSYEEGLGMLRLLTPEIVSSAARNEIQSGERVCLNWELENLNPP
GFGRRPFNHLIKVVDGSNGVALDDEYHFNPQQSSQWDGLRHHSAPWPDKENPDRKLWYGGTTVEEI
MNTDSPRIGIGHWAKKGIAGRGVLIDYASWAERTKGLKPDYALTRHKITLDEVLTIAKESKIEFKQ
GDILFLRVGLPQTWSAMSAAERKTYSEQAVPQHAGIEQSERVLKFLWNNHFSAVASDAVSFEVYPA
MEKDFDLHHHILAGWGMPIGEMFDLDGLAELCKQHNRWTFFVSSSPLNCARGVSSPPNCMAIF
>g2297
MSKISRQNMASLQNEEQTHILDAIDEMRNFGVNEDISIPQIVVVGDQSSGKSSTLQAITHMPLAVD
SELCTRFATQIVLRRTTESTVKVSIIPSSRTDEETKLHLLKFTREFSEEEFNEDGVFGEVVNDAAK
HMGLPTPGAESKEGFDKRFSGDVLKIEVFGPKQPHLSVVDVPGLFHNDTIYQTKEDLEIVRRLMKD
YMDNPRSIILAVMDARVNLSNQEVFRLAREADKDGSRTVGVITKCDALQRGDEKEVLNIAQNTVEK
LNHGWFLVKNRSTQDLRDGVTLQERDVREKSFFDTISPWNVLSKERVGVEPLTRFLSNLLLSHIRK

EFPVLLSEIEALVTKTQEQLKGYGEPRQTLSQQRRFLRRMASQYQKLVEDSLNGVHLSDTEPGDPT
KLRWRIRTANEAYARSFRRNGHRWVFKTAENTYDSDFVNVFEPLSYVDEESKEEDSDDIYNRIRQV
HNQTKGIELQGTVHPQLIKNIFRQQSQPWEGLSRNHLTNVKDIVDQYLKFTFQKVARDPYVFQNLE
RFLRPRILEGHSKADEELRKLLEDERGGILQTVNHYLAETLTKIRSDRVIRRLKDAGYEEGHEYKV
KFKNLAATSNISNDDSAIYDIHDTVKSYYKVAMKRFLDNVIIQVVERHVLGPNAPLRLFSSDFVDD
LEDDEVKFIAAESSTTSMTRSDLTMKLGRLQKALDIAHGVSLSY
>g2298
MRSLTAISFMNSSTYKPHITSSRPAMDNSYEVPGLKRLKTYDNLHENLALRPRHPKPRIGWNSLPA
ELRLEIYAYLIPRFDHAINVSSPRFENRFFREYYESMKDNVSEEEKANRLPWTKIFLINKQISHEC
LDILYGKNSFQIEMHKTGEADIRANFTRENRHRVRYITVAFLGTEGATFPPGNLIPDRCLWADILP
NIREMWLVTVQPMRPLIPEIIQPGDEFFAGESAIRFDQFFEWLPPYLRCFRELLRPDTILYSMSGQ
DERETQQIVSGYFTWWY
>g2299
MAFLSTLSDLSALSSLVSWKTALIVAFALVNRKSLPFAWTYNVFYQIYRNLRRDPLKLNFTPKSPP
VDLQGRPTHPVFAAASINSYVSLLETDYNIHKSNSTYFADMDHSRTSAVTPLYTPGAGIVSKELDA
EIAAAAIAEGKPAPKKKLPMFIALGATYCSFKREIKPLQRFELRSHVAAWDEKWMYIVTVFLIADK
KKGAQKGDKTVAAIGLSKYCIKKGRLTVPIERVFRASGLLPPRPDNTASPTGPAVTTAVSSAVDTP
RTLDGLPDTTLDEVKLVQEVSKLGDQAEEVLHRQKQQNAASWSSEEWTWERIEEQRKKGLEMVHGY
IALENNLFADWKN
>g2300
MKILTKEEEAAHYRAVLQGGTFGTVIGLVGGWAGVMAASRRFHTIRNLTLPMKAFLVTSSGTFVGI
IAADHSSRQYEAERNVGLKYLGEREERLRREELAQMSLGNRIGAWAREEKYKIIGATWIASIVGSF
VLVGRNPYLTGQQKIVQARVYAQGLTLAVMCASAAFEIHDQRKGQGLLDTAKKGREAVKEAKNQPA
ELHHQRRDENTDLWQDMVAAEEQKLKSRHQPLYEHEEKKGAVTEAVKAKKEEKAPKEAPKEEAKEE
KEEKEDEKEEPEEKDEKKESK
>g2301
MSASPEPQADSPAADRDELQAELDDLNDDGGDGAADAGLSDDESVLSEVDEAQFEDFNPDDVDIED
RPALDVDAENLKLIGRHKRKRRDDEDGEPRRTREGRREKKKRSQKDSDAESGDGKAVRRKAKPRRE
ATPEDDETLSPEERRRRALDRAMDAAMKQKTRRRRKADGIDLEQMADAEIEDMRRRMTDAARLDAE
ARRQGQPAMHKLKMLPEVLNLLNRNQYTASLVDPEINLLEAVKFFLEPLDDGELPAYSIQRDLMDV
LGKLPINKDSLVASGIGKVIVFYTKSKRPELKIKRQAERLLADWTRPILQRSDDYSKRVYEEVDFD
PTRLVNRVNIAQQTAEEARARELLPPRLANRARREMGHTSYSIVPRSTVVQDNKFSRPVGASGEDR
FRRMKARQLNAGKPSRR
>g2302
MSLFGAQTQQQQSGGLFGNTATKSPSLFGTPQQQQPAAGGSLFGNTQNKPATGTGLFGSSTTPQQP
AGGGGGLFGASAQTQPQQQTSSLFGASQNKPAGGGLFGGQQTQTPAQPQSTGLFGATTQQKPAGSL
FGATAQQPQQTGTGLFGATNTAQQQPQPQQQSMFGGGSMFANQQQQQQPQQQQQQPQLGQSLGQSQ
LGSSLWSPGRAITGVHRTVPAQMEIIKDKWDPTVLSSPFRAYTYNHPGEDQAPFFQPTSDDDPTKW
EEALKSRPGPGYVPVIIKGFMELGRRAARQKDYLSAIQTRLHEINNSLTDLLSRHDLKISVRIADC
RRKHLVLSQRCLSLAAKTQILRNRGYAMDDAEEELKKKLIQLERAIFDPSITGRAEEIWARMLAIR
EHSRRLAAEIEKAGTAGSGKTDGEDTLDENAIKTAKKILEDYASQINHLNKELASIRKELDEAQKL
AGTNSP
>g2303
MAAREPQFSQHILVDTTPMPNDIPKVQEIGATSAPLMSASFFIGDRCRAYNDDYMKCKSDANGRGE
LDCLKEGRKVTRCAASVIKDINTHCLKQFNAHWQCLENNNHHMFECRKPEVDLNQCVFEKLGLKKT
IPGTPENVTPVHLRPKQIYAQYPGNQY
>g2304
MTAQEGRHPLTKQDVTEAVQSFMELYKQLVLEKSFKGYDTKLLTEDLRFIDADPSGSCVWEMTVDE
TWCNMNGVLHGGAYGVIFDMCTAITMQTISRPGYWEFLAGVTRTLNISYLKAIPLGTTIRINCRVE
QHGKTMALISAYIESPDGKIKYATVEHHKVHVPAIKEFAERLQALKVARRRDRVRL
>g2305
MTAIIEEKVTTGTDNGFSTPSDPEQVVAADNDHESITEKEKALLQRKLDWVILPPITLLFFLSFLD

RSNIGNAKLDNLTTDLNMTGEQYLVTLSVFFIGYSAFEIPSNIALKLTSPRIWLPSLMLVWGVVAT
LMALSTSFGGLLAARFFLGATEAGVFPGAIFYLSMWYGRKALVRRVTLFFTSTSLAGAFGGILAYG
IGHMSGVAGKHGWFWIFAIEGIATVLIAGICFFLIQDFPAQAKFLSQRERKTLNALLFEDNDALRD
EPWSWAEVRRTFRDVKVWLYMIAWMGQALPLYTVTLFLPTIIKNMGYSAANAQLLTVPPYAFATII
SICIALLSEKYQTRAPFIVGSCIASIIGYILLLSDARVGVQYLGTFFVCAGIFPSAALIFTWIASN
VSGQVKRCVAAAIQISFGDIGAVVGCQLYRPDELPRYPLGHGMALGFVVGTLVTTLLLWHLLVREN
RRRDEITGGPPKDNSFLHNADFEGDDDVRWRFIA
>g2306
MPSSVRPYINKSSASRTFSKSDHDRLATLSRESQSSEPSPPFNTSNVTTFPQPETTDTVQSFGDLM
NKDEDHRAETFASGGATTETPAALPIFNSPSVSIDTVHGVITTEVGRFPRNSKFAGLSSPQVFAKS
AEEHFKGIAPHINVMDFFCPTMSFAEELPNQPTEHRPLVVKEIADVCIQRFLSTYNQLFPILNSRA
FCEAYLQFYDAGKCSDSAVVACFYFAIALGSEDEEIGDLHSQAACKLQGELLARPYMSSVQALILM
VRSPLIIAISEVAN
>g2307
MNSHHLQTADNGSDINSDNQERLYILQLMIERQQQIENLQELLSVSQLENLQYRLLELKEAHSDK
VALYNDLEQRILEQDREISGAYEHQLLQRASELDAYGDQNRLFSPSRKLIDTSLVVHDADIKLDME
AVTYVEGRNQHRIAQVTRGFELRYSMLVTDYYEYELCDAPDAVIDALNKKSDFEILPRYRKLVDED
WKLQAIAICESVIRQWKAFNAPSVDSSGFSIAAAERQMERVNDMVEILDNELQVSSTDDTEFSDGS
SIF
>g2308
MPAPCQIAQKQIDPPSIGRNGYQGFLNKTEILAKGSAPFNSRKLPCDILVEHDVGLKVHDGAKIYV
DIYRPVDESRKVPAIFSWSPFGKKLNGLKFLRMMTPYNMGLKPNTLSGLEKFEAIDPADFVLRGYA
VINADTRGVGDSDGMCVIMGTQEGEDGYDCIEAIAAKPWCNGNVGMAGNSHLAIAQWFIGQLRPPS
LKAIAPWEGCGDLFREQFVRGGIYAGNFFDHISQVMIQGYNGMESFKEMYRRSGGVANPYWEDKRP
DITKINIPTYITASFSSALHTMGSIRGFYDVDCPKKWIRFSPYQEWHDIWAVPKYTQELLSFFDKY
LKGIDNDWELTPQIRMTTLRFGDQDAVVDEPIASFPPPQVQDRQLFLLDNGVLNPNGSPSSTSVST
YNSEDGTSHASFTYTFENKSRLCGIPKAVLYMSCDARDDLDVYVVIRKLDKNKKSVLALNVPWDSI
PPNSIEEIPEDKATDLLLYMGPIGMLRASHREIDPSRSMHPNYPFHPHARTQAVPAGEVVKLDIGI
FAMSVEYEAGESVQIQVSGQTPQIHSFKELKDKLGNQDNKGSHKIHFGGEYPSHVILPFV
>g2309
MAPGRVSPTVQDEPINPYNGGYLKTSHGINHDYGTTKAGGTTDILEKQMSQKFGDWRDDFFTNGYT
VVKGAIPRERAIQYRERAMEWFQKFDFGFDINDKSTWTQEHLPVMMNGGMIMGYSATHEKWVWEAR
CEPGVIEPFAKLWGTDELLVSFDAVNITLPGRTDVRWTPWPHIDQSPTRKGLVCAQGIINLSEAGP
MDGGLQLLQGSSELFERFFIENPPRPKAKDAPGQFDWYGFTLEEVEWFKNNGCKLIKVEAEPGDVI
IWDSRTVHYASLPKSEIIRTIIYATYTPANLATPEDVALKADIFRRWEGTTHWPHCNIFAQGKAMR
GDILCPGERDEPLEKPELTDQVLKLAGVLPY
>g2310
MASNKQSATVGPELARVLPVLDKPWYRTPHLLKLNILLLLVTLSATGMGFDGSMMNGLQSLSTWTD
YFHNPSSYLLGITNAVLNLGPILFGGPVGWLSDRYGRRVAIQLGCLLIIILAAAVQTASQNLGMFVA
ARFILGVGIEFCIIPSPVLTTELAYPTHRAKITSVLFTFYFIGSILSSWTTYGTYRMSQSTWTWRI
PSLLQLALPSVEFIGLFWVPESPRWLVSKGRVQEARTIFVKYHANGDESSSLVDFELEEIHQNLRY
EAGASTLGWKEVWKTAADRKRLILMVYLSFMTQWCGNNVISYYLSLILDSVGVTSSDSKTLVNGIL
QIVSWIAAVIGSMLVDRLGRRFLWLYSLTVMMLSLAIAVLALIFLFQIHYSFGITPLSMSYPVEIL
PFHSRQKVMSLYYIGNSCANLFNNFVTPVALDAIHWKYYLVFIVMIAQFVVVVYFFFPETRGYGLE
QISGLFEGDSLFIGKTVVQHSDDSIETLEKAQAELKDDRVEQIESVVAMNKIG
>g2311
MASSTQDIDIIQFPTSSVAEKDCYIPPATPGQQRILKAGTVQIDGHRPLTKDIILDDDIPIVVRDG
VTIFADIYRPSFTASLDAAKIPAILVAGPFGKNGGSNKNNFNKWPWRFGCPRIATSGLEKFEGPDP
DYWCYNGYAVVHTDCRGTWKSGGDVIVPSQKEGEDNYDIIEFIAQQDWCNGKVTMAGNSYLAMTQW
FTGVERPPHLACLAPWEGISDTYNNNIRRGGVPNPGFMQGMLKGDVVSFTGNKGIDMHAMCYKYPA
WNSFWDSLCAEVEKIQCPLYIVASWTNFLHTTGTLRAWEESGSSEKWLRVHNTHEWPDLNDPYNVE
DLRKFYDYYMKDIQNDWPMTPRVRLSVLNAGGKDMINRPETSFPLEREVPFKLYLHPDSRSLSEAQ

PSAQSDVSYDAVTGDVVFSWPIQETVEFTGYINLHLWASTTDSNDMDIFVKLRKIKATTKETLESV
LVDVGRLTPNPEKERQELLSKHAADPKFGAAFFNSGPIGCLRASHRALNPEKSTLLRPVYTHAEEK
LLKDGEIVPLDIAIWPYGMICNPGEELQLIISGYHPEPHCRPTDPLPELRNEGVHTILAGGEYDSY
LLLPLIPSRKA
>g2312
MSISDEYDEEVPILIIGGGPSGLLLAYMLDQLGIRSLIAERYATRLAAPKAHALSPRSLELCRQFG
LDVNEIRKVGTCRSDAFWVNFITSLAGEHVGKLPYERMDPEVLDDTPTMIHNIPQPNFEQLIANKL
ANSSNVEIRKNHTFVGVEQFSEFVITTIEDRSTKKTYRVKSKHMVACDGAKSAVRSHLGVETEGED
SYETMMTIHFNADLRPIVKENVGMLHWVMDPLVSGFIIGYDLSGNQVLICNFDSKKYPVESWNEQH
CRKVVDAAIGTKAKYDVLSFRPWILSRKVANTYRVGRVFLAGDAAHSFPPTGGLGLNSGLGDVHNL
AYKLSFVHKGFAGSGLLDSYTLDRRQVALVNSQQSVKNGKKIFNLLKTLGTTDPDVEVARQNLHAN
IKDAKVMIDIQEGIEGQREHFDNLGLHIGYVYGDEKIPDNASIYQPVCIAGARLPHAWIQIPSTAA
TQTHLPAIDSSYVDELTPELIAQKQFSTLDLCRFDSFTVLVGENHADKIRTIVGEALDQIPSDISL
PVGIVVQGVDFVLEQGYEKRWSELTKLNEGQAVLVRPDQHILTVFGPEVESGDVVEALKRHLMW
>g2313
MQRAFSSRSALTRAPLSKLRTSGVSVQQQRFAHKELKFGVEARAQLLKGVDTLAKAVTSTLGPKGR
NVLIDQSYGSPKITKDGVTVAKAVVLQDKFENLGARLLQDVASKTNEIAGDGTTTATALARAIFSE
TVKNVAAGCNPMDLRRGTQAAVEAVVEYLTSNKRDITTTEEIAQVATISANGDTHVGKLISNAMEK
VGKEGVITVKEGKTIDDELEVTEGMRFDRGYTSAYFMTDAKAQKVEFEKPLILLSEKKISAVQDII
PALEISTQLRRPLVIIAEDIDGEALAVCILNKLRGQLQVAAVKAPGFGDNRKNILGDLGVLTNATV
FTDELDIKLDKLTPEQLGSTGSITITKEDTIILNGEGSKDSIAQRCEQIRGVMADPSTSEYEKEKL
QERLAKLSGGVAVIKVGGASEVEVGEKKDRVVDALNATRAAVEDGILPGGGTALLKASANALTNLK
GANFDQQLGIDIIRKAITRPARTIVENAGLEGSVIVGKLTDEYASEFNKGFDSSKGEYVDMIEAGI
LDPLKVVRTALVDASGVASLLGTTEVAIVDAPEEKAPGPAGGMGGMGGMGGMGGMGF
>g2314
MAREEKKALNRPSSRKLVRWNDDLDELLLLTIQSISNKEGIRLPWTEIAMSMGNNVSEGAIVQHLA
KLRARRVEKKKQVPPPLRRGGGYTGASKTSEAPVTPQSPKEKSDVTERRVNQRSQPKIKVEKRSAS
KITDVSSDSDEEWTSDTPKNKKSKQKRAPKKRKTTPVDPEPPVDYVVSDDDATNETTESSQSDGKV
AVGADFLEFLGDDDEAEEEDRAVTPSENEDQAYTQQSLVVKLKLGTDNMCRLKYKGTGVFQDRNPE
QRWEIPPPGPNGESWGFSRDYYGPIPQSAQHSHNPGLLRGEWPREVAMRQYEAPQAVTWSHETYHP
DPRLVHPALFLEDPNCEPQLQFHRKLRTAEESDPVARALDQTSPAYQAYATTHRELPTYRDSSWTT
TTAQPAQHLSLRDSGYASDFDTTRPHTEMKLDTRQLLDTRQLPGTSDDELPPGKIFTDGMVDETLV
SEAATVDWWQH
>g2315
MVAAWKAAGLTYNKYLAVAARAVRRSLKDSQRLNAERRGQSDLKFAKWENGKQGEVKQLVDANAEA
QAAHAEK
>g2316
MIPNDPARVPHRLIPAQQMVARDITREFVNAASKLKQGELVKDADFTLFEAVGALEIMDSKMDSGY
LGPGESHAEALEYDYDVMRELQPEEVLGLMDQLLCHEMAWHMGHPLSQTLFTSIYLDKLLWPIPKS
FDEYTFHRNNQTQSNPEQEQPLIHVVLRAYCLGLVKACDLVHRRITQEYYYEVGINVFVSRGKSVS
DAVQEEDFVPQLYNRNLLSEFDIEPVQNVLQTAVEYLQELQEDRISTAVREALIDRLRLRKQFLEA
LSKDLEAGFSQDNEPFKACLTLIHPVEQSVTYGKPIPESFTLKIQRKLASTIPPRPMVTIDIRAAI
DHLKRLFQDAIDLHEILNYSSPSDLRVCVWTIASRKPNPGIYIRCLMQAFIVNEMKILGSKSVKQL
FYDDLRDVVLPLSTLLDAENEECEVPSDPRFQIHQSMESFVTRVAHPFVDTFRTSCLNRSRVRRAL
CHSILEWDNLQIEAEELDTHLQTLTGEVPLTLENGQSTYAYPLGSWVYHEKLKQMQLIFQMGFELS
IYAPEELGGMYWYLSHLCSSHQLHIERIQAFVQADGRRSKRFRGNEATFERTLTTLGRHSLWLIAT
ETLSLGLQALYVFLERHGVLPHAASQSAYSSARLRYELRMKPFLPISLPELVSYDQYENNATLKGQ
ADNELLKRASGAITDARKAWEAVLAQGAFVTTTQQKQNSGEAVRRSAIEADWQRNTKNSLRACIGA
SIAIQTVTKAFSKLSLSTPNTTARKSLPVKVEIPEVGSTGRYHAWWAVPRISEVESG
>g2317
MSEKIQKIHSNYYIRAYYQYFHDAHPLLLPLQALEGPLGTEYVPEKIFTVMKYIGSHFLTINNTTT
EQTDSRDHWRRISAGPTPDKNDTTKHALETEAFSCLFRPEEDGYQVQCMLLLSITAHAHGKFLDAI

RIINSATSLALKLGMNQHSFSVVHGYGSPLLEEMWRRCYWELFVVQSLLCALIEESGELYGVSSDV
PLPCDEGMTDLHVYDAPPPGRHTIHDLLSHCQNRAQLVDPLPSFAYRIAAAHFLGSVLTSQSSTTA
TELERFYPRLIEHVDHKSFIGGIASQPNHIAQMLSQTTVIACSAMIYFYRQTSQLTPIKLQRNLFY
DQSPFYSSSFSMPVFPSSFILQSKHPTSNKNNNSDQFKSNPTSDPSSIHLISTAKHLATQLNNPRL
VQGTSPLIIDAIALAMLVEMAALLIPSLDAAQKERLENEFGNSLGVLKKFAIRWPLAKTVRDLLVD
EYENLFRPERAR
>g2318
MIICKGLSYILLLSRLVTASHSPRYHAHHARSDVDYDYVIVGSGAGGSPLACRLAIAGYKVLLLDA
GTDEGNSTQQMVPALQLQSAEYEPQKWDYFVHHYTNLTRQMEDSKMVYNTTSGDLYVGLEPPTDAT
PLGILYPRAGTLGGCTTHNAMITVTAEDADWQYIADITGDSSWEPAKMLTYFERLEANRYLPSSIV
GHGYDGWLMTTLTSLSLVVEDQKLLSLIVSAATAAGKSLLGIIIDTVVGLAGVLLQDLNAPDRSSG
VGPYQVPIAVKLPDSSRTGPRDFLMSTISATNSDGSRKYHLDIQLETLVTKVVFDQGGSTPRAVGV
EYLQGQSLYRADLRSNRAGPGIPGAVNASREVILSAGTFNTPQLLKLSGIGPSEELNQWDIPVVVD
LPGVGTNLQDRYENTIIGEASTDFYITAQCTFLRTSPDPCLEQWKNNKLNPGTYATNGLAVAILRN
TSTSDASYPDVFVVGAPANFMGYYPGFSNDSLADAKHWAWIVLKSHVRNNLGSVTLRSTDPRDTPV
INFRYFDEGVTEGGADELDLQAQYEGMEWSRQAFADLIPLDGSFTETWPGPNVTTEAEMKDFIKRE
AWGHHASCSCPIGNDTDPNAVLDSEFRVRGVDNLRVVDASVYPKIPGYYIVLPTYMISEKASDVII
AAAQSNATSS
>g2319
MRLETSILTALLAVSSVSAFPTTENLAQFAFKEAAEKRCPFSQVREGISNAIKKRNILDSLYSPIK
VTGEHAFVAPDFASGDQRGPCPGLNALANHGYIPHDGVVSLAEVVPALNEVFGMGFDLGLVLGVMG
TVWAGDPISLNPSFSIAGESPAIDNLLNDAFGLLGTPRGLNGSHNFIESDSSPFRDDLYLTYDAWT
LNLTKFDSFYAMSSNGTFGMDLIAEFAKDRFYESIATNPNFYYGPVTGMIARNAGYMFTGRLFANY
STEYPDGVLTKEVLKSMFGVNGSDDALQYNYGYERIPENWYRRPIDYGLVDLNLDILDFVAQYPEL
ASIGGNLGAVNNFAGVDITNLTGGVLNAADLLKDNNLLCLAFEVVKLASPNALTNIFATIAAPLEL
ITNVISAPLLNLSCPAFEDITYNGKPIWEGLQSAFPGAGWAQAAF
>g2320
MFAPAFDHSFNDLFNQYVNSDPSTSGDGSKDVHFGSDFDQFFSVGSFSSDCGELSPNGSKHFQQSS
PQSWRKDAWTVQQDSLNDLQLHRPKPVHDTVQPSDVVSGFNAVSLEESSPEALGLFSTSPSSPPTT
PSRKNHSKSVPITPKNVRVRKTNDRATLHRKQSSSPSLMRSAHLQRSKMAYPENWSSRLQQQFNSQ
MRADERLPLSPPPSDILVQNESMRRDSGIHLNSSSENLADFSPHYASTMYSSKLQQPASFINQRTS
TPPPVDDIFATPSSSDSQQLHAWHADALASTFQLTPDLNGHDGHWWSSPLPTRVSQPQNQNSYLVS
SLSQKSPMIHTNQNQQQHHHELLQNGLMIDFGSSFDLGASNDAFSSAAPLHSTTATHIPITSHQQP
TFSHHPYIPTTQATHQQFIAKPSRTPSLSPTNTTSPKSHPGMKNITPRRTHARKLSSNSTSAPKPV
KLSTPTKGGKGSSNVSFVNFTPSDSKKILTGVAPSGSSKTKARREQEAREKRRKLSEAALLAVRNA
GGDVEALEAVLC
>g2321
MVNSLWFKWKALKLPWRRSWVVGQDLSGNTFWEFRDALNANRNRRIVKYNPKAHYGDVKITPQWHQ
WLRYVRHQPPTIEEQQRDLVRQAQIRKLAQIADEKWAAKPSFLDKPKTQQPTPATRTSDQTVPADS
KVQGQPGVTKQINGQEEIQQEADQSKKTKGLAANVPTGPGEKWQPEAWTPDESSRVDATLSSKLEW
QQTTRRLTGSIQTDMYFLPVYTKTGK
>g2322
MASVTSLDKDLRNMRLSKYTPQAANEVRDWIEEVLRQRLPAGDLLDALKDGVALCKLVNLAVSPGV
KFKESAMPFVQMENISHFLRACQTAPLNLPPHDVFLTVDLYERKDPAQVLQCIGAFSRRANAIQPS
KFPRPIGGVKSKGSLMSPQGTGPNYGVGTTRSRGISNVSQTSSTTFDPTNRNNTSGRVSPHKTGHS
PASPPPTLSSWSKKSDEGTTAPAWNIHQYGYMGGASQGNQGVAFGARRQITAPSPAVPSLAEKEKR
RREAELEEERQRQEEELRRQQEEQRAREEEERRWAEETARVREKERQEIEKEKVRWAEEQRKWEEE
EQRRLREEKDAAERLEQERARKRASSDARLNGQFLSQYQATQKTEEESAESQRIKQLERELERARE
RERQYEQERLDLKAKEAARKARSRSRSRPRPVQPSYDFTSQEEERKLLRSEWKQQQQNVAPVMEQE
DLPPALPSRPLPNPVEVASPPSRPLPDPSKYSGGRDSRVDRFLASNPAPVQSRPVAHRPQDYSTTS
EVDAENDRRIAAQEKTKAGGWASKSLLEREMERERERQREWEENQKRTIEAAKRGVGAGTGSAPGQ
GAWDVHQYGYMGGDSQNRGGTGLGVSGPRRQIIGPRPPP

>g2323
MGFALTNSWWAIASSLGTGLPQGGTSGIVYGIILVFFFSSCVGITLSELASAWPHPGGQYYWTAQL
MSPRYRRFGAYCTGYISWFGALFTTSSVALAEGSSIVGLLKYTHPSIDIKPWMVFVASQVLNVVVA
LFNIYGKTLPPIGKICLWVTLISFLTTLFTVLACSSGNYNTPQFVFGTLTNESGWSLDGIAFLVGL
INPSWAFPCLDAATHLAEEVVNPERAIPIAIMGTVLIGFVTAFSYAIAIFFCAVNLDSVVSTVTGV
PILEVFSQTVIHRTGVIFLEILVILSGIGTLISSQTWQSRIAWSFSRDGGILGSRYWSRFHPHLHT
PIYSHLLFTASMAQETKSEYRAAKPLPYELRQHCAIYFEEKLYSQALTLLLNILSYGTSTHAPAYV
PAPQHLALAATLLVHPSTTTNAKSKEEKDSASAALQLLRLTLQLVGPISSKLNVAFTFTHFDSSRH
GGQWKGDEQITPVSGSDDDETPLNLDLAKSKSLWSRAEDFWHVVGWAFICSVLHPRRWERWQIWLE
YMCLAMEXDWIQREKMAAGTDKPEKILKDSLIWKFIDTDATGYGRERRILRAIFADGSAAALTEFR
EVFKNELKKQQQGEEEKNASNSRKRAEVNIDEDEYGDYLFQDDDESDIPEKSTRTRPKRARRGTRA
ADLTLVEEAAAKEEEQKAKKSADEVHAKGDIAAYGDFHSLELRQRLLSLLSKVSIAMPKTFMPTDA
LFHLFVENIRHLPLPIFQAFVSPPNLPWMTADAQSTLCETLILRMRESAAPNPTDPYLSQQELQEC
YLPYSANTTSAVDNTKYSILLEALIIILLARNDALHNTPLLREAAMTGIERRMAKAEGEVRRNKTSR
KMEGDEWSWLVESADRLLYLVEEILPQVDVADEN
>g2324
MFVYYDQRNTFTSVHKQDILQHSTRKKLVADRYTKSYVTKPNVADMIRQHAEVLMKKIQQQPIIDM
YLWLHYYAIDTVTNHVYGAYGTKTLEDEAHRDLVYDLSGVKHRSRLYFTFYFKDLLLLTETIKRFY
RWLRNQPTGFHVRGSRLNNYGLESVKSIGNDPDMHAESTAGKLYSVISGNPHGIAAECMDHLVAGV
DTTGDAMCILMWRISTPEFQHVQEKLFSELFSIDGEFDPRSGTAPFTVLDKLPYLDAVILEGLRWR
APVPMSLFRMCPPEGRIVEGHHIPGGTTVSCQAYSLHRIPEVFPDPDKYDPERWLTQDKEKLSEMK
AHFWPFSSGARACLGQK
>g2325
MPELRGARPYRSKRLRPCDTCRQRKHACQLQDKPPCASCRNLGVACTFKNPPSKRAQRNHPQNFPE
VGQPMMIQDLLGLQESSLPEFSDLGADIDFFEDIQLPQLSHPGLLRATPPPVAQNILCNAGENEYV
SERRQQNPRVSQVSLPSTVAPFAAGTIDSFPPTSLSSLFPGEGSCTDSIPAASRYLSAQAGDSEAS
RLKQSLDNAAGYSVQYSGLSGEMDPYLLRHFRFPDVGTAKFFKVHYRQLAPESPSDIPVHFRLTSH
DIADSLTPETSLSSGRELLENLVPVHLGVRLVGLFFRYVFPSLPVLSRTAFGLSRDTLIPDTHVLR
EMPSHLLAAVYASSHEFSSHDPVLCVSSTYQKVPAEKLWRLAYEGIHDDIHRAQLATLQTVLLYLQ
KPSLEGVTALADTPFHRSWVSLLACLATNLGLNLDCHRWPIPLWERRLRRRLWWAVYTETTWNSLL
MGFPHPISDDEWDVSPLDENDFVIDHLLLPNLESYNTKFPSLSPCVYCHGGYEFVFLASLTVIAYD
IYRKL
>g2326
MTSRALSTTARQLKQLNWSLHGTTIDVSWLIEQIERSIDEVPGLKARVVSAQVIGNPHPTPNRDDP
YHGSVVLLDKDGKRITSAHAYLNGFVKFSKEATFKPVKLPPMPGAPVIPPAGVATGKSGSTFESDQ
SNSRK
>g2327
MLSSTPNQADERTHLLSNGDEAATAKDKSRLRTQLVVILFVCVLYFNLYLCLAPEVNIREDIICKA
YYDALDNDEVSIERDCTVDDVQRELSLLSQVYVTISQLPGFILAFPYGALADRIGRRKVLLCSVVG
IVLCDAFKLLVMWQPNHFPLRVIWLAPLFRLIGGGDAVAATVVLATLADVYTEQDRVASMATACEL
FSPLIGSALMLKSSWIPVLLGFATLLVGTSVSLALLPETLAQRPRSGDPLDPARPKTHGPPRLGIS
GDQITAMLAVFRESITSLFATKNVGLLLFGFFAATVGTIAAAFELQYVHKRFGWSYPYASSILAIR
PSITLVVLLVIIPVASRISMENFGLSSARADLFLVRVSAALLTAGTLLLSVSEISSLAILSLIIFA
LGNAFTTIAKSLLTTFGPPEMAGTLLSAMNISASLGAMIAGPIIAVLFDWGLKQGGIWVGAPLFFV
TLLYTLTFCSVCVLRLPDRSEDGDDDDYEQDQQRAQSVLGS
>g2328
MSQSKGGRRRRSSSIIYQEPIESNEQMSDQAALPNLNANWVNAKGAWTIHFVLIAALKIFYDIIPG
VSQETSWTLTNITYMFVTFIMFHWVRGIPFEFNAGAYDNLNMWEQIDNGDQYTPAKKFLLSVPIIL
FLLSTHYTHYDLTSFLINFLATLGVVIPKLPIVSDSDFFPYDVLDADFPSLTLDSTGLKERFMMSL
LEEGNVFSGQNNPQHESSSSEDTEPKREDKISDAPENSVEWAWEDDPKNPYNWPTKLKVRQVVMMA
SAGFTTTVAVSILTPAHTQFMQEFGVSSTVAILPLSLYVFALGLGPVIGGPLSETVGRYPVYFGTT
LFGTLFSLGAGFCQSFAGLCVLRFLAGFSFGPSLAIGGGTINETFKPAQRAVPSTIYILAPFLGPG

LGPVIGCFVVDRKGWRWTQWTICFFGIFTMLTIMTCRETFHQVLRRRIAKAKGLSMPTPTPLSSQL
RLFASTALLRPIQMLCTEPIVALVCLASISWVSPVIAIMIFAWGNLCVFVSMIQYLVDTYHGQTVA
SAMSANSLARYGLAAAFPLFTIQMYSTLGIGWASSLLGFIAIALSPVPWLFYLKGERLRALSSFET
ATS
>g2329
MAGISDDEGDSRTASVGQARSQPNPNPSTSAKKKSRKSKKAAKKEPVDFVPQGAKFSTTPLEVDPE
NSTSSSGSDSSNSDNEPEQKSNKRQKRNAPTVSWNQASRSAIRTTLGAKPAAAAVKNQQAKSAFDA
VNDKFFRSRSASISEEEAHNKGDVGTTESSSKIDQEQKTYFVDDSDASDSGEVSEGDDSMMLNIGQ
QSNGHDLNDTQDDAVDLSTPPFIVDPLPSTQADKPSTQETGSVLSEPSITQLPPQSKAEAFADFAA
SYTTSPAILADLKVKDLEIQARFFFYNSNINELDLTRPISCTECLQEGHLSSVCPTKECENCGAWN
LHEDRLCPSVRRCQRCREAGHDAHQCPSSLKASAAETPCDYCGSDTHTEFDCDKLWKFPRVQPLDG
PIKVPTTSSTFSLKDYTPSVITNLNSVIGPKKADNPGMSIRPSRIPAAVEERLLLEELLAKIGVYM
ENSFLRHLLVSLHYLVNHHRHSSPEITGIEMMTSTTDNEGDLVPLRLVIGRRQEIFREEDPVLLLE
DVVMAEAAVVAAEEXTNTVLAGGKYARCCIILICE
>g2330
MASIQATPARPRRFAPLRQDADTSDAPKLKGIVFDVDGTLCLPQHYMFPQMRSALGIDQKTDILEH
IRNLPNEQARTEAVAKVQAIEREAMLAQQPQPGLVTLMNYLEKKGIRRALCTRNFEAPVTHLLQNH
LPNHIFEPIVTRDTPDLLPKPEPSGLLHIAEQWGLSNRADSMIMVGDSIDDMTAGHTAGAATVLLA
NERNTHLKEHVHTDICISRLDELVDILENGFVGDNCAVSGST
>g2331
MEATALGHLLDEKNHTPCLNCKSSRLMHLAFWTIYLIFLRALHFLPQCISLSPQWFQAHITKVESR
IVNIAISIVQIVSGREISYSSFCRNNYRSSRQRDGGYEGSIISTGPADNLMTPGDSSLETNFIPPA
MFAHQPDIAYLRPQAPLPPYLRRNYENSRRSSSLISPLPYAIHANGRRRNNAFQMQSRSLDRAVSP
ALFAEPIPRMPVPPIPAFIARMPFVSVSDSIYSSSCSTQLPAASSGVQTYIDLLPGRSFEDSDTSR
QANASAGNNAGGASSVPAHFRPDAQHGTLAAVAQHYTRRNYRRSRNARSSSAPVLQNTVKDSKL
>g2332
MGVNSRRPLLPAPTESIVDSRIDTTGSSLTTTDMATDVSRRTDKSSYSLPDDGSPITISTRRRSSK
KEDDDSSKLSRTSHHSQTSLLIEYFEREKTGSRLHSRPSVRVKVIPSGRKNREHGDTIQIREGGSS
SKRPNYTRRISVSTPSKKKTITEGNDDQSVTDDEQDGRPLEIEFLDRGNGSDLSNERYIQPTSEIS
SMPADSMLDGSMVSGVPRRKRSQSLDKARDTSDADLLKAPSRRRSRSLSRERIAYRVAQKLNAGDG
GRHNKELLAAESKPLKKRSHKYDEEEQPSIESSLLSGSNVSSRHKSGDAISFRSNTSKSSLNNTKF
LETVEDAIRRLILPELKELKKDQKVSDNKLKFERDTIESGSGVSSKDVPKRRLSKHSSAPDVSKSS
LFVASTERATPEKEEVGTPEERRRRYKERRREKETEREAGSASPEESLRIRKGSLPGNQREFTEEE
KLRRQRSKGLRDAAAAGIVGSALTAAALKHHDSKSSLGKKSRKRKSKSRSGSGSINDSDTELVFQK
HNVPPMPFRSDIDTELTRDSLLSQRTADTVDTETPTPRQIKEVIRGSPRGLESPSPGTPLGSHREL
ASRQSNRSDRDFEERTDSIDGDVFLEGAGGAIAAAAAGNLLGHHPDHDMLGPEDNIHHPRALSPIQ
SVASDHDLHETQKEEVFHDKEADDRRFSIQSLSSAPSTELARSTRPEGISFESRSEILKQHDEGAT
ELGYETDRNIEAEPGAEWNETSSYAGQHDDFHDEDYKADINRDTVEGDVYDEYDDSPREFNEGAAV
NPKFVTPVAVESAVASLLEPSVMDTRSGLSQPRSQADSLNRSVDGSHVEESRERDLAEGSRRGSPL
KYEYGLNNHDEKSFTKRLGAASPPQSVTQSDEDVDEHEERYPLDTTMVNNHENMHPDDAQYDEEES
EIDTNPSVIQGPIGSVPHGSRDHWPYNPTPPQARDIQSSPQHDLNPAAAAAIGGVLGAGLGLSAAE
HGAYNQGYDQGYRMYSTPPGAKDEGYISAANPMSPSNGTPEPRNKFSTLDHANPPLLFDNRDLEED
PFVGSGHQRHFSGYSHGIASPIYDSATGRGAESIESKDIIALMNHLTVRDSQRNARDTEILFTLVR
SAADMRSSFLEMKKFIAEQDEMILQANEKQHERTRAIGGPRPLPASRSNRQLAAADYGEEVQAKRR
NVFRRAMKSLSLKSSNDLTKIEDMLEQLLDEVEALRTAQGSVGTAPATNAASLNSNINAGAYPDGY
EPEGIAGTGSPDGQSGYTSNSSRPLADARPMNTRRASENRISTVLEGDEEVDAYEQPLLDREVPYD
ERIPDERGVPAHLGTPPRKPVPTGSGSQETTPRKSDEKARKHKSNSSSFFKISRWSKTTASSAGDN
VRNSVQTARKERPSSEMSRSGSDLANKDHYATGDFYDPHGDDRLRSTFTVDEEPQDNRPPSPLVPS
QVSEGPKYKAHRDSLNLQHPQPQKGPTDRYQTHLESQAQVYAPTSPTSERWGSQSSLPHYTNANRM
SSGNARLTPISDAGYSEVSSSRGPPRPPKVKDAGPLVPQRPPKIKEDAGSPHTDRFSARNSRGSAS
PVNAPPTRKPTGPRPITSSGQFSPSNIKRNQYRGSPNRIDYDDDEY
>g2333

MRLSSSLSYLLAVVAFSPFASALRLLQSTSLIECMDNSNFSASLFQVTFTPDNKTLSININGISSI
SGNVSAEIVVYAYGFQAVKEELNPCDYNLDGLCPMQTGSIPIDTTITVSDSVVKAIPSIAYSVPDL
DGQVFVYINSTDTGKRIACLEASLTNSQTVHQKAVEWIVAVIAGLGLVASAITSGLGHSNTAAHVA
ANALSLFGFFQAQAMIGDTAVPMPTIVQSWTQNFQWSMGIIQVDFLQTIATWYQRATGGTASTLLS
NLATTSVVVQKRSVDIGQQLYRRGLHYFGKRASETGTTVTVSGLERVGFKGDIEATNIFLTGLIWF
VIFVALVMIGVGAFKGICELLVKYNKMPSDKFQDFRNGWKIVMRGILFRLTLIGWAQMCVLCLWEL
TRRDSAAEVILAVMMFLSMATVLGWAAFKVITLAKRSIAMHKNPAYILYSDPACLNKWGFLYVQYR
ATAYYFVIPVLGYVLLKGIFIAFGQNSQVVQAVAFLVIEAAFLVAVSVIRPWMDKRTNIFNISIAA
CNFLNAVFLLVFSNVFNQPGMVTGVMGIVFFVYNAVFALVLLILVLIASIYAIVSKNPDMRYQPMR
DDRGSFIKSNTALNTELDALANTARGGSEMELKSTAYHPTYEDESHSISNGSGASINHRGQHDMNK
PPASPIDPSVPLFPSHAPPSYESNGYGRDIERSRSPAQIGDFNPASALAMQNYRQQHNASPWQRGA
GYDH
>g2334
MSVRKAHNAGRNHIRNVVDYYQQIGHEKAQSVIDSITSSYAAEGQANPLLQHPGAPGSFPPPPFGV
PGRPGQMPPPPFGIPPPGAPGGLPPPGGRGMPPFQPPFPIPGAPGGTPGGLPSLPAGMPPLPGNXP
FPPPAGLPANFQPPPGFPGAAGFPPVGPGGPAGHGPGASISPPPGAGGFPPGPPGGGYRPPGMDR
>g2335
MSARSAAYMVVEPHPSVPSTRRPALHTSRGGAGNVISLRDTKTTDSLTATGPASLTRLNSKTPLTY
KSGRGGAGNVHSSSERAIFSFDEELEREMRREKEIAPVFHVGRGGAGNLILNSERSSYGLERRESS
TSSSSNESLADRAKDRARQSLERTWSRLTGHN
>g2336
MRFSQLFINLTLSTSIAASTTNNYSTWMADSIISRGQGIAPETPTASTYLQIGIVQNALLQLIHSP
MVSHDAKEKYRAYVQQGTESILDRLLNASQDTLYPLDRFSLGRGLLAEYLDDQDKQNVKEGLDALN
ESIALQPRNQYGGLWYFTYPNWSYLDGMYSYASFASLYTTHFDPRSTLEASRDIIDQLDLLWSHCF
DNTTGLLFHGYDASKTAVWANPITGASPIVWGRALGWYFMALVNWLEANTSSQSDSSQWRHARSRF
VSLADALVKAADPVSGAWWQALNYPGREGNYIESSGSAMFVYGLYKGARLNYLPQQQVYRKIADKA
YAYLASTFVVNNHNGTLGWNGTVSVCSLNSTADYEYYVTQPLLYDSVHGSASFVLASLENEMQL
>g2337
MAPTPNLLSNTLSARQSTSDQSNNNSSIPVAAAIAPIIIGIAVFFFICAFCLRRRSRNAATQNAAT
TSSSANNSLALRRYGNNNNGGVVRNDTQNGEDGPPAYTSIPLPPPAYMPDQPPSNHTRMDSDGERE
PVHGENDDTTVRRDEEATEEGKEPEHTN
>g2338
MTQISIPPTIDYTITPVYNHVGEVISIKVRVELLVSPLSAGQVLLAVPLQIASVDVGCFVNDSIVA
FDAQGLLSLSRDAEKLGPPIAWQKWRVLRDTQGPISAEYEALPRRISTTTRNAPPFDLRLNFGGAI
SSGYGFLAAPLVEGFDQKFDVFVQWDLSKAPSGTRAIWTFGEGPDRVYRRMPCSEIQATYFAVGKI
VSHGSAQFGLYWLGNPPFDARLLSDDLCPIFMHMAKFFGDKEDDPYRVFIRYNIYRGSGAGTALVR
SFTFNYDDWDYKDPPSAEQQALFLCHEMTHNWVKLGDPETDNWYAEGLAEYYSLLMPTRTKLLTLE
QFRVALNYRLFAYYTNPLVHHSNDEVAKLTWKVTDAQQIPYGRGLLLAFAVDDLISSVSHGRQSLD
DLVLSILRRQRNGEPSNQQTYIEELGYLLGDDASSYIKARAMVAHMCAGKPMVPTVHGLARFGLKA
ERGEGAIWELGFDEAAAMDELIIKNLDPCSAAARAGVHEGDRIIKYTPWSNIKGKQDAMIELFLCR
RSGDEIVVSFTPRGSRMMVVWQYVSVN
>g2339
MSSAFPIRKVIIIGATGHLGSHLVSAFDADPHFEVSILSRASSDRSRFPSHIPVYCVGGNYDSSES
ELVELLKGQDAIISAIAAQGVPQQRTIINAALKAGVKHFVPSEFGHDTRNEQAARLLPPFLVTQKR
RIVEYLQSKEIEGLKWTAFVTGPFFEIAVPHYLGFDFARRQATVLNGYGDHHWSTTTLSTTALAVK
NALLTPVKTTNKYLFIESFNISQNGILSSLGTLTGKWDVSYQDAEEEKRRALAKLSHGDYSGLPAL
MRYVTCVEGYGGXYMKYEESANXLLCLPEERLEDALEKMVK
>g2340
MGLSELWKYFHQEPEFHALQTESYDVDEGDNESEKLLPRRSLARKSALPTACISLTIVNVFILSIT
IFLVFIARGNLSRNEKNAVLRPVSWWSPVLDDIEIPRYTTRLNGTLFALPEVSIAREEPSAENDAA
WAQYEKVLTHVVTREQIIKLGKDPETVARFDEEYWDMGDDAYMVQLDVMHVRAPVFWGAADRKLYF
VFTDLGNAQQIHCLNLLRKAAFEDYPGYEPKLDVKDKMWWIHLGHCTDILLQNLQCNANTEVLTLT

WVEDRQVPWPDFSVNRKCRDFNALVDWQRENAVDADKFDAMPVPTNAFVWPAPWKKQDYELGIKLG
DHHLQEGEELGDPHMHHNHQLEEN
>g2341
MRFLYYFAFLGAAGSALALPVSGNDQLERATEEVDKRFYYTNYAPPEKRDENLDKRFYYTHYVPEE
KRDENLDKRFYYTNYVDPEKRDENLDKRFYYTHYVPEEKRDENLDKRFYYTHYVPEEKRDENLDKR
FYYTNYVDPEKRDEEAVEAP
>g2342
MDQQIVRRTNAPAQKAPLRQQRTYEELLLKIHECNTLTARNQQLEQENASFRLQLQLQRAPADQGS
AGGRLESQLQDANTKMHNYITVMRAQLDASMKHIKEQQNTIEGMKLAIRRTRDFLNTEFNLDP
>g2343
MSERRSVRLKIPSERLALFQQSMDGVKKRKRVNWKPGEKEEMFALREKHPDMLLKDFQERFYPDRG
VKALSGIICQMRKDARKGEPEAKGTDDRRVSPKSPLCEINIAQDRVLMKMQSNTPRLPELKRPRKS
GPFEYSDSEGEESSSYEIIEDINPSGHRTPNKGLQFIKLSHSAEAQSPSTRHVEQQEPTIRSQTDE
APQVSKEDIALAYRPQTTPPETTIVAQHTGCLESSSNSTGMSPIVLDIPLEQPAECHGTEMQVDVA
KDRKHQQTQLQAPKQEAFPESYIRQGFAKEVTDIYTKLQSLCRFEEQLFSTVNQTRAYNSAVEKTS
HILKKDYDTIKTEFEAMKNEKDKEITQLKADWQAERDALVQENALLGKRIELMKSALYGNIEA
>g2344
MSRQFDPTFTPGVINAMGPKTPERARVVLGSLIKHIHDFAREVELTPAEWMLGVEFVNSIGQISTP
IRNECHRVCDVIGLESLVDEIANKIITEQGLSPTSNVILGPFWSPNAPFRELGDSIIQNTNPNGKV
TYMHGIIKDMETGKPIEGAVLDIWQASANGKYDFQDPEQTENNLRGKFVSNANGEFYWYCYHPTPY
SLPTDGPAGVLLNIMDRSPMRPAHIHLMITHPDYATIINQIYPSDDPHLTIDSVFAVKDDLVVDFK
PKTDDPKAQLDLEYNVKMALKKHHPNPNSAPPVSSFERSRKTAEEKL
>g2345
MERSPAQGDLNWRLSAHPITLLFFLGFRIGSLLMYLFGVLLIDNFVLVFIITLLLLSADFYYLKNI
AGRRLVGLRWWNEVNTSTGESHWVFESSDPTTRTITATDKRFFWLSLYATPALWIGLAILAIVLLQ
NVIWLSLVAIALVLTITNTLAFSRCDRFSQAGWLANSALSGGIAGNLASGLFGRFFR
>g2346
MASAARNNLRRALLYVPGSSQRFLDKSRSLAADCVAYDLEDSVTPHKKAEARQLVRRAIDEPAPSQ
IRERAVRINSVDSGLALADLTEVLQSPNLTTLVVPKVNSASDLTFINDVITHTLKEERKVPISILA
LIESAKSITNLNEICRATPLLQGLIFAAEDFALDLSLTRSPSLTEFLFARSAIVTAARAHDLPSTI
DLVCTGYKSESAKQLLEEESRGGKRLGFNGKQCIHPTQVETVQRVFSPEADEVEWAIRVVIADAKA
AEAGKGAWTLDGKMIDVPVAEKAKSIVRRAEACGLDVGELREQWKGQEPE
>g2347
MVSEELFELCQPVLQNTALDDEEQTEQLEDLLRSNTTLTGASLENAVLDILWRQRNQSKPNASPPT
RHTIIRRSSPAPWQVRSATPISSPSMTGTSPAGPGFHLTRAGLSRGPRSSTASPFTSPRPSPRLAF
AQPIPHSPNLNAYEFSDQNSPSSELYGDFGSESNVDWLVADDQLSVTSSVGGLSASAPEFVPDMSP
HDILRSVLGDKKSNDEIEAALEANSYDLGATIAALSEDPGKDGSQKSDEGRVVVGKSMTVDHSRPS
TPLNQARTPVVCKYWLSTGTCLRADCRFSHDLTNHICKYWMMGNCLAGDGCPFSHDPASLMGNLSI
TDGLNQQSPAQANFQFDNNQEAFPALQPAGAAGEQWYGRDMRKYTNHVLITTPAGNTSPLGVGRRN
GSSRPQSRPTSRHQQRDLNPSPLSVDDPEAFPALSAMAAKGSGKKHHGKRGGHNNKETVASSLADM
VRISSPGPGKGKNSKVGKEVKTRELSAAALAIPAPQNIPWLETGPRANQQYIKARTDAIRHGTVRN
KFLQSAAQAWNRNDARAAKALSLRGQAENEAMRRCHREAARQLYEEREQHLASSGLDGAVEELYVD
LHGLHPEEAVKYLDKILVKHSTEDRILYAITGTGHHSKNGKDKVGKAVKVWLNELKYVFREFSVPG
ERGGYVGGVLGIDPTNYDKEAAAKWRSEVDAQSETNDAQPVLSMGKIQLLKREDTNGK
>g2348
MPADYQTTARALSLPVSEPEERESPVFSGLRPPWSHSPTLSRRSSRRQSTMGEAVSFRDRMINQAE
SLYRTARERWEKMTLIQKIIYFTASLFLGALGVASLVLAGKIFIWLGPVAEEWGHSPWAFIIVWFS
IILVSFPPLVGWSTLGTISGFLFGFWKGWIVYASATVVGSTLSFIISRTVLSGFVKRMTEHDKRFA
ALALTLKYDGLKLLCMIRLCPLPYSFCNGAVSTFPTVQPLSYGLATLIVAPKFMVPAFIGSRIRIL
SENNEEMSAGSKAINIISIIVSVSIGVATGIYIYRRTLARAKELEAEERAGIRRSIQEDHAAGRPH
GDFSDDPDANAAAKTLARDEEARLGFYDEDNVDIDVEGDELAESRGNRSYRDEFTDNDSDVFDDGD
GDESDTYGLHTHVRQ

>g2349
MPTTPGKAKTMSSRLLTMKFMQRAAASSNPAPVEDPETPSPKRQRLSTEPGTPRTTSDLDAVNAAL
AAEEQRRADAIARQAAEAGESNWVLNFEGPAAKPAVSSQPMIVAADSLDAEDQELSNGGRKTYGGF
KSRSRRIVFEAEEEDLPADPKERAEYMAEKKRIKEKDERKADRVMQNATSISGGGGARTATVNDRD
KNKKHGGKSGKKRSK
>g2350
MFSSARGRIASVALSASRSSARVPARASYSITTPRWSDNASIPANDPTPKKPVPNVSATNAVPVDS
MGAFDAKLVEKPVEGEQNRQLQAPNRASTWAQSQKPREEAMTGPRFEQTIIELQPQPYAAIELIHK
QPVRWTKERVVSCDGGGGPLGHPRIFINTDKPEIATCGYCGLPFAHEKNKAYLKSLPSTSYPLEPT
GNPVEVEESQRITEGGYEQR
>g2351
MSWFQKQFTLPSRSRGSYLITDTVVNELPEIRDYKCGLLNLFIQHTSCALSLNENWDEDVRADMSD
ALDRIAPYDKKGNLYRHSAEGEDDMPAHIKSALVGASVTIPITNGRLATGTWQGIWYLEFRASRHS
RKVVATIQGEKA
>g2352
MTTQLESSGALTGTTPKYSRYRSVRRAATEAAGEASTAKPSNEAIARSMSRYRRPKTATTAPHAAN
CPPVPSLPASVQTHKAVTKGTVEVASPLSGASPRSEKIQNPFSDHDDELSEAEKARMREEAMRKLT
MAERPKAPATLPQARQENPVQAKFVPEKEKEGAGHSRRASWKDRLGLSHHRKVGPDALDDSSQNNN
SNGNGNNMTLPAPGIDAPVSAVNAGERRVLVKCKSSSLKLPVTPTTRVKDIIFSAANCFSERIDPK
NAIIVESFSSLGLERPLRRYEHVRDVLNSWSSDDANALLISDPPFDGATRKLELSAAPNTAPKDAK
FHVYHSSKPGKWDKRYITLRNDGQVVLSKKSDAKEAKDFTNVCHMSDFDIYTPTSRQLAKTVKPPK
KICFAIKSQQKSSMFLSTENFLRYFAISDSTVGQDFYDAVHGWRSWYLVHVLEVGKANEAANATVS
APISRDGPRTPSIRQQRHVSTDSIPYQLGSFKPLIDFGRNAESSVSIPEKATEETTASSRDMFLRK
KKARDHAPPPSSFPVHLPALEIQKESLSPQDDDSTFSPDSLLGRTYSQKQAAQREREAREGNEHDP
FSSHGLLKHLSPTSTDQASFQSMPFSNPSSRGNSRSNTVRSNHRPAEGFSHSRSNSVRKGKPLVDL
TPTYREPPQHARKGHGVKIDSGMPLVEAATGLEAIPGIPNIPSATTWRREEAASAPAPPGRRRANT
VKGQHKNHTPILDPFASPSDAAPFIPNGLVALSANTPSSQAGPGVGRGVATGDRHATRPLLDMTPQ
SQFAEGSLLRSIEKL
>g2353
MSAPRSVAAALRRTRLSSRCRFTPQTIIAPISVNTARSFSITTTNRADASRKIKLTAASYPQLKRN
PNFAEVTKEHVQYFKELLGTDSALIDGVNTDATDDLAAFNSDWMRKYGGQTRLVLKPQTTEEVSKI
LKYCNEHKLAVVPQGGNTGLVGGSVPVFDEIVINTARLNQIRSFDEESGVLVADAGVILEIADAHV
GEKGYLFPLDLGAKGSCHIGGNVATNAGGLRLLRYGSLHGNVLGLEAVLPDGTIIDSLSTLRKNNT
GYDFKQLFIGAEGTIGIITGVSILCPPRAKAVNVAYFGLESFDQVRKAFREAKGQLSEILSAFELM
DGRSQALVKESTGNKAPLEGEYPFYCLIETSGSNAEHDTAKLESFLEHVLGEGIVADGVLAQDETQ
IQSLWRWREGITESLSHLGGTYKYDVSIPLPELYQLVEDTKTRLTEKGLVGDDDSFPVRAVVGYGH
MGDSNLHLNVAVRQYTKEVEEAIEPWVYEWIAKRNGSISAEHGLGVAKKEFIGYSQNDTNIKLMKQ
LKDLYDPKMSHTALITGATGLLGREVTKSFKQAGWLTVGQGFNRAAPPTILKANLEDPADVKRILD
EAKPQVVVHCAANRQPDACEKNPEQARKINVEATRILAEGTNARGILLIYISTDYVFAGREGEAPY
ETDAKTSPTNIYGEMKRDGEEAVLEATKESGTGVVLRVPVLYGPTEYNGESAVNTILDAVEKSKDA
NANLKMDDWARRFPTNTQDVGRVCNDIAVRYIKDKHNLKNLPKILHFSAEEDMTKYEIAQRLAKIL
GVEIPGMVANKEGNDPNAAVQRPYNTHLSTKVLKDVGINVQAMKFDDWWKAYLKK
>g2354
MASDGDMASRYQVMEELGSGSFGTVYKGIEKSTGEIVAIKHIDLESSEDDIAEIQQEISVLAACAS
PFVTQYKASFLRGHKLWIVMEYLGGGSCLDLMKPGNFSEAQIAIVCRELLLGLDYLHREGKIHRDI
KAANVLLSESGKVKLADFGVAAQLSNIKSQRNTFVGTPFWMAPEVIQQAGYDFKADIWSLGITAME
LANGEPPHASTHPMKVLFHIPKAPAPRLEGNEFSQTFKDFVAQCLVKDPDRRYTAKELLKHKFVRN
AGKTETLQEIIQRKQDWDNGKGHPDDTRYYAETLTTFSSNEEDDDGWVFDTVKASTVKRPVIAEDF
CEDPFVKTEEMKSMPRPVANSTVRRTPVAERSPSVKRSNTKRRSSGVKHPLGLNLSYGNSPSTVRQ
FRRVSDKGNNDQAQHYNYPPGLDENVAAKTLFTDPVPNSKEAQLGRRAYSRGIGLACQEILANTGD
QEKREAISRLAEAFSDLEMVDPEGLYHILKLSNEKMRADSKLSALLPEPTPTTPQKQKLVLAQNNP
HLKSHRRRQSEQIQVEHIGSPQLSSSMPGQHVPGMEHTKQLADVLYQRWSEGLRNRWPAI

>g2355
MEEPVSDHGPSPEAPLPPKTSTKDLERSAVPSRPNAAARHAKRLTLNFPIAPALNLRPEQSSPSVA
TPATETSPRMMSPGQSVPDASILGEALNHEPTDLLTAIASQERKVLELKEELYKAEAELTSLKKQW
VRSERQKKQTEISHHAEAMRPIRPSMDGSSDLQSPRNSENTAAASMATTPIDAVPVQVRRSKELER
RNSIRSAAKGEALISANGRRVFAGSKHTRTLSLLSPDMGVVRPPFPMPSPSDSTQSSESQATRHPR
SATLPSVXXADTSKTVEASDKSTTENEWRRSLPPPSSEALLRTGRQMASDLREGLWTFLEDIRQAT
VGEEGISATRNRTLQPPTSGTPRSRSGQRSERSVTPVRRRDQNGNSIGRTSSSTSLSKDASASRKT
GNNNSSSPSPPTGTEVSFWSEFGVDTPGQKLKPKQATAVAASNTQNGQKGSANSSQLEVEEDDDWN
DWDTPQPNKKSHTPSSSRSTVTSKIDQSPSTQLSSPRTSASFGERNTDEAERPRTENGIPWPALSK
FTASQLTRTASSLMDEWEKSLTSASSSAKAEQDDWEAF
>g2356
MSFPGFPPMGGAGAGPNAGMSEQEQNMIKMMNAAAESCPFKIALSGGAGFALGGAFGFLTGAMAYD
VPLTAKGTEIYNLPWREQLKHGFKDMGTRAWSSAKTFGYIGAIYTTAECSVEGLRAKNDLTNHVVA
GCLTGAFLGRNAGPQAALTGFLTKGDATYDRNHGPIPHLNADTHTTTINGQVPNPLKLSIHDLQTK
FPQHEVICALQCAGNRRHTMRTQLKEVQGIDWLDGAIMNCKWKGPRLRDVLITAGVHPGQEGAEKK
KKKKKRYVAFECYQVECQDDTWFGGSVELERCMSVDDEVILALEMNGNPLSPNHGHPVRVVIPGVA
GARWVKWLDRIIIQDQHCQNHYQQYDYKVLPPEASDSEKAKEYWMKVPPLLDMPINSVIAAPDDGE
TVVVQVGRVGVKGYAVPQGRDGPVTRVEVSGDGGRTWIDAQLETXECESFNDTWCWKLWKASVPME
RGSGKLILSRAFDAAGNTQRESCPWNLRGVAYNGYGMSKDLNIV
>g2357
MFKHGLRTFATTALRSAEASTAYNTKVSSAQGWVNGLTEAIGNTPLIRLKRLSEETGCNVLGKAEF
QNPGGSVKDRAALYVVKDAEEKGLLRPGGTVVEGTAGNTGIGLAHVCRSKGYKLVIYMPNTQSQGK
IDLLRLLGAEVYPVPAVAFDNPQNYNHQARRHAESLDNAVWTNQFDNTANRQAHIETTGPEIWAQT
EGKIDAFTCATGTAGTLAGITRYLKTVSDGRVKSFLADPPGSVLHSYIQSGGKLAERAGGSITEGI
GQGRVTDNLKPDIDLIDDSLHISDEKSIEMVYRCLDEEGLYLGASSALNVVAAKEVAEKLGKGHTV
VTILCDGAYRYADRLFSDKWLKSKNLRGVIPKHLEKYIVLP
>g2358
MPARATSGPVSTGSRKTSGQSAVSSKRASSATPSVAVPPEPPLPTTSSHLRTDVCAIFADSQRSTT
GHRKLVVRLRKVQEISCGITPPKKSKDGQIEEGREIPSSDETFGEQEFNVEVTRCILRILPIKKTE
SVGDRVIRFLGLFLNHASEKDGEVFNPTDVDETQMFPETPTTRLTMAIANMVVPLLASKDKIVRYR
STQLLAHLVNSLDSIDDEVFRLVRTGLLKRIRDKEATVRVQAVLGLARLVGDESEGGLEDSSAALL
EKLLDILQNDATADVRKTLLINLPLIPTTLPYLLERARDLDAATRRALYSRLLPTLGDFRHLSLSM
REKLLRWGLRDRDENVRKATGKLFYERWIEDCAGNKPSAEGNTMGQISAPNTSALLELLERIDAVN
SGMEGGVAHEAMRAFWEGRPDYREEIVFDEDFWQGLTAESAFLARSFNDFCRVDNESKYGSLADDK
MPEVTALAYFLGKYSSALLAKLKQASASGENEEDTMELEFVVEQLLQIALTLDYSDEVGRRKMFAL
LRENLAIPELPELCTKLHVEVLRNVCGPEAAGESEFCSVVLEAVAEVHDAIATEDSFVSARSELSD
DGSSRASRDRSMTPASDVPFDKEKAKAKVLREIMINMKCLHIAQCMLQNVEGNLQQNMHLVTMLNN
LVVPAVRSHEAPIRERGLECLGLCCLLDKTLAEENMTLFIHCFTKGHEALQVTAIHILADMLTAXP
ALLLPVVQADSETVTPPPFQKNIFKVFAKALKSTSPADVQSAAAVALSKLLLVNIFTPSGPSIPQA
IKDLNESSIEALLQSLIVAFFHPRTRENPVLRQALTYFFPVFCHSRLANTQRMRKIAVPVVRSVLN
AAEEFFSLEAEEDSDGDIDDSVGEREIKALMTQVIGMLVEWTDERRVVGLGGENVLNGGTASSNAH
GYVHLALVNDILERILGISAGPNKCSKEEKKLLMSMLSKVYISAPVLPTSTAPGSRAGSRAPTEAT
EDAFRFSNRSSSVREEIEPEIVALAETVKELLNDALEEAATDAASRNAIVKVKNTILKILAYASQQ
NTSSRDTNRNTRASSVDSSYSHAGPGSRQASVEPHSTRAGTTEVAIDETIMEIDEEEDHDDSRTTV
IKNERNSEEPE
>g2359
MAAVPSPIPKLDRYIVIHVATTCDEHGVYVTKDSAEVIELGWILLDTKNCDELHRESILVKPVNTP
ITPLCTSLTTLTWENVRNAGSFRDAVNRFDAFAQEHLISKNLEFSFVTLDSWDLRVQLPREARDKA
VVLPPYLQHSRTFDLRTEYQRWQTHHPESLPFGPSSLANICAALEVEPVQSSAPIKHNLPFHLQAL
APASPRRAMEEAITLSRVLKGLIRKSQPAHEHPEILTRPMDARADVRAFLAERSKVLHMSGLPHDT
TQSELESWFTQYGGRPIAFWTLRTPDQHKPTGTGFAVFSSHEEAAESLCMNGRALNEKAIEVSPSS
SRVLDRAAEILTPFPPSKNRPRPGDWTCPSCGFSNFQRRTACFRCSFPAVGAAPDPYGYGYVPPSM

MPPMNPHGGHGMGHSRVGGNGVVPFRAGDWKCGSEGCGYHNFAKNTNCLRCGAPRSGAAVVADSAF
PSPMEPPSGFGMGPGSMAGTPAPAPFPTAGGGGAFGGFGQQFGGPPSNYGLPSGLGNTPGPYPPMG
QINAGYGSANAGHSAASFGNPATQAAFTGADHGSHSNALNGNLYTTDGASDPFAFLSTGLGGLTVN
DDNHGRRNGAGANKSPA
>g2360
MTDKAPVVKPTIVFVHGAWHTADCWSLVTTQLKKYGYSSQAIQLPSAGGDLATTVQDDAAHIRKTT
SELVAAGAKGLLKKDREAELKKGGINSIVYVAAFLLPLDASLKSFLGDLAPWIIFEGEKMTIINAD
RIFYNDLPESGMDTLISSLKHQSKASFFTPLTYAAYQDVPVSYLLCENDNAIPFQAQQAMVGIGGP
NVASHICAAGHMPMIQMPERVVNVIREAAXEVVA
>g2361
MPFPSEPLTLRGGCNCTAVRWRMSLPDASRRVANPYHTPGTSIGDLRLPTAVVCHCDGCRRATGSL
GAYGFASDMALLELSILPRGTIPDQREDQDDDGRPSYAPAENLMDGDTKELDTLWLTHYESSPDRH
RWFCGRCGTQIAIAASKDAIPPELGWPRVVNIWAGTTDRNLLENDWCRPDHINNCSVAIPWVRDHV
RNGAKGSEEHPSFMIDWHMTDDIRPHIEMLNAMGMNLNVTMWK
>g2362
MKLQDTRITPSKIGLIDTHVCLAFAGLNADARILIDKARLEAQSHRLTVEDPVTIEYITKYVAGVQ
QRYTQSGGVRPFGISTLIVGFDKGDGTPRLYQTEPSGIYSAWKANAIGRSSKTVREFLERNHKDDM
DREATLQLTIKSLLEVVQTGAKNIEIAIMAPGKPIEMLPTEQIESYVKNIEAEKQEEAAKKKTGRT
PGTGSAAILTRSGATGEGSES
>g2363
MAEDRKKAEGTDEAPLKPVSSLLSHFEHLSYSRNQSPTPGSNDERTSRQRFDHLDDSQPIVRASLD
LPRPQIPWTVSGGSDNGPISAVDRTSNPPWMDSKREKSPGSRRFGRPLSMSFRTSPQITPSLTVES
PRSPPRGANGQNGGHWPSFNRGNSDLTAIAKPRSRSPPGPVRPKDPVSPSARSVGAPRTSREPSPT
GSVKSSSVPPPVNRGGKPKIPAKPANISHLDVRALTPTHEPTHSTAQISPFSTPPSSPEKLQTKTD
QPRKSPNISRSLSTTPEPFSLPPPVDRSTRPLPTTTQSTDTKSRPPAIASHPNDARELGFTRRNTG
ISPSKHSRSASHIVSPTRDSRLPEPARKLSDEDRPELRPSLPPRDNPSGRRAPRSVVPSELRNPDL
PTRASLDLPSRTPRIPTSSRLPTLEMNTQFAPPPRRQTATVGGKLAPDTPQTPKLRQSPSPITNNV
TSKPTSRSDPRDSEDEAYPEEAPVSRADYPDSSQVNRRPPVFKTGPLTIPTRYDTREFDVCGRYVC
TTGYLTRVWDLTTGEQIMSISHGETVKAHTVAFKPGTGLEDEGTRLWVGTSVGEIHELDIGTQSIV
TSRSYPSRREVIKIYRHKKEMWTLDDEGKLLVWPPDETGTPNLQYSYDHPQDRVARGHTFSMVVGD
DLWLATGKEVRVYRPSAHGDTFHVLKKPLGANHSGEVTSGTHTTKDGGRVYLGHADGKVTVYSSTD
FSCIASVNVSVYKINSLAIVGDYLWAAYKTGMVYVYDVSTNPWTVKKDWHAHESPVCGLVLDPSSL
WTMNRLQVVSLGTDNYLRLWDGMLEDDWLETRMQGRDVEYCNFREIRATIMTWNAGASMPRDLHGN
AFIENAIHAEQPPELLVFGFQELVDLEDKKITAKSLLLGSKKREHNEKEHMSRQYRVWRDHLAACI
NNAMPLEESYVLLHTSNLVGLFTCVFIKQKERDRVRNISAAEVKRGMGGLHGNKGALILRFVLDDS
SMCFINCHLAAGQTHTAHRNNDIAAILETESLPAEHSLNTRTDLFVNGGDGSMILDHEICILNGDL
NYRIDSMSRNVVIDAVRQNNLSKLLERDQLLASKRKNPSFRLRTFTEAPITFAPTYKYDVNSDEYD
TSDKKRSPACSGYNLPTTTDNAYLVLSWSQAEGNSLIFDLADHFSQDSPSPIASDHPESPSTTTSA
SVDLDVLIPSPPPVILQQRDSFSQYTGYSPPPPLHPASNGNGVDTNLSSDITSATVSRLRRRRRRS
EISSSSSSSSASETSDIDGLRDVGSSSARPPLATNVSSAGTRGAQRSTTGCAEGHPPSPKRRRLAN
MRPNNGDSVTTANGRSDASNGIFSTSSRGKSSVNGKSSNHLHNGQSNTNGSARSSAVSSSYFGHDR
EEVTRILIQGLYDLGYNGAASALTRESGYELESPSVVAFRSAVLDGQWAEAESLLLESFEDGHDAS
NGEYESPSTWGKLALAETADKNEMLFLLRQQKFLELLEARDLGAALMVLRHELTPLNHNIAQLHAL
SSLLMSPVEHLRELSGWSGSVALSRELLLSDLSTFISPAVMIPDHRLASLLDQVKSNWVNQCLFHN
TAQSPSLYSDHMCDRNAFPRHSSMQLTRHSNEVWYVEFSHDGTKLVTTSKDKSVIIYDATDRFSVI
HRFYEHHDAVVFATWSPDDTKLITCSQDKTAKVWDVQTGHCYMTIEHHSDIVTAAAWAPDGETFVT
ASMDIRAPLCYWGLRSRNPIHVWRDGFRAQDCAITPDGTRLIASDNEDKLYVYDLASRAEEYCLSM
KNHITSVSVSNDSRFVLLNLRNNQIQLMDIETTEIIRRFDGQKQGEWVIRSRFGGAGENFVVSGSE
DSQIYIWHRENGALVEVLEGHTKGCVNAISWNPKNPSMFASAGDDCVVRIWTRDNPSESSLSKGRF
SKTSEFTRTSALRSTTSQL
>g2364
MADLSKGLVQPFLGALQACISVLLTLLYGVITRHVSLINDETIDQMSGICVKIFLPALILVKLGSE

LSLDIALHYVPVLVWSILYTLLSICIGHVLSRCLRLPTWVTPAIAFNNTSSLPLLLLQTLQSTGSL
KLITMPGQSESGCMNRAQSYFLVCAVVSKSIAYAVAPRMLSHADSNGRKGDDRETRHDRDEGLNVS
VQPQNQFRHDITNTSEDQQGEEEEEEEVNEQTSLLPRPIRTVRHNFFLKTHHLFSYLPRPVRRRID
ALDSPFLDTAILCAATGVLLGLVPKLHRAFFSPYEEGGIFNAWLISSIKNVGTLFTSLQVFLLGCK
LGASFERIKRSPSHSPSESSLEENDDEEESHTAIIPHDPVLWFALMVIPTGPPALLNSGLADMSAE
VTKGDKMAIAKMLAVRPSHFLSS
>g2365
MLGLGLRPRLKVRPSLLDLLSHNRTFIELADSAAEEATTASPPLDHLPPLPATPTPTSTSTSSTLL
NDNGSPSSPTHISRLTADNLPAEIIVELQPSSPLPSLSPAPPPVMAPSDKKQNDDEQLGAVFSISG
PVVVAEKMIGCAMYELCHVGYDHLVGEVIRIDGDKATIQVYEETAGVKVGDPVTRSGKPLSVELGP
GLMETIYDGIQRPLKAISDSSESIYIPRGIAVPALDRVKKWDFKPTKKVGDMITGGDIWGIVYENS
LLDEHKILLPPRARGKITKIADAGSYTVDEKILEIEFDGKKSEHGMMHTWPVRVPRPVNERLASDS
PFIVGQRVLDSLFPSVQGGTVCIPGAFGCGKTVISQSVSKFSNSDIIVYVGCGERGNEMAEVLMDF
PELSIDINGRKEPIMKRTCLIANTSNMPVAAREASIYTGITVAEYFRDQGKNVAMMADSSSRWAEA
LREISGRLGEMPADQGFPAYLGAKLASFYERAGKSVALGSPEREGSVSIVGAVSPPGGDFTDPVTT
STLGIVQVFWGLDKKLAQRKHFPSINTTVSYSKYTTILDRYYEKEHPEFPRLRDQIRELLSNSEDL
DQVVQLVGKSALGDSDKITLDVAALLKDDFLQQNGYSEYDQFCPLWKTQYMMKAFMGYHDEAQKAV
AQGQSWAKVREATADIQTGLRNMKFEVPDDEKAVSAKYEKLLSDMSERFATVSDE
>g2366
MATAPSGYSINVNDPGLISLVNKLQDVFSTVGVQNPIDLPQIAVVGSQSSGKSSVLENIVGRDFLP
RGSGIVTRRPLILQLINRPPTKQVNGTDEDKAVEGTSDSAANVDEYGEFLHIPGQKFYDFNKIREE
IIRETETKVGKNAGISPVPINLRIYSPNVLTLTLVDLPGLTKVPVGDQPKDIEKQIKDMVLKYISK
PNAIVLAVTAANQDLANSDGLKLAREVDPEGQRTIGVLTKVDLMDEGTDVVDILAGRIIPLRLGYV
PVVNRGQRDIENKRPISYALEHEKNFFEGHKAYRNKASYCGTPYLARKLNLILMMHIKQTLPDIKA
RISSSLQKYTQELSQLGDSMLGNSANIVLNIITEFSNEYRTALDGKNQELSNIELSGGARISFVFH
EIYNNGVKAIDPFDHVKDLDIRTILYNSSGSSPALFVGTTAFELIVKQQIKRLEDPSLKCVSLVYD
ELVRILSQLLNKSLFRRYPMLKEKFHNVVIQFFKKSMEPCNKLVKDLVNMEACYINTGHPDFLNGH
RAMAIVNERTQSSKPTQVDPKTGKPLPPRSSSPSLDTVPDANKEGGFFGSFFASKNKKKLAAMEPP
PPTLKASGTLNERENIEVEVIKLLITSYFNIVKRTMIDMVPKAIMYTLVQFSKDEMQRELLEMMYR
NNELDDLLKESDYTIRRRKECQQMVESLSRASEIVSQVQ
>g2367
MAPVHRVAVIQWHIKNLDPEYNHSQAIKHITSAAADGATLAVLPEYHLCGWAPESPEFYTQAFQNQ
PYLDAYCRLAEKLQINIVPGTIIEAEPKNPAATNETEKDHALYNTAYFISSSGTILGKYRKKNIWH
PERPHLTSSSTDPHEVLDTPVGKIGMLICWDLAFPEAFRELIAKGAEIIIIPTYWGKHDANEEALR
LNPNTEQLFIESTLTSRCFENTCAIVFANAAGPADFFLGMSRVVVPFIGPVGDKKSMEAEEEGAMV
VDLDLSVLTLAEENYKVRQDMASEGWHYSYRHDAVDKRPAEKA
>g2368
MSSLAGNAIRKRSASVDSPFTDINGDPNARCNLSHEYLKTDPAFNPSHVARPKSSAISTSANVKPR
LPRSRVAKIASKELANHIIHPRRTIIQHFESKTAKSLSKATRPYITPQADREFLAAHDALFEAEKD
EEKPSRRNRDRRGTEKEPGQRGRITDSEERTSPETDTEVQRRKVELLEAHRQSILVAWITSRHMKS
VRVTPSHIADFPNLDDEKFIERNGDGSEVNFRFLPLQLLLYYSQPFMARYVDVDDWKSPLDIVTLA
ERLVMATEPWQEWWMGIRDLYRWEKPRRTAFWYGVFSILWYTQHVVGFLYAYILYITLKNKFYPSD
VVSMRESLDRALDRGAQAYRFGELVDRYGREKWLEPLLDQIAPFIRLQLGDLVAFLEITRNFYYWR
DPRKTAATLFFFFSCLLITLLTDMEFCMKIVSFIVINVFFLCWPIASRYPRYRYLVSPFRWAFWDI
PSYPEWAIRFLQENEALRRHEIKTRGLAHNESCYSEECQTCRETIEGDDFQDATETQDTAKDSTAS
KNIQTAYHDDDTTSPYVQSVFFVTFHNGHEGQLILTSEGIRFVEKPHLLTTRHKQKPLLDREPRWS
YTYGQLLQMTKQHSPGLSKLTGLDRKLERLDFEFLVDPDMSASQEVFKGSVWDMETSYNTTTGAKQ
RTKIESVDVNQAERDEIFNLVVGWSKSRWQATSGQGDSTR
>g2369
MAYHGQSQYPKSDAPPYADNIALSSGINNYSPARFSQYSRFDPRGWRTRTQLIAAGGVVVILIAII
VGAYEGVKANRYPNYSALSYSLKDTYEGENFFDNFWYWSDADPTGGFVVYLQRANAVWSNLTYATN
ESAVIKVDTTYSDTTGGRNSVRIQSNNTYNDGLFIFDILHTPYGCGTWPALWLSDSSNWPTNGEID

IIEATNNGTWGNSMTLHTTDSCSMGVKRKETGTVTSKNCYNGTNSNEGCGVSSTVASYGPEFNSNG
GGVYATELRDAGIRIWWWPRASIPSDITNGSPDPSTWGEATADFPSTDCSISSHFRNQSIIANIDL
CGSLAAADKYYIDEAQCPSNCTAYVAANPSAFTEAYWEFKSFKVYQSS
>g2370
MTSNTYGSSYATDPYGTSTYGDTSLGGANRTTYDPTTGAYDQHIDYDADASTRRHHREQLDDSGRY
YHRDVDRDTSGLDNTTRVHKEYSNPNTGTSYTRDSAYDRNNLI
>g2371
MFSSIIISALIAAPLVAGHGKVTVLTGDLGGNGTALGIEGGSVANAGQNYQTEVDTTVFWSKDINT
DDDFGWTESSHGNLGMDDLVKAMALSGDTLPQVSQGGSINATLHIVTSDGAGPYSAIIDPTASGKF
SSAVNMEVTTQVPGEGGWIDTTPSAAALAAGNGHIIQKRATRTLERRASRTRGKRETATLVDKDYS
LEIAIPSGVTCSGTANGQSNLCAVKVSNNNENGPFGAVMIVQMAS
>g2372
MSAAHVPLTSPENLEGLYDGPPRNVASIDHLKFDDKLQPKNYSIFGTSPESKILITDVWIIDATGN
DPYHGDVLIIGERFAHVGKVPEKDSYLRDPKVRVFHGNGRTLIPGLGDAHTHMSWNGGDLGRLGEL
GVEEHTLLTARSAQCFLDSGYTMCFGAASAKKRLDVAIRDAINSGDIPGPRCLANGQEMAKRGGEL
VGGITAFANGPDEMREVIREHVALGVDQIKLSMSGESITEIRDAEECYFTNEETQACVDEAHKHGI
RLCAHARARDSVRQCLDHGVEVIYHASYIDGESMDILEKKKSQHVVVPAINWLVATLYEAGAFGYA
TEVAEKAGYKKELDTAIIGLREMHRRGIVVLPGGDYGFAWTPHGTYARDLEHFTKLLGFTAHEAII
AATYGVAKLFMRSDEMGQIKAGNFADCVVVDGDPLKDITILQDHAKLDIIMINGRVHKASRKEVTL
QPDQRVSDKIAKREVLEKVGVTVPMQAAH
>g2373
MSSKDYAIDADMHRDPAPSTNHLERLTTAGGHTNDRSQIPLPVVHRTFANPSPLGLISFAADIFLI
SVYGLHARGIQSPNAMIGMLVFFGGTCQFIAGIMEFITGNTVLSFRFWVV
>g2374
MPFNYCAYIDPSREHRIGHLDLNTEQIQPLSFVSGTRLSNLYEVIEAGEKNIAASQENSIPLSDVK
LLPPISGRDILAVGKNYVEHAKEFNSSGFDASDKNDQPTLPVIFTKRATSIVAHGEPVLLHPGFTE
TPDYEGEIGVIIGKAGHKISEADAMEYVWGYTIINDFTARERQRDHKQFFIGKSPDTYCPIGPVAV
PKEYLPANLRVQTFVNGEERQDATIDQLIFSVPHLIACLSQAQTLQPGDTIATGTPYGVGFGFRPM
KFLKAGDEVKVSVTGLGTLKNRIASLDATNFTVDRVKMQSSIPVFNQRARSHNGLVKIGNKELFYQ
FKGQTDGPQIIFVHGLGGSSTYFSPLYEKLQATHGLHLIDLEGHGMSPTSALSNLTIESFASDIRE
VYKRARPDSKPATVIAHSMGCLIALKFALKNTSLISSLVLMGPPPTPLPQAGSTGSFARAETVRSR
GMFAVVDAIVNAGLSSKTKASNSLAVTAVRLSLLGQDPEGYAKACMALARSAEETLDVAQLPAECK
TLIITGTEDAVSPPTVCSTYGQSIKSSKVNVLDDVAHWHLFEDVKGVSEAIYSFLGVA
>g2375
MGGDSDLLNAMTNSPPAPGFPFFSEQQIPVIDELLDMLPPLPHLEELKSAFFVVFSPLFHILHDPT
FHLQYQNFRTNPKSTPLSFLALLFVVLSLSVTALDDGHPLLSDLGHDADPGTNVRNLSKKYRLAAM
RCLVADNFMFCHNLRTVQCLVLLIYALNHAQGPAWSLLGTTLHIAISIGCAVDPAGLDVDRVEAEE
RRRCWAGLRMLYTIQNTCLGNLMPFRMDAQVALPLDVEDDHVFFSDHAAVLPAMERPYDQPPTKMT
YLLYKFRLYDLAFDICQLSSNPLEAHDRHRMETLDQKIIHESQCHIERFASSPIHTLELHHQAHFH
ILTIYTCHLTLLLHRPWLNDATNTGSGEISSSAQRCEIAAETIISSFERLCSTDTQFTRYRWYVDG
LGSFYAFFAITTLLVLHSGGTTGPRGGSRTSPSLEMIPRCVHFFVSNASRSDVCSKAAAILEPIMS
MTIQQPTMTDREAGRIHHDIGQLDESLGDDAVWGSFPELEDLFYGVPPEQWLMPSVALWNASPASR
NDNNLALCQRA
>g2376
MSPLQAVFTTKAPKPLPQFSQAVKFGEIVYCSGNIGLNPESWTLVKGGVREETKQALLNLSAILEE
AGSSINNIIKMNIFLTTMDDFALMNEAFDEVVKSETKPCRTCVAVYQLPLSAAVEIECSAFLNT
>g2377
MEIPRVKSTTRFGASVLKHLQTFFGIVLANVAFMHAKIPYLREIKYNFITFHYVYLITWTFIGSIV
LSASGSIPYIDALFFSSGAATQSGLNTIDVNLLPTYVQVFLYIITMWCNPIVIHTFVVFVRLYWFE
KRFQHIVRDARALRRTRTRTFTTGREQQDANREEQGIRGQSIVVLRNEQGEARDGVEDSHKAETDA
NSETGSSNSTSNDRRGPEESPQVGPPSPLTDTEGMRLPTQRSPEHHIAFLEHQRTDKGTLRIPSPR
EFDRGGVPQALDEAIENNPTSPKSPPVEDEQRQEGGNELGNHITINEPDIDRFRARTGPTATFPRM

ASQRTTFRDEGDNEPTRVRTRRGTFNGIFRSMTQEQDKDTLPYLSWNATVGRNSAFVALTEEQREE
LGGIEYRALKTLALVLVGYFVGFHVFGIISLTPWILLSKTYGPVVTSIGQGRAWWGVFTSQSAFND
LGFTLTPDSMNSFNEAVWPLLVMSFLIVIGNTGFPCMLRLVIWIISKFTRNGTALWEELRFLLDHP
RRCFTLLFPRNATWWLFAILVVLNGVDLIFFIILDLNDSVITAIPAGYRVLDGLFQAFSTRTAGFG
VVNISALHPAIQVSYTIMMYISVFPIAISMRRTNVYEERSLGIYGSAEEEEEDVKEPSYIGAHLRR
QLSFDLWYIFLGLFIIAIVEADRLESSADSYAFSLFAVLFEIVSAYGTVGLSLGYPNTNASFSAQF
RVISKLVIIAMQIRGRHRGLPYALDRAILLPSESLQQKEQDDAERRLRRRESNVSNMTGAFGQSSG
IYRTSTYNSMGSRARSRPRRSVDMRD
>g2378
MRDDAKDNPVSSSNSHLDTSFGDDPSSRHMHGDDSWKFRAPYQIQKDDEFGPVKWEAMCHCGNVQY
QIKQERPLAAKYCHCRACQVLHAAPFQWCAIFRKTDIRFKKGTDSLMFYSSHEKTALASTALAVTS
ISDDEMTTYLNDGAVDLAYNYAPMWFFGQAMDEAPCYPVWAFGGNVSTPDIYDPADHTPLAPQCEY
PDVGCGCRQPDVPINNPGPAFPIYYTFEQCNATEVRVVYNLFYQKDGAEVADVIDTGHDYDWERVI
IIHSKDTASNTWAPSRALLSAHSGYHDLAWGDIQNTLTTDQVNAGDAINPNGVQNNDHPKVYVSWS
KHANFDTRNTGWNDPISQSTDNAFRSEDWWHFVDAQYYIRSDNSXAAGQALGSVDWGSASSNPPSV
QATLCTQQALIAQAVKNS
>g2379
MASEPEKNKSGGTESQGDSSLDADIHRIHSQIELLLEGLRIDGNSSASHSIVQAIQDGESLESVLS
IAMELVTQDGKKKKNQKP
>g2380
MSAKQRFPKPLVVGPLKQPLKQTLIWLHGRGSSGPEFGPDLLTHAINPNGSDTLVTRFPHAHFVFP
SAPLSRAHVYNRSLTHQWFNNWKLDPPAQEREHLQVPGLQETTKYLHTLIAREIEMVPGGAGSVIL
GGLSQGCAAALVAALLWEGDDLGAVVGMCGWLPFAGRMQDVASTDEDDLFERDDNAEDKAQDAFNT
AVEWLREELDIPARSKSDMLKRTPVFLGHGVEDDGVDVWLGRKAASCLKDLGVDVCWKEYEQLAHW
YSENMLGDLDAFLQSRSVAQ
>g2381
MDVHIPGHEGIGTIVKLGSDVGNERALGQRVGIKWIHETCGDCEICERDSTACPNQHNSGRDRPGT
LQEYVTVPAKHASPIPDGISGHIAAPLLCAGLTIFSAVNKTNIREGDFLIITGAGGGLGHIGVQIA
NRRGFKVIAIDAESKRQFCIDNGAQHFIPFTHPRIEDEMQKITGCGGAHAIICCAGAETGYNQAPR
LLRRGGTMVCVGLPANTAYKIPIGPFDMVVQGLRIVGTSVGTEAELQELLALAEKCDILPVTHVLP
LEKFEEAIEAVKTSAISGKMVLRMAS
>g2382
MEEALPDTILMEHIPGTEIMRDVGKIHFVHGPKSHQVLIPYPSENADDPLNWTLTWKYITIGVQII
YTWVTVASALSMAPMFPLLQEEWNLSQTQLNLITGSCVLALGYANFIIVPISNIFGRRFTCIIMAM
LGAGSCIWEARAGSYSILIGARIVNGIATATSETLMVQVICDVFFLHERGVWMGVYFCTYFLGLFV
GPIISGAIASKHGWRSFFWLSMALSLLNIITLIIFFPETRYHRTNQLPTVVATPTVSPEEGLDKPP
SPHEIEYSASSNVIGTGRPTKSQFQLIARPDARWKSLIFKDIIFSGKAFFFPIIIWAGITVGGYAN
LLLYWNITESSILGAPPFNFSVSAVGYSNFAFMVGGLVGLATGGTVSDWIAQRATIRNNGVREAEM
RLPALIPYFFLTIIAVTIGGLAEKNSWSWPVLVVVGYGISGMCITATPAIAVAYAVDCYKPIAGEI
MVVATVIKNTCGFGMSYWVPQLTTSQGIIVPTMIQLALTAGPLLLALPLWLWGQKLRRLTRNSGVH
KLEEEF
>g2383
MNSKSISPPLKVIIIGAGIAGLSAAIALGKQGHDVVILEKSRFAKETGAAIHLPPNCTALLTWMDI
NPEDFGGTLLREMHRYRHEGDLKYIKDFAEMRKAWQSEWYLVHRVDLHNHLKGVALETTVIHTGCK
IVDVNTEDRRVILDDGREFTADLLLGADGAHSTFRSHIAPGSANPYPTGKACFRWLIPVQSLAQSE
YTREFVQKPGVFIEWAADDRRLVAYPCSDNTVFNLAGFVPTAEAGVQDMSEEKGWQITGSKDALAQ
SFAQFSPRVRELINLAEDNLKVWQLYDMGSLTTWVKGRAALIGDAAHPFQPYMGQGGAMAIEDAVS
IAILLPRDTTIDDIPARLRLYELARRSRVEKVLEYTRMNGRDENDLTGPRMSPADMVQFLRMCFAH
NEIENSGDLLARYRAGQLSI
>g2384
MEASEKVEPTCHVELSTAGLEGVDAERQAMSIEFASRDTEWHKRATRKLLMKIDLHLLPWVVLIAL
AQAKLGTLTKDLGLKGTEYNTITSILFIGYILFQLPSNLLLTRVRPALYLCGMMALWGTISALQST

TKSYVGELLCRLFLGAAEAPFFSGCIFLMSSWYRADELAHRIGILYAGVALANMFGGLIAAGVLAD
LNGAHGLAGWRWLFIIEGSATVGIALIAMWIMPNYPSTTRWLNIEEKHYAEWRLAQDAAGEIDDRY
AITPMQAVRMAFKDWKLYLFMIMHHLNLLSQSFTYFFPSIVKSLGYNNTTTLLLTVPVWFATFIVV
LLVSYHSSRTKERSIHIACCMLVGAIGNIIMITTNNLGARMFAMYLLPIGVLPPFQLILAWITSTF
ARPLAKRSVVVAICGMFGNASSIYGSYLYPDSQAPKYVVAGVVLACTCCGCAVMAIIIRFVLKMEN
KKLDEQEERNGVTVRGFRSANALVLPDNVGRLPALGWNSWNAYGCDVNETKIVTAATQLNTSGLQA
LGYKYVNIDDCWSVKSGRDNVTNRIIPNPDTFPNGINGTAQQVHDLGLKIGIYSSAGYETCAGYPA
SLGYETTDSQTFAEWGIDYLKYDNCNYPSQWDDEYNACIPDSDYPGVNPNGTCPGLADPAPAGYDW
STSNTTKRFNIMRDALVSVQDQRVILYSLCEWGSADVPSWGNGTGNSWRVTGDINATWDRITAIAN
MNAHELSSVDFWGHNDPDMLEVGNGNLTIEENRAHFALWAIMKSPLIIGTDLSTLPETHLSILKNA
DLIAFNQDPVIGKPALPYKQGYNNGTYNPHYPPEYWSGATSHGWNLVLLFNSEDVAASRTAVWSEI
PQLGKHANRYRVEDVWTGKDLGCVHKEYTVQLEAHDVAVLKVTGTC
>g2385
MAEGIDRRADERMEFTTSKEVTVAPTFEDMHLKENLLRGIYAYGYESPSAVQSRAIVQICKGRDTI
AQAQSGTGKTATFSISILQVIDTVVRETQALVLSPTRELATQIQSVVMALGDYMNVQCHACIGGTN
VGEDIRKLEYGQHVVSGTPGRVADMIRRRHLRTRNIKMLVLDEADELLNRGFREQIYDVYRYLPPA
TQVVVVSATLPYDVLDMTTKFMTDPVRILVKRDELTLEGLKQYFIAVEKEEWKFDTLCDLYDTLTI
TQAVIFCNTRRKVDWLTDKMREANFTVSSMHGEMPQKERDSIMQDFRQGNSRVLISTDVWARGIDV
QQVSLVINYDLPSNRENYIHRIGRSGRFGRKGVAINFVTSDDVRILRDIELYYSTQIDEMPMNVAD
LLS
>g2386
MVLEDQRFIHEDLERLEQGIADRVAEEPRNIRERLARDHQIAGFLDRIEEQSKRLQDIYKDAEGLR
AQEIQSISTGDAFEEFYKRLDEIKDFHKRYPNEPVENLERAYKRRHPGEGESFAPEIDSMFTGEEA
YGQFLDLIQLHEDYLNIPGVKRLTYIQYIDQFDAFTPPQLPIKRTAKVSDRYFKYVGDLSSYLEGF
LKRVRPLENLPKLFASYDQEFDKLWAAQEVPGWGAEAAMTESVGPKTEGTGEGIWCADCEKEFKNE
NVYKNHLTGKKHIKAAEAKKANGNATPNGASLAGAIKDSSLKSLKERAVAEREHRVRCLTKTLQSE
REATRVNVERKQGMTERERQMEIEALMADTELSSGAFGREEESDEEGEDRIYNPLKLPLAWDGKPI
PYWLYKLHGLGVEYPCEICGNFVYMGRRAFDKHFSEARHIYGLKCLGITQQTNLFREIVRIEDAMR
LWEKLEQERKKEKESKDNVVQMEDAEGNVMPERIYYDLQKQGIL
>g2387
MGFKFSYVCDLLSGLERNRYVKATSAAKAQNPDYRTISSWCLNYSKRLHDKDTDLLAVLSCLFPEK
RPDRVYWLQHASLARIIGRCLYLGASRLKDLSQWQVSGNGDLGECVENVMSQAENQILPGQEVTVE
EIDNVLQEVASRCQFSGPAVRRKYAAVDVEEVLGSLYRRLSSRDAKWLTRMILKSYHPVQLPADYL
LTKVHFLLPHLLLFQNSLEAAVELLRSPPLKLFPPQPQSHFANLLSDKALEHFVPRIGIKIGRPEY
YKARSIKHCSNMIRQRRMSLERKYDGEYCQIHIDLTKRQDCVQIFSKSGRDSTLDRVGIHDVIKRS
LAIGEPHCRVSRQCILEGELLVWDDRDGKILEFHKLRKFLSRSGMFIGADLDSQLRRLLLKETIKI
SPGRAEIAEQEIIDFSRADAVRRLQLALSRSIAERWEGYVIKACDEPYFSVLSSQRSDGFSRWIKL
KKDYIPGLGDTADFALIGARYDAKDASSLSSMHQHSWTHFFIGCLDNKEDVVRLGALPRFRIIDMI
GTHSLSVNDLHVLNQWGKFVACDVDSNTAFECYSIHNSLPNMDIAFKTPFVVELLGSGFDKPSNAQ
YYTLRFPRVLKIHWDRSFEDAISFIELQELAQKARAVPTHELEEENAVWVEKVAGTSRYHEYIVDK
SQSTPTSSLTGNSPLSRSASLVSPRLTPPSVKQAPASLIRDVRPFSQSPTTNSFNVRKRTISSVYP
TSNLDFAAKLDLTSSNLNFTSPIFNTISSNLASSSCLYHLKISAPTSSTIMENSATMDLDQAPQQF
LRAPGSPFPGAWTSNVGGPASTDSPSTESSGCEQNPSGLGSGWTTPLREKLTVPSHQVSTRAAATL
DEALKFIEEAEAKLESSRFRLPDGTKNTAKCVGLLHEKEYDTEIRALTDPLQTHPVSPLSTIPMYC
GESGFQEDTLRQAPREFTFSAKHFVKSLGFQYTRRAVRLSNPAAVKNDMALGIVLVDITKPSTVLA
MQLYDMGNLLATELCQPKPDLQPQGKIFFLDSRILKAGGEAGIESKMFLLSDWWSEFGTHYFYATI
SWRPGTEATPIPVLSGPRRVIDQINEHDTRASSSSSRVAANIVNIYEPSEVELVGEWVSVDPTVHL
YGDRYYAPSGTVTFDFDLGLVRPEKPRCTTHDDQMDPSSAFI
>g2388
MAMTFTEDSIKELRLRLEDAVVKCSERCLYQSAKWAAEMLNSLVSTDGNDTDAESPMETDPQPNVN
PFSPQSDPTEATLELQEAHKYLLAKSYFDTREYDRCAAVFLPPTIPPVPLSTVSPNVKSRTSLTPQ
KGKRKSFVRPGSKAGQALPRNPYPILSQKSLFLALYAKYLAGEKRRDEETEMVLGPADGGMTVNRE

LPDLARGLEGWFAERRDRGLQDRGQGWLEYLYAVILIKGKNEEEAKKWLVRSVHLFPFHWGAWQEL
NDLLPSVDDLKQVAETLPQNIMSFIFQVHCSQELYQATDETHQTLNGLESIFPTSAFLKTERALLY
YHSRDFEDASAIFADILIDSPHRLDSLDHYSNILYVMGARPQLAFVAQLATATDKFRPETCCVVGN
YYSLKSEHEKAVMYFRRALTLDRNFLSAWTLMGHEYIEMKNTHAAIESYRRAVDVNRKDYRAWYGL
GQAYEVLDMCFYALYYYQRTAALKPYDPKMWQAVGTCYAKMNKIPQSIKAMKRALVAGAYYEQSAD
AASADHPTAGRKILDPDLLHQIALLHERSDNEDEAAAYMELTLQQESGEIERTETDSEDDDGDENS
DDDTTTQPRSRRRRRQKSSRDDDNEVEAVGGTGVTATTSKARLWLARWALKHGDLNRADQLAGELC
QDGIEVEEEAKALMRDVRARREGGGGG
>g2389
MIYHNPIVPGFAPDPSVVLVDGTFYLVNSSFHLFPGLPVYASRNLKDWVHIGNAINRVDQLSLTRA
GVDGFPIGGGRRIVAATGLFAATIRHHKEIFYIICTNVYKQGDRLNTSNFYITTLDIWSDQWSDPV
YFDFEGIDPSLYFDEDDHVYIQGSWHAGPLGNPMCSIRQFEVEISTGRPLSEIKTIWTGHLSQGDV
EGPHIYKKDGYYYLFVAEGGTWQHHCITIARSTNIWGPYESYAANPILTXDGKDEYIQHTGHGDLF
QDTHGNWWAVHLGIRNGEGGRAPLGRETFLTPVNWPKGGWPEIQQTSTSFESVEVGSSQGCTGPDI
SKSGTPVEYLHLRSPRLSDYTFLAGGSKISIVARGNDLSTRKGTASFLAQRQRNLTCNPTATLHLQ
RDRQDSVQTLLKAGFSLFKDDVQHVDIYYDFAKGIVELTHHDKIRHTTCNPVVVSQHPVSKDADQI
IFEIRATPNDYTFLFRGVTAGNKNLPGSTTEWVCAGSVDSLHMTALDFTGPSFGIFASTNIQDPEG
DDDVLDRKFRDVIFEDFAVY
>g2390
MRLFKKYCLPSHTEKSAGKKNKEKPPFRASSSFSSNSSKSTSRNSPLSPSHSASSYDPTKRLPPQV
LNRIFSYVCPHALDESYNSSEESITEDGCMLCDMRDLACCALVSKKWSTPAQKLLYRNVRIDAVHY
CELEIELSAKRKRRRSFVDQNGKRLDVPQSRLHLFMRTVRDSRQLAETVLSLRLPYMTREASKSDL
ARTISVLHSLRYVDLPVGFFTDDPSSYALKQELIANCPDVRRMTYAHGSEMSFSQIVGSSLWKNLE
SLELSNLQVEESTLRQVLSSFPALRNLKMENMPSIGDSLFTPNQYLPTFPPIAKLTVRNISTITAG
GLADHLSAPQTQHALKELVLVQCGVRPESLHTVFSRAPRLANLTLELEVDKSFLAQETPLLASRSL
KTIHYEITCSGSPTGPQPVTKSYYDYLMSSLKANSLPSLRNIYVLDSSFADLLLLVPPPRLFGGGE
NGLQSVGPGLQQPLTLYTKGMDEFEWNVTEFEPVSSGRRSSTTRPVSFHSAQLSPRWGGDARESVL
VGNGFGGYLAVPVDEGRPMSSSGVRGNDKRDSKYDLWR
>g2391
MLWSVPSGADPDFENAAAAAKNVLGYNEPDLGSQANTIPSVAAAGYQTYMQPLAGKTRIGAPAVTN
AGNGVLAYMGLGWLEYFFDDCQGCHVDFIPLHWYANDTAANFEAYLTQAYDRFQKPIWVTEFQLQD
SDANQVAFLQEVLPWMDQQSWIERYAYFGPFETFLINGAGNGLSDVGKAYVSI
>g2392
MFSQILKTVAVLAVPTAAQQGYYFVLGSGMPLTIERLDPLLSPGKALSQHVHSVVGGNGFASTMDF
AQTQTSTCSTVQPIADKSNYWMPNLYFQDPSNGSFIRVPEQPYHKIYYKYGTGANTYDTDIQEFPE
EFRMIAGDATLRAENATSMGPYGSELGWFCHGNGTSEYPDISTVGFPTGFIGCPNGLAAAITMPSC
WNGQPFNVSNPSAHMAYPVADYIDGCPETHRVARFPQIFVEYWLNINSFDGLYTAEDSPFMLAMGD
KTGFGFHVDFINGWEPGVLNATMPTCTPGNTGVPPLSDPTCFGNRGGIFTDEQMAACMMQPTTGED
SGWTYPDLDNPVGGVLTYGGVLSALPGCQTITTGPDRAVLTTCESTVPTVNG
>g2393
MASLFPDQEKGQDVGVNTSDRDSSDEEFVKDGDIAYKVAEGANTSIATYQDARGAPVEKQSPLGYS
VNSWVSLCLNVNQMIGTGIFSTPATILNGVGSVGLTMIYWLIGFLLSQSTLSCYLELSSYFPSRSG
SDVVYLEQAYPKPLYFFPTVFAIKHVIFSFSSSNAIVFAQYMFKLAGYTYTAWELKAVAVGAYTLA
TLVVISSTRWSLRVVLGFGFIKLATLLLISIAGLVVLGGHTRVADPNINWRDAWRGTSSATAYGAT
NAMVKIIFSYAGYTNAFGLVNEMKNPTKTLRWSAPSSLLVVTILYILVNVAYFSVASREAILSSKQ
IAAAVFFQNVFGTGGAARALNVLICMSAFGNLVAVLINSSRLLRETGRQGVLPYPKFWTYTKPFGT
PLGPYVFVWGTTMIMILAPPAGDAFNFVVDLSVYPSNIFNLLLVVGVLFIRRRRRHAGLPRPQYRA
WDIAIAFATLTNTYMLIAPWYPPLKGATGGDVSFWYGTYLVVGIGLLLLCGVYYYVWIKVLPRVKG
YELRQTVIEYENKSIVHHLVKVPKADVARWDEEHDAEGKLRRRAAHAPGSTDSQ
>g2394
MKAFYSFPLIFLPTLVTAIPSDLAAVELDKKACIPFKNPLTSVSGCCDTVSAELFWVDILLGIGVC
CPVGQVLDGFNCVAPTFTGKPVCANKTGTDIGIQYGHCYVLLSLNNNYLGHDSAEKYVVMGENPGV

VFRVCGDTTTCTTSADQYVGINGTWWMQDQFGDPNGSGFAWLGGSGDLSIVNSTDLALIMGGSPTF
FGGSCAICISFPPGGAHAPCPLSPGQSHLGIAANPNSCQNFYWQEVPCRSEK
>g2395
MATRITRPEFEAFIHPLIQDILAATEKYNLPENAKKWFSDSLNANVVGGKMNRGMSVPDTGSILLG
RPLTEKELEHLSILGWLTELLQAFFLVSDDIMDGSITRRGQPCWYRQEGVGMIAINDAFMLESAIY
VILKKHFRSHPAYADFLDIFHETTWQTELGQLCDLITAPEDNVDLNNFSMEKYTFIVKYKTAYYSF
YLPVALALYYIQVASPKNLKQCEDILIPLGEYFQIQDDYLDAFGSPETIGKIGTDIQDNKCSWLVN
QALTMVTPEQRKLLDEAYGRKDSALEAKVKALYNDLKLEQHYLKFEEERVGHLRQQIDSVDESEGL
KKEIFSAFLDKIYKRSK
>g2396
MASSNNTMKAVVIHTQGGPEVLKVETRPIPTASPGQVLIRVKAFGLNRSEMFTRQGHSPGVEFPRV
LGIEATGIVEHAPGGEFEKGQIVMTAMGGMGRQFDGGYAQYTVVPTNQVQAITVPTKLGWDVLGAM
PEMLNTAYGAIIKSLEVKKGDRLLIRGGTTSVGLAALAIAKGLGAHVASTSRRPEREQMLRDYGAD
EFFVDDGAIAAQIKDDSQKFNKVLELVGVVTLKDSFQCTSKGGIVSMTGIVGNKWSIENFQAMEYI
PKYRYLTTFGGWIEEFMSTPLNDIVQQVEAGTFKIKVGRVFHIDQIAEAHRCMDDNAAEGKIVVLT
D
>g2397
MASTNGSGTPRRDTRNHFLFEIATEVANRVGGIYSVLKSKAPVTTAEYGERYTLIGPWNRASAAVE
VEAMEPTNQAMIDTMASMKERGIEMIYGRWLIEGAPRVLLIDTGTGYGYLNEWKGDLWNTSGIPSP
EGDHETNEAIVFGYLVAWFLGEYIAHESKRAVIAHFHEWLAGVALPLTKKRHMDLTTIFTTHATLL
GRYLCAGSVDFYNNLQYFDVDAEAGKRGIYHRYCIERAAAHSADVFTTVSHITAFESEHLLKRKPD
GVLPNGLNVKKFSAMHEFQNLHSQAKEKIHDFVRGHFYGHMDFDLENTLYIFTAGRYEYRNKGVDM
FVESLARLNHRLKTSGSKTTVVAFIIMPAQTSSLTVEALKGQAVVKSLRDTLEMVEKGIGRRLFER
CLSWKEGDSLPDEKDLITSQDRVLLRRRLFALKRHTLPPIVTHNMVNDSEDPVLNQLRRVQLFNHP
SDRVKVVFHPEFLNSANPVLPLDYDDFVRGTHMGIFPSYYEPWGYTPAECTVMGVPSITTNLSGFG
CYMEELIENSSDYGIHIVDRRAKGVDESVNQLCDFMFEFASKSRRQRINQRNRTERLSDLLDWKRM
GLEYVKARQLALRRAYPTSFDTEEDFSDYIGSTAQKISRPLSVPGSPRDRTGMMTPGDFASLQEGR
EGLSTEDYIAWKLPEEEEPDEFPYPLTLRTKKPGDRPQSPLDSISLNGK
>g2398
MPRGADYDNGIPQSDNAIEAGPDKVHGTNPGSDVDRSDKAAALPETMGENKVASGGGSAGHSSGKG
GHEPKTLGEKKGLRA
>g2399
MDRRNPYTLSVLPPSHPENEDESRVVIQGRLREFILAFQIDNSFIYRDQLRQNVLIKRYLCDVDIA
HLISYNGELAHKLTTEPADIIPLFENALKQCTQHIVYPSQRDIELPPHQLLLHSSASHTSIRDLSA
TNLSRLVRIPGIVISASTISSKATVVNIQCRNCEHQDNISVDGGFAGLTLPRRCGRKTQPGEQQSE
PCPLDPYVIVHEKCRFVDQQLIKLQEAPDQVPVGELPRHVQISADRYLANRVVPGSRCTVMGIFSV
YQSKGGAKAAAVALRNPYVRAVGITSDIDHTSKGAATFTEEEEQEFLEMSRRPDLYEAFARSIAPS
IYGNLDIKKAIACLLMGGSKKILPDGIKLRGDINVLMLGDPGTAKSQLLKFVEKASPIAIYTSGKG
SSAAGLTASVQRDATTREFYLEGGAMVLADGGVVCIDEFDKMRDEDRVAIHEAMEQQTISIAKAGI
TTILNSRTSVLAAANPIFGRYDDLKTPGENIDFQTTILSRFDMIFIVRDDHDRARDERIARHVMGI
HMGGKGVDEHTEAEIPLEKMKRYISYCKTRCAPQLSEEAADKLSSHFVSIRKQVHRAELDANARSS
IPITVRQLEAIIRITESLAKLTLSPVATTAHVDEAIRLFLASTMDAVTQGENQGSKELMEEVSKVE
DEIKRRLPIGSTTSLATLRREFVDGRGYSEQALNRALLVMQRRDTIQIRFGGFGRLPSNDMASSPR
KSIDSLASVASTPSLQQLAQNTYDSPRIAPSQRIPQRRSSTASSLVSIGGILDSSTHRSSIAELGQ
NAISTLLQPPIVRTGLVAHTSIPATGYKPPTARDIPPVTLTNVPNVDSKAFQPYLEQVGSLYDAFQ
RAKEESGNDGNASNLVRNNVTLSPSSALSPRPDEFDGRRTPVLERRLSGVSTSSRAISPFDMRGRR
RTSYGKGPQAVTPLSTIPIVYFEEDFHLENPRTFDVVSEKSEIVRQPPKPTASNGAAIEPATIGRK
ALATNAILQEKLSWYMDTVEIHLISSISTASKSFFTALGSLRELHAEAADSVRRIQILRKDLNKID
KDMAMGGLKVVKLRQRRQNIRKLADAMSQLHEVVEAVTECENIIEQGDIEDAIDGLDEVNLLLSGE
QATRRRIQGVAEQRPPVDLRGLHALQGVDEELGRLRQRIGVRYENRFLDDLLTDLRRHISSVPQDA
TLQRWGASFQRNRGGHRAAPSISPAYMSFDAEFRSKLHAELVGLGRSHYTMPAATSFKTAVLREMK
ALIRKHLPSSSDDDNESVMSASTHGGRQLSSQEKSSILARNLRALDADSAYDMLKQIYTGVSESLR

RLGVQVKVLLDITSGLGAQPSPSVGRSSPRSPDPQNARGPRSQGSAAEVAAVQEEILQVLDMSSLL
GQAVDIVQSQVVKVLKVRSEQTSHLPIEDFLRYFSLNRLFADECEAISGRSGTSLKTVVDNQIRDF
IVRFSDNQRHQIVEVMDADKWDARDFGDAENAVLDRILRASTADVNLWLQVSKIWQPAEESPAPTS
KTENGAAVNGEAKEKEKVRHATFDEQRYVLPESAVTIMKSIEEFEYLMANLPSMTQDIGSNLVECL
KLFNSRSSQLILGAGATRSAGLKNINTKHLALSSQALSFIIALIPYVREFARRQSPSSQLMGEFDK
VKRLCQEHQNGIHEKLVDIMGGRSASHVNSMKKIDWDAEREPKAVSTYMETLTKETATLHRVLVRH
FPEMTVIMIMEPVFKSYRDQWTKAFQEVNVKTESGRQRMLSDVEFFKSKMGKIEGSGDLGDTLIQV
VNAKTILPEKTSAAEPAQTEAENEAPSPPVPEKDDNNQQSKDEKN
>g2400
MAPLIRVIGSLNADMVSVTPRFPEAGETITATSFTTSAGGKGANQAVACGRLSRSKPTETHSSKSN
NPVNVEMVGAVGGRDAHFQSLLGPTLEKSGLDTSRIRTVDDYTGVAVIVVDSSAGGENRILFSPGA
NYTGMQAIPEVLGMGLASPVPDVIVMQGEIPTETTIGILREIAKYKKTQRENGLKGIDAGPEVMFN
PAPAPPGGLPEDVYSAIDHLIMNETEAELMAPKTEKLLHVVPGAKGASLREQVAQYFHKLGVTYVI
ITLGAKGVWYSACDAGIAIAADETTRFTNEIPAAKVSLVLDTTAAGDTFIGGYATHVARWREQRRA
QGKSINELEQHEKKERYQVVMDEAMKRAGRASARCVERQGAMDSIPWEDEI
>g2401
MSAIAVSVAPAVHRQKLVPMARSRLRVLQEAKRNVQNLANGRVPSTPLKHTIFCDSDGENASPYTK
SEPYKRKRSVGDDDQLCESPKSLKASRTSFVIKTDDKSDKAFTSTAVSQALGRPIGRDSQVKSKPS
KAFGRRSSRIPPFIKEIVQKGLNKSKEPRKKTHPNSWLFNIHVDTEVEEATNLMQHSAGRLDISDD
EGKSRFDECDKENIPPSELGITLPTPAQPVAMASRKNMMAESRSPLGELVASDYYGPGLSDLSYEI
VQEEEYIKMSSAHLGYRIISLPSDADSMAKRATKICVDK
>g2402
MTENQNEPQYAPWYRLPTRHIVAVEHPGIVKNVDRAIKTLQGDVGISKAKDPMANTIDSFNRQTNN
ILLKVTVPKWTGRKRKRGSDVSFTDSHSTTPEEIEKERQSAAFRQRSLMDNAHKYSVEAVGTVTRT
HNFRKIPDYVYSTSASPFVQRFRETILSSDYEKMRQFDISMSRGETKNVDLVPPPALTKTFIPFQY
TYRQNPTVRLSQDTSGNVTAINTQKPNKVFTHLVTCDIPEVPSEPRAGIPPISSLDSELQQVIATV
QELFKERLAWTRRALLNAIAGKDQRYVLKYAISYVAYIFRSGPWRDAIVKLGHDPRKDIESRKYQT
FVFRVLPREAEVGRDGGGSARQSSTNATRNFSGLDISSIPTVTRNENPTSHLWTGKAPLALDGKIW
MVCDIVDPILKQILYPSNANNKTFLRSECEIFSDGWYGSGTIAKAKVIMRQKIHALTHEHREPSDA
EFKCLLGFPDHVIEADGMKLERFHLDTERATPRETMLATEVRATIKGAISWRMFGKGSARTTATNT
ASTLAPAEAEDEEEREATRRVIEEEEEVNEEMDVEQNE
>g2403
MKGFRQRVHEQLSRAKDSNKSSKKRDSNNTSNAGLSANHNAQSTSPNHVTPTSSTTSLNETRNTKS
ENVSPAAAGSPAGQNNQGAAAPAGAGQHFVPNAQAIGGNLGGQPGNVPGTPTRQGQQPITPSVVIS
PSAPHVPPPGAAETMPGDLAPPKKSHVFDRLHSTPKDISEGIRTPKRQHSSRFDISDQRQRELEKL
PGFHEVPPNRRQDLFMQKIDQCNIIFDFNDPTADMKSKEIKRLALHELLDYVANNRSVITEPMYPR
VVDMFAKNLFRPIPPPVTPQGEAYDPEEDEPVLEVAWPHIQVVYEFFLRFIESQDFNTNIAKQYID
HQFVLQLLELFDSEDPRERDFLKTTLHRIYGKFLNLRSYIRRSINNVFFQFTYETERFNGIAELLE
ILGSIINGFALPLKEEHKLFLTRVLIPMHKPKGLSMYHPQLAYCIVQFLEKDSVLTEDVVMGLLRY
WPKVNSTKEVMFLNEVEDIFEVMDPTEFAKVQGPLFTQLAKSVASPHFQVAERALYFWNNEYFCNL
VSDNVETILPIMFAPLYENSKGHWNRTIHSMVYNAMKLFMEINPQLFDECSQEYNEHQNGADEREQ
ARRRRWEVLAEQAEKRKQGLITDPVSSTNSISASKADDLDPVTQDSQKRLNALKLDETTTIKERAT
TSVS
>g2404
MFNRNNIPFASRSNPPPDPRRGGYDTPMQDGGIPPSRGGYGAPPPQQRGPPPPSGRRPVNDGATWT
LRSDKSPNTQYTYGNIVAVSTADFPPSAFGQEFYVLINDHYVLSARPLDGYQAGYIGFAERQRMWA
RIAVTDSVKVQIYDPFRQERKAYLGSADVEVGFAGKTRTEEPYDQNELANAVIEKFKNQILAPGQK
VLMDHRGIPLSLTIKTVELVDLMSEKQPQPSTEGVATDPTARGILTPHTIFNFYKDAKSGLNLKAS
DRRPAANSIIRPDFKFENMGIGGLDSEFSTIFRRAFASRIFPPGLIDKLGIQHVKGILLYGPPGTG
KTLIARQIGKMLNAREPKIINGPEVLNKYVGQSEENIRKMFADAEKEYKEKGEESALHIIIFDELD
AVCKQRGSGAAGGTGVGDSVVNQLLSKLDGVEQLNNILLIGMTNRKDMIDDALLRPGRLEVHMEIS
LPDEKGRAQILKIHTQKMKENNVMEPDVNLLELAQLTKNYSGAEISGLVKAATSFAFNRHVKPDTM

AGIKDDVANLKVRRDDFINALDEVKPAFGVAEEQLEDCIQQGITHFSERIKDILNEGNLFVKQVGA
DGGTPLFSVLFHGPPASGKTALAARIAMDSGYPFVRLISPEDMVGMTESAKVSHITKIFSDAYKSR
TSIVVVDNIERLIEWVPIGARFSNAVLQTLMVHMRKTPAHGRRLLILATTTERRLLKDLDMYSSFN
ADIEVPTVNTYDELRHILEGSETFTPQEIEGILGGIYSIDDDKKIGVGIKKILLGIETAKQDIDKA
GRFVNVINRLIDQDRGPAFS
>g2405
MTEAKIDTFVRNLTNEKWEVAKRANVAVELRDTMETFCSGPSYSIFLTKLWPVFKNLLKGDPVFTT
SSFDHKLRNCLLETLHRLPTASPDVEPFAVDMVDTLMQLVRVENEENAVLCMKTIMDLVRNQVKAT
ASRVQPFLELIQEMFQGMEEVVRDTFDTPAQGTTPGMPSTPGAVSQTFQSPRPSSPSTSVSELGSD
QQANHHLQKGMQSFKVLAECPIIVVSIFQAHRTTVNANVKLFVPLIKSILLLQAKPQERAHAEAHA
QGKLFTGVCKEIKNRAAFGEFITAQVKTMSFLAYLLRMYANHLQDFLPTLPGVVVRLLQDCPREKS
SARKELLVAIRHIINFNYRRIFLVTIDQLLDERTLIGDGLTVYETMRPLAYSMLADLIHHVRDHLT
RNQIRKTVEVYTKNLHDEFPGTSFQTMSAKLLLNMAEKISKLEDKQDARYYLIMILDAIGDKFAAM
NYQFDNAVKVSRANKERTDSTPENYLSDRDSPPDWDEIDIFTAVPIKTSNPRDRGGDPVSDNLFLF
KNLINGLKNIFHQLKNCNPTHIQIDPSNTPINWPEVSYGYNAEEVKVIKKLFHEGARVFRYYGVDQ
PAPDINYSSSFEYLASQYTAPMGPQEKELLESFGTVFHCIDTATFHEVFHSEIPYLHELMFEHPAL
IHLPQFFLASEATSPAFSGMVLQYLMDRIQEVGTSDMAKAKILLRMFKLSFMAVTLFSNQNEQVLY
PHVTKIVTKCIELSVTAEEPMNYFLLLRSLFRSIGGGRFELLYKELLPLLEMLLETFNNLLLGARK
PQERDLYVELTLTVPARLSHLLPHLSHLMRPIVVALRADSDLVGQGLRTLELCVDNLTADYLDPIM
APIMDELMTALWDHLRPHPYSHFHSHTTMRILGKLGGRNRKFLNHPPELSFQQFADEVPSFDVRLI
GPNEKRPFPVEIGVDVAYAKLMEIPKTPAAKASDAYYKQQAFRMLSSQLKLYIGYDNLPEDLASLI
RLQADDLLESKIQGPADIFDKSERSSSIPKKLIQEESLKKLLKACFFATSILDLEQTATSFVADVC
RHVVVVEVGRALAQARHTRRPFDVNSGEGPVYLDSRLLANVIVDCLSSDDAKMRDSAKRAMEDIKA
AAGVIFGGADKAAKLPFWQHLGRVFCHSCHSEEWFTKAGGSLGIHLLATELDLGDSWLFERQSDFV
RALMYVIKDTPADLPASTRIRAQATLELILRRCNKGISKDDLKNEKSRLYMLCGFFIYELSHMNKH
VRETSQKSFSTLAEVIGCEAHELMLPVKDRLLQSIFNKPLRALPFPVQIGFIDAITFCLSLHKNIV
VFNEALNRLMLESLALADAEDESLASKPNEFKNADMIVNLRVACLRLLSMAMSFPEFGAGPNASSR
SRVISVFFKSLYSKSPEIIEAANAGLRDVLTQTNKLPKDLLQNGLRPILMNLQDPKRLTVAGLDGL
ARLLTLLTNYFKVEIGQRLLEHMKVIADDATLQKVSFNLVEQNPVMKIVASIFNIFHLLPPAATTF
MESLVNKVIDLEEKLRRTSNSPFRKPLVKYLNRYPKESWAFFQQRFNDERYGRFFGQVLADSDSAV
LRSAVIADTEGFMNAAFGTEPSDEKNTAAINAIYVVHSVCTHEPMKKWLLNQPSLRPKLLSVGRDL
EIKLRNDKLPASERLRVEQAEDQLMDVFTIYLADSLQDLDFLFEVIGGLSADELKRTLAFPKFIYK
HIITNESIDYRRSVIMRCLDLYGQRTSSQRMKTYAFHNLVNPIFAMDVQANWNSPPNIPKLMDKSM
TESIQNRLWRPQVGDITEESNQPGVDHSRMELLQLSALLIKYHSQTVQESRKDIIKFAWNYIRLED
IINKYGAYVLISYFIAYYETPFKIVIQVYVALLRAHQNEGKALVTQALDVLAPIMPTRIAAPNPTS
QQAPDLRYPIWAKWPRRILAEETANLQQVMSIFQFLVRQPELFYDSREHFIPLIVPSLSKIAAPPN
PSSESKKLALNLIGLIWHWEEKRVRASNPGGTASESPNSRKRKLDETSQPESLSLPSKTEYSVPPE
MRAALIKYLITFITTLPERYPVPAFKLRDLTTVKPQPPVVTGDMVKKAMQLLTNLLQPEYWGDLDI
DLYPKVTEPILTGEKAEKPDEKQTTSMINTLQILRVLIASKPDEWIINRLPLIQKLFEKPLRSDNP
EIQDCLHGTDEDANLSPKISPPIKRVLDALPEDQPEEEDGMDMDSSPSEFVTFLSAIATEGLSNNN
YVSSLNVLWTLSSSRPAEIDAHIPQVMKAFSQKLAKEHVAAYATNIPSGQLPGIKQGENGTSDQQE
FEIGVDLILKTIDVISIRMSNLGDQRRPFLSVLAQLVERSQNIKLCSKVLGMVESWIFHSTESWPT
LKEKTAVLHKMLLFETRPDQTMLKKFLNLVIRIYEDNKITRTELTVRLEHAFLIGTRAQDVEMRNR
FITIFDKSLTRTASSRLSYVMTSQNWDTLADSFWLAQAGHLLMGSVDMNTPARLLPDDMTVYPISF
LFSAAEQDPRKADVIVDNQLESLVAECKRFAADIGDVRTRDLLEPLCQLQHTDPKTSYKLWTTLFP
TIWSTLSKDERIDLEKGMVTLLTREYHQRQIDKRPNVVQALLEGAVRSTPRFKVPPHVMKFLSRTY
DAWYTSAVYLEESANKPTIDTPVVRESTLDALVEIFAGLQEDDFFYGTWRRRCKFVETNAALSYEQ
HGMWDKAQQLYENAQIKARSGAMPFSQGEYYLWEDHWLICAQKLQQWEILSDFAKHENLNDLLLEA
AWRNIENWQSDNNREQLDSLIKSVSDAPTPRRTFFQAFMSLLNFHLKKDGIQEFNNICDESIQLSI
RKWHQLPKRITNAHIPILQHFQQLVELHDASVICNSLSQTNERNLDTKSAELKLLLGTWRDRLPNV
WDDINAWQDLVTWRQHIFQLINATYLGLLPPQTNNVASNSYAYRGYHETAWIINRFAHVARKHQMP
DVCINQLSRIYTLPNIEIQEAFLKLREQAKCHYQNPKELSSGLDVINNTNLNYFGAQQKAEFYTLK

GMFLAKLNNVEEANDSFGVALYYDLKLAKAWAEWGQFNDQRFKMDPTDYELAGNAVSCYLEAAGLY
KNAKSRKLLSRILWLLSLDNEEGRVSSAFENFKGDTPVWYWITFIPQLLTSLSRREARLCKAVLVK
IAKLYPQALFFLLRTNREDMISIKKTQEAKQEKLNRAKQQASPNTKVEAARPGTAAGEENPQSSPA
VANTATPQNAAAQPNGQPHAQASAPTQSPAQQQTPAQGQVQTQPQQQNQAQGQAPAQNSAQPANPG
EAEKEPLKKPWEYSEEIMAGLKTAFPLLALSMETMVDQIQKNFKCPPDEDAHRLIVALLNDGLAYV
GRTPVAYAQDFKLPPATEANITXFAETILPAHIRKSFEADFVVKKITMFEYVQKLRRWRDKFEEKL
DRRPQSQSLEVYSPHLSEFRFLKFEEVEVPGQYLLHKDKNQDFVRIDRFLPDVDLVRGIGVCHRRL
KIRGLDGSIHPFAVQHPAARHCRREERILQLFRIFNGVLAKRKESRRRNLYFHLPLMVPVAPHIRL
VRDDPSYISMQGIYEDYCRREGINKDEPVLFTMEKMRGLAEMKQSVSFKSELYKNLNSDISKRSME
QQHVLRTEILTAIQEKWVPNTVMLDYFQKTYPNYADFWLFRRQFSYQYATICFMTYVMHIGNRYPN
KISISRATGDIWGSELIPSINPTKAFFFNPEQVPFRLTPNIQTLMGPIATEGVFACALMAIARCLT
EPRHELEQQLSLFVREEMIFWATAHHRGNVSENQLRELVQSNSAIIVNRAISLASPPEGNLPANQT
TIDLISKAVNPQSLAAADALWMPYL
>g2406
MSERKVLTKYYPPDFDPSAITRTKKVPGAKQKLLTVRLMAPFSMRCTSCGEYIYKGRKFNARKETT
EEKYLSITIYRFYIRCTRCSGEITFKTDPKNMDYVCERGAKRNFEPWRNAQADNLNETEEETLDRL
EREENEELEQLERDKMAELEEKMLDSKREMAVADALDEIRTRNARIQRGEALGEDVALSYVKDDAE
EARLQAEKEDAEVARRAFMTSTGEKVKRIVEDEEETTAARKTEEAPAPAVTFTKARKPKKPFNAGL
GIKKKPALV
>g2407
MEKFLRGWRQDALNRGQNDSAIYIGDKVLALTNSDADAFWLAQVHFNNNNFTRALALLSRKDLVSR
STSCKYLAAHCYIKQNKFEAALNILGDQNPTHLITTAGHSRRKIQHLNGHSHVTLRNGKTAASRSD
RADRSEERDREDANNIRFEAAMCYLRGLCYAKQNAFDRARDCYKDAVRIDIQCFEAFDQLMKNSLM
SPAEELEFLESLDFDSVTVPSEPSKSQEAAHFVKMLYTTRLSKYTSPATLSDATETLSTHYNLATN
PDILLSRAEALYTQCRFLEALELTSSILTSPETAGAMTESSVIPGQNLGHSPAVYPLHLACLYETG
AVNALFLLSHTLADNAPEEPYTYLAIGVYYLSVAKIAEARRFFSKASLLDPHSAPAWIGFAHTFAA
EGEHDQAIAAYSTAARLFQGSHLPQLFLGMQHLALNNMSLAHEYLCAAYSMSSGSPSGSLTTIPKG
PSSGLPALGGDPLVLNELGVVLYHQGNFKPAAELFHQALALANSLQCDPGAWVATRANLGHAYRRM
GELNDALAQFDECIRVGAGGGATGYSAAIGGTSATSAGVAGYEDRGLIGSLHTSRGLVYLQLGRTL
DAVTVLHEAVRVLGVSGGDAAGGAGTAGTILARALELWSMENRIKAIKTANSGRQRRDAASRSRDD
AYGSNLTEEIIHAEDDVETELDAHAEGLLGTALNKVRPRRAGKKPQRSAGEMKRNFLVDAVRRDEY
NLGVLMVLD
>g2408
MASYYSRPDSFSPGVGRGLNTAQNPSISDLPQAQIDAIIRTKRKAREPKACYPCHTRKVKCDRNLP
CDGCVKRDHADLCSYERPSKKRQANTAYLPTVINDDLPESGIPSVESSGVQVKSETVDAEFDSGGR
ISVPREHWNNVCNKLKEMEQAISTLRNGLEQIDGGRASVAMAPGSVQSVDANNSSAXTSPEREGIH
TTHTYGEGTVHLGSRSVLAYILNKSGSLQAQALLEGGILPKLGLDNETATYPFVDLWSSDTSAFDV
NAVCGALPDDQQILEFFHYYRDIAGTLYPVLVDVPGFEKNLHTFLQNRNAAGGILKQGEDRGLSQR
PFGMNLAFIGLLFAVLASGCQSSDLPSRERELTSQVYVCCAYQCLRMCNFVSQPTLEAMKTLLIIG
NVLSYNMNPGVSYVLLGMTMRMGLALGLHVESNRFDPVERYTRQHIWWSMAWQDSHFSLSYDRPST
VAVSQPDISDRPESKPGNRDYFETMCRIISLTLEVVRGRMVHPHSHMSLKSMQTYKNRIQEFLADA
SPHLRERRYCQTPMDHLQRLALRVHSSYITSELCRPALKAEADKDDPLQPVVRRYCIEHLISTVES
YIELHSFSTHGSRAWITLQRAISCAFLLAVIEEGKTEPKVRELLHELEVIISERATAEGGYDSGTA
IVPPGVESMKSPGNVAHNGMATGYPHFDTIQGVTKAHLSVSDSYQGAQPASIPANITMDTQTQWAK
PLVRTLRALQKLNIAFDSYNGQPGSSHTTSLGSAETLSAPHTLGYRRTSSANSQPQAGYTPNVGSL
PPPTPESSSSGDWTYPNLLDRAAEYIHPPLWG
>g2409
MSTLGGEAPPQQARMRSRTLSLGYDDGDVDPTGLPFGHLNEGADLEEYVTETRTGNIVRHTRSNVT
GEVADWKLVTFTIDDVENPKNWSKAYKWYITAVIAFTCFVVAFASAVITAGLDGPAKEFHVSMEVS
LLTITVFVVGFGVGPMVFAPLSEVVGRKPVYVVTLFIALIFEIPCAVAPNIGTLIVCRAIDGIAFS
APMTLVGGSLSDLWKNEERGVPMAAFSAAPFVGPAIGPLAGGFLYDAAGWRWLYWIQLILAGVTWV
LITFTVPETYAPALLKARAKKLRKAENDPKFVTEQEIDSRPLGQKIRIFMLRPFQLLFLEPIVLFI

SLYMSVLYGLLYMFFVAFPIVYQEGKGYSPGITGLMFIPLAIGVVLSATCAPFVNKHYLKMHAKYN
GKPPAEIRLIPMMFSCWLIPIGLFIFAWTSYPRLHWIGPTLGGFPVGFGFIFLYNSANNYLVDTYQ
HQAASALAAKTFLRSMWGAGCVLFTEQMYHRLGYQWASSLLAFLGLACCAIPYVFWFYGERIRSFS
RFAYVEDEETKAVPYDSEH
>g2410
MVASNEQTYLAIPGPVAFSKSRGRAIALEIGAIDVRAQWVHYVHTSQPLQAAQEAILEQLLRYGDI
LDISPTFEPTDGDVQNFHVYPRVGTISPWSSQATGISHVCGLATFVKRIERGIKISALFSKGSTPE
EGYLDHLHDRMTQIISQEEPDLAKMFSDHPPLPLETVPLDAGDKSPTEILQEANKRLGLALDQSEI
DYLVAAYASNGPVPRNPTDVELFMFAQVNSEHCRHKQFNASWVIDGKQMPNSLFSMIRNTHKKNPE
YTVSAYSDNAAVLQGENAGFWAPDPATREWTQTKEQVHILAKVETHNHPTAVSPYPGAATGSGGEI
RDEGAVGRGSRPKAGLAGYCVSDLLIPDLRQPWELDVGKPHHIASSLDIMLEAPIGSAAFNNEFGR
PCTAGYFRTLLTELDIGDGQKEIRGYHKPIMIAGGVGTVRPQHALKDPKLVKPGSFLVVLGGPAML
IGLGGGAASSLASGEGSADLDFASVQRGNAEVQRRAQEVINACVAMGEDSPIKFIHDVGAGGLSNA
LPELVHDAGLGATFELREVDCADRGMSPMQIWCCEAQERYVMAVGEEGMNKFTAICKRERCGFSVV
GRAEQSQQYEEKRLVLLDRDSKEYPKPIDLPLSVLFGKPPKMTRQVDSRKLKLPAFDASLSTYLPA
APSEQHLLEEAASRVLSLPAVGSKSFLITIGDRTVGGLTARDQMVGPWQTPVADVSVTATSLTQGI
RTGEAMAMGEKPTLALISPAASARMAVAESLMNITAADLFDRLSRVKLSANWMSASSHPGEGAAIY
EAVEAIGLSLCPQLGISIPVGKDSMSMKMKWNDEKSKEAKEVTAPLSVVISSFAPVEDFKKTWTPA
LRSVEDVGETVLMFVDLASGYKALGGSAVAQVFKQVGHESPDIRDVELFKDFFDATQQLHEQGIVL
AYHDRSDGGLFTTLAEMMFAGRCGVEIMLDDLQKTGDIQSTVETLFNEELGAVFQVRKADEMQFRS
CFATCGPPPGLIKRIGRISEKPKQNLTIYHKHKLIYRQPRGKIQQIWSHTSYHMQKLRDNAACADQ
EFANILEDTNPGISWNIKFDPKDKCLPILTSLMSPFNNKPRVAILREQGVNSQAEMAFAFNLAGFT
AVDVHMTDIISGRVSLAAFAGLAACGGFSYGDVLGAGQGWAKSVLLHEETRKEFKNFFERPDTFAL
GVCNGCQFLSRLKELIPGAQSWPSFERNASEQYEGRVAMVHISDPDPSAPSVFLHGMHGSSLPIAV
AHGEGRASFAMTKNVTAQDFVRHNLAPVRYVDNATLKPTMTYPYNPNGSPEGIAGVRSPDGRVLAI
MPHPERTVLGGVASWLPADADKWGDVGPWGRIFYSARRWVG
>g2411
MARSAAIGTLIDELIVALRQSDKTVPTFSATKKRCEAILKDSSTWNRTDPFAVEKQLDGLQEKFRV
LNKDDLADALGSRLSELEVQRSSWTPEYLSLLLQLSDRPADVSSVPKFETSSYDNASVQLKWSEID
AIGAEFADEDIWGDVDYGAASSDSETSIASSDISIPRILPQGAQPLRDEFVIPNELLTSGEDADLV
KTIKSGHFWEYDSAGIREFAQSHTRFITELQAAREILFMLQGLPTSLFWQIDNSIAVDRRYALNHA
SIAAFLHMLRSYADIGVEIGKLRTFVKIPQCVVFLQTFTRGVEKELTDFDAFLSRMQLSYLSPGKT
VTVSMLQLTEEVQKEVRVLLLLADLVVKLGSAPPEQSFLCLDLLYNLVCVNQASGNKHEHKSLAKL
FFSCFEAYARPLRLWLRSGDLEASQDHFFITDCRHDHDMQTLWHDWYLLRETSGHLHAPTFIKPSA
RRILTTGKSMVFLRHLNVAIDELESSHWQPLDFDAVCPDDSTWQLLPFSSLLDQAIGNLISTNHIL
ASSMLREQLNGQCGLWTSLSALQYIYLCKDASVSTTIDSKIFASLDKRGGVWNDRFLLTELVQSAF
GETDYVDTSRLIVRSARKTFHDFESQSRKVKILKSISIEYVLPWPVANIITKPAMSMYQRISTFLM
QIRRAKYILERQRLLKRNDADDNDEDEDDGMGYIIRHNLLWFTNILYGHITDMVIATNTETMEKTL
AESPDIDSMISVHEAYMSALEEQCLLSHNLAPIYQAVIALLDLCIHFADIQAARHGETNRYDTSHR
SSTVLKGQHSKRRRRRKRTEDSSDSEDDDDDDNDEHEQDGEDNEYYDEGMTSISFIESSYSHRLGH
VMRQFHRSLAFVIAGLRGVGRVDGQQSWDILADRLSWNAQSKK
>g2412
MAIARPIRLLGAVGILGVLLVLYRLSQTSQIXSITGTTGTVDTDKITDGMKRDPLLDPTGEPEGIL
WRAEDHDYAPDSENSARINATLLLSLVRNEELSELVTTMKQLERTWNHKFNYPWTFLNDKPFTEEFK
RKTQEVTKAKCYYEQIPADHWEAPEWIDDDLVKESNKLLEEQGLQYAGKASYHKMCRWNSGMFYKH
PALANTRYYWRVEPKVEFYCDVDYDVFHYMQDHNKTYGFTINLFDAPESLPSLWPETERFLAEHPE
YIADNNMMEWLVDDKLRPEHNKKANGYSTCHFWSNFEIGDMNFFRSEAYEAYFNYLDRAGGFFYER
WGDAPVHSVALGLFEDASKIHWFRDIGYRHIPYFNCPNSPKCSGCTPGKFYEGASFLAKEDCRPNY
FKYVGTH
>g2413
MASTTLFRLATRSVRPARSFVRASSYAPRSIRVTPSSFAIANNFSTSTKLRSGHEEETYEEFSARY
EKEFDAVQDVFELQRNLNNAFAYDLVPSVEVLSAALRAARRVNDFPTAVRVFEGIRAKVENEGQYK

QYLEALEGLRTELGVPLQEELYPKNE
>g2414
MSFDLKQIPLPTIDRPFGIELWPFFNKAFDAVVGYPADDFRFVNGETPMSTFKETAIAMVTYYTII
FGGREVMKKLPAFKLNALFMIHNFILTAVSGILLVLFLEQLIPTLYHHGVFYAICDHNGGWTQPLV
VLYYLNYLNKYLEFLDTVFLVLKKKPLTFLHTYHHGATALLCWTQLIGLTSVSWVPITLNLLVHVV
MYWYYFQSARGIRIWWKKYITILQIIQFVIDLGFIYFASYTYFSSTYFPSAPNMGTCAGEEFAAFA
GMGIISSYLVLFISFYLVTYKKAAKTGRPRSNTGRQALIDMKDAEVPSPIANGRKAANGATTTGRQ
GPVTRSRKA
>g2415
MEPSMASELVVKPDQNELLQMHSMPPWKRYESEEEDVSETGSQSVFSPTEGDLTHESDADSVNEAE
YVDAEMLAPAVYQKLTSDDSSSLYSHRLSIITAKRSSAATCVPTQHGQDTSTDDSEDVSPLTEYMT
PPVSPIYLSAVCYVPDGSESSSRDESSADAEDNDEDEVHEVSEAKQVFFTSPASKPSIVMIQSPTT
EAILTARMGNPVQSQQSTDQDKQEGSPFNLRKRSSTKRSSSASGMTTPMDRSKRYSAMFYRDFTQN
GEVTYSDARSHTRAQSSHSPARNSSRPRVGPRKDSLISNATDSSETRQSRPRRIPSQRSSSRGSRY
APDYESPRNYSRPRSIRSSSVASDMTSASSASWKRIHAHNWSNSSLQYPSSSSSIADQPSLSSSSS
SSPPQSAGENESKQTPHPLSIITTPTSPTTASPDFQQSRFTPQQQQPQSAITSHSSDIDWSPVEY
RRSSTTPYQKTFPYSRPRNPSLSLTSALATASSENLTEYASSPRSRRGSVATTSTTTTRTRKSSIS
AVMSAAVGSSKSSGHSTSTMKMLMGGFRGLGKQRTNRLS
>g2416
MDEKNQVRQFIYSNSSISTENGQHLYYIKTSKWPSSRPDVALHFGSNKNSPTVAVCDFGKWSGDKI
ALGDPGKTGLKWAPFERHSTFSPEYQWSFNVPGVGQQTFSWKRTHSEGIDGHKPSGFSSGNYKMVE
TRTKRVIAVYNNGGGVFSGRSKKFQINVNYGRDFDSLVLLTGCAMLEKARRNASRGAAAGAAGGGA
AGGAAGGGGFVPYQSLTVGIPRETFPNERRVAITPQNVALLLKKGFSRVLIESGAGAEAQFTDEAY
RKAGAIIVERDVVWSQSDILLKVRAPSIEDGTKNEVNLMRNGATIISFLYPAQNKPVVEALAGRGI
TSFAMDKIPRISRAQVFDALRIVDDQFNGQYCWIPPSKVLVIGAGVAGLSAIGAARRNGAIVRAFD
TRPAAREQVQSLGAEFIEVDLEEDGSGQGGYAKEMSKEFIEAEMKLFMEQCREVDIVITTALIPGR
PAPKLITKEMVHAMKPGSVIVDLAAEAGGNCEATVPGELAIYDGVKVIGYTDLPSRLPTQSSTLYS
NNITKFLLSLADKDKGFNVDLNDEVTRGAIVSFNGEILPPPPPPTPPPTPTAGTVQAQAKEVKAVE
VTPWQKATRQVATVTAXMGSALALGKLTGPAFMGNMFTFGLAGLVGYRAVWGVVPALHSPLMSVTN
AISGMVGIGGLFVMGGGYLPTTIPEALGALSVLLAFVNVSGGFVITKRMLDMFKRPTDPPEYPWLY
AIPGVIFGGGYIAAASTGMAGLVQAGXLVSSVLCISSISGLASQLTARRGNILGILGVSSGFLASL
LAVGFSPATLVQFGAVAGLGGLLGAVIGRRITPTGLPQTVAALHSVVGLAAVLTSIGSVMAEIAHV
STLHMVTAYLGVLIGGVTFTGSIVAFLKLAGRMKSTPTILPGRHVINSSLLGANIATMSAFVTMAP
GAPVIAATCLGANTVLSFLKGYTTTAAIGGADMPVVITVLNAYSGFALVAEGFMLDNPLLTTVGSL
IGCVAMNRSLTNVLFGGIAPIQASKKIEGEVVTTSVDETVDALASAENVIIVVGYGMAVAKAQYAI
SEITKMLRSKGVNVRFAIHPVAGRMPGQCNVLLAEASVPYDIVLEMDEINDDFNDTDVTLVIGAND
TVNPIALEPDSPISGMPVLQAWKSKEVIVMKRGMSSGYADVPNPMFYMDNTKMLFGDAKSSCDAIK
SALESRK
>g2417
MASQHPYQKLDIDERVKRVLLRTPLIDGHNDLPQQPRAIFHGKIHNNPKFDLEKGFQRGMTDIPRL
REGAVGGQFWSVCVPCLRSAENFSTPEYSDMARDAIEQIDLTLRLIESYPDVFQLVNGPGDVKSIY
ESGKIACSIGIEGLHMAGNSIGIIRAFYRLGVRYCTLTHVCNNAFADSSTSKIGPVHGGLSTLGKS
AIVEMNRLGMIVDCSHVSENCAKQVLQLSRAPVMFSHSNVKSVFDCPRNVPDDILDMVPINGGIVM
VTFVPEHVATRRKDATMDMVLDHLFYVAHRIGWDHVGLGSDFDGIASVIPGLEDVKCYPHLLKAIL
DRGATEEQLAKVVGENILRVWRGVEDVRDQMKVEGVLPVEDVWEDRTWWRYDGYYQMPDPDPEDKL
GLDWYGVPPPEEGLYLDP
>g2418
MSGNLERSQKPGKEALIDTEPAKALELLREGEIVENVNSKEAFTKLRWKLDLTLLPLLCLTYALQS
IDKTTLGYAAVFGLQTDLHLAGTQYSWLGAVFYLGYLVWEFPTNVLLQKLPINRFMSGTVIIWGIV
LMCHGAAFNFAGEAVARFFLGVFEASINPGTMLLFSMYYERHEQPLRMGIWIGSAGLGYVIAGIAS
FGIGHIQSSIASWKLLFIIWGAITTAWGVVLLVLLPGSPLTTKFLNEHERTLVINRVKDNGTGIEN
KAFKWIQFKEAVLDLKTWLLFLFAVTSNSPNGGLTSFQSLIIKGMGFSTLKTTLIQMPSGAVQLVI

CPLACYFASRYRNARLAIMLLCLIPFLVGIIGLWLIDKSKPYGRLVCLWISFSYTATWTLSMSIAT
ANTAGHTKKITTNALLLIGYCLGNFVGPFFFKTYQAPEYPLGVAMMFFCIGVQVLCLVGIWVLLWM
RNRSRKCLHADEQTTEFHTMAYERGLMDETDLQNKYFKIQQLLRTRLEARQPSKESDHQTKTIDDF
LADDASVQLLVHEYLAKIHGRPHSIFHAATLWKDIRERRVSKALILAICAMGAHVAAQPNLRSLEP
LLTAESKRLLQIDLERVCLENIQTCILVANLCVAHENPSSEFLFFQPRTGQEEDNEVFHELRIRIW
WALFAADNWCSSSLGFPRQMKDWPRPARLPMEECLFAAMGPDAPSTGPNEQCRNPGLWAHMATLIE
VFSPIQELNWRAANRDGLHPNHIEEDTHRLAQLLDNWETSLPHDVQLTESNLVEHSKQGTGGIFMG
LHLGFHHYATLLFYQYLDKRSIMTLRAKEFSTRCKHHALSYSTWLARGRRQSGCEAVYPTIAHMAI
VSSSVLLHTLLFGEENELPQSRQCLEANFRALLELEEYWPIVKTMVKLPSS
>g2419
MILASIFWFAASVAALTLDVASSGGNRSSSLLYGLLYEDIYHSGDGGLYGEMLRNRAFQGSSSNGA
ASLARNTDYWYPVGNVSLTIDTSSPSLSSSLPYQMRMDVAVGTTGTVGFYNEGFWGFNVDAAKRYI
ASFYMRGNYNGTVDCYFHSNTTDQVLGSSSMSIDQTSSEGWVQTFSTTFQPSTTGSDANNTFYFTF
DGSKLAGQSVYFNILSLFKQTFDDRYNGVREDLAEAVENMNAKYIRLPGGNNMEGNGSPFEWKWNE
TIGSLVDRPGRPGTWGDIKTDGFGLLEMMKMSQDLGLEVILGIWTGLYLNGEIISEANLQPYVDSV
MNELEFLLGDKSTTYGAQRAALGFPSPFRINWIEIGNEDYLNGGTSSYYSYRFMAFYNAIHAVYPS
INLMSTINPNPVKSDGSSLDLHVYGNEDYFVSLFNTFDQASRKYPVFVNEYAATNTGSNNVGQVGA
QTLGMSCAEAVFLLGCERNSDVIVGSAYGALIKSYNEAPDTVAVIKHTANEILYSISYYVQKLFAE
NMATETLPVTATNGSFGPVYWSATANSSSTILKLVNYDGMTGSSNAVQVNIEGSSKTTATLIILTA
PNSTSVNNLPSLGGESSTITTTTLSGSSGSFFVSFSKPYEIVILVV
>g2420
MAELTALRFEQHHSGLGVQCSSPRISWTFSAVDESISDWQQLQYEICVRRETGTPLYFVIQSPDSS
LVPWPSTPLQSREQAQVRVRVCGSWMDRNTRRAHSGWSAWSSWATVECSLLSAQDWIAVPISSPIV
PLSHEDESLRPLLFRKTFELPATFDSVQRARLYITAFGVYRAYLNGCRIGDHEMSPGWTSYRYRIA
YQVFDVTSLITTKGSNVLSIEVAEGWYAGRLGFDGGKRHIYGDRIAVMAQLEIDGLAGGRFKVASD
ISWKFHPSPIVNSEIYNGETYDARVEGENWNQQCPSNVDTDWETVSVLDFPRARLYAPNAPPVRIT
EILAPQKISTTPSGRIILDFGQNLVGKLLVRSMTLPVDGTVNFIHAEVIEDDGELGTRPLRGAKCV
DKVISSGQPLIDWSPKFTFHGFRFVGIDGWDPRSEKKSWKSNIQALVIHTDFKQTGQFECSNIAIN
QLHQNACWSMRGNFLSIPTDCPQRDERLGWTGDIQIFGPSATFLYDVVGMLSEWMEDVACEQSDHN
GVPPLVIPNILDHVWPSIPQALWGDVVIILPWVLYLSSGDRDILHRQYSSMTSWLDQGVRRGRDGL
WDPEIWQLGDWLDPQAPPDEPGDCRTNGTLVADAYLVYVTSLMAKIAFVLKREGEGTHYSYKAQCL
RALFQEKYITSSGLIVGDSQTAYSLAIVFGLLSTQLQLDTARCQLSARVRMAKFRVSTGFAGTPVI
LKALTETDNLSLAYRMLLEDRCPSWLYPITMGATTIWERWDSMLPNGSINPGQMTSFNHYALGSVV
NWLHETVGGLRALEAGWKTALVSPQPGGSLTYASVSYESVYGHWSCRWKLQFDGDRNEAMTLCLDL
IVPPNCRAVVRIQADEDGFSKLDESVGSGKHHWEIPFFPWSWPPQATAPFITNPGKHID
>g2421
MLKEEMGFPPKVYQFLVGTFAALGSLLYGYDLTIVAEVVSSGSFLSYFNPTTTEIGLVASLLTAGA
FVGAGLAYPCSDYFGRRATILAGGRFIAGVSIGVLTMIVPIYQAELVHPNIRGLVTGLQQFMLGIG
GVCGSWISYGTYVSFADNRQWRIPLGIQIIPAALLSGLIFLFPESPRWLISQGQLEKGLKTLARLH
ANGDTSDPWILAEYEQIKAQVAAEKEHSQVKIYEWITNKSNFRRLLLASAMQAATQMTGISAIQYY
SVTIFKQIGIDGTDTLRYQAINSIIGLLGEFVLMMVVDKIGRRKIVVGGNLAMCLTYVISTILLAQ
FPPETNNTGAHWGFIIMTWMYNFCFSSSMGPLSWMIPAEIFDTATRAKGVAIGCMVSFAFNTMIGQ
VTPIGMANSGWRFYILFVVCNFANAIFFWAMLPETKQLPLEEMKKLFTESPWFIGNSTKSKNQVTE
TSILAERIKNEGLEDKRGMTEQMEVTA
>g2422
MTSSPRRIAAKARHINTTSSSPRGQIPLTSTPQVETSHSHNFQGANGDFDPQHSLTARAPIGSYNL
EPSYTQILGSEDTPSNLRGIVAETGENSSHIISPAVADDSEFLESYLSTIPDAGRTSLIRTSVTAS
RPVRPVLFSTVPRRPLGVSSNQSLPATKCEIIEKYLEPAVKDVVNLFFEYANICFPVFDEASFKDV
YFTHKDKISPALLSNLYANALIYWDNSPKLSSVRSPDIRYIWNQANEALHSELFLSPGISTVMAII
LNVCGRPSTSIFGNGGMVGTAVALSNALGLNRDPSNWNISPLEKRFRIRIWWLVVLHDRWCSLAYG
TPLQIHRAQYDVAFPTIDDLCTTDSTQVETAAASVFIALTTLTEVLAQYLEHVYNVSNMEEFSSSK
MSAMDLERALNEWEESLSDNIRRIVVRGTNLNAPGAANFRLAYLAVKLLLRRLQLDADKPSSETVH

NSDDDYGTCSPFYIQAQRAAEEIVHLMQELDDSHFRGFWIPVNALCLTSATTFLLRSALRKRGSAT
TLNAPLNISRDMIQTLRSHRQRFSWDLADDCLATCGDLVDKITSYPNMNNGTMCVDLTMPSLQDCG
DISQSVLDELFVEFPGLVDSIGI
>g2423
MASTDSNSVFFSRADKYLMKTGVPFSPVVITKAKGTRLYDASGRQILDFTSGQMSSLLGHSHPEIV
EVVQRYVAELDHLLSNMITQPVVDLAERLARILPAPLEKSFFLNTGSESTEAALKMAKLYTGKFEI
VAFAASYHGLTSGSGSATYSAGRRKGGPVMPGQLAFPAPYAYRSPFRKADGSYDWETEMDFAWSMI
DRQSIGSLAAFIMEPILSTGGILDLPRGYLKRMEVECKKRGMLIIMDEAQTGVGRTGDMFAFQKDG
VVPDILALSKTLGCGLPLASVSTTAEIERGCTEAGFLWLTTHLNDPLTAAVGNKVLEIVERDGICQ
RATERGAQLREGLLKLQQKYWCIGDVRGRGLLQGIEIISDATTKAPGPDLGQAVSDRAMACGLSCN
VVNLPGMGGVFRLAPPVTVSAEEIEEGLRILDESFAHVLNLSTL
>g2424
MRSHALAPFTFELLPLGSVQPRGWIRDQLKLCAAGLGGNLYNFHRYVKDTSWLGGDVEYSPLRESA
PYWYNYIVPLAYILDDDDLKKQANEFLQRTLKNQHADGWIGPEHTKHGRGIWARCLVMMGMVNHAL
ADPSQYDRIVDSLLRFTRLINSMLKDNYQGLIPHPGDEFDEFCITRAHELSTSLQWLYEQVKNKRD
KEMIWETMDLAWEATRVGDRDWSKFFTEEEFPKKPAAPGSTLIKHVVNVSEALRYMPQLYRMNHDH
ELIAKTRQSVDMSFKYHGTTFGALAGDEFLAGVHPKRGAELCGTAELIFSLSYMYRLFGDNSYADR
AELATFNGLPAGMTPDWWAHNYLTQSNETWARNLENWPFYNCGGRALVYGLEVNYAMPKFAMNAFV
ASADLRSISHQFLVPAEVTVPVKGEKPIHIVSETHYPFDERITYKIETSRPFNFYIRVPEWATKGT
TVNKTLDTDEVREETVEVDANSLYKIAISPGKTAFRITLNAEIRVVPRANNAVAIYRGALLYAMEI
PHKAKVGPPTHFAEWKPLPDADYSSKLRDVEYIPAADWQVAIDPSQAHFHRSEVKGDLPNPIFESG
APPVTISVAGTKISNWKLEGDCAGLPPADPSPTGKPFTVKLVPFASAKLHISEFPVSKATLQSTQS
KKESGSDAKIEWVGCDMGSLKQVKEVFTKMREREERLDLLVLSAGINANQYRETDDKIDSHFQINW
LGQFYAVNQLYPLIRKTSKLPDTPAPRIVWESSELHRLSPSDVKFESKEEINNPNIGSAELYARTK
LAIILGVDFGLVQRVIKPNHDNVNTAMQQQWKEAYPGLLGKLLTTTMIAMSRDPEQGSYSALYAAV
SPEVEEKGWNGRYFTDPGQLGQRSKQASDPVLGDNLWKLSAKLIRDVVGEDAMVDWNSS
>g2425
MSQLIQQDDVPVPATVEENSRDPSTIDEDAALLETLGYKQVLHRSYTFLENFSTTFAALYFVGGVR
VTFSTGIASGGNLAYWTSYLVTLVFSFITAAVIAEVCSASPSAGSIYLWAAESGGPKYGRLLGFVV
AWWSTTAWTTFCASNTQAAANYMLSEITVFNLDFPTDPSDVKFRAVQWACTEVLLALAAVINFMPP
KYFRWVFYCSSAMVLLDFLLNMIWLPIGVVNTWGFRTTHDAFMTTYNGTGAPAGWNWCLSYLATAG
ILIGFDASGHVAEETKNASVVAARGIFWSTVVSGLGGLIVIILFLFCSPDPDTLFSFGAPQPFVSL
YAVVLGQGGHIFMNIVCIVALWFNTAIAIVAASRLVFAVARDGVLPFSGWVSQVSAEGQPRNAVIV
VWGVAALVSCTILPSAVAFTSLISAAGVPSAAAYGLICLGRVFLTPNSFPKPKWSLGRWSKPFQII
SIFWNGWVVAVLYSPYQFPVTGQTLNYAPVIMAGVTIFAIISWWFTPEDAWLPKKRIQRFVESKGE
ARETTTEITE
>g2426
MTELTNQRVYLSASLVAIPLFVLWSWRQRRLNIEAAEMAQTPTKTVVIIGASWAGIPVAHGLLKDV
PNAKVVLVNPSEDFYFNIASPRLSTKPNDIPREKYIYPIAPLFDKHANAKKNFEFVLGKATSIDLQ
SKNVIVQDVNSGSTKTLAYDYVVIASGSTSNATTGTDSLQVPFKESGTTKLEAELKAAQEAIKSAK
SIIVGGAGAVGVEFAGEAAEAYPSTQVTLLTNSDNVLTGLREPTRQKAAKLLKQKGVKILTDKTVT
SASRDAAGKWNVVTADGQTLTADIYVSTTGVLPNNEFIPASLLNKDGWVEVDSHFVSKADPSVYAV
GDITQYSARLVSRISGQVSVLLSNLKADITGKGKRGTYKDDSSLIVVMPIGKSTGTGQLGSFTPPG
FMVSFIKGKDYFTGSGKKFIAG
>g2427
MEYRHLGNSGLLVSVISLGGWLTYGGHVEDEKTFSCMKAAYDAGINFFDTAEGYAGGQSEIIMGKA
IKHFGWKQRDLVISTKLYFGQDNAKDPENPLNNRGLSRKHITEGLNQSLARLDLPYVDIVYAHRPD
RTTPMHEIVRGFNHLIDQGKTFYWGTSEWSASEISEAWRIADKLGLVGPVVEQPQYNLLTRDRVEK
EYRWLYAEHGLGLTVFSPLKQGILSGKYNDVEAPPPGSRVKEGDGKLAYVTQFAKTWGDETWQKNL
AVVAKLKPIAEELGVTRAQLALAWALKNPNVSSLITGASKPEQVFENVRAVEVAQRLTPEILERIE
KAAETAPAAEPLRY
>g2428

MKSSILICMASAVSASNFWRLPCRSRTAAARIDPIMAPGEPNSHLHTVFGSGGFSSNATPDDLLQS
DCTSCAVTQDRSAYWTPPLMFMFPNGSTTMVNQDGGMLVYYFVYGSDPQPFPQGFRMVAGDQYLRN
FTGPVPDPPTSEWSADDKTQFSLSQKALGFNCLHYDTGSDEDSLYRHTLPDKQFVDENCSDGLRLE
LMFPSCWNGVDVDSPDHKSHMAYPDLVKDGQCPEGFGTRLVTLFFETKYDTQDFRGVDGQFVLATG
DPTGCGYHGDFIQGWQPDFLRRAMNECSNPSGEVADCPLFTLQSDEDASACRAPVPEELWHEDPFF
NGDGLLGDVPIQQGPEQAAPYGVELGLMDHLPVPLPLPSIGVVGTVEDGVSRVVDSLIPTATPSRT
RFEPEAKRCDMDMGTVYETVTVQVTHSPASSSASALRKRGHGHRDHHDRRQAR
>g2429
MTQQLHKTIAVVNATGRQAASLIRVASALGYGVRAHTHTLKGVIAEELANLPNVTLLRGDLMNNPT
FMDEIFKGTQLAFINTVSQAGDEVVIGRALADAAKRAGTIQHFVYSSMPDHSVHGPWPSPPMWACK
FTVENYVRQLGIPATFVYAGIYNNNFTSLPYPLFQMELQPDGSFEWAAPFNPDVPLPWLDAEHDVG
PALIQIFKDGPKKWNGHRIALTFETLSPIQVCAAFARALGRRVKYRRTSKIEVKVRIPDGYREQLE
ALEELFGRHEAPYFPQPEFNTPAAGSPKGLGPAGGKGAGAGMMQGPGGVVSQRVTDEARKLWQGWR
DMEEYAREVFPVEEEANGLQWML
>g2430
MTRFWNRNADTPWGFAIYRTVYTEQSSQVWKDVVEKLESYLFYSVKRDIEVKARDMYDELEDTYGS
VLFNDASQFDGMSIDRVRRHWLAMQGRKVDPDPDSDADDDDDDDDDDDKVEKSKDDENGEAEFEIS
QEEIEDFRADHTNLEFCILIDEEVLQSILSAPATPKEAAKIFPKKWFESIGYVKVVDQQSGPGSSH
DYPGWMTVDLTCLWEIYGMDDLESQYPYEDPRTGKRIVYTGIPPTDPTREYHYGGVSLGRAM
>g2431
MSSITSQTQEMDTHQISSSIPESQVLIIMTGGTICMQRSPSGFVPARDFQQTCLAKMPIFNDGSRP
SVSMDVVVNSSGEKKKYASLRTPPSLYDRQVRYTVFEFEELLDSSSIDSKGWAEIAQTIYNNYTLF
DAFVVLHGTDSLAYTCSALSFMLQNLGKPVILTGSQAPMLELQNDASDNLLGSLVVAGHFMIPEVC
LYFNYKLFRGNRTTKVAASDFAAFDSPNAPPLAVTTSMRTHVNWDLVHRPTSIENFSIRTNLDTSH
VACLRIFPGIKPEMVDAVLQLKGLRGLVLETFGAGNAPGGQDNAMTKVLASAIQRGIVIINITQCL
TGHVSPVYAPGMTLFRAGVVAGQDMTTEAALTKLAYLLAMPDSTPESIAKDMAISLRGELTSHSQT
VFRHPEGGLSERTTTLTALGYAIAQGDLDKVKDLLKGANDWVVNDSDYSGNTPVHIAAHSPSLEIL
RYFLLQGASVHLRNSAGRTPLFLAASAGLMDHVVLLRKSGAHLHADERAVAEMQARRRPGIWALAG
VGARSMKERDELENSNRDDGIEHANSNAGWTGNVQTLAGSAPA
>g2432
MSGDEAPSLFERITLPSIPKEVGRKISQLEQEFVRAEIEQLRKQIPVLRPLYAKRNALVASKDLQE
ADFWPRVFSNAPQEIDEYILASDAAVIGQCLKNLTVERCDVDENGNGGDPRSVRFTFDFATGEENP
YFTNAQLVKEFHYRTKITKSPKGKRHVWEGYVSTPVRINWKKDQDLTKGLLDAACDLYEAEQKNGA
GVDRTSLPEYEKLVTKLEEVEAEADALVGDDDEEADDDTVPSGVSFFNWFGYRGNSVTAEESAQAD
KEEAERWQKIENGEKVDDDEDDEEDEDEDDDFDEDEESLSTVEIFPDADDIANTLAEEVWPNALKY
YVQSYSVADDFDDEMDIEELDDDEDEEDDEDRPAKKVKT
>g2433
MSEDPFLQVQSEVLNTLQTIRPLYSSFLRIRSLTTSPSNPELLQSRTELESTLEDLRADLADLRQS
VRAVELDPYRYGLELDEVERRRGFVQDLGREVDDMAAEIGKQPTTTSTTTTKGKNALPNPSEFDAL
SPDMDDKFGGEGDDDYYANFEQQRQVEMMAAQDQQLDGVFRTVGNLRLQADTMGRELEDQAVLLDD
VEGLADTVGGKLASGMKRIRTIVRKNEDTYSTCCIGILLVVLVLLFILLLVL
>g2434
MYGERPVIFGGVPDVLFLILSMGDASTIPVLCLLNKATYETIKSHEAFLCTSYLRLHDFNPPLDKI
LTMDPYTGHRAPLSFVNLQKFVFRRQTXLMLASRVARSGWGAWWAIKGGAGEMDAEAEMFRQRVER
GLYVMFHMSDIARDVERIKLRPHRVACSIYRVLLLNWLPCVRFEATGNSRNWWFPRHMSHIYVVLL
RMTQQREIGKQRWRFRQMLDLERRVDFHIALQMLRQFLETIIFAHCPDDDASISSLGSEDVDITNW
FLLRQGAHSLEEIFLELPDRDCCSIEDRARKQQDGVPPVCDYSEALKEYWDARNGDETVDCKQCEA
WLRSSSTQSILNDFFGLHALDLGKEWVRQMRGEGELFEG
>g2435
MGPSLPLPLHEEWKDVDSYIEALLSFATSTPLFLNLCGGVHILDFLTSEPDMYSTLFPEDWRSFFH
EHDLYDILDLVLTDDLSPFRSPNGAGWKTLEEREEWKNGPSPPPSLLDYIHDIRRLSLRRDFTSTI
PKNTSAIPQRLAIGMKDKKLHEVEHFSKYVDSFTTSVSESRKEPITHIADFGSGQNYLGRTLAHSY

NRHIIAIERKHAYIKGAQGFDVSAKMAKKKEVRMYDKKKEQLRNGAATEDICPTCPPEVQQGEKER
KEEDGAVFSVFKELDISAEDLATATSNAPHKAEEEDLEPHGTMDYIEHNIKDGYLEPIIRHVVNPD
PDEDSSQVATKQNNARVLVVSLHSCGNLLHHGIRSLILNPSVVAIAMIGCCYNLLTERLGPPTYNL
PVLRHMHQRLSIGATAKDPHGFPMSNLFEHYPYSGGEGIKFNITARMMACQAPYNWSREETKAFFT
RHYYRALLQKFFVDHGIAPKPELATIDVLTRDQPPVEKDNSWETNPPLVIGTLRKTAYKTFITYAQ
TAMNKLAKEPAHRVKLAPILDNEEATVAELERYVAEFEHAQKNLSIVWIMMSFSASLVEAMIVIDR
WQYLREHMASGVVKDCWVEPVFDYAESPRNLAVIGIKK
>g2436
MATLLTRSSRPVIHNFRQLTQSRLVSTLKDHPYIYTFPDDARPGNHVLSLLSSEPPNTDLAIGTTS
KLPPTPDSFTQNSKFLKILDEVVAEHAVEDPEVQSQAQVMVSTAGANLGSGGFFFAPQHNQRKRSG
YGQGLNTGGDSAGGASGQGGAGSGGRGGWIHVSDNRRPPEYGRIAWPEDIFGSVEVDGNGNFVGNN
GNYQSSGTYRIITRDGILGLSPFLREKLVERLRKAG
>g2437
MATPSRPNPGATPTHLTSSPHPSAVPMGRGLSHKSPSNIKTPSASGTVHGHHLSTSSHQGATPLTA
TAGLDDPVSLSSPSALLALGGFTGMTPALNVQDGLVSSGMNDSDIRALAFGLGGSRNHEEERRKRI
EEVVQILRGRVAGRGVSREAVERLGRLEGFESIWQDDSLSIAGNSVDLEIEFYPGEDNVKDVSLSY
ASLEGESESERRDEATSVLKSDLIQTPEGRENRTWKSLKGFQNNLERLAKLDHLSQEINCFEAVES
LFDSLQKVWDAENRRGNFYSVFDHICAGSIGRPSLHKRGRVGMGLEYWVDGHQILNANHIKKSEDS
MAVDTDDNDADIQDKLRSVTIECEEGFPSLRISKEWVGTEVLSTVETNDTANSSETVVNWIEPPPI
LQPSVSGLDTGMVGSGTANRRFVAKLEPHIHVPLLIANDIYRHLGLAIPQEYRTTTYDDLLVSGWT
SGSMDSNESMASESLEMPEKRRRTAVLSFNEKNEPVQRNHSYSFQAFEAAPGMTIREFPFSHPRQL
ADIFPILRQYSLLATLLQKVFPRKEIISRSAQSKGSSKASDTLSMSGNSTATTEQISMLSNGDTNE
DLLNSLLRESTDSTGTISIYNDPQTGDNSDIRVDITLRTQVGQAPLIILSITLPETRQKASVSFEI
RLNGRVNVIQVSDLAGSESQNDQSLAKIHKRLNRVLEVSEDLSILVEYVVGRLRGMS
>g2438
MAESTIVSNTENLLKYLSLDQKGKVLAEYIWIDAEGGVRSKTKTLSKAVTSVDELPEWNFDGSSTG
QAPGDNSDVYIRPVAIFPDPFRKGENILVLCETWDSDGTPNKFNHRHEAAKLMETHASQHFWFGLE
QEYTLLGPDGWPYGWPKGGFPGPQGPYYCGVGTGKVYCRDIVEAHYKACLYAGINISGINAEVMPA
QWEYQVGPCEGIQMGDHLWISRFLLHRVAEEFGAQISFHPKPIPGDWNGAGLHTNVSTDDTRAEGG
MKAIEAAMKKLEARHVEHIAVYGEGNEARLTGRHETGSIDKFSYGVADRGGSIRIPRQCAKDGKGY
FEDRRPASNADPYQVTGIIVETLCGGV
>g2439
MGHGGRSYLGLRGTRLNIAIGVIAGLDFLLFGYDQGVMGGLLTLDSFITQFPDLDTSSASFKAMSP
SAQMHRSNIEGTAISAYNVGCFLGSIICIFIGDRLGRRKTIFLGSAIMVIGATLQCTAFALSHFII
GRIVTGIGNGLNTSTVPTWQSECSKAHRRGQLVMVEGSLITGGICLSYWLDFGFSFLDPSSVSWRF
PIAFQIFIALIIILAFVMFLPESPRWLIFQGREDEASEVLSALLDTNREDTVVENEFTAIKDTVLEA
QNTSWRDLFTMDENRHFHRVVLAYVNQMFQQISGINLITYYAATIYQKSIGLSGFLSRILAACNGT
EYFIASWLPIFIIEKAGRRPLMLIGAAGMSLSMVVLAISTSFVGQTKPGIVAAVFLFVFNTFFAWG
WLGMTWLYPAEIVPLRIRAPANALSTSANWAFNFMVVMITPVAFQTIGYQTYIIFAVINAFIFPVV
YFFYPETAYRSLEEMDTIFHKTKSIWSLVRIARDEPRRYGKNGELLIDYTQTDEHFHHVGGVADAE
KRAVSNKDHANPQYHIENGDAGHVSESS
>g2440
MPVLEIVPPRTSTTTSSDSHPSKEYIDRTIEYWKTLDRSPVVLQEEVTGFVANRLSFALFREAIHL
VNSGVVTAAEVDRIVEESMGPRWAVKGPFWSYHAGGGKEKGLQGFFDKIGDTIQACWDDAGSPSLR
READASVASWEGSLCEQVEKAYGMLEDKDLRNRDEKTKQVLNITRQDWENEGANDGHNWGGPGPST
DSATLKEQNDFVGEDSETPRDDTCRNCGQSGHFARECPEPRQGSSGGCFNCGEEGHNKAECPHPRV
FKGTCRVCNEEGHPAAECPQKPADICKNCRGEGHKTSECKENRKFDLNDVRDETPEDAWAQLKKAD
KERDLEDFRDAFKVYTKAEPSKTFVDIEKQLRDEGANVYLIALEKEKEGFENYTFIDLQGKLDRTY
VVKFFFGPEPRRNHLKERWPATPEENIERLADAGFEYDRMIPKCINCGEMGHIAKSCKQERVVIER
VEIKCVNCSEVGHRARDCTKQREQRGKFACRNCGSPDHKAVDCTEPPNLDHIECRRCNQTGHFAKD
CPNTPKFAKACRKCGAEDHLSRDCDQEQNMDLITCNNCDQTGHYARECPKPRDWSRVKCSNCGEMG
HTVRRCSKPSAEEEIGVSDFGGQAGGEAAWGSAGPAPIDEEVGGWNDDVDKPAELAPAELAW

>g2441
MTENTLQFRIATPEDAPAIRQLVQAAFQAADTRQDWTADMDLNIRFRLTVEEVMPRIISPDGAIVM
AFVTTDDNGDESLVASVEVAKRSNDLARLSMLAVDQQHQRGGIGREILAHAERYCQQRWGVTKFGL
DALSTRQALIAWYMRRGYQKTGETVPFPVEKFSDLALPEDLCFVQLEKDLSLSA
>g2442
MTDASLHNVPIVIDNGSGTIRAGFAGEEIPSCYFPSFVGRPKHPRVMAGGLEGDVFIGQKAQDLRG
LLKIRYPLEHGIVTDWDDMEKIWHHVYESELKTLPEEHPVLLTEPPLNPRKNRDIAAQIMFETFNI
PALYTSIQAVLSLYASGRTTGVVLDSGDGVSHAVPVYEGFAVPNSIRRIDVAGRDVTEQMQLLLRK
NGYVLHTSAEKEVVRMIKEKVCYVSLDPKKEEKEWINSYHKSESKTIEYVLPDGHKMKIGQERYRA
PEILFDPEIIGMEYPGVHQIVQDAITRTDLDLRKALYLNIVLSGGTTLCKNFPDRLMREIKRLAVE
DMKIRISAPAERKYTTWIGGGILAGLSTFRKMWVSADEWHEDPEIIHRKFA
>g2443
MTTPRKVCFVTIGATAPFDALLSNVLDQPFLEALKTHGYTALLIQYGKEGQAIFDSFTKIKPPGSP
GRCDLDIKGFGFKSEGLVQEMRSTKANPSQNVVEGMILSHAGSGSIMEALRIGVPLVVVPNPALQD
NHQEELARQIAKNGWAIAGKLDRLAESVQRAETLRSALRSWPPKNSGALKDSRGLAGVVEDELGFL
D
>g2444
MAGTAKALSRAAFMLSQRSHVSVSSAFRTRTAPICLQQTTRRFSSSPFTYLPKDADEPQETESFRL
ADIPEYSVENFTDAEKSMYELMSSEERAVFDAENKRIVEMWNDPVRRAKEEEAIDILADQVEKESK
MRFEDVREKNRGFWAEEEEDEFANAEDGDDTFNDDEITSMAHAEMELHREVREYARIAAWDMPLLS
NLTKPFELPKENQILRFRYTSYMGEQHPAEHKVVVELSSKDLVPKHLTEQQRLTFLKLVGPRYNPD
TDIIRMSCEKFSARAQNKRYLGDTIKNLIKEAKEGESFADVPLDLRHHKPKVHYTFPDSWKMTETR
KRQLLADRQLRLEGEQQRITIVDGKETILHAIRTLPALSQNPHLHAAQEKGAVKETAARARGIARP
RR
>g2445
MSEIRPQFFLVRENDAIVPLIALDELPANITIPGVSRNLGMADTGGMTSVGKISSRGAVYVVNGVS
EGNDDRFPPSNGKGKGRFGDKKQTGVPVDGNGRKIYCSFWLRHGECMFEQQGCQYKHIMPQDLDTI
RRCGXHDIPAWYQKAYGVSGLLRPTGVNQNNWRKPRGAWKAPEHNDETINGNGNGAPAHRKDNRIT
ATPESSNKTDGPVSGVRDDAAEASNGANLATDKGKQPVVATTATTKKGGANPVTPTHGNRIVDFER
IGSASFGYKYDAPAPQTPTERLPMRARRLFEARGPNNDYSTSPDFSTPSMTLTGNSFSSFSSLGGT
PRSTSEETVKVAARVNNKPIAVIDGDIEDADDTLDNLNDELAKFTANEPPYRSISELLPTSLPEDR
FGNLILI
>g2446
MSSLERDFSSGSEDADELVADLLQQFSSVEDPAIGSVIRHAKLPPRKSVTRTVTSASFPPSVSPGS
EIPYVRASSTSKRPQISAVEVPPLPENAEDYEFLPGHSTAWRIWRQKRVRGKVLYEVELATTEKEW
LKESFVKALENGSEAITNFKQNGPPKAKRVKSAITGGYRSGSYTESGTEKSQLSEHEDEEEEEEDD
DDDDEIVVVSSTKRRRGRPSGSRRRKIRGRYDDETDSEDSDSRPVRRSLRAPKPRRYLKGDDQVDK
LHSVQQDAYGNINTKTAIRNHADAGCDHRPGPLNLTEETSERDKKMTSLKWRLHSQRRDSQEPRRN
FIGFLVTIPSENDIGKYVIAAIPRAMIAKKALFQREHLVTKVGWDHFVLQCRRCIGTSQKKDVTAP
HLGHCTACNEENPACRPFRQRLNPKQEQQQRENNGGEDPITTVNDDLINNVDNLLFRTDPNPDETD
TDNPDPDADLATLRLKEYSRRWQCHTCISAPGEIEGLVGWRPVDAEAYIPGYTANDMKETEKEYLV
KWKKKSYYRTTWMEAFLKSPKCVKPQMTTEDAIPEDYLRVDIVFDVKYTNIVSAHSKEIDMARVKD
VKEAYVKYKGLLYEDAVWETVPSSTEMERWNDFKAAYDDWVLKDYDFQTLAKKTQPTCLTGGKIMD
YQLDGLNWLYYKWYQGQNAILADEMGLGKTIQIISFLSTLVEDHKCWPFLIVVPNSTCPNWRREVK
KWAPSLRVVTYYGSAVARKLAHEYEMFPKNARDLRAHVLVTSYETMIDDKARRVLAGIPWAGLVVD
EGQRLKNDRNLLYSSLSSINIPFKVLLTGTPLQNNIRELFNLIQFCDPTQNAQDLEAEYETLTKEN
VPELHAMIRPFFLRRTKAQVLTFLPPMAQIIIPITMSVVQKKLYKSILAKNSKLIKSIFQSNQDAQ
NKQNERHSLSNILMQLRKYIIEDFLDGLGLLYCRLDGSMGAHEKQKKIDAYNESDSKIFAFLLSTR
SGGVGINLATADTVIIMDPDFNPHQDIQALSRAHRIGQKKKVLVFQLMTRESAEEKIMQIGKKKMA
LDHVLIEKMDADEDEGLDLESILKHGAEALFEDDTTRDVHYDAESVEKLLDRSQAENTQAGDDASA
ESQFSFARVWANDNAALEDRLGDSEAATPVNTTLWEKILAERQKAADEEALANAQALGRGKRKRAV
VNYSAKDKEDEQLSPGGTKRTKLQVESDGEFHAREASSESEDEEALLELEQTRKVKARPFRRVDPN

KLTPDPSNGNTGLPKRPVDRLLPCLACNQMHPVGYCPLKLAGVEHCGLCGIAHLGYSRVCPHLNME
EQILSMLQSLRQSTEPKWLVDAARHYLYTVKGDLVKRKKAKADPNRPTSATRPMPVQPNTSNPISQ
NGRAFGAPASNIVYPAPSASNSYSSPYPPTSSTL
>g2447
MGRGKNFNVSDSSSQKSGKGANNIKRRRGKQHEPTDAVPERVKPHVTVPETIPYTTPASEFIYGLS
AVEAALRCTRRKLYMLYIYQAAGEELSSEKIALRKLALARGIQVKMAFAGWDKILDKMSTGRPHNG
LILEASPMPQIPVSSLQPVSLPTDEYFEVGLDKQSVEEAEVNGSSNRIARVERFEEVAKSRYPLVI
LLDGILDPGNIGAVIRSAYYLGVDALVLTGRNSAPLTPVTIKASAGAAENMTILKANNEIDFIRRS
RENGWRFYAADSPQNVADLAATHGTLADDTASIAANEINHGASLLATAPTVLMLGNEGTGLLPRIK
AQADGFVSIPGARIRPNLGINDAARVDSLNVSVAAALLMEMLMRVPLRVASAVPVASAGSTKTTKE
TTPRSFDFN
>g2448
MASKVRVLLNPKYKRNGTKSYVHLLRKYGFDTTLDGPYFHASHVHQQGKFTAAGQKPVGGRAHVHR
TLQKKVDGQSGDVPADDVQNDTMYLAQVGIGTPAQTLKLDFDTGSADLWVWSTELPSSTQSSGSSH
TIFDPSKSSSWNSSSGQTWQISYGDGSSASGDVGTDTLDLGGIQIKNQAIELAKDLSAQFQQSEGD
GLLGLAWGSINTVQPQAVQTPVQNLISQNLIPSDAQLFTAHLGEAAVKSDNSFYTFGYIDQDIVSA
TGNQIVYADVDNSQGFWTFQSTSASVNGKTINRSGNTAIADTGTTLALVDDATVKAIYDAIPGSKY
DSSQQGYTFPSNTSADKLPTVTFAVGNAQIAVHKSALAFADAGNGYVYGGIQSRGSNPFDILGDTW
LKGLYAVFDAGNTRFGAVAKPDPDASSS
>g2449
MSFGYSIGDFVSLLELTNRIRKRFVRAPAEFKEISEDIKKLGALLQDIDDFESEQGLDTRQKASIN
QMSKSCREVLTELESVLDKYQELDHDEKNLGKKTRRVWKRLQWDQSKIMDLRKRTDSHVNWFSNVI
ARLNGSVILAVKDGVDRLCQYQGDEEKQKILDWLTPTDYTAQQNDILRLREEGTGKWLLEKAEFKS
WSSTKQQTLFCLGIPGAGKTTLVSIIIDHLEQKFRAEPDVGIAYIYCNFQSSSESNIQAFLAILIK
QLVQRQSELPVAVVDMYKNHEKKNTRPRTDELSSCLRSVTAGLSTAFIIADALDECRTANGLRVIK
ITKSVDGMFLLAKLYLNSLRGKSSPKAVRTALSRLNTGSNAYGHAYKDAMERIEHQGTEERDLAKN
ILSWITCAKRRLTVNELLHAFAVEIGLSYLDEDNLSDMQYMISVCAGLVAVDEESGIIRLVHYTTQ
EYFERTWQIWFPGAHRNIAIASLTYLLFDQFATGPCGNWEEYRDRLQENPIYEYAARYWGYHAKEA
YSEVKDLVDKFFQRKSTLASVAQVLSLERKFRSCIPLAHKGISGLHIAAWFGLNDKVKEILKGSQS
HNAADKKGRTALHWATRNAQAQTVDLLIQEGASANATDKRGMTALHYAALQGDNHLIQLLVKNGAH
LEVVDESGQTPFLTAAGYVKIASVQELLSYDTAINAVNPEHKNALHLAILAAKDETPQLVEMLLSH
GVCPTLCDVKNMTPVHYAVAIRNLEIMQLFIEAXVDINFGIERRRWRETTKAGRSFYVLDQPPERA
NANSRDAVGLTPLHFAAYTGYRIMIEYLLSKGADPNACCCSDGDTPLHIAIRRGLLTMEYEGDPWT
DITWTVETDFTTTDETETDGEYSQWHMANERWGVIDMLLATTNIDVNIQNAHLESSLHCVHYGGSH
SDTIVAKLLQKGADPLKINKKGQTVFHLACEATDLEAIDHILDAGGSTTIEDAKGLTALHYAVRAN
GYETAKLILDRNGDTATSLCLAADKKGRTPLQHHLQFDPHPQVEMITLLLDHGANVNHTDLDGNSP
LSIYLQAPTYGDRTAITRFLLEQGANPLWKDGGGQTLAHVTMNNNHKAEVGVLELLYSHGVDLSMKD
KQARSFLHYGAIYGSLHDEVIPFLLRNKFDGIYERDLEDKSPLMHAEAAEAAKEERRRTGKYHGPR
WRRSLKILKQMENNKLIQCDIQDEHAISVDLEELN
>g2450
MAPSRNLPDSDASSMVDTPMTDGNESVARIPVDEPDYAMADYQDTPDYTDSDTNPNTTSSSIAGDV
AHTDGRKRRSEAIQLRKSVLGKQHGRLDESKEDDSIRRFRYLLGLTDLFRHFIETNPNPRIKEILA
EIDRQNADEPTKAKGSSRKGGASNERRRRTEKEEDAELLKDEKSGGEAATIFRESPAFVKGEMRDY
QVAGLNWLVSLHENGISGILADEMGLGKTLQTISFLGYLRHICGITGPHLVAVPKSTLHNWKMEFA
KWTPEVNVMVLQGSKEERHELITERLEKEDFDVCITSYEMILKEKSHLKKFAWEYIIIDEAHRIKN
EESSLAQIIRVFHSRNRLLITGTPLQNNLHELWALLNFLLPDVFGDSEAFDQWFSNQESDQDTVVQ
QLHRVLRPFLLRRVKSDVEKSLLPKKEINLFVGMSDMQVKWYQKILEKDIDAVNGAAGKRESKTRL
LNIVMQLRKCCNHPYLFEGAEPGPPYTTDEHLVDNSGKMVVLDKILNRMKKQGSRVLIFSQMSRVL
DILEDYCVFREHKYCRIDGSTAHEDRIAAIDEYNKEGSDKFIFLLTTRAGGLGINLTTADIVVLFD
SDWNPQADLQAMDRAHRIGQKKQVVVFRFVTEHAIEEKVLERAAQKLRLDQLVIQQGRAQQQTKNA
ASKDDLLGMIQFGAADVFKNKSGTLTGGAEMTDDDIDAILRKGEERTAELNKKYEKLGIDDLQKFT
SDNAYEWNGEDFTNRKKDIGISWINPAKRERKEQFYSIDKYYRQALATGGRTADPKPKVPRAPKQV

SIHDWQFFPPRLHELQEKETAYFHKEIGYKAVLAEGTDEDLSEREANRDLEQQEIDNAVPLTEDEQ
AEKARLSEMGFSHWNRRDFQQFVNGSAKFGRTDYEGIATEVDSKTPQEVKEYAKVFWDRYDEINDY
PKYIRVIDQGEEKLRKSTHQRKMLRKKLDMYRVPLQQLKINYTVSTTNKKVYSEEEDRFLLVMLDK
LGKEEDTEGIQLFERMREEIRESPLFRFDWFFLSRTPVELSRRCTTLLNTIAREFEPESKANGESK
GRVRDRADEDADEEEDEAPAKKKSKNGAVVNKKLKGVKASSKGSSANTSRAASVSSNVQTSKSKSK
KK
>g2451
MDSMRSLNTSLPSSTSHVQPPEQLLQAFKAAALSVTNLYKSAVTDQAQAKQLGYQEAIEDLLHFLD
RKHLGLGDGEGWQVRQWATERLDGVPSNGDSDDEDKPTRSASPVVNRKEHHEIDQPRHIPEPTQPT
DEQRQQTETTQSTXPADTTFAKPPVFTFTSGPTFPVFAPQDVDMQADTVSVTSTSQDEAGISMFPR
NTRASGRLNNNTRRSSTHATSLGTKRKLQVPDFFDLSGLNGREFFGGGKRGRFT
>g2452
MNGAIRALYIYDEYNVPILEHVYRSRPPSASSLISLFLAHPAPRPSLLYLTETSPAVTVFSISHSN
LLFLSPSDVDTEPLLVLEFLHRVVDVLEDFVGAPLLSTKLQSNYDVVAQLLTEMCDAGLVSNTEIN
ALQENVETAGWMNKFLGGVGLPRAASPSTASLNPLKPQLSNPRATSALGPAIPWRRPGVRHTSNEI
YVDIIETLSVTIAPSGRLLSALASGTIACTAKISGVPDLLLTLTTPGGQRAIDQKMQLPVFHPCVR
LARWRERPGELSFVPPDGKFILAGYEADLLPIDPSIDEPPRHMEKLFLPAFVAIEKSLGPSGKDFE
VRLTLNTSFPGVRPLNTGRTGSGSSTPSFLGGGGNSSSPSLPVLDEIIVHVPIPQAVRNITDMRPS
RGDADYSPGDDHLEWRIYTKDSGSVSGTATLRCTVVGYQDDLDATEAEIAAGQELLQGYYDADSYQ
QDPTTYTSESYPTGVSSKSRKPKSTKTKKKKSKSKKESKANTAVEGQSTLIPDKPTSSSQTSTPPP
STTPNPTNNPLFKESASRKEQIHSALMPKSATVSFSVKGWLPSGIRVDSINIDPRKSRGLGEGVRP
YKGVKYLCVSRRGIERRF
>g2453
MVVNDESIKIRPLEYTPVETIQKKVAVARDAFYDSKTRSVEFRKLQLRKLYWAIHDNQDRMCEALA
RDMGRPSYETQLAEVVWLLGEILFTLKNLDKWLKDESAPDVPLIWKPLAPKIRKDPMGTVLVIGAC
NYPFQLTLSPMIGAIAAGNTVVIKPSEQPVHAAQVTQEIVESALDPSCYTVVQGAVTEMQALLAEK
WDKIFFTGSQTVGRIVSKAAAPHMTPVVLELGGLNPAIVTRHADPRIVARRLLWAKTINGGQTCTA
QNYILVEKSLLPALVDELKKAYKSFYPSGSSKGPDFTRVVNEKAFQRLKAMIDNSKGKIIMGGEVD
EKERYIEQTFIEVDSIEDSMVINEAFGPICAVMTVNSVEEAISLARKVQDTTLGVAVFGNSSDHQK
VMAGLRSGSVSLNDAQPHVNTLAFGGVGESGTGAYRGRSSIDVWTHSRPYTNTPKWMESLLDIRYP
PYTSSKLRMLQLSMGTTPDFDREGRQSRFTWLKYIIGGTPTSAATRVAAYAVNDQSTTTNGTNGTV
NGTTNGTISGDTNGDSQAK
>g2454
MAIQELFDTLQDRIAHQSGDHRLIIGVLLLPLFYVILNEVVRSKARIGRLNGPKGLPLIGNLYQIR
VNAAEQYRQWSKQYGDVYQIQLGNIPVVVVNTAAAAKVIFGNNSQALASRPEFYTFHKVLSNTAGT
TIGTSPYSDSLKRRRKGAASALNRPSVQTYVSHLDIESKDFIKELYDYGKGGSLGVDPMPMIQRLS
LSLSLTLNWGIRMDSQKSDLFHEITHVEEEISRFRSTTAKAREMRDRRDAYLTALNKGLDDRIAKG
THKPCIQANVILDKEAKLDKDELISISLTMLSGGLDTITTLVAWSIGLLAQRPDIQEEARKAIEEF
YSTKQPLCDALDDQKCKYIVALVRECLRYYTVLRLALPRASIKDIEYNGVRIPKGSIFFLNAWACN
MDDNVWDDPEVFRPERWLEQDDAPIFTYGMGYRMCAGSLLANRELYLIFMRLINSFKIEKETEFDA
HPVSGNMDPTSLVAMPKKYYARFVPRDDAALRRALREFVVVET
>g2455
MPVTTFASPDRYIYQNGFQSYHETEAIKGALPIGANSPQKPPYGLYTEKLSGTAFTAPRCENLQTW
LYRIIPAAAHSNFVPRQLDSAEEGPVKFHQIPNQLRWDPFDIDESVDWPSSLRLVAGAGSPELKHG
LGIYIFSAGKDMGKNDAFYSSDGDFLIVPQHGELDIQTELGYIHARPNEIVVIPRGIRYRVTLMAX
PVRGYILELYKGHFQLPELGPIGSNGLANARDFQAPVAAYDNDTTSEWTIVTKFGGQLFEAKQKHT
PFDVVAWHGLYYPYKYDLGRFNVIGSTSYDHPDPSIYTVLTGPSDHVGTAVADFVIFAPRWLVQED
TFRPPWYHRNNMSEFMGLIQGEYDAKTGGGFKPAGASLHNIMSAHGPDAETHERASNADLKSQKVG
TGSIAFMFESCYMLGVTDWGLETCQKVQKEYNKHSWETLKSNFAPPTEV
>g2456
MSTPKSWLPIAANSPFSLANIPFGIISTTSNQDPRPAVAIGEYALDLSIFSQNEGFCASPTIQSKT
AVFLQSTLNAFAASGRPFHREVRTYLQNVFTEGSPYSNLLEANKELQAKALIPLEDIKNHLPLAIG

DYTDFFAGKNHAFNVGTLFRGPQNALQPNYTHLPVAYHGRASSVVVSGTPLRRPWGQILLDSTAEP
KIPTFTPARRLDIELELGMFVGIGNELGEQIDINKAEEHIFGYVLMNDWSARDVQTWEYVPLGPFN
AKNFGTTISPWVVLADALAPFKTTGLKNETPLQSYLQEDEQNNALDINLEIDLTTSSGNTTTISRT
NSRKLLWSWPQMIAHHSVSGCNLRPGDLFGSGTISGEEPGTQGSLLEQIQGGKVAIKLNGGEERKF
LQDGDTITIRGWSGEEGGLVGFGDAVGVILPAVERV
>g2457
MPPSGHKRHRDEEIRHGSSVQKREKYTAIAXPEKDLDEISDQYKKTLSILRMILASVSVLEARGKT
AFAQRLVDTVEDEIGTILRQSEVSLPDLEILTLTSIYYFHCDEPVLAWRRIGNAARAAIEMGLHRR
ETLEKNFLDVNMQAQALKIFWCIYVLDRRWSFGIGMPFVLHDEDIDPALPTRHCEPYLRCMISYGQ
ICSKVWKAVAGFTDINATNKDTVNYLDFQIQQWLETIPSSLHLIHPRLGDAAQNQPKVLQMLRTFL
YIRANHMRIYVHRHNILSPSGIAQNPSAACLVIDIAKDTIHVLVHLRETSTVYDTQQVPFNYFLVS
AFSAIFLGVCHAPERFSHTCRTEFYSALELLYTLSARSSITRRLWKGIKGLRHIAPRLGLSPTKSP
EDGALPSALDNSIYDTIHQVARTSSPSFPPYRQRRATIPSYISNVQHIADAPGPITGISRPVLTPQ
SISGEFPQTTSTQYLPITNSTTTETASTLEDPMSLSTVSDVYEMGNDLTAFFEAFFSQNQQLATDD
QGAGHMDSSGLVGAGLDLLQDREELSQLFETFL
>g2458
MATNFELEDQKYLEDVNAVKQWWTDSRWRYTKRPFTAEQIVAKRGTLKIEYPSNTQAKKLWGLLEE
RFKAKTVSYTYGCLEPTMLTQMIKYLDTIYVSGWQSSSTASSTDEPSPDLADYPMNTVPNKVNQLF
MAQLFHDRKQREERITTPREKRGSVQDYDYLRPIIADADTGHGGLTAVMKLTKLFVERGAAXIHIE
DQAPGTKKCGHMAGKVLVPISEHINRLVAIRAQADIMGTDLLAIARTDSEAATLITSTIDYRDHDY
LLGSTNPSLQPLNDLMVAAEQSGKSGDQLQAIEDSWIAQAGLKKFEDAVIETINKGTSANKKELVA
KYLSSAKGKSNTEARAIAKGITGVDIYWNWDAPRTREGYYRYQGGTQCAVNRAVAYAPFADLIWME
SKLPDYKQAKEFADGVHAVWPEQKLAYNLSPSFNWKAAMSQDEQLTYIERLGKLGYCWQFITLAGL
HSTALITDQFASAYAKQGMRAYGELVQEPEMDRKVDIVTHQKWSGANLIDHSLAMVTGGISSTAAM
GKGVTEDQFKS
>g2459
MTSQERLQRAISIASDPFSEPEVYYGEPSTLDGPDGHHHRRTFSADLRSVNRDDIKEFLGKLPARR
GSHDESTLTSRKFLIDVDKTIASLLEREDTDQNMEITIEDVGPKVLPVGTAASHGYNKFDVRGTYM
LSNLLQELTIAREYGRKRIVLDEQRLSENPVSRLSRFIKKTFWNALTRRIDGSNIAVAGLDPKDWT
GDPRPRIYVPPGAPEQLEYYTRIAEENPEMGLDVQKLEGPITPEYVKSLNAKPGLLALEMEKFIDP
KTGKEDLRGVPFVVPGGRFNELYGWDSYMESLGLLVSDRVDLAKGMVINFCFSIKHYGKILNANRS
YYLTRSQPPFLTDMALRVYDKIKQEPDALEFLRMATLAAIKEYYSVWTAEPRYDPATGLSRYRPEG
LGVPPETEASHFLHLLTPYAEKHGMSFTDFVAAYNDGVITEPELDEYFLHDRAVRESGHDTSYRLE
RICANLATVDLNSLLYKYEVDISRIVRTYFKDKLAIPSEFRTPQTANVEFETSSVWDRRARKRKLT
MDKLMWNEEKGMFFDYDTVKKHCIQYESATTFWTMWAGLATPEQASKLVRQALPRLEEAGGLASGT
EESRGEISLTRPNRQWDYPFGWAPQQMLAWTGLLRYGYQDDAERLAYKWLYMITKAFVDFNGIVVE
KYDVTRPIDPHRVEAEYGNQGSSKTGFGWVNASYVYGLDILNAHQRRALGAVTPYETFSKAVDQQF
PY
>g2460
MDIQNLFNVKDKVVLVTGGAKGIGRMISEGYVTNGATVYISSRDAKACEQAVGELNALGKGKAHAI
PADFYKEEDIKKLVEEFGKRENKLHVLVNNSGSTWGAPYDEYPSAAWTRVLTLNLHRVFDLTKLLT
PFLEKGASEGDPSRIINIGSVDGLRVPPMETFAYSASKAGLHHLSRALANNLGKRNITSNTLACGP
FRSKMMAATLREREAALVRDIPLSRIGKSEDIAGACLFLSSQAGSYVSGATITLDGGMVVSPKL
>g2461
MEDLQAKHRKEQRDLQARITQKKKSATKKTRKGVNDECDRLQQELKAQQELEIAQLNGDAVAPTTQ
NLDNLTLDDDQEPTPANTTTEASDVNELPTPTPAPPQGDNETTRSKKPNRQKARLARREAERVAQA
EIAAEEAAKQTDHRGNEKEVMEAAFKRLGLQEVEINPDGHCLYSAIAHQLDNLGLGLKPDPARIVL
QPSTLSRVDTVSSPKHDGYRAVRAVTADYITEHKDDFVPFMEDPLDEYVKKIRLTAEWGGQLELQA
IARAYNVEINVLQGDGRVEKIESGSETDDDQVKRIWLAYYRHTYGLGEHYNALAQKTVAA
>g2462
MPEQSLPPSSLRDHMDKYAGETYVEGWAELWNNKQEKEPLPWDRGCHSTALEDLLMKWKQSGEGVF
AQKPQGGRRRRALVPGCGTGYDVLLLASFGFDVYGLDYSRTAVEYAKAYAAENAEKYPVKDAELGR

GNYVYVEGDYFKDDWLEGLGLPNNSFDLIYDYTFFCALQPWLRPRWAMRHRQLLAPAPTGTLICLE
WPRLKDPKLLGPPFASPSSAYFEHLTHPGEEIAYDSDGNVIVDPARKPCPIGLVRAEHFQPERTYT
TGKDEKTGEVHDRIAVWRRQDFSDD
>g2463
MHPPSKDQEPGGKNIEPSEITDNGRNSSEKEVHEEDQHPETEHEAIVEEDEHSDASTISEAANNSN
DNKDNKELEGTSEVVRRITTEIGPPVTVPRLKRRGLFAQLALIPEIENGKTYPRRTKWYITAIAAV
GGVAAPLGSAIFLPSLHQVAADLNSTSTIVNLSIALYMLAMSIFPLWWSSFSEAFGRRSIYIVSFA
LFVLFNVLSAISHSISVLIVMRMLAGGASASVQAVGAGTIADIWEIRERGQAMGYFYIGPLCGPLF
APIIGGALAEKWGWRSTMWFMVIFGGVVWVLILFGLPETLAVRKSPILQSDPATEETLERSTSRVS
SKVAQNSAKLLKTLKMTLVDPLSIILYLRFIPIALTIYYAAITFGSLYVLNISIQETFGAAPYNFS
TVILGLLYLPNSLGYLLASLFGGRWMDKIMHREAVKAGRFDEKGRPIYIPEDRMKENAWLGACIYP
AALLMYGWTVEYHVHWIVPMIANFGFGIGCMLIFGLVTTMLTEILSRKSSAGVALNNFVRNIFSCV
GTVVTSPIISGIGNGWLFTILALVSFISGVTVLLLMKKYGPRWRERGIDH
>g2464
MATMKYAQVPIFSYQKFDSNHHYQTFDRQTLSFTYHPIVPKPSVSLPLEDEKKEEKEIAADTEPEK
GGVEQTTEENSPKPPEESPSPDDDTPTPNPPAETKDEEPAPQNEEKTEDQESGGDAIANGDSTEAT
DAPAEPVEKEGSENDTPPEAPADEDAPKEDTPPAEDAPPKEDPPAEDAPSTEDAPPTEDSLSAEDV
PATENASPVEDAAPTEAEPAESASEETPTPEQTSEETQKEAAVPVEEQPAEGNPPEDAKREEPAPT
DDAAENPSAEEASTETPPAASPVEEAGPEAEATKAEEDQPAEPVKPEETPAAEPTTEAPKDGTTTD
EPPAEEETPKAEERAETEQPVEATEPEPVNEESETAEPAKSEPTGDNSKKNKRKEKKDKKNKKKNG
GSSPPVESQPEPEPAPEAAVTPKEPPVEEAPAVDLLVEETAVEEPPKSAEEWESFGGSKKSKRKNA
KKGKRKDQVQATPVEEPPEEAPPEPAPAQEPAAESPAVEDTGQTVPEDPPVSEEPDTEEKPSTEEK
VESSEQEASSPNEVPKDVPSEDPVEAAPTSEAVVETAVEEAAPSQEEAADSPTPEAPAETSEETPA
EEATEEVKEEIANSTPENLAEAPTEETVEEAKDDVFSDASADAPLDDTPATPAKEDLEERDPVDDI
LDDWELDDEYDEDSSPTHDEKANDNDDDKFSEIDFSAEQDSIEAEKEAEARQAEEDAEVDAFEPAL
HEEDHIDVHDDGPTVAEVEESAAAEPEAPEAAAEVEEVVEAEESVPATEEAEPEVAVEPAADALDP
EGESTPAEEIAVAEETVTDNQSADDSLAEENPAEAEAVSEEPVEAPTEAEAAEEPAIEDPVPEEAP
AQEASKEEVVEEPTPEPAAEEAIVVDEPKEEPVEEPAPAEEAVDALAEEPAEEPATDSAPEEPAAE
EPALEETPASEPVAEEPAAEESPAAEPVKENPTAPDAPKEEPAVEVVTEEPQAAEPQPEQAPAEEA
PAEEQAPASEEVAEQSAEAEAAEAPPVKDVPAESPLEPASDEPVAREIPVEEPTEPAEPTPEAEII
DRVPQEVETVERVPQEVVEASPGESAVVDDDQPVVERGPSSRRRRRHTSGWSRDRPSRTSMEASRG
QSDRPREANKLQLERPKGDSGLLKNRWAMALEEARRQQEVKQRKEAKLKLERAQEKQRQEEVARRA
KLEAEKENLREAERRKSKDISRSSGRTRRQSSDHSSRDSRHKRREASPKPESEPIARKVIESLTTG
ESDTNGPLLKINATKAVAAVPPLEPKQMQRRRDETDRIALNVERRSLQKKDDDITTSVEEMTVLLN
MSAVLVDRSQNRTQSQDPVLGRDLD
>g2465
MLSAKAVYPASTPRYADFASRRSTVHSTNGIVACTQPLAAAAGQKILSQGGNAADAAVAVAAALNV
TEPTSTGIGGDMFCLYYNASTKKIHSLNGSGRYAANASLEKIREDLGLSANDPGAIPLNSALAATV
PGAAAGWIDTIEKFGSGRLSVQQILTPAIELAEKGFPVSEFASNFWHNGEKLLRDASPNFKEMLKH
DPSAPDGVRAPNPGEILKNPSLGRTFRTLAAEGKKGFYEGRIAEEVVKVLKDLGGYLALDDLKNHA
DRGSQETDAISLIFRGQGVGKQGVASDGSEGGVEVWEHPPNGQGIVALMALGILEELERTGKIPHF
SLEQHNSAEYLHAVIESLRIAFADASWWVADPDVEKVPTEELISRPYLTERAKLFDPSKASDILDH
GSPAHNHCDTVYFAVTDKDGNGISFINSNYAGFGTGIIPAGCGFTMQNRGSNFSLQAGHPNVLAPN
KRPYHTIIPAMITNLDGSLHSVYGVMGGFMQPQGHVQVLLNMLAFGYSPQAALDSPRICIGAGMPE
EGKVLDRTVYVEEGISDETVNGLRKLGHQVEVLTGHRRXMFGRGQLIRVHYDDGQLVYSAGSDPRG
DGLAIPL
>g2466
MSSDAPPQPSLPLKPQRVLACAQCVPATLARRRKRRRFPERELLDRLHNYEELLRQNKISFDPIHS
DAIVGKARLDVESDHEMERLVPETGRADSVPSTPLGSEKSYGAKNIWHAMSRGFRDPENDSESSHD
YVRETEVKRAWEETTQKDDNLLFGSRDPTVDLSVIHPDAIQIFRLWQXYLDNVNPLLKVTHTPTLQ
ARIIEAASNLKNVHPNLEALMFGIYSTAVTSLTGDECQTMFHSSREGLLLRFHFGCQQALLGCGFL
RTSDRDCLTALYLYLVSVSFSTVPQSLASMLGIAIRIARRMGLHSETNLAECSILEAEMRRRLWWS

IITFDHRINEVSSSRSSALDPTWDCKVPLNVNDSDLRPEMKEPPVMQATSTEAVFAVARSGFWDLL
RHTIFHLEFTNPALKPLAKRHESSSEDGEIAQIEKIIDCRYLKFCDPENPLHFMTLWSLLGQMAKC
RLLEHLSSHSNSTQTDLQHDTATFHALILIECDTKIMTSPLTKRFRWFNRFYFPFPAYLQISQDIK
LRPGNKQTPQAWKYLSDNHEAWFENQPEEVNPVYQVFAKVVLQAWEACEANFIQSGRIMATPKIVL
SIRHIQAEMSRRAQKGTVEYANTNVTTAIDSLAISTTMPNCTVKPPRASMAKNRLRRLCKTCCAAP
IAYHLFPAGKLPTMQLPTSQQKKHAFTFSAFARTPCRCGDTLWLCEICGLALNKNDSTYRRVWTWR
TRYGAYLGTGLGTGIGEGCQGVKCGKGEECLAASAIELEVDCEADDSAITIQHHHNYQDGHLSGYA
ASPPKIEGLSLNERPDGHQHDDEPGYLRQEIVGIGGVVKRKVKKRVIVGACVEEYDDERETGKYLQ
READGLERSWCGWCWRVIPGRKDWEQGVVPI
>g2467
MASSFTVNCLTPAEVVETVTVAGAIKGNMRLDKVFFSGVSAGCLLAFACATALSTNTAPWWQENAP
GLIRTISALVFPYGLCMIILTGADLCTGSFMFTTMAALQRRLPWYKMLIHWFVTFFGNLCGSLFMV
AIILGYGEVFSADPYKSEVITFATKKQVTPEWQTVFLRGIGCNWLVCLACFFGMQGRDLASKVIGI
WWPVFAFVSLGFDHVVANMTFIPMAIWLDAPGISVGLYIWKGIIPAFIGNVLGGGLFCGMYYWYMY
ALEDDFSLALQTIKGRRQNVNSDIEANAAAAAGAGIKEGPHSD
>g2468
MMAAERPFTDLTFYNAIWKDKGQSDSGMSRQWRHQSKSASITSAPTTMITSMPIDVTSASYPASQV
STPLSSASPVIGSAIHPDLAGDYSLPWNGMEIPLQPRPTVFGSHVRDSPDDLPFYSSPDTCGSPAS
DAAGYVLPPHPSSAPTSVMEPFTYSDLSASPLQLPTATREWDGLDIVSSAPNMMPLALDGNQMIHP
VEPALPIPLSNLDGDEWYALRRELTSAPGVLSGDDGMEIIDTVKWQDCFECYWRNFHRLFPIVHRP
TFFTTKPSPLLSGAMVAIGSQFDTRPNAKEYSLALLEACIKLLAKRPAITSRSRVTDIQAVFLLEY
LSKFRSRKADVQISHRFRSLYGSFMQDRHWVHVNPLAVLNTLPPESSHDSLKKSQKFWVEHETRRR
VLQASFILDVYQSKLFGQPSVSVQPSSNPFNTRPQVSQANKIPFPCSAELWECNDSAQWLSLARSY
VSLSLPSAAVSVNRLSGLRPEEIDQFQASLLLAYGLASYAVTEDDRSSNDPLNALIISLQDRNGAD
MATSSHSQTLFTYHTLMAVQYTPLHTLLTVSGESWLFNQKLAQEGEFRSAKMKLREWVSDTDDVKK
AVWHAVRALNYVVESTEWHSSTITSATANPVWSNSVPLIKTPAAGPSIMLQATWSVYICVLICWAY
GFDPSNIVRSEAEHQPDNQANEARAYIQNMVSLAPTWRHISQTNIPATGRVGGLLNEANRVLARLS
EPRGARGEIRGMWSF
>g2469
MSMASEGQKELPVRPAGDAKMEAKSDSLPEIFIERNQLFEELWQEHLKXLAEKPRTDITITLDIGD
GKPSTITGKAWESTPGAFLKDIPKEISANIVIAKIDDKELWDLNRPLEKDCKVSYLPFDHPEGRDV
FWHSSAHCLGEACELEFGCLLSHGPPTPQGFFYDMAIPNGRVVLPSDWAPLDKKAAQIFKERQSFD
RLEVTKENLKKMFAYSKYKLHYINNLVEGEKSTVYRCGTLVDLCRGPHIQNTNKIKTFKIMQNSSA
YFLGDQSNDSLQRIRGVAFPDKNGMKEHLKFLEEAEKRNHHKIGKEQELFFFDDVSPGCPFLLPNG
TKIFNALQKLLRSEYRKRGYQEVQTPNMYDVSIWKTSGHWAHYSDDMFKLDVEKREWALKPMNCPG
HFMLFGHRERSYRELPLRIADFGVLHRNEASGALSGLTRVRKFQQDDSHIFCTAEQISSEIEGLFD
FLKAIYGLFGFTFKLKLSTRPEKYMGELEAWNYAEDQLTQALNKFMGDDWEINPGDGAFYGPKIDI
TIADALKREFQCATIQLDYQAPINFKLEYITNEKQTADAKVNKDGAEKAKESNVNRARPVVIHRAI
IGSFERFLGILTEHFAGKWPFWISPRQILIVPVMPVLNDYAKELQQILLADKLNVDVDVSGNTLQK
KIRTGQLAQYNFIFVVGAQEKESRSVNIRNRDDPASHAKGELISLEEARVKLRALRKERRLANAL
>g2470
MSFRGGGRGRGGFATGANRGGRGGYQQSYGPPAEVLEMGSFMHACEGEMVCESINPKIPYFNAPIY
LENKTPVGKVDEVLGPINQVYFTIKPQEGIVATSFKSGDKFYIGGDKLLPLEKYVYNINHSYSDHR
SDSFPSPRHLPVAKLENQQEVEEEPPEVVVEDEEHQEVVLVVDVEPLVASAVEVVVPVPLEAVAAS
HEEDLELVEALAVASVDVVTNFDIMSRRNPSHSAPEPSIANGVGGDVSQEKQKSLLQADTGHFTMV
RTLHLADLITELNGFCGFMSVLSSMRYLSEPERLNNIWFALAFMPFGLFFDFMDGRVARWRKKSSL
MGQELDSLADLISFGVAPAVAAFALGMRTFADHLFLSFFVLCGLTRLARFNVTAAVLPKDKSGKSK
YFEGTPIPTSLSIASLMAYWVSQGWIHENLPWGTVLQESVLEFHPAVLLFALWGCLMVSKTLRIPK
P
>g2471
MIFAASNALFAASSLLLLAPSPVNAITYNASTHVDCYSSLPSNFIDNGTWTYQSSGYCQEQCVPMG
YSVMAMTNGNDCWCGNELPANSTKTDTCTDTCDGWQADACGGKNSYDVYLTGLKSDVPIADSSSST

TTSSATTSTTAGVTVITQAGQTIVVTASSDASATAKSSGGGTSTAGIAAGVVVGVIALAAIIGAAV
FFLRRRQKQRVEEEYRRNAEISAFTQKKPSSMSSDARWDSDFMAQRRQSNGSIADAGDFSRRILQV
TNPDR
>g2472
MADIKQVPHRHPKHAIEQNQKHPESDTWFNEANQHVTSQLDGLGDDAPYFPNDIEPGHSGEKHSSP
NRLDDPVLSYMRANDGERENEELRGIIDDLTLDIKRLKQKLRRYKERDPPRLQGDKLFDVRTYGLP
RHRKRQLEQILQQFAAEISGSSRLSRNRPARAKSKPMRDNALISEDDARKALVVMLFKQHLGLSTA
KASIQHLANTSPPPNSWYDLNETLGSIKQMVSTIDVEFSREALKKYGAALELSADGHCVRQQGSDP
QGLTRPSTGSSVVYDHPHFHHTNDLLKKRSTHTETVLGLAESKTTSLHQSVGTDYVPYTLTTHRNE
AEDEILSLTSSDSEMSLHVPISDGHGHVSKPQTHKTSHERLTYYKNATFYIDCFGDSPDVMNLQPW
KSSLTESVGEKRKRQSSPSLLSQQQRRRHTNRTPADRFLSYPNEKDSVLNLSQCSSPVLSLAEEPL
ESVAVGHKAFPKELAASGVGDVTPHDNFAVHVVMKHVPVSSSDDTGGVINNHIKKAETRVPDHSFN
KSIPGHDQRNGRINDRNEKLFKTYVVTATHEELPPSSLPPATCGFFSSASSDDDSNGDEGREDGDD
ERDHVHEDCSGVNRHDAPPDVLARVGIKNGESPASVHSIEKWNADVVASLGNHEGNGFDGVAPQRA
SGSIRTR
>g2473
MPSQIRSCLRVARQVATRQNKHQQFLARAFSSSVRRCEINKVFPSAEEAIKDLKSNSTVLAGGFGL
CGVPDTLINAVRAKPEITGLTAVSNNAGVDGSGLGLLLASKQIKKMVASYVGENKTFERMYLSGEI
ELELTPQGTLAERCRAGGAGIPAFYTPAAFGTVVQTGELPLKHNSDGTVALYGAPRDVKVFDGKSY
VMEEAIKGDYAFVKAWKADKLGNCQFRYAAANFNGAMGRNAKMTIVEAEQIVEVGEIEPAAVHLPG
IYVKRVIQSTEEKRIEKRTFAKEDGDDMSALGKGDTANKRERIVRRAAKEFKNGMYANLGIGMPML
APSFVDPSVEVMLQSENGILGLGPYPKQGLEDPDLINAGKETVTLLPGAAVFGSDESFGMIRSGRI
DLTILGAMQVSARGDLANWMLPGKIKGFGGAMDLVSNPEATKVVVTMEHTDKKGNPKILKQCEFPL
TGKACVSRIITELAVFDVDFTDGLTLIEVADGVTVEEVKAKTGAPFKVADDLKTML
>g2474
MSKTPQTISSRLAHLIKAWPTDPIRPASVSVQSYLQARLPKTETEAPQISQSSVIALTNLLNDQYS
KQYPLPQKLRHPASNPTHYDDVVREFEEAPNRNWWGRVQKRLGGMLRLK
>g2475
MTSQSYQRVNTRDDDDGQVEAFPNLTAPVPASPPPSFHSRSSSPSSRRLLHEDPIRDGPDNALEDA
FDDGNDSDDADEADDRQRLMRGDPTTPTTPIITESNSASVQPSSTQRRGTILPTFTSTASSRVVGT
GSSNDGVFANLNAKPERAEKQEEDLPPSYEQAAADATPPYWETTIMAPGMSSDEVYVDGLPVGSVF
SFAWNGMISMSFQLVGFLLTYLLHTTHAAKNGSKAGLGLTLVQYGFYMKGSGVSRPDSDSDSYTAP
PDPNSHNFDPNSIGDGSAGGGQNQGGNAISAITTSEWISYILMIVGWFILIRSVSDFLRARKHEQL
ILQSPERGLSVPVIAEGEASENVV
>g2476
MYFKNLVMLAMAGAAVAQRPSNVSICDYYTTALLKDNNATNQYTLLALLVNTAVIGNYTQPNVGVA
VPGILNPNGTYNGTSVNLVPYFDGGLASTNNGGNAGVAVNFLDGGGAAPLMKNLPAEDNTSKQYFL
LTHLYEFFGSLLGCSEVGMTGFSAYGGDASMYEVHKFMVLDPYQIGYFITQVGLSAASFGVAESDI
SAVATALESLFAYRCAPKTTVITAQGPQYQSTCTDETCPLAANSTCADQPNIDQPLVANATLAMGE
GRNASSSSSAVAPSSTSSPSGSSTGSASGTSSGTVPAQTKSSGSQLSPSMGVLLGLLFIGAFAF
>g2477
MAATNNTNGTTAVDSYSIVDETAKVFRKSIFENPLVKQHLLKDLEKYAEPISFAGSPKPVLPVNWR
FAESVSSLKALEGTYVNAILDKVYGVSPQKAVINTDHALLFIMSLMLWSLEPEGRNVTFADTRTDP
AGKALYNSLFADYDVHGSLSGAYRCCTSNIYKTKDDKFYHIHGSMNPDVILDMLSLPRVPEGEDTF
ERHLPAFIDKIAQYESTELDHLSNVVLKQAGSISYPKEGYAQTEHGKANAHVGLWETWKANEHQAP
SWWTHDSDPSRPLAGVKVLDATRIIAAPIASRGLAELGASVLRITSPSVPDATLYHPELNWGKWNA
SLDFKKPEDRETMKQLIMECDVFISGYRPGALAKWGFDADDVLEMTKGREKGIIVVRLNAYGWNGP
LKDRSGWQQISDAFCGISYEFGRAMGHDEPVTPVFPNTDFCAGVSGICSVMDALIRRGEQGGSYKV
NLALDYYANWLVKSVGTYPEPVWKEVWQKHGAPVFRHYDHMLAMLFKVMGMLKKNSPELFDDSHFE
IRHPSHLNISYRTVKPVLQFPEGLVKPGFTVGTRGNGVDKPRWPKDLKTEVIV
>g2478
MAFPPEPSASINWKSLGFSISDVNGHIEAKWSSKTGSWGPPTFVTDPYIRLHGLAPALNYGQQAYE

GLKALRTADNRILIFRPNKNAARFAYSASFISIPPVPEELFVECVHAAVALNAEFVPPHETGAAMY
IRPLIFGSGPELRSQPPAEFTLCVYVNPVGTYHGAHPVKALVMEEYHRAAPKGTGSAKIGGNYAPV
MRWQRQAAEQXYGITLHLDAKTDSEIDEFSSSGFIGVKKDKENNITLVLTNSKNIIDSITVDSCLA
IGKSLGWSVEVRPIKYEEIGSFSEVLAAGTAAALLPIRSITRPSTSETISYIDDSSEEPGPVCSTL
LSTLKGIQSGKIKDNFGWTFPVNGVDPKQYAA
>g2479
MIKSPEDVSTTTQVVRGMESTEQMLEVDDTPSSTSTEDPPNGGLRAWLHVVASFCIYFNSYGISNS
YGIFQTYYTEQLGFDAPKAAAIGSIQSFLMFFGAFLAGPFYDAGYYRYVLVTGSIFITFGTFMQSI
STQMWHFVLAQGICIGFGGGIISMLGTSMLSTYFTSKLPVASGIGTTGSGIAGILLPVLFNNLQPR
IGYGWTVRVFGFXSLATLLVPVLVMRARVVAKKKSAIIDMSVFRDHPFLIFITGNVILILGAYIPF
NYVESFAVDEKITNANIAFYILAMMNFSSLAGKVLPMLLAPRIGPFNLLLFFGVATGASSLALLGV
KSLATVIVVSVIYAFLCGAFFALQPVVVINLCPDPGLIGKRIGMAFVGLSFGLLATSPIAGAFQDS
VGFNGVWLWAGLTAEIGACIMIISRGRKAGWALKTKI
>g2480
MGISTVVIDKSHKTKQAKYGRAATVYPRTSEMFDQLDVLEPLVQIGYVSRHYVNYKNGKRSGPGGY
NMLLSQMHDTYADFVLNIRQKYVEETVQEAFEIQGGKFYEGTELVDFLLDVEDPDFDDYAGTATVK
DISNESQRQISFKYILGADGTRSIVRKLADIPFVGDSTGMKWFRIDAVIKTDMPDHRSGLLSIESQ
AHGNVLWMALDHGRTRIGFALPQKLFEKYGTDLTEEQAKEEAKKAVAPFTLEFEHVDWWTLYSVSH
RVAETFRAHRRVILVGDACHCHSSGTGQGMNTGVHDAVNLSWKLGGVLKGWYDDSILDTYEVERKP
EAQRVIDNDKTVSALITGKIPDGMGSPDANPNELLQTFLDSINRFSIGLAIHYKPSILNQHSHVGR
VPSGHRGPDLLLARPGTRTPVRLYQITKNVGKFYAVVFSGNSALTIGGLRKLRNYLDSNAPSADEY
LGVKRFGPAFYDTNLAATRYGFSPAAGGIVILRPDGIVGFSAQLEEGAEIGAYFQGFVNMEREKKT
LATKSSKKVPETGMKEVEFDMETTRSF
>g2481
MAEIPKTMKGVVIEKTGGTEVLEYKTDLTVPTPQEGQVLIKNSFAGVNFIDTYFRTGFYPLPLPGI
MGMEGEGTIVALGSGNTYDLKVGDRVLWFGAMGGYGEFAAVPSLRVHVLPKEIPEGFGAAAAVQGL
TALTLIKEAYPVKKDEWVLVHAAAGGCGLWLCQFLREVGAKIIGTASTPEKLELAKKNGAQYLINY
AEEDVVAQVKAITGGEGVAVVFDGTGKDQFENDLEVVVRKGTVVSFGNSSGGVPPVPIFKLMAKNV
KLLRPTLQNYVFTRDEVDHYVGQLLDFIASGKGKVHIHKIYPLEDIATVHTDVVSRGTTGKLLLKH
>g2482
MGGTILRVTMFKIPEKENQLKLAENYKRLSTEALKDGKPYILSLQAGPTIEDPRSNGFTFVAKTEF
ASLEDMKYYDDEDQVHLSLKKTALQLNIQEIVILYAEPLVTL
>g2483
MSTETQEPPKYEWLIVAPDYADVLEKRMAVRQSHFEGLQPGLKSGFWQMGGAFLSDVPKEGEPLKI
IGSAMVALAATKEEVVERLKADVYNANGVWDLSKVC
>g2484
MEAAELFSVKGLVAVVTGGGSGIGRAMVHAFATNGAEKVYILGRRLEALQECAAFNPNVIIPIQCD
ITSKSDLQKSADRVQQEVGYLNVLICNSGTGGPESTKLGPTSTLDEFIKINWEHEVEDYTKTFEVN
TAGYWYTSLAFLKLLHLGNEKKNISQRSQVIATCSTLGFGRLAPTARFAYGQSKAASTHLMKQLST
SLVPFGIRANILAPGLFMTEMTSVMTSTSYDVGKLVPEGRAGEDQDIAGMILYLTSKAGAYLNGSV
LVCDGGRLGVVPSSY
>g2485
MGSTKRNQSVILYFGDQTEKNIPFEELFAYSQESDRTRQFLQNALRSIQLVTETLNEPERSKYKFD
SFEEVSKRLAADSSPDVVLRTIVLCAAQLGYLIAVLEKDEVLRDTWAEQKTIIVASCAGQLPAAIA
ASSHSLDELVDLAPETVAIAFRIGMDVDRRTASLGDDRSQSWAKAVFGVSAPDAQRAVDKFLLSEV
SRFTTCRASLADLKWLN
>g2486
MSGSPTKTVDVTIIGAGPTGLASAVLARQLGLTVCIVDQGDEPLKVGRADALNSRTQDYFDMLGIL
PSLQELGLKCNTSSTFANGEFISRQSHWWTSLKHTTYPNFLMLGQPVIMQHLAKILDTPVIYKSQL
QDLIETEDKVTAVLDNMIVESKYVIGADGARSKARQILNIPFEGTKPEMVWAVLDTFLDTDFPLCP
EIITFQVDGQARVSWIPRERGLARFYILLDDEITQENSEKEIRKFLAPYRVDFPKTEWFSTFEIKE
RLARSYFSPGGRIVLAGDAAHVHAVNGGQGLNTGISDAFALVWRLYLGTKGYESLVKTYELERRHI

AADVLSVAAKLVRSTLRTATEYVEIVEKNAAYITGGGIKYLRDTKTVSGADADPFVPGYRCPDLTL
TRGDGATVRLYQVLKYSSFTILRVNNAEVKIPEAAVPFSNVWDLIIDDKAADDHTEEQEVASKTLS
YNGTPLPEWVHGVVIRPDAYVGYVGEDAGQYLSGIFQN
>g2487
MAPELMMHTTTDVAHIDTKKDVKNSFAVASVAGPTDAEVLKGLDTAALLLSNKIFELIDSYRQPLP
GDMPDLRSQGRALFTQVIYRRVVKGEKIPMVLPAFPFKSPNSQAKVLGVLPDKAEEVALQCLDGLC
KNIERIYAPGAELFIVSDGIVYSDILGVPCKDVWNYGSGLRSFAERKLDNIHFVRISTLTGSEEPL
TGDEYEAQSPKLREKLIQLYLPSDYNVQDQKANDNNVLATYRGYCKFLLLDLANNPEFAGLGKNAM
KKKASILAEKMLIRGKAFAAAIRDNFTEHVRLSIHPSNGQDKVSISVIGQTTGLAMSPWHGTLLVS
SDGSFSIKHRQELESDPNYELVYQNGRPSYFREKSDIYHWGNDEIDFEPAYPCGWYITAPASSGLT
FDDVDMKKLRQLGELNSPVVLRGFKDTNNRELFEKKAEEMGNVQSWIFGKVLEVQDGGSETGGLNN
VLSAEAMPMHFDGLFNTKIITDEAGNEKRVSAYPRFQMFSSLVPAPKGTGKTLFASSRRLLQVLER
DYTVDQLTAVTWECQTDAFNATVLREMPLWVPHPENQKLCVRYHEPWPKSRTKFDATEVRLNNAPE
DLGKKIDAALYDSRVCNYLQFGENEIIVSDNILMMHTREAYPPGSPRRFWRIHVN
>g2488
MVSSGSLSHVFAQDEKFPESSTLVAFIGVASIVLFLLRSLYRFALPKPFPGIPYNEASAHRLLGDV
PDVISHIKNTDGTFITYLKESMIKLNAPLIQVFIKPLSTPLLILADFREAHDLLITRKEFDRSPSL
SDLVKGLVPEHHIHLPTEGGWRAQRRLVQDLMGPSFLHNVAGPVIYQRVELMIELWYSKCRIAEGR
PFQANEDINHVSLDAVLAFTYGEDFEHGMTKPNFEAVKNLDSKDIEKLRICTDLNEPIEFPEGKLD
EVIQATVDLTETVGEIQGKPIPALTRTLVSLRPGVRRATKIKEEYVAKELRGAVKRLHEAGSGEDV
VIKSAVDHLVMKERSFAEKEQRKPDYFSRIMIDEIFGFVFAGYETTSTTVSWALKFLADEPSIQTK
LRTAMLDAYVDAKVEGRNPNIQEITSKHVPFLNATMEEVLRCSGTAPVVDRQAVVDTEILGHKIPK
GTIITCLVTGPSMMSPGFEIDGKMRSSTSQQTIREGRDRAWDPEDVTLFRPDRWMVNDDFDPTAGP
QMAFGLGTRACYGKRLVYVEMRILLVLILWNFELLPCPASLSSYKSVLIMTNEPRQCYVRLKELDH
VMKD
>g2489
MAETASTQSPVEARSLSSITSIAANPPAYPRNPAQAKLDRVELYIVKVPGSKDVVLTPLKPPTKSN
ISIEAINSSLYYLHVATEEDENVLRSIELEQREHPHDALATTRLSNETVLRLNDFHRKPVRGQDAS
TATPYPPPPPPPHLEGATSYQGHSVSEEDTPRIPRKNVHDSYCPITQPKETTAMRDFAIPRRPVSS
GGGTLLTPTLTELNLGQRLSYDGPRPSIENIRPHQSHVSKNLSPGDILTDQNLGSPFRITLIRRDA
ASGNQWNVATMSSSKEDKGVIHVEIFNPGYGKFMNNVHLSSNNSGQHSTSAASEGQNAVEVLKRMA
QNMMPDDSIGKKVFYRDITPVRHDLQSHSLSEVLFNRHSGSAETPKLAQYVSSKVNRGYYTFKSPW
GGTCSFLASVNGGSLKCKHTIPGPAMSSEAXVSSSGQNPEVTVSELRYNIPFALEQSFRPPSILNV
NGNNKSPEEKGKGKRAALSQLITTNIQKVQQHARSRSHSQGGTPVPILSVARGSGDEQSPASTVPA
DDEDRLDFSLAREHGGGGMSGKSAKLGKLIIEDEGIKMIDLVVAASMGVWWRSTYQPS
>g2490
MSDLQGRKVFKVFNQDFVVDERYNVTKELGQGAYGIVCAAVNTQTNEGVAIKKVTNVFSKKILAKR
ALREIKLLQHFRGHRNIACLYDMDIPRPDNFNETYLYEELMECDLAAIIRSGQPLTDAHFQSFIYQ
ILCGLKYIHSANVLHRDLKPGNLLVNADCELKICDFGLARGFSIDPEENAGYMTEYVATRWYRAPE
IMLSFQSYTKAIDVWSVGCILAELLGGRPFFKGRDYVDQLNQILHYLGTPAEETLSRIGSPRAQEY
VRNLPFMPKIPFQRLFPQANPDALDLLDRMLAFDPSKRISVEEALEHRYLHIWHDASDEPSCPTTF
DFHFEVVDDVGEMRKMILDEVINFRAHVRQQQQAAYQNQQSQQVAQNVPIPEHNQGPWKQEDPRPQ
EAPLPGSVQNDLEASLQRGSDFLH
>g2491
MPSSYKRAGKTAPRFLADINLYDPVHLPTSGRNLLVDVPPVFPDGYDGPPEWLSPSACNHVYNTKP
NQTLVIPPDQRQGRGAHSKVCAICSKCRLHTQVVVNYPGNVAPTSTHLHHLVYQSSSHANGTVTPK
GQSLETHYYRCSYTACGITASVQISSPVLNPDFLQLLTDVEVVNERADEAIAAHPERLEGIARPQP
ITVLDNLRTYLRNSLQDPQRSKPISAVNKRFVVCFGVDGKPCKDLLEFLEFSYRDEGFWDPPNPNT
AEDLPFQNELNIFIDNVIHELSILIEQRPVAEKKTPYALNYSTSPASIDFLLALEAANYARSTRLI
EFEMPAQPAYEDLGTLEDMASDLIIEAYHRQVAVDPERGPHYLECLNSIAQWRGGPDRDTINEAVV
TAYSKGAYTTHDILKAYEYFGLSPNDTSLTEEVIVGRFHAYLSSTTQETEARQQLWRIGDARKSER
IKSAAEDRISTAEQALVYLGVEENTPDDFVITMFTTKVNDSPSSKEISEKALSLIAEKRNSETLRH

FLKTGETGAGEMDVGDAYRLLQIPDRTVGDDAILAAYTICVDEAPTQVETYTRALTIIAREKDSPM
LSSFVSGSTTHSDRNLSEWPVGLQNIGNTCYLNSLLQFYFTVRPYREMVLDFENNKTDLDEESLRQ
KKVGSRKVSRLEVERSQKFLRELRGLFHNMITTPRGFVRPEQELARPFGLGEINGAPVAGPLGPPE
SLKSCEKEDVVDHIAIPAKENPVKDPQVSDIDSEATLVSEHEENQPLPVHAASDVSGNSEKITSTP
SQPQTVTKDDPPSRPPPEPPRPAEADRKRQLIEEVELGAQQDVTEVINNVLFQSECAIRPRGIAPD
GEQLDQIKDLFYGKTKSYITAQNGTRSKEELWSDIKVDVATGSRDIYSAIDGAFDMQEVSVDDSLV
EQFGAISKLPPILQIQVQRVQFDPVKKSSFKSTHHLDLKETIYLDRYMDTQKQDIVNRRRDCWAWK
RKLVSLQNRKAELLRLNDNDGQNMPALFKNASNLLTELAAIDSTEEGAGDTSGINAELAAEMEKLS
QITQAELAHIEQETKDIEGMISTSFNDYTKLAYRLYAVFMHQGSVEFGHYYIYIYDFKKDVWRKYN
DNDITEVKDTAEIFRNPNRANPPTPYFLVYVNAAMKDRLVEPVCREIPSEGSTAENTATTVVPQTD
GPMDVDVDTEMKPPSYFDLKTKGESEISSLDPTSPLSPTAVPLKRKGSVDQMSITMGAPVDAS
>g2492
MRRRHLAIFKAAHSRRAPQTTMAQQYTEEHTYHPQDAIGAAFKATALTGGFGLFASAVQNTLAKQN
VGPLGIFLRSGGTIAIFASANLRETNDTYNTALGGFFAGSILGLRARTFPSVFGYGTALAVTLAAF
EYTGGSLFGKEKDHNLDKYAELERIRTNYRTPAEQTFAELGEGRGIYGPGYAERRRERIKEAYGID
VPATPPPAS
>g2493
MAMPQSPPHEGYGNIPYPNDPTQLSRGYGMSTDSRPTSYIANSIVPSDFAVAQPTLGTNPSQTAYP
SRFNEEIDMSQRGSPIMMDGANGGTGLQRSESQMSHNSIAPSRSGTLKKKASLSRKGSLKRSGSRR
SLRAGSVRSLNLGEREKYGSDDPNSAFYVPIPTSGSPTEELANRFQAWRKVLKDLIVFFKDIQKSY
ETRAKLLLSAGNIMNNTTLPSTFLDSGGLGDATEILRDYHKQGLIEANKAKEVESEVILQLTGLRS
DLQQKTKEIKGLSGDFKNSVDKEVDNTRKAVRNLQESLGLVDVDPSATSGRHDPFILRLQVDKQVD
KQIEEENYLHRAYLNLENSGRELEAIVVGEIQKAYNAYASILKREADVALDAVEKLRAGPISMPKD
HEWNNFIAESEQLVDPRIPVRSFDNISYPGRDHPAAAEIRSGMLERKSKYLKSYTPGWYVLSPTHL
HEFKSADRVAFQTPVMSLYLPEQKLGSHSQPDSSSHKFMLKGRQTGAMHRGHAWVFRAESHDTMMA
WFDDIASLSEKTGEARNAFVRNHVRSLSGNSVRPSSISSDGVMDEDEADRTPYSSQSAVLRRASSV
KAETPAQRPSPGGRFPSDVQIDRHLQATMSTSSGESTGVREALADAVALPGSGILYETSQQQPQEM
TYSSLPIDGQLAYRPADVNHETHGTWSGSANQNPGYVQDHNFGQQEQVYRQHPVTSPSQAPIVVAS
EITDRRDVNSTQTPANGAIYAVEAENASSLNRQESQRSRQTHSTYSLMASTPASEATSPLFDNANG
LVSKRPNPQSKESASTIDMLKMPGRYPRQMTG
>g2494
MGCDYCGPPAPPHEADVTPTAAPNGKTSSCQNSCCDCDDAEPLDTAASGSKMPTLEKAGDCCPPDK
CADNKTENQTDTPDCCRGKVSPCCDITCLNRLAMRECEMSAMATPAPNSQPKTCGGATVRKPCSQH
RLSTLDRYGATLQALGCICRALIALGRETCCELRETQSLDGKQCSTKSSARSLRTSLESYSSNGSV
TEDQVGQDPLRPQKSSLENTRSSKKVPTDACFASCCSKEKPAKKCPVKSKCLKPYCSEDTPAEVPF
VKDNCAKACDSLENSAKQPAAQTGRLDSCCSETLSIEESFPKIGCPGSCCPKDQAVKDNDAKSIYA
DPCCSKIEPIVEPATGVCARSSCGSKTMITIEPLNLVSSGSCCKLPKQDIVKTPIASCGGSRYTEK
QPPSSQGPCIDACCSSVVPNAGLEIVKASIQAVTDIENQGTGKEHVVLSISGMTCTGCETKLNRSL
VTXPAVTDLKTSLVLSRAEFNIDLRLGSVEEVMKHLERTTEFKCERVKNQGSSLDLIVPDQPLKFI
SQTWPGGILDMALMDKQTVRVVFDPKIVGARELAEKVWGTPIELAPPRGDPSLEAGSKHVRYVGYM
TLLSTVLTIPVLIMAWAPLPERKVAYASASLALATIVQVVIAGPFYPKALKALAFSRVIEMDLLIV
LSTSAAYIFSVVSFGYLIVRRSLSTGQFFETSTLLVTLIMASTAILVDQKSGTEREIDARLLQYGD
IFKVLPDTRIPTDGTVITGSSEVDESLLTGVMTIRLSRLPNENTINTISAMVDEAKMSKPKLQDLA
DQVASYFVPVVLALTIITFVIWFAVGTAIRSYDGSEATIEAITYAITVLIVSCPCAIGLAVPMVIV
VVSGVAAERGIIFKSADAIEVAHKTSHVVFDKTGTLTRGKLSVVMEHCDNVDKLPLLLGLIENSRH
PVSVSVAAHLKDAGVIASLVPEPKSLTGKENEAVYSLEDELRHDAVGTIKTLQQRGVSVHVVSGDD
DGAVQSLASRLSIPGGNVRSRSSPADKKDYIQTLLDTEIDHKKPVVVFCGDGTNDAVALAQATIGV
HMNEGTDVAQSAADVVLMRPSLAGILTMMDASRKTVNRIKFNFAWSFVYNIFAVLLAAGAFVNARI
PPEFAGLGELVSVLPVIAAALLLRWSKI
>g2495
MTSVPRSYCEYSDEEVEAARRYEGAQRALWEFRVDEYAREFYEDVRSPTPDDPFELTCTVTLDGEQ
YKCTFDVELWEIDPARSRSRVNWDLPGKVEVTYIADGSIRRHKTIDLILEDQYGSGTVGIFIDNWD

DMEMDILWNDYLLIFLKFDENTIXYHNPDTPYLLEVVEDVVKRHFEIYASPQASRGREFEGYGFVN
NEEEADEIPSLSQSDASSVSY
>g2496
MLPAVSKYLSTNSSQRLSKVGLAPGLALYSPEIPVHRDRSLTSRTYKRSVGFVRDRTRSPRGDRGR
RSRSPRRSRRSYSRSRSLSREDYHRSERRDRSPMSGAGGASGGHAGSTYGGRGGPPSGRSLEDRSV
AREHMMQSVRDSSQQDRRVYVGNLSYDVKWHHLKDFMRQAGEVLFADVLLLPNGMSKGCGIVEYAT
REQAQHAVNTLSNQNLMGRLVYVREDREAEPRFAGQQRDAGAGRGGPGGAGGAAGRQIYITNLPFN
VGWQDLKDLFRQAVGIDQKIFTAQQGAVIRADVLSDPSGRPKGTGIVVFESADDARVAIQQFNGYD
WQGRILEVREDRYAGGGGGGGGFGGGFGGGRGGFGGGFGGGRGGFGGRGGFGGRGGFGGPGGYGGR
GGFGGGGGGGNYGGGFDSAPAPPPGPPDPFTDYATSGGDKSAVIYVRNLPWSTCNEDLVDLFTTIG
KVERAEIQYEGNGRSRGTGVVEFDSPETAETAISKFTGYQYGGRPLGITFVRYVHPPGSGEVGDVG
DESAGMTQDQMM
>g2497
MIIAAPKAWMSLPKAMATRSSSRNINTRQYQTTCRQTGCDRMSIRSLPPTTRSYHSRFHAPLPTHE
YTNSQSTILAAALEHIPEHGFTKDALIMGARDMGFLDVSIQLFTRQEMDLVLYWLASRRGLLRAKV
QNGLIEGKQMSVDEKIKTLVIERLRMNGPIIHRWQAALARMSLLGNIPLSLSELHSLSSDILYLAD
DTSLDSSWYTKRLSLSAVYASAEVIMTQDTSPGFASTEKFVERRFEDSQYLGQKVSDVQSYMGFMA
GTAVGLGRSWGLKI
>g2498
MPRSQIEEISEFEKQRLANIAERDALLKKLTLEAQSSGLFAKASPKPSSKDTTSRPKKKPAPRVKK
EDEAPIVRRMSSRLRGIQADSEVAKRKADEAFEQAQKVEREKRLRKSDFFTLDNMLVSGQKLTGDA
LIGIDVVTKGVAKPYERTFGDEDVKNTKDKDLKALREEMNSLELWDQWTPGRIKITPERIYTMTFH
PSEAKPLIFAGDKMGHLGVLDASQKRPKFDEDDDDYDDPDPAITTLKPHTRTISSMVIPPQKMTRL
YTASYDSSIRELDLEKMTSEEKWAPATTAADEPVSSIDMAFDDPNVLYWTTLSGLFNRYDLRASHK
PSSVQEWQLSEKKIGGFSLLPSHPHFFATASLDRTMRPVGEHESRLSVSHAAFNSVGQVATSSYDD
SIKLYNFGSKGIASWRPGHRLNDKEMKPDTVVRHNCQTGRWVTILRPHWQQTPQSAIQRFCIANMN
RFVDIYTSNGDQLAQLGGDDITAVPAVAVFHRSKNWVAGGTASGKICLWM
>g2499
MEQERSNGFRRNSLQHGGIHSRPVERRPSKKSSSKDRHGIVYPESFQQGSIRTVTPDSASEVGNQS
FVSESDHLSTNPTVSPRSTARSRNTDWERRREFSPYQSAADDDDLSSESKSQRARSRTTTLEDQRS
EISPHFLSRARNRIGSINTSVPSTDSKDSEDSVSSIGYPSVVQSPTHAGQSGSRQRLPKAPSGGPA
LITGVSRNPAAFVSPLSNADSAKILQLMKTTCGRMHGILSFRTATNAQWTSGYCAINVATGSLIYQ
AKGEVAMAKTLIPDLRGCHVRTMFDTELQSTYLSVSTFSSGLGIQLKPHVNETFDSWLAALLCWQP
IRPKGVQNKRTKPQSVAIGDRRIADRRRNSESTVQKEAAIIKVGKMLLWEKPSPSGARPESGWQAS
TQHRNHRTHSTAWQRVSCTLQENGYFKLFTEADVSLLAIIHLSQLSRCAIQQLEPSVLGDEYCIAI
YPQYAALSVSETASRPVFLSLESRVLFEVWFVLLRAFTIPELYGPETAASEDPSRSQTLINPSAGS
TTDMFRIERVLSLRITEAKLTGNKNKTEESPKSKKPFDPNEKTFVSDYYTEVLLDGEIRAKTAVKQ
RTSNPFWREEFAFHDLPPILSNASILLKTLNPAQKDWTLIAHGPYSLDAQSNPLSVLDDLELSSHD
ATSGRVDLRLDELETGVDHEKWWPIIDDRDQVMGEMYMKVRLDEMIVLMSHEYEPMSEILHMFTNN
LTINMIQLVPTELTRISEILLNIFQVSGQAADWISALVEDEIDGFDRDTTSTAHHRFRYGTRIHSN
DSKESGQDREVIVRDLGRSATIEANLLFRGNSLLTKALDMHMRRLGKDYLEETIGDRIRDLDESDP
ECEVDPLKLRYNRPEDLERNWRNLISLTTLFWKSIAGSASRCPPELRRIFRHVRACAEDRYGDFLR
SVTYSSVSGFLFLRFFCPAILNPKLFGLMKEHPRPRAQRTLTLIAKALQGLANMTTFGSKEPWMEP
MNKFLLSNRTAFKEFVDSICAIPAERPAAIVTPQYATPIQILGRLPPTSREGFPSLPFLIDHARSF
AALTKIWLLKAPENLNEMEDVEPSVAKFHSLALELQRRTKECFSKAESAERPSGNLEMKWEELVES
MDRSVTFYDEASKPATPAAETPLTAPAVVSSHRNSIGYFAPKPSLPRRSTDTTADGDDETPPSSSS
ATWEHYRLPFAVGRSSDHRESGTSSRNSSQYSLENPDSSKSRQSTIGKEASSKLRLLDFVPGPSRR
KNKDSFNNNSNNTP
>g2500
MMNSNLLSRMLPPAGSPSIYEAMRNDDEASGSSDIEERAAMALDEENLQGSLHKYDPDDAGNSQIT
TQSTAFLGQGRPRKLSDTYSTKAGGNGVRTGRPRWLQHLSPRVAEADEGDDDDVPASLLIEGHDVE
DMPKPPAPPSHGMHYHDDASPGAGPSSTRSRKRWESPRNRRRLDQFLPTPIVQQTQKPGGLSGLAS

AGPKDKAMWRWANVENLDNFLKDVYVYYLGNGIWSIMMSRALNLLTLGFVVGFTTFLSSCVNYSLI
RGSNSLQNVLVPKYISDPEIQTISWQQVVSRLMNLRDSNPSTAAAISARHRRFMGSQSKQRMDAHD
IANRLMRKENYLIALFNKDILDLTLPIPFLRNRQLFSRTLEWNLNLCILDYVFNEQGQVRPLFLRD
THRRALSEGLRRRFIFAGVMNIFVAPFIVVYFMMHYFFRYFNEYQKNPSQIGSRQYTPLAEWKFRE
FNELWHLFQRRINMSXPFASRYVDQFPKDKTVQISSFVAFVAGALASVLALASLLDPELFLGFEIT
QDRTVLFYLGLFGSVWAIARGLVPEETDVFDPEFALLEVTNFTHYRPNHWAGRLHSDEVRQEFAML
YQMKIVIFLEEILSMIFTPFVLWFSLPKCSDRLIDFFREFTVHVDGLGYVCSFAVFDFKKDAQTNP
QGRVQTGPAGDLRDDYFSTKDGKMLASYYGFLDSYGANPKAAAAPGTRRRFHPPPPFPGLGSTHPT
DATPRAERWDQMAPRQSPMVGPQIHHSTLRGSRMGGVPGYNSPFHSMLLDPHHQPSASALRSAPRA
GAQSRFRPSRAGINDGMDDVKELSDVEASTKNAAGQQFGQIGDDSTGAVATSDNNLEGSWRINLAG
EADDSDSVEEGDEVEAIVNGPGGVLGLIQQFQKANNEGRAGGATVNI
>g2501
MPRSRRYRSFVIFSVIFGLALLHFVRSREWSSEWAQPAANPKVAPATIPHDSSPHEPILPHDHDAA
AEIKFDSTPRIEVPPAPKEQPLDTKKPSTQETAKQSPNSEKSKPNTIKPPTRPASDDTNSIDTPSV
ADSSRQSSGDKSYDETTRKPDSPAKVQPDVFAQTKPTKHWEKLPEHFPILESDLIPLPTGEPKKLP
KLQSVTKTETTAQSILREQRLAAIKEEFEHAWSGYTKYALGHDEVRPVSGSFRDPFAGWGATLVDA
LDTLWIMGMKDEFSDAIEEVSKIDFTTSFRSDIPLFETVIRYLGGLIGAYDVSGQKHTVLLDKAVE
LAEILMGAFDTPNRMPVTYYQWDPEHVAQPHRASTRVVLAELGSLSVEFTRLAQLTQEHKYYDVIA
RITDELETMQNNTRLPGMWPLQVDASGCKKAAPRVPSLTTLGQKSGVKVNEDRGLGTPTPSPTPKS
IHKDTAIPKDLEKREGGLELDAEPADYESETKTTTTANLATFPKEECEKQGLASSNAKFDRFGLGG
QSDSTYEYLPKEYMLLGGLSEQYRSMYEISMEPVRETLLFRPMLPEQRDIRFVATVQVSDPGPKDD
ADGFHIQYTYEGTHLTCFAGGMFAVGAKLFGIDEDMDIAAKLTDGCVWAYESTNTGIMPEGFELLA
CDDPLACPWNETRYLDVMDPNEKSRIQQAEAWYERELLNAKQAQEVSLEKRPKIVSPVRVLESEGS
GASSEKTYAGIAKREASNSGVDDYDEHAHLSTSKPSVEKTSVGTQDTPKSRLDSKVSPDTKYQADG
LPIKPTVLSHKDYVANRLEQDHIPLGYTKIQSRKYILRPEAIESVFIMYRLTGDDTWREKGWKMFQ
SIARYCSTEIADSAISDVTSTAPELLDEMESFWLAETLKYFYLLFSDPSVVSLDDYVLAGGESRIP
RSTSRVQVTRLPLTLLTSIVSCSFQITPNHSPTGTRRDECAIKWEALVFMQEMELEDVSYSLENEQ
QFWVELEKILSEQCDTHALIDNVLRAYLGFTAQFKGEYLVNEYDISRCSYKLFSSNLFAQHADYIR
RQIIYSLLQEDDPNILHFIVSFLLFDGRENDSTFQLMNEEGAFPRLLELVHFVGNQDNDAAGLHRL
LMDLLYEMSRIQRIKIEDLVLVEDDFVRYLFQIIEGLSDDAHDPYHYPVIRVLLVLNEQFMVLAHD
PATGTPSNPLTNKVMKILSVHGNDYKTFGENIILLLNREGETSLQLLTLKLLYLIFTTPSTYEYFY
TNDLRVLVDILIRNLLDLPAEAVALRHTYLRVLYPLLAHTQLKYPPHYKRDEIRRTLSILVHSRIE
GNEDDYEKIMHFGEADETTKRLVSRCSQVGWLLDTEPELRNTPDLPEEDGPSDHTGVPIPAVLHEK
AADLAGSPVSQSSDATSPTKLEPASPNTSFSIDKKQIEKLGMHHEPAAASTLSVGEVAVQTEKPGV
ITPSIKNTRSPGLTSETSPTVKVKLVPQPPKARRSRAHRNTEAHHDSRGRSGLDIHHESPQEGLPV
PHLSSTSRSTSRSAPALPPPRRSFHHTSPNATISDHHAHPPLPIKSNIKPQPPKARRSQRQHVARL
HNELSGADDERSASPASVASSVPPKDLDESHHSYDSSASIEQAMDRTNLQV
>g2502
MKYGVLGIAALALLHFTDGHVHHHQQLSKRALRQQAANQPCPKVTLTNTVVVQLDQAGHTVDVQVQ
SARPSTTARFSDAASGQRVASTSSTSPATATATPKPAQSDRHVIPKPSALSHLPDPLSSNLLPDTS
STFLGQRSNYGISYSPYRADGTCKTQEEVNNDIRQLRQFSFVRFYGIDCNQAETVTHAARQHDMLV
FAGLYDIDDIETDLQKIIDAADGDWSTFHTVSIGNELVNRGSKTPGEVVGTVNSARSILRQAGYEG
PVVTVDTFNQMIAHPELCDESDYCAANCHAFFDSNQTPENAGPYVLEQARHVSKATKYGNKQVIIT
ETGWPSAGEANGDAVPSRQNQLKAFKSLRQSFPDGGIVFFSAFDDKWKQDNAWTFGTEKHWGLYGL
AKTASGFPDLIGPLEDEV
>g2503
MDVHEVQRVLTDFLQELSTLFHRVPGSAILLRYVKSSYQNDPIRSAVELFLFLFAVRYLLAPKYST
KPGIVKLSEEEIDDLVDEWTPEPLVGTPTALEEAEVEKRTVIVGPTGPKVKLSNGRSVMNMASYNF
YNFTSNESLKEKAIQTLRTYGVGPCGPPGFYGTQDVHIKTEADVAAFLGTAACIIYAQAFSTISSV
IPAFSKRGDIIVADKGVSYAIRKGIQISRSTVRWYEHNDMEDLERVLEKVTREQARKPLTRRFIIT
EGLFESIGDMVDLPRIVELKFKYKFRLILDETWSFGVLGRNGRGITEHQNVDATEIDMIVGSLAGP
IIAGGGFCAGSEEIVHHQRISASAYTFSAALPALLSTTASETINMMQTNPDLFVQLRSNIKTMWSQ

LDPRSDWMYCTSAPENPVMILAIKPEIVSSRRLSYEDQQFLLQDIVDETLANGVLITRLKSLPDSV
TPSQQVLPSLKICLTTGLTKREVEKAGTIIRHAITKVLGKKR
>g2504
MSAGHYDDGYGHQGHADHQGHGDAYYQDDQHGYYDQNEYSNYGDGYYDQSGYYGDNHGEHHGEQHG
EQHAGGGYYEGGQDNYYNEQYYDQGHGGHPDYAQNGRSRRRGDSEEDSESFSDFTMRSETARAADM
DYYGRGDERYNSYNESQMGGQGYGYRPPSSQISYGGNRSSGASTPVYGMDYSNALPAGQRSREPYP
AWTSDAQIPLSKEEIEDIFLDLVNKFGFQRDSMRNMYDHFMTLLDSRASRMTPNQALLSLHADYIG
GDNANYRRWYFAAHLDLDDAVGFANMQLGKANRRTRKARKAAKKAAAENPENEQATLDALEGDNSL
EAAEFRWKTRMNRMSQHERCRQIALFLLCWGEANQVRFTPECLCFIFKCADDYLNSPACQNRVEPV
EEGTYLNNIITPLYSYLRDQGYEIYDGKYVRRERDHAQIIGYDDVNQLFWYPEGIERIILEDKTRL
VDIAPAERWEKLKDVNWKKVFFKTYRETRSWFHMITNFNRIWVIHLGAFWFFTAYNAKSLYTKDYQ
QQLNNQPPGSYSWSAVGLGGALVTLINIFATLAEWAYVPRRWAGAQHLTRRLLFLLLVFAVNIGPA
IYVFGVSKDGTDTIAYVLGVVQFFIALASFFFFAVMPLGGLFGSYMKKNTRQYVASQTFTASFPAL
SGNGMWMSYGMWVCVFAAKLVESYFFLTLSFKDPIRILRPMTIQHCLGDKIIGDILCHAQPQILLG
LMFFTDLTLFFLDSYLWYIILNTIFSVARSFHLGVSIWSPWRNIFSRLPKRIYSKVLATTDMEIKY
KPKVLISQVWNAIVISMYREHLLAIDHVQKLLYHQVPSEQEGKRTLRAPTFFVSQEDHSFRTEFFP
SQSEAERRISFFAQSLSTPIPEPLPVDNMPTFTVLIPHYSEKILLSLREIIREDEPYSRVTLLEYL
KQLHPHEWDCFVKDTKILADETSQFNGEYEKSEKDAARSKIDDLPFYCIGFKSAAPEYTLRTRIWA
SLRTQTLYRTISGFMNYSRAIKLLYRVENPEVVQMFGGNSDKLERELERMARRKFKICVSMQRYAK
FNKEERENTEFLLRAYPDLQIAYLDEEPPANEGEEPRLYSALIDGHSEILENGLRKPKFRIQLSGN
PILGDGKSDNQNHSIIFYRGEYIQLIDANQDNYLEECLKIRSVLAEFEELSTDNVSPYTPGVSTPK
TDPVAILGAREYIFSENIGILGDVAAGKEQTFGTLFARTLAEIGGKLHYGHPDFLNGIFMTTRGGV
SKAQKGLHLNEDIYIGMNAVLRGGRIKHCEYFQCGKGRDQGFGSILNFTTKIGTGMGEQMLSREYY
YLGTQLPLDRFLSFYYAHPGFHINNMFIMLSVNMFMITMINLGALRHETILCRYNSNLPITDPLMP
TGCANLVPITNWVNRCIVSIFIVFFISFVPLVVQELTERGVWRAATRLAKQFGSLSFMFEVFVCQI
YANAIQQDLSYGGARYIGTGRGFATARIPFGVLYSRFAGPSIYLGARCLLMLLFATATMWTAALIW
FWVSLMALCISPFLFNPHQFSWNDFFIDYRDYLRWLSRGNSRSHNSSWIAFCRLSRTRITGYKRKV
LGVPSEKLSGDVPRARITNIFFSEVLGPLVLVAVTLIPYLYINARTGVTENNPQATNALIRIGIVA
LAPVGVNAGVAGAMFGLACCMGPLLSMCCKKFGSVLAAIAHGIAVIMLLAIFEVMFFLENWSFPRC
VLGMISMAAIQRFVYKLIIALALTREFKHDQSNIAWWTGKWYNMGWHSLSQPGREFLCKITELGYF
AGDFVLGHLLLFAMLPALCVPYIDRFHSVILFWLRPSRQIRPPIYSLKQSKLRRRRVIRYAILYFV
MLVLFIVLVVAPLVVRRLNINLPTIPDSLMQPLDGGSLHNDTHTYYTGSGLPAGFVAHSGSDSSSS
SSTAGAKMMFMF
>g2505
MARLNASNPTVVYGRQSPNASRTASTTSSTFYRTTISSPLKPKPKPKPKPKKNNGFSDFKIFEDEE
ESDHDEGERADPFTESPRTNQKNSKAPLKLTHSNSITSGLSKRASQDSSGSSSRRSRSGSESEEEE
NSDKENLFFDDEAEEVSDNSEEEDDEEEEETGLHEDTNLEADNTLGENVYDGRSESEAQEPSFMRY
REPKLKEESDESEPEVSDNSDGYNSLDDFIVSDNEDLSYHDDGLEDEDEDEEEQKVKLPTPKPIRR
RLLRGRKPRTSSISSQTQKLNISNEAHSPATTKATPPHRSSHKPTTPRFSSPFTRLLPNESLDEEG
EPTKYIKPKRSPLRESINSITSSPGSTRKQQVNKMQTPPCSPTKTLRPSPSKTKIPPSPHRPSSDA
FWSQELTNAWNDQWSPPKQKNRSRALERLLALEEVEKADFRADTDNMPVDKDLSETPVKSPSKTAI
KKAEIAQRKAAAARKKEFDDRKTSLAQEFLLALDEAITGGQINELAQSTGGVKIEWSKTLRSTAGR
AKWKQEKFKQADGGFRYIQHASIELAERIIDDENRLLNTLAHEYCHLANYMISKIRDQPHGTSFKE
WGRKCVDAVQQHPIYKHYPIEVTTKHSYEIDYKYVWLCIDCGHKYGRHSKSIDPTKARCSLCKGGL
QQVKPKPRNVSPKKKTPVSDNASPQKVTVLGENFDDMLESLGNAHLN
>g2506
MRLTLGKGATAIYRARAGASAYRRHGVRNVQVKAAPLGEPTVVNGVNLPMSSTPSSASSADAKFHV
VGTPYSMLSVSLSASQNLYTRRGTLVGLSGKADNVISTLSVLEPFRRAVVGIPFLYQKVSSPSPIK
ALVSVQSPVTSFAVVHLDGTVDWMVAQRRALLAWTGHSLNIKPRINTNLSVTNWGSSEVTGRGLMA
LVGRGQVYSIELKAGEQYIAHPSNIVAYTLSSTPPQPYRFKSTTLRFQIPGLNIPELLLKSRYIRD
FTASDTWKFSMRIIHNFRTWARRTIWGDRLFLQFDGPATLLVQSRGARVRDIMSDREVNEIADXPA
GATYNALNKLTGATDQSDYRRAAAEAVSEAPVPSRTVEELTQELKGVSQTIATIRDGRVEFEQFRQ

QNTDNTRK
>g2507
MFARAVRSSCRRLPQRPQCLYGLARINNQNQLRILTTGRALRDNQKTNGNSPLKSKIAPIATDTTN
KDDGPGGQKLVAEAKSTPKKKTEDILNTSAATNKEQRKADWAIMKDLARYLWPKGDWGTKLRVGTA
LTLLIGAKVLNVEIPFYFKNIVDSMNIDFATVGGTAYTVAGXMIIAYGATRIGATVFQELRNAVFA
SVAQKAIRKVAGNVFDHLLRLDLNFHLSRQTGGLTRAIDRGTKGISFLLTSMVFHVFPTALEISMV
CGILTYQYGFRFAAITAVTMVAYTAFTITTTAWRTKFRRQANAADNRGATVAVDSLINYEAVKYFN
NEAYEVSRYDKALKAYEDASIKVTTSLAFLNSGQNMIFSSALAAMMYLAADGVATGQLTVGDLVMV
NQLVFQLSVPLNFLGSVYRELRQSLLDMETLFNLQKVNVTVKERPDAKVLDLSRGGEIRFENVTFG
YHPDRPILKNASFTIPAGAKFAVVGPSGCGKSTILRLLFRFYDVQEGRILIDGQDVRDVTLESLRK
SIGVVPQDMPLFNDTIEHNIRYGRINATSEEVQKAAERAHVHELIQRLPDGYKTAVGERGMMISGG
EKQRLAVSRLILKDPPLLFFDEATSALDTYTEQALLQNINSIIKEKQRTSVFVAHRLRTIYDSDQI
LVLKDGHVVESGTHAQLLETKGVYAELWHAQELSLAQDADLERNLEVGPDSELAENVAPTDSAQPK
K
>g2508
MGFIEKLQSKLELYRLXXRYTRRKHRTTFYGDAQYVDGEYVYVNSPTSPAISKNSTGSHWRPSWGR
SATSDSRNS
>g2509
MADQSKAEILLVGCGGVGTMCAYNLEVGSQANVTAVLRSNYDAVDKTGFSITSIEHGEVAGWKPSK
ITRTISGQDSTPFDFIVVTTKNVPDQPPTIAEVIAPAVTHGHTAIVLLQNGINIERPLFSAFPDNI
VLSGVSMISATETEPGKIRQDDPDILTISPFHNPRISADKELAAAQRFVDLYNASGKVSCILEPDV
ADVRWRKLIYNAAYNSVCAILDLDTTSIRYAKQPLNDLVRPAMWEVWNIAKAAGHPLPPDIVDEKL
DIDTWSFFKPSMAQDMSKARNM
>g2510
MSSGIPLGITSEDSYIVLQGSQGEGNGHYTNGAVIHHRQVTMTHPRAPFHRPAQKPEEGVTSFSNR
PYKSEALLQHVQSNIARPLAYRPRERPSDVTWRAVIVAVVGAAKKSTDSLVSERRLDSLKVPIVVQ
IKPIFSILVSLN
>g2511
MSQYPVPPSFDGQHNYAPPWPVRLPADSRESKQSAANFFAMNNSPIDFATFAGFPLGSVAPIQLPG
LGMAPNATSPYQPQHFAYDQTSMPFYGTFHTPFDASNDPMHSALENTVANPSKKKRKALPIRRPSK
SPKHHPPGAINETDREEGEVSDGSGHESSHRHSRRKMYTRKITPAGPEASLSRSRSALDTDTNLSR
SQTPPRNNSVSNLVPASNLRAYNLPVTVGATEVSQVKPEQADGLQMDKNRITPHNIRFSELVKEDI
DPTVLKQLYDDIGIKVTPVVPKTDSAKVKITEPTIDKLESRAATQEKSQVAPSASAKPLERKDVIA
RMLAEKAKKKATAVNADSVLDATTLRNGSSSALGIEKSADVLKSQPPTQTPSQTKEKNKALSELAR
QRIEQLKKMGLKRQQSNPESTNVPLVPASSLSDTTIVRPEATTPLHHPLPERPPVAPSSPKPSTPQ
HPESSIPSESRDTSSPSIVENRSMGASTPFKNSLGKRPRASDFDEPIPEIKKHSMNDRLVIDISED
ESLDEAEDIDMSEASDPTMNTHPLKTGDSIPRATISRNASSASGTPQSRASELESLRQKNLEIQAM
RRRIAEWEEKNAKKTKAVPPSATVVGNGSPAPASVNIETDEDKFQVNANTAQNLATPLEKPSPQRL
QRSPSVQSLASMDNSDLDRIRQKLLRKRVIESGLPNLDAELMKYEAKLAEYKREEEILLSEIARSR
EERKKLGEELESLGMETEGLTLEELRAAKEDIERGIAIEVGFETHGVVTPESMLPSKPLQAATTVV
EPVQAPQIPALPSDPPIPEPTLAERQGVPSITELDSSGLESSGSSMDESTSSSASSSMDQDSGSEP
EQEVAEAPFEPSIPAATEDTPAHNLSLIEASQTATLVPTNVTSTESNAMDVDPAVDLSAAADRLSA
SRESSVLSDNYEPPEPEESDEAYSPKLSPKDIEESEIEPAPQLMVPNDANVTLTRKPQDFVVIASK
GDALDNTPLGGDTVSKFSPYQSPLKYFKAYRYHPNYLKDVKDGYRSLTYSHQINSQQPFCPYEAAG
GVCNDVTCDFQHLRDIVMPDDKILVDMSNQREGKTPEEREAYIEGLKDLINSMQRDQVKDFPTIAG
EIAAYRRRFLSDPSRILPL
>g2512
MQPSSFNSSPRRANPFNRHSPSPSPSPQVSTIRPKSAVFPTTFGESEKKPHTRNSSLSHFSAVALE
SENRPRSSSLRHSVHASGTFAPQFIKTEELQRGANSIHNLEGDNDFSGKRYVWLKDAEKAFIRGEV
LEDRQDGNLLVQCDDGSQREVSSENADKVNPAKFDKADDMAELTHLNEASVVHNLHMRYQADLIYT
YSGLFLVTINPYCPLPIYTNEYVRMYKGQSREDTRPHIFAMADQAFRNLVEEGENQSILVTGESGA
GKTENTKKVIQYLAAVATPESSGSRSTGKHFSNLSQQILRANPILEAFGNAQTVRNNNSSRFGKFI

RIEFTRSGQISGAYIDWYLLEKSRVVKPGTRERNYHVFYQLLQGCDKSLRNKLLLGDMQIEDFAYT
RDGNDSIAGVSDVEEWKALMEAFQIMNFSENDRMCILRTIAAVLHLGNITVTKERIRGDQAALTPE
AFDSVQKACQLLGVDTDSFVKGLLHPRVKAGREWVEKVQTPEQVRLALDALAKGIYERGFGDLVNR
INNQLDMSSSASDDTFFIGVLDIAGFEIFESNSFEQLCINYTNEKLQQFFNHHMFVLEQEEYAREQ
IEWQFIDFGKDLQPTIDLIELTNPIGIFSCLDEDCVMPKATDKTFTEKLHSLWDRNSPKYRASRLS
QGFVLTHYAAEVEYTTEGWLEKNKDPLNDNITRLLSGSTDKHICNLFSDYVDTDDEQGFSRSRVKK
GLFRTVAQRHKDQLSSLMNQLHSTHPHFVRCILPNHKKKPKQFNGPLVLDQLRCNGVLEGIRIART
GFPNRLSFNEFRQRYEVLCRDMPKGYLEGQSAAHIMLNKLNLDKAWYRVGLTKVFFRAGVLAELEE
KRDALIREIMTLFQAEATRIIQKNFHPLLGATRTAAEVKKRDEKISALEAKAQQDLQDRHKLEEER
RRAELEINRVKKTLESERALALDKEEIFKRLQIREVELSEKLAEAIADQENLEGQLDELIEAKRKA
EEQLDARKIQLEQAGQIIMKLEADKKQLQERLSTMDSTLKEMENTHSKRDSQVQDLTQEMKMLQSQ
LNLKERKLQDLETKLLKSDQNLDVKLAKTSKELEASRIQVKNLFDENKAIRQEIAELSKTSTGYED
LIRRKEGELVVLRNDAKRHEEAKKSLEAEMDATKSEKLTLEREAADVKKMLEAKMTEDAEAGHSRR
LLEQQVQDLKAQLFQLQADLSRERQSRDDVQMLGEHKLAELKGKYENLNEAKITIEKEMYIQQDTL
RRATEARITAEQARKDLQAELIRLRERFANAEKARLDAESHVERNLIKQANEKQAALRDELDAELT
QMLADSETFRERHDQQKERLERELVTIQQKNLEISRSNSKASDSQRSRIVQLQNEKAKLEEEQKKV
QQDLNHEVAREHKASRKADAASSTAQANTRQLQAALDQAHKEIEGCHQQLMLLHKVFDPETNESNE
SPKSWEAIQPDLSKKVDLAQLLELAHSDLRVAEERYSRAENNNQVAAPRSPTHIRKNSESAKRFSN
PSTPNRRFNLNEKRMDMATELEELQNKLQMTEMQNRHLQNQLELTTPNRDMWQDDSPSIRRVQLLE
RENGRLHEKLDDSAKKVSALERMIQTGELSLRDEQSRKSLLNFDKLKRSKAALEVELRDARSDLQD
SEYSREQDAASRNQLLQEFSDLQIRLDAEESKVIDLTSSLSLYKSRADEYFSKLEQAEIAVLKAAR
AEQFAKSQAKEAEDTCATIMADRQRMDSLVEDLQRQVQSYEERVEDLSADLEGALQASEIEDKEAS
LEQTRKKYQREFATITNELEIERENLLHVRDENSRLREELEDLRSKWDNEVLNSSTWAKEKSRLEM
TLQDVSSSRDEAVKAHNDAQSKVVSLLSQVRNLRTSVDDITSERDFLAKDKRVLESRLKEATDRLE
DLANGDSPSMRDAAAQHEDVSAAAVGKMRRAEALSVEMQKELIAEREANATLFKDKASLEKQLKEV
QLRCVDLETKGYSSASQDIKFLHKRVKELEIQLEEQESKYSAEQRSTRNVDRTVKDLQSQIERRDK
MNAQLTDDITKSRDRIERLLQTIDELQSTDSQSQLQAKRAERELREEREKALRLERELEGWKQLRI
ERGSIAGRANGLSDVASRRGSGVYVANGTVEVPQRKFSNTKGFI
>g2513
MARNSEKAQSMLFRFRAAQAADLGILDIARTRRPKGITQIDSIPTAEKWRGQVLKEISRKVTRIQD
PSLSDYQIRDLNDEINKLMREKHMWESQIRNLGGPNYMRGGGRVYDDEGREIPGGGKGYRYFGRAR
ELPGVKEMFEAAARKNRPDEEETESGKKAAELFRKHVDAAYFGYGRDEEEDALVAYEQKREKEAIE
NMIKNGEDDAEDGWEPLPGDAGDGVEWRLPTLEEVQEELVDRRRRRLLDKIS
>g2514
MTAKFVPRAVFPQYDSIPRSYFLGHHKAGLEKMKSMISSIDYIIECRDFRVPITSINPMFEEALGD
KQRLIVYTKRDLGGAADAVTRKNKELMRKLDPKTTVFFTTSTSADSVSTVLRHLKSDKSTAHRIAA
CRAMVVGMPNIGKSSLINTLRNQGVHKAKALKTGEHPGVTRKIGTPVKVVEREDGSSIYVLDTPGV
FVPYMPDAERMLKLALCGCVKDTIIPPVTLADYLLYQINLHDPKVYEKWSEPTNEVMPLLTQFAHQ
VGLLSKGAIPNIEQAAINIVQKWRSGELGKFVLDDVKEELTARKEGTLKEFGPSRTQRLKAITLER
KQKQQERIETSKSSTAMKIISSSERR
>g2515
MSLITAFRPRSTRISSALRHRSERARRRHQASSPGDQTSVPEDGEQGEDLDAHSIITPSKHTPKPW
RALKGSVARGGPLRPFRLLKQDIVNVRRRYLSDWTVFNQQIFASAVYYVFFTNLLPGITFASDLYVL
TGANWGTIEVVFSTGLCGIIFALASIQPLTILGVTGPFSVLAENIYALCIDSFKIPFLPFMAWSLI
HAAWMHYILAIVNAHDWTMRYVTTFSTEIFSLLNSVIYFHKAIQELQRAHDSLSFAAFLYAVIGAV
GTMLLAIFLSTAESWRPLFHRYIRLGLAEYAAAISIIIFIGMPHVGELANLDKMTLNVSHSFRPTS
PERDTFFVHFWTLPVGWILAAILPGLIITVLFFFDHEPGGFAWDIVLLGTTTAICGILGIPPANGL
LPQAPLHSESLMYSEHEQHTVIVDGEEKVESREVRRVYEQRWSAFLHAGGIMLFVSPPFMTVLGLT
PTSVLAGLFLFMGEQSLSVNPILYRFFYILTPPSELPQLPAALHNKDSPSERPSYLPIHYYTILQI
VLTAAVFVLTLTKAAPAFPVLIIALVPFRLLFMKRWWNREVLRFVDAWACREGTPEDDEDQQAQSK
MMSNAAEGAVFTADAYPLNNGRNVNAESYVARREDAGARETSDTDHAQEWIELDLHEPRAPADEEI
GAPTDKNFDA

>g2516
MEAFTFATSTQVDFPVQVKIGALEGKQKPIPYSILLKDPELRHLGSNQSPASDLFVTVQLWSDSKP
LGVPMQTSYKSFKTSRTWNEWLQMPICVKDAPLGSQLAITVWDLSPLGTEASNGHGVPFGGTTISL
FDTDGKLRMGRQKCKIYRNKAADGFSSSTTPAIPPAQRRKTHGALPLTQSPEEAELERIEGLIKKH
EMGEMTRVDWLDQLVFRQLEKIKTSAEEAAKKRATIVQATKQKPNQRENGVHEDDSDTESLYEENF
LLYVEFPRFDHPIVWDDYEYPPPPVSTHPSLVQNNPTATLKPLPEVRLGPGIEGPDDAGTYRVVRI
YDPEVGQTGNPCEDKHRRLVRSHRTGIMDRDLKPNPKIRDELNFIMSYGPTHELTAEEKDLIWRFR
YYLTRDKKALTKFIKSVNWRDLGEARQAMEILPKWTEIDVDDALELLGPTFDNSTVRAYAVERLRK
AGDDELLLYLLQLVQALKFEKTPEETAEEVAHDSSLANFLIARAANNFVLGNYLHWYLMVECDDTG
IANLASHRRLFARVEYYFMTELEKVHPENRKILLRQGELITVLTRITKELRFSRENRAVKIDKLKK
YLKDPKNEIITIDPPLPLPLDPSVIITGCFPDESNVFKSSLSPLLVNFRTSDGRKYPVFFKVGDDL
RQDQLVIQIITLMDQLLQKENLDLKLTPYRILATGATAGAAQFIPSTSLSAASAKYKGSILAYLRA
NNPDDSEPLGVRKETMDIYIKSCAGYCVVTYLLGVGDRHLENLLLAPDGHFFHADFGFILGRDPKP
FAPMMKLCKEMVEGMGGANSQHYLQFKQHCFTAYTTLRKSANLILNLFSLMVDANIPDIRVEPDKA
VLKVKERFHLELSEEEAIRHFEQLISDSVNAIFGVVIDRLHDFVQGWRT
>g2517
MALDTSATGSSSSTKRVRLAELQGNAESTQSSQPRASQRAVNDYYDPDQDVAERRKIRKGLRDLQR
EMNDCRGEFLQTDNKGILNTIQKANEYFAQVKQTSDATVDSALLVNAADLSYKKAARASDGLAAGI
DVDEFVSKCLSFMRQTPANGSSNGLGTQRRTQNRGNADESDDEGVDDTLNWDLLGRAACFPYNTRP
SLSGFLLGPLSVQKRTRQLTQRRAANQIDRTQVVRPQDLEEQDLDRQESSNLTVMCTKISKLLKEV
ADARTNTVDEELRALGREPTTEEIEAAMDKHQISDIGGILLFPFAINPKSFGQSVENLFYISFLIR
DGNVGVGQNRHGLVTIQPSTPYAPLEAQQRGIQKHQLIWSLNFEIWQQLVETFDIKESIIPHRNDE
EFQRQNQHGWYS
>g2518
MSDEWSPSPTFSIPTDRLHISYFQVDNPEHSDFLVRLWNTDDFIKSEGNTGLDTLEKAAAFIKNRV
HNDYLRNKHGQFLVSLKPHPDASLAESRLIGVVSLMKGEQPNAYYAPDIGYTILPEESGKGYATEA
SIGLLDYARRELGINDVFGFCGHNDARSARVLQKIGLQFRGNRKLSFFGGAHSAVYAWPHMSQDLT
VYGIDD
>g2519
MSSKLFPGDPKKVMVIRQVTSDITTFSVPFNRFGLIKFGGRGTLVKLATGNMLVVSPVALTSEVEQ
TIATQGGRIKYIAAPDMEHHIYLTAWKKAFPDAEIIAPEGLYEKRQSNPDQKDTQFSHILTKANKH
DIHISEEFDREIDIEYVDGHGNKEIVFLHKPSKTMIQADLIFNLPANEQYSRTGESPRSGIITKLF
APLISANPPPATWHKRFVWYVTANDRKSVGESVARMDKWDFDRIIPCHGDVIETGGKALFQNVFEW
FLEGKNKKQQRQQ
>g2520
MYFSKFILFPLLLAAGSLAGEDTCPAGADEVVVVKTITETYSVPVHPSSIAVSSVSPVSAVESTTS
RTPAAASSTQAAWTTVGLLTSTTSVSTVQPISSSSSSSSSSSSTTTTATSAPTASTSETFVGLGTR
YGDDCTEEDCWQGGACSFVDYTLPAGIDGSTCVSQDIWNNGANCGGCISVTYKGKTITVMVTNETG
GNATHLDMTPATWSKLTNGYSGGGVDGIEWEWITCPIATTTPLEIRMHGGASKWWFAATVENARLR
TAKLEVSSDSGATWTAAVLENYNMWILDGTLPDDTAWVRVTSIDGDQVVVENVALQSGVVTQATEN
Y
>g2521
MPVISRLFSVVLRIAELACAAVVAGIVGYYLHIWHEDHLSAWDQGRWIYTEVIAGLSLLLGLLWLI
PTAHTFFIWPIDLIIALCWFAAFGLEVDALNGLPCGGAFDWGYIGDNDLCGRWKAAEAFSFLSAVI
WLVSAILGIYFVWRVDHRRRNYYYGRYAV
>g2522
MSSLFQLQSLSSLTVSSFLPPLPLVGLITSTNALIYAYCEHIFLNPLSDRSVPPATIRRVWQKCFP
PGFVLVATSRIGSIASGVAGYRAADPDSAAGTLYAAAIAFTVVHFAFLPWARDHLLPILDKNPTKE
SDEKIHEGNKNFIRIDIYRTLASEVPAFLCFLGATLEYLKL
>g2523
MQMLDNNLIPHCLLLIVLATGLRCLEPDSALPDRWAEECRHLVIPDIFSRASTTNLQTLLLLQRYE
WHRGAHLSAYFVAALATRLATALQLEAEPSANRSDAPPLPATVMETRRRLIWSCFTMDSVPDGSTS

PLKSALDPFSIEARLPCDERSYWLGLDAVTDTFSKIKESGNTTGLGSSSVAGYIILMAGIRLQILQ
YASTFSPKNKELLLPEIPWHSDSKFQHHKRLMDDFLVKLPHEFQWNEYTKLNPEAWQSTRSTLCTL
HVMYHAAYTDLLRVALFLERAQTVFQSKSLFANSPFANISDQDLDPFVSVGACVSIRVLILERGPG
DAESLGISEAAVRNALDVATTCAKRTARWSQPMRKMLHALAERALQYGIRLDVPEIFLPASPTSHL
PSRAHSPSLRTYGTQGSIHRSLASNKDDTQIFLDQQNHVLTAHTMVDSGKKTSLQEPTVVTTIPQS
AQNPGISMESLSLLEASTTGITGNAILNSGNSGAPGLNSPFLHSYEALSPESLRIASNWSNGTYDI
VTSDLSFAMSPGDTFLDFNSDDYQLFNTDIGNNNIWPT
>g2524
MAQSRPNILVILADGYSDIGCFGSEIRTPNLDELARNGARFTDYHTASACSPTRAMLLSGTDNHIA
GVGIMSEQKGRDPERWNVPGHEGFLNHDVAALPEILQDNGYHTLISGKWHLGLRPENNPAARGFDR
SFALLPGTSNHYGYEPQFGKDYLKFFVRIPPLYTEDGKRYEIEPNTTHDKRGFFSSDFYTDNLIQY
LKDRDPETRQKPFFAYLPFSAPHWPLQCSKEDRDRYKGIYDDGPDALRLKRLARLQDLGLVPKNIR
PHDVIVGPMNTEWDMMTDYERKCSARSMECFAGMVDNIDQNVGKMVQYLKETGEFQNTLIVFQSDN
GAEGATYEAIPTMGADLMRVINEYYDNSLDNIGNANSFAWYGTRWAQASTAPSRLYKMYTTEGGIK
VPLIVHYPGFAKEKQNGSILQPFSTVMDLCPTILDLAGIKHPASDGKPGTFRDRSVAPMRGRSWVP
YLSSASTHDNDSMDSIHGNDGMGWELFGRGAVRKGKYKLVYIENSVGGRGSNDRDGSQDGWQLYDL
SQDPGEIDDIAENYPHVVKELLQVWDQYRTETGVVWGTPIRFVGEEWDGYSEENIIGGDAISQTTA
WMRVRKNEAPPSRAL
>g2525
MPQSEDSLKGGRQTLGSMLKKMTPALLSACFITAMLNMLFGFDTTSFSGVQSIPAFERQFGTPTGT
GGYALSGSRASFMSSVAFSGKFFGTLTMWILCMISFTGIVIECTSHKVVQFVLGRIIVYYSVGLAE
NTSTTYQSDIVPAGLRGAIVGSIQLFIQFGQIFASGINERFSTETRPKGWIIPVAVQAIVPVIVVF
GAFLIPSAPRWLISKGRKEDAIRTLEKVRPKEDVAAGVCKDEADAIEEALNSNVDKGPWIDLFKGT
NLRRTSIATMVFIFQQFTGQGFVSQYSPRFYKTVGLSKNAFLYNIISSVVAWVGVLIGMPLSDLIG
RRDLLILGAFFQGIFLFSMAGIGTKSSFTTSDANGLVASVMLFNFSFAMQDLGAYRICTSEIGTAS
LREKTMAFTSTINVVAAFIVAFCVPYLLDDIGANIGWLFGAISFVAAIYAYFFVPEIKASDYIHFK
SVPKLTYELFENRISARKFAGTQTHGAGKRVAELENRINLHTTHHQTSEGGKIGTISKN
>g2526
MEQHQLEHLGHAIVGLCMMFFCTSLVHVYLTSKFWNHDGPRTHNDDGLLVLATITLFTVIPALWTT
GLVFWIDHLTYVSVIQRISSIIGGIGAANIGMVWACLWKSSATINTYLAYTLWPAYLGLIFGAYMT
STSYFCSDNDTADNNDSLLLKDLESGRSGIEFDSHEAPQEPDRSDDSLDLPEDEVGQGLLGGRSTR
KQYAETRPSQSGWFGSFLKGPDPPRKHHISPIFEGLQTAPTRVVKHLFPSLRSKLIALIVFHALWA
AIFLTILNWSVVGPEIPDYGIPNRLSCGARLWHNGTLCGIDGNACRPFDEQSFAFKCPAGCRDTIL
LEPYTVGDQELNYRSLVIGGAPESNASFYRGDSFVCAAAIHAGVINNAAGGCGILRRTGEQLGFSG
AKQNDIESVSFNSYFPLSFDFERETTVVCRDPRWLLFAISLVFTSVISLCTTSPSVFYVSVWFISW
FQVALASDPPFASDYLEVVSIGIGRLLPAAFVGWAIYYFCVRETLSGLTAQFEKTMLWLGPFWVGA
LNTDTFDRIPISRLTPHDIQQQPGAIPALIIIVAVIVVTMVTQALAIWSAGKLPRYLVIYGVMAGA
LMIMIILPQLNLRIHHYIISLLLLPGTALQTRPSLIYQGLLMGLFINGIARWGFDSILQTPNALLG
DAQLGSVLPNIKVPIISAAGDNLTFWFSALPSDIAGIAVMVNDVLRANIYRAAPKTAEDGAANKTA
ADMSFEWTRIHQNHSEYFRFAYMKNSALGGLWYEDFGKAGTWTEDGDWIPMDQGH
>g2527
MARDRTHSFGYPPKPTSFEMDMDNITVSSSSDDDDAINPHSRNAFQSFPRGGSTQRPLISFVTNKW
ETSRSSRGRSSSSDSSASYDWHRVPWLGFIASIVMAPKFRRYLVVYAILLVFAYTGWEAVLQPMIK
EHEEILTALNVDTLEKVGGWFGTNVRPEFVDVVPMKTLDPTLLPSPERIQSLKSSRRLVIVGDVHG
CQDELEKLLKKVTFDPSSDHLILTGDMINKGPKSSGVVDLARELGASCVRGNHEDRILLERSDMKL
HTNEASDQEYTVVSDTAERRVAKSLSDDQAAWLQDCPVILKVGVIKDMGEVTVVHGGLVPGVPLER
QELSSVMSMRTIDLDTHVPSASKDGIPWFKLYNQYHKLLASSHEDVDSSHPPKTMTVIYGHDAAKS
LNIRQYTKGLDSGCVYGRKLSALVIEDGGKNEVVQVKCDEYVKS
>g2528
MSSWLQRQAGSHNVQLAAAAVVSGAAVAAGIFGYQALRRHEAVETLKASIPQLDEQHHAETLNEFG
TAGPLPTLSKEDERSAALARRAQQGDYDEDLILEQLARNRVFLTDEGLKKLRSSFIIVVGCGGVGS
HATAALARSGVAKIRLIDFDQVTLSSLNRHALATLADVGTPKVHCIRRRMEQIVPWVHFDCRNELF

GASVADTLLAPWTGKESNGQKPDYVIDCIDNIESKVELLHYCHKNSIKVISSMGAGCKSDPTRIGV
ADISVSTEDPLSKSTRRRLRVLGVHSGIPVVFSVEKSGPGKASLLPLAAEEFAKGKVGELGVLPDF
RVRILPVLGTIPAVFGYTIANHVICAVSGYPIDYSLAAKKREKLGETMYTSLQGTMERLVRAEVKD
NELVGLRIPLGKEEIGYIVDEIWRGKSVISGLSNRLVLVPWKRPAEGFRVNPQWEKDGQKLVVMDW
RDLVCMTKEEAVQHEREILSGSKTVEELYDDKVLQLIDARLKEAEIYEQYR
>g2529
MMSLRRAVRTASSSYLRTTKFAHQKNISRIQPLSSTPWPTADFQWRRWTQSAAAAREQPDSSNPAM
SFPCLDAQEAKTVSLSRRSMNTGPEPSYTTGHHEKFHCENPLLLDWGGVLPEFDVAYETWGTLNAD
KSNAILLHTGLSASSHAHSTPSNPKNGWWEKFIGPGAPLDTNKYYVICTNVIGGCYGSTGPSSIDP
SDSKRYATRFPILTIEDMVRAQFRLLDSLGIQKLHASVGSSMGGMLSLAAGVLFPERMGRLISISG
CARSHPYSIAMRHTQRQVLMMDPNWQRGFYYDSIPPHHGMKLAREIATVTYRSGPEWEQRFGRKRA
DPSKQPALCPDFLIETYLDHAGEKFCLEYDANSLLYVSKAMDLFDLGHAHQVATRERREQNQKNMA
LGNIERNDASCSLTLPDKPYEEQPAALSSLTPMNEPATVESTDGPPQDLVRGMSPLKDHPVLVMGV
ASDILFPAWQQREIADTLRAAGNQKVEHFELGEDLSLFGHDTFLLDLENIGGRVGKFLG
>g2530
MKDLESRYHPARTSTMAPIRRAIVAFALLVGIILLFRSFSASEAAYRDDSPQGQKGLRIPPQARGS
AGTWQSQLTVGDAAYPSLLEKLRYQYPYDLDSKFPAYIWQTWKYSVDSGSFPNHLRSLEASWTSQN
PEFVHEVISDESADHLIQYLYGAVPEVVEAYNSLPAPVLKADLFRYLILLARGGIYSDIDTEALKP
AIDWLPDDMDPSSVGLVIGIEADPDRPDWHQWYSRRIQFCQWTIQAKPGHPVLRDVVATIVEDTLR
MKKKGVLTMSKMDKSIVEFTGPAVWTDAVFRYFNNPDYFLSEGTRNVTYEDFTRLTRQKKIGDVAV
LPITSFSPGVGQMGAQGPDDPLAFVSHQFEGSWKPENERIY
>g2531
MATEIVEPTIHEHEPTRNPIVVPKVQPTKSEDVTPSSAPNTESTTPKHNTISSEDDFSAPDLGQPS
RNLHVDDFELIKTLGTGTFARVWLTRLKNSPKRDSVYALKVLRKADVIKLKQVEHVRNERRTLGAV
AGYPFITTLITTFSDDQSLYMLLDYCPGGEIFSYLRRARTFSDATAKFYAAEITLTIEYLHDVQGV
AYRDLKPENILIDIDGHIKLVDFGFAKQVGNRETYTLCGTPDYLAPEVIHNSGHGIAVDWWALGIL
IYEFLVGQPPFWDQNVMRLYEQIVEGRLRFPHNITMAARDIISQLCKTDVTQRLGHIQGGSKRVKS
HPWFDSVNWDDVYHRRVKGPIIPRVEHPADAANFEEYPPPPDSAQLEPYTNDMREKYEPLFDDF
>g2532
MADNEYAPKFAPFFSFAGVAAAMIFGSIGAAYGTAKSGIGIAGVGQYRPDLIMRSLIPVVMSGIIA
VYGLVVAVLIANAMNPPPGQNTSLYTSFMHLASGLSVGLAGIAAGYTIGVVGDAGVRSYMQQSRVY
VGMILILIFGEVLGLYGLIVGLILNSRSGG
>g2533
MADLRSQQGRNYQANPYGQAPNPRRDAAFSDIFGSSAHAGRSQTMTSQTPPFPNERARTMTSHIPP
PQMQMQNRAPPPPGGGVRQLPNGYGPGPVNGYGGHHVPNGNVAPSHQQQHPQNYPRQYPGPIREYA
RPGPHYTNPNVPVPHGRPPPALNSDTSRSRSMASVVRHPPSYTQPPSNYQPPGNTFRSQHYNHIAR
TPQGRPVPEKNEDPRTMSLSSYSSSHDHAQTAYGRTVPTKRPPSGPDHIPPQLLPAQAQVQPQVQA
QAQPHIFPEQQGQYEDNYRPRRPSEASVTSRTMSMASTLIPDRTMSLQSQPLQKQTTQSTTATAVA
QARRSKPLVYPALLSRVADVFREGMPLAEYQKNGLSYQNAFPGSKAVDLIAHIIRTQDRNLALLLG
RALDAQKFFHDVTYDHRLRDSSVEIYQFKETMTEESPTSDVNGVFTLLTECYSPTCTRDNLCYSIA
CPRRLEQQARLNLKPQPGLRHSSSKGSLHADDDNDDQKLWINMVPKEVSDTLEDREKKRQEIIFEI
MYTERDFVKDLEYLRDFWMRPLRSAGNTSLSPIPEHRREKFIRTVFGNCLEVLKVNSTLCEALNAR
QKEDHVVRTVGDIFLQHVPRFDPFIKYGANQLYGKYEFEKEKASNPAFARFVEETERLKESRKLEL
NGYLTKPTTRLARYPLLLEQVAKNTADDNPDKQDIPKAIGLIKDFLSRVNTESGKAENHFNLVQLN
AALKFNPGDYVDLKLTEENRQMLTKMGFKKGPTDSSEVTAYLFDHAVLLVRIKVVNKREEYRVYKK
PIPLELLVITQMDEVIPRLGLAKRSSSSLIPGKVIPTNTPAPKEGLPITFRHLGKGGYDLTLYATS
PTQRKKFIEMVEEQQRKLRERNSNFYSKTILCENFFTTANRVNCLVPTDGGRKLVIGTDNGIYLAE
RWPKDKSAKPRRILDATAVTQIDTLEEYQLVLVLANKTLSSYPMEALEVNEGQNPLARRPKKIQGH
ANFFKVGIGLGRHLVCSVKTSALSTTIKVFEPMENLARGNRKPALGKMFRGGQEALKPFKEYYIPA
ESSSVHFLRSTLCVGCARGFEVVSLETTESQSLLDQADTSLDFVARKENVKPIHIERMNGEFLLNY
SDFSFFVNRNGWRARPDWRISWEGTPNAFALSYPYILAFEPNFIEIRHIETSELIHIMTGKNIRML
HSSTREILYAYEDEEGEDVVASLDFWNKATVK

>g2534
MMSSPLNAIRRKKNVKKGIQFCLMVCGASGTGRTTFVNTLCGKKVLEHKDADDAANAHIEEGVRIK
PVTVELELDEEGTRISLTIVDTPGFGDQIDNEASFSEIVGYLERQYDDILAMESRIKRNPRFRDNR
VHALLYFITPTGHGLRELDIELMKRLSPRVNVIPVIGKADSLTPAELAESKKLVMEDIEHYRIPVY
NFPYDIEEDDEDTVEENAELRGLMPFAIVGSEEVLEIGGKKVRARQYPWGIVEVENPRHSDFLAIR
SALLHSHLADLKEITHDFLYENYRTEKLSKSVDGGAGSNEDSSMNPEDLVSQSVRLKEEQLRREEE
KLREIELRVQREINEKRQELLARESQLREIEARMQREASAHLAATEETNGDAAEA
>g2535
MNTRQVIDDSAGPVSLSAAFNSDSSCFSVGLDTGFCVFNSDPCELKVSRGVPKLILCCIGVAEMLG
QSNYLAIVGGGKQPKLPQNKMVIWDDVKQKSVITLEFRTSVLRVRLSKSRIVVALLNSVHVFAFST
PPEKLSVFETTDNPLGLICLGKKLLAFPGRSAGQVQLVELETGNVSIIPAHSSSLRAIVLSPDGEV
LATASEQGTLVRVFSTRNCAKTAELRRGVDHAMIFSLAISPSNTYLAVTSDKSTLHVFDLPHPRTL
SRRSQSPSSYSEDGVNQKWGILGKIPLLPRVFSDIYSFASAHFEIGDDSLGGTPYIPPLGTSVGRP
SKGVLGWTDDHTILIIGAGKEGRWEKFVIREGEDGKRFCIREGWKRYLGG
>g2536
MNSIRQIQALNKRELENAVTPEASWHADYRDTAYIYIGGLPFDLSEGDIVTIFSQYGEPVHVNLVR
DKDTGKSKGFAFLKYEDQRSTDLAVDNLGGATVMGRVLRVDHTRYKRKDEEGEEDNVARIMGPTTG
ADEDTDTEERRKSRRRDGDKSDVEERRRPMLKEERELAELLRDHDDEDPMKEYLIQEKKEEVARAL
EALKASEKKSSSRRKDSRERSHRHRQHRRRRIGENDIGIGQGLLVEDETDQENDLDANEIKNGI
>g2537
MVGIGSRMSIGTQDSGGPGSRPSSKDGNKKNIWSSMLDSVASGKRLPEKNMIVLGGTPDSQREFLD
TLSSDPSDPRLPDERRRGKVAPIANEFALGYTYQDVLDADHEDILARVSIYLLSDPSPSFAPLLRP
LLTPQSVPETLIVILLDWSSPWTWVRQLREWIRLLRSVLISLEDSTKIVMEEVMTEWKDKKRGLDP
NSATGVTNSGGPVSIPLGPGEWDEGLGVPLCVVCQGANKIEQLEKENGWREEEFDFILQFMRTILL
KHGASLIYTTPFLANSLQSLVHSTLGIHSLLRRQSLKHNVIDRDKVLVPANWDSWGKIRIIREGFD
IEGVGDAWSIEIQQSTRHLVNGGSQDEDAETKNAPISPIAEDGTSAVAIYEQTIKDPKEDSAVPGS
SVSANRNKIEIETEDMQKFLGQQLTMLDQLKAEDEKNREAEKAPKPLEMSPHLDESGYVNEHIGPV
QFNMGGIQVDADDMLRKLKEREANRTPKKEITSSTGPTDEKAQNQALASFFAGLVKKPGGSPRSSP
APSSNQ
>g2538
MSVAEQVIEAAAPSTASPPFIHVDGVPNFRDIGGYRITDFSSTRRNFVYRSALLTRITPTGLQTLT
QELKITTVYDLRSNAELRKDPITSSPLDNHEAITVIHTPVFPERDSSPEQLAKRFANYMSDNGSEG
FVAAYAEILRDGVDAYRKVFEHVRDRPQDAFLVHCTGGKDRTGVLVALMFLVAGVRDRDLIADEYS
LTEKGFDGSVKARLAEKIIKDLGVDKANRAGIERLLSARKENMSATLEYIDRQFGGAEGYLKDQLG
FGDEDVERIRKSLVVEDRGLF
>g2539
MLEAVRLVESVPELPRKEDQDSNALVIGLGIGTTPAALIQHGIETTVVEIDPAVYKFALQYFHFPP
NHTAVIDNAILFIETARHESKKYDYIIHDVFTGGVEPVELFTLEFMQGLDALLQDDGVVAINYAGD
ISLPGAGVVIRTIKAVFPECRIFRENDGSEKPTEESLDFINMVVFCKKSRTPPLTFRQPNENDYLG
SQARKSYMYPRREIDSSVFDIVDDSTKTRILEVGKTSILEANHVTNAIGHWTIMRDVLPAKVWENY
>g2540
MSTGLTQQDATDAAANQLKPPVSVQAKNRRKRYLDQHPEYFSADLEMADPLLYDRLIRRFQTPAER
ETEGRTKGFSGILQADLLRSEAKMDALAHPDPNAMLSYRRGPNGEIVAEDKDDIPANKEEGEQRWR
WEMEMRFLKGADYDFDYETVDENDEYDDWNEEQERYFDEEEPEWILDKEEGNIQKEKLTGETGRQI
FTKGGVPPLLFLSSEGGCQPAERYHHTSNEKLHGKMLFGKNQNYPDEGEELNHKHGKPLSCMIFFF
ASQMLSNLETPAASTGSTDESRMPRTRAHRLDVLLAEVGEYANRSPYVTRFEPIPVEMSPPGRPTG
TSRDEMHDVLLLASFKDKTAWQKWIETPEWQRFMQRTENEAVFRRLPHVRCAHSHKGLRNTLEILM
A
>g2541
MLNTPVIYFSEPRPATAEQGTSYKRRRLTSTTNEYNAVLNYGCTAFPSSWVGLGAIQSAQMLAAPS
FPMFAPEIAKRPRLTFIKTSRSRVGSTATSSALLSTPVKAARNTSRARHAPITSPSSSSTSHASAA
TSIKSLFRRAGFTHHPSRMPEDPAIARNDRTRDIASGHGDAQELQCIPTSDTQDAPPSPVGDSHFS

VHHDNTLLTIPKSSRLQKVTSLLSLRERITFTGFPHPKPSRKKLFRSPSMGHSLRHRDDPDIAIPA
KFGAGLKSLRLGTALPSDFNVEYCELGDEFCSRRKIPGKSGKAIGRGATAIVRTMYRKGGSKTDLY
AVKEFRKCGRNEDQVAYENMVKSEFSIAKSLHHPNIVETVRLCKHSGRWNHVMEFCEYGELFLLIK
QEYLRDIDNLCFFKQLLRGVAYLHQNGIAHRDIKPENLLVTSEGQLKITDFGVSEVFAGIHPGLRM
TTQTDIKQEKTKEVRKCSPGICGSIPYSSPEVLAESGDYDPRALDVWSCAIVCFTLFVRGSPWKAA
KPEDPHYKKFLSGWHKFLLRNPDGIITETESPTCGRIFTTFPKRGLNHLILKMLHPDPEIRISIED
ALNDRCIQTIDCCSPDIEQEAPQKIAQDARMQSVFDVVNEDSLRMARKKAHRHLPPGGKKRHGL
>g2542
MMQPPLIEQTVGEHFASIVRKFGDRTAVVARHQKGRFTYDELDRKSNALARGLQSISVQKGDRVAV
MLGNSLEYASLTYALFKLGAILVPLNPSFNAAQVVSALSHLGASHLIISAEANLPRREPRDNTPLI
RHVVPDLDTVLHVSSELIPSLKQVVVVDNSAGRYDGSSYRALTSFSSIVSDLRADEEALPNQGLSP
HDVVNIQFTSGTTSMPKAACLTHRSILNNGAQIGDRMLLTENDIVCCPPPLFHCFGCILGYMATAT
HGSAIVFPSESFNGLASLQAVREERCTALYGVPTMFIEELGLLERGEIPSDGFQYLRTGIAAGSSI
PAELMRKLHKTLNLTELTICYGMTETSPVSAMTTTDDPIDKRIATVGRLMPHVEAKVVDPVDRSKT
LPIETKGELAVSGYLLMKEYWDAPDKTAEVMLADEEGKIWMHTGDEASMSADGYITITGRIKDLII
RGGENIHPLEVENCLLAHPAVKEVSVVGVPDEKYGEIVGAFVVLSPKTKSSDQEKQQDILKEDLRT
WVREKLSSHLVPKFVFFLASSDMFPKTASGKIQKFKLKELAIKMLKENQ
>g2543
MAAEPSEDDIGNFISFTSLPREKAVLFLKANNLNLEKAINAYFENPDGVPTEPQDDWTSFNNPSYS
QDNQSVPTFHIENSDTHPGYSVAPSRPPSRMNVSETLSDRKYEGGQDTTGAEEIPRQNLTSAQLEE
RDLQQAVAMSLGQDYGKQESGVITQAQDSHFGPATQEFYDETNWGMTVANSGTREEIISPDPEDRL
RKDDEPAFLRPSQEAQYIGGFLTILHSIPLAREALLLREKTASDYGYDSQWWNGQSIKIPRIVSLD
NPYADDQDWDDIIYEAQRLMAFLDGTRRAFGSTDALAGLRSLYGYSGDGGVERFLEAWQEASVRAT
PANQLSMVFSSCAMKQEMSEMDSPNQRDFYVLNTYVETGQGQTLYDILDDAIWPDAPGEELDDVWL
DHVGEILTIKLESYASEAMNVKIPSIFYPDRYMESRRDTVRELRLKRLDAIQKMNEIESIAARYRC
TETASRAGMSNQELLEKAAKAITIALPKHVPEGTGDIDDARAQQVTEQLRAIAGLIKEKVDELEAE
KQSARDILREQSKILTESDISSDDPPHYKYTLRGFCTAPHITYVLRKDTPIQTEEDLIEMEDKEPE
EWHWWRISFSTDDAKTQQAAKPENRRIKSSERNNADVIGYTAIKVREIEALRAARESRSLLLVYAN
NNAVEFAHHPAQPPLQEFVNTDNITFQAELDAAANIYNGEEGSGESVDAMMEDWPTIDAEDEIASA
TTVNAAKVNVFDYEVDAFDEVSSHPGQEMQEMQERGGKSLLH
>g2544
MSTETDAVASATIELLEGRLHRLEYLLTGETQWTGQPSAAPKTESLDDTVARRLDKLENTLNSLSK
SNPAVRDVLQLYSRFPDLFPGTSSAQNESIPADLTTQNLASIVLSYATAFPETASRLSSLKDLPIP
DAQASTALIELQPRLQKLLSIQERQAIEISELRARSARLLQRWYELGVLENSECWAEWESRLEDIE
REVKRREVIRDRRAREI
>g2545
MARFSASNILGDPFALATVSISILAWLISFIGSIIANVQTDFPNYVWWAVAYMICVIAGIVVVMGS
DTSLAYGNAITGYLSAGLVFTTLAVNSLVYQPQPSKQAAAAGFILLSMIIIIWIFYFGSTPQAGHR
RTIDSFALNKEGNSYHNSRPQSNAFGTRPGTTVSQPPQMYTSAQLNGFETSSPMSGYPGGPPGSDK
RNTTATNFPTSGAVDSSNEVSQPTEYPYKAKAIYSYDANPDDANEISFAKGEELEVSDVSGRWWQA
RRANGETGIAPSNYLILL
>g2546
MATENIPVAANVLGTIGTVFWCIQLVPQIWHNWRHKKTDGLPASMMLLWAVCSVPFGVYMILQNVN
IPLQIQPQIFGFFGFVSWGQILYYHHGHSRLKATLLVIGSCLLCAGLEVMFILTLRIPYRKGITWP
DLIFGVIAAIMLAVGFVPLYFEVWKRRGRVAGVNWVFLSIDSLGALFSLFALAAQGTFDILGGILY
LVVFLAEVGLFTSHVIWRLRHRQIVREAKESGKSVEELLAEKEDGRDRENKTNTSSVVQTPVEQGV
TDNNETVTLAREERDLERGPD
>g2547
MAPRRIWVKRPDASATLVSMPEDSVVDELRDHVLRKYLNSLGRTFDSPDLMVRIIPRATPKGAVVP
DRFLSPEESLFALIDQHYPGGQTVEEALIIEAPTRRTPIKPSPRQSYYYHAEPGEHGEYFPLMPVP
INVATPPGHPASSSNSSAGAAHQXPAMSVLTTGKVPPLPSPGSRPSRHPRRPPFARHTTASPTLVA
SSSMPKDPTLAVNGPVGSSQQSLPTPPAPAPPSESPTHAALNPVARTASPRPARRAKKIASPNTGA

FGGLIEGTVPPINVLIVEDNVINQKLLEAFMKRLSVRWQCAQNGEVAVKKWRQGGFHLVLMDIQLP
VMNGLDATKEIRRLERLNGIGVFSKTASGRSSATPSSSVPADSPLKEEDTLQDLSLFKSPVIIVAL
TASSLQSDRHEALAAGCNDFLTKPIGFPWLEQKVTEWGCMQALIDFEGWRKWRGYEDSPRSIGAAL
EETTSTHSPSRPRNNTTIHAVKVSKDDSPKKDNAQISIIEPDTSESSSGKDEDIEVPVPQEVAHEM
AHEIAQELQTAESSTNGV
>g2548
MTDFQSTFESSLDFTKPIGFAAEMSDNIDPSNLLTYQPSDAEWLTKDPFEMLPWDSFGADQTFNPF
TEAAGLVELDPRSSYATSNHGSPAGYGGYTIKTEDAMSDTSPLSDPESEMWSPSHSNDAKGGSLSP
VHTIGVTSRTSSQRTSHNPRKPSNASTSSSISSPSSPLASPKSLSVSSSKKSARSILAQDTDAAAV
MKRKKAAHNAIEKRYRTNMNAKFLALGNAIPRSGGFTGTQLASNRGPRKNSVCQPRGGGADNSASG
RRQSEQQQQNKSEILTNALQYIHELHDENSRLKSELLVLKENLLPRGGNMMWRR
>g2549
MPPRIKTNRILSSISSSSSSTSSTITTSYNTSKLQCSSRRHLSSTRVAQTRLRENMFDWLNGPGAE
LKHHIPGSTNYVTQLRNRRGGDDKESSSRRDNDAEDDGGLKTPFPLNPQFVSEPILSEELSNEIYK
RVVEQKKSVRSVSVELRVDMRRVGAVVRLKELEKRMKNEGKSLAVPYSRAIHEMVPTTPLAEKGDS
QPVHESINDLPVHRLTAPQIFYPVSESRQFTRVDAGRVFSAAPALEHGEEAKVNVETLITPEQRRY
EKVGKGDEEQQVLLPADARIPHPQLIALERDKISHSMERREYMERYQERLRQSDKLEQERKRIAKE
KAEKKLTRLQPENSRFEFRFKDVVVSKETTGPDGRGHIAPGQRYGVPTYDRKKGQVKIPTKVEV
>g2550
MVGSSTTQSSRTSPGSFGTNQRGTSVSSSTSSPQSLSQGPSGLHNAMSTDVYNSAISQPPASLSSS
FTYPFSPIGTSSSFDSLGASGMRLTEADLSPPMNGFHRMGSGAAILMRRLPQNTSREALRSMFLFA
KDFVDADFVPSEFAEDGKFLSAIARFNTVAAAEEARAMLDGKPNTSNEAIMIVEMFPGPLPNRRNT
IDHTATRGLSGPTARQSSRFNGTFQTMANGASQQPSTNEALPAPDTNSRIHSLFSPRSPIGSGLGE
LPRVSGKSVIDQDLDEETGELLKDPVGYAQNGHANMPLTRRSTNPSIPTTRFANLSLSTNVTSPPI
QGYSNGTGGRMGLPTPASAMPPNMNGNSPFHYPNQHNPRHSLPAANPNDLNPPCNTLYVGNLPADT
QEEELKALFSKQRGYKRLCFRNKQNGPMCFVEFDEVAMASKALNELYGYKLSNSVKTGIRLSFSKN
PLGVRSGQPGSSVNPPTPLSPQTPIHGGAGIGPLPNAMFSTASGPPPGLSAPPGLALPVGGRNGPM
PPPNSHAQMANGNAFAPNNGLGIRANGLNALNPAPFGNGIPGNAQGPGMGGFSSVYPDYMMGR
>g2551
MVVKIRFARFGPRHSPFYNIVVAQARSARDSKPLEVVGTYNPLPQKPTNLSEEEAKVAKAFKEISI
DRSRVKYWLGVGAQPTESVWRLLSMAINIRICDLERLHVKAPEARGLGRSDQIRQSSITLTKLSDS
KITDMASSNTSKPFHTEVDLRYTALLLSDIQNEILARFPADKREQYLKEVVSLINLFRKDIHQRRT
APAETKENASGAYVGIPLIIHHVLPYGYNNNAFISPYNKLSSWVKSLQERGLFTVTEKAKNPETPV
YSIPEALYPQGPQGGGFGGSHIDEILLPKFQTSSFGSSDLLGYLRARSIRHVVLCGLTTVGAILGS
ARHGADLDYHVIIPRSGVMDDEDDVNDFVLQRILPKFVDIVDMKDVKDLFASATD
>g2552
MSLKAISSKDAAALDKDLMDVGGFSLDQLMELAGVSVSQAVYRFHPISKGKNVLVVCGPGNNGGDG
LVAARHLVHYGYQPSIYYPKEGKNELYQRLKTQLQNLNVPFVPDFTDALRSADLVVDGIFGFSFQK
PLREPFPSIISQMEDTKVPVLSIDAPSSWDIQEGPPKEGPGAKFMPGALISLTAPKPCVKWYQGRH
FVGGRFLTKAIAEKYDIDIPDYPGVDQIIEVGVNADEKL
>g2553
MSEYKLSASLEGHSDDVRAVAFPNTKSIVTASRDATVRIWKQVSSPPPTYDYTISSHGSSFVNAVA
YYPPTSEYPEGLVFSGGQDTIIEARQPSKASDDNADAMLLGHGHNVCSIDVCPEGGWVVSGSWDSS
ARLWRIGNWETDVVFDGHQGSVWAVLAYDKNTVITGCADRMIRIFNTSGKLLKTIKDSRDVVRALC
RLPAGHPSGAQFASAGNDGIARLFTLNGDIIGELHGHESFIYSIAAIPSGELVTSGEDRTVRIWRG
NQCVQTITHPAISVWSVAVCKETGDIVTGASDRVARIFTKDTSRQADPMVIQQFESAVKESAIPQQ
QVGDINKEKLPGPEFLKQKSGTKDGQVQMIKEDNGSVTAHTWSSATQEWIAVGTVVDSAGSSGRKT
EYLGQDYDYVFDVDIEDGKPPLKLPYNLSQNPYEAATKFINDNELPIGYLDQVANFITQNTQGATI
GQTADTQQAAPGSDPWGQERRYRPGEAAAPHPPSIPETRQNVLPQKSYLSIKTANIKVIVKKLTEL
NEQLVSSGNKEHSYNPPELDALLRFCNYLENTASFPKDPADIETGIQLVMKAALKWPPANRLPGLD
LLRLLAAATPVTAQIRFDGQDIIDAVQQSGTFQSPISVNNAMLAIRFFANLFDTPLGGDLAASKFD
NILRMVSSATTAVGAPSNRNVTIAATTLYINYAVYFTAQGRAQSAESAEGALQIIEELSKIFTTEK

DSEAVYRGLVALGTLVIASGEEVKSAAKEIYELEKILSKVSTSSAGREPRVKGVIGEIRDALR
>g2554
MEVLQRPPSASSFTPLAEHQSRTPQSFYGGPPVLHYHSSRCKVVILESDLSASPALNTLRGGAAVV
NGSSGAAHESQERKDIVIDGVDVFVTSDKFLLYRPEANSGIALPYPVISLHAIQRLRLPDTPEDEA
EVQGLYMQIANPTPADGMGAEEDEEEDSITLTLVPTSVTESATSALGSYSAGTETAALIAEEMAAE
QGNTEIKPEETATQALYAAVSACSNLHPDPVVPGEEGDEDYDGNYEDSTEQPGFGGTLQAAGLVIP
GASDGGLPPPVDGSSGWITAENMHEYFDEEGNWIADSEPPTLPGLGPGAGNVHTRNDDDQVDNGNG
EENGDEETKWRRTD
>g2555
MPEEPEEVLDLEKDLVKPTQGRQSNQSPTAKGSVARPGSPEKQHLRHSRHRSTGSIKEESTHLSES
SPDTNSRIPRPRSSRKRARSGTESGDDVFTSENRVMIPNNIFSRSPDEGQLLTTPSPERHEPLWSL
PRSDEFSSSSPKFPANASTGSPRRATPKGPRNQPDRQQRTTEPPSLTIDTSYDVSPGSESPTKRGV
NRSQTLAASPTKDVRKSITLLRRMNSEACDEDSRSYRRMGRNTSFIQHSDRSIRTPPSKRNSAISN
DSLTIWEDASEDDIVISPAVRQKNLPFKILERIDSEGTVEENLNAIVENQKLLTIRQVPPSSSIIT
SEVGDRNNNSDSPDEVAPKRSTAMQTPNSKVRGYASVAATPGSLYDRDGFLKE
>g2556
MATTLGQPPFFTYFAWDSSSNAAQLEGAMNGLFQAGGPDTPAASAAVLFLFQHIASMLLFRLLRFY
LTASFIFVLSRDIAKLFDNSVEPANVKAVTKQIEVV
>g2557
MAGSPLDQNAVFVHDNSVPPVHNSLFSLFSLKGKTAIVTGGGAGIGLAVAQGLAEAGANIALFYNT
NDKAPERAAEIEKQYGVKAKAYQADLRDQKKAEATINEAVKDFNGRLDVFIANAGIPWTKGATVDG
PIQHYLDVVQTDLDATWYCARAAAAHWRRQKEEGTDLNGNKLENFTYGSFVATASMSGHIVNIPQL
QAPYNAAKAGVRHLVKSLSVEWPKFARANTISPGYIATEISNFIPKETHDLWKSKIPMGREGMPEE
LKGAFLYLASDASSYTTGTDIIVDGGYCAP
>g2558
MIQETTARKRSSGSTSKRKKSQRLKINPPKPPRLILHPPKPPQQKPTKLKMVLRPPPKSLSLEVDH
SPPRWKEVTLLGNGEDASYDYGDICKVQSIMKVFNGTLSATRKVESNEKKLSRDPLLPSDVTTTET
DTMTEDMRAKLEMNDILQEALKCHHCEKTMRLKPEAMESDILHGIYSCSEDPPFLISQPCDEDDVA
PDEMPKAVSEPGYLHLATTAQQEQYLQLVEKFITRNRERKVFRNMREGVDQGYESKFAFYCVDYHI
HQHFIHRRSILNYCRSNEEMDEVQTWKKDLAKWAAKNNAQLASQQREMQDESEFTVTTSSTTAPIQ
VARKANPGLFDPFSTSTTFSGEESSSSRNSSIFSSSMSHSSPSTAASSPPKAGSLPNTPPPITIPW
PKEQAAFSNQISLHRHLTNSHERAARVLQVRERYHRTMRKRAEQRRDGLIIANYEAAFALQKWQRG
RGGPLAMQRQRSIGELLNEYNR
>g2559
MMDVDSNGVDDRARRATSVLSMDDIEAAQALEGLRSDFAYTPPHPTALSQSHSRQNSIPATQSEQS
QAEPLISLLTSTHPLISSAINSSMSAYTSSKSYSSRFKYGAEFLERNIGSPVANTVGSVSRKTGVE
GGLRWALQRRDTNESTDRSKRRKTNPNSTEVDENTDIEKALPPLSEMHRNRSMSETSYSETLPPYD
DQKSPNYEEVQQHSRPSNTSEQNKTWQKRLMISTSGLGVAMSEESLRRLTYCLKWLKWANGRLNSA
VLALKSMLEKWEEEKLKNSTDENSSQNRALLLQHMQAVKEDVLRTLKQVVNIVSKYTGGALPENAG
TLVRSHLVSLPQRFKIASTSNSSPDSAGSGSEEASSAHRVLVLAQEGLEMMSQVHRVVDETLISAQ
TWCERLGRKLPDLKDDESHENPPEDRSNGAHQYGQAAVLADFKQPIVNTSNVDVEMTGTDAV
>g2560
MTSVFRTPSAKSVEVLEREIDSLNYEFALGIPNPKWQSPSSRPMAERSKEEQCRSWMKELFWKNPE
AFERACHDFRELVRSQIRQGKDWVHKLHQEHGTVPSREDFLKTNINYSNQVSDDERMKRVEALRKA
IYSELWIVRGGENPLEGTQSTPRRQNLANGPHRASLNGNHHESPMATTPQAKRKASNNQEVFHTAP
SSPVLPFEGQYPSLGNLDEYDDPMRSEPNLNADTSHGADKLKGETQRSRSSNKRQSKITDHMLITK
TSRPPARKPPPSLSDKATSSLRLDGSFSSPEVSFATTVADSVFDRSEVPRFLGTSFETTITEPCDE
TVTHPDSVDELLMSEEFSMMLKSVEEKASTSFETERRAPYIEQEMVEELIRNGPFSKDKEHFSEKV
PLRARYELERVKQAWDISPKDILVGDKPYEKHVDFWQWLGNVGGRKNKPLPEKPSIKAWNAAIGRF
EGDRHSEAVILSGSLDWCEKGEPGIFKLRLNPLKVDRTCRFYRRFGSDRFLSITMPSPSQPPSHLR
LKDHVSLREALTDWLVDNNHHLLNRTWKAFYVDDFKNKSKSKTGKPIVRVDFFAVDGSDFTTRLSP
PTLSPINETSESHTRMTVDQLLQWHIPLEDNHSQTDCKLFSRIGLALSKTWATVVIQPHQFLKLPD

LPGKPVMNDGCALMSRPLARKICEELGIDGITPSTFQGRIAGAKGLWMVDRDRDDDERIWIQISDS
QLKIKPHPCDFSGQVDTEKITFEVASWSKPLRSSELNTQLLEVLNNRGQVRARVADIARESISDVY
KEFESVMESNSIPLARALIQRIRPTTDEGSRTNIRRIDQWLSQTIESVIRFLEAGFSPRDFPPLRK
RMTRCLIDTLNRYVNELHIPIPLSTYAYCIADPYGVLAEDEVHFAFSGQWRDCSYFDDAMVDGVDV
LVGRTPAHCPWDIQKRRAVWKSELRHFKDVIVFSTRGTIPLASLLSGGDYDGDRPWICWDQGIVET
FTNTVPPLVEPGPEHFGLVKHARPMSTVGSVRDLLQNAFRFNLAMSYLGRCTVEHEKLAYEEKEGI
SSPLSIELATLLSHLVDSKKSGLQLTEEQWAKYRKSISPRERGVPAYKDLGAKEGKQDNINDFLKF
EVAFQEREKALMQFNQRFGDSEDNWDESLLRPWKAAVARAEREKEEDPRTNGHSNTALAKGPALYE
VLKRIEAQINEGYRKWIDGCHFHETPGTSTGFKIQLAAELLNRIQPPNICHPLVHTWQNSEFEWRM
LLASGAYYFRWRSLFPLYAAGETLCWIKTGKEPARLIRDQVYVSMKVSSSAARRLAGEEAEELANE
DLEGNAESEYNDSEEFDRESVIDALDEIDRMYQILHS
>g2561
MSGSGVESVLPFRVKSGESFEDDQVSETSYSNDSTYDASANGQLARLPEDLIRQIAFFLEDNKXPA
DLCHFAALTPNNWTVLKNDCHQFALKVAEPTQEEFKKYLTETGDASRFDVWEEVQESTDIGDRSKF
SEAIAGGRVHAVKSFLQHGVDPNTFSIVGNRPLTLAATCSRPVEMSELLLEHGSDPCKADILTDIF
TPLYTAATMAKDELVHLLLDHGANLKAKGIVQAICQVCKMDTIQRAFKMGADINAIDESGNNLLHF
AARNEDSEVLRLIFDLGFPVANINQQNNGGKTPLMKAVKIGSTENVLALIARGANPDFVSTNGATA
LHVACSWDHAEIASLLVNAGANIEAVDSNLMRPLHHAAYISSTKMLRVAVENGADISATTRRGDTA
LHLLLADLTEKSGKKYVARILGGVKILIAANANPAAQNEEGETPLDLAMNCGQYEIAHVLKTNDPH
AVDGKPPAGRNWREYLRDRVYNGQVA
>g2562
MPLQILNFQWLVGVALIGTTVAYIAFTRSRGGRARKASTAKTPPRSLSPEKKQPSSPAGSATAYKD
VLPPLRRDAMLSMQSSIPKTASTHINEEHVLKNILPMTQNYETAEDGLFTCMGFSVQEVKEMGDLP
DYSTLSGVPLPNAYEQFKIEKALPRPYRPFRWNYHQTMSLKKLETDWWVELENTYKERIAQRKELY
ERHGRDVLDWMPGSELACKELMEMVLQFICARYPQYFSIRNNRILVNKILETEQDIRSKHPLEILM
DNVPEDFAITLRDDKTGYYVFRAGVICSSLGWNVGTKIGLQLHEIHKIVPDYKEKMKFSMDRFFTK
MPADKPIQRGSWGLEIDQPLYMPAGHPHEKLRLSQNPDLALADCFLRVDWQTLRRLPISGAIVFNF
KALFTPVQEFRDEPKVPALIAKVLRGGKENLMKYKGTWHVEHVMLPALDKWNQEQEDNDIIERNWE
VTTLDEDPWFKGWEEKWHRQQGF
>g2563
MASHRADASAFLDNRGYTGPLVRGVNPVTLFEKAVRDRITDSYYWKEQCFGLNAATLCDRAIELTS
IGGTYGLSQKPTPFLCLAFKMLQLAPDKDIVLEYLNFTDPGSGDEENPEDREIDGEVVKGRGDFKY
LRALAAFYIRLTFEAAEIYKYLEPLLLDYRKLKRRMRENYVLTNVDQFIDDLLTKDRVCATSLWKL
PSRQMLEDLDLLEERVSPLADELEELDRESEASYHSRQDDDSDRGSDVMREA
>g2564
MVLLSSSTVATLFISVITLLCTFGIFIAGYALQQHSVRNIERDFRSLDSMQADYEHMTYDTNKEQS
ILDPETGELETWNQGNFAYLQLLSEPNPASICSTIALYKKLVEGGSTIQDRLFMYPQEWDLIMSTD
PVVNRALSFLREASKLYKFWLLPIDMNLVIEQGYSLTDSKLLRIGELQFMAYDSVLYLRTPGLLLD
AQELDKMILTRPLPMKYEPKRQESYRNAAWIGMPLRAHDEAKIPPVYLLSVNTMRNRVEARTHVPN
VALRGFGDLAAGPNFVKVTGKDPAYVFFDWDEDGRIQQENNTYYELWTQEVGEVCVGLDLSLY
>g2565
MPSLADDDDDREIIGSQDGSSDNEDEQMRDVDDNEGEGENDNDNDNDNDADQDDQEQDADSPSNNS
QTSDGPGNDAQQDDQNVPMDSLDAYRPTVRPECLTATTYDIVPTTAAPQSTSINAVTSTGDMRWVF
SGGSDGYIRKYNWVDSINGKLMLTVAQRHPFVDSVVKAGVLMNYWENMDGNVLSPVYSLAAQNEGL
WLLSGLESGNIRLQTVRHEEGREIALLKSHTSAVSVLNLAPDERSLLSGSWDKRVLDWDLNTGQVK
TAFGISSGQISAIEIRPDSALPVPQDTQEAQYEKSFSSNNHLPPSALVNGTASHGDAGQNDDAPAA
SPADSLFGGADSLFGDGDNNMVDGGEESGDAGNVGENALFGGSLDMDIRPSEEVKQESENISQPNG
TDNWAPPAAESNGVQKAASPKLEEAQSNGQMEQRTEPAVNGLPHAESLEDIANTKESQTSEGTARI
TSDTTFLASSFDGTIRIWDRRQPDPVARITPRNVPPWCMNACWSSDGNYIYAGRRNGTVEEYSLHK
GLREPERVFKLPQVGGAVTALKAMPNGRHLLCASHDILRLYDLKGQPHKHSPVPFLVIPGHRTGVI
SQLYIDSACRYLISASGNRGWEGNTTEVLLGYEINA
>g2566

MTSQLKNVIVVGGSYVGRATAQELARVIPTTHRVLLIEPHSHFHHLFAFPRFAVIPGHEHKAFIPY
SGVFNSVPNSSLHVVVQARVLSIQPKYVKLDREWQGSNQIPFEFLTLATGTNLAEPGSMKHDDKAS
SVQYLQSHQQRVKEANSILIVGGGAVGVQMATDLKEYYPKKEVTLVHSRPNLMPAFHSEIHNIIKK
RFDELGVKLITGSRVVVPSDGFKNDGRHSEVQLNNGTKLTTDFVILATGQKPNNSLVNDLISSNGE
TLINPDNGFVRVRPTLQFLDSKYPNLFAVGDIADTGMHKAARPGAAQAAVAAKNIQALIEGRPPQE
QFSWSPAGIHLSLGLKKNILFRNPNVQEGQTEPNVTYRDDGQDDLGVERVWERMNVTVDGPSRYHL
>g2567
MHLLIMKMKIPKAPKNRPRGGSFGAAPTRPQTVNKANPDIRKYVQKTLKLDNRESWLSYPEIPTAR
EIMGQTNAIDLEENIIDLPTNQIDEPWESTKTYLSAHYELLREDSVALLRDAVALVRDEPSMTDTQ
EICVYEKVYITGITLSPKGVATKISFSTARAGKNIVWEYSSRLITGSMVALSPAEDSFQTQCLVAI
VAARPLEQLKNRPHQIDIFFADTNNAPFDPQQEWIMVQPRSGYFESNRHTMKTLQVMNSEVFPFSE
HICSLKPEIGRPAYVKENSRMKLFTNEYIDILSDWPGKPQNNLDESQWEALHQILTKRLAIIQGPP
GTGKTHVSVSALRLFLGNWRPNDPPIVVAAHTNHALDQLLNHVSAFVPDYIRLGGRSLDENVKKRT
LFEKLDNLRDAMAELLSAFSGEEVXEPLPAAFFLQHGAITEKQHELLCEGASQWAGSSTEAINIWL
GDAFTNHRVSYDDDGLKQEEDEADLEYEQLKELEQEQDYLDDVIEAIRGTFLPLREPFVGLDNSDE
TENIEVDIEDLWQIPPGRRVFRHHTLKYNAAATDRQVGKWEHDFVVLKDAKLIGMTTTGLSKYRAL
VSALKPKIIMIEEAAETIEAPITAACVESLEHLILVGDHKQLQGSCAVRELEDEPYFLNVSMFERL
VKNGVDFRTLTLQRRMAPAIRCLLTPIYDSIQDHETVNDLAKYNQNEALMVATFFVYLVLAGVDSS
HITVLTFYNVRSSEQNGIGFLKVENRVCVALSRARCGFYMFGNAEHLAGESPLWMEVIRIMGEADP
ATTCIARYIPLICEKHGNEEFVELPEEMAELTGVNPSFRGQKPVLPTTSTPHQQANHGTKSSVNYA
EKVRLAAYQQFANGGVKDHDRSLAETMQAESAQDMLARLDAEAEANLFADDELEDFEVDVVTEESA
AGNASEAEREMNSNSMNSSPSRRRWVDSYNPSGGMKKMAVSTSQMSLLDLSD
>g2568
MGLECHGPIWVIDDLTHCFQRKSLANGSADPDTIEEDDLEDVDDLILHKSASRISHGDFEVSYPRG
QLLLVTSELAGLLVEIAVCITVLVTQSWGHKGTIASIAKLASWSYIFLLAAVRFILSALDKQIRPR
LWNHTASLYGVQWLLIVFVFRSEIIHPHSQRALILSSIEFAVCTLLVLLATTTRRGNKPVLLQRED
GLEPPHQPYASLLSLLSFAWIDPLIWKGYRQTLELDDVWNLTVSRHAGTILNDFRHKYRSYQLIWR
LLRYFDRDLFVQAAWTVFSNLFTFIPTLLLKAILEHVEDPRSSPPNAAWLFAILLFVSGAIQGIAD
GQALWIGRNNVGLKLRAIIIGEIYSKALRRKAGASTETEEEKDDSKDKKDKKKSDDKKEDGSAPMT
RIGTIINLMAIDSFKVSEVCAYLHFLWASVPVQVIIAVALLYRVLGFSSFAGIILMVLILPVNVYI
ARSFRRIQNEILTKTDARIHTTNEVLQNIRIIKYFAWERRYNEIVNEKRKAELRSLRYRYIIWSFA
ATVWYGTPILITFTSFLLYTVVEKKRLSPSVAFPALSMFSLLRVPLDQLADMIAHVQEAKVSIDRV
EAFLNEDETDKYNQVLSDTDESGESKIALEKATLTWGGNSGTQDLTDAFRLIDVDVSFKLGRLNVI
AGPTGSGKTSLLMALLGEMKLLDGRVYLPGVTTDRAELPVDPETLLTDSVAYCAQEAWLVNATIKE
NILFASPFDQQRYDAVIKACALERDLQILEAGDQTLVGEKGISLSGGQKQRISLARAVYSRARHLL
LDDCLSAVDSHTAKHIFREAFTGPLMAHRTCILVTHNVALAAPEAHYIVVLDNGKISSQGSPDEVA
ASGALGDDLMKSRPTSRPGSKGPSRTSSDLEEQIERANGETNGNTNGNANGTSKPVPDGKAPAGEG
SASNLTESKATGSVKWSTIQMYLASMGPWYYWIAGGGVFVAQQIGNVATNVWIRQWANAYNVEQID
TTDVSGYALMSNFKLPSFVTGSVSHPNVWNGIQASSVRADTGDHGVNVAYFLGVYALLGFVYVLIS
MLRELVLFWGSLHASWSLHQKLLKAVLHAKFKFFDSTPLGQLMNRFSKDVEAIDQDVAPIAIGMLH
SLASVIMIVILISIITPGFLIAAIIITLLFTAVGAIYLNASRDLKRLESVQRSPLYQQFGETLTGI
VSIRAYGDAQRFMIDNHRRIDSYNRPHIYLWAANRWLAFRVDMIGALVAFLSAAFVLMNIGRIDAG
AAGLSMTYAVTFTENVLWLVRLYAESQQSMNSVERIREYLEVEQEADQVIEERRPPSGWPTKGAVQ
FFDYSTRYRPDLDCVLKEVSFSVSAGEKVGIVGRTGAGKSSLALALFRGLEAVNGKIVIDGIDIST
IGLQDLRENITIVPQDPTLFTGTIRSNLDPFGLFSDEQIFVALRRVHLITDTASGTATPSTLAALE
NSVEDVSAAPENKNVFRNLDSPVAESGSNLSQGQRQLLCLARALLKSPRVLMMDEATASIDYATDA
KIQETLRELKESTIITIAHRLQTIIDYDKVLVLDHGRVVEFDHPWTLINKEDGSFRSMCENSGNMD
VLLEGAKGAWKSKRLVETD
>g2569
MAERILMNEFKALSKEKWVHIELNNEDIFCWNVALIVLNPDSLYYNGYFKAVMEFPKNYPYSPPKF
RFNRSIWHPNIYTNGNLCISILHTPGEDEQSGELAAERWSPAQRVESVLISILSLLDDAECSSPAN
VDAGVQLRMDPNGYRAHVRRDVELSKQDIPEGFVMPTFESTLAKPNAEKDDVKEDMDFWADSDVDE

DPFGGSDSSDMGGDDDDDMEFLDDDDEDEDEDESDD
>g2570
MRFFSRKHQQAEAADNNDDQPPAEGTETKGDLNKIAAENDLAASANQAAGTAWLAGHLNHLTPEQE
QKLVEFKALVEEKGYYKPKKEGTDVPSHSDATLLRFLRARKFDVQGAYKQFSETEDWRKENKIDDL
YENIRLESYERTRQMYPQWTGRRDRRGIPVYLFEVKHLTNKNISQFSQEVSEQGASETHKDSAIPA
RLLCLFSLYENLLQFVHPLCSALARPNPETPIVSSNNIVDISGVSLMQFWNLRSHMQDASVLSTAH
YPETLDRIFIIGAPSFFPTVWNWIKRWFDPVTVSKIFILSTAEVKSTLETFMEPSSIPSQYGGTLD
FKWGDLPNMDDAARALAGGLESPPATEGGKPIFLKGPVRFFNDHMEVLGSDQGKPRRETIPVPPTA
AVTTAVKLEEPAVVPAAETGATTAPIPDEKIEPVEPAAAAPVETTAPAA
>g2571
MPFEARVKQVLSGDTLVLGHVTNKGQERTLSLAYVSAPRLRREGDEPFAFLSREFLRELLVGKVVQ
FQVLYSVPTGREYGVVKLPGTEASLPELAVQEGWARVREEAGKRGDESEESLTLLGNLRALEGQAR
DSNKGVWGSDPRGSLETEYAIEDVKSLVEDNKDRQLDAVVERVFGGDRLLIRLFLEPTRHLQTIIA
VAGIRAPSAPRVAADGTQQXGEPLGAEAQQFVEARLLQRKVRSQLLGATPQGQLIGTVLHPNGNIA
KFLLEAXLARCFDHHSTLLGAEMATFRQAEKTAKDKKLGLFTGHVATKGPAGGDSDYIVGRVLNAD
TIFLRNKAGAEKKVSLSSIRQPKPSDPAQAPYAAEAKEYLRKKVIGKHVKVTIDGKKPANEGYEER
DVATVVLGNTNLALYLVEAGYASVIRHRHDDEDRSSQYDALLAAEEAAKNEGKGMWSTKPPKAKQY
QDYSENLQKAKMEVSILQRQKRVPAVVDFVKSASRFTVLVPRENAKLTFVLSGIRAPKSARGPDDA
AEPFGNEAHEFANRRVLQRDVEIDVENIDKIGGFIGVLYVNRENFAKLLVEEGLATVHAYSAEQSG
HGPELFAAEKKAKEARKGVWHDWDPSKDVEEEEEVVAGNGAAEGNGEGATERRKDYRDVMVTHIDP
ATAKLKLQQIGGGTSALTELMNSFRSFHLTKTNDTPLPGPPKAGDFVAARFSEDNEWYRAKIRRND
REKKTAEVLYVDYGNSEVIPWSRLRPLSQQFSVQKLKAQAVDATLSLLQFPVSAEYLADAVQFIGE
QTFDRELVANVDYVSPEGTLFVTLLDPRQSQNLEQSINAEVLREGLAMVPRKLKAWERASADALAH
YKKVEEEAKQNRRGMWEYGDLTED
>g2572
MVKAVAVLRGDSNIKGTVTFEQADENSPTTISWNISGHDANAERGIHVHQFGDNTNGCTSAGPHFN
PFGKTHGAPTDDERHVGDLGNFKTDGQGNAVGSVQDKLVKLIGAESVLGRTIVVHAGTDDLGRGGN
EESKKTGNAGPRPACGVIGISA
>g2573
MSSSLDQLKATGTVVVCDSGDFATIGKYKPQDATTNPSLILAASKKPEYAKLIDTAVEYGKQHGST
IDEKVDATLDRLLVEFGKEILAIIPGKVSTEVDARFSFDTKASVDKALHIIELYKSIGISKDRILI
KIASTWEGIQAAHILQSQYGINCNLTLMFSLVQAIAAAEAGAFLISPFVGRILDWYKAANKRDYSA
EEDPGVKSVQSIYNYYKKHGYNTIVMGASFRNTGEITELAGCDYLTISPNLLEDLLNSTTPVPKKL
DPSGAASLDIPKRAYLKDEALFRFDFNEEAMAVEKLREGISKFAADAVTLKDLLKTKIQA
>g2574
MDGSPAPAEQRSSPASLSTSLEQVVADLTPILRSIQEIIDRRPFDIDCVVRSTECTLTLRHSLIDT
AKPREAKDAFRNLGGFQTLLSLIRQLAELYNPRKLSNEDRKNLLTLWKDVLAVLSECLRDHTGNRR
YFAKKIVCDRAWSVEESLRLFVENTDGEHLPDNDVDQLYGGLLAAALCQESTAGIFTALRTKYVSG
RPEPTASSIRLDLEKALSSTETVELHEFIGPLLRTWLLQKPHSAQYSSQRLAIPACLCKLASNSQR
NVIAIHSTGILSSIVRFIFSNDLSDDEKTLYQELGHLLCTEGLPNLDDVIYLYRKACGSIEASNFL
LRAMQSSKGPPCIQFDMTSHGYSSIELPTLGRPFPPVSSSGYTLSIWARFDHFDPQSHTTIFGAFD
SRQACFVLAYLEKDTRHFILQTSINGPRPSVRFKSIAFKQGQWYHICVIHRRARATAPSRASLFVN
GEFVEQLKIDYPTVPVTRPTAKYPRVQAFLGTPQDLAVRIGKGVCSTKWSVANAILSEETFSDDLI
SVFYHLGPRYHGNYQDCLGSFQTYKASAALNLRNESLHPGKEEQSDIVTAIRQKASTLVHESAFLL
NISPTTVLDDDDSNNVDESQLVKSLSRQAAKSLHQAINAGGNAIAINGAIPAINDALTSSSGMAIL
AGDPVVSVPQSLDDASWRIGGCAIVNLNMLHAAKCPESTILAVEILFEAIEGNWRNSEAMERDNGY
GILALLLREKLGFPQIPQSGGSKTSTVCSDNRERSSLAIELLRQILSFVGYKFEDPAKSIITNPLA
YRVLLVDLDIWRYGEPSLLQMYYSQFSTFATESQFHRFNAKRLSRMRVNKRFLDALKGEDFTEETL
SLFIPAFRSLFESCVSAELLRSLSLYITFALHEEKVPSLHKKKSMRFNTRARQLSSASDSSPKKAM
PKKQIGLELLRMYTEYLCTPSDFINIKRFAXAVTNKWLLYLMCEDTPEAVTMATKILARLIVVHGG
SYSKKLADKTGGYVIMKNRLKRWWNVFALWPTCFSILFGLDVAEVDIDDLTDLSSLLDVLVYKKRL
DVAFPEVLPVIAEMLKNGLEDSMFDNALTKDRAVMSRSVTPLPVLRTSRAIPSESTSEAQGSLRRS

SSFVLISSDKSHYAASSAHLHRASIGSIDKDVSYENDKLVLETRSVILKIFVSHVIDRKDFTGLGL
FLKTPPSNLEHQAYFLSWLLRGTITELQATLSANEKLLQEPRILTNFSRQTLDLAGFVLEYLSRPE
IASLKIIRLCNQAITTIRSVLFKIILLELSEQDDNDSLNFLNRLGYWQTIILATGESEAEYLKLFF
YQLYIKSVSEKNEPTEMASILSHAPLSLQRRLTGGFEELASMDDAAFLNWIDDQREDLDAFFFGSL
SKFWDTFVQEENNRTQESSRNRVGKRKDRLKQWLHEENFAEDITRKHNTTFDHWTSNIFASEFLKH
QRLLQDQHDNYTFMWSLFSRLSEDLYRFGGVLEKKGEKRWRLDQTEGRSRMLRVIPDDSDDRQDY
QPKRKASEPPVMKITTQLQQTTISEPLGNAPTTSVFVGGTGDAEGDGEGDGDGDGDDKSALEESFE
MVDDPRVDIDEYEDKNRKVLRSLQRGDQVQSVCNMSCIIGLEACEGLLIIGKNAIYILDNYFQRAD
GEIVNVWQAPIEERDPFVRMISGRESNERKSREHEVRSWKWQDLISVSKRRFLFRDVALEMFFTDG
RSYLLTLISSRARDELYSQLCAHAPQFGGNMNLTRPEDIWRFEMLRVQDDAPQSLGSKFASVFGQA
SLNPATRKWLKGEMSNFHYLMLINTLAGRTFNDLTQYPVFPWVLADYTSNELDLDNIKSFRDLSKP
MGCQTPTREAEFRERYKSFAEMGDENAPAFHYGTHYSSAMIVSSYLIRLQPFVKSYLLLQGGTFDH
ADRLFYSIGKAWESASRGNLSDVRELTPEFFYLPEFLVNTNKYDFGLRQNVTKAIDSVELPPWAKG
DPKIFIAKHREALESPYVTEHLHEWIDLVFGFKQKGEAAIEAANVFHHLSYQGARDLDNIEDPVER
LATIGIIHNFGQTPHQVFQRPHPRRDDPSRREGRLDMLAESLTRVPLSLLDIQERVSSLSMKNERL
LCSPAFRLNVPPNFDKFLEWGYSDGSVRFYAADSKKILGHFEHLHVGQLSSFSTLLSASPDGQVIL
WDLNRRCFVRELPAEGPVECARINDRTGTVMICRGGRITLYSLNGALLVDQVICEGEDDYIISCAF
YEGESNEWLERELLFTGHKKGVVNVWSKVIKNGRFDLELIRQLHHVDQNKDSGVNVSAGISCILPL
PHSVYTGDEAGRVYEWDCIQRRS
>g2575
MKGVLSLSLLPLLTVASPVMPSTIHNNAAPILSSSNAVEVPDSYIIVFKDHVDSTSAAAHHNWVQD
IHSQHSELRKRSQFPFADDAFAGLKHTFDIAGSFLGYSGHFEENVIEAIRRHPDVDYIEKDSLVHT
MEDPALEKNAPWGLARISHRESLSFGSFNKYLYAADGGEGVDVYVIDTGTNIDHVDFEGRASWGKT
IPTDDEDVDGNGHGTHCSGTIAGKKYGVAKKANVYAVKVLKSNGSGTMSDVVQGVEWAATQHIKKV
KDAKAGKAKGFKGSAANMSLGGGKSVTLDKAVNAAVDAGIHFAVAAGNDNADSCNYSPAAAEKAVT
VGASTLADERAYFSNYGKCNDIFAPGLNILSTWIGSKYAVNTISGTSMASPHVAGLLAYFLSLQPA
SDSAFAVAEITPKKLKENLIAIGTQGALTDVPSDTTNILAWNGGGSANYTDIVAQGGYKTKTVSSE
VDELINKLEVVNEELGAIYSHIKDAIVA
>g2576
MWFKTAIVLLASCAIPSSAVFVDEVDHLDFHHALLGTPSPQSTFFHQPSSSSNASLLYTLSEKSII
GAVNPRDGAIVWRQDLTQLGSAQVEGFLRASDGEATVVSAIGEKVAAWGASDGKLHWERKVSGTIV
KDLELLELEDGRTNNHARDVIVLSEGKGGIVQRLDATTGTVKWEFKDESGDVPFQVSSSATEVFYI
SLQSALLKGYKIKITTLDTQTGKQKSHHTLASENDVSGPESILLVGSNTASPFIAWTDKSSKTLKV
NIIGSKNFVSLDIENTSNEEIRDIKIYGPTGLNCLPHFLVGYKTDSSSWAEVYHIDLQTSSISQAY
RLPRVSGNSVFAVSHRAANVFFTRITETEVAVFSSTSHGILGRWPFTKSGDGSLSSAVAEVISKGD
SAAVRFAYVLDSGDWQLNRNGGNEWTRHESLSGAVAAAWAEPNISEDLVHELELEGHEDVLSAYIH
RVKRHIKDLEHLPTYLQELPQRIFSSFLSAEVTNLDSFGLRKLAIVATEKGRVIAVDTGNHGTVVW
NVKVVDTPNWKVKAVKSDLSTVTIYVEDGSTVSLNVSSGSVVSQTPSGPLVRSVAVVPTETGINTL
AVTIAEDGTPLKSFQGAGYLVTLSEDGRVLGWDLSDNKVPVWQFLPPPGERVVTATSRPAHDPVAS
IGKVLGDRSVLYKYLNPNLALITSIGPTTATFYLLDSVSGQVLHSSTHTGVDVTQPITSIISENWY
AYSLWADLTDDSESKGYQLVVSELYESPLANDRGPLGAAGNYSSLDGTETYPRPHVVSQAFLIPEP
ISHFAVTQTRQGITIRQLLCTLPESNAIIGIPRHVLDPRRPVGRDPXPAEVEEGLFKYSPNLEFDG
RWYLTHSRDVAGIRSVIVSPTLLESTGLVFAFGGDVFGTRVAPSQAFDILGRNFAKLQLLGTVVAL
AVGVWFLAPMVRRKQINLLWKR
>g2577
MSVTQPVKGKTAIITGAGSGINLAFAELLLKNGCNVVIADLGLRPEAQAVVNKFNGRSGAARAVFL
KTDVTDWVQLEELFKVAEREFTEIDIVCPGAGVYEEHWSNFWRPPGSPESRDDPRGGRYRLLDINI
THPIRVTQLAISHFLRHRNSSKRKHIVHISSIAGQNPSMAAPIYVATKHAISGLVRSLAKLDQKFG
IRVTAVAPGLIKTPLWMEHPEKLKMFKEGQDVWVTAEEVGEVMLALVQQEEILILMNQKLIKGSRR
RGKRDVFHGAASWTSSVINLVNTIVGAGVLAMPLAIAHMGITLGVLVVIWSGVAAGFGLYLQARCA
QYLDRGSASFFALSQITYPNASVIFDAAIAIKCFGVGVSYLIIIGDLMPGVVQGFVGDAPTSDFLI
DRHFWITAFMLIVIPLSFLRRLDSLKYTSIVALISIGYLVILVVYHFTKGDTMADRGPIRVIHWAG

IAPALSSFPVIVFAYTCHQNMFSILNEISDNSHFQTTSVVFASIGGAASIYILVAITGYLSFGNNI
GGNIVSMYPASVSATIGRAAIVVLVMFSYPLQVHPCRASVDAVLKWRPARTSNGNDVSPHRFPLLP
RANRGGPEPMSDLRFAAITSTIIVLSFTTAMTVTSLEAVLAYVGSTGSTSISFILPGLFYYKISSP
DSAAHQQLMKEDDEVHDDAISDDEVSGDDQQQGLLSGSGILSSSGILPGSRLKKTLLRRLSLALSI
YGIVVMVVCLSMNIFFHASK
>g2578
MAANTRYQPAPQRDSFEEAQYAQAPPSYDATAEAPRSEDDNVPDDFKFGGTVAEGTLPIRMQFIRK
VYSILTVQILLTTVLSSISFFSDSYRIWIKSNLWLMIVSLVGAIGFMFLTYWKRKSYPTNLLFLSG
FTALEAYSISVVTSFYDARIVVQALVITLGIFVALTLFACQTKYDFTHWMPYLFGGLWLLIIFGFM
AAFFPYNSGVELVYGGVSALIFSGYVLVDTQLIMRHYHVEEEIAAAISLYLDIINLFLSILRILNS
QNNN
>g2579
MKFAKELNEELVPEWRAKYLNYKLGKKKIKAIQRALQKANRTPIRRQDTFTNTPSQRLPPNASTSV
NQFPLDYNGSPSRKDIPEQRPLRTPGSRFSNVVGSYGSIVSEPNQYDSKQLPNLELPSPAIGLDRT
AKKNKNLQIGPPIYDSGSPQAQRTPGRPRRYASSPAIFKKGNTPSGGFNAGGSDGYSKTANFLKRV
FTNSDMDSPGKSYQTNISSEVTKKEDDFYDFLDAELDKIETFYRQKEVEATERLQQLRRQLHMMRD
QRTTEMMEAERSGSTQGTGNGNYLNVFPKTKWTQAITGKHHFGKNSRALANMQTPKTPMGQELSGD
WRDFVRRPESAHVPYRTAKRKLKLAMQEYYRGLELLKAYAYLNRKAFRKINKKFDKAVDMRPTLRY
MSEKVNRAYFVQSEIVEGHMVVVEDLYSRYFEKGNRKIAVTKLRGKRRSDDHSPSTFRIGLFLAAG
LVSGIQGLILAIGLLNDANSTVQVQTSYLLQIYGGYFLIVFHCILFCLDCMLWARAKINYSFVFEY
DTRHTLDWRQLAEIPSIFFLLLGIFMWVNFSWVDSMFLYYPVVLIFITVVMLFVPLKVFYHHSRLL
WAVSNWRLLLAGLYPVEFRDFYLGDMYCSQTYAMGNIELFFCLYANFWNNPPTCNSSHSRLLGFFT
TLPSIWRGLQCLRRYRDTKNVFPHLVNFGKYTCGILYYMTLSLFRIDRDARYQILFIVFAFINAVY
CSIWDIAMDWSLGNFYASNKMLREVLAFRKAWFYYVAIVIDVIVRFNWIFYAIFTNDIQHSAFLSF
AVAFSEVFRRGVWSIFRVENEHCTNVNLFRALRDIPLPYQLEEHTLDEDFADHPSRIQTRESEMAR
EEEEIRGGEHPTDPTPMVANDVDLEAASIAGKTPTTLRARRPTISHAMSRFGNLVATAHSHDFQRR
RRIDPLSGETTNAGLQYMDDDSTDEDDDEDDRGDGSSTNNAARFSDVELPSQTTHIDSSQERSNS
>g2580
MPKDSHVLPSKPIETHPVPKKAFGRVLDDDRPIDDFENTVRAEEAQKHAEELGPSPPNRSAARHPP
DRPHAPHQPHGREGTTSSKYRSVSKH
>g2581
MPRVPFLGRLAWAEYLALFASLSLVFCEGFLRLVTLCLPDPIIRFFYQQSKALFNALASTQTRGFR
AKKKSVAESIAQTVDFADLCSIYGYETEEHIVQTKDGYLLGLHRLPYRRGEEADYVNAGENSTRKK
VVYLHHGLLMNSEVWVCLTDEQRCLPFQLVERGYDVWLGNNRGNKYSKKSTRNSPASTDFWNFSID
EFAFHDIPDSIEYILDVTKQPSLSYIGFSQGTAQAFAALSVHPTLNRKVDVFVALAPAMSPAGLYN
GIVDALMKASPQVVFLAFGRRSILSSTAMWQAILXPPIFVRIIDISLRMLFNWTGENISQHQKLAA
YPHLYSFTSTKSVVHWFQIIRNQAFQMYDDEVGGPLAVGNNSRYYKPVRYPTRNIRSPIVLIYGGS
DSLVDIGVMLRELPRRTTVREIPRYEHLDFLWASDVDELVFGHVYDALERYAGGQSTVEVPLQITG
PETDFNGDQYSLSTAKGEVERNVPSRRHLRTDSLNSLNGRFSPTNLTYGDQDWRIDPSKAPEDVDL
DTLT
>g2582
MESVRKTFFDPLATTKAQIRGILAELPVGSAHPYSPESILLCRTDHGIDEIDISFYPADYFFNAVD
LGEMGEVLSRRMANFASNLEPLKDFQIQQAPASTFRVPIVSGQITILAVSERNPTYWDLCFANAAY
TVSDWSRLSTLPGIDHSGDKNYGSDLLHHATISKQSLPEEEICVFISGYQSTQITRFLGTHPAMLV
ANHADKVLYIVPVPLDAATIGDAVVCCPLVIKRDGDSLVCSILASATTDRKAIHRGQPSPVISEAI
QRAEFTISQPNAATREPKKILVKKHTTLDSESFRQITAKENVVIATNSATLPSLLSLENTQIIRFG
TGISFELQNGLRNSDTQQSTVVLPCIYGSQLQGPVLLATGTTPSNVPFLDEIALRDYYKQLNDIVV
IVDDRMTDNARNLAPSYRINALFILQLNPENEPKSTEDILELMNSANINAVTALAGPQSLVLVQIF
DKYYFYRGLANRARLNSSGLAFDPRVMRIVDLDEDSNTIILPTSGDFVRPQDLQRIFEELPIEKSL
LSQKDLQELSTALVSTLSAKINDVTAPLRDMYLTFLAREYNVEDTESTTSKRTHDIKRLVRQAQIQ
GNVEAAKTMTFDTLAGYLETYAADMGVMLLNIDTVPYRQLLGNLKNTTIDASKCCELDSRVLYLEG
FDAGIIIEQSQNKHNGPLRSQNGHSSQPILAVPYLNQGRGYDGSMLAWVCWDEFVNLENPYTVRWM

EKCNEAHIAALRIIMRSTLSQAVASREHNLQPASSETGQLMSALLMAAMSKLAAMRTTAPIVVSGQ
AEDTITRLMRGLFGNFLTIAGSGVRPLSMVWQLFGSNPQYDIPTSHTDWVWQFLENLEKLLDKAII
RVVTKNEDMAAIKKSRTEEMIRYCKLRNIQLAHCRTIITIFMQMFTTEKLDVVAAAARLLEQLPRK
LEKQTQSYKRMINYLEHLANGGQPRVNDNIIAACVYTKRSAAFSELKTKISEACVSEDWLKVKQSC
QALIDKHTETATLWRIPPESLHIQNMKVYKEALDTDVNTGQENIAKLAGQIIGDAEKNRVPWQVGK
AGQFGDNIEHLNEAFLHEILTGEKLDERNIDEEFAEFGVTVQPSFISTMQKDLSAEDVCTIIQVPV
STMRIFIKALNPDFAWEDLGRNFKTVILGLLRERSNRAESRPVKKLLALGVENKALQIQSWN
>g2583
MGSISQETVLQSPALSTNISIFEPFSLPSSNQRVLSAPHPQDTVIPLALRPSGSNPSLDVAVSAVE
SLQKDHDGYLTKKLATHGALLFRGLPIHSADDFSRFAHAFGYSPHEIIGIVVDRPLLAPNVAPANE
APKEVLIYNHNESPQVPHAPEYVFFYAHKAPAKGTGGESAISSSLEIFNRANREIPEFIDALAEKG
ILSQVTYKVEQQYAGGSTLRQAFGKEIIEGDDLETRKRKIEDQIKRYGRGEFTTWKWEDDDQTLVV
THRLPAIRTQQSTNLPTLFTGLAAYYKNTFGTGTGKRKNITQTLYGDGTPIPEKYLAHLASITDEL
RVLHQWEQGDVLVFDNVIAQHGREPWTGDQKDRVVLASLFDAESLPGRYGYANWAQVVQASS
>g2584
MSAENESQKIKTVGSKNDNHPIQVYADEEPTSLTETKSKPQFFRSTLFQILVVGLCAFSAPGIWSA
MNGLGVGGSQSPNLVNAANALLYAFMTVTCFAGPFITNVIGFRSTLALGSVGYPLYAAGLYTNNRF
GTTWLVYFGAVACGLSAGFFWSVEGAIATGYPEQHKRGRYIATWFSFRNMGNVIGGAISLALNHNT
QHKGKVGYQTYLGFIAIQCLGLVFGLLLSNPEKVQRDDGTKIEAPRGIDWRTEVRAMWRLARSRSI
LLLVPLFWYFGWMQAYPGTYLATYFTVRSRALGSFMSAIVGTFATWLSGVLLDVPWHKDRNVRAVS
TYVLIAALNSATWIWAVYLQNEYRHTQPTLDWSNQAAFARGFGLYMFERVSLGMVENYIYWCIGNL
SDSPGDQIRYSSLLRGIETAAVAVSFGVQAVPTALIVTASINFGFWFVALPFSYYATIQVVRKFRS
S
>g2585
MVRSLFVPLALSAFVNTALAAPATSYATVEGFDSIPSGWEQVADATPTKLLNLRLSLAPANTDNFL
NTVLGLSTPGHASYGDFLSPGALQDLIAPASTASDAVTSWLDQSLSANAIQNNGNWLALQLSVEQA
ENLLQTKFYDYKSTTTGEIRTLTLGYSVPTSLTEFIETIQPTTDFSEVRGLPQIQAYGPDLSNLTT
EATSSECNTQITPACLQSLYGLPTASVSNSSPGIAVPGFINEYANYNDLSLFLKQFRPDLNPPPSF
TVESVDGGLNDQSQPGVEANLDIQYTVGLANGIETTFISSGLSTVQGFLDVISYVANLPTPPTVLS
LSYGFDENTLGTSNANSICNAFAQLGARGVSVIVASGDGGVAGSRPSNSCTTFVPTFPASCPYITS
VGATAGVPETGSTLSAGGFSNLFSRPVYQELAVEAYLIGLQSEYSGRFNTNGRGYPDVAAQGERIV
IELSGSGTLVDGTSASAPIFASTIALLNSQRLVPLGFLNPWLYLESAVLNDITTGSNPGCSTSGFP
AAAGWDPVTGLGTPNFGKMQLLI
>g2586
MVQRNSPNDCLRPPLSDDLPVRCQQLSKALSDCKRGLVDMRKRFRGNAPISVSREIEGGGGSSQSS
TQAPQQLYAGKPAYTSVKEISGNEVQMDPEKTRGL
>g2587
MRSFLSFLLLGIWGACVHALSSSGSRLLVVLEEDKSLYSNLWADLEGRGYDITFESPKSDQLSLFA
HGEKAYDHLILLPSKSKGLGPALTPKLLLDFLNDEGNVLLALSGKSATPSSISSLLLELDLHISPD
RSSIVVDHFNYDTISAAEKHDVLLLPLPDPVRSDVKRYFSGEGVLALPRPVGQSLGNASPLLAPIL
RAPETAYSYNAKDDSLSVEDSFATGSQLALVSALQARNSARFTVLGSAEALEDKWFAASVKSPKSK
SAVTAANRQFAKQLTGWTFKELGVLKVGKVEHYLSDEFGNIEGEVNPTIYRIKNDVTFNIELSEYE
FDKWVPYKVPANDELQLEFSMLSPFHRLRLLPQSTTENSTIFSVTFTTPDQHGIFSFRVNYLRPFL
TNIEEKHEVTVRHFAHDEYPRSWEITGGWVWIAGLWAVIGGFVAFVLVWLYSAPVPTTEAKKIK
>g2588
MSAPQFWSTPLRYLRWASHEKPAIFYSIVLGSLGPVCLVAIPPVRRYFGDVDPEKIPLSYPVPKGP
RVIPKGFDDE
>g2589
MAELSSRENLHAPSEFPNFHNGDVMVVIPPTEIYQLHSDVLRRCAPHHFGQLVAPANAANLIKAAV
NSGRFTRYKLVLRPSERHQTGAFRREEVDKYGRTTGDTDCHSMLENVYNAKLPAYAPEAWKNLFRA
FYNLDPDLSGSSLQTVLEKAVHLMDAAESVGAAAAVRAHIDNSLMRHGQLLYRAILALPILWGNLA
IRVRSPAIFKDAVIHVVGKWNSLTNAEKRNMNPHFRAICEQKAAELYKIKGAVEIRILGHHPQVLW

RNVGSASATSRANYANDVYMWMALNIHRQWFFQVTGMERRGRDGPDGGAELYHAIHEGGTTYLVAQ
DYACFHNTCPMSKKARTILYDKVRQMKFEVRKYAKCLTVNRTLLDLKDGLQVNHLLCTQVMDHDLP
WNRTDLARLEPSYLKDDGDEMVVVEGGIPSDVSDISVYGTEIHRPDGQVDV
>g2590
MTGRAYERPERHNEYFIPGDGISREVIQADICRYLGNDALVKPGTHQGRSGYLIRAYRNLTSEMIA
DLKADSARWEQEVERRANMGYSRGSYVSPTHELRSQAGPSPPSYAAAPAPYVDPYAQQTQYGAPQG
QPYATPSYSTSQSPGYNQNPYASPPQQNPFSGPGQGQPPISAPDAHQPSYTYSSNPSYGYERGNAA
PRYPGAGYDNEPEYSPVTTGMGYPAATAPSDARLGMDPRYTPEAAYDHRTGASRPQQNRDPHRRPR
>g2591
MSQTQTVIRIHGPRNSIHNLKAFKEEVPIPAKHEVLVKVHSVSLNYRDIGVATSGYPFPVKDNVVP
CSDAAGVVVAVGEGVKNLSKGDRVISTFDPTNLYGQQMDWLNGHGGPVDGVLREYFTTPAAAAVKL
PESSPQSFSEWSTLVCSGVTVWNALYALTPLKPGQVVLALGTGGVSVTGLLLAKAAGCITIITSSS
DEKLNAVKSKFGFDYGVNYKTHPKWSKEVLRLTNGEGVDYVLENGGSGTIAESINSIKMGGNVSVI
GFLSQAKEMPDVASLALSKGAVVRGVVVGSTQLLQDLVRFVARKGLRLPVEKEFGFSAEDASKAYE
YLMSGSHVGKVCIKISD
>g2592
MKSSRYTFRHLHLLRQNSPSSPLRVIAHIDLDXFYAQCEMVRLGTPRDTPLAVQQWDSLIAINYAA
RPFGVTRMLSAIEAKKRCPELVLQHVATFREGEGGKWAYRDDASYNIKTDKVSLDPYRAQSRRILS
TIKEEVSRQRDEIISSKDVPLEFQAARIEKASVDEVFIDLSSLVYAVLYQRYPELRRTPTASDKTT
SLPTPPTTALEWFPEDGLVDLDEEETEEDDPDWDDVAMLIGAEITRSIRAAVWDKLSYTCSAGIAR
NKMMSKLGSAQNKPNKQTIIRNRAIQKFLAGFQFTKIRWLGGKLGERISAEFGTEQVKELLNVSLD
QFKAKLDDDTAVWVYNIIRGTDDSEVNPRTQIKSMLSAKSFRPSINSTDQARKWLRIFAADLYNRL
VEDGLLENKRRPTTLSLHHRMGAQVHSKSTPIPGGKMVTEDILYELSSNLLNQVIAEARAWPCANL
SVNVTGFEEGPVNNRAIDSFFTSGQSRPSAEPSFPKKVAEPADDAERAAKVRKVAVKGIGSFFKRD
SPSLVSSENDKDSDDRSPPLFSTPDIVLMKNPEDDTNTVQEIDKDISAQHGTFTCPRCNEQVPEDQ
REEHDDWHFAKDLEAQEVRADQTSRNIAEQSSRRTASDRKKTGPGRPRVSGTKTEKAEKGQKRLFF
G
>g2593
MSRRPPPDDVYEKDNFYEEVDRRRRSKDDRYFDPDFGVRRRRLTRSPPAVREIDERVRVRRRDSSA
PDFIRDRDRDRFLPPRDEDVISPRRRERERLDDLRLQRETEELYIRRGTPPISRHRPRRVSRDEVI
LDEGDEIDDYRRKPREKEKIIFEERRESSRPPREEDERDEVVIRRRDRSLPPRRMREDEEDIIIEK
DKKRINAREELDDEKIVFRHRDRPRREDDFDRERIARHRRSMPPLEDFRAEEEEVVIRHKHDSPGP
RRRHRHRDERHLPDEDEREEIIIRERERDRRKQEEIEIRREERSPPGRRRDDDIDDILIRRRSRSI
PPLRDDDRDEIIIKRKNRSSPRRENDREEVIIRERDRSAPRRDDDLDEIRIKHKDRSSPRREDNRE
EIVIRDRSAPRREEDDREEIRVRHRHHSSHHRDDDLDDVVIRRRSAPALDDDDDREEVVIRKNKRG
RRKSISRDIEEDELIVDDDDRGRDRIEFRHVHSPHRSPRRSPSQNRGKEEIIIRENDKRKPNEASR
EVIIRKEESSSSESSREPSIVRKPPILQEVITHHRHIDSYEVIPPVRRDSPEAESVSDEVDVIDIK
RRGDKHGRPYEEEIIIDKGKERSLSPRGRPPISEPENELIITRTKSGDHGVEKEIEIRERERDKEI
RDLELEREIIIRERERREEIESIPEAKEGVRVHKRYTDTRRKRDKLWTEISKDLVVREALERAGYE
FDETENFYYVFDYLRYDDVERLVDMSEDIRQFRRERIRGMRRERERLPPLPVIPGPPPIPARPPLM
LERAPFREPYPWEERERYRERDVIIEEGRRPRRYREI
>g2594
MEDRDTYKTQRIINSVFFEHANWWIAERLAELMAGLMMNSKYSDLTLECDGETFPVHKAVVLTQSV
VLEKACQEPWKEGVENIIQIKEFDAQTVRRMVEFLYTGDYDQGKPEFVNPTTNGESADAGDEAEDV
DSNCGDDFHPLFERCRSNLDANIQSLEAGISNISLNAYQPSSPSVSASERNEWGPDDETAANDFFD
AFSLQLGVHAIADYYMIKVLKRKAIEKTKTLLKKTLWSAKGFLAVAKEAFETTTPSPASTAAAEKE
QDAETLRDVIIETASDHFTEIIDSHDFARSDLNPEFAAILLQKAAKKHNDIRAELKQAHRDNFNFY
IVNSHRTQELMKNISYLQAQTAAFEDCVQRVQELSTCQVCGCAYNALIREEGENNTDDDDGYDADG
SVDGSATGVGAQERKVKKYVIECKKCNLHSVRSGDLLRGDMSFFGGDELYQGVAELSVDELPAEPW
LQGENDQFAGTLYPDADADDV
>g2595
MMAFREMARLGVAALLIGSSSVAALTIEYCSAENTASSSTENTDIYQSNGACNTQCSADYAFAVLQ

GQDCWCSNYIPASTVDVSECNTSCPGYPSDLCGNTDKGYYGYIAMSGHKASGTATGTSTATATGSS
SSSSTSSEGTATETSTGPETVTVTESASTGTTLATSVSSSTSTTSSTTTTAAPTVSVQTIAGQPVT
ITVSNSPSATTSSSPVSTENKGSSLSGGAIAGIVIGALVGVGALAALVLWWFFFGRRKGDERSEKT
GSPIGGGDGSVTGGTVDTRRQSRGSQMSFMHNFIGSETSPTSPNDYLSPNTAFTDNRMKKDAVLYP
NGDRLSAVSLRDEEDYSRPVLRVRPFIPACEMHANLVCLNSSRIQIKIDCVIDTLFLIAFACTARH
SAATTVLIHDTRPVARNFSFTPLQRLTNLVLIPISEATPCKRHHNVLILAYTYCFFLSFPRFPVCE
FSRRLGLV
>g2596
MANITVYRGFPATANYVWSPFVTKLEARLRFAGVSYTPEQGSLIKAPRGKIPYMSIATPKTTSEPE
FLSDTQLISDQLTEHGILPDLNDRLSPTEKAVDQALRALLEDKVYFYNTNERWNENYYTMRDGVMA
AIPYPIRVIVGYLAWRKNNAGLHSQGTGRFSAEEIHSFRDKIWHSLDDLLAESRHKSLSGQKLFWA
LGGEGPTEADTSLYGFVIAGLVCDAGPDSKKLIRTLPNVVEYARRIHGEFFADYAAPVWE
>g2597
MEFVQTSPEAKIHLHLVKESSISDSSPLLVFLHYWGGTSSTWHKLTSPDSPTSLSSRYASVSITNR
GWGLSTGPTAENGATGKDYSITPLASDLAAVLQSLATNQTTASIVKNNGIVLVGHSMGAKVALAAH
ESLSDEILPLIKGVVLVAPAPPTPFALPPDMSEQQKHAYDTTEAVQFVISNVLSSPDLLTQDDIEM
LVKDSLAGCVLAKEGWLEHGMQEDLIPVLDRISKLPETQNVKVRVLAGEFDVVEQKDRVQSEVVQN
LVARGFNVAFSVVKGAKHLIPLEDPAAVARAIEEVSA
>g2598
MDGQTSGTSSAGNSSDFVIFVHPSDYSIFADNAIARINTYMLARMLEDPSYSDIVRWGDENDSFVV
LECEKFTKTILPKHFKHSNFASFVRQLNKYDFHKVRQNNEETGQSPYGPNAWEFKHPEFKANNKDS
LDNIRRKAPAPRKPAQMNEEAVPTQQIDMMNTQLLAQQHQIQQLSERNTQLTVDSQMIMQEVMRLQ
KTILQHENVIHNFMNYLFTVDARHRRDSRANGAFAQDGSAADDLPASPLQQASKILSDMTSSQMPP
NMVQFDSMNDPTKAQMIQTPPLGAGARNGLARASNSAGSNASMGFAKMNGELETVVYPVGATNGID
PMYSEHVNNIPYSIQPKDIAAPDPVAQQQQIQRPFSESRKKNTLVNPGWIKQPNILLVEDDDTCRQ
IGGKFLGSFQCQIDNAYDGLQAVTKVQSGKRYDLILMDIIMPNLDGVSACHLIRQFDRTPIIAMTS
NIRKDDIELYFQHGMDDVLPKPFTRKSLLDMLEKHLMHLKTVPATAMDPVSVTPMTTHTSAAQSIK
EDSSPGQSPAASMNNWQSPGQLQGMSPVHLNVQQVPQYMQPMTPTGPFIDQNGVQYAAPSALSQPP
VRGQHRRQVSDMSSAPDAASYAKRQRMYHNTQMMPQMAGQQNG
>g2599
MGPKQKGGGGGNKQKGNTAEEVEETLQAVVFADTFETRFEPFTLEKPRCLLPLANTPLIEYTLEFL
ANAGVQDVFLYGGAHSNLLEKHISTSRWKASSSPFKKLTFLKSTSTSVGDVMRDLDGKHLITGDFI
AVSGDVVSNFPIDEALSKHRARRQTDRNAIMTMVLREAGAQHRTKSSSVSPVFVVDPTKDRCLHYE
EIDHHSHTDGPARLTIDTELLTTHAELDIRQDLIDCNIDICTPDVLSLWSDSFDYQAPRKHFLYGV
LKDYELNGKTIHTHIIKEHYAARVRNLKAYDAITKDIVTRWTYPLCPDTNLLPGHSFELRRGNIYQ
ERGVILARSCVVGRRTVIGQGTSIGDKTTVTNSVLGRNCRIGKNVILDGAYIWDGVVIGDNTEIRQ
AIVAEDVVVGDNCKVESDVLLSYGVKISNGITVAEGTRVTVAPREDGSVPEDDQQIVGSEGRGYEY
VGEEDEDDEDVRSESSGLIYNMANLSLSTDSISTLSSEISDDGYFHGQRSDSFGTSASEDDDHDHF
QHDASNNLYESMRDGVSPDVVALELVSLRMSANASDHQVRHAVASSFMKRITQLVTESGKSAGDAV
HDVFTRYKDVIDRCLFDKNKEQKTDQIDMLLQIQQDLVHRAKGDTILLFAAKELYDLDIIEEEAFE
AWWADERSTATDEMKTVRAQTQQFVDWLANASEEEDDDDESEDESGEDDDEEDEDSDE
>g2600
MTRAFKSTNQPFFRPTYILNHAILLLQWNIPVSCASDIAELADTCEGSAADGTSVHPDIAVLSLDV
TSTPLVTEAAKAVSESGRGLDILVNNAAFGYTTPVLDIDIDEAKKVQDTNLCGSVRTIQVFKDLLI
ASYGRIINFKVITAVFSLPWDAAYKIERGSLDVRFTFLSGRLD
>g2601
MVGFKILACLTLIGAVQAQMPWIPLNIPIPKPAPAPAPAATQAPAPAPAPAPAPAPAPAPAINPTV
VSQAQQACTNAFPGTEPVFRADGTPYGCTPNICPFSSPVLSAQILNSLCLSPDMVRLSFGLLVVLG
QRSLAQNHGIAASSSLLGFNPTFSSADTEDPTASMVFVEPDISILCPVQPFQSFLLSENPTVVYIA
SFVSDAEAQHLIQESEPVFAPAAVYGSGQTQVDLSSRKSLTALPGRDEVVRCLEQRARTNQDWELG
MTIEAISVQRYEENGFFRHHYDAFGESYRKDRKTTLNVFLHANCTGGGTHFPYLPKPEDNRWCEFI
DCDSDQPGVTFKPIAGNAVFWENIRPDGSVYAETLHAGMPVLSGSKIGLNIWTWTKDRRM

>g2602
MIPKDASFRSFFAYLLFCRGVFSQNQPVFPQPGGSQQLVFSQPNQGQQPVFPQPGGNQPVFSQPGG
NQQPVFPQPGGNQQPVFPQPGGNQQPVFSQPGGNQQPVFSQPGGNQQPVFPQPGGNQXPVFPQPGG
NQQPVFPQPGGNQQPVFPQPGGNQQPVFPQPGGNQQPVFPQPGGNQQPVFPQPGGNQQPVFPQPGG
NQQPVFPQPGGNQQPVFPXPGGNQQPVFPQPGGNQQPVFPXXGGNQQPVFPQPGGNQQPVFPQPSQ
GQLPGSNPQPVVPQPHQGQQPSSSQYTSLPTCPQGHDLIYPAPDGGFFKLQCNYHGWHGTLSSTTA
SSYQDCIDICSATNGCLAINWDSLNGSGCSLMKSDQPGDTPCPNHDFAYAVTPPTIPQTDDLTPRC
TTECPGADHRQHTSSSGEVFKMNCDPPDNPRNTFTTPPTTRKRKAVEEAATTKTKTTNSERKVNGR
KSIPEATRSGSRSGLGPGSGPNKTLRLERSVSVHEEGEQSSQVSSENSKDSDSKEFPLSLRKKGSP
LVTPTTTTRSMDSDDDFMTDVSSREEDFLDMQDSEDESLGEDFGDDFGDSFSYDKDLVQKTKKPYE
VEFKVLSPADIERDQNSQITEVSSILGLPPESAGILLRFARWNREKLIESYMDRSEEILEEAGLGD
SFEVNPRTEVVPGFMCEICCEDGDDLQTYAMRCGHRFCVECFRHYLSQKIKEEGEAARIQCPQDHC
HRIVDSKSLNLLVTDELKDRYKMLLTRTYVDDKDNLKWCPAPNCEFAIDCGVKARDLNKIVPTVHC
ACKHSFCFGCGLNDHQPPPCSLVKMWLKKCEDDSETANWISANTKECPKCLSTIEKNGGXNHMTCR
KCKHEFCWMCMGLWSEHGTSWYNCNRYTEKSGSDARDAQARSRASLERYLHYYNRYANHEQSAKLD
KDLYLKTEKKMTSLQSQSGMSWIEVQFLDTASQALQQCRQTLKWTYAFAYYLARNNLTEIFEDNQK
DLEMAVESLSEMFEKPIAELAELKVDILDKTAYCNKRRVILLSDTAENLKQNEWKFNVDL
>g2603
MAEHIPGQEYEYEALPSNYGXGHNMLAGAFAGIAEHSVMYPVDLLKTRMQVLHPTSGGLYTGITNA
VSTIYRIEGWRTLWKGVSSVIVGAGPAHAVYFGTYEAVKEFAGGNEDGHHPFAAALSGACATIASD
ALMNPFDVIKQRMQVHGSVHKSIAQCARTVYRTEGLQAFYVSYPTTLSMTVPFTATQFVAYESISK
FMNPSKEYDPFTHCIAGGLAGAVAAAITTPLDVIKTLLQTRGLATDQEIRSARGLFNAAAIIKRQF
GWAGFLRGLRPRIISTMPSTAICWTSYEMAKAYFKNQMSDK
>g2604
MMRSKPSSSMQKTYDECYLMCSTAVYFEGQNNESEALRSWRSALDTIYYHNAYRVPPNYVPKSETE
KALQDSLRQLELQCKERVDLLEALQESRKEAAAENESTDEKDSSGNRPMGPRPPKLGKGKKPLMST
NASSNSPQGFIGNGTIPAVDYMDLSKPPPLPVRPSSAAVSVSLTPAPSLELKEPAPSSSSPHRLSP
SPVPAAVSSPGTPLTKESSRGSSPERRKIMLTTLRQKKDPKKSKYKSSSSSRSATRPAASKAAGLA
WGTTRKPVPLAAESSDSDLRKETESRQRLSSEDVSRRNGQQKVASSPFSSDYLPSSTWREPAYPDQ
APSPGLTSPQIDSAAAAASKSLTADASNKPPPTAPKPSIKSLPPPRYPAYRKEYLSHPATAPSERN
VRTPPLNRSTSATQNSTVSPSTGETSEQPARRTFPKSQSTPRALSSTGANRASVSSISGDEPARPP
PRRSDASFDGAAASAGRKPKPPAPRRKPRITPPSSDQDSAREDSSSEREEDPKLRDIMKKLPRGID
VNAARQILNDIVVRGDEVHWDDVAGLEAAKKALKEAVVYPFLRPDLFMGLREPARGMLLFGPPGTG
KTMLARAVATESKSTFFSVSASTLTSKWHGESEKLVRALFGLAKALAPSIIFVDEIDSLLSTRSSG
TEHEASRRSKTEFLIQWSDLQRAAAGRNQSTDKSHDGGGDPSRVLVLAATNLPWDIDEAARRRFVR
RQYIPLPEDHVREQQIRRLLSHQTHEMSNEDIEVLVKVTEGFSGSDITALAKDAAMGPLRNLGEAL
LHTPMDQIRPIRFEDFEASLYTIRPSVGKDGLKRYEDWAREYGERGG
>g2605
MPSEEQPQTEESRLLLVSNRLPITIKRSDDGKYDFSMSSGGLVSGLSGLTKSTTFRWYGWPGLEVP
EEEIPVVQKRLKDEYGAIPVMIDDELADRHYNGFSNSILWPLFHYHPGEITFDEYAWDAYKKANRL
FAQAVAKDVQDGDLVWVHDYHLMLLPEMLREEIGDTKKNVKIGFFLHTPFPSSEIYRILPVRNELL
LGVLHCDLIGFHTYDYTRHFLSSCSRLLNLQTTPNGIEFQGKIVATGAFPIGIDPDKFKEGLLKEK
VQKRIEMLEQKFQGVKLMVGVDRLDYIKGVPQKLHALEVFLSDHPEWVGKVVLVQVAVPSRQDVEE
YQNLRAVVNELVGRINGKFGTVEFMPIHFLHKSVNFDELIALYAVSDACIVSSTRDGMNLVAYEYI
ATQHKRHGVLVLSEFAGAAQSLNGSIIVNPWNTEELAGAYQEAVTMSEEQKALNFSKLEKYVSKYT
SAFWGQSFVSELKRISAQSKQKVSVRKASLLVPGVSTPVMNGDGEQTAPDEVVVVSSEGEAEEEAG
VRRLEQNLHLPSADEFXRHKMAPSNILRGSLLLSSTVARSATRMNAFQSQTRYAGAINASGSRRSI
WNFLTGEQQKNQAAKKGEEENQQQEQEAENEVEEQQSTSLDAQKQQQQLDAQNLASASTFSETQTS
PTPIIDQVKEAEQEILPSEQQETSWPSDVPQPSIRQYPYTLKRGTVTSVGLMSKTVRVAYQHREWD
RKIRKYYPKTTTYLVHDPRNSLREGDVIEFSSGAPRAPSVRHVVERIVAPFGSAVEERPAVPTRAE
REEEMVAKRMVKLMRQASRKAAEEGESDVGKVMRRVVESKEHVGRIRGLVIERAAAQSQASA
>g2606

MATQLVPLPEVERLSTTVIRILAGNPGKFTLQGTNTYLVGRGPRRLLIDTGEGRPRWATLLQSVLK
EENAVVHEALLTHWHPDHINGVPDLLKICPQATVYKHQPKDGQADIEDGQIFRADGATLQAFHTPG
HTVDHMSFILQEEDAIFTGDNVLGHGTAVFEDLSTYLSSLKKMKDSVRGRAYPGHGAIINEASAKI
GDYINHRQQRENEVLRVLKFGKLDVAAAESSPERVSSWTPIQLVKVIYKDVPESLHLPASHGVIQV
LNKLEAEGKVEHDGDTGEWQLTAQRPAL
>g2607
MISTRLTRLGAVGTKSRVLLGSRGMATVSDSPLDKKVEMTNWEKGNYINYKKFAENLAIVRGRLNR
PLTFAEKIIYSHLDDPHGQEIERGKSYLKLRPDRVACQDATAQMAILQFMSAGMPSVQTPATVHCD
HLIEAQLGGDKDLARANEINKEVYEFLSSACAKYNIGFWKPGSGIIHQIILENYAFPGGLMIGTDS
HTPNAGGLGIAAIGVGGADAVDVMAGLPWELKAPKVIGVKLTGQLSGWTAPKDIILKVAGELTVKG
GTGAIIEYHGPGTESLSCTGMGTICNMGAEIGATTSVFPFNDRMYDYLKATKRQQIGDYAREYAKE
LREDEGAEYDQLLEINLSELEPHINGPFTPDLATPISKFKEAVETNGWPEEVKVGLIGSCTNSSYE
DMSRAASIARDALNHGIKSKALFTVTPGSEQIRATIARDGQLQTLEEFGGVVLANACGPCIGQWDR
KDVKKGEANSIVSSYNRNFTGRNDANPATHAFVTSPDLVVAMSIAGTLKFNPLTDKLKDKDGNEFL
LAPPTGEGLPERGYDPGRDTYQAPPANRDAIEVAVSPTSDRLQVLAGFEPWDGKDAEGIPILIKTK
GKTTTDHISMAGPWLKYRGHLDNISNNMLIGAINAENGEANKVKNAVTGEYDAVPATARDYKARGI
KWVVIGDWNYGEGSSREHAALEPRHLGGLAIITRSFARIHETNLKKQGMLPLTFTDPADYDRIPPD
AKVDLLCTELAVGKPITLRVHPKDGSSFDIKLSHTFNESQIEWFKDGSALNTMARKSGK
>g2608
MGATRRLKTKRRTRDYDQIRQDLTSRKHLQDFQDTKDAEDLPGLGQHYCVECAKWFETEHNLVAHR
KGKNHKRRLRILKEDVHTHKTAEQAVGLGVDNGKRDQTLMDTDEPEA
>g2609
MTDQSSENSTGHESVRLQVYLDWVDAWVSSSKTITKTAILQPEDPTTLTSEEVFARQLEDSADLAP
GDPIEIPSLSTNEMAAYFLEEXLPYLLNPDDLKAIIHAFRQQLVHRRIKVGGRLPPSCPETDVFSE
QYDPRVECKCVGNTRDNKQTMTDLASWQDSECRAIRRTVKAAQTVISRQAEYKSQGIFTSQGLTDA
AIELFLSGSDLQPIPDTCQGSGITDPIPEIKAPDRRPSPTCDSDPDIYDSLFPTIEKIKLCADAKY
FFAMACGGSLVDEGLLRAIADSGNDILIGDYCDAADEQTLNILQKNGAAAVAFLKLCTLADVVTPV
QFDNLVASLIQFRVLGYYRDHTRTELVGGLYGSYYTGLPGPRYIDLAIWVGMMSASLATGEKITEE
MYLRLATVCTLLNDLVDLRSDTMRKQRENLILRGVRGCLCGYLDSCISHCLTLACEALESDRLSGL
VVLGFCNWAVMASHHKVYEVVNGVQEVKRYPTCKYGSIEDETKYNNLLKALEPYGTLREEGRHIYM
KRATMDQLYAVNRLKSETHLAWMADVTRSLLQPETLRKIVDVVHFEWRGDVGNREYCP
>g2610
MSSDFVAGYISGAVGILIGNPLDLVKVRLQARKASDTLRASQQSYRYFESTSSLIKGAAAPILGYG
ALNALLFVAYNRSLKSLDPSVDDPINLDANVSLVNIWLAGAAGGMASWLISSPTELVKCRAQLSRN
QNISSWSVFRDIIRAHGVRGLYFGGLITSVRDSVGYGFYFWSYELCKRSFSSLNESNQQEALKVLL
CGGLAGIITWASVFPLDVVKTRLQAQPIIAQTVTEVADAQQRPLLQVSSHRPRTLNSLEIARYAYR
SEGLSVFFRGLGICSIRAFIVNAAQWAVYEWLMKEFNKASAAPHVSI
>g2611
MLTRSRQRPRPQATISKQTPQKAARLHGPSDQSAERLSKPFKCPGQARTTIASDRPSRKRRKVDYA
GADGSVDDDYNRPWTTEERLALANRDVNKFGIFKVKDKEVTIKQRFSVPLMNKNSGAYDPSRPAPT
LGMRRGATFVVKPLHDPSGEFAIVLYDPTVDDKPVKKITETEPTAEEPPKLDEPLVHKSLADILGL
KKQVEDRPKVPVVIDPRLSKILRPHQIEGVKFLYRCTTGMIDENANGCIMADEMGLGKTLQCISLM
WTLLKQSPEAGKTTVQKCVIACPSTLVRNWANELVKWLGPDAVTPFVIDGKATKAELSSQLKQWAI
ASGRSVVRPVLIVSYETLRLNVEDLRDTPIGLLLCDEGHRLKNKESLTWKELNSLNVSRRVILSGT
PIQNDLSEYFALVHFANPNLLGSQNEFRKKFEIPILRGRDAAASDEDRKKGDERLSELSAIVNKFI
IRRTNDILSKYLPIKYEHVVFCNLSDFQTNLYNHFLSSPEIRSLLRGKGSQPLKAIGLLKKLCNHP
DLLDLSRDLPGCEQYFPDDYVPPDGRGRDRDIKSWYSGKMMVLDRMLARIRQDTNDKIVLISNYTQ
TLDLFEKLCRSRAYGCLRLDGTMNVNKRQKLVDKFNDPNGEEFIFLLSSKAGGCGINLVGANRLVL
FDPDWNPAADQQALARVWRDGQKKDCFVYRFIATGSIEEKIFQRQSHKQSLSSCVVDSAEDVERHF
SLDSLRELFQFKPDTRSDTHDTFKCKRCRPDGTQHIKAPAILYGDTSTWNHFVNDGEKGPLGKIQD
LLLRQETTEKDVSAVFQFISS
>g2612

MDQSVQRLLNDKLYDKRKQGALELEKIVRDATIKGEHDQIREIVDQLCHDYAYAVHQPHARNGGLI
GLAAASIALGSEGVAPYLQEIVPPVLACFSDQDARVRYYACESMYNIAKVAKGEILPFFNDIFDAL
CKLASDSELSVKNGAELLDRLVKDIVAESAASYVSVLQLGEKSLQDLDQARDIDETSVDLPTAFSL
SMFIPLLQERIYVLNPFTRTFLVSWLTLLDTIPDLELVCYLPAFLGGLIKFLGDPNKDVNVATQGL
LERFLSEIKRIARIKKGIAESKKSKEDIAAATVASENTSVVTGNSDELEGSDDIIADSVSADANDD
EQSIAVDGDWVPGQDAYVDYPKILEILVGFVDTTYDEEMQLTALRWIDAFFEISPEDILQFVPALL
IQVLPAISSGSDQVRQAATRVNTSLMEYIVSLSDEANESYPSSASKGTQSSAVKESDERRASVQSG
KPSVDNQSIDAPASSPPSDLDYAAAVNSLTLQFLNENEGTRVAALSWLIMLHRKAPRKVLAFNDGT
FPALLKTLSDPAEVVVTKDLQLLSQISRNSEDGYFTSFMINLLQLFSTDRKLLEVRGNLIIRQLCM
NLSPERIYRTLADCLEKEEDIEFASIMVQNLNNNLITAPELSDLRKRLRSLDSREGQTLFVALFRS
WCHNAVSTFSLCLLAQAYEQAYNLLQIFAELEMTVNMLIQIDKLVQLLESPVFTYLRLQLLEPDRY
PHLYKCLYGVLMLLPQSSAFAALKNRLNSVSNIGLLQGPRPPVAASGGYERPAGSRLKNREDSSVR
WTELLDKFKNVQEKSKRHQRASQRQFETDSTGANFKQPSLIGALSVAAATDHQGGRDKVLPDVPRS
GFGNAGLGISGRSDGAARDGRSTPTNKSRSSLSHLGRLGIGALKELFRFDDVDVNSTCLLEILEVR
RYYAMREMRIDTYPGLFHGLYWELDYLINPQAHHYNREPRVGAGIAIIVVFYVLLFPAIVTYIRLY
LVVAFNPDYLPRGVNWVPTETEHESWLSKRRRKTEKSKSDHGNGIMTGSKEKVQRTAAESADIARQ
TGGVAFPLDENGQEEFWMKDIYFKTDRSHHNRDADRCVRKLDHFCPWVGGVVSESSYKFFLQFVVY
TAFFTCFCLIVLAIFVAEHRADTGRTNAHWAVGLGLAALFLLFSGGMSGTTLQLASINTTTIENLS
RRSKVWMFAIYISPQQYQRMTSRQNGQWALTFPTVTFPPQPPSASSTTPTARAEAQPTATNGVTSN
TTYNTNYTDQPAKDIRMFAILRTQPGENPFDLGDPIQNLRQVMGHTLWDWLLPLRVAPCVDHSSTV
SAYPMGPVVERLKREAGIIFEDDEAEDVHNAHQQPNVTHQKSVDGNETQNR
>g2613
MTHASRYRSSSPGSRRLVDPMRASTGTVAAEDFYASPTSYHAYDGYLSPKVAERRGGFFSVDSGDR
LEAHPISTKTYRDANHSTKLKTSYAIRPRSQTTTDSSRRPLSLHIPSSTXSSSRKAPVVTSAYERS
ASPLPPRASHVRDEPERYVSPATSGRSHKRILSSDYTSDSGYGKSIEKHRPRETRYHVYRPAGVSR
YQAYGDPRQWDDSKYYDAYSYTSPRENFEKESAARSSQRTYGRVGRPTSMMATSSALVRPAHHKES
KRLPSSHRGSERHPAEDRLRITNGRDYTDSETYRDHQRYPHQQHLVVHQDREDGYQSFTDDHKHSS
RHRHHSRPRESRKKHVEDYLAPVLGGLATLGLASGYSDDGREADRAGRSERHRSREVGPDDHDRDY
YKDREREKEAVSGDERVRHRRRRHRDRSQRPGSSSDSEDSSSEGHSRRRRRREKSTSRKKHDSSSSE
GTRDRRSKQDSDKLLSVRKDDSSRRSVDEESVERPRKPIAVEPPVVKEPEAPPKGILKAPREKFPE
DENHIREGVAPLKDAQKNGIPPGARWTKIDRRLVNPAALEVGHERFEERAEYVIVLRVLTKEEIQA
YAVKTQEIRDARYKVAQEERRKLREERRRNGQIDISSSDDEEDDEEESAPLAIEPAPAKDDFPVPR
GRDRVAPEIVAEVKK
>g2614
MAATTQMQRPYPPIYQTTPQSNSPASVTSSQTHDQHGRSLYAQSPQIPSQMYGYQPYSPINPVHPA
YNPQPPAPQHPLTTQSLMMPPQTAPIQHTIPSHQSAMANSTMSNNSPRLKVDPLPQQHTPIQNQRP
PPMPASTLTSPNTPVQPLSANNVHVNHPAGANSTNAAPGPIPATTPQLVRQDSNGVQWIAFEYSRD
RVKMEYTIRCDVESVNVEALSADFKTENCVYPRACCSKDQYRGNRLVYETECNAVGWALAELNPPL
RGKRGLIQRAVDSWRNSNQDPRLRSRRVRRMAKMSRRQANQTPNHQLGANAPVPHLLTQNPAALAP
NARPPSAPLTLAAPPLQHHGHHADGQAGNEEVSGSTDFSNGAHRPPYVPTSAKPPISGAQEPTPDF
RAGQVYQGMLPYPATVHAPSGLGTPSMAPPLHGGGFDTIGRHSVATAASQRHDADDSYETEAVTGK
PGDKVLFGSXPAGKRRKFILVDDHQRGGRARVKVTLDRVNMNEIPDSYRLTNAVFPRAYYPIQMRG
SPGHTIPDSRYLEDNDADDNNNDDDDGYEFDNGNEPVTVGRTIVNVPTIEGLSPAVPQLTRSRHRK
DKIINDFGYRMSWSQSRVFANRHLFLQRSLDAYRNKMRGSMLTIGQEPASIASQFETRIGKRKFSS
KIHRRRNESSSMTAIRREAEEVEA
>g2615
MSRQPGSSYPRGDVSPIISDVQSTSSQISPVSDLPTSRSFRNHPSGRQEGRGLNRAAPSQPGNIDT
DLPPEPPQHRIMGSPPGIADNNRPQIDRSTSLRTNTTATPGADNMSAAAIGGGISGIAYDVASRNQ
RESGLEAVRTIRQGQQGHSYYDGPSDRNYSDVPYGATNNRHSQVDPSGVHYSAGLPLGGTALAAGT
PPQVGTPSLSASNSSHISVGELYPRYPSGGLYDGPYHSYNSHDPTAINPNDIADDGDDDIINTPLQ
QQRRSWFGKKSNQGLAAGAAGGAAAGGVFSAVNSLRGGPTTYESVPTGAGGVGQTPYEKDAFLTRQ
ADIAAAKKRKRWLLTILVGLAIVAIIVGAIVGGILGSQKHSAAASDSNKGSSSSSDPGTNINTAAG

DLATNGDLNKNSPEIVKLLNNTDLHRVFHGMDYTSWGVQYPLCLEYPPSQNNVTRDMAVLSQLTNT
VRVYGTDCNQTQMVLHAIDNLELTDMKLWMGVWVNSNETTTKRQIETLYDVIDNMNDTSIIEGVIV
GNEVLYSGGFSDQTTAQTALITYMEDVRSNLTAKGLKVPVATSDLGDNWTADLLAASDMVMANVHP
FFGGVPVDQAASWTVDFFNTHDVSLTSGTSKEAIISEVGWPSAGGNDCGSVNCTDSTSGSIAGIDE
MNQFLSDWVCQALTNGTKYFWFEAFDEPWKVIYDTPGKEWEDKWGLMDSARVLKSGLKIPDCGGKT
V
>g2616
MSPKAPRIAEEIWERHRDEIIDLYCAPKGTLKKTMDMMKDKYGFQATKSQYTTKFEAWGIQKYADN
TTWLYAYQKIRKRKLEENKDTGLITHGRKRTRKEVEKEIARNVTSRTQLQLLEDIPTPEGVEVFTP
GAEVFSTFEVSSPSVTLPKVCWNNLPSVQLQKEIQMLLLKDPRIFNFDAFRVFNTSDPSSSQLVIR
NNLTSEAGSLQKALEDAVNSAKSSTPQDFLSKKSIPLTSYFPPDETDNDCMLGSYGLKGFGIKLLQ
YFIVLTNNGAISGSKLHQGFRAFMRKILPTVVDLGNTSMVKYILKKHLDILNETNHFRFSPRILGP
YALFPARELPWDTGRLETLQTLLSSGAHPECFIVGKSRGFPLVDAAKSGSLEAVSILLRHGSGVNC
YVKNYYGTALQAAVWAGHLEMVEFLIACGADINAPFEASWSMMTPIQIACAKDNVPLVVLLLSRGA
DVNLSPLSQVDLSAQEGRYIFSHRDDEFMFSSIDYSPHYDEAQYFTAFQHSVRNGNTNLVQLLLLS
EANINSRATSHLWEDTPLQLSVRLDHSNIARILIESGAHVNASPGKFNGRTALQAAAERGNIHIAQ
ELLRRNADINASPAYEKGANINAPPAPEGGLTAIQAAAETGDLNLVNDLIRLGAHVENAIAGTKAM
LASISHEDLPILELLVKCGAPINLQEDDNYMPPIVASARKGWVDGVLYLLDNGADINSYYYDMSIC
SNLSSLSWSIITRDIRMVDLLLKRGASLRFPDAKYPFNDSLCQALRQGCPAEIIDRLLDQYTEAYP
LFLRQEVLAEAVCNVFDAFDDKDVARTARLLSIISKLPKTLYMAQVMNAWDKLLGDHHCWIDLAEF
LIQEGAEIQVPSGEDIGTPLQEAVQKNETDFTHFLLERGADINAAPAEDGGATALQAAAKNGNRSL
VKFLLERGAEINAPPANFSGVTALQAAAIKGYIDIAVDLLRHGAHVSAPCAAENGRTAIDGAAEHG
HEDMLQLLLNHYDGCENLSAVCERAANYAKEEGHGEIASWLRQYPNLSTEIE
>g2617
MPNASRKQRKKFTHPRRIEVTDEDGWTHVSNTHSVPRSSTSTTRKIMTDGAADDEDIPDSNDKSTM
TRLQLAPSEPPRKLNLAELRKQFTAHLETWESSQTWQHLKNALHKDLLSSQGQLGIENIVCIGLGS
PSGFVQGGWVDRRSVAMYQLAALTTIIDCVKHRAAAIPTQQESTDLKIIAQDPVFNTLDIELLALL
GITAVESPEGFNAVSNKTFLFGPGAERRHLRLMLPSNPVVVFGGPFEEGPSLTARGFNDDEDINEK
DDGSKFLADFVAQTRSVKIQEFEARSEAFWRMRIYWREEK
>g2618
MHFSLSQWTALAVTILSCANWVNAHTIIVYPGYRGNNLHTNGSVEEADGLGAAFVNDTTIYPYGMQ
WMYPCGGMPTSTNRTKWPIQGGAISIQPGWFQGHATAFFYFNLGLGAIPPNMSNPMLPPFQIIGPS
ANPYPGTFCLPQVPLPAGLTVNVGDLATIQVIETARHGAALYNCVDIEFAKPEDCVEVTQDNCFNS
SDIAFADIYEVNTTTSAAPPSLMAPGYAPISFVTLVTMLFSGLIL
>g2619
MAHLATASDPAPLPLPPGITSRQIDLRAYGHLSYHILEAGAPTDPLIILLHGFPDLAFSWRKVILP
LANADGGGRGRRAYHVIAPDQRGFGRTTGWDSRPYEQVDLHNFATTIYVRDVIALVYALGYSKVEC
VVGHDAGAVTAAACAVIRPDVFKSVALLTHPFNGVPRLPFNTIDTQGSSSFPPPSAGIAKPSINDK
LRVHNRKHYKWYYSTHDAIGDMAGPTTAGGLRGFLRGYFHVKSGSWKGNNPYPLIKKGESSRQLEW
PIDEVVKMPEYYIMPLDATMPQAIEMLMRDESQDASKDWLSDEDLEVYVNEYARTTFQGGLNWYRV
QTDDGGKKPELKHDLDIFAGKRISIPCAFIGGDKDWGTYQQPGAIEKMTGEEKEVCDDFRMLRMVE
GAGHWIPQEKPDEVVRAILEFIDF
>g2620
MFRRRSSSRHQPLNTTPSTSAQTAASRAFTANRDANASLSSAAAAAALRSHTPPPTSPGNVQTKRM
LQRQLSNSSRASSAGGLRHGSQPTLRRTPSSGSMTSRTFREPSPARSQSTAGNTSTHXPVPPIPQS
LSNRSKPSQRSASAQPNRGTTSPLRRLVGGRGASLDRGPGSLTASPTTPRNISQGNSLELDRPGSR
TSINFSYPMNARANSPPQSPVEREFQRSPIDKSPSPPSLSPVETADIQQSVNSAANRKVKLKPKTA
APGSKEGSHFAHKTMGGRPTGTALQDAVEKDEQQSHESASPQSPGVSTTDFATTQPATEDSVKSQP
PVAFEDDITHSPRPKPKKRPSMVMEDHEGEEMAEAAEAAQEDFAVSPKEPSSPQTENINKTKPTRE
ALVIYPPKTNQTVVHPYTRPSPSTSSTPSSPGDRLTVDSSIDGQLPQRQSSVSPNRSARFSAQLTV
GSESPLHEPPPRSISPVKPALKSSSSPDRRVYLGQTPSEFSDATSVASDDGSRTGSKRRVAKVSFD
DEAEVVGVAASPPTSPEPMNRAQSPVEKVKSRKWYSIGKKKTTSKDVADDDDFESMLRPRPALPSF

GSIRGLRELEETTALAINGNDSDSSSDDDFDARDFGASSDHAIGGILVNARKDAQPQTQPLAEPFA
PELGLKDAQLESPDEVETLNSTRDIEHLPTVQEESSSATATPSEAAEALPTETPKIALKEETIVEQ
QVDALKEPLPTIAIQPATPGEEPRNSIDLHNMPGGFPSYIAERVATPEPTQTTIEAPQAANPPVEE
ADGDDSGDSIYSDAAEDPDDFQGDVFGSINAIVDSPLPPQSELPKEVPESPTRTLLQPDPLTDISE
PIPSASLEAINPRMPATPSGPTQTPTAASVLEPVEEPQKKQQKQSPAPAEPSWPFKTDPTPIAATR
VQQRPKAQAADPHQGSHLRKALGKGDIQQPMAKQSLRSASGPDPSSPTVAKTRRTQTASPAQMNNK
PPRKQADKTLPHSMTFPAAATKPSANDSDSDSSFKRTRRPSRVSAGGQYSMKKTMRTTRPMSMVNS
DMGVERTTSPPRASSMRTTMRGPASERSSGFSSLRDKPRPRSSVISSKTRSRIGDSDDEGKGGRNL
FQSRFADSSDEDEPLPSNLTPVRGIPRRKGRYDGESTDLDDSSDEETKRQTRNGATKSATQMSPEE
LEAILSQPKKKGLFSRLRSPTRSSGKDGKVRKSLMESPARRDTPLERSRIELAQMRENATSPKLHK
RQRAVSAYTDTWPLATGQTSTLAAPATSTTRPSTSDGIAHSQDKDNGRPLFNRHGTSDSIDSRVTS
TASEVVVGRSGKKKRFPMLRRAFGLRD
>g2621
MTSKAIVLITGANRGIGYGVARRLVREHPNYHVIIGSRDATQGREAVSTLLAEEASSSPSISSVEL
DVTSDTSISAAAQSIEASHGRLDVLINNAGIALDVKEKGKISMREMLQRTYDVNVFGASVVTDIFI
PLLEKSDNPRIVFVSSSLGSLTYAADLSTRYSKERFLAYKSSKTALNMVMLYYNSLLHEKGFKVNA
ACPGYIGTNLNGFHGTGTVEEGSVNVVRLAVLDKNGETGTFTAKEGERPW
>g2622
MATSAPPAAVSDGDRVDPLGSSPEDQKGVLPNAYHVLFNLDFEARVPIPFSVFPSSYRSDAVSETT
QTRVEGEVDIKTTSRVGREDTAGAPLPDPRVYGKEEVDVRIRTDDRRPHSRYEETRVYEERDRFYD
SRQNYPEVELSRERFREPIGRYEEHPTSSTTTVTQDRAYETQLDVTERDFRRRIQPTYEVDVTYDR
RYQPETAAYRADAYDVTPEGRNPYNRSTQVSVTKETIREEAPKSKMGYYDDEGHYHSFRRGVERAA
DRLLHPFHHEREEVVVEGAPRAREEVRIIEPRGGVSSKESVPIPVHFIRVGDLLILQGRPCQVIRV
SVSPQTGQHRYLGVDLFTRQLHEESSFISNPSPSVVVQTMLGPVYKTYRVLDVHDDGFITAMTETG
DVKQSLPVISQGGLFQRIRQAYSDGRGSVRALVINDGGRELVVDYKVIHGSRL
>g2623
MPASQTSVECASVGDDIIKEDIIQTEMEESGNGLEQVIPKPESIRDMSPEELQRLETRMVRKVDMV
IMPIMGVLYLLNYVDRSALASAKVYGIMTDLNMTTNDFASAISILFAGYIPFQIPSNLLMSKISRP
GFYICAAATVWGVVSGCTGAVQSYHGLLAVRVLLGATEAVFFPGVIYLMSAWYTKKELGKRLGALF
IFQSIGSAFGGFIAAACLTLDGRYGIAGWRWLFIVEGATTVGCGLVSAMFMPEYPHKARLLKPIER
DYAVWRLEMEAGAGEANDEITVWKGFKMAFSDPKIWALVWCMGMSQAMGSTGNFFPTLVQTLGYNK
NITLLLTAPPYVVAAIASWGAAYISDHMNIILPIFLVSLGLAVLAYVICLATLTLGARYFAMMLMP
SVIACPQAMLFKTLNIHMARPYPKRAAGVAMINAIGGISNVWASYLYYDGPHYYAAFACTIGCAVS
FLVTLVVYTWHVRRLNRLLGGTPEEQRRAMKHGVTQQQIDLGWRFLGF
>g2624
MSPRRSSRARTTQPSPALLQHTNSNSSGNSLTRGERNTRSNNKHPSPQGSSTQRSQSIDDSDGKPD
LPQTRQRHRAQDDDNDELSQANDDEGDEEENDEEEITRCLCGQQEYPGLPPSRRSATGRHHHQAGG
KDNTILAKDEADPLSDDIGSMFIQCDSCKVWQHGGCVGIMDEAMSPDEYFCEECRKDLHKITAEPN
GSHSSQYLPVAPVFSPESSSRESSSDNARRSRDSRSRQADPPKRRSTMNSRDAAYDEEELLRRAIE
ESKEDNRMSNDETSSRRTKRSRSDDEVNKQAIKRPRTSSPSPSAVSKLSLPPSQTVSEDESKPKTN
GANRPKRGVPGRNQKDKEVKEPEEQPEEEEEEMEQPEPITRRKSRSEKKREEESDHETEASPTKPPA
PAPAPTEPEPLEQTPESPAPAAEPAPPPPPPPRQSTRKSGRPPARRGGRLGRNQYTRDRDLNGDSVN
SPNSPHGNHFRDNGRDSPSIGGPNGMANGADSKGKGRHLNPHRTSMNEMKRRVAAILDFISRMQVE
MAASGENVTPPDNNATNGNNGGAASRASLIKGVQDQLGSLLASANSEGASVASSAGSIADFDAMKE
KEKDFGDLTSIEMMDVLTRHLLKWQQEYGKYGEK
>g2625
MAATLSGTYSATPDTPSPIYPDRLIRPLPKRPLRSRLSSEAAESILFPPAPPVSQLFHGSYSDHHE
RQDSKYLMRQHSVDPYSHDHSPDRDHRHPYENGVSEVESGDESGSVVVRRSGGFRGSSLPPSTPYT
NGQTHDTLQTKSSPVGLDGYDAFENTNNKKKRKIPTSGTLSSHHSNLTTDLANMGLSGSTPGSPGG
IGDSGTGTYYGSGSPAASNAGSGISGPGRGRYGRHAPRVSSGRNAVPVHPQGTWFGGRPGGTRRDS
TPSSHIGTGEIGAKPDQGIISTAIANAAALSPSPRGPNNVSLLDQQANRSSPTKTQFTFTCESDSS
KSIAMHSTNPYPMSHPRMPNSTLHGPPNSRDFAAQGTQTSPNMGAIGGQQLPPTPAAGTPATNPGQ

RPKRSRDSIYALAARRRKIQQHYSNIHNPPNPEEFWMCEFCEYEAIFGEPPRALIRQYEIKDRKER
RRLAEKRRLLEKAKMKGRKGKKATKNAAKQAGTQQPAQHNHTPEHAPMDPPPPDDYLGEYDDAPPM
PTSAPPLPQNTANHGPVSIPNAGHSPPIGGSAQIKGGIGDGATNRPV
>g2626
MAAKILVPSIETGIKTTIHEITSRRKKSYKDEQRAQDFYNGHLEQQDMTNFSRPSARPKPPHIAKT
IANNFLRFSQFALALTVAGLYGVDLNNAHQKNVYIDAKWVFAEIVAALAAFTSAVYLVIWCCVHRQ
ARNARALTPFNSTNLLLWLWDMFMWLIWLTLFGIFGKMYFPEDPEGDSGIVRMKNAVWVDLVNWLL
WTVTMVWTGLRWWKGRGGKNKEAGSSEMTKESDSIDGVGAGGRVTPPVASGAAPLAMPNPTSYEMR
SALSDTPQHDVSPLVSRQNSRVNANAYSQERAVSPL
>g2627
MAYEFPADVVKTTDKFSTELRQFDGISVDDVARLWKAYTTQSAVVRNVVVSRLENLFWRIWGNASI
QQSLTGRVLATLFMSIHEDLSLHITDHPRSLNIASTTSNNVTEYQQDHNNSSPKNERKNPLPPILK
KSNGTKSVPPSEPSSHKTTRILVPEKPSEAPSQKVKDTTSTENGTGNKLPTSTSAAQKPVRKKPNF
VATRAASSRRRGVLIRRKSSQTSPVTSPTVEETEDSLQEQIQAQEEARQEEQAEETTAPPSPTKEN
EISWRSMPYLKKFDEDTRNVPTEREADPSGTSPPPIDFSVSSVRHHRSPEEHFXPASQETAQNGMV
SDKALVEQDFRTNFVQRQRQGSAFATRNNSAASSLTTRLSSTNFREMLNAAEAAAAMQRKDSGDAA
RSLSPGPRYILLPKHDGPISVSSTMTSPTRRSDGRDRSRRPIIINTSSSSKTPQERRLDDKQPGSR
YPTAVAASESVISTSQQTTNGVFSVPKESQELLGEQLTNLIKGKGKDNKMKRGQRVGS
>g2628
MSDDEFIYDDDDDWYWYEDDNMGLGDELAENAVHSPIMVEDPSLETVDTYSDWEYYSDDYYDDDPT
ITTKHETNGERRNKRRKLSSIENVPNLSLGSSIVDPSTSMANSFRGVLWRVPVKTDDQIELYEPGK
GEQVALLSDWRKLFQTPLHIPDGTKTGDHEINGDVKNGVSAISAKLPQRLDLESVVDGKHGRYSPP
PLSIENLDTTISRQRQKLSQPEMQIAPENVPAETVIVADSEDEEILDEIDETTIAVAEMDTVDDDK
ENTVLSEKEDQIPHHPTIRVEIPTLAAAMKEDALNQNTPPRKKGPKPKTATTEQVLSNGTKKAATT
HTPTKRGRKRALDADKEDEESPEASKRRSKRVASSATTKATKGNESDKKRTRTNLIDSPREERGKR
RRT
>g2629
MPLSSLLRTGSRLSQIAVLRSRTSSRAASTIAAPAENSTILSSILIANRGEIALRVGRTATQHGIK
VTTLYTNPDSKAQHALSSPYAFNLGETAAYLDGDRIIEIAKREGCQAIHPGYGFLSENSEFAQKCA
SAGLVFIGPPPKAIEDMGDKSRSKEIMTAAGVPCVPGYHGTNQDPLFLEAEADKMEYPVLIKAVKG
GGGKGMRIVRSKAEFQDQLRSAKNEALNSFGNDHMLVEKYITTPRHIEVQVFADKHGNCVALGERD
CSIQRRHQKILEESPAPHLPDAVRKDLWEKARAAALAVGYEGAGTVEFIFDNDTGNFYFMEMNTRL
QVEHPVTEMVTGQDLVHWQILVAEGAPLPLTQEEIEANIAVRGHAIEARIYAENPDQGFIPDSGRL
LHVRLPKETEDVRIDAGFVAGDEVSAHYDPMISKLIVRGANREEALRNLSVALEEYEIAGPITNIE
FLKAVCKSADFASGAVETGYIEKHREELFVKETASDEVLAQVALAIFTKRAASGPELTSSGLKGSQ
LGFSSSFQQRQFRFAEMSSSATPDAANPTHDVQVRQLSSDSFRVTINGRTFEKVTSQIDGESSQIT
SFFPHTRLDTRVIQGEDTIIAFQRGKQYRFSTERGKWMEKALGIKDVANSVLAPMPCKILSVEVSE
GQTVEKDQPLVVIESMKMETVIRSPHNGTIAKIVHRKGDQCKSGTPLVEFAGDD
>g2630
MATIFKPTAARQAVRAVNQVARPSIRAARPALRINTNTRALSTTPARFQDDEKKRATTAAPLGASG
THEGAFARTDDSLVIEHPKDEEMPRSPVVQGRGGMHFKRTLASFSLENKVSVVTGGARGLGLVMGQ
ALVASGSDLAIVDLNKDEAQEQAEKLVAQFQKENPGLEELPRVTAHYADVSDPDSVNNVLANIIEQ
HGKIDNLVTSAGFTENFDAISYPYDRIKKLWGVNVDGTYLFATGVAKHLMERQAPGSIVMIGSMSG
AIVNVPQPQAPYNAAKAAVRHLAASLAVEWAGSNIRVNCISPGYMLTALTKKILDENPELNRKWTS
LIPQGKMGTPEDLMGAVTFLLSDASRYVTGADLRVDGGYTVT
>g2631
MKSLTTERARKIIDEVRVYNGGITEEERRDTSEIVLQALENVRQQLGAVTRTLAQDLYTSESRFVY
ELIQNAEDNYYSSAHNDSPYIKFTLMPEEIIVENNELGFNEADVAQKVRIQSGPFDFYFEHLPSGN
GMGMITPFNEEENVGVYEDGTRITLTLIQPDDFNKRSEDLLGIPDALIIFLPKLQKIEVLIHGENG
DSSRTVHESHENGQNSKLIIRVDDTVSEQFFHVERETFQGLPSHTARENRDRAEVVLAFPITSDSR
PVVEQQFTFSFLPVRKFGFCFLIQSDFITAANRQDILQCRWNDALLQHVSEVFVNAVIKFCSRPVL
QYEWLSYLPGNSIAHEFWQDLHDMIIKALAKHPILLTRKGHGEPLFEDLQQEVYLSSDYSTEHRKY

LQELGVTDLSWNNIIARITPYLRGENPRFLHPDQDDDWHTKLPLIPLRDGSLSNNGSKTIYFPSDV
LGNHVPSDLGMQIVDDDALENNAQKSLYEKLGVQYCSPAFVARQIVKRYNPPQRINLAESVSHLKY
LYESLPDEALNKCIFVMNQSEIPIYRKFVTFGRSIIVDDLYFDTIGDFGTRHLAQELREVTGSSCI
NILNDAYLGAISLTDEINGRSWKQWLEEKILIRKVPRLQHPSTTGISLLFTKIIEFRPQLLAGVLK
RYWSSYQSQKTVAIEQAIRNAEVPCHGTNTHYPLKATYLPTPRLMEICQHARINDYFDLFIDFGSS
SEGSETGLAQWEFLKVFGVGITPDINFFRDVISALIENTTGKKLKDGLFYVYEQLFEEISYADGDD
ISDMLYDLEAVCLPETTVSCTTLDTCLWSGLSGLHLTDCLSQYAEYAGNTRVSFLFRDILSLGDAS
YQTYLEELTYLRKHVSAVYINFEHMCAVYAELLVTCTTVEASGAIRDAFTKDKLIFLPGEQIWVAP
HSCVWAETSNVGGQYGIGAVYPDLAGLFTEKLRIKAPTIATYIEQLRLLAAKDNDESLEEMMQLSC
LPVRAPGSTLKLMSPSEVFFVPDRREYGELFEGKVPVLDFSLEEIHALHPFLNALGVTDRYMSVAA
KETTKVQRPSETPSKELSQAFRAKSKAFYRCALHYRSTKAQTNSQDIYHKLRHALVYESDGFTKEI
RLSYGGLTVTALSDRGTFHLEEKEGILNLFVPRDNVQREKCYLTQLPNELRQYLLLMENSFGASKA
FLMVIQASSIYVLDEVLDDEGIVRIPDSDLEAFEQAEEGSTSLGEDFHIPAGRTESPRDNSLPETQ
VDIENRAYTPSSEEEVHSVTSHSRSSTQYIPVHQTEHVHRYNTPGIQDEAPAPPLAEPSQYVRVLE
KVIQVAQRTNIPLRHAGREASAARTGGFTSEEHVSAFGARSQNQLAHDIKIGAAGELFVFELLRNL
YLPDFGRDNWKSTIRKEVAVHPAYADLAPWNGVETSDIIYYDSASRLTQILIDNGYLPSHQWQYRI
PRYLIEVKSTTGNCDNAFFMSKSQYRRMQNQTFDDQTLQEFRTIYVIFRVYNLGKDDMSLRIYVDP
DALRRQGVLRFTPESYSVTPGDDVEGDGN
>g2632
MNSSSRKRGALSTTTRSITTSSRPSDEISKQTATHDREEVPERTSIQISKDQQRIEEKRLHGPIEK
EQEAKIEHLQACMKQLTIEQSELQFKLGQAQKREQDLLRDFRTQLENHQLENQELSQHLKATHEES
KRLKISNASLQGVLDDIQERAFRSMGKGGWSAPEDGKVRDNFLRLQEKIKKWAKTNALQISNGKDL
DHLKIDQKRDIIKSLSGYCVPGSWDDIIQMMTPSVARRVPYIFTHALLSKDIFGGIFENPFLTLEV
LEGASFPAASQLTGLYQAMKEIDATEAHIWRAQMLRVLQTPYRVSKSDITFVGRLEVNLSRMGDLR
SEQFLSGSVGCLLGRPGGGEQLAKKGLADIYIFAAQLSLSVWTQRSFLECPKLHQDSRFRNGDELM
SAHAMHHLDDEEDTRLDGQPIIAIIQPPVLAFGNDDAEHYEQYKIWAKAIVLIGEKT
>g2633
MLPDSALISVRAAYVNSIDDVDLCIINTWPGTGRDTENVLKTPSTIAYPEENKKIGTRRWGFGVEP
GHKFYSWTKLLLDKNAPLTKYDDPILEQATKSGILQLPANKDAVTVVADYLTGIYDHIMLTLEKQL
TEPVLRVSPIEFWFTMPAIWSDQAQAATREAAQRAGFGSRPGRPLDAINMITEPEAAAIAALTRST
TDIVGVPVKAGDGVLVCDCGGGTVDITTYLVTETQPTLAFEELCTGIGGKCGSTALDRNFYKLMSE
RFGPAFDKLPMKRKGPGSTFMSCFERIKRNFGHNSEDTTHDIEFPMRSLEVDPQYYDDDDQAVIIS
DEDLRDIFDPVVNQIIDLIKTQIKDANKEMEHGTINRIILVGGFGDSEYLRKAIKKTFGSNEKMAI
TVPSNPQGAIVQGAALRGLHGIRATARRCRRHYGFDWDLKFREGIDDEKHSYWDRYRGQKTVAGIM
KWMIKKGEKYNEDYSMTVNICSSHWPSYSLNRYTNLYASDLMEAPERIENPGVKYIGRIKSDFTGV
DLNQFDSKFIDGEMVYELSYSVKVVIGAQEGILKFETTSKGKVIGKSSITFAKASYY
>g2634
MSSKQLSRLLLRPSKSVVACGARPLHSPAWTRHHSTKHPKGFVPPTDDDLVQLRESVQDFARREIP
EEVAARTDQQNEFPAEMWKKLGEAGFLGVTADEEYGGLAMGYQAHCTVLEEISRASGSIGLSYAAH
SQLCVNQLSLNANAAQKAKYLPGLISGEKIGALAMSEHSAGSDVVSMKTTAKAVDGGWLLNGTKMW
ITNGPDADFIVVYAKTEPEKASKGISAFIVEKEFKGFSCARKLDKLGMRGSNTGELVFEDVFIPKE
NLLGELNRGVKVLMEGLDLERLVLSAGPLGIMQSALDLVLPYTHTRKQFNTPIAHNQLIQGKLADM
YTKLAASRAYTYATAKSIDESSVTPSGTLIRTQDCAGAILYAAERATECTLDAIQLMGGNGYINEI
AAGRXLRDAKLYEIGAGTSEIRRMVIGRAFNKEYA
>g2635
MLSSRPVLRQIAEKEFIPRLRPGRCLCTIAGQRRVTPSRKHVFNKTAVVRSSSRPVSNYTFPHHAN
AISVLPTAVDPESTDFKENAAQMNELLEHMTQLHDKIAQGGSEKAREKHISRGKMLPRDRVTSLID
PGTSFLELSPLAGHEVYPGEDVPGGGIITGIGTVEGVTCMIVANDSTVKGGTYYPITVKKHLRAQA
VAQENKLPCIYLVDSGGANXPHQADVFPDKDHFGRIFFNQARMSSLGIPQIAVVMGPCTAGGAYVP
AMSDETIIVENQGTIFLAGPPLVKAATGEVVSAEDLGGGHLHSTISGVTDYLAVDDAHALILARRS
ISNLNYPKTSIPLTGLSKDTDIKEPLYNPEELNGIVGTNLRRQIPVHEVIARIVDGSEFAEFKRDY
GSTLVTGFARIYGTQVGIVANNGILFSESSLKGAHFIELCAQRRIPLVFLQNISGFMVGADAEKGG

IAKNGAKLVTAVACADVPKFTVVFGSSAGAGNYGMCGRAYSPRLMFMWPNSKIGVMGSEQLSSVME
AVGRTADPELKSRIDQESTAIFSSARLWDDGVIPPAHTRRYLGMGLMAAVGGRNKVDETETKFGVF
RM
>g2636
MQPRREYHIVVLGAGGVGKSCLTAQFVQNVWIESYDPTIEDSYRKQIEVDGRQCILEILDTAGTEQ
FTAMRELYMKQGQGFLLVFSITSMSSLHELSEIREQIIRIKDDDKVPIVIVGNKSDLEEDRAVSRA
RAFALSQSWGNAPYYETSARRRANVNEVFIDLCRQIIRKDLQASQLRSLELQSRKREMAASNGPRK
RGERRSRRRGQCVII
>g2637
MFAARRFTAAVPRTLGTQSALFHATRPAFVKVGDAIPNVDLVEDSPGNKVNIAKELTGKGVIVGVP
AAFSPACSSTHVPGYINHPALKNAGKVFVVSVNDPFVMKAWGATLDPTGKSGVRFLADPASTFTDA
LELGFDSAAIFGNQRSKRYALVVEDGKVKEAHVEPDNTGVDVSAAEKVLA
>g2638
MADSASTSGVTADGLKEKLSNQLDAEFVEIEDMSGGCGQAFQAIIVSPHFDKKNMLARHRLVNSVL
KDEIAAIHAWTPKCYTPEQWQKLQDERA
>g2639
MAQTTAISPFSQAVVNAMRKLYPESLADKSFDNTGLLLESPYNPNSQLKNTVLLTTDLTKAVADEA
IARRDSIIVTYHPIIFRGLKSLTLQDPQQKSLLRLAQEGISVYSPHTAVDAVPGGMADWLCDIVSG
KFVNSSSATTTAGTKKSGKEVYSDVIYPQPTGNATTPSVDAHTREPVHPSPAPVPTGFEGAGAGRT
LTFATPQSLATIIERIGAATGLPGSVSVAIPQGQSISSITIRTVGVCPGSGGGVLMKGDQLPDLLF
TGELSHHEALAAIERGSAVVALFHSNSERGYLWDVMRQKLEDGIVQELGAAAATEQKGGLVAVSKA
DRDPFGVVVRK
>g2640
MTSYFANSNHFADASNAISPPGGANVAPRSTALSNRLTSVLSASYADSDIRDALETLDKRGIKNTA
ERRRQLRLDIQKEVLDCNGEIVADFGKVAEQLNRIGTVISNLQQTCAEMRKHITLAKQDTAPVLDE
ASSLLEQKKEAETKQRLLDAFTAHFIVPEEDINALTLYEEPVDNHFFAVLSRVKQVHNDCQVLLGG
ENQRLGLEIMEQSSKQLNMAYQKLYKWIQKEFKSLSLEDPQINVSIRLALRVLAERPSLFHSCLDF
FAEAREYILSDAFHYALTDAASGAVGERNVKPIEFSAHDPLRYVGDMLAWVHSAAVSEREALEALF
VYEGEELARGFQAGISSEPWSRVDGEGEEAPVFDGQKALNDLVNRDLTGVSRSLRQRVELVIQGHD
DAVTTYRVVNLLAFYKLTFSKLVGPESSLVEVIAGLVEFTFKHFEGLMKDQLHMHSADAHSSSPPD
DLSPPQFLVDQLEVLTSLMKEYNASVGAASDVEDAEENGFTPVLRVALDPFLDIAKNSAQEVDDHK
ASTIYKANILLAVRSTISPFIFACATHLAPISTVLSNLRIELLDIQRQYLLETSGLQQLLTALESF
SPSSTSTNKNQAESSNAHNPNLNLAEIADLPAFQPPTLSMISQQLDDFLPSALMDATDNLKQIRSS
ALVKSVTEEAIEEFCRDFEFVEGMIIGADEARGTKIDMSRIEMESDIVSVSVRGGQHLGGDESEGK
YSLRALFPRTVGEIRVLLS
>g2641
MDSLISPTKARQAAIQAKDWAYVNSWLNRKYAPHPVPAFERNEDTLRTLLAIAAANDAADEESSLL
HRAREEVVRALKEREKVEDARKRDILEEIEARLDSQGEESLNDLAETVVLLGDDSTDITELAHAFI
ELTRDEFELANQAQLVQELQRYLDKEIVLVQEEIETLKSHAAYKTPSDLTTQTVEWTRSAKLLGAK
IAEYRDKITSLQRNIGIDGPTIETVSEEERNVVQLKEMVKQLESKIKGYRDLPPHLEDAKNEYKRL
EKQLGRLTQERDALFDHALAQK
>g2642
MSDAGRKDFSTKAKEELTPDSSKSTQQKVKETVTDTTDRLARGVQPDDSKSGTQEAFDKTQRSHDN
TAHGGASNSIGDKIKDTLGLNN
>g2643
MQSTYESSTWDFTAVIDLLHAPTRPASLNGSSVPTPSVIPDVSQHNPGEQGLGDFTALWDFLHPSS
VTAESPSKDAPITPSRKQSRDKSRLSPSRPISILKRTTKTITQNNTNSSGGISDSTAESDGDLSVF
DSAVPSQPALSFVPSQVSRTDKAQLLTPPSSCEEDSALSAYTPKAKKPTKVTSRYKSAVERKAGLV
SKLSKQFLDFTIKPSAVSETSKLSVHVFVDASNISIGLHDSYKIQHGIPVTTHIKRLPLSFHNFSL
ILERGRPVAKRVLAGSDRFAAINEAEKLGYEVNILSRVHKAKEYTPRQLKFRNALGPGGSSEMGSS
PSERWVEQCVDEILHLKMLESLIDTDEPATIVLATGDAAEAEYSGGFLRMVERALQKGWSVEVVSF
AMNTSFAYKRKEFRAKWGDRFRMINLEDYVEEMLDM

>g2644
MENNISSRPQLPPVFLLKTPSSPRDLYEEYFFKNAFAFRSRQQSNPPTDTTDAHDSEHIKYDPVFV
PVFSHSFHTQNLDIVKSYFMPTTTENDRINGKEASLRNAFSGRGGIYGGLIFTSQRAVEALGHILE
HGGIPAEIMSSTSEDLILYTVGPATTRTLTPLRDKFLPFATIYGDEAGTGENLAHLILKHYNKLYP
STAEDGNQKPGLLFLVGEQRRDIIPRTLMSSTLDENQRINIDELVVYETTQMEGFEPAFQDVVRKA
EESLNDERTPLWTVIFSPTGCDAVLRTLNILDENNKSTGIDPSKRKCRVITIGPTTRDYLITKYGF
EPDVVARKPTPEGIGEGIKEYLLAMKV
>g2645
MNRTQKITLSPPPLSELAETLQAPLSANYEHSSVSVVSCPNLRESPFLLAHEGLSGDEKVADVGGQ
THLYPRPMMDKTYQLHQIAREMEMVVDDGPYPPPAVTFLGAGAGAYDLVGQNCEMVINFGMRYATN
MSFLTSNNGRYVKIDPETKGVLVQNTDQHKWSFGLMGNVFASRGESGDVLRITARKRIGEEGSFTE
CIRKALRDIYGDSQIISLGGVFAIKSGKARYHIMPDFPPEEGLPFEDREQFNNWLTYHDFRAPMTC
MTVLHSADPKKNLGLRMEHTHAFSTVGENTGGHYHYDLDDEEEIEYEGYFNTAKVLYRIDKPEHSL
EEILHR
>g2646
MAQAQNTTHGGSRKISFNVSDQYDIQDVIGEGAYGVVCSALHKPSGQKVAIKKITPFDHSMFCLRT
LREMKLLRYFNHENIISILDIQRPRNYDSFTEVYLIQELMETDMHRVIRTQDLSDDHCQYFIYQTL
RALKAMHSANVLHRDLKPSNLLLNANCDLKVCDFGLARSAASTDDNSGFMTEYVATRWYRAPEIML
TFKEYTKAIDVWSVGCILAEMLSGKPLFPGKDYHHQLTLILDVLGTPTMEDYYGIKSRRAREYIRS
LPFKKKIPFKHLFPKTNDLALDLLEKLLAFNPAKRITVEEALRHPYLEPYHDPDDEPTADPIPEEF
FDFDKNKDALSKEQLKLLIYEEIMR
>g2647
MASPQGGYPPQEGYGQAPAQPPAPGPAGGKKKRAYAGEAFDLGSGANAALGGQQKAGGDYGAYAAQ
AASVGYPQPGYGAEPAAGYAAPQPPAVGGYQPPVAGYPGPATAGVAQITQQFGQMGVADPQAQPVP
PQQVPRAVPLNQLYPTDLLTQPFNVAELDYPPPPIILPPNTSIYPSPYANCPPKYVRSTLNAVPTN
HSLLKKSKLPFALVIQPYAALHDAEDPIPVIPDQVISRCRRCRSYINPFVTFLDQGHRWRCNMCNL
TNDVPQSFDWDTALQKPADRSQRPELNHAVVEFVAPQEYMVRPPQPLVYLFLIDVSYASVTNGLLA
TAARCIKESLDRIPNADRRTRLGFLAVDSSLHYFTIPRDGSENSAPKMLVVSDLDEPFLPIPGDLL
VTLSECRENIESFLDKLQEMFQNTQNGGCALGSGLRAAYKLISHVGGKITVLSSSLPNIGHGALTV
REDKKILGTSKESSLLQTGNSFYKSFAVECSKAQVSIDMFLFSSQYQDVASLSNLPRYTGGQTYFY
PGWNAARTEDAIKFAREFSDYLSSEIGLEAVLRVRATTGLRMSTFYGNFFNRSSDLCAFPAFPRDQ
AYVVEVAIDETVTKPVVCMQTAVLHTTCNGERRIRVLTLALPTTQTLADLYASADQQAITTYFSHK
AVERVLSNGLEPAREAIQSKMTELLSTYRKELAGGSVGGGGLQFPANMRALPILFLALIKNLGLRK
SAQIPTDMRSAALCLLSTLPLPLMIQYIYPKMYSLHDMPDDAGLPNEQSGEIVLPPPVNLSSERLV
PYGLYLIDDGQTQFIWVGRDTVPQLLLDVFGVADRNQLRVGKQFLPELDNDFNERVRAVVQKSKDF
KAKGAGSIISPQLYVVKEDGEPGLRLWAQTMLVEDRADQSVSLQQWMGSLRDKVVQ
>g2648
MSVRVVARVRPLWKTERELDVIVRPGNVSTDALKVKKDSPSAKAAVKERDTLIRIPNPKNEGEQYT
FQFNAVYGGEATQQELFEAEVAPTVKHLFNGFDVTIFAYGVTGTGKTHTMRGGKSLQDRGVIPRLL
SAIYRRSRKIERESEGASKVEVLMSYYEIYNDKVFDLFEPPEKRTLAGLPLRDNHGKTVVVGLTER
PCPSLKDFESLYDQANTNRSTSATKLNAHSSRSHAILCVKLVVTNGDRTRVSIASAIDLAGSEDNR
RTENGKERMVESASINKSLFVLAQCVEAISKKQSRIPYRESKMTRILSLGQNNGLTLMILNLAPIR
SYHLDTISSLNFANRTKKIEVREVENEPMFKGPPRSTRLASAPSQRQPLRSLTASVNVNMPVNDEK
KADDKKVDEKKPPKAFYVYSDKQPVKPHRTSLKRSSPLKENTPNVKRQSYSKLSKETDKISVSADT
IAEMVEKKVEQMLAARALDDSHQHQAQLVQMNEQMQRRLEVLERRIEGTEDARAEGLSYLLMAKQH
HGRKEYSSALRMYKLALPFFPRNQKLAVKIASLEEKLQPMAKPQPEPTVTLPGRGSMLSISRPTKM
ARTNSSRDFSDDEYQESDNEFAEDDNYTDSFTGLKRKRAAADTKSSSLFGVQNDLSKGVGDELLIQ
SPRTANLLSVINSRDVGQIKLLKGVGAKKAEAIVDCLCEMDTVHESRSGQAQVRSLAELSRLKGVG
LKTVQNMRSGVLV
>g2649
MTQIEERELFGGAIAGAVPQGWIDGSTIREVPDHQEMFLSPTTLSNLIFEINEYVPRSSDGGLNGV
ASGLDVELPTAINIGADSEDVAAAMYHILDICDDDDAMDVIREPRRVPLKRLTASSPLGSKASAYA

GLVKFTTPRKQRREAAGTQRVAATTEQNAASGSTPPPPDRSTNNCHFLLVRLPDQKTDLLVFVNVP
QDEFLARGDTGLLAKEEELADEIIGKFAEVIDVKDWGLFGSD
>g2650
MAQSHHSHHSHFTEVNRQYFDKTARDYDTGFEKGISMMREETLRHRLWISSRWTDTAEGKGKEIRL
LEYACGPGHVTRTLEPFVTKCLGLDVSENMVAVYNQRVEEAGIPSEKMSAKVGDLLAENVSEDLQG
PEYRDLDIIIVSMALHHFSDPQLALKRLADRLQKGGVLWIVEMLEEPHAKDEHERVSPETAQIVHK
HGFGVDEMKDLFLGAGLGDADVKILDRRLFKPHTSPHLRSTHATIVSWRAGTESIGQSSFTTVFWG
DRSTMQKCRAFNGALGRKALAPTPAIGRVQFQVQRRNIQDVHITRTGKPILKIQGGRSSLGGHTAT
VFGATGFLGRYIVNRLARAGCTVVVPFREEMTKRHLKVSGDLGRVVFTEYDLRNTQSIEESVRHSD
VVYNLVGRDYPTKNFTYEDVHVDGAERIAEAVAKYDVDRFIHVSSYNAAEDSPSEFFKTKGWGERV
VREIFPETTIVRPAPMFGFEDHLLHKLAGVTNLLTANHMQEKYWPVHAIDVGTALERMLYDDNTAS
QTFELYGPKQYSTAELAEIVDKEIIKHRRHINLPKAVLKPAAYYLNKYLWWPTMSADQIEREFIDQ
HIDPTAKTFKDLDIEPAEINSLTYLYLMGYRSSQYYDLPPATEREQREEKKYLHVLDDQ
>g2651
MDADKIPDFLADQRDQAPSESQALFLDFEDYWERKLWHQLTNSLVEFFRTPASAPQRLPIFKSFIL
SFADRINQLNFVSLGLLASTQCGDDQERLAFLTSLADRVDKPDSQDAYVYAVADVAGVKLRLQDYE
GSRKDLDKXQKILDTFDSVETVVHAAFYKVNADYYHAKEEFASYYKNALLYLACVDLEDLSQEERI
SRAYDLSVAALVSDSIYNFGELLLHPILDSLTETPHAWLRDLLFAFNRGDLTAYDVLAGNISKNKL
LESHRQFLYIKISLSALTEAVFRRPPHDRTMTFTTISAETKVQPDEIEHLVMKALSLGLIKGSIDQ
VAQIARINWVQPKVLDMKQIEGMRNRLKEWDTGVNQLGHWIEGVGRDVWAA
>g2652
MRYYRVSDEEVKQWQXVSDTIQGIFRAAGLKVATQNYEYVSAGNVHKGQNVYSIIHAPRGDATEAI
VLVAAWRTIEGELNLNGVTLALTLARYFKRWSLWSKDIIFLITPDSKAGSQAWVDAYHDMHSSSAQ
PLPLKSGALQGALVIEYPFEHRFESLHIVYDGVNGQLPNLDLINTAVSIAGGQMGIGTSLQEMWEH
DYSYEKRLETMLRGMAKQGLGLAAGAHSSFIPYHIDAITLQTKGNGWQDEMALGRTVEGLCRSLNN
LLEHLHQSFFFYLLMHTNRFVSIGTYLPSAMLVAANFTIMAVALWLRTGYEDKLATKKQAPKESKG
EATTTASTGLNGVSERKLSVPLTVVAVLHFLGIVPLYILTNVSSQFFLPVTYAILLLTLTLPFLIS
LALCRLFTPQPQTFYLLKSFSLLLLGLFLSALATLNFSLSFIIGLLCAPLSFVPTPATTKKSPATL
VFSSMLFVLLNLLSPPAVLLAACTYTGISVEDVLSQAAFGWDVWGVWTPVVVWCVWWPAWMMGAVS
VGSSFFV
>g2653
MSSSSQPSALPAFSLNIAPLRSYLYRLPLFTRAVLLIIVAFWLLELQSVWDVVQWGALIPDEISIA
TLYRTNTYPVIHNGFFHMLLNAVALTPLMERFEAEHGTLTALLLFVGPLSTFPAAIYVLIEKYILY
SNTAVVGASVWVFLLLASESIKTFRTNPYFQVGTLKVPTWTTPLFVSFVVAVLLSNTSFLGHICGM
IFGYLLGLGYLKVFVPPEKILRFLEGKLNLLGRLPHYVSVDQKTHGRYGVLPTTSSGESGVAMSSY
IGSSQRLGP
>g2654
MQQSKSLRLLAAAFLLIAVATARLDGDHSHEASGGMDMGMDMSQKDSMSKPEEDWELSYWAYGEHS
ASITAHIILEVLAWCFVLPVAVMLSVAHSRFTIPTQFVFLVVNGVALFFGLIYNASTPDLYQNNAH
HKIGWIASSVVVAQFVIGIIYAYSGRRTKTTTSQYERATFFPVSTENMMEHQQLYTPVAEYRWSGD
SGQGTSCPTSPDEEHHNFTKPEEDDDVEVPTPLARGWLRSTFLDRFFTSRVPGMLSNKVLRALRLV
YNIIDRIILPFGFVALATGGVTYGGIFKAHSVFSGLAHFIKGGIFFWYGLVVLGRYMGCWANWGWS
WNLKPANTRAPTGEFVESALIFTYGSTNVFLEHLGGWGKGWSPQDLEHVSISVMFFGAGLCGMLLE
SKRVRSWLKQSILGASRNRSNEDEEPSSVSFNPMPALIIFLLGMMMGGHHQKQMVSTMVHKQWGSL
LSGAAMARGATYVLTYVRPPTSYLPARPPTELIGAFCLISGGLIFMLSASDIMDVMVWYDLDAMFT
FNIAIGLSCFIMAWVIVVIAIKAWAVKKENTQSHARSWSSA
>g2655
MAAAVASLATVIQVNILTEAEYGCEHLSVVLKDDSRGSNFIVSYMKQAAMRMTKNNRKHDGANGIK
ASMSQRLSLKPVYICIDCSEKHTNGNRKVHSEKTGHQFYIDSRTGALFCQSCQDFIYDHELEKIRI
RAAGLWSKDPLDRGQDGSRVTSSIESAVQDAYSQAKKANSIALSQDNPLSKPSRYILDVLSQLSNK
TRKRKASEMSIGESNGTRDHTFLNGVTTMEVPEADETYIKTNATKRTCQSEGVRGLFNLGQTCYMN
VVLQTLLHEPLLNAYFLGNGHKYYDCQVDGCVLCAMSRAFAEFNGCDKKDGFTAETMLYSTWKNHP

PLAGNHQQDAHEFYQFLAGGLHTASSDENDQLKCNCFFCKALFGKFQSTLTCPICRHTSPTEQPLL
ELSLDVQLHSKKRQMNGKLHGSVTPTLAGCLQSWVTPEELIMEGYQCQGCHSYPDKLTKQLRIKQL
PAMLCMQMKRFEQTDSETRKMQGKIDFPLEINMQPYTVASNSSSSSKKGGISRSDIYMYDLASAIV
HDGAGLNEGHYIAYIRQGDRWCLFNDDKVTLVSEGDVLNADAYLLFYTLRSLSFGK
>g2656
MDTAPRSSSRPRPTSRPTTPLRPSSRSSIRNSHGYSDSLNAAGYEEPAINSLEPQFAELADSMADL
EANFMHLQLMHESLSRFSESFASFLYGLNMNAFCVDFPEAPIPESFKRALEAKAQQESLSHKTGQD
IDATFLTTDTSFVDNPPATVSSKATPKKAPVSSRGRGSTRGTASSRYATRGSGTARGRPSGLPRGS
GLPRGRGNR
>g2657
MNSNEIPDGHLGNLTKEQEVKLREMWAVLFKLFGIKLEGDQLEKTPTNPPESPDSKRKSRRGFFGF
GAAKKEETAAPDVGGLTLSDQDDKYGQNKEFKQAVADLKPEQLRESLWSMLKADHPDALLLRFLRA
RKWDVNKAVVMLVSTMRWRLVEMHVDDDVMEGGEAKAIEMSESSDYDAKKLGGDFMAQTRMGKSFI
TGIDKQGRPICLIRVKMHKIGVHSEKSTERYTVHMIETARLMLPRPIETAVILFDMTGFTLANMDY
APVKFIIKCFEANYPESLGAVLIHQAPWIFSGIWKIIRGWLDPVVAAKVHFTNTTEDLEQFIDRSR
ILKEFGGEDEATFEYIEAQPGENDAMKDTTKREEVLSKQRALAQELQDATKHWIEAANKNDEAGVE
SWKAKREELAQKYSKGYWETDPYIRARSVYDRNGVILGGGKVKFYPEQEPKDTTALAPSTAEPSTA
ETEEKKVETNGTAPAPTVETEKKAEIAVTPEVNGVEAAAPAVAVSAN
>g2658
MTSPACIFCKIIKGDIPSFKIFESDKVFAFLDIQPLSRGHALVIPKYHGAKLVDIPDEYLTELLPV
AKKIVVATGAEDFNVLQNNGRAAHQLVDHVHFHVIPKPNETEGLGIKWPAQATDMDKLKALFEELK
SKM
>g2659
MVVDKDNLEQLPRITDLPFVPPTVVRPNNMLYLVFTSGSTGMPKGVVITHSNFSSALVYQTSRLGF
RRTSRVLDFASYAFDAAWYNTLHTFYAGGCLCIPSENDRRNDLTGCISRLRPNFANLTPKLCEFLD
TPSLQALDMIELAGEAPDERQVGRMRTVTKVRFAYGPAECSILSTVTDEHAVSSKIGNGLGVRTWV
VNADNPDVLSPIGSVGELWIEGPLVGRCYLNNPEKTAAAFVNSPAWLLQGAPGFEGRSGRLYRTGD
LVRYAADGSLLFVGRKDGQVKIRGQRVELGEIEHHIRQSLPENTSYSVVAEVISPLKSDNAILVAF
IENTDSMEAVMTTLESKLAKLLPSYMVPTAYISVDRIPVSQTGKTDRQQLRRLGATYTLERLAALN
LARTKGRPPKTAIERLLQRLWSSVLKINSNAINAEDSFLKIGGDSIGIMRLVAAAREEGLLLSVGD
VFRHPRLCDLANIVRKIPLEDVRSEVIPPFSLLGGQDKTTIRKSVAHICGIEASQVEDVFPCTPLQ
QGLLALSARRADQYVVRSVHELQPSVQLSQFRAAWEAVVSKSPILRTCIVDLDDQGLMQAVTNIPI
EWSLDADTTHNETIQSDVMGLGTPLARFALQTSPDGVRLFTLTMHHAICDGWTVPLLCHMAEKAYR
EQTLPPSPPFQAFVRHIQQLDGADAAIKWRKYLEGTEAQVFPRVSQAAQQPKANTEMMHSVSSFRW
PRTGVTRSNIVRAAWSILVAQYTATNDVVFGATVSGRQAPVPMVEQMTGPTIATTPVRVVLDWEED
IDQLLKRVQEQAVEMAAVEQMGLQWIRRVCDDAEKACQFQTLLVIQPPENETDRKSSLFMPTTDNN
WKLEAFNPYALLLECSLADEGIQFRISFDSTILDEQQANRMMQQLEQILRQLCTPESGAKKLSTMD
IISEQDSNDIWRYNRNVPAAVEGFVQDLILKTMWEQPSAPAICAWDGDFTYEELHRHSTRLANTLL
QHGVGPAIIVPVCLQKSKWAPVAMLAVLKAGGAFSMLPPSLPSGRRESMISVIQPMVVITSTQYVH
YFPNVATIFPEEYENVSGFDNSKYCALAQKVTPSTLAAVLFTSGTTGNPKGIMLDHRCLSTTAQYM
ARKFNVGTHSRVFQYASYSFDVSIHETMMVLSSGGCICIPSEEDRHNDLAGAVLRLEANWASFSPS
VARTISPENVSTLETLVFAGEALTDRDVLPWVDKLTLFNWYGPAEYSLSATSLVQLPGWATGTLGF
GSASTCWVVRPENRDALAPIGAIGELVVEGPGLMMGYLHDIKRTNAVVMTNPSWLNRGALSSPGRS
GRLYQTGDLVKYNADGSLTYIGRKDAQVKIRGQRVELEEVEHHLIKQLYTMGATSSQAVVEVVTPV
ASASPKLAAFVKGGGSQFQLNELEKRLACVLPSYMIPSLYISISDIPITANGKTDRKRLRAMGNSL
TREQVTSLRHTPGKQLHIPARTIIEKKLQELWISILGLDSIISIEDHFFEVGGDSIAAMRLVTAAR
QHGLSFTVADIFNRPLLGDLASIARPVMTAANNVAPRPFSLLDHNAQTFVRTKLAHMTSVSDVFPV
TRWQSTCIDLALASPPRLLDYFFIDLPSDSSLEEVTRGCSDLWREMDILRTIFIPWRDQYLQLVPE
QSNLPVQYRSADEGNIEYLTEKIYEDDVQQPLLLFAPFTRFYIVSGSNGAMRLIVRLSHAQYDGTS
LLNLMQCLRASFNKEQFPDKSPFTAFIQHTRSNQLAAVKYWRTLLEGSSFTSIAADRIISDFSKPL
IPIVVRQLVELLPCNDRFTPATTFTAACARFLSQITGSSDVTFGRLVSGRAMLPVQLRNAAGPCVN
IIPVRVHDTQSLEQXRQSVHEQLIDSLPFDTLGAKEVIENCTNWPRETRSFGFVTSYQDLGDAEEE

IKGVSCRLRCLEDIRPGMRMMEDEAVMIFAKPMGDYLQITVNADGRVYTEQTVRRWCDMLAASIED
FRLLEPSH
>g2660
MGSISPTSAIPTVDITDFLEVSDPRLRKQKADELVESLRVHGTCGVVGHGISVEKLRTAFEWSRKF
FDLPMEEKMKANHPDGIVPHRGYSGFGREKCLIYTEEELEKMEGELSAEFKKPLDWKEHIDIGSEE
EKVHHNLWIDEDALPGFRGFTTTFFWELEHLSSEILNAICLGLDTDEETAKYLRSIHTGQQNGLRL
IHYPAALESTIDRTSTTWCPTHTDFTTFTLLFQDENQGLEIEDRLNPGTFXPATTEIEERLYLTIG
DFGEIWTNGYLPASKHRVMIPQPPEGRRLTSKRYSMPYFITPEHHGIMRPMETGKPGTMPNGKYKT
TTVQQHIEFRMKFQY
>g2661
MPANMTAFMDKTKNDDDNVTEQVRLEDQHETVVAAMEGKLLYAPVDLSKAGLRILDSACANGRWIV
DLQKAASPAEHDYRGTDVVESLYPKPPPKRTHFQDQSIKEPFPSEWQGTFDVVHQRLVMAAAAPEQ
TPEFVVQNLAGLLKPGGWLQLVEVITTPVPENPVSQNQYIDMLDSLYKNLYAGTKYAKSNLMADLP
QEMEESGLRNVQKAEVLVKYGAATTDPSIREKSLRTAVAGVPNFLTVLKEMSGDVWKEEWNDLPQR
LQRDLAESGGYMKYIVCWGQK
>g2662
MTKDDQDADFHIPSEPEGFKNLLKGVSLKNTIHELHTSELDSGSDVTNEQFALLRAYYPPYVKRTA
FGKYCKKELDLMKARASARELLDASLSYGNMLRVEGLNYPNAFVRFLQQLRGLSLTKADKKAMEDP
AEPRPDARRKAQSPPAERIETKSRRRRAARIVRYFEDILSQEGDEMSETSNGSQDRNVESRKMASN
PDGHPQCADESIVNACCINLLETLAQLIPSKRSNWTLARLSFTAEFERGEYTAITDGALYTKDKHI
VQAIVEVKPHIRSSILTKVEKQEASEIVAWMKKSNGDGLPQLGGHRLLVGQNQHEVYLMFAKPDEE
YQAYLDSSHNVKNDQGFLSIQACGPWSLTDRGEDLDELCEILLAIIIRASAEVV
>g2663
MKKSNFFTRLSFGSDRKSAEETIDNSDRESLEEVIGDPDRNLVEQTIEDLYQKIDNTSESVLEDQH
ITELPISISELDMTSQESQNNTTELLSFFKPMSDEVYNGYTIFEKDDASETIETLGEPYPGLSTIA
IADGLSSDLAWFVNCAHRNEIPRDEYGFMSTLGSLTGWLFQHSLYDYGIDVNYFHHIFNSRKTIPE
TRYQGHWRRFPNADTEIKPHFMVTISIDLKKEEKLLRVEALAILAAMKGCFQDRRLKKHVVFPIML
FSFMGEYGRVIQAYYDGQRFVIRKSRLFSFRNSTITTLDLFVRYVANTPIGNTKGLLKQISTQPNC
SEVDSNVETDIRKEHPKIHTNDSPKRRSIKVLITSVPPGLKRKRDSVFHRHKGPTDYTSKRAIAQE
LM
>g2664
MRRSTRYRFPERPADFQWLENGIALKETLHTIDPTILKSGSEVTDRQYALFRAYFPRIMKPKIEFK
DFLKNELKLENAKALAVMILSGCKAYANMLQVVGAGLKDVDNLDHHSIDSPYHWIPFLQFLQDIAI
TKFQKNKLEDPSHDRKRALSPSRNAQGSMASKRQRPSMTQSESSGQAAGAIPNTIPQMTVNAQEYP
ICADESIVNADCLLLLKALASMIKDNPSKWALMRLDFEARFDDKRSFKAMTDGVLYARDAMVAQAI
VEVKPEIRGKILKKLQMQEAAEIVAWFKNSDEHLLPDLNGYRFALSLQRHQAHLLFARPVGDYRGY
INGTSKGGYLCIQVCGPWDITDKESLKKLCELLLAIMIQGARAAGLNVF
>g2665
MPLKIFHSGRQSRLERSKVEKAIEELYEAVDSMDVTVLDGQQIAKLAVRVYDLEIDDQDAGSSNSK
QLSFFAPLSQELYKRYPLGEKEERTFHNSLHNFFKIPPGKSSAINLADDLTTQFAWFVDGHDRPDI
PINGSCFKPTLGGLSGWGFQEGPFDFETDKDNDDYVFDDLVTEPPCLRAFFLDRYPGYEEEEISHF
MFGLKIDCENGHELFKGEVLQILMAMKGQLKLRELEKHVVFPLLVFSFMGRQGRIIEAFHDSSYLN
IHKSRLFEFRNFEEAPFDLFARYFANTPIGDTRHLPVPQQIHTEATSAVDSTMKLHDSGTVRQQRL
TKQGENTLERQPSLRNFWKQRLSVRSKKDNRRGDVRTTFPDQVEE
>g2666
MSRLIPKADRKKIHEYLFREGVLVAKKDFNLPKHGEIDTKNLYVIKALQSLDSRGYVKTRFSWQYY
YYTLTPEGLDYLREWLHVPAEIVPATHIKQQRSHAPPRGMLGGEERERRPGGRGGPRGDRDGGYRR
REEGKEGGAPGEFAPTFRGGFGRGRGAAPPS
>g2667
MAQQINILLSSFPGLALPPTLSFSLPITSTVTDLTERIEHYLPESLKRSAANLTLTTIGNKQLLPE
SSLPISSLFSTSQNALSEYNLLPLRLSVPLRGGKGGFGSQLRAAGGRMSSKRKRNQGENNGSNRNL
DGRRLRTVNEAKALAEYLAVKPEMDKKEKEERRRRWEAIVEMAEKRTEEIKNGGGKNRLDGQWMED

REEMSEKAREAVLAAMKDGGWNDSLRDAIMGGSSTSASEGNSSQEDESESAEDEDEDKPESSAPAP
SSSKPATSRRFIGFDDDDEFMSSDEDEGAEDGKDKGKGHSNEPGFYSRTRKKAKQQWHKKQQAIAK
EQRENSAQDGEGTDDGATSAALDTETTSKDLEDEGTYLAATEYIQSTAQHTQICDRCHDLLHHNKG
VSIPSPSIDSIAAYLDESPHRMNRVYHVIDAADFPMSLLPNIYEALDLQDQRSRNRRSKTEKYKYG
KRMTTINFVITRADLLAATKEQVDTLMKRIREIILKKMDMRREDVRLGNVHMISAHRGWWTTQVKE
EIRQHGGGVWIVGKANVGKSSFIESCFPKDSKNLDKIAHLLESRKEEDALKTPNDHTPETTPYLDP
DALLPPAPREELYPTLPVVSSLPGTTVGPIRIPFGRGHGEMIDLPGLDRGTLQDFMVDEHKSDLIM
TKRIQPPEQLSIRSRQSLLIGGGLVRITPKDLPEDCTVLAACFVPLETHTTRTEKAIEIQSGVRKY
NTGGGDRGNSVIKPEHLADEATTIRSAGTFTLSTDVTLSHLPSMIRKKWDDHGIRPNLDTLPYRVY
ATDILIEGCGWIELTAQVRTRRSSLQNDLPQIEVFTPLGKHIAARQPLETWNFIRDKRKAEKRKRF
TSGAHRPRRGNISHKKRTLHGGKGA
>g2668
MSRALRSVKNVTKGYSSVQVKVRNATSNDPWGPTGTEMGEIAALTFNNPSDFYEIVDMLDKRLNDK
GKNWRHVLKSLKVLDYCLHEGSELVVTWARKNVYIIKTLREFQYIDDEGRDVGQNVRVSAKELTAL
ILDEDRLRSERSDRKLWKSRVSGIDEGIQGYGGSSAAGGQRPNRDRRERRGREEDDTEYQLAIEAS
KAEAEEDRKRRERAARDDDDDDLAKAIRLSKEEEELRRRELEESNAKSLFDDTPVAAAQPTGYNQG
YQQQPAVDWFGNPLDAQQPLTTGYLNNQYAQPNGYQPTGQINAYGNGFQMQPTGYDPSQQFGQPQN
NFYQNQSLQPQQTAFNNNNPYGTNPVYGQVQQPQQDNYLQPGSHNPWATDKQQGLDSLRPMPTGSN
NPFASAINRPVSQPSRPGPPTLNTLAEERATTAFNTMPSGPNPIANYQAPNPIQASATSAKPTRDD
GQYSRLNALLASGEGQDTFGNTGDLRVPAHHTAPGQFINSAGQGFGPQQTGSNPFFHQQFTGVPQQ
TGFGQSNNPFGAPGHQQQQQGGSLIDL
>g2669
MAAAAAEAAALDPSNSSKNTLKIENFDKRNALQAIEKKYQQQWRENGVFEVDAPSLQEVPAGAMSA
KELRTKYPKFFGTMAYPYMNGTLHAGHSFTASKVEFMTGVARMQGKRALFPLGFHCTGMPILACAD
KLKDEIKLFGKNFEGYKPEDVVPEQPPAPTQEISVDPSKFSGKKSKAAAKTVKAKYQFQIMLSIGI
PLEDIHKFADENYWLEYFPPLAIRDLNSLGAKIDWRRQFVTTDANPYYDAFVRWQMNRLHELGKIL
YGSRYTIYSPKDGQPCMDHDRTEGEGIGPQEYTALKVKVTEWSQAAADLVKGKVEDDANVYFVPAT
LRPETMYGQTAIFLGPKITYGIYKVSEEYYVVTKRAAWNMAFQGVFFDSEHFPKSQDQLPLVAEAT
GSTFVGTSVSAPLALHDTIKILPMETVSATKGTGVVTSVPSDSPDDFATITDLAKKADYYGIKKEW
AELEILPVIETPTYGNLTAPALVKQFKINSPKDANQLAQAKDLAYSEGFYKGTMLVGEFKGEAVQT
AKDKVRQALIRDGLAFAFADPSGKVVSRSGDDCVVAYLGQWFLNYGENDAEWQKTTLNHVVNDLNT
YTPETRNGFEANLSWLNRWACARTYGLGSKLPWDQQFLVESLSDSTVYMAYYTIAHLLHGDRFGKT
TGVLNIKPENMIDEVWDYIFTRRELDDEVIAKSGISKESLQVMRREFEYFYPLDVRVSGKDLIQNH
LTFFLYIHIALFPQEYWPRGVRSNGHLLLNGEKMSKSTGNFLTLRDSVDKYGADATRIAFADAGDG
IEDANFDETVANSNILRLHALKDFLEEAIKDETLRTGPADTFWDKSFNNTINVIVEEAKKQYENTN
FKLALKSALYDLVNARDVYREATLGAGLGMHRDVLLRYAEMQALIISPITPHWAEYIWLDVLKKPE
TIHRQLFPEVPEADVGLSAAATYVRDISSNITSAEATFIKKLAKGKSLSFDPRKTKKVIIYAAKSF
PAWQEKYIDLVRESFDSLNLKIDDKDLNAKVGKLGEMKKAMPFVQGLKKRLIGNKESPETVFDRKL
PFDELEVLAEMVMGLKRVTGAKEIEIVAVEEGGKTGTVVATGEKREGLNAENSVPGQPTFDFVNIA
E
>g2670
MVNLLKRMRKLNQRLINIRVGTGAATLPSLSNPFNDLPVVTRMHLTYGRSIGKGHIGAHKFWRQCL
ARLKYHNPAIQMSVKPYEGTQEKLAPALTIFFSGAQPKKAQAYHDPALKDEFAPKPTETEKAIVLN
LKDYNFEEIWQQVKELTGAVEVAATPEDIEQIQKFKQMEIKSESDKIRVAGIRQAKKDQERMLQEA
RGEVEKLREL
>g2671
MPIRINLPPATRALLISQLALSFLYNVARWRQLDDGPPSSGAQSTPIVPYLTLVPSMFYYYPWTIL
TATFVEQNIFTVLINGATVFYGGKYLERAWGSKEFGKFILAIALIPNALMIVIYIFWGAVTGSSVR
GYAFWFSSDDECTTSCTMPADDPIIRSLTQICGGVALQASFLVAFKQLVPEHTVTILKGIIKMRVK
HFPAIFLVLNSISGIIFGTDTAAILAWLGLLTSWTYLRFFKYQPDLTGTSTNGLGFKGDASETFTF
ATFFPDAIQPPIAFVTDQIYAFLVAVKAITPFSAEDIASSTENAVARGQAGLPSLLSNPGSSARSA
GKREEAERRRALALRALDQRLQAATASKPPALSSQTAAPSSTNAPTVAAGQNMLGETTYTPDNS

>g2672
MPPVNKAVPEPSVATNGHPVTEAPLPPLNVKKGRKSHRAEPTTNGDDSAMEIDTNGIGHDRVSAPP
TVKSPSSAGLADAEGDIDMDLAPAVELHHEKQETPIDTLTAGRSVGVQYVSAKVSDLSADTTILDV
DVAREDHVTRSAWRPFDSSIFAAAGLSFCSLYKLAPSSTEKIYDSKDDGSWVTAVSWEPSGQKLAL
ATYNDHRSSIQMYDSSGSVVDLLPTASGMINGLHWAPKGTHMVVCSSNNVSSELSLWDDSIKPEEF
PSYQTIDAHVHDITWAGDNHVFASGLGSVWQCEIDSTLQMQIVNRYQSRESNTEWSYIRGVQTGSG
PVAVAAASSACSLWIPTHDTLIEDAHRADITGIEIRPKHQDQDANTSTSFTFASASLDDTVKVWTF
DVESKKITCLHTLFLSPGLPALALAFSPDGWAIAAASTDRLFVWNTDRGDEPIATWVCPADSATGN
PERPVNGENGSGPVEVYRTIGWDTNGKKIAMGYGKKVITYFLNKNEKE
>g2673
MTAETPLVRGHRLTPSFTDSAIDVELTEEYAHENGDEDVIATTAPSKGNSNSLNERIVHIAQLVAN
NDALRGLSQDDCAAVNAYLDNIEEIINLRRDNSPNVAPNRPASPTGNKTPTTKSPLAIADSRRKPI
PKGTTPSQSQIVSRDLTSILQELTTVNSELRQRYLESRHIHDVFIVKCEGLAQRIIELEEEVHELK
SDILEDTIELEGLRGTVRGLDSWVNRWQRQRESSLPSHKSDIRKSKGRSYWRRKSSTVASNEEEED
DGNDFNTFLDGVLAWMRGWNDVEEGFLIRARRRKLRRNRKELGLLDTQAL
>g2674
MSFPNLPATVAPTIVVARDVIAQSRNAPNPPNLLPLMTSIPAELLTPTLAYLRIAAKSKLSFLYES
AATTETIGRYSFVGADPRKIIKTGAGHGPECDPLPILEKELAQFRSATVPALKLPPLTGGAIGYVG
YDCIRYFEPTTARPLKDVLGVPESLFMLFDTIIAIDHFFQVVKIITYIPIPDNDADLEAAYLKGQA
LLQEKIDILIDPHVPLPPQGPIVPNQEYTSNIGQAGYESHVTKLKQHIQVGDIFQAVPSQRLARPT
SLHPFNIFRHLRSVNPSPYLFYIDCEDFQLVGASPELLVKEEKGRIITHPIAGTVKRGQTAEEDEA
LAAELRGSLKDRAEHVMLVDLARNDVNRVCDPMTTQVDRLMVVEKFSHVQHLVSQVSGVLRPDKTR
FDAFRSIFPAGTVSGAPKVRAMQLIGELEKEKRGIYAGAVGYFGYDKASIDGSSTIPGDMDTCIAL
RTMLVKGGVAYLQAGGGIVFDSDEYDEYVETLNKLGANIACIKGAEEIYMQLEKAVASRGTK
>g2675
MSEQDYMTAFCTALAAKASLDPGLPVPNPTSSYWQSSPHALSTHQSAHLPSTVDVAIIGSGMTGIS
AAYHLLQHQPDLRISVLEARSLTSGATGRNGGHCKEDPYDDYEDLKELYGKEGAKRVVRFRLAQLD
ALVDVMRELGRDAEKESCLRRVDGLDAFYDRDAWETVKKKVEDYLEDFPEERNVWIVHEGDDINER
FGTINAVGCITGPAGAMWPYKLVGAVTSHISNTQKYPNLSIETHTPVGSITSTDPQEAKYPFLLST
PRGDMKATHVIHCTNAYAAHLLPGLKGKIYPIRGQMTRQSAPPGFPRLGDRRSWILHYDFGYDYVT
QSPSSSSSTEDIYLGGGFLKALVTGQVSVEDADVGSTRDDLQNPTAXATLQDAVEKRLQHGNGTKI
VDQWTGVMGFTIDNNPVVGKIPYEISRRRPADAQIEGREWIAAGFCGNGMVNCWLSGKGIAQMLLN
GEEAVRGWFPVDEYACTTERLEKMTLVERFLEFVKDAV
>g2676
MADAEEDFSSLPLPDRFTHKVWKVRKEGYEDAAKQFEKTPDESDPVFTPFLQDPSLWKGAVADSNV
AAQQEGLAAYGAFLKYGGVQACTRTRGVTVSAIAEKGLPSARPAAKANALEALLLLIELDAPGPVI
EDLIPILSAKQPKSIAAGLAAITAIYHNYGCKIVDPKPVLKILPKVFGHADKNVRAEAQNLTVELY
RWLKEAMKPVFWAELKPVQQQDLDKLFEKVKEEAPPKQERLTRAQQDAMASAPAPGEDDVGGEAGD
DYGEEEGEMEPLDLAEPVDVMPKVPKNLHEQLGSTKWKDRKESLDELYKALNVPRIQEGPFDEIVR
ALAKCMKDANIAVVTVAANCVDLLAKGLRSGFAKYRSIIMAPIMERLKEKKQSVADALGQALDAVF
GSTGLSDCLEDIFEFLKHKNPQVKQETVKFLTRCLRTTRDVPQKPEVKSIAEAAIKLLTESSEVVR
SGAAEILGTLMKIMGEXAMNPYLDGLDEIRKTKIKEYFDTAEVKAKDRPKPIVAPPKPAPPAARKA
APGAKKPALGVKKAAPAAAPPPPVEEASPPPKPKAVARPGLARPGAPKSGLATPGGGLKMQRRIPG
VGGAAAGSPQRRVSSPPLEEPVAAPPSQPKFGLGRGLTGRPISKNAAPAEVAPAAAPVMSGLAAAE
RAELEELRIEKERLWKLNEDLRTEKTRLSSQVTELQNQNAQLIEDHTRDVLSIKAKETQLVRARSD
AETAEQTVQKQQREIERLKRELARAVRASTMSPPVLADGLNTGMSDNGPLSPDIGTYSSNGSRAGY
MSSRLDTGRPRSYVSSASEDKENNGFGSPTAETNLDSAFGRRKFSPPMGQTLSSRGSPVRSTARYA
SAGSTHSGDDQPSITSRSAEPAENWKRAAEVTSQLKARIEQMKARQGLSRPPPSTHS
>g2677
MLYSLPATFAGLPSESFLRTKGLFNDDEPTETIEIFKPFISYDDEFTSLLADDASLELVYTDPDNK
PIADEMGIWVWDHNQVWMASSSVENVSYVSILDMDTRSVKPLLVSSNGVPILNPNGGGYFNGKVYI
AGTGNATIPPTIYEVDPVTYETRELLNSYFGLRFNGPNDLTWAKRGDKAWLFFTDDPLSYYYNNGD

YPTIPDATWRWDPIEKTLLPVIDRTDILVPNGIRVNKNSTKLYITDTPSFTNPGSGFVTISSTEFY
YGSDSAAIYVFDLSENGFPHNKRLLGIAQRGIPDGLHVDDDERVWTGESDGIVVRSSAGKILGMIN
ALSIQGEAVVDSDQAPLQNFALAGDRVIILAYSKIYQVRLSRAIVENMT
>g2678
MGVIRDILSTALGSDQVKNGFNSRFSPSSDQYSTNQCQSSSLRQNLPNQVDYNMGRDMRMQTQSPN
YASSRNPIYDRQYYSRQDNEEDHNYDHIFGAVPPYSESTKYQSSTEDQGASGVRRKYNSNGGSTCH
GQVHSRRTKSRPLALPQIDYGDGQPFLRGYSQELGQSGISFEDFMQALDAINVAIIPNPEAAIFQK
GANIAGWFLPGAASIGLTIGQIGVGLGTAMGHSSTVAKALSKANMEIFVPNGLEICIARTKDVDAE
VGIAPSESRGIVSLSPDERVAWYGDLLAPLTIVLPPLQDSGRQDAIAALGQRFGDKSTEKKVRKAQ
KKLEKGDTKQFASLEGDLKWTNLSFCDGSLAESIARE
>g2679
MADPRVVNMDGFTSTSHSNITPTLSPSLPSNRLKRPFTVCVIGASGAIGAGVAKSYALAGCSDLIL
AGRNSDTLKRVSEDILSSSDESDNEGVKTRIHIQHCDIASHDSVRKLAEFIGSSFSGVLDAVVLVS
GISGPVELRITDGSPSDGHWAEVFNTNTLGTYHVAHYLVPLLLKSDTKALLVIGSIAACITKGMIA
NTKYCVSKLAQARIVECVAEQFAEQGLLAVSVHPGAVGSENALATAPKEFIKYLTDDPNLCGAFCV
WLSRDTARFSWLNGRLVSATWDPEELLAKKEDVVKGDLLKFEARTTLDL
>g2680
MPPKGKKVAAAPFPQGKAGAKKTKNPLFEARSRNYGIGQDVQPKRNLSRFVKWPEYVRLQRQKKIL
NLRLKVPPAIAQFSNTLDRNTAAQTFKLLNKYRPESKAEKKERLHKAATAVAEGKKKEDVSKKPYA
VKYGLNHVVGLVENKKASLVLIAHDVDPIELVVFLPALCRKMGVPYAIIKGKARLGTVVHKKTAAV
VALTEVRSEDNSEFSKLVSAIKEGYSDKYEENRRHWGGGIMGAKANDRQEKKRKAIESAIKI
>g2681
MINAVLVFNNTGQPRLTKFYTQLDTQIQQKLIKQIYDLVSQRPASACNFLPLPPLLSQGASSKAAS
GPSDAPTQITYRTYATLSFIMISTSTESPLALIDLIQVFVEALDRLFQNVCELDLIFGYETMHATL
SEMIIGGVVVETNTDRIVAGVKAQEGSRGKRMAVDAASSVGQLSIPGFGPWR
>g2682
MWKPHPTSPYRLCQTLLSQQQRCHLQTLSPNLQHTNPNNKPTTSTAHRTSFDDLQPTDLAAYWDTQ
PPNSTQLKSASRFFAHTRHSPVKLYSAAQFRTIPHDSREPEVAFLGKTNVGKSSLINALVDHEICR
SGDKRGKTTEMNAYGIGGTKGGESKIVLLDMPGYGTGSKLEQGEEIMKYLQRRKQLRRTFILLDLR
HGIKPGDQQILTLLRQYAIPHQIVVSKVDRLLCDRVQDLKHWHSNQIVRNSKLAKLQIDLDALRAM
DQIPSDSNPSDPQYEFAVRLKETKGPPPLGEIICVSAMKDVARNLKKSHLNIDALRWSIMQATGFD
GSFEGVCP
>g2683
MPFGPRNPFLTTEDDDIPLDTFDRELLAQLGERDSQPVATGRGALLRRFTEDSVSPVSPAIGQYSS
SPDTSFQSSPLLQYFRPSQPQIASTPGFSEYQYVPAQLSDRSEHTKDSYSSRKGPSTSPSLPRQIS
FSHAPPVVQGIHLIETKTLPDRFRALFPYPLFNAVQSKCFHHVYDNDYNVVVSAPTGSGKTVIMEL
AICRLVNNLKDGLFKLVYQAPTRALCSERFRDWSKKFTSLGLSCAELTGDTDYAQSRLVQTASIII
TTPEKWDSMTRRWRDHSKLMQLVRLFLIDEVHVLNETRGAALEAVVSRMKSVGSNVRFIALSATIP
NSEDIATWLGKNDTLQHLPAHKEHFGEEFRPTKLQKFVYGYPCTGNDFAFDRLLGSKLPEIISKHS
NKKPMMIFCCTRNSAISTAKELAKLWLNTIPQRRFWAGSVRMPVIKNTDLKALVASGVAFHHAGLD
SDDRHAVERAFLEGKLSIICCTSTLAVGVNLPCYLVIIKNTVCWQEGGCKEYTDLEMMQMLGRAGR
PQFDDTAVAVILTKKERVPIYEQLVSGTMQLESCLHLNLIDHLNAEISLGTVSDIQSAIKWLAGTF
LFVRLRRNPTRYKLKENADHRDEDEMLQQICDKDIKLLQETELVVKQGPIKATPYGEAMAKYYLSA
VSQADEFHELRLKAAERPFYREINRAHGIRFPIKVELVQNAHKISLLIQSELGAVDFPSAEQFQRH
RMQFQQDKAIVFTHINRLLRCIIDCQIHNEDGVAVRNCLELARSFASRVWENSPLQMKQIDQIGVV
AVRKLAGAGINSIQALEETEAHRIDMILSKNPPFGMKLLARLAEFPKLRVTIKLLGKASKHGGAVR
VNFKADIGFLNEKTPTTFHKRPVYVCFLTELSDGRLLDFRRLSAQKLQNDQEILLSAEIKSPLQHI
TCYVMCDEIGGTCQYAELKPDVPDSFFSVDIPMKTSRRRNGDGPRLKTIVQNEAFEDDDIPDDAFL
EAVHGLDDHPQNQGSVDRASNNSRKNERHTSDLREDTEELQPVMLENGKYNCNHKCKDKSTCKHFC
CREGLDKPPKVHKNARHNERTKSNIQLPLINNGIPKTPIKGSQRNGPSYDTIDYLDLTQPTKEEQH
KQHIAPESKAKRMANLDFASSQAMLPRATVRKIARNRKRSNNRRRRSSSEYDDSSLEEFFSPTQHA
LTDMTNGDSPTIGTSLDLNDDSPYTGLVAKALGPQDRLSYNAPKLSFKRNVGPMSDATNISNASKR

PKIDMQSSSNGDADTIHDPVNDLKNHSPNQPESQNEYIDALLLQEFGDIVNFSGI
>g2684
MHRLSWVMPTVAGKRYVTSTAVFLTEAIKLAISLTMALYEISKHAPPSMPATSLFSNLSNTIFAGD
SWKLALPACLYTLSNSLQYVALSNLDPATYQITYQVKLLFAAVFGLVLLQRYIPARNWGLLLFLAA
GVVLLHAPGHESDRLVARDESAHFPRSLEEWRQKKGYSPMKIAKRSASYEGIEEDMLLEHPTLDGR
VGLMATLCACVASALAAVSFEKVIRDSAAKTSLWVRNVQLALQSVVPAFFVGVIFLDGEVIAKQGF
FAGYSWIVWVIIFIQAIGGIGAGYAIAYADKTAKTTATGFSLVVGMLSSLSVFDLELSANFSIGAA
IVLIATFLYGTTLSPSPLARMRPPPIRIESFDEDPNRPGVSPPNDFSIKLPNTPLLTAGLSTSRPA
SPSHSRVKSNQSGSGYFLEPDE
>g2685
MPMENPQLHHSGSTAGFARDLNFDEYAFDTRSQLSLAFGVVDDMRGIGFPQLDQNSKSPKTAKPAK
PTTPKGDRRAEDVPKKKPSRLDLSLLFPKPKPAAAPLLSPQRYTNSPSPVTSEFSVKPQPAKLTKP
RSRGNMRASIKEAPAPIPEEPPIPRKRVSTRRPAVDWFDVPLEQIIRLGETLEVDEEEAKQPDRVL
DHAKPVKEAKHXKPITLAPAPAIIKPVQPPVQNGIAKTSSAVDSRRSSRAPSRVSSHTSRGSRGSE
NHYYRQPLPPSYTNPPPRASLQTWQSEGDLRAAHQKPAGRLVKKKSSSTFHTKDLTKNSVLSLSSS
EDEDTDADEDYGEQLVRKAAPSRGPRDSFATNDYVAPEICHAEAAVATKGYQLTRLGRANSNVSSV
SSDSRSTQRRPIPSRADSLSSMSRSSVSTKIYGSSAHFDVPLIEEPEEQAEEASNSRRPRLSSQSY
SDLHSAKRRSRIIAVTRQEESLLEAMRLRNGRITPSIFKDIEPGAERAAENHEVSEPPALSSPEPV
QTQFDTSFLRLSAAMRIPPIAAPNLAESESLIDKDPYLPYRTASNAEQKTDHNNNNNNFTASPRLS
LAYSETPSSSSTMGHASPVTPTLQPIHRFSHQLAAPPPSHALPPLPDEVATRRHSRRRTDSSDAIV
LEEGETEKLSSPPVYPLWAVKWGRDPNDVAIAH
>g2686
MPVITEPDPIAVIGFDVRLPQDACSAQEFWTMLYEGRNANTHVPKERVNMDAFYHPNPNRTDTANV
RSGNFITGDIAAFDAPFFSIQQIEAESMDPQQRFLLETSYRALENAGAPLDKIQGSKTSVYVAAST
RDYEVILYRDPELTPKYMGTGIGTSLLANRISWFFDFRGPSITLDTACSSGLAAVHMACQSLKAGE
SNLAVVGGVSLILAPEVNMNQLSTLGFLSPDGRSYSFDSRANGYAKGEGVSVVVLKRLSDAIRDND
SIRGVIRGTTVNQDGRTPGITQPSKHAQTENILQAYKDANLDVQDTNYFEAHGTGTAIGDPLEAEA
IHNAFQRPTEKPLWIGTLKANIGHLEAAAGVASLIKAVLVLENGIVPPNADYRVPNPNIPIGKYNF
KVGTLLIIHLLSISFAFVSDVVLFQFLTSPMPFPAEGLRRISINNFGYGGTNAHAIVDDALNYLRL
NGLEGKHCSVNQVSKSLTEGHRIQGQLPGEDTYGISPELNGNGVSRQLSFKQPIVLPFSTADESGI
ARILEIYQHYFRALKSDYLRPEEQQRYLQNLSYTLSTRRSALPWKSFVVVDSIEQLQAEELANLAS
KPQRSSSEPLVSFVFTGQGAQWFAMGRELSLYSAYRESLEKSETYIKGLGCEWNLIDELNRDKSLS
RINEPEFSQPLCTAIQIALVDLLASWNIRPTAVIGHSSGEIAAAYSAGGLSHKSAIQTAYFKGLLV
SKLLHEQPNSGAMISVALSESAIQKYLAEITEQIGEPLVVGCVNSPDNVTVTGGTRAVTELAQRMQ
QENIFGRVIPTGIAYHSSFMDAISDEYASKLGDLEPRDMHSESPRFFSSVHGVEVPVQDTCTSGYW
VDNLVGQVKFSSAFGEMVSHLLEERKKGGINKSGRDIMIEIGPHAALQRPIKDVIKDIPAAKEIEY
NSTLERNVHALKAMCGFAGRLRSSGVPIDLLHVNFPDVASAATQVLTDLPAYSFNHDTKYWVESRL
SRNFRFRQHARHELLGSRETDWNPLRPKWRNVISNVNFPAGLRIPENTDSVEAQVVFHPSQQSGGG
LPDQCRVHDFQIFAYFSDAWHELCYGTIEIEVSPGSDGITAANTADSSRRWENVNSKEFYDTINEH
GMSFGPAFQGLQDIRVSDRQATARIELDRWHNTLNENNKFPHTPHVIHPTDLDVIFQSSIASISQG
GRQPIPVLIPTKIRSLWISNDLLTRGSRNVLDIHNQIDTEAYREAEFSIAVSDEHRGRAQVRVDGL
SLTFVKDAKWMERADEEARRTCWSLVWKPDPSLLTKNDIVSLSSSSLNDDENYGLLAAATFVELFA
HQNPLVEIIEAGDVSLEASRSIRDRLMNYGLSSGDGYGLPLFRRYTITRSDDAEEVNDHEGPFSDR
VDRKTDTEGKDEEESVDGLYDILICNSRNYLLDNSPKSPYNLLKEHGKVLCLDSLESFTKADLAKL
TDAESIALSTSKPNGTNGTTSSDNTVIIVIHPHSVLQKDVAGLIQTRLSAIPTSIVPLDSIDVQQY
KGSHYVSLLELDASFFSDVNDEQWSIFKSLLSTAKSLLWVTKDQTPDKDLVSGLGRVIRSEYLDLR
FVELALEPSTSIDKTAGHIDSVLLKTLHIADDAVDFDSEYREKDGRLCIGRVREATSVNATIRNMT
SPSRPKQQKFGAEPERALSLSIASPGLLNSLHFEDDPDYDLALAPTEVEIEVRTAGMNFKDILIAM
GQISGNRLGFECAGIVSRAGSEAGFAPGQRVACCTLSGAYKTFVRADASWVFDLPDHIPFADAAGI
PLVFATAYYSLVEVARLSEGETVLIHSGAGGVGQAAIQIAQRHRARIFTTVGSAEKRQLLQDLYGX
PXSHIFNSRDTSFAQQVKSHCPSGVDVVLNSLRGELQEASWKCIAPLGRFVDVGKADFESSRKGLN
MKPFLTNVSFSSVDLSVVMRNAKPTMTRIMNALKGYLTDSVPAKSPQPLQVYQIENLQDAFRQMAS

GKSSGKIVVDLSPSDEVPVLPSRKSRYNFHPGATYVIAGGLGGLGRTLVDWMIERKARNFILLSRS
GSENNEAARKMIETYATQGIVIKAPMCDVANEQVVRQVFEDLAQTMPPIKGCIQGSMVLRDGLLDS
MSTVDWNAVLRPKTQGTLNLSTHLPSDMDFFVLLSSISAISGSRCQANYSAANSFMDALARKLAAE
GRRCLSLNLGVIKDVGIAAQLGTNAALRRDGFEGLSTPELLALLDYACDPDCSRAIDPQRAQLIAG
LGGAMTLLPEHFESVYWTGRPIFRQLQEVNRITFQDAHGSGKSDSKEESYGRLFIKAADESAAAEV
ALKGLVAKLASLLNVPVTDIEVRKPLHSFGIDSLVSLEIRYWISKELKAEVSIFDIMQASGLAELA
XXXVEKSELRK
>g2687
MVVLRETGRLTVRLTIAMSALAILAVVVRLIARNKVKLTFNLDDVFILLGLLFFLGVEARIYTSGG
TLDESQMNLNELINLFKYIYALESFVTTSITAIKISILLMYRRIFPIAEFQRYALVVGVLAAWQLD
LLMDGQATCLTYGHFIIGYEISNMLLDIAILMLPIRVLRTLQLSMTKKVVVGAIFILGGFIVVVCI
IRTVYLSNATLENPVALYNGLNWTSVELAVAVLCACIPTYGPLLPTQRAVRALSSWGSHNKSRAGN
RMFKSFGYERQSDNNELEHLSEPSTQA
>g2688
MVDNWDPAKPLVARQPFKLIYALYYILSSAASVPAWAILYILPHLRPVSSWPYKTAVTNKISYYLL
NFFSKIRIPTPQPISPGKETDLFVALKPANDPRKTYAGLLDNPQIQPTTIGGFWVPSLKQLATVSS
ATNDNWVVLHFHGGAYVLLTPRDPTVQRGPKQLCEELPADAALCVDYRLSSNPKSSHPAQLQDAIT
AYNYLIQDLQISPSRILVSGDSAGAHLAILLLRHLTTHPEIGLPLPRALLLHSPWLDLTPKGTSVD
SMPGGRDYISDAFLQWGATTFTPKGSSRDADFVSPFYHPFASPIPIWVQAGGVEKLYSTIAEWVGK
MRDAGSTIELYTMEDMPHDVFHFSSDMDLEAQARDAIRAAKGFLERTQ
>g2689
MSSDRLHGLTAVVTGSSSGLGRGIALALAAAGARLVVCADLKETQDVRSTMDPNVKASKGADMASK
ALEVSNNSSENEADLPTHELIRSLYGEDRALYVRCDISLEQDDVGNDVYSTDYAIQKAVSKTGRLD
FAVDVTGTFLATKFAMDQFLKQDEDEHGFRGNIINISSIAGSSGLPGCSAYCAAKSAILGFTRSVA
VEYGERRIRCNAIKPGYIMTPFLQRVIETTGDVTWLEKASPFNTLGQPKDIGSAVVWLASGRESGX
VTGIDLSVDGGFLAR
>g2690
MSRSLSPTNSNRTSPVDDLEKQDASSTDKVPSHQGKPPGPGDQYAPRTAKFWTIMLCNYLSLFLVA
LDRTIITTAIPRITDEFNSLGDIGWYGSAYMLASAISQPLFGRIYKYYDMKWAVNGMIFGITSVLG
PLIGGGLTDGATWRWCFYINLPIGAVSWTFMALVWHPEKPKRESASTATHIKRLDPLGMSLFAPAV
ICLLLALQWGGSTYAWNNARIIALFVVFGVLFLGFVAVQIWKPETATIPGRIIKQRSIISTSIYIL
SVYSTMMMLIYYLPIWFQTVKMVSAVKSGIYTIPLVLSLMVSGFIAAFMTQKIGYYVPSMYLCPCF
LAVGTGLMSTFTISTGSSHWIGYQFICGFGLGLGTQNAALVIQRILPMTDISIGFSLMLLLQQLGG
AVFTCVGETVLNNVLISQLAGVPGIDTNAIVDQGITDLASVVPAQYMIVVQEAYNKACTRIFLVCM
GLTLVGAVSSLGLEWKSIKKGKNGQKAGDGPTEKTVSSDGKDEENDQKTETEK
>g2691
MSDETVVLITGANRGKYISILSLSTQCLQGIPLKSISTNTFSGLGLGLATLYLNQHNTRVILTART
DDPSSILKALPNTYPGTLHGIYNLDSASTTDTIALRSSLLSQEHKIPKIDIIVANAGVGAPFDTIL
DAPIDALEQFYRVNALGPVRLYQQLWKDLLEKSANPRFILVSSVLGSLAYVDSTPCGGYGASKAAG
NYFVRKMHEENGRLVAVAIHPGWVKTDNGQAYADAVNVPEPPTDVEASVNAIFQLVSEAIREGTSG
KFIDVMSGSEIPW
>g2692
MARLNYTARKISGVKAGLAVKKDLNKRIPSTPNFKIRDPILEIDVSGKDLTDDGFNEFILDLQIAV
EYKSPHCPQGINKLQELHLQGNRLTVNTLVELAKVINLSSRDLKELDISNNEIKIVTASDSQKWRY
FLQSFGQCCVLKKIDFSGNALGPLGIELLARFYIRSELDFVEIVEDGEGDGDVPEDLMREHSQHED
EPGVAGHETKSARQVSPQTTGYKHHTHTDAELRRYACTRGLRSVPYLIVQDVGIANGSIVHLAEMV
RLHRAPENLLSFLPGGKPPSLPPIEQGRCGIYWQPNNGLGVLSHRLMKLTEEIKLFTTEADSEDDL
VEDFEGLDIDFDHEEIERAQEERERRRRLLQKLTVDLGRTISQQRIHVLGTEGLRKSILWHCALRM
LAIARTILLAPENRPPSAEDELITVRRNGVTAAQTRVPQARSPFSNPITTRTVLGAPSNHVYAGTR
ERSGRSTEAVLSVADLQFPITQPSARRQHCHAPPRGGALISSFRFDPQSSTFDVMFPAIHRPFENP
LIQIHPPPVAAVMPNYPQEDVGEDSGRNGSKQSNTTVKTTENYRSKYRFGLSLHLWQRIISESMNL
KPILHPDQQSKVINYAASWGALEAEMSINGAAENQQIWKILDSIDCFTYKPLS

>g2693
MEVSGSRSGVRDASPILGPSDPTSRPNSSKSTRRREVEKHREVTTTVGPTPSIGQFSFAPATRTTV
VTTTTTTTTNFPPLIFNPPRSARELNPKLYPLASYPTPSALRNFQFELDGQSVVFNEPEDTDAALH
EIHEQHVALKSSKGVLTSVAGYDGSGQNASPRASISRPSSVTASKRRAVSPFTYPEASGKRTRSLM
VPLDSRGQQRKSRLGGIASATNSSVAGLATPETETTRFRFDQHAALRKRLHTDKVGQSVTSAASSQ
GDAALSQTSTPSQTFASKTRVRHQSPVETTPVLEDQSSSMTHVLSDYHGQDPQSATEDDGSGETLA
ADNEGTQEPESFQQTAPTALSTHGSVLGQDMCLPSPSLSPVTAMNTHNPDYFLEFPDDQGTDTDSS
LDNHVPILSNTLRQKTMRSVSSSQTSTNDTAVQSRPTTLMDIPGTLDFFEAIPDEMKTYMMYQLLK
RCPKPTLQFVADIVNPALKCDFLALLPAELTLNIVKYFDYQTMCRASQVSKKWRHIINSDERHWKE
LFETAGFKLPPGELQQAIREGWGWQFPNGPEDYEKDISTPNINIDTDYFNQTSNLSSSFLSNSLSI
DTVSPRRSKRKANRTPSRKAVRRKLTSSRRIGSPASDSDWINELSLIEGPTAAANAAAAAIPYPDV
GLGSLRSLHLYKSLYRRHLSIQKSWMRSDLKPKHLAFRAHDRHVVTCLQFDSEKILTGSDDTKIHV
YDTKTGALRSVLEGHEGGVWALEYHENTLVSGSTDRSVRVWDIEKAKCTQVFHGHTSTVRCLQILL
PAEVGKNPDGSSIMMPKEPLIITGSRDSNLRVWKLPKPTNPYYLDASSHAEDTDCPYFLRTLSGHQ
YSVRAIAAHGDTLVSGSYDCTVRVWKISTGECLHRLQGHTLKVYSVVLDVERNRCISGSMDNMVKV
WSLDTGALLYNLEGHTSLVGLLDLKGDRLVSGAADSTLRIWDPENGQCKNTLTAHTGAITCFQHDG
EKIVSGSDRTLKMWNVRNGECVKDLLRDLSGVWQVRFNERTCVAAVQRDGLTYIEVLDFGAFRDGV
PEYRLGKRILVDRFGLSVDNDDDEDDYDATE
>g2694
MLRRQKSHQSLPPEEPSNAPSTFNDKLPLPKLIVFDLDYTLWPFWVDTHVSAPVKPKDNNSRCVDK
WGESFAFXPAVSSILHSAKAHNIPLGIASRTHAPDLARDMLKALHVIPTFTDNPAARDTRRRTVRA
LDYFDYIQIFPGDKTQHFTKIHQACNIPYDQMLFFDDEARNRNVQKELGVTFCLIRDGMTKEEVDR
GVWEWRRNFGIRPGDQPGDIEEREVSRFDE
>g2695
MAPDLESTPEKPPISEDGHDQQSSEPDQTATDQETKDNVVDWDGLEDPQNPRNWPVSKRVIQVVLA
TSFLLTANLAATMFAPGAAALAHEFHITSSTITSLTVSIYLAGFAVGPMLIAPLSELYGRLVIYHI
CNIIYIGFIVGCALGKNTGMFLVFRFLAGCAASGPLTVGGGTVADVVPPAQRGRVMSLFFVGPLLG
PVLGPIIGSFVSASIGWRWTFWLILILAGVAFTLSIFFLRETNAAVLLEWKAARLRKETGNAALVS
KMDRGLTPRQLFLRAIIRPTKLLILSPIVLLLSLLCAFVFGLLFILFTTFPTVFEEQYHFSAGVSG
LSYLGVGIAMAISLGVFATVSDKLQKAVGDSPKPEERLKPIMWVMPTVPIGIFWYGWAAEKQTHWI
VPILGTAFFGFGFLWVTMPIQLYLVDAFGPEAAASALAANVILRLLAAAFIPLVGPSLYAALGLGW
GNSVLGFIGVVFLPVPFFFYRYGGWLRERFPVEL
>g2696
MLNVRVRLQIGAETKSSFPVTHADAGTFSPNSVVAPGQSTSLHGRIAELENLVVTMMQDHTLPTPP
ASNSLSSTDVFKNFQDGRASLADPGTLELRRSGASYVQSVHWEAILTRIRGLKEDLVIDSKAPPGS
HLFYGPNRHASQDEVLAAVPPRPVVDRLMALHFDSYIVTQYLIHRKKFLREYEAFWDNPSATSIAW
VGLMFSMLYISAQLQTFAIDTTDGRAASLKTECLNMKDAFRERAIQCLILARYTTGGPYILESLIT
ILTGEFVLLKDNATDGWLLVSMILHIAVRMGYHRDPDHFPGISPFEGEMRRRIWTTILQLDISLSL
EMGLPRNATDTNIDTKPPHNLHDCDYDEDTIKMPPPKSESEWTPVLPLIARARLITALGLICDLNT
HINPPSYDEVIKVDTLLRDVYSRSIPPLLRWGTGPHPITDSPNIVVQRVSVETTYHKSRILLYRRA
LINYPVGQTQERDRESVGICLDSALEILSFQQMLHDESQPFGRLSQLRWKVAHIFNQDVLLATSVL
CLYLQDVDKFELPTNAMGQPACPPRAEEIRQQLIKSHKIWLQMSTTSAEAGKVAKALSIVIGTTEE
SIEDNSGPTFDNFSTDFDAIPLNGFGASFNNHYFHPGFYSPLAFFDNVLETEGV
>g2697
MSSISGQQDFDNVAKDNLSHAVNVEESMGRSLGISRAKLLRKIDLILMPMMLLTYGMQYLDKAVLG
FAAVYTLQKDNHLIGQDYSWVNSVFYFGYLGFQYPLLALVPKFRIERYLGFVIAAWGLTLMMTNFS
HSFAGLVIARIIMGALESIVAPSFLIIVSQWYTRDEQPIRQSVWFCGTPLFAIFGGLIGYAVGHTN
SLSIASWRVMFLIFGGVTFVWGVIFAFIFPGDPDHARFFTPSEREALSLTVKSQATGGKGPWKWSQ
AKEAMMDPKTYVFFTIGLCNTIPAGALSGFSSLIIKNLGFSAVDTQLIGIPANVIQLASLIFAGIM
CTKIPNLRLYAMSLCCIPSIIGTVLLHTLPSHAKWGRVVSLWIIYTNSVGLSVSFAVIGGNVTGAT
KKATVTFLLFLGYCAGSIAAPQLFIASEQSRGYPTGIKAMLGCFCVLFIMPFILRGLYTWENKRRD
RAAMEGEVQPDATHFEDATDIQMRSFRYVL

>g2698
MAPVKITETHMDSRNQGPQNGGIDNGLGSYTYPVYKNLDPSTLTEGKVVKFNLVLTANALETVNVP
VLAYRSRKAGPTVGITCAIHGNEINGIPVIQKLFEDINNNSLELLSGTVIGVPVVNVPGFLASIRQ
FDDESKQDLNRLMPGKQTGAAPEQYAHAIFNKIVSSFDYVIDLHTASAGRQNSLYVRADMSHPTIG
GLARLMQPQVLVHIATKGSLRGCAQSQGIHALTVEIGNPSVFQPNFINATYAGIRRVIASIGLTSE
YEPTPSIIESIRKITPEGTAVCTRSFWVYSKLGGILKVFKKPAERVHPRELIGEIQTIFGDPVEKI
YAPDFETVVVGIEANPVAKSGNRVVSVS
>g2699
MLSWSSASLCFSSKAGDLDDSLQHYSDCISHMQKEYGFKPEDLERIWPCAYDPIRDDAFGRASADD
LDAIFVGYFFLALSDLMAARSRQLRNILRFIAHLLRIPKIKSGMEGVRSRVATWFYILDAKASAFQ
PGEGAVLQAMGGQNGVVADLNASFFTLPQAYNLTYPDEEYERDKAQFPFLGRMLYLVSMLHDITRL
KHRVDCSAEIAKLRQDLDEHRDAIQSLQKAHNFQSRTRATFFVLSALYNTVEISFSRALGPCQSRH
SASAFALDIIRLSQKLNTLYKTHFKGSPSSPPPTKIWPLPLVMAAIEVDDDVYREWVIDRLKMYEE
TGGDHYKQARKFVEVVYEKESQAACRLDWEQIMVEYNDGLVI
>g2700
MLNSKTLDLAPERRRNLGTTQESRGEESRSPMSSPDNETDQITRQNYSGNFTGSNLIIGNQHATGD
VNNIAGPRQHTPIDWSQTNHSRSAPSPPSRTIYYARASHDFQPTQANELCFKAGDIIELTQGDVPN
RDGWFMGRVEGRPEEEGLVPEDYVVVTR
>g2701
MDVKPAVFEAAAAHRKHGKELIGCLLESRQKHQIQITANVLEAAARNKGIGKEIMELLLDRLGNKA
EISENLVKIAASNWKNTELMALLLKKHRNLIKITKDVVRVARKSKNGEKMMDLLMSELERRSQFDG
QHGPQTAAWNGIKKQLLADQASANCANRWGWTPLHAASWHGDNEAIELLLSKYAVDTLSVNHDEWT
ALDAAFAKQHMASAQLLLKHDYTAAGNFTPTGFSETRKSNALKLSDDKLEVNYIGRGKGEGE
>g2702
MQPTNSLASLSASLGRLRLQVEDLKICSGFDAQRITLLMQLAEQIDGELMRWPDYVPPSCQPKKAV
RDFYPNYPGWEDIYPSLAVAQDWNHYRYLRIMANIITSRCAALLRLYSQAAAADQVCEQMVDEVCA
SVMYYIGRLKIDNDESEDSNDDEDDEDDMQRKTTGPVEEKYAIAAIGAMLLLPKLRWILQAEPSLR
PKQKSCLELNVKKILRFYQIHGVENADIPDEESFDIL
>g2703
MSSTYLKPPPSASTSTQRQRISSSSHQHSGPAPPPRAPVSVHSTTTVSETATFHGTYPITIGSGTV
IHPRAKLYSFEGPIQIGDGCIIGEKTIIGDNDPNASSPTDGRDGILTRISSSVVIXPQATIRTGAY
IRSAAVVDSLAVINRNVTIGSHTKICSSCEIAEEATVEDWVVIWAAGTGIGQRKRRRFIGPAAEEQ
GHSHRPNAKRIEDARLLVLHKEREGLAKMISLSATGGTKRR
>g2704
MASATIEIPFLASHYGIAEATLTTLLQAPTVDLVNQLLESITVKAREFDDLKSDKLRLEVELENAV
RASESKVKVLKSSVEKGLNEMATLRTRLQESENARSTLESEITTLKSSTTSNESEVNGLKSRISSL
ESSNRDTLALLESKTTAYDKLSEELTTTHQKTVELRRQLSTAEQNLQSANSASTSARFREQSLQQE
LELTKKNNEYFETELKAKSAEYLKFRKEKNARVAELQREIEEASSTIDQLRRSENALKSRLDDVEH
KYEESIAAMQSLREEATSTSESLRIELDSANRLAELQGNAAATAKQRVQECQIALEKARDDAAEEI
ARLRAEIETEHSDKEAAERKVAELEAKMSQVETQAPFSPGPMNGGPSTPIRASTPVGAFSPRTSRG
RGGLTLTQLYTEYDRVRSQLVAEQRNNAELKATLDEMVQDLESSKPEIDELRADHARLENAVVEMS
EVLETSGKERDEATREARKWRGQVEGYERETEILRQQLRDLSAQVKVLVLEVALSKEDLEYDRDEL
EKLARNEIEESMVSLNATGRFITQNLTTFKDLSELQEQNVTLRRMLRELGDRMESQEARDRESAYQ
QDQEELKELRIRVQTYRDEIMNLTAQTKSYVKERDTFRSMLMRRGQAGGDTSIFSQSVPLGSAPPT
IQQQAADGTDYADLLRKVQAHFDTFRQETATDHSALKQQVNDLSRKNSELMNEASRLNSQLGAAAQ
RAELLQNNFNLLKNENVEIQKRYSNLLENVNRQDIKTQQAAEDLVAAKGMCDSLQRENANLKAEKD
LWKNIEKRLIDDNESLRNERGRLDSLNANLQNILNEREHTDAESRRRLQHNVESLETELQSTKRQL
NDEVEDAKRAALRREYDHEQSQKRIDDLVTSLSSVREELIAVKTTRDHLQSKVDELSVELKSAEEK
LQVLQTKPGTAPAPTAATADTEQAPTSGLTREQELAIEVSELKRDLELARTDLEHAREQVEDYKAI
SQATEERLQSVTETQEQYQEETERLVEEKDKKIQELEKRVEEISSELTTTNNELSKLRDEQSEVSR
RVEEQKTTYEAEISRLKDEAERQRENARTYEQSVQAQAEIAQQAQQNYESELVKHAEAAKLLQTVR
SEANQLRLEIVEVKTQAENAKKDLIQKEESWGEQKDRFEREISDLQKRREEVLHQNTLLHNQLENI

TKQITALQRDRANIVAEEEEAEGTNSSLESLQEVIKYLRREKEIVDVQYHLSTQESKRLRQQLDYT
QSQLDDTRLKLEQQRRATADNDHQNLSHNKLVETLNELNVFRESSVTLRNQARQAEAALAEKSARV
DELVQQIEPLQDKIRELENLVETKDEEMKLIHTDRDRWQQRTENILQKYDRVDPTEMENLKETLST
LEKERDEAIAARDSLQQQVDGFPEQLAKTAEDSKAELRGKLTEQFVKRSKELSGRISAKQSELNVV
QQEKEIIQQELTKAQEELNQLKSKPVEQQTTPMEVESAAVTPAVPQVVPPTTPGGDEEKIKALEEK
VARLEAALAEKEAQFDTRLKERSDKMKELFNSKLVELRKNHQQELENAKAQQQAPTEAGQPAELTI
PGTPAKGDTGELPELTDSQAKDLVAKNETIKDIIKRNIQNQVNKVKAQLQQEARAQAAAGPSPEA
TAELEIKLAEERERLKKASDEAIEEKVKSAIELNDKKIAVKISMLESRSKTAMAKIQIVQKAATET
PEKPVREVWEIAKDAKAPPAAPATPAKPAATAPAQAPISTPAATNNVAPAAAAATPAAASGPAPVS
APAAAPTAAQPPAQQQAANPFAAIQGQQALQAQPAQAAQPQNVSSPFAVIKQDGQTQASQPNQQAS
IPPKPPAGAVGNHTGPGPAALRALQSGLPIARGRGGGRGGAAGHQQTEQQPQQGQAQAQRGTGMQR
GRGRGRGGQQHVQTNVPQAAGQTQDSPGGNRGALNAGARQFIPQGNKRPREDGGDTVTGDAGAGKR
IRGGGHNRGS
>g2705
MSYLQQQINSFNSSVVSSAGRLPQQKRIIGSGQPVSSTSSQPTSTPGTPGPARRHEPANVVYSQPA
DTGTGKDIMTNVLYAIQKMKEKNAPLTFEDIVGYLSLQERRHDQGYVHALRSILQVHEKVNYDPSG
AAGKGTFSFRPPHNIRSEEQLLQKLQSQTTMTGILVKDLKEGWPGVEQAIDKLEQEGKLLVTRNKK
DNHPKMVWANDPSLMHKFDDEFRQIWEKTKVPDQQQVIEDLEKAGMIPTNKNKVVKPRPKIEQKKT
KKPRRTGKTTNTHMMGILRDYSHLKR
>g2706
MDKLPVEILTKIIDSETKTLYYHTDQSVGNIDLNPREQVKLQLVSRKFFELSRDNLLWRLHCYESS
GKANGNGFTPAHISGARPLSSITPLSTLGPRTLRTLIQPHPQNISGVNNSHDYALPSIGQRSRAAA
EWDPSYETEDVDWYSEYIARNGPISFSWTQRPVTGGPNRMLRDAMGMGLLRDWSFAREDRVIAPLD
DGTICVWDMNYSHSNSANSLKGKITGMSRPGVLMTDMSKRSDKTPPRSPPLEFLNVGECVSVDSMR
RRAYIAVGNILNEVDLETLHVVHQQRYAWSIFALSQETDYSVPLTLATTLSLQIYDARLSSPEEEA
EISLRSIPIVRSYSYSQTDNHLNAFEALVRIRKVRIMRRCFNLGLCRYYILRLRMLIPSFWQEDFL
VFSVTTGALFPGYKLPFTLEVGKHTIVACGEYNGRGSLELYDLNFHGSDPQHEDSESHMQSARYQN
RQSAASSKLLSVASHGARIVFSDSNGNVKWVERDGQTQVRHWNINTSRRQSRIASPGDVDESDQVR
GLFFTSDTSMRNDVVRKILPTDANLNGDELLLWSGDRIGRLRFSNDSDEGTDDEMNDWMSLDGEEA
DITEKQARRQSKELEKRREQEYAQRIRRALERQADETEYGITNPGFIGSLYRTPAAIYSKRSLLNI
HQRRYINKYLKIRKQRHIRSPWNNYTSIISIMPSFTEDNLTFDPVEDAVAAFRNGQFVIVLDSTDR
ENEGDLIIAADAVTESQMAFLVRYSSGYVCAPILPSHADKLLLPQMVINNADPLITAYTISIDSND
ASVTTGISAYDRALTCRQLADPNIKAENFRRPGHILPLRAREGGVRERKGHTEAAIDLCRLAGRSL
AGVIGELVEEGEVVDGVAEVRGNNGMMRRDACLKFGRRFGIKVTTIEDMVAYLEKVDPHGRFDFVN
GKA
>g2707
MDAFTVVICHGSYHTPIPYKPFMTALQKQRIEAYCPQLPTADLIKLNVSPSPNGSPDFDSPPPATG
YPQPADDAIVINELLEKLIEKQGKNVLLFGHSSGGFLATYVARRKYQKKARKENNLEGGIIGIFYA
CAFLVPVGESCHSFFQPKDGSEGVVPPFCRFHGNGFEGIASTVDAAKYFFNGLPEDEAKYYESTMT
ASPVLRTVLDHDAYRELPCAALICENDLALPHAYQEMMVAMQTEREETHFTVYKCDAGHSPHLTWT
DRLVECVQEWAVSIM
>g2708
MHFFKAVLAAVFVTGAAAFTLPANLQDGVYRAYYDEDGREVHELVQVVHPSNGTLRRGITDSEEPS
LAKRIPNPNTYYSCGCGFSLDHGNCDNAVVGLKNFLDQHADPNFSSGCYDLSPYSSAYCYVNDVVV
FLCNGEQDYGVCSNDVTNSLAAITNHCGWXIAGTEAAEAWFPNLDGSIEVDPPYFTGYMQYGPGLN
FCANDYLAGDNEHC
>g2709
MEVTVHKHIGDHRNIISFFHTGEDTTWRWIAMELAEGGDLFDKIEADAGVGEDIAHFYFTQLISAV
SYMHSKGVGHRDIKPENVLLSGNGDLKIADFGLATVFEYNGKMKACTTLCGSPPYIAPEVITCSTR
GQTKGSGYRADFADIWSCGVVLFVLLAGNTPWDSPTEESFEFSEYLEKNARTTDELWQRLPPSVLS
LLRGMMKVDVASRFTMEDVRRHPWFTRPNKYISSEGKLRDQLNLATSMFESLHIDFSQDPLAPPHS
QSQRSRQNGPSSSAMDIDMQENGDDLQSRLSSTQPEMPMGNVAIDWDTPLVNAFSSTQPTDHTMST

TQDYQLADRLEDEPAMSQFSQNPTVPLSRTQNAQNFHDIIPSRTLNRFYSTWGLKLLVPFLSEALH
RLGVPVPNQIAGIETGGPVVIRVATKDQRLCLLQGNIIVESVSEGLFEVEFTKVKGDPLEWRRFFK
KVAVLCQDAIYRPDQ
>g2710
MDYAMEDTQNTAPQAHEASKLGAPAPRGDTQSVTKRLQSELMQLMLSPSPGISAFPNADGNLLSWT
ATIDGPADTPYENLVFKLSFAFPNNYPYAPPTVLFKTPIYHPNVDFSGRICLDILKDQWSAVYNVQ
NVLLSLQSLLGEPNNSSPLNGQAAELWDSNPEEFKRHVLARHRDIEDDE
>g2711
MPSDPTFTTYTPTQARTYAQHRLSYPSKLYDTILNHHISTGGQLNVLVDVGCGPGRATRDLAAFFE
VGIGLDPGEEMIRTARAMSEEEGFKESGVREKVQFAVCGAEDCAKGVREVLSTLKNGIDDGDSGEG
RVDLLTAAMAAHWFSMPEFWDQAAEVVKPSGTVALWTCSSLYCHPSTPNAAAVQKALFHLEREVLA
PYELPQNRISRDMYDDLVLPWQATDPTTTAKGNLGEVFPESDFVRLEWDRNGTISDGSDFFLSSNS
EKKGETTLNDLAGGLGTASMVTRWREANPELVGTDRDCVKEMCAEIRRAMGVSMDENPTLKVAGSV
VLLLFKRK
>g2712
MRNPIKTIRDGYSPLKLRSSVRRVKSCPGPWSLFFFVFWPLPWRYRIQSPLLPVEAVGQRLRIINE
RQAAILQLRSIPIFRMRDTPLRSLYRLVEDVCACDFIMMGYECTYFFFHKESRWLLSAVPDPQDDD
PVRYAMLASMVEALVDAFNWRLELGIRRDNTLDKSEKRATNFEREVAPSWTAKVEGIQNKLIFNEE
GWTESDMTEERHFWKRNIKVPNGYMYTV
>g2713
MSLKGRNVLVTGASMGIGEAIVKKAAAEGANIALFARSKDKLATITEYLQDRYSSSKFIFRSVELQ
SYEAVESVVQDVVNELGDIDILINNVRSISSTALACPYYSQAGLALGAPAEFPNLKVADIVTMNGT
NINGMIRKEGTILNITSVTGLEVPPFPGEAVYHTNKAAQEAFTNALRNELTGTNIKVLALRPGCVA
ANFHSLRVGHDKEMYDSFFEGYTPLESEDIANAAVYMLSQPLNISVKAMDVVPSAQRSLNVFDRAW
NERNGL
>g2714
MATSLHPVFRKGATALITGAASGIGLATAKLCYSQRMNLILLDNNKDNLSKLSTEFPSGSDATTST
FEIDVSKLQDWDRVQRDVLALHPGGVDLLMLNAGNSFKPAAGKTTWQDPEYFQKTFAVNTFGYTNG
IAAFIDDVTSNKIDPRAIILTGSKQGITNPPGNPAYNASKSAVKTIAEHLSFDLHASNPNISVHLL
VPGWTYTGLTKGSSSSKPDGAWTSEEVVNFMSAKMAAGQFYILCPDNDVTEDLDKRRITWGYGDII
HGRPPLSRWRGDWKEKAAEGIAGLQIPPQ
>g2715
MQLFDVILNNTGSSTWLTSTDELQIFIESANLTTVQPAVIKRLQPGGSATVQVGVQSAPGVTENTQ
GPATAAARWRKGQHTTSLEFNATYGLPTYEASPVSVNLHEAPDWFRGAKYGIFIHWGIYSVPAYGS
TGVNESYAEWYWNHQMNPNDKTKTYQYHLEKYGADFLYDDFIANFTVEKWDPKGWVDLINDAGAKY
FVPTSKHHEGFALFDMPSNVSERNSVKQVPHRDLIRELFDAAKQXQPHLHRGTYYSLPEWYNPAYS
KYDTGTFGVGPPRNPITNKTVPYTGFVEVDDYLTGIQLPQMNILAYNYDTEIMWCDIGGPSLSDEF
AANWLNYAFQHNKPVTFNNRCGKVNGVQIKGDYKTPEYASTNTLSPQHWEACRGMDPFSFGYNAFT
PDDEYMNASTIVTTLVDIVSKNGNFLLDIGPRSDGTIADIMQTNLREAGLWIKAHAESLFDTKYWP
MGAGSGNLRYTTTDDAFYIHLLKKPDGTVRITDPVPYLEGDKVTVVGGEKNGSVMGSRLDGGSLVL
DVTDEIASADEYVWTFKITY
>g2716
MLPTTILLLLPVLQLALAAPVVDLESRQAPCDSVHIFLARGTDEPYPGRQSSLVEAICFGLPSCGY
EDIVYPAVYSPYCNSVSTGVSDGTYQVAAYASRCPGSKLVLSGYSQGAQIVGDILGGGGGTFFNNC
TEPTTSGIGPGTFPGNQIAAALLFGDVRHVPYQPYNTGTGAGANGIYPRTDAQLFSLNRFSNILRS
WCLATDPVCARGLDGNSHTTYFDLFSEEAGAWVKSKL
>g2717
MRSKILISAAVLSPIALAQAAVFIPDSSCATCAGLGSLPVPYNLSTSQTNAIDEGASNSFWISSFL
RGSNGHQYFLLSHVLDGSPASYRASVLDITNPSIRAQFSTRDTANXSLYSADTGVFNFTTSNFSFG
ATSETDGLSKMRTWSNYRGVQFDLTFETSSPVLLNGGTGSFEATGGTGYEWSMPAGKTTGSLTLDG
TTITIDSLHSKTWYDREWGNVAESWYWFEIHIEPMPARYPNGAVLSVWNWKDDISGSKNFATVRDG
VTGTQSVVPVTNFTLNTDSVWTSDNTGFKWVQDFELELSDGTSLHISSIRDDQELWDEADAAADSG

FEGYMTVTGSYKGLSGACGFAVTSGVSTPLRMGIDRGASVKEFLTVYNTLRYERTSAAQLMGFMTH
QVHHETDWEAVAKDVDPFILFGVKHVIYTDSKTSARFYIHPNPSPVKLQINNIPKVSLYCILL
>g2718
MSERMNDAPEPIAIIGTSCRFPGDCDSPLKLWELLKQPVDLVAEIPDCRFNSKGFYHENPEHTGTT
NVTKAYLLDADPFVFDNDFFGISAKEAESMDPQQRIILEVIYECLESAGYSISQLRGSSTGFFVGQ
MSDDYREMILRDVDCHPQYTATGSARSILANRVSYVFDWKGPSLNIDTACSSSLAALHLAVQSLRS
GETDMAVVAGVNLVLNPETFSFLSSLHMISSSAQCRMWDASADGYGRGEGFAAVVIKKLSTALADG
DDIESVIRNTAMNQDGRSAGLTVPSAIAQTDLIQSAYAKCGLDCANEEDRCQYFEAHGTGTPTGDP
KEAEGISMAFFPHQKRVESENAKIYVGSIKTVIGHLEGTAGLASLLKASQAVKHALIPPNLHFYQL
NPDIEPYYQYLEVPISLKPWPKLRPGTPRRADVNSFGFGGTNVHTIIESWDSSSRTAKSLSEQEPT
WGPFVLSAHSYKALVGTVVSLSAFLKTNADIDLHRLAWTQQCRRTHFKYRVSFSTNSKNELIDRLD
LALKNNEKSPLATQATKVPKISILGIFTGQGAQWSSMGKGLFLHSVSFRRTITQLESILKDVHNPP
SWSLAEELLRADDPGRISSAEISQPLCTALQVALVDLLKQCGIIFSAVVGHSSGEIAAAYAAGIIS
ARDAIIIAYYRGYHCRKVRDSSGRSGKMMAVGMTLQDAQRFCSQPCFLGRIVVAAENSRSSVTMSG
DFDAIDEAKITLDQNVVFARILKVDTAYHSHHMKTVRDQYFTSLRKANIQPLRNCFAGTCNWYSSV
YCSSNRKDVSTPTAFEHEYWVDNMVNPVLFYQAITSAVQKEDFELGLEIGPHPSLRGSAIETIKSV
SGRDILYRGVLERNKDAFDCFTNLLGYVWKNFDSETRIIDFAGFQEACNGPNWSMPRVHKDLPTYS
WDHDKPMFKESRISKERRMRKTTFHELLGRPISSRSSREVRWRNILKFEDVEWLQGHQFQNQILIP
ASAYLVMAADAAAHLFGQGLTIQLIELQDILIHNGVTLKEGCSGVDMVFDIKLIHEDISRKVAEFS
CVCRNVDAASTEFEKEVFTGRVMVTLGPVTENILPKSVASSLPMTDVNIDRFYFWMQKAGLQYLDP
FVLQSIVRRLNCATVITKRNAAGQYIVHPATLDSILQGLYAAFSYPGDGRMWTTYLPKSFRRVRFA
MNGYGKLYNEANSQLTANCYLSESSARSIRGDIDIFCQNGHTEIQIQGAFFSSLDVPSAANDRTMF
WKTTWKRGLSSIVELDSMKESGSGEFQLHEICERTAFYYLRKLCEEVEICDIGPMDWELRSLTQWA
RDYVLPQIRGGRLHKCQNSDQIDLKLIHHLGSKFPNILRGEEPTLQILKEDNMLDTLYTEGLGFRQ
SNQHLGNILNLLSHQYPRMRVLEIGAGTGGSTATALRHLSSSFENYTFTDISPAFFSVAQTRFAEF
EDAMRFQILDVERPPAEQGFQPQSYDLIIAAHVLHATKSIAHTVRHCRELLRPGGHMILLEITNPK
TLRIPFLFSTLPGWWLGREDGRNQGPTLTEAQWNSFLRDHSFSGVDCSFRDFHDDNMQTFSVMVTQ
AIDDRVCLLRDPLSLGNSVVRIENLLVIGGHTLVLSKLATKIRTLLSPFVEQIVVLQNIEEVPQNG
LKYGSAILSLSGLEEATFSQMNKTRLSSIQKLFRDAKYILLATRGCRDEDPYASMLVGVARTVSRE
AEHLKLRLVDLDYLNTQNYQSVAIMLSEMLLRMIFLDLPGYSEIFWSDETEVAIEEGAVLIPRVVP
DDKSNDIFSSARHKNTRSVCPTSHPIQLHIGDNGVHVTAEIEADKKQEDSNISHVLSSSLFCFVCS
DDDQPFYVCLAQAAVDDTSKLILTLSETNASIIKNKDSSQIAYEGYACADDFLSWILTTICCESIL
LSGTGTIWIHNADDYVGELICNLANRGKIPVSLTTSHITSRLVSIGKASYIHPRATERDCRSRFPR
DLGRFVNMGTDTDSLVEFVTSSQGQIIAVERGIHDVSVKEKISLSQNKFALLKSLEYYCSNSDSLN
VLGHLETKSVVKASQLEKFSKIVNATSVISWTGVQSVPVPITPAVDRACFAADKTYFLVGLTGDVG
LSLCQWMVDHGCKYLAIASRMPNVPLEICKNLKEQGITLRIFTLDVTDMTSLKSVHRDIVSSMPPI
AGVANGALVVRDRPFDSMSLEDMMTVLKPKVIGSDNLDRLFFSTALDFFILLGSVSSIVGHTGQAN
YHAANLFMSTLAKRRRKRGLASSVMHFGMLLGFGFLRGPAEASLEARFQREDLHAISEPEFHAVFA
QAIISGRPESGLDSEIIVGLGTEVHTPWRSIPRFGHCRVKSNEDRRGKSSSRHEGHSMKSIKDDLR
AAVNEKETLFILKEAIACRLSLALGSPSGKIDNKVGLIRLGIDSLVAVEIRSWLLRILQIDMPVLK
FLSGSSLSDICHDVLNELPDSLKPWVNEKDDDINRSDKQVRGLNLENTAMDSRISPTVSDIDKTKT
ERTDATLVEESTSGLDVALNLSSQATMANGKSQEAVKIKYERIGEMSHAQAELYFLHEYLQSNAYN
VAYYGRFNGQIDLKRLERALWLVGKLHEALRSAYFLDKTTAKSVQAVLSSPRIMLTHTTVSGNNDV
QKEISAVKDFQFEIEKGIVMRVVVMSHSPTVHTLVISHHHIALDGLAWGVFIADLAQAYAGCLSKK
STQIPQSIDIARRKSKENTPEILQDDLIFWEDIYRTIPEPLPLFPFARHKTSPVIADHSVDINDFS
LPIEMRTMVEITAARIGVTPFHFHLATFVTFLSHCLGVEDVAIGVVDANRTNAEDMETIGYFLNML
PVRIVLDQSEPFDVVAKRLFNATLTATTHSHVPLDAIIGKLGVPRLESRHPLFQVAINYRKSLFDE
IELGTDGKIQWDGGVPGGNPYDMLLNVVATPNSSIVSIITQRNFYRSSDGALMLKWYTRALEAVAR
DPQCEIGRCPISNEFDISDAIELGLGDRMEVSWKGKITDRIDAVAAALPEAIAVRDEDGHCLTYSE
MTERVTEITRQLFSLVSGSYVAVLLDPVADAVCCVLAILRLNAVWIPLDTRNHPRRLQAVVEESRP
QFLIYHDATQEVAKQITRSLNRAHLLNIDGKKLSHVESKDNLTTLTNESDGGGTWNGQLAMILYTS
GSTGVPKGVMLTHEGLLNQIYGTTTTFNLGREITLQQSPLGFDLMLDQIFLALTNGGTVIMVGKPG

RGDPTHIADLIVRHGVTLTHFVPSEYRALLNYGKHILTRAHSWRYAMSGGEKLTSDLRRAFRNLHL
DDLKLINVYGPAEITLACARGIIPYEDVDDSTDHLRPSPNYGVHIMDEDMNIMPVGFPGEICISGR
GVGIGYLQRPEESAAKFVQKSPDMLPSPSVKLYRSGDKGRILPNGTLEVLGRQDGDSQVKINGFRV
ELDEIANSIVRISNGVIVSAATSLRTGQPSDLLVAFVVFDIEYTGDMTEFIEGIRSDLPLPPFMRP
NFIIPIDKIPVTTNGKTDRCAIDVLPIPELLASTNVKSVTHSLTSSEKLLKEVWEEVLSTQIAHLL
GNTPHQQLTIHSGSDFFQIGGSSILMIKLKALVELHFGVMISMPELFRASTLHGMATLLGAFLGKR
QGVSSVQTEASFLSPQGNQSRMDWDLEISSLVDGLNVPYSVPSPKKHSNGEGGLIVVLTGATGFIG
RHLLSKLVQNIKVAQIHCLAIRPDTTGARRHVSVKSDKIVEYSGDLSTLTFGLSDSEFSFLMSNAD
CIIHNGADVSLLKTYQSLRRANVISTRTLCQMAIPRRIPLHYISTASVAKAIKNDKQEPLYEVPAS
PIVTDILNSIDGYAASKWVSETLIDKAAADGELPAYIHRLAHVVGNDVSDLDAVGMLTKYSVMMKT
LPRIEKGRIKGQWDFVTVQEVARHIVESAIGSTDSIFSKTTFMNHCSEVKVPHESLKAYLEEFSGI
GLEEVHMKEWLRLAQEKGLHPLVYEFLTALEEGQGQMVLPMIAKGLGPGKSNGRVTI
>g2719
MAELREGQRLKYTVTHHRQPHLTHEEFMKWIVDEHLPLAMPVFKKYGVLAYTLYDXPASMNEPLKE
AMIKVRPTWDFADYDCIIEYLMPSPEAMMNMQQDPEWQASIKDQEKWVNVSKALLTVGYAVPYLTE
SGEVMNMTK
>g2720
MTAQSPPQRGWRFWAIMTSMAVASLLAGLDATAVSTAMPSIIYDLGTSQGYMWIANAYLLTTAAFT
PIFGQTANIFGRRALTLFAIIVFAVGSAIAGPSPSLGALVAGRAIQGIGCAGINTMVEMVVCDLVP
LRERGKFMGFIFAIYAISTTIGPIVGGAFASSVTWRWIYYLNLPLAGLTLVVVLVSLSALPSGSSM
SMRSLKRVDFTGNALLVGSITSILLAMTWGGGEQPWSSWRTILPLVLGLLGLPLFLLYEIRVPEPT
TPLRLFANRTSLVGLWCAFIHNMLVYWILFVLPIYFQAVLRFSAFRSGLSLLPTAAVCMPFTIISG
GIMSKIGRYRPLLIAGFALFPIALGLFTRLDGHSTTGYWAGIQIIGAVAIGIGIPVTLPTILTPLP
ESDIAVATATWAFMRGFGTIWGVAIPLAIFNTKVNSLAAGRLAENWEARTKLTNGGAYTLAAGGFL
YPELGLDGDADLEATVKSIYVDSLKLCWQVSLGFALLGFLLTFVAKELHMRTELETEFGLENESDK
KETVQAAA
>g2721
MSSMLLLSLLPLLPRQAIDLDGPTIPPPSGSVSNFDSPPNGNHIAIPIIILCAVISAISYSIRIYA
KYVMKKVNTSDYLAVIAFPLYWVYIYYSYRLSETSGYLVHEWDIRLKDISAFSYICWLATLLYLWI
IALVKCAIMLEWTRIFAPEGTNTYLSWSWYATGAAISLLSTIIFIMDLVNCTPFSGNWDVLVAGRH
CRFSVPAFGLASSIVNLTLEIIPLVLVQKVIWGLHMSWKKKLGWLRCKYRQIILRNPILRLK
>g2722
MGRSEEDIAWEKSEGIPQVEDPFIQKYLNGRDALIEQEHKQRHDFAFRSSMSAVAKEACRIVSTIR
EKELREIWTKDNKGPDDELLYPGMMFTVARERMEQTKLWKILEQMPKGALLHAHMDAMFDLDFLID
QAFETPGIHMSAPAPLLTKDDLEIAPFTFQYLPHQLEDSDNLEPTWSKDYKPASFIHIQKSAATFP
DGGEAGFRQWLKNRMMITADHSYYHHHGVNAIWEIFVRVFPILQSIIFYEPIFRTCFRRMLAQLAK
DGIRYVDFRAAFVFEWRKTGSEKTESDFLEFFRVFKDEIEKFKASEEGKGFYGARMIWTTVRAFSN
RDIVDSMEQCIIAKHEYPDIICGFDLVGQEDNGRTLVDLVPLLFWFRKVCAEEGVEIPFFFHAGEC
LGDGDETDKNLYDAILLGTRRIGHGYSLYKHPLLINLVKEKKILIECCPISNEILRLSSTIKHHSL
PALLSRGVSVSLNNDDPAILGHGSNGLTHDFWQTLQGLENLGLSGLATMAENSIRWSCFEDQTTAE
WQTDIKQGIAGQGIKAKRLQEWYQDFEKFCQWVVLEFADEIDD
>g2723
MDYFLPSFVQKRLLRYALSRLELVDTEAIDLDSLGIRWGQRSTFELRELGLRLDKLTSILRLPATC
KLLKARASLIRVTIPADIYSSGIIIEVNGVDVHIQLLSGSELGNDTPTPGESDQSDGPALPTTNDL
AQSFIAAEPREERAEIEAAISSQSQYLQRSTTSLSDEDEVGLGNEEVSLPSFISGFLQGVIGRLQI
KVNNVNVRVDMDLPQDGPIKRIPTDKPGQLTVSLVVGELSIGGVTQQEGQTAPPNATGRKITLDSI
GVFLISDSVVFSNYARFAAAPSSPSTTTSNPGHNIRQARSVSPSQSASISEGSGLGLAQSMMLGRS
QVLERSATPTASGRRSLENSINSLDGRFSDADSEAGYGYQGQSHSLSVSRFDGFNDEVLDNPGYLD
EVFHSQFVDDPEDSTELPPRSPEDTGTPRPQSPQIDSSQVETWHDFDEPQSQSQHEYTVEDTHFYD
PLGQSAQIESFPEATHSFSPEPIQQSYYADLPTTPSSPEEDVKSQSSQSSSHSGSLPAELSESKLF
SHEEASSMYMSAVSGTSSHGEPVMPGAWDSRNFESPTHEGETVDESVTRSNQEAGASTPKVGVESP
SFINKNNDAPRTPTRESYQPDFQHHTSAAVKTHTEELEVTRRIIDISRIVVWVPENVDNSQQEPQE

QLQPQYPGTTMAQSIDSDALGISRMDFSTSMRRSSITSSIAIDSGEQFGNRNADVAGPLSFSNAPR
AICVDVGETVVQFDIATGWLLVKASQKVITTFSVEHSEANVSKNNQKSSSTEYTHFKFSLQSCSVK
FVESLAGGSYYTRVKAPESPQSTSSTGEDVILQVQILSITSNYLSANDTMKFQLNVTKFVLGCATE
PLISFNKDLKMRESVRDVRTPVPRDVTLTIIKTPSSGKVEFATLPLHINFNIQKLEEIFGMFGGLS
TLMELGSSMASVSTVRGDKSDSIKKSTRGVRFEGVPSPKAPSPITTTSAPWKINARLDGIILDLVG
ETCRVELVTSAVKAVSRFEGIGIQIDKANVVGPLLLDNDADRPATLSIGNIRIEYLYSPKEVDLDR
LLSLLTPSKDKYEDDDDIMLDTLFRQRRQGGVLRVTASTAKLTIPDIEGLKPLSDLPNEIARLSSV
TKYLPEDDRPGVMTLVLIREFESHTHVNERVGDLRLATKDTELAQIGIPSLVAAQVKSISLSRNGT
EELVGEALDIAAGQSHISSSLPMLMVRFIADEMDPTVKVKLHGFRVEYTLPSIIAFSGLGNDATSD
DLASKMASSVANLTEISAPQFHGPLFGEFGGSEPDTKPFKLAMSLRDCVVGLNPRDMPAKGLLVFT
YAKFECTNQDKGPFEANFEIKKATLMIIDDVQMIGSSDSTGNRTIGESQEEQVNKLGKMGFVPVSY
ISSAVLSINVQPPDSVGDKSVDVEFRDDLLILETCADSTQTLISLLNGLSPPSLPSASLKYRTEVM
PLQEMLSSFTGDAFTTDQVTNAEEISVHEAEENFEGALDDEVDYVSEFYPQEPGFEGPISASMMDP
MADMSRSRQLMDSFHSQYTVSTSVTGLEFQDDHFAKKSTVGGTAHRWNTTQNTYGLANEVKLHGSP
LRVRVRDVHFIWNLFDGYDWQRTRDTISKAVKDVEMKAAERRAKTASRLSPEDEEEESVIGDFLFN
SIYIGIPANKDPRELSRAINHDIDDMASETGSYVTSTTVTGYTSHQNRPSSTRGKKLRLSRSKHHK
MTFELKGISADFVVFPPDAGETQSTIDVRVNDLEIFDHIPTSTWKKFATYMNDAGVRESGTSMVHL
EIINVRPVAELAASEIVLKATVLPLRLHVDQDALDFLSRFFEFKDESAPAPSSPGDTPFLQRVEVN
AVRVKLDFKPKRVDYAGLRSGRTTEFMNFFILDEADMVMRHVIIYGVSGFERLGQTLNDIWMPDIK
RNQLPGVLAGLAPIRSLVNVGSGVKDLVVVPMREYKKDGRIVRSIQKGAFSFAKTTTNELIKLGAK
LAIGTQTVLQSAEDFLSTPIPGAAHGEGDGDSTDEEEKKQISHYADQPVGVVQGLRGAFRSLERDL
LMTRDAIVAVPGEVMESTSAAGAAKAVWRRAPTVILRPAIGATRAVGQTLLGAGNSLDPTNRRRIE
DKYKRH
>g2724
MEATTTMASLAISRKSANDLWAEAVEQLNDSDKSAVNFSHDKLTVLVKLQLLAEKSKDECIQRRWK
YTRKSGETVILRDVFEKMIAWIKTFEKVGSSAVQYDPVHAALPWAGIRFILQVAINNHDKFGEVME
GLAEIAEIICRHAILEDLYCGQMSIAAGELQRALVVLYATVLKYLSKARAYVKQRSIERMLKSGFL
PTSELDDYLEKTRKAEINVRSCASLVDKQENTVHHSKMIQLLEYMETPLRKMDENLRHIEDNLQES
KRRAILEWMSQEPYIQHHEQNKQGVLSGTGQWLLADSEFVKWKNERASSILWLHGFLGSGKSKLVS
IVIEDALRGFRSGQTIRPVYFYCSRNKAEPGRSDPSNILASIARQLSSLEPGTPLLSPTLDLYRKK
EVEGFMSGPMGLEESCSLIIELTARYSLTIIVVDALDECERQKKYDLLKALERILQESSGLVKVFV
SSRNDQDFVVRLKHYPNLEISSEKNGADIALFVQTETERLVREEGLLQYSDHPIEMKNLIVKKVTD
GARGMFRWASVQLQFLCSLNMDVDIRNNLGRLPPDLDTIYAELYCILSEKSGEMEKRLFKNVLSWL
LCARLTLQTSAFLEAVSVPTEGNDSRVRVTKELVLKICGSLVVFDAELNTFRFAHLSVREFLERRQ
EYTTTATNALIAGNSLRRLMSANIPSEVDGLLSKHGLYSNHDVPDRDRFVQYSYMYWCLHFKLAGH
RRISGDLKETFQRFILSADIPSGPIKSWLDTLPGVLSERYHCTQNEIKLRDTITTSGSTVSGVLFL
ACIFDFFEVLEVLKAEWMKSGHFLNAQNRTLFDVSVSYGNCGSLKFLLDAASNMNVSENLVETAAW
SESNGEEIMRILLDKRGRKIQITPKIVQTAAQNWQCGEEIIRILLDKRGTEIQITPKIVQTAAKNW
KNGGKIMKLLLDKRGTEIQITPDVVLAAVDNRGNGEEIMKVLLDKRGGEIRITPDVLRVVARNRGS
GEKIMRLLLAKRDSKIQITSDFILAAARMRVPERIMNLLLEKCDSVIRITPDMVRAAVENGRIGEK
VIRLLLTEHFSEVQITADIVQAVARMSGSEKFFELLLDKRGSEIQITPKIVQAAAENRQSGEEVMR
ILLDKRGSEIQITPDVVEAAAKNWQSGKKLVELLLDKRGTEIQISPDVVLAAANNGLHGEQIMRLL
FDKCDSKIQITLEIAQTAARNWRNGEKIMEILLDKRGAEIQITPEVFRAVPFNWEWELGLWL
>g2725
MYLIVVSAWIFACVAASGKGLIHFPRNDSKPVQSAPRTVGYFGNWDIYASSPYYITNIPGSNLTHL
IYSFANVNSTTGEVYLSDTWADLQYPYPGDDKTATGNNVYGNIKQLYLWKKEQRNLKTMLSIGGGT
YSTNIVPVLANDTLRQKFADSAVTLLANLGFDGLDIDYESVSDSVQAEQFVDLLNKTRTALDTFAA
NISAAPFSLSFASPGGSSFYDLLNFSAMDQYLDFWNFMGYAYTGSWNTYSGHQASLYNSSKNPLST
PVDTHTGILYYISEGVTPDKINLGCPLYGTSFNNTSGPGTTFDGIGTLGTDGAAGLWYYNSLPVPG
FNATTYNLPDIGASYSYDPVKKYMISYDTAKVAAVKAQYVLDMGLGGTMWWEVSQDKTDDSSLIGT
TVETYGGQSALDQSLNHLNYSTSVYDNLRAGFPGETIANSTATSTSTTTTATPSWTSSVTSSLVEP
STVPDCTEYHLVVTGDTCSKIESEYGITNEEFAQWNPYSGSTCENLWLGFYVCVQGPTSTTSSTPA

ISSATTTAAPSPAEPSTISSCTSYHLVVSGDTCYSIETEYGITVNEFSEWNPYIGADCMNLWLGFY
VCVQGPTSSTSSSAPVTSSASAISSFTPVTMPATTTTSATTTISTTTPPSPTEPSTISSCTDYHLV
VSGDTCYLIEQTYDITADQFNEWNPYVGSDCSNLWLTFYVCVGAPATSSSSSLSRKWRPIRNFASH
SRTSSDSTLPSTSAPTFKVKHNGAEFEILNPKSTSSFDFGEVSTYRLSNLFKNTMEEESPDISPTH
SFMSYALPDDMIRPLSASESAVLSKYTTPPLSPGLPQSNHSFAALPPLKESPSSPVSPRSPKTPAR
TLKQKVSRLFGGSDKRSSPRYEIDLNEKFDYRNEQDVPHSPLSYHWEAEDSVKILHYPGTAVDTTS
SDYEGNNSSKHDSEDVHSRRRPTWGPHSSNRHQTLQDALRQLETDNNGLTFEDIMRYIDAEQCEDR
AGNGDGDQALDEKVERPWQPDTWNEPTLLRMPQNRPSNSNLSTNAPSTRASIRPYVERAAAFMAGG
ADGXSSSSSSLDPAEETHEALSKNVIHGNDYGKDEDEEEARTEDDYEPIPPLRRSISRRARPLSSN
PPDLDTPLLFESPSSSSSSSLSLVSNVRAITPPPRRTATPQPLTRISWTPEIPARHPDHRLVRFEY
DATTINPRAGTPLPPLISTTSPQAAGPRVVTTSMAPRKKAKKHYLKKSFKV
>g2726
MFENFSFPSPSSTAEGDDRLMLDCDSTTISPLSSRCPSPSSYLSRRSRPLSRPRSPYHRRPQQPPT
SMPSNYEQFQRRISVSTLTEKLNAHTLDQNTTPVLAHAPYESPLSPLSPTSFSFSVTKDSASSRSS
VRTLLTPPGEYEDEGYYDQLTPGWEPYHPGHSPTSIPLEHDGFLDVPHPRFPRDPSLRLQRQQMSR
AQCNIDVLKLALLAEDFTRRGSESVGLSDEECHPSSLPPNTSPPSSLRRASIKPRGGSAASPAGNA
RYRSRTSVDPGMLRRRSTSAGTFASSTRIAKPRARELLSKKSEQTLRRKSLVCATLASAMCEVPTS
RSSSPVQ
>g2727
MTTLLKQPLKLALIQLASGADKSVNLSHARVKVLEAAKAGAKLIVLPECFNSPYGTQYFPKYAETL
LPSPPTEDQSPSYHALSKLAAEAKSYLVGGSIPELEPETKKYYNTSLVFSPTGALIGTHRKTHLFD
IDIPGKITFKESEVLSPGNKVTILDLPEYGKIGLAICYDVRFPELAMVAARKGAFILVYPGAFNTT
TGPMHWSLLGRARAIDNQVYVAMCSPARDINASYHAYGHTFIASPNAEVLGELDEKEDIVYADLTG
EAIDSARKGIPVYTQRRFDVYPDLDDDDNKRRQSGTSTKMSSTTKGKKSGRSAIADVVSREYTIHL
HKRVHGVSFKKRAPRAVKEIRAFATQAMGTKDVRLDPQLNKKVWEAGIKGVPFRLRVRISRKRNDE
EGAKEKLYSYVQAVNVKEAKGLNTTVVEDA
>g2728
MVESLIEAGFLQQQERHAAISRLLLEGLERRRSQNLRALQVSXATNTRVNGGNTLSASQPQPQQQP
QQQPQQQQQQQPQQPQQVGPTASQVPEGPVEFSDSEDESDYEDGSDVDGESDSDTSKSATTPKATN
AKEQSETRKFGESEESFCLIKDLNKRRARPNQPSAPQAPFSFGETSGATAASSSSTASNGFSFGQS
QSFPAPSASGATQNAAQSLSFGAGAPASFNFGGFGATPASNPFANLNGGNTQTQTQTQASNVFGAQ
SSTQPAQPSGQGIFGQSSNTAFQTQPASTGANPFAQSTATAPASPSPFQTQPASTGSSLFGQTKPA
STGANLFGQASSAAPASTSTATTTAPGSLFGQQSASAVPSAPLFQSKPATTAGSSLFGQNPPATST
GTMNMFGQSVGASSAGATSATATSGPANLFGQTPSVAGSPLFQPKPATTGTTNMFGQTDTASSPGA
SLFGQSAAAPSTPNKASASLFGNAPTTASNPFNFGQTTSTTQKPLFNVNSDTDDMSTSPDGKRKVS
EQPSAPLNIFGQKPAQTSTPSENSRPASSLFGAPTISATANPPSPSLSTSSNIFGQQQSRSVSPAP
NGVTTAPSSVFAPTTTASAPSNPFGSLFAGSSKSKTTETSPASATSVSAAAPAQSFFAATSKPATT
TTPATTAPAATSTTQSLFAAASQPAQAKPLFAPQSSPDKRLGGAIMDQAISKKAGVAQAPAEASLE
SDKRAQIIDLNRGFKEHLASYDPETQSLDSIIIYYIKMRKVIGAPVGSLTNKPGKRKLDDTHGIES
PVQRPTAKKSKAQEATPEPELPSVRHSTADKRKSSEDLDDSPNGKRRAQPYVRPTTQGAGKSETAS
IFASSFSAPKAAXPASTASSSSNLFSVGSTASPAKETLRAASPQKEEPAAKPAFEIPKFGNQNFAG
QFGKGSFQASSEAPAVKLDLPKFQVSGQSFMNQFGQQAKGDDDDTTDEDSDSDAEPVVEKKVEKKV
EKPSSAQPQPNFFSGSVFDSKPGTPSIGTNNIFGHLNTSTATSDAAESSDDDLTEQLQKRAKKSQP
DAETEPKAASSLFGRITRPDGTPVNLSSDEEKAETPKPKPLFGQSTSSPFLAAPNNTPLFSSTSTP
KSSSNIFGGSSTPSTTFNPFSSNLNPAAQFLAKEGTATASVTSDSNAEDTDTETVKDTQLNLLTNA
GEEDEDCLYEVRCKGLKMVEKVGDSGKIEKVWEVQGLGPLRVLVNRETKRARLLLRADPSGKAVLN
TSINRSIDYKVQPGSCSFLAPRTDGSGFDSWALRFRKEWTAEFENAVKTAKAGLPN
>g2729
MGARTFEGGDHHEDATNALSTKSNXVGGKPRGANMLEDGDGEFGSDDDDDGDGQDPSLAMVNPEDG
SKPKKRKRSKKKKSNKKKSAAGAQKQTSPPRVPLSQLFPDGKYPVGQMVEVPDENLRRTTDEELRY
LSRGTVTNDEALSDYRKAAETHRQVRQWVHETVKPGQSLTELATGIEDGVRALLGHQGLEPGDSLK
GGMGFPTGLALNNCAAHYTPNPGQKDIILKKEDVMKVDFGVHVNGWIVDSAFTVTFDPVYDNLVAA

VKDATNTGLRCAGVDARVGEIGGYIQEAMESYEVEINGKVHPVKAIRNITGHDILPYRIHGGKQVP
FIKSKDQTKMEEGEVFAIETFGTTGKGYMMDGPGVYGYSKEPDARNVHLPLASARALLKTINQNFG
TIPFCRRYLDRLGLDKYLLGMNSLISHGIVQMYPPLVDIPGSYTAQFEHTILIKSSGNEIISRGDD
Y
>g2730
MSREKFNEEHDVQIELASTPGADMGRPLNEQVEGSEHHGITAKIIAVYLSVVIIAFSGLVNIIGAG
AYSRDVAASLGGSSISIWLSQVSAITITILGPIIAQAADYWGRKWFVVLTTLLGFVGCIVVSRANS
MGVAIVGQIIAALGYSSQALLNAIVSEILPRRLRPAAQGGLGLTSGLGAAFSLIFGFHMVVVSTDG
WRIYWYVTAGLMFIAVILFVIAYNPSPRPLQAALTTAEKIQRLDWTAYALLAAGLVPLNMALTWSD
NPYPWSDAHVSATFAVGMVAFIAFGIHQTLIKKDGLCHHDLFRKDRNCAISFLCIFMEGLSFFSVN
NFFPAEMSVLYEPDTFRVGLRASITFFAAVVSAFAVSVYSTITKDIKNPIIVAYTLIVLYTILMAT
ATLDSATAVWAYPLFLGLGFGAAISNVTTAAQLSAPPSLIAVTSGLIAGTRSLGGSIALPVLNSIF
QSTLSKHMGANIAAAVLPLGLSSENLSQFITALSSDDQAALASIPGVTPATISAGLEALKATYLVA
YRYVWITAGAFSAVAVVAACFLINPKEDLNTSIDAPLQDENQEQLRNSPRSKTLVDDIHNRHRSRR
RPAHAAAEEFLSSEKATEGSCVPEPPTTNSADISIPRANSADQSPSTLTERLQNVHKGLVLSLSSV
VTSLSLETVVARCVDMYMQNTFCLHPIVHEGSLRAVMNLDTLVVQEVAAQPRTRSRDGSLHLLQSY
ALVTALCAATAYLLSPQTDVDGRLVGPVFLRVSRETLSIYHDLDLQQPESSSLAIRMFQAAALHTD
GKHELAWHLFGEALRLADQMRLYDEQSFGLLDPLEGRLRRTAYWDLTAYAQYHHILEEYSLVTHTW
NRERTTAMRLDYGPTDMFEEQLFAGFFAFHRLWEAASGLLLDLESYARVRRLTADAITLLEIQQNG
LTESYVRFLTVLDHLPACIHYPDAGVNTENANITLSQRRQFWIQRTNLFVTYHCLRMVLVTRISHL
GLGVLIGIQDAEPMLALRKTEVAHDMICLITSVPLEILRVMGETCISKIRHVGASLLEIMNQVPAT
PATVRAKSLLPVLLDVLTRLDSRRNVSHLVNLNHSPIV
>g2731
MSELEANVPYRNSALRVEERVDDLIGRMILEEKAGMMFHDLIFAGPDGTLAGAIPQFDFPSSEEVI
RQKLMNHFNLLGPISDARAAARWYNTVQREARKTRLGIPVTISSDPRNHFTDNVGTSFSAGVFSQW
PEMLGLAALRSADLVERFGHIARQEYLAVGIRLALHPQVDLATEPRWARLNAGFGEDADLSSELVA
AYIHGFQNGAVLNEHSVSTMTKHFPGGGPQKDGEDPHFVYGREQIYPGNNFGYHLKPFQAAIDAGG
AQIMPYYGMPVGTKYEEVGFAFNKALMTGLLREEMGFQGIVCTDWGLLTDKKILGQDMPARAWGLE
HLDRSERLLRILDAGCDQLGGESCVELLVQLVEAGRVSIDRLDQSVKRLLSAKFLLGLFDNPFVDE
EAAAGIVGNSQFSAMGYDAQCRSYTLLTNRQSCLPLKPTSKSQLGETKTLKVYVEGIPIYIVQARG
MTVVAAPEDAEVALLRLKTPFEPRPGPFEALFHTGSLEFSPEEKIRQSRIYDTVPIVIVDIYADRP
PVVPEIIEAATAVLISYGSTGDAFLDIVLGISAPEGQLPFDIPSSMDAIRQSKSDTPFDTENPLFR
FGHGLRYGMQEV
>g2732
MQLTILSDKLPTLSETQFAHEFRTVHAQQTRAIAINLGIILKYVQGLALPTVGRPKLINLPLEELQ
YRSFAQLTWPSLEVLQGSFSTEDYRQSAAKHQFARPFQLFLTERDESSISASLNTSSTTLTEIEKT
ATRLVVTVIPKTLSDNENTKFEEQWSRHAQWVCSLGASYTRYRVIPLDETRSRKIFDGTPFDPRLV
VTAGGYDEFMFQSQENAAQFLAEHGPQLRSSYLEFVDIDHSHAYAFDHVVQFGTEDRGTWQTIFGL
LVGSALRVKVFFGI
>g2733
MRWSSILSSALLVAGASAFPHHTHKKPTPSPSASSSVSVSTPSTVPIKAAAAATDPSYWLADITHQ
GNAPFAGSGYTVFRNVKDYGAAGDGVTDDTAAIQSAISDGDRTAPSSNTTTTKTPAIVYFPAGTYV
ISSPIIDYYFTQLVGNPNSPAILQATSSFTGIGLIDGDQYQSNGKEGWISTNVFLRQIRNLVIDMT
NIPGTTAATGIHWPTSQATSLQNVVVNLNADEGTQHTGIFIEDGSAGFIGDVVVNGGLQGLNIGNQ
QFTMRNVTVNNAVTGITQLWDWGWTYVGLTLNNCQIGISFSSNTAQLVGGVNIIDSTISNVETFVN
TSWTPTSSPAGAGNLILENVVLDTVPTAVAGPGTVYLEGGSTTIASYGQGHEYTPTGPSTLSGTYA
AGARPTGLLASGTSNYYTKSKPQYEAYTTDQVVSIRSAGAAGDGTTDDTAAINSALTSAAASGSLV
FFDYGVYLVSDTIYFPPGSKVVGESYPIIAGSGDKFSDASNPYPIVQVGKSGDTGTIEWSDMRVGT
IGGTAGAILIEWNLAGELGSGAWDVHSQIGGWQGSDLQLSQCPTTAAVSTACEAAFLNVHITSDAS
NVYFENNWFWTADHDIEDPTNTQISVYTGRGVLVEGSNIEMWGTGAEHHGLYQFQFNGASNVVGGY
FQTETPYYQPKPDATSGPYQSITTTPWSDPDFSTCLSGNCDALGLHITDSTDITMYGLGLYSFFND
YSTSCSDAGNGENCQSEIFRVDGSVSGLNVYGYQTVGTTNMITINGTSAALYSDNLSVYPDSIALF

SY
>g2734
MSQSFTKFWSTDLTRDSLLSFLDTSTLKSLRLVNRAFAATTSRQLFRDVSITFRSSTFTKPARMAA
LERIGWNIRTVTFVLNHGPEMFLPPLLDPLTGEEVCFVFTPCVRTTPTSSPTACSSSPTGEKEGVY
GTWEMTDLLVKQYPPLFHAAMNVSSFVAALTHMPALRHLKVSCAGQESTHRYRRSVVDYALSSLRI
AIEQAPLPRLEALSLTPIHPGGIQYLCPMAGIGSLPNSCRRWTQIRRLEIHMDTFPFDKGQATDHL
KLLHSYLQAFRALRDFKFRWVGDGQKGPSPLSLATEPALCELAARSQACPKTRHYLRPLKFPNLQY
MVLGNAVLDASQVSDFLTEHRHSLLEFDFEQVSLRSGTWDDALAPWRTLSSAEYDAAWGEKQAVEV
EVMEVPFVLGPSPDEESTPTSSPEQHSKRRKKKKKRHAKSSEVAINNDLFASTAAMLLEEEPVHR
NLNFKTLREGLSNWARSKRILWGSGHSHGHDYLHKAVQSPILV
>g2735
MSVKDDSFPASAAFDAINAALQSDADRKDAIKQGAGVYAFTLTNSSGQQASWHIDLKEKGTVGKGL
GAKPDVTLVLSDDDFASLVAGKANAQRLFMGGKLKIKGNIMKATKLDPILKKSQTKAKL
>g2736
MPAFPPPRNGMAFSNQPGPVGAIARFWARTYAADYVALGVVATGFILIRLFVTPFHRMFYLDNMAI
QFPFAKSERVSMPWCIVYAAVFPALSLLLWAVISRPSAHKLHVSFLGLAVSLITTPVLTDIIKNAV
GRPRPDLISRCIPEAATPEHKLVTFSVCTQANEHILQEGWRSFPSGHSSFAFAGLGFLSLFLAGQL
HVFRPRADLGRCLFAFLPTLGALMIAISRCEDYRHDVWDVTAGAFLGSTVAYFTYRRYYPSLRDRR
CHVPYDLDVNGDGFAKLVDDEERLLHVNALGSERGSDQESPILMDAPRI
>g2737
MDMSGMTMTTTTSATTMAMSMPSSTSTSSSDMSSMSSSDSMSMVMTFFISARSSLFTTTWTPTNDG
QYAGTCIFLIVLAVVLRFLLALRPILESRVWNDHARVTHGGHVLHDDAHAEQQSLKDGGLARVGQV
RYDFSSRWAEWRVNAAAGRATYELLVAGVGYLLMLGVMTMNVGYFLSVLAGIWLGTFILGGAAADN
QWAHC
>g2738
MPMLPWLTSPVMLHSSRDPGECTMTPEQCAYKQRYWVYWYEADHRYSLPTVALFLVAIALFTLARL
ITSLAPRSWKRLSGWTRLTALFRTASYKKVWFLWSAQSVGALMLASVGIIFFLAMTLXPQPYYWPN
TMEINYGNSPPIATRAGFMALACMPFIYMLATKANPITLLTGISHELLNNWHAWAAWAMFVLALVH
TFPFIVYHIRMGDIVEQWNDGGIWVTGVVALLAQAWLTFASIPWLRNRYYEFFKSTHYIAALIFII
FFFFHCDYTLSSWDYFIATAVLYTLSWCYSQCKTYFEYGIGHKARLQRETNDTLKITINTSKVIWT
AGQHVYLRFLAGGITHMLTAHPFTICSMPQLEMTGKTSTLVFYIRPRGGATKFLMAHAINHPNADI
PVLLDGPYGGIPSTQLNLSDSSVVVGGGAGAGFTLSVIEHFLQRCTTTQNRDRILRVVVATRQSDT
RLWYIEALRDISHRYPRFNEAVAGISIDFHETGQSGPEQKTGGAQVDVEKALDPTTVQLNNEDSQS
DSQNPSIAEVFNVSFHRGRPNLPLTIQTFVAGQEDVTVGILACGPSSMSHDVGEAAVAAQQEIIKG
PIKAREVWYHAEDFSY
>g2739
MADAQRALTAAFPPPPPPFWKHFTTDNIEKLEKIKKESASSKSSTGKKWTPSDLRALDVSPELRYLI
PPEAPTEGTYSVFGELQNLSTNLPSLNEQGIEQLYPDVPTDGKDDQNGGQPLNHAFYLLKISKSLL
LNFLEFVGVLSIAPEEFEAKLGDLRNLFINAHHLLNLYRPHQARESLILMMEEQLESTRNQIQEMD
DVKNRVERLLEQLTAEGLDAQQGASQEKDNAGNSAQFDSKTVEEARHLWELLKDEDE
>g2740
MEEDYNHPLSSPTEKMEATSSPSEGHSLNRSQTHHWPLKHKVIATKDREARSGFKARELGVTWQGL
NVDVVSADAAVNENALSQFNIPKLVSESRHKKPLRRILSDSHGCVKPGEMLLVLGRPGSGCTTLLN
MIANRRRGYTSVSGDVRYGSMTPQEAEAYRGQIVMNSEEEIFFPTLTVGQTLDFATRVKIAHHVPQ
DVESQEALRLETKEFLLESMGISHTHDTMVGNEYVRGVSGGERKRVSIIETLATRGSVYCWDNSTR
GLDASTALSYTKAIRAMTDVLGLASIVTLYQAGNGIYDLFDKVLVLDEGKEVFYGPLKEARPYMES
LGFVCRDGANIADYLTGVTVPTERLIRKGYEHTFPRNADMLLEAYKKSDIYPKMTAEYDFPETQRA
KENTETFMEAVAHDKHPQLPKSSPLTSSFATQVKAAVIRQYQILWGDKASFLIKQVSSLVQALIAG
SLFYNAPANSAGLFVKSGALFFSLLYNSLVAMSEVTDSFTGRPVLMKHKAFAMYHPAAFCIAQIAA
DIPIILFQVSIFGIVLYFMVGLTATASAFFTYWIIVIAASMCMTALFRAIGAASATFDAASKISGL
IVTATLMYNGYMIMKPHMHPWFVWLYWIDPLAYGFEALLGNEYKGKTIPCVGNNIIPTGPGYTDSV
YQSCAGVGGAVQGQTFVTGEAYLNSLSYSSSHVWRNFGILWAWWALFVAITIYSTCRWRMSSENGP

SLLIPRENLRTVQQKNILDEEALPQSADSGVSSSANTLAEKTADRSSQPDIDNNLIRNTSVFTWKN
LCYTVKTPSGDRLLLDNVQGWVKPGMLGALMGSSGAGKTTLLDVLAQRKTEGTIHGSVLVDGRPLP
LSFQRSAGYCEQLDVHEPFATVREALEFSALLRQPSDVSRDEKLKYVDVIIDLLELHDIADTLIGK
VGCGLSVEQRKRVTIGVELVSKPSILIFLDEPTSGLDGQSAYNTVRFLRKLADVGQAVLVTIHQPS
AQLFAQFDTLLLLAKGGKTVYFGDIGDNGAIVKEYFGRYGAPCPPEANPAEHMIDVVSGDLSQGRD
WNKVWLESPEFESTNRELDNIIAEAAAKPPGTLDDGREFATPLWEQTKIVTQRMNVALYRNTDYIN
NKFALHIFSALFNGFSFWMIGNTINDLQMRLFTVFQFIFVAPGVIAQLQPLFIERRDIYETREKKS
KMYSWKAFVTGLIVSELPYLCICAVMYFVCWYYTVGFPSDSNKAGATFFVMLMYEFVYTGIGQFVA
AYAPNAVFASLANPLLIGTLVSFCGVLVPYAQIQEFWRYWMYYLNPFNYLMGSMLTFTVWDSPVHC
AEAEYAVFNTPNATTCADYLAEYLSGFGAAAFLENPDATSGCKVCQYSQGSDYLKTLNLKEYYYGW
RDAAIVVIFVLSSYAMVYGLMKLRTKASKKAE
>g2741
MDISLETHQSYIGQPVEALPTPALVISKPVLENNVKKLHDDVEQLGISFRPHVKTLKSIETTRIML
GNGRYKKIVASTLCEIRGCLPLVKEGILDECLYGLPIRPSVLADLDAFRTQHGLKIVLMIDHTDHM
RILEEYNTRTGRTTPWPAFIKVDVGSNRAGIENSSSRLDEVIHTANSSDAVSIYGFYCHAGHSYAS
KTVGDAVKVLNDEINGVLIAAKKLIDPKTELVLSIGATPTAHVVSKLKANLPENASLELHAGNFPA
NDLQQVATGLVSISDQAVRVVAEVCSVYPERNEALINAGVIALTKETSSFTGLARWTDGLDWSIVR
VSQEHGIFGWVGDDSKKEKAEDRFKIGQKVLLYTQHACITSAMFHAFYVVDEQDVVCETWVPWKGW
>g2742
MRHTKTFTAASLALLTSAAAQSADPGLGVNSNAQGATEQVTPGAGPNGSEDWLNTGIDGDGWNPPF
MALGDIIHVDLNTFYSGFGSPCQQYDQYFQSAGSQYNVDPVILAIIAMQESSCNADAGGPTPGLMQ
VSCDNYPNGVCTDSIQDNVNAGTNYLVSQISAFNGNAIEAFGSYNGWFTAGNGLNNNQGLTTSYPC
SSEGQSNGEPQNLDYLQQIFNGWMMGLDVYGDDSWIGEYQCAGQCNGNNLC
>g2743
MLNKAAVAALTTALFSRAYAIDAEIKVDLQTRYQSVDGFGCSQAFQRAEDIFGKYGLSPKNQSYVL
DLMYSEERGAGFTILRNGIGSSNSSISNFMNSIEPFSPGSPTSKPNYTWDHYNSGQFPLSQQARAR
GLPYIYADAWSAPGYMKTNQDENWGGFLCGIEDETCPSGDWRQAYANYLVQYIKFYAESGVPVTHL
GFLNEPQEVVSYASMGSNGTQAAEFVKILGQTLEREGIDIELTCCDGVGWSEQEAMIPGLQVVGSD
GKSAEDYLSVVTGHGYSSAPTFPLSTKRRTWLTEWTDLSGAFTPYTFFADGGAGEGMTWANNIQTA
FVNANVSAFIYWIGAENSTTNSGMINLINDEVIPSKRFWSMASFSKFVRPHAQRVKATSSDASVTV
SAFENTNGVVAIQVINNGTSATSLTIDLGQTHKKVKTVVPWVTSNDYDLEELTKIDVKQNSFVTSV
PARSLTSFVTECD
>g2744
MRLFFCLLALLGSMLATAAPTPGQFSQVTGSWGSNPTNTGFYIYVPKNLASKPGIIVAIHYCTGTA
QAYYQSTPYAQLAEQYGFIVIYPSSPYSGTCWDVSSQAALTHNGGGDSNSIANMVTWTTSQYNADT
SKVFVTGSSSGAMMTNVMAATYPELFNAAVVYSGVAAGCFYSASGQADAWNSTCAQGNVDATPQAW
ASIVKNMYPGYSGSRPRMQIYHGSIDTTLYPENYNETCKEWAGVFGYTYTAPQQVETNTPQSQYTT
TIWGSNLHGIFATGVGHTVPVNGARDMQWFGFA
>g2745
MGSTHHPLDWSSYANVIDGRLSSTAQVRHSINPATGEDNPDVPVSTQDDVEEAMQAAKRAQVGWAA
ISYEDRRKALLAFADAVEAEFKAFSDMLIREQGKPRQFADFEMNIAIKWIRTMTNLDLPTHKIEEE
GKTIVTRYTPLGVVVAIIPWNYPIFLATGKIAPCLVTGNTMIVKPSPFTPCGGLKLVELGQRFFPP
GVLQALSGDDNLGPWLTSHPIPAKISFTGSTFTGKKVMETASKTLKRVTLELGGNDPAIVCEDVDI
ADVAPKIAQFAFLNTGQICLALKRVYVHERIFEDFKAAMIAATRLLKVGEGNEPGVFMGPIQNKMQ
YERVKGLYEHVKEEKLDISLEGDIPSGSGYFINPTIVDRPPENSRLVAEEPFGPIVPLLSYSTYEE
AISRANDTHYGLGASIWSGSSERANELAQKIQAGTVWVNTHFHLDGRVPFGGHKESGIGTEWGVSG
LVSYCNSQTLFLEK
>g2746
MSASHVVLVTGANRGLGLAIVHALATRAPGSTICLGSRSIDAGVKAVQELRQKGVDSIIEVVELDI
TKDSSIKSATTILRQKYGKIDVLINNAAIAGNPKPDLSDFREAYTSILDTNITSVALVVSHFLPLL
SASVEPRVINVSSARASVALQTSGKLPPTASIPYSISKTGLNILTLELAKLYENIRFYCASPGHCR
TAFNNYRGKKDPIDGARVIVELAVSESGRYDPGFWEFENDSMSPVAW

>g2747
MEEAVNVPLPSNEGIATTEEFSKPQYATSLHSFISDNQSVNLTDWSPTVESRRPPGYRSYEEPYDR
SNAPSRHFNKDVTLQDFPLPAFHSVNGHEGRNTPRPASRTEPENITDHNATRFDTHEPIEELRRKI
ADYELPLLQQVNENNNDPSFDPIIEEEHGEQQLSYDLVAPHQNGEAPLHSLEKKTELMFSRAHMLA
ILSNPRHLFKFREFLNAERPGSMPVLMYYLSACKALKAIEYANAVAQLLMPLSEIEASDEHIPNAR
NVKLEQQAQNALHALVIDELPAFITSICVSLVSRVVEEKIKGTLPPKFQGTTDALAEVFCLTDPSK
PDNPIIFASEEFHRTTQYGVDYVLGRNCRFLQGPKTNKHSTRRIREAIEAGRQHSEVFLNYRRDGS
PFMNLLMCSPLCDSKGKVRYFIGAQIDVSGLIMDDARMDSMTELNTSGQSLPNGQSDLXXEPQQKG
HINGHRHNGSVNGNLYNSSETPRKPGEARNNLMEFAELLSPGELSMLQEHGGSLFEPVVPRRDRPY
MGHTKPRIMLREGRPPSRDKLHTINKQPNLPGVYHNYLLVRPYPSLRILFTSPSLRIPGILQSSFF
SRVGGSPTVRDELLRAFIDGRSVTARVQWLNRFDSLGRSRWVHCTPLLAHTGGVGVWMVVVVDDDE
ADWRAQPIME
>g2748
MAEEISIGQYIFTRLAQLGVKCVFGVPGDYELVILDMIPAAGLTWRGNANELIASYAADGYARVNG
IGAFVTTFGPGELSAYCGMAGQYAEYVPVVHIVGYPSTSAMKSRAIMHHSLGNGEFGMYQKMIKHI
TVADTVLGDVHSAASEIDRVLEAMLYHSRPVYIGVPVDIGPKTIPSSHLSKPLITTLPSNKEEEET
EIITKIANLLKTRKDPVIIVDGGAVRFDLLEETRGIIESLRVPYFSTGMSKGGISEQLGNGKLFGG
VYSGGASPDTIRAAVEKSELVLLVGNYPSDFNTGEFTTQLQDQNIVDFQRFSVKIAGKSYNVTSKY
LLQNLVRHLSSLSVTPREVTWDPYPNDNYENFGSLVQDFLWPTLGRYFRANDFIIAETGXSSYGIP
AANLMAIDNVRMYNQTVFGSIGYATGAALGSFVAGKEDGSIKRGILVTGEGSLQLTVQALADYLRW
ELNATIFIINNGGYTIERLIHGMEASYNTVPVWKYEKLCETFGSNYPSRYHRVETGAELVKLLNDE
QFNAADCTQVVELILGKHDAPLAVKMASAAVEAFNSRQ
>g2749
MAPAVTETTESTIPIREHVTAKGLKQAPLKYSGALDVYEYFDLTPTIGREFPTASITEILRAPNSD
DLLRDLAYTISSRGVVFFRKQDDVTDEVQKEFNQRLGELAGKPKTSGLHIHPVINSGRENGAKDDE
ISVISSIEREKLLDYRNARAQSARIEWHSDITFEPIPSDYTSLRLITLPKTGGDTLWASGYEVYDR
ISPPYQKFLESLTATYAQPRFEEIAKEKNFELHPGPRGAPENVGNILKAIHPVIRTNPVTGWKSVF
AVGTHVQKVNGVSKEESAHLLKWFIDLIVENHDLQVRFRWQNPNDLAIWDNRSVYHAATWDLEGQG
PRAGHRVVSLGERPYFDPESTGRREALFGSE
>g2750
MIPSARPRRLQGRFIFFSAIASLFLLSLFLWRTADPRIVDPRLSSAVAHAEHTIGNEHSSHSLIHV
SDEYLDDYETLTNSQDKNIGLKPTPIVHPFSLSLPPASSSVSTPSSSAVQSQDAIPTEPAQTPVAK
VSPSINSGETVNTSQNPRIPGKPNNLTISGFVFYGRRDRVESMHCYLERNLITHGGWLDNVIWVVN
TKDEADLAYLDKILARSPLYKTHVLSEQVGWPDYAKIWRMVNRESMYIKIDDDIVWFADDTIERLV
KRKLERRDALAVSANIINNPPLSFLHYHTGALHPYFPDLPKDFDYSTLEPPPPEPSEEEAKYEEIL
VDAELQPIEETVETIEIQPEELQTEENEILFEEVESEKEHTALSKRLAILGNKTPWRPSEDPHWTG
PDDFEWSLDWSPPPFPNHRWLRVPDDKALNRTPVAHLTYEVWGPTYESWAIASQQHYSLLENIENE
KLDLYKFDHPWDMKGERIRINFMAILGSDVLDTDVFHWPDNQGDEDMLVLTLPHDTGRPILIEGTA
LAAHFNFQHQGGVTTTDLLSRYKSLAAERACLVPSPSAFD
>g2751
MNEYTRQKPIDNGRPVRVIIIGAGVSGIALYIRLLQYVPNVTITIFEKNAGLGGTWFENRYPGVAC
DIPSHVYQYSFEPNTRWSQLFASGPEILEYVKGVASKYRVAEKIKYNSKVVSATWNNKKGVWLVIT
MQNKSSGESEVRETEAEIVISAVGILNNWKWPEIEGLELFEGKLLHSANWDTTWDYEGKTVALLGC
GSSAIQILPHLQRKCLRVHNFIRGGTWISQPFGSTFTENILTNSTEPGNYSYTNEELQRFESDTDY
YQTFRREIESFINKDFPCLFPGTPEEIESTQKLKDNMRAKLASTDGVYEAIEPQFTPGCRRLTPGP
GYLEALTRDNVELVKKPIARFTRRGVVTTDGTEFEVDAIVCATGFDCSYKPRFPVIGKRHQNLSEK
WQRRASAYLSHSTPGFPNYFTVGGPNSATGGGSLLLILESVMGYVVKAVQKISRENIKSMEVKESA
LRSWQTHLDQYFPKTVHVDSCTSWYKIDGKIIGLWPGSSLHAMKTLEHPRWEDYEYELVEKEDELS
WLGNGWTIEDVNRGNLGYYIDFADMPPIPSEELLARQP
>g2752
MDKMDRTKYLDSLKGLAAVIVAFDRLFLNDLNLAFRSYWAEPASKNRVWLQLPFFRIIFAAHAMVT
LFFVISGYVVSINLLRLRERRHQADFLGQVASAATRRVFRLFLPVAVIACVSQFLFWVNLYSWTSV

EIKQFVAEHKIRPWSAPGGHLAFLFRYLTDIVNPFGALQYKDVNHGLNEQFWTMPLEFRGSCLVYF
LVVVMAPWRAQRRLIVLGALAVYFLWYGMWDIATFVAGLWLAEMKVKQEAEHGYPHHPQHHPDFED
DSDEENVNGTLQPPFSWSTTSSLSSTLSSLSTWSSSFRADSKKTRQIMTPVSITNIILFVLGIHLM
CLPSGSTGGNDGHLTTGYRILLILQPSTWKTWDVVHHSWKAIGSVLVIHSINNSPRWLFQRPLETS
RTLQYLGRISFPLYLVQQIVYSLWREPIKNWIWKDLTGYAYPGGQLADRERFACVVMWMGATMILG
PVLVYVSEEVGRYIDGAGLVVAREVERWLV
>g2753
MATTNPNEDDHVRDLEQSQNEPEPSLPPSIPSHLAYRLYTSHFLSTWNSRLFEFGSVLFLAAIYPQ
TLLPMSVYALVRSGAAIIFARVLGAWIDRGERLSTVQISIIGQRLAVAASCAIFWVLQQEYDTLKG
GKARDGLFAVTVVLACVEKLCSVLNLVSIERDWIVVITEGNEGVRRVMNARMRRIDLFCKLMGPLT
ISLVALGSTLIAIRVTLAMNVASVLVEYICIAQVYKRFPQLRRNEAERQQRTIDDTAQRSAKTTIV
QCLKAIFPISSLHFYINHPAFVPSFALSLLYFTVLSFSGQMITYLVSVGYSTLYIGIARTVSTALE
LSATWIAPRMMKRVGVVRGGIWSLCWQMAWLGAGVTWFFADYNREGRDAIVAATGLAVGVALSRVG
LWGYDLCAQNLIQDEVEDSHRGDFSTTEAAFQNLFELLSYASTIIFSKPSQFQWPVAISTAAVYMA
GGLYAYFVRRRRGHLFHPPEWMLIKRSEAV
>g2754
MTIQAKWSKLLDVEVLQRSSQALSVVDDKVYVFGGEFRPREPRDNVVHVVSLNSQATLQSIPSQSD
APSPRVGTATTTLNGDIYLFSGRGGVAMAPINEKGAVWKYTTATGQWSTVSPVDTANAPEPRSYHC
SCSNQKDTIYVHAGCPEKGRLSDLWSFNITTKQWKQLTSAPDPSRGGASITFANGKLYRMNGFDGK
TEQGGSIDVYTPETDSWETHTYKPDNESGPIARSVCALLPVAVGEKSYIVTLFGESDPSNLGHQGA
GKMLGDVWAFDIVGKTWHKVDTRGDAVPDARGWFGADVVGKDTIVVQGGLGEANNRLGDVWSLSFV
QSN
>g2755
MTSKIPQPPGVPLLGNVFDVDPSNTWGSLNKLAAKWGPIFKINALGTQIVFICNAALLEEICDETR
FRKCVNGPIVEIRQAVHASLFTAYESESEKVWGPAHRIMSPWVSDESNKITFNDQRDGIAAIVPAW
TSKPGQEVNVADGLKRLNLQSVVWSMFHQKGKWMSGPFPAFLTAIDKSTMEAVKRPTRPKLLNRLL
YQRGYDKDIKTMRSFCSDVIAKRRKEQGTIEDMLNVMLNGKDPQTGVSLTDEQVIDEIINIFIGSA
TSPCFVSFALYYLLKNPEYITKAREEIDSVLGADGELTYENLQQFQFCDAILREAMRLSAVAPGFN
IEPRPDVNTGPTVSLAGGQYQVPSNQALIAVLGAVNRDPAVFDDPEAFRPERMLGEAYDRLPSGVK
KGFGNGKRQCFGTKFAWQWSWITLVTVLREVDLELADKNYTIDGDVNGAFCVQPWHFVAKVEKRKD
RP
>g2756
MAGPHNSRPGPAATPAKVLVAQLSSPTLNIRSEFELAYKISKCTTSTAVKQLATTLCRGDIAETTS
RLTQNRSDLNALIEVAQEQVEKVKKMERDWRPDLPSKTKEFATDLVAGKNNDMREAMKLFDELRWE
YERFIKYLDWIEELKVEREHLLEDRKYMCGHFD
>g2757
MVYKHKLVNELVDNLLDKLVYQLVDELLNKLLDQHNNHYWISHHINEHFYLFYTHYSDNNKYFYKD
YSNYYSFHCFIINYSNHIFLVNLYEFINHFDHIFLTNIFLVNLYEFINHFDHIFLTNIFLANIFLT
NIFLTNIFLTNIFLTNIFLTNIFLTNIFLANIFLIIHNYYFYHTNLYLNINHSHYIFLATCINLYF
NINHPSHIFLVICFNLYLYFNVNHPNHIFLVIHISPYFYIDCFIHLNPYFYIDCFTHINLYLYFYH
DDHIHNHIDINRPIHVFLVIHINLYFYIDCFAHINLYFYFYFYHNDHIHNRIDINSHAQSN
>g2758
MLRFAITLIAWTLAESTTGLEWQNEVNVSMCNWAQFRANVIRDTIYLDGGELWWQAGLSDGTYASP
QNDGGINGVVYEFNLGQPFNHSTNITSLFKTLSKTGGLASNNIAPNYDDGAMFANDDELITFGGLV
DATNSSSAPAENAILGYEAYQYGPARQSWSPGFVNKQLPTNITRYVTNGASASAPSENMGYYFSGI
RAADWGPIFNDGESANTLANSLISVNMSVMREETWANDTLPSFVPARANAELAWIPAGEYGVLVAI
GGVIDPVSLTAAQELNSSQTNASSQTSPTFMETVSVYDIANKKWYQQNTTGDIPPQLTMFCSAVAS
ARDDSSFNVYIYGGYDGINQSSIPSDDVYILSVPQMIWTHAYTGQSNHGGIYENNPVICLQAGLAE
VFNVNNLKFQDSYDPRVWSEYMVPDVVTAKIGGNGNGSATVQSPASLSDVFASVYTKPITTYYPYA
LQSTNSTNTTNPTPGPTAITVSGGGGLPNWVGPVLGVVLGLIVVAAAIIFFLIWRRRKSGRSTRVS
ETQASTTGIRGFVLRWLYGTAAQDTKSQPDLAVADIDGATVVSSKTHERHVSEAGSVALHEIDNIH
TPVYEMXPNDQGLQWHETHWSQPQQSPPLVSPNIDGMMYSSQSPTRPTHQNTRHTSNMSTGYRSDI

SEIGSSDPGRPAHNRQVSDNSVSDVTSVPGSQDGSNAETSNREYTATNSATMSGRITTVREDEEHN
ATEAVVSPLVEEHDEIQRQRNHLTELE
>g2759
MQLFKAYLLTALVLVAPVSTATIQKRDTVDVFIWGSTSFQDYTETYTFNPGDCQAITAPASGSVGS
ATVDENATCNVYASSDCSGGVAGTFSAPGISDTGSLGSKFGSVSCTIPDSSGSATTTSSAVSEPTD
VCDA
>g2760
MTSTHTIPEVHIERTRRTILISKRRYQYDERVRFPSPNRTVSRGTQTLEMSDYPSALNMMYRTLSP
APGSGRKTRSNHHHHRTEETKRNRKHPKSSTVITTTMTTTEIHEADATLTATESITTSPTATKRTP
AVLDVGKIVGISQSDGTERLAMISEMTTFHDSRIVIVCAWLYSRADINEDLMDNAGFSSEESHKYL
QSMWPIINKDGTLMTSTGRSQCDYMVSTKRTIIVSDAMGSSLNAVSPIVATRVCRDAIYHADSVER
RICDVDEGSCYWLKKIL
>g2761
MATNEVKIYKIASIPGDGIGPEVVNVTIDVLESLQEILGTYKLEFTELDWGTARYKKTGKYMPAGG
LDTLKTFDAGLFGAVGAPDVPDHISLWELLLAIRGPLQLYANVRPIKTFPNVPSLLKDVRDGDIDW
VLVRENSEGEYCGQGGRTHRGTPWEIATDISVFTRVGVERIMRFAFETARSRPRKLLTVVTKSNAM
RHGMVLWDEVAAEISKEFPDVKWDKMLVDAMTVRMAAKPKSLDTIVGTNLHMDILSDLAAALGGSI
GIAASSNLDPTRKNPSIFEPVHGSGFDITGKDVANPVATLWCASEMLAWIGEKKAAAILLKAVEDT
CSQGICTRDLGGKAGTDLQANSTYLPHLTGSFGAFPIEVRDAQRVFQDEQESRPDVFFIISHAEHV
TESRKAIAKFVHAPVDECVFVKNASTGINTVLRNLEFKHGDVIVYFATVYNAVEQALESLTESTPV
QFRRVDYTFPILHDEIVRRFLNVVKQMKSEGLNVRAAIFDTIVSIPGVRFPFEKLIKACKEESILS
VIDGAHGVGHIPLNLGDLSPDFFVSNCHKWLYTPRGCALLYVPKRNQHLLRTSFPTSHGYTSPADR
GRGTHVGKTDFEILFEFVATADDTPYMSVPAALDFRKRICGGEEAIYSYLHTIAQEGGNVVAQIVG
TDVMQEPGLTNPIEQSDIRRCAMVNVRMPLAFKDDKDVNPHVPDPSSSPFPLLSIKDATPVCEWMH
AKLIKEYDTFAKFYPHGGWLWLRLSGQIYLEMEDFEWIGHIARSLCERVGSGEWLPKSRI
>g2762
MRVTASLPLIVLSTASVGNALSLHRRDAPAVLAAPLRRRQPATLSKRDTSPLNVSIINNQDSSYLL
NLTLGSPAQNFTLALDTGSSDLWVIGGSKEADNVYVSDKSSSYKSLNSGYNATYADGTTALGVYAT
DTLGLGDATVKDFQFIVVNQTSSDVGIAGVGYNISTYDAEHQNKVYNNLPYALTAAGITKSTAYSL
WMNDVNAETGTILFGGVNTAQYTGELQTLPVVPVYNNYYSLALALTEVIVQTGNSTSTTTNLPLAV
SLDTGSTLTSLPEALFKDILNALNGTYDDNAGLGRVDCSLMETNYNVTFSFSGAKVDVGLNQLVLN
QGVTGWPRSSCLLGVVPSDPGVNILGDSFLRSAYVVYDLENNEISLANTNFNPGKDDILEIGTGAG
AVPGATLVPSAVSTATGNGVQTATTGVPTVTGTSITTASATTATGSATTGGSAKSTGTSSGIATLP
TGNAKHLLSGLAGAGLLLMI
>g2763
MANETPKDVEALGTVESKHVADLGITDTPTIKDSDYEHHNVGTLQRRLKSRHVQFLALSGAIGTGL
FVGTGQTLSLAGPLSAVLAYLITGFNLYAVINSMGEMATWLPLPGAVPVYAARYVDPALGFTLGWN
YWYQFAIGVPIEISAAALVIDYWPNNVPTVVWITILLVAVIAINLFPVRYYGEIEFGFGTIKLTTI
CGLILLMLIITLGGAPNHDRIGFRYWKNPGPMLEYLEPGALGRFLAFFKVFVSATFAYGGSEMVVV
AAGETENPRRNIPKAVRRVFWRIAIFYVLSIFLVGMCVSATDSRLLNAINSSAPGVGASPFVIAMQ
NGGIKILPSIINAVVLTSAFSAGNSFFYASTRVLYSTALDGKAPSFLKYEKFGVPYACVGITTLLS
LLVYLNVNSSASDVFFWISNISSVSTLIVWTSVSITYIRFHKGMKHQGIARSSLPFRAPFQPYLAW
FSCLFSATVAFFNGFDCFFPGKFSAKSFVPPYIDIPIFAALFFGYKIYYRTSFVKLDEMDFFSGKE
EADRLEDTWEEVKPRNIFRKSIV
>g2764
MSAGSNSQPGSLYPYYPSHALPAVFATLIGISLLIHVYQNFRYRFWSTTFWMSWGGAVFTVGWIAR
CISSYHTSNVNLYIVQTVFVYAGPPIYSATAYNIIGRLLHYLPMHAPLHPGRATIFFIYLGAAVES
LTAAGAARMATNTDNLSVYKSGGTLISTALLLQGVIEAMVVCVVSLVHYRATHASHPPPPNVRRLC
ITLYGTSFLIIFRCICRAIESFTLYTQIESCFQGHCSPIITHEWFLYSFEAAPMVLFTYWLNIMHP
GRLLPRQKTRYLDLDCQTERLGPGWIDKRSKVQTVIDPFDLSGMIAGKPNHEAFWLWPEKYPAATD
GSFALGTASNVRNSGSKAAEKPTSAHEMLLTRSA
>g2765

MPGVPSGKACEGCRKLKKKCDEKTPSCSRCIRLGLECVGSGQQRYKFQSQQAFVVARTKKTNSQTK
ALSTTSQPNPFCNEADMVASFLRLPPSSKTTILTSQYVQAIQLTTNLKYNLAWAYGFFMFEIPRRM
NENEALDTAVNALICSHSNFCARQAVSAETLSSYSRALRSLRHCLDDPGKASQTETLCAVMLLLIC
QNLNGAEGQRWSGHCEGAAKILKARSFRKPRDGFEAQLLLSLRGSVLFEGLFNENIELDPQDWISL
VGSDLDQDLPEGKLMRYLANVPSILRRARAATNADEIAILRGEVKPMYEASQKLLLQVHKQWTDGR
TLDANNVLRNVILHAYFQRAYGIGLLIVIILNWVLRAFTPPFDAIELIADSTNLVTQTLELAQAAN
IYRPLGAGYMLIVLSAAWIAASNDNERVTVNDLLQDYRQDFPVRATDKFMIELRWMAEQLQYLPVG
CA
>g2766
MAAISEISAGIAKGTLTGTEDSLLATVIWLCVYENSRVEARRQSSIHAAALRRILLLREPMDYRHA
SMSELIWERMCVESYIYHSAVTTLFDGHTNLIEDTLVVLKKFGSRMSAALPDVDTLQTALDGNASS
QAIIQSPVLGAPYHMFLFLMEGTQLAREISSGYMPDEILAWSRYYEVQSMQFTLEKTASSYYGDSR
RWIGKLYAVGIRLLFLYIISASRHTNDEILEHDLSWYTEFESTLNEARKLLITTEDDIDRTWGKFF
LWPLAIIGAVLYDANDILLVRSWLDRVIRKSNSSSVLVIRKVLEERIWTVKLGSGAVNDAHGDHAQ
GLSIILDTNIMNRAAGSLLNQMPYDFSCRQIR
>g2767
MPVKLRLAEDSDIPRIAESALAAFESDPLHMAMLPREHTNHIMEWYQGRLRSALSDPNMRSIVAVD
SRQDADGVKEIIAGYSRWLLPSKSSEANNAVENERPAAAFPELANKPLVDFYLQQSDDKRGKQMDK
SRDVVLYVLATDPAYQGQGIGRMMLEWGINTAATLAESGQNTKPRILLEATPLAYPIYKKFGWKDY
ESITIDLKQYGIANGTEHTTVCMIRE
>g2768
MISDKLYQFAVSDKSVSSELAENPNQSPGEIVSRLFKHKQEAAAETSQQPTHEQSDEADLQRALEC
GNWGGQRPSNLFLKCYYNALRALENQPLAGLVSPSLMGTHGVVPLTIISTLPDINRHTANCIVRAE
KEVFLATNFWIHSDASIIITNALRELSKRAGERGDKVVVKIIYDRGSVKQLYENHQHVSPKEYTGD
KIQLPAPEDVPNLHMEVVNYHRPVLGTFHAKLCVIDRKIGLLQSNNVQDNDNLEMMVHLEGPIVDA
MYDTLLISWHKHLDPALPMIGNPAAKQPIPTHDNLASKAELTDAAASGSHQKQMEILPESTSSEPH
YDQTFAEEAKRVNSQLEARPDETRRDAATRHLNKRTLDHNQQALAAKSLPHPDGPEITEEDKMTPY
IVLPKXEPVPMALVNREPNGSPGNSNYPVPQNAAWLAAINNAERTVFVQTPNLNAEPLLEPLLNAV
RRGVIVTAYVTLGYNDAGELLPGQNGTNEMTAHRLYSSLTSEEKSRLRIHNYVAKDRRSPVHNKFK
YRSSHVKLMIVDETVAIQGNGNLDTQSFFHSTEINVMIDSPLICKIWREAIERNQNTSQFGLVSSE
DGCWHDPETGAIAEGTIGPDPGKFSWAKGIVGAVQRVRGAGGF
>g2769
MRVAIIGAGPGGLAAAKYLLAEKAFSKITIFEQRDEVGGVWCYTSHNAVEENFAIPHTKPTTTPEK
PVSAKQTNGDIIFQSPVYDLLETNIPHSLMGYSDWKFPEGTSLFPSHQAVKQYLQDYAKELLSSIV
FHTQVVDVRLRDETAESVWKVSVQNLRTQLRSTYEFDAVVVASGHYNDHYIPDIAGLREWNRAYPE
SISHSKHYRRPEQYADQKVVVVGNSASGIDVSVQIAAVLQQPLLLSARSESPPYLSDNPQIKIVPE
IVEFITADRAVRFADGHVEKDIDHVLFCTGYLYTFPFLSSLTPPVEVPNGSRPNNLFQHIFYYPQP
TLTFIGLPLKIIPFPLSEAQAAVIARVYSGRLPLPALDEMKAWETDWIARHGADKSFSVLGFPADA
DYLNYLHRWSLQATRKGGLDNDGQGKIPPVWGDEEQWVRKLTPIIKAASQALGSKRKDIQTLEELG
FSYDKDTKSKI
>g2770
MKFTVLSTLLLAGLAVAAPLTEKRLARRAARRSNPAYAPNAFGVEQLNITGNKNVEYSSNWAGAVL
IGTGYSAVTAEFTVPTPKVPSGGSSGTEYCASAWVGLDGDTCDTSILQTGVDFCVQGSSVSYDAWY
EWYPDYAYDFSGISISAGNVIKITVTATSTSAGTAVVENVSTGTTVTHKFSGESDKLCEYNAEWIV
EDFEEGSSLVPFANFGTVTFTGAEATKSGSTVDTSGATIIDIEQNSVLTSVTASGTTVTVKYV
>g2771
MGARTSSFRRGTTYSLFGAPDGISGATAATTTGVSYTSTHTIFSLTAGAVDITLDFFSPILPGPSD
YAEXSLPYSYLTVSATAAEATDVQILSGIDYTWTAQGGAAGLNYTTSGTAGYFWFYNPDQTYYTES
SDMATWGSILFGATTGENLTYACDKAANVYSNFTGNGILSNTGTCGGSDLAALSQDLGTVTSGQVT
FAVGFQRDLAINYLGSSQTGVHRSRWWLISEAIEYFLGNYNDALVKSQAFDSAVRTKSEAVSSDWG
SQYADIVEASVRQTFGATELTVPINDTTISPQVFLKEISSDGNIQTIDVMFQTWPVYYVSLNPEYLK
MLLQPVLAYLATGAWPDAWVIHDLGTHYPNAIGHSDGVAEEMPLFETSTVFILLYAYQKYTGDTRF

AAQYHSLLTGYADYLRENALYPASQLISVDAITAQANQTGLAIQSAIGLKAASLVLGDSKYSNTAH
KIAEIVYNDALGLDGATLAESKHFTYYYGQDDTWNVLFPAYSDVLLELRTFDPAAWAMQSTWYSQQ
MEEGGLPFAGPITDTSYTGSGINWGLADWNLLVAATVSADVQAKIVNTTHTFLTNGLNSIPFGTKY
YVEGDQVGIWIGNEARSTVGLSFGLLALNQGYWGNSY
>g2772
MYLRVAHAEANIALLQEFIKQNPLGILTTAIKSAVHPFIQSSHIPFILDVPELNDTNRNSTDDGLE
IYGTLRGHMAKQNPQAKVLMETLAATQNEHGDDTIRELPDEVLILFNGPHHHYVTPKFYVETKPTT
GKVVPTWNYSAVQAYGKIKVYCDSKSQETSSFIQSQIEALSRQAETSIMGYTGAERPKAWEVSDAP
NSYVDLLKKNIIGIEIQIHRLEGKFKMSQEMGKKDREGVIQGFNNLDSDVGRDIAKMVKERGDMKD
AQK
>g2773
MGSVLEAWEACSKIDTVFILVCCVFCWPIIPAVGLGYSGYSTRRSGLASFYPGLIAVIVCSIQWWI
IGYSLAYGEGNAIIGGFEKAFHRGVLADPVGTIPEILFSEFQLIFCATVCAIAVGGACERGRLLPL
FPFCFLWSTFVXEPLAHMVWSDTGFLGNLGVLDFAGGTPVHICSGASATALSVYLSYPLFRSRKSG
SRTPQHLKLHRPHNSMSQLLALVIIWNSWLAFDAGTTLSLNFKSVMAACVTNICASAGAMTWAFIT
YLVSGVWSLDSTFMGAIAGLVLITPSAGFIDMTTAFFFGVLGAVACRQALRIKFTKKAAIWRWVDN
GDTFATHCIGGFIGTIMTGLFAQKEVAAYDGVTYIAGGVVFDGNWKQLCIQFLEAVIGFSWSFIGS
YAIYALIDCVPGFEVLATDSEVVSGMDASQMDESLYEAQWEGEADYHPFEGSVRLD
>g2774
MSGSDRRTRPKSTDEQLTLTAAPSVETSAPIEPASGPDEEEEDEFETIDGIDATSSGSASVTSSIY
AHTYENGRRYQCFKNGRYPIPNDDSEQDREDMKHAMMLELTDGELFYAPIGDHPQSIVDIGTGTVG
DRYPSARVRGIDLTPIQPTWVPPNVDFLIDDCEKDWLMEDCDFVHLRFMIIILRNVPTVMENAFKS
LRPGGWIELQELCAEPLCDDNTMPDDDPVKYMYDLAGQAFGKFGMDVTLPKRLEPLLHNAGFRNIQ
VVVKKVPIGVWAKNKTLRLIGLYQKMAILDLMPALAGRPFSALGLSPEESQVTLAFARKGLDDYKR
HRYFNYYFWYAQKPMS
>g2775
MAENTVDDSQEENWQALPAVPTRVRSIQMKACLACRRVKMKCRLQSGETTCARCAQKSIECIVLEH
RRGRKPGTRITRKSVVGNNDKISPNSAMTSRSGAEEHGVAQSRFDAYSKPQFSAETSGLQPSELLN
HEAMRGKFSMQRILDTQLERADNAMPERSFHQPAQDAVEVGLVSQSIAEHLFKSFMDILNPYISQL
DPDLHTFSYVRHKSPFLLGSILAVSSKMFNPSLYKRLYRYAEDLFLENFRVGLKSTETVQAILMLT
YWKEPEDTRSWVNLGYAIRICMDLGWHKISPRTIRDRSGMNSTELREARNIERTWFVLFVYDRSLS
LQTGKPWMIERSGFIESIEHWSKEQTATANDTLLAAFVTLRLLTSEVFKLLGPSRHVSSFANTKSF
LTIIGNRIDEWEDKWIQACGDVFDGESSLDSRPRDWTILLHWALPILTDLLNDDVKNDLPSAICNP
YLDFNADVECLPCYNGNTGELLFKSPLPGSRRISRQRLRKVLSKGLDIKWGKKVVQIEFPSEDENG
GAKYESPVQLVFDDGNTDAADFVLAADGASSTIRELLLGPEAARVQLAGFMFATGVTNYHDADKVA
AVVKAHPVAAITLGMESVAGCGVLHVEDKDDMSTWTTFWTKIWRGTSAELPKGKNMVEYIKDNTKN
LCEPFQSLIDWTPPDSACYIDEMKSWVPVPFDNHAGSVTLVGDAAHPMLPYRGQGYQHAITDVKNY
VDALVKIRDAPEINDRREVMSAFDSEMIERTGNAVKQSLQEAEYSMNIETVSKMLMATHGHGRSA
>g2776
MAVAEPLSKAQAAHVNMMTDTIIANLPPAALRSIVRAVLASYPKFTAAFEEETRNHLSLTTPTTEI
SVVTKGKLVRDEFSLLQSRIRCMLGCGLCYYSLPLLGRIVDESKNINGNTDGIYDDMNLIASIDGD
IVQAVTAVQKTLAVSTGARELSDDEMALLDTLYESLTASRTYFDSSEYDFPFQRGLVAISSLLGRT
LPLSAAKSTNGTKTVNDTFLLAPREVKETFILGEIKLPRIFCGLWQLSSPAWGSAPATKIMTGFAR
YVQRGLGAFDMADHYGDAEIMFGGFRSAYPYQDALFAATKYCVFHPMTATRERVEANITERCQRLG
VNKVDLLQFHWQFYEDNQYLDVVKYLQEDERVGMLGLCNFDTEHMQRVIDSGVTVYSNQVQFSLID
SRPVVKMGEVCINHNHVSLLTYGTLCGGFLAEKWLGKPEPDLYGTEITPSQRKYFTMIRSWGGWELF
QNLLQTLKLIGAKHNVSVSVSNVATRWVLDFPYVGAVIVGSRMGISEHVDENMAAFGWSLDDEDQEAI
ENVLKKSRRSEMFQSMGDCGGEYR
>g2777
MFEDINNSGDGGIHGQVLKNNGFQGTNPGLTAYAAIGNVNISQDTSNPLSTAITSSLKVSVTSGTT
GYVGFANTGYNGVPVLETTYANYFWIKGTYSGTITLQLVGSESGIVYASHNITVDSDPTTFTYYET
SFRSTESPDGNNEWQLLFDATEVAGSSLNFGLVQLFPPTYHNRYNGLRDDVASFLAEIEPSFLRFP

GGNNLEGASPGDRWKWNETIGPVVDRPGREGDWTYPNTDALGLDEYLYWCEDMGMTPILAVWSGLT
LGGGIISGSALGPYVDDILNELEYLLGNTSTTYGALRAKNGRVNPWPVSHIEVGNEDNISGGCGTY
ASRFTQIYEAIHDQYPDITVIASTTSSSCLPATLPSGVWTDIHHYESPNTFVSLFNEWDNWSRDHP
IFVGEYASTTGNDGSTTYWSNMQGSCGEAVYMIGLERNSDIVKMASFAPLLEHFDLAEWSPDLFGL
DSSPDSITGSTSYYVQKLFSTNRGSTVLPVTSDSAFGPVYWVASVDKSTYYVKLANYGSSEELVTI
EIEGTTAGTVQLLSGEELDSNYPHNVTITPQRSDVSGSGSFNVTLPAWAVAVLAVS
>g2778
MPFEFIDNNAFIDRKTRKRIRSLVATGKNAGKTVVRPSRIKAFKEQSKYPTAFSSVPGTVEIRRQD
LSSSSPDETTSSPEIERPIGDDLFFTFTSNQLNPASRYLAKRAISFLMAPRHASELNEAFENSLTA
GPALAKMMFVDETFFHCAIALSTTAIDSLSEQSSPSRDAIHHLCHTLRLVNKKLSESKLASMSTIA
TILMLAQYERHQSEQYQGLVHLNGLQRLISMRGGVLQVQKEMPILMQKAFRVDLDFALYMGTATTF
NVSHVLAGRMVIFGPAIDPLERLRSQVISLPGLSEQLGADLHNALLEAKALAGQLNDATSSNTRKV
NLYTFNANILLLGYRVVYISSLSQSYQLSRIENAIHLGLAIFVTTFMNGLDRKIPNMPFMSELLRA
LVRFGFQDDHTVLLWLLFLGNATVLDFTDYDWLVPMVATTALELGLKSWAEVLKLLVRLPWVNALY
DRSGQSLWLQASTYYATP
>g2779
MSDKVSNQRLNLPLAISPEKCSGGTYIVTGANTGLGLEAAKHLVAVGAAKVILAVRNIPAGEAAKX
RIIKETGITGTAEVWALDLSNYDSVKAFAKKAITELDRIDAVIENAAVATSQGKAEGHGLCMTVNV
YSTILLGLLLLPKLKENAKRFGITPHVTIVTSGASFDVHEKWSEIKDDPLVKADDISIGLVSYPLS
KLFEIFVVRELATLLPVSETGVVLNLVNPGLCKTELSRNAPPEFQKQLADMHAKFGRTAEDGSRTL
LHGAVGGENSHGSYLSDCEVADFKLPSWITDEQGKKDQELIWNSLAKELEVISPGSVKSIL
>g2780
MAPSLQTESTLNPLSPGAGGHWRRISGGLHDFDSVSNGAARSVVSRRISRKAAGAGESSNISNVVY
SLKRRSGDELGVTTSSKLLNANHATLLDWIRHQRMSKIPPEGSSYDKVLAWAQLFVERLHSFDLAI
EHFAGDSYLAAQLAYGYCAILLELGEENSSALRISFGFFYSISMVLVNLLERTELFAVSQEIKEQL
VLALTDLVTLVASVSTYFHKALRGLTKPSISVNIYGTFSGQIESFRGRCEKISEAMWKHQLLKDDL
DGERVSEIKSIRQWLAPEDRVLANVAENTSHLAHDREELTCLWLSPYLTRFLKSQQLKGLSISGAP
GSGKTVLASVIVDHLQHPIGGVAYNTIFVPINARVPVEAEPQAVVKSILRQLFEKRIGNTQLFQIL
SNAYDRSKKAVGIDEYEKILWGALDASLGSPLRGAKPVVLVVDGLDETSGSEAILLQRLQEVTHKT
ANFKLIVLGSQRFEASRELTSINITSELIFDDIAAVVRNGYETYPVYTKLSETEQEILIEQITQAA
NGSFLWAKLASKLARNAETSEALQKSVNSLATSKFTIVDFVAQFIQSPDLTEEVKIMLVWLATANR
PLSQSELSTLLSVQIDKATVIDRKIDVLXLLKPVNSLVYLEDGYVYLRHGQVRSAIIDVFSKGKLI
PSVKDRHADLLRRLFIYSRFAITEDSEPSLSSFDRFEKSKLLDKFVLLDFSLRYWFTHLRQTTAFT
TDGENGVKKEFGKLFPTNTTFFLLEQAVWEHRPVPTLLSWYTAVTKLTRNILTPNHLATLQSILTL
ALFYRQINRYPEAGQLFYEATSISRTVLNSQHIITTQVASLFLEVTAEKVTETKTEIMTRREEVLL
LLVDAYKVHYGATSEIVISTLTQLVEHYQLIKEESKAREIIISLKSATIQQYGADSQEARDIGGSL
NVHLTGHATQIETDTQTTLLLDIEERDELIESETQDFWSLLKKAEEYSSKGHFRLAEQIFVQIWQR
ASRECRMHYSSEWEERKFKAVLSYSQFLKTQKREYEASSLLTTTWQEYEQTSVATETSVSYISEMA
NVMKTVGLASLALSVYKRVAQFYESTSRTQTSTYKEIQQSIQITSQEVMKVVSSSSSVTSESTLEE
IVYEASSSITKVDQTSITAVSSLYEMYISQHRWHDSTRLIKRVLHGIWPNLFAPSLQDVTLPGKHV
DHCIELAEKLSQCYHARRRLGKEEDIRTRIYRAVRSDRQVGDKLLDRVTTELLRLFERTSQTDKII
TLRQELLSDYIGKYGLHHSIVITNLWALAELTRPRPIFVDYYRQIVTAVNKDSDISLPDAFEPLVI
VATELWTQGRYSDAVHYYQILYKTFLQQPKVNPKFEDQEFVKSLFTRYTYSLRALQTDFAIIHKIT
VDYQAKVKATFGATASITIQATLTLAKICQESKRLEIEAIHLYEELLKIKSEEIDYQEITATLDAI
YEEQTAIVTSTELESVSSAQVERAVSVLKRRITTIRKSHGWAHEESLSKMQEMVSFYSKRNETETV
LKELKEATVQILTTETSSTKLIASASAIATSYIASNQIHKATELSHEVYRQIIMKDSANVKSVSFD
VTSKERQSLVFLAQLEYSLRQNSSVTLNEIFSALTTEYIYFEEYRGQLKSTSTTLQSLTLTAARLH
AFLLSRERQSIATQVYEDFLNYFLSTEGKRIKLTEISQARIFTLTLLNYFSLHETKNFLRSVGIAS
HEQTQQLIAAKRYDVAADLALAAFRYISAHAEYRSAAILKFVFQLGLTVAGRNAVPQSDEAARSKL
LKISSVILQEAFKVTKELKINLTQLSLANLNNIIALLGEQQDYVSLAWLLTLLWNSREAQRTWQSS
VTFALGRRFILVRYLVGEVSGALRLAEDIVYNCRRVHGPRHPSTLEMSILLSQLYTSVAQRYQSQK
SGHEIAHRYYKKAAAVHENILRVFSDPSFAELEGGDMSMSMDGSAYSLELVNVATSNWTEGQHVRQ

HLDLLKLAVERLGDWPKDYSEFERLNADIFREFKEDLNGVEGVEKWTPKKFGSGKAERNDDLLDTN
IKSWEIVETSDTNGFHAEEEEEL
>g2781
MRYYWQLLIKHDIHRMSSRRQYSSCDSCRRLKRKCVYEETTRRSGLVCLRCKELGYNCTFDYISAR
LEQKKRKRHSTASDYKNYQSQSTELMSIGEPVDTMDWISVLQPYDGDVSIEFDDLNNDIAAEYLNL
EGLDREWQDLTMAGCAQSSILDTNGRSSDYTLQVSPKPKNSNYSGPRKQIERAQLFASWKGSPTQL
LNSTFTARSLGTCLYHTYAAMMHGMESRYLEYECNPFGPTHRYKFDLEDVSLVIEDNRPLNELPGP
RKTDSPTMSTLQSLPALKQIHEEGLKQDTPLEDTIKITFVGLAKFLDHFGHFYGNKLDRRSKAEDE
RMLIAAQQVFALQWAVEGSREKDTSFPIIHRSDGSSTEDLHAFASSWFKARSLILNATPGCSFTRV
YAMILFHTCVAPEEAKEVAGQLNNILDRCLHQFEDLKSMVDEYTKLLSSTSTYRNLLQSSVKAFQW
FAYIKDTFAALLTERDLILTENPLNLSATGLRLTXQPVDNLVDADIPFVCQRAVLLIYRILRQTAK
VKQFVRDIASTEDSPQLDGTLDYAIGFVEEFNHTYDDFLNRCMADFQALSSPSRLSFGYLVLFWSL
GTLTLAESIERVRCSYLRQGTNIGEFAIGKGKQYESAAISSVIAISKHIADMSAENASISISGDSG
MKLPFISHHASLAPIVAVLSKSINHMLQRVMDSENPHDGAHWMLSIKPVLTCLLDMRSTVAGSRSI
QSPLSRLVASHGDLIMECWTVDDPNELLDFPLSTST
>g2782
MLTVHHLECSQSERIIWLCEELGIYYELKRYNRNPVFAPPELKALTPMGGAPVITDSTFDPSNPLT
LGESAAIVEYIIHKHGDGRLILPPSHKDYTHYLYWFHQSNTNLQGAIFRALLVGQLKLAPENPIQK
SVNAKVKAVLNMLNDRLNRVTWLAGDQFTAADIMTVVSVTTMRLYYPIDLTGYEGILSWLQRVGDR
PAYKSAMQKGDPEMAPCLGAATPDLFPAWAAIVQ
>g2783
MLTSFYCTSVQATGSAFTQFTLVVSASDTSINGTALVATNNNEIDIMGTLEPVQENELPSIFYFDS
STGRLVTDSALSNEERFAYTLYGNYTSSLRFDIGSNISGDIAYPIFTVSDDGRVIPPGNLTWAWCP
DEFNVGNGYYLPGSITLGDIAEGCEAINGLTAVDAAV
>g2784
MFFKVIASSTILAAYALAAPGKDIQDGAPHELVSDPSSQQVTSSEAFTTGAGNYDNGNIYDGTGNA
GTDQYNCYYGGWENFPPSSQWVEFDAMWNYSKSAMSTSCSDLGVGSCSDGAGAFGDGDTGEQIGLI
WNAIQQVAEASLVDHRFILATILQESSGCVNVCASTNPDPSQPDNPGLMQSDGGVTFIGNSASDSN
QQASITQMIIDGTQGTADGDGLVQCINQYGNIYEAARCYNSGSVDSSNLNNGEGATDSYVNDIANR
LTGWLYANSNYNNC
>g2785
MVLENAETAFLNEPSVAVATPSIEKSDSGTRSMEKKVAVLNGTTDVESGDIIQQSDYTEEQYKKLT
RKIDRYLLPMMFFCYGIQQTDKTSTSTQAVFGMQTALKLHGQEYQWSTTIFYLTYLICEIPSVYLL
QRYNVGRVLSIYMTGWGISLICISAVQNWSSLMALRALQGVFESNISAGFLLITGMWYRTKEHADR
SLFWQSSEGFFSIICNLILYGIARYASSNGGIAAWRCISLFLGSLTLAGAGASFMLLGTPHEVRWL
NEREKRMAVARTLDNYTGQDITGREWSWPQVTEAFKDPQLYFSFLNSFLANIPNGGITTFSSLMYE
TFGFSSWESMLYGCPRNAVYVVVFIAVGFFTRKFQNQRMWIMIASCIIPFVGMLVMSLLPNTAQYK
WIKWGMYVMTVVFSLSIFIGWSLIPSNVAGRTKTTIISSMTMIAYCVGNMAXAQVFQTKDAPRYVK
GTIACSVCFGLQSFLILAWRFWYMWENRRRDRLAAESGLTKEEQEEKAREYGENDATDLQNPYFRY
TM
>g2786
MAKPSSAPVPDPTLPFWRTETHELDCHRSTPDLPEECDILIVGAGYTGAAISHFLYKDNPSPPSVV
ILEARQACSGATGRNGGHLKPDVYFGLPKYIKKYGVKAAVDVARFEASQVHAVKELVEEEKIDCDF
VLTRACDVILDPGLAAETEAAFTEMANSGVANLTDVHFTKRKDAERVSGIKGALNCFTFTAGHVWP
YKMVMHLLKGVVDKGANLQTMTPVTQISEKQLSDGRWLVTTGRGSIKAKKVILATNAYTSHLAPQF
TNHIVPVRGICSRIVVPPHKVPPFLPQTYSIRYGPSMYDYLIPRPDGSIIVGGAKPHFWSDHSHWY
NVTDDSKLIEPAKSWFDGLMQRTFVGWEDSEAYTEKVWTGIMGYSSDFMPYVGEVPEKPNQLVLAG
FSGHGMPLILLCAKAISEMVRSGTTFEETEVPSLFKVTKERLASEKNEILEENNRGNLIAKL
>g2787
MSFPKPLPDWNNLKVIHKGTLPPRAHFYSYVSHEKALTLDRQQSEYVSLNGTWKFRHDESPFEAPD
WENADPLSWDDVKVPGMWQLQGYSHPTYTNVNYPFHVNPPHVPFLNETGSYWRQFVTPHKWEGQQI
RLRFEGVDSAFHLWINGKPVGYSQGSRNPAEFDITTFLHPAGSANTIAVRVYEFCDGSYIERQDQW

LLSGIFRDVGLLAFPMESIIDFCAVPTLSDDLSSGELTISVKIQGNEGDLQAKLYRADGTLLKDSA
FSTTESHKIQVLGEDLELWSAEHPVLYTLLLSFKGRTIPQRIGFRRIEQKDSNFLVNGKPIILYGM
NRHEHHHLHGRAVPYESMRADLILMKKHNINALRCAHQPNDPRLYEVCDELGLYVIAEADLETHGF
DPVERNNIKNQHLMTEYEIQETSYKMAKKWTSDNPAWKDAYLDRAVELVERFKNFTCIIFWSLGNE
AFYGHNHALMYKWIKETDPTRLVHYEGDRDAVTADLYSSMYWSLDTLKAHIKKKTDKPLIQCEYGH
AMGNGPGGLKEYIDAYRSEKLLQGGFIWEWCNHGLLKRDGDTSYYAYGGDYGDEPNDADFILDGMV
FSDHSPTPGLVEYKKVIEPVTVQLKGNQLELKNHFDFSSLDHLTVSWHLVNETGKTEPISWELPEI
KPGQSKLVDFPQGVSLGSDASWLALNFCLRDSTVWAPAGHEIAWAQIPLFKDQDLPIPSFSGSAKS
TLSISEGPGRLYISSEAFASRFTYDLVRGNLSWSTDTGKIFNSGPQLGIYRALTQNDLGLEGPHIE
WERFHVASTRMLVQAVTWRCNDDGTVDIKTNVRVAPKVLEWALEATLSYTITDSSVNLHVKGEFSG
TYPKYIPRLGLTVRLPRRYDASTWFGRGPGESYRDKKSAARFGKYTASIQDGLQTPYEWPQENGNR
TDTHWARIHSSSPDVSYAASAEALTPVPGLEVRMDSPFNFSLRQYSITELDRAKHPHELSALVDES
ELNIDFAHHGIGTCISYDAFYDAIEARIGEKLLAKNIGSRLFGYAWMEAGEREYTYLSMSGHYLKF
FDVFRSIFYRTLWQAGIAEPRKFATDDDREFLLNSYRELKPRPGIHECFSRLRAAGFTVWALTAGD
TARVAGYLAQGGVEMPEDNFFSCDTIKIGKPAPESYHYILDKLEKEGREAWFAAAHMWDAAAAKRT
GFKGAWVSLWEKETCSDIFGELDVIADDLPSLADGIIAASKKQ
>g2788
MRSKGIVGCAASLLLATTVSAAQITTHLSKNAKDLFDWSIYVNDLRWDDSYKYIWYSDNGPWSTRF
TAWYVAGLLYRNQGEDLSNAKAAIENILSCQMTESYDSAWYGTFKLSPDEPYPTPQSDLYSPSIYN
TYDPNWREFIGTQLVQIVEEFSDMLGDSLVSRIEDSLEIAAVGSMRRNGSFPEGDNLTPAYSNPGL
MRAWYVSWIGERRNNETFIDYANQQGDILLDLFKSTGSNVLSEYNAPTYYGMDIWALAGAIKYGPK
NATMTQNAHIMLTDLWEDIADHYNPYLARMAGPYDRAYTRDMVTNSAVIDYFWWGLYGYGNGPQPN
KLEADLLYDIAQGAALALVVDVVADHISKENATWLASREDWQGDRMITKSIPDALSDDAEVRIATT
WISSPLMIGAEQVNETVNRGVQYVPAIVQWAGDRDHTPQPYMTFFSLYPTASTIKAVAGPRSLEIS
YPNTTQDGTDIFTYALAQVPPSWTLNKKKVVNGLEDVPCLDVNITADGLVKQPVIYGATVEDNRVY
NVSYIVPSNFTGIPKMSFSFKYTC
>g2789
MVPSTPVTESPLTVGALKNKGTTFFWLSNGGIIKLNLMLALFQISSYATGYDGSMMNGLQSLTTWQ
TSFNNPGASELGLLNAIQNVGQLITMPVCAYSCDRFGRRPVLFVGAFILVIGVALQAAAQNVGMFI
AARGIIGMGLVLNITAAPLLLLELAYPGQQGPQVAIYNSLWNLGALVAAWVTYGTFRIESTWAWRI
PSVLQGLSSVIQIGLCFFVEESPRWLISKERDEEARQLICRYHANGDDSDPLVTLEMEEIRTALRL
EAEARRNTSYLTFFQNKANIKRFFIILSVGFFSQWSGNGLISYYLTLILDSIGYTAESTQTLINAL
LTLWTLLWSVLFATVMNKFGRRTLFLISTAGSLAVYIVWTGLEATYEKQTALEGTGDDGYAKGVLA
MIFLFNFFFSIGWTPLQVTYCIEILPFDLRARGLVLYNLFVALASIFNQYVNPIGVTNSKWRYYIT
YDVWLLFELIVVYFLFVETGNLSLEETAVIMDGEEYENKLIEGAASVAEKNLQKDDVAVESKALPG
ES
>g2790
MDATAAGTGFVVLIWVLTAISLLIVGLRVVAKVKINHFGLDDVLMIIALILGIVAAALNTNGVHYG
YGRPDETVPEPYAVLARKSYIIGQAVLIACTAMGRAAFVVYLLAILGGQKWQRIILISLAVMELIF
NLVAIVLIFGGCSPPEFLWDHTIGGHCWPGDIQVYYGYFSSVFNVFIDLYLAVVPTYIFWHLKLKV
AIKLSLIGLMSCGLVAMAAALVKTIQLHEINNGTLGGTMNLIRWGYIEAHLVIITASVPCLRSLIL
SGFHYMNSSGQRSRSYELGAAFTGTRRGTTTVTAHNNSQHRRADSRLRTMLSNRSNGDDGASAHHI
LESRTSMDAVKFSEGNSQDLRTRPFGISKQVDVTITMHNHSPDDDDGFKQ
>g2791
MKLLKNLALSIVLQRTVAAIGFTTYKEIDEGRDNSGEIAYSELFNRSSTLFLYIQITPDGADHSNI
IDDFTTLAQNYGSQGVSVIPRVRYGWPNGTIATEPEESILMNDVSTFATVFQNIRGTINIPVIQAG
FLGEWGEWHDGQYCQFQGTADTAVDLAIKKNVVNTLLSTGYKVALRYPADHQALFNGSRAVTIHDD
CIFDGGFNGTDGGTWPSDDRQTWIDYTIEVAGNNTFGGEGCNDAGDATFDWSDYTALCGSDGIAAY
INTLQIAYMNYDYPADYEDLIEDSGESTCVDAIQAALNSWNYALTSGAIACTNLTSSLGSGKVSSN
PLDLRYIASRGDYWNALQSIYQPSCIVYPTSAQDVSIVLQAIRVSNSRFAIKAGGHNTNNFFSSVD
NGVLIDLSSLNAKSYDPSRTLATYEPGSTYGELYDYYAQLGRTVLGPTLAGVGTGAALGGGLSYLS
PQYGMACDDFRELEVVLPSGEIVVASPESYTDLFFGLRGGGGNAYGIVTKYTVQSRPVGQFFAGNL

VYIFEQNDAVADAIQSFMQYNPDPKASIVATYLKLLTPDVNLNLDEAILVFLVYDGPHAGDVFQNF
TSIPYLLNTMSIKNYPEVINMPIPFLAELTRGDNTVRVGVHRVIDDDSYKTALDAWRNWAETNKGS
YELLTFDYEPVPKSLTDASNAQNGGNAMQMPDGPWYWISYILETPPGMSQVDYDAVQTSFANMVDS
VGNAKDLPLFVNDASFDQNPLATFSTYSDLQQIKAKYDPDGFFSQKTGGWSFK
>g2792
MSSRFPKPKLGPVLLTGGNGFVAYHIIAKILSEEPNCIIHSLDVNVDRNRHPNSNVHYHQADLASA
ADVERVMQIARPVTIFHTASPEFSNAPVSSYYHTIVDGTEHLLKAAINVRTVRALINTSTPGVIND
NHSDLIDATEDLPILRYPQQKRIYCLAKADAEEAIQAANRNGGHDDRGLLTCSIRPGLVFGEHDAG
SLGKMFAVARQGRSKFQIGNGQNSYDFVYVGNLADAHLLAAHVLLETWGKPRPTDSTVRVDGEVFN
ITNDDPWLFWDFQRAVASETGNPVLPEHIITIPKWLGLTMGFVSEWVVWAISGGTRAPNMTREGIR
FSTLIRTLNINKAKQVLGYRPQVNMQEGIQLGVRWFKEQAKKDT
>g2793
MASIKLNSAHLEKISAAGKAQVPTYRRDGAVKEGIVHIGVGGFHRAHLAFYINQLMQKYGISDYAI
CGVGLQPFDAKMRDALRSQDHLYTVIERSGKGNSAHVVGSINSYLFAPDDREALVAKMANPDTHIV
SLTITESGYYYNENTHELQVDHPDIQFDLDPANKKTPRTTFGFLYAALEQRYQQGLRPFTVMSCDN
MQKNGSITRHMLESFAHLQNPEVAKWISECGAFPNSMVDRITPQTSAIDITKLAEEFGIEDAWPVV
TEPFMQWVVEDQFSDGRPPFEKVGVQVVRNVHDVELFEKHKLRLLNASHSAIGYPGQLAGFKYVHE
VIQNPLFYNLVWQMMQQEVKPLLPDIPGVNVDEYCITLMERFSNPTIMDQLPRICLNGSGKIPQFI
MPSIAEAIWVTGPFRRLCFVTAAWFCYVKGVEDSGENFVVDDPMRAELQEMAHQGGNDPSKLLSIK
SLFGDDLRNDERFIKEVAMAMEAINRDGVMKTLPKYIDQK
>g2794
MQWWKSVFALLLTISPALAIWPAPMNITTGKNVLWLEDDVLVTYNGYPLMKEAMSEPSSNEEFLYA
GVQRTLDNIFHNSYVPWKFYDRNALESTEPSVEAKKTYIRLLNITLAGKQDIDESYELTITSNGSA
SIEANSTFGVLHGLETFSQFFAKHSKSGIYTIQAPVRVFDKPMYEHRGVLLDVSRSYYKTDAIYRT
LDAMSWNKLNRLHIHVTDAQSWPLEIPSIPSLSNEGSYSPETVYTTTDIENIQKYGSLRGIEVYFE
IDTPGHTSSIAFSHPELVAAFSAAPYILYCNEPPCGTLRLNDSAVDIFLDKLMGDLLPRLSPYSSY
FHTGGDEVKYNAYTLDPTVGTNNSDVINELLQRFYNKQHARVRAAGMKPMVWEEVPLNYNVTIGSD
VVVQTWLSADSVKAVTSLGNRVIDSGYDYWYLDCGRGNFLNFNNGEDYQMNYPFPSWCDPFKNWIL
VYSHDPSAGLTKNESSLVLGGEVAVWSESIDDVTLDSLLWPRASAAGEVLWSGRIDPATGQNRSEA
EASIRLSQFRETMVARGVRASLVVMPFCTQGMNGTICEVTT
>g2795
MNRIILPVATLIASAYCAPVSAPLASWFWNTVMITNSANVSNILEFAQVVGLTHIYAQIDSNIPNA
DWEKFIGQFNASGIIVDALLGAADWVLEAGDLETELQWIEQYQNSVSITSRFTGIHMDVESLGMPV
AADLPFWCYLINSTTAGQTMDIWILDRLDSANFMTYRNTSAGIESLASRPLQAGEKTGKGVWLAAE
TGEVANEAETSFYGQTFEETQSALAAVAEVESSNTYFAGIAIDDLTSWMALPGSGX
>g2796
MAVPPADVVESQLSMIDLLIAMFPSPSELEISESTREWLTKLHDCCQEQTEIPAGIPSSLSLAVHL
AVAQEKSIQLNISIFFSESNGSVRPSSYALRQPEWMSKAEVASIAAAMPSDDVFVAIEYIREEAMQ
ILKKRETLTKTVATRSTEPLVRVWFYFPSLSTRGKRNDLVNYASDYSLTGFVLAGKPGILCLEGDS
TNIDSYMSFIRTESWGDIPSHQKKVSERFRETLDVRRVFSKMEEITDSVGERSGQRANRGDMQALV
AWLAARGLQEAFGKVIF
>g2797
MAPRYLFGALLLLRNTFATQQLPLFDPSSLSHSPVTAKELVSSESLQDRIHADSLLERAKALYSIA
ELGEDEYNHPTRVIGSKGHLATLDYIYSTIAELGDYYVLSNQSFSAVVGNVFESRLVLGTEVPTSA
IAMSLTPPTKDKEPVMGPLTLVSNVGCSESDYPDLTGKIAFISRGECPFGTKSSLAGRAGAVAAVI
YNNEKGPLGGTLGTPSSDNIATFSISGSDASPYIKKLKNGQPVDASAYIDAIVENIETTNIVAQTT
EGDPNNCVMLGGHSDSVAEGPGINDDGSGSLTLLEVATQLTHYHVNNCVRFAWWAGEEEGLLGSDY
YISVLSAEENLKIRLFMDYDMLASPNFAYQVYNATNALNPIGSEELRDLYTDFYTSYGXNFTYIPF
DGRSDYDAFIRHGIPGGGIATGAEGVKTKAEQQMFGGVAGDWYDPCYHQLCDNLENLNLTAWEFNT
KLVAHSVATYAQTFDGFPERKGFEAADVLPSKYHGPKLQL
>g2798
MSSSSCIESNSTVIKPPKDNVWEGLTGEETASVVKWLFQQTDLNLTTSEKAGEWDNTIRYAHVVLD

NRASINPHFADILVGPLPITNQSVWAPLEFPYTRKSQGQVRNLNADYDTIYSEWLYKISASIADIT
IELFNGTALGLDNDTIEIWGIDPNWQDDGRIIRWDTFWNYPTDEFDSGTLLPLGLYFKSDVTGRDS
SLWKLEGWLYNDVFYETTDAFREAFFSPGFVKLRPNTEGAWARTDRLGPLLPHDEQQPPQMVAPSG
ARYSVDADSKYVTWMDFSFYISFSRDTAVSIFDVRYKGQRVLYEMGLQEALAHYAGNDPIQSSTAY
LDSYYGFGPYSFELVKGYDCPSYATYLNSSFYVTETTHTHIDSLCLFEYDADYPIQRHSTNQYVSN
TKNVYLTLRSVSTIGNYDYMISYTFYMDGTIGIEVRASGYIQSAYYAHNEEFGYHIHDSLSGSMHD
HVINFKADFDIMGVNNSIEITTIAPTTRNFTWSNGKSRNSMSLQRKILQSEDEGRFNWDPNAMMHV
INQDARNPYGEYRGYRISPAFGTAHLAVLDSSNLVNAAHWAEYDVQVTQQHDYEPRSAHPYNNQDV
YDPPINFAKFFDGECLNQTDLVVWLNLGMHHVPQTGDLPNTVFTTAHTGVQFSPVNYLLGDPSRQT
VNMVRVNYANGSATAVETFGQANGLRAVAVEDVEAELWQYHGDIVVRKFPYDPNDPYYEMNEDA
>g2799
MGLSSVFVAVSALLAGGAYALPTADPITFDIRDENITLARRSEAVNYNQDYIASGANVQYSPNLGA
GSFSINYNTQQDFVVGLGWQPGDTNPITYSGSFSASGVGILAVYGWTTNPLVEYYVMETHDGYQTV
GNHLGTVTSDGGTYDIWEHQQVNQPSILGTSTFNQYISIRQTPRTSGTVTIENHFQAWANAGLQLG
TFNYQVIAVESWSGSGSGQISVSKGTASPTTTSPGGGTTSTTPPSSGGTGSAHWGQCGGQGWTGPT
TCQSPYTCTSINPYYSQCL
>g2800
MAQAHEHFIRSLCNLQVPKTVQLSPDRSTVLYSTELSWGHRKGKHPVSTVWLASTGRADSSRQLTR
GLFRDYAPAWSPDGNAVAFISDRADAGSKWAIYLLPLREGCEAYPITPATSASPIEAFSFSPDGSR
GAFLSADEDIDLPSAGEGPPDVRPWGEAWRFTRLRVVDLRTKAVRCAKLDRHVTSVCWSPDGTRLG
FSSCLSPDIEEPHITGMTVSTIDVDLSSVEDVCHFPKMVVDLSWAADGSLYFCGGVPPPKTFAGYG
VYRTSPATESHHYEWVGFGVEDDSIGLVQRGSGTIFVKVQHRLESRVCLLDGTVLYSRKEELEAFD
SAIETDGKFVLAVATSNINHPTEVFTTLSYGSGAMTRMSNHGREFAGHRFGTCSFLTCTSADGEVE
LDALYLTPASSSDGANRHALATVVLIHGGPNTRLTNAFNTYYYMLTPYLLSLGYGILLPNYRGSSG
RGRHFASYSIGGLGVYDYSDVIALTQCAIDQGYADKERLLVGGWSQGGFLTNLCCVRNDLNPSGWR
FRAAVSGASICDIDAMALTSDLGCAFEPELSRDGVAWNMDYDDTRNRAPSALWAFKDAARRASLKN
APVVPPMLILHGEADARCPVSQAWGLRRALRSYDLPFELVTYPSQGHFFEEQKYWVDMALRIGRWC
QKYIGDALT
>g2801
MTASVLGAPHPPPPPPPPNSGIFNNITVFAPPESWSSHSTSYARSLLLDQNCETENVLLASWTFSPP
DGPYLPIAKSTDLGHSWNEISRVYFTNRNYTGGIILQPFLFELPQKVGQYPAGTILATGNAIPADF
SSTNIEIYASKDHGLTWEFVSVVATGGAPNTDNGATPVWEPFIGIYNNQILVYYSDQRDPLHGQKL
SLQTSYDLSHWGTVINAVAYGNYTQRPGMATVAQIGNGKWMISHEVGLAPQGDIAPYAVHYSIADS
PLEFDSAPSHLLQARNTGTISSAGPYTVWTPAGGPSGTIVVSDSTYSQVFINKNDGDPNMWEEIET
GLGVSYTRGLRVMPDESVILFLNGGYYGLPAATVTAGEWKVPGPSSSRDTISNCKNPXKP
>g2802
MLIDSKRGKYIRKNIRHSPTQRAWSSTITTDGQATSHASQSLTAKFSIDDILRVSDSSFSSFAAGE
HIATTTSADSERKVVDGSSNAEILPAQGPSEIAAEQNPEQQKDKEGSEMLYLRLCESYLSPPRPSS
TTPPGSHRVVYMGESYTFSYLVQKINTPSNISSPVPTTPFQPPKRKHPQPKLQWTVPASVDDKARN
IGYDPQLDLGEVDFLNSVGAFSVPERKISDQLVKTFSDYVFPIFPVFDSADFLRQYDTGCLSLLSL
NAVYLLAVTLCSDDLIAQAGFSNRFMARRTFHRRVKALYHLDYETDKIAIITSLFIISFCWNGLMD
EKDMWHWLGAAIGLAQAQGTEIMETDMVVYIRISPPHTKGRPLRIRDEDCDVEMLQIEDFEGTESP
NSAIFSTPHRVHILSSIHLAKLSVVFGQIILVKFTVQRSIMTELEKAELTKKLETWQRELPRELTN
QHCANQTAESQFCVNIIDILYSHHLILLHRPNCTDTNSEAFSAKSPAFSAASSVTRGIENLMMSDT
LRQAQLHIIPSIFAALAIQVLVIRNTKHDIQKRLAENHARQCMLSLGEMQKSWPVCSWVLKLFEQL
LSQKHGPETTSSSGSLITGARNSDSPVQEKNGGTGXDPTLRGTNVEQTGSRLYQNDVDGVACDLRE
VGRRQDEEDSSNIDLMQPFANNRSFALDNTFSAGDYGFDLNDNTEFLSYLLAQAPPT
>g2803
MVPKVLNHSSELQISRSCIILGGSSAGANLAAVVTRKAIAGGIPISGTLLQIPVVCHRNCYPSEEY
ELESMRQNEDAPLLSRAALDQFWAYYNPPNITDLQVSPLLAKDFTGFPRTFIQICGLDPLRDEGLA
YARKLWNFDVPCSVVVYPGLPHGFNAFTELSAARVYHEDMLKGLDGLISGEIAGGIRNYHGK
>g2804

MGQVSGFNLSIVLALCLGSLTYGYILAVITNTLGQPGFYTYFNLTTDITETEKYGYTNRIIGAING
IFSAGGVFGAIFSAWMSEVHGRKRTLSITTIVTLIGGALQTGSVHVGMFLVGRFVSGFGAGMLITI
VPVFQSEIAPPENRGMLVSLNGVLIVVAYSLAAWIGVACYFSTNVALQWRFPLAVQILWPLLLLAT
LPIVPESPRWLLANGNTQAAWEVISKLHHESAEGLARAEFEQMLQQIRADIAAAENESLLDLFRKP
SYRKRCICAFLVMYAGQASGNLVIANYSVIIYKGLGQTGVIPLILAAVYCSVSVFLNYLCALFIDR
IGRVTLFVIGLTGCLVCLCVESALLAEYIASSNQGGLKAATFFIFFFIVWYGGCIDANTYVYCAEI
FPTHIRPRGMALSMVTLFVTSTPFLEAAPTAFATIGWKYYLVFIVLTAINIPIMYLYLPETKGITL
EEISGKFGDHVIIHLNESRGKVDLVETENVDKGDGVRNGNVAGESLSV
>g2805
MQSNDHLLLNEVTRPMFPWRPAKGLLSYGCAFTELLHTNLTRMRKERVFVVVSKSLAESTNELNRL
KECLGTRLVGIKIGFPSHPPFEDVLKLSDDIQXADADAVVTLGGGSIIDAVKLVALSISNGVHDFD
SLEGLSKATAYTMETENDFANYTDCPDIKPAALTKISIPTTLSAGEYSPYAGAVDPRDGVKKLFIH
QSLICDVVILDPELTKTVPEWVWMSSGVRSIDHCVELLSSLRQHETQTEESAKRGLVMVARGLLKL
RLNPQDVESRLETQIGSNYAMDGLSRGIHLGASHAIGHQLGTMNVAHGHTSCILSPAVMKYNASAN
AHRQKRALDTLWGDPYIHDVLERCGLIHSQSDLGDTLHELFGQLGVPRTLKDVGVSGEQQLETLAQ
RSLEDPWCRTNPISLTRPEQVMEILRKVEG
>g2806
MDTEKKGDISPPSVAYLNSLPDKYGWQRENEHGYRIKEQLYGTQRPLRVIYVGAGASGICFSKFMQ
DRMKDVSLVCYDKNEDIGGTWLENRYPGCACDIPSVNYQFTWARKPDWSHFYSYSEEIWQYFKDVV
DRFDLTKFFRLRHQVVGAYWDDEKGVWDVHVKDLTNGKTFVDSAEIFINGGGILNAWKWPNIQGLS
DFKGKLCHTANYDTKIQLHGKRVAVIGIGSSGIQVIANIANKVSQLYTWVRSPTWITAGFAQKYAG
PNGANFEYTPEQKQDFAERYLEYQDYCKAIENELNQRFKFILNGTPEAAESKQFAIDQMKEKLHGK
LELAEKIIPTTFPVGCRRPTPGNGFLEALTLPHVHTFTGEVQKITPKGFVDHFGVEHEVDVIICAT
GFDTSWSPRFPVVANGKNVQDLYAEKPISYLAIGVPEIPNYWTMGGAYGPVGHGSFLPIIELLSTH
ILSIIKKMQTENIRSLAPRRERAEAFVEHADLFLKRTAWSSGCSSWFKRGRNDGPLTMFPGSRCNP
FEFLGTGFTIGEFDGSDLSYYLGTKENPGALLPL
>g2807
MELSSCVNIHTLSPTHFLPRAAAIEPEATAIHHISSSKNVIRRSYKDFARRASGLAYFLRRGGFQR
VGILATNTPAFLESIFGIAGAGAVNVAINYRLQPDDIKYIFEHAEVDMIIVDHEFAHILNDFRLAH
PQVPLLVDSDTDGISGPFDMAISEGQAIDLRNGDMGWSGLETQVPQEHAMLAISYTSGTTSRPKGV
VYTHRSIYLAALSNVIESGLYSDDRRCKYLWILPMFHAMGWTFPWAVTAVRGTHYCLRKVDYPQIW
SLLIHEHLTHFCAAPTVNRLLCHTKEARRLPDPVSVTVAASPPSAALFEQMTALNLKPVHAYGLTE
THGPITRTYYMPQWDKLQANVRYQMMARQGHGVVAGLPVRVIKPWLPEGELVDVQRNGQEIGEVVF
QGNLCAHSYYKDKEATRKLFAGGWMHSGDLAVWHPDSAIQILDRAKDIIISGGENISSVAVENLLL
DHPDIIEASVVGVADEKWGETPKAYITVVPGSKVTPADVISWARNKSKISKFMMPREVEIISELPK
TSTGKIKKKVLREAARKASRKSSKL
>g2808
MMGKRIFHCGPQGTGLAAKLANNYLLAISNIATAEAMNLGMKSGLDPAVLAQIINAGTGRCWSSEV
NNPVPGVQSAAPASRKYKGGFLVGHMNKDLRLAIAASSEAKAPLLLGPKAEEIYTTLENNQETRSC
DFSVVYRLLDEGVWYETAASKI
>g2809
MFESTSDTWYDVCDPATNKVVTSVPQNTDEELEAAVLSAQEAFPKWKATSIMTRQQIMFRFLSLLR
KSWDRLAASITLEQGKTFADAKGDVLRGIQVVETACGITSQLTGDILEVSQDMETRTYREPLGVVA
AICPFNFPAMIPLWSIPTAIVTGNCLILKPSERDPGATIIILAELMRGAGLPNGVLSIIHGKHKTVD
FILDHTLIKAVSFVGSNQVGEYIYTRASANDCNKKDTLNAVTAAAFGAAGQRCMALSALVTVGDSR
SWIPDIAARAQALNVSGGFENGADLGPVINPESKQRIEAIIGNAEREGATILLDGRNYKVPGYPAG
NWVGPTIITNVKPHMECYREEIFGPVLVCIELNNLDEAINLVNGNEYGNGAAIFTASGLSAQKFQT
LTEVGQLGINVPIPVPLPMFAFTGNKRSIAGGGANTFNGKTALSFYTQQKTVTSAWKKNDALEGNK
ADLNMPTQS
>g2810
MTEHKEKSSSEGPEGTREELRFLRRCPEDRVINYSLLWYRLVNPSEAELAFLVCSRCYTDHIQNTS
LVAEFEPVLQSEQFAAACSFWIPSVHDCFWPKALSTGNTVVFRKYVSRWVESPPCPGVQGSRDFGS

TWYGMVNGDVDGFMICEGCYVDNVLGTTFQNKFTTYSSEWESDTWACDLRFAFTSRALRTYTKLND
WNSFVFAAEQRHGLLECTGDEVYANSNTWFYPRHRPISNMRICAACCMDHWALTQFDEEFQQEYRG
PPVGSAEYMLFMTERWRCHVGINLPVLMTIQEAKAENNFNIFTEAIKVILESPSCSENGMTEGEGW
TLLGGCEGFTICYPCYSGIIWSTGLFIDFFERISWPKDNVKICSFNTSAPRFELVLNKLHESLDRQ
DFSFFSSFVKQFSSLPVCPRNTVYIGRPWWGYPQARCCAECWVDWLSHTPLAMTMPLRGAVFEEGA
ICQMWSPRLRTLWLSVCAEGEPGSAKSEAAVTKFCEIARGREEIFWKTIPAIKRLRSLKYAENLNI
QVQAMASVNYAGMKSLSVVSGMTDGNRYGNGSIGWYDTTSGAAAAQARNSMNQSMRAVSSRDAELY
RLQASWREVE
>g2811
MAEHPSFTLAALLPAGGVLGYLKSRSAPSLIAGVGLGLSYGYAGYLIKENKDYGTELALVNSVLLL
ASAIPRVIKTQAKFPMPIILAVTGSLSTFYYQKKAREFRFGV
>g2812
MHFLCLHGAGTNSRIFEAQTAALRYELGGDHTYDFVEGVMQLPKASAIDQLVPGDLGYFGYYKPND
CTSFHKALDDLELFIQKQGPFDGVFGFSQGATLASALLLRHSGSSNGGLDSPWTPKANDPLFKCAV
FLSGYSPLDWTCMHVQHMQGSTISVPTAHIWGMNERGDFGGPTALKQLCDPQTVYLHEHEGGHEVP
GAKNKKDLIQSANAIKRMLTSL
>g2813
MHLHIKVFCLWGVLAVKGLADDIDFSAAAQSISATLADPSFVPYTKPSQKVEEFIALGDSYTAGTG
CNGQDEKLSGEAFRGQRSYPMQMSQDKDNWAFINGGDETIPRFSFHAYTGDKSEDLVAKQLTQGDY
KDGNMSPRGQPFGSPQVAVLTIGGNDAMLSDILNNCIFRAWNPGNCQSKLDALQQEIDNGNLRDKI
NYALYEVARAGREAGGANPRESFQVYVLEYITFFDDAMSTECNDYTWAFFPWLSEPKLTQALREQL
NDKTRQVNAVIKAAAQDLQRMGVVFVEGLQDVYNGHRYCEAGATPRQTEYKVWFWTTYGITSTTSE
GPGDPNDVAAIDSTDPAQQLLDFVFPGQNKTVAANSAGDTAPWEWDGAEAYPDYQSLLNAISAAAD
DANTDSWIPFTLLRSFHPKGTAYGEHATALFAAIADNRVAITSNGTGQQIAVASYINPLADPSAWN
RLIDYPLAKMPILIANVVNGPDSAVDDSWTDVISRASASGKTVLGYVRTGYLGVSDQKFLTRLGSG
DLADWTAQIEEDVDMWYKLYGSNIGGIFFDEGWPECGDNNQYADLYKYINDYTKRAHPGAYTILNP
GSPMASCFEDTMDTLLTFELDYTAYTSSYTPNDWVPKDPRKLWHIVYNVPESAIGEVVKLANERGA
GFLQITDDTLPNPYDTLPSDSYVQTLLDAVAGGSLSKSDASAWASGGDAGAVSGLSVSFSDYSSAK
LSWSATTGAFGYHVYLGDTVVASVPSSMTTITIGGLQPGTSNALSIRAVGGGGNTGSASNTVTVST
ESLPLGKTITDYNVTAGASSTTYHADILVPYAFVRLYLWDSVECDFDNDPGWSVNFVTDDYVCTHY
MVEGTTLYKYSGTLPSGSTAPPWSWSEVGPITLSISGYTYSWTLPLGTSTTDTNKFVIQAQGYNPF
INVFDPDPSAYDCKGSTLCTTPDLLKWCDHAVNSLERNDLLTYGTK
>g2814
MLKSNVGQLLLLCTTALAILGSSTEIAQQKVLVGVDIMDEQEKVRGHNEAVYDAVPKEDQLFRVEF
LEIAPTPIIADRVFFVYLRGDIPESRKKELGLPDNGLVDATITVSSSVVYPDGSYDDEQSVTLPLK
TTPYNDIAHLLIRDADGNQADYLPSRRQSDIIFDFQIPTMFLKGGMWTFKADARAGDKNNSCLFAM
SLTQWLQP
>g2815
MSAEEVKTTNGNADAATDVQNLLAELKEGAEKPETENPVEEDEAAKEARIIAEANKLGKEQESKEE
RKPADRPRRNYRENIKSDLTAQEVTDDHNEIRKQVEFYFSDSNLPMDNFLLKKVGGSANHPVELSI
LHSFKRMRRFQPFSAIVEAMKTSTVLDLVDNDTKVQRKVPLPESVNDFLDPNVVKVFENEAMSRSA
YVKGFGEEGPKTQFEIEAFFAPFGPTKAIRLRRAFDKTFKGSVFVEFDTEETLKKFLALDPAPKWN
GKDLIIKSKKDYCDEKVKDIQSGKIKAGHRNGGHQGGRGGRGGRGGRGRGRGGRDNDRRDWRERRD
EDQKRGFREDKPAVQKDARGIPVIKSTEEPATSGQKRAREENGASEHAAKKVDAKEG
>g2816
MADSVDSAPPRPDQWSVADVVYITLVTPLILAAFAEWFLWLGAFLFGLVNVYRKAEHWTTRAIAVF
IMILFSMLRSIFLPVMIVTLPLPNNITRHFSWALVNVLQWFAFYMFSVLLLVPWALCLYRLITSEI
GRSKRIKDVLHDTAAPKVVVVMPVYNEEPGILIKAIRSVVGSDYPSSCLHVFLSFDGEPSDQLWDS
IAMQLGIPLGVSKRAQSIDAIYHSVRVTVSRFPHGGKRHCQKRTFKLIDRIYADYLARHDDLFILF
IDSDCILDKVCIQNFMYDMELKPGSGHDMIAMTGIITSTTKTMSLITILQDMEYIHGQLFERSVES
TCGSVTCLPGALTMLRFSAFRKMAKYYFEDQIDKVDDFFDYIKCYLGEDRWLTHLFMVSTVKRNQI
QLCTGAFCKTQAVQTMSSLIKQRRRWFLGFISNEVCMLTDVRIWRRYPLLCIIRFMQDTIRTTALL

FFIMILSVSTTSTSLASLPLGFIAISLGLNYALMLYFGYILHRFKAWLYPLMFLLNPFFNWLYLVY
GCCTAGKRTWGGPRTNAPKADVHTTPREATEQAEAQGDDLNVDVSTFREYGNATIGVPLHPTESVT
NRLANEPNYDGSCDFNLESELSLMEHDREESAIPEIPLHPRASFDSTTTDSYPISLPLPVESLVEE
ERKLQDLRDFDKQTLDSDSGVQLQTPVRKPNYRRDSLSFPGRQRDFGPRSSTTLSAMSRIDSSGEI
HMDPMRQLAPLHLTPSPLGQSCMQNTVPEDDDHEPTVRAPLFGGIGRRSIKKGRLLFTLGRKSGSQ
>g2817
MSVIGIDFGAQSTKIGVARNKGIDIITNEVSNRSTPTLVGFGPKSRYLGEAAKTQEVSNLKNTVGS
LKRLIGRSLSDPDVQAEQEYNTATFCEVEGQVGVEVNYLGKKDKFSATQLVAMYLNKIKQTASKEI
KLGVSDVVISVPAWFTDSQRRAMLDAADIAGLKALRLINDTTAIALGYGITKSDLPGPEEKPRRVA
FVDIGHSNYTVSIVEFRKGELNVKATAWDRNFGGRNFDKALTDHFADEFKEKFKIDIRSNPKAWAR
TLTAAEKLKKVLSANAQAPLNIESLMDDIDVRAMVKREEMQEMVKPLLERVSVPLEQALAEAKLKP
EDIDSIEMVGGCTRVPIIKEAVTNFFGKPLSFTLNQDEAIARGCTFSCAILSPVFRVRDFSVHDIV
NYPIEFTWEQSPEIPDEDTSLTVFNKGNVLPSTKILTFYRRQPFDLEARYVKPEALPGKVNPWIGR
FSVKGVQAQADNDFMICKLKARLNLHGILNVESGYYVEDVEVEEPVEEGEKKDGDAMDTDAADGEK
KTRKVKKQVRKGDLPISAGTTGLEEATKSAWTEKENAMTMEDKLVADTDEKKNELESYIYELRDKV
ETTYAEFSSEEEKEKLRAKLTEAEDWLYEEGEDTTKAIYVSKMEEIRFIAGPIIQRYMDKIENERL
SKLQAQEEEAAKKRAEEDAKRKAEEEAKKAAEGQKTDAPDAEMKDAPAEGEAKNDLD
>g2818
MAVDRLSSLLNHLKPGNSGIAAITTKNPDDVVVTLAIRTPLTKGFKGGFKDTSLDYIVYTVLKQLV
ERSQIDPALIEDVSLGNVGDGRAAYLGRAAALAAGIPHTAGASSVNRFCASGLKAIHDIANQIRTG
AIDIGVAVGAESMTFGGRGVDTFHEEILKNQEAADCLQPMGQTSENVGDDFGITRQMQDEYSVESF
RRAEVAQKAGWFDDEIVPVTTKVKDPKTGEEKTVTITKDEGPRYGTTYEGLSKIRPAFPQFGNKTT
GGNASQVTDGAAAVLLMRRSKAIELNQPILAKFCGATVAGVPPRVMGIGPTAAIPKLLKQFNLSVS
DIEIWEINEAFASMAVYCLKNLGIEHEKLNPRGGAIALGHPLGATGARQVVTILSEARRTKKKILV
NSMCIGTGQGMAGLWVNEQV
>g2819
MSDHERSTSPGRNDSTIDITVHHHGAPYTFTLPATATLDDLATSISSSPDLQIPIQNQKYMIAPGK
LGLQKPPFNTTTPLPEYLPLSSPKLKITLLGARAEDIESMNENISSTKQRLAARAQARRQPHPPIT
RRPPGGIHTLSSPSSSTYTFGRCEPLSYLPNPSRSLQFLERLRDDPGIKFAMAKHRFYVPLLTEMN
PAEHTTHESRTLGLNRNKGEVIELRLRTDAYDGYRDYRTIRKTLCHELAHCVHSEHDRDFWELTKQ
IEQEVEKGDWTRGGHKLSEQEFYNPSDWEEEGQRGDEEWVDHGGWTGGEYVLGGGQAGGQTVGQAA
GGLSRREIIARAAEDRLKKEQQRGRGSNAEGET
>g2820
MRIQRILFGLAVGSTYLSQIVTSAPIDDNSQNYEPLPPPVLDGMSYLTLNRSLEDKYFHEPVGHEQ
GADDRLGHYDVRYFHGMVSEEERAESLHHMVIAYLDFFRLNKLETWIAHGTLLGWWWNGELLPWDW
DVDTQVLDTTLARMARDFNRTVTSYTSTDKKFKRKYLLDINPWAYFRDRGPGHNIIDARWIDTSNG
LYVDITALSRFNAEDEPYTWECKNSHVYRTNDLFPLRQSTFEGTKALVPFRYQELLTEEYSVKALT
QTKFQK
>g2821
MPQETISTVFGDLHAIVQGNLASDKTLLLLHGNSSTWKAFEPILDDAVLKATYKIIGFDLPGHGDS
ADAPDPQLSYNIPAYAKAALEVLRHFRVKSYVAFGSSMGGQVAWSMIELAKSSPKEFDIRGVMTSG
SVPFGNLRDLQESFNIDSQDSVATTGSMTDEQAEWAVKHAYGGTPQPFMYESLKRTDPRARPLMFE
HIVKGESTDQRRTVRETKIPIAIVNGDMDVFIKLDVFDRLEYGNLWKDKVFTIKGSGHCPYWDNPK
EFLPFLTEFAADCYSA
>g2822
MPAPLAKGIIIGVSVLVAAGIAVYESPQFRRWVNQSRRKVAVALYNLGDEIHPREAHSVDISMTEE
TGAAAQERRRHAREEIERRREFNQARRRSTQSSSAAVSFDTLVDQDGRLKSEDGEDDPRGIIMNDD
HATAQSTALDTSNLTSLHKRGGSPEPEALTNARLQAQHEMLEAMERDRLHLTLPSETPSHHPSESL
IDLTPTSEFPESNQESTSNRGQMENSEYFSVANLSNSTHASEIGQSEYFYAHPNQSSSSTNSSIPA
NPFVDHDVSTASSIPGSLENVHDELSSDTLSEWGQPTDGILTPASWSEVGSVISSDDEHHHQQ
>g2823
MSSSTQRNLTLTEELERLEQSITLTLQEIDSNFSRAHRIVTTSILPIVEQYAEHSKDVWEGAKFWK

QFFEASANVSLSGYEEMPNEDTTTTDHEQTVTEDSTNLTQTSLDDEAASYQTPSSEHLDLGHHHQH
DESELDISSLTISPSHSTPRPQTYLKGGETPTAGGYPSAYGEDDTGRVGYEDSALAEGNDDNTAPV
TPGRNTTRFQPNTNETPMSSPFIPPPTISRTTQLSTSKKTTQKNYQKPTDPVLHHVLDKTYRVQAT
PLSKGYKPTKFSVSTPKEKTATTASEGKHAYDDSPLSSPELEAPKLRSELFSYRGAQNTPGTNRKT
RHHRSPEKRERTTPRPGVSVLTPAKPRLSLVSGNMKTTRGGWESDEDDEDFDAYDPEDTAAALGFS
PPKTMQFHIPQSRLMKTPAKEASKRIVSDLLATAGGTGTTADDDMTEDFYHRYRAGPEEDEHSPTV
VRSGGRLEDDTF
>g2824
MEDTRSNRDVETLSAREYPGSPEVPTRGYGTEERTSSRSRAEEPPAAQLEPPQNQDNLILPSSAYR
PDSSKRQSTMSTKSEVDTGEFLIPVAFDPTPDERVSPHNEVPGLEQARHQPQDYFDHSRPATSSRQ
TSVDRTETTQASAPHIAFQQKERQASNPVADQNRWRQEPTPKVAQTKAESDGFKLQEAPRGRKSAS
THSSKSDLNAAKEGTSVSTGSPGAVTAASSESSKAPQPFTEKPTSPPISIASPPVRSPDQNRSSYV
EIEHDRADTNMTLPKSSTVQALPKRGDSLEHFVSSQIQRKDVGSTPKASPSGRGDHRDSKESADVS
RQLTHNAETELSKEVSADRTNVPISLDNYHGRNGSNAPSLSDAIKNNLASSPALLRYSGGGDFSLD
EDLARILGPEAQTGKSESAKRPMSSSVRHGRSYSDKGSIMSKDGKWPRSPVNGNTFAQDPSSPDTP
SPSEQREDISWLRGELRRERQRVMERDQKIAELERALNAQADIKKANTELREKRSTMVVLDTKKEV
VMRELSAITEHLDREKHGAAPMDLAKLTNNVVRNFAEKLQQLKDSFAPQIEELMQKRNDIVEEVAN
LSRMKDKSFQEFEQLSSKNAQLAELNNQLVNQIQGLYKASSATESNRSTNGLGIYSHNKEKSTGSV
DASKTSDLASSVSTMTIQDDGDATIIPGPQVVSIRKGQVRKFNWKRGGQSVAKGVSKGLKAFSSER
DMIGPDGTVQQIEVNPSLPRSQTQEPRFGFFGNQKRQAAWKNQTNGSSPALADATVNSESLFGVDL
EQRLELEKSIIPSIVTRCIQEVELRGMDEEGIYRKSGASTVTQFIREGFEQANDFDISDPDLDIHA
VTSALKQYFRKLPSPLITHEVYDSVIDTFNIPEHSTRIEAMRNSLDGLPRVHRDVLEFLVFHLKRV
VEHEKTNLMTSQNIAVVFAPTIMQPKDIAREMTDVQKKNEALKFLVENCQEIFM
>g2825
MEARATELLGTLKNPNVSIDAKIAALTNVKSDIKQKNVPEPAALPLFEALRLAISAQHSSLSSAGF
STLTHFLKRLYIQENHHLVLFHARQLYPICLERLGDHKERLRAQASQAFTDLWIAAPQDVEAHVLG
TAMVGKNARAREMSMIWLATMTREHNILFRAHVPSLVAALEDADSGVRETAKTTIIEIFQNAPSRA
KSDLKRQLSENGVRKSIVAAILSNLGLSDHDVLASSRSETRRPGSSLAVSRXRDXPPRPTSVLSQR
PQSRAAIAREDSEHTLRPAPKVDLHSGLAHSEYVEPIFVDSNRDIDDMIRNMQPHFEGRETEHNWI
PREKSIETLRKLVRGNAPQAYSQHFLAGIKSLLDGILKVVNSLRTTLSTAGCLLLQEIAKICGPGI
DNMVELLLQNMIKLCAGMKKISAQNGNATVDAIIQNVTYVPRILQHLSFACQDKNVQPRLFAAGWL
KTLLKKQARHKSSIEHGGGLELVEKCIKKCLGDPNPGVRESMRGTYWTFHQIWPDRAEEIMSSLDA
KSKTLLEKDPHNPSAAPAPEGRPRPGFSQSVSASTQRPSLKETIAAQKRAAAAKNIPTQNIPPRPE
SAQSTFADTKPTRPTARTPAATKSTSSVRTVPTGTHLSSLSSAPMRPAVKPRRPELARPATADPYS
DRRPGSAASHVKPLSPEVSPAKARPKSVATPSARSPARPKSRAGGAPASSTARSKPKRLDISSLKN
GEFRSAAGPRSDSDDIIKPQPTIQNEIVESSPRYEETTQYSNVIALHDHDDMPTTPITPAMPATHT
HDDVMSLEEPQVIVDEEPTSSNTITSPSRVPLIHSRTNSVETNGTSSSVLVSPRPRHSRHGSEDRP
SHIPSPIASRSLVLEEPRSVGLTVYENPETNVSPETYSEKLSKSPEVPKKPVLEEIPVNEPSAPTP
RIAKQQSMPVSSLNGNIASPNALENSHRILGKIGVERRRSLSPRSKDPTKAKEMIDKGIARIKAKT
LDVHGYRKLQGLIKYHDSIFIDESKYDEMLLGLLDALEVPTDEKRTSVPRSMDLKIQILVTIRLMF
AYNGAWFAVHYPRVMTALLNTRKNYELTHHIVSGLEETAEDIIASCEAVDVIFAVLDLIETEKHDD
EGNRAITMGIYILSGLLKRLNKDHRTLNEGEIERLGRFANACLAQSQPDIRRAITEFSVELHDMVQ
NEEVFWRMVNPAGNDQRNLLTYFIAKKTSQAVRA
>g2826
MAPIHIGSLVYNYQVLDVLGPFDLLGSGGKHILNAVAYYTNIDKNILNRAPEFVFHHIGETLDPVP
LLTSSVIIKPTDTIDSCPELDYLLVGGPDVKDFELSPRYADFIRRHVAAGKTVFTTCTGAGVLAST
GVLNGKNATINNLEYDWVAKKYPKVKWTKARKWVVDGNIWTGGGAVAGMDMFAHWFKENFGLDMLI
QGAKGLDYEPRDSDGLFKVFPQRYDEDGNQLPTLVLP
>g2827
MEGSNTVDVSWLHHSHRAEQLLRTKAATSSVHEKTNGPADSKLERSGSNKETSQRSPDEPPQSTGT
SQPKTNGPHTEQPPSNMRNAPEKKAAINPPATPVKGSAPKAVPGRRNSWISSISSKFTTGTTPPGQ
GAALHVAPKLSPPVKPDINPFGASFSPKDKDPVIGESPNAFTSTTSSPKNPSFLQNAFRKFSSGGG

GMGKATNNGGICERRIMNVDPNRDRCKVPELSQGKLKRVAFCVDVEIAGFASREDDEVTKREPTNV
PTSQASKLKAKEKSEGSALKNPEASPEITNEPKPNDVDQPSPTLGVNGDATADVRTKEPTKKQEKK
KRSEEERKERKERKRRQAEENGTIPMQFRQGEDEQPAVPTAAPSGTRSKTQSHPTTDPLVEQMTSP
SSTLVEAPGTVAVLDLSDFPMTLQDIVTFNEARNRRRSTKEKLASVPPLFGVVEKVSLKNNTKIGP
EAIDLSGIPFPKKIANGSSPLKAGDLGITLANSLTTRFGGDYLEELLLSECQPSTEDIEKICNAAA
TIKLRRLGLANNNMTREGLEHVVRYLRCGNCEGIDLGGNELHDHLNLLTDAIDSENPLLALSLADC
SLTPATVFPLLQAFPTLPNLRFIDFSHNPALFTTQPDCLSMLRKFLPRMAALKRIHLADVDLSPDH
AIALAEVLPECPSLSHLNILENPRIASLGSSSDPADQEEACAVYASLMAAVRVSRTLIAVDIEVPT
AESNEVVRALASQIVAYSLRNLGALEELSDASQVQRSEVPIPEVLQHLVGHTGPVLSTEDEPAPNE
DYVIGGTGVVKALGVCLGNLDRQQAEIGGGSRPGSGRSTPVPQVTSSMQKTKKARDMSKNLLGSAR
NIQARIKIALVREDRAGNDANYRRLQFLDQTLQKIITRFEEEFPECRVSADSQTEETSSQRSSEDS
HEPITAVDSAVYVPDISTAEDEEADPFAIRLSRRGSNTSLHSRALTSEEGRLHRISQHLRRDFLNP
SLIAPEDGGAVSGDDSHINALREKLQNLKGDEKMGTHPESVGADKAFHELGSTVEELWIIQQQDQD
AFERWKQSQIAAQINAGLRSPPFQAGESSSSQPSK
>g2828
MPFTKTVKNSAYYSRYQTKYRRRREGKTDYYARKRLITQAKNKYSARKHRLVVRFTNRDIITQIVY
SEITGDKVIASAYAHELKRYGIEHGLTNWSAAYATGLLLARRLLKKLELDETFEGVEEADGEYTLT
EAAETDDGTRRPFKVFLDVGLVRTSTGARVFGALKGASDGGLFVPHSENRFPGYDIESEELDAETL
RKYITAGHVAEYMEGLADDDEERYKSQFSQYIDDELDAGDLEDVYLEAHKAIREDPFKKDEDAGEK
KTKEEWKAESKKYRTGKLTKAEKDARIQEKIRELAA
>g2829
MARPAQPRALLFDVGGVCVVSPFQAILDYEKSNGIPPGWINFSISRTAPNGTWHKLERGEILLDAD
FFKGFKRDLQQPHLWNEYQQRLRQKDGNSSSTDAPLPNIDAEALFWEMMRVSREPDPFMFPALGKL
KASGKFLMGALSNTVIFPPGHPFSEDKIGLRKQFDFFISSAHTGLRKPHPDIYAHALKTINKIAAQ
KGQPPISASEIVFLDDIGENLKAAKKAGFGTIRVVLGKSQNAVKELEDITGLQLLDGGDRPKL
>g2830
MEKSRTISEIKTSFIRTQIRILSASIAPQEGWRDFAPDTEDDLSDKVVDEVVQKLNTTLKQHNRVV
YSSQAIQHVARQIENLYWESVKSEVLSPSQNSTGVEIGSDLSNNSVIEKLPSQYRGDDLPPEEKER
YKMLYERLVALDNRRQEKQKQLAQYKQLEALLEPFKNPQENIQPNLLTRDGELVREIDKMRIYHVT
LNELHLDLTHLLEGAPARETSRAHRESMTTNGNNAAAMGNYMENSPNVVNMANMSRQSMSPSPSQM
NGNGGGGGGGGAGGGGNGMGNNNNNMMMNGLPMNAGHQMDLNNLYDMVVEFSDILKNNREMTRGIV
QSAEEIMRRSTVEGTSPDIHQVSGEISAARIAELERALARERRTVEALEREQIENTSLIGEFETSM
GIMVEQIRNYCQNNNMYFLAQKKEFNALLQAERDAHLSSRLDRDHWHAQTMRLAEMLRTAYRLRCE
EDQAPTTIIQALQSEVRALRSAVGLEPEKPEEETGYEFLKDLSPPVVD
>g2831
MNLFRRIRAVALWDSYASGKTSFLAKPEWRGLSDTPYDLLLDILVRLTALLSKADHIDSMAASGDD
AGATQAAQKLMWKCHSLRDDLVTWYADFQTKETGPLFYLEPEEQHPYLDPDSDLNDVFPDSVTFQS
PYIAQLLLLYWYGEVVVHSAMSTAHRHLWGQSPNIETPSPSSTSISSPSTTASVTTERLTEARQQM
TMLEGETLANIETIGEYFAAKICQAMASIGRNSLRGYGFQIGMVPLWSAQQYYQNRSARKYMWCRT
VLMNFGRKGFVVGQSLGTLALRQYPGRYVEDSSSSSIEELTS
>g2832
MPPYNMLTSAFKAPEVNPLTFKALSIPIFNPINRYGRVFFFSWLGFFVAFLSWFAFPPLLTVTIKK
DLNMSQADVANSNIMALLATLLMRFICGPMCDKFGPRWVFITVLLCGAIPTAMAGLVTTPSGLIAL
RFFVGILGASFVPCQVWTTGWYDKSVVGYANALAAGLGNAGGGVTYFAMPAIFDSLVKHQGLSDHK
AWRVAYVVPFIIITAIAIGMMFLCDDTPTGKWSERHLGGTDTPMMTSSNPDSSAFSSGTVTPRIAN
EKIDGKIASTSSAGSDTEAQVDNSEGDLIDEMIVAPTWRETLQVVFSLPTISLAALYACSFGAELA
LDGALGSYYTKNFPKMGQTKSGEWAAMFGLLNVVTRPLGGYIADVIYRRTSSVWAKKIWLTFLVIV
TGVFLLAIGLTNPTSESTMFGLMAGMAVFLEAANGANFALVPHVYPFANGIVSGTVGAFGNLGGVI
FNIVFRYNGTHYERSIWIMGVITLGIGVLQSWTRPVTKEVVVSGKH
>g2833
MAPLMHEDPSIEGVTADADNHHESAAETVSSRKKIVIVGLGMVAISFIEKIMKWDAERNQYDIVVI
GEEQHLAYNRVGLSTFFEHRKIEDLYLNSQEWYSSFTDGSFNYHLNTLVTEIDSTKKQVQTSAGDW

IPYDLLILATGSDAVLPESTPGYNANGVFVYRTIDDLERLIKFASHHKGETGVTVGGGLLGLEAAK
AMTDLQDFGSVKLVDRNKYVLARQLDADAGSLVTEKIRELGLDVMHQKKVARIETDEGNNVIGITF
EDGEKIDCCCICFAIGVRPRDELGATAGIECAPRGGFVINESLETSVSDIYAIGECASWENQTFGI
IAPGIEMADVLSYNLTHRGEVPKRFHRPDLSTKLKLLGVDVASFGDFFADRDGPKFLPGRRVSAEH
VRPFEASEQRKTSTNKVKALTYKDPFGAVYKKYLFTMDGKYLLGGMMIGDTKDYIKLNQMVKSQKE
LDVPPSQFILGSQTGGEENADDLPDDTQICSCHNVTKGDVVESVKNGTCSTIAQVKSCTKAGTGCG
GCMPLVQSIFNKTMLEMGQEVSNHLCSHIPHSRADLYNIVAIKQLKTFEEVMKACAKNPESLGCEL
CKPAIGSILSSLYNPHLMDKPVHELQDTNDRFLANIQRNGTFSVVPRVSGGEITPEKLITIGQVAK
KYNLYCKITGGQRIDMFGAKKQDLLAIWTELVEGGMESGHAYAKSLRTVKSCVGTTWCRFGVGDSV
GMAIRLEERYKSIRGPHKFKGGVSGCVRECAEAQSKDFGLIATDKGFNIFVGGNGGAKPRHAELLA
KDVPPEEVVPILDRFLIFYIRTADKLQRTARWVENLPGGLKYLQEVIVEDKLGICADMERQMEELV
GSFFCEWTETINNPERRKYFEQFGNTEETVETVEVIKERHQSRPAYWPKDGAEVDFKGHQWSSLSW
QPIIQASHFSVSKPEVSSTNIKRGDTQLAIFKIKGKYYATQQMCPHKRAFVLSDGLIGDDDAGKYW
VSCPYHKRNYDLNGEQAGRCSNDESMNIATFPAEEREDGWVYLKLPPTEELDALLGTEKWKIKSTE
SEDPFEKVDRIYKGTRGEKIGDVKVAPKEVSCGGAGSNIDW
>g2834
MATETLGYKEEMLEYSLPVSSESDNGSTSQSDSDSASTVSDLLPDIPLPPPSKAPTEILSVDLDTP
DNHVPRDPRLIRLTGVHPFNVEPPLTTLFKEGFLTSPELFYVRNHGPVPRVRDEDIPTWELSIEGL
VENPITLDFRQILQEFDQITAPITLVCAGNRRKEQNQVRKSKGFSWGAAGLSTALFTGPMMRDIIK
RAKPMRRAKYVCMEGADKLPNGFYGTSVKLNWVMDPNRGIMLAHKMNGEDLRPDHGRPLRAVVPGQ
IGGRSVKWLTKLILTDAPSDNWYHIYDNRVLPTMVSPEQSAKEPKWWYDDRYAIYDLNVNSAVVHP
QHDEVLRPAKAGPTATYTAQGYAYAGGGRRINRVEISLDKGKSWRLANIEYPEDKYREYESNLFGG
KVDMFWRETCFCWCFWNLEILISDLTESDAILVRAMDEALSIQPRDMYWSVLGMMNNPWFRVAVIK
DSDTIRFEHPTQPALMPGGWMERVKKAGGDLTNGHWGEVMSNEEVEANNGKAVEEINMKKEGLDRK
VDFAEFKAHVSPEEPWFVVNGEVYDGTKFLEGHPGGAQSIVSAAGMDVSEEFLAIRKFHFLYLDGI
NTNKTTDSETAKAMMPDYHIGTLEKASLKVLHDEAQNQSSLGTPDEKYLQPKVWKKAELSTKRSIS
WDTRIFTFKLDYDLQPLGLPTGQHLMVKLKDSTTSESIIRAYTPISETNQQGTLDLLIKVYTPTPT
EKGGKMTMALDKLAIGDNVEIKGPLGRLTYLGHGKVLLNDKQRDVKSFRMICGGSGITPIYQVLRA
VVTNAQDPTHCVVLDGNRMEEDILCREELDSYAALNDERCTIVHTLTKPSGDWKGLKGRIGEELLK
QYAPPSADGDSLALICGPEGMETATKAGLLKMGWDENDLVFF
>g2835
MNAVDDIEDPFKLKDLWQTSNFALETLPKLESAHFKLAISDIPNGIFNSSLHNFDKKDSLLYEINV
FGADNTENLSIELPDAAQSTTDGDKTDTASSIEEQEQDSPWNIESLLEYLHEAPKDGPNLASWDGF
LNRNYKEPASAYLSEFGNRGFDAAISHQATVSGLENAGRIMRSSVFLDCLIRLGLGWNSMLFHYNE
QKRVFEKSIKDIRITGLSLMALDGMISDISRCGTGVQRMRKFVRANPLVTEQPSALSSLASAVSVL
LYSLEKQLSGHLRVNVSLIQVHNLFQKCGHLVSTLVDIITAAEKALSESDIISVLLSKSEDLEQRV
PWLSNTLNELIQRCTKPWLSSVETWIGLKSEKPLLGQVQLTSNSFVEVEHSQKITNIALASANVDY
LYHPEFVPSSLPTEQAHLLFETGRALRLLKKNQPDHPISRADVIEAAEAPSLTCAFNWAHIEQIQR
RADEYENRLRQEITRYNNGDTSRSRPGTRHGKAERDREELFADDVVDNNFELIDFDDEKHVTGLLA
GNAHMESDRLYQDTEESEFFSPQTLIQSDTAFGPPLGSVLYLSLAPTLAVQAKLVDYSCLHLLFKK
HKLRYHLTLQWRFQLLGDGTFTSRLSHSLFDPEMTSGERKSGVARGGFHTGLRLGNRDTWPPASSE
LRLVLMGLLSDCYDAGENDTFLSEQPETKEKELSGNLSFAIRDLTGNELIKCKDPNAIEALDFLRM
QYKPSTTIESVITHRSLEKYDQLFKYLLRLRRMVSVVNGLIRDSTIRTSLSGDTHNVFQQFRIESH
HFITALNDYIFQVAIGTNWQRFENTLSAIESRIDHDDVDGTIEIAGSLHRLRKYHEDVLDQILFSM
FLVDPKDPKSQSRRSAPLKQINKILEDIFGTILAFMPLSRLDGKT
>g2836
MPPRKRPKLSPRASSSTPQVETTKQPSSAHSTTDSAQKSDTEYDLVNDPWTDEQETALLKSIVRWK
PVGMHKHFRMLAIAEYMKSQGYAPAEEEHTRIPGIWKKLGSLYNLPALDEREDSIISQDADDSEES
EYCPFELPEDEYGEMMFAKRLAAERSESPETSVHPESRRGSTVADTDEPRSSPAPSRGRRGKPTRA
STRGTRSTRLQVEIERPSSASVGTEDEQMEDADVDDDGEDEGESDAGDGDDNDAEEGDVETASSPA
TRSTRTQTAKSKKEKKGATATSTTTRRTGRRR
>g2837

MISARAFVAPSRQCLRGLRTVPTQASPFSQARGYAAAAGGNRVGAFKGQKGSDGNYTVTLIEGDGI
GPEISQSIKDIFSAAKVPIKWEPVDVTPILKDGRTTIPDEAIQSVQKNYVALKGPLATPVGKGHVS
LNLTLRRTFNLFANLRPCRSIAGYKTPYDNVDTVLIRENTEGEYSGIEHVVVDGVVQSIKLITREA
SERVLRFAFQHAQSSGKKKVRVVHKATIMKMSDGLFLNTARDVAKEFPDIEFDAELLDNTCLKMVT
DPTPYNDKVLVMPNLYGDILSDMCAGLIGGLGLTPSGNIGDECSIFEAVHGSAPDIAGKGLANPTA
LLLSSIMMLQHMGLNEHASRIQKAAFDTLAEGKFLTGDLGGSAKTHEYAGAIIKRL
>g2838
MAEDIDVXSHYGIQNPFPDQWPAELDEGDVSETDDVATGAGPKSNRRSTRYSALAGINSRNLRTGS
VRGDNVSVKDEPDPLGSSDSVMYILKQKRIPVDEDPRLRNRFLLSSTSFSPALFLAQAHQNASTDS
LLEGLEFLSRSIDQKSASLKVLVEANFERFVRAKATIDSVYTEMRDQGAAKARPQSHRASAQYRYS
LNGAAPPAPAPAAKKTALTKEAEYGVKGIRTPLVEASVKAEELWGPALGGRDREQNLYALVNAVER
NRAVYEVGGNIARAIKQRDYQTVFDEYRRAKTLRNEARQLADRATTSGRQLTDQESHTMLATGRMW
VDVEQQVEAFKRDLWHRLSDVNTSSSHMTAAGPVEEHMELIGALLELGVEDNPVWIWLLSRYDLLK
TSIVAFCERSSVEIEILRRRLALGEKPSTQVMTTFLRVTSRDGAESQDRLDTDQIIELWECIHTYL
TKLLSMQSGLLGEVTDFWETAQSFINGSKQKMLPAGFEGESRKHHRLSSDGVRQLQEGIIELVDLI
RKSVLSLFVDPPPEDISLLASPIPPSPSTPISRITPTESRFKLDPKNLPPPSPRRGEAWEEFAFWP
PYSNSLSSVHYLSKFLILIGTAASDMSAMSPIETGSTSYDRLKNLVSFARERCVRAACAAWNKDAE
NCKMLEDWTRDPERRELTKMPGFFVAFESSVLSGMQKILYISEAMSKAGSVDVVTPPPAKLLQMVR
TQFVTSVYKALSGLVENAERPVKMGDEEDWTLKPTVPVRSIDAASSIITTDNVDARNRNIRMLLTL
SNLKALQIDFVPQLVSNFETAFSVKLTDEANTIRDVLGQIDDRLFQSYTNQTSAALHSVIQEGITA
ADWVPTTSRPEQVKPYVYTTMLTLVLVHTEITTTIPVQPGTSLPSTSSSSAQANPLLTSVLTYLVT
KISTSLLTAFQSRPKYTLPALMQATLDTEFIAQTMAQYVTEEASSIQSQIYLELDRRTNNEARTKL
QAELGEMRGILKKLREHTKGEFACFRKVRSGVKGTTHT
>g2839
MSTKRTRDADDAVDAKKPSDNQDTRKRRKGFTVGPENLPDGTYRRKAQKIKRDLIQKAKVKKAYAK
IKAAEEESAPAVRSLYYEDEEITAATKDESADPSTGLELHPDRQAMLDAPEAPTTTERPKITREWN
RERNQRKRRPKPSAFQKELDIAEKRRKEQEARQKERELKAKERQAMAKARRPDQFGKRRLGRESTV
LLSKIQRTLGSQ
>g2840
MPIFHYPERSTTPPDRLVNSETGNQRDYPTVLKWKKDQFKEIHTGESSPQNSSAYDTKFLPPRPRS
REELIQSIKRGESPTWAHQNSGRKPTNQATRSNLSLEFEKKYDSTSDMDNVRPHVSGVDERSELAS
PSEIERPRSALHSGDFREGAAQNQETKFPDFGGYNSSPQPPSFKRYGSSPTTPWFNHVTSVRHTSR
DAEASPLSGQHARSVLGARSRAPSLGSFSSSYVLKAPTSPLVNQANNTDLDFSTKREFTDDFAKSI
KKSRRRTLPPETFMQLRNSPPNQLRISNLSRSFASAHGSEDSRLQAYQPHRSSFYSLQPASSSPQT
PFRLRRPSLASDSSPRIHASMVGSYEESILRGRMSTTPSQPLDFTAQIGVLGRGKCKSNLRCPPHI
TVPFPAVFYSYPSSGPGRTISDDSPSPYVGLIDLQNAQLRDAPSKRSRAQKTPTYDNSGSSTPIPS
PDCNEHQRREKRSVRSEAVKAPPGGSYRIPQRGQLQIVIKNPNKTAVKLFLVPYDLDDMESGTKTF
IRQRSYSAGPIIDMPLTARRNLGTDRPEASLNPTSDPKDKPTLRYLIHLNICCPSKGRFYLYSTIR
VVFANRVPDGKEKLRNEIQHPEPRYSPYKPLKDSNNSSSNLSAIDKGHWRRSTGVGFASSQFFDDK
LYPDAPAPSNHKQVQPVPFRLSISNAREPSTNDAGPSLFSNNDIWRTGNSSTKRSLSDEMSLDSPL
PPKSADLGDIIDDDAEMNNDCEKYGNYHQLATSPTSYSSVGESLLAQRLRDWSCSNEQTSDSQ
>g2841
MRQSMSPFFAAALAFTAVHALESNNFPALSPLQQPEAMNATEKPTGPLPWGNVNFLHTSDSHGWMD
GHHNVPSWGGDWGDLVSFTARMRELAVQKKVDLLVIDTGDLHEGNGLSDSTDPKGAVSDTIFKKID
YDLLTIGNHGVRSGAEALNIRANISSYYGERYLTSNMDVKTDVGWESIGHRYRSFMTKNGLRIVAF
GVTTNDTFQRDTKSTLYYSAAEMVGKGWFKKAMNIHADLYVILGHSPTYEPCKSMATGSPLVCLRD
YIRSLKSNTPIQVFGGHAHNRNFTCYDDKSSGIESGKYGDTVGWVALNKVSSSTWNGTSISILQDD
SRPKKSCNQSRDFFLDRRYLDWNRLTFAYHSVGLDNNTSNVPAARFDTPLGLNTSRAIHDTRLNLN
LTQFLGCATRKYCYNCKNASDDGNIYKLFKAALANTVVNESRRQNPRIILANEGSIRDHIYKGPFY
RGDAYAIVPFPNTFQYIANIAFEPLVKGVLKNLSIPYPAPVDEEVVFINGEMTKDMAGXGETRSEL
RKRSFTQQTLLSSSENKEREQAEPSPGYTTHDGLGDDGDDTKHSKVIENKRYHKYYYAKANFPSQQ
EEDRATKDPDFVDLVFMSHMKTNVLQALESIGSNKYSDADVQLYMPETYNSRYVLPDYIEKAWEKN

CT
>g2842
MAGNQMHNMTTLIKRLEAATSRLEDMAMSFEGASQANHVNLTTPVQQGAIEPSIQPPSPPTPTPDP
DPPQIEDFDALIKSDVQAFVDLGKQIGGLVGEQAEAVLQAFQAERTFILVTTKAKKPEPLAPELMT
ELHKASDTINNIRESNRSSPFFDHFSAIAEGIVALGWFFELKPATFVNEMVGGIQYYGNKVLKQYK
DKEPLHVQYIQAYYKVFKSLAAYLNKHYRTGLTWNNQSDLSALDALKQIQSGSTKAAPAAPAQAAG
APPPPPPPPPPPPPPPPADLAVPSASQPKGGDMNAVFAQLNQGDSVTSGLRKVDKSQMTHKNPSLRA
GSTVPERSDSQSSLGSPINRGKSPAPGKKPKPESMRAKKPPRKDLEGSKWYVEHFDSVSDVIEINA
QQNQSILISRCSKTIVKVNGKANAISIDNCSGLSIIVESLVSSLEVIKSTKFALQVDGVVPTLLLD
QVDGATVYLSPASINLEIFTSKTTGTNILLPPKEGTDDDTTEVPLPEQIKSYVKDGVLVSEIVEHA
G
>g2843
MSVDGHGIDKREVDVDFYDIEFKPPTPSVYGIIGGAGSFAVVGARMVAGNDHGKAVSWIVDAGSDF
PPDMTNTILSWNTGCVIREDKGRLTTRAWNGYSENEKRDFKYLTPKLRLEPYMLSDGQLFSKTFHM
VCSSERCIHIVQEILQRRGSRSSSQSTRPIFVWEPVPDLCTPEEQEKFLAACRVVDLVSPNELELG
MMFGRPGWHEADEGDRKLVQKILESGIGPEKKGALVIRAGKDGSYTYSKGRKGLWLPAYHQSESDQ
VSKVVDPTGAGNSFLGALAQGMISKDREPGEIMDEILATSDNWDKIKHKWGNNGQIPQALICATVA
AGFVVEQIGVPTISTTNEYETWNGIGFNERLRNYTQRLCKTLEQSPQDYEWVID
>g2844
MGGGGKIPYPKEVWSPAGGWYAQPGNWKANTAVMGLAIVGIVAATWSLSAEREYRDRMPEPGRFFP
SRNWSKQIIEHDRQNATKNES
>g2845
MFCFESVRKARDLAPDEEIAAPAMEPLGPVAVVENHVGWSFALAEEITPSAEPQILDTFKRAGFQK
PSPKVYALHSRLALPKKFPLETLARTLRDASSDPHPKFNNSSLALLGHDLLASYTTEHIICTYPRL
PMTVLYAALYAYIGPRTLAAIAGEWGVELAVAPGPEVDPGLLQFKRVQPGTEIVQEARRTTKPNTH
WRKSIASSVVHGDIFGDPDLRTKNPKYVESKIPDIKSIPGVTAEKATSTFVQALVGGIYLHAGRPA
AKRFFLEHIVSRHLDMTKLFSFTQPARDLATLCHRENIEPPVAKIISETGRHSRSPVFNVGVFSGK
DKLGEGSGASLVEARTRAAVAALKGWYLYSPLEVRVPSSMEEEGAKPWKPVYVDYGEIFV
>g2846
MTTTKIPAPSPLPVNTRPHPHQDDNETQPTKKETIPRPKFRLRLEDVRHSAAASFFGLIPDLEAVL
DSALTAIVEQLYTSPRRARPHNNNDENDASKAPIPSFIPFIPPTRSVTIILQDFNGVAYTNGTDLD
DDHKEIHFSLSYINHVCNNDTQPLSELKGVLTHELVHCYQHTAPAEGEVPRPPGGLIEGIADFVRL
KAGLGAAHWQKPKSALDRASDWDAGYQHTGYFLSWLEDVRFGKGAIGLLNDRLLRTGYLGEEEEEE
EEEEKKKDKKEPGFWRDLFGVEVDVLWEEYGRYLDKEV
>g2847
MRITRAKTAGSGFTTSEALTNIIGLLSRSSAHHGGVHSSVETGPEQPAIPGDFRSSPHLTAELMSQ
GCLADPQSNEDPNLAVHQNDTKSALEISCGASTTVDAAWITDLGLTPVVLDYLLDKFRGMASYFPF
VQLSETWTAALMAENRPFLLLAAVAAASSKHCHLQDALTRQFKESLSKRVVMAGEKDIDLLQGLLV
HLAWFQFHFIPGSQQDYLYVQIAISMVIDLHLDQEAADLLGQRTNLGNIYTREACRAYLGCYYISG
IITMSSGRPNNLQYHKNMLSCAVMLQQKPEFETDLLIHPVTKVLQFAEEVCETYRSEGLHGPCLYI
HAERFTTRLEDWWSSLSTDLRNTG
>g2848
MAALSKIRLPERSIGQTISHMTSLYVKHRTRISRAVYLALFLALAKRIHNAIGEQKAASRRQAEMR
ERPGTSSLDNNNIENKNADEKSASRKKVEINLEFFRNLLRLLRIVIPGWKSKEFRLLLSHSVFLVL
RTILSIYVAELDGKLVSHLVRGKGKEFLKSLVWWMTVAIPATFTNSMLSYHQCRLALEYRRRLTEH
VHDKYLSNMTFYALSALDDRIKNPDQMVTVDISRFSDSLAELYSNLAKPVLDMVLYNYQLSKNVGM
EGLFLMSLLVQLSANMMRALTPPFGKYVADEARLEGEFRFQHTRLIDYSEEVALYHGHEAEKDNLD
KGYFTLIKHVNRILRRRLYHGFMEDFVIKYFWGALGLMLCSVPVFFKIPGQISQTMGDRTESFVTN
RRLLLSSSDAFGRVMFSYKEVSELAGHTARVSSLLDVMEDVAAGRFEKKLVSSASTEENAAVLAGR
GTVTESPSIEFTDVPIVSPNGDVLVRKLSFTVHPGDHLLIVGPNGCGKSSLFRILGGLWPVYGGTV
KKPSFDDIFYIPQRPYLSRGTLRQQVIYPDGVKEMRAKGVTDTDLFEVLSILEISNIVEREGGWDA
EEEWRDVLSGGLQQRIAMARLFYHKPRYAILDECTSSVTLEIEKVMYETAKQLGITLMTVSHRRSL

WQYHKKILQFDGQGGYIFTGLDWEKRLKLEDEKEDLDLQLRAVPDIEKRIAELSAA
>g2849
MAEFVRAQIFGTTFEITSRYTDLQPVGMGAFGLVCSARDQLTGQPVAVKKIMKPFSTPVLSKRTYR
ELKLLKHLRHENVISLSDIFISPLEDIYFVTELLGTDLHRLLTSRPLEKQFIQYFLYQILRGLKYV
HSAGVVHRDLKPSNILINENCDLKICDFGLARIQDPQMTGYVSTRYYRAPEIMLTWQKYDVEVDIW
SAGCIFAEMLDGKPLFPGKDHVHQFSIITELLGTPPDDVIETICSENTLRFVQSLPKRERQPLAAK
FKNADPAAIDLLERMLVFDPKKRIRAGDALAHEYLAPYHDPTDEPVAEEKFDWSFNDADLPVDTWK
IMMYSEILDYHNIDQSADPTQVPLQFVKRGIILSVRPNFIFLRTELRICNRERTPHKSVLSSMTQG
SIMRQDPIVLVIDFHHARGPEIELCFADEGSNPAVDNDWSLLPFMALSDGAHASTEDFSYFTLRRE
ATSTQNATSLFGISCTRQIPASSLIVRSPDVTRSTVQKAVVVVTDGPQHFGPLREKLSMVTSAWFA
QRDFSDVDILKKFRESLIASLKNEEAQRDIYLVLAYMGLPLQLFGKGSIFGPYTPLQQLDLLADHG
TKSYVVGSTNSLLLQQKDRYSDILINLDEGTISIHSPSLRAALALTAADRRWVDFLTQTINETWDP
AHPEQPKTHGYVGSEEFIRLQFEEYLLALLASTNHRQHVQPTSPGKAAEVRSKGQSTDAEGDPTLD
FNTDFVTHWQTTPNYALFNQLTSDALLFSIVEPRHPYAGGLTIDDIQRRLAQQVSELHLDERVREG
REALNKTLASGQKKVSSAFNSFWADIEALREAQRKKNEEKVGITDRSSSSDRNVSNESVQSARNDA
ASPTNESQSSISSWFGSRRSPSVDITQAQASVNVASQRAGAYISSWGTWASERRKEWQDRRSTTTT
NSTISSPSSSETTTQKPSGLPSVNEKTELSDQPNCDHNISTTNQLRDASSLRESEDSSFPSSTTLS
RSMSRRKRWSSVLRVKDHRRSDSDSESSTSRKTSLDIPVRRGSASSTSVSGSMGAGDGIPKSPLGQ
RQMIVGDDVVPVAAPESIAAETGEKGVDVSPIPASGPEAE
>g2850
MYDTTNMAHTATSVFRTVPSGTDVRISQCPMHARYSTTTTITLTTYHLPPTTNHHTTKDRFQPFSA
ETVNIERSYHTATFTATFRFHVGFAPHVRPPNTTPHPYSVAKAQAFRPSDICVITTKTLTSTTLAA
DDNYFKRLISNS
>g2851
MGGCVSSESNADLEQKKRSQMIDRKLEEDSRRLRRECKILLLGSGESGKSTIVKQMKIIHQNGYSV
EELTMYRLTVYKNLLDCAKSLIEAYEHFDMEPTSQKVRDFITYISEYNIDPDPSIPLDPKVGDAVT
FIWNDPCTASVMEHQSEFYLMDSAPYFFEHAKRIAASDYIPNEADVLRARTKTTGIYETRFTMGQL
SIHMFDVGGQRSERKKWIHCFENVTSIIFCVALSEYDQVLLEESNQNRMMESLVLFDSVVNSRWFM
RTSIILFLNKVDLFRQKLGRSPLSNYFPDYSGGNDVNRAAKYLLWRFNQVNRAHLNLYPHLTQATD
TTNIRLVFAAVKETILQNALKDSGIL
>g2852
MSSKPLARERKPSIGVPVAELQAVGPGFSRPKHKRTFTGFGPAEIKAVEASIPEHLRETWRKYSAT
GFENKEEFEREFVRHIYTTLARSLYNCDDLAAYSGTALAFRERLIIDWNKTQQRQTLVDQKRVYYL
SLEFLMGRALDNAMLNVGKKETAKEGLEDLGFRIEDVINQEHDAALGNGGLGRLAACFLDSMATLN
YPAWGYGLRYRYGIFKQEIVDGYQVEIPDYWLDFNPWELPRHDITVDIQFYGQSVRHEDEDGKVTY
NWQGGEIVKAVAYDVPIPGYGTTTTNNLRLWSSKASSGEFDFQKFNSGEYESAVSDQQRAETISAV
LYPNDNLDRGKELRLKQQYFWCAASLYDIVRRFKKTKRAWFEFPDQVAIQLNDTHPTLAIVELQRI
LIDLEGLEWDEAWKIVVSTFGYTNHTVLPEALEKWSVPLMEHLLPRHLQIIYDINLFFLQDVEKKF
PNDRDLLARVSIIEESRPKMVRMAYLAIIGSHKVNGVAELHSDLIKTTIFKDFVNIYGPDKFTNVT
NGITPPSKLGGYDFLTDLTLLDGIERFVDDKEFRREWAEIKTENKKRLANHIKDTTGYAVDPTALF
DVQVKRIHEYKRQQLNIFGVIHRYLKIKSMTPEARKKLVPRVSIFGGKAAPGYWMAKTIIHLINKV
GQVVNNDSDVGDLLKVIFIEDYNVSKAELIVPASDISEHISTAGTEASGTSNMKFVLNGGLIIGTC
DGANIEITREITESNIFLFGNLAEDVETLRETHRYKGFTLDEDLAKVFESIRSGTFGDAKAFESLI
ASIADHGDYYLVSDDFKSYIQTQELVDEDFRKQDEWIAKSISSVARMGFFSTDRVIDEYAESIWNV
EPLVVE
>g2853
MGLTTAAFAAPAISLRRSDRLRKYPPLTPKMASLQKDDVQDTWTYLENGINSVMLKLEEGVDMKTY
MGLYTAVHNFCTSQKAVGSTGGLQALRGAHLLGEELYNLLGQYLSRHLENVYQASETHTEEALLGF
YIREWDRYTTAAKYVNHLFRYLNRHWVKREIDEGKKNIYDVYTLHLVKWREDFFKRXQEKVMAAXL
NLVEKQRNGETIEQSQIKSIVDSFVSLGLDENDSTKSTLEVYRFYFERPFIDATRVYYENESRQFV
SENSVVEYMKKAESRLDEEKARVGLYLHPDITKRLTETCLDVLVSAHSSLLRDEFQVLLDNDRQDD
LARMYHLLSRIKDGLDPLRAKFEKHVRNSGTSAVEKVASEGESFEPKMYVDALLQVHSRYQTLVNA

AFAGESEFVRSLDNACREFVNRNSICKTGSSKSPELLARYTDSLLKKGSRATEESELEEMLTQIMT
VFKYIEDKDVFQKFYSKALAKRLVHVSSVSDDAETSMISKLKEACGFEYTNKLQRMFQDIQISKDL
NASYRDWQEKIHDEEDRKKMVDPHFQILGTGFWPLNAPSTEFIPPTEIVKTAERFQHFYFDKHSGR
KLTWLWQLCKGEMKANYIKNTKVPYTFQVSTFQMGILLLYNEHDSLDFDEIQKATKLANEVLEPNI
SLLLKAKVLLASPEGSKPAPGVSFTLNHNFKAKKVKVNLNLQIKSEQKIEADDTHKTIEEDRKLLL
QSAIVRIMKSRKKMKHVQLVQEVIQQVKARFPPKVPDIKKNIEALMEKDYIERLDNDELAYIA
>g2854
MGLNPPSAIKVSRHLIPAWHSIPNTSIQNKPLLIYHCAFEKSVSASQISNNLKKVDVVVPQWQYSM
YRQTHFHSTTHEVLSVVSGRARLCFGGEDNPERVEPVIEAGDVMIVPAXVAHRLLEDLSGGFTMVG
AYPKGKNWDMCYGAGDENENEIRSAISQLTWFDQDPIYGRDGPASQA
>g2855
MAPDARESLLVPPIVQNTPINASAEQDSLLPPRTSATNRTQSSFAVTVNSHDTPTHTWSPPPVRTL
PAWVRSYDVPEDETDASSRLLPTEIDDALVAQHNHSPYQTSKPNKNLTRGTNDELDGTIPGRESRW
KRFAQTAAYPRTGVGAGEKRVSFEWLNNNLGDYSLPWGTSHDGSDAELGRATWSPRHRNLIERFQR
HLLRSPMVPLILRLTVWIFSLCALALGGSLRMLANRSHRDTGPSADMAIIVDAVALVYLVYITYDE
YASKPLGLRSPKAKMRLILLDIFFIVFDAANLSLAFVSLDDVQGTCTNAVINDVQDDRDDALCVRQ
KALASVLLVALIAWLLTFCISIFRLVERVSK
>g2856
MRFFLVPISTKRAFIYCRPPKTIKTGYVDRVTNKAAEIWAGWEKAEKGWKKQLVGYGHVILQRIPY
EEWGLKSIPPLSQSLQIQEAQQKQKIDVMFPKNAIKSEDVFGILRKLGTERQELHRRRMWWCIGIA
PLTAPIAIIPVVPNIPFFYLAYRAWSHWRAWSGSKHLIHLLDLNLISPHAFPELESFYATRLLKNG
VPGHQTKSNMVTKDTNKLTEKETAAPKDEAAAEERLLLEMKDGEELGKILETPAIAIEVERAVLQV
GQKLKLNEEKRQEKNAQ
>g2857
MSQHPPTIQSTLAQDTNVAYIDTIQAYDQWADVYDTDGNFLQALDTIEMQWMLPELWAQIKAQTRQ
IGRQEYKLVDLGCGTGRNTLQLLDTISQDSLQSNVQIIGVDASNGMLDVARQRIDAVNRPADIEVE
LAPFDLLADIEAPSNRRTLNLVRDADGVISTLVLEHIPLNNFFGAAASILRSGAYLLVTNMHADMG
KRSQAGFVDSATGKKVRPSRSYAHEVGQILAEAERWGFEVVQLPTGKAARSHGCVDGVIEREVTPD
MVERLGPRSQKYAGVCFVWLGVCFRKK
>g2858
MPSQTRVNAATPAGTEHDTEPPRPATPSVVRPKKLFSVPAPVKQIFDKFPLTTYEAEDIPGNVHNS
GSSGRNRLFVFIDPKQAATGAPSFNPQCLKWQAYLKFVGIDFDLVPSNNHASPTGALPFLYPALPA
ATRSPIPSNKLQKWAIEQVHCEEEQQLNIRFDVYASLIDHRIRNAWLHQLYLNELNFKQVARKRYI
NPATSSSAVRTALAVQLQSAARDELLKYSDYIDVNTLEADADSAFEALSTLLGENEYFFNRSQPGL
FDANVFAYTHLILDENMGWKYNRLAHLLSKHENLVRHREVLLQKYFS
>g2859
MAETNETKPTVPVARSTKPVSEALLNEKWDRAISSLIVRSSIGLGFGVVLSVLLFKRRAWPAWVGL
GFGAGRAYEEADSAFRHGDSPIANAVRQSRS
>g2860
MAPSKEIAGPSQEALLKLEKSTEDSDRQLAAAAQILKLDLSDTSSPLPSSNTIELITRPAPKEEWV
LRWLLKRLKTTTYRVHPRSYILLQQLLQRISRRAIASTFVEYKFAQILKNIVDDLNNAVFKSLSDG
ANRISSDSETSETVQGSPVRSKKRKRGNDSADADNDIVMYDAGNRESQSERAILAYGRFSDAFYVL
MNLVNTQSDRGDVSQFHLQQSLRLDPAIAAEILGKLIRLFAGLIVEYAKEKKSDQLQHLSKTATVY
FSLWDLKKVGINGIDKSSINTAFSESCFLEVLRLHRLVVSSGAESDDARFITRSIEKLIALHVILP
ARSLFMERGGSGIDYSKAEDPDWSAVQPVTATFKPLFEDRAPIDIKCNGTDIGSVDENSCSFPATW
KPAELIPTFYDIVARSVPRDSFRRQNAEASWLETVFVALAELAYSSTQQLGGNTLGFISLLEQLFI
VVRARKISLSPNTFLTHASYTGLLKGKEDPQQVRWSLTILLMELESDIFLPNSGFKDSSRLLDALF
LSLHHIRGNMSDQKTYQLVKEKVVIPLIRAFAGARDLPTFAGFWYEQLREIEVARNDLAYSVWEDD
DVLLIETAISTVTKSGHISKAPDSYAYFVILEAGTRIWGQSGLPGLRIDLVTGLMESLHKTLSKRK
GDRHWLWRLKSTFNTPNELADKFKREALVVANFSTGPTKLRSGWNGSSSSIFEAKLEGWNGRYKDI
NGLAQQATAVIECLLEHPDLLSELVFEDLKCFVKFFWDLASSSGQDWKHQIPEITEYTPSLASGVA
RSLCELFMEMVESEQTQPVIVRYLPGIVQDLPGIPVRHQEVPKFKDTIEALLSKVDYPVDFVPHMV

SLINGWCQESRQVGSEILEWITSDISLQDSPMDMRTIESFRSLIRTAFECEYKACKSPAETGKFLK
RFYKLTTSRVESSTKQLESSVKNKDQPSTLAPIFVGASLRLLWEKREEMSKTMDDIARQREKIFGL
LLSRLDKCTKTLRRKSHPEAAIILATTHQMMEDFDDLAAQKTELDKASTDLGENDPVLINGIKYIT
QRRKVARAATSDSLGFNESTLLQGIDLCRMQACEQTYLVREFTTKLKDMSNADRIKAIGEFKTAGF
ECSEGASRLLLSGLAASALNEITEKDSAEARGVSSLCTAVTHVLQHSIDDIERFSLAAENVDIILR
LHSRGVTQYNIDSCLAAVTATMSKIHSLRETNHISTKSQLGGRFHLLIPALQSLLKALFAPSQKRY
QKGGAALGISHTVQFTRLLTSLCDPTVSAVSRPSRPTGNSNNSLIDQTKKAKVIAGQHLRILIESY
AQNMLEAPLQPEIKTALAPGLYAILDAMPAESKRGLNAGMDISSRAIFKTLHDDYVRFGKWNSKG
>g2861
MSVRRFVDKIAVESEPGLSNAQLMLTNDDLRPVPPERRKWGWWNFVAFWIADSANINTWMIASSFI
QSTNGSNTVDLAWWQAWICVWIGYSISAILIVATGRIGAQYHVSFPVVSRASFGVWGSLWPVFNRA
AMACVWYGVQAWIGGECVTVMITAIWPSYANIPNHIPASSGTTTKDFVSFFVFWLCSLPAIWFPVH
QIRHLFTVKSIYTPIAMIAFFAWAISRANGIGPIIHQPTALHGSALAWAIVKGIMSSIANFATLIV
NDPDFSRFSKTKTSALWSQLFAIPVGFAITSFIGIIASSSSTVIYGKTIWSPLDLLSEFLNGASSG
QRFGIFVIALGFALAQLGTNIAANSVSAGTDMTALLPRFINIRRGGYICAAIGLAMCPWKLTSSSN
NFTTYLSAYTVFLSSIAAVIITDYYFVRKGYLELRELYSARKRSPYYFTFGFSWRGFTAYISGILV
NIVGFAGAVGANVPIGAQYIYNVNYFAGFIVSGTIYYLLCWAFPIPATSDTWNEVDVDVDHLTVAY
GQEVVTDEESRSIPEEDTLAYNKKDKSQGYSTF
>g2862
MTSTSALPLTLKSISSTKLEELSKQRALFNQRKSEILEAANAVPDNDLRRKVQILLEGVTRLAGRP
EDAFDREDDVGSSSSPSSLADLSSPDWKRSRDVNIRRFLFQSQYDRSISDEVLTGWIKSLESDFEM
LSVKYEHATFYSDMVTEWLSNFEEKGSTNKNLDGIDEEDDTKSDKSAFENVGRAAMHEQRTTWEHL
VFSPADVDSQSIETYLERLFTRTKLSQQALRDLREQVKTFGMQFRSEAKDSFDIELLKNVSRSLLG
SDLLSPEKVMILKEFLRNDEVAQEVADVLNLRFVALDRWNWSTEGVPVEMRRQLNGKYRVFMDEDL
IDALLFHYIGLKWAVELRHVFLQFFDSSAWKSNAKTIPKAWVDRRRYFTGQSTKAYSVDAIRKKMY
REDYFMSQLPESLQEGKRGYDDDGDDDDHATTERKSAIETKHSLLHLLITESIIHNRIRGQFTVIR
SDFKWFGPSLPHGTLLAVLNFFGVPQDWLEFFKKFLECPLKFVHDGPQAEIKTRKRGIPISHSLSD
VFGEVILFCMDYAVNQHADGAFLYRLHDDFWFWGEEKTCRKAWRAMTDFTAVTGLEFNEEKTGTAR
IGRMINSQGQKQRQRHEEDSSSSDEDSKDEEEEDSESDAEHFMDDAFDPLPRGDIRWGFLRLDPER
GRFLIDQDQVDAHIEELRHQLSACKSIFAWVQAWNSYFSRFFANNFAKPSFAFGREHIDMAVNALS
RIEREVFAHAASKASGNNGVVDHLRRVIAERFDVHDLPDGFFYFPIEFGGLELHNPFISLLAMRES
ITQTPQRILENVYLQEEVDYEAAKKRWQEGDHGSTFHSGVAWKRDDEDAPEKFWSLEEYTAHLETT
SNALRVAYDNLLRVPNEVQVGKSVGLGSGHLPPADKNKPTQRGSKWRKQKDRIGSDWKQMSPYWKW
VAEVYSAGMVQRYGGLSAVDRALMPLGVVKVLRQGKVRWQG
>g2863
MSSDTSSSVTEELTKLLPKIELHAHLSGSITRQCLHEIWYRKKAQNPNFAIEDPWVLMPPGKVNYG
LDVFFDVFSKSIYNLVNDAETILYATKSVLNDFRADGVRYLELRTTPREIRSEDGHVLISREEYVD
IVLQGIQEFKQEQQNDIKQNPDMSVYLILSIDRGRDTASSAEDVVNIAVRHYRTLNPVIVGIDLCG
NPLKGTVSTFRSAFELAKTHNLGITIHFAETIYSNENDAEELETLLSFEPDRLGHVIHVPDEIKGK
IVEKKIALELCMSCNVHAKMIVGGGGFEDHHFGEWWLRTECAVSLCVSLSLSIVPVLAAEYR
>g2864
MTDLDNAEYTLYSSPFSLYSMMVRHTIQLGRASNNARPPKSITLHFINHRKDKNLEEEYLKVNPKG
QVPAMTGNVLEHSLTDSISISLYLAENHYPALLPAEHAAVIKDLLQRFHSIYGLSFSNPKPTAEMK
EYNPSPVEGLLKRTDISPEYRKLLEGKLKFHNEHNATAFHPNVVAKARSDLQTIFAEIVEHRKQSG
TFGKHNEWTFGPAVGPTLLDSHFLPVVLRCIEVGNAELVPEELQLWSETKSKSPSWLKVMHGKPTR
YDPSMGPVEDMQEMMSL
>g2865
MGGDEEHSLPELRTSAPDAEQTRARELESKRTKISVLVGSGIIQLPIWGFAMSYGIFQEYYFNNWT
LEGDRTITGVIGTTANGVMYLSMPFLFALFTRRWARWRQIAALCGTLIACVSLFLSAYSTTVWHLV
ATQGIASALGCALVYSPMTLSLGEWYTSSNRAVAYGIVLSCKNIVGTSCPFMIRALIDAYGYKTTL
KAWAAIVGGTSLFAILMIPTHPSKISIHKTRARKIPWHFLGHRSIYFYSIAIIFQSSGYGIPQSYL
STYARDVTLLSQTSGTLMLALFNAPGIIASSFFGWLSDNQKVRLSAQTVSAIPPVACAIFTFFFWG

LTTPGNLGLLLVFSMTFGFFSSGYSATWGGMLKQMERESAERNEAVDPGMLYGLLNGVRGIGYASG
GLAGVGLLNAGTISASPRFGYGTSYGPLIVFTGVSSLLGGWVVLWRWNPKRLLPLISRS
>g2866
MGDGTDYPSPRSEGPTGPSALLIAAPESLSPPMKMADPLSSLANVSPDGTRPRLSVRRSRDPPKNQ
SNQIFCDHPDCQANPPTFRRPCEWNKHMDKHDRPYKCMEPGCDKIQGFTYSGGLLRHQREVHKKNA
TTKKALMCPYTDCNRSTGNGFTRQENLKEHLRRRHMHTEGGNSPELPVLTAADVEGLKSTFLDPPA
GLKRKHDDMTDPELNGVDTIDESNGVDLHNEVKRLRREVEEKDRRLEELEKIVAGLQQSIPHHALP
VVHVPGAEL
>g2867
MADDTPMSNMQGPTAVQLPANIQQNSIPQGPGELYTPAHDYHTSVPISRQKTLVPPLTLGAYSDDN
LILEPPKEHSFEIGEFVPPGFFDRAHHTWFSHHASTADWTYAQRREAQQILPFLYLGPASMARDRD
FVRSEGITLLLSIRDKRSAIARLLSGAKVAEELGIQADAIDVDSEQELISLYPSIIRGINQHISSG
VSARPDLPPRVLVFCESGNGRSASVVIAYIMSMMNLDMVSAMWAVHHRRFCIDVDESMQTLLQSYQ
AILDAKREVAKARRQEVTTSSSLGIQPSSRPGGQLAKKRSFIEYDCDNNTPIHSGHNGFSSQKTVI
QANDDAEHLKQKPAPFIERAA
>g2868
MRLRKTIRVPSRYDDECELSTGVSPRRLPRRRKAAVEAGAGAEEEEEEEERAIPYVDYDPNQPPAAF
PTLEHPLPAGQTYHSLPTATPPPAAAANASPDTDDADLDADRNHHLDAFNSLYRGIPPEKLDNFIA
SNGPQNPQYKRNMALLAREDDDFNLEISDDDDGRGLPGSENAGAQWIHREEPIPNDPQWADISLSL
QIEIAENMLETHRLSTIQLLLGLSQSDTEQLTRNLRMHNADLEYENRVLAEMRNKQMQVLMCLDNS
DLKQHSVPGRLVMSKKVWNHCHRVIQKRSQMRYLLCQAGDLLLARKYLSKRGLPMRYAGEWDNQFV
ATPSISSTGGFVGILKWKSSTESQSGASTTAAGDSRLLPKTRIGAQDPSPITVSETLIVQLPAAQR
YEPMPRVSTPQASTNRDVQLVLHNQALVRLRIGRENAARIHIEYPATPSRRHMDALERFLPTPRTR
TRAQTREPNSDDTIEELPSESDRIEGTSRLQPPELSFSTRPRPGSFNNIFPAGPPIAKPKARQFDQ
ATRISTDQSSTLDDAHSFDSSPPQPSDLSALEGVVHGNSQNLTARNAMPTPAASSPNYSTTTKLTA
HHNHNHFPKTSVETCLATPESYRTDVGERQLKSYISAYNRSSMPSNGNLGSATASFSRSDTMVNHV
ELKKDGVAQADAGVFSPSKAMQGLNLLPSLSMDFGVFVGHQEGPGTNHNSLAEEAPPGHANRITTP
QAAKSAKYINPLFGLDSFDIHADHEEASQNVKTSESSHSRASTGQGQTVAGIEEPPSINTENANGN
QRAVEDIPNNHLSAISTNVTVDVMPLPRERRQSVRKQTAGTKRGASTEMKAAHPKRGKFPDPASTP
TTRSATNATSSHTTNPPVPRGRGRPRKYPIQQ
>g2869
MSGVTTPTTLPESLDEATTGASSAPSRQQSSPPTSPATKSESEDVIMKGTEKESELEKTAVTEGEG
EGDEDEDTEGMDMKARALTKLLQTSSVFVAIMSDKMKAQQKQQQEAAKKAAAKKQTAAAAKEAPES
TTGRRSSRLKTGEKEEATDQKQSKKATAKKGAKKNAANGKNISSYFKKADVEVSGDNPSVQEALRQ
AADDLEAKPSALGEQEGLVATEQPSLVTGGKMREYQLEGLEWMKSLWINGLCGILADEMGLGKTIQ
TISLIAFFKENNISGPFLIAAPLSTVSNWVDEFQRWTPSIKTVLYHGSKPERAELRKQMKLKDQKE
ADFPVVCTSYEICMNDRAFLGQFSWKYIVVDEGHRLKNMNCKLIKELLTYHSANRLLITGTPLQNN
ISELWSLLHFLLPEIFNDLDSFESWFDFSSVLDNNGQRDVIERRKRNLVTTMHSILKPFLLRRVKT
DVETSLPKKREYILYAPLTSEQKELYREILSGTGRQYLENRAIERIEARSGRVSRSTSLKRKADDS
GSMTPSGKSVKSTRSSTPGSVSGRTRKGRQSYREIGDREFNAQLRNLENGIEDEEEKDESPGETEQ
EEIERAKTIKLAKKEIGSKKLQNPVMQARLACNSPHNFYWPWGDDSSTVDETLVTASGKMLLLDRL
VPCLMKKGHKILIFSQFKTQLDLIQDWATQLRGWNCCRIDGGVSQVDRRAQIKAFNSDKNFKVFLL
STRAGGQGINLTAADTVILFDSDWNPQQDLQAQDRAHRIGQTRPVIVYRLATKGTVEQTLLEKADS
KRRLERLVIQKGKFKSLLDNNTTSNDMEDIRKALGEDEFEQFHIQSDDPESILSQHDLDILTDRSE
EAYLRAEKGLEESNGRAFQAVETKEDKGIMAELTVR
>g2870
MAKRIEQWEVERYWEIFSSLANGQPRLNNSQAASVLRNSRLRDDQLEKVWDLADVDGDGELDFEEF
CVAMRLIFDLVNGEYQEVPRTLPDWLIPESKAHLVQASAALTGRQEQFERIEDEDDTPGLKDGFDW
YMSPADKAKYEEIYSANKNHRGEITFDSLQPLYDSLDVPDTDIRSAWNLVNPTASSSINKDASLAF
LHILNFRHEGFRIPRTVPPSLRASFESNKIDYQVDNVRPAQKWGADGDTETTTGRKAKFGDTYLSR
LGVKSTYQPKATDFSNTIQDEEWEKVRLRRELAELEQKLEAANRASEERRSGGGASSSAHHDGRPN
WTVIKKEALQLLEYKERELREMREGTGRTKDGQDLERLREDIKTVADQVDGLKDHLDRRNEALAEL

RRQIEDEKASR
>g2871
MEALLTHSFDYLSSYEPNKIRKGLRQVEGLLAQICLSRSKQTAAEKRRSMIPLGQAPPAPKALNEL
ADDPAFREFFKLQEGFQWNVAIRLVTCLEHLLGRGSNGANDLLIISTLDLIQGILLLHPPSRALFA
REIYMNILLDLLDPINCPAIQSSTLLTLVTALLDQPANTRTFEALDGLLTVTSLFKMRATSREVKL
KLVEFLYFYLMPEMPTIPAIAASAPNTAVLGLHRSPSKLSGAFAKSYSSAGDTNQSKRETRTTEEK
QKLLGRYLSNVEDLVEDLKETAPFGSTVY
>g2872
MTSEVNPPQIPQSLQSPTAIPPRTSSNGAVNGIAASAQPVAPGLASISESEWMSSSKRKSLSTTSK
HGRNRSSMDWTKAKEVMWTQEKESIIMGPYDYISKQPGKDIRKQMIAAFNVWLKVPEQSLAIITRV
IAMLHNASLLIDDVQDNSVLRRGIPVAHNIFGTAQTINSANYVYFLALQEVRKLNSPAAMEIYTEE
LLNLHKGQGMDLFWRDTLTCPTEEEYLEMVDNKTGGLFRLAVKLMQAESETGKDCVPLVNVLGLIF
QICDDYLNLSDKTYNKNKGLCEDLTEGKFSFPIIHSIRADPSNVQLINILKQKTKDEEVKLYAVNY
MESTGSFAHTKKVVSQLRDKASKLIDEMDGIHDGIEATDSPRDGRYVREILEKITEVTLKDKERAV
>g2873
MSQSQQSRGEDEDEDMHETTQYDLQSEEDITRHESSPTTTPLSFSITKSPSAADILQPLSTLAAQR
SFPLPTSGSLASLARGHQRDPADGRRIDHDFASATSGATSISTGTGSSWMHERLLQSDSTVDATTT
TDLDDVGASSITTSAAVRDFTTMVWASSTAAAVAEETKEAAESTTESKPIHRSVSAYSSSPFSSPG
SSLVEGSSGPFEEEQDDQTIFLSPFASIFPDGANTITPLFLQRFDDDSNNDDAADADQRTILKYTF
NHDQSSQSEEEYDRLRGLAREEANKRNNCFERSKQAYENGDGARAKQLSEEGKEHGRRMQDYNKQA
SEFIFRENNASGRVAEDTIDLHGQFVEEAEDILEERIKYARQHGQTHLHVIVGKGNHSANHVQKIK
PRVEQVCRELGLQYATEENAGRMYINLTGGPAEMPPYQQQQHQQHQQQHQQQPHYGGGGYPGQQQQ
QQQYGQQYGQQQGQQQGKQDDIEALVEKVLPRVLRKLGDCCIVM
>g2874
MATGTMKQWVLHGKRGIDSLILEETPIPQPGENEVLVKLHAVSLNYRDLMIANVSTTYLAPTFERL
DVLTYNAKGVYYWKQSDSVVPVSDGAGQVISIGAKVKRFTPGQRVMPTFFQGYISGNLTEEHMNSA
LGALPDGVLREYAVFNEDSLVRIPPSLSYEEASTLPCAGLTAWNCLYGSRSLRPGDTVLTQGTGGV
STFALQFAIAAGAETIATTGSAEKELRLKQLGVQHVINYREDPEWGTTAKLLSLDQKGANYVVEIG
GPGTLHQSSRAAAVGGEVAIVGMLSVTNGNGKEIAPWNPHAAAHSTRRIAVGSRLQFDEMNRAIEV
NKIRPVIDQVFDFKEAAEAFRYVWDGKHSGKVVIKIAEDGEIDKTNTYIMLRPRALTLVTVREELV
DLVTRCALSLDVDDREPFLSVWTISDPNSVDATVAGVTTKGVDKLCRTYFNLVGPMDTHQSIASIR
VDIHDGGKTAQITCTTLASISVLVML
>g2875
MYSKKHGRQYDIIVLGATGYSGHMTAEHITINLPSSLKWAVAGRSADKLKQLVSRCKDLNPHRIQP
AIEICNLNNEEISVLANKTFCLITTVGPYALHGECAFKACAEAGTHYIDCTPEVPWTLEMIKKYEA
TAKASGAWMIPQCAMESAPSDILTWAVAEEVKSKFSSQVGDVVMDLHQLTSVPSGGTLATILNLFS
QYPLKTLLQSIEPYALSPEPNNHPLPKRSFWSSLTGVYKVPGLPLLSTSISGKANEAIVFRTWGLL
KQDPNLQKEFYGPKFTYREFMYAPGFVRGMLMHYFIVIGGYLLLLAPVRTLVRRFVFKPGDGPDTK
KAKDEVIELQAVGKPVPTTEENKQVLGKLRYHGSMYYLTATFLAEAAATMLEEDESVRLTGGIYTP
ACLGERRKINNMARAISPPQPIHSDQQPSSSDTDTYTIDIINLNNLLARLEQNIFLSSSAGRQLSQ
QSHLECVRIGANIDYAQTLIKKIERSLPAVKAPAIRHERTTDLTKKRGRYEKVKERFDWIKEDVER
SADREEEDVQEADEEDDIWEGLAPVENGAAAAAELESNNLILSEETTTIRQRRSDKTSIAPATTSI
AAAMASGTSSSTFLAPKPTPATTAPPIDTKETALATHRSEQESLTTSLLTLASQLKSSSEAFQTSL
ENEKGILNRAVEGLDKNMTGMEAAGKRMGVLRRMTEGRGWWGRMMMYAWIFGLWIVALAIVYLGPK
LRF
>g2876
MGCGMSTEDKEGKARNEEIENQLKRDKMMQRNEIKMLLLGAGESGKSTILKQMKLINEGGYSRDER
ESFKEIIYSNTVQSMRVILEAMESLELPLEDPRAEYHVQTIFMQPSQIEGDSLPPEVGGAIQALWR
DAGVQECFRRSREYQLNDSAKYYFDSIERIAQPDYLPSDQDVLRSRXKTTGITETTFIIGDLTYRM
FDVGGQRSERKKWIHCFENVTTILFLVAISEYDQLLFEDETVNRMQEALTLFDSICNSRWFIKTSI
ILFLNKIDRFKEKLPVSPMKNYFPDYEGGADYAAACDYILNRFVSLNQAEQKQIYTHFTCATDTNQ
IRFVMAAVNDIIIQENLRLCGLI

>g2877
MAPAPEAYFASLDKCFSGDAQLLSWRRVFLALTSPPDSSNDADYITSFLSQPESVRLLSNCFTPFE
SPSAKSKSEFESKTAAIHVDTSPQAAYQIDEIKADTLWLSQKAGIDEVAALRIIVQEWQTRPNAQL
LGRFSEEEATSLQDAASVDSFRVSLAGPQLKEVLKKAHGGSEKAFFSEKSRRIRIQNIYLSEKSHI
VKTSRKLLGAFLHGKVPADVRLVPLTHDAEQFVRKDGLARLGEQLFKPRSGETESIRFLLDAIKAI
EKRLSDFQTEGGWLSAAESNFDTENFWRTSLVDEVVHVMQIIYLHVQSCDVVTSGEVVLAWLRLMA
EYSFMEPVSPPCEDPIVLRLSLQVLASATTLGLLKLPESLALVLKNGSLSQEEALSGSKPYFLSRT
HITEINEVLLNAIDMGILSASPAAFAWGAILYAMREIAXAAKDTREMEQFHSAVDSFQSNQPTAAV
ARSLEQTFYEDLLDMARVPAFGDDYVGVLTVGAMEKGQVFEVINNIASNVGSVSAIDDLVIDLWIR
NSLMGIIRVSSHVTDYSPELVAATLSILNASKNTLQAQKADHITQPSDPRFVFLHDEDLMSRIFHI
ARSRFPYESIPFLQLCRALASKDQINGSGPVRILNELDAMETFTQMVSPDFQGYETIREDENANFV
SLVQPIPMIASTFAKLTDNTDAQTALVVASSLQLQPTTTGQVVSESKPAVIMWYHNYSSITFLGSW
LEEWANKGGRMPDSDDEAIAEIIGLLTDLMAACQLTDDETGPKRILEMASESLNERSDIVSVIINI
LERSLESPGAQSGTSDSLDITVACMRFLRVLVTFMPSRTWPFLSRSSLLGPGGKGSRFSAIVSASE
VTSGNYPFLLSCVDLFEAIVEDAISNAAIRKITTRTVFKAADAFDRSAGVPSHVISKVLCNFVRSM
IEVYNSSGNWRFNQPEQRLQVDASLARTFERIIYFTYGIDETDSHQTKITAIFQESADYILDVLRP
QSKEELPLNPVLRIILGGLQTPTSTVYLRQLQHTERQLITTLDLATRLIQAAQLSGSTMSLLEDQL
FKAAPVLVKLYSCHDQFRLPVVTLLELLLTKAALDGEKEPASFLGHLGSESTCLFLDVLAQFDRPL
PNLTLLVSIWRLLSTFVGKRQQWVAIYLLTGSSPRDSMKLRNGNEKASRMRNTPFLKTALDSLANI
DHLEPIISLSLLEFISCAQEHWPWATPQLGNHSNFFSSIVNYVSHLKIASESVTTQISLIQIAAIT
ANLCAIYLHSAKEARDKTFVKMLVPLVFWYAENAVDVSGYNASLHANLKKNFEMKYAGCHLQQFKR
TVLEIRPLGKGYYYDLYLADKLLSYDFSWRGKKDQGFASEFERANLNLSVVEAQVTLFNSWKFFAI
EHASDFSPDQEIQKSMALIVRNCLLANTRPTPEEPIFSKLQKSRVDFALSMLQRLVEFGAEGPEMT
GLLAVVWEAMRACGTTYENALIHDNTDYYRGLLNVLFLALQFHVSKSRSAAAEPANKKKKFDLSSD
LAIVLEVIKVVIAQGFRSLTTYLHDEPQRCSPKDFAIITAILQTALRVKDAERLYEHIVFHIEDND
TARYATTLFSWADQLTVEGDPVYGELSIIFLVELSAIPMLAEYLAVEAVLMKLSTSRLTRILSQAK
TFGPFDPVPRLYAIWREGFLPLCLNLLFNVSRAAPEVAAFLNQFEGRLTRAAESFASIHAGTASSA
SSQPEKRISLSMASEAYSLSLMSFILERYRAAGPSAGVDSQAIQDLKWDKSQIKEDIEELLSRRSS
LRARMVATNETEAELARQQPLHASSSGAEDRLEEKIVYELRAALTCLSGSDEA
>g2878
MENGLVDYSPHHPAEVKKLDSASNVVLIDNYDSFTWNVYQYLALEGATVTVVRNDQITLEELIAKK
PTQLVVSPGPGHPETDSGVSRDAIKYFSGKIPVFGVCMGHQCIISVFGGKVDVTGEILHGKTSVLT
HDSKGVYTGLPTSLNITRYHSLAGTHPTLPESLEVSSWANIDVGRNPIIMGCRHKEYATEGVQFHP
ESILTEHGRSMFRNFLKLRAGTWKENEAFLPAVSSGTKPDKKTSILEKIYAHRKAAVAAQKLIPSQ
TPEDFQASYDLDIAPPQISFPSRLRKSPYNLALMAEIKRASPSKGIIAASVCAPAQARKYAVAGAS
VISVLTEPEWFKGSLDDLRAVRQSLEGLPNRPAVLRKEFVFDEYQILEARLAGADTVLLIVKMLSV
ELLTRLYKYSQSLGMEPLVEVNTPEEMKIAVELGSQVIGVNNRDLTSFEVDLGTTSRLMDLVPDTT
IVCDLSGISXPADVEAYKKDGVKAILVGEALMRAKNTENFVSELLGGNTASKSSKNLPPLVKICGT
RTPEAARAAIEAGADLIGIIQVEGRKRTVPDGVALQISKVVKTTLRPVSNQTVDTDASIYFEQSAK
LLRHPTRALLVGVFAGQPLSYILEQQRKLELDVVQLHGDEPVEWASLIPVPVIRKFSPGGPSLALQ
AYHALPLLDSGAGGSGELLDLSSVKKDLESNDGLRIILAGGLNPENVADVIKDLGKSASKVVGVDV
SSGVETNGAQDLDKIRSFVAAAKGL
>g2879
MGNGFKKVKASFVGSHKNSDKIPQWLRAHGGEYSREIDESVTHLIATESAMENNGAVERARQLKIK
IVNYDWLEDSLMSKPYRPKREGPYLWDRILRERDDSYRVTKDGKKGRRGVDIEKILGEFQKEAQAV
ISDMEKLGYHLYVDRSTGSSYSATLVRHTLIKNRSRKETFVARVYETNKEPHTYTTFVKYTRVGVA
SKDMLAPKGSSLEQALFAFKKFFRVKTGVEWKDRYDDSKLLVSPKASTNGESLLQPSEGAWFRYER
PGGLMGALNNDAVSVNDQDVQDDEVSRAKAEDVEENKEDDTTQSVY
>g2880
MIRRQARERRDYLYRKALTLRDASIAEKRAKLKAALASGKPLDPSIANDAELREDFKYDESLPSKG
QGKDDEFMDVDDEYALTSGLVDPRPLVTTSRSPSARLSTFAKEIRLLLPTSIRLNRGNLILPDLVS
SANAAALTDMVLLHEHRGTPTAITVSHLPHGPTASFSLHNVMLRADIPDSSRGTVSESYPHLIFEG

FTTNLGKRVVQILKHLFPPREGGGKLGNRVVTFQNREDAIEVRHHVFVRTGYQDVELAEVGPRMTM
RLFEIRGGTLEKNAGGDVEWALTQYTRTSRKKDYL
>g2881
MDAFTNIGNHMVSDSASTINADDGYSTVDPDESLLSLAGGARRRRHDDDDDGSDAFDDDELESLAS
VPVGGRLQKQKYEEEKELPPHACAYCGIHNPSSVVKCLSCSKWFCSARGNTSSSHIVNHLVRARHK
EVQLHPSSSLGDTILECYNCGTKNAFLLGFIPAKSDTVVVLLCRQPCAAMPSSKDMNWDTSRWQPL
IEDRSFLPWLVSTPSDQEQLRARHLSPQMIAKLEEMWKDNSAATIADLEKANSVDDEPAPVLLRYD
DAYQYQNVFGPLVKIEADYDRKLKESQSQDGLIVRWDLGLNNKHLASFVLPKLELGDVKLAVGDEM
RLKYTGELRPKWEGVGYVIKIPNNQSDEVTIELRAKGDHKSVPTECTHNFTADYVWKATSFDRMQY
AMKNFAIDEMSVSGYLFHRLLGHEVAAAPMKTQMPKKFSVPGLPELNGSQINAVKSVLQKPLSLIQ
GPPGTGKTVTSATIIYHLAKINGGQVLVCAPSNVAVDQLCERIHRTGLKTVRVTAKSREDVESPVR
FLSLHEQVRMNDSNIELNKLNQLKSELGELSSQDEKKFKQLTRAAEREILTNADVICCTCVGAGDP
RLAKFKFRTVLIDESTQSAEPECMIPLVLGCKQVVLVGDHQQLGPVIMNKKAAKAGLNQSLFERLV
ILGCSPIRLNVQYRMHPCLSEFPSNMFYEGSLQNGVSMAQRLRRDVDFPWPVAENPMMFWSNLGNE
EISASGTSYLNRTEAANVEKIVTRFFKAGVQPSGIGIITPYEGQRSYVVSSMQATGTFKKEHYKEI
EVASVDAFQGREKDFIVLSCVRSNDHQGIGFLSDPRRLNVALTRAKYGVVILGNPKVLSKHPLWNY
LLLHFKERKCLVEGPLSNLQESLVQFSRPKQAYRGPQRFHMAYNHASSMASGMSNVRNGNRPEYHD
TGSVVGYIPDDVSSVHSSAIGGVGVPSGYPPMFQNFAEAWPSLQGGHRANGARNKGAPSVAGESVA
ATESDMTSSVIDGKGGQGGVSLAGLSINDMTKQPSLSQSDRLKRYVESGGRDGYKSGVNDSGSIFG
GSSASIRVPRGPGLAQDDDDARSVSTAFASQIGGNYD
>g2882
MFCNINIPDTSLKMPIEYIYSSRQSGSDKSHRPALRSRTQTFVDLSSRFSALPVTSSSFSSSTIYK
REDAGRITTAYAITVTAINNGKDNYPRKREDKSVTYVTETGNTATAAVRVVTIKKPYAVDKPLPLT
PIETPVNAVDGLRKRLNHISTEEKRIKPISRSSRVPRTSTKSVIEEAGRLDIVDIDGNKFPFRNLY
SSSSEFPFREKEKLEKVMTVFIRPSFCCRSAKMIKEVNPLKYDGGKPEIGTAFLFESAETVKMSWS
YRIAGVSAQSEADVLRQALDIRSCAEEKEISQICPSPNRVLYRRVASMATANFLSCLLSLMMLFQD
ECYDNDVKGGVL
>g2883
MPALMRLVTAALAVAIPLAAAQTYTTCDPLNGTCPADPGLANYTFYTDFTAGDSAFEFWNTTAGTV
NSTSQGAAFIISEEGDSPTIQTAFYFFFGHVDVKMKIANGTGIISSIVFLSDDLDEIDWEGIGTYN
YEIETNYFGKDNTTSYDRATYPNVTTPSDEFHTYSVDWTPEQIQWFVDGDLKRTLAYDDALNGENF
PQTPMVLKLGIWAGGSSSEGEGTIEWAGGETDFSDAPFTMYVESVNITNYYPAEYYTYGDTTGAYT
SIVLSNSTSTSNSSVTTTTTKNGTSTTTLVSNTTSSNTSSSSASASSSAVISTAGAAVAELAMNPV
VIVSAVVLYLFM
>g2884
MRLIFATLLLSLALAFVQPEDILSTWPTVASSNNNTECSNPRPNDCSFYAQCLQTRYNCAPSDYPL
AYGQKYCQKFVADASLLSPKGQTWMQTVMLCLQRDLVPYALGGPQAFANCSALSDYAFNTHPGCYV
QSGLCTLPVHDWEIIVFRIVGVETLFDSWDAVKATVEAAGGCAEFYLWALEKGVF
>g2885
MNSTTSSSSALNGDNSVSSKVSPSSLARGPKLHNVLTSASSGIATNINAAFSASSVPSSAITSSTT
TSTAPTTTSTTSNSSPNFAPRSSFSNASSPIGTPYMLAADSGRGRYLSNTLAPPIPLELDETLDNT
MAVNGIPSVAPQPIDQVSSTTKGTTGLMRRISRGAANKLTRRRQSSTQRDKRERSSGPVIMRRRSD
SKTQPRTGRESTLDWSFDDEDNDVYESFTNGWAGSEANSISSELPGTLLPREIGAIAPKVDSVLQR
GTLLTKVTKRKRKQVRFFLDLDAAKVRWDLSNPSKKFYIDDILHIHVGADARNYREEYQVSAEVES
RWITIILDDPDRSKQRPFKAIHLITPDDYTLELWKATLEHVVRYRVGLMTSLVGPSQNETSLRAHW
QREMSRLYPHGLQPGETESLDLPAIESVCQSLHINCSKDMLRTQFAKADITGKGNLNYSEFKEFIA
GLKERKDLKHLYKQLAVSSPEEGLSLDEFLEFLRDVQREDIVRDRDYWSSIFARVVRRTRSRAQSL
PDSPDADLPRMNLNALTSFLQSEANGIYSSHAPESRFDRPINEYFISSSHNTYLLGRQVAGASSTE
AYITALQQGCRCVEIDCWDGSDGRPIVSHGRTLTTSVLFADCITVINRYAFISSNFPLILSLEVHC
NPEQQSAMVKLMKETFGEQLILEPLLTNCGILPSPEELKNRILVKVKTCDEPQSAFLPPIDGISTP
TPTFHHGRKRSSSTPFLRSAGYESLHGAIDIPLSSSPTIGPPLDASMPMPIIAPGRRSGTTTSMSS
ATEDSDSALINANREKKRRQRSKITKALSDLGVYTRGYKWHSFAAPESKQYNHVYSFAERAYESVC

RDAESKALFEIHNKRFLTRVYPSAFRLKSSNFDPISFWRSGVQMAALNWQTYDIGMQINQAMFAAG
TDRTGYVLKPEAFRRPSSALDALSEGKLATTEREIVRLSVDIISAQQLPRPRSMGPGDNINPYIEV
EMFSADDRGQCVALGGGGQDVSAXNGMSGLGLPHRRRTKIEPSNGFSPVFNEKLWLAVETKYPDLV
FVRWTVWSSPDGRTGGGSGAIQLASFTAKFSSLSQGYRYLPLYDDSGDQYLFSTLFCQITKEELVP
VQRLDLDELKAERKGLLRQISQTVKRTRSKERDRALKTGGDKFDSESIRSKDYSPFLRPVASTSPT
IPPLNALS
>g2886
MTKKGLPEQQDQSQEEAQGQLNQNAENHEGDNDNNNNNIFDQTFTSEPPPYSSPVSNTAIPRRPIR
TTSSPATSTSTSATFPLIDFSKYTIPNSSLSKDGSTISTYHPAFSTNPASLVRFIQEQAALPPLPY
IHILSDDNGGLQRDFDIKINMLPLFLNQTATSNQDRWNYVKLVADGESAYRGRSDAGISPTVKGGL
DEWAKRFCKEGSAVKSFTLTRQLSNWNTSYIEGQIRSLIASTGYNKPITITFPVKYSKIVVYPPRS
NSFNSFFTTLVSPLLDKKRYEVVRAVWPYASLPPGPAANPNRVPVVQTEEMWWEEWRDVLRWAIFS
KRVSGWVSVDDMLDFRMAPT
>g2887
MDPFSAEGELVNIHNAFHQGQYQTVIDFDTSSFSPENALPSRILQLRAKIALGQTEDVLADIEGEA
DDIPDLAAVKALAQQVAGNTEDALALAQKLAETQSENATVQVLAATVLQAQGQTEEALALLGKHQG
NLEAVALSVQIYLQTNRVDLALKEVAAAKRWAQDSLLVNVAESWVGLRVGGEKYQSAFYVYEELAS
NPNSTAPLSIVGQAIAELHLGRFPEAEAALTAALERYPDDVELIANTIVLNVLTGKDATELTSRLE
SLQPSHTLITDLAEKSSFFDTAAAKYVPKVSS
>g2888
MQVPLLRLQCGVNSYDWGKVGQQSAAAKYAAATADSNFSIEEAKPYAELWMGTHPSLPSKDVETQR
TLLDLVQDNQALMGQEITQRYGGKLPFLFKVLSINKALSIQAHPNKKLAEQLHARDPRNYPDDNHK
PEMTIAITPFEGLCGFRPLSEIVHFLNRVKPLRSLVGQQAAAQFEQIVKGSENAEDEATVSRNKDA
LKAVFTSLIESPQDKIEEAAKELVAEAQNSPHSFAIDPSSETNPNGASELSELVVRLNGQFPNDIG
LFILFFLNFIKLSPGEAMFLKADDIHAYVSGDIIECMASSDNVVRAGFTPKFKDVSTLTSMLTYSY
APIEEQKMQPTEYPYATLNTVAYSSGSSTILYDPTEIEEFAVIKTDLNKKGAKATFDPIPGPSIVI
CTGGQGKISVGPAKTEEVKEGYVFFVGADAECVIESTTEDDSQQFTTFKAFCELTGREDLPNGH
>g2889
MQLRSGSGLGGEVEGEPQSPATTTTATPPINAEGDKHSGSQCTVSPEEDSFPVTSDVKAGVVGASK
KKKKKKGQKSKKNKRSKAVPEVDEPQPAAAVPTNQQHHSYNLRLSASIVDLDQVSKWKALRRENTN
AREESASSSQTEPAQSFIMSRNRPRGPPTNRDNGIAANEEIDMWNKIIQDIRKAKEKNDKQKVIAE
QISALNEKIAREGNKPTLADINQLDGWYRQVLKLSEEEKAILLEEPSDVIKNLGLLTALRSASEAE
TSISRATSLPKSSKQSFARDGTAGHAATASNSALLPDKLNRVKGSTQRSSSVSSSGHAKEGRDSVS
VKLEDVAEGAAKGTIAERSGQLVIGAEVVYKHNKKQGAEGEGIQCIIKSIYGDGYKKKYDVQDPEP
NENGEEGAIYKTTAAWLIPIPQIGSTLPSFPVGKQVLARYPDTTTFYRAEVMGTKKDVYRLKFEGE
EDDKEMEVDRRFVLDIPSK
>g2890
MAEKSPYIAPQPRHPNASFDGLPSSIPTESHDITSHRSLLGRALASENTSSNATPALPESIISPAI
TPPSTPGGTIIRTQQAFQVEPHDARTHKHVHKPPKLMDRLPNVECIVRARIPTTTGAEMFLHLYQN
DADNKEHLAIVFGNNIRSRSLDRVQEGETEMDRMIRGAYVGRLHPGRVSSRFDAEGGLGGEAADKL
RKAQQAPLVRIHSECYTGETAWSARCDCGEQLDEAARLMSMASESSSDLTMDKDGPVIDNTTLSSS
ITEPTSGNDGPGGVIIYLRQEGRGIGLGEKLKAYNLQDLGSDTVEANLILRHPADARSYGLATAML
VDLGLGADAYPEGIRLLTNNPDKVLAVEGPNREVRVKERVAMIPIAWRTGGQKGIRSREIEGYLQT
KVSHCNFL
>g2891
MQSPNVKPEQASAESIGGLNHTMDPMDMSNPHHNGNVVAGTKRKINSASPRGVANLTPEQLARKRA
NDRQAQRAIRERTKAQIEALERRVHELTSQQPYQDIQNLVSEKERIMRENQDIKRRLSAMMNILQP
LMDRDDMPTISPTNSQLAMTTAPLANNSAIPPTNAGPIPAETTHPSPFTDRSHDTPVSLESHQLDH
SWNESIPGNLSPTNVELKCAPRPDAVSVMSIIANPQLTTQEFGEKLILNFLLDNPRQIPKIRDIPK
LSAEHSSSPEPPKLESSSSTGAVAGAGAAANDHRQAHLIAVQNLKPTCPLDSIFLDFLKNRRGELS
SGIQQRMAYPSVSSLLNPTENSTYSYSISKVFSDILRTFPDISSLPEQVGTLYTMFQIMRWQIYPT
QANYDRIPHWLTPRPAQLFNPHPAWMDYILWPKVRDRLSYAYRDYPFENWFVPYTRTISCNWPYEP

TDCLLHNTDSDELLINPVFERHIRDLNNWSVGTEFAEAFPALVDAYGLGSMGLAMASNLQRHLSAK
QGLGLLYSNRTLSRGEPLQQLGAVPQPNFGDLVSKSNIIFTMVSNDAVLESLINDVLKSGESLDSK
IFIDCSTVHPATITSISSQLSSKSASFLCAPVFGGNPVATAGKLVFALAGATPEVRQNIIKPLIQD
VMGRKVIDCGEDPAKSSLLKIAGNIVTVSLMETVGEAQVFAEKTGLGTGAMEELIDEAFGPIAGGY
SKRLTTGVYAPPLTSRPGFGVSLAIKDANHALSIAKDNGVELPSVELARDNMSSAREYAGECLDSS
SMYGILRQKAGLAFWNEKSRQGGK
>g2892
MTDAPIVLDGGTGFLKVGYAAQSAKNFPEFQFPSIVGRPILRSEEQGESDIVLKDIMCGDEAAAAR
SMLQISYPMENGIVKKWEDMQHLWNYTFYDKMKIDPTDRKILLTEPPMNPLKNRQTMAEVMLEGYG
FGGVYVAIQAVLALYAQGLSSGVVVDSGDGVTHIVPVFESTVLNHHIRRLDVAGRDVTRNLIALLL
RRGYALNRTADFETVRQIKEKLCYVSYDLELDQRLSEDTTVLVESYTLPDGRVIRVGSERFEAPEC
LFQPHLVDVDQPGMAELLFNTIQGTDMDVRSSLYKAIVLSGGSSMYPGLPSRLEKELKQLWLTKVL
QGNPERLNKFKVRIEDPPRRRHMVFLGGAVLANLIADKEDMWVTKAEWQEEGVRALEKLGPRQ
>g2893
MPFRSPFHRMNDAHSQQPLLKEDGPQRESNTFAESHERILERPHMSRRSTFRSRTPDIEDKNLTRR
KYILASGFLLLSLISFVIQTETAVYIQHELHWDKPYCMLYLTHGSWSLLWPTQLLILRIQKRKLSW
DAFWRRHVFLLRTTAQMVERQDLHLTTRDDHRSPVPYMLKTTAFITSALTIAGGSWYVAVNMTTAS
DLTAIYNCSAFFAYAFSVPLLKDKLRFDKIFSVLVAIIGVLVVAYGDSGPEAGESSGSADNRTLGN
IVIGVGSVLYGLYEVLYKKLACPPEGTSPGRSMIFANTVGSLIGTFTLLVLWFPLPILHWTGLETF
RWPTGEAAWMLLISVIANATFSGSFLVLISLTSPVLSSVAALLTIFLVAIVDWLRTGKPLSVAALS
GGVLIIVAFLLLSYSTYREVDEERRRRFAEDGPDSDSDD
>g2894
MPGDEYSAISGGGKLKLKGSKVKDGRVDKTKKKKKKTDQDGATAETRAKSTEAEPEKQEASERHFD
TVDDKLDDELSESGEKRVVFKTEAERKHEEQRKKRLNDRLRREGVKTHKERVEELNRYLSTLSEHH
DMPKIGPG
>g2895
MPARKPRCNFKECKNAAQRIIGDCTFCNGHFCDKHRMLESHACSGLEDCKKESHARNADKLNSERT
TVIKGV
>g2896
MPAVDIKTTGAKPDLSAKLAELRASKTRRSPPTVRQQQDPPPVTPALPSPIDLSSHTFSRPARRIL
SPKDLDAFASSPAYTLILAFIFGLSDKVRGRAVTDVQNDNVPDNIENILRIIDLLGSLVEKNPAED
QGGSRFGNPAFRALFDDTASQSPEWHREILGIQNTEAVDEISTYLIHSLGSRDRLDYGSGHELHFM
MWLLCLYELRIVSITDFPMLVFKVYYSYMQLMRRIQTTYYLEPAGSHGVWGLDDYHFLPFLFGASQ
LVEHPYITPRAIHSSAVLDEEGDKYLYLDQVRWVDSVKTVKGLRWHSPMLDDISGAKNWSKVESGM
KKMFVKEVLGKLPIMQHFLFGSLIAASPEMGQLSDEATSSGQQDQQGHPHDHNHTHGDDFWGDCCG
IKVPSTIAAGEEMRKRMGGGGLRPIPFD
>g2897
MDGSFRNTQYQSTGQLSREDGTTARNSWNDEYNRDEARQNGPQQEPQHPAPRPVNNRKSNDSTNRN
VAVTNDNTNSNGDRYNNNNNQAQNQTEEEDAAAHQPWFRRVAEKYGTLELENKGSVARDHLALERT
FLAWLRTSLSFASIGIAVTQLFRLNSSVSCSPSQPTSTASYVDNYYNNNNQIQNPLQNPLRSSPSL
LEVLLTPPSYQSSLDTIGLSATTTTGNRGQYRNIGKPLGATFIGIAILILVIGFHRYFESQYWIVR
GKFPASRGSVALVSFVAAGLVISSFVVILVVAPNSTEL
>g2898
MKEQDPASSSSFRVLIINPNTSTQMTSALKPIIQGLNYTDIHFDYFTAPNQSVTLPDGRVIEPVPS
INSGDDSVQSAQHCRPFVEPLIPQYDAFLVACYSAHPLVGMLRSTIRALENKALQSDSGTLQVFAD
GQEINPSKGKKYVTGIFEASVTTSLMLISSFHLLADWSHHKTQSQDTFGIVTTGAVWKKELTKAVS
AMINGPDESEEKKRDLISNVVDTSPTPSPPAPVSVPRFAGVETTGLTAVELHETPPEEVRRRMIEA
TEKLLKGTAHPVRAVCMGCAGMVGMEEAVRAGEIITIQAGQCGNNVGSQFWQQLCLEHGINQDGNL
EEFATEGGDRKDVFFYQSDDTRYIPRAILLDLEPRVINAIQTGPYRHIYNPENFFIDPQGSGAGNN
WAAGYAQGEHVHEEIFDMIDREADGSDSLEGFMLLHSIAGGTGSGLGSYMLERMNDRFPKKLIHTY
SVFPDGQAADVVVNPYNSLLTMRRLTQDADSVVVLDNGALSRIVADRMHVQEPSFQQTNQLVSTVM
SASTTTLRYPGYMHNDLVGIIASLIPTPRAHFLTTSYTPFTSDNIEQAKTVRKTTVLDVMRRLLQP

KNRMVSVTPSKSSCYISILNIIQGEADPTDVHKSLLRIRERRLASFIPWGPASIQVALTKKSPYIQ
HTHRVSGLMLANHTSVATLFKRIVQQYDRLRKRNAFLDQYKKEAPFAEGLGEFDEAKAVVVDLIKE
YEAAEKENYLNPDAGQKEAVAA
>g2899
MLLYIISTILFAAQTMALPTPANTSVNPAAITNTTCTAPGVTLNSHDINVAILSICGGIAGTIEQC
QGQPTTTTGASGDAQLNLQVETTGTNIIITKGRWEGCMRAARAVCGDSPFSSTCIGGANDNKNNVL
FQLVQQ
>g2900
MPLIASTGKPRVILGLMTYGPDLKTGARITSLDEFNQHLDYLQSQGYHEIDTARVYVGGQQEAFTR
EARWKERGLKIATKVYPTEAGLHKPENLRRLFELSLKELGTEQVDIFYLHAPDRSVPFAETLEEVN
KLHKEGKFVELGLSNYTAFEVAEIVTICNERGWVRPTIYQAMYNAITRSIETELVHALRRYGISIV
IYNPLAGGLFSGKIKSKDIKPDEGRFSDVAISGQRYRDRYFKDETFRALELVENVAAKHNLTLLEI
AFRWLRHHSVLKWDNGGDDGVILGVSSFDQLKNNLADLEKGPLPQDVVDALDEAWLICKATTAPYW
HNELKYTYDTQAALFKK
>g2901
MGFFWKLRLTIIVFFLRLIHRRTQKRPQANPDFVLRIPSRDAGRSIKAHVYRPTILQTPSPVLLNF
HGSGFIMPLHGSDDFYCRRVSKETPFTVLDIQYRLSPQNSFPAPLHDIEDVVKYIKSSPTDTNNGI
TYXPSRICIGGFSAGGNLSLAASSMILPPNTFRSVIAFYXPADLATPPDAKSPPEPTDKQIPPWMA
KVFDGSYFLDRDARDPRISPFYAPAENFPDNVLIVTAGQDYLADEATGLAERIKKEARDKHVVSLR
VEGCGHAWDKNIGEEDTHRAKKRDEAYTAAVEILNRSIS
>g2902
MRDYTPAQLPAFSFNPGNIHASTPSTSTVTAPRPIGHRRRPSELIGQDGPPSPRMMGTSPGTADGE
NATVPAPGMSAPSRGPGRRGHAHRRSQAMSSMDLTALTLAAPPIAPQILTVGSAPTTPADSKHDFQ
GHMSRPMSRSAVDLLPQPSPPVSPAKQPMTLRPDFTFHDRPRPVSMISTETSSSLSTVRPNHSRVS
SAAPSNKIEGSSSTETIKARPKTADAGVLMSKLSRPSEHFSEMSFLRRSSVGMGPLGLGRQSSETV
DMSDTESIKDKHSSKKNKKKKKRRKEEGNATFEEQKRRWNSLSTERTLDITQDPSAEKWESASSFN
SRSGAEDSHPRRGDDCHGKKQKKVRNWAGHIFPLKNKRTHVKRPLSRRSPTPPPILTRTNSDLGSI
EVNFDEDNTVIIRTPTNPNIPKQTTQDTEGESDKAFEKAWKPRSFYEQGVENDTFSPVIDLDAALG
PFNTPEMGAGRIAGSAFSQATKRMYSGGRRGEFVGPEMRYHRRAESAPEMPPFDRSALIGFPRLGS
DNAMASVDVFYEEEEDAFLAEHQSPTMKDQAQADVEQANAELSSVIDEESEGDMLPSSSETLQAVQ
RPKSALVLSRDDGLGIQVADDTVVEDGPGDAVEEEEEEEAKENETTLSHHQTQNKNSVEIVEADQW
AHPRVPHHANHSPELSPHMLAIDKRPCSSPLDLNSGFSQLTLPSRHTSSSAFPSPDPSNMSFDGPR
SATASSMTDHTTFNQSLHDQRQSSFEDVPSLSSSASTRTNPRGRISSSFYPHSSSERRESFNALPP
RSSHSNSAKRSSLVSLSRLVGASYGEKSKLSHEEKPPADEAEKTRRKSNRVSRLMFWKSKDKQNDS
>g2903
MGGGGNGKEKWKVGPTKKRGSPQRQKQAAKKTPPSSRKEYTTTAVNNDHKMEDENEHIDLSASAKR
ISPDLQQLLLNVFKYALFSSLEQVNRDVDNTEEGEGEEEEEKDISTQIQQLKTHLFNRDFESAFTD
ASPSLLRAYALRWSASRSLAYCGLFKGVIDCALESGHGLDDGKEDVGVLCIGGGAGAEIVALAGAW
RALQDEQNIIKDTDESLPLEGVENLSLDEATSPKQKRRRKMGITAIDIAEWSDVVSRLTAGIHSPD
VFGTKTFVAPLISGQEDEGIDISFQKMDILSLSEEGLKSLLHRQRVISLMFTLNELFSTSISRTTQ
LLLRMTDIASPGTILMVVDSPGSYSTLSFAKNDSTETQQQQRQYPMKFLLDHTLLSVAEGSWEKLV
SQDSRWFRKDANHKLHYNVELEGGQGYIRLEDMRFQIHIYRRLSSSNQKE
>g2904
MASPNALLRWFALPIAGALAIDASMYDVKGGSRAVIFDRLSGVQEKVIGEGTHFLIPWLQRSIIFD
VRTKPRNISTTTGSKDLQMVSLTLRVLHRPEVPNLPKIYQSYGTDYDERVLPSIGNEVLKAIVAQF
DAAELITQREAVSNRIRTDLMRRAEQFNIALEDVSITHMTFGKEFTRAVEQKQIAQQDAERARFIV
ERAEQERQANVIRAEGEAESAEIISKAVAKAGTGLIEIRRIDASKEIAATLASNPNVTYLPGGGEG
KEGKGTSLLLGLRS
>g2905
MVGKADPKNGIFAGGFGALGCPTPQRGIAAYTISPNRQNPLAGTLHAAIFNTFRRFRHQVLYFAPP
FIIAYAAMNWAVEENEYLNSKPGRLAHGGEE
>g2906

MSEITHPTIKDGWFSETSNMWPGQAMTLKVKEVLHHEKSQYQDVLVFESTDHGTVLVLDNVIQCTE
RDEFSYQEMITHLAMNSHPNPKKVLVIGGGDGGVLREVVKHETVEEAILCDIDEAVIRVSKKYLPG
MSIGFQHPNVKVHVGDGFKFLADYKNEFDVIITDSSDPEGPAESLFQKPYFELLNGALREGGVITT
QGSENQWLHLSLITDLKKACKEVFPVAEYAYTTIPTYPSGQIGFMVCCKDATRNVKEPLRSWSREE
EEKLCRYYNADIHRASFILPNFARKALE
>g2907
MTDSNISTTTPTGSATAKHPSCILCRKRKVKCDRKYPCSNCIKANAECIFRESIPARHHKRRAEHA
LLSRLRHYESLLRQHGIDPGDDATTSGGAGGSEVQHGMSALKLMESKPTLGTLEDSDCISDDSDAE
NADYLSPKHESEPLDHGYNILFSSPSNETLTSLHPDPVHIFKLWQVFLENVNPLTKIIHAPMLQQQ
MLNAIGELNSIGKGMEALLFSIYSCALLSMTDEEAYKEFGREKSSLQARFRMATQKALANAGLLKT
TDLVLLQAFCLYIVSCRSIYDRRTIWCLSGIAMRIAQQMGLHRDGSHLGLSVFETEMRRRVWWHII
YMDKTITRSSGFISAPLPANDTHYPLNVNDSDLHPNMKEPPIERNDICTDMIFILLIQELGKWQER
QPKFVESMFSGPEEKAKIREEKAQRRQKAVDDFRAVIHDKIIRFCDPSIPLHIVVNASAHTIVSTI
QLVAFHIAIMGYTEVDQLAINTIRVLAVDATSKANSGHPGAPMGLAPASHVLFSKFLNFNPKNPKW
ANRDRFVLSNGHGCMLQYALLHLFGYDLTIDDLKAFRQLDSKTPGHPESHDTPGIEVTTGPLGQGF
SNAVGLAIAQAHTAAVFNKPGYDLFNNYTYTIFGDGCAMEGVASEAASTAGHLKLGNLIAIYDDNH
ISIDGDTKCAFTEDVLKRFEAYGWHTLEVKDGDHDLQSIEEAIHKAKQVKDKPTVIKVTTTIGFGS
KLQGTGGVHGNPLKADDAEGVKKAFGFDPSQSFVVPQEVYDLYNKTASAGAAKEQEWNNLLQKYAG
EYKSEHADLTRRLAGKLPEGWEKSLPTYKPTDAAVASRKLSETVLEKIYSVLPELLSGSADLTGSN
NTRWKAAVDFQPPEYGIGEWSGRYLRYGVREHSMGAIMNGLAAYGTVIPAGGTFLNFVSYAAGAVR
LSALSQVRVIWVATHDSIGLGEDGPTHQPIETLAHFRALPNLMVWRPADGNETSAAYYSALTSKHT
PSILALTRQNLPQLQKSSIENGLKGGYVAVDAPDAAVAIVSTGSEVSIAIDAAQYLKEKHGVSARV
VSIPCFEVFDAQSKEYRLSVLPDGIPILSVEAASTLGWERYSHEQFGINRFGASGPYKKVYEKFEI
TPEGISKRALATIEFYKGVKPRSPINRAFQQLI
>g2908
METILIQQVNLMPSAIAVVDETHTISYRELIMRADILVDLLQKHSLEPEEPVCTFVPTGFSQVLAQ
IAVLRAGGTCVPIDPATPTDRLKGMLEDLATTLIITSGDFEPLALNMNVVPIEAALDARVDIDKDQ
EISIRANRPETHRSHIIFTSGSTGRPKPIQVLSSSILHVLRNLPTGPLSSSDRMTTMLNPGFDLSL
LEMWKPLLEGATVVHVPNIIRNDPFALQSFAKQQKITFTIVPTALFEVIALTAPAAFHGMRHVIVG
GEAVSSSAMHSVLREMPPENLWNGYGPAEATIFVTMARVDHQEVQRSRISIGQTFGDTRIFLFGDD
LQTIHEPRVTGQLWIAGPQISPGYLNRPEENKKSFVSVDLAGTCIRMYRTGDLAQWRDKXGXCLDY
IGREDDQIKISGYRVELGEVSRVIEEHPHVYSCVVDYHQENEVETLEAYVILADGELNINTEELID
WTKNKLPPYMVPSKIYKKRTIPLTVNGKVDREALASAKSDHETTNTPHMETPDENDGHTGDLSTRL
ISALQPMLPSWRGNPSDNFFSLGLGSLQAARFIGQIRRGQGKAITLEQLYSHPTIKSLAEFLADPP
VEVNPDSSDWRGADEGRVFLTGATGFLGAHILQQLLHLPGVKQIACLTRSQSGISASARIQQTLEK
YNIWDSRLEKTEKIMVLEGDLTDPNLGLGERQFTWLTNWASIVFHVGARVNWCEPYEAHYEPNVIG
TSHIIRATTLGRRKTLHYISSIDVWSVSGFINNVDRVDEDEPLMPHMNAVPYDTGYSASQFVAEEL
VQRTRARGLPTAIYRPGFIIGHSEIAIANANDYFSRLIMGCIQIGYFPDLATQYLEYVTVDYVASA
ILRIASDMGNLGRAYHIVPPDRAESVTYNDIYQMLQDLGYHVEKIDYQDWVDKVRESPGTPLEAMM
PLIEEPVYEGMTRLETSRNTPIYDPRNTSIALRDSPDVKYVALNSKLLQQFIQNWLDQGDFTLDYD
RTTRG
>g2909
MGDDSDSSEDHHVRDFLYKRVWENFCINNDHFVFGFPDTDVDLSALHPESADMLKLWQIYLDNVDP
LLKVTHARSLQRQVIDAASNRRKLKPAFEALLYSIYCAALKSLAPEDDIEPIFGASKSDLLARFQW
GCQRALVKCGFLQSKDRDCLTALFLYLVSIDDKIGPWSTSSVLGIAIRIAQRMAIHNESALVKCTV
YEAELRRRLWWALALFDARISEISGHKCTTLEPSWDCRIPLCLNDSDFGPEMKEMPIIQKPVAETL
FIVIRSELGDFVRRAKFHLEYTNPALKPISDAHHETAPESTKLDALEKAIGGKYLDSLDPEVAVHS
FTICFTRLMFARLRLLQLHSTLSSRTEAQEEVLVSHSLQMLENDTKIVTSPLIRGFAWLTNLYFPY
IAYAQLLLYLRKRPTGKQAEQAWEIMSANIEAHMHTQIAPFVETFTKMVLFAWKAREAALQVPPVP
PKIISIIRQKDTEQYHNDADSKNCGQKTENASFNVAVNESDGHSLANEQERPNDYTNSESYFLNIC
EQSQLDFNNLREFDWANWDWGNNAEMGTDEFASF
>g2910

MANATRGEDYSTQRLRQRYYFHDSNMDLFLVGALGWGPAGGLSIGEAYHVASQITDGDADSWSQAF
ENQGIAQSRQADEWLRRGAVRSAGEMRLKAFVCYRMAWQFVAPGERFIALYCAHEKLFDQAMTELG
LPVTRFAVNYGGGNLPGHFFQAADPTTPTVLVIGGADTCHEDRFLSQGRYLLERGYSVALVDLPGQ
GRVQEQGLHWEPAAEKPIAAVIDELINSFGVEPRKLALLGMSLGGYFACRAAAHEPRLVAVIATTP
LTCPLELFLSVGKRAAEATISQAEEKNMEVIMWKAGVSGLSELTQRWEGMVAEPGKVQVPFLSILG
EQEGQVWKEQAWAWHETIPSSKKSFVTLDAQSGADIHCQGSNPLRLTQEIDAWLREVL
>g2911
MPGILPMKVIKMGSNAQSRIAQACDRCRSKKIRCDGVRPCCTQCKNVGFECKTSDKLSRRAFPRGY
TESLEDRVRALESEVRDLKNLLDERDEKIEVLSRIHSFGTPRQKVQSTSSPASTSGSVSETPRSPL
SEDATASPIDTERIINVQHPTKSSGSSPFVGPSSVRAFSSTLTSKLEARGISSSSFSTKALTAFPA
RNYQRKASSLQSPPRLVSDQLVNIYFQEWAPLYPVVHRSTILKAYDRYLADPSLLKDQPLTIIQLN
LIFGIAALSSMSRTNQDPKLFENNWYTPLESLSGEMSVPAVQCMVLAQMYFLTKGDYQSLLRYRAL
SVGTIQSLGLDQSQQDFVRDPLLHETRKKVFWCQYMLDRFAAAATGLPVMLRNDRIGTEMPADIDD
ENITEEGILPGLPDECTRMSSALALIEASRILGKALEILYPSANAQVSVTKLHGLSEELDTWNKGL
PTHLKLIFIQDRPSTNVTGSRSPLLSLVYYYIRILIHRPAACFGTPDIMNPALLTVSDSSKHIIQI
LQLLDERRLSLSLAVNRLEVAYLSGLGILWQDMTLERGSKLVQESRSLLLDVKVQLEPESNTAATE
FRILSNLITGADNQRSLKIKKALQTSISRAERGSEAKPAKDGMLSRRNTISNLNAAHRSSQQTCQR
PGTMEPPQTMSRPRSFDLSSGVNVDYYPLNADSLRSVSTTDVSKMSLSAAEWECILSDLDHGNLNI
FNGIYGGQEGADQPSSQGPIDNSYSPHQTATAYAPIMQQHVRLSPQDTSSEAWSACSSGDISYVHD
GVPGYPAENGINTDDVLSFKDFDLPQSLQMAEAVRGIMIPSADEDYVDLGIFDGWDRSLIA
>g2912
MLNRPFLPIEALPAWARLNGIKFNDIEFQKLEFGSGIVAKADKEYSSAQEAEDKPEILMTVPPDMV
LSIDLVHEFAKSDPYLRAVLEASGDFGWTARGAILIFLLCHITYASNTHAKIGVTNPWSEYIKFLP
SETLLPTLWTEDELVLLYGTSLKDAVDHKLSALEAEFDRLRDATQSIAWCEREWWDEETGQLILDD
WKIVDAMYRSRALDLPGSGHVMVPCVDMANHASGEETVALYETDEERNAVLQLRWGKKLQRGEEVT
ITYGDEKGASEMIFSYGFLESSVEDARQLFLPIDIPDNDPLKKAKKRISAKRTAPGLRLALENGKT
VWESDFIFWACVNEEDGLSIEMMQPIEGPVELKALWKGEIEIGHVVPGASSKSNVKELRNILSQDP
LWDLFQLRAAVLVQQCLQSRLEMLGGEMEMAFEGVEHDADGSQTGVRSAVYEMIRKLRQGETALLQ
GGLRDLANEIEELMTSETVQKFLKQQQQQEEEEDFS
>g2913
MRRSLIPNPPRPYFFPLSAILQPASFHTPHSVRAQFSSNSDRTQQQQSTSEPRSSRSSDVKGHKFQ
EWKGSSTEDHAVNRAAKNDITDPEVEGVATGRAEKQRNSGIADSAMSGATTERDLGQNAKRAKEEH
PKSPEPIIGMNDERGEVSFLT
>g2914
MPRDPLIGLVGKVCWTISTPQHLRADQTEGRRSVPIELLDVAGLVPGAHEGKGLGNKFLDDLRHAD
ALIHVVDVSGTTDAEGKETRGYDPSQDIVWLRSEIVRWVQGNLMEKWGSIKRRHQATKANPVETLQ
AQFSGYGSTSQTVARCLDRLNLKEPLENWSNETVEQVVNAFVDEKFPTVYALNKIDHPDADKNISK
IARMQDAKSIVLCSAISEVFLRRLAKQKYVKYVEGSEFVDTREDLIEMGDPDGGGLKEMDEKLKQR
VENLKDMVLYRFGSTGVVQCLSRAAEVLGLVPIFPVRNIHTFASGSGSNAVFRDCVLVKKNSTVGD
VARKVMGDVPIAYVEGAGGVRVSEDEMVAVGKHDVLSFRVGR
>g2915
MSSSKFHGTTQELRKLESKVSRAHGGKTPADSDVSLMKSVLDSGTNKQKKVEEAQKNLPLPEQPPV
PSDWNSADQRAVNVGSGRVEGPVSGEGNSALRGPATVASSARVDGEEFYKATEPLSGVGRQGQEGL
DHLPKDALSR
>g2916
MDMQGKPIGNSTGGTKVTTDPASNNPRQEGTGAVTNDSLAAESVRQGGAFSQNPNSEPLGVSGPNS
TFNNTDTSGANTLRSAAHAGDRDTDRKERYPDALGGQGKYPGAHLPESGYAGGSTQAKRELGIGGH
QHQYHTTERATAGAAAGGSGGFRSKHNGGTAPSYVTPVVQNVGNTKPKGVNIKEGGFDSNSKNNAS
FNSDIGTKNDPGRLAEQKFQKVTAESGANAAYTNPTMDANQPYGNLESDQRI
>g2917
MAPSRINVEMVSERQSEVVTKLSTDAGQQIPLSEEDDKMETNSIDQDEIQEDAGFEVEEEEEEISL
DNYEQLKELYRNCHSSSGGNRYSYLVNCSPYNRQNEHPHSNIPKHHIATHSQSRYLNALQSAAAHS

DGLGDMFHRAPGTVTFTFVALLIIAILLVEMVDIFCYRRRRRSRDEEEEEEDRHRRVRRRRLRTRA
VRIPTVTVVDVSQQPSAVSAIGPTTPTILSTSLTATKDTSFFDTLTLNSHHRPITFYDKVQRIITM
AKAKKTGDTISSRLALVMKSGKVTMGQKSTLKSLRSGKAKLILIAGNCPPLRKSELEYYAMLAKTP
VHHFSGNNIELGTACGKLFRCSTMAVLDQGDSDILSQEA
>g2918
MGKDKSEKKDKKEKKEKRRSETDGVHKSSKKEKKEKKLSEVAEKELTAQVLEGLENKDAVEEAVAV
NGEAAETPIGALVPFANPLAEEKTAKKVFKTVKKAAVNKSLKRGVKEVVKALRKSPTPAANAPITE
PIGVVILAADISPLDVISHIPVLCEDHGIPYVFVTSRAELGAAGATKRPTSVVMVTPKSGKKSKKS
EDGDEEDFSKVYADLVKLALKEQEKVKV
>g2919
MASNPAFRQSVRPSQPVKNDLENEELRVQVNTLRYELDNLKQERDLTELRHEKELRDLQLRADSDF
RKAQGAEAASNRANQKSEALAKELKETQESALSEKASSERRIRTLQDQNQSLQEEATEVEAQRADQ
ERQFKRQITELDTLRITLAQTLQDVQQDLQNVQITLHSANAQVTRLENDVATLEAENLRLRAEGNG
AEELAVLKRELSEQVAHIKNLESTNREQSTELRHLRKVQKNVEVVEEQKKSLENQLQLMSTLEAEL
GNLQIQKRVLEDERRSWSSMLESNGEFADFDSPEAVVKALVEERIEKLTLIDKLGSVEPQILEKDE
IIQGLETEKRQFKQEIEKLRSAAAAPGGTMDTRAKLRLERQRALAVKEVEYLRAQLKAFDTEELTM
HEEGSHYDEQKNQQIEKLEKLVDEYRNELHKVHEDLSALEKASSKDEEGTAATTSRGIKRPLSPAD
SDAESERISILTRKNRSLQEALSKSEQSIKVLRHELDATKSHLSSLQEQSRTRILELRANPTAEAE
NLKMSTLRTLQAENRDLLAQLRGDNAKVARVVPVSTLDSLKLEMQEMERTVAEKEKRMRRLKEIWT
AKSSEFREAVASVLGYKLDFLPNGRVRVTSMFHLSPAYRHGASSTASTGPGSMGNGEENSIIFDGE
NGTMKISGGPNSLFALEIKHLIKFWVEERKDIPCFLAAMTLEFYDKTTRAARV
>g2920
MQTALYSFQPAIKRGVRVVALAELQETSDVACDTGSDVADLKREFDDYQLGPATPVVDLSLVPEDW
NKKVPTLIGRWAPSSDALVSRARAARQWLRQRPEKEVVAICHGGFLHYLTQDWSGIKAEEHASAWE
NCDFRTYQFDDNLDDATMVETDESRHARGVADQKVPTKEKQYVLYLQAMQTWEDRGFQNPLKLNKQ
FLTAP
>g2921
MTEHHHLVHLQAGLFDPPHQFAAPPGIKLTQEHHPTTTPEQLHARIRDATIIILSYTRIDAEALSE
AVSPKLKLIAVTAVGTDSVDLEACRKRGIRVTNCPGSNVEAVSNHVMALYFAARRNIVRMDRATKI
GTWVKFRSSLSDGMADRDGNWCLTCDEEVMGILGYGYVGKRVAQMAQALGMKVLVAGRKGEESPPT
NGDTNIQRTAFDEVLKRSTVLVIAVPRIPETMNMISTAEFAKMSCKTVLVNVARGGIVDEVALLQA
LKDRTIHGAATDVFAVEPASAETSPLLAEGVDELNLTASPHLAWNADKTAKNYARMSPENVMNFLL
GHPTNIIV
>g2922
MHSPASYSPLDSHNGHYVRPPISPTPSEQALLQPYESRDHGVSLDDPITPTESMRRYTLNDPGATI
FAAPPYQESDSASIHDRQGAQTPLSRNGSRTDALRRYGTRKIKLVQGSVLSVDYPVPSAIQNAIQA
KYRDVEESQDEFKFMRYTAATCDPDEFTLRNGYNLRPAIYNRHTELLIAITYYNEDKVLTARTLHG
VMQNIRDIVNLKRSEFWNKGGPAWQKIVVCLIFDGFEPCDKNTLDILATIGIYQDGLMKKDVDGRE
TVAHIFEYTTQLSVTATQQLIRPHGDDATNLPPVQMMFCLKQKNSKKINSHRWLFNAFSRILNPEV
VILLDAGTKPGPKSLLALWQAFYNDKNLGGACGEIHAMLGPRWRKVLNPLVAAQNFEYKISNILDK
PLESSFGYVSVLPGAFSAYRYRAIMGRPLEQYFHGDHTLSKRLGKKGIEGMNIFKKNMFLAEDRIL
CFELVAKAGFKWHLSYVKASKGETDVPESAAEFISQRRRWLNGSFAAGLYAMMHFGRIYSSGHNII
RLFFLHIQMIYNFCQLIMTWFSLGSYWLTTSVIMDLVGTPSTTNKEVGWPFGNTATPIVNNFLKYG
YIFVILLQFALALGNRPKATRIPYTLSFLYFSIVQLYILVLSFYLVVNAFTGQVLDFNLDNGIGAF
LTSFFSSSGSGIILIALASTYGIYIIASVLYMDPWHILTSSWSYFLGMTTSINILMIYAFCNWHDV
SWGTKGSDKADALPSAQTKKDDDSKKVFIEEVDKPQADIDSQFEVTVKRALEPWVEPKEDDQMSLD
DSYRNFRTTLVLLWVFSNLLLVLLITTNEIPWQNTSTTRTSWYFEVVLWTTAGLSIFRFMGSMWFL
VRSGILCCVSRR
>g2923
MGDSTIKTIVVVGATGNQGGSVARTFLKLPHWKVRVTTRNPSSPAAQALKVLGAEVVQADLSNPDT
LARAFDNAHAIFLNTDFWVTYRALAATVMAQEVCPSDEEKPDPSRVAYQTEISHGKNAAECAAKVP
TLERLVYSALPATRKLTGAKYQSNHADSKGAVVDYIETELPHLARKTSFIYLGAYNTNPLLSPRLD

LSDGRYRYFLPLSKAARMPIINVKESTGPFVRALIEDEAPGTKLLAYDNNSYLAYGEICDIWSRAS
GQEADFKTVTVQFLHETMKIPMEFISSLPALEEYGYTGSMRVIQPGDLKTDVRTKSWEEWMMERDR
TAILEA
>g2924
MATTAADSDDNATANIDPNMGSSNGDNIAPITSAQSFTPRSLLVGLGIGTLIIFSNTYFGLQTGWM
SLMTMPSSLIGFAVFKSLSKHLSFPFTPVENVLIQTVAGAVGTMPLGCGFVGVIPALEFLLKEGED
GKLGDGGTGEGGPLNVGFWKLVIWSLGVCLFGAIFAVPLRKEVIIREKLKFPSGTATALMIRMLHG
SADEKSDAQHQRASSVTNVPTSRPVSSASTTRDVQVNGDGRRDWKSKIRLLIGAFAVSAFYTLFSY
FVPQVRDLPIFGLSLAQNWLWTLNPSPAYVGQGIIMGPSTSMHMLLGAVLGWAILSPIAKHKGWAP
GPVDDWANGSKGWIIWVSLAIMLADSIINLGWLILRTSYGSLPWLKAKLQALKRKGGKGVISEQRR
RSHVQYSALHAPTNAEELNIEEDEGDLEEEESDAPPSELISNRTIFIFLPLTLVLNVICMHIAFGD
IITPFLSTFATLLAVLLSIMGVRALGETDLNPVSGISKLTQLIFSLVTPASMHTRRSAVVTNLLAG
AVSEAGALQAGDMMQDLKTGHLLGASPKAQFYGQVIGSVFGAVVSTAVYKMYVNVYPVPGDQFQIP
AAFVWIFTARLVTGQGXPPMAWQVAMLFGAVFVGTTVLRIVATVRRTQQHQGSPPPSWISFVPGGI
AVAVGMFNVPSFTLARAIGGVIAWWWARRNSSAPVPLPSSSDQGHIVSPTTTDATDADESTNESTS
SSPLISASVELQSAAKSQPSPLDPSQEDTSSSTVVVLASGLILGEGVLSIANLILASFKVPHL
>g2925
MMASHWAELGCSVGQSQPGLFFLRMNHPLPNQQTLSVMYAMAEDWRASYGFSKRLQRMSEITSAYR
NLSPSSTFVEAQPHSAPLEREAYAKATSEAEYDRLCQEIIDGFEAQHADVPVIDTPKAISSEEAYI
EPGEAANAVKIGAYENCVHHADGLMSTVYRSKSSDGKLVALKVTTPHQMSPPHDSKREARILRWTK
DQKHIITLLEAFELAGGRFVLVFPFMRYNFAEVLHQNVLTTLQIKIILRDMFRGLEHTHALHIIHR
DVKPSNILLDSLDGPAFLADFGISWKEMDEGSEAANEKIIDVGTTSYRPPEILFGSTEYDTSLDMW
AAGCVVAEAVDMRHRQLFDSGDLGSELALIKSIFSTLGTPNAEVWPESAKLPDWGKFQFYEYPVKP
WEEVLEGASTQGRDLVSKLVCYESSSRLTAEQALRHPFLPQNNQSEQDEPYPAGYRFNDDIIQELV
MSHAHFHRHERRDIISAVESFLGIDNNKGEQQSTVTETASVVYVTASPTFTGAIGGYVTGSDTTTT
ADPTTTGKGAPVAQSTKTTTTSQSEKSSTTTKAPETTTHTTSTQSTTSTSLSDLLTTSSDPSSTTF
LTSSSTQSASSTSSTGTAVDQFSSSTASATTSAAASASNGLTGGAKAGIAIGVLGGIGLIAGLVLF
WLHKQRKNREEAAAAAAAAENEKFARSQPPPMTSAPPPQSMAAYSSAPSTPATAPQVSLRPITQFN
PMLTQSGGANPYAAGAAGAAAGAAAGAAAAGGLQVARSAERPYGGSAHVPPQSPRQDPFTDPVNPF
DNGAQAASPPMPPAKDASSPIRDLTPSPPGSVHNLPSPITEEGSSAGSLEAAAAGAVAGAAVGAVP
VAAVAAAKSNDNPAEPRTPSPEYVEHAGSRPQSPVVGGAGAPVSNVHRVQMDFTPSLADEMELRAG
QLVRLLKSYDDGWTQCSSMDGSVQGIAPRTCLSARPLQPRRPPGPNGPGGPNGPGPRGPPIMGPNG
RPMSPAGGRMGPPPQMRGAPPRFYNDGRPMTPTGSGMPHFPPPPGTPRSMSPGPGRGADVPHPLTP
GGSRPASPAGGRSRSNSASVAQMGPRPVVHRGGQSPLAPPTGPLPGPPQQPVQSPTAPAPSPQVQN
DASPESPIERKPVPGQE
>g2926
MELSRQEYPALLTSLQPNQATAVLNDRIRLINKVNTDIADWLQERRRVEELYVQGLRKLARRPQLD
GGAALGIFQLPWNRITAATESLAASHEVLASKIEEDVERPLREYGSRNRDLSSMPSIHSDLVNLAK
GLDAAQKKLEKAKEKGPKGADKVAAAVTSVTEATQQWESRAPFVFEQLQAVDESRLNHLRDVLTQL
QTHEVDQIERSRQTAESCLNVLLNVQTEDEIKTFAAKLNGGREPVLTRNSSVAAAAPVTPVPAPVP
SSEADLPPPPRIQDDAASQHSNRSGRAARVPPPVPEPRHTPRMGGLRRLGTVMNRRKSIVIGSGTS
LPSEKKSRPGFGFRRGDSSSNVHTQIPSTPPGRDTPSIASESIGSPPPTGSTANDYVPSEDAITPI
AETTETTATTNGLASNHTEPVASPVAANQIQIDSEGYSERPQLVDEITQAQREASGIEDTGLNLTI
RDQPIPEDETQAKQAMDEMANTLRMQGLQVGLRRNAGTIRGRRDVRNTAFISPSTAAEIIPAALLG
TPTGTSSSGQLESPVPVSATPSHEDRAMSDTTSIHSSHTLQNISGPVSHPELHEPGLNASIIETVS
SWFENGTVTKSFVIGELALAYNAIPGTTSENVVRLDNFALLEKVAANPHFVTEVSKTDDEKRGEYN
VATEKIARAIPTVAFKYQLHLSPPDLSSYSPVLFRPVWNLEEFQASVIVFYSVNPEFVDKAASGSL
HLKNVVITVNLDLSSEDETTKQPREVVRATGAVMYPNTGALFRRKHSAVLWKIPELEVRAGSEEKL
LARFTTSASWPRKGRVEAKFEHHTTDSASRLGVSLAETAASGTEHDPFADDGSTPPQSASLATSWK
PVHTVRKLAAGKYVAS
>g2927
MRSSTILSALSLTLLYSLPTLALTSGKAWVNFYKDCPSEFVEVEELTFIVSSLKQSTGNRAQASSS

PSPVHSIKASPSSSASHFPSSSPALASASASGINPLSTSSTSVLSKKSQFRSALAARSTERTTTRS
PSVNITQGQCQAVPIVTARHIDSSSVSVGAELSTITPFQECNITVHEVPGCVDEPLLVAPVKNREA
ESSCTPRNFGAFNDVWVRLDCSEVGTGSKKPVRRVF
>g2928
MRAVLPGRPESKLRALSTALWDGLRIVAYISGHALVILTGAQTLLQTIYVDDSESLETIAIDESSG
QIAVSSGPDLYIYRPNGFKGESLKWSLSVTFRAKDDDEKVNTLSWGSSEELLVANSCLSVWFLNDE
PRLMWKKKLASPTKFAEFSPDAELVATTGKYDRLVKLWRRLSFGADDVRFEVFYLPHPATVTGIHW
RKPRHREQSMENALYTLCVDNKIRIWTVSDHQDLSAMHLWTEIDMNASIQPRDNSQHFSSRRYGFI
IDSRDFAAATERAVQRNTEKNNLALEHLVDVATKNPEICAVIDDQGHMCAWALENVGGKTKSSTDV
FNILHIEGVQFSFPKGVSLDEDYAQFYAFGASTAMDSLSLLVHYFDGRIEWFDSQIDILFDPTPRK
HRLKSQGIWSGHTEPIKKIVRNASGRVLVSRTDDNNAMIWRQKMSGSQSILATQSSLSSEEHIHRT
CVIEEGSFLVNLHHKGLSLWNIRASPAVKISSCEFTSASKPLCVLTVPNPEPFDRTVYVVMISADM
KGIAWELELPSKQCPNATISLRQYCTFDMGLDTEVSYILAVDPAGQMARHSGFLDLFAADIALSYT
EKGTIHTWAAKVDKTNRTLDWLLTSTVETGIQTPSLASGSAIRKAALVDQERTHLTIWDTSGAQLE
FEERFPDHDMIRDLDWTSTPDQQSILAVGFSHKVVLLSQLRYDYLDAGPSWTAVREIRIRDLTPHP
IGDSCWSGSGNLVVGAGNQLFVYDPAVEVDSHLVSKLRIPARGGSNVNLFEIVSRLNGPLPVYHPQ
FLAQCILSGKINLVHLILMNLHKKLKFYTEGDEIDTLLGIPVEEFYRDDDELRNSAWKEMQASQDY
IEDEPRVLDETVAVLLNENLTRFSLPQLSSQEQFRLVDTIECVAMVEKHRRSMDANAARYLLFFRQ
HMLRRSQGVAHKNTVSWREIVWAFHSESQDILTDLVSRQFGGKLLWKAARESGIFMWLSDITAVRA
QLEIVARNEYTKTDERNPVDCTLYYLALRKKNVLQGLWRMATWNREQVATTRLLANNFQEARWRTA
ALKNAYALLGKRRFEYSAAFFLLADHLREAANVCINQIGDIQLAIAITRAYEGDDGPVLKEIMEET
VLAEAATEGNRWMASWAFWMLSRRSSAVRALISPVDTLIPPTPASPGSPGMIPLQARSYLSNDPAL
VVLYRQIREKSLQTIRGATQVAPADEWAFVIRNARLYDRMGCDLLALDLVAHWEFLRTPPRRVPTT
DFTPKAEPDYRKLLRRHSSLVIADMPVRLPPEMAPRGNESTDKPAKPQAKPTTFQEPDANSLLDSF
GF
>g2929
MSPIAAQRPTVLDRDRALSDQDVGLLYQIITRAEQNPDVDRLPYRALFAAYDEVLAEHGGSEADPD
QICMHFLFKMGTGGLANQSLFDRFENILQRMGIVLSFDDSASEEIGRYTLPPIQEIEQEPQFTDRI
GEDTTQRSQRKRRASFNSMYDVNDDATQRYGNRPSSRSSMSRLDPGKPDFMETDDEPRNRRVQQGP
ATMLDKNQLLAQFLEMGRRLMGGLDPTRGEQQIPNNDPPVQTAADGHLSSSPRPASRSDSDSLRQM
DGNQSSQNDRNLYPEDMVVFDDMNPKPSLSDMLRDASSFSTYRKRVTARNIISYWLHQATRSQQRN
REMEALAINQDRLALLRQALDLWRAELERKRQNSRTKRFFRHLEKRAAKARDLYLMTKAFSHWAQL
TSDELSKASTARGHILSIKYFNAWREITAVNGMKAQRFAVQKPLDGWRRRIQQIRELNEHAIVTHE
HNMKRRFFMLMRSNFYFEHRAPEWSDFRLIKGSFLAWIRALRTQREREADLDLSNRQELLHAALKT
WSYRLRSVNAAHIDAETSHRNKLLGEQVEVWNAQTRLQPAAIQTATRIDTRVLKTTLTQWRLRNNN
IQQAKEADRLRVLSNAWTSWNDHLRCYALKERIGERLKKEAIYRWVLAERYRLAQRIRDQRIQREA
FAQFIFTVRDTSKELLQREEQYTRHYNEDLARSALECWRNKLAVQRQREYAAMEFYVPRIEQEAIT
IWGSKYQHLQNLDKWSRDARFYFVTTKFVKLWHNATLDASKKRRQDAYVKIRRKVKMNIAASALRV
WYDKSRRMTNLHKQANELHQTKLIIYSAELIGRWSDKTAKAIQDTQDAEVYYHRQLAYHQLTRWMD
EYETYRGMEEQADGMNLIHVSGVASASLRKLSLRIFQIRSAAEMAESMHDRVSRKHHRNMLRHWSA
KLRARRIEQEAYESIGIDIATHEPNTPGTATIPESADETLNMSELGSLPNFRLASTTPTATPGYLA
SPSRRAARARMLAQMSTTPATPLFTPFASRLRAALDGDRYDSENKTRSRRGSMATMVRFALEEPES
PSEGRKSLTRRD
>g2930
MSIDFPAEEESVLKRWREIDAFRRQVELSRGRKPYNTHGVPIEHEIDKKLGMSGSEAVEKLGIAQY
NAECRAIVMRYAGEWRQTIERLGRWIDFDNPYRTLDATFMESVWWIFKQLADKDMVYRGYRVMPYS
TALNTPLSNFEASQNYKDVTDPAVVVSFPLVEDPNVCLLAWTTTPWTLPSNTGLCVNPEFDYVKIL
DEASGKHYILMETLLRTLYKDPKKAKFKIVDKFKGSVMKGWKYEPLFDYFADEFTETGFKVCNDGY
VTSDAGTGIVHQAPAFGEDDYRVAVEHGVITDKKLPPNPVDPTGNFTSEVRDFVGQHVKAADRAII
KHLKGTGRLIVDGQITHSYPFCWRSDTPLIYRAVPAWFVKVGPVIPTMLQGIEDSHWVPSFVKEKR
FANWISNARDWNIARNRYWGTPIPLWVSDDFKEVVAIGSVAELKELSGYEGELTDLHRDKIDHITI
PSKQGKGTLRRVPEVFDCWFESGSMPYASVHYPFERQEEFQNAFPAQFIAEGLDQTRGWFYTLSVL

GCHLFGKLPYQNVIVNGIVLAEDGKKMSKRLKNYPDPTLIMDRYGSDALRLYLINSPVVRAEPLRF
KEAGVKEIISKVLLPLWNSYKFFEGQVALLKKIENIDYVFDPKAEVTNTNVMDRWILASCQSLLKF
VNQEMSAYRLYTVVPRLLELIDNTTNWYIRFNRRRLKGENGVEDTQHALNTLFEVLYTLVRGLAPF
IPFITDTIYLRLLPHIPQILRGEDDRSVHFQPYPQVREELFDEVIERRVARMQRVIELGRVSRERR
TLALRTPLKTLVVIHQDQQYLDDVKSLESYIVEELNVRDLVLSSDEEKYNVQYSVSADWPTLGKKL
KKDAQKVKKSLPSLTSEDVKKFVAEKRIVVDGIELAEEDLIVKRGIKEDETSQNMETNSDSDVLTI
LDVTVYPELADEGIGREIISRVQRLRKKAGLQTTDDVKMEYKVLSDPDEIGLAKVFQTQSAAFEKT
LRRPIDHHVITQVAGEIPKEAEPGLIVEEEQEVQKATFLLRLLKL
>g2931
MFRILESQAPAKQTATDAISVLSNRLQSATLLEDRRAAILGLRSFAKNYPASVASGSLRDLIGSVR
KDAEDVDTLKVVLETLLMLFSPDENSPEASEEIVLWLADEFTQRQDNITALLDLLETKEFYSRLYS
LQLISAICAARPDRTQECIFTAPLGISRLVGVLSDVREPVRNEALVLLIALTPSSAELQKLVAFEN
AFDRVFELIEAEGSLSNGSEVVVDCLSLLANLLRLNVSNQTYFRETGCVKKLAALLTDVTNPPAPQ
EELSAWSIARRDKNLWGLLAIIQLFLVKGSILKTNQTAFWQAGIMEKVLFVAFSKNFDVPVIAKAL
ETCAGLIRGNSSLQEKFADVDVSWNTVSVVSGTSNGTSKPNKPERINVLEALLKLTLEPAPVFALE
ARLAACECIQAFFAKHNGIRLHFLGRAIDGHMSGNDQIPNIMTTLLETPESRTRVDPYGNWMASVL
MFHLLFEDPEAKALALKVTEGNAEKGEEVVTCIQTIAGNLTTGLQRGDDDRVSVAYLMLLCGWLFE
DPDAVNDFLGEASIIQSLIREIKQSGVGNILVPGLSCVLLGIIYEFSTKDSPIPRETIHNLLNSGL
GREQYIDKITKLREDPLVRDFEVLPRTGRSDRDGALPEIFFDAVFIEFLKDHFSHFLRAIDREPGM
EVPVMTNGIQKGISRELVDSLRAQVEQHNQTIQKLELDLLNLQQKLEQEQLDHRRTKESTSHEMAR
IKNINDSLQKNHEAEISRMEEYYKGLNDSVQKNHRMELSKLEEHHHQELSKLEDRHQQTITGLQKD
HTSEVSKLEDQHRQGRNDLLKQHDEQLRAIDSQLKGTTADYERKSAKLRQRHEDEVAGLKKSISDL
TENLDKVSNDHILELQKIRDEHSTRYADFKAQLKQAAERASEAEQRAQEFEKELSEAKEAAQRAES
MATDAENARKEAQGELEDLLIVFGDLETKRKQEKQRLKELGEEVSEDEDEDEEGNEDEEEDDD
>g2932
MGVPALFRWLSNKYPKIISPVIEEQPYEVNGEVIPVDTTKPNPNGEETDNLYLDMNGIVHPCTHPE
GKPPPANEQEMMLEIFRYTDRVVNMVRPRKLLMIAVDGVAPRAKMNQQRSRRFRSAQEARENDQKK
EEFQKLLAKQNAAKGIDPDKDIKEQVIQKTWDSNVITPGTPFMDILSASLRYWISYKLNTDPAWEK
MKIIISDATVPGEGEHKVMNFIRSQRQDPSHNPNTRHVLYGLDADLIMLGLATHEPHFRVLREDVF
FQESRPRTCRLCGQPGHKAEECRGEAKEKNGEFDEKQKAAPLKPFIWLNVSVLREYLAVELQVPQQ
RFPFDLERALDDWVFMCFFVGNDFLPHLPSLDIKENAIDLLISIWRENIPLMDDYVTKDGIVNFNH
AQLILNGLGKQEDGIFRRRRQAEERKAANEQRRKEEEKARNAERSQKRRRSSPDYTPGEPGLSAPP
AKGRKSGEGFIASTEVTLITPSRGHLDKQTRQLTHSMIVNRSAIYKANEANKSAAAALKTQLLKGK
VPASDETETTNSEVPSGEEQSLETLPPALGKRKAEDLTDADNGTPGRDTPVAAGPAKPSDEMPPDT
VRLWESGYTERYYEQKFHVDPSNVEFRHQVARSYAEGLQWVLLYYMQGCPSWTWYYPYHYAPFAAD
FVDISSMKPNFVKGTPFRPFEQLMGVLPASSNHAIPEIFHDLMSNPDSEIIDFYPEDFPVDLNGKK
FAWQGVVLLPFIDETRLLAAMEKKYPLLSADEHARNTVGTDVLLLSDQHPLYQELAANFYSKKQSA
SKFALDMRVSEGLAGKVERDDNHIPHSALEPPYDVDMPSLDENRSMMVNYEIPKSSHLHKSMLLRG
VRFAPPALDQGDISAVRNKSRSNGRGGFRPNNGGGRGGNQYNQYNVPRPGMNDRANPFAAHLDPSF
VPPPQGGAGGWAPPAGGAGYSRGPPPPPRGGSYGYQNPAQGYNQSGYYGQDNNYQRGQNQYGYSAP
PADYNQQGYYNAPRGGGGNNRGDSRGYNNNRGGYQSQGRGGYNSRRNDSYDRY
>g2933
MAAPTKISILGRESIVADYGIWGTYIVQDLLTNLASTTYVLVTDTNLGSLYREQFAKVFNEAAAAL
SPAPRLLTKEIPPGENSKSRQGKADIEDWMLQQTCGRDTVIIALGGGVIGDLLGFVAATYMRGIRF
VQVPTTLLAMVDSSIGGKTAIDTPLGKNLIGAIWQPHRIYIDIDFIDTLPEREFINGMAEVIKTAA
ISDEKEFAALEQHADAILKAARSKPGKGRFDSVRQVVKDRIVASARHKAFVVTADEREGGLRNLLN
LGHSIGHAIEAILSPQVLHGECVAIGMVKELELARYLGILKPVAVSRMVKCLSKYGLPTSLKDSRV
RKHTAGKHCSLEQLMANMALDKKNDGPKKKVVLLSAIGQTYEQKASVVSNEDIRAVLAPSVEVIPG
VPKDLNVVCAPPGSKSISNRALVLAALGSGTVRVKNLLHSDDTEVMLNALERLGAATFAWEDEGEV
LVVNGNGGKMQASPTELYLGNAGTASRFLTSVATLSGKGSVDYNILTGNNRMKQRPIGDLVDALTV
NGAQVEYLEKAGSLPLKIAASGGFKGGRINLAAKVSSQYVSSLLMCAPYAKEPVTLKLVGGRPISL
SYIEMTTAMMRSFGIDVQKSTTEEWTYHIPQGSYSNPPEYVIESDASSATYPLAIAAVTGTTCTVP

NIGSASLQGDARFAVEVLRPMGCKVEQTATSTTVTGPADGVLRPLPNVDMEPMTDAFLGASVLAAI
AQGEGGNHTTRIYGIANQRVKECNRIEAMRVELAKFGVVCREHPDGLEIDGIDRSTLRHPAGGVFC
YDDHRVAFSFSILSLVAPTPTLILEKECVGKTWPTYWDALKQKFGVSLRGKELAESEIAHGPADRS
NASIVIIGMRGAGKTTTGRWAAKALNRKFVDLDVELEQTEGKTIPDLIKERGWQGFREVELTLFKR
ALTERPTGHVFACGGGIVEIAEARKILIDYHKNKGNVLLVMRDIKKVMEFLNIDKTRPAYIEDMMS
VWLRRKPWYQECSNVQYYSRHSSSPELALAMDDFDRFIQFVSGKTDHLSAIRRKRLSFFVSLTLPD
LRDSGDLLRTVASGSDAVELRVDLLKDPSSDSVIPSAEYVAEQISFYRSRVSLPIVFTIRTVSQGG
KFPDDAHDAALELIMLAIRSGCEFIDLEITFPEDLLRKVTEAKAHAKIIASHHDPQGKLNWANGSW
IQYYNKALQYGDIIKLVGVAETLKDNTALREFKDWAEQAHPDVPVIAINMGDKGQLSRMLNGFLTP
VSHPALPFKAAPGQLSATEIRKGLSIMGEIPAKKFAIFGKPVSASPSPPMHNTLFEQNGLPHVYTR
LETDQAQDVKEFIRSPDFGGASVTIPLKLDIIPLIDEVLNEAEIIGAVNTIIPVEGKDGRTRLIGR
NTDWSGIARCLREAGAHSNEGKSSALVIGGGGTARAAIYALHHMGFSTVYVLGRSPEKIQNMASTF
PTGFDIRVLENASDIENIPRVAVGTIPGDRPIEANMREILCTIFERGGRTGQSAAVLLEMAYKPSV
TPLMQLASDSGWTTIPGLEALVGQGVYQFEYWTGIVPVYEVARNAVLGTSK
>g2934
MLLYPTVRNKDLITHAQWPNTTTDLDLLQDGQLPPDILAHNHLYSQDISQQTYHSQLQSPFSSPRL
QLHPSSGSSSPTFSSSSPAFSSTRSPVTNFSGDFPIFDGSWSEAPSMSVASSYNPAFHIAPPAPSP
YPNAQYSTPQYMTQHSPMLAQSGIATNTHGSWDTDANVQLLSPAHAQKGLARGAHKRAASGSSGKT
GSTSPQAAATFAGQYQNSNPKKSLPTPVQTPVQNSFLAPAFQNYDPLTHNGDTAAAEQAMRQAVME
QHSHGSNHNHNNSHSTAEDDTSFHYSLAPSVSSLSHHNSPVTPQTTYDEFDDGSKAVVHGEDRSPD
FDRWMGDYLQFDANPDFNSQTAMSIGVPKLNRTISDIYQDELYNTTVVPTSSQQMRKANTNMGSQP
YRNLFADRLNAANQGHLSARSQSPAGTSMNRERSPFRQGSPLAADFRQQARLQASGLGSALQLPGA
QQDAQGEIKTISPKDALLEYNEGPDDLAHGLPSLFPANDIPISSNMTSRRQSATSAFQPTLAYNSM
ETFPTQYATQAAGLQQPMNNFMQPQLPSAQDLHANNTLLQQTPGFPASLPTMESTNSDRVMETATK
RPSDTSSDSGTYTCTYHGCTLRFETPAKLQKHKREAHRQTMAGQNNEAASSLALRNSQAGPHKCER
INPSTGKPCNSIFSRPYDLTRHEDTIHNARKQKVRCHLCTEEKTFSRNDALTRHMRVVHPEVDWPG
KQRRKGH
>g2935
MTGRGKGGKGLGKGGAKRHRKILRDNIQGITKPAIRRLARRGGVKRISAMIYEETRGVLKTFLEGV
IRDAVTYTEHAKRKTVTSLDVVYALKRQGRTLYGFGG
>g2936
MARTKQTARKSTGGKAPRKQLASKAARKAAPSTGGVKKPHRYKPGTVALREIRRYQKSTELLIRKL
PFQRLVREIAQDFKSDLRFQSSAIGALQESVEAYLVSLFEDTNLCAIHAKRVTIQSKDIQLARRLR
GERS
>g2937
MAETNNLEKRDQPLEAVTPKRISYWRKIVDQAGITAEVENHPYPGSGTEEDPYLISWIPNDPRNPM
LFSVFAKWFITMMMAVCTLAVALGSSMYSGGIAQIVVAFGISEEIAILGISLFVLGFAIGPLIWAP
MSEMFGRQLTFILTYAMLTAFNAACTGAQNSYTLIIILRFFAGAFGSSPLTNAGGVIADMFAASDRG
LAMSFFAVAPFLGPVLGPIIGGFLGESAGWRWVMGFMAAFSGALWILGSTLIPETYSPVLLRQRAK
KLSKMTGKVYLTEMDARRGKVTLSQALKTSLSRPWILLLKEPIVLLLSIYMAIIYGTLYMLFSAFP
IVYEGTRGWSQGISGLAFLGVMVGMLAAVAYSIWDNKRYLRKAEEEGGFAAPEARLPPCLVGSIAL
PIGLFWFAWTNYPSIHWMVSIAAGVPFGFGMVLVFLSVMNYLIDSYTIFAASVLAANSVLRSLCGT
AFPLFTTYMYNNLGIHWASTIPAFLALACVPFPFLFYKYGRQVREKCKYAAESAAFMRKLQESAQE
EETDGEKNDEVNFDRDEAPAPHDISSESEGEEETAPARRMYSRASTRTATSSHRGKVEYDGNPYDI
DRVNTRDSDFSFVSPKKSAPQ
>g2938
MRRVISDLLISAGRRPGNNLSSLRNRTTNGNKSQRRHNSNKPIEKNDLTPTKESSPPSETTTSPST
VPPPPTGSVTSSAGAATSPLPAARSSSSRRGIGELIKSGPIGRFGNWYTNAQHKRPYVTQLASSFI
IYLAGDLSAQLLFPSEVKTAENKDGKEGEESVATVGYDPLRTLRHLTVGLVSSIPSYKWFMFLHHN
FNYTSKFLSITTKVAVQQAVFTPVFNTYFFSMQSLLAGASIFETWERLKLAVPNSIKNSVKLWPAV
TAFSFMYIPPHFRSVFGGTIAVGWQTYLSWLNQKAAREVAAAEAATNALKSTTLQSADGNVAAIFN
LAKQNAMQPNDLDDDSSDYGSDFTLDEQELLSELLARVVAPEHQTSTVTTTTAEETTTATTSESQQ

QLLAELLLDDLQPLSDPLIVSDIEDYELPHSVRVPRVLGREAWSPATKRVWQRQQNQTGGTITRRL
WPSQLTAAVNVRSQIGEREGSPEGRTRDRERQRDREKEWTDAEGRTEAKTEPQTESTPANVVNNED
GTTAPSVEQKSPIERFRKPPNKAFSVSDLISPAWCELQYWYTLTKHGRKRRTPAMMQGTAVHKVLE
DEVHTTVPVDITTKEDGWALRIWNVIQGLRTLRTYGLTRELEVWGLVEGEIVTGIIDQLSYICPDP
KLEASAARHYADAEAARAALPEYQMSISDYFMASGAGKPLDEMWKQPEEPAPTDDDYGTDLDQSYL
DVPRIYITDIKTRASRSVPTVKSTGFRPTHLQLQLYYHILNRSVTSDDVTMEAIAERYDLDPQRTF
SDAFIAEVGGLNEEFFDAASSPISSSDPDFVPVGSSQDSVSILLSHNNLHSLWDLMKKHLRYTFLP
TSQNQNIAPSIPAAVQPDSLALYPTVLSPVLTAKYLSSTKMSEDGVPDELGSRSFLFDPSDLTSYT
SDQIKWWRGDRAPRGVEVFDAWKCRICDFREECDWRISKEYEFATRRRRLSKTAIEMLGPV
>g2939
MSSSEDEHSDDSHSSHSDSGRKRPPSPFYLPLNVAFYAFLTSNAVAAAYAPIQDCDEIFNYWEPAH
YLDHGYGLQTWEYSPDYSIRSWLYVSLHAAVGMITRIWSSNKKTQFYAIRAVLAFVCAACETRLYS
AISRTLNKRIGLLFLIIIAFSPGFFHASTAFLPSSFTMYTSMLGLTAFLDRRGRNKIAEGIMWFGT
GAIVGWPFSGALILPLLAEEILTSIFSGHWGNTLGQIVNGGMRCIVILGLEIGVDSLFFQRFTFVP
WNIVAYNVFGGEGRGPNIFGTEPWTFYIQNLLLNFNVWFILAASIAPLLILQALFGGRTSSSHTLL
RSLAFVSPFYLWQAIFTVQPHKEERFMYPAYPFLALNAALALHLILAYVGTNKPGTLMGLIPGKVK
FIAVAAFMLLSINAGLLRSVGMVTAYGAPLKVYDALDASGVAQEGSVLCLGKEWYRFPSSFFLPQS
MRAKFVKSEFAGLLPGEFPEGNSIQALLAGTSAIPTGMNDRNEEDLSKYVDISQCTFLVDSHFPSQ
PPTDLEPEYIHDQKDWEVLACHQFLDASETPTLGRVLSELLVIVIVLGTTYGVTFGQQIATDTARM
SSTYIYQIQQFLLEDRAQIHDAVFATTIIASLTWVLYIALQALKSRYQKTETTRPSTPDVEKNKRK
YGEWTPSSFKRPEPKPYEGWDVETTKPLLYRPFRYGPKYFVTMGLRSMKWDEWIELDNHYPRYHAD
KARRIKERASKCCHTAPEAMDGAIELLEELASYLPARYPTLFRATPVGIDNLLTGESFNIVQRPLA
EDPMITCARLVQDDLALMFEKPDGQYYLLAGAILLAGFWRLEDKLGMPLSEIHTSGDVPQFKEKLE
KGMMNFFRRLKPEDAVLRNNYFLQVDDQLAWSPSIGSEDEPGISWNTAQKNRAIEHHYFRSERQSL
RRLPRSGAVVFTIRTYFEPITEIVKEPYVPGRLASAVRSWGDDVSIYKGKEKYAEVLLEYLDKKHE
EQVANGLDLEKEDEVRAYPY
>g2940
MEEQSDLQMHLGEGHQQPKSEQPETSIPVSTTPLGASSDFYNTPLTGQTPVYPAENTTTTSNVYMN
EPDALTLAPVIPGLSVKNEGDKLAIPTEGPNTDNHQANGDAGNSVNDLAQQAASAQTVEGALADAM
ETDQTAADQLMTTEEEHPEWEVDSSPYESSSDSSTDSSDSDDSDEDEEDYPILSAEETARILMQAE
GGSDDEGDNKAGGGSHLRTANEQIEDAPPIPDIKVTEDMKIVPLGYLESMVENLILIKATVSGDYQ
VLESNSLLCLEDRTVIGVVADTLGRVEEPLYTVRYQDPAKIQDLGLEKGKHIFYVESHSEFVFTQP
LKGLKGSDASNFHDEEVAEDEIEFSDDEAEAEYKRRLKQKKKEKKDNRGDGGKPKNAPGPSPLGRG
ELNYDDDGGDRAAEDGYTPLARPKNYHEMMNHPEAHTEASVSFQNNRGNFRGRGRGRGGFDRGSGR
GRGNDRRGRGNYHNNLSQDNSHRSDYSRENPAQSQYQNYNAPSAAIPAQQYAQYPSFPQQHHQQSQ
QQPQFPQFGQAQTAGMPQFPFQMPYQQNHYQQNTYQPNPYQNVPAGAHINPAFFAALQQQLQQQQQ
QQQQPAGPPQNQPAANNQATFNQVQDILRQLSGGGNVGNPPR
>g2941
MADVSLKRPHPEDEELNAQKRTRSNNGSPMPGNTGAAPKSKVDIERMVAEARAKAEEVRARLNMAR
GGAASASASPSPAPPATSTGSAMSRIEQMRARVAAATGKVGSTPTQRPTPTPAPPASVEQPANRFD
DDLSKARGGLGVGLHPALLADTAQDQRLSKGRGAAQPKFATTMANRRSATPVSGKKLDLSGPSLDE
IRQNPYYDDSLHATSKPRLSKQLVFNQKGKYIAQAAALRRQAQMEEMKKRIASMARQAGVDEELSL
EKAFEVPAPPALEWWDEGLVNGDSYAAVDDPKNIKVDTDDSLVTIYIQHPVLLDPPQEKLVPQQKP
MYLTSKEQAKLRRQRRMADLKEQQAKIRLGLEPAPPPKVKKSNLMRVLGEEAVKDPTAVEARVNRE
IAERREKHEVANEERKLTKEQRKEKLALQQEKDAEKGIHVLVFKIDCLANGRHRFKISKNAEQMAL
TGVCVMHPKLNLVIVEGGSHSINQYKKLMLNRIDWTENSGPGPQREGANENRESSTSWLNPEDEKG
ELRDLGSNTCTLLWEGQQKSRAFRKWLGARVCETDGAAKETLARAKMENFWVLAKTYKPAAF
>g2942
MSSFARPVASELASVDFSVYTSEDIKKISVKRIFNTPSLDSLHNPIPHSLYDPALGAWGDHICTTC
RASSWSCPGHPGHIELPVPVWNVTFFDQVYRLLRAKCDYCHRFRISRIEIHTYICKLRLLQYGLVD
QTAEIDLIGEDPQDVAVDADEKGNIDPQTKMELRNKFVKRCIREAQKQGDNDAFFGGAKNPVAAEQ
RRELIKEFFREVAKNPQKSCSNCHYVSPGYRKDRYAKIFRKASTPKALVANQMAGMTAPNPVIVLQ

EDKKLLNKYKEQEKQNGTTVGDVTELHGAEEEVVRANAAIDQGSLAGEKIGGQQFVSAPEVHAAMC
LLFEKEREILNLVYSSRLGFKINPDMMFVRNILVPPNRFRPPAQQGGQIMEAQQNTPFTQILKTCD
LINMISHSRQTAETDGARMREYRDLLQAIVTLQEQVNSLIDSDRGPGGMAAARAANGIKQLLEKKE
GLFRKNMMGKRVNYAARSVISPDPSLETHEVGVPMVFAKKLTFPEPVTSFNFHELREAVINGPDKY
PGASAIENESGQVVNLKFKNLEERTALANQLLAPSHAKMKGNRNKKVYRHLTTGDYVVMNRQPTLH
KPSMMGHRARVLPNERVLRLPYPNTNSYNADYDGDEMNMHFPQNTLARSELLMIADADRQYISSTD
GKPLRGLIQDHITVSTFLTSRDTFFDEEEYHQLLYSCLRPENANTVTDRIQLMEPAFIRPRRLWTG
KQVISTVLKNIMPPNRRGLNLQGKSSTPGDRWGKGNEEDKVIFKDGELLCGVLDKKQIGASGGGFI
DAIHEIYGNTVAGSLLSILGRLLTRYLNMRAFSCGIEDLRLTPEGDQKRVEILKKASELGKSVSLK
YVTLDQNPVADQDAELKRRLEDVLRDDQKQSGLDSVYNSQTSQLTSEITAGCLPHGLIKQFPWNQM
QLMTTTGAKGSSVNANLISCNLGQQVLEGRRVPVMVSGKTLPSYRAFETHPQAGGYVCGRFLTGIK
PQEYFFHTMAGREGLIDTAVKTAKSGYLQRCMIKGMEAVKVEYDSSVRDTANGGAVIQFIYGEDGL
DVGKQVHLQNFSFLAQNYVSTMAQLNMGSDFRMLEKPEVTDWHKDAMKQVKRTGRIDAKDPALSRF
QPGGHFGSVSEAFSQALKKYEDSNPDKLLKDKKAGIAGSLSKKAFESVMHMKYLNSVIDPGDAVGI
VAGQSIGSQTTQMTLNTFHLAGHSAKNVTLGVPRLREIVMTASKKPMTPTMTVEVIEELSEEKGQS
FAKGISRLSIAEVIDTLQVRESITDIKGKKFKIYDINIKFFDAKEYEEEYAIKKRDLVRALQDDFL
PIFMKKIGTEVDRRQAEQNLSAFSAAQPEIGVSIGTTERFEGAVRETQAGGAGNDDDDDEDNEDDE
EDAKRASSKANRDNQVSYEAPDEDEDMIRKEQDESDNEDDDEDNENKQGQTTDDSSESSDSSDEEE
MTEEAKLDRTDAAERTKEILGKYPQIASFKFNALEGNSCFIRLHYDISTPKLLVLPLAEDSARRAV
IQAVQDIGACLYTPADPEKKEPAKIDIQGVNLMAMRDYQEYINPHTIRTNSIHDTLIYYGVEAARS
TIIREMSDVFTGHSITVDNRHLNLIGDVMTHSGGFKSYSRNGLIRESNSPFSKSSFETSIGFMRDA
VLERDFDDLKSPSSRIVVGRLGNVGTGAFDILAPVA
>g2943
MRISISSELEVLFPYPKIYPEQWQYMCDLKKALDAGGHCVLEMPSGTDKALHELKALMKYRASQLG
QTEDFRGLGLSSRKNLCLHPSVKREKSGTVVDARCRSLTASFVAQKKERGEEVETCTYHDLPYCNV
IIYSYHYLLDPKIAERVSRELSKDCIVVFDEAHNIDNVAIESLSIDLTEDSLRKASRGAKNLERKI
EEMKSSDAEKLQSEYTKLVEGLKATEEARDEDLIMSNPVLPQDLLSEAVPGNIRRAEHFIAFLQRF
IQYLMTRMKVTHTISETTPSFLQHLRELVFIEAKPLKFCSERLTSLVRTLELMNIEDYQPLQEVAM
FATLVSTYDRGFLLILEPFESEAATVPNPVLHLACLDAAIAFQPIKERFSSIVVTSGTLTPLDMFP
KMLNFTPVLQESYTMTLARRSFLPMIVTRGSDQSQISSSFQTRNDPSNLRNFGSLLLDFAKIVPDG
IVVFFPSYLYMESTLHVWSGMGILDMIWNYKLILVETPDAQESSLALETYRTACCNGRGAILMSVA
RGKVAEGVDFDHQYGRAVICIGVPFQYTESRILRARLEFLRENYGIRENDFLSFDGRVIRGKDDYG
IMVLADKRFARKRSQLPKWINQTILESEVNLSTDMAVATAKNFLRTMAQPFKAKDHEGISSWTPAQ
LEEQITKRKIEEERIERGEELVAAPQRNGGNGVVGGQADKDEFDDDIDDELMMLDA
>g2944
MSKKFKSQASSSXAATGGLGGFSNTFGGFSNASPQTTAPSSLSYVAEPPDLSRISDPNLVVAFKNL
TKKDDITKTKALEDIKETVLSLGNRTDDLEEGFLEAWTKVYPRNSIENARRVRQLAHTIQGLISSL
AGKRVARHLPKVVGAWLAGLYDNDRLVSRSVVESISQVFTTDEKRNGLWKIFQTSILEFVDDVILH
QTVLTLSDERIVKPDDAEAKYARVSATAILLFNRILSTASPEHIAKDRSTIHKILNSKNIWSFTHH
ADPFVRRSVYNLLRSSLAQDIEDLDWRIISNSIISQALPTTQLGSATEFSETLLQLSQSRPQIWTT
YFSGKTDSSKRLHQYIKKGSQGASELYWTNLVELLQTVPIEIISRYGSKAGDEPSETGLTQVKALM
NDLLTGLTSRDEPRHNLKTGWAAYYDIGIWLSTLIPETERSELVTEYLTKIFDPYVNGDGEDQWIL
PPSIASATCTVAFLRLSSHGFEKELTKAWENVTQKLLQAVKLSLPEQSKDFRSSQDGVCTKATQYF
KLEAAILYNLPPEDLKTSRLFEETSLLLLKGSLNLLQVRNGKPYGAAAVVEEAIRHVPEFIKDSEE
VVNSLKDAIPQLLPTPSADRIMSVILMCSDWNGFESVFDTSLQTMTEATMQDSSGSALQKLLSTVD
FQKVQDPSHFTSIVSSSVEKAITGTASQWPFVFSVIENKTVTDAMIDQIVVLLVQGLSSDDDTIVT
TLSGLSGLEGQKPEALKRLRNGKEGSRLVSRLLYLTESPIEEISQKAEHLEKXXXATVSADVTSAS
TREILTQELQEVGPQSLSVDVLSGMAQDLIQNTEAQDLESLLADIFPSCGGAVYLVNNDSSMNGRP
DLDATLRDSDGASSAFRLTTYVVKILSSADVLKSLHAKQREGLFYHLPLALQLMDDDASIEGSIGL
IGPGLREDRDEILETVSEGRSLIRQWIDLDTHLDNGRSISEDLLALWDQKVAELDNTSPESYRIGQ
AYAKIKSEVDPTNSSDALTALAREVRKYNPIRAAAELAVWGPALAASSSGTRLCNELIADATGFKP
SRNPSEGPRSIVLFNILAESMREILDTIPTQRMVFFVKNLVQVLQSNSDSTGIRTEVFKALSMVLR

PLSEIYGSHWTVSIETLSSTWKELGGGDAGLPVLHSSLRLFAVLRDLVKNDANDDLVDAWRDAKQD
LFESLVLTLNKLDSTSAVYLPRDITADLLRRELVHVPIESLANKTYMFSLLAVDCKAIQQTAFELL
HRQIPQAQEQISFDVALSKTTVNLPDELLSLLLEVPQANLVSSLSDAKTLLRIRSYLMSWKLVFDH
FSSSASLPVQENYAENIKEHQILAPFLEFTFDFLQKSHGKLIDASRFDIRSWEPTDEPSERDTQWL
LIHLYYLSLKHLSLFTKNWWIDSKKRIKGPVETWTQKYITPSIIEDALTSVSTWIESQEDDDERPL
TVKVSHRTTELVASIPVDEDSPPVAMAISLPPAYPLQPAIVSGRSRVLVDEKKWRSWMLIIQGVIM
FSNGNLVDGLLAFRRNVQGALKGQSECAICYSVISTDMQTPNKRCATCKNAFHSTQMSANQIPNLN
TLRRGGGGLRGRLRGRGGPGFAAPDDQSHAGNNTAAAKDKIIQGTDNDASVSRLSAVELGYLDDAF
ARALTVAXGGPGSRRFPIINRGTYVRTTAIDELVDRFLGEDSEPGHATKKQIISLGAGSDTRAFRI
FSKKPSIRLIYHELDFSVNTSAKIKAIRASPLLQRAIQVDREALPDGSEQNLTGTSLHLPNYHIHP
IDLRTLVANSTGTTDSAKNNSQQSTQPHEEKARISLPGLDPTLPTLLISECCLIYLAPTAADAVID
YFAASLFPPTTPLGLIIYEPIRPDDAFGKTMVSNLAARGIQLQTLHKYSSLPAQRQRFVDHGLGAG
QGAVDIDFIWSHWIADTEKERVAALEMLDEIEEWRLLAQHYCVSWGWRDGGDKSFASWRGLRSE
>g2945
MSDSLAALRKNRRDELAKLADQHLQHDLRESDRNALKTAATRVSTWTLVGSAAGIGLGFFVAFRLR
SARKAMFEAFKAREKPVSVVFADGRTESIPDITPLLRPSTWGDFATYFFASAGGLFLGGELGLLGG
SASASRSITKDPEMRKRIENAFRKFRADVLRREADELDRSDSVWDKMF
>g2946
MLPSVNWILGLGLLSANVARSVELSEHAQALFDQSMSFQDQIYDPEASYLRYFYYPLAAGPHETRS
TVWYSIGLLQRNHGDDVKEAVKILENVIGDQEKNSSVQWYGDYTVYPEQPTVGSAAYAPDIYNSWD
PNWRGFIGTALIIIYEEFGNLLPTKVQNLILESLHNNTVGDTYRVGGVDGDNLYPAYSNAWLMRTV
ASSWTGLKLNDSNMTTSGDKWAQDFLDLFDLNNTLSEFNGPTYAGVSLYALTIAAKYLSSTESVIG
QNAQRVIQDIWDYESLLWNPNMRNFAGPWDRSYGYDMNKYVAIMSEWIWALIGKEHVWHYSSPIGT
MTHADDFELAPVIAVLSEFHKSLLPKTVISRLKSFVGEHTYTGHTFAPPADYEPRNITTWVSANLT
IGTDSFNQSVVGGYSEDSTSFSPSVVQWIRPGGSIGYFNLHPSETALKADVAPYALNLTYPLGNET
SSFTFVLASNPLGSKRDITGFEDVDGLKIDVVGGTVDPVPEISFCGLVGGTCSIVHNFEFWNLTFS
MPANSSDVPSIQFKFTLE
>g2947
MAEYTTKDPVVQAKGEPDMNADELRLAQMGHTQELKRHFSILSLIGLASTTTISWTGLGLGIVTEI
NAGGPGAIIYGFVLVTILQSFLGASLAEFVSSYPTEGGMYHWIAAIAPKKYSAFLSFITGWLTVCG
WIFTTASTNLIFAEVVQALYALYHPDLVIKTWQTFVIYQILNLLTAGVVLFGNKIIPALNKFSLFY
LQIGWLVVLITVVACAPTHQSTDFVFRTWINNTGWNSNVICFITGLVNPLYSLGGLDGVTHITEEM
PNPSRNAPLAIAITLTIAFMTGLTYLIALMFSVQDFAALSTTSTGLPLAELFYQATQSAGGAFGLT
FILFVALGPCVVSSQLSTSRVLWAFARDGAMPFSDVWAKVSKRFGIPFNSQLLVAAANAALGCLYL
GSSTAFNSMLGAAVTINNVAYLIPIATNMIMGRTGMHKGEFHMGRWGWLVNGVTVAWLLFAIIFFS
FPYSMPVTVQNMNYTCVVVGGLPILILIWWLSEYLQNPQHHQHGPRLTATERINAFAPTAERPFVL
GLPTGSSPLGIYKALIEAYTQGKISFRNVVTFNMDEYVGLPREHPESYHSFMFSNFFSHVDIDSKN
VNILNGNAPDLREECISYEAKIKALGGIELFLGGVGSDGHIAFNEPGSSLASRTRIKSLAHETIVA
NARFFNNDLGLVPRMALTVGVQTIMDAKEVIIIASGTAKAPAIQQAIEGGVSHLCTLSCLQLHPCS
MVVVDEDATMELKVKTVKVKQTIVILV
>g2948
MATPGMSGQIAGDARILPALSPRISQTRRDSAFNSDIARTTPDFAGKPTTALSNMSANNKRPRVEY
SNGRSVHEEEYGAEQRARKACINCRRQKMKCLIEDSSVECRRCRRSGLPCIFVPRANAATLPELKN
GSREGGFRTDVLRRLQIIEDALGMSETSNERLESVDAGDNEGFDEDEETSDDFNGLGALWDAAVIL
QSAPCSVPRTIWRRRTVRDLWLSFHDRMPGLHFMPRRQTFSAPQPILLASILYCSSMRGPPDMADI
APHYFTVLCNAIAQLCIPGSAIGQTPADSEEWAFQTVLGIVIAGLLTEATVRETGIWISIAYRLIL
EHCPAHIDETSREWRKLFSGVQIIDLEHASLHLSCPVIPIESPLPGLRTSPRDQLYRLSRMMRTGL
THFTGRGLPTIWSCFTTDQPPAAVALANNFTAVDAAVIRDWARQLDEWLVDFSQGLEGTDQDLKVV
FRQYVLHRLVVLSIYHPGRGCDLWSNSITPKEQYELLLSARATLKLHSHDDSIWSNWDLIMITWAA
LIVVQGIEGGAGDPEELSLTNVHTPASADFRQQQQNGYDDLTMAAPMDLSWQIFDRSSLQQMMYTD
WPAPSVGDEEAGLG
>g2949

MSPVTNDHQTGLLAQAQRIADDFNFGPADVQRVTGHFVRQMRDGLVNHRAWQLPSFLRSVPTGAEK
GTFLAVDLGGSNCRICLVDLLGDSTFTVAQSKHRVPPEVMVCQSYKPLFEFIAGKIADFLAEKSTA
DTKDYDDIRERRTAPYRLGFTFSFTCEQTSLASGTLIRWDKGWDIPEAVGRDPCVMLQEAIDKLKL
PVLVTVLANDSVGTLLTRSYTAGQKDSTLAAVIFGTGTNAAYVEKMTNVKRLGATNTIAGQIMVIN
TEWGCLDDKMEVLPRTVYDDQLDGESIDPGSQMFEKRISGMYLGELLRLVLVQLQQGXAFDMGFDD
ESPIFESQGIDSSFLSDLALAGSEEYSRSSALIERKLSVKGVTVADVKAIQILSAAIVRRAARLAG
ASLAAIIIQSGRLEPSLAELKYPDTQVTERETSETANSPISSVQRLRQKLNSLWRRIIKLFARLNG
SSSPSGGMLPQYSKHEESIPETSIIDIGADGSLFELYPTFESDMRGALLEVQEIGPAGEKLVRIGL
AKDGSGVGAALMAQAALEMEKRGEQ
>g2950
MVTFNIEVISDPICSWCFIAHRALQKTIRLYKTTIPNGSADIFNITYLPYYLLDPALTSRDKTDFQ
GVITPRKHQNLTYTRITQIGRAYGIMFNFWEGKIGGTREVHRLIQLAQSRKGAEVRDGVLDEVFSA
FHERAIDITDREVLRSIAIEAGVXAEEVDEWLAKENDFGGDYLESQLEKARKLIGTEKKGVPVFMV
QGNYRFDGAPDISELLGTLVGIRDGKMPDEGNEIQEHTNSNAC
>g2951
MQATNNRAEGASSIASAFASLSNGSTELPPRFVSLKRDIISDEDAVLAGWSRLLERVSLKKLQNFD
TTMIPEVEFRDIAANNGKIPKDIVKRIRECGTIVIRGLVDEAXAMKWKEQIRDYLNKNPQTKGFPA
NDIQVYELYWSKAQLEARAHENMLMAQVALNRVWSAKPEDPVDLEVPLTYCDRVRMRKPGDRSFNL
GPHLDGGSLERWEDTEYRKCYSKIFSGDWENHDPFDVTHRLKATVDMYNGPGGCSVFRSYQGWLSL
SDCGPGSGTLRVMPDLVASTAYTLLRPFVRQTPNGLGWELNLDTPQFHGAAMGAGQELAITTHPHI
NPHGFVSIPRVRPGDAVFWHCDVAHMVEAEHRGMSDSSVLYIPSVPLCEVNSKYVKRQRENFEQGI
APPDFPGGAGESQHKGRGXSEDIKSTPGRRAMGLERFEVSEYVTTGQRIAVDVANAILGFEVSD
>g2952
MSDQASFWYLYTADERLDLMIIEANNNFAERKVWIVPEADDSDILNTPTKKQTKKFHQTWPKDFHV
SPFNSMHGFYSISTLDSFPPGTSLDAADTVDITITLLTSQRRPKLVARVWSVSKPLVTSRVSAPYR
FAFLASWCLSGPLIQPRTLYQAFTLYRKNKLKIRDRPEPDGKVFPRHSTPAERCIREIFTDYLQQI
MKASTKEFRLRLCDLSDSNLKITFSTPTADQKSPCYSISVFTPKFYATALAWPSIDTFLHEALLHK
NEERRTACTDAAEPEAFIQLFKCAADSHYRTCHTPGVTSSTSNTSIEKRRIWILRPSKFAASLLLW
MLWSCVTLVRAIFLLKMSGEVSGSSSIKGTKYQVNNKFFLQKYTCFKRVEVSTNLGSFLDQYVHQN
CGFGLTLRYLYANLTTVVRERILGIVGGG
>g2953
MFEFLQKSKARIAVVGGGVSGIACLWGLRDTDHEVHIYDADTQLGGHAHSHTIINNGIVVTVDTGF
IAMQDDCYPGFSTFLADLDVAMIATDMSTSVTEQNGPMAWGSTSLWNFIGSWTRLFSPWFWRFMFD
IMRFNFCATDIFAEKSHKSRHDKAGTNDRLESIGEYLDRKGYSEQFKKYYLIADVAAIWCMSMADV
FEDYPAEALIHFMSTHGLLNTITESLKWTTVKNGSKSYVDAFLRTLPKNHHVHLETKIRRVRRETD
GSVSLVFMDGSVENFDHVVLAVHANQALDLLGDDATVLEKQILGSFQTSRNDVALHLDPTVLPAKK
SAQAAWNAVIPSMEDNRVRAKVDFGNDEQTERLLSKSYYSKRQICVHNDMNRLQSIPFPGRPGSPG
RVIVTLNPLQKPEAIQCQRTYYLPIISSAAVQASRHLDLLNKSDNISFAGAWMGFAFHEDGFIAGL
AVAKKIRTGVYENPTRFRFGEELKEAVPRLSLKIQLLRFAIATVQFGIATVEFLASRKKSD
>g2954
MKSLSKHLPHFTGSSDQKQYHQGVRSTLNPDDSNMNTMDNSSDTKQQKKPLWGMHRQGKSQQQPRK
TSTGSFSDDDIAALPVEKQLETDYDQTGHLVPDTEHFTSEQDMVEASNEEFHHPTM
>g2955
MSVTPEITDAQLEEIYQFAIDLGKKAGARLMDGIHARIGGTISDISSNVASNANVTSNLAFQEKDN
AVDIVTQVDEVERFIHEAISARYPAHKFIGEETYAKTSSSAREYLITSDPTWCVDPLDGTVNYTHM
FPMFCVSIAFLVGGKPVIGVIYAPMLNQLFSSCLGRGAWLNETTRLPLIRNAKDGNVTNDNKDNYG
VPPIPDNAPSKCIFSCEWGKDRRNVPGGNLRKKINSFVNMAAEKGGDNADGLRGMVHGVRSLGSAT
LDLAYTAMGSLDIWWEGGCWEWDVAAGVAILLEAGGLVTTANPPKDPDTANIEDVRLGSRLYLAIR
PAGPSATETGRQSQERAVREVWKRVNHLDYSRPGA
>g2956
MNSPAQHSLAGKPIGLNGLGLMRLSWHIAPLPDSQTFPILKTALTAGMTVWNGADFYGTPENNSLH
LINRYLTAHPEDADKFVLCIKSGLLDHATFKMDCSPEGLRVFAHRSLEILRGTLTKIDIFGLSRVD

PNVPVEESVKALAQLRDEGKIGGIQLTEVKAETIRRAASVTKIDMVEAEISLWSTEVFSNGVAEAC
AEHGIVLVAHTPLGGGILTGKYESWDDLPAIMKLRPRFVPENFENNVKLIKKVKEMASSKGCTPAQ
LALSWVKKKGTEPGMPVIVPIAGARAPETVLENVQDAELTDADMKQIQDILQSFPVQGDRWPAGPA
KLNEY
>g2957
MSQQTAEMSTASAPESRPAKLTGREFYKSIGSPKYIVAPMVERSEFAWRMLTRSFMEKDQPQRVLA
YSPMFHARLFKESDGHRAQCIQPMRHDDQTDTDVPFLDGNPAIDRPLFVQFCANDPNDFLEAARLA
APYCDAVDLNLGCPQGIARKGHYGAFLQEDWDLIYKLINKLHTELPVPVTAKFRIQETKEKTLEYA
KMILSAGASIITVHGRRREQKGHNTGVADWSYIRYLRDNLPPETVIFANGNILGQGDIERCLEATG
ADGVMSAEGNLSDPTIFGKVPEIGQEGREYWRGRDGRGGYRVDAVFRRYLNIIHDYVLRNPVPERK
PLFLPSDPVDNTPEQTKNTQNGTENSEHINGKRKAEDDGHEKATEPPSKKQKRNNNVRVSDPNLTA
MQGHLFQLLRSLVKEHTHIRDMLARTRAGDIARFEQILSMVEDVVKEGLLAYEKNPEAFDKKPQDA
ENLQGSQATIAEYGRPWWVCQPYVRPLPEEAIAKGAMRVSKKDQKKAAEAAEAAAHSVFSKTEATP
SAIST
>g2958
MPLMISPEPAGDDIAVIGLACRFPGEASGPQEFWNLLCKGRSAYTSASKRWNVDAFHHSERGRLGT
SVARGGHFLEQDLAAFDAAFFNISQNEASSMDPQQRLTLEVSYEAFENAGVPIDKLSNSQTGCYMG
GFTSDWREAMTRDAEAAPMYTGTGVGSEFISNRVSWFFNMKGPSFTLNTACSSSLVAFHLACQSLR
TRESNMAIAGGVSVHLNPDFFMYLSNQGFLGQEGRSKTFDAKGDGYGRGEGCGVVVLKRVEDAIRD
GDNIRAIVRGTGVNQDGKTKGITLPNAVAQAELIRSTYASAGLNLGDTQYFEAHGTGTQAGDPTEL
EAVSLTIGAALPADKKILVGSVKPNIGHVEASAGIAGVIKGVLALENGVIPPNILFNTPNPKIQFE
KWRVQVPLALTAWPSDGVRRMSVNSFGAGGTNAHAVLDGAEYYLRERGYQLTGTQDIRPGTKRTPH
APRLFAISSHDQDGLARQRESLAKFIKSQVANIPSAKKDIYLDRLAYTLSERRSTLPWKSFAVAAD
ADQLVEELQAPMPPSGRTKEGLRLGFIFTGQGAQWAQMGMELRQYDVFQSSLESAERFFKEKLKCP
WSVIEEMEREEGDSHINSPEFSQALCTVLQVAIVDLLSSWGITPVAVAGHSSGEIGAAYCLGALTR
EDAWSAAYWRGILSSQIPTLNPNLKGCMIAVGLSEDEAEKYIARVSKGVISVACVNSPSSVTVSGD
TTGVDELQELLREDGVFERKLKVGVAYHSSHMQTIAAQYLEAISDIETADSHPDRRMYSSVSGDFV
EADELGPMYWVRNLTNPVRFSDALESLVLPIDDESGERQKTAAVDILIEIGPHSALGGPVKQILKE
IGVKDVEYQSILSRGKSAVQTALRCIGTLSVRGVPVEIKEANQYHYSATKLTPLHNLPSYAWNHTR
TFWAEPRVNREYRFREHPRYSLVGAPLPRMSENERDWKGMLRISEEPWIRDHRIQNLVLYPAAGYI
AMAIEGARQLADKSREISAFKLRDIQIGAAATVTDDADLEAMLHMRPHANGTRDKAFSWWEFSIST
CAPGQELRQNCAGLLLMEYKSDEGSAMSIENEIAAIAAAEEYADIKASCTAQEKPAKFYEDLASLG
LNYGPTFSLVTEIRLGKGVSCCTVEVADHGSKVIPPASVKRPYIIHPTTLDAMFHAVFAALKGPGG
VLKGAMVPKSIDQITIAADIDIAEKSLFHGFATASKHGFKEIMSDVHMFDSSMSNHSVSITGFCCA
DVASSVELTDATADAVNLCTTSTWLPATELLSSEQLQKVVEDLAASADVYALSTAYNEEVEKVESR
SMVHQIIKLIAQTKPESTIVEIGQIGLPLLTGISASNIGSPLNYTVASGNGEWLKEVEENLKDSSL
SPQFTLLDAGKDLPRSSFDIAMSTLISEHLNELDAVLKRLSSIAKPGAKVFVIDDVPSESLDRHAV
FLKHGLSVDFITTSRQTNLTVASVPASNANGIVTDEIIILEPAIPSSISNTIATQITAQLDAASIR
SKRTTLQAPLEELKGKRCISLIEADTSFLEDLSESGFESLKKILLETSSLVWFVGVDGPAASLIAG
LARTARNEIAGLELRTLQAPPGSFRSYENLASAIVKVATSSIRDNELRIKDGILQISRLLEDNGLS
GAIEGLQRGEKVLTPLSQTVRPQKLVVQNPGLLDSLCFELDESLESEIADGEIEIEVRASGVNFRD
VMTAMGQIPDETVGLEASGVVRRIGKNVTRFQIGDRVCCLAHAAHKPLIRVPVVQCERIPEHLSFE
EGATLPMVHYTAYHALVNLARIRKGQTVLIHAAAGGVGQAAIQVAQHFGAEVFATVGSDAKKTLIK
DLYGIQDDHIFNSRDISFVKGLMRMTKGRGVDCVLNSLSGEMLRQTWYCLAPFGVFVEIGMKDILS
NTGLDMLPFKKHATFCFFNLENIATINPQLIEEIGKGVFGLLQKGFLKPVAPVTVYPVSQMESAFR
LMQAGRHQGKLVLSYSPEDVVPMLRRADDALRLNPNATYVLVGGFGGLGRSLLRMFAASGARQLAV
FSRSGATSAAAQICVAEIEQLGTKVQVYQCDVASEAAVKEAVKHMKTNLPPIRGVIQGAMVLRDVL
FEQMTYQQWIESTRPKVQGTWNLHQFLPNDMDFFITLSSFAGVFGNQGQANYAAAGAYEDALAYYR
RSQGLKAVTIDLGVMRDSGVLAEQGTTANLKDFEPFAISDATFHAMIKKVIVDEVSGVSTPAQIIT
GMPTGRSIQAAGISRPYFFDDARFAILSLAGEQGEEGAGXEVSIEAQISRVASREEAVPLVQEALI
NRVAKFLQTSPSEIDASKPLYHYGVDSLAAVEMRNWVFREIKSDISLLEVMSASPISKLSQDIAMR
SKAIVA

>g2959
MSSTPTLGDLQSGCQAWRDDVLNIYHYVPSLAAGVVFTILFLLSAAGHLARFWMVRVIPSALLAIG
AILELVGWAARIWSSKCPYNDSAFKTQVTLLVIGPVFFTAAIYLFLSQLIRHYGEQYSLLKPKAYL
WTFITADVVSLLIQAAGAGIASSQLSKPDGSAKHGSDVVAGGMIFQIVAMTVFVVCFAVFLKRMYA
SLSMRGIRRKDNFLIASIIFSLIVIYIRNIFRAIQMKQGFTSNLFIHERYFIALDGVMMFLAVAVF
NVVDFLPAPEKLEQLPSGGSESQPHELRENGGK
>g2960
MAVPEEMTLKTLAGQFVPNKALSDDVDSSLALQGVPWLLRKGISLASPKFTISHSSDDDGTPVVNI
QVSGVGGNGVTEKRYLNWKPVSGKNPLYGKTESKDFTSPIQCTFLTDCILAQSRLYGXSXERSALH
TDEDYPFLNGETTKDGSASCWAEDADGQHLQCVITNSDAGWTMEQTWGFENIDGKRYHTRRMVVRK
GDQVERGRLVYNYNS
>g2961
MTILSAERAPSRRILAVVTVGRYTCAAPILEICRILHARGHTIEFACLDGHQGLATPHAFVSKIHV
VGRNMTVEEDKALYRLFDESDASNAEGRKGTFRALMKFHSWWPETYRNLKALVSDPAYRPDFILAD
LLADACIDIMNEFEIPLAVHYPQMPIQMAPQKYIPGMPGAQLKHLSSEHASLWDRLQEEYHVLQLI
FAMKDYILFQRKMRREMGLGPRPPPRKPDYLVLVNSFFGLEVPKDLPPLMIPIGPVLADTFTPLDT
TPELLNFLQVHRKVIYVAFGSHVELPAWRVRRLIKGLVQALESGDIDGVIWAWKDRASVMKDLESD
PSSIDDAKVEYSAILDNRNEGFKIVGWVPQRAILDHSSVCLYLSHCGASSTMEAVYHGVPVVAMPV
YGDQLANGKRLEAAGVGINMNRKNFTAEELSANIKTLVRDEQGSFARNVLRLQRIATANSRRKYVA
ADRIEEVMYDAELLSSDEAVVSRPMHLQTPDSRMSWFKANNLDLYLVYIALFAVPVGAVRWISNNW
L
>g2962
MVYPPDFTAQPTEHVVVSADEENTQNQSHILSQQIDSAEVNIGFFSLYRYATLSDRLLLLLSVACC
VIAGAAVPGLTIILGGLTEKIRDFVVGGVTVQDFKDDVSRYSLYFVYVGIGEFVTVLIGTAGFVYV
GERVTGKTRERYMQAVLRQNIGFFDKLGPGEIANRITVDTHLMQTAVSEKVGTALTSVGTFVTALV
ISLGYSWRLALISFSSVVAIVLLMGNVSRFIVKFNQRAQEEFDYAVSLAEEAIGYIRIVSSLNARD
QLSDRFEDHLEQSEKWGRKVRTLLGVSIGGLICIVMLNIGLDCWEGSRLLVKSQITQGDILTITLS
IVIGAFSLGYVAPNIQHITAGIAAAAKIFGTIDRESPIDPLKDGGIILNSISGQIDFKDITHIYPS
RPDTVALKNVSLHIEAGKTIALVGRSGSGKSTFINILQRFYTPIAGNISVDGHDIAQLDLSWLRQQ
MSLVSQQPSLFSTTIFENIAHGLIGTSNENASRETKALLVIQAAKTANAHSFIQSLPDGYDTWVGE
RGSQLSGGQKQRISIARAVIRNPKILLLDEATSALDSNSEHLVQEALDRAAEGRTTIMVAHRLSTI
RGADRIIVLDHGQIVEEGTHEELVEKQGAYLRLIEAQRIRQDIGDEQYGSISPISLNTDDVSISRF
GFGSLTDVHLLPLSLNEKQAEMIMDETVHEQPSLLSLVRMVAMLNRPEAKLLILGLCCSILAGGGT
PTHVVFLAKNVEALARPPTEYSELRSDVNFWSVLYLALGLGLLLIQGTQGFALGFCSERLLRRARS
TAFNSILKQKMTFFDQKDNSTGSLVSFISMQTVNLVGLSGSTLGTILTGATTMIAAICVSIAFGWK
LGLVCVALAPVLIACGFLRFYLLSRYESQSKILYERSAGYACEAVTDVRTVAALTREREICTEYGQ
QVQGIIAKNLLSVATTSILYALSQSLFFGCTALSFWYGGNLIADGKYTLFELFVCFIEIMFATQSV
GTIFSFAPDMARAKEAAINLKNIYEQQPETSEGDPLSLNKVQGRITLENVSFRYPTRPTKYALRDV
NISIEPGQHVALVGSSGSGKSTIISLLERFYEAERGILTLDGKNIKSFSASQYRSAFGLVSQEPTM
LRGTIRENILLGLDQHVPEDSIMKACKDANIYEFIQSLPDGMATMVGTKGVLLSGGQKQRIAIARI
LIRDPKILLLDEATSALDSESAVMVQKALEKLRQGRTCISVAHQLSAIQDADQIYVLHDGAVVERG
THEELLRRPAELILLVENDPSLFSSSSKVTALGLCTGLLPAAALAGSRNVTELILISTETIAVCFR
LALELYRRTRRIEDTPGHWAYTVLGVPVREMDVILEEFHASLSLPTHRQVFIGVEDESWLTLFGPP
PELDRLFSYSAKIEAAPKLKLAAYGAVHSPHLPIPDLEAVVGNSPILNRSIQSQVQVLCTSTGIPY
STSTLRDLYLSVCGEIVSSRLKMGTVLSAAAQGLQRNIPVRLNVIGLTQATPIVKRVLDSHKLSVT
LHDRPAALSEQSEGLRDGSNAIAIVGMAXRFPGSESLEEFWQSLMDGLDAHQEIPGDRFDIEEFYD
PTGRNKNSLSTRYGCFLRNPGAFDHRLFNVSPREAEQMSPLQRLLLMSTYEALQMSGYTSNGTLST
QSQRIATYFGQAADDWKDGSRIAGIDLYYVPGLQRGFTPGRLNYHYKWEGASYSVDSACASSASAI
GLACSALLSRECDTALAGGVNSITSPEPYSGLSKGSFLSPTGGCKTFQDGADGYCRGEAVGVLVLK
RLEDAFHDNDNILAVIRGHGRNHSAHASSITHPHAETQVRLYNDVLQKSGVQAEEVGFVEMHGTGT
QAGDAVEMSSVLEVFGRNRTKTNPLIVGAVKANVGHTEAAAGAVSMIKSIMALQKGVIPPQPGVPF
KINHNYPPLKQLNVRIADDKINFHKPFGGNGKRKVMINNFDAAGGNSCFIIEEAPQAGPKQQDPRS

HHTVAVSARTLLSLKKNKEALLEYLTAHTEIGLADLAYSTTARRIHEDIRVAYSGASVESIVQQLR
GDLSKPAAAQRGPQKKSMVWVFSGQGSQYSGMGWDLFHTNTTFRNSIESMQRICDTQELPSFEELI
SNKNTDFTQKTTVQVQLAVVAIELALAELWLSWGVKPDVVIGHSLGEYAALCVSGVLSVNDVLYLV
GQRALLTQKRCSEGAYAMLAVVAPAQALEEYLRRPEYQSCCISCYNSPSATVVSGPISELQTLEQL
MRAAGTVCTLIRVPYGFHSPQMDSILDDFERLAQTVRFQAPKIPIVSTVTGSIVRESGFITPQYLA
RHARQPVEFYKALAACRVEGVADAHTIWLELGPDSVCGSMVKATLGAMNVHSSIKSRESNWKTISA
TVATLYTAGSSPNWCDFHHEYTRSLRLLNLPKYAFDTKNFWRVYKEDTVNQVETALKKPTITKISS
SLHTIKEQLITIDTIKIVFHTSLADPELYEAVRGHQVDELPLCPAGVFCDIALTAAKYVYSKVDKT
EGSQKHLQISNLELHHPVVASGDPKQILQISAERETKTGHQVKITFHLQDGSATQEIGGCQVNTYT
SQEWENDWSKTPFFVKSRMDSLVESVKAGRGDHLRRSVIYKLFASLVNYDPKYQAIEEMFWDEQTN
DAVANVSLKPYNGRGKFECLPYWTDPLVHLAGFVLNVNLTSASDDVWLSGGVARMQLHEELSADTQ
YVSYVRSHAPDEHGVSLSDVYVFSEKRFVGFCSLVFQKMPRIVLHRLLHKHDSRAPAKVSGSTHTV
AVSKIEKQFTDDHQSPVAMPPPTSVEVENNLADRLIAIIAEETGVDMPDMVPTADFASMGVDSLMG
ITIIDRVQKELDVQLEASFFQENLTVADAQKALGYDDADSESDQGANAGSEDNSPSGGWTPISPPE
SDIEEILVTPNKLSEAIQIATEAPLTPPTEVLSSPAVDEISPSKTPPPAEYKSNVVLIHGRKKSNK
IPLFLITDGAGSATAYIHLPRFASGMPLYAVESPFVRCPLEYRFSVEETAKMYIAAIKKIQPEGPY
MLGGWSAGGAHAFEVSRQLLEAGEKIKRLVIIDMKIPKPMPEGLEVTMDFLDKVGLTTGINRAGPA
LAGMSERLKQHLASTIESLMFYTAREMDPSRRPEKSYIIWAEYGLAEIIGDAAFKDVAEMMGLKED
VDGNPMEDDTGLASWFYSRRDNFGPNGWDKLLGDVVCRSLKADHFSMVTPPAAPAASTGGKKQKKK
WSKGKVKDKAQHAVVLDKTTSEKLYKDVQSYRLITVATLVDRLKINGSLARKALADLEEKGQIKKV
VGHSSLNIYTRAVTAEE
>g2963
MAPKLRNRRAQAEDTAPRVVEVDTDEEVEITRTAEQQRERNRLEKDNKKRTVKVDDDDDEEHFYIS
LLDVLRVIVTFVVLSVGLSYYVTSGESYIWGFERQRPWWMRYNDIKQFLQGPINLTPSQLALYDGT
DPSLPIYLALNGTIIDVSANPGIYGPGGGYHFFVGRDATRAFVTGCFQEDLTGDMRGVEEMYIPVE
DADESHRERTLTAAEKKRRHEKEVQEAREKVVAQVNHWVKFYSGHEKYFAVGKVVPEKDGQEESPK
EERRLCEGAQKNRPKRSELNKQL
>g2964
MWNEYSIIPTPNKWPKVSPRQDRYVFAESSMLHGQPAQIKTRAQLSLEIIIQDIQRAKQTNQAQVK
AGLVPNPSDNKFRDSKPRLLLMGLRRSGKSSISSVVFHKMPPNETLFLESTTRIQKDSIHSFMDFQ
VWDFPGQLELLDPSFDLESIFGSIGALVWVIDAQDDYLESVVRLNRTILIVLQYYPNINIEVFIHK
VDALADEFRTEVFQDIVQRISDELSDAGYENAPVHYYLTSIYDYSVFEAFSKVIQKLIPQLSTLEN
LINILANNCGFDKCYLFDVLSKIYIASDTRPVDMSCYEMCSDYIDVIVDISELYSWEHADRKAKGE
QMREAESHVILQDRSMIHLMEMNKYLCLVSVIRNPDANEKKGLIDMNCRVFQEALNEVFTRPWEQP
EDIGAIEDAHEEQ
>g2965
MATLAELNHSTASPLPNFNSTHDLEFDSFLNLDQTAFPASESMKSNPSLISQPSMATPETSIADLG
PSYSGPSHQYGDHQQQTGFPPDAVAHAMAYNDNSLGALRAGHQGFPVTDGNYMHLKREDSTVDFGT
APSRSHSEMDIEPDNKSFFLPHSNIVRNQQFIDPTALGGHEMPMIAHPAPQAGRVYPGMHQQQAAM
ARAAQQQKQQAEIIRQQQMRQRQAEQFVRQTPAQPLPLTRAVRPTDPVVEERISRLLQQMRQNSL
GQDNRLGTPSSLLPQMAKQKKDEEDMDEDERLLASEEGKKLTSKERRQLRNKVSARAFRSRRKEYI
TQLEGEVASKTSENNELRLQNHALAEENSRLTDLTRILLSSPHFSDVLNDLSANGLPAQLQTAAQV
PQPQHQPMVSQAPIPTSVAGQVNMTMVPDNRMDVYGXGWNSGIDMNYNPSVFAVFEVPEPLIEVEA
LSGKSTSFETSLEPSGSSKIEVPQLERPVTPSTVVDTCGVAATEELDDTDPCFALFRDDIPVSSAP
PAALFERLESEKPCQYTLVVDQESQDVSESSIRDFKRLCSSMDAAFERVCRMTDHLL
>g2966
MQKKTVLITGCSQGSIGASLVKAFQAKGYHVFATLRNKSKADPFFLRSDDISVVDLEVTSAESIKS
CADYVADRTGGTLDVLVNNAGTGLCRPLLDTDIDEAKKVYDLNVWGVIAMTQAFSSMLVKAKGVVL
NISSVAACFTFAWSGKGIYNSSKAATALISETLRLELAPLGVRVITAMVGGVNTTFFADRADLTLP
QGSYYQPILDKINHENKGLQYTPSMKQDVDLFARNIVSDVVGGKSGLVWRGKSSSMAWLASALVPG
SMLTSMMNGDKGLEELARAHRQ
>g2967

MLPHFPFFHLPSALTAWQLRIERPVLFQAIITVTTLSTQRKLLQAENFKNLIFTSALTEAQSNIDL
LLGILTYIAWSTDAFLGRANLLSRMMMLAISLVYDLRLFKPSTPDVQLIVRLTQGYSEDALRWTPQ
MEEALRILERNKPYPTDEAFIFQVRLQLLTQRAAHIREQHEADRARIATAAAAPVPAYLYLKSLQK
QLQDFRDALSPELREQGELVFQYVELYIDQASRTLTPDAPLGSPSTVQTIDTSTLLPGYERIDSLW
KSVESIKIWLEVFYRIPPSDCIGLPFHFWSQVIRCTAILKHLSTLNDPGWDCHAVRKTVNIFSVLQ
WIPEKLDMTSKEAGLQSDDDLFKLLSKLLTRSREWVAARWNISSERHENDDVQVVIDHHAPRANVG
FEASASSANVDGIPYIDGIPWVQTMDLESDKWFEDVLGWSPPPLLATDSYHVGFPARYRKGRLPRN
NPPNTTSYDLDISSKKRNSSSDREDTPLAEQQPLLPGVLLVPAIPTLKGPGTKSRYLIFKSDSGGS
INSIRRPPSCDTPLAHPMKYFYQVLSXPTADSPGSTIVLNYPNRKYVFGHLAEGTQRAFIENGFSF
SYLNDLFVTGKTNWANYGGTLGMILSLADSRARGVGGLMEESSKKSDKLQSILSCLTIYGGRNISH
MLAAARRFIFRKSLPISIKEYDSKNLARDQQGSGGGEDPFETPTFADKQIRVWAMALKPSFERRFE
SRSGFKSPRKRSLNEFEELDRNEYKTADTLAKDQTLRDNIVKDMFDSDWKLDALYEERLYDVKQPA
VMFVRNPETKDLERYQGRLPDDTGSVPNIKVLVRKPWPAATLDSLPATTPSSESICYFVRHHDVRG
AFDSKKAAELGLPSGPAYGKLAGGETVTAENGTVITPDMVMGPTRRGKTVAFIDLPSNFYIDDLIS
RPEWKSPTLTETLASVFWILGPGVAQEPRLLDFISSLKNCEHVVSSTDVCPNRLTMVTAAKSAIRL
ANINSDNFIVPYHDNVKVPQAGTPTEHQVGVPPAQKPRWTVSQPGLLLNMEPKFGLDESELIASLE
NGDVAPSLPKSVQRRLKVIRRTLTNHKVISEVMKLRDELPPLALEAEVITLGTGSSSPSRHRNVSA
TLLKVPGKGYYLLDCGENTLGQMKRTYPPEQFREVMRNLRMIWISHMHADHHLGAVAVIREWNEIN
YGSTSGTATLSESIGDILKQKRLAVVGDKLYIHYLEEYSGVEDFGFEKVLPLSVMPPTKDKSFTSV
IYRSVRPDGIVKDDPNNSTWIRLDHPESHLSRLLRASTGLTNLNAVFVNHCRHAMGLVLEWEDGFK
VSYSGDCRPSRDFAEMGKDSTLLIHEATFQDDLKGQALAKKHSTISEAITVGKWMNAKLVLLTHFS
QRYAKISNMRDTGRRPGPQPGNAREAFKAQRGTLDVPDDDDYVDYHTENLINQASEAEFEAATMPV
CMSFDYMKVKLRDMPIAQMYMPAFEKLITVLDRQADEESLVAQEQALRYLESKQAKNGKKKNIQKL
PTRNFSTVAKKSAWDASESESGWTDESDKEDEPTQKGSEAL
>g2968
MSRLLTIRQAEELHKSIIAYLSANNLSNAASALRGELGLSEEVFDTATMVKYETLLEKKWTSIIMD
LEARCGALQTELNNATPTSLSKRNQDPASWLPKVPARYSLESHRNTINCLAFHPKFSSIASGSDDC
MIKIWDWELGELETTLKGHTRAVLDVDYGNTQSGVLLASCSSDLSIKIWNPLEDYKNIRTLLGHEH
SVSAVRFIPGRNLLTSASRDKDLRIWDVTTGFCVKTIQGHSGWVRDVCPSFDGNYLFSAGDDVTAR
LWDITNISNPEAKLTMVGHDHVIECCAVAPQTSYQYLAPMAGLKKEALSKMSAVEFTATGSRDKTI
KVWDPRGSCLMTLVGHDNWVRAIVFHPGGKYLLSASDDKSIRCWDLSQDGKCVKTLSDAHGRFVTC
LRWAPSVVKEVATNAENGNGQTNTNGLPKDKDSQPNMQIRCVVASGGVDQKLNIFAN
>g2969
MDFEFGKAVQELYPLKQTQLAEKASATGCSRPKTGPLVTSPLTTTAVNHPGTFLLLLGDSSSQQLR
KGLPSSCSIDIAAMTSIKFREPYSQEELDKLYPPHLKLQLVQVFMRHGERTPVSARFQNAGLPSYW
PYCAVARRMTSMVATNKDLSTWDTLQWRRKIETFGHDDEAVPTRGPAGETEAICHAGELTDKGRQT
TFELGQRLRHLYVDQLGFMPQIKSNAEDMYLRASPIPRALESMQQAFMGMYPASARTADFATPAIV
TRAFADETVYPNDANCRRFRQLSRLFAERAAQRWNETDDMEYLTKLFTKWMPSSSPRVAVDSRPRL
SGIMDTINATLAHGAATKLPAKFYDEKALKIIDKIAVEEWYAGYKENEEYRRLGIGGLMGDVVDRM
VSAALEGGWRSEVASSITSGSKVPIKFGLTGCHDTTLAAVLSSLGAFDNERWPFFTSHISLELFST
NAETQNTTVNRDSSSSSQTKSTGLFSFFSKPSVPSLQTPAPKSSVARIPLSDLEESSRETLRKYYV
RLRYNDRPMRIPGCVSKTANHLPGDDSFCTLEAFKEIVDKFTPKQWQSECSQNIGEGIFGKDGKDK
SVAGY
>g2970
MEFSKYGEPASEWTSYVLAHPIINQPWTRPSEEESLTDMHIAGNNARAALDQLALRARDLEGKFSS
QDFMIETRDGSSIPLRIYTPNDTLSPSEGHPIYIYFHGGGFLHGSIDTERSVCASIATKLNIMVVH
ICTRHVHEAKHPFLHYDSLDATKWLLEHATIHGGDVSNIVIGGISSGANLAAHVVQQFPTLSAAIN
NGNNAARLKGQVLVVPWLIQPDAFPYDRFFEKSKTSLVQCAEALALSTKRLEWLSSLLQAEDITDP
TINPALADDKILISLPKTAILVAGGDPLRDDGLLYATRLEKAGVKTKVHVFPGMPHHFGVYQLPHP
HVAYELGSATIYHKRIFESIEWAFNGGDENASKGWTVEEKS
>g2971
MTANRPVGFAVAEPTPAPRPSRITIPGRTVTLEPLRASHAEELFPHIGGDKHSWLWDYMLGGPPLT

VAELQSYLVGFEKSESSILWSIRVHDGEKDRESKLMGYIGLLNIVPAHRSIEVGHVMYSPALQRTT
PATETLFLLARYAFRDLGYRRLEWKCNSLNESSKRAALRYGFTFEGVFRQHMIVRGRNRDTAWYSL
LDSEWAEGGVEAGFEQWLDESNFDEQGRQKRRLELIKAT
>g2972
MWGRYLESGEYSDFLVICANREFHVHRLVICPRSSYFRLMCKDNFKEGITQKLELVDEDPDVFQSV
LTFLYTGIYETCKITGSEEIESAPSTDQTCNSSEDSENDDENQHKAGGRPATTEDILAHVQLYASG
DKFDIRDLKEVSSKMFRNQLHRTSVEKLDVPDIIRAVYTSTPHNDAALRPVLIDYCVQNLNALLVQ
PGMAEVLSDFGECAFEILRSYKDEADLRSRKAVEDQRTAVLEIKKAESQQLRESLAKLQKRDTHLM
EMLASHHNCRNCGKVFGAGPQVYGPMSDRVMLRCKGCYTKHS
>g2973
MARPLVSKRGVARLCHTPELPHPFSRSSTVTHRPLRRQSTVAATTTIEENGPIPAPHKPTRRFEAI
KSAKPFSDFLTDTFHRQHDYLRISITEKCNLRCLYCMPEEGVPLSPPAHLLTTPEIVYLSSLFVSQ
GVTKIRLTGGEPTVRKDIVPMMQAIGDLRKDGLKELCLTTNGISLHRKLDAMAEAGLTGVNLSLDT
LDPFKYTIMXRRNGFEAVMKSIARIEEMNRAFGAGIKLKINCVVMRGLNEHEIVPFVEMGKEKDIE
VRFIEYMPFDGNKWSEQKMVPYQEMLDIIRKSYPEIEKVAGHKNDTSKTYRVPGFQGKVGFITSMT
HNFCGTCNRLRITSDGNLKVCLHGNAEVSLRDLIREENNGLPIDEEAMKTLDLLESGRLASRIQSE
ENLLSRERKLLDVIGLAVKRKKAKHAGIGKLENMKNRPMILIDNPVLNLSRTRMNNLIPTHLLHSS
HTHQLRAYTTKRQPADDKEYKVKPNHPVLPHLNTAGHMHMTNVGSKPSTQRYARASCVVHFTNEIT
YTTLTSSSESSTGGMTLAKGDVFAAARVAGIMAAKKTPDIVPLCHPSIGVDSVSVDISLIPPSTSS
ITENGGEGGFGAVEIVATVTCEGRTGVEMEAMTAVMGAALTVYDMCKANDKGIVIDSVKLLEKRGG
KSGIWKR
>g2974
MTSTTPNIIATCKQTRFHFLDDRPSREVDIQGLNIVVHSPQHQASSEIGTAKSKGKSKSKADGLEI
TSNADLKLKAGFHYGLVGRNGTGKSTILRALAEKLIPGVPYSTRIAILQQTDAEIEESSKNDSIDG
LADSLQQNGSISTSSASQKTALEQVIASDTMRGEIERKISVLSKSLEQEDPLVPVLTIRKLRHEQL
AEQLFLAQKNASIRSGARGLQARKELKAIEIRVATSTELLQEEHSSIDAEAIQVDTQAAIDILQDL
QSQYEIMKVVDVEKQARKILTGLGFKEEMLSRPFTTLSGGWRMRCMLAGILIQDPDIMILDEPTNF
LDLLGVIWLENYLKQMRQSSGTTLVLVSHDRSFINAVCEEIIIILRDQALTYFKGNLAAYEENFESQ
KLYWGRMKEAQEKQIAHMEATIRETTKAAKKTGDDNKLRMAKSRQKKIDDRMGIQVSATGGRFKLN
RDLVGFHLKARAEIEVPKDERGTTIQLPDAPDLRFPGPLISLENITFRYKRTRPVVLDDIGLTIHL
GDRVGIMGLNGCGKSTLLQLLTGEFTEPTKGKVTRHPRLKIGYYAQNSVGELQKEGRTEPALTALG
YILRDAGGQLSEGEARGLLSSLGLAGRVASDVPVFRLSGGQLVRLALAKILWNPPHLLVLDEITTH
LDFYTVTALASALSTFNGAILVVTHDRFLVRSVVEGKRDVDAPLDDDFEAESDGEIGIDETPRRRT
VYVLKGGKLVEQGEGVSQFEKSLEKRVVKMLAT
>g2975
MSLLNQQAAPYCTATLIKVLYAHSCRFLRDTDTVWSLDHHGETFRQVVDRLTSEAKVALAMETLNQ
PCIPTIQALLQQSAREIACGNSSAAWLYSGMAFRMAIDLGIHVSPDILQSHTSSFSPEDVEIRKRL
FWSLYTWDKNMSLYLGRMPNFLLGADTMSRDFLDDFSEQEPWTPFYWGVPEASELPAYPPVPGQVM
SCFKSLCTLCKILARIMLELYSFPTNNDKSAPQVSKAKELSFVSIKDAVEIWWSVLPPSLRILTNE
LPPLSPPPHITSLNLMYNTTLILLHRPRVGGDSQSVQPAVRQSWETCRNATTAIYRILRLYVNTFG
FQYITYMNSYCTYTAATTAVYQLETSQKTSWPPPSEDVTWTELRFFLDILQRSATAMPGLQRSIQI
IHTRVKKIFDRQVQTQLKSLFGMNANTPSRKDKGSTNQDTNTMTPFAVPDSQFVPNHNQTGIPSID
SSAINQHAGQTNELWSLEPWLPAFPGQDTSQGAKIMLDLHDMPSIEAPMFIGSNLDSYMRLDTSLT
YDMDPNYGSYTNNDYIVDQ
>g2976
MHIEKQTRHLESLDEVRRVDNKTETDDNVDLKSELVSETAANATGYQEYLEGLNLELTPKENAKIR
WKIDLIVLPIFLVIQALQFMDKTALNYANLFGYQQALGLKGQEFNYLSAMIYAGYFFGQYPCSLLI
ARFPAQRVLGVTAFIWGLTVIILTQCKTYSSALGVRFVMGMFEASVTPGLTLMTGFWYTRNEIPLR
QCIWYSALGWGGIAGDYISLGINTLPVEFTPARWQLLFYILGAATCFWSLVVFFVLPDSPASAFFL
TKRERVLAVKRVAQNQMGIKNKAFNKKQALSTFKDPKAILLFISVFAAAIPNGVVNSFSTVIIQDI
GFNTAKTTEMKSVGDAMVIISLFIGGAITLNVPNSRLATATLANVLCTICAATMAYLPQHNKWGRL
VSFWMVNTQSVGFTISLVTITSNMAGYSHKAAASLLVFTAYCWGNFAGPFVVKASQAPEYRGATIG

LLIGYLIKMITHLLLWAYLVYMNRSRDKRYGPANKTRSNEAGMQDKTEFENKDFRFVY
>g2977
MAHAGKAFDFDGEVAIVTGAGSRMAGEIGNGRATAILLARQGAKVALLDFNVEWAQETKRMIDEEG
GVSEVIQADVTDEESCKQAVAKTVELFGTVHILVNIVGVGGAMGDATTLDLSAWERDFRINVTSMV
LMSRHVIPEMRKNGRGAIVNMSSVSGLLGGNPSLLYPTTKGAIIQMTRAMAAQHGPENIRVNCVCP
GMVFTPMVRGRGMTDDMRQARINQNLLKQEGTGWDVGYAILFLASKEAKWITGLVMPVDGGTTAGK
ADRPALKADTLAEKITGVPNQSS
>g2978
MRLPYAPSTADNQTLEVQDIYARIAARRLPRPLIPLDLTLLHAPPVADGYNSFVGALRTKTVIDAG
LLELSISRVAILNQAVYEWNIHAPLALKAGIKPAELNEVQKLPSYAELSNVEDVWKAWQETCLDVR
QRAVVAYTDEMTQNVAVKDETFKLLRDVEFSDREILELTTTIAGYNCVSRILVSLDVGENNGKEMK
SVESMVN
>g2979
MHFSTLVSAGLMATTAMAYPHVLNRFSAAKRQASGAQNVVYWGQNGGSAVENNDLSTYCTADSGID
IIVLAFLYEWGNGVTIPSGTIGQSCYIGTDGTGQNCDDLATAISTCQSNGIKVILSLGGASGAYSL
SSQDEATTIGQNLWAAYGSPNATSSTSTTVPRPFGKTFVNGFDFDLEANSGNEYYQYMISTLRTNF
AKDPSNTYYITGAPQCPIPEPNMQEVITNSQFDYLWVQFYNNEGCSTDTGTNLNDWITNIAGTPSS
GAKIFLGVPASEDGATGTASGAKYYLAPEDLATLVKQYSNNTAFGGIMMWDAGFSDGNVNNGCTYA
QEAHSILTTGSAC
>g2980
MVRIEQFAVERWMDDYENDAKYNLAETCCASISLNDLIAFAGKSDQSCNILDFSQKQVYGAIRGSN
ALRSNIAALYADKSSEQLSSENVLVTNGAISANFLGLYVNVGPGDHVICQFPTYQQLYSVPKSFGA
EVSLWKSKENDNWKLDIEELKSLIRPNTKMIIINNPQNPTGAVLSRENLQDIVNVARDHGLIVHSD
EVYRPLFHSWSGDEAPPSILSMGYEKAVATGSMSKAFSLAGIRLGWIASRSSEIIEACASARDYTL
ISVSQLDDQVASFALSKPTVQNILQRNTDLAQQNLKLLDAFVMEYSWACRWVRPTAGTTAFIQFCD
RSGRAIDAVEFCKQLQAGTGVMFVPGSRCFGNDVEYKGFVRIGYVPEHKVFADGLQQLMKFMETNY
AQLPPA
>g2981
MGLVWSLIFWGSASPAQSVSYTDSLFLCVSAMTGAGLNTVNLSTVNSFQQAILFGLIVLGHPILIS
STVLFVRKRAFETRFRSIVQAVARNRSTSHQGIHLSRIAKKALATGDNGHASGNKDSAADGATVDH
PAPTASGITSLNNETRSHSNLNDIDAIRWVDDDQITVQSHTRNKTRHRVFPMAGVGARPDARDPKD
AAPSFVADEERRPSLIESITKGSQRFFLSKGLVSRNSQFHGLTAGEREKLGGVEYKAICLLSVIVP
LYFVLFNMLGFLGLGTWLAVNRPSTARENGLSPFWTGSFLAISAFGNNGMSLLDLNMTALQTCSYV
LLTMGFLILAGNTLYPCFLRFSIWTMRHILPRTKWCEEWKIVLDFILDHPRRVYTHLFPKRHTWYL
LATIILFNGIDWAGFELLSIGNTAVQSLGGYRILDGLFQALAVRSGGFYVVNIAELHQGLLVLYVL
MMYVSAFPVLVTIRNTNVYEERSLGIYANDHLAEDQRHHASLGSFVSHIKAIISPPSIEKQDQHRG
TSQSDENNTSRSYFVRQQLRSQLSHDIWWICLAVLFITIAEAPHFTDDPVSYSTFNIIFEVVSGYG
CVGISVGLPDQNYSFCGGWYTISKLILIAVMLRGRHRGLPVAIDKAVMLPDESLAWAEEEDAAMRL
EGARSWNAAHGSAYDGKNDNIV
>g2982
MAMKKSTKKFQQRKLGDVLERRKEFAKIKQRHQVNDKRKARNAERREKAEAEKVVDPEEQAKEQAF
AEMNVDDFFAGAFDIADPKNDKGTKAQKKNVTPKIGKRKRVDESAKDDEEEAASDSDEQSDASSVG
DFEDHKGQLESLKEKDPEFYKYLKENDAELLDFEDHGDLAEVDELSETEEGPAKKKKKSKGLESEA
MKDNIVTISEVKKWQKSLQEQNSMRALRQVVLAFRSAAYVNDADNQDTKYTISDPDVYHQVLITAL
EMVPKVLAHHLPVKETAGGRIKLAAESQKFKTLSPLIKSHTSSVHQLLTNLSDASTLKLTLASIEP
MLPYLLQFRKVLKVIVKMVVSIWSDNTNSEAVRVTGFLIMRRLMKIGDAGIREAVLKATYEGVVKG
SRNTNVHTLAGVNLTKNSAAEIWGIDQNVSYTTGFSFIRQLAIHLRGSITNKTKDSYKTIYNWQYV
HSLDFWSRVLSQHCNALVEAEAGKQSALRPLIYPVVQITLGAMRLIPTPQYFPLRFQLARALLRIS
RATGTYIPLAPALMEVLNSTEMRKSPKSSTLRPLEFNTSIRAAKSYLRTRVYQDGVGGQVVELLSE
FFVLWTKHIAFPELALPVVVMLKRWLKEAGSRTGGNKNSKINHLVLLLVQKVELNARWIEERRAKV
NFAPKDRANVENFLKETEWETTPLGAFVKTQRKLREEKAAILEQGRQDEEKQRQRERDNADEDVLS
EIESEDDSDLIEDEDDEE

>g2983
MHSQRPLSYAPTPYSYTPNPALSATINLDEEVKLASSSAERDLYESLAEIYSIIVTLDGLEKAYIR
DAISESEYTETCARLLKQYKSTLGDDTVAREFVDLETFKRTWQLECPRATERLRIGLPATVEQASH
GAHTPSGGLPTNAPAAGASGSLILTATENFITFLDALKLNMVSKDALHPLLSEVIQSVNKVTDRDF
ESRGKIIQWLITLNQMRATEELSDDQARELSFDIEQADIAVLDLPQLYTKPSAIEILKALDHLAIQ
PKSFGLGVRDATRRKIDPTGVTEYLTTIISSSLKWLESDDLREQIWDVAAARLSERAGRTAMPAFT
RLFNIPTSSGEEFTLNLHEPSLTSDNLGMKTWVSSYLLSRRLDSIFEAVPELVRSESTTSSTSTRL
RALELGSGTGLVGLSFAALRGASASIHLTDLSVIVPNLAKNVELNSELLNKVNAKVTTGMLDWSVP
PAPHPTKDDLFDVILAADPLYSPDHPKWLVETINTWLSKGLDARVVVELPLRDAYLPQVEEFRRRM
ENIGLAIVEQGDETGYDDWEDADGQSMEVHCWWSIWGWSEKI
>g2984
MAASDENEKYIDPRMGKTDLTNGQYARYQILLKSLGGLKSLFSPKRFQIIRWLWENQYLNSIEPNA
FASIRGDMFETFLVAAEKVMSLRKEKGSSNQIINIASEYIITDKFIWSDYNVWENGFVNYFLGQVL
WCFYEAYPKFFPVKTSDKEPLEHAVTCMLALPNSDIENLTTSIAILVSNVRNKGIDDLFGTWVDGK
LVYLSRRILNFRMHDRKESKKLLDAGRENGNTINLRLELARYVDGSSRRKLLANSLDMAKALYGEC
HAVTWHCTEQLGDFYLKYADEYTLQYYFSKLDGDKVRSVQDIYEDFLNRADVLGRSKSLGGSEREA
LSALLREKVVPTVVNIYKRNGKESEARRLLWKTVKDSSSQIYHANWVPFGRGSGKMRRLMTEIHSL
ENLEEFTGYSDLKYSSLELVVFPFDEISLKSVTPSDEEKARKWHFVFERLPNGDIHAREITDWSPG
LEGGYEPKTIQKTDEAENKEKMILELPYIPYAHFKTSKAYREGSTYDFYRKGSFNQQILDWMKKNE
GHYILSFGHDDYSDYIAYADLWPSVGGALVDSEKGQVERFLYDRETGSVKMTNETTAAPKELENPP
SYKTLDIGQCKTQIPGFFNSTPGDKAAIDDTGTVTQGFNLVSAEWYDWKRFILHPCSSSTEKQPEL
VSTDGSPIPVLGYIYGTWTDDKLHKQWRGLQIWIVQATFKLTPDSSQVERSCHFVLNANDICKSRE
HELSLSETRAICTLLNPSLLRVMNHSMFTPFMCTMGEGIPDDFAGVTKKIDPSMQRDLIAELSFRD
AWDTEFAYKWWNRTWGDARKKIMCRQLKEETARRREWDAESVPVFKQWVNSHK
>g2985
MVIGLLAITAIPTVAGVAFGVSEQRKANTRMQDEKRMAKFYMQVYCEGDSEKAQSLNGRRAVLRDN
KVYLDHADPALRKIPSHTVGAFFIAYPDDDNKPGDGLVSTISDNPPMLNWIYVDKDTLELRYGNRT
QSLPHIVGHWDWTEDESGVLLKNKELWAAVEEEEDVWALYFDKNEDGLEGVVDPSKTVIDISIDRD
LIEKPSENGGENKT
>g2986
MDAFKLLTRSTKLKPSTSNTTSRLPSAGKAANPQLFGSAEDAQSSKKRKRQNEAAEETGEKNDDFD
ATQRLLFGGGHSTSVTATTAVKESKKSRKSKKASIQIDDTQAENESDSEQQAQGDAMDESERRTIL
KTHKIKVVDLRDLEDILAEKVEDQDDDSKSKKKKKRKISKEIALLSKKRQKEARQLYPEPLTSFQD
LRKKYQISRKLADNISEQGFTVPTEVQMGSLPLLLGNTSSAKDEQEPDLLAVAPTGSGKTLAFLIP
LINKTVGYHRENPGQREISSIVVAPTKELVGQIVNEGRKLAVGTGVTITAMRKGMQVVARAEHDDA
DEDSENSASGSESDGDEDADKSAKSKDSKNTDALTKSDILVTTPLALVNALSKKNAEEKESLPSVR
YLVLDEADVLLDPLFRDQTLAIWTACTNTHLRISFWSATMGSSIEELTKSTLESRHELLDAEKRPL
LRVVIGLKDSAVPNIQHKLIYAATEQGKLLGLRQLLHPTAAIAPNEVRLRPPFLVFTQTITRAVAL
HSELLYDIPVEAGGSSRIAVLHSDLSETRRSEIMKGFRKGEIWIIITTDLLARGVDFRGINGVVNY
DIPTSSAAYVHRVGRTGRAGREGGVAVTYYTKEDIPYVKSIANLIHTSEKLQGKEQSVQKWLLDSL
PDLSKKDRQDLKRHGVKARAASAKLKHDTEKGGKARISTKSGYDRRMENKRKGAISASRNRTQQPS
TAKDAADSGDDEWNGLDD
>g2987
MSPMATRAALRQSRFLIRRTAIRHNSSTTEAANKAKQATEQATSKASEGLSKVSSSAGPAIAGAAQ
NVGNALRKVGGPVGKVVTFVERMIPPTVYYARVGLELGKIVFHGQKMAPPNLATFQSYFQPLINAF
RXPATIKNLKFSPQSLIGRARNLSTKEWALVGVTTAEVIGFFTVGEIIGRRQIVGYQGEPAHAHGH
>g2988
MSDLIVLPRPGTTKSNDNPKPIQAPSEVAFIETFGQLLPPASYFQTPIGRVAYYSLPPSSTPTNSK
VERVVLVHGVQTPALGLHPLATALKSRFPSAHIVLIDLWGHGLTDTPFVPHEPSLFHSLLDSLLVH
LNWPNAHFVGYSFGGATVAPFASAYPHRVESFALVAPAGLIRAASFGELGNSYLRGGDGVSEEKVQ
EWVLETLEGGPLTVPADWKEKVSRGEVVAEAIKDWEIRHHEGHMASVVGIFRDGGVLDRQNEFVKA
ARSGVRNVSVVGEYDDWSTEKDLREVGFEDVVVVLGVGHGVVRERVPEVADVIERFWRGL

>g2989
MHSFLTSSAALLLAAAMPLASAQTYTTCNPLTSSCPSDPGMGATVDTDFTAGASKDWTVAAGSISY
SGNGAGFTVAKEGDAPTIQTSDYFFFGTVEVTLQAAPGTGIVSSIVLESDDLDEVDWEALGGDTTQ
IETNYFGKGYTGSYNRATYVGVSTPQTTFHTYTVNWQRDSIQWLIDGSVVRTLTAAEAGDYFPQTP
MRLRIGIWAGGDPSNAPGTIEWAGGQTDYSAGPFTMYLKSVKITNANPGSSYTYGDNSGSADSIKI
DGASAAVSTTSTVAPTSTHTSTSSSSSSSSSSSSSSSTSTTLTSTSPASSTGADTTSAPSSTVSS
TKSTAPSSTFSSISSSNPSAVTNPISGITSSNSTLPGSNSTTPSGSTHPTSPSSTSTIAPKAGAAS
QTSVTSSVVALYCFLFAIVAYVL
>g2990
MAVGTTTVSKPNIGVYTNPKHDLWIADAKPTLEEVQKGEGLNPGEVTVEIRSTGICGSDVHFWHAG
CIGPMIVTDDHILGHESAGVIIAVADDVKTLKVGDRVAVEPNVICNKCEPCLTGRYNGCESVEFLS
TPPIDGLLRRYVNHPAVWCHKIGNMSFENGALLEPLSVALAGIDRAGVRLGDPVLVAGAGPIGLVS
LLCVRAAGASPIVITDIDEGRLAFAKELVPDVRTYKVQFGKNAEENAAGILAALNDGDADAVDAIR
PRVAVECTGVESSVASAIWSVKFGGKVFVIGVGKNEMQVPFMRLSTWEIDLQYQYRYCNTWPKAIR
LIKNGVIDLRKLVTHRYPIEEALKAFETAANPKTGAIKVQIMSSEEEVAAATEAAKNL
>g2991
MALPQDSGGNTGTTAAEDRDALSRIFTHEDFSVGEGVPKPFYSNVYGRMDLANEGLNTQASITADG
RVDIDIAEPTSRLRSMFKLPAFGPRSRATTTSAPIPPSLDRRRDQKPPPLNIVIQVVGSRGDVQPF
IALGRVLKFSHGHRVRLATHASFQGFVEENGLEFFCIGGDPMELMAFMVKNPGLMPGFDALKNGDI
HRRRVEIAAIISGCWRSCFEAGDGTGVPVSDRTADLDNFKEGHIPFVADAIIANPPSFAHVHCAEK
LGIPLHMMFTMPWSPTQAFPHPLATINRSKADIGITNYVSYAIVEMMTWQGLGDIINRFRERSLGL
APISIMWAPSLVSRLKIPQTYCWSPALIPKPNDWGRHIDISGFYFLSLASDYTPPDDLAAFLAAGP
PPIYIGFGSIVVDDPNHMTQLIFDAVRLTGQRALVSKGWGGLGAEQLGVPEGVFMLGNCPHDWLFH
HVSCVVHHGGAGTTAAGIAAGRSTVIVPFFGDQPFWGAMVAKAGAGPQPIPYKELTAEKLADAIKF
ALEPATSEKAASLAAIIEAEHGADDGAISFQNHLNVDKMRCSLDVDRVAVFRIKRTQIRLSALAFA
VLNAEGKLGVDDVKLYRSQEYMTEPDPTDPVIGGGAAMLDSIEEFLMGFADLSMDVKRSLNSRISR
SSSRSSTNRKSGSQSRTGSPTAAARAKILSDDDSSLKHSTETSSISTKESVSMPLAGDAVTSLPAA
DEVTEDSTQLKAPTLASTAEETRTRKHARTINDIRDFDFDGFLTAGKSAAKVVNVGLRAPASFSMA
VAKGFHNMPLLYGDDTVREQSKVTGIKSGLKAAGKEFGYGFFDGISGLATQPVQGARKEGAVGAIK
GFSKGIGGLILKPAAAVWSLTGYTMSGIYQEIQKRFGESVENYIMASRCAQGLNDLSTCTTEEKEN
ILTNWSLFEADVEAKRNARRNRYKVDGDGKRVRTRTVSERSKDAGKTQEEQNAESIKAAERANRRE
SLRQLFRGRASSSPPPKKSTVDSALPDEYEHAIRHSVLATSQGNDAEDEMIERALRASMTELKNAK
AAGEEEDRAYDLAVEASIREAERVIEEKKREHDERSAVGHDEKSNYYIHQPQGXPPPYSVDQTDQN
KPRRPPTLPPRSPIPIPTDHETELQNAINESQRSYDDNLRREQREKEEMDIIMEYVKRQSLAEAEQ
QRRQQEQQEAKQEKALEL
>g2992
MQLPSNLSLLLPLTILLTTASCQLLPGITASPSHAPSKQSKIGPWKCDLAQDGSGLPQQPYCCDEK
SVKSRYQDGRILGVTDCTAVPADQNVQCAAAANVWCCYSLNASDPNQGAEWVCTSVAEWKPS
>g2993
MSLNLYYHIVLLLLSGFLFFAVWQSRKSSSKLPPGPRGLPLIGNIHQAPRRDPWKTFNLWAEEYGP
IFSLKYGQRIVIVLGSHKTASDLFSARTKIYSSRPIIRIQEYLTKGMHMGVMQYTPEWMKLRKTHT
FLLSPHYCARYRQLQDLESMQLIYDMLETDSFSKQFRRYTASLGFALAYGTRIQRDDAEEIDQIER
IAKVTDRFFSHGISLVLEMFPCVHDLWPVMLPLQKICDAFHQSTVEYFTTCMRAALQKGSWNWINA
ALEFPDTQTMSEITRVYNIGVLQEAALETTQSALNAFVLASVLNPAAVCKVQEELDKEVGDSRLPG
FDDMTSLPYTQAFIKETLRWCPVAPLGAPHSNTEVDEYMGYEIPKGAVIVPNIWGMHRDPEIYHRP
NDFLPERWLEQPDLPLSVFGFGKRICVGQELAMNSLFIIVSRMLWAFQISPASVGGRNVAIDPENM
IQSIISGPSPFEASFTVRDSLRAQIIRVKWISSTAALDVPNFPLMD
>g2994
MKTKAHPATVHGLDQKAHPTLHSTKSANRISKATNSNSPMSQRTRTPYAFQFERQTPTAVIYGECS
HSLPVDEMAKIYQTYGWNVPMTIYTRSQDRSRERNEVPGCKFRKFFGTDEGEAIESLQQKQFVGLT
VFMREDDKEKLDPKIYVEQSIIQDGKEKLIVHELNFRGLVQTGIEYMFRGLGDEHPEFGKGDLRVR
FLSVNNESFETYTSGAGYYGYDQTVYAITNRKMLDRGQAISTKQSSWGNELHQYSVAFDPADLRIL

LDTKQKTEMKKKQGASGELELLKLCIHKVFPSEDGTKVGDLNVRVVYKP
>g2995
MYECDNCSDRFHSRDDLDEHMDVWAHWECETCSRTFGSQHSRNQHMNAIDHWAKRYGCETCAMQFR
SEIAAEQHMEVNGHYENYCKDYAPFLNREKILRIVRRQDPNGLITNKQIEWHKEQTSEYEVTDQAY
NGNAWECYLCHRQFQMRQGLTQHLNSPVHKQQVYHCPNFKCPKQFVTLAALFNHLESEQCEFMKFQ
SVQKHVHGIVTGQRLISF
>g2996
MRPTSFLCRRQHLRINILRPRARILPDAASIRHCSCHASPLTPKRPASSAPSSLNRPKSTDASNIH
SNTPAADHRILGTAQELFTSSPYSPGSPLFLPNGTHIINKLIDFLRAQYRQYGFREVLTPNIYKKS
LWEISGHWQNYKDDMYEVRGRGATGVTEGELGEDEQYGLKPMNCPGHCLLFKSQNHSFRDLPVRYA
DFSPLHRNEISGSLSGLTRVRRFHQDDGHIFCRPQQIGKEIDLALAFVNMVMKTFGLPKYNLVLST
RPEKDFIGSLELWDSAEDQLRQALDKSGLPWELNEGDGAFYGPKIDIQLQDSDGKYHQLSTIQLDL
NLPQRFELEYQVAEGEPDYDPNTPGKATPVLIHRAIFGSLERFLALLIEQYAGRWPFWLSPRQGII
LTVQNDEPVLRLAEEAAAKLSGYDTIVPRLHSEDADLQQPAPLTPDPTFLIDIDSSSRSLSKKIQR
AKSMKYNLIFVVGSKNLADQTIDVDISGQLQGSTEDNASKFQSVLTEVVGASSQGNSRALKMKIDD
VRDLLLEMERKFV
>g2997
MASQQAGNPLEDSWVVSELEASSYSQPVIVEGTESAPASFPTEDLSPVSSFSESDLIKDYEEEDPP
ISISTADLPNPTESTATLASSVSSGPELIMPSIMFETRHSEHGSWVIPRKRSRQSLYESRKAPRIP
KPRSQTTSRERQVYRAQKQDDPPAAPTTDTSMRHHFAQVKDYFSEDINRYHFFRLILNSLLVLMTM
HLLIFPELVHQVPIFCKIPGISKVYSQTCTKSNSTMSGISSLSTSYQSAIRTQNQLQNYLNQTIQD
MAPLDASLKDTDTSLRAVYTNIRREYGSARHEIELEFEGAWAASRSISRTLMNLKVDMRSTVDGFQ
APARRLEYLKGAARTSRKNADSIFGRLTGRKRMKSDSEEDSGILAMNQFDRLSQELDLAIARLAHK
TDTLLVQLARLDDHLQSIEQLTVREEDRLNSRPTKDQKQNIWSALNEFIQGAKIPESTNNGEQART
MLEQLQQVASYHSLMADVVGKLDRELKALQKIRSIRT
>g2998
MGASREDIALEPITNPEDFYHAFEICAKAFGEQTADGIWTAMNPGWDTPEGKLQGAARMEARWRAT
KDAKNTHFLKASVSSTKGGGGPPLIVGVAIWVHASLIPGQGDAPSPLEFGDLYPNDERMTRFLTQL
IGSMQTRRRQVLQEKAGPQSAQKSVMILDLCAMDPEFQRRGIASRLVEWGLGEARRLGDIEAITEA
SRMGRSVYKRLGFQEIEEIQYEVDAEFRGRSLPSNVFLRTRPPPSSS
>g2999
MQSYHIKDYAPPQGYELGQLPRPEIENAAEVLIKVHAASVNPIDVKMASGAVKMMTSFKFPYKLGY
DCAGTVAEIGARVTRFKVGDEVYVRLPESTRGSWSEYCKAPEDTISLKPKNATFQEAASIPLTGLT
AYQSLKLAGDLTGKTVFVPAGLSGTGAYCCQLAKNVFKAGKVITTVSTGKIPKVPELLGEGVVDEI
IDYTKSDPKTEIPAGSVDFMFDTVGIAMDCLHLMRPNTGYLVSISTTPSGDYLVEAGNMQIGFPFR
HILNLVDSVRVWRAKRWNVKYRYVFMHPDGKDLEELKKHVENGKLRAVVGNTVHYKDVDAVKEACD
VVFKAKGGIGKHVISFE
>g3000
MSSSSRELISAITDFYKLVTKLPFIPPNALVHPSDRGWPDINAEELRKRSRTEEVISLLRHLPYLR
RPDSGKSKKGWMIGPDTHAIAYCDGEIYDEDLDRIQPTPGHCIWLANAAETEDGAALLLDVENSTI
TEYSVRNNRNVCLDADEYDSLPLEDRWMAHSTLPATEFIRTWTNKYKKLEWMPFLKKEGEPGTSIW
FINSARNENVSDDDYSEDSDESYEPSSEESDDDDDDYDSDDYDSEDDPYEYDSEYESDDLDDIPLE
HDALESAVQELKMNDDESTKLSSTTERLEHEVRSGDGKVLAADKPTLERAVNEAYSIYTQNGWPDA
FDRDECQRQLSDLFKLIDK
>g3001
MRGFAQLAAALAAFMVGQTLASPVARSLPGPVQVTPKKGDVVITPQNFLNGTWKGNETAKSDALNT
AAISGTLPIEIVNNLAGSASGSINAYIQGQDTTGAIVFVLADGSFYYPPGTSSGVPVQISENLAIP
LGAQGTTTTLTIPNYLISGRVYLAVGDLAFYMVSGGLVQPSATNPSDASANVNWGFIELTNSPTAG
LFTNISYVDFIGLILGMSVTSSDGTVQTALGLTSSAVQDICTKLASQTASDGYPWASLCQTDTSGA
YLRVLSPNDYISTDGSAFSDYWTSYISQVYSTYASSPLTINTQNTPGDVTCTTGGSAILTCEGSDV
TFAEPTAGDIFGCNSGPFAVTGNAVDTAIVPRLCAAFNRGTLLLSGGNVQPSLSADSYYTTSPCNY
YSKFVHQNEVDGKGYAFSYDDVNPDGENASGELTSTDASLLSITVGGPST

>g3002
MSPSSILIGAILAATGPFLASGQALDKPALTSNLDYLEQGSLDNLAPTQSTWDQWGAGWIPLDCKN
IANNEGKSPDDFEIYNVHYTDCGDAWVFCRHKDTPLDLVSTIDIFGRLPVAMRSWVRHVITIPASS
NYAYNSNGNIAFFGTTSGNMDVAIHETGHSLDLLGAYGEQLSASQDWLDNYNQDPNVPDDYAQTNQ
IENVAQNTVVGVYDKVVPGGFGSVQPNWNDIFHQYATLQWKAGDQILPGGTCDRHLINSETVSTSS
AAAAAIVNGPKKPDTSFKEKNRYRKYNSSLEQVLQNYPEKSCRRLVMFAERSVSDGSPHSAELDSF
FPPSFWRIVEEDLNGAFGLERYEEESDFTYDTWSTFKMKHVYQGNGIAGIKYHWSQVSVFTRQSKN
KQFVLFVDCPKAVVDQIVRNCLSGPSRPVGLAGHVVSACRDPFIWHALLIGGLESEYESDYWRLRD
IVRKWERERHEEPDFPLLHDLGRHLIHAKEIFEVATNTIDSIVYHHQSLYSEFETMGLITTEEQKP
RQKLFRIIQEKLYISDKDMAAKKARATSLNERLHNEINLVSISFHLVMKSFEVFNCFANDEIIALK
AYHVVTQKSNQLSINLASIARRDNATMKALALIGVLYLPGTFISGIFGMTFFNYNPPNNSSSADWM
MSDKFWIYWAITIPVTIFTVLLWAMLDETVEMYKKSMEKVKVTLQDLWNELPRFNRREEKEETIIN
DQSLRSKV
>g3003
MTPLSLQEVRSMLVDHDLDPHGNTILVLVNPETDHAKHNGIDSAKIDVHSSSSMVVCCEQPSGSEE
HGGEGVPPSNTQTADSEKAEAASVSYRLKGQQLCLVSRYFKNLFTGPWFENAHFQRDGGIRLYLWE
EKWDPVALLILLRIMHHQLCDIPEIIDLSLLGKMVVIAEYLKCIDFLSGFLRLWGLDALSQYRRHI
AFGIDDDTVVRDGLLCTFISWKLRLRGEFRLATKFLILWAQAPIHDPRASIDEYIVGALNIERERM
LGMVIEPLKTEIRNLARYSLYALQQNPRTSLLGFAASARYVRMLLGLSDLSNLPRVPYTGYNIIFV
DRFDHLLENFPGLSLDDF
>g3004
MADSPELPTSYAALPTKSIKLSHVPADSPTVTKVVVVTLYRPTKHNAWTSEMAQELRTVYNLFDVD
DRVRAIVLTGSGKMFCAGADLEVGFAANGSKDEIEPGGARDHRDGAGWVTMAMHRCRKPTVVAING
SAVGVGITCTLPAAIRVAYKDAKIGFVFARRGLIAEGASAFFLPKLVGHSRAMHLVTTGSVYPASH
PLLSNLFSETLPSPEATVARALELATDIAENTSTVSTAIMRDLLWRTPHSAEETHLLDSRLIWDMF
GKGDNMEGVLSFFEKRKPDFKASFNNNGEIPAAWPWWTPVSFEKWMRKSRL
>g3005
MADNQKTEDLESNNLATTPTYETKKQPAQDLSNEDLSIQSPSDLVNVQLDSAKEKKLLAALDLHFV
PIIMFAYLTCFLDRGNIGNVKVAGMPEDIGASNAQYSTAVSIFYATYVTFETPLAMLMKKITPRNL
LCGLCVVWSLTTIFTGFVTSVGGLWVYIIEGIFSIIVAVWIWFGLPNDPTNAYFLTPEQKWMMQVR
NEQSRHYLGPEKFTWDQFWIEVRDPKLYFSAATQFCQDILLYGFSTFLPAILKSSGYDTLRSNALT
VPVYIWGVISFTLLAWWADRITRFAPFILGANAVSMIGYILLLAVKTNNAVRYFATYLCVVACYLG
PGLNLAWLNVNVAPHHRRAAAVGLQQTIGNTAGIVAGQVYRTSPYVLGNSFSLGAVCLAQLLVVGH
TLYIRREIALKESIAEGKVEDKRNVTTGDGAVDYKYFY
>g3006
MAPIETKTYDYIVIGGGSGGSGSARRASGWYGAKTLIVESGRSGGTCVNVGCVPKKMTWNFASINE
NLHAARHYGYDVQDNIKFDYAHFKSIRDSRIQRLNGAYENNWGKEGIDLVHGRARFVEPKVIEVTS
NDGSETKTRYTAKHILIAVGGHPVIPGIEGAEHGISSDGFFEIEELPKKWAVVGAGYIAVELAGVL
AAVGVETHMFIRGDTFLRKFDPMIQETMTARYEAAGIKIHKQHKGFQQIQLLKEGKGAEKVLKLIG
VEGDEIEVNELLWAVGRAPEVEDLQLHIPGVKQAHSGHIIVDEFQNTSAEGIYALGDVTGQAELTP
VAIAAGRKLGSRLFGPPEFKTAKLSYENIPTVVFSHPEVGTIGLTEPQARQRFGDDKIKVYKTRFT
NMFYDFFPDEEKKQNPTQMKIICAGPEEKVVGLHILGLGVGEMLQGFGVAIKMGATKEDFDSCVAI
HPTASEELVTMR
>g3007
MEENQKMPVVEDVPISPASEKEPGSPVSPSERSPAKSLPSYRPGSKGNMTESSTPLGQVNAARRGV
VPPPRPLASLGGQSGGLNQDIMARMKAFSLSRQGAPAGLAHTTSTGGIPSSSPGTVPTNGVGSPVH
GSPHAVPVGAFAGGLAGRLPPTARPQAPNWSSSPVVPSTSPRPGGLAAKRLKPGLKLSDATASSPI
TRTSPAASESGAQGSDGATESAFSKYSEFIDTKAGTLSFKNKAVIHGNGIEFSSGQSFSISLDEVD
RLDELGKGNYGTVYKVRHSRPHLRKPGLGLSGIVSRATAPNDGSSPVLRPNDNLTGVVMAMKEIRL
ELDEKKLTQIIMELDILHRCVSPFIIDFYGAFFQEGAVYMCVEYMDGGSVDRLYDGGVPEDVLRKV
TLSTIMGLKSLKDDHNIIHRDVKPTNILANTRGQVKICDFGVSGNLVASIAKTNIGCQSYMAPERI
AGGGMQQAGATGGGTYSVQSDIWSLGLSVIECAKGRYPYPPETYNNIFSQLHEIVHGEPPTLPDEF

SDNAKSFVRACLDKNPSNRPNYAQLLRHPWLTSLMEPPTSTPPADDDQSVSSDQSAEPSPQPSPNE
TEDTEVAAWVSGALERRLHNLMGSPEKPALHAVALDAVGGSPLLEEPSTISPAV
>g3008
MAIFTAPTSFRSALERRYLSNDGIPQIVASAATHSSHPSFGNLLLLVLEAVLEVVCVSLPGYIAAR
SGMFDADAQKLVANLNVALFTPCLVFTKLASQLTAEKFTDLAIIPAIFVVMTAVSYMCSYIISRLF
RFKKRQANFVTAMAVFGNSNSLPISLVVSLSQTLQGLHWDRVPNDNDDEVAARGILYLLIFQQLGQ
LVRWSWGYHVLLAPKERYIEEGDVHTVQHGQEHYLDNPQQTDPDEPLLRTRTGDSEHDIVHTTSNS
VTFESGAQTPVTDRNLSYTKLPSHGSDQSPRHRDIVDSDSDDESDEDRSLPVIGLPPNGPFLPRQS
SQGHILTFPDVEVRRSSTPVATGIRGFFFRWRRSLNRSYARMGKYVDTKRNQIFEHLPPWLQKFLT
VTATWAGRFLRGAWEFMNPPLWAMLVAVIVASIPDLQHLFFNEGTFVRNSITRAIDQNGQVAVPLI
LVVLGANLARNTLPKEHLEDGVENSKEERNLIIASLVARMLLPTLIMAPLLALLAKYAPVSILDDP
IFIIVCFLLTGAPSALQLAQICQINNVYMGAMSKLLFQSYVVWILPSTLMLVMTALEVVEWATGGN
>g3009
MVETKDDASLAVQQTAAATPSAPSSAVTSSAEKCVDVNALVQKEHNPPAPYDTDQPLQRSTVHDDT
DIDIIWHNLHADSDFTAFRKKSDGPMERILVDFARVVEAGVFEEVVSVTASKYMFNVTVVCSKGWD
EVSGFIGAIL
>g3010
MPSPAPEATGHETDKSVDNISSESRTNDNTVSVSHRGISPTDPGDNTGLVATKQFRAPIAKRISAR
SPILQPSPVYSLQSQVSSLRLDAHEDDDETEITPASRPSEQALFSQVLTWLQQEQSRQRGSIDGTV
AASSNGSLHLEGKRHSVGSEKGLALDQLETILQHYAAATKDQTSPLQHRRLKKHRSRPKGLRRGSG
SDSDYFDDAVVPGVDAVLDNSKTLAYTGGAVSTDQLTLTSDHRNRYMKDQHHWLVFKAEILRLIHT
LRLKGWRRVSMDDSADLEITRLSGALTNAVYVVKPPPPSPPTPNADGTEKLAPRRPPPKLLLRIYG
PQVEHLIDRDGELQILRRLGKKNIGPRVLGTFKNGRFEEFFNAHPLTPQELRNPQTSRHIAKRMRE
LHDGIELLENERLNGPAVFRNWDKWVDRCEQVISWLDKEILLDRQDRSEPWRKRGLICGVPWATFR
KAVENYRLWLFDHLGGIKEVKRQLIFAHNDTQYGNLLRLEPEEQSPLLLPANSHKQLVVIDFEYSA
ANVPALEFANHFTEWCYNYHDAERSWACNTRAYPTPEEQYRFISAYVYHHRPQQQGMGTSPLATPD
IAPSSIPTPRLAPFSLDAPAMSLSATTSDGPQDRQLYEETSEAEVQQYMRQTRLWRVANSAQWVAW
GIVQAKVAALEEEEAAQAAADNASTSAVESTPDPSASQEFLQKIVEEAEESEFDYLAYSQDRALFF
WADVLSLGLIRENELPDELLEVVKPRMINY
>g3011
MADGGGWSTIESDEGVFTSLIENLGVKGVQFEELISLDADTIRSLSPVYGVIFLFKWIGGPSHNST
SPADGTYDQTASSEQGLFFAAQTIQNACGTQAILSVILNQDTPASGTSYPIEIGSDLSDFKSFTQG
FPSDLLGEALSNSEPIRTAHNAFARASPFVDETQRTAQDEEGDVYHFIAYTPVNGKLYELDGLQPF
PISHGECDNESFPEKVIEVLQRRIARYPAGEVRFNLMAAVRDLRIRAREFGDAELLERENAKRRAW
AWENTLRRSNFVGFIGEVAKGVVQKQVKDGKYEEWIEGAKKETLRRAEARRKGY
>g3012
MFGEKEGARVITTFKDKSPIDQRSKRRVDNINILVSPHWEELSKENQDEFQRLIDTVQNDDYLVVN
LYTAGELEENSRAVYKDRLSAKESHVFSPSFSSLYSKCSNALLLNAIKEKYTLVTDHAVPSILHQD
EILIEVSAIGLNPIDWKAPAFNFGIPSLPWVFGRDLAGTVIKTSSSSSRLQNGDLVLVPSTDYRDI
RKAAFQEYAVTTHYNAAKIPSTTSIHGGASIGVAYVAAVLALGVSFGLDFGLAQSVPGPDLRTILK
AVDAEKIPEDIRSECLPSSEAGAERVQRGDWIAIWGASTTTGYIALQIAKLCGLKVICIADIARHG
RRLHALGADILVDRHDTDRAVDIIRGVTGGQLRFAIDIVGRETATILEKALSQPNEGDNRPRSHLL
GLTGSPKEKKAGIVYHTVPIKIFHESPQVGEGLVRWLEELLESEELIPPEVVIRDEGGLSGVNDAL
ELLRSGEVSGKRIVVDLRKGDVKGTAAR
>g3013
MALLQENPVSGAVLNGHTIDSNEIKTAFKTSVSPLSDPKVESNGTTESKPKFEIEEHPIDQVRDIR
VGIIGAGLSGVTAAILLRAKLPGINVTVYDKNGDVGGTWYENTYPGVRCDIPAHVYQSNFEPNTQW
TEEFAQGHEIRNYWQGVAKKHGVYDRLKTKQKILKVEWINEEAKWKLTIQDLNTDKLYEEKVEFVI
SAIGHFNAWKLPDYEGIESFKGPLFHSSNWDHSVDLKGKRIALIGNGASGLQVLPSIQPLGSHIDH
YARNRTWVVDSFGATGVRRLEPNLFSEEDKESFKDPQKYLEYRKKVEADYFRRFGVIFKNDPQNAE
LRDKWTELMLRRVDPTLADKIIPDFPPNCRRPTPGPGYLEALSKPNVSYIQDRIQKFTEKGIVTVD
GIEREVDVVICSTGAHIDMAPHFPIVANGVNLQDAWKPDGLYGYPLTYLGITAPGAPNIAWILGPA

SAGFSGTVPNSVENQVTYIAKVIRKLRSQGIASIAPSFDATNEYQEYAEQFYQRTVWTGNDDSSEG
NKNCSSWYNGGIPGGRVVGLFPGSAAAVNYILRDPRWEDYEYTYTSRSGNRFAWFGNGWTTRESSP
NSSVNWTPHLKDPAAIDLRTHFEGWWDV
>g3014
MLSENFVPLSQGAGYGVVVGIGLAFGLGMVLVNRFLERYLNEKSDHTEMFMVANRSVGTGLTASAV
VSSWMWATAIIWTTAQGYLYGIAAPFWYAAGVSIQIALMTVLAIHAKVKVPNGHTVLEIVRLRYGM
AAHIIYIFLCFVTNLLSVTSMILGAAGVITALTGMNIVASTFLLPLGVIVYTVAGGLKATFLTDYV
HTLIVMVILAFLTIKVINNPAITGLSSFYDVLVEADKSRDIAGNYQGSILSFKSKSSIIFGLVHSF
GDFALVIMDTSFWQKGFAADTAATMPGYMLGGIAYFAVPWAVGTTTGLAAIALEKSPIFPTYPRLM
TTEEINAGYVLPYTVMAIAGKGGAWALLLVVFMCVTSTTSAELIAVSSIFSFDIYRTYINPKAHDR
QVIRVSHWTVVIFGVFASAFATALHYGGIDLNWMGYFLATIICPGMFPLAFTILWKKQSAAAAIIA
PLLGLPDNFDWRVFLLLSEVRDESDDDNSTNNLVNEIKDLDNVTHPYSPQELKRMNRAATLAAIFS
AVIGLVTWVVWPLPLYRDYIFGKSFFMGWTVVSEIWLFFCILVAVVYPVIDGRKVLARAFTLFFQS
LTGKKDAKSANSQNETFTSTTEKKEASTESLSVQNRSGVLEKSE
>g3015
MASVTKSLGFGVTASTTYELSPYALARKFSTLDHLTQGRIGWNIVTSFLDSAAKAYGLDEQIPHDE
RYTRADEYMELTYKLWEGTWQDGAVKKDPETKVYSDPSQVRGIEHKGKYFKSTAANQLPPSKQRTP
LLFQAGASSAGKKFAASHSEVMFLPGLEPEKTRLIVDDMRKQLVDVGRAPNSIKFVAGMLIVVDET
DEKAQAKYEEYLSYADFEGTATLFGGWTGADLSKYEDDEDFAFTSVAGIQSLIQSWSKTIPNSNGL
KWTKRRILQELSLGGVHPRAIGSPSTVADILQKWVDVGDVDGFNFSYAISPGTFEDMIEFLWPELR
RRGVIWDEYQVPGGAARENYFLDGKGPRVREGHPARNFRWE
>g3016
MEWQPQEEPLRQLASYLRDSLNAYDRNLQKQAELMLTRATASPDFVNYLTFIFCTETPPPAVSMDR
DTYNIVRFSAALNLKSKIHIAYNGIPPESLVFVRRATLLGLRDSNQQIRQSAGSIITELIQKGGLL
AWPEVLQELLGLVANVSGDVTQSTQEAAMSAVAKVCEDNRKILDKDYGGQRPLEVIIPRLLEFTGS
DSSIIRSTALSTIQIFLSMKPQALISNFDTFMNNLFRLASDPSTDVRRTVCQAFGQLAEIAPEQLI
PHLAGLVDYIILQQNNNEDPELALDAAEFWLTAGEQPILQQPLGPFLPKIIPLLLRSMIYDEDEVE
RLAAENDDAEEEDRAEDLKPRFAKAKGSRLEGSTDPNGKPELVEEREDGELSEEESDYDDGDPEDA
WTLRKCSAAALDIFSNVYHSPVFDLILPYLKETLRHEQWPNREAAVLTLGAVADGCMDAVTPHLPE
LIPYLISLLNDPEPVVRKITCWCLGRYSEWAAHLEGPNQSAQFFEPMMEGILHRMLDNNKKVQEAA
ASAFASLEEKSDASLIPYCEPILRQFVECFKKYKDRNMYILYDCVQTLADCVMGELAKPHLVNLLM
PALLDRYNRISDQSQELFPLLECLGYIAAAYGDVFTQFAAPIFQRCSRIVYDNIQQSVQAAQDSSI
EEPDKDFLITSLDLMSSIIQAIPSDEISQLVANSQPKFFDLLCYCMNDSNNEVRQSSYALLGDCAI
HIFDQLQPYIPTIMPVLIKQLDLESLVDETTFSVVNNACWSLGEIALEEKADLGPWLEKVYPALLN
IISNEMVIDSVNENAAVALGRLGISKSELLAPHLQQFAQEFIKSMAKVDFSREKATAFLGFNRVVM
HNPQAMESCLAEYFSTISSFPQKSLVQEEYRDLHQSFQQVLKGYRDMIPGFDSFLAQLPPQVSQKL
KSTYQI
>g3017
MPSLVRKLVIFAAVDGLILQQSDRGQRGSINGNLSVQIDYKTRKITSLSQARSESNAKSTSPCLEV
YGLVGLLNIASSSFLVSITQRKEVAQILGKPIYAITNVAIIPLTSQDDANRAIFQAQNTAKQGGDE
SDQNTNDDTSSSEAGSEADSGDEGIVTTPAVYLDDQQEPPHNSVAQDVIGNRGRNNRFTLNWFSRK
RWGASGGSPHSKSDPSPSETERAKEASPEKVEPLRESEEVRTEAEAAPVVEKAAEDVESQSHLPSS
KAIELMPKLLRYTKLLFASSNFFFSYDYDLTRRYNTQDPRTAQLPLHVLADRIYFWNKHLIQPFID
GGDTARSFVLPLIQGFVGQREFTVASVKDRVEDLENIQAPDDVLPSPATVVKEETNEEKLAEDPKK
QNFLLTLISRRSIQRPGLRYLRRGVDDEGNTANTVETEQILSNPGWDPARKVYSLVQLRGSIPLYF
TQSPYSLKPMPVLHHTYETNQLAFARHFRDLSRKYGKIQIVSLVDKHGVELKIGEAYESYTRAFNE
NESSNGLNLGFEWFDFHHECRGMRFENVRCLVDKLVDITMDFGETIILGNSLLKKQTGIIRTNCMD
CLDRTGVTQCAFAQRSLEQSLKSEGYTIDLLGDASTHWFNTLWADNGDAISKQYSSTAALKGDYTR
TRKRNYRGALNDFGLTLSRYYNNIVNDYFSQACIDYLLGTVSIQVFSEFQDNLKTIDPGISIAKLR
QNAIETCSRLVVDDQTEELVGGWAMLSPRQPNTLRTFPFEETVLLLTNAALYNCRFDWNTDKVLSF
ERINLSSIRHINHGTYITSVLTDAQTDEQRNVGMVIVYQDSETNAFRVNTRSLQSSFNQDLQDTPE
KKEEWNLYSWLSGTKRTSMRLMAFKALASSTSVTVSNNTISETSISERVAIRSICEEIERAIKAAQ

IMKDGEESDKESIIEEKEIISLAEAKKRTGYLEQLVYDVKKLVWA
>g3018
MPNLSDLPYEIRLLIWEALLHQPRTVYLNMREFPPKEPDADAYVCGGYNQESVYQCLCSTPAPILL
HICHETRALALGHYELAFGWAHCPPLRQAGVVLHHGFSAKGQEYPTTTIESNAAEVAKHCAKVYFN
FDHDTVVFGRFLPSSSSSNGVSRRAYFPFGEQFDVAMLARIRNLAVNIDIASSVLPFLNLSPGSER
FSSLRNLYVYIESDDPGRRPRRVVELVDLTEDREVEDGRRDLTQRLLERFKNVFGRRGVTVALKTI
CKDGLGFWRYLGLRDSLLKNGGDPDKLLKCVWLREMDTT
>g3019
MEDPFTDNMEMASPYNGHVDDFEIDIDIMDDQPANVDNDFDLQDATPDGASGELTHDADMMDDVPE
MTVTDTTTYNNDGNMQYTDNTFPTTQEPVESEMVDEEYEENTEAISTSFETFSAPQGGARPEETVE
VVNEQPVIVEEREDEKPQNAEEVVEEAQTDVEGTFNQELTQEGSTEAKLAAPHIAVVPQIEAAAEH
HFENPADENEVVEEIKEHATVTEIGAEHETADAVDVVDNDGLSNLPPAGETEEVVENEHDSTEQTP
YLHQVKVIYQESEISMFPPRTGNTSETYFLADEGLAHESIGRLFKECRAILGEHASSEDSLVFHID
SLGIELCEDNTHVTEITLSQIVDTYLHLCRNSGIEQPEPLYLGLNTRPHLSSELAALLNAAKEGKG
ISDIQTWEEEYEHEETEDAGGSSRAELSKQHDDERDQGEHTHLPEEELQNESLDNEPAKSQIHETE
VQEEPQPEGGLVDVHELRQAGVDSTDFQQYHDEEEDGEPNTAINESHQLAEAQADMERPDDVEEKE
PLDTSNQEPTTEAKYQQQEGSITGPGVDEFVAVDASVVGHENQEFLADEHDFASDSQVEQDDRQEQ
EASTEDNVDAAELTEVSENATATEQYNETGGYEDEEEIVGQEESVEQEEGEYDEVAGDENYEDYAE
EEYHQDDLQHEDDEELAGDEHTEELQEGQNDGSYAADQFDEASSKTVSASNEPVDNKDDLLDDLQA
GTKPDPTGDLDYAQEIPDLPDLPDDDLLDLDDDIFADTEQATQRETENGNDHDVSAEQATVTNDDQ
QNAQLKRQGSTVGKRPRSDEEEEIDFDGGLLHASETHGFEGEGFSYLRSPIWWAGIATLAIGEVAN
FAAYAFAPAILVTPLGALSVLIGAVLGSYFLNERLGTLGKLGCAMCLLGSVVIVLHAPPDKPVESI
EEILGYALSPGFLLYCVAVAIFSSVMIYRVAPIHGKKNPLIYISICSTVGSVSVMSIKAFGIALKL
TFNGNNQFTHASTYVFAIVTGFCILTQMNYFNKALSEFSTNIVNPLYYVTFTTATLCASFILFKGF
NTTDAVNTISLLCGFLVIFSGVYLLNLSRHDPDGRQLLASKDDEDGVPTDAIASFSTRRSMQARRS
TDPHRRSSSSIAFLNGNSDREGLMRSYDAENGVIGLTELREEDDGAPGPTRKSHVDDNPPFHTKQD
DR
>g3020
MVVLAASICTRGGKAVLSRQFREIARTRIEALLASFPKLADSGTQHTTVEQDNVRFVYQPLDELYI
VLITNRQSNILQDIDSLHLFAQVVTSICKSLDEREITRNAFELLSAFDELVTLGYRENLSLTQIKT
FLEMESHEERIQEIIERNKELEASEERKRKAKQLELQRKEAARTNRGIPSRPPSYPIYTPPTRPAV
TETIDTYEAEKKKTFAKPMPTRGKGMQLGKKSKTTDIYEKVREDLGPEVEETAPLVSPQAATPVVP
ESRASLSSERAGVQITVSETISAKLTREGALKSFEVKGDLQLRISDPALTKLKLDLLTNTSHGAQF
RTHPNVDKALFTNSNIIQLKDTTKRFPANNAIGVLRWRVASNGSEHADVLPITFTVWVNKGSDSTT
VTVEYELTGTDNLRDVSVTMPYGATEPSVSSFDAVYEVAGDSLEWNIGSIDTSNASGSFEYEVVDL
DVDENEFFPMSVHFSKTVPFVEVDVSSVSLLEEGQEVNFAKDVKSVSDGFTIE
>g3021
MSVASKNTNEQIILSKRVKEVFGVEAYVRGTRNLDFLLGVLVFATWDRRLGFDRSILTSVVQLAIA
ILCDLELDRSHSTDIGFTLMRSVKGETVSPSQAAKVPTMEERRASLGCFLLSTLSPPLRKGQSLRW
TSHFNDCLRVLQEEKACENDLILAQMVKFRLISEKVIDFTFRDLTRQGHYTTIPASFYVKSLEAEL
RECIASIPSGLLDNKVLLLERYYTEFRIYEIGLSQSSDIFTEQPNLRLDCLFACLQATKSHGATLM
NVDPNQFPGLSALVTGQMTNCFACLCRLSMCEQSGWDRDFVNSTVDVSFFLQELEKKYAQVREAVG
IDTDNSGGSDYFTIKASKLRSMKASWDALTLTAIPLQELESFSIDFWDACNW
>g3022
MSSKGWLDIHAHFYLPQSPSEAEALAKAFRDVCFMIDRPVTWNVQSILDYNNRANIQMQMLSYIPQ
NNPEKLIAANNYAARLVKENPDRFGLLGALPTDDPDLCLKEIERLTALDIPADGFATSTIFNDVGL
GDPRLDPVWKDLNARKAVVHIHPNAGSQPSHGRPSPVIEVAFDTARTVVDMLYNGVFRRYPNIKFV
LGHCGGALPVLSGRLSLLGTESWVPNPNKLTRREIEQQLGRLYVDTAATAKTGLSPAVQMVGVDHV
IYGADCGVPCSTELTMEENKTDVLDWELKNDVPKGTILTNGWPLFPQAAIRAGIEL
>g3023
MFVEHASHAGRERQNAQGISPGAAIVGKQESYAATLMGKGRSQRAKVQTYEEVIRKMSSRFGVSDE
QLMSNAISSDPKQAILGDQSDDGSTFTKGRSILAGDGSQRRSQSPSARSFAGTDRVEEDFNKDSKS

RATGYIGNSSEMFWLQKLRKKVNGDVPETGQSSPPLTPPKKNDDGLIASINFYADNKDFDLTEEVQ
AAAMPPKETATQLLRAYLRSIHPSFPIIGKSTFQSQYNMFFSQPGAKPGNKWLAILNLIFATSAVY
ARLVGADWKMELEHHSVYFKRARILSIRDTFFDHPDLQQLQIEGLTSFYLLAIGQVNRSWKISGSA
VRGAISLGLHLRNIGTTTSDTSKEIRYRVWWSLYTLDHMLNVITGRPSCIIDGACTTPIPIPFDEN
DFQKPEAAQMLSNHTRKGSWIQDWKPSSSSLPSPSQNGECSADNTVVIKTETESDGIDWLKTLPIT
MSLYFFYLATLASISKRANLKLYSGEATQAPWTSLEFTIQSLTMEIDVWLATVPEAFDFRVFSTDK
STITQKMGLAFAYYSTKISISRPCLCQLEKDCQSDGVYEFCSKTAAECVDAATQMINMLPATMEAS
ALYKISPWWCTLHYIMQATTVLLLELSFRAEHVPERAVEVSQALKKAVKWLFELSHSSDSAHRAWK
LSDEYLHNLAPPLNLDISDLPDSEILMDSSNNFVSVLENSLLDHQVPTLMPYLETDPLGFLDTQQT
ESETQGSLQNNAFDDFLPYDPNTGQITGSFFPSSASDIDMDFAHMWENSAF
>g3024
MESDFPPLPSHSARHSANSSQKRDSVSASANVTSGYRGLEIAENGSKIIHKILTRDGDAILEYVDT
DSRKATTHYRWQVSSDRLRENSPFFSALLDPNKFSEGRRFVESKSRSKGSSATTRDTRVADGFAIE
STERLPLVGLNLTRLMGKHRIESLELFLKVLLSAYDVDLDTIMGTDIVHQSVPMIAGLIEIADLFS
SSDVVNDALKHVNYRPSIKGIQSFEVFASSLLKLSDERLRQIIYIAMFIQHDTIFRVASHALVLAG
SARWSDGGIGINKSGRHRWSYFDNGIEGTF
>g3025
MPGKTLPSFTEAEVRSHNSSKSCYVTIDSKVYDVTDFLEGHPAGEEVILEWAGKDIKNILKDGSSH
EHSDSAYDILDEYHIGFLSNVVSKSESSQANGNSQAIYEKTGMANENDLSIETDIASDYKTHKFLD
LSKPLFPQIWFGGFSKEFYLDQVHRPRHYKGGESAPLFGNFLEPLSKTPWYVIPILWVPCVAYGTM
IGAAGLNNSTASVGYFVLGLFVWTLLEYGMHRFLFHIDKWLPDNRVGITAHFLLHGIHHYLPMDKY
RLVMPPTLFVALALPFWKVAHTILYFNWYAGLLGYCGGVAGYIMYDMTHYFLHHRNLPAYYKQLKQ
YHLQHHFADFENGYGVTSRFWDRVFGTELELPPPKVIKTQ
>g3026
MSGLDVEALLESTAASLNEAADKDREARSKVDSHDRHDRDRSRDKDRRRKDRSRDRRRDRDDKDDD
LRSEHGSANGSHRSRRRSRSRRRDRHGDRSGGDYYRGGGRARTRSRSPDDDRYYRPSGGRSRRDDR
DDERPARRERDRRRSRSHGHRSKTPEEELNEDERDKRTVFVQQLAARLRTKELIAFFEKVGPVKEA
QIVKDRVSGRSKGVGYVEFKDESSVPLAIQLTGQKLLGIPIIAQLTEAEKNRLARNPEASSGQSAS
APFHRLYVGNIHFSITENDIQNVFEPFGELEFVQLQKDETGRSRGYGFVQFRDPNQAREALEKMNG
FDLAGRPIRVGLGNDKFTPESTANLLQRFQGREQNQQFQGSLFSGHGGRGAQAGGTPSNFDRAGGR
DTDKGTGGASALDDTDVAGVNFNNYSRDALMRKLARTDEPEPSADEKLQPLRPKTEPKPLPVNVNM
ASRCVLLRNMFDPNEEEGEAWIKELEDDVRAECEEKYGHVVHISLDPNSQGDIYLKFDRVQGGENA
IKGLNGRFFGGRQISAQPVVDAVYSSLFSRTKAI
>g3027
MSLQQDLASAQEDLDSAQESLASTEHDLATAQDEIKQRDNQIKLRDGQIKVRDNEIRARDKELKSR
DDEIRNLQAQIRRLNAHIEKQDITIRTQSRTIQDPRSLRQRRMEETSSSLSATARPWSPQNAQSLV
GPIQSLSLGGPQQFVAGNSDSTQYYPPHGYAIGLAPRMQYQMPYPVEPTVPPMGTQFELNGTTAGV
RLQGLFNPRPSVGGPNQMHMHPQSTMHQAAFNTPASNSDAYGDTAYSGIEFEAKTTDFGGRFRSLW
AKIDTFGRIYVIKNELHPKDIPAPLKEYMMLDPKTAVAVRYLNHAITKPMCVAKVINFYLSKKVLK
YTEIIKNLSPNIDSEIFIAKRKMTMAAIEIPNSARHCLLEKVADQMAIARQKPNFAEFCSINRENY
VAELLEMLKPLVTSMEDNNSMHESLKDIVTEAQRIGIDLYSYPYDVRVHFPELNETYDPTVMVNLD
PSLNANLLRARVGMSVTPYIRLGFVNCEPIRMRIVCSAKVFTGVPGEKTTQKHISGSSS
>g3028
MINRNMVIGGSVSTSFLTAAYCPQIPWIQNDTIRNNIIGASRFDQTWYNFTISACGLEEDFKTMSG
GDLRMAGSDGSSLSGGQKQRVALARAVYSKLGVVLLDDCLSGLDSKNVSLILDRLFSDQSYFKKAG
ISVILSTTTYQLLPFVDEVIVLDQGEVTAKGSYDEIITQKPEISSRFQTIRNDSPQLSDNSELPLS
LKQEEAHTDNEQLTITRKEDQSRQQGSWSVYKYYFQSAGYTLLFFFLASATIECFCTSFQNLLKTT
LRAPVSFFQGIDTGSMINRFSQDLELVDIMLPMYAVNFVMSLLLVFINTVIISALGKFLGASIPLL
GIILYILQSYYLRTSRQVRLLDIEAKAPLYAHFLETIRGISSIRAFGWEGHFSITSSKDRFNTASI
GVALTLLLTLNQNLTQTIKMWTMTETSIGAVSRIQRFIEDTPSRRSVSSPPPPDWPSRGAVNFHNI
TAGYDLATNPIIRNISLTISPGEKIAICGVSGSGKSSLLMAMSQMLEVHSGHITIDGQDVYRLEKK
AIHSAINVISQEPLFLPGTLKFNLDPHQRVSEKRLIAALEKVGLWSQISAKGSLNMTFSDADWSVG

QRQLLSLARALVVKAPILVLDEATSSVDWDTETTMQSIIEQEFSEQTIIAAIHRFRYIRQFDRVVL
LQHAKLLEVDEPDVLLSKNSEFKKLYQTFNKGSEICRDQANQRYFHDYFVTHLPSSSLHPDSQGPP
SFHKLPRSASTPHTSESSRLSPASIQTPIGLTRDTTVVRIPLRSAKHHFGASVSRGSRKHNEDTYQ
AGVIEVPSFAKRSPLSLTIRRASGAESTAVTSGADSASGDPQVFYFGVFDGHGGSECSDFLRDRLH
EYIQDSAAEFEIQSSLKDKDSSIERKRERRGRQEAGQHGLHRADLPIIQSANIQKIEKLETELVEG
WKSLVGGYFRRFKPAYFSCIGRESAPRDLSQTLGSNQSRQASIDGVSMEEVMEYAFLKADLDFVSA
QAAKKDDDPVRSDQPLNNDDILYSPSQSRREYIGGPKRFPGGSTCSVAMISTPTPTPFWNPATPSS
LLVSHVGDTRVLLCDTSTGAAIPATTDHHPSSPIEANRLRRYAATFVTDSFGEERVSGLANTRAFG
DIHSKRIGVSAEPEIRRIEMAPAEFSFLVLMSDGISGTLHDQEIVDIIKEARTPEQGARDVVNFAT
EISKDADNATCLVVRLGGWERRLEGGLGSMGTKEARDFRRQEATDPRRSRT
>g3029
MSVRTRRQKAAAAAVDEQPATTANGIANTTSTPKRAEKGPQENIFLFWPNLVGYARVVLAVASLYY
MPLHPRTCSFLYSISCLLDALDGYLARLFHQSTTFGSVLDMVTDRCTTACLLVFLASAWPRYSIVF
QGLISLDLASHYMHMYATLSMGGAGKSHKAVDSSRSWILHKYYTSRTVLFIFCALNELFFIGLYLL
SFSSPKLSPSLLQPPPHTEPMTPQPGNPAHQPPGTLFASPWSAGALELARANKIDSTVPWIITGIS
FPIMAIKQFINIVQLVKASNWLAEGDLAKRRADRAEGKLA
>g3030
MAPYNIVVFAGDYCGPEVTAEAVKVLRVIEKHRSDIEFNFQEHPLGGASIDAHGEPLTDAALTAAK
NADAVILGAIGGPKWGHGTVRPEQGILKLRKEMGTFGNLRPCSFAAPSLVDISPLKADVCRGVDFN
IIRELTGGIYFGDRKEDNGDGFAMDTEPYSRAEIERITRLAAHLALQHDPPLPVWSLDKANVLATS
RLWRKVVTEVITNEFPQLKLGHHLIDSAAMLMVKDPRKLNGIVVTSNLFGDIISDEASVIPGSLGL
LPSASLTGVPDGKSKVNGIYEPIHGSAPDIAGKGIVNPVAAILSMAMLLQXSLNXPAEARIIEEAV
RNVTEAGVRTGDIGGKSTTVEVGDAVAAELEKLLQ
>g3031
MPRQPPRRAPPAPATPSFAXPPPVIARPPVVTTPVFATPQDFQRDEVLPPNMPAWQIPPPQQQQPR
RFNQPAPTMSEPLEDSDIENDPVDDDRVRAIDFDALDDDDGEDDDEDLDDELDDEDVEGEDDENEE
MHERRKSPSPLPPDLREISSLASWTVSTHKPGCGVAALRNPSPQQFWQSDGPQPHTLTLHFFKVVA
IVKIRVYLDFDLDESYTPTKMMFFAGMGGNDLVQFATWEDDTPAGWVNIPLTGVGGKIRRRRKSRS
MGSSSSRRRTGNDGDLYDDSLDEEGEDDPDDPYAGNVLRAMVVQVRVCENHQNGKDTHVRGFQVFA
HDDDRRRIAAVKATAGAANAGQQGHNRRKSVQGDSQDGFGGAEDEETKGKRHGLDEPDWMGEPVIR
>g3032
MATCAGQNAVCRSLRRVRPQRSLRYTPKPATRTRQFHSSRARREGHEDNQKNDKNQSFGSRLGRAW
GETKIEWHPIPIGLGIGFLGLWQFYRARRDTERLRDQEIEEDGKPPRRKRIRPSGPWQVQVMSTLP
LKALSRLWGRFNELDLPMPLRIPGFKLYSWIFGVNLDEVKEPDLRTYANLASFFYRELKPGARPID
QDPNAIVSPSDGKILSFGMIERGEVEQVKGMTYSLEALLGEASPSRAAIESHGIRPADMEHEPGNV
AMEEEFATVNGISYTLPRFLSGSKEKPKKEAAMDASTESGPSSEAKVKADLALGDGRPWYAPSASN
NALFYCVIYLAPGDYHRFHSPISWVVESRRHFAGELYSVSPYLQRTLPGLFVLNERVVLLGRWRWG
FFSYIPVGATNVGSIKLNFDAELRTNSLTTDTAADRAAAAAAKRGEAYTGFAEATYYNASRALHGH
PLQRGEEMGGFQLGSTIVLVFEAPMGVRKSFDEGWDGGREGGWTWNIKQGQKIKVGEKLGHIEL
>g3033
MDGTQPEGSAPTPEETSTISLRILSPSFEATQRLSFEKLSLSTTVGDVKNLIAPLAPNHPTPEQQR
LIYRGRPLLNNSAALESVLEPPLNTVHTLHLVLPPGPELSNNIYVAPDSGLRYRHPPGRSPNLNPN
IFADTAATATGAQTPGQTSVPPLSGLNNPTQTSTPSPIGQPSNAETMRQALEQHRRRTEELNQVVD
RARALINTHTSRMRFQNLNVPRRDNHGHPAVTTTPLGTTPVPNAHAPNVNYRQNQGNAQSLLVAQI
TSWIMLLELEVDRGIAPSIESIATVRNQLYAILDARYRHPSPGPRGIEVEALLLRLQNLDARAFQL
RQSEHVRSFSGVNTSAAGSSAQTNQASEYYLITTPSGEQMILTPPPPSTTQNIPETSLVFPEQQPA
RNEIDQLGMIPNNALHNVVRQAVLNQRPVRNNNDQFAQNLRRFWLFMRLYFFCYLFTDSNTWERYL
FVGVAAIFALFSESNITSRLFSIIVQPIQRHLEGLIHGEPPAQTQQSTNGTNNVDTNQPEQNRAPT
VAAQGSFRRIERAFALFIASLIPGLGERQVEVRNAAEEAARIAREQEQVRIDEANAQNEEQSQGEP
AVNEGGAPPAVADAAANVEAPQQQPPAQQ
>g3034
MKVKNKSVCQTCRARKLGCDGNRPECTQCVHSGRACDGYQTSWTFVASSTSTSTTKQRKKVSIHSS

GQARKGAQRHLWSPLNLAPSQASILHRTSQDDFIALVVRNYVPQDTVTAISNEWICTKQPRICGAW
VEVLPNLRNGGGTPDLVLSAAIEALATSITTQKLHPNSDNVESFQSYELALRSLRKSVVVDGRFDA
ELLASIMCLSLVELMMPSTSAALEAHLKAAGQLFQAYGPEACSGGILHTLFAGFRPLLIFEAFQLR
QPTFLASTSWTDVPFSKFSASPMQSLLNKAAIIPSLMSQSDLLLDKVHQANFGFELKDLFNRFADA
LIDLERWEMDYCQEADRWCYWPSNIAVSPATEHYISYGKPLWYTNVTMANVYTNLWAIRIICFSEL
ERLAAHLPSCQVTQDIPLLRPLKLKHIHTYKTALAKQICLSMEYLLRDEMGLFGPASTYFPLQVAY
ETFLEDEGTMRKEGVAFVQSVVLRLVQKGLRSAPVLVFTRRTLRV
>g3035
MASTKASLSITHIGTATSIIDIDGVKLLTDPFFSPAGTEWDVGATILKNTDTPALSLETLPPIDAI
LLSHEDHPDNLDELGRRLLDGRHVLTTIDGARKLAPRPGVRGLKPWETVALVAGGKTFRVTGTPCQ
HLPGGECTGFIVEYDGFGTSSDGRPNAIYISGDTVYVEELARTIPPKWNITVAILNFGKAQVPTSD
GGSLQITMDGKQGARLFRELGADVLVPMHFESWGHFTQFGDELRRDFEEEGVGKQVYWLTPGQPKK
LL
>g3036
MPLDTIYLIRHGHRMHWTIDYATGTYHSTYPTPTGNPADPTLTAHGVRQSQELANYFISENFPGPR
PWRIYSSPFYRCLQTIRPSVEALDGRDSGAGNTSNVWRREKGVRIENGLGEWFGSSTYFDHPPPAS
VSELYQHFPSIIPDPSTGPENIIHVIPSSRGETIPQLHNRVATALAAIIADADADIRADEEQETAK
DKNSKPSSKAILICSHAATLIAMGRVLTGNMPDDPNTEDFHVYTAGLSTYVRRESKQDNEYYTEKE
GLSLIAAGTKLIPEPEVRVPVWKDGKXVGGGWDCVGNCDCSFLSGGEERGWHFSGEESFDTGSMGA
ADEAAKL
>g3037
MVRTSVLNDALNAINNAEKAGKRQVLIRPSSKVIVKFLTVMQKHGYIGEFEEIDDHRSGKIVVQLT
GRLNKTGVISPRYNVQLRDLEKWVVKLLPSRQFGYVVLTTSAGIMDHEEARRKHVAGKIIGFFY
>g3038
MNVTQVLEGTLSPDANTRQGAEQQLIHAAEVDFPAYVITLSEALANEESPSHIRIAAGIALKNSLT
FRDVTRLREVQARWVQQVPPQTKAQVKALTLQTLGAKDVRAGHSAAQVVTSIATIELPRNEWPDLM
QALVQNVGSGTDALKQNSLATLGFICEERDADMRTALAGSSNAILTAVVQGARRDEANADVRIAAL
QALADATDFIGSNMGNEGERNYIMQVVCEATQAEDTRIQAGAFGCLNRIMASYYDKMRFYMEKALF
GLTIMGMRHEEEDVAKLAIEFWCTVCEEEITIEDDNAAAPDPSMFRPFFNFARIACREVVPVLLKL
MTKQDEDATDDEYNVSRAAYQALQLYAQCVQGDIIQPVLTFVEENIKSEDWHNRDAAVAAFGAIMD
GPEIDVLEPLVKQALPVLLGMMNDQSVIVRDSTAFALGKICEACPTXVDIDVHLQPLIAALFGGLA
STPKIAGSCCWALISIAENFSLQGDGSTNPLSKHFEESIKSLLALTERHDTDNQLRTAAYEVVNAW
VTNSANDSLAMVASLSDVILQRLEQTVTLQQQVVSVEDRIALEEMQSSLTVVLLAIVQRLEGEIKP
QADRIMHTLLQVLSTLPPKSSVPDIVFAAVGAVASALEEDFLKYMESFSPFLYKALQNHEEPGLCA
IGVGLVGDITRALNEKVQPFCDTFMNQMLSILTNSGLRDTLXPAVLVTFGDIAQAIGVHFQTYLSV
VAQVLQQASSVTLASDVDPDTVQYVISLREGIMDAWGGILLAYKGTPQIAALQPYIEPIFQLLHLI
STDSQRSEGLMRSTMGVLGDIADSFPNGEFAAFFRNDWVTSFVREVRTNPHYSQRTLDTARWAREQ
VKRQINLATAAAMS
>g3039
MTQPTPKDAHNTLSLQQQEQPPALTSVAEKTLPSKCKDGSLLAYCPTMDLVALATEDEQLYVYRLN
GQEVLSADLAGDPYLDEVRGEIRGVRWKNDGRLLAVADAENKLRIISSYTGKTVHHFSCLPHDEEG
HSSSPNESIKDPKDFRITCIGWGVNFTDSRAAQHHLRDGNGQISVEDLLAPDTDPIKAALQLKADL
PRELALLDVESSLPRLSTLPGSGSDEDLFSSRASLDAIFQSFNRMTSDAVDVLFVGLNDGRAHLRI
FDCFEIGNFSVTASKSPAATHFETLAHSSHPMSSTHAIVLSEKADGASNPALKVMSLDLRFITKSG
RYLSLLAFKITQLQNLLRYINQTQRQIALEWKNVYELPARFMRSIADELQEKCHCDFVTALYHLVV
TGNCFPPVKDFLVDIVGERGHKRWEKTVSTGYENVRRLTHECLLPALQRCEVLLSRLIGISKFHKL
NHILGLETRDLQEVVETLDCLHLLSHSILTKTTHELHQFLEFARWLRHEIDIQTAEPMSQTLEELL
EKSDLIDHATTLEYIEGALTKSSLRRYISSTPGTNTTAGDAQKRVSDGEKLSFYQAFKNTLAAEED
EKSRERNNSNKNESSRPALPQLNDLIARLESQCGKVFGKIALTQRRSILHHCLLVLHPDCDSNVID
TAMKYESLDIEDEYAIYVASRLRSSPNTLLIYKITIQCTNGVSSTKNIAVASLRLPGGEIKQAQFV
EDGIIMLLYTEQDRSYLLDFPISSSNTDNEDGFRPEYTIITDSQTLPTPTDLNTFILHMFPAAGTK
SKPVRMNVNGRKERRAACILSADMMHYEILDIDSHVEPEDED

>g3040
MLSTTLITFILRAPSDVKKVELLGSWDNFSRSYPMERDRRMGSGHWRGCHTFTDIVRDGTSPSQSP
GRSGGLKMGGTYWYYYRLDGDIEFYNEVEPVTSLCPLLPGQPVNILNVPVILPDTDYLHRRDNSTS
STKSEKRTMDPEDKYMNPRTPPKPRPALARLKTSPPFYQLTTPSSSSLSVSSSNEERPAMSQPSSS
THGPMYRLVPRVKAERSLSPPRSRGLRAALLNIAGARSPGDHAEDRGINRPLDAHPVDAFAPSTSA
SNPSSNYSSRLTSPMSSPVELDGREIGRLPFRHPQDEFEQPQGPPSIRVRRASKSSRDNTPLALSL
ESHSSRETNKAEISGFSRTAPLETLEEALSQQATPMAVNEHTPVFLDLREKRLPTLPNSPSSVLDA
ELRAIEARYKPLDMDLLYSHFSDDTSFAVSKLYDSPQEPPEKSRFSEWSTDTELVSPASMTSSSTF
NADQLSNASPLLGHEYQHNATTTYNDYTLDAPIPTIAISGPTTPVLGDEAEVVLSPTEVDSDHSPL
LISAHSSSAAGQLGSNYVHEPFEFSSLQIEDYDDASFHEPDPKRQGAILPLSVVTPLSVDESSYRL
TSPTRKEHQYRLSDDYTSQSAAMQELMDEIGYLGDMISSGL
>g3041
MESRPHKRPRIYSSSSGEDRPPKRPAYSEPTVRLQNGKQRDYADYTVAIVCALDFEMSAVRFMLDR
QHRPLPEKEGDRNSYVLGELNGHNVVIACLPGTQGKSAAAHVAADLERTFPHVQDRLLVGIGGGVP
SDRHDIRLGDVVLSMPDGQYNGVVQYDLGKDTEDGFSLKGSLLPPPRTLINAVALMKSDHYAKQNR
IAEFITEMTEREPKLLNYYQRPSSDLDILFRSGFRDTLGRDTYTPSNQSETIQRNSRLPSGPHIHY
GLVASGDRVLKSAHKRDSIRDRIGDILCFEMEACGLVTELPYMVIRGISDYADSHKNDQWQHYAAA
VAAGCAKELLTYIHVHNTPVTVSLDSEEDVNASRHAVGGTIKFTEEQRHKLLESLEFDQFNARQKT
IKLAHKRTCRWLLSRDEYKNWLDATKYKDHYGFLWIKGKPGTGKSTIMNFAHNEAKRKMKDKIVIS
FFFNARGADLEKSTMGMYRSLLLQLLQRFPKLQDVLLSCLETLGMKIGTTAEHLQFTINTLKYIFR
EAIHSLDRHSIVCFIDALDECSEEEVRDMISFFQEVDEYGENLDEDLHFQVCFSSRHYPHISIDRG
LRLVLEGQEEHNQDIADYVIDKLKIGNSKAAQDIRDLLREKASGVFMWVVLVVDILNKEYDRGRIM
GLRNRLQDIPSDLHELFRDILRRDSNDSDKDVLLLCIQWVLFARQPLTPEQFYFAMHSGVAPESLC
EWDTVEITQDVIKRFILDSSKGLAETTRSKNPTVQFIHESVVDFLLKKNGFADIWNDIGTNFRGLS
HERLKSCCINYINIDFSGYIQPNTEIPEASSKQAKSLRESVIGAFPLLKYALQNVFYHSDCAQVAG
IPQGESLEEFCVPKWVLLDNVIGKHHIRRHAQNVSLLYLLAELNAPNLVEVHPLVMSYLEIEKERY
GTPLFAALAMGNRAVVRAFLRAHAAQRPSSRHLSDLYDLYCQDDGTRSDFGRNFSFLKLKGILFHL
VEFDHEIIFQFAMDIYPDMLGDNYYKDRSPLSWAVKHGCYNTIKLLIEAYEVNISTDNTPNRNGEI
PLQYAEERGFGGLVELLLKNNFNANTVDPRDRTLVSYAAGYGHDALVKVLLENYEIDINLADRAGR
IPLSYAAEKGYFAVVRLLIEKADVNIADIDGRTPLSYAAIYGNSDIVKLLLAKANPDLKDHQGRTP
LSYAAENGYAYIVKLLLAKANPDLKDHQGRTPLSYAAIYDHSDIVELLLEKANADLEDTQGRTPLS
YAAGIGYAYIVKLLLAKTNPDLKDHHGRTPLSYAAENGNAYIVKLLLEKANADLEDDLGQAPLSYA
AGIGHAYIVKLLLEHVKVDSDNLKTTMSQAMAGGQFGVFMLLPRNSETAVKMLETPENASISYAAR
KRYDTMLKELFAEANTDDILQMSIDQRLLTIAAKLGDYSLLQLLLKERQVDVNGDNSGGRTALSAA
VLHGHESIVKLLLERSEVNVNAEGSMGTSALPKAAPKGPENTVRLLLEKKEIDVNATDLDGRTALY
SAVRNYNVPIVELLLATDGIDVNIPDNDGCTPFSFAAERGYKEIGRLLLLSTRVDPNIVQQYPDFT
STIRMHT
>g3042
MSLFNKISTSSLSKTLSFRSHSSSSKSEETRMGSGHSRPSKHGNAISRGFSDNNNAARPPYPVDMY
PSPAKPRQCDVWSPPPYSPTPAAAQIPYSPDSVQSPPYPVNNAAPLPSSSTSANNQISDDSEYSFL
KMFDTIFLVDDSGSMAGRSWWEAEEAIAAITPICTQYDQDGIDIYFLNHCSTLDKIGGYPKIKSPG
AVQEIFRSVEPRGMTPVGHRLRQILLPYLRRIEKMAANTDEFGCVLNEALIVKPLNIIVITDGVFS
DDAESVILKAARTLENCDAAPWQIGIQFFQIGTDIAAQKYLEQLDDELGRAVKTGHFRDIVDTVPW
KGQTGRTLSGDGILKVVLGAVNKRYDRQKGQHS
>g3043
MVYCGKPSKGCGACRARKVKCDQATPSCQRCIKANRTCPGYRDQLSLLFRDQSSSVAKKAKTNKTS
PHSPSEVTTTTSPSSGSWQDHANTPTDYGLIPTSSWDSPLDTSYRESYDSYDSYDQFIDAENFSLL
PAFTDRRQQAICYCLNSFVWLNGSLIKGFDYDADISSTAPMAQKAMMKGILAVGMANLSRTGARSQ
SMKLEAQQEYQKALKFTNMAISHPKQATDDATLTAILCMSLFEILTSKKPEKVDSFIQHTRGAVAL
LEMRGEKQIIQTQYLQMFEFMRSEIMVSCIMRYYPVPKTLLTISERVASLPNMAFSSKLSHQITVL
ICRINELRIENKDEKNSMDFASVIISRAFALDAELETMIDTLPPEYAPEVHTLAPGQYFEHPTLRI
RLTPLNGVYHKYQSFFHCSMQNVSRYGRIFAIELILNRLERMTLQPDFIPTTEFKETCHRLCNISR

QQAQAICATTPFLCGFLGDQQKTGKSPFLTNPAGGLASLFPLWTAVSVDGHGSATCRWIEKTFEMI
GREMGIDQALALRQITFVEKGTTLFVDRL
>g3044
MVDYRQGLSILEMIVYIPALFMALFMGFRHGFGRQAGWYFFILFSLARLIGNGCYLGTINNPNNIN
LYIAYAVCNSVGLSPLLLGLLAGLSRVNDSIQRKTGTAYWPIIFRLIGLISLVAMILSIVGQTSND
SSLEAGYTSSEVKAGIALFIVTWVATVFLTIMIWFRYEGIENGEHRLLWAVTISIAILFIRLMYSI
LSIFKHDSDFNLFTGNVTVFLVMDVLEEIIVVYIMMLTGLTLQLREKAVYDADKELQPTSGSAAAE
NTSQYQNVGLQEQGYQQGVSASGGAAPRPKRKYRGGPISWLVIFILNKVEGRK
>g3045
MTITMKASLGDFVLPRGAGGPPKGDPSSEPALPLKSKAKESAPFMGSRHNPQSRGQKRDWYNKRRN
DTGKHSQRAPQPENGHRLGEDQHQGYQDQRQYQPLYYAPANVVLEHPDYFNYNPSNDSHTPGRIYS
DAYGFDPQLLPMLPASYPIFMSIPPSPPRMGQLFPSSMTLAALTAEFEKHRVWDELEEVASMWSPP
LPSVDPFVDPPKSWTKMRRSQAGYHHQRSTLRPWANEFVPGRLFHPILGW
>g3046
MSSDTSHNVDHSRDSVSSNNETPGLGVDGNQDKPRLTEQEKKNNHIASEQKRRAAIREGFDRLTEL
VPGLEGQGRSESVVLKKTVDFLRLKLQERRELIAEIERTGGHVEESYRTR
>g3047
MPEKGPLPGSALPDPPTYEDATATTTTTTTTRPNQPRPPFPLALPILHQLRSKRVILASQSPRRKQ
IMSYLGLPNLEIIPCTEPEDFPKTMGPIDYVLATATKKAQIIYRQEVDNAEKGEPGLILAADTIVV
DGAHGGAILEKPRSEAHHVAMLKDLRDAGRHMVYTAIVAMVPLASARMPGYAIESHVEETSVQFDS
GVTDELIVAYVKTREGADKAGGYGLQGLGSILVNKIEGSYDNVIGLPLKATLQVIQKAVLKADDDD
LLPEEEVDDDEFEG
>g3048
MAAFIRPSSGAMMGTIMRVPAVYSTKSAPLAMTIARQAQITQFHSSAKRQILTPLPLNDAAPVPHS
SPSHGSYHWTFERLIAAGLVPLTIAPFIGGXLNPVLDSILAAGIVLHSHIGFQALIIDYLPEYRVP
KTLALFKWGLRAATLTVAVGLYEFETNDVGITEAIKRVWTA
>g3049
MSEPSIEDYTIGWICALQEEYEAGCRMLDVEYDGPETSEINDNNTYVFGCVGGHNVVIGCLPDGRC
GTSSAARVARDIVRSFPNLRFALMVGIGGGAPTPERDIRLGDVVVSVPQGKLGGVVQYDFGKRLSN
SRFQRTGQLNAPPEVLLGTIPEIRRRHNDPRKSDKISEYLRLMDDMPDYQRPADDRLYRADYAHKG
GKNCVTCEADGLAERPQRKNSREVTIHYGTIASANSVMKNATERDLYANDPELNVLCFEMEAAGLM
NNFPCLVIRGICDYSDTHKNDEWHKYAALTAAAYARELLHVLKPRKVAGLPPWAGKMEGLLADIQE
RVSGIARKADEIIRHQHGQEEEKILEWLTPTNYAPQQNDLIRKRQSGTGQWLLDSAQYQNWLETNK
QTLFCPGIPGSGKTIITAIVVDHLYKIFYSDATIGIAYIYCNFKRQEGQTADFLFLSLLKQLVQQS
PSIPDSVRALYDVYVNKRTPLSFDEVSRAVYSVIATYSRVFIVIDALDECQATDGSRSRFLSEIFN
LQLKSGASIFATSRFISDITDRFKGNINLEIRASDEDVGRYLDGNISKLPAFVGRNQDLQNKIKTA
VIKAADGMFLLARLYLDSLDDKLTTKAVESSLKQFQGHNLASDEDERLKLLSQAYEQAMERIMGQK
PGFQLRHALAVELGTSELDTKNLPEIEDMISVCAGLVTVDEESNIIRLVHYTTQEYFKRTQGTWFR
DAETRITTTCLIYLSFSLFDSGVCQTENEFEKRLESNHLYGYAAQNWGHHARISSIEDRKLILDLL
ESETKISACIQAMMVLRDYKGYYLEVPMRMKGVHLAVHLAAYFGLVESMTDLLEKQYAPNPKDSSG
RTPLSWAAENGHEAVVKLLLSDKRVDPHSKDKTYGQTPLFRAAGNGHEAVVKLLLSDKRADPNSKD
KSGWTPLLLAAENGHELLDGKRADPNSKNTYGKTPLLRAARNGHEAVVKLLLDDKRVDRDFKNSDG
YTPLSWAAENGHKAVVKLLLDDKRADPNSKDKSGWTPLLLAAENGHEAVVKLLLDGFYSWYSDYSQ
TCLITGNCQLAGVEIAQSTDIWLYHIVIKGMVEMVSPVNEYATLAANNVNGFVSSILAWVRETNAT
IGAYTFSGFDIYDFDDLDGLVTSTCQTAVAQTIRCPKYLTIFDVQFYLWSQPPELVAFSDANSMES
MPTNELCSWCWTQMVIMMQNSSYSAFNDYYQSGMQYMVSECSLDAPTTMPESLIQAQPTVTPMCLS
GVNYTIQEGDTCDSIAIANHVTSASILTNNRMIINSCSSLIPGRTICMPLSCTPLYSLQPDDDCFT
IENANRLRDGTLHQYNPWINYFYSNLQESTDILGHTLCLGPQAGKFVSNSRLHPNPPASSVPSTGY
SQYVTAPPINATVALGTTTYCGIWYTVNEGDTCAQICVMESIPSSLFLAINPSLSGTKCDTSLVVN
TTYCVAPMFSWDDPSYWADDDPPSIT
>g3050
MPENRALFFSDIPSGLPVAGKDLTLESVPYPEEAPADGIILKNIYASFDPYLRGRMRSPEKKSYAP

PFELHKPIANSQIALVTKSNNANFKEGDHVVGMLPFQEYVALSGQQLAALKPLQNPLGLEDIRVFL
GALGMPGLTAYSSLYEIGKPKKGDTIFVSAASGAVGQLVGQLAKHEGLRVIGSVGSDEKLEFILKD
LGFDAGFNYKKESPAQALQRLAPEGLDIYYDNVGGDHLEAALDAMKAYGRVIVCGLIAGYNQKPEE
IHPLRNYGQILWKRLTVRGFLVGDKGMGDKYADEHRERVSKWIKEGTFKAVTWEVQGIEQAADGLL
ALFTGRNFGKAVLKY
>g3051
MHFSGLIFASLATVAVAAPTSSVFNEVYNFDSSLEEFYTKVSTHIAQYANGAAPTCDMSKVSLPSS
KLPAPTGSLKYVAIGRGTQNYTCADSTADTVPAQIGAVANLYDASCVAANYPDLLDLITDIVLNYS
LPSANVQTPLAPANLYLLGHHYFETATEPAFNLNTTPEKQLGLALTAKQNSTAAPSGSVLGQFNVG
YGAVPWLYLSTVTGTTDGFTAVYRVNTAGGAAPTTCAGQPAAFQVQWHSHPVDEEVPLEVDAVALE
EAVVVLEVVIVVVVVVVVAVPPEAEAVLAVDVVLLEVGNNQTQSKYPQNTNVGKGGRGGRGGPPRG
GARGGQKAIIEPHRHAGIFVARGKEDMLVTKNLTPGDSVYGEKRIAVEAPAEGDAPAAKTEYRVWN
PFRSKLAAGVLGGLDKIFIKPGSKVLYLGAASGTSVSHVADIVGPTGTVYAVEFSHRSGRDLIGMA
TKRTNVIPIVEDARHPMKYRLLVPMVDVIFADVAQPDQARIVGLNAHMFLKNEGGVLISIKASCID
STAKPEVVFAHEVERLRAEKIKPKEQLTLEPFERDHCIVAGQYMRSISRTFIFVIEEATDKMAESI
KDLRIVIVGAGMGGLASALALAKEGFKKIDVYEYAADLGFVGAGIQLAPNMARILDRLGVWEPIAK
EATNIQATSIRQGSTNEELGHVELGYIEKTYGYPHMVGHRFSLANSMYQGCLKESAITFHFNTSIK
SIESFGPNPVVLAEPRDGSSEPYKVEADVLLAADGIKSNIRVEMLKLLNVDAQIIDTKQAAYRIMI
HRDQIKDDPELLALLDSDTVVRWIGAKRHIIAYSVDNKNIYNLSTTQPDTNFAAAPSATYTTRGSK
SAMLGVFEDFCPMIQRILNHVPEGEVCEWKLRVHAPLPTWVHKTVALVGDACHPTLPHLAQGAAQA
IEDAAALAASLSRLPDTQPSTINKALRVYERIRKDRAYALVEMAAASGRTLHLGDGAAKEERDKQF
AALKQGNGKVPDKWADADVQKLIYGFDTTKETLENFDEIFNGLEEQAVNGVNGH
>g3052
MPSILITGAGGYVGQELAAELLRTSSPDTTVIIADVQEPPVPASASEHASRLKTVAADLTSPKVVD
ELINPSTPYESVFLLHGIMSSGSEANFDLGIQVNFDSNRYILDRLKDTMPGVKVIFTSSLAVYGPT
KAGFVINETNLPPVPLSSYGSQKLMIEILLNDYSRRGFLDGRAVRLPTVTVRAGKPTQAASSFVSD
IIREPFHGKKAILPVAKDTPVWICSPYTIVKNILHARDVPGEAFGDSRSVNLPGLLVTVQEMLDAL
EQIGGPEKRALVEEKYDADVDRIVQSWSPLFDSARARALGFADDISMVENIKRKTKRREGPERFAK
RVIRPPNLNDHPRLSSFPLQLSLSPHLHISQLALMASRRLAQQFNQALRSRAALKAVQPVKRGFAT
PVGLPSRTQSTTLSNGLTIATEHNPYAATSTVGVYVDAGSRAETDKTNGTAHFLEHLAFKGTNKRT
QGQLELEIENMGGHLNAYTSRENTVYYAKSFNADVPKAVDILADILQNSKLETSAIERERDVILRE
AEEVEKISEEVVFDHLHATAFQGQSLGRTILGPKENIQSIQRDDLVNYIKTNYLAEKTVLVGAGGI
EHDALVKLAEQHFGGLPSAPPSSAAAAIAAEQKRKPEFIGSEVRLRDDTIPTAHIALAVEGVSWND
DHYFTALLAQAIIGNWDRTMGNASFLGSKLSNVVSHHNLANSFMSFSTSYSDTGLWGIYLVSENLT
NLDDLVHFTLREWSRLSVNVTEAEVERAKAQLKASILLSLDGTTAVAEDIGRQIITTGRRLSAEDI
EATIGRITAKDVMDFANAKLWDKELAISAYGSIEGLLDYQRITNDMSRNLA
>g3053
MDAFAVSENILAEQEKPDVNGSQNTEETNKFQRAISSWRGIDLTNTIPKLDATASEIVANQRDALV
QRKDLAQKTKDFRKLDDASKLTEYKGLLKAYQTFIDLLTTHGKTSSSAFLQLYSPLSEAPDPYPLL
EASVDSLVASEDTVPKLTAEKEHLQKSVAQLTSQLETTEKRLEEESEARKRLEDSQESRIKEIESS
WEAVLSEKSNNWEXKEKTLEEKIENQDRLVKELKASLEVSQRLDRDEDVDSARNTATAAELEIVVS
DLEKTSLRLAEVEARNEQMRLELAQAASHSQAEQRPIEDDPSYLRLQSENSALIRKLDAARFDRDT
ERHDWESKVRQYERTNSKLNAEKDEFRSKLDKYADYEDIKRELEVIKSIEFSTGDDDDAGTLQEEL
TSTNGTSKSKENSLEQLLMARNKKLSNELTVLRVSHRDLQSQIETLQEDSSTTKAELEKSQQLCAT
LENDLLRMQKSTNALPSAAMSVAGTYTSRFPSSRRGGGTSPTSSIISGFDQVAASANTMDAIRAGE
AVGGGSGILPMIQAQRDRFKQKNAQLEEELSKTYATVKSLRSEVASLQKDNLNLYEKTRYVSTYSR
NQGGSASTSASSYANRPSATSVYASDETPSGVSLDKYQSAYEAQISPFAAFRGREATRAYKRMSLP
ERLVFSITRVVLANRMSRNIFAAYCFALHVLLFVMLYSMSAVEIEKHHIDNLANGAAAAAAAAAGG
GGGGVGGSAAGKLHGDDWQQEGFSN
>g3054
MDGSAGSRKRRKLSASQAAPYVLRQLLDNVPIEPESVEADVHITCVEFWSEYDVLSFLREELTLMF
EDDNLYIGTSAAEILHFVSIPPEPTDETNEPTFILASRLQITGKAKKKNGIQQILLLPTVSKACVL

CNGVVTFYSLPELSPAFENTKVSSQWIGGVDLNQNETDNAANKPVIMIALSDRLMLVRIGDVAHRL
RYIEFPGCLVGARRDTIACVADGTAYSLLEVEHQQKIPLFPISSTSEVFESGHVEDIPQSTSNLKR
SSSTSHANSKADKRSEHSRSTSLNTFAGGLGANNDSLHPSLNDRSNNSTPGPSDNDTPRRSVSRDR
RDIDTSKDLPTPPKETAEDKQKPLPPAPKLGIASLKPHIVSPTPSEFLLVTGTQESEPGVGMFVNT
DGDVVRGTIEFQRYPEAVIVDAPSESHHSSGNPGSSEGYVLAIINLKEEDSVNKYLEIQRWDVEPG
EYGRQKTLVQIPAVDGFEIAHVGIKHTTSSSKLDFYELGETLRLVRLKTPHTGEDAPEETDPRTNA
TIEQVRKEKELFESQELTDSESGKKAGSTQTLELERTKEESAFAHRLGYIQTHLVLWSGNRIWRVT
KNPLPLQLDGLLQSAQTWEEGQFKSVDRDAVIDLLNSIKSTDPTTEAEFYGLEYIKQKAGLILFAD
LLAMDPNNRSESTIRVTEEVLVESNLDPRLILFMVPFLAEEVLQSKKGIWVHRGLAREAEFYLDHL
AESKAMPDIPDEALLNMVKRYLSAWQRKRGYGSVTDESNVFDSVDAALLHLILEQEARLRRASQPV
TRATRNELNGLVDDWKGNFDRAVSLLERYHRLFILSRLYQKRKIYGKVLRTWQRIANGEKEDDTDI
TIAVVEVHVRKYLPKIRDPQLVEEYGSWLASRNPSLGIQVFSDDSAKVKLEPDKVVQLLKKRAPDA
VQDFLEHLVFSKNHTQYADDLIAYYLDTVINVLQASSEARISLRESYSTYRALRPPKPTYLTFINE
NAPQESWWQSRLRLLQLLGGPNTVFTSSTGASNLSYSVSAVLARIEPFQNELVSESIILDGRQGRH
KEALRLLTHGLGDYDSAIRYCIFGGISSSQPAATISLPPPSDDVAYTEPALLFKHLLSEFLQIEDV
SDRIERTSDLLAKFSRWFDVNEVLATVPDDWSVDILSEFLVHVFRDLVSQGRETRVQKALSASLNL
RVSVEYSEDVEKRGGWIEDDQGLRSLKVAVPGQTDDDADDFGEMIDAR
>g3055
MDPSLVKIPPLRDLTIDNITENVILINSLCEDARMKYVLERLVTHLHDFARETRLSFDEWMAGIRF
LTETGQICTDVRQEFILLSDVLGLSLLVDSIDHPKPPNSTEGTVLGPFHTHEAPQMSHGDSMSQDT
AGEPLLVVCNIKDRTGHPIPGVKIDIWETDSTGHYDVQHADRSGPDGRCVMHSDAKGEFWFKAIKP
VPYPIPHDGPVGKLLKKLHRHPYRPSHMHFMFEKEGYDHLITSLYLRNDPYETSDAVFGVKDTLVV
DLGVVDATIAAKYGVKEGTLLMTYDFVLVTDAETKALREHNSQVALDKLGRKVKIVNGLPIPDLD
>g3056
MPKTQSNSLHINYLARQYPDNLPLVSTDDSLPNILRLIGEYDGVLQRHESIAGNLGACPLGPILMK
RFERLFEGPPRVLKSNGKEGTNVTWLDVVEFAKSKPEQFNLEKTRNGVRVCQFYTKQCRVEISEED
YVLIASGMPQKLIPPQPIDEDEEKEVGVLEILEKNLQQITAMADSAAARARQLSYRLKSRRNAIVT
RRENDRNKYSQSPPLHEHGSTDTAVNGTGFNPPSPPGGFVAVNAGRPVDAGDEHPSLSSQFMLNHP
NTDNITIINGTNIKGASPTTRAELLKKFLTTADRTGRSGQSEAEDSSLTRQSSRTGPRRSEASEMP
PVMFSNSTGTVAIPNTPPSLLPQPKSVNHHERDDGGPFKLEMVARMEDLQRGERILPPCDRCRRLH
MDCLKNLTACMGCTKKHAKCSWKDVKEEELRDLQPRQPRAHVDEQRDFARPAAPDSNNHQDEHSAD
HSHPHHSNLYTPHDNHSEPHPTPHKALVLAQSEPPSSHPYSQHQAPREHATAPRRANSDVHIMLNH
DQHPDTHHQHHHHQPQHQHQQHPPQPQHQHQPQHDRRSSFHHRNILHDNNSVNNANTHHRNNHLRN
ETDNDPDANSRLMQAIKDTVDHHSTRVQKHIRDGE
>g3057
MSERGSFRGGGHGGRGPRGGGGGGGDRRGGGSTRGGARHQGGDRHGGDRDQQEKPKKENILDLTKY
MDKEVIVKFNGGREVSGILKGYDQLMNLVLDDVKEKMRDESGKEATRSLGLIVARGTLLVLISPAD
GSEEIANPFVQAEE
>g3058
MASTAKSGLDDSRRNLGSPKLRGRENAKDTLCRNVTIYGKCRYEDKGCAFNHDPLKLQNQNQVDRK
GLNVDSPSFTPTLLAANGSTPKKPNTISPKAASAAPFLPKGPGSSAQSTRSDAGSSEWSVAEIADF
VPQGFDGGQVVPLQGNGNGVLSPAPPFDPFVTPQSSIAGSSAVTPHPVPNPYSHEAAVAASMGGAF
FAGQTGFQQPVQYHLYAPIGPHSTNISGYQRNVHDLFLPNDFREELQKKAAATLQTLPNSSLPAHV
DHFHSLVPLDLTHQKNAAMFGFPSWVYKAQSNKDGNFYVLRRLEGYRLTNEKAIRSVQAWKRITNG
SVVTVHDAFTTRVFQDSSLIFVMDYHPLSKTLAEQHLGAGNRFNARSNAHIPEQVMWSYVTQIASA
LKAIHSSGLAARIIDASKILLTGKNRIRLNACAIMDVVQHDTQRSVQDLQLQDLVNFGQLMLNLGV
SAAGALGSPSKAMEHFTRAYSPQLKNSVFWLLNGLQKDQERSIDIFISGISSQLISTFDSALQLDD
QLTSDLSRELENARLVRLITKLNFINERPEYEHDRQWSENGERYFLKLFRDYVFHQVDAQNNAVVD
LGHVLNCLNKLDAGTEEKVTLISRDEQSCFVVSYKELKKALESSFQALLKPVRRLH
>g3059
MSSQDGGADIGELSSTQQEALQTYMNVTGQEPDAAIPLLQRSQWNIAISKFFDGEGPDPVEEARAA
LNAPQDIRRTQNLMYDIDELPPRASFSASSSSSSRPAGLEPAPRIETQPEDQPAFRPPFILSLLFS

PLNLIYRLLYNSFRLFGTLFPFLPRWLNVTTGSSPLQRNLNTSGRRALAPKDTAARFIREFEEEYG
SHSLPFLENGYNMALEKAQQELKFLIVVLLSPEHDGTSDWVKETLLSRQVVEYINDPNNQILLWGG
NVQDSEAYQVANSLRCTKFPFASVIVHTPNVGTTAMSNIGRIPGNTSASEFLTKLRTAISQNKEPL
DRVRARRTEQQASRTLRQEQDSAYERSLAQDRERARQRREAEAERQRAEAEEAARAAAEQKRAADL
KQWKNWRAQNLAPEPEAQDAIRVSIRLPSGERVIRKFAPDADLEEVYAFVECYEVLTAAEDEEEQE
SEKLATEPVGFEHKYGFRLVSPMPRTVYEVEAGGSVRERIGRGGNLLVEMIENDDEDSDEEEEGNV
NDNESL
>g3060
MVVIKALPGEACRVTFHLPHRIDVKPGSHVYAFFPTIAWWMSHPFSVAWADPSTCVTAPSHPTAAW
NEAYNPYERKNLSDSLGLSDLEKQDYMINELRQGNKRRTSLSLVIAARTGMTRKLYNKALACPNST
LRTYGFIEGPYNSHTCPMGSYGTVILFSGGAGITHHMLHVRDLLLRAEEGCVATQRIYLIWSIRST
EALNWVREWMDAILQLPNRRQLLVIKLFISKPKTQHEIKSPSETVQMFPGRCRPSVVLEEALLRRV
GATVVSVCGPGAFTDEVRAATRECMGQGMSLDFVEESFTW
>g3061
MASLFSLEGRTALVTGGTRGIGQAMAIALAEAGADVVLVQRDESNTATRDEVAKLGRKALIHVAEL
SDREAVKRIIPTLVQQGVQPDILLNCAGIQRRHPSEKFPDEDWDEVININLTSVFTLTREFGANLL
SRDASEFPRGRRGSVINVASLLSFQGGITVPAYAASKGGVAQLTKALSNEWVAKGINVNAIAPGYI
DTDMNVALINDPNRNAGIMARIPAGRWGKPEDFKGVVVFLASQASGYVSGEIITVDGGWMGR
>g3062
MGPAKIQKDFASRQHRKLGPGQRVSRACLFCRQRKSRCDLDSSGNPGTPPCQRCVREGRECILGTS
NRGGRRIRKSKGVDAASKDDETSAHKTPENQSSISDYPEHNNGSSFSAPGYPITGLYVNQTGKPAK
SITADVNDQDQEEEQQYEDDDNESVSSSDSAIDSGVPRNPSDAWQCLTGIAKRGEETEEFVLSTES
VTQQRPLAVHVQDGLTGMPSKTPVASGIGAYELVKNGWLDPELIFNLIKRYQVHFHPYQPLVPRRY
FERGMLDAFASNEKHLLTAVLTIASKDLLEMPHIHEYCSKYMHELVASIAAGTECDVEAVESLILL
AEWEPQGLRPNIERVGRGEEDRAAWMHVGLALRSGYFMGLDRTSFRGDPTGEDCDARKRLAWTSCY
ITDRLISVRIGRAFWSRGPGPMTGFVSQDFPSLQPLTEGDEDYAKIIQATLDLTQLYGNVHEILYS
GMRTSNQMMLMGDYIKYADDFRAAIHRWNQTWGGLNCSPNIKLTLQMSYEYLRLYTNAFAFQAAIS
QAIVRNSKTADQHHQKEHLRATFSNVASMSDARFIYESLDAARSYLDILARSFGVMSPKEEIEVRE
LVSRTTETLHRASAGPDDMGSRYARLLELLWKPRSSGSVASSESRRRNELSIASLANSNNNTTMSP
GGVIAAGGAGVSGVSGGAPHQHQQHSPQQHHHQHQQAVHQHQQSTHFDPANDFSWLDLGAVGDYVS
GDQMTAGMLGFDGALQDGMYSTDQQQPQQQQQMWQTPFWTGDSMSTSLFF
>g3063
MSELNNIKGRLALITGASGGIGAACARQLALKGTNLALTYMSSLSAIESLVTDLHTLSPNLKISIH
KVDVGKKEDIDQMFSEIDTQHGHRPDILISNAGYGKRVPQIWDITLEEFDYTINVNLRASFILVKG
CVEYMREQRWGRIVFMSSIAAQGGGINGCPSKGGMSGMMRNLSTRLAEYNISVNDVAPAMIGETGM
IPNGAAFPGLAESIPLKRLGVPEEVANTVTMLVTTGYMTGQSLLLAGGLK
>g3064
MTTITTSKALILHGAKDLRLENRDIPAPTGNEVQLAIRATGLCGSDLHYYTHGRNGDFVVQCPMAL
GHESSGVVTAVGPDAKHLKIGDRVALEVGLPCRKCLYCLSDRYNLCVNMRFRSSAKSFPHLDGTLM
QFTNHPEDMCHKLPEGVSFAGGALAEPLAVCLHAIRRXHXPTSEEAALAKSVGEESAAMIFGAGAI
GLLMAAALAVSQNFSSIVIADIDAKRLKVAEDLGLGIKTFLIERSATPPPPKDAPLEEQTSYAMKS
AQETANKIKKTHNIPNGFARVYECTGVPPCIQAGIYAAAAGGVLVQVGMGAPILTLHHGAAMLREV
DVVGVFRYDKYAYPAAIELLQKEAFKKVEDKIVTHRVDLSVEGQGERAFTLAAKGVDEEGVPVVKV
MIESSL
>g3065
MHKSRSHQRLAHIAGQLAVMPSQAALLIGEITHARPEWEQLSSTLTLKEFPSGTREQFLTNCKNGE
YDDVVVIYRSNASTKHTGRFDKELVSALPKSVKYIVHNGAGYDNIDVPVVTEAGIAVSSTPVAVNN
ATADVGIFLMIGALRHAHVPLTAIRAGQWGGKSGLGRDPKGKVLGILGMGGIGREMAHRAKAFGMT
IQYHNRSRLSPELEDGAKYVSFEELLSTSDVFSLNLALNASTRHIIGAEELAQMKDGVVIVNTARG
ALIDEKALVSAIESGKVGSAGLDVYENEPQVEEGLLKSDRVFLLPHIGTATFETQKDMELLVLENL
RSAVEKGGLVTQIPEQKK
>g3066

MVRYAAHTIPNAKSARARGSYLRVSFKNTRETAQAINGWKLQRALVYLENVINHKEAVPMRRYAGS
TGRTAQGKAFGVSKARWPVKSAEQLLGLLKNAEANADTKGLDTSNLVVKHIQVNQAPKGRRRTYRA
HGRINPYMTNPCHIELILTEGDEVVQKAPHAIAKDSARLSSRQRGAQIRRAITEA
>g3067
MKRMFTGLQRRVTSSPSSAQDSPSPTSSGSDSPESIISREISNDYLHLPAIVEAAESSANAAKEAA
GRIRKYLSSPSKAQGNVQYNSIMLIRILSENPGHTFTRNFDTKFVTTVKELLREGRDMSAQQMLRE
TLEMFETQKSWDEDLAGLIAMWKKEKVKIGKYTRSPPPPGQAPFQQPPYPHAAPPRRGGQLPPPDE
LASRISEAKTTANLLIQLAQSTPAAEVQSHELMREFSQRCQAASRSVQAYMAVDNPPPDENTMLTL
IETNEQLSVALSKYQRTVLNARKALGTSTPVTSPGSNGNSSNVHSPPPGPPPGLPSINGQTNGNHV
ESGILIPELGSQSSSPANAHESRSLPPLASLNEPFSLSRFDDPIEVAADVPATNTRTVSSISNQPA
AKRYEYNADEFAVENPFADNAAEDAHDADHDRHEPSRRAV
>g3068
MDDSTLRHSITLHPHPFNLSREDARRSVSHPRTPVTFSSNEAAVVSGVERVTDTDIVTSDEERNSP
PRYTPTNDPLQLASKLKTEEEIKQIRANTSRKRDRVTPSFIGDRFGITSSGKLQEFYQVQNENIER
FLTPVEEHVRAAKEFNNSNQLKFKIAVWGSFAANVILSVVQVYGAVSSGSLSLFTTMADAIFDPMS
NITLLLSNKAVTRVDPRKFPAGKARIETAGNICFCFLMTAVSFIIIAFSVKELVDGSTEETTKFHL
TSVIAVAVAFCTKLSLFLYCWALRNQFSQVRILWEDHRNDLFINGFGILTSVGGSKLRWWIDPMGA
ILLSCLISFLWLRTAYSEFMLLIGVTADTQMQQLITYISMTHSPAITAIDTVRAYTSGPRLVVEVD
VVMDPNATLMATHDVAEELQIKLESLPDVERAYVHVDYETTHKPEHFLKKEL
>g3069
MDATAQARFNLIRENLDEVLNPEIVEAILAEGRNPRIYWGTATTGRPHTGYFTPAIKIAQLLAAGC
EVVILLADVHAFLDSMKAPLELVENRVKYYEKVIRAILEAVGVSTEKLEFVLGSSYQRNSDYVMDV
YRLAALTTEHDARKAGAEIVKQSNNAPLSGLLYPILQVLDEEYLKVDAELGGVDQRKLFTAATEWL
PKLGYRKRAHLVTPMVAGLNGGKMSSSDPDSKIDLLDPPEAVAKKVRKSEAIPRTIESNGVIALVE
YVLLPAAKLNGKNEFRVERRDAEPLIYTDIQQLKDDYMNDVLTPQLLKPAVAEALTSLMAPISEAY
QKSPEWQEITLKAYPPPVVQKKQKKIKDKGSHYPGGNKKITTDNAENLKDKTDHTEAEA
>g3070
MADALLTEPVIIVGGGTFGTTTAFHLAKKGYTNVTVLDRFPVPSLEAAGNDINKVVRTEYPDPLYT
KLATDAKNIWSNPNGLLSGLYHPSGWIIGASDRSMPFVEASIKSAKTVGTEPPKLVSTEEVHQKWP
MINGDLPGWQSYWSSGGAWVNAREGITRMARKAINSGVKYISGDAGYVKQLLFDANSTCIGVRSAD
GSNYHGTKIVVAAGAAAGSILDLKGQIVAKGHTIGHIQLSPEEIERYKDMPIIDHLEGGILFPPQE
DGIMKIGAIHFVTNYAKSYHGMSLPRYRSDNPGDDIPAEIEARLRRWMSEFVPELAQREWVETRIC
WDGDMPDYHFLITPHPTHKNLSLAIGGSAHGFKFLPVLGKYIVEMMEGTLDPAISAKWKWRPGAKL
SAYEANPHPEVPVDLSSLQGWETPPKSLL
>g3071
MDVAKDKVTTSESPAEKYSDTEVGETTLINNAGFHRSFSPRQIHVISLGGQIGAGLFITTGSILRD
GGPGSLFLGFVVVCSCVWAVLQTVSEMTIAFPTSGNFIDYADRFVDPALSFAAGFSMWIGWTAIIA
SEATFFSVVVNYWAENRVNEAIWLTIFLLLMFVIFSLPNTVFGWFEYVNSILKIVTLILFVIVGLA
MVFGAGPDGTVHHGEAWQDGSAFLNGFKGFGNGVLLAILSIGDNTFTGFLAGESKSPRYSVAHAVI
LIPIRVTVFYLISVVLIGILVSPTNQNLFGSSGVAASPFVIAIDQAGISVLPDILNAVIITAVAAI
AAESFFVASRILQSMAQQGLIHSYIAKVDSRGRPRVSLAITGILAIVLTYINLSAGGTTVFNWLAQ
VASTGYFMVXPADWTEAVDKSRNSGVGWLR
>g3072
MNFNRKTPTETTEAQDTSDISDIRNTEDKDGSEIEKNLPTPDQSSEDNKLSGAKLLILGFSLCFMV
FLIALNGTVVATAVPQITNQFNSLNDVGWYGSASLLTNCAIGPLTGKIYTFSPLKATFIIFLSIYM
LANLLSAVAVSSNMLIIGRAVQGIGGAGVMNGXFTIIAAAVPRDKKPIYLGVGIGISSIGAVIGPV
VGGPLTQHASWRWCFYINLPPVGLTILLLLFLRIPEQTAKKPVSQNIMPLLRELDIGGFVLFAPAC
IMFLLAMNWGGSTYSWKSATVIGLICGSFVTACIFAVWQRRRGDRAMIPPTIISNRLVLFGCLVSG
LQMGALSLLAYYLPLWFQVVKDASPTKSGVMTLPSAISQALGSLVAAKVVQIIKRCTPWAIFGSVL
SAIGAGLMSTFRPWTGAGSWIGFQILTGTGRGSVMQMPLTAIQSFLPGNQVAIATSSIFFFQNFGG
ALFLSLAQTIMDNSLPSTLNKYAPNADAQAIISAGATGFRSLTDKSDLTGVVLAFNSAITNTYYLA
AGGSCAAFLACFGMGWQKIEKSAGKK

>g3073
MAMLMDLGLHRASGIRGTTGLPLHYLRTFYSEPEVPRQRTLEERRAYIGCYHLLTTIALCNREMSV
LKYTKYTDECLVTLGEMKEHPSDEYIVYLLRLHRIGEKIRRTLWDDPIDIAWGLAAPVGMCVRYLE
TELRQFMELIPIDLAQNTLLRSYVNTLEISLYGIALSDNFNDGDYGEYQLTRFNILRSCLIATKAL
LNQISDLPLEVRICTPSTCWSQFGQAVVVLSKLSLMENELWKGIYEQYALDFPQEIDRIAEKFSKL
DSWTQGVDSRLFITEAYLKLGSRLKLMKETHEKIRVTQVERARDLTEENFGLDLGDLLPMPMLDLL
DGEFWQQVL
>g3074
MLLSPPQFHFSEASTFGDSTYSEVDALFGFTAHSEQPADQTIVDSAPIPKQSRMLPDAKGKLIYIG
QSASLSYLHIVQRVAADCIGSCAFTDDPQQQYILEKEFTSHLGNCPEPEPDLERSLALAEQYELAV
SGVFDFFDISYLQNAIPPWVADPSRRDRPETPVIFLALAIGALGRAADDKDEDIAETYFNYGRQQA
VIYLMDDPCLMTVVAFSLISYYMLASCRRNGAFTNIGIAARAAYALGIHRHETNRAFGGGARERAW
KSLRVCDLFLSASMGRPPATSEADCNIPWSKSFAKMNNGDYKDDRSVPTQEDSAIFRICLIFERIL
VEVFSRRAVSLDLAGSISQQHRQWTEELPSMLKIDGLLRNDTSTKASIQKLGTHIVTMAYYYSITL
LCRPFLSFHVGTRLKKAGNYQDPEAYSDFITTYADACIDSAVKGIALAHEIVFDESMPKRQPLIIN
SVFISALCLGHAIFGDYDQRGWPLNYNIDTAISILRKLGLKNPQAARYCEICCYLKEVVGTYTHRR
ADTLLRSPSSNVRSMFGDIQAIRINTPHFSKDADADDFHDRNIERFSIGQNHALAAQNKIINCNDN
NINDFAGSTILPPNSISTPESLMHLASLPQDDNEFHEAVNKHCDQNLDVESEYTPTMNNFAFSDTL
PLFSLIDGINPSLGADHSVLLGYI
>g3075
MPVFQETAHSSRDLRVRRSRARPLPRLSMQPEISRSNGHDGDEKYEVVVIGAGPAGLFLTLLLARY
GLSNSLACFDKKPGTLKAGQADGLQPRTLEVFSTLGLHHEILDEGCHMSEVAFWNPSSTKKGIERT
AFVPDVVVPARYKHEVTIHQGRIERILEEDFHAYSPDRSIFRSTGFVGFTLDASTDPEFPVRIEID
VLGDERTSPQRKIVRAKHLIGADGAHSKVRRSMGLQLHGDTTDHIWGVVDFVIDTNFPDIRKRCAI
HSDTGSVMVIPRERIASGHYLTRLYVQIKDVIPAAEEGETLDEKARSSKRRAAITLESILEQGKRV
FAPYNIAIKSGTDIDWWAAYQIGQRMTHKSTLKDENGVERVFLVGDACHTHSPKAGQGMNVSMMDS
YNLAWKLAHTLHGLSPEPQKANKASESNTPTTVLNTFEHERLTVARQLIEFDSKFSSMFSGQIGAE
KSVEGLTHDEFLKVFSDGSGFTSGCGVEYAPSIIVDQVSDPETIAHVDAISFVKKDAYLSGVLRPG
RRLLDTIVRRYADANLRHLHDDFLSTGRYRILVFTTDDLVDRQKVTTHKAETSISAQALETICREI
IPAYPRDTIELVVLHPFHTRDFEWDQMPDCLKDFAEMTFHGPTGPESFYEIYGVLESRGAVVAIRP
DGYVGVVVKSLADAKSLGDYFDNCLVRVDV
>g3076
MTLVETIASPPVFPILTNGESTHTNLPNGLDSKINAEIPGLDIKDTPVENFRKMRVVVIGAGFSGI
YLGVRIPQRLRNVELAIYEKNEDVGGTWYENRYPGCACDIPSHSYQYTFAPNPAWSRVYARGPEIH
QYLKSVVKRYSVDRFVKLSHQVLDVEWHEEVSKWYITIQNHATAEKFVDKADVVISARGTLNDISW
PDIPGLKDMNIPVMHSAAWDDRQEFEGKRIGIIGGGSSAIQIIPALQKVKDAQLTCMIRSRIWIAN
PFGAEILKDLGVENTEFTPEQRARFANDPEYYLKFRTTLERAANLEHSVTLKDSEMQRLAREGFTS
LMQERLSKKPEILNHLLPDFGVGCRRLTPGPGFLEALVENNVTVTNTPIKVAHKTGLELQDGTRLD
FDILICATGFRTSAPPPFRVAGVAGQLMSDKFHPFPETYMSLATDGFPNYFMMLGPNAAIGTGPLT
TMMEMTGDYIVKCIRKMQKENILRMEVQHRRVRDFSAVAENYFKKTVYLDKCSSWYRNEGGKGPKI
SGLWPGSALHAMETIRSPRWEDYNYKYEGETEHGEEVNRLAWLGNGWSVTQTGNGEAGELAHFLQP
AFVDVPAEPLPEETFMYKQKPFSH
>g3077
MLFVKILLLLGFSALLQAAPTPQASTAASSSGFWMANIQRQGTVAFGNSSSYQIFRNVKDFGAKGD
GSTDDTDAINSAISSSDRCGLGCDSSTTTPAIVYFPPGTYVVSKPIVQYYYTQLIGDATDLPVLKA
AAAFSGMAVIDSDPYTDTGANWYTNQNNFFRQVRNFVIDLTAMPQSSGAGIHWQVGQATSLQNIRF
EMVKGGGDANKQEGIFMDNGSGGFMTDLTFNGGNYGMFLGNQQFTTRNLTFNGCNTAIFMNWNWAW
TFKSVTVNDCAVALNMSNSPSNQTVGSVMMLDSTLSTTGQAIVTAWTQDSIPIGGGDLILDNVDFT
GSSVAVASIGGDTILAGGSVVKSWVQGNTYTGGSTSSKRDLESTNLEDDTCPAPEVVTVTVYQTLG
NGASEATSIPVASAAAGTSVASRLSPAQSDSASGSLSGAAASSKIPSSATGSPSTGKASQTATTGT
PASGTSSPSSGSSARTSALSSKTHSAVASGSTCAAPSATKSRVQATRAAASKPAALVDSSGKVFER
SKPQYETVAASSFVSVKSAGAKGDGTTDDTAAIQKVLDSATSDQIVYFDHGAYLITSTIKVPKNIK

IVGEIWPLLMASGTAFSDEKNPIPMLQIGESGDTGNVEIQDLILETKGSAPGAILMEWNVAGSSQG
SAGMWDVHFRVGGSAGTGLQSDTCSKNPASTHAANNACIGAFMLFHATQSASAYIENCWFWVADHE
LDLADHNQIDIYNGRGVLLESQNAVWMYGTASEHSQLYEYQLSNAKNVFMALIQTETPYWQSNPTA
LTPFTAQTNFNDPDFSTCTTDSCRKAWGLRVSSSSDTLIYGAGLYSFFENYDQTCLATESCQENIV
QIDCSSISIYGLSTKASTNMITSSDGTSLALQEDNTSSFASTIALFEQ
>g3078
MLPMRVQVDRLQLVSHFLPYVREWSDRVISSHLDLEDIRSLNAGAEVATGFQTVIRFLEGRLEWHK
QSSSPNYGDKPGRSSQYSLIIYCSRYGNALSFLAEFNQTILPTSTVRLLIDDLEHAVWQLINGRDD
QALRLDSLEQLGPHARRQLLRYNRPLAPKLNSFVHDIIEQQVSELPQAAAIDAWDGQLSYSQLWER
SRALAHHLQRLGVGPGVLVPLIFEKSSKSVIAILAVLQAGGATVALDPSVPVVRQHTIIRDVGAFV
VITSSTHRDKVPAHITIASLKYNVVLDDDLLRQLAGVTESGLIQHTPVSTPDDPAYVIFTSGSTGK
PKGVVISHSAFVSSSAGYSKAIYLTSPRSRVLQYSSFTFDACMLEILTTLTVGACICIPSEEDRLN
DLAKCINSFDVNWTILTPSVASLLDPKDVPGLEVIGMVGEALPFHLADRWAGHAKTINAYGPAEAS
ILTVISEPRIAGSGPVYLGRPPNCAVWLVDPDNFKQLVPFNTVGEIVLEGPVLATGYLNNRELTEA
AFVEDPEFLRPVIGASTGGIVPRVYRTGDLGQMHADGVIEFLGRKDTQVKIRGQRIELGEIESNIK
KLLSDESDDSLEIVVEVLWPDSKDRKQNQGDTGAMVLTCFACKKNTSPPDANSLALFSDHKDPGHS
ATEISKLMKRTMERLAGILPTYMIPKVILPCQAIPHTSSGKTDRKRLRALGAQMSTDILLTLGIPS
APSAAVSDPIEALSETERILQEIWAKLLGLPQQSIGPNDDFFKLGADSMVAIKMVAACRKAGLKIT
YATIFGKPGLREMAAVCERPKEATTDGLTASHQIPAPVPSQIRPFSMLKPQDILELTEEAAIQCNM
SKDSIEDLYPCTYLQEGLMAVNLTRPGAYTAQMVHELPRGTDMVRFRHIWETIYAACRILRTRFVM
TLAGSLQAVIKGRIQWNAADSLESYLKRDEGELMVPGVALSRFAIITGPDGHATHFVWTMHHMLYD
GWTLGLLLRKANAMYRGVKFSIIDDFCHFVAYTKSLDKDSMGRYWQQELKDAPASTFPVSSRLGYQ
SIARTIIRDRLELSHSPPAGLTLPSLIRAAWAIMISHFSQTDDVVFGAIVSGRSTPMPNIELIVGP
VIATVPVRLRVQPDQRVLDFLRHVQNHSIQMLHYEQAGIQQIRQLNSHTKRGCEFQNLLAVQFDWE
EDNGLLAKPQYREEFTTYPVTCEVKVIQSRKAIEFATHVDEEVTSRLVVAQAIGQVKTILHQLVRL
SAESATTLSEIKVDDELGEYDDGDKESAEESMPLSSSSLPPQLPLPVTVRACIHDLIREVVERQPN
AEALYSSLLSVSYWELDDFSTKLAAHLISVGVAAGTLVPLCFEKSVWTVVAMLAVLKAGAVFVALD
PSQPLTRLKSILQDTETSVLLTSKLHARVTILPVANVIVVEKTLLQRLTRPEERGISLKNRASPDD
LAYLIYTSGSTGKPKGVMISHSAYVSGALDHGPRQGIRQQQSRVLQFASYSFDASLVEILTTLVHG
GTVCVASDKERLEDVHGFMQRAQIDYAVLTPSVVRLLRPADLPLLRTLVLVGEAADDELVKRWLDA
AVDVFNGYGPSECSIVVAVQKMAPGMNVRNIGLPVAARAWVVNPNNVDELLQDGEDGELLVEGPTL
ARGYLNNREATESVFIWNPAWALQEGEENSGRRMYRTGDMVRRNPDGSLYYIGRKDLQVKVNGQRI
ELGEIEAHLSRCSQVKNGLVLFPSSGKFAKQLTAVLELEGNNYKFSEQVVHIRNYLSTRIPPAVVP
KHWLDIHSLSDGGLPLLPSGKVDRSTVVSWLTKRVSIDADLFMGTREAALERETAATTIQISENPA
AELAAKVSSMLPGDRSLALQHGEGFRDAIFSQSGLDSLDMMSLIHYISGRYRVHVNMQLLMDKCTT
IRKLAQLITESQSSGWEKPNDVTATATPIDIMAEINKYDARVAAVQLSSSVLSSKLSEETLINRSA
RHKSGSNGHSLYRGRLQQRSRNSSHGQSSTSSNGSTSSSSGFNILTGSTTDSKPAMSSLLKPDHLI
VLVTGASGFIGTQIIRQLLEKPTVAQVIALVRDDEHQPARTRTIASAQKALWWTDLHAEKLEVWAG
DLXKPHLGLDASRWGLLNSSDNSKRVDVIVHAGAAVNWTRSYVDLEAVNIGSSVELLQLAVTGSRP
RLVYVSGGRQWKSEHECDEDVALFLANDIGYSQTKFVAEAVIRRAALRTSPDCSNISIIKPGLVIG
TAQEGVANIDDYLWRLTAANIAIGAFDSAQAREWLHLSDVTTLARLVVETAIDPGLDQNPQIPVQY
IRDGMTWGALWDIIRNLGYQLEAKSTAQWYAAIRADLIARREQHPLWPLFHLFGEETEDSEDTANS
LVTADEVPVPPLHLRIAVVKNVEFLAQIGFLPASPQSQIHGPRSLLGQPFSRSR
>g3079
MATETSEQPPEMVKTTEQSDSSENVTISESSLEIKLKLFQERVERMSAWEVFLVWFALALSNLCVF
LDEGIISTAIPRITDKFQSLGDVGWYGSAYNITLSSFQLVYGRLFNEFPLKVTFLAALAIFEIGSL
VCATSPNSVAFIIGRAIAGLGAAGLQSGNFALLAAALPPKKLPLYMGALGMVYGIGAVLGPVMGGI
ITNSYLTWRWCFYINLPIAAPTAVATIFLIKVPPTDKGNKPLLKKIYNLDPLAVVILLPCIISLIL
ALQFGGALYSWNDGRTIGCFVAFGVLLLVFLVEQWWMGDGALVPPRLVRNPVVLFGALYAFCFDSA
FYSLVYYMPLWFQAVEGVSAEDSGIRYLPLVLSLVMTIFLGGWAVTKVGYFQPFMLLGTALLATGA
GLLSTLVPSSGKGSWISYQILAGLGIGVATQQAVVAIQAILSGDDVNIGIALAIFTQCFGPTIFIT
VSQAVFEGTLTSGLNDHLPGLISPSQIKNLGATQLRDLVSPPQLPIVLEIYSYALSRTYLTASIMA

ALSGPLIFCMGWRKLPSPPEESGEYQGGNNEANTEANTEAKSDGGETSLKAEAAKEATSITASQ
>g3080
MTTETVVATGSEGHHGNGLSTGVTGINPSSEPQLERPLHANRHLRVVCIGAGASGIMLAYKLKRHF
TDFTLHIFEKNSDVGGTWFENRYPGCRCDMPAHNYVYSFEGKPDWSASYAGAPEIYQYFSDFVEKY
DLKQFIHCNREVHRAEWDEVASEWVVKVLNTSSAFSREIRCDFLVNAGGILNNWKWPDIPGLHDFH
GDLIHTAAWDDRLDIIGKRVGLIGSGSSGVQILPWLQENAAHITTYIRSPGWIIPTHDGEYHHFSD
DDIARFKADPAYHLATRKKVEQEWGGEFAVLHRKSVAQQAIREQLSAEIRKSLNDDRLADKMVPQF
AYGCRRGTPSPGYTEALVQPNVTVEFGGIREVTPSSVISETGTATELDVLICATGFDTTFRPRFPI
VGRRGNLADFWKTEPRNYCSVAVAGYPNYFMFLGPNCTISNGPVLFAIEVEAEYMAKFFNRWQKEG
VASFDPKQDAQDDFMEHKDLFMEKLSVWGDNCPSWYKDRVTNKVTAVWPGSHLHFMETLSVPRFDD
YDIRYHNRNRFAYLGNGFSQIELNPHADIAYYVRSADAGDFVSRSTMSTFNAKDAGEKLHSKITPT
V
>g3081
MNPAAYTTVYDILNFLINRTRNEYEPRILLNDLEGALSMLAKLVRDVRTMDRGYSEADLQHCLEEM
QRRHEWAEFNSYMEGRSAPPPLILWRAHNFDTPYEVIDENTSNLVRNFASDLHKWNEMESASEITV
TLPSFHELDFTLGDLANELSSHLKKTQINQQRSDRGKDRLHTNLVSLSGDLRWTMHYTSQKAKAAL
NHDEVGIAAFDTKALRSMGIEIWRVKDIIEFLESKPGLLPGGLFDKYAKQWAKNADEYVIWDYIGR
DCLINFCSWVSLFQQPGIDKEKFLLEQFVDSKNLGYFAKDYGWRPMTLKLEEYVDRAADFAQALLG
GDRIITPDTIRITASPLSRPSDWGYRIRDSCQITEDLIMQKIMAEIMKNFVPEDATTPAEDRTVSG
AEDHDDIVSNADHHRIPYIIEKAQNLTIDDADSGLER
>g3082
MAIFSKALRVKDFKAAGDKPEAINLISSDTASFSMFTAVNYILPFSLIKFLFAVLFLLRLLGWQST
LMAVVATMITVPVQTRVIRNERAANKRLTVARDRKTKAITEALQALRQIKFSAIETEWEERIELCR
RAEIMEMRKRFLAISIRSAWKVASPFIVTAAAVCSYVYIHDEVSSSILFTTIELLPHIQGALGMVP
RVLQDYFGARANAKRMEVFLKMPEVRQILSESPNGGVSFRNASITWPSDEGQDLKEKQRELPPPFI
LHSLNVEFPAGELSVIHGETGSGKSLMLAAIIGEVDLLNGRIETQSIGQPVAFVSQTPWLQNDTIK
NNILFGSPLDDIRYQKVLRACALDIDLAALPKSDETYVGLRGVRLSGGQRARVSLGRALYSSAKLL
VLDDIFAALDSHVSNEIFQALTGELCIGRTRILATHHVSLCLPKAKCFVHVRNNTIENLHDVDLVK
SRLENRESSIAIELNRVAEKEPKGKPKITATKTGSDFGFYKSYFVAAGGLLFTTIFVLGLLGKQLM
IALTTWILGCINSKRREISNDGLTNADTETNLWFYLYLYLLGSLSTIVLEFLLNMHIFAGSLCASK
ELFRTMTAKVIRMPLLWIDTTPLGVMLKRFSGDARQVDDLLLEVMREFADCLIRLVVVGCIGMNNS
IYTACLTLALLFWCWEVGKGYAMARKPVKRAESESNAEILEYFTAVTAGLSTIRAFRAGDRFTDQM
HLHVDKLSIARRYFWIFNRWLGLQMCLMGIIFSTGTGIILLFSKSVVDASLMGFSLTFSMGFAHAG
FAAVNNFGMLESYMNAAAGIISYTQLKPEEQSGNDVPADWPSKGEIKIKGLNVAYSADLPLVLKNI
SFCVEAGQRIGIVGRTGAGKSSLTLALLRLVHYQSGSIWIDGIDISTIKLQSLRSKIAFVPQDPVL
FSGTVRSNLDYFKQVPTHKLNDILRRVKLLSAEDSKHAGLFTLESPVSPGGTNMSQGQRQLLCLAR
MMIKNPRIIILDEATSAMDNESDSLIQDTIRNEFNCTLIVVAHRLRTIASFDKVIVIDDGCIGEFG
TPAELLRAKGLFRDFVEKSEDKDFVLNTVFQKSNII
>g3083
MSREGSNVAHVETIALDGETNLKIKKVDKNVAETCSTPAKLANCSVNFNIEDPHSDLSSFRGRVIV
NGQDSPLNNDNVVYRGSVLRNTSEAIAMVVYSGQECKIRLNANADGQAHIKAPRLQYMVNKVVFVV
ALFVLGLSLFNTLAYQIWKSDIEKKLFYLSGATVPNHESIFGFLIMFATMVPLSLYISLEMIKLGQ
IFFLTSDLDMYDEASNMRCEARTSTINEELGQITHIFSDKTGTLTDNEMIFKKMYVGGSIWSDEDM
GYLDKEVHNQAAEQPKRFHPISEQRMESERSTKELIGQLSENPDSTLGRKAMLMLLCMALCHSCVP
EISQDGVTIDFQSSSPDELALIRAAQAMGVYMRVRDHDSIVLSLSTSNDASVRNQTYKVLNVIDFS
NDRRRMSVIVKFPDGRTCIICKGAESVVMERLRLSIQRTGEQVDHANVGALMDGDGRSDLEKCGLS
VTRFANESLRTLLYAYRFLDDTEYEEWKIKWDAATASLDENKDMKEQVAELIETNFELAGATAVED
KLQCGVPDSIDKLVRANIKVWMLTGDKRETAINIGHSCGLIKEISTILDLSNDKESRDTTDAIESH
ILALKSAQHSVVVIDGRTLTTIFNNSIGSAEERFLDLAVLADSVICCRTQPSQKAQVVRGVKARMP
NAVTLAVGDGANDIAMIQEAHVGIGITGKEGFQAARNSDYSIAQFRFLPKLLLVHGRWNYVRTCKY
ILGTLWKEVVFYLSQSLFQRWVGYTGTSLFEPMSATQFNTLFTSLSVMCIGVFDKDLAASTLLAVP
ELYISMGQRNGGFNIWIYLGWTAMAVSEAFIVFFSMLLLFGRAETTKDNTLFSMGDLNFVAVVIIV

SLKLLVLEMHNKSVVAGITLFLSVGAPFLWNLIFAAAYPLTTAYHVRGGFFIGFGQNPTWWLTLIF
IIVCVYMYELSISALRKAFFPTNTDIFQELEQDKTFMARYNGAEAGATVTALEEEWFSDRYSDDVT
IVCENEEIS
>g3084
MAAAVQTAPGLATNQGIEALSQPSKPAKHDVAYDMMYFALEKTPPPYYMGRPGTVLGPMTTMPVIV
HDISGETEKYTLDTHGFQFVKHESKEKDFFDVEKIKAEYYPETEELVKKVTGATRTFVFNHAIRRQ
PVEAADGSVPGRGPTYNAHIDQSYADAPNRVRRYFPEEAEELIKKRYQIINVWRPIKTILRDPFAV
CDARSVDDKDLIPVTMIYPNAIDEKVACKKNPNHMWYYKYAQRPDEPMLFKNFDSVTDGRARRNPH
SAFQDPAHKDDYMRESIEVRVLAFFDE
>g3085
MGSPTDARMMGNITDSVLGEIANARPHPRLSPRSSEDLSTGGETIDLDYPFSSTPGSQPPGGEDTG
LFGDALLGWSSVLDSGXNDNYGFDERYAADILGTLQDTQQELSHQQQPRQQLSPISQGYPQITPLP
RFSNTTNTTNSLNASDIESGYHERSLSPRSRTHHNKQFPLQSPPTITPPQTMVPDSGRNSQCIIVC
SQIISSLEKYQADKLKVLDLILGIVKRVTERLDPLVNGQFEFPNTKCISLFGIILYQLVEILEVGC
ANFLAEAANTPGSISSDILEAGFHEFDFGGLGIGFKDQERFQSQVILEVIRLVFKIIQKVFFISTK
AGLDKDNCYHDRDSRLKTLVEKVKKRREGAGGAMITSLNRRSDAVIY
>g3086
MGIRLLAEPPIPERRKAIICEDVGKLTIQNDRPMPVLKPDTVIVKVHAVGLNPHDIKGLDYSAAPG
TVHGGDFCGTIVALGEKAAAEARFVVGDRVAGLVXGENKLAPEKGSFADYIEATPHTMVKVPEDMS
DEDASTLGVGIATATFGLYNELKIPGGFERLLSGGGNLNEEDREFVLVAGGSTATGTRTLQLLKIA
GFRPIATSSPANFDLCLRYGAEKVFDYRSPTCAADIRAYTGNELEYAFDCIAEAETTQLCYGAMGR
AGGRYVTVEPFRDTVVKVRSLTIQPSWFMATQIFGEDIAMDGVYGRKANLDDREFGTEAFLVFQSL
LNRGLVHAHPVKSMPGGWEGVMKGVTSMRSSPPSGYKLAYKV
>g3087
MSGKAVQFPLIDISGYINPSSPEERAQVVAQIRDAFKTYGFFQCTGHGVPVSLQREMFKSLERFYN
LPKEEKMEISVHKSPCRRGYEASGVHSRDGKAIPDAKESYYFCEDDPKVEMGGIYGPNQWPSLPED
EFRKPIWEYYRKTSAMGKLLWEILIEGLDRSPSTMERFSKRPLIGMNLLRYPAPADTLEGQFGLGA
HNDWGGITVLLQEPGKEALEIWLDDEKEWVSVPAVEDRFVINCGDMLSGWSGNVYKAVHHRVVNKA
PVERYSAVTAWHGDIFATNPFEEAGTSKETVGHLFVKRFQKQMKPSKQLLAAIGVEA
>g3088
MAHPSVRAEPIAIVGSACRFPGSASSPSKLWTLLSQPRDLGFEIPADRFGKGFYHPNGAHHGTTNV
QHAYLLEEDIRVFDASFFNIHSKEADSIDPQQRLLLETVYEAMESGXHTIEGLRGSDTAVYTGTMS
VDWMDQGVRDFDTMPQYFATGSNRAIISNRISYVFDWRGPRESRVAVACGTQVILNPEQFIAESKL
QMLSPTGRSRMWDKDADGYARGEGTAAIIMKKLSDAIADGDYIESIIRETGTNQDGRTPGLTMPSS
EAQSALIHTTYKKAGLDPVNNPRDRPQYFEAHGTGTQAGDPREAAAIYESFGRHISAYETPLYVGS
IKTVIGHLEGAAGLAGLLKGSASIQKGYIAPNLLFKNLNPKLEPYYKSLQVPTELTPWPAVPEGVP
RRVSVNSFGFGGSNAHAILEQYTGSSGAETPTSSDATTFTPFVFSAMSENSLVAQLQAYSDHLKTH
SDISPHDLAWTLQARRSHFPLKIAFSALTIEQLASKIDAKLETLQTKPGTALGVRASSRQTNGAPH
ILGVFTGQGAQWASMGAQLIRSSEFVRNRLRDLEKSLASLPADDQPKWNLTEQILAGKETSRIAEA
ALSQPLCTAIQVVLVDLLRTAGINFTTVVGHSSGEIGAAYAAGFLSAGDAIRVAYYRGLYAYMADG
GSGQKGAMLAVGTSWEDAHELINLRRFKGRLAVAAHNSPASVTLSGDADAIAHAKRVFDEEKKFAR
LLQVDTAYHSHHMLPCGDRVTGSDKPMTAVDELQDEYWRDNMTKSVLFSEAVQNAFASDDQMHLGL
EVGPHPALKGPVAQNVNDDRSTPFTYTGVLSRGQNDIEAFADGLGFVWTQLGAGGVDFQSYEKAVT
GSKDSKYTQPKLVTGLPLYQWNHSKSYWSESRRSRKIRSRAEPNHEILGSLSPESTPQDMRWSNIL
KVSEMPWLSGHQIQGQMIFPAAGYVAMAIESSKILAADRSVKTYELYNLSIPRATVIDDADNASVE
TLVTLTKIQYHQDQTATADFSCYALAVTSTGSKQEMAINATGTVKIIFGEPDVDAISSTPLEDYNL
RAVDSDEFYEFLSKLGYGYTGPFRALTSTKRRLGHSSGLIDTYAYADSEPSRYLIHPSTLDVAFQA
SILAYTSPTDERLWSLHVPTVIGTIRVNAATCALPLPNGSRVPVYSVLEDSEAFSGFIELLSEDGQ
HSMVQVEDLVIKPLAPAAESDDRVMFTHTKFAPAVADGAIASDGQQPSSYELDLSLICERLCYFYI
SKWASELTDEDWKNGPAHFLHLRNWVKHSKSLCIVPTLFNCLAMFPSESFTMVLMLAFNVVLLNVA
KGKNPHIKREWSKDTPEFIEKLVSPYEKDGNVKIIRAVGDNLPASVRGETTILEHMIKDDMLEQHY
KKSLGAEVANEFLANMMKQIIHRYPHAKILEIGAGTGGATKAVLDTIEGAFSSYTYTDLSVGFFGL

AEKMFEAYSDKMEFKTFDVEKLPKSQGYEPNSYDIVIASNCLHATRSLQTTLEHTRQLLKPGGYLL
ILELTMPIEVIRTNMIFGVLPGWWLGVDDGRKLSPTISRGGWHSILRKTGFSGIDTINTDVSPITW
PASVVVSQAVDDRVQFLRRPLSAPATSAPIYIESLVILGNGTLETARVAEELQDHLQRFCGEITLL
DSLPTEEDAFSLNPMSTFINLVDLDSPIFKDITEDNMEGVKRMSELAKNILWITQGALAXEPYHMA
SIAFCRALRTEAPHVSLNNLDISDIQQDNISKSIAEHLLQLYALEEWEQEPQPLLWSKERESFIED
GKLKIPRIVANNNLNARLNSSRRLVTKQKIEDSDKVSFLPSSDTTPASLVNIASLDEHESSIKVET
SSAMAIKVDLSTTLFIAIGREHGSTESTTVLLSTTNTNKQSPVVQTTVPEFSSMSSEKLLILVASE
LLAESLTNNLSSGNHLLLHCSKEDRFLVEALSRRIANQNVRLSITYDSAEKVSSLQGPTWIKIGER
TPRHFLRKALRVAKPSHYLDLTATSSSGQSGGHLGARIAQDLPAGCKKIDASTLFHPESSLPVSYN
ESTLVGHLEGAVTKAITNSATEKEFQSLVVELSQVEEPSTQYYATSAVRWPSDGLVKTKVRPLDVS
GFFSKDKTYALIGLSGAIGQSLAEWMVSNGAGCVVLTSRRPDIDPRWIDSFKESGATVKVLPMDVT
DIDSVESAVKIIRDTCPPIAGVAHGAMVLHDSLFARMSADKMQQIIGPKVDGAINLETAFSEDDLD
FFVMFSSVSMVCGNAGQSLYAAGCGYLNGLARERRKRGLAGSTFDIGRVVGIGYVESAAQVVRDQL
IGLGLKPISEADLRNAFAETIRAGFPQPEDKDTLPEAVVTMGVRHFREDEDVKGPWFTNPLFSHCI
IEKPSSQSSSGDQRKKTSLPLSQQLAQVESKKEALEVIIDCFSEKLGVILQLGDQAIDRDAPLNEL
GIDSLVAVEVRAWFLKELKVDIPVLKIIGGASPTELCQKALELLPDAVLSGIGASKAEVPKSAAKL
DLKVPSQQQVLDSGSSSNTSKANSTPGSMSPTITTPSTAQSFVGSEAGDEVPTVAESQKANAKADA
FPPERRPQKFLKSVPISLAQSRYWFLQHLLADQSTHNVATYYRITGELRVNDLTRAIRLVCSRHES
LRTCFVADQTAEGEASQKIMPSSPLRLEVKKINSEEDVATEYAKIRTYELNLETGDLLKLVLLTLS
PSVHFLLMNYHHIIMDGISWQVFLADLQKAYNGESLGAPPRQYPEFSVTQRKALESGAMDEQLNYW
RQMFPTNDPPTVLPLLPMSRVSARKSMKIYDTHQVEFRLEPDLLAQVKSFSKANGASPFHLYLAAF
KAMLFSFMDAPDLTIGVADAGRSEPNLQGSIGFFLNLLPLRFRRQENQQFTDAMIEARNVHYAALE
NSRVPFDVLLAELNVARSSSYSPIFQAFLDYRQGFPTKHPFGNTEFEIKTVNHGQSAYDITLDVTD
SAVDTIVMIRAQKSLYDKMATVLFSETFTHFLEILTSDPSLTLKNTPLYGKKQRNNAIHVGCGPRL
ISDWPATLPHRIDQVAQDNGNKVALTDGFGNELTYKKMTDRIEAIAEVLITKGVGTGSRVPVFQQA
ASDWVCSMLAIMRVGAIYVPLDLRNPLPRLASVAKNCEPAAILADATTVHDVPQLGVVDAHVIDVT
TVSSKASGPITNSAQPDSPAAILYTSGSTGTPKGIVVKHSGLRNEIEGYTKMWKLGAERTLQQSAF
TFNHSSDQIYTGLTNGGSVYIVPWSARGNPLEITKIMHEQSITYTKATPSEYMLWMQYGGDQLRLA
SKWRSAFGGGESLTTTVTNEFAKLDLPDLRFYNSYGPTEISISSTKMEIAYREKQELESMGRIPCG
YSLPNYDTYIVDEEMRPVPIGAPGEICIGGAGVSLGYFKNLELTEKHFVQNPFATPEDIANGWTRM
YRTXDIAHLREDGAMVFHSRIAGDSQVKIRGLRIELSDIETNIIAASGGAINEAVVTLREGDPEFL
VAHVVFAPNHGVADQETFLERLLSNLDVPQYMVPVVAIPLDKFPLSNHSKVDRKAIKELPLPKKAQ
AVQEDDIELTETMVQLRGVWREVLGKSIKDLGLEIGPSTSFFFVGGNSLLIIRLQSQIRITFNTAL
SLIDLLSANTLSEMAHKIDESASVAPLDWEAETAPPIIPAFLNGVSKTRATAKERITGKTVVVTGA
TGFLAQHVMPQLAARSDVETIHCVAVRDPAKLTLTSPKVRFHAGNLSAPLLGLSTEDFIDIATQVD
VILHMGAARSFWDNYNVLRPSNVHPIKELLKLAAPGKVPIHFMSTASVFGGNDIDTVSAADSVPPL
DGTDGYSATKWASERILERAGATLGIPSSIYRFCPATTHEAAPKELLDLFAHFVEITKAMPDASGW
AGRMDMIPAANVASWLCESTVANVTNNGINFTHYPGKVSVSVDELMPHLENQMSGRDDLTRIPLLK
WTGVIKRAGFEYFMMSHEASIEKEAKLEMRR
>g3089
MLLSRFIQKTTPYATSLLVKQQYRYFSATVPAMTLISQTITHDHRELENAYNKILSATTDDEKVRW
QNQFTWELARHSIGEELLVYPAFEKNLADGKQMADKDRAEHQKVKELLYKFQGLKPSDHEFTTTLQ
SLWSDLSQHIKEEEEEDLVKLESALQQSESEKLTKSFTRTKKFIPTRSHPDAPDKPPFETVAGLMA
APLDHLSDLFRKFPAESKSELPP
>g3090
MSNSPPGKTKDNHNSDQTQAKYPIDLFRGWPNPALLPVDSLARSAATVFSSPSIYEPGLQYGPDEG
YAPLREHIASWLTSFYRADSGDSSTPNDSTPITAERICITGGASQNLACVLQVYTDASYTRNIWMV
TPTYYLACRIFHDAGFMERLRGVPEDEEGIDLEFLERGLIEAEDKAAKEGNIWPKYTFPRSWSNIY
KHIIYLTPTFANPSMKVVSLRRREGLVRLARRFDALIIADDVYDFLQWPADPNSPHIRETASIPRI
VDVDGYLDGGPKNRFGNAMSNGSFSKLIGPGMRVGWAEGTEKFAYGLSQAHRGSSRSGGAPSQLSS
TFVDQLLLSGILQRHIKQELIPAYRARYNRLMSAIKEHLLPLGFHVPVDHQPISGGFFIWLNLPLP
FKGDLLAQRALEEEKLIVGSGTLFQVQGDCSEDFRTFEHSIRLCFAYERYELLYEGVERLAAAAKR

MLSSEDV
>g3091
MAGTSTSETLSVSSSTTNVTRTVQDLDLSEKQIGDTESSHSKESKDEVRSIQGWRWLAVCISVYST
CLLYGLDTTIAANIQSNVVETYGAVEKLAWLGTGFSLGSIAVIMFVGKAFGIFNLRTLYLTCLVMF
EAGSALCGGAPSMNALIVGRIWAGMGGAGIYLGALNTVAIFTTNAERSIYIGLIALFWGLGCILGP
IIGGAFADSAATWRWAFYINLVICGVFAPTYYFMPSHDPQPTKSLRDKLRDLDWVGTVLNAIVYVT
FVLALTFGGATWRWGAGGTIGLFVAFGVSLIAFSVQQTFSIFTTPENRIFPVDLLRKPVMILLYVV
TACSATALSLSIYYIPVYFQFAHGENGITSAVRLLPFIILFVLFVMLNSIVMPRTGWYLPWFIFSG
IFITIGASLMYALVDSQTSNSAIYGYSVFLALGVGSACQAAYSIATAKVDAHRTSDAVGFINSAQI
GSITIALAISGTVFQNVAYQNIADATAGLGFSSADIEAAIAGAKSVIFSDTTAEVRAAVVEGIVNA
IRDVYIMAIAAGXLXTVAAFLLPRERLYMEMTAGG
>g3092
MLEISKAMAFSLTGISPGSTSILYVAVLICSALVLVFYKSCYNGHTLPLPPGPAPLPIIGNLHQLT
GNPYEKFEKWHKKYGPFISLRAGQRTVIVIGSYETAHALLDKQSAVYSSRPELHIASKHLAKNMSM
ALLPFSKKWQVHRRIVVSLLNSAVNKRYQYVQDIESKQVVHDLLTSQDFSSSFERYSTSLVFTLAY
GIRLESIIRPEISEMRFVIHCLLEGLKNITMAVVELFPVLDYLPGFIAPWKQFWQERHNRTIQFLR
KNLHYALSTQKSSWTWAREVQQALHNAGETDDAQVAYLVGTMNEAAVETTLGTLRTAIKALLSNPA
VAKKAQQEIDSIVGSDRLPQFEDLPKMPYMEALMKEVLRWQPVTPLGAPHSTDTDQEYMGYRIPQG
SIIMQNFYTMVHDSNVCPEPYQFKPERWLETPDLQVFSPFGYGRRKCPGEYMGKNSLFIVVSRILW
AYNINHMIRDGQTVEVDEWDIEHHFTSAPAPFEVSFEVRDERRREIIEREFQGIEKDASRILHEIA
PECND
>g3093
MVKPMALISTLLLTAHDAISKSKLSPTNRVSLKEKCAIAVNKLLHGTSYQYPAKVCFDQTLKKKVE
NSMLKAGLSSETLARIQPYIDSSVNISLTCYAHTSPEVREFVAIYTSYAITVDDLGHEFPDDMKGI
VSSLLNGHTIENTILRGYFDLMRDHGSQFGQFGSDMIAKATVDFVCSCYVELELEQRKGNANVNGD
HHHTVTAPEFAEYFRIKTGVSEPYAFFAFPEHLAREDEWLHTYLPAITYMVKFFNYANDVLSFYKE
SGAEEKANYISNQAAAHNISRFESLEMLCKRTIEVFHTIDAILAPNEDLRRKVHQWMHGYLAYHMV
SARYRLDELGIPAVVEARQLFFGKD
>g3094
MPSLDKLPTEVVNLICENISSSIQCQLISTCRRLKTQVVPILYRDVTLDVREEDQKSVDSQIACFF
RTIRDNLSLALCVRDLSVKGHRPASIEVGYIPIFENDEKDTTDQQTHQPDTRKLVYETCDTNSVIA
AMLHKLENLETLHLEYQFWSENGFLPVIYQNCLKKLRKINLGMMFHPYYTGREGELWERMQGSDQM
DMSRLRSLMSLPLIESITCVAANDEKDEEIEFASMLPAQNLTYLKLLRSKMTPRGLGKILSATPNL
RQLEYEFWIDMKINEDPTVYYDGTVLDNALKPVRNVLERLRLNIRHNGDYKFNGCSWRTEVRGILR
SLASFTQLTHLHLPHLLLLGRSIEEANAVKFVNMLPRSLKFLSVFADSLPPLRPFLPVDFTNEKLL
DRLYEYHRMES
>g3095
MATSAGKTNTIYQTSTIRPTITATPPPETTSGIVTAWIPLTTVAPPPFDACWNMSFTNDYDTAFDS
VPVAFDPDFAFQIGTEVVGCQPTEVTMWFNHATPASGTNTLSTVTSIQPVVCPSMWFTAHTSTVRN
SVQVTCCPSGYTADPNDDHGVQANCASSMTPGQTFAYFNSNKSTITTYFSTSITVAAVAVKGWNSI
VEASATTTTTTAGYTSSSLTSSSFTEPSSSTIDSQTSTGSTTGPSNVSNSSGLHGGAAAGIFVGCF
AAVALIGAGVFIFLRWRRQSLATGDVSGENSNRFDLKSGQYVNVPQNASHRLSELPTASNTPELYT
DYDPYSPRWNSAEIHEMQ
>g3096
MLSAPVKSARRISSLFNMKTNRDSASSSTSSGSPKQLPSSTHLSVDHRRARSHSRPAVRHASSPQP
QSTEALPTTSHGFDPDHLDLNTPLPPPPSLYAINQDLGDSGPSQRSHSRSASATRGAGARSRTPDP
HSHQRRRSWMPGKSNSIVIDAPIASDQYDLNAFDAWVAGLDQKIPYDLTRLMQGEQIPELWNEQGD
TFVYLFPQGTQRPPSFRIDSTLFSDSPSLNFLARGTDAKMEALRHSARTLTITDPDGATAQEDQWV
NDDVSNGSNRMALVEDQPEEVQELHLYLPVPLHGDVSSVDSPLSIEDSDTLILFRNLFAFLLGQAL
IATPRYPSLFAVLLEISNLLRRFEFSNFDGSNFGEIATSSFANYCDELHLADVRKSREKTIEAVIL
GESLRYYPLYVEGFVHGVGKMEELRSLQSPKFNLINPLTQKRLERGAIDLQNRLTIMRIKLDEFDF
PALFSGIANSNSAAESKIVRFKQWKLAHLALRKHVLSFYRQKYGSWPPKANSKKNEFEESGLNRLL

LKEVYEDFTDLYDMLVDRTSLTTRTTDMATEDLDVPDPIEAVTRAMRRMMSEYDRSTPPVQPPIPF
DIPQLPSLQALRRKPVDAKNIAKERQKKLHDSEINEVLMRSYTREALKPTPFVESFMQFERRSAHG
KSLDDLMDNRIGQWLFIYAVLQSLPLLVIDVLDLRFTDGVEYFLCVAPRGGAPWIQNDGKSGRSWF
GVAGGQGVVSLPSDVVNNATDAVYRRSHCWQVASQWAERNLNSPITTEDASSLFSAPPAPQSSIGS
ISDVQSMLSPGTNSPHTVPRTNSPGFASHGHRGSIYNTLEALPLPAGVIPVEPPAKPFARMNPTMS
FDDILKDIPATATKGGKKK
>g3097
MPSPQTETALEGGTPSDATRIRRIVRKIDWRLIPLLFFTYMLNFMDKTILSSASVFGLIDDTHLVG
QQYSWVSSIFYFGYFFWEYPTNILIARLPVAKYLAYTTFFWGAVVALTAACVNYGGLLAVRFLLGV
AEATITPAFMFITTTWYTRDEIPVRTGIWFSGNSIGGLVASLLAYGVGHIEHPLHPWMWMFIILGV
LTFLWGFVLLAFLPDSISNARFLDAEEKEIITKRAIIKGTGRTEQVSWRWDQTRECLLDPKTWHIF
CITILTQIPNGGTQNFANLVIKSFGFTSLQSTLINIPASVVSAASIALTGWLAGRYAHLNNILIIV
VVALAVTGSAIIYSRAHIPHKGAPLFGYFLLETGPAILPLVMSLVQANYKGVTKKQTMTAMMFIAY
CAGNIAGPQFFKTKEAPVYATAFRAILICYVLAAVLAGSLRFYLAWVNKRRDREEGVVVEDTEGGE
MRVQGKDVDAVVVTDVTLQAEDYDDVTDWNTVGFRYRL
>g3098
MNGLEISPFIAALPKVELHIHIEGTLTPALRWKLAHRNNIPLPYETYSELVDSYKVTYNHRRELNG
DNGAPTFLETYFAGCQVLCTEDDFYELAMDYFRKAAEMNVRYVEPFFDIQAHTKRGVAAEAVLDGF
LRAQRDGAERCDVRSNWIFCFLRDESVESGLEAYRTARPWATTKDGKGKGLFHAVGLASNEYDRPP
LLFEEGFRLAKEDGLHVTMHCDVDQKDAVDHVHEAIFQVCAGQGTERIDHGLNAIDRPDLMQGLKE
RNIGLTLCPHAYHRRQATETLFPKIRKLWDEGVKICINSDDPTYMHNVWIDGNMMKVYEYCRFSEG
DMIQLVRNAVEMSWAEDEVKQEILKELEEFAVSS
>g3099
MSRSRSRSSAGSEHGDRLEPQLLRAATNNDLTALEQITHLARQKGQLNPTFLGIGLVKSCERGCVE
ATQYLLKEGASVEVGGNRAPPIFRAVERNYVQIVKLLLDAGASPEFRDREGRTALMMAAWKNQWHV
LQLLITRGADVNARDNRKRNILHNLAADKQCDWGDDVVNLLLRTSCEIDARDDLGRTPLHWACTTG
KTHFAKLLLSRPQGCPTDYIHATELRNKTALHLATAHDREDVVELLLEYGADVNARSDGGWTPFHN
ACDKGCEKIVRILLEAGADFNSQLLNGMTPLHLAAQAGHTDVVQCLLEQPSLRRRTRDTFGSTPFL
RAAQFKRKDIVQLLAPFNHVEALSADAEGACKGFDATIVDFGNFHNENRVKKVSVYELLYGRDPKN
PRKQAITTLPTNVKASEFRWIHLPANNMAWVEALMTKSFIEEGASDVEGFKGLEKSFGHQHRGQRP
HSHFMRPLCQSTPRSLRGNDEVEKKEEHAPPEKSPLPQVVINGPVVTKPAEFSPTPLRKRETGLSK
DSYSQDDDSVSGRKPKAKHKNSTNNKSGKDNSKNDKTKSSGHGPDKHARSATPHLARDMSFAAKCN
ICVFMPYLHFETAQSQQKMQQTIKQAETFVSAPQPGLTRASTRDEMLIRAHINSSTASLHVRRTLD
QFFYPNIDTETRDGDQVVYRYQTRWTEHKAKDPKIFMVDQLWMWVLGKNLIVTSFPQRWEQPKNDP
LNVLDGIIEDINSKTREPVRSVYELATVITARCSGMFDRHRMGDEDYQFLDMFESCIGTATDRETV
LFREFNVASAQASEWLKHHRRMRRSIVENISAKEDDSPKGKGDGDGNNDKEDSQPLFVDKLLDIGQ
ETDLLAEAKDIRDELNMIRTVLEYQKQVLQDLQDAICEIYYDENRSQNDIKKKFREQQRNIDMHNK
DVDRMDKQTERIYNSITDLLDLKQKHANAFEARFARDQAAGTARQGKTIMMFTIVTIVFLPLSFIA
SFFAINVAEFPHDNQSGNTSLPISYVARYIFGIGFSISIPLILFALSVDDIRLVSRVVMRRIREWR
STHSSSSSKHNAQHNRDVSHSTANQQRMISQELEIQRILCRSKASSTRRLSEEELDNYFGTGLLPV
ATRNTHASSSVAATGRPRSSIHTYRSMGRSNGIGRDGEKGFRFG
>g3100
MSIFFEPRSHWGHPASFMQQPTPANAEDQPSDDNAERPGPRCGGPHHHRGHRGGPHGGPRGHRGDP
FGMRGGYSHHHGQRGFEHGHGPGPREEEPFGPMRWGRGGLFGHRGGHHHPYHGFHHRRGGRGGVRV
VPIPLGGPATGFDFLNRFASEMGNGFQDFLAGNREANDDNKTQVDFEPHADVFNTANEYVIHVSLP
GAQKPDISVEYDAQKSTVRIAGVVHRPEFSEELNAAMVWNERRREVGVFERSFKFIPSGSETVDVD
GENITAKMVDGVLVVRLPKTQQEQEEKQNKKIVINEDDDLYDADPVEKETNVERNTEDVPTDEKHA
SVVEVQDEMDSMHITSDTEAXDLLEEADHIIYTPSHLSEEDAEEAAEYVKVDAK
>g3101
MSPTPSSTTGRPEATPSSAEPDDIRHEQEEYNEQHEAALAAEFLEKAEQARIKHIWRLCFTIFALV
QAGNVVLQPPLVQLKELSSCMSYYGPGWSLGRDCRVEYVQNELETLIKWQQLLDTAPGILFGVFYG
MAADRFGRRPVLGLSLVGILLAAVWTQVVLFWPTVFPTRLTWLSVVFQLAGGGTLVANAMIFAMVS

DITPEEKRASKFFRLYGVALLSDMVLSPASGALASIQPWWPARFGLIAFIMAILTTVLVLPETFVK
PEALSSTNLAQTTPVEEEDPEDEEDDPVLYADAPKHTPWRRVRKVVSSLRDLRYLIASRQVLLLVP
LLSVGQLYDQSAGFFLNYVSKRYGWRISQASFWLTYRNVVNLVLLSTILPGVSALLLKRGFDAGKK
DLWISRASIVCLALGAFVIGLAPTLTIMVTGLTIFALGHGFVPAVLSLATPFIEPGHVGMLYTAMT
IGETIGKIANEPLLTGSFELGMRVGGLLLGLPFVATGVLLTITALGRPPSPFHLVPVLDGSYPQLD
FWRVGDIYETMHDNNRKRVHALLSLQVWHSTKSSR
>g3102
MTEATKQIHIALDNTGDGPLRVPGFANIPIHYELPSNARFAHGVVEWRQAPAVTARELTMVAVMNH
LTDRPSWYLEISDDEVVANWKQETFDTTPLMSEKAWEWCVRELRDKAVDLRESRQIHIRVLDTGSC
VCKADAETLDSLSAAFRESMPALLEEQQEQRILDWQSETVLIVVDPLLFPLVYGRSLVLVDGGQVD
FDNVLGSYRDATVAPKPYDRRTNSESLQMKIDRLGTPCWGIGDPDGDKFEFYHWSANYQCLPCEVK
FLHDSGTEVQITSYINNLHPAHEGLYQAIEKLISITIKPWNDCLVKGQIGWDDDSNQGQLGPVPLR
IITYGAEWENELPEWALTFRVPTEHRIKKYHKFQEMLQSNKDDQTRNGRRKYRHAQGQLRRLRDVS
DNLDKKLPPPESDLWKRAKEYLELPENGSNTPVAAPDGWMEGDSKPWWSIERKLERLLRFKHPEPG
TAFSYEEWKTNRHNDKAIIDIVRHRNNWAPDRQPFKPIIPPHSPYTISLQDTFRKQGLQIIVKMEN
IELTPQNPTYTNDSWQLDGQLNEHIAAVAIFAYDVSNITSPKISFRQNTTLYGDFYQILEELYTTG
NYDARTLRAHRCGKGYGQDLWAVGSILGYENPWHLSIDYNGMLSYQDIGSVATPQGRLITFPSVLE
YKAEPFQLLDATLPGHYRSIKMFLIDPHYRVCSTRNVPPQQHHWWTQEAGKELTSAGLPYELVDEI
FRGTEDWPMGREEAKQHRHNLTREHIWNETARFNRMPGPGF
>g3103
MASVLTTTGGDEYVGRVAEVIAAAFSNSIFTAYLHRTSESTWPSTQVPIEILRPYFEKSIAHRVLH
GAELVEAGNWAAVAVWVPPGISIPASGALDPRIHEYRDRFAEVKKEYMQDRQYWYLNLIGRHPDRT
EPGVVRALIDPYLHRARAAGIPVWLEAISEHGRQVYEHLSFRTVAEVRLGVGKVNGNGELDKKGEG
LVVFGMMAE
>g3104
MTDPSVSKSLPATVTTSSADSLGDSAAETIRDVSGDIIEDGLRRGLKGRQFVIIALGSIIGPGCLY
GLGLGFSETGPLGVLIGFVITGISMWILMQSVGEIATLFPVHGGFVEHCGRFVDPALSFSMAWMYY
FMWSVFLAADWNNAAVILQYWVPTTAVPIWAWYLIFWALFSTITTLGVGFYGEVEYFFGMFKFIAL
AVLFFISILSNVGAFGNGYVGFRYWGEPYGPIKNGINGFGQVFVLAAAFYVGTEIVSVAAGESRNP
RRDVPRATNSIVWRILFVFIGLAFFQGLICPSNSPDLLSADSTVASSPFTIGFVLAGWKSAGQFVN
TIILICFISAANGVVYIQSRTLYSLALKNKAPKFFAATTSRGVPYRAILFSNLWGFLSLMSLSTSA
GEVFVYITDFGGTAAYIAWAAITFTQLRVRAAAKLQGIDRKTFPFVAPGRSWIYWANFVFNIFILL
IQGFTVFETPFNYKTFIACYISIPLFFVMFFGYKWWFKTRWVRLDEISFVDRRIYDESTFEVDNRT
WGRKILDFLKN
>g3105
MNNIIVVVSALGTQGASVISACLSSPVLSNYKIRALTSNPTCEAATSLAASNSRITIHGTDLNSLK
SVTQAFENATLIFANTAFDPQTFIASGPTAAQTREETQGLNIARAAAQIPSLKHLIWSTLVDTDSI
TDGKIVTPHFQSKIPAEKYLRNPENGMADRTTFLHVGLYASVFQRMPYTPIFVKEAQKYIITLPCA
TQTKIPFVGDETLNIGLIAHSIFMQPEKTLGQTVSGAIEYLTGPEIALAISNALKKIASQHPGAIQ
STQVAYIDTTLESYEALWGSVGLETGLMFKFINDFPSIAESYGSDGRSKSLLTPAELGLKEELRLL
EQRLEDLDWKTALA
>g3106
MRVPYVPEDVPVQNQTESEILARIVERRGSHGLLPLDRALLHSVFVADGWNTFLGSIRTKTSLHPI
LSELLICRVAILNKASFEWDHHAPILVDAGFPSDSLHVLKLDSFENSGPELQANQLLSPSQVAALA
YADQMTLSVKVSDDVFERLKQYYNTREIVEITATVSAYNCVSRFLVALDVDERAGEEDASK
>g3107
MLLLVSTTVEFLSGLFSWSNADEGTPADGQGLMVDARLPELWFEDIPHCTLRCLYGPALACGCAYS
DFDCFCRAFSEIAGNAEIKNCITACPHNEDGRWGPDRILDFCQLVTGDDIQLPNYMNQYAGVHRRQ
AIPVTESQKGGTYYPSFPFISTISTISTSAQPLAAATGSVITSATSFITTLEPTTTISTGVAITSA
TTTSSSSASSSAPPPTGGLSTGAKTGIGVAVPLVVISILLGIFWFRRKQARDKKVHQGTGSDDIPD
MSKQFWPRNLSSTASRPLPAEADSNPIHEPDSNAIHEFDGNPVSSSRHLRSSDTPSPIVYELNAQP
SVRAPPPAVLASNKWGESIHDAAFTEDLSTNETLSKASLSSPVFVNDPKPATSREVDAAMPGKTTS

APAHLHHQSSDNNQEQTQLSPAKPEKNIDTDLLDLEAEIVRVREQRERLQHLQMLEDREEQLRKEI
QARKSGLSKSG
>g3108
MKAVISPSAWIGRCGRRYLSFISTTPFQARNNTFNATTKAQSFSTSPLTAASPVSSPEICLTPTQT
TYSQATHPSSHPSTNPKILVLGSGWGGYIFSRKINPAHNNCTVISPRSYFVFTPLLTDTAAGNLDF
SSIVEPMRELKSSVDFIQAAARKIDFTRKRVLCEASIVKSGVTESPRVEETERQFEEGPETGSMRG
KEHLRGWERGQLFEIPYDKLVIAVGCTSQTFGTPGVRENAMFFKDIGDSRRVKRRVRECFELAALP
TTTAEMQRNLLHFAIVGAGPTGTELAATLRDFVSKNMAELYPALKDKTKISLYDVAPTVLSMFDKS
LSQYAIETMSKEGIDIRTSHHIQELRWGMPNTEGPHEMDPKGCLTLKTKEQGDVGVGMCVWATGNT
MNKFVKYSLNEIDEFPTDSALLKLPSASSSSSASDSQIIEAKGWKIKKAPKVGALLVDSHFRVQLE
NPGTGQVAVLQDVFAIGDNAMPETGAPPATAQAASQEAKWLAERLNRDDFDSVPGFSFRNMGTLAY
IGSSNALMQIPHEKTAAPGGGDEKRRNPYLPEGLTGRMAWLVWKVAYLSMSISWRNRLRILFRWMI
NRVFGSDVSRF
>g3109
MFGRRTRTTRTTKPTLMTRLKGPNARHKTYKTEVTRHGGHGHTTTTGTTTTRRGYGTRSTRAAAPV
HHHRRKPSIGDKISGAMMKMKGSLTGRPGEKAAGTRRMRGTDGRGSRRVY
>g3110
MPFSSFTSVSFSASASATRNGETRNWGHRSAREVYRDEDNTRHERSLSHTLGQEPVYEERHYDAQG
RQVIEGRPQQQQQQQGRIEDVSEREK
>g3111
MALLRRGASRASNCTSKSLTRPLAIYTPIHGRRHISTTNTEKPPFNPNQPIKYTQTPNPTWKLGDG
AIDNNENKTKKHIPINPSDPARAPSKNYKFLISSIVPRPIALISTLSPTGKNPNDGKAKLNLAPFS
YFQVINHDPPMFVVSFTPPGSRSPKSQKDTLRNLTETGECVINVVSEHMLQAANATTINAEYGDSE
FDYSGLTPAYDTETVNGVPRVLESIVSIEGHLIETKEFESRVAPSDPKKKKGRTTLAIIEGTRFWV
REDAIDATELAAVDLQVLRPVSRLGGDAYGRTTEVVEIERPIKRS
>g3112
MAAPQLPPIPPLVPAEHDWQTSSTDSTIHQRRAIGAENIVGLEKSNAAGANDLYLISSVQFADSNL
TLSKLKGKVERALVKHRYEHPEIAQTGLWEEGQLSALIQYKPLTSNEEALTWAKGIVHVRAGKETG
LEIRTEHEIKRFQEKTGSAKAVVVDIVAPVDGLDAPLGSTNVEFVFHSNHLYFDGISKRIFVGDFF
RYLADTDDELPDLKWGEEVKNLGAPVLSLLKDGTPTSGPEFDGTFGQYAGGAMRAMNNYGLDPKVG
YGLPRVIFHTFTTAESDAIYKSVKSKVDPKASPAHLGHAAVLLALLKAKPTTAETPDSQVYMSSSP
VNGRRFLQDAKLGKTYYPLVQASCPVTFENIKQYNLVDADKETIKKYLTQASKVAQDSYDVWLKNP
LTLPVAISFHALVTMLIASGQVPETGLTAPLYTSDGVNETYIDREISDKSGNVAITVNTVRFFLNQ
YMPFLSIRLDSFRGRSELSLSYNDGQYTEEEASAFLKDIAFFMLQFIE
>g3113
MTASQQSAPESLTLWGWLCLLAKVGRIVAGLIFKAVKYPLLSKKLKTKRSFHHYIAYEGMIDFQSG
LSSVEKHAVLPSTAWQCTSFAHRNNLPYERLTLQDGTLAFMLNGSSSSTKHALGLNPFTRGSKKAA
ALMEEMIVAKQKANKAIVYFHGGGYVAPILPQHLHLIYSFEDKPRYRDGVVVYVLAYSLVDEHANH
YPTQLRQAISLLDHIINTENIAPSNITLMGNSAGANLLLSVLLHLSHPNPDVPPLNMKDDEQFTAA
VAISPWCNMDTSAESMTTNANRDVLAASALEYWGGNFLGGRPLDPWNSPLQAPAEWWTDLKVGELL
MIYGEDEIMKDDTEILCKRVQSVHPQTTVYGLQGESHEQMAMTKLLLLPWISDSEKIYTAWMKERF
Y
>g3114
MSNPSPGSKKIGASSDIFRATPNTTTTTTTNNVNININKNAPPPPRQIRFVSSDGQPQTKRRRVTA
AYVCVALVASERFDVRVNNPCLGYNDPDKPERSSVPGLMKNGLSDTTYTDNGGDDRSLSRSPTTPR
VTLAVPAAPVPKTSKSPQPKTIKLESPLPLTKDTLNKATRDQDKLAAVASPESNSIRSLKSPPIAA
TGPSLVSTLAEGESNSIPFYDREDSLPTSNVVTHLCELFFVHLGCSFPFLQRNRFMADLKEKKIDS
ILVDAVCALAARFSPHPLLSPAQARPIDGEELQPDLKRSDHGQPFAHRAMTAVVNALSCPTLSVVQ
ACLLLAYEEFGSNHDSGLWMYLGIAIRMAQDLGMQKLQGLKHRYGRTGLRPKAIITGQAGKLNVEQ
SEGPQIPAKRREGGEGRGEDSQRAMEREQVDTFWSVFFLDRVISSGTGRPVTLRDEDIELYFPLQS
ESILPNGWPAPFPPLIRIIHLYGRVTDLINAIQEDNHIDAETLKRLAGMENDLTSIYQRLSPKLHF
NTMNFQTYVKAQEGTNFILLHFWFHTLIVLLHQPTLLNSFGGRIQQLYPNSRELSMSSAKTIADIL

SFSELIDAKSFIGNPFTSQPMYIAACAFLMESAYYSSPSSRSGSPPVLVTDQSSAFTIPNMDSAHT
SERKPNTKHSLLASAAKENYQRCYRALRTLQTYWEGTKYILTVLDQKAKGIGDPLLYTTEEMESTT
EVAYAQSLASPNWQPTLQPNTGELAGKESESQAAQSEMGSPKIDPGQAIGWALTGETNSAQPNLSL
LYQLPAPQLDEASGGQVPVKDLPTVETHRAGHSYPQSIPTYHSGPTNSSNILSPPVPKFSGPGNNN
IIASNPYATTAPSHAVFSSALTPSQIESPYTFSSTSYHQHQQPPQPYTTHNQYAPSHLSDTLIESQ
DIDMAAFHNQAEFPFIFSNDFNPYLEYLPPDVINYFGDSSSSAAAAAQQFGAAAALLSPDEEQRAS
GQQRL
>g3115
MRWGGHYSAINSDPDGDHSEPLEMDNIMREEARGFESIPQSEELSTKGPNDSTAATKPSPLRKRWS
DPVWWCVSGNSIILIFNVIFIIVATARAGSRYGGVAFGSNILYEGSCTTARDMKIGIHLIINVFSV
ILTATSSFSSNILMSPSRADVDRAHSERNWLTIGVFSIRNIKSLRWPHRTMWVVLTVTSLVVQFIY
NSVIYSSLNANTYAALVASHDFVSNSSIPDSEFEAQSCFAESDLAWNVSALRTSVLNGEFEVLDPQ
ACIDAYAVEFLSDRRTVIAVTSDPMTDNSTRLYISGYGWPARIYDAIPNNTIEKSGLLTEQYADET
VDSSSYNTGDPFKWICSGNDWNGTYKYQSCTKSQAQDMSPWTISGMAWAPFTAIQQPPNTMLDWMT
VAWETDTDLDSIWTGLSDTERTDLRAKLEANPTLEQMQSFVQGRTWSNDTFGDALVINASCSDKEW
LPSDDFLVTAPQRKMAFPIDHCLSQYVDEQCQLLYNMPIGIVIIICILLKVICLSFLVNIDRRNLF
LTVGDAISSYLQSPDPLTKGWCMLSKKTVKRNKECPWNPKCEQEVRNAPLAGHPHPENPPTKRRIW
SNACKPIVTLILLPILVIYIAMTQILPQFAAAAAQGIAYGDISVSQIWDIKGFGAVQSNALLTNLA
RTYVGMQLLANTPQLLVSLLYFLFNDHLTRMLHAADYNQYALTRRPLRVSFPQGSQRSTFYLSIPY
RYSLPLLFTFTLIHWLISEGIFYVQLLPYDLAGNPILTSKLMTCGISTIPLEIAMFLTIACLVVVW
GLSAREFGCAKMPFALGCSVAISAACHPPKDDRHAAFGPVMWGAANADTDEVAHCCFTSFEVTEPL
KDRLYA
>g3116
MAAQSTRQWGVTPPISVALPTPEELAANDDLIAELKAQNNFELPSETERRQQTLQLIQKVVIEFVK
KVGQRKGLSPAALEAAGGKIFTYGSYRLGVYGPGSDIDTLIVAPKHVMIDDFFSDFPPILERMSPP
GAIEKMTPVPDAFVPIIKLEFSGISIDLIFARLILPSVPLNLDLKNNDYLRGLDDKEVRSLNGTRV
TDQILELVPQQKTFRIALRAIKLWAQRRAIYSNIVGFPGGVAWAMLVARVCQLYPQATGSVIVGKF
FRIMTKWAWPQPVLLKPIEDGPLQIKVWNPKIYHGDRFHLMPIITPAYPSMCATHNISLSTKAVIL
RELQRGGDIVDKIFAKHLTWNDLFTRHTFFSKDYKYYLQITASSKTKEAESVWSGLVESKLRHLVG
ALDRKNIIAVAHPFPKGFERIHVVKDDAEAEQVKNGSTKYQAQGTKTETTDEIKDPAHTAAAENGA
QDVQVPDPDTNGTVDNRTIYTTTYYIGLELKPLEPGASRSLDISVDANIFKSTCTSWQGFQEGIND
LSVTHVRSFDLPDDVFEPGEARPIRPKKKVAKPTTAAAPPKRSIDAVDGTIHPEAKRQVSANGFNH
TATPA
>g3117
MTITISPVEADDIKALIRLNEAAFSWTYSLLFDGPLKESTIDAIAEKRIKALHEQDEKSGSQGQEH
PANKSFDFKAVDDSTGEIVGEAHWMIYYEDEELTKSIEDEVEDRINPPIPQMQVQPTAEFGRILAT
TKRETLAVPSETGETTADPGAPIKLRKRVYLGVLVVHPNHQKRGIGRQLLQWGLDEADRLGLVTYL
EASTDGVRLYESAGFEKLKDVFMNLRPFGAKGDLPIRLMIRQPKPTN
>g3118
MAKEAEAQVQLQDFQNIFSLEGKVAVVTGGSRGLGLHAASGLLQAGCSKVYISSRKAAACDEAVAA
LNALPNKRPGAQAIAVPADGSKIADLDRLVAEVSKTTDHVDILFANAGATWGAPYDEYPENAFSKV
MDLNVKGVFYTIQKFTPLLEKRATLEDPSRVIITASVAGIGIGTVGPHGTYAYSASKAAAISLAKT
LAVELGPRHILTNAVAPGFYPSKMANGLMELAGGVKTLAAQSPNGRVGHPEDIAGLVVFLSSRAAL
HINGSIITTDGGRLLGDNKL
>g3119
MSSYAVLPAAAKDEEELYKKMASTNSTTSPSRGYFLVFFLSIITSLVFGVDMGWQLSAHYAAKEQI
LSRSSGLLSDLGGVKTVFKYDRIYAEMPTNESDAAWKEIFPARGGFFKHPQLAPGGSGLAVFHQLH
CLDNIRRGYWSLTDDGRHVHHISPAHIRHCIDYLRQSLMCHADTNVETLDEMLHGVHGFGVEHKCR
DFEGVQSKLVRHGTTSARGEEATADEDVTWNVDLYQPGTGERRSIVDARGEEETADEDVTWNVNDY
TGGSADDILVALNCMILVGSGCLTIVGATIGGIGANLAGVDADTIIAFASEFFYIFTVGIVKLSLL
LYYRRIFVLSLVSPGFRRMNDILIGVVGLWMLAFFITTIFQARPISWNWTEIGSVSNLHDFFISEA
ITNIALDLIVLCMPMLIVYRMHMSLRQKILVVGIFGLGFFCVIASAIRLYYVTKFFAVSIQAHPKQ

FEVNSFRAIFSLRSDSSSQDQVNGNRPPNVQDSVKLNTLQAAEQLSRNDSHSQDDAYHPKADQMLD
YEERGLRNHTGITRTSSPDSIILRIFTPLDPIPELRGIILDNTPQKLKGYQDIQLTTTMLYLYLII
FCVGATALWKSKQNLKIKHGKPIPGPRGKPIVGNLPDIPKIHSWLKFKEWSDEYGPIFQLNIAGRK
HVVVSQEKIANDLLRERGSLYSSREYLPFASGLLSNNLRPLLLPYNDLWRRGRKLMHQLTTSTVVN
SYQPVQDYESKRLLASLLEAPRAYEQWFERYASGVVYRLGFGRWIETGEETDFKRIVCVGKELERV
ASPGQYLVDSFPFLVFLPSCIAPFKREAARLQAEELGLFRKLQQDVRDGIQSGNTAKSFTRTFLEN
QGAFRLTNDEAAYVIGTLFEAGTGTTAAAMMSFCLAMCHHPEWQTKIQTELDREVGDRMPEFYDIP
KLPTVRAVIKEVMRWRPVTAGNVPHQLIQDDTYEGYLFPKGTVFHANQWAIHRDPELYPDPENFHP
ERWLDASFPTYREPLTQFPNMQNYSCFGFGRRICPGQSIAERSLNILTARIAWAATLSRKKDAQGN
LMPLPLYDYCPGFNVQPNFFEFDVTARSPQRAEQIAMAYEEARLTRDQILT
>g3120
MAFLSQILYLLLLGVAILTLRRRITQSRFIRKHGCKEAPSLTGNKLFFGLDLQFLFLKRSSSGLEK
LPLEKQFEVLGSTFRTQMAIKPVIRTMEPQNMQAVFSLDADSFGNRPLRHFAFSPLVGDGVMTLDG
ARHERARALIRPTFSKTHIENEEAYDLHVQNLIKLLPTDGSTVDLQPLFERLDLDSSTEFIFGESV
QSLHEDDSLTSPHKFPAAFDVAQQGMGARFRMLPFNFLHRDEKFWGACSTIRQFVAESVEKAVARR
QKPKEQTAKSYILVDNVVQETSDKTEIQDTLMNVFLPGHDTIAILLSNIFFHLARNPHVWQKLRKQ
LLSLSKITTVNLRRLDYIHHIINETLRVTPPVSNMTRIAVKDTTLPLGGGSDGLSPLFVPKGTVVA
SSFYNLHHRKDIYGSDADIWRPERWEELKLSTLTWKFIPFGGGAHVCPGQNLAMNRVAFTVARIVQ
TFDKIDNRDPVAEFVPLYKLVTASQNGVKVALHNAVDS
>g3121
MQPAIYAAFIGLLIAVYILRQRTRTPSLPLPPGPKPLPLIGNVHQAPKSHAWKTYREWSKKYGSIV
HVNMMGQSVIIVSTSEVAHELLAKRGAIFSDRPRLFLATELALKGLNVLMMNYTKQFQQHQRLQVS
VLNATPAAAYFPFQALESRQLMHDLLTQAGGSGADVRGIFLRAVASTIHSLLYGFRIRELNDPVLR
AIVRLNDEFSDFIQVGAHIVDQFPVLNHLPGFLAPWKVKAENHYKTKYELRDKNFKEGLESDAWNI
SKHLKKVVEKDDALDMPLHELAFELGTMIDAALDGTTDSLIWFVVACITQDQNFITKARKQLDAVV
GRDRLPGPDDKPNLPYITAIVEEIFRWRPAGPEGVPHLNKEETIYKGYRIPKESVIVPNVWTISRE
ESLFGPHDPDEFVPDRWLEADGTTFKALPAAAFGYGRRTCPGRYFARNGIWIVVAQLLWAFDIKAG
LSDETGESVDVDPIACTYGLVMRALPFKARFVPRGPWVSEIVGRDGDTYGVDYEDLLNEIGLEFDK
Q
>g3122
MSEPPAYISISTRKWEQVSNLIPAEWRLDGKYVPRAMRVSPLESVHQLDTFEHEYETNLLDVPREC
GILSAKEVNITEGYDVKGLLREIAEKGFSAEEVALAFCKRAAIAQQLTRCLTEPLFTSALQQARNL
DTHLARTGQPIGPLHGLPISIKDSFNIAGVDSSLGVAALCSKPASGSDASLVQLLRSLGAVIIAKT
NIPQTLGLLDSVNHVFGRTLNPLNQRLTPGGSSGGEGVLVAMRGSMIGFGTDVGGSIRIPAMCNYI
YGLKPSVGRVSYEGQTGFGLNGTRAVALKAVAGPIARXVADIDYVMREIVPRGERIANDCIPGTWP
AASTQFLGKRGKLRVGILRNDGLVTPLPPISKLLEEVSRILAGSKIADIEVIDIPTPPIMKQCFAN
AGRLMSVDGGVPMLDLISSTDEPLVPSLKHTKRRKPYTVEKLCALNAEREDIEIAMRKALWSSGNV
DAIILPVAAHPVPRHDQYGCVSYTSAFNLLDYPAGIIPVRKMTQADLELEIGGEKVLSRWDADNKK
LWDRSVYLDSPLSVQVITPKLHDYELCQAMGIIDETLKDYYARNRAERAML
>g3123
MASSAPVQAVKSAVEPEKLSGVGLYSRFAFAGAVCCSVTHGALTPVDVVKTRIQLDPVTYNRGLFG
GIRQVVANEGAGALLTGFGPTAAGYFLQGAFKFGGYEFFKQQWINQLGLETASNNRTAIYLASSAT
AEFFADIALCPLEATRIRLVSDPKFASGLLDGFAKILKNEGVGAFYSGFGPILFKQVPYTMAKFVV
YEKVAEAIYGVVDKNTLSDGGKTAVNLGSGLIAGFAAALVSQPADTMLSKINKTKGLPGEGTMTRL
FKIGKELGLRGSYSGIGARLFMVGTLTAGQFAIYGDIKRLIGATGGVEIAPK
>g3124
MEPRIDPETIVESLENISNNGEDLMSAFAMAEIPPDVSDAPHHETRRNDPDDAMAEHSESSTSSSR
RQSEVEGYLQIEDGPTGAKSASSRSSLSSIPDSVLVHPKDQSVYGRDSIGEIYNDEEELASYGNPV
YQKIRDRNSPFRHPSSVKAIQMQTEDEEDEEIMSPSRRKAMFRGSPASVRSLGSPLLRRHGYHSAG
NTPKRASPIKRENPLVLLHCTVLQPTVILGPGVTLPDRQILAKVLPPQYARRWKLLEEKISGSGLL
RDRGLLIQHPQEDYGLLEERLLESLELQKPRLENGHYINHEASDSENESGEEKLEHSIADDKSTCL
DCGCHTTHQDHRRKWDVKFYAANGLMKEGAWTAAWKEIERVDVQISLWLPYEVKSEVEKKIKEEIA

VAAEMEKIRAAESIMQEPQPSRQNIVSQDEIDGLDDRPDTTKSFTRQEKQTLQHETPVHERPRTKN
RNTDPDLQTLIINSIRVLASDKRNIITVISMLIAVLAVVFGGRSTKRSQYGYEMAQFGDFPAVSTQ
VYHTASTSVLTQTSTEVPICESESRVEGAGEIQRVAEGLLGEAKPKVAQPVEQKTVEIVFDPMFEG
LETALPPITQPDEPKYKSAAEVDLPVASLDTQEDDSASKEVDDSAMAQIDDSTVDPEASAIIFNND
QVPAQANDLAMTEENDSSITHNDDDAFSQTDGPDSPRDISAIAAQGDDSTTTQEDDAPIIKTEDSA
VTGDDEATMNKIEPPAIADNKHLTTGDNDSAISEDEGPKHPNRRCYHH
>g3125
MAMTQDTITKPKGFNTSYDNSPDMEPGIQAAGEMEEIRRDMKSRHINMIAIAGMIGTGLFLSSGSV
IATAGPAXAFIAYIIMGFVTAGVSYTTGEITSFMPSTGGFVRHATKFVEPALGAATGWNFWYTMAI
SVPAEISAAATVIQFWNASINSAVWISVFLVLIILLNLCGVKLYGESEVVFASLKIMLIIGLIIGG
LVIDLGGAPNHDRIGFRYWNDPGAFNTYIDTGSTGRFLAIWSVLISAAFSYGNIQVVAISGSETQN
PRKIIPAATKKTFFRVFFFYVLSIFIVGLIVPYNDRQLQISTGTAQQSPFVIAFQRSGVSVVPSII
NAIVCTSAFSSGSACIFIASRTLYGLSQDGHAPKIFQKCNRFGVPHYAVGLTCVLLPLVYLNVGSN
TSVVFGWFVNITTVAGLIGWIVIEVTYLRFYYGLKAQGISRNDLPYRGPLQPYTAWATLLMVFLIV
FFSGFSVFVKGNFTASGFLTCYLNVFIFIALYAFFKIFMRSKIIPLSVLDFQTEFQSIREEEQFRS
QTAKIEEDEKSSTILGHKAKDIENQTSWTSPNENTCMLEAFEWYTPPDGRHYQRLQSALSDLKEIG
VDNLLLPPGCKAMQPTGNGYDIYDLYDLGEFDQKGSVATKWGSKQDLIALAQAAEELGMGMYWDAV
LNHKAGADRKEKCLAVTVDPKDRNIDLTKPQEIEAWVGFDFASRGDAYSKLKYGWEHFNGIDYNDL
DSTKTAIYKIFAPGKDWARDVCTSENGNYDYLMFANLDHSNPEVRSDIMGWAEWIGGQLPLKGMRL
DAVKHYSAEFQKNLVEHIRQSGKEWFFVSEYWSGNVLEIQDYLKKVDYKVHAFDAPLCQRLSAVSQ
TKGADLRLVFEKTLVKFEPEHAVAPISPTFKPLAYALILLRKDGYPCIFYGDLYGICSSTPTKTGT
LKNRKFRHPHVPKELERSLPAIILARKLYAYGEQQDYLPSRNCIGFVRYGNARHPWGMACVMNNGL
TPVKLRMFVGKRHAGQRWSDVLDFDQALDESGDLNGKSEQTKRGGKMKKKRIVRISAKGYAEFPVA
SMGVGVWVNESAEGRERFGTCL
>g3126
MTQGNTALETDGCIGYVSGNDTLGIPSICMGDGPEGVGNNLNNVTTFPAPVLITATWDEDLNKEFG
EALAQEHRSKGRNVIFAPTINIVRSPLWGRAAESMSEDPYLTSRMTVAVVQGIQSQQMVACPKHFA
AYNQETNRFGLAPEFDAIDAIVDERTLHEVYLPAFKAAVQEGNAGSVMCSYNKLNGIHSCENPWLY
ERLKDDWGFSGFVVTDYYFAQRTTVSAALAGLDNSQPXGSWVDYYGLPDFFGDLLVEAVDNGSVPF
SRIEDMVARLWRPMFESGVIDRPVLGDENSVARTSAHLDLAQKLVEEGAVLLKNDNETLPLTGSKY
KSVAVFGADATNQSQVTELHGGFVLDTTMVVQSSVEYIKNRGQQENISTSYSEAYPGTGIFPTIPS
EMFGAAGLNVSYWTTRDFSGPINRTLHVENITGSTYPSDLGAVWPGVFSARYEGTFYPNTTGLYHF
SIYGNGGAILYLNDDVVANLNGSNFGMVVQGIANLYADTPVSIRLDYSMATSVDPGAITVAEKADV
SIIFVNDEHTEGLDSKLFLELPGDQDDVISLIAAHSKKTIVVLNTNSAILMPWLNEVDAVIEAFYP
GQQVGSAMAALLYGDVNFSGKLPVTFPRQYADVPTSPEERFPGVDLKATYSEGLLVGYRWFDEHEI
EPLFPFGHGLSYTDFEFSDLRLNVAHAKKANLSLNATFKVQNVGEVFGKEVAQLYVTFPARANEPP
KVLKGFRKTESLEPNAEVELSFSLAKEDLQIWDEKSKDWRFIPGLYTVSIGKSSRDILLTETILLA
>g3127
MKNQILAAAGLLFTSALAQDEIYSGSGFGTYYYDIANANQCYTDFSNANLGQVECSLTQVWTLENV
NSDYLVAMNHTQLIEDMGTYCGKRVIVSVNGVPSDLPLFIGDGCERCATGSSTSTTWAPGGAPGLD
FSYTVAEQLSSEACDAGHIEITWEIVDELIYQFDYDGTGGSQGFVSTTPTGTTTSTTATSTTSPTG
TTTTPTTTLVTTTATPTSTTSAPCSDNAWQCTPDGTSLEQCINSEWVIRAVCSSGLSCHGTDNPYC
A
>g3128
MASNDHIHADEPQLSELSQTVTRTSMLPELEIIASHSGSETTTLQGSTADECEHHEENNAVQQRYA
NPSSYRFRRLASKTIVSFGPDDPENPHNWSNGKKIFVVISGMMQVLNSTIGSSIPSGATKYIAADF
NVTDKTKLVLPISLFLVGYVLGPLLYGPLSEQYGRRTPLLIGYGLFNIFVMACALAPTFNALLVFR
LLNGMAASAPIAIITGTFSDVFDNPTVRGRTMAWYMSCTAVGPIISPFMSGFISTVSWRWSFWLAL
IIAGASFPLVIFYPETYGPVLLKRRARKLRKETGNKSIVAPMDLTPHDLRAVLTVTLTRPIRMIAQ
ELMVSLTCLYCALAYAIFYLYFQAYPLIFEGIYGMSTGIAGLTYLPIALGCFLACGIFIWYDGFLA
RAKARGASWAQNEDYRRLPLACVGGPLYVVSLFWLGWTASPDIHWAVPMSSGVIFGLAYELIFMAM
LNYLSDAYQQFAASAQSASSCCRSIFGAVLPLAAQPMFSAVGIDWGCSIIAFASLGVSVIPFAFIY

FGESIRRGSKLQQHLQALREEELRVWAAEALEGQQQGVEVDVEKRVHASVDSV
>g3129
MTGGIPHYGVWVGQPTRYTAQTLEQDPKSPHIYLYFTDSSDTSSSSKEIEAAINVRSTDKDSRLVY
WLTRNFSNAITQQLDSLSQSFHLLSDYAHVSNRTRNNTRHGHSHNNSHHHDSRHNNDAATNLQGLD
FIRTQGLVDIKSGSVLPTEAAGPNNDILDELEPVLTDAINQKATIYIFGSSYGTGIHDVHMNQGSQ
PSYENGIYEDGALLFKFADGHWEAVFLAFASQRIPTDDKGEYESNSQSLAAILGQADSSDQ
>g3130
MFVDSEAMRVGNREGLGLMEQMAKISSDLRAQKKINKEQKLTNERLDMVNGQQKLTNEEQKLVNEQ
QKLTNEEQKLRWCMVVYTEIEQKAHPQTEEAILARRERNQIIHGGNIIDHLEYIGFGKNKIPPGRH
DSVRKAFEIWYEVPFRYKERIDHAPELVVRTFNRLADTKSLRVWSNAPTVHEVQKICRGIISRWLE
WVDAGEGEYPDAYIRREFEKLESLKSG
>g3131
MSSILKSLGVRFSRSLRLPSPSTTNKSDATFFSPVWQSALLKEDIIHDWKQRRVGNKEGLGLIERL
DRIESILDQLARDLKMQKTVNDQLAKERKAENESQSLYLTAHSGIFSRRRENVIHGGDILGDLEVL
EWAKDKLPWRYDGGREGFESKYCISMQYKDQILVAPEPIIKAFNILGDMTTHIGWCTSSLEGMAYA
SCRIIIDKWLEHINTAGREYLEDVIKREFEILDRIKNGKNKT
>g3132
MVFLSTTTPGDSGSTMKPMGSFVYAMPDRTNPKSTISTILCNSAGSIEYATRTAKVLARRTALPVY
VGCNVDPVSTGTTVEEEMEGFKKIIDAVMARWEESR
>g3133
MADLEQPEAPQPEDAQSWQGDFDPFADEEERRVLFAAFDSFRLYRRTAHLNTTHHRRQSFYALPSA
HWQLLSEPPFSLLDTFNQVDDAIDANADIADEILATGLQSFGLSPDPDLDPNHDSQQSSWHGTANS
TDVGKAHSTIRQFYRDWSEEGRREREVCYDPVLAVLNQSFGGDSSSRPRSDYRVLVPGAGLGRLVF
DICAAGYAAEGNEISYHQLLASSWVLNHSRGAKQHALYPFALHFSNIISRKQQLQKIMIPDVHPAS
SMAQAQNEDGSTSFGSMSMSAADFIPLYSGESQRHAFDAVATVFFIDTSPNLIRYIETIKNCLKPG
GLWINVGPLLWHFENEKRRTTAADQNGTEDAMNEENALTLSGHHDGIGEPGNVELSEEEVFHLVRR
MGFTIEAQQHPEERPYCGYIQDPESMLQNLYRPSFWVARKNNTE
>g3134
MTSRLKNFTFSSKRKNSNNSTLSQTTPPAGNTFXPPPASSASTSPPPPSSASTTSLPMNAQNPQGR
PPSYTYAGRTSPMPPPPLNTAVQPGQYAQQPPAAANPPGYGYQQQPGPPSQQSQPSQPLHQHGIPS
ALGPAQGLRNPAAEVDGTNRNKSQLIVGIDFGTTFSGVAYAFATNNEAREDIITEWPGAGTHTKQK
IPTVLYYDQYQKVVGWGPDIADALAHTGYPKQGVQKVEWFKLQLMLSGNTYIDPINLPPLPPGKSE
IDVAADYLFKVRQAMRAQLQKTLGEVFNREERNIRYFLTVPAIWNEAAKAATRAAAIQAGFLRDEN
DNRLTLITEPEAAAMFCAKTGLLNLKVGDAILIVDCGGGTVDLIAYEVEEENPFTVAECTAGSGDS
CGSTALNRNFSNILRARIRKMNLPDGSRTAGRVYAKCIMDFENRIKADFRNNGQKWAVDVGIEADY
PDAGIEEGYMTFTNEEILQCFEPVVNRILELVRNQIIAIQAQNRLLQNVLVVGGFGASEYLFQQIK
LHVPPQFQSKVVRPMDSVAAIVKGAVTAGIAERVVTHRVARRHYLMATLQPFKEGYHPEQYRVPSL
DGKDRCKYTRQIFVQKGERVKIGEPVKVSFFRQVAPGATLMYEDILYACDEDVCPEYVKDPRIKEV
VTLTSDLSRKNLEKDFERMDTPNGTFYRVYFDIYLTLDGSEFNAELVCQGEVMGRCTARFK
>g3135
MMNRFISLTSTALLAQLAWTSPNHRNIIRTIDLSLPSTPAPGRQVVDAAYQSFSIEFCYMADYAGD
NTNSNKFSRQVIQNLHDISGAYPIFRIGGSTQNSAVYFPNQTDIAIIDPFDSEASDQPSHSYIGPR
FMESFGQFPEGTRYIYGLNFFKPENETLFDVGDGLGQCVLEAHAAYTALGESLYAFEIGNEVNSWA
GGSRRPANWTQQDYVDQWNQYATATSQNLTGRESMQLFQGCAYTAPRNVGDNMTIWNIEHAEEDGM
RSNKAKTVSDHDYMGANCDYTGAGPTIETSLFDRTNMLSRVRYHDYLGNATENSGIEYVLGETNSI
SCQGAFNISDVMASAVWAVDYVLYLSSLKVSRVHFHMGTRYRYSPWQPIYYNDTEAHVKPIYYGNM
FNAAVFAGGDKQTEVLANETNFGAYAVYNKGKPESIVAVNLNMWNSTMDAADRPYTALSLPGAWNE
ARVSRLTNPGVDTAANITFAGQYVDSEGRIVGEKSFDKVIDGTVHVGAGEAVLNSK
>g3136
MVYSLSFVVFTVVGVLFQWLLKLFHPSQGKVPEKSKVQDATRKAPYPCKAIKGNQKFRITMGLRRL
DEWNWLTVDKNYLKEHDVRASLLQNQRSQVIQSLPESKAACAEGLEVVAEFLCERYPDMFHMEEEQ
DMRKILNSKTGESFVIGDENSATDPLETAVRLAMEDLSILLMNEEGEYYLAASATLFPVGWTVQDR

IGWTISQMHGPVPDWKDKIGHSVNKFFCRLTPESPMERSNYFLETKEPGEDLGDTLFRPMALNEDQ
PGLSIDNILLRQERQTFRRLPRTGALIFSVKTTLNTFDELPVEQLQALATEIRSWPEDMAKYKGRD
IWGQLVLDYCIQRSIVPA
>g3137
MISGGNQNPTEYGYEESIRDGYTPSMADADTLHSSQDRLVPSAPLVGTPSDVPHLVSSLPNTEQHL
IYSLRQLKLTGWNSGDKSVFSMNKTEQDIVNLAFNMAPTEQLTEFERSVLAKVRGWQSATLPPLSL
ATHAQRTFINAIREWRRTQRPQPFIKRIFRR
>g3138
MSDAPVVLRSRISKSCDECKARKVRCIRARPDSLQCNNCLRRDQTCHFSHTKRKLKVKDTEDPYSR
STPQSPVEAEQHDTPPNSKNQPPKLYVDRLLENRDTHGIGELRFEACVIKEKVSVKYLPFEFGITL
SDCFCAATSSSLAFFSESRIRSLTSRIGHDRLREIVEAIGSLINARMNRKDEDTKTMFRFSPITFK
GPSTPVEVAPEAARAYIEAYFKHVHPIHPFLDRVEFEQKALSYDLLHHLSSNAAFSALYHTVLAMG
AQYTEGGSFEAGQGKPWKLYQVALGLFSDILLPRESLVNLQALTAMIISDSDIGCPIPEIPEAVHG
GVDWFYLFARFARLISRAYEMLYTVSATTKTPEEFYEAIDMVYQDLENWRLSVPKDLRPGDPFRPE
SCKSPWTVTVSLKAHFFYASLVMSLCRLTLHLGAGTESPRMEEAKKRLMYTARHIIGLTHYIDLQP
HTSIWILGVMPLSALFVLFDFVVHNPYHPESSTNLTFLDVVGAYFTRLEYSTGGSLPSSLVAEFAL
IARQFIRDIQLGNSNRNGSAIANTQYQLPARDKQYDEPIRPYDQVRAVSQPLIYQQSQMPQIEQMP
AAAQQVVTEDSMGYVDQLFYPTDDLQPFITGDLGSGFDITGLFDTMLPNFQI
>g3139
MAGGILPFLLGLLLLAHCASASTHRSNPKQNTEKQKRDSLPTNYTTPDYYPTPNGGWDSNWSAAYA
KAQKVVSNMTLAEKVNITSGTGYLMGPCVGQTGSALRFGIPRICLQDGPLGIRNTDNNTAFPAGVT
AGATWDKDLIYARGVAIGEEARGKGINVQMGPVVGPIGRKPRSGRIWEGFGADPSLQGIAAAQTIQ
GMQSTGVIATLKHFILNEQEMYRMTSVVQVGYSSDIDDRTLHEIYLWPFAEGVKAGVGSIMAAYNH
VNGSLSTQNSQILNGLLKDELGFQGFVVSDWYAQFGGVSSALAGLDMAMPGDGAIPLLGDSFWNYE
LSTAILNGTVPLERLNDMVTRIVATWFQLGQDDDYPEPNFSTNTEDATGPLYPGALFSPSGVVNQF
VNVQGNHSIIAREVARDAITLLKNVNQTLPLSKNASLSVFGTDAGPNSGGLNSCSDMGCDNGILTM
GWGSGTARLPYVITPQQAILNNSANTQFYVSDSFPSVTAAADDIAIVFINADSGENYITVESNPGD
RTTAGLNAWHGGDDLVVKAAAKYSTVIVVIHTVGPILMEEWIDLPSVKAVLVAHLPGQEAGDSLTD
VLFGDYSPSGHLPYTIPHNEFEYPASVGLIDQLLGQIQDQFTERIYIDYRHFLQANITPRFPFGYG
LSYTTFNFSDATLTTGTSLTEYPPARPAKSPTPTYATTIPPASEVAWPTGFNSIWRYLYPYLDDPA
AATSTAPYPYPTGYKTTPQPAPRAGGAQGGNPALWDTVFTVDLKVTNTGTTSGRAVAQLYVELPGD
TLGVDLPPRQLRQFEKTSILAPGDSETLSLPVTRKDLSVWDVVVQDWKAPVNGEDVKFWIGESVAL
EDMQIVCTVGQGCESA
>g3140
MTESKPNRQLHLTAFMRPVSLHTGAWRYPGSYPDANFNFKHISHFAQKLEEAKFDAFFMADHLAVL
NMPIEALKRSHTVTSFEPFTLLSALSAVTEKIGLAATASTTYDAPYHVARRFASLDHLSGGRAAWN
IVTTGNPESSKNFGFDEHMAHSDRYKRAREFYDVVTGLWDSFADDAFTRDVETGQYFDPEKLHVLD
HSGDELKVRGPLNIARPPQGWPVIVQAGQSEPGRQLAAETAEVVFCSPKDIEGAKALYADIKGRVE
KAGRKREHLKILPAALIIVGESKQDAQEKRLKLDSLVHYDSAISSLSIALGVDVSHLDPDGPLPED
LPETNGSKTGREGVEQLAKREGLTIRQLAQRYGGYSGLAFLGSPSEIAEEMETWLREEASDGFTCT
FPFLPQGLEEVTDKLIPELQRRGLFRKDYTGSTLREHFGLPRPDNQFFTKS
>g3141
MEKEDWWRDAVVYQVYPVSFQDTTGNGRGDLNGITSRLQYLKDLGVNVVWLSPIYASPMVDMGYDI
SDYRAIHTEFGVMEDWERLVDEAHKLGLKIVMDLVVNHTSDQHEWFKNAQSGGNKKDWYVWRDKNP
EGKEPNNWGAIFGGSCWEWNEQLQQYYLHVFDVSQPDLNWQNPEVREAVWDVMRFWLDKGCDGFRM
DVINCISKTPGFPDAPVADPKSAFQYGLVHRFNGPKVAEYLHEMHHRVLKHYPGTFTVGEAPGIKN
AEQALPLIQNGTPLQMLFHFEHMYIDHQPGQQCFNYRPWKLTELKDILGNFMEFNQNHNGWDSLYL
ENHDQPRITSRWGSDTTYREASAKMLALFHATGRGTLFVYQGQEIGMVNPPSWTFDELRDLEEIEY
YNGVNERNGDLNDALRQIQRIGRDNSRTPMQWSADVNAGFTTGSPWIKVNTDYFEWNVAAQLENES
TSVLGFWKKLLSIRKMHPGLVRGKFQMIDYENQSVYAYTRTDEVAQYLVVCSFSDKEITWVCPVQT
GSLLLANYPDGDRQELDGMKLKPYEGRLYIYAKNQH
>g3142

MSEDNKITPVEGVAFDREAIDVSIVKQANDARLAEHNLTLKEACIHYRAAIGWSFLFSLGVVMAGF
DPQLVGTLVAIPQFQHDFGVELADGSFVVQAKWQSAFNLGVPVGQVLGAFGIGWPLEKLGRRWTLA
ICCTITLITVALQTSAQNRPQILIAELINGIVLGAYPVIAPTYISEVTPVVFRGIAAAFVNLSFVI
GQLVASGVLAGCQSRGDRWAYDIPFATQIIFPVIILALLPFCPESPWWLLRRGDVEKAQKSLQRLV
DKSIDSSTLLAHIQLTTSIEDIETEQARFIDCFKKTDLRRTIICIMVYAIQPLVGNFLVIGYAVYF
FELAGLGTSASFDLGVGLLGVGFVGTVFSWVLLDKFGRRTIYNQGLCVLVILVLLVGVLDVVPGYG
TTNTAVVWAQCSMMVVYNFIYDLTIGPLCFVFLAEVSSAKLRDKTIALSTTVNALINVACAVGIPY
AINPDQGNLRGKLALIFLGVSVPCVIWCFLALPETKNRTFEELDLMFQRRVPTNKFATYDFQEDME
ERD
>g3143
MTIDEAVRRSHILHRALDRTPLNVASGSGISFTLNSGKEIIDAASGPSVACLGHNQPEVTKAILDH
LNNNIAYAYSGSFFTNDASEELASLLLTHQPGGLSKAIFVNSGSEATDAALKLAVQYWHEKGEKKR
THFIARRQSYHGNTIGALCVSGHDSRREFYKDFMSSNVSFVDPCYAYRLKERNERDEQYAQRLAQQ
VEDEILRVGPEHVAGFIAETVSGTTLGCVPAVPGYFSKIRDICDKYGVLLILDEIMCGMGKTGTMH
AWEQEAGFRGPDIQTIGKALGGGFVPLSGVLLHKKIFEALSNGSKVLVHGHTFQAHPLACAAAVAV
QKIIKRDNLLENVKQQGVLMEQLLKDKIAPLPFVGDVRGRGLFWAVEFVRDRGKRVPFSPEDKFCE
TVVDKALELGLAILGNLGRTGEHYVDLAILSPPYIVRSDEIHRIVDLLAQAIESTSANFL
>g3144
MLPPEDIGDTHAEYRKDLLPHLDECLAVCPVEIGYFDSKLGRFQLSSAKFIQQTLAFIVRDQAMNA
AKCGYVYAERGRFSEAAFYLSMVKDVLVQTLGYEHEKTMITMLGLAAVYWGLGRLEEAIALQKRVV
KARSKVLGPEHHDTLIAMDQLGRSFWLYGQYQEALQLQEVTTTRMKATLGDEHPDTLAALDNLGVT
LGSWHRFQESREIHEQVLRIREKKLGRTNPDTLTTLNNLAMALLDLGKLSEAKGMMQEVVEQRQLK
LGKEHPWTLWAICNLAKVNIEIGYLDEAEQMLIDGIAAGKRSLSDEHLGVLMGCGQLARVYARQGR
LEEAEKLTLDVISRVEKSRGEEHPDCVYGMFKLAQLYELQGKIENAIDTCETALRKAGTRLTNQHP
LYKKIDTYIRTLRQSFTPKQNQGEPDPRDKTPLPHYSKNTQRTQTW
>g3145
MSNEAQVLVKPTRPEEAGLDLIVVHGVTTLGLPRLPQDWYLVLGKILPENSSIISYDADLRVDNKF
RWQDILARTDTLLNSIWLKWNSGKPVDERLSTMTAHNLNTVAIDTSLASLFDSVDCVIHQHVRTFL
QDILGNLQKEQQVKDDTESKILDAVVDMTLSPRSEAQGILSTRDSLGLSLPLSISPEPNPFTANSP
PENSVQHPSSSAASDRSYLVVPEPNDTRVPDSVEYIQARSYSQPNSDYCGHEVVFKKMQNSLLDLA
NIPDMESPNSQFDVKIWIQANSKDSLFVAFKEISARLKLESKDDASDDILSRELVKGWMAEPFQNF
KRGTGKLMKWLLVIDNADNPDDVLDFWPNCGQGSIVVTSRDRQAMTKNYFGECGTELSVLEEDDAI
SLLQGLLKRHHRPTESVDVLRQVVTTLECWPLSIAQMASIIGRRKLSLSKFLEVYKSDKDRANYHS
KRSGKLDGYPLTLSAAWALKDMSLGAAQLLSVISLLAPASIPEEILTKKPELAQLPSYPLESDRYY
REIEKIESCSIITQQRGPTAEDPMELSIHPLIQEVVRGQLLNNNDQIVSVFNATVRLMTAVWPFET
LPFYGFHEFNRVQRWDQCDKILPHASQLRQLYEAFSDDIRQRCTTRDFLNILNEISWYLFQRSNYD
ASLDCIKMILKTLKIDSEDHSQIEAGLYGTWSGIAVETNQPQVALEKRLENLTILERIFAETGDVN
SQIAACYSETARSLIMNGMFTRAGEFIDKSVALRKKMPHFSRLQLYSPLYYTALIHIHERKFEDAE
SVLLEALRDREVKYGKDDCESKRLVRALPGSSMRKSWNHGADSGRAGILLFSLGNVLTKQGRYDES
FDYHQRALSQFRATGNANDLDVANAYYKLASHYLRYKSLDLAKFVPFHCSSEGLEKINC
>g3146
MEDSDTSIRGSFNFMKQAELYDNVKPYSIDNEIKRWVGHVPRSNYQNSAVENVLVKDLRGREAEFT
FEKNGFAIIEMESAMTYEDFDDPEKFSGIYLQEVAACLIQYFDARSVHIFNTVRRRRHPDFPNVAV
IRPGAATQPAARVHIDSTRQSLDQLVRELQDDKLNINGRHYIYLNVWKPLTGPVHDWPLAFCDART
IQSKDLEPHDQVFHDVVRENVMVYHNPKQEWYYLSGQMPSELLLFRQADSQNNPGVAHTAFEIPSP
PLHPVITRESIEVRVIVFLD
>g3147
MNIYMHGLRMGKGWRPFHSIVRSSEIHDSSRFSIEQFVTIHITRNQPSSDKWTVAIWSDAGADLSY
SPAVPWLGPTKNAHFRPVILHGPGPEVMAAKETAFSPSGISGGLPQSLSLLRSAYGASLDPVEMCQ
NPIYALDELFRFHSASEKQYLDMIASVLETSIAEDYRTNPEGLSNTRTILLHSHRMLQRRQSHIAE
MLNFLRFPAITDAADASPRAKSAVRSLCVDLEYLQHNNRELMSRCRHELDIMTNEAMFEDARRGIV
LSRNSQKFAAFAAFYVPFSFTCSIFGMNFVQVDSIGKGFELWIAVVVLTSSRESLLARDLSRHVFA

LDLRNHGDSAHHPIHNYMQMAYDVEAFIKTHGLKNVSLIGHSMGAKTALTLSLRAPDLISNVVAVD
NCPVQLPIPADFEKYIDALAEVAKARIRTHSEGERILAQYEESAAVRLWLLSNFVKDRQHAPYLKL
RLPLDTLRNALDALGDFPYPSPPSESGSESEAVKFSKPALFLRSYKSHYIPDDALPLIRSFFPQSR
VVDFDCGHWIVQDRPEEFRRVVVDFLRDGR
>g3148
MRFATRLCVRPTSYSRLPSLTPSGVRGINSYANFKIPTINNEPNKHYIPGSADRRGLELALAQVNQ
SNIRNIPLFIGGKEITTTKTSEQLNPATHKPVAYYSEASKANVEAAIEAALAARGDWAAMPWSDRA
SIFLKAADLISTKYRYDIMALTMAGQGKNAWQAEIDSAAELCDFFRFGVKYAEEVYSQQPAHNSPG
VWNRLEYRPLEGFVYAISPFNFTAIGGNLAGAPALMGNVVVWKPSPSAIASGHLVHQILLEAGLPR
GVIQFVPGDAEEVTSAVLNHPDFAALHFTGSTTVFRSLYGKIATGVAEGKYKGYPRIVGETGGKNF
HLVHQTADVRNAVVQTVRGAFEYQGQKCSATSRAYIASSIADDFVAQIVEEVRDLKVGDPADFTNF
CGPVIHEGSFNKLTKVIDEAKNDSELELLVGGNYDSSKGWFIEPTVYLTKNADHPLLSRELFGPVL
VLYAYDDASPSSLADICEKIEQTSEYGLTGSVFAQDRSAIRFAEDALRNAAGNFYINCKSTGAVVG
QQPFGGARASGTNDKAGSANLLSRFVSLRSIKEEFTPTYQVAYPSNFN
>g3149
MRFSVLLALVASTAALPTPDTVYDDDDVCYVEDDLVELILSLPEGPEFCRDLIHIPTDVTTITKPA
TVTVAALTSTITSLFTTTLPAETDYVTITTTDDNGAPITITDVQTALVTDVVATVALTDAVTQTIV
NDLTSAVTETSTFLTTGTVTEFATETITQPFTNTVTEYATILVTDSVYSTWTDVSTQTLTNSHTET
VVLSYTDTIFVTNTLDVTATETYIPTSVHSDWETVTDATTATSTDLLTSTITSVLTAGTTTTITTT
ISGMSVLKRALTESAIIPTPTALTTYLPDELSSACSELNLQPETSTEVVTSTETTTIYSTATGDSL
SYITQFVTVWETITATGSELMVTSTDVVNNPSTVTLDVYSTFETDISLTQTTTATATVTEDDVTTI
TDSATVDITTWATTVATVDVTTDETLTATVEASTTLTNTNSITVTLEETATATADVTVSTTQSTTF
DVTDVLTVTSTSSLTLTVPTNIETTITSTVSVTGTAVVTAVVDVTATQTTSTTSTVTATATANCGV
NLVTNPTFNVNNAFSLSGWSASGRSLSVGSSSGSAIGTTYVFYYAGTIPQTYTLSQSLSTLPTITY
TVSFWWRTGAQSASLIVSLGSETVTYHSPINKLGVWIQETFTYTATASSSQLTFEFVPSTLSAAYA
GTNEIDLRWIAWSNWTYTSHPVKGLLDNASSSWLVFNGLDTFATIELCNQVVGTANNQFRQWQFDI
SDALKSCKGDPVISINFGSAPEIANAIANETGQETWPVGVQGLFEFDNRWFIRKEQSDFGWDWGPA
FSPTGPWQPAYLVQFKPNDGVYVLNTDIDIHREGQVNWLPPDQSKPWVVNASIDYLGTLPKGASLT
VEIKDSQNKTTIASGSLKNVTTAGNSISGITTVGANIPKLWWPTSMGDQNLYYITINVVDSNNKTL
ASVTKRTGFRTIVLNQTNITDAQLALGIAPGANWHFEINGHEFYAKGSNFIPPDAFWPRVTEGRMK
RLFDSIIKGNQNMLRVWASGAYLPDFIYDIADETGVLLWSEFEFSDALYPVDQVFLDNVAAEVTYN
VRRVNHHPSLALWAGGNELESLELLLVKETDPEEYPRYVGEYEKLFISLIFPLVYDNSHSISYTPT
STGNGYTKIDFSLPVPMVERYENVTPGEYYGDSDYYNYNSAEAFDMSHYPVDRFANEFGFHSMPSL
QTWQQAVADEDLQFNSSVILHRNHHYPPGGLSTDTTLSAKGMGEMTIAVEQYYPIPSKSDPIENFS
AWCLATQRFQADMYKSEIQFYRRGSGHPERQLGSLYWQLEDIWQAPTWAGIEYDGRWKVLHYVAKN
IYEHVIVAPYWDLTTGQLNITVTSDLWESVSGSVNMTWYDLSGKPLEKNAGIPSSAAFEVGPLNIT
DILTTNINDLSIPDIKDAVLVLSLSAKGHLPNSRTTTQFSHENVFTPVFTNEAKLVDPGLKLSYDN
RTAKFTVEATKGVSLYTWLDYPAGIVGHFDENSFVLVPGQPKEIGFTVQEGSLTEHVVKNVTVQSI
WDQAERT
>g3150
MSFDILPQTKDDAAEVFAAARKAFTRFNRILFDPFPLSEESMDILSKRRVESFNKDPLAKSFKAVD
RETGAIIGAARWSIHAEVETIEKTVEEETAQGLGAFGVPELRVDVARAFYSGLLEGKREIMGIKEE
DGRVFKLKPRVDLESLFTHPEHQGKGVGKALLQRCIQDAEASGLVAYLEATEEGFPLYAKNGFETI
RTNVFDAKDFGFEGTHAYTYMIRPSRATE
>g3151
MSVVTEPRGFIDFYNKPGSSNGAKIAIILNELGLTYRYHEIHTPVSPLDKDTYHSLSPNGHFPVLT
DIHPDGFKVSLNQTGAIAQYLVSEYDQIDNAISFPRRSPEEIEAMNWFFFGASRVAPAHGEAVHYT
KKEEGPGSEAYSVGRFRSSTTGLFFALEQRLKETGDYLVGHKFSIADIAQIPFVVAAEEAGIGIEI
FPNLTAWYNRVLSRAGVKKGLTVAGIEFLA
>g3152
MASLFASSRIALRSINRHTRFRHALVRWESSISQRPGSDTVRFPGAVDSKFTSEMGFSRPSSLPAM
PTYRVMDSDGVIVDTKHEPSDVAIEEVITWYKNMLTVNIMDVIMFEAQRQGRLSFYMVSAGEEGIA

VGSASALKDHDVVFCQYRETGVFQQRGFTMKQFMAQLFANKHDSGRGRNMPVHYGLEYPRIFTISS
PLATQLPQATGAAYAMKIQALQNPNTPAGVVACYFGEGAASEGDFHAALNMAATRSCPIVFICRNN
GFAISTPTLEQYRGDGIASRGIGYGIDTIRVDGNDIFAVREATKEARRMALEDGGRPILIEAMSYR
VSHHSTSDDSFAYRARVEVEDWKRRDNPIIRLRKWLENKGAWSEELEQQTRADLRAAILKEFNAAE
REKKPPLKDMFVDVYEELTEEQEAQREELRKHLEKYPGEYNINDHENGLKGL
>g3153
MSNRNQIDSVIDDDDEFCPLCIEEFDLSDKNFKPCPCGYQICQFCYNNIKTHSEEGRCPNCRRVYD
ESTIQYKVPDVDEFKADLALKHRKAAAAKKKEAEKREIEASSRKNLAGVRVVQKNLVYVIGLNPTI
RDEGQLLQTLRGPEYFGQYGEIDKIVVSKAKPGGNPNQGIGVYVTFSRKVDAASCIAAVDGSTNGD
RLLRAQYGTTKYCSSFLRNEQCNNRNCTFLHETGDDNESYSRQDLSSMNTAQRPHYTNGATNAGTR
PFTQPLQSQPMRRQGSKDEGGKPLPDGPALPSSASWANKDAPFNRARRLSAGSRSSPSPKPANVPL
AKSEELKKNFPTAQESRRSTPAPAPAPAPVSTPAPAPVPVAAPTPAPSQGLPPAKPATAPAPVKDG
LLESLLNAVNSPGFRFVFSTDGLSDDDLKYIENGPSLIDPYGGAKRRAMREKVEQERAKQEAETQS
LLQSAAADDENRESGSLQLGGEPDDVHPSRNTVSRNRDTHGAIQPPSQQTTNANSLVGSPVSISQQ
FHNLNTNTRSLTPLQQQQLMLLKSGAGQQGGLTSNTRLFGQQNLMQTGNPGQLAAPAPQQGQFYTS
SVQGPPPGLKTAGTPPISGGGMFAQGHGFTSNTNLGLGSNVGKQESNPDLMRELLRGRGVSAGTGG
LQGHESAKREFLLPFIQQHHTPPPLTPATGLLNAFYGSQNGALTDFGPQKQKKKGKKHRHANTSSG
GGGVVDLADPSILQARMHQVGVGSTGQVLYGSQGQVDDEEFPPLSVGPKSRLEHAFRPTLTQESRE
HSRAGTPSLPPGLPLPHGHPPAAFFSDKKSLDSPEFPFSLPTHGPPGLPASGRATPLQKSLDTVSR
QQTPMVPDVSDENAQVEKIRNFGDISLGSPKARSARKPNAAAPTDLPASNKSTVTKGSQNKKHKPI
KLDLSMPLEEVPAASPVKESPASSNVTPANLPQSNVAANSRPGTPLTGISRNSDSSGPRQPRVLRV
VDTPKAETPPPPSAAPSLSSVVQAKIRSRRPSISSADRPETPGDIGSEFEFTSASASRANSPPPSK
IGSAPVRSMTKNQVKKERRMKAKQAEEAIKEEVATAIPEEPIQAPIIGRKRKTKKPPTASEVAAST
STKEPSDAENVPTPKVEAKADVSTQKPKPSPEKVPDVSKPVLDAEVVEPPADEPWRANNTIAQLIE
DAKATGTSIKDLFLERTAPLHVLLAQMQSKGDIDLNNHPLFNPPPLSQRTDMKSEAEDYEFFKLPL
YISEENKKILLSGQPLRINHGVDNLKVRCMITPKGRVLRHLSDEEEDRYLELESHVDAGTWNEYPS
TNVPGLDVTNMNGGLDALFLTPGRFGVALSEPPSPRMSLAAGGAVVTAEDQFALDPPSESGPTMGE
TDHTHKLPESARYPPIIPEMDNVFGMSNKELRAFIEQSQRELESSRKDFDAVDKKLAALVKRNKKL
AQQALGSVVEVGK
>g3154
MAATRSLMRLGSSRSLGAASRSAVCVRTFSSTHFRQLDKEDPVPNMRHAARPPQGELRAPVVNPAD
KYKDQADAMHKYGQYIMSCLPKYVQQFSVWKDELTIYVPPSGLVPVMTFLKYHTAAEYTQISDITA
VDYPTRDQRFEVVYNMLSVRHNSRIRVKTYCDEAQPVPSMTSLFDGALWYEREVYDMFGVFFSGHP
DLRRMLTDYGFDGHPLRKDFPLTGYTEIRYDEEKKRIVYEPLELTQAFRNFEGGSSSWEQIGSGTD
RAPDSFKLPTPKPEEKPAEKK
>g3155
MATVSKSLLKPHDYTVAWICALAIEMAAAEAMLDERHSLPTISGDDNTYVAGRVGSHNVIIACLPA
GVYGTTSAANVASQMRLTFKSIRFGLMVGIAGGVPSADHDIRLGDVVVGKPTRDFGGVIQYDYGKA
VAGGYLERTXVLNKPPTILLTAMTALQSAHIANKSRIPDLIADLAERHPRMKEKFTYDSRREDLLF
ESQYHHSDRATTCDECDRQRLVTRAPRDRNDPVIHYGLIASGNQVMKDAQNREKLARELGILCFEM
EAAGLVDTFPCLVIRGICDYADSHKNKQWQEYAAATAAAYAKELVTMVHALQVVDTPSVMPITDVV
HKSKAMAKLRGSSTWVAKPNGELDETMLRRISTYEHEKIHQRLARKRVMGTAQWFLDHPDFQAWLK
EKRFSRLWCSGKIGSGKTTIATAAIDEAKYRLSNNLASTAFFYCESDRPDELNGSYILSSLIRQLC
NYLRVKNRRCPGDVADALNRFFGKDRVFPDFEDLKGLMFQLFKLVPDTVYIIDGLDALNPSQSKDL
FEFFRSLFCGSGSPPESRILFFSREQLPGYTGIPLFMAGARQISTTSNVMPDLQVYIDTSVTDKLM
TRKLTDDLALLDEVKRVLLEESSGMFLWVYLQLEILWQECFTDNRIRSALRDLPTDLEETYRRCVR
RMTFANSYALRVLQWVSFAARPLHIDELREAVAFDLEDMTWDAGKIPQADVVIGCGANLVAVDPSD
QCVRFAHPSVKTYLLKDSDSASLIPRYPKSDRQGELQCGEFCVAYLSFSNFNLALVKPANETEVVK
VPNAQLLAGDALTSPFVRFFWGRGKNPKPPTQVQFRRIGSATIPDRSQYKFLDYAVTHWALQTKEI
TFKSLVWEKFERLSLSFNETWNFHPWVIGGRSQISRLHGLFGWAVENEHLPLLALALNSERDIRLI
CNLPLIDERLPALHLASKMGLVDVVRLLLPVCQLNLQDEEGYTALHHAASKGHVRVVVILLNKANI
KIGIPSKTQVTPLWLAASHGHCDVVQALAERQADFEAEDSEMRRTPLIMAASNGHRAVVDYLVSNR

AYLEAKDAGGRTPLSWAITNEHVDTIELLLERGANPACKFANKGQLLLRVAQTGHAAIMHLLVKDL
TTNLNTTDDNGLTGLRWAVQNKNLEIANILLDAGADREIRDARGQTPLLWAARNNHEALTDLLLKK
GAVVYAQDKEKRTALFWAVKNSREGLVKLLLDRIGDQHAGRKRLLNAIALRTAQMRTQDTVESSLG
ANGDFEDKTPLLSAVRKSHEWLTQQFMEIVPNVDRETILKEAASMWSTVNGQMSLLEMLVGKKSKD
DIDESYMRTSLLQAAEKGDEHRAKLMVAKGADIHGRNLYGETALWWAARNGHASLVEFFYDKGADI
ESKDKFGLTPLAWAVEKGYWDVVKLLVGWGADIRARDKSGQTPLTRADGKGMRNLLVGE
>g3156
MPKRKHSELSGEDASDNFNRVKATRLGHKFERGTQLLFRALKTSRGFERQKLGRRQKTAKSEKDAK
TLERLEKEVHVLKALDLEETAQRYLLKQLNKTKRIAEAPEFVRLEKSINASINAGPRDPAEANVTA
RLFKSNPVQNALPEILNDIRTLLGVDETSTKKQPQKKADQKATQKDESMEESANEPESTPDEVSSK
SAKDEKSKAEYLNDINVDDVSDGESMDLSYFDARLASASDGSDSDDDDISPIKPALNSNKITPKFP
DNISISSAEEASSISEYSEPEPESESDSPPPLTKSKPSKQDTNVADKPKDTTFLPSLMMGGYWSGS
DSEPEEDDEVAAAATGSGKPARKNRMGQQARRALWEKKFGARAKHIQEEQQKKGSRNNSKNSGWDA
RRXATDDNDNGPRWGGAAGRRQYTGSSSRNGDGGEKDVRKENAIAANKKKAYDGLHPSWEAARKAK
QEKAQASFQGKKITFD
>g3157
MSTTEEPIATAEDVELPATEAPAVEPVAEEPSESAPETEIPPPQTPVDAALLSGEESKSEAVTSPE
VGKTSASPAKKSTISSTTKRPVTSSTATKRTVGSTTTTGTKPTSRTSPGGSTLSKPPTRPTTISTS
ATRRPPSVSTTATHRSRASISSSADEKSSKVASSGDEKKGISSAAKRMSLAGTPSSRAPTKATSTA
DRXASVIGSAADKKPTSTTAARSTTSRPSTATTASRTPTTATTRTAVRPSTTAPARPTTTATKRLS
TAPEAKAAAADAGKLAELQEKLSESETTIASLKAELEATQEKMTTFANSAEEESSKVDTAVEAIRA
EHVAKIDKLSLDHAQEVQELQGKLEAAETRHKDLEAKSLQDLEVAKKSALDQNDSQTAAVLEDLKA
THLAELQRLQTELETEKASAAETAAQITRLTEELESQKSALETASKAAEEKEAASNEKHLSIVDQL
ERELKGRDQVVENLEVELKKLSESKDKEIAAEKESSTEKLSSLEAKISELEGKLAEAQSAHGDSEK
AAKILVEKENEIAEQTEVIKALKAEIDQYKATTESLTVEIESLKTASGDSEAQAKELGASHERNIA
ELKAEHESNVAALIAGHEQALSEKTQSAETLQSQHQQELEASKALQTELEAKLAEANATIDNLKRG
YEEGISSANTVAGQEIESLKEKVATMEKSLKLDAEKIKALETDLSSKQEELDLLQEKLKEEAAAAS
QSADALAQESSKLSEEHARAIEALKGGHSSELETLSSTHAEALQTLQSSYDELLRSKEALESGHAE
KLQALESQLATSQQEDSSKHASQLEAHQAELSATKKEYEEKLQALESQFAAIQQETSSKSASQLEA
HQAEIDALKKEHAETLQALEIQLAAAQQETSTQSASQLEAHQAEIDALKKVHEETQAKLKEEWEAA
QASKAAETDSDHSQVIEELLTAHESKLTNLRNELEASHQEKLESLQKTHDTVLANVSEQLNKANEA
AADTSIIDGLKAQIADLTAKLNAAEQNLAAAEEKSSSSTLELTGKHAKELSKVQQEKTELEQKHQA
VSTQLEEAHKSLGEHQQTQSRLESAIQQLTAVQDELDALKKAHEQTNGDLESAVARNKTIEEQIAK
GEKELNEQIEKNMSLLTQLTDVDSNISNSRKRIRELEAELAALKESSKDTKPAETSNTLLASRWAT
SEEETEEKGGASAEGEELGSSIEGTMASIQEQLKQIRAASDDWYDEHRRIVGELVQASRGTTPDPS
SKEPSLKSPTSITQTITTTVGGAEDETA
>g3158
MARNTRRRAARQKGEKALDIINSVAEDREDGDDSRATNFERRIDTELVDHEHVDEQRQLRRSARNK
RTAHGMEGRFSPLFVPQDEPQGKTKEAADKDINGSEEADELESEADDAADAEISEAEEAGEDEEDR
EVEEDGDSVDSEDDFSMSDQQQLLQEQEQAIVNSQQEQQEEEREQVLVNSPVLVVIDRSKERVSKE
RKNAATAKAAAPETARAKADHAHFLGEDGEFEDSESEQEDENVDGAGSELDYEDDEEVPSDDSFDM
DDTQHDAGTALKSLFDWGDEQPAEPVQGSRTNDQLETQSPRPRESSSRKRRRASNTSTDEPNRSRR
RIDLQNQNQVAARRSTPFALPADLEATTSPEMHDRTRRRMETPNQGIERAPRRRPPVARDSPEVAD
WEATILEKENQDDVIIVGESMSYDDATKIQDLQVTWKRLIKYCQKLKDNGVRRMRRYVGIEAIVER
ISDLLKDYKMMQRKRSRGRPIDAITWKRTRQEVNYISEDAAKIRNVLVRRAKEGKESNDLQTQADA
VEYAELIVQHVMTSLCELALECLKTYYSVEDKWLLNGGFGAVLDIIDIVGELDLTLTSLHNCAMVM
LSPYPGRPIRQELRSIRGSLARAEADEQRRER
>g3159
MASVLERIDKTHHIFAVPTKKIHDGGDVQTFFSTKAYRDIMSFLLQLNRAMFPRNIDNTVQTWQLN
SHEVEFSEPVRRIQLLLSRLESIIEEAPPDTGPRRFGNISFRKWYQLVEQRASDLLDEALGFDILG
TSQDSSSEQVPARKELEAYFLGSFGSAQRLDYGTGHELSFLAFLACLWKLSVFPEAGPGVEERGLV
VGVIEPYLELVRNLIKTYNLEPAGSHGVWGLDDHSFIPYIFGSAQYSPAIAETDETPTEGSLPDTP

DPPSVVKDNVVQRERKVNMYFSAIGFIYDVKKGPFWEHSPMLFDISGVKDGWGKINKGMIKMYNAE
VLSKFPVVQHFPFGSLFSWDRDPSATFPPPTTHSQQVNRAIVPPATQSAGTRAPWSQPGTSIPGAT
AAPWATSTARNPQPSVTGAPTATRMPVRTTLPDTSRNPPGPMTPTRAPWTKPS
>g3160
MQGFNMGRYIPPDQEGLTTSNKLANKHPLGNRARHLQTKGALIVRFEMPFAIWCTTCKPHETIIGQ
GVRFNAEKKKVGQYFSTPVYSFRMKHAVCGGWIEIRTDPKNTAYVVTEGGRKRDTGEDKDGELGPG
EIRIKLHAGDETDEKKDAFARLEGKVEDKRQFDTAATRIGELVKRQDRDWDDPYEQSRRLRKSFRT
ERKRLEKVGQATEALKDKMSLGIDLLEETEEDKLRAGLVDFGPANTLLDGSNALATMSQTRPMFDN
GTPIKQSSGFQRKEESRKHKAKLKAEQLAAERKALLSNELRGNTRAAVDPFLNEGAKIWQPTLLKT
RKKKFTDADTSTERGGQAEEAEPVASTTAPSKTPAPIALVTYGSDSE
>g3161
MRSALVLSFLLPLALGCNNPANHPCAAVYTASRSSASEFCATFTASTITATTGLPTWASACANEVK
QLSSACSCLDTTWTTTTGVVAASTSVAATTTPVVVSISTPVAETTSTAVAVVSSIKAATSATSVRA
TTLATSTKTTASITSAVDTAGACVVTSYAGISSAVKSCTNIVLSNISAPPSSTIDLQSLQTGATVT
FAGTTSFGTTLDSDFDPIVISGTDYTITGADGHIIDGNGANYWDGEGSNGGQAKPDHFIVVKDSYN
AQITNLNIQNWPTHCFYINGVQGLTVSGLTLDNSAGDEPNSLSGSDPAAHNTDGFDISGSDTVTLQ
NIKVYNQDDCVAVTSGSNIIVSDTYCSGGHGLSIGSVGGKSNNTVAGVTFENSQVVNSQNGCRIKS
NSGTTGSISNVIYQNITLSSISDYGIDVQQDYLNGGPTGDPTNGVTISGISFIDVTGTCTDDGTDY
YILCGDGSCSDFTFSGVSITGGVYGSRDVKDYTGSVSAKAALYAFLSIVTIIGFTKQGTSHGPSGE
FYAAKAQSYLVDIVQESTMDGLQALTSLILATSFSGDYKKAAMFVAMASRIAYALGGHKNPFESQS
SVYDTSRPECHIRNLFWLCYTFDKELALRTGQPPSISDDNCDLTLPPGYVELQNSNICCPDVVIDN
TTVPLYPWDPRLSVLKSRTYMLLYSESALRKSKSEILKHIRELDDTLEQWRLSLDREFRPTLAFSE
DMLFPSALATQPVMLRLAYHHCVIIIHGASNRCRDPSGDLDMQEIGVASSLGLSVEASRSSLLYLQ
AALPNIAGECFWHILFYVVSSIVTILGHMISNPNDPKSISDFELLLQVSDMVQSFPITELSAAELI
QMQLVKDFSQELVGITGMATFDHKVRIAVIGGTGLQDLPGFTKVASLDIETPWGKPSSPITIIHHQ
SSASGKDVPVAFLSRHGTHHQIAPHEVPNRANIAALRSLGVRTVIAFSAVGSLQEVIKPRDFVVPD
QIIDRTKGVRPFTFFEGGVVGHVPFGDPFDENVAKIVRECGHSLEGDGVVLHDRGTLICMEGPQFS
TRAESNMYRSFGGSVINMSALPEAKLAREAELAYQMICMSTDYDCWHESTADVTVEMVMGHMKANA
VNARRFVGAVLDALALDENAETVNAKHLEGGIKFGMSTPHTAWNEDVKKRMEFLFPGYF
>g3162
MASTQAEPPAKPMSEKQQLKLEKFAKKKEKQEQEARLKQQQGIGASKGKKPREQVAPAAETKDWVE
VTPAGHRKILKSLDDDFHKAYLPKVVESAWYSYWEEHXFXKPQTEEDGSLKPKGKYVIAIPPPNVT
GKLHIGHALALALEDTLIRWHRMRQFSTLYIPGCDHAGIATQAVVEKQLAKTPKNGKSKRQDFTRA
EFIQLCQDWKEDYHKSINNTVRKLGVSVDWSREAFTMSPELSKAVNETFIQLHSEGLIYRANRLVH
WSCRLSTALSTLEVDQKELEGTTKLDVPGYDRKIEFGTLTYFKYQIEGSDHTIEVATTRPETMLGD
TGIAVHPEDERYNEFVGKNALHPIIRGRKLRIVADEYVDTEFGTGAVKLTPAHDHNDFNLGKKHGL
PFINILNEDGTFNSNAGPYAGEKRFNARYGVIEELKKLGLYTRQEPNKMVVPICSRSGDIVEPLLK
PQWWMKMESLTKPAIDVVESGELVIRPEIQKRSYLQWMRNLQDWCLSRQLWWGHQIPAYFVTVDGE
TNGDDSDDSYWVCGRTESEAQEKAEKKFPGKKVTLKRDEDVLDTWFSSGLWPFSALGWPEKKEDFE
RYFPTTTLETGWDIIPFWVSRMIMFSLKLTGKVPFTEVYCHGLIRDSEGRKMSKSLGNVVDPIDIV
DGISLGALHQKLLQGNLAPREVKTAEKYQKKTFPHGIPEIGADALRFSLINYTQSSGGDINFDVKT
MHGYRKFCNKIYQATKYVLGKLGDDFVPRESSALIGKESLPDKWILMKMNTAAKEINRALEEREFS
RSTQISYRYLYDELFDVYIENSKAIISEGTPDEARSAKDTLYTALESGLRMISPFMPFLTEELWQR
LPRRPGDNTPSITIANYPEYESSFDDPKSEVAYELILGVSKGIRSLLSDYAVKDKGLAYIMPLNQF
SHDTISAQLSTIKSLSGKVPVDILILKVGEISPEACAIFSVSADANVYLDIKDRIQDTKKEVEKFK
GKLVDAMKDQEDVESIIAELSKVHDKDVADALRSADRRKRDVSASLRALEDTVAMFEKMTI
>g3163
MDPLTAIGLAGNIITFIDFSYKVISGVNKVLSTPNGMTPENERLSVLVEDLNAVTHDLVADVPART
ENERQFCALAANCHALSGELSQILRNLKVGDRKSKWQGLMVKWDSMRKEKEIIAIEQRLNGYQSQI
LIRLQVMFNQKTDEQHFLVNSQLAALRSEAKELQNQAVSQLDELYQTILTLVDSVRSAPAQENSRG
PKEGVLAELGVSLFKFRNVITTISQENKILERLAFPSIYSRENSIDNAESGTFAWIVDEESQPADD
WSDSFVVSEENHEEQREKRQYEEEKKKQEALRKCTREKFLTWLNSGRHIFHISGKAGSGKSTLMKF

LCKSSRVKSGLESWARGRPLIFVRFFFWNSGDEIQTSLEGLYRSLLFETCRQMPSLIPRLFPELWG
SSTFGFAPIRFDEVRDAFNRLIQEASSSKSYFCFFIDGLDEFKGDEVDHWRFSRDLQSWTGQAENI
KLCVSSRPHVPFVQSFANDLNHQISIHDLTREDIFKFSVAMFEKDPNFHRIKNSYADLVIEVVNAS
DGVFLWARLVVRSLLKSIGYRGSEKDLKRKLHLMPKGLDDLFDQILSSIDPDDQLLSDQLFLLTTP
NFCRWQPTVRNAITYSWLEDLDNPHFPYELPMRPCTETEINERLERVSHALDHLSRGLLEMTRKRS
RERDGHDYFTYEVRFLHRSARDYIANTREAQMRARIPDFDVYSGIIRLLLAEFKFARFTLHDTKPR
FCGYDVPSRFFEELSHIIQHHTHTGGSRTQVPPRNEILEPKGYHSPDFLCEQNSTSGPNLLLTAAT
RSEDLGFVQGLLREGRTPIEMVPMEPIFPLDRTNFESSTTIPAPQATISVWLIWLYCFVENYFMAG
CSSEPQNRCLEEFLQYDVDRNVMFVVRIFASELADNDQTADETSGESDQRVAFELLEFLDLVQPSN
METLRTKLSSKGTREVRPEVTPIYGPPIPHNRGSLSEALEEIVTAGKINRLCAFGLCLCLESVITP
SERLDVPFSFRVT
>g3164
MLASVSLAVAALVGASLVSASHHHSVTEPKATVKNGSYHGLHNDFYNVDYFLGMPYAQPPTGDLRL
ALPVSLNSSFDTRNATELGPGCIEFSIAGYDRPLSEDCLSVNVYRPSGYENEKLPVLVWIYGGGFV
QGLNADPRYNLTFLVKNSVDMGKPIVAASINYRLNGFGFLDSPQIREAGLSNLGLRDQRLALHWIQ
ENIDAFGGDPSKVTIWGQSAGAMSVGSQILAFGGRNDSLFRAAIADSGGPLGAQPPTANASLATWN
SIVNLTGCTSAQDRISSYFPVYDGDFLPTYNSVQIKSGQFLKVPLLIGSNADEGTDFVGGAPYIGA
LPSKSYPDKTSFLNVAQGYVRNSSQLDAALAALSVLYPDIPSIGTPSTYHGRLNATFGAQYARVAS
LAGDLNIHRGRRLSAQSWIKYGLPIYSYHFAAWPIGGLPDYTGTTHFTEIQLVFDNEYGNGYLYPW
YPSGSEFAGQGKSLIALAKLMSRMWISFVNDLTPNNHGLKHDSNGHSIPEWPLYAANVSDALNGYG
VNYRFDQAEKGLAIIEPDTWRAEALAYLIDNSDNIFGS
>g3165
MSQTFTNCDSGTWYAFNKTNLNIGDTVQVQFGAFDNTTNLNIYLTRPGGSLVQSLVLNAGFSRNSN
TYRLYNSSSTCELDQLNLTIPSDAGVSTNDVQTFQFGVFNSSVPLGQDGVPLSGFLAWSPDFNVWN
NVTTTTSAAPTTSATTATTTTAGAAVSATTTPASNNNSSSGLSSGAKAGIGIGVALGGVLLAVLVV
GAWLWNRRKQRAAAAAGQPDEPMESFTKPPAPGSNTTVGQEYSANNQHQGLHELPPAANQQTDVKG
NYQSIPQQSAVHEMPSSHQAYEMA
>g3166
MNIVSLLSAGSVLALLVSAAVPSKRDTASTIWSDIENATTCTGCESLLVVLQALAHLGNDDFTSVI
TEVCIDAGVEDSDVCTGAIGLDGPILAHDLRQMTVGTHTAQLFCNTIFGLCPFPAVTAYNVTFPSA
KPATSRPAVSGKTPIEVVHYSDIHVDLSYETGSSYNCTKNICCRPYTSADAPGNTSYPAGPYGNHA
CDSPVTLEESMYAAIQKIAPNAAFTLFTGDVVEGAVWLVNETEVTNDLKNAYTKMTALGQVYATVG
NHDAAPVNSFPPAAVDTTISSQWVYDTLSSLWETWIGSTASTTADTNPGAYSVLYPGGNLHIISIN
TNMYYKENFWLYEATMETDPSGQLAWLVTELQAAEDAGERVYIIGHMPMGAGDAFHDGSNYFDQIV
NRYEATIAALFFGHTHKDEFEISYSDYTAQTYSNAVELSYVAPALTPTSGMPAFRVYSVDPETFAI
LDMTTYIADMSDSTYQTAGPTWTKYYSVKEAYGPLVTPPLTSASAELTPAFWHNLTEVLSSNQTAF
NEYYARKSRGYDVSSCTGTCVTDEICQLRAAQSQYNCVTVSPGINFKRDGTQTSVSDGAHRDSCEG
SPAKSILKALLTKKDVKEEIEKRTSILAKRKAN
>g3167
MPHVDPIQEWKVIGVVGAGSMGSMMAFAFAELGLDVSIWDVAGKNVDKLLENVEKSREKLQGKING
FKKIDEFMTSLEGKGTRNLFLFSITHGEPADSVLHQMKGKLKSGDIILDGGNEYYRRTERRQQELK
PLGVSWIGMGVSGGYQSARRGPSLSPGGDKKAIEQVLPLLELYSAKDKKTGKPCVTYIGPGGSGHY
VKMCHNGIEGGLLSTTCEAWDIMHKGLGMSYDEIGDIFKAWSSHGELRNNFLLNIGVDICHRKKSA
QGDGRGEGVDPAGGHVLDDVLDKVVQDDDNTEGTPLWSIMESALRHVSSPSLATAHYMRIASGNRN
QRVRVAKKLDLPEPKRIDIKDKNDFLEQLRRAVYASFLCSYCQGLELIARASADERWGVDLGKCIQ
IWRAGCIIQSEAIADMLQPILAQDVQIMNIKLIDEVSRDLRNNFESLKEVVLRGTESDACIPSLSA
SLEYLKYESSTKLPTQFMEAEMDYFGAHAYNKPCKPGEDPGPVKKGPHHYEWKPA
>g3168
MVESSKINLSSLRRIPLSYASCSIGKVTDSLSQRLEAITNAGFVGIELSFPDIKQYAEQLLDKKVG
PDDYDDLCRAAQEIGKLCQQLNLEIMMLQPFANFEGWPNGSPEREEAFSRAKNWIKIMKACGTDLL
QVGSTDSSDEDRITTDRNEIVQDLQELADMLAKENFRMAYENWCWSTHAPTWKAVWEIVQQVDRPN
VGLCLDTFQTAGSEWADPTTASGLIEEVSKTELEKRWKDSMEELASSIPAEKIYLLQISDAYKPNT

PIERKIKNDLLPRGQWSHDFRPMPGWFSMEIFDSGPDGTGKEYDVGKFAKSAMENMQRLFKNVADA
SA
>g3169
MSKTALLVMDYQAGIISRLPLPTDHLQRLAKTIDTARPHVKVIYVTVAFRPGHPEISASNATFSAA
KQNNLYISGSPETQIDPAIAPKEGDILIEKKRVSAFTGSGLDLVLKGLGVETLVLAXISTGGVVLS
TVCEAADKDFKLVILKDLCADGNEDLHNTLLNNIFKKRGEVLEAEEWLQRFDA
>g3170
MISKSFLLLFSAFVPFIQAAFVHPGAMHTAADFTRIKNYVAAKSTPWYTGWELLLNSSYAQSTYTS
HAVATIYRGDDGIHAQNYALLYDDAAAMYQLAIRWRISGDATYADAAVNLLNSWGTTLKLISGNPD
QWLATGLYGYQLANAAEIMRDYSGWSSTDFATFQHMMEDVFAAQNEYFLLTHNGSANIPYANWDLC
NMASMLSIGILNDNATKYDWVLDYFYNGWGHGAVGNKTNFIIANFTESGSSKILSQGQEAGRDQGH
ATLDFTLAGVLAQQAYNQGTDLFAAVGNAFLKGSEYVSKYNVGYDVPYTTYVSYEGTQSVISDSAR
GDVRPGFELLYAHYNDLKKLNASWTGRYRDYVNSNISAGVEGGGGLWHTHDDLERIRNGVLNNEEP
WKSAYANFSINSYSQANYTMKGPYSVLCRGSCSNYTSFSQDVRAAYQNALMWYITKEQGHWDRATT
ILDSWGSNLTNIIGTDTSLLVGLEGDFFANAGEIMRWEGNWTEKGAAAIGTSGFSNQLYWLFARQS
VVIGQANYGMVSIKGLLSFGVYLDDVQMYNYALHAWQNDLCAGIYGNFDPATGQSAETGRDQQHSQ
GGLGWTAEAARVVQSQGTDLYSFDNNMLLKAAEYAAQYNLGYDVPYDEKFYRCEAILVNGPWSGPS
PMGRGYQSSPSVWDILYYQYVVKRGVTAPWLTKIKKAIDSIGGERIGTTNIDDHPSWGDLIWSYPG
KGSFLNNDSYTIWGGGEIGQNGTGNLNLT
>g3171
MKATNSLLRISWLILLSNVVQATNDPLVYREITGFVPAASANVTTLMEFIAGRPDLSTLAKNIGEV
GGFLEAFNTTPNWSFTFFAPNNDAFMSHTGEYFNTFQATPKGKWQLGNTILHHYVPNTKLASTVFN
STYQRIQTATYLYVGTQAENGTVVLNQVANIVEKDISVTNGVVHIIDRILDPSAQIFEAELPKISQ
AFIPGSCSNAALAYC
>g3172
MEVKRHFSWRSLVTIAALCLFHVTAGETTLGQTPQMGWNSWNTFKAIINESVIHETARLFGTLGLQ
KAGYNYVVIDDGWANHSRTADGYLQANVTGFPSGIKALADKVHAQGLKLGIYSDSGILTCDFKPGS
WGYEERDAQTFASWGVDYLKYDNCGGFEGMTEASQVRFGTMKNALELSGRKIFYSVCEWGFQFPWR
WGGGIGHSYRISGDITASFTNETGCACKTAYCLNTGYAGCSVLTIINKMREISQYGVQGHWLDMDM
LEIGVANMTLYQQQTHFTFWSALKSPLIIGADLSRLSNESLAILTNKNIIALNQDSLGKPVSYVET
LSEDGLWQVWAGNIESGYVVLLLNEKSYPQTISLQLSNLGLGTPRKIVELWSEKTLKTNAFNGTLQ
PYQTLVFKIDTA
>g3173
MPSLPVLIDNTADFDFSPAQNVTQKRSLLLAPPSIAAHEEKLRDIFATFDRSVTDLQMLDRLSAGF
VTLPTSTYDLVLVLTDTNGARRNEALGLLTRDVFNVLTPAMKPSAQLKLQDGPFQSTEGREAILAG
LVEKDGAFEKPQYQEAAVPLRFGLNKKKNKVAPEPVKVETVGFVDNYDDDELIDEDDLLAEEDLGR
PVQQPAECKPETAKKRRRACKDCTCGLAAQLEAEDAERREKANANLNVLKLNTNELNDEVDFTVQG
KTGSCNSCSLGDAFRCEGCPFIGLPAFKPGEEVRILNDMAQL
>g3174
MASTATPVASDEKQQKLDLAITLALNTWPALTLAVQSSWGGQNSSDKRDWLCGAISDIISTRPETD
SYDIEDVLVQVMTDEFDVAVDDGSAXVVADQIMTLKGKIELGDYEDVDRMWAEYKERTERKGAEQQ
AAIFKRVETKDEDQETDGDDDDDDDDENEDEEMGDAPALVSRPPRERVEPEVDEDGFTKVVGKKKR
>g3175
MFSMLDGDWDLNDVSQITFRKINFSMDAAQWTVACFLRQAWTFVPPTPDCKDVGPVMKRYQDLPEE
EQAKLPLNSDTKTLHNPWRNEIYWLWNHLFYDVGNFLDAAYGMGKFYPPNEDVFGLEFPKPHPDEE
SENVRVLAKVWETEVIFLPTYLAPGVTVRVETFGPGEENGVLRNVVKLRGKFSNEKDKLAQAWYVT
KGLETRFYIEGGDNLTTDGWAAVIVYGKIVEES
>g3176
MADQTPDNWEEXLSRQADNMNLNNGNNNFQAQAASFQPGAASFQPGANTFVPGQQFQQYGYPQYQQ
QQQGYYPAYNQQQQGYNQYGGYGQQPGNYNQIYNNQYSGGGYNQYSQQQQPQARQAPAAQRAPEPV
AAAAQAPKPATTTAPKAKVLSLGDSTGAPKAKVLSLGTPAATKPTKDETAPGDSKGAAAAEAANKV
TAAKAIEKTEKKAEAKAASSGKASPAPSSGRNSPGRSSPSRSDGPKATRDADAVSREQKADVDEAT

LKEIYGEKREHVNLVFIGHVDAGKSTLGGSILYVTGMVDERTLDKYKRDAKEAGRETWYLSWALDL
TNEERAKGKTVEVGRAFFKTTYQGPDGEVERHFTILDAPGHKSFVPHMIGGASQADVGVLVVSARK
GEYETGFEKGGQTREHALLARNTGVKKLIVAVNKMDDPTVEWAKSRFDECTVKIAKFLENLGYKKT
DLTFMPISAQRTLGIKDRIPKDICPWYDGPSLLEYLTEMKMPERKINAPFMMPISAKYRDMGTMVE
GRIESGVLKKTNNYIMMPNREEIGISALYGETEDEIATATCGDQVRLRLRGIEEEDILPGFVLCSP
KRPVHCVSAFEAKIRILELKSILSAGYNCVLHVHSAIEEVTIAALLHKLEPGTGRKSKRPPAFASR
GQTIIARVEVTGSAGAVCVERYEDYDQLGRFTLRDQGQTIAIGMITKLLLNEDAA
>g3177
MEVFLSKITNQAMNYAIRSGISITATYAIQQSTRLLKNVGGTERTELLALQQRLQSKITVISPAID
MIELIAARGNTSLESAVSLTKAIRHDIQSLARRLEKAASAEELAHRGALSSKARAQNEIEIKLIIT
DIRRVLERIEDAVPLMNLAITTSGARLSTTLPSTVSPSRLLQASTFLSACDTQYSIKPFRAVQVGP
TFTLSMYMLFSGHIRHFDEENIRETTWKEVIHKARVKLRRVPMDVFADTSEQKSNKSDLPATARVD
EFAYQILIVEDLDDDRVHNFEDNEAHPAPYEGVKLAGLREIIPVHQISKIFYADTGKMLNIGSEGE
TNSPVLLLKRDINAIPPRRMIDRNETMYSELSEEALVEETNEKQAARDELQEQLDAQLNATQPEEE
ELGYFKSLSERHIPAPWRLPPGLDPEWVAFEVYQEEDKDEDETDVDDEEEATDAASQMANLKLDDQ
KDSSTQQISFSSHDLSASTASNPLFNNIRTSLSLLETLLRLTSLQQFQQQSHLSITDELLNFFLEE
SSSTGAGGDEQHRQRVRAEARRKVGWDPYDESPLKRRGEDYQYQYNDDGHWTPTGTPARYAYRESS
VDPASPGASEEQYQTRSSREQTPPLSSPGLPRQTVRQGLAGGKKGIVPRVSPLSRRPTTALTDEGL
GTSPSVGQESATQ
>g3178
MDFAYDHIQEEILASNQSDKHEDGNPSTDSKNIDLGAEFEETFRAFSNSPWGAKIGGLWSNVRKQG
ESYYEGARQEYAAVSEEATKGFSGLRDSIVDRTKGISLGGALSAISPGNEEASGDSTTKTADDENK
DEGESKDQTQHEEAEGFISRFRAEAAKRLKEIEKAEDAADEALIRFGTNVRNFLRDAVSIAPPEQG
SDKVLFESKDSEGKRVFHATRLEAQLHAIHTNLEGLKKNPDTEQWTEFNKEFNVESKTDDITKDLE
QYPELRRAMEKLVPETVDYSEFWSRYYFLRLAVETEEQKRKELLKGAHKAADEEEVGWDEDSDSES
ESPSTPHVKSKPSAASSQNLTVVNDSNLLSEPRRSHDQHSQPDSESSYDVVSGATSQXPASPREKS
SAKVEESDEDWE
>g3179
MTSSVLYSNIANPPLQITRADGHYLFTEDGRKIFDATSGAAVAALGHNNAEVKNAIVEQLETLDYA
YLPFFTSAAAERICKFLTDSTGGEMEKVFIVSSGTEAVEASLKMARQYFVELGQTSRTKFIARKQS
YHGNTLGSLATGFHKGRRAIYEPILATNVSHVSPCYSYRGRNEEERDEDYVKRLAQELEDEFQAQG
PGNVCAFIAETVSGTTLGCVPPVPGYFKAMREVCDRHGALLILDEVMSGMGRTGTLHAWQQEGVVP
DLQTVAKGLGAGYMPIGALMLHKKVVDVLSKGSKAFVHSQTYQGHPVACAAALAVQNIVVRENLLE
NVRVQGELLGKLLQERVGGHKHVGDVRGRGLLWGVEFVKDKKTKEPFPAHEQIGAKINAKGLEEKY
SISLMPGPGIVDGRDGDIILLAPPYTISAAEVENMVNNVAEIVSEVGLGIRYRFNGSLASRGKLVG
ETIPSRLSYAGETDANSSSLIISSTNASHKNAQLRRSERKCAARHDEELRGLSDDPLATHIAPAAA
VIDRGWNGFRDLLLPYAETEPFVRSAIAAVSRQHIILRVGGDFTSDIREYNSLIAELVARSKVFPP
NQDIDSLVVLLLLHYRETVSGGTGFKYLYGSLKALPDLTIQNGLSXQLLRLFAEPLFDEAAGKQYL
SVHRTRCFEFLRFCQTLHPEYEELMRLLFAAVTLACEAYVRRAYFDPPSSEVAHLIETFRQLVRRV
KCYGNVVGQNMLAWPYFVMAAESSTEEDREFFLNELQALYQATGYRSALRGVETILNMRQAGSNIR
WTSLLGGYDQFLIM
>g3180
MSSRKTHQHQPRDNNDLPTTPAESTTTNATNGRRAATTSKASRKDRPIASSFLTNPKRRKFPSSPD
WDSQITLTQLVPKAETEAPDGGLFEYDDDGTEGKFVEVIDLADDADENDASWRPSTNRRPPAKRRT
NGGSRSPSSIPTTTFKKRKSSVLISGGPKISKSIRRNNKGSAQKETKRNKTLTQMDFVRRFIPLPD
SDEEDLNLYENPLAAKEEDDKTKGHTVEKDDLEDLTPRKRRKLNDENITPSPAIKSKVSPAEKKPT
LLGVPGVQPKTPQKERRFEIPSSQTPETPQETYVASPSIRQVSRFSIERLSNTIDTNISGSSLREA
PEETQDQQDDTRPPDLEVAGDDIVASTMISSSQLLLSGTKEQTTIQETDAMKQTSQPPEPPPLSSP
PMRTVVYDTDAETDYGELEDDNLPEISIGAKALEEWPEPIDNSSRDHTSQNCDYSDDLPPVPNSGT
DLEIIGNFLSDTTLPSESSVYYRRPARYTQYPNEPVPMMNTQQVAKLFPVIEDHDDSASTIQTSIP
STRDPAASTCHEHSNVLSSEMETPTQDDSVNRSIQVVPESSPITRSYDAARSASNTMAPPPARESI
VLVESSQLVDRLNRQLDSVDAGKSLRRLFSTRDFLTDSVMESIPAPPGMASQDSIGEPYPENDGHG

YS
>g3181
MPRTTLPRRGRVRPGNFQKPRNHRDREEEIPQVYRELLAEADARAGDEPQSERPIKRRRVGERAQH
THNVQTDTDIQDSPSSNNVIDLTQAQTIYDLDAEDSSEDESDMEWEEVIVQQPATGIQNSVHTGGD
ESLDITLEEPTVRRTLVRRRKPVTGSEKRLRLDIHKAHVLCLLGHVYTRNLWCNDDDVQKYLKQML
PKQTISLLNPRESMPQFQRSTTFIDGLKEASEAFVRRFKVTAPGLRRPYWAENVQECKDRAASIMR
DGEIISTREEFREQAKKLHGSRDFGAQLFCALLRSAAVEARLVCSLQVLPFSGVAKGISPEKPKRD
YIVISSDDNLSSSDDKIVAQSSSLPAKGRRLGQPSFKTQVRPSSSNNLHRPRSFTSSPYPVFWVEV
FNEAVQKWIPVDPLVTKTVARPSKFEPPASDRHNNMSYVVAFEEDASARDVTRRYAKAYNAKMRKT
RVESTKDGETWWTNVMNYFEKPFLEDRDQLEFSEFTAKSAAEPMPRNIQDFKDHPVYALERHLRHN
EVIHPKRKIGQVEAGKSGSKKGTVVEPVYRRADVHLVRTADGWYRLGRDIKIGEQPLKRVAASQRR
DESDDDPDAISGAERTLYALYQTELYKPPPVVNGKIPKNAYGNLDVYVPTMIPPGGFHLKHPEAAR
AARILGVDYAAAVTGFEFKGRHGTAVVNGVIAASEHREALEEVIRCIEDERIQEELDRRTEEALRL
WKHFLLKLRIAQKVKSYVIEGEASEGEEEVLEKDGHVSDDASDDPDFADGGGFIADDDVGESASGG
GFIPEDNETSSADGGGFIPEDDGHDLSLENNDFGNSLDSENHEGGGFIMNESDGLNSESRNDGGFI
TDEHSPATDRDSPYNKSVEDTNNVQQSAVTERNVPRSKKNIPRYKLIVIPNTTTIEISSSTSVQSA
KKAAEEKDVAQASMNISDGVVPADGLSEAAPIQLDSSSDQASAAMSVDLNSQGPEEPKSPEQFVQP
ADSESDSALEKESLLSHDPEDEDAEPEWLLSD
>g3182
MGTFSSQRRSLARADGGSSQSSGDDQIINVSIPKDSFDTYNIDPPPYSLETSKNELRQLYKDMTTI
RRLELLSDQLYKEQKIRGFCHLSTGQEAVAVGIEHAITKSDPLITAYRSHGFTYMRGGSLRSIIGE
LLGKKEGISYGKGGSMHMFARGFYGGNGIVGAHVPVGTGISLAQQYSEKRTMTIDIYGDGAANQGQ
VHEAFNMAKLWNLPVLYGCENNQYGMGTSAERSSAMTDYYKRGNYFPGIRVNGMDVLAVLSAVKYA
RRLITGEEGSHEGPLLYEFVTYRFAGHSMSDPGIAYRTREELKDARKQDPLMVLKELLIELKVNTK
DELKAMEKEIRGHVNAEAEAAQKMDNPPPTQDTLFQDIFVRGAEPPYMRGTTVDNTYYYPH
>g3183
MAPRRPPLLPIPGNNQKIQKTQSFEERLKQFFTSSYNTLVSDLLMDLDHPFIIPIELYLSVRQPTV
SLEGLDYSRHDPFDFKINFYIATFVDPASDDIAALLQASLGDTSSVSVSFEWAYVGAIEVMPTVYI
EVPPETVTDWADLESGIRAVLARNASKMGIVVDVQFRVKSSQNRAIEIT
>g3184
MTVDPQSILIRKKVGTSVKPRPRKRQQSLSSSDTTTSIQATEMTGSIGNMSVLSFREARRKRTPTG
TYQITIEDYINRNPQPNNFHKTLPTSVLSAVATSKAVDDAYGYLFTHLKGQCHGLNVITYPRVGKD
GNRAQPKSVDGSDISSITHFAILVYIQPGTICDDWEKIEEGLVHIFKEAAIKKDGEEVPKWRLKGV
VFELWTTKPAAWEFRSGHL
>g3185
MKLFPLTIAAVLKECVGARILGQRDASTDNSSFNVLDYVDPLIGTANGGHVFAGASLPFGMAKAVA
DSSVDNQGGFASDSNSISGFSHMHDSGTGGGASLGNFPIFPNPGCPDDDLNQCVYRYQDRSVGFSN
ATLIAQAGYFSIDLDSGIKGEITVTNHTALYRFTFPSDVTTAPMIFADLIDLPQSRSNGTASVDDK
TGRITGSGTFNPSFGIGNYDLHFCADFSGADIFDTGVFVNDRAGNEPKTVQTLSDGVNDDESYSAG
AFTRFKAGTTSVIARVGVSFISVDQACSNGEKEIPDFDFDSTKQAAQNIWKEKLGVISVDATGVST
DLQTVFWSGAYRALLSPQDYTGENPLWESTEPYYDSYYCIWDSYRAIHQLITLVDPLSQSRMIRSL
IDTYRHAGYLPDCRMSLCKGYTQGGSNADVLIAEAYLKNITDIDWATAYEAVIQDAEVEPLNWAVE
GRGGLTSWKNLGYIPTDDFDPYGQGPLTRSISRTVEYAYDDYCIALLAKALGKTDDYEKYIKSATN
WHNMFDANTTSLGYTGFLQVKYSNGTWGYQDPQFCTPLMNFTSCYLNPDGHETYEGSTWLYTFYVP
QDMASLISALGGRDTFIDRLTYFHNSGLLYVGDEQAFLTVYLFHYGGRPALSTKQVHAYIPSQFNT
SISGIPGNDDSGAMGSFSTLSMIGLWPVHGQDVYLINPPFFKEVNITHGITGNVATIRNVNFDASY
ADFYIQNVTRNGEVWTKNWIQHDFFEKGGVLEITTGASESAWGTKAEDLPPSMDSYAF
>g3186
MGWVYNLDTPNAPTNAPRLIAIVSVFSVAALLXVLLRFYARFFTNRKPGIDDYTALVSVLLLIVYS
GLAIGQTRWGQGLDPAAFPPEDIIPFGKIQYAGGPVYTLALLGFKLALLTSYLRIGGFVQFYKKIL
YGLVILCICNSITFTLLICLTCIPVAKIWDASVPGHCINAIAAYYAIAGSSLALDVLIIIILPIPVL
LKLKLNTRQKLVLVGMFATGFFVTVIQIIRIFTIKNLQSYTDSAAIIIWSAIEISLGIIVPCIPTW

APLVRSMALASGIGSSSGPSRPWQSHNTPQRSGGGTGLGGGGKRDKFSAIHSTDMPYDMSAFATVT
THHNDGDGDRSSEEAIIHRAANDAAPQHADDASREDRFQIHTTTTVKVESHQLEEDVDEESDFSR
>g3187
MHWSAVICSFLLSSVPAKAAASSSSSSTCRCFPGDTCWPSAAAWAKFNQSVDGTLIATVPLGTPCH
APSYNAAECDVLRNGWLLPEEHYESSSSIMAPWFTNETCDPFHPVSQPCTLGNYVVYSVNVSKPEH
VSKTLAFTKQNNIRVVVRNTGHDYNGKSTGAGAIAIWTHHLKTREVIDYNGTYYQGKAVKLGAGVQ
GIEAYELANANGLQVVGGECPTVGIAGGYSQGGGHSALSSRYGLGADQVVEWEVIDGTGKFRVANR
EQNSDLYWALSGGGGSTYGVVWSMTSKAHASTPVSGLNLTFTNEGVSQDTYYKAVGIYHANLASIV
DAGAMSVWYFTNTSFAISPITGPNIPVAKLISLVKPFTDALTELGIKYTTYSQQFDTYLDEYNGMQ
GAIEVGIAQYGGWLIPRSVVETNNDNLTDVYRYITEDGAQFIGVGVNVSKALVGNDIYNAVLPAWR
DTLIDTVITTPWNFTAPKSDMVAEQLKMTNDYIPRLKALAPNSGAYMNEADFRQPDFQEAFFGANY
QQLQCVKAKYDPEDLFYAITAVNSDQWTELENGRLCRT
>g3188
MAKRFLANHGLANNKAITADFRRDLQQLIAKAELASQKDTELVKLRRENSQLMTEVFRNRVRSESQ
QSQQSQQSQQSQQSQHSPVYSAQQAASATPSCPPSRTEISKLDVSDKSWDRIISEAF
>g3189
MPGVPSNKACDRCKKRHVKCDEGRPECQRCIAANVKCPGYSQDRKFIDQGATVRRRYAPYQREDSR
SQASSQHSARAGTEEESSNVQPGKVQQEIDTREASGQDPAPGSSRSSTTEDQINPSGSGQPDSLSR
NQMSLDVEPNIATTAPYANIALPANFLDTAQQPLSDLPNPENLAYTNTFGLFNSLQSPMFFGNNSG
NSENLNSTSTSNYAYTMPDARNVNAFGDLYSELMINSDREIAFLKRHYVEFISPWLDLHDSEKFFM
LNVPKRSLQVPFIQYATLALAAKHLARINGVRSCRTTALNTTTTETYPNAGQVDWLFKATNYYYQA
LFHLKQLLFGQADFQQLDAATPPIQILCQDLEIDHTSEGARRAVLPPSFISYMDDILPGVVILTIF
DIIDKPGTEWEKRLLGLRHLMQSIMALTTSPSKSTSLSYALQVSFWNLAYLDCFASYANRRRTCMD
AYNFDLFRAAGLAIDDAGIFLSPTPPSSPRRDDTFLRGITFIALKLMNFIAEFKDIQQSATTMSSP
VPDFLSNISVPQSSGLAVTWHQLVQELSNWHKSLPDTFEPYIRLENPHDLVSPTPSATSLPFPEIV
FSSATHAATVALHDFARIILLLNKPHDSQSVARDRLVEYREITKEVDIRAREIAGIAMGRTASAVQ
IHLVQPLYVVGLCDDRPEARQKVVELLRIIRAEAGWETEYKIAQLHELWKKS
>g3190
MRAFDTDPVFMCASIRTDRGITTDLKTVARDLTELGDMAAAFSKADGGRMLKIGYEAFNVLAVEFA
DPYNPAGHGRIYPTIEESIEVLAASMASLAATVPGDRIDFFQCGDAELMNPHNFKRPEDLKTPALL
PWSRGHRLYPFEHSKSAYMPVELVTAAVLAIGYTGPISLEVFNNSLDKPGADVPTMRCIRPLGQPP
KNPCERCSRNDRTYTIPQRKPQGRKPGARGRYQGIEKAYRKMQSELQKAKTSFDSARQMQELTSNA
TPGDDDTEILQMLLAENAVTYRRGHSRIVTKVGMRKRMYL
>g3191
MGFKNILVILSIATSALAAHTVGSGPWTSVTASTTFNVQECAGGVVDGDTFSLPTSPDGSTSGSGC
SNGHLRAERRYLNDYTSGVHQFGGNFQITSLTGTNIALKQTFNGNTGPYFIMGVKSNGDLYDVEGG
GIIASGVATVGATVTINTIHDANNQLYYVYVNGEETFNITAPGGSFYDKCGAYTSDSGTGAITVVW
SDISFWTQ
>g3192
MASSQPSTTLGKNVESDAATLNNHIKTEAVTAQPPKPTGPPPPPNGGTKAWLQVLGAFFLNFNTWG
LINTFGTFQSEYSTGLLRDSSQSSIAWIGSLQAFLMLVIGVLCGRALDAGYFYVDITLGLFLEVFG
MMMTSLCKEYWQVILAQGICVGLGAGMSFIASVTIVGTYFSTRRTTAQGLAATGSSIGGIIYPVVL
RRLIIQIGLPWAVRVMAFIMLGTLLISVAVMKPRLPPRKSGPLINVESLRDTVFVLWLFAAFFIFT
GLYIPFFYVGQYGLDLGMSQDLSFYMLIIMNAGSVPGRVLPSIIADKIGNLSVMIPSVLLTGILML
AWISVKSQSGLIAIAFFVGLTSGSIQAVLPATVAFLCPDLSKLGTNIGVTLFASGLGLLIGSPVAX
AILDHQRTSGGDVFWGTLTFSALFILAGGVLLAIARVFKVGFALKKA
>g3193
MLTFNHLNADAFENSKKPNGRKRPRIREPVACNHCRKLKVRCDRKSPCKQCRDRGMDTECAYSRSK
SPVVSVSGSVRPLVSPPSTEIDVLTAPASDGEVPRNVSTASHGNGAFRGSDYKTRLIGGTHWLAAC
NDLPVVEAMLKKTVDFQPTWSNFAEVKSLLRVANSVPAGVAGAGNTKLWHLLPDRPTCEKWIRRFY
ETYGRIYHVIDQNFLIGQLQRVLISPVDANEVYILKILLVITIAKQTDKSERLRGRLIMQEAEWHI
YTSPPFQKPCIGVMQVLLLLIILKTINASDTDSIHGLLGIMGLTTQMALSMGLQRDPALFPNVTPY

YAEFRKRLWACFFRLNLDYCIRSGSQFSIRLEDVDCPLPSSIDIQTLDQGTAREPVLLLSQTQQAS
DQAFNIAAMKLAMMRAPLQQQLCSTTPQLSSEVRDQVRASCRKILRELPPNLQEGASSCSPIEKLQ
QAILTAHVHGSMIIITHNNVLGVLPHKSQRDDLYEAWDNSVPILNQLQEVLQGDSELSSVAYHFLW
SDLARAALTACLVVFRLRSFTLGTIVSNCPPPTLVMFQQHLSKYLEYFSHILAGRYHLGPVVAKTR
LILAVTTSITSNLFRDFGDIQHDSKFIKLGIAAAEETVTEMQRSLRREYQVSAPLLLEFSDTARIE
TALTAPQPLITNWMDPTQISDPLTRTLFASDCDFGLGSKSPDLDLESSLYAMAPFEPTLTNQSTTD
SLWEDI
>g3194
MLNDIELEKLNSDLTSLTQTSKTYQNNLQLALESFHDLLERYKSLKSDYEEEKAGRERYKRQAKAR
SLIQERNPFVLVLIDGNGYLFKEDLVRAGAEGGLRAAQLLNNAIKAELPPDIPDNCRIMVRIYANI
LDLSAVLARGDLVGKEARSLSMFTASFTRAQNLFDFVDVDAKKGGAKSKIKENFRLFIDNHQCKHI
FLGGCHDREYMPMLALYRDEKNRVTLIKSANTYPEYQTFGLSMKDLPSVFMSTFIEDRPSPPIDPN
KPVCTHFSKGICKFGVSCTKSHLSEEFKIKSSEDPLFKSPPKNLPSLPIHKKCYAILLPKASGNTT
SRIPVDKEGGRIDTYTPRPSDDSFQRYKDLSLRLCNNFDTRVARWXKPDDDAEDEKFEETRTDDTG
SSNQTNAKDKGIEEFEAMWNGTDVAQTDNESSQSFSVANSTNEDTSPDLLEVEETASNFSIEW
>g3195
MSRNITSQLLDLELPTSVKLSPDGQSVVYSAKSNWKKDDHISSIWIGEINSPKSAYKLLDGTFNDY
EPRWAPDGQSIAFLSDRGARGKSCAIYTHHLNSSQEPKALTAATNTQTIAKFEFSPDGNFIAFLVP
SEEEKNPVVHVWNENWIYTNLKLLDVRSGDIRLISANSLHVVDFSWSEDGSQIAYATHRSPDIESE
WLHGTSIWTTEIVDTDVQSRKVCHIPREVVDINWLNSELYFIGFSTLTDDNSARSVYYIDMQDPEG
RIIKAAHGDDDCAEGLRRVGSKLLVYVQKGMEDQLRLLDSDIIYKEKKCILAFDAIMDTAGKIWVA
FVQGSVNRPQEVFEFLPAGELIQLSDHGRAWTTDFCTRKFIECPTLDGSEKIEGMFISPIAKAGQK
MPTVVLLHGGPYLRVTDSFDFLNHFFMPRLLLEGFGILLPNYRGSCGRGDKFARYTTAGVGVYDAP
DIIAMTQHAINLDLADPSRLLVAGKSQGGFLSYLLSVRNGAHGLGWHFKAAIATAGVTDWDAMAMS
SDIGYIEAGMAXGAPWRLSKSDVKTRSGSALWEFRDAAEKERIPPILLIHGENDIRVPVSQAIGFR
RALEEAGLEFQCAIYRGEGHYFQKRASAEDLVERIVRFITKYLV
>g3196
MPALLKKSTALQPGELVLVTGANSYIGSHVVDLLLSLGYIVRGTIRAEKPWLDEYFTTKYGTGKYE
SVVVPDVAQKESLVKVLGGVSGLIHVASDLSLSTETDKVINNVIAATRSMLEAAAEQKSIKRVVLT
SSSSAAILPKPNVEGIVITEDSWNDEIVKIAWEGTPETRNPMYVYGASKTEGERFAHKWVKENNPP
FVFNAVLPNMNIGRFLHPNFNGSSGKYILGLVDGNRLLMDLVPPEYYVNVEDVARIHAIALLDPEV
KSERLFAFAHPFRWTDIINLLRKYRPDSDKIPNPPENEGHDLSVVPPAKRAEELLKEWFGQPGWIS
LEQSVKDGLETYSS
>g3197
MSKASESPFNSLLVSPTELHSALSNASSXPRRIVPVAVGRESGLKSCEAKQIPGSVYEALSTKLES
IYHVRDTTSAYPFMLPSPSHFAVSMTKLGIRADDILVIYDTLETGFYFSPRVAWIFRHFGHGDVHV
LNSFPLYVEQCYEVLEGQNLGVLAAGETYPVRALLVPNDVVDFDELRTYLSAGGRTESSQFQILDS
RPEGQFSGSDTGGAIVGHMPSAVNVPLGSLLSTDKSLLPASQLKELFEAKGVKETKPVILTCNSGT
TAAALGLALDVSGYQMSKRLYDGSWFEWRDKVTKNEGLIVVD
>g3198
MPHPSHDGVPDGTTSAHVADGDFEKQESEDGRDDASRRVADEKRFTFPFWKKWGILSVIFLVQVSM
NWNASFYAGAITGLSKHFHISKQAARVGQMIFLVCYGFGSELWAPWSEEMGRWPIMQLSLFFVNIW
QIPCALAPNFGTIIVCRALGGLSSAGGSVTLGMVADMWEPDDQQYAVAFIVLSSVAGSAFAPIAGG
PVSEWLSWHWNFWIQLIIGGAVQAIHLWVPETRCSILVTREAQRRRKRGENVYSYSELENTKFQWR
PIVTVWLRPFLMFAREPIVLWLSLLSGFSDSLIFTFLQAYQPVYQQWGFGDVRVGLCFIPIIIGYG
IAYLSFLPWIYKHTKIRKRDPDALQPESRLYWLLWTAPLETIGLFGFAWTSLGPPHVHWIAPMIFS
TCIAIANYAIYMATIDYMVASYGPYSASATGGNALARDFLAGIAAMYSTPMYENMGTTLHLEWPTT
LLGCLCIFITAPIYIFYWFGPKIREKSKFAQVLASDRRAKGARSVRCGSETQGAEGTYLGSQNAHT
GSFRGTRASGSGCSA
>g3199
MGTNHDGALHHLEEELHTKIYPGTEIMTDVGSHHFVKSSINSKQVLVPQPSNEKHDPLNWNRRWKF
AIIALSTTLSFAQGLGPLALAPMFGELIEAFHSDLPSVVRFTGVCILVLGFSNFIWVPMAQTIGRR

PVAIFSSLICLASMIWRAKATTYSSFMGACILNGIGAGPSESLQPQVIADTLFLHDRGAYNTLYFT
FYFGSLMVGPIISGSMAYHVGMQNFWWLNVALFAVLIILLIFFFPETKWHRDQPLEADTVTPTSTE
LQTPPNPKGEITQPTEIESTKNEAEPVDEWLHRGSPAKSQFHFWKLSPEPVKTMAITLWIPWKLHF
FPIVEFAAFVVSWSASVYLTVNLTQSQVFAAPPYNWSSQSIGFTNFAVFIGALIGLATNGPLSDWI
SMRATRKNNGVREPEMRLPAMIPYVLIAILGNFIVAFGYQHSWDWRIIVIIGYTCSGIQVAALPAI
ASTYAVDSYKPVAGSLFVNITINKNLWGYGFSEFITNWIDMSGFLSPIMLNMCLHVLWCSFGIVFY
FWGKQCRKWTAKSSVHQMSDI
>g3200
MNAQLSSALDGLENKLNSLLTSLTTPAATGAPAAAIALLEADDVVSSALDTLRIHQANYAKILHLR
TEAQNLEERIKTIVRDISGAGKEIAQATGENDDDDYEMDTDDEDEDNESDTDTESENGDTIGQQKR
PQKGRKKEVDYKLLLEFARRISKYNIQAAADATSGVLGTTGSTTKKLQEKQLEDAKMIDANGLAEQ
EQQQQQPGGEQEEVTVGTVTKEATSWLEESADADRQYFMIPYPNEDRIRMGLMGQLQVAAVDGNAD
PEKEAERMVQEAEGIAGVASGPADVQPRPQTLADEAGMAAMNAGAHAVARPAAKPAAPAQPRPTLD
LDLYDPEDDD
>g3201
MSLVSGEKSNFQYIIRLLNTNVDGKQKIMYALTKIKGVGRRYSNLVCKKADVDLSKRAGDITSEEL
ERIVTIIQNPTQYKIPAWFLNRQRDITDGKDSQVISNGVDSKLREDLERLKKIRSHRGLRHYWGLR
VRGQHTKTTGRRGRTVGVSKKKG
>g3202
MRGLATTLLIGAAAAFSPAQQVLKAPEHVEKTHKTSSPSLAESIAQPLHELNEELKSLTHDAEEVW
EQVSNMFPGALDNVPFFSSPKKHTRRPDSHWDHIVRGADVQNIWVENANGEKEREVGGRLEAFDLR
VKSVDPSSLGIDPDVKQYSGYLDDNENDKHLFYWFFESRNDPKTDPVVLWLNGGPGCSSLTGLFFE
LGPSSIGKNIKPIYNPYSWNSNASVIFLDQPVNVGFSYSGSSVSETSAAAKDVYALLTLFFKQFPE
YATQDFHIAGESYAGHYIPSFASEILSHKKRNINLKSVLIGNGLTDGLTQYEYYRPMACGDGGYPA
VLDESTCRSMDNALGRCQSMIQSCYDSESAWTCVPASIYCNNALLAPYQRTGQNVYDVRKPCEDSS
LCYADLEFVSTYLNQAEVLKAVGAEVDSFDSCNFDINRNFLFKGDWMKPFHKLVPGILDEIPVLIY
AGDADFICNWLGNKAWSDALEWSGHEKYAATELEDLEIVDNKHKGKKIGQVKSSGNLTFMRLFGGG
HMVPYDQPEASLEFFNRWIGGEWTQ
>g3203
MPINQPSNQIKFTNVSIVKLKKGKKRFELACYKNKLLEYRSGVEKDIDNVLQIHTVFLSVQKAQTA
PSADLAKSFGAGVSQDEIIQEILRKGEVQVGERERKDILERIQKEVVDIVSGRLVDPTTKRVYTPT
MIEKALDQLSATSGQQQQQNGEETPEHGATPGEPRKPLWKGVSTTKSAKSQALEAMKALIAWQPIP
VMRARMRLRVTCPVSLLKQSVKGAASAGGAPKEEGKPSKGGNKKGQKGKGKKHQDSEDEDEAEDQE
AAPKAPSNVKEKILSFIESVESQEIVGEEWETVGFAEPGAFKGLSEFVTSETRGRGRVEVLDMAVT
HED
>g3204
MSSNTKTILILGGSYAGVGTAHYILKHVLPSLPEKDDYRVALVSDSSHFLCRPASVRALVSEDAFP
GNKRLFVPLSEAFERYASTSMTVYHGTVAKLDHLTRTVTAIPAEAGKAEIKLEYHALIIATGVSTQ
SPLMGLSGDHQNTIEAWTSFRKKVSTAKSIVITGGGATGVETAGELGQYLNGNVSGKPKVTINLVT
KSNQLLSYLPLSLAQKAEEQLSKLGVSVIKNRVVEKVVSVDADAVNGAGQSLERLTDAVIVHLDHD
QTLQADLYIPATGVSPNTSFLNGQLLDDEGYVIVHPSSLKVESAGPRVYALGHVCSSNPRAIHAIM
KQVPVAGENLKRDLLAAENSNQTHDAAYRAYEPESKVTQLVVIGKKGVGMFSGWRVPSFFVWLIKG
RDYWVDMTPPMWNGKAFAKAI
>g3205
MVHDLSGLDEDLPDAPTVPERETGRSNEGDDRGNDLARKRLDQIIDIWEDFVTQVPQPPVFHTSAQ
ASINGMSSSSSARVEPTHTDAASSVGELQLSIPVMQRVALTKILAALDEFSKARSHQDWSVGSRAD
AAYDKSSYDLTPSDIINVLAAPHPLISSFHWSNSTLATSRSTETISMELLDGKMQNESKTLPYKII
LNFQAAMLLDRQEELPGGIREHIELIKRRKFHDVLSKLDQISLRTPPSLHMLRAQCMGVVILQLFG
NVPEAWSMMIAASRTFVGLGYDAFQVDGQISNEDREACIVWCYQLDRSMSLLLRRPPLLPKSAIPP
QFSRIPLDDGIDWSLQFIQLSLDLTHIQGLISFLTSDSGKKMHWTDLEPLHNELNKLWERLDTWEV
ESPASVSDVFVHDLDHLRFRGYATQTILLQTSTDVAKSSLLQRVAVDCARKCFESLRDLTRNEQKL
EMIDLSWDALLYSISPLCVLSYNIMRTRDVTDLKLLEDFARFISVRIIGTSTDNNPLATRIYSLYR

SIINTCRPLVSAAPPPTTAGQPAYSTNHTPHRKNFMNFEHFFGGIGSATGSSLSSSTKEMSQNRNG
RSATNAIVEGNGTQIDDGIEDANVTEGRFMELLMYRVKIGWSD
>g3206
MTFREKVKKAFRRSSDKDKNNGKPKIEYYRRGECPRSKYRGPVDPEHRRRLYDWNFETATADRDRT
FELDLSPCTSLPDQPRDGNDSDATTLEDVSENEPELVGPQHIQIIGSGSITVAHHEVAFSGSFDTN
SDQSTAVVSSCHSSLLTLKESWDWPVTKRDALGSLKETLAMTAPMTRRTSAPEKRLPFDPQELSMA
LNAVRIVS
>g3207
MAVPTSVIVNTEDISSPSLAEELISPVTTDSMVTVPLSDRQSLSESIATEFDSPATPIDEKADQIS
FMGTATQVDLTPKSDVTGKVVETSMAGEMKRSSSISNEAANNTQDGYESSSSTESTGGVDWEKLDK
SEEQEPRTQGTDESTALLLARLEQENNALATNPKSGLAKKTHRKSRPPSIQHLQKLVNDPHSSLRY
SQLQPPPMTELEFWAALVADYPSTAQRLPTLTSHKIRAGVPPPLRGVVWPSIAGARDTLLIEEFAR
LCNESSPYEGLIGKDIGRSFPNVEMFRDPNGEGQQMLARVLKCFSLYDTKIGYCQGLGFVVGPLLM
HMTDAESFCVLIRLMEHYDLRSCFLPDLSGLHLRIYQFQXLLSRHIPALFAHLQSLNIEPIYVSQW
FLSFFAVTCPLPMLLRIYDVLLLEGASETLMRVALSLMQRNEKKIMGFTEFEDVLQFLLSRSLWDT
YACNADDLVNDFVSLTPLVSRESLQALEASYSQSQGVPTGISFPQMQATASRFLGRLWAGSHRSLN
PSAARPESTVRRSPSKQSMASTLNSMETSVSDASTAPTEASLGGEQKVRAKSAISHKDRDLHGQIE
DLLTALSDMQREQAELARDLQREREEREEDREISRALLSHIKQSEINEDTEELLSKAEERFSSVNK
RDSILQTKHQLRDEAGRWKEKHEMEVARCADLTRQLDDHESENTRLKEELREARSRIHDGYREKQK
LERTVQDLRARKGVVADNSSQDTSGAASDNEAWPTSSTGLREFKLGRSKTEVGRFPKRSSSLGLQT
LLSQSTGQSTESDNTTTTNAGSEDSLLLELVNAKTAEAVAKQELEEVKAKLDSLRRLMNGHSRVNT
STGEMPTNFSTPTNKLSPESIKATPTGGGFFGWGRRSASTVGVTDSK
>g3208
MDSLLDDSFNEEEEEEEVSDFVPDEAPKPKAKAKPAATKAKKLTQTTIKTNPAAKATKKKAKADSE
NEASDRDDIDDFLDSVMSTTPPSKPKTKKAPAKKAGSKPLADVANESFGGDGADDVDDEAPKQTGA
GDKYQKLTQYEHIIRRPDTYIGSIEKATQQMWVYDSATESMTFREVTFVPGLYKIFDEIVVNAADN
KQNDEKMDEIRITIDRESGVISVWNNGRGIPIEIHSKEKIYVPELIFGHLLTSSNYDDGQMKVTGG
RNGFGAKLCNVFSKEFSVETVDSRQGKKYVQTWTDNMSKVGKAKITSAKGDDYTKVTFKPDYTKFG
MEGMDDDFEALVKRRVYDLAGTAKVAVKLNGSRIPVRNFKKYMEMYTKAIKKERGEEAANDKSEIV
TASPDPRWEVGFAVSDGSFQQVSFVNSIATTSGGSHVNYIADQICNRLAEAVKKKNKQGATLKASQ
IRNHIFIFVNALIVNPAFTSQTKEQLTTKPSQFGSKCVLDDDFYKKVLKTEVMTNILHFAEKKADQ
LLKKTDGGKRSRMNNPKLTDANKAGTKDGHHCTLILTEGDSAKGLAMAGRAVVGPDLFGVFPLRGK
LLNVRDASIDQISKNTEIQNIKSFIGLQHKKEYTDTRGLRYGHLMIMTDQDHDGSHIKGLLINFLQ
VQFPSLLKIPEFLIEFITPIIKVWKGDPKNPTKSKSFFTMPEYEQWKEEHKNDRNWQHKYYKGLGT
STTDDAQIYFRDLDRHLKEFHTMQDHEAQLIDLAFSKKKADERKEWLRQYKPGTFLDHSTKQITYT
DFINKELILFSMADNQRSIPSVVDGLKPGQRKVLYTCFKRNVKKDMKVVELAGHVSGMTAYQHGDI
SLQQTIVGLAQTFVGSNNLNCLEPSGNFGSRLQGGSDCASARYIYTRLSPFARRVFNQQDEPLLTY
NEDDGEKIEPELYVPVVPLILINGADGIGTGWSSSIPNYNPEDIVANLKRMMDGEEPEPMHPWFRG
FHGDVTPQGPDRYKFSGRIQEVNEKEVEITELPIRTWTQDFKDKLEDIIKAEKTPSFIKDYKDYNT
HTKVHFVIQMDEKHLKSAVEEGLEEKFKLSKTIATTNLVAFDPEGRITKYATVSDILKEFFAIRLK
YYEKRKQHQLSELQKDLDKLTNQARFVQMIIDGKLVVSKKKKSILITELKEKGFKAFPKVAEAVKA
GETEQVVEEEGSEEEATTEVASNAYDYLLGMAIWSLTQERVEKLLRQIGEKETEIDELIKLTKEDI
WRRDLDEFINEWRFQLEEEHRRERKVAGMGRRQSSKLMTAASKGAGRKRKNDDDDDYAAPKSKKAA
TSKKAVAQGSLMSYLKSSPKPKKIAQDDGAEDDDMDFDDDFEAEIIPKKSRGAAKPKVVPKEEEDD
QFEIEAEALPKKSRATSKPPIAPDVEEKEDEGDDLVEVTAASKAASKPAISKPAAKKAAAKEPKEP
KSRTTSKKPVSYTVSSESESESEFGDDLLDDVSKMVKGIGDSNGKALISEFTKPGSSAGLKKSTPRLS
KISSEFDPDETDYSKLVPQSSPRRSLLVKQKEKPITDDDADESPKPATKAQPAAKAKPAASTAATK
SRGRPKKDAAAPAAKKAQPAAKGRGKKKVVDDLSDDDIDAMANDILDSDGGAELDDEPVKPRPSRR
AATTKAKKYVIEDDDSEIDDPAGDDSDAFDDDSE
>g3209
MASIESLLNPLPDRTGSKNHHEDDANISYFHRSSITSLPDRRMSNNTFSQSQSQITSNKKPRVAKD
APIFIPGSVRGEVRYRPCADQVEATMMEEHAKFSIYPPIDEIADYPRHIPYSSEKKSLMEKTGRES

FEVFQYTFKLPGDEKVWTMMWDYNIGLVRITHLFKCLDYPKTTPAKMLNSNEGLRDICHSITGGAL
AAQGYWMPFETAKAVAATFCYNIRFALTPLFGTDFPSQCIQPHERAFGKMTIDAKIIKRATELSRR
YCQMEFEAKSIVHSQNRPMSPNDVATGLHGAAGVWSEGNTCSSNNTFTPINMSSDQTSLPRVSPQS
KILKSDTLPSIKEALGVESFEYLCANPLKRRILNDVHVHDDGQQLKITSRPLINMARLPISLRKRC
AESGDDEDGCFTDEASLDNSASSYISESNTDSSESDSDAISELDISSECSILSKATQSRHDKKHIR
KEKRKRKTTSKRSSRHQLQQKQVVSPFSPPPTAPLSSSSASPTPVKHRHNHHSKTKRHSRGDELTA
AETLLALRSGSSTLSENESEDERVSAKNAKKRCRHSHQHRQRRHKAGAQAEIERFSLAVNSQSDLI
ERMAKRLRRASF
>g3210
MASSGDVKYPAVNGPASVADNVAVEASKTTAELRGLKDAKVTPSTRTTNGQQLTHYHSLVYSLLSW
ERPRATAISFASVVFFILAARFLPLLRWVFKFLYISLGVTAAAEGAGKLIFNQGLTSSFRPRKYYT
LPKETVEGILEDLEQLADFFLIEFQRILFAENVLHTAAAFIAAFLSYWLIKVVPVWGLALLGTTVA
YLGPLIYISNREFIDEQIAQIQQLVNAQANHAKELANQQTARATGLVKQYANDYSSKAHEYIGNRR
SASPEAVKAPAPAIKVEPISPPKFQTTDFPKAPKTEPVVDAAEPEPAASTEKQEPLLAL
>g3211
MAAGGTKAIGSAAWIAAEKENVTRLLEQEMEEVEYPVRHEMDWLNEHMAEIFSTNQFNMMEIFKTP
GKLRGRTPYTARKRNPDEARVPLSNLFSTANQSLRSPSKYSKSPEKPSSSRIANAPAAESALPRKL
VNDQPQGPIQTPVGNPIDTAPEKPISYPQLTTVDIKGKQPIRDVPLNKALNSFSTFNTDSGYHGLG
DDDELVLPSTQPLPETQVSTTTDMQVPSMLPLNSGMSNRKSLSVDRRTTEGSFVSAQENIVSRGQT
VEPMDVEEDLPDIADEETPRPNVKSVHVPSAFSEKPQDNQVASPVKDDLEKDGDVIIDDNFDDIGS
PSDGSTPARPAMRKNSSFGFASLPPRDPLIKKSMGPRVSRTSHVEMIKSTTLGRQSYFTGHMEGSK
SASQNTATEKEELQQDNTKTKQSQQGETEQAYDTSVLHNKLNTQRLHDKISMLGETRAPRTTKTAP
LSQAAIPQAKYPELPDIKNTNADLERIPTLASTTQEDWIKPLSSPQRPVITESQTADVMEGVADNE
TIVNLERGKMTRTETFHDFSEKGSRTPTKRSIFSAFDQHKPVSVSTPPPRRMDSPAQPTSNTNAGS
TTSLASPRRIDGTPKSRFQSIMDSAKSLFSSSAGLSAAAKLETLSSPSALRSKPNPQLVEISPERY
SPSPERLPARDQVTNESKGKQATKPIRLEDPFEAEERIRPTAKATVPSASIESRQKAESKERDIPA
SSTRANAKIAQPVQLHARKDPGTAADLEPKFPLPPTTNYSQSQPTRPRPVKPTREPPQKQRPQPVS
IRLGTRMPIPSVAPNSQEPSAAPSSVPAKQPTLAKNPSNSSLHTTSSSTSLKSSTSTQSQRRPHAA
AAAEAKKQEEREAMFHKEEQKKRAALLKQQQEEARRQERERSVAEENKKAAQRQQAEQRRLENSRL
RQGSQPPKPANDLESTSQQENATHRTDVGPARPPSRLGSSVRAYNRSINQPPPNPAKPPKKRPLED
EPRSQAFVSQAKSQVVEPKRRKTEDEYNQQQYNQTAPLKQQPIRKENGKLSLLGHTTYPQAPPPAA
HHHTGSMYKTGPVPLGTAGQPLKHAAPMDMAQFAVGKISFAPAHATQPGTAHKTPSHKTPKAGPSA
QRVPAKPSPQYPPSESIHLPEPPTDSEEEDSDEDMIPAPNWAQGEELHAALASQEDVDPAGIFGDI
PIFTLEDAFKNDKKIKKFRERTSSANWGGPDGLTQEEINRDLAARRVMRANGGXSFNLS
>g3212
MSFLSRDPVTGKNEIDALVQRRQWKDAESILLRILADADAEAADQVSPSNRRTTPFDDSPEVQKNK
LMLAHIFRQIGRPQDAESIDRQVLETRQRLLGPDAQDTLLVMYHLATDIKLQPERLLEGLAIEESV
LEAAAQIYWPDEFEDSPTRNSSGSVAAGPSKSGIEHSPAMDILLRTCHVADTFFMQNLPAQAARLH
ERVLRLSTTALGPGHPYTIAVMDSTGRDYVSQGRLPEAVRLLQDAAEAGRVHLGSRDPTXRRCIVH
LAEAHGRMTAEGDGKVPDAKTIAILEQAIEILEESIGSDETDTISLKYYLAVAYARLDGRFRESEA
LQNQVLDWCRRQLGDRTVTANLMVRNLVLIYRQLGRMDKAREIENEFGRIRRSIS
>g3213
MHFLLLGASGRTGQHVVSELLSQGHTAVALVRTSSSLGPRPGLNIVIGSVLSKSDIQSALYATPSL
LPSAAIITLNTVRKSDSPFAAQVSPPRFLADSCANVCEVLEQAGIHRVVIMSTAGVGDSWARLPLL
SKAFMSWTNVKYALEDHGLIDKEIRETKMDWTLVRAVRLEFDDPKPTKTKREVRTLGSKGDGMSLT
DSVGIASVASFLVKVAVDGLFIQSAVVVAD
>g3214
MPAFILHGARGSTNTDRVRLTLAEGGFTDYELVFLNLQRGEQKSEEHMKHHPWGKVPVLTFPDGFA
LHESRAICKYLARKYSFPLLPPDSDVEAAALFDQAQCAEMSYFSEPAGKIAFETFVKKFIGLSPDE
VIVSDALRSLEMFFDVAERLLLERKYMAGNDFTLVDIYYIPMIQRLFVCGYGDIVLSRKAVNAWWK
RCVNRPAIQTMLTTDKEAAIAASR
>g3215

MAPQKILVIGAGELGFQVLHALAAHPSXPATIAVLLRPGSINSTSRQKQEQNNTLRKIGVQLIPGD
IVENSEEILSSIFASYDTVIGCSGFVSSKAIQTKLARAVIAAETPRYIPWQFGVDYDAIGRGSPQD
LFDEQLDVRDLLRAPGQTKTRWTIISTGMFTSFLFEPSFGIVDQENGVINALGSLENRVTVTTPED
IGVLTAEIVMRDLFDNQPIFIGGDTVSYERLAQLIEAKTGKPFKRNVLEVENVLAALSKDPDNGLL
KYQAVFGQGRGVAWDLASTWNKRLGIHTESVEEWLTKNWA
>g3216
MKFSATIIAFFAAGALAAPSGHGLAARTEARAVGRSRLSNPRRPGSGTLNNELTGEATNTTNVDYS
SNWSGAVLTSPPAGATFTSVSARFTVPTPKAVNGKAGSSSAWVGIDGDTYSNAILQTGIDFSVTAS
GAVSYDAWYEWYPDYAYDFSGISISAGDVIAVSVVSSSSTAGTAVIENVTKGQTVSKKLTAPSSSA
KLGGQNAEWIVEDYEEGSSLVTLDNFGTVTFTSASAGLSNGNSVGTTGADIIDLEQNGQVLTSVTI
PSSSEVVVKYV
>g3217
MTVPHEEIQYLDLIREIFDQGERRPDRTGTGTLSIFAPRPFKFSLNDNGRPILPLLTTKRVFLRAV
IAELLWFIEGNTSSIALNEVGVKIWDGNGSREFLDNLGLTHREVGDLGPVYGFQWRHFGAEYKDAK
ADYTGEGVDQIADIIHKLRTNPYDRRMILTAWNPKDLKLMVLPPCHMFAQFYVSFPGQGLADGTSE
NKPKGHLHCQLYQRSCDMGLGVPFNIASYALLTHMLAHVCDLVPGSLTHVMGDAHVYLDHVDALKV
QLEREPRPFPELEITREKGASIDGWKAEEFVVKGYDPHKAIAMNMSV
>g3218
MADKPLKSTCHCGAITVIVPRHPEEINECQCTICRRYAAAWGYYKVDEVKFDIKEGAKLSKYVWGD
KEKSFDSCATCGCVCYWWPFELKNPDGTAAEFGVNTRNMDPLEIYHVNRKIERAWLFAPINNKTAA
HADDQAKY
>g3219
MAGKSDPPPKYDTFVSSATESEDGHTTFSERKNPFQDPEVAEHWRLTYEKSQYECRHVYDPSLTWS
EEEEKRLIRKLDWRICLWACVMFFGLQVDRNNLQQAVSGTFLKDLKLNTNDYNWGNITFRVSFLCA
ELPSQLISKKIGPDRWIPIQITLCFFWATLSLTGIVTSLLAFAIFHLDGVHGWAGWRWLFLIEGII
TLSVGLGSFFMMPASAVQTKSWFRPNGWFNDREVSIVVNRVLRDDPSKGDMHNRQAITPRRLWNAV
TDYHLWPIYIIGSVGFSTFTTNLLVIPSSVFHIVTLLLLTRLSEWVDERSLVAMLQPLWSLPCIVA
LRFWPGVIDNAWGTYALVTVLLSYPYCHAIVVGWISRNSNNVGTRSVSAALYNMSVQVGDVAAFFI
YREDDKPKYHRGNTNLLIINLVVILVFISTKVYYYVYVNRRRDRVWKVMSEEERTRYIKDTKLQGSK
RLDFRFAH
>g3220
MNGEARPKRKHSPVSASDRPAKHLKPESTVLTPGDETPANGTVYDVENGEDITPIVAMGAAQADSP
EWQATIESVVKSVVSIHFCQTCSFDTDLSMCSQATGFVVDAQRGYILTNRHVVCAGPFWGYCIFDN
HEECDVRPVYRDPVHDFGILKFDPKAIKYMDMTELKLNPDGAQVGVEIRVVGNDAGEKLSILSGVI
SRLDRNAPEYGEGYSDFNTNYIQAAAAASGGSSGSPVVNVAGHVVALQAGGRADGAATDYFLPLGR
PLRALQCIQDGKPVTRGTIQTQWMIKPFDECRRLGLTPEWEAYVRKSAPKETGMLVAEIVLPEGPA
DGKLQEGDVLLKVNGELLTQFVRLDDILDSNVDQSIRLLIQRGGEDLEVECTVGDLHAITPDRFVS
VAGGTFHDLSYQQARLYAIACRGVYVCEAAGSFKIENTLSGWIIDSVDKRPTPNLQEFIEVMKTIP
DRSKIVISYRHIRDLHTRGTSIIYLDRHWHPKMSLAVRNDETGLWDFSDIAGPLPAEPPVPMKADF
IQLDGISQTAAAEIVRSFVRVSCTMPLKLDGFPQAKKTGYGLVIDAEKGLVVVSRAIVPYNLCDIN
ITVADSIIVTGKVIFMHPLQNYTIVQYDPSLVQAPVRSAQLSTENIKQGQDTIFVGFNQNLRIVVA
KTTVTDITTVAIPANASAPRYRAINLDAITVDTGLSSQCTTGVLLREDGVVQALWLNYLGERTGNS
HKDVEYHLGLATPSILPIISKIQQDEIPKLRILNMESYVIQMSQARIMGVSEEWINKVAEANPSRH
QLFMVRKLDSPPPKFTHKADALKEGDIILTLNDKLITRVSEFDIMYDNEVLDALIVRNGQEMHVKV
HTVPTEDLETDRAVVFCGAVLQKPHHAVRQQISKLHSEIYISARSRGSPAYQYGLSPTNFITAVNG
VQTHDLDSFVEEVKKIPDNTYFRLRAVTFDNVPWVVTMKKNDHYFAMSEYVKDASAPAGWKVINYP
KKDESADQGNLTADAMDEGGEGGGSDAEPDC
>g3221
MNSNPTGYPTTMQQHANPMLAATAGYPVQSGSPSNQQFPYYAANPMAAYPPQMQQPSQARPGQMQQ
HSFNPMQIQTSGPGGAMMPANMVALGFVFPLLLIRLSAAVMALRQLAYLQPLQSLQEDWLGLSNFV
LLVYCLANQSPHANMAYSSPSPYHPTSIPTTSASPAHSQSPAIAASVSMQMPSQNMTAVSNPAMLL
AQQQQQQQQQQQQQLQLQMQQQQQQQQQQQQQQQLQQQQQQQQQQQRATPQTNQTPPGQQPQQPQQ

PQQQQAAPQSPAAAARERARVTALLDINSALLQELLNLQAAGKSGGAPQQPSTSTPEQSSSPGSAT
EPASASSDAPRKPSQEYVECLKRLQSNLVYLASVTDRNKKPVSVAPAILSPPPHMGNLNEQYAKLN
ELFAGAPRPAAAHQQRPSQSGAAAETAV
>g3222
MARVSQFTEEFDATFTSFPGLERPITLVDEKNALRTSTESVANTKTTTNVFAGAFKAARRLCASYK
CHFNELLTCNFRQGNTHDDDKSIEAGSIFTASYGAAPTSAEELEQQSRRNKLSAIFGLLLDDDSSA
FTAYNAKKQPTTAITKTQATATLRTPDAPTFWGQVCVTKGDTAEVIMTGYESEIENIDNNTNAELE
FLAYLGVVDFKTGKY
>g3223
MGQIEELPDDYDESQPPATTTTPPAETPSIPPSLLSNDNIPFPIIPEKVKNADPLAPELPPAMASI
RSYTPDQLADMMKKTPLFMTDLDNAGDEEGENVMLEAIRALQYEGTRGDVTLSFREQGNEAAKLKN
WTDAKEFYTKAIAVLNVKKEDDKWEKPTDLEKEEKMLKEAREACYANRALCNLELKNYRSTTLDCA
QALKVNPRNVKAYYRSSMALLALDKIAEATDTVLRGLAIDPNNKSLQQMSERIAARKEILDKIAAR
KQKEEETKQKQKLLLSTALQARQIRVRKTDAKPDMEDAHIALTPDPLSPESTLVFPVMFLYPMDAQ
TDFIKAFSEMDCINDHLEYLLPLPWDTKQEYQAAMLDCYMETVTGGLIKVGKKLPLLQVLSGGKVE
VVDELVRIIVVPRGKTQKFVEEFKARKGKA
>g3224
MVHLASVKKEHEVPKFEPRQLQRIETLDLNAPGADDYSTSVYGTRYAAESLPSLEMPEREMPREVA
YRMIKDDLSLDGNPMLNYNYLSNGHQEDEAEKLMTEAFSKNFIDYEEXPQSADIQNRCVNMIARLF
NAPTGADSDNAMGTSTIGSSEAIMLATLAMKKRWQNKRKAEGKDWSRPNIIMNSAVQVCWEKAARY
FDVEEKYVYCTDERYVIDPKEAVDLVDENTIGICVILGTTYTGEYEDAKSINDLLIKKGLDIPIHI
DAASGGFVAPFVNPNLEWDFRLEKVVSINVSGHKYGLVYPGVGWVVWRSPEFLPQELVFNINYLGA
DQASFTLNFSKGASQVIGQYYQMIRLGKRGYRSIMLNLTRTADYLSSKLQDLGFIIMSQGRGHGLP
LVAFRLNPDREHFYDEFAIAHHLRERGWVVPAYTMAPHSEGLKMMRVVVREDFTLPRADLLVNDIK
LALQTLGEMDKGMMDKYQKHVKDHVVNSHKSHHNHPAYKKEKHSLQGKHGKTHAVC
>g3225
MPSFFSKKTRSQQASSAGPAGESPSGSAVPLPPGPPPPPLPNDPYQLLHQQQQSLEEFADKRFEAN
RRPSEPFPTHQFHGHRPHSPHSPYPAVDLRYGREEDHQQQQQYRHPAHNRSQPSIDRPTISVVTPS
QQQQQIDDSGYQSPDRARESIDQQQWPTSAHNTSTPDLLQDSGKLQKKKKGFFSRNQSVASSSPAT
KQSGKKEKSLGRSASIKHSFGRDSTVPPSQSSSEYGRRVTTLSTSTHSLDQNQSRSTLDNASQPFE
SEASLRPPTAETPASPNRPSQPHTPLENTQSPQRLGVDVSDRSHLGWQHRRSESFESIPSQASSRR
PSIHTSVDPSVSDMATSQQPMGTRPTESQSQIPSRKDSLATPSGQPYLQNAAQGSFKNASQQNLND
MQGRETPPSAQLNSKVREELENLDVGALIMRHEELQAKYQKVKRYYFEKEAQVTALQNTVAHQRMA
VSRTVLDDNEYTARFQRLDGAIKELAFSIRKDWRAIPDWLHPFVNDDAVTVGTKEMTGVGRAVITR
WVVEDVFNRYFHPGLERSFSERLKAIEMNLRRQQTQVFNDDDKENQVARISNWRRTTLDGLADVLQ
AKTTQENMDQLVEYLVEKLSATLQCDLKEPAPPELAHYTRMIVENAINIAEKIPQEARDIVVDYIQ
PGTFVSEINMKVETGLPALTRPIAEARDDIASEGNEEMETDNKEQPTQPQQPDTQGKYIPQNREQR
KKYPFGSLMSKKSAASPNPAPAQSQGQTQGLATSPDNATSNKERDEQLQQQDKRSRVRLSTFMTVE
VKGRGPNNILVQAPVYTIE
>g3226
MWTSSSIRRAFSRMDIDVPILLRMMKAALPATIALAMYQSDLIAQEYATMAYLMPVAAMFSRCSAP
RGKYFHFLVQGLVLVCFGAASSLLMVYCCVKAREHTSGPLPAGISTAEILSVPHNTAASVVAAVWL
FFQTYIVNTLRGEDPQLGIPVQMHCMLFFVAVTEAPDYVTVSDGFALVKRLLLAILTGMALATVTH
ILIFPDTCRDAVLDVMGEYIRAMQHTLRVHESYFQSMEQLDMFVRVQTRNPTEEKKLYRAKADAVK
NAARRVSQMHASLTNELPYAKREIAYGKPSVSEFGMIFKQLRATMVPIVGLSTVVDIFDRIMEWNN
WIKPVEEGGVDPKNDAVHARMVRDWNAIMASTHDHFAQIIDVMHDGLEHVAYQLKLAKRKRQGVDK
DIETASSHAGPGEEGFAQYLEAKCEVFYKARLVTLREWCEQKGIELPEDYFYHPRADQPIQLPENV
PGALPRDRTERQLYILLYVEHMLYSSSLAILDFVRFADRRVASGKYSHSRLIVPGSKEVRKWIESL
YRNHQSESTGEDVYRRRVILDLGEAYHRRKDPEHLPPENFMQSMGEWLRVIPSFLRSSRSLYGFRV
ACAATSIGIIAYIEPSHAFFVRQRLVWAMVMQTISMGPTAGQTVLNFALRSSGTVIGMIGAMVVWY
IPVGHIAGVIVLFFIWTTGIWWIPLKKAVLQQAGIVSLLTIIVIIASEMEVVKLGEQAVEAGGQPY
YPTYLLAVYRLAVVLGGVVVAFIWTIFPYPISDHSVLRQNLGASLYLLAKYYSLTHETLIARARGD

EGDMTLKSSAGRRLEKARHKAYANQSMAINGLRSYADSIKWEIQVGGRFPRDQYERIIDCMQNIMN
YINLAEYASKTFSSSLSVHTPYNGPSKQTEWYTDFTKLITTTHVTSHQITSILALLSASIVNAQPL
PPYLQVPGPDAFSERLKEVDKDILNICHAAEPGYAAFSVLHLASRCIVGDLERLIQSVRDLVGELD
FSFHVISRLSEKEVEKRIA
>g3227
MDSNLGSVTTTKDARAVIEEVARRKGSFTHAFRLEAEEEARNGRSSMLQLIEGGEEIREDLAQTLK
IISTDLYTSRARFLMEVIQNADDNKYAEGETPTVSITVTPQHVKIGCNEEGFSRENILALCRSGRS
SKRPGQGYTGEKGIGFKSVFKLANRVHIRSPPYYFQLDQTRELGMITPQWDEDYFDNHEKEYQTTI
VLDHICDQSTNFSTALEDDIDAIDPVFVLFLRRIERLDLTLFNSLSRDEPVISKSFQLVNWTPGSG
IVSIKDENANTMRHLYKHQFTIKYEGTETRREDITETDIVLAFPVKKESGTYLASIRKQNFAFAYL
PLGDFGFKFVIQADFLTTSNRQSVNEDKDWNKNIAAAIPHAFEAAIDEFNFCSSNSDLEELSKTWP
LYLSHNFHVSSPYWRNIADNINEHLSRALIVKDRTGMVQAPKSLMFLDWAHDRNGSVREALMSLGI
TAPDWKWLCSKIQELHNTHMLSIKMQSKEWCSDLAKVILEPQESRGDSNYARDLKDIPLIPMATGT
WKCPPSADDPIYFPASSGTIIPPGLPLSLVDEEACACSERRKLFRLLGVKDCNVPNVVKQILDYHS
KLNLAVLAHVIAQLKYLYKMRQHLRPGDMDKVYFACSASEPLERGTEVYADITVDSKLKQLFSAFS
DAHFLNSNYFVQLEPFERAELAEWLNDVASVALAPRLISTTSYGLHRDFEWLLSNQREHVLDILHQ
HWCLYKKCMTSIAKDTLAGHEFVXKAQEFAYAEYCNFLVLPNGDPSDWTFLSDLGVGQDEGLAFYL
WILNQPGFKRHMDINRSKNLYLAIQSRAFSPIEKVMVKSLDSCIWVGPKGLSSKPALNPIYGHELD
RLFREILEVPNATSAEVLDYLEQLRHNESTTMTDVAEVYVFLQTHYADKFSVNNQTPCIAVPSMAG
SAIEWKTPSQCVWNDEEFSQNELKLESKTAIRRIVEQHAPAAKAFFTDILKLPNAGVNELLADLAL
MQKENRDDPKRVHRLYERIESCRRSSPAAIKIAFKKAPLVFLRGINNQRGRWLSLEDCIWTRSVLR
NKYALMPSLNDYRDLFRFTLKVPNASVDMLVTDLLESLMYCPMEDEDDYQYVKKLLQEIARLWKDN
KDLERLHGTNCWPCHAPLRQRELCSIGSFYVNDRQDLFNIFSNSYTFLDFEFDISKRLADLLRNLG
CDSFLSDQVVIDTESREPLDFDHDLTSEFRGRADAIVKYFEHVECKSPYEPRRLFENATVWISADI
KTHYTLGDTTVTRNEGGSSVKVSLGEDDTEKLEIFVSANKQTRDCALLVDLPEQLVAAMELEPADL
PDLSYLLSVPLASLKVLLVKKGFTGGDAADENEEHLVADPVNEESRAQRGNSSDDGGDDLWMTFRS
GVRSETSSPQVRLQNHQNLLSDESPRDRPFTPHSTATGLYSSINRNQNRTRIESFAQNADPVSNSR
RGIFQFGGGGGMFNMSTLREALDVNEPASVSTPIQANVSPRRRPNPIRNRNEEEVARDFEVGFLGE
QFVYTLLHDTLQLPDFTGEDNWTSSLRSRAGFSTFGIREVSDFTYKDTQGVLTRHFQRMQHPYIKP
EWLFTVCDNGNVPLYRLEVKSTTSQDPTTTFYMSGHQHKLAEKLRITSAMPSEVYAVLRISGLDAL
ADGASHQPQWRVYLDPYTRGEEGLLNFSVPTYAVTAQR
>g3228
MAATNPCKDCLKREEAAQARQKIQEILIAFPLFSEPSGEQQSLQEIVQILPNLEAVRFSLPSNSFL
ETFRGELDGLESLVLRHKSGFWGDEETLCAFDATAEELRQNYTSFIAALPPLHELSISGMGRPLNM
TPILETHGASLEKLSIHEFEHDCRYETGNATWVRSVLSVSEVEQIVLSAPNLKELTLDVYRSSNKW
PTSMLKTLSKFPHLARLTMNFNLEDPSTSKYAELCLVNEQARDEYCTIHGLMEPQLNYTTASEIFH
TIRQYQADTKLRHVTISAGDFGRRSPILDINGERLYDQEYESILHAIVRCNDVANLNCYIKQRPLD
GIAPCDSYPYDPIYVAAQSGSTDSLRVLLDLYEAEYLQIYASNKNGSELSLLNAACANGELETVRF
LLDRDPPLGTAYAGDRDGGQALVYAGMSLEVPRAFDNERDNDTSKGDFNQCIAESEDMLRLLLDRG
ACVRNAIDIISLNHGHKEELRKLPQSQQPIVTVLGQAVSRASHELISRLIAEGADVHQRQAIESNR
VFCIMDITALHIGSGYWNAAGIKALFDNCGGEFPLVSLFMTTHPSDPKSLLPATMPANPEAHPDQE
QHIPLYRGSCHCGFVSYEARINFKEPTAEEPDYVLCKCNCSYCHKAGLLIANPLEESFRLLTPVES
GDEKLSLPVYKFGSGAVSHPFCPRCGVTCYYYGSYKTDEGKIISFTRVNVHTIDGRVDGTQMESLK
EIKTKYWDGKTNGWDKGASEEPWDGGVW
>g3229
MQPIKFYGKCPSANPFKVEIFLKELNLSYESILIPFSDVKKPYFVAINPNGRLPAIHDPNTGITLW
ESGAIIEYLIEQYDKDERRLSFAPGSAEAYHAKQWLFFQVSGQGPYYGQFGWFVKFHAEKIPSAIE
RYVGEINRVSGVLDSWLAKQQQEFADADLTANGPWLVGNRLSYADLAFVRWHMIAGIFATKEQFDE
DNYPYLKAWIAKMVARDSVQQTIQENPNGE
>g3230
MLNVNDYNAQSLAQPTNLRAMLRSGELLWGTSCRIASEEAARIVATLPHHFCFIDSEHSPLNATLL
VSLVKTIQQYSEGRMVPIVRIAPNSTDLINYVLNAGAGGIVMPHVQNAVQNAAQAEAFVRLAKFPP

LGERSYPPMALLGAQTRTRDGQTVYDIWNEHTAIFCQIEDVEGIKNGEEIARVPGGESRL
>g3231
MPHAVNSNWTSVEKDYQQQLQIVEGPDEPIIRFPLVLQPHEPAKDKAEVLHGISAIAAQSTETNEQ
SKLRQLLDANGGVVHFKGLPLKTPEDFSDFLVSLAGKGDHAWKPHTDVGMEVLRRPRAKHVLTTNE
GPPSHFIGWHNEYAVSPVHPGYLILFCEVPAQSGGQTSVVSSLALYDRLKKEVPEFVEGCTSKGLV
YQTPHTAKQVGGIVGGNGLYKASAFGPTDGSPPADTEEERRKAVESRVIELAERGGWYAGIDKTDE
SIPAWQRRGFSWNWQDNGDLEVIHRVPGVRLYPTQHIPTIFNAMSTRYTNAKVNNTWEPPFTFKNA
DGTEGIAVPPHFSGIEKDTIIPKEWLERMDYYQHKLTADIKWDVGDVLVIDNYAAQHARWAWEGER
KVLASFWDQPGISAQPVPSV
>g3232
MDNPILHDEEIAPIPNSPAVELQTSATKTAKDSDPFLVEFRLPYDPENPLDWPTARKWRVTDILSA
TGFNRIMVSTIMAPALSTIAQELDMNSTESAMSLSIYLLATAFGPLFIGPLSELYGRQVVLHASNI
WFLVWNLVCGFAHTKGTLIAARFLAGFGASAIYALAGGVLGDIWRPEQRGRSLGTYLLIPLLGAAV
GPIIGGFITARTTWRWMFWATSIFQTVMIAVSFFSFHESYGALLLRRRADNLRRRTGNTQYYTAAE
RLDGNRSAIGILWKALTRPLRLLLFHPIIQVSATLSGLNYGIMYITLSTFSDLWTSQYHESIEISG
LHYIACASGELVASQIGGPLMDFLYNRQKNENPSPESRLRLMYPGILAAWLGVLLYGWAADYRLFW
LVVDIGVVIMMFGMQLGGMPMMAYVIDTYGEHTSSALAATQFVKSLTAFLFPLFAPAMYQALGFGW
TNTVMALIGLSIAAPLPFVIWKYGPKLRAKAISTY
>g3233
MQDSLPVGILAEYLDTITTLLDTITELFHTRQQSPKSLAEARALFVLQAFTWATWYQTTLLQLWR
DAKYHMKWRLSGLQGQRQPMIVPKREVVEKVRPNYMCKWAFELLRSDLSSVTQDFRKLFDVYNVHF
GDREPRCNLTAAADSSGKVRACDGKAPGNCQRFESDGVQIQSAHDVQCPGLDACTLITWDEESYKS
AKGARAVSLEDTDDKYIRYCPVTTETLAVSHVWSHGQGGRPETGFNLCLHRRYTKLARSLGCNSYW
MDSPCIPTEMELRYEAMGHINHNFINSKVTLLVDRDLMEIDIDPPSLEAKEAILATLVVCDWNVRA
WTLLEGMRGCRNLHILGKNNHIISLIDVFNDVLAYSNLSLVSPCLALQHYMPQVGYRPIEGEVPSV
GLEQATCLMNHRHATKDRDVTLIWSLVCGSKLIKASVDFWKSQVGQPLATGFLLSSSPRLEGEHGF
SWAPSRPNLLPPTADTHWFSSNITSKGEKKKLWPAYNGEGSSIGTITERGLKAEWLVCRIYRNRSL
LPTRLGGFAFLDQEKLSALQVHYFVYNEGGLDRMDRKSIYKLGSLVSPIFKKGFQYVALLFPVMTG
KGSDGGPRPYQYQGEGVKGPLMVIAGSNDFEEWEWQFVHEWDTSVLLPQFIRREDILLV
>g3234
MPKKGGKKNNKKKGAGGGGAKVPDVKNVISPGHKEEDKLLYGEGTAAETTTETGQATASGSAAAED
AAPVATALETQEASAQDITKTEPTAAVPEKEVALPVREKDIVSTEATNEQRGERTEALAAAAPIGP
TEAAPKEAPKETTIAEPELEHPATSRSTERADLVAATQGKAEPAASTTTDVSEAIPEVTKKEQVPI
EKTAGLVTAGGAAAVGATALAGVKAEEGRTQPAPSAVDKAETANKTPAPLAEEKTQGTEVATAAPL
VAPHIKRAHEVPAFVTEERDQPSKKPRVDEPVEAQQKLAIPGRFPSPSVAGSTASGTDSAIASEAQ
NISKGETVAGKPETAGIVTAAPSTQPAFRKGPTEATAVAPAAVPVTTKDTKTEQISTADTGASAGT
AVSAASPAGEISAQREPALPATPQKVDTTAKPTSPAAAAAAAAFRAQPTPEQQQKQQEAAATRTAE
EVTHSKQAQQAPAQKEAKKGGFMAWIKRKFKGEKPTTATTNGNGH
>g3235
MDFARASNSGAACTCVVTEYHFHDRNDYSIEVDYFSLQELRTQFEQLLSSLRDYESTTASALAAMA
AEDRENLKRKADLATSTFRASFRERLDNNTAVLDVMVFDEAIDTMMTWVLQVLPELGTGESVILSR
ESFEDINQCSNRLKSLTSEMEERDQACLWPFIRKLKVYMKAYILSRGLILADLPGLRDLNSARQNV
TERYVRQCHQIFAVARIGRATTDTGVKEVFELARRAAMSNVGVICTQSDDIRPNEAREDWPSERTR
ITKLEKDIVDQRHVIAALDEDIREFTEEFLISTEDAQELLEIQQHRDRAKKLLEKFEFELSHHIIT
FRNRKVSSQLKNIYRSYPGGDHLQTFCISNTMYSKERDKPAQKALPFLTLSGIIELRRYCIGIVAE
SHLRATTEYIKDEIPALLGSIELWIQAGSGDVNAERKEQILDMVSVIQRELDGLTSPVSQINNVSR
ILISKFDEQLRGYMRQRAQQWSASAGQASMLWDSFHHSSYAAFCRKYGDHTTGKVGHRCWNEEAIR
SMRSDMLGLWETFDTDIDTFLNQISMSIEEVFRNGIRATGGIANSDRCLQDRLNPLIATLHHRETL
LLGSVETVIEDFQIKLSSVQTDAFSPIRTSIVGQRMEGIYDAANKENGTGSDRRKKNIIESGFSSS
SLFVDHRRLFKEKFLAISETLQQELKDAVTHQLSFIAADLDTLRSENVVLESERNPEYRAHLTTEL
TRARENMEPVRSAMDNLLNLAREEVVMEG
>g3236

MADLTGSDDYNLQAAWDTVCRSFAKTTSVDLTATPKYTIDQVLDQIRQKQDDDEQRNAKYKVAKDV
ISKTLSCIEVLGGIAAQGASMVFGPSALCFNAVSYLIQTGAKFKGIFSSLAELFKRISDVLERCKI
YLRMPPEDVDRSLRRIINDELVCFVEVCALSMKVLKGHKILIALKVFAFNSDEGVTGQLNRLAELV
ERESQMRGTLGFESQKNSEKNIIETRDGTKKINVGVDKLLELQQKTEDGITGNKQLQDVDTYLDNP
SESFAKMRGKYRDLRSRKVDGSGQWLRNDLAYTEWASCEEDSPLDVLCISGSEGCGKSYLFTEVIR
DLQERISNATDNLSRKSIAYYFFEQGSPSRGAGQFTPNDTPSLVKVLKTVAYQLASHDPVYRKELA
SSAKSLEANQVTQLWNALFGKSYRGDSTFYILLDGAERIVLEDLKQLLNVLADLQKISDTMGRFRL
RILFSARDETVEQITNRLNEGISTINVSSKNKDDIELFINDRLGSIDILSSGSSLVEALKKEILEG
LLKEAHGDFVNIGLLLNEIGEKQRPGEIRDVISRSGEKRSNTIAREIERLNQTLGEDNISDLNELL
LWVMNARCPLRLYELEAVLYVKNGEPSLRPLVDEINGRFSALLRIEGEIDLKTKSVPPTATVSLVS
NSIEEYFRLKAATEETEADDGQLEWNTTGDVLESEVKIVRRFLELVCEPKLFKKFEFEAFFERKLT
KKTARLGVDTDTAHMRIIMACLRAVSEKTDESSPLVDYATFYFEDHLRLIDLSLIQPQIKAIVGPQ
LVKMFTDENIFRLRWTESRLSMRRSWIYEDDCVDVVLKWLQDSSVTKKLPEKDRAWVKSLSSKSQP
DADLLEHIAKFMAHKWLQAXDWDYTELFYWLNAYLNKIANRKDPNVERETKDLESKKITASRIYEI
ADWGRQRLGLDSLGYEETRNVAKTLREFGKLDDAVETFKRASSLQEDNWGARMGLARAYDYQGQYA
LALETLEALKASIECGKSKPSDPKYYLHPITQHLAEVNEKVGNAEKAWDIYESILQEHPDDYDTAY
AMTLLLHGKGNYKQLVEFLEGLKDSTDEITGLDRRTQMFHAYFWNSDFHGTIAAAGNSLNGNDITN
LKGSYRMAVDXAEKKLQDQLGREEETYREEIMAMLMHYCAKFFYNHSTCAEENEMALSMWVRIIHL
DETERNSYIGRAKLFATRELSSHYFEKALQAGHGTQLAAKYIDDLEHLATLESDGNPTDQTKEKYP
QQLLARYYGLCGNTEKAKNVIRAFVKLNIDLLSDDDPSNDWQGFMRLAAHFMFAGQDDNSLAAWSL
IIPNQEDEDTHATKETNRLESTNKATKELEGPLKFSCDGKCGTIWTYANDMYVCRVCADVQFDESC
LRKLQEGTLGLQVCSKNHEMLHVPKYDEDKLKNIGKGNVMVGQEVMAVEDWLQKIKQDWGLSTE
>g3237
MKNLTVAVAQSRTLDITCSTLAALERTXRHASSRGVHVLLFPEAYLGGYPRTCAFGTAIGARDPQG
RDQFLEYFNSAVDLGDTPSGAGDDWVERKLPCAKGKEYRGDGTREFLERVASETNVFIGVGLIERA
GGSLYCSVVYVDPKRGVLGKRRKVMPTATERLIWAQGSPSTLKAVTTEINGVNLIIGSAICWENYM
PLLRQSLYSQNVNLYLAPTADGRDTWLPLMRTVATEGRAVVLSANQCVRKTELPSWITGAPSADST
STEEHTKEPQEITWPQSAGEQLHPDKKRDQMYASLAVEDDAGAISELELKKKGLAQRSVLSNSASK
KDPATEEYICHGGSCIIGPLGQICGGPIWDVCTDDNDDSSAVGDGLVIAEIDFDDCTRGRLDLDVA
GSYSRNDAFKLTVEGLDLNPPPF
>g3238
MATTSNMFMYSLTLQNPTAVTQAILGQFAGTKEQQIVTASGSKLTLHRPDAAQGKVTPIYTQDVFG
IIRSLAAFRLAGSNKDYIIIGSDSGRITIIEYVPSQNRFNRIHLETFGKSGVRRVIPGQYLAVDPK
GRACLISSVEKNKLVYVLNRNSQAELTISSPLEAHKAQTLVFALTALDVGYDNPIFAAIEVDYSES
DQDPTGRAYEEVEKQLVYYELDLGLNHVIRRWSDVIHRTSNILFQVPGGADGPSGVLVCAEDSITY
RHSNQDAFRVPIPRRNGPTENPERKRSIIAGVMHKMRGAFFFLLQTEDGDLFKLTIDMVEDENGQL
TGEVKRLKIKYFDTVPPAANLLILKSGFLFVASETGNHHFYQFEKLGDDDEETEFLSDDVSADPAD
SVKPVYFNLRGAENLNLVESINSLNPIMDCKITDAIGEDAPQIYTICGTGARSSFRTLKHGLEVSE
IVESELPSVPSAVWTTKLTRNDEFDAYIVLSFTNGTLVLSIGETVEEVTDTGFLSTAPTLAVQQLG
EDSLIQVHPKGIRHILADHRVNEWPAPQHRTIVAAATNERQVAVALSSGEIVYFEMDTDGSLAEYD
EKRQMSGTVTALSLGEVPEGRVRSSFLAVGCDDSTVRILSLDPDTTLENKSVQALTSAPSALNIMS
MIDSTSGGSTLYLHIGLYSGVYLRTVLDEVTGELSDTRTRFLGAKPVKLFGVSVKGQPAVLALSSR
PWLGYSDVQTKSFMLTSLDYVNLEWGWNFSSEQCLEGMVGIQGQNLRIFSIEKLDNNVLQESIPLA
YTPRHFVKHPEQPLFYVIESENNVLAPATQARLLEDSKAQNGEAVIPPAETFGYPRATGHWASCIE
VVDPVSAKAVLSRLELEENESAVSIAAVSFASQDNETFLVVGTGKDVVTYPRSFSAGFIHIYRFQE
DGRELEFIHKTKIEEPPQALLAFQGRLLAGIGKNLRIYDLGMKQMLRKCQVEAAPNLIVGLQTQGS
RIIVSDVQESVTYVVYKYQENQLIPFVDDVIARWTTATTMVDYETTAGXDKFGNLWLVRCPKKVSE
ESDEDGSGAHLIHERSYLQGTANRLNLMVHYYTQDIPTSLHKTNLVVGGRDILVWTGLQGTIGIMV
PFASREDVDFFQNLETQLASQXPPLAGRDHLIYRSYYVPSKGVIDGDLCETYFLLPTDKKLMIAGE
LDRSVREIERKISDMRTRVAY
>g3239
MADDASAAEPTSITFSVKASNDAKYTFTLPDSTIVLDLKEKLSSSEHADTPAERQRLIYSGRVLKD

NETLASYKIKDGHTIHLVKSAASNQRQNPPPQPASTAAGTTPSSVPGVPTNIAAGTGNNPLAGLTG
ARYAGFAQLPGAGMFGPDGGMGPPPDTDQMLSMLENPQVQATMNEALQNPALIDLMIQQNPMLREM
GPGVRQMMQSPEFRRMLTDPAAIRQMAQMQRAFGGGGMGFGGAGNTAFPAPGVTNTTPEENRGQQQ
DTTQTTPPINPFAAGANPLAGLFGMPPQPPSTAVQQNTTATEPTTTSGQATTDSAAQNQQAQNPFA
ALFNPALFGAPSGQAGAGGAAQPNLNNMFNPQNNPFMRDPALLQNVMQAMNGGGAGAGAGAENPWA
SLFGAPPAAPSPVDNRPPEERYAEQLRQLNDMGFYDFDQNIAALRRSGGSVQGAVNFLLGG
>g3240
MADAILCLPVILHPSYQDNEPIPLLVNKIFSDHTQLQYAYFDLPFVCPPTGQKHGNSPFGSGHSLS
LNLGEILRGDRIMTSDYELVMKQDVGCRALCTREVDRKAVKWGRQLIKDGYVAEWIVDNLPAATSF
VTVDRSRKYYSSGFKLGYQDFSPAEGLGRIYIHNHFTIVVRWRKAPGKAGEEGKSVILGFEVYPKS
IGYENRDEDGCPYDVHADNSHLELFITPNNKEVEEKYSDSSYIPQRGEDADDGATMKIPYTYSVYF
REDTSIEWSNRWDLFFTDQVDSSMTHWLAIINSLTISGVLGVTVIVIWSRTVQSTTKGREDGVLEE
GKLKKRRSKSPNKDKKSSEGVLTQEGEGDPLSDDELEDVAGWKLLHADVFRLPAHSGLLAPLVGSG
TQLLFVAIGLLILSSLGVLNPSFRGGFVTVGFGLFVFAGLFSGYFSARLYKTLGGQNWRKNTIITA
SLFPGLTFCLIFVLNLFVWAQASSTALPFGTLVGLVALWLLVQVPLCYAGSWFGYVRAEPWQHPTK
TSPIPRQIPRQPWYFRGINGVILTGLIPFAVLFIELLFVFKNLWQDKSGYYYVFGFLSVVSTILIV
TVSEVTIIATYNQLCAENYHWWWQSFLTGGSSSFWIFAYCIWYYTYKLHITGFVSSLLFFSYSFLA
CAVYGLLTGTVGFLTAYAFIRRIYSAVKAD
>g3241
MAAKRPNILYIMADQMAAPLLSIHDSNSRIKTPNLDRLAKEGVTFDNAYCNSPLCAPSRFVMVTGQ
LPSKIGAYDNAADLPADVPTYAHYLRREGYHTALAGKMHFCGPDQLHGYEQRLTSDIYPGDYGWSV
NWDEPEVRPDYYHNMSSVLDAGPVVRTNQLDYDEEVIYKSTQYLYDHVRHRGDQPFCLTVSMTHPH
DPYAMTKEFWDLYEDVEIPLPKTAAIPHDEQDPHSQRVLKCIDLWGKEMPEERIKAARRAYYGACT
YVDTNIGKLLKALDNCGLTDDTIIVFTGDHGDMLGERGLWYKMTWFENSARVPFIVHAPNRFSAKR
VKENVSTMDILPTFAGLVGAQLEPGLPMDGVSLVPYLTGGEGLKTDTVFGEYMAEGSQTPVIMIRR
GKWKFIYSLIDPPMLFDLEADPLETTNLAAGLPIPAPKTKEIEIKATGLPTPPNGVAPDAVTFVST
EKSFFPKIPAKVVPTPPRTPSPSFPLVLPEQDDPETVYEHFIDEVHSRWNLRAIHEDVVRSQRRRR
IVYSALIKGVPTIWDYEPRIDPSTQYVRNQSKGNLDNVEYLSRWPRVANAPAPTNQF
>g3242
MPSLPMRNSRLKVDIALNGQVKGLVPSYTTFDKIEGEVHIQAERDTAFDHVQITFEGWSPTHPEMD
SKLIKAGISKTIIQRQSPVAHITNDLNAFHPFLRLRQPVDPNTYPESREFKPLETYIFPFTFVVPE
HLLLHACSHFTTNPHLKAVHTQLPPTLGDAMLSDDGKTMINDMAPMMSEISYKLKVAVMTKNPPAS
PKPFYAITSVAKKVRIIPATTEQPPLEVGCYSTDGYRVRREKHVRKALLGGKTGRLVVSAAQPKPL
QLSAPGSTGFKAPVSSHVKLHVRFDPETENEQPPRLKSTSSKLKVSTFYATQPWSDFPSQAVAEAY
NHTTHGVYSETINLSSLCVASAQWKKHDGTELDDSRRSSLVSESSSSLAESLIHHPSASSSYSGKT
FYTASILVPVNLTANRAFVPTFHSCLVSRVYTLDLSLSYQPASTKLLLAPSISLHIPIQITSAPGN
GLAALEDMSDLLLPETQIEEEFYRPRNVAPPTEEDYQDHYTQRSSSDSFQDAQSHTGSSDYAPPEY
STVTQSGAGVRTSNPLSPGWMLDSLQ
>g3243
MALALTAASRLKPEIRLAXAVSEFEADLTMEQKIRFNSYKTQVHKSPPEIHDVMRLTAEIDQKISP
KIGRGRCYGPRLTNFLQGVQQFVGLGDIIVGGSQNLVACGVWSLVRMSLMSLVSSASYLEKVSRLL
MIVGRGAPRYQTLAAIYPRSKELQANLGEYFIVVVRLCHHILKFTQKSTVMQLTSTMNTSTLESFQ
PELEIWATAIKEEVNLLMAKRSEDEADKNSKFRDWSLGFRTLDSHQRQLAATRRVLDLCSTYDHQA
KWKQIRKVGNATVFQSDEKYQNWKSFPKSATLMYTGRLGSGKSVLMANIVDDLNMNLQDNILVAYF
FCRHDEAESLKAKTILGSLARQLLVAAKTSAVELKRFDENKLKDVEDLMVMLDTTLPAEHRAFFVL
DGLDECGHAVAKKVIRHLRATQVNCNLHLCISLRLDPNQLLRYPPEDFAICTVISVPVENPDIEAF
IERELENRIQLNELIIGNPELILDIRDSLMAGSQGMFLWVALQIDILCTMKTDDGIRQALADLPKD
LSTTFTQILAVAQRPLTSDELREVLSVTPSDTSWDPGKLLNDVKSVLASCGSLVIIDEEDSTVQFI
HHSVKQFLIEEYRDSSGKEFVIDDANLNMARLILTYLSYDRFDRQLSTSVVPRMDVGSAPYKIVKS
TIKSQSGVQEAALKLLRLKTRRDQDIGRTIQAMNARHHEVKEYDFHFLNYAKLYWIHHLCATSASE
IPELSLLAKILERRAVSIDEAVAYSETSPGSQIIESRSKTPRNLVEVAPGGQNSVYRPKDRRGITL
LFWACGLEELSLLRRLIQIDGIEPDHRNMQGQTPLLHAAQCGYKVAVRELLLTKRVDLVARDLHGY

TPLIWAARRGYRDIVYDLLTRHGLTAAEHPETLIANKVIKDGSWSLDASRTAAQLGSIDFRDSRQR
TALSYAAGIGSSECVCELLVLGADRSLADVLNRTPLMHAALSYRANAVQQFYDFPDPDYDRDFPDN
KDFEGRTALSYAAQYGTLPMVQIFLRDKRIDPDTRDNKNRSPLSYAAQEGNPDIVEALLMNSQVDA
DSRCAVGLSPLSWAIEKKIPLAKGDGISSKEEDTQRSIIRAFLDSGRVNINTKDNAGRTPLIRAVF
HQRYSFVALLLDYGCDIHIQDNGGKTALDWAQWNGNEKIIQLLREHTSGSSGKKMFTSYGHLTHRF
ND
>g3244
MASSLAAQLAQVAAKSTNPLDLKAQRIAHSKSLIFEYKVAKSQDFDTLYQICNDAFQELCRLDARF
TEFERTIFSVHSKTEDRMEMTAAQNKELDVALEAFMCLVGGKLQLTPAVKALEWLIRRFRVHEFNT
APLILTFLPYHSLPIFLNLLDILPDDLTPTFNFLQPYKTERVNPPMAAIVRTAAKTPSFSSALNNY
TLQVSKQGAHYHGLLAFWSGVMTQACSEMLVAGRAGRRDVEKKNHEDIFIRLLPVLNDGLAMKKIS
ELVIGCYMIIVVFVTKAALTEEVLDTLMTAVAGSWTQETVNSAVVTLAVLAEQKTSPTLPKKVIRS
ITKLDNPINLLSDVASQHRASRLSLGLISGCLERLQKDSSQKSLKLVSQIFQQGLLDEAASKQALK
TIFQSTMDAQKSDNLSLDVQTQISEIVQTLNASETYRPILQAMTTEIDMDMDTLEHSLQTTIRTQP
ALPTSEDVVMIDLEQEEEVDSFTPLIASLAKEPLASTSFLSGEPSSLFEQIIQAFTIAGQSSDKLK
QISDLPLIGKSQFLQKPQYLSFLARVFSGPYAIRTKVAAIEMMSSALNAEQPTADLQALLPYIIAA
FADPSQAIRAEAAKLFSKLIWTQRKAKKESDTLSPWAKESLYDQRKDVQPVWLSTSDTQKVIERIL
LDGLEGFVHDSRHVISKIQNALKGSSSDSEKDTGTELKKSLRQNLLQFLCYHIVATPLFQVKLNLL
KIVNGVGRVGSVTRTKLLAPFIDSWRILSQAQAAQICDREHISLSELEQEVAAIISPKDSDAEKVL
FTNLENSIVRPTFVSAVFTRVEQIWNKLGEDRQNATARQIFEFSFTGPEVVQSYAKETLRNVSLSG
PVLVGFLNKIPESVATLESHSPSPKRRRTNNNNAVATLSDSPELDEVMGKVTFVLELVDNSAPEDH
PVLAAGLFKSLAALHHLKTRIQSSLGYLLSLVLGSLLAIVNKAKKSSKPSFDPAAIRTDLVVDCVR
SSESPQVQNTALLLVSGLAVIAPEQVLHSVMPIFTFMGSSVLKKDDDYSVMVIDQTIDQVVPVLVQ
SLRNQKRDVVAGTSELLLSFTAAFEHIPSHRRQRLFQALVTKLGAKDFLFAVLAMLANLHGLDKNV
SVLMTYLASNLEPQDQLITFQRYLSLVSDALKPKPGISQILLGVGNEGARDRHVVAEGLLRSLSYC
LKHSSLKAQMAIIFNSDAEDTVTQVAGLFSQILEQVLVMSDAVQDVKPLGNACSETLGSVLGVLSL
VDLLDTVEQLLQRPSVDLRRKVLRLLETRLRQQPERDGSAQNRVLEFISFLNQVVETSDDILLKHA
AIACIDRIAEKYGRKNPDMILPAAMVISSDSCLGQEDDRVRIMGLLCLASMAEVLGEGIIPALPEI
LKKSVAFLESSMEGTINEQLHNAVYSLLSALLVQVPFMLSGKTLDTILRLSFKSAELELSEEGNET
RRDALKLIAKKFDVKESYSAVERNWTIAVSSGPIATKEILDVVILAIDKHPKSATSKNVGFLSKLL
YRAFDLRREQQSEETSAQFEETQLQAIEDLVNNVAIKMIFKLNDTIFRPLFIEIVEWATNGVSKSD
AKGRILRLTSFYKFLQKFFGTLKSIVTSYSSYIIENVIEVLKFARPNVKESKDLWLSAVRTLNAAF
EHDQDAFWQSPSHFSGISKALISQLPRATNSSTATIIIDEVVPALSELAVATDSPDNYKEMNTTLM
KYLRPSTSTATEGGDSAHTRLAALKAEQALTDRLGEEWLTLLPEMLPYISELMEDEDEMVERETRK
WVKGIEDILGEKLDDMLT
>g3245
MQEAKPVPKFASFKAKPAPPIEDVKKSRRTEDEHRDERSRHRSRHNSHRHRSQSRDRDSSRRSHRE
ERPTRKVSPAPLPTKDESQDIYTIDRKGDIHNVVYGTIHRYSIPQYRRAGRGSIVGLPSRFRIDRA
YDGEDYIVIRTGGWIPDGARHKTKSVLAKTDTKERNIFRVRQKFIPDNEDDVLKGFIPLSHDGSRK
RRKTSGGYVSSDVSDDEAEKYGYRSIHGKAKPEEDVPSDMEASLESDTGDEGSALKWDREANKKNS
ELLRRAEDKPGDVDTWLEVIDYQDILLTGSDSTRQLTAAEKRSLADIKISLYEKALKKVGKHSEKD
RLLLGYLQEGATLWESKRLTEQWHTILKHNPGYINLWVRYIDFRQTAFLEFSFERCKSVYLECMNL
NASSENNAEQEAIQAYLFLRLTLFMREAGYSELSVGLWQAVLEFTLFRPETYSTAAKADILAAFTG
FWESEVARIGEPGSKGWKSNKSSDLEFVTDHAQTQLKVEELFSSWSTEERRLSCGARLPARTVDTV
ENDDPFRVVLWSDIEPFLSFFFSWKDESTLIQEFLKFCYLPPLRQASTSISGGNAFLRTELVHISD
SSIVTMVNASSEEETQPNSSTVFSTAPLQNMIHSVDTLFTNGNWVQSMSLWKEIIMHDKTIIDAEW
VRRCLRLLVGAFFQNDELAEFALAVEYAINPTEGKKYAKSLLKKRSSSLRLYNTFALMESCSDNFS
AALHVWATTFSMAAILSEVQRLEFGTLLRSWVWEFLVQSKPESAIQVLKSIPDFTINLEILQQAKQ
IDISPAQRLKTQRFLMESCDHSLSLQNLASFTAWIDLTALLHYLIGSYDLSSALEIYSSTFSRLET
SSLVTENFKSTALELLHQSRSRLMYHHITTKRTYKPSIIRELLIESTTLFPHNTLLLSLFTWNESR
FRVDERIRSAIVPKSSPTTERPITTSLLSILAELTRPIYTGSTIHSARAAFERALQPSPSGNATPS
PSLWKLYILFELHRAKDISAAQKLFYRAMRACPWSKDLLMLAFPSVPSSDNEAKGRLFVDDSGEED

WWELRRIYNVLLDKELRIHVEIDDDLLEDGERMWIEREQRRIKSRGRDSKQKGKVKKTGKGREVVY
LPDDADTDHSV
>g3246
MEINWKSHLAPSKLFFYFIFWGGHIGLFALGWYLQATNPKLAPLNTLKFSVWISRGAGLCLSVDGA
LILLPMCRTLLRYIRPKIKWLPLDESQWFHRQVAYSLLIFTIFHTSAHYVNFFNVEKAQLRPQTAI
QIHYTQAGGITGHIMLLCMLLMYTTAHHKIRQQSFETFWYTHHLFIPFLLGLYTHATGCFVRDTAQ
PFSPLAGSIFWQHCIGYEGWRWELWGGGFYLIERLYREVRARRETEITKVIRHPYDAMEIQFRKPS
MKYKAGQWLFLQVPEVSTTQWHPFTITSCPFDPYVSVHVRQVGDFTRALGNALGCGPAQAKDLEGL
DPMGMYEIAMQNGQTMPKLRIDGPYGAPAEDVFDNEIAVLIGTGIGVTPWASILKNIWHLRAGPNP
PQRLRRVEFIWICKDTSSFEWFQALLSSLEQQSAITAGAQGGPEFLRIHTYLTQRLDQDTAANIYI
NSVGTEVDPLTELKSRTNFGRPDFKRLFGAMRDGLIDQTYLSGLESSITTKVGVYFCGPNSAARQI
RQAAQDSSTREVKFNFWKEHF
>g3247
MGRLIKNHWARLIILSAAGWQVGASIEGFFWPKVFWDFITHNLDAAVKPIPILQIINLILGIAALA
WEWPLKPLAGTLPHRSIELRLLVYPLISPGQTASTEQVHAWFTTTKSNIAGLLTATTYSALDEQKP
EYLAVYELEDYSNINLSRILQDAGTKNFDSVELRLYNLFSEKTSPKYALANDVAGDNGERIFRTLA
LQPGPELSVQDYDDWYEQEHIPLLSIVPGWLKSTRWVLKEASSSSNQRNEGQKKVSNFLAIHEWES
LASFKTDEFKQATNTPWRDRIIPRIDKTDASADVDIKNEYDHDVDRARSFEGRLNQFLHKADTTST
MTKTTTPVLRRRITRSQSSSLSPSIITQSESIKSPSSSPSKTPTLQEQARSPIRKRKSKSRVTNPT
TATTTTIYSPPSRLRDSITPNLILLLIGVNPGLMTGSTGYAYAHPSNLFWKLLHSSGITSIRHPPS
DTYKLPELYSVGNTNIVERPTKDASMLSKAEMDAGVPILETKIATSRPEAVALVGKSIWESVWRAR
HGRNIKKEEFRYGWQDDEENMGKCEGWRGARVFVATTTSGLAATMSMQEKQEVWNELGAWVRRRRE
ERSFASQS
>g3248
MAXGNDQVTKVFYHGKADDFVIFIDDVAAAHQWRKDRTVPLAQVVSGWKVFVTHKQGAQGILDGAS
KAMLENEFGTTRDEDVVQQILEKGEIQETTNRERQGDRNVMNGGGFVNTFQV
>g3249
MSECTEDPAITDGEKEEQTLEEFDSDAETESCELLHSPPSGYLPLAPSVESSATYPWSKWVDSLRL
KKRGYQPETYVDGWFDGPSVDREDIASTLSNKRFWDQVSGGTASILGTMKTASMSVPPSSVPRSRT
NTITSNSMAVSRQSMESTRSSIYDAITQVSKVMAIRRRHVLHEIYTSELNYVDGLRTVVQILSTYL
TMRPAILRDLQQLYDMHDAFKKQLESVSPQSAEAWLNGSPLRKSTILQKFQNQSLLARRRINSRIG
KEGADPSEALLVAKEIDKLTAKFHLYEAFIRSYDVLSEDLTLLRQSHPADGFWTEGIEALQKSVHS
TQKRKENQKKAMTLDDLIAKPVQRVCKYQLLLTELLRTVPETAYPLTYSGIKKVLEKNTQAIDRIN
TVVGDPNLRIRIKKTISLHERLDYGDQNALENVYQELGPLILCGVLHVVYQLPSGITGQYLACILF
PGYMILAKPGSDSRRLALIASIYLSDMTVDGSMNGQGLACLDTPFTWKVIFQYNQKHYEFIFSASS
SAEERGWKTETLKASVMPPNEVPNISLEPRRFSCVCMALQPLESDGQSLLLKRRGSLRISSPRPVR
IQQNQVTIRKTHNPIYDTEVRLLQEAEPLRSHSLAKEAAFAPTVLIPARSHRIKLERNISSIYSQE
LLPYPGMTLGRGEYMSQTMVGKTVMRGLSIRGVFHSRRSSSLSKATSISLVGSLEQGDDGQHGGMG
SEKATTSKVPCEALCKSRDLMEDEKLMATKTGFGSNRQRKTRSTSDNGIEEGNVAKTSNKPRVLWK
RWLPPSIVGYLDITREVT
>g3250
MNRDIHAPTVPSGPTTAGTGFRDLSKLSMVELMTEKDRIEAELSAYSSVLTSQGVTMDTSLTTFDG
FPRDDIDVAQIRTTRARIIHLRNDHKEVMKHLEKGLHAHFEALQQAQASTIPTTSTPAQQQNGTHA
TQNGIIETPFARVNSVASGSPADQAGLKAGDIIRSFGTVNWVNHERLTKVGEVVQQNEGTTFVKRH
LTGEFEKKYIATLGVEVHPLGFTTNLGPIQFDVWDTAGQEKFGGLRDGYYINGQCGIIMFDVTSRI
TYKNVPNWHRDLVRVCENIPIVLCGNKVDVKERKVKAKTITFHRKKNLQYYDISAKSNYNFEKPFL
WLARKLVGNQTLEFVAAPALAPPEVQVDPEVMAKYQQELTDAAAQPLPDEEDADL
>g3251
MASFSDDMSIEDIIDELDLNKALLESLIETRPDELEEREELQATVMDLERKLAERRGVPYNPPGTH
PSDSRSRGTFQYDGAGSPQESGVHKGHDDDGATFSLPHRDTPQRKRNLNPSDIGDYSGSPVSKRAR
SRHSRSPALTGPSGSSSSRHDYEPLNDYFQDDDIDFREGQYEAEKWLEERRLQELKDEEFARRLQE
SFEEELSYVPSKQAEPFQPSQPEPYNAYFSSSTNPVKPEYSANPLKPLLNTQSPFMQKPTAPSSSR

DQPAFGTTGFGHPSSFQYPSNGPSFGFPSQQPQYPTLGAPSGYQVIDSSDEDEDFAEIGADDFYGN
GRVPILTSKKTKTSMGTGVGRYGSEMLNRNSALDYSPIGRSPKTWMQNMIEMPAVPPWMNVEDETV
DGKQANQELKSLLEGLRPDFDITRETTPEQLNFPLFEHQKLGLAWMKAMEEGQNKGGILADDMGLG
KTVQALSLVVARPSTDPSRKTTLIIAPVALMQQWKREIERLIKPEHKLSVFILHGEKRKTGFDKLK
RYDVVLTTFGSMGTELKKREQFDDMRRFAQNNNNLVAEARGLPLLGPDSTWYRVIIDEAQCIKNRN
TRAALACCSLNATYRWCMSGTPMMNGVHELHSLLRFLRIGPYNSLSLFSSTFTRPLKGSSKADREK
ALVQLRVVLKAILLRRTKFSKLDGKPLIQLPPRTTEKIHAVFSEDEQQLYDSLESKTQIQFNKYLK
ANSIGKNYSSILVLLLRLRQACCHPHLIRDLSVDISGITEQADFTENAKHFSPDVVRRLREDPPLE
CPVCIDAVQNAVVFFPCGHATCAECFARISDPALAVQQGVDGSTDAKCPNCRGKVDPKKVTDYSSF
RKVHFPELAIDGEELPEIAASPEDSDSDSNDDSDDEDDDEDDDEDSDLDGFIVPDNYDEDDNCDDD
NKVSKKKHDDSDYKPKSKSKPKPKVSKAKGKGKSKEKKKKTLAVLRREGQRNASAKRKYFKRLEKN
WMTSAKIEKAVEILEEIKGSGKGEKTIIFSQFTSLLDLLEVPINRRGWKYRRYDGSMNPRDRNESV
LEFSDKPDCDIMLVSLKAGNAGLNLVAASQVIIFDPFWNPYIEEQAIDRAHRLGQIRPVQVHRILV
ENTVEDRILELQDKKREVIEGALDEHAASQISRLGVRELKFLFNVQ
>g3252
MDMITNLYRRACDESDPNSAACAKPTSQILLNAVPAAIVGAMLVIAGIVFFFIARKRRRQFVAEAA
KERESFEIEIYEPTANNRNRNHNPAAYADPFNDPHGLSRDPDYNEFSLAPPQSRRDHSPSRSSVAT
ESTYAPHPDMSAPPPAYHEGSVGASVRRPSDNKPFLTYCELMKTEMRVAEPYWLQRQMSRHIEQSD
SDENNSSGTTTSSDSKRPPIPRLSPKSRSLSDAAKHGALQPSALRDFPPSSKDDIGLTHTTTNKTN
SPSTPQKTSQPRLSTLNGLSLQLPPQSTPSFSTPSANRIPLSPKLDAAPIYGXPASAIPRRSRGLD
FARACTNLHHSTLAESSPDSSPNVGGRGVNIPQRRGGTSSAFGSPGTSLTQPSGIGSANQTTMSSS
LSSVNMLESDTSSSEEDDDEPMNGDRDEMMITTTPQASKMHNGFLSNPFGGAVPSPGNDWMGTYSS
AATSLASFQRARFRKSRNSRHSSSSASGNSNKPSPGPLSPPVLKSIEVSNGGYFAREMSKSALQSR
RESLSLGTGDLQLSDLSDDGEARAFRADSPGANAETGPRGVVRRAVTRRGNLLPKTKTFARIRAAL
MEESAPLDTEAKKEAEVIRQVRESDPSETKAPSFPLEQLEELTSTTSVSENPSRSVPETAFSNQAS
LNSGGTKFWETFDGRYRTPPPPGRLSTQSSVMSEDTNMDTPVSNIGREGRWRSPTPQAVTSALATE
IRRKRRRDDGFDAESFKRRAVSPGMSVQSSPVLQSTAKDSNGLNGSWIIPSKAASALLTEQTHSSS
SNSTPTGIKRIGLQGINETHDGLMNMSIE
>g3253
MDQPLIDTNTQYISFFFDLVTSSVSPFIQLFSFASIGKLLHTNELVQDTVNLVGKAYVSQGSYGST
SWEPTSRLKYDSRKLLARLRVNMLRLLKAPSTTPQDSTGSLVAACLLQFVELLLYDSGSTWGSLVY
QVLSTVSPKDDSAPGGDDYFSSLNRRLRLFLRTSGALWAICFDHDCFWSTSEQKMPDTLPPEQDLS
VNNMLDKVDRQWDDMASALEESGKLQYKLLLWIKDSVHTTPDATIDALHRLNGIELVTRATHMQQR
IISTIVLYSTSYSEDIFCRSLLPYYYWLLTSLSHLFTHPSWQTLAIPLPVMPQSLARQQAERAFEY
VERNIHSYKLEAVAYAPILHVVAQQISVDNRERDRVLAVVESIRSSGYSVADSLEKDLQCHWAGME
TRFSCRDGM
>g3254
MQDFEDTFATVAPPPDDSWLVIMIDPLGLGFTAAAAPYFDGGEAGADTIKYITYASISFGAAIAGA
ALPGDQLYLLFNGSESAVDLLTTLVANSNFVAGMDGAPLGEYNATTQGTDDLGGYIGKAFFGFSIP
VAWTLSGTSAFVLDSGYSCGLPSDSTAKIDSINCVDCDPPPPECNPIYFTAPTGLDRIDGANWGYI
TISDLIVGAVNTYTANGNSNGGSLADPMNQRFL
>g3255
MDFISNNTALITEFINTMTYPEGYWTFHWSLQIDTCLQPGNSSFQNFQPIYVNSSTVFTISKTGQA
PNVVAATSSDTCGLASAFAFNATSFEEFEFCGWLGSTPTTNPCSATINATVASSILAEGTTTACAL
KNPNVTCPAYHSSSASSADLNGQFAVVTASALVAMLVIVIHL
>g3256
MSAVEIASSFIEGAPPGELSDVVADIKALTSEGPDILPDLEPAFKSYNEKQLATVKLPGSSQQVIV
SEFNRLDGDRYFDVESSTSFEFDHITQTASAAQSYPLDSQNADLVKSLLRSLSTHAKEHYPSSSYG
VYPIENDSAIAVVLVANRYSPNNFWNGRYRSIYQIPISSSSTTITGTIHVDVHYYEDGNVALSTTK
PISLSLSNISAETIMAKIGAAERDHQEELNRAFSRLAEGAFKSLRRQLPITRQKVEWERVGGYRLG
QDISGGKGR
>g3257

MATPQKTAITTTKNIKVAKPSPPPPPPVWKQFSNFTNAHPLGIEKTLRFIQAVCTILSSSIFISYV
PNSVYAVKSALAKNQIALARRYLRFFKFIDCFDAGYKAWLVPAPLEADAVKKIAAVGKWSFLGVYF
FMEDLTILDAMGIWPTAWAQETFLECHKWWFFGLALSIIGSTVNLFFPASEPPAAKQSDEKTKPAE
KQKKSDLTPLVKNLIVDACDIVIPGSVLGWIPASSTQVGIVMIVSTLAAXGDXXAXANRA
>g3258
MVAISEIRQQITSVKNLGSEAKKVGGTSGIGLSTAREFTRYAVAPHIYLVGRNEAQASDIISELKT
INESATVSFVKSDVSLLKNVDEACQEISKKESKVNLLFLSAGIFTTKGRTETSEGVDKKLSLHYYS
RIRFIQNLLPQLGQASSSGGLARVVSVLGPSTEGKMIEDDLDLQRNYSLPRAATHAVTMTSLSMIH
LAASNPSISFIHSAPGGVKTNLMRDFNPIAKMLANGAFALLSPFGSKIGLIGLQESGERHVYASTY
PKFAPRDQKVETVGADGEKGSGAYRVHFDSSIVESKQDVIQEYLDRGVVKTVWEHTTGVIDKVVGK
>g3259
MPSYLITGASRGLGYGFLRQISADPNNTVIALVRDKPATDARVSAELSDRKNIFVIHGDLTDVQSL
KTAVEETSKITAGSLDVLIANAAYVGNYARFDGMGDLDVEPLGADLRQHFESNVIGNINLFNLFLP
LIRNGNLKKIVAISSGLSELDLIKELRIANGAPYSITKAALNMVVTKFHAQYADEGILFMAVCPGS
VATGNFDNLTPEEEAKAGVMFQKFMKYAPHFTGPAPVDDAVKDVLQVVENATIETNGGTLVSHFVS
RSKGKNKKNLLKLPLIGDLHSSPIETPLSNWDAWAQENGPIVVPRLFGLIPIVVLNSFDAVTELFS
RRSQWYSNRPGSVSMEMITGAKLGQCKFTLMHDYDDQLKLHHRILSPSLGAPAVARYQPLIELECK
QLLFDLCKTAAEAPLDGIISTKTVYPLLERTQSSLILALHYGLRIPSYDEKILQEVFDTQTQVTHL
AANPRLPDMIPPLRHLPAFLSPWKRSADKLHATQVDLNMRLLRHGKDSAGWNATKQALATAKKHAT
NEIPELDIAFTLATSVQGGMETSPRQLLWLFIAALHKPSFMGKAHAILDDVVGRERLPRFSDRSNL
AFIDAITHELFRWRPIAPGSIPRRADKDDELNGVKISKGWTIMANAWAIGRDEKVFVPSLGDLHDF
VPERWLQKVEQDGKTKLRTDLPLPVFGQGRRTCQGKRVATDGAFMLIASLIWAFDIEPVNGVIVNP
WQMVVAGFMTMPGEVNFKLRPRGTWAVDVIRREWETAEKALEKIMGRANDVEQ
>g3260
MSLETTIPQLRGLPIAFHGTMIHTLDVTQLEILQNCLLIIDDEGKIEKIYKDVPIERVNDFISEAG
HTPDVFPVKYLKRGEFLIPGFIDTHHHAPQWAQRGMGRDQTLLDWLNNVTFAHERKFENVEYARKM
YTACVQAGIKQGVTTASYYGSVHVEATKILAEICLEQHQRALVGKCNMNRSSPEWYQDASAEQSLK
HTKELVAHVRRIDSEGKLVKPILTPRFAITCDDKLLSGLGEIVKQEAPPNSIHIQTHFNEAADEME
FTRQLYPQFKHEADLYDEFGLLGPHTILAHSIFLEEEEMDRIKSKGCGIAHCPISTATLGEFMMAP
IREYLRRGIKVGLGTDVGGGFSSSMLEIMRHAFIISKARETMTKGADPALKLHEGFFLATLGGAQV
CGLEDKVGNFVEGKEFDALEIHAIGPDPYSSLGVMSPIEDDDSIHVIFEKFLMTGDDRNIAKVYVA
GRSLKETVL
>g3261
MAEFVIKDEELNSVKGKVVIVTVFINAGTSSGIGLATAKLLLSLGASVVGADIQPSTLTESSPAWF
FVQSNVTVWSDLSNLFKKAKEHFGRIDYVFANAGIGPRANYLALEMDEAGELKEPSYDVLDVNLKS
VMNTATLAVHYLKQQPEGGSIVLMGSSTGLQPLRAPDYSTAKHGVLGFGRGIARLVEAAGLPIRVN
TLMPSWTSTNILPNLDGLMQGISHEAQPGLIVARCATYLMVNPSKHGEVIYVSDGKYTEIEKAVLW
PAAQTIIGEGNPSDDQILERIFALAA
>g3262
MVKPAGDGVALLYTAIVLLTLSWVVFVPRVIVRIWRKALGLDDYLMLCGLLLYSVTASLCIVCSFL
GSXQLAAALTPAIIQKGTKLFFIAEFFYASGAAVIKCSIAVTLLRIAGSRPVFTWSIIAIMFASGA
AAIIFMAGVANICHPITTLWGETTTGSCNLKLDSDISFFFSAIEIVTDFALAILPAVLLWNIQMKR
KVKFSVSIILGMAAFASCATIVRLRYLSLYSNPAEFMFSTGAIGLWSVIEEGIGIIAGSLPALRPL
LSLPIFSRSSAGESNSTPYAPYANSNNQRRSRSGHHPLGSGVKMDTLQRLNSADGDGDGDGGSERH
ILKQTEVAVTSKVRSTTPGAWERSQVLGFKTRN
>g3263
MSSPIKVAILDDYQDIASSKFEHLVKSNKISLTLFPQTLNIRNADEREAQIKRLQPFEVISTMRER
SIFNADLLGSLPNLKLLLTTGKRNFSIDHEFAATRGIVVAGTDRIAQDGAAGGGAGPDPTTQQCWA
LILGLSKHIARDDSALKSDKSYWQGDSLAIHLPGKTLGLVGLGRLGTASAKIAILAFGMKVVAWSS
SLTQERADEAATEIGLPAGSIQVAASKLDLLRRADIVSLHYVLSDRSRGLIGREELAAMKPTALLI
NTSRGPLIDQEALLETLKEGKIRGAALDVFDVEPLPADSEWRTTEWGKNGRSEVLLSPHMGYGVEE
YIGGMYDQNVVNLERYLEGKELLPTMAELTIRSYDNDSDAANVSTLWQNTFPQYPISPQHLEKLLS

LSIGSHFVALIENKLIGFCATYREPLKDGETGYLAILAIQSEFQSKGHGTKLLEHAIEHLCKSFKQ
VKVGSSIPRFWPGVPTDLNIKDQEFFVKRGFREGTKCKDLYQPLSTFKAPQYLLDRATSSGITFAP
LKSSGADECITAQELIFPQWAGGYKMLHSEKLYDEIMVAFDQNGSRQVGWTLMLSPGKSRLWHGFA
FLPVVGGEKDGGTGGDGKTGLIAAVGVRDDVRGKGVGLGLICAAMEEMRRRGGLDGVFIDSVVLDN
FYEKVGFKIWREYRVFVMDG
>g3264
MTNSTAAARRLSDKGASFRGQLGRFRLNPTVSSSQPPPPSQSQSQSQSQSQPSNGHPTPTASHRTE
LSAGSVSSTSTATIRRRASRSDSPETGAAGNDRDGHVSKSKRVSTACEFCRKRKKKCDFRYPNCSA
CTRAGVVCTVLTLGQSVAHQPVPRDQIENLEKRVEWLEELLRTHANIDVSSKATGSIVDDHQVESP
VAEQWFNVPIMLSQPRAGIGLTAVDDTIDSANPTNSPTTLPSLDSGQLPNIAEIFRDKLENRRSVV
PRPASSIIPPVRRLSSWEEAEQLVSKYFEGIGTQYPFIHKSDFMRGMRAIYQNKSVPPDMQNSYHI
TMAVALLTTSYDIQQATAFYRIARETLTPTLQNEDRVSLQALLSLALYSCSSPAGPSIWQVLGTAM
RLATSLGLHKARPASTSAKDMVDHEMDKRTFWSLYNLDRLISVTLSRPLGISDDDITVDIPIEYDE
NWVEAPRNCQMSISVQVVRLRRIYSRIYRCFYNPHAKPTADVGPLLQGFAQELHDWRTSAPVIQTC
LHYSTAYYDFLYYGALLVLYRPSVLNPHPDSSCIIGCGDASILVIQSYWDNYSANKIKWLWITLCH
IYSAGITMLWCLEQDIRAVRQGMSPLWGNPRVYSSLEFVHTLLDEFCAKRKGADRLAIQFKTQSQE
VTRRMALATAELQRQQQQMESAQQQMQQPIFIPAAVDPSMMVHPMFYNYGWAGQEIASFYGL
>g3265
MAEIQKTARNPVATEDDHSSGNVAGYKWHRGVFFQATVVGITAFAAPGLWNAMNSVGAGGQQSPYL
VMAGNAILFALMVISCLTGSLIANRFGLKAALIFGTTGYALYSAALYTNNRYGTVWFIYFGSAACG
ITAGVFWAAEGAIMLSYPSVETRGRYLSYWLAYRNSGSILGGIIQLAFNYSGVKTGKLDWRTYIVF
VVLLGCLLSPPEKVLRRNGTRVQAPERISDIAELKALGKLAIRKDFLLVIPFFMYVTWELPYSSAY
LSLYFSVRARALASLVSAISQVVTTLAFGAFLDQKRISLNKRAQIGYIFIMALVGGCWIWGTIVQT
GYQKHKPALDWVDRGFGRGWALYILWQVNFSLLYNFGYWLVGFMAKEPAEIVRYTSIARALEAAGQ
CIASGISSTSAPLIASLGVNFAVWGIAVIPAYLVVRQVGIVHVGINHEQTTTSETERKLSSTDEDI
HGED
>g3266
MATIKFEPFTLEGQQKSPNTGLPFPLALKTKDNWSPTVQEAVIAIQELRSENKILDLVQQHGGAIL
IRGLPIKTPDDYSKIAHAFGFKAHEEVGRPPLRTALAPNIKTANEGPPELPIWPHNEYGWSTINPA
WIMFSALKTPNSGGQTPINSSIDLAAAIQYQAPEFYQKLLVKGVKYVYRYSVKEVKSNTGTSLFDA
YGQNVLPSDSPETTRQKVEAEVRRHSNRFEWHDDGSISVTHIVPVIRKHQPTGLTTFFGNVTSAYG
RSKHHGATEPPYLGDDGSYHPLPTYGDGTPIENKYLDLALSLAESSQFLLTWQEGDIVLLDNYAVM
HSRSPWTGQRTVLAALWDEDGRIDDFEEGKEILKSSPRVPPVS
>g3267
MPTPFAQRPVIILGAGIIGCAAAYQLLQNGFQVTLIGEYLPGDKSILYASAWAGAAWHAAAGLDGD
QKYIQAVTHRHLLKMALDDSSSGVCVVKGKEYLDQVPGKNSSAWGKTVLKNFRTLKPGEYPDIFAT
AWEYDCLVVDPTLHMPWIGAKVEALGGKFVRQRVSSLGDLYNMYPESSVFINASGWGSRDLTDVLD
SKCFPDRGQNVRLRSPTYDTMYFRNGKEYTYIIPRPMSGHIVLGGHNSRDNLSGEPDMDVVRDEIR
RAHDLVPDLVPAEPAEEDISYIIGIRPAREGGFRLDSQKIGNRIVLSAYGFAGGGYAFSYGIGDAL
VKMVQQAEFDNVIIPE
>g3268
MDEKIASQSIMTSPPPEKGIRDNGDEEQTSHEATSPTDKNNPNSSSSSDEMIIDDDEARKTLDYDA
NDSPYPEVRAVVPAKDDPTTPTNTVRMWVIGLLFTIIGSVLNQLFSLRQPSVTISAFVGQLLAYPV
GVAWARVMPIGILNPDRHFNIKEHALITIMANVSFGSASATQVIEAMVKFYNLGNNPGFGILFTIA
TQLFGFGLAGLFQRWLVQPAAMIWPSVLSNSALLMALHSRTNALADGWTISRRKFFLIVFSAGFVW
YFLPGFLFKALSYFSFICWIVPHNVVVNQLFGQVSGLGMSVLTFDWAQVVYANANPLLTPFWAGVN
VIFGFVFFYWLICPIIYYTNTWYSAYMPMMSSNTFDNTGKSYNTSRIMNADATVNVEKYEKYSPMF
LPAGYALTYGIAFANLTGIFVHIALYHGKEIWTIWKGNGKKDVHARINEGYRQVPWWWFASITLGM
WVLSIIVNEVWHTGLPVWAVLLGFLLPLVYFLPIGIIKALTNISTNEINLITEFIGGYAFLGTPIA
NMSFKFLGYAGVAQGLEFIADQKLGHYFHIPPRTVFFAQGIATLVGALVQSGLTIGILEGVDNVCT
SKQSGGYTCPHGTVTYSSSLIWGALGPGRNFSPGQIYGNLLWFFLVGPLVVLLTWALGRKWKFFNY
IAWPVVFGGMSLVPPATGINFSSWFAFNAIFNGIIKRRRGAWWSKYNYILASALDCGVAVSTVIIF

LCIVLPGGTLQWWGNTVYAKTADGLGTPWKSLAPGQTFGPSSWH
>g3269
MNMLVSTLAVSASLIGLAKATVQGFDISSYQPSVDFSAAYSSGARFVIIKATEGTDYIDSTFSSHY
TGATNAGFIRGGYHFAHPDSSSGATQAKYFIAHGGGWSGDGITLPGMLDIEYNPSGATCYGLSASA
MVSWISDFVNTYHSSEGVYPLIYTTNDWWTTCTGDSTAFSSTCPLVLARYGSSAGTIPGNWGYETI
WQNADSYTYGGDSDIFNGGLSQLKAIASG
>g3270
MPARKGGKIILNPPTQTEIVEDETIEDDENIWSPLQVASRDGNLEEARKLIESLTSDESKKHIINE
SPHGWYGQTALQSACANGHLAIVNLLLDAGAEVDAPGGNNGGRTALTLAAINGHLPIVERLVEAXA
NVNIPPHKYYGRTPLQGAAEHGHVEIVNFLISKGADINAPVANNAGVTVLTGAASGGHVALIDMLT
EKGANVNPPLMRYKGLTPLQAAAYHGHMNAVERLLQAGADVNAEGCRYNGYTALAAAAEQGHADIV
ERLLDAGADVLQTSGNKHWTAYQCAYFVGQQDIAERLWKLERNKAPS
>g3271
MKATTASALLAALSATTFTAVNAAPKPDVDETYPYNGPAIPVGDWVDPTVNGNGKGFPRLVEAPAV
QPKHKNPTNNINVISLSFMPKGINVHYQTPFGLGELPKIKYGTDPKKLHQVATGYSHTYDRTPPCS
AVAAITQCSQFFHEVQLRDLMPSTKYYYKISAANGTTESDVLTFTTSRPAGTPGEFSLAVLNDMGY
TNAGGTFKQLQKAVDDGAAFAWHGGDLSYADDWYSGILPCADDWPVCYNGTSSSLPAGDYPDSYND
PLPEGEIPNQGGPYGGDMSVLYESNWDLWQQWLLNVTTKVPYMTVVGNHEAACAEFDGPGNPLTAL
LDSGELNGTAASAELTYYSCPPSQRNFTAYQHRFWNPGNETGGVGNFWYSFDYGLAHFITLDGETD
FVNSPEWPFARDLKGNETHPKENETYITDSGPFGRVSNYSDNKAYEQYQWLKADLEKVDRSLTPWV
FVMSHRPMYSSAFSSYMTNIKNAWQELLLEHQVDAYLSGHIHWYERMFPLTANGTVLESAIVNNHT
YYTSPGEAITHVVNGMAGNIESHSTLSSSQKVQNITAFLDQTHYGFSKMTVFNETAVKWEFVRGDD
GSIGDYLWLLKKESDTTPPDDTC
>g3272
MYTLKNLPRAVALSAFAFSTLSSADICSTVQTETNAVQEPLSIAYESELQDYWSEACSALRPTCIV
FPTSAEEVSEIIATLQGTDDLFQIKSGGHMPNNGFSSIQGGLLIATKNLNNGVAYDAETQTAKIGP
GLTWSETQDGLAGTGRTVVGGRLGGVGVGGYILGCGLSFLSSQYGWAANNVVDFEVVLANGTITHA
NNSTNTDLFAALKGGGNNFGIVTQYTLQTHPISDQVWGGVLTFSYDKSSQLLQAIRDFTEYYPDDK
AAIIPTCEHTALFTEWFVFVFYDGPTPPDGIFNNFTDIGADLNTAQTWDSYYDLLQDNDNYDLHGQ
RYAIATETTPLPNATVGVEVMTALFETFYNTTSEVLLDTGVIGTIAFQPMPRTITSKAIALGGDLL
SFPDTNDYIIMELDISHSLASDDDTTEAAIQQLYTALDNHIQNFIAEGVLPDVYRPLFMNDAHGTQ
DYWGRISTADEARTVREKYDPELFWQKRTSGGFRLG
>g3273
MAPAKTVKQWTVKGNVNGFDELKLDTAAFPECGENEVLVKLGAASLNYRDLMIPKAGLSLQKVMQY
EEKLTYKSEVVEVGSKVTKWKKGDQVVTLFNQGHQYGPMTVAAASTGLGGALDGTLREHAVFNENG
LVRKPDNLNTIEASTLSCAALTAWNALYGLKPLKPGQTVLVLGTGGVSIFGLQFAKAAGAKVIATT
SSEAKAEKMRKLGADHVVNYKTDANWGETVRKLSVGGEGVDHVLEIGGPATLKDSLRAVKFEGVIS
IIGQVGSSKREDVPQIIDALSNVCIFRGVYVGSREQMEDMVQAIEMNNIHPVVDDEVFDFEKAREA
YQYQWDQKHFGNVVIKI
>g3274
MEEIRSTSQAVKDTVTMIVKALLRGNLQLSRQLETLIDAAETCKNAAVKMDEELEIWNKYVMELHE
ACDATDQSTKSKYQIALSDEKARKVEQDLMNKSIADAEKWVKEREEQVKEMKDLVKEELKNYPTGN
QLMVMDLVQTLGNAAASASNILAGAAASRIGRGAAFNINTKDASFSSGGDQGSEGDEAADEVANDP
AATQLPFAVMAMEQLYGLVCDKTGVQWDLITNQDSTSGKGGQLDLSGVAGSLKRRRTYLEKNKNTK
SSKVGKALLKSIGEAETVISELKVERTKSRNMGDTWKKPAKDSKQVTDWQAVVKKTKKDVQKLDTK
NKALTSGTKNPPLPGANLQAAASTGSKTASQMAVETASFKVSTAREMYKSSLTSADTARTQLLSFQ
SQMNNIKADLDSLKEDKLTLEAVKLRLFFTKLSVQIKTLVSSNVIPFKGTVQDAISSPNALIDRMI
LQQYTVAIWAGFDQFKEIAEFYKSVHDPYIINGLNLVNKMTLQTNVAAGYKEIETYSQASQDAVKN
LINAETEKFKTRIEKTYKEIMELEKTALPATEDEQNLIGSAVQNAEGVIKDQLDAKSGPVHYLVQP
REKRDEQADKLSNALKPEILQQQADALADEGFFEQNSLEYLGLDD
>g3275
MVFRRHYAIEYKEVEDVSSLKDVDEVDAALKQMVTEGVNISATIQRQNENIKIGSELMIHAVYDSL

LDLSLQLIIKTYPGGLEGLIDSASHAYTSFPPEMEALVNQVRDGLRSYLLSSMHESSLTEAVNMTT
NLFGLVEKGHSELGILFDDHQETIAEKVQEMHSLLKRAEKGNKYLDTSSEHERSNLTSDTKTMDSE
KAELETLKSEISSLTQETQNFRSKRTRLEKPQNFGEGLAWGFLGIGAGVAAGKRASLSKSIESNES
KLGEERTLQATHQTRYNEAFGRVEKAKLALIDLNSHRTDINKHIQSLKDVQTSSTEALRFVVDRKN
TLTRIHTDLVNMTRFATRYTTHQYDEQVSAKFLAEGIIDVLDVTPDWPGTTIPIMLAVILLRWPPS
LVATSDADVLGEERDGKSDFEVKFDRVIEGANRNYRKFTETSSSAPRLDDYLRDVEFDWEKTIDAW
HKMISDAQDTRIRPAPVVRQY
>g3276
MYSSNSSSLTDVESGGCQAQKRLTLTFRNINVRVTAPDAALGDTLLSVADPRQFIKGFYKTQQPKR
TILKDISGQVRPGEMLLVLGRPGSGCTSLLRVLSNDRDSFDEIDGETRYGSMNHLEAKRYRQQIMF
NNEDDVHFPTLTVNRTLKFALRTKVPNERPENVEKQEYLQEKRDSILNALGIPHTKKTKVGNEFIR
GVSGGERKRVSLAEVMAGQSPIQFWDNPTRGLDSKTAVEFAELLRQEANDFGKTIVTTTYQAGNAI
YDQFDKVLVLAEGRVIYYGPRSSGRSYFEDLGFVCPKGANIADFLTSVTVHTERVVREGMEGKVPS
TPDEFEAAYHASKIYSSMMESIEPPEKLQNEKDNLIMAVNNEKKKSHIPRGHSPYTTKLMDQIISC
SIRQFQIMMGDKLSLTIKVASAIIQALVCGSLFYNLQPDSTSIFLRPGVLFFPVLYFLLESMGETT
AAFMGRPILSRQKRFGFYRPTAFCIANAITDIPIVLIQVTCFSLILYFMSNLQRDAGKFFTFWIIV
IVNTLCSMQLFRAIGALSRKFGNASKITGLLSTVFFVYGGYLIPFEKMHVWFRWIFYLNPGAYAFE
ALMANEFRGLELECVAPDYIPYGSGYSDTISQNRGCSVVGSSNGIIDGEAYIGQQFHYSYHHIWRS
FGVIVGMWFFFIFLTSLGFELRNSQSGSSVLLYKRGSEKKRHLDEEKGSSSTAAEVVLSGSVKQST
FTWNHLDYHVPFQGGKKQLLHQVFGYVKPGNLVALMGSSGAGKTTLLDVLAQRKDSGEIYGSILID
GQPQGISFQRTTGYCEQMDVHEGTATVREALEFSALLRQPSHVPRKEKIEYVDQIIELLELSDIQD
ALIGVPGAGLSIEQRKRVTLGVELVAKPTLLFLDEPTSGLDGQSAYNIIRFLRKLVDGGQAVLCTI
HQPSAVLFDAFDSLLLLAKGGRMAYFGETGQDSSKVLEYFSKNGARCPPDTNPAEHIVEVIQGKSQ
QKDVDWVDVWNKSEERRVAIEQLETLNRENIAKPRTDEDESDYATSHWFQFCMVSKRLMVQLWRSP
DYMWNKIILHIFAALFSGFTFWNMGNSSFDLQLRLFAIFNFIFVAPGCINQMQPFFLHNRDIFETR
EKKSKTYHWIAFIGAQVVSEIPYLILCATLYFVCWYYTAGFPNVSSIAGQVYLQMIFYEFLYTSLG
QGIAAYAPNEYFAAVLNPVIIGAGMISFCGVVVPYSQMQPFWRYWLYYLDPFTYLVGGLLGEVLWD
VKVECTASELVHFAAPANQTCGDYMADFLSTAAGYIVDANSTDTCSFCQYSTGADYAKTFNLQEKY
YAWRDTGITALFCISSYAAVFLMMKLRSKKTKSARSE
>g3277
MATSFETLPIIDFQALGIPATKSEALEKLKHALFNVGFLYLANTGLEKLIEETHETLPEFFDLPES
VKQSCNMLNSPSFLGYTSLGAETTARKTDLREQFDFGTPVTQLWKENDLPWQRLEGPSQFPNKQVE
RLVTRYTSALSTLSGAFLRAVAECLSLPPNTFETFLGNMHRLKFVRYPRSEPGSQGVGPHKDSTGL
FTFLSQDPVGGLEVLNKSGQWISAPYIEGTFVVNVQQGFEAITGGLCPATTHRVIAPTTSTRYSIP
FFQAVRLDLTLFFLKEAAADIVRRIPTQNVTNIEHITIPSEFLSPLFSCFGEAQLRNRIISHPDVG
RRWYPDLYEKYSQQSLS
>g3278
MDQQRFLQQLQIILNPSTGDLKEATGVLQKEYYNKPESFLFLLQLATSHDSDDLRQLAAVEARGLV
GKFWLKVPANQKPQIREQLLRGTMSSNSELVRHAIARIVSSVAKIDLQDGEWADLPNFLLQAAQSG
NKDERAIGVYIFFTILESLGEGFEDKFQDLFTLFSKTIRDPESAEVRINTLLALSKLAMHLDSDED
PAPVKAFQQVFPDMVRVLKDAIDTTDEDRIMQAFEVFQTLLGCDPALMNVHLKDLITFMNEVSANT
QLAEDTRTQSISFLMQCVKYRKLKVQGLRVGEQLTRTALHIATELDDDDDEDEITPARSALGLLDM
LAQSLPPSQVVVPLLQALGQYFNSENPDYRRAGILALGMCVEGAPDFISSQFNEIFPIVLHLLSDK
EPKVRQATLHGVARLADDLAEDVGKEHAKLMPLLVQNLASAMESYKGEESGPTVNIMKAAVSSIDC
VVDGLDEKDVVPYQDELVPLLHKLFQHPDFKIKSLTASAIGSLASSAGEAFLPYFEKSMHLMQEYA
TKKESEDELDLRASIIDAMGEMSAAAGPQHYQPYVEPLMRASEEALHLDHSRLKESTYMLWGSISK
VYGEDFKPFLDGVFKGLSACIEQEEADLEVELGDAAKDLVGQEVTIGGRKVKVAEASDDEDGDIED
IDLDDEEDWEDFTTVTPLALEKEIAVEVIGDLISHTKGAYLPYFEKTIELVLPLTEHPYEGVRKST
ISTLHRAYATLFALAEENGQMPKWQAGLPLKVQLPVEVQKFAEILMTATIKLWGEESDPATVGDLC
GNLAENLRYTGPALVADESVLTNVVQQVTDLITKKHPCQQEFAEDEELQEAGDETSEFDWIVIDRA
LDVVSGLAAALGPDFPQLWKIFEKSVLRFVSSSENIERATAVGTLAEVITGMKDAVTPLTGRFLPL
LLKRLDDEDPQTKSNAAYATGRLIEATNDASIVSQFPTILQKLEPCLQQQVSRLPDNATGCLSRMI

LKQHDKVPIADVLPAIVSILPLKNDYEENEPLYKMIAQLYKWEDPTVRQLTPQLLPIFQAVLSDDD
QLEDDRRKEVMELVSWLNK
>g3279
MKFSNVSLVWSALASVALGLTAAEWRSQSIYFLLTDRFALTSNSTTASCDVADGLYCGGSWQGVIN
HLDYIQGMGFTAIWITPVTENFEGDTSDGEAYHGYWQQNAYATNSHYGASSDLLKLSEALHARGMY
LMVDIVVNNMAYDGAGTSVDYSIFNPFPSKSYYHSYCLIDYSTYNATDWNVCWEGDNIVSLPDIDT
TQTYVKDTWGTWVESFVANYSIDGLRIDSAGHIQQDFFTAFEESSGVYCIGEVDYGDPAVVCPYQN
YLSGVLNYPMYYQLLYAFESSSGSISNLYNMINTVKSDCADTSLLGNFIENHDNPRFAYYTSDYSE
AKNVISFIFLTDGIPILYYGQEQHYNGGNIPLNREALWTSDYSTTAQLYTHTKTSNAIRSLAITKD
SAFLTYKNTPIYQDSNTIAMRKGTTGLQLVTVLSNLGASGSSSTLTLSGSGYTSGTVVTELYTCTN
VTVSSSGTIAVPMASGSPRAFLPWSSVSGSSLCSGSGSSCTAASTVAVTFEEVVTTTYGQEVYISG
SISQLGNWSTSSAVLLSASQYTSSDPVWTVTISLPAGESFQYKFIIVNTDGSITWESDPNRSYTVP
TGCAGLTATVDDTWR
>g3280
MRGLQNLAAGLLCSTLVSTVAAQGITALHEAGRCAIRGHCGKKSIFGGELPCPDNGLAREPDDAVR
EKLVSLCGSKWSEGPVCCEGEQVDALKKNLKLAEGIIASCPACVDNFFNTFCTFTCSPDQSLFVNV
TQTEPKGDKFLVTEVDNLWSEEWQSGFYDSCKNVKNGASGGKAIDFIGGGAKNYTQFMKFLGDKKL
LGSPFQINYETEPRNPDHGMKALTISPKACNDSDPAYRCSCVDCPEVCPTLPEVKSQEYCHVGLMP
CMSFAVIIIYSVFLSLVVSISSFVAYREHRYRKPERVRLLQDPVQSDDEDDGELVHGAGYLETPHK
HYKLNGICDKAFNRLGSLCARFPAITISASVVVVGLLSLGWTQFTVETDPVRLWVSPTSDAALDKE
FFDDNFGPFFRAEQAFLINDTGPVLSYDTLSWWFDVESRVRRMISLKQGSTLDDVCFKPGDACVVQ
SLTGYYGGSFAGVTPRNWQKTLAHCTESPGDVSCLPDFKQPLQPTMVLGGYEDTDNVLDASAILVT
WVVNNFLPGTEGESKAIDWEESLKQVLGVVQEEARERGLRLSFNTEMSLEQELNKSTNTDAKIVVV
SYVIMFIYASIALGSGALTLRSLLTNPSNALVQSKFTLAIVGIVIVLMSVSSSIGLFSALGIKVTL
IIAEVIPFLVLAVGVDNIFLIVHEFDRVNISHPDEEIDERVGRALGRMGPSILLSAITETVAFAMG
IFVGMPAVKNFAAYAAGAVFINAILQVTMFIAVLALNQRRVESLRADCFPCFTVRRATSSGLPEGV
AYDDTAGESFLQRFIRRVYAPALLDRRVKAAIIVIFLGLFTAGLALIPEVELGLDQRIALPTGSYL
IQYFDDLADYFQTGPPVYFVTRGVNITARSHQRQVCGRFSTCEEYSLPFVLEQESKRPNVSYISGS
TASWLDDFFYWLNPQSDCCKEDKKICFEDRTPAWNITLSGMPEGEEFIHYVEKWIESPTDASCPLG
GKAPYSNAVVIDSKHLTTNATHFRTSHTPLRTQADFINAQASARRISEYLSKEHDIDIFPYSKFYI
FFDQYASIVRLTGTLLGAAIGIIFVVSSVLLGSLATGAVVATTVVMIVVDIIGTMAIAGVSLNAVS
LVNLVICAGIGVEFCAHIARAFMFPSRNMLEKSPKLRGKDARVWAALINVGGSVFSGITITKLLGV
CVLAFTRSKIFEIYYFRIWLALVVFAATHALIFLPVALSYFGGEGYVDPESDGGLEDNLAARGYRS
LLMNDDYDSEEED
>g3281
MPWIQLGNFETASSKTPPPRPYPAQSQPAERAAARFGVHGNAISRALLEHGLTGLALFDLPSAIEK
SENAIEALQTEFPSTKIITQACDVTDEKGMIEATRSAKAQLGEINILCCFAGMVGVVPSAEQSVNH
WRKIIDVNTTGCWIAAQAVGREMIASKHGGKIIFIASISGHRVNYPQPQAAYNTSKAALLHMKNSL
AAEWTRYGIHVNTISPGYMDTVLNEGDDLAPWRQIWAERNPMRRMGSPEELTGPVILLCSDIGGSY
INGADIVVDGGGLVF
>g3282
MAKTQQPEFPIQINLGAALGTFFKSQLFVTPPYPEASFTGKTVIVTGANSGLGLEAARHFYRLNAT
KVILAVRTVSKGEAAKEDILTSVKTRNDTNAIEVWSLDQAKTSSTLAFSERAHTELSRVDAAVLNA
GINTKNFKLVEGYEHVTQVNVLSTFMLALALLPKLLDTKTRFLESTPHLTIVSSEAHHLTKFEEIN
APNLYEKLNEEQSYAQQPRYQVSKLLEILLTRELVARLKANPKFDTNPVIINLVNPGLCLSSLTRD
DVKPSLFFRIANKLIARTTEQGGRCLVLAASAPEDSHGEFQSDGKNQDVEAWIYKDLGEKVQKKVW
EQTVPILEARKPGLLSAVGL
>g3283
MASNSDLGIVVVVGGCGYLGSNLVKALQAESTCTAVHVISRKPDQNLYPNVTYHAGDISDAQQVAD
LFTKINPRVVFHVASPKDIAPEPLLYKTNVDGTRNLLKVARETENARAFIYTGSDSALEQMPGVKQ
TEETAKLYTESSKGANPYAKTKAIADAEVQAANTPPSLTTAVIRIPGLYGENDDNCVGTILSTIKR
DEHKVQLGDNKPVFEFVYVQKACEAHILAAKRILEGRLNGGQAFFISDGVSLPYFDFVRKLHAGAG

HPVPPDQIKIIPLWLVVGIAQLGEWLYWAFTFNTKFPEMRSQRLAHLAGGCQWDISKAKEQLGYEP
VKDQDEVLTKVAQAESLRLGIKK
>g3284
MDTIEEYMQRATASGSDQKIPGAVLIVANKDGITYSKSFGSMSLDPASELSTKPLTPDTTMWIASC
TKLMTAIASMQCVERGLLNLDEDVSSILPEWKNPQILVGFEDDSGKPILKDAKNKLTLRMLLTHQT
GLGYGFAQPELMRYCKYANIPPIGEKRIVCIRRSAWTYGVGLDWAGQMIERVTNKTLGSFMEENIW
KPLGMEATTFRLQERPDIISRRADMSMRSAEGTIGPSPTRFFPDNASDDQGGGGLFSCPADYAKLL
ISILKNDGTLLKPSTMDDLFAPCLTPDGTASLRENRAAGYKSFRESKAGSEATKQLIPQYEMNYSV
GGQISEKGWLNGRKSGSMSWGGLPNLMWVIDRETGVAFFYGSQLLPPGDSVSRAAFERFEYAIYNG
ELGDLGLKAS
>g3285
MSRNICVSTAEGNTGFLIAKLLLTDPKFRKNVDRVTALAINTQSPYVQELEKLGAVVAQHRPGRVS
DTTQALRYTEADTLMLIPPAHVDKIDITLEILEAAKRVNIGSVCLLSAAGCDMADRDAQPRLREFI
ELEVALMKMKGDPSTRTGYSNCIIRPGFYAENLLLYTPQAQAEGLLPIPIGRDHQFPPIALKDVAE
LAAHVLTSKGKHGFSDRHRGQLMTMTGPMLVTGDELAEIASKTLGVKLVFDDVSGNEARRLLRFQT
THDEAERQYLLEYYSLVREGKTNYISTNCFHDVTGSHPIEPPQFFDIYKDQFMPKMALQKVESDGA
>g3286
MYTKAPSTLLLALLALLLVGVNALPRVLHANVTPTPTTTLPSASFSMQASSQVPSSMAVQTPETQS
TLPGIPDAPMAEFEDGSETEANLTASSTTPSPSASATSLASSLSLLASMTSWLSSLTSSLAGLYGL
A
>g3287
MLSPVTSLNVGVFQTLESVSETTILIRDQLYGDHIISEPILVELLRSPTVLRLTGICQYGVTALLG
FTPRVTRFEHSVGAFLLVRKVGASLEEQVAALLHDISHTVLSHVVDWALKNPPGDESGTSFHEVHK
ERYIKTTPLPEILTRHGFGDLKAFNEDLYPLVEMPSPHLCADRLDYALRDATALGKLALDDAQQIF
ATLKAFPDASATRRLLVLEDKHLALILSRAYVATDRDVWSNRAHVNLYQRTGQLIGDVVRRGAVKE
EVLWTVSDQEFWELLRNSADSNELEILKQLESEDLPEEDTLQLPRGAKIRTIDPGIWIRGTEQPSP
LSVVSPQWALERQEYISSREATRA
>g3288
MAGGNKHNSNASVPENEMFACFTDTHGVITSTMNDIPGYKVTKVLGTVYGLTVRSRNWGAGLGGVA
RSIVGGEIRIFTKLLYTARNEAVERMVGECMSRGGNALVAMRFDVVDMGGFSQICAYGTACIVEKL
DD
>g3289
MSSEPNSATTANXLLNLPLELLLTVMDQLDNIYTLNALLNAFPDELLPLFKTYSKSILNTIFWRPV
QHYNDDNGVVYRWMIRELKLGYLFEPSKEVESKGANNGGTAVGSRNGEKVRGPQTDYREDMCQESR
RKARDKFILSENNDID
>g3290
MASDVAALEAEVKEFKLQLETVQSSLQVDPDNAELQSLKAELEEFIGLTEASIAELKPATPTPAPQ
PPTKEKWSRESHPAFQSGYRKATDSAPPDEPAAPISFSVNDSVLARWKSGDGSYYPARITSITGSS
SNPVYIVSFKSYGTTETLSARDIKPISSGADSRKRKADGTPGTPSSQSSTPQHTGIISAAADINPT
LADQARNEPSKVSDGPARPNKVPRKVKANKELEAGKSKWQDFTSKSKFGKTAKKESMFRTPEGVNA
RVGFVGSGQAMRKDPTRSRHIYQQGEEEGY
>g3291
MSAPTKVTFPSFMLQISGELYAPVAGAPNRNGAAVVISHPMTGVKEQTAADHARLLSKAGFYALTF
DAGYQGESTGQPRGLEDPHQRVEDNKAAVSYLSTLKGKVDPGRIGVLGICASGGYTSYAAQSDSRI
KALATVSAACVGRMTRNGGLHKENNKEVPEAIAGALQAAGQWRTSVANNPNIEAPPMFSSDISQIP
EDADSFFKEAASYYGSKRGNHPRSDQKVPPSSYDLMIGYDSFNLQHLISPRPLLMVAGSKAETLHY
SENAVAAAREPKELFVVEGKNHFDLYDDLTVSGPKLVEFFGKNL
>g3292
MSDFNSLYQQGLYLSPEQQVLLMAALNSNNPSQNQVENKKDAQNRSRTQSSSNDTSPSNSNAFDPA
SSSYFESPLLQDAPGSGQLGFGTDESPFLDFDPDVDFDFQGTDQLIGDLPEFENHESGDKRKSFDG
DEDDSSSGKKRRESDDKDKSAKKPGRKPLTSEPTTKRKAQNRAAQRAFRERKEKHLKDLETKVDEL
EKASQTANQENGLLRAQVERLQVELKEYRKRISWLSGGNGLSTMAAMSNMNSRNLSNLNNNDFYFD

FPKFGDLPGKHMFSNGGQNKQKSSTPSTTAVSPTAQVPENGRNSLNSKNLSKAAKANGMTNGQTYQ
SRASASSAASNTDSPSASSDSQHGQSSSIGTSPEPSLNSPNVGKMGDNSFEFYGNEAATNYGTIDG
EKSFCEKLGMACGNINNPMPAALNKNNDNPQLLGQLQPAAADQSLSFDWLAQQNGGSFXPVLFNDX
REPQDAILSQDFGAFFNDAFPLPDLDSPFGDANDVTTSPKDSVAAKKGPAQAGRKQDDEEEVVPGE
DKAQIMSCTNIWDRLQSMEKFRNGEIDIDSLCTELRTKARCSEGGVVVFRKDVDDIVGRAGNEMQS
H
>g3293
MASPRPPHSFGISSPLPNGAAPSGPVPGATPLLPNNGRVIQNGPVRVLCVADVRGNLKSLNELAKQ
ANADHIIHTGDFGFYDDSSLERIADKTLKHVVQYSPLLPDHVKRSISQIPPQQPVKARFTSDQLPL
SELPQLLDKRLTLEVPVYTVWGACEDVRVLEKFRSGEYKVDNLHIIDEANSRLLDIGGIRLRLLGL
GGAVVMHKLFDNGEGKTTIAGGQGTMWTTLLQMGELVDTANRVYDPSETRVFVTHASPAREGMLNQ
LSVTLKADFSISAGLHFRYGSSYNEFSVNPTLDHYRGKLAASKASFNDVWDTVRSEVESAISSNEA
QKTLLENALDIVDKMPSVANGGNPFGGPVAAANAGGQVDESAFKNMWNFNLADAAFGYLVLEIDNG
RIGTEMRAQGFNFSHRTGKPGVAAPAATQLGGGPGGPQFGQAPPAGRAAFPATQAKPQAPPATTVS
PAPVIPKPATPQPTTAEKEANGNKDGEKTSESPAFKGEKKHSNGLFISNVENEVAARDLFPEEDQG
KIVKVEKWGKFGHVVTFGTPEEAKAALDRQPIDHKKPTPQGQPRKPNIKFFEDRGSRREGNAGTWX
ASSRGGASAPTRGGYQSGASDSETGRGRGGFGRGRGGRGTDRGGRGRGRGGFSRSGDSPATDKPAA
AGSTGGES
>g3294
MAVKKSGFKSLADQLADLEDPTPKDFDPEDEDNGNQGSGDESGSDMDEDAGREHYEKVGKSKLRKP
ETLSLGKQYAGSHVSRDALDDSDDENPFGRGSDEDEDEDEDDEDLEGDEDEELSGSEDDEDELEEE
EDEDEDEDDISDDDDDAAEDGDENQESLSDERAELRRLMATDQKSVAATISQAAKADAAKGFAVRQ
QRVAFDALLNARIKLQKGFTAIINSSEISWEEAQENSDAIKSAESAALALWSTLEDLRLTLADVQT
KEGAKKRKRPGPVSHITTTESLWQRMQDLESDSQTHRRAVLEKWNHKVRGTNASLPNAKGKLIANS
DNSITAVLDAHVATELSERSTKKQRTAESSDSVYDDTSFYQTLLRDLVEQRMSSSDAVTNGLDSLH
LQIPSHPTSGMRKDKIRKDVDTKASKGRKMRYNVHEKLQNFMAPEDRGTWTDRARDEFFASLLGKT
ASGLLGEDEDEQINGAVSDEEDLEEGGLKLFRS
>g3295
MRLRRFFSTVRILQHDNPLGLPKSGTPPTFKSRRGLPQKRRIRDVQKVVAVSSAKGGVGKSTIAVN
LALALARRGIRTGILDTDIFGPSIPTLLNLHGEPRLDSNNCLIPLTNYGLKSMSMGYLLPQASSSS
SSSSDTELTTSSPPPMDTTPISWRGLMVTKAMQQLLHSVSWGPLDILILDLPPGTGDVQLTIGQEI
ILDGAVIVSTPQDIALRDAVRGFGMFQKMDVPVLGMVQNMAFFACPNCGHETKIFSHSHSHAHDAE
GGVVAQCQRLGIEFLGDIPLDARVCEDADRGMPTVVAEEGDPNSVRRKAFMSIAEKVVRGVGMDWL
>g3296
MYIDRKPRHQKPTAHISYDEGLNIVRAFLKHAAHHTVEDLQAFTAQKIPSPTWVKVQEQVIPNAHL
TEAADHVNAQLGERGIKRVGGRTWWQWRGLQGDLKCEWIEMKSDYKARKQANAHCKRVMLYVHGGA
YFFGSLETHRYQMQRHARKLKARVFARKSEYRLSPQFPFPCGLQDCLAAYLYLLTLQDPTEIILAG
DSAGGGMVLSMLVILRDQGIPLPAGAILVSPWADLTHSFPSIARASNFDYIPEHGFLHRPSRSWPP
PNSDDLAYMYQNANQKKPGEIGSHVQTDGIGEANDNDQQTAVQGFTVREENTAGVNHTYPGLHSGI
NRVGTADVGTAHADNIVVPMADNTVVEIKDQIQMYAPNQMLSHPLVSPVLQPTLGGLPPLLILTGG
GELLRDEQIYLAHKAADPLSYLPADIFLDEHDPDRILIHKYGPTNVQLQVWDDLCHVAPTLSWTRP
AKFMYRSIAQFGAWALAHAQHEEIDIPEDDASSISSESSSTSEIEEPVASVGKAGDPLPAFRKHMI
RQRVDRHGIVYHLDSPQSLPALQHPRNKIGALNPDIVKKWMGAKKTWDEKFAKEKLTVQRRRIKDF
EMGYFRFDGELPPACAMASRRTEKDLAGAKVRKSYGMMMWSLLASKHDKDIVAREAREDAIPHITN
KPLKGKEGQGDTDIVAQKTSSGREESISAPDRRKLSPFPVEERSHSRPRSRSRIVSDAGQVGEYGT
DQSEQHSTGVSTPIVLIPGVDINTDTQSTGKTPDNVSTIAVLNADGVLSTTDTTTVKSDDSIVLRP
SSLSDQNGTLSDSTPHTPSIITTDTATELGVHGSNASTVAVRNVPYILKEHEEEKQKRPSTVDEVS
PHAERDAVVSNGAETPKANGQDKDAFSFHAEKERPEMPDRDEFVTAEEIQA
>g3297
MTDNSSQIPSIMTAWLWSSTSGGLQNNLHKSDTAHRPGQTLRPHQCLIKILSSSLNPADYKVPEDT
PSLLWKCVVSMPASPGMDFSGQVVATGAKVTNVRIGQRVFGSLPLPRQFGALAEYSVADAGLVVPV
PEGVSADDAATVGIAGQTALQVIKGKVQAGDHVFINGGSGGVGTFAIQIAKAYGCHVTATCSTRNV

DLLKSIGADEVIDYTAVDVVDYLRKQGPLFSLTLDMIGKPDNMYRESHNFLLPGKPYLQVGNESHF
STAGRLITPRILGGGQRPYQLVFFKNRYEDLKEIADMMQDGKVKPVIDTVFEFDDAVKAFERLRTN
RARGKVVVRVAPVS
>g3298
MGVLDNIRGRDTSQLSAALXRSEPADVPDEKSPGQAQDISSSDSDTLSLEARNEKEIRLHPDQITA
NAELGVQKAEAVTLVWGKKTVYAVYAWIWICFFMLAFQSAVGNNLLYTAYSDFALAPAVSTASILA
SVIGGVLKLAIGKTLNLVGRAEGLLFFVGVYILGIILLASCNGPDAYAAGYVLYWIGYDAIYLILD
VFMADTSGLRNRAFAFGFVSTPFICTAFTGSLAAQSFINTSGWRWGYGAFAIIMPFVFVPLAVVFK
YYERKAIKLGLFKRQDSGRTTMQSILHYFHEFDMIGAFLLMAAFVLFLLPFSLQTSNRITYDSAAF
IAMVVIGILLFPVFAVWEAYFARTQFIRWELFKNRTVLGACICAATVNFAFYCWDLYYYYFVMVVY
DLDVSLTGYMTQIYNVGSCFWGPVFGMYVRYTQEFKWFCFFFGIPLLFLGAGLMIHFRGQDGSINY
IIMCQIFIAFSGGTLVIGEDMAVMAASDREGVPMMLSLVSLFSSIGSSIGSAVSTAIFSSTFPRGL
LQALPEDQKANATAIYLGGYTTQLLYPVGSPTRDAINYAWGYSQKYGAIAATCVTILLIPAVGMWK
NYRVDKAQNKGTLF
>g3299
MMCLQHPYAWLFCGFAALGAWLYGYDGVYFTGVTALGTSTYGKCTYNQGASDVFSREFGTQLSDGT
YAISPASLSLMTSMINLGELVGSLSSATFNDYLGRKGSWLLGALFLSIGVVLQLVTSSQTSFISGG
RAIMGFGVGIFCATSPLYIAEVAPTEVRGPLLMFWQLTLSVSQIVAAGINRGVSMNETNFAWRFPT
GFQLLFPFFVFCMVWFIPESPRWLVRKNKVAKAEHSLRRIHREKENYDPASDIQQINDTLQQEIHL
SGQGSWSSLIKDPIERRKVLYSAGALIAQQINGIQWFYYFGTVFSKAIGLSDPFLMTLIVFIIQLF
VVLAALLLSNRIPRRPLMLTCTCIMMLSIFIVGCMGIHSDGNYVAPIYGKVIICFVIVEIVAANFS
WGPLGWVIASEMAVGPNRNKIYAISVACFWISIWVTVFTLPYLYYSANLGPKTGFIYTGLCGISLS
YVYFCVGEVTGRSMEEIEEFFQKGIPVTEWKNQPRRQNPQLHSSHEDPEMTGGENGAREMGKHESE
AKEIELA
>g3300
MSAISLTASFSRPTGYAAAGDTIPFLQDDTYHLFQLSSPPDTLYHPPRVRCTWTRQRSQDLIQWKR
DEMPVISPGQAKTDFDSDGSWTGSAVLGPDGHMHIFYTGYNLLENGKQVILHAKSTDRQGTQFTKP
EAPICIESGSLEQYEATDFRDPYVFFHEPESQYWMLVATRLAKGPYWTRGCVALLTSKDLKAWTVA
SQPLHSPNNIFCPECPELFTLGNGKWYLLYSRFSAPHPGTLYMIADNPRGPFHLPRDCSGGVIDGR
RWYAAKSCARAGDPSKRIAFGWVHDYVEADSKWLWGGSTTQPREYSARMDGTLQCSVLPEIEKRLQ
EAGTMFNDHSKQLILNGPGTTVAKAVSTPQCDFYLTFALPKAEANSFGLMLDYDDDMRGYRISFQH
VASQYYSVTLVCDMPPFDDFWADLTSHSIPKLVDGPELVRHGAVRLDQPMTLIRSGDIIEFFVGGK
VITYRLPQRSKTSAKMKNSVGLFVEDGEIEFNLLRCVPVRYL
>g3301
MEAEYIRVANIIHTNAKNNHRGRYVVAIAGAPGSGKSTMADGVLRHIRNLSPKVQAVAISMDGFHF
PRSVLDQFPNREEAYTRRGAPWTFDAARCVDFVRRLRVFADTVPIGDGKSKEVLYAPSFSHADKDP
VSDAITISDNYSIIIIEGNYLLLDEPQWREITVFVDLRIFIQVDLREVRERLARRHVDSGIGKCHQ
DALLRVDGNDYVNGVTIHEKLLVPDIVVTSIQEALMGG
>g3302
MEYPVVFHMVSDSKAVSSVGDYTEADAIESMIRPCGPRLIRLFWLVVQPSYPLLDKRRFMELYNIS
YRKIGAALLGAVYLNAIQWWNYDSELSLQPPPNSTRLRKLTLDTIMNEFHRPHLSSIEAMLLYLQR
TDYEPLTSDHTFSRGLTSQMLAVAEAIGLHVDASEWTIPEWERAQRRRISWALYMQEKWTAISYGR
PSHILEVNWTVKDPCLLDFEPCDGPCAGLFFPLNGWGAGFTSPLHFFLSSSKQLSXCIMNTKGSSS
RKGHSQAQTPTKCATRACDQCRLRKTRCSLSRPCETCILNGFECTFLNPQKKRGPPAHRVTQIKER
QGQARRSSGSADSASLASLHGTSVSATVDLPTFDDSQTPGEQDFGFGLLLDKNATEATWNSPSHPD
SWLPDNFNSQTGSLFDFPGANIFVKQALPPIIDDVNLNFDESMPFMGGDINAHDTQSVNSMGAPNA
STSKTEFWPPYIQETNLIPWIDVFFDRLHPTLPVLDRSSLFTRLFQREHRQNPQFGAMILSLCAFA
LTQPIDIIERPSSSSRADHARMMMTEATKMHSSSDFGEHPTVESVLTSFFLFGFLFGSNQHNAARL
RLREAVDLASILGLNDPSTYTNHSSEYKGQLLRIYLVLSVTERAYALQRRLPVNFVNPPSNPIGSV
DEMDNTTRRLVSGIIVHNEKDAAAMMGLTLLMEVFDSIDEAIIVCWNSRCSIITTGKCQTLIESKA
INMYRILAQVSNVNRYMQRKNKDHFIVDSDPDLPKQNGGDNTSQSVSEELNVVRVMRDFLSETQCA
DVLVTQKWVLDRLWNLCFSHGLLRQHSEQPELCFRFAFDNGVKTLELCRSLRISAMEAHGVGIVSI

>g3303
MSFTSLTSTQQELEVLHLRNRLFIHKDRNNEYRTPISSNWRPTFHVTAPNGWLNDPCGPGYDPSTK
KYHLGFQWNPKGNDWGNVSWGNATSYDLVSWNRSAEPMLSPTTEYDHSGVFTGCLRATDIDGRSSG
ALTCIYTSVSHLPIHYTLPYVRGCETLSLAVSHDSGKTWQRLECNPILPSPPQNLEVTGWRDPFVG
PWRRTEPDQQLYGFISGGIVGQTPTIFAYTVNPKDLREWMFIGCLVDVGLNFRPSRWSGDFGVNWE
VANVFDLVSQEEEVVSRTFMVVGTEGCLLVKDDDRVMARTARTQRQQLWMALKAKPTIICDEENND
PSTNTQALATYSFAGIFDHGCYYAANSFYDPVTAQHVVYGWITEEDLPDDLRHGQGWSGLISLPRT
VHLQVLQNVVRARNSELQSITSMETEVDSRGTHTVRTLGIRPDDRLRKLRDKARWVHTMQSPSLLS
SQSSQSVIPLRTSKWEVEAEFAVQKQCTRVGIQISHSADLKSQTVISWDSCTETFTIDRPYPTYPG
INHGVESAPHTLLTFRNDQGDEVEEPLRIHAIFDTSVLEVFVNERTAISTRIYLDEDRCFQLAFFA
GRESNDEKCDSGAHAVLLRAQAWDGLET
>g3304
MAAQNSERQPWKEAVVYQIYPASFKDTNGDGWGDLPGIISKLDYIKSVGADTIWLSPIYASPQDDM
GYDISDYRSIHAPYGTLEDVDHLIAELRDRDMKLWMDLVVNHTSDEHAWFKESRRSYDSPKRDWYF
WRDPKYDKKGVRKPPNNWKGMFGGSAWTFDEATGQYYLSLFLPSQPDLNWTNSELRQAVHDDIRFW
LDRGVDGFRIDSMNLMSKHPDLPDGEITCPDEEYQSGDKWFASGPRMHEYLQEIRTKVFDAYDAVT
VGELGFTKDEESVSKYVAKSRHEINMLFTGDIIDMDFGPNGKYTPGGGFHPRQLRMLTEKWQTAMP
RFDGWNTLYLDNHDSGRSVSRYGSDDPRYRIYAAKMLATYLTTLSGTLFLLAGQEIGMANLPKDMG
IEQYIDVEGRNFYNQILQSRGGDHSKMADVMYELQLKARDHGRLPMQWNAGPNAGFTVPEARPXMT
VNPDYTLGWNVEAQQDDSNSVLSFWRESLDFRKKFADVFIYGSFEMLLESATHEKVVAYKRKDYGS
DKEALVLLNFSDENSTITIPWLSGWTVLRGNYSTPPSFSEMIVLRPYEAIVSCLGFPV
>g3305
MEQTMTNCSDVGPEDAAYRQSDARDATETERHMSIMQAFKLYPKAVGWSILLSTAIVMEGYDVSLL
SNFYALPQFNQKYGELQPDGSYVIPAPWKSALSNGVIVGEILGLYMTGIIQDRFGYRKTILGALVL
VICFIFVVFFAQNVQMLLAGEILCGIPWGVFQTLTTAYASEVCPVALRAYLTTYVNLCWVIGGFIA
SGVLRGLLELPSSWAYRIPFAIQWFWPTPLIIGCLFAPESPWWYVRHGQIENAKHSLRRLXSRSDT
TFDVDKAISMMQHTNELEKEITSGTTYWDCFKGANLRRTEIVCVAWLVQNLCGSTFMGYSTVFYIA
AGLSESSSFDMTLVQYAVGALGTIGSWFLMTRFGRRTLYLYGTFALFSLLMIIGFISFASQTNSAA
NWAIGSMLLVFAAVYDSTVGPVCYSLVSEISSTRLRAKSIVLARNLYNIAGIVANILTNYQLTSTA
WNWGAKAAFFWAGCCFLCLIWIFFRLPEPKGRTYAELDILFAQGVSARKFKSTKVDLFHRVGPAIV
EHEDEKAQVVLKESC
>g3306
MAILPLSIALKQIRHDSDFKPDHILRITAENITVACRTRLSAVVNGTIPGPALYLRENQTTWIRVY
NDLHSDNATIHWHGLSQSVAPWADGTPQASQWPIKAQHFFDYELRPRIGEAGTYFYHSHVGFQAVS
ASGPLIVDEADGNIPYKYDEERTLFLSELFNNTDKTIEQGLTASLAHFLWPGESESILVNGNGFSA
LLGNGTSASMPPEDAPSNSSDEKCHPEIIQVEANKTYRFRAIGGVALSPLAFAFEDHDNLTIIAAD
SSYTQRTETDVIQMAAGQRYDFLLHTKTEEELQAIGKTEFWLQIETRYRELNDTFYAVLSYAQNNA
STISSSPPTQPPVSIPYFLQDWLEYTLEPLMPNNFPGSDQVSRQVIMNAAQLNVDSGQYWTINNHT
WTETNQHEGDTPFNDTTPTADTPYLVNVYKEGERAIPDYQKAIQNNGWDPELNVFAAEIGEIVDII
LVNEPAGVVGGYDAHPFHFHGAHVWDMGSGSGIYNATANEERLKDYNPIVRDTSLLFKYGDPHYGD
GRNYTSQGWRAWRLKVTDPG
>g3307
MRVRLDQEASSRLPLRPASMTQRSSTSDKKLKGLKASLLIPGRGEPIRNGALVIDGPKIAWVGEQS
AIPNKYQDVEFNEVPVLMPGLWDCHTHYSGMEDDSDNIQNFFGSPALQGARAAKELETTLLAGFTT
IREVGGIAGEIWPAIKKGTLVGPNVYSSIGVLGITGGHSDIHDVPLDVVTARRSTGTYVVCDGVSD
CVKTVRLMVRRGATLIKICATGGVGSLLDDPEDAQFSPEEIKAMVDEAARTKRIVAAHCHGKEGIL
NALNNGVHTIEHGSYLDEEVAALMKEKNAILVSTRLIVEEGLKNPSIFPPTSYQKLLKVSEAHRKA
YALAIKSGVKVVLGTDWTAGENGKELAYAVEAGMSPLEAIEASTARCPETLGSHFAPLSGQLKEGY
DADVIAVASNPLDDINILGEPERITHVWKGGKLYKGSL
>g3308
MNKFIIEAEQVNIKMPRPANPKPGFSLLRIHSSQKPHNIANLSTKAKAELINSIAQDIEGCIWAIG
HYSKLGVLDSTHTLGFDQVINDIHNDERHENKEVLQRTLRKLERYKKRAKAQKKRCKRLEAKLQKE

RAKHTEEGSNDSDTSTIYSEMASMQTTFSTRDGKSNEAFRSAINGGDGTGQALQGTAPIQSVESPD
EPMTSPPPAFPSPPRRSMEVDYEKTVHFQLPYEEQTWQSILP
>g3309
MVSQPGDYAAVRKDIAAQLEKPDYDDGSAGPVFVRLAWHSAGTYDAETDTGGSNGAGMRYEAEGGD
PANAGLQIARAFLEPVKERNPWITYADLWTLAGVVALKEMGGPDVKWLPGRTDYVDDSKLPPRGRL
PDAAQGSDHLRHIFYRMGFNDQEIVALTGAHNLGRGHIDRSGFEGPWVNNPTRFSNQFFRLLLNLE
WKPRNLSNGVKQFSYSDPDASEDEKEEPLMMLPTDMALISDAGFLPWVKKYAEDRNLFFQHFADVF
AKLLELGIKRDEKGNVTNTDNVLGGYISAPKKSSAPTGPLKSEEQRWEVVAKARL
>g3310
MELAAAQIRTLAQTADKAGRQEILSTLNKLQSEIEDPMEMLMRLSNFNILIAMFRVAVHVQLFQAL
DKSESLDIDEIAKATGVTSELLTRILRYLASMNVIKENPNGRYTANNITRFFTNSRVQGQVLHGFE
TNIPVARELFDFLATTNYREITDNKHTPFQKAYNTNLHPFEWFAQNKKRFEHFHEGMAAMASAAWI
DVVEVFQKAAEQIPHATPKADEVPFFVDVGGGYGHQSVLVGRLYPNLLHRIVFQDLPQTLDQVSVI
EGIEAQAHDFFTPQPIKGAKFYYLRRVMHDWPDADALSILQHLTPSLSTDSRILIDEIVMPDQSAH
WHSTMIDLSMMMAFAGKERCAQQWKDLAARSGLRVEGIHTYDAASYASVIVMVSTRQL
>g3311
MATTSRQTLRVPHLGGTAVGYRTSTPNFDSTKPTVVLVNPFTATVDYYNAEFESHELTKAVNLIAI
EPLGHGATVTKSETWTYWDSASVNLQLLDALGIDRVFAVGTSQGGWIVTQMALLAPERVGFFPPTM
LQISSTVLIVDLQSIKIQGLVVIGTSMDSESPRSRDLGCWDCPTATAGFVQLAADLTPKDDFEPGQ
DYYDYLMDIGHGKDADAPTRAFWAEIIRKTYQGDEGKKKICMAAINLVSRDGLYERLPYIKCPVLW
LHGTADVVFTVANAQEGISHFVNSTDAQLVIIENGVHFLGASHQKEVQGHILDFTSRWK
>g3312
MTPNVSVLLQRWKVADVIGENLVQFEELNMRRKDGTLVGHTDIATIERSLKRPWWVVHRAHLHEGL
VTIAKRNGAIIHIASRIAEVDSSNDKPTVTTDKGVKYEFDLILGADGINSIVRKKLFPHVTPEPPT
TNCAYRAIVPYEQIRKDPVAKSIVEKLTMEVWMAPGAYIITYPISAGKGFNLVLSHHRPEKLRATQ
NEVPIEEMREEYKDFDPRIQRIVDMIPETSRWPLMVTGPLESWSSANKKVVLMGDAAHSMVNHMAQ
GAATSMEDGAFLAKCIGAVVQGKIDLKDAVSLYEKERMPKAYSKQQVSYINGAIWMLPDGPEQQAR
DSSMAPELAGKYFVRSANLYGDPETVLDVYGYDVEAHADAAVARLINKGKEPAHLVTGVTPEMQEK
YMDWFLPSRTESKL
>g3313
MARSKKEDVAWEKHRSELKALYERLTVDQIREYMFEQYSFSKSNEQYTRQFTKWSFKKNLTSEDWK
FIAHRREKRKREGKDPGPVRKDGNMIPEPKVRKAIARHVLPTAQYLETVNGDIGPPTPEGIVVGTP
KPDVQGTDSSSLRWDWFPNPLESLVGPDVGRPSFEAATDNINFASPIDVSELFTFPTHQTFDQAGL
DIVTDTDIGPASTTTHVPPGAFGYCDFENLNKEIMEGTGELFSFSKPVRNLSDLSESLEPITMEYV
HSDLIRKAKNIFQSDRSNNVTHYLSLCVYLSSNNLLSPLAMNKLVALLAKIKFQAILGDFLQSANT
TNEIFMSNCLASAAELGEIEIVRTLINYGVDVDATRGNLVRRTPLSLAIIHGNIECVRLLIENGSD
LNPVTADETPLQLACNTYKCSIEIVQLLLESGAHVNPPQDSSRLTALQLAVSQSRKDIVSLLLEWK
ADPNTFTTSESGTALQIACRRYSGAEIPELLIDKGAGIDTHAVRHRNDDKSGLTGSESESDVDSND
ENTLFRFKSPILIAADHENWETVQVLLEEGADVNLSMSAFPLKALKMEFEEADWDEMVVLTPLQAA
VRAENITMTRMLLSAGAHVDARPKGKCGHTALQIAAMVGNLRLVRILLLKNADIDAGASVLSGGLT
ALQAAARHSDTELLSFLIDEGADVNAPVSSDNDKSALRVAIAKGNIEAVRILLDAGASVNVNANCK
EGLLAIQESIRICNLKVRNEMVSLILRAGAPFVPPAGLEHFGALHAAVYMEDFDMTERFLKRGAKP
NIGYSRISPNYTPLQRASSEGNERLVKLLISNGADVNAPPSSSYGRTALQAATECGHSKMVKFLLD
SGADVRARGDYRNGTTAIGGAIEKCNQEMVQMLLDIHPDLITSDPNMRRGALHRALRSWKCNASFV
RFLIEHGAEVNDSPGLESSLQIAAKMGDFEMVQCLLNAGADVNHRWVSEWKATKVTALQTAVDSRN
IDIARLLLERGADASAPANSVAGGKTALQIAVSMNYFEMVTLLMEYKADINGLPSPRGGRTALQQA
ASSGYLKLTQYLLQHNADVNAPAAQFNGVTALQGASISGNIRIVMMLLAARADINAPPALENGRTA
IEGAAENGRLDTLHLLLNFHPYTEDFEIKKKRVVKLALANGHLAIGRFLMAYRKNFRSDDFSTRDR
LI
>g3314
MTLVHIVLFKFRPEVSDEHKKTFVTELKKLKNLSCVKDGRLIVGGPSVTDPIDRSKGFQIALVSYH
EDLAALTVYQASQEHHWVTSTYMFPYKEDLVRFDFEVDPEDEEMSESTYISNFEITPTMLSEPHPK

AVRQLAFNRKLCIFQSRLRQDHSTFSMLFNQSLYMISVNITYQFYGPELNEKWIPQKEVGTEPHAD
VASGGKSAYIANMENRIKWLESIIREKCSTVDLDTGNDRVPDFSDERPDISPLVEARGLNNPQTQP
TQVIQDEIQFPDQMRQQSSMNNEAPLAQDETQQAHEIGLVSLFRGEDPRYIGPSSGYFFAKRIFSN
TGWRGRHSFPXISATGKVSHFPVELLNTPISLPQQKEVAVKLSIQYFRSIHLFYPFLHEQTHMQMI
DEVYRSQEDADPISSFQTYMVLAIASLNLSRQCKQFLPVDGYYASAMKHVDYICNHGSMAALQCLL
LLMIVVDLGLQRDLRASPNYPMSVFKQEMRTRVFWVVYTIDRTLCTMMGRPIGVRDEACEMRFPLD
ISDAELITATANQSASDINPSHMSISIRLSKLAQLNSEIKYVMHSICRDVPSYAYPPVKDIFAWQR
DMMATLEGWYYDALQHTGNDDSGTKEYCIAKYHELMVLLLRPSPAIPDPADEIFDTCSEHAFALLQ
CFGDLYEKGNLLYSRFIVHSVFLGTLILLHCIWKFPRTASKFSIDQLMIKFNIAQNVLSSIGEHWA
EALSARDCIAQLSTVTIQRLLKSQPTTCSTLQSGGRFSAVSSRHGNMQPTNLERSSRDFDQFTVTP
SSNIQGQTSAESIGNVSIGTWATIQPYVSTQNPPSEYPNLFDDLLQTDLGGWNGMSDIDGLLNDLL
HSSHY
>g3315
MENTKPTNQDYDLQQSIISTTGLRRGLTSYGDAHFSLFLRKVFIKALGYSEDALSRPIIGIINTGS
GFNPCHGNVPQLIEAAKRGIHLHGGIAIDFPTISLHEGFSYPTSMFLRNLMSMDTEEMIRAQPVDA
CIMIGGCDKTVPAQIMGGISANKPVLPLLTGPMMPGSHRGKRLGACTDCRNNWASYRAGKIDMEEI
GAINEELAPTIGTCGVMGTASTMACITAALGLIPLQGASAPAVSAGRLRIAEQTGANAVAAAINQR
TPLDVLSSDSFYNAAVVLQAIGGSTNAMVHLMAIINRHPAVSGSINLGTLDEIGRTTPLLVDLKPS
GDNYMTDFHNAGGMLCLLHRLRPLLRESAKTISGETLGELLDKSPFRDFEFSRDIIRTLNNPLHPS
SSLVVVKGNIAPNGAVCKASASKDKRLLNHKGPAIVFENSHELALRLDDPDLDVSAESILVLKGIG
PIGNPGMPEAGMIPIPRKLAAQGVTDMLRISDGRMSGTAGGTIILHVSPESAIPNSSFGVIQTGDT
IICDIANRLLQLDISDEELQQRITQRQKRIEKEPDSIWNARGTRRGYRGLYEREVNQAHDGADFRF
LTAKGPNF
>g3316
MVSVLTHVQNVWETQRSNSPIYAFLLTDVTIEDASEGTIRARLLLNQNHTNSKGSLHGTMTACLID
WIAGMVIASYGSTYTGVSTDIHVSYLTGAKEGEVLEITGRSMKMGGTLAFVSAEITKIQGGEKVVV
ATGLHTKYVRQKS
>g3317
MLRLDNKVVLITGLGQTTDEGWGIGAAIACNLAKQGAVIFGGNRSLASAQRTKSRIESAGGTCDVQ
ETNVTDSASVKALVDACMQRHGRIDILVNNVGKSEPGCPVTMAEEVWDSQVDLNLKSVYLTCHHVL
PIMEKQATGSIVNVSSIAGLRYIGKPQIAYSTTKAAIIQFTKATAVIYAGKNVRLNTVVPGLIYTP
YTREMAKRYAPGEDEKEYMRKRDEQVPMNRMGDAWDVANAVVFLAADESKYITGQELVVDGGITSS
TGRV
>g3318
MTEPNTQQVHHIQPAELQNFVQRVLVGNGVPDDHATIVARCLIQADLRGVDTHGSNRIPSYMKRIR
QKVLDPSAIPELKQVTPAAAIVDAKNGFGFVAAHMGMQKAIEMAQQIGIGMVSIKHSNHFGMSAWL
VQQAIDAGMMSLVFTNSSPALPVWGGKSQLMGVSPIACGAPAGKERPFILDMAPSVAARGKIYKAL
RRGEKIPTDWALDKDGNVTDDPARALEGVMLPMGGPKGSALSIMMDVFSGVLSGSAFAGHVTGPYD
PSKPADVGHFLVAIKPDLFMDLEEFKSRMDYLYQRVVTSDKAAGVDKIYYPGEIEQITRDRRLTEG
IPFTEVEIANLNEEAELVGVEKLNI
>g3319
MADEITIASEGQDNENAVPESPLLTNKEAKSKLRDKYSKEIDKLHRNFKKDYYQNYKDPEENDSQI
IDLSQLQDPPDDFKITNLDARHPEMADGFALVVHHFWNINPQIFPRNARDRITLFRKLAYNQDEQY
RPRDLRELSDLSRDAGPRLLKPEILRRLGKLPISRHYPEFKEEADWEEQRTRPIHLLQHEALGENA
GDGGGDGPDALALEETPPNLDLIRECLERLLIQLRYRQLEVVDAKRGEELFHALIDTSESSRRKHL
PLRKVPLTLDNSHNVEKAFGGFTQPEYPSRERGPACTEFESASNSKFDCLMMVGKLLDAGITKIDV
DHLHSHSGQETNAHRNFLNMIQADWKQGNGTRSDEARRYFHRLLNDLHAKDFRDLVFKSTLLRAQF
NHTVSRTIQCDNDHDMPISSNTAAPSLIIFDGPYIKEDGTGVTMQEVLQTSFPQQEPPQGYGKMKD
HACTVKACEGNPKKTIIRRSMPLRMAVSLSSQVSPLEHTSNDIRVKFHNDARGGAEDVAVYRWIGG
VYANTQIEESMTRYRVYWTDSLRGGKPSETVSIYDPVQAGGQVIEGVFPASQNEPIPAGWWQDKFK
PVLFYERVFNPDLLDITLAAAILGDMKHTLDNSQYILQLHDPQWNLSQPPDDRSVDYGASNPPRPI
TPPTSGNPSGSLVPQSIGGYTGAAFETQPKKLDSRDVQMQDGNDYNEPSQSRSDFSQNIGKLGVGV

TGYNLNQSVRSMPPPAQPPPDNHGMFRAFNSDMSQVAPDRPRTLLTQHYNQTATTNTSISNFAQRD
LGQRPQFIPVQSSNVQGSNFAPQNIQTNTQYKPIGFGNANMGLSQPINTLNRAGNSNIGNGYLSPA
LDIDYNEYLRMTPQMSNMNLPLPDTQEDVGAQAPTVEKPDPLFDFDDIMVENPQ
>g3320
MKAIALSAAVLATSVVAANISSCAQTCYNNVVGLASSLGCEANDLACLCNDKDFGYGIHDCTVEAC
PGDNVQDVQSYASSVCASATASSGSGSTTXPATTTGTGSDTSGTASGTGTSATGTGVSSTPYSTEA
VLTTITSDSSTITSTVGSTTLYSDVSATSATSSPYSTEAVLTTITSDSTTITSTVGSTTLYSSVSG
TGASESAASSSAYTTEAVETTISSGSEVATSTVGSTTLYTAVSETSASTTGSGSSTGTGSGSSATS
SGASGASASSTTSHAAAAAMATVGSGLAGAVGIAALLAL
>g3321
MWLRSSASLLLAACGLPYVVADSYLSSTSLATCQEDSLITASLFDVTFFPGNDTIYYDINANASVT
GNVSLTLQVIAYGYYLQPIDINPCDGSSLSTNFCPIETAGEPIDLQASSPVPADIVSRVPGIAYTV
PDLDGKVQIFIASTDQNTTVACLQAELTNGKTVYQKAVGWTLAVIAGLALTASAIASGLGFSNTAA
HVAANALSLFSFFQAQAMYGMTSVSMPPIVEAWTQNFQWSMGIIHVGFLQTLSTWYQQATGGTPST
LLASLSVQSVSVQKRSLDLAKRALNTLTARAADDTMYSGNTVIKGILRVGFRAQIEPSNIFMTGLI
FLSAFFIIVILLIFVFKLYIKVAIKWGWMPSGGFQDFRNGWTSVVRGILFRLILIAYPQMVVLSLW
ELTRRDSVAEVILAILMLLSMTAILLWAAFNVHRLAKRSVVMHQNPAYILYSNPKFLHTWGFLYVS
YRATAYYWVFPTLFYIFIKGAFIGLSQSSPITQTIGLLCIETINLIFSSVFRPWMDKKTNTFNIAI
CAVHFVNAVFLLMFTSVFDQPAIVNSVMGVVLVLYNAIFALVLLLMVLVSAFYAIFSKNPDLRYEP
VRDDRGSFIRSQTHLDVHKELDALGATARGEEKSIY
>g3322
MEMPQDGKIGKPPAVVIIARHGARIDAVDKEWHLTSPTPYDPPLTYGGWIQGRALGARIASLLKAR
EDSVHDETIPTTNSSASVASNSTSEPTSPAEMHLKPPRKTSVAISAGMSQFYRAKNEHSNDQTPVL
SPLASEEISPMGTPLRSSTPTSSDPWNKLNKKDKLNHGKSTKVAPRTRLRIDAFLGEWLSPDYFDQ
ITPPPGSVMMVAGAKAELLRRVEIPQRANNYGSNSFSGNFPGGWKSNQTTSSDTPAENTSFRMSSL
ADSLPSEGQLGTEDGLRFSRPVKAITIRDVLSRSPSHSQEDLSAGYIPPIPSYAISTSDPIPTGYV
SHARDACVDVDYQWDSMKDPQNWGDGGEYDEEWSAMHKRFRNGLIKMVDWYSHEKPHKSHRHHHHH
HHHHHHSHNHDDDEETDTVLVLVTHGAGCNALIGALTNQPVLIDVAMASLTMAVRKDIFKARTWDY
RSDDLLNNDHTENHPSPSTLPLYHLYDLSLIASTEHLRVTSNPLSLPQLPSPTLERPRSISVYRHR
TGSSPLRFSVNTLDNQDNKAARSYGLQRSATTNSAHHHYTSRSISGLWASKEKENSIDEIPGYQFE
QRSAETQDSNMQPVADARSMLPSFQEQKKNDEERIAEQPKSQLGLWGSVAISQAREPALKRRWTVT
EQHGR
>g3323
MLGNSGLINDETRTGRHNEALRWYSRSLTNLQRKINQGTADIVVVLVSCAIFSIIEVLQGNLWAAV
MMQKHATDLIINTLTRAEDTKSTRYAAVNKMVVPFFMRMDAFGLILNDDLVVSKNTTRISSVLKLE
TGFTTLSGAGTALYALVAEWKVFNQDAFRHFRGVVDAQTAEKAYLTARQAELENRLLCWHRQLLSL
EAMNQFQNPQSRGDSLTRKTGGAIALLLMVYTSTLIHTQTALSDNETDYDRYQADFAQIIDYAPIA
LTATAVDDDGDLQPHFAFELGIGSPLFNTVLKCRSPSLRRQALRYLAQTPPIQSIYTGIHAADLLA
ALVAIEEDKSLYEATITESHINGKVILDTILATPGRIPTLTERVRDYMLLPPESTAETQMYFWVRY
TRNEANGRDRKWQLVEEESRILSRLQSRYK
>g3324
MATPMLDQFLAGVADLVSKRQGENLQDYLQVVPEQMRDGYRHMAVELQNTYPKGAGDDALLKRCEA
LVPKSADGTTWPAFPLFIRSYLAYLRDGNVSNLLEAYKALSGLLNQCLQALGDTQMGAIVLRTVVY
LSQIVAGLAMTLERNPQLLAQLRLDRPQDAFERTSLVEDAANVVREGFIKCLSDRGGAAGPKGKPE
GKRAGIYLMANHCLKLLHKCGKLRSADTIFKSISAQSPPLEYYPAAHRVTYLYYLGRYLFANNNFY
LAQNALQEAYNQCHIQCLKQKRLILTYLISSNIIMGRFPSLQLLQKPEAQGLYDVFYPVCLIIRSG
DYLAFRKHFDVGSSTGQWFLQKGLLYQMRNRCEVLVWRSLIRKVFILGGFHGDPSAARGPPPILHL
SKVETAVRLLQRRHGMSASSDVSETKSSEINFITIRLPDDSDYDGIAGFVLNTSDPEVDDYLFSQE
YYDENGEMIKNSGGQPVPGPEYGQYTESLDDLYPEIYGRYTETDNTPKLLQEIESIVASLIKQNLL
GGYLTHNPARFVIPGAKHVGAMAKGFPNIWQTISSQQHNYGENVPAWIIAKQPLPTQAAFPAIGAP
GGGRVINLKGAKAVAPENNEGLTITYSSGRDGAPDLRLIHYNDVYHVEPGSSDPIGGVERFQTVMK
DYRTAPQYKGLSDLITLFSGDVFNPSLESTVTKGQHMIPFLNKIGTDVACIGNHDLDFGVAQFRHL

RSQCKFPWLLANVLDPALGDDVPLANCEKTVMLTSSNGIKIGIIGLGEREWLGTINSLPPDLIYKS
ASQTAIELVPELRKQGAEIVIALTHQREPNDIKLAEKVPAGLIDIILGGHDHFYAHSLINNTHILR
SGTDFRQLSYIEAFRKPDSKGWDFNITRRDIVRSIPVDPDCQVLVDKLTSTLKAKLEKPVGYTASP
LDGRFTTVRTRESNLGNFICDLMRFYYDADCAIMAGXTIRGDQVYPPGLLRLKDVLNCFPFEDPVV
VIKVTGRDLVEALENGVSQLPALEGRFPQVSNITFEYSASSPPGSRVNWVKVGKKPVDYKKKYTLA
TRGYMARGKDGYTSLLVQSEGGEVEEVVSEENGMLISAILRQYFLSLKVMGKWRRMSVSLHKHWDS
VHSRLHAQIGGSWIKTPSPAVERREPNMLNNDPFNSATTATHPKTTTTTTTMFERRIHHTHKKNKP
RPHLRRYGRYYEPRRHHTHAGTSISRTSTPLSIYSSDDSSISRQSVDSHLDSDSDSEPEILVSTHE
ETNYITTTATSPAEEEQRIRLARKVLKKWMRLAGVQGKTGVVDEAGDEFTPPWTLGIAPRCEGRIL
VRTRVVEDGGAGLGEGKRTENINGGSVPIH
>g3325
MEETINHAKSILTFAITKPLSFIYLVIQQVLNLVFAPAPPPPSEKLHRPRVAVIGAGLTGVSSAAH
CVGHGFDVKIFEARSKEKGLGGIWSRVNSTSALQIHSIMFRFHPSVKWDTAYPTQEKIKEQIATVW
KRYGLDKKTVFDTPITSVKKNSSGQWIINDNEAEHGTFDGIIAAVGSCGDPQMPHLPDHEQFGGPT
FHSSELDGKDVKGKKVLIIGGGASAVEALEFAVRNDAAEIDVLSRSDKWIIPRNVFVDVLLSLNIF
GGETAFSWIPEWFLRKFFYRDLQDISPAKKGLYTDTPMVNSELFEQIRAGKARWLRGDIIACKEEG
ILFNHRAQGVPKGGPGHESIVNGDVIIMATGYKRPSLSFLPDYVFEKKYEPPNWYLQVFPPQDPTI
CATNSTYLNAIGTVGNYHIGIYTRFLLMFLVDPLSRPREGLMKLWIDFTRVVKKFAPTGAFDFFTY
AELIYWYCFVSIINPFRWKWAPFIFFGLFRSLPLGIVEGEDRFRSNGKDK
>g3326
MSTSTSSFAGETKNGGTRQRRQVDTSNKIETNEKSAARKNKRKHRPTWKRLARFSLKHRWTIPLVP
LVAFILAYLLNPTESNIVHRFLFLSYKFQQPGTKNPQYGKGLWDIAFVLFYTIFLSFTRELIMQEF
LRPLSVHYGIKSKAKQLRYMEQMYTVIYFGIMGPLGLYVMRHSVPEVWYFNTPGMYASFPHRSHDA
MFKFYYLFEAAYWAQQALVMILGLEKPRKDYKELVVHHVVTLALIGLSYRFHFTYMGIAVYVTHDI
SDFFLAVSLLSPPVLEREWSWLIEVDEFQVGKSLQYTDSDFVPPAFAVCVSAWIYLRHYLNLRILG
SLLPGGAFQTVGPYELNWETQQYKCWISQIITFGLLACLQALNLFWLYCLGRSVYRFLVYRVAKDD
RSDDEEENVTPGN
>g3327
MDGKKTAKFSWLPTTRRSKLLWIIVGFIVILAVALGVGLGVGLKGHHNPNPAGRRNPAAFCSNAAG
SYKVDNITAPSKFNVGSNTNINTVPSWPTNTNITAWNLTVDDSQSGYKQIVAGFGATVTDATVSSL
SSQSNETQKAILQQLVTGDGANFTLIRHTIGSSDLSTEAYTYDDNNGTTDTDLSNFALGNQGTAMA
QLLAQMKQMNGNITILGSPWSPPGWMKINGQLIGNTTDNNLDDGYGTSKGLGSTGHSRAFAQYFVK
YIQAYADLGVNVDAITIQNEPLNSQAGYPTMYVEADESGKLINGYVGPALQKANLSTTVWALDDNT
DDADYASTVIGYAAQYVDAVAWHCYASTLNWTVLTEFHNQYPNISQYMTECWTPTNLSWTHVVNFT
MGPLQNWANGVTAWTLGTNDDAGPHLESGGCSSCDGIVSVNSTVSADSSSSNTTDYTFNLSYYIMS
QFSKFIPPGARILQTDGSAVDDNGDGIQAIASLNPDGSRTVVIENTFANDVNVTVYLESGVGWVGN
VPNSSVTTTPTPTAIPTNPLARRDSSTSTTSTRTEFSLSLSLDLPTDTCTPTIAPDKNGYVPPTEC
NAMYNYYPSFSAALAFTVIFGMLLLAHSVQMFMYKTGFVWVIIMGVAWEFVGYLTRTFSTRNQQSE
GLATITQILILLAPLWVNAFDYMVLARMIYFFVPEKRIWPFKPSHLAIIFVCLDFGSFIVQAAGGT
MATPGASASTIKTGLDIYMGGIGVQQFFICCFLVLTVQFHRQMLRLERLGALQGDKTRWRGLLYSL
YFSLIAITVRIVYRLIEYTSGVGLNNPITTHEWFMYVFDAFPMLLAAGVWCVMHPGRILTGPDAKL
PSSGLGRILCCGYCCCRCCGGGRKKKVATVDSERSSQEELRPVERDSYASSSPMWRDDRTGSXEPY
RSQMV
>g3328
MTDNSNPGNFSNRPREEVQNIGKKGGQPSHSGGFASMDPAKQHDIASKGGRASSGSFKPGDPKARE
AGRKGGSRSGGGYQQSFDDLEYPEE
>g3329
MSNIVHKVKEAVTHHHDKPSTSTNAGPHDSNIANKADPRVDSDRDHRAAYSAAGTGNTTAGTGISS
GTGTGTGTGTGAYGAGTGGYTGTTTAGPHDSNLANKADPRVDSDRGQYGTTGGLTSGGAYDTNPNS
TNAGPHSSNLANKVDPRIDSDLDNRARHQNLGGGVGAPSGSSYTTPGSGSAQQTAGPHDSNIANKL
DPRVDSDLDNSRTIGGDATRY
>g3330

MAPMQQDRKRLNFKNLLLPSSQSGLSTSIVLLEQAIFVNASLSSPSVLRGCVNLDISKNVQLRDLC
INFTGVNSVQKFGRLYDKERVIDQTWLVPRLEARXSSSQVHTIHPHETDHKKWPILEPGHYVYDFE
LSFNEPLPETFNVGGCKLNYHIQAIANSGLRQQISPKQEVTVVHCPHDDIYLHDSNQVTLSRIWNR
QIMYNVELADKGAAIGGNIPISVRIGCSDIMYLAVQVYLAQKIKFPGIPGRQSQLRKKLLLKSKCN
DLSTGKFSETPSLNLDRESNLIIIAGSVPLLDEPSTQLRLHPDVNFTKVTASHTILFLIDITIPNP
QDSKKTTVCRLTAETPFRVRSSQTHLCDSSVPQYSESHNLAVDSGDFNSSRLLPPPFRSESLSRHS
SEVSATDLEGDRFSRPTSTCSLSTEELSTIPIYPPPAYSSIAAS
>g3331
MAPGRTNTTTGTAQAHIQERLPPKSQTNTHQRFREFDLQGKVFVVTGGGRGLGLAMAEALVEAGAQ
VHCVDRLATPDDEFYKAQKRANPAFEGSLHYRRIDVRDKENVAKTFAEIAELNNRFDGILAAAGVN
HVDKAMSHTPEEVNEVMSINYTGVFICATEAAKQMMKYKCRGTILLVASMSGMIANKGMFSPVYNS
SKAAVIQLTRSLAMEWGKVNENGEGGIRVNCLCPGHIITPMVQQVFDKEPESKELWQAENMMGRLA
QPEEFRGAALFLLSDASSFMTGASLVIDGGHTAW
>g3332
MLFYAHFHHPSLSRDDLILTGKKAQRAIKKVQRDLRTKFENGEPFFSKDAIRDVRDQLRQSTETGH
KITVRGLNNMPIDFILATGDICPRIVDDSSDDDDNEDDENSENGDVKHILTNPCIRYRTLQALLDY
PKPLIDMHSAYCNMQIAHHSVLKNKFTGKILDGSDAIPPSKKFAKRFFEKNLRTWKDGQQCQSLLS
ILKAKQDYFKNGGVSNIISFGSGTFTANVRSDWKTHSTFQHASLLTLRDFLREVLGDTNDTKILAQ
DPAYIQRDKDILTEQGITILEDPDGFLELDDRSIVFSCAPTGTNHADCDGSVSAIDAC
>g3333
MESAVLPAIHGRKAFLRGRSASLSSKTSRDGILISSLLPLSFFTTSQRRSILLEETRRYKDTMSSQ
DNISSSDYEVNKASLPGSHKGEKDIQPHVPYDHELGEMTEEERRGHAKFNRLGWKRLTVVLLVEAI
ALGSLSIPSSFATLGMVAGVICCVGLGLVAIYTSYVVGQVKLKFPHVAHYPDAGRLMFGRFGFELI
NAMLICILIFLVGSHCLTGTIAWMNITSSGVCSIVWAVVSAIILFAVALPPSFAEVAILGYVDFAS
IVIAIGITIIGTGVEATNSPGGLSAVNWSAWPKEGTTFTDAFIAVSNIIFAYSFAMCQFSFMDEMH
TPKDFVKSIWALGIIEIIIYTVTGALVYAFVGQDVSSPALTSAGHLLSKVAYGLALPVIFISGSIN
GTVVGRLIHGRIYKNSPIRFINSKMGWLTWIALIAVITVLAFIIAEVIPFFSDLLSIMSALFISGF
SFYFPALMWFILIREGPWYSSKNLPWAIINGITFIIGAITLVGGTYASITDIINNYNNGSVRGVFT
CASPE
>g3334
MTKGLPVIDTTKDLSALFIKQVEVSPNAVALEDGDVTYTYAQLDAEVDTLARRLRGYGVGRNIQAG
VLLPRCAHYVIACLAILRAGGAFLALELAYPPELLSDVIEDAKPVVVITHSSEKSKVVKQVPVICI
DQPLLEPNGNAKDPGPLPADDDLDRLCFVSYSSGTTGKPKGIANPHRAPVLSYNLRFLVQDVKPGD
RVACNVFFIWEIIRPLLRGATVVVVPDEVSYDPAALVDLLASKHVTETLMTPTLLATVLASAPDIR
TRLPELRTLWFNGEVVTTDLAGRSMKALPHTRLLNCYSACETHEIACGDIREMYDENSLYCPVGPP
MDLEHTYILDEQGKRMTTNEVGELFIGGSMLAWGYINRPETTAEAFTPNPYDTTPGARMYKTGDRA
RILPSGILEISGRVGTMIKLRGYSVVPGKVESEILKRFAACACSVVAHGEGLERQLVAYVVRDRDA
SSGRPPIVIDESGRSPEARRALAPYLAHYMIPTLWVEMDALPTNEVSGKVDIKNLPPPPKPAQANG
NHKRTLSDSVSFKDVAAFWAAILKVPQESLKAEDDFFILGGQSLMLADLSSRLSKNYGFRVSIARL
AEVPTLAGHLTAVRAARDGHSAAVQAELPSILRKDSTLDDDIRPHNVNLRPIDTAETVLLSGVTGF
LGAFLLNDLLECTSAQIICLVRFGHPTLDGQPEAVARIRKNLLDLGLWRDSIMERVEILPGNLSLD
KFGLSHEEFDDLASRVQVIVHAAATVNLVYPYAALQKANVEGTREIIRLACKSGATLQYVSTNGVL
PPSGKDGWPEDTILAVEDVPEKLPDGYGQTKWVAEQLVLEAGRRGLPVKIHRAGTISGHSTTGASN
AWDLLSAIIVESIRLGYAPDVDGWRAEMTPVDFVSKSIIHLSSQTAAQQTIFHLGDPDPITIRSVF
DQLEQLGYPTKPLGWDEWVTLWNEKRGTVKGGDGAFTVDILRSGMPTVDFLKGIVVLNNAKTRPFR
IAVERPKVDTALLSTYTRHWFARGWLPRAPRKGVAMSTKPANRSPLYGRVAVITGASSGIGAAVAL
ALVKQGCHVALGARRLEALEAVQRNFGVAEGKSIIRATDVTDKKQVEELVRAATDELGPVDILVSC
AGVMYFTMMANVQTEEWERTVDVNCKGLLHCLSSTVPGMLSRGSGHIVAISSDAGRKVFPGLGVYS
ASKFFVEATLQSLRLETAGKGLRVTSVQPGNTATDLLNMSTDAEAIKKYGEPTGAQILDPSDVANS
IIYALSQPSHVSVNEVLIEPRDEPI
>g3335
MYVDVSKPPPPLPVPRCTTISPGVSLLQPLSRRGTGPGMIIVVPDSTQSNLSIIDGVPSPLIKWAE

ESYTVVEIQESALSANILQRAIEAVFESGSCVTKEKVGLVVYSGHLWNLVAPLLSSTTEISGVVIY
ANSAEEPSLSESPILCLKHLSGKPSSTSDNIPTNRLYTYPSAKSSSFALPFQDDFSYSLEALSHTR
NLTFLKPLINGPYFDLEQIWDEHTYFEFENRSVEWTMSTMVQEPYVNHIPTMTGGVGREKLSHFYA
NHFIFKNPADAEMEVISRTVGIDRIVDEFIYKITHDCEVDWLVPGIPPTGKKLQIPMTAIVNIRGD
RLYHEHIAWDQGTILSQLDLMPEYLPFAYPLVGQEAKEGKRLEIKVPVAGVTTATKMRDRNAVESN
DLFEFKVREVEN
>g3336
MAAAMLPSAVLAQNNQTYANYSSQSQPDLYPQTLATLNLSFPDCVNGPLKDNIVCDTSANYVDRAE
GLIALFTLEELINNTQNSGPGVPRLGLPPYQVWSEGLHGLDRANFAKSGDEWTWATSFPMPILSMA
ALNRTLINQIAGIIATQARAFNNVGRYGLDAYAPNINGFRSPLWGRGQETPGEDANFLSSSYAYEY
ITGLQGGVDPDHLKIAATAKHFAGYDLENWGGNSRLGFDASITQQDLAEYYTPQFLAASRYAKARS
FMCSYNSVNGVPSCSSSFLLQTLLRENWDFPEYGYVSSDCDAVYNVFNPHGYASNQSAAAADSLKA
GTDIDCGQTYPWHLNQSFIEGSVTRGEIERSIIRLYSNLVKLGYFDGDKSEYRQLGWNDVVTTDAW
NISYEAAVEGIVLLKNDGVLPLAKKVKSIALIGPWANATEQLQGNYFGTAPYLITPLQGASDAGYK
VNYALGTNISGNTTEGFADAVKAAKNSDVVVYLGGIDNTIEAEGTDRLNVTWPGNQLDLIQKLSQT
GKPLVVLQMGGGQVDSSSIKSNSKVNALVWGGYPGQSGGKAIFDILSGKRAPAGRLVSTQYPAEYA
TQXPATNMNLRPDGASNPGQTYKWYTGTPVYDFGYGLFYTTFKETAQKLGSSSFDISEIISSPRSP
SYEYSELVPFVNVTATIKNTGKTASPYTAMLFANTTNAGPAPYPNKWLVGYDRLASIEPGKSVDLV
IPVPIGAIARVDENGNRIVYPGDYQLALNVDRSVVWDIKLTGDAVTIENWPLDEQEIKPDST
>g3337
MVTITLTAASGSSTSSAAVSTRSPSSSIAVPAPSQSVVSSSSTTSTLISSTSSSSSSSSSSSSSAV
IIPSSSSTSSTSSSTSTISSSVSAVASVTSTISTASSSISTAQSSTVVPSSSSQPIIPSSGSSSSS
STSTTGISSSSRPIIPNPSQTSTISSSGGVLATTSYAVSSGTSTITPVFQQTTYSQYSTLSGTVTS
STTESNGHIVPLIIWPRGLGWTCILFCGGGGGGGGAAFGGDIPFPTDPPEEPNSTPTNSDTQPTST
GKSTASSSSSSSSSSSSSGSSSSAVSSSSSSSSSGTFSISVYPSGIADTFEYFYDVSVTKSDCNSH
HANLYNYALVVGYDCIRYEHTISIEHGRSFYIGFIFSSTDLGTYTVPGSASTTVDTYCQCGDTIAG
IGTTTSGSVVLAGCEIATPWPIVSTISTIPPPPYQTGTCDLHVFEASESYAHPLYVQLNITDGANN
LLASQDFQMKWGDNATVSASDSKLPYDIDVDFLRSTSESSKKKRVIVPPPTNTVDWEDWILAISAG
GTSWTDKETDSSNLPYCSVGGWDNGNFWDWLDSVVTLGGDEHLPNRQMDCHWAC
>g3338
MRLFNDYFLLSALCLIVSYTPLVTATTTTTAATTTTASPVGGVWTTIPSPQSGNCGGYDVDTMVND
AKTLAQNAITAIQKMINGGLKNTDKTLVETAFTSWGVNYRQISLLNKIWITSGQDTLQTALSNYQT
VLNILDGSTSTRARLMCTDAGWTYATKLGDVGQKPADDPLPGITTNQPGFSWNPYFSVLIPDIEYD
SAGQYGSSICTPYNANGQQLARPDGITWWQANLIMMCAGAFNGATLSDRISGQSSLAVGTTLDSQQ
IPGGVFLHEMMHFIGQNIIDVVITMDNGSKFKAYGFDGSLMMAVNPDYAGKAVSNADNYRNFAMAV
TLSDVDWSCVPILLNLDSSLARLFLTKDGGAHVSITSFRIPASIYAADPEIKERIHDVLQALALGD
PRHGSHHIPPSEEDVPIARGIGTATSNWDSAWVDCLLPKKENSNTTVDKDWIACIQWERPHLEAEF
KARRERHFNLRLQRINGVRGLDNQDSSADASLEEEKDAVAATAGFWRPITETWDAQIEEHHVVFYE
INKYRSIAGREGSWSSCIVQ
>g3339
MTEFPEAISTYFASPNTDNDAASIWSDTAIIFITDVLGIYINAKLQADLFAQTFKCTVVMPDLFNG
GAIPADAFDKGPVDLKSFLERHTVDTVDPIIEKTIKYLQEDKKIQKIGAVGYCFGGKYVVRFLAEG
RTSVFKAGYIAHPSFVTEDELAAIKKPLAISACETDAIFTRDLRYKSEHILSKTGQPYQMNLFSGV
SHGFAVKSELTSRHEKWCKEQAFAQAVTWFKEYL
>g3340
MYRLFALTYLLATHIAGSTAFPPSKTIEKRAVCTPSSYGNTSEDDTPAIAAAIASCGKGGTIVIPS
GSTYSIRSTLEFTGCSGCELQLEGTLKASDDYEYWSDYKAIILISDITGATITSTTGAGVIDGNGQ
DAYDAFAADSSLSRPTLVYITGSSDINMSGFNVKNPPNVFFSNTGSSSNVEYASLTMTASSKSTNA
PKNTDGFDVGTSTYTTIRNVYVSNQDDCIAFKPGANYATVDTLTCAGTNHGVSIGSLGESGDSTVK
NIYLTNLSMSGCSKAAGIKLYASGPNYGTATVSNITYQGVTVNDCDYGLQIQSCYNGNTTYCAEYP
STASLTDVYFNDFSGTTSGKYGSVVANLDCSADMTCDVYVEDFTVKPPSGTAEVLCANFDVSPGVT
CTSGASG

>g3341
MTTLYTPPYNLQELSMANNDFLLYDNSVDNILGSSNNATVDQMEDLLWNFDYTNFAINDDADSKLF
DTALYNVSTENQCDTANEWDYAMQTYLSSPDMSTSSPSLTHSSSRRSSRSSSQSISRSTSPASESL
PTVVKPKRKRSPVVNKAAHNKVEKRYRSNINAKFAALNNILPVFEAVQSLLKRQDGTDESVQQHRN
KGEVLSEAMRYIKQLEERSRVLEGEVRILKDNLLPRKRKN
>g3342
MLSAQDILRPENNGIPQLFHVLLITHHLQKDPNAVIQKLRVTGSYTSLAAAKTAAYSCLFDAGYEK
EWFTEYTVHHDSNNGIVVSATSPDGTTFSVQILNSVLDDDEPQDASKLWNYNIEEHDQRISTELYY
VVQVKTHFDDEQLREINIEGTFLTYNAAREYAKGVLLSPTNGIDTESYAQYDEAGLDQRDCGFGAN
VIVHAVGVNGENFIVSVVKSQTMESVRLAEAAMRIS
>g3343
MSSSDLTNPAPAWLDKGDQAWQLTAGTLVALQSIPGLAILYAGFVKKKWAINSAFMVFYAFSATLI
CWVIWAYKMGFGEQWGNFPLVGAPGPVLTMQQNLGQTHLPAAGLTTNFNLSTMIYFQFVFAAITVI
ILGGSLLGRINILAWMVFVPLWITFSYTVGAFSIWGGGFLYQLGVLDYSGGYVIHLSSGTAGFTAA
YWLGPRLQRDRDSFYPNNILLMLVGAGILWVCWNGFNGXDPYAASPDAGVAVLNTNICTAMSLLVW
TTLDYIFFKKPSVIGAVQGEITGLVAITPAAGFVTGWXAIVIGACSGSIPWISMNVLGRQKWMKQV
DDVMGTVHTHMVAGFTGGFLTGIFATIDGCAAFGLTNPGGAIEGNGKQVWLQIVGALFIIGYNLFM
TSVICLFIKYVLRIPLRMSDEHLMVGDDAVHGEEAYALFFEGERSHLSLHGAGLESGQITEGQLPH
TSPTSGHVSPAEHSSSRDKDEVKMD
>g3344
MAESQINTTGHHAVVFGCSGINGWALVNQLLSGYPLAGSFSKITAIANRPFTAHEARWPDDDRLQI
VSGIDLLAGDDAQISKALADNVSSVETVSHIYYAAYRASDVPAEECRINKEMLRAAVQSIESLSPK
LSSVILITGTKAYGVHLLDKFPFRGQIPLKEDLPRIPAEYAKDLFYYHEVDLLHEISEGKSWSWCE
VRPDIIVGVAPFGNANCMAQTMGIYLGLYRALEGEGARVPFPGNETSWKLTNTDSNQDIIARFCIH
ASLQPREKVHTRAFNIADGATPVSWLQRWPILAKYFGLEGVGPDSSSLHPTEYMDRHWGELEALCR
EQGLKEDVIYRSMHNTGARMGSLRLMDFDRPFDLGRAKALGFTEEIDTATSWYTAFDRCDRKKKTN
PPHRAIIDFTLXMMPNTGSWRSRSLILSLLLAPVTQAITIDTSNRQSVIDAASEAAYGMMTWYAGN
ETGQIPGKLVDTWWEGGAMFMALIQYWHFTGDTTYNDEVTVGMEFQAGNGDYMPSNYSNYIGNDDQ
MFWGLAAMTAAELAYPEASSGYSWLSLAQGVFNTQIARWDTTACGGGMRWQIWNWEAGYTMKNSIS
NGGLFQLAARLARYTYNETYAEWAEKIWNWTIASPLIDTATWEVGDSVSMGDNCTTVDSTRWTYNY
GTYLSGAAYMYNYTNGSSEWLTAVNGLLTATWDTFASDTYGNILTEFECETTEICNRNEVLFKGLT
AGWMAFTALIVPSTYSTIVPKLQTSSAAAAASCSGDSNNTCGVKWYTKSWDGQIGLEEQMSALNIF
WSNLIMTTNASSYAPVTSRTGGNSTSDPTAGTSDSSDTTSTLKAITTADKAGAGILTALFAVAIIG
VVYWTVAGSE
>g3345
MVDETSAEFLAESKVQSIIVCNAILIACSGVGIGVRLFVRSRFLSGIGWDDIFCLIGYIFTFVECL
VCILMTNYGYGHHYQTIQNDLYKQNMFLKLDFVTEITYLIALWAVKVSFGLFYLKVFPGRWFRIIC
WGLIVLVTAEWIEETFVVIFQCSPVQKAWDASGSAAGKCLSLLSFYYISFAVRLATDLVIFALPLP
ELLRLNMPLGKRLGLVFMFGLGLMIVVTSIIRATYLNNFSIDHTWTLVNPLNWSSVEIGVGVFIAC
VPSFKTLITFCFPSLKHILGLSSGRSYGPQYELYGASGRRTEDPRSWNGKSHNHTKMSTITRTNVE
ASRNTSEERIISDNPVGIQVTTDVSVNRVTKPTDPHLHPWNQED
>g3346
MTDTKTVTAEDTSVPTTPTMSSSQEAMDFDQLPNGYYRSKNFIGSLVAVCLMAISLYLGYVMPVNS
LAAINKDLGPDPNYSLISTMFTLISGVAVLLVGRFGDIFGRRYFLIGGQFLGLIGAIVCATAKNVP
TVIGGSALCGLAAAVQLTFSFVIAELVPNKFRPAVNAGIFLTTFPFAAFGGLFAQLFIANTEKSWR
WSYYLNIITCGISVVLLVFCYFPPGWDTKHKGVSKMDGLKKFDYIGFVLYAGGLILVLLGLSWGGS
SYAWSSGHVVASLVVGFVALIAFVLYEIFVPMEQPLLPMSLLKNRGYSATVCSALVGNMVYFSMSL
LWPEAIAALYTTDTIKVGWLSVSTGTGVIVGEIAAGILMKPLGYSKYQLIFITVAITAFSGALAAI
NQNTQAMGLAFTAVGGFFVGYLELITLIMCPLYCKPEDIGLASGFLGSAKQVAGTIAVAIYVAILT
NRLTVNLGEIPEVAISAGLPASSVATLMEDIAGSLPISTVPGMTDKISAIVANAVQTAYSNSFHTV
FLASIAFGGMAIIAAVFTIPVDDKLDNIVAAKLAGTGASDEALNDIEKAHQ
>g3347

MPIKVGELSELLNARYVIHLSDGKRLVLFRLPSIDSSHQKPHESSDGWSYYAMEAECPHAGGPMEE
SSVDIEDSAYVVSCPWHAYDFNVETGESSVGIKTCTYPIRIQDQTVFLNYTQGEHDVRLIKLEAVS
ENIKLKHHQDSDNFTTTNTSIITSRSLDETSSFCDWAVEILETADPERKIELTHRLYTIFNNQETS
SSPMALGKGTVVPPDQPPRQGLTEVKPWQSQYHGRGGTVKSRIAMLHALANIELWAIDLAIDICVR
FATFHTEQNIHELPRAFFQDWLKVANDEAKHFSLLRTRIEELGSYFGALSVHHGLWESATRTAHDI
RARISIIALVHEARGLDINPMTIEKFRKSGDLESVKALEIIHHDEITHVTTGHRWLVWICDQEETD
PIQVFRSNVQKYFRGSIRGPFNTEARLQAGMDQRWYNIDDAVKT
>g3348
MISFITLLAASAALASPIERQVNTAVLPLKRVSNFTSVKNVVSKGQARLNKVNGVAAINAVSAVSS
GSVTNEDVSYVAPVTIGATTYSLIVDTGSSNTWCGAQSSCEPSSTGKSTGGTVSVSYGSGSFSGNE
YKDKVSFGGLTVSSQSIGAATSSSGFSGVDGIIGFGPVDLTEGTVSNANTVPTFLDNLYSQGSISQ
EVLGVSFKPESGSDTDDTNGELTLGGVDSSKYTGSLTYFSKTSSGDASYYWGISIAKFTYGSTTXA
SSASGIVDTGTTLIYIPTSAYNKFLSAAGGKTDSSSGLAVFTTKPTSNFGITFGSTTYTLTPSQYL
VPTAQYSYYGLSSGKYYAWINDGGSSGVNTIIGQKFLENYYSVFDTTNSRIGFATAA
>g3349
MATQSLQPNDVAGLSPGYAELTINAERLISTLEKSCSWGNTPDGGMNRLAANDDDKKVRDWFIAET
AKYGCHHKIDEMGNIFAIRPGKNNTLPPIGLGSHLDTQPTGGKYDGILGVISAIEVVKVLHQYNIT
TYAPVAVVNWTNEEGARFPPAMLCSGVWGGEFTVDWAHARVDPEGRTLKEELQRIEYLGPVPCSYD
ANPLLAHFEVHIEQGPILDRAEQPVAVVKGVQSIRWYNVQITGREAHTGTTPMDSRSDALLGAAKM
IVATNQIVTEGAIFERGGRATFAVIDSLPQSINTIAGNVRLGLDIRAPTDSDVKLIESLCRKQFQE
ICTKHGLKMEIDNFWVSPALNFNPTMVQCVRDSCGSVGCDSIELVSGAGHDSVYTSRKVPTAMIFV
RCRDGVSHNPAEYSRPEDCAAGAQVLLGAYLRYDELIQKSVKAKA
>g3350
MSIKVADYLFTRLRQLGVDTVFGVPGDFNLRLLDFVEPSGLRWVGNCNELNGAYAADGYARIKGLS
ALVTTYGVGELSAINGIAGAFAEKAPIVHIVGSVPRPKQEARIFVHHTFGDGDYRHFASMATHVTV
AQANLIDPRLIPDQIDWVLKQAILHSRPVYIEVPDDMPDVLVETKNLEDVIKLDPGRQTESDAVAL
EQVTERIYGAKQPLIIVDGETRAMGISTDAENLVKLTNWPTWTTMFGRALINEQLQNVYGIYTGQF
GDPSWKKYHDTADLIIIIGPHYSDTNTSIFTALPNAAVTVAFSPNTIQIGDDIYRDVSKSFLNRLV
SSLDASRIAKVEGPSYSIRTNKALDPSGLITQEHFWEKMNGLFQEGDLILAETGTSSYGSQAFSVP
KNARLFNAVTWLSIGYMLPATLGATLAQRDRTKDDTTRSFLFIGDGSLQMSVQEISTMIKENLNVI
IFVINNGGYTIERAIHGRTQRYNDVAVWRHTQATSFFGANESHSVNNNFVARSWSDLERILSNESI
QKGSGVRLVEVFMERDDVCGILLPLMKTRIANGD
>g3351
MSDVESSEHTPLLSPSSSASSNVISPDAPDRSVEHDTIPETATYGRNISWSSAYILVISRVIGXGI
FATPGAIVKSVGSIGLALSLWVVGTILSACGLAVSLEYGCMLPRSGGDKVYLEFTYRYPRFLASTL
VAVQAVLLGFTASNCIIFSKYTFFAFNVVPSDLQNKILAIGLLTLITIVHGCFMRTGIAIQNALGW
VKIFLVVFMALSGIYVILFHQGEDFTGALTRNKESDLWKDLWADSDWSWNTISTSLFKVFYSYAGL
SNANNVLNEVKNPVRTLRGVSLAALLTSCLLYMLANVAYFLVVPLDEIKQSGELVAALFFERAFGP
RWGRTFLPLMIAVSAAANVMVVTFALARVNQEVARQGFLPFARIISSSKPFNSPFWGLIIHYIPSV
LVIGLPSQGAVYNFILDVEGYPGQIFGLAVAAGLVLLRRRRPDLKRPFKAWLPAVWLKIATSVALL
AAPFFPPPDWKGDVDFFYATYAIVGVSIILFGILYWYIWTILLPRFGNYTLEEEVEVLDDGTSITK
LVHKSKS
>g3352
MKLLYPTSLKLDIQSLKGFAVTLHPYDVKIPIPEDLIDAEIMVTWSNNSENLTDAAKRMKNLRWIQ
SLAAGPNDVLAGGFDTSKIIITTGSGLHDGTVAEHTLGLLLSGARRFYEMRDYQLQGKWPGHLGGS
YPRSDFTTLRDARILIWGFGNIAKKLTPYLTALGSHVRGVARHAGVRSGVEVLTEDKLPELLPETD
ALVMILPGAESTRDALNAERLKLLPKHAWVVNVGRGISIDEDALVDALENGEIGGAALDVFKTEPL
PESSRLWKAPNLILSPHAAGGRPEGCTDFIADNLRRYLAQQXLRNRISE
>g3353
MVVFRSLRRCSPFSSSLRRLEKLSSIRNARGLATRATTSKLNTGDNIPALGFGTFQDADAQEDAVN
RCLKAGFRLIDTAQVYNVEREVGRGIKKSGVPREEIFLGTKLWCNTFHPEDVEAALEYSLQELDTS
YVDLLMMHYPVVFARGEERFPRDSEGKMILGKTTYLDTWKAMEKLLKTGKVKALGVSNFSKGEIQT

LLDQGSVPPAVHQMEVHPYLQQNDFNQWLRSQGIHVMQFSPLGNMNPFYRETGWSKDIAHTMERIT
ESPLLLELGRKYNKTPVQMALAWGVNNGRSVIPKSVIDWQIEQNLEADFEMDEEDIERIKFMDKKA
RFNDPSADYQYRLYGDLEGIEGTRDGRTH
>g3354
MAEQGKLSDPVVETYSAHPSPVIDPNERRRQALEQIDNAEFGWRHVRACIVAGVGFFTDAYDIFAI
NMASAMLGVVFWVDSNKGKIPTNSDTAIKVATSGGTVIGQLAFGWLADHVGRKRMYGLELIIMIFA
TLAQSLSSDSPSMSIVGVLIFWRVIMGIGIGGDYPLSSIITSEFATTKWRGAMMGSVFAMQGIGQL
VGALIALIVTAGFKESLETASTVAKCTGVCNIAVDKMWRVVIGFGAVPAVFALYYRLTIPETPRYT
FDINRDIVQASQDISAYLEGSREGHPDQVQRAAAIQKDSERLAAPKASWSDFIAHYSQWKNGRVLL
GTAGSWFFLDVAFYGLGLNNSIILSAIGWSGGSNVYHIFYNTAVGNLILICAGAIPGYWVTVATVD
TIGRKPIQLGGFIMLTILFIVIGFAYDPLKNSHNGLLALYVLAQFFFNFGPNATTFIVPGECFPTR
YRSTSHGFSAASGKVGAIIAQCVFGPLVHRGAPAGSSDSPWLNHVMQIFALFMLCGCFTTLLIPET
KRKTLEELSGDNLYWDSTPTLANHEKTAEEGLAGNGHDASH
>g3355
MESSTTTIVAPEIAAAEKQIPALITFQTQPDNDEHQTDRINALRLIADSVAQQRQEASRAIITHPV
TIAIVLALCAVISHYINDLATIVTTTAGCLMAGMAGVGYLTYGYLDLAEKTGTWSFLRPDANSDKE
DILIVTKYGDDIIAALVLRLLPLSETKKNPKLKAVIRAWTVKQRYRNKTFGTALLEEAVALCKKNG
WKGPEFSPDHANSKRILPGMFHKNLDRKEAKARKLLERTMETMK
>g3356
MRVLVFGGNGRIAKAMTSLMLARSWQVISVIRNPSHTKSIQSLGDDQKGAVNVICLDLNDLQDSGD
VQRRILDNHRPDCVVFAAGSFSDVYGIDRDAAKIVIKAVTSHPSVKKFLLISFPASRRKPAPWWNR
QDIRDYTQEQRSYPEIAEAKLQSDEYLVAMSRKRNLQDSAGFQAISLRPSWLLTRPGTGKVHLGKT
RALGQIPIQDVAAVGVSLLSREDTNGWYDVLQGNDAIDEAIQKVVRDGVNTIEGEDVEQMLGSSGL
KVSKVVLGSALPLIKHAYDQDINTWDVADTYSNGRSEEILGSALKKYNIPRSKMVIMTKCFHAVDE
SRGPLNAAEFAINDGPRVNQCGLSRKHIFDAVDRSVERLGTYIDVLQIHRLDRDTPMEEIMQALND
VIASGKVRYIGASSMAAWEFQMLQNIAEKNGWKKFISMQGFYNLLYREEEREMFPYCNATGVGLLP
WSPLAAGVLAHTWTDRSDKREQQDVFLKALFRSQEDKSAEEIVGRVNSVAEKRGISMAQVATAWVL
SKKNATPILGLDSIERID
>g3357
MTIISKFAIGVLIAVVSKATAQQTVWGQCGGIGWNGPVTCVSGSYCAPGNPYYSQCLPGSGPSTTT
AITTTTLITTTTTKSSITTSVSTTATPTGKVQFAGVNIAGFDFGMVTSGTQDLSQVVDESVDGVTQ
IRHFVNDDTFNIFRLPTGWQYLVNNNLGGQLDATNFGEYDKLVQGCLSAGAHCIVDIHNYARWNGA
IIGQGGPSDAQFVDLWTQLATKYKAQSNIIFGIMNEPHDLNITTWAATVQKVVTAIRNAGATSQLI
LLPGTDYTSAANFIENGSGAALVGVVNPDGSTHNLIFDVHKYLDSDNSGTHAECVTNNIDAFTSLS
TWLRSVGRQALLSETGGGNVQSCATYMCQQLDFLK
>g3358
MKPTLFIVHGGWHVPENYERLISALRADGYEVHCPRLPTTNQSRPPNADLYTDSDFMRSYVSSLVQ
AGREVVAILHSYGGQVGSDALIGLGTATREKEGLSGGVTHLIYMAAFAVAEGVGMMDKVREFGHMN
LVPIAFDFAEDQTCLNNDPRNLLVGPSDLAQEEVDKYLAKLVRWNGKAMYLPIRHAAWRDIPVAYI
YCTADMTVPLDYQESFVADMEKVGRPVRTFELATGHSPNLTATAGVVDSINQVVIS
>g3359
MPRTEITGGPSLQIDVAKPPSWTFTHGDTVIGTVIRRTPIVAPSATVTITLFGRTKTKITVKKSNG
QSTTTHHYRGRWSLIGTESTQILHQGPLHLAPGQASGSNYLSWPFSVTIPTKPPDTLARDHPQEQS
YIPVTNLANHQSPGTFYSSGRSGRTRSEAFVEYYLESTLRFNRGGSNESFTATLPISVASFSSGSE
PPINREPQQAMFYSTVQSQRLLPGMEKAELTFKQKTQKLFSSSKVPTFGYKVVVQVPRQIQLGDQS
PFPFVIQFHANEASISPSIKDTTQKVQLDWVRMILKSDTDLLAPGKFSHSRTHTHDHSLKQDLGLT
KAFSNLENRVVFQSTEGSQKVDIGAMFQLILHPSALCLGNRQLTVGPSIYPDFVTYNIKHLHSLMI
ELSFTVALETKVHKMDVPVRILPPTRLDPSPPEYSKGQ
>g3360
MGTFRLLVHAGYLLDRVIHLVSDQDIKGQTFIPRREQLYRTMTALMSVLEVEEKCSTASVRVARNV
LNSGLFVLFPIEDSLDTGLGALHDHKHPLNISARTWRSRYRMYLEGSLGGIDEIYPFLTHWAVCVF
RYHQFQYDRDGRVENMNALVDVRSALEALDGRWRLAGVYMSLLNATQVLTMTVSVAKYSEDEPAYG

QRIFVKLIDDIALNEPCRTLLSIPCSADPRDGWQDLSFGMFANAVNRLSHWITDQVGLAQEGTFPT
VAYIGPNDVRYLILLAATIKTGYKAFFISPRNDMKSQLSLFESTDCHWLCYAPEYASKVQPWSDIR
PMKSNAIDAIKDLIHEKPAPLFPYKIPYEKAINEPAMVLHTSGSTGTPKPIICRHGILTLMDAYHH
IPEFSGFPCFFKAFSDKTTKVLCTMPFFHAAGLYFFLGLSIYWGTPIALPIPDKPLTADAIRTFIH
ESYSDAAFIPPSVLADMSYEEDDIQALQSLNMVTTGGGKLDDEAGDRLVKAGVKLTIAISTTEYGL
YPLYIQPDVKLWKYFVFDDETFGTHYEPSGENAFVQVIRRKEKNPGLQGIFYTFPDLAEYNTNDLY
QPHPTLAHHWLHVGRADDVITFSNGEKLNPVTIELTVGRHAEVNQAIVVGQGRFRAALFIDPVVVP
ESEDEAKALINRVWPLIERVNKDTVAHGQIAKNMVRLCDSAKPVPYSSKGSLQRGRFLKLYAREMD
TLYDTEGNHEAIVLDFTSKESMFDSIKSIFKSLSITTDLDPETDIFVAGIDSLGAINLAKKLTDGL
QHAGIPTEKSKITAQTIYRNPTLARLTEFILLRGSKKFSESVSISKAGDTEAAATILKKYSIETAL
KTASEKPSPQKEGQLVILTGSTGSLGSYLLDFLERSPLISYVFCLNRAADGGLEQQLLSNSQRNLT
TMFRKTKFLQADLXKPYLGLDSATYRELLDSADRIIHNAWPVNFNLSIESFEPSILGVKNLTLFSA
QAQKQVPIVFISSIGVATQWPHLETIPESPLHDLRVASPGYGQSKLISEHLLAQATEKLKVPASVI
RVGQIAGPRGEKGMWNKQEWLPTIIASSVRMRKLPYELGSLDVVDWVPVDDVAAIVLEIAGATYPW
SSSTGRCNYYHIINPAKTPWKSLALSVKEFLKEQGHDLDLVSLQEWVDACEESLETDVENPAVKLL
DTYKSLIHSNRVSFSTNETCAVSSTLCNLGSCDSQMMRRWCQQWGL
>g3361
MMSLESDPKTTPISCCVFLTGVTGFVGGTILSSLLKAHPSVRVKALIREERDAKELQSIYSNLTPI
IGELSSHSLLRSTAAEADFVIHAGGDNVPAVCAIIDGLAFRNTTGSPMPRLISLTGPRSLIDLSDP
ITGNLKASSRPWSDITDAHIILNVPKDRMHAGADQAIIAHSTAKGVGTMLVSPGQLWGQGKGYLKK
ESNSAFYYATVKSRGRAFVIGEGTATWSWSSIGDLGDAVVFLMEQALKSGSERRGQVGVNEDGYYF
VRTGDLSMMERAKAVSERLGLGEVESVSVDTAREIHPFGPIMWGCGERTRSDKLLELGWRPKETDW
KALMEEGEGERA
>g3362
MIPLAPALLLLLPVVSGLALPTDETAQNTTEARAGCALPSSYSWTSTGPLANPASGFLALKDFTHV
PYNGQHLVYASDVNNGGNYGSLNFALFSDWNSMGSVAQHTMSTGAVAPQLFFFTPKNIWVLAYEWG
ASPFNYRTSSDPTNANGWSSPQSLYTGSTGSSTGPIDPSLIGDSQNMYLFFAGDNGKIYRASMPIG
NFPGNFGSSAQVVLSDSTVNLFEAVQVYSVQGQNQYLMIVEARDPSENRYFRSFTASSLSGSWTPQ
ATSVSAPFAGKANSGASWTNDISSGDLIRASADQTMPIDACNLQLLYQGRATNSGGSYNDLPYRPG
LLTHK
>g3363
MKVLATLAGVSVASASDLGYWKLYCGDSCSAGALISQGDFYTNVSTACTSLGDTYAYCYLEVPEGF
TAIYHSSLSAEATCGTPTILNSGDCAGAGSWISYEMIYSA
>g3364
MTDLTNSENHQHADNFEGLALKDVISNRRKPFYKDWTLIKLNTLLLCALLTQIASGYDSSMLNGMQ
SLPQWRKYFGSPTGTRLGAMTFGPTGGTLISVLVSSQLCEKFGRRYPICGGSLIIILGSILQTCAI
NYAMFVVSRFFVGFGLGIVAVAAPPLLSEVAYPSHRGKLVSFYLVTWSLGSLIAAWITYGTFKMTG
SSWSWRIPSVLQCFFSLIQAVLSLFAPESPRWLIYNGRKKEALEIFNKYHADGEPNSRLVRFEMAE
ITATLEMEKVQKMSRWSEWLATKGNKHRLFLALYIPAMLQWSGNALTSYYLSKVLNTINVTDSRTQ
LIINACLSIWSFVSAAFFATLVDKAGRRRLFLCGMGGMGVSYVIWTICSAINQQKNFKDTGYATAV
LLMIFIYGACYHMCSPVAPTYIMEVVPYSLRAKASMLYQLTGNLAGIYNSFANPVAMDKISWRYYI
VWCVVIAIHFTLIFFFFPETKGLGLEEVAEIFDGPNALSGVNAMRETGLDVHADKVLAFGNEDLSS
PIKGSVSYVERA
>g3365
MNLSSGSENETSSQGEITPSDCSYGDFISSRLVAAQACSMLPELLEIYSENVLAKLLRVAQEALEN
NNPPIAYPEFVPQRGKDIGKYFLREKYFWTCGFFPGMLYTLLERAVRYPSVFPFYGHEKATQSFDN
AFLRQQLASLCDHWKGPIHEMASRTDTHDLGFILQPSLRKDWELTGSEKSLQCLLSGARSLASRYK
TNVGAIRSWDVLNQKDVTINSMTDDFLVIIDSMCNLDLLYYASKHLGDPNLAEIATTHARTLIRSH
LRPEHITGEFEHKGELVYSTCHVVNFDPRTGLIKDRRTAQGYHVDSTWARGQAWAILGYAQTYNWT
KDKQFLWVASGLAEYFIWRLESAPVCVEMTYTGTNRRENVGRYVPLWDFDAPIEDHTNPLRDSSAG
TIAANGMLALSQSMAALGNMVLAEKYRSTAIKIVTDTLEFCLSREKARIVQRKNGLHVENVEEGSR
FDSILKNATANHNAYDLDRYSDHGLVYADYFLLEFGNQLLRAGFI

>g3366
MPPLSGFSDNPLRNRDDFIAAAIALVQPLHRHFSPGKATIRLAHSTGAHFDEGAARLEGFARPLWV
VATLLHSLKHDDDHPHGPIIESLAKPWIEGICIGTDENHHEYWGTIQDGDQPLILVAGIPKDELQG
EIDNDMAVLDTFDIGEGWSSDGPWLTAEQEAEEECESARTGRYDKVGIGRQADYYSGSFAIQFSQI
LYSRFAAELDPERADMYRQRSREYGATFWRYFDSNGASIPFGRSLTYRFACGGYFSALAIAQVSEM
PAPLSSAGAVKGFLFRHLRWWARHSEETFYTDGTMNIGWLYP
>g3367
MEELNQGDPRPACKACRTQCAYDNDREKPGLKTGAVETLRRRVDALEDAIYQQGRAFDHEPSHVCQ
ESRSAAASLLQEGLELLLEKLHSQTTERTAGLDTSHSTYGVSRQVTQETEYGPRIPTHNTEAPVSN
KRKRMDDTTLVDTNDWANLSSTLPPLEVLKHIDPRELSVDIVSIESICERARKVVILTGMEDMYVE
NLQALIIVCFEDIGSGRVSRAWPIVGSLTRTVEYLQLSVESENHDKGPVLQPRPSLPCAKSWVEEE
ERRRVFWTVFNLDRFCSVTTGWNTSLTSNDVHRRLPADGGLWHNEEAVTTPFFGIWDKSAGRIGNL
ITFLPTDYNATEPPRSRFQSGHLPTVPDEKVDMSTIGAFAYRVEATESLSRVTTFFLKQKINYRDR
QEMGSWLMRFKELDLRLVSWKMLLPQKWRDSNISRQPTVITMDPNLTLAHITHNTSMILLHQRIAY
PPLEWHEVLKLPTLSSAETCETAAAETANITKKFLDYSSPQSIVDSQFAFCVFVSARVLLVHWRYY
KTDLSPDFWTLMEALDVMSVRWSVQREEQNAPYRKNAAAKYFSQLNVIYGRCQNDESFKLDVLGYP
NEIEWAGQLFSALKDGD
>g3368
MAPTPDQGWKIYVGTLTTIVPATLAVGARFLARWIGGIRYWWDDWMVLIALIDCWAMTGLRLTAVK
YFGHGKHAADLPEETVIKFDKSFIAVQMLYFVDVVATKAAFLFLYYRIFGVNVWFRRALYFLAFLL
VAFLIACPIVAVAGCHPVSYYWNKNQLGSCINEVEFYRGNGIANVILDFIIFCLPVPMVLRLKTTV
RQKLLISGIFVLGFFVCVISIIRIVAFNGSNPTDMTWSTVETAMWSSIEQAVSIICTCLPTLRPLF
SRFHRSTTSSSEQSNNYPTEPRGKTSSNNITLSQFTNSGEGDSTAEFARLSDHYDNDGDDVDDEQ
>g3369
MLSANFRLATVVLATGVIFEITAQGQHLNFFDYGQRGPLLASHFGPPATNATFDYVIAGGGNAGLT
IATRLAQTGASVAVIEAGGFYEVDNGNLSIVPGYATYFTGSDLNNYSPLVDWGLATAPQSALADRQ
LHYARGKTLGGSSARNYMLYQRATVDSMQQWADEVDDQSYTWDAFLPYYQKSVNYTPPDQTLYTNS
SNKQDPNAFNTTGGPLKVSFSNFVDPFGTWAQQAFIMANMSEIKGLNSGKLLGSAYATLTIDPRNA
WRETSESSFLNYALNAGLPLTVYKSSLAQKIIFDSNNTATGVLVSAAGFFGTPPINFTLTARKEVI
LSAGAFQSPQLLMVSGIGPCGELAAFGITCISNLTGVGQNMQDHPIFGIAHRVNVNTASAGLNNAT
LSALGIQSYINNATGPLSIFGPGIYGWEKLPEPYRSQLSNQSRSILDSAFPSDWPEIEWLPVAAYN
GYNLNKQTADPRDGHNYATLNTALVAPLSRGTVKLQSNSMTDLPIIDPNWLNEPTDLDLAVQSFKR
QRQLWSILADLGVADATEAFPGSNYTTDGQIKQIITESMTTVYHASATCKMGKKNDTMAVLDSHAR
VYGVQNLRVVDASSFPFLPPGHPQALVYALAEKVADLISTQ
>g3370
MASTQGSPDLSLGSFDTALCIVPPTHLTHDINRLRALYDKAYEKWPPHVNLIYPFINPEALPHVVE
LIRSRFVQNKVEPFRFTLDKAGFFTHKHHRTIYTTSSGNGRKEVENIRRSVLSTLGQLDTHFQPHL
TIGQSEANVESDLDFLMSKVDLLPPVEWEVSEVVISIREKAATFSSTFNQLRVWGKLDLSSEYALN
YPSATPLSNNITTLSDSTRLESTFHFDSTNWDKREAPWKRTQSIGTSSEAKHTQLSLSISSYNVLA
DAAHPLSTERYPVLLENLLQSSSIADILVLQEVSDSFLTFLLHDENIRSQYPFSSHGPPDQRDIGP
LPSLRNIVVLSRWNFRWEWLNFEKPHKGSVVLQLEDIGVFRRSEFVPLIVAGVHLTCGLTDASIVA
KHAQVRTVIEHLQTRYPDSNWIIAGDFNIPSSSYTINAALKRNSITARGAATLSSLDILFAESKLV
DCYFGSRATGSNSVDYSTNYKENLRTVYEGEEGATYDPIANSLAAGNSGKSYHCRPQRYDRVYTKG
EEFRVTGFNLFGLPSEETEGRLGSDHWGVRATLDLNDAPTTGAIGKHNVVMFFKRATQHITSDTLF
NTTLHDLNMFPSEEEFAHRQQMFELVKSAIQQRNLGTSVETRLNISFVVVPVGSYGLGVWDTSSDI
DCLVIGTISPKIFIALMIQKLRRPEWQEIHILRKVKAASGTMLEIQAGGVRFDLQYCAATSAAERY
VLQIATGDMIDADLISWPSALNLPSSDSIFDLPMQSLLKLNSIRDSEYLQRTIPDLGTFRKAYRFI
KAWARCRGIYSSKLGYLGGIHITLLLARVCKLLFGAAGAASVSDILSTFFSYYANFDWKNDMIYDT
TFYTARPGYHRSYREPMVILSIHPPKVNVAHASTMLSLKTLEQEFKRAARLISDTSSTWKDITDPM
SIIQFFEGYHSYIKVDVHYWAKAAAKGRALFGWLEFRCVSLLVDINRGFPDIHARIWPARFTEMEE
DVLEGMEEYQGCLLIGLSRSEKPSTKSLNESDRQSAQISISSLLKEFAEKIRADETYFDKSCSWVD
ISLAKSTDLQNLRLDTSDWSRTTGEQVEDFESDDGFEQDHDDSVTFQTAGDTESMEQWIRPARSSR

PPPAVQILGGPRLRPAGDVLNRLRWDASFDSSDYLVGYVDRFVGEKEMPIDRWKSEQTHEEFIPMH
RVSYFKRKSDGERVWDREKRVDLIFNTGATNSGV
>g3371
MNVQAALDSGMSPMIHDGDYNTQPPSNVSDRDLLHATRQQHEKTLDASPESSNQSSLQSLLAKSLP
LRLQVTTLMNSLQDQTTYDHILAVGKELALASGEITAKLDRATSGINGLPATQFAQSHCSHLIRRL
PMCLHFVYAIKSKKNPLYSHSQKVCLEAALDIISLLDDDLYSRLLLSGGGMFRDVSTRAAILIFME
LSPDAEAEVSSLTKRRYQERQSFLLQYAQRLVQYAEDRIREGETSVKTYVFLRLMMARAEARFKDC
PFTENDMTEALREGFDKCQTMLKDRLLNVNQANVHDDAVPGSPLGAWTPNELNEPSYSDFPTDNFS
LVNDVNIDFDFLSDQLPSQWVDQTWF
>g3372
MAGESEHAFRGSTDRRSETEEQRQYEQEILARQHYLPQSDDSEAEDEKQTQPHRFTGKVRRFWDKK
VKPAFPEAIADISLVKDVFTTESLIHKDIHDAIRYPEIKQVAQVRQGLDLGPEEKAFLLKRKMHVR
VHFARYLGLDPEQVHPDDIPVIAFGGSGGGYRAMIGYMGYMTEMKKAGLWDLITYVAGVSGTCWSI
AAYYTFADTTIEHIFDHFKSRLSPYHPLSDPAIRTTLTAINGPYNTLGPLILKQISNLHVVLMDLY
SVFTTGYLFLHHSQESASIRHKPNADRGWWKWSSAQTWLTEGQEPFPIMTAIRHERPWKDWVDKEH
PFKDDNPLSKEHAETVDAWYQWFEMTPYEIGCDELESWVPTWGFGRPFDEGNSTMQLPEQSLALLL
GLCTSAPAXPLDAYLATIKRNLPSGSLGNAVHELALKITHMWGKQKTKVFQQHHPLHACNEHNYMF
HMTPVSKGEARPPGIENSPRIHLLDSGLDNNCPTYVLLRPEREVDVIIHMDASSDVHKDVFQERVD
QIGSRRGMRYTKRTSDAPSNTPPTSSSPDTDGKRSDEKYGQIYDGVLCDRPAHVTDSYGHTVTNPP
APVCHKDNTIVYMPLLSNEDAVPDFDPSTAKWSGSYNLVWTSEQVEILAKVSAANFAKAQSNIKKV
LLEVYEKKKASREALNP
>g3373
MRTSVLAQCLMVTSSLAGRVLKRDSVTAVVNFGNNTGTPQHLASGLLYGVPDTLDQIPSTFYSDIG
YNYERAGGAQVPAPGRGWIWGLTEYKNRFASALSNYKTAREHGANFIFLIHDLWGADGSQNSSAPY
PGDDGDWTSWDDYLAQLFSDIKANSMTESLIIDIWNEPDGSGFWNRAQSQYLEMWGRTYYKFRAEF
GTEVLLSGPASAGEPLASNTWWEAWASYVASNDSIPDQYAWHMEGGGGDLLSAQAGLQHWLSTYGM
PSKPININEYAVFSEQVPAGSAWWIAQLERINAHGLRGNWLSGYELHDFMASLLSKPDATSDYSAT
ATGYFPNGDYQVYKYYYQNMTGYRVGTTPSTDLKLEAYATVGTDGYARVLVGVRIEEGTWELQLDD
LTALGLPSSGTLNVHTWGFPVNSTNAHYGEINAPTDLGWYGHAYSGGTVTFPVYQVDTTTAYAFEF
AI
>g3374
MNQHRLVSALRELLPLSDGELEQIILHASELDDAEATQHFGSLLGDSPKSLEFISYFLDARKTLSV
RQDIDAGDSSSFLATDSAHMNEETGVNGSQLTTSGLDSKLVDQKSASQPPPYAASPGSQTQEHRIY
NSEIEPEHDTGYPCDCSIHRYKLKKWVRLGIQERWAKAVMYPGERFYSDCVTGLLFGINQYQLTVV
SPYGNQSTVYYSRGPQASYHARFVRKTNELNRRLNREAQAKVDALEPKQCLWDQPELLSSMSRVTL
GEHKPAGDDKSIGEQDAEDSVNGDNLHASAKLKAKSKLSSFRKAIGVRSTEERKNERSSILRVEIL
KEELGRWPDNQWRQLVAEYQERIGILKKVTELRNQCPLQYLHLLKAGYFEPIPVEWAVHPSNPLKF
KIEAAAGWRGITPAWRGFEDLAEERLYWVLNHPPFEPHDRPELPTDDTMILLDVSGSMSFEPPKNK
DVAKSIIRRFTEALSHGDQHHVDGYDLVTFASEAEYMGTINQRNFDEVWNNITFGGTTRVMAGWQQ
VKELHFQKHSQSAYRHPVYGWQAGPETPMLRLLLLLDGEAVDMDEFELDLLSLNWAHVTIFLIGVD
GCPHHHRHANELQRISDVNRHVSFIDAQGNIPERYVTHELLKRHLGYDLSMDDFRRMEGLPTYSEI
>g3375
MSSPNPSTRCLNSNKIKVKNPVVELDGDELMRVIWKDIKNRFIYPYLDIDIKYYDLGLPFRDETNN
QVVQDAAEAIKQYSVGIKCATITADEDRVKEFNLKQLWPSPNIAIRSAIGGTIFXEPLIIPRIPRL
VQGWQKPIIIGRHAFGDQYRAKERVLDTAGKLEMVFTPEGGEPEVVEVHNFSQRGGVGLAQYNTTE
SIEEFAHSSFAFALSKKMPLFFSTKNTVLKKYDGRFKNIFQHLYEENYQEDFEAAGITYEHRLIDD
MVAQMIKSEGGIVMALKNYDGDIQSDIVAQGFGSLGLMTSILVSPDGRTCMTEAAHGTVTRHYREY
QKGNETSTNPVASIFAWTRGLIKRGELDGTQDVVKFAKALEQACIDAVDVDGIMTKDLAYACGKSD
PSSWVMTNAYLTAVEKRLKTYFEP
>g3376
MSASLERTDTPRPLGDEFQPCFALQLREKLSHNIKTTTEVGYKRENPLEVFEGTYDFSLNLENDFP
PYDRRNLHVRVMRKAPQSPGLIPLYHEDLPHTLALIFQSDDTSEKEETKQCHAINDSTLFLGEHTL

RLGKIKVMIVGVIHLDFMDQMDLDDDDPCLTQTIDVNLAFWIRIVRPQV
>g3377
MNNTGFMEPGAPSTVGVSGPPTKKQKRTSPPDTVSVVPMSRKGKACSSCRKLKIKCDSADRDMGSC
SRCLRLGVQCIRRQPWIHTENGEGWQQQTNITIFKLERALEDVLEKLNMPALDMYAQTPIVEPVQS
PRSTRQNSQEPAIAIPKRGDRDVSPGPMQSLIEATRLNGLFSQLRSGKQRRKGGMRRMDTDLISEK
VITYEQAEEMFEVFKALLSPRLYSATIPPDATLETVRTSSTVLFTAIILVSALHISGYEQIHEICH
GRFLGLVSSAIFDRFHTLDDIRGLCIAAFWRPDLSWKLSGLCIRMATECHLHHAFYEAFYTPDITE
DALRESLEKTRLWYLLFVLDHQSSIAYGRPPVMAELRPIKDYEVLLNSPWCTAQDRALIAQVTGFV
ILSKAFETFGLEPRRTMSGDDASVLTHYRFTEDVRAWRERWQTLRSVESYNGKNQLPSGEIDLHFH
FSDLVLNSLVLRGRPLANLHDLPTSLRPLTLRAIEAAHALLRHFIEDPGSREGLVGMPLYLHSMIA
FAVVFLVKMSHCWHIIGITIDPAQQTRPLIEETIKFLRSCKAGANHMVFSMANGFDRMLKQASRQG
RMQPNHGDIPAARTMQWAKDSELDRNRSSWGLPDESSLNQSHASPTSAYLLDDQRRVAYNSNAPPN
DTAQTYHMQQNSEVSPYENWDFQDEELWSLAMGYDLLAPGGEGLPNTDFPYLFVR
>g3378
MSTNNTSFRPRIAITLGDPAGVGPELGAKLLADANNRARADLIVLADRSEVEAAAAVAQVTIPIAE
QAGPDSVQVLDDGTAPTTPIERGKISVEAGARTLYQLQRAVALVNAGKADAIVFTPLNKTSLHMAG
MHEEDELRWFAMHMGHEGTTSEINIIPGLWTSRVTSHVGIKDVSARINKKGVKDAVELLHSLLSDF
GIPNPRIGVCALNPHNGENGIFGREEIDEIRPGVEMARSTGIDAKGPFPSDTIFVGRKNYDGIVTM
YHDQGQIALKIIGFDGGVTVQGGLPVVIATPAHGTAFDIAGKNIASIRSSQNALDVAIAVASKKVA
REHAARISEVPLKASVKQVEVAAVAV
>g3379
MATIKGMETLNPPPSSQNCVRVRMVDTTSVMSLHAEGFVYPVMPGHEIMSVTTMAFLIENERLGKK
AMFDLGTRKDYWNSPPMTLRRIEDAIPGVRIEKDVTEILQEKGMALGEINDIIWSHSHWDHMGSPN
LFPHSTNLCYGKGTGVFPAYPESPNSNLNAADFVGRNCIEIEGKDLQIGPFPAHDFYGDGSLYLLD
TPGHWPGHLCALARTTPTTFLFLGGDICHFSGDFRPSEWIPFPETIPEDAFRGRAGKYPMPCPCAF
FSDHHPQLHNDEVDSSTVKNNEEPFYHLSTHKHSSYKDPALAKVTTAKMQQYFDSDPNVLVCLAHD
TALVDLLPVFNNEPEKDLNNWQELGMKEKCHWGWLGELPRYDKDGKVIGQGYREIPIVEGLWKEGR
RITSFNE
>g3380
MATTLEKTVEVSANSNLSQAEMATKKDVTPMEIEDARVVTEDEVQKYSKDDDEAMAAYAKYHGPPL
VLDEATNKRLLRTIDWHLMPILCLVFGLNYLDKTTLSYASIMGIKTDIHLVNDEYQWLGSIFYLGF
LIWEYPTNRLLQVLPLAKYSAFCVATWGGVLALSATLTNFSGAMAIRFFLGMFEAASMPGFALLSS
QWYTVREHNTRTGLWISSNGWGQIVGGLVAYGISRGTTDHPSALAGWKIIFIAIGCFTTLVGILFF
WVIPDNQLNCRWLNERDRLLALERVRENEQGIGNRKFKWYQFREALLDPLTWALFAYGVLSDIPNG
GITNFFSQLIVNFGYTPQQSLLYGCPGGAVVIIACISNGLAXDYFKQRTLIACLPMLCAAIGMLLI
IALPLSNNVGRLIGYYMTQALPATGATVLSLISSNIAGYTKKTTVAALYLIGYCTGNIIGPQTFRP
KDAPRYVSAEITILVCFGLCIVDLLFINWWCRRQNRRKAAIRASPEYVRVENQG
>g3381
MSLTQDLQETYINFKETAPEAVLNTLGSGVQELKNVFDPSKTIQVGQGFPSFELKDATGKLVSKDA
LLEDGALLISFYRGEWCPFCNLELRALQKRLPEFKAKGVSLVAISPQLPDNSISTVEKHELQFTVL
SDVENKLARQLGIVWSQPDTFRPLFQGLGVDWQKLYGNENLEVPIPATFLVDKDGVVRNMFLDPDY
TKRLDPETALEWATKL
>g3382
MLFNKANFCIAGALLGLSATALPLDARGESSKASHGAKAIYFLTNEDSNSVVAIPIGRNGTLSGGA
TIATGGKGGSTVNATGGANGPDALGSQSALAVVDDFLFAVNPGSNTLSMMRISKTNPTDLNLVGEP
ATLPGQFPVTVAASSKNSLVCVGTTGAQAGISCASYSVKGLGDMSPLTTFNLNQTTPPSGPLNGVA
QTFFSEDESRLITTVKGDPTRNNTGFVSVLPIENFYSYPGAHDTRSSPNGTAVLFGSTVIPGTSTI
FATDASFGAAVLQVNPFTDEVSLVSKQTLEGQKATCWAAYSEQRESVFVTDVALNRIVEMSAVDAQ
IISTIDLDNGDPGLIDLKISGRYIYALSPGNGTTASAVTVVDSFVGKQIQNFPVGSLGASRSSQGM
AILE
>g3383
MFTPAETRIRDDDWTGTTDPAERRRRQNRLHQRAWRRRQKLQVKSENIEDTKITAPVPSLVNKDLL

QKVALDHISGRQRIPVTLKQIYNWQAFGAIMEKTLGESAATFAVWAELKAWRRWELITDLSPSDLS
RTLTLYPSPSREGTPNELESPPSPESVDSLWFLNDKYSLHGSEFEVEFPISLDNKLFVLIQHNSLR
GMLANMAILIRLSGRDFQGWDEFYTEDLPTPSEDSPPSLQFTDLQKATSHEAWIDIIPSATMRDNV
IRYQDNIDIDDLCSDFLGGAFEGANDMHKRGMILWGEPWSSDGWELSENFMRKWWFLLQGCADMLT
STNIWREARGEKKLAFKL
>g3384
MPNLEQYVNFNPEKEIPELDGKVIFITGGTSGLGRASVIALAKHNPAHIYFTGRNNKAAQELIDEV
QTGKPSVKLTFVKMDMTSLVSVKEACKEFVHDRLDVLMCNAGVMFIPAGVSKDGFELHFAINHLAH
AMIIQELMPVLKNTASQPNSDVRVLCLTSTAWKGHSSDGITFSTIRTPQKGFMGSSFRYAQSKLAN
IMYAAELARQYPNTNIKFISVHPGAVNTHLTTSIPWHLRLITSIVLLFMGVTFIEEDQGRLSQLWA
AAGAKENELVNGGFYMPVGRFCNDRLDKTANSTELSQRLWTFTADVLSKF
>g3385
MKYLAVAGFLAAQAAALRNVMYVDHLPSSDLVSSVTHAIMAFAPSENFNSGSTFTPFESIDTFRAR
FPSTTKIMVAIGGWGDNAGFSTAAVSETSRSTYAQNVAAMLQSTGLDGVDIDWEYPGGNGADYKTV
PNSQRADEVTTFPLLVQAIREAIGDDKVLSIATPGLQRDMIGYTADNGPTVFDTVDMVNIMSYDLM
NRRDNVTKHHTSIKGSLEAVNNYINIGMNATKGNLGIAFYAKYFTTDPASDCATNPIGCATVQLED
ANGQDTGKSGAVTFETNPEVSSSSITSSWSKAKANGLTDEDAGGQYYWDSDAQLFWTWDTPDLITK
KFTDIIAATGLGGVMAWSLGEDSLDWSELKAINSGVAAAQ
>g3386
MVQSKLTLLALAGTALAGVVRREEAEVDHLKPEDLSYHPPTLDWMTPTPHHFEGTDPSPTSFVQAL
SEHSPEPHSTYLPSGLSSHFPQPTEEALPPFATSFPGDGEFAHTKDSPRPTSLSPTISHSAPPGAS
YDGPPPNFSEPVKPEEPQLNSPSPVFQASSSHPPIPSSHAETTYTSPTQPPSFTEAHPRPSEPVSV
KPTEPWDGLDAAKPTSSPEIPSWVSHLPSPSKVTPAPKTSENGHDTAPSAFPEAYQGPPPLPFNSI
PSEPHGSSPAGPHYTPSPGESEQEPHPLPLESASGKPPGPASSSPAAVSQTLXPHESYQGTPPLNS
GSSXPTGESGLXRPXSSPPAAVSQTLPPHESYQGTPPLNSGSSGXTGESGLPRPASSSPAAVSQTL
PPHESYQGPPPLKSGSSGPTGESYPPAGPHTITPAESYQGPSPTLTMPGEPSQPKGSPSAGLYQIP
PPTESHHESSPPPFESTSAEPHDPKSSSPEPYQLPSLSSPQSSHPSDADHPTEPATPHEGPSPHYG
AAPKSSCTCEEHPHPGESNLPPFNPPSEHGEVPHELAPPHAIPTPEEPSDTLTNAYVPHVSKPEPT
VNEAPSVSHVAVPLNMPPNCTEVHSKPAIPSPTSGSPQPQGPIGEHHIYESPPKIYPTEATSVAKQ
PSATRPAPEPSKTGVPKAAESVGHYSSFAPGSTVPITALLGAVSTLVFFFSLL
>g3387
MHHQKLATLFGFIAGIAQAAPSSAAAATTGYSSECSDITLNTSWLVANCPNDAGTSISSGVYLPSH
VTNTEGKLEWKTDGAYEESCSNCFLLDSGATLQCYCEGTYTYDSGNTTLNLEEYIVNYNGHLLSSL
EGTPTVPNDASLAVPSDVVLSLTVYDGDNACPSTPGGTLSFAGPETCWDLILAVDPIVWTELEATS
NEGWSVSVYNESTCTGTPLVTFDQTRVNECITVGQNGGAYLSLTPLWNYD
>g3388
MLPTNTDRAIDLAELVIYGILTIPTIYCMVRHGKQGLLGWLYIFLYCTIRLVAAGLKYKSDQDHKV
STGALIVDNIGLSPLLLGAAGILHEARYARHAIKNSKIEWLCVLQYHMLVSAGLALIAVSVTNLVK
PGSSNSGTGLLKGGFAILFITWVILAIATLFSMKRPSSYSQLTNDKSFRDGTLLLNAVVFSLPMSL
LRAIYGAINLFGSGSSIGTSTFSSSVAADIXPAGTDPYFDLGSYHRPITTKSANAQTWFDRCAIAY
WGLAYAIGPNYNKPWDTFDGQDLHISLREAQRAAGHAKDEAVDSTTPAERALIEAIQHRYPSDTIE
DVGNEANEQIFANWNLNYAEDMGVAYRNHSDDLDVAALYADALMNLTPWKLWNLKTGQPSPGSRTL
EAKKSNTRAIQADEKYFATAGSLRFYTLYRAHNYHFRLYAAMFAGQVQVALDTVENLEHSIPEELL
RVESPPMADWLEGFMGMRVHALIRFGRWEDILALKLPHDQMLYSVTTTMLHYAKGVANAALGRIEG
AENEREAFKEALLHVQPSRTIFNNSCLHILQVADAMLNGEIEYRKNNVDQAFIHLSQAITLDQELP
YDEPWGWMQPPRHAYGALLLEQGRFEEALDVYLTDLGLNDVLPRAMQHPNNVWSLHGDGRKISWGL
HDSAIVVLMGLIVVFEVAYLIMLFLQCRPLKALFDLVPTYHHVCTINSHLLTYAASIANTITDFLT
TLIPVTLIGKLHLPYRQRIVIMGLFALGATVNIAGALRTYYLHTSMLVKDLDRSWVGWPTCISGIV
EIGLGVIVTSVPALRPLIGRFWPYILESRRKYRYQGDDESPGISFGSEEKPRPSRSIFDLKTFDKS
WGKDSLVMSKISTTQIEMNSTQLTEQPRDAINVTQPWRQDWLNITPWPESRDSISRVGSVDEHVND
DYERFRFDVRSEEQSMTRFYEDDIGNRHP
>g3389

MNFELPPDLVSYLKDLDEFIDKEITPLQESNDNQRFFDHRREDARTNWAAGGTPTEEWEELLIEAR
RRADKAGFYRFSLPKKYGGSEGSSLWMAVIREHLAAKGLGLFNDLQNEASVVGNFPVVEMFLQFGN
EDQKRQFIWGQLNGHVLMTFGLTEPHHGSDATYLETAAVRHGRVGQAGWLINGQKMWQTGQHRATH
CIVFARTAGKAGASSGITAFIVPANSEGVHIESYQWTFNMPTDHATTSFTDVWVPNTAILGQLNQG
LSVAQCFVHENRIRQAASSLGAANYCVRESIEYAKSRKPFGRPLIYNQAIQFPLVRLATQIEMLRL
LIYKTAWQMDRTEKSRIEEVLTDRIAMCNYWANALCCEAADRAIQVHGAIGYSRHKPFEHIYRHHR
RYRITEGSEEIQMRRIGAKLASLNQSGRKFEAKPKL
>g3390
MSPAKPTSFNGIHHLKLPANEIRKTEAFYTQVLPFKSCPELDHYTPDGKLFAVLLTYNPASLMLEI
RHNPEQARIQKGWDPVTWSVDSRKDLEEWGAWLDEKGISHSKVLKGMKGWVMAAEDPDGKIVRLYT
VEDHEWTTDVDKDAYWLR
>g3391
MAPAPISPSITDFVVQTKQDNHGLEIPPATIERLTKAGIDLSHGYPYNPAKPLYLDDVYKIRSEPW
EHVDAGSRADPDKKALLGAAKEVIHLTRHIGTEIVGLQLKDLTNQQRDELALLIAERSVVFFRDQD
LSPQQQKALGEYYGEVEVHPQVGQVPGVPGVTVLWPALQAVEREPNFRKTGGASSWHSDLVHESQP
AGITHLHNDTIPSVGGDTLWASGYSAYEKLSPSFRKFIDGKTAIYRSAHTYLDRENPHAGPQFVER
EHPLVRTHPATGWKALWVNRSMTVRIVGLDKAESDLILGYLYDVYERNPDIQVRFKWSPRTSALWD
NRITIHNASWDYEGLEPRHGTRVTALAEKPYYDPKSPTRRQALGLLGPSEIKELEQYA
>g3392
MTGPSNTIDRVAVIGAGISGVVTAAHLLNAGLEVTVFERNQEVGGVWLYDDRRPVESRYPATKPSE
GERTGHYEEIQESDRLVLEHAPPGPCYVGLQNNVSTPLMRVTLNAWPDKTPDFVSHKVMNDYIIDT
SRKSGVHDATVFGAKVTHLEKVNEQSKWQVSWSELEDDGAGKVKELKKEELFDAVVVASGHYHAPR
IPDIVGLTDIKRLWPSRVFHSKGYRRPDSYTGKNVLLIGGGVSSTDIARELGPVARTIYQSTRNGL
YDLTEKMLPDNGTRVAEIASFEITGDAGAEELLTVKSRLPIRVHLKSDDKDATIEDIDYIIICTGY
HFTLPFLNRFHEDDTDPTAASDTVLVTDGTQVHNLHKDIFYIPDPTLAFVGVPFYTATYTLFEFQA
IALTEVFAGIARLPSGSNMREEYRAKVEQKGVGRNFHSLKGEEEGYVQELLDWINFFREKVGLPLI
PGYTETWHEAKKVQRKLIEQIFEGK
>g3393
MIATAGSGNERTSSELSDTVIQSEVKKKWTSYVWDTLDKSPEERRLLFKLDTALLTFASLGYFIKY
LDQINVNNAFVSGMQEDLGLYGNQLNYMTATWTVGYVIGQIPSNIALTKLPPRYWIPAAEVIWSVL
TMGLSRANSARQIYIMRFFIGLAESTFYPGMQYIIGSWYRKDELAKRSCIFHTSSGLGSMFSGYLM
AAVYHLGGRGGFKGWQWLFIIDGVISLPVALAGFFILPDVPEISNPWYLTEKEVALAKKRMELEGR
ENRQPYTKAKIKKIFSTWHIYLLTILYISFNNANGGQPVLAQWLKSSTHPKYTVPQINAYATTPYA
VQVITTLIYAWTSDTIFNGRRWPPILFGALSNIVCGASLLAWDIPRGWKWFCYIYSGAGYGLSGLC
MAWAHEICTEDNEERALTVGSMNEMAYVFQAWLPLIVWQQVDAPRYHKGWITMITMSCILITTAFI
IRFLHKREK
>g3394
MAARPLAFAAIAALFHQATSQQAPTPDNLVSLPTWKCTTSGGCVQQSTSIVVDWVYHWLHTVNGST
SCTTSSGLNSTLCGTEEECYTNCEISPATYDSLGIKSSGNSLTLNQYVTSGGTTSNASPRVYLLDP
AGKNYEMLQLLGQEISFDVDASNLPCGENGALYLSEMDATGGRSQYNPAGASYGSGYCDAQCGVGT
WFNGSINSGGLGSCCNEMDLWEANSEATALTPHPCSVDGPYACSGSACGSTGVCDKNGCGFNPYAL
GDQSYYGPGLTVDTSKPFTVTTQFVTSDGTKTGTLTEIRRSYTQNGKVIANAVASSSSGFSGASSI
TESFCTAMDSEAGTLGGLPTMGEALGRGMVLIFSIWNDAGGYMNWLDSGSSGPCSSTAGIPSTIQS
SDPGTSVTFSNIKWGDIGSTGSGTGTTSSSSSSSVSHTTSSTTTKTTTSATTTTTSAGPTQTHYGQ
CGGTYYTGPTVCASPYTCQVQNPYYSQCL
>g3395
MVKDDNTIIEEFNDLVNMSAPELREWLEEGVSQSSGWHKSDSSDTETVGHESGRKIVSILEHNPDK
DPSQYDSNDLQHMRRVVAYCKRHLAQEETAKRNTNSKSYKSLKNWGHDPLKT
>g3396
MISRDNHMALWQKFKDTNPGTEFVLVQFMGYNANLLVRAIPVARFEDLIKKGRTFSLTRAIFYLLP
HDHLAEGGEPVGIFYLKPDLSTLYVQPGSCHRAVVVADGVQTDGDTAISECPRSRLKYLSGLMVQE
TGHYPLVGFEIEVVFMTRVKNADGSVAEYQLTNKDHSFQSLTTDDEQQMPMVEGIARSLHAAGIRI

EQFHAEAAPGQWEFVLPPARPVDAVDTLLKARGIISRVAENYQLRATMFPRPSELHAGNGAHVHIS
CNQDRSSPVHSQTNKTQDRMQESFFAGILEHLPAILAFTLPQQESYARVQSGIWSGGEYAAWGWEN
KEAPLRRIEDNHFELKLMDGFANPYLALSALLAAGVDGMKKGTQLSAGPCSKGAGLLTPEERKKLG
IHTLLPKTLSESLDVLTSNTVICESIGQAIIGPYVAIKRGEIEQVKTWTEKQRRNYLISRY
>g3397
MPRKVNRFVIQSAIFIGVLLLILYINRPQSATDKLLAWTEIRYKPKSVAEQLESRGACPSLAGSKK
PALVVARVAADGDASWIDSLSKKYHTCVYTVDAPIDHTSKYLQVPTNRGHEAMVYLTFIIDNYYHI
PEAGVVFVHGSRWAWHNDEPTYDNSVLLKALDVHAALEPWGYHNLRCDWSVSTCPASAKPQGSMET
SLQAIVSPWDARAVSDSYLPGAFENLFGKGDGKLGNHHTVRSQCCAQFIVGRKNILQHSLEEYIAL
RQWLLDGTRDAAPRDDRVSGRILSYLWHILFIRKQEIEENGAISLDKLNLAACPRAPDCYCRLYGL
CNLDKCTAGHCEGQYEIPPDYKIPADWAATHS
>g3398
MTATNGTNVPLAPVICVSHGGGPMPILGEPGHKNMVNSWQTRVRKILKLGTPEAPKAIILVTAHWT
TDRPTISSGAKHELYYDYYNFPPETYKIKYDAPGAPSIAKDIYNRLQKAGFSPKLDDKRGWDHGVF
IPMKLIAPDESIPIIQMSVLESEDPEEHYKIGQVLAELRKENVAIVGSGFATFHNMRLFPRGSNFP
PDTAGKLREWSNVLNEAVMTQDADARIAKLKHWRDFPHAYTMHPPHGAEHFLPLIVTAGAGASSKG
GLYGDEFRGADMFSFYWE
>g3399
MASAPTPSQANAGQIRQFVPTLHTDVYNAIDPSSIKLPQPFTAVILGGSGAVGSGLARSYARAGAT
GIVVAARRLDEIEKVAKEAKAINPSLETLALRCDVSSASDIAAVANATKAQFGATVGAVIVNAGYS
GPFVTDITQERAEDFQKAFNVNTLGPAYAAQSFIPLLNESAAAAPTSFQGLFIAIGSMAGPTVTAP
LTDIHYNASKFAQSRIIELLHEERLRLKGENDGVFFASVHPGGIYSDFAKNSKVPDYIIPLLTDSP
DLAGSFIVWLTRNQDRVRGLSGRFLSCKWDVDELLSKMDDIVDRDLLRPRFAV
>g3400
MASGSVSSALKPYTPPPQTSENLDWADLEGLDISLLDQPGGIEVLAEQVLKFINTNGFFYVTNHGL
TNDQINRQYAIADAFFELPLEEKMRYLSNTAAGDFRGYKPRSTGELSSRDNDERYNIPKFTKEHER
PHPQLIKDYYEEIKEFSLHIHNKILLPLLRLFAYVLEVDSETLVQRHRYEENGLEYLRYMKYHPRS
AEEDAKAGNIWAKGHTDYNTLTFLFHQPVAGLQVLQNPDDSSSWQYVKSEPEAIIVNIADALEYLS
GGFLKSTVHRVVRPPADQAEKPRLSLIYFARPEAAVRMEPLQSPLLQRLGIQVPEHYLKALEGVTA
EEWARARIAKDHRFRTGIVNQPEREILAGVPQRYYD
>g3401
MEPKHLNVAKTGTVVDENQVSDRSYVLEQSIINNEPENDQKQVPLTFRRRLKQIIWDTLDYTPEER
KFVSKIDFFILQVTHLITWASFSYFSKNLNSNNLSNAYVSGMKEEINVVGNEYQTFTTMWTIPIVS
LVSFLGTPMGGCNICVFIRQDATPTLRLSLFXLAEGTFYPAVHTVLGAWYTKREIAKRACIFFGSA
FVGSMFSGYLQAALYTGMNGVGGLSGWRWLFIFDGIITFPMAIWGYIALPDLPTNTRVFWLKPHEK
ELARERIKKAGKAIGEPVTWAGTKRVLGKWHFWVYTAYYTFFICSENIGTYMNLWLKSLNRYSVSN
INTYPTVTNAVTIVTSLAYGWTSDYIKLRSPIVFFSLTVCFFAAVNLAVWDGVPFGLKWASFYLTG
FAQGSGPVFLTMVNEVCAGDSLERIIILGSTNSIAYAFNAWIPLISYNTTYAPRFLVGNSITVALI
VCAATTLALAVYLERRDKARKNDLVEQDPTTEPIVAAKAGNGEIKGAQEELTGAARV
>g3402
MYEINKIRRKEYPSLNKTCYLDYGGATPYAKSLIDVSAKLWKTDLFGNPHSKSASSLRATEYVNKA
RRRVLEFFHADPDDFDVVGFWYGYHTDAHTSLVGVRELADKGYHCFSSDKELNQWIESSKGEADET
YLPVDFDGRVIDNGLNKLIGYPAQSNMNGHRTPKHWAQRMRQKSYTTAGRLYTLLDAAAYCSSAQL
DLSDPDAAPDFISVSFYKIFGMPDLGALIYFGGGTVDMVTAFGQFHAKKSDHIHEALEDGTLPFHN
LLMLNTDLSRLQHANGRMVCEIYKDASSKYGDSETQGPTVAFTIRKSNGAWVHYDHVEALASACNI
QIRAGGVCNPGGIAEHLELASWELRRNYSEGYRCGEPFKVRGGKPSGIVRASLGAMSNKRDIENLV
AFVKYFFISNDHQEGSHDITPETSEKGQRQCVHPHLPLKHTTAYFDFRKNDLVMVRDEGCGSEVIF
RQHTVAVMPDDKFIFVNAPTIINAGPRDARRQLRSQLMRRVYLKKYKVSPSTVEDDGVSVAERDSS
SSPEQCHCSFPSVSSRMSPPDDTQHERSKAGKKTRNGKSRIPSQSKLPTPPIDDDEVVEVCESCGG
LCHSLKQGPRNRRTEPTPKEHMQGDKRIMNGDPKMTLGVSIINPFEQKFSNQKDCPNSNALIQHFA
QTLIPTLRSGQAYNQKVFTDSSVHLDKLMLWNGVDSDHGRRELEQSHYRYNALRELRRAVVGPKIG
TPEFDGILLSICLLAVSDPMGELPPSINKMDYNPFHHVLQPLGGLNIYGYQPIHPVHWKGLLTLID

QHGGXNQIQLFGAKWKISYTALKYALHTSTKPVFPICDHLGTLLIDQEPLNILGLSPSDLPPLTGT
GFSNLETFSIHESVQQVFVEISQVAQGMNILMPQHENRDLKDCMADARNLVQYRFQNLPTLTNDPS
LIVDLSLLEGSKQSTAYARSIMEAASTVYGMCWLVAYLFTTHVTFPVPSSRRFRLKTVYQIRDAIS
NCGYALQHNTWILKLQLWSVVIAGIATEDIDAELRQWMALKARDLCTKLCLQRWDEVVDVMTSFAW
MEVACTRGARKFWAQVQSNDG
>g3403
MKTTTLAAAACPVLLGTGAAAYTLQWDVNSSNFLDYFVFDTETDPTAGFVNYVDQSTASSDGLYST
SNSQIYLGVDSTTVLDSSSTGRNSVRVYSQDTFSNGILITDFAHLPVSVCGIWPAYWTINNQANPY
GEIDIMEAYDDVAGAYVSLHTGNTCTLSTTDFTGTDIRTDCTLSNGGGCGVQSTSSQFGAGFNSAG
GGVWVLSLEDSLQLWVFPRDEIPADITNGNPEPSGWGTPLFEFDSNNGCDVASNFIDQTVIFNLDF
CGANGAGGQEWSDWTDCATTTGQSTCNAYVAANPSAYSETYFSINSIKLYQ
>g3404
MFSTIKLERTALLLTSLIAILSPSLSAVALANQTCYYPDGSIAQGDKPCGNGTDVACCGSGGICLS
NGLCMDVAQPNTLARSSCTDKSWTSGNCPNYCLQETSFNKSGCSIILFSYLNSVGEYCCNNMDASD
NTPICAYNLSTFQIPDSSIVPGFALLSDYTTISNATSSTNATSTNATCSTENNSNSDHHTVIGVGV
GVGVSLGLVALASIAWALWERQKLNRYKTETLASAKASSPYSDAAPQQQAYNMTPMDTGAYGQPAP
TYNHSNNQYNSALLSEMGTRGPVELDSRK
>g3405
MIPPLRLLRDTPIPGINEFVENAIANIRCVEDITPELIASIRREIRRNLAIPAFPVTMNKFLEWQA
KQPPGSLQGFDYDARQGRLLLRPQEPDPACGALKGILTWFHTAIDRLGDDNSRSWSIVQGEPYMLT
NNIDMIKRPQTALINANQRWPLMVVHVENNETEEEVLDNAKQWLYGTNGEVQLVIVITIHEQDAPP
YDVRNFLVGEDFGKPTDIDLGMMNHAACGYTAEQAVLLRKYHPIGKIAYHPDYHGNFLLTALDCSN
QEKPEEWKFQIQHCWWGPPYLDDLKDPKTRLEFYKARCFKMCEPFRTAGVALPEDEILPLDQSQQW
APIQWNNHHGTVTLAGDAAHSMLPHRGQGLNNAMRDVAELFCAIKKTILGQVTLETGVTSYETAMR
PRGVRDVELSLETAKKMRLSDLMESPFVKMGFAKQETWKDNRDES
>g3406
MTITMTSYMVLFLAALSTARPLQRREVPQEHSHQKFLTTVAASLALNNPDKIVDPVFGLLGNAAAA
AGAGSVTDLNCLQQAVADQAFTNAKAAGDVNGMTAALVYRTLERNTGAVGTASALCTSVTAVNPEI
AALQQHQDPASTGAAATNKKIALELATQIASIGGDPTTAIESGTFAPGTIGDPTAKGKSCDDQNDP
TGCIFTQNLLVADVTADEINAALASAGVSGSATAAASATATAAAASSTVVAAAATATSTASTETCS
GSTAVVSDSGSTNTTSTSATSTTSTSTTTASTSSLDLGSCSGSSPFIVFGAGFDGRNTDSFEPADK
TTFNHGSALGIAVVADFICGQLNSPCNAPQSTIDKCNSAASAASSLSGQAAADSFNSALA
>g3407
MREGPPAVKMSHRKYEAPRHGSLAYLPRKRAARHRGKVKSFPKDDPKKPVHLTASMGYKAGMTTIV
RDLDRPGAKMHKKEVVEAVTVIETPPLVAVGVVGYIETPRGLRSLTTVWAEHLSDELKRRFYKNWY
KSKKKAFTRYTKKHTESSARELERIKKYCTVVRVLAHTQIRKTPLKQKKAHLMEIQVNGGSVADKV
EFAQGLFEKTIDIDSIFEQNEVIDVIAVTKGHGFNGVTSRWGTKKLPRKTHKGLRKVACIGAWHPN
HVQWTVARAGQMGYHHRTSVNHKIYRIGKGTDEGNASTEFDVSKKTITPLGGFVRYGEVKNDFVIV
KGSVPGVKKRVMTLRKTLYPQVSRKATEKIDLKWIDTSSEFGHGAYQTPAEKRAFLGTLKKDLVTT
V
>g3408
MSNIITVFGATGNQGGSVIRSLLADPVLSKEYKIRGITRDITKEAATKLSSQGVEMVTADMSSPSS
LQAAIKGSHTVFLVTNFWETMNKDIEVAQGKAVADACKETGVKHLIFSSLRSISEITNGKLPNVSH
FEGKAEIEQYIRDSSIPATFILPGLFMQGLSGGMIKKRDDVYMLALPIDPEKAKIPVFEVEEDTGK
FVKAVVKNYPNTLGKRILMATDYLSPKQLTDEIAQTFGVKAAAVQLPEDTFKSFLPPPVAQELLEN
MLVMDREGYFGGESLKESHSLLEDNLTSWKEHILRHKSLFA
>g3409
MENINIRPATIEDAPSISQIQYEALKQFHNFYSGFLATHPRDILPITTKTAISDPKNIFLVAVDAA
TNEVVGFVRYAIVPEKQAEEEKHEKEEEKEDAKSQLDRSQPVFANLFAPKDHVKELWEEFSTRDDE
MDACYEGVVKGQKHYCTAPTTESMK
>g3410
MASDSDYMSFLDKANKQRDAGKDEAHTEESAQQSKQVRTETVEKGVKVPEQLKKVDAFYVSETDEP

FEPVALSWKGASSGKWPGNDEFASLIASGSKPDIETLSEKSFDPRSQYSSVIKAVRAAVAASDSKV
SESDVEVKVYRVEVGHSRVEYWVIALDGAEGKIVGLRAKAIES
>g3411
MAKPKVCLAYSGGLDTSVILKWLIEQGYDVVAFMADVGQAEDFAAAEKKAIAIGAVKFVVEDLKQE
FVEELCFRAIQCNAIFEDQYLLGTSLARPVIARAQMRVAQQNQCVAVSHGCTGKGNDQVRFELAFY
AIQPTIKVIAPWRLPEFFERFAGRNDLLDYSAQVGIPVTSTKAKPWSMDENLAHCSYEAGILEDPN
ITPPADMWKLTDSPQNAPNTPEEFSLSFEKGIPTKLVTPSGQTVTGSLDIFVTLNAIGRKHGIGRV
DIVENRFIGIKSRGCYDSPAMTILRAAHVDLEGLVLDREVRSLRDQFVTFNWSKLLYNGLYFSPER
EFIEKSVIASQDTVNGTVRLCAYKGAVSILGRYSETEKLYSAEESSMDTLTDFSPVDTTGFIGISK
IRLAKYGQAKAESGESLSRA
>g3412
MSSTDTGAGYRQAKDWDANQYLKFEQERTRPVHDLLAQVPVSSPRRVVDLGCGPGNSTEVLVSKWP
DAQISAMDSSPDMVEKARARLSNVEFELGDLNTWTPTTEEKVDVFFSNAVFQWIPSEKRLPLISRF
IEMQAPGGVFAFQVPDNSLEPSHAMMRETASEANKPWTEVLRNRQPALARFQSTQEIYDALIPLCE
SINVWHTHYYHVLENHEAVVEWVKGTGLRPFIDPLSEEDRKGFVEAYLERIKKVYPVSVDGRVILK
YPRLFVVAVRK
>g3413
MAPTYAGMSGKRLSLSISAIGTLGFLLFGYDQGVMSGIISDAAFNDVFTATKGDSTMQALVTAVYE
LGCLAGAIFALFRGDVIGRRRMIMIGASIMILGVIIQVTSFPGHVPLAQFMIGRIITGIGNGMNTS
TIPTYQAECSRTTNRGLLICIEGGTIAIGTLIAYWIDYGAHFGPSDLVWRFPIAFQIVFGILIIVG
MYHLPDSPRYLITRNKIEDGEYVLAALIGREVTDHETQIQKQLVLDSVRASGALDEDVRYKDLLTG
GPTQHFRRMLIGASSQIFQQLSGCNAVIYYLPVLLQQSLGQSTNQALLIGGINMICYAVFATFSWF
FIEKIGRRKLFLGGTFGQLTAMIITFACLIPNNSQVADGAVFGLFLYMCFFGATWLPLPWLYPAEI
SPIKTRAKANAVSTCCNWLFNFTVVMITPVMVSSIGWGTYLFFAAMNASFIPFIWLFYPETSGRSL
EEIDIIFAKGYSEKKSYVTAARELPKLTDDEVDAQAVEFGLARNSEEETEKHIEERQSSEGAPGTV
KPVVYP
>g3414
MSARTSTIFVTGATGCQGGAVARLLLSSGHTVHAITRSPSSPLSLRLQSLGAKLFTGSLDDESALV
AGMQRCTGLFLVVPPAPHETTIKYTNTILSAARSVETITHVVVSTTLGTDRPERMAAWNPEGGMLA
MVIKPKIAMEELVRAAEFKNYTILRPGNFMANFIAPKISVVNPDLEKTGVFRTSYTRETRIPMIDE
ADTAKFTVAAFVDPERFHGKAIALASQLCTVDELVTCLSRVSGKQIKAQYLSDAEIKEEASKNSPA
SWHRFLSDMDRLVDMDEVKAWGIELNTFGQFLERQSEIVRDTFSQVA
>g3415
MASRLRITLDVLQNAHRTMVLENQTPWCHPLLYKNGMPRVMQDAYACCSLYMNRNPTNSKTILSII
ENRVAELLASEQSNDAWDCLARLHALILYQTIRIFDDDASARVAAELTMPALKATLSHFEENVFLE
DTLLCMSGLPINCLIDDPATSGAKDYWHNWIFEESARRTLFLSYLLIRIWEVSQYVNMPQTITTST
AKSQRVGEKNKLTCDGRLGLSNSWWYWYLSAHLWEAPTRYDFALAYAEKNRFVIKNLDFSEFLALG
QPEDVDLLGKMALSTLMGNEKLQDWFFERGAMLMVP
>g3416
MMQLVYRPSPAELRGSASHPTAQTRLSQQTSGSSGNPALCAIAPLANQQGFKYVDQYPIDKHAGPM
FIHKKQNSFCGICGMCITLEDEQAVSRVVNPFTTTGPDAAQHADAGNEEFEELQQGEDNSIDCDAA
NERKFGVMPRPGWTGMYRVVVRQKKNHHGKEEPHYYVSGLGRYHLWDTDPKARKMYNKCTYEAKEP
VFTVPREQGDSLIDRRHFYNKKLTKKQKQNTTQYPPVKRVSVYPRHYHDDKNEKFFPHGFPIHDNC
WKMAVKIIGESRLQSDLNLFIETLQDRFIDEAMFTEGRQISDWIVNETGNCGTWHQANRTADDVNR
ILIAMHDPVHVDEVEDLIAESARRYTTMKQKLHDAGATTSASNQNGAHDSVMNMLDEMHHSEVEAF
LNQTKVVVPWTYWVRRAPYDIIWELESIEEYSTPLDWQYLCLHAERLCEESLGIINRSRICNILRE
VKHSMFDSMSDDDDVSIVERSSSDGASTLAANTKSTEKELVDLIDSDWEQDAFMEDE
>g3417
MAGFLLGPVFQPPLGLLSSPPMRAVTTNLSNALMDTIQHGYSNFGNFSANSSAISITALSTEDDGN
SPFFDFQFSSPFLNRSAGSTEIVNSTSMYRIGSISKLFTVYTLLVNYGWEHWDEAITQYLPELQDV
TNLPGDTSLTSVDWSKITVGDLASQLSGIGRDYSYGDVATLDIPWNESGLPPLPSENIPRCGGNSS
APPCDRDEYFHGLLQRAPVILPQTTPIYSNTAFRILGYILGAISNIPFDKLLDSTVLRPLNLTTTS

YVIPAEGSWVIPNGDSAWYQNTSDETPTAGMYSSSSDLANFGRDILLSKQLSTLETRRWMKPNSHT
STLSYSVGSPWEILRTKSQISHGHTIDLYTKSGSLGQYNSLLVVSQDYGVALSIMTAGSSSSAVME
VVSEMVLQTLIPALENQMLLEECDRLCGIYSSSDQNKNSSLTLSVDTNGLQIERWINRGVDFLAAI
QTYASQTNSPPIQRVLLQATNLESPSDGQDGGFRRVRYRAIFETSTSERAGPTRIMPQSANQWATI
DTPMYGEIAFDDFIFHFDHAGNAFAIEPRVMRDTLLTADRESSNFSRFTYRMGPES
>g3418
MPVKRGLVRESCDFCYRRKVKCDRLSLASQGIATCSQCALRDLECRVKDSDDARIRQRNRLISNSR
NHNGGLNADTVIRNSSPRSEIIPCPAFVPETPVTHAIPSRSGQCKAQDENTTPLITPEGLPVDDTF
GLSLDNMLFLDQVFLNEGSFDWSMMNNQDGISLSHEIDEQTSTTCSSALHRFQTPDNLGESSTLAA
ALHSYFNLAALHLPILVEDAFWQDFYSGRCSPALIYAIACRGMPFTTVENKLDLEQRYARQFRKAF
LEARSAHSGNGTVRLDELEALALMINYDYEENENDDESALHSKLGTLFLTHDALILLMLQNQICDR
PSSTTGLDSSPESLANAAQRRMLLYWHIYGLDAFHCLDRKQSSHIHIPNIDSDNKISNVEIRDYFD
AMVSLATIAREIIQKLCTISAKRQGVDPKHVHGLYDQLHHWSKTLCPRHLRRNYQEDGELKPLDQR
PQDEPAQPSSSTRTTRKQKHTHLRRAVLWALEFHCFLQIEAIVADYGIKHDEKSLLEADVLASRIE
YESIRILNGMASVCRWINQYEIEDDSGHTKRHSLVDLAPNILRNICAGMCYWSAQRGIELCQRGPS
ELLRVRHRSTEKTDGGDMRYQIVSTYIKTASLLRDTAATAISHNQTKQVLERIDHQLVLVGRHFEN
LRGPNC
>g3419
MEQQNQSVDYYGYLPSKSAALFGIIFFGVSTIICLLQVVFGPYKHYWMLTVALMASGEAIGWGARL
WAHNSPTDWMPFMIQICSLVISPVFISAADYVLFCKIIEKSGRRLFSIPSKLFWIGFIIFDIISLS
IQIVGGVEVSSAQNFDDLNHGSAVMRSGIIFQFSNTVLFAVLLLGATVSLKRRDIPLSSVAKWPII
VTLCLSTLMILIRNAYRILELGDGWNGHLMRTEIYLIGFDMVPMAVAVGVCVIFSPSFFLSPMDKE
VALSLHSLA
>g3420
MGLLPARKAVALQISAGQAIKIINTHGKQVVDFWAFNPDDTHDYLSMVHTRTVLRKVAVRLGDSLY
STRRKPMLTIEEDTTKGVHDIIWSACDAERYRMQGVQGYHDNCSENMHTALKTGFPDWTGIPDDWV
PDPFNIFMNVAIDHHGGLDIREPTSEKGQYVILRSNVDLILVMSSCPQDIEAVNGGMPTDCEYEIL
EKEAAGETQVAKILSPVKALSTRPQRRVKVALSFDFDAVSHWLGTGCHPDNNMADYSSGIFAGQVG
AIRLLNMLKKFDIADKVTWFVPGHTMETFPDTVQQVVKSGAEIGLHGYAHEGIYQMNVDQERDVLE
KCIEVATKLCGKPPRGYRAPMYTIRETTVEMLRKHKFLYDTSLMHHDSQPYWTPSDPPIEHIDFTK
DASSWLHPSPIAAQIKPAEGQHPLVEIPCGWYNEDMMPLQYLPHLANSNGYVSTRVVEEMWKDKFL
WLWENAKGEEGSSTTDFVFPILMHPDTSGMAHIIGMSERIIKWLKSWGDSVEFYKHEDIAREWLAD
QK
>g3421
MPAPPRPEIIDGVKTFIPLENNPDVLEILCENLQLSWDLAFHDVLSTSPDFLQDSHFRPCHALIFL
ADQSIYRAARSAVEPTISEYKGSGPSEPVVWMKQTIGHACGLMALLHVVFNLEGGRYVRPDTAIDA
LRQQAILLGPTERAQLLYDSSFIEEAHMDAASRGSSNVPSPRDDNRHHFLAFVQKDGKVWELNGGM
NGPLLRGELGEGDDILSERGLELTVQDFLTAAEQTGCNEMSIVAVTGAGAVA
>g3422
MTATEPISETYDVVIVGGGPVGLLTAYQIARFGLSVCVLEKYDKQTQDAFGRAIALFPRTLEQLDQ
LDLIEPMLQKGFACRTSVTYKDGVQQIPGRVWTFMENIKDTAFDFVLVLRQMHTEEILRDKLRSVG
AVYRESTECTGFEIEDGFASSDAYPVKSTFTSTKTQEKIVLRSKYIVGADGGRSFVRRHAGIPFEG
DTSEDKWIRIDGIVETDMPIKRAYGAIESKTHGNVLWAPLDHGATRIGYAFTPEIAAKYPSGVTEE
VAVKEAIESMQPFNVKFTEVHWWTLYTIGQRIAKNFSTKDRIFLCGDAAHTHSSGAAQGLNTGIHD
SVNLAWKLASQIHGFTRPEVLQTYATERRTAVEKLINYDKDISLLMTHKWPSWYTGDPAADPYLVL
GQIFEQAASFNTGLGINYPANILNQFPAATSLKIEPGNRAIDVDLTVPGINRKVRFNEIARNLGRF
WIIVFAGRIESTIPSLQALRKYLVDRNELYSHPATKWLTISRDAGCSPYETLGMAPFGDTYYDIDG
LAHEKYGIDLSVGGIVIIRPDGLVGTGGAIEGSLIQHYLETVLNL
>g3423
MTLSEVDSIMTSAQSGGSLDFSTFYNVIDGKLSPTQKIRHGINPATEELNPEVPVSTLEDVDRAVE
AGQRAFRSWSKTTYEERRKALLAFADALEAERDKFANLLVKEQGKPTRLAHVELGFALSTIRETAA
LEMPTEEIVEGDCKMLVRYTPLGLAVGIVPWNYPVLLCCAKLVPCVLTGNTIIIKPSPFTPYGGLK

VVELAQRFFPPGVIQALSGDDNLGPWLTEHPGPAKISFTGSTVTGKKVMMSASKTLKRVTLELGGN
DAAIICEDVNIDEVAPKIAEVCFLNAGQLCLAVKRIFVHESIHEQFRDALVRCTKALKQGPGTEEG
IYLGPVQNAMQYERVKTFFGDIEKDQLTVAVGGKLPDGPGYFIPPTIIDRPAKNSRIVIEEPFGPI
VPILTYSTDEEAIERANDTKYGLGASVWSSNAQRANSIAEQIEAGCVWVNMHYVLDPRVPCGGHKE
SGIGVEWGVGGLKAYCNSQTLSRKELDLTLQNQSQTLSKPSLTNHKMPSNTIVEQNGRKYRSKKQR
PCDLCRSRKTHCRITGSESICELCKKLGRQCTFVQKPLRRAQNPQANGENIDRTSPPQPNVMSVSN
SQHDPGLQDLPLAGNDDTNSTPIESSSFWLPQMDWSAMNLSSIGLANSGMSPVIEHSIDYALHQDV
DMSNAIPGRSPSTTTPRESPNNVQDAGNGSINQSSEMGSEAYASSSVGSARVITDIRQDGLEDWPS
EFSLDTRKGYSNQLIGLSCESDPYLLRNYYYNKLDTYPMFRLDFRKVKDDASVQPFLETSAYGNSL
LPADSPPVQFVMTHEDIWKDSQNFVEAIQSGGNTEESDLELLKKIVPADLGPRLLKLQSLSDINKD
TKVYFEASLRLSVIELKYSELEKDINLSASAARLVSEPDQLKIRDLLFDIYTVLIEANIVALMKEV
SSKVNAWLYAHLIWRTTSMVASFLRIQPASTVRPSKNNQYLEHHAAVRSIWTMRELFNSDQDALYW
NLGYFQSFFTPCLGILFGTILTITSKSDAAEDLALTLWVQTVVSRPPATIPSSGKKDSSAAEYVLT
SLDQVVNWARQGSLWPLSFGLACCAIEMMHLSMPRYDQDRLGIIFRASPRQADVMIVAGTVTNKMA
PALRQCYDQMPDPKWVISMGSCANGGGYYHSYSVVRGVDRIIPVDIYVPGCPPTPEALMYGIFQL
QKKMKATKVTRMWYRR
>g3424
MALSISSFPGLFSAATGVALHHLLFRHGEWDNNAPTIIGSYAAVFAALNVFKSAGPVVGLQNTNVY
YLLACHFLGLFSSIIIYRISFHRLRKFSGPALAGVTSWYVNILSAKKLNNFEIVDKLHRKYGDYVR
VGPRELSIKDPRALPFIYGPASQTTKGPFYDGAQPYISVHSTRDKKDHARRRKAWDRAFSSKSLRD
YEQRVSKYAAQLLSVVADNAGQPIEMSRWFNYYSFDVIGDLTFGKSFDMLITGKDAYMLKTLHEQM
QSLGPFLHSTWILALFKLIPGLNSSYLEYFVWVKTQVENRIKNVPENPDIFIQLETDFRKGKQTNI
DRLHLDGEVNTGIVAGSDSTASTVTNLFYELANAPEYTRLLQSEIDSITEPTYQELSQMKLLNAAI
DETMRLHPAIPSGMQRVTPQKGVMIDDIYIPGDCLVQIPLYSVFRDERSFVKPNEFIPQRWIDRPD
LLKNPSAFTPFGLGSFACAGKQLALMEIRRITRDILCRYDVTFAKDQTREAFYDGQQDAFTLVCGK
LRLVFTERKSMGV
>g3425
MSPSLPHESQQAAVDSPVSPQSSSGLFPPYYPERTTSLPPSDTSSTGQRSQSQASNKTSSQVYTQE
AIGAVAGDSTPKRKSLWQKFTYFFLVVLSRGWCIEYISCVISVAAYVSIVLVLNRYDQKAMPSWPM
GITLNTLLAFLTAVSQTCFVQPVVQGLSQMKWNWFASKSRPLADFESFDNASRGAWGSILLVFSTK
GRLSVLLAAVILVTSAVTSTITQAALSQDTRYTPGTGNATTERLTTDYGLQMGTINEAMAIMMNAM
YTSANSTYEFDTPLCSSGNCDWPTFGSLAVCSQTNDITDQLRTLTGEAQCCWQNSTANKLEPWNWG
PATCQNCTYSGLPNGFYLDYFASGNMANTSNIWLSAEGNGSAWSNVYSFTNETFGPNALNAVQVVY
LDSTDESYYNISIGLPNSTVLLNHSRAAETLFYLCAQTYDVSTTNGSATTTVNATLHSINDTQAVT
VTDGSHYNIDTFHTFILDGQNYSYYTVDWNLGQMLSSFTSGFYNYDVTGDGLDVMTPFSYAVGNTL
YHTVDVSTSSATTDGLMRQALASMVDNMAKGLTNWLRSKGTAAEGQANTLEIFVVVKWDFLIFLGV
QVLLSAVFLVWIVADSRIRKTRILKESALATLLAIPGSDREYLEGLLSATEEGEMGAERREDIEQN
SRAKLIKDTITGQWSLKVVDQKV
>g3426
MISVARPSVLRAVRSQIPSSRQMFDVRLSALTRLLSTLAILEQRGGKLSSASLSAIAAAQKLGGSV
TGFVAGEGVKSSAAAEAAKIKGLEKVVAVDNAAYEKGLPENYAPLLVDNIKSGGYTHVIAGHSAFG
KGLLPRVAALLDVQQISDITAIESEDTFVRPIYAGNAILTVQSEDPIKVITVRGTAFQGVETEGGS
AEIVDGADPKAESTTEWVSEDLAKSDRPDLATASRVVSGGRGLKSKEEFDRVMLPLADALGAAIGA
SRAAVDSGYADNSLQVGQTGKNVAPQLYLAAGISGAIQHLAGMKDSKVIAAINKDPEAPIFQVADV
GLVGDLFEKVPELTEKLKK
>g3427
MVAATKALTTKVASGTPYQLDDSQVLRASNALLRHIKSEQKDQEETSVKKTLIGDDDDESDAEGSP
ATSELVWLXMTTKKHVVDKNRLKPSKISLPHSLNKSSSLNICLITADPQRSVKDIVADPAFPAELS
SRITKVIGLTKLRDRYKSFESRRQLLSEHDVFLADDRIIMRLVETLGKIFYQSSKRPIPIRIEQID
KVDGKRVKKDLKSKQSKEERKASFASPQVVAKEIEKALSCAPVYLAPSTTTSVRIGSTKFTPEQLA
ENLKAVVNGLTEKFITKGWRNVKAIHIKGTSTMALPIWLADELWLDETDVLEKEEEEKKAIDNDKS
GNKRRNPEENEXAQGQSKKTKKIKAAADEDEVSLKEKLQKRKSQALEEGKVPVKATKKK

>g3428
MKLSATVAIAAAGTLAAAQGHHHAHHHAHKRDTVATSTVDEVVTVYQLDGTPVPYADVCQGIKNGD
YKWAEGAPLGACATTTSTSLSVKAAEFIQTTVSISSSTSSTVITTSAVPTTSATPTTSSTSTTPTT
SSTPAATTTASSSSSSGATGVDASFPDGEIDCTTFPSEYGAVSLDYHGIGGWSGIQYPTYNALETL
VETIVTAVTGESCTEGAFCSYQCPAGYLKSQWPSTQGATGQSVGGIKCSGGKLHRTNTDYETLCIA
GVSGVTVKNTLSSVVSVCRTDYPGTESETLPTVVEAGSSSPLANPDGSTYYQWEGKSTSAQYYINP
AGKDETVACTWGTSGSDVGNWAPMNMGLGFKDGLTYISIFPNTPTTTATLDYNVKIEGASGPCYWD
ASAQTYYQGTTASSTGCTVSIPEGGSAEVVFY
>g3429
MESSPPRATMNGVGPAAGVKRPASLLPAFEPLSSSPALPRPQKRMARDVGDYPTPVPTSSTHIMSS
SPPHMSMGRPKLQRSVFSSSTTERAPLSTVPTLTLPESGESLLMGRSSASCHHQLSANRLISRVHV
KATYKPAGNPFDRDRVEIMCTGWNGIKLHCQGKTYDLAKGKTFTSDIKDADIMIDVHDARVLVQWP
RHERKETSSTEPELAWDASSPSKSHRDLQQSPIKEAPRLVSPTSPSPLARNRIPPSSPLLSPSRLH
STISIYEDEPLSPTPANRESKTLDASITTTFDHSLNDSHLSALTDTFEDFSDHDEENDPIVHSFGP
FGENLLPRMAQFRAGDSPIRIARPRTQPEPLRSVHSPPQPVAIKEEETQDKKKDEQLEEKPKIDER
RAAAIAAIQNHAANQLAFSRLSSTPLSTILQNLPASLWKSDSNVFERFTRHEIRAVLEDTNCIGEV
IREGKDAAGKALESEFYYIPDNDDDEMRRQAVVNDLRKPGELRDLNERAWAGESDTFEVSKSLDSS
LKKNTAFIKRLRTGISASALATFIADIRTLSLHKYLSEIISACFEGLCKLKSPGEVAAGVEIVSAL
HQRFGPHEFTKQIGWLLGRGLSSPDKSQLKALSQDVREREEKDRLSRHRVLLKVVTELWLVAVLKT
LDDIERPDDLGAKNKDASSSAGGKSLDGPSRVKSAAKGDPEKESDPYPLEVLKDLLGHDRDHTNLP
LAVLFVKSFSWDILGVKTAEEGRKTVDADGATTAAPDSGNLPEETNGDAAAEPPLASEKLQLRFKN
ILTRYLEDVKAHVVRDQKALTAQSRRNAEAYVKSGEIFEDRQANFEKQSKAHEKLVANTQAMCEIL
GVEMPSLVDKEAADASTTGGIGLVKTAEYLRGQGDGAGIWEDEEERRFYENLADLKGKVPAVLLED
GKKKKGDADEQAKGQDATEKADADVSADAKAAAEADNESVAIVNKTVGAQVDALLVKLPDLQTKDQ
VDQLALDFCFLNSKASRNRLIKALQDIPKGRTDLLPLYSRLVATLGQHMPDIPQGLITYLDEEFRS
LQRRKQKEFLGQVRMINIRYLAELTKFGVVPEHVIFHCLKVCLDDFSRTNIEIMGNLLENCGRYLL
RNPETSPRMSSFLETVSRKKAATHLGQQERMIIENAMYYVDPPERPAIQQKERTPMELFIRKMLYL
DMNKRNYTKILKSIRKLHWEESEVVDILERVFSRPHKVKYGNIHLLAILASALYRYHQEFVIGVVD
NVLESITLGLELNDFKFNQKRVAEVKYLGELYNYKMIDSPVVFDALYRIVAFGHENGTPSPNKINL
LDMPDDFFRIRLVCTVLDTCGVCFDRGSAKKKLDFFLTYFQYYIFTKDPLPMDVDFLIQDTYSLVR
PQWKIAGDIEEAGRLFSEAIAQNYKVQDGDKSAEVDDDEVESISSDANGEEGLDEDGVADIDDGQS
SAEEAEPFAQNGDEEGEVADSESEDEKIFVARQEEERDPEAEADFDRAFEKMMAESMEGRRFERKA
LFDIPLPMRPSRREQTASDEMPIQAPQAPAKTMAFSLMTKKGNRQQTRTIELPSDSSFAIAMKTQQ
QADREEQQRIKNLVLSYDPGDVEQPEGEQGLSIITQEPIITRGKRKPRSPYRSTNAKTSEIVSEKR
PSRDNRMDKSNRPGFRSRKLQLSDVDW
>g3430
MFRQSATRRATLLLRNTKPSTTTNSITNRFATAQRRFASTEGGDTIVPNPPSQKSRSWRNRLVRLG
LAGGVIYFYNTSIVFAEEPTLSLRPQPLDSEDAPTLNTLGQKVGLKARDEPPAPHSTDAQVVDPRQ
QQQEGGLEGLEQEAKHEGAFNPETGEINWDCPCLGGMAYGPCGEEFREAFSCFVYSKEEPKGMDCI
EKFKGMQDCFRLHPEVYGSELQEDEVDEQLTEQIAQRDREEQSIKSQKEQQQQQQPPSAEDVKITD
PAVLSLNDAEKHAETEELRRADEAAKVANQREEELVPRAWHESEGQSKTEK
>g3431
MSSSRYRVVDVKYRSSLDGRNIRLARYGVLNEQNDLHHSTTTGSSTSQTSAFSMLQSMSSLSLQSD
MDPMDFTNWELPAATNGSAVAPGEKPTSPIPSTSSKAASPFVERSNPMGGSSTAQTVSSKDPKAAA
QAASDLRNVVRRKLTGYVGFANLPNQWHRKSVRKGFNFNVMVVGESGLGKSTLINTLFNTSLYPPK
ERKGPSHDIIPKTVSIQGISADIEENGVRLRLTVVDTPGFGDFVNNDDSWRPIVENIEQRFDAYLE
AENKVNRMNIVDNRVHACVYFIQPTGHSLKPLDIEVMRRLHTKVNLIPVIAKADTLTDEEIALFKQ
RILADIQYHSIQIFEGPRYELDDEETIAENQEIMSKVPFAVVGANAEVTTPDGRKVRGRRYPWGVI
EVDNEEHCDFVKLRQMLIRTHMEELKEHTNNVLYENYRSDKLTQMGVAQDPSVFKEVNPAVKQEEE
RALHEQKLAKMEAEMKMVFEQKVREKESKLKQSEDELYARHREMKDQLERQRQDLEEKKARLESGR
PLEKESKRKGFSLR
>g3432

MAPPPPATLPLGERIKALAQTLQFGWFVGHLTLLLSVFRYGLSYIVFNYYSRWAKISYRLAFISAA
ATYGIVVYKQYIARGKLSGSPLQIAVKLLSDENVQYLGMALVWLYSRQIPLALLPFSVYSVFHVAT
YTRTYLLPTIQPAPQGATGPASPGGSRPAAKQSPLAESIGKFVKQYYDSSMYLVASLELGLLFRLV
LSALTFSKGSWVLLIIYLAFFRARYSQSSFVQSAVTRFTARVDTLVSHQNTPPQVRQGWEAFKGVT
FQVYEASDVKRYLGGGPAAAGKKPQ
>g3433
MSLDSKAPDIYAPATSPSSSSSSSSSSPSSSSFRGDHRNDATQLNNEQDTGSRHILQQFVSRRSTS
SESRAMDMAAGSAVVPVDDYEHETRPPYLHSMIAGGIGGXSGDMLMHSLDTVKTRQQGDPHIPPRY
GSTSASYIKILREEGFRRGLYSGVSPALMGSFPGTVIFFGTYEWSKRHMIDSGINPTLSYLSAGFI
ADLAASVVYVPSEVLKTRLQLQGRYNNPYTKSGYNYRGTTDAFRTIVRTEGFGAMFSGYKATIFRD
LPFSALQFAFYEQEQEWAKNWIGSREIGLPMEILTATTAGGMAGVLTCPLDVVKTRIQTQQNPDAL
PKKSSTQASISSKKDSRPHAQHSQHTSSSTIPSSSSSKTHSRPISTSSPSTSTPQPGAPNLHTSSV
FTGLRLIYKTEGLAGWFRGVGPRAVWTSVQSGTMLVMYQYLLKKLETMSESEAVPVI
>g3434
MSSSSTGPYLRALRKTELAELAEISDLKDYADLKKAELEAALDSHLRANSSIFSGEKRLADYYRRL
SQPARLSSPVKKEPKSESSLISVGSEEKRVSRRTRKASTEAEETESEKTQTPASTAVATRTPARSP
LSFAALPPSPAVVTDAIDRQTTIVREKVSDAWTKSGISERSDALRATLSSVKSIETIFVVLELIGL
ARELIPLRYLTTVPAVETVNTPELSVKIPDLFVLITGTFWAPFLLWATTSLVLPLTLAYFFNLSLH
AQASTHSHNTRRASSTVQQQVASFDPLVYNITKALISYFVFANHFTFWSTFSHFSIEKVNVAIPGQ
WPGVLTGSAVGVLVSLYDAVLRK
>g3435
MADDDRTIYEARLDAFELYHKYEYPERPKPNVDWKTAALRGPALPALGHAVAGAAGSAVSNVATYP
LKLIVTRLQMQRLAARKKQSSGDKEADRKTGYASIQDAAQKIYAKEGGIQGFFTGVGDDTWKTIAD
SFLFFLAYTILRQRRLNSKASNNNGKKRAVLPILDELAIGVVAGAFSKLWTTPLANIVTRKQTAAL
DNQSKPISTKQVAAQIRTEKGLIGFWSGYSESLVLTLNPSITFFLNELLKYLLLPRHKRKNPSATV
TFLLAAISKAVASAVTYPVSLAKTRVQADSSSSEDTKPSNTFAFSLFTSLQSIAAEEGISALYDGL
FGEVVKGFLSHGITMLTKDIVHSSIVQTYYTLLILIKKYPSPEELIVRARLQAEEYAEVAREGAKE
VAESVKEGVSSVTSPNVSVDMSSNAPPEYSPFEETNELAEIVGEYVEDDAAEWRSIFSEILQAELQ
GMTQSDMEWHPASFFRTDIIPTTPFAILILNQPINENAFDAVRRHACYTLVADGGANRYYELMKSR
SIENIDLPSCILGDLDSIHPHVLTHYKTHHVPILHDPDQYSTDFTKCIRFLHAHAHSILARENPTV
GVDNHDLYMTSHLTLPSATAKKEQLDIVVLGGLGGRVDQGFSQVHHLYAAYEEKRQSTTNTTTPIS
QAQAGDLYLLSEESLSFILPPGKNVIHTPFTHQQVPSHHHDEEEERKEVFSENVGIIPLSGPTLIS
LTGFEWDVTRWRTEIGGQLSTSNHIRADRLEVDVDVDINRAVLFTVELGERFKRIVNS
>g3436
MTPNPEQRRRQECIKFQPTKPYTRERLEQAIRVFGRHKPVATRKQSYAIGDFAACYNLRKRNASQL
REIDMDIPDDDENDDNYEPSTPRTRRRRAIQPQSSTPVEPAPASRIVKLSFSSVRGKSLLLQLKHA
DVFRDFVDSDDDEIADAPSSTKNDSKDTAQEDVSMEDSEGIERIIITAFAHPIDCELGNNCGSPCD
FCTDFTFGMFGLGIKTVSVIDHVTWFEELCGGHRDDGQEPTRMCNVCALTRLHIMNCGGHQVTAIP
NYDPYTFNIQTAFNSLGGRRLRGRRHQINEWCSFCINPAFFRCSTTQIIDIFANDIEDPSSPEAQG
CGLRLCQGCKDLMDEHRNDFDRVIAALPGATEKRADAEFLVNGSDLNRYFSE
>g3437
MESNDGPGPSGDVIDLTSFSSPEQASQDTEVARPALQIISIEKSQHDTKNIVMSPDKTSLFRPPAF
TLDLFKECVDEDDDKTREEEPEVLSSRQKSDSRTLLPGFTTPEQTFRNTATSDDTELFVRADNYDD
DDVVGVFAPRIKRPKPLRTSLKLLTTQRMPGLTQQGQRLPQHLRNGYRRGKQDIIIGGESYKDLDH
LAGASQRHISTQSYGHVHTALPAANMIPTDGRGYIVTNSFMTGAQRKRLEQQERLERALADLEECV
TANPTDVKLQGRLLHLRALQNRLEQNENTFLKSAEVGIVKEVERMLSRRRMHMVTAEFENQAKAMV
ESMVMSSSAGRKGGKKRGFDDGSMSPQSSSYRSEAGGTSERSSFSHPSTGGKTVPQSYYQRHKRER
FTHEIEGDDNDEDEDVNLDDGQERKTYGGKTTAQDSLDYDTAKVLVKAMEGEEGVKDGGEVEDSSD
DVKDTSDDNTDDGNGGANE
>g3438
MHTHSPSSETSSASSSHSSHSSHSVTHSHPQRPTLPRSSFSSQSYLRRHRRSPSYSKASLPSVGEG
NSTDRTSLSMTVDPHASLRQSPPPRNNAIIPSCAVISPPESAGNSSDEDTSPTDLRHVDLIDAAIR

TLNAQKKNEGQEEKKETSAMTSVVPALTPEARKISHSRSSTEGSLLITDSIETSSESDRDEDEEGP
KPPMVRKKSGELVRPALRSRRRPSSMPGTPTFSKNVHFDAQLEHIRHFLQLDKPLAVSANTSPVET
YSEEHEYPFKRPQPSAEWEIKXPNFPFNPAIRKQQPVRLERVFLSSDHKNLVGVVSVANLAFHKHV
VAXFTVDYWKTVSEVTAEYNHDIRKKQAHDGFDRFDFSIKLSDITDLGSKTLQVCIRYTVAGQEYW
DNNDNMNYQIQFVKSEKAQPAQEQAPTRDRARALPRSRHNPSTAARPRSMPPASFDDFGPSFDGSD
RHDDSLGFRLTTLDSHDLFEAPKPREKPGRQAFGNRYDFGLSLSAARSSGATEDRTTLSAKAKSQS
SRGESLTTVHAHPRDAFQSPAIGKPHLESPTYKELVDKYCFFGSPKVEGSLNSSKSNSGSVSPDTM
PRVDSGVAMNLVTESEPAKVGMNSFNASPIIFNPVYRGHISRSGFESQPAAILG
>g3439
MTGRGKGGKGLGKGGAKRHRKILRDNIQGITKPAIRRLARRGGVKRISAMIYEETRGVLKSFLESV
IRDAVTYTEHAKRKTVTSLDVVYALKRQGRTLYGFGG
>g3440
MFGLDAPKITRREFDGSDPQLQLAMAYAASKKSASTSTSAVSSARPSVDEKRNLSTSSGKSTSSLT
DGQKKRFSFFRATGIVLSLK
>g3441
MGSIQLNRHDQVYAAPQARWDLKFTIRNLNLYRLEDKKPFRPDDEKGARARINQFFEEQTHGLLPK
SYSYQDLRDFLALADIGMFDQNMSTCLNEQDMARIVLLDERMDDQSSMSRQWKEYEQYPPRGTAEC
TTVLNSRDCYEKLIQTREHLGRSSPERRSIYIQDLSPSCAAGLILAVSRRYAVFLRDFLSRYMTNR
VYFGVTMDYPIQPPSPYALEFHIPYFALRKSFSTSEKLHSNIVGRRSGVFADSFTRNCEPGQKEFF
HEAQVSVLLVGIDEWVWTLYCLVESQFEGSRDPAELEKYVINMRDAPSGNYASYKMPIWNPREYFL
LVLCRRMDQATLEWLNLVETLDQRLKDLENDTFVKPSAESFAGHNSDISRINDYTWAIQVLQLFTN
TLIKTIESWQVFSQIHLPMFLPGDGEPEYSSTVPSYLQRLDRDVSELRYFHTLLGQRIAAFESRRS
MILNTSALVETRISNTQAADIGLLTKVTVVYLPLGLVTALFSMSFMQIVGSFIQSAIDLLCFVMDQ
PQNQDDAANRQ
>g3442
MQRRSSDNSDGTDHSLKTRPPLRLSTDHGHGASTNTANSDTDQASQSQIASTPVTLTNPDHRFNNN
SNSVVSPRSRNSSAVRTSFSSLASASHASPSPTPSPKPTAAMMPLASRQLSFESSADTSQRMRHHR
LRSPWSISILALSTALAGVFFLLYIAHSFVNRPVGADGCQVPMMSPTYLRMVGFDAEHTRFASKYN
LYLYREEGVDHYSQENIGLSGAPVLFLPGNAGSYKQVRSLAAEASRHFHDVISYDKDRLDAGIRNL
DFFMVDFNEDMAAFHGQTILDQAEYVNEALAYILSLYHDPNRSHRDPSLPDPSSVILIGHSMGGIV
ARTVLTMANYQANSVNTIITMSAPHAKSPVSFDSDIVHTYKQINDYWRGAYSQEWANNNPLWHVTL
ISIAGGAKDTVVPSDYASLASLVPETHGFTVFTSGIPDVWVGTDHLSITWCDQFRKVVIKSLYDVV
DVHRTSQTKPRAERMRIFKKWFLTGMEPTAERALAQQEPNYLLTLESSTNKILPQGERLVLRELGN
HQDSVVHLMPVPPQGVPGKKFTLLTDQPFDEPDGKLEVLFCTVFPMQAGRGAALFSMRMDLSRGGS
GSTRLACKNAAPDRIHLPASTSSSQKAFSDGQPFSYLQYELEELSEHQFVAVVDKAQTRTNGWVVA
EFSDSSDALIPTRVGLGRLLSAGLHVKLPANRPMLTEVRIPSLHSSLLAYKLSVHSAGCSDSSPLF
TPLLRQSMPEPHESKFFVNVHDVDVNLHGVAPYMPPPLREEAAYGVSFHLWSDPTCASSVELSLKV
SIPHSFGELVMRYRTVFAAFPLLVVALVLRKQFKIYDETGYFITFMESLNLCLRSSLPIVIGILSL
LASSLASSRRLPSNDDPFHWRRNSTETAVDFTKYDLLLGAQDAFFWFLVPLFGLVSIGACVLINYA
SLLILSLLSSLYGMLRTKTGYIRREDRNGPIFSHPSPRRRIINATILLVLVATVIPYQFAYMVACM
VQIATCVRALWHVRETRLAKSASFFNYAHSIFTLMLWVLPINILVLVVWVHNLAVHWLTPFSSHHN
VLSIMPFLILVETMTSGLMIPRIFSRAKHITYILLFCLAGYSALYGVSYAYLLHHVANIFSAWLVI
IYLASGGFSVQRFIRILETDDYHLNSNTMSGGGNSGLSEKDTGKHHIKKKP
>g3443
MGAIWTQMYPPSPHFTEKDLPSLVGKVFIVTGGNAGVGLELVKILFSKGGTVYMAGRSATKIAAEI
EAIQAAAGTELTEGKLKSLIVDLSDLSTIRSCASTFLSQESRLDVLFNNAGLSRQPPGSVTAQGHE
LHMGTNCLGPYLLTKLLLPILIETARTAPKDSVRVVFTSSGIIDLLGPPGGVSFAELEPGKHSKDM
NRNYSASKAGNWFLASEFDKRYAKDGIVSVVQNPGTIKTKGWDGTPWLVRQLMRPLFYPPRMGAYT
GLWAGLSPDVKSGDGQKFINPWGRWYETPKKEIVESLKTSEEGGTGLATEFYDYCELHTKEFAI
>g3444
MQSTTHNGQDLWQRAFDSLPESLQGTLKQANDWKLDKLHAALKVAEDKRQLSFKKRWKKKRPGGGE
DIIIRDIMEKIVQWIRRFRDVGDVIVQYDPGYSALPWAAVRFLLQSAINDIDVHTAMVEDLEMVTR

LLARYDQVERHYLAQFGNLYDQFSDTIVAVYAAVLDLLGKAVEYFKETAADRFFKAPFRSVNEGSR
DQILAKDAELVKLASLADADRLANIETQVLRMADLSLVALKQVGENLYVDVLRWISTLEYPRYHAD
HSTARSLGTCQWLLQHSEFLTWESSSTCSLFLLYGIPGCGKTKLSSAVIDRYLEAAVKNPNSVPVA
YFYCCDSHAEEERRTTNGLLRSLARQLIAFHSQGPSIHSAAMSLYGRLTDEAKEQGFLAKSPNTAE
CIDLILAVVDDNPVTIIIDAIDELDHPKEVMDALQIITQKAGNVVKIFVTTRESASIMTILSTQHK
IRVTAETNNADMRRVTTEAVDKAVNNCELLSGRISQSLKDEIIDVLASSAGEMFLLIKFQLLRLSN
FEHEDDVLAGMANLADSTLEALYKEAYDTILKAGDTARDIAIHVFSWLLYAQEELTLDALVVAIKK
KQVSIDRILAICRGFVYFDGQSKVIRVVHHSVQEFLRLQQALQVDKAQELLAMSCLSICQDPPGED
VTKLKPSDRFYDYAALYYGNHCYAAQSSDQHELLFDELEDFLFGSDSPYSPARLWLDSVKTAYDSL
PNEHPQKTAMELVSAEECAPLFVICTFGLTKVLQRHPWPVDFDWDQQNDYGHTALYAACYNGHTDV
AAFLIDRGASVNIQCGRLGSALHCAAFRGKFDIVKMLIDRGADLRFGGKFDSAVHAACRGNHEEIA
LAILDKDVILSLAEFDSILEAVLXVGFPXATEKLYNLPWRNAPTPNMGK
>g3445
MCNVQATQAIIGGKLGILQRLLRKSSAADLLPNGSLALAALHGKEEILRFLLDQKLNIEEAGELGT
PLRCAVLNGHSDICRLLVDLGADLNGNGRLGSALHAAAMKGRLQTVRLLIQLGADVDINGGYYGTP
LQAATCLGHAETVRLLLSAHASPHKPGLSKDAFHAAAENGQYHIIQILLSAGFQPPVPISWGKTRA
RRRSPPQARNLLRESSPSVKKEKRLLKWSHRVRAWRKGHNVRGLPDDSDDISSDDDEKLTDLTQEE
PEIHPKPYRPLRRKPSDANFNAPDGNYPFEAAAELGHLETVSMMLENRMFFDLTISAVEQALSAAC
RSGHKQIVKKILEWREDIPMDLGKPLSEAVSCGHVNVIMTLLKDGTPINNKKKYLKMALESAIYPP
RAWDTQPRPEAIFHEILNSANEEFPKLEITDLILACLDEALGADRADIVKVILGRSTDLSYERLLN
MFCQTLESAKARTAAVIYDALQERNFSPSRSQFHQFILLSAGNGLAQLTSQLLRHRDICFDCDGQL
LQEALNIAAHHGHHEVCTYLLSKGVDPCLSAHIITSHEVPSRKYGLSHTIFESFRSENLPSEGTIS
AEKRVTALASSLSGYQRFRPGNREHRNANAAWRKADEKTQADTIRLTLDHIPSMKGMDLAKDICCA
ATCCPPDILQLLIEKGADPLAHIENVSVLQFAARRERLSFMVSRILIEAGADKNMTESEMQLLLDE
ALKHFRAPSHSREDERNDRSARLPRSFSNTLYKDYGKLLTETESLEDVFITGPGALVYYLLGRIPG
FRLDLDPAGILLQCAAAAGHDNLVEILVEHGADVNWTGSYYGTALQAASRFGHAQIARVLLQSGSD
PNLNGGRHGTALRAAVLARSLEIVTLLLQSGAYEYDQGTDLAIALQHALHNKDTDIALTLVAAGAN
VVSEDQSAQPLFIQACSTGYLPLIEALLNIGTNVNVYGIKSSTSIKQETGSPVHSAIHIQRTDIVK
ILLAKGFELESDFGDFDHPLCFAIRKGDLQTLDILLDSMSECSSKVLREAIEMAIMMSDLAAITKL
IQYYEANFSSENNFDFVSGLLKKACGAQKIQVTKMLLDWLSQCGMVHEEIIRDVLKEACARQNXPA
TEMLLGWLSERGAVAAICTTLLRDIPSTSAEIYEILL
>g3446
MCEYESVVQMLLSRGAEADTPTGHFGTALSLTAYMGLCNTFNSLLQQGASLNASGGFLQSPLAAAI
LGGHSKITKHILSLGPGGRPEALCFACKKGNLPVVKALLDSGVQSAATPADGQTALQLALAKFNYL
SVSYRTHEDVMTGDEEEILNLLLDSNTTKKISDQDLIAATQIQDDSIRERVLDIMISRAISPYFPE
EGLIQLLHHDRYHDRYNEHSTIEKLLQDHRIQEIPIRVLLAAGNIDSIKMLVAYDPKYNTAPAAFD
ATMTEGRYGPELCIEKVVELLLYENEAIDVSESDILAALRLRSGFSFQVDRSHILKIMFENNPELR
ITEEMLKAVQTTHDLDLLLAHTSPEKGLVTPAVMSAVAESCAEEATEMLRMLHDFQNTAITIADL
>g3447
MSFREKRSWMTARGTWKDRKGSGKPPLSSTSPPPLGEILATIQLEDLESGKTETDNYARITNTQYL
TSYNWINGNSPQIMVPGEPPAWTPLSKPVQLRGDSGLYYRDKNAARYPLYPLEPMIRAIMMDKPEF
SLESLDIIGCGSTLGNLLHFARGQGRPFRMLIEVVGNTVFFIRRENSPTETIPDVRGYGHAFPEAY
TTWSKSTRGSESHQRLITYDFAGMSCLVRFEADGFLPNMIPDGLRTDPEHTAGTNDIDANDLLSSI
EAVTVSTMLPITTATKSTTLEISKGGCYIPQCAVFDLKTRSIKRSNVDTLSEELSRLWIRQVPNFV
LAYHTFGNFNDIRVRDVRQELMQWEHAQKPGLLRFATLLQMVVSFARSVEDGKLEVEFEQDSNILN
LRSPGGVVGSVLPSSITDKWDLYHDSGA
>g3448
MTMSAKTRLDRLAKYLGAVLHGKQELRNLSDFKRFIEAILSQQDPSITIERLVTSPSALGALQTGL
RFNLTTAFINEYTSKFINFLDNPGVKALCNGLFLQQILLIILEPRSLWNALVDAFHSRMLDGDATQ
TFCWLVTELLSLPASCAVDITVDVQTIINDDLMISSPSASLRNLGHKMKYLLEMKSSASVLLSSEH
TAGGRHDNDFSDFRQVAILPTAGEMSCVECPFYRRVDEISQLSGNQRIAAHVDNQFRLLREDMLSG
LRDDIQVARGAKKGRRSALRLQGLSLARISCVSANLRFLNPCTIGVTAKSGLKRLESLSEQEKKTH

LKQNPDFIRHHAFGCFLRDTEIVAFATIERRIDDLISTPPIVMLRIAGEEALKKCILFLKLYDDVE
FLVVDSPTFAYEPILKCLQERTDFPLTEELFLYKKDEPVKESSLAPWVVIQELREKYRRNIQNILQ
TSRPITLDQSQLDSLLKGLTQRLSLIQGPPGTGKSFIEALLAKVLHNQTKDKILVMCYTNHALDQF
LEDLLDIGIDGSHIVRLGSKSTSRTQPLSLKEQKINLNRPHTTWQTINSLRNEGDELRTNLDESFK
RYQAMSTKSSAILEYLEFEEPAYFEAFTVPDDDDGMNVVGKDGKVVHKDYLYHRWIKGQSPEPFSN
ILPAQCRDIWATEQRLRDEKAQSWKRALLNEQAASLAVQIRLFDQCQEKLSGVLGERTREILKRKQ
IIVCTTTAAAMYSQDIQNASPGIVLLEEAGEILESHVLTAISPQTKHLIMIGDHLQLRPKVNSYAL
SLEKGDGYDLNVSLFERLIQEGYPHTTLTKQHRMCPEISSLVRSLTYPGLEDDEKTRNRPNPRGLC
DRVIFFNHSNAEEDFAEISDRRDEGAKQSKKNIFEVEIVLKIVKYLGQQGYGTDKLVVLTPYLGQL
SLLRQTLSKQNDPVLNDLDSHDLVQAGLLSQASADHSKRPLKLSTIDNYQGEESDIVIASLTRSNN
KGDIGFMAAAERLNVLLSRARNILIIVGNSNTFVSSRKGRDTWRPLIDHLKDNGHLYDGLPVRCEQ
HPDKTAILRTKEEFETETPDGGCAAPCGVKLSCGIHDCPSKCHQIVDHSKMLCTKIVKWNCPRGHT
LSVRCSSVKGACRFCNQEDAIKERKHERDQNLEAERQRKQAAYARELAELQDEASHLRRLRSDVLA
NAEQDRVIKQYRDEIEALKSPVKPVETIVNSRSVGKIAIVPDSPEPTTTEKNTEYIKPSRDESHKK
IKVSDPMPTQAPSEAKADWDYQKTYHNSQSPEIDQLMNMVGLESVKTKFLDIKQKVDLKIRQNIDL
SSERYGTVLLGNPGTGKTTVARLYAKFLAAVGIIPGSKFIETTGSRLSNEGVSQCQKTIESLLKQG
GGVVFIDEAYQLVQANSSGGGQVLDFLLAEVENLTGKIVFILAGYQRQMEQFFAHNPGLPSRFPHQ
LKFEDFNDTELMLILEHWIEKTYKKQMKIDGGPSGLYCRIVARRIGRGRGSDGFANARAVENAVTK
ITERQAKRVARERRNGSAMVDDFFLDKEDMIGPDPSQSLNSSRVWQKLQGLIGLAEVKKTIEALVD
TIKYNYRRELAEQPLVEYSLNKVFLGNPGTGKTSVAKIYGQILVDIGLLSNGEVIVKNPSDFVGSV
IGESEKNTKGILAATLGKVLVIDEAYGLFAGGTSHSTGARSDPYRSAVVDTIVAEVQSTPGDDRCV
LLLGYKDQMQEMFQNVNPGLSRRFPIDQAFVFEDFTPKELDMILDLKLSEQGYEVTDRARRVVLEM
LERARNRPHFGNAGEIDILLNAAKMHHQRSLSTKNLMSSVPNAILDAIDFDENYDRVEQSNLSVSK
IFEGVIGCENIVSRLEGYQQVVRNLKKLNMDPRLQVPFNFVFRGPPGTGKTSTARKMGQVYYDMGL
LNSTEVIETSATDLVGQYVGQTGPKTQLLLERGLGKVLFIDEAYRLAEGQFAKEAMDELVDCITKP
RFARKLIIILAGYDADINRLMSINPGLTSRFPESLQFEPLSPGMCFKLLEGLLSKQKKELLEQSQT
KFDITCVQCPDPDLSEKLTQGFDTLSKTASWANARDVETLATSIFRKTITTMQDSSASGLILNRDS
VIEEIQKLISERQSRENFQSQHLLPQVNRNSLFSAPLRTQNTNTPILNLQSQNAKSNAVPDAKTNE
AVESGIPKQNMAETDHRDDGVTEEVWAQLERDKAKANTEEKTYLRVCHDEEEQKREIQRLQDQEAR
SIREADEAKKREDEGAKKRYEQERLRHELERRRLETLARDLEKKRKALAEARRKEQVNQEKLRHMG
VCVMGYRWNKQSGGYRCDGGSHWVSDAELR
>g3449
MGDLTRGLFEAMDRGATYPFLTDGDASLTEGSGLNTVLVKNGVLYTPQRGILEGITRKSVIDVARM
NGLKIRVELVPVQMAYKADEFFTCTTAGGFMLITSLDGKPVVNGKIRLNREEDMRWVLGNSLR
>g3450
MDKINTSNVVHRFEKRSLLIVINCMAGLSILFFGYDQGMMGGVNTAYDYYTLMGFGHRGPDGGVVV
TNSLLQGGIVSVYYLGTFVGCLVGGSIGDRFGRIKTIFIGALVAVIGASLQTSAMNHKWMICARLV
NGWGTGILNAIVPVWATETAEHKSRGQYIAIEFTLNICGVVIAYWIEYGCSFYGDGTSSFIWRFPI
AFQIPMLLGLMAGISFFPESPRWLVKMGRNEEARYILGRLRGNTGEECEVAEAEFQEIVASCELER
ANFRRQTYFHMLLGIGSGKLHTRRRVQLVIWLQIMQEWVGIAGVTIYAPTIFGIAGIGPEKRQWIS
GLNNIFYMFSTLICVFTLDKIGRRWTCYWGSVGQGIAMALAGAFSYLGQEATKEADFTKATQYGNA
AVSMVFIFTFVFGATWLTVPWLYPAEIFPLEIRAKGNAWGVVGWSIGNGWLTLLCPIMFQQLGEKT
LYIFAACNAITIPMVWAFYPETNQRTLEEIDLLFASDSIWNWEAEKNFARLHGEMTAGMLDSDSKG
DAEQGYSQNQSLTCDMNLHVEHSKV
>g3451
MLIYNQNISFVLRGINDVAFENRPMPKIDDPYDVLLQVEMTGICGSDVHYWSNGRIGPFVVEEPMV
LGHESAGIVVEVGAKVRSLKVGDRVAMEPGISCRRCTRCKEGNYNLCAEMAFAATPPYNGTLAQYY
VLPEDMCYKLPENVSLEEGAMLEPLSVAVHIVRQTNIKHSDSVVVFGAGPVGLLCCAVAKAHGASK
IIAVDIQKSRLEFAKSFAATSTFEPGSGSAQEKAQQLIQENDLGSGADIAIDASGAAPAIQMAIHV
LRHGGSYVQGGMGSDEIAFPIVAACTKEITFRGSFRYSSGDYESALHMVAGGRVNVKELITGSFAF
EEAEQAFQYVKDGKGIKVLIEGAHFFH
>g3452

MVPLSSDYYIGIDVGTGSARACIIDDTGDIVGLAAEEIRTWQPKPGYYEQSTDNIWLCICKAVKQA
VAESRIDVHLIRGIGFDATCSLAVFAHDSDEPISVTGPNFENNSHNVILWLDHRAEEEVRKINSTG
HNLLKYVGGKMSIEMEIPKLLWLKNHMPKELFNRCKFYDLTDALEHLATGNEKRSFCSVVCKQGFV
PLGVDGSTVGWQEDFLKDIGLEDLTDDNFKRLGGVHKINGEYLSAGDLAGHLCQRAAQELHLPAGI
PIGSGVIDAYAGWIGTVGAKVNDLAEDDLDICLSQSFSRLAAVAGTSTCHLLMSPKPVFVDGIWGP
YRDCLIPGYWMAEGGQSATGQLLKHVLETHPAYKQAIFQANSTNMDIYDFLNTHLAGMKETTSAPS
IAYLGRYLFFYGDLWGNRSPIADSDMSGSIVGLSADVSLENLALYYYGTMEFIALQTSHIVAKMNE
SGHDIRSIFMSGSQCKNNILMSLIATACKMPVVIPQYSHAAVCHGAAILGAKAATADNTGKTEMLW
SIMKRMSKPGRVIKPTQTPGVKDLLEVKYKVFHEQCDRQRAFRMMVDSILNDAL
>g3453
MPRIGRTLYLSGSVKLRVILERLDYLTTLVELDRLDRGADRSKLEPGNHNGGLRRSLSPLLDRREN
LQDVSNPASLEDAVSLTTLGCRMRSDIYFGSSEDILEWPILGGKHDRRQIEALIFDPTLISDISNG
VAPSPRVTDDSLRNEYEDPRHSSNIGRGVREEDVMQLVEIFLRNVHTKNPIFDPKYLRNMARSVVK
NGFDWEASSCLVLIACALATISSSFARQPLVGAENFEEAEDSLLNTPAYYTAESYYTASRKRMGIL
TNTILATECYFLTGVYEMYSLRPLQASISFNRACVSFQTLTWMKPEYYIIEGQLAKAHASRLYWSC
LKSEHEISVELRFPSSGLTGLNYTNSFPTPPVAVAVEQLYETPSVIDVESASLLSTDVQQEFEKGW
YYYLADIAARRILQRVVDTFYNKYEITWSTASLPKLIQTARELERQLSQW
>g3454
MKSPMYQLLLALLVSLSTAEPLLTVRSDTENATYVDLVVRKGPSQHVASGFIYGIPDTFGQIPSHF
YENIGFNYGRAGGAQEGAPSRGWIWGLTDYYGRLNSTLTDYKTCREFNAGFILLPHDVWGTDQANS
STVWPGDNGDWTDYDNFVTTLMSDLAANDALEGLVWDIWNEPDGSWFWARSMQQWIDLYIRTHKLI
RANSAFDGVKISGPTLSASPYNPDIWWTSWLSQIAGNDTIPDQIAYHLEAGPGIGPWQSSYDLTNT
YPSVQALLRSYGIPNRPQVINEYASYEEQIPSGAAFWISRLERYEVQGLRGNWQSGNTLHDLMANL
LTKSDPFNYAATDYMPGPEYSVYEYYYQNMTGCRLNTTGSVNLQFDVYATVDDVVRILAGGQTATG
NWTVTVENLTEVGLPQQGSVSIRTLAFNGNGHYVPTYGPVDLGVSNYSYSGNNLVIPIDQRTNYTA
YAFEFTGGHSRRVW
>g3455
MPRFSSLRDFSLLCLGLALLHTRSFVMAVNPIIQTRFTADPAPMVWNDTVYIFCDHDQPGAATGFN
MIDWRLYSTTDMVNYVDLGSPLALTAFSWASYNAWAPQTIERNGKFYMYVAAGQSNGNMAVGVAVS
DTIEGPYTDPIGKPLVATSTGNIDPTVFIDDDGQAYLYTGNPVLYYVKLNEDMISYSGVVQNVSLT
TQGFGARPNFLYNAEKPTTFEEGPWFYKRNGLYYLMYAADCCPEDIRYSTSNGPTGPWQYQGLIMG
EQGGCLGNHAGVIDYKNKSYFFYHNQGNSGGGQYDRSVCVEQFEYLSNGLFPTIEMTYTGAPQIGT
LNPYQRVEAETMADSFGVQTQPCSLGTQNIDALEDGYWLMVQGVNFGRGSGARNWTASVASNTNGG
TIELRIGSLTGTLIGSLTVPSTGGWQNWQTLSTSVSGATGVQNLYLVFRGEPGFLFNVDWWSFSK
>g3456
MTEIPMSDAAAPAADVSSPPAPESSGLKKRRWRSPSQEPPRTGKKLKKNESESTAAPASAPDPAPT
LAAAAAAAPAPAPAPAPSRLSGVHLRTKAELFELAAKREDEWKTRQIQLADDKHALEYYKKRVEET
ERAIAAAPAKIAELKKEMKRLKSAAFAAKK
>g3457
MPAVDVSSVPAVSGKETANSVAVVESLIQNLSISKTPDEANTHANNLATLLNGPIEEKTVPLKAVE
ALKKQLTNKKDANARERAVEAIRAIAEHSSVAPGVEPHLVSLLGPTLNAVGDKMTNVKNAAQVAAI
SLVKAINPNATKAALPSIINSILSAQKWPEKMTGLQCIEALVETAPAQLAFLVPDLIPVVSESMWD
TKPEVKKAAYGTMEKICGLIVNKDIERFIPELIKCISKPENVPETVHLLGATTFVTDVHEPTLAIM
VPLLERGLAERETAIKRKSAVIVDNMCKLVEDPQIVASFLPKLMPALTKNYETLADPEAREKTKQA
LDTLVRVGDVKDGKIPEVSHAGDVATVSAILKEILEPKYKAQIANADAAINYISAIAGQLIDEKVV
EVVSWTENTTPYLVALIGEADAKSVAETLRKRASPTASEEEELNSDEEEGEDLCNCTFNLAYGAKI
LLNQTHLRLKRGQRYGLLGPNGSGKSTLMRAINNEQVEGFPKKSEVKTVFVEHDLDAADTEQTVIG
WTQKKLESVGINLAQKDIEAKLLEFGFLQEQFESPITSLSGGWKMKLALARAVFEDPDILLLDEPT
NHLDVKNVAWLEQYLINSPCTSIIVSHDSKFLDNVIQHVIHYERFKLRRYRGTLSEFVKKVPSARS
YYELGASEMEFKFPEPGFLEGVKTKAKAIVRVSNMSFQYPGTPKPQIRDITFQVSLGSRIAVIGPN
GAGKSTLVNVLTGELIPTSGDVYQHENIRIAYIKQHAFAHIDNHLDKTPSEYIQWRFQTGEDRETM
DRANKIVTEEDEKAMDKIYKIEGTSRRVIGIHSRRKFKNSYEYECSFALGDNIGMKNEKWTPMSTQ

DNAWIPRTEILASHAKMVAEVDQKEALASGQFRPLVRREIEVHCANFGLDAELVSHSRMRGLSGGQ
RVKVVLAACSWQRPHLIVLDEPTNYLDRDSLGALSKAIKTFEGGVVIITHSREFTENLTEEVWAVM
DGRMTPSGHNWIQGQGAGPRISEKADDGEEQRDALGNVVAKVEKKKKLTSAEERKKRKDRMARRKR
XEEVFSDEE
>g3458
MADTKEYTEPSHEVSPSSFPEENMSVGRYLATRVTSLRPPMRKAPNPFKALMLLNKQQWLFFLVAF
WGWTWDAFDFFSVSISLTSLAKYFDRPNSEITWGITLVLMLRSVGAIIFGILSDRYGRKWPFVANN
VLFIILELGTGFCKTYKQFLACRALFGIAMGGLYGNIAATALEDCPPEARGIISGMLQQGYAFGYL
LVTAFARGLVDTTSHGWRPLFWFGACPPVLFIIFRLCLPETQAFQSREQVRKSLAGGVAGNFLSEG
KVAVKNHWKLLIYLVLLMAGFNFMSHGSQDLYPTMLSAQFGFSANAVTVTQVIANLGAMTGGTLCG
WASQIFGRRFSIIVISIVGGALLYPYDFVSNKSVMAAAFFEQFCVQGAWGVIPIHLMELAPGPIRT
FAVGTAYQLGNLVSSASSTIESTIGERFPLPPSKTGVKRYDYGKVICIFMGCVYAYVIIITFFGPE
KLGREFDAEHDSDLNEVAARRMYEKDTEKGTAQHAE
>g3459
MDSTYTIIRGEGPDLPIDDYETSNSSTNKSISPPKWHTIDNNKYDLVPTVAMAADESNDRDAYGKT
EMQSKKILTGAERRDSLLSVDSESGTGLQCRLTIIFGIVCSLACIACSIGYWVTSSGPANIFIHSG
LPGEAASFIVNLILTQCLEGLAYVHSVSLRWALLKEKRLVFNTNIRLITSSNRSRPNSWYINAISA
ALLILCYAATSMLFIPRVNMDYGTLYETHVNLLALLALGLALLGQTVVAIWCYYSNLRDIPTWSSN
PLDTTVLMLDRQLVRHREGRCMYSMQVRDILGEEQESAPKLPSPQQXSQWQLIPTTRHALIFIWIL
AGLSFVWFLTIVLVARGNMIGAINQIGHLPNATWHFSMTWNATSNPVVDDLGGTYFFNAVFFSLDW
AQINSDMPFVPALIVNLLFVCAIQGLQTLGLHFSELVINLSRDEDVWRSLDAYGRSKGVKTSVLEK
PPFLAALMSWKYIVLVIFKSLLHWLLGQSSQASVELLEDRVWLTMNYSRLFIYAICATAFASVITF
IACMKPKGPQPATYGHIQTLADVIDDWKLDDDKRFWWGDKGFSGMESVRHAGMSRNKHYLGEIQMN
ELYAG
>g3460
MAAVATRQPFEPAPGFVPTETQATGSFLHSTLDGAPAFVDISAGGSNVKSIEHSLKVINLRDYQPQ
VKLDVEGVEYVPFSSSLQEESLLHPDKAEIKSVVEKVYWPECAELVKKHTGAVEVVPYHWRHRQTP
KNMQEQDASKGVSSKPVTHFHIDNDASTAEGSLKAALGEEEASRWLASGHWGIINVWKPIGDPAYQ
YPLAVVDVSKVNWQRDVEVVLTRNNYKNSINGLRFRDGFDYYYVKDLRADEALLFRNFDSQDGRGI
GVPHAAVDDVNTPVDCPPRRSIEVRCLVLYGN
>g3461
MVESQNPSEQSTRESSVILNDNLAVDMNDEGEIGHGDDSTSVGGETLNKVMDVRDEKVITDIKIVS
SDLSSSSSDLEAAVKTTDEDDQQNRDPNEVDWDGPDDPECPMNWPRWRKSPFASSMMAPAILNIHV
EFHSNSVVLDTLSISIFMLGVGAGPLLFAPLSELYGRNLIYHLSNFFFTISSVACAKAPSLKTLIG
FRLLAGVAGSAVISNGGGTIADIVPKKSRGLITTLMVLGQLIGPVIGPTVGGYVSQGVGWRWIFWL
LTIVSGVFTFLGFIFLRETHAPTLLKRKAAKLRKETGNELLYHKGKSSVPPRKQFMDACQRPLKML
FTNPVIFIVSIYIALILGYAYILFTSFAFVFQQRYGFGEGTTGLLYFGIGIGMLVALVWMSRYSDR
IFAQKEEQTGISKAE
>g3462
MAKKQAAMEQHAESHTVTRQFLSSSDSHKEIFYEYDDAQPETGTSAAASPEKTVPPAKSPAPSMVV
ETSQRVDTGSPTIMAAGTVDDIPMTATDIILAVVAQKLRKQFDQIPISKSIQELSGGKSTLQNEIV
GDLGSELGSLPDGSEDMPLAALGAALAGGFKGRPGKQVSSLISKLISSRMPASFNQQAIRQYLSSR
WGLGPQRQDTAISYSITLEPSSRLTSVDAAKEFFDQVVQRYAAFANFSLTSGSAGPSVAQAPLVDA
KALEAAQREHQVALEEIYEVIGNLIKRRDGDETTNCGKDSDDTLLETLGHWTSEFGDDLYAGTKPI
FDTNKIRVYDSWWNWVRVDLIALKQELQIGRLDSGDFKDRVRMILNRWTPTCDDILTQFPKNHNLL
KDLRPNKPSLQTRSPVFIYSKPAQGPRTRINESGKIIYEELPRNMLGQGCSFVDIINYGLTGSTSG
GLRKPVIQLKQQVGTEWKYSSEWTDNFMNHLMTGETSGLSFSGNTVLVTGAGPGSIASDIVRGLLE
GGAEVFVTTSRTPPASTTKFFNEMYKAHGSKXSKLTVLPFNQASKQDCEALVNHIYETKDGDLDVI
IPFAAIGETGQLDGIDAKSELSHRLMLTNLLRILGHIKQQKQKRSYNTRPTAVILPLSPNHGTFGG
DGLYSESKLGLETLFNRFHSESWSDYLTICGAVIGWTRGTGLMNTNNVLAQSIEAENALTFSPAEM
AFSILGLIHPDIMAAMENEPVLADLSGNLSAVQNFKELISGIRQQLSNRSRLQQALNEEDGRLDAL
CRGQNSQHSSSKKTISLQTRRVNLQVGYPVLSDFQTTTSGLPNLQGMVDLQKTVVVVGFSELGPWG

SSRTRWEMEHGGEFTAGGMLEIAWIMGLVEHYDGQIKGSHYVGWVDSKTKEPVPDIEVASRYGKYV
LEHTGIRQIEPEILDGYNPDEKEFLHEVVMDKDLPTFEASKATAEAFQLQHGDKVSITPIPNSEDY
QVKVKKGARLLIPKAVSFDRKVAGLLPTGWNAATYGIPEDIISQVDPVTLYVLCCVSEAMYSAGIE
DPYEIYSHMHVSELANCIGTGAGGVSALRKMYRERYMEHAANSDVFQETFLNTMGAWVNLLLLSST
GPIKTNSGACATAIESLDSGCESILLGKSKMALVGGTDDFREEASYEFGQMKATVNTDEEVLQGRA
PSEMCRPSTTTRNGFMEAAGCGVQIITTAEMALKMGLPIYAIVAHTQMASDRIGRSIPAPGQGILT
AAREAPDAAESPLLNLEYRRERLNEAIQEIDSWRARQLQLIKTMPEDDIDEMLQLLDRLRDRKLSD
ARNMWGSSFRQQNPQIAPIRAALAVWGLTVDDIGVASLHGTSTKANDKNEANVINQQMIKLGRTAG
NPLLTISQKWLTGHPKGAAGAWMFNGGMQVLQTGIVPGNRNADNIEIALQPFEHLIYPSKTIQTSG
LKAFMLTSFGFGQKGVLAIGISPKFIFATMTEAEYQQYRDTVQSRVQKAELAFIRALMTKSLFKPK
KESPWKDNEQAVLLDPRARAFWDSEMSEYFITA
>g3463
MSPAAIISSDDTVKSVDAKTSTKTAVASISRNFLHVIDERTGQYYQIPVRHNAINASEFKKIKAPD
TEYYADQNENGIRVLDPGFTNTAVVESKVTYVDGKRGKIQYRGYDLADIVANGKKFIDTAHLMVFG
FWPSAEEGAGFQRKLFDAMHIDQCVFDTIHAFPPTASTTLMLTAGLAAVQATQMDKIPAHMAKNLY
LGNPTLVEEQIIRLMGVLPIVSAVAYCHHTGRAFKSPRSDLTYIENFLYMCGHVLEETGLPNPRYV
ENFERLWSLVADHEMTCSTAAFLLTASSLPDPISCIISGIGASYGPLHGGAIEFAYKDMADIGSVA
NCQTKIDRVKSGKERLFGYGHRVYKVTDPRSTHIQAVLDTLKEEIDNDPLLKVAFELNRIAQEDEY
FVSRGLKPNADLFAAFTYGAMGFPPDFILTISTISRTQGLMAHWKEAMSGKPLIWRPTQIYTGKLD
QKMGE
>g3464
MEHFSPSTTPDTIQSSASTPSMTYITPSTDVEDDPFCPRVTFSVVYKNLKYSTSLSSNHTIDLFQH
KDVFLSSLEESSDKPKEALDTIESLLIRFFRFLLDRQVPTTQILPLVVAFRRDFLLSNDIHSLIAS
LQEPIEVQRDLLCTYYTTILRCNLPPDTHIRESALLEAAKKGNVKLMAIFGGQGASNPRCFRDLQD
LYNTYGPLLDDLISTVDNALYELCVVPETRDFFRGREIRLLSWLKNPQRLPDEQFLATAAVSFAIH
GITALAHWCVTCKVLGKHPGALRSLLDGTSGHSQGIIVAVAISASNSWSSFYQNAVTVAKLLFWTG
YESHQAAPRLALTSTIVHDVENRGEGQPSHMLHVSGLSSKHVISLVEKCNQDLPLEKRVYLGLSNS
RSSFTIAGPASSLVRFSGLVHDIGVDRGVDQSRIPFDKRKPEVETQFLPISSPFHTPYLATTAQII
KSRLSDEAFNSEQLAIPVYNTRDGSNMAAVTAGENLLPLLIDAVTIHHVDWPSVLNAASYTHALVF
SKGLSDMVASNTDGKGVQVVSATELTTTNARFGSKSSIFSLSLSESFLYPQSWENQFRPRLSRSSN
GSIQIETKMSKVLCAPPLMVAGMTPTTVPSDFVTAIMNAGYHAELAAGGYFSAETMSKAIETLSTN
VQPGMGITCNLIYASPQSMAWQIALVRRLARNQRPIDGLTIGAGVPSLDVATEYITSLGLRHISFK
PGSEDAIEKVLEIAKAHPDFPIIIQWTGGRGGGHHSFEDFHSPILRLYGKIRRYPNVILVAGSGFG
DATGSYPYLTGSWSVEYGYPAMPFDGILLGSRVMVAKEAHTSQTVKQLICNTPGTHESNWTKSYSG
PIGGIVTVQSEMGQPIHKIATRGVLFWKEMDDKIFSLPRAKQLDAIKVRKDYIIGKLNKDFAKPWF
CRNSNGDNVELSDMTYLEVLSRLISLMYLPTKSRWIDPSYQVLVHDVAVRILERLDADCEIDDSCK
PDQLEKLFLQKCPQSKTSIIHPEDLSWFLERCRRRGQKPVNFVPVLDENLATWMKKDSLWQSENIE
AVIDQDPGRVCVLQGPVAVKFSQRVDEPVADILNGIKNSWIEMLTDGFYRDEDVPLQRPTWKPNFL
PSIQFNIEVIEHDHAVTVQAIRGRALPTRKEFLHYLASKTHGWMRAILLSDLVLQGQAKRSNFLRD
CFQLDEDKIVEIDKINGKMLLKKSIDGSKHQRMLAEISSPDGLSIAAQISHYSPGRDTPTVLHLKF
RYDPQNSLYPLTEDMRDRNMQIKSFYSRLWLXSEIESSSFGGVHSTFGGYRTVLSKEVLSGWISSV
GLSHSNDRLVSPNSELFPLNATIIPAWEALVQPLMVPEIDGDLLRLVHLSIEFELFSGSKPLCVGQ
TVEINSHIKSITIDDKGKTIAVRAVISRDGLPTVAIISKFLIQGSFSNYKDTFQDNTEPDMILSVS
SPIDDTILRTRPWFQLDDETIDLVGKTLCFRLSSFCTWKAQNVFDVLRVTGDVYVQDRRDRLRRIG
SVDFDATNASGNPVVDFLTRKGKPAIERHELEKPGWNGPSTRTIKMPYDNEVYSRVSKDCNPIHVS
PIFSSIASLPGTIVHGMYTSAVTSAILDHVVNDFNHLRTRRYTVSFVDMVLPGEEVELKCSHVAML
QGRLILRIQAFKAGTGDKVLEGEAELEQVPTASSPVAKKIWDDIDQYLLENYGWSILKMVQENPKR
LTIYFSGKRGRAIRDRYLSMTISSIGPDGRIISTPLLKGITPNTRSFTFEDSKGLLYATQFAQPAI
AVLEKATFEDMRANGLIQEGALFAGHSLGEFGALACQVEFLPFQQQIDIAFYRGLTMHNAVKRDST
GATDFSMVAVNPGRVKKPSFDEVALRRLVDIIATESGKLLEIVNFNVEGEQYVCAGHNANLYALAQ
ILNEVSRMPTEQVAIWSRENVSVRGSQQQNETYSPQTAAINTLIKDAIQSAHALPKPIILSRGQAT
IPLQGIDVPFHSAQLRSGVAAWRKFLLSRIQPEDIQPNDLLDRFVPNVMGRPFSLDREYIAEAMRV

MGSETLQAVLNEGVVH
>g3465
MLDLLGLKSLAARLDSIDSTILSLVGQIPTPQRSSSDASNPRLRSESSPSTPEALCNDADDIDLIF
SSALDGQDYWSRHAELHKSGPEQEPQTISGTSIGNRALLEKVRQAVHNLLQGNRSRKPSPDSTSGD
TNNVSTCPMLSNAPPSALSELKTIYDTFPTFDDASAVSLLPGGQFVSLPPQSILDTSLQLFLDKFN
AVTPIFNESRLREAIKEQYSSGPDSKTDEAYNLCFNNIIVLSSGLRARLARLDKSYSKGMNEELLP
AFLANSFRALQHLKFFLQPRHVNLQALATLALVAREYHESCVFDMICHMACDLFKAMDSEQSPMSK
VDHPNFEEWRTLYWTLFVMDKQRTSVNGLPFDLYFYHSDLPLAPEGGTISLGQQSWMMHIYITTIW
EETYISLYSARAARKGSSYRESQIARLDKLAQKWHSRTLQLFPANLTDHDSDSSNVNRLVEDNWQI
ELEYAYHIAQVLIHSRSDTANSKEIELENARAALKIINRVVNQTPTEGSLALLCRILRSYPLIAGH
SICMRFFENPSIHMLEDLDLMEGVRDAYKILVDPNITICSTGKTFMGISWCLEIAKACRRILFEVS
ATPPSSELHHPGLSRKRFSEGLPSSSLPNQSLPKKMKLSLVDSGQADYSNLSDFPVEKDYSTGLQD
RNNSGAISTTEDLNNLGHHPYIINQQPQQQRQQQRLHHQHNEPPGLSSASNIQSGSSSTEELSLVT
DSIPALSLGTAATPNYPITQFPTTTGATIAATTPMMFYTYSDDPPADLCNTNSITNFLDCDNFLQH
EDHDIPNHYMSDDISNWLLQIAQKEVR
>g3466
MSIDLSEAGVMTPFWPGTNLTSEHDGQCYGNVTLCLNNDILCTLSTCDLTLAHFDYIPNLGANAFL
AALFGVCALVQFGLGIRYKTWGFLVAAIFGLALEIVGYAGRIMINQSPFDKNNFLIYLVCLTIAPA
FLSACIYLCLARIIVVYGENLSRFKPRFYTIVFCTCDFISLLLQAIGGAIASIATDYTTTQLGIHI
MIGGLSFQVFSLVLFIVLCGDFAYRVWRSPFQWDTTRKEVFESSIFTFFLGGLALATLTILIRSAF
RVAELSGGFHGSLANNQATFMVLEGAMIAIASLCLTALHPGVAFKGTWADADFNFFHSKKNQYDAE
TETERELETKAPSTPSEAVQHSNEFADRG
>g3467
MAYNVFSSPLSINSVLVILLALAVALASPIHQTIINSPKVFVVGLPQTGTKSVGDALEKFSFRRPE
LKDFYSRYLYYMFSANYTAPLISYAQEYDILEDIPWAIAYKELATAFPDAKFILTLRANESAWLES
IDKHTHRHKWKANKRIFGCKKVRGEECIRKYLDAYRAHVEGVRDFFDKEGNGRLLEVIIDAPMEMR
SIKTGGEDTTVVDDKWLSLIDFLQLEGRVERLGGWGNLGLFPESTASNKAGKTWSKLQHAWDRMLN
ACENGIWKGVKSTVKVVKVFGFLAARGL
>g3468
MLAFTVRLCFSLISLWRQATMPLQQARFHGNDKRQDTGLILDSTYANSISPGSLDNSFSAPSSPRP
MPKYADVGIYSLEDQYLTPAPPNNELIHAIIVPNYHEDLHTLFTTLSVLASHARASSQYEIFLAME
EKENEAVEKAKTLISSFGNSFYAIHATFHPVGLPGEIAGKSSNVAYAAREITNLHRHELSEDNCKV
VITVMDADTHLLQEYFLEVRRLHYEHIDTADRQLYSCPILFDRNSSETPVLVRCADIMWAFAGIST
MYPGSPVSIPTSVYSLPIQLANQVGGWDSDPTAIGEDMHMMLKCYFKTGGSVITRAVHVPASQCNV
SSDCPIKGWRRTLDTCSNRYKQALRHMWGALDTGYALRNSMPFANIKSKRTQLWAFPNMLQPLALV
HLLWEAHFLPCHLTVLLIFSVIYTAFSPPETLHPTLVRAFSLANLIRTLSFIGMNINLTIYDQWHQ
TWLCARRRDMAESNLKDCGFSSRTWWHVAQLKERICFPVSGIIFGALAMGHAVFSHFWTDRLVYRV
SKKPTFANPDHYSLA
>g3469
MKFHQTTALFLSTVFSLLTSTVCQNDESAAVYISSLRTSSGNLTFALSVADPTVSQDMYFHISGPS
SYSYIGIGTGTVMQDALIFVIYPNASGDGITLSPRIATGNKEPVLAPSLDVEVLPGSNTSNGLMTV
NARCKNCLSWSSGALDLTDMAYPWNYALGPNTAQYVRIKSNSLSADLEMHVEYGLFTLDMTRATGG
AGALPTSLTNPIGSNATRPRVLTSNYPTIIHAVLATLPLVLLLPTGVIFLRFFPGSVRWHWVSQTA
SSVISVLGIAVGIYLSTWFNKSKSFGSGHQIIGYIICAGILAQWFLGFWHHRLYKKIQRPTNYGFV
HRNFGHLVFVFAIVNAGIGLSWSQAASPVIIGYSVAAGIVAIVHFSLVAWKRWERRKLDASEPKPE
>g3470
MPYSVAFFGATGGCTNACLASLLLFSEYQAIALARTPQKLLDSLKKQPGITDAILAQRLSIVKGDA
TNVADVKRTLLFQADDNKNSNAEPKLVDAIVSGIGAYPQRKKGTWLTFEMDNRNITEQSTTALVSA
MQEIRAQQQQQEHKPVVTVISTTGLAIPAGSKEDVPFGLQTLYHSLLAEPHKDKKRMEEIISENA
ALFQGVIITRPTLLNGDGIVKVDGVRKTVRAGTEDAPAKGYLISRSDVGGWIWENVLKPTEHGKKW
YGRRVSLAY
>g3471

MASDYLSSDFDPALYLHSELSNTLLRSEIEFTSFSDDIEALQQRRSEIAEENRRNGVVIQSRAWKL
SDVFRSDDDSQPLTPTKSSNLPSLSQGIIIDNYLPSTPPAAMAPFMSTQSTITASPPSADSRKRKL
ESLLSTEDTKRPKTSDGAADDVDNTPTSTSGIRTLGENDRVPRTIGGFIDEEDDDEDALAEFQGGG
YMTYSTQAITTRCETVRPQQNTLSQETRGPFIDEDDDKDEDNLDMLRGLSTNNDAALLQIISQPTS
IEKSQPDTTSIDETLHDPFRSVEESSVNPINLPSDRKGLITIRTCAGRAHRVFSRKATNTTSYERL
VASRSKTAEGKAQRSYYGIEIHRLLDDAACELKNAKQKEASIHIERSIETPIYETGGKKKPTPMWT
EKYRARRFAELIGDERTHRSVLRWLKGWDHIVFPGIAKSKHARNVEEEGEFAHRKILMLTGPPGLG
KTTLAHVCAQQAGYEVLEINASDERSSHVVRGRIRDAVATENVKSISAGADSSKVNRSGRPVCVVV
DEVDGVVGGAGSGGEGGFIKALIDLVMTDQKNAAARPSQNSFSGRKRKGDNFRLLRPIILICNDAY
HSSLRPLRTANVAEVIHVRHVPMENAISRLKLILTKENIQFDADGVRRLCEASWGMSAKKDRTERN
RGVSEGDIRSILVSAEWVARKLRAETAATARLTKTWLEDNILNDAESGSRGLGRGGVREITDRVFI
EGAGFPAAPVGANSFSDPFDQERTKTPVGVAELRRRHASSKLREMIDASGEYDRCATDCFIEYPTR
TYQDDTFLSKPNAAYDWLHFHDMISSKIYSNQDWELNPYMSQSILAFHHLFAGNGSSSNKKKFDDD
KNEEEEHPFSGPRADFAAFEATKQNTSVLVGFQSTFSAPLLRLFRSVDSIATDLVPNLMRMLSPEV
KPVIVRGSEQTSVASVRKESERALVRAAVRVMAGLNVTFEKIRIGNEGAHAGWAYRMEPPIDTLVG
FSKTKGTVLDTGSLAPVRYAIRQVLDQEYRKETMRKQGETLLGISGPGRKSSKNKQLSNGDDKENV
NGSRPKVGPDAPGVKRDFFGRIIRNSETAPTTTTSIKQRQSGHKRNSSGNDHDRKVWVTFHEGFSN
AVRKPITLAELLSEL
>g3472
MEDTHNVRSGSGEEHQPQEVVQPVGETTKPVPEQDASVQNTEAASDNPLDAPASPRPQDNDAEPED
EEMGGTETEAKKETDNDNDIGADDGTEAQGADANADASADDVQEKSALEASARNHLVSQTHAIILP
SYSSWFDMHQIHPIEKKAMQEFFNGRNRSKTPAVYKDYRDFMINTYRLNPIEYLTVTACRRNLAGD
VCAIMRVHSFLEQWGLINYQVDPQTRPSNIGPPFTGHFRVVADTPRGLHSFQPGSNHLVTPGKQLA
ATDRAASGTPIKTDLNLEIRRNIYDDKGKEITPAVEDKEKQTNGDTPAANGTSGDSTNKALEAAIR
EPRKTYNCWSCGIDCTRMRFHYAKSAPVSASNNAPDRKYDICPNCFLEARLPASHNAADFVRLEEA
DYTRNEDKAAGWSDSELILLLEGIETFDDDWQQIADHVGTRTREECVMKFLQLEIEDKYVEEIDGS
QDATVRSLQGRDPISQLDNPVLSVVAFLAQLAEPSVAAAAAGRSVDQIRKELRDQLNKAPSAEKGK
GKEGEVKAEDSMEIDNTREAESTGATEKRESLATIALAASAARAGALVSHEEREMTRLVSAAVNVT
LQKFEIKLAQFNEMEEIIEAERRELELTRQQLFADRLAFKKRVKDVQDAIQQISLQGPGEDTTRAL
HDVADISMANRYQFQAQGDVSNGIQPLSAQPPADYKTLEL
>g3473
MSAAADSEPVADSDEEKIFGPPEYYPVLSSSESWHESEEPSSQSDDEETAEDASEEDQIKSEDEGS
QSESDLKKGKQRPASTKSQLSFDKSAVQAYRTLLKDMHVDADSKTWNKQQNVSLSTVGLVQWTGKE
NDTFFTALARKGRGNVGEIAQQIKSKSRLEVADYIEFLEERLQRHRLSEPSLLEAEVPAAVEISEE
LEEVLNHFAETVALEEQRKDLFDAEKEHGLSEYWILDRAKADQIEELLASDDEEALPEAISLSAGL
LDLKNWVQLSQKVFMNPGKSRPEDSWKNVAQKGEMPSLTADALGEFYDLTVDLVIRLMEEAHKTAL
DRLDKERRHRQVPVVRKKDVQRALTTMGMKHNTFEYYVNLPRRLKLDVTRGKRKGKAKNYVSWDKV
ERMLSIKRQKKAKGSSDSSSDDSSEEDAENDEKADDDSQEEDLDKFGSSEDESDIANQDDQSNPEEE
EEEEEEEEEAEEVSSADEANEANEDNNVPAIKTEPTDDSQYEGISESDYRQLEIEQRLDDYNDXLD
RQASHKEEARIRVLLDAPAIDSLVPIVKKGKEKQEEPPEITEDMRREIMQNNVVVDWRKNLVYQDE
WETYGMKTRDVEKDIAANRNKRRRLE
>g3474
MQRMAFFLRRKSRSLLCSWGRNTPWQQSIPARSPRKLLPTTVLAHASTKAEDINPPKIQLREYQEE
SIQSVLENLEKGHNRLGLSLATGSGKTVIFTQLIERIPPRNGYADQTLIIAHRKELVEQAARHCRL
AYPDKTVEIEMGATKASGAADITVASIRSLASKGRIEKFDPSYFKLVLVDEAHHIVAPQYRQALEY
FGLTEPSDNAPALVGVSATFFRFDGLKLGSAIDHIVYHKDYIDMIGEKWLSDAIFTTVKTNVDLSR
VAKDSSGDFATRALSEAVNTATVNDVTVRSWLAHANDRRSTLVFCVDTEHVRQLTEAFRDNGIDAR
YITANTPRQTRNEELEAFKQGEYPVLLNCGLFTEGTDIPNIDCVVLARPTRSKSLLIQMIGRGLRL
HPQKENCHIIDMVSTLNTGIMSTPTLFGLDPDEALNTRSVQEIRKQKEHKGFDSSLPDAGYVRPLG
DDEVDVTFTTYDSVFDLLNDERVNRHIRSISRYAWVRVNTDRYILADKSGWMTIEKSDQDIDGPWV
VDLVQKYELATRTVHTRPRVIARAPDFEQAVHSADTFASTNFKDFTINSSQSWRRAPASESQLKVL
NSKNILDRTITSGDLTKGQAADMLTKLRFGTKNRFQDEQKKRKKEEKMRKEFEKQMSQGDVRVGPL

SQQDL
>g3475
MSQASLAPPEVWRHLLRTTLRECSYLPDPIARKYMHKYVLERYRKKVSAFAPDEQKDTKPRRTSAK
HQERLNAMSLEKQKKLQKSARSILSLIRRGNQGYQLPLDKILMLAYGRVGAKKYTLLSEIVPNGRG
GNKVFNSSADIEKYLEQKKKESTGDDWQLPEVLHALIKSQRNDSFVRSAHGKRFRAGDTIKIPRLN
SWMKPLPECRRENIRKRILHRSKFAALXPADPDEVNTLRGLIDGTEPWKPPIRRVNVLPAAKKSAL
ESLIHEGPQGGLSFKQAYADGRPHRLTRKFMRSIWKKVYKLMPVQGINLEGRPKFDFPTFKGRRYT
LTMGKEEFQSILNNDEHGHDIASLATNSEHREETTDRGAESSIQ
>g3476
MSNSFNGYERTNGYGAMPPHQEEYKPQIYRAVYSNVSVYEMEVNGVAVMKRRSDSWLNATQILKVA
GVVKAKRTKTLEKEIAAGEHEKVQGGYGKYQGTWVSYQRGVELCREYQVEEALRPLLEYDMDDGNS
RRASLETPTKEQAMAAQRKRLYNAENRGISQPAQATFFQNMSRTAANAVNAISKARFESPNKRRPS
LMRNSSQQMSSQESFNAPGGTQTSLYSVHSDSGFASQNAHGRNDHTLDQENGLEPPRKRVRSSSNQ
ARSFGANSTMSVYELTPTEPNDSFYQQADDSMAVDDINGRDPLPPPAHPDRFQKMKLIMTLFLDKR
MKDFSSHPALVQLSGEDLEIPLDEYQNSALHWAAMLARLPLLLALVEKGVNIYRLNAAGETALQKA
VGTRNNLDYRTFPRLLEILGATVDMVDHSGRSILHHIAIMAATGGGGQVAAKHYLESLLELVVRHG
TSGQENDKPVTNGENEQSQGEIIGLGRFVSELVNLRDDQGDTALNIAGRARSVLVPQLLEVGADPQ
IPNHTGLRPADYGVGVDMVNESSQSQLDEEKKDTFIDQLAKTKKEILDATLAQIGSLVEETLGGID
RDLTSDLSKKQAEFDHWHAKIREIARARQIDQSELESLKRKASDRSSVGRRIKNLEGDAEHLVASL
KGYFGDDFDVSATCPVGYADADSGIDVAEFSALFSNFDPSGEMSADQKKFLTALPSAATLESRLDA
YQSLNKDIHDEIKLLKSRNVVLGENYRRVVMACTGFTAEQVDEAAEGLTRHIKDLNDNPVSEEEAI
EILMKDRGQDW
>g3477
MQNFKAIALFTLFLHGFSVYANPLLDSNLDVPLRLRRLDRPLIQRRTSIDFLSHENASDIKTEIIT
IPAERLLARADSTSVPTFDETAFNQSVTTACANAVNSYAEAINPSGLVACYNIASFDNATGIFQTD
VRLYQKSAATGSFGGLIPSDLSMSFSIAQATLSNPMLVSTNGSITASTAASGQFVMGFQNIGQLST
SLTFSKLSVDDLRILMIPSISLGADSQTGGTVNTSLSSDTLSYVAGELVQANGTPTNITTPQAASL
ASPIVAAASVFVLPGTHIKIAPVGLIITCIWTGLLLLAVGGGTIGRYQFRVHYRQRVQRALVNQSI
PY
>g3478
MALDAPVSSTKPAHFATLYDDATTKGNFDLIRIRTTDQLLHDPLAQTILNITTNSEEALHKGEISY
TDILVDRVRKRVSESAGKEHELLSQILHIGFAALFYFLQSNVTGPPLEFDSATVVFGETIAGNRET
LNSIRRRMLRDLSMDGEAVYPLTPNIELFCIAKAILVEADVLVTGDAPLVAWTGRMRVNFLHQKML
SEISGTLQKSIYDDLDRISTTVLAEGSNSNGKLQTAFLLERAAIHTHHGFDAKARSDLDKAAVASG
FEYALTGKLGKRTKFQDRDITQLVVLAVSSEMDPASDTAASKQVSETSAPKALDLNDDTLLETIAF
AKEGAQPEKSTTVQDESSLPPALASIDPSNQPKLNPLDSAILLGMASAITNNSPEHGLTREETLPY
AVRVIESGSSNWQIYTQALLVRSRCEAFKSRTVERGVLQMQALVDQVIADTATSNAAVVTTHTVTN
DQQEQPTTFLPRPQESESAPAEERLEYLWILDFQTRWNLEAELAQKWVSLGGLRTALDIYERLEMW
AEVALCYAATEREEKAKAIVRRQLYEPTNADAVDDDNERYEGPELAQLPTDAPRLFCILGDIDSEP
TMYERAWEVSGQRYARAQRSLARLYLKAQPPAYEKAEEAYKKSLKINPQNHSAWFALGCVQLNREQ
WFEAVESFTRTVQIEQDDAEAWSNLAAALLNIPSNKNKPSVDANADTTLSEENKDIEDADGERYEE
DPYKHTRDALTALHHAARLKQTDHRIWDNIITVGTSLPPQSIPYKDIVFAQRKVIELRSPKKGEKA
IDLTVLDMLVEHLIDNYDYDNLLINVDGTDQKIVRSGTVAGQIMTLVDDVVVPLITHSAPLWLLVA
KIELWRHRPSKAFAAHEKAWRATVSTCTQGAFQMGDEKRWNQIVKATERLVRDGYAKIGGMTKERA
DGSIPEGEEGGEEELVAKDWRFKSKSAVRGILGKGKDWEGTEGYTQLKELLTEVDG
>g3479
MSARYSDHSTPICRRCKSKGIANKCYYHPAPLTRPKNPNDRTNPLSSTRRNKTKVHVRPSRAEETI
AARTLTDIRKRANSFTGSNTKESNHSNYLGTTSYLSVFKETASWTPHASWNCSLHAEFEHWRRDHA
YTSARLVRLLCAVGFYRKQIKWFYARGRFTKIPGPLVLDPLRRVQEYIEKNAWIQERNWERIHNQI
TAATSHSLRLTSETSPDEFYSLFTGENLRWEFVGLIFALAGNSVRCRYDESYIVDLGNGEEMDAYT
FSKEMVLASNACIEICRQYEHVNDLLIWMYKSHLYLGSEVLGETSERLYSMFGEMVSGIYAMGLHR
EHHSINVPFFLSETRKRLLAEIHKSDKTVATLYGRPPRLPYHYCDVALPLDLSDDQLFLDEQSLEV

VLNSLDSEGWNLQGQFYPATVIRMRQIMSALGEKMLELSLGSRTTSYHNDLIKTYKLCQRTWDQIP
VRFRYSADCWEKGDVVEGLAKTIVYLEYLLSVFQIQRIRRQEDPEATKDLIDTSVQLLSAIVGMTK
HYQHGDVQRNFGWIFGAFGSAAAGVLVTEVRRYTISNLPLPSSTPRSEIIRTLSYLISWFQSVKMP
PDLAGTVCTEFIKVISRLLDEALDYQQPPPSSVARPITPNPHEFPEEADIPASQQQQQIQGLMLDG
GPMESLWFGMGMEYGVGDDAFHGLGRGLVSDGMNWLDELGLNTDLDDGGIGS
>g3480
MRGVQIFSGTSHPTLTESICERLGAVPAPAELRKFSNGETLVNIGASVRNQDVYIVQSGSSKINDS
LMELLIMISACKGGSAKTITAVMPQSKKKSHRGAITARMIANLLTIAGVDHVITLDLHSALQASQM
QGFFAKPVDNLFAEPLIARWIRMNVPSWRDAVVVSKNAGGTKRVTSLADTLKLNFGIVTTDRRRPK
IQMNMTDSMVFFDTIEKGNHSAGRAINFLNANPDSAVDDYFLSRSADTLRPETPPSARRPSELEAA
EYTTDERAREVITGRIVQGHLVDDDFVDGSQVATPLPPTGSESGDSTNNDHMTASFMSVASSHEDH
ALGGTYDAGESDDDDPPALAANNDEKTITLVGNVKDKAVFIIDDMIDRSGSWITAAETVVRRGGAK
TVYCIATHGLFGDNCLEEMEQSDAIDHIVVTNSFPIPSEKMGLSKKLTVIDVSALLSESIRRHHYG
ESISALFQLTD
>g3481
MSQKFPEVQGGGSLIVAWQIRNKHVLVVGGGEVASGRILHALNADAKVTVVCPNSGLNEEVAYRVA
QNQVSHIDRNFEPHDLDNADMVLCAIDDPEASTQVWKLCKERRIPANIADVPPECDFYFGSMHRDG
PLQIMVSTNGNGPKLANIVRQKIASTLPQNTGAAIENVGLLRKKLREVAPDTQEGPKRMKWRERYP
VLRTFAEHQDDTLYLLFWYYVLGFYQLVGRLVIGFRASQLTAIFPVYASFVAPPSIRGSFGGLQML
E
>g3482
MADTEHEVAEVTTQADANAPNDEIVSPQPLRADKAQLPSGPEWKEIGGMEGTGSHESEGIESVPED
APAEEAATSETKNIGEGEIDTVDQDDSSQHEEPQSITEVPFTPQVPTAEAVPEPRARSDSRSTTAT
QATQATHRSTPVSSTVFVVTALEAIAASKDAKRNKELEDAVQKALVNIKQSDTKPLDPEVIFLPLQ
LATKTFSIPLQVTALDCIGKLITYSYFAFPSSRPSQDGTDPAIKELPLIERAIETICDCFENEATP
VEIQQQIIKSLLAAVLNDKIVVHGAGLLKAVRQIYNIFIYSKSSQNQQIAQGSLTQMLGTVFDRVR
MRLDLKEVRLREPEDQHEDPVESTIVDAGEGSQLSESLEAESTVSDQPTQKEPTQKLTLQSFESSK
DDTMVTDNAPTMVTRARPTHRSTRSVSNVTDEKEDEGSEDEVDEIYVKDAFLVFRAMCKLSHKALT
HDQQQDIKSQNMRSKLLSLHLIHHLINNHIAVFTSPLATIRNSASSADSMTLLHAIRPHLCLSLSR
NGSSSVPKVFEVCCEIFWLMLKYMRVMLKKELEVFLKEIYLAILERRNSPAFQKQYFMEILERLSG
DSRALVEIYLNYDCDRTALENIFQNLIEQISRFVIMPVPVMGQHTFQETRVKSSNTPDWHQRGTLP
PNLTTASLTNSVAPSVQNGSSELSMKYHALDCLVEILRSLDSWSSQRLPSSTNGTHDDMSRKSVEN
YRESIDAPSFAAALPSPYIDSGGTGTGRSTPAVEDDPNEIEKARQRKAALSHAIQQFNFKPKRGIK
LLLKDGFIRSDSPKDIANFLLRNDRLDKAGIGEYLGEGDPENVAIMHAFVDSMDFAKRRFVDALRQ
FLQSFRLPGEAQKIDRFMLKFAERYLAGNPNSFANADTAYVLAYSVIMLNTDQHSSKLKGPRMTKE
DFIKNNRGINDNADLPDEYLNSIFDEIAKNEIVLDSEREHAANQGIPTATPAGFASRAGQVFATVG
RDIQGEKYAQASEEMANKTEQLYRSLIKAQRRTAVKDELSRFIPATSVRHVGPMFNVTWTSFLSGL
SAPLQETQDIEKIRLCMEGLKLAIRISCSFDLETPRVAFVTALAKFTNLGNLREMSSKNLEALKIL
LEVAVTEGNHLRDSWREILTCVSQLDRFQLLSDGVDEGTLPDVSRTRIVPSNSTDASRRSTQSTRR
RQRSTASTLSFRPEIALESRSAEMVHAVDRIFSNTANLSQEAIVDFVQALSEVSLQEIQSSGQSES
PRTYSLQKVVEISYYNMTRVRIEWSRIWEILGQHFNEVGCQSNTNVVFFALDSLRQLSMRFMEIEE
LPGFKFQKDFLKPFEHIMANSNTVTVKDMVLRCLIQMIQARGHNIRSGWKTMFGVFTVAAREPYEG
IVNMAFEHVTQIYNTRFGVVISQGAFADLIVCLTEFSKNIKFQKKSLQAIETLKAAVTKMLKTPEC
PLSRKHXPATEATDVTGSILKHQLNRQTQEEQFWYPVLIAFQDVLMTGDDLEVRSRALNYLFETLI
RYGGDFPQEFWDVLWRQLLYPIFVVLQSKSEMSKVPNHEELSVWLSTTMIQALRHMITLFTHYFDA
LQSMLDRFLGLLTLCICQENDTIARIGSNCLQQLILQNVTKFSDEHWGKIVGAFVELFDKTTAYEL
FTAASPLPPRTPTSEVPKSNGDATSNGAVSVSETSVVASDDEHAINGAQTPIAENGDAGETSNGAL
HDTAAAAELEDYRPQAQELTQQPAAVTVARRRFFNRIITNCVLQLLMIETVHELFSNDNVYDEIPS
PELLRLMGLLKKSYQFAKKFNEDKDLRMQLWRQGFMKQPPNLLKQESGSAATYVNILFRMYSDERE
ERKSSRAETETALVPLCADIIRSYVRLDEETQHRNIVAWRPVVVDVMEGYVNFPQDNFTQYIDTFY
PLAIDLLARDLASSEIRHALQSVLRRVGEVKLGLTFPHPAASAEPSSSANSLRTHSRGH
>g3483

MRTPNAMLSRGAALMKQRPQTVYRLTDAARPAVSALARYYASKSFPPHTIISMPALSPTMTAGNIG
SWQKKAGDTLAPGDVLVEIETDKAQMDFEFQEEGVLAKVLKDSGEKDVAVGSPIAVLVEEGADVSA
FESFSLEDAGGDKXPAAPKAEETKEEAPKPAPETQDKPEAVEPEVTGERLQPALDREPQISPAAKV
LALEKGIPIKALKGTGRGGQITKEDVEKYKPAATAAAAGPSFEDIPLTSMRKTIAARLQKSTQENP
HYFVSATLSVSKLLKLRQALNASADGKYKLSVNDFLIKACSIALRKVPAVNSSWTEENGQTIIRQY
NNVDISVAVATPSGLITPIVKNAHNLGLSSISNTVKDLGKRARENKLKPEEYQGGTFTISNLGMNS
AVERFTAVINPPQAAILAVGTTRKVAVPVETEEGTSIEWDDQIIVTGSFDHRVVDGAVGGEWIKEL
KKVVENPLELML
>g3484
MEQPSPSHSDATDDEENIIKWKQTSPKIIQDLYHSLLHEQRSTIQPFHLWLRLLEHNLMPNARDLT
VHYHTVLSLQPEYDLVRIWLNANPDKGLNRKMRVQILAFSAATASAAPISASGNEKKEIPWNLLLR
DLRHQHDIMRDKNLLLGDGDYDCFIAYEQYARLYSVQSHADPECPIIKECWLNFGSEMAQQFGLQP
LWKDSNDKGPVWEIKTHFGVICQLLRAFAWNVNIPYVYG
>g3485
MDALREKLHHTKLHDAKAKASHLKHKIGKLENLFNPNHRHDEAHEQEVDRQRTAIAESHRFESFAP
EHDGNKVKWYVDASDYMWAVSVALDRAKETIYIADWWLSPELFLRRPPNRNQEWRLDQVLKRRAEA
GVQIYVIVYKEVSQALTCNSAHTKHALRNLCPEGSKGAGNIHVLRHPDHNMFENLGDMTFYWAHHE
KFVVVDYELAFIGGIDLCFGRWDNHQHPLADVHPAGIENEIFPGQDFNNNRIMDFQSVEDWQTNEL
NKTDYGRMPWHDVAMGLMGPCVYDIAEHFVLRWNCVKRDKYKRDEKVDYLLIEGRTGEDEGLIAVQ
RPKFPCGEYIQHPLTPLSTKPHVEQGTVHAQINAYSEIISKAEHYVYIENQFFVTATGDQQHPVQN
TIGSAIVEAVVRAGKEGRKFRVMIVIPCIPGFAGDLRENAAAGTRAIMDYQYKSILRGENSIFEQV
RAQGVDPTEHIFFFNLRSYDRINKTKELVEQEEKSGVHYQDVQRANAEQIMGESVHPEVGEEGDIH
EHNHHISKKEQESKIDSVRRFEEQREETYDGDREIKSSDSIAQNAMLNQRKVSEEYWGDEDPEKEK
ENFVQEELYVHGKVCIVDDRIVICGSANINDRSQLGDRDSELAIVLEDTDIIDSTMNGQPYRASRL
AATLRRHLWREHLGLLLPQELDSSNNPNAQPPNDCPNSNGEGPEYEFVTDPLGEELWETWTSQATT
NTEVFRFLFRADPDNNIKTFEEYDHFAPRKSIKQGHLHDPFLPVSIVRENLDKIKGHLVWMPLDFL
KDAEMAEPGLSVNQITVSIYT
>g3486
MDSSRNIYKREIDFRALALTSPEFAKRLKSNDQLDFSDPDSIRQLTKSLLERDFKLVVDLPNDRLC
PPIPNRFNYILWLQDLLDTSSRTGTDQYDPNRQVLGLDIGTGCCAIYPLLGCSSRPRWRFIATDID
SKNISSSRKSVLDNELDDRVMIMETKPSDPLIPFDKLDVDRLDFVMCNPPFYESEDELVSSADAKS
RPPFSACTGAAVEMITPGGEVAFIESLIKQSLVLKTQVLWYTSMFGKLSSVSVILQKLLDIGIHNW
AVTEFVQGRGTRRWAVGWSFSDWRPRSDVSRGIASLPKHFLPFPSEYDFEIPGRRSGSLEHNIGKI
NTELSPLRYFHYTWYTKQSCIGYAREDVWSRKARRKFAREQHDGDSISHYEKALDVEMAALGFRID
LSLQADHSVDVVVRWIKGSDQHEWIPGDDVNGIKLGLGQPRAATGKEPAFVGWQVAGALYINLLNP
PSKCLMLKLLPRAPLNHLKRREYSQNYASSIPRTSANSSSRAPVPREIEKTESVSTASLIDVDAPS
VQSVHSDFLDQEVKTTTQAERIEREAEEAALAREKAAAAAASKKSKKSKAQGLSKNAQNPVYLGNA
FIITTLSAVLGYGAYRKHAEGKLSWEVAGVWAGAVGLFAGADYFNSLFNSALKQSTAIRHDLDTFA
ESPATTSPALQGQISASLASFSRTIDDYAALTKSELIPEKKEKAVEREKSFRASLLDYRQQFERLR
KEREETVSVTNRSELFGRRPHHAATPENPYADHALPRSSAFGNASSSSNSAGGLSFGAGPGTYDRE
THALREQSFFAQTNTQLDEFLDRGRAVLQDLGQQRDILKNTQRRLYSVGNTLGISGDTIRRVERRA
KEDKWIFWGGVVVFFLFCWLVIHFLKS
>g3487
MFAARQSFGVFQKRAFSASARQASKVTVLGAAGGIGQPLSLLMKLNPRVSQLALYDIRGGPGVAAD
LSHINTNSTVTGYDPTPSGLKEALSDAEIVLIPAGVPRKPGMTRDDLFNTNASIVRDLAKAAADAA
PNAKILVISNPVNSTVPIVAEVFKSKGVYNPKRLFGVTTLDVVRASRFISQVKTTDPANEEVPVVG
GHSGVTIVPLLSQSNHADIEGETRDALVNRIQFGGDEVVKAKDGAGSATLSMAFAGARFAESLLKA
AQGVKGVVEPTFVDSPLYKDQGIEFFASRVELGPDGVKEILPVGKVNAYEEKLLEACLADLKKNIK
KGVDFVAQNP
>g3488
MQSPDRTASTSSTVPSPFFTPTSETSSQQRDEYNNQNRLNQSNMPENPVTDHQEEQEQPQQHQAEP
QEENTPSETTTSSTVAVASPGTSTTGKSLEPAATTSKEEVAKVSEAKASKKSKCKSHKHHSATKKA

PSKKNKKKKHFKVESSSEEEETSSSESETSDSTESEESESEDSDSEYSETESEPERHRRRRNKAKK
AKKLKKKSRRHRRGEESSDSDSTSSSDEDALADQKALKEKIARMAKKQRKAKKMAKRYLSDEDDEE
DVPEPDPVRLRRQLANLRLLQGGNRRRLKIKESKGEKGKRGKSKRASKVAFKRVDELWDSTIHNFK
LRETLDDPDADEFDQYIFNVRRKFDWENKYVDTWVDVKSKLLREALATVMDGVKGVSLVAETPVID
PNMLFLYLEETRDHMKALKKVVKTEKKKKARKMAALKVAHLKVLIKYLDTDYAETKKTLYPLLESN
MITFDLLWALFKPNTIAYTTTYGHTDEPRAFKIEYATKESSFMKGSWYMVEGKYLEYDGKTFGMGS
MVADVESFKGARKITSLACYPLKYHRESEALKTKLIERGKHFVALRGMNYRFHKGMAFFKKKRSVI
KVNINGRVMIDPALHRRINPNYPISTVRPKDPDVLESESGEDSDECGCGGSSSGADHCGDLSHDSE
PPRPVYKIMRDKKGKAHVVQVEYDENGNEIGKKEDLELLDADNDEKNGESSPEFTEEELLIASPVV
LGFAFSEKLWLEFSVSGVNEIEWNDGAFDSLVLPDNQKSIVKALVESHTFNAAQNIDDVIQGKGKG
LVAVLHGPPGTGKTLTAEGIAELLKRPLYMVSAGELGTDSRSLEGELNKILDIAHSWGAVLLLDEA
DVFLEKRTIQDIHRNALVSIFLRLLEYFQGILFLTTNRVETFDDAFQSRIHIALRYGDLTTKAKRS
VWKMFLEKVKLIDGVETETFTDKDFDILARHNLNGRQIKNSVRTAQALAVNEKLPLSMSHIKRVLD
VAETFDHDLKGGTGYMDAMRSYT
>g3489
MPESRGSYARLMAMCAVSALRIKNGAVLNERSVPDDLNPRLYFDETLQAIPHNVNEFKEFESLQAT
GLACLTALHYSDGPLLHQILGLYHAVVAEHGFSDEKRWPHGLSEIEIEERRRLFWHMYRLEVHTSL
VIGHVVRCPELQSSVAYPTIQDIDFTKSEDYNVHQSEWFSGWNFVTDLYRGIEHVVAQFKYRRASA
NIDRRSLSTAFILDYDPQKKILDPLAAARDDLPDRFKKALPVSSDTCRNRCGYQTANIACTYQLLR
MVTFSAYHTTTLYEACQTVLELIDEISNIPIEYLRAMGLAMLQELSGFGHILSSFINEGLSRSDYY
HLRTVMLCMLELLESLSSCMATALEASQRLRAYVENIDQLLDIPQTPDRQTPDEEDGQSQDDARAL
DILTQQLMVPLDILQRIPWPAAWEESVGLF
>g3490
MSTTVTSTDNTPKTIRLTNGKDFTFVSNAATTQETIPIIDVSRMHSPNLADRQALAEEIREAAHII
GFFCITNHGIDMNLASNAMEQARKFFALPEAKKMEVSSELIPDEYCGYHGMQRYNPNGWKYRDLYE
AFNWNYNPAKDPDYPDSSTPQINLWPKDMPAFEDKLSAYQTEMIRFARRLTRIFALALHVSEDFYD
ENVKQPEAGLRILHYPRQEACRDEQNGIGAHTDVEFFTVITSDAEGLEVLSKSGHWIKVKPIPGCF
VVNIADCFMRQTNDFFVSTVHRVINESGRERYSLPFFWGIDRKTLLSPVPTCVSDENPNKYPVMTA
GDYYLWRTRKQKRSWKVGENASETVKE
>g3491
MHALQKFIKFIEVKDGRQDDNEGQQGLKSLNADTRPLEPARRTFGPWEFVTLWVITGSFNIGGWTM
GSSLIAYGLNVWQAMLTVIIGNIIVGFLCVMTGAPGGKWHIGFPIIQRASWGVNGFVFVVIQRVFL
ACIWFSTQVFWGGQCVKIVLTAIWPSFARVDKPLANGTMTTGDLTSFIIFTVLYYPFIWIKPEKYK
LPFLISCVTVIPTIIVTWIWFMAKAQGAGTLVTDVSSVSGVTQATGSHLGWMMVLGIFTNINSMSV
HVYVQSDYTRYARKPKDQILAQLVMVPMGTIVVALIGIICTSCAAQIFPEQEGSLLWAPYDLYAAL
QAHYGNSSHSRAAVAFGGLSFMIAQFGMVVANNGVAAGLDLSGLFPRYFTIKRGMILMSILSFIVQ
PWTLLNGATKFLTVLGGYGVFIGPMTGVMFSDYFLVRRQILKLSDLYNYNPNGNGIYWYYKGYNWR
ALLAWMMGVWITLPGFVRMVQDGSANPWAGWSNLYDLSYLLGCLLSCGTYYVAHRISPIQGLGVQD
DEDYFGTFRRASVIDGVDYVTGSSLLEIDEKAKSAV
>g3492
MTALSASQIAAQAAMQSTQQHHVRHRSQTVPIEEGLKSRKGSRDSPPPAHLVAHTTTSTSAAGAGA
GGAGFYSNTYQNGLMGSRSAAATTAANIAFPRQQAGTEVADPKQKADKTKRTKFFPKPKHIGISSS
RDWIGSSKDKAQSSPNKLGFGSSSGLSRMVSASTTNLTEVSSNNSSMYSLSNASASTVVPLEKGEK
EKEKDKEKNHKHHFLSRQKMKLKEDLHNLHLSSANSNSRPLDPNAPQSLYNFTPSSPGPSSTSFPK
SGLDLLHGGRALREKRREEKAMVFDANRDSADSDWPLGPSSNVSGTATPYGNPSLGNLTSVISNDS
VKETLQGFGLHNMTAEDVWDFLKAKLLVIFDGGDVRIAVEDLNKLVVIHIQLCVQKHMPTIVEDLR
ELLHTGFSTLNHSISGIPDEKLVPHLMQIWLLAFGTILPFIQAVFLPLDLEFKGRGSVMSAREARE
FWGALPTNGDSEESSLGDELDVRMIVLTAFRDTVILQRYEGLNAVFSRLSLDTINATIGALSSTSN
SSSSGGRPGTAASLDPTYSSFNSQQSSLLNIAGSYSSDSASANRSRATSNTSSNPDQLIFQSFTSP
SQRQLQTLRNNQNADSSHLITDTVGRMLQCFSVLASIQSGDQAQERVEALTKTLKHNWLGRGRTGR
DRRGFVGTKVKPVMLPRQNSDDSSSDRVTMTNGSSNGNAASRDSLLAGGHRNMGMSTAG
>g3493

MTSKPAISALYSSPQPPHTSYTFRHEIAAPFPAVDKAQESVATKKAYLSELRGLVILVQAEVNVFL
TERMEEDKQRAGASKDAEQEAKEEENYGEENVEDDA
>g3494
MLSAFKPRPLVELKQRDKSKIESILAYGDRLLVGLNNGSLRVYRVNEAVTESAAENGKGNGHALEN
AAKDETQQQQQESSTVKSVDLLREKEKFTRYKAEQLAFIKEANILVVLSGSYVSLHDLQSYELQEQ
LAKTKGASTFAVTSNVINDIETDLPSIVSRLAVAVKRKILLWTWKDMELVPEITEITLVSGIKTLT
WVSSTKLIAGLGSNYVLVNIETKELNDIVGPGSIGGGPGQESSRLGGVGVASMSYIGMGGMVPKPL
ATRLSEGQVLLAKDINTHFIDIDGKSLGKRQIPWSTAPEALGYSYPYLLALQEPSKGTLEVRNPDT
LSLLQSISLPSASLLHMPHPTISLAHAGKGFLVASDRVIWRMEALSYDDQIDGLVENGYLDEAISL
LNMLEDALLKDKAGRLREAQLQKAQKLFDLRKYRDALDLFAEVSAPPEVVIRLYPKVIAGELSTVE
EDSDRTDEEEPSTPKGQNEVQAGTDGPSIVDASPAKASIYAPSLTSFLRTREEGSDDGSVRGKSSE
ILEPDKKLEGKDLKNAVRELQGYLADVRRRFQRFINPDGTLRTESLHQDSANNEFLQSVRMLLGLS
QNAEDIEFGDRLRENAKLVDTTLFRAHMYATPSLAGSLFRIANFCDPDVVMEKLEEHGRDTELIDF
YYGKKMHRRALELLFKFGQAEADVEKEEEENPILAQLRGPKRTVAYLQHLSPEYIDLILEFAEWPL
KEDSELGMDIFLADTENAETLPRHQVEEFLEKIDIALAIRYLEHVIDELNDLSPDLHQRLLHLYLE
RLKAYEKTGEEDEETYSSWQTKFLEFLKSSTQYSPAKMLNLLPRDDPNFYEARAIVFSKMGQHRQA
LEIYVFKLQEHEKAEEYCNHIHITEETSTAEVAPSQRLAPTDADNTKPTIYLTLLSLYLSPPHDYK
PQYGPALDILAKHGARLPANSTLDLIPEDIAVRELEFYFRNRIRAASSIVNEARIVANLQKVQNVK
MQAQLLVGEGLSNDNKARSRHVTITDDRACGICYKRLGGSVISVFPDNTVVHLGCANRMTSNQVAP
>g3495
MSSGESGRTESASKTFYASSDDTKICVVMVGLPARGKSLIAGKALRYLAWVGISARIFNVGTYRRK
MSPHPLANFFDPHNSEGEKMRWAAAEAAVSDMLKWFKESNGVVGIFDATNSTKQRRAWIHKTCTEA
GIETLFVESSCDDEDIIMNNILEVKTTSPDYKGQDPEIAAQDFRNRIRNYEKVYETIDDNEKAYTY
VKLINVGSTVIINQIKDYLSSRLVYYIQNLHIKPRSIWLSRHGESEYNLTGKIGGDSDISTRGEAY
ARALPGLLKQSGVPPGTKLTIWTSTLKRTIQTARHLAAETGYDKLEWKALDELDSGVCDGLTYEEI
AEKYPEDFAARDEDKYNYRYRGGESYRDVVIRLEPIIMELERSENIMIVTHQAVLRCIYSYFHNMS
QEQSPWMEVPLHTLIKLTPRAYGTEEQRFKANIPAVSTWRGKGSSAKHQEPAEGSQPSH
>g3496
MADTEQTNEQQPWTPTKANGVRTNGRAFNSSNWRVKSDDSPSTNSPRFNNQSGNNSPRSPFNKSWQ
PVPQSITEGRRLYVGNMPYTAKREDVETIFEGGEYSIERIDISIDPFTGRNPSYCFVELASKEQAE
RAMVELDGKDLQGRPIRIKPGVAKSAQDRSTTARSPQSSPSRLNDNAGSPSTLVDRWQRKDTAFSN
NKPNDIENSRRLYVGGLPKIMDKQALDADVQSFFKGYNLEAISKLITPHPSKRFEPGDHYYLFVDF
ATIEEAAAAQTALDGQDGPWGGKLRLXRARGESTKMISDRQKLASQTDDLEDAAISAA
>g3497
MPSTFGWDHFPLPAADKRHHHTHTTNTAATTTDFVAQQDTLHTITRTRTNLTAAERARRNLNTKLA
NPLAGFTHDELKKQGLRFSITHQVGDEEDIRAFEFGAVLAQAPEKFEEIEGLTEEELQVLRQEFTN
RWSQPVLMYVVIGICSICAAVQGMDETVVNGAQVLYKSQFGIDGNDPRSTWLLGLLNSAPYLCCAI
AGCWLTIPFNHWFGRRGTIFITCVISSLACIWQGFVNTWWHMFVARFVLGFGIGPKSATVPIYAAE
TSPPAIRGALVMQWQMWTAFGIMFGYAADLAFFKVPDPPHICGFNWRLMMGSACIPAILVCLLVFV
CPESPRWYMSKGRYDKAYQVMCRLRYTKLQAARDVYYIYTLLEAETSMRLGQNKVLELITVPRNRR
ALLASEIVMFLQQFCGVNVIAYYSSQIFLEANFSPISAFSASLGWGVVNWLFAIPAVYTIDTFGRR
NLLLSTFPMMAISMFFTGFSFYIPETHHTARIGCIALGIFLFGVVYSPGEGPVPFTYSAEAYPLYV
RSYGMSLATATTWFFNFILAMTWPSLSSRFTTAGGFAWYGAWNVVGWWLILMFMPETKGKTLEELD
QVFSVPTRFHAEWGLRQIPYVIRRYLFRQKHLKPEVLYEMEDVNDGGGGSGGGPRGEEDGYRELGL
I
>g3498
MWKASDKLMATIKHYASFPATGVSLRQMVQFGETPSPGTLFRASQFLSEELPIRLAHRVEELNHLP
DGLNEMPSIKKVKDWYAQSFEEIINVPKPSLSEEVKAHLIRPMRNHGKEIKPLSETTWNPSIREGQ
YRSSPDSAIALNGKENHDAQHHKGTNGNGKGKLILSRRYFXPADDYADWPPEIDAYNQRFGKTLQQ
IKRRHDGVVTTVAQGILEYKRKRQRLQIDSRIQAFLDRFYMSRIGIRMLIGQHIALTEQTHAHHPN
YVGIICTKTNIREVAMEAIENARFVCEDHYGLFEAPKVQLICKPDLNFMYVPGHLSHMLFETLKNS
LRAVVEAHGAERDDFPVTKVIVAEGREDITIKISDEGGGIPRSSIPLVWTYMYTTVDQTPSLDPDF

DKSDFKAPMAGFGYGLPISRLYARYFGGDLKLISMEGTRSTPHIRLNSGRHEPFVDMEDDGRERLK
HTLSARSMRRSPVPAIIRHSAAPTTAASTLVEGAGGGALSDGGLISPPLISPTESSELLFPPPRKV
RRSRKSPDRSKSPSVMSYRRTSWDDSHSDLSRDSRGFPFDPFADSRAPSHASSDDENVNTQTVSEK
YNIMPTEGLLLFPEDVEKDDYLHNPDPADRERDCDICNTRGLVNVGGLALLVTGIVALFIIYPVTT
FVDGVIQGARDPCTLDXMCISMTRPLLKNIRTGLIDPTTPKSAMTKKAADGTEWQLVFSDEFNTPG
RTFYEGDDPFFQGVDLWYGVTQDLEWYDPDAISTSDGVLEIQFDSYDNHNLGFRSGMLQSWNQMCF
SGGRLEASISLPGRGDTSGFWPGFWAMGNLGRAGYAATTDGMWPYSYDDVCDVGITANQSSTDGLS
FLPGMRLPACTCPGTDHPSPGRSRGAPEIDVIEASVTVLDEMQDRIGVVSQSLQLAPYDIWYLPDY
DFTAVYNPSVTTINTYRGGPFQQVASGLTNLNNNWYDGKEYQIYAFEYAPGSKGNVTWFVGSEKTW
TIDAQAVRPNGNIGQRVIPMEPMYPILNFGISPSFAMLNWTGLQETWPATMRVDYIRIYQDPNEIS
ITCDPAGYETTEYIKQHANAYHNSNLTLWSQTGYDWPKNSFVDGC
>g3499
MTAPIKFYYSPGSCSFAVHILLFEANVPFEPARIEMGNFPQEFLALNPKGRVPVIILKDEYNNDQV
ITEAPAILTAVSLQVPERKFLGSNNLETVRVYEWMCWIAGVLHGQGFGGFWRPERYINDPTDEDAL
RLRKKGLETVLSSYDTIESKLNASSAISHSPFAVGSAMTVVDPYLAVFYRWGFRNLELDMTQYPKY
TALWKELEKLDSFQQALERQTGSW
>g3500
MYFSKFQFLTLLYATTPVLAKDVIPPSQRRNIVQSFNPHRNASSATTPLQVGNGNFAFGADVTGLQ
TFAHFATQSTTAWHNFSLPVTPGQTAPDDFTGLDWWTHGRLVNYDMPNPAENNISNWLIQNPQRLS
MANIGLYFDGENVTEASLLDKSQDLDLWEGTITSSFQYNGSNVSVQVWCHPSEPVLGIEINSGLLT
AGDLGVVFDFPYPDVNKFDAPYVGVWDTKATTNNTVNVQSTSSTASFKHTIDNNTVRLDAQWSTKK
GSITGHLAGTNKFVLTSSETETLHLVVSFPADPNTSKKSLPSYNAIASASKAWWYNYWTSGAFIDL
SATDNANATELQRRIVLSQYLVAVNEASDNPPQESGLVNNGWYGKFHLEMFLWHSLHFARWGHYDL
LSRNAPGTYQRFLQSSMDRATAQGYAGARWGKMTDPTGRSAPGEINALLIWQQPHVMHFAEYFYRA
FPHKKTLQEWDEVVTATADFMASYAWWNTTTKVYDLGPPMYPVSENTSPNVTINPTFELAYWRFGL
DVAIQWKERQSLPVPKDWTLVRDFLAPLPIIDDAYAVYEGIPNMWQNGTTTVQDHPAMTGIYGLLP
PPSSGSPLNLTVLQHTADLINELWDFADVYGWDFSMVAMNSLRLGNAEQAVAYLLDEYYVFDDAGY
PEGGNRVATPYFPDAGGLLLATAMMAGGWDGDEGPHFPEGWDVKVEGFTPSL
>g3501
MNQITISSDSDSDASVFATSTTTNRHPHPQSSHHHHHHYTQHPTLPINTANNNNNNPTSDKPDKRK
TTTHHTHETMHLSHTILSSKELLLLHSLAANESVPRTRRRLWAQVIEADDPVKAEKLLYDGAPSLG
PSGLSGAGGGGGGGGEGGTTVVGGLRVAAGNVVDVVEMGDGDGWERLRVKKSASRSPIVNKTSSSP
GGSSKKGLQSRRVVSGERSGSPLASPVVRRGGRRIEDDGDVDMDIS
>g3502
MAHPQPSPPGGVPPHHGPLAAHPQLNGHIPVQSAGQKGPPLTTAQKIAALNEQVWLQIGSLTELMG
DLDGGMAAYEQALRHNQWSIPAMTAISQIMRTREQFPKAIEYLQQVLKLDPQSGETWGSLGHCHLM
MDNLQEAYTSYQQALYHLRDPKEPKLWYGIGILYDRYGSLDHAEEAFSQVMRMAPDFEKANEIYFR
LGIIYKQQQKFNQSLECFKYIVNDPPRPLTEEDIYFQIGHVHEQQKDFDAAQAAYRRVLDKDPNHA
KVLQQLGWLYHQQSTSYASQEKAIEYLEKSVSADNNDAQSWYLLGRCYMSQQKYPKAYEAYQQAVY
RDGRNPTFWCSIGVLYYQINQYRDALDAYSRAIRLNPYISEVWYDLGTLYESCNNQTADALDAYGR
AADLDPTNVHIKARLQLLQGQAQGQTQGSAPAPVPQDIHPQAYQGPGVGAPPAPQWGAPAPTGGPP
PQAPAPPRQIADWNRGINDIQNAPQHPPQPSAGYEPREGLRGPAPGQHPSPRQDLRSAFPEPRGPA
PRSPAPNAYPQSHTLPQLAAPAPGPHDRPPNGAGFRGGPLGPNGAPAPGAAPPPGYPARPFSPPTE
IRPLREISDRPSSPGPTYPHQQFHHGPSIQGGAGIAGGAPPPASAMTAADAAARDRVDDRPTSAMK
RGRDWDSVEGPNKKIANEESRLRQDEIGRRATPPLELHRRSSSEARREEQRRANENYHPSEAAHHP
PTLPSIQHMQQPAGPSTIPPMSEGPQPASNPPSAGPDNQAPSVKEEAGRKEPAPLHEPAARKMDVD
EDYDDDGDDDKRAGAGLKGSPRGNGAGGSTNGNFANGNSSSAKSEAT
>g3503
MKTSIIATFVGLLASNALAANCEGSHYAAWGGTTWDAINAAIAQTCNSQPYNSGIKYVEDIQGYDV
AAAEADVGTGGSPNCWAAMYQIRDQCLDTGNGKYATQGWWQYGDEYYFLWAWSEADCRGQASLSGY
SNSGC
>g3504

MRSSSMPTSMFMSQERDASGLTPTSITIADDTTTPTPEDYYNYAHVAKQPHCFPHYSTDPVNATYY
YPSSPETLISMSGSSTGSYAYSIPIQSPHRNGNWDSAVAMSSSPVPPPAAVLDAHAEASRAEAGFI
TDNYKTGINSGLSWSAEGSLSDGGHGHGRNSSRGGVHDVPNTVAVYTTPEDKEGERNREPNAVLVL
LLFSAPIPLFSICTSIYTFLAVFLVILTTPLRLCPPTAFFRSTTFSKQLCQLLAPALCRHDRLAQS
SXSHYNTHQPNSPSNSTDAFSAPGLILVLTLAPFLCFGLLLAVWIAAFFWIFAMILGNPDGTERKD
DGRAAVLGVNRWWQTWLRKGRKYS
>g3505
MATLAEKFEKIKSPKLQNQHHTAVVLNAVEDTLRDQKADFSATAYFAALLALLSQALNTTHGVVNK
DLATSVVYLLDITAEEVPAPLLRSKFAQILSSLVPALTLADVEAPLLRPSIGILETLLVAQDANSW
ALPHTQIGPRRATAGLLNLAVDHRPKVRKRAQDALVKVLKNPPPSPSLDHPAADMCAETALQTLTE
SVAAAGKIKKNKQKEKDSSHHEPAIIHALHLVKTVAAASGGWPSKKIEPLCELLMNVSRSSNEYIT
MGAFEVFEVIFAGMADEFSSSKLPRLLEAIQELRPAQNDSQLLPPWIAVLSRGYDVSAQINPEDTF
EKLPGLFELIASFLSSPSKNIRISASECLISFLANCVPDNVILEPSVYDEKTLEKLAKFASDLLSV
KYQAAWVEVFNVCAAMFEAFKWRSSPYLVNIVRTVGELRSNDSFQGKKEADEVLGRAVYAMGPAEI
LRILPLNIIEQKAGQPGRVWLLPILRDNASNTELAHFRSEMVPLSEALYQRVLEFGQAEKTVEVKI
FETIIQQIWSILPGYCELPLDLETAFDQDFAELLSNVLYKQTELRVDVCRALQNLVESNQAIVEVD
TGAEGENLVLQRRISKAAAKKNLAHLSGFASNLLAVLFNVYSQTLPHYRGFILQCVNAYLSITPEK
ELEETFTRVTGLLESELPSEEQAAKQGNLQKGTDNKMPPTSHTLIDLLIAMSIYLPRTSFANLFRI
AAVVLNRDASDPQLIKKAYKLIPRLASTETGQSALIERSEELQALLLSTADKTPSPARRDRLLAIH
ELVTYLPTSDLHFIPSILPEVILGCKESNDKARTAAFDLLIHLAKRITDSERNPVGTVIRNSLVPH
MPNDTPDAAANLEEFFTMVSAGLAGTAPHMVAASVTALSRLFFEYRTELTSETLSNLVETVELFLT
SNNREIVRSVLGFAKVAVVCLPEDMLRPRLNSLVPNLMVWSKEHKGRLRTKVKGILDRLVRRFGAP
LIESLVGEADRKLVVNIRKERERKKRKKKEGAENDDEDDETKAGGGGFSNEFDRAVYGSDISSDDD
DSDSEVERQQKRGGKRNGPRGGEQYIRELSPDSNPLDLLAPDALASISTTKPSLRFLDNGVGKNRK
RHAKVDVDGKLILRDGADKHDGNDYEMTGAGAEDTGSSGINAYLDAVSGPSAVRRGQKGRLKFGKK
DNNNDGMEIDDDDEGDNNTKSKTGGSGLAAGRRGLGMPKTHGASTAANKTKGRIEKRRMSGSGAGR
GGAGRGGRGGFGARSRGRGGRR
>g3506
MERLPIRESAGSPRQQGQNQQSGPPPAPQGPPQLPPQMFTTAAQLLDLTDKKLVLVLRDGRKLIGV
LRSWDQFANLVLQDTVERIYSGNLYAEEHVGLYLVRGENVLLLGEIDLDKEDDLPETLIQVSMKEV
RELKAKEEEERKRKDKKSVGKLQTHGFEPEHSGEVLF
>g3507
MTSAIPVPSLSGGSQSSISDNSSTASQALSKEMKPRVNVINILETDSTGNLVASPPAQSALPSAEE
QIVYHCLFYILPCDHQSHNIDSWKTHILAHFRGTRLPPTAICQWCPLEFKQPTTSNSNAHTHQIWD
TMLTHVAHEHFEKGHTLAASRPNFELMKYMYNSKIITEDQLKAIQLCPSPDSPAYHPSQDPVRASI
GSADEPYCSTYSPRREKRLREQRRGISVV
>g3508
MAGDGDADDNGALDGISGFFNTKNTWLAIFLMFFLLVSIYNALELVVMILVSFRRFRGLYFWSLLL
SALLGLIPYSVGFFIKFFTHVSPWITCTVLTIGWWMMVTGQAMVLYSRLHLVLRDPKRLKQVLYMI
CFNALVLHLPTTVLTYGSNDPDASPKFITAYNIMEKIQLTGFSIQEAIISGLYLYETYKLLHTTHR
NQAQRWIQYQLIAVNVLIILMDLTLLVLEYANEYAIQICLKGAVYSVKLKLEFAVLGQLVDFIRNK
NGNYHHHDDNPTNGTGIDMGDGNMLSSAKRRYAGQDAGLPSTRADDPHFYGKTTTMIHPNEPTNSS
EERLNLPSGIIMTKTEFVQRIDHKRDNSDELE
>g3509
MTLKYDPEFAEILQSFSERVPEVPSADTTGFEAKRRFYADLVFELESRRHMPPDVEISRFEAVAVD
GFRVPVYRFFQPAKHNAGRNDSTTALASAILYCHGGGMVLGSALSEQKSLATKAFSTGVQIFSVDY
RLAPEHPHPTLVEDCYAALCWLQANAREQFGIDPARIAVMGDSAGGGIAAGVALLARDRKLCPPLA
KQILIYPMLDDRNTIPNPILDPFAFYKTADNKINWTALLGAQTGTEGVSPYAAPARVESVEGLPPT
YIDLGDLDIFRDEDVKYASKLILANIQTELHLYPGVPHGFENVAPEITITKQAVENRKRAMQSF
>g3510
MALQDVTFPVKPIVKIHFIINCVLVFITLVVVGLRILSRVITNAGLGWDDFLILVATPEAIGMLVI
QGLYLPMGVGYPINETLPNLVVILKLMVSYALIYSFCISAIKMSVLCFYLRIFVNKYVRIATMVVA

GFVLTWTTANILLLFLICRPFRANYDPTLATGHCGNQVTAFISIGVFNIISDIMICLLPLPTIWGL
KARQKMKLSLTGLFTISLAVSAVAAGRIAALTHLELVNLTGTMVWVDFLSTTEISLGILCVSLPML
GPILSKVRKGGHSRYGSNSWQKKYPNAASKGSASKFSSKKRQPGADTIILNSIDAADEHDHDPINY
PGNAETSSQHGSEASLNPAYKQAQIGVARV
>g3511
MGSVDEFDYIIVGGGTAGLTVADRLTEDENVKVLVVEAGEDRSKDPLVLTPGLVAGMYGNPDYDWN
FNSVPQPGLNNRKINQPRGKQLGGSSALNFLMLVYPNRGSIDSWKALGNEGWGYDDLAPYFRKFAT
VHAPPQAAKDVVGLTYHNESLIGDGPVQVSFSEGYGPTNSAWMETFAKFGLEMTADMRSGDALGAF
QQPGSIDPATKTRSYAATAHYTPAIASRPNLTILTGTVVKKIIFDTTGPEPVATGILAQSKDGSEK
TLNAREVILSAGSLMSPQILELSGVGSKSLLEKHNIPVVIDNPSVGENLQDHAITCQSYEVNPDIA
SSDMLRDPAVLNALLGMYQASRDGPMGQSNISVAYAPLTDASGISSAETKKSLFAAHDQHTQTLDG
QIIRSLLEKAGGPTAQYLLFPGQANTYLAEPTSMMDYLAPSQPGNFITIMSLLNHPFSRGSVHINS
ADVHQLPTWDPNFNSNPLDLEISALHAMFVEKFTQTAPFSNLLKPDGARIPKGKADSVEAAKEVIR
ETQVSDFHPSGSCSMRPKDKGGVVDTRLRVHGAKGLRVVDASIFPFVPLGNIQSVVYAVAEKAADL
IKEDRLNAA
>g3512
MSPARCNLREVSFSHLFPLEAARALSGGLAMAVASAHSRGFVHGDIHLRNVLSPLPNAANSRFLPT
FRPPLVMRPPEARFAPLSPLSYSADIWSLAFAIWEIIGMKAIFSSEFATADDVFAQQIDVLGASSL
PPEWWDAWEQRSNFFSSSSDQKIKIPREDRYVWPPLDEAFEEGVQKYRRQSQKVKEFDEEEMTAIL
ELMRRMFGFRPEDRLTADEVLQSNWMVKWALPEFFKTLEMM
>g3513
MSLDNSFLEDPVPQSTQEAIANYLWNNSRFQLQPYFRYYTDQCRFAVQSYSGYLPVKTHQHLINIA
LDILNGYPHHEVKARLVARRTQLGETDSEESLNGSIDLTLRLIYMVDVGVFRNAYSGRERLLWDTG
TSKTFIENKVFRAEMRREHSGIKLEPVFNVCNIVRIGGFRVELTSNLSDHLLLRDTGVEKSITIFH
HASFLTGQKQSSLFPDGLVDDTLRTLALLFPQGNSTTARWYGKHVQDNIEEPDFNVLKCGPAHRHI
DGYKYWHNRLVELKEAFDQARPSTLTQWWNDRRDGVQWYTLWIAISLTVFFGLVQSIEGALQVYKA
YHPNS
>g3514
MKNGKNCWIAKGPAQEAFQQISSEIKEVLEKDCGPVPGSHWVLFDIFMIGDAPATAIPHIMFSCPK
PGPRKEARTAVRNSDVLSHCTPGIALGHWDYPPHIKSPQPLASSLTPVESHETKDVNQCYLTPIFD
KHQPRKVPAIRLGLHSSFDEAKPFAKATVGSIVEVDEKRFYLVPAHVFSKRIPPQDVTADGKMQES
DSECEFGGFDDGDEDFADDGEVTFMSQYSITPEVSDLEVRSDPGGSQSSSDEVREDTQLNQHQEEI
NHDARSSPLLGRLPPKPVKPERVDVSGKDHTIRLEIKSPFFTSTELDFALIEVSKIDYLTASLPFL
RNDTIASVNMLADVIVTTMTGSGKTLTGKLSGRPSFIRLPNASKYQKILTVDFEGCLQIGDCGSIV
RNASDGKIYGHIIAGSVESRIAYIIPAVDVLHTIEQRFNLKSGSDVPSSASLPASIQDVNRRYTVG
WICSSSTEFRDAWIMLDERHLTLPRFAGSSVSYILGKIGQHNVVIACPSERSNAAAWVASQILLDF
PHIRFCLAVSVSSGISTLPLGNIQLGDIVVGMPGGSRPGVVQYKTFNDQDVIRVKGKVNLAAPPKP
LRAAVTRLKNAHKMGKDHFLTSRSRPVPNNLQSLPSSSEPISEHFNARVYNQTAQPLEANFRHQCL
TEHCKHVPKVHHGLIALITELLNEEDLRRLVHNDSDEFLCLEKEVERFPNRFPCLFVRGICDLVDS
PLPTKEKAEARVYAATTAATYARDLLLTLPEHKVLKMPLASKVINDWQRGVWPMIQQAQVRSQPLP
RLRAPIHAFVEMTQPVLRRISPPLTDSSDHQIIQPVIHELSPPETILTDFETDLNVHSIILEDSDS
HLSDALDPAPLDTVASRNWDGDANDGKPVPNLRRRLRRLRNNAGKNPN
>g3515
MAIESPREAIKAPPTLGEPATVTTIKPRAYQQEMLVESLRQNTIIAMDTGSGKTQIAVLRIQEELS
RCPPEKLIWFLTPTVALAEQQYAVISKQLPLYQSRLLLGSDNVDHWSTQNIWDKILLNIRIVISTP
QVLLDAMCHGFVKLTGLSLLVFDEAHRCVKDSPFNGIMRLFHHARSSGVDELPAILGLTATPATKA
TKEAVKILEDNLHAICKTPVVQREELLKYTHRPELFVVTYSRHLEETTQTLKSLDIILQMTLADIE
NDPYVKSLRAKKDDEKSRGMLLKILDSGKTFTRKEILSLAQRARVIHEELGAWAADVFVKTCAEKF
IEASVKRSDNNIFRQWEDAEKTYMMQYLSMMPAIAETRTWGSLPDNTSQKVELLITTIADTYNTGY
RVIVFAEQRATVIMLAHILSVHPLTKDIVTKYFLGNSNYANRKSAITELSTLSDQKDVLTELRVGK
TNVLIATNVLEEGIDVPACNMVICFDPPKDLRSFIQRRGRARDRQSRFVLFIDQNDDDGLAKWASM
EKQLKEIYADNMRELEEIKALEDIVEESAEIFKIPSTEAFLNHQNAHSFLAHFCATGSSAYTTDKA

EYIISRQELALGTVQITAKVVLPSYLDPSLRVACSKSTWKTEKAAKRDAAFQAYVALYEAGMVDDN
LMPLHRRKYADSSIPPQEKQAKLIQVSECWNPWHDIAQLWRDDLPLIPTKVQFEQPNSLSVPDMTL
LLPSRIPCGWEFELFWNQNVTLKARISSQRDMKLDFDTKQAARFTQELFLTVYQNRMNSSEVDFLT
LFWPTQAVKSPVEEFLASVKGKIGPLTVSVNQLTIEQIQELGLARTTERYPRPFIIKSIVETTSTD
VDLQDPDTPADTIEPIKLEPHFQGTTLPKRTDFLHQIPQSERLPLAHTAQQLEPVRICHIDRMPAK
FAKFALFIPSITHKIEVLYIAERLARTVLKEVKFSNLAHVLTAISASSAHEKDDYQRYEFLGDSLL
KLITSIHLVVNKPLWHEGLLSSAKDKIVSNGRLCRTALELGLDRFILTKSFTGRKWQPSYVSQFET
VPDEKRELSTKTLADVVESLLGAAFLDGGYEKLEACAQIVLPGRAWSSISDDVRILYEVAPDSNQA
ALHPKLPQMQEMLGYKFNKPSILFEALTHPCSNDGALTYQRLEFLGDSLLDHFVVQEFFNHPEKLS
HQEMHLMRTAVVNAHFLGFLCQRLYITEQRTEAASPGKAHISTVTTDHKLYLWQLMRHDASWEIVN
AQQETKKRYEAWGSAVHDALARSKTYPWAALFRINASKFFSDIIESILAAIFVDSKGSLDTARQFL
ERIGVLPCLRRIIREKVDCLHPFNQVREAAGSEKVEILSSAEKRVVTLEDATAPSVESEMVHTCTI
RVAEEEIVRVEDGVNKLEAEARAAAMAAEILRARMKAS
>g3516
MADRYSFSLTTFSPSGKLVQIEYALNAVNQGVTALGIKATNGVVLATEKKSSSALIEAPSLSKISL
VTPDIGMVYAGMGPDYRVLVDKARKVSHTGYKRIYNEYPPTRILVQDVARIVQEATQSGGVRPYGV
SLLIAGWDEGVEPEVAEQTEEGEKKATGKTGGILKGGPSLYQVDPSGSYFPWKATAIGKSATSAKT
FLEKRYTEGLELEDAIHIALLTLKETIEGEMNGDTVEIGIVGPPADHLLGYQGLEGARGPRFRKLT
KEEIEDYLTNL
>g3517
MEQCQYEMEQLQLDISNDHLDCSGSGSESDSTVGPSPRRSSTRSNPFDIELPDDFAPRSLEWFLSP
RPDPKKPISMFWSDPAGYYAPPTSSPVTEINDEQSEPGNVKLGTMGSLEQWFVPRRRAKTDRPNWT
IGAPLPDLAFRRVSLNASDVSQFQLARPPTPPRKRSRYPDTMSNKPLPPLPPRRFVSEGNAPLLSA
HPRSRSIPEFALSRWSESSESDNAPEEKAQSVADSHLEAPQESEVRAPEAMNGESVVENTLSTGPH
LLVRLNQYQENQMAAVTQMENTLSNPHIPDALREKFMGWSQQLIDLDCPFRVKVQPGVLEEVKVTE
KDAHRVVDWAIEVGTETNSIHDQINTYMNAADLVICDLREMSAGVNMHNGSLLPVEELYHKMTDIV
RKRCRAPRLHEVLLLRSFLNLYEHLLYTPFQKVKDRVKVVHDTYPHNPVSIALNIDTPLSLLKRVL
DAAYIAHGEYTALTHRMRQEFFVPIEERIPGVSSRTDGYLNNVVGPANVI
>g3518
MASNDVYQTPLNSRYSSNEMKYLFSPRNRFSTWRSLWTWLAEAEKELGLPISDEAITQMKAHLIIQ
DDEFAVAAEEEKRRRHDVMAHVKAFGDVAPAASGMIHVGATSCFVTDNADLIFLRDGLDILIPKLA
TVIDKLAAFAKEYKDLPCLGFTHGQPAQLVTVGKRAALWLQDLLMDLRNLERARKDLRFRGVKGTT
GTQASFLQIFNGDHAKVEKLDELVTEKAGFDSAFIISGQTYSRKIDVDVANALGSFGSTCERIGGD
IRHLAMLKEVEEPFEKDQIGSSAMAYKRNPMRSERLCSLGRHLQNLPKDALDTYSAQWFERSLDDS
AIRRISLPELYLCADACLILLNNVTSGFVVYPEVIRRRVQEELPFMATENIIMACVAKGLSRQDAH
EEIRVLSHQASDNVKKQGKSNDLIERIRRTPFFEPIIAELDSLLDPSTFIGRAPQQVDKFVDTEVK
AALAPYQKSLVKEIAELHV
>g3519
MVGTKNTTYAEESAEVEVLYANLDKLKILTKKIQGSLDRLETSGKVVKDAIGPIYSNTQTLQITNS
NIDRVIEAIDKLRKPLDAKGKEEGIIRAGPQSAGLSQYLAALKRIDYALQNLNSTNLKSNQQAITE
FTSLLSFGSQKLQDLFRSMLKQNIETIEPLHYLTKQLPFPSIPEETIAELAPIANAITSAAAQLPQ
RGPEDPAVAIYAETRGPYITSSLQNLAMASISTAKRRAGDGPYKQGTNGIGVYSSALEGFIVVEHD
AIVQIYTGENQGRALQATCRSALAEFSKTLRELNQYIKANLMTDCFLAFEIIEIVTAMSYRIDAKT
GELKSLFIEALRPIRETAKSSLSELLEETRKKAAAYTVLPPDGGTVPLVNEVMSSLTTLTGYSGPL
ASILTSLGDGNWRNKSQSASTTPLDVSPDSQTLFSHFILDMVEALMNSLEARGRATYRSKAVLGVF
ISNTFCVVDRSIRSSSDLSRYLASPDSIARIDAFRKRGTSAYLEAWRETNQYLLDVQYTSRGGPRP
SSTQQTDSAQVVKSLSSKDKDAIKEKFKSFNATFDSLVTQHRQLHMEREVRGALGREVQGVLEPLY
GRFHDRYHEIDKGRGKYVKYDKAAFSAQLAALC
>g3520
MHSAAALRLRWGVPPAFLAAWGSWNYTSRDSLRADGSSRAWNQKLTYSTQQAQRKNETEITNTTSV
NAQEAQTAWALFTKKFAVARDSFASVELGNIGDNIKNFILPDWARFLPATIQKLQRELSMAPGSLA
DDIWKEAHDPDINPEIAIEAKVRVGDTLCPEEVRFREKRQKHVVKALAKYLDLNEEDIHPDDVPVI

AMCGSGGGLRALVAGSGSYLASQEAGLWDCVTYTAGVSGSCWLQTLYNSSLGNCDFDRLVGHLKSR
LGVHIAFPPKALNLLTTAPTNKYLLSGLVEKLKGDPDANFGLVDIYGLLLAARLLVPRGELDVSDR
TLKLSNQRDFVQDGNNPLPIYTAVRHEIPVPDDTKGRRAVEKVKEEALKEAWFQWFEFTPYEFFCE
EFGAGIPMWAVGRHFYEGKDQPPNINPVPELRIPALMGIWGSAFCATLAHYYKEVRPVVKGLAGFG
GIDSLIEGKSDELIKVHPFDPASIPNYILGMKGKLPSTCPESALSDPHLQLMDAGMSNNLPIYPLL
RPGRDVDIIVAFDASADIKQENWLAVVDGYARQRGVQGWPVGAGWPPADLQPEETANMISEVNQLS
ESKSDEKLHEAQEHDPNRNNRDSPLSDTLMKSDARYDSTSKKSGSSPSDLTYCNVWLGTKQEMTSK
KEPPSSKRLFHPSHGDENNSDFHLMQPDAGIAVVYFPLLPNPSAPDYKTQSIDRSQIARPRKSTTA
NDDVVVTTKPKPKPNSINPDVDDFLSTWNFVYTPEQIDSVVGLAKANFAEGEAQVKRVVRGVYERR
KRDRLQREKLAKEAEVDRRVDELKRRHEGFTPIY
>g3521
MAEQGITADSATLHAALEVLAVHPDYLLRQEVIEALRARWLSLSPAGWHHVIAGLLRENQLELALD
RLDQMERKGILLEQWLHSLLVYKLCDANEFSEALRLIRSRPNKAKSISSAMWLYLLRAAIRKSHFE
TVEYVWKNSVDLGYQELSVADCRDLLGFAAQHGDAILAEAIFRHLSTLQAHLTTYEYGKLVQVYAG
NADIKSALGIVCQMHKNNILIESESAQPILKSMEITSSDPTTVWKEIRQLGNEEFGIPINLANIVL
EHIGTLFKGDSFSASRAIDVAVSIYKDLFDVCTSGANTDTFNHLFSICHQTRRPDICTFFAKEMAG
LNVPPDQRTLETLILICLESGNRDSAEKYLADLKAHGWELSETVEEQIRDKLEHASLLRQIVAGPR
LQHPEAGLDLCYVTDFLIATSGPSSKYPKRAYRNPTDALVRFLDSKHDTDWAIWEFRAEGTGYPDS
EVYGRIHHFPWPDHHPPPFALIPAIMASMRNWLNEETPNEAGGNKKKKRVAVVHCKAGKGRSGSVA
CSYLISQEGWKMEDAMQRFTERRMRVGFGDGVSIPSQVRWVGYVNRWANDMGKVYVERPVEILEVH
VWGLRDGVKIALEGYVDEGRKIKCFHLFRRKERIVIDDGKTALPLPHDDLIAKERLEKKERAANSS
PSPSPSSSANSATIIQQPSMTFTSSTSTSKNIGENNKKISAAILRPDKPVILPTSDVNIDFERRIK
AAYTGWAVVTSIAHVWFNAYFEGGDKHDSGIFECDWEALDGIKGSSSKGTKALEKVKVVWRYASKE
QLAAEGKQEPAEIAKLPEERVVTLPKPGEPVQESHAADWHGENVVREDEPGMGFEGHKTVVNPTAS
PVRRHPGSEHPPSSTEAEVEEEEEEALNGTTHPFIAQASASTAAAVTSAVKRVRRELGLRKQTAAS
AEVSLANSSAEDLSSPPSQHRRNEQEQAKDENGNGNESDMEGVKSCMDREGNGGTNTSTAKDL
>g3522
MLPLGLLTAAQGHPMLVELKNGETLNGHLVNCDNWMNLTLKEVVQTTPEGDRFFRLPEVYVRGNNI
KYLRVPEEILDIVKEQQQNQPSGRGGRGGRGDGKDRGRGGRGGRGRGRGRGG
>g3523
MRGGVAQASIFLLSTLAPALSYSLPTLQTSTNAVTQDDPVLLWRDSDGVYVQFEASCASCSLDDGD
SWPINLFIGSTNGLCDNATVSVNGQELSHRWEGQFATGSGVIPSSPYNGSTELRATWHSTCITAPL
GAHHGQNAHVFTFIFDTDRSHDLEDDIGFTISFGNDTCKDDGIKVLRLSNFPVKFGDAARWGEWND
ANDATDSTTGSRLDPGSVSLEVQLEAEIQRLQVLQYELQQLHNSVALQEKKIFHMLKQDCKPLFAK
WEQCDDFFCYLKSSWQKVPEFYWSMRYRFGLLAPGRVIPICRLAPEKPSDDKTDANRPSALPSHSI
PTPSKHAVPTFETSSSSSSTSSIPTTTTEPLMPVTTPPPSKPDSHLDTHPNHSDITEVVLPLPIQS
PTKHFIKSCAIILLIALIISLFFRVVRHSTAFRRRRRDIASRREELRAQRAYRNAARRLRWRQWWE
GRSYFQAASVISSHSLPEFQHARGAANLGDENNRDISNHDSASQSSDTYDEEPQQRMMQAEILGLR
RVLEYVGQLVGTDETTTTSTSSSSARRRARSRSRSVPPPYEQIMRYNRNASVGGGGNNPSRNHNTL
EIRRSATPGGVISGGPSSPRASTMFSLETGSLVTLETLDTLESETAPPSYHP
>g3524
MADSGDWTCDANDAIQLTLVKPGEQKPTTAEIFHPQFTYPIFGDEEQIFGYKGLIIRLRFATHDLR
THVHISFDEKFKAVGDAAAVDLNKTLREWVSESAFTKLSDYENSVQNDPKAKDFTPPGKLVHSYKS
KNRNYEIWAGSLADDAVRNLLGRAQVFIPFFIEGGTPLILDDPEWTLERWTVYFVYEKTTPTTPTE
SQYSFVGYSTVYRWWFFPEQHGDKVVKNDTFPYPEELRFSQLPSRSRIAQFLILPPHQGSGHGSQL
YNVIHKACIADKTVVELTVEDPNESFDVLRDSADYHNLYHAFLDEDININPNPYADLKVSGKRRPR
NMPTSSLIPTQKLAEIRTKYKIAPTQFAHILEMYLLSRIPSRKRGGTNMVRLLIKKHNAADEDDRR
YYWWRMLVKQRLYKRSRDILIQLELQDRIQKLDETVTNVEEGYDQLLTVFDAREKGRATFAPDVDS
DADAVITAGGITTMHALRAKRKYAIEDEDEDEEDDDVELIEAGQAAKRPKI
>g3525
MGFFTLIGQMEIPSWPILFLDASGLYTLHLAVTAIYNIWFHPLAKFPGPKIAAIGPWYEFYYDVIK
DGRYLWEIEKMHDRYGPIVRVNAHELHIKDVTYYSEVYSGSTRKVNKDASSTDGFGIPTSAATTID

HDHHRARRSYINKYFAKRNMTALEPIVQERLDRLCSRIDEKLRSGETLNLDGCFSALTADVITVLF
YGNNFDYLGSPDFRFVIRDAFLGVSKMYNLARFIPLAVKALKSMPLSVIRLIAPTVAELERVRESI
AENGYRKFHAGKWDAEEKKSVIVSALNDESIPPAERTVDRLVDEGTVILFAGTDTSARSLAITMYY
LLSSPDCLARMRHELETSLPLNKDHVYSLAQLEKLPYLTAVLQEGFRLSFGPVSRMPRIATQDSLE
YKEWSIPPGTAVSMSTMFMHLDENIYPNPTKFDPERWIRATEQGVNLNKYLTTFSKGSRNCLGMNM
AYAEFYMTLARIVLTYDMELYNTTDKDIEIHHACMVGYPKKSKDPDFGQVIVKVIGRRALPDTI
>g3526
MATKEKSLPFGKIEPYSTKYFTSCALGGIIAEKGPTHTAVTPLDLVKCRRQVDPNIYKSNLSAWRS
IFAKEGVRGVFFGWSPTFVGYSLQGAGKYGLYEVFKYWYGEQLFPNTNRTLVYLGASASAEFFADM
ALCPLEAIKVRMQTTLPPYASTLREGWSKAVAQDGFGGLYKGLYPLWARQIPYTMTKFATFEEVVN
MIYRGLGGPKESYSRLTQTGVSFAGGYIAGILCAIVSHPADVMVSKLNAERKAGEGAMTAVSRIYS
KIGFSGLWNGLPVRIAMLGTLTGFQWLIYDSFKVFLGLPTTGGH
>g3527
MAARYTPPPSLGQRRASLRDAAAGDHENNIPRSVPQSLTDMPSRMPAGGKDISSDNNTTSHNNNHH
PKPLPTPNKSASPLPSNLLPSAPASPPTPAPSPTPHHTVSNWQSSLGGGGGHEVDDEDEDDSLLLK
AQIHFSSMGSAQKQKFLVDILNLCDNQQLSFISSFISPRLRKDPFLSFPNEICLRVLSFVDDPKTL
ARASQVSKRWHELINDDITWKHLCDKHAYVYRRPSDDDRDFVDPFHSGRRLSFLSSISDYQQQPFQ
PWRRRKSTSSAADSVSSMSTDLPLESSWPPPFSTSNRKRRVQPASYRTHFKKKYMVESAWNKGGHC
TQKHVTPDQGVVTSLHLTHKYIVVALDNAKIHVYDTNGGNQKTLEGHVMGVWAMVPWDDLLVSGGC
DREVRVWNMATGQIYAIAFDGRRIATGSLDTSVRIWDPNTGQCHAILQGHTSLVGQLQMRGDTLVT
GGSDGSVRVWSLTRMAPIHRLAAHDNSVTSLQFDSTRIVSGGSDGRVKVWDLKTGQLLRELSTPAE
AVWRVAFEEEKAVIMASRSGRTVMECDEARPGCRNCEIYGKTCPGYRPPVVLQDKSFASRKWRSSL
IDAQQQHQYQMNVGSEASGDNDNEEDGLALIPKPRWLADASMDDRALCYFFDQYTTQRTPDSLPGA
LESIPLLYVLCRENEVAGSPSSCLRWAVEAAALTSYANESHDRHLALKGRQRYGLALHGLKEALSM
PVEKVQDSTLATILLLVIFEDINGERAQLESSHTAGLELVARLHTYRRLDGKYSRCLFNFAYTQLQ
IQILGLGAQPKLDLHFLIDLFDHSDPLQSLMGIVTKLSQFVLDVPALLSDGNPRYNIADLSLEDLV
NDLSRAQSLDEELSLWCRCVPDQWLPRVVYSTTGEALITCVSVSAATLWNFYRCSRIILQTIIEKI
HTRMEAVNEGGLSMWHTADIIDMTNPANTPIYSSQTSASASPSSSILSQSTPYEIVQNMIVDICRS
MPYSLGDVDSSGVPFPQTHNENSNTRVKAIEGYELLWPFWHVLTSHFSTPKQRLQAREALYRLDAM
GIKLASKMAGAVDLQQQEQSSFTYI
>g3528
MEGLFTLLILSIVMAITSFLVGALPLTFTLSSSQSRFISSLGMGILVGTALVIIIPEGVETLYSSQ
NYRSSSTLQTRGLDIRWQHKSSPVNILPPRGIPEIDALPGPVVADDTSAFGDAAPPKTPTVPGEVH
ALNLDGGSNVGDSHDHNHEDENRALHAWVGISLISGFILMYLIDKLPSFATPPKPRRLPYHISLDN
LGGSGLRRSSSPNRDGGLLDQDSNNHAAHNPHTFAMTTGLVIHAAADGIALGASGSDSSLSFIIFL
ALLVHKAPAAFGLTSVLLKQGLSSRVAGRHLLVFSLAAPVGALLTFLIVHTLGSGFGGDEASNRWR
TGVLLLFSAGTFLYVAMHTIQDIAPSSAPSHSHSNGYADSREPPKPKGSIQDLAVSVVGMILPLFL
QIGHAH
>g3529
MDTSAPSTTTNNHNTPNPPSIELAYKKKCIQLKKRLNEIEAENDLVRARNKRAKLYVAKMRLETCI
MLERLATVTGMLDEAASTSAAAGQQGAAAGGQISAELRAKAAAIVTSAHAQSTQGVMDDETEGSSE
EQPPTPQERPLRVKRSRKSNIAFEELEAEAAMQYEANSPEPRGGNDSGYNHNIDVDRDSRNIGTGD
EQDVNDRDLSQGADRERSLLTAGGAVDGDERRSSGFRAVNARFDSQGAVPMDVDEKPARGES
>g3530
MATTETSPLLPQYQRPAANPATTSPRSDRRTVTFNPLTTISTYNGTSQSDSIIQPLQASSPPVRHT
SLNPVGQPMLSALNSKLRRRNSAGSPVTAAAGTIHAPKIGPQRTSKNTQKLKLLPDPVTGNDEEVI
DDGFPRDVYTQISRIKEPTARRAAARLGKADRDRLPRVTAYCTANSYRLEGVMRFLKSRDKTRGAN
PKLFDECVYSPFDYQYEKKQAEQQAQTRNGQHTEGPSQSQQQRLPGERRYSDSAVEVEDHGKNIRE
DLIDFRDDETTRPQSTSDINKSVTQDEEGLDLDTTVHTPEVFLFDYGTVVIWGMTPTEETRFLADV
AKFATAILSTEDRQVENFNFYYARDYQARIYNDFISLRDPRNHMIKLAISHALSQSVKTSLFEDLV
SETIAATAPFPAQIAKTGSVNMTRKQINMQIGELFILRINIHLQGSVLDSPELMWAEPQLEPVYQA
VRTYLEMDQRVGLLTERLDVIADLLAVLKDQLSHRHGEYLEWIVIVLIAAEILVAAINIIVDLYAG

VE
>g3531
MVTATRQGNVASPRTNGSESATNTKRKTMESKTNGHSNKRRRLSPEQEDTVIEVLSSQQEVPADNV
IIQIPATQQRKIRNGDNSTSESVSTSLDRSAKEPSNHIRFGSEEPALPIRTAKEIDIEAVQPAQAQ
DEEDSESDDDDAPEVVDNSAQLLALKVKAQKQKEAKRRDEILQKEKRRLQDEKQKAQAKVSAAAKA
KAEKLQKQSSFDSKLISDDDIVSESTATLQGSVVRESGRRVLPALLPDEILNAEPVHRLPSPPPEE
QPKINTKQHKFFKEDEPAKDIRRGDVTIRVLETKKEMLPPKSSAVGRRVKAAWQAGQRGRNGNAAG
GLKRVGGGSKGFVRR
>g3532
MADPFEVRMRFTMQLQHLSAAVASSQRAAQYALKYRDMDEDLHSCILEQLERNNMNNRANIMYFIE
HFCELASKENHLNYVRMIQRDMLTIVDSVAPADGSGAANVKHVRRVLQGLQDKDFLSNETVTEIDA
ALKERETNPSNVLDSEAPEDSGRTPSRGSKTNGSTIRVDKRQIEQRIEEDRERNKRLRESMWTVSG
DNKGEFQQCWDEISDIGEDDYLGAEEEFGERRLIVTT
>g3533
MLILRAGNLCLAVLTGILSSISEPSTSEEKLSFQTPTKFIACIRSERSAARIRSAVEPYPKFPVKV
LQNDNVAGVKEADVVILGCKPYMLKDVLDVPGMRDALKGKLLISVLAGVSSQQIEDTLYLEGSEPA
SERCTVVRAMPNTAAMVREAMTTISIPSPALPKEWDDLISWIFTRIGKIVRLPPSQMDVCTALAGS
GPAFVALIIEGLADGAVAMGLPRAEAQLMACQVLKGTVGMIEGGEHPSIVKEKISSPGGCTIGGLL
VLEDAGVRGTVARAVREATCVASELGQGTKLVNGTRR
>g3534
MSLATPISGDVGLSSSARHAYANESEDDDPVSNYEQDEAIFAEDPFAQNDPEPISFKRKQKQPARF
SLSNVFSGSGSSTSHIPAASLNQDANLNDWSNFADDPTAAATNGGVGAFKEDGPLDWYVEGPGRRL
AYDDMTAIDWVFEYTKERQRKRLLFSTGQGVLGHLRQLLDGSHVWVVLVATGIAVGLLAAGIDIAS
DWLSDIKTGYCKGEEGFSQFYLNRGFCCWGHEDFAECSGWIPWRKAFKVTSAGGGYVVEYILFLMF
AVLFATCACILVRSYAPYAKHSGIPEIKTVLGGFVMKRFMGGWTLAIKSLGLCLVVASGMWVGKEG
PLVHVACCCANILMKAFDTLNNNEARKREVLSAAAAAGISVAFGAPIGGVLFSLEQLSYYFPDKTM
WQSFVCASVAAVTLQALNPFHTGKIVLYQVTYTRGWHRFEIIPFMILGIVGGLYGGLFIKLNMKIA
RWRKSRGWAFPLLEVALVAVISSLVNFPNKFMRAQSSELVYQLFAECATTTDDQLDLCKTGAASFG
VIALLLLAAIGGFCLASVSFGLEIPAGIILPSLAIGALFGRALGIAVEMWQAAFPTFVLFESCQPD
VPCITPATYAIIGAASALGGATRMTVSIVVIMFELTGALTYVIPIMIAVMLSKWCGDIFGKRGIYE
SWIHFNEYPFLDHKDDRPPPDVPVSRMMTNVDDLTVIPAVGHTIESLRSLLAQTRYRGFPVVLDTS
NPILLGFITRNELSYALESSLSSSTRNLGPETQTYFVHQPFADPGDTLDLRPWMDQTPITLNSHTN
FSIVLGMFQRLGLRYVLFVHKGNLRGLLTKKDVWFILNGIENRREKDFEEGPRDTNRPEAMGLLVD
EVSQGNTEDIGLMEGRNSMQSL
>g3535
MEERPVYKLELSEAATLRRFPHELAEQLRNASGSQFLDALALAALEPGNIEYIFATYEALIVDLAS
RWLDLDPQLHYLNILSAFSRILPFAPHLRSFARRYAQMHLESFSSSILSFDTTTIRSALLCIFRLS
SFDVESFSTVISPIQLQSLFKHPDNSVRYLAVRCFCYYMRSGDAATQRMVTTHIGAGAIDGPWENT
TVDYRLLGLWEERRWQFFKTELRTKRTKSTADVAPKLVEVYMDSLSHRAADIGGVLVPKVRWDIPV
PESSLVKTETVTDNLTRIATALLEQKPLLLVGLPNSGKTSLVNDIARVMGQGPSMVTLHLNEQTDA
KSLLGMYATSSLTGTFSWQPGVLTKAAKEGRWILIEDLDRAPSEVIGLMLPVIEKGELIIPSRKER
IKCADGFRIIATMKSSLNSKGEEVAPSHGLLGARLWNKIQINSLPLSEVRQVLLEKYPLLATRVPT
LIDIYDRLCSAFHSSFAARSAQGRTPGLRDLIKLCNRLHKRLLRLGVTRGNEATPESFQDEIFLDT
VDAFIRYLPDRDFGHRLSSIIAEALQLSPQRANYCLQERTPPYADHEDRFSVGREICTKMKVTKTT
RARFNKLSSRFAPTRAALRTMEQIAAAVQVSEPVLLVGETGIGKTAVIQQLANLTCQKLTVVNLSQ
QSESTDLLGGFRPVNLRSMAVPILDEFNQLFEQTFSAKKNQRFLSSVTKSLTAGNWPRLVNLWHEA
VRMADSVFCVPKESEQNAEEQPAKKRKLDSPKYAALRDKWETFKVQLSDFEAQASRGDSKFAFAFV
QGKIVRALRNGEWVLLDEINLATPDILENIASLLHHGDEGLPSVLLSEAGDVERVYGHSGFRIFGA
MNPATDAGKRDLAPGLRSRFTEIYVHSPDVDFDDLLGLIQTYLGNLAQSDLRLASDISSLYLATKK
LTLENKLTDGAGQKPHFSIRTLVRTLVYITDHVHSYGLRRAAYEGFCMSFLTLLSQESEKLVVPLI
EKHVFGSTKHAKSLLGQIPRAPEDGSEYVQFKHYWMKKGPFIAEKQPHYIITPFIEKNLKNLLRAS
STRRFPILLQGPTSSGKTSMVEYLAKVSGNKFVRINNHEHTDLQEYLGSYVSTDDGSLKYQEGVLV

EALRNGYWIVLDELNLAPSDVLEALNRLLDDNRELFLPETQEVIQPHPNFMLFATQNPAGLYGGRK
VLSRAFRNRFLELHFDDIPEDELEYILRERTQIAPSFCSRIVSVYKKLSVLRQSTRLFEQKNSFAT
LRDLFRWALRQADDRETLAINGFMLLAERVRNSSERAAVKKVIEDVMKVKIDEDAIYGLAELERRA
EGKSSLSQNIVWTKAMRRVFVLVSEALRNNEPVLLVGETGCGKTQICQAVAEIYGKEMFIVNAHVN
LETGDIVGAQRPLRNRSAIERQLHDDLASVLSNSPHTEPETSTTSLEDLKTAFAQLSPEILEEVPS
ELLSRIKTNTTRSQALFEWSDGSLITAMKTGQYFLLDEVSLADDSVLERLNSVLEPHRSLLLAEKG
PVDSLVVAQDGYQFLSTMNPGGDYGKRELSAALRNRMTEIWVPQLSEEEDILPILKNKLRSMDEQK
TKAMLQFAKWFKQVFQGSSTSSVSIRDLLGWVEFINKCPPSNPDFAIVQGASMVYIDTLGANPSAM
LSTSTDSIAQSRQMCLEKMASLLEIDAVRIYNEKATVSTQDGSLCVGPFRLPVSSNSSPDPDFVMD
APTTVANTVRIARGLQTSKPILLEGSPGVGKTTLVASLARALGKPLTRINLSDQTDLTDLFGSDVP
VEGGDVGQFAWRDAPFLQAMQKGDWVLLDEMNLASQSVLEGLNSCLDHRQQVYVAELDQTFKRHPD
FVLFAAQNPHHQGGGRKGLPASFVNRFTVVYADSFSDTDLNSICTKLFPRIPADQIFNMVEFMSKL
NWAIDHDREFANIGGPWELNLRDIIRWFQLAGREDAHIPSGYFLENIISHRFRTEKDRSLISSVFE
QSFKIPVPEKSFYHNLTAEKFQVGLATMPRRQLEQNITDLDIKILPAHLPILESLIFCIERAWPSI
LVGPSGCGKTTTIRTLASIQGAELVELALSADTDTMDLIGGFEQIDYRRQTQSLAKDVVKFIHRQI
MHSVTSESATNSNTYLLETYHICSNPEINTETLAHVINALQPHFDTQAFKWLAERCTALVQTYKDA
QKVGFEWTEGALTEAIQLGHWVVLDNANLCNASVLDRLNSLMEPNGYLVLNEQRTEDGSARTIVPH
PNFRLFLTMDPRNGELSRAMRNRSVEVCFLSESPVAASSKHTPSFTLESALYRLRYVWHSDAESKS
VVANQSLGIRLDHLEMSDLSCFYNSLRQLAASFPVTEQLNAAMEVLDRYAKLVEDNSQWKLLSSLR
SGTGGQGAKLVHPLIDEPRLTRHPNLADRSAIISRLQDIKLSLHQLQQRLAHSHQNSQHLKPSQMT
RLERSFASNRISSLMKDDTQAVSSFLTDFVQTSYDFIQGLESDSEFVSGAVSALKGFTRFCWDIYT
VTQVRELNEGVFQIYLQIGQQILSSAAETQSALSPLFSSLAQLLGRFRSTWALTTGLSMRRIWDAW
RPVTCTDIEQLHSLLDLESVVSEFNKIALQTRLKVSQLGLVRKSLIEAQNAMLLNNADGKLLVPSL
KQQVTELVSATQLYSSSQPPHFLQVFESICEYHDLPMLWNESQIQQHGYLNETLPLLAGRPAYSTI
PFNEKSPVSSILHNLALYNGSEGSAGAVSPWSSLFSINVLQHVQRTSEVLLGRLDFFQSELHALVE
AVTYSSREIALSQSLLLVRVFARVILEILSLHQDFLNTVILEDVVVALRKVEKTGTFHRPVPQLEP
AIDLPPNHFFRQFVDDLSKCVQTLASIQDMSSVSILYELGYVCVQLSVICLRLFVPDKPYDPSLGL
VVERQRHAEQVLQLTTEDEALATFEQEFSGRTSNLRRRLIKEELQKLGSMPPASPVSRPAKSRHME
LYGELMNLHNSVLSRNPETFGAAEFKQYGKLLKDNIRLICARLRENFRVYDDITVLVTRFLAMLDL
GASLCLGATSKSQSKISKIITQTTPFIGGKTHATTIFTAGSSKNQIKVQMHQLSSLVLHHGANSSS
LASEAGMSLLKDILQSLHYVWKTQLMEDQKEAAEKSSTYRYKGSYEDSEEVDEMELRELFPVYDGN
EQEDSDKSRMDIQSISLKLTELHAAIFAPGDIEANLRQFIVESTRLLGTLSSGEESCSAPKSYLPG
ILLVLKDELKSYENVSPRHYNFYLDANIAEAKRLVTLVLSVRSRFVQLQAAWPDHAAIHDVLVCCQ
EILQFKHLEPVAKYITKLEKLHGLVHEWQLVASKEYTAASLYDEITALTISWRRLELSTWAKLLDL
EFEKCEQAVSTWWFVAYEAFIALPLQKTENGEDLKDYTTEALHTLESFLKSTTIGQFSARLRLIEQ
FRSLLLLFTKQNPVLGCIVSALENFLYHYRPYEPVVNKTLEEKRKALDKDVKEQIQLASWKDTNIT
ALRESARRSHYKLFKYVRKYREFLAQPTDPILQQGMPELQGQVVDTQPANPLSIALWDPSDALAVC
EQSDLLWATRAPRFKDPSTTAANMLRVYTSSLSDLDIPSQLSSFVTDVLETVKSFKSETPGTLTEA
NKEDVQHLKTQKRRFYAETLRQLHFMGIRRNVSTVFIEEQATVNQVLATTPNIILENGPAASITQS
ADSYFHRFLDVLPNVRKYSREYNEDLNPTEVTRSIGSAEGFLVLIRKQRESLAPTIKHLVDLKHTS
TGLTERETAFKRVSWLIPLLGVGKEIVEIHSLYSGSASVAGTFGLWKDALACLKNEIEQLPRLPEG
LTSQVTIDTGREVMQSFTRLSSEFTALIEDRPDLSFILEQVISWLEIPVPVDAPSTVCDLMIDSLN
DHYLSAVDKILVALQGVKGSLASLPSDMEDKDWLSRTDSSLSRALRDLHIEEITTSLNSVLKKLQS
LQNAGSSLPIASAIIASLLPITEQFYNICEDMNSRYLEVHREVCKMSYVLSKSFTQIASEGFCSPR
EASNTEESGSGKVESGTGLGEGEGAEDISKDIQDDEDLSELAQEKNQEKSKDDVENSEDAVNMDQE
DLEGEFDEEQKEEEGEDKEDGSNEEEEDDEIDEEVGSVNDLDPNAVDEKLWDGAHDEQQKETENEE
GKGQTESDEQAAANDKKEQDLKDQEEKGEDEPMDGEEGDEGEEAPEDEEETGGREELDVTDPHAKE
EQTLDLPEEMQLDGDGQEEDDQDMSDDGMDELSDFEPATEDKVEDMAEDDAERSATPDPGDIDNME
EDDQNEAESGEQEDQQEANIPGEEEIAEEEAEQKDNKNLEKDENTATGENPEQSDEITGGIGLDTD
TKEEKGSSGNAQQEGGAEEAGSEEQPNGATEEDADRKKADSSAGGTGEDSQDDPQSEAFKKLGNVL
EEWHRRQREIMNSSKDKEEQTLPPDTDMADAEFEHLADEDDAADTQALGQASEEQAEALDQQKGVE
TEKPTATDDVPPEVMDGEKNNQSDENLLDDTAMDLDIAEASATEGAQTAKRTLVGETNKEQDGEQP

TEEELDEVDTHLAAIHLSSANAPKTSIDEARKLWAHYEAVTNDLSLSLTEQLRLILAPTLATKLRG
DFRTGKRLNIKKIIPYIASQYKRDKIWMRRSIPSKRNYQIMLAVDDSKSMLESGSGQLAFETLALV
ARSLSMLEAGDLCIVGFGDEEHVRVAHEFGKPFSSEAGTQVFQQFSYQQTGTNVRKLVADSIALFR
EARARRSSSGGNSADLWQLELIISDGICEDHETIRRLVRQAQEERIMIVFIIVDATSGSSILDLTQ
ASFEPDDSAMGTGEMKLKMKKYLEGFPFAYYLVVRDVRELPAVLAMALKQWFAEVVDTSS
>g3536
MGLVQYSDSEGSDDEKQPIAPAVSEPQTIAPDTKSSQPDQKFSSIVDRGNPRKIRVALPSIKPETS
AEGPEDDQDGPARKKPRTTGGGIFSGFNSILPAPKRANLPTTSSGSGSKNTKVFNLKTGAGPGFDR
QADAEWRQEQTFTQASTAGSEDDTIPKAGSLRNEAKEGSLLKEEDYKKKGNAMVFKPLSVARNAPK
KKSAAFVAARAAAATPSAVQKTNSSDNLSSEKDISKSAASIPAAAAPAKPKVSLFSFSSEEKTTPI
TTTNAYDYSPEFSDLPTTAEQDISTLEQHTQPSNSYPTPSTHQQSTLDAIANDLNLSRAQRRQLLG
RNPGTAAQANSRILTFNTDAEYASNQEMLSKTTEEELAAQQHNPVRAIAPGKHSLRQLVQAASSQK
DALEDSFAAGRRNKKEAGSRYGW
>g3537
MDGTAQSNSEHVSDGCNPLREEEARDTKIGINDSEYKSSRSSKFRFKSSKSRARNHSSSHDSSDAT
HHEEYRSNRHRHRHHHRHHHSXRHSPKRQKTEGEPTLRSPSGRRSLSPDTAFRESLFDALGDDEGA
MYWESVYGQPIHTYAIPSVPKGPDGKLERMTDEEYAEYVRARMWERSHEGILHERERQRQEKAKNK
KRAETEHRERARFDEALEESLRRGEKRRRTKQWKEVWAKYSESWDELDNLAKTPPSETEKKFFIRN
HIHWPVESGKRRDISRDEVREFMQHSPAEDLLNTLKAERIRWHPDKMVQRYGGLGLGEEKTLVQSV
TEVFQILDDLWIEQKGRQSS
>g3538
MWFAQWAASALALASVTAADTKYWQDNTDVKMLPPYLDSDMHSRWWDFGGDTVIRTDQYVRLTSER
QSQQGWIFSRVPLTATNWEIEFEFKISGSGHFHGDGFALWLTKNRATPGPVFGSADKFEGLGIFFD
TYKNNRPGTSFPYVMAMMGDGQTTYDQDHDGKANELAGCSARGLRDAAVPTKARLTYFQDRSLTLD
LQYKSDGAWTECFSLSASDTNIAIPAVTYLGFSGETGEVTDKHDIISVTTHNLYDSSTSASRNRGG
ASPSRGRGGRGRKKATSSGGWLWFFFKIFLFLGAVGGAYVGYTIYRSSQRQSRF
>g3539
MSFFGSSSSSTSPASEPSSTELKNALIQQVQAEAATANTRILITKVNENCFDRCVSTPGSTLSAGE
STCLTTCMEKYTNFWNATGRAYVSRVATESKRMGAGADVLGALGASE
>g3540
MPTFGSLLKKKKTHSTGSKELSTSSATETSASDKPGDETSEQHSKESKESNDSNNSYNSAGSTGTE
TNNIKTGSXDNQMTLTSSTNQQNAQGANNSSSHNHHHLPNIGSIKSIINPTHHQHGNESQAQGQAE
NNNNNSVSQGQLPKSQSARQTKGKYSLEDFSLQRTLGTGSFGRVHLVQSRHNHRFYAIKVLKKAQV
VKMKQVEHTNDERRMLQRVKHPFLITLWGTFQDSKNLYMVMDFVEGGELFSLLRKSQRFPNPVAKF
YAAEVTLALEYLHAQHIIYRDLKPENLLLDRHGHLKITDFGFAKEVPDITWTLCGTPDYLAPEVVS
SKGYNKSVDWWSLGILIFEMLCGFTPFWDSGSPVKIYENILRGKIKYPPYMHPDAVDLLSQLITPD
LTKRLGNLHGGPEDVKNHPWFAEVTWERLLRKDIDAPYVPPVRAGQGDASQFDKYPEESEAYGSTG
EDPYHNLFTEF
>g3541
MPDIRNFFGAKAGSGTAAPAAAKPAPKEDPTAARKKRSRKVVDDSDEDDFEPQPSPKKTPVKKHTK
DEAKGEPTSTSDYFASNKKRGRPPKNAEAKDTAPATASPVKNGTSTPKAKPHKEVLASTEKKVKTR
ASKSVSKVLDDDEGLGGDDIFATEYGKSGRRDDDYVDDKDASDSEDLIVKPANSRKSKLDDEVEML
DTPPKAKPGRKRKADADEEDNKPKKSAGPAVKKQKSATKLQEPENKEIQDIFDAIPTVRPPTPPPT
VPGAKFNFSTAARSRTPPAAGSAEIPVGAENCLTGLSFVFTGVLDTLGREEGQSLVKRYGGKVTGA
PSSKTSYVVLGSDAGPSKLRKIKEMNIKTIGEEGLFELIKRLPANGGSGKAAGQAQAKKDAEEKKI
RAMAEEIEQEEKQKAAEAKAKSTPQKTSTKTSTETATATATPASSQPEADGRLWTTKYAPSSMNMI
CGNKGQVEKLQAWLRNWRLNAKRNFKMPGKDGFGLYRSVMIHGPPGIGKTTAAHLVAKLEGYDVVE
TNASDTRSKKLVEGGLLGVLDTTSLQGYFSGEGKNVDSQKKNLVLIMDEVDGMSAGDRGGVGALAA
AAKKTNVPLILICNERSLPKMKPFDHVTYELPFRRPTADMIRARLMTICFREGLKIPPQVLDSLIA
GTNADIRQLINMLSTVKLDQKTLDFDQGQQLSKAWEKHIILKPWDIASKILNAQTFSQSSKSTLND
KIELYFNDHEFSYLMLQENYLKTQPTLASNYSGKERKLKLLELADNAASSISDGDLVDRMIHGSQQ
QWSLMPTHAAFSFVRPASFVFGNMSERTSFTSWLGNNSKFGKLNRVTKEIQGHMRLRASGDRDEIR

QQYLPALWDKMVRPLMVDGKDAVDGVIDLMDSYFITREDFDSIVELGLGPMSESLVNIHAQTKSAF
TRIYNQRSHPLPFMKATNVVAPKKGPKVKPDIEDAIEDSEDEIVEDAKEEDEEEELDLKKDKYVKV
PKAKKAAGKGTAAKGKKAKAKDDDEDDEEEKPKKGRKGKAKANYYVDIDFGVEWTLPTTYFRPRNI
VKIPARLELALYSTMSLLQSEDFTIWQLRTSYLSTIKDGIGDRLIDVNNSVLNTPGFRAAGWVPTS
ANATTTTQESGSLVKRTYSPPIPTTANVASEYYRFARHTELNDGDGLSIDDAEEDEGGMVTGGGGG
STYALGIKNHGKSARKNRRRDRQIPVIQRVGEDEDSSDLSDESDDEGDFARAAQQIKFSKMPVRNR
AGSLPMEDEEVQHAGSDANLGRGYRRSSMGAVEVAQGRPRGDTVTSSDMSEGDNPALFRRRQIQFS
SKHQVIEEPADDGQANSEAPSGELGDPDEDSAAGSVDSDLSSDFGVTAGSASLLAGVPLNDTLDSS
PAMMHKLPNANSQGASPRKAKVTTPGLQELPPPRPISSVQCVSLLSEKLNAKKAIPTNPVERFGPL
WAADSNRALNIKIYAPFSEDPETPYNMPIFRDFRDQGRTGPVTVTEAIGLALWRYMKEGLKPAIDP
GKMNVNRWTLRMVEDGEVDFDFPALGRHLPLADFTSNNNRAAGMRGRSRGKQYDEFALVEATDAEF
EEYSRLYPQFNPEPVVETSEATPQTSSTNTPQTKAIPSAVRLNPILGLPFTSALNGSTSKPADMPA
PTSHATPRLGVQKTLKVRFVNIEGSTQTTTVNTATDSYIAEILDSVCKRWGLDKGNYLLKISGTNT
EALQDRTVEVLGNVSELDVVRRRFGAGASFAGSPGSASPNAPLQIGGTTSVPFKKGKKHQMLHPLA
QPQKQDLVGGYYRRYYVYRKQSMSFTASNQRVLVLDNDYLHVMPGETAKGVFDTTGKTRSINFNEI
VGTKVSRRHPKSFQVVVLRGADAHEQKRYDFEARSEAEAAEIVGEIKKNMEQYRVW
>g3542
MLAKSQFGLLRSYNVLLQSQITRCFSTTSPAMDWLTPKFAEKSKSPKGRPKMHTGGSMRGTTVVHG
DYGLRMKDHDRRVAASSLKIGEDTIRKRLRGMNYTLYKRVSANIGVYTSGNEMRMGKGKGKFDYWA
ARIPVSRIVFEIKGDIHEKIAREAFRLAAHKLPGMWEFVKKGDPPVVGITKLGNGVTLESLKRARR
EVPLDVKNSLNPPKTASTSTSPTQ
>g3543
MHVTDWLASATQKSTISPLKTLSKDESEEQRDNGNQASKDPALTGPQTAGMTSGVSGEVDKDLKPD
AEGIDEEARRTRTAQRLGPGSGIGA
>g3544
MALLVDRLRPRSLDSLTYHPELSARLKSLAQSGDFPHLLVYGPSGAGKKTRIIATLKELYGPGVEK
IKIDARVFQTTSNRKLEFNIVASVYHLEITPSDVGNYDRVVVQELLKEVAQTQQVDQSARQRFKVV
VINEADHLTRDAQAALRRTMEKYSPNLRLILLANSTSNIIAPIRSRTLLVRVAAPSETEICAVLKS
AAAKENWAPSDALNMRIAKESGRNLRRALLMFEAIYAQNEKVSDKTPIPPPDWEALISVVADDIIA
ERSPARILQVRERLYDLLTHCIPPTTVLKTLTFVLISKVDDALKPEVIKWSAFYEHRIKLGSKVIF
HLEAFVAKFMRIYEGYLMGMDF
>g3545
MDMRRLRFLGRYRKTCHPQQSFSHLTTNGTRRFSQRSNLRQQASSQGNADSKQKNTSTLYYTASII
VGTLALAYGSVPLYKMICQQTGWNGQPVQVHKVGDGDTASRLKPVTDSRRLRITFNGSVSDVLPWK
FTPQQREVRVLPGETALAFYTAVNKGPQDIIGVATYSVTPGQVAPYFSKIQCFCFEEQKLNAGESV
DMPVFFFIDPDFAKDPTMKNIDTITLSYTFFSEPPFPRFSSPNALFLYGAVY
>g3546
MSTKRKRSNDSNEDTTNPARENKRFTWLKPQVKNVSKHTIKSKWSSLPEPAQSKVRDLFRSLERPV
IVRQRDERKRIEAQVALGGVVKTLERRLPRMPFPPLTRDAGFDHESVLNEHRVLETQLSTTRNTID
LLKLEIEREEALLAKETKYVEEMEKNAKKAEVERRRQMKNEHPVLRHIDDHPGHDNISTPSAPFVV
IDRSKEQTALCEIEPDREIESIVMQLNGHLNSMQNNIAPFVELREAITEAQAALDFLPLPPD
>g3547
MAHLNTDNFLAANEPPYDVGPAPPPGPSLLDDTESNMLESFFTTLNTSQFNVNDAWFQDLSHDKGG
GAFGLDWTEGLPPNLEGSTTSLSQPPILSMNPQKNNNMMGGMGPDSDILAAAQMLYGNGANTMNFA
TGHLFPAGMPEMAPNHMPHPRIKQEQTHHPPANHNDPRQMMPQGQHTSVFNPEQPAIPVDPHTTIE
VQRLRWGSDAGFVDQGYRRPAEMENTDEVTKNLLENMKCLEPQSSTTNTRAPTPTKFETPHNREWN
NGNGVSTSQSSDTTNYDEGDGSSRPRKRSKIKMQEDEDSDDNSGTPRIKKMKGASGGKARRGSSES
SSKRTKAQQAKAARENLTEEQKRTNHILSEQKRRNLIKQGFDELCALVPDLRGGGFSKSAMLIQAA
DYLEEVLIGNNELRKQLSQLKTVNGFMIPR
>g3548
MAVPRERAVHPHLIQPILKAYGLGYLALTTPRLLGFIPLLLISKNANYRAKLEKLYRILRGGFGLYK
FSTFCAVLVGGSTWLPFLFFQCIARSRRSSSENLRRIVRFVATCLSGWAAFHLLNKKGEDQGSIRN

SDFDQKLMVERQSDDIKTKEYRPDLAGRSLDLTLFSFTRAAGVIASLAWTEWARRRRSKGRWSRVE
DIAPRLADAGLFAGSAAIVMWAWFYAPERLPRSYEKWIGQAAQVDGRLIEALRRARRGIYVYGKDT
GQAPLLESMCEDYGWPITWGDPAVTVPIPCEMVHMGCGPNCEWHALSRFMRSFKFAMLTNLPLQLL
LRLRAKQPATAFNRAVKDATRSSTFLALFISMFYYGVCLSRTRLGPKIFSSNTVTPLMWDSGLCVG
AGCFMCGWSILAETASRRQEIALFVAPRAAAAILPRRYERKYLKREQIAFTLSAAVLLTCAQERPD
LIRGVFGKLLSTVFV
>g3549
MPETIATAAPDPRPHALTGTGGAEEDVKPNIENASSSQGVNNAGKGSSTERHQHHPAKADAAAGQA
HASPKKRRKVNHACVYCRRSHMTCDSERPCTRCIKRNIGHLCHDEPREPAKKNSRSDHEDSAVVGN
DFANPHNLADTGNQSMLSDSSLSIRPSTIDPSQAVQPAAIQPTAGPGNNQQVFGYNTGGSGGGDWA
MNGQSNGFQDMHTFHPSYMFNAPEVTNEYNLLGDFLNNSLLDDNGMYGNEELQGLFADSSLINMST
NINSNAVPFSQQQPQQQHQQQLAPLAPQQATNPQANAVQRLATSAVTTDKAREIYYMTAADPSGMD
PPEERMNKLLKAKYEAGMLRPFNYVKGYARLNQYMESHMQPALRHKILRQLDKFRPKFRERMQSLT
DIELVLSEMWFERSLMEYDRVFASMAIPACCWRRTGEIFRGNKEMAELINVPIESLRDGKLALHQI
ITEDQLVSYWEKFGAIAFDGSQKAMLTSCTLKSPDDDDSTKGIPCCFSFTIRRDPNAMKKDKILTN
PFNNTPDLSTMDIDEEENAISICAFTADIMSRLDNDNASLQSFSAWMQEADISHTLSTTMQSLTTS
ASSLRFSSRSLRLVDCSATALWNAFVRFLRRVGVDDEVKGGLVTVPLLVCRMLECCCSEDGESGWQ
TFQVLVRSAEICLGSSMIVEATDLLQQAALKVNSIDLGELADWDGQDVIARYHFLCAWMALKNEDI
KEFDAHFACLPQDVAKMSLGSQEQYVSIRLSAFRLAVKHGLRELAESLLHNLSETFSILRQCHHEV
PEYKTLVSGFFYVLEDMSKDFNRLRSELLEITEQDLDGPLGLFLQLEVASRQEVFDRIIFFQILTK
YIETMEPTKDKFKRKSMDCSFQTGDHTEVKLTWELLSKAERDQTTIYIMYQVSLRDSDINTAASLL
ASLIELANGEHRYVLAAVAAAVTVSSQLRRKALQSAQALVEYSIVSKKKPINLDLHRALIRLIVYE
LRDDPEDVETLKELMCKLLTWVSSAISQEPSDSNDHDWFVRCAYVLLLESRDWIARHQLLVLGVCF
KLIDMYPNDVGNDVKYELNCRRIACNLLGMLIYTNMARSEQNVDMKVSNMHLYQNMIIDSVKRTYY
LVMTGRYTDIQKFVATSTDAPNITKYLDASMIEICYEILCFYLEAVIYLEQWPDVEAFIKTASTID
SDHVHSIMVDMILTAKKMPLEYALRSLQELLNALQCIDTKRFSLIRCIFDLCLQHRRNDIRMCEIV
LNQALITAQDTTASTEIKDDELEYISTKSFNYAVDLYLSGQQTDSQRWARKAIELSQLMREDCGSL
ALILQSKYEKWLTYDMVISDS
>g3550
MASTPWNTKSKIPGSGATLKITPANSPVLRPGTRSPSKPSPHQSVLSLQTVIGTTTATPNGFSSHE
PSRSFALCVGSAAILADIDIDGTVNQRFFRARPTATPVNPVVSFYNQPASPTTPDNRVRSVASRVG
NGGGISPAADWADSSGARTWTSRERIKAVTSVAISPNGRFLAVGETGYNPRVLIFSTAPDAPRDIP
LTALTEHTFGVRSLAFSSNSQYLATLGDMNDGFLFVWQINLRTGAAKLHSTNKCTSFIRDMCWMGQ
TLITAGVRHVKVWRLDSRPGSPSKLRPNSDNFSSSFSGNPKALSGRNCLLGSLGEHTFTCIASVSD
CEAIICTDSGAVCLLDDTEGNQKLTLVKLVPFGLTSVTVAPESGTVWLGGRGRKSLKLRMEDIREW
TKLRSPSPSSPVDTINPKGKVPTFISIAYLATHIVTVDSTKAIHVCPVDAFGNGDDEEILRDSLVP
AHRDQVLGVGPLKLPNVYMADFFTWSCEGSVNFWDMQGRCRASKKVELEQLVWGEDEVANELKVIR
VAEDTNTFVSGDRYGVLRVLSAEPWKCLNEVRAHASSGRDRMVQLFKKTEETFELIQTMDDHVGAV
GQLLFMNDGERLLSCSADRTVIVREKATRDVDSSTVVAYLMSKVITLKVSPLSMTITPGDPDNLVL
STIDRHIQRFDVTSGRHVHSFKATDFETNDAVVMSSLTVASEIPGQSPRILVGVSTTDKSIRVYDF
ERDALLTKEFGHSEGVSDIILLERRKDNTHEVGRTLVSTGLDGVVMIWDLSIQQQQAQELSQGNIR
DEEETPSKELVAAKPPLRRILSRSELAGFRQDGLMATPTPIREQSPPRIRKKTSRYTLTPSLPRNI
GRNDSPPPLPNLRRSPAPVLDPRRSPSPPSPKTRLINGVNHRSSINNLRNSSIDFRSSTRSRNSTP
SEFGSLNMSTEQVCRTLRAYRKKLHSSTEYPRGAKELERELNLTVHALSERAARHSSSPDTEIDSS
EKENKDNHTSNGIAVHKQSGRKQASSSSSSTNASLLAPSAKLTKVARRMPSTPLLSTKSGTRQVSR
SRSLDADGEG
>g3551
MKASSAVLLGAAAFARHAAAAAVFAHYMVGGITQDHANTDIQDAINVGFDAFALNLMSTEDWSTEA
VASLFTAASGTDFKLFFSFDMGHFSDPSQFLPILEQYASHNNYYQYDSKPFVSTFDGGKLTFGSSS
PNAGWQSTFKDAASAAGVDVFFVPDFDDASGYPDSFFDTFSVVDGAFSWESAWPYEDAGFANVSDT
VDSTMIDAAHNAGKIYMMPLSTFQFKHLDTSDNWYRRGESNLYVRMGQILDLKPDLVEVITWNDPG
EGHYIGNFWNESLGNTNIYEYAGGFDHTGWQKVISPFITAYKNGTSAISEITASSNVGTMWYRTLL

TTASCSDDSIGLPRSHENAQDAINYAIILPDSGSDYTINVYSNNNLIGTAEGVTGLNGGSVLGLAA
XSADTQRVEVVKTSSDGATTTILAATGTKNVGCKCCWLFGSCFDGGSSGINLEFVGTLHNFINVVC
FFTSNFITNVTYTGNSSSIHCTNDLINVNLNNCTNHHLTQNNYNSNYNNCLFLPTRQIRVPMGPFR
RALRQRNSPGRAQPRIQQTRSQRWLLNYPSP
>g3552
MAVCAASRRLTLSNVLQGVYRTELVRQHADHSVINLYTHRVTRPSLSSSWLSLSNRSFSTSQASSG
ANFVIYDAIATKAPDRDVLTELTSTKTETPGETQENPKSTSEKPKSDDKKKKGTKKISTKKAELET
LGFAKKVKRPHWQTQKEALEKKFEEGWHPRKKLPPDSLDTIRHLHATKPDVWTTPVLADQFKISPE
AIRRILKSKWQPTEEERERREARWEERYKKIYSHMAELGLRKKKGEWVARASDARALGLEEKTIRR
NVRRRPQVAPSEPSEGTKVRFTRPDRGDAPKESASS
>g3553
MEGLKITSLLQRFAYILPLALLSIGVVKADNPFVQTIYTADPAPIVYNDRVYVFMDHDNTGATYYN
MTDWHLFSTADMANWQDHGVPMSLANFTWANANAWAPQVISRNGQFYFYAPVRHNDGSMAIGVGVS
STITGPYHDALGKPLVENNEIDPTVFIDDDGQAYLYWGNPDLWYVKLNQDMISYSGSPTQIPLTTA
GFGTRTGNAQRPTTFEEAPWVYKRNGIYYIIYAADCCSEDIRYSTGTSATGPWTYRGVIMPTQGGS
FTNHEGIIDFQNNSYFFYHNGALPGGGGYQRSVCVEQFTYNSDGTIPTIEMTTAGPSQIGTLNPYV
RQEAETAAWSSGVSTEVSSEGGIDVGYINNGDYIKVKGVAFGSGAHSFSARVASAASGGTIAIHLG
STTGTLVGTCNVPSTGGWQTWTTVTCSVSGASGTQDLYFVFGGSGTGYLFNFDYWQFA
>g3554
MKCLTIAILASTATALNNGLAVTPQMGWDDWNAFGCSLSQSLILNTASVIVKTGLRDLGYHYVILD
DCWSSGRGSNNSIIPDSSKFPNGMAWLGNQLHAQGLGFGMYSSAGTKTCAGYPGSLGYETVDADTF
ASWGVDYLKYDNCNNNGEAGTQAASSARYEAMEKALAASGRNILYAICNWGQDSPWIWGPSVGNSW
RITGDITDSFNTENSVCQVPNYGGYGCSVTQILSKQATISSYSVKGGWNDLDMLEVGNGGMSDSEY
VAHFSLWAAAKSPLIMGNDMTKLIASDYSILANPAVIAVNQDPLGVAATYRWTQNNIQLWSGPLVS
TSGSSVNDQVVALYNNGGSSATVSASISDIFGSSSVPSVQFEIRDLWGSRLNDSQAQAILTNGAAA
NPSLLYNATAKSYASGLAQGDSKLLGTSIGSVTAPGGSISVSVPADGCRILRLRAVTSSSTSSSTS
ISTTTGTSTTTTSPSQPLQTHWGQCGGQGYSGPTQCQSPYTCQVENMVQVFAATVVAAAAATDLSR
RQSSSTTAVVDLSTNRGATKHLASGFIYGIPDNETQIPDHWYTNMGFEYNRGGGAQMDAPNRGWVW
GLNEYKGRFQSTLSNYQVTRKFGGTFIILPHDIWGTDHTNSSTHWPGDNGDWSSYDAFLDQLLSDI
KASNMVDHSVFDIWNEPDISIFWARSSAQWVELYTRTHQRIRSDSALNAMQISGPTLAYRPSSSNS
WWTSWLQAIKSNNAIPDQYAYHLEGGTTAVDNDLQTSNQTLGALLSQYGLPERQININEYANTGEQ
VPAGAAWWIARFERYDAIGLRGNWRGGCTLHDLFADLLTKTSNPNSCGATDYAPAPEYPVYEYYNL
NMTGHRVATQGSGDRQIDVYATVGSDGVVRVLCGVRINTGTWYITIDNLSSVGLPTSGTLNIQTWG
FPGGSSVFSTASGLQNKGVASHSYSGNSVTFPVYQTDTSTAWAFEFRAGSGTSSTTTTSVSSTTST
TSTSPSAATQTHYGQCGGEGWTGPTQCAPPYACESENTWYSQCL
>g3555
MVAMPSDLQVFVKWKEQTVFAGEDVECTITFKNVAEIAEEKANNPSHQQQHQRRVSRPLNALNGSK
NDGYFAAKSSPTFFFNGSRRSVPSSPRRPTLGSHRVSASLSSPLTGSHSFPPLHTPSQLNNGAPNS
HKHKRSVSILSIESDNGGALEKSPATQFSRSRPWGHGRSASLQMPPSRSNGLDEFLGNGRNSARGI
PKESALSPRLEAEKTGRTIPTSPSNASHLRSPRTRPPSFPTDFKFPPSPDPAAETDGNTNGAPAAA
NDGPASRPLSKQPPSLTQQAHLAPATRILSSSSANGSNRSSGEFYAASNNSTDTLESEYTNYGGNR
SKANLVKHRRHYSSLEPIGRKRDALTLLMGYAQVSASFTVDGALINQSAFEEVKRKGVVGGQTSGR
KQEKPRRTGGFLGAFGWDAIEESISGLLSNGDLEGLRDMRGVTSSNSIPLLSTPQSLLFVDLRLGP
GEEKSYSFSFTLPRGLPASHKGKAIKISYNLVIGTQRPSGPKESQQVNRINVPFRVFSGVNDQGDV
LGHDLMQPYVLLRDEARVQKVSSTPRPPQKDKSISGPTWATAPEFLSYVDEILEKHETQHLRTPGP
PLGTPLERRRSSTGQGPPPTCKEAIEFAILRSNHVNTSNRSANRFDIARNGRRIAVVVLNRPSHRL
GETILATIDFTDATLPCFSLRATLETSEKVDPSLALRSNTSIHRATRRIYASYFENTLFSTRVVFS
PAIPISATPTLLTTGIKLDWDLRFEFVTTHSKHDTNVSLSGNGLLELLGQDERGSVYSALENLPSE
SFEIAIPLTVYGETIREPLAEEVEGYSI
>g3556
MSTLSATAAAPSSGLTPSDSTQTIKARHHYLPIEPPSYLSTAVRALKGTIPILTPPSNARTKGPFK
PRSSRGTTSYQLRKFAEETLGSGSLRKAVKLPEGEDVNEWLAVNVVDFYNQINLLYGSITEFCSPQ

SCPEMKATDEFEYLWQDSENYKRPTKMSAPAYIEHLMAWIQGNIDNEQLFPSRTGVPFPKTFPALI
RQMFKRLYRVYAHIYCHHYHVILKLGLEPHLNTSFKHYVLFIDEHNLASGKDFWGPLGDLVESMLK
SD
>g3557
MLSARVAARAGLRNFAVPRSVAVGGLRTYAAAATQQDVRPPVALYGVDGTYANALYTASAKTSSLD
AIAKSLSQLGEVFKKDPKLTTILNAPTLSVSDKQQIIKELQTVAGGDKNEVLKNFLSTLAENNRLG
LLEGVIEKFETLISAHKGEIELSITSAQELDNKAIQRIEKAVSKSEFSQGKKLKVVTKVQPDVLGG
LIVEIGDRTIDLSVSSKINRLNKALTDAV
>g3558
MVVVNDVEYLCDEERRLKEDRERKRYWKKWGPYVAERQWATVREDYSENGDAWSHFSHDHSRSRAY
RWGEDGIAGVCDTHGQLNIAFSFWNEKDDFLKERLFGLSNPQGNHGESIKECHFHLDNTPSHSYMK
YLYKYPQKKFPYQDLIDENARRGRQDHEYQLLDTGVFNESKYWDIFIETGKEEDDEEEISFRVIAY
NRGPEPAPLHIVPQVWYRNTWTWGWEDKSKKPIIKQESPLKVKTSHHALGDRYVTLSPSPSFNGSE
EDIQPRMLFTENDTNNEALYGTKNDQPYVKDAFHRYIVDEEKDAVNPDNEGTKFAAWYAFDEGDGV
PPGECAVVRFRFSRKNEEYVNEEELDNVIEQRRSEADDFYYRINPLPMAEDLRNIQRQALSGMLWT
KQYYHFIWDQWANGDPAMIPPPPGRKNVRNQQWRHMYLDDILSMPDSWEYPFFAAWDTAFHCIPLA
MVDPDFAKKQLDLLTREWYMHPNGQLPAYEWNFGDVNPPVHAWAVFRTFKIERKMYGRQDLDFLER
VFQKLLLNFTWWVNRKDADGKNVFEGGFLGLDNIGLFNRSEPLPTGGVLEQADSTGWMAFYCLSML
NIALELAKHRRTYEDIASKFFEHFILISDAMSFKNKDGETSLWNEEDSFYYDSISYGGPWSQQLPV
RSLVGLIPLYAASTLEPEIINMFPSFKRRLNWFIENKNDVAERNIASMSNRGKDDRLLLALVSKDR
LEKILKRMLDETEFLSDHGIRSLSKYHEKNPFSMDVNGQKFTVGYVPGDSDSGLFGGNSNWRGPIW
ICVNFLLIESLLRFYMFYGDSFKVECPTGSGDYMHLGHVAEELQHRLQHLYVRDYDGRRAINNGND
LLDFDEHWRDYLWFYEFFDADTGRGLGASHQCGWTGLLAKVIHDTGLNCRLPQTPRTPTTAAAHYF
DDVFSHPGRPPKGGRRPSRLLRRSSTSRSIGNRSDFTASINGAGDEENEDATSTNGADDHMQENFT
TYIADQLNRVRSSATLGTYEDEFTTEVDRNGTNGTNGH
>g3559
MSDTEQEWKPNPRRPQSTMAQAFSLALDSAFMLDNEVNDLTQSIELKKQRMTIQTRELEALQARIR
ETEERLQREQAAKEAAKNAENNTLQGTDSAQQGRGESDLARALSPTSSTLSDEHQSDEGATDSDTS
AATSNDMDEQEDDGKGKPRA
>g3560
MPPLSAYTPFESLLFFQCLASLNTRPANFAAISDTLRKNQFIKEDDAFDTNRLTPQALEELYTTLL
QEGIDESLDKNGLQDGGSTNPKKRKIISGVGASQSHTAIIPELVTQLYARYKDRVTKEIREEEKRY
KDISQEIEKIQKDALQEQAVPAAPEQKATAXLAGAAAEQVSDETDLDIKEDAKLVEKAAPTVAKIT
EKARVDVQVPAPGPLEQKPAPPPTVATTPLPPKPELAPPTPDLSKQQVPLPPVPLPQVAPQLQTPQ
PTQPPKIPTPQPQQVSPKQATPGGHIPPVTSTPPIRSASNGKPPPLAPQPPAKQQLSPNIQVKVPP
PPQPQRQSQPPSRGAANRNQPNLPPGSTIVFQAQQGPSQPATPTSQRQAHAAGPGLGRGTPVPVGS
PAFSQLPPGQQQFQQWVPHPQAGVFPATPHAPPPYKNQPMMPKQGTFVPHDSTGKPVPIAPQGHGP
ATPGPFPHPMQTPMAQIHPQATPITATPSLSGMKPRPHRPSMDTAGSLTPWKRTPNLHINIPETPG
SPPRPRPEDVSPISERAPSPTEPVAPTVKEQKRRGRGRKQSVPPEAEVVATKAVTATSGTKRAGST
ASTRSRARSIMSRDDESASELATAQRRIKHEAPPTPAGVADDVEVETRAGTRRKGVAGDDTQSKSR
GKRKRGASEALEADMIQPTPSRGVSSQLVYCTRNFTRTGAPIMNDVAAHKHASIFAKPLTEREAPG
YKDLIYRPQDLKSIRGALSQGNRAVAAATEAAAAEGESPMPSGTPSKNTAWLAKTSELIPPKAIVN
SSQLEKELIRMFANAIMFNPAPDAERGFGPSFRMIRHGQSSATTRSSSHPWDMDEGGISRDTREMC
DDVEKAVTKWRAAERTTTTDEQANKSMLSLRGSSGDSNVDAMDEK
>g3561
MDGIEDTTSRPATRQPKKRFVGRRTADAQAQTDSTNTDTSIEKATPRKPPRTLNHIPPSISENPDI
LAAVSLLPSNYSFEIPKTIHRIRTLGAKRVALQFPEGLLLFATTISDILTEFCDGVEILIMGDVTY
GACCIDDYTARALGCDLLVHYAHSCLIPVDVTTIKTLYIFVDISIDTTHLIATLERNFQAGKTIAT
VGTIQFNATLHGLKPVLERAGFKVIIPQIMPLSKGEILGCTSPQLSDKEIDYLLYLGDGRFHLESA
MIHNPKIPAYRYDPYSRVLSRETYEHEEMHTLRRDAITAASSARKWGIILGSLGRQGNPHTMSLIE
SHLNKKGIPFVNLLLSEIFPGKLASMPDVECWVQVACPRLSIDWGYAFPRPLLTPYEALVALEARK
GWDEGNGGVYPMDFYGKDGLGRTRAEQVSV

>g3562
MQKFKSSLRLTSALRTAGPRTTPVRHFATEARLTTDHVRIVEVGPRDGLQNEKKSISLETKLELIN
RLAKTGVTTIEAGSFVPAKWVPQMASTSEILQYLIKTPPQSQNQIAYNYLVPNIRGLENLVKVMEA
EGATTVGSKDDPKASTTTEISLFAAATESFSKANTNCSITESIERVRPIVALAKEKDIRVRGYVSV
ALGCPYEGPDVSPGKVAEITATLLEMGADEVSVADTTGMGTAPRTLELLQALSAAGIANSDLALHF
HDTYGQALVNTIVGLEHGIRIFDSSVGGLGGCPYSKGATGNVSTEDLVHTLHGLGMHTGIDLEEMS
RIGAWISEQLGRANDSRAGKATLARIAGEKS
>g3563
MKLSQFLIIPRAVAHANRRVFLSSPRRSFTSSYNRRVADDDKASPFGSGPAPPRLPKEEQEIFERL
QKESTGAFSTPRTPPKVNQSPDSQVSTTTTTTTSTTQQTSSNGQPEGELFPHMRETVKPEFEGDTN
PKTGEVGGPKNEPLRWGHEGDWSYNGRVTDF
>g3564
MTIRAAARRLQSLKSVQPLLTARSFSSTSYVCSLPRDEKARILKESSLPTPDPGPDSSTAAFLRDR
TPYMVPTYVRPTPVFVKGQGVYLWDMENRRFLDLTAGIAVNSLGHCDPEITKVISEQSETLIHASN
LYHNPWTGALSKLLIDETITSGAMRDASQVFVCNSGAEANEAAIKFARKVGKTRDATGEKVELVSF
QGSFHGRTMGALSATPNPKYQQPFAPMIPGFKYGAFNDVEQLETLITEKTCGVIVEPIQGEGGVNV
ATPEFLIALRERCDQVGAVLIFDEIQCGLSRTGTLWAHGNPVFTPKDGSKKAAHPDILTTAKALGN
GIPIGATIVTENVSSAIKTGDHGTTFGGNPLVCRVAHHIVERLAASELQNGVEVKSAQFIAGFKAL
QEEYPEIISEVRGVGLILGVQLSEKYASKATDVLTAARQRGLLVITAGEGCLRFVPALNITEDQIE
AGLKVLRDSFAAVFTE
>g3565
MEATKIDEALQRATNEFGLCPNRVWAIGHNLEDWEKKLPDLIPTNQRANSMGIIGHEGHDRCTFEL
CEYSILNYTSVIQRHEYGVASCREEKRCDTLKGLFSVRKLDEASLAGRPTAWRLDGETTIDVAEPY
MAISHVWSDGTGNGVSANGEVNECLYRFFERIARRWHCTGIWWDAICIPKDKAAKDKAIRRMEHNY
EGARITLVHDCYIRSWKWEDSETACLAIILSPWFSRGWTALELARSTRVKVIFKGNVIKDLDDDIL
SKSDSDSACHRFATEAIRGLRRKRVENLNDLLTVLGPRSTSWARDMASISALLVGTPKSSGGEKQH
VIYQQVLYTIGWVAHGHLFHRSATMAGKFSWCATNLLAMPMASRTQERLKIEHDGSITGRWKVIKQ
NSVPRERYVLQNGFPLVDMKVNVTLEAGDKSVLLGEPDAKALTRAIAAKVLDEAEHHYEFMGPVYF
NPPLNLDEIHWENTGNVTISGRQEDTNSSQKIPFEPELLYIPDHLRYLIDSVPTVMSAAFRRKYRL
ARAQLEGDHEKASNILNEGPVGAMPRVDTSISKGNRLVRAVMEGDENAAHKVLEDKFIDFSFKDDA
GWTALHYSVRRGYNSIIEKLLYNMRYGRGDTAVHVAVQDGLGQQCIHLAAERGDCELVKSEEELLK
VHKECKDGQTALHRAVLGGSLEMVRLLPDAFLEAAKDAQDLQGRTALHLCAELGQGKMFTALLSIK
ANPLLKDKGGRTTLHYAAMDWNHTTDVDMVSILLNYEELNIPDNDGRMPLHYAAEIGYPRAVKLLS
RKTNLAVADSRGQTALHLAVNGGHLEAVNELLDYDMEKHELLQLALDTALHLAASLNHLKIVQALV
HHGASIWYRDASDNKTATEVAVEQKRAPLTMYLMELVLSSQRHEEAPPGWIEVASTCSLEKNNDAS
ALHHAVNRNYQKVAIALALGGDDVFVTDGFGKTLISLAVAKLAKSVVYALAYSYGQAVFRAKNAEQ
GLQKAAYNSGKPLDSYTIELGEVWRDLFGATLSDSWTAVKHLEQAYQSTDNPEMQALLHPDWEVLR
LEFPNVDLGEAKRSQDQLTPLRLHGRSIGRLEPHVREVLDLYRMDAQTLGLHAWQYVARNRSPSGQ
YELEDDEEEEEEEEEEEENDENEIDDDDIDEEEEDEGDDYSSSDADLYSPRRRRRGRKEDSEEDPD
SD
>g3566
MAPLAVTDPLASMPLEPVAIIGMGCRWPGDSETPSELWEFLEAKRNSYSKFPTDRINGEAFYHPDP
NRPGSFKTEGGSFLKSDVRQFDANFFGIHPKEVLTLDPAQRKLLEVVYEAFESAGVPLHKLSGSNT
GTFIGNFNYDHQLMQYRDAENALPYSVTGSGITILSNRINYIFNLKGPSMTLDTACSSSLYALHLA
CSAIQTGDCTAAVVGGSNLILTPECQIFSSVLGAVSPTSVCHTFDSRADGYARADGIGALFIKKLS
QAVADGDPIRAIIRGTSFNANGRTGGITHPSPDGQEACIRRAYERAGGLDVSLTGYFECHGTGTPV
GDPIEVSAIGRVFADAKLPSQPLLIGSIKSNMGHSEPSSGIAGVIKAVLAIEKGIIPPTIGIETLN
PNIDLKDGRLKIVTESTLWPDLPVRRASINSFGYGGSNAHAIIESPESLLPGYRARQQASSDSFSI
EAFASANGVSQLASPAENRQTNGHTNGHSHAQASADGSSSGNCVLKDNGNRSKLDKTRFLLPFSAH
DEKTLRANYKSLVENIEKWNVEDIAFTLSARRSLLTYRSFVVTSSDNTRHDLPEENELTVAKRSSN
AHPFLGFVFTGQGAQWPQMGLSLMKEFPSFLSTIRRLDGYLDDIGYRSWHIEEVLSQDPEQNPVYN
AELSQPLVTALQIALVNLLSLWNIKPHAVVGHSSGEIAASYAAGMLTEREAMVAAYLRGVAVAQNN

QNGLMMAVGCQLSYIQPLVDKFDGEIVVACHNSLESYTLSGDADAIVQLKKTLDEEKIFCRLLSTN
NNAYHSHHMKAFGSQYESNLEVCSPKHVDSYKVKDAKTWSTEAVFFSSVFGHATPRSLLGPKYWRQ
NLESPVLFHQAVTDMVSRVPVDALVEIGPHSALRGPLRQMSKIMDSGSKFPEYFTALVRGDDNVKN
ILTLAGSLYNRGYEIDLTRANAIEDTGGDQCQFGKVITDLPRYQWQYSKDILLYENRYTREWRLRM
HPRHDILGSRVPGGNRTEPTWRNLLSVKNVPWLNDHKLGTECVFPSTGYLCMVLEAAIQVVELDGR
ISTDVVYYDIQDVSLSKALIIPEDDHGIETLFSLRPASLNANSRHQWLFEFTLTSVSIEEGRDIFS
EHCRGLVEVSFERHEFPESQMMKDIQDAATVRKTINAGQWYQTFARVGLFYGPTFQGLSSIFAVGE
KTMVEARVDLEPAAKIMKGESRYLIHPATLDASMQLSILAAHKSDTTKFQRAFMPTAFDSIRVWPN
MVGQGHGFATSYASAHLKGVRGLSSDIVLLNAHHQRMLEVNNVLLTASDQSAPKLIENSSPYTRII
WKPDFDHLSTTILEKIFKPVVLSDDAVIPSLNHLALHQLIHFKLTNQEIFEAGSEKWHLQRLLDWT
TEKLDLAKLDPNSHAQKIMSYSNKFRAEEINRLSDILKPQSSEARLMCHLYDNLHEIYSGEKTGIG
VALQNNLLQDYYETGQVYREGNRRLAAIVSLYAHHSPGLKILEVGAGTGSATNGLLPALKGDSVWR
QYSEYRFTDTTTSFLAEAKDRFCQYGGMTFGSFDMEQSPDSQGYERDWDLIIASNVIHATSDIKKT
LTNIRSILKPGGKLILLELTQSQLSATLVLGTFSDFWKADYDPTYPRYDGPFLSQSLWRTVLLDAG
FSGLDFLLDDYAAENVSTTVLCATAIEPQSHVTLSTGISGHEGISFIYRNSTEPFMQSLASHISSS
RGVPIECIPLRAIDSARYQRFVFLLETTDPFFVNVTTEEWTAFQLGLKRSASSLWITNGDLMTGKE
PLHAMISGLVRGMKTEDSSLRLSILDLDKTPQASDTKLFDVIEELEARVANITAVDDDSEFRYKDG
TLYISRLAPDEDLNEKYNEAAERSSGFEKIALRDLKTLPLQIAIEKPGILSTIYFKPDPAFSNLLA
ADEVEIEVKYAGLNTKDIAVLSGRHHSNSFSDECSGIITKCGRDVQNLKVGDQVYCQSFSKFGNYV
REKAIFCQRLEQGDTLEGTSTLPIVFSTALYGLTDLGRLARGESVLIQSATGGVGLAACQVAHMIG
AEIFATVGTEHKKKELLSLGYGIKEDHVLWSREKSSAKKLLEMTEGKGIDVILCSARGDFMHEYWK
CIATCGRFVDIGRTEVLDNGRLSLDVFRRNATFTSFDLEVLSKTKPEVTASLMKRIQQLKSQGHIR
PLPCQKFHISEVEQAMKTFARGSHIGKLLLQYDDQSDLGLNVRRDPFKAQFDPNAAYLLVGCLGGL
GRSFSSWAVSRGARNLIYLSRSGAGNEIAKSFIDGLIAKDINISVIRGDVTSLQDVRTAVKSTKLP
IKGVIQGALTLHDGLFESMNLEKFNRTVRPRVIGTLNLHEALKDCPLDFFELWSSWTVVFGTAAQS
NYLASNAFLDAFARHRQAAGLPCTSLALSQVLGIGIVSYMPEYQQAMIRNGFYGNDEEDFLKYCEA
GITTQNMKDHPTFRYDPQTTSHLLAGIEPAGLQNVGHQYPLSEMLWYRDPRFKNLTQATNLLSSDS
KSKPLDQGQEGCALDRIRMKISRLLYVPLDEVDVGKPVNDYGIDSMIAAELRNWFFASFGKDISLL
KLLGATMTIQKLAEEAEQEE
>g3567
MSGIDPNARKEVNDAIINAGSAAVGQMIDTANSSVEKRRKNRDERAKREAEEKAAKDKADAAVRTP
PTFWGFGALFLRACNMLTKTFNRKTTPLLRKIRTMDTNALVDIIHEVQAQFWVDKVNETHRTGRLC
KWVSTFHPDRLPCQLDGTFHHGAFNAGMKMVFSDNTAWMVRFPRVGMVSDDYADEKVAMEVAALNL
IASRTSIPVPRVQAWGPAISNELALGPFIMMDFVDGVSLSELLRDPNAERPTRLMREDISDSDIES
IYRQLATFLLQIFELDFDQIGSLPSPEAGAASPTPRRPLTFKAHSILQNGGVDTFGDRSQGFATVN
EYFQYVIGQDWEQLVHQPNSIFGSYDAQNKYMAFKALRNSIPDLVHEQYDKRNFKLICDDLGLANL
IVRSKEDLTVVGVVDLEWSYIGPAQLFGSAPWWLLQDRPVNSSWDYTGDEPPKIADRYLKYLNIFI
RVLEEEEARMSEHAEKELSGLVKWSQASGAMWLHMLISSGFNDHRSFPFTQLRRHLGPAKWAQYER
GCGSAEELDAFAARKVIQLDEYDEALEKKEEEKALVDSGKMTKEAFIANALSLSDEPCHSDITTPV
KIEMGRREGLIHRVAIWLGLRAA
>g3568
MVITLLWLYVARNFSKIMGFESPEPLANLYSRSYFRATWITTALDAGFWTAMKVRPKWLKDIASMV
FTAYYLIAAEQAEEKVRRVRATLTVEHLRVSWNKATTPYLKFFNGLVRPKYTKYAPRQIRIARPKE
SSYREPVNAWLYFDGTLAELREQTQLVLDVPGGGYVAMDPRTADDKLLAWAGKTRVPILSLDYKKA
PEFPYPYALNECFDVYRAIVASRGRCAGLGGHSRPHIIVSGDSAGGNLATGMTIMILQSGRTEARK
WLGEVPLPPPNGLVLIYPSLDMNIGSWMTEEQMSLIQDRGMRKTNERVLRRKSEDYSRLTPKSSRS
LSSDYHDQASPVKDYFSNRPQNKDSEKSVTKIDPTAQNQPTKIKTRIAVSSMISYFGDRILTPEMM
RAMIILYIGPHHRPDFKTDFLLSPILAPDAILATFPKTYFITGERDPLVDDTVIFAGRIRQAKLHR
FRERQDLALEKSRVEFNEKEHVEVNLIPGISHGFVNFPGIFPDGWKHIHKCARWITELLELAAERE
LRNSSSSIASTVEKSLRASDSKDFKQQGKQQDSRRSSLAESSADEDHPLEMSVTRFTPIKQSVVEG
NEGNSKAPSRSDAQNKSATDRSKRKDSTPSAQQPRARRPSLSTSNLKPLSFPLHLPLLKTVKSSVN
NTDTNGFQVESVDDDDDGTKSAVSENGKTFTRLPSEEDLIGRRMDGLAGGLMGLGEGARTP

>g3569
MFNAAQDLVGLVGLLWPAAREDLYAQAEGDPELKKVLQAIKKPGRDPTKELSKLSSSHLARIELMN
TESLAKIFGYRENLHLRVAARFQPLLDLICIQRSQGSHLKTHNGESVKLGTLFKKIHYCIAKAEFH
PAEEVEYQMWHRAAANEYVSAGEKTDIKIAPPTAAVRKSSRDQKDFPVSTIPLRKLAMANFSTKLA
RLNAIMEAIGGDTHVATLRLWRAKGINAEMIHRITRGKGERLAATAEELLSFFSEGSPALRVAFQE
VLKAKVIEQLNKAQFNQHQKLIIGEVIPANAFFLETVLRALLIDARVFHADLSHQAKGQMVDLFND
AKSTLKVLIMLYDVGAVGLNLHKACNRVLISSLPRSRCQESQLAGRALRITSEFPLTVVRRWTPNS
HDQFRGARQAEKATLQLAANAQDRSIQRLIIQLLQALQPEIDQCHQSAEGKDLLREMTARNQKRSE
AATEVIREKRSRRAPERYASLKYDSRGGILPHSSSSETAGLDSDDESIVHEEDRDEEYEIPDDIDS
EDEMLDTNWEEEDLNTDLEVDNDVANFVNENHPDDETRHKLALLQLPPHKTWTEKDLENELFMRVG
LHLLYNKIHGGSSLHLERSIHIAYLQFPQKVIDTMKGYTEATEKQMETAMSI
>g3570
MASKRGSSSDQGPSETKKVRKGITGKDAINDCPGRSSDPSTFIETEGNEQKTAAYVGYSQVAHLRQ
LVISWPLMKSYPRFDAVCRGLSRKSAFLLLILSNEDAYTGVKPDQKWPLGDNPDSTIRSRHIAAILL
DVEKDLKDFRFAPATNASKNSRFRNEFTQKYPDLTIATYNRKCPPPELGPLEKEHRLESINRCWAL
LRYMRNCIQSKCWDSRFGNDGVTVKVSVAPSHIPGWMRAADTLNPPEIAPQGEVATADNATPIVVT
VVWERTKKFVGYDELMTVLETAEEKEITIPDDIYQLPTNADPRLFKTIFQFKDCLRRLYRCPEIGM
DIRRLHLQYTITSSEEQRMVNILIGDWDKTLSIFDNKDFSSFTVKVLFAAVEDNSVSLFESFEPLP
ALASFFTTAESDVTVNVQNVNKNLIEYLESIPASNSDEVGLPPEPKAFDNKNDFLAYYSGFDVETE
EGKRSFQLKVLSNLSGNANSATVTQEKLPRDLANADRQIISNIQEAIRHDSIEEESYDEKNDKLMQ
AQYYTMQRAFSGTQRRAGPPLDVCMRLLLATKQPNGLYKSELLKDFVKSELYNYQLCGAVGVVLKL
YGYVDAELLLAGTGQLDHPQAADVRVAASQLRDICIHGALLADETGFGKTKQALLAAVLHTFLYTE
FDDDGEQCYRPILIVLPPTLIRQWAVEIFNEWPLFRVFLSYEDMTLRSSVDIDQIPHIAMR
>g3571
MRVTVGVDHIDDIRQRICILFPDCLPIFLELLLCSPVRPINSAINPDDLALLDVHQNVSPEFPSSK
DPRLDIIIVKPQGHVQCDESPYIGPQDRICARSGTVAVLANMVDAEKIIHVRGTPSSGKSTLALLF
RDFLRQNDRKVFLISGWRRCLEDLDGEDPWAKFAEVLRVKYRNVHKIKEFFAKDTVIIIDEAQGTY
RDTTFWEDIVKDIRGEKRFEIQLCLFCSYGSPAAGTDYDPIDNRTPINFTAKQTVSLTPLVADGIG
LFYDRHEFDDVVTRICSWDATQRYSIEAGARDYIFGLTNGHPGGVLSIVKYIFSVFRSEIKHGRKS
TITTADVVDVLAKDDEVFEKIEGTVVTRSFPTRGGFTPQAKNVLCKILEEGFVPFDRSDETDPGLR
VCYENGWIQRALRDVGSKETEYIAVLASRLHEKYVEYLIGTSPSPFPANDFPNILILCTQILQNFS
PKRLRHCSAGKMSTAARLRPVEAQYQDEFYRAFNKVVGRGVPISSEWSPGSHGRVDFWIPEPRWGI
ELLRDHSRLDQHCNRFKPGGLYHPWIVNGMINDHIVIDCSTSFPRNAHSDPKLIRAVFTQDYSLLT
ILDHNNNPLGHPIVLTN
>g3572
MALAMKLSDVVAKHDSHGNAKSPGAELKKIKPLATLNEQRLQYDTFDIQHPVTNSEQLWKRAFENL
NATDKDSLVQDSSSQDTPSLEEIIQYVKLQQTKCMEKRWKIEIKGKTVYLRDLCDKIILWVNKFKE
IGDIVMQYDPGHAALPWAAFRLLLQVTVNDNQMFSSMAEGLERVARLLAVYSDTQYVLQGYSTSAK
LVDALIRLYGTILTYFANAIRYYGSGALTDLWQLEERLVKSASVSGIEPYLQRMDDAERHVRSLMD
NLNLEYQVDSRKNMNFVLQYHHETFRELKLTSNRISRDSLRRWLHCVSTEDRYEQVLRSTLEKTCT
WFPDRNEFHNWVTSDPDNGRKVLWIHGGPGHGKTYLCASLIRHITSKKEEYGLGEIASFFCSSDFE
SHRKPEMILRSWIAHIITVNNDAFDIAADVWNGNDIRPATTDEIWKVFRLIIREIRHCIFVVDGLD
EWIGADPDEEYEIVEFFERLWDESMDCECRFLLCSRDDPTIRLCTKLPSIRSSELIHYAILSTDTD
TDIAKFAKNTVTKKLGSYNDQKFLDEISEEATKRANGMFLWIRLLEQRLHRGENKKQLRNAVSAPP
KMEKVLERELQRIMQLEDTEKSRAIAILRWILFAVRPLTVRELTEAMITDEASSFDYPYDDLPEAW
DDDHIDRQIRRFCPSLVEIRGDSTDIQSHTVHIVHSLVREFLMKDVRKNPIWDISFKDEERQHTYL
AKTCLQYVRFDDITEGYQSSATHQENLLQKFQFLSYAAEMWAVHRTTSNEYASEAESLLQETFDPA
SGRWLVCHEIFEKKVEKFDVYEKQSLNMPPNALYYAALMGLCDVISWLASIGCDVNSVGGRYGSAI
QAAVVKGRKEAVEILLRHGANVDQIGGEYHSPLLAAARKCSDKTTEMILDILIDEKADFECADKDG
KTAMHYAATNGSQRAVQKLISNGAYVDCEDNEGSTPLHLACKNGHSAVARLLLEKGADVNARSEEG
RTPLHLASGNGHHDTVDILLKRKAEVSALDSSGSTPLHLASQNGHHIIVEILLRHQAELNMLDNDG
WTALHAASGKGHQTIVQLLLKRGAEVNIADNQCQTALLIAGSHGHTDIVRSLLEKNADVKARHRDG

FSTLHIACQEGFQPIVELLLSSGADADEIDNDGYSALMVAAQNGHQSILELLLREGANVKTQSSSG
WSAIHAASQSGHHQIVQLLLKEGADVNIAGNEGHTALIQATHGGHSDTVQFLIRHDADATAQTIHG
YTALHSAAQNGNQTIVQLLLDKNATAKAQTDDGCTPLHLAAQNGHQTIVQQLLDNAANVKAETDDG
WIPLHLASQNGHLNIVKSLLEAGADVDSETKARCTPLHLASQNGHQPIVELLLELGADVEARTDSG
WTALHLTSQNGHQKAAQLLLDRKANVGALTNSGGTALHLAAQNGHEKVLQLLFERGADANIQMKSG
TALNLASQNGHLSIVELLLKEGADANAQVPRSGYSALHLASQNGHKAIVQLLLERHVDVNSQTYEK
GRTALHLACANSHEDIVQLLLEHHADPNPHPRQSWTPIFAAVQNGLYNITKLLLANNANVNVHDDL
GFSPLLVACSTGYYSVVKLLLENHADVHASELQRWTSLHLAAQNGHYEIVKLLIEAHIDVRAQTID
GRTAVFIASFKGYHSIVKLLIDNNIDVNISNENGWTPLQVACQDGHYSIVKLLLENGASVNAPNEN
GWTALHAASLHGHYGIVKLLLENGGNIKAAYQNGWSPLHAASLHGHYGIVKLLLRNGADIKCPTED
DWTPLHVASQNGHYGIVKIYVEHNADVNAADDDGDTPLHVAARNGRVAAAQLLLQAQANPRALEKN
SRSPLTYAIYWGHLHVAIALLDHGVSSGVGSFDNRSWSAGICKGDSSVVSHYLQQGYDPNTEVCPG
VSALLAASCLGHGHIVKLLLDKDAVFDNQNSHGWAPLWGAAFNCKTDIVHTLLQHHVPVDATYVKQ
RSALHAAAFHGHDSVARLLLQNGASVDIEDSQYSTPLHEACREGKEEVAKTLLDYKASINMCDVHL
HTPLHLAARKGHESIVKFLVEKGADLDSKDENGQTPQLLAEKDGQTSVAELLKNLKDEDEDDE
>g3573
MASSSSASAELSADGSAPMPSATTNLAIQDLKQSLFDAITTASDSRSRAVENQFVTMEQLKELIQT
TLDKLIEAKAESTAEDEEEDKETEDDKKKSSDAASSTSEIKIVNEIWDRDTWDFVLVDGSSTVKTD
ELPPFGFQVRKRVDRENVVTTFIDLKSPDLRDILRDVFKDIRSVDLASQTPTIKADVLYHFLPELK
ELSQTGQSDEDKKEYFQNLGKICQYLEEAYRVTTDRVESLIRGGYISYDLLWALFKPESLSYCSCP
ATDLPRCIKYIEGYTQKPNYQEDKEFFKIRACFLNVDGGSVRETLKTYEIEKFNGRKRIEDLPIYP
LQYYPDQGIKEFLISRGRKFVSLASNRCHRRYSGNIFFEPEDPNEIKLARKFVDGRIMVDSVAFRK
MVPRYRRLRSETPASYSMFWSSGGTPAKVAGRNCLDPEDLQDDDFLICSPTVLGFALDDKKWGELS
VSEVEEIQFSASSFGKVLIPEKQKDVILSLTASHLVSTSDKEFGDIIDGKGRGIIILLHGPPGVGK
TVTAEALADHLRKPLYSVSAGNMGEKADEFEAELTRMFRIASDWESILLIDEADTFLQRRDQNIQH
NRIVATFLRTLEYFDGILFLTTNFVNNFDKAILDRVHLKVRYEGLDQSKRKNIFIHFLQSINADVK
DADLDRFCEVRLNGRQIRNIVRVAHSLASHQRLKTSAAHIQLALESNGHYVRA
>g3574
MANKIRVTADLGEVENPPIYDGQGTEEDPFVVEFIKDDPGNPMNWGSSRKWLITAIATVSVFAITL
TSSAYSVSANEIFQDFHDISSEVFTLGLSLFVLGFAIGPALWGPLSELYGRQKLWVITHIAMVAFL
GGSAGSKNVATLLILRFFAGTFGASPLVNSGGTIADLFPPAQRGLALTVYAVAPFLGPLLGPVMGG
FISENIGWRWVQGVCTIFIGVVGVLGVILIPETYGPVLLQKRARQLSKANGRSQIYISVLEKSQGT
KKPSEVFKRALFRPWILLFQEPIVMIASIYMAIIYGTVYMFMGAMPIVYSEDRGWSEGIGGLSFLG
IAVGIICGLVYAIWDNNTRYMKLVVSKSTAPESRLPPAIIGAVALPIGMFGFAWTNYPSIHWSVSI
ILSAPFGFGCVSVLLPIVNYLIDSYTIYAASVLAAAAIFRCVVGAVFPLFTTQMYDNLGIHWASSI
PAFMTLVCLPFPLVMYRYGMQVRMRCKYSFEAAETMKKMQMQQAQATQAKVEERNSASE
>g3575
MYGANAGDIDVPDRPFDEQLERSLPTSEVLISADYVPLSLAWPVRLAHKLISSNFILTASAQDKQL
ALSTVQWADSSLCLRECPPLSGMSDSSDAETARPAMPLPLPGSPGSSWAVYREKFKTLFHDADPRV
CAAFWFFGLINNVLYVIILSAALDLVGSGFPKGVVLLADVVPSFVTKLTAPYFIHLVPYPIRVLIF
VALSVCGMLIIALTPAHNDGGSISTKLAGIVLASLSSGAGELSFLGLTHFYGRFSLASWSSGTGAA
GLVGAGAYAVATTSLGLSVRFTLLASACLPAIMLVSFFVILPLGPLSLAAREATQYRAIDDDHDEE
YEARHGTREDETNEQDGLLGPEHSRPYAKAAERGKVSRAWASFQENLNRSKGLFFPFMLPLLLVYV
AEYTINQGVTPTLLFPLKETPFDQYRAFYPAYNAIYQAGVFISRSSSPFFRIHHLYLPSFLQIANL
AVLTLHALFNFIPTVYIIFFIIFWEGLLGGLVYVNTFAEIADRVPAKDREFSLAATTVSDSGGICI
AGFLGMAFEVWLCRWQVSQGRKYCTMS
>g3576
MESRPPSIPHPPFPRPSLFSTAPQHSRQYGSSQPQTSRPEARSHTAYESLRRPADEPARTTPTYGY
AAHSYNPDLRAQARSTSPTRFGAMQSDAAKRSALASPVKNNGSLGKDSAIYRDASGMRRTSSPRDQ
ESRWSANRGRNLKSPTAAATSESSAHTIGNRQMPPPSPPQPYPARNSSASLSQTSSSLFSRDNGFG
SIHRPGTSMSISSMLGSDPDRPSRDHPTSLFSRPSVSSPFGTSRNSTGAMSPPTAPARPASSIDHP
LMRRSETPDKPVAKDPLSRQSSRSDSGGFSDSTKFSFGRSSFSQYPEKPTTTQKSPRNSTASDPPF

GHSRRVSLNSAIQRPNSQPQQEESRAPAYSPRTRASGDSIFGSAHRNTSYLDDTRPFARYGGLYSD
RAREEQLAREREKERNTPQETERKSAFGLLHGRYGAPANRDEDRPPNRPSWEIGRSQPKSPEARRF
TTASESGAGSGFGFGAIQSYTKSLGSQLGASRSAPATTPSHSNFSLHSRQSQPTPPPNEQSFLGKP
QPRPRPLSVTSVPSASQSTLGALGSAAADEQRRKGSDDLIQHRTLLGIGADGKRAGRASPLPQAVQ
GAQAQFIGSAGETGLKGELGRVFAGIGSGVGTGPSSSTGSGPPTPMTASPFKRDSFGASDHADEGK
GGRGGAALGRKRSSKDEEPSDLGEQAQELRGTPSTRGGSRRGRHVHHHHHQYVTLNDILWGRTNHE
NYSHHHHRSKNEDESVAPRSNIGATSVLGKAPTPAEPVAPVATTLPAHHHHHHHHHHHAPRNASSN
ITSAPAVVTLRDTRTLVNIKPLLESVAQFPRHHLGSTLYAPRIGVPSASSSGESQKFGYTSTPVPI
PRFEGKENCTFTIRVPRFRIDPSHREEICARRAVWGTGVYTDDSDPVAAAIHSGFIRGEWGEDVDI
SMLDLEIKSDYHHAPQPTADSSATAKPSIPPVPPPNKDLHITLLILPRLERYESTLMFGLKSRAWE
GNHDGMSFKVERIEWVDEGSTRGEERSGAARRKRLRNMMQTGRICTGPGLLRLRGGPGLLLGSTTD
LVAGQPASAPVQPVS
>g3577
MSSVLITGSSRGLGLELVKQLLLLPPTTISTIYATSRTSSPPEPLSRVITASNHRVRHVQLDVTDP
ASITAAADQVTRLSEGKGLDILINNAGTQSERPSSTSGMSLSDLESTMRINVTGVHLVTTAFLPLL
KASKTNSSQTKKIVNITSTLGSITWSHKSDIAPVPAYKISKAALNMLTVQYATELAPKKFTVFAIS
PGWLQTDLGGGYAHLKPEEGAREVVRILLEATHEKDNGVFRDICVKGFGGTYSGVNPPW
>g3578
MDWRFLSLQLFFSALLLTARAQTDNLGLKDGYISFSTANFDVELVKDAQVLASLKPKGSSFDFLPF
DYLPFRAGNGQYHNGDITFRYRSTSDQTWIDGDSSQARQSVLAITNTNGSTLAAADLKPTLPKGSP
LQITRQWLEXDGDLGLSFTIANQGSTSLEIGSLGFPTEFNSIFTNRTAEDIQAYCSLTDPYIGLDA
GYLQVVPVSGNGDALIVTPLRNTKTPLEAWRNLNEPDVYTADAYGSQVFEGFYEWQTYSLAYEENE
WVNATPWNTATSTVLQPGRSITVGLRFSLVSGGVRGIQRAVQTTGTPLTIGASYVVPLDLTANLFI
FHNASISNVAADSNAFKIVRQSKYLQLTPTDAVQGRTKITINYSDGKTQTVHYFITASGPDVLKTL
GNFTTTEMWFDDSSDPFGRAPSVMTWDDSVQAIVDQEPRVWIAGLSDEGGTIHLASTMKQFAQADV
AEVGQLEQFATQVLSTTVQNANNFTVRKSVFYYDPTVLPNYAYSSSIDWGNWWSWSQAAAYSTDRA
YDYIHVLGSYWALYRAGRDNPALFPNFDWEWYLAHAYNTTITCFATDSNGDGLVGYSRLGLMGETV
VGELLVDLRREGWTDEAAQVEAAMKLRADTWDTQAVPFGSEMAWDSTGQEGVYYWSNYFNLSSTST
KAIGTILGYSPTVSHWGWNGNARRYWDFIYAGKLQRIERMLHHYGSSLNALPLLAQFRQTPSDTYL
LRVAYGGITGPLSNVRADGSMYNAFHSFPDTLEGDAYSGDYGQNFLGVMLGSGTYVVHDPDVGLIA
YGGNIAVAGNTVTVNPRDPVRRRIYVAVLGVYVTISAGQIDKFIFASNGQPGSVQLTIVPGVSGAT
EVIVWVETPGTTDTYSVTTAGGQRLRGGTHFDLPSSGLQVTVAKSK
>g3579
MSGKIDVHHHYYPPTFTAALERAGGDPSGWFVPTWTLEADKEINAACGTGTTILSMTAPGACIEKK
PTAAAKLAREANEFAAAIRDAEPKSYGFFASLPSLLDTRESLEEIAHGLDVLKADGVILFTRYGED
NHYLGHPDFHPIWEELNRRKAVVFVHPTHAVDTNLVNKNLPQPMFDYPHETGRTAMDLITSGNLRR
YPDVKIILSHAGGTLPYLINRAAGMLPYTPDDIGLSAQEIIDEARKFYYDTALSTSHETLDLLYKV
AGADHVLFGCDFPNAPVPSIHFFRDLFDTYKVDGSRDQVDVTRRNALKILSGLRERLAS
>g3580
MSTQPGQFLAGKRIIIAGGSFAALSFILALNKLWNPSLKHPEIIMYERDDRDKSIEKDPYTLNING
FSQDGGLVAIRDLGLLDEVCKHGTLNSGDIRVWSDNWKWLSSINPSPYGDLPAATVRITRGNLKRI
LIQTAEETKTTLHWGLACTSAESLPDGKIRITISDGTTQDCDLLVAADGADSSMRACFRPNNMKTV
YAGATQIGGISRLTGLLPKPIDKDYVLQMSSGEGVCCIYNPFDENTIAWAVSTIGPERKAKTNFTS
EEFDALKTEALKTASMFQEPFKTVVEATLPDTAFIRPAKEKYAFRHDDSNPKGVLFIGDANHILSP
YEFVGANLALKDGWDLAEQICRNASMDAAVASYDRISIARFDPVFKFSHERIGFGHATGKKWLFYK
YGMAAQRRLKQVH
>g3581
MEYARLGNSGLKVSKVILGCMGFGSPEWAGWVMDEEKSLPLIYHAYQHGINTWDTADCYSNGVSEI
IVGKALKKYNIPRQRVVIKTKLYTGVDDEGGQTPIHLAQNNDGPWVNRVGLSRKKIMDAVDESVKR
LGTYIDVLYIHRLDRQTPREEIMRALNDVVESGKVRYIGASSMRAWEFQALQNVAIRNNWHKFIVM
QSYYSLIGREEEREMISYCKDAGIGLVPWSPLARGVLARPWTTRDSNRETTDLAMKALVRMRESEA
DKAIVDRVERIANAKGVSMAQVAIAWLLHHGTFPALGLNSPERIDEAVGALQVKLTEEEIKELEEP

YLPKGLALIEM
>g3582
MPFSLDGLVSTSISCVEMHTSGEPTRIILSGFPELTGTLMEQRAQAKENFDQYRRFLMLEPRGHRD
MYGAILRPETELVKVGEADFGVLFMHNEGFSTMCGHATIALGRFLVDCDDEQIFPRRKLLKHDTET
QTTAIKLHVPCGVVDVSVPTLSKGKSDPSRPVSFVSVPSFATGISVTIYLPKENRWPELGGRESIT
ADFCYGGAYYCVISVSELGFPQGISEDHIQKLSHATALLKKTVNENSDLSYLTRHVQTAEQGVLYS
VLIVDDTKRENVLSDSEIGLCFFANQQIDRSPTGGSVAARISLAYAKGDLLIGEQRKFDSIFTASG
TGLKGFIGSAFEEVKNEGEADGSRKNTCVRVKVEGYANYMGYSTFVAEKTDGLGVNGFFI
>g3583
MAPQRRKQSSVPEGYVEDLSKGRTLRFEASLPKLPVPTLEETAKRYLKSVHPLLSTPEYERTTKAV
QEFIKPGGQGETLQKRLIARRDDPKIDNWLIEWWNFGAYLGYRDPVVPYVSYFYSYKDDRKRRGNP
AARAATVTLSALEFKRQVDDGSLEPEYLRKIPMAMSSYQYMFNCSRIPAQPADYPQKFSPEENQHF
TVVRKNQFFKVPYVVNGQKLTSGELQKQFEVIYERAEHPVTPVGVLTAANRDHWTATRAKLLEVSP
ANAKALKDIESSAFLVCLDDAAPVTLEERAHQYWHGDGCNRWYDKPLQFIINDNGTAGFMGEHSMM
DGTPTQRLNDYVNSLIFGNKLEYDNTPVRSTLPPPAPINFELNVEVEKAINEAHQEHKSLIAAHEL
RVQAFLAYGKGLIKKFKCSPDAYIQMVIQLAYYKMYGINRPTYESATTRKFKEGRTEVIRTVSDES
TAFCRAHQDPNVPREEVVKLFREALKAHTKYTMDXSDGKGVDRHLLGLKKLVQEGEKLPEIFDDPT
YGYSSSWYLSTSQLSSEYFNGYGWSQVIDDGFGIAYMINEDSLQFNIVSKRLGCERMSFYLNEAAL
ELRDVLMPDLLAEKEKAKL
>g3584
MEKCWFTLAQNHYPPSTPTQKGPIQLGHLLSSLVPLPPPINTASGPLPLPAYMPIYPSRLTDLRWQ
SATKSSVSASATAQLPIPQAMGLVSAGGGLSIAFKKSTARFWCFDAVDVEIIQPDEEYIQDSLEIE
CVKAWIERKRKKSLTGTWSMFMITGLAIARGIKGDEKNGMVEEKGWGVELGPQVSVPGIVDVDISG
GISRTGTQKMTFSGCSDFVWAIQVMRISKRFMSSGLNFEPYVKGATYAVEEEEDIEKMLAGEAVPF
ESIHRDGDDIFVVTLQKDQDVVED
>g3585
MCGHPKAVQFLLDKGANLKARTKDGWTPLHAASYYGHTSVLPILLTQGADIEAELPFGSHPLHKAC
ERGYIEIIRLLLEHGAVIEAQAKNHQTPLYTAAFYGHPKAVRFLLDKGADLEARHVDGCTPLYAAS
RENHPEVAQLLLEKGADSEASSNTGYTPLHVAAEKGYEKLARLLVEKGRMRDLDKMTKSGDTALHL
AARNDHAEIVRYLLAHGADCDIPNKNGWGPLHTAAYKGHSSIVAVLLTASQGDLESQIRMDGYRPV
HKAAEAGHVDILLALLNAGAKINSRTTLGLTPLHVAVKFGKVATAKELLEHRADVHSQTYEVDGLR
TPLHFAVQNGDQRIVSLLLTYGANSRRLDKEGTSPLAYAEELKDEKMLALFNTTVETTVAGNGEDF
ILSVRGKTMTETSSTSSKAWFDKLDELRSVLLRPRGTVHKPVQFAVLDSGVEPAHPNKMMIHGYRD
FVDDSEEWKDNSGHGSTGVDLICKVVKMPKIYVARVFETSSGSAEVQDRIAEAIKHARTEWKVNII
ILASGFNRPHENMEKEILAACAEGILIFAAASNSGNSDFITFPANIPEVICMFATDANAKAITSGI
NPSPSLRGRPNFAILGHDVESRPRTKPHTGTSMSTFIGAGIAGLILEFSRQSIVSDIIGTDWKRYL
QTVRGMSAIFELMAEGGHEGYHCITPWKIRPIFQKSRELEREFIANAIKHALERCIRGS
>g3586
MNPNLNPASSGSNDLSHRSSLSGHRSSPQSTRPSISEGRRRSHLNLNDSPSALASAVNAAGGGIEH
YEHRSPSFSNYFRTASPSSLGGSPIIATGDPHHQRAPSLGELHQELEQEQEAQVNRLLQMIRQQQL
QLHQLQLQQQQGGQQPSSAIIDDNHNNNNNSNLTPSSERSHHQFPTLPPLPTPGISRSSTVHSHSH
SPFSTRRGSRGSETAAPAYLQSFSAAATPGESVFDLNDLVDRDRQHDTSVAPARRSSLDDSAYYQA
QVDSLNRDNRMLRARIRVLERQIAELHSSAAAAGGGGGAAADTETATAAVTGDGATHDEPAAEKNV
SG
>g3587
MSGSYTSDFSLLNSTFWVDLLSQRDVSSQVPINYVTQPSIALTAACFGDNQYLGDGGSLCLSDLLT
IGYRRFIIDVYWSNSNQQWLLCPISIPSTTTLDDESPYKLGNYTCSGSFDLTTIMDVLRSYIQSSS
ADIDSTLLFVVFNVHAAADSSNPDQPASVLSGVELPPTSHLLGNIANNALYGLIYTPPELAEERSN
LNGSWYHGTSSRTTVLQKYFTTTTNADNILSTPDGWPCQAYLINKLAKRFILGRGSIDPQLQGYNA
SGDDPYIFPADYIEDVIGVSTTSDGNGLQSGCFYSSQTTSLKNMNNSWALSTLDGTTANTSSSAAL
LLQNYTGCGISPIINQTLGGQTANIEVDPYRNMSISSMWSWAMGEPRNASSLPGYEQIPVNSDVLR
CAMLDPTSNGHWRAGNCSNAYRAACRVDNNPYTWIISDNKQSFSDSSNVCSTGSVFDVPRTGLENT

FLYNTILSTSNTTTTTVDEPVWINLNSIDVQYCWVMGGANATCLYVGDSDSVARRIILVPTIAAII
ILVITASTLFVKCNSNRRISRRKRVSQGWEYEGVPS
>g3588
MNAPPGMSPQQMAAFQQHMQQQVAAEAAKRGMTPEAFVKMQREQVAAEAAKHGLTPEQYVQGLRMR
ALQQRAAAQQAAQAQAQQQGQPGQPAQPGQPQQQPSQGAPGQPPQVPVQQQVQKQVNVNPNNPPDP
KAVALAKWLRTQDLKTRTCILDGQRKDMFKVKRAIRAIESPAYAKAAAKSKGLLPPVTDRASAENV
FKLLPLSLLALRVTKQDPHAGHNHGKTKRVKGLWTVKIEQHQETDPMMHYVFLYEGSQWKQKAMAV
GVVLAIFAVVLFPLWPIVLRQGVWYLSVGMMGLLAAFFAMAIFRLILFCVTVFVVPPGLWLFPNLF
EDVGFFDSFKPLWGWQEKKKKKVKSAKKGKSAAADXPAASEKAEATTTTVTPAVPSAAPATDDSKR
VLAASVEDAEEE
>g3589
MAVLSRLLLPTAIAALGVSGSPVSRSSCESIEDGYTCFTDVSRNWGMYSPYFSLESESTICSEVPS
GCDVTFVQSLXRHGARYPTASKNTAYAKLIDAIQTNATKLEGDYTFLKTYNYSWPAATLTPFGTNE
LYQAGIKFYDRYESLARDIVPFVRASSSARVVASGEAWNSGFQATKDADRHADKSQAAPVINVVLE
EGATFNNTLDHSRCTNFENSDLADDVQAGFAAIFVPKILTRVQAHLHGVTLTSTDIIYLMDMCAFD
TVARTSDASELSPFCDLFTESEWKEYNYYQSLGKYYGYSAGNPLGPAQGIGFTNELIARLTGIPVQ
DETSTNHTLDSSATTFPLNATLYADFSHDDGLEPIFAAMGLFNGTSPLSETKVESIEQTNGFSAAW
TVPFAARMYVELMTCEGEKEPLVRVLVNDRVIPLYGCEHDSLGRCTLDDFVDGLSFARENGNWASC
FN
>g3590
MADKLNNHSAQLLVRLPPAAGLESLPPVYTLANAQDERAKTLLLLLEKGHATVAPLREPKLILHSH
LPHLLGSAYFLGASAQQLEELYAHEITTLKEIDDTFITGDAIRKENWREFLGQKSYTVAYVQYFDK
[truncated: 450,888 more chars]
